# An Epithelial-Mesenchymal Transition (EMT) Preoperative Nomogram for Prediction of Lymph Node Metastasis in Bladder Cancer (BLCA)

**DOI:** 10.1155/2020/8833972

**Published:** 2020-11-03

**Authors:** Rui Cao, Bo Ma, Gang Wang, Yaoyi Xiong, Ye Tian, Lushun Yuan

**Affiliations:** ^1^Department of Urology, Beijing Friendship Hospital, Capital Medical University, Beijing 100050, China; ^2^Department of Stomatology, Beijing Shijitan Hospital, Capital Medical University, Beijing 100038, China; ^3^Department of Biological Repositories, Zhongnan Hospital of Wuhan University, Wuhan 430071, China; ^4^Department of Urology, Zhongnan Hospital of Wuhan University, Wuhan 430071, China; ^5^Department of Internal Medicine, Division of Nephrology, Leiden University Medical Center, Leiden, 2333 ZA, Netherlands

## Abstract

Lymph node (LN) metastasis is a lethal independent risk factor for patients with bladder cancer (BLCA). Accurate evaluation of LN metastasis is of vital importance for disease staging, treatment selection, and prognosis prediction. Several histopathologic parameters are available to predict LN metastasis postoperatively. To date, medical imaging techniques have made a great contribution to preoperatively diagnosis of LN metastasis, but it also exhibits substantial false positives. Therefore, a reliable and robust method to preoperatively predict LN metastasis is urgently needed. Here, we selected 19 candidate genes related to epithelial-mesenchymal transition (EMT) across the LN metastasis samples, which was previously reported to be responsible for the subtype transition and correlation with malignancy and prognosis of BLCA, to establish an EMT-LN signature through LASSO logistic regression analysis. The EMT-LN signature could significantly predict LN metastasis with high accuracy in the TCGA-BLCA cohort, as well as several independent cohorts. As integrating with C3orf70 mutation, we developed an individualized prediction nomogram based on the EMT-LN signature. The nomogram exhibited good discrimination on LN metastasis status, with AUC of 71.7% and 75.9% in training and testing datasets of the TCGA-BLCA cohort. Moreover, the EMT-LN nomogram displayed good calibration with *p* > 0.05 in the Hosmer-Lemeshow goodness of fit test. Decision curve analysis (DCA) revealed that the EMT-LN nomogram was of high potential for clinical utility. In summary, we established an EMT-LN nomogram integrating an EMT-LN signature and C3orf70 mutation status, which acted as an easy-to-use tool to facilitate preoperative prediction of LN metastasis in BLCA individuals.

## 1. Introduction

Bladder cancer (BLCA) is urogenital malignancy with high mortality and morbidity, which is usually divided into two major subtypes: non-muscle-invasive bladder cancer (NMIBC) and muscle-invasive bladder cancer (MIBC) [1, 2]. About 70% of new BLCA diagnosis cases are NMIBC, which are not that life-threatening but have a potential to recur and progress even though receiving repeated transurethral resections of bladder tumours (TURBTs) and intravesical therapies with Bacillus Calmette-Guerin (BCG) or chemotherapeutic drugs [3]. Moreover, patients with pathological T1G3 NMIBC or contaminated with carcinoma in situ (CIS) are more likely to develop into MIBC or exhibit lymph node metastasis [4]. Although only 30% of BLCA patients are diagnosed with MIBC at first diagnosis, they are responsible for the vast majority of BLCA-specific deaths. The standard treatment procedure for localized MIBC is radical cystectomy (RC) accompanied by cisplatin-based neoadjuvant chemotherapy (NAC), adjuvant chemotherapy (AC), or in selected cases trimodal therapy (TURBTs, chemotherapy, and radiotherapy) [5, 6]. Moreover, patients with advanced or metastatic BLCA are recommended for cisplatin-based chemotherapy or the novel immune checkpoint inhibitor (ICI) immunotherapy, which were found to improve the prognosis of patients with advanced malignancy, such as non-small-cell lung cancer (NSCLC), melanoma, and BLCA [7–10]. But we have to notice that the response rate of immunotherapy is only modestly above the historical 10% response rate to traditional chemotherapies.

Lymph node (LN) metastasis is an independent risk factor for BLCA that patients with LN metastasis demonstrateds a poorer prognosis compared with patients without lymphatic spread [11]. As pelvic is the most important route within the draining lymph nodes of BLCA, mounting evidence suggests that addition of pelvic lymph node dissection (PLND) and/or extended lymphadenectomy to RC showed substantial oncological benefit compared with non-PLND cohorts, irrespective of pathological nodal status [12, 13]. And PLND was highlighted by providing prognostic information such as tumour burden, lymph node density, and extracapsular extension of metastatic LNs, which can guide subsequent treatment management [14, 15]. Currently, various PLND templates are used in clinical practice while extended PLND templates were reported to provide optimal recurrence-free and cancer-specific survival when compared with standard PLND and superextended PLND [16, 17]. However, the potential drawbacks, such as increased operative time and adjacent visceral, vascular, and lymphocele injury, must be weighed against those potential benefits. Moreover, nearly 50% of patients with MIBC already exhibited occult distant metastases at the time of diagnosis [18]. Peyton et al. reported that NAC followed by RC could induce downstaging and significantly improve prognosis compared with RC alone for patients with BLCA [19]. But Cha et al. found that LN-positive patients previously treated with NAC exhibited a strikingly poorer prognosis than LN-positive patients subsequently treated with AC and should be considered for regimens using trimodal therapy approaches or novel agents [20]. Therefore, accurate evaluation of LN metastasis in BLCA patients is of vital importance for disease staging, treatment selection, and prognosis prediction.

As the bladder has a multitude of primary lymphatic landing sites, lymphoscintigraphy and the sentinel LN concept have limited value for the detection of regional LN metastases in BLCA, which has been confirmed by several clinical studies [21, 22]. Currently, many imaging techniques could help the surgeons to remove suspicious nodes which could not been resected in a standardized template. CT and conventional MRI, which mainly rely on morphologic criteria including LN size, shape, and internal architecture, are widely used for preoperative detection of LN metastases in patients with BLCA. Until now, there is a consequent lack of consensus regarding the normal limit for size in the diagnosis of pelvic LN metastases as the size of nonmetastatic LNs varies widely and may overlap with the size of LN metastases [23]. In BLCA, their diagnostic accuracy is less than optimal as CT might miss 30% to 40% of LN metastases, resulting in upstaging which pathological N+ is frequently found despite negative preoperative imaging [24, 25]. Furthermore, positron emission tomography (PET) depends on the increased metabolic rate of specific tissues and their volume to improve its diagnosis efficiency of malignancy, which is different from conventional CT or MRI just relied on traditional parameters such as size or shape. But a meta-analysis demonstrated low sensitivity rates of choline PET/CT (pooled sensitivity and pooled specificity of 49% and 95%) in the setting of LN staging prior to RC [26]. The main cause of low sensitivity might be that only small amounts of the tracer are accumulated in small metastases, which are probably insufficient for positive imaging and are likely to be missed. Moreover, some groups even used molecular markers, such as MUC7 and uroplakin II, to detect occult LN metastases, but the accuracy efficiency is not that stable [27, 28].

With the rapid development of next-generation sequencing technology, we could comprehensively investigate the transcriptome and genetic alteration in individuals to identify the difference of BLCA in terms of LN metastasis status at a genomic level. The last decades have witnessed a transition away from multiple biomarkers from individual analyses to combined analysis of a panel of biomarkers for construction of a signature, which was recognized as a promising and powerful approach for clinical management [29]. Kessel et al. even established a gene signature to predict LN metastasis in MIBC before RC, but their model showed low AUC with no clinical applicability [30]. In our previous study, we found that epithelial-mesenchymal transition (EMT), which is a multistep process where epithelial cells lose their epithelial characteristics and gain mesenchymal characteristics, such as motility and invasive properties, is responsible for the subtype transition from NMIBC to MIBC and significantly correlated with BLCA malignancy and prognosis [31]. Therefore, in the present study, we aim to establish a nomogram incorporating an EMT-LN signature and somatic genetic mutations for preoperative prediction of LN metastasis in BLCA.

## 2. Materials and Methods

### 2.1. Data Collection and Processing

The TCGA-BLCA dataset, which was utilized as the training and internal validation cohort, was obtained from the TCGA Genomic Data Commons (GDC) data portal (https://portal.gdc.cancer.gov/). The Level 3 transcriptome HTSeq-counts and fragments per kilobase per million (FPKM) data of the TCGA-BLCA cohort were downloaded from the TCGA Genomic Data Commons (GDC) data portal (https://portal.gdc.cancer.gov/). Since multiple ENSEMBL IDs mapped to a single gene symbol, the highest expressed ENSEMBL ID was used. Transcripts per kilobase million (TPM) values were transformed from the FPKM values to represent the gene expression, which is more similar to gene expression from microarrays and more comparable between samples [32]. Detailed information of clinicopathological characteristics in the TCGA-BLCA cohort can be found in our previous study [31]. Then, we have enrolled 128 samples with LN metastasis (LN+) and 228 samples without LN metastasis (LN-) as the entire TCGA-BLCA cohort. Three independent microarray cohorts including GSE13507, GSE31684, and GSE106534 were obtained for external validation from Gene Expression Omnibus (https://www/ncbi.nlm.nih.gov/geo/). The raw data were processed via RMA algorithm background correction, log2 transformation, quantile normalization, and annotation by the package “*Affy*” in R [33]. When several probes mapped to a single gene symbol, the highest expressed probe was annotated as the gene expression. Data were analysed with the R (version 3.5.3) and Bioconductor packages.

### 2.2. Differential Expression and Functional Enrichment Analyses

Differential expression analysis was performed by comparing LN+ and LN- samples in the TCGA-BLCA cohort through package“*DESeq2*” in R, which contains multiple testing with an embedded Benjamini-Hochberg procedure for *p* value adjustment. The significance criteria for determining DEGs were set as falsediscoveryrate(FDR) < 0.05 and ∣log2FC | >1.0. Then, gene set enrichment analysis (GSEA) was performed to investigate the difference on biological processes between LN+ and LN- samples through package “*clusterProfiler*” in R [34, 35]. We also utilized a single-cell gene set enrichment analysis (ssGSEA) algorithm to validate the difference of EMT pathway enrichment in individuals based on gene sets of “h.all.v7.1.symbols,” which was downloaded from MSigDB of Broad Institute (https://www.gsea-msigdb.org/gsea/msigdb/index.jsp). To further investigate the relationship between EMT pathways and LN status, more EMT-related pathways were used for GSEA analysis, and EMT-related gene sets were collected via key words “epithelial-mesenchymal transition” or “EMT” in the GSEA website (https://www.gsea-msigdb.org/gsea/msigdb/search.jsp).

### 2.3. Somatic Genetic Mutation Analysis

The TCGA-BLCA somatic mutation data processed with the MuTect2 algorithm were downloaded from the Genomic Data Commons (https://portal.gdc.cancer.gov/) using the package “*TCGAbiolinks*” in R [36]. The significant tumour mutated genes (driver genes) were identified through “*MutSigCV_v1.41*” (20) (http://www.broadinstitute.org), which was a reliable algorithm for mining the driver genes in disease development and has included the following features: high mutational burden relative to background expectation, accounting for heterogeneity, clustering of mutations within the gene, and enrichment of mutations in evolutionarily conserved sites. The significance levels (*p* values) from each test were combined to obtain a single significance level per gene [37]. The procedure was processed with default parameters, and *q* value was set as <0.05. The oncoprint of mutation landscape was visualized by package “*ComplexHeatmap*” in R [38]. Then, all the significant frequent nonsilent mutation genes were validated by an independent test between the LN+ and LN- groups with *p* < 0.05.

### 2.4. Generation of EMT-LN Signature

Total 228 LN- and 128 LN+ samples in the TCGA-BLCA cohort were randomly separated into two datasets based on 10-fold stratified sampling, where the training dataset included 9-fold LN+ and LN- samples and the testing dataset included the other 1-fold sample. Then, the EMT-related genes were screened out through the DEGs and identified as the candidate EMT-DEGs for further analysis. Least absolute shrinkage and selection operator (LASSO) logistic regression analysis based on the package “*glmnet*” in R was performed to select primary predicative features and build an EMT-LN signature [39]. The optimal values of the penalty parameter *λ* were determined through tenfold cross-validation error for dimension reduction to reduce noise or redundant genes. The risk score of the EMT-LN signature for each sample was calculated via a linear combination of the selected features and weighted by the corresponding coefficients. The formula of riskscore = ∑_*i*=1_^*n*^(coef_*i*_ × Expr_*i*_), where Expr_*i*_ is the relative abundance of the feature in the signature for patient *i* and coef_*i*_ is the corresponding coefficients of feature *i*. The correlation between EMT-LN signature and LN metastasis status was then evaluated in training the testing datasets by using the Mann-Whitney *U* test.

### 2.5. Development of an Individualized Prediction Model

The EMT-LN signature and significant tumour frequent mutations were selected as candidate features for univariable and multivariable logistic regression analysis. Then, features with respective *p* < 0.05 were retained in the prediction model. An EMT-LN signature-incorporated nomogram was generated with the packages “*rms*,” “*nomogramEx*,” and “*regplot*” in R as a quantitative tool for clinicians to predict the LN metastasis probability for individuals.

### 2.6. Validation of EMT-LN Signature and Nomogram

After construction of the EMT-LN signature, internal validation was performed in the testing dataset of the TCGA-BLCA cohort based on the formula of the risk score established in the training dataset. Then, the receiver operating characteristic (ROC) curve and calibration curves were used to detect the prediction accuracy and stability of the nomogram by using packages “*pROC*” and “*rms*” in R. Moreover, external validation for the EMT-LN signature was also tested in the three independent GEO cohorts (GSE13507, GSE31684, and GSE106534). Since the mutation data were absent in these cohorts, unsupervised clustering was harnessed to determine if the EMT-LN signature could help us to distinguish LN metastasis status and whether it was associated with prognosis in three GEO cohorts. A supervised hierarchical clustering method (Ward.D) for analysis of the EMT-LN signature was applied to identify clusters with *k* = 2 based on 1-Pearson's correlation distance. Before supervised hierarchical clustering, expression profiling was transformed by log2(*x* + 1) and median-centered.

### 2.7. Statistical Analyses

The statistical significance of continuous variables between two groups was estimated by Student's *t*-test or Mann-Whitney *U* test when appropriate. For categorical variables, Fisher's exact or *χ*^2^ tests were used. Kaplan-Meier curves and Cox regression for survival analysis were generated which is performed by package “*survival*” and “*survminer*” in R [40]. The significant differences between survival curves belonging to different defined groups were determined with the log-rank (Mantel-Cox) test. Multivariate Cox proportional hazard models were used to estimate the hazard ratios of variables and determine independent prognostic factors. The oncoprint was used to present the mutation landscape of significantly mutated genes in the TCGA-BLCA cohort via packages “*maftools*” [41] and “*complexheatmap*” [38] in R. A nomogram and calibration curves were built with the packages “*rms*,” “*nomogramEx*,” and “*regplot*” in R. DCA was performed to determine whether our established nomogram was of clinical usefulness according to Iasonos et al.'s suggestion [42]. The package “*pROC*” in R was used to plot and visualize receiver operating characteristic (ROC) curves. All statistical analyses were performed with R software 3.5.3. Statistical significance was set at *p* < 0.05.

## 3. Results

### 3.1. Demographic Characteristics

**Figure 1:**
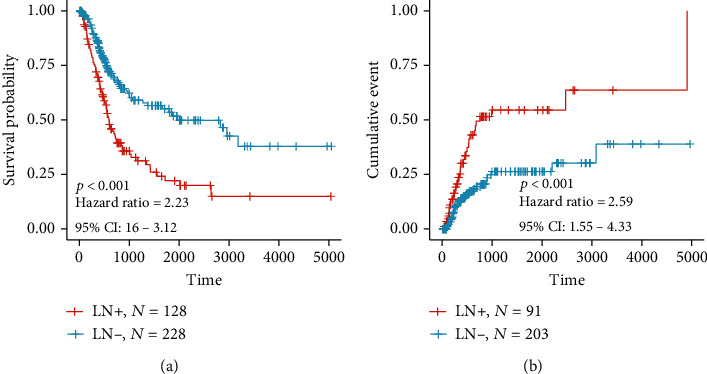
Association between LN metastasis status and prognosis of BLCA patients in the TCGA-BLCA cohort for overall survival (OS) (a) and for recurrence-free survival (RFS) (b). The difference in the prognosis of LN metastasis status was measured and visualized through Kaplan-Meier survival curves and determined with the log-rank (Mantel-Cox) test.

The design and workflow of the study are shown in Figure S1. We comprehensively evaluated the difference in clinicopathological characteristics based on LN metastasis status. The distributions of gender and age (dichotomized by median age of 65) were not different between LN+ and LN– samples. But we observed that the histology subtype (*p* = 0.0237), grade (*p* = 2.92*E* − 22), lymph nodes positive by hematoxylin and eosin (HE) (dichotomized by the number of 0, *p* = 6.41*E* − 36), lymphovascular invasion (*p* = 5.40*E* − 14), AJCC pathological T stage (*p* = 1.07*E* − 06), AJCC pathological N stage (*p* = 8.35*E* − 50), AJCC pathological M stage (*p* = 8.91*E* − 17), AJCC pathological tumour stage (*p* = 9.765503*E* − 71), extracapsular extension (*p* = 0.002231268), and tumour status (*p* = 1.05*E* − 13) were significantly associated with LN metastasis status (Table 1). Moreover, the LN metastasis status was also significantly correlated with survival-related parameters, such as the primary therapy outcome (*p* = 1.13*E* − 05), additional treatment outcome (*p* = 2.59*E* − 06), and vital status (*p* = 2.02*E* − 07) (Table 1). As expected, Kaplan-Meier survival curves demonstrated that patients with LN+ exhibited poorer overall survival (OS) than LN- patients (*p* < 0.001, HR = 2.23, 95%CI = [1.6‐3.12], Figure 1(a)), and a similar tendency could also be found in the recurrence-free survival (RFS) estimation where LN+ patients presented a higher recurrence rate (*p* < 0.001, HR = 2.59, 95%CI = [1.55‐4.33], Figure 1(b)).

### 3.2. Differential Expression Genes (DEGs) between LN+ and LN- Tumours Are Enriched in EMT Signalling Pathway

**Figure 2:**
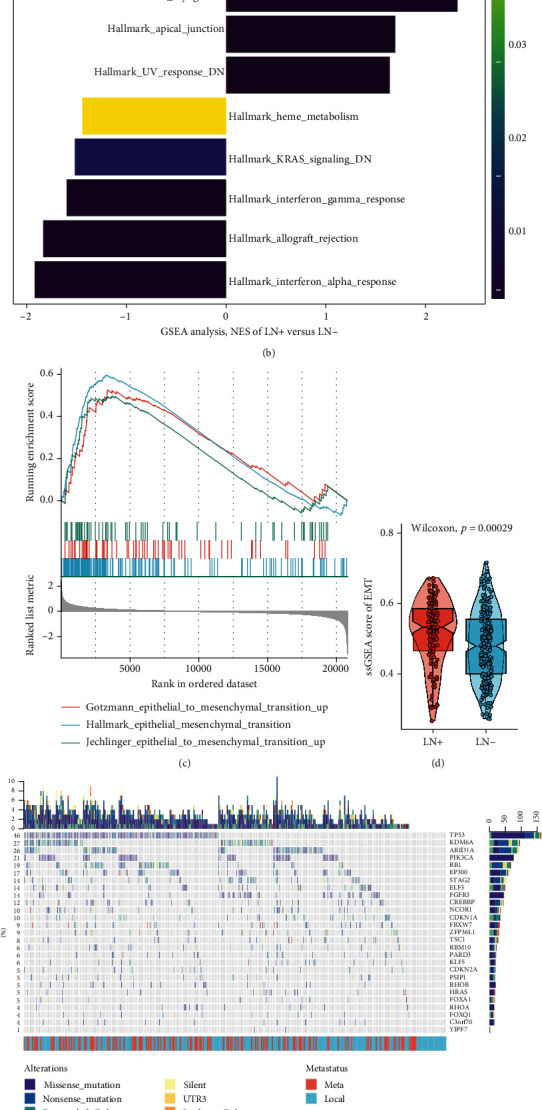
Overview of the molecular differences between LN+ and LN- tumours in the TCGA-BLCA cohort. (a) Volcano plot for differentially expressed genes. The cut-offs were set as follows: log2(foldchange) > 1, falsediscoveryrate(FDR) < 0.05. Red indicated upregulated genes; blue indicated downregulated genes. (b) Bar plot demonstrated the enrichment of hallmark gene sets. The shades of the bar represented the adjustment *p* value. (c) GSEA showed the high regulation of EMT-related pathways in LN+ tumours compared with LN- tumours. (d) Boxplot showed a significantly higher ssGSEA score of hallmark EMT gene set in LN+ tumours as compared to LN- tumours. (e) Oncoprint showed distribution of SMGs identified by MutSigCV in BLCA. The genetic alteration types were indicated in the waterfall plot annotation. The number on the left and right bar plots showed the mutation frequency of each gene. The LN metastasis status was shown as patient annotations in the bottom. (f) The heat map showed the difference in the number of patients with mutation of TP53, FGFR3, and C3orf70 between LN+ and LN- tumours. (g) The stacked bar plot showed the difference in the percentage of patients with mutation of TP53, FGFR3, and C3orf70 between LN+ and LN- tumours.

The cut-offs were set as follows: |log2(foldchange)| > 1, falsediscoveryrate(FDR) < 0.05, and 352 differentially expressed genes (DEGs) were screened out between LN+ and LN- tumours by using the package “*DESeq2*” in R (Figure 2(a); Supplementary Table S1). Then, GSEA showed a striking higher enrichment of “Hallmark epithelial mesenchymal transition” gene set in LN+ tumours than that in LN- tumours (Figure 2(b); Supplementary Table S2). Moreover, we enrolled other gene sets associated with the EMT signalling pathway including “Jechlinger epithelial mesenchymal transition up” and “Gotzmann epithelial mesenchymal transition up” and found that they were also highly enriched in LN+ tumours compared with LN- tumours (Figure 2(c); Supplementary Table S3). The patients with LN+ demonstrated a higher ssGSEA score of “Hallmark epithelial mesenchymal transition” gene set than LN- patients (Figure 2(d)).

### 3.3. Somatic Mutation Landscape between LN+ and LN- Tumours

After filtering out nonsilent mutation, 27 significantly mutated genes (SMGs, *q* < 0.05) were identified through all the 366 samples by utilizing the “*MutSigCV*” algorithm (Supplementary Table S4). Then, oncoprint visualized that most of SMGs were the driver genes in BLCA development, which has been reported before (Figure 2(e)) [43]. Moreover, we found that TP53, FGFR3, and C3orf70 within the SMGs were remarkably highly mutated in LN+ tumours when compared with LN- tumours through Fisher exact tests (Figures 2(f) and 2(g); Supplementary Table S5). Therefore, mutation of TP53, FGFR3, and C3orf70 was selected for further analysis.

### 3.4. Establishment of EMT-LN Signature

**Figure 3:**
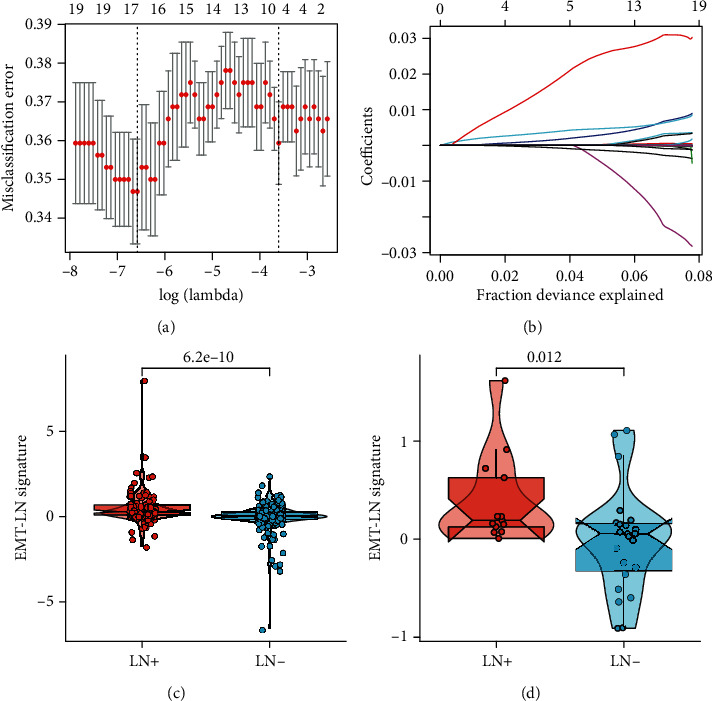
Feature selection using LASSO logistic regression model. (a) Tuning parameter (*λ*) selection 10-fold cross-validation error curve. The misclassification error was plotted vs. log (*λ*). The dotted vertical lines were drawn at the optimal values by the minimum criteria and the 1-SE criteria. (b) LASSO coefficient profiles of the 19 candidate EMT-related genes. A coefficient profile plot was produced against the log (*λ*) sequence. A vertical line was drawn at the value selected by 10-fold cross-validation, where the optimal *λ* resulted in 17 nonzero coefficients. (c, d) Difference in the EMT-LN signature risk score between LN+ and LN- tumours in the training dataset (c) and testing dataset (d) of the TCGA-BLCA cohort.

As the EMT signalling pathway was highly enriched in LN+ tumours, genes within the “Hallmark epithelial mesenchymal transition” gene set and the DEGs were merged as EMT-related candidate genes. Then, we submitted them to the LASSO logistic regression analysis and construct a signature (EMT-LN signature) for distinguishing LN metastasis status in the training dataset of the TCGA-BLCA cohort (Figures 3(a) and 3(b)). The coefficient of each feature and formula of the EMT-LN signature are shown in Supplementary Table S6. Moreover, the EMT-LN signature was strikingly higher in LN+ tumours than that in LN- tumours both in the training (*p* = 6.2*E* − 10) and testing (internal validation) datasets (*p* = 0.012) of the TCGA-BLCA cohort (Figures 3(c) and 3(d)).

### 3.5. Supervised Hierarchical Clustering by Using EMT-LN Signature

**Figure 4:**
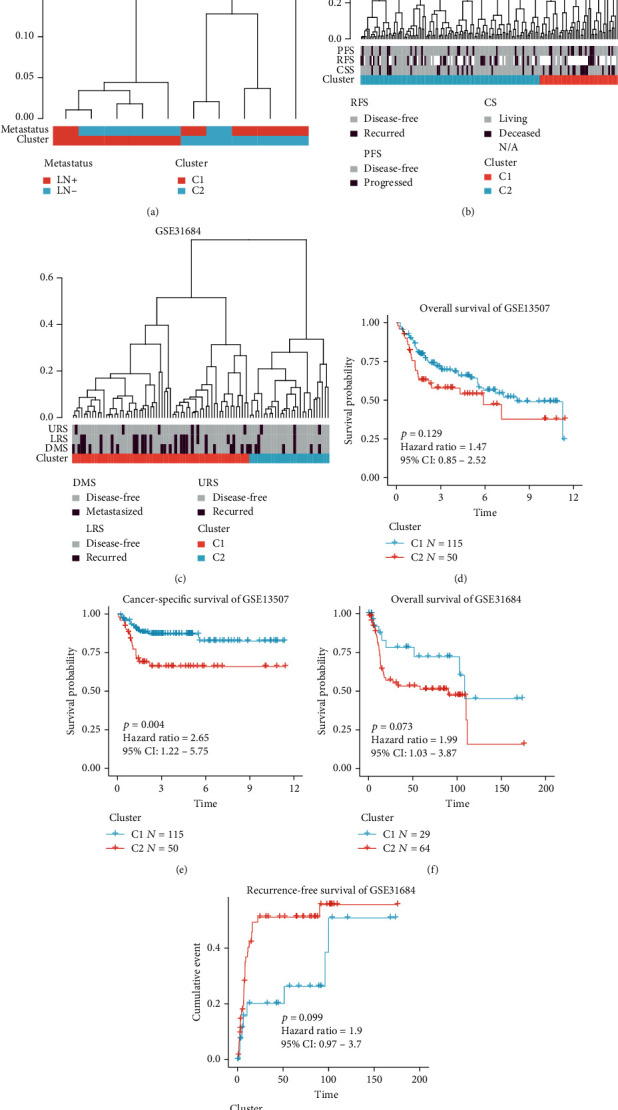
Supervised hierarchical clustering for EMT-LN signature. (a) Dendrogram showed that two clusters created by supervised hierarchical clustering could significantly distinguish LN metastasis status in the GSE106534 cohort. (b) Dendrogram showed that two clusters created by supervised hierarchical clustering were strikingly associated with OS, RFS, and CSS in the GSE13507 cohort. (c) Dendrogram showed that two clusters created by supervised hierarchical clustering were strikingly associated with DMS, LRS, and URS in the GSE31684 cohort. (d, e) Kaplan-Meier survival curves showed the difference in OS (log-rank test, *p* = 0.129, d) and CSS (log-rank test, *p* = 0.004, e) between LN+ and LN- tumours in the GSE13507 cohort. (f, g) Kaplan-Meier survival curves showed the difference in OS (log-rank test, *p* = 0.073, f) and CSS (log-rank test, *p* = 0.099, g) between LN+ and LN- tumours in the GSE31684 cohort.

Supervised hierarchical clustering was performed to validate whether the EMT-LN signature could distinguish LN metastasis status in independent cohorts. In the GSE106534 cohort, we found that cluster C1 was almost accumulated in LN- tumours, while cluster C2 concentrated in LN+ patients (Figure 4(a)). Moreover, we observed that the EMT-LN signature was significantly correlated with recurrence-free survival (RFS), progression-free survival (PFS), and cancer-specific survival (CSS) where patients with cluster C1 were more likely to recur, progress, and decease in the GSE13507 cohort (Figure 4(b)). In the GSE31684 cohort, the similar role of the EMT-LN signature in disease metastasis survival (DMS), lymph node recurrence survival (LRS), and urinary tract recurrence survival (URS) was observed (Figure 4(c)). Furthermore, Kaplan-Meier survival curves revealed that the EMT-LN signature was associated with OS (*p* = 0.129) and CSS (*p* = 0.004) in the GSE13507 cohort (Figures 4(d) and 4(e)), as well as OS (*p* = 0.073) and RFS (*p* = 0.099) in the GSE3684 cohort (Figures 4(f) and 4(g)).

### 3.6. Development and Validation of an Individualized Prognostic Nomogram

**Figure 5:**
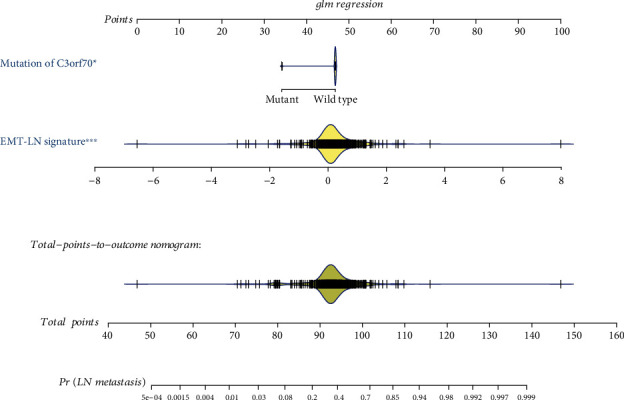
Development of preoperative EMT-LN nomogram. By incorporating the EMT-LN signature and genomic mutation of C3orf70, the EMT-LN nomogram was built in the training dataset of the TCGA-BLCA cohort.

**Figure 6:**
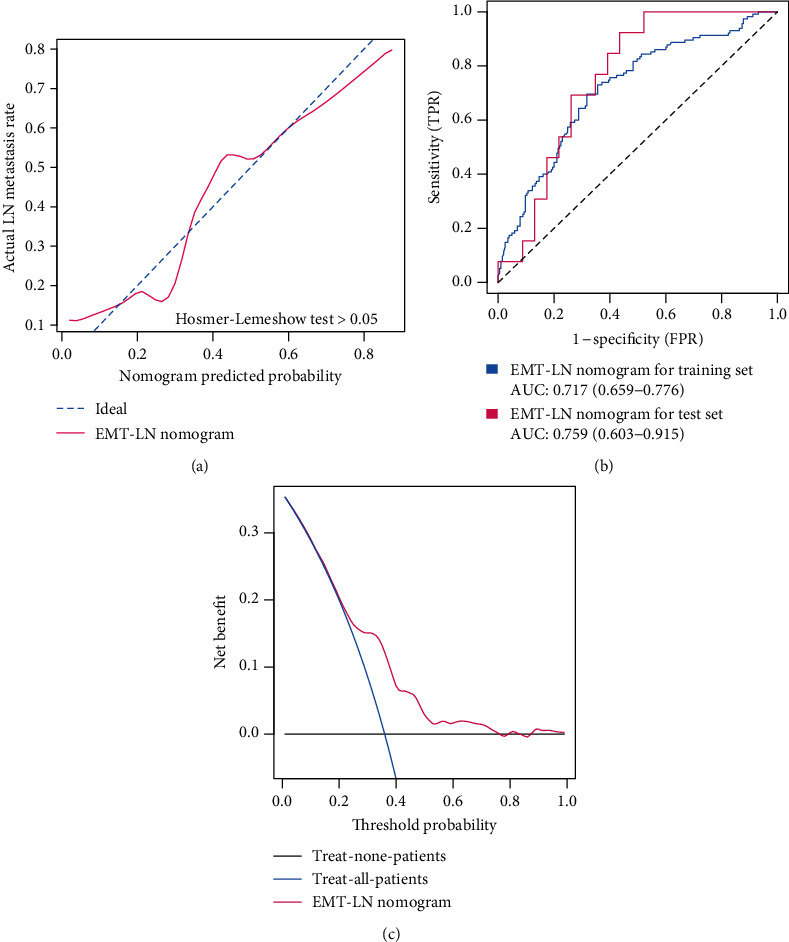
Clinical utility of the EMT-LN nomogram. (a) Calibration curve of the EMT-LN nomogram in the training dataset of the TCGA-BLCA cohort, which depicts the calibration of the fitted model between the predicted risk of LN metastasis and actual LN metastasis rate. The *x*-axis represents the predicted LN metastasis risk, and the *y*-axis represents the actual LN metastasis rate. The pink solid line represents the performance of the EMT-LN nomogram, of which a closer fit to the diagonal dotted blue line represents an ideal prediction. The difference in the two models was measured with the Hosmer-Lemeshow test. (b) ROC curves showed the prediction accuracy of the EMT-LN signature in prediction of the LN metastasis in training and testing datasets of the TCGA-BLCA cohort. (c) Decision curve analysis (DCA) for the EMT-LN nomogram. The *y*-axis measures the net benefit. The pink line represents the EMT-LN nomogram, the blue line represents the assumption that all patients have LN metastases, and the black line on the bottom represents the assumption that no patients have LN metastases.

Logistic regression analysis was performed to identify the preoperative features in predicting the LN metastasis status. As the pathological stage and detailed TNM classification should mostly be detected by biopsy postoperatively not preoperatively, we just enrolled the EMT-LN signature and mutation of TP53, FGFR3, and C3orf70 as the candidate features to establish the preoperative nomogram. We found that only the EMT-LN signature and C3orf70 mutation have statistical significance in the full model with *p* < 0.05 in the logistic regression (Supplementary Table S7). Hence, we integrated these two features to develop an EMT-LN nomogram (Figure 5). According to the nomogram, every patient would obtain a total point value by adding the point for each prognostic parameter. And higher total points correspond to a higher presence of LN metastasis. Then, the calibration curve of the nomogram showed that the performance of our model was similar to the ideal model in the training dataset, with a Hosmer-Lemeshow test suggesting no departure from perfect fit (*p* > 0.05). ROC curves revealed that the EMT-LN nomogram was efficient in predicting the LN metastasis status, with high AUCs in both training dataset (AUC: 0.717 (0.659−0.776)) and testing dataset (AUC: 0.759 (0.603−0.915)) (Figure 6(b)). Moreover, external validation also obtained AUCs of 0.64, 0.612, and 0.619 in GSE10634, GSE32684, and GSE13507, respectively, when deploying the EMT-LN nomogram (Figure S2). Then, decision curve analysis (DCA) demonstrated that the EMT-LN nomogram exhibited a high net benefit over the “treat-all” or “treat-none” strategies, which indicated that the EMT-LN nomogram had high potential clinical utility (Figure 6(c)).

## 4. Discussion

Bladder cancer (BLCA) is a lethal disease that little progress has been made in the past decades to improve the clinical outcome until the development of immune checkpoint inhibitors (ICIs) targeting immunotherapy [44]. Moreover, many clinical trials revealed that the patients who received ICIs immunotherapy did not all respond to it and non-responders will finally recur or progress to upstaging or even lymph node (LN) metastasis [45, 46]. A considerable amount of research demonstrated that BLCA patients with LN metastasis exhibited a poorer prognosis compared with patients without lymphatic spread [11]. Several lines of clinical evidence supported the idea that LN metastasis played significant roles in systemic dissemination of cancer cells and has been recognized as a robust independent prognostic factor for clinical outcomes of BLCA patients [47]. Furthermore, the addition of pelvic lymphadenectomy (PLND) to radical cystectomy (RC) has also been reported to improve oncological outcomes even in patients without LN metastasis preoperative and would give a privilege for adjuvant therapy (AC) [48, 49]. These findings not only emphasized paramount importance of LN metastasis in BLCA development but also outlined the necessity of standardization of systemic therapy including PLND in the treatment of BLCA. Recently, neoadjuvant therapy (NAC) was widely used to improve the survival rate of BLCA patients. But some study demonstrated that preoperative utility of NAC in BLCA patients with occult LN metastases would induce a poor prognosis compared with patients treated with AC [20], which indicated the complexity of BLCA treatment and the extreme essence to know the LN metastasis status when applying distinct regimens.

Several histopathologic parameters are available to predict LN metastasis postoperatively, but none preoperatively. To date, medical imaging techniques have made a great contribution in preoperative diagnosis of LN metastasis, but it also exhibits substantial false positives that LN metastasis is unexpectedly found in 25% of BLCA patients undergoing RC and PLND who are diagnosed as stage N0 preoperatively by computed tomography (CT) or magnetic resonance imaging (MRI) [50]. These findings indicate that we could not just depend on the imaging techniques to predict LN metastasis.

Over the past couple of decades, researchers have paid increasing interest to molecular markers at achieving better prognostic stratification of cancer patients. Some groups even found molecular markers such as UPII and MUC7 to detect LN metastasis and prognosis in the setting of a positive marker with a negative lymph node at pathologic examination [28, 51]. However, some argued that qRT-PCR is oversensitive to achieve positive results even in the absence of viable cancer cells [52], while others proposed that protein detection including immunohistochemistry (IHC) is not capable of reliably detecting somatic mutations. In addition, Malmstrom et al. even reported the inconsistency of IHC and quantitative real-time PCR (qRT-PCR) that only 50% of positive p53 patients with PCR had detectable protein expression at IHC [53]. In addition, with the development of new technologies such as microarray and RNA sequencing, we could easily evaluate multiple molecules at the same time instead of detecting a single marker using qRT-PCR, which broadens our eyes in biomarker finding and improves the sensitivity of previous assays [54].

Epithelial-mesenchymal transition (EMT), which was first observed during embryonic development [55], now has been mainly recognized as an essential process in neoplastic progression [56, 57]. With the genetic and epigenetic alteration, neoplastic cells induced oncogenic EMT to favour clonal outgrowth and localized tumours development. Many reports also demonstrated that induction of EMT was of vital significance for LN metastatic in different types of cancer, including gastric cancer and head and neck cancer [58, 59]. Moreover, our previous study revealed that EMT is essential for the histology subtype transition from NMIBC to MIBC and strikingly correlated with survival in several independent cohorts [31]. In the present study, we found that EMT was significantly enriched in LN+ metastasis tumours compared with LN- tumours. Due to the aforementioned aspects, the importance of EMT in LN metastasis in BLCA is partly confirmed and we aimed to establish a diagnostic nomogram incorporating EMT for preoperative prediction of LN metastasis in BLCA patients.

For the construction of the EMT-LN signature, we firstly screened out 352 DEGs between LN+ and LN- tumour samples, merging them with genes from hallmark gene set to obtain 19 EMT-related candidate genes. Then, we performed LASSO logistic regression analysis to establish a 17-gene EMT-related signature for predicting LN metastasis status by shrinking the regression coefficients for dimension reduction. The LASSO algorithm could not only reduce noise or redundant features but also combine all selected features and assembles a single signature, i.e., marker panels instead of single predictor selection based on strength of univariable regression analysis. Recently, multiple biomarker panels have been merged as novel biomarkers for outcome prediction and are proved to be more effective than single biomarkers, owing to the high disease heterogeneity [60]. The Oncotype DX genomic testing is the first clinically used and validated multigene assay, which can quantify the likelihood of breast cancer recurrence [61, 62]. Chen et al. demonstrated a 10-gene biomarker panel based on the consolidation of data derived from transcriptomics and proteomics to predict the occurrence of BLCA with diagnostic sensitivity of 79% and specificity of 79% in a multicenter cohort [63]. Recently, our group even constructed an immune-related lncRNA panel to predict the immunotherapeutic efficiency in BLCA [64]. Similarly, the EMT-LN signature established in the recent study showed a significantly different distribution in LN+ and LN- tumours in TCGA-BLCA training and testing cohorts. The EMT-LN signature was also presented as a prognostic independent factor for prognosis in the TCGA-BLCA cohort. In addition, the EMT-LN signature could also allow us to distinguish LN metastasis status through supervised hierarchical clustering and was highly associated with patients' outcomes to some extent in several independent GEO cohorts. In total, the EMT-LN signature was a noninvasive factor for us to predict the LN metastasis preoperatively.

The landscape of somatic mutation was evaluated across LN+ and LN- tumours that mutation of TP53, FGFR3, and C3ort70 was differentially mutated between LN+ and LN- tumours. Moreover, multivariable logistic regression analysis demonstrated that only mutation of C3ort70 remained as a significant variable in the predictive model. Recently, C3orf70 was reported to be involved in neural and neurobehavioral development [65]. Ward et al. establish a 23-gene panel containing C3orf70 with utility for noninvasive diagnosis and risk stratification of BLCA [66]. There are no other reports about C3orf70, which push us to investigate its underlying role in BLCA development in the future. Then, we integrated the EMT-LN signature and C3orf70 mutation to generate a nomogram, which allowed the clinicians to predict LN metastasis risk in patients with BLCA preoperatively. The nomogram is an easy-to-use quantitative scoring tool that the individual will get total points by a plus single point of each feature within the nomogram, which is calculated through a specific algorithm by considering the contribution of features. Then, the total points could be transferred into the probability of the individual clinical ending event, such as LN metastasis. Next, we found that the EMT-LN nomogram was effective in discriminating the LN metastasis status with high accuracy in both TCGA-BLCA training (71.7%) and testing (75.9%) cohorts. The calibration curves with the Hosmer-Lemeshow test showed that the EMT-LN nomogram exhibited no significant difference with the ideal model. Moreover, DCA curves revealed that decisions based on the EMT-LN nomogram are superior compared with the treat-all patient scheme and the treat-none scheme, which give a large threshold probability and yielded more favourable clinical consequences. All of these suggested that our established nomogram could be of a high potential for clinical utility.

In summary, we establish an EMT-LN nomogram integrating an EMT-LN signature and C3orf70 mutation, which act as an easy-to-use tool to facilitate preoperative prediction of LN metastasis in BLCA individuals.

## Figures and Tables

**Table 1:** Summary of detailed clinical information of the TCGA-BLCA cohort.

TCGA-BLCA	LN- (*n* = 228)	LN+ (*n* = 128)	Total (*n* = 356)	*p* value
Gender				0.8016887
Female	61 (26.8%)	32 (25.0%)	93 (26.1%)	
Male	167 (73.2%)	96 (75.0%)	263 (73.9%)	
Age				0.9572861
≤65	93 (40.8%)	50 (39.1%)	143 (40.2%)	
>65	135 (59.2%)	78 (60.9%)	213 (59.8%)	
Vital status				2.02*E* − 07^∗^
Alive	153 (67.1%)	47 (36.7%)	200 (56.2%)	
Dead	75 (32.9%)	81 (63.3%)	156 (43.8%)	
Primary therapy outcome				1.13*E* − 05^∗^
CR	157 (68.9%)	43 (33.6%)	200 (56.2%)	
PR	9 (3.9%)	10 (7.8%)	19 (5.3%)	
SD	12 (5.3%)	11 (8.6%)	23 (6.5%)	
PD	25 (11.0%)	33 (25.8%)	58 (16.3%)	
Additional treatment outcome				2.59*E* − 06^∗^
CR	132 (57.9%)	28 (21.9%)	160 (44.9%)	
PR	2 (0.9%)	3 (2.3%)	5 (1.4%)	
SD	5 (2.2%)	6 (4.7%)	11 (3.1%)	
PD	42 (18.4%)	43 (33.6%)	85 (23.9%)	
Subtype				0.0237^∗^
Nonpapillary	142 (62.3%)	95 (74.2%)	237 (66.6%)	
Papillary	83 (36.4%)	31 (24.2%)	114 (32.0%)	
Lymph nodes positive by HE				6.41*E* − 36^∗^
>0	166 (72.8%)	2 (1.6%)	168 (47.2%)	
0	0 (%)	115 (89.8%)	115 (32.3%)	
Lymphovascular invasion				5.40*E* − 14^∗^
No	96 (42.1%)	16 (12.5%)	112 (31.5%)	
Yes	53 (23.2%)	85 (66.4%)	138 (38.8%)	
AJCC pathological T stage				1.07*E* − 06^∗^
T2	87 (38.2%)	17 (13.3%)	104 (29.2%)	
T3	105 (46.1%)	75 (58.6%)	180 (50.6%)	
T4	22 (9.6%)	33 (25.8%)	55 (15.4%)	
AJCC pathological N stage				8.35*E* − 50^∗^
N0	228 (100.0%)	1 (0.8%)	229 (64.3%)	
N1	0 (%)	45 (35.2%)	45 (12.6%)	
N2	0 (%)	75 (58.6%)	75 (21.1%)	
N3	0 (%)	7 (5.5%)	7 (2.0%)	
AJCC pathological M stage				8.91*E* − 17^∗^
M0	129 (56.6%)	45 (35.2%)	174 (48.9%)	
M1	0 (%)	7 (5.5%)	7 (2.0%)	
AJCC pathologic tumour stage				9.765503*E* − 71^∗^
II	99 (43.4%)	1 (0.8%)	100 (28.1%)	
III	125 (54.8%)	1 (0.8%)	126 (35.4%)	
IV	3 (1.3%)	125 (97.7%)	128 (36.0%)	
Grade				2.92*E* − 22^∗^
High grade	209 (91.7%)	126 (98.4%)	335 (94.1%)	
Low grade	18 (7.9%)	0 (%)	18 (5.1%)	
Tumour status				1.05*E* − 13^∗^
Tumour-free	152 (66.7%)	30 (23.4%)	182 (51.1%)	
With tumour	58 (25.4%)	79 (61.7%)	137 (38.5%)	
Extracapsular extension				0.002231268^∗^
No	48 (21.1%)	35 (27.3%)	83 (23.3%)	
Yes	20 (8.8%)	48 (37.5%)	68 (19.1%)	
Mutation in TP53				0.027352667^∗^
No	133 (57.5%)	59 (44.5%)	188 (52.8%)	
Yes	95 (42.5%)	69 (55.5%)	168 (47.2%)	
Mutation in FGFR3				0.037310566^∗^
No	190 (83.8%)	117 (89.8%)	306 (86.0%)	
Yes	38 (16.2%)	11 (10.2%)	50 (14.0%)	
Mutation in C3orf70				0.013517206^∗^
No	214 (93.9%)	27 (96.4%)	241 (94.1%)	
Yes	14 (6.1%)	1 (3.6%)	15 (5.9%)	

^∗^Fisher's exact test *p* < 0.05.

## Data Availability

The datasets generated during and/or analyzed during the current study are available from the corresponding author on reasonable request. The data that support the findings of this study were derived from the following resources: TCGA GDC data portal (https://portal.gdc.cancer.gov/) and GEO (https://www.ncbi.nlm.nih.gov/geo/).

## Supplementary Materials

Supplementary 1 Supplementary Table S1: differential expression analysis based on LN metastasis status by DESeq2.

Supplementary 2 Supplementary Table S2: GSEA based on preranked gene list calculated by DESeq2 for hallmark gene sets.

Supplementary 3 Supplementary Table S3: GSEA based on preranked gene list calculated by DESeq2 for EMT-related gene sets.

Supplementary 4 Supplementary Table S4: significantly mutated genes identified by MutSigCV.

Supplementary 5 Supplementary Table S5: differentially mutated genes based on LN metastasis status.

Supplementary 6 Supplementary Table S6: selected features with nonzero coefficients in the LASSO logistic regression model.

Supplementary 7 Supplementary Table S7: multivariable logistic regression analysis for preoperative features.

Supplementary 8 Figure S1: the design and workflow of the study.

Supplementary 9 Figure S2: ROC curves showed the prediction accuracy of EMT-LN signature in prediction of the LN metastasis in GSE13507, GSE31684, and GSE106534 cohorts.
