# Supplementary material for: An Epithelial-Mesenchymal Transition (EMT) Preoperative Nomogram for Prediction of Lymph Node Metastasis in Bladder Cancer (BLCA)
Source: Dis Markers. 2020 Nov 3;2020:8833972. doi: 10.1155/2020/8833972 (PMC7656235; doi:10.1155/2020/8833972)
Supplement: Supplementary 1 — Supplementary Table S1: differential expression analysis based on LN metastasis status by DESeq2. [file 8833972.f1.pdf]

SYMBOL	logFC	adj.P.Val	P.Value
GC	2.478	6.887E-03	1.970E-08
KRT38	2.409	2.950E-05	9.504E-03
TMEM247	2.130	1.476E-03	2.470E-12
CST8	2.050	4.680E-03	2.540E-09
SFTPA1	1.969	4.320E-05	2.196E-04
CHST8	1.961	2.040E-08	1.200E-08
SSX1	1.936	1.533E-02	3.615E-04
ASZ1	1.884	7.962E-03	5.069E-04
SFTA2	1.869	3.320E-06	3.030E-06
SCGB2A2	1.844	8.365E-03	4.110E-05
GPR12	1.840	2.376E-04	1.520E-09
MYPN	1.827	3.930E-07	7.810E-14
SOX1	1.798	9.058E-02	1.010E-08
SOST	1.795	1.251E-04	2.936E-03
PPP1R14D	1.789	4.810E-08	5.320E-15
MUC21	1.758	1.420E-05	1.225E-04
SIGLEC6	1.753	5.400E-08	1.200E-17
TAC3	1.729	3.843E-04	7.073E-04
NOG	1.727	6.040E-07	1.890E-14
KERA	1.703	2.030E-06	1.050E-10
MESP1	1.656	1.270E-09	3.730E-13
PCP4	1.650	1.219E-04	6.230E-08
OR1N2	1.649	1.554E-01	3.680E-10
DLK1	1.644	2.688E-02	4.840E-12
ATP6V0A4	1.639	2.880E-07	1.410E-06
SPINT3	1.607	6.230E-02	2.000E-05
CALCB	1.603	1.650E-06	3.983E-04
MYH2	1.599	5.560E-05	2.920E-10
SHISA9	1.595	5.848E-04	6.290E-07
PSG8	1.579	9.747E-03	1.310E-02
CLDN9	1.571	8.800E-07	4.721E-04
LCE1A	1.559	1.258E-01	3.770E-08
AC018755.2	1.557	1.159E-01	2.881E-02
OR3A2	1.537	3.340E-02	1.491E-03
UGT2A3	1.527	5.266E-02	2.330E-08
SIGLEC5	1.508	4.580E-05	4.670E-07
FXYD4	1.500	5.058E-04	4.920E-09
CLDN3	1.487	1.710E-05	2.050E-07
KRTAP5-2	1.482	7.945E-02	1.050E-06
RARRES1	1.451	5.040E-07	7.660E-12
MAGEB16	1.446	6.857E-02	2.077E-04
ERVV-2	1.435	1.073E-03	1.037E-03
HMX1	1.434	7.371E-02	9.030E-13
ANPEP	1.431	4.270E-09	5.650E-05
MAL	1.419	8.500E-05	1.390E-05
C1orf105	1.409	4.113E-04	1.773E-04
MYBPH	1.402	4.460E-05	2.760E-07
GABBR2	1.391	4.698E-04	1.260E-06
OR8S1	1.391	1.254E-03	1.530E-07

CLDN6	1.386	9.223E-04	1.090E-08
SPNS2	1.374	1.080E-12	3.170E-06
SLC39A2	1.362	7.540E-05	1.580E-05
LYZL6	1.349	5.335E-03	8.160E-07
GPX6	1.333	1.473E-01	2.640E-06
SCRG1	1.332	3.859E-04	3.960E-08
PIWIL3	1.329	2.043E-02	2.500E-03
SCGB1D2	1.323	1.283E-01	5.920E-06
REG3A	1.315	4.107E-01	5.570E-05
DCX	1.300	2.106E-03	5.690E-07
TMEM215	1.295	7.671E-03	2.003E-04
FOXI1	1.293	1.532E-02	1.240E-08
MRGPRD	1.282	1.045E-02	1.038E-04
GUCA2A	1.275	2.139E-02	9.020E-07
TMPRSS3	1.259	5.090E-06	1.470E-14
KCNK13	1.254	2.990E-07	2.420E-08
EFEMP1	1.245	6.120E-06	3.670E-05
CGB5	1.244	9.216E-03	1.994E-03
PNMT	1.244	1.251E-04	3.820E-08
PSD2	1.243	8.290E-05	8.440E-06
DES	1.238	3.638E-03	3.340E-05
SEMA3D	1.237	2.470E-05	6.083E-03
MRGPRX1	1.228	3.770E-01	1.260E-05
GPR1	1.227	4.460E-05	2.660E-07
BHLHA9	1.223	8.943E-02	4.230E-08
ACTC1	1.220	5.419E-03	1.342E-03
CRP	1.211	1.779E-02	1.766E-04
MSLNL	1.208	1.709E-02	1.520E-06
TRIML1	1.208	7.280E-02	7.009E-03
PGC	1.202	5.335E-03	1.600E-08
IGFBP1	1.198	3.391E-04	1.373E-02
ETNPPL	1.195	1.141E-02	4.989E-03
WDR87	1.194	3.613E-03	3.640E-05
TM4SF4	1.194	1.447E-02	1.592E-04
STEAP4	1.193	3.230E-07	1.140E-06
C5orf67	1.188	6.332E-03	7.273E-04
CACNG6	1.183	2.299E-02	1.311E-02
KCNG1	1.180	1.950E-05	4.365E-02
KCNF1	1.174	5.830E-04	2.930E-06
AMOT	1.159	3.960E-06	7.170E-07
ZMYND10	1.150	9.840E-09	5.513E-03
MIR4666A	1.147	5.765E-04	4.780E-05
ACTG2	1.145	2.686E-04	2.170E-08
SMIM2	1.143	2.764E-04	1.920E-07
SYNM	1.140	6.120E-05	4.286E-02
IZUMO2	1.139	2.906E-02	1.023E-04
HS3ST2	1.135	3.730E-06	3.430E-06
FGF16	1.134	1.108E-02	1.368E-03
RGS9BP	1.132	1.440E-06	2.330E-07
KRT72	1.126	2.350E-03	1.100E-06

PHOX2B	1.119	1.816E-01	4.550E-05
ALB	1.113	3.274E-02	9.130E-09
GALNT16	1.113	9.010E-06	9.665E-04
KRT24	1.109	4.091E-02	6.440E-05
SLC2A4	1.103	2.870E-05	1.174E-03
SLC16A12	1.102	1.226E-04	4.290E-06
PHYHIPL	1.100	3.843E-04	5.680E-05
CHRNA1	1.099	1.510E-05	1.462E-03
LECT2	1.098	2.345E-01	1.970E-07
PSG11	1.098	2.474E-01	3.550E-08
SUSD2	1.096	4.810E-08	8.120E-05
NCCRP1	1.091	8.385E-04	5.750E-04
KRTAP3-2	1.084	7.665E-02	3.150E-07
CASQ2	1.084	7.123E-03	1.050E-05
SYT5	1.079	9.378E-04	2.050E-07
MYL2	1.075	2.058E-02	3.515E-02
C2orf40	1.075	4.680E-03	2.117E-04
RBFOX3	1.075	1.907E-03	1.670E-06
C15orf48	1.074	1.450E-05	3.090E-03
RASGEF1C	1.061	2.205E-03	8.240E-10
KRT33B	1.060	5.848E-03	1.306E-03
FAT3	1.058	3.037E-04	5.112E-04
GPX5	1.053	2.419E-01	1.673E-01
PRSS27	1.052	4.000E-07	1.379E-01
CPM	1.051	1.290E-06	2.750E-07
RGS7	1.051	2.750E-02	2.512E-02
SLC22A25	1.048	1.076E-01	2.930E-07
ATP1A2	1.045	1.618E-03	8.260E-02
EFHD1	1.041	1.510E-05	2.081E-02
GALR2	1.037	1.172E-04	2.050E-05
ZIM2	1.035	4.395E-02	1.638E-02
MGAM	1.034	4.530E-04	1.077E-04
SYCN	1.034	9.485E-02	1.133E-04
TMEM151A	1.034	1.367E-03	1.090E-04
PRB2	1.032	4.662E-02	1.095E-02
MYT1L	1.030	3.714E-02	2.770E-05
SULT2A1	1.024	7.382E-02	1.823E-04
CIDEA	1.021	4.921E-03	8.630E-06
CLIC3	1.018	4.341E-04	3.154E-02
ITLN1	1.017	4.928E-02	1.230E-06
PSG5	1.016	4.133E-02	1.660E-03
KRT23	1.014	4.421E-03	8.521E-03
CST6	1.009	5.111E-04	7.243E-02
STS	1.008	2.870E-08	5.497E-03
HGF	1.004	7.320E-05	5.543E-04
GDPD3	0.996	3.020E-05	1.380E-05
ENPP1	0.995	2.530E-05	3.200E-07
SLC9A4	0.995	1.138E-02	7.660E-05
CPNE4	0.991	5.519E-03	3.000E-01
MUC16	0.989	4.068E-02	1.780E-02

PSG4	0.988	6.380E-02	2.685E-03
CST2	0.988	2.943E-03	8.970E-05
CEACAM7	0.987	5.160E-02	5.160E-08
PCSK2	0.985	3.715E-02	1.070E-05
SLC2A14	0.985	7.034E-03	1.522E-04
SSX3	0.984	4.435E-01	3.190E-05
KRT4	0.984	4.560E-02	1.855E-03
MUC1	0.978	5.740E-05	1.392E-04
LHFPL4	0.975	3.375E-02	1.560E-05
ARX	0.973	3.259E-02	1.650E-06
TEX101	0.969	1.286E-02	2.506E-02
LIPH	0.964	9.620E-05	4.430E-05
XCR1	0.963	1.293E-03	2.243E-04
HYAL1	0.963	8.100E-06	2.060E-09
GABRR3	0.959	4.417E-02	3.320E-05
MUC15	0.959	7.123E-03	1.317E-03
SLC1A6	0.958	5.550E-02	1.665E-01
FTHL17	0.957	4.752E-01	2.472E-02
THSD7A	0.956	1.890E-05	6.218E-02
ACKR2	0.956	2.931E-03	5.340E-09
TRPC5	0.955	4.151E-02	1.520E-05
HRASLS5	0.955	1.184E-02	1.150E-06
C7orf57	0.954	8.500E-05	2.194E-04
ITGA11	0.953	8.200E-05	8.322E-03
KRT77	0.953	4.040E-02	9.580E-05
SHCBP1L	0.951	7.379E-02	6.050E-07
CYP26A1	0.950	6.419E-03	2.805E-03
PATE4	0.948	3.267E-02	1.140E-03
SPINK8	0.943	9.663E-02	1.210E-04
BEAN1	0.934	1.207E-04	5.766E-02
COMP	0.933	9.633E-03	7.060E-05
TACR2	0.932	3.492E-04	1.113E-04
NPTX1	0.931	1.083E-03	2.991E-02
GAS6	0.930	3.320E-06	5.528E-04
VEPH1	0.929	6.574E-04	1.198E-02
FGF18	0.928	3.920E-05	1.050E-01
SPP1	0.927	6.793E-04	3.479E-04
GPR37L1	0.927	5.110E-06	9.325E-02
PROK1	0.926	2.266E-02	1.890E-06
MUC22	0.926	5.494E-02	3.577E-03
WFDC6	0.925	2.291E-01	2.334E-03
KISS1	0.924	2.284E-03	2.129E-01
SORBS2	0.921	1.860E-05	2.040E-09
PGLYRP4	0.916	1.743E-02	5.460E-06
TMEM88B	0.914	1.392E-02	1.317E-02
GCKR	0.914	4.281E-03	2.070E-05
KPNA7	0.913	1.251E-04	5.190E-05
GALNT5	0.913	4.983E-04	2.680E-02
AARD	0.907	1.172E-02	2.087E-04
CNTFR	0.906	2.871E-02	4.258E-03

ASS1	0.904	8.500E-05	1.254E-01
SULT1E1	0.904	5.610E-02	2.997E-01
FGF22	0.901	1.251E-04	2.268E-03
GSTM1	0.901	9.628E-02	1.256E-03
GABRA5	0.897	1.310E-01	1.290E-05
GJB1	0.895	4.994E-02	1.823E-01
TPO	0.888	2.583E-02	7.320E-05
PRR15L	0.887	1.665E-03	9.060E-04
FOXR1	0.885	3.244E-02	1.001E-02
MS4A15	0.884	4.994E-02	1.668E-02
KRT79	0.882	3.460E-02	7.824E-02
SSX4B	0.879	NA	6.650E-05
SLC10A1	0.877	2.363E-02	4.625E-04
SEL1L2	0.875	1.471E-02	2.398E-02
TPPP2	0.873	2.595E-02	6.080E-03
HBE1	0.870	1.239E-01	5.780E-05
SNAP91	0.864	2.703E-02	2.920E-05
MIR4728	0.862	5.335E-03	1.940E-05
AL035460.1	0.860	2.147E-01	3.260E-07
HSPB6	0.859	1.129E-02	1.493E-03
ALK	0.858	1.644E-03	1.078E-01
FGF10	0.856	2.391E-02	2.628E-01
AKR1B15	0.855	1.797E-02	2.179E-03
COL4A3	0.852	3.614E-03	7.170E-04
PROM1	0.852	3.623E-02	6.004E-03
PAGE5	0.851	1.396E-02	8.710E-06
LCE2C	0.850	5.309E-01	5.952E-03
C10orf67	0.849	1.497E-02	5.563E-03
MGP	0.849	1.212E-03	5.334E-02
FAM180A	0.849	4.121E-03	4.790E-05
IL34	0.848	1.670E-05	9.166E-02
MSLN	0.846	4.923E-02	2.821E-03
TH	0.846	3.937E-02	8.190E-07
SPNS3	0.846	1.122E-04	1.556E-02
LCN8	0.846	1.291E-01	5.092E-02
KCNK15	0.845	1.748E-03	1.432E-04
FGF1	0.844	2.370E-05	3.760E-07
GRIFIN	0.843	7.785E-02	3.642E-04
CACNG4	0.843	2.595E-02	6.050E-08
C4BPB	0.842	3.030E-02	4.530E-02
PCDHA12	0.842	1.233E-01	3.144E-01
ERBB2	0.840	8.150E-06	6.030E-04
:PPIN-WFDC	0.836	NA	2.081E-04
HPSE2	0.836	3.243E-02	3.090E-07
HSPB7	0.834	1.342E-02	1.093E-04
GRID1	0.832	1.557E-04	3.450E-05
THBS4	0.832	5.740E-03	3.350E-06
ATP2B3	0.830	1.634E-02	5.070E-08
TMEM130	0.829	1.839E-03	2.317E-01
CNTNAP3B	0.829	6.801E-03	9.158E-04

FLNC	0.826	1.009E-02	1.440E-04
IL17C	0.825	4.451E-02	2.480E-06
CHRDL2	0.825	3.085E-02	6.420E-06
MESP2	0.824	7.247E-03	1.770E-07
PIANP	0.824	3.843E-04	3.458E-01
CYP2B6	0.821	1.351E-01	2.196E-03
CNN1	0.820	1.911E-02	2.026E-04
SLC30A2	0.820	3.818E-02	2.230E-03
UPK3A	0.819	6.530E-02	5.790E-06
C5orf38	0.819	1.497E-01	9.940E-06
ST6GAL2	0.818	6.843E-03	3.609E-02
SPATA3	0.818	3.735E-01	4.000E-06
CHRM2	0.817	8.185E-02	4.559E-04
GABRB1	0.814	1.658E-01	1.180E-05
EPYC	0.813	1.032E-01	1.752E-04
A2ML1	0.812	2.354E-02	4.611E-02
SLC19A3	0.811	4.225E-03	9.680E-05
DMRT3	0.809	3.800E-02	1.090E-06
KCNK3	0.809	7.868E-03	6.910E-05
CEACAM6	0.807	5.670E-02	1.642E-01
PROKR2	0.807	3.340E-01	1.084E-03
UNC5C	0.807	1.251E-04	8.730E-05
PTN	0.805	2.623E-02	4.301E-01
HSD3B1	0.805	1.833E-01	8.280E-06
MIR4257	0.804	1.179E-02	1.023E-01
MTRNR2L1	0.802	1.462E-01	3.989E-03
SCIN	0.802	2.603E-03	4.071E-02
C15orf62	0.796	1.220E-05	1.042E-02
C1QL2	0.794	1.844E-01	3.808E-03
NCAM1	0.793	1.985E-02	2.290E-03
DAB2	0.793	7.900E-06	2.480E-02
RTL3	0.793	2.434E-02	1.146E-01
MYOZ2	0.789	1.252E-02	7.820E-05
WISP2	0.789	2.361E-02	3.352E-03
DEFB119	0.789	5.243E-01	3.143E-03
PGLYRP2	0.787	1.572E-02	3.916E-04
CBLN2	0.787	6.251E-02	3.829E-02
ANTXRL	0.786	3.573E-02	1.386E-04
ADRB3	0.785	4.479E-02	8.835E-04
CYP4F8	0.783	1.435E-01	1.654E-02
RGS4	0.782	2.931E-03	2.010E-07
CAPSL	0.782	9.131E-02	8.278E-03
C7	0.780	5.732E-02	1.801E-03
CALB2	0.779	2.829E-02	7.073E-03
OGN	0.779	6.634E-02	6.621E-02
JPH3	0.777	2.492E-02	1.374E-04
LRRC26	0.771	2.904E-02	7.444E-04
MAGEB1	0.770	6.329E-01	3.540E-05
BLID	0.770	NA	1.273E-03
GPR88	0.770	1.545E-02	3.548E-04

GABRA3	0.768	1.173E-02	1.307E-02
REEP6	0.767	1.770E-03	1.170E-07
NDST3	0.767	2.174E-02	1.392E-02
BARX1	0.767	6.613E-02	2.428E-04
GPHA2	0.766	9.333E-02	2.049E-03
KCNJ18	0.766	2.551E-01	8.810E-05
SCT	0.765	1.260E-02	8.879E-03
OLFML3	0.762	1.942E-03	1.290E-05
HYPM	0.762	4.201E-01	9.120E-07
REEP1	0.760	9.308E-03	9.781E-03
NTRK3	0.760	1.074E-02	1.010E-01
SLC46A2	0.759	1.887E-02	3.870E-06
TINCR	0.759	1.118E-03	1.264E-02
CSDC2	0.755	1.339E-02	3.624E-04
SAA2-SAA4	0.755	1.524E-01	5.463E-04
OR7C1	0.755	1.173E-01	3.144E-02
SYNPO2	0.755	1.254E-02	2.047E-02
LOR	0.754	1.576E-01	3.061E-03
PDK4	0.754	2.078E-03	8.430E-06
ITGBL1	0.752	4.494E-03	1.917E-04
LIPN	0.751	4.852E-02	1.645E-04
MAN1C1	0.751	1.492E-02	5.120E-03
PTX3	0.751	1.953E-02	2.459E-02
FAM19A4	0.751	1.560E-01	1.660E-06
AKR1B1	0.750	6.230E-05	5.030E-06
ERVFRD-1	0.750	2.437E-02	8.054E-03
MORN5	0.747	9.369E-02	1.900E-05
TRDN	0.747	2.041E-01	2.669E-01
STXBP6	0.747	1.985E-02	1.054E-02
CASP14	0.745	1.489E-01	1.169E-02
C5orf46	0.745	4.588E-02	3.499E-03
KRTAP17-1	0.745	1.786E-01	1.380E-05
PATE2	0.745	1.445E-01	7.926E-03
C2CD4B	0.743	2.901E-03	6.205E-04
KIAA1257	0.742	9.865E-04	3.805E-03
STOML3	0.741	7.080E-02	6.200E-06
CFAP77	0.740	5.875E-02	6.250E-05
NUPR1	0.740	1.805E-03	1.567E-02
DKK2	0.739	4.578E-03	2.253E-02
LEFTY2	0.739	1.196E-01	4.473E-02
AC008982.1	0.739	1.193E-02	6.287E-02
FABP3	0.738	1.401E-03	1.950E-05
SLC5A11	0.738	2.448E-03	3.604E-04
MFAP5	0.738	5.095E-02	1.082E-04
AMER2	0.737	1.702E-01	1.082E-04
MARCO	0.737	3.948E-02	9.925E-03
LCE2D	0.736	6.454E-01	1.950E-05
ARMC4	0.735	2.433E-02	2.180E-07
ADAMTS8	0.735	8.746E-03	3.341E-02
FBXL16	0.734	5.935E-03	1.509E-03

ADAM29	0.733	1.316E-01	2.109E-04
PRRG3	0.733	8.198E-03	1.217E-01
PIWIL2	0.732	2.862E-02	4.470E-05
TAGLN	0.731	4.103E-03	3.330E-05
HSD17B1	0.730	6.490E-05	3.033E-04
TNXB	0.730	4.762E-03	9.350E-05
C4A	0.730	7.658E-03	1.654E-01
CCR3	0.729	4.318E-03	6.157E-04
HSPB8	0.728	5.665E-03	4.965E-01
GATA5	0.728	9.781E-02	5.911E-02
SERPINB7	0.728	1.410E-01	1.191E-01
EMX1	0.727	5.580E-02	8.648E-03
OPTC	0.723	9.644E-02	9.474E-04
GRM6	0.722	3.638E-02	1.322E-02
CD164L2	0.719	3.472E-03	1.377E-02
HIST2H4B	0.718	2.097E-01	6.790E-06
KCNG3	0.718	6.259E-02	1.681E-01
CLIC5	0.718	5.848E-03	8.162E-02
DRGX	0.714	2.206E-01	6.346E-03
NTNG1	0.714	6.067E-02	3.250E-06
PTGIS	0.713	3.595E-02	3.517E-03
GRAMD2	0.712	1.942E-02	5.551E-04
ST8SIA3	0.712	2.348E-01	1.360E-01
TDRD1	0.710	7.499E-02	1.697E-02
ELN	0.710	6.668E-03	2.911E-04
PLA2G5	0.707	1.092E-02	7.043E-03
SGCA	0.706	2.944E-02	7.720E-05
TMEM78	0.706	1.132E-01	9.980E-06
OR7A5	0.705	7.797E-02	1.560E-05
VSTM2L	0.705	4.852E-02	2.323E-04
MYH11	0.705	7.102E-02	6.410E-06
PTCHD1	0.704	9.503E-02	2.830E-07
GALP	0.703	4.252E-01	4.160E-07
EPHA3	0.703	1.271E-03	1.126E-02
NDRG2	0.702	2.712E-04	1.150E-06
CDHR4	0.702	3.519E-03	1.020E-01
ROPN1L	0.701	2.166E-03	1.512E-01
MT1M	0.701	3.460E-02	7.198E-04
PYGM	0.699	5.211E-03	2.046E-02
COL8A1	0.699	2.461E-03	5.860E-06
TRIM63	0.698	5.801E-02	2.518E-02
POTEH	0.698	3.558E-01	9.814E-02
KLF17	0.698	1.026E-01	1.400E-05
OR3A1	0.697	NA	8.045E-02
ASB5	0.695	2.526E-01	7.304E-04
P2RY6	0.695	4.243E-03	1.170E-06
BHMT	0.695	1.852E-01	1.595E-03
TPM2	0.694	1.910E-03	8.185E-02
HIST1H1T	0.692	6.634E-02	4.661E-04
DMKN	0.691	1.573E-02	2.200E-05

ATP13A4	0.690	3.318E-02	1.140E-02
LMOD1	0.690	2.938E-02	8.426E-03
ADCYAP1	0.689	3.150E-02	3.161E-03
WBSCR17	0.689	2.646E-02	1.659E-02
CNDP1	0.688	1.256E-02	2.021E-01
ISLR	0.686	1.927E-02	5.985E-02
ATP6V1G3	0.686	4.053E-01	2.459E-04
VSIG10L	0.685	4.221E-03	2.924E-02
TAS1R3	0.685	3.615E-03	2.110E-06
CGB8	0.684	2.338E-01	2.364E-01
COL10A1	0.683	7.146E-02	6.664E-04
SNTG2	0.682	2.903E-02	6.980E-06
RASGRF1	0.682	9.308E-03	3.730E-01
INA	0.681	6.194E-02	1.068E-01
CFAP47	0.681	8.830E-02	4.487E-02
PLIN1	0.680	3.773E-02	6.070E-04
ZDHHC15	0.680	1.911E-02	2.493E-04
COBL	0.680	6.781E-02	1.111E-02
FBXO15	0.679	6.574E-04	3.302E-04
WFDC10B	0.678	4.431E-02	8.270E-13
LCN6	0.677	7.118E-02	2.492E-04
SLC28A3	0.676	4.575E-02	1.500E-05
FAM189A1	0.676	7.872E-02	1.570E-01
ADAMTSL4	0.674	8.705E-03	5.073E-03
MFAP4	0.674	1.066E-02	1.070E-05
LMO1	0.673	1.599E-01	5.267E-02
ASPRV1	0.673	2.263E-03	3.123E-04
LRP2	0.673	4.427E-02	1.938E-03
NXPE1	0.672	1.562E-01	2.289E-01
SLCO4C1	0.672	7.483E-02	1.198E-04
AMH	0.672	2.014E-02	6.330E-03
C10orf10	0.671	4.113E-04	7.570E-05
IL17F	0.670	3.045E-01	2.030E-05
CSF2	0.670	5.835E-02	4.523E-02
MYL1	0.669	NA	2.670E-02
CCL11	0.669	2.413E-02	2.949E-01
SGCD	0.668	1.778E-02	3.771E-02
CFAP45	0.668	2.235E-03	5.590E-05
CYS1	0.667	5.710E-03	3.894E-02
SMOC2	0.667	1.057E-02	4.560E-05
NRK	0.667	2.783E-02	4.100E-04
APOBEC4	0.665	1.495E-01	2.266E-03
PSD	0.665	6.843E-03	9.680E-05
ANKRD35	0.664	1.618E-03	3.592E-04
WNT9B	0.664	4.395E-02	2.228E-04
SCGB2A1	0.662	2.107E-01	8.785E-02
PEG3	0.662	3.149E-02	2.320E-02
STUM	0.662	6.453E-02	1.050E-05
IL11	0.661	1.102E-02	4.647E-02
PSORS1C2	0.661	1.555E-01	3.294E-02

SPRR3	0.659	9.247E-02	1.159E-02
DAND5	0.659	8.128E-03	6.987E-03
PRLR	0.659	6.012E-02	2.685E-03
NCBP2L	0.658	3.389E-01	2.507E-03
RGS16	0.657	3.822E-04	1.889E-02
OLIG3	0.657	5.978E-01	2.333E-02
SERPINB2	0.653	1.816E-01	2.461E-02
PADI6	0.651	1.326E-01	3.173E-03
GNA14	0.651	8.073E-03	7.794E-04
JSRP1	0.650	2.001E-02	2.444E-02
SOWAHA	0.650	1.503E-02	2.330E-05
GRPR	0.649	1.705E-02	5.870E-03
SFTPA2	0.649	1.912E-01	7.480E-07
MYL9	0.649	8.087E-03	9.638E-04
CRABP2	0.648	1.024E-02	7.432E-02
IGFL1	0.648	1.936E-01	1.068E-02
RBP7	0.648	2.021E-03	3.572E-04
INSC	0.648	4.702E-02	6.090E-02
MEIKIN	0.647	1.746E-01	7.940E-04
FOXG1	0.647	3.973E-01	4.497E-04
WNT7A	0.646	1.100E-01	1.296E-03
OCSTAMP	0.645	1.627E-01	8.290E-05
PGM5	0.643	7.338E-02	2.725E-02
TMPRSS12	0.643	4.079E-03	1.089E-02
GFY	0.642	1.461E-01	4.966E-02
UPK3B	0.642	4.418E-02	1.151E-01
MLN	0.641	2.131E-01	9.278E-02
BPGM	0.641	4.699E-04	4.084E-02
SCEL	0.641	1.368E-01	1.683E-04
KRT40	0.641	1.190E-01	1.060E-03
ZBTB16	0.639	3.966E-02	7.460E-05
HCRTR1	0.638	3.691E-03	1.322E-02
VCX2	0.638	3.154E-01	7.000E-05
PODN	0.635	9.376E-03	1.230E-04
SYPL2	0.635	3.867E-03	5.533E-02
RLN3	0.635	4.120E-01	1.810E-05
ERP27	0.634	2.391E-02	2.090E-07
PCDHA11	0.634	2.356E-01	5.446E-02
SLC2A2	0.633	3.388E-01	6.503E-03
TTC9	0.632	2.764E-04	1.322E-01
MXRA8	0.632	4.225E-03	3.515E-03
TMEM210	0.632	2.026E-01	7.450E-07
PEX5L	0.631	5.001E-02	1.738E-03
MKX	0.630	5.099E-02	9.279E-02
PLN	0.630	8.286E-02	9.700E-05
MAP7D2	0.629	6.763E-02	2.317E-03
MB	0.629	8.925E-02	9.320E-05
PIP5K1B	0.629	1.710E-02	2.086E-02
KRT80	0.628	9.396E-03	2.690E-05
RYR1	0.628	3.537E-02	7.441E-04

C1QTNF7	0.628	1.076E-02	6.776E-02
C1orf194	0.626	4.625E-02	5.242E-03
GPLD1	0.626	1.285E-02	1.133E-01
DCLK2	0.626	1.442E-03	1.370E-01
KLHL38	0.625	3.887E-02	7.160E-05
DRC1	0.624	2.696E-02	1.631E-03
ELOA2	0.624	1.233E-01	1.338E-02
ZNF729	0.623	4.891E-01	9.561E-03
ARHGAP40	0.623	2.117E-02	6.386E-03
SPACA3	0.623	2.043E-01	4.287E-02
TRIL	0.623	8.136E-04	5.260E-05
C5orf49	0.622	2.394E-02	3.462E-03
ADGRE1	0.622	5.489E-02	1.422E-04
FREM2	0.622	2.029E-01	1.649E-02
PCDHA5	0.620	1.489E-01	8.881E-03
CLPSL2	0.620	1.369E-01	1.247E-02
ACTA1	0.620	1.073E-03	3.980E-02
LINC00890	0.620	2.149E-01	2.982E-02
OXTR	0.620	4.964E-03	6.394E-02
CRYAB	0.619	1.324E-02	4.405E-03
INHA	0.619	1.670E-02	8.965E-02
VIPR2	0.618	3.806E-02	6.472E-02
RPRML	0.618	1.799E-01	5.120E-05
CACNA1B	0.618	1.672E-01	9.137E-04
RSPO1	0.618	5.594E-02	6.639E-03
C1QL1	0.618	4.869E-02	4.163E-02
ANXA9	0.618	9.723E-03	6.903E-01
DACT3	0.617	1.396E-02	6.840E-06
PNLDC1	0.614	7.144E-02	1.335E-03
KNDC1	0.613	3.578E-02	1.977E-04
CBLN4	0.612	1.448E-01	2.240E-02
C6	0.612	1.748E-01	1.010E-01
PPY	0.611	1.686E-01	1.423E-03
AC093155.3	0.611	3.119E-02	2.846E-04
PLEKHG4B	0.611	1.283E-01	7.103E-04
RANBP3L	0.610	6.956E-02	5.936E-03
C11orf97	0.610	NA	6.284E-02
ACER1	0.609	1.029E-01	1.098E-04
SLC2A3	0.609	4.811E-03	3.544E-01
TEX44	0.609	NA	1.745E-04
NETO1	0.609	1.381E-01	2.700E-02
VASN	0.607	4.965E-04	8.640E-05
HIST1H4H	0.607	1.619E-02	8.984E-02
TCEAL2	0.607	1.421E-01	3.407E-02
C16orf90	0.607	1.665E-01	7.776E-02
ACKR1	0.607	7.581E-02	5.612E-02
ASPG	0.606	7.146E-02	9.973E-04
ADTRP	0.605	3.499E-02	3.125E-02
CST11	0.604	1.349E-01	4.552E-02
KCNB1	0.603	1.003E-01	1.355E-03

HOPX	0.603	1.001E-02	1.057E-02
OTOS	0.603	3.524E-01	1.015E-04
MYLK3	0.602	2.781E-03	3.563E-04
CWH43	0.602	2.479E-01	8.186E-04
FAM131C	0.601	1.685E-02	1.250E-01
ERBB4	0.601	1.439E-01	9.241E-02
AEBP1	0.601	2.175E-02	2.190E-05
OTC	0.601	4.243E-01	1.320E-06
CNGA3	0.600	5.843E-02	1.065E-02
C9orf135	0.600	2.107E-01	1.212E-01
AC091167.7	0.600	5.610E-02	4.404E-04
ABCB5	0.600	1.540E-01	4.401E-04
RUNX2	0.600	6.574E-04	2.506E-01
IL1R1	0.600	4.460E-05	1.785E-02
WFDC10A	0.600	3.393E-01	8.542E-03
NLRP2	0.599	1.562E-01	7.446E-01
CYP11A1	0.599	1.137E-01	8.999E-04
PDX1	0.597	2.155E-01	1.824E-02
UPK1B	0.596	1.515E-01	2.410E-05
PSG9	0.596	3.044E-01	7.002E-04
PGPEP1L	0.596	5.088E-02	6.826E-03
ACTA2	0.596	1.664E-02	1.408E-01
RDH12	0.595	5.312E-02	3.772E-01
RBP2	0.595	5.256E-02	1.506E-01
TGFBR3L	0.595	2.837E-02	1.509E-02
DAW1	0.594	2.917E-01	9.080E-05
TNNI3	0.594	1.427E-01	1.985E-02
MTNR1B	0.594	3.003E-01	1.822E-01
SPATA4	0.593	5.858E-02	3.023E-02
PSG2	0.593	3.736E-01	1.451E-03
MGARP	0.592	9.460E-03	1.116E-03
RBPMS2	0.591	1.798E-02	2.007E-01
VTN	0.591	5.941E-02	2.632E-03
PPFIA2	0.590	4.256E-02	4.677E-01
KCNQ4	0.590	6.178E-03	1.279E-01
RAMP1	0.590	4.299E-02	2.650E-05
DHDH	0.590	9.308E-03	1.464E-02
FXYD6	0.588	3.244E-02	2.425E-01
OR1J2	0.586	1.705E-01	1.057E-01
MGAT4A	0.586	4.929E-04	6.721E-02
ONECUT1	0.586	2.055E-01	2.383E-02
KIAA1462	0.585	9.098E-04	4.240E-02
CYP3A43	0.585	9.109E-02	2.925E-02
C3orf80	0.584	7.698E-03	1.490E-05
AMELY	0.584	5.543E-01	2.555E-02
MYO18B	0.584	1.295E-01	3.204E-01
PTH2	0.583	4.913E-01	1.730E-05
CXCL17	0.583	9.426E-02	6.247E-02
KCNMB1	0.583	3.296E-02	1.579E-02
TNNT1	0.583	1.013E-01	6.878E-02

SLC28A1	0.582	4.442E-02	3.951E-04
TNMD	0.580	2.236E-01	4.984E-02
MYL7	0.580	2.922E-01	8.090E-05
GBX2	0.580	1.100E-01	6.552E-02
MYEF2	0.580	4.027E-02	6.250E-05
P2RX1	0.579	1.076E-01	2.007E-02
ARHGAP29	0.579	2.649E-03	9.170E-03
BOC	0.579	2.354E-02	1.232E-03
LRRIQ4	0.578	4.578E-02	1.494E-02
GOLGA6A	0.577	NA	7.516E-04
HIST3H2A	0.577	4.522E-04	5.172E-02
SLC6A4	0.576	7.319E-02	4.315E-03
FGF14	0.575	1.753E-02	1.130E-01
KL	0.575	5.242E-02	5.319E-03
TNF	0.574	4.253E-02	5.752E-02
TBR1	0.574	2.058E-01	1.006E-01
TRIM17	0.573	3.595E-02	2.843E-01
TACC1	0.573	6.481E-04	1.620E-05
KRT20	0.573	2.645E-01	6.992E-03
C1QTNF3	0.573	8.198E-03	4.588E-02
RAB11FIP1	0.573	2.827E-03	6.667E-02
SLC9A2	0.572	8.521E-02	3.247E-01
RHO	0.571	2.357E-01	5.138E-03
ABCC12	0.571	5.102E-01	2.335E-01
`ON1-GTF2A	0.571	1.009E-01	7.566E-02
PTCHD4	0.571	4.300E-02	3.561E-02
SLN	0.571	1.013E-01	1.073E-04
KLHL34	0.570	2.569E-01	1.236E-04
FZD10	0.569	8.528E-02	4.513E-03
ABCA3	0.569	1.857E-02	1.540E-03
FNDC4	0.569	3.181E-03	8.850E-02
PENK	0.569	1.666E-01	3.975E-02
CYP8B1	0.568	1.485E-01	1.460E-03
ST8SIA6	0.568	5.975E-02	2.382E-04
SFRP2	0.568	1.769E-01	2.085E-04
VTCN1	0.567	1.435E-01	2.118E-04
TXNDC8	0.567	4.932E-01	2.298E-04
LKAAEAR1	0.566	9.645E-02	6.660E-07
FAM71F1	0.566	1.899E-01	3.831E-02
GUCA2B	0.566	4.709E-01	1.852E-03
FSTL5	0.565	3.384E-01	2.010E-03
NANOS1	0.565	8.083E-03	2.030E-04
ODF4	0.564	2.444E-01	9.041E-04
GEM	0.563	1.697E-02	2.115E-02
GOT1L1	0.563	2.755E-01	6.340E-06
TMEM61	0.562	9.271E-02	1.773E-02
PTGDR2	0.562	5.744E-02	1.743E-03
KCNE4	0.562	1.184E-02	1.366E-03
POTEM	0.561	5.749E-01	4.010E-06
AC112484.1	0.561	4.318E-03	8.525E-02

TIMP2	0.560	8.650E-03	1.463E-01
GABRR1	0.560	2.135E-01	3.271E-01
MTUS2	0.559	2.060E-01	1.261E-01
GH2	0.559	6.039E-01	1.088E-04
NMNAT2	0.559	6.757E-02	6.366E-03
PPP1R14A	0.559	1.100E-02	3.419E-03
TSPAN2	0.558	1.916E-02	1.287E-01
TSNAX-DISC	0.556	8.244E-02	1.772E-02
RTBDN	0.556	1.137E-01	4.115E-02
APOBEC3B	0.556	1.184E-02	4.553E-04
PCDHA10	0.556	1.192E-01	4.050E-02
TCEA3	0.556	3.471E-03	2.117E-03
FADS6	0.556	2.392E-01	5.926E-03
ABLIM2	0.556	1.569E-02	2.862E-01
MIR3164	0.555	6.072E-02	1.912E-02
GABRG1	0.555	4.980E-01	7.440E-05
SSC5D	0.555	3.113E-02	1.327E-03
SERTM1	0.554	4.157E-01	2.038E-02
FAM78B	0.554	7.167E-02	7.867E-03
ABCG2	0.553	1.541E-02	5.976E-02
WFDC13	0.553	1.897E-01	1.818E-04
CFAP74	0.553	5.411E-02	5.940E-06
OR1J4	0.552	1.349E-01	6.684E-02
FGF5	0.552	2.154E-01	5.707E-02
TMEM139	0.552	8.849E-02	4.980E-03
CH25H	0.551	1.620E-02	4.440E-05
FGF4	0.551	6.329E-01	7.900E-04
P2RX2	0.551	1.595E-01	8.562E-02
CPXM2	0.551	4.667E-02	1.840E-01
KRTAP4-7	0.551	7.283E-01	2.014E-03
GSTA5	0.550	NA	3.410E-01
MAPK4	0.550	1.724E-01	4.800E-02
ADPRHL1	0.549	1.033E-03	1.296E-01
SEMA7A	0.549	5.304E-03	4.997E-04
ADAM33	0.549	4.814E-02	9.017E-02
CHI3L1	0.548	1.141E-01	1.748E-02
SLC15A2	0.548	5.419E-03	1.418E-02
GABRQ	0.548	1.926E-01	2.902E-03
FBXL22	0.548	8.304E-03	1.987E-04
CILP	0.547	1.926E-01	9.629E-03
MIR4636	0.547	NA	2.885E-02
LCE3C	0.547	NA	1.926E-01
CNNM1	0.546	1.096E-01	2.282E-03
C2orf50	0.546	2.724E-02	8.927E-04
CCDC105	0.546	4.171E-01	1.164E-01
CD55	0.546	1.073E-03	3.447E-01
IFNB1	0.546	3.438E-01	1.399E-01
SELP	0.546	4.982E-02	1.341E-03
KIRREL3	0.546	8.336E-03	3.974E-01
APELA	0.545	2.017E-01	4.244E-02

HAND1	0.545	3.698E-01	4.858E-02
FAM92B	0.545	2.113E-01	8.475E-02
ETV1	0.545	4.871E-03	3.895E-02
MYO1H	0.544	4.954E-03	4.815E-03
STAC2	0.544	1.089E-01	1.358E-01
ARID3C	0.543	1.541E-02	4.330E-02
CMYA5	0.543	1.798E-02	2.336E-02
CCL2	0.543	2.724E-02	1.067E-02
ROPN1B	0.542	3.448E-02	4.441E-02
TPTE	0.542	6.048E-01	2.289E-02
SBSPON	0.541	5.234E-02	9.850E-02
TM4SF1	0.539	2.123E-02	6.954E-02
NOTUM	0.539	8.246E-02	8.096E-03
SPEF2	0.538	3.515E-02	5.269E-02
MRVI1	0.538	1.342E-02	1.882E-03
TNFRSF19	0.537	1.137E-01	3.148E-03
OR56A5	0.537	4.667E-01	3.934E-04
RHPN2	0.537	3.613E-03	5.159E-02
FAM166A	0.536	4.195E-02	1.023E-01
FAT4	0.536	6.967E-03	1.981E-04
SNED1	0.536	3.691E-03	3.470E-02
MUM1L1	0.536	1.020E-01	4.721E-02
ZNF703	0.536	7.652E-03	4.045E-03
COL8A2	0.536	2.432E-02	2.111E-01
CDKN2C	0.536	1.976E-03	6.666E-02
TNS1	0.535	2.581E-02	4.521E-02
ISM1	0.535	2.912E-02	5.286E-02
TMEM200B	0.534	1.057E-02	4.573E-04
TRPV2	0.534	1.306E-02	3.897E-02
COL6A2	0.534	2.595E-02	5.172E-02
VGLL3	0.533	4.285E-02	9.985E-03
ANKRD66	0.533	3.897E-01	2.539E-02
APCDD1L	0.532	1.422E-01	1.880E-01
PPL	0.532	2.045E-03	1.480E-01
PIP	0.532	3.353E-01	2.713E-04
PRELP	0.531	7.266E-02	1.087E-04
FBLN7	0.531	2.436E-02	8.363E-02
TPM1	0.529	3.019E-03	2.892E-02
WBSCR28	0.529	1.001E-01	1.737E-03
IRX2	0.528	3.338E-01	1.060E-05
RGN	0.528	6.306E-02	3.560E-03
ALPP	0.527	2.763E-01	2.812E-02
MMP23B	0.526	2.856E-02	1.480E-05
CSRP3	0.524	2.045E-01	1.198E-01
RHCE	0.524	1.087E-02	2.255E-04
FOXD1	0.524	1.684E-01	6.510E-02
SOD3	0.524	4.299E-02	3.400E-03
IQCJ	0.523	4.077E-01	4.054E-03
PTPRT	0.523	2.280E-01	5.959E-03
SRRM4	0.523	2.122E-01	5.530E-03

AP003419.2	0.523	4.579E-01	1.975E-03
LY6G6C	0.522	1.637E-01	2.087E-02
PDLIM3	0.522	5.985E-02	2.806E-03
CST5	0.522	3.789E-01	1.340E-01
DOK5	0.522	3.887E-02	3.109E-01
SORBS1	0.522	7.663E-02	7.659E-02
PRRT4	0.521	8.600E-02	7.217E-02
BHMT2	0.520	6.772E-02	1.035E-02
PACSIN1	0.520	5.244E-02	7.390E-03
CLDN4	0.519	1.290E-02	4.999E-02
FGF7	0.519	1.200E-01	1.358E-02
GRB7	0.519	8.072E-03	7.191E-02
TCEAL7	0.518	1.298E-02	1.332E-01
CACNA2D1	0.518	4.573E-02	3.969E-02
COL4A4	0.518	6.270E-02	7.436E-02
AMPH	0.517	3.904E-02	7.827E-04
CREB3L2	0.517	4.460E-06	1.770E-03
SUN3	0.517	9.054E-02	8.014E-02
C20orf85	0.516	4.934E-01	1.126E-01
BMP8B	0.516	1.164E-02	6.680E-08
MAGEE2	0.516	3.274E-01	1.288E-03
ITGA7	0.516	2.740E-02	4.241E-03
CIB4	0.516	3.638E-01	1.135E-02
F2RL3	0.515	8.782E-03	4.739E-04
SFTPD	0.515	7.442E-02	3.394E-02
PDZRN3	0.515	4.017E-02	1.340E-03
CLEC4M	0.515	2.288E-01	1.653E-01
SLC24A2	0.514	5.841E-02	7.055E-02
ANGPTL1	0.514	1.297E-01	3.320E-01
NEGR1	0.514	7.581E-02	3.311E-03
COL6A1	0.513	2.906E-02	1.107E-03
FBXO32	0.513	5.710E-03	1.889E-01
SPARCL1	0.513	1.286E-02	7.790E-02
EMILIN1	0.512	3.714E-02	9.340E-03
ERVV-1	0.512	2.510E-01	7.880E-03
TMEM229A	0.511	3.489E-01	2.306E-01
HTR1D	0.511	1.381E-01	3.384E-04
CTAGE6	0.511	1.495E-01	4.918E-04
HTR2A	0.511	1.389E-01	2.286E-04
DCLK3	0.510	2.469E-02	2.325E-02
ASPN	0.510	9.793E-02	3.583E-01
PRR9	0.510	4.222E-01	5.255E-02
CKMT2	0.510	8.198E-03	1.682E-01
RAPSN	0.510	1.006E-01	4.760E-06
LCN10	0.509	2.917E-01	1.343E-01
TPBGL	0.508	5.998E-02	1.169E-03
OTOR	0.508	5.697E-01	1.447E-02
SFRP4	0.507	2.583E-01	1.368E-01
SFTPC	0.507	3.839E-01	3.610E-04
RNF222	0.506	1.104E-01	2.717E-02

LAMA2	0.506	5.617E-03	5.219E-02
FHL1	0.505	9.606E-02	1.541E-01
LYPD3	0.505	4.505E-02	7.330E-06
KLK15	0.505	4.962E-01	3.259E-03
POPDC2	0.504	4.155E-02	6.702E-02
KCND2	0.504	3.559E-02	1.955E-01
TBC1D3K	0.504	4.299E-01	1.464E-01
AADACL2	0.503	4.739E-01	1.809E-01
OR56A1	0.503	NA	7.036E-03
ZNF716	0.502	5.641E-01	2.472E-01
SCD5	0.502	2.595E-02	7.750E-03
ECM1	0.502	1.537E-02	8.147E-02
STPG4	0.501	1.899E-01	5.827E-02
GADD45B	0.501	4.641E-03	1.238E-01
TNFSF4	0.501	3.122E-02	1.035E-03
CCR10	0.501	3.375E-02	5.714E-03
OTOA	0.501	1.949E-02	1.311E-01
TMEM45A	0.501	1.300E-01	1.259E-03
TGFBI	0.501	8.675E-02	1.950E-02
MIR151B	0.500	NA	2.050E-02
BNC2	0.500	6.431E-02	5.520E-04
VCX3A	0.500	3.884E-01	5.879E-02
ICAM5	0.500	6.660E-02	2.575E-03
COL6A3	0.499	3.722E-02	1.466E-01
JPH2	0.499	8.682E-02	3.002E-02
OMD	0.499	2.574E-01	1.206E-03
P4HA3	0.499	3.647E-02	3.498E-02
TRPM5	0.498	2.027E-01	5.888E-03
LRRN4	0.498	1.379E-01	6.068E-02
POU1F1	0.498	9.171E-02	8.026E-02
HELT	0.497	6.436E-01	9.001E-03
SOWAHB	0.497	3.078E-02	1.049E-01
ACOT4	0.497	1.948E-03	9.384E-04
C17orf64	0.497	1.132E-01	3.890E-05
OMMD3-BM	0.496	2.185E-01	2.672E-01
EPB41L4B	0.496	4.680E-03	8.632E-03
FAM20C	0.496	3.536E-02	3.156E-03
LUM	0.496	4.300E-02	3.254E-01
TMEM74	0.495	1.706E-01	4.546E-02
SLC1A2	0.495	4.694E-02	1.254E-01
CDC20B	0.494	1.309E-01	4.256E-02
ASB10	0.494	3.527E-01	1.450E-01
EFNA1	0.492	1.156E-03	1.572E-01
RSPH1	0.491	2.750E-02	6.864E-02
AKAP12	0.491	2.998E-02	2.629E-03
RGS2	0.491	2.481E-02	8.911E-02
GAN	0.491	4.423E-04	7.430E-04
POSTN	0.491	1.253E-01	2.090E-02
QPRT	0.491	7.926E-02	2.548E-01
CRAT	0.490	1.697E-03	6.688E-02

GAS7	0.490	3.147E-02	2.196E-01
HUNK	0.490	1.042E-01	4.198E-04
PPEF1	0.489	4.689E-02	1.950E-01
REG1A	0.489	5.991E-01	1.641E-01
PCDHA8	0.489	3.842E-01	2.375E-04
NTM	0.489	3.739E-02	1.585E-02
BPI	0.488	1.341E-01	1.465E-02
TRIM24	0.488	1.659E-03	3.107E-02
GABRP	0.488	3.840E-01	4.678E-01
EEF1A2	0.487	2.427E-01	6.113E-02
GDF5	0.487	1.937E-01	8.114E-02
XKR7	0.487	3.205E-01	6.664E-02
GRID2	0.487	4.543E-01	1.723E-03
OPRL1	0.487	4.406E-03	2.193E-01
KALRN	0.487	3.205E-02	1.829E-02
TBX5	0.486	7.483E-02	2.530E-02
DNAI1	0.486	1.707E-01	1.786E-03
NALCN	0.486	9.863E-02	5.785E-02
UBXN10	0.485	4.853E-02	1.987E-01
MMP11	0.485	1.404E-01	6.218E-02
HHLA1	0.485	4.415E-01	5.494E-03
CSF2RA	0.485	4.560E-02	2.355E-02
ENPP5	0.485	3.639E-02	5.850E-02
COLEC12	0.485	9.960E-02	1.006E-02
TEKT1	0.485	2.044E-01	6.044E-04
IL36B	0.484	3.581E-01	3.484E-04
CDH11	0.484	4.662E-02	4.843E-03
KLK4	0.484	2.853E-01	1.199E-02
GNG4	0.483	2.286E-01	5.340E-03
TSGA13	0.483	2.469E-01	2.134E-04
SLC8A2	0.483	1.117E-01	1.155E-01
E2F2	0.482	7.447E-03	1.181E-02
NLRP2B	0.482	1.407E-01	3.394E-02
PCDH10	0.482	2.176E-01	4.890E-05
PRND	0.482	2.013E-01	6.493E-04
ZNF728	0.482	4.150E-01	4.746E-02
ASAP3	0.482	1.033E-03	5.789E-03
CDA	0.481	1.340E-01	1.072E-04
OR2AE1	0.481	2.491E-01	1.815E-01
PRSS8	0.480	2.574E-02	2.497E-02
SSTR4	0.480	6.338E-01	8.082E-02
PYCR1	0.480	1.586E-02	2.471E-02
MNS1	0.480	5.442E-03	1.803E-03
LIN7A	0.480	5.457E-02	1.095E-02
SLC45A1	0.480	1.141E-02	2.749E-03
MUC20	0.480	1.040E-01	3.026E-01
MAGEB10	0.479	6.813E-01	1.225E-02
ZSCAN5B	0.479	2.233E-01	1.001E-03
S1PR3	0.478	2.835E-02	1.878E-02
TNFSF15	0.478	3.749E-02	2.602E-01

EDAR	0.478	1.734E-01	7.794E-03
HAND2	0.478	1.656E-01	9.986E-02
SULT2B1	0.478	1.595E-01	8.101E-04
SLC44A2	0.477	5.710E-05	2.044E-01
SEMA3A	0.477	6.772E-02	1.404E-04
TGFB1I1	0.476	6.603E-03	1.827E-02
TMEM190	0.475	4.738E-02	3.883E-03
MRGPRF	0.475	6.427E-02	1.099E-02
HIST1H4E	0.475	7.953E-02	7.307E-01
ASIP	0.475	3.536E-02	4.784E-01
SCN11A	0.474	1.552E-01	4.317E-02
PRORY	0.474	7.475E-01	2.228E-03
YBX2	0.474	1.983E-01	5.219E-02
ADGB	0.474	3.558E-01	1.836E-02
CT45A3	0.473	NA	7.286E-03
PLA2G16	0.473	9.639E-02	6.150E-05
GMNC	0.473	4.971E-01	8.422E-02
HIF3A	0.473	2.091E-01	2.680E-03
SPEG	0.473	9.752E-02	7.783E-02
AC092143.1	0.473	3.289E-01	7.246E-02
OR10H5	0.473	5.246E-01	3.654E-01
OR7D2	0.473	2.701E-01	1.842E-02
C1QTNF1	0.472	3.656E-02	1.725E-02
CERCAM	0.472	8.964E-03	3.418E-02
NEU2	0.472	6.304E-01	2.858E-02
HS3ST4	0.472	4.002E-01	4.632E-03
PTPN5	0.472	9.109E-02	7.961E-01
N4BP3	0.471	3.891E-03	3.375E-02
AKR1D1	0.471	2.122E-01	5.908E-03
MIR4756	0.471	NA	1.336E-02
ENDOU	0.471	1.169E-01	2.106E-01
EGFR	0.471	5.632E-02	2.874E-02
CHIT1	0.471	2.334E-01	6.735E-02
NT5M	0.470	5.073E-02	2.494E-02
ZNF521	0.470	3.001E-02	3.999E-03
CCDC158	0.470	2.687E-02	4.611E-03
NAV2	0.470	4.794E-03	1.502E-02
FUT3	0.470	9.208E-02	4.196E-01
HIST2H3C	0.469	5.170E-01	7.505E-04
BAMBI	0.469	1.389E-01	1.370E-05
ZNF860	0.469	7.915E-03	3.147E-02
TMEM239	0.468	4.739E-01	1.601E-04
FAM49A	0.468	1.572E-02	1.423E-01
MC2R	0.468	5.024E-01	2.832E-04
DEGS1	0.467	5.121E-03	1.521E-02
CACNG1	0.467	4.014E-01	9.693E-03
RAET1E	0.467	1.309E-01	2.517E-01
AC015688.5	0.467	2.356E-01	5.110E-05
ADH1B	0.466	3.572E-01	2.316E-01
HSD17B6	0.466	4.092E-02	5.737E-01

DHCR24	0.466	1.220E-02	7.833E-03
GATM	0.466	1.244E-01	9.159E-02
MIR3616	0.465	4.330E-01	2.234E-03
CNIH3	0.465	6.259E-02	1.204E-02
CMA1	0.465	3.029E-01	5.531E-03
KIAA1549L	0.465	7.483E-02	1.244E-01
C2orf54	0.464	1.510E-01	3.003E-04
F3	0.464	1.357E-01	3.844E-01
MSRB3	0.464	5.524E-02	2.577E-03
LRRC3C	0.464	3.517E-01	1.631E-04
KRT37	0.464	3.256E-01	5.637E-03
TEPP	0.463	3.451E-01	1.150E-01
GPR156	0.463	1.285E-01	7.218E-03
FN1	0.463	1.125E-01	5.998E-03
PCSK6	0.462	3.292E-02	3.084E-01
MEDAG	0.462	8.912E-02	1.511E-02
ADCY10	0.462	1.179E-01	6.001E-04
CRYGN	0.462	1.491E-01	5.130E-06
METRNL	0.461	6.392E-04	4.980E-05
PDE1A	0.461	3.880E-02	5.645E-03
SPSB4	0.461	2.113E-01	2.435E-01
FIBIN	0.461	1.523E-01	3.707E-03
44088.000	0.461	5.194E-01	7.236E-03
MAGEL2	0.461	1.376E-01	7.970E-02
CDRT1	0.460	3.662E-02	5.982E-02
DEFB1	0.460	2.443E-01	5.184E-02
PARD6B	0.460	4.039E-03	2.710E-02
FADS2	0.460	7.652E-02	5.398E-03
SCN10A	0.459	5.013E-01	1.830E-05
KLHDC7A	0.459	2.511E-01	2.861E-02
ERV3-1	0.459	5.907E-02	9.590E-04
STPG3	0.458	7.420E-02	1.608E-02
CCDC114	0.458	3.887E-02	6.293E-02
AOX1	0.458	1.796E-01	5.186E-02
PLEKHF1	0.457	3.198E-02	1.114E-03
CTHRC1	0.457	8.842E-02	4.416E-03
FKBP10	0.457	4.147E-02	1.106E-02
SAXO1	0.456	5.207E-02	2.297E-02
FAM25A	0.455	2.641E-01	1.458E-02
CDR1	0.455	3.288E-01	5.546E-02
OSR2	0.455	5.764E-02	6.318E-03
RNF223	0.455	8.534E-02	1.878E-02
ART4	0.455	2.924E-01	8.769E-02
FOLR1	0.455	2.962E-01	1.102E-01
APOC2	0.454	1.290E-01	3.305E-02
OR1F1	0.454	6.122E-01	2.734E-02
AK5	0.454	1.336E-01	2.312E-02
KCNE5	0.454	3.200E-02	3.153E-01
UPK1A	0.454	3.559E-01	5.485E-01
PCDHB2	0.453	1.291E-01	1.702E-01

KLHL41	0.453	9.109E-02	1.883E-01
SPRR1A	0.453	2.755E-01	2.266E-01
FILIP1	0.453	7.355E-02	4.923E-02
POTEG	0.452	2.772E-01	6.380E-02
TCP11L1	0.452	1.748E-03	1.040E-01
NCALD	0.452	1.617E-02	4.060E-05
PAX2	0.452	3.995E-01	1.988E-02
LACTBL1	0.452	NA	5.713E-02
MMP9	0.452	1.722E-01	5.540E-02
GPX3	0.452	1.101E-01	2.891E-03
IZUMO1	0.452	6.453E-02	2.110E-06
SERPINE1	0.452	6.902E-02	3.512E-03
GRHL3	0.451	1.321E-01	1.279E-03
SRPX	0.451	1.168E-01	9.860E-06
DENND2A	0.451	1.769E-02	1.776E-02
CCDC80	0.451	1.134E-01	3.464E-02
CAMP	0.450	2.214E-01	8.067E-04
NPAS3	0.450	1.411E-01	1.933E-01
RCVRN	0.450	1.346E-01	4.922E-02
PLXNA4	0.450	1.584E-01	1.416E-02
INMT	0.450	4.468E-02	1.311E-03
MYOM1	0.450	6.581E-02	1.097E-01
IL6	0.450	2.122E-01	1.036E-01
RSPO2	0.449	4.171E-01	1.006E-02
SPON1	0.449	1.086E-01	1.618E-04
BICC1	0.449	7.558E-02	1.093E-01
PRLHR	0.448	5.024E-01	7.010E-06
COX8C	0.448	1.997E-01	1.594E-01
C4orf26	0.448	2.819E-01	2.293E-04
EMILIN3	0.448	1.113E-01	1.898E-02
ADRA2A	0.448	8.643E-02	2.221E-01
COL14A1	0.448	5.331E-02	2.711E-01
TMEM252	0.448	4.243E-01	1.010E-02
CT55	0.447	4.920E-01	4.770E-02
RELN	0.447	1.586E-01	2.495E-01
SYNGR4	0.447	4.603E-02	1.219E-03
BEND7	0.447	7.800E-02	1.556E-03
ADH6	0.447	2.140E-01	1.663E-01
HSPA6	0.447	7.090E-02	6.015E-04
WFIKKN2	0.447	1.880E-01	6.237E-03
ALDH3B2	0.446	8.185E-02	5.400E-03
BVES	0.446	9.529E-02	1.650E-05
RD3	0.446	2.624E-01	4.733E-03
IL1RL2	0.445	2.392E-02	3.338E-03
DGKI	0.445	4.304E-02	3.970E-02
ACP7	0.445	2.416E-01	1.259E-02
GDAP1L1	0.445	8.925E-02	9.143E-02
DIO2	0.445	1.360E-01	2.685E-02
NKAIN4	0.445	1.876E-01	3.563E-03
TTLL7	0.444	3.868E-02	2.008E-02

WWC1	0.444	5.207E-04	3.885E-03
DDX53	0.444	6.324E-01	1.103E-01
KATNAL2	0.444	8.189E-03	9.822E-02
RFX8	0.443	8.374E-02	6.868E-03
EMP3	0.443	6.544E-02	8.569E-01
SRRM3	0.443	4.986E-02	1.117E-02
ARSE	0.443	1.435E-01	6.502E-03
KIAA0895	0.443	1.375E-02	6.779E-02
SPTLC3	0.443	2.798E-02	1.574E-01
LARGE2	0.443	9.590E-02	8.982E-03
USP6NL	0.443	1.220E-05	8.540E-05
HEPACAM2	0.442	3.688E-01	1.485E-04
NTN5	0.442	8.336E-02	1.717E-02
DCN	0.442	9.911E-02	4.146E-02
HIST1H2BB	0.441	3.286E-01	5.333E-04
GPR21	0.441	3.489E-01	7.912E-02
P2RY2	0.441	1.697E-02	6.828E-02
NAV3	0.440	1.332E-01	5.034E-02
FOXO6	0.440	1.090E-01	1.270E-01
HPGDS	0.440	5.254E-02	6.140E-03
NMT-MINDY	0.440	4.565E-01	3.410E-05
DUSP10	0.440	3.213E-02	2.942E-03
GRIK4	0.439	1.491E-01	1.510E-05
MIR4645	0.439	1.764E-01	1.881E-01
NANOS3	0.439	3.200E-02	1.206E-02
RCN3	0.439	5.883E-02	7.753E-03
FP565260.3	0.439	8.669E-02	1.537E-02
MYLK	0.438	1.056E-01	8.725E-04
GPR139	0.438	NA	9.227E-02
C9orf129	0.438	5.109E-01	9.564E-02
FXYD1	0.438	8.556E-02	1.574E-01
MAB21L3	0.437	1.724E-01	4.000E-04
SPINT2	0.437	6.274E-04	1.125E-03
TNFSF11	0.437	1.359E-01	1.487E-02
TMPRSS2	0.437	2.399E-01	5.419E-01
RHOU	0.436	6.630E-02	9.216E-02
GPR152	0.436	3.286E-01	5.249E-02
RPEL1	0.436	6.506E-02	7.463E-02
CFAP221	0.436	2.007E-01	1.420E-02
GLT8D2	0.435	4.625E-02	3.642E-03
FUT6	0.435	3.231E-01	2.109E-03
EML1	0.435	1.695E-02	2.905E-02
PAIP2B	0.435	3.300E-03	1.616E-02
LIMS2	0.435	4.869E-02	2.560E-03
GIP	0.435	3.543E-01	4.427E-02
MALL	0.434	1.560E-01	1.223E-01
TBL1Y	0.434	5.299E-01	2.116E-01
RHOJ	0.434	1.375E-02	4.906E-03
CDKN2B	0.434	1.471E-01	3.059E-03
PPP1R12B	0.434	3.720E-02	4.001E-01

HIST3H2BB	0.433	5.983E-02	7.164E-03
LMCD1	0.433	3.887E-02	6.774E-02
SLIT2	0.433	1.365E-01	2.364E-01
C4B	0.432	1.817E-01	3.718E-02
FBN2	0.432	2.453E-01	2.014E-01
PSG3	0.432	5.839E-01	3.922E-01
SLC2A6	0.431	3.450E-02	1.268E-01
SLC35F4	0.431	1.653E-01	2.715E-02
TMEM97	0.431	9.259E-02	2.207E-03
NOV	0.431	8.842E-02	3.684E-02
SLC25A31	0.431	3.308E-01	6.514E-01
CALD1	0.431	3.200E-02	1.970E-06
GGT5	0.431	4.572E-02	3.035E-01
FAM81B	0.431	2.862E-01	4.990E-03
ACOT6	0.430	3.056E-01	2.148E-02
GAS1	0.430	1.896E-01	1.203E-01
SLC38A11	0.430	1.333E-01	1.004E-01
TMEM254	0.429	3.492E-04	3.821E-03
SDR9C7	0.428	3.278E-01	5.070E-02
OR51J1	0.428	6.826E-01	3.583E-02
CCDC155	0.428	4.168E-01	7.929E-04
TNNT2	0.427	1.637E-01	2.222E-02
AC011346.1	0.427	NA	2.365E-01
L3MBTL4	0.427	1.680E-01	5.406E-02
CEP295NL	0.427	1.782E-01	5.582E-03
DUSP15	0.427	1.363E-01	4.134E-02
PNMA5	0.427	5.307E-01	2.720E-03
MAMDC2	0.427	1.702E-01	8.132E-02
KCNA10	0.427	5.592E-01	6.492E-03
ANK2	0.427	1.560E-01	4.811E-02
TAS1R1	0.427	1.406E-01	2.925E-03
DSCR4	0.427	6.663E-01	3.714E-01
C10orf107	0.427	1.162E-01	1.029E-03
KCNK12	0.426	1.471E-01	2.810E-02
CTGF	0.426	7.953E-02	1.050E-05
TUSC5	0.426	5.490E-01	4.762E-02
AGBL4	0.426	2.329E-01	2.904E-01
LYPD8	0.426	4.281E-01	3.259E-03
SLC6A2	0.425	3.256E-01	1.201E-04
LGALS1	0.425	3.745E-02	5.316E-03
GNAO1	0.424	1.530E-01	1.526E-01
SMC1B	0.424	1.692E-01	1.214E-02
LMO3	0.424	2.161E-01	1.159E-03
PCDH1	0.423	8.919E-03	3.152E-01
FOXL2	0.423	2.855E-01	2.436E-03
SUGCT	0.423	9.005E-02	1.393E-03
OR10Q1	0.423	NA	1.785E-02
SPRY1	0.422	6.005E-03	1.873E-03
MAGEB4	0.422	7.904E-01	9.981E-02
COX6B2	0.421	1.351E-01	5.573E-02

KLK8	0.421	5.887E-01	4.477E-01
RNASE1	0.421	5.895E-02	9.690E-03
OR1N1	0.420	NA	1.216E-01
GPR62	0.420	1.207E-01	2.532E-01
AKAP14	0.420	3.590E-01	1.514E-01
ACER2	0.420	1.896E-01	3.720E-02
STX19	0.420	2.004E-01	1.406E-02
RASL12	0.420	6.515E-02	3.442E-03
WNK4	0.419	1.667E-01	8.761E-02
EFCAB8	0.419	1.221E-01	9.393E-03
LTBP2	0.419	3.450E-02	6.241E-02
HLF	0.419	1.786E-01	4.295E-04
ENTPD2	0.419	1.316E-01	4.894E-03
MISP3	0.419	3.578E-02	2.012E-02
GNE	0.418	1.663E-02	1.060E-01
PIWIL4	0.418	5.740E-02	4.543E-01
IGSF21	0.417	1.390E-01	7.430E-05
ABTB2	0.417	1.032E-02	1.002E-01
TESMIN	0.417	3.974E-02	5.250E-03
TMPRSS11B	0.417	7.188E-01	2.196E-01
HPR	0.417	4.320E-01	2.088E-02
NUTM1	0.417	1.278E-01	3.460E-02
GSTM5	0.416	1.991E-01	7.020E-05
SCNN1B	0.416	2.627E-01	6.540E-04
NEXN	0.416	6.991E-02	3.240E-01
CRISPLD2	0.416	5.071E-02	8.924E-04
NRXN2	0.416	1.358E-01	2.581E-01
WNT16	0.416	2.035E-01	9.132E-02
XKR9	0.415	1.050E-01	1.609E-01
SLC27A6	0.415	3.965E-01	2.868E-03
MIR193A	0.415	2.939E-01	2.000E-01
IRX6	0.415	4.500E-01	1.948E-02
LRRC53	0.415	NA	2.347E-03
PDGFRA	0.415	5.929E-02	3.271E-01
SCARA5	0.415	2.960E-01	1.350E-01
RERG	0.415	6.858E-02	9.542E-03
S100A12	0.414	2.829E-01	2.721E-02
DPYSL5	0.414	5.373E-01	4.252E-03
ARMCX4	0.414	1.685E-02	8.016E-02
DISP3	0.414	2.131E-01	4.380E-03
WDR93	0.414	5.051E-02	3.667E-04
ARRB1	0.414	1.286E-02	1.503E-04
PPP1R1B	0.414	3.624E-01	9.503E-02
BGN	0.414	6.648E-02	5.731E-03
STAB2	0.414	1.336E-01	7.360E-01
TRIM71	0.414	3.855E-01	1.368E-04
OSBPL10	0.414	4.743E-03	2.137E-04
CA4	0.413	4.415E-01	2.297E-03
HIST2H4A	0.413	9.038E-02	2.016E-03
TMPRSS11A	0.413	3.617E-01	7.888E-03

CRLF2	0.413	1.509E-01	6.282E-03
C1orf87	0.412	6.272E-01	4.625E-03
APOA1	0.412	3.093E-01	2.264E-03
CD70	0.412	2.173E-01	2.557E-01
GCGR	0.412	2.836E-01	1.564E-01
KRTAP5-1	0.412	2.628E-01	1.712E-01
HAS2	0.411	1.786E-01	2.385E-02
BEND6	0.411	5.163E-02	3.039E-01
NECTIN4	0.411	5.457E-02	6.505E-01
ADGRG4	0.411	5.088E-01	4.286E-02
RIPOR3	0.411	5.620E-02	3.291E-02
SLC23A1	0.411	1.252E-01	1.355E-02
TTC23L	0.410	1.346E-01	3.762E-02
PEX11A	0.410	3.691E-03	3.574E-03
MOXD1	0.410	1.515E-01	7.635E-02
COL11A1	0.408	3.228E-01	3.601E-02
SH3RF3	0.408	2.451E-02	1.658E-02
XPNPEP2	0.408	3.587E-01	4.599E-02
EPHA5	0.408	3.810E-01	7.345E-03
ILDR2	0.408	1.929E-01	2.081E-01
KLF14	0.407	3.446E-01	3.501E-02
NXPH4	0.407	2.101E-01	1.302E-01
EPHA4	0.407	5.436E-02	1.038E-01
OR5AU1	0.406	6.199E-01	2.077E-02
SYT12	0.406	2.551E-01	2.884E-02
CPT1A	0.406	1.052E-02	4.536E-03
GTF2A1L	0.406	3.328E-01	8.782E-02
AVPR1A	0.405	9.216E-02	1.292E-02
UNC5A	0.405	1.743E-01	7.789E-03
ADRA1A	0.405	3.823E-01	3.184E-01
HIST1H2AC	0.405	3.079E-02	3.378E-02
TEX14	0.405	1.853E-02	1.841E-02
GSG1L	0.404	2.831E-01	6.195E-03
CRISP1	0.404	6.118E-01	1.093E-02
DCST1	0.404	1.404E-02	4.891E-04
PLA1A	0.404	1.410E-01	2.410E-03
LALBA	0.404	NA	3.439E-01
SLC30A3	0.404	2.389E-01	8.100E-05
NEK10	0.404	9.913E-02	1.496E-01
ARSF	0.403	3.833E-01	2.288E-01
GREM1	0.403	2.747E-01	5.314E-04
LRRN4CL	0.403	8.343E-02	1.652E-01
ANXA3	0.403	1.644E-01	1.370E-03
PCDHGB5	0.402	2.134E-01	3.783E-03
A2M	0.402	5.331E-02	5.378E-01
FAM110B	0.402	1.013E-01	2.021E-03
NRTN	0.402	1.195E-01	1.176E-01
MMP21	0.402	1.584E-01	7.637E-03
TMC1	0.402	2.044E-01	4.330E-03
CYR61	0.402	7.144E-02	7.172E-01

AP000721.1	0.401	2.490E-01	2.388E-02
SAMD7	0.401	4.593E-01	4.569E-03
FOXP2	0.401	1.850E-01	2.089E-02
OR2B6	0.401	1.964E-01	1.765E-01
SLC39A8	0.401	3.695E-02	1.552E-02
ASB18	0.401	4.438E-01	3.488E-02
TCP10L	0.401	1.121E-01	3.405E-04
RIMS1	0.401	3.996E-01	1.233E-01
AL355987.3	0.400	9.882E-02	6.014E-03
OSCAR	0.400	1.132E-01	7.304E-03
SHOX	0.400	4.378E-01	2.341E-02
TNNC2	0.400	1.399E-01	4.995E-02
MYADM	0.400	5.610E-02	8.747E-04
PDGFRB	0.400	4.417E-02	2.260E-02
FAP	0.399	1.713E-01	1.479E-01
KRTAP1-5	0.399	3.241E-01	1.144E-03
VWA1	0.399	1.015E-02	2.181E-01
FAM198A	0.399	1.231E-01	2.445E-02
PAX6	0.399	7.971E-02	2.538E-02
OR8G5	0.399	6.672E-01	2.812E-04
NR3C2	0.399	1.449E-01	1.076E-03
FAM184A	0.399	8.949E-02	3.608E-04
AS3MT	0.398	1.830E-01	7.250E-05
AC005324.3	0.398	1.097E-01	8.179E-02
MEGF10	0.398	1.816E-01	8.935E-02
GLIS1	0.397	1.517E-01	1.660E-06
WWC3	0.397	1.151E-03	1.494E-02
CYP7A1	0.397	3.617E-01	4.612E-02
UCMA	0.397	NA	7.362E-03
AKAP6	0.396	1.331E-01	3.456E-02
TSHB	0.396	4.669E-01	1.903E-01
GLIS3	0.396	6.660E-02	5.861E-03
AC008878.1	0.395	3.195E-01	1.347E-01
TMEM178B	0.395	1.974E-01	6.360E-07
GGTLC1	0.395	4.902E-01	5.129E-01
BSPRY	0.394	3.952E-02	1.596E-01
RALBP1	0.393	2.142E-02	9.182E-03
KCNK6	0.393	5.053E-03	2.352E-02
WNT2	0.393	1.734E-01	4.654E-02
PLAT	0.392	1.894E-01	9.615E-03
MTRNR2L7	0.392	NA	8.988E-03
DIO3	0.392	3.406E-01	9.775E-03
FAM196B	0.392	3.422E-01	1.189E-01
VCX3B	0.392	4.310E-01	1.035E-01
RGS11	0.392	2.420E-01	8.242E-02
DEFB124	0.391	3.308E-01	4.122E-02
PDE9A	0.391	1.259E-01	1.523E-01
SPOCK1	0.391	1.586E-01	1.346E-01
CRIP2	0.390	2.517E-02	1.515E-01
NPR1	0.390	5.746E-02	9.560E-05

PRDM13	0.390	5.330E-01	4.500E-05
RCAN1	0.390	1.804E-02	4.598E-03
SSPO	0.390	1.110E-01	2.226E-01
MYCL	0.389	1.612E-01	8.265E-03
GALNT12	0.389	5.422E-02	5.695E-02
WDR38	0.389	1.952E-01	1.879E-01
TGM7	0.389	5.309E-01	1.043E-01
GUCY2F	0.389	4.506E-01	5.495E-02
MEOX1	0.389	1.604E-01	4.490E-01
BPIFC	0.388	5.120E-01	6.129E-01
FSTL3	0.388	1.363E-01	2.362E-02
C16orf82	0.388	NA	2.345E-01
CRYBG2	0.387	6.003E-02	3.183E-02
MPIG6B	0.387	1.238E-01	4.505E-02
IFNA6	0.387	NA	2.669E-02
ITGB3	0.387	1.515E-01	6.047E-02
BCO1	0.387	2.173E-01	9.390E-03
EYA2	0.387	2.229E-01	1.473E-01
RNF157	0.387	1.788E-01	1.383E-03
C11orf96	0.387	1.107E-01	2.300E-02
HIPK4	0.387	3.121E-02	7.005E-03
NRP2	0.386	9.092E-02	7.943E-02
RNF113B	0.386	4.006E-01	1.813E-01
ADGRE2	0.386	7.285E-02	5.162E-02
ATOH7	0.386	1.533E-01	1.597E-01
RABGAP1L	0.386	1.394E-03	6.573E-02
PCOLCE	0.386	7.926E-02	1.156E-01
MYH14	0.385	7.775E-02	7.281E-03
LINC00238	0.385	3.751E-01	1.500E-01
CALCR	0.385	1.686E-01	1.108E-03
C1orf116	0.384	8.684E-02	1.362E-01
ZG16	0.383	4.756E-01	1.459E-03
SNX31	0.383	4.216E-01	1.045E-02
KHDRBS3	0.382	1.584E-01	5.411E-03
ADGRD1	0.382	3.057E-01	6.212E-02
PTGER3	0.382	2.693E-01	2.818E-01
PAQR7	0.382	2.237E-02	1.659E-01
ADAM9	0.382	1.065E-02	2.013E-02
CDR2L	0.381	5.935E-03	8.880E-05
PKD1L1	0.381	2.313E-01	1.244E-01
HIST1H2BK	0.381	5.559E-02	1.011E-03
HIST1H1C	0.381	9.873E-02	2.524E-03
MACC1	0.380	1.333E-01	3.008E-01
OCLN	0.380	5.575E-02	1.253E-02
WSCD1	0.380	1.705E-01	5.927E-02
AL163636.2	0.380	1.289E-01	4.264E-04
INHBA	0.379	1.983E-01	1.096E-01
NIPAL1	0.379	7.596E-02	3.072E-02
APOE	0.379	1.714E-01	7.444E-03
RASGEF1B	0.379	8.185E-02	9.383E-03

ZNF813	0.379	1.447E-02	3.676E-01
CD24	0.379	1.182E-01	2.291E-03
BHLHE22	0.379	1.012E-01	2.781E-01
PAPPA2	0.378	3.378E-01	8.162E-02
IFNW1	0.378	5.210E-01	2.223E-04
KLHDC9	0.378	1.050E-01	3.455E-01
LINC00675	0.377	4.280E-01	1.560E-03
VGLL1	0.377	2.419E-01	2.536E-04
OR13J1	0.377	4.891E-01	2.605E-01
AP002990.1	0.377	5.430E-05	2.746E-02
EPN2	0.377	1.710E-05	1.199E-02
RTL4	0.377	NA	1.160E-01
ADAMTS1	0.377	8.457E-02	1.271E-02
WFDC3	0.377	1.595E-01	1.384E-02
KDELR3	0.377	3.695E-02	1.009E-01
ATP6V1B1	0.377	2.690E-01	2.913E-02
ADAMTS12	0.377	1.679E-01	2.277E-02
HIST1H4I	0.377	4.511E-02	6.196E-03
SLC1A3	0.376	1.258E-01	1.586E-02
PDGFB	0.376	2.142E-02	8.161E-02
LCN2	0.376	3.770E-01	1.268E-02
SERTAD4	0.376	7.934E-02	1.691E-01
NPTXR	0.376	2.135E-01	1.546E-02
C8orf88	0.376	2.288E-01	3.301E-03
NEURL1B	0.375	2.401E-02	2.696E-01
NRIP3	0.374	4.344E-02	1.008E-02
CIART	0.374	1.028E-01	7.971E-03
CSRP1	0.374	2.294E-02	5.996E-02
SRY	0.374	5.847E-01	1.134E-01
MMRN1	0.374	2.570E-01	1.255E-01
NXNL2	0.373	1.891E-01	7.344E-01
NPY5R	0.373	5.348E-01	2.382E-01
AGXT2	0.373	5.662E-01	3.755E-01
DEFA6	0.373	6.794E-01	3.686E-02
FAIM2	0.373	2.835E-01	2.067E-03
CXXC5	0.373	2.142E-02	1.030E-02
CACNA1C	0.373	1.134E-01	6.304E-02
SKIL	0.373	2.951E-03	2.463E-01
AC137834.1	0.373	1.063E-01	1.220E-05
ARMS2	0.372	2.917E-01	1.103E-03
ASIC3	0.372	8.374E-02	3.073E-02
CHRNA2	0.372	5.344E-01	1.345E-01
CAMK2B	0.371	2.301E-01	9.471E-03
FOXO4	0.371	6.165E-03	1.885E-03
RGS22	0.371	2.356E-01	4.585E-02
CHST4	0.371	3.439E-01	4.933E-03
SLITRK4	0.371	3.314E-01	4.730E-03
TNFAIP8L3	0.371	1.536E-01	1.000E-02
DMD	0.370	1.724E-01	1.308E-02
INSIG1	0.370	2.589E-02	4.275E-01

SCRT2	0.370	7.094E-01	2.082E-01
TTC39A	0.370	4.569E-02	1.654E-02
SLC8A1	0.370	6.119E-02	1.316E-01
MIR6829	0.370	5.596E-01	6.393E-02
EPHB2	0.370	6.581E-02	3.542E-01
C2orf71	0.369	4.014E-01	3.901E-02
CD99	0.369	8.128E-03	3.930E-03
SPINT1	0.369	6.958E-03	6.087E-02
PRDM6	0.369	1.680E-01	3.660E-03
DNM1	0.369	7.926E-02	9.126E-03
HS3ST6	0.368	4.617E-01	1.117E-01
TAS2R40	0.368	NA	1.918E-02
FETUB	0.368	5.713E-01	5.062E-03
ZMAT1	0.368	1.443E-01	2.407E-01
AC004691.2	0.368	2.293E-01	1.852E-01
DDR2	0.368	1.172E-01	7.210E-02
CYSRT1	0.368	1.761E-01	3.021E-02
CNN3	0.368	8.422E-03	1.997E-02
NECAB1	0.367	1.902E-01	2.200E-02
AC138894.1	0.367	2.393E-01	1.412E-03
CLCNKB	0.367	1.818E-01	1.980E-02
DOC2A	0.367	2.934E-01	3.534E-01
LEPR	0.367	4.300E-02	1.193E-02
SBSN	0.367	5.276E-01	1.280E-01
DSCAM	0.366	5.299E-01	1.729E-01
LBX1	0.366	6.465E-01	1.126E-03
ADORA1	0.366	1.111E-01	6.325E-04
CSPG5	0.366	1.493E-01	1.906E-02
ARMCX1	0.366	8.809E-02	5.031E-02
TACSTD2	0.366	3.638E-02	4.442E-01
DRD5	0.365	4.471E-01	1.184E-01
RNF133	0.365	2.359E-01	1.529E-01
ERLIN2	0.365	8.919E-03	1.460E-02
EHF	0.365	1.011E-01	1.400E-03
OXT	0.365	2.565E-01	2.086E-02
SYNC	0.365	1.472E-01	1.637E-03
CD44	0.365	1.169E-01	1.599E-01
AMOTL2	0.365	2.058E-02	2.399E-01
RAB42	0.365	5.256E-02	1.044E-02
GDF15	0.364	2.756E-01	2.567E-02
RARRES2	0.364	1.764E-01	2.848E-01
TMEM71	0.364	1.264E-01	7.542E-02
ACSL1	0.364	2.069E-02	1.255E-03
PDGFC	0.364	7.601E-02	2.697E-03
43900.000	0.364	1.539E-01	2.309E-01
PRUNE2	0.364	3.483E-01	2.681E-04
CCDC74A	0.363	3.896E-02	2.040E-02
PTPN21	0.363	8.685E-04	2.110E-02
SLC16A11	0.363	9.578E-02	2.517E-03
MYO5B	0.363	4.479E-02	5.854E-01

DPYS	0.363	3.238E-01	6.451E-03
PMEL	0.363	3.536E-02	1.339E-01
OR6C70	0.362	7.837E-01	7.899E-01
ZNF761	0.362	3.054E-02	6.031E-04
TMOD1	0.362	2.225E-01	5.651E-02
CRYM	0.362	3.056E-01	4.387E-01
FAM171B	0.362	5.509E-02	6.547E-03
AL691442.1	0.361	5.607E-01	2.138E-02
SMPX	0.361	3.977E-01	2.757E-02
MGLL	0.361	8.590E-02	3.220E-02
PDZD4	0.361	1.674E-01	6.766E-03
TWIST2	0.361	2.490E-01	1.617E-02
TWIST1	0.361	2.044E-01	1.656E-04
SLC12A5	0.361	1.427E-01	2.760E-01
VASH2	0.360	1.714E-01	7.147E-01
KCNJ6	0.360	2.753E-01	2.073E-03
EMX2	0.360	4.429E-01	2.704E-01
GRIN1	0.360	1.905E-01	2.563E-01
ALPK2	0.360	3.054E-01	6.642E-02
VCAN	0.360	1.866E-01	4.405E-02
CKM	0.359	2.038E-01	6.182E-03
GULP1	0.359	1.146E-01	4.527E-02
CCL28	0.359	1.659E-01	3.309E-03
RNF182	0.359	3.714E-01	3.545E-01
MATN2	0.359	1.775E-01	2.372E-04
VCX	0.359	5.025E-01	2.226E-03
CDKL5	0.359	9.218E-03	8.113E-03
CTXN3	0.358	7.251E-01	9.107E-02
U2AF1L5	0.358	2.825E-01	2.099E-02
PSMB11	0.358	5.568E-01	3.564E-02
7orf55-LUC7l	0.357	3.056E-01	2.977E-01
KLF9	0.357	4.702E-02	7.001E-01
DLGAP2	0.357	2.689E-01	1.132E-03
MIR365A	0.357	5.293E-01	1.566E-01
CAMK2A	0.357	3.099E-01	6.881E-02
TMC5	0.357	2.715E-01	1.727E-02
AKT3	0.357	7.536E-02	2.292E-01
CCER1	0.357	NA	7.969E-02
HIST2H2AA4	0.357	5.316E-01	6.960E-05
ZCCHC24	0.357	7.637E-02	2.576E-01
SLPI	0.356	3.832E-01	1.953E-01
HSD3B7	0.356	2.141E-02	2.507E-04
LRRC20	0.356	6.831E-03	2.536E-01
CAVIN3	0.356	8.413E-02	1.421E-02
MIR1915	0.356	5.776E-01	1.426E-01
TFAP2C	0.356	3.715E-02	1.328E-02
KRTAP29-1	0.356	3.914E-01	4.302E-02
NNMT	0.356	2.358E-01	4.917E-02
ZNF750	0.356	1.526E-01	5.844E-02
CPXCR1	0.355	7.077E-01	2.155E-02

TUBA1A	0.355	1.028E-01	9.252E-03
LRRC17	0.355	2.329E-01	5.240E-03
VIM	0.355	8.449E-02	1.641E-02
WT1	0.355	5.001E-01	1.797E-04
TMTC1	0.355	1.861E-01	6.127E-02
MIR548V	0.355	NA	8.277E-02
NEBL	0.355	1.160E-01	5.347E-02
GLP2R	0.355	3.475E-01	1.341E-01
FRMD5	0.354	1.560E-01	1.619E-01
OR9G1	0.354	NA	3.257E-02
RGMA	0.354	2.864E-01	2.229E-03
RTKN2	0.354	6.285E-02	6.995E-02
MRAP	0.354	4.490E-01	1.609E-02
NLGN1	0.354	3.340E-01	9.240E-07
RRAGD	0.354	6.295E-02	5.214E-03
ACTL8	0.354	5.330E-01	5.036E-02
PTGS1	0.354	2.843E-01	1.542E-02
PLPPR5	0.354	4.181E-01	1.027E-01
AL080251.1	0.354	3.373E-01	1.931E-02
TRPS1	0.354	1.909E-01	9.634E-02
CNIH2	0.354	1.745E-01	1.043E-01
LHFP	0.353	4.089E-02	1.278E-01
FBXO27	0.353	1.738E-01	4.218E-02
SHANK1	0.353	1.865E-01	1.994E-02
TIMP3	0.353	1.469E-01	1.060E-01
FAM129A	0.353	1.097E-01	1.286E-02
PEA15	0.353	3.492E-04	9.550E-04
BST1	0.353	1.760E-01	3.557E-01
SPTBN2	0.353	4.114E-02	2.759E-01
FZD8	0.353	8.842E-02	1.263E-03
PRRG1	0.353	3.611E-02	1.966E-01
C3orf70	0.352	1.336E-01	5.304E-04
C1orf204	0.352	6.530E-02	2.036E-02
C2orf73	0.352	2.628E-01	2.472E-03
KRTAP1-1	0.352	6.336E-01	2.106E-01
YIF1B	0.352	7.178E-04	2.462E-01
MIR5195	0.352	4.220E-01	2.882E-03
GRIN2B	0.352	4.292E-01	1.774E-04
FBN1	0.352	1.611E-01	1.052E-01
AHNAK	0.352	1.615E-02	6.812E-02
MIR125B2	0.352	NA	1.878E-04
44077.000	0.351	2.853E-01	1.669E-02
MYT1	0.351	5.289E-01	1.036E-03
KAZN	0.351	9.834E-03	4.723E-03
ADAM32	0.351	1.046E-01	1.695E-01
NR2F2	0.351	1.558E-02	2.817E-01
SYDE1	0.351	2.056E-02	1.344E-01
SLC6A11	0.351	3.468E-01	8.080E-01
ROBO2	0.350	3.231E-01	9.322E-02
DPT	0.350	4.250E-01	8.258E-04

RNF165	0.350	2.735E-01	1.317E-01
TACR3	0.350	5.244E-01	2.829E-01
RUNX1T1	0.350	1.930E-01	2.243E-02
ABCD3	0.350	1.290E-02	1.771E-01
EPPIN	0.350	5.764E-01	3.484E-03
TDRD5	0.350	3.676E-01	3.483E-02
C14orf132	0.349	1.830E-01	1.372E-01
SEZ6	0.349	1.980E-01	5.095E-02
HTR2B	0.349	1.498E-01	2.049E-02
HIST2H3D	0.349	1.470E-01	8.655E-02
HIC1	0.349	7.447E-02	2.830E-01
TNFRSF12A	0.349	4.954E-02	2.510E-01
EFNA2	0.349	4.950E-01	1.096E-01
SMCO3	0.348	9.192E-02	3.381E-02
EFHC2	0.348	2.836E-01	4.778E-01
EPO	0.348	3.403E-01	2.538E-02
DIXDC1	0.348	8.102E-02	7.185E-01
SMIM10	0.348	4.852E-02	4.003E-02
EVA1C	0.348	1.285E-01	1.244E-03
ZNF454	0.348	1.975E-01	4.730E-06
CLEC12B	0.348	4.425E-01	2.736E-02
TTC7B	0.348	5.193E-03	3.747E-02
GBA	0.348	1.251E-04	1.555E-01
SLC16A5	0.348	1.530E-01	5.885E-02
CHGA	0.347	4.939E-01	8.138E-03
GLB1L3	0.347	4.944E-01	5.121E-01
HRASLS	0.347	3.993E-01	9.647E-02
CPT1C	0.347	1.369E-01	1.248E-01
CAPG	0.347	2.361E-02	3.154E-01
ZBBX	0.347	5.706E-01	1.555E-01
ADAM30	0.346	NA	2.516E-01
EXD1	0.346	1.621E-01	1.648E-01
ACTN4	0.346	6.640E-05	1.478E-04
PLAU	0.346	1.461E-01	5.513E-02
DBNDD1	0.346	8.518E-02	7.689E-01
ERRFI1	0.346	5.005E-02	1.362E-01
CARTPT	0.345	7.538E-01	9.907E-02
MZB1	0.345	3.714E-01	1.869E-02
CTXN1	0.345	1.595E-01	1.192E-01
PQLC3	0.345	1.700E-03	2.635E-02
AC003006.1	0.345	3.468E-01	2.827E-01
ZIC2	0.344	4.031E-01	5.300E-03
TPPP3	0.344	1.757E-01	1.294E-01
FGFR2	0.344	1.576E-01	4.277E-04
CAPN6	0.343	4.839E-01	6.049E-02
HIST1H3D	0.343	1.418E-01	1.804E-03
CDKN2D	0.343	1.505E-03	9.174E-03
HIST1H2BD	0.343	8.294E-02	8.180E-03
HIST2H2BE	0.343	1.009E-01	2.400E-01
C9orf47	0.343	2.708E-01	1.257E-01

GRIK5	0.342	2.077E-01	2.030E-01
RGSL1	0.342	5.140E-01	1.213E-02
SIPA1L3	0.342	2.603E-03	9.858E-02
IRX3	0.342	3.213E-01	1.801E-01
XG	0.342	2.547E-01	7.757E-04
CD59	0.342	2.727E-02	1.465E-02
MIR3939	0.341	5.686E-01	2.933E-01
ANKRD53	0.341	6.546E-02	3.689E-01
KCNMA1	0.341	2.257E-01	1.191E-04
LHX2	0.340	4.664E-01	3.934E-02
TMEM31	0.340	2.843E-01	1.440E-02
PRKG1	0.340	1.309E-01	7.504E-01
GRP	0.340	5.546E-01	1.934E-01
ZFHX4	0.340	2.871E-01	1.432E-01
AC120114.5	0.340	4.603E-02	2.270E-02
ALDH1B1	0.340	3.647E-02	1.298E-01
TDRD15	0.339	7.029E-01	2.994E-02
DOCK3	0.339	2.466E-01	3.664E-02
GCOM1	0.339	2.044E-01	6.481E-01
COX7A1	0.339	1.090E-01	7.009E-02
SLC50A1	0.339	1.110E-03	1.194E-02
GGN	0.338	2.597E-02	4.598E-03
NR2F1	0.338	1.152E-01	5.705E-03
MYADML2	0.338	3.080E-01	4.895E-02
CORO6	0.338	2.665E-01	1.885E-02
IGSF5	0.338	4.080E-01	3.332E-03
B4GALT6	0.338	1.341E-01	3.068E-02
MAPK8IP2	0.337	2.566E-01	1.985E-01
CORIN	0.337	1.693E-01	3.743E-02
MTAP	0.337	1.699E-01	7.788E-01
AXL	0.337	1.799E-01	1.144E-02
IGDCC4	0.337	1.132E-01	4.373E-01
ANO1	0.337	2.006E-01	3.262E-02
MIR558	0.337	NA	5.198E-02
C3orf52	0.336	4.068E-02	2.700E-05
APBA1	0.336	6.072E-02	1.641E-01
MT1A	0.336	4.071E-01	1.644E-01
SLC6A7	0.336	2.323E-01	1.743E-03
TSPAN4	0.336	6.230E-02	1.053E-04
HSF2BP	0.336	5.493E-02	3.976E-01
BARX2	0.336	4.279E-01	1.106E-01
LYVE1	0.335	2.092E-01	2.844E-04
ADAM11	0.335	1.955E-01	4.022E-02
APOLD1	0.335	5.393E-02	9.004E-02
MTRNR2L5	0.335	NA	5.220E-02
LOXL3	0.335	2.939E-02	6.186E-02
NTSR2	0.335	6.118E-01	1.007E-02
CCNI2	0.334	3.638E-01	1.223E-01
COL1A1	0.334	2.474E-01	1.611E-01
STKLD1	0.334	2.134E-02	3.872E-02

ZNF229	0.334	2.250E-01	2.851E-01
AC099518.3	0.334	4.107E-01	2.209E-01
PLPP4	0.334	4.020E-01	4.142E-02
NPC1L1	0.334	2.536E-01	5.235E-03
AFF3	0.334	2.871E-01	8.660E-02
GPR37	0.334	3.839E-01	3.753E-02
CALU	0.334	3.181E-03	2.575E-01
TPRG1	0.333	1.249E-01	4.049E-01
GSN	0.333	2.530E-02	1.798E-01
SLC17A5	0.333	8.473E-03	2.163E-03
ADAMTS6	0.333	1.285E-01	8.261E-02
RORA	0.333	6.084E-02	1.224E-01
PHEX	0.333	2.396E-01	9.959E-02
HMCN2	0.332	2.437E-01	4.138E-01
KIAA1161	0.332	2.992E-02	3.249E-01
MIA-RAB4B	0.332	6.701E-01	3.550E-02
POU2F3	0.332	2.437E-01	7.197E-01
SGK2	0.332	4.374E-01	2.459E-02
S100A7	0.332	6.218E-01	6.212E-01
IZUMO3	0.332	NA	1.796E-03
SELE	0.332	3.253E-01	1.152E-02
ACOX2	0.331	2.004E-01	3.140E-02
ANTXR1	0.331	7.464E-02	3.980E-06
SPATA46	0.331	3.227E-01	4.089E-03
DPRX	0.331	6.721E-01	5.594E-02
ENPP4	0.331	7.519E-02	9.504E-04
WNT4	0.331	2.312E-01	8.180E-05
PROSER2	0.330	1.054E-01	2.371E-01
TNFAIP6	0.330	2.640E-01	1.584E-04
HRASLS2	0.330	4.569E-01	6.093E-01
DPYSL3	0.330	2.120E-01	2.214E-03
PIH1D3	0.329	5.984E-01	2.025E-02
WNT6	0.329	3.903E-01	1.727E-01
TRIM6	0.329	1.483E-01	9.340E-06
PLP1	0.329	4.521E-01	1.184E-01
DRP2	0.329	1.615E-01	9.597E-03
SYNDIG1	0.329	3.874E-01	3.351E-03
KRTAP9-1	0.329	6.413E-01	4.012E-03
HIST1H2AD	0.329	1.922E-01	6.102E-02
PRR32	0.328	7.918E-01	2.273E-01
TSC22D3	0.328	3.885E-02	6.180E-07
SH3BGR	0.328	3.326E-02	1.852E-01
CHRNA3	0.328	1.662E-01	3.565E-02
HTRA1	0.328	1.149E-01	8.453E-04
SLC35A2	0.328	2.082E-04	1.508E-01
TTYH1	0.327	1.782E-01	3.374E-02
PPFIA3	0.327	4.569E-02	4.471E-01
CCDC110	0.327	2.225E-01	3.862E-02
NPM2	0.327	2.551E-01	5.917E-01
CTNNAL1	0.327	9.856E-02	1.097E-02

NCEH1	0.327	3.450E-02	2.723E-02
PGR	0.327	2.195E-01	4.287E-01
TAF7L	0.327	3.118E-01	5.487E-01
CLIP3	0.326	1.903E-01	9.904E-02
ELOVL5	0.326	1.753E-02	4.244E-04
MYH3	0.326	1.794E-01	1.425E-02
B4GALT1	0.326	2.341E-03	5.235E-01
DAO	0.326	4.928E-01	1.201E-01
MELTF	0.326	3.175E-01	2.478E-01
HMOX1	0.326	1.760E-01	4.908E-02
MOV10L1	0.326	2.118E-01	1.052E-03
CACNB2	0.325	1.738E-01	3.205E-03
DPP4	0.325	3.221E-01	4.766E-02
LAMA3	0.325	4.256E-01	1.443E-03
KANK2	0.325	5.073E-02	8.669E-02
KLF8	0.325	1.491E-01	2.567E-01
LZTFL1	0.325	1.897E-03	2.205E-04
KRTAP4-1	0.325	2.448E-01	6.280E-05
RCAN2	0.325	1.297E-01	2.718E-01
ADAMTS17	0.325	2.019E-01	4.758E-03
TMIE	0.324	1.592E-01	1.417E-01
RGR	0.324	6.708E-01	1.470E-02
EFCC1	0.324	1.512E-01	3.137E-01
TGFB3	0.324	1.629E-01	2.497E-04
FBXO24	0.323	1.420E-02	2.880E-06
IL17D	0.323	1.152E-01	1.001E-01
PPM1H	0.323	1.984E-01	4.720E-03
SPECC1	0.323	1.160E-01	4.597E-01
AC004233.2	0.323	4.634E-01	3.107E-02
RGS10	0.323	3.375E-02	4.013E-04
ZNF99	0.323	5.346E-01	5.551E-03
SCD	0.322	1.732E-01	3.715E-02
HHIPL1	0.322	1.523E-01	5.679E-01
XKR3	0.322	6.278E-01	2.721E-03
CFAP58	0.322	1.076E-01	2.612E-02
DIO1	0.322	3.548E-01	1.425E-01
OVOL3	0.322	7.433E-02	3.771E-01
RDM1	0.322	1.252E-01	4.877E-02
KLHL35	0.321	1.603E-01	2.343E-01
CAVIN1	0.321	1.384E-01	1.312E-02
CLDN5	0.321	1.728E-01	1.339E-02
AP001458.2	0.321	NA	1.402E-02
ZSCAN10	0.321	5.163E-01	1.718E-03
CAVIN2	0.321	2.482E-01	8.266E-01
SLC5A6	0.321	1.736E-02	2.274E-01
PSMD8	0.320	1.695E-04	6.592E-02
HTR6	0.320	3.292E-01	1.100E-03
DAAM2	0.320	1.174E-01	2.088E-01
ARHGAP28	0.320	2.149E-01	5.950E-05
EMP1	0.320	1.339E-01	8.056E-02

UGT2B7	0.320	5.998E-01	4.529E-02
TRIM64B	0.320	NA	4.316E-01
COL1A2	0.320	2.536E-01	1.756E-02
UFSP1	0.320	2.051E-02	2.090E-05
ARHGEF26	0.319	1.394E-01	4.119E-01
ABHD4	0.319	5.670E-03	1.062E-01
C1QTNF2	0.319	1.342E-01	1.071E-03
ADGRA2	0.319	1.283E-01	3.920E-02
LAMC2	0.319	3.548E-01	1.247E-01
CNPY4	0.318	2.985E-03	4.392E-01
GREM2	0.318	4.646E-01	3.217E-02
MROH9	0.318	4.588E-01	3.570E-08
AP004243.1	0.318	NA	1.852E-02
DCSTAMP	0.318	4.420E-01	5.675E-01
ANGPTL5	0.318	5.293E-01	9.270E-05
MEST	0.318	2.440E-01	1.560E-01
ARID3A	0.317	2.439E-01	1.042E-01
TLCD1	0.317	4.750E-02	6.512E-03
SRGAP2C	0.317	3.441E-04	1.257E-01
OR52N4	0.317	4.607E-01	6.460E-03
MIR6740	0.317	3.914E-01	1.780E-03
ANKFN1	0.317	5.208E-01	5.037E-02
PLPBP	0.316	1.442E-02	7.316E-02
PMEPA1	0.316	2.013E-01	1.717E-01
SORCS1	0.316	4.770E-01	3.433E-02
RTN3	0.315	1.898E-03	2.059E-04
PAX1	0.315	6.743E-01	1.897E-01
CALML6	0.315	2.444E-01	2.790E-05
IFNA21	0.315	NA	9.612E-03
GRXCR2	0.315	NA	6.291E-04
HIST1H2BC	0.315	2.197E-01	1.962E-02
FO681492.1	0.314	2.582E-01	3.247E-01
STC1	0.314	1.906E-01	5.560E-03
TIAM1	0.314	5.317E-02	4.780E-02
SPTSSB	0.314	4.242E-01	2.861E-01
HMCN1	0.314	2.359E-01	6.657E-02
OR5M1	0.314	NA	7.743E-02
HIST1H2BN	0.314	1.330E-01	2.114E-02
ITLN2	0.313	5.577E-01	1.248E-04
GPR137B	0.313	4.273E-03	2.059E-02
ZNF365	0.313	3.567E-01	1.374E-02
RAB9B	0.313	1.899E-01	3.110E-02
DNAH12	0.313	1.419E-01	1.752E-02
METTL7B	0.313	3.464E-01	6.068E-04
OPN5	0.312	5.633E-01	3.032E-02
VEGFC	0.312	1.881E-01	1.942E-01
SPESP1	0.312	4.641E-01	4.140E-02
AL355102.2	0.312	4.526E-01	2.512E-02
IDI2	0.312	5.194E-01	2.118E-02
PTGER2	0.312	1.901E-01	4.296E-02

CCDC177	0.311	4.607E-01	1.271E-01
HIST1H2BF	0.311	3.216E-01	2.911E-01
ACSM3	0.311	2.917E-01	1.338E-01
EDN1	0.311	2.252E-01	1.892E-02
BIRC7	0.311	3.736E-01	5.621E-04
AC011530.1	0.311	4.250E-01	1.120E-05
KIAA0408	0.311	5.559E-01	7.006E-03
CT62	0.310	4.669E-01	1.218E-01
PLAC9	0.310	2.207E-01	4.586E-02
SOBP	0.310	1.862E-01	3.842E-01
CACNA1H	0.309	2.497E-01	5.941E-04
SYNGR1	0.309	1.560E-01	1.649E-01
MIR593	0.309	3.202E-01	2.021E-01
PAQR4	0.309	4.553E-02	1.154E-01
ADAMTS9	0.309	1.249E-01	9.525E-02
CROCC2	0.309	4.098E-01	7.503E-02
SH3KBP1	0.309	9.202E-02	4.362E-01
C4orf17	0.308	7.426E-01	2.520E-02
SYP	0.308	1.396E-01	5.368E-02
AC131097.2	0.308	4.521E-01	6.515E-02
CLIC4	0.308	9.521E-02	2.222E-01
HIST1H2AG	0.308	1.613E-01	5.727E-02
TLX3	0.308	6.609E-01	2.393E-03
HERC3	0.308	3.245E-02	4.545E-03
SDS	0.308	2.041E-01	6.330E-01
SMCO2	0.308	1.933E-01	1.673E-04
HIST1H2BJ	0.307	2.357E-01	4.100E-02
AMPD1	0.307	4.735E-01	1.575E-02
CCL21	0.307	5.348E-01	8.352E-01
UPK2	0.307	5.241E-01	5.778E-02
CHRNB1	0.306	5.670E-02	1.034E-01
PTRH1	0.306	1.378E-01	1.748E-03
F8	0.306	4.838E-02	3.781E-02
AKAP3	0.306	1.075E-01	1.091E-01
AOC3	0.306	2.435E-01	2.678E-02
ITIH5	0.306	2.393E-01	3.549E-02
MAP6	0.306	2.473E-01	5.838E-02
FIGLA	0.306	6.663E-01	1.313E-02
GRIN3B	0.305	2.357E-01	2.845E-03
PLS3	0.305	2.740E-02	1.070E-02
ZNF385C	0.305	1.371E-01	4.890E-07
AK7	0.305	2.543E-01	6.795E-03
SCN7A	0.304	5.367E-01	4.359E-02
NXPH3	0.304	2.937E-01	7.782E-03
GADD45G	0.304	1.435E-01	7.480E-02
SVIL	0.304	5.149E-02	6.398E-02
SYNGR3	0.304	3.117E-01	2.325E-01
FLNA	0.304	1.592E-01	2.108E-02
SLC52A3	0.303	1.073E-01	4.292E-01
PEPD	0.303	1.682E-03	3.018E-02

MPZL3	0.303	1.205E-02	9.410E-02
LGALS16	0.303	NA	4.014E-01
HFM1	0.303	3.766E-01	1.040E-03
ENTHD1	0.303	4.040E-01	4.635E-01
MYH10	0.303	2.551E-02	1.552E-02
AQP1	0.303	9.639E-02	2.333E-01
RHGAP19-SLI	0.303	5.685E-01	1.227E-01
PCDHGB3	0.303	2.548E-01	3.081E-02
CPPED1	0.303	1.494E-01	2.202E-01
GPC5	0.302	5.047E-01	1.039E-01
UXS1	0.302	4.243E-03	5.859E-02
NT5DC3	0.302	3.999E-02	3.349E-01
PCDHB7	0.302	2.418E-01	1.330E-02
SSC4D	0.302	8.951E-02	1.018E-02
CSF2RB	0.302	2.838E-01	2.800E-02
DGKB	0.301	5.246E-01	2.630E-06
ARHGAP36	0.301	6.860E-01	1.693E-01
WFDC1	0.301	2.053E-01	2.814E-02
CMTM8	0.301	7.644E-02	3.535E-04
COL3A1	0.301	3.084E-01	6.216E-03
CELA2A	0.301	3.538E-01	1.636E-01
SRGAP2B	0.301	1.186E-02	4.582E-02
GLIS2	0.301	1.289E-01	4.136E-03
CXCL3	0.300	4.120E-01	3.931E-02
TMEM52B	0.300	4.633E-01	1.100E-01
RASSF8	0.300	1.016E-01	1.840E-05
FLRT3	0.300	3.219E-01	6.497E-01
GPR160	0.300	2.357E-01	1.453E-01
CSGALNACT	0.300	5.387E-02	2.756E-03
SLC30A4	0.300	9.760E-02	1.414E-01
RNASE13	0.300	4.563E-01	3.855E-02
SULF1	0.299	3.499E-01	5.816E-04
CYTH3	0.299	1.690E-02	7.399E-01
LPAR4	0.299	2.480E-01	1.236E-01
TNC	0.298	3.796E-01	1.255E-02
JDP2	0.298	7.389E-02	3.866E-02
ZNF479	0.298	8.383E-01	2.839E-02
IGSF23	0.298	4.461E-01	2.197E-01
CLCN1	0.298	3.338E-01	3.951E-01
TMPRSS13	0.298	3.889E-01	7.341E-04
COL22A1	0.298	4.747E-01	5.708E-01
NFIB	0.298	8.837E-02	8.142E-01
GPR87	0.298	2.007E-01	6.008E-01
STBD1	0.298	2.889E-02	5.085E-01
CYBRD1	0.297	1.731E-01	3.223E-03
EGR4	0.297	3.918E-01	6.865E-01
H2BFWT	0.297	6.211E-01	1.020E-06
PRDM14	0.297	NA	1.660E-02
LGI1	0.297	6.088E-01	3.261E-02
SLCO3A1	0.297	1.285E-01	5.495E-02

KCNH2	0.297	3.972E-01	4.208E-02
PPBP	0.297	5.299E-01	3.092E-03
SMIM5	0.297	3.392E-01	1.012E-01
TWSG1	0.296	2.958E-02	5.446E-01
HAP1	0.296	3.558E-01	2.635E-02
TYRP1	0.296	4.675E-01	1.689E-01
TXK	0.296	2.038E-01	1.077E-01
RCN1	0.296	1.569E-02	6.140E-04
ANKS1A	0.296	4.300E-02	4.564E-02
FBLN5	0.295	1.664E-01	1.329E-01
SLC6A9	0.295	9.259E-02	4.751E-01
ATP10A	0.295	2.143E-01	1.174E-01
PALLD	0.295	1.470E-01	2.792E-01
SPRED3	0.295	8.172E-02	7.159E-03
SPARC	0.295	1.586E-01	3.246E-01
DHTKD1	0.295	9.196E-03	4.085E-02
AC004076.1	0.295	2.338E-01	8.348E-02
OXCT2	0.295	1.964E-01	2.699E-01
RTN4RL1	0.294	4.652E-01	2.814E-01
GFPT1	0.294	2.931E-03	1.456E-02
FAM129B	0.294	1.140E-02	8.286E-04
MYH4	0.294	5.633E-01	5.002E-02
GHR	0.294	3.047E-01	3.603E-01
APOC3	0.294	8.265E-01	5.180E-01
FMO1	0.294	3.443E-01	6.082E-03
CTSK	0.294	2.510E-01	5.133E-01
FILIP1L	0.294	1.916E-01	6.782E-01
MIR3944	0.294	5.965E-01	9.120E-02
FGFR4	0.293	2.819E-01	1.369E-01
PCLO	0.293	3.235E-01	1.207E-02
SMKR1	0.293	3.221E-01	2.994E-01
MIR3197	0.293	5.426E-01	1.491E-01
SOX17	0.293	1.489E-01	7.991E-02
LRRC10B	0.293	2.763E-01	6.618E-02
RAB3IL1	0.293	1.954E-01	1.040E-05
AC139530.2	0.293	4.247E-01	6.231E-03
KAT2B	0.293	3.647E-02	1.538E-01
GALR3	0.292	4.433E-01	2.687E-01
MIR548I1	0.292	NA	2.603E-01
DDX43	0.292	4.657E-01	1.620E-02
TRIB3	0.292	1.177E-01	2.102E-01
HAMP	0.292	3.430E-01	3.730E-02
GUCY1A3	0.292	1.538E-01	1.392E-04
COL16A1	0.291	2.550E-01	3.980E-02
FAM107B	0.291	7.285E-02	1.746E-04
ATP6V0E2	0.291	9.619E-02	5.049E-02
ANXA6	0.291	2.023E-01	1.373E-01
VWA3B	0.291	3.194E-01	1.763E-02
CGREF1	0.291	3.708E-01	1.069E-01
MIR548AT	0.291	NA	1.473E-01

TMEM52	0.291	3.080E-01	1.550E-02
MAGI1	0.291	1.152E-01	3.160E-05
AP002373.1	0.290	4.905E-01	6.409E-01
MYOCD	0.290	4.577E-01	2.825E-02
SLC17A3	0.290	7.397E-01	4.103E-02
NFKBIB	0.290	3.822E-03	5.450E-02
PRKAG2	0.290	8.478E-03	7.450E-03
PGK2	0.290	4.968E-01	4.276E-02
MFGE8	0.290	1.205E-01	8.331E-02
PKIG	0.290	6.672E-02	5.604E-04
IDH2	0.290	5.710E-03	2.360E-03
CFHR1	0.290	6.298E-01	4.777E-02
FSTL1	0.289	9.695E-02	4.214E-01
REXO2	0.289	1.697E-03	1.530E-02
FANCD2OS	0.289	2.438E-01	1.987E-01
EXTL1	0.289	2.463E-01	2.980E-03
AM47E-STBI	0.289	4.260E-01	2.859E-02
PAMR1	0.289	1.623E-01	3.211E-01
SGIP1	0.289	1.891E-01	1.356E-01
PDZK1IP1	0.289	5.246E-01	3.445E-02
SCN8A	0.289	3.506E-01	2.213E-01
COX6A2	0.288	6.931E-01	3.508E-02
TSPAN15	0.288	5.929E-02	1.845E-01
TMEM79	0.288	5.331E-02	1.418E-01
SCARA3	0.288	2.623E-01	1.463E-01
LDOC1	0.288	3.042E-01	3.076E-01
ADAM12	0.288	2.987E-01	2.362E-01
TMEM132B	0.288	3.003E-01	3.570E-02
ARMCX2	0.288	1.757E-01	1.927E-02
CCDC3	0.288	1.366E-01	6.813E-01
MGAT5B	0.287	4.006E-01	1.281E-03
AL713999.1	0.287	NA	1.249E-02
GRIN2C	0.287	2.074E-01	3.058E-02
CPED1	0.287	2.843E-01	1.530E-01
GLRB	0.287	3.339E-01	7.560E-05
HIST1H2AE	0.287	3.208E-01	1.964E-02
CCDC169	0.287	2.917E-01	2.266E-01
IRF5	0.287	8.486E-02	2.429E-02
PPP1R15A	0.286	3.578E-02	7.318E-03
WDR31	0.286	2.017E-02	1.130E-03
EBF1	0.286	1.961E-01	8.906E-02
OLFML1	0.286	1.789E-01	1.208E-01
HIGD1C	0.286	5.003E-01	8.115E-02
SLC39A7	0.286	1.156E-03	5.847E-03
TCEAL3	0.285	2.703E-02	3.934E-01
STMN3	0.285	2.551E-01	4.775E-03
MPP7	0.285	1.132E-01	4.194E-02
ACOX3	0.285	2.322E-02	6.831E-01
ADAMTS4	0.285	2.388E-01	1.436E-02
ADPRH	0.285	6.965E-02	1.628E-01

POMC	0.285	3.472E-01	4.558E-02
PIGM	0.285	2.017E-02	7.758E-04
HEYL	0.285	1.212E-01	1.386E-01
GFPT2	0.285	4.197E-01	2.690E-01
DKK3	0.285	1.469E-01	2.448E-02
MEIG1	0.284	2.013E-01	6.752E-02
MAGEH1	0.284	6.625E-02	1.185E-01
UBL3	0.284	3.205E-02	2.658E-01
GPRASP1	0.284	1.688E-01	8.393E-02
KRT78	0.284	4.889E-01	5.251E-02
NKX2-4	0.284	NA	1.527E-02
APBB1	0.284	1.954E-01	4.445E-03
PRG4	0.284	2.520E-01	2.778E-02
PTGES3L	0.283	1.255E-01	5.741E-01
DAPK1	0.283	2.116E-01	6.913E-01
FBXL7	0.283	1.693E-01	5.860E-05
STOX1	0.283	2.219E-01	4.449E-03
AGT	0.282	3.920E-01	9.018E-02
KIAA1211L	0.282	1.609E-01	1.111E-01
ST6GALNAC	0.282	4.115E-01	6.206E-01
PTGER1	0.282	2.934E-01	2.350E-02
BCL9L	0.282	4.462E-02	2.005E-03
SPEF1	0.282	2.890E-01	5.621E-03
DPYSL2	0.282	1.070E-01	1.891E-02
KLF11	0.281	3.362E-02	1.810E-01
EFEMP2	0.281	1.432E-01	2.117E-01
SVOPL	0.281	3.375E-01	7.159E-03
CDH15	0.281	3.793E-01	4.412E-01
MAGEA2	0.281	7.046E-01	2.035E-01
SIGLEC8	0.281	4.302E-01	1.532E-01
COL5A2	0.281	2.735E-01	6.095E-01
MIR203A	0.281	6.311E-01	4.246E-02
ADIRF	0.281	NA	4.112E-01
HSPB3	0.281	6.198E-01	1.803E-03
FOXL2NB	0.281	6.001E-01	3.613E-02
MLIP	0.280	5.184E-01	7.348E-02
DSCR8	0.280	7.892E-01	9.256E-03
HDGFL1	0.280	5.915E-01	1.549E-01
ACOX1	0.280	1.118E-01	1.526E-01
AL645922.1	0.280	7.408E-01	2.533E-02
MEOX2	0.280	4.840E-01	5.309E-04
HACD4	0.280	1.637E-01	4.540E-05
THY1	0.279	2.081E-01	1.899E-01
PLPP7	0.279	3.054E-01	3.101E-01
UCN	0.279	2.194E-01	5.764E-02
C1orf115	0.279	2.329E-01	1.448E-02
PLA2G2C	0.279	5.312E-01	9.360E-03
XAGE2	0.279	8.084E-01	1.452E-01
AC091551.1	0.279	4.261E-01	3.032E-01
PGM2L1	0.279	8.970E-02	1.082E-02

ACAT1	0.279	4.226E-02	8.257E-01
AL359736.1	0.279	6.413E-01	6.311E-02
AC090227.1	0.279	NA	5.745E-01
ATF6	0.279	1.975E-03	3.830E-01
STMN1	0.279	6.666E-02	3.622E-01
ADAMTSL3	0.278	4.080E-01	6.518E-01
PCDHA2	0.278	6.048E-01	6.428E-02
DEFB4B	0.278	NA	5.987E-02
BTBD19	0.278	1.277E-01	7.820E-02
ITSN2	0.278	1.584E-02	2.104E-02
HIST1H3E	0.278	2.478E-01	1.106E-01
TMEM187	0.277	1.186E-02	3.718E-02
MEIOB	0.277	4.953E-01	3.353E-01
AC022826.2	0.277	NA	1.011E-02
ACBD7	0.277	3.178E-01	5.140E-05
OPHN1	0.277	2.750E-02	8.804E-02
HRG	0.277	6.826E-01	2.933E-02
LRRC43	0.277	1.978E-01	1.261E-01
RETSAT	0.277	4.281E-03	1.889E-01
IDI1	0.277	1.341E-02	4.696E-02
ASAP2	0.277	1.107E-01	5.257E-02
ANKRD34C	0.276	6.722E-01	3.997E-03
TCF15	0.276	4.234E-01	4.767E-03
FRMD7	0.276	5.805E-01	1.516E-02
TAS2R1	0.276	7.214E-01	3.915E-02
MME	0.276	4.218E-01	4.421E-02
FAM219A	0.276	4.921E-03	6.988E-04
GLB1L2	0.276	3.914E-01	4.065E-02
DMRTB1	0.276	8.141E-01	2.237E-01
DOPEY2	0.276	5.051E-02	2.837E-02
AC010327.1	0.275	NA	6.958E-04
SLC19A2	0.275	9.425E-02	8.085E-02
AC112229.3	0.275	3.056E-01	6.290E-02
LY6G6D	0.275	7.446E-01	2.500E-02
AC008758.6	0.275	2.396E-01	2.795E-01
CACNG8	0.275	4.358E-01	2.636E-02
CLLU1OS	0.275	6.401E-01	4.200E-02
ELAVL2	0.275	5.710E-01	9.177E-02
SORT1	0.275	3.818E-02	9.093E-01
PANX3	0.275	7.488E-01	6.936E-02
AC008770.2	0.275	3.689E-01	3.671E-02
CPNE9	0.275	2.764E-01	3.039E-01
VENTX	0.275	3.303E-01	2.974E-02
TMEM255B	0.274	6.893E-02	2.408E-04
PVR	0.274	3.815E-02	7.490E-05
TCTEX1D1	0.274	2.349E-01	3.972E-04
JAM2	0.274	1.940E-01	5.449E-03
AC020914.1	0.273	NA	3.320E-06
JCHAIN	0.273	5.013E-01	4.919E-02
TAF1B	0.273	3.822E-03	5.730E-01

CYP2S1	0.273	4.555E-01	5.202E-03
KIF6	0.273	3.793E-01	1.960E-02
ULK2	0.273	3.326E-02	3.736E-01
THBS1	0.273	2.449E-01	1.408E-02
PIRT	0.273	6.824E-01	1.083E-01
ACSM5	0.273	3.660E-01	5.548E-03
FBXO3	0.272	2.228E-03	1.017E-02
RERGL	0.272	5.325E-01	3.429E-02
EPHB3	0.272	2.440E-01	1.068E-03
TNNC1	0.272	5.076E-01	2.635E-01
C12orf75	0.272	3.413E-01	3.685E-04
DHCR7	0.272	4.172E-02	3.619E-01
ADAMTS16	0.272	4.640E-01	7.900E-05
MAT1A	0.272	5.131E-01	8.269E-02
FAM69C	0.272	5.691E-01	4.827E-02
ABCC9	0.271	2.766E-01	1.178E-01
MAPT	0.271	3.839E-01	7.439E-03
MIR3143	0.271	NA	6.344E-01
THSD4	0.271	1.500E-01	3.508E-03
MIR548H1	0.271	NA	2.023E-01
ZNF385D	0.271	3.966E-01	3.238E-02
ADCK2	0.271	1.494E-03	1.241E-01
NPY4R	0.271	5.412E-01	4.138E-03
SAMD11	0.271	3.446E-01	2.646E-01
OR8D1	0.271	7.709E-01	5.330E-04
ATP6V0D2	0.270	3.996E-01	5.215E-03
C1QTNF9	0.270	5.380E-01	1.085E-01
SGMS2	0.270	1.380E-01	5.221E-02
VWA3A	0.270	3.696E-01	2.266E-02
PBX1	0.270	1.390E-01	1.531E-01
WASF3	0.269	1.906E-01	8.726E-02
MAN1A1	0.269	1.444E-01	3.917E-01
RAB23	0.269	1.742E-01	1.459E-01
CHRDL1	0.269	6.252E-01	9.824E-02
PRRX2	0.269	2.986E-01	3.059E-02
TTC25	0.269	1.565E-01	2.344E-01
PRR19	0.269	5.628E-02	1.021E-01
AL591806.3	0.269	1.782E-01	2.557E-01
CNTNAP1	0.268	1.190E-01	5.223E-04
MORN3	0.268	3.332E-01	4.388E-01
EPHA7	0.268	4.715E-01	3.006E-01
EID2B	0.268	1.118E-01	5.292E-02
KIAA1958	0.268	3.983E-02	1.976E-02
STXBP1	0.268	1.764E-01	1.311E-03
ASGR1	0.268	2.016E-01	2.356E-01
PLEKHD1	0.268	3.745E-01	3.040E-03
FZD9	0.268	4.148E-01	3.018E-02
ZNF467	0.267	1.714E-01	4.450E-03
OPN1SW	0.267	3.477E-02	8.008E-02
AC008687.4	0.267	3.345E-01	4.660E-04

PTH1R	0.267	2.934E-01	3.345E-02
ATL1	0.267	9.171E-02	2.712E-01
TTLL9	0.267	2.754E-01	2.231E-01
OSBP2	0.266	3.080E-01	3.501E-02
CATIP	0.266	2.746E-01	6.753E-01
ANO2	0.266	2.238E-01	1.212E-01
FMO5	0.266	2.346E-01	2.260E-02
TCHH	0.266	4.938E-01	2.108E-01
MIR601	0.266	6.061E-01	1.421E-02
CHRM5	0.266	3.460E-01	1.090E-01
CLEC1A	0.266	1.627E-01	5.418E-02
EFCAB9	0.266	6.582E-01	4.239E-02
PRDM8	0.266	2.965E-01	7.730E-02
TUBB8	0.266	4.053E-01	5.157E-01
MAGEA2B	0.265	7.364E-01	5.903E-01
HIST1H2BO	0.265	4.150E-01	5.411E-02
MIR4701	0.265	3.482E-01	6.419E-01
IL17RE	0.265	1.991E-01	1.951E-01
VLDLR	0.265	1.940E-01	3.865E-01
IFNA13	0.265	NA	4.400E-05
WIPI1	0.265	1.286E-02	5.773E-03
TUBB3	0.265	3.525E-01	7.604E-01
SIRT2	0.265	2.078E-03	3.830E-01
AL590560.1	0.265	6.048E-01	5.442E-02
SH3TC2	0.265	2.483E-01	2.311E-02
GPIHBP1	0.265	3.882E-01	1.568E-01
KRBA2	0.265	2.479E-01	1.077E-03
CAMK2N2	0.264	3.966E-01	7.236E-02
PRR18	0.264	6.784E-01	3.331E-02
MIR149	0.264	6.889E-01	5.309E-01
VWA5A	0.264	2.243E-01	3.380E-01
CFL2	0.264	1.560E-01	2.803E-02
SLC12A8	0.264	1.584E-01	4.496E-02
TUBA3D	0.264	2.648E-01	5.889E-02
NFIX	0.264	1.187E-01	1.388E-02
NPY1R	0.264	5.228E-01	5.883E-03
FAM155A	0.264	3.558E-01	1.280E-01
SLC25A43	0.264	7.609E-02	1.377E-01
CPS1	0.264	4.667E-01	7.665E-02
PILRB	0.264	2.109E-01	3.535E-04
METRN	0.264	2.253E-01	4.048E-02
CD5L	0.264	6.543E-01	1.933E-02
NRGN	0.264	1.298E-01	5.538E-02
PTP4A3	0.263	1.195E-01	1.324E-01
FAM110D	0.263	1.867E-01	3.142E-01
MATN3	0.263	2.244E-01	5.290E-01
FOXS1	0.263	2.155E-01	4.182E-02
CAP2	0.263	2.154E-01	6.467E-01
LONRF2	0.263	5.455E-01	2.150E-01
PCDHA3	0.263	5.316E-01	7.117E-02

ZNF883	0.263	3.244E-01	9.659E-04
PRRT2	0.263	2.062E-01	2.187E-01
MCIDAS	0.263	2.565E-01	9.148E-02
NLRP6	0.263	3.402E-01	6.639E-01
CYP27A1	0.263	3.056E-01	1.100E-02
CD109	0.263	4.159E-01	7.959E-02
TLL2	0.262	4.522E-01	1.433E-02
PLIN2	0.262	1.658E-01	3.762E-01
CHSY3	0.262	2.511E-01	1.055E-02
CD300C	0.262	3.945E-01	8.124E-01
RDH13	0.262	5.858E-02	8.109E-01
EDNRA	0.262	2.294E-01	7.677E-01
FAM205C	0.262	6.591E-01	3.059E-02
RORC	0.261	5.070E-01	6.240E-05
MIR4444-2	0.261	NA	2.530E-03
ARMCX3	0.261	8.189E-03	4.540E-01
SEMA6D	0.261	3.267E-01	5.932E-02
CLDN23	0.261	2.819E-01	3.735E-02
TAGLN2	0.261	6.772E-02	1.442E-02
ALPK3	0.261	1.775E-01	1.147E-01
CDS1	0.261	5.167E-02	9.494E-02
MAS1L	0.261	7.583E-01	2.918E-01
SMARCA2	0.261	2.469E-02	1.450E-03
ITGA10	0.261	2.032E-01	7.502E-03
IGDCC3	0.260	6.417E-01	9.709E-02
PXMP4	0.260	8.351E-02	6.859E-04
MIR4787	0.260	NA	3.470E-04
NKAIN1	0.260	4.841E-01	4.557E-03
LCNL1	0.260	5.105E-01	8.214E-02
CRYBG3	0.260	1.824E-01	2.008E-02
PMVK	0.260	1.642E-02	5.359E-02
FAM102A	0.260	1.664E-02	4.545E-01
NOX5	0.260	3.878E-01	3.852E-01
CAV3	0.260	6.829E-01	7.729E-02
PCDH20	0.260	7.285E-01	1.177E-04
RADIL	0.260	3.729E-01	8.235E-02
CD300LG	0.260	5.935E-01	1.384E-01
TRHDE	0.260	5.702E-01	2.912E-01
DTNA	0.260	4.560E-01	7.801E-02
AQP9	0.260	4.500E-01	2.386E-01
PPP1R3G	0.260	2.356E-01	2.111E-01
SLC35C1	0.259	9.832E-03	6.295E-03
CRABP1	0.259	6.336E-01	6.460E-03
POU6F1	0.259	7.661E-02	7.788E-01
NECTIN2	0.259	1.033E-02	4.126E-02
GGCT	0.259	2.322E-02	1.808E-03
SPTBN1	0.259	3.893E-02	2.166E-01
TNS2	0.259	8.409E-02	8.206E-02
WFDC8	0.259	7.036E-01	5.986E-04
PARD3B	0.259	1.985E-01	5.421E-01

PLAGL1	0.259	2.349E-01	5.319E-02
TOB1	0.259	1.568E-01	5.142E-01
ADAM17	0.259	6.321E-02	3.940E-05
ZEB1	0.259	1.963E-01	7.490E-01
AL160275.1	0.259	3.953E-01	6.962E-02
XPR1	0.259	3.198E-02	7.001E-03
TEKT2	0.259	4.236E-01	3.868E-01
PROCA1	0.258	1.878E-01	2.794E-01
PTF1A	0.258	NA	6.250E-07
MIR4802	0.258	NA	2.964E-03
ZFPM2	0.258	3.107E-01	7.809E-01
OOEP	0.258	6.655E-01	7.744E-01
TCAF2	0.258	1.250E-01	2.787E-01
C1QTNF6	0.258	2.246E-01	2.074E-03
GK3P	0.257	4.569E-01	1.067E-01
SH2D7	0.257	2.901E-01	1.654E-01
ARHGAP20	0.257	3.472E-01	2.912E-02
MIR126	0.257	4.522E-01	2.743E-01
HCN2	0.256	2.858E-01	7.875E-01
MAP6D1	0.256	7.926E-02	2.039E-02
TBC1D1	0.256	4.318E-02	8.421E-03
NAALADL2	0.256	3.013E-01	3.600E-02
DCAF12L2	0.256	6.145E-01	3.797E-04
TULP2	0.256	3.973E-01	6.921E-01
KCTD8	0.256	5.996E-01	4.370E-05
MREG	0.256	4.852E-02	1.590E-01
ISLR2	0.256	3.755E-01	2.373E-01
ASRGL1	0.256	3.670E-01	1.059E-01
44079.000	0.256	2.835E-01	2.728E-02
ETV5	0.256	1.810E-01	7.980E-02
PLXDC2	0.255	2.396E-01	6.229E-02
C7orf61	0.255	1.721E-01	5.672E-01
SHMT1	0.255	7.092E-02	1.208E-01
ARL4C	0.255	1.694E-01	2.159E-03
TMIGD3	0.255	3.041E-01	1.239E-01
C2CD4A	0.255	4.427E-01	1.863E-01
CFB	0.255	3.675E-01	5.599E-01
PNPLA1	0.255	4.709E-01	9.764E-02
AC068580.4	0.255	2.616E-01	1.456E-02
KCNIP3	0.255	3.286E-01	6.975E-01
SLC25A41	0.255	4.607E-01	5.306E-04
CPN2	0.255	6.122E-01	4.818E-01
PCDH18	0.254	2.462E-01	6.419E-02
EDEM1	0.254	2.194E-02	6.015E-01
KCNC3	0.254	3.084E-01	1.970E-05
ACTL7B	0.254	6.392E-01	3.780E-01
EXPH5	0.254	1.586E-01	7.144E-02
H2BFS	0.254	NA	4.317E-04
FNDC11	0.254	3.630E-01	1.639E-01
FAM124B	0.254	2.305E-01	3.021E-02

GLRA3	0.254	5.630E-01	1.341E-01
ALDH1L2	0.254	3.743E-01	8.252E-02
EPB41	0.254	8.505E-02	1.248E-02
CNGB1	0.254	4.891E-01	1.666E-01
PTGS2	0.254	4.281E-01	7.254E-01
IL18R1	0.253	1.877E-01	7.920E-05
P3H4	0.253	1.005E-01	3.373E-02
KRTAP5-4	0.253	7.217E-01	2.154E-02
MIEN1	0.253	7.699E-02	6.233E-04
ACO1	0.253	3.830E-02	4.911E-04
MIR3135A	0.253	NA	3.738E-02
ROR1	0.253	3.600E-01	7.436E-02
RIIAD1	0.253	5.407E-01	9.639E-03
HSPA2	0.253	2.399E-01	3.091E-04
CALY	0.252	5.737E-01	1.281E-01
NRCAM	0.252	5.114E-01	5.784E-01
CLMN	0.252	1.456E-01	2.620E-02
ZC3HAV1L	0.252	9.092E-02	7.804E-02
AD000671.1	0.252	3.314E-01	8.917E-04
SCCPDH	0.252	1.877E-01	7.444E-04
CLMP	0.252	4.031E-01	1.295E-01
DC169-SOHI	0.252	5.749E-01	2.450E-01
ANTXR2	0.252	1.958E-01	1.767E-01
CDKN2A	0.252	5.808E-01	5.400E-02
PLCXD3	0.252	6.413E-01	1.553E-01
LRRC46	0.252	7.921E-02	4.053E-02
HOMER2	0.252	2.755E-01	3.492E-02
TCEAL5	0.251	6.231E-01	1.187E-04
DSTN	0.251	6.005E-03	9.208E-03
FAR2	0.251	3.369E-01	1.315E-02
FERMT2	0.251	2.405E-01	1.991E-01
ZP2	0.251	7.026E-01	7.861E-01
GLMP	0.251	1.104E-02	1.549E-02
C1R	0.251	2.989E-01	6.107E-02
FAM83D	0.251	1.377E-01	1.270E-03
FCN2	0.251	6.949E-01	3.570E-02
C1orf185	0.251	NA	2.310E-02
SLC25A52	0.251	4.652E-01	9.825E-02
SGSM1	0.251	4.645E-01	9.735E-03
GPR176	0.251	3.238E-01	1.199E-02
CYP46A1	0.251	2.770E-01	9.964E-02
NAP1L3	0.251	4.170E-01	5.861E-03
ZNF835	0.251	4.518E-01	2.338E-01
KIAA2022	0.251	5.373E-01	8.254E-02
ACOT1	0.251	1.195E-01	7.865E-02
OR5T2	0.250	NA	1.023E-01
XIRP2	0.250	6.991E-01	6.320E-05
MIR3690	0.250	NA	3.709E-02
PLOD1	0.250	3.078E-02	6.860E-05
DSEL	0.250	2.878E-01	2.236E-01

MIR4263	0.250	1.174E-01	3.810E-06
HTR3B	0.250	8.107E-01	8.060E-05
USP2	0.250	3.093E-01	1.854E-02
TMEM147	0.250	2.106E-02	2.771E-02
SPON2	0.250	3.104E-01	2.903E-02
MIR33B	0.249	6.726E-01	3.427E-01
TRAM1	0.249	2.341E-02	3.313E-02
FTCDNL1	0.249	4.331E-01	2.339E-01
MIR4726	0.249	NA	2.286E-04
FGF6	0.249	NA	1.226E-02
FMOD	0.249	3.362E-01	8.240E-01
FGF12	0.249	3.996E-01	3.202E-01
PRPH	0.249	5.860E-01	6.955E-02
SFTA3	0.249	7.770E-01	5.063E-02
MAGED4	0.249	7.656E-01	4.404E-02
ECH1	0.249	3.267E-02	1.959E-03
FAM81A	0.249	2.193E-01	7.546E-02
HCAR3	0.249	4.137E-01	9.989E-02
TRPC1	0.249	1.892E-01	2.447E-01
CLEC1B	0.249	5.703E-01	1.237E-01
TIMP4	0.249	4.362E-01	8.250E-02
CAV1	0.249	3.982E-01	4.334E-01
C5	0.248	1.273E-01	3.113E-02
SMAGP	0.248	1.146E-01	7.908E-03
F7	0.248	5.010E-01	9.159E-04
LRRC32	0.248	2.496E-01	9.573E-02
CGB2	0.248	7.722E-01	1.335E-01
PRPF18	0.248	1.770E-03	4.201E-01
ZNF704	0.248	1.974E-01	2.349E-01
PRADC1	0.248	3.649E-02	1.385E-02
ALKAL2	0.248	3.923E-01	1.313E-01
LYG2	0.248	3.469E-01	3.440E-05
PPP2R3A	0.248	1.278E-01	8.667E-01
XAGE1B	0.248	8.831E-01	2.423E-01
UGT1A3	0.247	5.592E-01	5.253E-02
UBE2E2	0.247	3.075E-01	4.823E-02
SHTN1	0.247	1.734E-01	4.654E-01
ANXA2	0.247	1.328E-01	4.614E-01
COL12A1	0.247	3.375E-01	3.595E-01
TAB3	0.246	8.128E-03	3.123E-01
SCN2A	0.246	5.749E-01	5.310E-02
CLEC11A	0.246	2.760E-01	2.862E-02
SCARF2	0.246	3.172E-01	6.036E-03
VGF	0.246	3.696E-01	1.171E-01
RIPPLY3	0.246	3.743E-01	1.770E-01
GABARAPL1	0.245	3.880E-02	8.571E-03
S100A10	0.245	3.071E-01	1.485E-01
AC002985.1	0.245	6.478E-01	8.694E-02
PGBD5	0.245	4.806E-01	5.901E-01
MTRNR2L4	0.245	3.048E-01	1.007E-04

AP3B2	0.245	4.930E-01	6.204E-01
TMEM109	0.245	3.071E-03	6.794E-02
GLYATL1P3	0.245	6.527E-01	1.361E-03
CATSPERG	0.245	2.583E-01	8.577E-01
KRT3	0.245	5.936E-01	4.981E-03
MIR5094	0.244	6.846E-01	6.584E-01
B3GNT9	0.244	4.224E-02	5.025E-02
LRRC73	0.244	2.498E-01	1.735E-02
KRT10	0.244	2.141E-01	2.399E-04
COPA	0.244	1.221E-02	8.080E-02
MSR1	0.244	3.853E-01	2.303E-01
TUBB2A	0.244	2.914E-01	5.600E-05
MCEE	0.244	5.126E-03	1.097E-04
NEK11	0.244	2.178E-02	2.352E-02
ESYT2	0.244	8.653E-03	8.469E-02
NR0B1	0.244	7.970E-01	1.104E-03
RENBP	0.244	3.165E-01	1.433E-02
BMP4	0.244	4.028E-01	1.624E-02
ABLIM3	0.244	2.818E-01	7.117E-01
EFHB	0.244	2.565E-01	1.313E-01
TGM5	0.244	5.829E-01	7.485E-02
WDR62	0.243	1.195E-01	3.580E-05
AKR7A2	0.243	3.777E-03	1.454E-01
BCAT1	0.243	4.422E-01	4.725E-02
FXYD7	0.243	4.823E-01	4.040E-03
CCDC103	0.243	4.986E-01	1.119E-01
IFFO2	0.243	2.116E-01	4.710E-01
SLC34A2	0.243	7.251E-01	3.343E-01
RECK	0.243	1.964E-01	1.549E-01
RHOV	0.242	3.031E-01	2.538E-02
PHACTR2	0.242	4.494E-02	2.774E-01
CUEDC1	0.242	8.608E-02	3.702E-01
MINDY2	0.242	6.633E-02	5.493E-03
SOSTDC1	0.242	6.641E-01	4.370E-02
CFAP54	0.242	2.688E-01	4.174E-02
E2F1	0.242	1.738E-01	4.471E-03
MAML2	0.242	7.483E-02	3.976E-01
CSMD2	0.242	3.221E-01	7.524E-02
GDPD5	0.242	1.443E-01	1.475E-02
MMP8	0.241	6.336E-01	2.246E-03
LGALS14	0.241	8.479E-01	1.318E-02
SLAMF9	0.241	5.413E-01	2.530E-01
EPHX1	0.241	2.095E-01	5.682E-02
UNC5D	0.241	7.426E-01	4.761E-02
SLC9A3R2	0.241	9.253E-02	7.806E-03
FRMPD2	0.241	5.433E-01	1.518E-02
CXADR	0.241	2.547E-01	1.915E-02
MSX2	0.241	3.902E-01	3.373E-03
PLK5	0.241	5.157E-01	1.907E-02
DAP	0.241	4.689E-02	4.890E-05

RTL8B	0.241	3.745E-02	8.272E-01
MED8	0.241	7.403E-04	7.685E-01
BBOF1	0.240	4.917E-02	2.029E-01
TACR1	0.240	4.808E-01	7.997E-04
PDGFRL	0.240	4.080E-01	3.954E-02
DHRS13	0.240	1.616E-01	1.038E-01
GOLGA4	0.240	1.520E-02	1.307E-01
HACD2	0.240	3.251E-02	1.674E-01
CYB5A	0.240	2.645E-01	7.292E-01
KANK1	0.240	1.296E-01	3.329E-02
ZP3	0.240	2.035E-01	4.397E-02
MAGI2	0.240	1.657E-01	1.709E-03
ARHGEF25	0.239	2.501E-01	1.528E-01
ACTN1	0.239	1.682E-01	1.615E-01
TLR7	0.239	4.497E-01	2.421E-01
TTC7A	0.239	7.029E-02	1.599E-01
HTATSF1	0.239	1.215E-03	1.919E-02
HCAR2	0.239	4.150E-01	9.219E-01
MIR567	0.239	5.100E-01	1.308E-03
CNTD2	0.239	4.907E-01	1.790E-02
MRPS12	0.239	5.593E-02	3.474E-02
TMEM47	0.239	3.509E-01	5.132E-02
CAMK2N1	0.239	3.339E-01	6.510E-01
RASD2	0.239	3.465E-01	1.702E-01
MIR657	0.239	6.198E-01	9.184E-02
UPP1	0.239	1.603E-01	1.967E-01
MIR4767	0.239	6.839E-01	2.609E-01
KRT18	0.238	2.580E-01	1.524E-02
FSCB	0.238	7.752E-01	2.201E-02
SLC16A6	0.238	3.102E-01	6.484E-01
LRRTM4	0.238	6.632E-01	1.372E-02
CLEC2A	0.238	8.050E-01	6.801E-02
LY96	0.238	3.545E-01	3.198E-02
CHST3	0.238	2.785E-01	4.607E-02
GRHL1	0.238	2.770E-01	2.766E-01
TRIM16	0.238	1.232E-01	5.659E-03
BTC	0.238	3.396E-01	4.239E-01
KIF26B	0.238	3.268E-01	1.666E-04
PCDHGB4	0.238	4.999E-01	6.463E-02
HIPK3	0.238	2.298E-02	5.123E-01
FADS1	0.238	2.903E-01	6.640E-02
SFRP1	0.238	6.004E-01	1.281E-01
GXYLT2	0.237	4.725E-01	6.368E-01
AC005779.2	0.237	4.562E-01	8.320E-01
MIR345	0.237	NA	1.420E-01
DHDDS	0.237	2.444E-04	1.353E-01
NME9	0.237	2.139E-01	2.397E-02
RCC1	0.237	1.563E-02	5.530E-02
RAB31	0.237	2.487E-01	1.156E-01
LINGO1	0.237	3.219E-01	4.435E-02

GGCX	0.237	2.277E-03	9.931E-02
PLSCR4	0.237	2.575E-01	3.572E-01
AC010422.5	0.237	NA	3.351E-04
CYP24A1	0.237	6.067E-01	5.677E-02
TEKT4	0.237	5.026E-01	8.295E-01
ZNF573	0.236	6.300E-02	8.225E-01
PHYH	0.236	9.786E-02	6.947E-02
TDRKH	0.236	1.603E-01	1.225E-01
DMRTC2	0.236	7.257E-01	6.441E-01
KCNJ8	0.236	3.201E-01	5.567E-04
PDZRN4	0.236	6.535E-01	1.134E-02
CNRIP1	0.236	2.989E-01	2.707E-01
TGFBR1	0.236	1.472E-01	1.723E-04
KCNMB3	0.236	8.175E-02	1.983E-01
KIAA1755	0.235	4.888E-01	1.050E-01
MINDY4B	0.235	5.691E-01	6.393E-01
TMEM266	0.235	8.627E-02	2.056E-02
KCTD3	0.235	1.298E-02	8.593E-01
ZNF117	0.235	3.755E-01	1.020E-01
ATP11A	0.235	1.814E-01	7.814E-02
FBLN2	0.235	4.289E-01	8.454E-02
C2orf72	0.235	5.788E-01	8.908E-02
CSF1	0.235	3.182E-01	1.474E-02
ANXA8	0.235	4.282E-01	8.544E-03
OTUD7B	0.235	9.642E-03	5.583E-03
NET1	0.235	1.579E-01	1.551E-03
GRIK2	0.234	5.299E-01	5.232E-02
CALM2	0.234	2.296E-03	4.514E-04
OLR1	0.234	5.772E-01	2.252E-01
AUNIP	0.234	1.221E-01	3.561E-01
MMRN2	0.234	3.816E-01	2.112E-01
TUNAR	0.234	8.327E-01	1.227E-01
IFRD1	0.234	3.170E-02	9.166E-02
GDF7	0.234	4.921E-01	2.423E-02
ZNF706	0.234	1.176E-01	3.994E-04
GPRC5B	0.234	3.342E-01	6.834E-01
DCST2	0.234	2.517E-01	1.524E-01
ITGB1BP2	0.234	2.171E-01	7.588E-02
BFSP1	0.234	1.458E-01	7.878E-01
DACT1	0.233	3.998E-01	1.849E-03
STK3	0.233	2.032E-02	3.347E-04
LRP1	0.233	1.656E-01	2.367E-01
MIR4706	0.233	6.822E-01	6.542E-01
AC135178.2	0.233	4.075E-01	6.673E-03
EFNA3	0.233	2.479E-01	2.493E-04
GLA	0.233	4.851E-02	3.561E-01
C15orf59	0.233	4.833E-01	7.460E-01
MIR3909	0.233	NA	5.460E-07
ANKRD13A	0.232	3.200E-02	1.485E-04
CASC10	0.232	4.426E-01	1.695E-02

PRDM12	0.232	4.033E-01	3.032E-01
SETBP1	0.232	3.294E-01	1.670E-01
CSMD3	0.232	6.822E-01	7.639E-01
UBTD1	0.232	8.396E-02	3.482E-01
DZIP1L	0.232	2.202E-01	1.954E-03
PALM3	0.232	5.829E-01	7.427E-02
SLC22A8	0.232	NA	6.210E-02
CEACAM16	0.232	7.193E-01	6.344E-02
ART1	0.232	6.631E-01	1.022E-01
SIAH3	0.231	6.036E-01	3.865E-02
CFAP52	0.231	2.885E-01	1.954E-01
WFDC2	0.231	5.024E-01	2.463E-03
GALNT15	0.231	4.745E-01	6.577E-02
ANKRD34A	0.231	1.365E-01	1.322E-01
DAGLA	0.231	2.746E-01	4.031E-01
CLPSL1	0.231	6.749E-01	6.058E-02
CCDC178	0.231	4.737E-01	5.474E-02
SPAG8	0.231	1.662E-01	2.086E-02
SLC6A6	0.231	1.601E-01	1.352E-01
CNTNAP3	0.231	5.673E-01	4.603E-04
MYL3	0.231	5.194E-01	7.982E-01
FGF2	0.231	4.302E-01	4.544E-03
MIR5188	0.230	6.029E-01	3.914E-01
B4GALT4	0.230	1.417E-01	7.876E-01
LAMB2	0.230	1.221E-01	6.487E-01
RNF144A	0.230	1.436E-01	3.918E-04
FAM106A	0.230	6.235E-01	1.771E-01
VAT1L	0.230	4.317E-01	1.022E-01
SEC13	0.230	1.045E-02	3.912E-01
MAL2	0.230	2.608E-01	1.243E-03
C14orf37	0.230	2.664E-01	5.671E-01
OR52E6	0.230	8.587E-01	3.494E-01
ACPP	0.230	3.855E-01	2.351E-01
CADPS2	0.230	1.836E-01	5.089E-01
CLLU1	0.229	6.311E-01	9.210E-03
FAM171A2	0.229	3.996E-01	6.537E-03
MIR378G	0.229	7.278E-01	4.504E-02
ARMC9	0.229	7.581E-02	1.416E-02
HIST1H3G	0.229	4.889E-01	4.561E-03
THAP8	0.229	3.200E-02	1.435E-01
PXDN	0.229	3.576E-01	2.837E-02
TES	0.229	6.228E-02	3.113E-02
TEAD1	0.229	6.012E-02	1.952E-01
PCYOX1	0.229	7.698E-03	2.050E-01
ULK4	0.229	8.038E-02	9.970E-06
SYT15	0.229	3.056E-01	5.739E-01
POGK	0.228	8.038E-02	4.218E-02
GPR26	0.228	7.162E-01	5.831E-01
AC006269.1	0.228	7.525E-01	3.889E-03
LAYN	0.228	3.061E-01	2.090E-05

MPC2	0.228	8.468E-02	5.021E-02
GATA2	0.228	4.413E-01	1.607E-01
C10orf55	0.228	3.561E-01	1.601E-01
FRRS1	0.228	3.782E-01	6.847E-01
CALML5	0.228	7.423E-01	2.390E-03
AC106782.1	0.228	3.974E-01	3.190E-02
GPR137C	0.228	3.623E-01	2.443E-01
MIR6165	0.228	NA	3.195E-01
ARID5B	0.228	6.732E-02	1.381E-01
MYOG	0.228	7.488E-01	3.059E-01
POMK	0.228	2.607E-01	9.270E-04
CTF1	0.227	2.039E-01	8.150E-02
PLCD1	0.227	3.046E-01	2.389E-02
MMP16	0.227	4.352E-01	4.031E-04
RAB6B	0.227	3.446E-01	4.460E-02
TSHZ3	0.227	3.673E-01	1.219E-01
SUCO	0.227	4.198E-02	1.398E-03
PRR34	0.227	1.805E-01	7.870E-04
CTH	0.226	2.666E-01	2.745E-02
PDZD2	0.226	3.859E-01	1.014E-03
DHRS7C	0.226	NA	1.528E-01
ZFP92	0.226	4.170E-01	5.635E-01
OXCT1	0.226	4.025E-01	4.114E-01
ABHD1	0.226	3.218E-01	3.365E-01
WNT8B	0.226	5.815E-01	5.415E-04
LOXL2	0.225	2.833E-01	1.242E-01
OTOP1	0.225	NA	3.639E-01
TSPYL5	0.225	4.547E-01	6.727E-01
APOA2	0.225	7.793E-01	4.610E-02
MDFIC	0.225	9.760E-02	1.175E-03
FAM189A2	0.225	3.905E-01	3.685E-03
NT5C2	0.225	1.639E-01	5.424E-01
BACE1	0.225	1.021E-01	6.101E-01
OR4E2	0.225	NA	1.523E-01
GUCY1B3	0.225	1.876E-01	6.099E-04
SGCB	0.225	1.310E-01	7.162E-02
LPCAT1	0.225	2.566E-01	2.258E-01
LINC01314	0.225	7.011E-01	1.136E-02
FNDC3B	0.225	5.068E-02	2.577E-01
DUSP3	0.224	9.832E-03	6.129E-02
PLEKHB1	0.224	4.428E-01	8.648E-04
DKKL1	0.224	5.731E-01	1.739E-02
MYZAP	0.224	3.798E-01	5.696E-02
IL20	0.224	6.951E-01	2.240E-01
COPZ2	0.224	3.547E-01	8.792E-01
SCN3A	0.224	5.426E-01	7.180E-02
RAB11FIP4	0.224	2.463E-01	3.932E-01
MOCS1	0.224	2.420E-01	5.258E-01
PDIK1L	0.224	2.804E-02	3.508E-02
LAMA4	0.224	2.462E-01	3.542E-03

CACNA1F	0.224	3.809E-01	2.074E-02
FBXO47	0.224	6.515E-01	6.654E-02
NCSTN	0.224	2.621E-02	4.055E-01
CCL26	0.224	5.496E-01	8.158E-01
CEACAM5	0.224	7.086E-01	7.737E-02
SIGLEC9	0.223	4.302E-01	9.045E-02
CLDN7	0.223	1.940E-01	1.311E-01
TYROBP	0.223	4.378E-01	6.308E-01
ARMCX6	0.223	3.166E-02	5.851E-01
ZAR1L	0.223	5.434E-01	2.760E-01
TUFT1	0.223	1.398E-01	3.512E-04
KIAA1644	0.222	5.460E-01	5.142E-02
ADD2	0.222	6.184E-01	8.065E-02
INPP4A	0.222	1.238E-01	6.435E-03
ABI3BP	0.222	4.737E-01	4.377E-01
LRP11	0.222	8.242E-02	4.104E-01
C2CD2L	0.222	8.650E-03	5.537E-02
ARHGAP33	0.222	2.051E-01	1.130E-04
KRTAP4-9	0.222	NA	2.841E-01
MPP2	0.222	3.286E-01	9.606E-02
LRFN1	0.222	3.729E-01	1.062E-02
CLN3	0.221	4.575E-02	1.109E-01
MAGEC2	0.221	8.270E-01	8.863E-02
VSIG8	0.221	4.376E-01	7.629E-01
LHX4	0.220	3.511E-01	4.408E-02
CTSL	0.220	2.608E-01	2.941E-01
BANF2	0.220	NA	8.899E-01
SELENOM	0.220	3.740E-01	2.693E-02
LRFN2	0.220	6.432E-01	1.695E-03
HSPB9	0.220	2.920E-01	1.531E-01
SPTAN1	0.220	1.690E-02	5.480E-03
DGAT2	0.220	3.925E-01	5.894E-04
TMEM132C	0.220	6.924E-01	2.195E-01
FAM217A	0.220	4.511E-01	1.445E-01
PCDHA6	0.220	6.853E-01	4.161E-01
AC092042.3	0.220	5.413E-01	1.446E-01
C9orf92	0.220	7.559E-01	3.580E-05
LGALS3	0.220	1.532E-01	2.303E-02
GDF2	0.219	NA	1.743E-02
NR1D1	0.219	1.811E-01	1.960E-02
PDLIM7	0.219	2.195E-01	1.585E-01
C8orf86	0.219	NA	9.423E-02
CLIP2	0.219	1.705E-01	2.133E-01
DIRAS1	0.219	5.999E-01	8.146E-02
NAT2	0.219	5.970E-01	5.789E-03
NFATC4	0.219	2.372E-01	3.543E-03
AC142391.1	0.219	NA	6.081E-01
CPLX1	0.219	3.084E-01	1.193E-04
TC2N	0.219	2.561E-01	5.762E-02
RHEB	0.219	1.214E-03	3.186E-01

RASAL2	0.219	7.296E-02	1.950E-05
SNX24	0.219	6.222E-02	2.602E-04
DNAH14	0.219	2.185E-01	6.688E-03
CRYBA4	0.218	6.126E-01	1.022E-01
MIR554	0.218	6.188E-01	3.909E-02
DENND4C	0.218	1.116E-01	8.807E-02
SLC39A1	0.218	4.137E-03	1.074E-03
THBS2	0.218	5.086E-01	6.085E-01
CELF5	0.218	6.113E-01	3.360E-02
HSPA12B	0.218	2.598E-01	2.553E-01
SLC22A18AS	0.218	3.495E-01	1.493E-02
ARHGAP22	0.218	3.588E-01	6.938E-01
CPLX4	0.218	6.598E-01	2.391E-02
RBM46	0.218	7.551E-01	2.548E-04
KHK	0.217	2.378E-01	6.996E-01
CFAP65	0.217	6.887E-01	4.635E-04
IL17RD	0.217	3.313E-01	2.560E-03
UGT1A5	0.217	7.046E-01	1.459E-04
XKR5	0.217	5.455E-01	5.150E-05
TRH	0.217	5.749E-01	9.218E-02
KANK3	0.217	2.141E-01	6.464E-01
TUB	0.217	4.257E-01	1.389E-04
MID2	0.217	7.523E-02	1.756E-01
CCDC160	0.217	5.285E-01	3.395E-02
SPATA32	0.216	2.917E-01	3.940E-02
KLHL10	0.216	3.550E-01	3.637E-01
CD14	0.216	5.026E-01	2.588E-01
DIAPH3	0.216	2.299E-01	5.241E-01
MAB21L2	0.216	6.991E-01	6.211E-01
F11R	0.216	1.232E-01	1.348E-01
TMEM245	0.216	1.636E-02	1.494E-02
OSBP	0.216	3.894E-03	1.456E-01
MT1G	0.216	5.577E-01	1.266E-01
TMEM214	0.216	7.442E-04	3.304E-03
C4orf47	0.216	3.138E-01	4.265E-04
UHMK1	0.215	5.598E-02	5.026E-02
ADAM15	0.215	7.126E-02	2.750E-01
BRSK1	0.215	3.916E-01	3.820E-02
TMEM11	0.215	2.888E-02	2.467E-01
HABP4	0.215	1.345E-01	2.815E-01
OPN3	0.215	2.712E-01	5.305E-03
ST6GALNAC	0.215	3.136E-01	2.395E-01
WDR49	0.215	6.099E-01	1.046E-01
POPDC3	0.215	6.620E-01	7.130E-01
FAM107A	0.215	5.093E-01	4.427E-01
B4GALT3	0.215	1.032E-01	8.067E-02
GALK1	0.215	6.364E-02	7.418E-01
FTL	0.214	1.625E-01	6.820E-02
CHST7	0.214	2.038E-01	6.652E-01
S1PR1	0.214	2.730E-01	8.058E-01

CHODL	0.214	6.272E-01	2.486E-01
FSF12-TNFSI	0.214	5.516E-01	2.729E-01
NKX3-1	0.214	2.821E-01	1.329E-01
WRB	0.214	2.708E-02	1.427E-01
C1orf198	0.214	4.997E-03	1.869E-01
P3H3	0.214	3.608E-01	1.843E-01
POU4F1	0.214	6.846E-01	6.466E-01
TMED3	0.214	3.430E-02	1.050E-01
SERPINB12	0.214	8.017E-01	1.212E-01
COL19A1	0.214	6.761E-01	6.118E-02
DMGDH	0.214	2.848E-01	1.163E-01
HTRA3	0.214	5.361E-01	7.316E-03
FAM72B	0.213	2.411E-01	9.828E-03
CPZ	0.213	5.347E-01	7.433E-02
CBY3	0.213	4.182E-01	1.162E-01
PBX3	0.213	7.466E-02	3.092E-02
DEFB126	0.213	7.135E-01	5.317E-03
KLF6	0.213	1.464E-01	3.473E-01
GATS	0.213	1.831E-01	1.691E-01
ITGA1	0.213	2.201E-01	3.078E-04
COPG1	0.213	4.831E-04	2.137E-02
DUPD1	0.213	8.069E-01	3.626E-01
BHLHB9	0.213	5.644E-02	9.088E-02
APOC4-APOC	0.213	6.106E-01	2.206E-01
EDIL3	0.213	3.892E-01	1.199E-01
CDKL2	0.213	4.889E-01	3.739E-04
BCL6B	0.213	2.375E-01	2.492E-01
PCDHB8	0.213	5.754E-01	3.834E-01
MX2-CTNND	0.212	NA	2.186E-01
PAK4	0.212	3.305E-02	2.729E-01
PDE4DIP	0.212	6.262E-02	1.664E-01
FGD3	0.212	3.286E-01	9.324E-04
CMKLR1	0.212	5.243E-01	9.729E-03
NBL1	0.212	3.042E-01	5.238E-02
RBM24	0.212	5.469E-01	1.310E-02
MCOLN3	0.212	6.016E-01	4.625E-01
OXR1	0.212	9.201E-02	3.617E-01
IFNL3	0.212	7.224E-01	5.410E-05
AC136428.1	0.212	7.325E-01	1.996E-01
STK31	0.211	3.995E-01	2.587E-01
PLA2G15	0.211	5.418E-02	7.427E-03
SYNDIG1L	0.211	5.853E-01	7.484E-01
PTCRA	0.211	5.678E-01	4.233E-04
MROH2B	0.211	6.915E-01	6.199E-02
LAMP5	0.211	6.038E-01	2.008E-01
MROH6	0.211	3.339E-01	1.443E-01
NPFFR2	0.211	7.464E-01	3.929E-01
PGA5	0.211	7.488E-01	8.330E-02
BSCL2	0.211	1.943E-02	2.626E-01
OR1L3	0.211	NA	3.894E-02

PIM1	0.211	2.131E-01	5.531E-01
CHAD	0.211	4.562E-01	4.358E-02
PPP1R42	0.211	5.810E-01	4.411E-04
HPCAL1	0.210	1.414E-01	1.266E-02
HSD17B7	0.210	8.905E-02	1.490E-05
ANGPT1	0.210	4.897E-01	3.240E-03
APIP	0.210	3.200E-02	2.196E-02
CLSTN2	0.210	5.434E-01	1.413E-02
OR1L6	0.210	NA	7.547E-02
PARD3	0.210	3.272E-02	1.315E-02
PXDC1	0.210	1.827E-01	9.482E-02
SLC10A2	0.210	8.599E-01	6.753E-02
CD33	0.210	4.746E-01	2.904E-02
PLCH1	0.210	5.277E-01	7.639E-02
VSTM4	0.209	3.379E-01	5.320E-03
ESR2	0.209	2.956E-01	3.963E-02
SLC11A1	0.209	3.894E-01	6.926E-01
ANKRD24	0.209	3.686E-01	9.758E-02
RNF225	0.209	5.764E-01	1.722E-01
TMEFF2	0.209	6.930E-01	3.487E-01
SEC22B	0.209	4.508E-03	4.150E-03
CGB3	0.209	7.089E-01	2.860E-01
ENPEP	0.209	3.054E-01	5.784E-02
C1orf74	0.209	3.359E-01	2.036E-03
CA3	0.209	5.386E-01	6.677E-01
LCA5L	0.209	1.145E-01	1.081E-01
SH3BGRL2	0.209	3.098E-01	8.345E-02
PSMD3	0.209	1.620E-02	2.450E-01
PNMA8B	0.209	3.841E-01	3.846E-01
NEU1	0.209	7.065E-02	4.414E-01
RD3L	0.208	NA	2.492E-01
MCUB	0.208	2.565E-01	3.238E-01
NKIRAS1	0.208	1.457E-02	5.568E-02
LRRC31	0.208	6.431E-01	3.525E-02
L1CAM	0.208	7.050E-01	6.045E-01
PPP6R3	0.208	2.054E-02	2.663E-01
KDELC2	0.208	1.668E-01	7.235E-01
PRTFDC1	0.208	4.120E-01	3.734E-02
AAK1	0.207	1.298E-02	1.589E-02
HFE	0.207	1.926E-01	7.171E-02
ROR2	0.207	5.373E-01	2.143E-03
OR51E2	0.207	4.885E-01	1.575E-01
DYNC1I1	0.207	4.802E-01	7.650E-03
GPX8	0.207	3.356E-01	6.065E-02
SCUBE2	0.207	6.413E-01	6.899E-02
44078.000	0.207	2.173E-01	2.558E-03
MISP	0.207	5.569E-01	3.084E-01
C16orf46	0.207	9.753E-02	1.606E-01
TRIM47	0.207	3.059E-01	2.084E-01
ITGA5	0.206	3.973E-01	4.570E-02

MIR3202-1	0.206	6.985E-01	1.825E-01
TMEM62	0.206	5.256E-02	5.808E-01
ANKRD1	0.206	6.817E-01	7.804E-02
PLA2G4C	0.206	4.137E-01	1.357E-03
CAV2	0.206	3.736E-01	1.165E-02
RUSC2	0.206	1.830E-01	4.767E-04
GPM6A	0.206	6.338E-01	6.575E-04
HIST1H3H	0.206	5.181E-01	5.601E-02
RETN	0.206	5.806E-01	2.005E-01
GPR17	0.206	5.380E-01	2.306E-01
TCTEX1D2	0.206	2.356E-01	5.905E-01
XYLB	0.206	2.195E-01	2.949E-01
ANXA5	0.205	2.226E-01	4.595E-02
PANK1	0.205	1.378E-01	1.634E-01
LRRN2	0.205	5.579E-01	1.338E-01
KSR1	0.205	2.455E-01	9.309E-03
GSTM4	0.205	5.434E-01	4.634E-01
DPEP1	0.205	6.063E-01	2.390E-04
GUSB	0.205	6.675E-02	5.503E-03
CHST1	0.205	4.271E-01	7.506E-03
EPDR1	0.205	4.913E-01	3.578E-01
ANKRD7	0.205	6.575E-01	1.706E-01
FNDC1	0.205	6.532E-01	4.378E-02
SIX3	0.205	7.257E-01	1.556E-03
HSPB2	0.205	4.408E-01	2.399E-01
TCF21	0.205	4.747E-01	1.589E-01
ATP6V0A1	0.204	1.451E-02	1.492E-02
MYH15	0.204	5.896E-01	2.657E-03
COL5A1	0.204	5.056E-01	1.364E-04
ZDHHC18	0.204	1.834E-02	8.941E-01
TREM2	0.204	4.723E-01	1.297E-01
FUT5	0.204	NA	3.081E-03
SLC46A3	0.204	2.836E-01	5.571E-02
TOR1AIP2	0.204	8.087E-03	1.000E-01
ANLN	0.204	2.763E-01	1.213E-01
PDE1B	0.204	5.209E-01	1.376E-01
RIBC1	0.204	1.689E-01	5.451E-01
MIR4754	0.204	5.415E-01	7.138E-01
MIR1289-1	0.204	7.800E-01	3.109E-01
ICAM1	0.203	4.895E-01	6.141E-02
TXLNB	0.203	4.517E-01	5.220E-02
SLC16A13	0.203	1.380E-01	3.530E-03
PLK2	0.203	3.566E-01	4.864E-02
HEATR9	0.203	5.350E-01	5.550E-01
HFE2	0.203	6.609E-01	2.179E-03
ADAMTSL1	0.203	5.209E-01	1.997E-02
CENPVL3	0.203	9.095E-01	2.033E-02
RASGEF1A	0.203	5.322E-01	2.652E-01
IRAK3	0.203	3.699E-01	3.238E-01
SCN1B	0.202	4.261E-01	8.511E-02

MFAP2	0.202	4.196E-01	4.097E-02
ZSWIM5	0.202	4.306E-01	7.695E-01
MXRA7	0.202	2.051E-01	1.442E-01
IGFBP5	0.202	5.442E-01	1.155E-03
ANXA4	0.202	7.388E-02	3.881E-01
JADE3	0.202	7.447E-02	1.621E-03
MFSD11	0.202	1.342E-02	8.202E-04
TULP3	0.202	2.434E-02	1.420E-02
AC010326.2	0.202	3.733E-01	6.045E-02
CDX1	0.202	5.708E-01	3.925E-02
VIT	0.202	7.404E-01	4.635E-02
ABRA	0.202	5.684E-01	2.385E-03
AL355916.3	0.202	NA	2.267E-01
MORC4	0.202	8.449E-02	4.466E-01
ACTL7A	0.201	NA	6.793E-01
LILRA5	0.201	5.508E-01	6.720E-05
PRPH2	0.201	4.592E-01	7.399E-02
HMX3	0.201	8.397E-01	1.559E-01
PPP1R3F	0.201	1.804E-01	1.048E-01
OR6T1	0.201	6.964E-01	1.120E-07
UPF2	0.201	1.217E-02	3.660E-01
DAG1	0.201	7.916E-02	1.237E-01
RRM2	0.201	2.653E-01	6.796E-01
DEDD	0.201	7.242E-02	1.278E-02
ANKH	0.201	3.578E-01	2.061E-01
MIR4698	0.201	NA	1.830E-07
FAM149A	0.201	5.196E-01	4.186E-04
POMT1	0.200	4.379E-02	6.638E-03
MAP1B	0.200	4.660E-01	5.260E-01
NAGK	0.200	4.228E-02	1.564E-01
PTGDS	0.200	6.105E-01	1.787E-01
AC003002.4	0.200	3.525E-01	1.634E-01
PHLDB3	0.200	2.670E-01	3.941E-01
CSPG4	0.200	4.735E-01	4.718E-03
PPIL6	0.200	2.814E-01	2.612E-01
MAP7D3	0.200	3.057E-01	1.674E-01
RASA3	0.200	3.385E-01	1.197E-01
ACTN2	0.200	6.647E-01	4.780E-02
MYH13	0.200	6.324E-01	2.145E-01
CCL17	0.200	5.208E-01	8.821E-02
C11orf74	0.200	1.145E-01	2.054E-01
CHRM3	0.199	6.244E-01	9.586E-02
CRIM1	0.199	1.786E-01	3.470E-01
BMP6	0.199	4.814E-01	5.361E-02
MMD	0.199	3.115E-01	3.110E-01
BBC3	0.199	1.734E-01	1.229E-02
MSMO1	0.199	1.810E-01	2.642E-01
PGA3	0.199	NA	8.190E-06
LCE6A	0.199	NA	9.328E-01
MALRD1	0.199	6.425E-01	1.763E-03

KBTBD13	0.199	7.827E-01	2.630E-02
MC1R	0.199	2.717E-01	3.804E-02
CCL19	0.199	6.915E-01	2.794E-01
MRAS	0.199	3.002E-01	4.568E-03
RHBDL2	0.199	3.229E-01	1.430E-01
CDSN	0.199	7.907E-01	2.829E-04
OTOF	0.199	5.831E-01	1.488E-01
CCDC144A	0.199	6.815E-01	3.270E-05
RIM6-TRIM3	0.199	NA	2.668E-03
RSG1	0.198	4.681E-02	8.085E-02
ODF3L2	0.198	5.411E-01	1.038E-01
LPP	0.198	1.878E-01	7.892E-04
PKD2L1	0.198	5.065E-01	2.936E-01
PIK3CB	0.198	5.183E-02	1.238E-01
NOTCH2	0.198	1.492E-01	3.637E-02
SULF2	0.198	5.559E-01	3.155E-01
EIF2AK3	0.198	7.104E-02	1.323E-01
CPAMD8	0.198	5.556E-01	2.126E-01
PROZ	0.198	4.763E-01	5.584E-02
RND1	0.198	4.310E-01	6.223E-02
MIR6078	0.197	NA	3.776E-03
RRM2B	0.197	1.573E-01	2.817E-01
SEC24D	0.197	1.058E-01	4.680E-01
MYOZ1	0.197	5.191E-01	1.783E-04
TEN1	0.197	2.029E-01	3.911E-02
TBXA2R	0.197	3.174E-01	2.967E-02
EXOC4	0.197	2.017E-03	6.423E-01
KIF3C	0.197	1.794E-01	6.686E-04
NCOA2	0.197	6.544E-02	3.152E-01
MIR1276	0.197	7.084E-01	8.416E-01
P2RX5	0.197	5.776E-01	6.857E-03
TIMP1	0.197	3.966E-01	1.797E-01
MIR548K	0.196	NA	6.589E-03
FAXDC2	0.196	3.080E-01	3.779E-02
44085.000	0.196	4.589E-02	2.920E-01
NFATC1	0.196	3.958E-01	3.381E-01
KRBOX1	0.196	6.310E-01	1.529E-03
AMER3	0.196	7.754E-01	1.796E-01
AC087632.1	0.196	3.059E-01	1.262E-01
SCML2	0.196	5.829E-01	5.373E-01
NREP	0.196	3.489E-01	5.300E-06
ATL3	0.196	8.550E-02	6.295E-01
POR	0.196	4.395E-02	1.775E-01
PDE11A	0.196	5.772E-01	1.190E-05
FSHR	0.196	8.541E-01	3.954E-01
GJC1	0.196	3.396E-01	5.559E-01
ZBTB38	0.196	7.699E-02	2.070E-01
SERINC5	0.196	1.906E-01	9.707E-02
RAB5A	0.195	9.859E-03	4.367E-03
C4orf36	0.195	4.975E-02	1.928E-01

LETM2	0.195	3.217E-01	1.936E-01
DNAJC5B	0.195	5.933E-01	7.658E-02
SLC9C2	0.195	7.185E-01	1.655E-01
ADAMTS2	0.195	5.290E-01	7.624E-02
RGS5	0.195	4.490E-01	2.277E-01
FAM114A1	0.195	5.040E-02	2.071E-01
B3GNT2	0.195	4.004E-02	5.733E-02
OR5I1	0.195	NA	7.188E-02
MMP2	0.195	4.720E-01	5.313E-01
L1TD1	0.195	6.081E-01	1.001E-01
GLIPR1	0.195	3.916E-01	1.602E-03
DCAF6	0.195	5.244E-02	1.728E-01
PHKG1	0.194	2.817E-01	9.476E-02
C11orf80	0.194	1.530E-01	2.933E-01
TMSB15B	0.194	4.746E-01	6.843E-02
TMEM136	0.194	2.606E-01	1.570E-02
VMO1	0.194	5.306E-01	3.406E-02
ABCA12	0.194	6.398E-01	3.330E-05
RFTN2	0.194	2.113E-01	1.407E-01
PRSS3	0.194	6.842E-01	2.164E-01
NXF5	0.194	8.360E-01	1.491E-03
CRLF1	0.194	4.891E-01	5.473E-02
NKX3-2	0.194	6.059E-01	1.060E-03
PLAC1	0.194	6.324E-01	1.189E-01
SEMG1	0.194	8.070E-01	8.769E-03
NFIC	0.193	1.219E-01	2.267E-03
STARD13	0.193	3.172E-01	1.172E-03
ZMYND12	0.193	4.010E-01	8.015E-04
SVEP1	0.193	3.904E-01	8.172E-01
PODXL	0.193	3.025E-01	8.680E-02
NOTCH2NL	0.193	2.916E-01	6.180E-04
SPATA17	0.193	4.522E-01	2.509E-01
TNFRSF11B	0.193	5.993E-01	2.778E-03
B4GAT1	0.193	6.472E-02	4.157E-01
JADE1	0.192	1.621E-01	4.252E-01
FGFR1	0.192	4.756E-01	8.960E-02
ZMAT3	0.192	1.258E-01	9.229E-02
ADAMTS15	0.192	5.959E-01	1.808E-02
AL138752.2	0.192	4.608E-01	5.727E-02
PALM	0.192	5.413E-01	1.734E-01
TNFRSF11A	0.192	4.553E-01	4.558E-01
HIST1H2AM	0.192	4.895E-01	7.065E-01
C17orf50	0.192	5.896E-01	4.081E-03
FAM198B	0.192	3.100E-01	5.380E-01
GPSM2	0.192	1.984E-01	1.046E-04
NMBR	0.192	6.324E-01	1.914E-01
PCSK7	0.192	8.486E-02	1.815E-01
TGIF2LX	0.192	8.834E-01	3.712E-02
FGD1	0.192	1.898E-01	6.266E-01
PFN4	0.192	2.392E-01	1.092E-01

MIR130A	0.191	NA	7.402E-02
CT47B1	0.191	NA	9.520E-02
MAST2	0.191	9.095E-02	7.488E-03
MCFD2	0.191	5.331E-02	1.816E-01
ZSCAN1	0.191	7.152E-01	2.035E-01
PLD3	0.191	1.091E-01	5.095E-02
NWD1	0.191	6.419E-01	2.840E-02
NIPA1	0.191	2.501E-01	3.413E-03
CBX6	0.191	3.075E-01	4.307E-01
AC008750.8	0.191	3.472E-01	2.513E-03
HEPH	0.191	5.168E-01	7.891E-02
CTSA	0.191	7.551E-02	9.331E-03
MIR6512	0.191	7.347E-01	6.520E-01
EPAS1	0.190	2.370E-01	1.827E-01
HIST2H2BF	0.190	5.201E-01	1.155E-02
BASP1	0.190	4.988E-01	3.217E-02
ZFHX3	0.190	2.000E-01	2.485E-01
MBTPS2	0.190	4.399E-02	1.654E-01
FEV	0.190	7.781E-01	5.235E-02
IGFBP7	0.190	2.814E-01	5.200E-02
MPZL1	0.190	6.891E-02	1.050E-05
MAPK3	0.190	4.188E-02	3.590E-02
ADAM19	0.190	5.467E-01	5.424E-01
B3GALT5	0.190	7.037E-01	3.332E-01
CXorf58	0.190	5.711E-01	2.314E-02
FCGR1B	0.189	6.058E-01	8.190E-05
ARF4	0.189	2.551E-02	1.065E-01
ELSPBP1	0.189	8.539E-01	1.714E-01
RPTN	0.189	8.217E-01	1.612E-02
PLA2G1B	0.189	5.624E-01	3.205E-02
SLC16A2	0.189	4.617E-01	1.122E-02
GUCY2C	0.189	3.251E-01	2.481E-01
ADRA2B	0.189	5.455E-01	7.006E-04
FBXW10	0.189	6.136E-01	7.909E-02
CDKN1C	0.189	4.803E-01	3.980E-03
CD9	0.189	1.811E-01	1.754E-01
DUSP12	0.189	8.209E-02	3.738E-01
ZNF329	0.188	1.186E-01	1.836E-04
KDELR2	0.188	1.186E-02	1.705E-01
MAATS1	0.188	6.351E-01	6.611E-02
GRM7	0.188	7.010E-01	4.526E-01
USP43	0.188	2.120E-01	3.984E-02
DNMT3L	0.188	8.201E-01	3.108E-02
DNAJB9	0.188	4.402E-02	6.989E-01
CDIPT	0.188	1.558E-02	9.067E-02
CDC42EP2	0.188	3.213E-01	4.960E-04
MIR6821	0.188	NA	5.715E-02
HIRIP3	0.188	7.098E-02	1.249E-01
CCDC121	0.187	1.234E-01	1.291E-01
PNMA2	0.187	5.508E-01	4.427E-02

DYSF	0.187	3.139E-01	5.330E-05
IL1RAPL2	0.187	6.419E-01	6.099E-01
FOXF1	0.187	4.933E-01	9.275E-02
SLC12A3	0.187	6.116E-01	4.036E-01
NUCB2	0.187	7.921E-02	4.489E-02
CFAP99	0.187	5.540E-01	4.446E-01
CRY2	0.187	3.081E-02	6.718E-01
AK8	0.187	4.327E-01	5.844E-03
ZNF382	0.187	4.577E-01	1.794E-01
AL928654.4	0.187	6.396E-01	3.450E-01
XK	0.187	5.593E-01	6.026E-03
SMPD1	0.187	1.362E-01	2.907E-01
SYT1	0.187	5.762E-01	3.656E-04
ECM2	0.187	4.392E-01	1.082E-04
QSER1	0.187	1.227E-01	7.863E-01
SLC22A15	0.186	3.056E-01	5.306E-02
CCDC87	0.186	3.736E-01	2.652E-01
ZNF185	0.186	3.946E-01	4.498E-01
DDAH1	0.186	3.922E-01	3.049E-01
ADGRB2	0.186	4.633E-01	5.784E-01
NOS3	0.186	2.848E-01	3.670E-02
RELL1	0.186	2.836E-01	1.500E-04
FAM124A	0.186	4.681E-01	1.121E-01
MIR641	0.186	6.664E-01	4.550E-01
A4GNT	0.186	6.513E-01	1.776E-01
NXF2B	0.186	NA	1.909E-01
MIR6855	0.186	NA	1.750E-04
GPRIN2	0.186	5.856E-01	3.493E-01
ESPN	0.186	5.783E-01	3.828E-01
FSD1L	0.186	4.629E-01	1.164E-02
MICAL2	0.186	3.280E-01	8.398E-01
TMEM41A	0.186	3.999E-02	9.398E-03
MMP17	0.186	5.229E-01	9.404E-03
FASN	0.185	2.556E-01	1.500E-01
ERICH5	0.185	6.231E-01	1.627E-03
NUAK1	0.185	4.159E-01	6.686E-01
PLSCR5	0.185	NA	1.201E-02
RPN1	0.185	4.774E-03	1.795E-03
ZDHHC9	0.185	3.292E-02	1.191E-01
VSX1	0.185	7.415E-01	3.956E-03
TMEM158	0.185	5.194E-01	5.707E-03
TSPAN9	0.185	1.615E-01	3.078E-02
CAST	0.185	1.452E-01	1.194E-01
MIR6783	0.185	NA	8.030E-06
ZAR1	0.185	7.172E-01	6.471E-01
GNAT2	0.185	NA	1.207E-04
ITGB1	0.185	2.134E-01	2.724E-01
RAB20	0.185	2.419E-01	7.509E-03
CATSPERD	0.185	6.728E-01	7.741E-01
RNF148	0.184	5.551E-01	3.774E-04

P4HB	0.184	2.178E-02	4.964E-01
AMIGO1	0.184	2.543E-01	5.756E-01
EPHA10	0.184	6.339E-01	9.350E-02
SMIM3	0.184	3.081E-01	2.113E-01
NKAPL	0.184	5.070E-01	3.525E-01
LRRC2	0.184	6.425E-01	1.417E-01
RNASE9	0.184	NA	1.100E-01
CFD	0.184	5.664E-01	6.566E-01
MIR199A2	0.184	NA	5.577E-01
EXO5	0.184	7.118E-02	2.717E-01
SEMA5A	0.184	5.765E-01	5.562E-01
ZNF727	0.184	7.283E-01	8.755E-03
LDLR	0.184	4.216E-01	8.274E-01
ADAM8	0.183	3.973E-01	7.167E-01
EPHA2	0.183	3.504E-01	6.738E-01
PPP1R36	0.183	4.388E-01	4.697E-03
ATP6V1C1	0.183	5.303E-02	2.195E-01
ENC1	0.183	2.104E-01	1.543E-01
FIBCD1	0.183	6.384E-01	7.208E-02
GNAQ	0.183	1.142E-01	1.528E-01
ITGB5	0.183	2.684E-01	3.372E-01
PDIA6	0.183	1.108E-01	1.447E-01
APOC1	0.183	5.239E-01	1.548E-03
AC135050.2	0.183	3.954E-01	2.274E-01
CDK16	0.183	3.122E-02	8.907E-01
SOAT2	0.183	6.285E-01	3.184E-03
HESX1	0.183	2.310E-01	3.785E-01
ZNF347	0.183	1.896E-01	1.440E-06
CAPN5	0.183	5.412E-01	1.089E-01
GPT2	0.183	3.172E-01	2.254E-01
KCNS1	0.183	6.609E-01	4.915E-01
NECAB3	0.183	1.571E-01	4.251E-01
BRK1	0.183	5.676E-02	6.539E-01
ANKRD31	0.183	3.840E-01	3.419E-03
GPR150	0.183	4.920E-01	8.940E-05
C1S	0.182	5.336E-01	3.838E-03
OTUD1	0.182	2.615E-01	3.946E-01
C19orf68	0.182	4.698E-02	7.324E-02
FN3K	0.182	2.598E-01	3.810E-01
CNTLN	0.182	4.804E-01	4.550E-03
LOX	0.182	5.201E-01	4.089E-01
TCEAL4	0.182	5.089E-02	5.271E-01
CXorf56	0.182	7.409E-03	1.034E-01
LRFN5	0.182	6.634E-01	6.441E-04
PBXIP1	0.182	1.132E-01	2.468E-01
TMEM253	0.182	5.208E-01	7.742E-01
SNAP25	0.181	6.198E-01	3.530E-01
CPEB4	0.181	1.810E-01	7.491E-02
CAPN2	0.181	2.575E-01	3.813E-01
GRK5	0.181	2.497E-01	2.066E-01

C16orf96	0.181	5.829E-01	3.979E-01
AZIN1	0.181	1.090E-01	3.340E-03
S100A9	0.181	7.080E-01	2.020E-01
LRIG1	0.181	5.402E-01	7.060E-03
UBQLN3	0.181	NA	4.067E-01
NOX4	0.181	4.576E-01	3.650E-01
IFT122	0.181	5.280E-02	6.713E-02
KIF19	0.181	5.467E-01	2.870E-01
CPE	0.181	4.746E-01	2.370E-01
SLC39A13	0.181	9.353E-02	4.148E-01
GPAT3	0.180	5.643E-01	3.326E-02
LRRC59	0.180	4.428E-02	2.540E-01
DHH	0.180	5.236E-01	1.543E-01
FCN1	0.180	5.996E-01	8.522E-01
TPSD1	0.180	7.280E-01	7.789E-02
ACSBG1	0.180	6.931E-01	4.218E-01
ACBD3	0.180	2.342E-02	3.538E-01
DUSP8	0.180	4.366E-01	1.094E-01
LINC00694	0.180	6.088E-01	2.474E-01
SGCG	0.180	7.172E-01	1.319E-02
EVPL	0.180	4.293E-01	8.569E-02
TIGD4	0.180	3.493E-01	7.780E-02
SYCE1L	0.180	5.169E-01	1.007E-01
TBX4	0.180	6.732E-01	3.318E-01
BRF2	0.180	2.202E-01	6.074E-01
ADCY9	0.179	2.640E-01	2.347E-02
MMGT1	0.179	6.893E-02	9.797E-03
C7orf73	0.179	2.828E-02	1.434E-02
CXCL6	0.179	7.125E-01	7.930E-01
NPW	0.179	6.378E-01	5.895E-02
EPN3	0.179	4.321E-01	5.188E-02
TRAM2	0.179	2.162E-01	2.473E-01
LDHAL6B	0.179	5.030E-01	4.023E-02
FAM43B	0.179	5.732E-01	1.784E-01
STK32C	0.179	2.551E-01	2.224E-01
CRHBP	0.179	6.128E-01	1.504E-01
ARHGAP23	0.179	2.717E-01	8.920E-02
HOOK3	0.179	6.902E-02	5.565E-01
MIR219A1	0.179	5.568E-01	1.110E-03
GPD1L	0.179	4.312E-01	1.252E-01
AC013394.1	0.179	2.889E-01	7.782E-02
METTL24	0.179	6.300E-01	1.681E-03
HIST3H3	0.179	6.842E-01	3.321E-02
DIP2C	0.179	1.655E-01	6.998E-02
CYB561	0.178	5.841E-02	7.657E-03
TBC1D9	0.178	2.255E-01	2.080E-03
OR1M1	0.178	NA	6.124E-01
ALDH3A2	0.178	3.365E-01	5.810E-01
ZNF436	0.178	2.571E-01	6.106E-01
PAFAH2	0.178	4.258E-02	2.913E-04

ARIH2OS	0.178	1.494E-01	1.507E-01
ELOVL1	0.178	7.028E-02	3.749E-01
GABRB3	0.178	7.404E-01	2.471E-02
FUT9	0.178	7.174E-01	1.555E-01
PIAS3	0.178	1.134E-01	1.834E-03
SCPEP1	0.178	3.970E-01	2.347E-02
AF130351.1	0.178	6.387E-01	5.333E-01
AL162231.1	0.178	3.878E-01	8.974E-04
TSC22D1	0.178	2.061E-01	6.599E-01
GOLGA2	0.178	2.178E-02	7.541E-02
ICMT	0.178	6.005E-03	7.617E-02
KRTAP16-1	0.178	7.867E-01	6.192E-01
NCAPG2	0.178	2.135E-01	8.373E-01
MINOS1-NBL	0.178	6.336E-01	5.170E-06
FOXD2	0.177	4.931E-01	5.961E-01
LEP	0.177	7.656E-01	4.086E-02
DMRT1	0.177	8.001E-01	4.751E-04
FRG2	0.177	8.599E-01	4.738E-02
FAM46B	0.177	6.298E-01	2.151E-03
CACNA2D4	0.177	3.815E-01	2.809E-01
HIST1H2AI	0.177	5.985E-01	6.695E-03
C19orf33	0.177	4.905E-01	5.947E-02
TFDP3	0.177	NA	1.221E-02
TNFSF12	0.177	2.632E-01	2.917E-01
MIR125B1	0.177	NA	6.971E-01
STX3	0.177	1.985E-01	4.704E-02
BRPF1	0.177	3.570E-02	1.908E-01
STT3B	0.177	3.668E-02	8.671E-02
CPXM1	0.177	6.002E-01	1.234E-01
ADARB1	0.177	3.968E-01	1.255E-01
IL17RC	0.177	1.458E-01	6.528E-02
PDRG1	0.177	7.723E-02	1.603E-01
44086.000	0.176	6.647E-01	5.339E-02
GATA6	0.176	5.414E-01	1.448E-02
BAIAP2	0.176	3.060E-01	4.649E-01
PRSS23	0.176	4.170E-01	9.372E-01
PRR16	0.176	5.316E-01	9.217E-01
WDR78	0.176	3.187E-01	1.525E-03
PMP22	0.176	5.058E-01	4.750E-02
IL31RA	0.176	7.377E-01	5.822E-02
PRSS54	0.176	7.759E-01	8.225E-02
ESYT3	0.176	5.436E-01	9.791E-02
CTRC	0.176	5.402E-01	5.180E-01
EBF2	0.176	5.516E-01	7.142E-02
MIR3149	0.176	7.029E-01	2.640E-02
CAPRIN1	0.176	6.530E-02	1.967E-04
KIAA2012	0.176	6.004E-01	5.494E-01
TMCC1	0.176	2.542E-02	3.699E-02
YPEL2	0.176	1.675E-01	7.452E-01
LDB2	0.176	3.433E-01	7.520E-02

HRH1	0.176	3.217E-01	1.095E-01
ZIK1	0.176	5.064E-01	6.965E-02
MRPL33	0.176	5.331E-02	1.649E-03
CD300LB	0.176	5.473E-01	2.938E-01
STON1	0.175	4.345E-01	4.037E-01
SRGAP2	0.175	5.998E-02	5.027E-01
DCBLD2	0.175	5.263E-01	2.647E-01
EPHA1	0.175	3.483E-01	7.244E-02
CFAP43	0.175	4.360E-01	8.090E-01
C3orf20	0.175	6.076E-01	5.359E-02
CASC4	0.175	6.012E-02	8.748E-03
SERPINF1	0.175	5.490E-01	7.236E-02
TALDO1	0.175	1.435E-01	1.782E-01
WDR34	0.175	2.575E-01	3.575E-01
EFCAB12	0.175	4.611E-01	3.801E-04
MAGED1	0.175	2.511E-01	7.275E-03
FCER1G	0.175	5.764E-01	7.946E-01
CLEC2L	0.175	7.806E-01	2.287E-03
ARHGEF10	0.175	2.556E-01	6.836E-03
CCDC102B	0.175	3.227E-01	4.327E-01
PROS1	0.175	4.173E-01	3.533E-02
WLS	0.175	4.065E-01	4.022E-01
ATG7	0.175	7.285E-02	1.055E-03
TMEM108	0.175	6.559E-01	1.830E-02
PLA2G4A	0.175	4.931E-01	2.239E-03
C11orf91	0.174	5.272E-01	1.716E-01
BRDT	0.174	7.953E-01	4.691E-01
DEFB125	0.174	NA	1.827E-01
LURAP1L	0.174	4.398E-01	2.051E-01
DSCR3	0.174	1.654E-02	2.034E-01
NR1D2	0.174	9.503E-02	5.929E-02
ZSCAN23	0.174	5.942E-01	1.453E-02
VAT1	0.174	7.388E-02	3.516E-01
TCEA2	0.174	1.616E-01	1.155E-01
FAM84A	0.174	5.997E-01	1.255E-01
BDKRB2	0.174	4.560E-01	4.318E-03
DUSP23	0.174	3.267E-01	5.990E-01
EPS8L1	0.174	4.889E-01	1.090E-03
PHF13	0.174	1.030E-02	1.762E-01
PDLIM2	0.174	3.278E-01	4.389E-01
BCL2L1	0.174	1.742E-01	7.098E-02
TPGS1	0.174	3.314E-01	2.427E-02
CLDN16	0.174	6.096E-01	3.201E-03
PRDX4	0.174	1.459E-01	1.743E-01
ADCYAP1R1	0.174	6.513E-01	7.211E-02
HMGN2	0.174	1.132E-01	2.502E-03
CCDC186	0.173	8.507E-02	1.185E-03
CHAC1	0.173	4.216E-01	3.256E-03
GLCE	0.173	1.947E-01	3.857E-01
CENPA	0.173	3.348E-01	3.832E-01

CLGN	0.173	6.543E-01	6.110E-01
ZNF418	0.173	5.586E-01	7.033E-04
PPP1CB	0.173	1.690E-02	4.395E-02
C10orf128	0.173	4.778E-01	1.950E-03
SC5D	0.173	3.524E-01	7.328E-02
AADAC	0.173	7.827E-01	1.429E-01
FRZB	0.173	5.032E-01	1.280E-01
COX7B2	0.173	8.772E-01	5.841E-04
TRIM9	0.173	6.234E-01	5.910E-01
NKX1-2	0.173	8.092E-01	3.257E-01
HIST1H1E	0.173	5.412E-01	1.382E-01
FZD1	0.173	3.500E-01	3.765E-01
MIR4653	0.173	4.017E-01	4.030E-01
CCSER2	0.173	3.638E-02	9.621E-03
BLVRB	0.173	2.482E-01	2.922E-01
CDON	0.173	5.484E-01	4.715E-01
GPRC5A	0.172	5.576E-01	1.953E-01
PSAP	0.172	1.620E-01	4.536E-01
OR11G2	0.172	NA	4.675E-01
LRRN3	0.172	5.753E-01	1.389E-04
SLC17A7	0.172	5.404E-01	1.945E-02
GOLGA8M	0.172	6.251E-01	6.048E-01
SLFN11	0.172	5.208E-01	1.028E-02
TMX4	0.172	1.394E-01	5.968E-02
CAPZA2	0.172	2.965E-02	3.938E-01
USP46	0.172	1.678E-01	7.065E-04
TNPO3	0.172	6.005E-03	1.171E-02
ADM5	0.171	4.405E-01	1.363E-01
BCAR3	0.171	3.126E-01	9.326E-03
TEX49	0.171	7.609E-01	3.230E-03
RTL8C	0.171	1.243E-01	8.349E-01
LATS2	0.171	2.328E-01	4.069E-03
KLK7	0.171	8.470E-01	2.426E-01
GOLM1	0.171	2.574E-01	1.192E-01
WWTR1	0.171	2.648E-01	9.504E-02
ATP11B	0.171	1.704E-01	3.309E-02
COL5A3	0.170	5.387E-01	3.804E-01
TEX261	0.170	8.207E-03	3.145E-01
C11orf71	0.170	2.260E-01	1.636E-01
NUCB1	0.170	3.871E-02	2.545E-01
SV2B	0.170	7.057E-01	5.991E-04
FBXO43	0.170	5.102E-01	1.571E-01
OPRPN	0.170	NA	7.972E-03
BCL3	0.169	2.641E-01	2.348E-03
C20orf144	0.169	2.508E-01	8.057E-01
SLC35F6	0.169	5.768E-02	1.532E-02
CENPU	0.169	2.575E-01	8.573E-01
ARHGAP10	0.169	2.167E-01	3.989E-04
MS4A10	0.169	7.489E-01	7.231E-01
POLB	0.169	2.356E-01	2.300E-05

DIRAS3	0.169	5.616E-01	3.428E-02
SPACA9	0.169	2.105E-01	7.284E-01
RASSF1	0.169	1.314E-01	5.152E-03
MET	0.169	3.603E-01	2.902E-03
MEIOC	0.169	4.473E-01	1.279E-04
CTSD	0.169	2.715E-01	2.433E-03
SREBF1	0.169	3.507E-01	5.262E-01
DOCK11	0.169	4.738E-01	1.614E-01
TMEM86A	0.169	3.211E-01	6.588E-04
MYO1E	0.169	2.384E-01	1.705E-01
CHRNA4	0.169	8.107E-01	1.706E-01
VANGL1	0.168	1.103E-01	3.234E-02
NEO1	0.168	3.573E-01	2.331E-01
STRA6	0.168	5.867E-01	3.371E-01
HILPDA	0.168	4.819E-01	1.138E-03
SMIM11A	0.168	6.708E-01	6.502E-01
MNX1	0.168	6.257E-01	4.525E-01
AL121753.1	0.168	4.329E-01	1.934E-02
ZNHIT6	0.168	8.783E-02	3.988E-04
PRAG1	0.168	2.760E-01	2.381E-02
TMEM212	0.168	8.405E-01	5.641E-01
PIAS1	0.168	5.331E-02	3.070E-02
SYTL2	0.168	4.930E-01	1.319E-01
AC098850.4	0.168	7.907E-01	4.462E-02
DEPDC7	0.168	6.336E-01	9.150E-02
PODNL1	0.168	5.753E-01	2.702E-01
KRT12	0.168	7.390E-01	5.411E-01
TEX30	0.168	2.160E-01	1.649E-01
ANKRD28	0.168	2.319E-02	4.838E-01
OR14K1	0.168	NA	1.167E-02
C16orf87	0.167	1.954E-01	1.918E-03
15orf38-AP3S	0.167	3.887E-01	1.478E-02
PHF19	0.167	3.520E-01	1.176E-01
TRAM1L1	0.167	5.686E-01	1.990E-01
PACSIN3	0.167	2.049E-01	7.639E-04
DIRC1	0.167	7.418E-01	9.356E-03
MAMLD1	0.167	5.062E-01	1.364E-02
AC092718.3	0.167	3.215E-01	1.808E-01
NFIA	0.167	3.590E-01	1.792E-01
PCDHGA2	0.167	5.156E-01	3.194E-02
TDO2	0.167	5.676E-01	1.555E-02
CD244	0.167	6.364E-01	6.526E-01
RRBP1	0.167	8.812E-02	1.303E-02
CCDC136	0.167	4.961E-01	2.241E-01
NAJC25-GNG	0.167	NA	1.245E-02
FKBP7	0.166	2.545E-01	2.680E-02
MAP7D1	0.166	1.190E-01	3.643E-02
AL807752.6	0.166	NA	2.160E-01
FAM47E	0.166	5.064E-01	9.283E-03
TJP1	0.166	9.344E-02	2.784E-03

ESAM	0.166	3.228E-01	6.447E-03
SLC31A2	0.166	4.537E-01	2.650E-02
ZNF607	0.166	2.990E-01	3.946E-01
IL10	0.166	5.867E-01	1.175E-02
YIF1A	0.166	1.031E-01	1.855E-01
C1orf112	0.166	3.056E-01	6.047E-04
PRDM16	0.166	6.794E-01	7.379E-03
MIR4734	0.166	NA	6.052E-02
HCRT	0.165	7.845E-01	6.944E-03
HAL	0.165	6.285E-01	1.318E-01
SPATA16	0.165	8.973E-01	7.681E-02
IL17B	0.165	4.575E-01	3.189E-01
HSPG2	0.165	3.840E-01	1.926E-01
TEX36	0.165	NA	5.891E-03
TMOD3	0.165	1.082E-01	5.795E-04
RR5-ARHGAI	0.165	4.895E-01	1.977E-01
LRRC74A	0.165	6.932E-01	3.215E-01
ATP6AP1	0.165	5.003E-02	1.398E-01
MIR6761	0.165	NA	1.431E-01
STMND1	0.165	8.298E-01	4.191E-01
MIR3168	0.165	NA	2.794E-03
C15orf65	0.165	2.718E-01	6.200E-05
43898.000	0.165	6.726E-02	3.049E-01
OR4F15	0.165	NA	1.847E-01
HOXA7	0.165	5.099E-01	3.660E-02
MIR6509	0.165	8.171E-01	1.593E-01
LRRC3B	0.165	8.168E-01	4.329E-01
C10orf35	0.165	2.639E-01	2.809E-01
CCNE2	0.165	4.425E-01	3.524E-02
RNF130	0.164	2.614E-01	2.070E-05
CGN	0.164	5.559E-01	1.013E-02
SLC5A5	0.164	7.295E-01	1.299E-01
MIR548AR	0.164	6.599E-01	9.553E-04
GPBAR1	0.164	5.389E-01	5.766E-03
SELENOS	0.164	2.163E-02	1.375E-01
SUN2	0.164	1.398E-01	4.933E-03
ELOVL3	0.164	6.580E-01	6.837E-02
FAM216B	0.164	7.583E-01	3.059E-01
ARRB2	0.164	2.462E-01	1.209E-02
TM7SF2	0.164	5.180E-01	5.691E-04
SPAG9	0.164	5.800E-02	3.900E-02
PNPLA2	0.164	2.543E-01	1.258E-03
TFR2	0.164	5.496E-01	7.615E-01
MIR3129	0.164	NA	3.629E-01
THRA	0.164	2.713E-01	5.332E-02
KCNK2	0.164	7.339E-01	6.999E-02
ATP2B4	0.164	3.714E-01	5.329E-01
SLC25A25	0.164	2.790E-01	1.208E-02
HPN	0.164	7.478E-01	5.663E-04
C11orf87	0.163	8.208E-01	5.570E-02

MELK	0.163	3.954E-01	7.727E-01
SOX3	0.163	NA	1.396E-01
MIR4714	0.163	NA	7.472E-01
PTGES	0.163	5.629E-01	5.648E-01
ZFYVE19	0.163	5.457E-02	5.675E-04
LY75-CD302	0.163	6.805E-01	2.430E-01
TEK	0.163	4.495E-01	2.428E-01
TBCEL	0.163	7.924E-02	3.114E-01
MYCN	0.163	7.467E-01	1.846E-04
PXYLP1	0.163	3.552E-01	2.065E-03
PRSS35	0.163	6.834E-01	4.177E-03
SDHAF1	0.163	9.109E-02	4.455E-04
GPR107	0.163	5.559E-02	8.039E-01
UNC93A	0.163	8.507E-01	2.457E-01
EHD2	0.163	4.463E-01	6.375E-01
C6orf106	0.163	1.118E-01	1.710E-02
EVA1B	0.163	4.000E-01	3.351E-02
SDHC	0.163	1.433E-01	2.675E-03
LDHD	0.163	5.922E-01	1.781E-03
STX5	0.163	5.302E-02	1.790E-06
MAPK7	0.163	1.024E-01	9.004E-02
POMGNT2	0.162	1.492E-01	5.097E-01
HDAC5	0.162	1.060E-01	6.305E-04
PITRM1	0.162	6.245E-02	6.731E-01
CDNF	0.162	3.330E-01	2.142E-01
ERMP1	0.162	4.216E-01	8.461E-01
HIST1H3C	0.162	6.004E-01	3.014E-01
SHROOM3	0.162	5.176E-01	3.189E-02
PI15	0.162	6.609E-01	7.207E-01
TLE6	0.162	6.586E-01	3.248E-03
CLEC5A	0.162	6.109E-01	2.236E-04
HES6	0.162	5.102E-01	2.653E-01
IFNA14	0.162	NA	6.870E-03
MPL	0.162	3.923E-01	2.773E-03
MAFF	0.162	3.567E-01	4.654E-01
FAM156A	0.162	2.762E-01	1.810E-02
FJX1	0.162	5.000E-01	1.173E-04
WTH3DI	0.162	7.322E-01	2.554E-02
REPS2	0.162	3.385E-01	1.481E-01
MIR548J	0.162	NA	3.763E-01
SLC35G2	0.162	5.271E-01	4.740E-02
SMOC1	0.161	7.415E-01	2.938E-01
DLG4	0.161	3.493E-01	3.314E-01
TNR	0.161	7.229E-01	3.230E-03
AC083902.2	0.161	7.837E-01	2.109E-04
HHLA3	0.161	2.628E-01	1.362E-01
KDELR1	0.161	1.705E-02	7.340E-02
TMEM55A	0.161	2.786E-01	2.494E-01
MRC2	0.161	5.140E-01	1.627E-03
HGSNAT	0.161	1.479E-01	6.085E-02

NAGS	0.161	4.125E-01	3.251E-01
PINK1	0.161	9.861E-02	2.521E-01
YWHAQ	0.161	1.398E-01	3.934E-02
C3orf18	0.161	3.339E-01	9.410E-06
ACAP2	0.161	6.799E-02	8.949E-01
PRKCI	0.161	1.320E-01	3.778E-02
FAM180B	0.161	7.321E-01	6.149E-02
HENMT1	0.161	2.370E-01	3.503E-01
APH1A	0.161	3.346E-02	2.132E-02
TM2D2	0.161	2.021E-01	4.346E-01
PSMC3IP	0.161	1.738E-01	1.643E-03
SPSB1	0.161	2.206E-01	4.130E-05
NDP	0.161	7.159E-01	3.726E-01
MORN2	0.161	1.428E-01	1.618E-01
NOS1	0.161	7.460E-01	2.874E-01
PLA2G2F	0.161	7.624E-01	3.719E-01
NCOA1	0.160	9.720E-02	9.314E-02
IGFBP3	0.160	6.134E-01	4.987E-01
RNLS	0.160	4.056E-01	2.017E-02
ZNF415	0.160	3.956E-01	3.039E-01
FUT11	0.160	1.178E-01	1.872E-02
LZTS1	0.160	4.801E-01	6.000E-01
LCE2B	0.160	9.468E-01	7.103E-03
CBLC	0.160	4.968E-01	2.736E-01
PTGIR	0.160	4.968E-01	7.322E-03
TMSB4Y	0.160	7.787E-01	8.845E-01
SPG20	0.160	1.830E-01	4.230E-05
CLDN25	0.160	NA	1.370E-01
WNT9A	0.160	5.601E-01	2.237E-03
TRIM34	0.159	3.403E-01	2.634E-01
OR6V1	0.159	NA	7.555E-03
ASAP1	0.159	2.534E-01	2.673E-01
ALDH5A1	0.159	4.569E-01	8.991E-02
SKIDA1	0.159	5.651E-01	6.322E-02
RBP1	0.159	6.582E-01	5.758E-02
GGACT	0.159	2.563E-01	1.836E-01
43892.000	0.159	2.946E-01	1.242E-01
ALCAM	0.159	5.419E-01	8.467E-02
VPS37C	0.159	9.462E-03	2.522E-01
CACNA1G	0.159	5.887E-01	5.265E-01
YS1-DBNDD	0.159	4.164E-01	9.168E-02
AGFG2	0.159	2.082E-01	1.595E-01
DCDC1	0.159	6.735E-01	1.882E-01
LCA5	0.159	2.473E-01	2.097E-03
ILK	0.159	2.437E-01	2.565E-01
CMTM6	0.159	1.368E-01	3.147E-01
CCNB3	0.158	5.713E-01	1.410E-01
REM1	0.158	5.568E-01	8.664E-01
NBPF26	0.158	4.251E-01	8.484E-01
ACOT7	0.158	2.758E-01	5.288E-01

ANXA1	0.158	5.894E-01	8.372E-01
SNX12	0.158	1.668E-02	8.076E-01
CRYBB1	0.158	5.749E-01	1.044E-03
ZNF398	0.158	6.781E-02	2.359E-02
ZNF765	0.158	1.252E-01	4.958E-01
KLF12	0.158	5.202E-01	3.129E-01
HIVEP1	0.158	2.247E-01	2.006E-02
SPAG1	0.158	3.751E-01	7.629E-02
PTPRF	0.158	2.043E-01	2.716E-01
ZNF676	0.158	8.213E-01	4.435E-02
MSH2	0.158	1.713E-01	1.145E-03
CD248	0.158	5.211E-01	2.281E-01
MAGT1	0.158	2.349E-02	2.332E-01
SND1	0.158	3.758E-02	1.468E-01
RNF150	0.158	6.094E-01	6.391E-02
CCK	0.158	7.738E-01	1.516E-01
ALMS1	0.158	1.079E-01	1.172E-02
GPRC5D	0.157	4.622E-01	4.661E-04
PM20D1	0.157	8.249E-01	5.795E-03
STX2	0.157	4.595E-01	1.245E-02
SEC61A2	0.157	1.586E-01	8.036E-02
DDHD2	0.157	2.566E-01	5.604E-01
MYLIP	0.157	4.060E-01	5.584E-01
CDC42EP3	0.157	3.708E-01	6.259E-01
MIR4742	0.157	8.007E-01	2.870E-01
RAB11FIP2	0.157	2.253E-01	1.587E-04
FOXI2	0.157	7.875E-01	1.675E-02
GRASP	0.157	4.362E-01	8.283E-01
TBX20	0.157	7.723E-01	6.956E-01
FAM120AOS	0.157	6.061E-02	7.823E-01
CCDC47	0.157	5.741E-02	1.122E-01
TVP23B	0.157	1.132E-01	1.035E-03
ELL3	0.157	4.291E-01	5.748E-03
NFATC2	0.157	4.526E-01	1.626E-02
PPP1R21	0.157	2.363E-02	4.122E-04
MIR1283-2	0.157	NA	3.428E-01
PP2D1	0.157	3.372E-01	3.305E-01
APOBEC1	0.157	9.066E-01	7.613E-01
MBOAT2	0.157	4.900E-01	1.931E-01
CREB3L1	0.157	6.070E-01	2.272E-01
PLPPR4	0.157	5.902E-01	4.651E-01
TMEM169	0.157	6.030E-01	4.160E-03
MIR635	0.157	2.389E-01	5.894E-02
GPR179	0.157	6.288E-01	2.777E-03
MTRNR2L3	0.157	6.620E-01	5.216E-04
SLC35D3	0.156	7.538E-01	2.859E-01
FYCO1	0.156	2.035E-01	1.031E-01
NUMBL	0.156	2.839E-01	7.831E-03
MIR4259	0.156	NA	1.273E-01
ABHD12	0.156	2.956E-01	9.173E-01

CSF1R	0.156	6.298E-01	8.685E-01
MCAM	0.156	3.888E-01	4.646E-01
PACS1	0.156	7.692E-02	5.431E-03
KIAA1210	0.156	6.896E-01	5.557E-01
LGALS12	0.156	7.472E-01	2.496E-01
MIR5192	0.156	NA	5.080E-03
TAS2R42	0.156	7.699E-01	1.334E-01
EPCAM	0.156	5.032E-01	1.676E-01
MIR3684	0.156	NA	4.592E-01
RGPD6	0.156	7.237E-01	1.344E-01
SYNPO2L	0.156	6.099E-01	7.280E-02
EFCAB14	0.156	6.230E-02	1.677E-04
ECSCR	0.155	4.820E-01	5.504E-01
ACRV1	0.155	4.593E-01	1.600E-01
KIAA1841	0.155	1.719E-01	4.352E-01
SSPN	0.155	4.064E-01	1.638E-01
APOL5	0.155	7.159E-01	3.811E-03
CCL14	0.155	7.280E-01	1.094E-01
VAMP5	0.155	5.170E-01	3.079E-01
ITGA8	0.155	5.826E-01	7.349E-02
RNF170	0.155	1.366E-01	2.415E-01
HIGD2B	0.155	6.910E-01	7.210E-01
DGKG	0.155	6.268E-01	1.302E-01
IAH1	0.155	6.268E-02	1.749E-03
ANKRD27	0.155	1.145E-01	1.419E-02
FAM222B	0.155	1.237E-01	2.048E-01
PIMREG	0.155	4.525E-01	4.478E-04
ETV3	0.155	1.838E-01	2.629E-01
OR5P2	0.155	7.832E-01	6.109E-01
44082.000	0.155	2.036E-01	4.896E-04
ELK3	0.155	2.492E-01	1.651E-03
FAM26E	0.155	5.446E-01	2.312E-01
MYH1	0.155	7.635E-01	4.832E-01
LYZL2	0.154	9.038E-01	4.361E-01
TCF19	0.154	4.072E-01	6.489E-02
DNAJC3	0.154	5.607E-02	1.605E-02
MMP27	0.154	8.681E-01	3.108E-01
RASSF2	0.154	5.303E-01	9.331E-01
NDNF	0.154	6.860E-01	5.659E-02
YIPF2	0.154	8.185E-02	1.876E-01
INAFM1	0.154	2.497E-01	7.106E-01
RAD18	0.154	1.881E-01	1.454E-02
PRICKLE2	0.154	4.641E-01	3.263E-03
PPIC	0.154	2.516E-01	5.772E-02
ROM1	0.154	2.902E-01	9.084E-02
TRIM16L	0.154	5.746E-01	3.770E-01
TIE1	0.154	4.190E-01	3.274E-01
SPTLC1	0.154	1.710E-01	3.980E-05
ARHGEF17	0.154	2.648E-01	3.027E-01
SAPCD2	0.154	4.657E-01	2.621E-01

SKA2	0.154	1.365E-01	4.045E-01
ZBTB8B	0.153	6.985E-01	1.150E-01
C16orf71	0.153	3.358E-01	3.022E-01
LZIC	0.153	4.068E-02	4.514E-02
WDR54	0.153	3.307E-01	7.304E-01
SCAMP2	0.153	1.709E-02	8.146E-02
NTNG2	0.153	5.593E-01	1.913E-01
C11orf63	0.153	3.934E-01	4.070E-03
AC008758.5	0.153	NA	7.467E-02
SCTR	0.153	7.364E-01	3.546E-03
CHCHD3	0.153	5.384E-02	5.679E-02
LDB3	0.153	5.861E-01	9.062E-01
FSBP	0.153	NA	8.412E-01
PIGV	0.153	6.474E-02	1.879E-04
ARHGAP31	0.153	4.211E-01	1.627E-01
HS2ST1	0.153	9.566E-02	2.707E-01
MIF4GD	0.153	9.781E-02	2.769E-03
ARL2-SNX15	0.153	8.329E-01	2.276E-01
C4orf51	0.153	7.086E-01	4.269E-03
MAP1LC3A	0.153	5.131E-01	5.323E-03
MIR4690	0.153	NA	9.691E-02
RFFL	0.153	2.038E-01	6.490E-01
RPUSD3	0.153	1.461E-01	2.273E-02
TESK2	0.152	3.848E-01	2.460E-02
VCAM1	0.152	6.266E-01	5.039E-02
ATAD2	0.152	3.178E-01	3.981E-01
TMEM238	0.152	4.961E-01	2.663E-01
ZNF829	0.152	4.072E-01	1.556E-01
UNC13D	0.152	5.412E-01	1.605E-03
RNASE6	0.152	6.219E-01	2.616E-02
SLC35B3	0.152	5.895E-02	4.674E-04
RBM47	0.152	3.118E-01	1.148E-01
PRAC2	0.152	7.944E-01	1.255E-03
ITM2A	0.152	5.993E-01	2.230E-03
ZNF462	0.152	4.321E-01	1.383E-02
OR52I1	0.152	NA	7.726E-02
ZNF805	0.152	1.538E-01	4.107E-01
TMEM209	0.152	8.350E-02	5.470E-01
MIR32	0.152	NA	3.067E-01
AC002310.5	0.152	7.085E-01	2.299E-01
TPSAB1	0.152	6.743E-01	1.779E-02
ZYG11B	0.152	4.575E-02	8.627E-04
TMEM206	0.152	1.629E-01	8.641E-03
ZNF217	0.152	2.396E-01	2.846E-01
TFE3	0.152	3.888E-02	1.547E-01
CACNA1A	0.151	6.190E-01	3.102E-03
MARK2	0.151	3.200E-02	5.217E-01
ST8SIA2	0.151	7.696E-01	1.935E-01
SLC2A10	0.151	4.920E-01	1.419E-02
SNTB1	0.151	5.829E-01	2.770E-02

TMEM135	0.151	2.338E-01	1.654E-01
GLRX2	0.151	1.434E-01	1.866E-04
EPS15	0.151	1.541E-02	1.474E-02
TECRL	0.151	NA	2.748E-02
SIGLEC14	0.151	7.441E-01	9.972E-02
USP25	0.151	1.162E-01	2.712E-01
HYI	0.151	2.185E-01	7.374E-02
AC026464.1	0.151	NA	4.865E-01
ITGA2	0.151	5.780E-01	3.464E-01
SAMD14	0.151	5.125E-01	2.472E-03
DCTN3	0.150	1.380E-01	3.428E-01
NTN4	0.150	5.194E-01	1.957E-02
LRRC55	0.150	6.157E-01	1.726E-01
TPST1	0.150	4.187E-01	4.538E-01
TPP1	0.150	1.981E-01	5.793E-02
DNM3	0.150	4.931E-01	2.304E-01
MANEAL	0.150	5.303E-01	3.263E-01
MBNL3	0.150	3.970E-01	1.774E-01
GPR68	0.150	6.425E-01	2.362E-03
FBXO17	0.150	6.488E-01	1.144E-01
TMEM100	0.150	7.193E-01	6.015E-01
ASB2	0.150	6.513E-01	4.331E-02
OR6P1	0.149	NA	1.924E-01
SNTN	0.149	7.972E-01	5.500E-02
YIPF7	0.149	7.309E-01	2.460E-02
C12orf56	0.149	7.203E-01	2.579E-01
ST14	0.149	3.429E-01	3.661E-01
ARHGEF9	0.149	1.829E-01	4.510E-02
CCT6B	0.149	3.475E-01	3.856E-02
TINAGL1	0.149	5.662E-01	6.928E-01
NGLY1	0.149	1.438E-01	3.398E-01
LRP1B	0.149	7.921E-01	3.887E-01
MIR8078	0.149	8.248E-01	7.559E-03
TMOD2	0.149	3.714E-01	1.027E-01
HNRNPF	0.149	8.164E-02	1.091E-01
ARCN1	0.149	2.298E-02	6.195E-01
HMMR	0.149	3.920E-01	1.314E-03
GNA12	0.149	3.005E-01	5.365E-01
CALCOCO2	0.149	3.402E-02	3.067E-01
NLGN2	0.149	3.516E-01	5.789E-02
TOMM34	0.149	2.478E-01	3.968E-01
CENPL	0.149	2.191E-01	1.130E-01
GOSR2	0.149	2.357E-02	2.822E-01
SLC24A5	0.149	NA	8.786E-03
RSPH10B2	0.149	6.572E-01	2.720E-01
EDA2R	0.148	6.207E-01	5.744E-03
PCDHGA7	0.148	6.106E-01	1.300E-01
ZNF367	0.148	4.163E-01	1.143E-01
TMEM104	0.148	1.137E-01	5.173E-02
PIGC	0.148	1.013E-01	2.392E-01

TOR1B	0.148	7.581E-02	1.736E-03
CYP21A2	0.148	7.280E-01	3.087E-03
FRRS1L	0.148	7.668E-01	7.301E-01
CSGALNACT	0.148	1.966E-01	8.855E-02
TGOLN2	0.148	1.013E-01	8.681E-01
LMO7	0.148	5.047E-01	4.461E-01
ERICH6	0.148	4.223E-01	2.120E-01
ARHGEF33	0.148	5.844E-01	2.248E-01
EIF5A2	0.148	5.915E-01	2.684E-02
SAMD4B	0.148	4.419E-02	5.124E-03
YKT6	0.147	1.013E-01	1.608E-02
RNASE4	0.147	4.996E-01	1.224E-01
MS4A14	0.147	6.292E-01	8.552E-02
CHST15	0.147	6.189E-01	5.993E-01
AP2A1	0.147	2.335E-02	1.968E-01
TMEM240	0.147	4.433E-01	9.884E-04
NPEPPS	0.147	5.256E-02	2.200E-01
FFAR3	0.147	7.714E-01	3.988E-02
TBX19	0.147	2.821E-01	1.606E-03
UAP1	0.147	3.324E-01	8.849E-01
IGF1	0.147	7.513E-01	3.514E-01
LRRN1	0.147	7.322E-01	7.921E-01
MYO9A	0.147	9.314E-02	3.849E-01
CHMP4B	0.146	6.878E-02	8.332E-01
PKD2	0.146	2.449E-01	6.566E-03
RDX	0.146	4.589E-01	6.333E-03
CXCL16	0.146	3.975E-01	9.249E-03
TPSG1	0.146	6.764E-01	1.109E-01
OTUD7A	0.146	5.675E-01	3.099E-01
ZNF525	0.146	2.013E-01	5.749E-01
PIGS	0.146	1.625E-01	4.557E-02
C2orf91	0.146	7.869E-01	1.746E-01
NOMO2	0.146	3.401E-01	1.191E-01
WISP1	0.146	6.961E-01	4.781E-04
KIF23	0.146	3.393E-01	4.703E-01
TCTA	0.146	1.595E-01	1.232E-01
UBQLNL	0.146	5.714E-01	7.678E-02
SLC44A1	0.146	1.978E-01	4.255E-02
CYP4F22	0.146	7.728E-01	2.701E-01
DDOST	0.146	4.779E-02	1.758E-01
C1orf43	0.146	2.181E-02	7.744E-01
ANKEF1	0.146	2.839E-01	3.160E-01
JAZF1	0.146	4.627E-01	3.342E-03
AP2S1	0.146	2.009E-01	9.665E-03
HIST4H4	0.146	4.471E-01	1.424E-03
ZNF28	0.146	2.154E-01	2.992E-01
UBE2F-SCLY	0.145	5.778E-01	4.329E-01
SLC17A6	0.145	NA	4.432E-03
CANT1	0.145	1.264E-01	4.933E-01
DNASE1L1	0.145	1.443E-01	5.277E-01

GPR19	0.145	5.753E-01	2.851E-04
C16orf89	0.145	7.470E-01	1.286E-01
LRRC6	0.145	5.311E-01	2.388E-03
SPDYE3	0.145	9.119E-02	2.599E-01
SEMA4C	0.145	2.747E-01	4.085E-04
GCM1	0.145	7.254E-01	1.412E-04
KCNJ12	0.145	7.360E-01	1.608E-01
MIR548L	0.145	8.007E-01	3.861E-04
SLC35E1	0.145	3.375E-02	6.605E-02
MAPRE1	0.145	4.852E-02	3.983E-01
INPP5A	0.145	1.984E-01	1.541E-02
WTIP	0.145	4.053E-01	1.812E-02
RAD23B	0.145	1.078E-01	8.257E-01
BTG3	0.145	2.269E-01	5.249E-01
MINDY1	0.145	3.312E-01	3.214E-04
GCHFR	0.145	4.425E-01	9.484E-01
PPP1R2	0.145	3.647E-02	8.942E-01
TCF7L1	0.145	5.943E-01	4.702E-03
ABCG4	0.145	6.033E-01	1.444E-02
NPTN	0.145	1.249E-01	8.226E-01
KIF25	0.145	7.180E-01	2.564E-01
DNAJB6	0.144	8.038E-02	2.629E-02
EID2	0.144	1.195E-01	2.762E-01
PDIA4	0.144	1.622E-01	8.121E-02
AIF1L	0.144	6.430E-01	7.584E-01
MDGA1	0.144	6.805E-01	1.880E-02
GPR146	0.144	4.043E-01	9.291E-03
PAF1	0.144	7.092E-02	8.030E-01
PARVB	0.144	5.559E-01	5.764E-01
DYRK2	0.144	3.104E-01	6.467E-01
TBCB	0.144	1.091E-01	5.705E-02
THEMIS2	0.144	5.726E-01	1.055E-02
COPB2	0.144	4.047E-02	3.955E-01
DNAJC15	0.144	6.066E-01	3.722E-03
CMPK1	0.144	3.211E-02	8.600E-01
PNMA1	0.144	4.363E-01	2.329E-01
TMEM107	0.144	2.163E-01	8.776E-04
REEP3	0.144	1.783E-01	6.427E-03
CCPG1	0.144	2.474E-01	5.760E-03
ISYNA1	0.144	5.795E-01	2.661E-02
PRSS58	0.143	NA	4.797E-01
PGRMC2	0.143	1.488E-01	2.411E-01
SYN3	0.143	6.985E-01	2.528E-03
ZBTB21	0.143	1.615E-01	3.255E-01
UBR5	0.143	2.158E-01	1.055E-01
FSCN3	0.143	5.803E-01	8.336E-03
WNK3	0.143	6.884E-01	3.975E-02
LSMEM1	0.143	4.045E-01	1.306E-01
PITHD1	0.143	3.971E-02	2.184E-01
CRTAP	0.143	1.364E-01	7.726E-04

KIF1C	0.143	1.665E-01	4.216E-02
SLC6A19	0.143	8.998E-01	4.086E-03
RIMKLB	0.143	4.906E-01	6.750E-05
RYR2	0.143	7.056E-01	8.586E-03
ITGB1BP1	0.143	8.003E-02	1.875E-02
SUSD4	0.143	6.961E-01	3.270E-01
MLLT11	0.143	5.477E-01	1.374E-01
MYO5C	0.143	5.236E-01	5.091E-02
PIP4K2A	0.143	2.730E-01	2.617E-02
IKZF2	0.143	5.310E-01	4.071E-01
GPR157	0.143	4.968E-01	3.481E-02
THUMPD3	0.143	1.025E-01	3.280E-01
FOXJ1	0.143	8.031E-01	4.089E-01
S100A8	0.143	8.117E-01	3.709E-01
GAB1	0.143	2.751E-01	2.600E-01
PDHX	0.143	1.506E-01	2.161E-01
FKBP9	0.143	2.665E-01	2.034E-01
LDLRAD2	0.143	4.407E-01	9.495E-01
SLMAP	0.143	2.608E-01	9.495E-01
DESI2	0.143	6.638E-02	9.186E-01
F10	0.143	6.305E-01	9.183E-01
CNTN1	0.143	7.786E-01	9.121E-01
SNRNP27	0.143	2.025E-02	4.253E-03
NBAS	0.143	5.457E-02	5.907E-01
SLC5A2	0.143	6.127E-01	9.502E-03
CLVS1	0.142	6.620E-01	2.437E-01
MIR3680-2	0.142	NA	5.315E-04
TXNDC2	0.142	6.641E-01	2.798E-01
ZCWPW2	0.142	3.202E-01	4.396E-01
YIPF6	0.142	2.556E-02	4.458E-01
ABHD5	0.142	2.357E-01	5.586E-02
TRIM44	0.142	1.284E-01	7.544E-02
C1orf167	0.142	7.443E-01	4.743E-02
GMCL1	0.142	8.905E-02	4.060E-04
LIN7B	0.142	3.504E-01	2.937E-03
ANGPTL2	0.142	5.986E-01	7.895E-03
CBFA2T3	0.142	6.284E-01	2.106E-01
SYNGR2	0.142	1.796E-01	2.684E-01
PHF20	0.142	4.243E-02	9.396E-02
SLC27A4	0.142	3.201E-01	4.817E-01
ESRP1	0.142	4.196E-01	8.515E-01
GJA4	0.142	5.271E-01	4.505E-01
RAB12	0.142	1.370E-01	7.373E-01
STK32B	0.142	6.027E-01	1.258E-02
PQLC2L	0.142	7.327E-01	3.533E-02
BMPER	0.142	6.802E-01	5.748E-01
IL6ST	0.142	3.708E-01	2.818E-01
NBDY	0.141	2.753E-01	1.082E-01
STAG1	0.141	2.473E-01	1.720E-01
SLC46A1	0.141	3.856E-01	3.261E-03

KRT39	0.141	8.113E-01	2.137E-02
CD200	0.141	6.002E-01	5.427E-02
SRR	0.141	2.109E-01	3.138E-01
PTMS	0.141	4.064E-01	3.789E-01
ERLEC1	0.141	4.058E-02	2.317E-01
CLPB	0.141	3.410E-01	3.859E-02
CETN2	0.141	8.457E-02	8.280E-03
OS9	0.141	8.521E-02	1.375E-01
CA13	0.141	4.836E-01	6.501E-02
MYCBPAP	0.141	5.568E-01	4.488E-01
CREB3	0.141	1.514E-01	1.391E-01
LNPK	0.141	1.574E-01	4.289E-01
AK1	0.141	4.207E-01	2.145E-02
MIR6745	0.141	7.380E-01	2.603E-02
FHL2	0.141	4.705E-01	4.543E-03
SMTN	0.141	4.714E-01	8.469E-01
CAND2	0.141	6.602E-01	1.923E-01
RER1	0.141	2.930E-02	3.888E-01
PCDHB11	0.140	6.724E-01	8.758E-03
MAP2K6	0.140	4.646E-01	1.113E-01
TP53TG5	0.140	3.733E-01	3.777E-02
RMND5B	0.140	1.017E-01	6.238E-02
TMEM39A	0.140	6.947E-02	1.884E-01
OSMR	0.140	6.336E-01	2.166E-01
NLRP3	0.140	6.440E-01	2.175E-02
RARB	0.140	6.367E-01	1.589E-01
TSPAN6	0.140	4.529E-01	5.343E-03
NINJ2	0.140	5.732E-01	1.015E-03
AL049844.1	0.140	7.488E-01	8.372E-01
G6PD	0.140	5.696E-01	9.503E-01
VAMP8	0.140	2.460E-01	9.503E-01
ARL8B	0.140	9.654E-02	9.503E-01
PLPP5	0.140	3.818E-01	9.427E-01
WDR44	0.140	1.992E-01	3.809E-01
TMEM38A	0.140	5.411E-01	1.077E-03
SGO1	0.140	4.179E-01	5.305E-02
OVCH2	0.140	7.254E-01	8.352E-02
C6orf226	0.140	3.509E-01	4.626E-02
TMEM9	0.140	1.786E-01	4.566E-01
P2RY14	0.140	6.151E-01	1.181E-01
RAD51C	0.140	1.448E-01	3.477E-03
DEUP1	0.140	8.021E-01	2.591E-03
NRSN2	0.140	4.729E-01	1.798E-01
PATL1	0.140	1.351E-01	1.161E-02
EHBP1	0.140	2.843E-01	1.527E-03
MIR3613	0.140	8.235E-01	1.777E-03
LACTB2	0.140	2.781E-01	2.101E-01
PSAPL1	0.139	8.562E-01	6.932E-02
LRRC34	0.139	5.060E-01	7.166E-03
FOCAD	0.139	3.753E-01	7.431E-01

STPG1	0.139	3.833E-01	3.085E-01
MIR373	0.139	NA	4.057E-03
MIR4497	0.139	NA	2.109E-01
MVD	0.139	2.823E-01	5.935E-04
FAM72A	0.139	4.242E-01	1.441E-02
CCDC120	0.139	2.495E-01	3.327E-02
PCSK1	0.139	7.084E-01	4.401E-01
MANF	0.139	1.917E-01	1.058E-02
VKORC1	0.139	2.334E-01	1.785E-04
PPM1E	0.139	6.166E-01	8.803E-01
CLCN5	0.139	3.407E-01	8.697E-01
CENPI	0.139	3.906E-01	5.464E-01
UNCX	0.139	NA	2.954E-01
DLGAP3	0.139	6.369E-01	2.748E-01
TMEM211	0.139	8.044E-01	4.599E-01
TM9SF3	0.139	5.740E-02	9.476E-03
MAP2K1	0.139	7.953E-02	8.303E-02
AC244517.10	0.139	8.579E-01	3.375E-03
UGT2B17	0.139	8.186E-01	8.518E-02
SMYD2	0.139	1.989E-01	4.034E-01
IDS	0.138	2.189E-01	3.365E-03
CHRD	0.138	6.101E-01	3.483E-01
NOCT	0.138	4.522E-01	1.049E-02
SPG21	0.138	1.606E-02	4.921E-01
DDAH2	0.138	4.502E-01	1.061E-01
ERCC6	0.138	2.141E-01	1.347E-02
PDE5A	0.138	4.938E-01	7.299E-02
GDAP2	0.138	4.246E-02	2.461E-03
CASP2	0.138	7.505E-02	1.028E-02
ELF3	0.138	6.198E-01	3.122E-01
OAT	0.138	6.127E-01	1.160E-02
CCDC153	0.138	4.979E-01	3.764E-01
PABPC1L2B	0.138	NA	2.762E-02
MCUR1	0.138	1.984E-01	8.079E-01
XAGE3	0.138	9.202E-01	6.622E-01
RAD9B	0.138	5.086E-01	6.124E-03
SLC25A35	0.138	4.907E-01	2.833E-01
SBK2	0.138	8.876E-01	7.839E-02
SERP1	0.138	4.693E-02	4.316E-03
PRSS51	0.137	7.560E-01	3.515E-02
MIRLET7A1	0.137	7.981E-01	4.844E-03
POU6F2	0.137	7.883E-01	5.636E-03
C2CD2	0.137	3.701E-01	1.473E-02
SMG9	0.137	1.511E-01	4.740E-01
FAM196A	0.137	5.246E-01	1.246E-02
SHOX2	0.137	7.318E-01	3.660E-02
SMCO4	0.137	4.279E-01	1.181E-01
OR52B6	0.137	NA	2.919E-02
CDR2	0.137	3.215E-01	1.655E-01
CORO2B	0.137	6.483E-01	6.654E-01

IQGAP1	0.137	1.691E-01	1.473E-01
NEDD4	0.137	3.415E-01	7.725E-01
RNF5	0.137	1.119E-01	8.081E-03
GDF3	0.137	7.128E-01	3.546E-01
RAB11FIP5	0.137	2.288E-01	4.540E-03
ABHD11	0.137	4.132E-01	2.382E-01
AL355987.1	0.137	NA	4.221E-01
CACNA2D2	0.137	5.633E-01	5.469E-01
MLLT3	0.137	4.430E-01	3.581E-02
TRNAU1AP	0.137	1.401E-01	9.492E-01
TMEM106B	0.137	1.629E-01	9.432E-01
TMEM170B	0.136	5.241E-01	9.429E-01
BAG2	0.136	5.635E-01	8.880E-01
RAB3C	0.136	7.489E-01	1.612E-01
TAF9B	0.136	1.884E-01	8.242E-01
AC017083.4	0.136	7.722E-01	1.058E-02
ACMSD	0.136	6.701E-01	1.007E-01
CLEC4A	0.136	6.351E-01	1.526E-01
C11orf70	0.136	5.706E-01	8.207E-01
CDO1	0.136	7.723E-01	2.852E-02
NAALADL1	0.136	5.931E-01	8.229E-02
NAAA	0.136	3.466E-01	3.454E-01
TMCO1	0.136	1.954E-01	3.625E-01
C3orf36	0.136	6.231E-01	3.108E-03
ATP6V0D1	0.136	1.872E-01	2.383E-01
CREB3L4	0.136	3.836E-01	1.268E-01
WIPF3	0.136	6.847E-01	3.605E-02
STT3A	0.136	5.841E-02	1.578E-01
SLC25A18	0.136	4.496E-01	5.874E-02
PNMA6F	0.135	NA	4.670E-01
GMPR	0.135	6.393E-01	1.839E-01
COQ8A	0.135	2.757E-01	4.400E-01
INPP4B	0.135	5.549E-01	5.440E-05
ZDHHC19	0.135	7.261E-01	1.497E-03
MSH6	0.135	2.346E-01	4.137E-01
C17orf67	0.135	4.130E-01	3.132E-01
TSEN34	0.135	1.057E-01	1.735E-01
ZFP30	0.135	3.815E-01	2.748E-03
SETD7	0.135	2.434E-01	5.114E-01
MIR6780A	0.135	NA	2.524E-01
CEP89	0.135	1.091E-01	1.366E-02
FAM98C	0.135	2.510E-01	2.941E-01
C14orf105	0.135	8.904E-01	3.907E-01
RTN4R	0.135	5.731E-01	4.538E-01
CACYBP	0.135	1.325E-01	1.923E-01
ETFB	0.135	3.617E-01	1.641E-02
MYO18A	0.135	3.232E-01	7.331E-01
MUSTN1	0.135	6.683E-01	2.159E-03
SETD3	0.135	5.291E-02	4.140E-01
DERL1	0.135	8.769E-02	7.050E-02

GFOD2	0.135	1.666E-01	2.410E-04
LAPTM4A	0.135	6.877E-02	7.165E-02
TGFA	0.135	5.895E-01	7.564E-03
NAT16	0.135	7.364E-01	1.154E-01
C1orf21	0.135	3.906E-01	1.129E-02
STX12	0.135	3.314E-02	1.389E-01
ABHD15	0.135	2.747E-01	3.565E-03
SDF2	0.135	1.132E-01	8.003E-01
FAM19A5	0.135	6.856E-01	3.301E-02
CDK15	0.134	6.336E-01	3.232E-01
SPDYE6	0.134	4.835E-01	1.328E-02
MCOLN1	0.134	1.230E-01	9.277E-01
TMCO3	0.134	3.123E-01	9.222E-01
PRRG4	0.134	5.013E-01	7.571E-01
SOCS5	0.134	7.123E-02	2.440E-02
POLR2K	0.134	3.231E-01	4.680E-02
TBC1D8B	0.134	3.358E-01	3.567E-01
MIR199B	0.134	NA	8.586E-01
SEC31A	0.134	5.858E-02	3.539E-03
SPO11	0.134	NA	1.115E-04
IQCD	0.134	5.208E-01	1.094E-02
UBE2T	0.134	3.167E-01	3.509E-03
STOML1	0.134	2.178E-01	2.231E-01
PSORS1C1	0.134	6.207E-01	2.185E-01
SAP30	0.134	2.944E-01	6.123E-02
IER5	0.134	4.652E-01	1.531E-01
UGT1A7	0.134	7.696E-01	5.013E-01
ACSS2	0.134	2.045E-01	2.843E-01
CTTNBP2NL	0.133	1.724E-01	1.695E-01
MIR4295	0.133	NA	2.722E-01
MIR6505	0.133	NA	3.453E-01
STARD3	0.133	1.854E-01	1.907E-03
DHFR2	0.133	2.517E-01	1.701E-02
AC048338.1	0.133	NA	5.816E-03
LINGO4	0.133	7.776E-01	5.448E-02
SYT11	0.133	5.955E-01	3.288E-02
NES	0.133	6.128E-01	7.611E-03
TCAP	0.133	5.549E-01	2.761E-03
ZNF154	0.133	5.392E-01	5.198E-01
LEXM	0.133	7.295E-01	6.487E-01
MGAT3	0.133	7.417E-01	1.595E-03
PARP6	0.133	5.145E-01	1.993E-02
LMNA	0.133	2.722E-01	2.494E-04
STK24	0.133	2.962E-01	4.491E-02
PER3	0.133	5.316E-01	4.989E-01
ZFP36	0.133	5.500E-01	2.819E-01
HACD3	0.133	2.549E-01	2.997E-01
BANK1	0.133	6.244E-01	9.922E-02
ACAD9	0.133	5.209E-02	1.990E-01
SH3PXD2B	0.133	4.028E-01	3.394E-02

NRM	0.133	3.669E-01	1.378E-02
SPAG16	0.133	5.209E-01	4.304E-03
EXOC6B	0.133	2.154E-01	2.971E-01
PODXL2	0.132	6.534E-01	1.293E-02
ZNF567	0.132	2.664E-01	2.041E-02
IFT20	0.132	2.312E-01	2.317E-01
CTNNBIP1	0.132	1.691E-01	1.159E-02
PQLC2	0.132	1.501E-01	3.045E-01
GYPC	0.132	6.626E-01	5.452E-01
ZMYND15	0.132	5.366E-01	4.296E-01
LHB	0.132	6.663E-01	1.314E-02
CPEB3	0.132	2.755E-01	8.258E-03
COL15A1	0.132	6.108E-01	3.910E-02
IGFBP4	0.132	4.980E-01	3.953E-03
TNFAIP3	0.132	6.274E-01	2.769E-01
HAPLN2	0.132	7.409E-01	3.811E-01
HNRNPH2	0.132	2.673E-02	2.722E-01
FRMD4A	0.132	4.537E-01	4.592E-02
TEX26	0.132	8.249E-01	2.753E-01
LMX1B	0.132	8.333E-01	3.433E-01
EMCN	0.132	5.686E-01	1.626E-01
AFAP1	0.132	4.261E-01	3.022E-01
MIR580	0.132	NA	3.499E-04
MRGPRE	0.132	8.317E-01	5.747E-02
CLTCL1	0.132	6.276E-01	4.621E-01
GOLGA5	0.132	4.989E-02	3.271E-01
EPHX3	0.132	7.782E-01	5.636E-03
FNDC10	0.132	5.778E-01	1.005E-01
SH3BP5	0.131	5.507E-01	7.924E-02
CARHSP1	0.131	4.013E-01	9.462E-01
MGST3	0.131	3.489E-01	1.630E-02
C12orf54	0.131	7.857E-01	6.480E-03
SLC41A2	0.131	6.352E-01	3.883E-02
FLOT1	0.131	1.830E-01	3.506E-02
RNF11	0.131	1.107E-01	7.232E-02
TMX2	0.131	1.104E-01	8.753E-02
SYT7	0.131	6.904E-01	7.713E-03
CLPTM1	0.131	8.173E-02	2.280E-01
OSCP1	0.131	2.941E-01	2.481E-01
ZNF486	0.131	7.415E-01	4.871E-02
BMT2	0.131	1.658E-01	1.321E-03
ABCB8	0.131	1.526E-01	4.479E-03
GPC6	0.131	7.295E-01	9.246E-03
EPB41L4A	0.131	5.156E-01	4.833E-01
C6orf52	0.131	6.198E-01	3.740E-01
NDC80	0.131	4.667E-01	3.830E-03
RPA2	0.131	1.351E-01	1.233E-03
TSTD1	0.131	5.341E-01	2.489E-01
EDEM3	0.131	2.250E-01	2.523E-02
ADGRF5	0.131	5.106E-01	1.961E-02

LARP4B	0.131	8.636E-02	1.668E-01
LRRFIP2	0.131	1.016E-01	1.699E-01
FARP1	0.131	4.235E-01	8.868E-01
ACLY	0.130	1.635E-01	8.726E-02
REEP2	0.130	6.769E-01	8.001E-01
CLPTM1L	0.130	2.941E-01	4.781E-03
C9orf116	0.130	5.088E-01	1.712E-01
PRX	0.130	4.310E-01	3.642E-03
ATRIP	0.130	4.762E-01	5.045E-01
FOXC1	0.130	5.829E-01	8.387E-03
MAP1A	0.130	6.549E-01	1.671E-02
SFXN2	0.130	4.062E-01	5.609E-02
CLIP4	0.130	5.331E-01	4.415E-02
JMJD8	0.130	1.750E-01	3.635E-02
ELOVL7	0.130	5.552E-01	5.445E-02
SPDYE1	0.130	4.082E-01	2.965E-02
ARHGAP8	0.130	5.299E-01	2.198E-02
HSFX2	0.130	8.595E-01	2.693E-01
SHE	0.130	5.701E-01	4.083E-01
OR6C3	0.129	NA	7.485E-02
TSPY4	0.129	NA	1.576E-02
FDPS	0.129	2.392E-01	1.861E-02
SLC35F5	0.129	1.479E-01	5.606E-02
NMRK2	0.129	8.421E-01	2.314E-01
EZH2	0.129	4.198E-01	1.201E-02
AL772284.2	0.129	NA	3.690E-01
C7orf77	0.129	9.133E-01	1.853E-01
FAF1	0.129	5.387E-02	2.635E-02
SCAPER	0.129	2.746E-01	2.712E-01
OC1S5-TXNI	0.129	6.861E-01	4.283E-01
SHROOM4	0.129	5.439E-01	1.001E-02
SLC16A3	0.129	5.561E-01	3.819E-01
CABLES2	0.129	3.236E-01	3.107E-01
COG4	0.128	1.834E-01	1.956E-02
TMTC2	0.128	4.589E-01	3.303E-01
FAM76A	0.128	5.301E-02	9.536E-01
REXO5	0.128	4.083E-01	9.536E-01
AC093227.3	0.128	8.506E-01	4.584E-01
SPHK1	0.128	7.071E-01	8.179E-02
OR5P3	0.128	8.080E-01	2.051E-02
NPIPB9	0.128	7.757E-01	6.050E-01
P2RX4	0.128	4.137E-01	1.667E-01
TRIM46	0.128	5.668E-01	6.796E-01
CCR7	0.128	7.904E-01	3.357E-01
MFSD9	0.128	3.040E-01	1.081E-03
BMP8A	0.128	5.271E-01	2.309E-01
ANKRD45	0.128	7.472E-01	1.163E-01
ZNF25	0.128	2.278E-01	8.532E-03
SAA4	0.128	8.444E-01	3.774E-01
COL6A6	0.128	7.674E-01	1.868E-02

ZNF221	0.128	4.115E-01	2.092E-02
SFT2D2	0.128	2.200E-01	3.663E-03
MID1	0.128	5.032E-01	2.114E-02
RBMS3	0.128	6.383E-01	6.314E-03
CHMP3	0.128	7.169E-02	6.433E-01
ITPR1	0.127	5.601E-01	8.486E-02
AL121758.1	0.127	7.504E-01	9.842E-03
EIF1AD	0.127	6.895E-02	4.952E-04
RFWD2	0.127	1.717E-01	5.756E-02
SGCE	0.127	6.433E-01	1.821E-01
PTK7	0.127	4.663E-01	3.809E-01
PRDX6	0.127	2.792E-01	2.947E-03
LMO2	0.127	5.741E-01	8.887E-01
GAB2	0.127	5.114E-01	6.600E-01
RDH5	0.127	5.659E-01	7.761E-02
IER5L	0.127	5.012E-01	6.196E-02
SENP2	0.127	1.150E-01	2.185E-01
RTKN	0.127	5.314E-01	5.104E-01
STIL	0.127	4.170E-01	8.216E-01
SMARCD2	0.127	1.374E-01	5.735E-02
TET3	0.127	3.753E-01	6.039E-02
CCDC151	0.127	6.938E-01	7.206E-01
TPM3	0.127	1.536E-01	2.050E-03
RTN4RL2	0.127	6.333E-01	1.062E-02
LAMC1	0.127	3.888E-01	6.565E-04
ANTD3-TME	0.127	6.758E-01	3.758E-01
ARHGAP1	0.126	2.110E-01	7.380E-03
SYVN1	0.126	7.644E-02	2.146E-02
OSGIN2	0.126	2.627E-01	2.384E-01
ATP10B	0.126	7.874E-01	2.444E-02
NAGA	0.126	1.891E-01	1.548E-01
MALT1	0.126	3.460E-01	1.972E-03
AC007040.2	0.126	7.002E-01	4.936E-01
SCAMP3	0.126	9.854E-02	2.254E-01
GAGE2E	0.126	NA	6.498E-02
FOXR2	0.126	NA	3.744E-02
MIR1256	0.126	NA	1.351E-02
CABP2	0.126	NA	2.044E-02
MIR557	0.126	NA	9.030E-05
NEB	0.126	6.620E-01	4.408E-01
SNPH	0.126	5.613E-01	2.811E-02
CCDC9	0.126	8.141E-02	4.186E-03
BLZF1	0.126	1.926E-01	2.973E-01
AL021997.3	0.126	7.494E-01	4.260E-01
MORF4L2	0.126	7.388E-02	4.527E-02
SURF4	0.126	1.637E-01	6.357E-03
PLAUR	0.126	6.173E-01	4.316E-02
AL121845.2	0.126	NA	2.322E-02
ASH2L	0.126	3.331E-01	1.318E-01
CNBD2	0.126	5.616E-01	1.410E-02

MTPN	0.126	1.674E-01	8.446E-01
OSBPL5	0.126	3.091E-01	1.384E-01
PYGO1	0.126	6.776E-01	3.577E-04
MAGEA10	0.126	8.883E-01	1.110E-02
AC011479.1	0.126	7.294E-01	1.527E-02
TMEM57	0.126	1.334E-01	2.686E-01
URI1	0.126	3.056E-01	2.076E-03
CHST11	0.126	6.632E-01	2.945E-01
HP1BP3	0.125	9.219E-02	3.589E-01
MIR4322	0.125	8.436E-01	3.627E-02
CAMK4	0.125	6.816E-01	2.040E-01
AC010531.1	0.125	6.140E-01	6.525E-03
PLAC8L1	0.125	6.184E-01	1.089E-01
CCDC170	0.125	6.226E-01	2.353E-02
C6orf132	0.125	4.709E-01	2.610E-03
KIAA1614	0.125	4.631E-01	1.833E-02
KIFC3	0.125	4.944E-01	5.199E-01
WDYHV1	0.125	2.356E-01	6.014E-01
NEK6	0.125	5.822E-01	8.616E-02
HSPA5	0.125	2.121E-01	7.876E-01
TTC9B	0.125	6.270E-01	3.349E-01
EXT2	0.125	2.440E-01	2.259E-02
PPT2	0.125	4.270E-01	1.366E-01
ZNF695	0.125	5.559E-01	3.503E-03
PTPN20	0.125	7.944E-01	7.636E-04
MYLK4	0.125	7.460E-01	2.585E-03
RNF115	0.125	8.031E-02	3.878E-01
MAP3K13	0.125	1.992E-01	8.569E-03
COMMD9	0.125	2.205E-01	3.424E-01
PKN1	0.125	4.845E-01	4.929E-02
BECN1	0.125	1.257E-01	3.981E-01
HIST1H1D	0.125	6.958E-01	1.751E-02
CLTC	0.125	1.432E-01	3.599E-02
SBDS	0.125	1.162E-01	2.336E-02
C9orf50	0.125	6.398E-01	2.343E-03
POLA1	0.125	3.104E-01	7.390E-01
CELF3	0.125	7.927E-01	8.427E-01
VCL	0.124	3.468E-01	8.325E-01
EIF3K	0.124	2.931E-01	4.327E-01
LIN28B	0.124	8.819E-01	4.569E-03
SEZ6L	0.124	8.405E-01	7.118E-03
C5orf47	0.124	7.971E-01	1.811E-01
SORCS2	0.124	7.678E-01	4.038E-01
FBXO4	0.124	3.215E-01	1.456E-01
NME6	0.124	1.042E-01	1.965E-01
ANO10	0.124	1.651E-01	4.116E-03
SLC37A1	0.124	5.011E-01	8.311E-02
PIGO	0.124	2.290E-01	2.561E-01
ENTPD1	0.124	3.790E-01	5.105E-02
MMP19	0.124	6.303E-01	1.465E-02

SLC39A11	0.124	2.819E-01	2.130E-03
AC005697.1	0.124	NA	4.339E-01
AKTIP	0.124	1.983E-01	6.798E-03
SAE1	0.124	1.296E-01	1.121E-01
PEX19	0.124	3.239E-01	4.885E-01
HIST1H4K	0.124	7.120E-01	3.771E-03
APLP1	0.124	7.159E-01	1.407E-03
SMC1A	0.124	2.637E-01	1.467E-02
PSMD2	0.124	2.131E-01	8.026E-02
DCAF8	0.123	2.746E-01	1.101E-01
APOOL	0.123	1.583E-01	3.388E-01
NRBP1	0.123	8.131E-02	8.413E-04
SLC9A3R1	0.123	5.327E-01	1.460E-02
C2orf15	0.123	5.867E-01	3.927E-01
SMURF1	0.123	1.422E-01	2.516E-03
AKR1A1	0.123	1.714E-01	1.199E-02
TAF12	0.123	8.955E-02	1.190E-01
S100A16	0.123	5.943E-01	2.837E-01
AC013470.2	0.123	7.531E-01	4.882E-02
GNL3L	0.123	2.928E-01	7.507E-02
TASP1	0.123	1.713E-01	3.386E-01
SEPHS1	0.123	1.823E-01	3.909E-03
TMPPE	0.123	4.150E-01	2.851E-01
VEGFB	0.123	3.216E-01	1.945E-02
CEP85	0.123	3.555E-01	2.120E-02
EMC3	0.123	1.992E-01	9.485E-03
FBXO18	0.123	1.368E-01	9.156E-03
LAMP2	0.123	3.804E-01	3.427E-01
CIB2	0.123	4.891E-01	4.883E-01
FCHSD2	0.123	1.653E-01	2.530E-01
H2AFJ	0.123	4.931E-01	5.208E-01
C1QTNF4	0.123	7.020E-01	3.744E-02
EZH1	0.123	1.991E-01	7.448E-03
HIST1H2AJ	0.122	7.539E-01	1.433E-01
SHCBP1	0.122	4.868E-01	3.464E-01
MUL1	0.122	8.925E-02	1.522E-01
PGAP3	0.122	4.717E-01	5.214E-02
EHD1	0.122	3.735E-01	3.359E-01
POT1	0.122	1.902E-01	3.074E-01
KATNAL1	0.122	6.338E-01	9.380E-01
NLRX1	0.122	3.061E-01	3.062E-01
NSD3	0.122	3.478E-01	5.656E-03
AC004754.1	0.122	5.935E-01	5.366E-03
C20orf204	0.122	6.352E-01	3.705E-01
CD151	0.122	3.335E-01	8.496E-01
HUWE1	0.122	1.110E-01	1.087E-01
AC087289.3	0.122	6.116E-01	4.532E-01
SIDT2	0.122	2.438E-01	1.351E-01
DPAGT1	0.122	1.302E-01	3.827E-01
RANBP9	0.122	2.324E-01	8.112E-01

OR1D5	0.121	NA	2.223E-03
OR52I2	0.121	NA	2.101E-02
TBC1D3F	0.121	8.592E-01	3.947E-01
CTSF	0.121	5.411E-01	1.687E-02
NLRP12	0.121	6.616E-01	3.618E-01
MIR4482	0.121	7.010E-01	1.450E-02
P3H2	0.121	7.046E-01	7.809E-04
SFMBT1	0.121	2.808E-01	6.812E-01
JTB	0.121	1.685E-01	3.246E-02
TRIM8	0.121	2.922E-01	1.192E-02
TANC2	0.121	4.211E-01	6.629E-01
MORF4L1	0.121	4.852E-02	1.651E-01
CAMK2G	0.121	3.688E-01	3.132E-01
IGSF9	0.121	6.609E-01	1.269E-02
ADAM10	0.121	4.243E-01	8.735E-01
RAET1L	0.121	8.145E-01	8.177E-01
CFI	0.121	6.931E-01	1.909E-02
C19orf47	0.121	2.606E-01	2.495E-01
SETD5	0.121	2.463E-01	6.801E-01
ZBTB47	0.121	3.054E-01	3.078E-01
AC104581.3	0.121	NA	1.344E-01
STK26	0.121	4.186E-01	1.501E-02
CSF3	0.121	8.482E-01	3.969E-01
YIPF4	0.121	3.578E-02	5.150E-02
YY1AP1	0.121	8.498E-02	4.013E-01
ANXA11	0.121	3.649E-01	1.286E-01
BRAF	0.121	2.763E-01	3.566E-01
BEGAIN	0.120	6.843E-01	6.845E-01
RIBC2	0.120	5.829E-01	7.962E-01
SIK1	0.120	8.182E-01	3.289E-02
CFL1	0.120	1.583E-01	6.116E-01
PCDHA9	0.120	8.582E-01	2.284E-01
DAD1	0.120	1.237E-01	7.032E-02
CLEC3B	0.120	7.033E-01	4.373E-03
KLHL4	0.120	7.805E-01	1.050E-01
RNF19A	0.120	5.101E-01	5.305E-01
CENPF	0.120	4.966E-01	4.186E-01
HSPA1B	0.120	5.257E-01	2.823E-02
KRT8	0.120	6.298E-01	8.410E-02
NUP210L	0.120	6.000E-01	6.182E-03
YME1L1	0.120	1.233E-01	1.001E-02
BHLHE40	0.120	5.641E-01	2.522E-03
KIF9	0.120	4.075E-01	3.168E-04
SLC9A6	0.120	1.693E-01	6.048E-01
RAP1GAP	0.120	7.107E-01	9.735E-02
CADM3	0.120	8.278E-01	6.848E-01
IFT22	0.120	1.974E-01	3.471E-03
C1orf228	0.120	6.225E-01	4.607E-01
SRMS	0.120	7.227E-01	3.018E-02
MIR604	0.120	8.317E-01	2.576E-01

MIR1245A	0.120	9.011E-01	3.355E-01
FUOM	0.120	6.598E-01	1.714E-02
PLOD2	0.119	6.298E-01	3.794E-01
PKMYT1	0.119	5.045E-01	7.536E-01
SQLE	0.119	5.146E-01	9.562E-01
ASH1L	0.119	2.939E-01	9.339E-01
RASSF3	0.119	4.062E-01	1.335E-02
TRMT2B	0.119	1.502E-01	5.261E-01
SUSD1	0.119	3.760E-01	1.178E-02
AL672043.1	0.119	NA	2.788E-01
APMAP	0.119	2.122E-01	2.550E-01
IMPDH1	0.119	4.996E-01	6.574E-02
OR1J1	0.119	7.755E-01	4.156E-01
RAP2C	0.119	1.937E-01	3.900E-02
TMEM8A	0.119	2.675E-01	2.710E-02
SMTNL2	0.119	7.780E-01	7.477E-02
ITFG1	0.119	2.121E-01	9.576E-02
MIR942	0.119	8.635E-01	3.393E-01
CLIP1	0.119	3.294E-01	5.076E-02
PLXND1	0.119	5.516E-01	2.563E-02
DYNLL2	0.119	1.713E-01	2.455E-01
LMAN2L	0.119	1.724E-01	5.265E-01
ZFP82	0.119	5.455E-01	7.567E-01
SSMEM1	0.118	8.371E-01	1.525E-02
WDR76	0.118	4.959E-01	4.604E-02
TNKS1BP1	0.118	2.746E-01	1.804E-01
SLC13A4	0.118	6.514E-01	5.696E-02
BTD	0.118	3.051E-01	3.166E-02
SLC20A2	0.118	4.299E-01	2.318E-01
TBC1D29	0.118	7.618E-01	7.808E-01
KAZALD1	0.118	6.387E-01	1.815E-02
RANBP17	0.118	6.887E-01	5.028E-04
TCF4	0.118	5.856E-01	1.799E-02
AC010547.4	0.118	NA	3.018E-01
UBAC2	0.118	1.180E-01	2.654E-02
APLP2	0.118	2.616E-01	2.941E-03
UBE2Q2	0.118	2.676E-01	9.424E-01
BEX5	0.118	7.598E-01	1.343E-02
AKAP10	0.118	2.053E-01	3.723E-03
SMYD3	0.118	5.696E-01	6.700E-03
MGAT4D	0.118	NA	2.993E-01
KIRREL	0.118	6.352E-01	9.491E-03
FHL3	0.118	4.719E-01	1.132E-02
SEC61A1	0.118	6.444E-02	7.175E-02
PPP1R12C	0.117	2.462E-01	6.361E-01
VAMP7	0.117	2.221E-01	4.022E-02
AHNAK2	0.117	8.067E-01	7.661E-03
SLC2A5	0.117	7.010E-01	8.460E-01
FUT2	0.117	7.084E-01	7.225E-03
TBL2	0.117	1.174E-01	8.747E-03

ZFYVE9	0.117	3.376E-01	3.925E-04
MUC12	0.117	7.271E-01	2.730E-01
CALML3	0.117	8.632E-01	5.600E-03
PLCL2	0.117	6.769E-01	1.288E-01
TIGD3	0.117	6.669E-01	3.168E-03
NINJ1	0.117	4.891E-01	2.473E-02
ZDHHC14	0.117	4.871E-01	8.581E-01
NUDT19	0.117	3.293E-01	7.910E-02
OR52N5	0.117	NA	8.530E-01
MAP2K3	0.117	3.451E-01	6.799E-02
HUS1	0.117	9.936E-02	5.300E-03
TADA1	0.117	3.056E-01	6.927E-03
HYOU1	0.117	3.147E-01	3.113E-01
ZWINT	0.117	4.552E-01	9.319E-04
KIFAP3	0.117	3.473E-01	1.282E-01
HDAC11	0.117	5.024E-01	2.678E-03
C1orf162	0.117	6.708E-01	1.616E-03
C8orf44-SGK3	0.117	6.262E-01	5.730E-02
PLPPR2	0.117	4.555E-01	3.012E-01
TROVE2	0.117	3.138E-01	3.318E-01
BRCC3	0.117	2.765E-01	2.939E-02
ACTB	0.117	2.900E-01	4.422E-04
PCDHA1	0.117	8.534E-01	1.046E-02
PRKAB2	0.117	2.999E-01	2.373E-01
LIX1L	0.116	5.030E-01	2.978E-02
RAB1A	0.116	4.494E-02	2.949E-02
VGLL4	0.116	3.966E-01	3.267E-01
MAGEA3	0.116	8.903E-01	1.686E-01
SLC22A24	0.116	NA	1.898E-01
MIR4435-2	0.116	NA	7.019E-02
FAM50A	0.116	3.348E-01	5.889E-02
NMB	0.116	5.728E-01	5.634E-01
FAM131A	0.116	4.190E-01	1.311E-03
BMPR2	0.116	2.389E-01	9.520E-01
PLXNA3	0.116	3.905E-01	9.339E-01
ZNF469	0.116	7.319E-01	8.537E-01
TBC1D3	0.116	9.380E-01	9.872E-03
MYCT1	0.116	5.841E-01	2.675E-03
KCNJ5	0.116	7.354E-01	5.533E-03
SH3D19	0.116	3.533E-01	7.073E-03
ADCY4	0.116	5.436E-01	5.821E-03
UGP2	0.116	9.926E-02	3.019E-02
ACP2	0.116	3.272E-01	2.072E-01
RASA1	0.116	4.194E-01	1.103E-01
MT-ND1	0.116	6.571E-01	4.983E-01
AP000275.2	0.115	7.738E-01	8.186E-01
GPR20	0.115	7.402E-01	4.470E-01
TECTA	0.115	5.839E-01	2.491E-02
SRPRA	0.115	6.965E-02	3.974E-01
AL161911.1	0.115	5.003E-01	1.828E-01

RBM17	0.115	2.334E-01	2.376E-02
OR51M1	0.115	NA	2.779E-02
OXSR1	0.115	2.510E-01	8.197E-03
TEX9	0.115	5.199E-01	5.517E-02
HEXB	0.115	2.699E-01	7.533E-02
SNX8	0.115	3.815E-01	2.220E-01
PARD6A	0.115	6.044E-01	5.012E-01
CENPBD1	0.115	4.147E-01	1.222E-01
LMTK3	0.115	6.942E-01	1.923E-02
ZEB2	0.115	6.321E-01	3.226E-01
PGD	0.115	5.711E-01	1.517E-01
ARRDC5	0.115	7.280E-01	1.296E-02
MIR6781	0.115	NA	6.070E-02
SLC4A2	0.115	1.997E-01	1.407E-01
GOLGA8O	0.115	8.381E-01	9.558E-01
PECAM1	0.115	5.639E-01	8.970E-01
AC243547.3	0.114	NA	3.283E-01
MBLAC1	0.114	4.159E-01	1.821E-02
MIR4502	0.114	NA	6.936E-01
PLCL1	0.114	6.118E-01	2.378E-02
RTN4	0.114	2.304E-01	9.258E-01
MAGI3	0.114	4.492E-01	2.852E-03
C1orf54	0.114	5.088E-01	2.936E-01
CYB5R1	0.114	5.475E-01	1.455E-01
ZFAND2A	0.114	3.475E-01	1.592E-02
RNF13	0.114	1.642E-01	3.127E-02
SRRM1	0.114	1.444E-01	1.970E-02
DCHS1	0.114	6.408E-01	3.624E-02
CUL1	0.114	8.185E-02	4.417E-02
NEK2	0.114	5.114E-01	9.772E-02
GYPE	0.114	7.380E-01	7.078E-01
MIR4779	0.114	NA	9.373E-03
HIST1H4D	0.114	6.958E-01	5.197E-02
HSPA13	0.114	3.905E-01	1.266E-02
PDCD1LG2	0.114	7.857E-01	3.873E-02
ASAH1	0.114	4.756E-01	1.070E-02
CDKL4	0.114	7.671E-01	1.524E-01
DOHH	0.114	3.536E-01	4.492E-01
CNKSR2	0.114	8.288E-01	3.484E-02
NIPA2	0.113	2.334E-01	6.530E-02
TLN1	0.113	3.461E-01	3.886E-01
OGDH	0.113	1.818E-01	2.648E-01
BAG3	0.113	4.033E-01	1.300E-01
RUBCN	0.113	1.756E-01	9.996E-02
SPEM1	0.113	8.667E-01	6.968E-01
MAP3K20	0.113	5.818E-01	8.743E-01
FAM20A	0.113	7.551E-01	8.698E-01
SCG3	0.113	8.248E-01	1.515E-02
KCNS2	0.113	7.733E-01	3.325E-02
TRIT1	0.113	4.797E-01	6.253E-01

FITM2	0.113	3.735E-01	4.989E-02
CSRNP1	0.113	5.330E-01	5.445E-02
IFFO1	0.113	6.367E-01	2.482E-01
MED29	0.113	1.394E-01	4.601E-02
HLA-DQB1	0.113	7.649E-01	4.879E-02
ACSF2	0.113	6.384E-01	1.978E-02
THSD7B	0.113	8.450E-01	4.788E-01
NOL3	0.113	4.828E-01	1.521E-01
ZP1	0.113	7.892E-01	3.601E-01
CCSAP	0.113	3.735E-01	3.432E-01
FBXW12	0.112	7.589E-01	5.278E-02
ZMPSTE24	0.112	2.754E-01	1.106E-01
TUBGCP5	0.112	2.210E-01	1.074E-01
PDXK	0.112	3.743E-01	5.500E-01
YIPF3	0.112	1.977E-01	4.017E-01
DNAJC6	0.112	7.858E-01	5.839E-02
GLB1	0.112	2.456E-01	1.174E-01
SUPT5H	0.112	1.215E-01	1.464E-03
ZNF550	0.112	3.648E-01	9.158E-02
CD93	0.112	6.003E-01	4.212E-01
CYB561D2	0.112	3.723E-01	1.975E-02
WFS1	0.112	4.499E-01	1.468E-03
CA14	0.112	5.867E-01	1.938E-03
CYTL1	0.112	7.227E-01	5.081E-01
NAT10	0.112	3.219E-01	5.094E-01
KLHL20	0.112	2.669E-01	1.716E-01
H6PD	0.112	3.515E-01	1.046E-03
CCDC69	0.112	6.241E-01	3.206E-01
ZSWIM4	0.112	4.014E-01	1.555E-01
ATP7A	0.112	4.016E-01	1.717E-02
ZNF420	0.112	3.973E-01	8.295E-01
NPPA	0.112	7.138E-01	1.085E-02
C11orf24	0.112	3.572E-01	5.228E-01
GCC1	0.112	1.663E-01	2.794E-01
FOXE3	0.112	8.097E-01	9.501E-03
AC069368.1	0.112	7.391E-01	9.130E-01
PRKACG	0.112	NA	1.074E-03
HYAL3	0.112	5.194E-01	9.586E-02
CFAP57	0.112	7.085E-01	2.014E-01
FAM24A	0.111	NA	2.986E-01
GHRL	0.111	6.072E-01	2.269E-02
MB21D2	0.111	5.442E-01	6.524E-02
DPF1	0.111	7.325E-01	2.975E-03
KRT34	0.111	8.636E-01	1.451E-01
NEURL2	0.111	5.088E-01	6.371E-02
CILP2	0.111	7.614E-01	4.798E-01
COBLL1	0.111	6.269E-01	1.463E-01
SLC43A3	0.111	6.543E-01	4.873E-01
PRSS36	0.111	5.556E-01	4.645E-01
TM9SF4	0.111	2.159E-01	4.125E-01

RSPO4	0.111	8.459E-01	1.842E-02
FAM26D	0.111	8.971E-01	2.428E-02
AC012651.1	0.111	6.819E-01	2.547E-01
MFSD14B	0.111	2.558E-01	4.666E-01
DNAJB5	0.111	6.637E-01	2.359E-02
MIR4799	0.111	NA	7.836E-01
AC008763.2	0.111	7.946E-01	1.229E-01
WNT5B	0.111	7.660E-01	1.660E-02
EPRS	0.111	2.206E-01	8.106E-01
C8G	0.111	6.417E-01	3.504E-01
ATP6V1F	0.111	1.760E-01	5.954E-02
BMF	0.111	5.731E-01	2.117E-02
CNNM4	0.111	3.863E-01	1.224E-02
HM13	0.110	2.338E-01	1.915E-01
SLC22A4	0.110	5.829E-01	9.615E-02
PRKACA	0.110	1.001E-01	2.475E-03
HMGCL	0.110	2.746E-01	3.551E-02
AC005832.4	0.110	8.319E-01	5.064E-03
GINS4	0.110	5.706E-01	3.990E-01
SPI1	0.110	7.326E-01	1.724E-01
C17orf51	0.110	6.281E-01	3.454E-01
BOLA1	0.110	4.847E-01	4.689E-01
TMED4	0.110	1.986E-01	6.140E-01
OR7E24	0.110	NA	3.805E-01
OPTN	0.110	5.416E-01	9.082E-01
›6V1G2-DDX	0.110	NA	7.771E-01
FLRT2	0.110	7.857E-01	5.667E-01
IFNAR1	0.110	1.118E-01	1.497E-02
ARPIN	0.110	2.920E-01	4.556E-02
FOXRED2	0.110	5.516E-01	1.021E-02
EGLN1	0.110	2.195E-01	3.590E-01
SNAPC2	0.110	2.843E-01	2.501E-02
WBP1L	0.110	1.876E-01	2.550E-02
SDC1	0.110	6.273E-01	6.198E-01
MAGEA6	0.110	9.033E-01	4.202E-01
ENAH	0.110	4.535E-01	7.269E-01
C8orf37	0.110	4.130E-01	3.596E-02
EFCAB10	0.110	5.449E-01	3.393E-01
PDCD6IP	0.110	1.368E-01	7.304E-03
ZNF576	0.110	2.237E-01	6.209E-01
TSC22D2	0.110	4.340E-01	5.235E-01
GRK1	0.110	8.734E-01	7.094E-02
KLHDC10	0.110	1.458E-01	2.835E-01
GTPBP4	0.109	2.634E-01	3.936E-01
FANCC	0.109	4.466E-01	8.178E-02
RHEBL1	0.109	5.902E-01	3.947E-01
SPRY4	0.109	6.043E-01	6.992E-02
ARPC1B	0.109	4.301E-01	3.359E-01
CCDC63	0.109	8.706E-01	1.442E-04
STRN	0.109	2.324E-01	1.808E-01

REC114	0.109	7.816E-01	1.936E-01
MYH9	0.109	3.043E-01	7.018E-01
VKORC1L1	0.109	2.221E-01	8.209E-03
LAMTOR2	0.109	3.863E-01	3.733E-01
OR2M4	0.109	NA	4.149E-01
NATD1	0.109	4.734E-01	1.983E-01
EID3	0.109	6.724E-01	4.316E-01
C5orf42	0.109	5.070E-01	7.227E-01
ABCA8	0.109	7.909E-01	1.722E-02
GCNT4	0.109	7.441E-01	1.198E-01
RABGEF1	0.109	3.493E-01	9.522E-01
PHKA1	0.109	4.920E-01	9.339E-01
NGEF	0.109	7.209E-01	9.286E-01
MTA3	0.109	1.760E-01	2.627E-01
VRK3	0.109	1.351E-01	1.941E-01
DDI1	0.109	NA	1.345E-01
FICD	0.109	2.525E-01	9.147E-02
OR5C1	0.109	NA	8.131E-01
MPZ	0.109	6.594E-01	2.929E-01
CAPZB	0.109	1.048E-01	4.519E-01
CEP55	0.109	5.392E-01	2.680E-01
ARL3	0.108	2.970E-01	7.419E-01
GBF1	0.108	1.132E-01	3.499E-02
PALMD	0.108	7.171E-01	8.168E-02
FAM220A	0.108	2.086E-01	1.025E-02
TMED5	0.108	2.848E-01	1.494E-01
NPBWR1	0.108	8.450E-01	2.603E-01
ZNF488	0.108	7.904E-01	1.192E-01
AC011455.3	0.108	NA	4.615E-01
PIFO	0.108	7.696E-01	4.237E-03
ITPRIP	0.108	5.541E-01	3.715E-02
AC024592.3	0.108	NA	3.756E-01
MED1	0.108	3.558E-01	1.249E-01
SLC29A1	0.108	6.250E-01	3.237E-02
APPL1	0.108	2.647E-01	4.698E-01
TTC9C	0.108	1.335E-01	7.443E-02
GLRX	0.108	6.609E-01	2.811E-01
BEX3	0.108	4.306E-01	3.346E-02
TTC21A	0.108	5.747E-01	1.244E-01
BABAM2	0.108	2.444E-01	5.469E-03
LCE1B	0.108	9.105E-01	5.411E-02
:AB4B-EGLN	0.107	7.173E-01	9.868E-04
NUDT5	0.107	3.079E-01	7.852E-02
TMEM50A	0.107	5.781E-02	3.560E-02
CC2D2A	0.107	4.686E-01	2.084E-01
HLX	0.107	5.991E-01	2.922E-02
KIAA1683	0.107	7.442E-01	1.403E-01
MYO5A	0.107	5.889E-01	1.164E-02
PRC1	0.107	5.137E-01	7.933E-01
RAD54L	0.107	5.331E-01	1.751E-01

SCRN1	0.107	5.995E-01	3.966E-01
HEXA	0.107	3.147E-01	2.007E-02
NPIPB8	0.107	8.614E-01	2.445E-01
SHISA7	0.107	7.973E-01	5.177E-01
BARD1	0.107	4.384E-01	2.559E-02
PEX11B	0.107	1.816E-01	2.514E-01
GABARAPL2	0.107	1.384E-01	3.179E-03
C17orf58	0.107	3.793E-01	1.261E-02
BPHL	0.107	4.289E-01	2.050E-01
C1D	0.107	1.050E-01	8.511E-01
SLC37A3	0.107	1.515E-01	6.208E-01
MIR548AM	0.107	NA	7.813E-01
BACE2	0.107	5.038E-01	1.892E-01
TRPC7	0.107	8.711E-01	4.565E-01
EEFSEC	0.107	2.556E-01	1.043E-03
C10orf105	0.107	7.492E-01	6.006E-02
COMMD7	0.107	3.433E-01	4.779E-02
AC002996.1	0.107	NA	2.541E-02
RNF180	0.107	6.920E-01	5.598E-02
AARS	0.107	2.931E-01	8.836E-01
MIR6813	0.107	8.602E-01	3.052E-01
MBOAT7	0.107	4.876E-01	7.184E-02
GPR173	0.106	7.295E-01	5.009E-02
FAM221A	0.106	6.410E-01	3.302E-02
LSM14A	0.106	1.625E-01	1.896E-03
PFKP	0.106	5.670E-01	1.916E-01
PGRMC1	0.106	2.542E-01	1.249E-01
C1GALT1C1	0.106	2.109E-01	1.942E-02
COPS6	0.106	1.799E-01	9.948E-02
MAP4K4	0.106	5.012E-01	6.355E-01
RYBP	0.106	3.600E-01	1.202E-02
LRRC1	0.106	4.148E-01	5.867E-01
CCDC168	0.106	7.264E-01	1.703E-02
TMEM174	0.106	NA	3.261E-02
MED13	0.106	2.314E-01	1.575E-01
PCNA	0.106	4.284E-01	2.621E-02
CYP17A1	0.106	7.510E-01	1.507E-01
ZNF670	0.106	4.160E-01	1.995E-01
PSIP1	0.106	5.726E-01	1.013E-01
CYP1B1	0.106	8.054E-01	2.100E-03
PHF21B	0.106	8.462E-01	2.810E-01
EZR	0.106	3.874E-01	9.352E-01
RTFDC1	0.106	8.741E-02	6.611E-03
PPT1	0.106	4.257E-01	5.031E-03
TMED9	0.106	2.162E-01	6.867E-01
ZNF135	0.106	7.690E-01	1.313E-02
ZNF844	0.106	5.455E-01	1.255E-01
DARS2	0.106	4.216E-01	2.473E-03
PXDNL	0.106	6.938E-01	5.121E-01
SMC3	0.106	3.555E-01	2.829E-01

DCAF13	0.105	3.880E-01	5.128E-01
KIAA0825	0.105	4.924E-01	5.316E-02
UBA1	0.105	1.678E-01	9.510E-02
PTTG1IP	0.105	3.003E-01	4.222E-01
EMC1	0.105	1.995E-01	1.586E-02
TRIM45	0.105	5.672E-01	2.942E-01
KIDINS220	0.105	3.340E-01	5.228E-04
CD82	0.105	6.795E-01	7.109E-01
TM9SF2	0.105	2.618E-01	4.991E-01
ZNF114	0.105	8.245E-01	6.513E-01
ITGA9	0.105	6.348E-01	3.974E-01
NME7	0.105	4.077E-01	3.260E-01
CDH1	0.105	6.262E-01	3.197E-02
PARG	0.105	2.346E-01	4.158E-02
DBP	0.105	6.030E-01	9.185E-02
SLC26A11	0.105	4.577E-01	1.001E-01
ARIH1	0.105	1.585E-01	6.495E-01
TMEM125	0.105	7.385E-01	1.029E-01
SMS	0.105	4.729E-01	5.880E-01
FGGY	0.105	4.919E-01	5.286E-01
TRAPPC6A	0.105	5.996E-01	3.533E-01
LSS	0.105	4.361E-01	4.213E-01
ST7	0.105	1.491E-01	4.613E-01
FAXC	0.105	7.280E-01	3.788E-02
RPN2	0.105	2.734E-01	1.074E-02
TPPP	0.105	7.612E-01	2.582E-02
KCNE2	0.105	6.637E-01	2.231E-02
AGBL1	0.105	NA	9.559E-02
DSCAML1	0.105	8.162E-01	8.770E-03
SPNS1	0.105	3.288E-01	5.837E-01
C1orf216	0.105	3.793E-01	6.178E-01
C19orf73	0.105	5.676E-01	3.135E-01
MEIS2	0.105	6.116E-01	3.121E-02
SLC4A1AP	0.104	9.606E-02	2.547E-03
APOF	0.104	7.992E-01	6.673E-02
HDGF	0.104	2.746E-01	5.194E-01
KPRP	0.104	8.880E-01	5.467E-01
TMED2	0.104	1.739E-01	7.630E-02
ATP13A5	0.104	8.839E-01	6.493E-01
CCDC8	0.104	7.867E-01	6.965E-02
TMEM25	0.104	5.832E-01	8.914E-02
PDE1C	0.104	7.759E-01	5.796E-03
JPT2	0.104	2.051E-01	4.049E-01
B3GALT2	0.104	7.696E-01	3.415E-01
ARSG	0.104	4.593E-01	1.550E-01
VAPA	0.104	2.262E-01	1.385E-01
HTRA4	0.104	7.827E-01	7.028E-02
OGG1	0.104	4.047E-01	4.071E-01
GRXCR1	0.104	NA	1.163E-01
OR10W1	0.104	NA	5.911E-03

GTSF1L	0.104	8.541E-01	1.871E-01
LRRC58	0.104	3.520E-01	7.195E-02
KIAA0355	0.104	2.558E-01	2.314E-01
MAP3K3	0.104	3.178E-01	1.709E-01
ZNF701	0.104	4.868E-01	5.295E-02
DLEU7	0.104	6.732E-01	2.518E-02
PCDHGB6	0.104	7.002E-01	3.015E-01
OR52N1	0.103	NA	7.985E-02
MIR4652	0.103	NA	2.959E-01
METTL4	0.103	3.671E-01	3.561E-01
PEX13	0.103	3.958E-01	1.610E-02
MIR4525	0.103	NA	1.390E-02
C7orf33	0.103	9.441E-01	3.671E-02
TCTN2	0.103	3.177E-01	2.904E-02
RNPUL2-BS(	0.103	4.980E-01	1.024E-01
ALG3	0.103	2.545E-01	2.086E-02
DONSON	0.103	4.686E-01	6.398E-03
C5AR2	0.103	7.559E-01	1.904E-01
LSR	0.103	5.257E-01	5.426E-02
FEZ1	0.103	7.321E-01	9.516E-01
ZNF665	0.103	5.732E-01	9.466E-01
CD276	0.103	4.093E-01	1.128E-01
POC1A	0.103	5.089E-01	1.698E-01
RSU1	0.103	3.294E-01	8.655E-01
PRSS16	0.103	6.235E-01	1.622E-02
CPTP	0.103	3.582E-01	4.776E-02
ARL8A	0.103	1.946E-01	1.599E-01
UGT8	0.102	8.660E-01	4.225E-01
CEP112	0.102	6.543E-01	3.964E-02
FN3KRP	0.102	2.597E-01	1.479E-02
TSACC	0.102	5.855E-01	3.501E-01
RAMP3	0.102	6.657E-01	1.041E-02
HYDIN	0.102	8.053E-01	1.714E-01
RASEF	0.102	7.295E-01	1.668E-01
GPR52	0.102	8.088E-01	1.808E-02
SLC25A40	0.102	3.198E-01	2.821E-02
DET1	0.102	3.796E-01	8.014E-01
AHR	0.102	6.118E-01	2.160E-01
IFT46	0.102	2.688E-01	6.694E-03
SCGB2B2	0.102	6.356E-01	3.280E-01
PADI3	0.102	8.570E-01	5.583E-01
PNMA6A	0.102	8.086E-01	3.264E-02
SEMA4F	0.102	5.312E-01	2.071E-02
MIR3178	0.102	8.932E-01	3.644E-02
PRRT3	0.102	6.337E-01	3.184E-02
TTC26	0.102	3.558E-01	3.731E-02
GLG1	0.102	3.228E-01	5.479E-01
PIGX	0.102	3.500E-01	4.763E-02
UBE2A	0.102	1.900E-01	2.634E-01
CCDC107	0.102	4.729E-01	1.929E-02

HAUS4	0.102	3.489E-01	1.736E-01
SRGAP1	0.102	5.310E-01	8.242E-03
KRCC1	0.102	4.921E-01	7.547E-03
MITF	0.102	6.903E-01	5.161E-01
TTC3	0.102	3.356E-01	3.019E-01
IFT52	0.102	3.550E-01	3.683E-01
AKAP9	0.102	3.558E-01	9.095E-01
FHOD3	0.102	7.217E-01	4.228E-01
TMEM42	0.102	3.155E-01	2.307E-02
MS4A6E	0.102	8.615E-01	2.279E-03
CEP70	0.102	4.149E-01	3.117E-01
PWWP2B	0.101	5.895E-01	2.830E-03
EFCAB2	0.101	5.386E-01	7.131E-01
MIR6801	0.101	8.715E-01	2.374E-02
MIR3691	0.101	NA	1.641E-02
WAC	0.101	1.195E-01	5.138E-02
CDS2	0.101	2.141E-01	2.082E-01
WWP1	0.101	4.060E-01	3.418E-03
TBC1D24	0.101	3.027E-01	5.465E-01
PYGO2	0.101	2.051E-01	8.845E-01
KCNA5	0.101	7.851E-01	5.013E-01
PELI1	0.101	4.786E-01	5.446E-02
CIZ1	0.101	2.185E-01	8.086E-02
SLC11A2	0.101	3.770E-01	6.187E-02
KCTD7	0.101	4.489E-01	4.511E-01
LIPJ	0.101	7.745E-01	2.994E-02
MAGEB17	0.101	8.645E-01	2.602E-02
RNF208	0.101	6.430E-01	5.561E-03
DTNB	0.101	5.690E-01	3.953E-01
CDYL	0.101	2.976E-01	3.364E-02
PLSCR2	0.101	8.249E-01	4.683E-01
ZCCHC17	0.101	1.075E-01	1.079E-01
SEMA3E	0.101	8.201E-01	5.199E-02
ZNF845	0.101	2.931E-01	7.290E-02
SMCHD1	0.101	4.317E-01	1.446E-01
CSTL1	0.100	8.269E-01	1.274E-01
ROCK2	0.100	2.356E-01	2.736E-02
CDC42EP1	0.100	6.396E-01	6.148E-02
RAI2	0.100	7.300E-01	2.815E-01
MFSD12	0.100	5.024E-01	6.381E-01
CCIN	0.100	8.092E-01	1.399E-01
RGS3	0.100	4.082E-01	4.558E-02
FADS3	0.100	7.177E-01	9.901E-03
SOGA1	0.100	4.467E-01	4.550E-02
PDIA5	0.100	4.243E-01	3.398E-02
OR56A4	0.100	NA	1.133E-01
MIR4635	0.100	8.096E-01	1.156E-03
ANGPTL4	0.100	7.754E-01	5.925E-02
PBLD	0.100	4.954E-01	6.268E-01
HS1BP3	0.100	2.612E-01	6.577E-01

PHGDH	0.100	6.910E-01	1.531E-01
MEF2D	0.100	2.140E-01	6.562E-01
GOLGA6B	0.100	NA	1.025E-01
KRT36	0.100	8.192E-01	6.885E-01
SLC9A5	0.100	6.664E-01	8.418E-01
CRELD1	0.100	4.256E-01	6.508E-01
SLC29A2	0.099	6.496E-01	4.768E-02
ZC3H12C	0.099	6.137E-01	1.019E-02
TUBA1B	0.099	4.715E-01	9.870E-02
MIR644A	0.099	NA	9.348E-02
NAT14	0.099	6.114E-01	3.161E-02
PDAP1	0.099	1.770E-01	4.979E-01
ARHGAP15	0.099	7.443E-01	1.773E-01
CLCC1	0.099	1.856E-01	5.996E-02
WDR45B	0.099	2.580E-01	9.963E-02
C16orf86	0.099	6.025E-01	2.277E-02
LYZL4	0.099	8.880E-01	1.576E-02
GABRG3	0.099	8.884E-01	3.921E-02
HERC5	0.099	7.544E-01	6.140E-01
FNBP1	0.099	5.622E-01	1.048E-01
TUBB6	0.099	7.722E-01	3.689E-02
FAM3C	0.099	5.816E-01	2.019E-02
GK2	0.099	NA	4.203E-01
TIGD7	0.099	5.696E-01	9.759E-03
OR4C6	0.099	NA	3.340E-01
MYL12A	0.099	3.711E-01	5.238E-02
MEA1	0.099	3.355E-01	4.906E-02
PTGFR	0.099	8.283E-01	4.253E-01
PLGRKT	0.098	5.749E-01	4.170E-01
LHX3	0.098	8.998E-01	2.393E-01
ARHGAP24	0.098	6.860E-01	4.308E-01
PSMC2	0.098	1.257E-01	3.106E-01
INPP5F	0.098	3.493E-01	7.818E-02
C16orf62	0.098	4.726E-01	3.399E-02
RBM42	0.098	2.964E-01	2.114E-01
FP565260.6	0.098	6.501E-01	2.944E-02
FAM151B	0.098	4.244E-01	5.412E-02
AC010522.1	0.098	6.949E-01	5.020E-02
MIR3125	0.098	8.858E-01	9.468E-01
SYDE2	0.098	6.274E-01	9.375E-01
SLC36A4	0.098	6.431E-01	7.635E-01
PDE2A	0.098	6.874E-01	1.906E-01
ENO3	0.098	6.335E-01	2.458E-02
GANAB	0.098	1.972E-01	9.921E-02
KCTD10	0.098	1.910E-01	5.807E-03
MMP14	0.098	6.127E-01	3.581E-01
TLE2	0.098	7.568E-01	7.649E-02
OAZ3	0.098	5.745E-01	1.076E-01
AMOTL1	0.098	6.516E-01	1.935E-01
IQSEC2	0.098	4.715E-01	1.520E-02

NMNAT1	0.098	3.169E-01	3.741E-01
PRELID3A	0.098	7.402E-01	1.862E-01
GTF2I	0.098	5.785E-01	2.085E-02
RNF24	0.098	4.800E-01	1.434E-01
PTPRU	0.098	6.599E-01	3.134E-02
TOR1AIP1	0.098	2.414E-01	2.211E-01
GBGT1	0.098	6.655E-01	1.819E-01
USP32	0.098	4.087E-01	3.898E-01
MED19	0.098	2.694E-01	5.853E-01
SIK3	0.098	2.794E-01	7.782E-01
CCDC149	0.098	4.822E-01	1.368E-02
HECTD3	0.098	2.149E-01	1.659E-01
B3GALT6	0.098	4.237E-01	2.254E-01
BOK	0.098	5.793E-01	2.141E-01
ELK1	0.097	2.635E-01	6.035E-02
FREM1	0.097	8.217E-01	3.765E-02
QRFP	0.097	6.599E-01	9.020E-02
ATP6V1A	0.097	3.085E-01	3.927E-02
ATP6AP2	0.097	2.043E-01	3.903E-01
THBS3	0.097	5.990E-01	9.113E-01
LIPG	0.097	7.972E-01	1.101E-01
TCF23	0.097	8.603E-01	8.918E-01
LAMC3	0.097	7.353E-01	1.680E-01
DSN1	0.097	4.225E-01	8.897E-03
PDS5B	0.097	3.556E-01	3.277E-01
LIPA	0.097	5.533E-01	3.171E-01
REL	0.097	7.010E-01	1.025E-01
SAP25	0.097	7.492E-01	9.654E-02
ZNF350	0.097	6.324E-01	3.406E-01
CRTC3	0.097	1.814E-01	3.720E-01
LMAN1	0.097	3.524E-01	3.862E-01
CCDC6	0.097	2.937E-01	6.332E-01
TEC	0.097	5.162E-01	4.709E-01
HEG1	0.097	6.708E-01	2.151E-01
SUMF2	0.097	4.422E-01	4.382E-01
PRICKLE1	0.097	7.745E-01	8.184E-01
DMPK	0.097	5.228E-01	5.199E-01
STK19	0.097	2.421E-01	1.458E-02
ABL2	0.097	4.374E-01	6.629E-01
MARVELD1	0.097	6.462E-01	3.352E-03
LRRC63	0.097	8.174E-01	5.149E-01
SCGB1C1	0.097	NA	7.153E-02
FAM111B	0.097	6.777E-01	1.337E-01
P4HA2	0.097	5.651E-01	3.279E-02
CLCN4	0.096	7.172E-01	1.324E-02
MIR6127	0.096	NA	8.028E-01
EFNA4	0.096	4.934E-01	4.590E-01
CALR	0.096	2.650E-01	1.034E-01
MIRLET7C	0.096	NA	8.873E-02
MIR569	0.096	NA	1.173E-01

OR1I1	0.096	NA	1.665E-02
PTTG2	0.096	8.168E-01	1.095E-02
ZNF32	0.096	4.148E-01	4.888E-01
FEZ2	0.096	2.551E-01	3.117E-01
SH3GL2	0.096	8.903E-01	7.546E-01
GPR50	0.096	9.380E-01	3.905E-02
UBE2G1	0.096	3.154E-01	7.301E-03
CYP51A1	0.096	6.272E-01	8.305E-02
GRIN2D	0.096	8.055E-01	5.115E-01
TOMM7	0.096	3.841E-01	3.079E-01
AL117348.2	0.096	8.731E-01	9.838E-03
SWI5	0.096	4.076E-01	3.436E-01
RNF38	0.096	3.378E-01	8.887E-02
SLC1A4	0.096	6.657E-01	1.901E-03
UHRF1BP1L	0.096	4.301E-01	1.039E-01
C4orf45	0.096	8.815E-01	3.470E-01
RABAC1	0.096	5.104E-01	6.093E-01
RGS20	0.096	8.186E-01	9.388E-01
CCP110	0.096	5.516E-01	9.265E-01
CXCL2	0.096	8.333E-01	4.579E-01
ZNF425	0.096	5.956E-01	6.901E-01
QPCTL	0.096	4.744E-01	1.384E-01
CKAP5	0.096	3.971E-01	2.967E-01
FAM153A	0.095	8.535E-01	2.707E-01
TCTE1	0.095	7.877E-01	1.239E-01
POLR2I	0.095	4.475E-01	8.468E-01
LGR6	0.095	8.446E-01	5.510E-01
SLC22A13	0.095	7.247E-01	8.812E-01
LPAR5	0.095	6.030E-01	8.598E-01
AC091959.3	0.095	7.677E-01	8.555E-01
PAM	0.095	6.310E-01	4.239E-03
UBXN2A	0.095	2.551E-01	2.261E-01
MRPS14	0.095	1.814E-01	3.321E-01
RUSC1	0.095	2.986E-01	5.005E-01
SCGB3A2	0.095	8.623E-01	3.878E-01
CALM1	0.095	2.583E-01	1.320E-02
ORM2	0.095	8.675E-01	1.356E-02
MAN1B1	0.095	3.955E-01	2.016E-01
CCZ1B	0.095	3.828E-01	8.427E-01
TMEM159	0.095	5.217E-01	1.775E-02
CHN1	0.095	6.579E-01	1.111E-02
PSENEN	0.095	4.644E-01	1.352E-02
GOLGA7	0.095	2.963E-01	1.254E-01
ZNF781	0.095	7.764E-01	2.456E-01
PREX2	0.095	7.380E-01	6.158E-01
MIR4326	0.095	7.827E-01	3.578E-01
SLC2A1	0.095	7.380E-01	2.703E-01
RAB18	0.095	2.519E-01	1.283E-01
NACC2	0.095	5.920E-01	5.856E-01
EMC2	0.095	2.526E-01	5.064E-01

SNX17	0.095	1.615E-01	1.007E-01
CA5B	0.095	4.121E-01	4.611E-02
PIH1D2	0.095	4.835E-01	3.632E-01
RRAGA	0.095	3.489E-01	4.181E-01
GALK2	0.095	3.499E-01	5.771E-02
C5orf52	0.094	8.888E-01	1.007E-01
RLF	0.094	4.715E-01	4.504E-01
BRI3	0.094	4.251E-01	5.241E-02
LNP1	0.094	5.960E-01	4.861E-03
YIPF1	0.094	2.985E-01	9.300E-01
DDX10	0.094	3.189E-01	1.103E-01
CARD19	0.094	4.931E-01	6.776E-01
SP6	0.094	7.867E-01	3.535E-01
BEX2	0.094	7.402E-01	2.175E-01
PLXNC1	0.094	7.011E-01	7.407E-01
TMEM150A	0.094	4.351E-01	7.014E-02
ERAL1	0.094	3.408E-01	4.105E-02
SDF4	0.094	1.786E-01	4.892E-02
DHX8	0.094	1.533E-01	4.841E-01
CHPF	0.094	5.895E-01	7.243E-03
SLC51B	0.094	7.698E-01	1.602E-02
SNX33	0.094	3.714E-01	2.900E-01
MTMR14	0.094	3.678E-01	5.854E-02
CALM3	0.094	2.299E-01	3.049E-02
OTX1	0.094	6.807E-01	2.181E-01
ACTR1B	0.094	2.200E-01	6.305E-02
TRADD	0.094	4.517E-01	5.437E-04
OR6A2	0.094	NA	1.029E-01
PECR	0.094	6.511E-01	2.418E-02
DMWD	0.094	2.478E-01	3.107E-01
UBC	0.094	2.613E-01	1.045E-02
MFSD7	0.094	6.855E-01	6.840E-01
CCER2	0.094	7.874E-01	6.229E-02
SEC22C	0.093	3.408E-01	4.246E-01
AC008763.3	0.093	NA	2.934E-02
MIR3163	0.093	NA	1.285E-02
HRK	0.093	8.791E-01	6.729E-03
CSN2	0.093	NA	7.961E-04
ARPC1A	0.093	2.234E-01	2.690E-02
PTAR1	0.093	3.918E-01	4.201E-02
EGR2	0.093	7.758E-01	5.447E-02
SLC24A3	0.093	7.831E-01	3.004E-01
SARM1	0.093	6.599E-01	3.407E-02
BTBD8	0.093	6.179E-01	7.504E-01
TMEM167B	0.093	1.476E-01	5.017E-01
PMM2	0.093	5.187E-01	2.581E-01
WFDC5	0.093	8.861E-01	2.020E-02
RPGRIP1L	0.093	4.250E-01	6.427E-01
GMDS	0.093	4.765E-01	5.216E-02
FBXO10	0.093	5.855E-01	4.786E-02

UBR4	0.093	2.622E-01	2.939E-01
CCDC102A	0.093	6.476E-01	7.627E-03
LTBP3	0.093	5.782E-01	4.515E-01
ALOX15	0.093	8.292E-01	2.663E-01
NCOA3	0.093	5.032E-01	9.629E-01
ZFPL1	0.093	2.019E-01	9.623E-01
STIM1	0.093	4.210E-01	9.536E-01
ATP6V0B	0.093	3.620E-01	9.493E-01
MIR2116	0.093	8.317E-01	4.757E-01
ZBTB4	0.093	3.348E-01	1.298E-02
SLC9C1	0.093	7.402E-01	5.452E-01
CXCL12	0.093	8.040E-01	2.185E-01
MIR5692B	0.093	NA	8.619E-01
ASGR2	0.093	7.759E-01	1.793E-01
MIR8063	0.093	NA	1.743E-03
MIR1262	0.093	NA	4.803E-01
LYSMD1	0.093	4.145E-01	9.459E-03
E2F6	0.092	1.917E-01	8.545E-02
TIMM23B	0.092	3.525E-01	1.437E-01
CCDC152	0.092	7.931E-01	1.147E-01
HIST2H2AB	0.092	7.921E-01	3.591E-03
WASHC5	0.092	4.006E-01	6.736E-03
GUCY1A2	0.092	7.224E-01	1.966E-01
OTOGL	0.092	7.823E-01	4.914E-02
RIT1	0.092	4.064E-01	3.315E-02
MIR1301	0.092	NA	2.297E-02
PREB	0.092	2.516E-01	8.991E-02
TSPAN13	0.092	5.899E-01	5.161E-02
PCDHGA12	0.092	7.558E-01	8.557E-01
ZNF568	0.092	5.975E-01	1.752E-01
CD1E	0.092	8.184E-01	8.403E-02
SDCCAG8	0.092	2.613E-01	1.227E-01
MSANTD1	0.092	6.977E-01	2.207E-01
TRIP11	0.092	2.580E-01	1.107E-01
GATAD2B	0.092	2.696E-01	5.348E-01
NCOA6	0.092	3.139E-01	1.377E-01
HCRTR2	0.092	9.096E-01	3.379E-02
PGM3	0.092	3.414E-01	7.757E-01
FYN	0.092	6.722E-01	7.355E-02
C1QL3	0.092	7.547E-01	2.618E-01
FAM228B	0.092	5.436E-01	4.322E-02
SLC41A1	0.092	5.891E-01	3.906E-03
HECW2	0.091	6.300E-01	2.368E-01
RNF32	0.091	6.047E-01	8.701E-02
FSCN2	0.091	6.807E-01	4.834E-01
MIR548AJ2	0.091	NA	5.538E-03
OR2V2	0.091	NA	6.034E-02
MIR4319	0.091	NA	7.818E-01
COG6	0.091	4.242E-01	9.274E-01
KLRG2	0.091	8.391E-01	9.114E-01

GP6	0.091	8.191E-01	8.928E-02
VPS50	0.091	2.570E-01	1.507E-01
FAM72D	0.091	6.769E-01	7.985E-02
CDC123	0.091	3.256E-01	4.210E-02
LRCH4	0.091	4.415E-01	4.893E-01
MAGEC1	0.091	9.437E-01	5.271E-01
TSR2	0.091	2.718E-01	2.685E-01
KCNG2	0.091	7.869E-01	2.980E-03
PER1	0.091	6.826E-01	1.292E-01
AGPAT1	0.091	2.872E-01	2.748E-01
CHAF1B	0.091	5.981E-01	4.523E-01
PTP4A2	0.091	3.236E-01	5.903E-01
FP565260.1	0.091	7.084E-01	4.006E-02
PSMD7	0.091	3.080E-01	2.371E-01
ENG	0.091	5.989E-01	8.028E-02
SIGMAR1	0.091	4.968E-01	4.223E-01
SEC23A	0.091	5.157E-01	4.986E-03
LRTOMT	0.091	4.715E-01	2.370E-01
LGALS13	0.091	NA	1.927E-01
SLC22A23	0.091	6.714E-01	9.730E-02
C11orf95	0.091	5.089E-01	3.210E-01
LIMK1	0.091	4.137E-01	3.608E-01
VPS26B	0.091	1.681E-01	4.825E-02
MED22	0.091	4.195E-01	6.065E-01
GOLT1B	0.091	3.344E-01	1.759E-02
GRHPR	0.091	4.256E-01	1.469E-01
GNG8	0.091	8.203E-01	3.164E-02
USP48	0.091	1.719E-01	6.033E-04
SLC25A23	0.091	6.657E-01	1.091E-02
ENOX2	0.091	3.189E-01	4.255E-01
HDLBP	0.090	3.340E-01	4.331E-01
C9orf66	0.090	7.696E-01	2.063E-02
OR1G1	0.090	NA	3.394E-01
MIR5588	0.090	NA	3.775E-01
SLC25A51	0.090	4.629E-01	1.815E-01
PIK3CA	0.090	3.154E-01	1.426E-02
FMR1	0.090	3.403E-01	2.467E-02
WWC2	0.090	6.321E-01	5.941E-03
FXYD5	0.090	7.180E-01	7.227E-01
SIK2	0.090	3.276E-01	2.257E-02
DCTN1	0.090	2.634E-01	4.557E-01
TBC1D20	0.090	2.193E-01	9.637E-01
GSTM2	0.090	8.631E-01	9.637E-01
CLSTN1	0.090	5.047E-01	3.543E-02
PDK2	0.090	4.579E-01	8.732E-03
AC100868.1	0.090	NA	8.759E-01
ETV4	0.090	7.587E-01	8.727E-01
PPP5C	0.090	1.933E-01	1.153E-01
IGLON5	0.090	8.054E-01	1.169E-01
SIRPA	0.090	7.519E-01	3.436E-01

ADCY3	0.090	5.270E-01	4.194E-01
LRRC52	0.090	NA	6.134E-02
VWA7	0.090	7.060E-01	2.732E-02
SYCP1	0.090	9.229E-01	2.249E-01
ZNF674	0.090	2.890E-01	1.764E-01
TAF1L	0.090	8.604E-01	2.067E-01
CPEB1	0.090	8.254E-01	7.649E-03
MAP4	0.090	3.590E-01	2.891E-02
SFMBT2	0.090	7.290E-01	3.117E-01
QSOX1	0.090	5.516E-01	5.823E-02
OR8G1	0.090	NA	2.246E-01
PCDHB10	0.090	7.010E-01	4.773E-03
ZNF461	0.090	5.098E-01	1.553E-02
PPP2R5B	0.090	4.288E-01	1.846E-01
CLDN18	0.089	8.278E-01	4.220E-02
WDPCP	0.089	3.347E-01	8.121E-02
CCDC33	0.089	8.807E-01	6.483E-03
RAB6C	0.089	8.245E-01	2.164E-01
ZNF880	0.089	6.623E-01	2.000E-01
KCNJ16	0.089	8.882E-01	3.513E-01
PHC2	0.089	3.864E-01	5.670E-01
GRID2IP	0.089	7.333E-01	7.882E-01
LAGE3	0.089	5.673E-01	4.918E-02
GPR182	0.089	7.364E-01	3.970E-02
FBXO11	0.089	4.641E-01	5.023E-01
TAF6	0.089	2.794E-01	1.744E-02
TBL1XR1	0.089	4.582E-01	1.039E-01
KRTAP3-1	0.089	9.296E-01	3.423E-03
RXFP3	0.089	8.945E-01	1.139E-01
ZNF213	0.089	3.383E-01	2.862E-01
RSPH14	0.089	8.265E-01	1.382E-01
ITIH6	0.089	8.329E-01	9.293E-01
NFASC	0.089	7.598E-01	9.290E-01
C2orf74	0.089	7.789E-01	9.250E-01
RIPK2	0.089	5.271E-01	9.848E-03
GPKOW	0.089	2.750E-01	1.567E-01
COG5	0.089	3.676E-01	8.822E-02
TPST2	0.089	5.576E-01	7.053E-01
SLC25A4	0.089	5.179E-01	6.817E-03
RPIA	0.089	5.118E-01	5.532E-04
FNDC5	0.089	7.739E-01	6.411E-01
SEL1L	0.089	2.931E-01	1.678E-01
CUL2	0.089	2.450E-01	2.540E-01
CCNY	0.089	2.063E-01	3.243E-01
ZNF585A	0.089	4.773E-01	1.068E-01
BBS9	0.089	3.578E-01	2.025E-02
UPP2	0.089	8.369E-01	7.698E-03
ANO6	0.089	4.687E-01	5.524E-02
RAB6A	0.089	3.189E-01	9.253E-01
DYNLRB1	0.089	4.114E-01	1.703E-01

MCCC2	0.088	5.803E-01	1.483E-01
TPD52L2	0.088	2.924E-01	2.989E-01
MCMDC2	0.088	6.151E-01	8.352E-01
ZWILCH	0.088	4.070E-01	8.516E-02
KIF17	0.088	7.244E-01	6.406E-02
SEC11C	0.088	4.492E-01	1.299E-01
ALOX12	0.088	8.170E-01	1.177E-02
PLCD4	0.088	6.199E-01	4.953E-02
MAP3K1	0.088	5.778E-01	1.289E-01
COMTD1	0.088	6.686E-01	3.796E-01
DNAH5	0.088	8.088E-01	7.951E-02
PNMA8A	0.088	8.450E-01	3.638E-01
AGO1	0.088	2.980E-01	6.843E-01
GMPPB	0.088	3.238E-01	5.325E-01
MIR6871	0.088	NA	8.602E-01
ZNF260	0.088	4.789E-01	5.118E-01
ZBED6	0.088	8.015E-01	3.139E-01
NEURL3	0.088	8.577E-01	2.222E-02
SEMA3B	0.088	8.201E-01	2.849E-02
PCBP1	0.088	3.025E-01	1.241E-01
TRIM65	0.088	4.074E-01	3.381E-01
MAP3K8	0.088	6.428E-01	3.387E-03
ZNF619	0.088	4.153E-01	1.059E-01
USP39	0.088	1.810E-01	3.204E-01
RBM43	0.088	4.735E-01	1.260E-01
UBE2E1	0.088	2.772E-01	2.347E-02
EIF2AK1	0.088	2.038E-01	1.261E-02
TRAPPC5	0.088	6.126E-01	1.919E-01
ZSCAN18	0.088	7.441E-01	1.572E-02
C2orf16	0.088	6.149E-01	1.509E-01
INCA1	0.088	5.146E-01	2.522E-01
PARVA	0.088	5.237E-01	8.591E-01
CDH24	0.088	6.382E-01	4.124E-02
RHOD	0.088	7.810E-01	2.432E-01
DNMT3A	0.088	4.844E-01	9.215E-01
CYP2W1	0.088	8.858E-01	5.897E-01
C16orf70	0.087	3.377E-01	8.860E-01
PCSK1N	0.087	8.694E-01	8.825E-01
TMEM30A	0.087	3.540E-01	1.186E-01
TIPRL	0.087	3.116E-01	6.929E-02
XPO6	0.087	2.852E-01	1.644E-01
DNAJB11	0.087	3.258E-01	1.138E-01
MIR98	0.087	NA	3.587E-01
TMEM53	0.087	5.025E-01	1.856E-02
GADD45A	0.087	6.064E-01	1.001E-01
S100B	0.087	8.172E-01	5.745E-03
C9orf40	0.087	6.579E-01	7.427E-02
H1F0	0.087	6.402E-01	2.619E-02
GDI2	0.087	4.153E-01	7.444E-01
CHN2	0.087	7.539E-01	3.211E-02

UBE3C	0.087	3.403E-01	8.144E-03
HAS1	0.087	8.645E-01	9.730E-02
AL033529.1	0.087	8.531E-01	4.085E-01
DNAJC21	0.087	3.589E-01	4.214E-02
CENPX	0.087	5.272E-01	2.381E-02
SUMF1	0.087	5.271E-01	5.400E-01
EIF4EBP2	0.087	3.251E-01	7.889E-01
LPL	0.087	8.055E-01	1.354E-01
RPA3	0.087	5.010E-01	2.378E-01
MIR4530	0.087	8.906E-01	1.834E-01
PANX1	0.087	6.118E-01	3.665E-02
OLFML2B	0.087	7.879E-01	2.265E-01
GDPD1	0.087	6.582E-01	2.491E-01
MCM2	0.087	6.368E-01	8.503E-01
DNAJB13	0.087	8.582E-01	7.509E-01
RUNDC3B	0.087	7.326E-01	1.168E-01
PLIN3	0.087	5.088E-01	5.249E-02
MIR1972-2	0.087	NA	1.182E-01
RAB33B	0.087	4.150E-01	4.126E-01
IFITM2	0.087	7.415E-01	9.666E-03
EPN1	0.087	3.736E-01	2.242E-01
GNG11	0.087	7.062E-01	1.305E-01
MIR660	0.087	NA	9.431E-01
PTPN3	0.087	5.442E-01	9.389E-01
RBBP7	0.086	3.348E-01	9.269E-01
MIR4312	0.086	8.910E-01	1.511E-02
TMEM241	0.086	6.336E-01	5.343E-01
INPP5B	0.086	3.441E-01	3.926E-01
SYNE2	0.086	6.128E-01	4.783E-01
RFC2	0.086	4.398E-01	3.176E-01
HLA-DQB2	0.086	8.234E-01	4.627E-01
SLC25A32	0.086	4.535E-01	4.240E-01
PLPP3	0.086	6.122E-01	4.633E-01
SERINC3	0.086	3.708E-01	1.355E-01
SOCS6	0.086	5.331E-01	3.626E-02
DUSP21	0.086	NA	5.901E-01
CKS1B	0.086	5.985E-01	4.267E-02
LEPROT	0.086	3.637E-01	8.464E-01
SH3GL1	0.086	3.292E-01	4.412E-02
CTIF	0.086	4.833E-01	2.526E-01
CDC42	0.086	1.756E-01	3.646E-01
NCMAP	0.086	8.513E-01	5.080E-01
SSU72	0.086	2.999E-01	2.925E-03
AC245041.1	0.086	8.903E-01	5.314E-02
CDC5L	0.086	3.232E-01	4.396E-02
ZNF571	0.086	5.896E-01	3.220E-01
INSM2	0.086	8.815E-01	4.326E-02
TTC30A	0.086	6.134E-01	5.249E-02
SMC2	0.086	6.143E-01	6.737E-01
SOCS3	0.086	7.336E-01	1.093E-01

SLC41A3	0.086	3.465E-01	1.417E-02
CNOT3	0.086	2.728E-01	4.475E-01
HSPA1L	0.086	4.932E-01	5.282E-01
ATF3	0.085	7.946E-01	2.719E-02
CFDP1	0.085	4.082E-01	4.563E-01
GPR4	0.085	6.638E-01	1.191E-01
MXRA5	0.085	8.061E-01	5.096E-02
AC022384.1	0.085	NA	6.343E-01
PDXDC1	0.085	4.497E-01	4.769E-01
PGM2	0.085	5.436E-01	4.444E-02
DPH3	0.085	4.020E-01	8.926E-01
PAGR1	0.085	4.897E-01	2.130E-01
SPOCK2	0.085	7.874E-01	3.601E-02
WDTC1	0.085	3.384E-01	2.818E-02
CARD14	0.085	7.342E-01	7.620E-01
GLCCI1	0.085	6.988E-01	7.041E-01
CDH2	0.085	8.598E-01	3.570E-01
SLC38A3	0.085	8.529E-01	1.374E-01
MAMSTR	0.085	6.972E-01	2.889E-01
ZNF541	0.085	8.436E-01	5.411E-04
PLXDC1	0.085	7.026E-01	2.013E-01
CHPF2	0.085	4.145E-01	3.487E-01
AP3M1	0.085	3.696E-01	1.694E-02
P3H1	0.085	6.137E-01	5.652E-03
MIR1273G	0.085	NA	4.003E-01
ILF2	0.085	3.735E-01	2.987E-02
SSUH2	0.085	7.633E-01	5.490E-01
AC079594.2	0.084	8.560E-01	3.230E-02
FCRLB	0.084	8.488E-01	3.752E-01
CASTOR2	0.084	6.272E-01	1.373E-02
MOSPD3	0.084	4.802E-01	5.111E-01
HCFC1R1	0.084	5.629E-01	2.371E-01
CERS4	0.084	8.172E-01	2.449E-01
ZFP57	0.084	9.038E-01	6.372E-01
EEA1	0.084	5.545E-01	6.839E-01
FAAP24	0.084	4.798E-01	1.139E-01
CREG1	0.084	5.935E-01	2.638E-01
USP1	0.084	5.792E-01	9.894E-02
COCH	0.084	8.438E-01	2.824E-02
TMEM99	0.084	5.282E-01	8.545E-02
PPP1R1A	0.084	8.761E-01	2.174E-02
SLC26A10	0.084	8.007E-01	3.515E-02
ZDHHC20	0.084	5.407E-01	1.611E-02
LTBP1	0.084	7.300E-01	6.271E-01
OVOL2	0.084	7.152E-01	2.331E-02
RNF26	0.084	4.355E-01	8.654E-02
RDH14	0.084	2.327E-01	4.513E-01
SYNJ1	0.084	4.822E-01	9.049E-01
CC1L-ADOR	0.084	8.033E-01	9.246E-01
NKIRAS2	0.084	2.042E-01	1.899E-01

TMEM50B	0.084	4.588E-01	1.541E-02
ACVR2A	0.084	5.964E-01	5.347E-02
ST3GAL6	0.084	7.586E-01	1.445E-01
MLH1	0.084	2.921E-01	1.124E-01
SASH1	0.084	6.298E-01	6.392E-01
COL18A1	0.084	7.244E-01	2.707E-02
C21orf58	0.084	6.706E-01	9.946E-02
BRSK2	0.084	8.601E-01	5.828E-01
SPACA6	0.084	7.114E-01	3.297E-01
TMBIM1	0.084	6.519E-01	6.740E-01
FAM131B	0.084	8.184E-01	2.458E-01
GPC4	0.084	7.960E-01	2.953E-01
MECR	0.084	3.430E-01	3.405E-02
POTEE	0.084	8.579E-01	7.200E-02
CA11	0.084	7.171E-01	9.656E-01
SLC35B2	0.084	4.118E-01	9.656E-01
ADAMTS10	0.084	7.464E-01	9.571E-01
TIMMDC1	0.084	2.701E-01	9.445E-01
HAGH	0.083	4.218E-01	9.402E-01
C9orf64	0.083	4.663E-01	9.402E-01
PPP1R15B	0.083	3.996E-01	3.950E-02
OSTC	0.083	3.021E-01	6.400E-01
[ASEK-C17or	0.083	5.979E-01	6.295E-02
VSIG10	0.083	6.702E-01	1.109E-01
ZBTB37	0.083	5.426E-01	8.229E-01
KCNV1	0.083	9.479E-01	4.902E-02
CAPNS1	0.083	3.904E-01	1.555E-02
UBE2H	0.083	3.567E-01	5.711E-02
MIR6077	0.083	NA	7.060E-02
DUSP26	0.083	8.354E-01	4.707E-02
C1orf210	0.083	7.247E-01	6.502E-02
GPR85	0.083	7.738E-01	5.006E-01
MBOAT1	0.083	7.941E-01	8.909E-02
MIR4669	0.083	9.133E-01	1.458E-01
ATP8B3	0.083	7.675E-01	2.714E-01
HK3	0.083	8.402E-01	4.152E-02
ANK1	0.083	8.404E-01	5.089E-02
AC025283.2	0.083	7.903E-01	1.125E-01
TBC1D23	0.083	3.137E-01	4.082E-01
MIR574	0.083	8.741E-01	1.442E-01
GPN1	0.083	3.056E-01	5.102E-03
OR2V1	0.083	NA	4.151E-01
TTC29	0.083	9.164E-01	1.180E-02
BPIFA3	0.083	NA	2.573E-01
CDC73	0.083	4.445E-01	1.339E-02
TMEM248	0.083	1.108E-01	4.354E-01
EVI5	0.083	3.427E-01	1.084E-02
PRKAR2A	0.083	4.476E-01	4.069E-01
TFG	0.082	3.714E-01	2.743E-02
PEF1	0.082	2.252E-01	3.876E-01

TOM1	0.082	5.025E-01	4.407E-01
CERS1	0.082	8.655E-01	8.582E-03
INSIG2	0.082	4.582E-01	2.907E-02
PYGB	0.082	6.387E-01	7.546E-02
RSRC1	0.082	4.204E-01	1.355E-02
CACNB3	0.082	6.408E-01	5.768E-01
ZNF285	0.082	7.619E-01	2.476E-01
LILRB5	0.082	8.401E-01	3.001E-01
TYMS	0.082	6.884E-01	2.008E-02
YPEL1	0.082	7.909E-01	7.625E-02
PRIM1	0.082	6.124E-01	4.072E-01
THTPA	0.082	5.299E-01	1.446E-01
SPRYD3	0.082	3.460E-01	4.837E-01
DDIT3	0.082	6.609E-01	7.056E-01
RTP1	0.082	9.180E-01	1.521E-01
SNAPIN	0.082	2.895E-01	5.096E-03
KBTBD4	0.082	4.913E-01	1.086E-02
MIR5585	0.082	NA	4.002E-02
VPS41	0.082	3.029E-01	3.407E-02
FAM8A1	0.082	4.525E-01	9.289E-01
TXNRD3	0.082	4.912E-01	9.134E-01
TAOK1	0.082	4.752E-01	9.064E-01
KIAA2026	0.082	5.102E-01	3.194E-01
S100PBP	0.082	2.831E-01	6.046E-02
SMARCD3	0.082	7.093E-01	8.752E-02
CDK11B	0.082	1.838E-01	3.945E-02
STX1B	0.082	8.174E-01	8.084E-02
SEPHS2	0.082	4.715E-01	2.519E-02
ECHS1	0.082	5.559E-01	2.392E-01
NUAK2	0.082	7.952E-01	4.619E-01
VHL	0.082	5.404E-01	1.498E-02
AC004223.3	0.082	NA	1.087E-02
HTRA2	0.082	3.776E-01	5.289E-01
HLCS	0.082	4.396E-01	2.720E-01
CYFIP1	0.082	3.500E-01	1.493E-02
RAB11A	0.082	4.773E-01	2.646E-02
ARHGEF35	0.082	7.655E-01	3.268E-01
EIF1AY	0.082	9.001E-01	7.328E-01
XRCC1	0.082	3.067E-01	8.388E-02
PAGE4	0.081	9.480E-01	7.316E-02
RIDA	0.081	6.037E-01	8.218E-01
ANXA8L1	0.081	8.211E-01	2.730E-02
ENDOD1	0.081	6.769E-01	9.622E-02
VAC14	0.081	3.826E-01	4.873E-01
BAX	0.081	4.283E-01	1.764E-01
L34079.1	0.081	NA	7.042E-01
GREB1	0.081	8.006E-01	1.421E-01
SLITRK3	0.081	9.137E-01	5.767E-02
DEFB127	0.081	NA	5.065E-01
MIR548O2	0.081	NA	8.712E-01

KPNA6	0.081	2.345E-01	8.368E-01
SOAT1	0.081	6.036E-01	6.824E-02
KIF1BP	0.081	3.169E-01	2.759E-02
ACACA	0.081	4.930E-01	5.719E-01
RASGRF2	0.081	7.220E-01	5.710E-01
MYOF	0.081	6.305E-01	5.189E-01
RNF122	0.081	6.434E-01	5.260E-02
UBE2M	0.081	4.282E-01	2.259E-01
IFNA2	0.081	NA	1.082E-01
RAC3	0.081	7.659E-01	9.284E-03
ZNF487	0.081	5.467E-01	4.439E-01
SULT1C4	0.081	7.874E-01	2.681E-01
FAM189B	0.081	4.715E-01	1.139E-02
ZNF146	0.081	4.392E-01	6.094E-02
FAM222A	0.081	7.435E-01	5.426E-01
MLANA	0.081	7.037E-01	7.522E-04
AC011195.2	0.080	8.883E-01	8.521E-02
CD300E	0.080	8.498E-01	4.265E-02
TAF4	0.080	4.255E-01	2.875E-02
ABL1	0.080	4.497E-01	5.001E-01
GARS	0.080	4.833E-01	7.585E-01
SELENON	0.080	4.064E-01	5.314E-01
LDLRAP1	0.080	5.217E-01	6.922E-02
TMEM127	0.080	2.487E-01	9.663E-01
ZNRF1	0.080	5.126E-01	9.651E-01
SSR3	0.080	3.595E-01	9.538E-01
ZFP36L1	0.080	5.871E-01	9.504E-01
HES5	0.080	8.514E-01	3.271E-01
CNP	0.080	3.802E-01	1.375E-02
MIR8083	0.080	NA	3.787E-01
HMGA1	0.080	6.677E-01	3.557E-01
CBLN1	0.080	8.972E-01	4.895E-02
CNNM3	0.080	3.954E-01	3.182E-01
PLEKHA2	0.080	6.108E-01	1.772E-01
TMEM120A	0.080	5.037E-01	2.176E-02
KPNA3	0.080	5.876E-01	7.158E-01
GJA5	0.080	8.169E-01	1.060E-02
RNASEL	0.080	5.405E-01	3.874E-01
SNX2	0.080	4.437E-01	4.104E-02
TRIM37	0.080	4.822E-01	4.772E-01
HACD1	0.079	8.156E-01	3.423E-01
ZYX	0.079	5.951E-01	4.980E-01
ZNF667	0.079	8.569E-01	5.204E-02
PCDHGA4	0.079	7.807E-01	4.532E-01
DPM1	0.079	3.680E-01	5.024E-01
SPRY3	0.079	6.474E-01	7.433E-01
WDR7	0.079	4.085E-01	8.788E-01
CERS3	0.079	8.880E-01	5.542E-02
PSMC1	0.079	4.540E-01	3.438E-01
FLVCR1	0.079	6.942E-01	8.222E-01

ERGIC1	0.079	3.483E-01	2.553E-02
ZYG11A	0.079	8.549E-01	3.882E-01
ZBTB46	0.079	6.116E-01	1.173E-01
GPX7	0.079	7.459E-01	1.224E-01
RIC1	0.079	5.551E-01	1.452E-01
SPIDR	0.079	4.001E-01	9.930E-02
MTMR6	0.079	3.838E-01	1.049E-02
RLN1	0.079	8.447E-01	1.167E-02
PRRG2	0.079	6.842E-01	3.481E-01
HIST1H4A	0.079	8.668E-01	1.817E-02
PRRC2C	0.079	5.636E-01	1.708E-01
ATP1B2	0.079	7.851E-01	5.253E-01
AAED1	0.079	7.170E-01	1.519E-01
SP2	0.079	2.940E-01	5.125E-01
B3GALT4	0.079	6.534E-01	3.098E-01
CBX1	0.079	4.414E-01	4.482E-01
C2CD6	0.079	8.570E-01	2.994E-01
ARF6	0.079	3.558E-01	9.448E-01
AC133555.3	0.079	NA	9.154E-01
MIR520C	0.079	NA	9.121E-01
MIR941-5	0.079	NA	1.476E-02
PPM1B	0.079	3.654E-01	1.857E-01
EIF3C	0.079	5.633E-01	5.734E-02
NUDT3	0.078	3.250E-01	2.822E-02
USP13	0.078	6.666E-01	9.319E-02
EGFL7	0.078	7.427E-01	4.615E-02
ENSA	0.078	4.930E-01	1.024E-01
KLHL7	0.078	3.626E-01	3.519E-01
JAGN1	0.078	5.237E-01	1.387E-01
IGLL5	0.078	8.878E-01	8.511E-01
STRBP	0.078	5.568E-01	1.473E-01
FNIP2	0.078	6.036E-01	3.389E-01
GDI1	0.078	3.856E-01	4.180E-01
IARS2	0.078	4.715E-01	8.698E-01
ICOSLG	0.078	8.317E-01	4.471E-01
ATG4A	0.078	3.729E-01	1.350E-01
MIR1269A	0.078	NA	8.552E-01
SUSD3	0.078	8.227E-01	8.514E-01
OR2A5	0.078	NA	8.981E-01
MIR603	0.078	NA	2.037E-01
IL4	0.078	8.470E-01	5.403E-01
VPS25	0.078	3.446E-01	6.852E-02
CUBN	0.078	7.426E-01	4.797E-01
ZNF491	0.078	7.219E-01	2.058E-02
RAD1	0.078	4.631E-01	3.054E-02
SLCO2A1	0.078	7.800E-01	1.426E-01
ZMIZ1	0.078	6.091E-01	2.748E-01
ZRANB1	0.078	5.371E-01	3.846E-01
PRKCE	0.078	5.556E-01	6.940E-02
NRDC	0.078	2.396E-01	3.367E-02

AP3S2	0.078	3.156E-01	2.315E-02
ZFP69B	0.078	6.723E-01	2.351E-02
PPP3R1	0.078	1.658E-01	6.468E-01
C6orf89	0.078	2.498E-01	1.165E-02
MPP4	0.078	7.962E-01	3.781E-01
CMTM3	0.078	7.129E-01	6.259E-02
F2R	0.078	7.509E-01	5.453E-01
OSBPL2	0.078	4.705E-01	4.378E-01
C2CD4D	0.077	8.096E-01	9.955E-02
ECE1	0.077	5.523E-01	1.152E-01
SLC30A6	0.077	2.715E-01	3.120E-01
BATF3	0.077	7.918E-01	4.148E-01
MRPS33	0.077	4.708E-01	9.670E-01
COL4A2	0.077	7.295E-01	9.670E-01
AC244517.6	0.077	8.739E-01	9.670E-01
XXYLT1	0.077	5.204E-01	9.670E-01
BTNL9	0.077	8.566E-01	9.670E-01
KNG1	0.077	9.377E-01	9.670E-01
HECTD2	0.077	5.608E-01	9.646E-01
ANKRD37	0.077	7.364E-01	2.343E-02
CHSY1	0.077	5.024E-01	2.341E-01
MAFK	0.077	6.012E-01	4.759E-02
ZDHHC6	0.077	3.330E-01	6.744E-01
MAGEB3	0.077	NA	2.048E-01
C11orf52	0.077	8.249E-01	1.072E-01
POFUT2	0.077	5.194E-01	3.026E-02
MIR16-2	0.077	NA	3.652E-01
MIR4675	0.077	NA	1.959E-01
MIR4713	0.077	NA	4.196E-01
ACAP3	0.077	6.378E-01	4.165E-01
NCKIPSD	0.077	5.029E-01	9.134E-01
ZNF233	0.077	7.357E-01	9.026E-01
CEACAM19	0.077	8.196E-01	4.984E-01
CDK14	0.077	7.971E-01	2.229E-02
CAMTA1	0.076	3.973E-01	3.559E-01
PUSL1	0.076	6.037E-01	4.462E-01
B3GNT4	0.076	7.960E-01	2.986E-02
KCNK18	0.076	NA	4.183E-01
FAM57A	0.076	5.771E-01	6.973E-02
SLC31A1	0.076	5.246E-01	4.316E-01
CCND3	0.076	5.920E-01	8.395E-02
TEX2	0.076	5.214E-01	8.177E-02
CCDC43	0.076	3.354E-01	2.745E-01
PPP4C	0.076	4.672E-01	8.712E-02
SMG7	0.076	2.730E-01	2.989E-01
RAB5C	0.076	2.969E-01	7.512E-01
IGF1R	0.076	6.923E-01	1.286E-01
ZNF565	0.076	5.224E-01	3.467E-02
MKRN1	0.076	3.174E-01	9.235E-03
NPB	0.076	8.269E-01	6.014E-01

MICU1	0.076	3.216E-01	5.956E-02
EM189-UBE2	0.076	8.529E-01	4.996E-01
SYT14	0.076	8.918E-01	2.091E-02
AC012531.3	0.076	NA	4.277E-01
MIR3617	0.076	NA	1.485E-01
HMGB4	0.076	NA	1.006E-01
CTNS	0.076	4.561E-01	4.667E-01
TMEM2	0.076	7.048E-01	7.134E-01
B9D1	0.076	6.708E-01	9.441E-02
SPIN1	0.076	4.445E-01	5.084E-01
MKLN1	0.076	4.492E-01	4.621E-02
MAST1	0.076	8.245E-01	1.759E-01
ZNF473	0.076	4.737E-01	1.263E-01
CITED2	0.076	7.082E-01	8.148E-02
MSRB2	0.076	6.543E-01	9.321E-01
FAM229B	0.076	7.101E-01	9.217E-01
MCRIP1	0.076	6.533E-01	7.423E-02
SNX19	0.076	5.662E-01	2.489E-01
FTH1	0.076	6.142E-01	2.755E-02
AC073610.3	0.076	8.106E-01	2.975E-02
CDC37L1	0.076	5.024E-01	3.471E-01
CCDC85C	0.076	6.559E-01	1.076E-01
NADK2	0.076	5.889E-01	6.694E-01
CALR3	0.076	8.498E-01	2.502E-01
C16orf58	0.076	4.266E-01	5.760E-02
IL22RA1	0.076	8.091E-01	7.117E-01
NID1	0.075	7.583E-01	4.788E-02
DLC1	0.075	7.517E-01	9.724E-02
HIST1H3J	0.075	8.534E-01	8.592E-02
ITCH	0.075	4.034E-01	2.774E-03
LYRM1	0.075	5.711E-01	6.741E-01
EXT1	0.075	7.085E-01	5.307E-01
OCRL	0.075	4.412E-01	1.046E-02
TACC2	0.075	6.921E-01	7.755E-02
PCDHGB1	0.075	8.628E-01	7.899E-02
PHACTR4	0.075	3.379E-01	1.761E-01
COPG2	0.075	4.923E-01	7.292E-02
TRAPPC4	0.075	4.353E-01	2.793E-02
BLOC1S3	0.075	5.089E-01	7.951E-02
FKBP14	0.075	5.738E-01	1.863E-01
GNB1	0.075	2.483E-01	3.378E-02
MIR4261	0.075	NA	5.671E-01
PTPRH	0.075	8.599E-01	1.732E-01
SERPINI2	0.075	9.192E-01	6.006E-01
SLC35B1	0.075	3.985E-01	5.835E-01
HSPA14	0.075	4.231E-01	7.452E-02
IQGAP2	0.075	8.034E-01	7.839E-02
TSPYL6	0.075	9.128E-01	3.717E-01
GGPS1	0.075	2.545E-01	1.713E-02
MIR5685	0.075	8.880E-01	6.940E-01

SAR1A	0.075	3.240E-01	3.695E-01
STH	0.075	NA	4.773E-01
TMEM140	0.075	6.351E-01	4.779E-01
R3HCC1L	0.075	3.381E-01	7.606E-02
MIR193B	0.075	NA	4.863E-02
MIR2909	0.075	NA	1.897E-01
TMEM89	0.074	8.421E-01	1.524E-01
SPDYE16	0.074	8.879E-01	5.935E-02
SLC25A53	0.074	4.950E-01	5.169E-02
PFKFB4	0.074	7.247E-01	3.279E-02
PCTP	0.074	6.754E-01	2.725E-01
B3GNT3	0.074	8.136E-01	2.089E-03
SUN1	0.074	5.104E-01	4.694E-01
ADAM22	0.074	8.142E-01	1.591E-01
KLHL21	0.074	6.364E-01	4.680E-01
PELI3	0.074	5.475E-01	1.175E-01
FAM43A	0.074	7.769E-01	8.471E-02
ARPC4	0.074	5.713E-01	3.366E-02
ZNF165	0.074	7.192E-01	9.078E-01
KRT27	0.074	9.028E-01	4.486E-01
EFNB2	0.074	7.628E-01	1.409E-01
CUTA	0.074	5.433E-01	7.172E-02
S1PR5	0.074	8.261E-01	8.385E-02
NDUFS2	0.074	5.913E-01	3.247E-02
ZBTB8A	0.074	4.151E-01	9.860E-02
BMP1	0.074	7.334E-01	2.881E-02
CPSF3	0.074	3.555E-01	4.193E-01
PNPO	0.074	5.386E-01	9.438E-02
CKAP2L	0.074	7.283E-01	1.102E-01
COL13A1	0.074	7.638E-01	6.536E-02
ZNF644	0.074	4.004E-01	4.426E-01
EIF4A3	0.074	4.031E-01	2.927E-01
ARAF	0.074	4.204E-01	6.601E-02
COPRS	0.074	6.308E-01	5.198E-01
ST6GALNAC	0.074	8.421E-01	6.999E-01
A1BG	0.074	8.192E-01	2.793E-01
ARF1	0.074	2.197E-01	6.369E-02
MIR212	0.074	NA	5.244E-02
HIST2H3PS2	0.074	NA	2.749E-02
VCY1B	0.074	NA	1.099E-01
MIR1224	0.074	NA	3.742E-01
MIR4694	0.074	NA	2.107E-01
OR10G8	0.074	NA	8.589E-01
OR1A2	0.074	NA	6.537E-01
OR2G3	0.074	NA	3.925E-01
OR4K15	0.074	NA	7.782E-02
GRN	0.074	5.776E-01	6.738E-02
DYRK1A	0.074	4.004E-01	5.350E-01
PCDHGA6	0.074	7.247E-01	8.382E-02
ADK	0.074	6.387E-01	9.298E-03

SEC24A	0.074	4.803E-01	1.793E-02
DNAJC13	0.074	3.489E-01	1.882E-01
SNX21	0.074	5.835E-01	5.238E-01
IGF2BP2	0.074	8.739E-01	9.447E-01
SEC24C	0.074	6.795E-01	9.410E-01
EMP2	0.074	7.332E-01	9.406E-01
LRFN3	0.073	5.629E-01	9.340E-01
FAM91A1	0.073	5.238E-01	2.108E-02
TPR	0.073	4.701E-01	2.562E-01
NUF2	0.073	7.396E-01	4.007E-01
DMTN	0.073	8.038E-01	3.226E-01
DIRC2	0.073	5.986E-01	7.103E-01
CDKN1B	0.073	5.405E-01	4.359E-01
SERPINH1	0.073	6.620E-01	4.376E-02
CBX7	0.073	7.062E-01	1.077E-02
PHLDA2	0.073	7.649E-01	6.493E-01
KIF4A	0.073	7.027E-01	4.836E-02
NCS1	0.073	7.048E-01	2.350E-01
GLB1L	0.073	5.852E-01	7.242E-02
METTL13	0.073	5.088E-01	1.396E-01
CAMK1	0.073	7.204E-01	2.480E-01
NOL10	0.073	3.590E-01	9.471E-02
ZNF850	0.073	7.588E-01	4.742E-01
OBSL1	0.073	7.191E-01	5.038E-01
TK1	0.073	6.977E-01	5.670E-01
TBCD	0.073	4.115E-01	6.330E-02
PDIA3	0.073	4.306E-01	1.026E-01
RABIF	0.073	4.107E-01	1.651E-01
SLC12A2	0.073	6.813E-01	8.557E-02
TMEM208	0.073	5.228E-01	3.986E-01
PRRC1	0.073	3.776E-01	6.747E-01
RIN2	0.073	6.877E-01	3.184E-01
EIF1B	0.073	3.970E-01	2.490E-01
GKAP1	0.073	7.185E-01	3.879E-01
ARFGEF2	0.073	5.434E-01	1.777E-01
GOLGA8K	0.073	8.858E-01	2.151E-01
HMGCS1	0.073	7.325E-01	4.170E-01
GPATCH1	0.073	4.157E-01	2.466E-01
SMIM11B	0.073	9.003E-01	8.205E-02
MYL6	0.073	5.652E-01	6.276E-02
RAB5B	0.073	4.667E-01	8.673E-01
YAP1	0.073	6.390E-01	7.173E-01
POLE4	0.073	5.802E-01	5.109E-01
NUDCD3	0.072	3.558E-01	3.931E-02
USP26	0.072	9.450E-01	6.548E-01
RPS27L	0.072	6.088E-01	6.528E-01
RNASEH1	0.072	5.217E-01	3.710E-01
SPATA6	0.072	7.348E-01	9.782E-02
KIAA1522	0.072	6.603E-01	9.163E-01
ATRN	0.072	4.931E-01	4.650E-02

GMPPA	0.072	5.136E-01	3.573E-02
TMEM182	0.072	5.964E-01	1.482E-02
P2RX7	0.072	8.199E-01	1.304E-01
FUNDC2	0.072	4.808E-01	6.875E-01
ENDOV	0.072	5.985E-01	3.455E-03
MIR5700	0.072	NA	9.095E-02
GAA	0.072	7.149E-01	4.156E-01
CASC3	0.072	4.168E-01	7.337E-02
MSC	0.072	8.534E-01	8.073E-01
IPP	0.072	4.600E-01	4.626E-02
SDC4	0.072	6.931E-01	4.484E-01
KIAA1429	0.072	4.377E-01	7.391E-01
KCTD12	0.072	7.679E-01	1.068E-01
CIPC	0.072	5.024E-01	4.745E-02
AIF1	0.072	8.450E-01	2.205E-02
EDEM2	0.072	4.835E-01	3.931E-02
PEX1	0.072	5.434E-01	5.571E-02
ZNF98	0.072	9.224E-01	4.306E-02
CXorf36	0.072	7.415E-01	7.375E-02
SLC13A3	0.072	8.403E-01	3.493E-01
SGMS1	0.072	6.116E-01	1.764E-01
C1GALT1	0.072	6.020E-01	6.745E-02
USP51	0.071	7.551E-01	6.483E-01
PSMD10	0.071	3.656E-01	3.363E-02
SOX14	0.071	9.678E-01	6.244E-02
FBXW5	0.071	5.777E-01	3.188E-01
USP34	0.071	6.708E-01	3.842E-01
BAZ1B	0.071	3.479E-01	9.808E-02
USP14	0.071	5.131E-01	6.975E-01
AEBP2	0.071	5.643E-01	6.145E-01
CHURC1	0.071	5.847E-01	6.899E-02
CDC6	0.071	6.826E-01	7.156E-01
DDB1	0.071	3.131E-01	4.615E-01
ZNF772	0.071	6.972E-01	2.816E-02
H1FX	0.071	5.607E-01	1.084E-01
CALCRL	0.071	7.580E-01	6.816E-02
KIF27	0.071	5.999E-01	6.030E-02
MIR132	0.071	NA	4.925E-01
SEC61G	0.071	6.609E-01	5.104E-02
SNX29	0.071	5.942E-01	1.439E-01
IL1RN	0.071	8.246E-01	9.689E-01
ISPD	0.071	7.086E-01	9.539E-01
HAX1	0.071	3.981E-01	8.428E-01
MAK	0.071	7.482E-01	3.861E-01
SCGB1C2	0.071	NA	1.578E-01
MIR6809	0.071	NA	4.219E-02
PRR23A	0.071	NA	1.502E-01
SEC22A	0.071	3.308E-01	1.956E-02
MIR4744	0.071	9.238E-01	3.572E-01
PDLIM5	0.071	6.387E-01	6.414E-02

ZNF526	0.071	4.054E-01	2.898E-01
CCDC50	0.071	5.014E-01	7.673E-02
LST1	0.071	8.319E-01	8.844E-01
CDK8	0.071	4.360E-01	5.091E-02
C19orf38	0.071	8.026E-01	7.774E-01
RNF168	0.071	5.404E-01	4.245E-01
AKNAD1	0.071	8.772E-01	2.163E-02
PSEN2	0.071	5.804E-01	2.482E-02
SLC19A1	0.071	6.847E-01	2.903E-02
SYF2	0.071	3.253E-01	4.724E-02
SCYL3	0.071	5.449E-01	1.068E-01
PPP1R3D	0.071	5.686E-01	1.689E-01
GID8	0.071	4.278E-01	2.683E-01
SORD	0.070	7.307E-01	2.392E-01
C2orf68	0.070	5.003E-01	6.567E-01
ZNF747	0.070	5.312E-01	1.794E-01
CYB5R3	0.070	4.607E-01	3.625E-01
NR4A2	0.070	7.999E-01	1.450E-01
ZNF766	0.070	4.721E-01	3.674E-01
CDCA8	0.070	6.996E-01	3.649E-02
EM256-PLSC	0.070	7.810E-01	2.702E-01
C3orf58	0.070	7.639E-01	1.276E-01
DLG5	0.070	7.116E-01	2.370E-01
KIFC1	0.070	7.116E-01	7.884E-01
APBA2	0.070	8.504E-01	3.405E-01
IL11RA	0.070	6.842E-01	4.605E-01
GHSR	0.070	NA	3.975E-01
KIAA1024	0.070	7.623E-01	5.803E-02
UBE2S	0.070	7.084E-01	9.046E-02
BARHL1	0.070	9.065E-01	5.529E-01
CCNB2	0.070	6.923E-01	4.910E-01
COG7	0.070	4.513E-01	1.969E-01
ZC3H12B	0.070	8.007E-01	2.514E-01
IRGC	0.070	9.296E-01	4.560E-01
PPOX	0.070	7.203E-01	6.165E-02
MAP4K5	0.070	4.327E-01	2.335E-01
MIR4762	0.070	NA	2.204E-01
CAMSAP1	0.070	5.593E-01	6.144E-01
TCEAL8	0.070	5.299E-01	9.502E-01
KCTD13	0.070	6.310E-01	9.251E-01
YPEL3	0.070	7.382E-01	9.186E-01
DBX2	0.070	9.232E-01	1.504E-01
CBX4	0.070	5.305E-01	4.647E-02
AVPR2	0.070	8.306E-01	4.032E-02
CEP350	0.070	5.155E-01	6.471E-01
ZNF593	0.070	6.512E-01	7.534E-02
PRKAR2B	0.070	8.318E-01	1.078E-01
SIMC1	0.070	7.941E-01	3.207E-01
TPSB2	0.070	8.660E-01	3.453E-02
MAN1A2	0.070	4.931E-01	1.596E-01

CTSZ	0.070	7.191E-01	5.789E-01
RRAGB	0.069	5.749E-01	7.433E-02
HRNR	0.069	8.675E-01	2.278E-01
FNTA	0.069	5.607E-01	5.539E-01
TMC2	0.069	8.598E-01	7.622E-01
RNMT	0.069	5.030E-01	3.594E-02
KIF26A	0.069	8.096E-01	9.550E-02
AC006030.1	0.069	7.904E-01	6.199E-01
DHX32	0.069	4.207E-01	4.121E-01
RMDN3	0.069	3.991E-01	2.844E-01
TMEM64	0.069	8.109E-01	2.577E-02
PHYHD1	0.069	8.579E-01	2.335E-01
MIR215	0.069	9.257E-01	1.840E-01
IQCC	0.069	6.347E-01	6.878E-01
TSPAN16	0.069	8.638E-01	3.593E-01
FAM208B	0.069	5.490E-01	3.159E-01
RGPD5	0.069	8.931E-01	8.963E-02
SH3BP5L	0.069	4.027E-01	1.482E-01
POLR2G	0.069	4.801E-01	5.315E-01
TTLL1	0.069	6.263E-01	1.841E-02
MIR3135B	0.069	NA	8.453E-02
VTI1A	0.069	3.345E-01	1.038E-01
DDX60L	0.069	7.782E-01	5.725E-01
MIR1208	0.069	NA	1.179E-01
RARRES3	0.069	8.688E-01	7.565E-01
MOB1A	0.069	3.403E-01	3.137E-01
C16orf91	0.069	5.272E-01	4.170E-01
AKR1C4	0.069	9.128E-01	1.033E-01
PPM1D	0.069	5.706E-01	5.913E-01
KPNA2	0.069	6.763E-01	4.237E-01
MYD88	0.069	5.710E-01	6.477E-01
ZW10	0.069	4.600E-01	4.488E-01
MIR3180-5	0.069	NA	2.372E-01
TRIM77	0.069	NA	1.022E-01
MIR196A2	0.069	NA	2.917E-01
MIR4423	0.069	NA	5.570E-02
FAM126A	0.068	7.921E-01	4.444E-01
PGM1	0.068	7.329E-01	4.777E-02
AFTPH	0.068	5.030E-01	2.259E-01
ZNF324B	0.068	5.696E-01	3.788E-01
CES4A	0.068	7.951E-01	1.146E-01
ANKRD2	0.068	8.759E-01	9.696E-01
STARD9	0.068	7.624E-01	2.771E-02
TFPT	0.068	6.620E-01	9.592E-01
C11orf42	0.068	8.512E-01	2.863E-01
VAMP3	0.068	3.742E-01	4.215E-01
ECT2	0.068	7.162E-01	1.565E-01
MTCH1	0.068	4.106E-01	3.640E-01
TCAF1	0.068	5.404E-01	1.352E-01
KIF24	0.068	7.230E-01	7.165E-01

SUCLG1	0.068	3.911E-01	2.864E-01
PCDHGA1	0.068	8.146E-01	1.375E-02
JAM3	0.068	8.083E-01	5.483E-01
SRD5A3	0.068	7.067E-01	7.993E-02
ARFGAP3	0.068	5.593E-01	1.023E-01
RBM28	0.068	3.904E-01	2.289E-01
ZNF592	0.068	3.959E-01	1.142E-01
MTSS1	0.068	7.739E-01	9.129E-03
ACTR1A	0.068	3.624E-01	5.781E-01
ARMC3	0.068	9.212E-01	2.604E-01
SNTB2	0.068	4.980E-01	4.853E-01
MIR1299	0.068	NA	2.164E-01
KLK2	0.068	9.323E-01	3.103E-02
AL662899.2	0.068	9.468E-01	2.357E-01
NBPF10	0.068	6.807E-01	1.474E-01
PRKCH	0.068	5.209E-01	5.693E-01
GTF3A	0.068	5.749E-01	5.532E-01
THAP1	0.068	4.581E-01	2.004E-01
ZNF609	0.068	4.642E-01	4.640E-02
TOP2B	0.068	7.233E-01	4.007E-01
SNX4	0.068	4.931E-01	4.537E-02
KIF15	0.068	7.325E-01	1.038E-01
ZNF416	0.068	5.371E-01	6.021E-02
SATB1	0.068	7.734E-01	5.497E-01
C12orf66	0.068	5.568E-01	2.978E-01
KCMF1	0.068	3.138E-01	4.160E-01
CLDND2	0.068	7.941E-01	3.044E-02
TCTEX1D4	0.067	8.294E-01	6.014E-01
KCND1	0.067	7.502E-01	3.720E-01
PPP1R18	0.067	7.418E-01	2.029E-02
ENOX1	0.067	8.448E-01	2.796E-01
PITPNM3	0.067	8.452E-01	9.865E-03
PREPL	0.067	5.146E-01	4.463E-01
ZNF17	0.067	5.032E-01	3.463E-01
PDCD10	0.067	4.839E-01	3.256E-02
PTPN14	0.067	6.603E-01	3.247E-02
USHBP1	0.067	7.462E-01	1.550E-01
SURF1	0.067	5.533E-01	7.203E-02
HLA-DPA1	0.067	8.577E-01	5.781E-01
MIR1183	0.067	NA	6.021E-02
CGB1	0.067	NA	9.375E-01
PDK3	0.067	6.874E-01	9.342E-01
BTF3L4	0.067	3.489E-01	9.306E-01
SAMD4A	0.067	7.855E-01	9.276E-01
TPMT	0.067	5.891E-01	5.152E-02
MAGEF1	0.067	5.729E-01	7.497E-01
PLEKHA8	0.067	5.518E-01	2.258E-01
ELOVL2	0.067	8.575E-01	1.044E-01
SMURF2	0.067	5.381E-01	6.739E-01
UQCRFS1	0.067	6.108E-01	2.254E-01

ZNF331	0.067	7.909E-01	5.273E-02
GNA11	0.067	4.375E-01	7.432E-02
DCTPP1	0.067	6.079E-01	7.435E-02
NLK	0.067	5.651E-01	4.435E-01
SCAND1	0.067	6.764E-01	3.921E-01
MAPK8IP1	0.067	8.068E-01	6.666E-02
CDCA7L	0.067	8.676E-01	3.352E-02
CHEK1	0.067	6.702E-01	2.532E-01
CDK5	0.067	5.650E-01	1.842E-01
SLC10A3	0.067	5.711E-01	2.098E-01
GLT6D1	0.067	NA	4.641E-02
RNF40	0.066	4.913E-01	8.562E-01
S100A4	0.066	8.691E-01	1.944E-01
METTL18	0.066	6.278E-01	2.886E-02
TMEM132E	0.066	8.500E-01	6.585E-02
MFSD1	0.066	5.732E-01	2.687E-01
RAET1G	0.066	8.616E-01	5.625E-01
PCDHB15	0.066	8.333E-01	2.077E-01
AR	0.066	8.886E-01	4.170E-02
SPICE1	0.066	5.838E-01	3.709E-02
SIX5	0.066	6.513E-01	6.561E-02
GIPC2	0.066	8.681E-01	5.005E-01
ZNF843	0.066	7.829E-01	4.142E-02
TPCN2	0.066	6.877E-01	6.610E-02
CEP63	0.066	4.053E-01	4.114E-01
ADPGK	0.066	3.757E-01	3.436E-01
ADGRE3	0.066	8.781E-01	1.674E-01
GIT1	0.066	5.568E-01	2.415E-01
HBP1	0.066	5.696E-01	4.087E-02
SSX4	0.066	NA	3.839E-01
MIR4452	0.066	NA	7.104E-02
DENND5A	0.066	6.387E-01	1.875E-01
CSDE1	0.066	4.054E-01	5.917E-02
PICALM	0.066	4.729E-01	1.857E-01
TLE1	0.066	7.432E-01	1.659E-01
AAGAB	0.066	3.894E-01	6.929E-02
ABCB10	0.066	5.510E-01	6.747E-02
UBE4A	0.066	5.419E-01	3.526E-01
DDI2	0.066	6.157E-01	2.081E-01
POU4F2	0.066	NA	1.136E-01
NAXE	0.066	5.401E-01	4.437E-01
TMEM106C	0.066	6.823E-01	6.921E-01
H2AFV	0.066	4.937E-01	8.943E-02
UBXN11	0.066	6.956E-01	4.782E-01
IRF2BPL	0.066	5.986E-01	1.056E-02
IMMP2L	0.066	6.903E-01	9.710E-02
PRRX1	0.066	8.740E-01	3.784E-01
TOR3A	0.065	4.862E-01	2.063E-01
UCHL5	0.065	6.012E-01	1.079E-01
FAM72C	0.065	7.941E-01	8.007E-02

GPR89A	0.065	5.650E-01	9.704E-01
TP53BP2	0.065	5.210E-01	9.704E-01
ATXN3L	0.065	NA	9.704E-01
EAF1	0.065	5.749E-01	9.704E-01
AL049650.1	0.065	7.832E-01	9.704E-01
RRAS	0.065	7.715E-01	9.704E-01
PROSER3	0.065	6.106E-01	1.538E-01
C2CD3	0.065	4.967E-01	1.003E-01
ASPA	0.065	8.831E-01	7.947E-01
B4GALT5	0.065	6.628E-01	9.121E-01
ADIPOR1	0.065	4.131E-01	9.089E-01
AGPAT4	0.065	8.047E-01	9.058E-01
LENG1	0.065	5.769E-01	8.960E-01
TTLL10	0.065	8.712E-01	5.444E-02
SPECC1L	0.065	5.405E-01	7.979E-01
UBE2V1	0.065	4.731E-01	7.159E-01
ZNF575	0.065	6.030E-01	4.399E-01
RAPGEF5	0.065	7.818E-01	2.195E-02
TM9SF1	0.065	4.705E-01	6.921E-01
RHBDD2	0.065	5.607E-01	1.561E-01
ODF2	0.065	4.876E-01	7.347E-02
UFC1	0.065	6.985E-01	2.850E-01
STAU1	0.065	3.555E-01	5.241E-02
ATRAID	0.065	4.663E-01	4.023E-01
UBE2Q1	0.065	3.356E-01	1.410E-01
USP31	0.065	6.958E-01	4.017E-02
MAGED2	0.065	6.151E-01	3.443E-01
CMTM7	0.065	7.643E-01	4.950E-01
OAZ2	0.065	3.558E-01	1.128E-01
FAM122C	0.065	6.128E-01	4.536E-01
LRRC15	0.065	9.105E-01	3.064E-02
ZNF681	0.065	8.092E-01	1.880E-01
SEC11A	0.065	3.735E-01	1.433E-01
APP	0.065	6.696E-01	1.416E-01
BCL2L15	0.064	9.009E-01	1.503E-01
CLCF1	0.064	8.345E-01	5.527E-02
KCTD5	0.064	5.123E-01	1.783E-02
PRSS22	0.064	8.270E-01	2.915E-01
SMIM29	0.064	6.364E-01	1.167E-01
SUPT6H	0.064	3.815E-01	2.569E-02
HOOK1	0.064	7.528E-01	1.166E-01
EDNRB	0.064	8.448E-01	3.284E-02
MAPRE3	0.064	7.467E-01	4.609E-01
RAMP2	0.064	7.638E-01	9.975E-02
CDK2	0.064	6.598E-01	1.460E-01
B3GALNT2	0.064	7.122E-01	2.793E-01
TNFAIP1	0.064	4.934E-01	8.087E-01
MIR1269B	0.064	NA	3.213E-01
MIR3648-1	0.064	NA	3.140E-02
SCP2D1	0.064	NA	5.845E-01

WDR26	0.064	3.920E-01	5.852E-01
CAT	0.064	7.275E-01	5.002E-02
SOS1	0.064	5.177E-01	3.271E-01
MEX3D	0.064	6.336E-01	7.303E-02
TCEAL1	0.064	6.894E-01	3.322E-02
YARS	0.064	5.196E-01	3.850E-01
UCK1	0.064	5.651E-01	6.088E-01
MSANTD3	0.064	6.490E-01	1.433E-01
ZHX3	0.064	6.211E-01	6.637E-02
KAT6A	0.064	6.572E-01	7.228E-01
COL20A1	0.064	9.095E-01	6.518E-01
SIRPB2	0.063	8.544E-01	3.205E-02
DDX25	0.063	8.886E-01	9.486E-01
ALM2-AKAI	0.063	8.854E-01	9.395E-01
DEFB133	0.063	NA	9.172E-02
CU639417.1	0.063	NA	3.059E-01
TNFAIP2	0.063	8.284E-01	9.967E-02
AGTRAP	0.063	7.023E-01	1.282E-01
ZNF460	0.063	7.363E-01	9.614E-02
NYNRIN	0.063	8.189E-01	1.101E-01
TANGO6	0.063	6.218E-01	1.550E-02
IFNA5	0.063	NA	6.001E-01
B3GNT5	0.063	7.867E-01	1.629E-01
TBC1D17	0.063	5.257E-01	1.066E-01
BNIPL	0.063	8.799E-01	2.966E-01
ZNF22	0.063	5.953E-01	4.261E-01
TAS2R3	0.063	9.246E-01	6.780E-02
DVL1	0.063	6.332E-01	5.290E-01
ZNF148	0.063	4.525E-01	1.730E-01
PPP1R2P3	0.063	8.470E-01	1.850E-01
BMPR1A	0.063	4.931E-01	3.346E-02
MIR3115	0.063	NA	6.367E-02
NEDD8-MDP	0.063	7.926E-01	3.976E-02
NRAP	0.063	9.364E-01	7.616E-02
SLC29A3	0.063	7.944E-01	1.305E-01
NUP62	0.063	4.412E-01	4.516E-01
ZNF652	0.063	5.322E-01	8.292E-02
RHOA	0.063	4.577E-01	6.816E-01
NIT1	0.063	6.632E-01	3.317E-01
GORASP1	0.063	5.453E-01	1.293E-01
MPHOSPH10	0.063	5.268E-01	1.571E-01
SPDYE4	0.063	9.331E-01	2.501E-01
SERINC1	0.063	5.559E-01	8.037E-02
CEP104	0.063	3.456E-01	4.516E-01
C4orf3	0.063	5.436E-01	2.317E-01
SLC33A1	0.063	4.688E-01	1.328E-01
ANP32E	0.063	6.784E-01	1.972E-01
METTL23	0.063	4.416E-01	4.261E-02
ZFP36L2	0.063	6.826E-01	6.256E-01
PLPP1	0.063	7.921E-01	1.236E-01

FAM199X	0.062	5.110E-01	7.795E-01
CCNK	0.062	7.128E-01	6.076E-02
RIMBP3	0.062	8.602E-01	5.367E-01
B3GAT3	0.062	6.387E-01	4.114E-01
LY6H	0.062	8.918E-01	1.515E-01
MAOB	0.062	8.903E-01	5.958E-02
ALKBH5	0.062	4.588E-01	3.402E-01
TMEM222	0.062	4.847E-01	1.525E-01
FAM104B	0.062	6.582E-01	1.841E-01
PRDM2	0.062	5.307E-01	4.269E-01
BIK	0.062	8.320E-01	6.821E-01
HSFX1	0.062	8.999E-01	1.115E-01
CAPN7	0.062	5.305E-01	6.805E-01
RNF20	0.062	5.176E-01	6.487E-02
EIF3M	0.062	6.246E-01	1.037E-01
SSR2	0.062	5.348E-01	4.250E-01
PLEKHA1	0.062	6.807E-01	6.558E-02
GOLPH3	0.062	5.712E-01	9.142E-01
MEIS3	0.062	8.481E-01	1.175E-01
ASF1B	0.062	7.229E-01	4.864E-02
MIR5687	0.062	NA	7.867E-01
GALNT3	0.062	7.789E-01	6.062E-02
RNF6	0.062	5.711E-01	2.068E-01
STK39	0.062	6.899E-01	1.613E-01
MIR181C	0.062	NA	8.472E-01
TMEM87A	0.062	4.888E-01	6.683E-02
HINFP	0.062	5.199E-01	4.539E-01
SLC7A2	0.062	8.693E-01	1.008E-01
PRPS2	0.062	6.559E-01	4.219E-02
FAM182B	0.062	8.532E-01	1.828E-01
MICALCL	0.062	8.329E-01	3.338E-02
PPP4R3B	0.062	4.033E-01	2.676E-01
RIPOR1	0.062	6.369E-01	7.891E-02
MBNL2	0.062	6.333E-01	6.113E-01
GDAP1	0.062	7.868E-01	6.473E-02
ZNF705A	0.062	9.166E-01	4.426E-01
C3orf67	0.062	8.752E-01	5.408E-02
GPRASP2	0.061	7.280E-01	1.050E-01
OR2A7	0.061	8.429E-01	1.284E-01
RSPO3	0.061	9.056E-01	7.314E-01
AGBL5	0.061	5.636E-01	3.422E-01
ANXA7	0.061	5.299E-01	4.494E-01
SLC25A44	0.061	4.422E-01	2.591E-01
COMMD1	0.061	5.950E-01	1.495E-01
SH3RF2	0.061	8.791E-01	6.233E-02
ATP6V0E1	0.061	5.412E-01	1.570E-01
RHOC	0.061	7.149E-01	1.566E-01
MIR4764	0.061	NA	1.152E-01
MIR3140	0.061	NA	3.740E-01
OR5B17	0.061	NA	7.756E-01

CACUL1	0.061	4.577E-01	2.998E-02
UACA	0.061	6.938E-01	1.137E-01
RBL1	0.061	6.784E-01	5.180E-02
ARMC8	0.061	3.063E-01	7.888E-02
TAOK3	0.061	5.970E-01	3.646E-02
FBXO28	0.061	4.829E-01	7.322E-01
CDV3	0.061	5.852E-01	7.877E-01
WNT2B	0.061	7.885E-01	3.882E-01
USF2	0.061	5.054E-01	7.258E-02
TUBG1	0.061	5.825E-01	4.320E-03
AGFG1	0.061	6.777E-01	1.331E-01
TRAPPC10	0.061	6.679E-01	3.384E-01
SLC25A34	0.061	8.235E-01	8.062E-02
ACOT9	0.061	7.426E-01	4.553E-01
NETO2	0.061	8.374E-01	3.125E-01
GES3L-AARS	0.061	8.706E-01	3.951E-02
CDC27	0.061	5.304E-01	5.240E-01
MTHFD2	0.061	7.677E-01	1.275E-01
HTR2C	0.061	9.132E-01	5.609E-01
UBE2D4	0.061	5.966E-01	1.332E-01
RFPL4A	0.061	9.593E-01	9.708E-01
MIR378B	0.061	NA	9.676E-01
MAD2L2	0.061	7.347E-01	9.642E-01
MFSD4B	0.061	6.567E-01	9.540E-01
ZBTB42	0.061	7.214E-01	9.509E-01
MEAF6	0.061	4.374E-01	9.506E-01
RPS6KL1	0.061	8.421E-01	9.506E-01
LPCAT3	0.061	7.574E-01	9.478E-01
SALL3	0.061	9.475E-01	9.440E-01
ZNF625	0.061	8.227E-01	5.269E-02
EMD	0.060	5.455E-01	1.886E-01
RFXAP	0.060	6.812E-01	6.450E-02
TRAF4	0.060	7.354E-01	1.984E-01
RAD51D	0.060	5.890E-01	5.054E-01
ATL2	0.060	6.848E-01	7.711E-01
DHRS4L2	0.060	7.029E-01	1.486E-02
ARID4B	0.060	4.979E-01	7.472E-01
XPOT	0.060	6.607E-01	4.892E-01
SMARCA1	0.060	7.629E-01	5.457E-01
ZNF319	0.060	6.145E-01	1.032E-01
RNF111	0.060	5.568E-01	5.738E-02
AC009163.4	0.060	9.273E-01	6.119E-01
BCHE	0.060	9.085E-01	9.219E-01
DLG1	0.060	5.978E-01	3.983E-01
SFXN5	0.060	6.559E-01	4.585E-01
TMEM263	0.060	6.155E-01	5.086E-01
BCAS4	0.060	7.892E-01	7.569E-01
SFXN3	0.060	7.138E-01	1.475E-01
SKI	0.060	5.007E-01	1.797E-01
ZNF720	0.060	6.011E-01	5.285E-01

KMO	0.060	8.602E-01	1.251E-01
MOB2	0.060	5.737E-01	9.129E-01
SYTL5	0.060	9.080E-01	3.478E-02
MTX1	0.060	5.271E-01	4.185E-02
MED7-TICAM	0.060	7.962E-01	3.286E-02
S100A13	0.060	7.712E-01	1.662E-01
MCTS1	0.060	5.453E-01	1.145E-01
CDKN3	0.060	7.858E-01	1.002E-01
PHC3	0.060	5.788E-01	1.077E-01
AC008687.1	0.060	9.644E-01	1.634E-01
ADAMTS14	0.060	8.661E-01	5.215E-01
PPP2R5A	0.060	5.426E-01	1.095E-01
THPO	0.060	8.771E-01	6.548E-01
THADA	0.060	4.064E-01	2.795E-02
METTL6	0.060	5.143E-01	2.361E-01
TMEM55B	0.060	3.918E-01	7.142E-01
RNPEP	0.060	5.596E-01	5.787E-02
EIF2B3	0.060	5.773E-01	4.849E-01
CNOT10	0.060	4.756E-01	4.952E-01
TMEM17	0.060	7.074E-01	9.752E-02
CCDC15	0.059	6.960E-01	2.556E-02
CPVL	0.059	8.706E-01	2.000E-01
RBKS	0.059	7.244E-01	4.381E-01
HNRNPUL1	0.059	4.803E-01	9.477E-01
GLTP	0.059	7.759E-01	9.408E-01
MATN1	0.059	7.950E-01	9.288E-01
AC073508.2	0.059	9.153E-01	9.257E-01
CPNE2	0.059	7.148E-01	9.222E-01
UBE4B	0.059	5.579E-01	1.238E-01
WDR86	0.059	8.502E-01	2.635E-01
FP236240.1	0.059	NA	1.192E-01
MICA	0.059	7.519E-01	7.208E-02
TRIM29	0.059	8.502E-01	4.010E-01
NBPF3	0.059	7.754E-01	1.101E-01
CD99L2	0.059	7.618E-01	5.909E-01
C1GALT1C1I	0.059	8.500E-01	5.218E-01
SRF	0.059	5.596E-01	4.760E-01
AP4M1	0.059	5.990E-01	2.090E-01
TPRG1L	0.059	5.730E-01	2.851E-01
DPM3	0.059	7.686E-01	3.532E-02
C11orf68	0.059	5.871E-01	5.252E-02
PAQR5	0.059	8.818E-01	5.870E-01
DNAJB4	0.059	7.451E-01	1.317E-01
YWHAZ	0.059	7.117E-01	7.152E-01
FKTN	0.059	5.932E-01	7.082E-02
TPX2	0.059	7.812E-01	1.676E-01
NXPE4	0.059	9.277E-01	7.329E-02
ANKRD18A	0.059	9.003E-01	4.704E-01
ZNF273	0.059	7.426E-01	1.517E-02
ENY2	0.059	5.730E-01	7.834E-01

ZNF610	0.059	8.427E-01	4.648E-01
RNFT1	0.059	6.495E-01	1.207E-01
CXorf23	0.059	6.456E-01	9.409E-02
TCHP	0.059	5.693E-01	2.376E-01
KLF2	0.059	8.386E-01	4.596E-01
HYLS1	0.059	7.203E-01	8.338E-01
RBFOX2	0.059	5.776E-01	2.377E-02
DVL3	0.059	4.944E-01	1.753E-01
FARS2	0.059	5.682E-01	1.598E-01
CD46	0.059	7.078E-01	4.944E-02
PPEF2	0.059	9.014E-01	4.054E-01
MIR3171	0.059	NA	7.105E-01
FAM161B	0.058	6.904E-01	2.703E-01
EPHX4	0.058	8.598E-01	9.418E-01
NXT1	0.058	6.252E-01	3.582E-02
C1orf127	0.058	8.822E-01	1.307E-01
DDX1	0.058	4.497E-01	8.749E-01
RALGPS1	0.058	8.083E-01	6.932E-02
SYS1	0.058	5.928E-01	4.741E-01
RAB22A	0.058	4.777E-01	8.152E-02
MYBL2	0.058	8.249E-01	2.947E-01
ARF3	0.058	4.629E-01	1.399E-01
MS4A4A	0.058	8.822E-01	1.642E-01
ATP5J2-PTCD	0.058	7.855E-01	1.218E-01
MAPKAP1	0.058	4.709E-01	8.626E-02
ZCCHC6	0.058	6.414E-01	6.389E-01
ACADM	0.058	7.467E-01	3.898E-01
MORC3	0.058	6.496E-01	4.047E-01
MAPK8	0.058	5.426E-01	2.827E-01
OR51B6	0.058	NA	1.653E-01
MIR3679	0.058	NA	6.140E-01
TBC1D10B	0.058	4.765E-01	1.508E-01
ST3GAL3	0.058	6.946E-01	2.260E-01
LASP1	0.058	6.387E-01	8.212E-02
FBLN1	0.058	8.729E-01	5.490E-01
NPR2	0.058	7.691E-01	1.034E-01
PCDH12	0.058	7.953E-01	1.687E-01
SAA1	0.058	9.282E-01	5.661E-02
MOCOS	0.058	7.564E-01	1.186E-01
LIG3	0.058	6.579E-01	7.268E-01
TTLL5	0.058	5.155E-01	1.314E-01
RBSN	0.058	5.701E-01	8.724E-01
NDEL1	0.058	5.449E-01	6.987E-02
HAUS2	0.058	5.715E-01	5.346E-01
PLK3	0.058	7.636E-01	5.332E-01
SLC2A12	0.058	8.999E-01	4.518E-02
ZNF37A	0.058	5.841E-01	5.665E-01
NEK7	0.058	7.294E-01	6.453E-01
CSTF2	0.058	5.972E-01	6.380E-02
ZNF449	0.058	6.264E-01	5.362E-01

JPT1	0.058	7.024E-01	9.722E-01
TCEAL9	0.058	7.814E-01	9.722E-01
DUSP14	0.058	7.441E-01	9.722E-01
ITSN1	0.057	6.543E-01	2.721E-01
ARHGAP12	0.057	7.071E-01	9.655E-01
RAB7A	0.057	4.482E-01	9.653E-01
TGM2	0.057	8.880E-01	9.622E-01
MIR1302-8	0.057	NA	9.620E-01
BAK-RBAKD	0.057	8.519E-01	9.557E-01
ATP6V1E2	0.057	7.080E-01	2.935E-01
SUFU	0.057	6.343E-01	1.614E-01
MAN2A2	0.057	7.154E-01	2.205E-01
NBPF1	0.057	7.029E-01	2.913E-01
PILRA	0.057	8.658E-01	6.065E-01
CTRB2	0.057	9.319E-01	3.458E-01
GALNS	0.057	7.081E-01	4.405E-01
PGBD2	0.057	6.616E-01	5.420E-01
OR10D3	0.057	NA	4.700E-01
VPS37D	0.057	8.541E-01	3.216E-01
LYPD1	0.057	8.887E-01	2.545E-01
EBP	0.057	6.972E-01	4.406E-01
44081.000	0.057	4.789E-01	4.564E-01
COLEC11	0.057	8.728E-01	1.757E-01
TUBGCP3	0.057	6.286E-01	2.566E-01
LRRC8E	0.057	8.146E-01	1.590E-01
MYDGF	0.057	6.578E-01	2.558E-01
PIBF1	0.057	6.116E-01	3.314E-01
HIST1H2BG	0.057	8.921E-01	1.349E-01
SERHL2	0.057	7.299E-01	7.331E-01
SEC23B	0.057	6.199E-01	8.307E-01
MPHOSPH8	0.057	5.212E-01	5.220E-02
FOXJ2	0.057	6.496E-01	9.653E-02
NUDT4P1	0.057	NA	1.182E-01
DNAJC1	0.057	6.663E-01	5.188E-02
UCHL1	0.057	9.137E-01	2.082E-01
NYX	0.057	9.391E-01	4.374E-02
NSDHL	0.057	6.387E-01	8.991E-02
BCAS1	0.057	9.133E-01	1.078E-01
TMEM179B	0.057	5.732E-01	3.512E-01
HERC4	0.057	5.902E-01	1.359E-01
TGFBR3	0.057	8.541E-01	8.686E-01
ZNF585B	0.057	6.777E-01	3.426E-02
YWHAG	0.057	6.030E-01	3.874E-02
ALX4	0.057	9.208E-01	1.885E-01
CNOT11	0.057	5.759E-01	3.749E-02
SYNPO	0.057	8.067E-01	9.312E-02
ARNT	0.057	5.865E-01	6.859E-01
ANO7	0.056	8.046E-01	2.062E-01
SAMD8	0.056	6.128E-01	1.417E-01
MYRIP	0.056	8.997E-01	1.173E-01

PAOX	0.056	7.065E-01	6.247E-02
GSK3B	0.056	4.889E-01	2.071E-01
ZFP28	0.056	8.580E-01	5.737E-01
PRIMPOL	0.056	5.311E-01	3.099E-01
TAS2R41	0.056	NA	4.007E-01
SLC35A5	0.056	5.470E-01	2.440E-01
TLCD2	0.056	7.699E-01	9.486E-01
NXT2	0.056	6.769E-01	9.303E-01
DUSP19	0.056	7.442E-01	5.538E-02
DMRT2	0.056	9.296E-01	3.211E-01
DOLPP1	0.056	6.278E-01	3.027E-01
RAPH1	0.056	7.687E-01	5.745E-01
CASZ1	0.056	7.852E-01	2.333E-01
DHRS7B	0.056	7.247E-01	1.556E-01
TTF1	0.056	5.467E-01	4.175E-01
TAF15	0.056	5.251E-01	2.110E-01
F8A1	0.056	7.869E-01	3.538E-01
PDCD6	0.056	6.056E-01	1.997E-01
MED10	0.056	6.751E-01	2.580E-01
RGS7BP	0.056	9.092E-01	1.767E-01
ANKRD40	0.056	4.801E-01	1.177E-01
LAMP1	0.056	6.143E-01	7.492E-02
HAAO	0.056	8.413E-01	9.427E-01
PIGP	0.056	6.298E-01	1.426E-01
BCL2	0.056	8.177E-01	2.217E-01
MAEL	0.056	9.161E-01	1.205E-01
VSX2	0.056	9.356E-01	5.837E-01
CHCHD1	0.056	6.758E-01	5.092E-01
POM121L2	0.056	9.249E-01	2.576E-01
LSG1	0.056	5.344E-01	6.294E-02
JUP	0.056	7.677E-01	2.612E-01
PAPSS2	0.056	8.427E-01	5.904E-02
FLAD1	0.056	6.081E-01	8.875E-01
PSMD1	0.056	5.629E-01	3.700E-01
POLR2M	0.056	5.102E-01	1.122E-01
SCARB2	0.056	6.142E-01	1.146E-01
PIK3C2B	0.056	8.118E-01	9.318E-02
ZHX1-C8orf7(	0.055	7.362E-01	1.462E-01
KRTAP4-16	0.055	NA	2.571E-01
PSMG3	0.055	6.917E-01	7.841E-02
IL3	0.055	NA	8.799E-02
MIR3158-1	0.055	NA	1.436E-01
C21orf62	0.055	8.849E-01	2.951E-02
FES	0.055	7.971E-01	5.021E-02
GNB2	0.055	5.559E-01	8.694E-02
ZNF774	0.055	7.759E-01	1.252E-01
PRSS55	0.055	9.420E-01	1.809E-01
TAL1	0.055	8.463E-01	5.705E-02
GLUL	0.055	7.826E-01	4.932E-02
PCDH9	0.055	9.182E-01	1.085E-01

TOX4	0.055	4.377E-01	3.872E-02
ALDH16A1	0.055	6.840E-01	5.642E-01
SELENOT	0.055	5.194E-01	1.267E-01
KLHDC3	0.055	6.607E-01	4.491E-01
SCAMP4	0.055	5.215E-01	2.583E-01
SLC25A33	0.055	7.002E-01	2.086E-02
SNX7	0.055	6.879E-01	9.730E-01
C20orf24	0.055	6.419E-01	9.730E-01
CEP68	0.055	5.944E-01	9.730E-01
RAB3D	0.055	8.057E-01	9.708E-01
USP22	0.055	7.180E-01	9.704E-01
ACTN3	0.055	8.791E-01	9.674E-01
MTOR	0.055	5.412E-01	3.906E-02
LONRF3	0.055	8.528E-01	2.108E-02
PLEKHG6	0.055	8.492E-01	4.958E-01
LINC00998	0.055	6.409E-01	1.693E-01
DCXR	0.055	7.671E-01	7.576E-01
NBPF4	0.055	9.347E-01	5.059E-02
SLC39A6	0.055	7.300E-01	4.479E-02
C9orf78	0.055	5.521E-01	2.453E-01
BCL9	0.055	7.271E-01	6.770E-02
COASY	0.055	5.695E-01	8.633E-01
SPR	0.055	7.300E-01	6.392E-01
HEBP1	0.055	6.620E-01	3.358E-01
CRISP3	0.055	9.358E-01	2.242E-01
AVIL	0.055	8.074E-01	1.055E-01
AC097637.1	0.054	9.452E-01	1.049E-01
TEAD4	0.054	8.483E-01	6.304E-02
LRRIQ3	0.054	8.113E-01	3.246E-01
MIR4494	0.054	NA	3.015E-01
BICDL1	0.054	8.199E-01	6.269E-01
ST3GAL2	0.054	7.488E-01	7.166E-02
PHLDB1	0.054	7.638E-01	5.478E-01
CERS2	0.054	6.513E-01	4.528E-01
PALB2	0.054	6.632E-01	5.204E-02
PAPSS1	0.054	6.128E-01	1.023E-01
ALKBH3	0.054	6.978E-01	7.033E-01
MIR4679-2	0.054	NA	6.288E-01
MIR4262	0.054	NA	5.427E-01
MIR4274	0.054	NA	4.828E-01
NXNL1	0.054	NA	2.509E-01
RRP36	0.054	6.410E-01	1.621E-01
MIR3972	0.054	9.412E-01	2.362E-01
POLR3C	0.054	5.778E-01	2.673E-01
SYT17	0.054	8.453E-01	1.688E-01
XPC	0.054	6.718E-01	4.346E-01
C19orf54	0.054	6.884E-01	7.375E-02
PCP4L1	0.054	9.243E-01	1.416E-01
ACPT	0.054	8.645E-01	6.630E-02
TTC32	0.054	7.203E-01	1.275E-01

HSF5	0.054	8.983E-01	1.314E-01
CADM4	0.054	8.278E-01	3.269E-02
PHTF1	0.054	6.367E-01	3.131E-01
ANKRD12	0.054	6.977E-01	6.787E-02
ARPC5	0.054	5.425E-01	6.291E-01
PKN3	0.054	7.847E-01	5.292E-01
MYL4	0.054	8.415E-01	9.445E-01
MLF1	0.054	8.146E-01	9.386E-01
NCF4	0.054	8.580E-01	6.515E-01
HDDC3	0.054	6.784E-01	1.948E-01
DYM	0.054	5.847E-01	3.637E-01
PRPF31	0.054	6.257E-01	1.715E-01
NFKBIE	0.054	7.585E-01	2.346E-01
FCRLA	0.054	9.159E-01	2.281E-01
ZNF611	0.054	7.197E-01	8.031E-02
SELENOI	0.054	6.759E-01	8.710E-02
KMT2E	0.054	6.776E-01	5.396E-01
PIGU	0.054	6.674E-01	4.402E-01
RALB	0.053	6.748E-01	8.803E-01
SPTBN4	0.053	8.759E-01	8.763E-02
HIVEP3	0.053	8.397E-01	5.336E-01
LYN	0.053	8.145E-01	5.769E-01
FBXW2	0.053	5.970E-01	6.658E-01
ARSB	0.053	7.048E-01	2.999E-02
PPM1G	0.053	5.772E-01	2.408E-02
LRRC57	0.053	5.229E-01	1.000E-01
MIR6817	0.053	NA	5.812E-02
AL138826.1	0.053	NA	3.873E-01
MAP1-GIMA	0.053	NA	4.261E-01
LINC00633	0.053	NA	6.503E-03
TOR1A	0.053	5.596E-01	5.150E-02
FKBP5	0.053	8.693E-01	1.566E-01
S1PR2	0.053	7.433E-01	1.944E-01
PNRC2	0.053	6.158E-01	4.907E-01
MRPL49	0.053	5.511E-01	8.840E-01
DUSP16	0.053	7.580E-01	8.958E-01
TTLL11	0.053	7.256E-01	1.570E-01
ZNF669	0.053	6.814E-01	8.207E-02
USB1	0.053	5.845E-01	6.796E-02
TIAF1	0.053	7.630E-01	3.413E-01
NDN	0.053	8.658E-01	5.922E-02
ARPP19	0.053	5.510E-01	1.131E-01
KLF4	0.053	8.512E-01	5.681E-01
NPAT	0.053	6.650E-01	7.121E-03
PLEKHO2	0.053	7.864E-01	8.086E-02
PLAA	0.053	6.898E-01	1.600E-01
MAPK14	0.053	5.613E-01	3.534E-01
ESPL1	0.053	7.992E-01	4.320E-01
ABCF2	0.053	5.629E-01	1.564E-01
UBE2V2	0.053	6.058E-01	1.583E-01

USP21	0.053	7.559E-01	5.847E-01
RALA	0.053	6.571E-01	3.942E-01
ENKUR	0.053	8.912E-01	4.630E-02
NANP	0.053	6.387E-01	8.455E-02
SPPL2A	0.053	5.411E-01	5.813E-02
RAD21	0.053	6.927E-01	7.203E-01
UBTD2	0.053	5.734E-01	2.839E-01
ADAT1	0.053	6.019E-01	6.210E-01
RTCA	0.053	4.659E-01	1.001E-01
WBP2	0.053	5.233E-01	1.479E-01
CETN1	0.053	NA	9.737E-01
MIR3147	0.053	NA	9.737E-01
SPATS1	0.053	NA	9.737E-01
AC037459.1	0.053	8.736E-01	9.737E-01
SELENOF	0.052	4.778E-01	9.737E-01
TXLNA	0.052	5.293E-01	9.737E-01
C1QTNF9B	0.052	8.927E-01	9.737E-01
NFKBIA	0.052	7.851E-01	9.737E-01
RGMB	0.052	6.663E-01	9.737E-01
GAS2	0.052	9.113E-01	9.737E-01
PCDHB6	0.052	9.135E-01	9.737E-01
PSMC4	0.052	6.566E-01	3.397E-01
GLS2	0.052	8.668E-01	8.264E-02
COQ2	0.052	7.193E-01	6.326E-02
MAGEA12	0.052	9.626E-01	1.882E-01
PDSS2	0.052	6.923E-01	5.637E-02
KDSR	0.052	6.296E-01	1.208E-01
MCM7	0.052	7.152E-01	3.015E-01
ATP1A3	0.052	9.014E-01	3.178E-01
PABPC1	0.052	8.429E-01	4.823E-01
NCF2	0.052	8.829E-01	6.066E-01
FCAR	0.052	9.060E-01	1.010E-01
ANKRD46	0.052	8.147E-01	3.282E-01
BBS4	0.052	6.467E-01	8.235E-02
KDM4A	0.052	6.198E-01	2.661E-01
TBC1D5	0.052	6.589E-01	7.740E-01
IL36RN	0.052	9.356E-01	1.616E-01
IRF2BP2	0.052	6.426E-01	3.261E-01
WASHC2C	0.052	5.425E-01	3.167E-01
CAMSAP3	0.052	8.274E-01	5.536E-02
CDH5	0.052	8.235E-01	2.783E-02
CATSPER4	0.052	9.031E-01	4.959E-01
MIR4296	0.052	NA	4.543E-01
TSPAN5	0.052	8.514E-01	1.185E-01
PEAK1	0.052	7.029E-01	1.454E-01
PFKFB1	0.052	7.970E-01	1.863E-01
SEC62	0.052	5.436E-01	3.275E-01
DNASE2	0.052	6.497E-01	1.886E-01
HTATIP2	0.052	7.390E-01	1.285E-01
PIPOX	0.052	8.682E-01	1.110E-01

CTNND1	0.052	6.999E-01	4.880E-01
BCORL1	0.052	7.326E-01	5.278E-01
PDE3A	0.052	8.504E-01	1.744E-01
FNTB	0.052	5.621E-01	3.968E-01
APITD1-COR'	0.051	7.729E-01	7.000E-01
FANCG	0.051	7.347E-01	1.875E-01
PLD1	0.051	8.237E-01	6.446E-01
TIFA	0.051	7.046E-01	8.037E-02
ORMDL3	0.051	7.722E-01	1.240E-01
MED24	0.051	6.365E-01	5.798E-01
MIR1285-2	0.051	NA	4.191E-02
OR4M1	0.051	NA	9.424E-01
MIR548F2	0.051	NA	2.311E-01
ACSL4	0.051	8.107E-01	1.335E-01
NEK4	0.051	7.198E-01	1.526E-01
WNT3A	0.051	9.265E-01	1.479E-01
ZNF507	0.051	6.805E-01	3.644E-01
ADIPOR2	0.051	6.603E-01	1.544E-01
KDM1B	0.051	7.605E-01	2.598E-01
MIR563	0.051	NA	6.290E-01
SF3B2	0.051	4.584E-01	1.153E-01
IDH3A	0.051	6.884E-01	2.528E-01
TMEM14C	0.051	6.634E-01	1.690E-01
TYW1	0.051	5.126E-01	9.549E-01
MDH1	0.051	5.156E-01	9.490E-01
MOSPD1	0.051	7.624E-01	9.490E-01
LIG1	0.051	6.620E-01	9.432E-01
GON4L	0.051	6.000E-01	1.300E-01
ZNF773	0.051	7.598E-01	6.137E-01
ACP1	0.051	5.859E-01	9.438E-01
SUSD6	0.051	7.599E-01	1.032E-01
MIR3907	0.051	NA	3.017E-01
TM2D3	0.051	5.303E-01	1.864E-01
VWDE	0.051	9.237E-01	2.373E-01
FAM89A	0.051	8.198E-01	1.753E-01
PIK3R4	0.051	5.735E-01	4.021E-01
SLC7A6OS	0.051	6.632E-01	3.564E-01
AC009690.3	0.051	NA	1.946E-01
MIR1303	0.051	NA	8.999E-02
TRPM3	0.051	9.105E-01	6.790E-02
XYLT1	0.051	8.759E-01	8.067E-02
RGS19	0.051	7.319E-01	7.417E-02
PTPA	0.051	5.985E-01	2.130E-01
ING4	0.050	6.847E-01	1.384E-01
STARD8	0.050	8.354E-01	2.803E-01
ZNF572	0.050	8.317E-01	7.463E-01
METTL2B	0.050	5.924E-01	1.785E-01
PTEN	0.050	6.352E-01	1.382E-01
DCAF10	0.050	6.364E-01	6.408E-01
ZNF530	0.050	7.354E-01	3.166E-01

KIF16B	0.050	7.396E-01	1.681E-01
CHIC1	0.050	7.257E-01	3.601E-01
SLC25A5	0.050	7.082E-01	2.426E-01
ZNF75A	0.050	7.690E-01	1.582E-01
NRG3	0.050	9.359E-01	9.100E-01
MCMBP	0.050	5.749E-01	9.129E-01
LMBRD2	0.050	7.446E-01	1.032E-01
RNF2	0.050	6.430E-01	8.357E-02
KCNK17	0.050	8.903E-01	1.543E-01
SSRP1	0.050	6.273E-01	1.390E-01
CELSR3	0.050	8.736E-01	1.376E-02
PCDHB16	0.050	8.772E-01	3.908E-01
APEX2	0.050	6.440E-01	1.097E-01
MCM3	0.050	7.364E-01	7.778E-02
TULP1	0.050	8.425E-01	5.948E-01
LEMD2	0.050	5.596E-01	5.757E-01
DRD1	0.050	9.292E-01	1.530E-01
CANX	0.050	5.765E-01	6.392E-02
CENPB	0.050	6.771E-01	6.748E-02
NSMCE2	0.050	6.142E-01	7.847E-02
NID2	0.050	8.829E-01	3.240E-02
PEX10	0.050	5.729E-01	5.504E-01
ACBD5	0.050	7.481E-01	7.556E-02
PAK2	0.050	5.436E-01	7.433E-01
TMED1	0.050	6.713E-01	1.147E-01
MYL12B	0.050	6.603E-01	1.177E-01
RPRD2	0.049	6.946E-01	7.323E-01
ZFAND3	0.049	5.213E-01	1.489E-01
UHRF2	0.049	7.295E-01	2.173E-01
NADK	0.049	5.522E-01	4.050E-02
ARL2BP	0.049	7.684E-01	1.596E-01
CYP7B1	0.049	8.839E-01	2.731E-01
RALGPS2	0.049	7.794E-01	1.126E-01
DAPK3	0.049	6.953E-01	1.513E-01
FLNB	0.049	8.046E-01	6.021E-02
CTBP2	0.049	7.373E-01	2.194E-01
GSTK1	0.049	7.311E-01	5.248E-01
ZBTB7B	0.049	7.480E-01	1.626E-01
VPS53	0.049	5.583E-01	8.723E-02
C19orf12	0.049	7.533E-01	4.534E-01
MIR3619	0.049	NA	9.198E-02
RND2	0.049	8.932E-01	1.193E-01
VPS4B	0.049	6.732E-01	1.047E-01
STARD6	0.049	9.032E-01	7.000E-02
PRR11	0.049	8.198E-01	1.613E-01
REEP4	0.049	7.559E-01	3.648E-01
TRAPPC3	0.049	5.048E-01	1.243E-01
NTAN1	0.049	7.722E-01	1.134E-01
GORAB	0.049	7.084E-01	1.520E-01
NKD1	0.049	8.889E-01	2.467E-01

TIMM23	0.049	6.185E-01	4.421E-01
NUDC	0.049	6.490E-01	7.145E-01
EXOC6	0.049	6.826E-01	8.754E-01
ZBTB41	0.049	7.046E-01	1.336E-01
GTF3C1	0.049	5.480E-01	1.310E-01
USP9X	0.049	6.293E-01	8.160E-01
LCTL	0.049	8.242E-01	8.671E-02
MTDH	0.049	7.244E-01	6.406E-01
LINC01125	0.049	8.279E-01	1.228E-01
MIR4796	0.049	NA	6.871E-01
MIR8073	0.049	NA	2.960E-01
MIR8053	0.049	NA	4.689E-02
OR10G7	0.049	NA	2.874E-01
KRTAP6-1	0.049	NA	4.076E-02
MIR138-2	0.049	NA	7.637E-02
TADA3	0.049	6.886E-01	3.215E-01
TMEM230	0.049	5.996E-01	1.306E-01
GPR45	0.049	9.173E-01	2.073E-01
NKAP	0.049	5.050E-01	3.020E-01
DLL3	0.049	9.246E-01	9.593E-01
ZNF532	0.048	8.072E-01	9.569E-01
SLC35A3	0.048	7.161E-01	9.566E-01
NAGLU	0.048	7.214E-01	3.508E-01
ZNF354C	0.048	8.671E-01	3.977E-01
BOD1	0.048	5.826E-01	4.393E-01
CENPP	0.048	7.513E-01	2.920E-01
KCNA1	0.048	9.525E-01	6.793E-01
NBPF19	0.048	7.662E-01	5.422E-01
ITIH3	0.048	8.986E-01	5.270E-01
RALY	0.048	6.513E-01	5.894E-01
G6PC3	0.048	7.080E-01	1.523E-01
BCKDK	0.048	5.846E-01	3.294E-01
TRIP4	0.048	4.752E-01	2.304E-01
ATG101	0.048	6.938E-01	8.782E-02
OPA1	0.048	6.122E-01	7.451E-02
TMEM119	0.048	8.934E-01	1.243E-01
SCNN1D	0.048	8.667E-01	2.115E-01
SIL1	0.048	7.442E-01	7.813E-02
CHMP5	0.048	6.748E-01	1.214E-01
SRPRB	0.048	6.544E-01	1.694E-01
BRWD1	0.048	6.894E-01	3.352E-01
LSM1	0.048	7.426E-01	7.036E-01
BHLHE23	0.048	NA	1.766E-01
KCNK1	0.048	8.598E-01	6.827E-01
DHRS11	0.048	8.217E-01	2.707E-01
UBA2	0.048	6.620E-01	7.706E-02
SPEN	0.048	6.529E-01	3.975E-01
ACTRT1	0.048	NA	7.341E-02
PKD1	0.048	7.325E-01	3.169E-01
BCL11A	0.048	9.093E-01	5.450E-01

SMAD4	0.048	6.188E-01	4.136E-01
AZI2	0.048	6.860E-01	2.802E-01
FCGR1A	0.048	9.092E-01	7.446E-01
SH3BP4	0.048	8.292E-01	4.884E-01
TMSB4X	0.048	7.889E-01	1.077E-01
KIN	0.048	6.897E-01	4.655E-01
UBAC1	0.048	7.254E-01	8.262E-01
C7orf49	0.048	6.321E-01	5.756E-02
STAT3	0.048	7.024E-01	3.165E-01
PTPRN	0.048	9.248E-01	6.666E-01
C2orf42	0.047	5.127E-01	5.594E-01
BAG4	0.047	7.851E-01	1.396E-01
LBX2	0.047	8.385E-01	3.300E-01
TAX1BP1	0.047	6.321E-01	5.892E-02
AC114296.1	0.047	9.344E-01	5.604E-01
MERTK	0.047	8.506E-01	1.620E-01
C15orf39	0.047	6.816E-01	4.794E-01
PLCB1	0.047	8.181E-01	4.750E-01
POMZP3	0.047	7.902E-01	2.460E-01
XKRX	0.047	8.756E-01	1.229E-01
TMF1	0.047	7.172E-01	2.484E-01
COX17	0.047	7.191E-01	1.695E-01
EPG5	0.047	6.589E-01	2.689E-01
ZNF468	0.047	7.867E-01	9.264E-01
CHUK	0.047	6.335E-01	7.342E-01
BRCA1	0.047	7.802E-01	4.846E-01
DNAH3	0.047	8.822E-01	3.374E-01
SYPL1	0.047	7.446E-01	9.288E-01
MYCNOS	0.047	9.456E-01	9.263E-01
GNGT1	0.047	9.019E-01	2.419E-01
ANKRD13C	0.047	5.315E-01	2.481E-01
ICE1	0.047	7.354E-01	3.713E-01
VAPB	0.047	6.439E-01	7.939E-02
ZDHHC1	0.047	8.153E-01	1.634E-01
RPA1	0.047	6.877E-01	5.282E-01
C4orf19	0.047	9.033E-01	4.503E-01
C11orf65	0.047	7.971E-01	8.788E-02
NCAPH	0.047	8.172E-01	4.888E-01
RASSF5	0.047	8.167E-01	4.085E-01
SERTAD2	0.047	7.441E-01	1.867E-01
UMAD1	0.047	6.904E-01	2.416E-01
EXOC8	0.047	6.382E-01	1.510E-01
SHB	0.047	7.970E-01	2.277E-01
MAPK9	0.047	5.648E-01	1.925E-01
ZNF132	0.047	8.232E-01	2.536E-01
ADGRF4	0.047	8.772E-01	1.199E-01
TIGD2	0.047	7.873E-01	2.333E-01
LRP10	0.047	7.321E-01	6.082E-01
GCLM	0.047	8.765E-01	8.124E-02
MAP10	0.047	8.631E-01	8.176E-02

DNALI1	0.046	9.147E-01	5.736E-01
NHLRC1	0.046	8.495E-01	1.240E-01
WDR5B	0.046	7.010E-01	5.612E-01
ZNF584	0.046	6.927E-01	2.567E-02
ARID1A	0.046	6.937E-01	7.291E-01
NUP214	0.046	5.964E-01	5.096E-02
RPS6KC1	0.046	6.398E-01	4.701E-01
ANKRD26	0.046	7.322E-01	1.779E-01
AC104109.3	0.046	9.027E-01	3.071E-01
ZFP1	0.046	6.853E-01	6.672E-01
PEBP1	0.046	8.118E-01	2.266E-01
SIRT4	0.046	8.386E-01	3.255E-01
CCL16	0.046	9.274E-01	1.448E-01
43895.000	0.046	6.036E-01	3.677E-01
DOCK9	0.046	7.789E-01	4.897E-01
EPHB4	0.046	8.007E-01	1.211E-01
PCDHA7	0.046	9.212E-01	2.599E-01
APBB2	0.046	7.744E-01	7.656E-01
COLGALT1	0.046	7.479E-01	2.090E-01
DNAJC12	0.046	8.878E-01	5.381E-01
OR8A1	0.046	9.698E-01	2.787E-01
TTC28	0.046	8.446E-01	1.651E-01
MIR8068	0.046	NA	1.941E-01
MIR6799	0.046	NA	8.507E-02
MIR6731	0.046	NA	2.003E-01
OR2S2	0.046	NA	4.043E-01
ESF1	0.046	7.293E-01	9.755E-01
KIAA1217	0.046	7.436E-01	9.698E-01
RPS27	0.046	7.460E-01	9.697E-01
DENND1A	0.046	7.347E-01	9.694E-01
ATP2C1	0.046	6.422E-01	6.173E-02
TMEM231	0.046	7.805E-01	4.662E-01
USP37	0.046	8.421E-01	2.248E-01
AC233723.1	0.046	8.725E-01	1.267E-01
MIR30C1	0.046	NA	3.464E-01
ARR3	0.046	8.315E-01	2.427E-01
MCCC1	0.046	8.058E-01	6.247E-02
DCAF12	0.046	6.987E-01	1.613E-01
SMC6	0.046	7.192E-01	9.383E-01
EIF2B4	0.046	5.696E-01	2.041E-01
KCNH3	0.046	8.998E-01	5.251E-02
ARHGEF11	0.045	6.840E-01	1.157E-01
TRIM4	0.045	6.841E-01	2.371E-01
C11orf94	0.045	8.854E-01	7.660E-02
UBN1	0.045	6.609E-01	8.475E-01
MCM10	0.045	8.452E-01	1.763E-01
MIR599	0.045	NA	2.839E-01
TGIF2LY	0.045	NA	1.788E-01
SHOC2	0.045	5.744E-01	3.456E-01
ORC5	0.045	6.128E-01	1.369E-01

MAP4K2	0.045	7.333E-01	1.118E-01
SLC7A6	0.045	8.183E-01	1.730E-01
BCL2L12	0.045	7.558E-01	6.834E-01
CDK5RAP2	0.045	7.809E-01	2.978E-01
KRTAP9-6	0.045	NA	3.538E-01
DHRS9	0.045	9.397E-01	7.901E-02
GPR142	0.045	9.375E-01	8.242E-02
SHISA4	0.045	8.283E-01	3.348E-01
APOBEC3F	0.045	8.172E-01	2.133E-01
ADGRE5	0.045	8.397E-01	2.067E-01
ZNF212	0.045	5.996E-01	2.679E-01
MDC1	0.045	7.510E-01	6.495E-02
ZNF362	0.045	7.085E-01	5.595E-01
PLPP6	0.045	7.383E-01	7.938E-01
CDHR3	0.045	8.466E-01	1.694E-01
TMEM160	0.045	8.204E-01	6.625E-02
MIR4786	0.045	9.107E-01	6.427E-02
ZNF780A	0.045	7.692E-01	9.862E-02
TUBB	0.045	7.288E-01	6.115E-01
TPM4	0.045	7.656E-01	5.955E-01
MFSD13A	0.045	7.953E-01	2.694E-01
CTDSPL2	0.045	6.582E-01	1.693E-01
KLK3	0.045	9.729E-01	3.741E-01
STOM	0.045	8.720E-01	1.250E-01
PRKAG1	0.045	5.012E-01	5.252E-01
NUP205	0.045	7.298E-01	9.497E-01
TICAM1	0.045	7.510E-01	1.910E-01
MIR3609	0.045	9.380E-01	9.472E-01
UBASH3B	0.045	8.750E-01	9.417E-01
MED14	0.044	6.967E-01	9.392E-01
CDC42BPA	0.044	8.055E-01	4.691E-01
ANG	0.044	8.621E-01	8.457E-02
ST5	0.044	8.446E-01	2.397E-01
LSM11	0.044	7.412E-01	8.789E-01
43893.000	0.044	8.566E-01	7.739E-01
NENF	0.044	7.789E-01	6.504E-01
NUDT7	0.044	8.815E-01	1.592E-01
BICD2	0.044	8.084E-01	2.169E-01
MIR3151	0.044	NA	2.537E-01
FOXP4	0.044	7.271E-01	6.976E-01
TMEM269	0.044	9.075E-01	1.585E-01
SEMG2	0.044	9.623E-01	4.706E-01
CCDC30	0.044	8.361E-01	6.764E-01
DNAJC9	0.044	7.368E-01	2.830E-01
TRIM62	0.044	7.848E-01	6.298E-01
FPGT	0.044	6.802E-01	6.064E-02
LENEP	0.044	9.180E-01	2.795E-01
POU5F2	0.044	8.937E-01	4.543E-01
DNAJC11	0.044	6.612E-01	9.463E-02
ANKMY2	0.044	7.212E-01	4.323E-01

COX7A2L	0.044	6.030E-01	4.832E-01
CTAGE8	0.044	9.080E-01	1.266E-01
MAP3K6	0.044	8.023E-01	7.241E-01
MIR3134	0.044	NA	7.200E-01
PLEKHA5	0.044	7.794E-01	2.037E-01
CAMSAP2	0.044	7.193E-01	2.332E-01
SYN1	0.044	8.983E-01	1.306E-01
STMN2	0.044	9.452E-01	3.738E-01
MCM4	0.044	7.934E-01	1.222E-01
EIF4E3	0.044	8.195E-01	8.983E-01
TMUB2	0.044	5.699E-01	9.449E-01
PPP2R1A	0.044	5.985E-01	1.314E-01
SYTL4	0.044	8.458E-01	3.467E-02
BCKDHA	0.044	7.632E-01	1.372E-01
FAIM	0.044	7.203E-01	8.712E-02
CAPN1	0.044	7.222E-01	6.515E-02
AGAP9	0.044	8.533E-01	5.121E-02
DCHS2	0.044	9.314E-01	1.525E-01
ADGRL4	0.044	8.494E-01	3.390E-01
BCAP29	0.044	6.989E-01	2.575E-01
RNF167	0.044	6.612E-01	1.939E-01
KLHDC1	0.044	8.361E-01	4.071E-01
PPIB	0.044	7.029E-01	8.342E-02
NFE2L1	0.044	6.971E-01	3.474E-01
TRMT44	0.044	6.884E-01	6.479E-01
SPAST	0.044	6.455E-01	2.356E-01
NAB2	0.044	7.927E-01	2.447E-01
TST	0.043	8.661E-01	7.571E-01
FASTK	0.043	7.390E-01	5.837E-01
NUFIP2	0.043	7.152E-01	6.562E-01
FBXL18	0.043	8.171E-01	5.788E-01
RASIP1	0.043	8.883E-01	1.966E-01
LRRC42	0.043	7.874E-01	1.944E-01
GPAT4	0.043	7.526E-01	5.769E-01
MIR3200	0.043	NA	2.078E-01
YY2	0.043	8.164E-01	9.763E-01
ARHGEF37	0.043	8.791E-01	9.763E-01
RAB43	0.043	7.958E-01	9.763E-01
MT1E	0.043	9.238E-01	9.763E-01
ATP6V0C	0.043	6.954E-01	9.745E-01
SIAE	0.043	8.208E-01	9.745E-01
RETREG1	0.043	9.095E-01	9.741E-01
BUD13	0.043	6.362E-01	1.890E-01
MROH8	0.043	8.481E-01	1.751E-01
ALDH9A1	0.043	7.453E-01	5.946E-01
MIR378J	0.043	8.862E-01	6.899E-01
WDR60	0.043	7.125E-01	1.413E-01
TCF12	0.043	7.251E-01	3.487E-01
DPY19L4	0.043	7.475E-01	4.850E-01
HEATR5A	0.043	7.161E-01	1.448E-01

VPS13C	0.043	7.671E-01	1.490E-01
BPTF	0.043	6.983E-01	4.311E-01
TMEM43	0.043	7.275E-01	1.594E-01
ODF3	0.043	9.508E-01	1.003E-01
FAM168A	0.043	7.193E-01	8.714E-02
EPC2	0.043	6.559E-01	1.902E-01
TMEM101	0.043	7.572E-01	3.373E-01
RTL8A	0.043	7.559E-01	4.120E-01
ART5	0.043	9.272E-01	1.774E-01
MAZ	0.043	7.481E-01	2.477E-01
TRIP13	0.043	8.632E-01	1.256E-01
FOSL1	0.043	9.041E-01	7.794E-02
MRPS5	0.043	6.683E-01	1.964E-01
AL391650.1	0.043	8.448E-01	1.078E-01
ZSCAN22	0.043	6.840E-01	6.703E-01
SNRNP40	0.043	6.603E-01	3.519E-01
ZNF428	0.043	7.966E-01	7.444E-01
OSBPL9	0.042	6.261E-01	8.496E-02
ADGRG1	0.042	8.329E-01	2.248E-01
NIM1K	0.042	8.655E-01	1.684E-01
SPTSSA	0.042	8.296E-01	2.278E-01
NABP2	0.042	6.927E-01	2.989E-01
LLGL2	0.042	8.504E-01	1.184E-01
UPF3B	0.042	7.283E-01	2.142E-01
HRCT1	0.042	9.171E-01	4.395E-01
PIH1D1	0.042	6.962E-01	7.448E-02
CADM1	0.042	9.238E-01	5.495E-01
TRMT61B	0.042	6.935E-01	9.728E-02
GNG3	0.042	8.496E-01	2.463E-01
ZNF613	0.042	8.414E-01	1.621E-01
BROX	0.042	6.504E-01	3.729E-01
NOC2L	0.042	6.814E-01	3.181E-01
PTPRA	0.042	6.073E-01	1.984E-01
DHX57	0.042	6.516E-01	6.168E-01
HSDL2	0.042	8.416E-01	8.339E-02
ALX3	0.042	9.671E-01	1.699E-01
GMPS	0.042	7.299E-01	2.779E-01
L2HGDH	0.042	7.941E-01	2.582E-01
RNF207	0.042	8.643E-01	7.039E-01
ALDH18A1	0.042	6.486E-01	9.989E-02
SMG6	0.042	7.794E-01	5.039E-01
GRB2	0.042	6.030E-01	2.772E-01
CCL7	0.042	9.450E-01	1.041E-01
UBL4A	0.042	7.439E-01	1.156E-01
SLC25A19	0.042	7.535E-01	8.509E-01
DPP8	0.042	6.722E-01	1.069E-01
PTPRM	0.042	8.839E-01	5.604E-02
OR2J2	0.042	NA	7.556E-01
UHRF1	0.042	8.278E-01	1.273E-01
HINT2	0.042	8.033E-01	4.444E-01

PCLAF	0.042	8.350E-01	2.542E-01
TBKBP1	0.042	8.167E-01	2.378E-01
SPINK2	0.042	9.326E-01	8.592E-02
NECAP2	0.042	6.507E-01	1.454E-01
TMTC4	0.042	8.192E-01	4.009E-01
EIF4G1	0.042	6.713E-01	1.644E-01
EIF4H	0.042	6.239E-01	3.947E-01
SQSTM1	0.042	8.018E-01	1.353E-01
C6orf47	0.042	6.273E-01	1.351E-01
GOLPH3L	0.042	7.812E-01	9.539E-01
PUS7	0.042	7.851E-01	9.514E-01
TRPM8	0.042	9.346E-01	5.727E-01
DNAJC8	0.041	5.474E-01	1.489E-01
PFDN2	0.041	8.306E-01	1.427E-01
AC010325.1	0.041	9.218E-01	5.797E-01
MIR3924	0.041	NA	5.038E-01
MIR4293	0.041	NA	3.496E-01
MIR618	0.041	NA	7.552E-02
AL359922.1	0.041	NA	5.779E-01
MIR6823	0.041	NA	1.156E-01
MIR4448	0.041	NA	2.647E-01
IQCF5	0.041	NA	3.477E-01
LSM2	0.041	7.485E-01	1.239E-01
DPM2	0.041	7.656E-01	2.424E-01
CDADC1	0.041	7.627E-01	2.003E-01
GRIP2	0.041	9.026E-01	9.740E-02
SLIT1	0.041	9.081E-01	1.408E-01
SYNE4	0.041	9.094E-01	5.972E-01
HSP90AB1	0.041	7.267E-01	1.159E-01
ZNF730	0.041	9.379E-01	5.275E-02
ZNF256	0.041	8.121E-01	1.180E-01
HSD11B2	0.041	9.026E-01	3.013E-01
BCL7B	0.041	6.141E-01	2.751E-01
CLN6	0.041	7.754E-01	7.627E-01
MIR1273F	0.041	NA	5.424E-01
DICER1	0.041	7.415E-01	3.029E-01
DBI	0.041	8.184E-01	6.032E-01
AL162231.3	0.041	NA	1.268E-01
C3orf14	0.041	9.003E-01	1.598E-01
YPEL4	0.041	8.693E-01	3.835E-01
KRT9	0.041	9.344E-01	4.428E-01
PPP2R5D	0.041	6.516E-01	9.240E-02
ATP8B2	0.041	8.849E-01	4.099E-01
PPP2CB	0.041	7.274E-01	2.976E-01
CRTC2	0.041	6.603E-01	2.953E-01
ITGAV	0.041	8.317E-01	7.676E-01
IGHMBP2	0.041	7.587E-01	1.795E-01
UBN2	0.041	7.696E-01	1.632E-01
NAP1L5	0.041	8.494E-01	2.495E-01
STEAP1	0.041	8.937E-01	4.563E-01

SAR1B	0.041	6.630E-01	3.472E-01
TXNDC16	0.041	8.523E-01	6.297E-01
ALAS1	0.041	8.599E-01	2.334E-01
KLC2	0.041	7.363E-01	2.843E-01
AC005520.1	0.041	9.128E-01	1.992E-01
CBLL1	0.041	6.454E-01	2.787E-01
NQO1	0.041	9.018E-01	1.627E-01
GOLGB1	0.041	7.029E-01	4.306E-01
ZDHHC4	0.041	7.085E-01	9.771E-01
MCL1	0.041	7.941E-01	9.771E-01
UROS	0.041	7.295E-01	9.771E-01
OR52J3	0.041	NA	9.771E-01
MIR3161	0.041	NA	9.771E-01
OR2L5	0.041	NA	9.771E-01
HKR1	0.041	7.800E-01	9.771E-01
MASTL	0.041	7.783E-01	9.771E-01
HOXC5	0.041	9.394E-01	9.771E-01
SIAH2	0.041	7.203E-01	9.771E-01
AC099811.2	0.041	NA	9.771E-01
FLYWCH2	0.041	7.709E-01	9.771E-01
GINM1	0.040	6.776E-01	9.771E-01
MLX	0.040	5.935E-01	9.771E-01
GOLGA1	0.040	6.030E-01	9.771E-01
TFDP1	0.040	7.941E-01	9.771E-01
ANKRD65	0.040	9.095E-01	9.771E-01
TBC1D31	0.040	7.567E-01	3.558E-01
PAXIP1	0.040	7.064E-01	8.870E-02
ARHGAP35	0.040	7.756E-01	2.852E-01
LMBR1	0.040	6.912E-01	5.265E-01
ZNF433	0.040	8.512E-01	5.599E-01
PRPSAP1	0.040	6.222E-01	4.701E-01
ENTPD7	0.040	8.655E-01	4.545E-01
ADAR	0.040	7.251E-01	2.500E-01
CHP1	0.040	7.057E-01	1.538E-02
ZNF236	0.040	7.661E-01	7.443E-02
NPPB	0.040	9.640E-01	6.657E-01
SRRM5	0.040	8.628E-01	1.158E-01
VPS45	0.040	7.098E-01	4.977E-01
HERC1	0.040	6.834E-01	1.611E-01
IRAK1	0.040	8.107E-01	8.991E-01
OR13H1	0.040	NA	1.781E-01
ZNF514	0.040	8.024E-01	5.713E-01
NFATC3	0.040	7.710E-01	2.964E-01
ARL6IP1	0.040	7.771E-01	6.074E-01
KIF5B	0.040	7.301E-01	7.813E-02
CTNNB1	0.040	7.606E-01	1.772E-01
ASCC1	0.040	7.203E-01	1.208E-01
SLCO1C1	0.040	9.056E-01	6.284E-02
NBPF9	0.040	8.096E-01	2.986E-01
RANBP6	0.040	7.875E-01	7.788E-02

MIR6842	0.040	NA	6.767E-02
GDE1	0.040	8.103E-01	4.925E-01
WAPL	0.040	6.545E-01	8.981E-01
TAF3	0.040	7.563E-01	1.721E-01
DLK2	0.040	9.093E-01	8.573E-01
RAB8B	0.040	7.739E-01	8.886E-01
PLBD2	0.040	7.467E-01	2.946E-01
BRPF3	0.040	7.651E-01	3.716E-01
SNAPC5	0.040	6.840E-01	5.905E-01
PPP1R8	0.040	5.573E-01	5.057E-01
EFTUD2	0.040	6.453E-01	7.737E-02
VEGFD	0.040	9.218E-01	4.657E-01
ACD	0.040	7.551E-01	2.758E-01
CASTOR1	0.040	8.546E-01	1.179E-01
TCF24	0.040	8.999E-01	4.327E-01
VAX2	0.040	9.310E-01	5.941E-01
LIF	0.039	9.152E-01	4.948E-01
ZNF570	0.039	8.561E-01	4.656E-01
ISY1-RAB43	0.039	9.272E-01	3.522E-01
QSOX2	0.039	8.197E-01	4.016E-01
OARD1	0.039	7.339E-01	5.497E-02
HSP90B1	0.039	7.494E-01	1.734E-01
C9orf3	0.039	8.533E-01	7.364E-01
SMIM18	0.039	9.219E-01	6.654E-02
GPATCH8	0.039	6.910E-01	4.009E-01
NVL	0.039	6.720E-01	2.795E-01
C10orf53	0.039	9.725E-01	2.043E-01
GBP7	0.039	9.142E-01	3.179E-01
NUDCD1	0.039	7.883E-01	3.754E-01
BUD31	0.039	6.776E-01	8.411E-02
SPATA18	0.039	9.010E-01	1.257E-01
SCN3B	0.039	9.283E-01	4.273E-01
HACL1	0.039	7.883E-01	7.483E-01
TUBA4B	0.039	9.347E-01	9.721E-01
HPS3	0.039	7.627E-01	9.709E-01
AP5Z1	0.039	7.354E-01	9.639E-01
SECISBP2L	0.039	7.769E-01	9.636E-01
MSL1	0.039	6.813E-01	9.587E-01
OR11A1	0.039	NA	9.558E-01
GADL1	0.039	9.442E-01	9.532E-01
SLC2A11	0.039	8.513E-01	1.893E-01
FKBP1A	0.039	6.979E-01	2.029E-01
TXNDC9	0.039	7.598E-01	1.370E-01
MIGA1	0.039	7.868E-01	8.009E-01
PPP2R2D	0.039	7.441E-01	5.158E-01
WNT10B	0.039	9.190E-01	6.371E-01
CHTOP	0.039	5.895E-01	2.270E-01
PHF7	0.039	8.195E-01	1.436E-01
NAPA	0.039	6.842E-01	8.343E-01
SPA17	0.039	8.079E-01	1.522E-01

NOX1	0.039	8.973E-01	4.126E-02
IGFBP6	0.039	9.267E-01	5.342E-01
PSMC3	0.039	7.618E-01	6.558E-01
ATP8B4	0.039	8.945E-01	1.241E-01
ACAA2	0.039	8.908E-01	1.245E-01
KRT19	0.039	8.986E-01	1.869E-01
MYB	0.039	9.181E-01	7.617E-01
EVI2A	0.039	9.133E-01	8.136E-01
HMGB3	0.039	8.281E-01	4.165E-01
RELA	0.039	5.714E-01	2.210E-01
OR2AG1	0.039	NA	2.548E-01
MIR190A	0.039	NA	3.846E-01
MIR5739	0.039	NA	2.025E-01
OR5W2	0.039	NA	1.057E-01
MIR4433B	0.039	NA	2.154E-01
MIR551A	0.039	NA	1.306E-01
USP12	0.039	7.443E-01	3.516E-01
IRX5	0.039	9.292E-01	4.445E-01
IDE	0.039	7.294E-01	2.800E-01
PPFIBP1	0.039	8.383E-01	9.337E-01
FAM3A	0.038	7.697E-01	5.120E-02
TOPBP1	0.038	7.763E-01	2.248E-01
PSMA7	0.038	7.339E-01	1.640E-01
RAD23A	0.038	6.371E-01	1.800E-01
NOMO3	0.038	8.981E-01	2.112E-01
GNRHR	0.038	9.051E-01	6.105E-01
SLC38A10	0.038	7.559E-01	6.947E-02
KIF18B	0.038	8.544E-01	5.831E-01
RPS6KA2	0.038	8.620E-01	1.646E-01
TERF2IP	0.038	7.099E-01	7.288E-01
ZNF823	0.038	8.781E-01	1.003E-01
RAB1B	0.038	6.650E-01	5.545E-01
MIR550A1	0.038	NA	7.373E-01
TMEM45B	0.038	9.250E-01	5.815E-01
BCDIN3D	0.038	7.579E-01	9.366E-01
NBPF14	0.038	8.362E-01	2.539E-01
NXF2	0.038	NA	1.202E-01
PRDX1	0.038	7.998E-01	1.924E-01
HS3ST5	0.038	9.576E-01	7.960E-02
RNF213	0.038	8.534E-01	5.244E-01
SEC63	0.038	6.620E-01	8.021E-02
CKS2	0.038	8.473E-01	1.772E-01
ZNF497	0.038	8.345E-01	3.080E-01
NSFL1C	0.038	7.024E-01	1.752E-01
UBR3	0.038	7.415E-01	8.245E-01
AHCTF1	0.038	7.244E-01	4.777E-01
RAB29	0.038	8.167E-01	1.831E-01
CCDC85B	0.038	8.759E-01	8.946E-01
MIR624	0.038	NA	5.476E-01
PRIM2	0.038	7.513E-01	5.814E-01

APC2	0.038	8.694E-01	1.913E-01
TSHR	0.038	9.389E-01	9.531E-01
LIN37	0.038	7.795E-01	5.873E-01
TOMM70	0.038	7.029E-01	9.273E-01
KIAA1328	0.038	7.523E-01	1.368E-01
LDAH	0.038	8.055E-01	5.460E-01
CRCP	0.038	6.612E-01	7.743E-01
APLNR	0.037	9.095E-01	1.449E-01
MANSC1	0.037	8.592E-01	4.358E-01
SAC3D1	0.037	8.519E-01	1.828E-01
FHAD1	0.037	9.171E-01	6.721E-01
CCDC71L	0.037	8.886E-01	5.855E-01
MIR661	0.037	9.563E-01	2.252E-01
KIAA2013	0.037	6.662E-01	8.516E-01
LARP1B	0.037	7.722E-01	4.805E-01
HAUS5	0.037	7.902E-01	1.820E-01
UBAP1	0.037	7.575E-01	6.826E-01
KRTAP2-4	0.037	NA	2.811E-01
CORO2A	0.037	8.688E-01	2.982E-01
MIR4671	0.037	NA	2.558E-01
OXNAD1	0.037	7.364E-01	1.464E-01
RMI1	0.037	8.317E-01	4.711E-01
AC022400.6	0.037	8.540E-01	1.454E-01
SZRD1	0.037	5.896E-01	1.447E-01
ACVR1	0.037	8.135E-01	9.928E-02
RNF216	0.037	7.290E-01	1.537E-01
MAP2K5	0.037	6.754E-01	7.714E-01
SLC12A1	0.037	9.551E-01	8.232E-02
MAPRE2	0.037	8.772E-01	8.057E-01
F1E1-BLOC1	0.037	NA	5.475E-01
STRADB	0.037	8.235E-01	3.083E-01
ZFP91	0.037	6.949E-01	7.182E-02
ZNF777	0.037	6.387E-01	1.247E-01
LRRC71	0.037	9.192E-01	1.268E-01
MEI4	0.037	9.600E-01	3.373E-01
SETD2	0.037	7.114E-01	3.342E-01
PIGK	0.037	8.046E-01	1.392E-01
CRYBA2	0.037	9.656E-01	1.990E-01
PPP3CB	0.037	7.234E-01	1.443E-01
TSC22D4	0.037	7.494E-01	9.447E-02
PIK3R1	0.037	8.713E-01	3.855E-01
VCP	0.037	7.098E-01	1.368E-01
OSR1	0.037	9.333E-01	1.624E-01
HOXC10	0.037	9.579E-01	5.378E-01
CKB	0.037	9.207E-01	4.472E-01
RAB36	0.037	8.925E-01	2.071E-01
EPB41L1	0.037	8.772E-01	3.508E-01
NELFE	0.037	7.719E-01	6.613E-01
EIF5B	0.037	7.970E-01	3.944E-01
CLASP2	0.037	7.415E-01	1.385E-01

LRRC45	0.036	8.283E-01	2.108E-01
RAP1A	0.036	7.024E-01	4.570E-01
SLC1A5	0.036	8.427E-01	1.441E-01
GPR27	0.036	9.329E-01	9.782E-01
SYAP1	0.036	6.884E-01	9.752E-01
ALLC	0.036	9.575E-01	9.705E-01
KLLN	0.036	8.574E-01	9.675E-01
PSMD12	0.036	6.870E-01	9.675E-01
TAF1	0.036	7.770E-01	9.628E-01
C3	0.036	9.344E-01	4.474E-01
EFL1	0.036	7.244E-01	1.406E-01
MIR6832	0.036	9.455E-01	4.220E-01
NFIL3	0.036	8.662E-01	2.943E-01
RNF112	0.036	9.171E-01	2.648E-01
KIF22	0.036	8.297E-01	1.207E-01
KLHL33	0.036	9.519E-01	1.858E-01
IGSF8	0.036	8.480E-01	5.788E-01
ARHGAP21	0.036	8.349E-01	5.119E-01
ENHO	0.036	9.425E-01	2.933E-01
PIGW	0.036	7.725E-01	1.698E-01
DCDC2C	0.036	NA	1.419E-01
MIR6816	0.036	NA	3.134E-01
POTED	0.036	NA	5.234E-01
MIR4782	0.036	9.456E-01	8.013E-01
RND3	0.036	8.770E-01	2.135E-01
GSC2	0.036	NA	5.265E-01
SELPLG	0.036	9.225E-01	5.038E-01
AKAP2	0.036	9.345E-01	2.491E-01
GALNT6	0.036	9.103E-01	2.414E-01
KLHL9	0.036	8.715E-01	7.404E-01
ZNF790	0.036	8.145E-01	7.531E-01
KRBOX4	0.036	7.475E-01	7.399E-01
SMAD2	0.036	7.036E-01	2.879E-01
DYNC1LI1	0.036	7.222E-01	9.265E-01
ACTR2	0.036	7.291E-01	4.045E-01
AURKA	0.036	8.635E-01	5.160E-01
FOXM1	0.036	8.834E-01	2.330E-01
ZNF226	0.036	7.904E-01	1.448E-01
MPP5	0.035	7.691E-01	2.491E-01
SOS2	0.035	7.480E-01	7.608E-01
ZNF417	0.035	7.957E-01	5.564E-02
PUS10	0.035	8.278E-01	6.583E-01
POTEI	0.035	9.328E-01	2.162E-01
SCYL1	0.035	6.958E-01	1.898E-01
GPATCH3	0.035	7.474E-01	1.301E-01
HSPB1	0.035	8.876E-01	1.238E-01
NBR1	0.035	7.196E-01	1.135E-01
TMEM203	0.035	7.431E-01	2.864E-01
ATP6V1E1	0.035	7.283E-01	1.323E-01
FMNL3	0.035	8.545E-01	5.260E-01

AC003688.1	0.035	NA	7.329E-01
AC093893.1	0.035	9.534E-01	6.500E-01
ACSS3	0.035	9.314E-01	1.397E-01
PSMB5	0.035	7.536E-01	3.060E-01
RTN2	0.035	8.760E-01	2.783E-01
MIR6895	0.035	9.326E-01	1.908E-01
PAFAH1B1	0.035	6.641E-01	1.688E-01
UBQLN4	0.035	7.528E-01	6.442E-01
SNRNP200	0.035	7.041E-01	2.017E-01
POP4	0.035	8.153E-01	5.967E-01
PRAF2	0.035	8.512E-01	4.555E-01
TAOK2	0.035	7.122E-01	5.104E-01
ARMCX5	0.035	7.523E-01	6.203E-01
TMEM143	0.035	7.904E-01	7.348E-01
ZC3H6	0.035	8.168E-01	2.189E-01
WIPF2	0.035	7.225E-01	8.636E-01
PTBP3	0.035	7.912E-01	2.232E-01
EPS8L2	0.035	8.668E-01	1.501E-01
AL110118.2	0.035	9.092E-01	1.151E-01
ABCF3	0.035	6.409E-01	9.500E-01
RTEL1	0.035	8.552E-01	9.479E-01
SEMA3C	0.035	8.933E-01	9.410E-01
BTBD1	0.035	6.324E-01	4.917E-01
CREBL2	0.035	7.832E-01	7.734E-01
OR6C2	0.035	9.835E-01	3.254E-01
ARPC5L	0.035	8.046E-01	7.271E-02
GNPAT	0.035	6.664E-01	8.434E-01
PRTG	0.035	9.356E-01	1.924E-01
ITPKC	0.035	8.471E-01	7.011E-01
NEK8	0.035	8.574E-01	2.091E-01
KDM3A	0.035	8.054E-01	6.219E-01
GBP3	0.035	8.903E-01	7.132E-01
PUDP	0.035	8.494E-01	5.920E-01
M6PR	0.035	8.230E-01	3.730E-01
AFMID	0.035	7.867E-01	2.394E-01
GPBP1L1	0.035	6.321E-01	1.985E-01
SLC25A14	0.035	7.460E-01	1.910E-01
DPY30	0.035	7.296E-01	2.017E-01
SENP5	0.035	6.992E-01	7.002E-01
FGFBP3	0.035	8.962E-01	1.743E-01
GPATCH11	0.035	7.590E-01	6.797E-01
VEZF1	0.035	7.360E-01	1.294E-01
ERI3	0.034	7.441E-01	2.892E-01
NO80B-WBP	0.034	9.100E-01	2.750E-01
JMJD1C	0.034	8.127E-01	5.899E-01
CHRNA9	0.034	9.600E-01	4.200E-01
WIPI2	0.034	7.161E-01	7.922E-01
PRSS53	0.034	8.727E-01	1.501E-01
TDRD3	0.034	7.710E-01	1.181E-01
CTDNEP1	0.034	7.311E-01	8.152E-01

CMC2	0.034	7.722E-01	5.578E-02
BAHCC1	0.034	8.870E-01	2.173E-01
MPI	0.034	7.696E-01	1.262E-01
CCDC7	0.034	8.143E-01	4.635E-01
KAT7	0.034	7.486E-01	5.246E-01
MRPL15	0.034	7.759E-01	2.065E-01
GSK3A	0.034	7.177E-01	2.530E-01
KDM5A	0.034	7.387E-01	1.007E-01
KIAA1549	0.034	9.033E-01	9.049E-01
EML3	0.034	7.782E-01	9.200E-01
NAA50	0.034	7.583E-01	3.706E-01
GPATCH4	0.034	7.909E-01	5.229E-01
TMEM261	0.034	8.383E-01	6.503E-01
ZNHIT3	0.034	7.346E-01	3.730E-01
AGA	0.034	8.145E-01	7.667E-01
TEX11	0.034	9.246E-01	3.498E-01
FRS3	0.034	8.074E-01	1.563E-01
JOSD2	0.034	8.544E-01	3.772E-01
LRRC23	0.034	8.334E-01	2.192E-01
LRRC47	0.034	6.426E-01	4.366E-01
CALHM2	0.034	8.632E-01	6.985E-02
KCNJ13	0.034	9.499E-01	8.061E-01
B3GLCT	0.034	8.369E-01	2.272E-01
OTUD5	0.034	6.647E-01	2.703E-01
DPP3	0.034	7.627E-01	2.646E-01
BBS12	0.034	8.055E-01	1.512E-01
DYNC1H1	0.034	6.915E-01	8.837E-01
MIR5089	0.034	NA	7.146E-02
TNFAIP8L2	0.034	9.261E-01	9.789E-01
ZSWIM8	0.034	7.874E-01	9.789E-01
ABI1	0.034	7.382E-01	9.745E-01
FHOD1	0.034	8.512E-01	9.741E-01
PIGQ	0.033	8.007E-01	9.737E-01
WDR88	0.033	8.775E-01	9.715E-01
RALGAPB	0.033	7.513E-01	9.714E-01
ANKRD23	0.033	8.834E-01	7.438E-01
LELP1	0.033	NA	6.613E-02
KDM2A	0.033	7.364E-01	1.284E-01
NMI	0.033	8.590E-01	3.278E-01
CCNO	0.033	9.019E-01	6.133E-01
DAP3	0.033	7.325E-01	3.278E-01
ELF5	0.033	9.498E-01	2.819E-01
USP29	0.033	NA	7.869E-01
FAM234B	0.033	8.993E-01	4.636E-01
MIR5088	0.033	NA	9.239E-01
OR2T33	0.033	NA	2.942E-01
PRY	0.033	NA	6.413E-01
PRR30	0.033	NA	3.278E-01
CCDC97	0.033	7.496E-01	3.377E-01
AFF1	0.033	8.284E-01	2.042E-01

NRP1	0.033	9.013E-01	1.804E-01
SDC3	0.033	8.775E-01	8.014E-01
DBR1	0.033	7.269E-01	3.739E-01
C19orf57	0.033	9.100E-01	1.170E-01
TLR5	0.033	9.052E-01	2.023E-01
GCA	0.033	8.407E-01	1.534E-01
DCK	0.033	8.361E-01	9.241E-01
ARL11	0.033	9.122E-01	7.041E-01
C11orf45	0.033	8.986E-01	7.896E-01
INIP	0.033	7.464E-01	4.699E-01
PDLIM4	0.033	9.292E-01	2.914E-01
ARHGEF4	0.033	9.444E-01	1.938E-01
NCAPD3	0.033	8.172E-01	2.548E-01
SLC25A38	0.033	7.955E-01	3.845E-01
CNIH4	0.033	7.446E-01	3.109E-01
CRY1	0.033	7.967E-01	2.327E-01
TM2D1	0.033	7.594E-01	1.875E-01
GLOD4	0.033	7.247E-01	2.972E-01
C2orf80	0.033	9.621E-01	7.574E-01
MIR3122	0.033	NA	1.126E-01
SLC27A3	0.033	8.368E-01	8.889E-01
MEGF9	0.033	8.626E-01	1.763E-01
ADGRL2	0.033	9.174E-01	3.266E-01
TMEM204	0.033	8.843E-01	2.227E-01
KCNN4	0.033	9.237E-01	6.656E-01
DYNLT3	0.033	8.666E-01	2.930E-01
NKD2	0.033	9.427E-01	3.473E-01
AC068533.4	0.033	9.314E-01	4.926E-01
ZNF232	0.033	8.512E-01	1.736E-01
HSPA12A	0.033	9.188E-01	4.390E-01
PIP4K2C	0.033	7.906E-01	4.030E-01
GRAMD1C	0.033	9.054E-01	1.949E-01
ZPR1	0.033	7.489E-01	1.369E-01
AIFM1	0.033	7.280E-01	2.098E-01
PATE1	0.033	9.785E-01	2.852E-01
ELOA	0.033	7.280E-01	3.370E-01
C15orf61	0.032	7.902E-01	1.245E-01
ZSCAN20	0.032	8.090E-01	1.913E-01
EIF4G3	0.032	7.619E-01	2.363E-01
ARSD	0.032	8.890E-01	2.374E-01
CYP2U1	0.032	8.845E-01	3.925E-01
NUP155	0.032	8.502E-01	7.295E-01
TNFSF9	0.032	9.440E-01	5.002E-01
AC027796.3	0.032	9.133E-01	2.003E-01
FAM136A	0.032	8.067E-01	1.868E-01
SMIM12	0.032	7.251E-01	3.591E-01
RABGAP1	0.032	7.628E-01	2.510E-01
C11orf54	0.032	8.329E-01	1.794E-01
MPDZ	0.032	8.815E-01	9.733E-01
ZNF2	0.032	7.480E-01	9.546E-01

CUL5	0.032	7.643E-01	9.544E-01
CYP2E1	0.032	9.444E-01	1.363E-01
SCRN3	0.032	7.867E-01	3.346E-01
JMJD6	0.032	7.637E-01	2.983E-02
RPE	0.032	7.858E-01	2.395E-01
RHOB	0.032	8.878E-01	3.378E-01
SEMA3G	0.032	9.046E-01	2.711E-01
RBM41	0.032	8.210E-01	2.299E-01
AKT2	0.032	7.583E-01	7.224E-01
RNF220	0.032	6.935E-01	1.092E-01
TDRD10	0.032	9.317E-01	5.327E-01
COA3	0.032	7.904E-01	3.433E-01
NUP133	0.032	7.295E-01	3.647E-01
MAPK15	0.032	9.341E-01	6.662E-02
ABHD8	0.032	8.655E-01	4.964E-01
CAB39	0.032	8.220E-01	4.876E-01
FYTTD1	0.032	7.654E-01	2.185E-01
CDK12	0.032	7.953E-01	6.564E-01
TSPYL2	0.032	8.645E-01	2.283E-01
NAA35	0.032	7.618E-01	2.458E-01
EDDM3B	0.032	NA	3.605E-01
MYO1C	0.032	8.153E-01	1.132E-01
KIF1B	0.032	8.245E-01	2.502E-01
GCG	0.032	9.822E-01	3.247E-01
MIR1179	0.032	NA	2.001E-01
MIR3921	0.032	NA	4.730E-01
MIR4801	0.032	NA	6.190E-01
LPGAT1	0.032	7.970E-01	5.004E-01
PLEKHO1	0.032	9.079E-01	2.050E-01
KAT5	0.032	6.993E-01	2.077E-01
IMPAD1	0.031	7.722E-01	3.982E-01
AC099329.3	0.031	9.388E-01	1.461E-01
WNT10A	0.031	9.512E-01	3.116E-01
C2orf81	0.031	9.013E-01	7.680E-01
PIGF	0.031	7.959E-01	7.526E-01
DDX50	0.031	7.261E-01	2.565E-01
DENND6A	0.031	7.475E-01	5.934E-01
GNAI3	0.031	7.858E-01	2.553E-01
PDZD8	0.031	8.235E-01	5.040E-01
ACOT2	0.031	8.438E-01	1.082E-01
EID1	0.031	7.654E-01	7.046E-02
XYLT2	0.031	7.874E-01	3.580E-01
PGK1	0.031	8.667E-01	4.164E-01
POLR3A	0.031	7.720E-01	1.041E-01
TMUB1	0.031	8.285E-01	3.093E-01
NPHP4	0.031	7.955E-01	1.670E-01
NFU1	0.031	7.559E-01	1.760E-01
ZNF180	0.031	8.092E-01	1.562E-01
ERN1	0.031	8.223E-01	1.783E-01
ZNF283	0.031	8.127E-01	1.150E-01

EGR1	0.031	9.248E-01	7.459E-01
ZDHHC5	0.031	6.977E-01	5.659E-01
CTU1	0.031	8.701E-01	4.174E-01
TMEM189	0.031	8.217E-01	8.266E-01
SIGLEC7	0.031	9.380E-01	1.851E-01
TRIM50	0.031	9.475E-01	1.967E-01
CAVIN4	0.031	9.095E-01	1.597E-01
UNC45A	0.031	7.095E-01	6.278E-01
RARA	0.031	8.136E-01	6.156E-02
NOD1	0.031	8.303E-01	6.043E-01
RBBP4	0.031	8.013E-01	2.382E-01
RRNAD1	0.031	8.075E-01	1.219E-01
SLC25A42	0.031	8.672E-01	2.247E-01
EFNA5	0.031	9.174E-01	8.097E-01
OR6C75	0.031	NA	5.085E-01
AC124312.1	0.031	NA	7.667E-01
FAM71A	0.031	9.761E-01	9.797E-01
ZNF587B	0.031	8.264E-01	9.797E-01
COTL1	0.031	9.004E-01	9.797E-01
EME1	0.031	8.880E-01	9.797E-01
CEBPB	0.031	8.908E-01	9.797E-01
NBEAL2	0.031	8.787E-01	9.797E-01
AP1M2	0.031	8.880E-01	9.797E-01
PNO1	0.031	7.684E-01	9.797E-01
MTM1	0.030	8.315E-01	9.797E-01
DYNC1LI2	0.030	8.552E-01	9.797E-01
TKFC	0.030	8.227E-01	9.797E-01
TMEM87B	0.030	7.955E-01	9.797E-01
ZDHHC24	0.030	8.265E-01	3.174E-01
SFRP5	0.030	9.692E-01	2.253E-01
CLCN7	0.030	7.471E-01	2.216E-01
PELO	0.030	8.168E-01	1.842E-01
AL136295.1	0.030	9.574E-01	4.885E-01
ZNF112	0.030	8.469E-01	3.274E-01
FAM118B	0.030	7.918E-01	6.403E-01
MCM6	0.030	8.638E-01	2.742E-01
UBP1	0.030	7.433E-01	9.061E-01
ALDH7A1	0.030	9.429E-01	7.455E-01
INTS5	0.030	7.805E-01	1.814E-01
SF3B3	0.030	8.041E-01	2.637E-01
E2F8	0.030	9.007E-01	2.741E-01
GNAI2	0.030	8.054E-01	7.384E-01
FAM103A1	0.030	7.396E-01	1.913E-01
GNG12	0.030	8.713E-01	1.248E-01
OCM	0.030	9.380E-01	5.405E-01
FST	0.030	9.495E-01	3.160E-01
BAD	0.030	8.397E-01	2.364E-01
HYAL2	0.030	8.222E-01	4.286E-01
MRPL3	0.030	7.464E-01	1.327E-01
BZW2	0.030	8.328E-01	2.154E-02

QPCT	0.030	9.361E-01	3.261E-01
DTNBP1	0.030	8.340E-01	1.368E-01
PZP	0.030	9.518E-01	3.662E-01
SNRK	0.030	7.739E-01	7.348E-01
TATDN2	0.030	8.077E-01	5.512E-01
PRPF19	0.030	7.513E-01	3.217E-01
FLII	0.030	8.016E-01	5.790E-01
NRIP1	0.030	8.925E-01	2.310E-01
SPOPL	0.030	8.141E-01	1.895E-01
ARSI	0.030	9.517E-01	4.372E-01
MTUS1	0.030	8.886E-01	3.328E-01
ZNF587	0.030	8.512E-01	4.942E-01
TSEN15	0.030	8.245E-01	5.408E-01
RIOX2	0.030	8.183E-01	4.420E-01
HERPUD1	0.030	8.545E-01	1.709E-01
HIP1	0.030	8.703E-01	6.915E-01
SNF8	0.030	7.949E-01	1.333E-01
FBP2	0.030	9.487E-01	2.866E-01
CHMP1A	0.029	7.868E-01	3.673E-01
KPNA4	0.029	7.443E-01	3.767E-01
GON7	0.029	8.253E-01	1.905E-01
C1orf122	0.029	8.444E-01	2.934E-01
PSME3	0.029	7.443E-01	6.969E-01
DPY19L3	0.029	8.545E-01	2.902E-01
PIEZO1	0.029	8.711E-01	7.040E-01
CLOCK	0.029	8.242E-01	6.123E-01
ZMYM5	0.029	7.915E-01	3.354E-01
STAMBP	0.029	7.011E-01	1.812E-01
LIFR	0.029	9.370E-01	2.091E-01
FAM98A	0.029	7.764E-01	2.067E-01
TEX13A	0.029	NA	1.967E-01
NPC1	0.029	8.759E-01	3.676E-01
HIPK1	0.029	8.124E-01	1.434E-01
APPBP2	0.029	7.856E-01	2.625E-01
SLC8A3	0.029	9.483E-01	4.212E-01
TMEM98	0.029	9.202E-01	4.230E-01
NOD2	0.029	9.428E-01	1.410E-01
ZMAT2	0.029	7.488E-01	3.936E-01
MIR4427	0.029	NA	5.806E-01
SPATS2	0.029	8.062E-01	9.652E-01
RCAN3	0.029	8.583E-01	9.633E-01
ATAD3A	0.029	8.512E-01	9.629E-01
ABCA1	0.029	8.880E-01	9.610E-01
PHB	0.029	7.935E-01	9.608E-01
VAMP2	0.029	8.369E-01	9.589E-01
CD47	0.029	8.933E-01	7.634E-01
MIR6124	0.029	9.693E-01	1.347E-01
MYOM2	0.029	9.330E-01	3.363E-01
PQLC1	0.029	8.394E-01	9.606E-01
TRAK2	0.029	8.355E-01	5.963E-01

NMT2	0.029	8.998E-01	2.990E-01
ZNF134	0.029	8.369E-01	7.337E-01
MIR5584	0.029	NA	1.195E-01
MIR107	0.029	NA	2.424E-01
MIR526B	0.029	NA	6.996E-01
AL163195.3	0.029	NA	7.648E-01
LIMS4	0.029	NA	3.697E-01
MIR1238	0.029	NA	5.524E-01
IPO13	0.029	7.868E-01	1.925E-01
LRIF1	0.029	8.425E-01	2.126E-01
ZNF512B	0.029	8.602E-01	2.250E-01
ATG3	0.029	7.652E-01	2.360E-01
DAPP1	0.029	9.094E-01	1.336E-01
SHF	0.029	9.246E-01	7.142E-01
MYO6	0.029	8.797E-01	2.024E-01
SERPINB6	0.029	8.770E-01	5.026E-01
DOLK	0.029	8.080E-01	2.582E-01
NDUFB2	0.029	8.269E-01	2.052E-01
MSTO1	0.029	8.270E-01	3.850E-01
RPRD1B	0.029	8.044E-01	4.226E-01
ZNF318	0.029	8.303E-01	2.684E-01
PTPDC1	0.029	8.374E-01	7.022E-01
SLC9A8	0.028	8.282E-01	1.247E-01
ZNF274	0.028	8.528E-01	1.559E-01
PIGT	0.028	8.394E-01	5.439E-01
KIF20B	0.028	8.838E-01	5.616E-01
GPR32	0.028	NA	1.909E-01
NTN1	0.028	9.513E-01	8.039E-01
PRTN3	0.028	9.695E-01	4.751E-01
GSTA3	0.028	9.792E-01	3.493E-01
SIPA1L2	0.028	9.113E-01	1.385E-01
MIR550A3	0.028	NA	2.872E-01
HHIP	0.028	9.593E-01	4.011E-01
STRN4	0.028	7.558E-01	1.823E-01
HNRNPL	0.028	6.647E-01	1.477E-01
ADIG	0.028	9.728E-01	4.077E-01
STK10	0.028	8.635E-01	7.666E-01
AATF	0.028	7.998E-01	6.765E-01
OR2W1	0.028	NA	2.153E-01
RNF169	0.028	8.983E-01	3.328E-01
BET1	0.028	7.644E-01	3.922E-01
VAV3	0.028	9.302E-01	9.029E-02
DDX19A	0.028	7.798E-01	4.506E-01
CSRNP3	0.028	9.344E-01	2.825E-01
ATPAF1	0.028	7.793E-01	3.093E-01
MIR4668	0.028	9.549E-01	3.206E-01
CTNNBL1	0.028	8.031E-01	2.308E-01
NCK2	0.028	8.161E-01	7.350E-01
PPP3CA	0.028	8.532E-01	6.361E-01
MIR645	0.028	9.602E-01	2.665E-01

TPRA1	0.028	7.918E-01	7.101E-01
TGIF1	0.028	8.446E-01	2.716E-01
DCAF11	0.028	7.488E-01	2.156E-01
MUS81	0.028	7.826E-01	3.278E-01
MRPS10	0.028	8.204E-01	9.001E-01
DNAJC27	0.028	8.284E-01	8.511E-01
MIR4641	0.028	NA	7.084E-01
HK1	0.028	8.448E-01	3.065E-01
MIR26A2	0.028	9.650E-01	2.509E-01
UFM1	0.028	7.904E-01	9.804E-01
HIST1H2BI	0.028	9.479E-01	9.804E-01
TOP2A	0.028	9.043E-01	9.804E-01
PMCH	0.028	9.474E-01	9.804E-01
KIAA1107	0.028	8.854E-01	9.804E-01
WNK2	0.028	9.483E-01	9.804E-01
IVNS1ABP	0.028	8.599E-01	9.804E-01
PEX12	0.028	8.235E-01	9.804E-01
ZNF174	0.027	7.541E-01	9.804E-01
LAMB1	0.027	8.835E-01	9.804E-01
HSD17B12	0.027	8.352E-01	9.804E-01
NOL9	0.027	7.827E-01	9.804E-01
NR1H2	0.027	7.979E-01	9.804E-01
POLH	0.027	8.727E-01	9.804E-01
GTF2IRD1	0.027	8.472E-01	9.804E-01
SERINC4	0.027	NA	9.804E-01
ATP2A1	0.027	9.170E-01	9.804E-01
CCDC51	0.027	8.421E-01	9.804E-01
ABCB9	0.027	8.694E-01	9.804E-01
FDFT1	0.027	8.940E-01	9.804E-01
KRTAP5-3	0.027	NA	9.804E-01
KCTD20	0.027	7.939E-01	9.804E-01
RNASEH2A	0.027	8.692E-01	9.804E-01
C17orf47	0.027	9.460E-01	9.804E-01
EYA4	0.027	9.529E-01	9.804E-01
PRKAR1A	0.027	7.696E-01	9.804E-01
OSBPL11	0.027	8.318E-01	9.804E-01
FH	0.027	8.015E-01	9.804E-01
SLC22A3	0.027	9.587E-01	9.804E-01
GUCA1A	0.027	9.630E-01	9.804E-01
SPCS1	0.027	8.695E-01	9.804E-01
TXNDC15	0.027	8.055E-01	9.804E-01
CTAGE4	0.027	9.480E-01	9.804E-01
FBXL3	0.027	8.232E-01	2.984E-01
CBARP	0.027	9.229E-01	4.595E-01
BIRC5	0.027	9.079E-01	2.899E-01
PAAF1	0.027	8.450E-01	3.567E-01
ZNF672	0.027	7.962E-01	3.888E-01
TMEM165	0.027	8.383E-01	1.647E-01
RELB	0.027	8.979E-01	3.159E-01
LRRC74B	0.027	9.631E-01	6.491E-01

COPS7A	0.027	7.609E-01	2.259E-01
MEMO1	0.027	8.071E-01	2.940E-01
RNF128	0.027	9.521E-01	9.249E-01
MS4A7	0.027	9.442E-01	4.621E-01
RUFY1	0.027	7.865E-01	1.894E-01
ZNF732	0.027	9.580E-01	1.323E-01
MANBAL	0.027	8.057E-01	7.326E-01
TRAF2	0.027	8.632E-01	5.500E-01
CAMK2D	0.026	8.889E-01	2.027E-01
PDGFA	0.026	9.205E-01	9.441E-01
C21orf59	0.026	8.520E-01	3.734E-01
TMEM268	0.026	8.386E-01	2.512E-01
RAD51	0.026	8.879E-01	8.930E-01
SUMO3	0.026	8.288E-01	2.999E-01
LSM14B	0.026	8.107E-01	6.617E-01
DNAJC10	0.026	8.570E-01	1.558E-01
TRDMT1	0.026	9.009E-01	1.850E-01
FUT1	0.026	9.074E-01	2.649E-01
TM6SF2	0.026	9.457E-01	2.599E-01
AMDHD2	0.026	8.667E-01	1.624E-01
KPNB1	0.026	7.858E-01	3.116E-01
PUM3	0.026	8.729E-01	5.989E-01
UFL1	0.026	8.596E-01	6.627E-01
TOR2A	0.026	8.369E-01	2.119E-01
CHST12	0.026	8.765E-01	4.196E-01
GYG1	0.026	8.306E-01	9.066E-01
SLC39A14	0.026	9.169E-01	4.124E-01
SCML1	0.026	9.168E-01	3.319E-01
VPS11	0.026	7.903E-01	4.164E-01
MPRIP	0.026	8.693E-01	3.323E-01
PLEKHA4	0.026	9.295E-01	2.525E-01
BICDL2	0.026	9.457E-01	2.280E-01
IFT88	0.026	8.403E-01	6.682E-01
INCENP	0.026	8.846E-01	2.171E-01
GORASP2	0.026	7.442E-01	6.105E-01
ZNF471	0.026	9.475E-01	1.867E-01
KRTAP24-1	0.026	NA	1.930E-01
MIR549A	0.026	NA	4.009E-01
IFNA17	0.026	NA	1.712E-01
OR2M3	0.026	NA	6.768E-01
VCY	0.026	NA	2.481E-01
MIR4789	0.026	NA	3.521E-01
CMC4	0.026	NA	4.657E-01
JOSD1	0.026	8.153E-01	8.709E-01
FDX1	0.026	8.815E-01	7.216E-01
XRN1	0.026	8.486E-01	1.036E-01
PLLP	0.026	9.391E-01	1.690E-01
FAM168B	0.026	8.446E-01	4.509E-01
PCGF6	0.026	8.298E-01	1.884E-01
KIF3B	0.026	8.424E-01	6.739E-01

ZFP90	0.026	8.421E-01	6.501E-01
IKBKG	0.026	8.502E-01	5.765E-01
MIPEP	0.026	8.425E-01	1.470E-01
CUEDC2	0.026	8.463E-01	9.715E-01
KLK14	0.026	9.620E-01	9.714E-01
NONO	0.026	8.053E-01	9.714E-01
UNC80	0.026	9.578E-01	9.671E-01
METTL2A	0.026	7.970E-01	9.669E-01
SRP54	0.026	8.041E-01	4.863E-01
ARFGAP1	0.026	8.448E-01	7.708E-01
AGAP1	0.026	8.631E-01	2.908E-01
RNF121	0.026	8.165E-01	4.513E-01
CMC1	0.026	8.207E-01	7.897E-01
FTO	0.026	8.278E-01	3.250E-01
SLC37A4	0.026	8.655E-01	2.691E-01
KLC3	0.026	9.502E-01	7.795E-01
IFI27L1	0.026	9.117E-01	4.650E-01
S100A5	0.026	9.547E-01	8.196E-02
HCFC2	0.026	8.528E-01	1.582E-01
COQ8B	0.025	8.481E-01	2.302E-01
SSH2	0.025	8.344E-01	2.509E-01
HSP90AA1	0.025	8.571E-01	1.482E-01
PPM1L	0.025	9.257E-01	6.039E-01
OR2L3	0.025	NA	3.038E-01
MIR1271	0.025	NA	1.572E-01
MIR30B	0.025	NA	2.751E-01
AC010132.3	0.025	9.481E-01	3.233E-01
DVL2	0.025	8.402E-01	6.288E-01
ARNTL2	0.025	9.326E-01	5.970E-01
AC008878.2	0.025	9.680E-01	5.792E-01
TMEM185A	0.025	8.404E-01	4.517E-01
TINF2	0.025	8.167E-01	2.516E-01
ANKIB1	0.025	8.249E-01	1.490E-01
KLHL11	0.025	9.296E-01	3.022E-01
GLT8D1	0.025	8.545E-01	6.406E-01
CTSO	0.025	9.049E-01	4.380E-01
CCDC13	0.025	9.251E-01	3.612E-01
CCNYL1	0.025	9.005E-01	7.469E-01
CCDC189	0.025	9.113E-01	9.247E-01
PPP6C	0.025	7.715E-01	5.687E-01
GJB7	0.025	9.555E-01	7.394E-01
TMLHE	0.025	8.584E-01	5.979E-01
BORCS7	0.025	8.402E-01	1.608E-01
ADRA2C	0.025	9.641E-01	2.304E-01
TAF11	0.025	7.941E-01	2.035E-01
LINC00094	0.025	8.533E-01	5.937E-01
VWF	0.025	9.192E-01	9.286E-01
ZNF438	0.025	8.794E-01	2.716E-01
CYB561A3	0.025	8.570E-01	1.224E-01
TSSC1	0.025	8.177E-01	2.606E-01

COPB1	0.025	8.038E-01	4.005E-01
EEF2K	0.025	8.514E-01	3.414E-01
IFITM3	0.025	9.423E-01	2.277E-01
ANKRD9	0.025	8.964E-01	2.938E-01
PDCL2	0.025	9.770E-01	1.437E-01
TSPAN11	0.025	9.443E-01	4.436E-01
ADPRHL2	0.025	7.941E-01	3.033E-01
VPS35	0.025	8.381E-01	4.133E-01
CLEC4G	0.025	9.666E-01	1.395E-01
EHD4	0.025	8.667E-01	3.388E-01
PMM1	0.025	8.645E-01	2.048E-01
UGGT1	0.025	8.416E-01	4.718E-01
NEDD9	0.025	9.344E-01	6.392E-01
VMP1	0.025	8.755E-01	4.893E-01
ZNF71	0.025	9.146E-01	8.500E-01
MIR5697	0.025	NA	2.340E-01
SLC25A39	0.025	8.469E-01	2.856E-01
MIR3912	0.024	NA	7.277E-01
ATG13	0.024	8.469E-01	4.828E-01
CYP4B1	0.024	9.679E-01	2.981E-01
C5orf51	0.024	8.404E-01	7.143E-01
SECISBP2	0.024	8.369E-01	3.894E-01
FOXO3	0.024	8.632E-01	7.739E-01
CSTB	0.024	9.213E-01	2.517E-01
RASSF4	0.024	9.395E-01	2.625E-01
MIR3189	0.024	9.680E-01	2.850E-01
SEC14L1	0.024	8.535E-01	2.638E-01
DNAAF3	0.024	9.489E-01	1.653E-01
NDST2	0.024	8.356E-01	5.609E-01
AGK	0.024	8.044E-01	3.702E-01
SLC35E4	0.024	9.138E-01	3.828E-01
CLTA	0.024	8.693E-01	7.381E-01
IER3IP1	0.024	8.381E-01	3.767E-01
FADD	0.024	9.013E-01	2.478E-01
PRPF3	0.024	8.539E-01	4.207E-01
SAV1	0.024	8.620E-01	1.752E-01
TXNDC5	0.024	9.133E-01	8.405E-01
TRMT6	0.024	8.438E-01	2.852E-01
PVRIG	0.024	9.310E-01	2.510E-01
C18orf25	0.024	8.480E-01	7.601E-01
TCEAL6	0.024	9.721E-01	1.987E-01
SUV39H1	0.024	8.903E-01	2.809E-01
AL121845.3	0.024	9.020E-01	5.030E-01
H3F3B	0.024	8.224E-01	9.586E-01
AFG3L2	0.024	8.534E-01	9.481E-01
PROM2	0.024	9.452E-01	5.154E-01
RAB33A	0.024	9.380E-01	4.944E-01
STAC3	0.024	9.205E-01	3.510E-01
ATF7	0.024	7.867E-01	8.862E-01
CEBPD	0.024	9.193E-01	7.855E-01

HSPBP1	0.024	8.799E-01	3.600E-01
NSMCE1	0.024	8.735E-01	5.832E-01
CLIC2	0.024	9.334E-01	6.045E-01
TIMM22	0.024	8.421E-01	1.386E-01
MKI67	0.024	9.153E-01	3.032E-01
ENO1	0.024	8.880E-01	8.813E-01
TMEM35B	0.024	8.786E-01	5.094E-01
UIMC1	0.024	7.976E-01	4.098E-01
CDK2AP2	0.024	8.806E-01	4.791E-01
METTL9	0.024	8.164E-01	2.431E-01
MILR1	0.024	9.482E-01	1.476E-01
MINDY4	0.024	9.105E-01	3.920E-01
XPO1	0.024	7.993E-01	4.033E-01
LCLAT1	0.024	8.587E-01	2.351E-01
AC104532.1	0.023	9.545E-01	2.109E-01
SEC61B	0.023	8.574E-01	1.772E-01
ZNF92	0.023	8.965E-01	4.861E-01
NBPF15	0.023	9.100E-01	8.195E-01
UTP18	0.023	8.399E-01	6.596E-01
NEURL1	0.023	9.606E-01	2.874E-01
TFAP2D	0.023	NA	3.486E-01
ADNP	0.023	8.261E-01	7.001E-01
TEX38	0.023	9.380E-01	4.418E-01
POLR3GL	0.023	8.635E-01	1.847E-01
MIR4650-1	0.023	NA	1.607E-01
AC008758.1	0.023	NA	2.330E-01
T5C1B-RDH	0.023	NA	2.373E-01
MIR6504	0.023	NA	8.906E-01
OR6K6	0.023	NA	5.717E-01
MIR4684	0.023	NA	3.240E-01
OR10A7	0.023	NA	7.301E-01
MIR103A1	0.023	NA	2.178E-01
RAB13	0.023	8.621E-01	5.178E-01
WDR63	0.023	9.495E-01	4.604E-01
ACSL3	0.023	8.810E-01	4.042E-01
ISOC2	0.023	8.931E-01	8.570E-01
SKA3	0.023	9.092E-01	3.127E-01
ZNF556	0.023	9.695E-01	2.270E-01
CHRNB4	0.023	9.576E-01	2.937E-01
LRRC36	0.023	9.579E-01	6.138E-01
EIF1AX	0.023	8.413E-01	4.441E-01
LHX6	0.023	9.331E-01	9.771E-01
MIR30C2	0.023	9.783E-01	9.770E-01
UBQLN1	0.023	8.512E-01	9.769E-01
TRIO	0.023	8.937E-01	9.750E-01
VN1R1	0.023	9.298E-01	9.749E-01
RCN2	0.023	8.561E-01	9.747E-01
CPNE3	0.023	8.791E-01	9.728E-01
RINT1	0.023	8.276E-01	4.656E-02
EMC6	0.023	8.876E-01	8.156E-01

RIN3	0.023	9.092E-01	8.324E-01
SLC4A10	0.023	9.689E-01	5.465E-01
CD34	0.023	9.230E-01	5.327E-01
KSR2	0.023	9.643E-01	4.198E-01
CCT7	0.023	8.446E-01	1.204E-01
EIF3A	0.023	8.672E-01	1.522E-01
ZER1	0.023	8.199E-01	6.376E-01
FAM45A	0.023	8.325E-01	1.943E-01
CLPX	0.023	8.251E-01	5.140E-01
TMEM115	0.023	8.541E-01	2.129E-01
MIR4436A	0.023	NA	2.571E-01
OR2AT4	0.023	NA	1.899E-01
MIR2278	0.023	NA	2.009E-01
SNX10	0.023	9.379E-01	6.036E-01
NRBP2	0.023	9.107E-01	5.800E-01
CHTF8	0.023	8.278E-01	3.321E-01
PPM1K	0.023	9.210E-01	1.653E-01
EI24	0.022	8.436E-01	3.556E-01
C1orf174	0.022	7.926E-01	1.991E-01
IQANK1	0.022	9.338E-01	1.935E-01
MPV17	0.022	8.491E-01	2.305E-01
LPIN1	0.022	9.207E-01	9.335E-01
POTEJ	0.022	9.529E-01	9.397E-01
SKAP2	0.022	8.815E-01	2.275E-01
MAD1L1	0.022	8.663E-01	5.528E-01
CEBPZOS	0.022	8.526E-01	5.801E-01
PUM2	0.022	8.058E-01	2.546E-01
EBF4	0.022	9.456E-01	2.043E-01
PCDHGC4	0.022	9.587E-01	5.867E-01
ENOPH1	0.022	8.447E-01	4.094E-01
PCIF1	0.022	8.245E-01	2.470E-01
TBC1D12	0.022	8.635E-01	8.781E-01
CCDC126	0.022	8.744E-01	2.778E-01
PDGFD	0.022	9.458E-01	8.032E-01
GH1	0.022	9.760E-01	6.784E-01
SMARCC2	0.022	8.880E-01	3.584E-01
OR1K1	0.022	NA	7.108E-01
DDRGK1	0.022	8.735E-01	4.618E-01
FBXW9	0.022	8.693E-01	4.074E-01
KAT14	0.022	8.701E-01	9.039E-01
ERICH4	0.022	9.718E-01	6.856E-01
MALSU1	0.022	8.457E-01	4.842E-01
UBE2Z	0.022	8.183E-01	3.173E-01
RNF125	0.022	9.371E-01	3.607E-01
AC093668.1	0.022	NA	2.749E-01
RNF214	0.022	8.194E-01	9.522E-01
TP53INP2	0.022	9.272E-01	3.283E-01
ZC4H2	0.022	9.480E-01	6.007E-01
CUL4B	0.022	8.153E-01	3.819E-01
C2orf76	0.022	8.541E-01	1.296E-01

EXOSC10	0.022	7.993E-01	2.560E-01
COL4A1	0.022	9.336E-01	9.698E-01
MSL2	0.022	8.172E-01	9.640E-01
MESDC2	0.022	8.086E-01	9.505E-01
ZCWPW1	0.022	9.177E-01	2.412E-01
KIAA1211	0.022	9.601E-01	2.162E-01
ZDHHC3	0.022	8.735E-01	3.549E-01
RAB15	0.022	9.457E-01	2.747E-01
GAPVD1	0.022	8.454E-01	3.427E-01
TP53BP1	0.022	8.830E-01	7.212E-01
DCUN1D1	0.022	8.533E-01	1.522E-01
NDUFAF6	0.022	8.654E-01	2.956E-01
RFWD3	0.022	8.800E-01	4.145E-01
AKT1S1	0.022	8.514E-01	1.425E-01
CRIPT	0.022	8.517E-01	7.787E-01
SRP68	0.022	7.980E-01	4.644E-01
ZNF254	0.021	9.308E-01	5.909E-01
MIR199A1	0.021	9.711E-01	2.657E-01
ARSH	0.021	9.648E-01	7.523E-01
LRPPRC	0.021	8.540E-01	2.644E-01
BCAS2	0.021	8.242E-01	2.272E-01
AP2M1	0.021	8.369E-01	5.040E-01
RASGRP1	0.021	9.579E-01	2.259E-01
PGS1	0.021	8.324E-01	2.916E-01
DLGAP4	0.021	8.497E-01	2.385E-01
PSMC5	0.021	8.557E-01	4.472E-01
ALDH1A2	0.021	9.616E-01	7.794E-01
NSF	0.021	8.879E-01	2.642E-01
GPR180	0.021	8.830E-01	9.302E-01
THAP3	0.021	8.781E-01	2.282E-01
VAMP4	0.021	8.693E-01	2.692E-01
YPEL5	0.021	8.513E-01	3.153E-01
GZF1	0.021	8.446E-01	2.041E-01
IQCB1	0.021	8.726E-01	7.613E-01
DNAJC30	0.021	8.791E-01	2.739E-01
TRAPPC9	0.021	8.676E-01	2.873E-01
AKAP13	0.021	8.869E-01	2.249E-01
SPATA12	0.021	9.453E-01	5.205E-01
ARID5A	0.021	9.249E-01	2.671E-01
TFRC	0.021	9.394E-01	9.223E-01
TEX46	0.021	9.560E-01	3.242E-01
SWT1	0.021	8.719E-01	3.571E-01
MNT	0.021	8.667E-01	5.829E-01
SLC30A7	0.021	8.302E-01	3.163E-01
SCFD2	0.021	8.520E-01	7.472E-01
ZHX2	0.021	9.004E-01	5.662E-01
CROT	0.021	9.455E-01	5.362E-01
UBR7	0.021	8.755E-01	2.066E-01
STK17B	0.021	9.229E-01	1.759E-01
NBN	0.021	8.784E-01	3.720E-01

TERF2	0.021	8.242E-01	1.026E-01
SPATC1L	0.021	9.479E-01	9.823E-01
RNF103	0.021	8.566E-01	9.823E-01
DNAH7	0.021	9.354E-01	9.823E-01
MIR548AA1	0.021	9.604E-01	9.823E-01
RNF139	0.021	8.513E-01	9.823E-01
FNBP1L	0.021	9.208E-01	9.809E-01
AMZ2	0.021	8.361E-01	9.804E-01
MIR6880	0.021	NA	9.804E-01
OR10G3	0.021	NA	9.802E-01
MIR877	0.021	NA	9.787E-01
AC120057.3	0.021	NA	9.783E-01
OR2M2	0.021	NA	5.930E-01
WDR46	0.021	8.658E-01	8.892E-01
KRTAP10-6	0.021	NA	3.868E-01
ALAD	0.021	8.704E-01	5.817E-01
SMYD5	0.021	8.531E-01	2.908E-01
GPAA1	0.021	8.903E-01	7.732E-01
AC239799.1	0.020	NA	4.191E-01
ERAS	0.020	9.648E-01	3.034E-01
MIR1302-5	0.020	NA	3.700E-01
TLN2	0.020	9.280E-01	4.469E-01
MT1B	0.020	9.858E-01	2.138E-01
AAR2	0.020	8.248E-01	3.685E-01
HERPUD2	0.020	8.054E-01	3.745E-01
TAF5L	0.020	8.355E-01	2.969E-01
RECQL	0.020	9.167E-01	2.655E-01
MIR3136	0.020	NA	3.087E-01
STUB1	0.020	8.655E-01	7.395E-01
SAMHD1	0.020	9.404E-01	2.467E-01
MPO	0.020	9.626E-01	2.497E-01
ANKRD16	0.020	8.976E-01	7.146E-01
RAE1	0.020	8.533E-01	3.069E-01
TIRAP	0.020	8.759E-01	7.926E-01
SSR1	0.020	8.673E-01	8.805E-01
AP2B1	0.020	8.815E-01	3.314E-01
NCOA4	0.020	8.784E-01	3.306E-01
SARS	0.020	8.512E-01	1.659E-01
SLC6A3	0.020	9.721E-01	1.785E-01
DCLRE1B	0.020	9.003E-01	4.053E-01
BORA	0.020	9.153E-01	3.156E-01
ELMO1	0.020	9.462E-01	1.847E-01
CHMP2A	0.020	9.015E-01	2.454E-01
LACRT	0.020	NA	2.636E-01
MBD3L2	0.020	NA	2.080E-01
TRIM60	0.020	NA	3.855E-01
CNTF	0.020	9.333E-01	2.686E-01
COA5	0.020	8.786E-01	7.427E-01
C14orf28	0.020	8.844E-01	4.832E-01
CYB5D2	0.020	9.083E-01	2.343E-01

APOH	0.020	9.869E-01	3.025E-01
ANOS1	0.020	9.593E-01	5.389E-01
GOLGA3	0.020	8.421E-01	8.219E-01
NOL4L	0.020	9.297E-01	6.384E-02
URB1	0.020	8.998E-01	5.081E-01
PPM1J	0.020	9.512E-01	4.794E-01
ITPRIPL2	0.020	9.237E-01	5.192E-01
AC104581.1	0.020	9.117E-01	2.595E-01
ZFP3	0.020	9.525E-01	5.617E-01
VPS13B	0.020	9.094E-01	9.680E-01
GHITM	0.020	8.661E-01	9.680E-01
BICRAL	0.020	8.801E-01	9.345E-01
FANK1	0.020	9.326E-01	9.638E-01
LDLRAD3	0.020	9.494E-01	9.602E-01
HHATL	0.020	9.770E-01	9.582E-01
RUVBL1	0.019	8.896E-01	7.531E-01
ASB14	0.019	9.068E-01	6.422E-01
TEDDM1	0.019	9.733E-01	2.386E-01
RILPL1	0.019	8.671E-01	2.391E-01
PRPF6	0.019	8.529E-01	2.507E-01
PCDHB9	0.019	9.538E-01	5.674E-01
NUDT16	0.019	8.608E-01	1.576E-01
GPN2	0.019	8.325E-01	5.881E-01
RACGAP1	0.019	9.221E-01	5.401E-01
SSBP3	0.019	9.107E-01	1.703E-01
RPGR	0.019	8.951E-01	2.662E-01
NPDC1	0.019	9.484E-01	2.568E-01
KDELC1	0.019	9.272E-01	2.729E-01
MTFR1L	0.019	8.783E-01	7.053E-01
NFE2L3	0.019	9.462E-01	6.776E-01
SNX11	0.019	8.579E-01	2.403E-01
ZNF496	0.019	8.807E-01	4.309E-01
NUSAP1	0.019	9.314E-01	2.844E-01
MIR4688	0.019	NA	2.694E-01
UROD	0.019	8.772E-01	3.394E-01
MDM1	0.019	9.207E-01	2.185E-01
MIR6856	0.019	NA	3.066E-01
PCDHGB2	0.019	9.626E-01	5.752E-01
COMMD2	0.019	8.754E-01	2.187E-01
ATXN7L3	0.019	8.519E-01	4.466E-01
MRPL37	0.019	8.936E-01	7.131E-01
IL13RA1	0.019	9.128E-01	3.971E-01
CFAP20	0.019	8.500E-01	2.080E-01
MIR3978	0.019	NA	4.609E-01
ısa-mir-3119-	0.019	NA	8.917E-01
MIR1228	0.019	NA	5.424E-01
MIR4266	0.019	NA	4.186E-01
OR4D2	0.019	NA	1.955E-01
OR5D18	0.019	NA	1.012E-01
CXorf66	0.019	NA	9.252E-01

MIR4422	0.019	NA	3.735E-01
MIR518F	0.019	NA	4.968E-01
MFRP	0.019	NA	7.328E-01
OR4C12	0.019	NA	3.378E-01
ALOX5AP	0.019	9.626E-01	2.727E-01
HNRNPR	0.019	8.472E-01	1.826E-01
PDE4B	0.019	9.582E-01	3.186E-01
PARP16	0.019	8.773E-01	3.098E-01
TAF13	0.019	8.850E-01	5.353E-01
PPME1	0.019	8.858E-01	1.297E-01
CL2L2-PABPI	0.019	9.345E-01	8.772E-01
EPB41L5	0.019	9.127E-01	5.169E-01
ATXN2L	0.019	8.711E-01	1.473E-01
FAM208A	0.019	8.592E-01	2.447E-01
KLHL15	0.019	9.003E-01	9.830E-01
UFSP2	0.019	8.701E-01	9.830E-01
ELF2	0.019	8.533E-01	9.830E-01
CNTD1	0.018	9.347E-01	9.830E-01
SCAF1	0.018	8.532E-01	9.830E-01
ST6GALNAC	0.018	9.134E-01	9.830E-01
AMBRA1	0.018	8.597E-01	9.830E-01
PSMG1	0.018	9.133E-01	9.830E-01
MIR579	0.018	9.766E-01	9.830E-01
XKR8	0.018	8.799E-01	9.830E-01
FAS	0.018	9.520E-01	9.830E-01
LAS1L	0.018	8.779E-01	9.830E-01
ZKSCAN5	0.018	8.386E-01	9.830E-01
NPIPB4	0.018	9.508E-01	9.830E-01
C15orf41	0.018	9.133E-01	9.830E-01
SETD6	0.018	9.019E-01	9.830E-01
PRB4	0.018	NA	9.830E-01
RNF19B	0.018	9.272E-01	9.830E-01
FBXL5	0.018	8.799E-01	9.830E-01
MTMR12	0.018	9.040E-01	9.830E-01
ECEL1	0.018	9.759E-01	9.830E-01
CCL23	0.018	9.733E-01	9.830E-01
MTRR	0.018	9.043E-01	9.830E-01
TUFM	0.018	8.890E-01	9.830E-01
CEBPG	0.018	8.908E-01	6.428E-01
VDAC3	0.018	8.972E-01	3.097E-01
RAB10	0.018	8.889E-01	2.820E-01
MIR591	0.018	NA	2.770E-01
SCGB1D4	0.018	NA	4.117E-01
MIR147A	0.018	NA	8.607E-01
AC034102.2	0.018	NA	3.023E-01
OR8B12	0.018	NA	5.548E-01
MIR4771-2	0.018	NA	2.655E-01
USP6	0.018	9.454E-01	3.579E-01
PLEKHM3	0.018	9.296E-01	4.152E-01
DROSHA	0.018	8.986E-01	4.183E-01

DCLRE1C	0.018	9.032E-01	2.366E-01
AARS2	0.018	8.791E-01	3.873E-01
LRCH2	0.018	9.626E-01	2.352E-01
TMEM14B	0.018	9.001E-01	1.680E-01
TAS2R60	0.018	9.828E-01	4.081E-01
TANGO2	0.018	8.909E-01	2.818E-01
MED27	0.018	9.033E-01	5.999E-01
SPCS2	0.018	8.645E-01	9.444E-01
FOLR3	0.018	9.789E-01	2.777E-01
DLG3	0.018	9.190E-01	2.450E-01
CDK5RAP1	0.018	8.781E-01	7.643E-01
GFM1	0.018	8.833E-01	2.464E-01
ZNF768	0.018	8.945E-01	2.711E-01
IARS	0.018	9.095E-01	6.956E-01
IGSF11	0.018	9.712E-01	2.198E-01
XCL1	0.018	9.688E-01	4.300E-01
NUP85	0.017	8.693E-01	2.192E-01
HIST1H4C	0.017	9.626E-01	3.270E-01
ZNF724	0.017	9.502E-01	1.673E-01
IQGAP3	0.017	9.418E-01	1.333E-01
ZNF534	0.017	9.734E-01	6.232E-01
VPS26A	0.017	8.735E-01	7.673E-01
UBE2R2	0.017	8.815E-01	6.734E-01
MRE11	0.017	8.807E-01	3.051E-01
PURB	0.017	8.571E-01	6.149E-01
ZNF264	0.017	8.940E-01	3.269E-01
NIPSNAP2	0.017	9.105E-01	3.954E-01
LSM12	0.017	8.791E-01	6.712E-01
AP2A2	0.017	8.786E-01	8.224E-01
TUSC1	0.017	9.418E-01	3.426E-01
CCDC163	0.017	9.420E-01	2.880E-01
ENO2	0.017	9.579E-01	4.590E-01
DAOA	0.017	NA	4.801E-01
SOX18	0.017	9.508E-01	2.081E-01
MRPL35	0.017	8.423E-01	3.125E-01
HLTF	0.017	9.170E-01	6.062E-01
KLHL22	0.017	9.110E-01	6.009E-01
ST8SIA1	0.017	9.647E-01	3.240E-01
RRP1B	0.017	9.002E-01	4.304E-01
SNX30	0.017	9.151E-01	9.104E-01
POLR3K	0.017	8.993E-01	4.993E-01
JAK1	0.017	8.983E-01	9.492E-02
POLR2L	0.017	9.192E-01	3.600E-01
CELF1	0.017	8.312E-01	4.289E-01
PROSER1	0.017	9.132E-01	4.209E-01
AC024270.1	0.017	9.423E-01	4.733E-01
OCM2	0.017	NA	3.419E-01
LAPTM4B	0.017	9.435E-01	5.143E-01
NDUFB6	0.017	9.137E-01	2.117E-01
BPNT1	0.017	8.983E-01	2.940E-01

LRRC70	0.017	9.508E-01	2.934E-01
MARS	0.017	8.728E-01	7.180E-01
ARMC7	0.017	9.128E-01	6.022E-01
LEMD3	0.017	9.033E-01	2.788E-01
PNPLA3	0.017	9.747E-01	2.432E-01
ENPP6	0.017	9.721E-01	3.096E-01
SUPT4H1	0.017	8.671E-01	6.806E-01
LYSMD3	0.017	8.933E-01	3.409E-01
C1orf27	0.017	8.869E-01	3.473E-01
DHX38	0.017	8.841E-01	2.070E-01
NEK5	0.017	9.524E-01	3.447E-01
ZNF548	0.017	9.078E-01	9.806E-01
ZNF35	0.017	8.901E-01	9.749E-01
LIN54	0.017	8.880E-01	9.657E-01
ARL13B	0.016	9.237E-01	9.637E-01
FTSJ3	0.016	8.831E-01	4.601E-01
KIF3A	0.016	9.092E-01	3.015E-01
GNMT	0.016	9.579E-01	2.302E-01
XRRA1	0.016	9.320E-01	3.156E-01
SDE2	0.016	9.042E-01	6.738E-01
DHRSX	0.016	9.208E-01	3.337E-01
MRPS24	0.016	9.120E-01	3.274E-01
FAM114A2	0.016	8.604E-01	1.823E-01
TOM1L1	0.016	9.334E-01	1.732E-01
GALNT11	0.016	9.138E-01	3.141E-01
STARD3NL	0.016	9.056E-01	2.159E-01
GINS1	0.016	9.369E-01	7.884E-01
AC011462.1	0.016	9.725E-01	5.145E-01
ARPP21	0.016	9.882E-01	7.270E-01
ATXN7	0.016	9.152E-01	2.519E-01
ZNF808	0.016	9.100E-01	2.488E-01
UBAP2	0.016	9.026E-01	2.880E-01
MIR1291	0.016	9.692E-01	3.291E-01
ARFRP1	0.016	9.128E-01	6.284E-01
MOGS	0.016	8.853E-01	5.700E-01
RRP1	0.016	9.245E-01	1.730E-01
HLA-DRB5	0.016	9.721E-01	4.521E-01
MIR4759	0.016	NA	4.319E-01
MIR4464	0.016	NA	6.790E-01
MIR4695	0.016	NA	2.804E-01
MIR548B	0.016	NA	4.787E-01
MIR548H3	0.016	NA	5.469E-01
MIR583	0.016	NA	3.888E-01
MIR2115	0.016	NA	1.745E-01
MBD3L5	0.016	NA	6.904E-01
MIR1200	0.016	NA	9.354E-01
MIR1343	0.016	NA	4.356E-01
MIR4299	0.016	NA	6.976E-01
MEIS1	0.016	9.460E-01	5.058E-01
RLIM	0.016	8.853E-01	2.903E-01

STX4	0.016	8.903E-01	2.665E-01
MIR6865	0.016	9.840E-01	1.271E-01
ITPR2	0.016	9.418E-01	4.530E-01
BANP	0.016	8.731E-01	5.469E-01
HEATR5B	0.016	9.070E-01	2.672E-01
MIR548AY	0.016	NA	3.249E-01
ADAL	0.016	9.188E-01	4.120E-01
RHBDF2	0.016	9.292E-01	3.030E-01
PCDHGA11	0.016	9.548E-01	3.287E-01
SLAIN2	0.016	8.831E-01	7.601E-01
PSMB2	0.016	8.858E-01	4.751E-01
TRMT12	0.016	9.151E-01	7.446E-01
TARBP1	0.016	9.272E-01	4.590E-01
MOCS3	0.016	8.999E-01	9.838E-01
LRRC28	0.016	8.880E-01	9.838E-01
NPIPB5	0.016	9.640E-01	9.838E-01
SH3PXD2A	0.016	9.431E-01	9.838E-01
OSER1	0.016	9.110E-01	9.838E-01
PTPRO	0.016	9.640E-01	9.838E-01
NELFB	0.016	9.060E-01	9.838E-01
MBD4	0.016	8.781E-01	9.838E-01
C7orf50	0.016	9.270E-01	9.838E-01
RHOQ	0.016	9.202E-01	9.838E-01
AC026464.4	0.016	9.748E-01	9.838E-01
FBXO42	0.016	8.329E-01	9.838E-01
SLC22A9	0.016	9.811E-01	9.838E-01
OR52E5	0.016	NA	9.838E-01
LIMS3	0.015	NA	9.838E-01
MTPAP	0.015	8.914E-01	9.838E-01
PL17-C18orf3	0.015	9.770E-01	9.838E-01
DSCC1	0.015	9.479E-01	9.838E-01
C1orf106	0.015	9.579E-01	9.838E-01
SBNO2	0.015	9.213E-01	9.838E-01
FUBP3	0.015	8.882E-01	9.838E-01
MYLK2	0.015	9.587E-01	9.838E-01
RMND5A	0.015	9.121E-01	9.838E-01
KDM7A	0.015	9.435E-01	9.838E-01
BRD3	0.015	9.213E-01	9.838E-01
SDC2	0.015	9.635E-01	9.838E-01
SSBP2	0.015	9.517E-01	9.838E-01
ZNF385B	0.015	9.824E-01	9.838E-01
TMEM170A	0.015	9.058E-01	9.838E-01
ARFIP1	0.015	9.051E-01	9.838E-01
PRELID3B	0.015	9.105E-01	9.838E-01
LMLN	0.015	9.280E-01	9.838E-01
SHANK2	0.015	9.663E-01	9.838E-01
ERI1	0.015	9.270E-01	9.838E-01
FXR1	0.015	8.867E-01	9.838E-01
RAB14	0.015	8.890E-01	9.838E-01
INTS2	0.015	9.207E-01	9.838E-01

ASPM	0.015	9.567E-01	9.838E-01
RASL11B	0.015	9.729E-01	9.838E-01
ITGAM	0.015	9.714E-01	9.838E-01
BSDC1	0.015	8.635E-01	9.838E-01
PIM2	0.015	9.587E-01	9.838E-01
DDX6	0.015	8.746E-01	9.838E-01
RAB2A	0.015	8.898E-01	9.838E-01
UEVLD	0.015	8.997E-01	9.838E-01
NOL11	0.015	8.853E-01	9.838E-01
ZNF775	0.014	9.345E-01	9.838E-01
SLC39A9	0.014	8.829E-01	9.838E-01
TSNAX	0.014	8.879E-01	9.838E-01
RBM14-RBM	0.014	9.150E-01	9.838E-01
SUV39H2	0.014	9.203E-01	9.838E-01
ISG20L2	0.014	8.868E-01	9.838E-01
EPB41L3	0.014	9.709E-01	9.838E-01
TNKS2	0.014	9.174E-01	9.838E-01
FBXO36	0.014	9.448E-01	8.353E-01
TEAD3	0.014	9.321E-01	4.117E-01
CCNI	0.014	9.033E-01	6.139E-01
SMC5	0.014	9.128E-01	1.104E-01
ENKD1	0.014	9.494E-01	4.674E-01
PRCC	0.014	8.880E-01	3.005E-01
OLFM2	0.014	9.718E-01	4.475E-01
RAB40AL	0.014	9.683E-01	4.003E-01
C11orf84	0.014	9.159E-01	2.002E-01
WWP2	0.014	9.010E-01	4.277E-01
ZFAND6	0.014	8.759E-01	3.099E-01
DCUN1D3	0.014	9.238E-01	4.891E-01
PSKH1	0.014	9.261E-01	2.808E-01
ZNF33A	0.014	9.246E-01	2.153E-01
WDR48	0.014	9.116E-01	9.699E-01
NUB1	0.014	9.132E-01	9.609E-01
SELENOH	0.014	9.273E-01	3.407E-01
FLCN	0.014	9.164E-01	4.521E-01
METTL7A	0.014	9.709E-01	3.990E-01
TGFBRAP1	0.014	8.815E-01	3.985E-01
CUL7	0.014	9.246E-01	4.778E-01
DHX9	0.014	8.828E-01	3.231E-01
SUMO4	0.014	9.650E-01	3.205E-01
FOXK2	0.014	8.797E-01	4.628E-01
MIR1273E	0.014	NA	2.526E-01
MIR6768	0.014	9.845E-01	3.851E-01
FKBPL	0.014	9.369E-01	4.937E-01
NAP1L2	0.014	9.736E-01	4.788E-01
ORAI1	0.014	9.391E-01	3.186E-01
ZNF366	0.014	9.650E-01	2.342E-01
ACTL6A	0.014	9.144E-01	3.242E-01
GPR148	0.014	NA	2.280E-01
CHD6	0.014	9.361E-01	7.564E-01

TXNL4A	0.014	9.152E-01	7.825E-01
MINOS1	0.014	9.146E-01	7.037E-01
FAM110A	0.014	9.359E-01	3.618E-01
KLHL24	0.014	9.414E-01	6.963E-01
COG1	0.014	8.784E-01	5.251E-01
ZNF439	0.014	9.684E-01	3.603E-01
SQOR	0.014	9.579E-01	4.611E-01
AC055811.2	0.014	9.718E-01	7.788E-01
DIS3L	0.014	9.133E-01	3.519E-01
GPR137	0.014	9.198E-01	3.523E-01
ACP5	0.014	9.630E-01	7.765E-01
NDUFV3	0.014	9.238E-01	7.109E-01
TARSL2	0.014	9.327E-01	3.870E-01
PAFAH1B2	0.014	8.858E-01	3.501E-01
MIR4740	0.014	9.719E-01	7.507E-01
LANCL3	0.013	9.693E-01	2.933E-01
LRRC8A	0.013	9.545E-01	9.235E-01
MMP12	0.013	9.833E-01	8.979E-01
ZNF836	0.013	9.272E-01	2.959E-01
PUM1	0.013	8.886E-01	4.791E-01
MORC2	0.013	9.164E-01	4.666E-01
IGIP	0.013	9.334E-01	3.033E-01
HARBI1	0.013	9.165E-01	6.542E-01
OR52A1	0.013	NA	2.818E-01
MIR676	0.013	NA	4.598E-01
OR9G4	0.013	NA	6.760E-01
KRT13	0.013	9.861E-01	1.614E-01
NOM1	0.013	8.998E-01	1.869E-01
MRPS23	0.013	9.133E-01	7.429E-01
TMEM132A	0.013	9.579E-01	7.578E-01
KIAA0556	0.013	9.207E-01	6.493E-01
USP8	0.013	9.033E-01	4.902E-01
SLC7A7	0.013	9.728E-01	2.204E-01
ARRDC3	0.013	9.494E-01	3.296E-01
MIR4449	0.013	NA	4.138E-01
RGCP-MRPS	0.013	9.696E-01	2.895E-01
TNFRSF10C	0.013	9.733E-01	7.688E-01
PRR14	0.013	9.105E-01	9.779E-01
CNIH1	0.013	9.103E-01	9.779E-01
GTDC1	0.013	9.094E-01	9.764E-01
GPR42	0.013	9.893E-01	9.761E-01
ZNF678	0.013	9.288E-01	9.761E-01
SEC16A	0.013	8.986E-01	9.744E-01
YTHDF1	0.013	8.883E-01	9.729E-01
SMU1	0.013	9.095E-01	9.725E-01
TIMM10B	0.013	9.056E-01	9.707E-01
TEX29	0.013	9.718E-01	4.486E-01
CCDC113	0.013	9.579E-01	2.142E-01
SCAF4	0.013	9.080E-01	3.501E-01
GLE1	0.013	9.274E-01	2.137E-01

CALCOCO1	0.013	9.246E-01	7.023E-01
SRSF4	0.013	8.880E-01	3.694E-01
TMED7	0.013	9.180E-01	3.277E-01
NOTO	0.013	9.889E-01	7.592E-01
DCPS	0.013	9.238E-01	7.280E-01
ZNF263	0.013	8.868E-01	8.134E-01
GRHL2	0.013	9.612E-01	9.348E-01
ARL1	0.013	9.164E-01	6.910E-01
PPP1R3A	0.013	NA	8.445E-01
CLINT1	0.012	9.372E-01	4.210E-01
FAHD2A	0.012	9.272E-01	3.595E-01
ASAH2B	0.012	9.375E-01	2.091E-01
MIR6088	0.012	NA	7.147E-01
FANCE	0.012	9.524E-01	4.241E-01
TRPC4	0.012	9.706E-01	6.031E-01
CU639417.2	0.012	9.688E-01	3.316E-01
PCYT1A	0.012	9.192E-01	3.293E-01
ERC1	0.012	9.475E-01	6.308E-01
SNRPG	0.012	9.238E-01	5.346E-01
CEP295	0.012	9.392E-01	3.898E-01
ASL	0.012	9.412E-01	7.772E-01
HMCES	0.012	9.183E-01	6.146E-01
LBH	0.012	9.635E-01	7.073E-01
ZNF543	0.012	9.319E-01	3.452E-01
INTS8	0.012	9.246E-01	2.116E-01
TMEM41B	0.012	9.273E-01	6.280E-01
RAI14	0.012	9.579E-01	5.612E-01
TRRAP	0.012	9.208E-01	8.885E-01
SART1	0.012	9.210E-01	3.177E-01
CREBRF	0.012	9.344E-01	3.415E-01
KLF10	0.012	9.597E-01	3.041E-01
CD63	0.012	9.452E-01	6.730E-01
ALKBH6	0.012	9.488E-01	2.372E-01
CIAO1	0.012	8.882E-01	7.233E-01
TMCO4	0.012	9.484E-01	3.805E-01
MPP1	0.012	9.718E-01	2.633E-01
ADSS	0.012	9.131E-01	6.160E-01
UBE2QL1	0.012	9.785E-01	1.616E-01
HADHA	0.012	9.095E-01	5.483E-01
PCDHGB7	0.012	9.749E-01	7.627E-01
UBXN2B	0.012	9.289E-01	3.186E-01
TADA2A	0.012	9.251E-01	5.905E-01
SMG5	0.012	9.218E-01	5.027E-01
HINT3	0.012	9.190E-01	3.956E-01
ORC6	0.012	9.579E-01	4.193E-01
DERL3	0.012	9.752E-01	2.831E-01
SNAPC3	0.012	9.345E-01	4.693E-01
FTSJ1	0.012	9.296E-01	6.776E-01
IGLL1	0.012	9.877E-01	4.885E-01
ERGIC2	0.012	9.096E-01	4.261E-01

PURG	0.012	9.796E-01	7.758E-01
ST18	0.012	9.769E-01	7.557E-01
TMEM184B	0.012	9.250E-01	5.351E-01
JUND	0.012	9.582E-01	7.526E-01
TBC1D7	0.012	9.435E-01	4.915E-01
EIF4EBP1	0.012	9.654E-01	3.164E-01
LCMT2	0.012	9.384E-01	4.162E-01
PACSIN2	0.012	9.128E-01	6.603E-01
FMO2	0.012	9.828E-01	7.776E-01
SMOX	0.012	9.692E-01	9.119E-01
MTCH2	0.012	9.141E-01	3.005E-01
MIR6728	0.012	9.802E-01	7.476E-01
TJP3	0.012	9.780E-01	7.716E-01
CACFD1	0.012	9.547E-01	2.817E-01
BTBD6	0.012	9.371E-01	3.469E-01
CCL8	0.012	9.828E-01	4.081E-01
MSRB1	0.012	9.542E-01	3.247E-01
TAF2	0.012	9.489E-01	4.522E-01
AMIGO3	0.011	9.770E-01	3.557E-01
DTYMK	0.011	9.480E-01	8.070E-01
CYP4V2	0.011	9.587E-01	2.694E-01
ZNF865	0.011	9.380E-01	4.280E-01
ZBTB45	0.011	9.324E-01	3.431E-01
KIAA0513	0.011	9.652E-01	4.347E-01
SLC10A7	0.011	9.375E-01	2.833E-01
CLCN2	0.011	9.488E-01	2.675E-01
IFNK	0.011	9.945E-01	2.641E-01
EIF2S2	0.011	9.319E-01	7.439E-02
NUP210	0.011	9.742E-01	4.172E-01
SPINT4	0.011	NA	4.820E-01
NIPBL	0.011	9.326E-01	9.576E-01
HIST1H2AL	0.011	9.792E-01	2.201E-01
ZNF630	0.011	9.643E-01	3.180E-01
ZBED9	0.011	9.787E-01	7.754E-01
ZNF407	0.011	9.265E-01	3.587E-01
GRIPAP1	0.011	9.167E-01	7.487E-01
UNC13A	0.011	9.790E-01	6.475E-01
STK38	0.011	9.317E-01	3.248E-01
AMY1B	0.011	NA	3.826E-01
NRBF2	0.011	9.333E-01	2.454E-01
ATP5J2	0.011	9.435E-01	3.123E-01
ZNF429	0.011	9.638E-01	4.334E-01
ZNF41	0.011	9.483E-01	6.252E-01
HMGN1	0.011	9.282E-01	2.805E-01
PHLPP2	0.011	9.519E-01	8.630E-01
MPP3	0.011	9.718E-01	3.050E-01
SMG8	0.011	9.480E-01	3.100E-01
DCLK1	0.011	9.837E-01	3.973E-01
CD1C	0.011	9.809E-01	2.675E-01
MIR4479	0.011	NA	4.745E-01

ETHE1	0.011	9.604E-01	4.147E-01
AC010542.3	0.011	9.870E-01	6.128E-01
RYK	0.011	9.171E-01	3.699E-01
PABPN1L	0.011	9.916E-01	1.303E-01
CHMP2B	0.011	9.508E-01	3.188E-01
AC009133.6	0.011	NA	7.435E-01
TAAR6	0.011	NA	2.808E-01
DEFB115	0.011	NA	6.410E-01
SMARCC1	0.011	9.460E-01	7.965E-01
ZMYND11	0.010	9.443E-01	6.974E-01
MPPE1	0.010	9.466E-01	6.537E-01
SNX25	0.010	9.499E-01	4.402E-01
TPP2	0.010	9.188E-01	5.427E-01
MIR3653	0.010	9.715E-01	4.214E-01
NANS	0.010	9.544E-01	3.761E-01
ELAC1	0.010	9.361E-01	4.805E-01
STK4	0.010	9.313E-01	5.751E-01
GPR61	0.010	9.721E-01	6.071E-01
AC090360.1	0.010	9.780E-01	3.180E-01
AIDA	0.010	9.479E-01	4.755E-01
PCDHB12	0.010	9.749E-01	3.958E-01
UBQLN2	0.010	9.430E-01	2.729E-01
CBLN3	0.010	9.643E-01	7.721E-01
AUTS2	0.010	9.755E-01	8.373E-01
RAB25	0.010	9.718E-01	7.715E-01
AMDHD1	0.010	9.837E-01	8.171E-01
DTX2	0.010	9.551E-01	3.284E-01
BCL6	0.010	9.604E-01	9.849E-01
BIRC6	0.010	9.371E-01	9.849E-01
C17orf74	0.010	9.919E-01	9.846E-01
ERICH2	0.010	9.833E-01	9.833E-01
OR51I1	0.010	9.945E-01	9.830E-01
HDAC6	0.010	9.435E-01	9.815E-01
GNG10	0.010	9.520E-01	9.809E-01
IGFL4	0.010	9.874E-01	9.809E-01
MIR1207	0.010	NA	9.808E-01
ZDHHC13	0.010	9.585E-01	9.778E-01
RP9	0.010	9.463E-01	9.776E-01
STAG2	0.010	9.592E-01	9.774E-01
GPNMB	0.010	9.837E-01	5.695E-01
YWHAH	0.010	9.502E-01	4.184E-01
SPPL2B	0.010	9.585E-01	3.250E-01
C9orf43	0.010	9.654E-01	5.725E-01
TMC6	0.010	9.616E-01	3.139E-01
FBXO34	0.010	9.447E-01	4.210E-01
GGH	0.010	9.738E-01	4.381E-01
FAM89B	0.010	9.520E-01	6.446E-01
CFAP36	0.010	9.444E-01	5.201E-01
VPS4A	0.010	9.364E-01	6.947E-01
DCAF1	0.010	9.375E-01	3.760E-01

DDX27	0.010	9.264E-01	4.588E-01
TBC1D19	0.010	9.455E-01	7.054E-01
C4orf22	0.010	9.858E-01	3.628E-01
RAD51B	0.010	9.580E-01	2.840E-01
ZNF595	0.010	9.691E-01	4.837E-01
ADNP2	0.010	9.418E-01	5.945E-01
MRPS21	0.010	9.643E-01	5.663E-01
ZNF599	0.010	9.579E-01	3.359E-01
PLPP2	0.009	9.749E-01	6.372E-01
VMA21	0.009	9.377E-01	3.749E-01
FAM20B	0.009	9.367E-01	2.627E-01
RAB11FIP3	0.009	9.521E-01	5.235E-01
GPSM1	0.009	9.749E-01	8.324E-01
INTS14	0.009	9.133E-01	2.615E-01
UBIAD1	0.009	9.292E-01	4.152E-01
TSR3	0.009	9.458E-01	8.902E-01
ZMYM4	0.009	9.251E-01	8.497E-01
MRPS18B	0.009	9.468E-01	6.996E-01
NCKAP1	0.009	9.502E-01	2.523E-01
CCDC171	0.009	9.644E-01	8.206E-01
TMEM173	0.009	9.718E-01	3.956E-01
NOMO1	0.009	9.480E-01	6.435E-01
DNMT3B	0.009	9.759E-01	2.441E-01
SP1	0.009	9.359E-01	2.309E-01
GCFC2	0.009	9.350E-01	4.013E-01
MEF2A	0.009	9.542E-01	3.087E-01
3UB1B-PAK(	0.009	9.861E-01	5.567E-01
BRMS1	0.009	9.587E-01	4.881E-01
H2AFX	0.009	9.634E-01	2.541E-01
PPP4R1	0.009	9.586E-01	7.773E-01
FBXO48	0.009	9.468E-01	7.737E-01
KBTBD12	0.009	9.916E-01	5.165E-01
KRT35	0.009	9.946E-01	2.647E-01
PLEKHA7	0.009	9.733E-01	7.900E-01
MIR8069-2	0.009	NA	4.964E-01
MIR216B	0.009	NA	8.339E-01
MIR362	0.009	NA	3.379E-01
MIR517B	0.009	NA	3.058E-01
MIR586	0.009	NA	4.228E-01
POTEB	0.009	NA	2.872E-01
DEFB106B	0.009	NA	3.180E-01
MIR1265	0.009	NA	4.407E-01
MIR378D1	0.009	NA	3.874E-01
MIR4325	0.009	NA	3.844E-01
MIR448	0.009	NA	6.707E-01
MIR4486	0.009	NA	7.209E-01
MIR548AV	0.009	NA	7.659E-01
EDC4	0.009	9.483E-01	5.278E-01
MPZL2	0.009	9.749E-01	6.930E-01
THAP11	0.009	9.386E-01	3.477E-01

MAPK1	0.009	9.457E-01	4.057E-01
FOSL2	0.009	9.638E-01	3.192E-01
SSX2IP	0.009	9.632E-01	3.557E-01
GIMAP1	0.009	9.785E-01	5.796E-01
CSPP1	0.009	9.556E-01	7.046E-01
KRT222	0.009	9.877E-01	6.445E-01
SLC4A3	0.009	9.796E-01	3.454E-01
HNRNPUL2	0.009	9.325E-01	3.019E-01
CTDSPL	0.009	9.598E-01	5.266E-01
IMMT	0.009	9.313E-01	6.914E-01
MFSD14C	0.009	9.612E-01	3.388E-01
PCDHGA10	0.009	9.843E-01	4.023E-01
ZNF697	0.009	9.692E-01	3.083E-01
ARHGAP19	0.009	9.598E-01	6.186E-01
CEP41	0.009	9.692E-01	4.768E-01
OR6B3	0.009	9.946E-01	4.505E-01
CNGA4	0.009	9.809E-01	1.899E-01
CRELD2	0.009	9.566E-01	3.049E-01
GDF11	0.009	9.718E-01	9.738E-01
MLLT10	0.008	9.488E-01	9.677E-01
ACOT8	0.008	9.508E-01	3.012E-01
MAPKAPK2	0.008	9.473E-01	7.150E-01
EGFL6	0.008	9.790E-01	4.574E-01
TESK1	0.008	9.517E-01	2.973E-01
PCGF5	0.008	9.579E-01	7.494E-01
MEPCE	0.008	9.450E-01	3.983E-01
TDRD7	0.008	9.616E-01	7.674E-01
CEP76	0.008	9.586E-01	8.306E-01
GTF3C2	0.008	9.386E-01	3.803E-01
EP300	0.008	9.601E-01	7.063E-01
CPSF2	0.008	9.457E-01	8.999E-01
MASP1	0.008	9.861E-01	2.709E-01
GPHN	0.008	9.680E-01	4.327E-01
ARFGAP2	0.008	9.423E-01	4.751E-01
AC004997.1	0.008	9.792E-01	3.109E-01
NUTM2B	0.008	9.796E-01	4.870E-01
NDUFAB1	0.008	9.544E-01	5.718E-01
EPB41L2	0.008	9.759E-01	6.830E-01
ANO8	0.008	9.752E-01	5.472E-01
SLC25A11	0.008	9.513E-01	9.165E-01
TOMM40	0.008	9.600E-01	3.264E-01
B4GALNT3	0.008	9.874E-01	5.222E-01
ASNA1	0.008	9.380E-01	5.343E-01
CCT5	0.008	9.667E-01	7.625E-01
HTR7	0.008	9.903E-01	3.920E-01
OXSM	0.008	9.536E-01	2.535E-01
HBG1	0.008	NA	6.095E-01
MIR4774	0.008	NA	9.393E-01
OR7G3	0.008	NA	3.665E-01
MIR1537	0.008	NA	6.826E-01

MIR4725	0.008	NA	8.544E-01
OR6J1	0.008	NA	5.706E-01
GOLGA6D	0.008	NA	7.440E-01
CYP11B2	0.008	NA	4.046E-01
PCDHGA8	0.008	9.835E-01	8.367E-01
OR13C5	0.008	NA	6.450E-01
SF3B6	0.008	9.455E-01	7.469E-01
LHFPL1	0.008	9.895E-01	4.278E-01
STX17	0.008	9.580E-01	2.815E-01
PRNP	0.008	9.824E-01	4.106E-01
ZNF879	0.008	9.821E-01	3.413E-01
STYXL1	0.008	9.600E-01	3.518E-01
ARHGAP18	0.008	9.671E-01	4.434E-01
CCDC12	0.008	9.654E-01	6.378E-01
POU2F1	0.008	9.582E-01	3.847E-01
CENPS	0.008	9.619E-01	9.856E-01
RANBP10	0.008	9.508E-01	9.856E-01
MKL2	0.008	9.614E-01	9.856E-01
ZNF776	0.007	9.643E-01	9.856E-01
CGNL1	0.007	9.837E-01	9.856E-01
HSPA8	0.007	9.697E-01	9.856E-01
SLC2A4RG	0.007	9.685E-01	9.856E-01
KCNIP1	0.007	9.919E-01	9.847E-01
SYTL3	0.007	9.792E-01	9.846E-01
AP000311.1	0.007	9.939E-01	9.846E-01
EPC1	0.007	9.478E-01	9.844E-01
RAB17	0.007	9.843E-01	9.844E-01
OGFOD1	0.007	9.413E-01	9.843E-01
SCARB1	0.007	9.783E-01	9.842E-01
ZNF726	0.007	9.837E-01	9.841E-01
SOX13	0.007	9.756E-01	7.826E-01
FCN3	0.007	9.815E-01	2.878E-01
KIAA1143	0.007	9.481E-01	3.593E-01
AP1S2	0.007	9.749E-01	5.852E-01
SIX2	0.007	9.914E-01	3.633E-01
TBL1X	0.007	9.766E-01	6.001E-01
CASS4	0.007	9.832E-01	3.969E-01
HNF1B	0.007	9.925E-01	6.749E-01
PAIP1	0.007	9.600E-01	7.219E-01
ZBTB18	0.007	9.783E-01	6.930E-01
BIRC2	0.007	9.696E-01	5.023E-01
ASB16	0.007	9.753E-01	4.270E-01
SPRTN	0.007	9.544E-01	4.482E-01
PIGBOS1	0.007	9.587E-01	2.802E-01
MT-ND2	0.007	9.828E-01	6.733E-01
POLD3	0.007	9.600E-01	5.599E-01
ABCB1	0.007	9.835E-01	3.172E-01
DUS4L	0.007	9.590E-01	8.918E-01
POLD1	0.007	9.690E-01	7.786E-01
PRUNE1	0.007	9.652E-01	9.060E-01

KDF1	0.007	9.759E-01	2.338E-01
JRKL	0.007	9.644E-01	4.181E-01
IGSF10	0.007	9.930E-01	3.295E-01
CATSPERB	0.007	9.873E-01	7.826E-01
SLC4A5	0.007	9.767E-01	2.912E-01
MYPOP	0.007	9.598E-01	3.280E-01
CERS6	0.007	9.733E-01	4.268E-01
PHF14	0.006	9.624E-01	4.064E-01
BCAT2	0.006	9.837E-01	9.624E-01
ERLIN1	0.006	9.650E-01	8.920E-01
ZNF606	0.006	9.773E-01	7.932E-01
ATP6V1D	0.006	9.630E-01	4.402E-01
DRAP1	0.006	9.713E-01	5.967E-01
SLC22A17	0.006	9.832E-01	3.877E-01
NUP58	0.006	9.669E-01	6.481E-01
OR2T27	0.006	NA	2.922E-01
MIR4436B1	0.006	NA	4.263E-01
MIR548X	0.006	NA	5.221E-01
OR8H2	0.006	NA	3.815E-01
DAZ3	0.006	NA	4.940E-01
MIR1267	0.006	NA	5.514E-01
MIR3922	0.006	NA	7.612E-01
MIR4703	0.006	NA	7.872E-01
OR2T4	0.006	NA	4.919E-01
OR4B1	0.006	NA	4.306E-01
OR4D9	0.006	NA	8.737E-01
DEFB131	0.006	NA	4.071E-01
MIR605	0.006	NA	2.154E-01
OR10Z1	0.006	NA	3.270E-01
OR51H1	0.006	NA	4.256E-01
MIR3162	0.006	NA	5.416E-01
ZNF280D	0.006	9.586E-01	6.088E-01
AHCYL1	0.006	9.600E-01	5.529E-01
PPP2R3C	0.006	9.616E-01	2.402E-01
ZMYM1	0.006	9.632E-01	6.619E-01
ZHX1	0.006	9.747E-01	3.204E-01
ZMYND19	0.006	9.696E-01	2.247E-01
CHRNA5	0.006	9.783E-01	2.688E-01
BTRC	0.006	9.616E-01	3.502E-01
SLF2	0.006	9.602E-01	3.643E-01
USP11	0.006	9.694E-01	5.054E-01
PFKM	0.006	9.749E-01	2.524E-01
ZNF566	0.006	9.721E-01	8.290E-01
NSRP1	0.006	9.584E-01	3.339E-01
COA7	0.006	9.644E-01	4.311E-01
VPS54	0.006	9.545E-01	4.283E-01
MIR378H	0.006	9.925E-01	3.834E-01
EMC10	0.006	9.714E-01	7.345E-01
HOXC6	0.006	9.919E-01	3.216E-01
PARP2	0.006	9.652E-01	5.585E-01

RRAGC	0.006	9.714E-01	3.893E-01
SEC23IP	0.006	9.597E-01	8.373E-01
PLEKHF2	0.006	9.748E-01	9.741E-01
IPO9	0.006	9.663E-01	9.726E-01
ARHGDIG	0.006	9.946E-01	9.723E-01
MRPL13	0.006	9.689E-01	4.186E-01
NEDD4L	0.006	9.824E-01	3.051E-01
GNAT1	0.006	9.937E-01	2.635E-01
FAM204A	0.006	9.520E-01	4.149E-01
CBWD2	0.006	9.600E-01	9.571E-01
HEMK1	0.006	9.749E-01	9.728E-01
THAP5	0.006	9.602E-01	4.553E-01
KCTD17	0.006	9.770E-01	2.494E-01
CWF19L1	0.006	9.598E-01	7.310E-01
ZNF442	0.006	9.822E-01	5.272E-01
KIF2C	0.006	9.804E-01	9.775E-01
ZNF639	0.006	9.604E-01	2.540E-01
CKLF	0.006	9.752E-01	7.532E-01
ATF6B	0.006	9.630E-01	2.913E-01
AC068946.1	0.006	9.873E-01	2.895E-01
PBRM1	0.006	9.714E-01	7.509E-01
USPL1	0.006	9.669E-01	4.394E-01
DHFR	0.005	9.792E-01	2.948E-01
ARL5B	0.005	9.763E-01	2.366E-01
PDCD2L	0.005	9.752E-01	2.844E-01
NAA20	0.005	9.719E-01	6.026E-01
SYNE3	0.005	9.873E-01	7.330E-01
IFT172	0.005	9.733E-01	3.208E-01
DCANP1	0.005	NA	7.987E-01
MIR4637	0.005	NA	6.216E-01
MIR4518	0.005	NA	3.870E-01
CCDC106	0.005	9.847E-01	7.454E-01
PAXBP1	0.005	9.783E-01	4.446E-01
CRISP2	0.005	9.959E-01	4.017E-01
ADAD2	0.005	9.919E-01	8.274E-01
RAG2	0.005	9.945E-01	6.688E-01
TRIM32	0.005	9.729E-01	5.921E-01
NAXD	0.005	9.710E-01	7.908E-01
ERGIC3	0.005	9.713E-01	6.254E-01
FAM96A	0.005	9.618E-01	7.178E-01
GPI	0.005	9.763E-01	3.894E-01
MFSD14A	0.005	9.691E-01	4.536E-01
MRPS11	0.005	9.638E-01	3.086E-01
NFYB	0.005	9.624E-01	4.136E-01
DYNC2LI1	0.005	9.709E-01	8.066E-01
LGI2	0.005	9.919E-01	8.784E-01
EIF2AK2	0.005	9.749E-01	3.016E-01
BTBD18	0.005	9.889E-01	6.846E-01
NEK1	0.005	9.752E-01	5.920E-01
RBMS1	0.005	9.728E-01	8.300E-01

POLDIP2	0.005	9.689E-01	9.864E-01
THRAP3	0.005	9.537E-01	9.864E-01
ARF5	0.005	9.718E-01	9.864E-01
RETREG3	0.005	9.695E-01	9.864E-01
ACVR2B	0.005	9.865E-01	9.864E-01
PKIB	0.005	9.919E-01	9.864E-01
MANBA	0.005	9.789E-01	9.864E-01
C12orf74	0.005	9.945E-01	9.864E-01
RAD54L2	0.005	9.718E-01	9.864E-01
STAB1	0.005	9.915E-01	9.864E-01
PKM	0.005	9.824E-01	9.864E-01
CLEC12A	0.005	9.944E-01	9.864E-01
CIC	0.005	9.718E-01	9.864E-01
KCNJ1	0.005	9.928E-01	9.864E-01
DHX36	0.005	9.729E-01	9.864E-01
UBE2Q2L	0.005	9.946E-01	9.864E-01
ING2	0.005	9.769E-01	9.864E-01
SHKBP1	0.005	9.752E-01	9.864E-01
KIF11	0.005	9.845E-01	9.864E-01
AP002495.2	0.005	9.785E-01	9.864E-01
CCDC142	0.005	9.774E-01	9.864E-01
ZNF518B	0.005	9.881E-01	9.864E-01
SLC16A14	0.004	9.892E-01	9.864E-01
ATP11C	0.004	9.783E-01	9.864E-01
SAA2	0.004	9.949E-01	9.864E-01
HIST1H3A	0.004	9.931E-01	9.864E-01
TRIM28	0.004	9.761E-01	9.864E-01
XPNPEP3	0.004	9.749E-01	9.864E-01
ZBED2	0.004	9.946E-01	9.864E-01
DOCK7	0.004	9.766E-01	9.864E-01
HPD	0.004	9.919E-01	9.864E-01
DAPK2	0.004	9.919E-01	4.108E-01
AMFR	0.004	9.709E-01	6.613E-01
SLC39A3	0.004	9.792E-01	4.501E-01
AL049779.1	0.004	9.921E-01	4.771E-01
NFKBIL1	0.004	9.792E-01	3.469E-01
HOXB13	0.004	9.946E-01	3.797E-01
NEDD8	0.004	9.749E-01	7.453E-01
SNX9	0.004	9.749E-01	3.330E-01
STIP1	0.004	9.752E-01	6.726E-01
MICU3	0.004	9.926E-01	3.761E-01
TMEM220	0.004	9.911E-01	3.034E-01
CMTM1	0.004	9.874E-01	3.223E-01
CBX3	0.004	9.721E-01	8.727E-01
ATP6V1B2	0.004	9.811E-01	7.908E-01
PRKCSH	0.004	9.717E-01	1.997E-01
CABP1	0.004	9.936E-01	6.868E-01
MAP1LC3B	0.004	9.745E-01	4.500E-01
ARHGAP4	0.004	9.916E-01	4.452E-01
MKKS	0.004	9.749E-01	3.200E-01

ITGB6	0.004	9.928E-01	4.581E-01
ZNF662	0.004	9.946E-01	6.236E-01
ASB3	0.004	9.751E-01	5.115E-01
ATOX1	0.004	9.833E-01	3.195E-01
GLO1	0.004	9.811E-01	9.567E-01
RNF181	0.004	9.792E-01	3.334E-01
WDR53	0.004	9.783E-01	6.877E-01
CCDC184	0.004	9.926E-01	3.393E-01
DNAL4	0.004	9.821E-01	5.107E-01
FKBP15	0.004	9.752E-01	8.063E-01
LMOD2	0.004	9.946E-01	4.198E-01
AVEN	0.004	9.836E-01	9.125E-01
TCTN3	0.004	9.733E-01	6.560E-01
TLK2	0.004	9.719E-01	4.275E-01
HERC2	0.004	9.792E-01	4.947E-01
AK2	0.004	9.689E-01	9.305E-01
WBP2NL	0.004	9.919E-01	6.020E-01
CYP19A1	0.004	9.939E-01	4.976E-01
FGD6	0.004	9.905E-01	8.560E-01
STAT5B	0.004	9.759E-01	5.149E-01
POF1B	0.004	9.946E-01	9.255E-01
DUSP11	0.004	9.770E-01	4.002E-01
SMARCA4	0.004	9.792E-01	8.033E-01
ARL13A	0.004	9.919E-01	7.108E-01
ZNF346	0.004	9.783E-01	7.654E-01
SERPINI1	0.004	9.913E-01	6.017E-01
NELFCD	0.004	9.785E-01	2.813E-01
CDC42BPB	0.003	9.790E-01	7.035E-01
MIR7850	0.003	NA	5.930E-01
KRTAP12-3	0.003	NA	3.343E-01
OR8B3	0.003	NA	5.571E-01
MIR6726	0.003	NA	5.466E-01
MIR6868	0.003	NA	4.436E-01
MIR7975	0.003	NA	6.311E-01
SPATA31D3	0.003	NA	3.660E-01
OR10G9	0.003	NA	9.540E-01
STEAP3	0.003	9.905E-01	8.429E-01
ZBTB10	0.003	9.916E-01	5.669E-01
CD302	0.003	9.905E-01	2.873E-01
EIF4G2	0.003	9.779E-01	5.725E-01
TAB2	0.003	9.784E-01	4.715E-01
NF670-ZNF69	0.003	9.919E-01	6.735E-01
DNAJC2	0.003	9.789E-01	3.937E-01
UCK2	0.003	9.881E-01	5.844E-01
UTP4	0.003	9.815E-01	5.332E-01
SOD1	0.003	9.837E-01	4.814E-01
ILVBL	0.003	9.861E-01	8.750E-01
SYNE1	0.003	9.926E-01	3.163E-01
CAB39L	0.003	9.945E-01	7.636E-01
TMEM39B	0.003	9.801E-01	3.285E-01

TBCE	0.003	9.785E-01	3.355E-01
C8orf33	0.003	9.824E-01	7.007E-01
ACRBP	0.003	9.935E-01	3.549E-01
SELENOP	0.003	9.946E-01	9.844E-01
EMC7	0.003	9.786E-01	9.804E-01
OR10H2	0.003	9.973E-01	9.801E-01
GABARAP	0.003	9.822E-01	9.768E-01
LRRC25	0.003	9.946E-01	4.532E-01
C16orf95	0.003	9.881E-01	4.280E-01
TPD52L1	0.003	9.945E-01	3.725E-01
DEK	0.003	9.916E-01	3.642E-01
ANKRD36	0.003	9.942E-01	4.665E-01
RFESD	0.003	9.881E-01	4.069E-01
OTUD6B	0.003	9.873E-01	3.378E-01
TMEM18	0.003	9.832E-01	6.815E-01
CACNB1	0.003	9.930E-01	7.971E-01
UMODL1	0.003	9.959E-01	7.258E-01
RASGRP4	0.003	9.946E-01	5.543E-01
HAUS6	0.003	9.909E-01	6.582E-01
PSME4	0.003	9.809E-01	6.250E-01
AC115220.1	0.003	9.987E-01	8.454E-01
WRNIP1	0.003	9.847E-01	3.201E-01
ECE2	0.003	9.918E-01	6.187E-01
APOBEC3G	0.003	9.945E-01	3.152E-01
GNA13	0.003	9.861E-01	2.932E-01
ALDH4A1	0.003	9.942E-01	4.853E-01
ANKRD39	0.003	9.874E-01	6.882E-01
TSTD2	0.003	9.921E-01	6.459E-01
KRTAP1-4	0.003	NA	6.993E-01
MIR1913	0.003	NA	4.356E-01
JUN	0.003	9.939E-01	9.283E-01
PGP	0.003	9.905E-01	7.634E-01
ZNF45	0.003	9.877E-01	5.209E-01
KLF-CMTM	0.003	9.926E-01	3.284E-01
KTN1	0.003	9.893E-01	6.251E-01
PRMT2	0.002	9.833E-01	6.366E-01
PHF2	0.002	9.864E-01	5.205E-01
ABCF1	0.002	9.861E-01	3.507E-01
PTPN23	0.002	9.846E-01	3.789E-01
SLC25A24	0.002	9.916E-01	8.952E-01
ADAMTS5	0.002	9.946E-01	8.483E-01
SLC30A5	0.002	9.845E-01	9.472E-01
CNOT4	0.002	9.809E-01	6.284E-01
POLR2C	0.002	9.833E-01	4.881E-01
TRPM7	0.002	9.883E-01	9.585E-01
MIOS	0.002	9.873E-01	8.671E-01
RNH1	0.002	9.892E-01	3.606E-01
ERVMER34-1	0.002	9.958E-01	8.750E-01
SMIM19	0.002	9.928E-01	7.644E-01
PLTP	0.002	9.952E-01	5.020E-01

GPC1	0.002	9.946E-01	4.896E-01
NODAL	0.002	9.962E-01	5.129E-01
TAS1R2	0.002	NA	5.861E-01
HEATR1	0.002	9.919E-01	3.697E-01
BEX4	0.002	9.950E-01	3.966E-01
KCTD2	0.002	9.877E-01	5.615E-01
UBAP2L	0.002	9.874E-01	9.288E-01
TSPAN33	0.002	9.947E-01	7.648E-01
YEATS2	0.002	9.916E-01	6.572E-01
SPOP	0.002	9.892E-01	9.871E-01
CCDC40	0.002	9.946E-01	9.871E-01
TUBD1	0.002	9.918E-01	9.871E-01
BRIX1	0.002	9.926E-01	9.871E-01
DYRK1B	0.002	9.946E-01	9.871E-01
MUT	0.002	9.904E-01	9.871E-01
AGO3	0.002	9.890E-01	9.871E-01
PHKG2	0.002	9.926E-01	9.871E-01
MIR613	0.002	NA	9.871E-01
TMEM131	0.002	9.916E-01	9.871E-01
H3F3A	0.002	9.919E-01	9.871E-01
ZNF800	0.002	9.894E-01	9.871E-01
CBX2	0.002	9.963E-01	9.871E-01
ZNF787	0.002	9.926E-01	9.871E-01
KCTD6	0.002	9.941E-01	9.871E-01
RNF187	0.002	9.911E-01	9.871E-01
ATXN1L	0.002	9.942E-01	9.871E-01
TMEM44	0.002	9.946E-01	9.871E-01
PGLYRP3	0.002	9.982E-01	9.871E-01
CHD2	0.001	9.926E-01	9.871E-01
TMEM213	0.001	9.979E-01	9.871E-01
ASXL1	0.001	9.930E-01	9.871E-01
STK40	0.001	9.945E-01	9.871E-01
RAB9A	0.001	9.946E-01	9.871E-01
MIR6851	0.001	NA	9.871E-01
PEX6	0.001	9.949E-01	9.871E-01
PDF	0.001	9.946E-01	9.871E-01
CCDC86	0.001	9.946E-01	9.871E-01
USH1G	0.001	9.981E-01	9.871E-01
MC3R	0.001	NA	9.871E-01
SGSH	0.001	9.946E-01	9.871E-01
PDCL3	0.001	9.936E-01	9.871E-01
IMPA2	0.001	9.966E-01	9.871E-01
SUMO1	0.001	9.919E-01	9.871E-01
ESR1	0.001	9.977E-01	9.871E-01
SLC15A4	0.001	9.945E-01	9.871E-01
ORMDL2	0.001	9.946E-01	9.871E-01
GALNT10	0.001	9.966E-01	9.871E-01
CUTC	0.001	9.948E-01	9.871E-01
JAG1	0.001	9.971E-01	9.871E-01
R3HDM1	0.001	9.946E-01	9.871E-01

AP1G1	0.001	9.946E-01	9.871E-01
API5	0.001	9.945E-01	9.871E-01
FAM229A	0.001	9.969E-01	9.871E-01
ZNF555	0.001	9.956E-01	9.871E-01
R3HDM2	0.001	9.946E-01	9.871E-01
TRIP12	0.001	9.946E-01	9.871E-01
MRPL9	0.001	9.952E-01	9.871E-01
CD3EAP	0.001	9.961E-01	9.871E-01
PSKH2	0.001	NA	9.871E-01
ISY1	0.001	9.946E-01	9.871E-01
SNAP47	0.001	9.946E-01	9.871E-01
PTPRG	0.001	9.970E-01	9.871E-01
INTS12	0.001	9.946E-01	9.871E-01
FIS1	0.001	9.958E-01	9.871E-01
ATXN7L1	0.001	9.958E-01	9.871E-01
ZNF490	0.001	9.970E-01	9.871E-01
BUB1	0.001	9.971E-01	9.871E-01
HMGB2	0.001	9.971E-01	9.871E-01
FAM206A	0.001	9.962E-01	9.871E-01
MIR4472-1	0.001	NA	9.871E-01
MIR6087	0.001	NA	9.871E-01
MIR6133	0.001	NA	9.871E-01
MIR208B	0.001	NA	9.871E-01
MIR520E	0.001	NA	9.871E-01
MIR7150	0.001	NA	9.871E-01
OR6N2	0.001	NA	9.871E-01
ZNF749	0.001	9.968E-01	9.871E-01
PRKDC	0.001	9.970E-01	9.871E-01
PLCXD1	0.001	9.979E-01	9.871E-01
CNPY3	0.001	9.970E-01	9.871E-01
TBX2	0.001	9.988E-01	3.388E-01
LENG9	0.001	9.980E-01	7.616E-01
MFN1	0.001	9.961E-01	8.268E-01
AL159163.1	0.001	9.987E-01	4.068E-01
UNC13B	0.001	9.978E-01	2.963E-01
RIPPLY2	0.001	9.993E-01	3.707E-01
ARHGAP32	0.000	9.982E-01	8.722E-01
METTL11B	0.000	9.993E-01	5.407E-01
UBE3A	0.000	9.970E-01	8.387E-01
43896.000	0.000	9.976E-01	2.834E-01
ZNF524	0.000	9.982E-01	3.397E-01
LSM10	0.000	9.975E-01	3.507E-01
KCNN2	0.000	9.993E-01	4.532E-01
TRAPPC8	0.000	9.982E-01	5.509E-01
VBP1	0.000	9.982E-01	4.737E-01
CES5A	0.000	9.997E-01	3.329E-01
REV3L	0.000	9.989E-01	5.650E-01
DNAJC24	0.000	9.988E-01	3.103E-01
MRPL19	0.000	9.986E-01	8.201E-01
STK35	0.000	9.990E-01	3.318E-01

RWDD2B	0.000	9.990E-01	9.605E-01
PPP1R3B	0.000	9.995E-01	9.619E-01
GNL1	0.000	9.997E-01	5.295E-01
GAGE10	0.000	9.999E-01	6.969E-01
CCT3	0.000	9.997E-01	5.112E-01
H2AFB2	0.000	NA	8.471E-01
KRTAP19-6	0.000	NA	4.572E-01
TBC1D15	0.000	9.999E-01	5.880E-01
ALG2	0.000	9.998E-01	5.748E-01
GATAD1	0.000	9.998E-01	2.742E-01
ZNF771	0.000	9.997E-01	3.006E-01
ASMTL	0.000	9.997E-01	4.438E-01
LRG1	0.000	9.998E-01	2.846E-01
SUPT7L	0.000	9.985E-01	7.897E-01
IDH1	0.000	9.994E-01	3.486E-01
PATJ	0.000	9.991E-01	4.982E-01
RAB19	0.000	9.994E-01	4.849E-01
PCNP	0.000	9.978E-01	5.102E-01
PATE3	0.000	NA	2.020E-01
TAT	0.000	9.994E-01	8.156E-01
MRO	0.000	9.994E-01	8.369E-01
SAP30L	0.000	9.976E-01	8.608E-01
RPP38	0.000	9.981E-01	6.189E-01
PABPC4	0.000	9.987E-01	4.743E-01
NSUN2	0.000	9.981E-01	6.570E-01
CATSPERZ	0.000	9.994E-01	4.246E-01
RUNDC1	0.000	9.971E-01	4.213E-01
MYH7B	-0.001	9.982E-01	4.727E-01
MRTO4	-0.001	9.968E-01	3.891E-01
FAM149B1	-0.001	9.959E-01	8.126E-01
COA4	-0.001	9.968E-01	8.275E-01
ZNF341	-0.001	9.970E-01	6.238E-01
GLRX5	-0.001	9.962E-01	4.113E-01
CPSF4	-0.001	9.963E-01	4.288E-01
ACTL10	-0.001	9.982E-01	9.653E-01
PPIL1	-0.001	9.963E-01	5.440E-01
ANKFY1	-0.001	9.962E-01	8.779E-01
ADGRG6	-0.001	9.982E-01	4.495E-01
TMEM234	-0.001	9.963E-01	5.331E-01
LGALSL	-0.001	9.975E-01	9.395E-01
DTL	-0.001	9.971E-01	3.785E-01
EARS2	-0.001	9.961E-01	9.537E-01
USP33	-0.001	9.955E-01	4.157E-01
UBXN7	-0.001	9.958E-01	8.301E-01
DNAJC16	-0.001	9.951E-01	8.087E-01
PEAR1	-0.001	9.970E-01	5.748E-01
MMS19	-0.001	9.946E-01	4.748E-01
ZNF551	-0.001	9.962E-01	4.919E-01
NIPAL3	-0.001	9.958E-01	4.235E-01
FBXO45	-0.001	9.960E-01	4.000E-01

SGTA	-0.001	9.949E-01	5.993E-01
HELZ	-0.001	9.946E-01	4.230E-01
NRAS	-0.001	9.958E-01	5.781E-01
PABPC1L	-0.001	9.970E-01	9.843E-01
INVS	-0.001	9.946E-01	9.831E-01
DNAJC5	-0.001	9.950E-01	9.829E-01
PPM1M	-0.001	9.961E-01	9.816E-01
TMEM201	-0.001	9.946E-01	9.812E-01
MGST1	-0.001	9.970E-01	9.801E-01
KLHL28	-0.001	9.950E-01	9.800E-01
SMARCE1	-0.001	9.946E-01	9.799E-01
MIR3127	-0.001	NA	4.014E-01
H2BFM	-0.001	9.987E-01	3.196E-01
RARG	-0.001	9.959E-01	4.808E-01
ZNF830	-0.001	9.931E-01	2.641E-01
AZU1	-0.001	9.971E-01	8.812E-01
LRBA	-0.001	9.946E-01	6.983E-01
CAPN13	-0.001	9.982E-01	6.026E-01
TAS2R20	-0.001	9.962E-01	7.169E-01
TBC1D25	-0.001	9.928E-01	3.940E-01
BCL2L2	-0.001	9.945E-01	4.355E-01
TBC1D3C	-0.001	NA	3.725E-01
GLIPR1L2	-0.001	9.963E-01	5.317E-01
CEP250	-0.001	9.945E-01	5.095E-01
MRPL58	-0.001	9.931E-01	4.071E-01
MBD2	-0.001	9.928E-01	5.015E-01
USF3	-0.001	9.942E-01	6.268E-01
PITPNC1	-0.001	9.946E-01	4.493E-01
CMIP	-0.001	9.945E-01	4.642E-01
CT83	-0.002	9.991E-01	5.161E-01
PLEKHM1	-0.002	9.926E-01	4.012E-01
MIR6076	-0.002	NA	8.727E-01
PDC	-0.002	9.971E-01	8.156E-01
CENPO	-0.002	9.942E-01	4.158E-01
MDH1B	-0.002	9.960E-01	4.363E-01
LLGL1	-0.002	9.935E-01	8.306E-01
RNF149	-0.002	9.922E-01	9.513E-01
ZNF322	-0.002	9.939E-01	5.698E-01
LTN1	-0.002	9.930E-01	9.782E-01
SLC4A7	-0.002	9.946E-01	4.149E-01
SLC6A17	-0.002	9.970E-01	5.054E-01
SYT2	-0.002	9.962E-01	4.922E-01
XPA	-0.002	9.909E-01	4.254E-01
AIRE	-0.002	9.970E-01	4.400E-01
NT5E	-0.002	9.969E-01	6.483E-01
NGF	-0.002	9.968E-01	4.452E-01
CAP1	-0.002	9.930E-01	4.405E-01
ZNF878	-0.002	9.951E-01	6.494E-01
SEH1L	-0.002	9.919E-01	4.002E-01
MXD3	-0.002	9.946E-01	7.873E-01

CDC14A	-0.002	9.942E-01	5.966E-01
MTIF2	-0.002	9.871E-01	5.147E-01
TAL2	-0.002	9.967E-01	4.805E-01
BZW1	-0.002	9.915E-01	6.370E-01
BDKRB1	-0.002	9.962E-01	7.287E-01
SLC15A1	-0.002	9.968E-01	5.544E-01
MIR3908	-0.002	NA	3.602E-01
MIR182	-0.002	NA	7.683E-01
MIR4459	-0.002	NA	6.769E-01
MIR4772	-0.002	NA	3.887E-01
SCGB1D1	-0.002	NA	8.137E-01
ZC3H11B	-0.002	9.958E-01	4.388E-01
SEMA6B	-0.002	9.946E-01	5.022E-01
MIR4665	-0.002	NA	3.163E-01
KIAA0319L	-0.002	9.856E-01	7.428E-01
AL365273.2	-0.002	NA	3.815E-01
SMIM13	-0.002	9.911E-01	3.797E-01
CSMD1	-0.002	9.971E-01	5.718E-01
CEP19	-0.002	9.939E-01	3.941E-01
CEP192	-0.002	9.913E-01	9.054E-01
NSUN4	-0.002	9.837E-01	4.273E-01
CGGBP1	-0.002	9.875E-01	4.110E-01
FCGR2A	-0.002	9.957E-01	8.157E-01
PAPD7	-0.002	9.898E-01	3.894E-01
MRPL36	-0.002	9.919E-01	5.155E-01
PCID2	-0.002	9.837E-01	8.855E-01
ZNF227	-0.002	9.861E-01	6.436E-01
BIN1	-0.002	9.947E-01	3.252E-01
PABPC5	-0.002	9.956E-01	5.087E-01
C19orf48	-0.002	9.918E-01	4.536E-01
AP4S1	-0.002	9.895E-01	4.582E-01
ZNF197	-0.002	9.845E-01	9.667E-01
RGL1	-0.003	9.942E-01	9.669E-01
ZDBF2	-0.003	9.952E-01	9.681E-01
UTP14C	-0.003	9.889E-01	9.734E-01
SURF6	-0.003	9.858E-01	3.741E-01
NKRF	-0.003	9.833E-01	7.959E-01
HCCS	-0.003	9.847E-01	6.926E-01
RSPRY1	-0.003	9.815E-01	8.374E-01
ABHD2	-0.003	9.939E-01	7.449E-01
LHX1	-0.003	9.971E-01	5.133E-01
MUSK	-0.003	9.952E-01	5.784E-01
MIR6500	-0.003	NA	3.958E-01
EXOC3	-0.003	9.874E-01	7.200E-01
HACE1	-0.003	9.889E-01	4.999E-01
GLI2	-0.003	9.950E-01	4.181E-01
LHFPL5	-0.003	9.958E-01	3.808E-01
APOBEC3C	-0.003	9.911E-01	5.044E-01
MIEF2	-0.003	9.847E-01	8.324E-01
NPR3	-0.003	9.946E-01	3.451E-01

CARS	-0.003	9.821E-01	3.495E-01
G0S2	-0.003	9.951E-01	5.066E-01
APH1B	-0.003	9.877E-01	4.190E-01
ZBTB20	-0.003	9.892E-01	5.616E-01
BCAS3	-0.003	9.861E-01	5.132E-01
DHX16	-0.003	9.783E-01	3.330E-01
MYBPC3	-0.003	9.941E-01	3.516E-01
MZT1	-0.003	9.861E-01	6.696E-01
CDKN1A	-0.003	9.922E-01	7.087E-01
DCTN2	-0.003	9.751E-01	8.561E-01
NXPE3	-0.003	9.919E-01	5.300E-01
METTL27	-0.003	9.945E-01	5.299E-01
TRIP10	-0.003	9.869E-01	6.194E-01
CPA5	-0.003	9.946E-01	7.560E-01
WWOX	-0.003	9.885E-01	8.001E-01
MYO9B	-0.003	9.763E-01	6.232E-01
MFN2	-0.003	9.785E-01	3.816E-01
KRTCAP2	-0.003	9.843E-01	8.363E-01
GPR158	-0.003	9.953E-01	4.934E-01
ZNF626	-0.003	9.947E-01	5.184E-01
SARDH	-0.003	9.938E-01	7.426E-01
OCEL1	-0.003	9.881E-01	8.671E-01
OR2D3	-0.004	NA	9.428E-01
ZNF404	-0.004	9.911E-01	4.620E-01
ANP32B	-0.004	9.837E-01	6.024E-01
ATG9A	-0.004	9.796E-01	3.955E-01
KRTAP10-1	-0.004	NA	4.470E-01
LRRFIP1	-0.004	9.828E-01	6.698E-01
MYL10	-0.004	9.957E-01	7.876E-01
SOX9	-0.004	9.946E-01	4.838E-01
SON	-0.004	9.783E-01	4.356E-01
GPR84	-0.004	9.945E-01	8.589E-01
GJE1	-0.004	NA	5.003E-01
MIR3692	-0.004	NA	5.727E-01
MIR4309	-0.004	NA	5.242E-01
MIR4447	-0.004	NA	7.060E-01
MIR548AZ	-0.004	NA	3.056E-01
MIR6134	-0.004	NA	9.471E-01
OR4D11	-0.004	NA	9.883E-01
AC013269.1	-0.004	NA	9.856E-01
FAM236D	-0.004	NA	9.856E-01
MIR4760	-0.004	NA	9.854E-01
MIR4790	-0.004	NA	9.842E-01
MIR548I3	-0.004	NA	3.878E-01
MIR651	-0.004	NA	5.502E-01
MIR6724-2	-0.004	NA	6.762E-01
OR10H3	-0.004	NA	4.535E-01
OR10T2	-0.004	NA	4.834E-01
OR11H1	-0.004	NA	4.028E-01
OR1S2	-0.004	NA	7.684E-01

OR4C11	-0.004	NA	4.867E-01
ısa-mir-548d-:	-0.004	NA	7.148E-01
MIR1283-1	-0.004	NA	4.815E-01
MIR1302-4	-0.004	NA	5.551E-01
MIR516B1	-0.004	NA	3.879E-01
MIR6846	-0.004	NA	4.576E-01
MIR8075	-0.004	NA	8.081E-01
MIR873	-0.004	NA	5.587E-01
MIR5787	-0.004	NA	3.885E-01
TMOD4	-0.004	NA	6.193E-01
MIR938	-0.004	NA	6.405E-01
CTBS	-0.004	9.810E-01	6.690E-01
LRRCC1	-0.004	9.874E-01	6.752E-01
RNF7	-0.004	9.749E-01	5.662E-01
ZSCAN31	-0.004	9.919E-01	5.065E-01
GSTM3	-0.004	9.946E-01	4.889E-01
USP53	-0.004	9.867E-01	3.911E-01
TANC1	-0.004	9.828E-01	4.647E-01
CUL4A	-0.004	9.752E-01	5.171E-01
DEFB132	-0.004	NA	7.830E-01
LSM8	-0.004	9.749E-01	3.673E-01
FLVCR2	-0.004	9.910E-01	5.655E-01
NEMF	-0.004	9.826E-01	4.160E-01
GFER	-0.004	9.795E-01	5.686E-01
DIAPH2	-0.004	9.874E-01	3.713E-01
BANF1	-0.004	9.784E-01	5.285E-01
ACTR3C	-0.004	9.893E-01	9.314E-01
SPHK2	-0.004	9.874E-01	5.161E-01
TMEM186	-0.004	9.837E-01	4.183E-01
PPP1R3E	-0.004	9.856E-01	6.989E-01
SCNN1A	-0.004	9.946E-01	9.013E-01
WNK1	-0.004	9.804E-01	3.940E-01
Z98752.3	-0.004	NA	5.405E-01
ZNF383	-0.004	9.792E-01	4.844E-01
GTF2A1	-0.004	9.749E-01	4.233E-01
TOMM5	-0.004	9.828E-01	4.316E-01
TYW1B	-0.004	9.874E-01	4.442E-01
UBE2D1	-0.004	9.749E-01	4.763E-01
IL7R	-0.004	9.942E-01	4.866E-01
CPSF7	-0.004	9.614E-01	6.422E-01
ZDHHC16	-0.004	9.711E-01	5.447E-01
PIGN	-0.004	9.792E-01	4.565E-01
MIR553	-0.004	9.946E-01	5.534E-01
TIMM17B	-0.004	9.787E-01	5.518E-01
HMGA2	-0.004	9.957E-01	5.145E-01
THOP1	-0.004	9.821E-01	7.974E-01
C1QA	-0.004	9.942E-01	8.625E-01
NHLH1	-0.004	9.918E-01	3.707E-01
RAB40C	-0.004	9.813E-01	5.548E-01
NDUFA5	-0.005	9.692E-01	4.561E-01

TMEM59	-0.005	9.749E-01	2.297E-01
TMEM38B	-0.005	9.861E-01	5.962E-01
SUMO2	-0.005	9.641E-01	7.704E-01
RAB4A	-0.005	9.700E-01	5.311E-01
MIR548A3	-0.005	NA	6.884E-01
CAGE1	-0.005	9.945E-01	6.931E-01
AC233992.2	-0.005	9.916E-01	6.481E-01
MIR609	-0.005	NA	7.279E-01
MIR4655	-0.005	NA	5.375E-01
DNAAF1	-0.005	9.902E-01	7.829E-01
NR1I3	-0.005	9.885E-01	3.989E-01
INTS11	-0.005	9.713E-01	5.222E-01
TARDBP	-0.005	9.380E-01	4.828E-01
SPATA31D1	-0.005	NA	7.290E-01
UNC50	-0.005	9.667E-01	5.527E-01
CTCF	-0.005	9.593E-01	7.956E-01
NOTCH3	-0.005	9.837E-01	6.472E-01
WHRN	-0.005	9.849E-01	8.193E-01
LEF1	-0.005	9.895E-01	8.227E-01
CNOT1	-0.005	9.758E-01	7.239E-01
IFT74	-0.005	9.785E-01	4.259E-01
SRBD1	-0.005	9.680E-01	9.761E-01
AL662899.1	-0.005	NA	9.738E-01
TMEM183A	-0.005	9.580E-01	5.768E-01
NPIPB7	-0.005	9.928E-01	9.714E-01
CEP170	-0.005	9.809E-01	9.713E-01
MIR8064	-0.005	NA	2.927E-01
PIP5K1C	-0.005	9.642E-01	6.709E-01
AC126755.2	-0.005	9.909E-01	4.853E-01
ELP4	-0.005	9.714E-01	4.930E-01
KBTBD2	-0.005	9.600E-01	7.936E-01
TRPC6	-0.005	9.869E-01	4.500E-01
ECD	-0.005	9.612E-01	7.947E-01
FAM122A	-0.005	9.640E-01	8.911E-01
UBA5	-0.005	9.587E-01	6.655E-01
DCP1B	-0.005	9.783E-01	5.385E-01
GOLGA6L1	-0.005	NA	7.700E-01
ARHGEF12	-0.005	9.718E-01	8.609E-01
RGP1	-0.005	9.774E-01	4.623E-01
MIR548Y	-0.005	NA	5.977E-01
OLFM1	-0.006	9.918E-01	5.241E-01
DUSP1	-0.006	9.884E-01	6.837E-01
CDC34	-0.006	9.756E-01	5.580E-01
HAVCR1	-0.006	9.949E-01	5.736E-01
C8orf76	-0.006	9.671E-01	5.123E-01
HSD11B1L	-0.006	9.829E-01	7.580E-01
CCT4	-0.006	9.733E-01	5.612E-01
AZIN2	-0.006	9.828E-01	6.954E-01
HIST2H2AA3	-0.006	9.949E-01	8.835E-01
COL9A2	-0.006	9.905E-01	5.546E-01

TRPV6	-0.006	9.928E-01	4.829E-01
SRPK2	-0.006	9.676E-01	7.583E-01
PAPOLG	-0.006	9.601E-01	5.019E-01
MIR1255A	-0.006	NA	6.272E-01
TXNL4B	-0.006	9.692E-01	5.778E-01
SUPT16H	-0.006	9.669E-01	3.496E-01
PHF3	-0.006	9.705E-01	8.635E-01
C1orf109	-0.006	9.681E-01	4.745E-01
KDM1A	-0.006	9.711E-01	4.861E-01
SYCE2	-0.006	9.856E-01	5.809E-01
TIMM17A	-0.006	9.621E-01	6.521E-01
FAM78A	-0.006	9.874E-01	5.416E-01
CTAGE15	-0.006	9.936E-01	6.160E-01
GNPTG	-0.006	9.689E-01	6.244E-01
ZNF786	-0.006	9.673E-01	4.283E-01
KIAA0368	-0.006	9.626E-01	6.609E-01
MIR4663	-0.006	NA	5.537E-01
FRMD4B	-0.006	9.837E-01	3.372E-01
COX20	-0.006	9.689E-01	5.262E-01
RDH8	-0.006	9.945E-01	5.005E-01
VSIR	-0.006	9.825E-01	4.359E-01
TM6SF1	-0.006	9.847E-01	4.607E-01
MTMR4	-0.006	9.721E-01	6.153E-01
PIN1	-0.006	9.667E-01	8.967E-01
SERPING1	-0.006	9.881E-01	4.414E-01
ING1	-0.006	9.647E-01	9.743E-01
COX6B1	-0.006	9.743E-01	7.051E-01
RNF8	-0.006	9.587E-01	3.593E-01
CCM2L	-0.006	9.823E-01	5.934E-01
ATP6V1G1	-0.006	9.580E-01	4.917E-01
MAP4K3	-0.006	9.752E-01	3.100E-01
ZC3HC1	-0.006	9.568E-01	4.676E-01
PCDHA13	-0.006	9.946E-01	4.675E-01
ATP6V1H	-0.006	9.601E-01	7.063E-01
MED25	-0.006	9.619E-01	6.520E-01
SNTA1	-0.006	9.807E-01	7.700E-01
C17orf53	-0.006	9.763E-01	9.890E-01
KRTAP4-11	-0.006	NA	9.890E-01
MED30	-0.006	9.692E-01	9.890E-01
ZNF527	-0.006	9.691E-01	9.890E-01
PUS3	-0.006	9.621E-01	9.890E-01
HMBS	-0.006	9.694E-01	9.890E-01
MIR556	-0.006	NA	9.890E-01
NUDT2	-0.006	9.718E-01	9.890E-01
SCG2	-0.006	9.915E-01	9.890E-01
MIR3659	-0.006	NA	9.890E-01
MIR4716	-0.006	NA	9.890E-01
OR2AP1	-0.006	NA	9.890E-01
DEFB105A	-0.006	NA	9.890E-01
MIR3663	-0.006	NA	9.890E-01

MIR4529	-0.006	NA	9.890E-01
MIR5698	-0.006	NA	9.890E-01
MIR6802	-0.006	NA	9.890E-01
OR4L1	-0.006	NA	5.713E-01
OR52M1	-0.006	NA	5.423E-01
KRTAP13-1	-0.006	NA	4.427E-01
MIR183	-0.006	NA	5.930E-01
OR11L1	-0.006	NA	6.851E-01
OR2M7	-0.006	NA	5.034E-01
OR4C15	-0.006	NA	9.625E-01
MIR1289-2	-0.006	NA	5.613E-01
MIR511	-0.006	NA	4.893E-01
MFAP1	-0.006	9.489E-01	4.900E-01
NCBP2-AS2	-0.006	9.690E-01	6.964E-01
ZFR	-0.006	9.528E-01	8.504E-01
ABCB7	-0.006	9.619E-01	4.970E-01
HIST1H2BE	-0.006	9.881E-01	5.584E-01
CDK13	-0.007	9.506E-01	5.143E-01
MIER1	-0.007	9.508E-01	8.417E-01
MARCKS	-0.007	9.678E-01	7.287E-01
SIN3A	-0.007	9.454E-01	4.449E-01
MFSD6L	-0.007	9.921E-01	5.916E-01
HADHB	-0.007	9.598E-01	8.491E-01
PRR23C	-0.007	NA	3.078E-01
ACADS	-0.007	9.709E-01	3.683E-01
MAP3K14	-0.007	9.774E-01	8.264E-01
ANKRD61	-0.007	9.770E-01	6.707E-01
CEP72	-0.007	9.764E-01	8.082E-01
BTN2A1	-0.007	9.593E-01	5.047E-01
ZSCAN21	-0.007	9.586E-01	7.857E-01
DTX4	-0.007	9.828E-01	7.429E-01
MIS18A	-0.007	9.692E-01	5.065E-01
FNIP1	-0.007	9.567E-01	6.478E-01
ALKBH8	-0.007	9.619E-01	4.875E-01
AC096887.1	-0.007	9.733E-01	5.817E-01
CREBBP	-0.007	9.579E-01	4.440E-01
NOLC1	-0.007	9.612E-01	7.643E-01
PRR12	-0.007	9.587E-01	5.536E-01
EIF2AK4	-0.007	9.521E-01	9.727E-01
LMO7DN	-0.007	9.919E-01	5.868E-01
DDX59	-0.007	9.524E-01	3.552E-01
MIR2355	-0.007	NA	4.055E-01
MIR627	-0.007	NA	8.142E-01
ATP5C1	-0.007	9.631E-01	6.480E-01
COG2	-0.007	9.466E-01	4.878E-01
TCEA1	-0.007	9.523E-01	6.877E-01
GAL	-0.007	9.930E-01	8.670E-01
URM1	-0.007	9.592E-01	6.009E-01
USP16	-0.007	9.479E-01	3.973E-01
KDM4C	-0.007	9.579E-01	4.276E-01

AP3D1	-0.007	9.399E-01	4.903E-01
MIR4711	-0.007	NA	5.210E-01
AC110275.1	-0.007	NA	4.934E-01
MDH2	-0.007	9.554E-01	4.136E-01
MIR4659A	-0.007	NA	5.075E-01
RBM18	-0.007	9.483E-01	9.410E-01
OR51Q1	-0.007	9.946E-01	4.628E-01
TIMM29	-0.007	9.479E-01	3.691E-01
EIF2B5	-0.008	9.446E-01	7.217E-01
CC2D1B	-0.008	9.452E-01	7.884E-01
MID1IP1	-0.008	9.713E-01	5.639E-01
IMMP1L	-0.008	9.600E-01	4.367E-01
DENND1B	-0.008	9.601E-01	5.651E-01
STAU2	-0.008	9.600E-01	4.210E-01
WDFY3	-0.008	9.600E-01	6.914E-01
USP7	-0.008	9.600E-01	4.945E-01
TMEM105	-0.008	9.861E-01	3.939E-01
SF3A3	-0.008	9.446E-01	8.470E-01
TOX2	-0.008	9.861E-01	3.813E-01
USP17L2	-0.008	NA	7.633E-01
ETV2	-0.008	9.766E-01	4.883E-01
SPATA5	-0.008	9.558E-01	7.169E-01
ZNF106	-0.008	9.600E-01	5.426E-01
TMEM251	-0.008	9.527E-01	7.058E-01
LRWD1	-0.008	9.519E-01	6.924E-01
ANKRD36B	-0.008	9.792E-01	5.739E-01
ZBTB8OS	-0.008	9.453E-01	9.089E-01
NLGN4Y	-0.008	9.926E-01	7.916E-01
FAM83A	-0.008	9.900E-01	9.803E-01
MCM3AP	-0.008	9.481E-01	9.779E-01
C1orf123	-0.008	9.468E-01	9.767E-01
ZNF569	-0.008	9.751E-01	9.767E-01
LIPC	-0.008	9.890E-01	9.757E-01
NAT6	-0.008	9.641E-01	4.890E-01
SLC24A1	-0.008	9.621E-01	3.813E-01
UBFD1	-0.008	9.513E-01	4.682E-01
ATF7IP	-0.008	9.459E-01	8.705E-01
ZNF675	-0.008	9.749E-01	3.867E-01
INSL4	-0.008	9.946E-01	5.681E-01
MIR3126	-0.008	NA	6.376E-01
DIABLO	-0.008	9.381E-01	5.675E-01
NEK9	-0.008	9.498E-01	3.893E-01
KCNK16	-0.008	NA	5.868E-01
CRYAA	-0.008	NA	6.093E-01
STX8	-0.008	9.586E-01	8.492E-01
RBM7	-0.008	9.452E-01	5.972E-01
ANKDD1A	-0.008	9.749E-01	7.549E-01
INO80E	-0.008	9.579E-01	7.779E-01
ARHGEF5	-0.008	9.715E-01	4.298E-01
TBCCD1	-0.008	9.501E-01	6.925E-01

ATP13A2	-0.008	9.543E-01	4.796E-01
CENPE	-0.008	9.718E-01	5.826E-01
PLEKHA3	-0.008	9.373E-01	3.198E-01
RAB3GAP1	-0.009	9.349E-01	6.464E-01
AC005020.2	-0.009	9.861E-01	4.348E-01
CCDC90B	-0.009	9.444E-01	6.675E-01
ZNF687	-0.009	9.550E-01	4.002E-01
TMEM184C	-0.009	9.481E-01	4.575E-01
ZBED1	-0.009	9.586E-01	4.093E-01
EPX	-0.009	9.802E-01	5.985E-01
OR2A1	-0.009	9.919E-01	3.921E-01
NFKB2	-0.009	9.610E-01	7.273E-01
TACO1	-0.009	9.502E-01	4.781E-01
PARN	-0.009	9.310E-01	6.922E-01
NUDCD2	-0.009	9.326E-01	8.808E-01
SLFNL1	-0.009	9.779E-01	6.378E-01
SNRPC	-0.009	9.491E-01	6.789E-01
HDAC8	-0.009	9.325E-01	9.667E-01
ZCCHC14	-0.009	9.494E-01	6.472E-01
NGDN	-0.009	9.350E-01	7.437E-01
ARHGAP27	-0.009	9.669E-01	4.267E-01
UBE2D2	-0.009	9.164E-01	4.331E-01
FUZ	-0.009	9.675E-01	8.539E-01
ZNF3	-0.009	9.509E-01	6.189E-01
PPP1R12A	-0.009	9.507E-01	8.020E-01
ARHGDIA	-0.009	9.394E-01	6.768E-01
PROK2	-0.009	9.885E-01	6.053E-01
PIGB	-0.009	9.480E-01	7.686E-01
PROB1	-0.009	9.624E-01	6.025E-01
ZBTB5	-0.009	9.502E-01	5.299E-01
SREBF2	-0.009	9.568E-01	9.163E-01
WASHC2A	-0.009	9.321E-01	5.066E-01
MIR6089	-0.009	NA	3.485E-01
MIR4727	-0.009	NA	4.834E-01
MIR95	-0.009	NA	7.881E-01
MIR4474	-0.009	NA	4.668E-01
OR2T3	-0.009	NA	4.953E-01
AL592490.1	-0.009	NA	8.835E-01
DAZ1	-0.009	NA	9.897E-01
MIR652	-0.009	NA	9.897E-01
DMRTC1	-0.009	NA	9.897E-01
MIR921	-0.009	NA	9.897E-01
CFHR2	-0.009	NA	9.897E-01
ALG5	-0.009	9.448E-01	9.897E-01
CCDC22	-0.009	9.430E-01	9.897E-01
ATP9A	-0.009	9.639E-01	9.897E-01
OSTM1	-0.009	9.598E-01	9.897E-01
INPP5K	-0.009	9.467E-01	9.897E-01
ZNF83	-0.009	9.709E-01	9.897E-01
EBI3	-0.009	9.824E-01	9.897E-01

ZNF277	-0.009	9.460E-01	9.897E-01
ZFAND2B	-0.009	9.533E-01	9.897E-01
VPS72	-0.009	9.484E-01	9.897E-01
ZNF717	-0.009	9.663E-01	9.897E-01
NRN1L	-0.010	9.873E-01	9.897E-01
IREB2	-0.010	9.403E-01	9.897E-01
DGCR6L	-0.010	9.548E-01	9.897E-01
SH2B3	-0.010	9.692E-01	9.897E-01
AP3B1	-0.010	9.307E-01	9.897E-01
AC079447.1	-0.010	9.604E-01	9.897E-01
HOXA6	-0.010	9.792E-01	9.897E-01
HLA-DRB1	-0.010	9.828E-01	9.897E-01
REEP5	-0.010	9.394E-01	9.897E-01
ATE1	-0.010	9.488E-01	9.897E-01
H2AFZ	-0.010	9.513E-01	9.897E-01
DNAAF5	-0.010	9.403E-01	9.897E-01
GAGE2A	-0.010	9.960E-01	9.897E-01
FBRS	-0.010	9.380E-01	9.897E-01
ASB7	-0.010	9.158E-01	9.897E-01
OST4	-0.010	9.370E-01	9.897E-01
STAM	-0.010	9.443E-01	9.897E-01
NOSIP	-0.010	9.516E-01	5.643E-01
BEX1	-0.010	9.865E-01	4.561E-01
NUMA1	-0.010	9.378E-01	6.639E-01
HLA-DPB1	-0.010	9.807E-01	8.725E-01
NPRL2	-0.010	9.547E-01	8.126E-01
MCM8	-0.010	9.590E-01	5.101E-01
SPACA5B	-0.010	NA	8.192E-01
RCL1	-0.010	9.554E-01	5.947E-01
GAS2L3	-0.010	9.671E-01	5.781E-01
RBBP9	-0.010	9.458E-01	6.055E-01
CEP57	-0.010	9.302E-01	6.241E-01
PRKD1	-0.010	9.756E-01	4.407E-01
FOXJ3	-0.010	9.465E-01	7.474E-01
GBP2	-0.010	9.668E-01	4.977E-01
MICU2	-0.010	9.186E-01	5.340E-01
RPL7L1	-0.010	9.318E-01	4.153E-01
C1orf189	-0.010	9.792E-01	6.746E-01
CENPQ	-0.010	9.475E-01	5.797E-01
ERVW-1	-0.010	9.861E-01	5.626E-01
SDCBP	-0.010	9.468E-01	6.125E-01
LBR	-0.010	9.502E-01	8.305E-01
DDIT4L	-0.010	9.847E-01	5.179E-01
PPP3CC	-0.010	9.497E-01	6.786E-01
SMIM27	-0.010	9.580E-01	5.276E-01
ACYP2	-0.010	9.502E-01	5.415E-01
TMEM216	-0.010	9.385E-01	8.880E-01
TNS3	-0.010	9.671E-01	8.291E-01
MRPS7	-0.010	9.257E-01	8.032E-01
FAM175B	-0.010	9.096E-01	4.078E-01

GFRA3	-0.011	9.847E-01	6.712E-01
TRIM56	-0.011	9.381E-01	6.943E-01
THNSL2	-0.011	9.796E-01	9.762E-01
ARHGEF40	-0.011	9.598E-01	4.082E-01
PET117	-0.011	9.460E-01	6.111E-01
LMBRD1	-0.011	9.455E-01	6.156E-01
TCFL5	-0.011	9.464E-01	5.652E-01
DR1	-0.011	9.188E-01	3.786E-01
MIR6070	-0.011	NA	4.390E-01
FEM1B	-0.011	9.376E-01	5.167E-01
SLC52A2	-0.011	9.452E-01	5.155E-01
DYNLL1	-0.011	9.179E-01	7.149E-01
ZNF235	-0.011	9.480E-01	5.529E-01
CCDC82	-0.011	9.357E-01	5.807E-01
MTMR2	-0.011	9.280E-01	5.731E-01
HIST1H2BL	-0.011	9.785E-01	5.590E-01
OR1C1	-0.011	NA	7.050E-01
FAM53A	-0.011	9.667E-01	5.201E-01
TBC1D3H	-0.011	NA	6.797E-01
MIR4460	-0.011	NA	5.439E-01
MIR4484	-0.011	NA	4.211E-01
FAM120A	-0.011	9.180E-01	5.519E-01
HOXA10	-0.011	9.695E-01	4.553E-01
DBNDD2	-0.011	9.697E-01	6.595E-01
AGRN	-0.011	9.579E-01	5.120E-01
RNF135	-0.011	9.334E-01	7.663E-01
DLEU1	-0.011	9.583E-01	4.924E-01
AMPD2	-0.011	9.379E-01	6.890E-01
GPR22	-0.011	9.769E-01	9.059E-01
PEX7	-0.011	9.364E-01	4.782E-01
MFSD4A	-0.011	9.772E-01	5.959E-01
FAM210A	-0.011	9.456E-01	6.415E-01
IFNAR2	-0.011	9.483E-01	7.418E-01
HIF1AN	-0.011	9.167E-01	5.956E-01
CEBPZ	-0.011	9.152E-01	5.120E-01
PCDHGA9	-0.011	9.749E-01	9.569E-01
KRTAP10-2	-0.011	9.930E-01	6.017E-01
TMEM199	-0.011	9.200E-01	5.618E-01
KCNA7	-0.011	9.792E-01	5.888E-01
CCDC89	-0.011	9.759E-01	8.585E-01
SLC22A14	-0.011	9.758E-01	9.249E-01
SIKE1	-0.011	9.171E-01	4.714E-01
BNIP1	-0.011	9.257E-01	6.013E-01
TXNDC12	-0.011	9.027E-01	5.335E-01
SMNDC1	-0.011	9.033E-01	9.874E-01
CDK20	-0.011	9.639E-01	9.851E-01
OFCC1	-0.012	9.856E-01	5.597E-01
EXD2	-0.012	9.146E-01	5.567E-01
BOD1L2	-0.012	NA	6.482E-01
PM20D2	-0.012	9.601E-01	4.974E-01

PIN4	-0.012	9.389E-01	5.679E-01
AC020915.1	-0.012	9.263E-01	7.941E-01
GET4	-0.012	9.328E-01	5.933E-01
TRIB1	-0.012	9.616E-01	7.426E-01
EFCAB11	-0.012	9.349E-01	5.197E-01
ATRX	-0.012	9.324E-01	7.531E-01
MDS2	-0.012	9.759E-01	5.461E-01
APAF1	-0.012	9.500E-01	5.158E-01
OSTF1	-0.012	9.324E-01	4.618E-01
DRD4	-0.012	9.736E-01	6.262E-01
EDDM3A	-0.012	9.915E-01	7.969E-01
ARHGAP39	-0.012	9.454E-01	6.431E-01
COQ10B	-0.012	9.167E-01	4.327E-01
ZNF324	-0.012	9.277E-01	5.543E-01
RETREG2	-0.012	9.110E-01	4.896E-01
ARHGEF18	-0.012	9.463E-01	7.354E-01
DNAJC14	-0.012	9.340E-01	6.879E-01
FCER1A	-0.012	9.809E-01	4.664E-01
GEMIN6	-0.012	9.310E-01	6.253E-01
RING1	-0.012	9.182E-01	7.599E-01
CLIC1	-0.012	9.325E-01	4.033E-01
MIR634	-0.012	NA	5.335E-01
AC074143.1	-0.012	NA	6.890E-01
MIR4717	-0.012	NA	6.080E-01
EVPLL	-0.012	9.841E-01	8.967E-01
TMEM167A	-0.012	9.026E-01	5.631E-01
HNRNPK	-0.012	8.735E-01	5.019E-01
TMEM65	-0.012	9.569E-01	4.517E-01
STK16	-0.012	9.153E-01	8.519E-01
PRDX3	-0.012	9.186E-01	4.563E-01
NUCKS1	-0.012	9.282E-01	4.157E-01
ALS2CR12	-0.012	9.634E-01	7.319E-01
SF3B4	-0.012	9.202E-01	4.886E-01
IVL	-0.012	9.832E-01	9.629E-01
EBPL	-0.012	9.579E-01	6.672E-01
PHTF2	-0.012	9.173E-01	5.654E-01
TMEM218	-0.012	9.238E-01	6.622E-01
MAOA	-0.012	9.756E-01	4.833E-01
PPARG	-0.012	9.767E-01	6.557E-01
MN1	-0.012	9.783E-01	7.683E-01
SLC25A20	-0.012	9.506E-01	6.442E-01
C11orf57	-0.012	8.966E-01	8.771E-01
'RIM39-RPP2	-0.012	9.769E-01	5.685E-01
USP36	-0.012	9.004E-01	7.498E-01
SNIP1	-0.012	8.934E-01	6.156E-01
PSMF1	-0.012	9.028E-01	6.869E-01
UHRF1BP1	-0.012	9.331E-01	5.238E-01
INSL5	-0.012	9.890E-01	6.602E-01
FANCI	-0.012	9.435E-01	8.055E-01
TSNAXIP1	-0.012	9.650E-01	8.658E-01

GPATCH2	-0.012	9.146E-01	5.957E-01
FAM25G	-0.013	9.924E-01	7.317E-01
ZNF181	-0.013	9.372E-01	6.764E-01
DNAJB12	-0.013	9.058E-01	4.232E-01
LACC1	-0.013	9.482E-01	6.031E-01
ABCA13	-0.013	9.837E-01	5.890E-01
ZNF680	-0.013	9.481E-01	4.538E-01
DGUOK	-0.013	9.137E-01	5.682E-01
GALNT14	-0.013	9.769E-01	5.912E-01
ZBTB12	-0.013	9.528E-01	5.309E-01
SNX32	-0.013	9.632E-01	7.489E-01
FAHD1	-0.013	9.274E-01	5.582E-01
TMPRSS11E	-0.013	9.857E-01	6.738E-01
PEX2	-0.013	9.113E-01	5.009E-01
AC008770.3	-0.013	9.688E-01	6.313E-01
HID1	-0.013	9.749E-01	6.067E-01
EXOSC2	-0.013	9.252E-01	9.730E-01
APTX	-0.013	9.276E-01	9.731E-01
ELF1	-0.013	9.445E-01	8.882E-01
ADI1	-0.013	9.246E-01	6.590E-01
ELOC	-0.013	9.117E-01	5.290E-01
GALNT2	-0.013	9.435E-01	5.390E-01
OR4D1	-0.013	NA	5.319E-01
MIR3926-1	-0.013	NA	8.553E-01
NT5C3B	-0.013	9.414E-01	5.961E-01
SAMD15	-0.013	9.578E-01	7.587E-01
PCCB	-0.013	9.327E-01	4.265E-01
APOO	-0.013	9.282E-01	4.002E-01
POLR3F	-0.013	9.153E-01	4.297E-01
BMS1	-0.013	9.019E-01	8.578E-01
CSNK1G1	-0.013	9.105E-01	4.578E-01
RIOK2	-0.013	9.002E-01	8.898E-01
SCP2	-0.013	9.171E-01	5.910E-01
KAT8	-0.013	9.105E-01	6.398E-01
TMEM123	-0.013	9.463E-01	6.352E-01
MIR1972-1	-0.013	9.829E-01	7.408E-01
SPAG5	-0.013	9.518E-01	4.861E-01
PTPN9	-0.013	8.945E-01	8.179E-01
H3F3C	-0.013	9.521E-01	9.153E-01
EGLN2	-0.013	9.375E-01	8.002E-01
CREM	-0.013	9.437E-01	9.187E-01
AACS	-0.013	9.319E-01	6.796E-01
TATDN1	-0.013	9.218E-01	3.113E-01
ZNF638	-0.013	8.781E-01	9.665E-01
STPG2	-0.013	9.749E-01	9.685E-01
COL4A3BP	-0.013	9.208E-01	5.945E-01
LYSMD2	-0.013	9.423E-01	4.959E-01
C10orf76	-0.013	8.838E-01	4.619E-01
KMT2A	-0.013	9.112E-01	8.108E-01
PTPMT1	-0.013	9.248E-01	9.530E-01

HNRNPU	-0.013	8.557E-01	6.219E-01
PTGES3	-0.013	8.935E-01	5.806E-01
AC010319.2	-0.014	NA	4.511E-01
MIR1275	-0.014	NA	4.834E-01
MIR449A	-0.014	NA	5.319E-01
MIR4749	-0.014	NA	7.168E-01
MIR502	-0.014	NA	5.554E-01
MIR552	-0.014	NA	8.335E-01
MIR5682	-0.014	NA	4.270E-01
MIR617	-0.014	NA	8.602E-01
MIRLET7F2	-0.014	NA	5.667E-01
DEFB106A	-0.014	NA	5.719E-01
MIR139	-0.014	NA	5.778E-01
MIR548W	-0.014	NA	5.597E-01
MIR6862-2	-0.014	NA	7.477E-01
MIR7702	-0.014	NA	9.909E-01
OR4F29	-0.014	NA	9.900E-01
AL132780.3	-0.014	NA	9.887E-01
BPY2	-0.014	NA	9.887E-01
DEFB112	-0.014	NA	9.876E-01
LCE3B	-0.014	NA	9.865E-01
MIR1-1	-0.014	NA	9.865E-01
MIR133B	-0.014	NA	9.854E-01
MIR1471	-0.014	NA	9.852E-01
MIR299	-0.014	NA	9.109E-01
MIR3169	-0.014	NA	8.661E-01
MIR323B	-0.014	NA	5.044E-01
MIR3687-2	-0.014	NA	9.406E-01
MIR379	-0.014	NA	5.915E-01
MIR3910-1	-0.014	NA	6.153E-01
MIR3935	-0.014	NA	8.883E-01
MIR4288	-0.014	NA	4.989E-01
MIR4290	-0.014	NA	6.442E-01
MIR4318	-0.014	NA	5.607E-01
MIR4471	-0.014	NA	5.172E-01
MIR4508	-0.014	NA	8.157E-01
MIR6130	-0.014	NA	5.018E-01
MIR6795	-0.014	NA	9.455E-01
OR4C46	-0.014	NA	6.402E-01
TP53TG3F	-0.014	NA	5.942E-01
AC005670.2	-0.014	NA	5.439E-01
MIR1185-2	-0.014	NA	7.870E-01
MIR1298	-0.014	NA	6.447E-01
MIR3141	-0.014	NA	5.759E-01
MIR3648-2	-0.014	NA	9.455E-01
MIR4465	-0.014	NA	5.371E-01
MIR4487	-0.014	NA	4.535E-01
MIR4527	-0.014	NA	5.129E-01
MIR489	-0.014	NA	9.037E-01
MIR493	-0.014	NA	9.258E-01

MIR514A1	-0.014	NA	7.193E-01
MIR5197	-0.014	NA	5.561E-01
MIR548AB	-0.014	NA	5.651E-01
MIR548AI	-0.014	NA	7.951E-01
MIR6083	-0.014	NA	5.560E-01
MIR653	-0.014	NA	6.702E-01
MIR6770-1	-0.014	NA	8.756E-01
MIR8060	-0.014	NA	8.050E-01
MIR890	-0.014	NA	6.528E-01
OR51A4	-0.014	NA	6.043E-01
POTEB2	-0.014	NA	9.696E-01
RNASE12	-0.014	NA	9.717E-01
KCNC1	-0.014	9.780E-01	9.744E-01
SLC22A18	-0.014	9.530E-01	5.813E-01
DEPDC1	-0.014	9.592E-01	6.452E-01
PABPC3	-0.014	9.667E-01	5.529E-01
TRAPPC2	-0.014	9.182E-01	7.720E-01
IL3RA	-0.014	9.586E-01	7.446E-01
SHD	-0.014	9.828E-01	5.816E-01
CWF19L2	-0.014	9.296E-01	7.172E-01
SNX27	-0.014	9.151E-01	6.101E-01
XPO4	-0.014	9.113E-01	7.409E-01
TMPRSS7	-0.014	9.719E-01	6.318E-01
PLA2G7	-0.014	9.709E-01	6.553E-01
DSPP	-0.014	9.829E-01	4.865E-01
KLF3	-0.014	9.296E-01	5.984E-01
HNRNPLL	-0.014	8.532E-01	5.157E-01
MKL1	-0.014	9.036E-01	9.485E-01
TMED10	-0.014	8.997E-01	7.373E-01
IFIT2	-0.014	9.733E-01	4.892E-01
ATMIN	-0.014	8.936E-01	9.768E-01
FZD7	-0.014	9.587E-01	6.120E-01
PIP5K1A	-0.014	9.272E-01	6.618E-01
ZNF12	-0.014	9.096E-01	6.661E-01
SPATA25	-0.014	9.502E-01	5.399E-01
ZNF623	-0.014	9.135E-01	9.084E-01
PPP2R3B	-0.014	9.468E-01	5.554E-01
RIPK4	-0.014	9.597E-01	4.838E-01
RMI2	-0.014	9.541E-01	5.799E-01
USP42	-0.014	9.270E-01	6.010E-01
ERCC2	-0.014	9.190E-01	9.075E-01
USP20	-0.014	9.041E-01	6.573E-01
POLE3	-0.014	9.220E-01	6.565E-01
GLIPR2	-0.014	9.676E-01	7.691E-01
AC093525.1	-0.014	9.858E-01	7.273E-01
TMEM150C	-0.014	9.630E-01	6.637E-01
ABO	-0.014	9.768E-01	7.911E-01
MIR146B	-0.014	NA	4.687E-01
SCG5	-0.014	9.714E-01	6.576E-01
DTX3	-0.014	9.518E-01	8.340E-01

ZNF671	-0.014	9.632E-01	7.682E-01
CPA4	-0.014	9.835E-01	6.283E-01
C17orf62	-0.014	9.133E-01	5.947E-01
VAMP1	-0.014	9.441E-01	6.833E-01
TOPORS	-0.014	9.128E-01	7.994E-01
ARL5A	-0.014	9.151E-01	4.850E-01
SLC38A7	-0.014	9.108E-01	8.851E-01
FAM46C	-0.014	9.639E-01	5.831E-01
PSEN1	-0.014	8.759E-01	5.109E-01
GALM	-0.014	9.506E-01	5.774E-01
ARMC10	-0.014	8.857E-01	6.034E-01
DIAPH1	-0.014	9.210E-01	7.021E-01
PCMTD1	-0.014	9.335E-01	5.838E-01
ZNF160	-0.015	9.356E-01	6.313E-01
NTHL1	-0.015	9.356E-01	6.900E-01
DNASE2B	-0.015	9.792E-01	8.396E-01
MOB3A	-0.015	9.277E-01	9.839E-01
MIR4306	-0.015	NA	6.395E-01
AC134025.1	-0.015	NA	6.078E-01
KRTAP9-8	-0.015	NA	6.169E-01
USP27X	-0.015	9.128E-01	9.122E-01
FKBP4	-0.015	9.268E-01	5.658E-01
ATR	-0.015	8.997E-01	5.845E-01
TUSC2	-0.015	9.203E-01	8.298E-01
FIP1L1	-0.015	8.776E-01	6.401E-01
DNAJC25	-0.015	9.026E-01	8.819E-01
MIR4689	-0.015	9.861E-01	5.565E-01
MIR181B2	-0.015	NA	5.051E-01
DSTYK	-0.015	9.152E-01	6.594E-01
RPS6KA3	-0.015	9.238E-01	5.547E-01
FBXL20	-0.015	9.132E-01	6.044E-01
MIR374B	-0.015	9.822E-01	6.269E-01
SACM1L	-0.015	8.996E-01	5.315E-01
RANBP2	-0.015	8.880E-01	7.835E-01
PRPS1	-0.015	9.326E-01	6.596E-01
SELENOW	-0.015	9.292E-01	6.066E-01
TMEM219	-0.015	9.262E-01	5.317E-01
MAFG	-0.015	9.326E-01	7.327E-01
RBM8A	-0.015	8.931E-01	4.406E-01
LCMT1	-0.015	9.107E-01	7.753E-01
SLC6A13	-0.015	9.780E-01	6.326E-01
MFAP3	-0.015	9.450E-01	5.210E-01
NCBP1	-0.015	9.026E-01	7.286E-01
FBXL14	-0.015	9.326E-01	5.649E-01
WDR70	-0.015	9.041E-01	9.649E-01
ATP5SL	-0.015	8.815E-01	8.046E-01
MINPP1	-0.015	9.095E-01	5.574E-01
MTF1	-0.015	9.209E-01	5.700E-01
SLC35D2	-0.015	9.133E-01	8.205E-01
TTC39B	-0.015	9.447E-01	7.325E-01

PGAP2	-0.015	9.094E-01	4.850E-01
ZNF597	-0.015	9.513E-01	8.607E-01
AC008481.3	-0.015	NA	9.916E-01
ZNF143	-0.015	8.559E-01	9.916E-01
C2orf69	-0.015	9.013E-01	9.916E-01
DIEXF	-0.015	9.132E-01	9.908E-01
PCDH17	-0.015	9.553E-01	9.906E-01
ZFAND5	-0.015	9.100E-01	9.898E-01
LMNB1	-0.015	9.420E-01	7.844E-01
AC092835.1	-0.015	9.540E-01	7.204E-01
AUH	-0.015	9.105E-01	5.627E-01
NICN1	-0.015	9.237E-01	5.554E-01
CEACAM1	-0.015	9.692E-01	8.183E-01
ZNF268	-0.015	9.133E-01	6.223E-01
AK4	-0.015	9.718E-01	7.189E-01
GNB4	-0.015	9.621E-01	9.151E-01
OLFML2A	-0.015	9.630E-01	5.264E-01
EIF3CL	-0.015	9.494E-01	8.709E-01
FOXN3	-0.015	9.238E-01	7.497E-01
TRIM11	-0.015	9.137E-01	7.158E-01
RAB27B	-0.015	9.650E-01	6.885E-01
TEX19	-0.015	9.778E-01	6.792E-01
UGT1A6	-0.015	9.787E-01	5.319E-01
MIR26A1	-0.016	NA	6.615E-01
MIR6754	-0.016	NA	5.489E-01
CCSER1	-0.016	9.714E-01	5.531E-01
KANSL1	-0.016	9.037E-01	5.610E-01
SCAI	-0.016	9.081E-01	8.282E-01
LARP6	-0.016	9.475E-01	9.367E-01
ATP13A3	-0.016	9.235E-01	5.460E-01
FRAT1	-0.016	9.296E-01	6.141E-01
RBM39	-0.016	8.878E-01	6.747E-01
XIAP	-0.016	9.131E-01	6.337E-01
FAM19A3	-0.016	9.749E-01	9.495E-01
ATF4	-0.016	9.013E-01	6.628E-01
UBTF	-0.016	8.283E-01	6.381E-01
VEZT	-0.016	8.866E-01	5.425E-01
DNAL1	-0.016	8.858E-01	4.850E-01
RET	-0.016	9.692E-01	9.713E-01
DAAM1	-0.016	9.344E-01	6.427E-01
COA1	-0.016	8.760E-01	5.812E-01
NQO2	-0.016	9.282E-01	6.680E-01
ATF1	-0.016	8.953E-01	7.068E-01
MIR148A	-0.016	9.749E-01	7.630E-01
RAB41	-0.016	9.376E-01	6.382E-01
SETDB1	-0.016	9.079E-01	5.251E-01
EHMT1	-0.016	8.688E-01	9.267E-01
NHLRC3	-0.016	9.113E-01	6.313E-01
MIR499A	-0.016	9.718E-01	7.073E-01
CTNNA1	-0.016	8.823E-01	6.493E-01

FAM71E2	-0.016	9.890E-01	5.711E-01
FBLIM1	-0.016	9.324E-01	6.005E-01
KCNAB2	-0.016	9.516E-01	6.230E-01
MIR4771-1	-0.016	NA	5.904E-01
KRTAP21-3	-0.016	NA	6.875E-01
MIR3919	-0.016	NA	8.091E-01
MIR4311	-0.016	NA	8.349E-01
MIR500A	-0.016	NA	8.031E-01
MIR5583-1	-0.016	NA	7.714E-01
MIR6790	-0.016	NA	6.113E-01
MIR941-4	-0.016	NA	6.455E-01
OR8K1	-0.016	NA	6.182E-01
IFNA4	-0.016	NA	8.525E-01
MIR3154	-0.016	NA	6.329E-01
MIR375	-0.016	NA	7.188E-01
MIR4457	-0.016	NA	9.021E-01
MIR4488	-0.016	NA	8.133E-01
MIR4718	-0.016	NA	7.106E-01
MIR4735	-0.016	NA	6.592E-01
MIR545	-0.016	NA	5.809E-01
MIR5571	-0.016	NA	9.824E-01
MIR577	-0.016	NA	6.696E-01
MIR802	-0.016	NA	8.630E-01
OR2T1	-0.016	NA	8.912E-01
OR5T3	-0.016	NA	7.124E-01
SPATA31D4	-0.016	NA	7.004E-01
MIR8054	-0.016	NA	6.087E-01
FANCL	-0.016	9.128E-01	7.609E-01
TBC1D22B	-0.016	8.735E-01	9.843E-01
ASXL2	-0.016	9.033E-01	9.826E-01
LRIT1	-0.016	NA	9.807E-01
GOT1	-0.016	9.105E-01	9.799E-01
MIR101-1	-0.016	NA	8.501E-01
RIF1	-0.016	8.887E-01	6.426E-01
USP44	-0.016	9.693E-01	7.378E-01
RABL3	-0.017	8.858E-01	5.532E-01
AVL9	-0.017	8.908E-01	6.800E-01
DENND4A	-0.017	8.903E-01	5.935E-01
HMGCR	-0.017	9.238E-01	6.618E-01
CHCHD4	-0.017	9.152E-01	6.442E-01
MRPL30	-0.017	8.932E-01	6.737E-01
LMTK2	-0.017	9.206E-01	6.809E-01
DBF4B	-0.017	9.167E-01	6.709E-01
MRPL10	-0.017	8.559E-01	6.267E-01
PLXNB2	-0.017	9.128E-01	5.893E-01
FNDC7	-0.017	9.748E-01	6.444E-01
FBXW8	-0.017	8.934E-01	6.909E-01
RAD51AP2	-0.017	9.785E-01	6.226E-01
COX14	-0.017	9.198E-01	6.615E-01
VPS16	-0.017	8.760E-01	7.891E-01

ERCC4	-0.017	9.131E-01	7.548E-01
TRAF3IP1	-0.017	8.830E-01	6.880E-01
RNPS1	-0.017	8.481E-01	8.819E-01
TOMM40L	-0.017	9.290E-01	6.290E-01
ACAT2	-0.017	9.257E-01	8.498E-01
TMEM184A	-0.017	9.624E-01	9.201E-01
LRP3	-0.017	9.450E-01	7.672E-01
ATM	-0.017	9.096E-01	6.161E-01
SHISA5	-0.017	9.210E-01	7.048E-01
MRPL46	-0.017	8.873E-01	6.384E-01
GSTCD	-0.017	9.188E-01	5.651E-01
SCN9A	-0.017	9.730E-01	7.089E-01
NME4	-0.017	9.264E-01	6.140E-01
CAD	-0.017	9.104E-01	5.525E-01
HIGD1A	-0.017	9.128E-01	6.100E-01
COPS8	-0.017	8.723E-01	7.653E-01
EBAG9	-0.017	8.842E-01	8.794E-01
POP7	-0.017	9.047E-01	6.554E-01
EVC	-0.017	9.598E-01	7.129E-01
C18orf32	-0.017	8.972E-01	7.445E-01
TSPO2	-0.017	9.725E-01	6.866E-01
NDUFB11	-0.017	9.180E-01	8.108E-01
LITAF	-0.017	9.296E-01	6.470E-01
PCMT1	-0.017	8.880E-01	9.923E-01
BIRC8	-0.017	NA	9.923E-01
PLD2	-0.017	9.028E-01	9.923E-01
RPL21	-0.017	9.153E-01	9.923E-01
DNAJB8	-0.017	NA	9.923E-01
WHAMM	-0.017	8.852E-01	9.923E-01
MIR4662B	-0.017	NA	9.923E-01
MIR7853	-0.017	NA	9.923E-01
FAM71B	-0.017	NA	9.923E-01
PRAMEF10	-0.017	NA	9.923E-01
SKA1	-0.017	9.389E-01	9.923E-01
MIR3170	-0.017	NA	9.923E-01
SORBS3	-0.017	9.400E-01	9.923E-01
VPS18	-0.018	8.934E-01	9.923E-01
FRG2B	-0.018	NA	9.923E-01
RNF123	-0.018	8.969E-01	9.923E-01
SAP18	-0.018	8.695E-01	9.923E-01
MIR4673	-0.018	NA	9.923E-01
APC	-0.018	9.026E-01	9.923E-01
CASP7	-0.018	9.158E-01	9.923E-01
KLHL5	-0.018	9.458E-01	9.923E-01
C1orf56	-0.018	9.185E-01	9.923E-01
CCDC127	-0.018	9.095E-01	9.923E-01
FGF11	-0.018	9.655E-01	9.923E-01
MGAT1	-0.018	9.326E-01	8.630E-01
ANKRD6	-0.018	9.345E-01	6.562E-01
PPP1R11	-0.018	8.462E-01	8.661E-01

CBS	-0.018	9.733E-01	7.141E-01
HAUS1	-0.018	9.159E-01	8.981E-01
REXO4	-0.018	8.868E-01	6.053E-01
PKP2	-0.018	9.555E-01	8.510E-01
VRK2	-0.018	9.105E-01	4.973E-01
GPRIN1	-0.018	9.579E-01	7.631E-01
VDAC2	-0.018	8.972E-01	8.061E-01
AL139011.2	-0.018	9.577E-01	9.275E-01
CDCA5	-0.018	9.412E-01	7.920E-01
44076.000	-0.018	8.694E-01	6.233E-01
LCOR	-0.018	9.283E-01	5.615E-01
ANKS6	-0.018	9.361E-01	6.384E-01
AGBL3	-0.018	9.171E-01	8.420E-01
USP54	-0.018	9.238E-01	8.741E-01
L3MBTL2	-0.018	8.530E-01	8.145E-01
RCE1	-0.018	8.936E-01	5.907E-01
ANKRD13B	-0.018	9.475E-01	7.200E-01
SLC27A2	-0.018	9.598E-01	7.127E-01
CCZ1	-0.018	8.997E-01	6.420E-01
MIR1261	-0.018	NA	6.260E-01
CCDC92	-0.018	9.095E-01	7.486E-01
EDARADD	-0.018	9.587E-01	7.663E-01
CEBPA	-0.018	9.586E-01	6.106E-01
THAP10	-0.018	9.230E-01	8.669E-01
YWHAB	-0.018	8.743E-01	9.155E-01
ACAD8	-0.018	8.854E-01	8.764E-01
PARK7	-0.018	8.669E-01	6.839E-01
C8orf74	-0.018	9.785E-01	9.745E-01
NDRG3	-0.018	8.693E-01	9.777E-01
ATP6V1C2	-0.018	9.518E-01	6.578E-01
WDR81	-0.018	8.876E-01	7.027E-01
ZNF718	-0.018	9.394E-01	6.106E-01
CRNKL1	-0.018	8.628E-01	6.824E-01
EIF1	-0.019	8.645E-01	6.494E-01
NTMT1	-0.019	9.079E-01	5.555E-01
TIPIN	-0.019	9.028E-01	8.214E-01
FBXO8	-0.019	9.003E-01	8.105E-01
VN1R2	-0.019	9.861E-01	5.382E-01
SDCCAG3	-0.019	8.896E-01	9.001E-01
SNUPN	-0.019	8.587E-01	6.833E-01
DDX23	-0.019	8.092E-01	8.015E-01
RBBP6	-0.019	8.586E-01	7.888E-01
NFATC2IP	-0.019	8.903E-01	7.566E-01
HEATR6	-0.019	8.936E-01	6.013E-01
DTD1	-0.019	9.041E-01	9.093E-01
GALC	-0.019	9.265E-01	8.776E-01
C16orf59	-0.019	9.292E-01	6.625E-01
NKPD1	-0.019	9.631E-01	7.106E-01
GPR149	-0.019	9.877E-01	8.396E-01
TSR1	-0.019	8.828E-01	6.973E-01

WDSUB1	-0.019	8.693E-01	7.211E-01
RBAK	-0.019	8.759E-01	9.002E-01
IL10RB	-0.019	8.936E-01	5.551E-01
COPS4	-0.019	8.599E-01	7.200E-01
LCT	-0.019	9.680E-01	6.692E-01
TRMT1L	-0.019	8.775E-01	8.744E-01
HAT1	-0.019	8.852E-01	8.149E-01
MIR5590	-0.019	NA	7.974E-01
MIR181A1	-0.019	NA	5.924E-01
OR7A10	-0.019	NA	7.326E-01
NEUROD6	-0.019	NA	8.805E-01
OR6C1	-0.019	NA	7.116E-01
OR51V1	-0.019	NA	5.440E-01
KRTAP12-4	-0.019	NA	8.771E-01
MIR1254-2	-0.019	NA	8.509E-01
OR5J2	-0.019	NA	8.376E-01
CNGA1	-0.019	9.685E-01	8.543E-01
ZNF189	-0.019	8.775E-01	8.037E-01
SVBP	-0.019	9.080E-01	9.598E-01
ZNF175	-0.019	9.513E-01	6.872E-01
LIN52	-0.019	8.728E-01	9.693E-01
MIR3139	-0.019	NA	9.705E-01
CLEC14A	-0.019	9.378E-01	7.158E-01
EIF2B1	-0.019	8.135E-01	5.814E-01
MIR760	-0.019	NA	9.891E-01
MFSD6	-0.019	9.105E-01	9.888E-01
ATP5G1	-0.019	9.002E-01	9.858E-01
SLC27A1	-0.019	9.135E-01	9.850E-01
KTI12	-0.019	8.635E-01	9.849E-01
SPATA45	-0.019	9.600E-01	9.842E-01
AKR1E2	-0.019	9.610E-01	6.724E-01
DLAT	-0.019	8.769E-01	6.420E-01
CRADD	-0.019	8.878E-01	5.776E-01
UQCRHL	-0.019	9.095E-01	6.983E-01
ZNF250	-0.019	8.786E-01	5.831E-01
ZNF852	-0.020	9.033E-01	7.769E-01
TIMM50	-0.020	8.858E-01	6.539E-01
UTP14A	-0.020	8.569E-01	6.315E-01
LYST	-0.020	9.162E-01	7.312E-01
ULBP2	-0.020	9.533E-01	7.471E-01
ZDHHC12	-0.020	9.101E-01	6.292E-01
CD300A	-0.020	9.529E-01	8.313E-01
P2RY4	-0.020	9.673E-01	7.862E-01
BCL7A	-0.020	9.038E-01	7.319E-01
MTG2	-0.020	8.553E-01	7.003E-01
BMP15	-0.020	NA	7.546E-01
CADM2	-0.020	9.769E-01	6.818E-01
GLYR1	-0.020	8.504E-01	7.287E-01
NUBP2	-0.020	8.635E-01	8.382E-01
CARD9	-0.020	9.539E-01	9.090E-01

FOXN2	-0.020	9.135E-01	6.223E-01
CYB5R4	-0.020	8.797E-01	6.514E-01
TP53TG3	-0.020	NA	5.450E-01
SHANK3	-0.020	9.153E-01	9.446E-01
WDR11	-0.020	8.512E-01	7.228E-01
RAB3GAP2	-0.020	8.707E-01	5.828E-01
GPS1	-0.020	8.586E-01	6.537E-01
OMP	-0.020	9.671E-01	6.704E-01
RAPGEF2	-0.020	8.980E-01	6.111E-01
ZNF282	-0.020	8.406E-01	6.643E-01
UBE2B	-0.020	8.325E-01	8.133E-01
EEF1AKMT2	-0.020	8.690E-01	8.317E-01
DTX1	-0.020	9.586E-01	6.803E-01
RAB27A	-0.020	9.140E-01	8.995E-01
ZNF544	-0.020	8.824E-01	6.997E-01
ANAPC2	-0.020	8.561E-01	7.548E-01
MYO1D	-0.020	9.105E-01	5.602E-01
EMC4	-0.020	8.217E-01	9.545E-01
TCEANC	-0.020	8.765E-01	7.108E-01
WASL	-0.020	8.759E-01	6.429E-01
PPP2R5C	-0.020	8.306E-01	8.094E-01
HNRNPAB	-0.020	8.475E-01	6.576E-01
RHOBTB2	-0.020	9.508E-01	8.317E-01
ZCCHC11	-0.020	8.873E-01	8.709E-01
SERAC1	-0.020	8.711E-01	7.144E-01
CHD8	-0.020	8.348E-01	8.895E-01
NSD2	-0.021	9.026E-01	5.207E-01
ITGB3BP	-0.021	8.791E-01	7.759E-01
GID4	-0.021	8.693E-01	7.836E-01
CXorf40A	-0.021	8.694E-01	9.254E-01
IRF6	-0.021	9.452E-01	7.676E-01
TRAF6	-0.021	8.743E-01	8.113E-01
RUFY2	-0.021	8.525E-01	7.021E-01
EPS15L1	-0.021	8.189E-01	6.990E-01
PLCE1	-0.021	9.501E-01	6.420E-01
ZNF684	-0.021	9.003E-01	7.327E-01
DAGLB	-0.021	8.513E-01	6.517E-01
HADH	-0.021	9.105E-01	8.570E-01
SLC30A1	-0.021	9.081E-01	8.224E-01
C4orf46	-0.021	8.911E-01	7.612E-01
SLC12A4	-0.021	9.092E-01	9.931E-01
POLM	-0.021	8.896E-01	9.931E-01
C19orf35	-0.021	9.513E-01	9.931E-01
DHRS4	-0.021	8.933E-01	9.931E-01
UBA6	-0.021	9.076E-01	9.931E-01
YOD1	-0.021	9.093E-01	9.931E-01
SHISA8	-0.021	9.714E-01	9.931E-01
TPBG	-0.021	9.292E-01	9.931E-01
ELP3	-0.021	8.706E-01	9.931E-01
MOSPD2	-0.021	8.945E-01	9.931E-01

IRGQ	-0.021	8.712E-01	9.931E-01
PRR15	-0.021	9.619E-01	9.931E-01
FAM178B	-0.021	9.709E-01	9.931E-01
IP6K1	-0.021	8.487E-01	9.931E-01
KDM6A	-0.021	9.170E-01	9.931E-01
RHOT1	-0.021	8.799E-01	9.931E-01
NCK1	-0.021	9.128E-01	9.931E-01
SOCS2	-0.021	9.506E-01	9.931E-01
DOCK4	-0.021	9.345E-01	9.931E-01
HIBADH	-0.021	8.775E-01	9.931E-01
BCOR	-0.021	9.070E-01	9.931E-01
ZNF470	-0.021	9.406E-01	9.931E-01
SCN4B	-0.021	9.494E-01	9.931E-01
CNBP	-0.021	7.970E-01	9.931E-01
TMEM70	-0.021	8.541E-01	9.931E-01
ZFP2	-0.021	9.466E-01	9.931E-01
SLC17A2	-0.021	NA	9.931E-01
TUBGCP2	-0.021	8.346E-01	9.931E-01
TRAF7	-0.021	8.496E-01	9.931E-01
H1FOO	-0.021	9.846E-01	9.931E-01
SDHB	-0.021	8.138E-01	9.931E-01
FAM177A1	-0.022	8.586E-01	9.931E-01
SLC35F3	-0.022	9.749E-01	9.931E-01
NDC1	-0.022	8.758E-01	9.931E-01
LUC7L2	-0.022	8.197E-01	9.931E-01
MIR4755	-0.022	9.783E-01	9.931E-01
ZNF668	-0.022	8.377E-01	9.931E-01
ZMIZ2	-0.022	8.693E-01	9.931E-01
ENPP3	-0.022	9.621E-01	9.931E-01
CENPN	-0.022	9.133E-01	9.931E-01
CIDEC	-0.022	9.784E-01	9.931E-01
MIR1180	-0.022	9.770E-01	9.931E-01
KPTN	-0.022	8.948E-01	9.931E-01
DKC1	-0.022	8.692E-01	9.931E-01
PHYHIP	-0.022	9.631E-01	5.796E-01
RABEP2	-0.022	9.035E-01	6.764E-01
PTPRD	-0.022	9.681E-01	6.562E-01
MIR6777	-0.022	NA	9.234E-01
MIR562	-0.022	NA	7.988E-01
MIR4265	-0.022	NA	6.004E-01
MIR633	-0.022	NA	7.516E-01
MIR4736	-0.022	NA	9.803E-01
RIC8A	-0.022	8.384E-01	9.385E-01
MIR4435-1	-0.022	NA	6.444E-01
NPVF	-0.022	NA	7.108E-01
LCE4A	-0.022	NA	6.988E-01
SETX	-0.022	8.542E-01	7.760E-01
TRAK1	-0.022	9.334E-01	7.125E-01
GNL3	-0.022	9.022E-01	7.258E-01
FLT3LG	-0.022	9.188E-01	7.663E-01

RBM15B	-0.022	8.284E-01	7.515E-01
CABP5	-0.022	NA	6.762E-01
CMTR2	-0.022	8.810E-01	7.683E-01
TRIM3	-0.022	8.762E-01	7.475E-01
SCOC	-0.022	8.460E-01	7.660E-01
ZNF410	-0.022	8.476E-01	9.361E-01
FAM167B	-0.022	9.259E-01	6.259E-01
FPR1	-0.022	9.607E-01	5.156E-01
EHBP1L1	-0.022	8.853E-01	6.498E-01
SARAF	-0.022	8.735E-01	8.170E-01
CISD1	-0.022	8.911E-01	7.284E-01
TJP2	-0.022	9.003E-01	7.398E-01
VOPP1	-0.022	8.983E-01	6.437E-01
RC3H1	-0.022	8.311E-01	7.083E-01
PRPF8	-0.022	8.465E-01	8.166E-01
TAZ	-0.022	8.797E-01	7.712E-01
SPC25	-0.022	9.175E-01	6.170E-01
CRK	-0.022	8.217E-01	7.058E-01
DOCK1	-0.022	8.833E-01	7.139E-01
GRTP1	-0.022	9.267E-01	6.757E-01
ATAD1	-0.022	8.632E-01	6.548E-01
SHC1	-0.023	9.093E-01	6.620E-01
FKRP	-0.023	8.277E-01	6.738E-01
BNIP2	-0.023	8.123E-01	9.458E-01
PCBP4	-0.023	9.175E-01	9.843E-01
ZNF79	-0.023	8.570E-01	7.753E-01
TIMM21	-0.023	8.512E-01	7.214E-01
TARS	-0.023	8.969E-01	8.230E-01
HAUS8	-0.023	8.654E-01	5.972E-01
TMBIM6	-0.023	8.345E-01	9.645E-01
PRG3	-0.023	NA	7.862E-01
KRTAP10-4	-0.023	NA	6.860E-01
MIR548E	-0.023	NA	6.834E-01
TDP1	-0.023	8.357E-01	8.470E-01
SPIN2B	-0.023	8.547E-01	7.330E-01
CAPZA3	-0.023	9.861E-01	6.387E-01
FMO4	-0.023	9.248E-01	7.397E-01
C3AR1	-0.023	9.571E-01	9.232E-01
GCNT7	-0.023	9.412E-01	6.721E-01
ETS1	-0.023	9.128E-01	8.860E-01
NFAT5	-0.023	9.314E-01	8.864E-01
C6orf120	-0.023	8.472E-01	6.437E-01
UBE2W	-0.023	8.235E-01	6.533E-01
PSMD4	-0.023	8.656E-01	7.010E-01
SRSF10	-0.023	7.805E-01	6.315E-01
NAT1	-0.023	9.054E-01	9.429E-01
BABAM1	-0.023	8.465E-01	7.148E-01
H2AFY	-0.023	8.042E-01	7.306E-01
NASP	-0.023	8.674E-01	8.848E-01
CISD3	-0.023	9.160E-01	6.479E-01

FCF1	-0.023	8.328E-01	6.398E-01
UMPS	-0.023	8.455E-01	6.699E-01
HCAR1	-0.023	9.563E-01	6.457E-01
ALDH3B1	-0.023	9.455E-01	8.443E-01
LOXL4	-0.023	9.621E-01	7.339E-01
POM121	-0.023	8.632E-01	7.068E-01
MMAB	-0.023	8.878E-01	8.160E-01
FAM120C	-0.023	8.857E-01	6.779E-01
DNTTIP1	-0.024	8.575E-01	8.685E-01
CSN1S1	-0.024	9.847E-01	6.839E-01
IGFBPL1	-0.024	9.680E-01	6.226E-01
PPP6R1	-0.024	8.430E-01	8.408E-01
BAG5	-0.024	8.092E-01	6.958E-01
SMARCAL1	-0.024	8.055E-01	6.445E-01
PDZD11	-0.024	8.407E-01	9.391E-01
FAM117A	-0.024	8.948E-01	5.785E-01
CHMP1B	-0.024	8.570E-01	7.659E-01
SENP3	-0.024	8.449E-01	6.352E-01
TMEM63B	-0.024	8.549E-01	9.928E-01
CAMLG	-0.024	8.085E-01	9.909E-01
IST1	-0.024	8.132E-01	9.895E-01
HNRNPA2B1	-0.024	7.421E-01	6.587E-01
CYP2F1	-0.024	9.785E-01	9.046E-01
SCYL2	-0.024	8.384E-01	7.888E-01
STXBP3	-0.024	8.006E-01	7.652E-01
DHX30	-0.024	8.165E-01	6.785E-01
ACTR10	-0.024	7.982E-01	6.731E-01
ZNF583	-0.024	9.133E-01	7.214E-01
WDR82	-0.024	8.188E-01	8.942E-01
RBM48	-0.024	8.306E-01	7.127E-01
MIR6757	-0.024	9.749E-01	7.319E-01
GFOD1	-0.024	9.350E-01	6.387E-01
FAM35A	-0.024	8.067E-01	5.681E-01
TDGF1	-0.024	9.690E-01	8.891E-01
CCDC115	-0.024	8.401E-01	8.661E-01
DIS3	-0.024	8.513E-01	7.467E-01
MBTPS1	-0.024	8.050E-01	8.646E-01
C1orf158	-0.024	9.783E-01	5.958E-01
ZC3H14	-0.024	8.189E-01	6.991E-01
NFYC	-0.024	8.529E-01	7.383E-01
PAGE1	-0.024	9.861E-01	8.207E-01
SLC16A4	-0.024	9.520E-01	7.428E-01
EPM2A	-0.024	8.909E-01	7.420E-01
ZNF444	-0.024	8.750E-01	7.741E-01
ZFP69	-0.024	8.755E-01	8.923E-01
MARVELD3	-0.024	9.158E-01	9.648E-01
WDR92	-0.024	8.727E-01	7.024E-01
EFCAB7	-0.024	8.512E-01	6.437E-01
MAX	-0.024	7.874E-01	9.483E-01
CDK5R1	-0.024	9.386E-01	6.219E-01

CASK	-0.024	8.772E-01	6.994E-01
ZNF214	-0.024	9.394E-01	6.791E-01
OSM	-0.024	9.507E-01	9.268E-01
QRICH1	-0.024	7.691E-01	6.247E-01
TMEM9B	-0.024	8.165E-01	8.575E-01
ROCK1	-0.024	8.692E-01	9.401E-01
N6AMT1	-0.024	8.580E-01	6.638E-01
MIR6739	-0.024	9.529E-01	6.434E-01
ERMAP	-0.024	8.631E-01	8.150E-01
NIT2	-0.024	8.368E-01	6.126E-01
PHKB	-0.024	8.397E-01	6.387E-01
ZNF655	-0.025	8.873E-01	6.155E-01
UQCC3	-0.025	9.001E-01	6.765E-01
FCGR3A	-0.025	9.586E-01	9.491E-01
RAB2B	-0.025	8.276E-01	5.298E-01
MIR4676	-0.025	NA	6.339E-01
AC114783.1	-0.025	NA	6.276E-01
OR13C3	-0.025	NA	7.073E-01
ZSCAN25	-0.025	8.143E-01	6.882E-01
LRRC8B	-0.025	8.876E-01	7.109E-01
SH3GLB1	-0.025	7.762E-01	7.806E-01
MIR3177	-0.025	9.747E-01	6.806E-01
ALG1	-0.025	8.192E-01	6.229E-01
PDPK1	-0.025	8.497E-01	6.291E-01
EFS	-0.025	9.265E-01	6.848E-01
EIF3J	-0.025	8.054E-01	9.247E-01
CDK2AP1	-0.025	8.425E-01	9.625E-01
INHBB	-0.025	9.411E-01	7.116E-01
CCNDBP1	-0.025	8.394E-01	7.621E-01
P4HTM	-0.025	9.134E-01	7.667E-01
ZNF131	-0.025	8.545E-01	7.219E-01
RGL2	-0.025	8.697E-01	9.135E-01
SKOR1	-0.025	9.282E-01	8.772E-01
JKAMP	-0.025	8.198E-01	8.996E-01
SET	-0.025	8.346E-01	6.894E-01
DDX3X	-0.025	8.169E-01	6.672E-01
EHMT2	-0.025	8.210E-01	8.211E-01
ADGRG2	-0.025	9.487E-01	7.293E-01
DGKZ	-0.025	8.684E-01	6.205E-01
GUK1	-0.025	8.590E-01	6.908E-01
THEG	-0.025	9.758E-01	7.990E-01
ACTG1	-0.025	8.110E-01	5.851E-01
SIRT1	-0.025	8.520E-01	7.258E-01
PSMG2	-0.025	8.243E-01	6.523E-01
USP5	-0.025	8.189E-01	6.920E-01
CYTH4	-0.025	9.494E-01	8.497E-01
MIR6819	-0.025	9.493E-01	8.135E-01
TMEM258	-0.025	8.360E-01	8.687E-01
FOXA3	-0.025	9.671E-01	9.008E-01
TNFRSF1A	-0.025	8.735E-01	8.380E-01

BUB3	-0.025	8.165E-01	9.646E-01
LNX2	-0.025	8.693E-01	7.070E-01
CLSPN	-0.025	9.296E-01	6.717E-01
BMI1	-0.025	8.423E-01	9.840E-01
NHP2	-0.025	8.439E-01	9.860E-01
CTPS1	-0.025	8.989E-01	9.868E-01
MIR4470	-0.025	NA	6.698E-01
UBR2	-0.025	7.883E-01	7.251E-01
MTMR11	-0.025	9.169E-01	7.830E-01
GNB5	-0.025	8.631E-01	6.985E-01
SLC39A10	-0.025	8.918E-01	7.104E-01
PRAMEF14	-0.026	NA	8.243E-01
PAPOLB	-0.026	NA	6.330E-01
INPPL1	-0.026	8.369E-01	6.589E-01
MBD5	-0.026	8.292E-01	8.052E-01
CRBN	-0.026	8.249E-01	7.217E-01
DDX49	-0.026	8.381E-01	8.412E-01
TMEM81	-0.026	8.586E-01	7.579E-01
TOP3A	-0.026	8.541E-01	6.490E-01
VPS8	-0.026	8.016E-01	8.243E-01
KYAT1	-0.026	8.896E-01	7.517E-01
KCNJ14	-0.026	9.137E-01	6.908E-01
GPANK1	-0.026	8.557E-01	6.645E-01
XRN2	-0.026	8.189E-01	7.851E-01
FKBP1C	-0.026	8.735E-01	7.898E-01
SNX16	-0.026	8.671E-01	7.745E-01
LMF1	-0.026	9.138E-01	7.396E-01
ARFIP2	-0.026	8.220E-01	7.613E-01
CHD4	-0.026	7.321E-01	9.106E-01
GTF2E1	-0.026	8.074E-01	8.399E-01
MIR6129	-0.026	9.730E-01	6.928E-01
DNAJB1	-0.026	8.876E-01	6.900E-01
RFK	-0.026	8.775E-01	7.448E-01
NRIP2	-0.026	8.880E-01	7.537E-01
PPP2R1B	-0.026	8.384E-01	9.599E-01
RPS6KA4	-0.026	8.797E-01	6.606E-01
CCHCR1	-0.026	8.878E-01	8.266E-01
PPIE	-0.026	8.656E-01	7.931E-01
PJA2	-0.026	8.579E-01	8.542E-01
NDUFS6	-0.026	8.922E-01	7.060E-01
UNC119	-0.026	8.512E-01	9.942E-01
LRP6	-0.026	8.570E-01	9.934E-01
SP3	-0.026	8.192E-01	9.929E-01
KIF1A	-0.026	9.743E-01	6.765E-01
MIR608	-0.026	NA	9.923E-01
DNPH1	-0.026	8.797E-01	9.923E-01
FBXW11	-0.026	7.906E-01	9.922E-01
OIP5	-0.026	9.053E-01	9.921E-01
ASTN1	-0.026	9.672E-01	9.916E-01
ZNF764	-0.026	8.259E-01	9.251E-01

TTC37	-0.026	8.384E-01	7.959E-01
KPNA1	-0.026	7.770E-01	9.156E-01
DEFB128	-0.026	NA	7.317E-01
MIR1237	-0.026	NA	7.806E-01
MIR3938	-0.026	NA	9.459E-01
MIR4443	-0.026	NA	9.488E-01
MIR509-3	-0.026	NA	8.147E-01
MIR548H5	-0.026	NA	9.132E-01
OR4C3	-0.026	NA	7.332E-01
OR5B3	-0.026	NA	6.752E-01
OR8J3	-0.026	NA	9.479E-01
MIR1231	-0.026	NA	6.455E-01
MIR3668	-0.026	NA	7.434E-01
MIR3923	-0.026	NA	9.398E-01
MIR4752	-0.026	NA	7.138E-01
MIR7159	-0.026	NA	6.346E-01
MIR8080	-0.026	NA	9.621E-01
OR6X1	-0.026	NA	6.956E-01
TRIM49D1	-0.026	NA	6.700E-01
ELOA3B	-0.026	NA	9.740E-01
FAM231C	-0.026	NA	8.798E-01
KRTAP15-1	-0.026	NA	7.058E-01
MIR2053	-0.026	NA	6.107E-01
MIR4300	-0.026	NA	8.863E-01
MIR4418	-0.026	NA	8.679E-01
MIR4643	-0.026	NA	8.014E-01
MIR500B	-0.026	NA	7.477E-01
MIR5681A	-0.026	NA	8.565E-01
MIR8074	-0.026	NA	8.890E-01
MIR885	-0.026	NA	8.135E-01
MIR99A	-0.026	NA	7.519E-01
OPN1MW	-0.026	NA	8.231E-01
OR10G6	-0.026	NA	6.944E-01
OR13C8	-0.026	NA	9.309E-01
OR14C36	-0.026	NA	9.127E-01
OR5T1	-0.026	NA	7.362E-01
AC073610.2	-0.026	NA	7.801E-01
AC245748.1	-0.026	NA	7.353E-01
MIR2392	-0.026	NA	8.458E-01
MIR3179-2	-0.026	NA	9.322E-01
MIR450A2	-0.026	NA	8.053E-01
MIR5100	-0.026	NA	8.022E-01
MIR518B	-0.026	NA	7.778E-01
MIR6068	-0.026	NA	8.905E-01
OR5D14	-0.026	NA	7.714E-01
USP17L10	-0.026	NA	8.048E-01
IQCG	-0.026	8.853E-01	7.744E-01
ST20	-0.026	9.167E-01	8.033E-01
MDFI	-0.026	9.347E-01	8.088E-01
INTS7	-0.026	8.233E-01	6.992E-01

PTDSS2	-0.026	8.693E-01	7.894E-01
RBM15	-0.026	8.181E-01	8.877E-01
PTDSS1	-0.026	8.512E-01	6.438E-01
RBM3	-0.026	8.528E-01	7.016E-01
LINC00959	-0.026	9.116E-01	7.411E-01
ARHGAP11B	-0.026	8.880E-01	8.758E-01
AC020922.1	-0.026	9.683E-01	7.333E-01
ST6GALNAC	-0.026	8.972E-01	7.332E-01
CFAP157	-0.026	8.922E-01	7.503E-01
MED26	-0.026	7.356E-01	7.938E-01
DPEP2	-0.026	9.508E-01	6.702E-01
KIAA1109	-0.026	8.589E-01	7.361E-01
ERFE	-0.026	9.426E-01	7.654E-01
LRRC37A3	-0.026	9.213E-01	8.898E-01
HMGN4	-0.026	8.269E-01	6.926E-01
FAAH2	-0.026	9.106E-01	6.640E-01
ZBTB33	-0.027	8.184E-01	7.441E-01
LANCL1	-0.027	8.570E-01	7.455E-01
MIR3116-1	-0.027	NA	6.494E-01
RXRB	-0.027	7.637E-01	8.093E-01
MPHOSPH9	-0.027	8.815E-01	7.091E-01
JMY	-0.027	8.513E-01	7.561E-01
TNNI3K	-0.027	9.563E-01	8.173E-01
DHX40	-0.027	8.405E-01	9.048E-01
LARGE1	-0.027	9.213E-01	7.315E-01
IGSF3	-0.027	9.128E-01	6.668E-01
SLC25A13	-0.027	8.309E-01	6.804E-01
ATF2	-0.027	7.972E-01	7.847E-01
RPUSD1	-0.027	8.349E-01	9.231E-01
PPP1R7	-0.027	8.245E-01	7.138E-01
DFFA	-0.027	7.791E-01	7.355E-01
DCAKD	-0.027	8.635E-01	6.988E-01
RPE65	-0.027	9.766E-01	8.198E-01
BAG6	-0.027	7.528E-01	9.882E-01
SPTY2D1	-0.027	8.232E-01	7.517E-01
MPLKIP	-0.027	8.053E-01	8.162E-01
IL12B	-0.027	9.600E-01	8.956E-01
RGPD8	-0.027	9.249E-01	7.225E-01
PRKCZ	-0.027	8.671E-01	6.971E-01
GRINA	-0.027	8.416E-01	9.452E-01
RAP2B	-0.027	8.815E-01	9.819E-01
GK5	-0.027	8.516E-01	7.079E-01
FECH	-0.027	8.692E-01	7.966E-01
SMAD7	-0.027	8.989E-01	9.235E-01
IRS1	-0.027	9.212E-01	8.941E-01
GSKIP	-0.027	8.093E-01	8.079E-01
PGAM1	-0.027	8.773E-01	8.948E-01
C3orf33	-0.027	8.688E-01	8.214E-01
OPA3	-0.027	8.110E-01	7.101E-01
ERCC6L2	-0.027	8.245E-01	7.627E-01

ZNF345	-0.027	8.930E-01	7.957E-01
BLOC1S6	-0.027	7.793E-01	7.495E-01
KIAA1586	-0.027	8.511E-01	8.065E-01
LRRC41	-0.027	7.758E-01	7.666E-01
PTPN1	-0.027	8.181E-01	7.459E-01
AKIRIN1	-0.027	8.361E-01	9.110E-01
AP001781.3	-0.027	NA	9.087E-01
MIR6827	-0.027	NA	7.226E-01
MIR5189	-0.027	NA	8.162E-01
MIR584	-0.027	NA	7.892E-01
PRAMEF33	-0.027	NA	8.090E-01
WFDC9	-0.027	NA	9.621E-01
PPRC1	-0.027	7.782E-01	6.342E-01
TSPAN14	-0.027	8.596E-01	8.625E-01
MIR2467	-0.027	NA	9.772E-01
MIR3117	-0.027	NA	7.385E-01
BBX	-0.028	8.446E-01	8.906E-01
MIR3183	-0.028	NA	8.030E-01
FAM109B	-0.028	9.083E-01	7.476E-01
CNGB3	-0.028	9.334E-01	9.257E-01
LINC00672	-0.028	9.246E-01	9.950E-01
MIR4489	-0.028	9.319E-01	9.950E-01
ZNF66	-0.028	9.455E-01	9.950E-01
MYNN	-0.028	7.519E-01	9.950E-01
FAM210B	-0.028	8.574E-01	9.946E-01
AL136295.3	-0.028	9.468E-01	9.946E-01
MAPK13	-0.028	8.693E-01	9.946E-01
ZBTB17	-0.028	7.857E-01	9.945E-01
ROBO4	-0.028	9.043E-01	9.944E-01
SOX4	-0.028	9.133E-01	7.607E-01
ADCY1	-0.028	9.445E-01	7.476E-01
NDUFA6	-0.028	8.343E-01	9.248E-01
NBPF20	-0.028	8.622E-01	7.165E-01
COG8	-0.028	7.599E-01	7.788E-01
ADGRL3	-0.028	9.567E-01	7.439E-01
ZNF138	-0.028	8.630E-01	7.328E-01
THOC2	-0.028	8.504E-01	7.567E-01
AC025594.3	-0.028	9.257E-01	9.685E-01
CBL	-0.028	8.570E-01	9.156E-01
REV1	-0.028	8.296E-01	8.459E-01
FA2H	-0.028	9.457E-01	8.409E-01
CRB1	-0.028	9.601E-01	7.971E-01
ALG9	-0.028	7.910E-01	7.786E-01
POLI	-0.028	8.828E-01	7.726E-01
SHBG	-0.028	9.001E-01	7.138E-01
C17orf107	-0.028	9.070E-01	6.289E-01
CNNM2	-0.028	8.423E-01	7.141E-01
›PAN-P2RY1	-0.028	9.567E-01	7.713E-01
DNAJC7	-0.028	7.478E-01	9.652E-01
ERG	-0.028	9.113E-01	7.897E-01

GLIPR1L1	-0.028	9.162E-01	7.943E-01
SPATA2	-0.028	7.602E-01	8.025E-01
C14orf1	-0.028	8.217E-01	7.242E-01
TNIP1	-0.028	8.421E-01	7.209E-01
FBXO31	-0.028	7.970E-01	8.662E-01
ZMAT5	-0.028	8.530E-01	7.657E-01
MCAT	-0.028	8.269E-01	8.916E-01
GSG1L2	-0.028	NA	7.325E-01
MIR4667	-0.028	NA	9.494E-01
WDR13	-0.028	8.425E-01	7.282E-01
TRPC3	-0.028	9.454E-01	7.615E-01
C17orf98	-0.028	9.631E-01	7.411E-01
MYLPF	-0.028	9.399E-01	8.342E-01
ZXDC	-0.028	7.970E-01	9.048E-01
ADD3	-0.028	8.878E-01	9.052E-01
RFC4	-0.028	8.781E-01	8.064E-01
EEF2KMT	-0.028	8.075E-01	9.872E-01
SLC25A26	-0.028	8.181E-01	7.874E-01
AP3M2	-0.028	8.580E-01	9.078E-01
NUDT6	-0.028	8.622E-01	8.819E-01
ST13	-0.028	7.848E-01	7.784E-01
SGK494	-0.028	9.132E-01	7.027E-01
SEMA5B	-0.028	9.444E-01	8.357E-01
ABCC6	-0.028	9.272E-01	8.236E-01
UVRAG	-0.028	8.164E-01	8.594E-01
URGCP	-0.029	7.644E-01	8.446E-01
NARS	-0.029	8.057E-01	7.335E-01
MSH5	-0.029	9.015E-01	8.686E-01
TMEM179	-0.029	9.692E-01	9.017E-01
BRICD5	-0.029	9.095E-01	7.837E-01
ERO1B	-0.029	8.861E-01	9.030E-01
RBM23	-0.029	7.192E-01	8.087E-01
SAMD12	-0.029	9.319E-01	8.985E-01
PSMD14	-0.029	7.937E-01	7.643E-01
CMTM5	-0.029	9.552E-01	8.209E-01
AGTPBP1	-0.029	8.587E-01	9.370E-01
ZNF677	-0.029	9.394E-01	6.903E-01
TARS2	-0.029	8.259E-01	8.564E-01
SV2A	-0.029	9.380E-01	8.525E-01
MIR1202	-0.029	NA	9.205E-01
MIR1263	-0.029	NA	8.060E-01
MIR16-1	-0.029	NA	8.259E-01
MIR3646	-0.029	NA	8.780E-01
MIR466	-0.029	NA	8.206E-01
MIR548U	-0.029	NA	7.935E-01
OR2T29	-0.029	NA	9.898E-01
OR5AS1	-0.029	NA	7.337E-01
AC008537.1	-0.029	NA	7.829E-01
DEFB129	-0.029	NA	9.709E-01
ısa-mir-4773-	-0.029	NA	7.781E-01

MIR134	-0.029	NA	8.671E-01
MIR217	-0.029	NA	9.752E-01
MIR4280	-0.029	NA	8.651E-01
MIR4439	-0.029	NA	9.929E-01
MIR4445	-0.029	NA	9.924E-01
MIR4462	-0.029	NA	9.919E-01
MIR548F3	-0.029	NA	9.894E-01
NPS	-0.029	NA	7.609E-01
OR13F1	-0.029	NA	9.753E-01
OR2M5	-0.029	NA	8.199E-01
OR4S2	-0.029	NA	7.943E-01
OR52A5	-0.029	NA	9.833E-01
TAAR2	-0.029	NA	7.675E-01
TSC1	-0.029	8.206E-01	8.465E-01
IGFLR1	-0.029	9.113E-01	8.251E-01
PPARD	-0.029	8.444E-01	9.353E-01
CCDC112	-0.029	8.760E-01	7.835E-01
CPD	-0.029	8.582E-01	9.077E-01
AP1S1	-0.029	8.254E-01	7.980E-01
PTGDR	-0.029	9.464E-01	7.594E-01
CDC42BPG	-0.029	8.873E-01	7.376E-01
ZBTB25	-0.029	7.968E-01	8.130E-01
MRM2	-0.029	7.583E-01	8.083E-01
TBK1	-0.029	7.994E-01	8.406E-01
SH3RF1	-0.029	8.561E-01	9.362E-01
ZBTB11	-0.030	7.671E-01	8.266E-01
TMEM243	-0.030	8.164E-01	7.653E-01
MORN1	-0.030	8.539E-01	8.309E-01
CRYZL1	-0.030	8.007E-01	8.068E-01
CCNA2	-0.030	8.880E-01	8.753E-01
OLIG1	-0.030	9.652E-01	9.139E-01
TEAD2	-0.030	9.314E-01	8.395E-01
PLEKHB2	-0.030	8.092E-01	8.453E-01
HIST1H2AB	-0.030	9.475E-01	6.935E-01
RP2	-0.030	8.448E-01	7.572E-01
FMC1	-0.030	8.815E-01	8.142E-01
ABCA2	-0.030	9.002E-01	7.444E-01
FAM86C1	-0.030	8.291E-01	8.282E-01
BICRA	-0.030	8.054E-01	9.496E-01
EXOSC3	-0.030	8.466E-01	7.781E-01
ZCCHC7	-0.030	8.192E-01	9.393E-01
TMEM60	-0.030	7.837E-01	8.135E-01
VPS37A	-0.030	8.080E-01	7.086E-01
PPCDC	-0.030	8.352E-01	8.227E-01
ZNF184	-0.030	8.410E-01	8.296E-01
TMEM110	-0.030	8.369E-01	7.773E-01
MRPL57	-0.030	8.192E-01	8.195E-01
AC003002.1	-0.030	8.292E-01	8.359E-01
AUP1	-0.030	7.864E-01	7.725E-01
NDUFB9	-0.030	8.404E-01	7.736E-01

WDR61	-0.030	7.586E-01	7.625E-01
ADCY6	-0.030	8.725E-01	9.281E-01
SPSB3	-0.030	8.184E-01	7.698E-01
TMEM37	-0.030	9.315E-01	9.819E-01
MIR1229	-0.030	NA	8.085E-01
MIR1272	-0.030	NA	9.848E-01
MIR34B	-0.030	NA	7.955E-01
OR6F1	-0.030	NA	7.399E-01
TRPM4	-0.030	8.852E-01	8.130E-01
POLR1A	-0.030	7.959E-01	9.957E-01
CBFA2T2	-0.030	8.517E-01	9.957E-01
TAS2R14	-0.030	8.579E-01	9.957E-01
RP1L1	-0.030	9.516E-01	9.957E-01
MS4A6A	-0.030	9.380E-01	9.957E-01
KBTBD3	-0.030	8.345E-01	9.957E-01
MIR4692	-0.030	NA	9.957E-01
CDKN2AIP	-0.030	7.779E-01	9.957E-01
PEX16	-0.030	8.278E-01	9.957E-01
SMAP2	-0.030	8.570E-01	9.957E-01
EIF4EBP3	-0.030	9.128E-01	9.957E-01
GOLGA8S	-0.030	9.579E-01	9.957E-01
PLK4	-0.030	8.614E-01	9.957E-01
ETFDH	-0.030	8.123E-01	9.957E-01
SRL	-0.030	9.326E-01	9.957E-01
PNPT1	-0.030	8.026E-01	9.957E-01
RCCD1	-0.030	8.197E-01	9.957E-01
POMP	-0.030	7.972E-01	9.957E-01
BRIP1	-0.030	8.896E-01	9.957E-01
RSPH4A	-0.030	9.081E-01	9.957E-01
MIR331	-0.030	9.235E-01	9.957E-01
C20orf202	-0.031	9.105E-01	9.957E-01
SPATA33	-0.031	8.448E-01	9.957E-01
CLCN6	-0.031	7.847E-01	9.957E-01
LTA4H	-0.031	8.055E-01	8.912E-01
BAZ2A	-0.031	7.907E-01	8.519E-01
NDUFB3	-0.031	7.714E-01	9.710E-01
CHM	-0.031	8.217E-01	7.559E-01
SLC38A1	-0.031	8.549E-01	9.335E-01
TK2	-0.031	8.109E-01	8.108E-01
DSG4	-0.031	9.689E-01	7.312E-01
VPS52	-0.031	7.389E-01	8.027E-01
PLCG1	-0.031	8.162E-01	8.621E-01
PDCD4	-0.031	8.860E-01	9.401E-01
ENDOG	-0.031	8.545E-01	7.811E-01
HEXIM2	-0.031	8.333E-01	8.443E-01
POLR3H	-0.031	7.781E-01	7.445E-01
ZNF234	-0.031	8.184E-01	8.558E-01
ısa-mir-3130-	-0.031	NA	9.273E-01
FAM104A	-0.031	7.093E-01	7.625E-01
TRAPPC11	-0.031	7.509E-01	7.639E-01

ZBTB34	-0.031	8.162E-01	6.603E-01
MAPKAPK3	-0.031	8.298E-01	7.915E-01
MIR7849	-0.031	NA	9.402E-01
HSD17B10	-0.031	8.130E-01	8.140E-01
DZIP3	-0.031	8.374E-01	7.666E-01
RAP1GDS1	-0.031	7.994E-01	8.467E-01
NPLOC4	-0.031	7.535E-01	8.349E-01
FAM133A	-0.031	9.733E-01	8.819E-01
HLA-DMA	-0.031	9.198E-01	8.034E-01
TOMM20	-0.031	7.643E-01	7.962E-01
CBFB	-0.031	7.586E-01	9.322E-01
GRWD1	-0.031	7.558E-01	9.860E-01
SUZ12	-0.031	8.291E-01	9.878E-01
PRPF4B	-0.031	7.435E-01	9.500E-01
PLOD3	-0.031	8.402E-01	9.470E-01
EPM2AIP1	-0.031	8.450E-01	9.196E-01
NUDT17	-0.031	8.596E-01	8.167E-01
SGPL1	-0.031	8.786E-01	8.360E-01
F13A1	-0.031	9.426E-01	8.864E-01
DNAJA2	-0.031	7.397E-01	9.153E-01
FOXD4L6	-0.031	9.630E-01	9.135E-01
C9orf142	-0.031	8.615E-01	9.336E-01
TECR	-0.032	8.053E-01	8.155E-01
PIP5KL1	-0.032	9.146E-01	9.279E-01
SEC24B	-0.032	7.601E-01	8.354E-01
ALG6	-0.032	7.653E-01	8.086E-01
MVP	-0.032	8.625E-01	8.958E-01
PCDHB14	-0.032	8.978E-01	8.699E-01
AC009086.2	-0.032	NA	8.076E-01
OR6N1	-0.032	NA	8.982E-01
OR4A47	-0.032	NA	8.144E-01
KRTAP22-1	-0.032	NA	7.915E-01
KRTAP23-1	-0.032	NA	7.087E-01
OR5AC2	-0.032	NA	9.661E-01
NHSL2	-0.032	9.369E-01	8.907E-01
SRP9	-0.032	7.093E-01	8.056E-01
GRAMD3	-0.032	8.693E-01	8.748E-01
TLR3	-0.032	9.158E-01	8.154E-01
TTI1	-0.032	7.705E-01	8.308E-01
PPP4R3A	-0.032	7.618E-01	8.064E-01
FAM24B	-0.032	9.092E-01	7.543E-01
MIR1293	-0.032	NA	7.972E-01
IDH3B	-0.032	7.671E-01	8.527E-01
CCDC81	-0.032	8.671E-01	7.937E-01
ANGEL2	-0.032	7.357E-01	8.755E-01
BORCS6	-0.032	8.120E-01	9.474E-01
GNA15	-0.032	8.918E-01	8.096E-01
NMT1	-0.032	6.751E-01	7.771E-01
EVL	-0.032	8.693E-01	8.524E-01
SF3A1	-0.032	7.433E-01	8.403E-01

MAN2B1	-0.032	7.945E-01	8.458E-01
POLA2	-0.032	8.279E-01	8.524E-01
FAT1	-0.032	8.972E-01	7.679E-01
PHF5A	-0.032	7.433E-01	7.872E-01
CREBZF	-0.032	8.081E-01	9.563E-01
FARSB	-0.032	7.932E-01	8.146E-01
MON1B	-0.032	7.598E-01	8.355E-01
GNS	-0.032	8.278E-01	8.194E-01
ATP6V0A2	-0.032	7.247E-01	9.693E-01
GIGYF2	-0.032	7.610E-01	9.648E-01
CCL5	-0.032	9.475E-01	7.935E-01
HSPBAP1	-0.032	8.171E-01	7.479E-01
MT1F	-0.032	9.207E-01	8.292E-01
SPTLC2	-0.032	7.800E-01	7.876E-01
MAP3K11	-0.032	7.677E-01	8.414E-01
SLC6A14	-0.032	9.616E-01	8.134E-01
ZCCHC2	-0.032	8.015E-01	9.955E-01
LYPLA2	-0.032	7.953E-01	9.932E-01
ZNF304	-0.032	8.168E-01	9.924E-01
KRTAP12-1	-0.032	NA	8.434E-01
MEX3B	-0.033	9.257E-01	9.517E-01
ATF5	-0.033	8.460E-01	8.584E-01
COPS3	-0.033	8.109E-01	8.585E-01
PSMD13	-0.033	7.795E-01	8.547E-01
CCDC167	-0.033	8.528E-01	8.310E-01
RANGAP1	-0.033	7.923E-01	7.621E-01
PHF21A	-0.033	7.757E-01	8.890E-01
ACBD6	-0.033	7.290E-01	7.439E-01
SMIM26	-0.033	7.793E-01	9.172E-01
SBK3	-0.033	9.488E-01	8.098E-01
HCFC1	-0.033	7.093E-01	8.190E-01
ZNF43	-0.033	9.056E-01	8.132E-01
HIVEP2	-0.033	8.423E-01	8.734E-01
RBFA	-0.033	7.868E-01	8.150E-01
ZSCAN32	-0.033	8.090E-01	9.595E-01
FAM118A	-0.033	8.880E-01	9.158E-01
GJB3	-0.033	9.229E-01	8.917E-01
PFDN6	-0.033	8.269E-01	9.322E-01
RNF10	-0.033	6.967E-01	9.680E-01
TSSK3	-0.033	9.010E-01	9.450E-01
SDHA	-0.033	8.306E-01	9.151E-01
RFX3	-0.033	8.666E-01	9.641E-01
ANK3	-0.033	8.883E-01	9.240E-01
PSMD11	-0.033	8.261E-01	8.328E-01
KMT2C	-0.033	8.265E-01	7.970E-01
GOLGA8Q	-0.033	9.598E-01	8.270E-01
MYCBP	-0.033	7.909E-01	8.417E-01
TXNDC11	-0.033	7.771E-01	8.960E-01
PITPNA	-0.033	7.085E-01	9.073E-01
ANKLE2	-0.033	7.601E-01	8.806E-01

RIMBP3B	-0.033	9.600E-01	8.161E-01
DCUN1D4	-0.033	7.489E-01	8.883E-01
COPS9	-0.033	8.320E-01	8.070E-01
ANAPC11	-0.033	8.292E-01	8.421E-01
PDCD5	-0.033	7.978E-01	9.051E-01
AC012184.2	-0.033	NA	7.785E-01
ZNF451	-0.033	7.125E-01	9.369E-01
CDC25A	-0.033	8.720E-01	7.876E-01
WFDC11	-0.033	9.783E-01	7.961E-01
EXOC2	-0.033	7.321E-01	7.030E-01
TPGS2	-0.033	7.893E-01	8.470E-01
BET1L	-0.033	7.106E-01	9.582E-01
SLC38A9	-0.033	8.253E-01	9.310E-01
EVI5L	-0.033	8.113E-01	9.340E-01
MAP3K9	-0.033	8.741E-01	8.110E-01
CYTH2	-0.033	7.968E-01	8.190E-01
STAT5A	-0.033	8.602E-01	8.465E-01
CDC26	-0.034	7.782E-01	9.575E-01
ICA1L	-0.034	8.760E-01	9.047E-01
SMIM10L2B	-0.034	8.900E-01	8.255E-01
NGRN	-0.034	7.585E-01	9.220E-01
POFUT1	-0.034	8.196E-01	9.063E-01
MADD	-0.034	7.781E-01	8.944E-01
MIR8081	-0.034	NA	9.476E-01
MIR4757	-0.034	NA	9.964E-01
NPBWR2	-0.034	NA	9.964E-01
C22orf39	-0.034	7.931E-01	9.964E-01
AKIP1	-0.034	8.240E-01	9.964E-01
RNF144B	-0.034	9.092E-01	9.964E-01
AC007906.2	-0.034	9.450E-01	9.964E-01
SPIN3	-0.034	8.348E-01	9.964E-01
USP4	-0.034	7.606E-01	9.964E-01
HTD2	-0.034	9.032E-01	9.964E-01
TUBGCP4	-0.034	7.671E-01	9.964E-01
COIL	-0.034	7.607E-01	9.964E-01
CIT	-0.034	8.618E-01	9.964E-01
AL049634.2	-0.034	9.685E-01	9.964E-01
SPINK13	-0.034	9.582E-01	9.964E-01
PANK2	-0.034	7.299E-01	9.964E-01
GTPBP10	-0.034	6.848E-01	9.964E-01
ISCA1	-0.034	7.471E-01	9.964E-01
MIR576	-0.034	NA	9.964E-01
NAPEPLD	-0.034	7.745E-01	9.964E-01
ARL6IP5	-0.034	8.192E-01	9.964E-01
IQUB	-0.034	8.995E-01	9.964E-01
SNRPB2	-0.034	7.519E-01	9.964E-01
IFNGR2	-0.034	7.867E-01	9.964E-01
TRMT10A	-0.034	8.171E-01	9.964E-01
WNT8A	-0.034	NA	9.964E-01
RPAP2	-0.034	7.224E-01	9.964E-01

SOX5	-0.034	9.133E-01	9.964E-01
KCTD18	-0.034	7.329E-01	9.964E-01
MRPL20	-0.034	7.639E-01	9.964E-01
TMEM250	-0.034	8.192E-01	9.964E-01
RFT1	-0.034	7.203E-01	9.964E-01
DKK1	-0.034	9.502E-01	9.964E-01
PNKP	-0.034	8.004E-01	9.964E-01
SLC12A9	-0.034	7.852E-01	9.964E-01
MIR4792	-0.034	NA	9.964E-01
ELMO2	-0.034	7.244E-01	9.964E-01
RLN2	-0.034	9.394E-01	9.964E-01
UGT1A9	-0.034	9.610E-01	9.964E-01
TAS2R7	-0.034	NA	9.964E-01
INS-IGF2	-0.034	NA	9.964E-01
NKX2-6	-0.034	NA	9.964E-01
MIR548Q	-0.034	NA	9.964E-01
MIR5680	-0.034	NA	9.964E-01
OR6C76	-0.034	NA	9.964E-01
MIR5186	-0.034	NA	9.964E-01
MIR1302-7	-0.034	NA	9.964E-01
PRAMEF5	-0.034	NA	9.964E-01
PEX11G	-0.034	8.775E-01	9.964E-01
MRPL17	-0.035	7.633E-01	9.964E-01
CLDN12	-0.035	7.759E-01	9.964E-01
BLOC1S2	-0.035	7.719E-01	9.964E-01
SHARPIN	-0.035	7.684E-01	9.964E-01
PCDHB3	-0.035	9.367E-01	9.964E-01
SLC25A17	-0.035	6.632E-01	9.964E-01
FHL5	-0.035	9.226E-01	9.964E-01
TJAP1	-0.035	7.601E-01	9.964E-01
ZC2HC1C	-0.035	8.600E-01	9.964E-01
VPS13D	-0.035	7.435E-01	9.964E-01
EYA3	-0.035	7.084E-01	9.964E-01
COPZ1	-0.035	6.834E-01	9.964E-01
RPRD1A	-0.035	7.777E-01	7.783E-01
ZNF34	-0.035	8.050E-01	7.084E-01
MIR3165	-0.035	NA	8.380E-01
TXNRD1	-0.035	9.113E-01	8.104E-01
SRCAP	-0.035	6.915E-01	7.587E-01
MARK4	-0.035	7.180E-01	8.703E-01
ZNRF2	-0.035	7.868E-01	8.305E-01
MIR561	-0.035	NA	9.400E-01
MIR3945	-0.035	NA	7.486E-01
NRF1	-0.035	5.610E-01	8.324E-01
SARS2	-0.035	8.403E-01	8.509E-01
B4GALNT2	-0.035	9.646E-01	8.729E-01
ERBB3	-0.035	9.061E-01	8.617E-01
MIR320C1	-0.035	NA	8.927E-01
CCNJL	-0.035	9.145E-01	7.803E-01
SMIM7	-0.035	7.101E-01	8.919E-01

KCNAB1	-0.035	8.741E-01	9.583E-01
NFE2L2	-0.035	8.143E-01	8.792E-01
SERF2	-0.035	8.169E-01	8.086E-01
RPS6KB2	-0.035	7.850E-01	8.236E-01
KHDRBS2	-0.035	9.508E-01	7.891E-01
SMPD4	-0.035	6.884E-01	8.089E-01
CELF4	-0.035	9.351E-01	7.925E-01
XPNPEP1	-0.035	6.655E-01	8.198E-01
TIAL1	-0.035	6.410E-01	8.443E-01
PRPF38A	-0.035	6.630E-01	8.530E-01
RAB32	-0.035	8.838E-01	9.018E-01
MRPS34	-0.035	7.875E-01	8.690E-01
TRIM36	-0.035	9.340E-01	9.749E-01
TPRKB	-0.035	7.435E-01	9.914E-01
GIT2	-0.035	7.483E-01	8.721E-01
LONP2	-0.036	7.415E-01	8.115E-01
VPS9D1	-0.036	8.278E-01	8.371E-01
CCDC83	-0.036	9.502E-01	8.744E-01
MIR6806	-0.036	NA	8.432E-01
OR10V1	-0.036	NA	8.389E-01
PI4K2A	-0.036	7.945E-01	9.405E-01
NMRAL1	-0.036	8.109E-01	9.262E-01
ZNF343	-0.036	7.218E-01	8.109E-01
USF1	-0.036	8.369E-01	9.638E-01
ATG10	-0.036	7.624E-01	8.430E-01
KIF18A	-0.036	8.781E-01	9.160E-01
FAM185A	-0.036	7.428E-01	8.938E-01
NCAPH2	-0.036	7.851E-01	9.453E-01
ATAD2B	-0.036	7.692E-01	8.167E-01
CSNK2A1	-0.036	6.848E-01	8.834E-01
SLX4IP	-0.036	7.835E-01	9.606E-01
ATIC	-0.036	7.921E-01	8.839E-01
FAM32A	-0.036	6.607E-01	7.997E-01
ZNF518A	-0.036	8.384E-01	8.613E-01
NLRC4	-0.036	8.692E-01	9.785E-01
NF1	-0.036	7.874E-01	8.180E-01
CASKIN1	-0.036	9.394E-01	9.197E-01
SPIN2A	-0.036	9.094E-01	8.722E-01
C14orf80	-0.036	8.283E-01	9.710E-01
ADRM1	-0.036	7.380E-01	8.537E-01
RHBDF1	-0.036	8.446E-01	7.944E-01
TEX264	-0.036	8.006E-01	7.782E-01
RPGRIP1	-0.036	8.786E-01	7.900E-01
ACO2	-0.036	7.694E-01	9.452E-01
GFRA1	-0.036	9.455E-01	7.824E-01
SS18L1	-0.036	8.192E-01	8.890E-01
CRMP1	-0.036	8.880E-01	9.485E-01
DCUN1D5	-0.036	8.257E-01	9.449E-01
UBE2J1	-0.036	7.084E-01	9.424E-01
ARRDC1	-0.036	8.295E-01	8.358E-01

FBXO46	-0.036	7.674E-01	8.239E-01
ATAD3B	-0.036	8.328E-01	9.028E-01
TNP2	-0.036	NA	9.586E-01
STARD7	-0.036	7.122E-01	8.289E-01
RHOBTB1	-0.036	8.692E-01	9.162E-01
RBM12	-0.036	6.549E-01	8.441E-01
CS	-0.036	6.871E-01	8.260E-01
SYN2	-0.036	9.324E-01	8.201E-01
TAF8	-0.037	6.839E-01	9.006E-01
LEO1	-0.037	7.224E-01	7.395E-01
SAMD1	-0.037	7.656E-01	7.938E-01
NUTM2E	-0.037	9.545E-01	9.611E-01
SCNM1	-0.037	7.955E-01	9.953E-01
TRIM69	-0.037	8.452E-01	9.949E-01
EIF2S3	-0.037	7.443E-01	9.946E-01
ATXN3	-0.037	6.432E-01	8.520E-01
AC092587.1	-0.037	8.846E-01	8.254E-01
CNDP2	-0.037	8.070E-01	8.490E-01
REST	-0.037	6.949E-01	9.543E-01
PER2	-0.037	8.448E-01	8.552E-01
MMP15	-0.037	8.706E-01	8.792E-01
SNX3	-0.037	7.128E-01	8.101E-01
OMA1	-0.037	7.595E-01	8.435E-01
KIAA1191	-0.037	6.680E-01	8.226E-01
NNT	-0.037	8.186E-01	8.765E-01
CBR3	-0.037	9.041E-01	9.754E-01
FP565260.7	-0.037	8.076E-01	9.376E-01
ICE2	-0.037	6.915E-01	8.518E-01
TNRC18	-0.037	7.322E-01	8.875E-01
KRIT1	-0.037	7.535E-01	9.432E-01
CDCP1	-0.037	8.806E-01	8.519E-01
NDUFA4	-0.037	7.350E-01	8.572E-01
AFAP1L1	-0.037	8.755E-01	9.122E-01
MRPL55	-0.037	7.863E-01	8.532E-01
CACHD1	-0.037	9.027E-01	8.405E-01
ESYT1	-0.037	7.691E-01	8.649E-01
NDUFS1	-0.037	7.193E-01	9.094E-01
ZNF563	-0.037	8.211E-01	9.873E-01
MMACHC	-0.037	7.612E-01	8.182E-01
POLR3D	-0.037	7.633E-01	9.636E-01
OR2Z1	-0.037	NA	9.171E-01
MIR519B	-0.037	NA	7.956E-01
DHODH	-0.037	7.443E-01	7.941E-01
BBS1	-0.037	7.910E-01	8.395E-01
ZNF200	-0.037	6.988E-01	8.237E-01
GGTLC2	-0.037	9.680E-01	9.428E-01
TUBB2B	-0.037	9.356E-01	9.431E-01
BCAP31	-0.037	7.832E-01	9.510E-01
ARHGEF7	-0.037	7.645E-01	8.286E-01
AKAP11	-0.037	7.912E-01	9.802E-01

TEX33	-0.037	NA	9.408E-01
SENP1	-0.038	7.295E-01	8.196E-01
FAM217B	-0.038	7.874E-01	8.224E-01
PARP11	-0.038	8.750E-01	8.841E-01
PNRC1	-0.038	8.177E-01	8.772E-01
ZNF654	-0.038	7.874E-01	9.461E-01
RNF146	-0.038	7.046E-01	8.599E-01
SLC7A3	-0.038	9.590E-01	9.454E-01
IQCH	-0.038	8.452E-01	8.628E-01
BLOC1S5	-0.038	7.154E-01	9.039E-01
NUTM2A	-0.038	8.878E-01	8.545E-01
ACTR3	-0.038	7.295E-01	9.024E-01
IER2	-0.038	8.278E-01	9.292E-01
DIDO1	-0.038	6.861E-01	9.287E-01
IMP3	-0.038	7.580E-01	8.251E-01
USP47	-0.038	6.593E-01	9.895E-01
PHF1	-0.038	7.519E-01	9.901E-01
ABCD1	-0.038	8.403E-01	9.911E-01
IKBKAP	-0.038	7.353E-01	9.220E-01
ASCC2	-0.038	8.540E-01	9.924E-01
TRUB1	-0.038	7.628E-01	9.490E-01
YES1	-0.038	7.725E-01	8.439E-01
ZNF664	-0.038	7.778E-01	8.587E-01
RDH11	-0.038	7.585E-01	8.829E-01
PFN2	-0.038	8.590E-01	8.248E-01
RBMX2	-0.038	7.321E-01	8.762E-01
KMT2B	-0.038	7.325E-01	8.621E-01
ZFYVE1	-0.038	6.737E-01	9.852E-01
HNRNPM	-0.038	6.235E-01	8.267E-01
ECSIT	-0.038	7.671E-01	8.795E-01
RAF1	-0.038	8.006E-01	8.770E-01
ELF4	-0.038	7.972E-01	8.787E-01
ANAPC5	-0.038	6.482E-01	9.079E-01
PRAMEF15	-0.038	NA	9.523E-01
PFKFB2	-0.038	8.487E-01	8.638E-01
GLS	-0.038	8.354E-01	9.622E-01
RNF41	-0.038	6.477E-01	8.070E-01
MED12L	-0.038	9.081E-01	8.397E-01
ACAN	-0.038	9.202E-01	9.232E-01
PCSK9	-0.038	9.502E-01	8.643E-01
DCAF4	-0.038	7.502E-01	8.883E-01
LAMTOR1	-0.038	7.405E-01	9.034E-01
PXMP2	-0.038	8.398E-01	8.869E-01
DBNL	-0.038	7.334E-01	9.625E-01
CLDN1	-0.038	9.208E-01	8.878E-01
ZNF821	-0.038	7.981E-01	8.806E-01
RRP12	-0.038	7.653E-01	9.653E-01
MRPL53	-0.039	7.770E-01	8.820E-01
CDK1	-0.039	8.373E-01	9.509E-01
SKIV2L	-0.039	6.545E-01	8.506E-01

CARS2	-0.039	7.446E-01	8.261E-01
USO1	-0.039	7.193E-01	9.223E-01
RFLNB	-0.039	8.775E-01	8.796E-01
SRSF9	-0.039	6.751E-01	9.425E-01
DNAJA1	-0.039	8.062E-01	8.479E-01
GPD2	-0.039	7.939E-01	8.782E-01
TBC1D13	-0.039	7.759E-01	9.231E-01
BRWD3	-0.039	7.868E-01	8.984E-01
UPB1	-0.039	9.152E-01	9.929E-01
DPH2	-0.039	7.283E-01	9.061E-01
UTS2B	-0.039	9.127E-01	8.972E-01
C10orf113	-0.039	9.435E-01	9.538E-01
MTBP	-0.039	8.306E-01	8.625E-01
PEX14	-0.039	6.870E-01	9.660E-01
ARL6IP6	-0.039	7.666E-01	8.913E-01
MIR548AL	-0.039	NA	8.737E-01
AF241726.2	-0.039	NA	8.609E-01
AMY1C	-0.039	NA	8.207E-01
MIR3182	-0.039	NA	8.632E-01
MIR4437	-0.039	NA	9.906E-01
MIR4705	-0.039	NA	8.675E-01
MIR4803	-0.039	NA	8.908E-01
MIR548Z	-0.039	NA	8.852E-01
MIR6771	-0.039	NA	8.660E-01
OR4C5	-0.039	NA	9.579E-01
OR51S1	-0.039	NA	9.517E-01
OR5M3	-0.039	NA	9.102E-01
AC011452.1	-0.039	NA	8.484E-01
AC018709.1	-0.039	NA	9.276E-01
DEFB110	-0.039	NA	8.974E-01
FAM230A	-0.039	NA	8.675E-01
FAM231A	-0.039	NA	9.671E-01
IFNA16	-0.039	NA	9.214E-01
MIR154	-0.039	NA	8.845E-01
MIR3681	-0.039	NA	8.947E-01
MIR412	-0.039	NA	8.788E-01
MIR4276	-0.039	NA	9.570E-01
MIR450A1	-0.039	NA	9.307E-01
MIR496	-0.039	NA	8.800E-01
MIR575	-0.039	NA	9.076E-01
MIR595	-0.039	NA	9.172E-01
MIR6794	-0.039	NA	8.715E-01
MIR6798	-0.039	NA	8.751E-01
MIR6857	-0.039	NA	9.976E-01
OR14A2	-0.039	NA	9.976E-01
OR51F2	-0.039	NA	9.973E-01
OR5K4	-0.039	NA	9.973E-01
PGA4	-0.039	NA	9.973E-01
SLC36A2	-0.039	9.481E-01	9.973E-01
BUD23	-0.039	7.176E-01	9.970E-01

COQ5	-0.039	6.951E-01	9.970E-01
PDE3B	-0.039	8.999E-01	9.968E-01
POLR2B	-0.039	7.084E-01	9.967E-01
U2AF2	-0.039	5.956E-01	9.967E-01
WDR37	-0.039	7.257E-01	8.513E-01
MRGPRX2	-0.039	9.634E-01	9.004E-01
SELENOK	-0.039	7.421E-01	8.884E-01
NME1	-0.039	7.918E-01	9.140E-01
FAM209A	-0.039	8.882E-01	8.831E-01
KIAA1468	-0.039	7.937E-01	9.702E-01
TP53I13	-0.039	8.386E-01	8.660E-01
GNG7	-0.039	9.075E-01	8.892E-01
ALDOA	-0.039	8.032E-01	8.899E-01
NMU	-0.039	9.333E-01	9.238E-01
ANAPC13	-0.039	6.683E-01	8.219E-01
LYRM2	-0.039	6.632E-01	9.226E-01
NAMPT	-0.039	8.759E-01	8.576E-01
NCAPD2	-0.039	8.207E-01	8.863E-01
MIR4778	-0.039	NA	8.522E-01
HS6ST1	-0.039	8.400E-01	8.857E-01
ZNF19	-0.039	8.160E-01	9.473E-01
UGT1A4	-0.039	9.469E-01	8.525E-01
PAFAH1B3	-0.039	8.472E-01	8.490E-01
RPUSD4	-0.039	7.046E-01	8.881E-01
ZNF391	-0.039	8.666E-01	9.110E-01
CSNK2A2	-0.040	6.348E-01	9.768E-01
NDUFAF3	-0.040	7.893E-01	9.531E-01
COPS7B	-0.040	6.492E-01	9.047E-01
FXR2	-0.040	6.882E-01	8.837E-01
SKP1	-0.040	6.410E-01	8.977E-01
LBHD1	-0.040	8.329E-01	8.740E-01
ZNF394	-0.040	6.702E-01	9.654E-01
C21orf2	-0.040	8.202E-01	8.574E-01
ERCC3	-0.040	5.691E-01	8.615E-01
SRM	-0.040	7.789E-01	9.335E-01
MRPS16	-0.040	7.056E-01	8.962E-01
PIK3R3	-0.040	8.475E-01	8.876E-01
APEX1	-0.040	6.657E-01	9.326E-01
CERKL	-0.040	8.933E-01	9.022E-01
PTCH2	-0.040	8.815E-01	9.403E-01
ZNFX1	-0.040	7.883E-01	8.760E-01
VWA2	-0.040	9.311E-01	9.048E-01
ACYP1	-0.040	7.962E-01	9.911E-01
FAM234A	-0.040	7.816E-01	9.916E-01
UBE2I	-0.040	6.324E-01	8.871E-01
ZNF616	-0.040	7.688E-01	8.639E-01
KMT5C	-0.040	8.158E-01	9.928E-01
BOD1L1	-0.040	7.691E-01	9.851E-01
GMEB1	-0.040	6.307E-01	9.407E-01
CXCR5	-0.040	9.424E-01	8.783E-01

NUP93	-0.040	6.961E-01	8.908E-01
ZC3H18	-0.040	6.261E-01	9.135E-01
EMSY	-0.040	7.125E-01	9.177E-01
GOLGA6C	-0.040	NA	9.711E-01
SLC35G4	-0.040	NA	9.411E-01
OR1L4	-0.040	NA	8.806E-01
MIR301A	-0.040	NA	8.833E-01
OR51I2	-0.040	NA	9.728E-01
PYY	-0.040	9.013E-01	9.791E-01
UGT1A10	-0.040	9.600E-01	8.763E-01
MPG	-0.040	7.732E-01	9.510E-01
PSPH	-0.040	8.474E-01	8.921E-01
FAM84B	-0.040	8.541E-01	8.937E-01
FAM58A	-0.040	7.509E-01	9.087E-01
UAP1L1	-0.040	8.600E-01	8.989E-01
CASP3	-0.040	7.122E-01	9.445E-01
FZD2	-0.040	8.861E-01	9.867E-01
CSTF1	-0.040	6.261E-01	9.645E-01
AC093525.2	-0.040	9.296E-01	8.745E-01
VIPAS39	-0.040	6.364E-01	9.651E-01
MED12	-0.040	7.171E-01	9.084E-01
CRYL1	-0.040	8.469E-01	9.087E-01
DNAJA3	-0.040	6.593E-01	9.087E-01
TMEM185B	-0.040	7.671E-01	9.091E-01
SLTM	-0.040	6.717E-01	9.083E-01
CCDC174	-0.040	7.662E-01	9.319E-01
CD4	-0.040	8.999E-01	8.761E-01
ARHGEF15	-0.040	8.632E-01	8.746E-01
PLEKHM2	-0.040	6.142E-01	8.824E-01
RBBP5	-0.040	6.922E-01	9.866E-01
AL049844.3	-0.040	NA	8.842E-01
RB1CC1	-0.041	7.267E-01	9.912E-01
TXNL1	-0.041	6.533E-01	8.984E-01
CEPT1	-0.041	7.161E-01	9.949E-01
PMF1	-0.041	7.357E-01	9.953E-01
UPRT	-0.041	7.135E-01	8.668E-01
ASB6	-0.041	6.637E-01	9.026E-01
ETAA1	-0.041	6.387E-01	8.825E-01
AFDN	-0.041	7.909E-01	9.063E-01
AL449266.1	-0.041	9.400E-01	9.805E-01
SPC24	-0.041	8.448E-01	8.930E-01
RHNO1	-0.041	7.117E-01	8.799E-01
EMG1	-0.041	8.211E-01	9.157E-01
ITGB8	-0.041	9.131E-01	9.937E-01
AAMP	-0.041	6.454E-01	9.874E-01
DAXX	-0.041	6.012E-01	8.933E-01
CENPT	-0.041	7.816E-01	9.770E-01
SPPL2C	-0.041	NA	8.926E-01
CHST14	-0.041	7.589E-01	9.056E-01
IRAK2	-0.041	8.868E-01	9.112E-01

DPP9	-0.041	6.777E-01	9.024E-01
TIMM8A	-0.041	7.782E-01	9.366E-01
DDX19B	-0.041	7.116E-01	9.128E-01
GVQW1	-0.041	8.210E-01	9.002E-01
PHF12	-0.041	7.257E-01	9.275E-01
A4GALT	-0.041	8.755E-01	9.578E-01
TMEM249	-0.041	8.986E-01	9.048E-01
CDC42SE1	-0.041	7.245E-01	9.066E-01
PDE6A	-0.041	9.103E-01	9.442E-01
GINS2	-0.041	8.351E-01	9.458E-01
AC007192.1	-0.041	9.188E-01	9.466E-01
PANK4	-0.041	6.134E-01	9.309E-01
TRA2B	-0.041	5.577E-01	9.899E-01
IL1B	-0.041	9.311E-01	9.076E-01
SHPK	-0.041	7.852E-01	9.780E-01
GTF2B	-0.041	6.784E-01	8.765E-01
SLC7A1	-0.041	8.671E-01	9.242E-01
HTR1F	-0.041	9.270E-01	8.896E-01
C1orf35	-0.041	7.311E-01	8.888E-01
CD81	-0.041	7.624E-01	9.692E-01
MRPS18A	-0.041	7.191E-01	9.127E-01
DRC7	-0.041	9.317E-01	8.939E-01
AC010422.3	-0.041	8.500E-01	9.268E-01
COX19	-0.041	7.131E-01	8.854E-01
MGME1	-0.041	7.125E-01	9.983E-01
UBE2F	-0.041	7.643E-01	9.983E-01
MIR4254	-0.041	NA	9.983E-01
UBXN1	-0.042	7.235E-01	9.983E-01
ZNF579	-0.042	8.433E-01	9.983E-01
FLOT2	-0.042	7.860E-01	9.983E-01
WBP4	-0.042	6.769E-01	9.983E-01
MRPL45	-0.042	7.508E-01	9.983E-01
NSUN5	-0.042	7.715E-01	9.983E-01
AC010619.1	-0.042	NA	9.983E-01
MIR4277	-0.042	NA	9.983E-01
KRTAP10-8	-0.042	NA	9.983E-01
MIR4533	-0.042	NA	9.983E-01
MIR517A	-0.042	NA	9.983E-01
MIR526A1	-0.042	NA	9.983E-01
OR6C4	-0.042	NA	9.983E-01
OR6C68	-0.042	NA	9.983E-01
AL020996.2	-0.042	NA	9.753E-01
ANKRD60	-0.042	NA	9.338E-01
MIR1976	-0.042	NA	9.924E-01
MIR378E	-0.042	NA	9.284E-01
MIR6859-3	-0.042	NA	9.133E-01
OR2T35	-0.042	NA	9.271E-01
OR4K13	-0.042	NA	9.277E-01
MIR4441	-0.042	NA	9.945E-01
USP17L4	-0.042	NA	9.440E-01

TICAM2	-0.042	8.888E-01	9.538E-01
SESTD1	-0.042	8.235E-01	9.127E-01
44080.000	-0.042	8.544E-01	9.093E-01
PPCS	-0.042	6.958E-01	9.860E-01
TRUB2	-0.042	6.958E-01	9.005E-01
IWS1	-0.042	5.597E-01	9.415E-01
PCNX4	-0.042	7.149E-01	9.032E-01
SDHAF3	-0.042	7.864E-01	9.565E-01
PRKRA	-0.042	6.048E-01	9.055E-01
SAT1	-0.042	8.540E-01	9.383E-01
AL354761.2	-0.042	NA	9.189E-01
GABPA	-0.042	6.757E-01	9.693E-01
CDH19	-0.042	9.545E-01	9.221E-01
FAM186B	-0.042	8.005E-01	9.343E-01
CRLS1	-0.042	6.905E-01	9.626E-01
CARM1	-0.042	6.710E-01	9.571E-01
MOAP1	-0.042	7.214E-01	9.842E-01
ADPRM	-0.042	7.396E-01	9.265E-01
ELL	-0.042	5.846E-01	9.601E-01
MYO19	-0.042	7.589E-01	9.939E-01
AIFM2	-0.042	8.286E-01	9.145E-01
CD2BP2	-0.042	6.641E-01	9.236E-01
ZSCAN2	-0.042	8.065E-01	9.267E-01
PDPR	-0.042	8.007E-01	9.814E-01
SLBP	-0.042	7.503E-01	9.681E-01
IDNK	-0.042	8.235E-01	9.132E-01
PSMB3	-0.042	7.779E-01	9.637E-01
FUCA2	-0.042	7.101E-01	9.313E-01
PRKRIP1	-0.042	7.017E-01	9.251E-01
ELANE	-0.042	9.380E-01	9.301E-01
VPS33B	-0.042	5.966E-01	9.227E-01
WRN	-0.042	7.759E-01	9.276E-01
TRAIP	-0.042	8.083E-01	9.924E-01
KNOP1	-0.042	7.224E-01	9.094E-01
ZNF69	-0.042	8.835E-01	9.239E-01
RSF1	-0.042	7.084E-01	9.798E-01
AEN	-0.042	7.597E-01	9.736E-01
SLC22A2	-0.042	9.481E-01	9.875E-01
CCDC192	-0.042	8.495E-01	9.576E-01
MRPL24	-0.043	7.643E-01	9.365E-01
ZNF853	-0.043	8.834E-01	9.582E-01
AASDHPPT	-0.043	7.295E-01	9.231E-01
TSTA3	-0.043	7.729E-01	9.878E-01
CMSS1	-0.043	7.623E-01	9.424E-01
IFIT5	-0.043	8.425E-01	9.699E-01
GTF3C4	-0.043	7.381E-01	9.765E-01
CLUAP1	-0.043	7.887E-01	9.958E-01
TMEM138	-0.043	6.917E-01	9.465E-01
CTTNBP2	-0.043	9.370E-01	9.333E-01
POLR2F	-0.043	8.888E-01	9.966E-01

C5orf22	-0.043	7.480E-01	9.484E-01
CCDC191	-0.043	8.317E-01	9.053E-01
MAJIN	-0.043	9.516E-01	9.174E-01
RCOR2	-0.043	9.030E-01	9.114E-01
ERP44	-0.043	6.826E-01	9.666E-01
ZNF888	-0.043	9.026E-01	9.084E-01
XPO5	-0.043	7.159E-01	9.323E-01
MBNL1	-0.043	7.095E-01	9.742E-01
AL139353.1	-0.043	8.352E-01	9.178E-01
C2orf49	-0.043	5.430E-01	9.886E-01
MIR5095	-0.043	NA	9.542E-01
MIR6885	-0.043	NA	9.795E-01
BCAR1	-0.043	7.754E-01	9.032E-01
MIR6825	-0.043	NA	9.962E-01
OR14J1	-0.043	NA	9.247E-01
DCTN4	-0.043	6.625E-01	9.494E-01
PLBD1	-0.043	8.644E-01	9.428E-01
PALM2	-0.043	9.167E-01	9.721E-01
EML6	-0.043	9.152E-01	9.628E-01
FAM214A	-0.043	8.206E-01	9.604E-01
HSPA4L	-0.043	9.033E-01	9.068E-01
ERI2	-0.043	7.585E-01	9.222E-01
ZNF239	-0.043	8.672E-01	9.963E-01
APEH	-0.043	7.083E-01	8.634E-01
ME2	-0.043	7.364E-01	9.319E-01
STAM2	-0.043	6.398E-01	9.701E-01
NACAD	-0.043	8.969E-01	9.730E-01
NR2C2AP	-0.043	7.473E-01	9.973E-01
PYGL	-0.043	8.668E-01	9.974E-01
FAM173A	-0.043	8.211E-01	9.761E-01
ZNF649	-0.043	8.478E-01	9.838E-01
PLK1	-0.043	8.450E-01	9.942E-01
EFR3A	-0.043	7.430E-01	9.296E-01
ST3GAL4	-0.043	8.890E-01	9.178E-01
CBX8	-0.043	7.875E-01	9.649E-01
KHDRBS1	-0.043	4.755E-01	9.397E-01
SMYD4	-0.044	6.870E-01	9.278E-01
TTL	-0.044	7.598E-01	9.549E-01
BRD2	-0.044	6.156E-01	9.763E-01
POU3F1	-0.044	9.378E-01	9.830E-01
C19orf18	-0.044	9.042E-01	9.560E-01
SDHAF2	-0.044	7.313E-01	9.894E-01
SUPT3H	-0.044	7.579E-01	9.500E-01
CBWD1	-0.044	7.587E-01	9.867E-01
NUDT21	-0.044	6.873E-01	9.514E-01
SYNJ2	-0.044	7.525E-01	9.314E-01
TOM1L2	-0.044	8.182E-01	9.117E-01
JMJD4	-0.044	7.107E-01	9.827E-01
CDPF1	-0.044	7.029E-01	9.408E-01
SUDS3	-0.044	6.779E-01	9.680E-01

G3BP2	-0.044	6.669E-01	9.581E-01
NAE1	-0.044	6.607E-01	9.397E-01
MTMR1	-0.044	7.088E-01	9.512E-01
ZNF627	-0.044	7.493E-01	9.956E-01
GTF2A2	-0.044	6.364E-01	9.534E-01
SLC38A8	-0.044	9.575E-01	9.848E-01
GPR135	-0.044	8.512E-01	9.636E-01
SPATA5L1	-0.044	6.572E-01	9.599E-01
COMMD4	-0.044	7.322E-01	9.681E-01
TMEM196	-0.044	9.711E-01	9.717E-01
FBXW4	-0.044	7.413E-01	9.489E-01
SNX1	-0.044	6.162E-01	9.677E-01
SDHAF4	-0.044	7.695E-01	9.520E-01
BBS7	-0.044	6.551E-01	9.573E-01
SASS6	-0.044	7.674E-01	9.744E-01
SUPV3L1	-0.044	6.127E-01	9.404E-01
ARNT2	-0.044	8.969E-01	9.966E-01
SLC38A6	-0.044	7.328E-01	9.414E-01
PCNT	-0.044	6.936E-01	9.580E-01
NDUFB5	-0.044	6.338E-01	9.662E-01
FAM96B	-0.044	7.124E-01	9.870E-01
PNPLA8	-0.044	7.257E-01	9.768E-01
PLEKHJ1	-0.044	7.678E-01	9.402E-01
AURKB	-0.044	8.377E-01	9.677E-01
CARMIL1	-0.044	8.341E-01	9.545E-01
LRRC37A2	-0.044	8.172E-01	9.960E-01
ZNF594	-0.044	8.034E-01	9.990E-01
DRAM2	-0.044	6.321E-01	9.990E-01
POLD2	-0.044	7.627E-01	9.990E-01
APLN	-0.044	9.103E-01	9.990E-01
IRF2BP1	-0.044	6.882E-01	9.990E-01
ARMC1	-0.044	6.315E-01	9.990E-01
SLK	-0.044	7.599E-01	9.990E-01
NSMCE3	-0.044	7.073E-01	9.990E-01
MXD1	-0.044	8.590E-01	9.990E-01
MAK16	-0.044	7.107E-01	9.990E-01
MAFB	-0.044	8.880E-01	9.990E-01
RIPK1	-0.044	6.429E-01	9.990E-01
EIF2D	-0.044	7.084E-01	9.990E-01
MAD2L1BP	-0.044	6.848E-01	9.990E-01
ZSCAN16	-0.044	8.384E-01	9.990E-01
DUS2	-0.044	7.564E-01	9.990E-01
CIAPIN1	-0.044	6.314E-01	9.990E-01
MIR626	-0.044	NA	9.990E-01
MIR5704	-0.044	NA	9.990E-01
MIR8061	-0.044	NA	9.990E-01
MIR190B	-0.044	NA	9.990E-01
MIR6074	-0.044	NA	9.990E-01
MIR1250	-0.044	NA	9.990E-01
DPF2	-0.044	5.559E-01	9.990E-01

RYR3	-0.044	8.542E-01	9.990E-01
ZNF230	-0.044	7.382E-01	9.990E-01
MIIP	-0.044	7.722E-01	9.990E-01
FAM126B	-0.044	7.214E-01	9.990E-01
CELF6	-0.044	9.358E-01	9.836E-01
ATP5H	-0.044	6.708E-01	9.484E-01
MRPL51	-0.045	7.085E-01	9.850E-01
LILRA2	-0.045	8.997E-01	9.894E-01
CLTB	-0.045	8.345E-01	9.578E-01
POLR2H	-0.045	6.805E-01	9.957E-01
C12orf4	-0.045	6.374E-01	9.523E-01
HDAC4	-0.045	7.918E-01	9.605E-01
NUTF2	-0.045	6.915E-01	9.747E-01
MIR6877	-0.045	NA	9.963E-01
MYEOV	-0.045	9.400E-01	9.693E-01
CFAP73	-0.045	8.755E-01	9.818E-01
IFT81	-0.045	7.339E-01	9.872E-01
COG3	-0.045	7.161E-01	9.888E-01
SIGLEC15	-0.045	9.241E-01	9.615E-01
RPS6KB1	-0.045	6.262E-01	9.495E-01
FANCA	-0.045	8.055E-01	9.441E-01
PADI2	-0.045	9.060E-01	9.701E-01
NXN	-0.045	8.823E-01	9.849E-01
RX5-TAX1B	-0.045	8.034E-01	9.664E-01
TTF2	-0.045	7.421E-01	9.841E-01
MIB1	-0.045	7.422E-01	9.773E-01
DPYSL4	-0.045	9.344E-01	9.411E-01
ARHGAP30	-0.045	8.559E-01	9.782E-01
OSBPL1A	-0.045	8.345E-01	9.646E-01
COX15	-0.045	5.984E-01	9.824E-01
SRP72	-0.045	5.804E-01	9.482E-01
LRCH1	-0.045	7.301E-01	9.650E-01
INTS3	-0.045	7.644E-01	9.714E-01
DNAJC19	-0.045	6.578E-01	9.687E-01
COMMD3	-0.045	7.257E-01	9.883E-01
VTA1	-0.045	5.965E-01	9.561E-01
KANSL3	-0.045	5.923E-01	9.868E-01
OSBPL6	-0.045	9.218E-01	9.626E-01
C16orf72	-0.045	7.244E-01	9.746E-01
ATG16L1	-0.045	6.257E-01	9.898E-01
ALG12	-0.045	6.571E-01	9.881E-01
FIBP	-0.045	7.211E-01	9.708E-01
FUT4	-0.045	8.622E-01	9.676E-01
TCEANC2	-0.045	6.377E-01	9.979E-01
KNTC1	-0.045	7.761E-01	9.886E-01
IMPACT	-0.045	7.301E-01	9.939E-01
CASC1	-0.045	8.762E-01	9.821E-01
LRRC4C	-0.045	9.246E-01	9.553E-01
GLTPD2	-0.045	8.475E-01	9.625E-01
PITX2	-0.045	9.035E-01	9.590E-01

WBP11	-0.046	6.190E-01	9.640E-01
TRIM51	-0.046	NA	9.707E-01
MIR597	-0.046	NA	9.886E-01
RESP18	-0.046	NA	9.830E-01
MIR8086	-0.046	NA	9.617E-01
CCDC93	-0.046	6.886E-01	9.722E-01
EXOC5	-0.046	6.848E-01	9.764E-01
RAP2A	-0.046	7.275E-01	9.891E-01
VPS39	-0.046	5.735E-01	9.873E-01
PARP12	-0.046	8.189E-01	9.649E-01
KCNK4	-0.046	9.489E-01	9.606E-01
TCAIM	-0.046	7.321E-01	9.775E-01
CUL3	-0.046	6.324E-01	9.725E-01
SALL1	-0.046	9.133E-01	9.895E-01
CHRAC1	-0.046	6.511E-01	9.682E-01
NFRKB	-0.046	6.634E-01	9.602E-01
AC105001.2	-0.046	NA	9.750E-01
CENPC	-0.046	6.364E-01	9.819E-01
PTER	-0.046	7.719E-01	9.941E-01
TTC17	-0.046	6.245E-01	9.746E-01
PDE12	-0.046	6.262E-01	9.960E-01
TMEM121	-0.046	8.755E-01	9.636E-01
ORC4	-0.046	5.659E-01	9.970E-01
TPI1	-0.046	7.227E-01	9.847E-01
WDR73	-0.046	6.736E-01	9.899E-01
HIST1H4B	-0.046	9.128E-01	9.992E-01
MAF1	-0.046	6.321E-01	9.990E-01
LYRM9	-0.046	8.386E-01	9.990E-01
SHC2	-0.046	8.880E-01	9.989E-01
DCTN5	-0.046	6.058E-01	9.989E-01
RAPGEF1	-0.046	7.595E-01	9.926E-01
AC072022.1	-0.046	8.671E-01	9.912E-01
PTCH1	-0.046	8.154E-01	9.755E-01
DHX34	-0.046	7.063E-01	9.924E-01
SBF2	-0.046	7.315E-01	9.669E-01
ZNF549	-0.046	7.879E-01	9.829E-01
S100A3	-0.046	8.997E-01	9.853E-01
WRAP73	-0.046	6.410E-01	9.775E-01
CENPM	-0.046	8.217E-01	9.883E-01
PAK1IP1	-0.046	6.751E-01	9.802E-01
TTBK2	-0.046	6.787E-01	9.926E-01
CCDC117	-0.046	8.211E-01	9.929E-01
FRG1	-0.046	6.748E-01	9.933E-01
ZNF510	-0.046	6.620E-01	9.742E-01
BOLA3	-0.046	7.152E-01	9.913E-01
PSMD5	-0.046	7.250E-01	9.934E-01
ARHGAP17	-0.046	6.543E-01	9.848E-01
SPANXN1	-0.046	NA	9.805E-01
ACTR3B	-0.046	7.702E-01	9.821E-01
APOC4	-0.046	NA	9.783E-01

LTK	-0.046	9.208E-01	9.812E-01
ARL6	-0.046	6.714E-01	9.906E-01
PIP4K2B	-0.046	6.549E-01	9.827E-01
GRM1	-0.047	9.133E-01	9.937E-01
EPHA8	-0.047	9.436E-01	9.966E-01
THUMPD1	-0.047	6.416E-01	9.789E-01
FAM173B	-0.047	7.322E-01	9.980E-01
CINP	-0.047	6.933E-01	9.842E-01
PDHA1	-0.047	6.822E-01	9.858E-01
COX6C	-0.047	8.080E-01	9.830E-01
CEP78	-0.047	7.222E-01	9.798E-01
UBOX5	-0.047	6.471E-01	9.806E-01
STOML2	-0.047	7.280E-01	9.841E-01
LSM3	-0.047	7.433E-01	9.916E-01
MED6	-0.047	6.176E-01	9.974E-01
PPP4R2	-0.047	6.708E-01	9.842E-01
UBL7	-0.047	6.364E-01	9.798E-01
ACTRT3	-0.047	8.600E-01	9.911E-01
CLSTN3	-0.047	7.918E-01	9.960E-01
STARD5	-0.047	8.511E-01	9.869E-01
CCDC166	-0.047	9.473E-01	9.938E-01
KXD1	-0.047	5.140E-01	9.806E-01
ZNF622	-0.047	7.066E-01	9.904E-01
TSN	-0.047	5.690E-01	9.973E-01
C16orf52	-0.047	6.417E-01	9.974E-01
KRTAP10-9	-0.047	NA	9.861E-01
MIR887	-0.047	NA	9.881E-01
AC012254.2	-0.047	NA	9.932E-01
TAAR8	-0.047	NA	9.869E-01
MIR361	-0.047	NA	9.907E-01
OR13G1	-0.047	NA	9.880E-01
MIR5706	-0.047	NA	9.869E-01
OR4K1	-0.047	NA	9.933E-01
AMER1	-0.047	7.759E-01	9.898E-01
FAM160B1	-0.047	6.606E-01	9.862E-01
GRAMD1A	-0.047	7.902E-01	9.877E-01
TNRC6B	-0.047	6.410E-01	9.905E-01
ARV1	-0.047	6.609E-01	9.901E-01
AHCY	-0.047	7.151E-01	9.913E-01
KLHL42	-0.047	7.020E-01	9.866E-01
GAS2L1	-0.047	7.738E-01	9.935E-01
AC011499.1	-0.047	9.105E-01	9.883E-01
MIR559	-0.047	9.207E-01	9.900E-01
ZNF707	-0.047	6.549E-01	9.890E-01
C10orf120	-0.047	NA	9.909E-01
MED20	-0.047	6.234E-01	9.941E-01
SPACA7	-0.047	NA	9.947E-01
BID	-0.047	7.442E-01	9.918E-01
OAZ1	-0.048	6.823E-01	9.964E-01
NXPH1	-0.048	9.458E-01	9.914E-01

ZNF789	-0.048	7.519E-01	9.918E-01
MIR128-1	-0.048	NA	9.964E-01
SPG11	-0.048	6.647E-01	9.916E-01
INTS4	-0.048	6.513E-01	9.912E-01
PPP1R37	-0.048	8.059E-01	9.930E-01
AL096870.1	-0.048	9.192E-01	9.925E-01
MRC1	-0.048	9.127E-01	9.903E-01
TFIP11	-0.048	6.324E-01	9.927E-01
SNX13	-0.048	7.001E-01	9.965E-01
KMT5B	-0.048	6.594E-01	9.940E-01
LSM5	-0.048	6.616E-01	9.987E-01
PTP4A1	-0.048	8.265E-01	9.959E-01
CFAP97	-0.048	7.014E-01	9.973E-01
IL4R	-0.048	8.032E-01	9.958E-01
RBMXL2	-0.048	9.370E-01	9.950E-01
ZNF177	-0.048	9.245E-01	9.987E-01
ZNF737	-0.048	9.133E-01	9.988E-01
GABPB1	-0.048	4.407E-01	9.998E-01
PIAS2	-0.048	7.285E-01	9.953E-01
CEP97	-0.048	7.093E-01	9.988E-01
RWDD2A	-0.048	7.198E-01	9.981E-01
NHLRC2	-0.048	5.957E-01	9.988E-01
CLDN2	-0.048	9.003E-01	9.969E-01
SCFD1	-0.048	5.344E-01	9.995E-01
NAPB	-0.048	6.777E-01	9.993E-01
DDX41	-0.048	5.508E-01	9.994E-01
GGNBP2	-0.048	5.241E-01	9.996E-01
FANCD2	-0.048	7.738E-01	9.997E-01
LHPP	-0.048	7.827E-01	9.999E-01
FOXRED1	-0.048	6.879E-01	1.000E+00
IL20RB	-0.048	9.296E-01	1.000E+00
SRI	-0.048	7.292E-01	9.993E-01
LYL1	-0.048	8.502E-01	9.999E-01
LYPLA1	-0.048	7.418E-01	9.992E-01
OR10P1	-0.048	NA	9.990E-01
MIR6792	-0.048	NA	9.979E-01
MIA3	-0.048	5.792E-01	9.979E-01
HPDL	-0.048	9.153E-01	9.970E-01
TRAPPC12	-0.048	5.601E-01	9.975E-01
KIF13A	-0.048	7.671E-01	9.977E-01
DDX46	-0.048	5.804E-01	9.995E-01
KIAA0100	-0.048	7.010E-01	9.965E-01
ARL2	-0.048	7.572E-01	9.965E-01
PLCB3	-0.048	6.847E-01	9.985E-01
ZC3H15	-0.048	5.975E-01	9.948E-01
EMC8	-0.048	5.867E-01	9.964E-01
RAB35	-0.048	5.331E-01	9.949E-01
KLK1	-0.048	9.251E-01	9.936E-01
C8orf58	-0.049	7.494E-01	9.985E-01
DAB1	-0.049	9.435E-01	9.962E-01

WDR1	-0.049	6.156E-01	9.983E-01
ZC3H11A	-0.049	5.460E-01	9.953E-01
NDUFS3	-0.049	6.469E-01	9.972E-01
SLCO4A1	-0.049	9.189E-01	9.909E-01
MIR765	-0.049	8.854E-01	9.956E-01
C12orf65	-0.049	5.859E-01	9.975E-01
AC140504.1	-0.049	NA	9.933E-01
CXorf51B	-0.049	NA	9.955E-01
DEFB107A	-0.049	NA	9.934E-01
MIR4429	-0.049	NA	9.927E-01
MIR4528	-0.049	NA	9.998E-01
MIR488	-0.049	NA	9.998E-01
MIR548AE2	-0.049	NA	9.998E-01
MIR548AP	-0.049	NA	9.998E-01
MIR548H2	-0.049	NA	9.997E-01
MIR6515	-0.049	NA	9.997E-01
MIR6724-1	-0.049	NA	9.997E-01
PRR23D1	-0.049	NA	9.914E-01
TP53TG3B	-0.049	NA	9.941E-01
AC010615.4	-0.049	NA	9.938E-01
AC073612.1	-0.049	NA	9.932E-01
AL929554.1	-0.049	NA	9.899E-01
CFC1B	-0.049	NA	9.899E-01
CT45A9	-0.049	NA	9.849E-01
GIMD1	-0.049	NA	9.936E-01
MIR105-2	-0.049	NA	9.853E-01
MIR15B	-0.049	NA	9.867E-01
MIR2113	-0.049	NA	9.991E-01
MIR3152	-0.049	NA	9.909E-01
MIR3179-3	-0.049	NA	9.886E-01
MIR422A	-0.049	NA	9.868E-01
MIR4791	-0.049	NA	9.847E-01
MIR520D	-0.049	NA	9.892E-01
MIR532	-0.049	NA	9.929E-01
MIR548F5	-0.049	NA	9.840E-01
MIR7151	-0.049	NA	9.861E-01
MIR888	-0.049	NA	9.857E-01
OR4K5	-0.049	NA	9.941E-01
TRIM49D2	-0.049	NA	9.873E-01
AC046185.1	-0.049	NA	9.922E-01
AC083800.1	-0.049	NA	9.854E-01
ARL14EPL	-0.049	NA	9.835E-01
CU633980.1	-0.049	NA	9.951E-01
ELOA3	-0.049	NA	9.774E-01
ELOA3C	-0.049	NA	9.922E-01
GAGE12E	-0.049	NA	9.815E-01
KRTAP20-3	-0.049	NA	9.861E-01
KRTAP25-1	-0.049	NA	9.989E-01
MIR100	-0.049	NA	9.959E-01
MIR30D	-0.049	NA	9.863E-01

MIR3156-2	-0.049	NA	9.861E-01
MIR3167	-0.049	NA	9.877E-01
MIR380	-0.049	NA	9.978E-01
MIR381	-0.049	NA	9.846E-01
MIR411	-0.049	NA	9.841E-01
MIR4283-2	-0.049	NA	9.804E-01
MIR4327	-0.049	NA	9.954E-01
MIR449B	-0.049	NA	9.790E-01
MIR485	-0.049	NA	9.963E-01
MIR516A2	-0.049	NA	9.855E-01
MIR518A2	-0.049	NA	9.830E-01
MIR520A	-0.049	NA	9.747E-01
MIR520B	-0.049	NA	9.825E-01
MIR544A	-0.049	NA	9.794E-01
MIR548AS	-0.049	NA	9.917E-01
MIR5702	-0.049	NA	9.719E-01
MIR5708	-0.049	NA	9.775E-01
MIR6082	-0.049	NA	9.760E-01
MIR654	-0.049	NA	9.981E-01
MIR6760	-0.049	NA	9.794E-01
MIR764	-0.049	NA	9.709E-01
MIR891B	-0.049	NA	9.733E-01
OR4A15	-0.049	NA	9.853E-01
OR5L2	-0.049	NA	9.790E-01
USP17L3	-0.049	NA	9.764E-01
USP17L8	-0.049	NA	9.867E-01
ZNF705B	-0.049	NA	9.714E-01
PTGFRN	-0.049	8.265E-01	9.761E-01
PRB3	-0.049	8.870E-01	9.872E-01
OR2D2	-0.049	NA	9.678E-01
PHC1	-0.049	7.644E-01	9.686E-01
RNASEK	-0.049	6.384E-01	9.881E-01
DDX24	-0.049	6.147E-01	9.773E-01
TPD52	-0.049	7.812E-01	9.968E-01
TIMELESS	-0.049	7.300E-01	9.911E-01
LYRM4	-0.049	7.128E-01	9.866E-01
PHF6	-0.049	7.225E-01	9.884E-01
ASIC1	-0.049	8.868E-01	9.796E-01
MIR6746	-0.049	9.448E-01	9.896E-01
CEP164	-0.049	6.061E-01	9.714E-01
DYDC2	-0.049	9.400E-01	9.673E-01
MIR4507	-0.049	NA	9.698E-01
PSMB7	-0.049	7.120E-01	9.592E-01
SLC25A45	-0.049	8.090E-01	9.543E-01
ZNRF4	-0.049	NA	9.618E-01
ONECUT2	-0.049	9.306E-01	9.868E-01
CCDC70	-0.049	9.536E-01	9.760E-01
ATP5B	-0.049	6.236E-01	9.620E-01
ATN1	-0.049	5.975E-01	9.654E-01
KRBA1	-0.049	7.354E-01	9.658E-01

PLXNA2	-0.049	8.153E-01	9.589E-01
FZD6	-0.049	8.069E-01	9.717E-01
NSL1	-0.049	5.759E-01	9.793E-01
ZNF81	-0.049	7.357E-01	9.687E-01
TMEM200A	-0.049	8.861E-01	9.735E-01
MIR5691	-0.049	NA	9.822E-01
CSE1L	-0.049	6.708E-01	9.981E-01
NR3C1	-0.049	8.513E-01	9.986E-01
GALNT1	-0.049	8.199E-01	9.779E-01
FLI1	-0.050	8.449E-01	9.680E-01
LUZP1	-0.050	7.095E-01	9.828E-01
PKNOX1	-0.050	5.434E-01	9.654E-01
C14orf119	-0.050	5.650E-01	9.844E-01
TGFBR2	-0.050	8.031E-01	9.765E-01
PPM1A	-0.050	5.695E-01	9.625E-01
SLITRK2	-0.050	9.290E-01	9.971E-01
KIAA0040	-0.050	8.069E-01	9.541E-01
SRD5A1	-0.050	8.272E-01	9.742E-01
MCM5	-0.050	7.595E-01	9.853E-01
GNG2	-0.050	8.656E-01	9.902E-01
RIPPLY1	-0.050	9.041E-01	9.804E-01
DBN1	-0.050	8.414E-01	9.587E-01
MAP3K2	-0.050	5.851E-01	9.672E-01
UTP23	-0.050	6.470E-01	9.694E-01
ZNF275	-0.050	6.949E-01	9.830E-01
PSMD9	-0.050	6.100E-01	9.757E-01
DUT	-0.050	6.176E-01	9.837E-01
TOGARAM1	-0.050	6.640E-01	9.694E-01
POP5	-0.050	6.847E-01	9.861E-01
TRIM64C	-0.050	NA	9.564E-01
OR10A5	-0.050	NA	9.945E-01
OOSP2	-0.050	NA	9.497E-01
PRAMEF6	-0.050	NA	9.863E-01
TLK1	-0.050	6.008E-01	9.813E-01
TRIM2	-0.050	8.519E-01	9.569E-01
CERS5	-0.050	5.829E-01	9.493E-01
ABHD10	-0.050	5.759E-01	9.529E-01
IL23A	-0.050	8.863E-01	9.835E-01
KMT5A	-0.050	5.659E-01	9.792E-01
ATP1B4	-0.050	9.448E-01	9.650E-01
KLF16	-0.050	7.071E-01	9.599E-01
REPIN1	-0.050	7.442E-01	9.552E-01
NPHP1	-0.050	7.800E-01	9.691E-01
ELOF1	-0.050	6.363E-01	9.503E-01
FGFRL1	-0.050	8.369E-01	9.772E-01
CTDSP2	-0.050	6.988E-01	9.489E-01
FGD4	-0.050	7.722E-01	9.473E-01
SAP30BP	-0.050	5.373E-01	9.736E-01
UBALD2	-0.050	7.685E-01	9.761E-01
KLHDC7B	-0.050	9.274E-01	9.735E-01

CNGA2	-0.050	NA	9.991E-01
MRPL47	-0.050	6.116E-01	9.991E-01
PLP2	-0.050	8.108E-01	9.990E-01
MTHFD1	-0.050	6.776E-01	9.989E-01
MBTD1	-0.050	7.538E-01	9.989E-01
AMMECR1	-0.050	7.617E-01	9.989E-01
KLHL12	-0.050	5.820E-01	9.989E-01
FEN1	-0.050	7.860E-01	9.989E-01
MADCAM1	-0.050	8.533E-01	9.506E-01
ZBTB14	-0.050	5.312E-01	9.494E-01
PLEKHG2	-0.050	7.912E-01	9.750E-01
APOL4	-0.050	8.873E-01	9.485E-01
CAPN12	-0.051	8.570E-01	9.775E-01
ZSCAN5C	-0.051	9.081E-01	9.514E-01
RITA1	-0.051	6.410E-01	9.456E-01
MIR6499	-0.051	NA	9.857E-01
AURKAIP1	-0.051	7.317E-01	9.433E-01
RP1	-0.051	9.344E-01	9.736E-01
TMEM80	-0.051	7.193E-01	9.822E-01
TMEM117	-0.051	7.915E-01	9.777E-01
MIR6833	-0.051	NA	9.266E-01
GIPC1	-0.051	7.172E-01	9.514E-01
MAGOHB	-0.051	6.637E-01	9.340E-01
BEST1	-0.051	8.259E-01	9.370E-01
EDN3	-0.051	9.618E-01	9.597E-01
THAP9	-0.051	6.582E-01	9.864E-01
ELAVL4	-0.051	9.095E-01	9.413E-01
AGAP3	-0.051	7.289E-01	9.561E-01
SAP130	-0.051	5.864E-01	9.790E-01
ADAP2	-0.051	8.448E-01	9.480E-01
SUGT1	-0.051	5.809E-01	9.511E-01
MIR548C	-0.051	NA	9.547E-01
PLRG1	-0.051	5.689E-01	9.592E-01
RBMS2	-0.051	7.294E-01	9.411E-01
AP002360.1	-0.051	8.058E-01	9.851E-01
TTC23	-0.051	6.885E-01	9.799E-01
ASB8	-0.051	5.693E-01	9.396E-01
TRIM33	-0.051	6.787E-01	9.760E-01
ZNF484	-0.051	6.826E-01	9.386E-01
EGFLAM	-0.051	8.446E-01	9.815E-01
RFX1	-0.051	4.375E-01	9.328E-01
HRH2	-0.051	9.009E-01	9.547E-01
MIR3941	-0.051	9.436E-01	9.346E-01
CSTF3	-0.051	5.711E-01	9.680E-01
MGEA5	-0.051	5.533E-01	9.746E-01
STYX	-0.051	6.209E-01	9.792E-01
FAM160A1	-0.051	8.363E-01	9.416E-01
FBXO22	-0.051	5.804E-01	9.396E-01
TMEM236	-0.051	8.765E-01	9.403E-01
IKZF5	-0.051	6.245E-01	9.860E-01

NFS1	-0.051	5.942E-01	9.508E-01
OSBPL8	-0.051	7.037E-01	9.781E-01
GCC2	-0.051	6.826E-01	9.517E-01
SEMA6C	-0.051	8.289E-01	9.308E-01
EIF6	-0.051	6.508E-01	9.770E-01
MIR3689F	-0.051	NA	9.773E-01
DEFB104A	-0.051	NA	9.444E-01
HIST1H2AA	-0.051	NA	9.862E-01
KRTAP19-7	-0.051	NA	9.399E-01
MIR527	-0.051	NA	9.437E-01
MIR9-1	-0.051	NA	9.807E-01
AL355315.1	-0.051	NA	9.876E-01
ELOA3D	-0.051	NA	9.478E-01
MIR1193	-0.051	NA	9.714E-01
MIR1323	-0.051	NA	9.695E-01
MIR2681	-0.051	NA	9.466E-01
MIR337	-0.051	NA	9.492E-01
MIR4270	-0.051	NA	9.618E-01
MIR4310	-0.051	NA	9.414E-01
MIR4425	-0.051	NA	9.454E-01
MIR4438	-0.051	NA	9.855E-01
MIR4466	-0.051	NA	9.360E-01
MIR4490	-0.051	NA	9.797E-01
MIR4693	-0.051	NA	9.333E-01
MIR4747	-0.051	NA	9.834E-01
MIR494	-0.051	NA	9.569E-01
MIR5707	-0.051	NA	9.754E-01
MIR6769B	-0.051	NA	9.338E-01
OR10H4	-0.051	NA	9.839E-01
OR10J5	-0.051	NA	9.502E-01
OR2T6	-0.051	NA	9.774E-01
C21orf140	-0.051	NA	9.660E-01
MIR6749	-0.051	NA	9.285E-01
MIR6811	-0.051	NA	9.338E-01
MIR7-2	-0.051	NA	8.953E-01
OR5M9	-0.051	NA	9.267E-01
IL1A	-0.052	9.207E-01	9.357E-01
PLEKHG1	-0.052	7.855E-01	9.417E-01
S100A1	-0.052	8.429E-01	9.706E-01
POLG	-0.052	5.516E-01	9.407E-01
BTBD17	-0.052	9.460E-01	9.772E-01
MAP2	-0.052	9.095E-01	9.309E-01
FIZ1	-0.052	6.533E-01	9.140E-01
ATG4C	-0.052	5.585E-01	9.169E-01
NCOR2	-0.052	6.848E-01	9.272E-01
LRSAM1	-0.052	6.324E-01	9.439E-01
LURAP1	-0.052	8.604E-01	9.125E-01
ZNF253	-0.052	8.071E-01	9.319E-01
C1QC	-0.052	9.009E-01	9.311E-01
BRCA2	-0.052	7.856E-01	9.359E-01

IGSF22	-0.052	8.340E-01	9.843E-01
MSI1	-0.052	9.192E-01	9.776E-01
UCKL1	-0.052	6.419E-01	9.903E-01
TYW3	-0.052	6.783E-01	9.479E-01
ZCCHC18	-0.052	8.765E-01	9.624E-01
C3orf38	-0.052	6.309E-01	9.966E-01
ORC1	-0.052	8.049E-01	9.967E-01
PRKAA2	-0.052	9.053E-01	9.967E-01
DYNC1I2	-0.052	5.699E-01	9.371E-01
TTYH2	-0.052	8.693E-01	9.674E-01
ZNF799	-0.052	7.918E-01	9.341E-01
TBC1D9B	-0.052	5.342E-01	9.417E-01
LRCH3	-0.052	4.837E-01	9.439E-01
KLHL18	-0.052	5.442E-01	9.513E-01
NUDT9	-0.052	6.743E-01	9.225E-01
PHP3-ACAD	-0.052	8.791E-01	9.324E-01
MOB4	-0.052	5.402E-01	9.673E-01
TAB1	-0.052	5.415E-01	9.151E-01
NECTIN3	-0.052	8.806E-01	9.418E-01
RWDD4	-0.052	5.026E-01	9.100E-01
AC104151.1	-0.052	NA	9.534E-01
TRIP6	-0.052	7.402E-01	9.563E-01
TSPAN18	-0.053	8.688E-01	9.078E-01
DENR	-0.053	4.979E-01	9.457E-01
CHMP6	-0.053	6.861E-01	9.097E-01
ORCS7-ASM	-0.053	NA	9.405E-01
AC007731.5	-0.053	NA	9.091E-01
GTPBP2	-0.053	6.543E-01	8.923E-01
POM121C	-0.053	5.133E-01	9.816E-01
SPCS3	-0.053	5.552E-01	9.568E-01
PMS2	-0.053	6.574E-01	9.267E-01
MIR4515	-0.053	NA	9.850E-01
OR5B2	-0.053	NA	9.215E-01
MIR519A1	-0.053	NA	9.392E-01
MMP7	-0.053	9.246E-01	9.072E-01
RUVBL2	-0.053	6.324E-01	9.321E-01
LGMN	-0.053	7.626E-01	9.200E-01
EIF5	-0.053	5.706E-01	9.767E-01
SGK1	-0.053	8.701E-01	9.324E-01
COX8A	-0.053	6.278E-01	9.787E-01
AP1AR	-0.053	6.641E-01	8.967E-01
MIS18BP1	-0.053	6.664E-01	9.185E-01
DCAF7	-0.053	5.815E-01	9.041E-01
ZDHHC22	-0.053	9.202E-01	9.347E-01
BRD7	-0.053	5.012E-01	9.398E-01
BAIAP2L1	-0.053	7.250E-01	9.284E-01
DDX31	-0.053	6.100E-01	9.097E-01
GSS	-0.053	5.970E-01	9.119E-01
PPP2R5E	-0.053	5.191E-01	9.484E-01
NDUFAF7	-0.053	4.705E-01	9.290E-01

TECPR2	-0.053	5.696E-01	9.390E-01
ACSS1	-0.053	8.292E-01	9.160E-01
STRIP1	-0.053	4.881E-01	9.121E-01
C18orf54	-0.053	7.585E-01	9.090E-01
NIPSNAP3B	-0.053	7.959E-01	9.045E-01
IQCK	-0.053	7.503E-01	9.935E-01
EEF1E1	-0.053	6.622E-01	9.983E-01
MLST8	-0.053	5.749E-01	9.983E-01
WDR20	-0.053	4.582E-01	9.983E-01
ETFBKMT	-0.053	6.471E-01	9.983E-01
MIR4513	-0.053	NA	9.983E-01
ZNF133	-0.053	6.663E-01	9.983E-01
ZNF480	-0.053	7.151E-01	9.983E-01
BMP2	-0.053	8.997E-01	9.983E-01
C8orf48	-0.053	8.853E-01	9.983E-01
PON2	-0.053	7.575E-01	9.983E-01
TLE3	-0.053	7.167E-01	9.983E-01
TTC13	-0.053	6.262E-01	9.983E-01
PCK2	-0.053	7.364E-01	9.983E-01
MIB2	-0.053	6.628E-01	9.983E-01
MEN1	-0.053	5.425E-01	9.983E-01
CTSB	-0.053	8.211E-01	9.227E-01
MTIF3	-0.053	6.314E-01	9.588E-01
TTK	-0.053	7.879E-01	9.317E-01
ANKMY1	-0.054	6.748E-01	9.153E-01
ANKRD55	-0.054	9.023E-01	9.464E-01
MIR320D1	-0.054	NA	9.196E-01
UBTFL1	-0.054	NA	9.602E-01
CXorf40B	-0.054	6.465E-01	9.139E-01
PDE8A	-0.054	6.632E-01	9.368E-01
RAC1	-0.054	5.754E-01	9.075E-01
SNAP23	-0.054	5.415E-01	9.448E-01
LTC4S	-0.054	8.966E-01	9.673E-01
ZKSCAN4	-0.054	5.769E-01	9.802E-01
PTOV1	-0.054	6.571E-01	9.185E-01
PAPOLA	-0.054	4.803E-01	9.434E-01
C14orf180	-0.054	9.590E-01	9.199E-01
MIR4677	-0.054	8.936E-01	9.270E-01
FLG2	-0.054	9.518E-01	9.061E-01
ZNF746	-0.054	5.668E-01	9.596E-01
ZFP41	-0.054	7.635E-01	9.089E-01
KCNN3	-0.054	8.369E-01	9.579E-01
RBM4	-0.054	5.305E-01	9.056E-01
SLC25A12	-0.054	6.813E-01	8.964E-01
DNAJB2	-0.054	6.632E-01	9.419E-01
PRCP	-0.054	7.227E-01	9.290E-01
BLNK	-0.054	8.477E-01	9.486E-01
KCNK5	-0.054	8.632E-01	9.084E-01
CYGB	-0.054	8.374E-01	9.787E-01
UXT	-0.054	6.387E-01	9.587E-01

FCGRT	-0.054	7.904E-01	9.446E-01
KYAT3	-0.054	6.089E-01	9.752E-01
SUOX	-0.054	7.805E-01	9.408E-01
DDX52	-0.054	6.048E-01	8.836E-01
UBE2G2	-0.054	5.380E-01	9.553E-01
SOX11	-0.054	8.966E-01	9.423E-01
OR4N5	-0.054	NA	9.601E-01
MIR6839	-0.054	NA	9.480E-01
MIR1227	-0.054	NA	8.689E-01
MIR6786	-0.054	NA	9.613E-01
MIR620	-0.054	NA	8.823E-01
OR4Q3	-0.054	NA	9.820E-01
PRNT	-0.054	NA	9.512E-01
MIR320C2	-0.054	NA	9.767E-01
KRTAP20-1	-0.054	NA	9.258E-01
MIR3144	-0.054	NA	9.291E-01
IFNA7	-0.054	NA	9.604E-01
MIR3973	-0.054	NA	9.180E-01
OR2F2	-0.054	NA	9.117E-01
SPANXN4	-0.054	NA	8.895E-01
MIR525	-0.054	NA	9.129E-01
SDR39U1	-0.054	6.288E-01	9.037E-01
EPS8	-0.054	8.160E-01	9.204E-01
TRMT61A	-0.054	6.776E-01	9.229E-01
DHX29	-0.054	5.985E-01	9.084E-01
ANAPC16	-0.054	5.887E-01	9.560E-01
AIP	-0.055	6.507E-01	9.568E-01
PDHB	-0.055	5.315E-01	9.032E-01
TRAP1	-0.055	6.638E-01	9.721E-01
USP50	-0.055	8.773E-01	8.768E-01
FZD4	-0.055	8.195E-01	9.918E-01
43894.000	-0.055	9.244E-01	9.595E-01
LMF2	-0.055	6.444E-01	9.966E-01
SALL4	-0.055	8.759E-01	9.961E-01
MTURN	-0.055	7.710E-01	9.961E-01
ZFY	-0.055	9.334E-01	9.961E-01
PLEKHA6	-0.055	8.421E-01	9.954E-01
KDM8	-0.055	6.118E-01	9.956E-01
GYPB	-0.055	NA	9.956E-01
LRRC61	-0.055	7.963E-01	9.957E-01
CHERP	-0.055	5.024E-01	8.951E-01
HMGXB4	-0.055	6.348E-01	9.732E-01
PANX2	-0.055	8.858E-01	9.221E-01
CYB561D1	-0.055	7.448E-01	8.636E-01
IKBIP	-0.055	7.364E-01	9.680E-01
YIPF5	-0.055	5.747E-01	9.089E-01
MIR7976	-0.055	NA	8.905E-01
FDXR	-0.055	7.542E-01	9.414E-01
SPIN4	-0.055	8.421E-01	9.432E-01
KIAA1324L	-0.055	7.719E-01	8.965E-01

CCM2	-0.055	7.112E-01	9.523E-01
TTC27	-0.055	5.607E-01	9.509E-01
ZXDA	-0.055	7.021E-01	8.703E-01
CCDC91	-0.055	6.680E-01	9.247E-01
TTC1	-0.055	5.209E-01	9.646E-01
KNCN	-0.055	NA	8.778E-01
LXN	-0.055	8.544E-01	9.095E-01
PRPF4	-0.055	6.252E-01	8.645E-01
TEX35	-0.055	9.208E-01	9.045E-01
SLC34A3	-0.055	8.880E-01	9.121E-01
NUP160	-0.055	6.393E-01	8.754E-01
DPYD	-0.055	8.785E-01	9.027E-01
NAA30	-0.055	5.470E-01	8.915E-01
MLXIP	-0.055	6.880E-01	9.504E-01
COL23A1	-0.055	8.591E-01	8.811E-01
RBM34	-0.056	5.515E-01	8.897E-01
KRTAP10-11	-0.056	NA	8.851E-01
OR4D6	-0.056	NA	9.335E-01
RABEP1	-0.056	5.551E-01	8.999E-01
YTHDC1	-0.056	4.244E-01	9.542E-01
ZNF446	-0.056	6.829E-01	9.863E-01
RHOG	-0.056	7.035E-01	9.279E-01
TPK1	-0.056	8.030E-01	9.079E-01
PCGF1	-0.056	5.583E-01	9.279E-01
GHDC	-0.056	6.425E-01	9.614E-01
MTERF3	-0.056	5.859E-01	9.114E-01
LRIG3	-0.056	8.469E-01	8.495E-01
ZFP14	-0.056	7.398E-01	9.075E-01
TMEM128	-0.056	5.519E-01	8.521E-01
RAD50	-0.056	5.926E-01	9.523E-01
ABCB4	-0.056	8.192E-01	9.687E-01
DSE	-0.056	8.537E-01	8.987E-01
UGDH	-0.056	7.770E-01	9.494E-01
XPO7	-0.056	6.017E-01	9.160E-01
BBIP1	-0.056	5.686E-01	9.169E-01
DMXL1	-0.056	6.456E-01	8.777E-01
LRRC40	-0.056	5.948E-01	8.646E-01
SPDYE2	-0.056	8.477E-01	9.399E-01
L3HYPDH	-0.056	7.408E-01	8.841E-01
AC135586.2	-0.056	8.876E-01	9.976E-01
PRR5L	-0.056	8.628E-01	9.976E-01
MDK	-0.056	8.351E-01	9.976E-01
ZFAT	-0.056	6.287E-01	9.976E-01
ZRANB3	-0.056	6.311E-01	9.976E-01
OTULIN	-0.056	6.751E-01	9.976E-01
STX7	-0.056	5.675E-01	9.976E-01
PARL	-0.056	5.373E-01	9.976E-01
WDR35	-0.056	6.604E-01	9.976E-01
DNAJB14	-0.056	5.559E-01	9.976E-01
CCNC	-0.056	5.852E-01	9.976E-01

TAS2R50	-0.056	9.229E-01	9.976E-01
LRRC27	-0.056	7.185E-01	9.976E-01
C8orf89	-0.056	9.004E-01	9.369E-01
ASTE1	-0.056	5.146E-01	9.870E-01
DTWD2	-0.056	7.325E-01	9.759E-01
C11orf88	-0.056	8.908E-01	8.804E-01
CDH20	-0.056	8.932E-01	9.049E-01
ABHD13	-0.056	5.026E-01	9.163E-01
ZNF395	-0.056	7.120E-01	9.055E-01
XRCC2	-0.056	7.612E-01	9.653E-01
LINS1	-0.056	5.586E-01	8.961E-01
TFDP2	-0.056	6.417E-01	8.575E-01
GMEB2	-0.056	4.769E-01	8.591E-01
RBM33	-0.056	4.924E-01	9.431E-01
NFKBIZ	-0.056	8.189E-01	8.829E-01
LYPLAL1	-0.056	6.374E-01	9.334E-01
GAPDHS	-0.056	9.158E-01	9.185E-01
EDC3	-0.057	5.012E-01	8.880E-01
NLGN3	-0.057	8.513E-01	8.803E-01
KRTAP5-6	-0.057	9.314E-01	8.386E-01
AC105052.1	-0.057	NA	8.782E-01
C17orf49	-0.057	6.544E-01	8.460E-01
ZC3H7A	-0.057	5.109E-01	8.153E-01
NPPC	-0.057	9.218E-01	9.408E-01
FDX2	-0.057	7.378E-01	8.926E-01
BCL2L13	-0.057	5.126E-01	8.603E-01
NSUN3	-0.057	6.198E-01	8.622E-01
ELOVL6	-0.057	8.156E-01	9.404E-01
ZNF445	-0.057	5.832E-01	9.029E-01
SKP2	-0.057	7.679E-01	9.180E-01
PLVAP	-0.057	7.868E-01	8.695E-01
CEP128	-0.057	7.085E-01	9.272E-01
TERF1	-0.057	5.338E-01	9.033E-01
SZT2	-0.057	6.085E-01	9.642E-01
PCOTH	-0.057	8.541E-01	8.769E-01
ZNF785	-0.057	6.890E-01	8.407E-01
FCHO2	-0.057	6.209E-01	8.619E-01
TNFRSF4	-0.057	8.490E-01	8.598E-01
FAM135A	-0.057	7.353E-01	8.740E-01
CPQ	-0.057	8.192E-01	8.925E-01
APOPT1	-0.057	6.730E-01	9.378E-01
NUP188	-0.057	6.593E-01	8.748E-01
NFX1	-0.057	5.635E-01	9.121E-01
CHML	-0.057	7.722E-01	9.204E-01
SRRM2	-0.057	5.733E-01	9.043E-01
B4GALT2	-0.057	6.572E-01	8.880E-01
SDF2L1	-0.057	7.699E-01	9.600E-01
MIR6090	-0.057	NA	9.065E-01
OR4D5	-0.057	NA	8.793E-01
NKX1-1	-0.057	NA	9.043E-01

POM121L12	-0.057	NA	9.919E-01
PRM3	-0.057	NA	9.681E-01
PRAMEF9	-0.057	NA	8.924E-01
MIR3166	-0.057	NA	8.724E-01
KRTAP10-7	-0.057	NA	9.838E-01
OR2AJ1	-0.057	NA	9.591E-01
OR10K1	-0.057	NA	9.775E-01
MIR548AG2	-0.057	NA	8.611E-01
SPANXN2	-0.057	NA	9.101E-01
RPL22	-0.057	5.759E-01	8.978E-01
HOXD11	-0.057	9.139E-01	9.606E-01
CA5A	-0.057	8.896E-01	9.333E-01
FAM153C	-0.057	9.246E-01	9.422E-01
IFIT1	-0.057	8.901E-01	9.181E-01
ATP5J	-0.057	5.227E-01	8.715E-01
PCNX1	-0.057	6.377E-01	9.395E-01
ZFAND4	-0.057	7.024E-01	8.827E-01
PACS2	-0.057	5.675E-01	9.475E-01
ARPC4-TTLL	-0.057	7.949E-01	9.347E-01
ACBD4	-0.057	7.739E-01	8.494E-01
HPRT1	-0.057	7.063E-01	8.594E-01
SLC35B4	-0.057	6.209E-01	8.965E-01
NCKAP5L	-0.058	6.594E-01	9.326E-01
ARHGEF39	-0.058	7.509E-01	8.264E-01
IFT57	-0.058	6.748E-01	8.873E-01
CCNE1	-0.058	8.245E-01	8.798E-01
CRIP1	-0.058	8.504E-01	8.738E-01
ACE2	-0.058	9.038E-01	8.788E-01
TMEM88	-0.058	8.174E-01	9.965E-01
TMX3	-0.058	5.310E-01	9.954E-01
LY86	-0.058	8.759E-01	9.952E-01
ZNF628	-0.058	5.682E-01	8.894E-01
IK	-0.058	3.406E-01	9.758E-01
ATPIF1	-0.058	6.148E-01	8.237E-01
DYNC2H1	-0.058	7.984E-01	9.667E-01
AKAP1	-0.058	6.543E-01	9.099E-01
CPNE1	-0.058	7.017E-01	9.840E-01
DRG2	-0.058	5.177E-01	9.544E-01
ZNF562	-0.058	6.387E-01	9.602E-01
RRP15	-0.058	5.598E-01	8.833E-01
ZFYVE21	-0.058	5.552E-01	9.332E-01
C20orf173	-0.058	9.380E-01	8.920E-01
BCCIP	-0.058	5.307E-01	8.443E-01
NAALAD2	-0.058	8.070E-01	8.841E-01
PHF8	-0.058	6.686E-01	9.169E-01
USP38	-0.058	5.621E-01	8.721E-01
KDM2B	-0.058	4.935E-01	8.540E-01
R3HCC1	-0.058	6.272E-01	9.782E-01
GTPBP3	-0.058	5.732E-01	8.505E-01
STOX2	-0.058	8.208E-01	9.505E-01

KCNE1	-0.058	8.781E-01	8.224E-01
ZDHHC7	-0.058	6.043E-01	8.409E-01
CLDN11	-0.058	8.918E-01	9.500E-01
RBX1	-0.058	5.675E-01	9.182E-01
NAIF1	-0.058	4.641E-01	9.515E-01
MIR5580	-0.058	NA	8.527E-01
DNA2	-0.058	7.077E-01	9.926E-01
ATP2A2	-0.058	6.563E-01	9.383E-01
SF1	-0.058	2.444E-01	8.508E-01
SLC35C2	-0.058	5.141E-01	9.836E-01
OGFOD2	-0.058	5.177E-01	8.853E-01
MED17	-0.058	5.331E-01	8.618E-01
PRDX2	-0.058	6.702E-01	9.201E-01
FAM76B	-0.058	5.902E-01	8.517E-01
SNRNP25	-0.058	6.640E-01	8.634E-01
RBM4B	-0.058	5.195E-01	9.053E-01
HOXC8	-0.058	9.096E-01	8.832E-01
ERCC1	-0.059	6.017E-01	9.459E-01
TVP23C	-0.059	6.842E-01	8.854E-01
KLHDC2	-0.059	5.732E-01	8.961E-01
CENPJ	-0.059	6.481E-01	9.577E-01
AGPAT3	-0.059	5.942E-01	8.611E-01
PPIA	-0.059	5.316E-01	8.972E-01
AC073896.1	-0.059	8.897E-01	9.475E-01
ATP2B2	-0.059	8.775E-01	9.530E-01
FRA10AC1	-0.059	5.508E-01	8.174E-01
MIR4505	-0.059	NA	9.277E-01
TMEM59L	-0.059	8.933E-01	9.577E-01
FHDC1	-0.059	8.283E-01	8.134E-01
RERE	-0.059	6.554E-01	8.643E-01
CLP1	-0.059	5.661E-01	9.204E-01
CIB1	-0.059	7.148E-01	8.724E-01
RPS10-NUDT	-0.059	8.801E-01	9.037E-01
RANGRF	-0.059	7.454E-01	8.384E-01
LILRA6	-0.059	8.822E-01	9.454E-01
CPA3	-0.059	8.921E-01	9.522E-01
TIMM8B	-0.059	6.626E-01	8.069E-01
PNPLA4	-0.059	7.122E-01	9.684E-01
MIR4258	-0.059	8.391E-01	8.467E-01
IPMK	-0.059	6.972E-01	8.566E-01
ABI2	-0.059	5.329E-01	9.415E-01
PSPN	-0.059	6.579E-01	9.029E-01
SNRPB	-0.059	6.374E-01	8.271E-01
SLC9A1	-0.059	7.229E-01	8.325E-01
RGCC	-0.059	7.847E-01	8.385E-01
KLHL26	-0.059	5.680E-01	8.453E-01
DDN	-0.059	8.858E-01	9.398E-01
WDR91	-0.059	7.551E-01	8.064E-01
TRPC4AP	-0.059	3.867E-01	9.492E-01
SAXO2	-0.059	8.007E-01	8.142E-01

PDCL	-0.059	5.133E-01	9.337E-01
MAN2B2	-0.059	6.254E-01	8.845E-01
CCR1	-0.059	8.638E-01	7.686E-01
NCBP2	-0.059	4.840E-01	8.852E-01
FCHO1	-0.059	8.368E-01	8.758E-01
SYNRG	-0.059	5.542E-01	9.073E-01
ATG5	-0.059	5.888E-01	8.560E-01
DLD	-0.059	5.673E-01	8.310E-01
IAPP	-0.059	9.503E-01	8.306E-01
LEKR1	-0.059	7.643E-01	9.030E-01
PALD1	-0.059	8.088E-01	8.426E-01
PHAX	-0.059	4.530E-01	8.373E-01
FOPNL	-0.059	5.924E-01	8.509E-01
MX1	-0.059	8.549E-01	9.165E-01
ISH5-SAPCD	-0.059	7.998E-01	8.256E-01
C9orf131	-0.059	8.931E-01	8.685E-01
CHCHD6	-0.059	6.329E-01	8.655E-01
TSGA10	-0.059	7.451E-01	8.355E-01
GPR161	-0.060	8.107E-01	8.818E-01
C6orf62	-0.060	5.767E-01	8.273E-01
MIS12	-0.060	5.790E-01	9.263E-01
TLE4	-0.060	7.921E-01	9.303E-01
SETD9	-0.060	6.612E-01	8.933E-01
MIR6835	-0.060	8.590E-01	9.780E-01
PEX26	-0.060	4.500E-01	8.618E-01
SDAD1	-0.060	6.050E-01	8.424E-01
QKI	-0.060	6.593E-01	8.614E-01
PLA2G2D	-0.060	9.283E-01	9.839E-01
ADAMTSL5	-0.060	8.466E-01	8.217E-01
CTXN2	-0.060	NA	9.114E-01
KCNA2	-0.060	9.128E-01	7.644E-01
C9orf153	-0.060	8.668E-01	8.361E-01
COL4A6	-0.060	8.841E-01	9.706E-01
KHNYN	-0.060	5.753E-01	8.250E-01
NCLN	-0.060	6.144E-01	7.672E-01
RPA4	-0.060	8.529E-01	8.376E-01
GMPR2	-0.060	4.561E-01	8.429E-01
CARD8	-0.060	5.970E-01	8.029E-01
COX16	-0.060	4.568E-01	9.020E-01
TMEM67	-0.060	6.816E-01	9.332E-01
AC093899.2	-0.060	NA	7.993E-01
OR6K2	-0.060	NA	8.067E-01
OR5K1	-0.060	NA	7.689E-01
CCL27	-0.060	NA	7.859E-01
OR1E2	-0.060	NA	8.451E-01
RBMY1J	-0.060	NA	9.717E-01
MIR4674	-0.060	NA	8.057E-01
PRAMEF11	-0.060	NA	8.081E-01
OR6B1	-0.060	NA	8.089E-01
CLCNKA	-0.060	8.882E-01	9.371E-01

PBX2	-0.060	5.402E-01	9.290E-01
HPS5	-0.060	6.534E-01	9.776E-01
ARPC2	-0.060	5.771E-01	8.865E-01
PWP1	-0.060	4.645E-01	9.357E-01
FKBP2	-0.060	7.134E-01	7.953E-01
ENO4	-0.060	8.217E-01	8.301E-01
CHST6	-0.060	8.833E-01	9.026E-01
CD84	-0.060	8.755E-01	8.158E-01
AGGF1	-0.060	4.207E-01	7.880E-01
ASNSD1	-0.060	3.864E-01	9.654E-01
CUZD1	-0.060	8.383E-01	9.965E-01
HELLS	-0.060	6.898E-01	9.957E-01
TOP1	-0.060	5.316E-01	9.948E-01
TNNI1	-0.060	8.248E-01	8.221E-01
AP4E1	-0.060	5.533E-01	8.544E-01
ZNF827	-0.060	6.949E-01	8.219E-01
PCCA	-0.060	7.051E-01	7.696E-01
ZZZ3	-0.060	5.455E-01	8.424E-01
GCM2	-0.060	NA	9.590E-01
NC02210-CRH	-0.060	NA	8.968E-01
BRF1	-0.060	5.434E-01	9.282E-01
AP002495.1	-0.060	8.791E-01	9.341E-01
CTU2	-0.060	5.970E-01	7.628E-01
SUCLG2	-0.060	6.309E-01	8.530E-01
TMEM40	-0.060	8.570E-01	8.357E-01
STK33	-0.060	8.927E-01	9.151E-01
FAM172A	-0.060	5.987E-01	8.283E-01
MEX3C	-0.060	5.985E-01	8.157E-01
C19orf84	-0.060	8.936E-01	9.337E-01
AC010422.6	-0.060	6.910E-01	9.026E-01
RC3H2	-0.060	6.058E-01	9.256E-01
AK9	-0.060	7.389E-01	7.491E-01
GABRR2	-0.060	8.500E-01	8.691E-01
DEXI	-0.060	6.073E-01	8.590E-01
GSTT2B	-0.061	8.533E-01	8.181E-01
PRR36	-0.061	8.966E-01	8.192E-01
KIF5C	-0.061	8.826E-01	8.595E-01
43897.000	-0.061	4.735E-01	9.916E-01
AC011498.1	-0.061	5.731E-01	8.178E-01
ADO	-0.061	4.808E-01	9.196E-01
ANAPC15	-0.061	5.680E-01	8.654E-01
SERTAD1	-0.061	7.049E-01	9.376E-01
C5orf34	-0.061	7.162E-01	8.606E-01
CASD1	-0.061	6.293E-01	9.618E-01
RCC2	-0.061	5.013E-01	9.776E-01
ZNF891	-0.061	6.922E-01	7.516E-01
CCDC124	-0.061	5.696E-01	9.195E-01
CCDC137	-0.061	5.719E-01	7.567E-01
CCDC138	-0.061	7.044E-01	8.376E-01
IPO7	-0.061	6.395E-01	7.543E-01

CCDC77	-0.061	6.399E-01	9.310E-01
DPCD	-0.061	6.841E-01	8.217E-01
ZNF75D	-0.061	5.879E-01	8.425E-01
MIR7156	-0.061	NA	8.146E-01
MAGEB5	-0.061	NA	8.112E-01
XRCC6	-0.061	4.582E-01	8.376E-01
ELP6	-0.061	5.310E-01	8.401E-01
MICALL1	-0.061	7.125E-01	8.360E-01
RBM10	-0.061	5.596E-01	7.438E-01
TMEM164	-0.061	6.638E-01	7.911E-01
FBXO7	-0.061	4.380E-01	8.208E-01
PI4KA	-0.061	6.410E-01	9.254E-01
DNMT1	-0.061	6.807E-01	9.338E-01
SMAP1	-0.061	5.508E-01	7.679E-01
RRM1	-0.061	6.958E-01	8.865E-01
RAB8A	-0.061	3.096E-01	8.136E-01
LRMDA	-0.061	7.994E-01	7.953E-01
ZNHIT1	-0.061	6.514E-01	8.513E-01
KCNJ3	-0.061	9.237E-01	8.749E-01
CDY2B	-0.061	NA	8.242E-01
DEFB114	-0.061	NA	9.309E-01
KRTAP13-4	-0.061	NA	7.643E-01
MIR153-2	-0.061	NA	8.292E-01
MIR3943	-0.061	NA	8.188E-01
MIR4268	-0.061	NA	7.669E-01
MIR4476	-0.061	NA	7.568E-01
MIR4498	-0.061	NA	8.564E-01
MIR4666B	-0.061	NA	7.613E-01
MIR501	-0.061	NA	7.874E-01
MIR509-2	-0.061	NA	7.717E-01
MIR548AW	-0.061	NA	7.415E-01
MIR592	-0.061	NA	9.053E-01
OR4F17	-0.061	NA	7.224E-01
OR8B4	-0.061	NA	9.323E-01
OR8U1	-0.061	NA	9.358E-01
SPATA31A5	-0.061	NA	9.001E-01
DEFB107B	-0.061	NA	8.299E-01
MIR1185-1	-0.061	NA	7.705E-01
MIR124-3	-0.061	NA	7.641E-01
MIR1468	-0.061	NA	8.417E-01
MIR184	-0.061	NA	9.215E-01
MIR188	-0.061	NA	8.438E-01
MIR3180-4	-0.061	NA	8.093E-01
MIR378F	-0.061	NA	7.985E-01
MIR3920	-0.061	NA	7.997E-01
MIR4307	-0.061	NA	9.568E-01
MIR4475	-0.061	NA	8.915E-01
MIR4794	-0.061	NA	9.164E-01
MIR487A	-0.061	NA	8.316E-01
MIR521-1	-0.061	NA	8.722E-01

MIR548A1	-0.061	NA	8.119E-01
MIR548AE1	-0.061	NA	9.053E-01
MIR548G	-0.061	NA	7.688E-01
MIR5689	-0.061	NA	8.315E-01
MIR6131	-0.061	NA	9.029E-01
MIR6788	-0.061	NA	8.827E-01
MIR759	-0.061	NA	9.458E-01
MIR8062	-0.061	NA	7.748E-01
MIR8485	-0.061	NA	9.910E-01
OR4C13	-0.061	NA	9.837E-01
OR5D16	-0.061	NA	9.546E-01
OR5H1	-0.061	NA	6.674E-01
PRY2	-0.061	NA	9.392E-01
AC026786.1	-0.061	NA	8.291E-01
AP000322.1	-0.061	NA	8.417E-01
MIR3142	-0.061	NA	7.486E-01
MIR4281	-0.061	NA	8.000E-01
MIR4699	-0.061	NA	9.175E-01
MIR495	-0.061	NA	8.101E-01
MIR8057	-0.061	NA	8.717E-01
OR10G4	-0.061	NA	9.175E-01
OR1A1	-0.061	NA	7.682E-01
TRIM64	-0.061	NA	7.882E-01
ECI1	-0.061	6.893E-01	8.422E-01
CHST5	-0.061	8.760E-01	8.189E-01
MGAT2	-0.061	7.021E-01	7.627E-01
MIR3138	-0.061	8.847E-01	7.575E-01
PLA2G12A	-0.061	5.762E-01	8.973E-01
GLUD1	-0.061	5.530E-01	8.460E-01
ZKSCAN2	-0.061	6.516E-01	8.264E-01
ACTR5	-0.061	5.733E-01	9.833E-01
KEAP1	-0.061	5.175E-01	8.015E-01
KIF7	-0.061	7.539E-01	7.390E-01
DNAJC18	-0.062	6.846E-01	8.696E-01
YY1	-0.062	3.361E-01	9.610E-01
CISD2	-0.062	5.165E-01	7.724E-01
ZNF689	-0.062	5.410E-01	7.612E-01
ZNF287	-0.062	7.251E-01	8.786E-01
C5AR1	-0.062	8.505E-01	9.957E-01
NHEJ1	-0.062	6.708E-01	9.957E-01
MIR6858	-0.062	NA	9.957E-01
PTK6	-0.062	8.463E-01	9.957E-01
SLC35A4	-0.062	4.043E-01	9.957E-01
TTC30B	-0.062	6.321E-01	9.954E-01
TCIRG1	-0.062	7.539E-01	9.954E-01
TNRC6C	-0.062	7.049E-01	9.954E-01
CPT2	-0.062	5.307E-01	9.954E-01
BSN	-0.062	8.401E-01	9.954E-01
PARP4	-0.062	6.672E-01	9.954E-01
FNDC3A	-0.062	6.841E-01	9.345E-01

AKT1	-0.062	4.020E-01	8.369E-01
ACVRL1	-0.062	7.705E-01	7.613E-01
L3MBTL1	-0.062	8.090E-01	8.134E-01
CHD1L	-0.062	5.766E-01	9.276E-01
EIF4E2	-0.062	5.382E-01	7.938E-01
C7orf26	-0.062	4.973E-01	8.077E-01
PURA	-0.062	5.007E-01	8.192E-01
HSD11B1	-0.062	8.664E-01	9.696E-01
EHHADH	-0.062	7.313E-01	9.352E-01
CCL13	-0.062	8.999E-01	8.605E-01
TNPO2	-0.062	4.588E-01	7.994E-01
RBCK1	-0.062	5.636E-01	8.170E-01
VTI1B	-0.062	4.024E-01	8.545E-01
WIZ	-0.062	4.148E-01	7.177E-01
EIF2S1	-0.062	5.752E-01	8.118E-01
TMEM192	-0.062	5.812E-01	8.305E-01
FAM69B	-0.063	8.369E-01	7.973E-01
ZFX	-0.063	5.651E-01	8.511E-01
MAIP1	-0.063	4.762E-01	9.023E-01
HPS1	-0.063	5.516E-01	8.048E-01
LIPT2	-0.063	6.902E-01	7.575E-01
MIR4305	-0.063	NA	8.353E-01
FAM231B	-0.063	NA	7.682E-01
TAS2R39	-0.063	NA	7.721E-01
OR10S1	-0.063	NA	8.024E-01
MRPL2	-0.063	5.920E-01	8.933E-01
NUMB	-0.063	4.475E-01	7.592E-01
IFRD2	-0.063	5.943E-01	7.646E-01
44083.000	-0.063	5.882E-01	7.778E-01
SLC16A7	-0.063	8.407E-01	7.286E-01
ZNF408	-0.063	5.596E-01	9.348E-01
ETF1	-0.063	4.913E-01	9.390E-01
ABCC10	-0.063	5.293E-01	7.952E-01
SLC9A7	-0.063	7.917E-01	8.128E-01
RASGRP2	-0.063	8.607E-01	7.386E-01
TOE1	-0.063	5.434E-01	8.897E-01
GYS1	-0.063	5.436E-01	7.852E-01
DDX18	-0.063	4.531E-01	8.167E-01
UVSSA	-0.063	7.311E-01	8.724E-01
IRF3	-0.063	5.891E-01	9.753E-01
RFX2	-0.063	7.007E-01	8.703E-01
ZNF517	-0.063	6.593E-01	8.145E-01
C6orf48	-0.063	6.647E-01	6.646E-01
KRAS	-0.063	6.057E-01	8.275E-01
AIMP1	-0.063	2.828E-01	7.852E-01
TKT	-0.063	7.205E-01	7.867E-01
MIR191	-0.063	NA	8.190E-01
KNSTRN	-0.063	6.662E-01	7.891E-01
AFF4	-0.063	5.729E-01	9.190E-01
CORT	-0.063	7.909E-01	8.101E-01

GPR3	-0.063	7.943E-01	8.232E-01
CHID1	-0.063	5.622E-01	6.834E-01
NUP98	-0.063	4.490E-01	8.893E-01
AC133555.5	-0.063	7.060E-01	9.854E-01
ARSA	-0.063	6.893E-01	9.023E-01
SFXN1	-0.063	5.381E-01	8.698E-01
SCAMP1	-0.063	5.896E-01	8.696E-01
LEMD1	-0.063	9.167E-01	7.496E-01
PXT1	-0.063	8.354E-01	7.506E-01
RANBP1	-0.063	6.374E-01	8.086E-01
NFYA	-0.063	5.749E-01	7.795E-01
GAL3ST4	-0.063	8.000E-01	7.111E-01
ZNF629	-0.063	5.552E-01	7.534E-01
TFPI	-0.063	8.369E-01	7.549E-01
ADCY5	-0.063	8.880E-01	7.396E-01
SGPP1	-0.063	8.055E-01	9.373E-01
CSNK1D	-0.063	3.456E-01	8.696E-01
RARS	-0.063	3.947E-01	8.883E-01
FASTKD3	-0.063	5.749E-01	9.146E-01
MGRN1	-0.063	4.631E-01	7.325E-01
TMEM19	-0.063	6.657E-01	9.263E-01
MAML1	-0.063	4.638E-01	9.315E-01
TMEM176B	-0.064	8.496E-01	8.065E-01
UPF3A	-0.064	5.209E-01	7.801E-01
HSF1	-0.064	4.945E-01	7.579E-01
SF19-TCTEX	-0.064	8.868E-01	7.486E-01
OTUB1	-0.064	4.931E-01	8.269E-01
MINDY3	-0.064	5.281E-01	9.503E-01
AC008403.1	-0.064	NA	7.243E-01
NPIPB3	-0.064	7.904E-01	7.956E-01
WDCP	-0.064	5.316E-01	7.739E-01
CCNF	-0.064	6.481E-01	9.578E-01
SLU7	-0.064	3.958E-01	7.888E-01
ST7L	-0.064	4.820E-01	9.145E-01
ZNF512	-0.064	6.311E-01	7.506E-01
NAPG	-0.064	4.836E-01	7.951E-01
MRPL14	-0.064	6.572E-01	7.859E-01
ABHD3	-0.064	6.544E-01	8.467E-01
ATP5E	-0.064	6.036E-01	8.766E-01
IRF2	-0.064	5.155E-01	9.591E-01
MFSD2B	-0.064	7.598E-01	9.896E-01
POLR3B	-0.064	5.522E-01	9.896E-01
MIR5684	-0.064	NA	9.900E-01
CASP8AP2	-0.064	6.514E-01	9.901E-01
DPH1	-0.064	5.271E-01	9.901E-01
C7orf25	-0.064	5.316E-01	7.702E-01
EIF3B	-0.064	5.596E-01	7.837E-01
OCLM	-0.064	8.184E-01	7.736E-01
TAS2R16	-0.064	NA	7.705E-01
AC005258.1	-0.064	NA	7.976E-01

CPLX3	-0.064	NA	9.371E-01
DEFB103A	-0.064	NA	9.431E-01
DEFB135	-0.064	NA	7.968E-01
MIR129-2	-0.064	NA	7.520E-01
MIR325	-0.064	NA	8.424E-01
MIR377	-0.064	NA	9.753E-01
MIR4670	-0.064	NA	7.927E-01
MIR519E	-0.064	NA	8.153E-01
MIR551B	-0.064	NA	8.899E-01
MIR6765	-0.064	NA	6.770E-01
MIRLET7A2	-0.064	NA	7.482E-01
CDY2A	-0.064	NA	8.923E-01
MIR301B	-0.064	NA	7.521E-01
MIR3688-1	-0.064	NA	9.447E-01
MIR4536-1	-0.064	NA	8.155E-01
MIR4540	-0.064	NA	7.160E-01
MIR4661	-0.064	NA	8.160E-01
MIR4769	-0.064	NA	7.032E-01
MIR6715A	-0.064	NA	8.570E-01
MIR6729	-0.064	NA	7.035E-01
OR8H1	-0.064	NA	7.344E-01
PRAMEF27	-0.064	NA	7.897E-01
PHF20L1	-0.064	4.931E-01	7.152E-01
NEPRO	-0.064	3.661E-01	7.360E-01
SPDYE5	-0.064	7.326E-01	8.127E-01
MIR548AC	-0.064	NA	8.565E-01
YTHDF2	-0.064	3.399E-01	7.627E-01
GK	-0.064	7.457E-01	7.605E-01
ITPA	-0.064	5.596E-01	7.472E-01
ZNF384	-0.064	5.680E-01	9.038E-01
GOSR1	-0.064	4.150E-01	6.921E-01
MTR	-0.064	5.225E-01	9.143E-01
ZNF621	-0.064	5.851E-01	9.950E-01
SUSD5	-0.064	8.693E-01	9.950E-01
CORO1C	-0.064	7.190E-01	9.950E-01
AK3	-0.064	6.186E-01	9.950E-01
MIR5008	-0.064	NA	9.950E-01
DDX42	-0.064	2.938E-01	9.950E-01
NUPL2	-0.065	4.312E-01	9.950E-01
RPL30	-0.065	6.574E-01	9.950E-01
DNHD1	-0.065	7.508E-01	9.950E-01
SMIM9	-0.065	NA	9.950E-01
PRRC2B	-0.065	6.907E-01	9.950E-01
ZC3H3	-0.065	4.490E-01	6.964E-01
TNFAIP8L1	-0.065	6.387E-01	8.137E-01
LRRC66	-0.065	8.582E-01	7.789E-01
PNP	-0.065	6.802E-01	7.009E-01
PDE6D	-0.065	4.625E-01	7.424E-01
AGAP5	-0.065	7.739E-01	9.145E-01
CDKN2AIPNL	-0.065	5.568E-01	9.772E-01

SERBP1	-0.065	4.622E-01	8.299E-01
CARNMT1	-0.065	5.864E-01	7.465E-01
ANAPC1	-0.065	5.195E-01	9.784E-01
WDR24	-0.065	5.342E-01	8.499E-01
MMS22L	-0.065	6.664E-01	7.535E-01
EXOSC1	-0.065	4.883E-01	7.124E-01
PRDM5	-0.065	8.391E-01	8.790E-01
ITGA2B	-0.065	8.540E-01	7.490E-01
ZFP64	-0.065	5.227E-01	8.424E-01
SPRYD4	-0.065	5.056E-01	8.845E-01
CAMTA2	-0.065	5.163E-01	7.809E-01
KANSL2	-0.065	3.985E-01	8.096E-01
CDKL1	-0.065	7.489E-01	8.329E-01
ABCG1	-0.065	7.827E-01	6.667E-01
RCC1L	-0.065	3.810E-01	7.182E-01
ATP10D	-0.065	7.687E-01	8.954E-01
STRAP	-0.065	4.996E-01	7.025E-01
CTR9	-0.065	4.868E-01	7.287E-01
ZNF16	-0.065	4.858E-01	9.371E-01
RSPH10B	-0.065	8.182E-01	8.011E-01
PKNOX2	-0.065	8.806E-01	7.713E-01
MIR5693	-0.065	NA	9.458E-01
MIR326	-0.065	NA	8.861E-01
MIR5093	-0.065	NA	8.524E-01
KRT26	-0.065	NA	7.525E-01
OR1L1	-0.065	NA	7.266E-01
KRTAP9-7	-0.065	NA	8.055E-01
GSG2	-0.065	7.645E-01	8.935E-01
HBS1L	-0.066	5.088E-01	8.115E-01
FAM161A	-0.066	7.347E-01	8.352E-01
MICALL2	-0.066	7.054E-01	8.904E-01
RTTN	-0.066	7.271E-01	7.880E-01
ELAVL1	-0.066	2.225E-01	8.472E-01
PDZD3	-0.066	9.164E-01	6.825E-01
SH3YL1	-0.066	7.482E-01	9.067E-01
HECTD1	-0.066	5.524E-01	9.665E-01
AP4B1	-0.066	4.808E-01	8.037E-01
ARL17A	-0.066	7.084E-01	7.596E-01
AC138696.1	-0.066	8.755E-01	7.498E-01
MGA	-0.066	5.191E-01	8.541E-01
TUBB4B	-0.066	6.296E-01	9.837E-01
TMEM69	-0.066	4.490E-01	9.899E-01
UFD1	-0.066	5.134E-01	9.911E-01
TECPR1	-0.066	4.935E-01	7.920E-01
MMADHC	-0.066	4.176E-01	8.791E-01
SETD4	-0.066	5.651E-01	8.199E-01
SIPA1	-0.066	6.453E-01	7.898E-01
COPS2	-0.066	4.093E-01	9.344E-01
NXF1	-0.066	3.923E-01	6.988E-01
ZNF691	-0.066	5.778E-01	7.492E-01

ZNF624	-0.066	5.788E-01	7.546E-01
NLRP5	-0.066	9.468E-01	7.291E-01
XIRP1	-0.066	9.019E-01	7.437E-01
MON1A	-0.066	5.568E-01	7.036E-01
HCST	-0.066	8.590E-01	7.842E-01
SIRT3	-0.066	4.980E-01	9.146E-01
OSGEP	-0.066	4.949E-01	8.677E-01
PLGLB2	-0.066	8.735E-01	7.253E-01
AIMP2	-0.066	5.586E-01	9.531E-01
YWHAE	-0.066	5.155E-01	8.226E-01
BRD9	-0.066	5.467E-01	6.714E-01
GABPB2	-0.066	6.194E-01	6.226E-01
RAVER1	-0.067	6.136E-01	6.544E-01
RNF185	-0.067	4.562E-01	9.641E-01
CYB5RL	-0.067	6.805E-01	8.439E-01
TMED6	-0.067	6.149E-01	9.901E-01
LIMS1	-0.067	5.533E-01	9.191E-01
RAI1	-0.067	6.579E-01	9.780E-01
ABCA4	-0.067	8.906E-01	7.726E-01
DCLRE1A	-0.067	5.412E-01	7.028E-01
GPR75	-0.067	7.782E-01	7.349E-01
CCS	-0.067	6.308E-01	9.856E-01
CCDC179	-0.067	NA	7.260E-01
PFKFB3	-0.067	7.860E-01	9.912E-01
MIR216A	-0.067	NA	9.906E-01
HAPLN4	-0.067	NA	9.900E-01
MIR629	-0.067	NA	9.893E-01
MIR4743	-0.067	NA	9.887E-01
OR2T2	-0.067	NA	6.722E-01
MIR8067	-0.067	NA	8.304E-01
AC135068.1	-0.067	NA	6.995E-01
TAAR9	-0.067	NA	9.100E-01
H2AFB3	-0.067	NA	8.581E-01
IFNA10	-0.067	NA	7.097E-01
MIR542	-0.067	NA	8.827E-01
HNRNPH3	-0.067	2.964E-01	6.531E-01
ZNF77	-0.067	6.169E-01	7.492E-01
C10orf88	-0.067	4.062E-01	8.340E-01
EXOSC6	-0.067	4.470E-01	7.427E-01
DLL4	-0.067	7.464E-01	8.771E-01
ZNF320	-0.067	7.678E-01	7.901E-01
EYA1	-0.067	8.773E-01	7.010E-01
PFDN4	-0.067	5.659E-01	6.632E-01
SLC27A5	-0.067	7.792E-01	7.276E-01
PTBP2	-0.067	6.030E-01	7.370E-01
AP1B1	-0.067	5.730E-01	8.992E-01
MIR5692A1	-0.067	NA	8.658E-01
ZZEF1	-0.067	5.173E-01	8.382E-01
SLC12A7	-0.067	7.354E-01	8.763E-01
KDM5C	-0.067	4.705E-01	7.646E-01

TXLNG	-0.067	5.373E-01	7.296E-01
NIPSNAP3A	-0.067	5.238E-01	7.509E-01
SLC2A7	-0.067	8.854E-01	7.380E-01
UBXN4	-0.067	4.432E-01	6.872E-01
ZNF839	-0.067	5.650E-01	6.995E-01
PTPN2	-0.067	5.194E-01	7.329E-01
GTPBP8	-0.067	4.736E-01	7.488E-01
MAGEB18	-0.068	NA	7.570E-01
MRPL48	-0.068	5.141E-01	7.669E-01
APOD	-0.068	8.940E-01	9.121E-01
MACF1	-0.068	6.805E-01	7.084E-01
YAE1D1	-0.068	4.857E-01	6.958E-01
PSMB6	-0.068	5.418E-01	6.665E-01
HIKESHI	-0.068	4.715E-01	9.022E-01
SERPINB8	-0.068	7.240E-01	6.740E-01
TTC5	-0.068	4.729E-01	6.532E-01
RAN	-0.068	5.143E-01	7.052E-01
CD163	-0.068	8.800E-01	9.191E-01
DXO	-0.068	5.156E-01	9.313E-01
DCDC2B	-0.068	7.850E-01	8.486E-01
ZNF155	-0.068	6.935E-01	6.122E-01
LHX5	-0.068	8.936E-01	7.005E-01
CREB1	-0.068	3.587E-01	6.998E-01
HEBP2	-0.068	6.542E-01	7.523E-01
ZBTB44	-0.068	5.452E-01	7.252E-01
CNEP1R1	-0.068	3.735E-01	7.034E-01
TLR9	-0.068	8.631E-01	7.572E-01
KDM5B	-0.068	5.896E-01	6.892E-01
TRMT112	-0.068	5.246E-01	8.777E-01
ROGDI	-0.068	6.009E-01	7.395E-01
MOCS2	-0.068	4.591E-01	9.093E-01
WDR43	-0.068	4.893E-01	8.246E-01
MIR4316	-0.068	NA	6.277E-01
KRTAP10-12	-0.068	NA	6.408E-01
PRAMEF19	-0.068	NA	8.547E-01
TCF20	-0.068	5.047E-01	6.138E-01
MTA2	-0.068	3.398E-01	7.057E-01
FUK	-0.068	6.266E-01	6.368E-01
HPCA	-0.068	7.908E-01	8.830E-01
TTC33	-0.068	5.758E-01	8.422E-01
MIR769	-0.068	NA	6.449E-01
POLK	-0.068	5.246E-01	9.798E-01
ARHGAP5	-0.068	6.062E-01	8.611E-01
C19orf43	-0.068	5.670E-01	6.423E-01
PSRC1	-0.068	7.086E-01	6.895E-01
GPR34	-0.068	8.436E-01	9.338E-01
MIR598	-0.068	NA	7.345E-01
ATP1A1	-0.069	6.474E-01	7.330E-01
AC020915.2	-0.069	6.915E-01	7.398E-01
SLC6A16	-0.069	8.570E-01	9.520E-01

ZNF589	-0.069	6.040E-01	7.681E-01
KCNQ1	-0.069	8.397E-01	9.435E-01
ANKRD20A4	-0.069	9.081E-01	8.780E-01
GLMN	-0.069	5.282E-01	7.412E-01
NKX2-2	-0.069	9.458E-01	7.223E-01
GPALPP1	-0.069	4.952E-01	6.535E-01
SSR4	-0.069	6.494E-01	6.873E-01
LYG1	-0.069	7.816E-01	9.053E-01
C12orf76	-0.069	4.434E-01	8.773E-01
MPV17L2	-0.069	5.032E-01	6.237E-01
FMNL1	-0.069	7.772E-01	7.018E-01
PRR7	-0.069	7.585E-01	7.269E-01
PTS	-0.069	4.730E-01	7.544E-01
NOX3	-0.069	NA	8.942E-01
OR2AK2	-0.069	NA	8.298E-01
TRIM23	-0.069	4.765E-01	6.948E-01
QTRT2	-0.069	4.289E-01	8.550E-01
LIN7C	-0.069	4.367E-01	6.037E-01
ITPKB	-0.069	6.016E-01	6.854E-01
CMTR1	-0.069	3.918E-01	8.309E-01
ARL16	-0.069	5.795E-01	6.591E-01
EPOP	-0.069	7.771E-01	8.097E-01
GJC2	-0.069	7.925E-01	8.628E-01
PAPD5	-0.069	4.391E-01	9.808E-01
DERL2	-0.069	4.244E-01	9.848E-01
TSEN54	-0.069	5.059E-01	9.855E-01
ARMC6	-0.069	4.551E-01	7.055E-01
ZNF620	-0.069	7.309E-01	6.551E-01
BAP1	-0.069	4.299E-01	6.667E-01
GP1BA	-0.070	8.158E-01	6.460E-01
ZNF791	-0.070	3.735E-01	7.288E-01
PFDN1	-0.070	3.262E-01	6.873E-01
CD2AP	-0.070	6.582E-01	9.182E-01
HEATR4	-0.070	7.441E-01	8.342E-01
VDR	-0.070	7.538E-01	9.265E-01
NBPF6	-0.070	9.218E-01	8.050E-01
AC009014.1	-0.070	9.133E-01	7.659E-01
SRSF3	-0.070	1.846E-01	6.587E-01
MIR4304	-0.070	NA	7.504E-01
MIR4681	-0.070	NA	7.090E-01
OR8B8	-0.070	NA	8.474E-01
AC007375.2	-0.070	NA	7.790E-01
MIR4267	-0.070	NA	6.986E-01
MIR6766	-0.070	NA	9.482E-01
OR10AG1	-0.070	NA	8.536E-01
FAM207A	-0.070	6.779E-01	6.814E-01
PLEKHN1	-0.070	7.993E-01	9.030E-01
HMOX2	-0.070	5.436E-01	9.015E-01
CNPPD1	-0.070	4.726E-01	9.288E-01
CLK2	-0.070	5.624E-01	8.047E-01

SLC22A7	-0.070	9.347E-01	7.770E-01
ZNF48	-0.070	6.440E-01	7.538E-01
DYRK4	-0.070	6.904E-01	8.866E-01
DNAJA4	-0.070	7.334E-01	7.197E-01
SCLT1	-0.070	4.613E-01	9.917E-01
IBSP	-0.070	8.681E-01	9.916E-01
HMG20A	-0.070	5.729E-01	9.910E-01
HSF4	-0.070	7.719E-01	9.909E-01
AC003005.1	-0.070	NA	9.908E-01
UBR1	-0.070	4.841E-01	9.908E-01
HPF1	-0.070	4.798E-01	9.902E-01
UTP11	-0.070	3.855E-01	9.894E-01
MFSD8	-0.070	4.177E-01	7.229E-01
GNB1L	-0.070	6.761E-01	8.634E-01
SLAMF8	-0.070	8.514E-01	6.565E-01
IQCE	-0.070	5.957E-01	9.662E-01
WDR5	-0.070	5.374E-01	9.104E-01
GRK7	-0.070	8.279E-01	6.788E-01
FEZF1	-0.070	9.152E-01	8.077E-01
NT5DC2	-0.070	7.101E-01	7.823E-01
RNF114	-0.070	4.076E-01	7.115E-01
TRNT1	-0.070	4.524E-01	8.968E-01
NUP88	-0.070	4.820E-01	7.140E-01
KLHL2	-0.071	6.432E-01	6.137E-01
NEMP1	-0.071	6.348E-01	8.090E-01
MECP2	-0.071	3.665E-01	8.838E-01
EEF1D	-0.071	4.913E-01	6.385E-01
PPIF	-0.071	7.051E-01	7.531E-01
PPFIA1	-0.071	6.497E-01	6.117E-01
TMSB10	-0.071	7.280E-01	7.497E-01
ZNF804B	-0.071	9.474E-01	7.676E-01
GTPBP6	-0.071	5.533E-01	8.199E-01
YLPM1	-0.071	3.526E-01	6.830E-01
MYBL1	-0.071	7.932E-01	8.542E-01
TEX22	-0.071	7.793E-01	7.399E-01
RPS16	-0.071	6.057E-01	7.522E-01
GDF10	-0.071	8.984E-01	8.277E-01
AHSG	-0.071	9.494E-01	5.942E-01
DERA	-0.071	5.978E-01	8.295E-01
NCAPG	-0.071	7.171E-01	8.634E-01
PSD3	-0.071	7.321E-01	8.758E-01
PRR23B	-0.071	NA	7.039E-01
MIR3194	-0.071	NA	6.504E-01
GCAT	-0.071	7.232E-01	7.928E-01
CAAP1	-0.071	5.310E-01	7.728E-01
NDUFB4	-0.071	4.362E-01	9.345E-01
PCDHB5	-0.071	8.847E-01	6.934E-01
AGPS	-0.071	4.998E-01	8.488E-01
LTBR	-0.071	5.348E-01	6.858E-01
MIR602	-0.071	NA	8.153E-01

NECAP1	-0.071	4.053E-01	7.194E-01
TAPBP	-0.071	6.590E-01	7.049E-01
PBDC1	-0.071	4.962E-01	7.914E-01
MRM1	-0.071	6.215E-01	6.757E-01
RPH3A	-0.071	8.727E-01	7.330E-01
CSTF2T	-0.071	6.273E-01	8.170E-01
CARF	-0.071	6.151E-01	6.208E-01
FAHD2B	-0.071	7.641E-01	6.715E-01
NUP54	-0.071	3.918E-01	8.855E-01
UQCRC1	-0.071	5.136E-01	7.044E-01
TAF5	-0.071	5.348E-01	9.249E-01
PAN3	-0.071	5.124E-01	8.663E-01
RABEPK	-0.071	5.004E-01	8.316E-01
CYP4F3	-0.071	8.909E-01	7.220E-01
SMIM14	-0.071	6.324E-01	6.733E-01
GNAZ	-0.071	8.587E-01	6.800E-01
ZNF140	-0.071	5.478E-01	9.244E-01
TCP11L2	-0.071	7.083E-01	6.913E-01
ADAMTS18	-0.071	8.667E-01	9.779E-01
SS18L2	-0.071	5.353E-01	6.914E-01
LMAN2	-0.071	4.806E-01	9.667E-01
PTCHD3	-0.071	9.314E-01	8.183E-01
ZNF816	-0.071	7.125E-01	7.429E-01
ZNF423	-0.071	8.167E-01	8.559E-01
DNAAF4	-0.071	6.991E-01	6.820E-01
DPH5	-0.071	4.442E-01	7.242E-01
TWF2	-0.071	5.859E-01	7.277E-01
RAB28	-0.072	3.542E-01	9.219E-01
NBEAL1	-0.072	6.530E-01	6.756E-01
COA6	-0.072	5.533E-01	6.056E-01
TNK2	-0.072	6.776E-01	8.739E-01
TSG101	-0.072	3.603E-01	9.458E-01
MSANTD4	-0.072	5.436E-01	7.822E-01
STX6	-0.072	4.733E-01	8.704E-01
ZFP62	-0.072	5.887E-01	7.001E-01
ZNF580	-0.072	6.413E-01	6.199E-01
ZBTB6	-0.072	5.100E-01	6.414E-01
NIF3L1	-0.072	3.293E-01	7.102E-01
SHQ1	-0.072	5.213E-01	7.512E-01
PRAMEF1	-0.072	9.695E-01	8.276E-01
C3orf35	-0.072	7.865E-01	7.036E-01
KARS	-0.072	4.425E-01	6.060E-01
CLK3	-0.072	4.763E-01	9.176E-01
TMEM126B	-0.072	3.856E-01	6.794E-01
SPAG4	-0.072	7.889E-01	7.134E-01
TREM1	-0.072	8.735E-01	8.985E-01
KCNC4	-0.072	7.696E-01	5.714E-01
MIR218-2	-0.072	NA	8.106E-01
ZNF280B	-0.072	8.773E-01	7.902E-01
SESN2	-0.072	6.067E-01	8.387E-01

NSMF	-0.072	6.729E-01	7.982E-01
GABRD	-0.072	8.172E-01	7.066E-01
PRKD3	-0.072	5.521E-01	8.469E-01
PIAS4	-0.072	3.688E-01	6.546E-01
MAT2A	-0.072	5.004E-01	6.391E-01
TOP3B	-0.072	6.839E-01	6.800E-01
PRDM1	-0.072	7.767E-01	9.248E-01
NEIL3	-0.072	7.882E-01	8.527E-01
C9orf24	-0.072	8.092E-01	6.794E-01
LIN9	-0.072	5.722E-01	8.836E-01
AP003108.2	-0.072	5.248E-01	9.800E-01
SS18	-0.072	3.805E-01	9.816E-01
LIMD1	-0.072	5.539E-01	9.853E-01
SNRPN	-0.072	7.927E-01	8.393E-01
TIGD6	-0.072	3.675E-01	7.111E-01
SMG1	-0.072	4.997E-01	6.695E-01
DCTN6	-0.072	4.762E-01	6.936E-01
ELMOD2	-0.072	3.820E-01	7.020E-01
INO80	-0.072	2.575E-01	8.971E-01
CST3	-0.072	6.938E-01	8.114E-01
RHBG	-0.072	8.940E-01	8.881E-01
AP5S1	-0.072	5.194E-01	7.255E-01
ULK1	-0.072	5.733E-01	6.595E-01
SVOP	-0.072	9.003E-01	6.473E-01
CISH	-0.072	6.837E-01	6.386E-01
MINK1	-0.072	5.412E-01	7.215E-01
RNASE7	-0.072	8.893E-01	6.880E-01
CHMP4C	-0.072	7.105E-01	6.203E-01
MIR28	-0.072	NA	6.108E-01
MIR934	-0.072	NA	9.018E-01
AC068234.1	-0.072	NA	6.222E-01
AC187653.1	-0.072	NA	9.130E-01
OR11H12	-0.072	NA	6.904E-01
POLDIP3	-0.072	3.655E-01	6.839E-01
HAGHL	-0.073	7.752E-01	6.967E-01
CCDC180	-0.073	8.222E-01	6.827E-01
ASPDH	-0.073	8.369E-01	6.836E-01
SCAF11	-0.073	4.225E-01	6.628E-01
ECI2	-0.073	6.833E-01	6.869E-01
METTL5	-0.073	3.970E-01	8.654E-01
GATC	-0.073	2.983E-01	6.942E-01
CCDC38	-0.073	8.632E-01	7.546E-01
MIR619	-0.073	NA	6.367E-01
ZNF358	-0.073	6.942E-01	9.923E-01
MRPL11	-0.073	5.986E-01	9.916E-01
NAT9	-0.073	4.966E-01	9.915E-01
OSGIN1	-0.073	8.386E-01	9.915E-01
TAPT1	-0.073	3.996E-01	9.914E-01
ARNTL	-0.073	6.414E-01	9.914E-01
IMP4	-0.073	4.282E-01	9.914E-01

NAF1	-0.073	4.206E-01	8.819E-01
DENND2C	-0.073	7.970E-01	5.330E-01
MAP1S	-0.073	4.604E-01	7.599E-01
AXIN2	-0.073	8.333E-01	6.797E-01
SAYSD1	-0.073	4.735E-01	8.777E-01
RAB21	-0.073	5.012E-01	8.463E-01
RALGAPA1	-0.073	5.798E-01	7.329E-01
DPP7	-0.073	6.556E-01	5.996E-01
GTF3C5	-0.073	5.793E-01	8.228E-01
RXRA	-0.073	6.519E-01	8.229E-01
MIR4660	-0.073	NA	6.640E-01
POLE2	-0.073	6.582E-01	7.453E-01
TAF7	-0.073	4.535E-01	6.740E-01
CCND1	-0.073	8.298E-01	7.158E-01
EFCAB5	-0.073	7.899E-01	7.387E-01
UNKL	-0.073	5.613E-01	5.926E-01
PTMA	-0.074	3.879E-01	7.274E-01
HGH1	-0.074	5.093E-01	8.775E-01
CSRNP2	-0.074	5.146E-01	8.018E-01
RHBDD1	-0.074	4.181E-01	7.907E-01
CTDP1	-0.074	3.786E-01	7.106E-01
SRGN	-0.074	8.292E-01	6.308E-01
ALYREF	-0.074	5.500E-01	6.612E-01
C8A	-0.074	9.523E-01	7.669E-01
ATXN7L3B	-0.074	5.000E-01	6.770E-01
NDUFS8	-0.074	6.185E-01	7.011E-01
MIR4428	-0.074	NA	8.295E-01
TBCC	-0.074	4.415E-01	7.701E-01
PSAT1	-0.074	7.563E-01	7.217E-01
ALKAL1	-0.074	8.693E-01	6.234E-01
HMGB1	-0.074	3.438E-01	9.033E-01
CASKIN2	-0.074	4.242E-01	5.936E-01
JARID2	-0.074	6.915E-01	8.735E-01
MIR5696	-0.074	NA	8.928E-01
SEM1	-0.074	5.436E-01	6.255E-01
KRI1	-0.074	4.715E-01	5.564E-01
HPSE	-0.074	8.217E-01	9.156E-01
STXBP4	-0.074	5.655E-01	7.846E-01
C15orf40	-0.074	3.911E-01	6.761E-01
LAMTOR4	-0.074	6.321E-01	6.093E-01
PPP1R26	-0.074	6.027E-01	6.885E-01
COMMD6	-0.074	5.405E-01	8.026E-01
THNSL1	-0.074	6.897E-01	8.339E-01
DUSP22	-0.074	5.483E-01	6.515E-01
AC099850.2	-0.074	NA	8.832E-01
AC129492.3	-0.074	NA	6.230E-01
MIR218-1	-0.074	NA	7.337E-01
MIR3689B	-0.074	NA	9.153E-01
MIR498	-0.074	NA	9.054E-01
MIR5002	-0.074	NA	6.170E-01

MIR513A1	-0.074	NA	6.658E-01
MIR519C	-0.074	NA	5.690E-01
MIR526A2	-0.074	NA	8.942E-01
MIR548A2	-0.074	NA	8.787E-01
MIR5692C1	-0.074	NA	7.318E-01
MIR6892	-0.074	NA	6.054E-01
MIR7158	-0.074	NA	9.738E-01
MIR8059	-0.074	NA	7.796E-01
OR13C9	-0.074	NA	7.333E-01
OR2T5	-0.074	NA	6.830E-01
CENPVL1	-0.074	NA	8.232E-01
MIR300	-0.074	NA	9.594E-01
MIR3118-1	-0.074	NA	6.920E-01
MIR3925	-0.074	NA	7.382E-01
MIR409	-0.074	NA	6.122E-01
MIR504	-0.074	NA	5.880E-01
MIR518E	-0.074	NA	6.708E-01
MIR539	-0.074	NA	7.583E-01
MIR638	-0.074	NA	5.471E-01
MIR663B	-0.074	NA	6.521E-01
MIR892A	-0.074	NA	7.167E-01
NPIPA2	-0.074	NA	8.840E-01
OR4K17	-0.074	NA	7.614E-01
OR51A2	-0.074	NA	8.814E-01
OR6C65	-0.074	NA	7.976E-01
RNASE11	-0.074	NA	8.826E-01
OR5H15	-0.074	NA	5.999E-01
PCM1	-0.074	4.997E-01	9.196E-01
KLHL25	-0.074	5.566E-01	6.876E-01
KCND3	-0.074	8.558E-01	9.417E-01
IQCF1	-0.074	9.320E-01	6.606E-01
MIR8070	-0.074	NA	6.467E-01
PTPN18	-0.074	5.753E-01	5.877E-01
TEX10	-0.074	4.584E-01	5.397E-01
TBC1D10A	-0.074	5.213E-01	6.871E-01
RIMKLA	-0.074	8.569E-01	6.024E-01
POLG2	-0.074	5.166E-01	9.096E-01
ACKR3	-0.074	8.147E-01	7.031E-01
LILRA4	-0.074	8.704E-01	6.380E-01
PDP2	-0.074	4.767E-01	6.187E-01
BCR	-0.074	5.697E-01	8.976E-01
EBNA1BP2	-0.074	5.593E-01	5.832E-01
NDUFB10	-0.074	5.322E-01	8.559E-01
POLQ	-0.074	6.829E-01	5.841E-01
SNW1	-0.074	2.737E-01	8.478E-01
MRPL21	-0.074	6.348E-01	5.616E-01
SPRYD7	-0.074	5.803E-01	7.852E-01
PCF11	-0.074	4.582E-01	9.820E-01
IL32	-0.075	8.384E-01	6.145E-01
MIR4639	-0.075	8.878E-01	7.227E-01

LRP5	-0.075	6.476E-01	9.357E-01
PTPN4	-0.075	3.668E-01	4.288E-01
TAF6L	-0.075	4.148E-01	5.491E-01
NUDT1	-0.075	6.416E-01	9.068E-01
SIRT7	-0.075	5.373E-01	9.591E-01
SF3B1	-0.075	3.525E-01	8.108E-01
PRMT6	-0.075	5.964E-01	9.514E-01
C7orf31	-0.075	7.021E-01	9.287E-01
RPS4Y1	-0.075	9.084E-01	8.906E-01
ITFG2	-0.075	4.256E-01	9.552E-01
TREX2	-0.075	7.644E-01	7.586E-01
ESRP2	-0.075	7.519E-01	6.780E-01
NUS1	-0.075	3.789E-01	7.020E-01
FAM192A	-0.075	3.330E-01	6.600E-01
SIGLEC1	-0.075	8.528E-01	6.749E-01
GEMIN7	-0.075	5.188E-01	6.634E-01
CCDC84	-0.075	6.603E-01	6.207E-01
DDX21	-0.075	6.292E-01	6.581E-01
TTC4	-0.075	4.446E-01	6.578E-01
SERTAD3	-0.075	5.070E-01	6.267E-01
ASPH	-0.075	7.229E-01	7.461E-01
ELMOD3	-0.075	4.412E-01	7.516E-01
CREG2	-0.075	8.587E-01	8.374E-01
COX11	-0.075	3.460E-01	8.057E-01
PTCD1	-0.075	4.832E-01	5.625E-01
NOP56	-0.075	4.707E-01	7.173E-01
ATP5L	-0.075	4.546E-01	6.838E-01
ARL14EP	-0.075	4.278E-01	5.947E-01
AL139260.3	-0.075	7.583E-01	9.924E-01
EMC9	-0.075	6.147E-01	9.924E-01
NAT8L	-0.075	8.870E-01	9.924E-01
RASAL1	-0.075	8.513E-01	9.924E-01
AARSD1	-0.075	4.906E-01	9.924E-01
CNOT8	-0.075	3.245E-01	9.924E-01
C17orf105	-0.075	8.695E-01	6.737E-01
ALG10	-0.075	5.489E-01	7.878E-01
RNF217	-0.075	7.877E-01	6.714E-01
MIR875	-0.075	NA	6.779E-01
MKRN2	-0.075	5.837E-01	8.535E-01
MED4	-0.075	3.473E-01	9.281E-01
SP5	-0.075	8.861E-01	7.777E-01
MIR4500	-0.075	NA	6.737E-01
OR8B2	-0.075	NA	6.049E-01
FP565260.4	-0.075	NA	7.677E-01
KRTAP4-3	-0.075	NA	6.989E-01
MIR1255B2	-0.075	NA	7.133E-01
MIR1288	-0.075	NA	6.241E-01
UCP2	-0.075	7.778E-01	9.635E-01
SGO2	-0.075	6.361E-01	5.396E-01
PRDM10	-0.075	3.561E-01	8.712E-01

MTHFD1L	-0.075	6.606E-01	8.290E-01
?MF1-BGLAI	-0.075	6.594E-01	8.843E-01
AP5B1	-0.075	5.124E-01	5.922E-01
UBE2O	-0.075	3.598E-01	6.331E-01
OTUD3	-0.075	6.128E-01	7.441E-01
ZMYM3	-0.075	4.331E-01	9.685E-01
TSPAN17	-0.075	4.471E-01	5.788E-01
ZNF267	-0.075	4.500E-01	8.609E-01
POLR3E	-0.075	4.133E-01	4.739E-01
SLC48A1	-0.076	5.829E-01	6.020E-01
AL136531.2	-0.076	7.981E-01	6.531E-01
DMP1	-0.076	8.534E-01	7.360E-01
CBWD5	-0.076	5.408E-01	7.065E-01
DIRC3	-0.076	8.503E-01	5.334E-01
RPP40	-0.076	5.434E-01	6.865E-01
NSMCE4A	-0.076	4.251E-01	6.553E-01
DHRS12	-0.076	6.215E-01	5.337E-01
SURF2	-0.076	6.127E-01	5.944E-01
MTHFS	-0.076	5.367E-01	8.849E-01
C17orf80	-0.076	3.456E-01	6.071E-01
BDNF	-0.076	8.469E-01	7.348E-01
ZNF574	-0.076	3.994E-01	7.067E-01
ZNF600	-0.076	6.763E-01	6.424E-01
CSNK2A3	-0.076	7.494E-01	6.539E-01
MIR570	-0.076	7.169E-01	6.990E-01
CYLD	-0.076	5.803E-01	7.694E-01
KIAA1671	-0.076	6.722E-01	8.914E-01
HSDL1	-0.076	4.242E-01	6.349E-01
CABIN1	-0.076	4.770E-01	7.763E-01
LMOD3	-0.076	7.765E-01	6.171E-01
MRPL27	-0.076	5.706E-01	5.433E-01
MX2	-0.076	7.574E-01	6.263E-01
TEX45	-0.076	8.703E-01	5.751E-01
LENG8	-0.076	6.117E-01	8.919E-01
VCPIP1	-0.076	3.504E-01	6.689E-01
C14orf79	-0.076	5.744E-01	8.595E-01
WDR33	-0.076	1.152E-01	6.671E-01
SYMPK	-0.076	3.446E-01	5.361E-01
TYRO3	-0.076	6.644E-01	6.500E-01
POLRMT	-0.076	4.835E-01	6.783E-01
SMCR8	-0.076	5.656E-01	8.867E-01
ARHGEF6	-0.076	7.493E-01	8.636E-01
ZNF330	-0.077	3.331E-01	7.364E-01
SCO1	-0.077	4.261E-01	5.260E-01
PDIA2	-0.077	8.728E-01	6.224E-01
SFXN4	-0.077	5.241E-01	7.904E-01
TMEM223	-0.077	5.136E-01	6.204E-01
CGRRF1	-0.077	4.137E-01	5.852E-01
SSTR3	-0.077	8.765E-01	8.714E-01
MIR4733	-0.077	NA	5.317E-01

MEGF8	-0.077	5.241E-01	7.232E-01
ROBO1	-0.077	8.171E-01	6.025E-01
DNAJC4	-0.077	5.751E-01	6.914E-01
TMPRSS9	-0.077	7.122E-01	9.011E-01
BBS2	-0.077	4.170E-01	6.395E-01
MGAT4B	-0.077	4.660E-01	5.371E-01
DALRD3	-0.077	4.709E-01	9.278E-01
KAT6B	-0.077	5.670E-01	6.571E-01
DEFB121	-0.077	NA	6.081E-01
MIR1266	-0.077	NA	6.507E-01
MIR585	-0.077	NA	7.077E-01
MIR6879	-0.077	NA	6.650E-01
OR1D2	-0.077	NA	5.668E-01
PRAMEF25	-0.077	NA	7.680E-01
MIR3713	-0.077	NA	7.499E-01
MIR4765	-0.077	NA	7.735E-01
OR2T34	-0.077	NA	7.683E-01
OR51D1	-0.077	NA	7.901E-01
OR5F1	-0.077	NA	6.569E-01
OR9Q2	-0.077	NA	9.436E-01
HS3ST3A1	-0.077	8.878E-01	9.813E-01
SLC9B2	-0.077	7.274E-01	9.850E-01
HSD3B2	-0.077	9.107E-01	8.242E-01
RREB1	-0.077	4.705E-01	7.290E-01
MTFMT	-0.077	3.286E-01	6.261E-01
ANKRD42	-0.077	4.854E-01	6.166E-01
GSTA4	-0.077	7.931E-01	6.634E-01
HNRNPC	-0.077	2.176E-01	6.344E-01
SERP2	-0.077	8.086E-01	4.853E-01
LRIG2	-0.077	3.841E-01	8.063E-01
ANKRD49	-0.077	4.331E-01	8.817E-01
G3BP1	-0.077	4.182E-01	8.632E-01
MIR4642	-0.077	NA	6.494E-01
RTL6	-0.077	6.565E-01	7.345E-01
IER3	-0.077	7.677E-01	4.701E-01
FKBP6	-0.077	8.769E-01	8.379E-01
SPPL3	-0.077	3.180E-01	6.335E-01
MTF2	-0.077	4.090E-01	6.502E-01
PEX5	-0.077	3.793E-01	6.289E-01
TFB2M	-0.077	4.062E-01	5.494E-01
SSBP1	-0.077	3.944E-01	6.082E-01
ZNF70	-0.077	5.593E-01	6.607E-01
PDCD7	-0.077	2.989E-01	5.720E-01
MTFR2	-0.077	6.556E-01	7.277E-01
HYKK	-0.077	6.036E-01	5.954E-01
BRD4	-0.077	2.366E-01	5.627E-01
NDFIP1	-0.077	3.061E-01	6.859E-01
NABP1	-0.077	7.240E-01	6.522E-01
ZXDB	-0.077	5.038E-01	5.926E-01
IRS2	-0.077	7.864E-01	5.378E-01

PQBP1	-0.077	5.053E-01	5.071E-01
CD36	-0.077	8.801E-01	6.087E-01
IL19	-0.077	8.880E-01	7.916E-01
ZNF782	-0.078	5.613E-01	8.904E-01
TCF25	-0.078	3.638E-01	8.648E-01
PLSCR1	-0.078	6.393E-01	5.726E-01
ZFAND1	-0.078	4.485E-01	4.721E-01
CYCS	-0.078	4.444E-01	8.026E-01
DFNB59	-0.078	7.214E-01	6.104E-01
WEE2	-0.078	8.033E-01	9.916E-01
FBXL2	-0.078	7.004E-01	9.916E-01
LSM4	-0.078	4.378E-01	9.916E-01
GDPGP1	-0.078	5.732E-01	9.563E-01
RFC5	-0.078	4.988E-01	6.536E-01
RRP8	-0.078	3.149E-01	6.367E-01
RAB4B	-0.078	6.060E-01	9.580E-01
PAWR	-0.078	4.939E-01	5.573E-01
TRMT10B	-0.078	4.737E-01	7.243E-01
HRH4	-0.078	8.261E-01	6.078E-01
ZNF557	-0.078	4.622E-01	5.628E-01
PGBD1	-0.078	7.116E-01	6.498E-01
DISC1	-0.078	7.185E-01	7.254E-01
PSMC6	-0.078	2.288E-01	8.554E-01
SLC25A15	-0.078	6.104E-01	5.117E-01
CCDC61	-0.078	5.902E-01	5.997E-01
PNPLA7	-0.078	7.441E-01	8.503E-01
PAIP2	-0.078	3.080E-01	4.645E-01
SDK2	-0.078	8.635E-01	5.524E-01
CEP95	-0.078	4.756E-01	6.478E-01
ICK	-0.078	5.210E-01	7.979E-01
DNM2	-0.078	5.851E-01	7.666E-01
ANKRD52	-0.078	4.871E-01	5.927E-01
PTBP1	-0.078	1.417E-01	5.603E-01
C20orf27	-0.078	5.823E-01	5.939E-01
UBE2J2	-0.078	3.496E-01	8.742E-01
S100A7L2	-0.078	NA	5.611E-01
CLRN2	-0.078	NA	5.385E-01
OR4K14	-0.078	NA	7.547E-01
TIMM9	-0.078	4.107E-01	6.676E-01
C11orf49	-0.078	4.997E-01	5.590E-01
PLXNA1	-0.078	6.047E-01	5.055E-01
ZC3HAV1	-0.078	4.207E-01	6.247E-01
ANKRD54	-0.078	4.381E-01	8.149E-01
MAPK11	-0.078	7.214E-01	8.732E-01
SAG	-0.078	8.675E-01	6.966E-01
PCYT2	-0.078	6.351E-01	7.598E-01
HDHD2	-0.078	4.841E-01	7.705E-01
PEX3	-0.078	4.835E-01	5.576E-01
HECA	-0.078	4.261E-01	6.284E-01
TRIM39	-0.078	3.326E-01	5.993E-01

SLC25A37	-0.078	5.845E-01	5.096E-01
MRM3	-0.078	5.348E-01	9.493E-01
ZNF136	-0.078	4.511E-01	5.095E-01
CYP20A1	-0.078	3.504E-01	5.398E-01
IPO5	-0.078	4.306E-01	7.990E-01
MLKL	-0.078	6.430E-01	6.003E-01
WASHC1	-0.078	6.738E-01	8.266E-01
ETFA	-0.078	4.028E-01	6.984E-01
ARHGAP45	-0.079	7.155E-01	8.498E-01
KCTD9	-0.079	5.943E-01	8.710E-01
LRRC37B	-0.079	5.433E-01	7.273E-01
RBBP8NL	-0.079	8.317E-01	8.354E-01
XAB2	-0.079	4.674E-01	7.595E-01
TCTN1	-0.079	5.749E-01	8.241E-01
THAP12	-0.079	4.066E-01	7.205E-01
MIR4753	-0.079	NA	6.731E-01
C1orf131	-0.079	3.139E-01	9.299E-01
TMEM35A	-0.079	8.831E-01	9.135E-01
IKBKB	-0.079	5.469E-01	5.051E-01
BTBD9	-0.079	4.216E-01	5.541E-01
SIRT5	-0.079	4.794E-01	6.099E-01
RABL2A	-0.079	5.607E-01	5.341E-01
MVK	-0.079	6.288E-01	6.082E-01
NOL7	-0.079	5.032E-01	6.404E-01
POMGNT1	-0.079	4.292E-01	8.740E-01
NDUFB1	-0.079	5.415E-01	8.459E-01
SHPRH	-0.079	6.977E-01	5.362E-01
MIR6747	-0.079	9.003E-01	7.858E-01
PPP1R3C	-0.079	8.534E-01	8.121E-01
OR56B1	-0.079	NA	6.723E-01
CEP120	-0.079	3.745E-01	6.807E-01
ATP9B	-0.079	4.412E-01	7.986E-01
YTHDF3	-0.079	3.215E-01	8.076E-01
GRAMD4	-0.079	6.189E-01	5.077E-01
Z84492.1	-0.079	9.070E-01	7.474E-01
SNRNP48	-0.079	4.120E-01	7.916E-01
RPL26L1	-0.079	4.432E-01	6.606E-01
RRP7A	-0.079	5.851E-01	5.410E-01
ALDH6A1	-0.079	5.753E-01	9.629E-01
LAIR1	-0.079	8.176E-01	9.877E-01
SLC36A1	-0.079	5.438E-01	9.869E-01
MIR4632	-0.079	NA	9.838E-01
GGT7	-0.079	6.927E-01	7.868E-01
MLNR	-0.079	8.994E-01	9.469E-01
PRMT5	-0.079	5.316E-01	8.871E-01
ZNF554	-0.079	5.753E-01	5.166E-01
C15orf52	-0.079	7.991E-01	7.926E-01
FRAT2	-0.079	5.753E-01	7.148E-01
ZNF646	-0.080	3.379E-01	5.220E-01
RARS2	-0.080	2.278E-01	9.652E-01

C11orf53	-0.080	8.759E-01	7.582E-01
OR5H2	-0.080	NA	5.433E-01
KRTAP12-2	-0.080	NA	7.477E-01
MIR610	-0.080	NA	6.161E-01
MIR5096	-0.080	NA	6.990E-01
OR5AK2	-0.080	NA	5.242E-01
AL672142.1	-0.080	NA	5.955E-01
DEFB103B	-0.080	NA	8.401E-01
METTL14	-0.080	2.390E-01	9.643E-01
SLC22A12	-0.080	NA	4.620E-01
C5orf24	-0.080	3.721E-01	6.231E-01
TSSC4	-0.080	5.146E-01	5.588E-01
ZNF225	-0.080	5.204E-01	4.290E-01
SSH1	-0.080	4.729E-01	6.737E-01
AMBP	-0.080	9.095E-01	8.873E-01
PMPCB	-0.080	2.356E-01	7.220E-01
UBE2C	-0.080	6.716E-01	4.025E-01
TMEM68	-0.080	4.631E-01	6.683E-01
RPS26	-0.080	6.219E-01	7.062E-01
WDR59	-0.080	3.793E-01	6.258E-01
TMEM262	-0.080	5.968E-01	8.372E-01
B3GAT1	-0.080	8.878E-01	6.356E-01
HELB	-0.080	6.181E-01	5.191E-01
AC003002.3	-0.080	8.223E-01	6.761E-01
ZNF770	-0.080	7.372E-01	6.011E-01
KIF13B	-0.080	6.155E-01	5.523E-01
RBMXL3	-0.080	NA	5.817E-01
HBZ	-0.080	NA	7.956E-01
FAM160A2	-0.080	2.931E-01	5.249E-01
ANKRD17	-0.080	5.018E-01	4.954E-01
NAGPA	-0.080	4.470E-01	5.780E-01
LAD1	-0.080	7.385E-01	5.392E-01
ZNF337	-0.080	6.927E-01	6.353E-01
GNG5	-0.080	3.348E-01	5.688E-01
UBALD1	-0.080	4.606E-01	7.649E-01
SETD1A	-0.080	2.051E-01	5.724E-01
NDST4	-0.080	9.133E-01	5.143E-01
ATG14	-0.080	3.228E-01	5.706E-01
DYNLT1	-0.080	3.708E-01	7.382E-01
KRT32	-0.080	9.017E-01	4.933E-01
KNL1	-0.080	6.583E-01	9.089E-01
PGAM4	-0.080	7.843E-01	6.334E-01
ZNF316	-0.080	5.246E-01	8.076E-01
ACOT13	-0.080	5.398E-01	5.326E-01
PSMD6	-0.081	3.395E-01	5.212E-01
ACHE	-0.081	8.403E-01	5.538E-01
TMEM126A	-0.081	4.040E-01	7.820E-01
FOXL1	-0.081	8.515E-01	4.719E-01
SNX6	-0.081	4.403E-01	5.124E-01
PGAM5	-0.081	4.257E-01	5.356E-01

PPP1CA	-0.081	4.360E-01	3.886E-01
DPH6	-0.081	4.871E-01	5.941E-01
BICD1	-0.081	6.181E-01	6.511E-01
CMBL	-0.081	7.855E-01	6.952E-01
RGPD2	-0.081	8.582E-01	7.725E-01
TMEM176A	-0.081	8.050E-01	6.225E-01
GTF2H4	-0.081	4.500E-01	7.114E-01
AL109811.4	-0.081	8.521E-01	6.918E-01
NAA40	-0.081	4.278E-01	8.586E-01
MIR6826	-0.081	NA	4.309E-01
UBE3B	-0.081	2.414E-01	6.213E-01
EFHD2	-0.081	5.737E-01	9.596E-01
OR10H1	-0.081	8.908E-01	5.887E-01
HLA-C	-0.081	7.149E-01	3.890E-01
MIR8076	-0.081	NA	7.779E-01
MIR6854	-0.081	NA	7.076E-01
MYF5	-0.081	NA	3.999E-01
MRFAP1	-0.081	3.182E-01	8.050E-01
C7orf43	-0.081	3.708E-01	7.280E-01
SUB1	-0.081	4.682E-01	6.017E-01
CELA3A	-0.081	9.380E-01	5.020E-01
GATB	-0.081	4.628E-01	6.394E-01
C5orf58	-0.081	8.703E-01	6.002E-01
CRYBA1	-0.081	8.041E-01	5.445E-01
FERMT1	-0.081	7.285E-01	4.885E-01
NOVA2	-0.081	6.864E-01	6.985E-01
KLHL29	-0.081	7.879E-01	6.367E-01
ZSCAN26	-0.081	4.403E-01	4.776E-01
KCNT1	-0.081	8.192E-01	5.453E-01
TBL3	-0.081	3.968E-01	4.283E-01
CMAS	-0.081	4.256E-01	8.526E-01
EDF1	-0.081	5.289E-01	7.446E-01
SH2B1	-0.081	5.559E-01	5.462E-01
GNL2	-0.081	3.545E-01	8.496E-01
FFAR4	-0.081	8.222E-01	8.949E-01
HES2	-0.081	8.693E-01	9.711E-01
ACACB	-0.081	7.396E-01	7.067E-01
FZR1	-0.081	3.438E-01	5.100E-01
CDK5RAP3	-0.081	5.364E-01	5.312E-01
FUBP1	-0.081	3.154E-01	4.980E-01
ABHD18	-0.082	3.317E-01	7.657E-01
AQR	-0.082	3.404E-01	5.375E-01
GART	-0.082	4.244E-01	4.677E-01
HSD17B4	-0.082	4.591E-01	8.531E-01
TERB1	-0.082	8.272E-01	6.592E-01
HELQ	-0.082	2.051E-01	6.084E-01
MIR6814	-0.082	8.852E-01	5.385E-01
VARS2	-0.082	4.976E-01	5.681E-01
EIF2B2	-0.082	3.620E-01	6.001E-01
LNPEP	-0.082	4.798E-01	7.581E-01

DOCK8	-0.082	7.322E-01	7.228E-01
RASSF7	-0.082	6.443E-01	5.111E-01
MIR3666	-0.082	NA	5.015E-01
GPR89B	-0.082	5.381E-01	4.770E-01
KCNH7	-0.082	8.735E-01	5.377E-01
FOXN1	-0.082	8.806E-01	6.357E-01
FAM83C	-0.082	8.739E-01	7.367E-01
HMG20B	-0.082	4.895E-01	8.082E-01
SHMT2	-0.082	4.976E-01	8.565E-01
MEF2C	-0.082	7.240E-01	8.333E-01
TNFRSF21	-0.082	7.397E-01	9.560E-01
POLR2A	-0.082	4.560E-01	7.461E-01
CALCA	-0.082	9.070E-01	8.774E-01
MAP3K21	-0.082	7.378E-01	9.892E-01
MARVELD2	-0.082	5.631E-01	9.848E-01
NOL8	-0.082	3.576E-01	9.324E-01
RABL2B	-0.082	3.976E-01	5.731E-01
TMPO	-0.082	5.434E-01	8.707E-01
CEP126	-0.082	7.295E-01	6.704E-01
MSN	-0.082	7.764E-01	6.934E-01
MED18	-0.082	4.193E-01	8.921E-01
VCPKMT	-0.082	3.268E-01	6.625E-01
HEXIM1	-0.082	5.146E-01	8.234E-01
GPX4	-0.082	5.438E-01	7.267E-01
CPEB2	-0.082	6.599E-01	8.770E-01
PTCD3	-0.082	3.558E-01	5.043E-01
ATXN1	-0.082	6.807E-01	8.560E-01
MIR6737	-0.082	NA	5.797E-01
LRRC30	-0.082	NA	4.562E-01
OR13C2	-0.082	NA	7.116E-01
AC020909.1	-0.082	NA	9.014E-01
OR5AN1	-0.082	NA	4.579E-01
MIR4478	-0.082	NA	8.533E-01
SNX5	-0.082	3.610E-01	6.843E-01
DLGAP5	-0.082	6.877E-01	4.753E-01
PPP2R2A	-0.082	4.523E-01	6.604E-01
TRIAP1	-0.083	3.525E-01	5.292E-01
KIAA1524	-0.083	6.367E-01	6.105E-01
RAB39B	-0.083	8.289E-01	5.718E-01
MPND	-0.083	5.976E-01	7.464E-01
PKN2	-0.083	4.476E-01	7.763E-01
CDCA2	-0.083	6.805E-01	8.300E-01
CC2D2B	-0.083	7.475E-01	9.021E-01
TMEM33	-0.083	3.879E-01	8.539E-01
IPO8	-0.083	3.502E-01	4.857E-01
MMP24-AS1	-0.083	6.296E-01	7.364E-01
GUCD1	-0.083	2.819E-01	5.406E-01
CNST	-0.083	3.614E-01	5.032E-01
MESDC1	-0.083	4.366E-01	9.207E-01
PSMA3	-0.083	3.536E-01	6.213E-01

STK38L	-0.083	5.818E-01	4.874E-01
EML2	-0.083	5.847E-01	9.088E-01
ZBTB22	-0.083	3.356E-01	8.273E-01
RSBN1	-0.083	3.330E-01	3.970E-01
RSRC2	-0.083	2.396E-01	4.665E-01
FBL	-0.083	5.583E-01	6.523E-01
FAM227A	-0.083	7.545E-01	9.731E-01
SLC5A8	-0.083	9.094E-01	7.466E-01
VPS33A	-0.083	2.581E-01	8.978E-01
NXPE2	-0.083	8.896E-01	4.410E-01
RBPJ	-0.083	3.751E-01	5.108E-01
CDC16	-0.083	2.914E-01	6.343E-01
PPA2	-0.083	3.618E-01	3.426E-01
DGCR2	-0.083	3.628E-01	7.051E-01
AGMAT	-0.083	6.985E-01	6.655E-01
CSK	-0.083	3.645E-01	5.220E-01
SLC35A1	-0.083	5.045E-01	9.719E-01
NIPSNAP1	-0.083	5.480E-01	7.312E-01
CHAMP1	-0.083	5.311E-01	9.826E-01
SNCG	-0.083	8.421E-01	5.514E-01
TMED8	-0.083	4.144E-01	5.993E-01
PTPRJ	-0.083	7.063E-01	5.722E-01
MIR8087	-0.083	NA	4.304E-01
ARFGEF1	-0.083	4.709E-01	8.995E-01
MSX1	-0.083	7.806E-01	7.696E-01
CLEC9A	-0.083	8.360E-01	8.234E-01
IDH3G	-0.083	4.151E-01	7.008E-01
MARCKSL1	-0.083	6.708E-01	7.153E-01
WDR83OS	-0.083	3.621E-01	8.061E-01
EVX1	-0.083	8.911E-01	4.242E-01
LRP8	-0.083	7.535E-01	6.232E-01
PRELID1	-0.084	3.502E-01	5.445E-01
TMEM202	-0.084	NA	4.762E-01
CFAP161	-0.084	7.821E-01	5.839E-01
MIR4473	-0.084	NA	8.651E-01
ZFYVE26	-0.084	4.261E-01	7.335E-01
LILRB3	-0.084	7.782E-01	5.433E-01
AKR7A3	-0.084	7.545E-01	9.493E-01
MIR4286	-0.084	NA	7.439E-01
TNIP2	-0.084	4.156E-01	6.393E-01
MCF2L2	-0.084	7.042E-01	5.045E-01
MIR6069	-0.084	NA	5.290E-01
MRPL34	-0.084	4.053E-01	4.755E-01
NDST1	-0.084	5.579E-01	6.697E-01
UTP6	-0.084	4.076E-01	6.923E-01
MRPS22	-0.084	2.551E-01	4.253E-01
C1orf226	-0.084	7.383E-01	7.041E-01
EIF2A	-0.084	2.542E-01	9.015E-01
ZNF641	-0.084	4.278E-01	6.145E-01
SNU13	-0.084	3.258E-01	9.813E-01

RPS19BP1	-0.084	5.025E-01	6.724E-01
AC093423.3	-0.084	NA	5.518E-01
DLEC1	-0.084	7.782E-01	8.383E-01
CASQ1	-0.084	8.720E-01	9.388E-01
LAT2	-0.084	7.596E-01	4.293E-01
ERH	-0.084	2.924E-01	7.486E-01
UGT3A2	-0.084	8.672E-01	4.857E-01
COPE	-0.084	3.911E-01	7.195E-01
EFCAB6	-0.084	7.793E-01	7.063E-01
PPIG	-0.084	2.348E-01	5.493E-01
CELA2B	-0.084	7.970E-01	7.985E-01
NOP10	-0.084	4.526E-01	7.848E-01
MRPS6	-0.084	4.115E-01	3.951E-01
AGO4	-0.084	3.903E-01	5.185E-01
SSNA1	-0.084	4.705E-01	5.786E-01
SRRT	-0.084	1.961E-01	5.691E-01
SPAG11A	-0.084	NA	6.760E-01
ZNF284	-0.084	5.304E-01	5.116E-01
ZNF85	-0.084	6.781E-01	8.458E-01
TMEM205	-0.084	5.984E-01	5.336E-01
NDUFAF5	-0.085	3.410E-01	9.898E-01
RABL6	-0.085	4.582E-01	9.898E-01
FGFR1OP2	-0.085	3.022E-01	9.898E-01
C1orf50	-0.085	4.412E-01	9.889E-01
MAD2L1	-0.085	6.288E-01	9.887E-01
AGL	-0.085	5.032E-01	9.873E-01
WDR6	-0.085	4.652E-01	3.227E-01
RAD51AP1	-0.085	6.557E-01	6.599E-01
NKX2-3	-0.085	9.128E-01	8.148E-01
SLC22A11	-0.085	8.854E-01	8.252E-01
BYSL	-0.085	4.207E-01	7.857E-01
HSPA9	-0.085	3.327E-01	7.736E-01
CSNK1G3	-0.085	3.321E-01	7.801E-01
HLA-DOA	-0.085	8.257E-01	5.574E-01
SEC14L2	-0.085	7.447E-01	8.412E-01
SART3	-0.085	1.717E-01	7.839E-01
FBXO21	-0.085	3.735E-01	6.265E-01
CHIA	-0.085	9.230E-01	4.525E-01
EXOSC7	-0.085	3.600E-01	6.451E-01
URB2	-0.085	4.727E-01	3.423E-01
ERMN	-0.085	8.156E-01	8.271E-01
CMTM2	-0.085	7.322E-01	5.328E-01
MIR4324	-0.085	9.068E-01	5.945E-01
FAM200A	-0.085	3.504E-01	5.550E-01
MTFR1	-0.085	4.418E-01	4.703E-01
CHCHD2	-0.085	4.239E-01	6.993E-01
HNRNPCL4	-0.085	NA	8.736E-01
MIR4710	-0.085	NA	9.583E-01
MIR4646	-0.085	NA	4.481E-01
KRTAP10-10	-0.085	NA	5.794E-01

OR9I1	-0.085	NA	5.205E-01
MBD3L3	-0.085	NA	5.588E-01
AC010646.1	-0.085	NA	8.571E-01
AC078927.1	-0.085	8.780E-01	5.972E-01
UNC5B	-0.085	6.635E-01	8.446E-01
PSPC1	-0.085	3.340E-01	7.909E-01
DDA1	-0.085	2.615E-01	4.437E-01
P4HA1	-0.085	6.202E-01	5.973E-01
CENPH	-0.085	5.675E-01	8.175E-01
THOC1	-0.085	4.433E-01	9.352E-01
AC023055.1	-0.085	5.299E-01	4.369E-01
C4orf32	-0.085	6.414E-01	4.611E-01
ATP5F1	-0.085	2.288E-01	8.326E-01
ADGRF2	-0.085	8.713E-01	7.036E-01
RNF113A	-0.086	4.027E-01	5.407E-01
FAF2	-0.086	2.038E-01	5.185E-01
KIF20A	-0.086	6.419E-01	6.454E-01
SPTY2D1-AS	-0.086	5.986E-01	8.424E-01
RBPMS	-0.086	6.942E-01	7.091E-01
IPPK	-0.086	7.172E-01	8.193E-01
FIGN	-0.086	7.867E-01	8.489E-01
C9orf172	-0.086	6.680E-01	7.885E-01
ELP2	-0.086	3.225E-01	7.069E-01
CDK4	-0.086	5.259E-01	5.482E-01
GSE1	-0.086	6.942E-01	8.325E-01
SSB	-0.086	2.498E-01	4.053E-01
GPM6B	-0.086	7.251E-01	3.073E-01
CFAP61	-0.086	7.847E-01	4.520E-01
FANCB	-0.086	6.165E-01	5.623E-01
FAM109A	-0.086	5.441E-01	5.367E-01
AC009163.2	-0.086	NA	5.752E-01
HIST1H4F	-0.086	9.349E-01	8.052E-01
RBMXL1	-0.086	4.008E-01	5.501E-01
URAD	-0.086	NA	4.153E-01
B3GAT2	-0.086	7.154E-01	6.304E-01
TSC2	-0.086	2.312E-01	9.745E-01
RPTOR	-0.086	2.509E-01	5.961E-01
PPP1R35	-0.086	5.484E-01	6.296E-01
TRAPPC6B	-0.086	2.203E-01	7.993E-01
TBRG1	-0.086	3.268E-01	4.990E-01
RASA2	-0.086	4.076E-01	5.560E-01
AXIN1	-0.086	2.511E-01	5.133E-01
TMPRSS6	-0.086	7.951E-01	5.800E-01
PYCR3	-0.086	5.460E-01	5.706E-01
GRM2	-0.086	7.715E-01	6.179E-01
B4GALT7	-0.086	4.087E-01	9.433E-01
MIR5692C2	-0.086	NA	6.307E-01
PI4KB	-0.086	3.861E-01	4.511E-01
PRICKLE4	-0.086	7.193E-01	4.856E-01
BRD1	-0.086	3.193E-01	7.207E-01

KRT7	-0.086	8.317E-01	9.175E-01
TTC31	-0.086	2.445E-01	5.637E-01
RBM45	-0.086	9.259E-02	4.798E-01
C1RL	-0.087	4.986E-01	5.058E-01
SLC26A1	-0.087	6.855E-01	7.253E-01
FRMD6	-0.087	6.977E-01	3.774E-01
NSUN7	-0.087	7.635E-01	6.991E-01
FAM186A	-0.087	7.397E-01	4.614E-01
LIMK2	-0.087	5.348E-01	5.772E-01
WARS2	-0.087	2.708E-01	4.807E-01
NMRK1	-0.087	6.018E-01	5.506E-01
AL358075.4	-0.087	NA	8.066E-01
DEFB113	-0.087	NA	6.059E-01
DEFB116	-0.087	NA	3.573E-01
MIR510	-0.087	NA	3.464E-01
MIR512-1	-0.087	NA	4.435E-01
MIR516B2	-0.087	NA	5.704E-01
MIR5591	-0.087	NA	8.981E-01
MIR7160	-0.087	NA	4.990E-01
MIR920	-0.087	NA	8.661E-01
OR10C1	-0.087	NA	5.818E-01
OR2T12	-0.087	NA	5.843E-01
OR51T1	-0.087	NA	9.776E-01
VN1R4	-0.087	NA	9.788E-01
MIR668	-0.087	NA	9.835E-01
MIR8082	-0.087	NA	5.122E-01
OR6Y1	-0.087	NA	6.060E-01
OR8I2	-0.087	NA	4.839E-01
CHKA	-0.087	6.663E-01	4.760E-01
CDK17	-0.087	3.884E-01	7.924E-01
PTPN12	-0.087	5.293E-01	4.055E-01
ZBTB3	-0.087	4.368E-01	8.130E-01
STN1	-0.087	5.341E-01	5.216E-01
MFSD5	-0.087	4.801E-01	7.164E-01
SLFN13	-0.087	8.096E-01	7.014E-01
FKBP11	-0.087	5.943E-01	4.265E-01
WASF1	-0.087	6.871E-01	7.790E-01
AHDC1	-0.087	4.980E-01	6.952E-01
PNPLA6	-0.087	3.892E-01	5.688E-01
PSMA6	-0.087	4.109E-01	5.534E-01
DDX3Y	-0.087	8.880E-01	6.653E-01
CAND1	-0.087	5.449E-01	4.227E-01
DDB2	-0.087	5.474E-01	5.084E-01
SH2D3C	-0.087	6.499E-01	7.604E-01
AP1M1	-0.087	2.772E-01	4.121E-01
SSFA2	-0.087	6.769E-01	8.561E-01
GM2A	-0.087	6.278E-01	7.330E-01
GNAS	-0.087	3.841E-01	7.896E-01
CWC15	-0.087	3.356E-01	9.503E-01
CX3CR1	-0.087	7.922E-01	6.428E-01

GBE1	-0.087	5.530E-01	5.292E-01
ZNF788	-0.087	7.508E-01	9.308E-01
MIR6859-4	-0.087	8.858E-01	5.769E-01
INO80B	-0.087	5.248E-01	3.690E-01
POP1	-0.087	5.516E-01	7.939E-01
MBD1	-0.087	3.227E-01	6.327E-01
DAZAP1	-0.087	1.988E-01	8.627E-01
CWC25	-0.087	3.194E-01	5.076E-01
CGB7	-0.088	7.883E-01	8.057E-01
ARID4A	-0.088	3.558E-01	8.034E-01
CDK9	-0.088	2.873E-01	7.940E-01
FANCM	-0.088	4.381E-01	5.588E-01
ZNF419	-0.088	4.906E-01	5.631E-01
METTL16	-0.088	3.317E-01	5.392E-01
ATP1B3	-0.088	5.948E-01	9.890E-01
RCOR1	-0.088	5.146E-01	9.890E-01
ATXN10	-0.088	3.189E-01	9.890E-01
SLC25A2	-0.088	7.759E-01	9.890E-01
CWC27	-0.088	2.464E-01	9.890E-01
NBPF12	-0.088	5.965E-01	9.890E-01
:NF625-ZNF2	-0.088	7.240E-01	9.890E-01
SMO	-0.088	7.770E-01	8.322E-01
ZSWIM1	-0.088	3.588E-01	6.639E-01
VDAC1	-0.088	3.686E-01	2.976E-01
ME1	-0.088	7.722E-01	8.567E-01
MIR588	-0.088	NA	5.913E-01
MIR4323	-0.088	NA	5.861E-01
EIF3H	-0.088	3.479E-01	3.828E-01
SBNO1	-0.088	3.482E-01	6.174E-01
HOXA4	-0.088	6.367E-01	3.791E-01
TNRC6A	-0.088	3.906E-01	4.362E-01
CDT1	-0.088	6.414E-01	6.444E-01
SMIM10L1	-0.088	3.628E-01	4.106E-01
TATDN3	-0.088	2.648E-01	8.528E-01
HSPB11	-0.088	4.847E-01	6.392E-01
PSMG4	-0.088	5.027E-01	6.705E-01
ARHGAP42	-0.088	6.781E-01	6.192E-01
ZFYVE16	-0.088	3.364E-01	6.593E-01
STMN4	-0.088	8.822E-01	9.626E-01
FAM193A	-0.088	2.057E-01	7.589E-01
VPS51	-0.088	3.045E-01	4.373E-01
MIR181D	-0.088	NA	5.461E-01
C14orf93	-0.088	3.189E-01	5.323E-01
HGS	-0.088	2.989E-01	8.753E-01
HSPH1	-0.088	5.274E-01	3.573E-01
WIPF1	-0.088	7.240E-01	5.835E-01
ACTR6	-0.088	2.921E-01	4.087E-01
CD1B	-0.088	8.534E-01	7.238E-01
ITPK1	-0.088	5.070E-01	6.057E-01
BLM	-0.088	5.284E-01	5.128E-01

RRP9	-0.088	3.977E-01	9.232E-01
XRCC5	-0.088	2.603E-01	6.819E-01
SCUBE3	-0.088	8.015E-01	4.119E-01
ETV6	-0.088	4.359E-01	3.617E-01
C12orf60	-0.089	5.676E-01	4.483E-01
PLEC	-0.089	6.180E-01	4.104E-01
ZC3H13	-0.089	3.874E-01	6.815E-01
RPL23A	-0.089	4.945E-01	6.956E-01
DTWD1	-0.089	2.872E-01	4.645E-01
DDIT4	-0.089	7.559E-01	8.360E-01
USP19	-0.089	2.446E-01	4.683E-01
NBPF11	-0.089	6.743E-01	8.234E-01
CTC1	-0.089	4.568E-01	5.099E-01
CSNK1G2	-0.089	3.231E-01	6.972E-01
IKBKE	-0.089	5.489E-01	4.634E-01
AP3S1	-0.089	4.734E-01	6.625E-01
SNAPC4	-0.089	3.964E-01	7.256E-01
POLR2J	-0.089	4.705E-01	6.675E-01
MRPS18C	-0.089	2.632E-01	3.812E-01
ZNF492	-0.089	8.760E-01	6.098E-01
SH3BGRL	-0.089	5.985E-01	4.226E-01
B4GALNT1	-0.089	8.349E-01	4.519E-01
FAM133B	-0.089	3.056E-01	6.879E-01
UQCRB	-0.089	4.328E-01	4.331E-01
C15orf57	-0.089	2.510E-01	7.971E-01
RWDD1	-0.089	3.339E-01	7.218E-01
LHFPL2	-0.089	4.962E-01	5.387E-01
PSMB4	-0.089	4.078E-01	8.420E-01
BLVRA	-0.089	6.516E-01	5.494E-01
EL1-TNFRSF	-0.089	5.847E-01	5.511E-01
PARP1	-0.089	4.249E-01	4.167E-01
PIGH	-0.089	2.675E-01	4.975E-01
DLST	-0.089	2.924E-01	8.897E-01
WDR45	-0.089	3.839E-01	5.087E-01
CDC20	-0.089	6.664E-01	4.708E-01
TNPO1	-0.089	3.688E-01	5.649E-01
XRCC4	-0.089	3.620E-01	4.933E-01
TMEM154	-0.089	7.093E-01	5.377E-01
PDP1	-0.089	5.957E-01	5.058E-01
COQ7	-0.089	3.567E-01	4.892E-01
RORB	-0.089	8.460E-01	7.635E-01
CFAP206	-0.089	7.868E-01	5.350E-01
NEDD1	-0.089	4.053E-01	6.936E-01
FBXO25	-0.089	4.235E-01	3.936E-01
MIR4650-2	-0.089	NA	8.059E-01
MIR204	-0.089	NA	6.475E-01
MIR3121	-0.089	NA	4.671E-01
MIR509-1	-0.089	NA	8.358E-01
ACTRT2	-0.089	NA	6.085E-01
MIR4510	-0.089	NA	7.505E-01

MIR513A2	-0.089	NA	6.385E-01
RPAIN	-0.089	4.486E-01	9.789E-01
KCNMB4	-0.089	7.754E-01	5.448E-01
SNRPD2	-0.090	4.301E-01	5.956E-01
LGALS8	-0.090	4.330E-01	7.690E-01
NYAP1	-0.090	7.764E-01	6.403E-01
PCDH7	-0.090	8.259E-01	4.920E-01
PARVG	-0.090	7.795E-01	5.254E-01
NRXN1	-0.090	8.830E-01	7.265E-01
ZBED8	-0.090	4.931E-01	6.039E-01
VRK1	-0.090	4.827E-01	4.057E-01
CCR2	-0.090	8.211E-01	4.580E-01
AAMDC	-0.090	5.944E-01	4.692E-01
SLC30A9	-0.090	2.604E-01	4.762E-01
NPL	-0.090	6.471E-01	6.724E-01
DEAF1	-0.090	4.294E-01	4.241E-01
CHORDC1	-0.090	5.064E-01	4.716E-01
SMAD1	-0.090	5.861E-01	6.953E-01
C19orf44	-0.090	3.736E-01	8.499E-01
EIF3I	-0.090	1.961E-01	7.036E-01
PPID	-0.090	2.254E-01	5.015E-01
TFCP2	-0.090	2.675E-01	4.989E-01
ZNF710	-0.090	3.507E-01	5.594E-01
HNRNPA3	-0.090	1.552E-01	6.897E-01
PRDM15	-0.090	3.422E-01	3.520E-01
MIR4252	-0.090	NA	3.209E-01
MIR8066	-0.090	NA	6.451E-01
GTF2F2	-0.090	3.292E-01	6.048E-01
CAMKK1	-0.090	6.188E-01	8.761E-01
PUF60	-0.090	3.410E-01	7.851E-01
NRL	-0.090	4.800E-01	4.975E-01
ERP29	-0.090	4.427E-01	5.835E-01
GPX2	-0.090	8.622E-01	9.369E-01
SHISA3	-0.090	8.645E-01	6.178E-01
THOC7	-0.090	3.355E-01	8.029E-01
PERP	-0.090	7.084E-01	3.570E-01
FLT4	-0.090	6.677E-01	5.000E-01
EP400	-0.091	2.822E-01	4.241E-01
LMNTD2	-0.091	6.854E-01	7.791E-01
RPP25L	-0.091	5.299E-01	8.249E-01
SYNCRIP	-0.091	2.157E-01	4.465E-01
GP5	-0.091	8.097E-01	5.907E-01
TSHZ1	-0.091	5.327E-01	5.225E-01
GABRE	-0.091	8.384E-01	6.722E-01
RINL	-0.091	6.981E-01	9.137E-01
TUT1	-0.091	3.826E-01	8.021E-01
LRRC9	-0.091	8.506E-01	5.079E-01
WSB2	-0.091	3.408E-01	5.158E-01
BNIP3L	-0.091	5.024E-01	9.265E-01
WDR4	-0.091	4.600E-01	6.275E-01

SFR1	-0.091	3.836E-01	7.547E-01
GGT6	-0.091	8.224E-01	5.824E-01
MIR544B	-0.091	8.909E-01	7.097E-01
SSX5	-0.091	9.462E-01	5.284E-01
BCL7C	-0.091	4.874E-01	6.869E-01
CCNH	-0.091	2.624E-01	7.197E-01
ATP6V1G2	-0.091	6.970E-01	6.228E-01
MIR4308	-0.091	NA	4.688E-01
MIR1252	-0.091	NA	7.419E-01
OR2G6	-0.091	NA	8.637E-01
MLEC	-0.091	4.257E-01	5.429E-01
OR52K2	-0.091	NA	5.126E-01
ARHGAP44	-0.091	7.390E-01	2.525E-01
RTCB	-0.091	3.332E-01	7.373E-01
ZNF107	-0.091	6.190E-01	9.360E-01
GBA2	-0.091	3.972E-01	4.199E-01
NEIL2	-0.091	5.195E-01	6.078E-01
NFAM1	-0.091	7.874E-01	5.630E-01
ZNF300	-0.091	7.974E-01	5.106E-01
DNM1L	-0.091	2.900E-01	5.683E-01
ACTR8	-0.091	2.235E-01	8.096E-01
USP10	-0.091	2.281E-01	6.466E-01
ALG8	-0.091	3.973E-01	6.910E-01
KRTAP2-3	-0.091	9.026E-01	5.244E-01
LRRK2	-0.091	7.632E-01	4.839E-01
CDC23	-0.091	2.251E-01	5.429E-01
FOXP1	-0.091	5.181E-01	3.532E-01
THYN1	-0.091	4.471E-01	6.784E-01
WDR47	-0.091	4.555E-01	6.406E-01
AQP11	-0.091	5.826E-01	6.602E-01
GPR108	-0.091	2.582E-01	8.620E-01
LAMTOR3	-0.091	2.529E-01	8.666E-01
BUB1B	-0.091	5.999E-01	9.407E-01
WBP1	-0.091	5.475E-01	5.858E-01
MIR8055	-0.091	8.423E-01	5.492E-01
CSNK2B	-0.091	4.020E-01	6.424E-01
RFC1	-0.091	3.124E-01	5.177E-01
MON2	-0.091	3.504E-01	3.677E-01
PHLPP1	-0.091	5.928E-01	3.261E-01
PTRH2	-0.091	4.097E-01	9.630E-01
DRC3	-0.092	5.337E-01	9.682E-01
CASP6	-0.092	4.066E-01	6.890E-01
LCE3E	-0.092	9.299E-01	4.237E-01
DHX35	-0.092	2.483E-01	7.617E-01
NUP153	-0.092	4.840E-01	4.521E-01
NAP1L1	-0.092	3.190E-01	4.518E-01
C14orf166	-0.092	1.984E-01	7.879E-01
FURIN	-0.092	4.432E-01	3.205E-01
MIR4298	-0.092	NA	4.972E-01
GGA2	-0.092	3.863E-01	4.712E-01

PRRC2A	-0.092	2.195E-01	6.236E-01
ZNF529	-0.092	4.909E-01	6.649E-01
CDCA3	-0.092	5.935E-01	9.600E-01
MFF	-0.092	3.356E-01	7.379E-01
EEF1AKMT1	-0.092	4.705E-01	6.826E-01
MT-ND4	-0.092	6.947E-01	5.850E-01
ANP32A	-0.092	2.181E-01	4.019E-01
AC004832.3	-0.092	NA	5.192E-01
MIR7843	-0.092	NA	5.356E-01
MIR4314	-0.092	NA	5.320E-01
NMS	-0.092	NA	5.113E-01
HIPK2	-0.092	6.440E-01	9.862E-01
IGF2R	-0.092	4.381E-01	9.806E-01
DNTTIP2	-0.092	2.634E-01	9.804E-01
CHAF1A	-0.092	4.298E-01	9.778E-01
ZRSR2	-0.092	3.917E-01	6.872E-01
ZNF141	-0.093	5.293E-01	9.294E-01
AMACR	-0.093	7.478E-01	7.662E-01
ZKSCAN1	-0.093	4.499E-01	5.383E-01
PTPRQ	-0.093	8.876E-01	5.756E-01
PCMTD2	-0.093	5.093E-01	8.313E-01
GTF2H3	-0.093	3.274E-01	6.161E-01
ZNF793	-0.093	7.128E-01	5.826E-01
COMT	-0.093	6.128E-01	3.580E-01
MIER2	-0.093	3.620E-01	8.428E-01
MSI2	-0.093	5.737E-01	6.769E-01
SENP8	-0.093	4.721E-01	6.323E-01
RHBDL3	-0.093	8.328E-01	4.543E-01
ZC2HC1A	-0.093	5.570E-01	7.941E-01
CFLAR	-0.093	4.084E-01	5.160E-01
MT-ND3	-0.093	6.977E-01	3.989E-01
RPF1	-0.093	1.954E-01	3.494E-01
TTPA	-0.093	8.673E-01	4.649E-01
ZC3H4	-0.093	1.292E-01	4.636E-01
GPR75-ASB3	-0.093	7.643E-01	4.737E-01
ERICH1	-0.093	3.319E-01	6.953E-01
GPR183	-0.093	7.729E-01	8.184E-01
GTF2H1	-0.093	2.573E-01	7.214E-01
PCNX3	-0.093	2.565E-01	7.453E-01
HS3ST3B1	-0.093	7.955E-01	5.157E-01
FAM214B	-0.093	4.800E-01	5.952E-01
INTS1	-0.093	3.253E-01	7.467E-01
FER	-0.093	5.024E-01	6.515E-01
ZMYM2	-0.093	4.278E-01	3.151E-01
ZBTB39	-0.093	4.733E-01	6.102E-01
FBXL17	-0.093	3.717E-01	3.962E-01
BRI3BP	-0.093	4.896E-01	4.605E-01
RSPH9	-0.093	4.206E-01	5.325E-01
MIR103A2	-0.093	6.484E-01	6.391E-01
EBLN2	-0.093	7.124E-01	6.408E-01

ABHD16A	-0.093	2.690E-01	6.109E-01
ATP8B1	-0.094	6.828E-01	7.957E-01
COL24A1	-0.094	7.410E-01	4.570E-01
TAF1D	-0.094	3.894E-01	6.368E-01
RPAP3	-0.094	2.537E-01	4.041E-01
SMC4	-0.094	6.162E-01	5.205E-01
MYSM1	-0.094	4.966E-01	2.816E-01
U2AF1L4	-0.094	4.900E-01	7.927E-01
F8A2	-0.094	NA	8.774E-01
TRIM42	-0.094	NA	5.340E-01
VASH1	-0.094	5.847E-01	7.558E-01
ICA1	-0.094	7.091E-01	5.847E-01
FCRL2	-0.094	8.699E-01	3.904E-01
MIR1273D	-0.094	NA	5.941E-01
TIMM13	-0.094	5.193E-01	4.206E-01
INPP5E	-0.094	3.973E-01	4.971E-01
RMDN2	-0.094	4.968E-01	4.948E-01
DEF8	-0.094	3.302E-01	7.431E-01
AMN1	-0.094	4.941E-01	5.994E-01
AL031708.1	-0.094	7.021E-01	6.420E-01
SETSIP	-0.094	7.054E-01	4.486E-01
GANC	-0.094	3.874E-01	6.977E-01
RHOF	-0.094	7.638E-01	8.561E-01
MTERF1	-0.094	2.122E-01	3.884E-01
TMEM141	-0.094	5.774E-01	6.767E-01
NIP7	-0.094	3.544E-01	5.913E-01
CD86	-0.094	7.632E-01	2.629E-01
SLC13A5	-0.094	8.324E-01	8.380E-01
ARHGDIB	-0.094	5.662E-01	4.754E-01
DCTD	-0.094	2.095E-01	3.887E-01
OR11H4	-0.094	9.186E-01	5.061E-01
PRCD	-0.094	6.941E-01	4.596E-01
MCPH1	-0.094	3.056E-01	5.979E-01
RALGAPA2	-0.094	7.122E-01	6.617E-01
TMEM244	-0.094	8.760E-01	8.049E-01
WSB1	-0.094	5.305E-01	4.549E-01
ARL4A	-0.094	6.533E-01	3.737E-01
SPTBN5	-0.094	7.528E-01	8.052E-01
SGF29	-0.094	5.664E-01	5.167E-01
DAZAP2	-0.094	2.427E-01	9.143E-01
GSR	-0.094	6.387E-01	4.132E-01
UBA3	-0.094	2.493E-01	4.249E-01
ZNF688	-0.094	5.208E-01	6.494E-01
ESRRG	-0.094	8.421E-01	7.162E-01
WDR3	-0.094	4.070E-01	9.760E-01
GOLGA8T	-0.094	8.533E-01	5.305E-01
SLC35E2	-0.094	5.829E-01	4.593E-01
IQSEC1	-0.094	3.973E-01	4.150E-01
SMARCAD1	-0.095	3.279E-01	4.401E-01
CDAN1	-0.095	2.511E-01	5.332E-01

DHRS7	-0.095	4.530E-01	7.277E-01
MRGBP	-0.095	3.325E-01	7.782E-01
CDC37	-0.095	2.446E-01	3.668E-01
ADSL	-0.095	2.689E-01	4.652E-01
SLC25A1	-0.095	4.763E-01	2.295E-01
MIR621	-0.095	5.769E-01	3.371E-01
KIAA0232	-0.095	3.735E-01	4.823E-01
GATA1	-0.095	7.837E-01	4.116E-01
IGSF6	-0.095	7.402E-01	7.856E-01
LYPD6B	-0.095	7.508E-01	5.639E-01
ZFP91-CNTF	-0.095	8.402E-01	8.935E-01
SLC26A6	-0.095	5.422E-01	5.433E-01
TCN2	-0.095	6.741E-01	3.350E-01
APOL6	-0.095	6.389E-01	7.286E-01
SLC25A29	-0.095	5.976E-01	5.004E-01
MIR548S	-0.095	NA	4.933E-01
MIR548AQ	-0.095	NA	6.256E-01
MIR3927	-0.095	NA	8.374E-01
OSBPL3	-0.095	5.519E-01	2.563E-01
TTC12	-0.095	4.477E-01	8.239E-01
INTS6	-0.095	3.410E-01	3.535E-01
OSBPL7	-0.095	5.706E-01	4.583E-01
USP24	-0.095	2.480E-01	9.871E-01
PRPF40A	-0.095	1.706E-01	9.871E-01
ABHD14B	-0.095	5.373E-01	9.858E-01
OCIAD1	-0.095	1.857E-01	9.843E-01
CTTN	-0.095	5.455E-01	9.842E-01
AC011498.5	-0.095	8.446E-01	9.810E-01
DEDD2	-0.095	3.301E-01	6.596E-01
SIRPB1	-0.095	8.172E-01	5.043E-01
PPP1R9B	-0.096	2.666E-01	6.530E-01
MIR1205	-0.096	NA	3.852E-01
MLYCD	-0.096	3.417E-01	2.982E-01
CEP83	-0.096	3.460E-01	4.784E-01
MIR550A2	-0.096	NA	4.499E-01
NRG1	-0.096	8.589E-01	4.091E-01
MT-ATP6	-0.096	6.764E-01	3.734E-01
PHF11	-0.096	4.949E-01	7.702E-01
PFKL	-0.096	3.735E-01	4.831E-01
C18orf8	-0.096	3.154E-01	5.725E-01
CDK19	-0.096	3.324E-01	2.561E-01
P2RY11	-0.096	6.105E-01	5.495E-01
SPDL1	-0.096	3.863E-01	6.457E-01
SYNJ2BP	-0.096	2.356E-01	7.951E-01
TFAM	-0.096	3.003E-01	4.384E-01
DENND4B	-0.096	3.242E-01	7.774E-01
ALS2	-0.096	2.764E-01	8.882E-01
HDX	-0.096	6.848E-01	5.458E-01
NOC3L	-0.096	2.676E-01	3.083E-01
DCUN1D2	-0.096	3.883E-01	5.384E-01

CELF2	-0.096	7.386E-01	5.143E-01
ZNF605	-0.096	4.633E-01	5.230E-01
RPAP1	-0.096	2.149E-01	6.213E-01
NARFL	-0.097	3.724E-01	4.670E-01
IRAK4	-0.097	3.139E-01	5.836E-01
EXD3	-0.097	5.229E-01	7.588E-01
CASP9	-0.097	3.840E-01	5.669E-01
RSBN1L	-0.097	1.699E-01	3.023E-01
PIDD1	-0.097	4.606E-01	5.120E-01
PFN1	-0.097	3.686E-01	2.716E-01
DKK4	-0.097	8.693E-01	4.661E-01
PIKFYVE	-0.097	3.221E-01	4.227E-01
TMX1	-0.097	3.176E-01	4.363E-01
APOB	-0.097	8.758E-01	2.941E-01
EIF4E	-0.097	2.465E-01	5.387E-01
FSD2	-0.097	7.566E-01	3.240E-01
CNOT9	-0.097	1.491E-01	7.567E-01
SIPA1L1	-0.097	2.517E-01	3.692E-01
CHIC2	-0.097	2.307E-01	4.430E-01
TBC1D22A	-0.097	2.226E-01	3.291E-01
TWISTNB	-0.097	3.742E-01	8.458E-01
GLOD5	-0.097	7.744E-01	3.096E-01
DOK2	-0.097	7.869E-01	6.839E-01
TBCA	-0.097	2.508E-01	5.221E-01
BORCS5	-0.097	3.086E-01	4.043E-01
PC	-0.097	6.336E-01	7.464E-01
UPF1	-0.097	8.669E-02	7.401E-01
PI4K2B	-0.097	3.533E-01	3.828E-01
RNASEH2B	-0.097	3.761E-01	7.726E-01
TMA16	-0.097	2.606E-01	4.030E-01
CCDC25	-0.097	2.978E-01	5.339E-01
MIR596	-0.097	NA	3.267E-01
KRTAP10-3	-0.097	NA	8.590E-01
SPATA7	-0.097	3.819E-01	5.678E-01
PCYOX1L	-0.097	4.907E-01	4.826E-01
ADAT3	-0.097	6.057E-01	5.067E-01
CTAGE5	-0.097	3.959E-01	5.261E-01
PPP5D1	-0.097	5.662E-01	5.205E-01
TRIM61	-0.097	8.172E-01	3.941E-01
RNF25	-0.097	3.522E-01	3.927E-01
ZSWIM3	-0.097	4.092E-01	3.452E-01
GABRA2	-0.097	8.771E-01	6.655E-01
IFT27	-0.097	5.306E-01	6.713E-01
PELP1	-0.098	3.524E-01	4.533E-01
PPP1R14B	-0.098	4.552E-01	3.517E-01
WDR89	-0.098	2.443E-01	5.152E-01
USP30	-0.098	2.556E-01	7.438E-01
ALKBH4	-0.098	2.315E-01	8.087E-01
TIMM44	-0.098	2.732E-01	9.661E-01
VPS13A	-0.098	4.302E-01	9.675E-01

ZNF862	-0.098	6.024E-01	4.268E-01
NUDT8	-0.098	6.424E-01	3.252E-01
TTC8	-0.098	4.054E-01	6.456E-01
TMEM168	-0.098	4.841E-01	8.353E-01
ZSCAN5A	-0.098	4.200E-01	2.799E-01
NDE1	-0.098	5.516E-01	9.365E-01
CRLF3	-0.098	2.976E-01	5.552E-01
MIR4421	-0.098	NA	3.388E-01
MIXL1	-0.098	7.993E-01	5.547E-01
RABGGTB	-0.098	3.403E-01	3.539E-01
MIR3678	-0.098	NA	4.253E-01
OR12D3	-0.098	NA	5.584E-01
OR9A2	-0.098	NA	2.657E-01
MIR1278	-0.098	NA	2.628E-01
RFNG	-0.098	3.970E-01	4.273E-01
ZNF441	-0.098	5.015E-01	4.579E-01
TMEM267	-0.098	3.185E-01	2.156E-01
PRKCQ	-0.098	8.092E-01	9.081E-01
GALE	-0.098	5.091E-01	5.558E-01
FARP2	-0.098	3.762E-01	4.227E-01
PHLDA3	-0.098	6.101E-01	4.471E-01
DOT1L	-0.098	3.670E-01	8.408E-01
WDR19	-0.098	3.736E-01	4.983E-01
ARID2	-0.098	3.459E-01	6.310E-01
CHD7	-0.098	4.772E-01	9.864E-01
TSPY2	-0.098	9.756E-01	9.864E-01
FARSA	-0.098	1.734E-01	9.864E-01
RNF43	-0.098	7.770E-01	3.943E-01
CCDC28A	-0.098	3.319E-01	5.724E-01
SNRPA1	-0.098	2.551E-01	4.782E-01
DNASE1L3	-0.098	8.192E-01	6.525E-01
MRPL52	-0.098	4.216E-01	5.194E-01
ADH1A	-0.098	8.514E-01	5.734E-01
NOSTRIN	-0.098	6.387E-01	3.123E-01
NDUFA1	-0.098	4.042E-01	6.973E-01
SNAI1	-0.098	7.408E-01	5.169E-01
TRPV1	-0.098	7.120E-01	4.725E-01
FUNDC1	-0.098	3.108E-01	7.304E-01
USP28	-0.098	3.382E-01	5.252E-01
ZNF281	-0.099	4.230E-01	4.098E-01
TROAP	-0.099	5.752E-01	2.941E-01
NEMP2	-0.099	4.200E-01	6.105E-01
ELK4	-0.099	3.628E-01	4.761E-01
ZSCAN29	-0.099	3.232E-01	7.001E-01
EMILIN2	-0.099	6.853E-01	9.352E-01
TTC36	-0.099	7.180E-01	7.949E-01
MLLT1	-0.099	1.360E-01	6.523E-01
ABT1	-0.099	2.044E-01	6.585E-01
MTSS1L	-0.099	5.662E-01	2.923E-01
RAB3A	-0.099	5.744E-01	4.025E-01

TIMM10	-0.099	3.866E-01	8.585E-01
HRH3	-0.099	8.909E-01	4.205E-01
ACAA1	-0.099	5.162E-01	3.750E-01
ANKRD11	-0.099	2.581E-01	4.066E-01
AC114490.2	-0.099	5.758E-01	6.360E-01
COX18	-0.099	2.936E-01	8.000E-01
MIR3153	-0.099	7.945E-01	7.569E-01
NPAS1	-0.099	7.080E-01	6.846E-01
SEMA4A	-0.099	5.675E-01	7.918E-01
SMIM4	-0.099	5.516E-01	4.921E-01
SPAG7	-0.099	3.268E-01	3.854E-01
C11orf58	-0.099	2.066E-01	4.630E-01
USP49	-0.099	4.223E-01	9.145E-01
POLR2E	-0.099	2.210E-01	4.976E-01
WASHC3	-0.099	2.585E-01	7.279E-01
SGTB	-0.099	5.867E-01	4.176E-01
MIR4739	-0.099	NA	8.163E-01
MIR512-2	-0.099	NA	8.107E-01
MIR548AX	-0.099	NA	4.306E-01
MIR548X2	-0.099	NA	5.914E-01
MIR6815	-0.099	NA	7.766E-01
MIR3672	-0.099	NA	2.788E-01
MIR507	-0.099	NA	2.318E-01
OR4S1	-0.099	NA	7.400E-01
MIR369	-0.099	NA	9.615E-01
MIR6803	-0.099	NA	9.693E-01
OR8J1	-0.099	NA	9.800E-01
ZSWIM7	-0.099	3.670E-01	2.734E-01
OGFOD3	-0.099	2.922E-01	2.215E-01
IL5RA	-0.099	8.045E-01	3.560E-01
MR1	-0.099	5.338E-01	3.251E-01
ATP1A4	-0.099	8.625E-01	4.636E-01
SRP19	-0.100	2.107E-01	2.905E-01
MTA1	-0.100	2.624E-01	3.970E-01
CAPZA1	-0.100	1.839E-01	7.745E-01
MACROD1	-0.100	6.364E-01	4.181E-01
FCGR2B	-0.100	7.802E-01	5.493E-01
FAM122B	-0.100	3.822E-01	3.603E-01
FAN1	-0.100	3.794E-01	3.132E-01
ILDR1	-0.100	6.673E-01	6.135E-01
ABCA7	-0.100	5.996E-01	3.117E-01
MED13L	-0.100	3.842E-01	4.598E-01
ZGPAT	-0.100	2.558E-01	5.935E-01
SRSF7	-0.100	5.672E-02	4.292E-01
PID1	-0.100	7.691E-01	6.986E-01
PIK3C2A	-0.100	3.147E-01	3.008E-01
RTF1	-0.100	2.130E-01	7.261E-01
RAB30	-0.100	5.887E-01	5.592E-01
UBE2E3	-0.100	2.317E-01	3.371E-01
MAGOH	-0.100	2.576E-01	4.483E-01

RFX7	-0.100	3.803E-01	5.062E-01
GJA8	-0.100	NA	5.520E-01
MIR4514	-0.100	NA	3.009E-01
TMCC3	-0.100	7.138E-01	3.710E-01
ZNF280C	-0.100	4.301E-01	5.314E-01
BDP1	-0.100	3.796E-01	7.330E-01
OXA1L	-0.100	1.968E-01	3.097E-01
EWSR1	-0.100	5.352E-02	9.706E-01
CCDC88B	-0.100	6.626E-01	9.706E-01
RAVER2	-0.100	6.311E-01	9.719E-01
NPRL3	-0.100	2.107E-01	9.722E-01
C22orf46	-0.100	2.922E-01	2.732E-01
RBM27	-0.100	1.241E-01	3.298E-01
COPS5	-0.100	1.421E-01	4.786E-01
CD1A	-0.100	8.318E-01	3.500E-01
ULBP1	-0.100	7.754E-01	6.355E-01
RAB37	-0.100	7.443E-01	6.784E-01
RNGTT	-0.100	2.686E-01	7.437E-01
IQCF3	-0.100	NA	4.401E-01
ADAMTS7	-0.100	6.748E-01	4.321E-01
FMN1	-0.100	7.488E-01	4.437E-01
DDX55	-0.101	2.675E-01	4.416E-01
GCN1	-0.101	2.551E-01	4.981E-01
ORAI2	-0.101	4.735E-01	8.210E-01
DTX3L	-0.101	4.814E-01	5.528E-01
ARSK	-0.101	3.999E-01	3.808E-01
CLYBL	-0.101	6.094E-01	3.374E-01
RNPC3	-0.101	4.305E-01	4.448E-01
SCGB3A1	-0.101	8.232E-01	7.126E-01
MIR6876	-0.101	NA	6.358E-01
MIR450B	-0.101	NA	6.817E-01
ANGPTL3	-0.101	7.415E-01	5.904E-01
GDF6	-0.101	8.093E-01	2.923E-01
SMARCA5	-0.101	2.315E-01	4.699E-01
RELL2	-0.101	5.629E-01	3.946E-01
CIR1	-0.101	1.497E-01	6.594E-01
GEMIN4	-0.101	3.422E-01	7.852E-01
NDUFV1	-0.101	3.368E-01	4.989E-01
KATNB1	-0.101	2.282E-01	7.240E-01
CAMKMT	-0.101	3.571E-01	8.366E-01
NAA15	-0.101	2.435E-01	4.580E-01
MEGF11	-0.101	6.701E-01	4.368E-01
PPP1R13B	-0.101	3.968E-01	6.891E-01
UNC119B	-0.101	5.176E-01	3.305E-01
FAM227B	-0.101	4.833E-01	8.687E-01
LMBR1L	-0.101	3.874E-01	4.000E-01
MORN4	-0.101	4.997E-01	8.988E-01
EBF3	-0.101	7.083E-01	9.857E-01
RRH	-0.101	7.509E-01	9.857E-01
C14orf178	-0.101	8.092E-01	9.857E-01

MYH7	-0.101	8.533E-01	4.703E-01
ATG2B	-0.101	2.961E-01	3.678E-01
ING3	-0.101	1.978E-01	4.267E-01
NRROS	-0.101	6.846E-01	4.113E-01
TAC4	-0.101	7.547E-01	5.605E-01
SLC25A46	-0.101	2.393E-01	5.394E-01
MIR320B2	-0.101	8.081E-01	6.460E-01
HJURP	-0.102	5.516E-01	9.399E-01
STX1A	-0.102	5.732E-01	6.318E-01
THUMPD2	-0.102	2.206E-01	6.939E-01
NSD1	-0.102	3.005E-01	8.759E-01
POGLUT1	-0.102	3.017E-01	6.259E-01
MXI1	-0.102	4.697E-01	8.588E-01
NUTM2D	-0.102	5.831E-01	4.253E-01
CFAP46	-0.102	8.167E-01	5.130E-01
MIR3199-1	-0.102	NA	3.201E-01
FBXO9	-0.102	2.312E-01	5.081E-01
PLAGL2	-0.102	4.934E-01	3.021E-01
TULP4	-0.102	1.808E-01	5.131E-01
MARF1	-0.102	1.947E-01	8.569E-01
CSAG3	-0.102	8.831E-01	5.649E-01
TAF1A	-0.102	3.973E-01	3.999E-01
OXER1	-0.102	7.180E-01	6.949E-01
PCBP2	-0.102	1.137E-01	7.022E-01
CLASP1	-0.102	1.665E-01	2.907E-01
MIR374A	-0.102	NA	7.507E-01
TEX28	-0.102	NA	4.958E-01
KRTAP10-5	-0.102	NA	6.234E-01
AC005837.2	-0.102	NA	3.475E-01
MIR506	-0.102	NA	4.016E-01
MIR520H	-0.102	NA	4.573E-01
RANBP3	-0.102	1.132E-01	4.728E-01
DHX58	-0.102	5.071E-01	2.718E-01
TTC22	-0.102	6.820E-01	4.232E-01
IFT80	-0.102	3.355E-01	2.692E-01
HSPA1A	-0.102	7.666E-01	4.519E-01
MARK3	-0.102	1.658E-01	1.999E-01
PRPSAP2	-0.102	3.067E-01	8.255E-01
MRPS9	-0.102	1.914E-01	5.878E-01
NUP50	-0.102	2.085E-01	6.905E-01
CDK11A	-0.102	4.130E-01	8.699E-01
MDP1	-0.102	3.337E-01	5.777E-01
PARS2	-0.103	2.671E-01	3.060E-01
RPP30	-0.103	1.937E-01	6.110E-01
ADD1	-0.103	1.407E-01	7.603E-01
DTD2	-0.103	3.600E-01	9.517E-01
SIN3B	-0.103	2.692E-01	9.531E-01
PCBD2	-0.103	2.516E-01	4.623E-01
SERF1B	-0.103	5.060E-01	9.628E-01
ALG11	-0.103	3.642E-01	5.215E-01

GRK2	-0.103	2.790E-01	6.324E-01
SNRNP70	-0.103	3.175E-01	3.692E-01
YRDC	-0.103	3.132E-01	2.608E-01
PRKCA	-0.103	6.149E-01	3.131E-01
MIR33A	-0.103	NA	3.624E-01
LONP1	-0.103	2.125E-01	6.843E-01
DECR2	-0.103	5.106E-01	5.659E-01
CNTROB	-0.103	2.205E-01	2.846E-01
CDCA4	-0.103	5.065E-01	8.382E-01
SDK1	-0.103	7.280E-01	4.414E-01
HECW1	-0.103	7.795E-01	8.584E-01
KLC4	-0.103	3.264E-01	8.212E-01
SPATS2L	-0.103	4.662E-01	6.414E-01
GEMIN2	-0.103	3.587E-01	3.859E-01
OR52K1	-0.103	NA	6.814E-01
COQ9	-0.103	2.454E-01	3.693E-01
NDOR1	-0.103	3.221E-01	2.334E-01
SLC25A6	-0.103	4.071E-01	8.049E-01
PPT2-EGFL8	-0.103	5.070E-01	4.406E-01
EOGT	-0.103	4.375E-01	3.599E-01
MAST4	-0.103	5.999E-01	5.949E-01
WASF2	-0.103	4.525E-01	2.753E-01
TNFRSF1B	-0.103	6.566E-01	2.970E-01
PPM1F	-0.103	3.782E-01	5.051E-01
BTG1	-0.103	4.800E-01	5.915E-01
SLC6A12	-0.103	7.737E-01	5.674E-01
ZNF546	-0.103	5.526E-01	3.835E-01
NOXRED1	-0.103	5.701E-01	8.467E-01
SPOUT1	-0.104	2.044E-01	5.332E-01
OR7A17	-0.104	NA	4.426E-01
KRTAP26-1	-0.104	NA	5.695E-01
NDFIP2	-0.104	4.924E-01	2.974E-01
LZTS3	-0.104	6.413E-01	9.556E-01
CDC45	-0.104	5.596E-01	2.810E-01
ITGB2	-0.104	7.730E-01	7.127E-01
TMEM8B	-0.104	4.476E-01	2.409E-01
PSMA4	-0.104	2.195E-01	4.807E-01
RPS27A	-0.104	3.217E-01	7.874E-01
GEMIN8	-0.104	3.572E-01	3.677E-01
MYCBP2	-0.104	4.465E-01	6.211E-01
FLG	-0.104	8.438E-01	3.395E-01
ERBIN	-0.104	2.790E-01	6.247E-01
NAP1L4	-0.104	1.622E-01	3.284E-01
BRMS1L	-0.104	4.227E-01	8.306E-01
FUS	-0.104	1.658E-01	3.571E-01
SFT2D1	-0.104	1.538E-01	4.440E-01
MIR1290	-0.104	NA	7.059E-01
MUTYH	-0.104	3.489E-01	3.432E-01
BDH1	-0.104	5.981E-01	3.247E-01
NOL6	-0.104	3.056E-01	8.156E-01

SAMM50	-0.104	2.730E-01	3.970E-01
PMS1	-0.104	1.782E-01	8.653E-01
INF2	-0.104	4.490E-01	8.166E-01
AHSA1	-0.104	1.975E-01	4.307E-01
RICTOR	-0.104	3.228E-01	9.173E-01
ARHGEF1	-0.104	2.855E-01	4.096E-01
MRPL43	-0.104	1.980E-01	2.715E-01
MIR5000	-0.104	NA	3.751E-01
FOXE1	-0.104	8.693E-01	5.128E-01
FAM111A	-0.104	3.475E-01	7.758E-01
LMO4	-0.104	5.788E-01	3.662E-01
MAP3K12	-0.104	5.045E-01	4.190E-01
BCL2L11	-0.104	3.833E-01	6.358E-01
NPM3	-0.104	5.054E-01	3.843E-01
TUBA1C	-0.104	4.060E-01	7.384E-01
AFAP1L2	-0.104	6.871E-01	2.324E-01
SOD2	-0.104	6.751E-01	4.695E-01
AC009779.3	-0.105	5.030E-01	7.698E-01
TBC1D2	-0.105	5.706E-01	7.055E-01
SLC1A1	-0.105	6.977E-01	9.454E-01
NR4A1	-0.105	7.507E-01	4.245E-01
PTPN13	-0.105	6.751E-01	4.825E-01
CYYR1	-0.105	5.930E-01	8.509E-01
F2RL2	-0.105	7.944E-01	7.261E-01
RRAS2	-0.105	5.415E-01	4.763E-01
NDUFS5	-0.105	4.646E-01	4.925E-01
QDPR	-0.105	3.863E-01	3.916E-01
LGALS3BP	-0.105	5.435E-01	4.824E-01
AC010463.1	-0.105	NA	7.008E-01
MIR1273A	-0.105	NA	9.349E-01
MIR1251	-0.105	NA	7.680E-01
OR13C4	-0.105	NA	2.075E-01
MIR625	-0.105	NA	4.813E-01
LINC00116	-0.105	5.218E-01	4.145E-01
BX248409.2	-0.105	NA	5.328E-01
MIR513C	-0.105	NA	5.864E-01
MIR548AO	-0.105	NA	4.754E-01
GFRA2	-0.105	7.464E-01	3.817E-01
TOMM22	-0.105	2.225E-01	3.503E-01
SLC22A16	-0.105	8.196E-01	5.115E-01
PCBP3	-0.105	7.681E-01	3.367E-01
DDX56	-0.105	1.816E-01	7.884E-01
GCNT2	-0.105	7.442E-01	5.601E-01
HIC2	-0.105	7.451E-01	3.228E-01
SDHD	-0.105	2.356E-01	2.953E-01
MIR150	-0.105	NA	3.210E-01
DUSP28	-0.105	3.422E-01	2.654E-01
NLRP7	-0.105	8.663E-01	4.193E-01
RALGDS	-0.105	3.234E-01	5.502E-01
CACTIN	-0.105	2.073E-01	8.444E-01

KIAA1551	-0.105	3.966E-01	6.079E-01
MNAT1	-0.105	2.980E-01	5.681E-01
FNDC8	-0.105	7.558E-01	7.386E-01
SNRPD3	-0.105	2.578E-01	3.970E-01
KIAA1147	-0.105	4.715E-01	4.660E-01
RNPEPL1	-0.105	3.714E-01	2.306E-01
PHF24	-0.105	7.994E-01	9.592E-01
UQCC2	-0.105	3.918E-01	9.594E-01
ORCS8-MEF2	-0.105	5.325E-01	2.925E-01
GTF2H2	-0.105	6.444E-01	4.097E-01
CHRNA10	-0.105	6.329E-01	5.366E-01
AKR1C3	-0.105	8.167E-01	4.009E-01
NCL	-0.105	2.657E-01	2.253E-01
ZC3H10	-0.105	3.286E-01	5.920E-01
STXBP2	-0.105	3.905E-01	7.557E-01
SCRIB	-0.105	3.548E-01	2.191E-01
HSD17B8	-0.106	5.442E-01	6.548E-01
MRPL38	-0.106	2.569E-01	2.316E-01
MT-ATP8	-0.106	7.013E-01	3.457E-01
BIN3	-0.106	2.346E-01	6.273E-01
TPRX1	-0.106	NA	2.089E-01
PPP1CC	-0.106	1.582E-01	3.803E-01
CCDC172	-0.106	NA	7.162E-01
ERO1A	-0.106	5.204E-01	4.604E-01
UTF1	-0.106	8.570E-01	9.825E-01
ZGLP1	-0.106	6.454E-01	7.919E-01
PPP1R10	-0.106	1.917E-01	4.821E-01
SPRED2	-0.106	3.841E-01	3.188E-01
LRR1	-0.106	4.054E-01	2.462E-01
RNF14	-0.106	1.232E-01	3.302E-01
MIR5009	-0.106	NA	4.990E-01
MIR589	-0.106	6.897E-01	6.770E-01
PRR4	-0.106	4.844E-01	3.199E-01
NUDT16L1	-0.106	2.813E-01	4.058E-01
TBC1D2B	-0.106	3.267E-01	4.095E-01
FAM162B	-0.106	6.937E-01	6.843E-01
IRX4	-0.106	8.971E-01	3.241E-01
CWC22	-0.106	9.192E-02	5.306E-01
CCDC78	-0.106	6.979E-01	7.894E-01
INO80D	-0.106	2.425E-01	8.434E-01
ZNF485	-0.106	3.953E-01	5.862E-01
HSBP1	-0.106	2.375E-01	3.460E-01
RIMS4	-0.106	8.592E-01	4.878E-01
THAP2	-0.106	3.430E-01	5.346E-01
FAM57B	-0.106	7.819E-01	7.552E-01
ANAPC10	-0.106	1.984E-01	3.401E-01
LAMTOR5	-0.106	1.568E-01	5.672E-01
PRKACB	-0.106	5.348E-01	3.242E-01
B9D2	-0.106	4.499E-01	3.526E-01
ZNF474	-0.106	7.029E-01	3.095E-01

SOGA3	-0.106	7.364E-01	6.963E-01
MCEMP1	-0.106	7.850E-01	5.575E-01
RBM12B	-0.106	3.093E-01	5.768E-01
RNF152	-0.106	6.415E-01	6.967E-01
GLRX3	-0.106	2.399E-01	5.441E-01
OTUD4	-0.106	3.240E-01	9.585E-01
ZNF708	-0.106	5.133E-01	6.968E-01
EML4	-0.106	3.317E-01	3.080E-01
DEFB136	-0.106	NA	4.915E-01
MIR4731	-0.106	NA	8.199E-01
MIR6882	-0.106	NA	6.073E-01
OR10J3	-0.106	NA	5.777E-01
MIR6718	-0.106	NA	2.337E-01
PIK3R6	-0.106	6.730E-01	5.043E-01
TIAM2	-0.106	4.378E-01	7.354E-01
WDR55	-0.106	1.289E-01	4.680E-01
ABHD17A	-0.107	2.876E-01	4.816E-01
LYPD6	-0.107	7.175E-01	2.298E-01
PDZD7	-0.107	7.093E-01	3.299E-01
PRKD2	-0.107	2.101E-01	3.098E-01
OTOP2	-0.107	8.587E-01	1.653E-01
CCDC134	-0.107	4.833E-01	2.644E-01
REPS1	-0.107	1.364E-01	1.762E-01
C9orf152	-0.107	8.249E-01	7.462E-01
ENTPD6	-0.107	3.083E-01	2.924E-01
NRARP	-0.107	7.363E-01	7.264E-01
GTF2H2C	-0.107	3.894E-01	9.184E-01
PMPCA	-0.107	3.056E-01	3.313E-01
C8orf59	-0.107	2.924E-01	5.927E-01
APPL2	-0.107	3.174E-01	2.957E-01
TSSK6	-0.107	3.928E-01	5.709E-01
FBXO38	-0.107	1.502E-01	6.093E-01
MLF2	-0.107	1.732E-01	2.768E-01
PSMA1	-0.107	1.555E-01	8.029E-01
RFPL4AL1	-0.107	NA	3.255E-01
ZNF8	-0.107	3.335E-01	7.173E-01
SGPP2	-0.107	7.413E-01	3.551E-01
ZNF333	-0.107	4.112E-01	2.519E-01
CCT6A	-0.107	3.662E-01	4.907E-01
TTLL8	-0.107	8.829E-01	5.983E-01
SFN	-0.107	7.275E-01	3.706E-01
STAG3	-0.107	6.887E-01	4.168E-01
LRRC69	-0.107	6.393E-01	3.510E-01
MIR1273H	-0.107	NA	8.040E-01
MLXIPL	-0.107	7.977E-01	8.426E-01
ATG12	-0.107	2.091E-01	3.069E-01
TOB2	-0.107	2.976E-01	3.695E-01
CCDC181	-0.107	7.532E-01	5.591E-01
ARFGEF3	-0.107	7.125E-01	4.233E-01
IGF2BP1	-0.107	8.773E-01	2.493E-01

GTF2F1	-0.107	9.091E-02	4.763E-01
MYO3A	-0.107	8.895E-01	4.538E-01
TEX12	-0.108	7.250E-01	3.417E-01
RPS24	-0.108	4.359E-01	5.205E-01
WASHC4	-0.108	2.240E-01	4.479E-01
EXOSC5	-0.108	4.229E-01	2.960E-01
DCAF17	-0.108	1.986E-01	6.508E-01
MIR6752	-0.108	NA	8.817E-01
MIR5191	-0.108	NA	5.513E-01
MIR607	-0.108	NA	6.827E-01
OR7D4	-0.108	NA	1.996E-01
MIR1277	-0.108	NA	5.003E-01
MIR4450	-0.108	NA	4.177E-01
MIR4491	-0.108	NA	5.194E-01
OR5A2	-0.108	NA	9.773E-01
GAGE12H	-0.108	NA	9.065E-01
TIGD5	-0.108	3.453E-01	3.497E-01
SH2D4B	-0.108	7.642E-01	8.145E-01
TBCK	-0.108	3.368E-01	5.659E-01
ASPHD1	-0.108	7.380E-01	2.801E-01
FASTKD2	-0.108	1.674E-01	7.352E-01
EXO1	-0.108	5.622E-01	6.748E-01
ADA	-0.108	6.861E-01	5.306E-01
SRA1	-0.108	2.556E-01	6.519E-01
MT-CO2	-0.108	5.856E-01	4.325E-01
PTPN11	-0.108	3.452E-01	4.440E-01
SFPQ	-0.108	4.155E-02	3.348E-01
FKBP3	-0.108	2.410E-01	2.567E-01
LPAR1	-0.108	6.318E-01	5.975E-01
MIR6081	-0.108	NA	3.379E-01
AHSP	-0.108	8.693E-01	2.358E-01
PPIL4	-0.108	1.596E-01	3.501E-01
UNG	-0.108	2.526E-01	1.083E-01
FBXL4	-0.108	1.974E-01	4.448E-01
CBY1	-0.108	3.160E-01	4.650E-01
PDS5A	-0.108	1.421E-01	2.149E-01
IQCA1L	-0.108	NA	9.838E-01
SHROOM2	-0.108	6.190E-01	9.822E-01
COQ4	-0.108	3.184E-01	9.802E-01
ZNF354B	-0.108	3.796E-01	4.917E-01
PRICKLE3	-0.108	3.802E-01	2.908E-01
EDRF1	-0.108	2.194E-01	7.506E-01
ULK3	-0.108	3.696E-01	3.479E-01
SLC2A8	-0.108	5.084E-01	2.032E-01
PATL2	-0.108	6.822E-01	2.791E-01
EFCAB13	-0.108	5.895E-01	3.140E-01
UTY	-0.108	8.690E-01	7.995E-01
SARNP	-0.109	3.379E-01	6.847E-01
MVB12A	-0.109	3.006E-01	5.093E-01
UBXN6	-0.109	2.019E-01	7.422E-01

AL133352.1	-0.109	6.769E-01	8.222E-01
STX10	-0.109	2.246E-01	5.944E-01
AC107871.1	-0.109	7.972E-01	3.903E-01
ZBTB26	-0.109	2.526E-01	3.949E-01
PITPNM2	-0.109	5.177E-01	1.739E-01
GGA3	-0.109	1.515E-01	6.674E-01
ZNF292	-0.109	4.151E-01	7.031E-01
ZNF598	-0.109	1.738E-01	4.169E-01
RCOR3	-0.109	2.931E-01	7.326E-01
CMPK2	-0.109	6.849E-01	2.698E-01
GNPDA2	-0.109	2.292E-01	2.406E-01
AC006486.1	-0.109	NA	9.361E-01
ZKSCAN3	-0.109	3.863E-01	4.598E-01
FAM219B	-0.109	4.471E-01	3.223E-01
METTL25	-0.109	2.157E-01	5.826E-01
PCDH8	-0.109	8.993E-01	9.252E-01
DDX17	-0.109	1.899E-01	5.026E-01
RPL11	-0.109	3.219E-01	6.936E-01
MIR5688	-0.109	NA	3.224E-01
NUBPL	-0.109	2.264E-01	4.474E-01
AC018630.6	-0.109	6.620E-01	2.852E-01
HSF2	-0.109	2.985E-01	3.797E-01
TMEM242	-0.109	2.250E-01	2.673E-01
MIR3186	-0.109	8.407E-01	2.801E-01
RPL27	-0.109	3.118E-01	7.884E-01
PNOC	-0.109	8.417E-01	6.662E-01
DGCR6	-0.109	6.632E-01	2.804E-01
POLD4	-0.109	4.170E-01	4.197E-01
DPY19L1	-0.109	3.736E-01	2.378E-01
STYK1	-0.109	5.873E-01	2.158E-01
DRG1	-0.109	2.136E-01	7.724E-01
MAP3K10	-0.109	3.726E-01	7.754E-01
MYO10	-0.109	6.513E-01	3.972E-01
NPIPB12	-0.110	7.467E-01	7.174E-01
RAB11B	-0.110	2.159E-01	4.896E-01
GPR101	-0.110	8.940E-01	2.615E-01
CHD9	-0.110	3.314E-01	6.031E-01
LCN1	-0.110	8.072E-01	4.500E-01
CCNT1	-0.110	1.750E-01	2.028E-01
HDHD3	-0.110	4.338E-01	4.449E-01
TMEM14A	-0.110	3.446E-01	2.236E-01
HLA-E	-0.110	4.778E-01	2.939E-01
STEAP2	-0.110	6.839E-01	1.333E-01
MBD6	-0.110	2.551E-01	3.060E-01
LRRC29	-0.110	4.986E-01	8.537E-01
CCDC65	-0.110	3.803E-01	2.765E-01
ZNF721	-0.110	3.393E-01	2.957E-01
PRRT1	-0.110	5.791E-01	4.270E-01
ELFN2	-0.110	8.414E-01	2.149E-01
MTHFSD	-0.110	2.593E-01	2.824E-01

AMIGO2	-0.110	6.839E-01	2.024E-01
POLE	-0.110	3.409E-01	6.064E-01
ADCK1	-0.110	2.478E-01	8.599E-01
ZFYVE27	-0.110	1.680E-01	6.037E-01
DHPS	-0.110	2.222E-01	9.526E-01
ZNF500	-0.110	1.517E-01	2.450E-01
TBC1D16	-0.110	4.181E-01	6.917E-01
MGAT5	-0.110	4.021E-01	2.111E-01
INHBC	-0.110	7.415E-01	3.901E-01
PSMA2	-0.110	2.313E-01	8.376E-01
CD68	-0.110	6.626E-01	3.481E-01
DOK1	-0.110	5.463E-01	7.520E-01
GAS8	-0.110	3.041E-01	2.757E-01
TET2	-0.110	3.887E-01	5.941E-01
JAKMIP3	-0.110	7.904E-01	8.919E-01
GATAD2A	-0.110	9.309E-02	3.769E-01
NFKB1	-0.110	3.093E-01	6.436E-01
BBS10	-0.110	2.848E-01	5.217E-01
DDX28	-0.110	3.378E-01	4.752E-01
CLDND1	-0.110	2.918E-01	6.606E-01
ZNF443	-0.110	5.559E-01	9.613E-01
G2E3	-0.111	2.761E-01	9.665E-01
OR11H2	-0.111	NA	7.615E-01
MIR615	-0.111	NA	7.695E-01
SENP6	-0.111	1.481E-01	7.064E-01
SULT1B1	-0.111	8.234E-01	8.062E-01
PRSS21	-0.111	8.635E-01	4.183E-01
PRR13	-0.111	2.284E-01	3.763E-01
POLR1E	-0.111	3.598E-01	4.091E-01
STAC	-0.111	7.959E-01	3.156E-01
ADGRL1	-0.111	6.292E-01	2.695E-01
EIF4ENIF1	-0.111	1.512E-01	4.592E-01
VARS	-0.111	2.420E-01	3.909E-01
NAV1	-0.111	5.749E-01	2.675E-01
GPATCH2L	-0.111	1.933E-01	5.086E-01
C1QB	-0.111	7.805E-01	1.656E-01
'HURC1-FNT	-0.111	7.801E-01	9.831E-01
ZNF7	-0.111	1.461E-01	9.831E-01
ITPR3	-0.111	5.703E-01	9.831E-01
MIR3132	-0.111	NA	2.453E-01
PGPEP1	-0.111	5.299E-01	4.884E-01
UGGT2	-0.111	3.638E-01	4.218E-01
MAP1LC3B2	-0.111	2.919E-01	5.605E-01
CFAP100	-0.111	6.907E-01	5.365E-01
VPS29	-0.111	1.501E-01	6.879E-01
KLHL8	-0.111	3.206E-01	5.451E-01
MRPL32	-0.111	1.293E-01	1.579E-01
C19orf25	-0.111	3.208E-01	3.942E-01
PARD6G	-0.111	5.417E-01	6.580E-01
ALKBH1	-0.111	1.023E-01	2.624E-01

KDM3B	-0.111	2.044E-01	6.696E-01
VMAC	-0.111	3.708E-01	7.907E-01
TTPAL	-0.111	3.572E-01	4.342E-01
CNPY2	-0.111	3.597E-01	1.523E-01
INSR	-0.111	4.222E-01	6.290E-01
UTP3	-0.111	1.440E-01	8.089E-01
LYPD5	-0.111	6.848E-01	3.678E-01
NF2	-0.111	3.143E-01	1.407E-01
ADCY7	-0.111	7.173E-01	5.613E-01
KCNRG	-0.111	5.915E-01	3.703E-01
EXOC7	-0.111	1.691E-01	4.677E-01
TMBIM4	-0.111	3.561E-01	1.268E-01
IL7	-0.111	6.425E-01	2.219E-01
UNK	-0.111	1.984E-01	1.404E-01
NUDT4	-0.111	5.348E-01	4.711E-01
TELO2	-0.111	2.429E-01	2.399E-01
NAA10	-0.112	3.791E-01	3.110E-01
TMEFF1	-0.112	8.472E-01	2.247E-01
ZBTB2	-0.112	1.169E-01	5.870E-01
SRPX2	-0.112	6.747E-01	3.572E-01
CELA1	-0.112	8.596E-01	2.275E-01
MIR766	-0.112	NA	2.900E-01
MKS1	-0.112	2.027E-01	7.362E-01
MIR6780B	-0.112	NA	3.294E-01
MIR4776-1	-0.112	NA	6.214E-01
MIR4451	-0.112	NA	1.811E-01
MIR4511	-0.112	NA	4.262E-01
MIR3128	-0.112	NA	7.574E-01
CORO1B	-0.112	2.925E-01	2.482E-01
EIF3L	-0.112	3.753E-01	3.649E-01
AC006538.2	-0.112	NA	5.714E-01
KRTAP19-2	-0.112	NA	5.887E-01
MIR517C	-0.112	NA	3.269E-01
MIR518A1	-0.112	NA	9.655E-01
OR14A16	-0.112	NA	2.711E-01
OR51G2	-0.112	NA	3.153E-01
MAVS	-0.112	2.549E-01	6.059E-01
MPC1	-0.112	3.057E-01	3.552E-01
SCRN2	-0.112	4.466E-01	2.388E-01
MAGEE1	-0.112	6.234E-01	7.406E-01
TAF10	-0.112	2.869E-01	4.602E-01
UBE2D3	-0.112	7.331E-02	3.522E-01
LAPTM5	-0.112	7.046E-01	4.673E-01
WDR25	-0.112	2.721E-01	2.456E-01
AP000350.4	-0.112	8.472E-01	4.495E-01
TFAP2A	-0.112	6.776E-01	2.893E-01
MBD3	-0.112	3.156E-01	9.076E-01
CDC42EP4	-0.112	4.490E-01	3.709E-01
SH2D4A	-0.112	5.990E-01	2.917E-01
PKP4	-0.112	2.307E-01	3.008E-01

CTPS2	-0.112	2.346E-01	1.634E-01
ARGLU1	-0.112	3.923E-01	5.313E-01
SH3BGRL3	-0.112	4.192E-01	2.272E-01
FASTKD5	-0.113	2.129E-01	1.753E-01
RAP1B	-0.113	4.216E-01	3.970E-01
MAP2K2	-0.113	2.380E-01	9.714E-01
TM4SF18	-0.113	6.282E-01	9.716E-01
PTCD2	-0.113	1.708E-01	3.203E-01
HHAT	-0.113	4.895E-01	3.068E-01
NR2C2	-0.113	3.950E-01	7.485E-01
NT5C1B	-0.113	5.905E-01	1.541E-01
WNT3	-0.113	6.513E-01	1.752E-01
STRN3	-0.113	2.278E-01	4.571E-01
PDCD11	-0.113	1.721E-01	5.883E-01
RGS1	-0.113	7.214E-01	7.302E-01
IPO11	-0.113	2.265E-01	4.113E-01
E2F4	-0.113	1.615E-01	3.364E-01
EXOSC9	-0.113	1.728E-01	7.695E-01
KCNIP4	-0.113	4.484E-01	6.682E-01
SECTM1	-0.113	7.652E-01	7.191E-01
PPP1R13L	-0.113	5.191E-01	3.245E-01
CPSF1	-0.113	2.271E-01	6.962E-01
MLH3	-0.113	1.388E-01	3.834E-01
PTGR2	-0.113	4.301E-01	7.315E-01
FAM71C	-0.113	NA	2.431E-01
MED9	-0.113	1.843E-01	9.157E-01
TMEM54	-0.113	4.511E-01	3.222E-01
TRIQK	-0.113	4.170E-01	3.152E-01
ZNF211	-0.113	5.020E-01	4.348E-01
PACRGL	-0.113	1.519E-01	7.297E-01
MED21	-0.113	2.288E-01	6.249E-01
BATF	-0.113	6.348E-01	4.422E-01
MICB	-0.113	6.776E-01	3.156E-01
MED15	-0.113	2.444E-01	3.623E-01
CXorf38	-0.113	2.444E-01	1.997E-01
FKBP8	-0.113	1.987E-01	6.011E-01
MRPL23	-0.113	4.709E-01	3.653E-01
PLEKHS1	-0.113	8.369E-01	2.880E-01
EEF1AKMT3	-0.113	2.995E-01	7.117E-01
MICAL3	-0.113	4.715E-01	3.018E-01
RPS4Y2	-0.113	8.786E-01	3.240E-01
GPN3	-0.113	1.968E-01	3.418E-01
AL109827.1	-0.113	NA	5.919E-01
MIR4522	-0.113	NA	1.359E-01
OR6M1	-0.113	NA	4.567E-01
MIR578	-0.113	NA	9.568E-01
CCDC71	-0.113	2.149E-01	9.546E-01
CHRNA6	-0.113	7.759E-01	7.120E-01
SRPK1	-0.113	2.399E-01	3.034E-01
PYURF	-0.113	3.772E-01	5.359E-01

ZNF24	-0.114	1.492E-01	4.227E-01
ZCCHC8	-0.114	1.494E-01	2.368E-01
CNKSR3	-0.114	5.935E-01	2.549E-01
SLC5A10	-0.114	6.799E-01	2.646E-01
KHSRP	-0.114	7.018E-02	6.890E-01
RPS6KA5	-0.114	5.300E-01	6.877E-01
NCDN	-0.114	1.114E-01	5.296E-01
MIR3936	-0.114	6.169E-01	5.415E-01
HTR1B	-0.114	7.910E-01	6.621E-01
ESCO1	-0.114	1.435E-01	6.059E-01
CARD16	-0.114	6.551E-01	1.125E-01
RBBP8	-0.114	5.475E-01	3.067E-01
MIR26B	-0.114	7.560E-01	5.742E-01
CDC40	-0.114	2.149E-01	2.870E-01
ASB1	-0.114	2.986E-01	5.165E-01
APBA3	-0.114	1.819E-01	2.450E-01
FOXP3	-0.114	6.413E-01	5.031E-01
FBXL12	-0.114	1.842E-01	2.202E-01
CCDC122	-0.114	4.384E-01	2.153E-01
LILRB2	-0.114	7.586E-01	3.267E-01
HIST1H3I	-0.114	7.698E-01	2.710E-01
MBLAC2	-0.114	3.662E-01	4.475E-01
SNN	-0.114	4.828E-01	8.802E-01
HEATR3	-0.114	1.451E-01	8.516E-01
RPS6	-0.114	4.593E-01	9.444E-01
CLEC18A	-0.114	7.095E-01	2.496E-01
MFHAS1	-0.114	5.099E-01	8.314E-01
PPP1R9A	-0.114	7.759E-01	1.451E-01
PPP2CA	-0.114	6.706E-02	5.751E-01
METAP1	-0.114	3.169E-01	1.682E-01
ZDHHC23	-0.114	4.745E-01	1.641E-01
SNX18	-0.114	2.961E-01	3.093E-01
NCOR1	-0.114	2.546E-01	4.810E-01
DNAJC22	-0.115	7.551E-01	6.273E-01
MSL3	-0.115	8.669E-02	6.359E-01
SVIP	-0.115	5.364E-01	1.647E-01
MIR623	-0.115	8.599E-01	5.028E-01
TWF1	-0.115	3.480E-01	2.897E-01
PHLDB2	-0.115	6.098E-01	1.792E-01
ANKS3	-0.115	3.795E-01	4.937E-01
GLI3	-0.115	6.595E-01	1.530E-01
PTTG1	-0.115	5.364E-01	6.985E-01
MIR30E	-0.115	NA	4.254E-01
CCDC59	-0.115	1.356E-01	5.582E-01
DEFB104B	-0.115	NA	7.441E-01
RNF141	-0.115	3.175E-01	6.074E-01
GIMAP4	-0.115	6.236E-01	3.203E-01
S100A11	-0.115	4.715E-01	2.544E-01
POLR1D	-0.115	1.878E-01	5.172E-01
AC008764.4	-0.115	6.781E-01	6.031E-01

NDUFA10	-0.115	2.051E-01	9.706E-01
CMTM4	-0.115	5.419E-01	9.729E-01
SETDB2	-0.115	2.961E-01	9.728E-01
BMP2K	-0.115	4.841E-01	9.711E-01
LGI4	-0.115	6.817E-01	2.169E-01
AKR1C2	-0.115	8.210E-01	3.221E-01
ATP1B1	-0.115	6.609E-01	4.420E-01
PPP2R2B	-0.115	7.851E-01	3.218E-01
PRR3	-0.115	2.978E-01	8.138E-01
IL27RA	-0.115	6.560E-01	2.771E-01
AGPAT2	-0.115	5.633E-01	1.300E-01
MIR6736	-0.115	NA	7.310E-01
WDR18	-0.115	3.225E-01	9.497E-01
SLFN14	-0.115	7.722E-01	5.816E-01
RHBDD3	-0.115	2.986E-01	1.260E-01
MPDU1	-0.115	2.624E-01	9.174E-01
RSL24D1	-0.115	1.046E-01	3.816E-01
TRAPPC3L	-0.115	7.433E-01	2.335E-01
ANKRD10	-0.116	3.674E-01	1.734E-01
GNPNAT1	-0.116	2.726E-01	7.771E-01
ZNF714	-0.116	5.655E-01	2.991E-01
NAPSA	-0.116	6.048E-01	7.232E-01
PMP2	-0.116	8.448E-01	3.919E-01
FAM213B	-0.116	4.168E-01	8.395E-01
FUT8	-0.116	4.801E-01	7.124E-01
AK6	-0.116	1.296E-01	6.313E-01
ERCC6L	-0.116	5.342E-01	1.707E-01
KCNS3	-0.116	6.516E-01	1.869E-01
WISP3	-0.116	8.038E-01	5.998E-01
DBT	-0.116	2.837E-01	6.297E-01
MIEF1	-0.116	1.584E-01	2.545E-01
KLK6	-0.116	8.858E-01	7.163E-01
IFI44L	-0.116	7.591E-01	3.110E-01
PRKX	-0.116	4.733E-01	7.295E-01
CLN5	-0.116	2.444E-01	3.272E-01
NLRP14	-0.116	7.764E-01	4.970E-01
EXOC3L1	-0.116	3.840E-01	2.447E-01
OLA1	-0.116	1.547E-01	7.263E-01
SMPDL3A	-0.116	5.697E-01	6.060E-01
CYB5B	-0.116	3.056E-01	6.658E-01
MRPL4	-0.116	2.462E-01	4.111E-01
NKX6-2	-0.116	8.206E-01	2.655E-01
PITPNB	-0.116	1.693E-01	6.640E-01
R3HDM4	-0.116	1.844E-01	2.801E-01
USP40	-0.116	3.710E-01	8.345E-01
MIR7155	-0.116	NA	5.242E-01
CRYGB	-0.116	NA	3.872E-01
FERD3L	-0.116	NA	2.793E-01
B4GALNT4	-0.116	7.998E-01	3.093E-01
C9orf16	-0.116	4.644E-01	9.226E-01

DDIAS	-0.116	4.801E-01	1.963E-01
WDFY1	-0.116	2.179E-01	4.991E-01
HRC	-0.116	6.869E-01	8.470E-01
SOX12	-0.116	5.001E-01	6.159E-01
ROMO1	-0.116	4.334E-01	2.099E-01
SNCAIP	-0.116	7.143E-01	4.630E-01
SPINK9	-0.117	7.585E-01	8.006E-01
ZNF564	-0.117	2.742E-01	4.835E-01
SMIM20	-0.117	2.105E-01	5.580E-01
PHLDA1	-0.117	6.387E-01	8.518E-01
LAMP3	-0.117	6.904E-01	4.524E-01
FBXO16	-0.117	5.876E-01	6.972E-01
OR2J3	-0.117	8.999E-01	4.875E-01
TICRR	-0.117	4.840E-01	4.876E-01
B3GNTL1	-0.117	3.370E-01	5.607E-01
UTRN	-0.117	5.204E-01	5.859E-01
LEPROTL1	-0.117	1.597E-01	4.723E-01
GAREM1	-0.117	6.127E-01	2.265E-01
LMNB2	-0.117	3.623E-01	6.147E-01
RAB34	-0.117	6.081E-01	2.175E-01
METTL26	-0.117	4.107E-01	2.812E-01
RSPH6A	-0.117	8.262E-01	2.515E-01
TSSK4	-0.117	4.868E-01	5.138E-01
KLF7	-0.117	5.187E-01	1.924E-01
FOXO1	-0.117	3.970E-01	3.192E-01
SUCLA2	-0.117	3.043E-01	2.954E-01
NOP9	-0.117	1.867E-01	1.199E-01
RMDN1	-0.117	1.393E-01	4.532E-01
ZNF326	-0.117	1.265E-01	3.330E-01
MKNK1	-0.117	1.155E-01	7.467E-01
DUSP5	-0.117	6.616E-01	2.898E-01
SLC7A13	-0.117	8.932E-01	6.182E-01
DCP1A	-0.117	1.954E-01	1.985E-01
MIR185	-0.117	NA	6.660E-01
ATP5O	-0.117	2.487E-01	2.527E-01
FZD3	-0.117	5.404E-01	1.753E-01
NUDT22	-0.117	2.232E-01	3.975E-01
MFSD2A	-0.117	6.030E-01	8.359E-01
POLR2D	-0.117	1.090E-01	1.232E-01
FBXO44	-0.117	3.708E-01	9.503E-01
MCU	-0.117	1.738E-01	9.524E-01
TMEM144	-0.117	3.815E-01	9.546E-01
CDC25B	-0.117	6.300E-01	5.388E-01
MIR548N	-0.118	NA	1.701E-01
MIR3160-1	-0.118	NA	4.841E-01
AC138647.1	-0.118	NA	6.540E-01
RBM6	-0.118	3.607E-01	3.470E-01
COMMD5	-0.118	1.956E-01	4.234E-01
ASCL5	-0.118	7.154E-01	9.741E-01
RUFY3	-0.118	2.152E-01	3.538E-01

COX5A	-0.118	2.322E-01	5.520E-01
NFXL1	-0.118	3.065E-01	3.111E-01
SFSWAP	-0.118	1.368E-01	4.693E-01
AL669918.1	-0.118	6.912E-01	1.121E-01
METTL21C	-0.118	8.595E-01	4.501E-01
RPL15	-0.118	2.338E-01	3.373E-01
BBOX1	-0.118	7.998E-01	3.147E-01
MGAT4C	-0.118	8.505E-01	2.632E-01
CNTRL	-0.118	2.889E-01	1.351E-01
TBC1D30	-0.118	6.285E-01	4.356E-01
CCDC148	-0.118	5.682E-01	3.303E-01
CLPP	-0.118	2.195E-01	9.243E-01
TBRG4	-0.118	1.523E-01	1.470E-01
MMP3	-0.118	8.211E-01	5.483E-01
ESRRA	-0.118	2.307E-01	3.560E-01
NDUFA3	-0.118	4.067E-01	3.661E-01
MB21D1	-0.118	5.964E-01	6.798E-01
MED11	-0.118	2.435E-01	5.288E-01
UQCRC2	-0.118	1.761E-01	8.332E-01
SGK3	-0.118	2.051E-01	2.934E-01
ISCA2	-0.119	1.669E-01	2.364E-01
CCT8	-0.119	1.172E-01	6.886E-01
COMMD8	-0.119	1.873E-01	2.537E-01
DBF4	-0.119	3.043E-01	5.662E-01
BLACE	-0.119	NA	1.999E-01
CD163L1	-0.119	6.814E-01	4.641E-01
ARMC2	-0.119	1.838E-01	2.169E-01
CRNN	-0.119	9.098E-01	7.040E-01
NDUFS4	-0.119	2.427E-01	1.839E-01
FAM83B	-0.119	6.549E-01	4.917E-01
RIOX1	-0.119	2.750E-01	9.518E-01
ZNF207	-0.119	3.605E-02	3.518E-01
AC002429.2	-0.119	8.448E-01	2.342E-01
FAAP20	-0.119	3.408E-01	6.459E-01
MAPK1IP1L	-0.119	9.070E-02	3.494E-01
GOPC	-0.119	1.579E-01	2.637E-01
BTBD7	-0.119	1.844E-01	2.448E-01
MIR548T	-0.119	NA	4.226E-01
USMG5	-0.119	2.883E-01	5.195E-01
E2F7	-0.119	6.158E-01	2.720E-01
PRSS48	-0.119	8.240E-01	3.631E-01
BECN2	-0.119	8.249E-01	8.755E-01
CCDC66	-0.119	1.669E-01	1.406E-01
MSH4	-0.119	7.290E-01	3.326E-01
MIR4419A	-0.119	NA	3.978E-01
HTR5A	-0.119	NA	2.222E-01
PYCR2	-0.119	2.201E-01	3.157E-01
NOC4L	-0.119	2.576E-01	7.898E-01
SLC12A6	-0.119	4.948E-01	1.832E-01
HOXA11	-0.119	6.560E-01	5.165E-01

PDLIM1	-0.119	4.579E-01	7.627E-01
CBWD6	-0.120	4.082E-01	2.932E-01
SMAD9	-0.120	6.314E-01	4.964E-01
PRKAR1B	-0.120	4.976E-01	1.691E-01
C17orf113	-0.120	5.679E-01	4.571E-01
KRT74	-0.120	8.374E-01	1.552E-01
RPL3L	-0.120	6.395E-01	2.730E-01
CLN8	-0.120	2.642E-01	4.481E-01
FIGNL1	-0.120	3.916E-01	1.793E-01
TSPAN31	-0.120	2.964E-01	1.949E-01
NDUFA8	-0.120	2.211E-01	1.799E-01
SERPINC1	-0.120	7.685E-01	4.701E-01
VANGL2	-0.120	7.240E-01	4.970E-01
SLA	-0.120	7.013E-01	5.534E-01
FPGS	-0.120	2.823E-01	1.429E-01
AHSA2	-0.120	4.966E-01	4.993E-01
FAM13B	-0.120	3.544E-01	2.594E-01
NIN	-0.120	2.407E-01	3.380E-01
TM7SF3	-0.120	4.820E-01	4.786E-01
PREP	-0.120	1.893E-01	4.758E-01
ZNF208	-0.120	8.217E-01	5.621E-01
HDAC3	-0.120	7.416E-02	4.803E-01
CHMP7	-0.120	1.782E-01	4.250E-01
DUOXA1	-0.120	6.710E-01	2.899E-01
VWA5B1	-0.120	8.570E-01	3.520E-01
MEF2B	-0.120	5.713E-01	6.198E-01
ZNF44	-0.120	6.413E-01	5.261E-01
JAK2	-0.120	5.048E-01	2.244E-01
YAF2	-0.120	1.137E-01	2.739E-01
MIR548AK	-0.120	NA	3.131E-01
MIR519D	-0.120	NA	5.175E-01
MIR1294	-0.120	NA	4.459E-01
OR4F21	-0.120	NA	7.405E-01
CLDN14	-0.120	7.380E-01	3.361E-01
BLCAP	-0.120	4.223E-01	1.358E-01
NCOA5	-0.121	1.027E-01	2.337E-01
RAD17	-0.121	6.772E-02	3.973E-01
C11orf98	-0.121	2.611E-01	5.049E-01
ANKK1	-0.121	7.453E-01	1.718E-01
CEP57L1	-0.121	2.305E-01	3.302E-01
BSG	-0.121	2.508E-01	9.805E-01
PCSK4	-0.121	6.351E-01	9.805E-01
ATP4B	-0.121	8.245E-01	9.805E-01
PYDC2	-0.121	NA	9.805E-01
N4BP2L1	-0.121	4.522E-01	9.789E-01
ZC3H12A	-0.121	6.190E-01	9.787E-01
TTC14	-0.121	3.090E-01	9.787E-01
SRRD	-0.121	1.350E-01	9.764E-01
SRSF11	-0.121	1.984E-01	9.759E-01
GIGYF1	-0.121	2.222E-01	6.258E-02

BOLA2B	-0.121	5.299E-01	6.442E-01
KCNE1B	-0.121	8.514E-01	6.827E-01
COL21A1	-0.121	7.351E-01	3.869E-01
LCORL	-0.121	2.276E-01	1.866E-01
LAT	-0.121	5.429E-01	1.738E-01
TAS2R5	-0.121	6.769E-01	1.718E-01
ZNF57	-0.121	2.259E-01	5.605E-01
METTL22	-0.121	3.094E-01	5.100E-01
WRAP53	-0.121	2.818E-01	2.884E-01
TSHZ2	-0.121	6.579E-01	1.403E-01
TMEM260	-0.121	1.387E-01	5.178E-01
TOLLIP	-0.121	2.900E-01	1.828E-01
WDR12	-0.122	1.492E-01	3.441E-01
SLF1	-0.122	2.611E-01	6.566E-01
STRADA	-0.122	2.395E-01	2.930E-01
N4BP2	-0.122	3.560E-01	5.589E-01
RAPGEFL1	-0.122	6.816E-01	7.945E-01
GOLGA8J	-0.122	NA	4.973E-01
PRMT9	-0.122	1.523E-01	3.226E-01
FXN	-0.122	2.627E-01	5.794E-01
RBL2	-0.122	3.219E-01	2.978E-01
CD247	-0.122	6.960E-01	3.942E-01
RCBTB1	-0.122	3.835E-01	6.351E-01
CPNE8	-0.122	6.972E-01	2.610E-01
RBM14	-0.122	2.607E-02	6.344E-01
JADE2	-0.122	5.223E-01	4.410E-01
ANKHD1	-0.122	1.500E-01	5.049E-01
ZBED4	-0.122	1.973E-01	2.468E-01
WDR74	-0.122	2.131E-01	7.668E-01
UGCG	-0.122	3.736E-01	6.829E-01
CDC7	-0.122	4.244E-01	9.070E-01
QARS	-0.122	1.899E-01	9.091E-01
PPTC7	-0.122	2.476E-01	1.285E-01
MCOLN2	-0.122	7.044E-01	3.925E-01
SMPDL3B	-0.122	7.038E-01	8.823E-01
MT-CYB	-0.122	6.145E-01	1.192E-01
MIR5582	-0.122	NA	7.683E-01
ZCRB1	-0.122	9.960E-02	7.979E-02
FAM71F2	-0.122	6.167E-01	8.132E-01
PTRHD1	-0.122	2.516E-01	6.194E-01
TDP2	-0.122	2.446E-01	1.887E-01
TSKS	-0.122	7.794E-01	1.703E-01
AGTR1	-0.122	7.907E-01	8.276E-01
NHSL1	-0.122	4.024E-01	7.164E-01
SMIM6	-0.122	7.685E-01	8.243E-01
SNRPE	-0.122	1.526E-01	7.655E-02
WDHD1	-0.122	4.199E-01	3.256E-01
ETFRF1	-0.122	2.540E-01	2.596E-01
ALG14	-0.122	1.364E-01	1.980E-01
NME2	-0.122	2.418E-01	2.439E-01

FBXL15	-0.123	4.064E-01	1.283E-01
PSME1	-0.123	2.528E-01	7.209E-01
GXYLT1	-0.123	2.503E-01	4.627E-01
THAP6	-0.123	7.285E-02	2.024E-01
PGLYRP1	-0.123	7.426E-01	4.838E-01
IFNL1	-0.123	8.369E-01	3.223E-01
RBMX	-0.123	3.147E-02	1.740E-01
MRS2	-0.123	2.648E-01	7.040E-01
VPS28	-0.123	2.871E-01	5.436E-01
PSMB1	-0.123	1.880E-01	5.523E-01
DAPL1	-0.123	8.575E-01	6.285E-01
ZNF511	-0.123	2.096E-01	1.917E-01
SH2B2	-0.123	4.988E-01	6.960E-01
SLC38A4	-0.123	8.344E-01	3.416E-01
ZNF14	-0.123	3.056E-01	4.169E-01
SPRY2	-0.123	5.943E-01	2.537E-01
STK25	-0.123	1.415E-01	6.306E-01
MAP2K4	-0.123	1.351E-01	3.691E-01
NR1H3	-0.123	4.975E-01	3.166E-01
GATD1	-0.123	2.444E-01	4.135E-01
MIR6894	-0.123	NA	4.860E-01
MIR340	-0.123	NA	4.149E-01
NPHP3	-0.123	2.569E-01	4.826E-01
RPSA	-0.123	3.348E-01	2.283E-01
TBC1D32	-0.124	4.062E-01	6.539E-01
SUPT20H	-0.124	1.838E-01	2.084E-01
ZNF257	-0.124	7.274E-01	2.051E-01
POGZ	-0.124	2.819E-01	4.357E-01
CCDC144NL	-0.124	8.583E-01	4.787E-01
HAVCR2	-0.124	6.950E-01	2.137E-01
XRCC3	-0.124	2.931E-01	9.538E-01
CCDC34	-0.124	3.679E-01	9.601E-01
AC006978.2	-0.124	6.638E-01	9.604E-01
HNRNPD	-0.124	1.132E-01	7.754E-01
SMCO1	-0.124	7.209E-01	7.024E-01
DNAJC17	-0.124	2.135E-01	1.172E-01
GALNT9	-0.124	7.910E-01	6.578E-01
HNRNPDL	-0.124	6.757E-02	1.145E-01
NT5C3A	-0.124	1.552E-01	2.726E-01
TEF	-0.124	3.383E-01	4.630E-01
IQCF6	-0.124	8.794E-01	1.028E-01
GLUD2	-0.124	5.489E-01	8.986E-02
MRPS26	-0.124	2.675E-01	2.226E-01
PBK	-0.124	5.244E-01	1.265E-01
HSPD1	-0.124	2.573E-01	2.717E-01
ZNF205	-0.124	2.613E-01	3.448E-01
ZFP37	-0.124	6.669E-01	2.749E-01
PTK2	-0.124	2.354E-01	3.296E-01
ZNRF3	-0.124	6.572E-01	9.291E-01
EIF3F	-0.124	1.418E-01	6.089E-01

EVC2	-0.124	6.799E-01	3.581E-01
CDCA7	-0.124	6.019E-01	1.695E-01
ATP5S	-0.124	1.180E-01	6.109E-01
DOK6	-0.124	7.199E-01	6.452E-01
MIR1286	-0.124	NA	5.208E-01
KRTAP4-2	-0.124	NA	2.543E-01
GPR132	-0.124	6.557E-01	3.959E-01
PERM1	-0.124	6.582E-01	9.797E-01
BTN3A1	-0.125	4.320E-01	9.797E-01
CABLES1	-0.125	6.108E-01	9.797E-01
INTS9	-0.125	1.429E-01	2.690E-01
PITPNM1	-0.125	3.050E-01	6.545E-01
HOMER3	-0.125	4.442E-01	9.187E-01
TEX48	-0.125	8.371E-01	1.247E-01
MSH3	-0.125	1.876E-01	3.552E-01
TOMM20L	-0.125	4.808E-01	2.537E-01
TMEM5	-0.125	1.221E-01	5.221E-01
ZCCHC13	-0.125	NA	3.112E-01
MIR1305	-0.125	NA	8.624E-01
MIR4654	-0.125	NA	7.097E-01
MIR4696	-0.125	NA	2.702E-01
OR4A5	-0.125	NA	7.405E-01
OR5K3	-0.125	NA	5.122E-01
OR5M8	-0.125	NA	2.251E-01
RHD	-0.125	6.543E-01	6.027E-01
AP1S3	-0.125	5.617E-01	8.906E-02
SSSCA1	-0.125	3.184E-01	3.024E-01
TRIM5	-0.125	2.917E-01	6.297E-01
SERPINB9	-0.125	6.727E-01	2.246E-01
MIR4768	-0.125	7.528E-01	5.427E-01
ZNF586	-0.125	2.197E-01	4.898E-01
TMC4	-0.125	6.231E-01	6.094E-01
MAP2K7	-0.125	7.797E-02	7.734E-02
ERICH3	-0.125	8.204E-01	3.877E-02
NOP58	-0.125	1.224E-01	4.374E-01
TYK2	-0.125	1.212E-01	1.136E-01
KLC1	-0.125	1.523E-01	7.188E-02
UBLCP1	-0.125	2.347E-01	3.054E-01
ADORA2A	-0.125	6.288E-01	2.869E-01
SOWAHC	-0.125	5.246E-01	3.634E-01
PTPRK	-0.125	3.587E-01	5.844E-01
SMARCB1	-0.125	1.437E-01	2.192E-01
TGDS	-0.125	1.122E-01	5.010E-01
ABCG5	-0.125	7.057E-01	5.306E-01
RIOK1	-0.125	2.329E-01	1.327E-01
CABP7	-0.125	7.492E-01	6.917E-01
CDC25C	-0.125	4.427E-01	9.317E-01
SULT1C3	-0.125	NA	9.364E-01
MIR6779	-0.125	NA	6.508E-01
MIR4777	-0.126	NA	9.431E-01

ZDHHC21	-0.126	3.477E-01	1.517E-01
GPR35	-0.126	6.805E-01	6.654E-01
AC008575.1	-0.126	NA	2.259E-01
GSPT2	-0.126	6.571E-01	1.608E-01
BGLAP	-0.126	5.860E-01	3.580E-01
DZANK1	-0.126	3.661E-01	3.069E-01
BPIFB6	-0.126	NA	7.663E-01
RNASEH2C	-0.126	3.527E-01	2.738E-01
RNFT2	-0.126	5.985E-01	1.453E-01
MRPS17	-0.126	2.962E-01	2.445E-01
SRFBP1	-0.126	1.836E-01	5.308E-01
ARHGEF3	-0.126	2.790E-01	4.312E-01
SUGP2	-0.126	1.369E-01	1.197E-01
DDX39A	-0.126	2.091E-01	3.136E-01
LY6G5C	-0.126	4.192E-01	1.469E-01
UQCC1	-0.126	9.047E-02	9.640E-01
MIR151A	-0.126	NA	9.708E-01
MIR4729	-0.126	NA	8.922E-01
MIR516A1	-0.126	NA	1.406E-01
C17orf97	-0.126	5.494E-01	7.105E-01
QTNF3-AMA	-0.126	6.814E-01	2.026E-01
CDH6	-0.126	5.596E-01	4.138E-01
CNFN	-0.126	7.296E-01	1.013E-01
NR2F6	-0.126	3.733E-01	4.943E-01
ELOVL4	-0.126	7.390E-01	5.912E-01
SPSB2	-0.126	3.993E-01	3.141E-01
ELAC2	-0.126	1.036E-01	7.278E-01
MYO1B	-0.126	5.953E-01	4.525E-01
CCAR1	-0.126	7.802E-02	6.874E-01
SRP14	-0.127	7.296E-02	1.593E-01
44084.000	-0.127	2.914E-01	1.716E-01
ABHD6	-0.127	3.625E-01	3.049E-01
SCMH1	-0.127	3.293E-01	5.355E-01
HOOK2	-0.127	3.217E-01	3.660E-01
CTRL	-0.127	6.318E-01	7.073E-01
ZC3H7B	-0.127	1.289E-01	1.354E-01
SIGLECL1	-0.127	NA	4.657E-01
TMEM26	-0.127	5.909E-01	2.123E-01
FNBP4	-0.127	2.045E-01	7.679E-01
ZNF645	-0.127	NA	1.780E-01
LRRTM3	-0.127	8.758E-01	4.075E-01
PROX1	-0.127	7.228E-01	1.486E-01
ACR	-0.127	6.847E-01	5.855E-01
E4F1	-0.127	1.978E-01	7.100E-01
LTB	-0.127	7.196E-01	3.480E-01
CHRNE	-0.127	5.895E-01	6.373E-01
GYG2	-0.127	7.037E-01	2.353E-01
RSPH3	-0.127	1.713E-01	2.045E-01
NOS1AP	-0.127	6.311E-01	2.900E-01
LARS	-0.127	7.349E-02	6.397E-01

PPIAL4E	-0.127	NA	3.405E-01
UBE2L3	-0.127	1.161E-01	2.552E-01
LY6G5B	-0.127	4.076E-01	3.651E-01
ODF3B	-0.127	5.676E-01	1.677E-01
TP53RK	-0.127	1.906E-01	7.836E-01
DENND5B	-0.127	6.017E-01	4.323E-01
C4orf48	-0.127	6.235E-01	9.303E-01
RIC8B	-0.127	8.669E-02	1.570E-01
CCDC116	-0.127	5.749E-01	7.001E-01
MIR3159	-0.127	NA	3.401E-01
MIR3196	-0.127	NA	1.431E-01
OR8D4	-0.127	NA	2.628E-01
OR7G1	-0.127	NA	7.677E-02
C6orf136	-0.127	2.345E-01	2.721E-01
ANKRD29	-0.127	7.432E-01	1.761E-01
AC113554.2	-0.127	NA	2.361E-01
IFI6	-0.127	7.213E-01	2.088E-01
NUFIP1	-0.128	1.385E-01	1.907E-01
ATPAF2	-0.128	1.751E-01	6.960E-01
DUSP18	-0.128	2.636E-01	7.689E-01
SRSF1	-0.128	1.306E-02	4.771E-01
LILRB4	-0.128	7.509E-01	5.319E-01
SNAP29	-0.128	2.162E-01	2.010E-01
PWWP2A	-0.128	7.678E-02	2.260E-01
CCDC17	-0.128	5.184E-01	5.956E-01
RIPOR2	-0.128	6.777E-01	2.970E-01
RPP14	-0.128	6.772E-02	5.634E-01
OXLD1	-0.128	3.061E-01	9.600E-01
MIR4292	-0.128	4.715E-01	1.930E-01
PRKAA1	-0.128	1.985E-01	4.036E-01
IGSF9B	-0.128	7.344E-01	3.866E-01
CEL	-0.128	7.713E-01	5.012E-01
OR5K2	-0.128	7.719E-01	2.445E-01
CUX1	-0.128	1.352E-01	1.142E-01
RPL10	-0.128	2.323E-01	3.581E-01
TMCO2	-0.128	7.963E-01	9.105E-01
MIR181A2	-0.128	NA	6.919E-02
MIR152	-0.128	NA	9.604E-01
RSAD2	-0.128	7.257E-01	9.606E-01
GOT2	-0.128	2.097E-01	8.166E-01
BACH1	-0.128	3.579E-01	6.156E-01
PRSS57	-0.128	8.534E-01	8.381E-01
PARP14	-0.128	4.067E-01	1.930E-01
KCNV2	-0.128	7.114E-01	1.586E-01
CES2	-0.128	2.849E-01	4.429E-01
MOB1B	-0.128	1.345E-01	6.481E-01
OR52W1	-0.129	NA	3.581E-01
MRPL40	-0.129	2.422E-01	8.832E-01
PANK3	-0.129	2.612E-01	1.085E-01
SLC15A5	-0.129	8.693E-01	7.889E-01

SPIRE1	-0.129	5.749E-01	5.629E-01
EXOSC8	-0.129	1.319E-01	2.313E-01
LARP1	-0.129	9.109E-02	1.366E-01
ACADSB	-0.129	5.217E-01	2.012E-01
MIR6772	-0.129	7.415E-01	1.552E-01
SLC25A3	-0.129	7.458E-02	8.606E-01
PHF23	-0.129	9.114E-02	2.896E-01
HOXB2	-0.129	6.915E-01	6.076E-01
DNAH1	-0.129	4.868E-01	3.058E-01
DCP2	-0.129	1.278E-01	2.259E-01
EP400NL	-0.129	2.558E-01	6.411E-01
CC2D1A	-0.129	1.673E-01	4.622E-02
PAM16	-0.129	2.684E-01	4.850E-01
FAM200B	-0.129	2.154E-01	9.511E-02
NHLRC4	-0.129	5.689E-01	2.608E-01
AC008764.1	-0.129	NA	2.295E-01
OR4K2	-0.129	NA	2.430E-01
GPHB5	-0.129	NA	2.110E-01
METTL3	-0.129	1.532E-01	1.061E-01
POLR1C	-0.129	1.686E-01	8.957E-01
EAPP	-0.129	7.456E-02	2.431E-01
AMMECR1L	-0.129	4.617E-02	9.839E-02
RELT	-0.129	3.735E-01	8.251E-01
SYT10	-0.129	8.541E-01	4.237E-01
ITGAD	-0.129	7.085E-01	1.336E-01
GOLIM4	-0.129	3.618E-01	2.794E-01
GJA1	-0.129	6.471E-01	3.670E-01
STAT2	-0.129	2.479E-01	6.009E-01
COQ6	-0.129	1.107E-01	4.580E-01
KLF5	-0.129	5.503E-01	6.790E-01
PDXP	-0.129	4.961E-01	5.576E-01
SCN5A	-0.129	7.754E-01	3.998E-01
LARP4	-0.129	8.590E-02	1.866E-01
RECQL5	-0.129	2.463E-01	3.974E-01
TTC21B	-0.129	1.394E-01	2.155E-01
DHX37	-0.129	6.427E-02	7.255E-01
ADM2	-0.129	6.751E-01	2.539E-01
SACS	-0.129	6.313E-01	6.501E-02
RRN3	-0.129	1.349E-01	1.506E-01
PGGHG	-0.130	6.470E-01	2.072E-01
GRSF1	-0.130	7.447E-02	1.315E-01
EPSTI1	-0.130	6.708E-01	2.010E-02
KATNA1	-0.130	8.521E-02	2.051E-01
KLHDC8B	-0.130	5.062E-01	9.181E-01
ORC3	-0.130	7.867E-02	8.219E-01
SEZ6L2	-0.130	6.544E-01	9.494E-01
UBE2K	-0.130	4.620E-02	1.078E-01
DDR1	-0.130	3.611E-01	7.408E-01
SERPINB1	-0.130	6.482E-01	1.124E-01
UBXN8	-0.130	3.138E-01	5.996E-01

COX7B	-0.130	1.954E-01	6.800E-01
SPG7	-0.130	1.724E-01	6.794E-01
ANXA2R	-0.130	5.211E-01	5.656E-01
RAD9A	-0.130	3.635E-01	8.150E-01
STXBP5	-0.130	2.715E-01	6.060E-01
FLT3	-0.130	7.215E-01	5.749E-01
ZNF354A	-0.130	2.252E-01	5.051E-01
C5orf15	-0.130	1.862E-01	6.713E-01
PYM1	-0.130	2.370E-01	8.845E-01
KLHL36	-0.130	2.086E-01	1.414E-01
METAP2	-0.130	9.320E-02	1.641E-01
GNPTAB	-0.130	1.908E-01	8.825E-01
ODF1	-0.130	NA	2.925E-01
RPS21	-0.130	3.106E-01	4.348E-01
CHCHD7	-0.130	2.205E-01	3.511E-01
AC243756.1	-0.130	8.171E-01	1.056E-01
KIAA0753	-0.130	1.674E-01	1.712E-01
MIR505	-0.130	NA	2.218E-01
MIR3137	-0.130	NA	1.877E-01
CRYGA	-0.130	NA	8.844E-02
CDRT15L2	-0.130	NA	5.362E-01
MIR410	-0.130	NA	1.802E-01
OR8K5	-0.130	NA	4.945E-01
PDSS1	-0.130	2.398E-01	7.124E-01
NUBP1	-0.130	1.532E-01	1.318E-01
TAP2	-0.130	5.449E-01	6.089E-01
FAM19A2	-0.130	4.939E-01	6.429E-01
KCNG4	-0.130	8.369E-01	4.124E-01
ALDOC	-0.130	5.772E-01	4.873E-01
OPN4	-0.130	7.791E-01	7.171E-01
TMEM237	-0.130	2.917E-01	4.352E-01
NCBP3	-0.130	1.339E-01	5.045E-01
44166.000	-0.131	8.167E-01	3.857E-01
PAQR3	-0.131	3.136E-01	9.990E-02
DGKE	-0.131	3.617E-01	5.531E-01
SF3B5	-0.131	2.004E-01	3.493E-01
TRIM26	-0.131	9.271E-02	2.158E-01
MRPL41	-0.131	4.244E-01	5.516E-02
MIR4634	-0.131	NA	6.192E-01
MIR6508	-0.131	NA	2.353E-01
LCN12	-0.131	6.837E-01	6.844E-02
TRIM68	-0.131	2.664E-01	1.981E-01
ZCCHC3	-0.131	1.218E-01	4.921E-01
MIR6769A	-0.131	8.082E-01	5.130E-01
GAR1	-0.131	9.561E-02	2.901E-01
MTMR9	-0.131	1.562E-01	6.160E-01
CLEC16A	-0.131	1.160E-01	7.448E-01
HMGN3	-0.131	2.640E-01	2.778E-01
ANGEL1	-0.131	1.370E-01	6.859E-01
RPL39	-0.131	2.907E-01	3.318E-01

TXNIP	-0.131	5.136E-01	3.464E-01
PIGA	-0.131	3.162E-01	2.330E-01
CCNA1	-0.131	8.425E-01	3.043E-01
PLA2G2A	-0.131	8.156E-01	7.965E-01
LRRC39	-0.131	4.931E-01	6.406E-01
NME3	-0.131	4.840E-01	3.131E-01
KLRF1	-0.131	7.291E-01	2.336E-01
RNF4	-0.131	8.665E-02	3.825E-01
HLA-B	-0.131	6.200E-01	6.945E-01
CD164	-0.131	1.489E-01	7.088E-01
TMEM191B	-0.131	7.558E-01	5.597E-01
SRSF8	-0.131	1.418E-01	1.522E-01
DNAH10OS	-0.132	6.410E-01	9.458E-01
C1orf52	-0.132	4.924E-02	3.686E-01
METTL15	-0.132	9.883E-02	1.495E-01
NUP62CL	-0.132	5.957E-01	1.821E-01
SLC43A2	-0.132	4.397E-01	4.757E-01
SAFB	-0.132	1.054E-02	6.856E-01
KLB	-0.132	5.394E-01	4.694E-01
CCDC157	-0.132	3.509E-01	7.025E-01
TMCC2	-0.132	6.030E-01	7.163E-01
AC005702.2	-0.132	8.534E-01	4.714E-01
ACCSL	-0.132	8.857E-01	5.187E-01
FEM1C	-0.132	1.369E-01	1.287E-01
MRPS28	-0.132	1.585E-01	5.958E-01
ASNS	-0.132	4.552E-01	2.417E-01
MIR5003	-0.132	NA	1.730E-01
TXN2	-0.132	1.798E-01	2.940E-01
MUC4	-0.132	8.136E-01	9.254E-01
C20orf196	-0.132	1.954E-01	6.239E-01
SNX15	-0.132	3.240E-01	4.278E-01
SLCO2B1	-0.132	6.871E-01	1.778E-01
LACTB	-0.132	1.342E-01	6.068E-01
MT2A	-0.132	7.410E-01	2.451E-01
TMEM92	-0.132	6.807E-01	8.975E-02
TANK	-0.132	8.842E-02	1.340E-01
L3MBTL3	-0.132	3.080E-01	5.181E-01
FCRL1	-0.132	8.369E-01	3.693E-01
METTL1	-0.132	1.984E-01	3.683E-01
ZNF653	-0.132	1.767E-01	7.546E-01
SRPK3	-0.132	5.710E-01	3.425E-01
ZC3H8	-0.132	1.786E-01	1.185E-01
PHIP	-0.132	1.996E-01	7.728E-01
STX18	-0.132	3.245E-02	1.045E-01
FUT7	-0.132	7.271E-01	6.996E-01
KCTD15	-0.132	6.103E-01	4.280E-02
ZNF91	-0.133	5.013E-01	2.361E-01
CEP162	-0.133	1.789E-01	1.222E-01
DEFB108B	-0.133	NA	1.459E-01
UTP20	-0.133	1.752E-01	3.518E-01

OR2C1	-0.133	7.845E-01	9.497E-01
MTX3	-0.133	1.592E-01	2.835E-01
CCDC88C	-0.133	3.483E-01	1.502E-01
LILRB1	-0.133	6.926E-01	3.668E-01
PARM1	-0.133	6.611E-01	2.119E-01
SRCIN1	-0.133	6.921E-01	6.518E-01
FAM71E1	-0.133	5.673E-01	2.014E-01
DMC1	-0.133	7.046E-01	3.678E-01
BCO2	-0.133	6.352E-01	1.325E-01
NECAB2	-0.133	7.448E-01	3.906E-01
DARS	-0.133	9.171E-02	7.742E-01
OR7C2	-0.133	NA	1.262E-01
MIR543	-0.133	NA	2.947E-01
MIR3664	-0.133	NA	9.771E-01
ZMYND8	-0.133	3.409E-01	4.804E-01
PARP9	-0.133	4.698E-01	1.526E-01
ILF3	-0.133	6.924E-02	5.292E-01
CFP	-0.133	6.521E-01	1.825E-01
METTL12	-0.133	3.228E-01	8.331E-01
NARS2	-0.133	1.984E-01	2.594E-01
NEK3	-0.133	3.211E-01	5.168E-01
PPP1R17	-0.133	8.791E-01	7.132E-01
MIR3133	-0.133	NA	3.948E-01
E2F3	-0.133	4.976E-01	4.198E-01
ZBED3	-0.133	4.721E-01	5.967E-01
KIAA0930	-0.133	1.602E-01	3.664E-01
CHAC2	-0.133	4.159E-01	7.732E-01
TSKU	-0.134	4.847E-01	2.521E-01
ATAD5	-0.134	3.483E-01	6.229E-01
CXorf49B	-0.134	9.001E-01	1.198E-01
PCOLCE2	-0.134	7.489E-01	2.768E-01
LARS2	-0.134	1.233E-01	2.770E-01
DMTF1	-0.134	1.738E-01	1.428E-01
OVOL1	-0.134	6.484E-01	7.029E-01
ZNF430	-0.134	2.872E-01	7.242E-01
MTERF4	-0.134	6.198E-02	1.301E-01
N4BP2L2	-0.134	1.483E-01	5.468E-01
IL17A	-0.134	8.760E-01	1.829E-01
SNRPA	-0.134	1.310E-01	3.993E-01
PPIH	-0.134	2.444E-01	4.193E-01
DHX15	-0.134	4.979E-02	6.405E-01
IBA57	-0.134	2.922E-01	3.609E-01
MAU2	-0.134	1.052E-01	2.539E-01
PCDHGC3	-0.134	6.043E-01	8.365E-01
(NJ2BP-COX	-0.134	3.161E-01	1.202E-01
GTSE1	-0.134	3.906E-01	9.635E-02
LLPH	-0.134	2.346E-01	1.114E-01
TMEM256	-0.134	3.233E-01	1.394E-01
MIR328	-0.134	NA	7.334E-01
MIR4504	-0.134	NA	1.281E-01

GPR78	-0.134	7.840E-01	7.627E-01
CTAG1B	-0.134	NA	2.375E-01
PYCARD	-0.134	4.914E-01	5.859E-01
ACSF3	-0.134	1.717E-01	3.962E-01
OR1E1	-0.135	NA	2.738E-01
REC8	-0.135	5.011E-01	2.648E-01
HOMER1	-0.135	3.714E-01	3.699E-01
U2SURP	-0.135	9.398E-02	5.328E-01
EM110-MUS'	-0.135	4.269E-01	3.671E-01
NSMAF	-0.135	8.209E-02	5.492E-02
GTF2H5	-0.135	1.222E-01	9.240E-02
WDR90	-0.135	3.345E-01	2.280E-01
SATB2	-0.135	5.696E-01	4.491E-02
AKAP17A	-0.135	3.025E-01	1.829E-01
ZMYM6	-0.135	3.567E-02	4.027E-01
MIR6753	-0.135	5.853E-01	1.769E-01
CORO7	-0.135	3.321E-01	1.059E-01
CNOT6L	-0.135	1.378E-01	6.461E-01
MITD1	-0.135	1.178E-01	7.120E-01
IRF7	-0.135	5.635E-01	6.168E-01
S100A14	-0.135	7.295E-01	2.324E-01
OR52B2	-0.135	NA	8.371E-01
AL139392.1	-0.135	7.441E-01	1.190E-01
MTRNR2L13	-0.135	8.427E-01	1.034E-01
SH2D6	-0.135	6.096E-01	6.884E-01
ZNF26	-0.135	2.540E-01	6.280E-01
MT-ND4L	-0.135	6.245E-01	4.335E-01
TRIM14	-0.135	3.387E-01	3.305E-01
RSAD1	-0.135	1.920E-01	3.751E-01
ZNF335	-0.135	7.856E-02	6.400E-01
PRKCD	-0.135	3.403E-01	2.084E-01
C14orf2	-0.135	2.229E-01	5.045E-01
NARF	-0.135	2.635E-01	1.766E-01
MIR454	-0.135	NA	3.702E-01
AL353588.1	-0.135	5.489E-01	1.002E-01
ARHGAP11A	-0.135	3.676E-01	3.327E-01
BTBD3	-0.136	3.984E-01	1.799E-01
C14orf177	-0.136	NA	1.156E-01
ZNF783	-0.136	2.043E-01	2.235E-01
HDAC1	-0.136	5.952E-02	6.734E-01
NTPCR	-0.136	1.112E-01	1.421E-01
COX5B	-0.136	3.043E-01	2.313E-01
SAPCD1	-0.136	5.731E-01	5.628E-02
GAREM2	-0.136	5.025E-01	7.472E-02
SCARF1	-0.136	3.954E-01	1.008E-01
HMHB1	-0.136	8.174E-01	5.751E-01
SRSF2	-0.136	1.066E-02	2.621E-01
PAPD4	-0.136	5.524E-02	2.677E-01
PLD5	-0.136	8.540E-01	3.076E-01
EXOG	-0.136	2.696E-01	1.484E-01

PET100	-0.136	3.796E-01	9.094E-02
SETD1B	-0.136	7.009E-02	2.769E-01
OR7G2	-0.136	NA	5.824E-01
SPATA31A7	-0.136	NA	5.548E-01
MIR640	-0.136	NA	6.676E-01
ZNF396	-0.136	3.522E-01	6.572E-01
OPLAH	-0.136	5.361E-01	1.286E-01
RBM38	-0.136	3.162E-01	7.802E-01
MCRS1	-0.136	3.580E-02	8.466E-01
PHKA2	-0.136	2.987E-01	2.154E-01
DNPEP	-0.136	1.009E-01	9.340E-02
HIST1H3B	-0.136	7.227E-01	2.201E-01
PPA1	-0.136	1.679E-01	5.217E-01
MAN2A1	-0.136	1.911E-01	3.177E-01
SLCO1B7	-0.136	8.999E-01	5.176E-01
PPP1R14C	-0.136	7.685E-01	1.615E-01
COL4A5	-0.136	5.981E-01	7.685E-01
SLC35F2	-0.136	4.378E-01	4.257E-01
ARHGEF19	-0.136	4.593E-01	6.535E-01
EXOC1	-0.137	5.345E-02	7.534E-01
TSTD3	-0.137	2.565E-01	1.261E-01
ZBTB43	-0.137	1.738E-01	6.591E-01
FAM69A	-0.137	4.176E-01	1.933E-01
PSMA5	-0.137	9.314E-02	2.604E-01
DDX5	-0.137	4.427E-02	4.737E-01
PIK3IP1	-0.137	4.705E-01	9.113E-01
MRPS2	-0.137	2.109E-01	3.016E-01
MIR4477B	-0.137	5.613E-01	5.109E-01
SMAD5	-0.137	1.389E-01	1.429E-01
RCHY1	-0.137	5.929E-02	6.555E-01
TMEM86B	-0.137	4.471E-01	7.177E-01
FBF1	-0.137	4.067E-01	9.815E-02
AC036214.3	-0.137	4.705E-01	8.340E-01
TCF3	-0.137	1.346E-01	4.801E-01
RAB24	-0.137	2.172E-01	9.194E-01
TRO	-0.137	6.579E-01	2.766E-01
FLRT1	-0.137	5.905E-01	5.440E-01
PARP10	-0.137	3.213E-01	6.225E-01
TMEM134	-0.137	3.185E-01	5.003E-02
MIR1268B	-0.137	NA	5.534E-01
MIR664A	-0.137	NA	9.379E-01
RBM26	-0.137	6.508E-02	2.151E-01
ZNF76	-0.137	1.571E-01	2.560E-01
MIR508	-0.137	NA	7.213E-01
POTEB3	-0.137	NA	6.228E-02
MIR3677	-0.137	6.866E-01	8.507E-02
LIPT1	-0.137	1.191E-01	1.212E-01
C22orf42	-0.137	8.635E-01	4.959E-01
ACIN1	-0.137	5.067E-02	3.885E-01
GSPT1	-0.137	1.260E-01	1.299E-01

NUP35	-0.137	8.865E-02	1.134E-01
LAMA5	-0.137	4.137E-01	2.190E-01
FAM110C	-0.137	6.224E-01	8.764E-01
DLG2	-0.137	5.864E-01	8.739E-01
BAHD1	-0.137	1.124E-01	5.691E-01
C2	-0.138	6.683E-01	1.257E-01
CRISPLD1	-0.138	6.621E-01	1.156E-01
CNOT6	-0.138	7.477E-02	4.299E-01
RBM22	-0.138	1.128E-02	4.134E-02
METTL21A	-0.138	1.469E-01	6.465E-01
SLC35E2B	-0.138	3.654E-01	1.909E-01
MRPS35	-0.138	1.013E-01	1.259E-01
GBP6	-0.138	7.944E-01	1.859E-01
TAMM41	-0.138	1.891E-01	3.983E-02
PIK3R5	-0.138	6.063E-01	1.816E-01
ADAP1	-0.138	5.701E-01	2.596E-01
DECR1	-0.138	2.872E-01	4.364E-01
FUCA1	-0.138	4.280E-01	9.632E-01
ZNF516	-0.138	4.840E-01	2.258E-01
GTF3C3	-0.138	4.561E-02	2.658E-01
CNTN4	-0.138	6.602E-01	2.485E-01
TMA7	-0.138	2.551E-01	3.524E-01
SLC2A13	-0.138	4.840E-01	1.093E-01
CPOX	-0.138	1.687E-01	4.835E-01
CTSG	-0.138	7.847E-01	1.187E-01
MIR1285-1	-0.138	6.842E-01	3.513E-01
MTMR8	-0.138	5.246E-01	9.732E-02
NPC2	-0.138	3.392E-01	4.499E-01
TADA2B	-0.139	3.276E-02	5.949E-01
AC008575.2	-0.139	5.189E-01	8.929E-02
UBL4B	-0.139	7.402E-01	8.729E-01
GSG1	-0.139	7.125E-01	2.027E-01
MTHFR	-0.139	1.738E-01	1.201E-01
PASK	-0.139	2.545E-01	4.429E-01
C19orf60	-0.139	3.714E-01	8.158E-02
CHCHD5	-0.139	2.718E-01	6.440E-01
SPAG11B	-0.139	NA	6.776E-01
PRR21	-0.139	NA	1.895E-01
CYLC2	-0.139	NA	9.582E-01
AGO2	-0.139	2.153E-01	9.605E-01
ZCCHC4	-0.139	4.865E-02	9.633E-01
HLA-DQA1	-0.139	7.084E-01	4.475E-01
FAM60A	-0.139	2.673E-01	3.591E-01
ZNF615	-0.139	4.838E-01	3.860E-01
ATG2A	-0.139	3.888E-02	3.418E-01
THOC6	-0.139	1.766E-01	2.938E-01
ACP6	-0.139	5.114E-01	5.478E-01
GCSH	-0.139	2.430E-01	4.373E-01
MIR7845	-0.139	8.921E-01	1.308E-01
HDDC2	-0.139	1.526E-01	8.070E-01

CALML4	-0.139	3.515E-01	1.127E-01
IL5	-0.139	7.452E-01	8.818E-02
CRIPAK	-0.139	3.101E-01	8.227E-02
BTN3A3	-0.139	4.646E-01	4.701E-01
DENND6B	-0.139	3.299E-01	8.400E-02
DIP2B	-0.139	2.614E-01	6.427E-01
ARHGEF10L	-0.139	4.053E-01	3.507E-01
CDX4	-0.139	NA	5.490E-01
RPS25	-0.139	1.971E-01	3.429E-01
RPS12	-0.139	2.730E-01	7.840E-02
PABPN1	-0.139	1.205E-01	8.875E-01
DIP2A	-0.140	1.510E-01	1.761E-01
CEP135	-0.140	1.910E-01	5.874E-01
LDHA	-0.140	4.330E-01	5.018E-01
GTPBP1	-0.140	5.477E-02	1.720E-01
ZRANB2	-0.140	3.674E-02	6.032E-01
ZNF20	-0.140	3.175E-01	6.043E-01
NR6A1	-0.140	3.603E-01	1.385E-01
ZSCAN9	-0.140	2.640E-01	4.942E-02
NDUFA12	-0.140	6.846E-02	1.821E-01
TRABD2B	-0.140	7.062E-01	8.976E-01
PRDM4	-0.140	2.321E-02	8.976E-01
LIME1	-0.140	4.107E-01	5.781E-01
OR2A4	-0.140	NA	8.406E-01
CAPS2	-0.140	4.622E-01	6.201E-01
FSD1	-0.140	7.528E-01	5.270E-01
BTBD10	-0.140	8.382E-02	1.262E-01
AL513165.2	-0.140	7.026E-01	6.654E-01
OR4F5	-0.140	NA	1.448E-01
OR10K2	-0.140	NA	1.871E-01
MBD3L2B	-0.140	NA	5.412E-02
KRTAP19-4	-0.140	NA	2.548E-01
GOLGA8H	-0.140	5.163E-01	6.619E-01
PPP1R27	-0.140	6.839E-01	3.309E-01
REXO1	-0.140	5.920E-02	4.076E-01
AC113404.3	-0.140	5.436E-01	8.443E-02
KMT2D	-0.140	1.880E-01	3.383E-01
AHI1	-0.140	1.980E-01	2.306E-01
AC067968.1	-0.140	8.184E-01	1.903E-01
NAA60	-0.140	1.110E-01	7.632E-01
ADAMTS13	-0.140	5.070E-01	2.683E-01
MTTP	-0.140	7.224E-01	3.188E-02
MIR6738	-0.140	NA	3.728E-01
ELMSAN1	-0.141	1.532E-01	4.220E-01
KLRG1	-0.141	5.507E-01	5.886E-01
CCNL1	-0.141	2.027E-01	5.775E-01
GRK4	-0.141	1.736E-01	1.654E-01
MRPS15	-0.141	1.334E-01	6.424E-02
BCLAF1	-0.141	1.357E-02	8.786E-01
PRPF38B	-0.141	5.474E-02	7.506E-01

ZNF101	-0.141	8.327E-02	9.264E-01
SIAH1	-0.141	1.401E-01	9.291E-01
HOXA13	-0.141	6.616E-01	1.304E-01
LRRC8D	-0.141	2.193E-01	5.958E-01
PYDC1	-0.141	8.425E-01	4.545E-01
COX10	-0.141	7.285E-02	8.399E-02
MIR4737	-0.141	5.606E-01	7.619E-02
NMD3	-0.141	1.246E-01	4.111E-01
CCDC96	-0.141	2.970E-01	8.175E-01
LARP7	-0.141	1.057E-02	2.102E-01
CEP152	-0.141	2.206E-01	6.267E-02
SPAAR	-0.141	4.617E-01	4.476E-01
EIF4A2	-0.141	1.435E-01	6.712E-02
ARMC5	-0.141	2.115E-01	1.484E-01
ABI3	-0.141	5.405E-01	1.507E-01
ILKAP	-0.141	9.529E-02	5.692E-01
ARIH2	-0.141	5.740E-02	3.422E-01
CRYBB2	-0.141	7.086E-01	3.412E-02
SLC25A30	-0.141	1.906E-01	4.392E-02
IKZF4	-0.141	1.713E-01	1.304E-01
MANEA	-0.141	2.358E-01	1.978E-01
AL139300.1	-0.141	NA	4.739E-01
SPATA6L	-0.141	4.607E-01	5.271E-01
ZNF711	-0.141	5.996E-01	5.940E-01
CDX2	-0.142	8.391E-01	2.447E-01
FBXO6	-0.142	3.094E-01	1.157E-01
SMR3A	-0.142	NA	1.037E-01
MAEA	-0.142	4.479E-02	3.490E-01
MLLT6	-0.142	1.984E-01	1.904E-01
TFEC	-0.142	6.829E-01	2.261E-01
FBXL8	-0.142	2.931E-01	7.699E-01
SSBP4	-0.142	2.098E-01	1.365E-01
CLEC7A	-0.142	6.571E-01	1.358E-01
HSD17B14	-0.142	6.089E-01	2.364E-01
DOCK6	-0.142	1.835E-01	1.804E-01
RPLP1	-0.142	2.931E-01	3.711E-02
ANO5	-0.142	7.257E-01	7.890E-02
TNFRSF17	-0.142	7.808E-01	3.447E-01
BTN2A2	-0.142	3.379E-01	7.858E-02
LINC00854	-0.142	5.204E-01	5.237E-01
RNF175	-0.142	6.035E-01	5.880E-01
SLC18A1	-0.142	8.007E-01	5.239E-01
DRAM1	-0.142	4.938E-01	5.789E-01
FBXO5	-0.142	3.339E-01	1.025E-01
GEMIN5	-0.142	8.576E-02	2.204E-01
ZNF740	-0.142	7.551E-02	4.193E-01
MRPL50	-0.142	1.020E-01	2.720E-01
MIR3131	-0.142	9.273E-01	1.586E-01
PTPRB	-0.142	4.401E-01	1.678E-01
GRK6	-0.142	8.595E-02	7.295E-01

EIF3D	-0.142	8.457E-02	8.246E-02
PABPC4L	-0.142	6.314E-01	7.201E-01
SERF1A	-0.142	7.214E-01	1.038E-01
TRPM2	-0.142	5.795E-01	4.506E-02
CRYZ	-0.143	4.715E-01	8.035E-02
DCAF5	-0.143	4.108E-02	3.871E-01
TAAR5	-0.143	NA	5.658E-01
IFNA8	-0.143	NA	3.573E-01
ANKRD20A3	-0.143	NA	5.048E-01
RNASE3	-0.143	7.572E-01	6.873E-01
LCE1D	-0.143	9.296E-01	2.070E-01
TRANK1	-0.143	3.317E-01	5.964E-01
CPNE5	-0.143	5.208E-01	7.044E-01
C6orf201	-0.143	5.465E-01	1.691E-01
TP53I3	-0.143	3.524E-01	2.288E-01
MIR933	-0.143	NA	3.485E-01
DLX5	-0.143	6.884E-01	6.681E-01
MIR3661	-0.143	NA	3.017E-01
MIR521-2	-0.143	NA	6.430E-01
SLC7A10	-0.143	7.962E-01	2.611E-01
PELI2	-0.143	5.942E-01	7.543E-01
ABTB1	-0.143	2.987E-01	1.944E-01
TBP	-0.143	2.446E-02	4.300E-01
ZNF74	-0.143	2.711E-01	1.282E-01
SLC15A3	-0.143	5.905E-01	1.773E-01
CAMKK2	-0.143	7.007E-02	7.389E-01
PRAM1	-0.143	5.578E-01	1.598E-01
MIR4512	-0.143	7.294E-01	6.230E-01
SPATA2L	-0.143	2.596E-01	3.825E-01
ISCU	-0.143	5.764E-02	1.134E-01
TEPSIN	-0.143	1.665E-01	8.635E-01
MIR4783	-0.143	NA	7.728E-01
PRKG2	-0.143	5.613E-01	8.037E-02
FAM218A	-0.143	7.124E-01	1.824E-01
IGBP1	-0.144	7.483E-02	6.395E-01
HDAC2	-0.144	9.760E-02	9.328E-01
AMPD3	-0.144	3.217E-01	9.411E-01
SAT2	-0.144	2.708E-01	9.441E-01
SP140L	-0.144	2.980E-01	8.601E-02
NR4A3	-0.144	6.893E-01	2.453E-01
SAGE1	-0.144	9.080E-01	9.745E-01
MCM9	-0.144	8.468E-02	4.948E-01
FAM174A	-0.144	1.500E-01	3.156E-01
GNRH1	-0.144	3.460E-01	4.439E-01
CD58	-0.144	2.392E-01	9.032E-01
ART3	-0.144	7.317E-01	6.410E-01
BRAP	-0.144	3.540E-02	4.062E-01
EN1	-0.144	8.298E-01	1.995E-01
C12orf29	-0.144	8.982E-02	7.309E-02
DUSP9	-0.144	7.351E-01	2.731E-01

BMP10	-0.144	NA	5.758E-01
HDAC9	-0.144	6.730E-01	2.032E-01
AC090004.1	-0.144	4.579E-01	4.357E-01
NTN3	-0.144	6.985E-01	2.661E-01
TBX10	-0.144	8.124E-01	1.011E-01
TBC1D3B	-0.144	6.301E-01	1.383E-01
MAP3K19	-0.144	6.169E-01	4.507E-02
TLR2	-0.144	5.877E-01	3.891E-01
ZNF700	-0.144	2.855E-01	5.403E-01
RGS14	-0.144	2.963E-01	4.735E-01
PLEKHH2	-0.144	5.667E-01	3.033E-01
GNAL	-0.144	5.753E-01	1.404E-01
ARMT1	-0.144	6.582E-02	1.883E-01
PANO1	-0.144	3.958E-01	5.202E-01
MIR5092	-0.144	NA	7.175E-01
ITM2B	-0.144	3.093E-01	6.113E-01
IQCF2	-0.144	NA	3.526E-01
SOX8	-0.144	6.333E-01	5.524E-02
PES1	-0.145	1.669E-01	1.097E-01
PCGF2	-0.145	2.735E-01	4.220E-01
THOC5	-0.145	1.627E-01	6.489E-01
ABR	-0.145	2.153E-01	8.516E-02
PRH1	-0.145	4.566E-01	3.319E-01
CD83	-0.145	4.709E-01	1.892E-01
LPCAT4	-0.145	3.923E-01	6.021E-02
PRMT1	-0.145	8.643E-02	2.353E-01
DNTT	-0.145	8.934E-01	9.340E-02
KRT83	-0.145	7.806E-01	8.811E-02
BEND5	-0.145	6.329E-01	1.782E-01
DYRK3	-0.145	4.142E-01	4.296E-01
OMG	-0.145	4.652E-01	7.271E-02
LDB1	-0.145	1.460E-01	4.219E-01
UNC93B1	-0.145	3.598E-01	2.332E-01
PISD	-0.145	1.132E-01	3.311E-01
NOTCH1	-0.145	4.199E-01	5.269E-01
SLC35G5	-0.145	6.190E-01	1.142E-01
RNF31	-0.145	9.202E-02	2.258E-01
HOXA3	-0.145	4.289E-01	4.120E-01
SPATA1	-0.145	3.056E-01	3.262E-01
CIRBP	-0.145	2.482E-01	5.507E-01
TNFRSF13C	-0.145	6.036E-01	3.674E-01
EED	-0.145	5.001E-02	4.912E-02
CEP131	-0.145	2.913E-01	5.165E-02
SLC25A16	-0.146	1.258E-01	3.905E-01
MGMT	-0.146	4.253E-01	4.568E-01
PDYN	-0.146	NA	4.031E-01
AC025263.2	-0.146	NA	3.831E-01
KRTAP4-5	-0.146	NA	7.623E-01
ZNF169	-0.146	1.937E-01	4.664E-01
TTYH3	-0.146	2.019E-01	1.898E-01

CNOT2	-0.146	1.404E-01	4.101E-01
NME1-NME2	-0.146	4.528E-01	1.026E-01
FIG4	-0.146	1.531E-01	2.269E-01
TRIM13	-0.146	2.512E-01	1.362E-01
TLDC2	-0.146	5.605E-01	2.453E-02
PLA2G4F	-0.146	6.664E-01	4.550E-01
ZNF552	-0.146	4.868E-01	7.879E-01
PSMB9	-0.146	6.560E-01	3.271E-01
LAMB3	-0.146	6.620E-01	6.604E-01
IMPDH2	-0.146	1.909E-01	1.266E-01
RNF138	-0.146	1.984E-01	1.505E-01
EFR3B	-0.146	5.816E-01	8.760E-01
AREL1	-0.146	6.966E-02	3.205E-01
CRHR2	-0.146	6.586E-01	9.604E-01
RABGGTA	-0.146	1.110E-01	3.340E-01
DMAP1	-0.147	1.609E-01	5.725E-01
PCBD1	-0.147	3.004E-01	3.125E-01
ETV3L	-0.147	7.994E-01	5.474E-01
ABHD17B	-0.147	1.667E-01	7.692E-01
TRMO	-0.147	9.416E-02	7.681E-01
PTGES2	-0.147	1.196E-01	3.565E-01
RPL36A	-0.147	2.917E-01	8.223E-02
KIF2A	-0.147	2.143E-01	4.686E-02
ABLIM1	-0.147	4.076E-01	5.337E-01
MIR320A	-0.147	7.196E-01	4.339E-02
EXTL2	-0.147	1.469E-01	3.657E-01
PHPT1	-0.147	2.796E-01	7.746E-01
LRRK1	-0.147	2.901E-01	2.260E-01
MRPL44	-0.147	7.148E-02	3.686E-01
MRPL28	-0.147	1.877E-01	4.096E-01
FGFBP1	-0.147	8.227E-01	1.909E-01
NPIPA7	-0.147	NA	4.311E-01
KRT31	-0.147	8.450E-01	6.447E-01
TNFSF13	-0.147	2.963E-01	8.164E-01
MIR548O	-0.147	8.259E-01	5.974E-02
ZNF23	-0.147	3.742E-01	4.612E-01
PCDH11Y	-0.147	8.269E-01	8.388E-02
AL049839.2	-0.147	8.080E-01	2.471E-01
FAM92A	-0.147	2.131E-01	5.768E-01
MTO1	-0.147	1.736E-02	6.296E-01
TP53INP1	-0.147	3.152E-01	9.166E-01
GRPEL2	-0.147	1.531E-01	1.896E-01
WDR83	-0.147	1.823E-01	2.064E-01
AFG1L	-0.148	7.033E-02	4.954E-01
CROCC	-0.148	1.824E-01	1.778E-01
TMEM51	-0.148	3.548E-01	6.291E-01
ASB11	-0.148	8.162E-01	2.535E-01
CCRL2	-0.148	5.315E-01	5.131E-01
TMEM175	-0.148	2.174E-01	3.987E-01
USP3	-0.148	1.346E-01	6.988E-02

MPST	-0.148	3.366E-01	6.578E-01
CCNB1	-0.148	3.618E-01	1.957E-01
YBX3	-0.148	2.418E-01	1.007E-01
QRSL1	-0.148	1.978E-01	6.462E-01
HSBP1L1	-0.148	3.057E-01	1.913E-02
SUCNR1	-0.148	6.962E-01	6.547E-01
SGSM3	-0.148	1.523E-01	1.031E-01
SLC16A10	-0.148	6.372E-01	3.196E-01
CHFR	-0.148	4.872E-02	4.029E-01
SNRPD1	-0.148	1.305E-01	7.242E-01
EIF5A	-0.148	1.420E-01	5.758E-01
CXCR2	-0.148	6.834E-01	2.080E-01
TONSL	-0.148	2.185E-01	1.548E-01
PPARA	-0.148	2.558E-01	6.189E-02
PRPF40B	-0.148	2.027E-01	2.322E-01
PBOV1	-0.148	7.221E-01	5.912E-01
OPN1LW	-0.148	NA	5.573E-02
PRG2	-0.148	7.538E-01	1.059E-01
MAP3K7	-0.148	3.578E-02	1.757E-01
ZNF528	-0.148	4.490E-01	5.986E-02
ZNF559	-0.148	1.965E-01	2.266E-02
PYROXD1	-0.148	9.666E-02	9.445E-01
FAM175A	-0.148	1.232E-01	7.367E-01
NXPH2	-0.148	8.582E-01	2.883E-01
TRIM41	-0.148	3.187E-02	3.422E-01
MTCL1	-0.149	5.613E-01	1.824E-01
ZNF317	-0.149	2.757E-02	7.189E-01
CTDSP1	-0.149	1.209E-01	1.135E-01
ADAMTS3	-0.149	6.604E-01	5.008E-01
MIR4472-2	-0.149	NA	1.407E-01
TSPAN12	-0.149	6.134E-01	1.400E-01
MCF2L	-0.149	5.519E-01	1.268E-01
ENTPD8	-0.149	7.322E-01	4.602E-01
ZBTB49	-0.149	5.879E-02	2.438E-01
HPS6	-0.149	6.247E-02	2.807E-01
PGAP1	-0.149	4.028E-01	1.359E-01
ATG4B	-0.149	1.080E-01	2.273E-01
IL18	-0.149	5.133E-01	4.868E-02
C1QBP	-0.149	1.876E-01	1.366E-01
CDCP2	-0.149	7.548E-01	1.181E-01
ZNF182	-0.149	1.619E-01	1.408E-01
CLUH	-0.149	1.475E-01	3.910E-01
RNF166	-0.149	1.596E-01	7.775E-01
TMEM161A	-0.149	1.128E-01	2.813E-01
KANSL1L	-0.149	1.495E-01	6.278E-01
TIMD4	-0.149	7.624E-01	7.102E-01
SCART1	-0.149	5.754E-01	3.931E-01
DGCR14	-0.149	5.457E-02	8.773E-02
ELMO3	-0.149	4.589E-01	3.664E-01
OR13D1	-0.149	NA	1.566E-01

BCL10	-0.149	1.369E-01	1.161E-01
ATP5A1	-0.150	1.032E-01	6.280E-01
SDSL	-0.150	3.940E-01	5.120E-01
GIPC3	-0.150	3.999E-01	6.223E-01
RNF219	-0.150	7.273E-02	4.300E-01
TDRP	-0.150	5.970E-01	3.383E-01
ZNF426	-0.150	9.058E-02	8.314E-02
LYSMD4	-0.150	1.678E-01	4.148E-01
ANKRD33B	-0.150	6.603E-01	7.263E-01
ZNF501	-0.150	3.842E-01	5.057E-02
MOB3C	-0.150	1.878E-01	1.423E-01
N4BP1	-0.150	9.604E-02	3.787E-01
OR52E4	-0.150	NA	1.225E-01
CYSTM1	-0.150	3.976E-01	2.596E-01
PLEKHG3	-0.150	3.142E-01	1.785E-01
MIR6888	-0.150	NA	3.248E-01
MIR513B	-0.150	NA	5.154E-01
RILPL2	-0.150	2.023E-01	2.757E-01
PLD6	-0.150	4.251E-01	5.496E-01
MAT2B	-0.150	5.312E-02	2.234E-01
MRRF	-0.150	1.132E-01	7.987E-02
CLDN15	-0.150	2.962E-01	2.496E-01
SYNGAP1	-0.150	1.393E-01	1.148E-01
RPL41	-0.150	1.603E-01	8.734E-01
GFM2	-0.150	8.725E-02	7.894E-01
AC104831.1	-0.150	8.168E-01	4.447E-01
CDC42SE2	-0.150	9.559E-02	1.680E-01
KCP	-0.150	6.020E-01	1.480E-01
CD160	-0.150	5.455E-01	9.245E-01
APLF	-0.150	2.545E-01	8.713E-01
STRIP2	-0.150	5.516E-01	4.729E-01
GTF2E2	-0.150	1.278E-01	4.385E-01
RPS5	-0.150	1.992E-01	2.392E-01
H2AFY2	-0.150	3.994E-01	1.149E-01
CRB3	-0.150	4.774E-01	8.077E-02
ZNF84	-0.150	2.397E-01	1.381E-01
KIF14	-0.151	4.210E-01	7.056E-01
AASDH	-0.151	5.051E-02	9.524E-01
SCAP	-0.151	3.201E-01	9.650E-01
TNFSF8	-0.151	6.471E-01	1.748E-01
DDT	-0.151	2.361E-01	5.881E-01
ZNF837	-0.151	3.525E-01	6.550E-02
HCN3	-0.151	3.773E-01	2.164E-01
EGLN3	-0.151	5.888E-01	5.690E-01
SCRT1	-0.151	6.448E-01	5.577E-02
PHRF1	-0.151	4.994E-02	5.781E-01
ZNF302	-0.151	2.715E-01	3.359E-01
CKAP4	-0.151	2.351E-01	5.938E-01
VSTM2A	-0.151	8.217E-01	7.589E-01
DNAJC28	-0.151	2.479E-01	4.222E-02

MIR3174	-0.151	7.538E-01	7.679E-01
NLRP9	-0.151	7.347E-01	9.067E-02
ARRDC2	-0.151	2.290E-01	6.135E-01
AKAP8	-0.151	2.969E-02	1.624E-02
RPL36AL	-0.151	1.457E-01	3.661E-01
MOB3B	-0.151	5.631E-01	3.181E-01
NYAP2	-0.151	8.023E-01	1.563E-01
CAPN9	-0.151	7.795E-01	1.220E-01
STK11IP	-0.151	6.518E-02	6.380E-01
SLC5A3	-0.151	2.839E-01	8.919E-01
COX7A2	-0.151	1.181E-01	4.276E-01
UBA52	-0.151	1.297E-01	5.567E-01
LSM6	-0.151	7.399E-02	9.010E-02
TTI2	-0.151	1.633E-01	5.096E-01
PCDH19	-0.151	6.910E-01	3.237E-02
CADPS	-0.151	6.594E-01	1.986E-01
ZNF608	-0.152	5.996E-01	3.534E-01
FIGNL2	-0.152	7.414E-01	2.435E-01
NF816-ZNF32	-0.152	7.827E-01	2.273E-01
NLN	-0.152	2.970E-01	5.246E-01
TERB2	-0.152	8.218E-01	3.600E-02
BEND3	-0.152	2.922E-01	2.149E-01
WEE1	-0.152	2.763E-01	3.574E-01
SP4	-0.152	1.724E-01	6.929E-02
SRSF5	-0.152	1.479E-01	3.815E-01
NINL	-0.152	5.743E-01	1.774E-01
SOX7	-0.152	6.387E-01	3.922E-01
IFNL2	-0.152	8.232E-01	6.537E-01
CXCL8	-0.152	7.203E-01	8.428E-02
TMEM114	-0.152	8.570E-01	4.459E-01
GPBP1	-0.152	1.032E-02	1.257E-01
THEM5	-0.152	5.438E-01	3.175E-01
CETN3	-0.152	9.457E-02	5.309E-02
LGALS2	-0.152	6.600E-01	1.048E-01
DAB2IP	-0.152	2.831E-01	3.965E-02
DHRS1	-0.152	1.996E-01	2.440E-01
MACROD2	-0.152	7.299E-01	1.624E-01
TRIB2	-0.152	5.327E-01	1.403E-01
DNAI2	-0.152	7.295E-01	4.652E-01
ORC2	-0.152	2.915E-02	1.876E-01
PDE4A	-0.152	3.339E-01	2.960E-01
SF3A2	-0.153	8.443E-02	5.677E-01
AC234771.5	-0.153	NA	6.093E-02
HTT	-0.153	4.579E-02	1.910E-01
ARID1B	-0.153	5.413E-02	1.393E-02
MIR4440	-0.153	NA	1.620E-01
LDLRAD4	-0.153	5.311E-01	6.903E-01
SAFB2	-0.153	2.618E-02	6.226E-01
ZNF596	-0.153	2.941E-01	3.236E-01
TP53I11	-0.153	4.374E-01	1.834E-01

TNFRSF10A	-0.153	3.714E-01	5.429E-01
PRR14L	-0.153	1.311E-01	1.931E-01
ELL2	-0.153	3.809E-01	6.783E-02
C19orf53	-0.153	1.495E-01	9.349E-01
MED16	-0.153	9.918E-02	5.073E-01
DSC1	-0.153	7.816E-01	1.824E-01
TAF9	-0.153	7.950E-02	1.435E-01
ACSM2B	-0.153	8.145E-01	2.523E-01
ZSCAN12	-0.153	2.917E-01	4.450E-02
CRAMP1	-0.153	1.086E-01	5.820E-02
RECQL4	-0.153	3.331E-01	7.958E-02
PPP1R32	-0.153	4.263E-01	3.173E-01
HARS	-0.153	2.442E-02	3.758E-02
CBLB	-0.153	1.506E-01	4.003E-01
TAX1BP3	-0.153	1.935E-01	5.121E-01
PNKD	-0.153	2.396E-01	2.398E-01
PRKN	-0.153	5.552E-01	7.173E-01
MIR1226	-0.153	NA	1.007E-01
PRDX5	-0.154	2.056E-01	4.626E-01
PFDN5	-0.154	1.285E-01	7.833E-01
ZNF561	-0.154	1.878E-01	1.320E-01
NDUFB7	-0.154	2.146E-01	5.404E-01
ZCCHC9	-0.154	1.639E-02	8.059E-02
EVA1A	-0.154	6.873E-01	7.359E-01
B2M	-0.154	4.237E-01	2.553E-01
SEBOX	-0.154	NA	7.508E-01
POLR2J2	-0.154	8.044E-01	3.334E-02
MIR646	-0.154	NA	1.851E-02
DSG2	-0.154	4.444E-01	1.935E-01
STX16	-0.154	8.209E-02	1.563E-01
LTV1	-0.154	3.430E-02	2.873E-02
ACSBG2	-0.154	5.001E-01	4.518E-02
CYTH1	-0.154	1.290E-01	2.005E-01
ADRA1B	-0.154	7.333E-01	6.114E-01
RAP1GAP2	-0.154	4.930E-01	7.187E-01
ERCC5	-0.154	1.107E-01	1.131E-01
PRR25	-0.154	6.384E-01	3.531E-01
ETNK2	-0.154	4.053E-01	2.834E-01
EBLN1	-0.154	NA	2.638E-01
INTS10	-0.154	7.797E-02	3.416E-01
FBXO41	-0.154	3.555E-01	5.331E-01
CYHR1	-0.154	1.398E-01	3.528E-01
HSD17B11	-0.155	4.492E-01	2.234E-01
RPS11	-0.155	1.576E-01	7.340E-03
TMEM102	-0.155	2.518E-01	1.091E-01
SUGP1	-0.155	1.581E-02	4.950E-01
INO80C	-0.155	2.019E-01	8.091E-01
PIGG	-0.155	4.846E-02	7.430E-02
PIF1	-0.155	3.325E-01	3.264E-01
ESD	-0.155	1.517E-01	1.949E-01

MYO1F	-0.155	5.311E-01	4.235E-01
TIGAR	-0.155	2.445E-01	2.694E-02
OR10A3	-0.155	6.977E-01	8.582E-02
NDUFA2	-0.155	1.491E-01	3.800E-01
FAM102B	-0.155	3.332E-01	1.408E-01
DISP2	-0.155	6.204E-01	8.305E-01
TNKS	-0.155	1.615E-01	3.040E-01
ORMDL1	-0.155	4.108E-02	5.690E-01
OR13A1	-0.155	6.912E-01	8.967E-01
ZNF286B	-0.155	3.241E-01	6.001E-01
GIMAP7	-0.155	5.097E-01	6.887E-01
FAM216A	-0.155	3.694E-01	9.614E-02
TMEM120B	-0.155	7.182E-02	5.714E-02
RAB40B	-0.155	2.571E-01	4.945E-01
UCHL3	-0.155	1.560E-01	2.085E-02
S100A7A	-0.155	8.750E-01	1.029E-01
ASB9	-0.155	5.184E-01	2.888E-01
SLC35G6	-0.155	6.840E-01	1.846E-02
GPR63	-0.155	5.432E-01	4.212E-01
MIR298	-0.156	NA	7.851E-01
RFC3	-0.156	2.758E-01	7.881E-02
CCDC28B	-0.156	3.472E-01	8.955E-01
MIR647	-0.156	4.298E-01	2.756E-02
OPRD1	-0.156	5.125E-01	4.299E-02
MIR153-1	-0.156	NA	4.997E-01
OR6C74	-0.156	NA	2.437E-01
KRTAP6-2	-0.156	NA	1.361E-01
ZNF124	-0.156	3.251E-01	5.328E-01
ABCE1	-0.156	1.000E-01	6.010E-01
FABP12	-0.156	8.635E-01	2.580E-01
CCR8	-0.156	6.669E-01	4.375E-01
CHPT1	-0.156	4.358E-01	2.278E-01
TMEM145	-0.156	6.543E-01	9.711E-01
EDA	-0.156	6.184E-01	1.612E-01
CNOT7	-0.156	6.230E-02	6.364E-02
MAGEA9B	-0.156	8.526E-01	1.068E-01
ASAH2	-0.156	4.289E-01	5.868E-01
C22orf23	-0.156	4.128E-01	6.830E-02
RNF34	-0.156	4.318E-03	7.354E-01
ST6GALNAC	-0.156	5.832E-01	1.945E-01
PARPBP	-0.156	2.356E-01	3.587E-01
FOLR2	-0.156	6.367E-01	3.433E-01
GSDMD	-0.156	2.573E-01	2.063E-01
MYRF	-0.156	6.361E-01	8.510E-02
COL11A2	-0.156	5.331E-01	3.125E-02
LRP2BP	-0.157	2.378E-01	7.393E-01
SERPIND1	-0.157	7.378E-01	1.973E-01
LINGO2	-0.157	8.044E-01	6.439E-02
C18orf21	-0.157	5.764E-02	2.810E-02
KLRB1	-0.157	5.864E-01	2.954E-01

SIVA1	-0.157	1.585E-01	1.810E-01
SMYD1	-0.157	NA	2.119E-01
ADRA1D	-0.157	6.586E-01	7.955E-01
NANOGP8	-0.157	8.038E-01	3.828E-01
NLE1	-0.157	1.288E-01	4.758E-02
NISCH	-0.157	1.273E-01	9.589E-02
SLC9A3	-0.157	6.046E-01	6.251E-02
PPIP5K2	-0.157	7.165E-02	3.013E-01
APRT	-0.157	1.824E-01	9.979E-02
BIVM	-0.157	9.111E-02	2.042E-02
BMP3	-0.157	7.684E-01	5.923E-01
MIR3667	-0.157	NA	7.699E-01
KRTAP6-3	-0.157	NA	2.914E-01
TP73	-0.157	6.254E-01	9.114E-01
USP9Y	-0.157	7.994E-01	5.264E-02
ADCY2	-0.157	6.969E-01	2.378E-01
TNFSF10	-0.157	5.926E-01	3.838E-01
TUSC3	-0.157	4.013E-01	5.169E-01
PHB2	-0.157	4.599E-02	6.660E-01
VPS36	-0.157	1.573E-01	9.074E-02
FAM98B	-0.158	2.915E-02	1.725E-02
BCKDHB	-0.158	2.848E-01	3.353E-01
GALNT18	-0.158	4.261E-01	6.895E-02
LYRM7	-0.158	8.045E-02	1.613E-01
TMEM161B	-0.158	1.139E-01	3.244E-01
DGKD	-0.158	2.135E-01	4.297E-02
HLA-DMB	-0.158	5.972E-01	4.014E-01
MT1HL1	-0.158	NA	6.308E-02
FBXO30	-0.158	9.749E-02	5.636E-01
SPDYA	-0.158	3.302E-01	6.068E-01
OR6B2	-0.158	NA	9.034E-01
GALT	-0.158	1.226E-01	4.276E-01
FGF17	-0.158	6.949E-01	8.082E-02
LIPE	-0.158	5.032E-01	4.490E-01
DUS1L	-0.158	1.249E-01	7.833E-01
POLR1B	-0.158	1.046E-01	6.898E-01
MT1X	-0.158	6.521E-01	6.077E-02
CATSPER1	-0.158	6.935E-01	3.054E-01
WNT1	-0.158	7.114E-01	4.146E-01
PRDM11	-0.158	3.642E-01	3.822E-01
RPRM	-0.158	7.057E-01	5.569E-01
SP100	-0.158	2.223E-01	2.594E-01
SH3D21	-0.158	3.625E-01	7.230E-01
DDX54	-0.158	3.274E-02	5.167E-01
FAM212B	-0.158	2.256E-01	2.947E-01
CCNB1IP1	-0.158	1.195E-01	3.616E-01
MKRN2OS	-0.158	5.484E-01	9.421E-01
ZNF440	-0.158	4.398E-01	4.837E-01
SLC9B1	-0.158	2.843E-01	2.691E-01
PGGT1B	-0.158	1.666E-02	8.666E-01

MIR4526	-0.158	NA	5.255E-01
PIK3C3	-0.159	6.857E-02	6.299E-01
SCX	-0.159	5.299E-01	3.893E-02
RXRG	-0.159	7.371E-01	1.417E-01
DEPDC5	-0.159	7.451E-02	4.907E-01
PRB1	-0.159	NA	1.695E-01
ELP5	-0.159	9.975E-02	1.727E-01
C5orf56	-0.159	3.781E-01	1.725E-01
RASL11A	-0.159	5.697E-01	3.792E-01
BFAR	-0.159	1.641E-02	1.377E-01
SEMA3F	-0.159	4.000E-01	8.683E-02
EGF	-0.159	7.280E-01	1.175E-01
MT1H	-0.159	7.271E-01	5.024E-01
TRAPPC13	-0.159	2.481E-02	3.398E-01
ERCC8	-0.159	2.054E-02	2.090E-01
TMEM177	-0.159	1.097E-01	1.513E-01
NR1I2	-0.159	6.594E-01	3.543E-01
C6orf203	-0.159	2.760E-01	1.466E-01
USP15	-0.159	2.051E-02	2.323E-02
HAUS7	-0.159	3.218E-01	7.504E-02
PIM3	-0.159	2.113E-01	5.927E-01
NBEA	-0.159	6.419E-01	5.193E-02
PCDH15	-0.159	7.563E-01	1.532E-01
CNKSR1	-0.159	3.054E-01	6.321E-02
AL136295.5	-0.159	3.100E-01	9.117E-02
AHCYL2	-0.159	1.989E-01	5.536E-01
CRACR2B	-0.159	5.902E-01	1.521E-01
CD300LF	-0.159	6.057E-01	5.983E-01
C3orf49	-0.159	3.735E-01	2.357E-01
KIAA0319	-0.160	7.049E-01	6.886E-01
TRAPPC2L	-0.160	1.097E-01	3.965E-01
ASPSCR1	-0.160	1.902E-01	5.621E-01
C19orf70	-0.160	1.834E-01	2.910E-02
SLC43A1	-0.160	4.967E-01	3.945E-01
ADCK5	-0.160	2.027E-01	2.832E-01
ZNF784	-0.160	1.930E-01	1.523E-01
SH3GL3	-0.160	8.071E-01	2.329E-01
RPL31	-0.160	1.513E-01	5.740E-01
FBXL19	-0.160	9.191E-02	6.544E-01
MIR1827	-0.160	NA	5.481E-01
OR5B21	-0.160	NA	3.342E-01
SELENOV	-0.160	8.502E-01	7.276E-01
SPRED1	-0.160	2.922E-01	4.281E-02
ZNF736	-0.160	3.403E-01	1.739E-01
AGAP2	-0.160	5.146E-01	4.532E-02
COLCA2	-0.160	6.321E-01	1.337E-01
FOXK1	-0.160	2.257E-01	6.872E-02
ASCC3	-0.160	1.556E-01	6.611E-01
SBK1	-0.160	6.820E-01	1.392E-01
MOK	-0.160	3.736E-01	5.648E-01

ERF	-0.161	7.384E-02	2.297E-01
KBTBD7	-0.161	1.658E-01	5.924E-01
MMAA	-0.161	8.236E-02	1.642E-01
PXK	-0.161	1.978E-01	4.677E-01
CHD1	-0.161	6.799E-02	9.408E-01
AC008878.3	-0.161	8.553E-01	5.237E-01
SOCS7	-0.161	2.880E-01	2.619E-01
ANKRD13D	-0.161	1.791E-01	1.254E-02
NDUFV2	-0.161	1.592E-01	3.998E-01
OGFR	-0.161	1.095E-01	3.083E-02
EEF1B2	-0.161	1.458E-01	3.816E-02
EEF1A1	-0.161	9.483E-02	1.348E-01
TIPARP	-0.161	3.628E-01	2.978E-01
IL15RA	-0.161	5.436E-01	2.595E-01
BRD8	-0.161	3.200E-02	2.083E-01
AC117457.1	-0.161	NA	6.731E-02
FRMD8	-0.161	1.232E-01	3.663E-01
MIR4253	-0.161	NA	7.808E-01
UBL5	-0.161	1.038E-01	1.833E-01
CREB5	-0.161	5.758E-01	7.433E-01
OR2B3	-0.161	NA	3.144E-01
OR6C6	-0.161	NA	6.756E-01
STAP2	-0.161	3.558E-01	5.322E-02
PRR5	-0.161	2.158E-01	7.169E-01
SRSF6	-0.161	8.374E-02	5.096E-01
SLFN5	-0.161	3.729E-01	3.223E-01
ITGAX	-0.161	5.713E-01	3.885E-01
ZNF696	-0.161	1.078E-01	4.657E-01
HOXB7	-0.162	3.292E-01	1.822E-01
CD200R1L	-0.162	7.220E-01	3.588E-01
SERINC2	-0.162	4.664E-01	1.578E-01
GMNN	-0.162	2.462E-01	8.832E-02
AES	-0.162	2.042E-01	2.697E-01
CLNS1A	-0.162	6.228E-02	7.799E-02
LIG4	-0.162	1.359E-01	8.791E-01
USE1	-0.162	1.232E-01	6.878E-02
HIST2H2AC	-0.162	4.151E-01	2.516E-01
BHLHA15	-0.162	6.387E-01	9.753E-03
ARHGEF2	-0.162	1.269E-01	3.364E-01
CD22	-0.162	6.805E-01	5.272E-01
PRAMEF12	-0.162	9.149E-01	5.749E-01
LRRIQ1	-0.162	7.198E-01	4.179E-01
SH3GLB2	-0.162	3.096E-01	1.859E-01
ELFN1	-0.162	4.477E-01	1.210E-01
HINT1	-0.162	1.427E-01	1.705E-02
HIRA	-0.162	5.004E-02	2.940E-01
NEURL4	-0.162	5.646E-02	1.390E-01
POLL	-0.162	4.863E-02	5.203E-01
PHF10	-0.162	3.078E-02	3.367E-01
AKAP8L	-0.163	7.020E-02	8.635E-01

ANHX	-0.163	NA	5.152E-01
HIST1H2BA	-0.163	NA	1.642E-01
SPANXA2	-0.163	NA	7.136E-02
GAK	-0.163	1.904E-02	1.856E-01
GABRB2	-0.163	7.659E-01	3.238E-01
SETMAR	-0.163	2.122E-01	1.262E-01
CBX5	-0.163	2.359E-01	9.055E-01
REP15	-0.163	4.244E-01	9.153E-01
RPP25	-0.163	3.966E-01	7.728E-01
FAM171A1	-0.163	5.749E-01	1.687E-01
C12orf45	-0.163	1.406E-01	4.882E-03
STAT6	-0.163	1.416E-01	2.546E-01
GAB3	-0.163	4.841E-01	1.045E-01
ZBTB7A	-0.163	7.285E-02	7.041E-01
ZCCHC10	-0.163	9.234E-03	9.863E-02
RPS15A	-0.163	1.402E-01	9.201E-02
KHDC1	-0.163	5.792E-01	1.125E-01
CCDC68	-0.163	5.515E-01	2.133E-01
SLC35D1	-0.163	2.090E-01	3.815E-01
NRDE2	-0.163	2.194E-02	4.354E-01
MRPS27	-0.163	3.079E-02	2.535E-01
GSTO2	-0.163	3.378E-01	1.347E-01
TYW5	-0.163	1.526E-02	2.130E-01
KRTCAP3	-0.163	5.610E-01	1.130E-01
NDUFA9	-0.163	5.584E-02	1.078E-01
RBM25	-0.163	4.739E-02	1.707E-02
GPX1	-0.163	2.317E-01	1.331E-01
HNRNPCL3	-0.163	NA	8.485E-01
HRAS	-0.163	2.531E-01	1.224E-01
MIR606	-0.163	NA	2.395E-01
GEN1	-0.163	1.262E-01	2.411E-01
DNAAF2	-0.164	3.423E-02	4.316E-01
HES1	-0.164	3.384E-01	4.427E-01
TBPL1	-0.164	4.897E-02	7.220E-01
DNASE1L2	-0.164	5.228E-01	5.081E-01
FAM120B	-0.164	8.569E-03	1.280E-01
EIF3G	-0.164	6.515E-02	2.211E-02
ZNF202	-0.164	5.735E-02	4.834E-01
PDHA2	-0.164	NA	4.738E-01
C12orf73	-0.164	5.929E-02	6.246E-01
AGPAT5	-0.164	1.691E-01	6.362E-02
SULT1A4	-0.164	8.147E-01	7.526E-02
CLCN3	-0.164	2.395E-01	8.615E-03
SLX4	-0.164	7.652E-02	2.264E-01
BRAT1	-0.164	6.857E-02	3.928E-01
APOBR	-0.164	5.492E-01	8.486E-01
SKIV2L2	-0.164	5.457E-02	3.205E-01
ATXN2	-0.164	3.766E-02	5.585E-02
RPL35A	-0.164	8.534E-02	5.550E-01
PRAMEF7	-0.164	NA	8.721E-02

MTX2	-0.164	3.252E-02	8.415E-02
SWAP70	-0.164	1.322E-01	2.228E-02
TTBK1	-0.164	5.518E-01	8.884E-01
TRIM35	-0.164	1.029E-01	3.385E-01
SMARCD1	-0.164	1.002E-02	4.584E-01
YTHDC2	-0.164	1.036E-01	3.972E-01
MRGPRG	-0.164	NA	5.574E-01
MAPK12	-0.165	4.778E-01	1.942E-01
RIOK3	-0.165	1.470E-01	1.073E-01
INAFM2	-0.165	2.082E-01	4.117E-01
SLC26A2	-0.165	4.121E-01	9.070E-02
CCDC18	-0.165	1.834E-01	2.474E-01
CLASRP	-0.165	9.216E-02	6.743E-01
AC020636.2	-0.165	NA	2.958E-01
MIR6719	-0.165	7.397E-01	4.156E-02
APOL3	-0.165	4.935E-01	4.105E-02
MAPKBP1	-0.165	1.291E-01	3.014E-01
UQCRQ	-0.165	1.952E-01	1.225E-01
KIF4B	-0.165	5.864E-01	7.229E-01
UBE2L5P	-0.165	2.842E-01	5.881E-02
MAP7	-0.165	2.452E-01	5.810E-01
UBB	-0.165	1.340E-01	8.715E-02
SMDT1	-0.165	1.664E-01	5.017E-02
CD274	-0.165	6.549E-01	5.771E-01
EFHC1	-0.165	2.269E-01	2.507E-01
CAPN3	-0.165	5.034E-01	5.895E-02
ARL4D	-0.165	5.293E-01	9.574E-01
CSNK1E	-0.165	7.856E-02	1.542E-01
MYH8	-0.165	7.396E-01	3.289E-02
BORCS8	-0.165	8.513E-02	1.759E-01
OR2Y1	-0.165	NA	1.013E-01
KRTAP13-3	-0.165	NA	2.649E-01
HTR3D	-0.165	NA	2.558E-01
RUBCNL	-0.166	6.243E-01	3.858E-01
HMBOX1	-0.166	9.961E-02	4.218E-01
EMID1	-0.166	5.829E-01	7.912E-01
PLB1	-0.166	5.065E-01	6.656E-01
TIA1	-0.166	1.427E-01	5.024E-01
AL603832.3	-0.166	6.905E-01	3.776E-01
FBXW7	-0.166	3.617E-02	5.181E-02
AVP	-0.166	8.430E-01	6.594E-01
SRC	-0.166	2.872E-01	4.867E-01
SLC5A7	-0.166	7.934E-01	4.030E-02
AAAS	-0.166	7.463E-03	1.770E-01
ABCD4	-0.166	2.950E-02	3.145E-01
LRRC14	-0.166	7.090E-02	2.382E-02
FRYL	-0.166	5.207E-02	3.540E-01
CNN2	-0.166	2.615E-01	3.858E-01
ZNF248	-0.166	1.370E-01	2.757E-01
RNASE10	-0.166	6.574E-01	2.481E-01

KRT33A	-0.166	7.426E-01	2.607E-01
TAPBPL	-0.166	3.006E-01	5.852E-01
MED31	-0.166	4.195E-02	4.767E-01
TRIM66	-0.166	2.362E-01	2.398E-02
TDRD6	-0.166	4.738E-01	7.131E-01
PRR35	-0.166	7.975E-01	3.586E-02
PJA1	-0.166	1.502E-01	6.422E-01
PDILT	-0.166	NA	3.355E-01
CNTNAP4	-0.167	8.428E-01	4.526E-01
PLD4	-0.167	6.027E-01	9.191E-02
TEN1-CDK3	-0.167	3.383E-01	1.467E-01
CYP26B1	-0.167	6.417E-01	1.331E-02
TPRN	-0.167	1.968E-01	3.417E-03
ZNF578	-0.167	6.387E-01	6.937E-01
CYP2R1	-0.167	9.126E-02	1.853E-01
MIPOL1	-0.167	3.470E-01	8.808E-01
PSTPIP1	-0.167	5.577E-01	1.074E-01
TRIML2	-0.167	8.405E-01	7.545E-02
PLS1	-0.167	4.979E-01	8.358E-02
TRMT2A	-0.167	1.074E-01	4.640E-01
OR10A4	-0.167	NA	6.357E-01
MIR4424	-0.167	NA	1.897E-01
WDR41	-0.167	8.244E-02	4.238E-01
MEX3A	-0.167	5.629E-01	1.490E-01
ACCS	-0.167	3.916E-01	5.505E-03
PRAME	-0.167	8.298E-01	1.872E-01
ZDHHC17	-0.167	7.926E-02	1.001E-01
CSNK1A1	-0.167	1.492E-01	3.997E-02
UCN3	-0.167	8.413E-01	9.389E-02
DDX20	-0.167	1.342E-02	4.784E-01
ZKSCAN8	-0.167	8.955E-02	4.738E-01
HIST1H2AH	-0.167	6.073E-01	4.245E-01
LUC7L3	-0.167	9.856E-02	2.906E-01
OIT3	-0.167	6.367E-01	7.152E-01
SH3BP2	-0.168	1.135E-01	9.435E-01
DCAF15	-0.168	2.724E-02	9.444E-01
MIR4797	-0.168	NA	6.829E-02
CSTA	-0.168	6.273E-01	2.594E-02
STK17A	-0.168	3.478E-01	7.390E-02
PKDCC	-0.168	6.847E-01	3.660E-01
LIMA1	-0.168	3.070E-01	2.018E-02
PRAMEF20	-0.168	NA	1.251E-01
LY6K	-0.168	6.155E-01	3.928E-01
KRR1	-0.168	4.774E-02	3.463E-01
MUM1	-0.168	4.925E-02	1.350E-01
NRG4	-0.168	5.561E-01	1.082E-01
MIR5007	-0.168	NA	3.910E-01
SSTR2	-0.168	5.574E-01	4.875E-02
TNFRSF8	-0.169	5.832E-01	1.523E-01
FEM1A	-0.169	1.980E-01	2.262E-01

CRYBG1	-0.169	3.735E-01	1.441E-02
HLA-DRA	-0.169	5.935E-01	2.392E-01
AL365205.1	-0.169	3.597E-01	5.469E-01
ALG13	-0.169	5.835E-02	5.292E-02
KIAA0907	-0.169	1.621E-01	4.749E-01
CCL20	-0.169	7.357E-01	2.449E-01
ITGA3	-0.169	5.018E-01	3.847E-02
CACNA1D	-0.169	7.122E-01	2.411E-01
ZNF90	-0.169	5.896E-01	1.572E-01
POMT2	-0.169	6.722E-02	2.816E-02
KIAA0586	-0.169	6.857E-02	5.298E-02
RPS17	-0.169	1.140E-01	1.404E-01
TSPYL4	-0.169	1.087E-01	1.172E-01
EPHX2	-0.169	5.348E-01	1.825E-01
ZBTB1	-0.169	3.889E-02	4.550E-02
SAMD13	-0.169	5.858E-01	4.105E-02
HYPK	-0.169	2.457E-01	1.004E-01
C20orf194	-0.169	1.903E-01	5.455E-01
ATAD3C	-0.169	5.335E-01	3.471E-01
LRRC8C	-0.169	3.238E-01	1.451E-02
MRI1	-0.169	7.945E-02	4.112E-02
TEFM	-0.169	2.946E-02	3.003E-02
SLX1B	-0.169	6.352E-01	3.398E-01
MRPL16	-0.169	8.987E-02	2.829E-01
FBXO33	-0.169	1.415E-02	1.239E-02
SLC14A2	-0.169	5.859E-01	3.585E-02
ESCO2	-0.169	3.093E-01	9.070E-02
ZNF713	-0.169	2.551E-01	5.933E-02
PUS1	-0.170	9.013E-02	8.530E-02
SOCS4	-0.170	1.998E-02	4.930E-01
C2CD5	-0.170	3.806E-02	4.880E-01
TENM3	-0.170	7.122E-01	1.190E-01
RAPGEF4	-0.170	3.131E-01	2.578E-02
LETMD1	-0.170	5.511E-02	5.233E-02
SELL	-0.170	6.693E-01	8.231E-01
IBTK	-0.170	2.646E-02	9.450E-02
NDUFS7	-0.170	9.005E-02	3.863E-01
ATP2B1	-0.170	1.956E-01	1.561E-01
CECR6	-0.170	5.047E-01	6.674E-02
DENND3	-0.170	2.597E-01	2.523E-01
COMMD10	-0.170	4.026E-02	3.843E-02
HMGN5	-0.171	4.330E-01	1.832E-02
HUS1B	-0.171	3.631E-01	1.171E-01
SERPINE2	-0.171	6.435E-01	5.661E-01
ZNF296	-0.171	4.131E-01	6.110E-01
PA2G4	-0.171	5.672E-02	6.802E-01
METTL17	-0.171	4.300E-02	7.553E-01
KCNJ9	-0.171	6.680E-01	1.006E-01
MIR944	-0.171	NA	1.342E-01
OR4N2	-0.171	NA	6.039E-01

OTUD6A	-0.171	NA	1.108E-01
TRIM27	-0.171	2.814E-02	3.989E-01
LRPAP1	-0.171	1.219E-01	2.181E-02
COQ10A	-0.171	1.585E-01	3.033E-02
TRAF3	-0.171	1.012E-01	4.677E-01
SLX1A	-0.171	6.392E-01	2.454E-02
RPS19	-0.171	1.913E-01	2.376E-01
MIR186	-0.171	5.896E-01	6.297E-02
FAM49B	-0.171	6.424E-02	5.294E-02
FAM163A	-0.171	6.314E-01	1.220E-02
DESI1	-0.171	2.998E-02	5.357E-01
NOA1	-0.171	1.324E-02	6.064E-01
MRPS30	-0.171	3.638E-02	3.056E-01
IFT140	-0.171	3.500E-01	1.919E-01
TRIM52	-0.172	1.192E-01	7.406E-02
EGR3	-0.172	5.826E-01	3.823E-01
MIR4516	-0.172	NA	5.877E-02
FAM46A	-0.172	2.654E-01	3.345E-01
FAM170B	-0.172	7.910E-01	6.988E-02
WDR17	-0.172	7.352E-01	2.462E-01
VASP	-0.172	1.042E-01	2.192E-01
BTBD2	-0.172	3.021E-02	2.252E-01
ATP23	-0.172	1.224E-01	3.777E-01
SIX4	-0.172	3.075E-01	3.733E-01
DFFB	-0.172	5.366E-02	3.689E-01
SLC9A9	-0.172	4.430E-01	4.487E-01
TRPT1	-0.172	1.592E-01	6.855E-01
IGFALS	-0.172	6.045E-01	1.527E-02
PPP1R1C	-0.172	5.441E-01	4.268E-01
DIS3L2	-0.172	2.596E-02	1.076E-01
SKAP1	-0.172	5.191E-01	2.691E-02
PHYKPL	-0.172	9.219E-02	5.116E-01
RNF126	-0.172	7.551E-02	7.689E-01
RAPGEF6	-0.172	8.120E-03	4.435E-01
CBR1	-0.173	3.524E-01	1.100E-01
MIR6893	-0.173	NA	3.948E-02
CYC1	-0.173	8.826E-02	3.119E-01
CYB5R2	-0.173	3.430E-01	1.248E-01
CEP170B	-0.173	1.214E-01	8.327E-01
TNFAIP8	-0.173	1.764E-01	3.723E-01
WDR36	-0.173	5.292E-02	3.801E-02
CKAP2	-0.173	1.817E-01	1.071E-02
ZNF334	-0.173	6.626E-01	5.407E-01
ALDH2	-0.173	5.470E-01	4.975E-02
PUS7L	-0.173	4.068E-02	1.241E-02
RPS10	-0.173	1.368E-01	3.116E-01
AL021546.1	-0.173	6.752E-01	4.253E-01
WTAP	-0.173	3.181E-03	8.896E-01
SENP7	-0.173	1.118E-01	8.897E-01
KCTD21	-0.173	8.573E-02	8.938E-01

KDM4D	-0.173	1.621E-01	9.225E-01
OAF	-0.173	2.252E-01	9.448E-01
PLCB2	-0.173	4.709E-01	1.685E-02
GSC	-0.173	7.162E-01	4.729E-01
AP5M1	-0.173	2.515E-02	1.336E-01
43891.000	-0.174	3.770E-01	4.826E-02
RPL13A	-0.174	1.254E-01	5.958E-01
KANTR	-0.174	4.855E-01	8.470E-02
C17orf75	-0.174	1.149E-01	1.131E-02
GGA1	-0.174	5.800E-02	5.831E-02
BHMG1	-0.174	6.207E-01	4.415E-01
MED7	-0.174	1.190E-02	1.630E-01
EIF3E	-0.174	7.355E-02	3.902E-01
POC5	-0.174	2.583E-02	3.418E-02
SPP2	-0.174	8.627E-01	4.651E-01
MZT2A	-0.174	2.154E-01	1.538E-01
PLEKHH3	-0.174	2.091E-01	2.837E-02
MIR4804	-0.174	NA	1.842E-01
SHISA6	-0.174	7.285E-01	2.814E-01
S100A6	-0.174	4.622E-01	2.147E-02
NUDT13	-0.174	2.186E-01	2.524E-01
BAG1	-0.174	2.504E-01	3.163E-01
RAX	-0.174	8.282E-01	2.618E-01
MIR5579	-0.174	NA	3.258E-01
NUP37	-0.174	3.256E-02	9.329E-02
FRAS1	-0.174	6.257E-01	2.218E-01
NPAS2	-0.174	4.145E-01	4.035E-01
MAPK6	-0.174	2.095E-01	4.125E-02
MTRF1L	-0.174	9.912E-03	9.589E-03
MROH1	-0.174	7.007E-02	5.417E-01
TNFRSF25	-0.174	2.629E-01	3.103E-01
OPRK1	-0.175	8.188E-01	1.023E-02
IFT43	-0.175	5.273E-02	3.964E-01
TTLL12	-0.175	1.138E-01	2.549E-01
EIF4B	-0.175	3.724E-02	6.270E-02
INTS13	-0.175	1.132E-01	1.800E-01
RUNX1	-0.175	1.057E-01	5.654E-01
NUGGC	-0.175	6.222E-01	6.865E-01
ACAD10	-0.175	6.404E-02	6.613E-02
SNX22	-0.175	3.201E-01	5.373E-01
AC138811.2	-0.175	4.769E-01	2.769E-01
MAPKAPK5	-0.175	1.670E-02	1.066E-01
GJB5	-0.175	5.831E-01	6.002E-01
INS	-0.175	8.403E-01	3.172E-01
AC011005.1	-0.175	3.938E-01	1.815E-01
CTAGE1	-0.175	7.936E-01	5.774E-01
RAD52	-0.175	1.120E-01	6.268E-01
ABHD17C	-0.175	2.573E-01	4.814E-01
HOXC9	-0.175	6.887E-01	6.977E-01
MIR520G	-0.175	NA	1.779E-01

OR4A16	-0.175	NA	6.373E-03
EIF5AL1	-0.175	5.776E-01	2.160E-01
PRMT7	-0.175	1.090E-01	4.604E-02
AP000781.2	-0.175	5.606E-01	1.188E-01
NCR1	-0.175	6.266E-01	3.062E-01
PSTK	-0.176	7.090E-02	2.944E-01
ADARB2	-0.176	6.642E-01	4.553E-01
HCLS1	-0.176	5.210E-01	9.069E-02
ZNF286A	-0.176	1.316E-01	6.563E-03
ULBP3	-0.176	4.185E-01	8.158E-01
C12orf42	-0.176	6.141E-01	9.177E-01
TFAP4	-0.176	7.950E-02	1.862E-01
CXCR4	-0.176	4.774E-01	6.828E-01
ZNF100	-0.176	2.058E-01	3.199E-02
THOC3	-0.176	1.054E-01	7.156E-02
ENTPD4	-0.176	9.416E-02	9.658E-01
NUDT18	-0.176	2.453E-01	9.658E-01
AC010547.5	-0.176	6.482E-01	3.210E-01
IL16	-0.176	5.195E-01	7.156E-01
ZNF33B	-0.176	1.169E-01	2.794E-01
GAPDH	-0.176	1.411E-01	1.778E-01
AGAP4	-0.176	2.027E-01	3.051E-01
QTRT1	-0.176	1.595E-01	4.263E-02
DENND1C	-0.176	1.790E-01	2.833E-01
TLDC1	-0.177	2.058E-01	1.759E-01
IVD	-0.177	9.781E-02	4.158E-01
SLC25A36	-0.177	6.314E-02	3.355E-01
MIA	-0.177	7.244E-01	2.302E-01
STK11	-0.177	3.714E-02	1.952E-01
AL049697.1	-0.177	3.996E-01	2.594E-01
CCDC58	-0.177	4.428E-02	1.014E-01
LIPF	-0.177	9.060E-01	4.428E-01
C20orf96	-0.177	2.676E-01	3.794E-02
HSFY1	-0.177	NA	1.890E-01
EYS	-0.177	3.977E-01	1.766E-02
MCC	-0.177	3.939E-01	1.366E-02
ADH5	-0.177	1.091E-01	2.612E-01
TRIM59	-0.177	1.280E-01	6.491E-02
LCP1	-0.177	4.975E-01	2.842E-01
RPUSD2	-0.177	1.822E-02	2.755E-02
ARPC3	-0.177	3.843E-02	2.222E-01
MTRNR2L6	-0.177	6.190E-01	3.748E-01
PADI1	-0.177	7.084E-01	2.657E-01
SMUG1	-0.178	6.761E-02	5.121E-01
ATP5G3	-0.178	4.257E-02	3.380E-02
NACC1	-0.178	1.911E-02	3.874E-02
C19orf24	-0.178	1.901E-01	5.290E-01
SMN2	-0.178	4.966E-01	1.014E-01
GPER1	-0.178	4.735E-01	1.250E-02
MOV10	-0.178	5.961E-02	4.604E-01

NOTCH4	-0.178	2.684E-01	1.997E-02
MTMR7	-0.178	2.405E-01	2.006E-02
LSM7	-0.178	1.561E-01	4.581E-02
C17orf100	-0.178	1.660E-01	3.928E-01
SMIM8	-0.178	5.345E-02	5.923E-01
RPLP0	-0.178	9.631E-02	1.358E-01
KRTAP22-2	-0.178	NA	1.724E-01
ZNF846	-0.178	2.534E-01	3.004E-01
GRK3	-0.178	2.930E-01	3.304E-01
DDX51	-0.178	4.917E-02	5.816E-01
KLF1	-0.178	5.749E-01	5.774E-03
EDN2	-0.178	6.915E-01	3.832E-01
TRIM21	-0.178	2.094E-01	6.656E-01
HSPA4	-0.178	8.059E-03	7.490E-01
BTBD11	-0.179	5.521E-01	1.914E-01
RPH3AL	-0.179	3.883E-01	3.192E-01
RNF151	-0.179	5.902E-01	6.681E-01
KLHL6	-0.179	5.251E-01	6.545E-01
DDHD1	-0.179	1.831E-01	2.120E-01
TBXAS1	-0.179	5.218E-01	2.160E-03
AQP8	-0.179	6.259E-01	5.281E-01
NDRG4	-0.179	5.271E-01	3.831E-01
NSA2	-0.179	3.922E-02	2.496E-01
C21orf91	-0.179	1.665E-01	3.652E-01
TRAPPC1	-0.179	6.254E-02	9.129E-02
ZNF414	-0.179	8.443E-02	4.308E-01
KLHL14	-0.179	6.655E-01	2.865E-01
NHLH2	-0.179	7.528E-01	4.260E-01
ADHFE1	-0.179	5.547E-01	3.282E-02
ATG4D	-0.179	7.780E-02	4.024E-02
ZNF660	-0.179	4.778E-01	6.771E-02
TYSND1	-0.179	1.118E-01	8.656E-02
UQCRH	-0.179	8.361E-02	4.138E-01
DDO	-0.179	5.468E-01	6.043E-02
DCAF16	-0.179	4.151E-02	2.710E-01
CHMP4A	-0.180	8.675E-02	1.478E-01
RPL26	-0.180	1.530E-01	1.736E-01
CETP	-0.180	4.366E-01	6.187E-03
CCNG2	-0.180	2.913E-01	3.795E-01
SHC3	-0.180	5.497E-01	8.009E-02
TOGARAM2	-0.180	5.316E-01	2.999E-01
CES1	-0.180	7.203E-01	2.820E-01
MZT2B	-0.180	1.810E-01	1.024E-01
MRPL18	-0.180	6.176E-03	8.948E-02
RPS3A	-0.180	1.103E-01	2.645E-01
USP45	-0.180	2.178E-02	5.294E-01
NOXO1	-0.180	NA	5.120E-01
DCDC2	-0.180	7.351E-01	2.888E-01
MRPL39	-0.180	3.140E-02	3.046E-01
SNRNP35	-0.180	5.910E-02	3.678E-01

TTC39C	-0.180	1.502E-01	3.484E-01
ZNF506	-0.180	2.027E-01	5.805E-01
HSPE1	-0.180	5.003E-02	7.815E-01
ZNF397	-0.180	9.912E-02	7.847E-02
CTBP1	-0.180	2.451E-02	4.680E-02
RGS8	-0.180	5.926E-01	8.087E-01
GBP4	-0.180	5.218E-01	8.839E-01
PCDHB13	-0.180	3.973E-01	4.155E-02
CD28	-0.180	5.341E-01	5.544E-02
HOXD12	-0.180	7.221E-01	5.689E-01
NHS	-0.180	3.742E-01	2.210E-01
TMEM63A	-0.180	2.169E-01	1.015E-01
TMEM106A	-0.180	4.622E-01	3.758E-01
IMPG1	-0.180	4.681E-01	6.162E-02
TRMT13	-0.181	3.638E-02	1.141E-01
RBM5	-0.181	7.483E-02	2.708E-01
HNRNPA0	-0.181	3.653E-03	8.183E-01
PRKAB1	-0.181	3.647E-02	7.511E-03
OR2A12	-0.181	NA	9.188E-01
CCDC42	-0.181	6.027E-01	1.269E-01
JRK	-0.181	1.854E-01	1.185E-01
CCNG1	-0.181	1.253E-01	1.011E-02
MIR1284	-0.181	NA	2.527E-01
OR2G2	-0.181	NA	1.117E-01
MIR4417	-0.181	NA	2.369E-02
SLC25A28	-0.181	8.486E-02	2.364E-01
SMIM17	-0.181	5.720E-01	3.907E-01
MSANTD2	-0.181	1.506E-01	4.546E-01
PNLIP	-0.181	NA	2.082E-02
TMEM259	-0.181	3.657E-02	9.078E-02
CCDC88A	-0.181	3.533E-01	3.024E-03
RPP21	-0.181	7.637E-02	1.644E-01
TGFB1	-0.182	3.187E-01	5.222E-01
TRIM38	-0.182	5.883E-02	3.423E-01
ZNF792	-0.182	2.393E-01	9.538E-03
C12orf43	-0.182	6.024E-03	3.914E-02
E2F5	-0.182	2.367E-01	1.398E-02
TMSB15A	-0.182	6.535E-01	6.353E-03
PPIL2	-0.182	2.673E-02	4.330E-01
MIR4748	-0.182	NA	1.186E-01
ALPK1	-0.182	9.666E-02	1.445E-01
DZIP1	-0.182	4.276E-01	2.662E-02
CTNNA3	-0.182	6.848E-01	3.243E-01
FAM181B	-0.182	6.336E-01	7.729E-01
RNF224	-0.182	5.436E-01	7.800E-02
FLYWCH1	-0.182	8.636E-02	1.960E-02
CIDEB	-0.182	5.012E-01	8.469E-01
AC018523.2	-0.182	7.294E-01	7.369E-02
RSL1D1	-0.182	3.130E-02	1.579E-01
FHIT	-0.182	4.027E-01	1.867E-02

GATA3	-0.183	5.629E-01	1.810E-01
FGF9	-0.183	6.533E-01	6.934E-02
MRPL1	-0.183	2.082E-02	5.070E-01
ZBTB24	-0.183	2.235E-03	3.047E-01
AIM2	-0.183	6.748E-01	9.140E-02
IFI27L2	-0.183	4.168E-01	3.983E-01
ADGRB3	-0.183	6.953E-01	8.347E-01
RSRP1	-0.183	2.120E-01	2.087E-01
ARL10	-0.183	3.815E-01	7.467E-03
PRELID2	-0.183	1.586E-01	4.533E-01
IDUA	-0.183	3.356E-01	1.592E-01
EPPK1	-0.183	4.854E-01	4.740E-02
LATS1	-0.183	3.375E-02	3.782E-01
PEBP4	-0.184	6.081E-01	5.091E-02
SRSF12	-0.184	5.926E-01	1.341E-01
HELZ2	-0.184	2.580E-01	3.458E-01
RPL38	-0.184	1.378E-01	5.729E-01
MIR4682	-0.184	NA	3.217E-01
PCDHGA5	-0.184	3.736E-01	3.926E-01
PREX1	-0.184	2.931E-01	4.346E-01
KLHDC4	-0.184	7.338E-02	5.727E-01
MIR3155A	-0.184	NA	4.802E-02
SLC44A3	-0.184	3.863E-01	5.400E-01
ZC2HC1B	-0.184	NA	3.139E-01
TSPY8	-0.184	NA	2.742E-01
SPANXA1	-0.184	NA	3.776E-02
OC90	-0.184	NA	2.934E-02
ORAOV1	-0.184	1.582E-01	2.040E-01
GALR1	-0.184	7.539E-01	3.137E-01
RASSF9	-0.184	6.599E-01	9.363E-01
ITGA4	-0.184	4.854E-01	1.812E-01
DHX33	-0.184	4.299E-02	6.666E-02
NME8	-0.184	6.004E-01	4.897E-01
NAA16	-0.184	6.162E-02	2.047E-02
MIR302D	-0.184	NA	1.309E-01
IFI35	-0.184	3.660E-01	4.021E-02
CNBD1	-0.184	8.317E-01	4.084E-01
ERICH6B	-0.184	3.918E-01	1.170E-01
MTG1	-0.184	1.397E-01	8.054E-01
FAM83G	-0.184	3.227E-01	1.926E-01
BOP1	-0.185	1.601E-01	5.150E-01
SLC25A22	-0.185	8.164E-02	2.749E-01
BMP7	-0.185	7.012E-01	3.318E-02
DMXL2	-0.185	9.038E-02	1.498E-01
CUL9	-0.185	4.917E-02	5.971E-01
ANKZF1	-0.185	4.469E-02	6.124E-01
GADD45GIP1	-0.185	1.562E-01	5.497E-01
CDK7	-0.185	4.155E-02	5.222E-03
ZNF503	-0.185	2.516E-01	7.657E-01
MS4A4E	-0.185	5.330E-01	2.426E-01

NPM1	-0.185	5.170E-02	2.592E-01
PRSS38	-0.185	NA	8.289E-01
UMOD	-0.185	NA	5.242E-02
KDM6B	-0.185	1.353E-01	8.640E-01
CLEC4E	-0.185	6.211E-01	2.494E-01
SPATA24	-0.185	7.783E-02	3.674E-01
PCDHGA3	-0.185	5.620E-01	3.749E-01
NEU3	-0.185	7.202E-02	1.101E-02
NOB1	-0.185	2.910E-02	4.457E-03
RNF145	-0.185	2.419E-01	2.458E-01
MTERF2	-0.186	8.556E-02	5.986E-02
TXNRD2	-0.186	3.029E-01	2.093E-01
IGF2BP3	-0.186	7.152E-01	1.008E-03
ZSCAN30	-0.186	7.296E-02	2.106E-01
CLEC10A	-0.186	5.954E-01	3.644E-01
NUDT15	-0.186	3.551E-02	6.205E-02
MIR548AA2	-0.186	6.432E-01	5.281E-02
KCTD11	-0.186	2.635E-01	6.431E-01
C12orf49	-0.186	3.558E-02	7.125E-01
MAP3K7CL	-0.186	2.595E-01	1.204E-02
CDKL3	-0.186	6.853E-02	7.240E-03
SCN2B	-0.186	6.900E-01	6.209E-01
LOXHD1	-0.187	5.246E-01	3.949E-01
ATP5L2	-0.187	5.449E-01	8.944E-01
MIR6762	-0.187	7.538E-01	1.643E-01
MIR548I2	-0.187	NA	1.303E-01
MIR6796	-0.187	NA	3.716E-01
GIN1	-0.187	4.964E-03	6.873E-02
DEPDC4	-0.187	7.338E-02	4.942E-02
KIRREL2	-0.187	6.748E-01	4.834E-01
PPP1R16A	-0.187	1.513E-01	7.712E-02
BLMH	-0.187	8.925E-02	1.895E-01
ATP13A1	-0.187	1.119E-02	9.469E-02
CDIP1	-0.187	4.524E-01	8.949E-01
ECHDC1	-0.187	3.990E-02	9.631E-01
CHD5	-0.187	5.584E-01	1.590E-01
MIR302C	-0.187	NA	1.538E-01
TRAFD1	-0.187	5.695E-02	2.571E-01
CASP10	-0.187	2.346E-01	7.092E-01
AC068775.1	-0.187	4.517E-01	9.272E-02
MED23	-0.187	2.349E-02	6.522E-01
SNX14	-0.187	2.117E-02	9.588E-02
ZNF581	-0.187	7.785E-02	2.752E-02
THRB	-0.187	4.114E-01	2.273E-01
TTLL4	-0.187	3.001E-02	6.176E-01
ETNK1	-0.187	3.620E-02	3.093E-02
GIMAP2	-0.188	3.228E-01	3.702E-01
TRIM54	-0.188	6.873E-01	4.394E-02
MZF1	-0.188	1.838E-01	1.373E-01
BTN3A2	-0.188	2.605E-01	5.061E-01

PPDPF	-0.188	1.994E-01	1.848E-01
MRPL22	-0.188	1.666E-02	2.905E-01
GRAPL	-0.188	6.258E-01	5.297E-02
MIR7-3	-0.188	NA	1.581E-01
MIR124-2	-0.188	NA	1.637E-02
MIR541	-0.188	NA	7.520E-02
MIR670	-0.188	NA	3.203E-01
OR52H1	-0.188	NA	5.396E-01
TSPYL1	-0.188	1.613E-02	3.247E-01
ASF1A	-0.188	4.756E-02	2.115E-01
AD000671.2	-0.188	3.833E-01	3.945E-01
MIGA2	-0.188	1.160E-01	4.041E-01
LY6E	-0.188	4.595E-01	3.909E-01
SLC4A11	-0.188	4.227E-01	9.710E-03
MND1	-0.188	2.195E-01	1.767E-02
ZNF431	-0.188	1.629E-01	1.519E-01
MIR3682	-0.188	3.113E-01	2.206E-01
RNF215	-0.189	6.515E-02	4.375E-01
MYBPC2	-0.189	6.321E-01	2.489E-03
MECOM	-0.189	4.276E-01	4.224E-01
GPR119	-0.189	NA	2.256E-01
THAP4	-0.189	4.299E-02	2.247E-01
NT5C	-0.189	1.305E-01	5.239E-01
ELOB	-0.189	1.149E-01	1.869E-01
AREG	-0.189	6.647E-01	3.553E-02
ZNF577	-0.189	3.535E-01	3.330E-02
OR56B4	-0.189	6.817E-01	3.069E-01
PLEKHG5	-0.189	2.252E-01	2.366E-01
PAK6	-0.189	3.511E-01	1.339E-02
VPS37B	-0.189	6.259E-02	1.320E-02
CHTF18	-0.189	1.656E-01	1.306E-01
AL360181.3	-0.189	4.778E-01	4.165E-03
CYP27C1	-0.189	6.237E-01	1.762E-01
TBX3	-0.189	5.634E-01	5.823E-02
MICAL1	-0.189	2.023E-01	3.541E-01
SEMA4D	-0.189	1.146E-01	6.319E-01
DUS3L	-0.189	3.478E-02	1.608E-01
HLA-A	-0.189	4.006E-01	7.586E-02
CFAP69	-0.189	3.061E-01	9.112E-02
[illegible]PGT-TNNI3[illegible]	-0.189	5.316E-01	1.503E-01
CNTNAP2	-0.189	7.738E-01	4.466E-01
NDUFAF8	-0.189	1.568E-01	5.170E-01
RPL7	-0.190	8.201E-02	3.568E-01
MIR1268A	-0.190	NA	2.825E-02
ANKUB1	-0.190	6.829E-01	2.770E-01
MIR874	-0.190	NA	1.860E-01
ALDH3A1	-0.190	7.075E-01	1.163E-01
PDE6C	-0.190	2.810E-01	4.452E-03
CCND2	-0.190	5.188E-01	4.441E-02
AANAT	-0.190	4.522E-01	3.840E-01

YBX1	-0.190	6.050E-02	3.522E-01
ZBTB40	-0.190	3.251E-02	1.369E-01
FAM53C	-0.190	8.710E-03	1.434E-02
D2HGDH	-0.190	1.872E-01	5.335E-01
STAMBPL1	-0.190	2.463E-01	2.538E-02
DNMBP	-0.190	2.053E-01	7.007E-02
GREB1L	-0.190	6.794E-01	2.022E-03
GPAT2	-0.190	6.743E-01	1.164E-01
KRT86	-0.190	5.302E-01	4.282E-01
ZNF142	-0.190	1.042E-02	7.898E-01
CD74	-0.191	4.920E-01	4.251E-01
ACER3	-0.191	6.857E-02	3.673E-01
MIR7152	-0.191	7.848E-01	6.695E-03
TDG	-0.191	4.114E-02	1.435E-03
ZNF705D	-0.191	NA	5.821E-01
CSHL1	-0.191	NA	3.865E-01
MIR2276	-0.191	NA	9.009E-03
MIR515-2	-0.191	NA	8.370E-02
OR5H14	-0.191	NA	2.019E-01
TMEM233	-0.191	4.425E-01	8.662E-02
FXYD3	-0.191	4.591E-01	3.301E-02
CDK6	-0.191	5.093E-01	9.101E-02
MIR6514	-0.191	6.157E-01	6.322E-01
MIR421	-0.191	6.488E-01	6.187E-01
IQSEC3	-0.191	5.754E-01	1.789E-02
ZNF780B	-0.191	1.555E-01	2.485E-01
AQP6	-0.191	6.471E-01	2.807E-01
ZNHIT2	-0.191	1.310E-01	8.983E-02
TXN	-0.191	1.954E-01	2.737E-01
GARNL3	-0.191	3.509E-01	2.980E-01
POTEF	-0.191	5.749E-01	4.682E-01
EXOSC4	-0.191	1.059E-01	1.215E-02
IPO4	-0.192	6.427E-02	4.796E-01
PPHLN1	-0.192	2.607E-03	2.423E-01
NDUFAF4	-0.192	4.004E-02	7.878E-01
ZNF658	-0.192	1.738E-01	3.911E-02
WDR75	-0.192	1.337E-02	2.794E-02
ANKDD1B	-0.192	4.622E-01	4.025E-03
MARK1	-0.192	3.410E-01	1.746E-01
ARID3B	-0.192	1.680E-01	4.578E-01
BAZ2B	-0.192	6.285E-02	3.328E-01
ZNF493	-0.192	3.017E-01	6.070E-02
HIST1H1B	-0.192	5.737E-01	3.713E-02
POC1B	-0.192	2.953E-02	4.710E-01
GPR65	-0.192	4.891E-01	3.226E-02
CLEC4C	-0.192	7.057E-01	8.922E-01
IRAK1BP1	-0.192	1.325E-01	3.322E-01
NELFA	-0.192	4.986E-02	7.599E-02
COX6A1	-0.192	5.330E-02	4.382E-02
TRA2A	-0.192	1.461E-02	6.098E-02

AC011511.4	-0.192	4.797E-01	1.988E-01
MIR367	-0.193	NA	2.877E-03
NDUFC1	-0.193	3.766E-02	3.611E-01
ACTL6B	-0.193	6.374E-01	4.147E-03
C4orf33	-0.193	7.326E-02	3.236E-01
RGS21	-0.193	8.204E-01	1.108E-01
MIR3651	-0.193	NA	3.060E-01
C7orf72	-0.193	NA	1.003E-02
ZNF513	-0.193	6.772E-02	1.745E-01
ACADVL	-0.193	1.061E-01	4.470E-01
ADAM7	-0.193	NA	4.028E-01
CARD6	-0.193	2.565E-01	3.748E-02
CSF3R	-0.193	5.263E-01	2.272E-02
HIP1R	-0.193	1.314E-01	1.222E-02
C17orf78	-0.194	5.050E-01	4.957E-01
TCP10	-0.194	NA	2.428E-02
MTMR3	-0.194	8.936E-02	3.575E-01
MIDN	-0.194	4.395E-02	3.472E-01
MIR3611	-0.194	NA	3.533E-03
KRTAP9-9	-0.194	NA	2.035E-01
HNRNPH1	-0.194	7.561E-03	1.234E-01
MIR6750	-0.194	7.387E-01	6.576E-02
MIR3157	-0.194	NA	1.922E-02
TMTC3	-0.194	1.034E-01	1.751E-01
OFD1	-0.194	9.271E-02	5.252E-02
TLL1	-0.194	6.393E-01	5.507E-03
ETS2	-0.194	2.277E-01	3.112E-03
AKIRIN2	-0.194	8.473E-03	6.158E-01
MPP6	-0.194	3.856E-01	5.212E-01
SCAF8	-0.194	4.281E-03	5.060E-01
TRMT10C	-0.194	8.746E-03	9.082E-02
SNURF	-0.195	5.093E-01	1.499E-02
MTMR10	-0.195	2.851E-02	2.811E-01
HIGD1B	-0.195	3.384E-01	5.370E-01
PPP6R2	-0.195	6.863E-03	3.276E-01
CCDC60	-0.195	7.407E-01	3.075E-02
RHCG	-0.195	7.161E-01	2.478E-02
BAZ1A	-0.195	4.232E-02	8.610E-01
CCBE1	-0.195	6.681E-01	3.306E-01
AP001931.1	-0.195	5.492E-01	6.049E-02
MRFAP1L1	-0.195	2.014E-02	2.067E-01
CLK4	-0.195	6.924E-02	5.369E-02
NAA25	-0.195	3.536E-02	2.189E-01
ACKR4	-0.195	5.656E-01	9.560E-02
PAGE3	-0.195	NA	1.601E-03
DPF3	-0.195	4.061E-01	5.374E-03
TYMP	-0.195	5.203E-01	5.733E-01
SLC3A2	-0.195	1.748E-01	5.201E-02
UBAP1L	-0.195	2.487E-01	3.227E-01
IRF1	-0.195	3.308E-01	3.640E-02

SPATA21	-0.196	6.978E-01	1.293E-01
PNN	-0.196	9.682E-03	4.681E-01
FAM71D	-0.196	3.240E-01	1.297E-01
RNF39	-0.196	4.044E-01	5.950E-01
TBC1D14	-0.196	1.824E-01	3.760E-02
MIR140	-0.196	7.738E-01	5.383E-02
PLIN4	-0.196	5.597E-01	3.179E-01
IMPG2	-0.196	3.106E-01	4.422E-01
RPL7A	-0.196	6.761E-02	1.697E-01
SBF1	-0.196	5.280E-02	2.160E-01
PAICS	-0.196	5.387E-02	7.468E-02
XAGE1A	-0.196	NA	9.548E-03
CUX2	-0.196	6.291E-01	1.552E-02
MIR4499	-0.196	NA	5.159E-01
PITX3	-0.197	5.983E-01	5.433E-01
MIR3913-1	-0.197	NA	1.243E-01
LETM1	-0.197	2.183E-02	9.460E-02
AIG1	-0.197	1.682E-01	5.711E-01
AGRP	-0.197	4.640E-01	6.276E-03
CCAR2	-0.197	1.582E-02	2.042E-01
YJEFN3	-0.197	4.331E-01	1.760E-03
MIR187	-0.197	7.902E-01	3.738E-01
UCP3	-0.197	2.641E-01	3.850E-01
EPOR	-0.197	3.293E-01	2.507E-01
NDUFC2	-0.197	9.556E-02	1.758E-02
RPL17	-0.197	4.632E-02	4.755E-03
ERAP1	-0.197	1.332E-01	3.443E-01
TLR1	-0.197	4.705E-01	3.369E-01
VAV1	-0.198	4.536E-01	1.882E-01
HOXB1	-0.198	6.798E-01	2.560E-01
OR5V1	-0.198	NA	1.755E-02
NCOA7	-0.198	2.704E-01	5.143E-02
MIR3660	-0.198	8.680E-01	5.645E-01
HPS4	-0.198	3.362E-02	5.092E-01
EEF2	-0.198	2.319E-02	1.118E-02
FAM183A	-0.198	4.868E-01	1.759E-02
SIGIRR	-0.198	1.973E-01	2.111E-02
CCDC36	-0.198	3.171E-01	1.836E-01
MIR25	-0.198	3.403E-01	3.649E-01
RFX5	-0.199	4.979E-02	3.758E-01
PCP2	-0.199	5.217E-01	4.184E-01
PPAT	-0.199	6.528E-02	7.874E-01
ZBTB7C	-0.199	5.320E-01	1.791E-03
QRICH2	-0.199	3.147E-01	6.219E-02
FFAR2	-0.199	6.387E-01	7.615E-02
NIPAL2	-0.199	2.101E-01	1.378E-01
HDC	-0.199	5.568E-01	3.141E-03
LDHAL6A	-0.199	7.467E-01	1.049E-01
TGS1	-0.199	1.906E-02	1.656E-01
IGFL3	-0.199	8.035E-01	1.712E-03

DST	-0.199	3.078E-01	1.246E-02
AC005726.2	-0.199	2.965E-01	1.507E-01
RRS1	-0.199	5.883E-02	6.543E-01
CIB3	-0.199	6.321E-01	6.029E-01
SLC5A12	-0.199	5.867E-01	3.059E-02
KLHL40	-0.199	NA	3.166E-01
NKTR	-0.199	1.655E-01	1.348E-01
ZNF222	-0.199	1.814E-01	4.576E-02
GJB2	-0.199	6.116E-01	1.317E-01
LILRA1	-0.199	5.068E-01	4.991E-01
NFKBID	-0.199	1.258E-01	5.140E-01
EAF2	-0.200	2.038E-01	6.113E-01
AC025165.3	-0.200	4.278E-01	9.543E-01
GTF2IRD2B	-0.200	1.768E-01	4.114E-01
MIR425	-0.200	7.910E-01	4.976E-01
GIMAP8	-0.200	3.172E-01	4.089E-01
PPIL3	-0.200	2.828E-02	4.022E-01
SULT1A3	-0.200	4.078E-01	1.690E-01
RASA4B	-0.200	5.746E-01	2.799E-01
ITPRIPL1	-0.200	4.763E-01	2.446E-01
DEPDC1B	-0.200	2.102E-01	5.223E-02
GRIN2A	-0.200	6.848E-01	1.854E-01
KCNQ3	-0.200	4.014E-01	3.709E-01
C1orf159	-0.200	1.091E-01	2.692E-01
EML5	-0.200	5.898E-01	2.858E-02
MYBBP1A	-0.200	2.915E-02	2.626E-01
MANSC4	-0.200	5.935E-01	3.307E-01
FOXD4L5	-0.201	7.106E-01	7.875E-01
ABCB6	-0.201	2.435E-01	6.186E-02
KCNJ11	-0.201	5.210E-01	2.179E-02
CERK	-0.201	2.919E-01	5.328E-01
APOM	-0.201	1.350E-01	8.544E-01
PEMT	-0.201	9.042E-02	9.458E-04
DGCR8	-0.201	5.121E-03	3.040E-02
SRGAP3	-0.201	3.918E-01	6.892E-02
FAH	-0.201	1.356E-01	3.353E-01
DOPEY1	-0.201	8.844E-02	2.437E-01
CFH	-0.201	4.289E-01	5.689E-01
MIR4795	-0.201	7.363E-01	2.858E-03
UBE3D	-0.201	3.372E-02	3.741E-01
COX4I2	-0.201	3.769E-01	3.906E-03
KLHL13	-0.201	5.344E-01	7.188E-02
UQCR11	-0.201	6.453E-02	3.774E-02
RWDD3	-0.201	1.446E-02	5.022E-03
UQCR10	-0.201	1.021E-01	4.396E-01
MLPH	-0.201	4.756E-01	4.838E-01
SLC4A9	-0.201	5.568E-01	8.046E-01
FRG2C	-0.201	7.540E-01	1.290E-02
B3GALT1	-0.201	6.507E-01	3.428E-02
OTUB2	-0.202	2.497E-01	7.848E-01

DDX39B	-0.202	8.695E-02	1.960E-01
HDAC7	-0.202	2.724E-02	3.529E-01
ATP5G2	-0.202	1.848E-02	6.064E-01
RIN1	-0.202	3.178E-01	1.115E-01
NDUFAF1	-0.202	9.538E-03	7.587E-03
C11orf1	-0.202	1.310E-01	3.771E-01
FAR1	-0.202	2.183E-02	2.908E-01
PAQR6	-0.202	3.783E-01	1.768E-01
NCKAP1L	-0.202	5.163E-01	2.476E-01
TAS2R13	-0.202	6.392E-01	1.148E-01
ZNF814	-0.202	1.940E-01	3.820E-01
C22orf29	-0.202	1.258E-01	1.603E-01
C12orf57	-0.202	1.195E-01	3.139E-02
ROPN1	-0.202	8.217E-01	5.493E-02
LIMD2	-0.202	2.864E-01	7.033E-01
DPH7	-0.202	4.745E-02	2.632E-01
PGBD4	-0.202	4.125E-02	4.840E-01
MIR3176	-0.202	4.732E-01	6.676E-01
MDN1	-0.202	3.147E-02	1.671E-02
GPR143	-0.202	5.208E-01	1.613E-02
HSPE1-MOB4	-0.202	3.884E-01	1.872E-01
NIFK	-0.202	5.134E-03	8.680E-03
MIR6085	-0.202	NA	7.752E-01
MIR587	-0.202	NA	3.391E-01
SP110	-0.202	2.078E-01	6.556E-03
RPS7	-0.202	3.648E-02	5.221E-01
ZNF618	-0.202	2.449E-01	7.074E-01
HECTD4	-0.202	3.999E-02	1.665E-01
SLC40A1	-0.202	4.261E-01	3.093E-01
APOBEC3D	-0.203	2.569E-01	1.130E-03
TPD52L3	-0.203	NA	1.231E-01
MSS51	-0.203	1.816E-01	4.180E-03
BTLA	-0.203	5.522E-01	1.269E-01
JAK3	-0.203	3.181E-01	4.709E-01
CNTN2	-0.203	6.443E-01	2.798E-02
P2RY1	-0.203	5.118E-01	2.214E-01
CLDN34	-0.203	5.526E-01	8.586E-02
ZUFSP	-0.203	1.818E-03	4.094E-02
FREM3	-0.203	5.752E-01	1.856E-01
MIR6867	-0.203	6.219E-01	3.263E-01
LTBP4	-0.203	2.748E-01	9.874E-02
LRRC56	-0.203	2.098E-01	7.838E-02
COQ3	-0.203	3.479E-02	2.201E-01
CXXC1	-0.203	8.970E-02	4.794E-02
DEFB134	-0.203	8.570E-01	7.720E-02
CENPV	-0.203	4.216E-01	1.254E-02
MATR3	-0.204	2.073E-01	4.550E-01
MIR370	-0.204	NA	2.798E-01
IL24	-0.204	5.607E-01	7.303E-01
C9orf72	-0.204	9.540E-02	6.970E-03

ACAD11	-0.204	2.250E-01	1.769E-01
LBP	-0.204	6.711E-01	7.966E-01
ABCG8	-0.204	7.084E-01	5.599E-01
MYOZ3	-0.204	4.773E-01	2.951E-01
KDM4B	-0.204	2.228E-02	7.700E-02
OSGEPL1	-0.204	9.841E-03	5.483E-02
MIR3188	-0.204	NA	5.888E-01
MIR324	-0.204	5.127E-01	4.624E-02
MAP3K4	-0.204	9.308E-03	2.111E-01
TNIK	-0.204	4.441E-01	2.397E-02
PGLS	-0.204	8.844E-02	2.131E-02
FBRSL1	-0.204	2.322E-02	8.300E-03
ANGPTL8	-0.204	3.406E-01	3.821E-01
MIR1203	-0.204	NA	4.612E-01
GJC3	-0.204	4.895E-01	2.805E-02
TMEM198	-0.204	2.429E-01	3.350E-01
LSMEM2	-0.204	5.578E-01	2.668E-01
UBE2L6	-0.204	3.082E-01	5.095E-01
METTL8	-0.204	3.830E-02	4.931E-01
MCTP1	-0.204	3.902E-01	1.762E-01
COL17A1	-0.205	6.707E-01	4.134E-02
MASP2	-0.205	3.842E-01	2.164E-01
BTNL2	-0.205	7.566E-01	2.352E-01
NOP14	-0.205	4.791E-03	8.210E-03
ANKRD44	-0.205	2.416E-01	3.642E-01
SLFN12	-0.205	3.470E-01	5.035E-01
PROKR1	-0.205	6.894E-01	8.600E-01
RIMS3	-0.205	3.953E-01	2.548E-01
CCDC73	-0.205	3.458E-01	5.695E-01
MDM4	-0.205	4.605E-02	7.683E-01
HCK	-0.205	4.980E-01	5.374E-01
AC073082.1	-0.205	7.604E-01	4.594E-01
MAN2C1	-0.205	4.818E-02	1.142E-02
FMN2	-0.205	6.935E-01	1.630E-01
OR6K3	-0.205	NA	3.856E-01
KRTAP9-3	-0.205	NA	1.019E-01
PMFBP1	-0.205	3.365E-01	8.414E-01
RPS20	-0.205	6.012E-02	9.562E-02
MT-CO1	-0.205	2.975E-01	5.481E-02
FBXO2	-0.206	5.358E-01	5.466E-01
KIAA0391	-0.206	1.032E-02	4.251E-01
MTHFD2L	-0.206	3.148E-02	3.846E-01
STARD10	-0.206	2.122E-01	7.138E-02
HSCB	-0.206	4.055E-02	3.572E-01
PSCA	-0.206	6.938E-01	3.433E-01
SLC18A2	-0.206	4.729E-01	3.614E-01
C2CD4C	-0.206	4.284E-01	2.419E-01
MAGEB6P1	-0.206	NA	8.008E-01
VSIG4	-0.206	5.778E-01	1.277E-01
OPCML	-0.206	5.897E-01	9.412E-01

KATNBL1	-0.206	8.795E-03	1.810E-03
DOK4	-0.206	9.614E-02	2.112E-01
TEP1	-0.206	4.980E-02	1.937E-01
TRMT11	-0.206	1.634E-02	2.083E-02
OR51E1	-0.206	3.132E-01	7.248E-03
MIR5190	-0.206	NA	7.916E-03
CLRN1	-0.206	NA	5.728E-01
TNP1	-0.206	NA	1.987E-01
ZNF219	-0.206	9.798E-02	5.960E-01
ANAPC7	-0.206	4.641E-03	6.510E-02
IL27	-0.206	5.371E-01	6.160E-01
OR10A2	-0.206	NA	1.502E-01
AWAT2	-0.207	7.625E-01	1.757E-01
PCK1	-0.207	7.638E-01	8.134E-02
PKP3	-0.207	2.551E-01	3.608E-02
RTN4IP1	-0.207	8.161E-02	2.166E-01
KAT2A	-0.207	4.994E-02	6.549E-01
GCDH	-0.207	9.522E-03	2.185E-01
MRPS25	-0.207	7.826E-02	1.925E-02
HOXA2	-0.207	3.729E-01	5.746E-01
FBXL13	-0.207	2.226E-01	3.636E-01
RHOBTB3	-0.207	3.651E-01	3.711E-03
RDH16	-0.207	4.800E-01	1.781E-03
MPEG1	-0.207	4.735E-01	2.713E-01
C9orf85	-0.207	1.724E-02	4.109E-02
CCNJ	-0.207	1.795E-01	6.484E-01
WDR64	-0.207	5.213E-01	5.524E-02
PSMB8	-0.208	2.479E-01	1.536E-01
LPAR2	-0.208	1.107E-01	6.477E-02
SV2C	-0.208	5.628E-01	2.700E-01
C6orf118	-0.208	7.180E-01	2.437E-02
MRNIP	-0.208	1.226E-01	9.319E-03
TCOF1	-0.208	1.639E-02	3.266E-01
MIR181B1	-0.208	NA	1.079E-03
MIR138-1	-0.208	NA	3.421E-01
CDH22	-0.208	7.415E-01	9.151E-02
PRAMEF18	-0.208	NA	1.904E-01
MIR4294	-0.208	NA	3.069E-01
POLR3G	-0.208	3.561E-01	3.226E-01
DLX6	-0.208	5.643E-01	2.810E-02
MATK	-0.208	5.052E-01	7.387E-02
TWNK	-0.208	2.616E-02	7.590E-02
FERMT3	-0.208	4.223E-01	4.386E-01
CRYBB3	-0.208	3.840E-01	5.694E-02
ZNF763	-0.209	2.760E-01	1.837E-01
ATP4A	-0.209	7.397E-01	3.414E-01
BAIAP3	-0.209	5.011E-01	4.606E-04
FAM213A	-0.209	2.627E-01	2.918E-01
EMB	-0.209	3.323E-01	1.016E-01
RAB44	-0.209	4.163E-01	8.106E-02

ZNF699	-0.209	2.096E-01	4.198E-01
CDK3	-0.209	2.210E-01	3.154E-01
MIR6797	-0.209	2.730E-01	2.143E-01
TOX	-0.209	5.175E-01	7.922E-02
FBXL6	-0.209	5.883E-02	9.072E-01
SMTNL1	-0.209	5.133E-01	1.299E-01
GNPDA1	-0.209	2.727E-02	3.103E-01
LGALS9	-0.209	3.504E-01	8.752E-01
HARS2	-0.209	1.902E-03	4.580E-02
MIR4715	-0.209	NA	5.822E-01
PICK1	-0.209	5.768E-02	2.484E-01
MIR6859-1	-0.210	5.236E-01	2.483E-01
ABRACL	-0.210	5.667E-02	8.803E-01
TMPRSS15	-0.210	7.904E-01	2.334E-01
PML	-0.210	1.058E-01	2.223E-02
SIGLEC11	-0.210	5.540E-01	1.679E-01
LONRF1	-0.210	1.110E-01	1.755E-01
MIR4766	-0.210	7.202E-01	3.683E-01
EEPD1	-0.210	2.790E-01	1.652E-02
SIGLEC12	-0.210	6.169E-01	5.582E-01
ACVR1C	-0.210	5.373E-01	5.357E-01
DNAH10	-0.210	3.871E-01	3.024E-02
ECHDC3	-0.210	6.177E-01	4.930E-01
GUF1	-0.210	1.868E-03	6.301E-02
RHOT2	-0.210	2.220E-02	4.218E-02
CSNK1A1L	-0.211	6.701E-01	5.420E-02
GSTZ1	-0.211	1.145E-01	3.067E-03
DPEP3	-0.211	5.971E-01	7.063E-02
TBC1D8	-0.211	2.174E-01	5.156E-01
CCDC159	-0.211	1.629E-01	2.649E-02
CYLC1	-0.211	NA	9.372E-03
MED28	-0.211	6.392E-04	1.254E-01
MSRA	-0.211	1.670E-01	2.925E-02
CPSF4L	-0.211	5.010E-01	1.688E-02
MPPED2	-0.211	5.735E-01	1.806E-01
ACE	-0.211	2.161E-01	3.196E-02
TTC19	-0.211	2.204E-02	2.641E-01
TMEM191C	-0.211	4.979E-01	9.297E-02
ZNF738	-0.211	2.427E-01	1.122E-03
IL15	-0.211	3.493E-01	8.483E-02
RMND1	-0.211	5.562E-03	5.893E-01
MPHOSPH6	-0.211	3.460E-02	5.027E-01
A3GALT2	-0.211	5.788E-01	1.361E-01
RS1	-0.211	5.436E-01	5.288E-01
VAV2	-0.211	1.872E-01	2.769E-01
RFLNA	-0.211	6.211E-01	8.641E-01
GNGT2	-0.211	3.673E-01	1.288E-01
ATAT1	-0.211	1.289E-01	5.161E-01
MRPL42	-0.211	3.796E-03	2.512E-01
RPL28	-0.212	1.033E-01	9.179E-02

TMEM72	-0.212	6.941E-01	1.413E-01
PFAS	-0.212	3.147E-02	1.659E-01
IMPA1	-0.212	5.550E-02	9.155E-01
MS4A2	-0.212	5.153E-01	2.609E-01
FAAP100	-0.212	5.759E-03	3.653E-01
TMEM94	-0.212	3.474E-02	4.203E-01
MIR5699	-0.212	NA	4.236E-01
CENPK	-0.212	1.476E-01	2.674E-01
SYBU	-0.212	5.516E-01	3.544E-01
C11orf40	-0.212	NA	1.876E-01
CXorf21	-0.212	4.568E-01	1.247E-01
MIR3146	-0.212	NA	8.237E-03
C12orf10	-0.212	1.159E-02	1.876E-02
VHLL	-0.212	NA	3.248E-02
KCTD1	-0.212	2.733E-01	1.787E-02
NUP43	-0.213	1.781E-04	6.377E-03
RPL10A	-0.213	2.349E-02	3.449E-01
BLOC1S4	-0.213	3.078E-02	3.037E-02
NPIPA1	-0.213	1.519E-01	1.715E-01
STK36	-0.213	9.947E-02	2.044E-02
AC004805.1	-0.213	8.158E-01	2.061E-01
ARL9	-0.213	4.938E-01	6.979E-01
MIR3928	-0.213	NA	6.888E-03
THG1L	-0.213	9.293E-03	1.495E-02
HOMEZ	-0.213	4.068E-02	4.510E-01
COX7C	-0.213	2.595E-02	1.402E-01
TCP1	-0.213	5.081E-03	5.366E-01
SYT3	-0.213	5.612E-01	1.191E-02
VIP	-0.213	4.910E-01	2.086E-01
CYP2J2	-0.213	4.912E-01	4.468E-01
CPSF6	-0.213	1.084E-02	6.706E-03
OR52D1	-0.213	7.671E-01	3.132E-01
CXorf49	-0.213	8.773E-01	7.851E-01
WNT7B	-0.213	3.544E-01	8.004E-01
MIR3173	-0.214	NA	1.678E-01
SLC39A4	-0.214	2.051E-01	6.475E-01
GRPEL1	-0.214	1.083E-02	5.382E-01
RPL18A	-0.214	7.971E-02	9.314E-02
MIR99B	-0.214	7.441E-01	1.508E-01
ENGASE	-0.214	2.356E-01	5.184E-01
GTF2IRD2	-0.214	2.000E-01	8.894E-02
SPTA1	-0.214	6.086E-01	7.422E-01
CT45A5	-0.214	8.854E-01	2.996E-01
SREK1	-0.214	1.849E-02	7.966E-02
ZNF547	-0.214	1.664E-01	7.378E-03
IL17RA	-0.214	1.086E-02	2.678E-01
ENOSF1	-0.214	7.972E-02	7.534E-02
LVRN	-0.214	5.559E-01	5.525E-01
ARRDC4	-0.214	2.326E-01	4.194E-01
ISOC1	-0.214	4.527E-02	2.922E-02

SIX1	-0.214	3.511E-01	1.578E-03
FANCF	-0.214	4.986E-02	2.886E-01
RPL5	-0.215	1.547E-02	4.221E-01
IFIH1	-0.215	3.403E-01	5.118E-01
SWSAP1	-0.215	7.126E-02	3.060E-01
UBE2N	-0.215	1.116E-03	8.405E-02
TSEN2	-0.215	1.565E-01	2.034E-01
DNAJC5G	-0.215	5.310E-01	8.537E-02
DNAH11	-0.215	6.682E-01	6.395E-01
SALL2	-0.215	3.838E-01	6.401E-01
SLC35E3	-0.215	1.601E-01	2.605E-01
C12orf71	-0.215	4.176E-01	9.296E-01
CEACAM4	-0.215	5.551E-01	9.164E-03
UTP15	-0.215	2.456E-03	8.931E-02
FAU	-0.215	3.158E-02	7.775E-01
EPHA6	-0.215	6.579E-01	6.629E-01
APOBEC3H	-0.216	3.593E-01	8.759E-01
CHST10	-0.216	3.547E-01	5.817E-03
SPINK5	-0.216	5.706E-01	4.642E-01
MIR590	-0.216	4.348E-01	2.415E-01
FGFR1OP	-0.216	1.100E-02	1.933E-02
RPL23	-0.216	5.041E-02	7.907E-01
IFI44	-0.216	4.279E-01	1.596E-01
NFE2	-0.216	5.684E-01	2.147E-01
CTSS	-0.216	2.487E-01	7.839E-01
PHACTR1	-0.216	3.430E-01	3.842E-01
RPS29	-0.216	5.593E-02	5.902E-01
LNX1	-0.216	2.155E-01	7.454E-01
MKNK2	-0.217	5.800E-02	4.458E-01
ZADH2	-0.217	1.129E-02	1.078E-01
CCDC183	-0.217	4.190E-01	1.803E-02
GPCPD1	-0.217	7.844E-02	3.398E-01
SPAG6	-0.217	6.367E-01	1.041E-01
JUNB	-0.217	1.321E-01	2.121E-01
C19orf66	-0.217	5.331E-02	1.182E-01
CHEK2	-0.217	3.150E-02	1.560E-02
PINX1	-0.217	6.772E-02	2.574E-01
PCDHB4	-0.217	4.589E-01	8.259E-01
RILP	-0.218	1.596E-01	2.253E-02
RASGRP3	-0.218	1.331E-01	7.986E-02
SLC38A2	-0.218	8.161E-02	6.350E-01
SYCP2	-0.218	4.696E-01	2.476E-01
PCED1A	-0.218	8.408E-02	1.407E-02
C2orf78	-0.218	NA	3.940E-01
CEP44	-0.218	9.320E-02	3.182E-01
C1orf53	-0.218	1.478E-01	2.656E-01
JAML	-0.218	4.106E-01	1.428E-01
TSPOAP1	-0.218	4.128E-01	1.171E-01
C1B-GALN	-0.218	8.630E-02	2.573E-01
TIGD1	-0.218	6.795E-02	5.295E-01

WDR77	-0.218	1.324E-02	5.560E-02
SLC4A8	-0.218	4.366E-01	3.192E-01
SRXN1	-0.218	3.219E-01	1.380E-01
ZNF224	-0.218	9.054E-02	1.629E-01
RPS4X	-0.218	2.464E-02	5.094E-02
MRPL54	-0.219	7.805E-02	8.646E-01
AC040162.4	-0.219	7.085E-01	1.738E-03
AC092647.5	-0.219	3.914E-01	6.079E-02
MIR3145	-0.219	NA	5.254E-01
TMEM151B	-0.219	5.204E-01	3.278E-01
ITIH4	-0.219	2.758E-01	3.905E-01
KDR	-0.219	1.802E-01	6.006E-01
ITM2C	-0.219	3.432E-01	2.445E-01
ATP6AP1L	-0.219	1.441E-01	6.458E-03
IL1F10	-0.219	7.665E-01	6.803E-01
MYL6B	-0.219	1.233E-01	9.167E-02
ZNF18	-0.219	8.648E-03	1.401E-02
GPAM	-0.219	1.082E-01	1.965E-01
CACNA1S	-0.219	6.842E-01	3.807E-01
MIR582	-0.219	NA	4.355E-01
CAPN10	-0.219	1.718E-02	2.350E-01
KCNH6	-0.219	5.845E-01	3.397E-01
VILL	-0.219	4.799E-01	1.418E-01
ADGRF3	-0.220	1.490E-01	1.992E-02
PRH2	-0.220	3.989E-01	6.309E-01
PIK3CG	-0.220	3.987E-01	7.117E-02
TRMU	-0.220	1.191E-02	1.168E-01
BHLHE41	-0.220	3.864E-01	2.284E-01
B3GNT6	-0.220	7.396E-01	1.372E-01
NOP16	-0.220	2.889E-02	8.906E-02
NACA	-0.220	4.017E-03	2.069E-01
RFXANK	-0.220	3.230E-02	1.437E-01
CDK10	-0.220	4.652E-02	2.628E-02
MBD3L1	-0.220	NA	5.804E-02
MCRIP2	-0.221	1.538E-01	3.119E-01
PDCD2	-0.221	1.206E-03	6.996E-02
MIR486-1	-0.221	NA	5.400E-01
TARBP2	-0.221	8.648E-03	6.112E-02
PRAMEF4	-0.221	NA	8.196E-02
GALNT7	-0.221	9.243E-02	7.319E-03
HES3	-0.221	NA	7.778E-02
GMIP	-0.221	2.817E-02	8.554E-02
SESN1	-0.221	6.086E-02	9.107E-01
TAP1	-0.221	3.803E-01	2.608E-01
AMD1	-0.221	1.126E-02	4.798E-01
KCNT2	-0.221	5.030E-01	3.864E-03
GIMAP5	-0.221	3.897E-01	6.541E-02
PRODH	-0.221	5.641E-01	3.698E-04
BRS3	-0.221	6.253E-01	2.884E-01
PTPRE	-0.221	1.509E-01	4.067E-02

HHEX	-0.221	2.830E-01	8.587E-02
TSPAN10	-0.222	3.240E-01	8.187E-02
C19orf67	-0.222	4.908E-01	6.917E-01
TTC38	-0.222	3.999E-02	6.847E-02
SSX7	-0.222	NA	2.411E-02
KPNA5	-0.222	3.460E-02	5.563E-01
RPS3	-0.222	6.781E-02	1.119E-01
TUBA4A	-0.222	3.495E-01	2.018E-01
NOP53	-0.222	7.823E-02	2.389E-04
PRPF39	-0.222	1.257E-02	3.964E-02
MIR6804	-0.222	6.363E-01	2.543E-03
AC011511.1	-0.222	2.139E-01	2.910E-02
FUT10	-0.223	7.202E-02	2.708E-01
KHDC3L	-0.223	7.934E-01	3.801E-01
INTU	-0.223	1.087E-01	2.897E-01
NT5DC1	-0.223	4.243E-03	3.276E-01
PDE6H	-0.223	6.958E-01	9.104E-01
PCDHB1	-0.223	6.848E-01	3.733E-02
CYTIP	-0.223	4.310E-01	3.075E-01
ATP12A	-0.223	6.988E-01	2.986E-01
PPIAL4C	-0.223	5.425E-01	7.646E-02
ARL15	-0.223	6.902E-02	3.515E-03
C6orf222	-0.223	6.430E-01	1.728E-01
ENPP7	-0.224	6.212E-01	1.366E-01
ALOX5	-0.224	3.522E-01	2.909E-01
NRXN3	-0.224	5.739E-01	6.286E-01
ANKRD63	-0.224	7.850E-01	3.785E-01
SIRT6	-0.224	1.104E-02	7.467E-01
STAT4	-0.224	3.376E-01	2.549E-01
ACSL5	-0.224	3.590E-01	5.881E-01
ABCC11	-0.224	2.425E-01	1.384E-01
WDFY2	-0.224	2.492E-02	2.317E-01
MT-CO3	-0.224	1.786E-01	1.102E-03
GMFB	-0.224	1.254E-02	3.087E-01
MIR5690	-0.224	NA	4.149E-01
PKHD1	-0.225	6.276E-01	1.263E-01
SCNN1G	-0.225	6.298E-01	5.060E-02
C3orf62	-0.225	3.478E-02	1.875E-01
RNF44	-0.225	1.767E-02	1.334E-02
RPL24	-0.225	2.939E-02	2.080E-02
ZNF540	-0.225	2.399E-01	4.115E-01
HAPLN3	-0.225	4.040E-01	3.130E-02
DDX11	-0.225	3.448E-02	3.639E-01
ZNF519	-0.225	1.249E-01	1.236E-01
ZNF93	-0.226	2.379E-01	4.230E-02
AC008073.3	-0.226	6.737E-01	1.213E-01
SLIT3	-0.226	4.064E-01	5.200E-04
TAF1C	-0.226	1.680E-02	2.407E-01
AQP2	-0.226	7.900E-01	1.207E-01
ACVR1B	-0.226	4.155E-02	2.136E-01

TMPRSS4-AS	-0.226	5.984E-01	1.071E-02
RTP4	-0.226	3.339E-01	3.535E-01
DUSP4	-0.226	3.188E-01	3.721E-02
MAP3K5	-0.226	8.967E-02	3.470E-01
SPATA9	-0.227	2.131E-01	1.596E-02
DLX4	-0.227	4.425E-01	1.058E-03
IFITM1	-0.227	4.445E-01	2.014E-01
PITX1	-0.227	4.151E-01	9.139E-01
MKRN3	-0.227	6.970E-01	6.078E-01
TMEM225B	-0.227	4.001E-01	2.770E-01
NDUFAF2	-0.227	2.727E-02	2.448E-01
CLHC1	-0.227	1.042E-01	4.169E-01
SCLY	-0.227	1.650E-02	1.046E-02
MMP13	-0.227	6.424E-01	1.762E-01
CASP4	-0.227	1.568E-01	1.844E-01
AGMO	-0.227	6.680E-01	1.181E-01
CCDC57	-0.227	5.532E-02	8.063E-02
LANCL2	-0.227	2.763E-02	5.854E-01
XAGE5	-0.227	9.029E-01	3.290E-01
RPLP2	-0.228	5.756E-02	7.816E-02
LIAS	-0.228	8.365E-03	2.177E-01
CECR2	-0.228	6.032E-01	8.193E-02
SLC44A5	-0.228	4.087E-01	3.048E-02
ZNF483	-0.228	2.405E-01	2.990E-01
43899.000	-0.228	6.515E-02	7.828E-03
EGFL8	-0.228	2.007E-01	3.620E-01
HIST2H3A	-0.228	NA	1.985E-01
SOWAHD	-0.228	2.407E-01	1.354E-01
TRPV5	-0.228	5.146E-01	2.762E-01
MAGEC3	-0.228	7.229E-01	7.782E-03
CPA6	-0.228	6.435E-01	1.975E-01
GAL3ST1	-0.228	5.436E-01	2.975E-01
FAM160B2	-0.228	2.227E-02	6.458E-03
USP18	-0.228	2.351E-01	2.575E-01
POU2F2	-0.228	3.083E-01	1.173E-01
LRP12	-0.228	2.460E-01	1.320E-01
GOLGA6L22	-0.228	8.765E-01	3.953E-02
PDE4D	-0.228	1.612E-01	2.000E-03
CSAG1	-0.229	7.559E-01	3.545E-01
MRPS36	-0.229	5.335E-03	8.798E-03
YBEY	-0.229	1.317E-01	4.764E-01
TCF7	-0.229	2.390E-01	3.017E-01
SERPINB3	-0.229	7.335E-01	2.895E-01
ETV7	-0.229	2.885E-01	2.939E-01
DOK3	-0.229	2.580E-01	5.781E-01
CDKAL1	-0.229	2.109E-01	5.233E-01
SAMD10	-0.229	1.874E-01	2.570E-01
TSPAN3	-0.229	2.116E-02	1.154E-01
OR51A7	-0.229	NA	2.819E-01
KRTAP27-1	-0.229	NA	1.841E-01

BIVM-ERCC5	-0.229	6.937E-01	1.081E-01
PARP3	-0.229	8.949E-02	2.911E-01
ARVCF	-0.229	2.002E-01	4.288E-01
DEPTOR	-0.229	3.848E-01	1.148E-02
MYO15A	-0.229	2.257E-01	3.123E-02
IL20RA	-0.230	3.339E-01	1.688E-01
PHOSPHO1	-0.230	3.311E-01	3.542E-01
GRAP	-0.230	2.666E-01	3.584E-03
DYNLRB2	-0.230	4.256E-01	3.392E-03
PAG1	-0.230	1.861E-01	5.259E-02
MS4A13	-0.230	8.504E-01	8.667E-01
HES7	-0.230	5.419E-01	8.499E-02
NAB1	-0.230	8.038E-02	7.521E-01
LPCAT2	-0.230	2.134E-01	2.422E-01
KHD1-EIF4E	-0.230	5.457E-02	3.518E-01
CLEC4F	-0.230	5.310E-01	1.579E-01
ZBTB48	-0.230	2.667E-02	3.859E-01
CYBA	-0.230	1.872E-01	7.166E-01
BCS1L	-0.230	9.642E-03	6.150E-01
CCDC39	-0.230	1.708E-01	5.632E-01
ZNF280A	-0.230	7.875E-01	2.448E-01
HNRNPA1	-0.231	4.129E-04	3.906E-01
PRAMEF2	-0.231	NA	5.953E-02
HAO1	-0.231	8.033E-01	5.941E-01
CHADL	-0.231	2.478E-01	2.384E-04
NECTIN1	-0.231	2.463E-01	1.597E-03
MIR770	-0.231	NA	5.620E-02
BBS5	-0.231	1.486E-02	1.623E-01
OR11H6	-0.231	7.443E-01	3.972E-01
TMEM133	-0.231	1.222E-01	1.691E-01
RPS9	-0.231	2.391E-02	2.099E-02
AC009690.1	-0.231	3.782E-01	4.951E-01
DOCK5	-0.231	1.261E-01	5.274E-01
CCL25	-0.231	6.198E-01	9.887E-03
SNAI2	-0.231	3.714E-01	3.255E-02
SEPSECS	-0.231	6.967E-03	4.481E-03
GOLGA8G	-0.231	7.970E-01	8.165E-02
MIR222	-0.231	7.780E-01	1.273E-01
MIR4506	-0.232	NA	8.807E-01
RPS8	-0.232	2.598E-02	6.520E-02
ZNF121	-0.232	5.929E-02	7.467E-03
THEM6	-0.232	2.356E-01	3.854E-02
MAML3	-0.232	1.512E-01	4.523E-01
CCDC130	-0.232	2.263E-02	3.861E-01
ABCC5	-0.232	2.508E-01	9.809E-02
ADA2	-0.232	3.337E-01	1.954E-01
RPS13	-0.232	1.695E-02	3.069E-01
SPB2-C11orf	-0.232	NA	9.620E-03
IFI30	-0.232	2.717E-01	8.920E-02
TMEM56	-0.232	3.709E-01	3.677E-01

FAM90A1	-0.232	4.670E-01	8.032E-02
EVI2B	-0.232	4.090E-01	3.248E-01
SLC25A10	-0.232	1.691E-01	2.544E-01
NANOG	-0.232	4.956E-01	6.094E-02
KCNIP2	-0.232	1.975E-01	1.960E-01
CFAP44	-0.233	2.122E-01	3.959E-01
MIR648	-0.233	NA	4.891E-01
SCO2	-0.233	8.905E-02	2.360E-03
TNK1	-0.233	6.986E-02	9.581E-03
AC091167.3	-0.233	1.222E-01	8.544E-02
AC104662.2	-0.233	NA	6.278E-02
AL512785.2	-0.233	5.792E-01	4.384E-01
DCAF4L1	-0.233	2.931E-01	2.667E-01
PKD1L3	-0.233	2.508E-01	1.362E-01
MIR7111	-0.233	2.645E-01	1.938E-01
ARAP2	-0.233	8.457E-02	4.947E-01
FXYD2	-0.233	4.958E-01	3.631E-01
SLC24A4	-0.233	3.081E-01	1.990E-01
NWD2	-0.233	5.999E-01	1.770E-03
KLRC3	-0.233	6.598E-01	4.641E-01
ISL2	-0.233	2.509E-01	5.092E-01
IL9	-0.233	NA	3.534E-01
DRICH1	-0.233	3.761E-01	2.736E-01
CRB2	-0.234	5.026E-01	4.780E-03
DCBLD1	-0.234	1.513E-01	3.147E-01
NUPR2	-0.234	6.543E-01	1.562E-02
RNASE2	-0.234	4.840E-01	2.290E-01
STRC	-0.234	5.371E-01	2.969E-01
FER1L5	-0.234	2.299E-01	6.332E-03
TAS2R46	-0.234	6.106E-01	2.148E-01
FAM135B	-0.234	6.467E-01	7.735E-01
EXTL3	-0.234	5.642E-02	2.894E-01
MIR339	-0.234	4.407E-01	4.822E-01
TSNARE1	-0.234	3.536E-02	1.953E-02
EN2	-0.234	6.390E-01	3.353E-01
ADAM21	-0.234	4.471E-01	7.726E-02
UCP1	-0.235	6.261E-01	8.194E-02
TET1	-0.235	2.454E-01	4.488E-02
FAM3B	-0.235	5.994E-01	3.750E-01
OTOL1	-0.235	NA	2.708E-01
HS6ST2	-0.235	4.950E-01	2.090E-02
TSFM	-0.235	1.179E-02	4.272E-01
CELSR2	-0.235	2.980E-01	3.138E-04
MOG	-0.235	7.402E-01	5.988E-03
IFIT1B	-0.235	6.488E-01	2.699E-01
BDH2	-0.235	4.300E-02	2.338E-01
GPR15	-0.235	6.259E-01	8.318E-02
ZNF502	-0.235	2.424E-01	5.235E-03
TAGLN3	-0.235	6.367E-01	8.410E-02
TRIOBP	-0.236	3.849E-02	2.838E-01

YARS2	-0.236	3.160E-03	2.437E-01
BTAF1	-0.236	3.578E-02	1.596E-01
AC245033.1	-0.236	NA	7.129E-03
CA6	-0.236	6.620E-01	3.543E-02
HTR3A	-0.236	7.148E-01	4.236E-01
FOXA1	-0.236	4.709E-01	1.144E-01
MIR1249	-0.236	3.075E-01	2.907E-01
HMGXB3	-0.236	1.489E-04	4.847E-02
PI16	-0.236	6.234E-01	6.759E-02
PPIP5K1	-0.236	3.431E-02	3.841E-01
KRTAP9-4	-0.236	NA	2.738E-03
MIR4685	-0.236	4.729E-01	1.677E-01
SLC8B1	-0.236	2.136E-02	2.740E-01
SIDT1	-0.236	3.618E-01	2.013E-02
BFSP2	-0.237	5.846E-01	8.754E-02
MIR573	-0.237	3.739E-01	2.125E-02
LRRC37A	-0.237	2.399E-01	3.966E-01
CEACAM21	-0.237	3.762E-01	5.798E-02
CYBB	-0.237	4.634E-01	6.381E-03
NAA38	-0.237	4.395E-02	2.007E-01
CTAG1A	-0.237	8.369E-01	1.370E-01
CT45A10	-0.238	8.599E-01	1.693E-01
BSPH1	-0.238	NA	1.876E-01
PSME2	-0.238	6.515E-02	5.100E-01
TISP43	-0.238	NA	3.290E-01
CDH26	-0.238	5.213E-01	1.010E-02
MIR135B	-0.238	7.619E-01	6.401E-01
BSX	-0.238	8.261E-01	3.168E-01
CAMK1G	-0.238	3.091E-01	8.321E-01
KLHDC8A	-0.239	3.572E-01	4.023E-01
TAS2R38	-0.239	5.989E-01	2.582E-01
ID4	-0.239	4.527E-01	1.094E-01
SDR42E2	-0.239	2.796E-01	3.913E-01
CPN1	-0.239	7.744E-01	7.969E-02
SMLR1	-0.239	7.185E-01	1.596E-03
GTF3C6	-0.239	8.336E-03	3.048E-01
MIR4446	-0.239	NA	4.379E-01
AP1G2	-0.239	5.858E-02	7.301E-02
TRPA1	-0.239	5.221E-01	5.946E-02
SMN1	-0.239	3.848E-03	9.040E-01
VGLL2	-0.239	8.206E-01	4.948E-01
STIM2	-0.239	5.712E-04	5.735E-01
NPTX2	-0.239	3.245E-01	3.106E-02
HK2	-0.240	1.443E-01	3.716E-02
RPL35	-0.240	4.958E-02	2.691E-01
RPL18	-0.240	3.923E-02	5.289E-01
FAM13C	-0.240	3.240E-01	2.997E-03
CDC14B	-0.240	2.359E-02	1.663E-04
PORCN	-0.240	7.948E-02	7.404E-01
IL36A	-0.240	8.337E-01	1.833E-01

LYAR	-0.240	2.204E-02	3.781E-01
SLC17A9	-0.240	3.705E-01	8.067E-02
IEM56-RWD	-0.240	6.809E-02	2.732E-01
PCNX2	-0.240	6.807E-02	4.075E-03
ASB17	-0.240	NA	1.214E-01
KLHL17	-0.240	7.797E-02	2.190E-01
LTF	-0.241	6.273E-01	3.310E-01
KCNQ2	-0.241	5.964E-01	3.385E-01
NOP2	-0.241	4.924E-03	6.903E-01
AC026954.2	-0.241	4.256E-01	8.551E-01
IFIT3	-0.241	4.080E-01	4.115E-01
ZMAT4	-0.241	6.860E-01	4.310E-01
ZDHHC2	-0.241	3.131E-01	2.231E-01
HNRNPA1L2	-0.241	4.441E-03	9.539E-02
CSAD	-0.241	1.603E-01	9.615E-02
MIR34C	-0.241	NA	2.194E-01
SERPINA1	-0.241	5.551E-01	1.657E-01
GTSF1	-0.241	6.826E-01	1.672E-01
AC010323.1	-0.241	5.152E-01	1.525E-01
SLC51A	-0.242	1.994E-01	1.079E-01
C18orf63	-0.242	NA	2.362E-02
TUBE1	-0.242	3.613E-03	2.010E-01
CCDC94	-0.242	8.189E-03	3.287E-01
NLGN4X	-0.242	5.412E-01	2.788E-02
PDK1	-0.242	5.107E-02	3.092E-01
MIR3192	-0.242	5.923E-01	3.987E-01
SNCA	-0.242	4.107E-01	2.148E-01
YDJC	-0.243	5.019E-02	1.963E-01
OR2A2	-0.243	NA	2.688E-03
P2RX3	-0.243	6.783E-01	3.038E-01
LPIN2	-0.243	1.810E-01	1.190E-02
LIX1	-0.243	6.434E-01	3.659E-01
GNB3	-0.243	2.781E-01	2.452E-01
SUN5	-0.243	NA	4.017E-01
TCERG1	-0.243	2.208E-03	1.127E-01
GDF9	-0.243	1.794E-01	3.006E-01
MAGIX	-0.243	3.391E-01	2.386E-02
MIR3942	-0.243	3.331E-01	1.004E-02
ST8SIA4	-0.243	2.086E-01	5.718E-01
SELENOO	-0.243	9.916E-03	1.169E-01
BTG2	-0.243	2.746E-01	9.656E-04
C3orf84	-0.243	NA	4.717E-03
LGR4	-0.243	1.452E-01	6.462E-01
IFNLR1	-0.243	1.681E-01	5.875E-02
GDF5OS	-0.243	6.843E-01	4.738E-01
ZBED6CL	-0.243	2.278E-01	3.925E-01
SLAIN1	-0.243	3.987E-01	6.531E-04
LZTS2	-0.243	5.061E-03	2.242E-02
TNFRSF13B	-0.244	6.188E-01	1.260E-02
TMC3	-0.244	4.055E-01	4.494E-02

ALG10B	-0.244	3.943E-02	2.337E-01
KLHL31	-0.244	2.531E-01	3.684E-01
ANKAR	-0.244	1.252E-02	2.959E-01
ANKS1B	-0.244	4.966E-01	1.198E-01
HEXDC	-0.244	7.605E-02	2.181E-01
SLC20A1	-0.244	1.481E-01	3.127E-01
MGST2	-0.245	9.565E-02	6.489E-01
CHST13	-0.245	3.999E-01	7.049E-01
MAPK10	-0.245	3.038E-01	2.012E-03
RBM19	-0.245	2.951E-03	3.603E-01
TMEM30B	-0.245	1.211E-01	3.223E-01
C1orf64	-0.245	5.806E-01	4.772E-01
UGT1A8	-0.245	7.508E-01	2.969E-01
AC006254.1	-0.245	1.584E-01	4.362E-03
TRMT5	-0.245	6.793E-04	9.572E-03
GPR155	-0.245	1.978E-01	9.418E-02
TUBB1	-0.245	1.984E-01	5.813E-01
CLEC18B	-0.245	3.918E-01	4.710E-03
MIR5581	-0.245	4.968E-01	5.329E-02
FZD5	-0.245	1.485E-01	2.503E-01
C5orf30	-0.245	7.760E-02	2.699E-01
CCL22	-0.245	4.151E-01	7.062E-02
GFRAL	-0.245	NA	5.194E-01
GSAP	-0.246	6.782E-02	1.110E-02
BEST4	-0.246	2.526E-01	2.666E-01
TCF7L2	-0.246	2.944E-02	1.346E-01
ACOT12	-0.246	6.663E-01	1.089E-01
NDUFA11	-0.246	3.578E-02	7.751E-02
SMAD3	-0.246	1.374E-01	4.017E-01
LRRC75A	-0.246	1.954E-01	1.962E-02
COL28A1	-0.246	4.425E-01	4.922E-01
ZDHHC11	-0.246	4.137E-01	6.449E-03
BLOC1S1	-0.246	5.853E-02	1.489E-02
MYC	-0.246	2.833E-01	8.847E-01
GLDN	-0.247	2.440E-01	7.755E-01
IL1RAP	-0.247	3.079E-01	7.153E-02
PPIAL4F	-0.247	NA	4.854E-02
ARHGEF16	-0.247	1.615E-01	7.580E-01
APOBEC2	-0.247	4.137E-01	1.328E-02
C8orf46	-0.247	4.261E-01	3.893E-01
SAAL1	-0.247	3.552E-03	5.247E-02
KRT25	-0.247	7.477E-01	1.106E-01
NDUFA7	-0.248	4.003E-02	3.425E-01
MIR4672	-0.248	NA	2.415E-01
SPDYC	-0.248	6.867E-01	2.131E-01
CXorf57	-0.248	4.027E-01	2.910E-01
LY75	-0.248	2.090E-01	2.175E-01
SH2D3A	-0.248	1.033E-01	3.489E-01
SPTB	-0.248	2.770E-01	2.461E-02
CCDC74B	-0.248	2.725E-01	2.298E-01

MAGEA8	-0.248	6.620E-01	3.742E-02
KDM5D	-0.248	6.549E-01	8.674E-03
RPL37	-0.248	3.148E-02	3.529E-01
LEFTY1	-0.248	4.997E-01	2.106E-01
ANKRA2	-0.248	1.141E-02	9.941E-02
RFPL3S	-0.248	2.668E-01	1.058E-01
CSH1	-0.248	NA	1.641E-01
CASP8	-0.249	1.364E-02	1.722E-01
ALKBH2	-0.249	1.753E-02	1.144E-01
ASB13	-0.249	2.750E-02	2.335E-02
NPEPL1	-0.249	6.518E-02	6.252E-01
POU4F3	-0.249	3.238E-01	3.892E-01
SLIRP	-0.249	1.655E-02	3.604E-01
RDH10	-0.249	2.034E-01	2.411E-01
DGAT1	-0.249	2.077E-02	6.719E-01
MIR93	-0.249	4.593E-01	4.523E-01
SERPINF2	-0.249	3.558E-01	5.841E-02
HAUS3	-0.249	1.618E-03	3.989E-01
PCED1B	-0.249	1.532E-01	2.803E-01
SPRN	-0.249	9.390E-02	8.358E-02
ZBTB32	-0.249	2.327E-01	2.290E-01
FAM187B	-0.249	5.434E-01	3.392E-02
ADM	-0.250	2.824E-01	9.431E-02
TRIM72	-0.250	4.017E-01	6.254E-03
CLK1	-0.250	2.181E-02	1.650E-01
KCNMB2	-0.250	2.922E-01	3.625E-01
AP002748.4	-0.250	NA	2.264E-01
EPB42	-0.250	2.396E-01	9.397E-01
FAM209B	-0.250	2.091E-01	4.086E-03
HIST1H3F	-0.250	4.475E-01	5.884E-03
MCHR1	-0.250	4.733E-01	2.082E-01
USH2A	-0.250	2.479E-01	2.784E-01
HLA-DOB	-0.251	3.918E-01	4.964E-01
OGFRL1	-0.251	2.483E-01	3.460E-01
IL2RA	-0.251	4.289E-01	1.172E-01
SLC26A5	-0.251	4.281E-01	5.735E-03
MBIP	-0.251	4.192E-02	4.921E-04
GRAMD1B	-0.251	2.869E-01	4.896E-02
LAX1	-0.251	4.475E-01	1.144E-02
DCD	-0.251	NA	1.295E-01
HNRNPCL2	-0.251	6.374E-01	7.057E-01
RPF2	-0.251	7.465E-04	3.298E-01
EQTN	-0.251	4.078E-01	4.702E-03
GABRG2	-0.251	8.096E-01	1.346E-02
MRPS31	-0.251	4.214E-04	1.359E-01
UGT2B4	-0.251	7.472E-01	3.355E-01
SLC16A8	-0.251	3.348E-01	1.081E-01
SLAMF1	-0.251	4.111E-01	5.770E-01
CCDC125	-0.251	2.118E-02	3.872E-01
COL6A5	-0.252	5.853E-01	1.681E-01

NPAS4	-0.252	5.422E-01	5.048E-03
FGR	-0.252	1.629E-01	1.093E-01
SNAPC1	-0.252	5.399E-02	7.745E-01
HGFAC	-0.252	4.112E-01	1.686E-01
MRPL12	-0.252	5.546E-02	3.302E-01
MOS	-0.252	NA	4.833E-01
MAP1LC3C	-0.252	5.650E-01	8.825E-03
ZNF778	-0.252	1.205E-02	4.428E-02
OR6S1	-0.252	NA	4.660E-05
CATSPER2	-0.253	1.412E-01	1.145E-01
PLA2R1	-0.253	1.665E-01	5.456E-02
ZFC3H1	-0.253	4.964E-03	2.688E-01
GP9	-0.253	6.960E-01	1.872E-02
SULT1A2	-0.253	5.316E-01	3.547E-02
TOP1MT	-0.253	3.809E-02	9.280E-01
MFNG	-0.253	2.192E-01	1.953E-01
MIP	-0.253	2.053E-01	2.304E-01
GJB4	-0.253	4.547E-01	3.055E-01
IL10RA	-0.253	3.438E-01	1.006E-02
DLX2	-0.253	5.651E-01	3.060E-01
ANGPTL6	-0.253	1.595E-01	3.244E-02
BCL11B	-0.253	3.317E-01	6.145E-03
TBPL2	-0.253	5.436E-01	2.577E-01
ZDHHC8	-0.253	1.351E-02	4.775E-02
MAP9	-0.253	2.986E-01	2.905E-01
MMP25	-0.254	2.558E-01	5.194E-03
RPL14	-0.254	3.077E-02	6.888E-01
FASTKD1	-0.254	1.608E-03	4.484E-01
MYOT	-0.254	3.685E-01	3.357E-01
TMEM181	-0.254	6.843E-03	6.005E-01
FGL2	-0.254	3.592E-01	1.183E-01
DDX47	-0.254	4.300E-02	1.047E-01
WNT11	-0.254	5.299E-01	4.620E-01
TRAF1	-0.254	1.637E-01	8.924E-02
IL18BP	-0.254	1.314E-01	5.882E-01
MARS2	-0.254	2.338E-02	4.024E-01
MIR6722	-0.254	NA	8.038E-01
PPWD1	-0.255	5.783E-04	7.420E-01
ELMOD1	-0.255	5.141E-01	4.491E-02
TSPO	-0.255	8.071E-02	4.190E-02
RFPL3	-0.255	5.607E-01	1.114E-01
SEMA4B	-0.255	2.007E-01	2.346E-01
C7orf34	-0.255	5.942E-01	4.396E-02
MT-ND5	-0.255	2.843E-01	3.795E-01
GPA33	-0.255	4.450E-01	2.133E-02
ZBTB9	-0.255	2.696E-01	2.706E-01
CTAGE9	-0.255	4.571E-01	3.892E-01
PBX4	-0.255	1.878E-01	4.289E-01
RGS18	-0.256	3.250E-01	1.288E-01
CLUL1	-0.256	3.949E-01	1.079E-01

C9	-0.256	5.106E-01	9.418E-02
ZNF30	-0.256	4.402E-02	2.280E-01
MIR4284	-0.256	6.364E-01	8.688E-02
RASL10B	-0.256	3.894E-01	1.386E-01
IFITM10	-0.257	4.097E-01	3.648E-01
AC010616.1	-0.257	3.794E-01	4.425E-03
UNC13C	-0.257	7.396E-01	2.216E-01
ALKBH7	-0.257	5.426E-02	4.674E-03
ZNF276	-0.257	8.919E-03	2.695E-01
SMIM15	-0.257	3.500E-05	7.975E-01
NPIPA3	-0.257	4.463E-01	1.421E-01
PSMB10	-0.257	8.291E-02	1.035E-02
CCDC182	-0.257	NA	8.611E-02
HLA-F	-0.257	3.315E-01	1.188E-02
IGFL2	-0.257	6.116E-01	6.761E-04
DIMT1	-0.257	2.370E-05	4.685E-01
BIN2	-0.257	3.003E-01	4.466E-02
FAM174B	-0.257	3.789E-01	6.784E-01
GSDMB	-0.257	2.440E-01	2.163E-01
PRMT3	-0.257	2.603E-03	3.006E-01
AMY1A	-0.257	NA	7.412E-01
SLCO1B1	-0.258	6.802E-01	1.316E-02
C2orf70	-0.258	4.722E-01	6.602E-01
CCNT2	-0.258	2.849E-02	3.189E-01
WDR97	-0.258	1.841E-01	5.275E-01
AC092821.1	-0.258	1.668E-01	3.219E-01
NLRC5	-0.258	2.154E-01	1.519E-01
SPATA13	-0.258	8.846E-02	1.884E-03
NDUFB8	-0.258	1.389E-02	7.829E-04
DNASE1	-0.259	2.199E-02	1.083E-02
MAS1	-0.259	6.318E-01	1.072E-02
COX4I1	-0.259	1.736E-02	2.947E-01
BTK	-0.259	3.612E-01	5.578E-02
SPACA1	-0.259	NA	1.949E-01
AL662899.3	-0.259	1.712E-01	1.860E-01
AC008758.4	-0.259	2.634E-01	1.913E-01
IL1RAPL1	-0.259	5.728E-01	1.497E-01
MIR4730	-0.259	5.152E-01	2.824E-01
FLT1	-0.259	1.116E-01	8.061E-01
CCDC150	-0.259	1.104E-01	2.709E-01
PAK1	-0.259	2.688E-02	4.235E-01
HIST1H1A	-0.259	7.598E-01	2.854E-01
ZNF582	-0.260	1.899E-01	1.412E-01
RPS15	-0.260	3.647E-02	2.859E-02
TMC8	-0.260	1.980E-01	2.102E-01
SMPD3	-0.260	2.173E-01	2.415E-01
C6orf223	-0.260	5.607E-01	5.844E-01
FTMT	-0.260	8.586E-01	2.913E-01
C3orf22	-0.260	7.528E-01	4.012E-02
ASTN2	-0.260	3.513E-01	2.710E-01

CIITA	-0.260	3.277E-01	9.188E-02
PLGLB1	-0.260	2.922E-01	3.541E-04
ALS2CL	-0.260	2.051E-01	9.542E-02
TGIF2	-0.260	3.359E-02	7.255E-01
GLI1	-0.260	3.399E-01	2.268E-01
OR5AP2	-0.260	NA	1.242E-03
NR2C1	-0.260	4.225E-03	2.568E-01
ZNF251	-0.261	3.066E-02	3.424E-01
ZNF223	-0.261	9.109E-02	8.689E-04
RPS6KA6	-0.261	3.832E-01	2.291E-01
RIMS2	-0.261	4.876E-01	2.021E-01
PXN	-0.261	2.141E-02	5.067E-01
FMNL2	-0.261	1.782E-01	4.134E-01
HIST1H2BH	-0.261	3.989E-01	4.187E-01
MIR6793	-0.261	6.035E-01	2.431E-01
TP53	-0.261	5.858E-02	1.920E-01
RAD54B	-0.262	4.852E-02	9.081E-03
PTPN6	-0.262	6.005E-03	3.609E-01
GRM5	-0.262	6.927E-01	2.932E-01
IRF9	-0.262	6.777E-02	7.497E-02
GBP1	-0.262	3.462E-01	3.080E-01
DNAH6	-0.262	3.894E-01	2.851E-02
TOR4A	-0.262	1.832E-01	6.388E-02
CD19	-0.262	5.444E-01	6.250E-02
MIR491	-0.262	NA	6.995E-02
DLX3	-0.262	4.765E-01	2.878E-01
SOX30	-0.262	4.384E-01	3.332E-01
ZKSCAN7	-0.263	2.057E-01	2.678E-01
MTRF1	-0.263	1.190E-02	3.911E-01
CHD3	-0.263	9.665E-03	4.582E-02
ADGRA3	-0.263	7.797E-02	1.525E-01
MFSD10	-0.263	1.947E-02	1.816E-01
LCP2	-0.263	2.510E-01	4.489E-01
GGTLC3	-0.263	7.778E-01	6.285E-03
CDH23	-0.263	4.150E-01	4.710E-01
GRIN3A	-0.263	1.827E-01	1.693E-01
TAS2R30	-0.263	4.492E-01	1.080E-01
IL4I1	-0.263	3.855E-01	1.662E-02
AL357673.1	-0.263	5.583E-01	3.070E-01
GSTO1	-0.263	2.978E-02	6.144E-02
MIR1254-1	-0.263	2.338E-01	4.006E-01
IL17RB	-0.264	2.157E-01	1.268E-01
PATZ1	-0.264	5.929E-02	2.336E-01
CPO	-0.264	3.809E-01	2.404E-01
CRKL	-0.264	2.659E-02	2.872E-01
PIR	-0.264	2.395E-01	4.971E-02
MTCP1	-0.264	9.471E-02	7.448E-04
INSL3	-0.264	1.620E-01	1.803E-01
GSX1	-0.264	NA	3.924E-02
HIST1H2BM	-0.264	5.163E-01	1.467E-03

RTP3	-0.264	7.932E-01	4.789E-01
SLC23A2	-0.264	1.565E-01	4.032E-01
DDX58	-0.264	2.051E-01	8.740E-04
RGPD4	-0.264	5.728E-01	8.125E-02
FSTL4	-0.265	4.976E-01	8.229E-01
TARM1	-0.265	6.757E-01	9.954E-03
EFCAB3	-0.265	6.288E-01	1.598E-01
HLA-DQA2	-0.265	4.931E-01	1.023E-03
RHPN1	-0.265	2.604E-01	1.104E-01
B3GNT7	-0.265	3.060E-01	2.132E-02
TBX22	-0.265	NA	2.883E-01
FAM83H	-0.265	2.573E-02	1.270E-01
MIR320E	-0.265	2.101E-01	1.074E-01
MIR643	-0.265	5.360E-01	4.844E-01
CEP85L	-0.265	4.047E-02	1.532E-04
MTRNR2L11	-0.265	5.957E-01	4.638E-01
CD80	-0.265	4.010E-01	3.289E-01
CD72	-0.265	2.878E-01	1.489E-01
FAM47A	-0.265	NA	1.633E-01
PKD2L2	-0.265	1.678E-01	1.020E-01
ZACN	-0.265	4.745E-02	1.283E-01
HERC6	-0.265	2.480E-01	2.775E-01
RFTN1	-0.266	1.393E-01	2.290E-01
GCLC	-0.266	2.308E-01	7.167E-01
ZNF311	-0.266	2.825E-01	4.569E-02
FRK	-0.266	8.445E-02	2.970E-03
SOCS1	-0.266	2.463E-01	9.171E-01
C5orf63	-0.266	8.837E-02	2.933E-01
ABCA5	-0.266	1.169E-01	2.859E-03
CCDC14	-0.266	3.468E-02	2.417E-01
HDHD5	-0.266	8.882E-04	1.433E-02
PLXNB1	-0.266	8.233E-02	2.972E-02
ITGAE	-0.266	9.223E-03	3.335E-01
CYP2D6	-0.267	1.777E-01	2.138E-01
MIR6810	-0.267	NA	5.932E-02
ALG1L2	-0.267	1.663E-01	7.465E-01
FAM156B	-0.267	5.124E-01	1.353E-03
CCDC154	-0.267	2.601E-01	5.397E-01
AKR1B10	-0.267	6.353E-01	5.363E-01
SLC29A4	-0.267	3.614E-01	1.255E-01
DSC2	-0.267	4.388E-01	3.583E-01
SHROOM1	-0.267	2.187E-01	4.620E-01
IP6K2	-0.267	8.970E-03	3.183E-01
MIR3181	-0.267	NA	3.380E-01
DISP1	-0.267	5.829E-02	6.621E-03
ALOX12B	-0.267	5.078E-01	1.710E-01
CYP4X1	-0.267	4.375E-01	5.165E-01
ANAPC4	-0.267	2.233E-03	5.018E-01
ANGPTL7	-0.267	6.073E-01	1.519E-02
PNMA8C	-0.268	6.920E-01	3.555E-01

UBA7	-0.268	8.434E-02	2.972E-01
TNFRSF10D	-0.268	1.984E-01	1.067E-01
AC023509.3	-0.268	6.956E-02	2.744E-01
LZTR1	-0.268	1.526E-02	1.218E-01
ARSJ	-0.268	3.932E-01	8.289E-03
GFI1B	-0.268	5.227E-01	3.014E-03
DPPA4	-0.268	4.622E-01	6.015E-01
IFITM5	-0.268	6.278E-01	3.279E-01
TAS2R43	-0.268	4.977E-01	7.082E-02
SNRPF	-0.268	8.287E-03	2.408E-01
FAM50B	-0.268	3.253E-01	1.632E-01
OR1L8	-0.269	4.646E-01	3.650E-01
'ORO7-PAM1	-0.269	2.748E-01	7.065E-03
HHLA2	-0.269	6.082E-01	6.249E-01
CARNS1	-0.269	1.146E-01	6.480E-06
FDXACB1	-0.269	7.295E-03	6.276E-02
PAPPA	-0.269	3.970E-01	4.589E-02
SOX2	-0.269	5.943E-01	3.149E-02
ATXN7L2	-0.269	4.745E-02	4.903E-01
AC069503.2	-0.269	1.448E-01	1.089E-02
FAM167A	-0.269	3.199E-01	1.851E-01
C22orf15	-0.269	2.444E-01	2.849E-01
CCNL2	-0.269	7.002E-02	7.259E-02
ATP5D	-0.269	5.768E-02	2.861E-01
IL12A	-0.270	3.201E-01	9.615E-02
CAMK1D	-0.270	2.168E-01	4.418E-01
CYSLTR2	-0.270	3.277E-01	6.172E-01
NMNAT3	-0.270	3.006E-01	3.181E-03
SKOR2	-0.270	NA	4.529E-01
MRAP2	-0.270	4.171E-01	1.440E-01
TLX2	-0.270	4.207E-01	1.802E-04
MLC1	-0.270	3.588E-01	2.203E-03
ST20-MTHFS	-0.270	3.449E-01	9.459E-02
ANO3	-0.270	5.227E-01	3.013E-01
NGFR	-0.270	4.969E-01	3.825E-01
CLEC20A	-0.270	NA	8.983E-02
MIR4633	-0.271	NA	5.497E-02
GRIP1	-0.271	2.322E-01	3.757E-02
PAX4	-0.271	NA	8.468E-03
MIR4664	-0.271	2.556E-01	1.806E-02
MC5R	-0.271	6.769E-01	2.600E-01
MCCD1	-0.271	NA	4.250E-01
PKIA	-0.271	3.001E-01	3.642E-01
RIMBP3C	-0.271	4.871E-01	1.124E-01
ECHDC2	-0.271	4.741E-02	8.890E-02
RGPD1	-0.272	5.315E-01	2.729E-02
SLC25A48	-0.272	4.949E-01	2.430E-03
AC009119.2	-0.272	1.643E-01	1.651E-01
ENAM	-0.272	5.248E-01	1.013E-02
CD320	-0.272	1.938E-01	6.956E-03

C8orf82	-0.272	9.007E-03	1.125E-01
MFSD3	-0.272	3.515E-02	1.190E-05
SEC14L5	-0.272	3.355E-01	3.078E-01
CABP4	-0.272	3.631E-01	1.236E-04
FAM184B	-0.272	9.109E-02	1.040E-03
MYO1A	-0.272	3.475E-01	2.377E-02
FGA	-0.272	8.046E-01	4.718E-01
TOPAZ1	-0.272	7.381E-01	6.713E-02
PPIAL4G	-0.272	5.080E-01	2.541E-01
PRAP1	-0.272	6.095E-01	1.401E-01
CRACR2A	-0.272	1.672E-01	9.956E-02
AKAP5	-0.272	1.530E-01	2.780E-01
SERPINB5	-0.273	4.058E-01	1.104E-02
KBTBD6	-0.273	4.243E-03	1.910E-01
MIR101-2	-0.273	NA	5.981E-02
TSLP	-0.273	2.769E-01	3.268E-01
CAPN15	-0.273	2.157E-03	2.960E-01
ERMARD	-0.273	4.363E-03	4.783E-01
RCBTB2	-0.273	2.978E-02	1.235E-01
RPL4	-0.273	9.499E-03	4.585E-01
SSX2	-0.273	8.513E-01	7.095E-02
MAST3	-0.273	6.270E-03	5.483E-01
BHD14A-AC	-0.273	1.235E-01	4.218E-01
MIR548P	-0.273	NA	1.427E-01
LRRC49	-0.273	9.109E-02	1.363E-01
SNCB	-0.273	5.038E-01	8.317E-02
TAF4B	-0.273	1.522E-01	3.656E-01
GLYATL3	-0.273	NA	2.592E-01
OCIAD2	-0.274	1.666E-02	1.329E-04
XCL2	-0.274	2.693E-01	2.035E-02
PROCR	-0.274	1.665E-01	3.738E-01
RAG1	-0.274	2.960E-01	3.672E-01
KIF21A	-0.274	5.457E-02	6.192E-02
ODF2L	-0.274	6.387E-03	8.183E-03
AC008560.1	-0.274	1.073E-03	1.048E-02
BMP5	-0.274	5.680E-01	2.330E-05
NOXA1	-0.274	2.096E-01	8.096E-02
MIER3	-0.274	3.822E-03	1.096E-01
AL365232.1	-0.274	NA	6.833E-01
GLYCTK	-0.274	3.647E-02	7.071E-02
DDTL	-0.274	4.994E-02	3.500E-01
PGF	-0.275	2.569E-01	3.538E-01
PLEKHG7	-0.275	4.555E-01	4.072E-02
SLC26A4	-0.275	2.815E-01	5.923E-01
GRIA4	-0.275	3.826E-01	6.241E-02
ZNF841	-0.275	1.091E-01	4.497E-02
CENPW	-0.275	1.009E-01	8.115E-01
SCHIP1	-0.275	1.207E-01	3.403E-02
ING5	-0.275	1.690E-03	5.495E-01
CLECL1	-0.275	3.618E-01	2.910E-01

C6orf229	-0.275	6.552E-01	4.542E-01
TPTE2	-0.275	3.617E-01	2.098E-01
ADRB1	-0.275	4.634E-01	4.631E-01
TRAF3IP2	-0.275	1.112E-02	1.660E-01
AC040162.1	-0.276	4.851E-02	1.101E-03
DACH1	-0.276	3.743E-01	9.979E-02
THAP7	-0.276	2.279E-02	4.034E-01
B3GNT8	-0.276	1.335E-01	1.529E-01
KRTAP4-4	-0.276	NA	7.547E-01
XKR4	-0.276	5.436E-01	8.372E-02
GOLGA8N	-0.276	1.788E-01	1.375E-01
MIR4480	-0.276	NA	1.998E-01
RGS17	-0.276	2.706E-01	6.580E-01
GPRC5C	-0.276	3.106E-01	5.020E-03
ECT2L	-0.276	7.355E-02	1.516E-01
GPR82	-0.276	2.847E-01	1.218E-01
FCRL5	-0.276	5.340E-01	8.255E-01
CRTC1	-0.276	3.691E-03	5.572E-02
NF559-ZNF1	-0.276	2.044E-01	4.011E-04
GAL3ST3	-0.276	6.504E-01	1.268E-02
TBC1D3I	-0.277	6.726E-01	2.283E-01
LPIN3	-0.277	5.331E-02	2.399E-02
RCSD1	-0.277	2.556E-01	3.733E-01
RAC2	-0.277	1.966E-01	2.964E-01
TRIM25	-0.277	8.072E-03	2.922E-02
HAO2	-0.277	5.260E-01	2.932E-01
C10orf71	-0.277	7.805E-01	2.807E-03
RNASE8	-0.277	NA	2.000E-01
TEKT3	-0.277	3.418E-01	5.033E-02
CHRM4	-0.277	2.742E-01	1.946E-01
GPR18	-0.277	2.498E-01	4.842E-01
SPANXN5	-0.277	NA	2.996E-01
SPINK1	-0.278	6.454E-01	2.104E-03
SEC16B	-0.278	2.288E-01	4.118E-01
TTLL13P	-0.278	1.130E-01	1.575E-01
GPS2	-0.278	3.647E-02	7.534E-02
NF103-CHMI	-0.278	1.561E-01	2.331E-03
CD300LD	-0.278	7.395E-01	7.305E-02
OR2W3	-0.279	6.356E-01	7.547E-02
L36A-HNRNI	-0.279	2.396E-01	2.924E-01
KRTAP19-3	-0.279	NA	1.398E-01
CR1	-0.279	4.695E-01	4.668E-01
KCNE3	-0.279	2.082E-01	1.567E-01
HVCN1	-0.279	1.007E-01	3.243E-01
C8orf44	-0.279	1.447E-02	2.038E-01
ANKRD22	-0.279	2.171E-01	4.871E-04
AC134669.2	-0.279	5.066E-01	5.945E-03
DENND2D	-0.280	7.663E-02	4.739E-01
APOBEC3A	-0.280	4.715E-01	8.131E-02
FOS	-0.280	2.393E-01	2.616E-02

RPS18	-0.280	1.134E-02	4.758E-02
CCR4	-0.280	3.735E-01	8.899E-01
DUXA	-0.280	NA	1.777E-02
FOXI3	-0.280	7.508E-01	3.809E-02
RPL32	-0.280	3.742E-02	1.642E-01
IFNGR1	-0.280	1.754E-02	1.664E-02
PMAIP1	-0.280	1.835E-01	5.833E-01
PRAMEF17	-0.280	NA	2.127E-01
DSG1	-0.280	6.096E-01	7.999E-01
GAPT	-0.280	3.322E-01	9.522E-02
SERGEF	-0.280	1.054E-02	3.339E-01
C19orf81	-0.280	5.839E-01	1.174E-01
LGALS7B	-0.281	6.513E-01	1.733E-01
SLCO1B3	-0.281	7.659E-01	1.842E-01
SLC47A1	-0.281	3.739E-01	6.198E-01
USP35	-0.281	1.613E-02	1.610E-01
AJUBA	-0.281	1.349E-01	8.672E-02
TMEM129	-0.281	1.348E-02	4.470E-02
GINS3	-0.281	1.989E-02	4.383E-03
GLT1D1	-0.281	3.287E-01	1.481E-01
CCDC173	-0.282	1.802E-01	1.327E-01
SMPD2	-0.282	8.668E-03	1.979E-01
AVPI1	-0.282	1.234E-01	1.070E-01
WAS	-0.282	2.569E-01	5.504E-02
CEP290	-0.282	1.520E-02	8.361E-02
NFE4	-0.282	5.559E-01	5.398E-01
P2RY13	-0.282	3.521E-01	9.187E-03
ANKRD62	-0.282	5.689E-01	8.612E-01
AC092073.1	-0.282	4.929E-01	1.450E-01
TTLL3	-0.282	1.477E-01	1.696E-01
CYB5D1	-0.282	2.851E-02	5.301E-01
GFRA4	-0.283	NA	1.556E-01
HOXA9	-0.283	3.845E-01	1.410E-01
OLAH	-0.283	3.856E-01	2.025E-01
HOXA5	-0.283	1.937E-01	1.324E-02
TMCO6	-0.283	1.117E-03	1.082E-01
AGAP6	-0.283	3.885E-02	5.447E-01
UBE2U	-0.283	7.793E-01	4.936E-01
TAS2R4	-0.283	1.738E-01	1.222E-02
MIR4269	-0.283	7.732E-01	1.078E-01
TFB1M	-0.283	6.574E-04	1.222E-02
RIC3	-0.283	4.444E-01	3.293E-04
TAS2R19	-0.283	1.454E-01	8.194E-03
KIAA0922	-0.283	1.374E-01	1.419E-01
GRIA3	-0.283	3.600E-01	2.849E-03
THEM4	-0.283	4.300E-02	2.409E-01
RAB40A	-0.283	8.842E-02	1.116E-02
C16orf45	-0.283	1.814E-01	5.090E-01
HIF1A	-0.284	5.373E-02	1.712E-01
TMEM217	-0.284	6.743E-02	3.874E-01

PLCZ1	-0.284	7.277E-01	4.616E-02
TMEM63C	-0.284	4.820E-01	1.606E-01
MIR5091	-0.284	5.964E-01	7.142E-01
NLRP8	-0.284	NA	8.650E-02
ZGRF1	-0.284	1.834E-02	8.368E-02
ZPBP2	-0.284	6.338E-01	5.349E-04
FAM86B2	-0.284	2.960E-01	1.957E-01
S100P	-0.284	4.076E-01	3.787E-01
RPL8	-0.284	8.563E-03	3.051E-02
DPPA5	-0.284	8.215E-01	2.340E-02
KIF2B	-0.285	NA	2.984E-02
LRRC24	-0.285	6.340E-01	3.569E-01
TNFRSF14	-0.285	4.299E-02	1.945E-01
KRTAP9-2	-0.285	NA	2.691E-01
APBB3	-0.285	9.462E-03	3.177E-01
FPR3	-0.285	3.481E-01	3.661E-02
CCDC62	-0.285	1.132E-01	3.889E-02
DUOX1	-0.285	2.034E-01	2.283E-01
NT5DC4	-0.285	2.156E-01	4.403E-01
KRT73	-0.285	6.431E-01	9.065E-02
CD180	-0.285	3.437E-01	2.801E-02
MIR659	-0.285	2.269E-01	2.289E-01
PSD4	-0.286	2.910E-02	2.536E-01
GJB6	-0.286	4.312E-01	9.793E-02
ZFHX2	-0.286	1.657E-01	3.579E-01
SELENBP1	-0.286	2.961E-01	4.266E-01
MDM2	-0.286	2.216E-01	4.976E-01
NDRG1	-0.286	2.190E-01	5.807E-01
TREML1	-0.286	2.444E-01	3.352E-01
RPS28	-0.286	2.495E-02	6.279E-02
FOXB1	-0.287	5.953E-01	1.301E-02
CABS1	-0.287	NA	4.494E-01
WDR66	-0.287	3.942E-01	1.224E-01
KRT71	-0.287	5.533E-01	1.739E-01
GUCA1B	-0.287	9.883E-02	2.036E-01
RPS23	-0.287	4.412E-03	2.857E-03
KIAA0141	-0.287	7.540E-05	5.358E-01
PRM1	-0.287	NA	2.361E-01
CFHR4	-0.287	6.020E-01	5.479E-03
FCHSD1	-0.288	6.176E-03	7.626E-02
PAN2	-0.288	5.449E-02	1.770E-01
CCDC140	-0.288	8.248E-01	4.494E-01
TUBAL3	-0.288	4.838E-01	3.859E-02
GCH1	-0.288	7.804E-03	1.441E-03
PARP8	-0.288	2.402E-02	1.714E-01
MIR455	-0.288	NA	3.128E-02
BMX	-0.288	2.473E-01	6.130E-02
CD40LG	-0.288	3.736E-01	7.845E-02
SLC6A18	-0.288	8.423E-01	1.258E-01
RPL12	-0.288	8.649E-03	2.216E-02

SSH3	-0.288	1.248E-01	2.438E-01
KBTBD11	-0.288	2.760E-01	2.812E-03
WFIKKN1	-0.288	3.299E-01	6.670E-01
IL13	-0.288	4.526E-01	1.580E-01
PCDH11X	-0.288	6.020E-01	2.342E-02
CCDC185	-0.289	4.709E-01	3.297E-01
RPL27A	-0.289	9.355E-03	5.700E-05
CARD11	-0.289	1.983E-01	2.793E-01
VWA8	-0.289	2.139E-02	3.573E-02
ORAI3	-0.289	4.884E-02	4.749E-04
KLHL23	-0.289	6.409E-02	1.929E-01
SEC31B	-0.289	9.042E-02	1.151E-01
SDR42E1	-0.290	2.715E-01	3.314E-01
KLHL30	-0.290	2.624E-01	1.934E-01
ISG15	-0.290	2.511E-01	4.509E-02
SAMD9	-0.290	2.956E-01	1.540E-01
CFC1	-0.290	NA	7.416E-04
ARL17B	-0.290	1.122E-01	1.363E-02
TMEM225	-0.290	NA	3.198E-01
AC119396.1	-0.290	3.006E-01	2.407E-01
LRCOL1	-0.291	4.358E-01	1.251E-02
TPT1	-0.291	4.887E-03	3.176E-02
PCDHAC2	-0.291	5.208E-01	1.040E-01
KLF13	-0.291	7.656E-02	4.140E-01
RPL37A	-0.291	1.070E-02	1.163E-01
PPP1R2P9	-0.291	NA	5.066E-01
IL12RB1	-0.291	3.295E-01	3.669E-02
CTSW	-0.291	2.348E-01	1.672E-02
DEFB123	-0.291	NA	8.363E-03
OR10J1	-0.291	NA	2.541E-01
CYSLTR1	-0.292	2.078E-01	3.612E-01
ZNF195	-0.292	7.804E-03	7.891E-01
C2orf83	-0.292	6.030E-01	8.198E-02
SNX20	-0.292	2.914E-01	5.600E-05
FAM169A	-0.292	1.795E-01	1.175E-01
XDH	-0.292	3.189E-01	7.675E-02
LIMCH1	-0.292	2.044E-01	9.707E-04
ITGB4	-0.292	1.237E-01	5.967E-02
CCL3L1	-0.292	3.482E-01	2.560E-06
KCNA3	-0.292	3.578E-01	6.086E-01
MIR1295A	-0.292	NA	2.138E-01
DYTN	-0.292	NA	2.269E-01
VSTM5	-0.292	3.376E-01	3.545E-01
C1orf146	-0.293	2.284E-01	9.881E-02
USP17L7	-0.293	7.296E-01	1.714E-03
GOLGA8R	-0.293	2.244E-01	2.511E-01
HIGD2A	-0.293	4.774E-03	1.208E-01
AGXT	-0.293	6.610E-01	1.295E-01
MYL5	-0.293	4.926E-02	2.375E-02
HOXD13	-0.294	5.270E-01	9.228E-02

HBEGF	-0.294	1.835E-01	9.292E-02
CTSH	-0.294	1.479E-01	1.397E-01
ST6GAL1	-0.294	2.317E-01	6.218E-01
LCAT	-0.294	8.662E-02	4.268E-01
PRLH	-0.294	NA	1.107E-01
NR5A2	-0.294	1.170E-01	5.765E-01
ST3GAL1	-0.294	9.216E-02	8.221E-02
PTHLH	-0.295	5.102E-01	4.516E-01
SYT16	-0.295	4.331E-01	3.669E-02
CDH4	-0.295	2.763E-01	3.747E-01
TMEM150B	-0.295	3.982E-01	1.340E-01
GOLGA6L6	-0.295	8.228E-01	1.255E-01
AHRR	-0.295	1.964E-01	1.373E-01
ALPL	-0.295	1.554E-01	6.944E-03
GIMAP6	-0.295	1.026E-01	2.425E-01
SGSM2	-0.295	1.904E-02	2.606E-01
DAZ4	-0.295	NA	1.826E-01
ME3	-0.295	1.076E-01	2.340E-05
SPAG17	-0.296	3.842E-01	9.892E-03
PRIMA1	-0.296	4.903E-01	2.088E-01
RHOXF1	-0.297	3.864E-01	3.987E-02
ABCA9	-0.297	3.740E-01	7.750E-02
OGT	-0.297	2.295E-02	3.868E-02
TFAP2E	-0.297	1.786E-01	5.477E-02
ID2	-0.297	1.513E-01	2.578E-02
ZNF705G	-0.297	NA	8.572E-02
ATRNL1	-0.298	3.949E-01	2.934E-03
CRTAM	-0.298	3.411E-01	9.268E-04
PDPN	-0.298	2.986E-01	3.722E-02
SYCE3	-0.298	1.675E-01	1.776E-01
RPL29	-0.298	1.362E-02	2.343E-01
MIR7851	-0.298	4.419E-01	5.636E-03
AL365214.3	-0.298	NA	1.170E-05
SPATA31A3	-0.298	NA	3.693E-03
TUBGCP6	-0.298	2.078E-03	5.214E-03
DUSP6	-0.298	1.073E-01	2.658E-01
KIZ	-0.299	3.558E-02	1.639E-01
LRRC7	-0.299	1.076E-01	7.996E-02
GNAI1	-0.299	9.197E-02	2.930E-01
LAP3	-0.299	6.004E-02	1.535E-02
THSD1	-0.299	1.876E-01	5.304E-01
TRAF3IP3	-0.299	2.079E-01	5.755E-02
FBXO40	-0.299	5.018E-01	2.910E-01
VP23C-CDR1	-0.299	8.454E-02	6.932E-01
RB1	-0.299	2.735E-02	3.908E-01
ENP3-EIF4A	-0.299	9.045E-02	3.078E-02
HIST1H4J	-0.300	2.461E-01	1.177E-01
RTP2	-0.300	6.387E-01	1.095E-02
ABHD14A	-0.300	4.254E-02	4.211E-02
LOXL1	-0.300	1.729E-01	8.777E-02

FRY	-0.300	1.781E-01	4.282E-01
DGKH	-0.300	4.852E-02	8.986E-03
THRSP	-0.300	6.151E-01	1.360E-02
A1CF	-0.300	5.277E-01	4.883E-03
DNAJB7	-0.300	1.284E-01	1.062E-01
WDR72	-0.301	3.770E-01	5.135E-02
MIR6503	-0.301	5.819E-01	5.057E-01
MMD2	-0.301	NA	5.858E-02
ENTPD3	-0.301	3.125E-01	2.501E-01
SH3BP1	-0.301	3.982E-02	2.354E-01
SERPINE3	-0.301	1.867E-01	2.032E-01
MIR5587	-0.301	3.628E-01	1.165E-04
SREK1IP1	-0.301	1.690E-03	2.271E-02
TENM4	-0.301	2.453E-01	3.355E-01
RAB3B	-0.301	3.422E-01	8.119E-02
VPREB1	-0.302	NA	9.437E-02
ICAM2	-0.302	9.606E-02	1.735E-01
PDE8B	-0.302	8.905E-02	1.140E-01
RPL36	-0.302	1.690E-02	6.105E-01
PCSK5	-0.302	2.627E-01	4.019E-04
RAPGEF3	-0.302	1.224E-01	1.597E-03
FAM117B	-0.302	3.615E-03	1.870E-04
TRABD	-0.302	2.277E-03	9.931E-02
POU3F2	-0.302	4.762E-01	1.605E-01
PPIAL4A	-0.302	2.558E-01	6.796E-04
PHOSPHO2	-0.302	6.012E-03	5.982E-02
TMEM91	-0.303	1.006E-01	7.732E-02
PLAG1	-0.303	1.938E-01	4.417E-03
BPIFB3	-0.303	NA	4.530E-01
NAPRT	-0.303	4.560E-02	3.465E-01
AC233724.12	-0.303	NA	8.255E-02
MBP	-0.303	1.949E-02	4.130E-01
RAB7B	-0.303	2.316E-01	8.600E-05
PPP4R3CP	-0.303	8.041E-01	2.445E-01
HIBCH	-0.303	3.463E-03	1.071E-01
ADAD1	-0.304	NA	2.340E-01
SCUBE1	-0.304	2.671E-01	4.078E-01
ALOXE3	-0.304	3.678E-01	1.087E-01
AC138969.1	-0.304	6.424E-02	3.877E-03
AC005154.6	-0.304	4.075E-01	1.532E-01
CDH3	-0.304	3.215E-01	1.494E-01
NADSYN1	-0.304	5.114E-02	1.144E-01
LRP4	-0.304	1.380E-01	9.165E-02
RTL5	-0.305	1.995E-01	3.269E-01
MPC1L	-0.305	5.621E-01	3.601E-04
MIR8071-2	-0.305	6.049E-01	1.279E-01
AWAT1	-0.305	NA	2.669E-02
ADAM2	-0.305	6.940E-01	4.192E-02
MT-ND6	-0.305	1.891E-01	1.089E-01
ZNF215	-0.305	1.984E-01	3.091E-01

C1QL4	-0.305	4.906E-01	7.573E-01
FAM47B	-0.305	NA	2.516E-02
DGKQ	-0.305	1.653E-03	1.343E-01
CLU	-0.306	3.422E-01	1.659E-01
ARHGEF28	-0.306	8.702E-02	3.549E-03
DMBX1	-0.306	4.818E-01	5.180E-02
MIR6870	-0.306	6.585E-01	3.830E-05
CA2	-0.306	3.459E-01	5.727E-01
THBD	-0.306	2.841E-01	7.717E-02
MIF	-0.306	1.504E-02	2.521E-01
SYK	-0.306	6.057E-02	2.135E-02
FSCN1	-0.306	1.454E-01	2.502E-02
G6PC2	-0.306	6.686E-01	1.701E-01
CST7	-0.306	2.583E-01	1.987E-01
IFI27	-0.306	3.316E-01	3.213E-01
SLC36A3	-0.306	4.931E-01	4.831E-02
ARMC12	-0.307	2.921E-01	1.815E-01
SPATC1	-0.307	2.463E-01	6.225E-02
CCDC24	-0.307	2.500E-02	2.963E-03
SHC4	-0.307	1.788E-01	5.631E-01
LRGUK	-0.307	1.947E-01	5.797E-01
TLR6	-0.307	2.349E-01	3.389E-01
RAD21L1	-0.307	5.696E-01	1.712E-01
TCP11X2	-0.307	NA	1.272E-01
SH3TC1	-0.307	8.054E-02	6.925E-01
ZFPM1	-0.308	5.848E-03	6.642E-01
TKTL2	-0.308	4.805E-01	1.968E-01
CD200R1	-0.308	1.510E-01	1.767E-03
MIRLET7I	-0.308	5.896E-01	4.378E-01
GPR162	-0.308	1.393E-01	1.108E-01
PIK3CD	-0.308	1.356E-01	1.517E-03
AURKC	-0.309	1.051E-01	1.102E-01
RPL3	-0.309	2.943E-03	6.309E-01
BTF3	-0.309	3.361E-04	2.212E-02
CREB3L3	-0.309	4.688E-01	9.762E-02
RACK1	-0.309	2.643E-03	2.673E-03
CELSR1	-0.309	1.615E-01	5.809E-01
PNISR	-0.309	2.473E-03	4.718E-01
PLCB4	-0.309	2.961E-01	8.925E-01
GALNT4	-0.309	2.521E-01	2.272E-02
TRIM7	-0.310	2.583E-01	2.872E-01
PTPRR	-0.310	4.804E-01	2.103E-01
RIPK3	-0.310	5.027E-02	1.842E-01
GJA9	-0.310	2.917E-01	9.227E-02
JAG2	-0.310	7.945E-02	1.189E-01
FITM1	-0.310	6.957E-02	2.205E-01
ATP2C2	-0.310	2.891E-01	2.210E-01
STAT1	-0.310	1.211E-01	7.172E-01
ANKLE1	-0.310	1.579E-01	4.665E-01
PPAN	-0.310	1.859E-02	2.848E-02

SDCBP2	-0.311	2.165E-01	1.041E-01
TMEM232	-0.311	9.309E-02	5.841E-04
TAS2R9	-0.311	NA	4.756E-01
CNTN3	-0.311	4.367E-01	7.233E-02
DNLZ	-0.311	8.445E-02	1.276E-01
HOXD8	-0.312	6.743E-02	2.393E-04
RPS2	-0.312	3.822E-03	3.782E-01
U2AF1	-0.312	1.559E-01	9.124E-02
IFI16	-0.312	1.700E-01	1.190E-02
MIR378A	-0.313	3.777E-01	4.006E-01
GAMT	-0.314	2.017E-01	3.334E-01
ADGRF1	-0.314	4.071E-01	5.111E-02
MIR6763	-0.314	4.931E-01	3.272E-01
F8A3	-0.314	3.397E-01	1.738E-01
NSUN6	-0.314	1.298E-02	4.769E-02
C19orf71	-0.314	1.479E-02	1.235E-01
CEMIP	-0.314	2.510E-01	1.488E-02
AMT	-0.314	1.311E-01	3.119E-02
OR51B5	-0.314	7.761E-01	2.618E-01
SLC34A1	-0.314	3.377E-01	3.255E-01
RPL19	-0.315	7.317E-03	7.205E-02
SH2D2A	-0.315	6.909E-02	2.901E-01
AL512506.3	-0.315	2.444E-01	1.435E-01
STARD4	-0.315	1.621E-01	5.283E-01
BAK1	-0.316	9.859E-03	3.813E-03
RPL13	-0.316	1.148E-02	1.144E-02
POLR2J3	-0.316	1.152E-01	5.008E-01
GIF2-C20orf2	-0.316	1.525E-01	1.733E-02
RPS6KA1	-0.316	2.914E-03	4.767E-02
DMRTC1B	-0.316	NA	1.928E-01
FAM83E	-0.316	3.936E-01	2.411E-01
ADAMTSL2	-0.316	8.171E-02	1.333E-02
PTPN7	-0.316	2.583E-01	1.532E-01
DNAH2	-0.316	3.467E-01	3.133E-01
LRFN4	-0.317	1.428E-01	6.737E-02
PRSS42	-0.317	3.902E-01	1.456E-01
TCTE3	-0.317	9.087E-03	4.574E-01
PDE6G	-0.317	2.390E-01	3.610E-02
MIR4477A	-0.317	NA	3.981E-01
ARAP1	-0.317	1.080E-03	1.096E-01
NANOGNB	-0.317	NA	1.617E-03
AC012309.1	-0.317	2.233E-01	7.529E-02
CDH18	-0.318	7.076E-01	3.668E-01
PLSCR3	-0.318	1.042E-02	2.174E-01
OR3A3	-0.318	NA	1.218E-01
NEIL1	-0.318	8.977E-02	8.419E-02
CXorf65	-0.318	2.879E-01	2.253E-01
LY6G6F	-0.318	NA	1.482E-01
MIR4644	-0.318	4.199E-01	2.037E-01
LY9	-0.318	3.317E-01	1.066E-01

ACY1	-0.319	2.673E-02	4.034E-02
MIR23A	-0.319	3.268E-01	1.002E-02
OR2L13	-0.319	7.686E-01	4.139E-02
ITPKA	-0.319	2.193E-01	7.188E-02
GRB14	-0.319	2.545E-01	4.485E-02
C16orf74	-0.319	1.345E-01	9.533E-02
CRIP3	-0.319	3.025E-01	1.479E-01
GAP43	-0.319	4.841E-01	1.539E-01
PCYT1B	-0.319	3.833E-01	5.488E-03
RPS14	-0.319	1.942E-03	3.534E-01
ACTL9	-0.319	NA	1.695E-01
AC011448.1	-0.320	8.365E-03	3.695E-01
TRIM22	-0.320	1.585E-01	1.053E-01
C10orf95	-0.320	6.334E-02	4.598E-03
DUSP7	-0.320	1.117E-01	1.193E-02
LEUTX	-0.320	NA	9.664E-02
FAM3D	-0.320	5.243E-01	2.107E-01
CITED1	-0.320	1.395E-01	1.345E-01
ACSM4	-0.320	3.918E-01	9.872E-02
CHRNG	-0.320	3.043E-01	2.144E-01
ZNF614	-0.320	2.595E-02	6.475E-02
KIR3DL1	-0.320	5.293E-01	2.431E-02
KRTAP20-4	-0.320	8.911E-01	4.296E-01
MIR656	-0.320	NA	1.110E-01
SLC35G1	-0.320	3.267E-02	8.143E-01
ZBED5	-0.321	8.290E-05	6.476E-01
PCDHAC1	-0.321	5.299E-01	4.776E-02
PROP1	-0.321	6.308E-01	1.891E-01
CD101	-0.321	5.616E-02	2.844E-03
RBM11	-0.321	2.351E-01	1.676E-01
PABPC1L2A	-0.321	NA	5.127E-02
MXD4	-0.321	1.806E-03	8.400E-03
MIR194-1	-0.322	NA	3.533E-02
SORL1	-0.322	1.997E-01	6.954E-02
MIR8058	-0.322	7.507E-01	5.258E-02
WDR27	-0.322	1.126E-02	4.770E-01
CX3CL1	-0.322	2.576E-01	1.304E-02
CTNND2	-0.322	4.595E-01	3.915E-03
PTK2B	-0.322	2.363E-02	1.747E-03
TMEM221	-0.323	1.717E-01	5.246E-04
UGT2A2	-0.323	NA	1.135E-02
ATP5EP2	-0.323	5.331E-02	3.909E-01
TTN	-0.323	5.262E-02	4.876E-04
ZNF536	-0.323	4.525E-01	6.995E-02
ADGRD2	-0.323	4.859E-01	2.092E-01
TP63	-0.323	2.510E-01	2.136E-01
MIR5194	-0.323	5.415E-01	3.212E-01
SLC6A20	-0.324	3.525E-01	3.462E-01
CD27	-0.324	2.581E-01	1.517E-01
PIGL	-0.324	3.803E-03	5.103E-01

MMEL1	-0.324	2.498E-01	4.504E-01
F11	-0.324	6.293E-01	1.623E-01
OR2A42	-0.324	NA	1.070E-03
CKMT1A	-0.324	2.007E-01	1.047E-03
GPR153	-0.324	1.107E-01	4.555E-02
ADGRG3	-0.324	2.578E-01	2.611E-01
TFEB	-0.325	4.300E-02	2.373E-01
NUP107	-0.325	8.023E-03	7.959E-01
ZNF558	-0.325	1.231E-02	1.291E-02
MYO7B	-0.325	2.869E-01	4.013E-01
AL136295.4	-0.325	5.858E-02	5.466E-02
TBC1D4	-0.325	2.042E-02	1.925E-03
HEPACAM	-0.325	5.419E-01	5.524E-01
AOAH	-0.325	2.828E-01	6.119E-03
CTSV	-0.326	3.175E-01	4.352E-02
CFAP53	-0.326	8.930E-02	9.291E-03
MEGF6	-0.326	1.620E-01	9.447E-02
DOC2B	-0.326	2.131E-01	1.206E-01
GPRC6A	-0.326	6.851E-01	3.668E-04
DGKA	-0.326	2.953E-02	6.872E-01
METAP1D	-0.326	2.461E-03	2.582E-01
ROBO3	-0.327	1.438E-01	4.207E-01
FOXB2	-0.327	NA	6.693E-02
DUX4	-0.327	NA	7.503E-03
OR2T10	-0.327	NA	1.671E-01
JMJD7	-0.327	7.326E-02	7.813E-02
MIR566	-0.327	6.211E-01	3.876E-02
AC068896.1	-0.327	8.905E-02	2.105E-01
DPY19L2	-0.327	2.542E-01	1.834E-01
FAM166B	-0.327	7.505E-02	1.076E-01
CLDN17	-0.327	8.306E-01	1.298E-01
HOXD10	-0.327	2.758E-01	8.112E-02
CHL1	-0.327	4.624E-01	5.625E-02
NUDT14	-0.327	1.558E-02	1.194E-01
FAM159A	-0.328	2.384E-01	2.819E-01
GPR31	-0.328	4.578E-01	2.960E-01
TRIM43	-0.328	8.204E-01	6.045E-02
SULT1A1	-0.328	1.732E-01	6.055E-02
DRD3	-0.328	NA	6.462E-02
MIR7848	-0.329	5.638E-01	4.723E-02
H1FNT	-0.329	3.439E-01	2.272E-02
RGS9	-0.329	2.044E-01	2.584E-02
ID3	-0.329	1.082E-01	4.150E-01
ANKRD18B	-0.329	1.699E-01	6.512E-02
TXNDC17	-0.329	8.083E-03	5.824E-02
DSP	-0.329	2.484E-01	2.222E-01
TP53AIP1	-0.330	3.515E-01	1.123E-02
AP000295.1	-0.330	3.376E-01	1.407E-01
UNC79	-0.330	5.040E-02	2.730E-01
MMP24	-0.331	8.719E-02	2.244E-02

LSAMP	-0.331	2.122E-01	8.591E-02
FOXD4	-0.332	1.811E-01	3.591E-02
TAS2R31	-0.332	1.366E-01	3.501E-02
PLG	-0.332	5.852E-01	6.459E-02
GML	-0.332	NA	4.587E-01
RASAL3	-0.332	1.514E-01	4.449E-01
RUFY4	-0.332	2.405E-01	1.687E-01
ARL6IP4	-0.332	1.666E-02	7.628E-02
VSIG2	-0.333	3.798E-01	1.625E-01
TX16-NPEPL	-0.333	1.211E-01	7.653E-02
VWA5B2	-0.333	2.027E-01	3.592E-01
TRIM75P	-0.333	6.274E-01	4.880E-05
FSHB	-0.334	NA	1.385E-01
OAS3	-0.334	4.989E-02	7.294E-04
CBR4	-0.334	1.950E-03	8.143E-01
MIR1296	-0.334	NA	5.037E-02
LINC00282	-0.334	4.309E-01	5.290E-02
SMIM21	-0.334	NA	1.353E-01
DRAXIN	-0.334	2.019E-01	1.826E-01
C16orf78	-0.334	NA	5.221E-01
OR51B4	-0.334	7.321E-01	1.750E-01
TMEM246	-0.334	2.498E-01	1.027E-01
ADGRV1	-0.334	1.744E-01	4.197E-01
TMEM156	-0.334	2.021E-01	3.768E-02
HBG2	-0.335	3.077E-01	1.336E-01
INPP5D	-0.335	8.846E-02	3.607E-01
NR2E1	-0.335	4.523E-01	2.457E-01
SLC6A1	-0.335	1.192E-01	6.930E-02
LPO	-0.335	3.277E-01	6.443E-02
GJA10	-0.335	NA	9.069E-02
SLC35F1	-0.336	1.560E-01	1.093E-01
AC090527.2	-0.336	4.680E-01	9.117E-02
KAAG1	-0.336	5.210E-01	1.511E-02
CASP1	-0.336	1.517E-01	1.009E-01
GLI4	-0.336	9.522E-03	1.259E-01
MIR210	-0.336	2.549E-01	3.252E-03
TMPRSS5	-0.336	4.243E-02	4.115E-02
DDX60	-0.336	1.427E-01	1.823E-01
HEY2	-0.336	3.326E-02	1.533E-01
MIR6864	-0.336	4.827E-01	5.393E-02
MIR3685	-0.336	1.904E-02	4.319E-02
STX11	-0.337	1.097E-01	4.046E-03
RUNX3	-0.337	1.991E-01	2.044E-02
HMX2	-0.337	5.508E-01	2.261E-01
GHRHR	-0.337	6.736E-01	1.249E-01
NDUFA13	-0.337	1.032E-02	2.253E-02
FMR1NB	-0.337	5.045E-01	4.373E-02
MAFA	-0.337	2.959E-01	7.030E-03
ABCB11	-0.337	4.810E-01	3.523E-02
LRRC4B	-0.337	1.438E-01	1.038E-01

PWP2	-0.338	2.891E-01	3.802E-01
SLC44A4	-0.338	3.636E-01	4.231E-02
PPIAL4D	-0.338	NA	3.082E-02
AC005833.3	-0.338	2.676E-01	4.892E-02
FCGR3B	-0.338	3.742E-01	2.894E-02
ANKRD36C	-0.338	1.195E-01	1.495E-01
CAPS	-0.338	1.980E-01	2.861E-02
RGL3	-0.338	3.067E-01	2.878E-01
FAM13A	-0.338	4.689E-02	1.712E-01
AKNA	-0.338	1.043E-02	5.704E-01
AC069257.3	-0.339	5.146E-01	1.327E-01
CDYL2	-0.339	4.040E-02	2.907E-01
COL25A1	-0.339	3.321E-01	3.010E-05
TBX6	-0.339	1.491E-01	1.687E-01
MIR1270	-0.339	4.412E-01	6.040E-01
MBOAT4	-0.340	3.638E-02	3.278E-02
MIR23C	-0.340	NA	4.933E-01
NACA2	-0.340	5.835E-02	1.594E-01
HNRNPCL1	-0.340	3.222E-01	9.205E-02
AMN	-0.340	1.891E-01	3.198E-01
TMEM255A	-0.340	2.586E-01	2.632E-01
FAM193B	-0.340	2.914E-03	2.200E-01
MS4A5	-0.341	6.074E-01	4.342E-03
TNFRSF10B	-0.341	8.198E-03	1.308E-01
NKX6-1	-0.341	5.133E-01	2.586E-01
GSTP1	-0.341	1.664E-02	7.406E-02
KITLG	-0.341	1.538E-01	2.827E-01
[illegible]JD7-PLA2G[illegible]	-0.341	7.146E-02	5.390E-05
HOXD3	-0.342	4.869E-02	1.473E-01
FAM129C	-0.342	3.063E-01	4.497E-01
CFAP70	-0.342	8.812E-02	7.466E-02
RAX2	-0.342	NA	3.040E-03
LRRC3	-0.342	1.091E-01	1.563E-01
HOXB4	-0.342	4.686E-02	2.133E-01
ARGFX	-0.342	NA	1.189E-04
PARP15	-0.342	1.911E-01	2.526E-01
NOL12	-0.342	2.981E-04	7.355E-01
HNMT	-0.342	4.832E-02	5.033E-02
SPATA20	-0.342	8.642E-03	6.952E-02
MTFP1	-0.343	2.807E-03	3.786E-03
ATP5I	-0.343	6.024E-03	9.583E-03
PPFIBP2	-0.343	1.414E-01	1.229E-01
PTH2R	-0.343	5.540E-01	9.196E-02
LIPM	-0.343	1.603E-01	1.491E-01
LINGO3	-0.343	2.346E-01	5.396E-01
OR5M10	-0.343	NA	3.792E-03
NME5	-0.344	2.762E-01	3.221E-01
CKMT1B	-0.344	1.736E-01	3.992E-01
DOCK10	-0.344	1.316E-01	1.982E-01
MIR3065	-0.344	5.299E-01	7.181E-02

CEND1	-0.344	1.273E-01	5.339E-01
VIPR1	-0.344	2.204E-01	1.078E-01
GOLT1A	-0.344	2.558E-01	1.579E-02
TUBG2	-0.345	1.248E-02	8.566E-01
XKR6	-0.345	1.374E-01	5.914E-03
FOXD3	-0.345	4.690E-01	6.349E-01
LRRTM2	-0.345	1.703E-01	1.649E-01
SLC22A5	-0.345	1.252E-02	1.740E-04
SLC5A4	-0.345	1.792E-01	1.877E-01
BST2	-0.345	1.666E-01	5.002E-01
BNIP3	-0.345	9.191E-02	3.331E-01
AGBL2	-0.345	4.426E-02	1.889E-01
CSRP2	-0.346	3.952E-02	4.372E-01
ERAP2	-0.346	1.985E-01	2.009E-02
SLC7A9	-0.346	1.859E-01	7.779E-04
DLGAP1	-0.346	4.234E-01	1.416E-04
ZDHHC11B	-0.346	3.347E-01	5.200E-02
RNF212B	-0.346	1.401E-01	4.501E-01
ZG16B	-0.346	2.693E-01	2.271E-03
DEF6	-0.347	2.157E-03	1.148E-01
IKZF3	-0.347	7.123E-02	6.882E-01
C1orf94	-0.347	5.829E-01	2.470E-01
PPFIA4	-0.347	1.616E-01	6.533E-04
IL37	-0.347	3.100E-01	7.614E-02
CLDN20	-0.347	1.136E-01	9.902E-04
MIR6812	-0.347	2.512E-01	2.514E-02
FRMPD3	-0.348	2.142E-01	8.611E-01
TGFB2	-0.348	2.235E-01	3.592E-01
REG3G	-0.348	NA	1.776E-01
SPACA5	-0.348	6.079E-01	9.061E-03
OR9A4	-0.348	6.274E-01	4.510E-05
PRPS1L1	-0.348	7.737E-01	4.145E-01
LYNX1	-0.349	2.207E-01	4.005E-01
PRAMEF8	-0.349	NA	4.421E-03
LPAR6	-0.350	7.442E-02	1.454E-02
HMGCS2	-0.350	5.533E-01	1.914E-01
HMGCLL1	-0.350	3.625E-01	6.274E-01
ITGB7	-0.350	6.795E-02	2.812E-01
RHBDL1	-0.350	8.949E-02	6.856E-02
DCC	-0.350	2.097E-01	6.235E-02
CNTNAP5	-0.350	5.599E-01	1.074E-01
TTLL6	-0.350	4.244E-01	5.690E-02
RGS12	-0.350	1.976E-03	3.803E-02
ZNF692	-0.350	1.042E-02	2.371E-01
SLC7A5	-0.350	1.283E-01	5.279E-01
PRSS50	-0.351	3.736E-01	2.045E-02
SLC47A2	-0.351	3.771E-01	3.444E-01
KIFC2	-0.351	6.762E-02	5.982E-01
LIPI	-0.351	2.429E-01	2.680E-01
ZNF705E	-0.351	1.587E-01	3.541E-01

NUDT12	-0.351	2.361E-02	3.749E-01
OR2B11	-0.351	NA	6.258E-01
KCNH1	-0.351	1.746E-01	3.874E-01
SIX6	-0.351	NA	9.021E-02
DUSP13	-0.352	3.743E-01	1.946E-01
MEPE	-0.352	4.425E-01	4.781E-01
CARD18	-0.352	6.755E-01	1.562E-01
ODC1	-0.352	5.695E-02	1.280E-01
MIR3657	-0.352	4.532E-01	3.035E-02
WSCD2	-0.353	4.130E-01	3.086E-03
PDZD9	-0.353	1.066E-01	1.900E-02
ANKRD50	-0.353	4.913E-02	9.816E-02
CATSPER3	-0.353	2.547E-02	4.830E-03
F12	-0.353	1.223E-01	1.009E-01
SPANXC	-0.353	8.043E-01	1.800E-01
ZFR2	-0.353	3.110E-01	3.690E-02
SEC14L6	-0.354	2.735E-01	1.656E-01
IZUMO4	-0.354	4.243E-02	6.355E-02
ZNF266	-0.354	2.347E-03	4.029E-02
USP17L1	-0.354	NA	5.857E-02
DBX1	-0.354	7.733E-01	2.449E-02
ADSSL1	-0.354	1.359E-01	7.862E-02
ARHGAP25	-0.354	4.242E-02	5.090E-02
CDHR2	-0.355	9.219E-02	7.295E-01
C11orf21	-0.355	1.984E-01	2.408E-01
FOSB	-0.355	2.613E-01	4.009E-02
IGF2	-0.355	5.157E-01	2.870E-03
TEKT5	-0.355	3.232E-01	8.963E-03
MIR616	-0.355	2.308E-01	5.384E-02
AC068790.8	-0.355	1.255E-01	3.164E-02
ADCY8	-0.355	5.194E-01	1.861E-01
TGM1	-0.355	3.525E-01	2.317E-02
SLC45A4	-0.355	1.339E-02	1.427E-03
COL9A3	-0.355	1.824E-01	1.345E-01
PEG10	-0.355	3.886E-01	9.753E-03
FGD2	-0.356	8.912E-02	7.131E-02
KRTAP4-12	-0.356	NA	1.748E-03
FAM177B	-0.356	1.734E-01	2.090E-02
FAAH	-0.356	4.427E-02	8.041E-02
SMIM28	-0.356	6.359E-01	2.197E-03
NR2E3	-0.356	5.019E-02	2.190E-05
FBXO39	-0.356	2.565E-01	2.515E-01
CRX	-0.356	3.268E-01	4.410E-02
SYCP2L	-0.357	2.715E-01	5.120E-02
RGL4	-0.357	1.709E-02	1.428E-03
CYFIP2	-0.357	5.768E-02	3.418E-03
ADORA2B	-0.357	1.249E-01	1.328E-02
CYP2C18	-0.357	4.721E-01	9.960E-02
SLC2A9	-0.358	1.092E-01	1.166E-01
PDE7A	-0.358	2.826E-03	1.254E-01

SLC25A47	-0.358	3.742E-01	9.584E-02
KCNK7	-0.358	1.438E-01	5.664E-02
CD1D	-0.358	8.171E-02	5.607E-01
INPP1	-0.359	1.871E-02	1.720E-01
SSX2B	-0.359	8.413E-01	2.804E-02
HPX	-0.359	1.080E-01	4.288E-02
TNFSF13B	-0.359	1.579E-01	1.048E-01
NKX2-8	-0.360	4.833E-01	1.039E-01
C1orf141	-0.360	5.576E-01	1.067E-01
SPDEF	-0.360	3.642E-01	8.445E-02
AQP7	-0.360	3.573E-01	6.432E-03
CEACAM8	-0.360	4.173E-01	7.653E-03
NMUR1	-0.360	1.561E-01	4.641E-01
FAM90A26	-0.361	4.657E-01	1.371E-01
ALDH1A3	-0.361	1.544E-01	2.749E-01
GJD4	-0.361	3.486E-01	4.824E-02
BEND2	-0.361	5.701E-01	3.752E-02
ZRSR1	-0.361	1.380E-01	1.912E-01
MAMDC4	-0.361	3.276E-02	7.549E-02
GSTTP1	-0.362	4.362E-01	7.392E-02
OAS2	-0.362	9.233E-02	3.610E-05
HTR1E	-0.362	6.594E-01	1.647E-02
ACSL6	-0.362	1.878E-01	3.857E-01
SLC10A5	-0.362	2.828E-02	2.195E-04
ARAP3	-0.362	1.075E-02	9.073E-03
RBM20	-0.362	2.601E-01	2.297E-02
PLEKHG4	-0.362	1.755E-01	2.484E-01
GCSAM	-0.363	5.835E-02	3.870E-04
CD79B	-0.363	2.027E-01	1.187E-04
MATN4	-0.363	1.713E-01	5.265E-01
TAGAP	-0.363	1.370E-01	2.149E-01
AC243967.1	-0.363	NA	2.422E-01
FAM53B	-0.363	1.561E-03	2.152E-02
MYO7A	-0.363	9.309E-02	1.092E-01
CCDC54	-0.363	3.660E-01	4.250E-01
ZNF80	-0.364	3.045E-01	1.503E-01
CITED4	-0.364	1.015E-01	1.159E-01
CXCL1	-0.364	3.509E-01	5.426E-02
UCN2	-0.364	1.944E-01	1.627E-02
HDAC10	-0.364	4.921E-03	1.044E-02
HAS3	-0.364	1.680E-01	8.014E-02
TRAF5	-0.364	4.243E-03	3.424E-02
NTF4	-0.365	1.619E-01	1.317E-01
ARL5C	-0.365	4.518E-01	2.218E-02
PLA2G4B	-0.365	9.592E-02	2.214E-01
SLC7A14	-0.365	5.097E-01	6.442E-01
AL024498.2	-0.365	5.191E-01	2.042E-01
LGI3	-0.365	3.753E-01	3.537E-03
RBP5	-0.365	5.234E-02	1.901E-01
PCGF3	-0.365	6.793E-04	5.087E-02

TTC6	-0.365	2.279E-01	9.185E-02
RNF212	-0.365	2.546E-01	3.229E-02
THEGL	-0.365	3.688E-01	2.925E-01
TMEM132D	-0.365	5.494E-01	1.176E-01
SLC23A3	-0.365	7.965E-02	1.187E-01
CA7	-0.365	3.504E-01	9.446E-02
KIAA1456	-0.366	2.754E-01	3.495E-01
FEZF2	-0.366	NA	4.938E-02
CSH2	-0.366	7.301E-01	7.805E-02
Z82206.1	-0.366	5.147E-02	1.325E-01
TBC1D21	-0.366	NA	6.570E-01
GPR33	-0.366	6.058E-01	1.071E-01
SEMA6A	-0.367	1.933E-01	3.136E-01
ZNF10	-0.367	1.396E-02	6.160E-04
ITGA6	-0.367	8.974E-02	8.010E-05
G6PC	-0.367	5.313E-01	2.211E-01
SNTG1	-0.367	5.200E-01	5.132E-02
GALNT8	-0.368	2.605E-01	6.893E-02
ASB12	-0.369	1.708E-01	4.374E-04
ORM1	-0.369	5.732E-01	3.609E-02
SCAMP5	-0.369	5.695E-02	1.249E-02
BPIFB1	-0.369	5.299E-01	3.167E-02
CXCR1	-0.369	3.219E-01	5.995E-02
PFN3	-0.369	NA	1.538E-02
M1AP	-0.369	3.449E-01	3.900E-02
SLC52A1	-0.369	1.826E-01	4.556E-03
ARHGAP26	-0.370	1.139E-02	9.761E-04
RNF17	-0.370	3.056E-01	5.501E-02
PPARGC1A	-0.370	3.969E-01	2.228E-01
ZC3H12D	-0.371	1.316E-01	1.405E-01
DCAF8L2	-0.371	7.525E-01	2.535E-03
RRAD	-0.371	2.938E-01	1.292E-01
EEF1G	-0.371	3.364E-02	2.796E-01
TNFRSF9	-0.371	1.738E-01	1.692E-01
IL2RG	-0.371	2.038E-01	2.543E-02
RAB26	-0.371	1.495E-01	3.631E-01
HEY1	-0.371	9.749E-02	4.559E-01
TRMT1	-0.371	1.200E-04	3.764E-01
HTR1A	-0.371	NA	1.653E-01
RNASET2	-0.371	3.460E-02	4.179E-02
CTAG2	-0.371	7.274E-01	2.434E-03
TMEM257	-0.372	NA	5.363E-02
TSPY10	-0.372	NA	1.413E-02
LUZP4	-0.372	7.712E-01	2.292E-01
PPARGC1B	-0.372	1.290E-02	1.630E-01
OR52L1	-0.372	6.371E-01	1.253E-01
KCTD4	-0.373	4.635E-01	2.059E-01
COL7A1	-0.373	1.374E-01	6.761E-01
CD52	-0.373	1.657E-01	1.072E-02
FAM86B1	-0.373	8.185E-02	1.566E-01

KISS1R	-0.373	3.314E-01	7.639E-02
NEUROG1	-0.373	NA	1.102E-03
TACC3	-0.373	1.102E-02	1.365E-01
CD53	-0.373	1.655E-01	2.139E-01
MIR571	-0.374	3.567E-01	1.665E-02
ANGPT2	-0.374	3.136E-02	6.568E-03
CACNA1I	-0.375	3.627E-01	7.008E-02
PRM2	-0.375	NA	1.175E-01
EIF4A1	-0.375	1.039E-03	1.765E-02
CDHR5	-0.375	3.091E-01	2.194E-01
TNFRSF18	-0.375	1.669E-01	1.803E-01
GPRIN3	-0.375	4.040E-02	2.557E-02
CD37	-0.376	1.830E-01	6.519E-04
ADH4	-0.376	3.897E-01	1.393E-01
ARG2	-0.376	5.226E-02	1.096E-01
TPCN1	-0.376	8.083E-03	1.130E-02
MEP1B	-0.376	1.368E-01	5.583E-02
KLRC2	-0.376	3.973E-01	4.550E-01
RPL9	-0.376	2.487E-04	2.747E-01
TFCP2L1	-0.376	1.365E-01	9.553E-02
CAPN11	-0.377	7.148E-02	1.449E-02
C6orf58	-0.377	8.493E-02	7.291E-02
ZIM3	-0.377	5.826E-01	4.073E-03
CALHM1	-0.377	2.116E-01	1.381E-04
C21orf33	-0.377	2.986E-01	2.560E-01
LIPK	-0.377	4.641E-01	3.256E-02
IL18RAP	-0.378	1.444E-01	8.003E-02
SIRPD	-0.378	3.299E-01	3.191E-01
UGT1A1	-0.378	4.801E-01	1.632E-02
JPH1	-0.378	9.314E-02	2.014E-03
KRTAP19-8	-0.378	6.927E-01	1.743E-01
REM2	-0.378	1.636E-02	1.546E-01
POU3F3	-0.378	6.417E-01	8.848E-01
MIR106B	-0.378	2.813E-01	6.683E-04
GPR55	-0.378	1.427E-01	3.550E-05
MIR302A	-0.378	NA	1.906E-02
SPIRE2	-0.379	1.137E-01	3.629E-02
MIR7974	-0.379	4.791E-01	1.115E-01
ASXL3	-0.379	2.227E-01	6.088E-03
NOVA1	-0.379	2.452E-01	3.045E-02
ATP2A3	-0.379	5.998E-02	1.011E-01
NPIPA5	-0.380	1.777E-01	2.866E-01
COLQ	-0.380	5.511E-02	7.969E-02
RPL6	-0.380	6.910E-05	2.830E-02
C1orf145	-0.381	7.384E-02	2.252E-03
HOXD9	-0.381	3.198E-02	7.005E-02
VWCE	-0.381	8.443E-02	4.305E-01
SLC16A1	-0.381	2.113E-01	7.591E-02
AC009336.2	-0.381	1.573E-01	7.163E-02
GPR39	-0.381	1.331E-01	7.375E-02

TREH	-0.381	3.079E-01	1.912E-02
CARMIL2	-0.381	1.630E-01	5.458E-03
ZNF709	-0.381	2.322E-02	2.852E-02
PKP1	-0.381	2.980E-01	7.111E-03
ANGPT4	-0.381	2.100E-01	2.742E-01
KRTAP2-1	-0.382	NA	2.852E-01
STC2	-0.382	9.413E-02	2.280E-01
LTA	-0.382	1.712E-01	7.010E-02
TMEM116	-0.382	4.787E-03	5.000E-02
CCT8L2	-0.382	NA	5.512E-02
CYP27B1	-0.382	4.299E-02	2.926E-03
ENTPD5	-0.382	2.853E-02	4.237E-01
BAIAP2L2	-0.382	2.090E-01	3.703E-02
LDLRAD1	-0.382	4.667E-01	1.600E-02
PDZK1	-0.382	2.254E-01	2.104E-01
CACNB4	-0.382	2.009E-01	2.917E-02
KCNJ15	-0.382	1.279E-01	5.304E-01
AQP12A	-0.383	6.713E-01	1.520E-02
RBPJL	-0.383	2.456E-01	1.788E-01
TIFAB	-0.383	2.833E-01	2.430E-02
KIF12	-0.383	3.187E-01	4.965E-03
MIR4420	-0.383	4.408E-01	1.163E-01
MYOC	-0.383	3.914E-01	3.503E-02
APOL2	-0.383	1.119E-02	7.723E-02
MIRLET7G	-0.383	NA	4.708E-02
SP140	-0.384	1.830E-01	6.312E-02
RBP3	-0.384	3.286E-01	2.224E-01
TRPV4	-0.384	5.616E-02	3.058E-01
ADAT2	-0.384	7.139E-04	1.902E-01
KIF21B	-0.384	1.076E-02	1.526E-01
ABAT	-0.384	1.066E-01	6.503E-02
GNG13	-0.385	3.462E-01	9.960E-02
SYT13	-0.385	5.413E-01	1.172E-01
GAS2L2	-0.385	3.360E-01	2.174E-01
NPIPB2	-0.385	1.760E-01	4.870E-02
GOLGA6L4	-0.385	1.139E-01	2.693E-01
ARHGAP9	-0.386	1.277E-01	2.112E-01
GRM8	-0.386	1.721E-01	2.201E-01
MIR6859-2	-0.386	4.299E-01	4.793E-02
TNS4	-0.386	1.431E-01	3.137E-01
CDK18	-0.387	1.129E-02	1.339E-01
FOXD4L1	-0.387	1.012E-01	2.991E-02
TTC16	-0.387	1.453E-01	4.863E-02
PAQR8	-0.387	8.665E-02	1.414E-01
GPR83	-0.388	1.123E-01	1.406E-04
TRERF1	-0.388	3.924E-03	3.077E-01
ACSM1	-0.388	1.138E-01	2.675E-02
HHIPL2	-0.388	2.500E-01	9.172E-01
RAB39A	-0.388	2.288E-01	7.020E-02
RNF186	-0.388	5.361E-01	1.895E-01

POU5F1	-0.388	1.878E-01	6.409E-03
AASS	-0.389	4.872E-02	4.898E-02
SPRR2F	-0.389	5.732E-01	8.242E-02
TMEM74B	-0.389	1.091E-01	6.259E-02
ZFYVE28	-0.389	1.158E-02	4.677E-01
ZNF682	-0.389	6.902E-02	5.119E-01
LUC7L	-0.389	1.402E-03	7.422E-02
MYH6	-0.390	4.244E-01	4.395E-01
ABCC1	-0.390	5.484E-03	4.220E-05
KYNU	-0.390	1.539E-01	2.310E-01
OR10AD1	-0.391	4.001E-01	5.385E-02
VNN1	-0.391	2.422E-01	8.593E-04
GCSAML	-0.391	1.940E-01	2.708E-01
VEGFA	-0.392	2.349E-02	1.649E-01
FSIP1	-0.392	5.331E-02	2.174E-01
OR1Q1	-0.392	3.340E-01	3.970E-06
HES4	-0.392	5.358E-02	5.331E-03
CASP5	-0.393	2.991E-01	7.396E-02
PF4V1	-0.393	3.790E-01	7.400E-02
KIAA0895L	-0.393	3.240E-03	2.786E-01
MIR3929	-0.393	NA	2.031E-01
SAMD9L	-0.393	1.050E-01	5.772E-01
ZNRD1	-0.393	1.010E-05	2.158E-01
FBP1	-0.393	1.485E-01	2.206E-01
TAS2R8	-0.394	NA	8.320E-05
DMRTA1	-0.394	3.330E-01	1.399E-01
ITK	-0.394	1.667E-01	2.530E-02
PSTPIP2	-0.394	1.664E-02	2.718E-02
MROH7-TTC4	-0.394	3.031E-01	2.171E-04
SPAM1	-0.395	6.516E-01	2.908E-02
FGF13	-0.395	1.013E-01	7.933E-02
CYP2A13	-0.395	6.669E-01	7.007E-01
BCL2A1	-0.396	1.817E-01	3.537E-01
FPR2	-0.396	2.555E-01	2.705E-02
PIK3R2	-0.396	2.373E-03	6.017E-02
GLRA2	-0.396	4.497E-01	2.210E-02
GRB10	-0.397	1.690E-02	1.222E-01
FCGBP	-0.397	2.273E-01	1.700E-06
MIR3198-1	-0.397	4.729E-01	1.155E-03
GAGE13	-0.397	NA	7.650E-02
GOLGA6L9	-0.397	2.300E-02	2.662E-01
BCAN	-0.398	8.785E-02	6.248E-01
CD96	-0.398	8.521E-02	4.170E-02
PDE7B	-0.398	8.439E-02	2.872E-02
MIR6776	-0.398	4.808E-01	3.668E-02
SOX6	-0.398	1.902E-01	2.436E-02
SMCP	-0.398	6.219E-01	1.080E-02
ATCAY	-0.398	3.587E-01	1.400E-01
DOCK2	-0.399	1.309E-01	1.919E-02
PRR29	-0.399	3.111E-02	3.835E-01

CSN3	-0.399	7.691E-01	3.364E-02
PLCXD2	-0.399	1.615E-01	4.184E-02
PLEK2	-0.399	4.869E-02	2.848E-01
CLEC2D	-0.399	8.650E-03	3.108E-03
NIPAL4	-0.400	1.452E-01	2.195E-01
SERPINA7	-0.400	6.898E-01	2.172E-02
PKDREJ	-0.400	4.402E-02	5.244E-02
CPB2	-0.400	2.922E-01	2.155E-02
RPL34	-0.400	2.686E-04	8.454E-04
YEATS4	-0.400	6.176E-03	3.773E-02
IRX1	-0.400	5.633E-01	3.320E-02
P2RY8	-0.400	9.030E-02	4.357E-01
NTS	-0.401	3.572E-01	3.627E-02
RASA4	-0.401	4.420E-02	5.406E-02
GOLGA6L7P	-0.401	5.215E-01	2.583E-02
CHKB	-0.401	2.461E-03	7.459E-02
EME2	-0.401	5.710E-03	4.200E-05
MIR23B	-0.402	5.055E-01	3.243E-02
P2RY12	-0.402	2.666E-01	5.964E-03
ACOT11	-0.402	2.034E-02	1.909E-01
EXOC3L4	-0.402	1.877E-01	1.177E-01
FAM105A	-0.402	3.448E-02	4.210E-02
CATSPERE	-0.402	1.647E-02	5.866E-02
STAP1	-0.403	1.909E-01	4.301E-02
MIR7973-1	-0.403	4.962E-01	1.147E-01
HTR3E	-0.403	3.256E-01	2.443E-01
GABRA6	-0.403	NA	5.840E-05
TTC34	-0.403	8.864E-02	4.429E-02
ATG16L2	-0.404	8.983E-03	1.173E-03
C4orf50	-0.404	2.909E-01	4.881E-02
MIR6782	-0.404	NA	1.115E-01
ZSWIM2	-0.404	NA	6.239E-02
RAB38	-0.404	1.343E-01	3.208E-03
CD226	-0.404	7.490E-02	1.687E-02
GAGE1	-0.404	7.853E-01	8.029E-02
TERT	-0.405	1.656E-01	6.384E-02
SLC32A1	-0.405	6.805E-01	5.004E-03
ZCCHC12	-0.405	1.525E-01	4.376E-02
PCDHA4	-0.405	3.714E-01	2.150E-06
SEL1L3	-0.405	1.420E-02	2.144E-02
GPC2	-0.405	1.577E-01	3.887E-02
SLCO1A2	-0.406	4.184E-01	3.042E-03
ZSWIM6	-0.406	8.290E-05	3.891E-03
GLYAT	-0.406	4.729E-01	6.026E-01
MIR7856	-0.406	2.164E-01	4.958E-02
LPXN	-0.406	3.962E-02	4.007E-01
PTAFR	-0.406	3.540E-02	1.620E-01
SLC6A8	-0.406	6.898E-02	3.715E-02
ZAN	-0.406	3.231E-01	2.105E-01
SLC18B1	-0.407	6.373E-04	3.210E-02

SLC39A12	-0.407	4.017E-01	1.640E-01
CD6	-0.407	1.112E-01	3.516E-01
IPCEF1	-0.407	2.718E-02	5.875E-02
APCS	-0.407	7.962E-01	2.143E-01
GVQW2	-0.407	4.641E-03	8.540E-02
DGAT2L6	-0.408	4.840E-01	1.862E-02
RPL22L1	-0.408	2.951E-03	6.770E-02
LRRC75B	-0.408	1.543E-02	1.103E-02
SLCO5A1	-0.408	2.055E-01	4.370E-01
SLC7A8	-0.408	4.005E-02	1.180E-01
SFI1	-0.409	3.441E-04	1.088E-02
UNC45B	-0.409	1.461E-01	5.349E-02
SLC5A9	-0.409	7.742E-02	1.199E-01
RAB3IP	-0.409	2.359E-02	6.746E-01
AC022335.1	-0.409	NA	3.770E-01
CLEC2B	-0.409	1.258E-01	1.996E-01
KDM4F	-0.409	NA	7.384E-02
GPR171	-0.409	1.573E-01	4.346E-01
MIR4538	-0.410	5.733E-01	2.575E-03
MAGEA1	-0.410	6.534E-01	7.386E-02
SPX	-0.410	2.917E-01	7.181E-02
LMNTD1	-0.410	3.293E-01	7.962E-03
PRSS45	-0.410	2.584E-01	2.543E-02
MST1	-0.410	2.675E-02	3.863E-04
DSC3	-0.410	4.114E-01	1.988E-03
OR10G2	-0.410	NA	2.427E-02
RFPL1	-0.410	5.064E-01	9.070E-05
KCNN1	-0.411	1.623E-01	1.595E-01
INSRR	-0.411	2.665E-01	9.721E-02
PTGER4	-0.411	4.698E-02	7.935E-02
LPAR3	-0.411	1.721E-01	1.730E-01
ZIC5	-0.411	4.651E-01	1.794E-01
MIR8071-1	-0.411	5.348E-01	6.488E-02
CAPN14	-0.411	1.658E-01	1.545E-03
AL645941.2	-0.411	3.861E-01	7.598E-02
DPP6	-0.412	3.293E-01	1.460E-01
AC244197.3	-0.412	7.104E-02	3.664E-01
NEUROD2	-0.412	2.185E-01	2.504E-03
CCDC194	-0.412	1.980E-01	3.969E-01
SHISA2	-0.412	2.733E-01	6.578E-03
BACH2	-0.412	1.691E-01	5.098E-02
NUTM2G	-0.413	6.859E-03	5.742E-02
CEACAM20	-0.413	3.100E-01	1.926E-01
KLK5	-0.413	6.387E-01	6.762E-03
ASPHD2	-0.413	3.567E-03	8.212E-03
MYO15B	-0.413	2.321E-02	6.824E-02
PLET1	-0.413	3.906E-01	1.768E-01
C10orf99	-0.414	3.904E-01	7.943E-03
C14orf159	-0.414	1.370E-03	4.185E-03
ANO4	-0.414	1.978E-01	5.096E-01

PADI4	-0.415	1.495E-01	5.354E-02
ADIPOQ	-0.415	6.722E-01	2.094E-02
ASCL2	-0.415	7.699E-02	4.449E-02
TSPY1	-0.415	8.936E-01	4.048E-02
IRGM	-0.415	1.621E-01	4.030E-02
HORMAD1	-0.416	4.361E-01	8.134E-02
C17orf99	-0.416	1.441E-01	2.713E-02
MTRNR2L8	-0.416	3.536E-02	1.776E-01
ASIC4	-0.416	1.063E-01	8.661E-02
C16orf54	-0.416	1.906E-01	1.092E-01
ADAM20	-0.416	4.958E-02	2.155E-02
RBM44	-0.417	4.318E-02	2.078E-03
SPN	-0.417	7.926E-02	2.050E-05
CD207	-0.417	2.357E-01	1.955E-02
KRTAP5-5	-0.417	NA	5.540E-02
ESRRB	-0.417	1.602E-01	7.561E-02
FAM221B	-0.417	1.420E-01	4.762E-01
NCAM2	-0.417	3.402E-01	3.542E-03
ATG9B	-0.417	1.195E-01	5.074E-03
GLRA1	-0.417	3.595E-01	9.719E-02
NAP1L6	-0.418	4.289E-01	3.044E-01
TRIM49C	-0.418	NA	2.610E-02
SLC22A1	-0.418	8.925E-02	9.582E-02
CHRFAM7A	-0.418	1.670E-01	2.219E-01
CBWD3	-0.419	3.822E-03	6.530E-02
MRGPRX4	-0.419	6.140E-01	8.832E-03
MIR4648	-0.419	5.434E-01	3.192E-01
LRRD1	-0.419	9.005E-02	2.236E-03
CDRT4	-0.419	1.339E-02	7.997E-02
CLDN8	-0.419	4.117E-01	1.169E-01
HPGD	-0.419	2.900E-01	6.720E-05
GRAP2	-0.420	7.996E-02	2.640E-01
DIRAS2	-0.420	4.420E-01	5.674E-02
RFPL2	-0.420	3.340E-01	8.873E-02
ANKRD30B	-0.420	4.877E-01	1.743E-02
ISG20	-0.420	6.222E-02	1.279E-02
INPP5J	-0.420	6.728E-02	3.040E-05
44075.000	-0.420	2.361E-02	2.987E-02
KIR3DX1	-0.420	2.154E-01	2.368E-01
PLA2G6	-0.421	9.022E-03	3.560E-02
IGFN1	-0.421	3.396E-01	4.287E-02
C1orf100	-0.422	4.739E-02	1.738E-01
TMEM8C	-0.422	5.325E-01	1.342E-01
FNDC9	-0.422	1.316E-01	3.630E-05
HMP19	-0.422	3.240E-01	3.638E-02
IL23R	-0.422	1.057E-01	1.508E-01
MYBPHL	-0.422	2.909E-01	5.465E-01
SEC14L4	-0.423	3.493E-01	5.825E-03
KRTAP8-1	-0.423	NA	1.337E-02
GPD1	-0.423	2.042E-01	3.138E-02

LRP5L	-0.423	4.406E-03	4.640E-07
DNER	-0.423	3.914E-01	3.739E-02
ACSM2A	-0.423	5.003E-01	6.416E-01
EREG	-0.424	3.782E-01	3.215E-01
SIM1	-0.424	5.650E-01	1.802E-03
DACT2	-0.424	3.331E-01	2.820E-02
DLX1	-0.425	2.854E-01	1.709E-01
AKAIN1	-0.425	NA	2.462E-01
DLL1	-0.425	6.196E-02	2.459E-01
SPACA4	-0.426	2.122E-01	3.393E-02
CARD17	-0.426	3.782E-01	1.130E-06
HOXC4	-0.426	1.622E-01	1.903E-04
GPR141	-0.426	1.346E-01	2.035E-01
MVB12B	-0.426	3.881E-04	5.185E-02
GPSM3	-0.427	1.834E-02	2.541E-02
TLR10	-0.427	1.686E-01	5.495E-03
AC233724.6	-0.427	NA	1.234E-02
MIR3671	-0.427	1.284E-01	1.301E-01
MIR3649	-0.428	NA	9.544E-02
CD209	-0.429	1.526E-01	1.517E-02
SLC14A1	-0.429	3.084E-01	2.468E-01
KLRF2	-0.429	4.997E-01	5.698E-03
CELA3B	-0.429	5.327E-01	1.084E-02
CNR2	-0.429	2.664E-01	3.165E-02
AIFM3	-0.430	1.119E-01	3.273E-02
MIR744	-0.430	1.954E-01	5.219E-02
TMEM82	-0.431	3.507E-01	4.584E-02
TAS2R10	-0.431	1.556E-01	1.483E-01
ALG1L	-0.431	3.624E-02	3.289E-01
BTG4	-0.431	4.075E-01	7.350E-04
EDDM13	-0.431	1.186E-01	4.988E-02
ADAM18	-0.432	5.327E-01	2.745E-01
MIR4999	-0.432	3.430E-01	9.021E-02
STON2	-0.432	1.395E-02	1.903E-01
AC005943.1	-0.432	2.791E-01	1.185E-01
SSU72P8	-0.432	NA	1.342E-01
KRT16	-0.433	3.470E-01	1.471E-03
LGSN	-0.433	5.316E-01	9.668E-04
KRT5	-0.433	3.973E-01	1.842E-02
SLC26A8	-0.434	4.402E-02	4.882E-04
CARD10	-0.434	1.634E-02	2.783E-01
MROH7	-0.434	9.777E-02	9.641E-02
CCDC146	-0.434	5.134E-03	6.793E-01
CNR1	-0.434	2.321E-01	1.017E-03
BLK	-0.435	3.011E-01	3.695E-02
ANO9	-0.435	4.955E-03	1.938E-01
SIM2	-0.435	3.213E-02	1.878E-01
MIR6891	-0.435	4.944E-01	1.130E-05
CCL1	-0.435	4.522E-01	3.907E-01
ACAP1	-0.436	2.915E-02	8.479E-02

FAM26F	-0.436	1.117E-01	8.799E-03
AADAT	-0.437	1.042E-02	2.697E-02
SULT6B1	-0.437	4.330E-01	3.464E-03
DNAH17	-0.437	1.853E-02	1.417E-02
CLEC19A	-0.438	5.259E-01	4.252E-03
MIR4524B	-0.438	6.017E-01	4.296E-02
SYTL1	-0.438	1.547E-02	4.100E-06
PTPN22	-0.438	8.334E-02	1.977E-02
CLEC6A	-0.438	3.028E-01	3.163E-02
LSP1	-0.439	1.103E-01	4.715E-02
CEBPE	-0.439	1.738E-01	4.211E-02
PRSS46	-0.439	3.373E-01	4.210E-03
DPCR1	-0.439	2.993E-01	4.244E-02
MIR548AN	-0.439	2.852E-01	4.910E-02
SMIM22	-0.439	1.081E-01	1.278E-01
ANXA13	-0.439	2.965E-01	1.849E-01
MIRLET7D	-0.439	9.271E-02	9.583E-02
MIR221	-0.440	1.940E-01	2.832E-01
ATP8A1	-0.440	4.689E-02	8.352E-03
CAPRIN2	-0.440	1.105E-04	1.257E-01
SLAMF7	-0.440	9.557E-02	2.082E-01
F5	-0.440	2.356E-01	8.332E-03
HSH2D	-0.440	2.724E-02	9.691E-02
NCR3	-0.441	1.494E-01	7.667E-01
GRIA1	-0.441	3.872E-01	3.504E-02
CXCL5	-0.441	3.466E-01	1.348E-01
EFCAB1	-0.441	2.004E-01	2.173E-02
CCL24	-0.441	2.588E-01	4.816E-01
LAMA1	-0.441	2.170E-01	4.702E-02
TMEM229B	-0.442	4.990E-03	2.514E-01
TRPV3	-0.442	9.666E-02	1.317E-02
S1PR4	-0.442	4.700E-02	6.853E-02
PINLYP	-0.442	6.761E-02	1.796E-02
CDRT15	-0.442	1.152E-01	4.820E-01
SLC1A7	-0.443	2.113E-01	3.380E-02
CYP3A4	-0.443	1.911E-01	7.015E-02
ZBP1	-0.443	1.760E-01	1.649E-01
CD5	-0.443	8.486E-02	3.647E-03
ADAMTS20	-0.443	3.587E-01	3.302E-01
TIGIT	-0.443	8.846E-02	1.305E-02
OR52N2	-0.444	5.024E-01	2.812E-04
CRYGS	-0.444	6.184E-03	1.556E-03
TEX15	-0.445	4.310E-01	1.589E-01
MAGED4B	-0.445	5.065E-01	1.128E-02
AGER	-0.445	2.666E-03	2.820E-01
OVCH1	-0.445	2.543E-01	6.829E-01
NR1H4	-0.445	4.747E-01	1.107E-02
MNDA	-0.445	1.001E-01	8.910E-02
CD79A	-0.445	2.335E-01	1.105E-01
RETNLB	-0.446	3.796E-01	2.787E-01

TLR4	-0.446	1.710E-02	8.781E-02
FAM9A	-0.446	NA	5.249E-03
LIM2	-0.446	5.475E-01	1.076E-01
POU2AF1	-0.447	1.577E-01	3.066E-01
WDFY4	-0.447	1.134E-01	9.483E-02
STXBP5L	-0.447	2.752E-01	5.058E-01
BCL2L14	-0.448	6.181E-02	4.404E-04
MAF	-0.448	2.724E-02	2.538E-01
OAS1	-0.448	6.005E-03	4.353E-02
C1orf68	-0.448	6.004E-01	1.507E-01
FGD5	-0.448	3.518E-02	3.880E-02
TNN	-0.448	1.817E-01	2.137E-02
SH2D1B	-0.449	5.207E-02	5.494E-02
C6orf141	-0.449	1.788E-01	4.720E-02
FAM163B	-0.450	2.285E-01	1.074E-01
CHRNB3	-0.450	5.050E-01	4.889E-02
CLPS	-0.450	5.205E-01	2.039E-01
POU5F1B	-0.450	2.595E-02	9.112E-02
KRTAP20-2	-0.450	NA	4.112E-02
VAX1	-0.450	5.260E-01	3.018E-02
EHD3	-0.451	4.762E-03	5.529E-02
MMP28	-0.451	9.585E-02	7.689E-02
PIEZO2	-0.452	5.494E-02	3.948E-02
HOXD1	-0.452	1.486E-01	1.355E-02
ZNF804A	-0.452	1.352E-01	1.699E-01
IRF4	-0.452	1.146E-01	3.109E-01
CABYR	-0.452	5.379E-02	6.790E-03
OR2K2	-0.452	NA	1.077E-01
PTX4	-0.452	2.071E-01	6.383E-02
HOXA1	-0.453	1.128E-02	2.645E-03
OR52E2	-0.453	NA	5.892E-01
NOL4	-0.453	3.464E-01	3.341E-03
TRIM74	-0.453	5.331E-02	1.189E-01
KRTAP2-2	-0.453	NA	4.862E-03
POLN	-0.453	2.947E-02	1.615E-02
CHRNB2	-0.453	1.137E-01	1.440E-05
CD48	-0.453	1.118E-01	3.920E-03
CPT1B	-0.453	6.562E-03	1.354E-01
TAC1	-0.453	4.769E-01	3.432E-02
FRMD3	-0.454	3.088E-02	1.122E-02
DHRS3	-0.454	1.848E-02	1.483E-03
CCL18	-0.455	2.463E-01	2.479E-02
ZPBP	-0.455	5.346E-01	4.315E-02
SLAMF6	-0.455	1.292E-01	9.136E-02
GCK	-0.455	7.285E-02	6.278E-03
MIR4297	-0.455	4.876E-01	1.474E-03
IHH	-0.455	3.842E-01	2.196E-02
MIR135A1	-0.455	2.214E-02	3.176E-02
BATF2	-0.455	1.132E-01	8.116E-02
GOLGA6L10	-0.456	1.989E-02	1.749E-02

FAM159B	-0.456	8.987E-02	4.200E-04
PRL	-0.456	3.740E-01	7.019E-02
CRHR1	-0.456	3.231E-01	2.067E-01
TMCO5A	-0.457	6.779E-01	3.510E-03
TBX18	-0.457	1.940E-01	1.406E-02
HOXD4	-0.457	1.073E-01	3.251E-02
TUBA8	-0.457	1.162E-02	2.021E-02
SYCP3	-0.458	1.708E-02	4.064E-01
SCN1A	-0.458	4.020E-01	2.792E-01
SLITRK5	-0.458	3.375E-01	2.773E-01
BCL2L10	-0.458	7.926E-02	4.556E-03
POU3F4	-0.458	5.996E-01	1.009E-01
KY	-0.458	1.098E-01	4.533E-02
TMEM27	-0.459	2.002E-02	7.159E-02
ZNF831	-0.459	1.170E-01	4.875E-02
ICOS	-0.459	1.104E-01	3.831E-04
SLC16A9	-0.459	1.428E-01	4.304E-02
AOC1	-0.459	2.399E-01	2.205E-02
MIR1273C	-0.459	NA	6.690E-02
NANOS2	-0.459	4.889E-01	3.078E-02
ARTN	-0.460	4.599E-02	3.225E-01
TBC1D3L	-0.460	1.076E-01	5.077E-01
MIR7854	-0.460	3.122E-01	2.181E-01
ABCC3	-0.460	6.705E-02	1.193E-02
SEMA4G	-0.461	3.722E-02	3.976E-02
GPR25	-0.461	2.161E-01	2.898E-04
Z83844.1	-0.462	3.904E-02	3.598E-04
NUDT11	-0.462	1.664E-01	1.725E-01
DEFB118	-0.462	6.002E-01	8.894E-03
GJA3	-0.462	2.226E-01	3.097E-01
MOGAT1	-0.463	3.428E-01	8.018E-03
GSDMA	-0.463	9.728E-02	5.001E-03
MYF6	-0.463	4.490E-01	4.851E-01
IL31	-0.464	NA	2.058E-03
CCR5	-0.464	8.429E-02	4.157E-02
NTRK1	-0.464	7.090E-02	3.420E-02
GPT	-0.465	4.407E-02	1.236E-01
C9orf84	-0.465	4.527E-02	1.324E-01
ADAMDEC1	-0.465	1.830E-01	7.000E-02
GOLGA8A	-0.465	2.434E-02	2.476E-04
SMIM24	-0.466	6.852E-02	6.722E-02
PAEP	-0.466	3.472E-01	2.846E-01
PNMA3	-0.467	1.443E-01	1.297E-02
KCNJ2	-0.467	1.690E-02	9.952E-02
MAPK8IP3	-0.467	7.620E-04	1.886E-02
AC026461.4	-0.467	4.945E-01	8.170E-05
KRTAP5-9	-0.467	2.206E-01	2.098E-02
LRRC4	-0.467	7.212E-02	1.562E-03
BCAM	-0.467	2.595E-02	9.533E-02
DTHD1	-0.468	1.608E-01	4.457E-01

SPRR2B	-0.468	6.204E-01	3.166E-01
C20orf141	-0.468	4.854E-01	2.850E-01
OR4D10	-0.468	NA	4.827E-02
OR2H2	-0.468	3.339E-01	8.161E-02
CYP2A6	-0.469	3.154E-01	3.375E-01
KRT15	-0.469	2.143E-01	1.707E-03
ICAM3	-0.469	6.086E-02	1.797E-02
RASL10A	-0.469	7.108E-02	4.634E-03
LMAN1L	-0.469	5.197E-01	1.847E-03
TMIGD2	-0.469	3.515E-02	6.666E-02
ABCD2	-0.470	9.291E-02	5.952E-02
AL845331.2	-0.470	3.970E-01	1.195E-02
KLF15	-0.470	8.243E-02	1.549E-01
IL26	-0.470	2.570E-01	1.558E-03
STAR	-0.471	9.457E-02	2.457E-02
SLC3A1	-0.471	5.212E-02	4.911E-03
TMEM163	-0.471	1.196E-01	3.970E-02
HOXC13	-0.471	1.395E-01	8.790E-02
MIR3680-1	-0.471	2.934E-01	3.615E-03
SIGLEC10	-0.471	8.696E-02	3.641E-02
CD2	-0.471	8.247E-02	1.320E-02
MIR27B	-0.471	3.197E-01	6.616E-01
TGM4	-0.472	7.945E-02	7.640E-08
PLIN5	-0.472	1.349E-01	3.530E-07
SORCS3	-0.472	5.099E-01	2.410E-01
KCNAB3	-0.473	6.247E-03	7.298E-02
SESN3	-0.473	2.142E-02	1.927E-01
S100G	-0.473	5.927E-01	1.851E-02
GCNA	-0.473	5.740E-03	2.713E-01
RTP5	-0.473	2.570E-01	1.282E-01
C8orf22	-0.473	5.287E-01	1.751E-02
ENPP2	-0.473	2.764E-02	2.234E-02
CST9L	-0.473	6.128E-01	8.655E-04
SASH3	-0.474	6.804E-02	1.255E-02
ITGAL	-0.474	5.835E-02	4.115E-02
MIR1302-3	-0.474	1.442E-01	1.120E-02
ODAM	-0.475	3.085E-01	5.231E-02
SLC45A3	-0.475	9.007E-03	2.552E-02
TBC1D26	-0.475	2.131E-01	4.073E-02
CTLA4	-0.476	8.185E-02	6.910E-03
PRODH2	-0.476	5.510E-01	2.686E-02
PTGR1	-0.476	2.951E-02	1.691E-02
PYROXD2	-0.476	1.610E-02	6.888E-03
CYP4F2	-0.476	3.326E-01	6.776E-02
FAM151A	-0.477	3.144E-03	2.339E-04
AMHR2	-0.477	1.108E-01	5.501E-04
TLX1	-0.477	3.923E-01	1.658E-03
AC091980.2	-0.477	5.316E-01	8.806E-02
CDK5R2	-0.477	2.190E-01	1.079E-01
TESPA1	-0.477	4.805E-02	3.589E-02

SCIMP	-0.478	7.285E-02	1.875E-01
ATF7IP2	-0.478	2.050E-02	2.298E-01
CALB1	-0.478	3.493E-01	1.381E-02
F2RL1	-0.478	1.694E-02	4.003E-02
SPATA22	-0.478	3.705E-01	1.536E-01
CFHR3	-0.478	5.758E-02	1.158E-02
CCDC27	-0.479	3.189E-01	1.192E-02
GLDC	-0.479	2.269E-01	2.750E-05
NT5C1A	-0.479	3.841E-01	3.409E-02
ZNF157	-0.479	1.971E-01	1.046E-02
MIR6807	-0.479	4.134E-01	1.720E-01
LHX9	-0.479	2.538E-01	1.487E-02
ASMT	-0.479	5.616E-02	2.735E-03
ARC	-0.479	1.207E-01	2.004E-02
AC099489.1	-0.479	1.973E-02	4.405E-02
AIPL1	-0.480	3.661E-01	6.406E-02
EFNB3	-0.480	6.806E-02	6.139E-02
CAPNS2	-0.480	2.154E-01	3.334E-02
NEU4	-0.480	1.328E-01	8.509E-01
SLC26A9	-0.480	1.814E-01	2.400E-02
RPL39L	-0.480	1.475E-01	7.802E-03
GOLGA8F	-0.481	4.812E-01	5.002E-01
DEGS2	-0.481	1.227E-01	2.791E-02
OR5A1	-0.481	NA	2.623E-01
ZNF735	-0.481	NA	3.675E-02
DDX4	-0.481	2.038E-01	2.095E-01
KANK4	-0.481	2.994E-01	2.806E-01
PTPRZ1	-0.482	4.220E-01	5.078E-02
MUC6	-0.482	2.082E-01	6.628E-03
LRMP	-0.482	6.257E-02	3.700E-02
AATK-AS1	-0.482	4.672E-01	2.016E-02
CYP39A1	-0.482	4.318E-02	1.217E-03
AL121594.3	-0.482	1.973E-03	2.832E-01
MCTP2	-0.482	3.318E-03	7.259E-03
HIST1H4L	-0.483	3.728E-01	1.976E-03
SP7	-0.483	3.473E-01	3.604E-02
LYPD2	-0.483	3.735E-01	2.464E-02
NUDT10	-0.483	1.717E-01	1.232E-01
DHRS2	-0.483	2.582E-01	7.304E-02
NPSR1	-0.483	2.983E-01	1.952E-01
LHCGR	-0.483	3.238E-01	1.313E-01
HR	-0.483	6.975E-02	1.302E-01
ADRB2	-0.483	6.633E-02	4.316E-01
GCNT1	-0.484	6.902E-02	4.838E-02
NAT8	-0.484	1.846E-01	4.195E-02
AMZ1	-0.484	1.224E-01	1.830E-04
TDRD12	-0.485	6.731E-02	1.562E-03
MIR3193	-0.485	3.840E-01	4.000E-03
HIST1H4G	-0.485	NA	2.420E-01
LDHB	-0.486	8.290E-05	5.178E-02

TM4SF5	-0.486	3.473E-01	1.159E-01
TBC1D10C	-0.486	5.027E-02	9.050E-03
RFX6	-0.487	4.250E-01	2.223E-01
STK32A	-0.487	1.548E-01	2.677E-02
KCNJ10	-0.487	5.792E-02	5.133E-01
CCR6	-0.487	1.835E-01	1.445E-01
NPIPB6	-0.487	9.109E-02	2.160E-05
FAM162A	-0.487	1.680E-04	3.345E-02
C10orf62	-0.488	2.719E-01	4.519E-02
KRTAP5-7	-0.488	2.573E-01	1.978E-02
NLRP1	-0.488	2.748E-03	4.578E-02
NLRC3	-0.488	8.128E-03	1.447E-02
LTB4R	-0.489	1.666E-02	3.097E-02
SLC37A2	-0.489	6.831E-03	4.318E-01
TLR8	-0.489	1.284E-01	1.208E-02
FGFR3	-0.489	1.254E-01	2.372E-02
LMX1A	-0.490	4.617E-01	5.116E-04
PPP1R16B	-0.490	1.724E-02	1.192E-01
FAM46D	-0.490	3.671E-01	4.106E-02
SCN4A	-0.491	1.192E-01	4.746E-03
TRIM73	-0.491	1.457E-02	6.983E-03
HOXC11	-0.491	1.932E-01	2.928E-02
BOLL	-0.491	1.221E-01	1.999E-01
NCKAP5	-0.492	5.764E-02	2.091E-02
C8orf4	-0.492	5.099E-02	1.693E-02
OR5M11	-0.492	3.309E-01	1.712E-01
UST	-0.492	7.903E-03	2.054E-02
IL12RB2	-0.492	1.557E-01	2.046E-01
CCL4L2	-0.493	9.314E-02	3.613E-03
TECTB	-0.493	4.068E-01	5.130E-02
C8orf34	-0.494	1.132E-01	4.667E-02
IKZF1	-0.494	4.300E-02	2.163E-01
CR1L	-0.494	1.234E-01	7.690E-02
KRTAP21-1	-0.494	NA	1.016E-02
EPHB1	-0.496	4.162E-02	5.537E-02
S100Z	-0.496	5.925E-02	3.592E-02
TTLL2	-0.496	3.220E-01	2.273E-02
TRIM67	-0.496	2.556E-02	1.758E-02
MIR503	-0.496	NA	6.450E-03
SAMSN1	-0.496	3.814E-02	5.947E-02
FKBP1B	-0.496	1.957E-02	3.823E-02
MC4R	-0.497	3.917E-01	1.289E-02
IL6R	-0.497	1.042E-02	9.080E-02
MIR4539	-0.497	4.744E-01	1.590E-01
KDM4E	-0.498	3.697E-01	1.702E-02
TVP23A	-0.499	2.943E-03	7.134E-02
CCKAR	-0.499	5.909E-01	4.127E-01
XAF1	-0.499	2.867E-02	2.959E-02
GABBR1	-0.499	9.841E-03	8.340E-04
NPFFR1	-0.499	7.483E-02	3.665E-01

TMEM155	-0.499	5.212E-02	9.513E-02
TSPAN1	-0.500	1.046E-01	1.263E-03
FAT2	-0.500	1.526E-01	2.685E-03
TBC1D28	-0.501	NA	1.584E-01
KREMEN2	-0.501	5.998E-02	4.236E-02
IL1R2	-0.502	8.356E-02	1.525E-02
TBC1D3E	-0.502	2.689E-01	1.246E-01
TNFSF14	-0.502	4.299E-02	5.396E-04
CHRNA7	-0.502	1.387E-01	8.033E-02
SYT6	-0.502	1.131E-01	3.751E-01
FBN3	-0.502	2.986E-01	6.653E-03
FP565260.2	-0.503	3.406E-01	5.783E-02
SYT8	-0.503	1.560E-01	1.328E-01
MIR31	-0.504	4.490E-01	4.986E-02
NPNT	-0.504	5.782E-02	1.576E-01
VSTM1	-0.504	1.833E-01	1.425E-01
GAD1	-0.504	9.746E-02	1.907E-03
LRRC18	-0.504	1.585E-01	1.182E-01
NMUR2	-0.505	2.480E-01	3.336E-02
FCMR	-0.505	3.663E-02	1.135E-04
PAK3	-0.505	1.626E-01	2.732E-02
FOXQ1	-0.505	8.590E-02	2.439E-02
DRD2	-0.505	1.503E-01	7.016E-03
C15orf53	-0.506	1.757E-01	2.594E-01
MIR514B	-0.506	NA	6.705E-03
BPIFA1	-0.506	5.540E-01	6.110E-03
GDPD4	-0.506	1.234E-01	4.395E-02
ASCL3	-0.507	3.996E-01	8.482E-02
FAM47C	-0.507	3.795E-01	9.541E-02
AADACL4	-0.508	5.045E-01	6.143E-02
MAP4K1	-0.508	2.750E-02	1.102E-01
ZAP70	-0.508	4.340E-02	3.826E-01
SAMD5	-0.508	5.256E-02	3.308E-02
NTF3	-0.508	6.047E-02	1.445E-01
ABHD12B	-0.508	2.552E-02	1.473E-02
TSPAN32	-0.508	2.769E-02	2.027E-01
CTSC	-0.508	5.603E-03	1.642E-04
ABCC4	-0.509	4.225E-03	1.606E-01
LCN9	-0.510	NA	2.699E-02
CD3E	-0.510	4.974E-02	9.026E-03
TBC1D3G	-0.510	2.830E-01	1.451E-01
PRR22	-0.510	4.442E-03	7.311E-04
GOLGA8B	-0.511	8.189E-03	5.909E-03
PAX9	-0.511	4.197E-02	5.929E-02
FCER2	-0.511	2.550E-01	1.095E-03
OVGP1	-0.512	4.114E-02	2.873E-01
FCRL3	-0.512	1.343E-01	9.500E-02
CCL3	-0.512	6.877E-02	9.509E-03
CACNG7	-0.512	3.478E-01	1.022E-01
DPPA3	-0.513	5.749E-01	7.660E-03

IL2RB	-0.513	2.817E-02	5.275E-01
RGS6	-0.513	1.088E-01	2.091E-01
NCR2	-0.513	4.378E-01	1.012E-01
OASL	-0.513	3.205E-02	1.685E-01
LINC00521	-0.513	3.857E-01	1.853E-02
SMIM23	-0.513	2.755E-01	5.479E-02
NPHS2	-0.514	5.070E-01	2.775E-02
CORO1A	-0.514	1.479E-02	5.953E-03
EIF4E1B	-0.514	5.729E-01	9.808E-03
CARMIL3	-0.514	1.186E-02	8.473E-02
KLHL3	-0.514	2.680E-03	5.288E-02
MYO3B	-0.514	1.550E-01	2.518E-02
DNAH8	-0.515	1.132E-01	1.959E-01
FGF21	-0.515	4.634E-01	4.967E-01
ATP8A2	-0.515	4.702E-02	4.030E-03
CNTN5	-0.516	2.924E-01	5.231E-04
AATK	-0.516	2.798E-03	2.896E-02
SPANXN3	-0.517	NA	7.241E-03
SOX21	-0.517	3.028E-01	1.067E-03
RTL9	-0.517	1.108E-01	3.415E-03
MAP3K15	-0.518	4.502E-02	2.637E-02
COL27A1	-0.518	9.051E-03	4.396E-02
BEST2	-0.518	2.195E-01	3.212E-03
CXCR3	-0.519	6.806E-02	2.342E-02
ITIH1	-0.519	2.685E-01	3.920E-02
RASSF6	-0.519	3.595E-02	8.757E-03
KCTD19	-0.519	8.809E-02	5.348E-02
KCNH8	-0.520	1.616E-01	4.847E-02
LYPD4	-0.520	3.725E-01	1.428E-01
AMY2A	-0.520	5.020E-01	2.301E-02
ACSM6	-0.520	3.283E-01	4.936E-01
CD69	-0.520	6.938E-02	7.585E-03
KRTAP21-2	-0.521	NA	3.413E-01
PPP2R2C	-0.521	2.051E-01	2.754E-02
MIR6889	-0.521	2.920E-01	6.002E-01
CLNK	-0.522	2.427E-02	2.388E-04
GPC3	-0.522	1.023E-01	4.246E-01
ODF3L1	-0.522	1.985E-02	1.465E-02
MYO1G	-0.522	2.889E-02	9.651E-02
OR2A25	-0.522	NA	4.191E-04
CDH13	-0.523	1.740E-02	6.085E-02
RASSF10	-0.523	8.003E-02	4.025E-02
OLFM4	-0.523	3.736E-01	3.395E-02
RGPD3	-0.524	4.745E-02	3.692E-03
OBSCN	-0.524	3.835E-03	1.201E-01
MTRNR2L10	-0.524	3.478E-02	1.929E-01
CACNG5	-0.524	5.410E-01	6.856E-02
CLEC18C	-0.525	1.876E-01	2.991E-01
B3GALNT1	-0.525	6.793E-04	1.203E-02
ARL14	-0.525	1.368E-01	3.171E-02

NCR3LG1	-0.525	1.627E-02	2.181E-02
TRHR	-0.525	NA	4.308E-01
AOC2	-0.525	3.245E-02	2.947E-02
OBP2A	-0.525	1.887E-01	3.496E-02
TMEM200C	-0.526	5.351E-02	3.091E-01
PLA2G4E	-0.526	2.566E-01	4.062E-02
DQX1	-0.526	3.111E-02	2.804E-01
CAMKV	-0.527	1.719E-01	2.791E-02
KLKB1	-0.527	4.884E-02	4.378E-02
MIR27A	-0.527	2.019E-01	3.977E-02
TMIGD1	-0.527	4.836E-01	4.609E-01
CYP4A11	-0.528	1.178E-01	3.800E-02
GGT1	-0.528	1.354E-02	1.839E-01
CLIC6	-0.528	1.583E-01	4.501E-01
LRRC14B	-0.528	1.950E-01	1.213E-01
CACNA2D3	-0.528	3.836E-02	4.130E-06
LDHC	-0.528	2.927E-01	2.687E-02
CSAG2	-0.529	3.259E-01	4.845E-02
CLEC4D	-0.529	1.150E-01	1.076E-01
PTPRC	-0.529	4.561E-02	9.681E-02
CDH9	-0.530	5.535E-01	5.009E-03
CCT2	-0.530	1.940E-04	2.854E-03
SPATA31A1	-0.530	NA	1.232E-01
RNF183	-0.530	1.607E-01	1.417E-02
MFAP3L	-0.530	2.712E-02	1.537E-02
SLC7A4	-0.530	1.341E-01	3.849E-02
MIR6881	-0.530	3.356E-01	3.210E-03
KLK9	-0.530	6.348E-01	3.365E-01
C8B	-0.531	4.398E-01	7.487E-02
OTP	-0.531	1.616E-01	4.527E-02
MIR4285	-0.531	NA	2.801E-02
DACH2	-0.531	2.313E-01	3.900E-03
GJD3	-0.531	5.695E-02	7.754E-03
AKAP4	-0.531	NA	3.654E-03
WARS	-0.532	3.341E-02	3.172E-02
TRPM6	-0.532	4.508E-02	8.108E-04
MIR4537	-0.532	4.530E-01	2.793E-01
FAM181A	-0.532	3.293E-01	3.503E-02
ANP32D	-0.533	1.794E-01	1.395E-01
PIK3AP1	-0.533	2.978E-02	5.553E-03
CHI3L2	-0.533	9.582E-02	9.490E-05
TMEM95	-0.534	1.393E-01	1.110E-03
HSD17B13	-0.534	2.624E-02	1.184E-01
CXXC4	-0.535	3.714E-02	5.111E-03
CYP26C1	-0.535	8.846E-02	5.357E-02
MS4A1	-0.535	2.449E-01	8.726E-03
TRPC5OS	-0.535	1.984E-01	1.218E-01
KRT76	-0.536	2.768E-01	8.974E-03
RHOXF2B	-0.536	7.506E-01	7.206E-02
HSD17B3	-0.537	9.092E-02	2.780E-02

HLA-G	-0.537	1.707E-02	9.160E-06
CD40	-0.537	8.650E-03	4.154E-02
SPRR4	-0.537	3.807E-01	1.348E-02
TCERG1L	-0.537	2.839E-01	2.614E-01
C4BPA	-0.537	2.770E-01	7.825E-01
DFNA5	-0.537	1.456E-02	1.865E-02
OXGR1	-0.537	9.259E-02	1.376E-02
IDO2	-0.538	5.983E-02	7.565E-03
FMO3	-0.539	7.964E-02	2.854E-03
FAM25C	-0.539	4.223E-01	4.315E-02
CHCHD10	-0.539	8.957E-04	3.240E-02
SLFN12L	-0.539	3.252E-02	2.882E-03
ZNF385A	-0.539	1.644E-03	2.140E-01
FYB	-0.540	5.181E-02	6.268E-03
DUSP27	-0.540	3.358E-01	1.955E-02
CST1	-0.540	1.940E-01	2.682E-02
EPGN	-0.541	2.324E-01	1.115E-03
GRIA2	-0.541	2.531E-01	1.358E-01
ID1	-0.541	1.043E-02	1.218E-01
CYP4A22	-0.542	1.722E-01	1.700E-02
CYP2C8	-0.542	9.975E-02	1.287E-02
KBTBD8	-0.542	4.412E-03	1.490E-01
C2orf82	-0.542	3.055E-03	4.640E-08
CYP3A7	-0.543	6.633E-02	1.619E-02
NPIPA8	-0.543	3.807E-01	2.325E-03
NLRP10	-0.543	2.313E-01	8.096E-04
SIT1	-0.543	5.352E-02	3.048E-02
KIT	-0.544	7.581E-02	5.765E-03
RBMY1F	-0.544	NA	8.824E-03
HOGA1	-0.544	5.550E-02	1.296E-02
PAK5	-0.544	2.891E-01	3.668E-04
SERPINB10	-0.545	3.094E-01	3.891E-01
UBASH3A	-0.545	3.884E-02	7.270E-06
SLC45A2	-0.545	1.107E-01	3.859E-03
FATE1	-0.546	3.657E-02	4.759E-02
FAM155B	-0.546	1.332E-01	2.784E-02
TPH2	-0.546	2.757E-01	1.441E-02
FGFBP2	-0.546	8.207E-02	7.279E-02
AC097372.1	-0.547	1.679E-01	4.576E-03
BIRC3	-0.547	3.536E-02	1.837E-03
LRRC19	-0.547	4.407E-02	8.457E-02
FRS2	-0.547	9.516E-04	1.602E-02
CCL4	-0.549	4.798E-02	1.254E-02
SH2D1A	-0.549	5.236E-02	4.789E-02
APOL1	-0.549	1.128E-02	1.002E-02
TSPAN19	-0.550	3.931E-01	5.974E-02
CXCR6	-0.550	2.141E-02	1.166E-02
ARG1	-0.550	1.805E-02	5.075E-03
MIR649	-0.550	NA	3.603E-02
GDA	-0.550	1.877E-01	2.436E-01

PLAC8	-0.551	7.118E-02	1.896E-01
SYT4	-0.551	5.209E-01	6.449E-03
LRRC72	-0.551	NA	1.368E-02
BTN1A1	-0.553	1.069E-01	2.028E-02
GMFG	-0.553	5.848E-03	9.449E-02
PTH	-0.554	3.839E-01	5.318E-02
C6orf163	-0.554	5.061E-04	2.537E-01
SPINK14	-0.555	3.080E-01	1.112E-02
LFNG	-0.555	4.687E-03	1.943E-02
DNAH9	-0.555	7.104E-02	1.843E-01
MS4A3	-0.555	2.985E-01	5.891E-02
TP53TG3D	-0.555	3.086E-01	8.892E-03
CHDH	-0.555	1.904E-02	4.838E-03
CXCL14	-0.556	8.842E-02	3.593E-02
MIR342	-0.556	1.912E-01	2.736E-02
SYNPR	-0.556	4.288E-01	2.963E-02
SLC25A27	-0.556	1.372E-02	6.339E-02
USP41	-0.557	1.551E-01	2.099E-02
GSDMC	-0.557	1.020E-01	1.187E-03
GRIK1	-0.557	6.574E-04	2.193E-02
PTPRN2	-0.558	1.870E-02	2.652E-02
ADGRB1	-0.559	3.638E-02	9.614E-03
INTS6L	-0.559	2.053E-04	8.637E-03
MEI1	-0.560	3.221E-02	1.987E-02
ANKRD34B	-0.560	2.044E-01	4.147E-02
TENM1	-0.560	2.086E-01	4.811E-01
PAX5	-0.560	1.101E-01	1.710E-01
CHKB-CPT1B	-0.560	1.843E-03	1.019E-03
GAST	-0.561	2.988E-01	3.770E-05
GPR151	-0.561	2.225E-01	9.239E-02
C10orf90	-0.561	1.912E-01	1.062E-02
DEFA4	-0.561	4.721E-01	9.125E-02
PAX3	-0.561	4.225E-01	1.496E-02
CACNG3	-0.561	3.983E-01	2.562E-02
MMP26	-0.562	NA	1.408E-01
MIR4686	-0.562	NA	2.860E-03
SLC4A4	-0.562	1.211E-01	2.697E-02
SOHLH2	-0.563	1.346E-01	3.668E-01
TGM3	-0.564	1.507E-01	2.558E-02
FAM205A	-0.564	3.093E-01	1.024E-01
ACOD1	-0.564	2.687E-01	1.002E-01
SYCE1	-0.564	1.060E-01	1.342E-02
LAG3	-0.564	5.274E-02	7.300E-03
FCRL4	-0.565	2.045E-01	1.220E-01
SERPINA5	-0.565	8.983E-02	8.551E-02
S100A2	-0.565	1.205E-01	6.178E-03
MOBP	-0.566	7.532E-02	4.296E-03
FCRL6	-0.566	4.224E-02	1.417E-02
LRIT3	-0.566	1.697E-02	2.845E-03
UROC1	-0.566	1.975E-01	4.441E-02

ANKRD20A1	-0.566	2.205E-01	3.896E-03
GZMK	-0.567	9.556E-02	1.078E-02
ALDH8A1	-0.568	1.552E-02	7.604E-03
CPA2	-0.568	1.380E-01	7.814E-02
LTB4R2	-0.568	1.036E-03	2.527E-01
P2RY10	-0.568	4.798E-02	1.133E-02
ZSCAN4	-0.569	1.340E-01	1.289E-04
CP	-0.570	1.975E-01	7.418E-02
MIR5695	-0.570	2.141E-01	3.571E-03
SLC30A8	-0.570	2.543E-01	2.844E-02
IL1RL1	-0.570	5.740E-02	2.692E-01
NPIPB13	-0.570	3.756E-02	3.367E-02
TCHHL1	-0.570	4.919E-01	5.842E-02
TRABD2A	-0.571	4.922E-02	3.876E-03
NAIP	-0.571	2.106E-03	9.430E-06
EFNB1	-0.571	4.113E-04	3.241E-02
MTRNR2L12	-0.571	1.127E-02	2.226E-02
HOXB8	-0.571	9.171E-02	1.436E-02
CRYGD	-0.572	5.583E-01	2.999E-03
MEFV	-0.572	5.095E-02	2.065E-02
CD38	-0.572	5.732E-02	2.543E-02
RFPL4B	-0.572	3.180E-01	1.331E-01
KRT17	-0.572	4.917E-02	1.201E-01
PROX2	-0.573	4.719E-03	1.379E-01
PLEK	-0.573	2.992E-02	6.821E-03
PNMA6E	-0.573	6.287E-01	2.772E-02
INHBE	-0.574	7.145E-03	3.315E-02
APOA5	-0.574	3.326E-01	1.096E-02
CPNE7	-0.574	4.863E-02	1.061E-01
TMPRSS4	-0.574	7.953E-02	8.277E-03
KLRD1	-0.574	2.727E-02	1.965E-01
ADGRG7	-0.575	2.760E-01	2.415E-02
C12orf50	-0.575	6.012E-02	6.782E-04
TRIM43B	-0.576	7.730E-01	4.217E-03
AFM	-0.576	4.777E-01	1.416E-01
FAM212A	-0.576	2.155E-03	6.982E-02
DYDC1	-0.576	5.004E-01	2.611E-02
CES3	-0.577	6.938E-02	1.725E-01
OGDHL	-0.577	9.202E-02	1.864E-04
LCK	-0.577	6.843E-03	2.535E-01
MIRLET7F1	-0.578	NA	9.791E-04
TMC7	-0.578	4.668E-03	2.285E-01
PRMT8	-0.579	1.464E-01	3.415E-04
PTPRS	-0.579	8.385E-04	2.889E-02
SLC25A21	-0.579	1.306E-02	2.668E-01
MUC7	-0.579	NA	1.522E-02
IGSF1	-0.579	9.130E-02	1.680E-04
IL21R	-0.579	4.329E-02	2.174E-03
ESPNL	-0.581	3.952E-02	2.591E-03
FOXD4L4	-0.581	1.270E-01	5.963E-03

LRTM2	-0.581	2.305E-01	6.874E-02
VSNL1	-0.582	1.384E-01	7.044E-03
GJD2	-0.582	4.522E-01	2.198E-02
FTCD	-0.582	8.514E-02	4.117E-01
CEACAM3	-0.583	5.051E-02	5.889E-02
KRTAP5-8	-0.583	1.525E-01	1.231E-04
RBMY1A1	-0.583	NA	1.496E-02
TG	-0.583	4.344E-02	1.717E-01
MIR10B	-0.584	1.380E-01	3.864E-01
GLP1R	-0.584	1.536E-01	4.226E-04
TNNI2	-0.585	1.001E-01	3.559E-03
TRIM58	-0.585	1.046E-01	8.841E-02
ZNF679	-0.585	6.109E-01	2.747E-01
MIR642A	-0.585	2.407E-01	1.220E-01
LHX8	-0.586	2.508E-01	1.661E-02
TMEM171	-0.586	3.079E-02	5.737E-02
CCL15	-0.587	2.617E-01	2.487E-01
HS3ST1	-0.587	2.448E-03	1.633E-02
PKHD1L1	-0.587	5.759E-02	6.287E-01
TFPI2	-0.587	1.044E-01	1.406E-01
PRSS37	-0.588	1.980E-01	5.230E-02
PDE4C	-0.588	2.724E-02	5.377E-01
TREML4	-0.588	1.764E-01	3.470E-02
LCE2A	-0.588	7.046E-01	3.338E-03
RBMY1E	-0.589	NA	1.203E-02
CYP4F11	-0.589	7.499E-02	8.578E-03
JAKMIP1	-0.589	3.884E-02	5.295E-03
RBP4	-0.590	1.040E-01	2.701E-04
C2orf88	-0.590	4.273E-03	9.299E-03
MAGEA4	-0.590	4.435E-01	3.800E-07
SAMD3	-0.590	1.375E-02	3.639E-02
LRRTM1	-0.591	2.921E-01	3.863E-02
AQP3	-0.591	5.999E-02	4.196E-02
SERPINA12	-0.591	5.096E-01	3.698E-03
CA9	-0.592	1.060E-01	2.371E-03
SLA2	-0.593	1.596E-02	6.418E-02
ACOXL	-0.593	8.052E-02	4.481E-01
SLC22A6	-0.595	5.526E-01	1.826E-02
SLC26A7	-0.596	3.130E-02	6.180E-04
NR0B2	-0.596	7.483E-02	6.704E-03
RUNDC3A	-0.597	2.361E-02	2.041E-04
ALDOB	-0.597	1.261E-01	9.929E-04
SLC22A10	-0.598	2.558E-01	3.912E-01
BAAT	-0.598	1.272E-01	2.039E-03
SERPINA4	-0.598	4.137E-01	7.695E-02
UNC5CL	-0.599	3.613E-03	2.710E-02
IQCA1	-0.599	4.994E-02	1.820E-05
ABCC8	-0.599	1.873E-01	1.827E-01
SLC39A5	-0.600	1.184E-02	5.383E-02
ATP7B	-0.600	2.078E-03	6.156E-02

OR2AG2	-0.600	1.530E-01	2.952E-02
HMSD	-0.601	7.872E-02	3.158E-04
CLDN24	-0.601	3.189E-01	2.038E-02
PLXNB3	-0.601	5.603E-03	7.575E-02
VPREB3	-0.601	4.756E-02	6.646E-02
TRIM55	-0.603	7.945E-02	9.639E-02
MIR6730	-0.603	4.469E-02	5.168E-03
GFI1	-0.605	8.036E-03	1.423E-01
PDE6B	-0.607	1.129E-02	5.532E-02
ASIC2	-0.607	7.104E-02	1.713E-02
SPRR1B	-0.607	2.114E-01	3.520E-03
FOXD4L3	-0.608	1.225E-01	4.975E-03
SLC26A3	-0.608	4.852E-02	2.027E-01
CYP2C9	-0.608	2.191E-01	2.942E-02
DCAF12L1	-0.608	4.362E-01	5.512E-03
CHRM1	-0.609	2.101E-01	1.689E-02
PLCH2	-0.609	2.354E-02	4.081E-02
DMRTA2	-0.609	1.283E-01	1.095E-02
TM4SF19	-0.609	1.041E-01	2.867E-03
TEX47	-0.609	4.632E-01	1.477E-04
TBX21	-0.610	2.305E-02	2.124E-01
BPIFB4	-0.610	2.288E-01	1.165E-01
TEX43	-0.610	2.565E-01	5.022E-02
CRCT1	-0.610	2.167E-01	7.340E-02
OR14I1	-0.611	NA	1.596E-01
FABP2	-0.611	1.452E-01	3.470E-05
MUC3A	-0.612	9.005E-02	2.714E-02
HTR4	-0.612	9.456E-02	3.681E-03
SMIM1	-0.613	6.005E-03	1.599E-01
HBM	-0.613	3.904E-01	3.527E-01
GSX2	-0.613	4.308E-01	5.211E-03
RASD1	-0.614	8.919E-03	1.831E-02
SPZ1	-0.614	4.635E-01	2.774E-03
RPL10L	-0.614	2.293E-01	3.620E-05
AC011604.2	-0.614	5.032E-01	1.194E-02
ZPLD1	-0.615	1.132E-01	2.442E-02
PRR26	-0.615	1.298E-02	5.630E-06
IZUMO1R	-0.616	2.715E-01	1.938E-03
GDNF	-0.616	3.039E-02	2.585E-03
BSND	-0.616	2.332E-01	2.272E-03
PPP3R2	-0.617	3.805E-01	2.200E-05
CXorf67	-0.617	3.623E-01	1.387E-01
SEC14L3	-0.617	1.748E-01	2.611E-02
OR2H1	-0.618	3.339E-01	5.226E-03
ASB15	-0.618	1.822E-01	4.078E-02
OR2T8	-0.618	5.737E-01	4.554E-03
RGS13	-0.619	3.413E-02	3.335E-03
RIMBP2	-0.621	6.453E-02	1.611E-04
MIR8089	-0.621	5.175E-01	6.193E-02
PIGZ	-0.622	1.792E-03	2.089E-02

CD3D	-0.623	1.533E-02	4.390E-05
LHFPL3	-0.623	3.005E-01	2.676E-03
FAM19A1	-0.623	6.634E-02	4.038E-01
SULT4A1	-0.623	2.551E-01	1.511E-02
TOX3	-0.624	2.158E-01	2.854E-02
TRAT1	-0.624	5.040E-02	5.450E-02
PRKCG	-0.624	1.560E-01	3.538E-03
HBA2	-0.624	1.793E-02	8.186E-02
DAZL	-0.625	1.422E-01	1.380E-02
OR2B2	-0.625	3.647E-01	3.658E-03
TREML2	-0.626	2.992E-02	9.048E-03
CR2	-0.626	2.121E-01	1.627E-01
PRKCB	-0.626	1.066E-02	8.594E-03
IYD	-0.627	1.119E-01	3.774E-01
AC005324.4	-0.627	1.089E-01	1.378E-02
HOXB5	-0.627	1.849E-02	2.591E-02
CDH12	-0.628	3.418E-01	1.361E-03
FGF8	-0.628	4.962E-02	3.113E-03
RHOH	-0.628	1.634E-02	2.739E-03
FOXA2	-0.629	3.317E-01	1.585E-04
CNMD	-0.630	2.109E-01	4.857E-02
BRINP3	-0.630	2.569E-01	9.425E-04
SLC28A2	-0.630	5.983E-02	2.073E-03
KIR3DL3	-0.631	4.956E-01	3.145E-01
CACNA1E	-0.631	5.630E-02	6.227E-01
KCNU1	-0.632	4.130E-01	2.285E-01
OPALIN	-0.633	3.644E-01	1.942E-01
CCDC175	-0.634	4.312E-01	3.378E-03
NEUROG3	-0.634	2.296E-01	4.312E-03
CCDC85A	-0.635	3.478E-02	2.477E-01
FAM83F	-0.636	5.235E-04	4.900E-03
ANKRD30BL	-0.636	3.757E-01	1.623E-02
KCNJ4	-0.636	1.980E-01	2.145E-04
AC010255.3	-0.636	1.342E-01	1.997E-02
JPH4	-0.637	8.128E-03	1.594E-01
GABRA1	-0.638	3.502E-01	8.251E-02
AXDND1	-0.638	8.949E-02	3.206E-01
NRN1	-0.639	1.751E-02	7.786E-03
FGF19	-0.639	3.469E-01	9.951E-02
FOLH1	-0.642	3.887E-04	1.737E-02
43901.000	-0.642	3.960E-01	3.690E-04
ACTBL2	-0.643	1.984E-01	1.880E-01
KRTAP4-6	-0.643	NA	1.674E-02
KCNQ5	-0.644	1.215E-01	1.924E-03
CHST2	-0.644	5.848E-03	2.888E-02
SPERT	-0.644	9.859E-03	3.262E-02
AC007998.2	-0.644	7.868E-03	2.864E-02
CYP11B1	-0.644	4.765E-01	1.359E-01
AKR1C1	-0.644	6.043E-02	7.794E-02
LGALS9C	-0.644	6.786E-02	3.637E-03

OTOG	-0.644	2.509E-01	7.286E-04
CYP2C19	-0.645	4.234E-01	2.200E-04
JAKMIP2	-0.646	1.694E-02	2.456E-03
CCR9	-0.646	3.518E-02	1.117E-02
LYZL1	-0.646	NA	2.583E-02
SERPINB13	-0.646	2.368E-01	7.466E-03
FGG	-0.646	3.918E-01	6.659E-02
IRF8	-0.647	1.930E-02	4.104E-03
FER1L6	-0.647	1.530E-01	1.082E-02
CD7	-0.648	9.640E-03	4.112E-04
GAL3ST2	-0.648	7.661E-02	1.460E-01
GZMB	-0.648	4.109E-02	2.254E-03
NCF1	-0.649	1.880E-02	1.211E-01
PLCG2	-0.649	1.550E-05	6.340E-04
CYP2A7	-0.649	3.139E-01	2.453E-01
SLC17A8	-0.650	2.550E-01	1.478E-02
ERC2	-0.650	2.342E-02	1.210E-08
GZMM	-0.650	1.342E-02	3.933E-03
OSTN	-0.651	2.444E-01	8.306E-03
FCAMR	-0.651	1.679E-01	5.013E-03
AC068775.2	-0.651	NA	7.431E-04
NEUROG2	-0.651	2.396E-01	3.513E-04
COL26A1	-0.651	8.600E-02	2.284E-02
HP	-0.652	1.933E-01	7.349E-04
CYP4F12	-0.652	5.802E-02	6.252E-03
CXCL10	-0.653	7.239E-02	9.297E-03
TRIM40	-0.653	2.401E-01	1.358E-02
ASIC5	-0.655	2.483E-01	1.136E-02
NPY2R	-0.655	5.607E-01	3.898E-03
ZNF560	-0.657	3.465E-01	1.560E-01
ABCA6	-0.657	3.166E-02	7.760E-03
PAQR9	-0.657	1.132E-01	3.802E-02
NDUFA4L2	-0.658	2.517E-02	2.151E-01
HYAL4	-0.659	4.863E-02	5.893E-02
TRPM1	-0.661	5.073E-02	6.622E-02
ALDH1L1	-0.661	3.989E-02	1.774E-01
SLC4A1	-0.662	3.574E-02	4.536E-03
KRT6A	-0.662	2.253E-01	3.993E-02
C11orf16	-0.662	5.790E-03	5.703E-04
C16orf92	-0.663	3.252E-01	5.545E-02
SNAI3	-0.663	8.865E-04	2.398E-03
EVX2	-0.663	1.368E-01	9.192E-02
MAB21L1	-0.663	3.656E-02	8.374E-03
TPH1	-0.663	6.818E-04	1.253E-01
ADAM23	-0.663	2.319E-02	1.726E-02
AICDA	-0.664	1.389E-01	8.430E-05
PNLIPRP1	-0.664	2.607E-01	1.889E-03
GNRH2	-0.664	3.614E-03	1.039E-02
KCTD14	-0.665	6.624E-03	1.265E-01
IL22	-0.665	3.671E-01	4.750E-05

GYPA	-0.665	4.222E-01	3.004E-02
SLC35G3	-0.665	1.285E-01	1.636E-01
VNN3	-0.666	7.082E-02	4.515E-03
SLC13A2	-0.666	1.973E-01	2.074E-03
ANKRD33	-0.667	1.663E-01	7.496E-04
HOXB6	-0.668	9.424E-03	5.449E-04
PAGE2B	-0.668	1.991E-01	2.466E-03
CCDC129	-0.668	1.816E-01	2.045E-03
CACNG2	-0.668	2.288E-01	4.540E-05
POTEC	-0.668	4.525E-01	1.878E-03
NNAT	-0.668	3.983E-02	3.257E-04
PRDM9	-0.669	1.866E-01	1.776E-02
LRAT	-0.669	1.552E-01	1.137E-04
ST8SIA5	-0.669	5.335E-03	2.913E-04
FGF23	-0.670	2.961E-01	4.375E-03
TMEM235	-0.670	1.970E-01	1.074E-04
MIR4520-1	-0.670	8.578E-03	2.221E-01
NELL2	-0.670	3.406E-02	2.107E-02
KIF5A	-0.670	2.433E-02	7.821E-04
CGA	-0.670	1.986E-01	3.304E-03
CXCL13	-0.671	6.171E-02	1.270E-02
SERPINB4	-0.672	2.349E-01	1.534E-03
AC004080.3	-0.673	2.898E-01	6.231E-03
FAM170A	-0.673	1.202E-01	3.594E-01
CTNNA2	-0.674	1.796E-01	3.205E-03
CLEC17A	-0.674	7.375E-02	2.228E-03
AQP5	-0.675	1.219E-01	1.212E-03
PLPPR1	-0.675	1.768E-01	9.479E-04
CST9	-0.675	3.489E-01	1.951E-02
NPIPB15	-0.675	4.451E-02	1.768E-04
IL25	-0.676	1.663E-01	1.715E-04
SPIC	-0.676	2.627E-01	5.844E-02
NEFM	-0.677	1.899E-01	1.496E-01
TKTL1	-0.678	1.745E-01	5.399E-02
OR2L2	-0.678	6.387E-01	3.773E-04
LAIR2	-0.678	1.930E-02	5.753E-03
GKN2	-0.680	3.548E-01	1.322E-01
AADACL3	-0.681	5.920E-01	1.950E-05
PHOX2A	-0.681	3.202E-01	1.843E-01
PCDHGC5	-0.681	1.479E-02	2.224E-03
PHACTR3	-0.681	2.758E-02	1.972E-02
LCE3A	-0.681	4.279E-01	6.733E-01
ZNF648	-0.683	1.388E-01	2.312E-01
PNCK	-0.684	6.905E-02	4.971E-02
RTN1	-0.684	5.345E-03	3.751E-04
HNF4G	-0.685	3.230E-02	4.973E-02
GLYATL1	-0.685	8.844E-02	2.021E-04
GLRA4	-0.685	1.011E-01	2.123E-02
NPAP1	-0.686	2.735E-01	5.423E-03
ADGRG5	-0.687	9.981E-03	1.952E-04

OR9K2	-0.688	3.398E-01	1.820E-05
MIR141	-0.688	1.883E-01	4.154E-02
SPATA31A6	-0.689	NA	4.204E-01
SLC10A6	-0.690	2.551E-02	5.100E-01
TAAR1	-0.690	4.168E-01	5.175E-03
CTRB1	-0.690	2.643E-01	3.634E-02
SCML4	-0.690	1.351E-02	3.899E-01
HOXB3	-0.691	1.957E-03	1.023E-03
FBLL1	-0.691	4.560E-02	1.422E-02
ALX1	-0.691	2.085E-01	6.038E-02
PRSS41	-0.692	2.292E-01	2.742E-02
PLEKHH1	-0.692	4.550E-04	6.606E-03
DBH	-0.692	5.034E-03	4.851E-02
TSSK1B	-0.692	2.140E-01	9.479E-04
IL22RA2	-0.693	6.036E-02	1.022E-02
CPA1	-0.693	1.117E-01	1.832E-01
GZMA	-0.693	1.916E-02	2.724E-03
CD3G	-0.693	2.798E-03	2.497E-01
HCN4	-0.694	1.075E-01	7.941E-03
HSFY2	-0.694	NA	9.761E-04
SH2D5	-0.694	5.059E-02	7.154E-02
HBA1	-0.694	2.632E-02	1.011E-04
MRLN	-0.694	1.699E-01	2.258E-02
FAM228A	-0.695	9.403E-03	6.504E-04
FFAR1	-0.695	1.714E-01	2.107E-02
MIR6775	-0.696	1.019E-01	1.269E-02
MMP20	-0.697	3.384E-01	1.325E-03
HPCAL4	-0.697	3.578E-02	5.792E-04
TDRD9	-0.697	5.297E-02	9.236E-03
SMIM10L2A	-0.697	3.614E-03	3.584E-01
NLRP13	-0.698	3.347E-01	5.617E-02
FAM237A	-0.698	NA	5.949E-02
LRIT2	-0.698	3.642E-01	4.010E-04
SPATA8	-0.699	2.636E-01	1.438E-04
PLCD3	-0.699	3.442E-04	3.272E-01
SLC7A11	-0.699	1.954E-02	6.483E-04
NRG2	-0.699	2.321E-02	7.254E-03
CLCA2	-0.699	1.019E-01	1.541E-02
ZNF432	-0.701	4.778E-04	9.127E-02
SERPINA3	-0.701	1.138E-01	1.113E-03
PDCD1	-0.704	1.493E-02	7.423E-02
DUSP2	-0.704	5.421E-03	3.530E-04
IL33	-0.705	2.551E-02	1.287E-01
IL13RA2	-0.706	2.405E-02	2.253E-01
SPRR2D	-0.707	1.647E-01	4.863E-02
PAPLN	-0.707	8.500E-05	1.635E-02
MYOM3	-0.707	5.855E-03	2.451E-01
COLGALT2	-0.708	5.569E-03	3.043E-02
SIRPG	-0.709	5.519E-03	4.335E-02
ARHGAP6	-0.709	6.005E-03	7.734E-03

ASTL	-0.709	2.050E-02	3.218E-02
C10orf142	-0.709	1.680E-01	3.915E-02
GOLGA6L2	-0.710	2.703E-01	9.070E-05
TBX1	-0.710	2.129E-02	1.267E-03
ACADL	-0.711	4.285E-02	1.341E-02
TRNP1	-0.711	3.894E-03	8.142E-02
ADAM28	-0.712	3.613E-03	1.756E-02
KLK11	-0.712	1.576E-01	4.570E-03
CCKBR	-0.712	2.467E-01	1.743E-02
SLC13A1	-0.713	NA	2.609E-01
MIR200B	-0.713	4.642E-02	7.002E-02
RHOXF2	-0.713	5.732E-01	1.339E-02
SPATA31E1	-0.715	2.288E-01	7.590E-05
LCE1E	-0.716	1.831E-01	3.679E-04
GDPD2	-0.716	5.612E-02	8.570E-02
GIPR	-0.717	9.378E-04	9.600E-06
KCNC2	-0.718	5.342E-01	3.443E-03
AQP12B	-0.720	2.930E-01	5.607E-02
TEX13B	-0.720	2.095E-01	4.467E-03
CXCL9	-0.720	4.745E-02	6.246E-03
TEX37	-0.720	3.147E-01	1.172E-03
BMPR1B	-0.721	4.155E-02	1.053E-02
HORMAD2	-0.723	1.973E-01	5.810E-06
TSPAN7	-0.723	1.306E-02	1.518E-01
AL589666.1	-0.724	9.109E-02	5.029E-03
TNFSF18	-0.724	1.822E-02	1.788E-03
TRIM31	-0.725	6.727E-02	6.890E-05
LGALS9B	-0.725	1.107E-01	1.916E-02
PROC	-0.726	2.461E-03	4.119E-03
LCE5A	-0.726	2.263E-01	1.984E-02
CCDC141	-0.727	2.943E-03	1.001E-01
MIA2	-0.728	1.929E-01	6.434E-02
KLRC1	-0.729	8.605E-03	4.801E-03
BNC1	-0.730	2.334E-01	1.757E-02
TCL1A	-0.730	9.425E-02	3.003E-03
SERPINA9	-0.731	2.195E-01	1.580E-02
ESM1	-0.732	9.887E-04	2.431E-01
OR5B12	-0.732	NA	2.573E-02
UPK3BL1	-0.732	1.289E-01	3.042E-01
CDC42EP5	-0.733	8.882E-04	3.289E-04
IL2	-0.734	1.398E-01	1.495E-02
KCNB2	-0.736	1.744E-01	1.550E-05
FRMPD4	-0.737	1.233E-01	5.595E-02
MRGPRX3	-0.737	2.301E-01	6.640E-06
TUBA3E	-0.738	3.021E-01	4.073E-02
CRYGC	-0.739	4.907E-01	2.920E-03
DCT	-0.739	9.005E-02	4.482E-04
ABCC2	-0.739	1.358E-03	4.542E-03
FGB	-0.740	3.375E-01	2.501E-04
GAGE12J	-0.741	6.958E-01	4.475E-03

KRT2	-0.741	3.041E-02	7.681E-03
AL132639.3	-0.741	4.388E-01	1.211E-03
MYO16	-0.741	1.698E-02	4.595E-04
SLC10A4	-0.741	2.042E-02	1.525E-01
SLC5A1	-0.742	1.428E-01	7.513E-03
MIR483	-0.742	4.147E-01	1.047E-01
PVALB	-0.744	1.100E-01	3.014E-02
KLHL32	-0.744	3.181E-03	9.958E-02
PIGR	-0.744	9.168E-02	2.374E-03
TBC1D3D	-0.744	3.714E-01	6.282E-03
AC018554.3	-0.744	6.219E-01	6.400E-03
LY6G6E	-0.746	3.558E-01	1.070E-03
CNTN6	-0.746	5.526E-02	5.984E-03
FASLG	-0.747	1.252E-02	1.069E-01
MST1R	-0.747	1.249E-03	3.338E-02
EPS8L3	-0.747	1.040E-01	6.055E-02
PRSS12	-0.748	1.489E-02	2.111E-02
DAZ2	-0.748	NA	3.515E-02
MAG	-0.748	6.409E-02	1.293E-01
OR2A14	-0.750	1.023E-01	2.630E-07
LY6L	-0.750	4.993E-01	9.940E-06
ALAS2	-0.750	9.218E-03	3.436E-02
MIR429	-0.750	9.218E-03	6.983E-03
SPRR2G	-0.750	3.276E-01	5.813E-02
MOGAT3	-0.751	7.368E-02	7.472E-03
SMAD6	-0.751	1.382E-04	1.897E-01
CCDC188	-0.753	1.156E-03	1.589E-03
SPRR2E	-0.753	2.772E-01	3.063E-02
OR9Q1	-0.754	3.334E-01	1.095E-03
HAPLN1	-0.755	1.387E-02	7.627E-02
LYZ	-0.757	8.108E-03	7.360E-05
PYHIN1	-0.759	3.472E-03	1.383E-01
HBD	-0.760	7.336E-02	5.018E-04
NTRK2	-0.760	1.945E-02	1.730E-02
CDH7	-0.760	1.823E-01	2.745E-02
PSMA8	-0.760	1.458E-01	3.284E-03
FABP6	-0.761	1.592E-02	1.017E-02
MCHR2	-0.761	2.059E-01	1.117E-02
OTOP3	-0.762	2.508E-01	3.905E-02
PIK3C2G	-0.763	1.532E-01	2.997E-02
ZIC4	-0.764	2.730E-01	1.021E-03
ARHGEF38	-0.764	3.076E-03	6.720E-06
IRS4	-0.765	1.336E-01	2.864E-02
SLC38A5	-0.766	4.921E-03	2.988E-01
KRTAP7-1	-0.766	6.003E-01	1.691E-04
PDE10A	-0.766	3.615E-03	1.800E-02
CER1	-0.766	2.203E-01	4.079E-01
PRKAG3	-0.766	5.066E-02	9.728E-03
MIOX	-0.767	1.260E-02	2.531E-02
CDH8	-0.768	6.859E-03	2.731E-03

DUOX2	-0.769	2.014E-02	4.002E-02
OR2C3	-0.769	2.935E-01	4.252E-02
KRT82	-0.770	1.185E-01	3.091E-02
NKG7	-0.771	5.335E-03	3.418E-03
SSTR1	-0.771	1.926E-02	1.016E-02
ST3GAL5	-0.771	4.993E-04	8.276E-04
TSPY3	-0.772	NA	4.806E-04
DSG3	-0.773	1.725E-01	5.545E-03
ANKS4B	-0.776	5.426E-02	6.949E-03
ALDH1A1	-0.777	1.503E-02	3.942E-02
LEAP2	-0.777	2.156E-03	4.450E-02
ADGRA1	-0.779	2.246E-01	3.769E-03
GABRA4	-0.780	2.726E-01	1.082E-03
PRDM7	-0.780	4.217E-02	7.644E-04
MDGA2	-0.781	2.226E-01	1.783E-04
MIR200C	-0.781	1.417E-01	6.016E-02
CHRND	-0.781	1.680E-01	1.920E-05
TBATA	-0.782	8.165E-02	2.821E-02
AVPR1B	-0.782	6.577E-02	3.229E-02
TCN1	-0.782	9.706E-02	5.459E-02
FSIP2	-0.783	4.292E-03	6.753E-03
UBD	-0.783	3.611E-02	1.840E-03
ACY3	-0.783	2.191E-03	1.320E-05
PLA2G12B	-0.784	1.757E-01	5.524E-02
NLRP11	-0.785	5.305E-02	1.528E-03
TSPEAR	-0.786	6.885E-02	2.411E-01
NELL1	-0.786	8.521E-02	2.380E-02
KCNK9	-0.786	2.208E-02	2.187E-03
CD8A	-0.787	2.722E-03	3.901E-03
AL583836.1	-0.788	3.504E-01	2.880E-05
MAGEB6	-0.789	3.152E-01	7.260E-04
COL9A1	-0.789	4.700E-02	5.112E-03
MIR6071	-0.791	2.313E-01	5.625E-04
BAALC	-0.791	1.298E-02	3.344E-03
CA12	-0.791	1.369E-03	4.544E-04
KLK13	-0.793	1.132E-01	1.132E-03
KCNH4	-0.793	3.254E-03	1.440E-02
C1orf186	-0.793	2.791E-02	9.262E-03
CD8B	-0.794	3.340E-03	1.277E-02
ZIC1	-0.796	2.781E-01	6.014E-03
PLPPR3	-0.796	2.319E-02	5.810E-03
KLRK1	-0.796	9.300E-05	5.438E-02
PF4	-0.796	3.638E-02	1.368E-04
RIT2	-0.797	3.438E-01	3.020E-06
KIR2DL3	-0.798	5.352E-02	1.290E-01
DPP10	-0.799	2.027E-01	6.988E-02
APCDD1	-0.799	3.770E-05	1.835E-03
FABP5	-0.799	1.033E-03	1.803E-01
IL21	-0.801	2.044E-01	1.217E-04
THEMIS	-0.803	3.891E-03	4.530E-04

PPM1N	-0.805	2.678E-03	1.135E-01
MOGAT2	-0.806	8.461E-02	1.136E-04
EOMES	-0.806	2.802E-03	4.018E-03
MIR200A	-0.809	1.655E-02	2.253E-02
BTNL8	-0.813	4.794E-03	2.812E-02
SGCZ	-0.813	1.708E-01	6.210E-06
TYR	-0.814	2.781E-01	5.926E-02
NPY	-0.816	3.182E-01	4.751E-03
DGKK	-0.816	1.924E-01	3.138E-02
CLEC3A	-0.816	1.344E-01	1.058E-02
AC024940.1	-0.817	2.981E-03	3.766E-02
CCDC187	-0.817	1.890E-02	1.986E-02
GNLY	-0.818	9.329E-03	1.694E-01
AFF2	-0.819	1.355E-02	4.247E-02
LINC00371	-0.819	2.162E-01	5.251E-03
HOXB9	-0.820	8.120E-03	4.680E-05
CLC	-0.821	7.379E-02	5.012E-02
SLITRK1	-0.823	2.444E-01	5.167E-04
CHGB	-0.823	1.558E-02	3.550E-04
FOXF2	-0.826	9.300E-05	4.925E-02
SLC30A10	-0.827	5.033E-02	1.122E-03
APBB1IP	-0.827	1.214E-03	9.132E-04
SPINK6	-0.827	1.254E-01	1.410E-03
TF	-0.827	1.342E-02	3.517E-04
HBQ1	-0.827	3.425E-02	2.663E-02
KRTDAP	-0.830	1.258E-01	4.521E-02
RBFOX1	-0.831	1.219E-01	3.510E-01
OLIG2	-0.832	1.065E-01	4.560E-06
ELAVL3	-0.834	2.941E-02	1.290E-01
SPDYE2B	-0.834	3.079E-02	7.826E-03
SLCO6A1	-0.835	5.610E-02	3.613E-04
HS6ST3	-0.835	7.780E-02	1.533E-02
GATA4	-0.835	1.504E-01	3.480E-02
TMPRSS11F	-0.836	1.844E-01	5.819E-04
WNT5A	-0.836	6.793E-04	8.639E-03
GBX1	-0.837	1.214E-01	2.781E-03
AGTR2	-0.837	2.689E-01	5.813E-02
GZMH	-0.840	5.745E-03	4.698E-03
TTC24	-0.841	8.128E-03	3.340E-05
FOXH1	-0.843	3.340E-03	2.824E-04
CALHM3	-0.843	2.750E-02	3.263E-02
UTS2	-0.844	8.767E-03	2.310E-03
PRF1	-0.844	1.979E-03	5.451E-04
MIR148B	-0.844	1.663E-01	2.203E-04
ABCA10	-0.845	2.277E-03	5.430E-05
CCL15-CCL1[illegible]	-0.846	1.628E-01	1.194E-03
APOA4	-0.847	5.444E-01	1.861E-03
MIR708	-0.848	1.328E-01	4.281E-01
C12orf40	-0.848	1.816E-01	7.442E-03
NEFH	-0.849	1.104E-03	1.070E-01

ALOX15B	-0.849	2.826E-03	5.130E-06
AGR3	-0.850	5.280E-02	1.423E-03
SOX15	-0.852	3.831E-03	1.094E-02
R3HDML	-0.852	4.744E-01	3.513E-02
CXCL11	-0.853	2.481E-02	4.773E-03
AC110814.1	-0.853	7.384E-03	5.520E-05
ANKRD20A2	-0.855	1.986E-01	3.653E-02
MCF2	-0.856	6.842E-04	5.528E-04
BEST3	-0.857	4.603E-02	4.053E-04
TUBA3C	-0.859	3.803E-01	1.181E-02
VNN2	-0.862	8.885E-04	5.019E-03
ZNF683	-0.864	6.176E-03	1.010E-02
AKAP7	-0.864	1.700E-09	4.786E-03
GPR174	-0.866	7.710E-03	9.850E-06
UGT2B15	-0.867	7.551E-02	9.323E-03
BEND4	-0.867	6.176E-03	1.321E-03
MT3	-0.870	1.129E-02	3.732E-03
ISX	-0.871	1.810E-01	1.411E-02
GPR6	-0.872	3.043E-01	8.133E-03
TRIM49B	-0.873	6.181E-01	4.479E-04
GUCY2D	-0.873	5.060E-04	2.450E-03
PKLR	-0.876	1.203E-02	1.017E-02
ZIC3	-0.879	4.835E-01	6.891E-02
NPIPB11	-0.880	2.053E-04	1.097E-04
NKX2-5	-0.880	1.398E-01	1.358E-04
GRM4	-0.881	6.130E-03	1.750E-01
T	-0.881	2.141E-01	1.337E-03
DCAF8L1	-0.882	4.408E-01	7.049E-04
UGT2A1	-0.882	9.748E-02	2.131E-03
PPP4R4	-0.883	3.417E-02	2.785E-03
SOHLH1	-0.883	6.335E-02	2.497E-03
INSL6	-0.884	7.678E-02	3.030E-03
HBB	-0.884	1.156E-03	9.808E-04
CA8	-0.885	9.522E-03	8.997E-03
UGT3A1	-0.889	2.574E-01	4.270E-05
CBSL	-0.890	2.178E-02	4.673E-02
TBX15	-0.890	1.251E-04	1.384E-04
HNF4A	-0.890	1.286E-02	7.887E-02
SPATA19	-0.891	2.552E-01	3.218E-01
IL9R	-0.892	8.427E-04	1.792E-02
PSG1	-0.892	2.890E-01	1.407E-01
C9orf57	-0.893	1.278E-01	7.385E-03
GYS2	-0.895	4.530E-04	6.270E-05
AFP	-0.896	1.118E-01	1.912E-04
TCP11	-0.897	1.275E-02	3.231E-03
MIR572	-0.899	NA	5.190E-05
TGM6	-0.899	2.956E-01	1.033E-04
LAMB4	-0.899	1.251E-04	2.930E-06
KRT14	-0.901	7.620E-02	2.470E-05
C1QTNF12	-0.902	6.120E-05	1.905E-01

AMY2B	-0.903	4.580E-05	1.596E-02
MPV17L	-0.903	4.805E-04	1.005E-02
MIR6784	-0.904	5.909E-03	5.495E-03
NXF3	-0.905	9.116E-04	1.165E-02
PAX8	-0.909	6.574E-04	2.084E-02
SLITRK6	-0.910	2.866E-03	2.510E-03
SERPINA10	-0.910	5.632E-02	5.733E-04
GCNT3	-0.911	6.967E-03	2.735E-04
MGAM2	-0.912	9.894E-02	1.099E-02
AMELX	-0.912	3.493E-01	6.945E-02
SLC22A31	-0.912	1.068E-02	1.351E-03
C3orf30	-0.913	3.638E-02	3.440E-03
RXFP1	-0.914	2.934E-04	7.931E-03
SATL1	-0.915	5.288E-02	6.305E-02
CHAT	-0.916	2.570E-01	2.287E-04
DPPA2	-0.916	3.959E-01	8.282E-03
GOLGA7B	-0.916	4.983E-04	5.765E-02
MIR581	-0.919	8.674E-02	1.015E-04
KRT1	-0.919	8.461E-02	3.515E-03
NKAIN2	-0.921	1.541E-02	1.634E-02
RFX4	-0.921	6.455E-02	6.919E-03
MAGEA9	-0.922	3.240E-01	2.960E-02
MIR6774	-0.924	2.127E-01	1.670E-02
REG1B	-0.925	NA	2.166E-04
AC015813.2	-0.926	1.140E-02	1.442E-04
KRTAP4-8	-0.927	3.918E-01	3.263E-04
C1orf61	-0.928	3.257E-03	1.656E-04
SLURP1	-0.928	7.207E-03	4.490E-07
ATOH8	-0.930	1.618E-03	1.314E-04
GALNT13	-0.930	1.967E-03	2.404E-03
TCL1B	-0.932	3.284E-01	3.690E-04
NUTM2F	-0.934	5.562E-03	2.634E-02
MMP10	-0.936	2.098E-02	2.339E-04
DEFA3	-0.937	1.516E-01	4.081E-04
DOK7	-0.938	3.900E-05	1.881E-03
PLA2G4D	-0.938	1.073E-03	4.054E-01
OBP2B	-0.939	7.048E-02	3.988E-03
CYP4Z1	-0.939	3.613E-03	4.059E-02
TNIP3	-0.940	3.613E-03	6.000E-03
REN	-0.942	8.128E-03	6.170E-05
FOXC2	-0.942	1.073E-03	2.780E-05
SST	-0.943	2.203E-01	6.601E-04
RHAG	-0.945	1.806E-01	2.276E-02
STRA8	-0.946	1.190E-01	1.558E-04
FGL1	-0.946	9.374E-02	3.930E-05
TUBB4A	-0.947	2.461E-03	7.539E-03
P2RX6	-0.947	4.699E-04	9.979E-03
OCA2	-0.951	3.829E-02	1.720E-05
SYT9	-0.951	1.144E-02	7.850E-05
AC110611.1	-0.954	6.066E-02	3.084E-02

KLRC4-KLRK	-0.954	8.411E-04	4.398E-02
NPHS1	-0.955	3.637E-02	5.044E-03
ZFP42	-0.957	7.811E-02	3.231E-02
ASCL4	-0.957	7.325E-02	1.394E-03
FGF3	-0.958	5.170E-01	5.509E-02
AGR2	-0.960	1.067E-03	1.566E-02
SPRR2A	-0.963	6.144E-02	1.892E-03
PLA2G10	-0.966	4.087E-04	4.280E-07
TMEM178A	-0.966	1.680E-04	3.500E-02
LGALS7	-0.967	1.258E-01	1.365E-03
BRINP2	-0.967	4.630E-02	9.026E-04
MMP1	-0.970	4.745E-03	5.502E-03
HKDC1	-0.974	5.215E-03	1.525E-04
COLEC10	-0.977	5.848E-04	4.860E-05
CLCA4	-0.979	3.341E-02	3.561E-02
GALNTL5	-0.979	1.119E-01	7.027E-04
HCN1	-0.981	1.462E-01	2.208E-02
MUCL1	-0.985	1.058E-02	3.369E-02
IFNG	-0.986	8.139E-03	7.762E-04
GIF	-0.986	8.224E-02	2.100E-05
ISM2	-0.988	3.391E-04	1.293E-03
MSGN1	-0.988	2.936E-01	5.595E-04
BPIFA2	-0.991	6.977E-02	4.472E-04
GFAP	-0.991	1.098E-04	1.864E-01
ITIH2	-0.991	7.964E-04	4.360E-05
TFAP2B	-0.992	6.577E-02	7.099E-04
GBP5	-0.992	3.125E-03	8.040E-02
MIR196A1	-0.993	2.583E-02	1.558E-02
C14orf39	-0.993	6.060E-03	8.943E-03
SP9	-0.994	8.739E-02	1.588E-03
GALNTL6	-0.997	2.440E-05	3.840E-05
MIR6075	-0.998	8.422E-02	6.407E-04
FAM153B	-1.005	8.087E-03	9.524E-04
ASCL1	-1.007	1.862E-02	1.469E-01
PACRG	-1.010	1.682E-03	3.899E-01
ADH1C	-1.010	1.298E-02	2.451E-03
AQP10	-1.010	4.236E-03	1.948E-03
LCE3D	-1.011	2.280E-01	8.995E-03
MTNR1A	-1.013	5.646E-02	2.364E-03
FABP9	-1.013	2.361E-02	7.216E-03
CDH10	-1.014	6.259E-02	1.502E-02
EPHB6	-1.014	3.740E-05	8.000E-05
ESX1	-1.017	9.728E-02	3.046E-02
ASB4	-1.017	1.469E-02	4.858E-03
IFNE	-1.017	4.243E-03	3.397E-03
AC011473.4	-1.018	1.646E-01	8.251E-03
CAPN8	-1.020	8.563E-03	9.794E-04
NKAIN3	-1.020	3.403E-02	2.744E-01
UTS2R	-1.021	3.540E-02	1.801E-03
FRMPD1	-1.022	2.897E-03	7.006E-04

MS4A12	-1.022	3.175E-01	1.886E-03
LCE1F	-1.023	1.264E-01	2.570E-08
MYBPC1	-1.031	2.764E-02	1.395E-03
CCDC190	-1.032	3.829E-02	4.647E-02
LCE1C	-1.032	7.280E-02	4.680E-08
PNPLA5	-1.033	1.705E-01	6.710E-05
KIAA1024L	-1.035	3.948E-02	3.141E-02
SPANXB1	-1.036	2.961E-01	6.448E-04
KLRC4	-1.038	5.821E-04	4.597E-02
SMR3B	-1.040	NA	2.107E-03
GRM3	-1.041	3.105E-03	1.170E-06
KIR2DL1	-1.041	1.673E-02	1.257E-01
KRTAP1-3	-1.041	3.905E-01	7.113E-03
NCAN	-1.042	2.654E-03	3.386E-04
HGD	-1.045	1.240E-04	3.761E-03
KRTAP5-10	-1.048	3.340E-03	1.367E-04
C1QTNF8	-1.051	1.835E-01	1.788E-03
ADAMTS19	-1.053	4.924E-03	1.806E-02
KIR2DL4	-1.054	7.403E-04	3.875E-02
CST4	-1.055	9.405E-03	1.254E-02
CDH17	-1.055	3.442E-04	1.522E-01
DYNAP	-1.061	1.586E-01	2.830E-04
CD177	-1.062	6.574E-04	3.268E-02
GRIK3	-1.063	4.522E-04	8.800E-03
PI3	-1.064	2.724E-02	3.792E-03
F2	-1.072	5.419E-03	1.319E-03
MAGEA11	-1.074	2.703E-02	2.751E-02
DEFA5	-1.078	NA	4.620E-06
C2orf66	-1.084	3.420E-05	3.054E-04
SPIB	-1.085	4.805E-04	2.694E-03
CDHR1	-1.085	2.376E-04	4.815E-03
HEPHL1	-1.089	4.318E-03	5.075E-04
ALPPL2	-1.090	1.504E-02	2.793E-04
SPANXD	-1.091	4.778E-01	4.040E-05
IGFBP2	-1.092	1.300E-05	1.038E-03
CYP3A5	-1.092	2.996E-04	2.221E-04
IL17REL	-1.093	1.654E-03	1.520E-06
LUZP2	-1.096	3.615E-03	7.828E-04
SPOCD1	-1.097	4.920E-05	2.916E-03
MUC5B	-1.100	5.519E-03	2.351E-01
SDR16C5	-1.103	1.128E-02	2.740E-08
KRTAP3-3	-1.104	NA	2.384E-02
PSG6	-1.104	1.817E-01	2.619E-03
KIAA1324	-1.104	1.480E-05	2.072E-03
KIR3DL2	-1.105	8.169E-03	1.161E-02
NOS2	-1.107	5.700E-05	1.120E-07
SPOCK3	-1.110	4.314E-03	3.770E-05
CFHR5	-1.114	1.954E-01	2.562E-02
NKX2-1	-1.116	9.322E-02	9.440E-07
MYRFL	-1.116	9.980E-05	3.972E-03

KCNH5	-1.118	3.179E-02	5.648E-03
ALPI	-1.120	2.760E-01	7.300E-08
CLDN10	-1.124	2.051E-03	1.321E-04
MBL2	-1.124	2.001E-01	2.320E-05
CHST9	-1.125	4.872E-02	9.084E-04
FAM9B	-1.126	5.071E-02	2.806E-01
TSGA10IP	-1.126	5.989E-04	8.275E-04
WFDC12	-1.127	5.096E-02	3.482E-03
KRT85	-1.132	1.271E-02	1.088E-04
HEMGN	-1.133	2.688E-02	1.210E-03
TRIM48	-1.134	5.126E-01	1.834E-03
OR56A3	-1.137	3.056E-01	3.410E-05
CDH16	-1.137	2.078E-02	6.061E-04
MSTN	-1.139	5.050E-05	6.009E-02
C1orf168	-1.141	1.073E-03	1.977E-04
PON3	-1.145	1.457E-03	2.196E-03
INSM1	-1.150	2.323E-03	2.404E-02
NRSN1	-1.151	5.147E-02	2.069E-03
PNLIPRP3	-1.153	1.237E-01	8.855E-04
USH1C	-1.154	9.489E-03	1.809E-03
LY6D	-1.155	2.589E-03	5.070E-01
STATH	-1.155	2.513E-01	3.227E-03
PRAC1	-1.160	9.777E-02	5.783E-03
ANKRD30A	-1.161	7.090E-02	2.920E-03
TENM2	-1.163	1.495E-02	3.490E-05
PON1	-1.164	2.771E-03	2.051E-03
HTN1	-1.168	4.148E-01	5.791E-03
IDO1	-1.170	7.964E-04	2.616E-02
SERPINA6	-1.173	1.848E-02	1.629E-02
OPRM1	-1.178	2.750E-02	1.170E-06
FLJ22763	-1.181	NA	6.987E-03
GUCA1C	-1.182	3.339E-01	6.650E-07
KRT84	-1.183	4.116E-02	2.415E-03
VWC2L	-1.184	5.519E-03	6.391E-04
SLC6A5	-1.184	1.331E-01	6.780E-06
BTBD16	-1.185	2.166E-03	1.130E-06
CPNE6	-1.188	1.116E-03	1.415E-04
SCGB1A1	-1.191	8.449E-02	8.228E-04
CLRN3	-1.193	4.325E-04	1.163E-03
AJAP1	-1.198	1.110E-06	8.410E-08
HTN3	-1.203	4.551E-01	5.640E-05
NLRP4	-1.205	2.805E-02	6.680E-02
MS4A8	-1.207	3.664E-02	6.768E-03
STEAP1B	-1.209	1.864E-04	1.684E-03
AL035425.2	-1.211	2.791E-01	1.470E-08
IFNA1	-1.213	8.034E-03	1.050E-05
LRRC10	-1.216	3.006E-01	2.210E-07
CNPY1	-1.220	7.945E-02	9.780E-06
MUC5AC	-1.223	1.066E-02	7.780E-09
MYOD1	-1.224	1.419E-01	1.980E-05

CALN1	-1.224	3.653E-03	4.120E-06
SPINK7	-1.225	2.689E-03	4.731E-04
AC005885.1	-1.228	2.097E-01	1.030E-06
PAH	-1.230	3.849E-03	1.639E-02
F13B	-1.231	1.718E-02	2.962E-03
ADH7	-1.234	3.166E-02	2.619E-04
TESC	-1.236	8.200E-05	1.068E-02
RLBP1	-1.242	3.319E-03	4.134E-03
H2AFB1	-1.242	6.280E-02	6.070E-07
HTR3C	-1.247	5.710E-03	6.570E-07
NTSR1	-1.251	1.251E-04	6.503E-04
LPA	-1.254	8.987E-04	4.305E-03
KEL	-1.258	2.390E-06	8.000E-05
PSG7	-1.259	2.191E-01	8.660E-08
PRR27	-1.262	1.262E-01	2.295E-01
TRIM49	-1.263	3.934E-01	6.870E-03
GSTA2	-1.264	1.909E-02	2.990E-04
GLYATL2	-1.265	1.618E-03	4.748E-03
VSIG1	-1.272	1.110E-06	2.670E-08
NGB	-1.276	5.768E-03	2.520E-05
ISL1	-1.278	1.957E-03	7.790E-07
SLC17A1	-1.286	1.275E-01	7.307E-04
COL2A1	-1.288	1.680E-04	2.951E-03
TNNT3	-1.293	9.116E-04	3.110E-03
LRRC38	-1.297	4.243E-03	3.850E-07
QRFPR	-1.301	2.407E-02	4.976E-03
TRIM10	-1.307	1.570E-03	2.044E-01
CLDN19	-1.321	4.493E-03	2.945E-04
DDC	-1.321	6.842E-04	3.645E-03
HSD17B2	-1.323	1.310E-05	6.104E-03
KLK10	-1.325	3.167E-03	8.090E-05
SERPINA11	-1.328	3.079E-02	8.492E-03
SLC18A3	-1.328	1.021E-01	4.184E-03
KRTAP19-5	-1.328	5.502E-01	2.086E-04
TFF2	-1.330	2.847E-03	6.127E-04
IP6K3	-1.334	5.000E-05	1.240E-07
NR5A1	-1.336	8.385E-04	1.550E-07
CLVS2	-1.336	1.067E-02	5.230E-07
MORC1	-1.339	4.852E-02	2.600E-11
RXFP2	-1.340	2.407E-01	7.110E-06
PLA2G2E	-1.342	6.399E-02	1.491E-04
HOXC12	-1.346	2.238E-02	4.280E-03
LGR5	-1.346	1.306E-04	5.665E-03
DEFB4A	-1.348	1.466E-01	5.500E-06
ERN2	-1.354	5.956E-04	1.743E-04
GSTA1	-1.355	5.041E-03	1.069E-02
HABP2	-1.358	1.397E-03	3.641E-03
KCNA4	-1.361	7.483E-02	5.166E-03
UGT2B28	-1.362	3.615E-03	2.680E-05
FRMD1	-1.371	3.691E-03	1.592E-02

UGT2B11	-1.372	3.613E-03	5.850E-06
PASD1	-1.373	9.661E-02	4.703E-03
CPLX2	-1.377	1.110E-03	9.930E-07
BARHL2	-1.385	1.132E-01	6.390E-08
KRT28	-1.392	2.584E-01	3.340E-05
SFTPB	-1.393	4.710E-05	1.918E-02
NEUROD1	-1.393	1.277E-01	2.272E-03
ONECUT3	-1.397	4.540E-05	2.510E-09
F9	-1.399	3.519E-01	5.960E-10
MT4	-1.402	3.947E-01	7.295E-02
PRSS1	-1.403	1.015E-02	3.410E-06
VRTN	-1.406	1.902E-02	2.291E-04
KCTD16	-1.409	5.160E-07	5.310E-05
NAA11	-1.426	3.363E-02	5.890E-08
CASR	-1.430	1.681E-04	4.260E-05
C6orf10	-1.439	5.569E-03	1.613E-04
KLHL1	-1.442	1.562E-01	1.473E-03
DUOXA2	-1.444	3.740E-05	1.620E-05
VWC2	-1.446	6.793E-04	2.987E-04
BRINP1	-1.448	4.960E-05	1.137E-02
MAGEB2	-1.452	1.094E-02	1.370E-05
CLCA1	-1.453	2.944E-03	4.860E-11
RXFP4	-1.460	1.010E-05	7.599E-02
CFTR	-1.461	3.700E-05	6.922E-02
CRH	-1.462	2.051E-02	6.440E-08
KHDC1L	-1.463	2.294E-03	4.650E-06
HNF1A	-1.466	3.613E-04	1.070E-06
CYP1A2	-1.477	1.753E-02	1.420E-07
BTNL3	-1.481	2.513E-03	4.214E-03
C6orf15	-1.482	1.558E-02	3.070E-08
MEP1A	-1.487	3.730E-06	2.976E-03
ANXA10	-1.506	1.976E-03	4.520E-02
IL36G	-1.509	1.243E-04	5.880E-05
KRT81	-1.510	4.080E-05	2.920E-09
TCP10L2	-1.511	1.955E-02	5.868E-04
SHH	-1.514	3.037E-04	8.930E-09
KRTAP5-11	-1.516	3.493E-03	2.455E-03
DCAF4L2	-1.524	1.757E-01	4.170E-07
TSPAN8	-1.525	3.650E-05	3.030E-06
AZGP1	-1.526	7.040E-06	2.860E-06
PLA2G3	-1.527	2.051E-03	8.140E-06
SPHKAP	-1.530	4.853E-02	7.860E-12
PIWIL1	-1.539	9.040E-05	1.010E-08
C10orf82	-1.542	2.712E-04	8.320E-06
CT45A1	-1.545	1.779E-01	9.030E-08
UGT2B10	-1.559	8.437E-02	1.715E-03
PAX7	-1.571	1.286E-02	2.577E-04
GGT2	-1.579	1.251E-04	4.480E-10
CYP1A1	-1.583	4.650E-03	8.649E-04
KLK12	-1.592	1.700E-03	1.077E-04

ATOH1	-1.597	4.561E-02	6.167E-04
AMBN	-1.614	8.665E-02	3.880E-06
TFF3	-1.618	5.470E-06	1.972E-04
PAGE2	-1.618	5.616E-02	1.042E-02
CA1	-1.621	1.557E-04	1.129E-03
SLC6A15	-1.633	4.954E-03	1.196E-03
OLFM3	-1.638	1.931E-02	2.026E-04
CHP2	-1.639	1.140E-05	1.445E-02
ROS1	-1.645	4.460E-05	2.640E-07
FOXN4	-1.661	7.827E-04	1.548E-01
NOBOX	-1.666	5.147E-02	1.200E-07
KRT75	-1.668	1.618E-03	1.106E-04
FDCSP	-1.671	5.848E-04	6.360E-07
MSMB	-1.695	1.050E-05	1.170E-06
OTX2	-1.698	2.536E-02	4.389E-04
LCN15	-1.725	1.708E-03	1.830E-08
DMBT1	-1.734	7.590E-06	3.990E-10
SULT1C2	-1.747	6.910E-11	1.440E-08
LIN28A	-1.748	1.092E-04	1.552E-03
PHGR1	-1.749	3.529E-03	5.354E-04
VIL1	-1.757	4.480E-06	9.369E-04
AQP4	-1.759	5.380E-03	3.273E-04
LINC01207	-1.772	7.800E-05	4.970E-05
RALYL	-1.809	2.277E-03	1.793E-01
SERPINB11	-1.814	4.550E-04	2.532E-02
TINAG	-1.827	2.939E-02	1.453E-03
SPINK4	-1.832	1.080E-05	4.690E-06
PRSS2	-1.835	2.487E-04	3.179E-02
TMPRSS11D	-1.846	9.980E-05	1.981E-04
CA10	-1.855	9.188E-04	5.460E-07
FGF20	-1.858	2.872E-04	1.160E-16
TRIM15	-1.862	4.160E-06	1.590E-05
MPPED1	-1.863	3.010E-05	2.430E-05
AMTN	-1.867	1.331E-04	6.160E-06
TM4SF20	-1.884	4.540E-05	2.590E-07
RTL1	-1.889	4.955E-03	5.130E-06
TTR	-1.909	8.424E-04	6.700E-07
SCGN	-1.916	2.284E-03	2.730E-12
SRD5A2	-1.929	1.700E-09	1.066E-02
GHRH	-1.937	1.634E-02	1.930E-05
SP8	-1.955	5.519E-03	9.498E-03
MROH2A	-1.959	9.840E-09	7.790E-10
LRTM1	-1.963	1.219E-04	1.200E-02
WIF1	-1.971	3.740E-05	7.660E-08
CPB1	-1.974	2.250E-06	6.950E-06
GKN1	-1.980	6.640E-05	2.830E-07
C11orf86	-1.982	7.410E-06	6.303E-03
OR52E8	-1.992	2.077E-02	3.057E-03
KRTAP11-1	-2.003	1.387E-01	2.170E-02
NEFL	-2.016	1.050E-05	2.459E-02

SLC17A4	-2.025	9.624E-03	1.550E-09
TMEM207	-2.028	8.436E-02	4.823E-04
CTCFL	-2.030	8.290E-05	8.390E-06
CRTAC1	-2.040	2.490E-07	3.640E-07
GNAT3	-2.063	8.648E-03	3.430E-09
BPIFB2	-2.072	1.092E-03	8.213E-03
FABP7	-2.072	1.471E-04	3.550E-10
FABP4	-2.094	6.990E-09	2.177E-03
TFF1	-2.161	2.880E-07	3.476E-02
SSTR5	-2.172	1.500E-05	1.060E-06
MUC13	-2.175	1.000E-09	5.420E-13
PRSS33	-2.202	9.390E-08	4.330E-09
KCNK10	-2.204	7.100E-11	9.980E-10
FABP1	-2.210	1.257E-02	4.640E-06
REG4	-2.236	2.250E-13	5.750E-11
GAD2	-2.264	3.885E-03	5.670E-08
LGALS4	-2.305	3.330E-11	4.660E-11
VSTM2B	-2.314	3.251E-02	1.170E-06
KRT6C	-2.337	3.960E-06	1.480E-02
CTSE	-2.375	2.440E-10	5.660E-10
KRT6B	-2.479	8.800E-07	2.480E-06
SI	-2.547	1.862E-03	3.796E-04
GP2	-2.562	2.764E-04	7.740E-09
ZP4	-2.571	1.009E-02	3.440E-04
KRTAP19-1	-2.591	8.128E-03	9.393E-04
FAM9C	-2.632	4.460E-06	1.740E-11
NEUROD4	-2.744	5.710E-03	2.480E-07
MUC17	-2.859	1.290E-06	1.611E-04
NKX6-3	-2.931	4.220E-09	2.960E-05
KRTAP13-2	-3.290	6.857E-02	1.480E-07
PRSS56	-3.411	6.490E-06	2.865E-04
