# Supplementary material for: An Epithelial-Mesenchymal Transition (EMT) Preoperative Nomogram for Prediction of Lymph Node Metastasis in Bladder Cancer (BLCA)
Source: Dis Markers. 2020 Nov 3;2020:8833972. doi: 10.1155/2020/8833972 (PMC7656235; doi:10.1155/2020/8833972)
Supplement: Supplementary 2 — Supplementary Table S2: GSEA based on preranked gene list calculated by DESeq2 for hallmark gene sets. [file 8833972.f2.docx]

| Description | setSize | enrichmentScore | NES | pvalue | p.adjust | qvalues | rank | leading_edge | core_enrichment |
| --- | --- | --- | --- | --- | --- | --- | --- | --- | --- |
| HALLMARK_ALLOGRAFT_REJECTION | 195 | -0.524 | -1.835 | 0.000 | 0.003473 | 0.003 | 4232 | tags=45%, list=20%, signal=36% | HCLS1/IL16/RPS3A/CD28/TGFB1/NPM1/HLA-A/CCND2/CD74/GPR65/TLR1/IL15/UBE2N/CTSS/TAP1/STAT4/RPS9/IL9/ST8SIA4/CCL22/LY75/HLA-DOB/IL2RA/FGR/PSMB10/PTPN6/LCP2/CD80/SOCS1/IL12A/IFNGR1/WAS/HIF1A/CD40LG/IL13/IL12RB1/CRTAM/TLR6/STAT1/TGFB2/CD1D/IL2RG/RPL9/IL18RAP/ITK/CD96/EREG/CD79A/IRF4/CCR5/CD2/ITGAL/GCNT1/MAP4K1/ZAP70/CD3E/IL2RB/CXCR3/PTPRC/HLA-G/CD40/SIT1/CCL4/KLRD1/LCK/CD3D/TRAT1/PRKCG/PRKCB/IRF8/CD7/GZMB/CXCL13/GZMA/CD3G/CXCL9/IL2/FASLG/CD8A/CD8B/PF4/PRF1/KRT1/IFNG/F2/NOS2/MBL2 |
| HALLMARK_INTERFERON_ALPHA_RESPONSE | 95 | -0.597 | -1.919 | 0.000 | 0.003473 | 0.003 | 6240 | tags=71%, list=30%, signal=50% | CMPK2/IL7/IFI44L/LAMP3/PSME1/TRIM5/RSAD2/PARP14/STAT2/EPSTI1/TRIM26/TXNIP/PARP9/IRF7/TRIM14/RNF31/PSMB9/CCRL2/B2M/OGFR/SELL/MOV10/TRIM21/GBP4/SLC25A28/HELZ2/IFI35/TRAFD1/LY6E/CD74/IRF1/NCOA7/SP110/UBE2L6/PSMB8/IL15/IFIH1/IFI44/TAP1/RTP4/IFITM1/USP18/IFI30/PSME2/IFIT3/CASP8/IRF9/HERC6/UBA7/PROCR/TRIM25/ISG15/SAMD9/LAP3/IFI27/CASP1/DDX60/BST2/LPAR6/SAMD9L/ISG20/OAS1/BATF2/OASL/CXCL10/CXCL11 |
| HALLMARK_INTERFERON_GAMMA_RESPONSE | 198 | -0.456 | -1.598 | 0.000 | 0.003496 | 0.003 | 5550 | tags=48%, list=27%, signal=36% | PARP14/STAT2/EPSTI1/TRIM26/TXNIP/HLA-B/MT2A/IRF7/TRIM14/GBP6/HLA-DQA1/RNF31/PSMB9/B2M/TNFSF10/OGFR/IL15RA/CD274/RAPGEF6/TRIM21/GBP4/SLC25A28/HELZ2/IFI35/TRAFD1/LY6E/HLA-A/CD74/IRF1/CFH/SP110/UBE2L6/PSMB8/PML/IL15/ISOC1/IFIH1/IFI44/TAP1/STAT4/RTP4/CASP4/USP18/IFI30/PSME2/IFIT3/ST8SIA4/CASP8/IL10RA/FGL2/IL18BP/PSMB10/NLRC5/CIITA/PTPN6/IRF9/LCP2/DDX58/HERC6/SOCS1/TRIM25/GPR18/HIF1A/GCH1/ISG15/LAP3/IFI27/STAT1/OAS3/CASP1/DDX60/BST2/ITGB7/OAS2/SAMD9L/ISG20/SLAMF7/ZBP1/IRF4/BATF2/XAF1/IL2RB/OASL/CD69/HLA-G/CD40/CD38/IRF8/CXCL10/GZMA/CXCL9/ST3GAL5/KLRK1/CXCL11/IDO1 |
| HALLMARK_UV_RESPONSE_DN | 141 | 0.429 | 1.642 | 0.000 | 0.003496 | 0.003 | 3496 | tags=31%, list=17%, signal=26% | EFEMP1/ERBB2/DAB2/RGS4/AMPH/KALRN/F3/SERPINE1/HAS2/COL11A1/PDGFRB/ITGB3/INSIG1/PTPN21/MGLL/AKT3/KCNMA1/COL1A1/COL1A2/COL3A1/NFIB/FBLN5/SCN8A/COL5A2/VLDLR/CAP2/CAV1/ANXA2/MAGI2/MMP16/IGFBP5/ANXA4/MAP1B/NOTCH2/DDAH1/LDLR/GRK5/PIAS3/PMP22/NR1D2/CDON/MET/TJP1/ATP2B4 |
| HALLMARK_APICAL_JUNCTION | 198 | 0.424 | 1.697 | 0.000 | 0.003496 | 0.003 | 3151 | tags=31%, list=15%, signal=26% | CLDN9/CLDN6/ACTC1/ACTG2/FLNC/CALB2/AMH/MYL9/ACTA1/CLDN4/GRB7/NEGR1/TGFBI/ICAM5/CRAT/CDH11/SPEG/CERCAM/EGFR/MMP9/SLIT2/PCDH1/WNK4/NEXN/NRXN2/NECTIN4/SLC30A3/NRTN/ADAM9/INSIG1/CD99/VCAN/AKT3/FBN1/ACTN4/TSPAN4/B4GALT1/LAMA3/CLDN5/LAMC2/MYH10/KCNH2/COL16A1/CDH15/THY1/ITGA10/NECTIN2/ACTN1/LAYN/CLDN7/ADAM15/ICAM1/ACTN2/CDSN/PIK3CB/EXOC4/MMP2/TNFRSF11B/MPZL1/ITGB1/AMIGO1 |
| HALLMARK_EPITHELIAL_MESENCHYMAL_TRANSITION | 198 | 0.596 | 2.383 | 0.000 | 0.003496 | 0.003 | 3288 | tags=59%, list=16%, signal=50% | ANPEP/COMP/SPP1/MGP/DAB2/RGS4/PTX3/MFAP5/TAGLN/ELN/TPM2/SGCD/MYL9/OXTR/ACTA2/GEM/COL8A2/COL6A2/TPM1/SFRP4/LAMA2/ECM1/GADD45B/TGFBI/COL6A3/LUM/POSTN/NTM/CDH11/FN1/CTHRC1/SERPINE1/IL6/EMP3/DCN/MYLK/SLIT2/FBN2/CALD1/GAS1/LGALS1/BGN/COL11A1/GREM1/PDGFRB/FAP/SPOCK1/FSTL3/ITGB3/PCOLCE/INHBA/CD44/VCAN/MATN2/NNMT/VIM/TIMP3/FZD8/FBN1/TNFRSF12A/CAPG/CD59/COL1A1/CALU/DPYSL3/HTRA1/LAMA3/COL1A2/LAMC2/MEST/PMEPA1/VEGFC/FLNA/COL3A1/TNC/FBLN5/SPARC/COL16A1/FSTL1/ADAM12/EFEMP2/COL5A2/THY1/PVR/THBS1/MATN3/CAP2/FERMT2/PLOD1/FMOD/COL12A1/COPA/SFRP1/FBLN2/LRP1/FGF2/LOXL2/SGCB/ABI3BP/THBS2/EDIL3/ITGA5/COL5A1/NOTCH2/TIMP1/MMP2/GLIPR1/CRLF1/TNFRSF11B/BASP1/ECM2/ITGB1/ITGB5/LOX/SGCG/CXCL6/PMP22 |
| HALLMARK_MYOGENESIS | 198 | 0.581 | 2.321 | 0.000 | 0.003496 | 0.003 | 3711 | tags=52%, list=18%, signal=43% | MYH2/MYBPH/DES/ACTC1/CHRNA1/CASQ2/MYL2/NCAM1/REEP1/FABP3/TAGLN/HSPB8/PTGIS/SGCA/MYH11/PYGM/TPM2/MYL1/SGCD/MB/RYR1/ACTA1/CRYAB/AEBP1/TNNT1/MYL7/SLN/COL6A2/CSRP3/SOD3/SORBS1/ITGA7/CKMT2/LAMA2/FHL1/GADD45B/COL6A3/CRAT/SPEG/NAV2/CACNG1/GPX3/MYOM1/MYLK/FXYD1/TNNT2/GNAO1/TNNC2/CAMK2B/FOXO4/DMD/CNN3/ACSL1/CKM/COX7A1/COL1A1/GSN/SH3BGR/MYH3/SCD/CACNA1H/CHRNB1/SVIL/COL3A1/KCNH2/SPARC/MYH4/COX6A2/ADAM12/EPHB3/TNNC1/SIRT2/PTP4A3/CAV3/DTNA/IFRD1/MYL3/FGF2/MYOG/CTF1/SPTAN1/PDLIM7/PDE4DIP/HSPB2/ACTN2/MRAS/MYOZ1/IGFBP7/ITGB1/CFD/ITGB5/SGCG/ADCY9/BDKRB2/WWTR1/ATP6AP1/HDAC5/NOS1/IGFBP3/SSPN/MYH1/LDB3 |
| HALLMARK_KRAS_SIGNALING_DN | 197 | -0.433 | -1.517 | 0.002 | 0.010211 | 0.008 | 3388 | tags=38%, list=16%, signal=32% | PCDHB1/CDKAL1/MYO15A/CELSR2/SIDT1/KLHDC8A/KCNQ2/MAGIX/BTG2/MYOT/CD80/AKR1B10/ALOX12B/CAMK1D/PAX4/MAST3/SNCB/GPRC5C/TAS2R4/CD40LG/VPREB1/PLAG1/GAMT/CHRNG/PROP1/FSHB/ABCB11/TGFB2/DCC/TGM1/TFCP2L1/PKP1/KCNN1/CD207/CLDN8/KRT5/TEX15/CLPS/NUDT11/KRT15/TLX1/CYP39A1/FGFR3/NTF3/LFNG/PAX3/CPA2/MEFV/TG/PDE6B/GDNF/CHST2/PDCD1/BMPR1B/TCL1A/IRS4/SERPINA10/KRT1/P2RX6/NPHS1/LGALS7/IFNG/TFAP2B/IGFBP2/CDH16/TENM2/SCGB1A1/NGB/COL2A1/TFF2/HNF1A/AMBN/CPB1/GP2 |
| HALLMARK_HEME_METABOLISM | 191 | -0.413 | -1.442 | 0.007 | 0.041433 | 0.033 | 930 | tags=9%, list=4%, signal=9% | TRIM58/AQP3/ALDH1L1/SLC4A1/GYPA/SLC7A11/ALAS2/HBD/TYR/SLC30A10/HBQ1/HBB/RHAG/KEL/TRIM10/CA1/CTSE |
| HALLMARK_APICAL_SURFACE | 44 | 0.474 | 1.499 | 0.019 | 0.096154 | 0.077 | 2042 | tags=23%, list=10%, signal=21% | ATP6V0A4/MAL/SLC2A4/NTNG1/LYPD3/SRPX/GAS1/B4GALT1/RTN4RL1/THY1 |
| HALLMARK_PANCREAS_BETA_CELLS | 40 | -0.547 | -1.520 | 0.024 | 0.107689 | 0.086 | 2372 | tags=38%, list=11%, signal=33% | NKX6-1/SYT13/GCK/PAK3/ABCC8/FOXA2/NEUROG3/PKLR/SST/INSM1/ISL1/NEUROD1/HNF1A/SCGN |
| HALLMARK_XENOBIOTIC_METABOLISM | 197 | -0.375 | -1.315 | 0.032 | 0.135101 | 0.108 | 2541 | tags=21%, list=12%, signal=19% | XDH/LCAT/ID2/CA2/F11/PLG/TGFB2/CYFIP2/CYP2C18/G6PC/ARG2/ENTPD5/ACSM1/KYNU/VNN1/FBP1/SLC22A1/ABCC3/ABCD2/PTGR1/CYP4F2/GAD1/ITIH1/FMO3/ARG1/RBP4/IRF8/ABCC2/LEAP2/TYR/HNF4A/ATOH8/ADH1C/MBL2/SERPINA6/ADH7/DDC/HSD17B2/CYP1A2/CYP1A1/FABP1 |
| HALLMARK_SPERMATOGENESIS | 132 | -0.384 | -1.286 | 0.065 | 0.246291 | 0.197 | 2399 | tags=17%, list=12%, signal=16% | ADAM2/DCC/PRM2/MEP1B/GRM8/ZPBP/RPL39L/DDX4/IL12RB2/GAD1/LDHC/AKAP4/GFI1/TKTL1/IL13RA2/NEFH/TUBA3C/TCP11/PACRG/MTNR1A/NAA11/CFTR |
| HALLMARK_COAGULATION | 138 | -0.378 | -1.276 | 0.071 | 0.246291 | 0.197 | 2483 | tags=23%, list=12%, signal=21% | DUSP6/RAPGEF3/CLU/THBD/F11/CTSV/PLG/HMGCS2/F12/CPB2/MST1/ITIH1/KLKB1/C8B/GDA/PLEK/TFPI2/FGG/PROC/DCT/PF4/TF/HNF4A/MMP10/MMP1/F2/MBL2/F13B/F9/MEP1A/CTSE |
| HALLMARK_BILE_ACID_METABOLISM | 112 | 0.338 | 1.249 | 0.077 | 0.246291 | 0.197 | 2506 | tags=25%, list=12%, signal=22% | GC/HSD3B1/ABCA3/CYP8B1/CH25H/SULT2B1/AKR1D1/HSD17B6/DHCR24/FADS2/DIO2/APOA1/SLC23A1/PEX11A/NR3C2/CYP7A1/ACSL1/HSD3B7/ABCD3/DIO1/IDH2/RETSAT/IDI1/CYP27A1/AQP9/CYP46A1/FADS1/PHYH |
| HALLMARK_FATTY_ACID_METABOLISM | 156 | -0.365 | -1.248 | 0.081 | 0.246291 | 0.197 | 2400 | tags=15%, list=12%, signal=13% | CA2/MIF/SLC22A5/HMGCS2/ODC1/CD1D/AQP7/VNN1/HPGD/GPD1/AADAT/CYP4A11/CYP4A22/FABP2/ACADL/BMPR1B/ALDH1A1/ADH1C/ADH7/CYP1A1/FABP1/GAD2 |
| HALLMARK_CHOLESTEROL_HOMEOSTASIS | 73 | 0.363 | 1.264 | 0.084 | 0.246291 | 0.197 | 4454 | tags=42%, list=21%, signal=33% | FADS2/TMEM97/AVPR1A/TNFRSF12A/ERRFI1/SCD/TRIB3/IDI1/DHCR7/PMVK/ANTXR2/ECH1/MAL2/LGALS3/HSD17B7/GPX8/ANXA5/GUSB/CD9/FASN/LDLR/SC5D/TM7SF2/STX5/ALCAM/CXCL16/MVD/ACSS2/FDPS/PLAUR/SQLE |
| HALLMARK_TGF_BETA_SIGNALING | 54 | 0.384 | 1.267 | 0.108 | 0.300278 | 0.240 | 3449 | tags=28%, list=17%, signal=23% | NOG/LEFTY2/SERPINE1/LTBP2/SKIL/PMEPA1/PPP1R15A/THBS1/SPTBN1/RAB31/TGFBR1/CDKN1C/BCAR3/WWTR1/TJP1 |
| HALLMARK_KRAS_SIGNALING_UP | 198 | 0.284 | 1.136 | 0.118 | 0.311719 | 0.249 | 3363 | tags=28%, list=16%, signal=24% | PCP4/CIDEA/SPP1/SNAP91/GABRA3/ITGBL1/CSF2/PEG3/RGS16/WNT7A/BPGM/ETV1/SPARCL1/AKAP12/MMP11/CSF2RA/MMP9/SPON1/RELN/IL1RL2/MALL/TNNT2/PLAT/RABGAP1L/INHBA/EPHB2/SLPI/PLAU/ANO1/EMP1/ADGRA2/GADD45G/PPBP/PPP1R15A/GFPT2/VWA5A/ADAM17/ETV5/CFB/PTGS2/LY96/BTC/GPRC5B/CMKLR1/RETN/TOR1AIP2/SCN1B/ANKH/MMD/TMEM158/ADAM8/SNAP25/CPE/DCBLD2/FCER1G/CCSER2 |
| HALLMARK_MYC_TARGETS_V1 | 196 | -0.337 | -1.179 | 0.139 | 0.336724 | 0.269 | 8580 | tags=49%, list=41%, signal=29% | HPRT1/PPIA/NCBP2/PWP1/XRCC6/RRM1/EIF2S1/ETF1/DDX18/RANBP1/EIF3B/SERBP1/AIMP2/YWHAE/RAN/SRSF3/DDX21/NOP56/HNRNPC/G3BP1/SSBP1/PSMC6/FBL/ERH/MAD2L1/EXOSC7/CDK4/SSB/PSMA6/VDAC1/RRP9/AP3S1/CDC20/SNRPD2/HNRNPA3/SYNCRIP/NAP1L1/EIF4E/SNRPA1/SRSF7/COPS5/CDC45/PSMA4/SNRPD3/PSMA1/PSMA2/MRPL23/SRPK1/RPS6/COX5A/HNRNPD/HSPD1/UBE2L3/SRSF1/GOT2/SLC25A3/SNRPA/DHX15/SRSF2/GSPT1/HDDC2/LDHA/EIF3D/HDAC2/IMPDH2/SNRPD1/C1QBP/RPS5/ORC2/ABCE1/PHB2/EEF1B2/CLNS1A/PA2G4/CYC1/RPS10/LSM7/RPLP0/HSPE1/RSL1D1/NPM1/TCP1/NOP16/RPS3/HNRNPA1/RPL18/MYC/RPL14/RACK1/RPS2/U2AF1/ODC1/EIF4A1/RPL6/RPL34/CCT2 |
| HALLMARK_MYC_TARGETS_V2 | 58 | -0.418 | -1.243 | 0.142 | 0.336724 | 0.269 | 8252 | tags=60%, list=40%, signal=36% | AIMP2/WDR43/RABEPK/IMP4/NOP56/TFB2M/BYSL/CDK4/RRP9/NIP7/FARSA/EXOSC5/UNG/TBRG4/NOC4L/WDR74/HSPD1/UTP20/PES1/PUS1/PA2G4/HSPE1/NPM1/IPO4/NDUFAF4/MYBBP1A/TCOF1/NOP16/HK2/NOP2/MYC/PRMT3/PPAN/DUSP2 |
| HALLMARK_ANGIOGENESIS | 36 | 0.410 | 1.243 | 0.148 | 0.336724 | 0.269 | 3228 | tags=39%, list=16%, signal=33% | SPP1/VTN/LUM/POSTN/VCAN/STC1/COL3A1/FSTL1/COL5A2/KCNJ8/OLR1/TIMP1/FGFR1/CXCL6 |
| HALLMARK_ESTROGEN_RESPONSE_EARLY | 197 | 0.276 | 1.103 | 0.184 | 0.399702 | 0.320 | 3503 | tags=26%, list=17%, signal=22% | TMPRSS3/CLIC3/MUC1/KCNK15/CALB2/OLFML3/REEP1/HSPB8/ANXA9/ABCA3/PDLIM3/SULT2B1/NAV2/WWC1/P2RY2/PEX11A/SYT12/CALCR/TTC39A/CD44/TFAP2C/KAZN/PGR/ELOVL5/B4GALT1/TIAM1/SYNGR1/SVIL/SLC19A2/DHCR7/MAPT/THSD4/NPY1R/FAM102A/TOB1/MREG/KRT18/RAB31/GLA/CLDN7/SNX24/OPN3/NBL1/PODXL/ZNF185/FASN/LRIG1/ADCY9/PRSS23/BLVRB/SOX3/PTGES |
| HALLMARK_ADIPOGENESIS | 197 | 0.267 | 1.066 | 0.257 | 0.514425 | 0.412 | 4203 | tags=23%, list=20%, signal=18% | CIDEA/REEP6/HSPB8/CDKN2C/SORBS1/ITGA7/SPARCL1/OMD/CRAT/GPX3/MYLK/PTGER3/APOE/MGLL/MRAP/CAVIN1/CAVIN2/SLC5A6/RTN3/ITIH5/ACOX1/RETSAT/DHCR7/PLIN2/TOB1/ECH1/PHYH/LAMA4/ANGPT1/NKIRAS1/RETN/POR/DNAJB9/LEP/TALDO1/SDHC/SSPN/DNAJC15/CMPK1/ETFB/COL15A1/MGST3/RNF11/ABCB8/ACLY |
| HALLMARK_IL2_STAT5_SIGNALING | 197 | 0.267 | 1.066 | 0.257 | 0.514425 | 0.412 | 3587 | tags=24%, list=17%, signal=20% | ENPP1/MUC1/SPP1/CSF2/RGS16/DRC1/SLC2A3/HOPX/PENK/SELP/COL6A1/ECM1/GADD45B/TNFSF11/SLC39A8/RABGAP1L/CD44/RRAGD/AHNAK/CAPG/RORA/EMP1/TIAM1/PTGER2/ITIH5/MAP6/TWSG1/PTH1R/PLIN2/PLAGL1/IL18R1/GABARAPL1/TLR7/CSF1/KLF6/PIM1/SH3BGRL2/ANXA4/ADAM19/CDKN1C/LRIG1/WLS/BCL2L1/MYO1E/IL10/MAFF/ALCAM |
| HALLMARK_INFLAMMATORY_RESPONSE | 199 | -0.311 | -1.089 | 0.273 | 0.525726 | 0.421 | 3974 | tags=28%, list=19%, signal=23% | SEMA4D/CSF3R/IRF1/TLR1/FFAR2/IL15/VIP/PTPRE/ACVR1B/RTP4/IFITM1/CYBB/BTG2/FZD5/CCL22/MYC/ADM/KCNMB2/SLAMF1/IL10RA/LCP2/HIF1A/GCH1/KCNA3/HBEGF/PDPN/CX3CL1/BST2/ADORA2B/NMUR1/TNFRSF9/IL18RAP/LTA/PTAFR/PTGER4/EREG/CCL24/CD48/KCNJ2/GABBR1/IL2RB/CD69/GPC3/CD40/CXCR6/SLC4A4/MEFV/LCK/SLC28A2/CHST2/CXCL10/CXCL9/CXCL11/MEP1A/ROS1 |
| HALLMARK_COMPLEMENT | 200 | -0.296 | -1.040 | 0.375 | 0.694822 | 0.556 | 3539 | tags=24%, list=17%, signal=20% | CTSS/PIK3CG/SIRT6/GMFB/MMP13/CASP4/KCNIP2/LTF/SERPINA1/GP9/C9/LCP2/CR1/WAS/CD40LG/CTSH/DUSP6/LAP3/DGKH/CLU/CA2/CTSV/PLG/CASP1/DOCK10/ERAP2/CXCL1/KYNU/CASP5/F5/CTSC/ITIH1/CDH13/KLKB1/GZMK/CP/PLEK/LCK/TFPI2/CR2/GZMB/GZMA/HPCAL4/APOA4/MT3/HNF4A/F2 |
| HALLMARK_APOPTOSIS | 160 | 0.261 | 1.019 | 0.392 | 0.699734 | 0.560 | 3008 | tags=21%, list=14%, signal=18% | HGF/GSTM1/ERBB2/TNF/TIMP2/IFNB1/GADD45B/LUM/GPX3/IL6/DCN/BGN/AVPR1A/PDGFRB/PLAT/CD44/TIMP3/PEA15/TNFRSF12A/GSN/HMOX1/EMP1/RETSAT/CAV1/DAP/KRT18/CTH/SPTAN1/LGALS3/CD14/ANKH/TIMP1/MMP2 |
| HALLMARK_HYPOXIA | 196 | 0.250 | 0.996 | 0.452 | 0.761965 | 0.610 | 3084 | tags=23%, list=15%, signal=20% | IGFBP1/PYGM/INHA/SLC2A3/TMEM45A/TGFBI/EFNA1/AKAP12/SULT2B1/EGFR/F3/SERPINE1/SRPX/IL6/DCN/BGN/LALBA/KDELR3/PDGFB/CAVIN3/RRAGD/ERRFI1/RORA/HMOX1/TGFB3/CAVIN1/STC1/STBD1/PPP1R15A/VLDLR/PLIN2/DTNA/CAV1/ANXA2/CHST3/EFNA3/SLC6A6/TES/GALK1/KLF6/PIM1/COL5A1/NAGK/PHKG1/CDKN1C |
| HALLMARK_ANDROGEN_RESPONSE | 98 | 0.271 | 0.985 | 0.482 | 0.761965 | 0.610 | 3421 | tags=24%, list=16%, signal=21% | STEAP4/AKAP12/DHCR24/TMPRSS2/ADAMTS1/INSIG1/PTPN21/ELOVL5/B4GALT1/SCD/PMEPA1/HERC3/IDI1/HOMER2/ACTN1/FADS1/ARID5B/NKX3-1/ANKH/SEC24D/DNAJB9/GPD1L/TSC22D1/PIAS1 |
| HALLMARK_MITOTIC_SPINDLE | 198 | 0.247 | 0.986 | 0.492 | 0.761965 | 0.610 | 4938 | tags=24%, list=24%, signal=19% | SORBS2/ARHGAP29/RALBP1/ACTN4/GSN/TIAM1/FLNA/MYH10/PALLD/OPHN1/SPTBN1/EPB41/NET1/SPTAN1/CLIP2/RASAL2/ANLN/NOTCH2/KIF3C/CDC42EP2/HOOK3/ARHGAP10/MYO1E/SUN2/ALMS1/PKD2/KIF23/MAPRE1/NDC80/FARP1/MID1/VCL/SMC1A/RANBP9/CENPF/CLIP1/DYNLL2/KIFAP3/RASA1/NEK2/TUBGCP5/MYH9/CAPZB/PRC1/EZR/SMC3/ARL8A/CDC42EP1 |
| HALLMARK_P53_PATHWAY | 195 | -0.281 | -0.985 | 0.501 | 0.761965 | 0.610 | 5258 | tags=32%, list=25%, signal=24% | FUCA1/RPS12/DRAM1/ISCU/NOTCH1/TAX1BP3/ERCC5/HINT1/SLC35D1/HRAS/FBXW7/NHLH2/PRKAB1/CCNG1/TGFB1/NUDT15/TRAFD1/CCND2/SLC3A2/MKNK2/SESN1/TAP1/PTPRE/ACVR1B/ABCC5/IFI30/RPL18/BTG2/ANKRA2/TP53/SOCS1/IP6K2/SERPINB5/PROCR/FOS/MDM2/NDRG1/ITGB4/HBEGF/RB1/RPL36/RACK1/JAG2/BAK1/MXD4/TP63/DGKA/CASP1/DEF6/CYFIP2/RRAD/ABAT/FGF13/LRMP/LDHB/FAM162A/CDH13/KRT17/SLC7A11/CLCA2/ALOX15B |
| HALLMARK_PEROXISOME | 104 | 0.266 | 0.973 | 0.509 | 0.761965 | 0.610 | 2490 | tags=19%, list=12%, signal=17% | ALB/STS/CRABP2/CACNA1B/CRAT/SULT2B1/DHCR24/PEX11A/ACSL1/HSD3B7/ABCD3/ELOVL5/DIO1/IDH2/ACOX1/RETSAT/IDI1/CRABP1/ECH1/FADS1 |
| HALLMARK_HEDGEHOG_SIGNALING | 36 | 0.316 | 0.959 | 0.518 | 0.761965 | 0.610 | 2315 | tags=25%, list=11%, signal=22% | AMOT/CNTFR/UNC5C/NRP2/DPYSL2/THY1/OPHN1/VLDLR/NRCAM |
| HALLMARK_WNT_BETA_CATENIN_SIGNALING | 42 | -0.330 | -0.925 | 0.580 | 0.828311 | 0.663 | 5052 | tags=36%, list=24%, signal=27% | NOTCH1/WNT1/CSNK1E/NOTCH4/CCND2/KAT2A/TCF7/MYC/TP53/GNAI1/JAG2/HEY2/HEY1/DLL1 |
| HALLMARK_TNFA_SIGNALING_VIA_NFKB | 199 | 0.236 | 0.942 | 0.655 | 0.875056 | 0.700 | 3561 | tags=21%, list=17%, signal=17% | PTX3/CSF2/SERPINB2/SLC2A3/TNF/GEM/CCL2/GADD45B/EFNA1/F3/SERPINE1/IL6/SLC2A6/RCAN1/INHBA/CD44/KLF9/PLAU/TNFAIP6/B4GALT1/PMEPA1/EDN1/CXCL3/TNC/PPP1R15A/GFPT2/PTGS2/TUBB2A/SLC16A6/CSF1/OLR1/KLF6/ICAM1/PLK2/LDLR/CXCL6/TSC22D1/BCL3/MAFF/FJX1/SPSB1 |
| HALLMARK_MTORC1_SIGNALING | 195 | 0.236 | 0.941 | 0.662 | 0.875056 | 0.700 | 5175 | tags=27%, list=25%, signal=21% | NUPR1/SLC2A3/DHCR24/FADS2/TMEM97/INSIG1/ELOVL5/SCD/STC1/TRIB3/NFKBIB/PPP1R15A/IDI1/DHCR7/VLDLR/EDEM1/BCAT1/FADS1/IFRD1/GLA/SLC6A6/TES/CTH/IGFBP5/RRM2/CD9/RPN1/LDLR/SC5D/SYTL2/TM7SF2/YKT6/MLLT11/G6PD/SERP1/CACYBP/ACLY/HSPA5/SLC9A3R1/BHLHE40/PLOD2/SQLE/MAP2K3/RAB1A/GLRX/PHGDH/PDAP1/PSMC2/CALR/CYP51A1/SLC1A4/SLC2A1/RIT1 |
| HALLMARK_PROTEIN_SECRETION | 95 | 0.253 | 0.911 | 0.673 | 0.875056 | 0.700 | 6006 | tags=42%, list=29%, signal=30% | EGFR/ATP6V1B1/GOLGA4/KRT18/GLA/SEC22B/CAV2/SEC24D/RAB5A/SSPN/ARCN1/GOSR2/YKT6/AP2S1/COPB2/YIPF6/RER1/STX12/SEC31A/SCAMP3/CLTC/LAMP2/ADAM10/VAMP7/ATP7A/GBF1/SCRN1/PPT1/TMED2/LMAN1/SH3GL2/PAM/SNX2/OCRL/ARF1/ARFGEF2/SGMS1/ZW10/VAMP3/ARFGAP3 |
| HALLMARK_ESTROGEN_RESPONSE_LATE | 197 | -0.260 | -0.909 | 0.683 | 0.875056 | 0.700 | 2868 | tags=16%, list=14%, signal=14% | IL17RB/FRK/PLXNB1/FOS/ID2/CA2/SLC22A5/HMGCS2/SLC7A5/SLC16A1/PDZK1/ISG20/DHRS2/HR/FGFR3/CXCL14/SERPINA5/IGSF1/TFPI2/CYP4F11/SERPINA3/DUSP2/KLK11/CA12/FABP5/AGR2/ASCL1/KLK10/TFF3/TFF1 |
| HALLMARK_IL6_JAK_STAT3_SIGNALING | 87 | -0.270 | -0.856 | 0.743 | 0.928697 | 0.743 | 4104 | tags=29%, list=20%, signal=23% | ITGA4/CSF3R/IRF1/IL17RA/ACVR1B/IL2RA/IRF9/IL17RB/SOCS1/IFNGR1/IL12RB1/STAT1/BAK1/CXCL1/IL2RG/IL1R2/CD38/INHBE/CXCL10/CXCL13/CXCL9/PF4/CXCL11/IL9R |
| HALLMARK_REACTIVE_OXYGEN_SPECIES_PATHWAY | 49 | -0.276 | -0.795 | 0.805 | 0.981653 | 0.785 | 4025 | tags=22%, list=19%, signal=18% | TXN/MSRA/JUNB/SRXN1/HHEX/GCLC/MBP/ABCC1/IPCEF1/LSP1 |
| HALLMARK_NOTCH_SIGNALING | 32 | -0.277 | -0.741 | 0.846 | 1 | 0.800 | 3619 | tags=19%, list=17%, signal=16% | FZD5/TCF7L2/DLL1/LFNG/WNT5A |
| HALLMARK_UNFOLDED_PROTEIN_RESPONSE | 108 | 0.222 | 0.817 | 0.904 | 1 | 0.800 | 5404 | tags=28%, list=26%, signal=21% | IGFBP1/CCL2/KDELR3/ATF6/WIPI1/EDEM1/TUBB2A/EIF2AK3/DNAJB9/PDIA6/CHAC1/YIF1A/DNAJC3/GOSR2/SERP1/ATP6V0D1/SEC31A/HSPA5/HYOU1/SRPRA/BAG3/WFS1/PDIA5/CALR/SLC1A4/DDX10/PREB/DCTN1/CKS1B/ATF3 |
| HALLMARK_GLYCOLYSIS | 198 | 0.213 | 0.853 | 0.912 | 1 | 0.800 | 5504 | tags=34%, list=26%, signal=25% | CLDN9/CLDN3/NDST3/TGFBI/EGFR/DCN/GNE/KDELR3/CHST4/CD44/VCAN/RRAGD/B4GALT1/STC1/CACNA1H/GPR87/GFPT1/STMN1/VLDLR/PLOD1/CYB5A/EFNA3/B4GALT4/CTH/GALK1/GUSB/CHST1/COL5A1/CAPN5/TALDO1/GLCE/CENPA/MET/SDHC/IGFBP3/HS2ST1/TPST1/HMMR/COPB2/G6PD/ELF3/TGFA/SAP30/SLC16A3/HSPA5/EXT2/AKR1A1/PLOD2/UGP2/NOL3/SDC1/GLRX/PFKP/ANGPTL4/B3GALT6/P4HA2/PAM/GALK2/CHPF/PMM2/HDLBP/QSOX1/GMPPB/PGM2/CHPF2/GPC4/PYGB |
| HALLMARK_UV_RESPONSE_UP | 156 | -0.221 | -0.757 | 0.951 | 1 | 0.800 | 3836 | tags=17%, list=18%, signal=14% | PPAT/APOM/GRPEL1/JUNB/TAP1/AMD1/TUBA4A/NPTX2/BTG2/DGAT1/HLA-F/FOS/GCH1/CA2/BAK1/CTSV/SULT1A1/FOSB/RRAD/SLC6A8/AQP3/NKX2-5/IGFBP2/COL2A1/CYP1A1 |
| HALLMARK_OXIDATIVE_PHOSPHORYLATION | 185 | -0.198 | -0.689 | 0.994 | 1 | 0.800 | 7894 | tags=41%, list=38%, signal=26% | UQCRC1/MRPL11/NDUFS8/COX11/CYCS/TIMM9/ETFA/NDUFB1/ALDH6A1/GPX4/IDH3G/MRPL34/MRPS22/HSPA9/VDAC1/UQCRB/DLST/PDP1/TIMM13/NDUFA1/TIMM10/ACAA1/OXA1L/NDUFV1/SLC25A6/TOMM22/SDHD/PMPCA/MPC1/ATP1B1/SUCLA2/COX5A/NDUFA3/UQCRC2/NDUFS4/NDUFA8/FXN/GOT2/ACADSB/SLC25A3/COX7B/SDHD/COX5B/DECR1/LDHA/MRPS15/COX10/ISCU/COX7A2/NDUFB7/NDUFA2/PHB2/NDUFV2/NDUFA9/MTX2/UQCRQ/NDUFS7/MRPS30/CYC1/UQCRH/COX6A1/NDUFC1/ACADVL/NDUFC2/UQCR11/UQCR10/RHOT2/COX7C/GRPEL1/BDH2/NDUFA7/NDUFB8/COX4I1/MTRF1/LDHB |
| HALLMARK_DNA_REPAIR | 148 | -0.155 | -0.527 | 1.000 | 1 | 0.800 | 9582 | tags=49%, list=46%, signal=27% | ERCC3/MPG/GTF2B/ELL/POLR2F/DCTN4/NUDT21/GTF2A2/POLR2H/ZNF707/DUT/FEN1/CSTF3/NUDT9/NFX1/HPRT1/RBX1/ERCC1/CLP1/NCBP2/GMPR2/ITPA/AK3/PNP/TSG101/GTF3C5/ALYREF/RFC5/EDF1/POLR2A/GPX4/AGO4/DDB2/SNAPC4/POLR2J/GTF2H3/GTF2H1/POLR2E/GTF2F1/ADA/CMPK2/POLD4/TAF10/POLR1D/POLR2D/VPS28/NT5C3A/MRPL40/POLR1C/NME3/GTF2H5/SMAD5/IMPDH2/TAF9/ERCC5/RFC3/APRT/ERCC8/SRSF6/POLL/AAAS/RAD52/HCLS1/NT5C/VPS37B/DGCR8/TARBP2/TAF1C/TP53/PDE6G/ZNRD1/BCAM |
| HALLMARK_E2F_TARGETS | 198 | 0.091 | 0.365 | 1.000 | 1 | 0.800 | 18910 | tags=100%, list=91%, signal=9% | CDKN2C/STMN1/SHMT1/CDKN2A/DIAPH3/RRM2/PRDX4/MELK/PSMC3IP/MSH2/TCF19/ATAD2/HMMR/STAG1/RAD51C/UBE2T/RPA2/EZH2/SMC1A/HUS1/GINS4/BARD1/PCNA/PSIP1/SMC3/DONSON/PDS5B/CCP110/RPA3/MCM2/RBBP7/RFC2/CKS1B/USP1/MLH1/HMGA1/RAD1/CDKN1B/KIF4A/TK1/POLE4/CDCA8/UBE2S/CCNB2/PPM1D/KPNA2/ZW10/DCTPP1/CHEK1/ANP32E/ASF1B/TUBG1/MTHFD2/CDKN3/MYBL2/JPT1/ESPL1/RAD21/MCM7/LIG1/SSRP1/MCM3/BRCA1/RPA1/SMC6/TUBB/NUP205/MCM4/TRIP13/PPP1R8/HMGB3/KIF18B/CKS2/PRIM2/KIF22/AURKA/DCK/MCM6/E2F8/TOP2A/RNASEH2A/BIRC5/SUV39H1/MKI67/XPO1/TFRC/UBR7/NBN/DCLRE1B/RACGAP1/MRE11/GINS1/DSCC1/ORC6/POLD3/POLD1/KIF2C/AK2/DEK/HMGB2/PRKDC/MXD3/CDKN1A/CTCF/NOLC1/CENPE/LBR/SPAG5/DEPDC1/PRPS1/LMNB1/POP7/TIPIN/SPC25/NASP/CTPS1/PLK4/POLA2/PHF5A/CDC25A/CIT/CDK1/NME1/NCAPD2/SPC24/TRA2B/SLBP/PLK1/NUDT21/AURKB/POLD2/CENPM/TIMELESS/CSE1L/MCM5/DUT/BRCA2/PMS2/RAD50/CCNE1/SNRPB/HELLS/IPO7/XRCC6/DNMT1/EIF2S1/RANBP1/MMS22L/RAN/NOP56/TMPO/DLGAP5/MAD2L1/RAD51AP1/CDK4/CDC20/SYNCRIP/BUB1B/RFC1/NUP153/NAP1L1/CDCA3/SMC4/CNOT9/ING3/BRMS1L/UNG/POLE/PTTG1/CDC25B/TBRG4/HNRNPD/DDX39A/SRSF1/EXOSC8/ILF3/WDR90/SRSF2/GSPT1/EED/WEE1/ORC2/RFC3/CBX5/LUC7L3/PA2G4/ASF1A/PNN/PAICS/CHEK2/NAA38/LYAR/MYC/TP53/GINS3/PAN2/NUP107/TACC3 |
| HALLMARK_G2M_CHECKPOINT | 194 | 0.103 | 0.411 | 1.000 | 1 | 0.800 | 6402 | tags=28%, list=31%, signal=20% | CDKN2C/E2F2/DMD/STMN1/E2F1/RASAL2/NOTCH2/HMGN2/CENPA/BCL3/HMMR/KIF23/RAD23B/STAG1/SAP30/NDC80/RPA2/EZH2/STIL/SMC1A/CENPF/SQLE/HUS1/CUL1/NEK2/MAP3K20/FANCC/PRC1/RAD54L/BARD1/MEIS2/PDS5B/MCM2/CKS1B/SMC2/ABL1/HMGA1/CBX1/CDKN1B/KIF4A/SLC12A2/CDC6/UBE2S/CCNB2/KPNA2/KIF15/CHEK1/ODF2/RBL1/CDC27/CDKN3/TPX2/MYBL2/LIG3/JPT1 |
| HALLMARK_PI3K_AKT_MTOR_SIGNALING | 104 | 0.156 | 0.570 | 1.000 | 1 | 0.800 | 2779 | tags=10%, list=13%, signal=8% | FGF22/GNA14/EGFR/TIAM1/TRIB3/NFKBIB/FGF6/E2F1/DUSP3/PAK4 |
