# Supplementary material for: An Epithelial-Mesenchymal Transition (EMT) Preoperative Nomogram for Prediction of Lymph Node Metastasis in Bladder Cancer (BLCA)
Source: Dis Markers. 2020 Nov 3;2020:8833972. doi: 10.1155/2020/8833972 (PMC7656235; doi:10.1155/2020/8833972)
Supplement: Supplementary 3 — Supplementary Table S3: GSEA based on preranked gene list calculated by DESeq2 for EMT-related gene sets. [file 8833972.f3.docx]

| Description | setSize | enrichmentScore | NES | pvalue | p.adjust | qvalues | rank | leading_edge | core_enrichment |
| --- | --- | --- | --- | --- | --- | --- | --- | --- | --- |
| ANASTASSIOU_MULTICANCER_INVASIVENESS_SIGNATURE | 64 | 0.819 | 2.774 | 0.000 | 0.004 | 0.003 | 3311 | tags=91%, list=16%, signal=76% | COMP/EPYC/ITGBL1/MFAP5/COL10A1/AEBP1/ACTA2/C1QTNF3/COL6A2/ASPN/SFRP4/COL6A3/LUM/POSTN/NTM/MMP11/CDH11/FN1/DCN/RCN3/GLT8D2/LGALS1/SUGCT/CRISPLD2/BGN/COL11A1/GREM1/PDGFRB/FAP/SPOCK1/PCOLCE/INHBA/VCAN/TIMP3/FBN1/PLAU/COL1A1/TNFAIP6/COL1A2/COL3A1/SULF1/SPARC/CTSK/ADAM12/COL5A2/THY1/EDNRA/RAB31/LOXL2/COPZ2/THBS2/COL5A1/MMP2/NUAK1/TMEM158/LOX/NOX4/SERPINF1 |
| HALLMARK_EPITHELIAL_MESENCHYMAL_TRANSITION | 198 | 0.596 | 2.389 | 0.000 | 0.004 | 0.003 | 3288 | tags=59%, list=16%, signal=50% | ANPEP/COMP/SPP1/MGP/DAB2/RGS4/PTX3/MFAP5/TAGLN/ELN/TPM2/SGCD/MYL9/OXTR/ACTA2/GEM/COL8A2/COL6A2/TPM1/SFRP4/LAMA2/ECM1/GADD45B/TGFBI/COL6A3/LUM/POSTN/NTM/CDH11/FN1/CTHRC1/SERPINE1/IL6/EMP3/DCN/MYLK/SLIT2/FBN2/CALD1/GAS1/LGALS1/BGN/COL11A1/GREM1/PDGFRB/FAP/SPOCK1/FSTL3/ITGB3/PCOLCE/INHBA/CD44/VCAN/MATN2/NNMT/VIM/TIMP3/FZD8/FBN1/TNFRSF12A/CAPG/CD59/COL1A1/CALU/DPYSL3/HTRA1/LAMA3/COL1A2/LAMC2/MEST/PMEPA1/VEGFC/FLNA/COL3A1/TNC/FBLN5/SPARC/COL16A1/FSTL1/ADAM12/EFEMP2/COL5A2/THY1/PVR/THBS1/MATN3/CAP2/FERMT2/PLOD1/FMOD/COL12A1/COPA/SFRP1/FBLN2/LRP1/FGF2/LOXL2/SGCB/ABI3BP/THBS2/EDIL3/ITGA5/COL5A1/NOTCH2/TIMP1/MMP2/GLIPR1/CRLF1/TNFRSF11B/BASP1/ECM2/ITGB1/ITGB5/LOX/SGCG/CXCL6/PMP22 |
| GOTZMANN_EPITHELIAL_TO_MESENCHYMAL_TRANSITION_UP | 65 | 0.527 | 1.789 | 0.001 | 0.004 | 0.003 | 3349 | tags=38%, list=16%, signal=32% | NCAM1/ACTA2/CCL2/GADD45B/FN1/SERPINE1/INHBA/TIMP3/NR2F1/COL1A1/TGFB3/EDN1/CHRNB1/COL3A1/TNC/COL5A2/FBLN2/CSF1/GATA2/TIMP1/IGFBP7/ITGB1/PROS1/PLA2G4A/CENPA |
| JECHLINGER_EPITHELIAL_TO_MESENCHYMAL_TRANSITION_UP | 67 | 0.498 | 1.697 | 0.001 | 0.006 | 0.004 | 3693 | tags=48%, list=18%, signal=39% | DAB2/TNXB/C4A/IL11/CCL2/COL6A2/COL6A1/DCN/GAS1/PDGFRA/PDGFRB/PCOLCE/INHBA/DDR2/SLPI/VIM/PTGS1/HTRA1/COL3A1/TNC/SPARC/FMO1/CDH15/VLDLR/UPP1/GALK1/MMP2/CXCL6/PMP22/BCL3/CCK/PPIC |
| LIEN_BREAST_CARCINOMA_METAPLASTIC | 32 | 0.618 | 1.822 | 0.002 | 0.007 | 0.004 | 3824 | tags=59%, list=18%, signal=49% | MXRA8/SFRP2/KCNE4/LUM/POSTN/PDGFRA/RARRES2/TIMP3/RUNX1T1/SPARC/COL16A1/THBS1/MMP16/THBS2/HTRA3/EDIL3/P4HB/HOXA7/PKD2 |
| LEF1_UP.V1_UP | 194 | 0.324 | 1.292 | 0.024 | 0.069 | 0.047 | 2930 | tags=31%, list=14%, signal=27% | KCNG1/KRT23/GJB1/TH/FLNC/ADAMTS8/IL11/MYL9/CRABP2/FOXG1/MLN/RYR1/SLC2A3/FNDC4/NMNAT2/TRPV2/COL6A1/FHL1/AKAP12/GNG4/CDA/EDAR/FN1/PLEKHF1/SERPINE1/EMP3/LGALS1/LTBP2/HAS2/PAX6/ADGRE2/NRIP3/TMOD1/CAVIN3/VIM/WT1/RRAGD/C14orf132/AXL/ANO1/ELOVL5/EMP1/PMEPA1/CYBRD1/ADIRF/WASF3/TCHH/ZFPM2/TULP2/CDKN2A/PRPH/TMEM47/FADS1/CTH/PDZD2/L1CAM/ROR2/MFAP2/MAP1B/PTGDS |
| LIEN_BREAST_CARCINOMA_METAPLASTIC_VS_DUCTAL_DN | 108 | 0.373 | 1.373 | 0.024 | 0.069 | 0.047 | 2098 | tags=25%, list=10%, signal=23% | SCGB2A2/SCGB1D2/EFHD1/CST6/PRR15L/ERBB2/CEACAM6/REEP6/FBXL16/SCGB2A1/MB/PIP/ACOT4/RSPH1/DHCR24/P2RY2/SPINT2/TESMIN/KLHDC9/SERTAD4/TTC39A/LRRC17/TRPS1/ACOX2/RTN4RL1/ADIRF/JCHAIN |
| AIGNER_ZEB1_TARGETS | 35 | 0.472 | 1.417 | 0.054 | 0.134 | 0.092 | 2729 | tags=40%, list=13%, signal=35% | MUC1/DMKN/SCEL/PPL/CDH11/OCLN/CD24/TACSTD2/PMEPA1/TSPAN15/CXADR/MAL2/CLDN7/F11R |
| LEF1_UP.V1_DN | 185 | -0.356 | -1.237 | 0.086 | 0.190 | 0.130 | 3680 | tags=29%, list=18%, signal=24% | MRNIP/CDK3/CYP2J2/VILL/STAT4/ACSL5/USP18/IL20RA/KLRC3/BDH2/FOXA1/KDM5D/PAK1/IL17RB/DDX58/HERC6/TRAF3IP2/RAC2/SELENBP1/SEC31B/SAMD9/LIMCH1/CTSH/ENTPD3/NSUN6/FAM83E/GRB14/SORL1/SLC6A20/ID3/OAS3/SLC44A4/ERAP2/RAB26/CLEC2D/ATP8A1/CXCL5/TSPAN1/ID1/KIT/PLAC8/RBP4/IL33/PF4/AGR2/CDH17/LGR5/ERN2/PRSS1/CFTR/TSPAN8/SULT1C2/TFF1 |
| ALONSO_METASTASIS_NEURAL_UP | 18 | 0.511 | 1.325 | 0.130 | 0.260 | 0.178 | 5091 | tags=61%, list=24%, signal=46% | MAL/PDGFRA/EMP1/S100A10/L1CAM/ENC1/PMP22/APLP2/MAGEA6/MITF/CHN1 |
| ALONSO_METASTASIS_DN | 24 | -0.464 | -1.176 | 0.250 | 0.455 | 0.311 | 2769 | tags=42%, list=13%, signal=36% | TCOF1/PAPPA/RGPD1/GIMAP6/NTF4/BCL2L10/LFNG/OLIG2/CDH10 |
| SINGH_KRAS_DEPENDENCY_SIGNATURE_ | 20 | -0.405 | -0.987 | 0.491 | 0.810 | 0.554 | 3038 | tags=40%, list=15%, signal=34% | FGFBP1/AREG/SH2D3A/SEMA4B/C6orf141/MST1R/SDR16C5 |
| ALONSO_METASTASIS_EMT_UP | 35 | 0.309 | 0.928 | 0.583 | 0.810 | 0.554 | 3786 | tags=37%, list=18%, signal=30% | LUM/VCAN/TUBA1A/EMP1/CLIC4/SPARC/TUBB3/L1CAM/ANLN/MMP2/ENC1/FZD1/HMMR |
| REACTOME_TGF_BETA_RECEPTOR_SIGNALING_IN_EMT_EPITHELIAL_TO_MESENCHYMAL_TRANSITION | 16 | 0.347 | 0.877 | 0.628 | 0.810 | 0.554 | 5131 | tags=44%, list=25%, signal=33% | TGFBR1/F11R/PARD3/CGN/SMURF1/PARD6A/UBC |
| GOTZMANN_EPITHELIAL_TO_MESENCHYMAL_TRANSITION_DN | 195 | -0.262 | -0.915 | 0.668 | 0.810 | 0.554 | 3400 | tags=15%, list=16%, signal=13% | AMD1/MMP13/RPL18/UGT1A8/MYC/GJB4/TP53/TRIM25/FOS/ITGB4/PTHLH/GNAI1/PTPRR/CX3CL1/GSTP1/PPFIBP2/CD53/GPD1/KRT15/CYP2C8/HS3ST1/FOXA2/ACADL/BNC1/SPRR2G/ITIH2/CYP3A5/CFTR/DMBT1 |
| LIEN_BREAST_CARCINOMA_METAPLASTIC_VS_DUCTAL_UP | 81 | -0.285 | -0.894 | 0.672 | 0.810 | 0.554 | 2001 | tags=16%, list=10%, signal=15% | ADORA2B/BCL2A1/HORMAD1/ENPP2/IL1R2/COL27A1/EFNB1/DAZL/HAPLN1/COL9A1/FABP5/OCA2 |
| JECHLINGER_EPITHELIAL_TO_MESENCHYMAL_TRANSITION_DN | 60 | 0.265 | 0.888 | 0.690 | 0.810 | 0.554 | 4255 | tags=32%, list=20%, signal=25% | GRB7/F3/INMT/TIMP3/FZD8/ACTN4/TGFB3/TIAM1/FLNA/THBS1/BMP4/PLK2/ITGB5/TSC22D1/EPCAM/HMMR/VAMP8/ZFP36/ITPR1 |
| ALONSO_METASTASIS_UP | 190 | 0.230 | 0.917 | 0.729 | 0.810 | 0.554 | 5554 | tags=30%, list=27%, signal=22% | MAL/IGFBP1/FHL1/LUM/VCAN/TUBA1A/HIST1H3D/SCD/EMP1/CLIC4/SPARC/TUBB3/CAV1/S100A10/AKR7A2/ARID5B/L1CAM/ANLN/ATL3/FAM114A1/MMP2/DUSP12/ENC1/BRK1/MMGT1/PMP22/FZD1/YWHAQ/HMMR/RNASE4/MAPRE1/VKORC1/PDE5A/TMCO1/ATP6V0D1/LAPTM4A/STK24/ASH2L/DAD1/APLP2/RAB1A/MAGEA6/DCAF13/CDYL/LIPA/RABAC1/RRAGA/ATP6V0B/CXCL12/ANO6/IFITM2/CKS1B/CDC5L/CDH2/TMEM50B/TMEM248/KIF1BP |
| SARRIO_EPITHELIAL_MESENCHYMAL_TRANSITION_DN | 149 | -0.252 | -0.856 | 0.780 | 0.821 | 0.562 | 4115 | tags=26%, list=20%, signal=21% | RSRP1/RNF145/FXYD3/EEF2/DST/IFI44/MKNK2/SESN1/MAP3K5/KLRC3/RPL37/MMP25/PLXNB1/DSC2/FOS/IFNGR1/CLU/CA2/IFI16/TP63/DSP/LPAR6/CXCL1/KYNU/VNN1/DSC3/KRT16/FGFR3/CCL3/BIRC3/AQP3/SPRR1B/SERPINA3/KLRK1/KRT14/ADH1C/ROS1 |
| SARRIO_EPITHELIAL_MESENCHYMAL_TRANSITION_UP | 178 | 0.190 | 0.751 | 0.994 | 0.994 | 0.680 | 4785 | tags=26%, list=23%, signal=21% | SERPINB2/ETV1/F3/CPT1A/VCAN/TUBA1A/TNFRSF12A/VEGFC/SH3KBP1/IDH2/STMN1/TUBB3/GGCT/E2F1/CAMK2N1/FGF2/ANKRD1/ANLN/RRM2/ENC1/CENPA/GPRC5A/CENPU/HILPDA/PHF19/FJX1/IGFBP3/MSH2/SAPCD2/HMMR/ZNF367/EIF5A2/FHL2/SMTN/UBE2T/NDC80/EIF1AD/STIL/PLAUR/PKMYT1/ZWINT/ARPC1B/CEP55/PRC1/BARD1/MBOAT7/PCNA |
