# Supplementary material for: An Epithelial-Mesenchymal Transition (EMT) Preoperative Nomogram for Prediction of Lymph Node Metastasis in Bladder Cancer (BLCA)
Source: Dis Markers. 2020 Nov 3;2020:8833972. doi: 10.1155/2020/8833972 (PMC7656235; doi:10.1155/2020/8833972)
Supplement: Supplementary 4 — Supplementary Table S4: significantly mutated genes identified by MutSigCV. [file 8833972.f4.pdf]

Gene	expr	reptime	hic	N_nonsilent	N_silent	N_noncoding	n_nonsilent	n_silent	n_noncoding	nnei	x	X	p	q
CDKN1A	904665	228	37	403704	133500	0	39	0	0	50	59	18520188	0	0
CDKN2A	225405	357	-15	849060	221076	0	20	0	0	50	41	22001156	0	0
PSIP1	200332	583	6	1453548	338556	0	18	1	0	50	51	20764412	0	0
RHOA	1623187	201	33	505164	133500	0	16	0	0	37	40	14298384	0	0
RHOB	621277	189	19	486296	144892	0	20	0	0	50	53	21143196	0	0
YIPF7	62950	1034	-6	727308	198648	0	3	0	0	50	54	20233972	0	0
ARID1A	975965	182	27	5695288	1710224	0	105	5	0	3	30	6070868	2.22E-16	5.24E-13
PIK3CA	401889	613	11	2810264	700252	0	81	1	0	19	25	9039552	2.22E-16	5.24E-13
TP53	2069567	213	34	1099684	303668	0	186	3	0	25	34	7496292	5.55E-16	1.16E-12
STAG2	338292	NaN	7	3391968	814884	0	50	6	0	32	29	10414780	1.11E-15	2.09E-12
ELF3	751933	168	31	971524	254540	0	61	2	0	36	34	14461076	2.11E-15	3.49E-12
ZFP36L1	543449	259	46	825564	264864	0	27	0	0	22	18	8559664	2.22E-15	3.49E-12
KDM6A	59340	NaN	36	3633692	977932	0	98	5	0	50	56	21128244	3.11E-15	4.51E-12
RB1	349930	450	41	2459248	628340	0	77	0	0	3	0	1821652	2.48E-14	3.34E-11
FGFR3	859388	222	42	2155224	666432	0	55	4	0	34	41	15698532	2.82E-14	3.55E-11
EP300	1167197	154	42	6166988	1698832	0	70	3	0	5	9	2911368	3.50E-12	4.13E-09
TSC1	1919595	461	33	2996096	826276	0	26	1	0	12	12	4879336	3.04E-10	3.37E-07
FBXW7	117295	501	51	2173024	582416	0	29	0	0	5	4	3387696	3.34E-09	3.50E-06
HRAS	1970020	236	42	550020	153792	0	16	0	0	50	66	20259248	2.55E-07	2.53E-04
KLF5	274388	328	28	1140624	339624	0	19	1	0	50	65	20677904	3.29E-07	3.10E-04
CREBBP	637890	242	39	6196180	1759352	0	42	8	0	21	38	9370632	3.58E-07	3.22E-04
RBM10	819569	NaN	38	2410832	666076	0	19	3	0	50	54	16576428	1.27E-06	1.09E-03
FOXQ1	114944	481	14	943400	351016	0	13	3	0	11	13	4454984	2.53E-06	2.08E-03
PARD3	139914	776	0	3538996	948740	0	20	1	0	8	2	4232128	3.62E-06	2.85E-03
FOXA1	67481	487	-58	1159136	360628	0	13	2	0	50	74	17440084	5.79E-06	4.37E-03
C3orf70	760533	598	32	636884	171592	0	15	0	0	50	67	24319428	2.00E-05	1.45E-02
NCOR1	954548	370	-18	6256700	1756504	0	38	6	0	50	65	20488156	2.43E-05	1.70E-02
METTL3	707390	470	30	1486300	422216	0	18	1	0	50	56	23682188	8.46E-05	5.58E-02
MARK2	1321109	201	51	2029556	575296	0	9	0	0	15	6	7966568	8.57E-05	5.58E-02
CDKN1B	791330	136	44	506944	139196	0	7	0	0	50	52	20311224	9.57E-05	5.92E-02
ERBB2	1284992	226	38	3187980	947316	0	49	7	0	36	47	13340032	9.73E-05	5.92E-02

NFE2L2	372300	145	36	1549668	409044	0	22	1	0	50	56	19076260	1.04E-04	6.13E-02
GPS2	1911735	206	36	866860	228908	0	11	1	0	50	69	22072000	1.24E-04	7.09E-02
ASXL2	602691	287	31	3585988	1061948	0	29	3	0	39	48	19503816	1.34E-04	7.43E-02
KRAS	259193	512	16	610184	147028	0	13	0	0	50	60	20519840	1.42E-04	7.63E-02
ASXL1	780777	242	57	3834120	1161984	0	25	5	0	50	62	21383852	2.04E-04	1.07E-01
TRAF3IP2	320530	201	32	1436460	411180	0	15	0	0	20	28	8850516	3.52E-04	1.79E-01
SSH3	1369264	177	44	1671776	498400	0	17	2	0	44	60	18796088	3.61E-04	1.79E-01
SPTAN1	1917687	197	43	6519428	1659316	0	43	10	0	6	14	3518348	4.98E-04	2.36E-01
KLHDC2	642923	342	12	1088292	267000	0	11	1	0	50	64	27191992	5.04E-04	2.36E-01
EPHA2	866090	197	37	2485236	713424	0	15	2	0	30	28	11814216	5.13E-04	2.36E-01
AHR	370010	410	2	2191892	571024	0	24	1	0	16	22	6917436	6.84E-04	3.07E-01
TFG	345618	509	16	1028128	286580	0	9	2	0	44	55	20652272	7.25E-04	3.18E-01
PHF3	256893	371	24	5253492	1344612	0	25	1	0	19	13	9175544	7.97E-04	3.40E-01
ZFP36L2	227876	208	47	1181208	409044	0	14	3	0	50	48	16953432	8.26E-04	3.40E-01
MBD1	229269	262	51	1710224	504808	0	11	0	0	24	19	9497724	8.30E-04	3.40E-01
ATM	415837	582	16	8044532	2014960	0	54	5	0	3	6	2869716	8.53E-04	3.42E-01
KIAA0368	366471	419	28	5208636	1470636	0	12	0	0	2	0	4241740	1.01E-03	3.89E-01
HES1	420702	294	33	690996	222144	0	9	1	0	50	60	20790044	1.01E-03	3.89E-01
RBM26	161846	521	30	2538636	692064	0	17	0	0	10	7	5247440	1.14E-03	4.31E-01
WAC	204806	355	17	1669996	463868	0	14	2	0	50	49	17826700	1.24E-03	4.57E-01
SIRPD	422390	424	46	499468	149876	0	6	0	0	50	51	20868008	1.48E-03	5.36E-01
DCLRE1A	264051	515	7	2668576	700964	0	16	1	0	40	30	16881520	1.57E-03	5.57E-01
TAF11	1099530	304	24	553224	145248	0	8	0	0	50	60	21156012	1.70E-03	5.94E-01
ERBB3	2693176	215	38	3546828	1009260	0	38	8	0	35	37	12635152	1.80E-03	6.08E-01
RIMS2	198819	1035	-8	3634048	996800	0	19	3	0	40	38	17603844	1.80E-03	6.08E-01
PTEN	259678	300	34	1077968	250624	0	9	0	0	50	50	18237880	1.89E-03	6.26E-01
E2F8	132848	504	16	2195452	632612	0	16	3	0	50	42	18586404	1.92E-03	6.26E-01
BAP1	1078407	229	62	1867220	540052	0	17	1	0	7	6	2946256	1.98E-03	6.33E-01
FLG2	387153	671	6	5887172	1785340	0	29	1	0	3	1	2462096	2.18E-03	6.86E-01
SLC17A3	313920	673	30	1283024	371308	0	8	0	0	36	28	16902524	2.26E-03	6.98E-01
RFTN2	647239	538	26	1297264	345320	0	11	0	0	50	83	26648024	2.30E-03	7.01E-01
CASP8	571730	386	37	1523324	344608	0	13	1	0	50	74	21850568	2.51E-03	7.51E-01
GOLGA5	360146	405	41	1898192	498400	0	10	0	0	15	17	9571416	2.93E-03	8.37E-01

RAI14	270712	554	-7	2628348	652548	0	18	0	0	18	12	8363152	2.93E-03	8.37E-01
C8orf34	36554	910	-22	1137776	284800	0	11	1	0	34	35	12163452	2.95E-03	8.37E-01
BTG2	574553	420	26	399788	116056	0	6	1	0	10	5	3111796	2.97E-03	8.37E-01
GPR89B	NaN	NaN	NaN	1211824	313280	0	11	2	0	50	118	39958864	3.30E-03	9.16E-01
TLK2	316879	370	-2	1995024	500892	0	13	0	0	11	8	6580304	3.70E-03	9.92E-01
TAOK3	635780	823	26	2390184	571380	0	15	2	0	28	21	12587448	3.77E-03	9.92E-01
EDIL3	161019	764	-20	1262732	321112	0	11	2	0	50	64	22025008	3.78E-03	9.92E-01
EEF1A1	716887	139	38	1177648	335708	0	10	0	0	20	16	7067312	3.79E-03	9.92E-01
ZNF750	711691	350	16	1758640	565328	0	16	3	0	50	46	16906796	3.89E-03	1
RXRA	300063	295	42	1179784	342116	0	19	4	0	15	20	4435760	4.09E-03	1
TIGD2	147602	529	24	1335000	350304	0	9	1	0	32	19	12300156	4.24E-03	1
PTPN3	352374	431	12	2404780	630476	0	9	4	0	50	60	22735584	4.24E-03	1
EXOSC10	577167	408	34	2341768	603776	0	13	3	0	24	27	10163088	4.24E-03	1
MECOM	215619	985	-10	3233192	820936	0	18	0	0	3	1	1971172	4.40E-03	1
H3F3B	1885370	198	27	343184	108580	0	5	0	0	50	68	21651920	4.45E-03	1
HIST1H4L	228884	634	40	249912	86508	0	4	0	0	50	48	14804260	4.57E-03	1
NRAS	600650	472	11	500892	129228	0	7	0	0	50	62	21029988	4.61E-03	1
NKX2-2	283479	694	-10	672484	209684	0	9	2	0	11	19	6145272	5.06E-03	1
IKBKB	514464	361	44	1998228	516912	0	11	2	0	24	26	9337168	5.11E-03	1
ABCB5	164218	648	4	3287304	898188	0	23	1	0	11	7	6019248	5.14E-03	1
DDX3X	627364	NaN	54	1747248	445356	0	13	2	0	50	78	21071996	5.37E-03	1
DKN2AIPN	929843	170	46	305448	80100	0	4	0	0	50	67	23256412	5.39E-03	1
ZBTB45	2673870	276	11	1217876	428980	0	9	2	0	30	39	15664000	5.40E-03	1
TOR1AIP1	470940	364	5	1489148	420436	0	12	0	0	33	34	13858724	5.42E-03	1
MED13	369807	275	3	5557516	1537208	0	32	5	0	50	54	24805368	5.68E-03	1
CD1E	135429	902	5	1009616	258100	0	11	1	0	50	50	16503092	5.74E-03	1
ATAD5	605762	209	25	4794964	1210400	0	28	2	0	2	3	2710228	5.76E-03	1
RAD21	482614	426	18	1673912	406552	0	13	2	0	50	57	17804272	5.79E-03	1
PPP4R1	454636	202	51	2475980	652192	0	11	2	0	50	47	19329376	6.04E-03	1
TPRG1	131839	421	1	712712	192952	0	6	1	0	8	6	2928100	6.33E-03	1
EPS8	259826	496	11	2157004	565328	0	10	2	0	50	53	19892568	6.36E-03	1
USP28	169458	534	10	2820232	738344	0	12	2	0	50	69	25564716	6.43E-03	1
CD9	2261351	277	32	610540	153080	0	4	1	0	50	53	24237548	6.48E-03	1

GABRA4	104768	839	15	1414032	398364	0	13	2	0	50	56	19046712	6.51E-03	1
MIPOL1	67481	526	-48	1188684	277680	0	13	1	0	41	62	15976924	6.81E-03	1
CD2BP2	2312884	165	47	873624	247776	0	8	2	0	16	12	6329680	6.83E-03	1
NOP14	680459	336	49	2244224	583840	0	12	1	0	50	44	17555428	6.97E-03	1
LETMD1	564607	155	40	928448	264508	0	8	1	0	50	43	19587832	7.21E-03	1
DCAF12	779457	142	39	1153440	335352	0	12	1	0	50	48	21010052	7.39E-03	1
FOSL2	465927	197	30	801356	259168	0	5	1	0	50	63	21832056	7.46E-03	1
ARHGAP3	265987	585	23	3598448	1078324	0	17	1	0	48	44	23357160	7.51E-03	1
HFM1	263616	615	-10	3829136	934144	0	17	2	0	30	36	16546168	7.84E-03	1
SUMO1	371912	192	41	284088	61944	0	3	0	0	50	50	19879040	8.22E-03	1
RBM17	576205	399	45	1052336	282664	0	9	1	0	50	55	22853064	8.33E-03	1
METTL12	2425755	248	51	580280	196156	0	7	0	0	50	68	20007556	8.56E-03	1
AGR3	360434	376	-15	457816	107156	0	3	0	0	50	33	20718844	8.97E-03	1
ITGAL	2276763	142	57	3024576	855468	0	17	4	0	50	70	22297348	9.13E-03	1
CARD18	46073	568	-18	242792	58384	0	3	0	0	50	51	19559708	9.25E-03	1
LRRC41	690472	205	31	1983632	661804	0	13	4	0	50	43	17617372	9.27E-03	1
NBEA	38939	1070	11	7627300	2058392	0	16	0	0	5	3	6061256	9.35E-03	1
GABARAF	1782958	316	21	312924	77964	0	3	1	0	16	13	4887168	9.45E-03	1
MYO16	108921	641	-19	4772536	1328948	0	23	3	0	12	14	9320436	9.50E-03	1
SLC25A30	680643	296	78	763264	210752	0	5	0	0	50	51	21491720	9.60E-03	1
KHDRBS2	1561	1153	-32	903884	251692	0	10	0	0	34	70	13196208	1.01E-02	1
LATS1	431427	210	40	2866156	783200	0	15	2	0	50	38	19087296	1.02E-02	1
UFC1	728669	234	37	444644	114988	0	4	0	0	50	54	22528392	1.03E-02	1
GTF2I	908884	151	45	2667152	678892	0	15	1	0	49	62	23995824	1.03E-02	1
RGS21	123814	860	-26	412604	94696	0	5	0	0	50	73	21356084	1.07E-02	1
MAN2A1	181420	913	10	2985060	773232	0	14	0	0	12	4	5517288	1.07E-02	1
VPS26B	270368	601	18	870064	231044	0	4	0	0	50	31	23855916	1.07E-02	1
MAPRE1	550127	293	48	708084	179424	0	5	0	0	50	40	20120408	1.12E-02	1
ZWILCH	530195	219	49	1583132	390532	0	7	1	0	50	48	19706380	1.12E-02	1
CSAG1	NaN	NaN	NaN	207192	58740	0	3	0	0	50	116	39704324	1.15E-02	1
GPR156	429121	146	19	2035252	610184	0	10	0	0	8	3	4501620	1.16E-02	1
BEND6	428244	271	23	741192	181560	0	5	1	0	50	65	21924616	1.18E-02	1
EPHA5	57073	706	-19	2666084	732292	0	19	4	0	50	76	22947760	1.21E-02	1

HLA-A	983517	596	36	935924	268780	0	5	1	0	50	55	18348240	1.23E-02	1
OR5M10	NaN	NaN	NaN	777148	235316	0	9	0	0	50	116	39880900	1.24E-02	1
JMJD1C	226859	712	23	6535448	1712716	0	23	0	0	1	0	1903176	1.26E-02	1
CD101	765941	540	24	2589188	725884	0	20	0	0	1	1	1387332	1.29E-02	1
SPSB2	2822601	232	32	644004	214668	0	6	0	0	50	62	20662240	1.30E-02	1
ABHD12	608597	348	-3	1092564	301176	0	6	1	0	50	54	19105096	1.31E-02	1
THSD1	384523	370	28	2122828	627272	0	13	0	0	6	5	5444664	1.33E-02	1
ARID1B	143122	621	27	5662892	1627276	0	19	4	0	37	30	16270980	1.34E-02	1
MYOT	946917	396	47	1284448	348524	0	10	0	0	50	72	24090520	1.40E-02	1
BUD13	419339	326	36	1587404	439660	0	10	1	0	50	47	21154588	1.44E-02	1
ACTG1	3582152	268	26	949096	276968	0	10	2	0	17	14	4681756	1.45E-02	1
EIF4EBP2	551720	338	46	310432	85796	0	3	0	0	50	51	22922840	1.49E-02	1
KRT222	953708	269	13	776080	195800	0	8	0	0	50	60	20121832	1.53E-02	1
RPL31	335036	406	23	378784	107156	0	3	0	0	50	36	21770468	1.56E-02	1
CST8	188567	750	-16	368816	97900	0	5	0	0	50	74	22064524	1.58E-02	1
NAP1L1	604083	438	17	1083664	236384	0	9	0	0	50	67	21773672	1.59E-02	1
SNRPB	652241	477	33	613744	197936	0	5	1	0	10	6	4985780	1.60E-02	1
OR4N2	275024	858	-57	754364	231400	0	10	2	0	27	46	10634076	1.61E-02	1
C5orf45	1610057	359	38	877540	252404	0	5	1	0	50	48	18056676	1.63E-02	1
CRISP3	67784	1246	-2	660736	157352	0	6	0	0	50	36	19283096	1.64E-02	1
WTAP	643775	604	19	1036672	267356	0	8	1	0	50	53	20025356	1.64E-02	1
SEMA5B	504904	328	34	2917064	867928	0	17	4	0	50	48	19651556	1.73E-02	1
MICAL2	319572	254	10	2909944	805628	0	16	1	0	13	8	6453924	1.74E-02	1
APLP1	1060028	321	47	1656112	501248	0	12	2	0	50	67	19297692	1.85E-02	1
POM121	641244	332	59	2391252	811680	0	8	0	0	12	2	4691012	1.87E-02	1
CD79A	1360684	454	23	580992	163404	0	4	0	0	50	69	18698900	1.88E-02	1
NF2	734742	149	38	1622292	400500	0	9	1	0	50	55	21913580	1.89E-02	1
DTX2	876888	220	58	1562128	472412	0	5	0	0	9	0	3267368	1.90E-02	1
CASP1	45435	622	-19	1064440	273764	0	8	1	0	41	32	14348580	1.91E-02	1
ZFHX4	72333	731	8	9103276	2528312	0	53	11	0	50	69	26998684	1.94E-02	1
HCRTR2	27686	676	-13	1131724	319688	0	7	1	0	37	51	19557216	1.95E-02	1
SF3B3	905402	131	40	3114644	894628	0	18	2	0	34	40	16234668	1.98E-02	1
HNRNPK	460176	343	2	1250272	338912	0	8	0	0	20	18	8075504	1.99E-02	1

HIST1H2AK	228884	634	40	312924	108936	0	5	0	0	50	48	14804260	2.00E-02	1
RUNX1	164915	429	45	1238524	384836	0	10	0	0	16	21	7080840	2.03E-02	1
CDC42	441481	155	28	572448	162336	0	5	0	0	50	69	23471792	2.04E-02	1
DDAH1	309815	308	0	729088	208616	0	7	0	0	50	64	26465396	2.05E-02	1
CSNK2A1	671817	229	40	1039520	263440	0	10	0	0	50	61	22906820	2.06E-02	1
GAPT	125347	446	5	397296	108936	0	5	0	0	50	46	16544744	2.08E-02	1
ACOT11	688014	415	18	1712716	478820	0	10	0	0	16	11	7164500	2.11E-02	1
IDH1	260367	539	17	1090428	273408	0	8	0	0	50	42	22991904	2.12E-02	1
TRPA1	19123	936	29	2968684	730868	0	16	4	0	29	25	12173064	2.12E-02	1
RIPK4	302894	182	30	1932012	615168	0	14	3	0	31	33	11818488	2.12E-02	1
SALL2	707507	470	26	2435040	798864	0	13	2	0	50	57	21821732	2.16E-02	1
ARIH2	1818938	241	37	1338560	304024	0	9	1	0	50	71	22662604	2.17E-02	1
PHLDB2	192496	160	4	3344620	885728	0	21	5	0	21	20	7718436	2.17E-02	1
SLA2	1019481	176	62	695268	174084	0	5	0	0	50	44	18338984	2.18E-02	1
CCDC88A	621554	341	27	4936652	1197940	0	27	4	0	18	13	6999672	2.22E-02	1
C9orf41	95299	649	-13	1067644	275900	0	8	0	0	50	48	22879052	2.23E-02	1
EIF2B4	1245306	182	52	1395876	436812	0	7	0	0	50	41	22639464	2.24E-02	1
TTYH1	807048	535	-7	1203636	380208	0	8	2	0	50	83	18359276	2.29E-02	1
BRE	536663	376	27	1191532	315416	0	7	1	0	38	31	12724864	2.29E-02	1
RIF1	228411	589	19	6382368	1690644	0	21	1	0	12	3	4857976	2.38E-02	1
ZNF385D	10169	1033	-21	998224	300464	0	10	2	0	50	97	23625940	2.39E-02	1
THUMPD1	179233	641	14	908512	238520	0	4	1	0	50	61	25750904	2.42E-02	1
HIST1H3C	408919	685	43	330368	110716	0	5	1	0	50	57	16881520	2.43E-02	1
ZNF626	222609	1011	-67	1421508	344964	0	12	1	0	14	9	3927392	2.44E-02	1
CFHR4	211450	853	-31	871488	215736	0	9	1	0	7	14	4122124	2.46E-02	1
IPO7	566709	145	21	2755440	676044	0	16	1	0	50	51	18961984	2.46E-02	1
ZNF383	228048	984	-52	1249560	294768	0	6	0	0	50	46	19357500	2.47E-02	1
TAF7L	404269	NaN	29	1248492	288360	0	8	0	0	50	44	21693216	2.48E-02	1
ADH6	167884	786	-3	971880	271272	0	7	0	0	50	33	21521980	2.49E-02	1
HIST1H4K	267617	634	32	248844	86508	0	4	0	0	50	56	15505936	2.49E-02	1
KATNA1	437046	210	41	1280888	336064	0	9	1	0	50	38	16434028	2.53E-02	1
COX6A1	1573849	216	44	278036	85084	0	4	0	0	50	64	21461816	2.53E-02	1
ZNF511	386585	472	13	653972	182272	0	6	0	0	50	46	19712788	2.54E-02	1

CNST	416573	346	4	1884664	498044	0	12	2	0	50	44	19481744	2.58E-02	1
NKG7	368509	610	-9	415096	129584	0	6	2	0	5	5	1191888	2.58E-02	1
BRWD1	383347	235	30	6273076	1606628	0	18	1	0	13	9	9850876	2.59E-02	1
KCTD21	551110	208	23	654328	186188	0	6	0	0	50	61	23537296	2.59E-02	1
SMARCA2	113887	805	18	4149180	1089360	0	22	0	0	1	0	1488792	2.60E-02	1
RBL2	237885	403	-2	2956936	785336	0	11	2	0	50	43	22041740	2.60E-02	1
CRABP1	505232	435	56	360984	93984	0	3	0	0	50	72	22705324	2.61E-02	1
DEPDC5	557139	336	19	4202224	1106804	0	15	2	0	25	25	10969784	2.62E-02	1
TMEM200A	126083	954	23	1226420	354220	0	10	2	0	50	47	21686452	2.63E-02	1
ARL8B	263029	325	22	499824	124956	0	3	0	0	50	50	21875844	2.65E-02	1
ZNF529	644122	889	-48	1483808	340336	0	12	1	0	50	67	17940620	2.66E-02	1
OR10R2	177013	929	7	820580	254896	0	7	2	0	15	9	5331812	2.67E-02	1
RXRG	291474	623	-10	1232828	351016	0	7	0	0	20	12	8966572	2.69E-02	1
SOX2	39313	1104	-14	787116	231756	0	6	0	0	50	87	28734896	2.70E-02	1
RPS13	511749	436	14	399432	113208	0	3	0	0	50	59	20773668	2.70E-02	1
FAM19A4	263741	842	-15	374156	94696	0	5	0	0	50	70	20173808	2.71E-02	1
PPP2R5D	1200368	259	40	1581708	416520	0	10	3	0	50	74	22706748	2.72E-02	1
PDSS1	339802	540	18	1082240	299752	0	8	0	0	50	53	26041400	2.75E-02	1
ZNF800	313691	807	-5	1702392	450696	0	13	0	0	27	30	12943804	2.82E-02	1
PAPD4	260678	684	28	1287652	326096	0	4	1	0	50	43	20299120	2.82E-02	1
CYP46A1	375738	250	21	1287296	377716	0	8	0	0	19	20	9925636	2.84E-02	1
DCLK2	88181	409	-4	1956220	567464	0	11	2	0	50	51	21157792	2.85E-02	1
TNPO1	469585	388	30	2386980	595944	0	11	1	0	13	15	5940928	2.85E-02	1
RAB13	2028800	222	34	548240	137416	0	3	0	0	50	66	18282736	2.86E-02	1
SLC25A27	141781	596	1	838736	233536	0	6	0	0	50	44	20688228	2.89E-02	1
TP53BP2	609494	361	4	2914572	794592	0	14	4	0	50	51	20773312	2.91E-02	1
C4orf22	46363	1141	-4	610540	162692	0	4	0	0	50	61	21150316	2.94E-02	1
DENND5B	300830	361	17	3290508	880032	0	16	2	0	5	6	3379152	2.95E-02	1
ARRDC3	51436	334	5	1070848	288716	0	6	0	0	50	38	19461096	2.99E-02	1
TNFAIP6	216177	589	24	735496	178712	0	7	0	0	50	53	23028216	3.01E-02	1
PBOV1	341233	545	21	345320	92560	0	4	0	0	35	48	15242496	3.01E-02	1
FADD	765202	286	34	509792	164116	0	5	1	0	50	82	26425880	3.02E-02	1
C9orf153	311398	417	8	273764	67996	0	3	0	0	50	58	21260676	3.05E-02	1

SESN3	349302	468	13	1283380	334640	0	11	1	0	50	40	18832400	3.08E-02	1
RSPO2	187803	942	-9	650412	148452	0	5	0	0	50	45	20618808	3.10E-02	1
ZNF513	1244404	179	39	1326100	423284	0	9	1	0	29	38	12368864	3.13E-02	1
THRAP3	742773	170	31	2424004	681740	0	12	1	0	14	14	6586000	3.15E-02	1
DCAF4L2	26672	994	-27	969388	301532	0	10	0	0	7	5	1813820	3.17E-02	1
PIK3CG	171312	700	32	2807772	768960	0	14	3	0	50	53	20751240	3.18E-02	1
LYG2	473611	633	32	559632	144180	0	5	0	0	50	70	26140724	3.18E-02	1
CSRNP3	104412	947	2	1491284	403348	0	7	0	0	19	5	6188348	3.19E-02	1
PLEKHA8	659459	466	26	1177648	288716	0	6	0	0	50	61	22901836	3.19E-02	1
CUL1	517446	532	0	2069428	505520	0	16	2	0	50	78	22507032	3.22E-02	1
S100PBP	1179496	180	20	1057676	285868	0	7	2	0	13	12	4292292	3.25E-02	1
AQP6	1033911	220	48	679604	239944	0	5	0	0	50	62	21015036	3.32E-02	1
ANXA1	247950	411	-15	924888	238164	0	6	0	0	50	31	18382416	3.33E-02	1
UBL4A	2163759	NaN	42	397652	123532	0	6	0	0	50	72	22102972	3.36E-02	1
APOA2	728669	234	37	263440	72980	0	3	0	0	39	44	15898604	3.36E-02	1
TMEM169	603452	419	35	746532	212532	0	5	0	0	50	58	22205500	3.36E-02	1
CYC1	2720766	211	27	813104	259168	0	7	1	0	31	38	13524084	3.41E-02	1
FAM177A1	640994	321	45	619084	159488	0	4	0	0	50	49	19980500	3.47E-02	1
USP7	401069	159	12	2978652	683520	0	15	1	0	12	7	4959436	3.48E-02	1
PIK3R1	48999	619	32	2020656	500892	0	6	1	0	50	30	20178080	3.48E-02	1
TRDMT1	558326	451	5	1031688	269136	0	8	1	0	15	20	7732676	3.51E-02	1
CDH17	474043	543	18	2153800	587756	0	12	3	0	50	64	21600300	3.53E-02	1
DDX52	585337	340	23	1572096	412248	0	8	2	0	50	41	21676484	3.53E-02	1
CPA2	424934	212	30	1099684	292988	0	7	2	0	25	25	7341076	3.54E-02	1
IKZF2	55860	1226	19	1386264	332148	0	11	2	0	50	51	19102960	3.58E-02	1
SNAP29	470051	216	39	667144	179780	0	5	0	0	50	35	16767244	3.60E-02	1
POLA2	3946871	242	49	1551092	440728	0	11	1	0	27	30	12483852	3.66E-02	1
ATAD2	544857	225	30	3628352	943756	0	24	1	0	27	31	10924572	3.68E-02	1
SMCHD1	502296	394	33	5273072	1354936	0	19	2	0	14	11	7646880	3.69E-02	1
FUS	1250318	351	26	1378432	369884	0	6	0	0	12	11	4995036	3.69E-02	1
HIST2H2BI	798975	560	17	312568	96476	0	6	1	0	21	23	6253852	3.70E-02	1
HGS	3745109	205	30	2027064	557496	0	10	1	0	33	25	10695308	3.72E-02	1
TMEM105	2765068	191	24	317196	107868	0	3	0	0	50	55	23441888	3.72E-02	1

FBN2	226750	605	9	7719860	1886800	0	29	3	0	10	10	6342852	3.73E-02	1
MEA1	1200368	259	40	475616	135280	0	4	0	0	50	74	22706748	3.75E-02	1
TIMM17A	751933	168	31	447492	127092	0	3	0	0	50	42	20179148	3.79E-02	1
OR4A47	99957	835	-45	768248	222856	0	6	0	0	18	13	5248508	3.80E-02	1
EIF5A	1911735	206	36	485584	130652	0	5	0	0	50	69	22072000	3.81E-02	1
RANBP3L	213853	402	5	1308300	324672	0	6	0	0	50	33	19018588	3.83E-02	1
ZNF710	848910	192	40	1681032	466716	0	9	2	0	50	48	18274548	3.86E-02	1
MYO1B	480504	416	4	3016388	754720	0	12	0	0	14	7	5458192	3.88E-02	1
GYPA	131210	1033	-8	401212	108224	0	3	0	0	50	46	20182708	3.88E-02	1
TNRC6C	620383	217	26	4337860	1281956	0	12	0	0	2	2	5069440	3.89E-02	1
MAP4K3	363056	435	10	2423292	587400	0	11	3	0	50	61	21055620	3.90E-02	1
OR2AK2	NaN	NaN	NaN	819868	255608	0	6	0	0	50	116	39901192	3.91E-02	1
AIMP2	NaN	NaN	NaN	807408	233892	0	6	0	0	50	116	39879476	3.92E-02	1
ZNF471	135553	817	-69	1649704	376292	0	10	0	0	14	16	7903912	3.92E-02	1
RAB42	816470	183	25	260948	78676	0	2	1	0	9	1	1974732	3.97E-02	1
SLC32A1	450934	551	36	1285160	404416	0	11	4	0	34	43	13522660	3.99E-02	1
CLIC5	62331	701	13	1112500	295124	0	7	1	0	50	51	20593532	3.99E-02	1
SH2D1B	363646	391	1	350660	88288	0	3	0	0	50	54	19589612	4.01E-02	1
C4orf51	221010	1168	26	533644	138128	0	4	0	0	50	39	20567900	4.01E-02	1
CWC27	132018	601	8	1270920	300108	0	7	1	0	50	53	20634116	4.02E-02	1
HIST1H4E	390169	544	57	248132	87220	0	4	0	0	50	61	13862640	4.02E-02	1
RGS18	96080	925	-20	624424	148808	0	5	0	0	50	70	20639100	4.02E-02	1
DCK	267678	690	22	698828	163048	0	5	1	0	19	19	7655780	4.04E-02	1
UPF2	353958	125	28	3358148	810256	0	15	1	0	34	30	17005052	4.05E-02	1
APOC1	1003719	196	6	221788	60164	0	2	0	0	50	42	17116124	4.08E-02	1
SETD2	593886	365	46	6529396	1776440	0	25	1	0	2	2	2358144	4.10E-02	1
RPA3	218130	434	16	325384	80456	0	3	0	0	50	58	20587124	4.10E-02	1
CD1D	120333	711	8	852620	245284	0	11	2	0	12	22	6894652	4.14E-02	1
TLR3	355117	414	-13	2296200	620508	0	10	1	0	50	40	21026072	4.17E-02	1
MSH2	793002	406	28	2430768	631188	0	11	3	0	50	65	26426592	4.17E-02	1
GPC2	1299015	205	52	1413320	483448	0	6	0	0	31	25	11569644	4.20E-02	1
GNRH1	178665	515	21	242080	67640	0	2	0	0	50	47	20796808	4.24E-02	1
TFPI2	228127	480	-12	616592	158776	0	6	0	0	50	63	21073420	4.25E-02	1

SLC24A1	501873	240	44	1216452	321468	0	6	1	0	45	40	18830264	4.27E-02	1
RIMKLB	302894	262	17	978644	282664	0	5	0	0	32	33	15036728	4.28E-02	1
ACSM2B	166410	726	-6	1492352	415096	0	7	1	0	35	30	15301236	4.31E-02	1
PCDH11X	123263	NaN	-21	3461744	1013176	0	15	1	0	8	7	4312584	4.34E-02	1
NEK10	107333	515	-15	1911008	471700	0	13	1	0	15	19	8062688	4.34E-02	1
PYGO1	306625	405	-9	1056252	297972	0	5	0	0	21	14	10290892	4.37E-02	1
PCF11	308252	305	10	3947328	1102176	0	15	2	0	17	9	6498424	4.38E-02	1
MORC1	88595	1029	-12	2639028	634392	0	17	1	0	41	52	19260312	4.39E-02	1
C8orf37	313002	375	45	555716	134212	0	4	0	0	50	56	18502388	4.39E-02	1
PROS1	79315	1341	-10	1771456	457460	0	12	0	0	50	58	24269232	4.40E-02	1
DDX4	331341	486	34	1953372	503028	0	9	1	0	50	43	18082308	4.41E-02	1
SND1	566402	650	22	2347464	669636	0	10	1	0	50	56	19265652	4.43E-02	1
EXOC1	579538	452	12	2361348	583128	0	9	0	0	8	1	2643300	4.47E-02	1
MS4A2	336373	691	4	675332	189748	0	5	0	0	30	49	15492408	4.48E-02	1
LIPH	953031	366	31	1179784	306872	0	5	1	0	50	52	20219732	4.51E-02	1
CPD	491081	265	22	3522620	989680	0	21	4	0	50	60	19058460	4.52E-02	1
KLF6	129035	476	2	734428	188324	0	6	1	0	31	21	8867248	4.55E-02	1
UQCR10	848649	195	37	201140	54112	0	2	0	0	50	54	19150308	4.55E-02	1
RNF216	1965381	220	47	2420088	608760	0	16	3	0	50	57	20102252	4.55E-02	1
ARPC2	724341	197	30	808832	200428	0	5	0	0	50	32	18184480	4.56E-02	1
RNF111	591201	230	31	2512292	705592	0	12	0	0	7	4	2675340	4.58E-02	1
FPGT	NaN	NaN	NaN	1516560	409044	0	9	2	0	50	118	40054628	4.58E-02	1
KIF21A	76823	675	-19	4381648	1143116	0	20	3	0	6	7	3931308	4.59E-02	1
TXNDC15	727517	696	49	909224	264508	0	4	0	0	44	37	19698904	4.61E-02	1
H2AFX	1390854	195	58	337488	126024	0	3	0	0	50	44	17195156	4.63E-02	1
RTP3	289420	565	32	600928	154148	0	3	0	0	50	68	24259976	4.63E-02	1
COPS2	317456	528	8	1221080	277324	0	8	0	0	50	56	21579652	4.63E-02	1
TRA2B	978158	213	30	744752	215380	0	7	0	0	50	59	19409120	4.64E-02	1
AMY2A	27205	1015	-20	1340696	342472	0	8	0	0	9	6	3662884	4.65E-02	1
CDK15	391799	588	32	1015668	273408	0	7	2	0	19	20	10323288	4.66E-02	1
IFT88	399247	434	27	2244224	538984	0	8	1	0	36	31	16798572	4.66E-02	1
NRSN2	879317	185	41	505876	159488	0	3	0	0	50	64	20950244	4.67E-02	1
AKAP6	127586	715	-10	5896428	1592388	0	27	3	0	50	70	29267828	4.68E-02	1

AKR1E2	287187	559	-13	851196	215736	0	6	0	0	50	54	17280596	4.68E-02	1
NKTR	350455	445	42	3775736	980068	0	21	0	0	1	0	1032044	4.69E-02	1
CACNA2D	58044	982	4	2945544	715560	0	18	2	0	50	58	24710672	4.70E-02	1
FCRL4	232460	820	28	1321472	378784	0	10	1	0	50	67	23680408	4.71E-02	1
RBP2	232582	754	21	363476	84016	0	5	0	0	50	63	21978372	4.71E-02	1
PHF6	271272	NaN	47	1072628	252760	0	7	0	0	50	67	21999376	4.72E-02	1
ZMPSTE24	627703	373	18	1237456	326096	0	9	1	0	50	58	21496704	4.73E-02	1
NMRAL1	751633	160	39	761128	223568	0	5	1	0	50	50	17444712	4.75E-02	1
PDE11A	320610	618	18	2566048	689216	0	16	2	0	36	39	16101168	4.77E-02	1
RC3H1	445943	353	13	2876480	835888	0	20	3	0	12	16	6946628	4.80E-02	1
C11orf88	401074	326	18	525100	131720	0	3	0	0	50	56	20209764	4.82E-02	1
FOXE1	468629	276	38	878608	319688	0	6	0	0	50	57	19671848	4.83E-02	1
SPTBN5	874211	259	40	9177680	2766832	0	16	2	0	4	3	5106108	4.83E-02	1
SLC27A5	2673870	276	11	1689576	562836	0	8	2	0	50	60	24216900	4.85E-02	1
ZNF620	195638	546	22	1100396	271984	0	8	1	0	50	48	20952736	4.86E-02	1
ARID4A	290710	552	36	3332160	796728	0	17	1	0	24	14	8645104	4.89E-02	1
CLVS2	138384	1106	-26	839448	228552	0	8	1	0	50	123	28645184	4.92E-02	1
ELAVL3	1255985	265	32	932008	272696	0	6	2	0	50	60	18512000	4.93E-02	1
EIF1	1617880	323	9	302244	75828	0	2	1	0	7	5	2311152	4.93E-02	1
KIAA1841	631979	302	30	2012468	485584	0	8	0	0	36	41	16855888	4.95E-02	1
GLIPR1L2	313411	606	-22	656820	171948	0	6	0	0	6	5	2737284	4.96E-02	1
EXOC3	787542	399	37	1919552	521896	0	7	3	0	50	58	23058832	4.98E-02	1
YME1L1	389022	551	30	2022080	538984	0	9	0	0	2	0	1180140	4.98E-02	1
OR4A16	1067	1102	-42	814884	237096	0	8	0	0	10	13	5818464	5.00E-02	1
NPAS4	1963418	182	55	1985412	617304	0	13	1	0	44	48	19173092	5.00E-02	1
PICALM	366028	313	11	1737636	465648	0	7	0	0	18	15	9425812	5.01E-02	1
NALCN	47011	804	5	4560360	1195092	0	19	1	0	13	11	7466744	5.02E-02	1
ITGA4	233378	518	20	2723044	702032	0	15	0	0	12	13	5814192	5.07E-02	1
GNA13	811904	401	12	961912	262016	0	11	0	0	16	22	5799596	5.10E-02	1
HMG20A	341694	645	30	920972	232468	0	4	1	0	17	23	9342864	5.13E-02	1
CAPN3	592239	284	38	2240308	549308	0	10	2	0	29	33	10407304	5.13E-02	1
ITCH	996942	155	37	2289080	574228	0	10	1	0	46	69	23745200	5.14E-02	1
CREB3L2	227556	553	20	1326456	389820	0	6	0	0	50	35	17889356	5.14E-02	1

SPO11	181292	604	9	1054828	270560	0	6	0	0	14	10	8165928	5.14E-02	1
MTHFD2	1026994	272	50	897120	259524	0	5	2	0	50	51	18634108	5.15E-02	1
CCKBR	466636	728	4	1071560	380920	0	8	1	0	50	57	22924620	5.15E-02	1
ICAM5	1802015	191	21	2226068	782488	0	9	0	0	3	1	1995024	5.15E-02	1
TTLL3	953772	264	43	1959780	568176	0	7	0	0	8	2	3292644	5.15E-02	1
PLA2G2E	437659	348	22	372732	100392	0	4	0	0	50	42	18701748	5.16E-02	1
FAM134B	203311	553	-11	1311860	359560	0	6	0	0	14	21	7376320	5.16E-02	1
ZNF585A	268806	1018	-60	1849776	453900	0	7	1	0	41	33	13166660	5.18E-02	1
DUSP6	236331	448	-23	953012	279460	0	5	1	0	50	106	30544088	5.20E-02	1
COL4A4	248623	465	5	4238180	1379500	0	15	4	0	12	13	6803160	5.22E-02	1
ZC3HC1	473377	146	38	1283380	368816	0	9	1	0	50	51	17580348	5.23E-02	1
F11	364737	606	-12	1641516	422928	0	8	0	0	12	2	3737644	5.24E-02	1
HIST1H3B	408919	685	43	331080	110004	0	11	2	0	9	9	1289432	5.24E-02	1
ATG4C	220392	415	5	1214316	299040	0	6	1	0	50	34	17931008	5.24E-02	1
C3orf62	1661046	201	34	687080	180136	0	5	0	0	27	36	12583176	5.31E-02	1
NPS	470657	703	-30	235672	65504	0	3	0	0	50	60	18654756	5.31E-02	1
KHDC1	742452	565	13	425064	116412	0	4	1	0	4	5	1331084	5.33E-02	1
SPHK2	1597109	242	31	1544328	575652	0	10	2	0	50	57	20781500	5.33E-02	1
EYA4	97438	409	-8	1775016	468852	0	9	2	0	50	47	20565764	5.36E-02	1
MEOX2	25585	1039	-17	770740	215024	0	5	1	0	50	72	22740212	5.37E-02	1
PAPOLA	328369	576	19	1956932	522964	0	9	0	0	16	10	10287332	5.39E-02	1
WDHD1	752481	255	35	2968684	758636	0	13	0	0	1	0	1163764	5.40E-02	1
ELAVL4	93599	824	-20	1067288	282664	0	8	2	0	50	66	19429768	5.41E-02	1
GLYR1	706891	195	33	1447496	393736	0	6	1	0	50	50	19489932	5.44E-02	1
CRADD	375586	385	13	497688	151656	0	3	0	0	50	75	24566136	5.45E-02	1
SLC2A2	167361	735	18	1337492	387328	0	8	2	0	50	50	21212260	5.45E-02	1
RBM8A	706346	230	49	467784	116412	0	3	0	0	50	50	21640884	5.46E-02	1
NDUFA8	220092	528	30	449984	117124	0	4	0	0	50	54	20193388	5.47E-02	1
PDCL	433740	487	10	787472	192952	0	4	0	0	50	48	19317628	5.48E-02	1
CACNG3	360425	348	8	797796	227484	0	5	1	0	50	41	20918560	5.50E-02	1
IFI16	97615	866	7	1891428	490212	0	12	1	0	50	52	19923540	5.51E-02	1
KIAA1522	1339617	195	26	2587764	948384	0	16	3	0	50	75	22599948	5.54E-02	1
TFAP2A	199695	375	6	1157356	339980	0	8	2	0	27	24	10300860	5.56E-02	1

HSP90AB1	1037256	312	54	1888936	478820	0	8	2	0	44	60	16584260	5.57E-02	1
NEK11	312654	797	12	1766472	388752	0	11	1	0	50	53	20648356	5.57E-02	1
TCF21	102722	388	-4	454256	131008	0	3	1	0	41	43	19368180	5.57E-02	1
PLD5	75320	854	-18	1173732	290496	0	7	0	0	50	63	20851276	5.60E-02	1
STAM2	394536	218	24	1385552	355288	0	9	0	0	6	4	2548248	5.60E-02	1
FAM120A	421454	547	35	2921336	845500	0	10	3	0	15	15	6881480	5.63E-02	1
TBL1XR1	135357	433	0	1363836	346032	0	7	1	0	50	51	21514860	5.63E-02	1
UBE2T	813304	325	32	524744	135280	0	4	0	0	50	57	21385276	5.64E-02	1
C12orf4	283278	908	27	1467432	359916	0	7	1	0	36	23	12163808	5.64E-02	1
ACPP	210154	765	13	1117840	291920	0	10	3	0	10	16	5055556	5.65E-02	1
TRIM13	337743	357	44	1046996	274120	0	8	1	0	42	44	16112560	5.67E-02	1
LRRFIP2	392784	376	27	1941268	487364	0	8	0	0	27	15	12139600	5.69E-02	1
ZNF343	652241	477	33	1538988	396228	0	6	2	0	10	6	4985780	5.70E-02	1
RHCE	667862	185	32	1091496	288360	0	9	1	0	50	47	19250700	5.71E-02	1
SHOX2	162819	946	-8	883948	278036	0	6	1	0	50	52	20953092	5.73E-02	1
FAM132A	2356409	196	27	747956	257032	0	4	0	0	50	61	26748416	5.74E-02	1
LYZL2	82218	425	28	512284	131720	0	3	0	0	50	34	19804636	5.85E-02	1
LMO4	279257	521	-10	433252	115700	0	3	0	0	50	62	24546200	5.86E-02	1
ATP10D	93423	528	29	3663240	1002852	0	14	5	0	18	17	8751192	5.86E-02	1
OPN1LW	2438903	NaN	17	928804	264152	0	5	0	0	50	67	30452240	5.87E-02	1
ZNF622	203311	624	-20	1240660	312212	0	7	0	0	27	36	14729856	5.87E-02	1
HEXIM1	806811	207	32	897476	255964	0	9	1	0	50	63	22248576	5.87E-02	1
SDHC	743182	293	35	557852	124600	0	3	0	0	50	77	27478216	5.91E-02	1
GDF11	1814311	384	26	1003564	314348	0	8	0	0	35	27	11847680	5.93E-02	1
PBLD	490115	250	17	825208	232112	0	6	0	0	50	54	21199800	5.94E-02	1
ASB8	511392	352	32	719476	219296	0	4	0	0	50	55	18729516	5.95E-02	1
PARD6A	1065785	147	41	832328	290140	0	7	2	0	21	38	13766876	5.95E-02	1
ZNF236	124234	597	17	4756160	1286584	0	22	5	0	28	33	10624464	5.95E-02	1
RPL23	1255539	159	41	368816	104308	0	3	0	0	50	69	22650856	5.97E-02	1
HUS1B	138359	674	23	671416	224636	0	6	0	0	50	46	20248924	5.98E-02	1
TPO	56524	599	-23	2349600	710220	0	16	4	0	50	55	17505232	5.99E-02	1
DNTT	780047	804	30	1331796	344964	0	8	1	0	50	45	24252856	6.00E-02	1
HPS3	382046	386	7	2586340	702032	0	15	2	0	50	63	24274928	6.00E-02	1

SCNM1	1562281	184	33	599148	168744	0	8	0	0	5	6	1818092	6.03E-02	1
RHBDD1	221611	573	11	823072	215024	0	4	0	0	50	47	18548312	6.07E-02	1
WAPAL	209036	398	4	3099336	793524	0	10	0	0	14	7	5913160	6.10E-02	1
CCDC158	539298	436	24	2926676	740836	0	10	2	0	50	46	20299476	6.10E-02	1
AGL	289704	595	9	4085812	1040588	0	20	2	0	44	41	19618092	6.10E-02	1
ITPRIPL1	904852	213	43	1413676	412604	0	7	1	0	50	63	24111168	6.11E-02	1
STEAP1	128669	1182	-10	877184	231400	0	7	0	0	50	52	25939228	6.13E-02	1
NCOA6	1006385	170	47	5138504	1530088	0	18	1	0	4	1	2424360	6.13E-02	1
TRIP10	736129	272	27	1435748	371308	0	4	0	0	11	9	6014976	6.14E-02	1
PYDC1	NaN	NaN	NaN	222144	72624	0	3	1	0	8	17	3998592	6.14E-02	1
SCAND1	1257811	144	50	419724	161268	0	2	0	0	50	65	22258544	6.17E-02	1
CIAO1	904852	213	43	875760	239232	0	5	0	0	50	63	24111168	6.18E-02	1
FAM49A	8240	1427	-4	867928	212888	0	7	1	0	48	41	20399868	6.22E-02	1
DYNLT3	105234	NaN	28	318976	72980	0	2	0	0	50	59	27275296	6.23E-02	1
NDUFS4	186422	619	5	461376	121752	0	3	0	0	50	47	20980504	6.23E-02	1
EFEMP1	376977	264	-15	1292280	333216	0	8	1	0	50	67	19595664	6.24E-02	1
KDR	175189	706	3	3533300	938416	0	15	2	0	16	15	7273080	6.25E-02	1
HIST1H2Bl	394491	544	55	312212	96832	0	5	0	0	50	53	13774708	6.26E-02	1
RNFT2	225255	411	4	1187616	350304	0	4	1	0	50	33	18818160	6.29E-02	1
ZHX2	426706	533	13	2077972	611252	0	10	0	0	6	1	2171244	6.31E-02	1
DIAPH2	56713	NaN	30	2958716	740836	0	16	0	0	9	17	6191552	6.31E-02	1
PRRC1	210691	763	12	1102532	360628	0	5	0	0	21	28	10516240	6.31E-02	1
OIP5	897414	237	24	578856	177288	0	6	0	0	50	50	15496680	6.35E-02	1
KIT	92734	649	0	2552164	665720	0	10	1	0	5	1	1811328	6.36E-02	1
CLEC4E	321790	564	13	588112	138128	0	5	0	0	50	64	21703896	6.37E-02	1
CASP4	45234	626	-20	995732	251692	0	6	1	0	38	35	14987600	6.39E-02	1
PON1	134258	327	-2	926668	250268	0	4	0	0	50	49	23047084	6.39E-02	1
SMC6	135213	1245	1	2918844	690996	0	8	0	0	11	3	5101124	6.41E-02	1
TCN2	723012	330	56	1091496	313992	0	6	1	0	50	37	15331496	6.42E-02	1
TGOLN2	975949	211	18	1095412	320756	0	8	0	0	9	4	2652200	6.44E-02	1
TMEM67	234269	416	15	2752948	707372	0	13	0	0	7	5	4090796	6.44E-02	1
BGLAP	2266205	211	33	265576	72980	0	2	0	0	50	61	22146048	6.46E-02	1
SUPT3H	68471	830	4	975796	238520	0	5	0	0	50	60	21955232	6.47E-02	1

HAUS3	836839	332	29	1565688	382344	0	11	0	0	26	24	12052380	6.49E-02	1
CD207	596622	629	24	848348	229264	0	5	2	0	33	30	10462128	6.49E-02	1
GAL3ST1	653792	330	28	1044504	322536	0	7	0	0	37	30	15384540	6.49E-02	1
TDRD3	69594	808	16	1929876	514776	0	9	0	0	16	10	6000380	6.53E-02	1
ZFAND2B	773232	177	36	674976	185832	0	5	1	0	50	56	19964124	6.54E-02	1
CCDC47	1259976	346	12	1298332	303668	0	10	1	0	50	91	25277780	6.55E-02	1
SLC30A6	387408	276	25	1200432	337488	0	7	0	0	29	42	15012520	6.55E-02	1
CHRAC1	432697	238	39	343896	87576	0	2	0	0	50	35	17934568	6.64E-02	1
TDRD6	141781	596	1	5267732	1463872	0	18	1	0	22	11	8910324	6.64E-02	1
ANXA7	847821	249	45	1263800	357424	0	5	0	0	50	67	24394188	6.69E-02	1
VIPR2	179290	348	-24	1135640	322180	0	4	2	0	29	35	14049184	6.70E-02	1
C14orf39	325446	618	0	1600220	356356	0	9	0	0	50	64	21583212	6.70E-02	1
CPLX1	694849	268	39	357068	88288	0	3	0	0	50	78	26245744	6.70E-02	1
EIF3I	1460758	218	29	871844	217516	0	7	0	0	50	52	21684672	6.71E-02	1
HK1	712074	229	47	2518344	692064	0	11	3	0	50	40	22123620	6.72E-02	1
RAI1	1199281	260	16	4631560	1495556	0	17	4	0	49	57	22510236	6.73E-02	1
NUCB1	1927185	211	43	1202568	328944	0	7	0	0	50	57	20562560	6.76E-02	1
CCDC102E	70753	1048	-8	1353156	323604	0	7	3	0	50	52	21477480	6.77E-02	1
FAHD2A	255441	536	19	802780	236384	0	3	0	0	50	38	18741976	6.78E-02	1
NIPA2	352778	529	-8	908868	269136	0	4	0	0	50	53	22668300	6.80E-02	1
CALD1	390559	513	17	2138848	519404	0	13	2	0	50	64	22455412	6.81E-02	1
ZBTB7B	1612870	224	35	1312216	426488	0	8	1	0	50	56	18488504	6.84E-02	1
BAMBI	204133	418	13	665720	179068	0	5	1	0	50	50	21099052	6.84E-02	1
LRRC18	NaN	NaN	NaN	657176	188680	0	5	1	0	50	117	39834264	6.92E-02	1
BRCA1	1466229	201	27	4921344	1219656	0	14	1	0	22	8	6972616	6.92E-02	1
NSUN3	85578	1378	-9	875404	238520	0	3	0	0	50	47	24136444	6.95E-02	1
MAGOH	545236	258	25	397652	92560	0	3	1	0	6	4	1742620	6.96E-02	1
RAB38	149319	616	-15	545392	144536	0	5	0	0	50	53	24409140	6.97E-02	1
COL25A1	157338	437	-5	1797800	542188	0	7	1	0	50	51	20741628	6.98E-02	1
AMACR	270768	793	4	1328236	268424	0	7	2	0	50	66	23758728	7.00E-02	1
ACP5	1152381	272	31	807764	251692	0	8	0	0	50	48	19593884	7.01E-02	1
IL10RA	581910	576	49	1450700	432184	0	6	1	0	41	38	14796784	7.03E-02	1
MFSD8	230666	331	9	1343188	370952	0	8	2	0	50	50	19138560	7.07E-02	1

ACTL6A	399933	477	11	1147388	290140	0	10	1	0	50	51	21358576	7.08E-02	1
POP1	635159	368	44	2616600	731580	0	7	0	0	9	2	5371328	7.10E-02	1
CADM3	99569	785	14	1109652	316128	0	4	3	0	36	28	11476372	7.12E-02	1
ACY3	1408104	424	60	799932	250980	0	7	0	0	50	64	18269920	7.18E-02	1
IVNS1ABF	325191	535	-10	1690288	425420	0	9	1	0	50	54	21252844	7.19E-02	1
CEP97	547972	421	9	2219304	600216	0	10	2	0	17	24	9166288	7.19E-02	1
PTBP2	334207	676	-16	1376652	383412	0	8	0	0	25	22	6910672	7.20E-02	1
ZNF582	164565	599	-49	1359564	317196	0	9	0	0	8	8	3522976	7.21E-02	1
C12orf45	514255	204	37	487720	125312	0	4	0	0	50	62	18692848	7.22E-02	1
SLC38A2	755445	311	14	1331440	357068	0	11	3	0	5	8	2807772	7.23E-02	1
BASP1	237391	349	-10	564972	169812	0	3	1	0	50	43	20754444	7.23E-02	1
CCNE2	424520	389	43	1084020	260592	0	5	0	0	50	43	20079112	7.24E-02	1
ARID2	820796	602	10	4610200	1357784	0	29	6	0	0	6	1357784	7.24E-02	1
NSMAF	200152	574	17	2589900	646140	0	13	3	0	35	26	10153832	7.24E-02	1
BEX1	456169	NaN	16	329300	78676	0	3	0	0	50	53	18791104	7.25E-02	1
THAP11	1039476	143	49	766468	242792	0	5	1	0	50	49	20248924	7.26E-02	1
LRRC6	188389	902	-4	1254544	290852	0	8	1	0	50	43	15828472	7.27E-02	1
ZDHHC21	203594	844	-18	695268	186900	0	4	0	0	50	72	22309808	7.29E-02	1
ITGBL1	26094	536	-6	1324320	304380	0	5	0	0	50	61	22001868	7.29E-02	1
NFKBIA	416091	125	31	808120	232112	0	6	0	0	32	27	13205108	7.30E-02	1
HNF4G	47353	914	7	1175512	294056	0	5	1	0	50	57	25542288	7.32E-02	1
ZNF26	NaN	NaN	NaN	1389824	333928	0	8	0	0	50	116	39979512	7.33E-02	1
CCNH	125767	837	8	857960	214312	0	5	1	0	50	65	21749464	7.33E-02	1
AUP1	1019591	315	50	1038452	325384	0	7	2	0	40	63	17658668	7.36E-02	1
RAB21	260669	431	-3	592028	157708	0	2	0	0	50	47	20123612	7.38E-02	1
BRCA2	221300	462	44	8815984	2249564	0	39	4	0	9	11	5327540	7.39E-02	1
EIF3K	1496245	363	27	591672	142044	0	4	1	0	50	65	19481388	7.40E-02	1
NOP16	576954	347	45	467784	121752	0	3	0	0	50	48	20914644	7.40E-02	1
EMD	2277674	NaN	40	652904	185476	0	4	0	0	50	46	20570392	7.41E-02	1
CEACAM8	495357	380	5	879676	265220	0	7	0	0	50	53	24055276	7.43E-02	1
PHF1	1441897	236	31	1522968	356712	0	9	2	0	50	72	19862664	7.44E-02	1
MRM1	468055	548	23	866504	284800	0	7	1	0	50	60	18803564	7.45E-02	1
QPCT	495490	182	32	925600	259880	0	4	1	0	50	62	19063800	7.48E-02	1

CX3CL1	749108	159	39	969744	316128	0	7	1	0	50	52	18373516	7.50E-02	1
TMEM50A	671522	270	35	424352	107512	0	3	0	0	50	57	20137496	7.53E-02	1
APOBEC4	NaN	NaN	NaN	930584	252760	0	7	0	0	50	116	39898344	7.55E-02	1
MCTS1	388105	NaN	7	502316	119260	0	4	0	0	50	56	20001504	7.56E-02	1
GAL3ST4	1299015	205	52	1174800	394092	0	7	0	0	50	42	20842020	7.57E-02	1
ZNF791	1975767	409	1	1501608	359916	0	9	1	0	50	74	27829232	7.57E-02	1
RSRC2	868107	154	39	1179072	291564	0	9	1	0	50	52	20672564	7.59E-02	1
MTMR4	855696	384	23	3060176	848704	0	13	4	0	50	77	27314812	7.60E-02	1
EIF4G3	661924	406	22	4090084	1119620	0	24	5	0	45	72	24004724	7.61E-02	1
TMEM120I	796879	190	43	905664	230688	0	4	0	0	50	58	17354288	7.64E-02	1
FAM76A	735205	210	35	911360	224992	0	6	1	0	50	50	19959496	7.66E-02	1
ENAM	262250	609	16	2914216	782132	0	11	1	0	17	13	9977612	7.67E-02	1
ZNHIT1	1022626	258	39	400500	113208	0	4	0	0	50	67	19705312	7.67E-02	1
POLQ	292766	680	7	6645096	1780356	0	26	3	0	9	13	4844092	7.67E-02	1
CEACAM1	495357	380	5	1417948	408332	0	5	2	0	28	26	11732692	7.67E-02	1
KLK15	655673	589	25	646496	196156	0	6	1	0	50	77	24644100	7.68E-02	1
AFF3	353071	868	12	3217884	891780	0	20	6	0	50	70	18120044	7.68E-02	1
ANKRD35	NaN	NaN	NaN	2493068	772876	0	10	2	0	50	118	40418460	7.69E-02	1
GIMAP2	682986	689	20	866860	224636	0	4	1	0	50	48	19609904	7.77E-02	1
FMR1NB	96935	NaN	-13	673552	170168	0	3	1	0	22	22	9148132	7.78E-02	1
CLIP1	885125	222	41	3714504	959064	0	18	3	0	50	68	24466812	7.80E-02	1
BMPR1B	119865	642	-15	1311504	342828	0	5	1	0	8	6	4890728	7.81E-02	1
PPARGC1A	19326	405	2	2061596	549664	0	10	1	0	50	46	18423356	7.83E-02	1
RFWD3	577167	151	31	1975444	558920	0	8	2	0	50	56	21806780	7.85E-02	1
PCK1	181790	562	-4	1603424	431116	0	9	2	0	50	59	19947392	7.85E-02	1
PDIK1L	783481	212	23	868640	235672	0	4	0	0	26	32	13104004	7.86E-02	1
SNX16	156346	680	6	904952	230332	0	5	0	0	47	61	25211564	7.87E-02	1
KRTAP20-	22916	1048	-79	143824	37736	0	2	0	0	50	84	16257808	7.87E-02	1
GPR85	110068	954	-8	912072	276612	0	5	0	0	50	63	21424080	7.90E-02	1
LCE1F	64902	818	18	295480	84728	0	2	0	0	50	55	20674344	7.90E-02	1
CPEB3	423765	469	33	1793528	513352	0	5	0	0	14	5	4158436	7.92E-02	1
FUCA2	338098	401	20	1202212	325028	0	6	0	0	20	21	11526924	7.93E-02	1
MBP	102723	561	39	868996	247064	0	5	0	0	50	56	17082304	7.94E-02	1

MUCL1	590746	746	-4	232112	74404	0	2	0	0	50	57	21088728	7.96E-02	1
KCNJ15	109140	246	56	939840	269136	0	5	1	0	50	61	21470360	7.96E-02	1
MNDA	97345	985	2	1057676	275188	0	8	0	0	46	54	16686076	7.97E-02	1
NBN	251472	419	35	1985056	498044	0	11	1	0	48	47	18562552	7.98E-02	1
C1orf61	2199280	201	27	407620	116768	0	2	0	0	50	86	24636980	7.98E-02	1
MAPRE2	261776	582	11	941620	233180	0	6	1	0	50	50	19558284	7.99E-02	1
AKR1B15	403585	319	21	916700	231400	0	6	1	0	50	49	23084464	8.00E-02	1
TRERF1	746644	342	44	3022440	881100	0	13	2	0	18	22	9631580	8.00E-02	1
PLN	133888	1029	1	136704	37380	0	2	0	0	50	48	19834184	8.01E-02	1
LTBP1	213347	605	2	4524404	1192600	0	16	5	0	50	51	19617024	8.01E-02	1
STAT1	529053	318	42	2023148	482380	0	11	2	0	50	32	18744824	8.02E-02	1
BTBD8	220071	406	-6	1002852	247776	0	4	1	0	50	44	20928172	8.05E-02	1
C16orf70	1093307	150	47	1128520	290852	0	5	1	0	50	53	22438680	8.07E-02	1
HOXB4	946208	524	29	616948	196868	0	5	0	0	50	60	17564684	8.07E-02	1
RNF141	755456	391	25	604844	156640	0	5	0	0	50	63	27469316	8.09E-02	1
ARHGAP9	1347802	194	32	1842300	575652	0	11	2	0	50	70	20818880	8.10E-02	1
OR4K5	431654	854	-49	793524	242436	0	9	0	0	50	78	18107940	8.11E-02	1
DTWD1	206183	390	-22	799932	194376	0	5	0	0	50	62	25111528	8.11E-02	1
CARS2	532346	216	42	1456040	416164	0	7	0	0	26	18	8707760	8.14E-02	1
CHMP2A	2673870	276	11	581704	156284	0	4	0	0	50	60	24216900	8.15E-02	1
CKS2	163101	627	15	220720	46280	0	3	0	0	50	61	18462516	8.17E-02	1
RPL26	772200	257	20	382344	100392	0	4	0	0	50	52	17300532	8.19E-02	1
HIST3H2A	626588	351	7	314704	109292	0	3	1	0	17	22	7678564	8.19E-02	1
CD274	348920	629	23	763620	194376	0	4	0	0	50	50	21714932	8.20E-02	1
FGF10	107231	886	-39	536136	142044	0	5	0	0	50	64	16624488	8.22E-02	1
LAMA4	131437	282	-7	4881116	1300468	0	21	6	0	50	50	21050280	8.26E-02	1
SPRR1B	687290	704	30	230688	61944	0	2	0	0	50	34	21429064	8.32E-02	1
BRMS1	1963418	182	55	782844	198648	0	5	1	0	50	53	20984776	8.32E-02	1
CT47B1	NaN	NaN	NaN	740836	228908	0	5	0	0	50	116	39874492	8.33E-02	1
FTSJ3	1353057	346	20	2207200	595232	0	9	3	0	50	79	25722780	8.34E-02	1
ERGIC2	100875	599	-3	1016380	250268	0	6	2	0	11	18	5749044	8.37E-02	1
CLVS1	491040	596	1	907088	247420	0	6	0	0	50	81	24640896	8.40E-02	1
ACTL8	249962	416	27	909580	274832	0	6	2	0	32	30	12207952	8.42E-02	1

NGDN	1220464	499	27	843364	220364	0	5	0	0	50	79	23877276	8.45E-02	1
IFI44L	161560	636	-13	1181920	303668	0	7	0	0	50	44	20899692	8.46E-02	1
SNCB	579630	359	40	358136	95764	0	4	0	0	50	54	21554376	8.47E-02	1
TNFSF13B	66992	899	1	728020	211820	0	5	1	0	25	16	9186580	8.48E-02	1
DCLK1	82515	1033	-14	1877544	534000	0	10	0	0	17	15	9027448	8.49E-02	1
PRPS1	226650	NaN	57	820224	227484	0	4	2	0	50	61	24225800	8.50E-02	1
WFDC2	1053862	382	36	325384	89000	0	2	0	0	50	76	17660804	8.51E-02	1
STAU1	877789	147	49	1499116	423284	0	9	2	0	50	77	23875852	8.54E-02	1
PAGE5	140886	NaN	18	345320	91492	0	3	0	0	50	55	17222212	8.54E-02	1
FLG	433645	548	21	10044184	2979008	0	61	11	0	27	45	14085496	8.54E-02	1
OPCML	9310	1074	-19	934144	269492	0	7	2	0	50	73	19704956	8.54E-02	1
CRHR2	484075	433	32	1074052	292988	0	5	2	0	36	33	13132128	8.56E-02	1
CRAT	1428394	206	30	1611612	452832	0	9	0	0	50	55	20767616	8.56E-02	1
RBM27	394957	229	31	2728740	756144	0	11	2	0	43	36	17421216	8.56E-02	1
TAF15	620019	235	8	1551092	415096	0	7	0	0	43	53	21666160	8.57E-02	1
HIST1H4A	408919	685	43	249556	85796	0	2	0	0	50	57	16881520	8.59E-02	1
MACF1	767679	401	25	19222576	5170544	0	77	10	0	10	17	8731256	8.60E-02	1
GABRA6	12083	986	-6	1168036	320756	0	7	0	0	34	44	11061276	8.61E-02	1
GRB14	71885	630	-5	1420084	368816	0	6	2	0	38	33	14674320	8.63E-02	1
MEF2A	197556	416	12	1417592	393736	0	6	0	0	50	51	22730244	8.63E-02	1
CADM2	8467	1155	-19	1180140	328944	0	11	2	0	16	20	5719496	8.65E-02	1
CTHRC1	428438	615	23	612676	184052	0	3	0	0	50	52	16150296	8.67E-02	1
NDC80	374218	390	0	1723752	406908	0	12	2	0	50	50	19166684	8.69E-02	1
CAMKK2	981640	258	42	1545040	419012	0	5	0	0	16	19	9214704	8.70E-02	1
SMG7	716427	385	25	3255620	887152	0	10	2	0	24	30	14903228	8.70E-02	1
HD1-EIF4I	688522	296	30	6630144	1934148	0	28	5	0	31	30	15168448	8.72E-02	1
SAMD7	400118	622	17	1141692	320400	0	6	0	0	50	55	20217596	8.76E-02	1
LRP6	412583	408	15	4144908	1120332	0	16	4	0	50	38	14295892	8.78E-02	1
ALG1L	213057	583	19	493772	132076	0	4	0	0	50	28	15956988	8.78E-02	1
RCOR2	1321109	201	51	1320760	405128	0	5	0	0	50	43	20743408	8.78E-02	1
OR2V2	978482	478	29	781776	229620	0	6	1	0	50	67	21549748	8.79E-02	1
PRKAR1B	543988	401	7	986120	280528	0	4	1	0	50	59	24878704	8.80E-02	1
OMP	437413	450	40	402280	122108	0	4	0	0	50	69	21014324	8.81E-02	1

C1QTNF7	144115	540	20	740124	220008	0	5	1	0	4	3	2249920	8.84E-02	1
MMD2	1539893	542	5	704168	189748	0	4	1	0	50	83	26270664	8.86E-02	1
PARP14	427462	423	47	4622660	1221436	0	17	4	0	24	26	11210440	8.86E-02	1
UCP1	125708	752	7	774300	233892	0	4	0	0	50	77	23962716	8.87E-02	1
NDUFAF1	897414	237	24	845856	224280	0	5	0	0	50	50	15496680	8.88E-02	1
GHSR	327135	420	17	953368	306872	0	5	2	0	40	55	19696056	8.89E-02	1
ENO3	1416161	231	30	1127096	315772	0	5	1	0	50	65	18681100	8.89E-02	1
C1orf35	702621	391	5	690996	186900	0	5	1	0	50	46	17014308	8.89E-02	1
ODC1	732059	270	47	1206840	316128	0	5	1	0	50	54	22328320	8.90E-02	1
IQCB1	371177	520	32	1570316	404416	0	10	1	0	50	52	21325824	8.91E-02	1
FAM78B	320493	733	-12	651124	192596	0	4	1	0	50	66	19477116	8.93E-02	1
CALM1	323571	599	21	410468	91492	0	2	0	0	48	43	20523756	8.95E-02	1
PRPF19	794900	522	47	1311860	370240	0	6	1	0	50	51	21913580	8.95E-02	1
CDK13	221361	636	14	3770396	1132792	0	12	3	0	12	30	12996492	8.97E-02	1
SLC28A3	396533	595	2	1804564	491636	0	9	1	0	50	70	22864456	9.05E-02	1
OR2T3	112460	903	-20	786048	236028	0	5	1	0	50	74	22093716	9.09E-02	1
PLA2G4C	983603	431	26	1479892	396584	0	9	1	0	50	83	25596756	9.12E-02	1
STON1	NaN	NaN	NaN	1868644	504452	0	12	3	0	50	119	40150036	9.13E-02	1
GMFB	466180	371	28	395516	90424	0	3	0	0	50	50	17319044	9.14E-02	1
FAM184A	115280	856	-1	3015676	712712	0	10	1	0	50	54	23034980	9.14E-02	1
CACNG7	813774	360	-33	703100	200428	0	5	0	0	16	28	7035272	9.14E-02	1
C8orf86	840384	454	46	553580	174796	0	4	1	0	50	68	24507752	9.14E-02	1
SKP1	766424	144	54	464224	107156	0	4	0	0	50	96	21985492	9.15E-02	1
ATP5S	666223	337	28	614456	145960	0	3	1	0	6	6	2954088	9.16E-02	1
MRPS24	872910	239	44	425064	128160	0	3	0	0	50	64	22627716	9.19E-02	1
EDN2	220093	381	32	451052	141688	0	3	0	0	50	53	21344692	9.25E-02	1
HSPA4L	222085	352	31	2226780	541476	0	9	2	0	50	48	19084448	9.26E-02	1
NHLRC4	1115707	213	39	294768	102528	0	2	0	0	50	53	16969808	9.27E-02	1
ERP44	246986	681	19	1090072	260948	0	5	0	0	50	57	21080540	9.31E-02	1
NAA15	389880	299	26	2323968	535068	0	14	1	0	50	59	24407716	9.31E-02	1
PRDX3	789279	149	29	665008	186188	0	3	0	0	50	67	23654420	9.32E-02	1
WWC3	169744	NaN	34	2771104	824852	0	8	3	0	29	31	14260292	9.34E-02	1
ERI3	758083	308	29	873624	243504	0	5	0	0	50	40	24339720	9.35E-02	1

UBE2L3	521773	194	37	411536	100036	0	3	0	0	50	70	19466792	9.39E-02	1
RAB3GAP1	358477	577	26	2588120	685300	0	11	1	0	8	9	4867588	9.41E-02	1
OR52A5	109516	1024	-15	783912	230688	0	5	1	0	9	5	3195456	9.41E-02	1
SDHAF2	1404745	245	58	438236	111784	0	3	1	0	50	41	15958412	9.42E-02	1
FBN3	914975	258	15	7301916	1968324	0	26	7	0	50	62	22031416	9.48E-02	1
RPS18	1456953	262	30	401924	113920	0	5	0	0	50	71	20945260	9.48E-02	1
MPEG1	542222	686	9	1766116	531152	0	8	2	0	50	72	23453636	9.48E-02	1
MLH1	392784	473	21	1977580	524744	0	9	2	0	24	30	11410512	9.48E-02	1
SLC27A2	308506	282	1	1579216	448916	0	9	1	0	50	55	27381384	9.50E-02	1
TMEM79	2266205	211	33	954080	324316	0	5	1	0	50	61	22146048	9.51E-02	1
OR6Y1	179404	940	1	804204	238164	0	5	1	0	50	67	22978376	9.53E-02	1
FBXO43	810757	396	25	1804920	488076	0	11	2	0	50	67	28712824	9.55E-02	1
ARPP19	469724	386	15	293344	77252	0	2	0	0	50	61	19857324	9.57E-02	1
RBM18	319049	597	19	499468	133856	0	2	0	0	50	39	24640896	9.61E-02	1
ELOVL7	188782	596	4	752584	180848	0	4	1	0	50	50	17872980	9.64E-02	1
PRH1	NaN	NaN	NaN	425420	131008	0	3	1	0	50	117	39776592	9.64E-02	1
FAP	70846	615	5	2052340	492704	0	11	0	0	22	37	12327924	9.66E-02	1
FAT1	351973	513	-13	11525144	3289084	0	40	9	0	8	21	5948760	9.66E-02	1
DHX30	843941	197	34	3001080	929160	0	9	2	0	19	19	8445388	9.69E-02	1
HIST1H2BI	390169	544	57	312212	96832	0	6	2	0	15	17	2533296	9.69E-02	1
LARP4B	378329	515	9	1895700	542544	0	7	2	0	50	47	18497404	9.69E-02	1
UCHL3	197113	362	37	623000	153436	0	4	0	0	50	50	20555084	9.71E-02	1
CCDC92	903375	539	36	839092	241724	0	5	1	0	50	46	18738416	9.72E-02	1
IFIT2	253000	263	27	1209688	310076	0	5	0	0	50	59	19597800	9.73E-02	1
CTNND1	921177	497	8	2485948	699896	0	7	0	0	9	3	2826640	9.74E-02	1
ST3GAL6	338260	516	10	881100	221076	0	5	1	0	50	47	21678264	9.77E-02	1
PSMA8	168448	577	57	672484	176576	0	5	0	0	50	53	16561120	9.78E-02	1
TGFBR1	201435	211	30	1294772	356356	0	7	2	0	50	60	19817096	9.78E-02	1
GFM1	197292	890	-9	1948388	535780	0	9	1	0	50	86	29038208	9.79E-02	1
ASPSCR1	3814891	211	28	1408692	434676	0	5	2	0	32	25	10015348	9.83E-02	1
MFF	251488	144	18	877896	255252	0	4	1	0	50	39	21090864	9.83E-02	1
DHX15	194657	491	35	2045576	560344	0	11	3	0	50	66	20769752	9.84E-02	1
SLFN11	761918	456	11	2280180	626916	0	11	0	0	5	2	2080464	9.84E-02	1

BANP	639210	231	31	1390892	385192	0	6	0	0	4	1	1412608	9.85E-02	1
KLHL12	422793	318	28	1467788	402280	0	5	0	0	24	6	8926344	9.86E-02	1
TEX14	735964	268	13	3893928	1022076	0	16	3	0	8	6	4752956	9.86E-02	1
ZSWIM2	278175	629	-5	1655044	412604	0	9	2	0	19	21	9005376	9.87E-02	1
SRI	157359	454	23	543256	137060	0	2	1	0	38	39	14524800	9.88E-02	1
REEP3	207194	412	22	672484	177644	0	3	0	0	44	48	18744468	9.88E-02	1
TMEM71	166227	872	-6	788184	198648	0	5	0	0	50	56	21469648	9.89E-02	1
C15orf52	872646	184	42	1346392	412604	0	7	2	0	50	55	20353232	9.89E-02	1
NFE2L3	910990	445	39	1737636	501960	0	9	1	0	24	16	8865112	9.90E-02	1
C7orf33	473795	593	-4	440728	138128	0	3	0	0	50	62	20687160	9.90E-02	1
VEGFA	1564130	278	41	1071204	307584	0	4	0	0	50	57	21752668	9.91E-02	1
GPR141	103891	630	6	764688	215736	0	8	0	0	50	75	22283464	9.91E-02	1
FAM83G	NaN	NaN	NaN	2028844	628340	0	12	1	0	50	117	40273924	9.92E-02	1
HNRNPA3	374611	145	30	992172	264864	0	7	0	0	21	19	9840196	9.92E-02	1
SULF1	137404	578	-19	2306524	568532	0	12	0	0	7	6	4212548	9.92E-02	1
CPA4	424934	212	30	1106448	290496	0	6	1	0	35	41	14766880	9.93E-02	1
RNF112	588566	414	6	1354936	415808	0	9	3	0	6	8	3064804	9.93E-02	1
SULT1C4	361799	483	-2	811680	184764	0	4	1	0	50	72	23161004	9.94E-02	1
C1orf54	1213150	197	38	357424	89000	0	4	0	0	50	58	21071640	9.96E-02	1
CD200	260383	621	20	753296	217516	0	5	0	0	3	0	1109296	9.98E-02	1
MGAT1	830596	566	25	1079036	349948	0	9	1	0	50	93	28405240	9.99E-02	1
LARP4	855863	125	41	1886444	502672	0	8	2	0	50	54	24607076	9.99E-02	1
CORO1A	NaN	NaN	NaN	1168392	353508	0	7	2	0	50	118	39999092	1.00E-01	1
BSX	698665	416	8	572804	184408	0	4	0	0	50	44	16918900	1.00E-01	1
TNNI1	857879	209	26	494128	138128	0	5	0	0	50	50	16783264	1.00E-01	1
SPATA12	418332	332	46	465292	146672	0	3	0	0	50	52	20246076	1.00E-01	1
C12orf5	295315	860	33	702744	189036	0	4	0	0	50	40	17136416	1.00E-01	1
ADIPOR1	421206	389	30	960844	273764	0	7	1	0	48	58	18106160	1.00E-01	1
ZNF35	434068	541	34	1356716	343540	0	10	0	0	11	9	4909596	1.00E-01	1
CNTNAP4	11522	975	-26	3401580	904596	0	18	3	0	21	34	11094028	1.01E-01	1
MSC	25834	905	6	499468	168032	0	4	1	0	37	37	15161328	1.01E-01	1
COIL	333715	254	-10	1459600	416876	0	5	0	0	50	46	20300900	1.01E-01	1
FXYD6	473498	601	41	273408	64080	0	2	0	0	50	83	23683612	1.01E-01	1

PLEK	208260	586	23	932008	226772	0	5	1	0	50	50	21405212	1.01E-01	1
GNG11	228127	480	-12	195088	46280	0	1	0	0	50	63	21073420	1.02E-01	1
CGA	209764	625	-3	306872	80812	0	2	0	0	50	50	20723116	1.02E-01	1
PRKCQ	235334	561	7	1887156	450696	0	8	1	0	50	62	22975884	1.02E-01	1
ZRANB1	287282	361	22	1819516	486296	0	10	0	0	27	27	13358900	1.02E-01	1
MEP1A	137754	431	2	1959424	491636	0	6	2	0	25	19	7767920	1.02E-01	1
POT1	34691	524	-16	1662520	436100	0	13	1	0	50	75	22637684	1.02E-01	1
PRDX4	494082	NaN	35	694556	206836	0	5	0	0	50	68	21432980	1.02E-01	1
CEACAM2	1044508	451	31	751872	215736	0	6	0	0	50	62	20279540	1.02E-01	1
NUPL2	509506	218	23	1077968	306160	0	8	1	0	50	52	17724172	1.02E-01	1
C1orf112	212980	614	8	2241732	590604	0	8	2	0	43	44	19315848	1.02E-01	1
HOOK1	59567	747	-3	1969392	456036	0	6	0	0	34	36	18682880	1.03E-01	1
AXIN2	212714	773	-26	2130304	612320	0	15	2	0	50	87	21610980	1.03E-01	1
GPR6	209151	624	13	846924	318264	0	6	1	0	50	63	24306968	1.03E-01	1
HMOX2	751633	160	39	821648	215380	0	4	1	0	50	50	17444712	1.03E-01	1
PDSS2	235480	588	33	1023500	288004	0	7	1	0	50	45	21604572	1.03E-01	1
GOLIM4	147581	681	-8	1869356	427912	0	8	1	0	50	63	26573620	1.03E-01	1
TAS2R60	371260	379	1	782844	238164	0	5	1	0	50	51	20287016	1.03E-01	1
CBFA2T2	613808	148	52	1567824	451764	0	12	3	0	45	60	15730928	1.03E-01	1
ARL6	89238	731	-5	506944	122108	0	2	0	0	50	55	23802160	1.03E-01	1
ZDHHC6	197289	325	3	1098616	266288	0	4	0	0	50	44	20520196	1.03E-01	1
RWDD1	254581	479	14	661804	145604	0	4	0	0	50	42	18121112	1.03E-01	1
GPD1L	466186	403	6	904952	252760	0	5	1	0	50	56	24088028	1.04E-01	1
TPT1	680643	296	78	471700	106088	0	3	0	0	50	51	21491720	1.04E-01	1
CDO1	384075	759	1	530796	130296	0	3	0	0	50	61	23231848	1.04E-01	1
IFNGR2	868693	182	68	862944	245640	0	5	0	0	19	14	6984364	1.04E-01	1
TIFAB	625030	509	47	388040	135280	0	2	0	0	50	53	24138580	1.04E-01	1
C11orf71	157610	477	-8	358848	121752	0	2	0	0	50	63	23313372	1.04E-01	1
VIT	577288	323	-8	1884308	534712	0	12	2	0	50	61	20722760	1.04E-01	1
RFC4	449980	384	25	951232	262016	0	7	1	0	50	53	20002928	1.04E-01	1
RAB8B	451708	214	44	559276	139196	0	3	1	0	50	44	17735564	1.05E-01	1
EZR	564251	163	55	1550024	386260	0	8	1	0	50	70	19554724	1.05E-01	1
SMARCA1	278446	NaN	-7	2781784	700964	0	7	1	0	9	11	4876844	1.05E-01	1

DZIP1	229772	438	45	2295844	566396	0	13	3	0	50	67	23081972	1.05E-01	1
HTR2B	NaN	NaN	NaN	1197584	355288	0	6	1	0	50	117	40000872	1.05E-01	1
PRUNE	1617972	165	35	1143472	341048	0	9	1	0	18	28	7487036	1.05E-01	1
ARMC3	164960	449	12	2269144	603776	0	12	2	0	50	55	20417668	1.05E-01	1
ZNF502	433000	541	28	1417236	337488	0	9	0	0	40	36	15331140	1.05E-01	1
SNRPA	1127178	201	27	725528	202564	0	7	2	0	8	14	2966192	1.05E-01	1
HTN1	59679	975	3	174796	32396	0	2	0	0	50	56	23451500	1.06E-01	1
FUT5	1217326	294	35	923820	281952	0	5	0	0	50	56	18189820	1.06E-01	1
CARHSP1	401329	247	5	365968	121040	0	2	0	0	50	59	23017180	1.06E-01	1
HOXA6	356045	385	28	589180	164828	0	5	1	0	9	4	2886804	1.06E-01	1
CDH1	924096	240	30	2241376	651836	0	8	3	0	50	54	18167036	1.06E-01	1
IL33	355118	432	5	726952	169100	0	6	0	0	50	47	16560052	1.06E-01	1
SF3B1	736523	276	30	3379152	930228	0	17	1	0	12	12	6941644	1.06E-01	1
CXCL17	704668	235	34	318264	79032	0	2	0	0	50	55	18620580	1.06E-01	1
TMEM143	1478750	217	36	1129232	376648	0	7	1	0	50	89	31278516	1.06E-01	1
HSPB3	61471	617	14	370240	113564	0	3	0	0	50	61	22644804	1.06E-01	1
HIST1H4J	267617	634	32	248844	86508	0	2	0	0	50	56	15505936	1.06E-01	1
RNASE10	464497	395	17	547884	146316	0	2	0	0	50	58	21385632	1.07E-01	1
TSPAN18	174144	622	15	639732	187968	0	3	0	0	50	60	17878676	1.07E-01	1
NOP10	542657	234	22	169100	45568	0	2	0	0	50	46	18844148	1.07E-01	1
CCND3	467876	186	41	724460	231400	0	5	2	0	48	56	17186256	1.07E-01	1
SLC23A3	773232	177	36	1559636	470632	0	9	0	0	12	9	4375240	1.07E-01	1
CD244	1118846	583	28	969388	253472	0	7	0	0	50	65	17825632	1.07E-01	1
LAX1	515575	280	39	1056608	284800	0	7	0	0	37	25	12651528	1.07E-01	1
GJA9	524940	458	24	1295484	362052	0	7	0	0	39	34	14577132	1.07E-01	1
CTXN3	284904	682	29	204700	62300	0	1	0	0	50	46	22653348	1.08E-01	1
SLIT2	44181	1366	3	4021732	1034180	0	12	0	0	5	1	3562136	1.08E-01	1
MPZ	735152	293	41	632968	188324	0	3	0	0	50	46	22289160	1.08E-01	1
CUTC	709789	139	18	709508	202564	0	5	0	0	50	46	22013972	1.08E-01	1
TECR	1008795	204	26	823784	217516	0	3	1	0	50	74	20546896	1.08E-01	1
CARD6	212186	526	19	2613040	723392	0	10	3	0	50	42	19365688	1.09E-01	1
GCSH	287527	327	13	447136	129584	0	2	0	0	50	44	21348252	1.09E-01	1
ELMO1	63863	859	16	1945896	476328	0	10	1	0	50	61	20144616	1.09E-01	1

APC	247811	399	28	7178028	1998228	0	22	4	0	50	56	24417328	1.09E-01	1
NDN	59699	961	-58	791032	242792	0	3	1	0	45	38	14448972	1.09E-01	1
GIF	366560	494	8	1075832	299752	0	4	2	0	50	49	19554724	1.09E-01	1
RND1	1655612	214	57	596656	168032	0	4	1	0	50	64	19943476	1.09E-01	1
EDA	135563	NaN	-2	1090428	336420	0	5	0	0	24	23	14862288	1.09E-01	1
FTL	1927185	211	43	452476	124244	0	3	0	0	50	57	20562560	1.09E-01	1
IKZF3	1607516	292	26	1344612	321468	0	5	0	0	15	9	5823448	1.10E-01	1
SOCS6	64018	264	7	1332152	385192	0	7	2	0	44	46	19295200	1.10E-01	1
MPP6	254420	405	18	1412964	367392	0	9	0	0	13	10	5969764	1.10E-01	1
CNOT1	660382	191	8	6340004	1713784	0	19	4	0	7	10	4862248	1.10E-01	1
RABEP2	1189140	257	55	1436460	443220	0	7	1	0	50	73	21972320	1.10E-01	1
CAMTA1	289717	462	23	4265236	1192244	0	16	5	0	50	74	23353956	1.10E-01	1
MAPK8	107152	633	31	1204704	297972	0	4	0	0	50	39	23296996	1.10E-01	1
NSUN6	74263	512	-4	1216096	332504	0	8	0	0	14	21	8690316	1.10E-01	1
SPDYE1	1042621	239	49	880032	225348	0	4	0	0	50	57	19388116	1.10E-01	1
SKIL	368934	202	23	1762200	453900	0	10	1	0	30	29	15265992	1.10E-01	1
NDUFV2	419199	391	53	655396	177644	0	4	0	0	50	61	21298412	1.10E-01	1
ZNF623	2198262	229	33	1371668	351016	0	10	1	0	46	47	19989044	1.10E-01	1
GYPB	146672	990	-12	245996	67996	0	2	0	0	50	58	19488152	1.10E-01	1
UTP14C	385945	461	41	1931300	526168	0	8	3	0	15	19	7166636	1.11E-01	1
SLC29A4	1791782	289	11	1328592	418656	0	7	1	0	13	15	8217192	1.11E-01	1
RIPPLY2	108781	870	-22	337844	88288	0	4	0	0	50	68	20592108	1.11E-01	1
MRPL44	314020	346	23	838380	243504	0	5	0	0	50	48	22850572	1.11E-01	1
RNF182	256934	590	20	609116	185476	0	3	0	0	37	21	15932068	1.11E-01	1
MBD6	1347795	303	44	2369536	894272	0	15	4	0	40	78	26763012	1.11E-01	1
GTSF1	642618	190	38	469564	102884	0	2	0	0	50	46	21238248	1.11E-01	1
LMAN2L	921353	219	45	933788	255964	0	5	0	0	29	30	14102940	1.11E-01	1
RNF2	346010	458	-1	886440	218940	0	4	0	0	50	67	23809992	1.12E-01	1
CCDC23	820426	426	30	182628	40584	0	1	0	0	50	65	22644448	1.12E-01	1
PPP1R11	1820240	496	30	320044	95408	0	2	0	0	50	54	18891140	1.12E-01	1
ATOX1	584468	406	46	188680	47348	0	1	0	0	50	66	24436196	1.12E-01	1
DGKA	2080397	229	31	1948388	508012	0	6	3	0	11	12	5452140	1.12E-01	1
PRAME	190814	641	23	1278396	377004	0	5	2	0	50	48	19743048	1.12E-01	1

AIM1L	1021101	217	22	1592388	452832	0	10	0	0	6	1	1611612	1.12E-01	1
PROL1	264684	915	25	602708	203632	0	3	0	0	50	42	17431540	1.13E-01	1
TGFBRAP1	191525	381	4	2180856	627984	0	8	3	0	50	40	19769036	1.13E-01	1
XRCC2	256955	444	6	719476	191528	0	4	0	0	50	42	19305880	1.13E-01	1
SAP18	447305	240	43	443576	125668	0	4	0	0	50	47	18943116	1.13E-01	1
TRA2A	432206	231	23	719476	217160	0	4	0	0	50	44	18255324	1.13E-01	1
SORCS1	6116	881	-26	3194744	907444	0	18	5	0	50	90	21258184	1.13E-01	1
ITLN2	777836	441	19	855824	220720	0	4	1	0	50	69	22066304	1.14E-01	1
ZFP2	95669	845	10	1195804	288716	0	6	0	0	40	55	18453972	1.14E-01	1
ZNF569	234564	939	-52	1805632	412604	0	8	2	0	48	51	15925304	1.14E-01	1
MTHFD1L	341712	438	21	2519056	733004	0	10	1	0	24	25	11947716	1.14E-01	1
GHRHR	390101	631	4	1129588	325028	0	5	2	0	44	66	19025352	1.14E-01	1
YWHAQ	700762	154	40	642224	167320	0	4	1	0	46	46	17670416	1.15E-01	1
C14orf2	694566	320	33	210396	45924	0	1	0	0	50	55	25165284	1.15E-01	1
NUDC	1025094	188	34	878608	219296	0	4	2	0	15	21	6155240	1.15E-01	1
SPARCL1	526761	537	16	1761132	412248	0	8	1	0	50	65	20563984	1.15E-01	1
CDHR5	1774224	236	57	2079396	690996	0	12	2	0	50	45	17867640	1.15E-01	1
DPYS	39710	972	15	1320404	384124	0	10	3	0	29	46	13340744	1.15E-01	1
MAS1L	404210	854	21	932720	279460	0	4	1	0	50	48	16616300	1.16E-01	1
RAB11B	726988	204	0	564260	156640	0	3	0	0	50	52	20477120	1.16E-01	1
DCPS	367696	426	30	855112	249200	0	5	1	0	18	19	8476360	1.16E-01	1
HIST1H2BC	228884	634	40	315060	96120	0	2	0	0	50	48	14804260	1.16E-01	1
CROT	222199	876	3	1694204	432184	0	8	2	0	38	52	15591376	1.16E-01	1
MFSD11	827288	206	22	1181920	315416	0	6	2	0	50	41	14517680	1.16E-01	1
PIBF1	258763	533	30	2018520	482736	0	12	1	0	50	81	24135020	1.16E-01	1
CLDN17	10231	994	-69	549308	175864	0	5	1	0	50	68	13239640	1.17E-01	1
ERP29	812811	317	45	662160	185832	0	7	0	0	50	88	27070596	1.17E-01	1
IGFL3	1177162	249	28	331080	87576	0	2	1	0	15	12	5112872	1.17E-01	1
ZNRF2	626711	388	42	603776	191884	0	2	0	0	50	64	24160652	1.17E-01	1
CYP2R1	353856	596	-13	1276260	349236	0	5	0	0	8	1	2338564	1.18E-01	1
GJA5	NaN	NaN	NaN	888576	265932	0	5	1	0	50	117	39911516	1.18E-01	1
MAD2L1BP	1424296	166	44	808120	241724	0	6	0	0	50	77	23797532	1.18E-01	1
GDF6	215566	720	-7	1103600	363832	0	4	0	0	20	17	11374200	1.18E-01	1

UBXN4	511712	403	25	1332864	349236	0	5	0	0	12	5	4843736	1.18E-01	1
BTBD2	1899295	239	37	1306164	413316	0	6	1	0	50	67	24113304	1.18E-01	1
MBLAC1	1299015	205	52	621220	238520	0	4	0	0	50	42	20842020	1.19E-01	1
PTN	189251	879	-26	449272	108224	0	4	0	0	50	83	20214392	1.19E-01	1
CAPSL	138289	614	0	548240	136348	0	5	0	0	50	43	19196944	1.19E-01	1
KRTAP12-[illegible]	819099	391	35	239944	72980	0	1	0	0	50	61	23447584	1.19E-01	1
TMCO6	774470	356	31	1237456	394448	0	4	3	0	50	59	20786128	1.19E-01	1
IGSF11	197243	714	3	1127096	341404	0	5	2	0	50	68	24048512	1.20E-01	1
ZNF548	451095	869	-24	1423288	338912	0	10	1	0	50	68	17938840	1.20E-01	1
ARPC4	953772	264	43	451052	111784	0	3	0	0	50	72	27173124	1.20E-01	1
DDX5	1171555	184	15	1602712	419012	0	8	1	0	23	22	8280916	1.20E-01	1
DNM2	2114424	208	31	2356008	672840	0	11	3	0	9	12	5271292	1.20E-01	1
C5orf58	97631	636	31	275544	67284	0	2	1	0	8	4	1936284	1.20E-01	1
BAZ2B	351229	414	23	5621596	1477400	0	21	1	0	2	2	2702396	1.21E-01	1
ZNF470	135553	817	-69	1862236	455324	0	8	0	0	14	16	7903912	1.21E-01	1
LANCL2	752223	455	7	1151304	327876	0	5	1	0	50	34	15986892	1.21E-01	1
COG7	521590	611	28	1990396	548240	0	6	2	0	21	24	11306916	1.21E-01	1
MTA1	773175	244	40	1860100	519404	0	5	0	0	15	12	8390920	1.22E-01	1
STRA8	425774	594	40	865792	226772	0	6	1	0	50	71	22560076	1.22E-01	1
BHLHE41	151502	396	15	1176580	388040	0	5	1	0	50	50	17846992	1.22E-01	1
OR6F1	191710	995	-32	750092	239944	0	9	2	0	27	75	17761196	1.22E-01	1
POLR2I	978272	326	6	348168	79032	0	3	0	0	50	68	15103300	1.22E-01	1
ATP7B	353147	461	28	3683176	1099328	0	9	1	0	12	3	7052004	1.22E-01	1
C2orf15	NaN	NaN	NaN	322180	90068	0	2	0	0	50	116	39735652	1.22E-01	1
SERTAD2	632067	332	25	775724	237808	0	3	0	0	50	44	26090884	1.22E-01	1
HIST1H2BI	408919	685	43	312924	96120	0	2	0	0	50	57	16881520	1.23E-01	1
EAF2	474007	550	30	688148	171592	0	4	1	0	50	52	19828844	1.23E-01	1
DENND4C	499163	277	13	4333944	1147032	0	21	0	0	6	5	3438248	1.23E-01	1
POU4F2	38882	987	-10	1007836	310076	0	6	2	0	50	74	23513088	1.23E-01	1
KRTAP5-1	NaN	NaN	NaN	685300	212888	0	2	0	0	50	116	39858472	1.23E-01	1
IQCC	1460758	218	29	1377364	394448	0	8	1	0	50	52	21684672	1.24E-01	1
SLC26A5	355264	620	17	1951592	553936	0	8	1	0	50	46	20792180	1.24E-01	1
GLT1D1	34971	1008	-16	693488	194020	0	5	0	0	50	72	20243584	1.24E-01	1

MRPS18C	330595	378	40	387328	94340	0	4	0	0	50	62	20397020	1.24E-01	1
CUL2	209586	319	7	2012112	463512	0	6	3	0	50	47	19089432	1.24E-01	1
LCN8	1898901	190	39	404416	111428	0	2	0	0	50	67	24888672	1.24E-01	1
RPS2	1747301	183	46	733360	236384	0	6	2	0	16	22	6444668	1.24E-01	1
CELF4	55294	588	-11	1278752	332860	0	7	1	0	33	54	17549020	1.25E-01	1
VPS37B	977561	184	37	704524	225704	0	5	2	0	10	18	4763992	1.25E-01	1
MXD4	836839	332	29	536492	159844	0	4	0	0	50	50	19201216	1.25E-01	1
NACA2	323585	358	-8	536848	159488	0	3	0	0	50	34	19804636	1.25E-01	1
FRG2C	NaN	NaN	NaN	719120	202564	0	5	1	0	50	117	39848148	1.25E-01	1
HSPBP1	1035412	209	5	900680	282664	0	3	0	0	20	16	7264180	1.25E-01	1
HSP90AA1	1488666	145	41	2225712	560700	0	13	3	0	50	85	29412364	1.26E-01	1
SNX12	790100	NaN	37	421148	118192	0	2	0	0	50	62	24805724	1.26E-01	1
ACSS3	26211	1037	-20	1760064	507300	0	13	2	0	50	86	22144268	1.26E-01	1
ITGA8	110825	853	-3	2792108	742972	0	16	5	0	37	49	14263140	1.26E-01	1
ZFAND5	314501	333	18	568888	141332	0	2	0	0	50	49	26527340	1.26E-01	1
PRDX1	809450	176	25	526524	135636	0	4	0	0	50	51	21873352	1.26E-01	1
ASL	477708	345	38	1217876	340336	0	6	2	0	18	14	7568916	1.26E-01	1
KRTAP9-8	996799	468	-7	408332	106444	0	3	0	0	50	60	17141400	1.27E-01	1
CHD1	133125	595	15	4542204	1085088	0	18	1	0	4	3	2563912	1.27E-01	1
DPF2	4112809	242	50	1035248	265576	0	7	0	0	27	30	12483852	1.27E-01	1
PBX2	2119974	392	28	1095056	320044	0	9	1	0	50	62	23774036	1.27E-01	1
RHO	779764	210	59	884660	252760	0	4	1	0	50	58	19116132	1.27E-01	1
CENPN	231500	284	4	1094344	267356	0	4	0	0	50	47	21594604	1.27E-01	1
AMOTL1	332670	472	-7	2428632	688860	0	8	2	0	50	61	25749480	1.28E-01	1
SLC26A8	876298	407	33	2501612	690640	0	12	1	0	42	46	19350024	1.28E-01	1
BLZF1	286300	491	7	1031332	283376	0	7	0	0	7	8	2818808	1.28E-01	1
CAB39	503356	395	29	912072	217872	0	4	0	0	50	74	23438328	1.28E-01	1
RPL5	582788	274	4	791388	197580	0	3	0	0	50	68	24206576	1.28E-01	1
NOL6	1001877	166	4	2865088	918836	0	11	4	0	50	43	17029972	1.28E-01	1
CDH8	3859	1301	-48	2037032	568888	0	14	4	0	10	32	8423316	1.28E-01	1
HRH1	181134	226	35	1220724	347100	0	9	2	0	50	69	19331156	1.29E-01	1
OR4P4	1295	1091	-20	788184	213600	0	9	2	0	38	56	13911412	1.29E-01	1
RAPH1	383558	333	19	3247076	997156	0	9	1	0	21	13	10516952	1.29E-01	1

PCDHGA7	NaN	NaN	NaN	2304388	727664	0	10	1	0	50	117	40373248	1.29E-01	1
ZZZ3	491029	297	0	2336428	606980	0	11	4	0	35	48	15649760	1.29E-01	1
GABPA	534601	728	31	1188328	307940	0	4	1	0	38	14	10888616	1.30E-01	1
MUC4	920866	365	49	3022796	846568	0	22	4	0	50	84	24431212	1.30E-01	1
NKAIN2	23367	993	-28	553224	142044	0	5	0	0	50	97	22319420	1.30E-01	1
NKX2-1	58829	624	-18	987900	310788	0	3	1	0	40	28	13382040	1.30E-01	1
KLHL11	1857838	191	33	1774304	503740	0	12	2	0	50	87	22638752	1.30E-01	1
YAP1	456821	327	-14	1289432	375580	0	5	1	0	50	52	20387052	1.30E-01	1
CCDC96	459652	426	70	1380212	403348	0	7	1	0	14	11	4743344	1.30E-01	1
DNAJC3	204387	552	41	1340696	324316	0	7	1	0	50	60	21194816	1.30E-01	1
CAP2	249798	454	-8	1249916	331792	0	5	1	0	50	46	20533368	1.31E-01	1
DHTKD1	341646	227	25	2358856	659312	0	10	2	0	1	2	1928096	1.31E-01	1
SULT1B1	14468	1020	1	799576	181916	0	6	0	0	50	65	21034260	1.31E-01	1
SHC4	398758	314	9	1616596	452120	0	5	0	0	42	44	22308740	1.31E-01	1
IQCF2	849777	453	39	426488	114988	0	2	0	0	50	44	18199076	1.31E-01	1
PNPLA3	155890	393	24	1217520	360984	0	5	0	0	31	22	12691400	1.31E-01	1
CTNNB1	305811	448	8	1992176	577432	0	10	0	0	7	2	2508732	1.31E-01	1
EIF4A3	674340	219	22	1079036	288004	0	7	0	0	36	55	18118976	1.31E-01	1
DSC3	204823	521	-25	2331444	641868	0	10	2	0	17	36	9788220	1.32E-01	1
SCAMP3	1574928	194	45	887152	262016	0	5	1	0	50	57	20795740	1.32E-01	1
NAPEPLD	594757	579	34	1023144	260592	0	4	1	0	50	70	23421240	1.32E-01	1
KIAA1324L	154470	1087	-7	2695632	694200	0	8	2	0	50	53	21563632	1.32E-01	1
MLST8	2179404	224	39	855112	226772	0	4	0	0	50	46	19527312	1.33E-01	1
TSPYL1	260958	593	22	1080460	322892	0	6	0	0	30	23	12449320	1.33E-01	1
IRAK4	132735	558	-16	1216096	307940	0	6	0	0	50	60	24506684	1.33E-01	1
BRF2	660779	379	37	1028484	330012	0	3	0	0	40	53	23201232	1.33E-01	1
FAM83D	263385	581	30	1509440	479176	0	8	0	0	4	2	1399080	1.33E-01	1
RNF25	1033606	269	43	1171240	347456	0	4	1	0	50	68	23381368	1.34E-01	1
ZIM3	311003	924	-64	1242440	290140	0	10	2	0	50	74	18807836	1.34E-01	1
IL18BP	747702	311	59	541120	142400	0	3	0	0	50	43	16615232	1.34E-01	1
STK38	942238	148	37	1253832	294768	0	6	2	0	50	68	21788268	1.34E-01	1
MEGF10	245811	814	6	2995028	763264	0	9	4	0	50	49	19436176	1.34E-01	1
CDKN2C	290894	417	2	422928	127092	0	2	0	0	50	60	22843808	1.34E-01	1

TMPRSS7	265735	336	21	1867220	497332	0	6	1	0	8	3	3330736	1.34E-01	1
TGFBR3	284037	252	1	2183348	610540	0	10	3	0	50	45	22245016	1.35E-01	1
SHISA5	1480689	223	45	605912	187612	0	4	0	0	50	66	22607068	1.35E-01	1
CMPK1	257134	210	7	600216	154860	0	3	0	0	50	56	21139280	1.35E-01	1
COX7A2	394127	423	-3	301532	85084	0	2	0	0	50	59	20882960	1.35E-01	1
IL17F	313097	427	23	415096	121040	0	2	0	0	50	54	21973744	1.35E-01	1
CST7	545344	676	-29	377004	103596	0	2	0	0	50	56	19251056	1.35E-01	1
TRIB3	879317	185	41	857604	303312	0	5	0	0	50	64	20950244	1.36E-01	1
CXorf36	72853	NaN	50	1129588	336776	0	4	0	0	7	3	2790684	1.36E-01	1
CACNG5	284959	536	-39	954436	284444	0	6	2	0	50	78	20115780	1.36E-01	1
CKMT2	182569	777	30	1071204	312924	0	9	1	0	26	36	11459996	1.36E-01	1
LIPC	441540	310	27	1285516	354932	0	7	1	0	22	22	7240328	1.36E-01	1
HIST1H4D	398813	636	53	249556	85796	0	3	1	0	50	45	13638004	1.36E-01	1
HGF	57326	604	17	1945184	480244	0	10	2	0	50	68	23461468	1.37E-01	1
DACH1	30451	1135	-22	1774660	539696	0	11	1	0	5	9	2877548	1.37E-01	1
TAS2R20	365490	676	-2	771452	221788	0	5	1	0	50	58	17850552	1.37E-01	1
DDR2	322980	293	9	2215388	595588	0	9	1	0	50	46	22589624	1.37E-01	1
COL28A1	220971	682	6	2915640	839448	0	7	0	0	3	0	1871492	1.37E-01	1
CCL2	26189	889	-12	262372	68708	0	1	0	0	50	80	29689332	1.37E-01	1
NUP93	706254	143	26	2136356	580636	0	8	2	0	49	45	20778652	1.37E-01	1
RPS28	778783	209	14	184052	55180	0	1	0	0	50	66	22945268	1.37E-01	1
SPANXN5	476232	NaN	26	194732	43432	0	1	0	0	50	80	25507400	1.37E-01	1
COQ5	1552470	216	46	854400	224280	0	5	1	0	50	64	22266732	1.37E-01	1
MGA	765304	256	41	7706332	2215388	0	29	4	0	11	15	7060192	1.38E-01	1
RPL37	212186	526	19	256676	72268	0	1	0	0	50	42	19365688	1.38E-01	1
KERA	21995	523	-17	896408	243148	0	7	1	0	50	74	21279544	1.38E-01	1
ARSJ	100364	497	15	1501964	424708	0	5	0	0	43	40	15174144	1.38E-01	1
ECM2	579226	474	38	1809192	472056	0	8	1	0	50	68	24917864	1.38E-01	1
ZNF273	217076	1017	-15	1472060	369172	0	5	1	0	50	56	19717416	1.38E-01	1
BRAF	305191	616	25	1975088	557140	0	9	2	0	50	56	23716008	1.38E-01	1
POLD3	540458	245	36	1225352	320044	0	6	0	0	50	51	22210484	1.38E-01	1
KATNAL2	120136	561	12	1229624	326452	0	7	2	0	34	45	13609168	1.38E-01	1
FREM2	173964	1042	-10	7899996	2354940	0	32	8	0	21	26	10410152	1.38E-01	1

METTL6	507426	256	49	744040	186188	0	4	0	0	50	44	18728804	1.39E-01	1
IMPG2	656520	580	-4	3199016	857248	0	14	3	0	17	20	8089388	1.39E-01	1
CASP5	45234	626	-20	1176224	299752	0	7	0	0	27	27	9984020	1.39E-01	1
MRPL21	594397	303	45	539696	156640	0	2	0	0	50	41	20150668	1.39E-01	1
RANBP9	277023	378	29	1863660	532932	0	6	0	0	50	47	23014332	1.39E-01	1
ATP11B	228613	559	16	3092572	805628	0	11	1	0	30	31	14612732	1.39E-01	1
APOC2	1003719	196	6	265220	74404	0	1	0	0	50	42	17116124	1.39E-01	1
HK2	707060	470	21	2355296	658600	0	9	0	0	6	2	2360636	1.39E-01	1
CCDC81	356405	283	12	1724108	427912	0	7	1	0	50	47	21887236	1.39E-01	1
HAMP	767246	472	7	224280	60876	0	1	0	0	50	41	16380272	1.39E-01	1
ASH1L	1960625	247	33	7540080	2075124	0	29	2	0	3	3	2931660	1.39E-01	1
MCM7	1503181	254	41	1829840	536848	0	8	2	0	50	81	27223676	1.39E-01	1
DNAJC6	269422	441	0	2353872	651480	0	10	4	0	12	15	6118216	1.39E-01	1
EDNRB	63780	954	31	1231048	345320	0	7	2	0	20	13	8276288	1.40E-01	1
DHRSX	NaN	NaN	NaN	841584	248844	0	5	1	0	50	117	39894428	1.40E-01	1
SNX10	918425	418	29	547884	124956	0	3	0	0	50	59	23097992	1.40E-01	1
ERCC2	1280459	215	23	2012468	582772	0	32	4	0	5	13	2335360	1.40E-01	1
C2orf16	1192670	306	41	4928464	1433612	0	20	0	0	0	0	1433612	1.40E-01	1
HEY2	229400	351	-5	834820	265220	0	3	0	0	50	45	22624512	1.40E-01	1
CORIN	113961	622	44	2758288	675332	0	8	0	0	7	1	2568896	1.40E-01	1
PRAMEF1[illegible]	141239	571	18	1196160	365256	0	6	2	0	50	46	17600996	1.40E-01	1
ZCWPW2	67695	922	-13	962624	215380	0	7	0	0	50	70	24680412	1.40E-01	1
SESTD1	66477	684	11	1843724	462088	0	5	1	0	50	70	24885824	1.41E-01	1
RAD17	467846	314	46	1801716	463512	0	6	0	0	37	31	16097252	1.41E-01	1
UROS	211688	280	-5	702388	188324	0	3	1	0	50	53	21904324	1.41E-01	1
YAF2	173442	585	2	472412	122464	0	2	0	0	50	47	20054904	1.41E-01	1
PTTG1IP	690574	213	62	475260	126024	0	3	0	0	50	59	16183404	1.41E-01	1
WNT7A	422569	498	34	886796	247420	0	4	1	0	50	39	18377432	1.42E-01	1
ACTA2	181406	267	30	978644	266644	0	7	0	0	50	47	17394872	1.42E-01	1
GSDMC	258154	428	13	1342120	344252	0	4	1	0	50	50	21604572	1.42E-01	1
WASF2	795528	156	26	1250984	381988	0	6	0	0	34	36	15755136	1.42E-01	1
TRIO	518529	557	29	7991132	2176228	0	29	3	0	9	5	3907456	1.42E-01	1
CCDC28B	1460758	218	29	516912	148452	0	3	0	0	50	52	21684672	1.42E-01	1

EEF1E1	566741	368	9	483448	132788	0	3	0	0	50	69	24927832	1.43E-01	1
INSL4	372067	616	20	347812	107156	0	3	0	0	50	47	18943828	1.43E-01	1
UQCRHL	821457	174	37	238520	58384	0	1	0	0	50	43	16225768	1.43E-01	1
TKTL1	2436137	NaN	14	1531512	432540	0	6	2	0	41	58	22788984	1.43E-01	1
TGFBR2	80018	417	-9	1523680	406196	0	5	1	0	50	58	21712440	1.43E-01	1
TMEM159	338008	549	39	410112	121752	0	2	0	0	50	53	20769752	1.43E-01	1
SYT15	165245	269	20	1111788	330012	0	3	0	0	50	43	19432616	1.43E-01	1
S100A2	1454773	186	26	258812	63724	0	1	0	0	50	56	19376724	1.44E-01	1
NUP133	249616	277	25	3000724	815240	0	9	2	0	34	29	14543312	1.44E-01	1
PTH1R	442069	376	41	1516916	446068	0	6	1	0	50	51	20073772	1.44E-01	1
BMP5	68578	483	-9	1173376	312212	0	5	2	0	50	68	24549404	1.44E-01	1
DRAM2	376648	708	13	694912	190460	0	4	0	0	50	69	22199092	1.44E-01	1
ANO10	106227	518	29	1723752	445356	0	6	0	0	50	53	23984076	1.44E-01	1
BTN1A1	426020	498	34	1324320	391956	0	7	2	0	25	17	8419756	1.44E-01	1
MUSK	254657	422	9	2252768	630832	0	7	3	0	50	44	23370332	1.44E-01	1
PRDM4	119393	805	22	2050204	562124	0	9	1	0	50	44	21404144	1.45E-01	1
KRTAP5-1	586193	626	50	399788	105376	0	2	0	0	41	39	13806036	1.45E-01	1
BFAR	519767	367	45	1166968	307940	0	6	1	0	50	58	18288076	1.45E-01	1
NAT8	876583	590	16	560344	174440	0	4	0	0	50	92	26607084	1.45E-01	1
DNM1L	307288	308	-5	1908160	534356	0	5	0	0	9	12	5362784	1.45E-01	1
KLHDC7A	276385	648	15	1870068	624780	0	7	3	0	40	54	22712800	1.45E-01	1
TGM5	825765	415	44	1863304	500180	0	7	3	0	50	75	24827084	1.45E-01	1
WWC1	413858	343	23	2868648	815952	0	10	2	0	50	38	18760844	1.45E-01	1
UTP3	262444	609	19	1202568	335352	0	5	0	0	18	11	7832356	1.45E-01	1
HCN1	80822	1235	-24	2233188	653616	0	19	7	0	18	33	6364568	1.45E-01	1
MYO1F	674374	214	-7	2854052	782488	0	19	2	0	50	74	21331164	1.45E-01	1
ZFYVE16	318922	590	33	3966552	1040232	0	11	0	0	4	0	1913144	1.45E-01	1
BOLL	639001	684	22	780352	210752	0	3	0	0	50	46	20752664	1.46E-01	1
SYK	126037	542	32	1656112	437168	0	7	3	0	16	20	8519436	1.46E-01	1
COX5A	792240	199	45	385548	115344	0	4	0	0	50	67	21522692	1.46E-01	1
TSSK3	1529661	216	41	677112	191172	0	4	0	0	50	65	23479268	1.46E-01	1
FABP9	191177	595	1	356000	81880	0	3	0	0	50	53	20427636	1.46E-01	1
LPPR5	37516	947	-17	816664	236384	0	6	1	0	50	72	19946680	1.46E-01	1

ADHFE1	259302	268	12	1200432	356712	0	4	1	0	50	51	20442588	1.47E-01	1
GUCY1A2	63799	885	-21	1850488	527948	0	10	1	0	19	16	6332884	1.47E-01	1
RPS12	131337	443	18	359916	89712	0	3	0	0	50	58	20879756	1.47E-01	1
EMILIN2	564910	468	37	2634400	772520	0	14	4	0	50	60	24026796	1.47E-01	1
HRASLS	141065	851	-4	433608	118548	0	3	0	0	50	64	18979784	1.47E-01	1
CORO6	719319	383	30	1211468	344608	0	8	2	0	50	60	20013252	1.47E-01	1
ANXA9	1621279	172	30	901748	258100	0	7	0	0	33	60	14315472	1.48E-01	1
GABRG1	36353	926	-2	1212536	314704	0	9	0	0	12	24	5916364	1.48E-01	1
IL21	126588	388	2	435032	108580	0	3	0	0	50	42	21396312	1.48E-01	1
C9orf43	390186	297	36	1229624	306160	0	4	1	0	50	42	19391676	1.48E-01	1
SLC5A9	27457	480	-10	1783204	543968	0	8	2	0	50	67	23994044	1.48E-01	1
FAM189A2	216587	539	0	1139912	347812	0	4	0	0	18	20	8594196	1.48E-01	1
B3GNT4	808864	187	12	923108	299752	0	5	0	0	50	65	20660104	1.48E-01	1
RPGRIP1L	248316	406	-15	3471712	855824	0	13	1	0	47	27	17714560	1.48E-01	1
BLVRA	690958	250	45	775368	208260	0	4	0	0	24	30	12240348	1.48E-01	1
CRLF2	NaN	NaN	NaN	673196	176932	0	2	0	0	50	116	39822516	1.49E-01	1
PREPL	433205	522	29	1917772	475616	0	6	0	0	19	7	6167344	1.49E-01	1
PNPLA5	157623	473	13	1088648	323248	0	3	0	0	45	47	20216172	1.49E-01	1
CRTAM	651341	416	7	1033824	267000	0	4	0	0	35	39	14183752	1.49E-01	1
DDI1	NaN	NaN	NaN	977220	294768	0	7	1	0	50	117	39940352	1.49E-01	1
KCND2	49438	962	7	1579928	463156	0	8	2	0	14	17	8073368	1.49E-01	1
UGP2	312741	380	10	1311148	358136	0	6	0	0	50	55	23432276	1.49E-01	1
H3F3A	866965	237	23	343184	108580	0	3	0	0	50	51	16052396	1.49E-01	1
SPATS1	993997	443	40	796728	200784	0	5	1	0	50	77	24910032	1.49E-01	1
ATP5F1	397613	348	23	663940	185120	0	4	1	0	36	30	14351072	1.49E-01	1
OR56B4	465433	806	-1	780708	242436	0	4	1	0	8	7	2668576	1.50E-01	1
GNB1	1492510	229	26	890000	245284	0	3	0	0	50	48	18853404	1.51E-01	1
GDPD4	448138	469	44	1380924	352440	0	7	1	0	50	69	22328320	1.51E-01	1
C9orf40	95383	649	-18	475972	153080	0	2	0	0	50	62	27519512	1.51E-01	1
DYNLT1	623771	306	49	313636	70844	0	1	0	0	50	43	18139624	1.51E-01	1
INSIG2	190880	537	8	576008	165184	0	3	0	0	50	67	25875860	1.51E-01	1
PDGFRA	51422	518	27	2835540	750804	0	16	5	0	17	21	7212204	1.51E-01	1
C6orf226	1086050	203	41	238876	90068	0	1	0	0	50	45	16793588	1.52E-01	1

OR7C1	963401	546	14	780352	245996	0	4	1	0	47	68	17530508	1.52E-01	1
DEPDC1	196163	601	-18	2113216	535424	0	8	0	0	4	2	2216812	1.52E-01	1
OR1S2	637499	828	-25	804916	238520	0	6	0	0	50	51	16635524	1.52E-01	1
TXNDC12	665927	315	21	466004	113920	0	2	0	0	50	59	26353256	1.52E-01	1
STATH	9753	991	-4	182272	40940	0	1	0	0	50	76	18672912	1.52E-01	1
ALDOA	NaN	NaN	NaN	927380	276256	0	5	2	0	50	118	39921840	1.52E-01	1
TRIM15	1932849	486	48	1178360	340336	0	5	0	0	50	60	20279184	1.52E-01	1
[illegible]MPRSS11[illegible]	211571	812	-6	1145964	301176	0	6	2	0	50	49	21367832	1.52E-01	1
PTGER4	210761	496	-8	1185836	389464	0	6	1	0	50	58	23764068	1.52E-01	1
GATC	1563159	216	45	349592	106444	0	2	1	0	23	20	7249584	1.52E-01	1
CDH16	942986	142	34	2093280	638664	0	12	4	0	50	65	21541916	1.52E-01	1
SCEL	193860	752	51	1908872	435388	0	7	0	0	6	2	2382708	1.52E-01	1
VPS28	2043455	200	36	693844	177644	0	5	0	0	50	64	16927800	1.53E-01	1
UBQLN2	126215	NaN	7	1530444	472056	0	9	0	0	11	9	5062320	1.53E-01	1
BMF	884609	226	39	492348	113208	0	3	0	0	50	64	21160640	1.54E-01	1
STOM	580458	388	39	738344	215380	0	4	1	0	50	65	22559008	1.54E-01	1
STK3	332778	550	25	1312928	306160	0	6	1	0	11	10	5637616	1.54E-01	1
[illegible]RTAP19-[illegible]	14979	1035	-68	134568	33108	0	1	0	0	50	77	14168444	1.54E-01	1
GSTM3	927739	201	22	611252	142756	0	3	1	0	50	50	14380264	1.54E-01	1
PRRX1	61224	921	-7	672484	195800	0	4	0	0	50	65	23701768	1.54E-01	1
NRP1	360090	488	-9	2410120	630476	0	9	1	0	50	62	25866248	1.54E-01	1
USP33	432858	192	2	2515852	616592	0	10	1	0	50	51	18491352	1.55E-01	1
IL7R	139461	511	5	1193312	312568	0	7	1	0	8	7	2180500	1.55E-01	1
PAIP1	482851	434	19	1238524	342116	0	8	0	0	27	30	9310824	1.55E-01	1
MX1	253874	454	53	1724108	455680	0	9	0	0	25	43	14717396	1.55E-01	1
ATP6V1H	263637	437	9	1293704	312568	0	6	0	0	37	35	16767244	1.55E-01	1
SLC17A2	392745	685	34	1114636	328232	0	6	3	0	16	14	5856200	1.56E-01	1
DMXL1	305800	381	28	7786788	2094348	0	22	3	0	30	19	12425112	1.56E-01	1
OR4F6	215780	643	-16	771808	228908	0	5	1	0	42	59	17722748	1.56E-01	1
GNG12	476287	228	-7	192952	49484	0	2	0	0	50	64	22043876	1.56E-01	1
PSEN1	474907	269	39	1211824	330368	0	5	0	0	50	47	17333640	1.56E-01	1
MRPS22	261288	671	9	937704	253116	0	7	0	0	50	56	21099764	1.56E-01	1
CD96	186134	406	-16	1540056	397296	0	8	1	0	50	40	20447216	1.56E-01	1

CATSPER1	3854361	208	57	2006772	542544	0	8	0	0	6	2	2582780	1.56E-01	1
GTF2E2	489611	228	38	767892	197580	0	3	0	0	50	30	17008968	1.56E-01	1
RAB25	2005405	213	39	541120	161624	0	4	1	0	30	49	13721664	1.57E-01	1
OR2J3	155147	738	1	760060	238520	0	5	1	0	37	37	16234668	1.57E-01	1
HTR1F	123008	898	-19	915632	264508	0	6	1	0	50	72	22331168	1.57E-01	1
SLC14A1	381180	401	25	1138132	329300	0	4	0	0	50	32	19872276	1.57E-01	1
CHL1	25264	796	9	3208984	826988	0	14	2	0	43	44	20773312	1.57E-01	1
ZMYND8	303060	186	44	3103252	802424	0	15	4	0	50	46	16443640	1.57E-01	1
PSPH	749444	642	7	582772	162692	0	5	0	0	50	72	22576096	1.57E-01	1
FMN2	55160	851	-26	4205784	1387332	0	24	5	0	38	74	17333996	1.57E-01	1
USP12	283040	221	52	984340	238520	0	3	0	0	50	50	20403428	1.57E-01	1
TRIM10	1932849	486	48	1294060	369884	0	6	1	0	50	60	20279184	1.58E-01	1
BMPR1A	379827	195	26	1388756	361696	0	3	0	0	22	15	11290540	1.58E-01	1
ZNF292	222401	547	19	6914944	1844792	0	22	2	0	5	3	3164128	1.58E-01	1
OR52K1	190769	879	-1	769316	244216	0	4	0	0	50	51	19817096	1.58E-01	1
OPA1	148817	456	12	2721264	662160	0	11	1	0	50	63	22583928	1.58E-01	1
OR10V1	431271	409	27	759348	233892	0	4	0	0	31	35	13655092	1.58E-01	1
FAM20B	352872	244	1	1042012	297260	0	3	0	0	50	55	25243960	1.58E-01	1
KLRG1	301954	416	11	499824	128160	0	2	0	0	50	55	24142140	1.58E-01	1
DFFA	829921	392	32	860452	250268	0	6	1	0	50	57	23206928	1.58E-01	1
MRPL50	112947	898	-22	402636	113208	0	3	0	0	50	80	21577872	1.58E-01	1
MTF2	611888	391	16	1568536	394448	0	5	1	0	33	28	13505928	1.58E-01	1
ERG	162262	418	4	1286940	337488	0	5	1	0	50	33	17654040	1.59E-01	1
NPM3	817474	335	37	466716	131364	0	4	0	0	50	56	20205136	1.59E-01	1
ST20	228288	702	18	207192	59808	0	2	0	0	50	55	18606340	1.59E-01	1
FAM160A2	466636	728	4	2391252	813816	0	11	2	0	44	49	19814604	1.59E-01	1
GC	17296	933	-6	1242796	330368	0	6	0	0	6	4	2399084	1.59E-01	1
THG1L	243382	987	31	783200	196156	0	4	0	0	50	48	18799648	1.59E-01	1
AFF2	97330	NaN	-13	3406208	941620	0	13	5	0	19	17	7347840	1.59E-01	1
TAF3	189040	596	-1	2349244	655040	0	9	1	0	47	39	18690000	1.59E-01	1
CPE	171042	494	-9	1230336	332148	0	6	0	0	27	36	14329356	1.59E-01	1
OR5K1	416592	885	-4	772164	220008	0	3	2	0	50	71	20173096	1.60E-01	1
GPR108	736129	272	27	1419016	398720	0	5	1	0	50	67	24524840	1.60E-01	1

C10orf67	77657	278	-3	491636	123532	0	2	0	0	50	48	21196952	1.60E-01	1
CRTC3	1079582	204	41	1566400	479888	0	9	1	0	50	48	18000072	1.60E-01	1
DCAF4L1	272281	261	25	974728	299396	0	3	0	0	50	58	22426220	1.60E-01	1
UBE2I	1150864	215	36	427912	107156	0	2	0	0	50	52	19119336	1.60E-01	1
TUBB6	492344	214	26	1130300	316840	0	7	2	0	37	65	15004332	1.60E-01	1
FGF16	547612	NaN	-7	293700	83304	0	2	0	0	50	67	19959496	1.60E-01	1
HTRA3	170889	242	52	1131724	357068	0	6	1	0	50	45	17282732	1.60E-01	1
TMEM213	352046	385	7	281240	79744	0	1	0	0	50	46	18605984	1.61E-01	1
NDUFA5	88676	779	-25	314348	77608	0	2	0	0	50	81	21308024	1.61E-01	1
RTP1	425258	445	28	666788	185476	0	4	1	0	16	13	7467100	1.61E-01	1
GYS2	510830	830	-17	1834824	484872	0	10	2	0	50	53	15575000	1.61E-01	1
PRDM2	68342	892	25	4400872	1213604	0	15	2	0	50	51	22361072	1.61E-01	1
CREB5	58752	518	26	1367752	360272	0	7	3	0	13	17	5782152	1.61E-01	1
NCOA1	343965	403	33	3720200	1035604	0	14	2	0	50	48	20224716	1.61E-01	1
TRMT112	1613996	257	65	323960	94696	0	3	0	0	19	29	6446448	1.61E-01	1
NR2C2AP	940952	232	17	367392	100392	0	2	0	0	50	65	20645152	1.61E-01	1
GSK3B	467052	421	19	1112500	325028	0	4	1	0	50	45	16033884	1.61E-01	1
HSBP1	298486	673	-13	214668	46992	0	2	0	0	50	57	20502752	1.62E-01	1
MMP27	530224	567	-10	1347104	342472	0	4	1	0	50	50	17183764	1.62E-01	1
CYB5D1	2148615	337	24	580992	165540	0	3	1	0	32	35	14422984	1.62E-01	1
CXCR4	431866	406	42	902104	262016	0	4	0	0	50	50	20390256	1.62E-01	1
TMSB4Y	0	0	0	121752	26700	0	1	0	0	5	1	910292	1.62E-01	1
IL31RA	319744	438	44	1994668	520472	0	7	1	0	50	67	24088028	1.62E-01	1
RSRC1	196207	1080	-3	879676	232112	0	4	0	0	50	36	16677532	1.62E-01	1
PRSS42	413835	495	38	741192	216804	0	3	0	0	50	53	17553292	1.63E-01	1
PPFIA3	2011861	267	47	3038460	918480	0	14	4	0	50	71	20637320	1.63E-01	1
CFL2	573593	145	24	443932	109292	0	4	0	0	50	48	18848064	1.63E-01	1
TECRL	2644	1049	-22	971524	241724	0	9	3	0	5	11	2044864	1.63E-01	1
CAMTA2	1416161	231	30	3164128	955148	0	15	1	0	33	38	12888980	1.63E-01	1
UTP20	261840	760	-5	7282336	1904600	0	20	0	0	0	0	1904600	1.63E-01	1
RPL7L1	1086050	203	41	639376	175508	0	3	0	0	50	45	16793588	1.63E-01	1
FAM188A	70308	903	-2	1188328	300464	0	6	0	0	50	52	20210832	1.63E-01	1
BCLAF1	286851	153	21	2366332	631544	0	7	1	0	50	51	25828512	1.64E-01	1

OSR1	63105	842	-9	658956	205056	0	5	0	0	50	87	28890824	1.64E-01	1
CLK2	1574928	194	45	1304384	343540	0	9	1	0	50	57	20795740	1.64E-01	1
NAPA	794875	276	44	799576	191528	0	4	0	0	43	60	18144964	1.64E-01	1
USE1	865138	212	38	673552	193664	0	3	0	0	50	59	18864796	1.64E-01	1
SLC30A9	270740	261	28	1494132	401568	0	5	0	0	9	8	3733728	1.64E-01	1
OVCH1	100195	539	-5	2973312	780708	0	10	4	0	50	62	21611692	1.64E-01	1
OR10Z1	179404	940	1	762552	242436	0	7	2	0	6	15	3888232	1.64E-01	1
MTM1	287123	NaN	16	1588828	406196	0	6	1	0	28	22	10031724	1.64E-01	1
CALR3	808967	218	35	1032044	239944	0	3	2	0	50	44	17139620	1.64E-01	1
NRNPA2E	915230	445	43	945180	236028	0	9	2	0	15	16	4912444	1.64E-01	1
COPS4	556523	169	47	1071916	270560	0	5	1	0	50	53	17290208	1.64E-01	1
FOS	855880	154	51	936992	296548	0	4	1	0	50	77	21924260	1.64E-01	1
TNF	2517285	220	35	581348	181204	0	3	1	0	15	15	4137432	1.64E-01	1
OGDH	1420793	179	56	2783920	773588	0	16	5	0	29	26	10196908	1.65E-01	1
NMD3	242883	581	-16	1355648	323248	0	7	0	0	38	35	14140676	1.65E-01	1
IIST1H2AI	228884	634	40	313280	110716	0	3	1	0	23	29	6014264	1.65E-01	1
SLC6A7	1002307	228	49	1615528	480956	0	6	2	0	50	53	19888652	1.65E-01	1
RPL36A	374364	NaN	37	294056	70132	0	1	0	0	50	61	21564700	1.65E-01	1
FAM181A	242993	645	15	865436	276256	0	4	1	0	33	43	18195872	1.66E-01	1
SAMD13	226331	769	-12	309364	78320	0	3	0	0	50	50	16186252	1.66E-01	1
ANKRD55	248990	402	45	1580996	436456	0	8	2	0	50	53	19831692	1.66E-01	1
FCGR3B	645754	503	22	604488	162336	0	4	0	0	50	66	18965188	1.66E-01	1
FERD3L	103347	812	-15	414384	124956	0	5	1	0	6	5	1796020	1.66E-01	1
JUN	300720	419	5	809188	254540	0	4	1	0	50	57	21345404	1.66E-01	1
ARL4A	120492	469	-11	508724	142756	0	5	1	0	5	6	1798156	1.66E-01	1
SUV420H1	1059031	161	51	2290860	597012	0	15	1	0	50	64	19828132	1.67E-01	1
MKI67	408820	641	-1	8126412	2368824	0	40	7	0	24	30	9684268	1.67E-01	1
SYCP3	344663	338	12	691352	138484	0	3	0	0	50	56	22774388	1.67E-01	1
STAC3	2162032	238	58	975084	240300	0	6	2	0	18	36	10611648	1.67E-01	1
MRGPRX1	561250	695	15	782132	254896	0	6	1	0	50	60	19607768	1.67E-01	1
CAP1	873408	387	23	1236388	339980	0	7	0	0	50	79	27832436	1.67E-01	1
PPP1CA	1269888	177	51	880032	241368	0	5	1	0	31	22	11381320	1.67E-01	1
SLC35B2	1037256	312	54	1053048	347100	0	5	3	0	44	60	16584260	1.68E-01	1

RNF144A	40925	1103	7	767536	201140	0	3	0	0	50	51	23655132	1.68E-01	1
DIRC1	124843	739	-7	264864	75828	0	2	0	0	50	51	22755520	1.68E-01	1
EIF4A2	449980	384	25	1076188	275900	0	7	0	0	18	26	8166284	1.68E-01	1
AHI1	152087	422	29	3220376	784624	0	8	0	0	11	9	6661828	1.68E-01	1
POLH	1221714	194	46	1810616	519760	0	9	2	0	50	87	24616332	1.68E-01	1
EHBP1L1	3945819	202	45	3750104	1209688	0	13	1	0	19	25	9711324	1.68E-01	1
ACOX3	138504	332	47	1803852	514776	0	8	3	0	32	35	12344656	1.68E-01	1
ZDBF2	328087	597	18	5995396	1562840	0	19	2	0	13	8	6398388	1.68E-01	1
TTLL5	415501	540	40	3334652	905308	0	16	2	0	50	52	18145320	1.68E-01	1
PPM1G	1244404	179	39	1414744	376292	0	6	2	0	50	61	21622372	1.69E-01	1
CORO2B	396006	603	25	1246712	343540	0	8	2	0	8	11	4091152	1.69E-01	1
ODF1	525392	236	55	640444	168032	0	5	1	0	50	62	22349324	1.69E-01	1
ATF6	407279	420	16	1735856	480244	0	6	1	0	50	46	16560052	1.69E-01	1
NDUFA7	778783	209	14	290140	90068	0	2	1	0	15	26	7223240	1.69E-01	1
RHOBTB3	310056	242	46	1601288	406552	0	5	0	0	50	55	19739844	1.69E-01	1
CD86	557964	715	39	869708	213244	0	6	1	0	50	49	18745536	1.69E-01	1
SGPL1	424452	579	30	1486656	396228	0	6	0	0	15	13	5503404	1.69E-01	1
BZW1	620967	155	38	954080	227128	0	4	0	0	50	50	20122188	1.70E-01	1
DAPK1	141078	494	26	3656476	1030976	0	12	1	0	25	15	11176976	1.70E-01	1
C18orf32	236267	352	55	203632	51620	0	2	0	0	50	49	16950940	1.70E-01	1
HEXB	356374	567	28	1450344	391956	0	3	0	0	9	3	4995392	1.70E-01	1
MED26	898005	258	42	1461380	472768	0	6	1	0	50	62	27848812	1.70E-01	1
ATE1	233024	427	-3	1486656	367392	0	6	1	0	50	41	19546892	1.70E-01	1
NAPG	191442	594	17	840872	208972	0	3	1	0	50	46	15692480	1.70E-01	1
PTGER2	405022	533	18	861876	292632	0	6	2	0	20	28	8124276	1.70E-01	1
[illegible]RTAP10-1	874480	461	25	622288	187256	0	4	1	0	50	76	22638396	1.70E-01	1
RGS10	825813	217	22	503384	110716	0	4	0	0	50	39	14537616	1.70E-01	1
NEUROD2	1164935	239	28	928804	302600	0	3	0	0	50	54	18916772	1.70E-01	1
WDR45	833689	NaN	41	945180	257388	0	7	2	0	14	22	6888244	1.70E-01	1
BLM	997342	176	45	3682820	950164	0	14	3	0	11	7	5192616	1.70E-01	1
IFI27L2	200295	605	47	327164	109648	0	2	0	0	50	57	19244648	1.71E-01	1
ANGPTL4	726988	204	0	1028840	300820	0	7	3	0	22	25	7295152	1.71E-01	1
SERHL2	969686	196	65	841940	216448	0	4	0	0	50	42	17941332	1.71E-01	1

HLA-E	2019724	424	30	918836	263440	0	7	2	0	5	4	1867576	1.71E-01	1
WDR38	1125524	207	41	808120	237452	0	3	1	0	50	59	18818160	1.71E-01	1
ZNHIT6	277976	411	-20	1250984	296548	0	5	1	0	50	47	21067724	1.72E-01	1
TNIP3	214178	900	4	894984	196512	0	4	1	0	50	60	19325816	1.72E-01	1
PPP4R4	204538	714	17	2370604	612320	0	10	2	0	50	54	19349668	1.72E-01	1
KPTN	797690	276	38	1112500	334640	0	4	2	0	50	64	22709952	1.72E-01	1
NGLY1	216966	701	-1	1799936	455680	0	9	2	0	36	50	16811744	1.72E-01	1
RAB6A	549152	224	37	652904	164116	0	5	0	0	50	53	22047792	1.72E-01	1
PEG3	NaN	NaN	NaN	3749036	951232	0	19	5	0	50	121	40596816	1.72E-01	1
SPIB	1038277	491	30	665364	200784	0	3	1	0	50	64	20883672	1.73E-01	1
RARRES1	197614	972	-6	758280	224280	0	3	1	0	50	41	18970528	1.73E-01	1
SUPT16H	713944	380	38	2787480	677112	0	13	2	0	17	18	10392708	1.73E-01	1
CNTF	284768	644	2	505876	144536	0	3	0	0	50	42	18244644	1.73E-01	1
OR8H1	10317	969	-43	769316	228196	0	7	0	0	50	80	18788256	1.73E-01	1
MANBAL	666037	571	64	217160	66928	0	3	0	0	50	57	14462144	1.73E-01	1
QRICH1	1848854	185	39	1962628	565328	0	11	0	0	31	38	14456092	1.73E-01	1
POLE4	642459	577	26	300464	92560	0	1	0	0	50	66	22434052	1.74E-01	1
HTR3B	182706	555	9	1142404	310076	0	4	0	0	50	57	22938860	1.74E-01	1
CYP11B2	447193	438	36	1275548	375580	0	11	0	0	1	0	749380	1.74E-01	1
DDIT4L	193061	795	-8	491280	138840	0	2	0	0	50	49	19714212	1.74E-01	1
MLF1	196830	940	-10	722680	169100	0	3	1	0	50	46	19960920	1.74E-01	1
TAS2R9	408176	517	12	783912	223212	0	4	0	0	50	54	19041016	1.74E-01	1
GAD1	352183	519	31	1600576	415808	0	8	0	0	34	37	16005048	1.75E-01	1
TOX3	62431	993	-53	1535784	425064	0	9	1	0	50	57	18957712	1.75E-01	1
STRBP	341358	431	29	1744400	480244	0	5	0	0	50	49	24462184	1.75E-01	1
SOD1	268523	621	34	402992	110716	0	3	1	0	41	43	10847320	1.75E-01	1
PURB	1107527	219	54	762196	242792	0	6	0	0	22	38	12614148	1.75E-01	1
ARF5	411207	619	-7	472412	131008	0	2	0	0	50	41	15048476	1.75E-01	1
BRI3BP	1000229	229	37	610184	205768	0	5	0	0	50	54	18508084	1.75E-01	1
AFF4	686574	200	66	3010692	804204	0	19	1	0	10	8	3264876	1.75E-01	1
MAGED2	257482	NaN	20	1544328	451764	0	6	0	0	14	13	5035976	1.75E-01	1
DEPDC4	184616	650	3	767892	196512	0	5	1	0	7	8	3126748	1.76E-01	1
EDN1	198728	410	27	561056	138484	0	2	0	0	50	47	20597804	1.76E-01	1

KRTAP9-4	1149433	468	-14	396584	102172	0	2	0	0	50	53	16834528	1.76E-01	1
ADM	860704	273	12	463156	145604	0	3	1	0	50	82	24173468	1.76E-01	1
PNOC	311812	229	35	457816	122108	0	2	0	0	50	47	16612028	1.76E-01	1
ZNF263	1397628	303	32	1732296	480600	0	8	3	0	50	92	23311236	1.76E-01	1
CLLU1OS	168575	582	19	269492	72268	0	1	0	0	50	43	17827412	1.76E-01	1
C1orf146	304492	524	-3	480956	120328	0	4	0	0	50	78	22918568	1.76E-01	1
NISCH	1096247	192	55	3772888	1134572	0	9	0	0	3	0	2294420	1.77E-01	1
DCUN1D2	806605	318	46	696692	164116	0	5	0	0	50	70	24417328	1.77E-01	1
DEK	467694	497	4	1021008	226416	0	5	0	0	50	49	19195520	1.77E-01	1
DDA1	842088	211	43	276612	70488	0	1	0	0	50	61	21639816	1.77E-01	1
PYDC2	101042	886	13	244928	66928	0	1	0	0	50	71	21387768	1.77E-01	1
CEP57	298879	467	2	1325388	322536	0	6	1	0	50	62	25030360	1.77E-01	1
CD160	NaN	NaN	NaN	472412	127804	0	2	0	0	50	116	39773388	1.77E-01	1
OR1J2	373314	646	3	768604	236384	0	4	0	0	50	63	22349324	1.78E-01	1
HOXA1	356045	385	28	852976	229976	0	4	1	0	9	4	2886804	1.78E-01	1
CLEC1B	305376	565	19	615168	145248	0	4	1	0	5	5	2335716	1.78E-01	1
LRRC57	534369	269	40	614456	175864	0	4	2	0	13	10	3998236	1.78E-01	1
DUSP14	541310	201	29	491636	150232	0	3	0	0	50	93	26971628	1.78E-01	1
GGNBP2	468055	548	23	1837672	449984	0	8	1	0	31	37	13224332	1.78E-01	1
CHIA	398726	559	15	1247068	330368	0	7	1	0	50	61	21076268	1.78E-01	1
GABRB2	11638	1049	7	1323608	358492	0	6	2	0	16	16	8430436	1.78E-01	1
TAF1D	290677	527	0	735852	179424	0	4	1	0	50	59	19845576	1.78E-01	1
ABCA12	526764	857	9	6761864	1808836	0	22	3	0	2	8	4606640	1.79E-01	1
FAM45A	721947	194	19	933432	249912	0	4	2	0	9	12	4359220	1.79E-01	1
RIT2	4853	1195	-57	573872	141688	0	5	1	0	29	63	13463564	1.79E-01	1
TOB1	1152451	161	15	874336	236384	0	5	1	0	34	34	10998264	1.79E-01	1
DTNB	367255	356	44	1666080	430404	0	6	1	0	50	49	21271000	1.79E-01	1
NPFFR2	17885	811	3	1338204	356712	0	5	1	0	50	55	21841668	1.79E-01	1
HOXA2	356045	385	28	935568	276612	0	3	0	0	50	46	22711376	1.79E-01	1
IL22RA1	812508	235	33	1427204	442864	0	6	1	0	50	55	19026776	1.79E-01	1
GPR50	215822	NaN	-7	1520832	465648	0	8	1	0	50	90	28179180	1.79E-01	1
MOBP	161535	694	18	225704	45568	0	1	0	0	50	60	18461448	1.79E-01	1
CLSTN2	93930	998	9	2475624	655752	0	12	2	0	50	68	26595692	1.79E-01	1

HNRNPU	695318	417	6	2147036	555004	0	9	0	0	31	26	10348208	1.79E-01	1
HOMER1	177305	593	37	930940	240656	0	5	1	0	50	51	17585332	1.79E-01	1
PLXDC2	19386	1014	-10	1386620	367036	0	6	2	0	50	69	22876204	1.79E-01	1
HOXA7	356045	385	28	577076	167320	0	3	0	0	50	46	22711376	1.79E-01	1
OR10A6	105341	679	6	775724	232468	0	5	1	0	50	63	24898284	1.79E-01	1
RPF2	343624	410	29	823784	202564	0	4	0	0	50	55	23354668	1.80E-01	1
MED27	593548	566	29	808120	223568	0	4	1	0	50	59	20495632	1.80E-01	1
STXBP1	1015071	168	40	1666436	440728	0	7	1	0	50	78	28271384	1.80E-01	1
GAR1	212757	462	28	556784	167320	0	4	0	0	50	47	18097260	1.80E-01	1
HEG1	367509	177	50	3421872	1076544	0	10	2	0	44	34	15323664	1.80E-01	1
BTBD10	285926	458	10	1232828	326452	0	7	1	0	50	44	22190904	1.80E-01	1
TBL1Y	0	0	0	1380212	355288	0	5	0	0	50	39	21173456	1.80E-01	1
ATP6V1G	2517285	220	35	312212	81880	0	1	0	0	50	55	21824936	1.81E-01	1
KIF12	244063	558	0	1300824	405840	0	5	2	0	50	54	18649060	1.81E-01	1
MORF4L1	482778	413	50	971880	242436	0	4	0	0	50	71	21518064	1.81E-01	1
DBT	314135	495	9	1250628	341760	0	5	0	0	31	33	14180904	1.81E-01	1
PRDM5	19279	961	-16	1697408	388396	0	11	0	0	8	8	2874700	1.81E-01	1
F11R	777836	441	19	778216	221432	0	6	0	0	50	69	22066304	1.81E-01	1
DPYSL2	207566	520	26	1473484	417944	0	4	0	0	32	23	11871888	1.81E-01	1
RBMS3	30243	808	-11	1199720	311500	0	4	2	0	20	30	11536180	1.82E-01	1
OR51B5	100115	1056	1	762552	240300	0	3	0	0	50	49	17770808	1.82E-01	1
MEIS2	74106	628	-7	1318624	332504	0	5	0	0	50	49	21327604	1.82E-01	1
IDH3A	395288	314	50	961200	259524	0	3	1	0	50	60	20999016	1.82E-01	1
FAM19A2	136152	930	-44	349592	89356	0	2	0	0	50	70	16949160	1.82E-01	1
ABHD10	239044	250	10	779996	222856	0	5	0	0	50	64	21098340	1.82E-01	1
OR4X1	400891	509	-27	754364	224992	0	5	1	0	50	74	19622364	1.82E-01	1
SKIV2L	2314693	203	34	3144548	970456	0	7	0	0	3	0	2076192	1.82E-01	1
CHIT1	507603	453	31	1210400	330724	0	7	1	0	50	72	22933520	1.82E-01	1
HIST2H2A	798975	560	17	311856	106800	0	3	1	0	21	23	6253852	1.82E-01	1
ATP6V1A	442365	364	33	1604492	435388	0	10	2	0	50	45	18104024	1.82E-01	1
CLIP4	383097	497	32	1823788	502316	0	7	2	0	40	30	13361036	1.83E-01	1
MCCC2	436559	600	36	1470636	404772	0	8	0	0	50	78	22903260	1.83E-01	1
CHD2	243747	332	14	4826292	1198296	0	18	1	0	1	1	1329660	1.83E-01	1

COPB2	246935	671	15	2382352	613388	0	7	2	0	50	63	23934592	1.83E-01	1
HBG1	109545	1056	-1	377716	109292	0	2	0	0	50	50	17996156	1.83E-01	1
SURF1	1827885	416	28	771096	233892	0	2	1	0	50	47	17145316	1.83E-01	1
FCHO1	1181745	246	21	2292996	684588	0	12	2	0	30	42	15709212	1.83E-01	1
NEDD8	944836	220	40	218228	58384	0	2	0	0	50	68	23143916	1.84E-01	1
SCRN3	385177	419	24	1111432	280172	0	3	1	0	50	52	25814628	1.84E-01	1
RBMXL2	354782	922	-11	932720	326452	0	4	1	0	50	59	17749092	1.84E-01	1
GUCA1B	524386	273	43	527236	129584	0	2	0	0	50	56	22267444	1.84E-01	1
GLUD2	339278	NaN	-6	1381636	411536	0	9	1	0	25	26	8703844	1.84E-01	1
VSIG10	637549	608	17	1378076	389464	0	6	1	0	28	32	10905704	1.84E-01	1
CYP4A11	492091	578	5	1341408	373800	0	7	2	0	50	61	22411268	1.84E-01	1
RNF213	718251	198	24	13542952	3763988	0	38	12	0	32	41	17457172	1.84E-01	1
C20orf85	372863	576	7	358848	98256	0	2	1	0	48	65	22614900	1.85E-01	1
DEFB128	215351	430	25	243148	62300	0	1	0	0	50	54	17273476	1.85E-01	1
TAC3	2026922	199	41	333216	82236	0	3	0	0	50	67	21271000	1.85E-01	1
NXPH1	85530	915	-11	690996	184764	0	5	1	0	31	51	17486364	1.85E-01	1
IPPK	587288	484	46	1287652	339980	0	4	0	0	22	14	9751908	1.85E-01	1
CSN3	201562	928	3	462088	141332	0	4	0	0	50	70	21109020	1.85E-01	1
PXMP2	724450	495	35	494128	153080	0	2	0	0	50	61	25012204	1.85E-01	1
ATP5C1	199228	475	16	796372	208616	0	4	0	0	50	52	19143900	1.85E-01	1
RPGR	141269	NaN	28	3501972	846924	0	9	0	0	16	11	9880424	1.85E-01	1
CEACAM[illegible]	1341589	499	16	865080	261660	0	5	2	0	29	24	8609148	1.85E-01	1
[illegible]RTAP22-[illegible]	22916	1048	-79	126380	29548	0	2	0	0	50	84	16257808	1.86E-01	1
EML1	528390	354	10	2174092	597368	0	9	1	0	11	20	7106116	1.86E-01	1
CNIH3	408298	633	-5	430760	106444	0	2	0	0	50	60	19671848	1.86E-01	1
PAK2	798731	446	41	1380568	361340	0	8	0	0	26	27	9472804	1.86E-01	1
CXCL10	489826	399	43	263440	68708	0	2	0	0	50	51	20872992	1.86E-01	1
GSTM1	927739	201	22	595944	137772	0	2	0	0	50	50	14380264	1.86E-01	1
HDAC11	370030	199	39	897476	262372	0	5	0	0	50	54	21713508	1.86E-01	1
ROPN1L	628188	219	21	598080	161268	0	3	1	0	26	33	10568216	1.87E-01	1
SLC10A2	199120	713	24	882880	256676	0	7	1	0	6	94	28179180	1.87E-01	1
C11orf63	675003	416	7	2063020	533288	0	9	2	0	43	43	15370656	1.87E-01	1
TUBG1	1884163	177	38	1166612	324316	0	6	1	0	50	68	23826724	1.87E-01	1

ORAOV1	441431	536	41	374156	85084	0	2	1	0	50	63	20424076	1.87E-01	1
BOLA1	798975	560	17	327520	118904	0	2	1	0	21	23	6253852	1.87E-01	1
GNG4	565883	457	16	199716	52332	0	2	0	0	50	72	21697844	1.87E-01	1
PENK	411547	624	-16	687436	181916	0	4	1	0	50	39	17028548	1.87E-01	1
ZNF485	310272	630	-6	1150592	282664	0	5	2	0	41	36	15733776	1.87E-01	1
AMOTL2	303429	189	21	1930944	609828	0	9	1	0	34	37	17612032	1.87E-01	1
GPR171	NaN	NaN	NaN	808120	221432	0	2	1	0	50	117	39867016	1.88E-01	1
BATF3	399829	364	30	325740	92916	0	2	0	0	50	43	21174168	1.88E-01	1
HMGN1	383982	346	44	276256	68708	0	2	0	0	50	50	21398804	1.88E-01	1
CIDEA	428253	350	20	668924	206836	0	3	1	0	50	53	21052416	1.88E-01	1
UMPS	307759	254	36	1202568	359916	0	5	0	0	27	17	9052368	1.88E-01	1
FAM174A	23650	1030	13	463512	156996	0	2	0	0	50	68	24788992	1.88E-01	1
RBAK	1723793	462	34	1874340	433608	0	11	0	0	12	8	4310448	1.89E-01	1
HDGFL1	11748	925	-14	629764	177644	0	3	0	0	50	75	24054564	1.89E-01	1
DEFB124	605825	450	-76	183696	51264	0	1	0	0	50	111	28209084	1.89E-01	1
GPR37L1	704755	156	27	1169816	380920	0	4	1	0	34	26	15189808	1.89E-01	1
IFRD1	166238	412	-4	1185124	310076	0	4	0	0	50	48	19834896	1.89E-01	1
RPS6	498767	339	12	644004	182628	0	5	2	0	12	30	7585292	1.89E-01	1
NPPC	893534	439	33	301532	109648	0	2	0	0	50	51	22395960	1.89E-01	1
C1orf186	755856	400	10	460308	115344	0	2	1	0	34	28	10062696	1.90E-01	1
KIAA2018	453036	419	24	5582436	1635108	0	27	2	0	1	5	2682816	1.90E-01	1
CPS1	158852	465	-1	3913152	1079748	0	13	1	0	5	6	3961924	1.90E-01	1
FAM175B	290823	200	36	1093276	275900	0	6	3	0	16	19	4564276	1.90E-01	1
TMX4	72842	609	-25	911716	239588	0	7	1	0	50	83	21755516	1.90E-01	1
B3GALT4	1456953	262	30	900324	313992	0	6	3	0	20	43	9065896	1.91E-01	1
RSU1	506025	792	1	734428	190460	0	3	0	0	50	53	22357868	1.91E-01	1
ICA1	176442	544	19	1296552	313992	0	5	0	0	50	40	19343616	1.91E-01	1
FAM166A	1724821	399	43	826632	220008	0	7	0	0	50	60	16636592	1.91E-01	1
PRPF3	1301004	182	34	1774304	481312	0	9	2	0	34	57	16988320	1.91E-01	1
USP48	510117	181	30	2778224	658600	0	11	1	0	18	25	8029580	1.91E-01	1
FAM104A	205112	251	-15	524032	157352	0	2	0	0	50	58	23675068	1.92E-01	1
SRD5A2	157835	693	3	639020	196156	0	2	1	0	38	33	15232172	1.92E-01	1
FAM111B	437114	741	-6	1898904	464580	0	10	1	0	49	54	18637312	1.92E-01	1

RAC1	NaN	NaN	NaN	550020	154860	0	4	0	0	50	116	39800444	1.92E-01	1
VTI1B	479937	260	39	598080	169812	0	4	0	0	50	42	17838804	1.92E-01	1
PTCH1	186893	510	53	3825576	1129944	0	18	2	0	28	29	12660784	1.92E-01	1
TMEM92	1297691	196	28	414384	121752	0	2	0	0	50	80	24625588	1.93E-01	1
ZSCAN20	349443	614	31	2659320	711288	0	8	2	0	32	41	18254968	1.93E-01	1
SLAMF8	802263	403	6	714848	218584	0	4	0	0	36	37	12550780	1.93E-01	1
ROBO4	175852	743	14	2494136	808120	0	8	3	0	50	63	23580016	1.93E-01	1
ZNF567	610659	922	-38	1597728	391956	0	10	3	0	50	74	18264580	1.93E-01	1
ZNF395	289053	229	44	1286584	398720	0	5	1	0	50	53	18820296	1.93E-01	1
TSGA13	278944	433	9	727664	182272	0	3	1	0	50	44	20053836	1.93E-01	1
COX7B	549293	NaN	19	215024	55180	0	2	0	0	50	74	23808568	1.93E-01	1
KDM5B	463071	378	35	3985064	1076188	0	12	3	0	50	50	19503104	1.94E-01	1
SKAP1	1097775	588	4	979712	224992	0	4	2	0	11	7	3179080	1.94E-01	1
MAF1	2720766	211	27	685300	168032	0	3	0	0	50	67	26365716	1.94E-01	1
SLC13A5	800049	424	7	1442512	429692	0	5	1	0	42	30	12306920	1.94E-01	1
GPR75	NaN	NaN	NaN	1331440	401924	0	9	3	0	26	43	11979044	1.94E-01	1
C1orf158	141239	571	18	502316	135280	0	3	1	0	11	8	2696344	1.94E-01	1
CYBA	802673	189	41	496264	157352	0	2	0	0	50	65	20750172	1.94E-01	1
ADAM10	506165	386	31	1975800	488076	0	11	1	0	12	12	6472080	1.94E-01	1
C18orf25	413073	142	58	1014956	296548	0	4	2	0	50	68	20965908	1.95E-01	1
HNRNPAE	672705	448	46	877896	223212	0	3	0	0	50	73	25444744	1.95E-01	1
KIAA1045	676166	394	23	1018872	295836	0	9	2	0	22	39	12320092	1.95E-01	1
DEFB123	605825	450	-76	182984	43432	0	1	0	0	50	111	28209084	1.95E-01	1
EXOSC5	1148529	249	36	590960	188680	0	4	0	0	50	39	16082656	1.95E-01	1
DDI2	677127	162	28	1035248	286936	0	5	0	0	9	10	4870792	1.95E-01	1
GIGYF2	325071	328	35	3479188	878252	0	8	2	0	50	41	18336848	1.95E-01	1
EPB41L3	57432	1036	-5	2789260	790676	0	15	1	0	1	2	1414744	1.95E-01	1
APH1A	1213150	197	38	672128	215380	0	3	0	0	50	58	21071640	1.96E-01	1
PVRL1	744818	555	30	1660028	493060	0	5	1	0	50	74	24278488	1.96E-01	1
GABRR1	398929	446	29	1253476	322892	0	6	0	0	50	42	25327620	1.96E-01	1
SPOP	943930	348	28	987900	252048	0	5	1	0	50	62	18842012	1.96E-01	1
MMEL1	323457	335	30	2052696	546816	0	6	3	0	35	31	12502364	1.96E-01	1
NFS1	1423559	169	53	1178360	344608	0	6	0	0	15	12	5442528	1.96E-01	1

OR4A15	1067	1102	-42	855468	248844	0	8	2	0	9	13	3819524	1.96E-01	1
GSG1	692979	203	5	1120332	212532	0	6	0	0	50	56	21266372	1.96E-01	1
MRFAP1	501655	278	47	322892	91492	0	1	0	0	50	50	19690004	1.96E-01	1
PDE5A	183496	900	-6	2349956	572092	0	9	2	0	50	49	20043512	1.96E-01	1
SNRNP48	825677	209	14	910292	215380	0	4	0	0	50	60	21797880	1.97E-01	1
FAM179B	231363	490	1	4331096	1259884	0	19	3	0	44	52	20638032	1.97E-01	1
ARGFX	289448	613	18	805984	223568	0	2	0	0	50	42	24871584	1.97E-01	1
MPL	1088321	252	27	1609832	477040	0	6	1	0	50	56	22185208	1.97E-01	1
DKK3	324993	243	11	902460	254184	0	6	1	0	50	58	21244656	1.97E-01	1
FAM3C	100604	535	-1	620152	148808	0	3	0	0	50	67	24128968	1.97E-01	1
MCF2L2	398145	606	24	2949104	749380	0	11	1	0	13	9	5241032	1.98E-01	1
PRR15L	1183803	203	21	263440	74048	0	1	0	0	50	60	21372816	1.98E-01	1
RAB5A	103261	609	-8	565328	148096	0	3	0	0	50	53	19967684	1.98E-01	1
CBR4	174340	258	16	606980	172660	0	2	0	0	50	38	16291628	1.98E-01	1
SLC39A8	275900	441	4	1253476	357068	0	4	0	0	16	7	5590980	1.98E-01	1
CRNN	387153	671	6	1243152	354576	0	3	0	0	3	1	2462096	1.98E-01	1
NT5E	393239	321	13	1462804	413672	0	8	2	0	17	13	5725904	1.98E-01	1
IL21R	228256	597	36	1359564	401568	0	6	0	0	23	21	9360308	1.98E-01	1
USP47	327690	317	17	3406208	831616	0	11	3	0	17	19	9430084	1.98E-01	1
GKN1	338803	222	16	532576	131720	0	2	1	0	37	38	15321884	1.98E-01	1
RNF220	742455	355	34	1458888	413316	0	6	0	0	42	42	17956284	1.98E-01	1
YIPF1	505734	319	24	801356	220720	0	5	0	0	22	25	7631928	1.99E-01	1
EXOC4	618702	965	12	2526888	679248	0	13	1	0	2	1	1171596	1.99E-01	1
HS3ST2	432670	708	14	896408	289072	0	5	1	0	50	66	21351100	1.99E-01	1
KCNQ3	163210	970	-6	2195096	661804	0	10	0	0	29	24	11279148	1.99E-01	1
UACA	247817	364	19	3764700	896052	0	15	1	0	19	17	7591344	1.99E-01	1
LMCD1	92154	581	1	934856	259168	0	3	1	0	50	59	25910748	1.99E-01	1
APOA5	419339	326	36	923820	269136	0	4	0	0	50	47	21154588	1.99E-01	1
XYLB	301536	437	24	1411896	385548	0	6	1	0	50	63	23628076	2.00E-01	1
FAM180A	470926	386	-1	434320	131720	0	3	0	0	50	45	16597432	2.00E-01	1
COQ3	259457	638	13	948740	264508	0	4	1	0	46	68	25937448	2.00E-01	1
PSPN	943949	407	31	368816	137416	0	1	0	0	50	47	18083732	2.00E-01	1
ADCY8	45406	771	-7	3152380	933788	0	19	5	0	37	42	16193372	2.00E-01	1

PRICKLE1	175176	650	6	2137780	561056	0	8	2	0	30	28	10420120	2.00E-01	1
SAMM50	155890	393	24	1232116	333572	0	3	1	0	50	44	19080532	2.00E-01	1
HSPB2	437513	221	22	443932	148808	0	3	0	0	50	49	18660452	2.00E-01	1
CCL7	26189	889	-12	263084	67996	0	2	0	0	50	80	29689332	2.00E-01	1
TP63	140525	403	15	1907804	503740	0	12	4	0	6	11	3055904	2.00E-01	1
CRYBA2	922162	386	35	514420	139196	0	2	0	0	50	52	18596728	2.00E-01	1
VAMP8	1158965	170	43	261304	70844	0	2	0	0	50	53	19662948	2.00E-01	1
CLN3	NaN	NaN	NaN	1120332	350304	0	8	0	0	50	116	39995888	2.01E-01	1
RASSF8	143649	396	4	1114280	294412	0	5	0	0	50	30	17014308	2.01E-01	1
IGSF21	55390	692	31	1171596	366324	0	4	1	0	50	52	21275628	2.01E-01	1
MEIS1	21299	641	-10	1046284	257744	0	5	2	0	16	24	8268456	2.01E-01	1
GTF2H3	871640	315	40	840516	205056	0	4	1	0	50	59	21342912	2.01E-01	1
CEND1	1788356	193	48	360984	123888	0	3	0	0	50	64	19157072	2.01E-01	1
ELP4	165949	879	-7	1092208	310076	0	7	1	0	50	64	23671152	2.01E-01	1
TMEM206	487387	298	17	915988	238520	0	7	1	0	50	61	19624144	2.01E-01	1
SCN1A	98532	550	-11	5186564	1362412	0	24	2	0	10	12	4749396	2.01E-01	1
TLR7	208310	NaN	55	2650776	721968	0	9	1	0	46	39	17239656	2.02E-01	1
PPP1R3C	276001	206	16	814172	208972	0	3	1	0	50	40	17426556	2.02E-01	1
NUDT16	274681	843	20	781420	262016	0	4	1	0	50	69	25513808	2.02E-01	1
RUVBL1	834133	419	46	1181920	325028	0	6	0	0	50	83	24660476	2.02E-01	1
GPR39	46530	849	3	1119976	338912	0	4	0	0	36	37	16968028	2.02E-01	1
TNMD	182582	NaN	-2	841584	202920	0	3	1	0	50	64	18681100	2.02E-01	1
HIST1H3I	228884	634	40	331436	111784	0	4	1	0	23	29	6014264	2.02E-01	1
ANP32E	1207965	197	39	725172	162336	0	4	0	0	50	67	20708520	2.02E-01	1
ZNF491	739660	633	-2	1133860	273764	0	5	0	0	25	19	8588500	2.02E-01	1
CRTC1	1398467	222	29	1631904	515844	0	5	1	0	50	56	20215104	2.02E-01	1
HARS	774470	356	31	1310792	376648	0	5	0	0	19	25	9696372	2.02E-01	1
CASC4	973993	493	37	1150236	288360	0	5	0	0	50	57	24795044	2.02E-01	1
C1orf168	39470	514	-6	1936284	480600	0	6	0	0	12	5	4710236	2.02E-01	1
PSG8	386041	417	-19	1100396	309364	0	5	0	0	21	21	9097224	2.03E-01	1
TAF9	445098	314	50	1140268	309008	0	5	0	0	50	60	21405568	2.03E-01	1
GRK5	831479	338	30	1561416	396228	0	6	3	0	34	34	12058788	2.03E-01	1
PABPN1	1171456	330	20	768248	241012	0	3	0	0	50	49	21656192	2.03E-01	1

ITSN2	380538	378	37	4505180	1154152	0	19	2	0	10	12	5090444	2.03E-01	1
ZNF583	135115	713	-50	1491284	352084	0	8	1	0	10	32	8144212	2.03E-01	1
ITIH2	199302	528	-8	2464588	655040	0	12	1	0	8	8	4807068	2.03E-01	1
TUBGCP3	273358	399	11	2363128	635816	0	6	2	0	50	58	24560796	2.03E-01	1
PDS5B	243309	471	50	3820236	964404	0	17	3	0	13	15	6774680	2.03E-01	1
CELF1	1011926	219	40	1362412	347456	0	4	1	0	50	65	23817112	2.03E-01	1
NDRG2	1005555	429	4	981136	274832	0	4	0	0	30	29	8819900	2.03E-01	1
HRG	508316	436	45	1350664	362408	0	6	1	0	50	77	27976260	2.03E-01	1
BRIP1	326357	412	-9	3243160	843008	0	17	3	0	17	26	8152400	2.04E-01	1
SYT16	215309	626	-6	1647924	443220	0	6	2	0	7	8	5262392	2.04E-01	1
RBP1	226923	848	13	566396	151300	0	3	0	0	50	54	17424776	2.04E-01	1
MYO3A	63308	873	-6	4247436	1074408	0	20	6	0	36	50	16311920	2.04E-01	1
KRT33B	1321356	424	-6	1031332	294056	0	8	0	0	18	19	6810636	2.04E-01	1
MAGI3	369997	623	2	3840884	1048420	0	15	6	0	50	58	21445084	2.04E-01	1
DTNBP1	118453	750	24	1015312	260948	0	5	1	0	50	141	46629592	2.05E-01	1
DHRS7B	409501	382	-18	818800	253472	0	3	0	0	37	39	16113984	2.05E-01	1
AGTRAP	606580	187	36	566396	71200	0	2	0	0	50	60	23033912	2.05E-01	1
RASL11B	170130	812	9	628696	181916	0	4	0	0	50	66	19346820	2.05E-01	1
MPHOSPH	170324	715	-10	440372	94696	0	3	1	0	6	8	2889296	2.05E-01	1
GSTA3	604568	455	8	590248	149876	0	2	0	0	50	57	21199088	2.05E-01	1
IER5	201966	253	23	780352	270560	0	2	1	0	50	33	19168464	2.05E-01	1
CSNK1G3	121115	474	23	1216096	296192	0	3	1	0	50	45	19783988	2.05E-01	1
RNF217	120614	269	-24	735140	175864	0	4	1	0	50	61	19568608	2.06E-01	1
HDHD3	390186	297	36	603776	203632	0	4	0	0	50	42	19391676	2.06E-01	1
PEX19	891852	358	25	790676	202564	0	5	0	0	50	67	19194452	2.06E-01	1
MIF4GD	1723762	171	31	668212	180848	0	5	0	0	50	81	19557572	2.06E-01	1
ASGR1	1682013	348	15	771452	198292	0	3	1	0	50	56	20137140	2.06E-01	1
STK4	938230	294	42	1287652	320756	0	7	0	0	44	73	25500636	2.06E-01	1
NDUFB2	329944	567	29	278036	76540	0	1	0	0	50	74	24895436	2.06E-01	1
N6AMT2	403677	429	44	565684	140264	0	4	0	0	50	55	22681828	2.06E-01	1
ZNF223	533136	558	-35	1261664	302956	0	6	1	0	50	58	16442928	2.06E-01	1
MRPS36	423022	261	47	275544	72624	0	2	0	0	50	52	19542976	2.06E-01	1
BLNK	817052	804	34	1214672	317908	0	6	0	0	19	18	8348200	2.06E-01	1

IFNK	NaN	NaN	NaN	547172	124600	0	4	1	0	50	117	39770184	2.06E-01	1
MPHOSPH	306203	602	18	2251700	564616	0	12	1	0	29	28	15106148	2.06E-01	1
HNRNPCL	NaN	NaN	NaN	746176	200072	0	4	1	0	50	117	39845656	2.07E-01	1
C1orf110	334195	540	-7	781776	201852	0	5	1	0	50	40	16887572	2.07E-01	1
MYF5	27810	974	-1	644716	184052	0	8	3	0	3	8	1275904	2.07E-01	1
ITGB8	207764	550	30	2011400	511216	0	9	3	0	46	49	19172024	2.07E-01	1
UPRT	145779	NaN	-5	796728	222144	0	5	0	0	50	51	18929588	2.07E-01	1
ELANE	2168802	198	28	657532	222500	0	5	1	0	50	73	22578944	2.07E-01	1
ZC3H18	626708	184	39	2418308	710932	0	6	1	0	46	36	19748032	2.07E-01	1
HOXC8	1185281	279	54	620864	161980	0	5	0	0	50	59	19280960	2.07E-01	1
GNL3L	302365	NaN	31	1530444	401568	0	6	0	0	50	56	24348264	2.08E-01	1
NPRL2	843769	206	51	998936	264508	0	4	0	0	50	68	20472848	2.08E-01	1
XAB2	611341	384	33	2208268	615524	0	6	1	0	16	13	8447524	2.08E-01	1
CLDN1	164468	348	3	534000	158064	0	3	0	0	50	47	21290936	2.08E-01	1
TP53I13	760947	353	26	953724	336420	0	3	0	0	50	54	24137156	2.08E-01	1
CENPT	1039476	143	49	1404420	447492	0	8	1	0	50	49	20248924	2.08E-01	1
SAMHD1	817783	225	47	1655756	421504	0	5	2	0	50	68	25624524	2.08E-01	1
LCN2	1762678	201	44	526880	138484	0	3	1	0	50	65	19259600	2.08E-01	1
AURKC	407201	924	-63	801000	228552	0	5	1	0	50	58	18412320	2.08E-01	1
C9orf156	468629	276	38	1114280	321112	0	8	0	0	50	57	19671848	2.08E-01	1
ZNF330	71072	671	-4	875048	191884	0	5	0	0	50	69	21972320	2.08E-01	1
KRTAP17-	1149433	468	-14	268424	73336	0	1	0	0	50	53	16834528	2.09E-01	1
SLC35F4	175308	918	27	1321828	380564	0	5	1	0	50	42	20255332	2.09E-01	1
CD5L	116471	778	28	890000	246352	0	6	1	0	15	13	6004296	2.09E-01	1
NPY1R	74427	1197	-18	975440	262372	0	6	1	0	50	78	21835260	2.09E-01	1
TRIML1	32696	595	-20	1201500	322536	0	5	1	0	50	51	18116128	2.09E-01	1
ZNF10	334913	834	6	1489504	366680	0	7	2	0	50	71	19917132	2.09E-01	1
TRAPPC6E	314417	166	13	429336	101460	0	1	0	0	50	41	19241800	2.09E-01	1
TAAR5	125852	605	19	833040	254184	0	4	1	0	50	67	19971956	2.09E-01	1
S100A6	1454773	186	26	239944	62300	0	2	0	0	50	56	19376724	2.10E-01	1
BCAT1	252342	905	2	1048776	288360	0	4	0	0	50	82	25417332	2.10E-01	1
RCOR3	279632	545	-1	1534360	320756	0	5	1	0	50	49	18467500	2.10E-01	1
CA1	99296	1463	15	689928	181560	0	3	1	0	50	52	18027128	2.10E-01	1

ZNF846	938482	600	-49	1386976	345320	0	9	2	0	50	135	30618136	2.10E-01	1
PAIP2B	349503	464	30	339980	70132	0	2	0	0	50	41	20361420	2.10E-01	1
TRIM71	507311	469	24	2113928	685300	0	8	4	0	30	24	11402680	2.10E-01	1
CLC	1286220	526	26	377004	96120	0	2	0	0	50	75	22079120	2.10E-01	1
MPDU1	1957426	194	24	624068	196156	0	2	1	0	50	81	21975524	2.10E-01	1
FOXD4L5	NaN	NaN	NaN	1001072	334996	0	5	1	0	50	117	39980580	2.10E-01	1
TRIML2	32696	595	-20	1010328	262728	0	5	1	0	50	51	18116128	2.10E-01	1
INO80	950881	181	41	4055552	1082596	0	18	4	0	14	15	5665028	2.10E-01	1
CD300E	876565	484	14	521540	155572	0	2	0	0	50	52	17749092	2.10E-01	1
NAGK	367824	470	34	1001784	289428	0	4	0	0	50	37	18057032	2.10E-01	1
TPK1	123029	998	-17	651480	164472	0	3	0	0	50	65	22342204	2.11E-01	1
USP4	1661046	201	34	2520124	660380	0	10	2	0	27	36	12583176	2.11E-01	1
KCNJ3	15788	1135	7	1258816	358136	0	4	2	0	28	30	12813152	2.11E-01	1
LCE2D	169158	818	24	280884	79032	0	2	0	0	50	53	20184132	2.11E-01	1
ODAM	181352	975	12	742616	199360	0	4	0	0	50	57	17976932	2.11E-01	1
FLRT2	18925	671	-45	1625852	491992	0	12	4	0	7	22	4594180	2.11E-01	1
PPCS	570562	583	27	762196	248132	0	7	2	0	2	3	1269496	2.11E-01	1
SLC48A1	395089	320	14	367748	111784	0	1	0	0	50	52	22689304	2.12E-01	1
IL1RAPL1	11003	NaN	14	1809548	468496	0	10	2	0	50	69	23568268	2.12E-01	1
MRPS35	348119	376	4	843008	227128	0	4	0	0	50	55	19941340	2.12E-01	1
SPATA19	270077	622	-14	448916	117124	0	3	0	0	50	42	17328656	2.12E-01	1
MDH1	328344	468	7	865792	243860	0	4	1	0	50	47	17987612	2.12E-01	1
PTPN13	239175	533	31	6454636	1727312	0	19	5	0	50	73	22785424	2.12E-01	1
PARK2	8197	1243	-30	1224284	315772	0	5	0	0	38	85	18210824	2.12E-01	1
SETD7	400132	191	15	954436	251336	0	4	0	0	50	48	20227208	2.13E-01	1
KCNJ6	253563	541	36	1074052	297260	0	7	1	0	50	41	17181272	2.13E-01	1
KCNE3	633655	283	23	258812	78676	0	2	0	0	50	65	23451500	2.13E-01	1
PTGFR	249935	671	-16	976864	265220	0	5	2	0	50	81	22653704	2.13E-01	1
FAM120B	366503	600	-4	2334648	626916	0	8	0	0	9	9	4145976	2.13E-01	1
TNFSF10	319022	263	23	743684	179068	0	4	0	0	50	57	25565072	2.13E-01	1
INPP4B	51592	807	10	2437888	619796	0	8	2	0	39	50	18969460	2.13E-01	1
SAMD3	84835	891	27	1391604	337488	0	5	0	0	50	42	21684316	2.13E-01	1
TMX1	338961	610	38	739768	190460	0	2	0	0	50	50	17363188	2.13E-01	1

THAP10	250598	339	10	637952	197224	0	3	0	0	50	54	19786124	2.13E-01	1
ARVCF	616246	217	37	2373452	788896	0	10	4	0	45	43	18624496	2.14E-01	1
CD28	320806	477	19	565684	155216	0	3	1	0	29	27	13344660	2.14E-01	1
MAGEB10	10745	NaN	16	870776	248488	0	5	1	0	50	64	18168460	2.14E-01	1
CTDSP1	734742	197	37	667856	197224	0	3	0	0	50	56	21809272	2.14E-01	1
ZNF714	164756	1079	-42	1441800	349236	0	9	1	0	26	42	10608088	2.14E-01	1
RHGAP11	244857	383	1	2641520	710932	0	9	1	0	23	25	11827744	2.14E-01	1
EVI2A	NaN	NaN	NaN	656464	185120	0	3	0	0	50	116	39830704	2.14E-01	1
HRSP12	635159	368	44	363476	102172	0	2	0	0	50	46	22628072	2.14E-01	1
CDK12	1409087	239	28	3727320	1105380	0	20	3	0	25	31	10126420	2.15E-01	1
POLR3H	1467336	183	66	540052	142400	0	2	0	0	50	56	20734864	2.15E-01	1
PXT1	968861	146	41	363120	82236	0	1	0	0	50	69	26742008	2.15E-01	1
FNBP4	979103	158	19	2590256	739768	0	11	0	0	7	3	3375592	2.15E-01	1
SHH	213448	634	23	1114280	379852	0	5	0	0	41	41	17509148	2.15E-01	1
TCF23	1214097	238	50	525812	175864	0	2	1	0	50	60	22743060	2.15E-01	1
YTHDF2	818065	208	30	1469212	410468	0	9	2	0	50	75	21917852	2.15E-01	1
ARL16	3745109	205	30	494840	158776	0	3	1	0	33	25	10695308	2.16E-01	1
ZNF37A	138629	916	-18	1468856	348880	0	8	0	0	12	19	5440036	2.16E-01	1
PQLC3	491115	255	25	521184	154860	0	4	0	0	50	57	18627344	2.16E-01	1
MRPS21	1213150	197	38	233180	57316	0	1	1	0	9	3	1880748	2.16E-01	1
HACL1	433102	235	32	1518696	404772	0	10	3	0	28	25	9571772	2.16E-01	1
KRT84	936407	213	42	1506948	454968	0	6	2	0	50	55	23702836	2.16E-01	1
STIL	321135	366	6	3315784	882524	0	15	2	0	50	46	17856248	2.16E-01	1
UBE2E3	114904	888	11	543612	144180	0	2	0	0	50	60	20862668	2.17E-01	1
DHRS9	303546	646	12	809188	233180	0	4	1	0	50	70	25775468	2.17E-01	1
WEE1	553054	158	28	1644720	470988	0	7	1	0	50	64	22006140	2.17E-01	1
COL5A2	237205	422	1	3796384	1236032	0	16	3	0	50	49	22283108	2.17E-01	1
SPANXN3	4577	NaN	-29	373088	90424	0	4	1	0	11	39	6381300	2.17E-01	1
KRTAP24-	6302	1011	-69	640444	178712	0	4	2	0	41	58	10000752	2.18E-01	1
DCAF8	866729	355	27	1551092	416164	0	5	0	0	50	59	21739140	2.18E-01	1
FGA	59962	1036	-4	2222152	624068	0	13	1	0	30	28	11140308	2.18E-01	1
MCCC1	275779	546	22	1896412	506588	0	11	3	0	22	18	8817052	2.18E-01	1
PGAP2	425863	258	23	1063016	166252	0	3	0	0	50	55	19846644	2.18E-01	1

KCTD6	567081	265	35	605200	165896	0	2	0	0	50	52	21882608	2.18E-01	1
GREM1	244472	383	5	459952	135992	0	2	0	0	50	44	17500960	2.18E-01	1
HSD17B11	502611	417	16	767536	222500	0	3	0	0	50	48	17617728	2.19E-01	1
SOX10	1370748	203	50	1153796	355288	0	2	0	0	46	33	17324384	2.19E-01	1
TRIM21	264090	862	-7	1218944	331792	0	7	0	0	22	33	12089760	2.19E-01	1
CIRBP	2214581	234	29	454968	124956	0	2	0	0	50	68	20485664	2.19E-01	1
MCM4	898350	459	35	2212184	624424	0	9	1	0	46	43	18109008	2.19E-01	1
C3orf14	213716	935	-3	343896	86508	0	3	0	0	50	69	21505960	2.19E-01	1
CCND1	441431	536	41	752940	212532	0	5	2	0	50	63	20424076	2.19E-01	1
PFN1	1416161	231	30	354576	105732	0	3	0	0	50	65	18681100	2.19E-01	1
TDGF1	277267	528	52	498400	134924	0	5	1	0	11	14	3646864	2.19E-01	1
HDGFRP3	299178	679	24	541832	133144	0	1	0	0	50	43	23314796	2.20E-01	1
HORMAD1	1611525	178	42	1072984	252404	0	6	1	0	50	66	20744120	2.20E-01	1
SSPN	157088	383	25	605556	186900	0	2	0	0	50	50	20347180	2.20E-01	1
CNKSR2	90158	NaN	-2	2712364	700964	0	10	1	0	20	26	12236076	2.20E-01	1
CCDC87	1343990	194	62	2077972	647564	0	8	4	0	21	20	8972268	2.20E-01	1
H6PD	954202	297	33	1957644	597012	0	10	2	0	50	78	21810696	2.20E-01	1
ELOVL1	1088321	252	27	734428	192596	0	3	0	0	50	56	22185208	2.20E-01	1
NPB	3798594	211	19	295836	114276	0	2	0	0	34	28	11428668	2.20E-01	1
SPANXD	NaN	NaN	NaN	258812	65860	0	2	0	0	50	116	39711444	2.20E-01	1
ZPBP2	1602321	292	18	896408	219652	0	6	2	0	33	61	15816724	2.20E-01	1
TPRKB	978005	466	31	458528	122464	0	2	0	0	50	67	22206212	2.21E-01	1
TCN1	337128	548	8	1132792	294056	0	5	1	0	50	56	21099408	2.21E-01	1
RNF181	1158965	170	43	410824	101816	0	2	1	0	27	36	10060204	2.21E-01	1
TCF12	203021	599	27	1913500	519404	0	7	0	0	25	22	11918880	2.21E-01	1
HIST1H2A	267617	634	32	313636	110360	0	3	0	0	50	56	15505936	2.21E-01	1
HAO2	409405	720	3	897832	259880	0	7	1	0	50	56	19659032	2.21E-01	1
RCHY1	461415	491	19	728020	145604	0	2	0	0	50	63	20447928	2.21E-01	1
F13B	220645	802	-30	1744044	426132	0	8	2	0	24	43	11316528	2.21E-01	1
TMED1	2054203	208	30	570668	172660	0	3	0	0	50	73	19425852	2.21E-01	1
NUP107	486884	294	31	2462452	619796	0	8	0	0	5	0	1368108	2.21E-01	1
SLC25A24	226531	454	-15	1349596	357068	0	3	3	0	43	37	13814936	2.21E-01	1
NGFRAP1	448080	NaN	32	292988	70132	0	1	0	0	50	52	16837020	2.22E-01	1

LST1	2517285	220	35	294768	58740	0	2	0	0	50	55	21824936	2.22E-01	1
GUK1	702358	341	15	564972	168744	0	2	0	0	50	43	17240012	2.22E-01	1
PCSK5	96278	886	-24	2410832	605200	0	11	3	0	37	45	14190516	2.22E-01	1
TSKU	287129	214	42	830904	307584	0	4	2	0	50	56	18200144	2.22E-01	1
ADAM21	423685	706	22	1823432	493060	0	7	1	0	37	41	19971244	2.22E-01	1
ANKZF1	733837	158	40	1821652	563192	0	8	0	0	15	12	5688168	2.23E-01	1
MED9	915530	220	-3	372732	104664	0	2	0	0	50	67	16916408	2.23E-01	1
ECHDC3	371900	163	15	753652	237452	0	4	0	0	50	43	21952740	2.23E-01	1
ZDHHC7	481076	273	28	889644	248844	0	3	1	0	50	52	20133936	2.23E-01	1
SH2D5	634204	208	34	1055540	341404	0	4	1	0	50	59	20255332	2.23E-01	1
RSC1A1	677127	162	28	1537920	445356	0	7	0	0	9	10	4870792	2.23E-01	1
HDDC3	NaN	NaN	NaN	357780	108936	0	1	0	0	50	116	39754520	2.23E-01	1
FAM3D	497368	503	38	609472	149876	0	3	1	0	50	77	24522348	2.23E-01	1
TFB1M	116637	428	26	891780	247776	0	4	0	0	50	38	20981572	2.23E-01	1
TUSC5	1447271	228	21	437168	141688	0	3	1	0	30	48	12364592	2.24E-01	1
AHSG	508316	436	45	920972	285868	0	6	1	0	50	77	27976260	2.24E-01	1
MYL2	907625	570	47	457104	105732	0	2	1	0	24	35	12869400	2.24E-01	1
ZFYVE28	845507	271	40	2399440	727664	0	6	2	0	50	76	28542300	2.24E-01	1
NUBP2	1604603	288	36	684944	214312	0	2	0	0	50	49	20717064	2.24E-01	1
NXNL2	130577	438	1	475616	150232	0	2	0	0	50	55	19031048	2.24E-01	1
CTTNBP2	96915	674	-11	4228568	1199008	0	15	2	0	46	63	26790780	2.25E-01	1
MYF6	27810	974	-1	610184	179068	0	5	1	0	40	75	17126092	2.25E-01	1
DAZL	140687	470	24	783912	207192	0	4	0	0	50	43	19332224	2.25E-01	1
UBE2A	663615	NaN	48	408688	102884	0	2	0	0	50	60	18555788	2.25E-01	1
OR2B2	228884	634	40	892848	258456	0	5	1	0	50	48	14804260	2.25E-01	1
LCE2B	169158	818	24	279460	80456	0	2	0	0	50	53	20184132	2.25E-01	1
SOX5	29515	973	-7	1986124	534356	0	17	5	0	10	15	3859752	2.25E-01	1
LACTB	451944	230	36	1389112	392312	0	4	0	0	50	52	20650492	2.26E-01	1
SLC30A1	380154	236	22	1249560	382344	0	3	0	0	18	9	7749052	2.26E-01	1
FAM57A	662733	483	10	645428	200428	0	2	0	0	28	22	9770420	2.26E-01	1
HIST1H2A.	267617	634	32	306160	109292	0	5	2	0	12	20	2202928	2.26E-01	1
CSPG5	888051	200	35	1329304	417944	0	7	1	0	50	63	22998312	2.26E-01	1
NPFF	1469374	191	55	290852	85084	0	2	0	0	50	47	18664012	2.26E-01	1

EIF3A	701340	194	9	3584564	936280	0	14	1	0	13	14	7547556	2.26E-01	1
LGALS13	1148741	322	25	368460	95052	0	3	1	0	16	12	4700624	2.26E-01	1
SLK	508085	271	37	3258824	780352	0	10	0	0	6	6	4493432	2.26E-01	1
PRR23B	336415	520	11	624068	228196	0	4	1	0	50	46	19800720	2.27E-01	1
C11orf85	3151458	209	52	590248	142400	0	3	1	0	50	83	23753744	2.27E-01	1
OR11A1	329776	872	7	763620	250980	0	4	1	0	50	77	21945976	2.27E-01	1
MED28	271066	989	32	458528	129940	0	3	0	0	50	47	19786836	2.27E-01	1
KCNA2	479017	640	21	1248492	353508	0	7	1	0	50	64	18776508	2.27E-01	1
HTATSF1	374989	NaN	23	1993244	467428	0	9	0	0	10	12	4821308	2.27E-01	1
CR2	447441	354	30	2810976	772164	0	14	2	0	50	53	20601364	2.28E-01	1
LEO1	575343	136	34	1773592	412604	0	10	1	0	50	44	18330440	2.28E-01	1
TLL2	798938	623	33	2653268	687436	0	10	3	0	50	52	19747320	2.28E-01	1
RSPH1	411865	374	67	824852	202564	0	4	1	0	50	55	18865864	2.28E-01	1
HNRNPD	651473	390	26	936636	238164	0	3	1	0	24	33	11107912	2.28E-01	1
PYCR1	3798594	211	19	863300	279460	0	3	1	0	34	28	11428668	2.28E-01	1
SPOCK3	7931	1043	-11	1176936	268068	0	5	0	0	50	80	23523768	2.28E-01	1
MED21	408250	320	26	375936	103596	0	1	0	0	50	53	21014324	2.28E-01	1
ACBD5	383472	555	36	1396588	339980	0	4	1	0	50	40	18981920	2.28E-01	1
TGIF2	1019481	176	62	578500	192596	0	2	0	0	50	44	18338984	2.28E-01	1
PRRG4	392152	375	10	583840	164828	0	3	0	0	50	60	27611360	2.28E-01	1
CCDC36	1812695	203	45	1540768	395516	0	9	3	0	10	9	3276980	2.28E-01	1
SFXN2	878626	180	27	852976	228908	0	4	1	0	50	59	20336144	2.29E-01	1
ATMIN	231500	284	4	2047712	607336	0	9	1	0	50	47	21594604	2.29E-01	1
PCP2	611341	384	33	354932	100036	0	1	0	0	50	46	20710656	2.29E-01	1
CHSY1	409968	290	26	2012468	568888	0	8	2	0	23	29	10393776	2.29E-01	1
HBD	109545	1056	-1	371664	111072	0	1	0	0	50	50	17996156	2.29E-01	1
NPSR1	106021	949	0	1055896	288716	0	4	0	0	23	26	11325428	2.29E-01	1
DPYSL4	147055	416	-6	1467788	423640	0	4	1	0	50	47	21665804	2.29E-01	1
YTHDC1	126481	485	0	1917772	483092	0	6	0	0	5	1	2249564	2.29E-01	1
GNAI1	94843	475	-34	936992	230332	0	5	1	0	21	32	7716656	2.30E-01	1
FANCD2	885389	170	41	3957652	1026704	0	18	3	0	49	52	22072000	2.30E-01	1
CYTH4	902502	201	61	1056252	260592	0	4	0	0	5	3	2555012	2.30E-01	1
SCGB1D2	3058870	450	42	237452	64792	0	2	0	0	50	51	16305156	2.30E-01	1

COMMD9	222571	248	-8	510148	150944	0	3	1	0	50	50	22389196	2.30E-01	1
CCBL1	1706086	201	24	1114280	292276	0	4	0	0	50	89	24955244	2.30E-01	1
AQP10	1653296	236	29	740124	250980	0	3	0	0	50	69	22962712	2.30E-01	1
ZNF133	575071	471	-1	1657536	454968	0	6	0	0	9	9	3711656	2.30E-01	1
TMCO4	668417	220	29	1594880	495196	0	9	1	0	25	31	10119300	2.30E-01	1
D4S234E	143657	525	46	478820	134212	0	2	0	0	50	81	25934244	2.31E-01	1
SLC25A13	149460	802	2	1744400	497332	0	6	0	0	12	10	5308672	2.31E-01	1
RABGGTE	152545	578	-9	875760	224280	0	4	1	0	50	66	20684312	2.31E-01	1
PTPN4	346126	446	25	2458536	622644	0	13	1	0	14	21	8307616	2.31E-01	1
RSPH4A	247712	479	10	1863304	455324	0	6	1	0	50	50	20262808	2.31E-01	1
MFI2	719859	308	37	2046288	600216	0	6	1	0	12	12	8851228	2.31E-01	1
TFF3	313101	464	90	243504	69420	0	2	0	0	50	54	17032464	2.32E-01	1
TNFRSF10(	648331	237	48	657532	192596	0	3	1	0	50	68	22960932	2.32E-01	1
HIST1H2B(	398813	636	53	312568	96476	0	1	1	0	50	45	13638004	2.32E-01	1
YIPF3	1278255	230	56	887864	270916	0	4	0	0	50	54	19955936	2.32E-01	1
IP6K1	1429151	191	43	1120332	312924	0	5	1	0	50	79	28414140	2.32E-01	1
RPL34	160719	574	14	312568	86864	0	2	0	0	50	46	20184844	2.32E-01	1
PHOSPHO2	345692	393	35	624780	156996	0	4	0	0	50	59	21154588	2.32E-01	1
ZNF580	1077402	254	-10	408688	149876	0	4	0	0	50	67	17041008	2.32E-01	1
RHEB	660831	196	32	498756	123888	0	2	0	0	50	53	18681812	2.32E-01	1
SCAP	875682	197	45	3170180	1024924	0	10	5	0	50	63	22037824	2.32E-01	1
IL19	466144	357	34	569244	146316	0	2	1	0	50	48	17546884	2.32E-01	1
BDKRB1	326763	501	16	865080	275544	0	3	0	0	50	56	21728460	2.32E-01	1
SPHKAP	93607	615	-9	4300836	1198296	0	24	6	0	9	13	4289800	2.33E-01	1
C3orf17	279709	639	18	1474908	381276	0	6	1	0	36	41	16854108	2.33E-01	1
PRKCI	361822	270	18	1599864	385548	0	6	0	0	21	36	12626252	2.33E-01	1
LGALS4	1843355	219	37	845856	232824	0	4	1	0	50	67	22791476	2.33E-01	1
PSMB8	1253457	401	26	812036	245284	0	4	1	0	50	63	19067004	2.33E-01	1
NIP7	1202748	125	20	464580	132432	0	2	0	0	50	55	18011464	2.33E-01	1
C9orf47	248480	646	4	501960	159132	0	1	0	0	50	43	20655120	2.33E-01	1
RABL3	307936	475	10	626560	164828	0	2	0	0	50	43	21403432	2.33E-01	1
C1QTNF2	197614	469	27	808476	262728	0	3	0	0	50	48	18013956	2.33E-01	1
NPBWR2	1032302	312	51	791032	278036	0	4	1	0	50	69	20843800	2.34E-01	1

OR13C3	146816	930	-33	859028	255964	0	7	1	0	50	86	17955928	2.34E-01	1
OR2B3	155147	738	1	785692	224636	0	2	0	0	50	51	24788992	2.34E-01	1
IL2RG	759088	NaN	27	962624	254896	0	4	1	0	50	74	25911460	2.34E-01	1
CADM1	29879	993	5	1134216	323604	0	6	0	0	50	65	28530552	2.34E-01	1
C4orf45	155458	337	10	497688	120684	0	3	0	0	50	75	21192680	2.34E-01	1
UGT1A8	320853	284	24	1342832	377716	0	3	0	0	20	18	9508760	2.34E-01	1
BMI1	195915	462	23	868640	217516	0	4	0	0	50	54	17596724	2.34E-01	1
PACSIN1	888245	207	32	1177648	286580	0	6	1	0	50	92	26125772	2.34E-01	1
ALCAM	165047	784	-3	1537564	397652	0	4	0	0	50	34	21561496	2.34E-01	1
TMEM204	1366033	256	35	553580	184408	0	2	1	0	50	86	26448664	2.34E-01	1
FAM49B	272194	242	6	866504	217516	0	3	0	0	50	51	22807496	2.35E-01	1
FAM101B	248876	591	1	356712	113208	0	3	0	0	50	51	18116484	2.35E-01	1
NTNG2	361444	509	37	1343188	383768	0	4	1	0	50	46	17994376	2.35E-01	1
HMGB1	243134	295	82	581348	127804	0	3	0	0	50	69	20293068	2.35E-01	1
BUB1	NaN	NaN	NaN	2866512	715560	0	13	2	0	50	118	40361144	2.35E-01	1
GRXCR1	32225	966	18	739056	206124	0	5	2	0	19	28	7365284	2.35E-01	1
TMPO	487754	218	21	2404780	712712	0	7	0	0	6	1	2386980	2.35E-01	1
B3GNT5	NaN	NaN	NaN	966184	248132	0	8	0	0	50	116	39893716	2.35E-01	1
ARL8A	649164	297	34	501248	123532	0	3	0	0	50	59	22559008	2.35E-01	1
ADAM23	290387	726	16	2213608	562124	0	7	2	0	50	64	22258900	2.35E-01	1
KAT2A	2171374	206	37	2141340	618372	0	8	0	0	30	24	12789656	2.35E-01	1
RAD51C	524193	222	17	971524	274832	0	5	1	0	50	42	19503460	2.36E-01	1
NT5C1A	974845	388	36	930940	272696	0	4	1	0	50	62	16757632	2.36E-01	1
CPN2	344707	269	34	1323252	430404	0	7	0	0	49	39	17287004	2.36E-01	1
PHF7	1078407	229	62	1030976	235672	0	5	0	0	35	36	12427960	2.36E-01	1
DIRC2	423454	412	37	1191532	377360	0	5	1	0	50	69	21578228	2.36E-01	1
TNRC6A	291928	342	19	5022804	1369176	0	16	5	0	35	35	15996860	2.36E-01	1
GEMIN7	1135715	208	25	318976	108224	0	2	1	0	12	19	4374528	2.36E-01	1
KRTAP6-2	22916	1048	-79	159132	41652	0	2	0	0	50	84	16257808	2.36E-01	1
TUBA1A	2141275	214	56	1134216	328944	0	9	0	0	37	60	18398436	2.36E-01	1
DCTD	204483	431	-12	514064	116056	0	2	1	0	21	22	8400176	2.36E-01	1
TNFRSF8	517022	191	35	1519408	452120	0	7	1	0	9	19	5359224	2.36E-01	1
FAM127A	304745	NaN	42	282308	85084	0	3	1	0	23	19	6299420	2.36E-01	1

EGR4	582263	454	36	1407624	489144	0	5	1	0	50	49	22596744	2.36E-01	1
PTMA	1003689	202	24	312924	63012	0	1	0	0	50	79	20680752	2.37E-01	1
NDUFB5	397411	520	10	496264	135992	0	3	1	0	50	51	19040304	2.37E-01	1
CYP2C8	318306	717	-10	1270208	339268	0	5	1	0	50	67	22179512	2.37E-01	1
PON3	135737	327	-2	925956	249912	0	3	1	0	19	20	9287328	2.37E-01	1
OR9I1	NaN	NaN	NaN	770740	238520	0	5	1	0	50	117	39884104	2.37E-01	1
C3orf20	540760	510	41	2299760	659668	0	9	2	0	50	61	24296288	2.37E-01	1
PYCARD	1203752	478	17	484872	153792	0	2	1	0	50	54	16147804	2.37E-01	1
CCDC140	235091	682	12	404772	120684	0	4	1	0	50	71	18734856	2.37E-01	1
TRIM62	756335	385	27	1190820	351372	0	5	1	0	50	70	26678284	2.37E-01	1
EIF4E3	74213	527	3	580636	168032	0	2	0	0	50	44	18272768	2.37E-01	1
WASF1	127917	636	16	1396232	432184	0	5	0	0	10	7	3756868	2.37E-01	1
SNRPB2	76240	856	-21	599504	150232	0	3	0	0	50	67	21170608	2.38E-01	1
ANKIB1	708388	452	30	2835540	737988	0	11	0	0	10	8	5061964	2.38E-01	1
TOMM70A	377740	361	20	1584912	413316	0	6	1	0	50	53	23216184	2.38E-01	1
METTL5	338901	489	35	600216	142044	0	2	0	0	50	36	15621992	2.38E-01	1
SFXN4	789279	149	29	901392	239232	0	4	0	0	50	67	23654420	2.38E-01	1
CCL23	779228	525	19	359916	97188	0	1	0	0	50	69	21358220	2.38E-01	1
TNFRSF1A	2670962	198	46	1164832	334640	0	5	1	0	4	7	2824504	2.38E-01	1
CCDC71	1855373	203	40	1103600	400144	0	5	0	0	50	67	23488524	2.39E-01	1
ARFRP1	1126597	202	36	534712	143468	0	3	1	0	15	17	5909600	2.39E-01	1
CCDC39	182757	1031	2	2516208	583128	0	9	1	0	21	15	9560380	2.39E-01	1
ARL6IP1	739644	279	45	534000	143112	0	2	0	0	50	58	19373520	2.39E-01	1
NCAM1	68889	524	-1	2392320	650412	0	8	2	0	50	65	23755880	2.39E-01	1
DNAJC5B	270769	377	45	524032	133856	0	2	0	0	50	53	18424424	2.39E-01	1
PDGFD	19576	992	-10	959776	254540	0	6	0	0	50	71	24416260	2.39E-01	1
STARD3NL	161770	578	11	624424	162692	0	2	0	0	50	47	19802144	2.39E-01	1
ZNF165	213170	562	23	1253120	316840	0	5	0	0	50	42	19286656	2.39E-01	1
GSTO2	293891	376	8	635460	167676	0	2	1	0	50	51	20513788	2.39E-01	1
PLVAP	842088	211	43	1125316	317552	0	7	2	0	50	61	21639816	2.40E-01	1
OR51S1	66543	987	-3	773944	263084	0	6	1	0	50	67	17575720	2.40E-01	1
BTF3L4	658095	315	18	423996	106800	0	3	0	0	50	69	22620596	2.40E-01	1
C12orf54	904892	390	11	361696	82592	0	4	0	0	50	73	15126084	2.40E-01	1

ARAP2	28107	1066	26	4464596	1130656	0	11	5	0	50	44	21372816	2.40E-01	1
NPC2	749329	384	31	400500	103596	0	1	0	0	50	65	22029992	2.40E-01	1
TMEM55A	104917	935	1	659312	192952	0	4	0	0	50	66	21354660	2.40E-01	1
SETD6	661173	320	1	1196872	353864	0	7	0	0	50	69	20724184	2.40E-01	1
MARCAD	128578	311	16	2741200	653972	0	11	1	0	10	8	3978656	2.41E-01	1
AQP8	189953	551	12	647208	215736	0	2	0	0	50	54	22679336	2.41E-01	1
ZNF615	444655	838	-57	1892140	466004	0	9	4	0	37	56	12206528	2.41E-01	1
FAM172A	264052	622	6	1106092	272696	0	4	0	0	50	43	18713496	2.41E-01	1
RBBP6	283287	204	25	4619456	1253476	0	20	5	0	21	22	8073368	2.42E-01	1
C5orf28	497364	369	1	542188	158420	0	3	0	0	50	51	21692504	2.42E-01	1
FOXN1	1517567	225	24	1601644	509792	0	6	3	0	50	49	17603488	2.42E-01	1
SMAD1	204089	1182	24	1200076	314348	0	4	0	0	50	39	19909656	2.42E-01	1
PLEKHB1	547370	311	38	627272	184408	0	2	0	0	50	41	17143180	2.42E-01	1
SNX33	586224	231	34	1451768	396940	0	7	2	0	50	42	19685020	2.42E-01	1
EGR1	1202314	205	51	1322184	425064	0	6	0	0	50	47	21712084	2.42E-01	1
PPP3R1	204289	594	23	465648	103596	0	1	0	0	50	48	21499196	2.42E-01	1
GINS1	611174	335	-5	523676	133144	0	2	0	0	50	58	19428344	2.42E-01	1
ARHGAP1:	57425	639	11	1269140	311500	0	6	1	0	50	64	21969472	2.42E-01	1
EPHX3	557083	381	20	904240	282308	0	6	3	0	9	17	5213264	2.43E-01	1
ZNF593	783481	212	23	336420	106800	0	1	0	0	50	65	22539072	2.43E-01	1
KLHL9	232155	466	-14	1555364	424708	0	7	2	0	50	45	18385976	2.43E-01	1
OR5H6	391500	849	-2	813460	231044	0	5	0	0	50	61	20695704	2.43E-01	1
GLOD5	823539	NaN	29	418300	112496	0	3	0	0	50	93	24512736	2.43E-01	1
CBLB	169365	628	0	2519056	707372	0	10	2	0	3	3	2723756	2.43E-01	1
OR5V1	329776	872	7	796728	236028	0	4	0	0	50	77	21945976	2.43E-01	1
MYOM3	817104	171	22	3715216	1045928	0	13	4	0	50	52	19825996	2.43E-01	1
L2HGDH	666223	337	28	1184056	343184	0	4	0	0	18	14	8109680	2.43E-01	1
NRXN1	8891	1372	-25	4160928	1171596	0	25	7	0	8	18	4028852	2.43E-01	1
GIMAP5	734961	689	14	776792	218584	0	5	1	0	50	53	19509868	2.44E-01	1
RIOK2	381706	614	28	1473840	364188	0	6	0	0	29	36	18157424	2.44E-01	1
KIF6	77217	940	26	2165192	542188	0	9	0	0	17	13	6753676	2.44E-01	1
DLX3	1160590	191	12	713780	217516	0	5	2	0	8	12	3368472	2.44E-01	1
FAM98A	116876	865	5	1317200	377716	0	5	1	0	50	53	17621644	2.44E-01	1

KCNIP4	35439	1264	3	787116	187968	0	4	0	0	50	41	19681104	2.44E-01	1
OR5A1	430021	507	-1	770384	242080	0	4	0	0	50	65	21241096	2.44E-01	1
OGG1	954590	264	39	1495200	394092	0	5	1	0	50	62	26339728	2.44E-01	1
LRRC37B	692401	356	23	2395524	690996	0	11	2	0	15	15	6585644	2.44E-01	1
C11orf49	1268399	373	31	1011752	282664	0	3	2	0	50	56	19008976	2.44E-01	1
OSBPL3	254504	312	28	2338920	595944	0	11	3	0	50	60	19819588	2.45E-01	1
CDCA7	255854	559	28	1175512	307940	0	5	1	0	50	55	21637680	2.45E-01	1
PPP1R3A	12173	914	-21	2856900	756144	0	14	2	0	5	6	2728028	2.45E-01	1
GBP1	337373	745	2	1543260	399432	0	9	1	0	50	62	23640892	2.45E-01	1
HOXC13	1257445	274	23	808120	256676	0	4	2	0	5	2	1519764	2.45E-01	1
SETD5	781227	404	34	3632268	1076544	0	13	1	0	24	34	13926720	2.45E-01	1
CXCL9	489826	399	43	332148	86508	0	2	0	0	50	51	20872992	2.45E-01	1
SEC61A2	334974	227	29	1286228	365968	0	5	1	0	16	22	10298368	2.46E-01	1
TCP10	254009	704	1	840516	239232	0	3	0	0	38	53	18767964	2.46E-01	1
CPB1	276750	681	-16	1087580	296548	0	4	2	0	50	79	23784716	2.46E-01	1
S100A5	1454773	186	26	299752	68708	0	2	0	0	50	56	19376724	2.46E-01	1
FAM150B	304765	624	-18	389464	122108	0	1	0	0	50	45	17740904	2.46E-01	1
IGFBP1	146330	623	8	652192	193664	0	4	0	0	50	55	20455404	2.46E-01	1
PRDM9	15618	1069	-19	2308660	601640	0	10	2	0	21	22	7730896	2.46E-01	1
LY96	250079	468	26	435032	102172	0	1	0	0	50	39	17338624	2.47E-01	1
STX3	395993	494	8	820224	205056	0	3	0	0	50	53	19654760	2.47E-01	1
CELF3	1210719	255	29	1220368	330368	0	7	0	0	50	45	17707084	2.47E-01	1
GPR107	622734	222	29	1581708	425064	0	6	2	0	36	49	13906072	2.47E-01	1
LILRB3	717623	458	-6	1604492	474904	0	9	3	0	50	58	18028196	2.47E-01	1
RAE1	181292	604	9	985408	243860	0	3	0	0	14	10	8165928	2.47E-01	1
PSD	975992	178	39	2525464	826988	0	11	2	0	26	34	12137108	2.47E-01	1
ENOPH1	684394	387	46	674976	185832	0	2	0	0	50	53	22010768	2.47E-01	1
THSD7B	11902	824	-16	4094356	1071560	0	20	4	0	50	68	22737364	2.47E-01	1
DAAM2	53428	914	26	2770748	753652	0	9	3	0	50	46	22158864	2.47E-01	1
HIST1H1E	398813	636	53	534000	175152	0	4	2	0	48	43	12614504	2.47E-01	1
XPOT	466767	402	-15	2532940	655040	0	10	0	0	3	1	1846572	2.47E-01	1
PCDH1	760355	281	23	3083316	987900	0	13	3	0	38	50	20134292	2.47E-01	1
RGS7	134467	834	-27	1325032	311144	0	7	0	0	50	74	19741980	2.47E-01	1

RYR2	86856	1024	-24	12993288	3368472	0	72	23	0	20	53	11085484	2.48E-01	1
MSLN	1187310	273	39	1569248	520828	0	7	1	0	50	70	23301268	2.48E-01	1
CEP76	453636	206	41	1687084	474548	0	6	2	0	50	44	16761192	2.48E-01	1
PTCD2	421218	342	27	1021008	265932	0	2	0	0	39	21	15306932	2.48E-01	1
ACD	1065785	147	41	1359208	452120	0	7	2	0	49	79	26733820	2.48E-01	1
TLE4	45472	956	-9	1998940	562124	0	6	0	0	4	1	1789968	2.49E-01	1
MMACHC	809450	176	25	698472	221076	0	2	0	0	50	51	21873352	2.49E-01	1
OSCP1	608987	181	29	1128164	298684	0	3	1	0	50	43	16456456	2.49E-01	1
CEBPZ	488101	253	42	2782140	666432	0	12	1	0	24	18	8787504	2.49E-01	1
GIMAP7	563029	671	24	772876	195800	0	4	0	0	25	19	9400180	2.49E-01	1
OSBPL8	586978	492	3	2366332	583484	0	8	1	0	42	41	16703876	2.49E-01	1
GNL3	1217701	272	47	1468144	362408	0	8	1	0	50	99	29167792	2.50E-01	1
ZKSCAN3	273677	592	31	1382704	365612	0	5	1	0	50	49	21622728	2.50E-01	1
SYT1	186847	899	-21	1105024	284444	0	7	2	0	23	42	9565008	2.50E-01	1
RIC8A	1463610	437	24	1348528	413672	0	4	1	0	6	2	2533652	2.50E-01	1
BFSP1	552334	239	-23	1674268	495908	0	6	1	0	49	72	20392748	2.50E-01	1
PDXK	919787	170	68	827344	218228	0	2	1	0	25	22	7822032	2.50E-01	1
GLT8D1	1217701	272	47	973660	256676	0	5	0	0	50	99	29167792	2.50E-01	1
ZNF483	437885	309	22	1961560	498044	0	8	0	0	6	3	2123540	2.50E-01	1
ASTL	821367	251	45	1082952	334284	0	4	1	0	50	59	19377792	2.50E-01	1
PTPN9	669446	257	47	1534716	419724	0	9	2	0	50	57	21914292	2.51E-01	1
NAT8L	717079	321	34	729800	249556	0	2	0	0	50	53	24191980	2.51E-01	1
ZNF429	164342	1162	-59	1746180	429336	0	11	1	0	34	50	11607024	2.51E-01	1
VSIG2	168652	622	14	816308	264508	0	3	1	0	50	50	19749100	2.51E-01	1
ITGB1BP2	728362	NaN	26	921328	238520	0	4	0	0	50	84	28751272	2.51E-01	1
SH2D6	1067736	180	39	436456	144536	0	3	0	0	50	68	23641960	2.51E-01	1
NPY	148162	478	6	247064	77608	0	2	0	0	50	60	18240372	2.51E-01	1
FAM26D	261450	479	19	329656	85796	0	2	0	0	50	57	23330104	2.51E-01	1
SLC35A4	706371	319	29	753652	287648	0	2	1	0	50	44	23563640	2.51E-01	1
NECAP1	142724	300	23	714848	201496	0	2	1	0	50	43	20540844	2.52E-01	1
PPP3CC	701231	142	41	1349596	349592	0	5	1	0	50	50	21519844	2.52E-01	1
LMO2	748742	394	32	571380	171948	0	2	0	0	50	74	25077352	2.52E-01	1
ZP1	794900	522	47	1605916	492704	0	4	1	0	50	51	21913580	2.52E-01	1

PIK3C3	38836	1300	13	2363840	585976	0	8	1	0	50	57	22353952	2.52E-01	1
ZC3H13	576966	679	49	4006780	1075832	0	11	1	0	20	9	6734452	2.52E-01	1
RDH16	2103486	286	26	793524	238164	0	4	1	0	50	85	24401308	2.52E-01	1
BAG4	705837	277	46	1140268	344252	0	4	0	0	50	54	23183788	2.53E-01	1
RANBP2	365106	418	20	8262404	2190112	0	25	4	0	38	32	17730580	2.53E-01	1
MRPL35	773440	425	39	483448	141332	0	3	0	0	50	55	19342548	2.53E-01	1
LECT2	489402	443	19	392312	107512	0	3	0	0	50	52	16320820	2.53E-01	1
GPD1	1021750	192	57	897832	255608	0	5	0	0	6	3	1730516	2.53E-01	1
CHPT1	347543	338	8	1073696	304024	0	5	2	0	50	48	22295212	2.53E-01	1
NADSYN1	546912	626	50	1851200	499468	0	7	1	0	33	22	10237492	2.53E-01	1
OR2T27	141426	710	-10	780708	237096	0	3	0	0	50	70	30005816	2.53E-01	1
RBM5	965735	196	35	2167328	549664	0	8	1	0	21	35	8909968	2.54E-01	1
DHRS7	277355	547	4	867216	247776	0	4	1	0	50	68	22287380	2.54E-01	1
UBXN6	2078211	278	37	1132080	328944	0	6	2	0	45	47	19025708	2.54E-01	1
MRPS34	1604603	288	36	541832	170524	0	3	1	0	50	49	20717064	2.54E-01	1
ASB15	86799	644	-7	1506948	418656	0	6	1	0	50	55	22390620	2.54E-01	1
PLAT	580110	382	45	1472772	386616	0	5	1	0	32	33	14971936	2.54E-01	1
LAPTM4A	520645	166	26	612676	162692	0	4	0	0	50	72	23124692	2.54E-01	1
SRPRB	513771	523	23	694556	202564	0	5	0	0	41	46	12998628	2.54E-01	1
DGKH	191275	407	57	3197236	840872	0	11	1	0	19	19	7155600	2.54E-01	1
OGN	537639	574	47	776080	207548	0	3	0	0	50	52	17802848	2.55E-01	1
CUL4B	416744	NaN	14	2436108	593808	0	8	0	0	5	11	5595608	2.55E-01	1
REV1	383815	639	27	3235684	869708	0	7	1	0	15	11	10603104	2.55E-01	1
AMY2B	31276	974	-19	1342832	342472	0	6	2	0	50	91	24336872	2.55E-01	1
OR51Q1	99042	1030	1	779284	243860	0	3	2	0	13	6	3056616	2.55E-01	1
TDRKH	1062482	255	29	1454972	401212	0	4	1	0	50	50	22423016	2.55E-01	1
SGMS2	144491	683	6	935212	254540	0	3	1	0	50	66	26688964	2.55E-01	1
TMEM62	843516	333	42	1657892	461020	0	8	3	0	46	58	20300900	2.55E-01	1
FZD6	428438	615	23	1796020	494840	0	10	0	0	6	5	2716992	2.55E-01	1
HIST1H4C	398813	636	53	249556	85796	0	2	0	0	50	45	13638004	2.55E-01	1
ZBTB12	2485614	188	36	1124604	353508	0	5	2	0	36	35	13374208	2.55E-01	1
ALAS1	1114737	181	52	1634396	462088	0	8	3	0	16	23	7041324	2.55E-01	1
DNA2	761140	294	29	2973312	787116	0	9	0	0	14	11	7662544	2.56E-01	1

NUDT11	118616	NaN	31	413316	120684	0	2	0	0	50	47	21852348	2.56E-01	1
POU3F1	448782	374	32	1074408	375936	0	4	1	0	50	48	18799292	2.56E-01	1
RRS1	259302	268	12	892848	279816	0	3	1	0	50	51	20442588	2.56E-01	1
ITGB1BP1	624421	340	37	532932	136704	0	4	0	0	50	57	22754808	2.56E-01	1
CFHR2	217857	853	-24	707016	180492	0	5	1	0	50	67	19868716	2.56E-01	1
HSPB1	850568	195	53	506232	162336	0	2	0	0	50	69	21293072	2.56E-01	1
CLEC4C	206421	467	15	575652	131364	0	2	0	0	50	61	21156368	2.56E-01	1
PKNOX2	412479	731	16	1224640	333572	0	5	2	0	50	54	21187696	2.57E-01	1
RAC3	3814891	211	28	493060	146672	0	3	0	0	32	25	10015348	2.57E-01	1
ARPC5	608114	407	24	390532	109292	0	4	1	0	1	3	1400860	2.57E-01	1
FOXP2	42204	828	-13	2027064	513708	0	9	0	0	50	83	28125424	2.57E-01	1
TNFRSF25	644634	203	44	1106448	304380	0	4	0	0	50	75	21467868	2.57E-01	1
PSME4	238848	489	9	4832700	1274124	0	17	3	0	50	50	20218308	2.57E-01	1
TMEM184A	595899	243	38	1044860	313636	0	3	0	0	31	45	14566808	2.57E-01	1
C4BPA	501293	142	22	1556788	406196	0	7	4	0	19	20	7289100	2.57E-01	1
PHLPP1	229775	442	34	4241028	1331796	0	9	1	0	10	11	6474572	2.57E-01	1
LIMS1	363356	408	30	878964	203988	0	4	1	0	23	24	9018904	2.57E-01	1
UBE2V2	890573	464	31	384480	98256	0	1	0	0	50	60	22040672	2.57E-01	1
CRELD1	950574	170	54	1271276	331792	0	5	0	0	39	56	16713132	2.57E-01	1
MASP2	639826	307	33	1766828	496264	0	5	1	0	50	54	26043892	2.58E-01	1
MRPS31	270527	470	44	1029908	268780	0	6	0	0	50	78	23838472	2.58E-01	1
FER	140251	741	20	2195808	520116	0	10	2	0	50	50	21075556	2.58E-01	1
HELZ	345587	399	-25	4992544	1360988	0	22	4	0	4	6	3179080	2.58E-01	1
ATXN7L3	930750	175	41	955504	231044	0	5	1	0	50	69	21793252	2.58E-01	1
GULP1	98119	869	-12	821292	198648	0	4	0	0	50	94	32568304	2.58E-01	1
SPP1	445914	367	8	833752	201140	0	3	0	0	50	71	27385300	2.58E-01	1
AURKAIP1	1736268	273	28	496976	159844	0	2	0	0	50	63	20986200	2.58E-01	1
PLEKHG7	244442	509	2	1008904	251336	0	4	0	0	50	53	21293784	2.58E-01	1
SLC7A14	372782	466	24	1918484	580636	0	7	1	0	8	11	6005008	2.58E-01	1
SEC16A	1413299	233	44	5891088	1792104	0	14	1	0	1	1	1960492	2.58E-01	1
LRRN1	61807	888	-18	1795308	501960	0	7	2	0	25	29	12360676	2.59E-01	1
OR2AT4	752176	556	48	777148	250268	0	3	0	0	50	54	23399524	2.59E-01	1
LACRT	615189	494	31	363120	103596	0	1	0	0	50	83	25232212	2.59E-01	1

TREX1	1480689	223	45	871488	316128	0	6	1	0	50	66	22607068	2.59E-01	1
FAM173B	623675	325	5	596656	172304	0	2	1	0	50	72	28009724	2.59E-01	1
P4HB	3798594	211	19	1323608	349948	0	6	3	0	28	25	7704552	2.59E-01	1
BUB3	213809	769	0	862944	231756	0	2	0	0	50	40	19819588	2.59E-01	1
LRRC17	749653	456	23	1158780	287292	0	6	0	0	50	62	21362136	2.59E-01	1
FRMD8	4112809	242	50	1168036	362408	0	6	1	0	27	30	12483852	2.59E-01	1
T6GALNA(	999845	191	31	1521544	438236	0	4	0	0	2	1	1460668	2.60E-01	1
NDUFB4	317876	449	0	358848	104664	0	3	1	0	13	19	6550400	2.60E-01	1
FLOT2	1834230	220	37	1103600	313636	0	5	1	0	50	67	23772612	2.60E-01	1
NR0B2	1025094	188	34	624424	206480	0	3	0	0	50	62	18463584	2.60E-01	1
OR2AG2	424776	548	8	765756	249912	0	6	1	0	50	65	19789684	2.60E-01	1
HEATR3	276539	476	3	1754724	487008	0	5	1	0	25	38	14902516	2.60E-01	1
STK32A	95084	839	-7	1100396	255964	0	5	0	0	14	10	4642952	2.60E-01	1
CGRRF1	559596	348	34	872200	218228	0	4	0	0	50	55	22288804	2.60E-01	1
PAX9	55912	494	-39	837668	270916	0	4	0	0	24	31	10519444	2.60E-01	1
EPGN	172790	402	18	353508	90780	0	1	0	0	50	53	17866572	2.61E-01	1
NDUFAF3	1799657	241	41	459952	149876	0	4	0	0	50	76	22684320	2.61E-01	1
DHX38	414449	479	9	3174096	871488	0	8	2	0	36	39	16830256	2.61E-01	1
SNRNP27	844060	221	37	412248	111072	0	1	0	0	50	49	17507368	2.61E-01	1
ZNF45	464210	419	-38	1768608	436812	0	7	2	0	50	78	19959496	2.61E-01	1
PPP1R1B	1164935	239	28	540052	142400	0	3	0	0	50	54	18916772	2.61E-01	1
GK2	112173	875	0	1373804	403348	0	8	1	0	50	54	22887240	2.61E-01	1
EMILIN3	383021	309	5	1836604	633680	0	10	2	0	50	64	25202664	2.61E-01	1
SFI1	741049	313	37	3233904	887508	0	10	2	0	6	9	5136012	2.61E-01	1
CTSF	1343990	194	62	1247424	359916	0	5	0	0	13	11	5250288	2.61E-01	1
FNIP1	346839	637	27	3029560	782132	0	11	2	0	8	10	5859048	2.61E-01	1
JAM3	270247	601	6	909936	267000	0	3	0	0	50	44	20916780	2.61E-01	1
CCDC33	604483	222	45	2161276	618728	0	5	0	0	7	5	4016036	2.61E-01	1
VPS54	352987	407	20	2572456	655040	0	6	3	0	50	46	23507748	2.61E-01	1
34GALNT2	868792	346	21	1454972	425776	0	5	0	0	29	24	11042052	2.61E-01	1
ACSM5	181483	823	6	1483808	430048	0	8	0	0	14	14	5134588	2.61E-01	1
PI4K2B	218037	523	28	1257036	325740	0	3	1	0	44	45	17922820	2.62E-01	1
HNRNPA1	753625	205	59	983272	254540	0	7	1	0	50	68	19974092	2.62E-01	1

SLCO6A1	132386	647	-12	1851200	511216	0	6	1	0	50	51	24155312	2.62E-01	1
HOXC9	1257445	274	23	650412	192240	0	4	0	0	50	62	22406640	2.62E-01	1
TNFSF4	293612	492	-1	469920	128160	0	1	0	0	50	68	25149620	2.62E-01	1
LTC4S	1610057	359	38	370240	132788	0	1	0	0	50	48	18056676	2.62E-01	1
RAP1GDS1	116633	909	6	1594880	416164	0	7	1	0	50	51	18446140	2.62E-01	1
CRYM	467861	575	37	804916	238520	0	4	1	0	50	59	19353584	2.63E-01	1
CAMK4	192731	767	29	1224640	338912	0	6	2	0	10	16	4991476	2.63E-01	1
MYL12A	551966	328	55	459596	104308	0	2	1	0	15	18	5606288	2.63E-01	1
LRRC36	1158788	125	49	1949456	555004	0	10	0	0	1	0	808832	2.63E-01	1
TCHH	527465	349	35	4997884	1234964	0	32	7	0	15	17	5681048	2.63E-01	1
SPRR2A	687290	704	30	187968	50196	0	1	0	0	50	34	21429064	2.63E-01	1
SPRR2D	687290	704	30	187968	50196	0	1	0	0	50	34	21429064	2.63E-01	1
SPRR2F	687290	704	30	187968	50196	0	1	0	0	50	34	21429064	2.63E-01	1
ICMT	627248	240	31	709508	222856	0	4	0	0	50	61	18409472	2.63E-01	1
CD2AP	203824	554	16	1680676	442508	0	9	1	0	50	53	21021800	2.63E-01	1
KCNC4	532510	400	38	1715564	548596	0	10	3	0	50	73	20847360	2.63E-01	1
NID1	555035	318	19	3159856	922040	0	12	4	0	50	45	23701768	2.63E-01	1
CDH7	4645	524	-49	2004636	558564	0	11	1	0	3	2	1550024	2.63E-01	1
RNF7	406166	406	29	323604	50196	0	2	0	0	8	12	2883600	2.64E-01	1
HOXA3	356045	385	28	1075120	351728	0	3	0	0	50	46	22711376	2.64E-01	1
RBM23	1177534	225	34	1138132	327164	0	5	0	0	50	44	16175572	2.64E-01	1
SIGLEC6	328446	627	-26	1178360	341404	0	5	2	0	50	89	22063100	2.64E-01	1
CLEC10A	1696302	360	24	844432	215024	0	2	1	0	50	49	16256384	2.64E-01	1
RPL22	627248	240	31	347812	80456	0	1	0	0	50	61	18409472	2.64E-01	1
ZNF225	554986	634	-28	1847640	434676	0	7	0	0	50	52	17611320	2.65E-01	1
NUDT14	872033	260	25	555004	178712	0	2	0	0	50	67	20410192	2.65E-01	1
AKR1D1	220333	478	16	862232	221788	0	6	0	0	50	54	18168460	2.65E-01	1
LRRC28	272870	380	-1	945892	271628	0	3	1	0	50	49	25107968	2.65E-01	1
ARHGEF7	405184	579	30	2331800	632968	0	8	1	0	25	16	11146360	2.65E-01	1
MTBP	193807	877	-3	2387336	606268	0	8	2	0	50	48	18343968	2.65E-01	1
EIF2B5	1453689	334	42	1861524	515844	0	10	1	0	10	20	5986140	2.65E-01	1
STMN3	1094905	193	54	473480	125668	0	2	0	0	50	79	24540860	2.66E-01	1
ONECUT1	294072	514	18	1151304	346032	0	4	0	0	50	46	21168472	2.66E-01	1

RAB11FIP4	501371	307	30	1650772	453188	0	6	1	0	50	60	20966264	2.66E-01	1
CTF1	1508459	247	46	475616	182272	0	2	0	0	50	71	20582852	2.66E-01	1
BOLA3	1023516	250	38	361696	75116	0	3	0	0	50	63	19889720	2.66E-01	1
SETX	888858	429	36	6867240	1815600	0	21	1	0	1	1	2003924	2.66E-01	1
TIGD4	209186	413	41	1286228	357424	0	6	1	0	50	66	22524120	2.67E-01	1
NCOA3	266824	287	49	3714148	973304	0	10	1	0	9	6	4190120	2.67E-01	1
PRKRIR	234429	366	57	1943760	514776	0	8	3	0	50	58	20234684	2.67E-01	1
ACMSD	275440	496	15	894984	225348	0	2	1	0	50	52	18304096	2.67E-01	1
C12orf71	489310	328	23	681740	189748	0	4	1	0	50	47	19871564	2.67E-01	1
MTDH	605177	163	38	1494488	420436	0	6	0	0	50	52	21745904	2.67E-01	1
PRM1	632841	308	37	138484	34532	0	1	0	0	19	22	7369912	2.68E-01	1
ITGB3	965617	188	19	2036320	553580	0	9	0	0	6	3	2795668	2.68E-01	1
LY6H	1104860	446	20	411892	124244	0	1	1	0	21	20	8005728	2.68E-01	1
CCL16	779228	525	19	308296	90068	0	1	0	0	50	69	21358220	2.68E-01	1
ROPN1	244828	450	24	543612	155928	0	2	1	0	44	47	15727368	2.68E-01	1
LRFN2	100435	887	20	1903176	636528	0	6	3	0	22	25	10950560	2.68E-01	1
ZNF514	223452	616	24	1040232	257388	0	4	1	0	48	53	21341844	2.68E-01	1
DCTN4	1015949	211	43	1232828	324316	0	5	1	0	50	65	23620956	2.68E-01	1
ZNF215	403341	827	6	1351020	325740	0	5	0	0	50	71	21769756	2.68E-01	1
CAPG	1067736	180	39	902104	254540	0	2	0	0	50	68	23641960	2.68E-01	1
HLA-DQB1	1292851	535	17	666076	192596	0	3	1	0	50	68	20377084	2.68E-01	1
APOA4	419339	326	36	994664	285868	0	4	2	0	6	4	2394100	2.69E-01	1
FHOD1	1109491	150	46	2881464	940908	0	6	0	0	5	1	3011048	2.69E-01	1
CRYGB	321897	440	5	462800	113920	0	3	0	0	50	50	16889352	2.69E-01	1
KDM4D	304973	513	-12	1294772	384124	0	3	1	0	46	53	20091572	2.69E-01	1
TNP2	632841	308	37	363120	88644	0	2	0	0	50	60	21937788	2.69E-01	1
HLA-B	2552688	347	28	924532	270560	0	3	1	0	49	61	27729196	2.69E-01	1
EMP3	1478750	217	36	423640	121040	0	1	1	0	50	89	31278516	2.70E-01	1
DDX10	250455	616	2	2313644	567820	0	8	1	0	24	23	11628028	2.70E-01	1
HMX2	213809	769	0	668212	213956	0	3	0	0	50	40	19819588	2.70E-01	1
IFIH1	70837	712	12	2680680	670704	0	8	1	0	50	68	24183436	2.70E-01	1
GNPNAT1	307859	213	26	487008	127092	0	2	1	0	13	13	4673212	2.70E-01	1
SAA2	601955	371	17	340336	92204	0	2	0	0	50	58	21345760	2.70E-01	1

UBE3C	389401	334	9	2808484	758636	0	9	2	0	50	55	24152820	2.70E-01	1
SPATS2	2172889	217	43	1419372	377004	0	6	0	0	50	59	22776524	2.70E-01	1
RFESD	307041	359	32	550020	143112	0	1	0	0	50	41	18933504	2.70E-01	1
COX10	96214	985	-54	1104668	343540	0	5	0	0	45	44	17054536	2.71E-01	1
THY1	1054477	263	40	406908	124956	0	3	0	0	50	80	23473572	2.71E-01	1
RNF168	1173736	125	34	1472416	383768	0	8	2	0	13	20	5821668	2.71E-01	1
NAB1	504177	232	37	1254900	338556	0	4	0	0	50	47	19300184	2.71E-01	1
CD63	1814311	384	26	623712	171948	0	2	0	0	50	44	15945952	2.71E-01	1
RPUSD4	601483	341	26	961556	279460	0	4	0	0	27	19	10030300	2.71E-01	1
HAND1	581156	416	29	531864	164472	0	3	0	0	50	52	20192320	2.71E-01	1
MAEL	264649	681	-11	1150236	292632	0	4	1	0	50	57	22892936	2.72E-01	1
AKR1C3	438967	678	-14	850128	224280	0	3	0	0	50	44	16064144	2.72E-01	1
GTF2A2	521844	172	33	293700	75828	0	1	0	0	50	69	21237180	2.72E-01	1
ECM1	1532790	163	43	1380212	391600	0	7	2	0	50	76	23299844	2.72E-01	1
PLP2	724395	NaN	44	388040	119260	0	2	1	0	42	35	15951292	2.72E-01	1
SERPINH1	546732	227	41	1046640	312924	0	5	0	0	4	0	1279464	2.72E-01	1
TBC1D20	726170	229	41	1028840	295480	0	5	1	0	8	8	2654692	2.73E-01	1
ITPA	997984	255	52	516556	138128	0	2	1	0	50	56	18980852	2.73E-01	1
ABCG4	1258103	195	57	1644720	488076	0	6	1	0	46	54	22055268	2.73E-01	1
CDH12	127633	1075	-20	2024216	565684	0	11	1	0	41	48	17551512	2.73E-01	1
FBXO48	212087	617	30	409400	98968	0	3	0	0	50	58	17461088	2.73E-01	1
SMC4	435184	513	16	3403716	824496	0	17	0	0	0	0	824496	2.73E-01	1
ATP5G3	343228	752	28	363120	114276	0	1	0	0	50	62	22706392	2.73E-01	1
NODAL	550900	338	42	867216	258456	0	4	1	0	50	41	18662588	2.73E-01	1
GGTLC2	179695	641	22	556784	164116	0	3	1	0	50	49	20114000	2.74E-01	1
RHOU	689585	450	9	647208	191172	0	4	1	0	50	48	17201920	2.74E-01	1
CLIC1	2513551	176	26	619796	176932	0	3	0	0	50	57	25759092	2.74E-01	1
HRASLS5	887496	459	31	731224	187256	0	5	1	0	50	61	22549396	2.74E-01	1
TMCC2	690215	169	34	1743688	548240	0	6	1	0	50	56	21454696	2.74E-01	1
RPS17	NaN	NaN	NaN	719832	194376	0	4	0	0	50	116	39839960	2.74E-01	1
FBXL7	13845	956	-27	1210044	379140	0	9	2	0	50	91	23858764	2.74E-01	1
CYTH2	1418188	209	41	1054828	276968	0	5	0	0	50	91	32574000	2.74E-01	1
RNASE3	1070609	561	16	399076	121040	0	3	1	0	50	62	17665076	2.74E-01	1

IL1F10	672307	479	25	399076	108224	0	3	1	0	33	39	12373492	2.74E-01	1
TRPM7	325708	250	23	4902120	1238880	0	12	2	0	20	14	10696020	2.74E-01	1
IGFL4	997099	261	31	331080	84372	0	2	0	0	50	59	22367124	2.74E-01	1
BCAS3	211130	576	-11	2405492	673552	0	8	2	0	48	52	19186976	2.74E-01	1
TSG101	623656	341	20	1014956	276256	0	3	1	0	50	51	24734880	2.74E-01	1
PPIB	743794	251	43	555716	156640	0	3	1	0	50	57	23116860	2.75E-01	1
ARRDC1	1197708	478	45	1077256	345320	0	3	1	0	45	76	25224024	2.75E-01	1
LCE1C	64902	818	18	300820	84728	0	2	1	0	13	28	5373820	2.75E-01	1
SLC18A3	118787	312	32	1248848	458884	0	4	4	0	21	24	8163792	2.75E-01	1
KIAA1614	232445	398	15	2882888	969388	0	8	1	0	7	5	3997524	2.75E-01	1
FIGN	20376	586	-11	1870068	569244	0	8	3	0	50	73	23692512	2.75E-01	1
ATP2B1	235578	418	9	3271284	890712	0	9	2	0	47	41	21802864	2.75E-01	1
SEC11C	280782	512	53	506944	134924	0	3	0	0	50	54	16867280	2.75E-01	1
AKAP9	674222	611	16	10258140	2472420	0	47	8	0	5	10	4494500	2.75E-01	1
SERPINB1:	190401	339	20	1025992	259880	0	5	1	0	50	49	19965548	2.75E-01	1
DOK2	402326	470	12	1018160	324316	0	3	0	0	43	47	20968756	2.75E-01	1
RLF	698659	303	25	4913512	1254188	0	17	1	0	15	25	11031372	2.76E-01	1
IRX1	7226	1155	-24	1166256	389820	0	11	2	0	3	4	1202212	2.76E-01	1
TMEM144	135911	684	-9	915988	239588	0	3	0	0	50	71	30278156	2.76E-01	1
SAA1	601955	371	17	321824	85084	0	2	0	0	50	58	21345760	2.76E-01	1
THTPA	1250373	553	35	568176	180492	0	2	0	0	50	82	25432996	2.76E-01	1
OR4S2	1295	1091	-20	778216	221432	0	5	2	0	38	56	13911412	2.76E-01	1
SLC9A1	886940	336	27	2034896	626560	0	6	1	0	37	39	15330072	2.76E-01	1
KLRB1	264317	618	7	597012	149520	0	3	0	0	50	51	22308028	2.76E-01	1
MAK	263773	468	14	1632260	418300	0	4	1	0	50	41	17401280	2.77E-01	1
KCNA5	160467	896	22	1474908	492348	0	7	2	0	50	47	23319780	2.77E-01	1
FAM71A	409070	224	26	1457820	448560	0	9	4	0	20	26	7435060	2.77E-01	1
MLLT3	85091	575	14	1510508	355288	0	6	1	0	50	61	22513440	2.77E-01	1
RBM7	157610	477	-8	699896	174796	0	4	0	0	50	63	23313372	2.77E-01	1
HIST1H2A(	227588	622	29	313636	110360	0	7	2	0	8	16	1721260	2.77E-01	1
FILIP1	441614	325	19	3068364	842652	0	17	4	0	8	7	3311868	2.77E-01	1
NCOR2	1239461	472	37	6345344	1934860	0	17	1	0	0	1	1934860	2.77E-01	1
CNNM1	270704	575	-6	2331444	761484	0	6	0	0	10	7	4751532	2.77E-01	1

STXBP5L	319082	785	-8	3095420	823072	0	12	1	0	32	32	16536912	2.77E-01	1
IRF2	207866	400	55	920616	234960	0	5	1	0	50	53	20572884	2.77E-01	1
REEP4	584373	219	34	661804	194732	0	2	0	0	50	59	21330096	2.78E-01	1
ZNF546	1181917	521	22	2173380	529728	0	11	1	0	50	77	22919280	2.78E-01	1
PAGE4	257202	NaN	7	276968	70132	0	2	0	0	50	64	19684664	2.78E-01	1
ZNF781	484296	888	-51	833396	217516	0	8	2	0	50	72	17622000	2.78E-01	1
MACC1	220237	676	13	2165904	584196	0	10	3	0	49	66	18401996	2.78E-01	1
ZNF544	1471648	581	-53	1841944	469208	0	10	0	0	3	3	1713072	2.78E-01	1
PPP3CB	966152	220	40	1383060	357780	0	6	0	0	50	62	23185924	2.78E-01	1
CXXC1	247194	379	68	1710224	467428	0	8	1	0	50	64	21669720	2.78E-01	1
MAP3K5	329812	448	31	3581004	948384	0	13	4	0	50	47	21842736	2.78E-01	1
PCBP1	686152	156	52	870064	273764	0	7	2	0	49	59	15290200	2.78E-01	1
FBXO34	744260	256	38	1789256	496264	0	11	2	0	50	67	23760508	2.79E-01	1
OR10J5	492825	646	-4	760772	230332	0	6	0	0	50	73	21442592	2.79E-01	1
SNRPE	527029	280	26	259524	57672	0	2	0	0	50	51	15071260	2.79E-01	1
KRTAP12-4	874480	461	25	279104	87220	0	2	1	0	10	14	5109668	2.79E-01	1
AGPAT2	1918290	190	56	698472	218940	0	2	0	0	50	46	19629484	2.79E-01	1
GPR158	43942	814	-21	3056616	884304	0	15	3	0	7	10	3603788	2.79E-01	1
BCL2L15	335231	545	13	424708	113564	0	1	0	0	50	60	19779004	2.79E-01	1
RPS3A	214920	335	26	700608	174084	0	2	0	0	50	51	18916060	2.79E-01	1
RASA2	405454	259	28	2262380	561412	0	8	1	0	41	57	22794324	2.79E-01	1
MFSD9	70590	913	-3	1163764	381632	0	4	2	0	50	67	19587476	2.80E-01	1
GTF2E1	307733	475	11	1123892	302956	0	6	1	0	50	50	20964128	2.80E-01	1
RFX3	159455	1028	3	2071208	564616	0	7	1	0	8	8	4106460	2.80E-01	1
PSMA6	452722	188	40	648632	168388	0	5	0	0	50	63	17921040	2.80E-01	1
LAMP3	296133	596	24	1035248	322180	0	3	1	0	50	61	27744860	2.80E-01	1
CLUAP1	805495	246	39	1110364	263084	0	3	0	0	21	14	7587428	2.80E-01	1
PKP2	292831	381	-18	2233544	647920	0	10	0	0	7	5	2854408	2.80E-01	1
PDCD1LG2	307589	556	37	708796	194732	0	3	0	0	50	33	17868352	2.80E-01	1
FOXJ3	541904	579	23	1628344	419012	0	5	0	0	22	14	8447880	2.81E-01	1
TFRC	959125	170	52	1984344	530796	0	9	2	0	50	73	20954160	2.81E-01	1
QPRT	NaN	NaN	NaN	720544	249200	0	4	0	0	50	116	39894784	2.81E-01	1
IFNE	242654	411	-6	530796	138840	0	2	1	0	50	34	19573236	2.81E-01	1

GPR151	377370	463	14	1038096	307584	0	5	0	0	48	40	17922108	2.81E-01	1
PDCD5	358893	455	-32	347100	80100	0	3	1	0	11	11	3467440	2.81E-01	1
CENPH	422005	232	38	681384	149520	0	3	0	0	50	37	19057036	2.81E-01	1
C6orf195	467575	342	25	315060	95052	0	3	1	0	12	13	3536148	2.81E-01	1
PIKFYVE	256462	539	10	5479908	1430052	0	17	3	0	29	24	13897528	2.81E-01	1
SNRPN	NaN	NaN	NaN	598792	199004	0	3	0	0	50	116	39844588	2.82E-01	1
[illegible]NFRSF13[illegible]	1335011	323	67	433608	169812	0	3	0	0	50	49	15999352	2.82E-01	1
GDF3	237142	672	14	911004	267000	0	4	1	0	50	67	22681828	2.82E-01	1
OR2B11	272980	927	-14	768604	249200	0	4	0	0	50	59	17172372	2.82E-01	1
CDKN2B	192919	212	-21	392312	145960	0	2	0	0	50	57	20206204	2.82E-01	1
FAM189B	1574928	194	45	1625140	565328	0	7	0	0	29	25	9535460	2.82E-01	1
WFDC3	742720	180	48	609116	159844	0	4	0	0	50	49	15404476	2.82E-01	1
GBA3	103435	746	22	1211824	313280	0	5	2	0	21	22	8754396	2.82E-01	1
ATG7	184628	425	30	1834824	497688	0	4	2	0	50	59	25932108	2.83E-01	1
NXF3	456169	NaN	16	1445004	342828	0	6	2	0	50	53	18791104	2.83E-01	1
LIMD1	340032	231	45	1686728	514420	0	4	0	0	9	3	4035972	2.83E-01	1
ZNF439	739660	633	-2	1299044	311500	0	7	0	0	25	19	8588500	2.83E-01	1
ATAD3B	1714469	248	27	1651128	494484	0	6	1	0	6	7	3193676	2.83E-01	1
ASAP1	264818	427	30	2938424	803848	0	9	3	0	50	61	26467532	2.83E-01	1
TSEN2	352597	317	36	1254900	324672	0	6	1	0	50	46	20198016	2.83E-01	1
CCDC83	361760	380	3	1200076	272696	0	3	0	0	50	54	19738776	2.83E-01	1
MYO1A	2026922	199	41	2718416	741904	0	16	2	0	50	67	21271000	2.83E-01	1
TUBB2B	451139	453	37	1130300	311500	0	6	2	0	50	57	17322960	2.83E-01	1
LHX3	799089	244	42	1066220	334996	0	4	1	0	30	38	15176636	2.83E-01	1
DCDC1	84163	938	-25	920972	246352	0	13	3	0	8	18	2942340	2.83E-01	1
PPP1R3B	159748	526	23	731580	189036	0	3	0	0	50	42	20661884	2.83E-01	1
GRIA1	67092	914	-3	2441448	655752	0	13	5	0	50	68	19361060	2.83E-01	1
POLE3	390186	297	36	393380	93628	0	2	0	0	50	42	19391676	2.84E-01	1
PARP8	76181	1215	14	2258820	591672	0	11	4	0	17	20	6479912	2.84E-01	1
AHSA1	504548	359	47	889644	230688	0	4	0	0	33	43	14040284	2.84E-01	1
BAK1	1252332	205	35	539340	161268	0	2	0	0	50	58	16890776	2.84E-01	1
FBXO28	638932	174	13	936636	264864	0	5	1	0	50	52	21216888	2.84E-01	1
PAPSS1	144822	679	4	1617664	433964	0	6	2	0	50	47	20551524	2.84E-01	1

LMNB2	1604277	267	35	1524392	450340	0	6	0	0	12	10	4611268	2.85E-01	1
BNIP3	123624	470	-21	513708	132432	0	2	0	0	50	80	22720632	2.85E-01	1
SLC29A1	1107034	216	51	1170172	348524	0	6	2	0	50	69	20304104	2.85E-01	1
PCMTD1	63777	905	2	939128	229264	0	3	1	0	50	34	16651900	2.85E-01	1
NME4	1549307	207	37	475616	145960	0	1	0	0	36	55	20238956	2.85E-01	1
ACVRL1	493265	371	27	1276260	377004	0	5	1	0	7	4	2520480	2.85E-01	1
PHOX2B	308280	376	22	766824	250980	0	1	1	0	50	37	23283112	2.85E-01	1
WDR17	133039	616	-8	3456404	910648	0	17	0	0	0	0	910648	2.85E-01	1
NKX6-3	434010	533	37	333216	108936	0	2	0	0	50	50	18020364	2.85E-01	1
CCDC77	409007	372	27	1294416	319332	0	5	0	0	50	36	19883668	2.85E-01	1
RPL21	284055	265	46	431472	107868	0	1	0	0	50	59	21531948	2.86E-01	1
LOXL4	225311	581	9	1936996	548240	0	4	0	0	50	44	22323692	2.86E-01	1
IRF3	1733181	195	36	1076188	327164	0	5	1	0	9	8	4296208	2.86E-01	1
SLC22A12	1970283	411	54	1363836	453900	0	9	2	0	50	67	19338632	2.86E-01	1
HIST1H3E	390169	544	57	331080	110004	0	3	0	0	50	61	13862640	2.86E-01	1
ZNF507	298401	596	-66	2436820	641156	0	15	4	0	50	116	28978044	2.86E-01	1
CCDC90B	308108	414	18	683164	168032	0	3	0	0	50	53	24526620	2.86E-01	1
ANKS4B	402663	575	41	1055896	289784	0	5	0	0	50	69	22447580	2.86E-01	1
OPN1SW	830283	310	35	885728	251692	0	4	1	0	50	76	22960220	2.86E-01	1
SNCG	401785	164	45	339624	87576	0	2	0	0	50	45	17084796	2.86E-01	1
RNASE9	637784	493	22	553580	136348	0	2	1	0	50	49	16434740	2.87E-01	1
FGD2	832054	220	41	1684948	480956	0	7	1	0	50	65	22074136	2.87E-01	1
NUP155	339666	151	25	3639744	969744	0	16	3	0	21	26	12461424	2.87E-01	1
DUSP12	447958	504	29	878252	237808	0	2	0	0	50	38	20965196	2.87E-01	1
PDIA3	784327	286	37	1334288	338200	0	5	0	0	17	14	6234272	2.87E-01	1
ENTPD2	1951939	202	40	1227132	398364	0	3	1	0	50	61	21392396	2.87E-01	1
GDI2	656468	303	43	1174088	297616	0	5	1	0	50	43	20856972	2.87E-01	1
CLEC6A	321790	564	13	564972	129228	0	2	0	0	50	64	21703896	2.87E-01	1
CNR2	923316	254	23	876828	284088	0	4	0	0	50	60	19342192	2.88E-01	1
H1FNT	809696	412	16	614812	205412	0	4	1	0	50	60	19819232	2.88E-01	1
GMPPA	559651	403	32	1089360	315060	0	4	1	0	50	45	17782200	2.88E-01	1
REM1	605825	450	-76	728732	242080	0	3	0	0	7	4	2020300	2.88E-01	1
ZNF665	392290	946	-49	1751876	436456	0	11	1	0	50	50	16685008	2.88E-01	1

FOXP1	99202	675	-1	1915636	505520	0	6	2	0	35	43	13423336	2.88E-01	1
ATP11A	585697	416	45	3179792	850840	0	11	3	0	50	74	25844176	2.88E-01	1
C10orf35	824126	519	31	299396	100036	0	2	1	0	42	47	15680376	2.89E-01	1
ZNF419	455231	801	-28	1329304	328232	0	3	0	0	28	22	9242828	2.89E-01	1
CETN2	201810	NaN	-3	465648	107868	0	3	0	0	50	44	16214020	2.89E-01	1
FGR	732210	217	24	1370244	374868	0	6	2	0	50	74	26421608	2.89E-01	1
PAGE1	355308	NaN	14	394804	99680	0	2	0	0	50	61	25040684	2.89E-01	1
IL32	1626425	234	44	510860	126736	0	2	1	0	50	77	22657976	2.89E-01	1
NEUROD1	210899	380	17	897120	250980	0	4	2	0	33	32	10958036	2.89E-01	1
GSC	195536	892	8	634748	200428	0	3	0	0	50	68	20770108	2.89E-01	1
PCIF1	725555	209	59	1810972	511928	0	10	4	0	10	8	3232836	2.89E-01	1
S100A7A	1140044	401	32	272340	63012	0	2	0	0	50	58	17066640	2.89E-01	1
COQ6	443041	158	51	1234964	316840	0	7	1	0	50	55	19172380	2.89E-01	1
KDELC1	195534	475	24	1317200	332860	0	5	2	0	50	40	16969452	2.90E-01	1
GINS4	325055	496	29	597368	150232	0	4	1	0	5	8	2418308	2.90E-01	1
SMEK2	597106	382	25	2223932	567820	0	11	2	0	27	31	12652240	2.90E-01	1
KPNB1	1198427	217	24	2281604	619084	0	10	2	0	21	30	9209008	2.90E-01	1
VTI1A	232217	459	7	579924	148452	0	2	0	0	50	51	21285240	2.90E-01	1
ZBTB47	359739	329	38	1880748	537204	0	6	0	0	15	11	6409424	2.90E-01	1
ARRB1	740999	252	46	1105736	300820	0	4	0	0	27	27	11840204	2.90E-01	1
NUDT17	NaN	NaN	NaN	822360	268068	0	3	2	0	39	100	30990156	2.90E-01	1
FTCD	742075	538	10	1362056	436456	0	4	2	0	50	61	21237180	2.90E-01	1
ANKRD54	1385064	203	66	759704	236740	0	3	0	0	50	52	18523392	2.90E-01	1
RGS5	194452	794	-8	484160	116056	0	2	1	0	8	2	2485236	2.90E-01	1
FAM127B	304745	NaN	42	332148	101460	0	2	0	0	50	59	20553304	2.90E-01	1
SP4	105280	643	26	1941624	594876	0	8	0	0	47	37	21302684	2.91E-01	1
RYBP	102513	607	32	561768	152724	0	2	0	0	50	38	23272788	2.91E-01	1
GABRA1	12179	927	-16	1179072	323604	0	11	3	0	17	32	7338584	2.91E-01	1
LRRCC1	99309	1450	-7	2742268	644360	0	15	1	0	41	44	19063800	2.91E-01	1
SIRPG	421072	486	32	971880	294768	0	5	1	0	50	34	16659020	2.91E-01	1
PRC1	753718	413	31	1637600	411892	0	8	1	0	42	60	20298408	2.91E-01	1
RFTN1	117533	528	29	1478824	414740	0	5	2	0	15	12	7128544	2.91E-01	1
GET4	737267	310	41	847992	239232	0	4	0	0	50	43	21549036	2.91E-01	1

DNAJC1	197451	608	2	1434680	390532	0	4	0	0	14	12	5599880	2.91E-01	1
TCEB3B	120831	639	34	1856184	559632	0	10	2	0	50	61	20147820	2.91E-01	1
PNP	464497	395	17	744396	206124	0	4	0	0	50	58	21385632	2.91E-01	1
FAM96A	740524	234	48	421860	111072	0	2	0	0	50	49	21541204	2.92E-01	1
PLAC1	268956	NaN	39	540764	145960	0	2	0	0	50	70	21315144	2.92E-01	1
DDX59	371965	276	13	1583132	433252	0	7	0	0	38	34	17241792	2.92E-01	1
PTP4A3	170603	382	51	448204	126380	0	1	1	0	50	49	19034608	2.92E-01	1
GLO1	280153	845	28	495908	118192	0	1	0	0	50	71	25337944	2.92E-01	1
TTLL9	1011028	241	37	1180140	291564	0	6	0	0	50	57	18029620	2.92E-01	1
TNP1	261601	642	23	150232	35600	0	1	0	0	50	44	18467856	2.92E-01	1
MED12	759088	NaN	27	5597388	1568892	0	20	2	0	25	26	13759400	2.93E-01	1
LGSN	255425	359	-9	1320760	328232	0	5	0	0	50	41	21634120	2.93E-01	1
POPDC3	108747	650	2	738700	205412	0	3	0	0	50	54	20440808	2.93E-01	1
SDHA	1040169	530	24	1692068	498400	0	9	1	0	50	65	18657604	2.93E-01	1
IPO9	764022	208	26	2691360	747600	0	8	0	0	19	20	11701008	2.93E-01	1
MS4A6A	336539	784	-2	681028	169100	0	1	0	0	50	44	22125044	2.93E-01	1
LASP1	1255539	159	41	699184	165896	0	3	0	0	50	69	22650856	2.93E-01	1
SYTL3	606618	188	51	1407624	387684	0	6	0	0	20	15	6931676	2.93E-01	1
TMEM145	792230	218	42	1267004	377716	0	8	2	0	37	50	17369596	2.93E-01	1
NUP50	395347	362	-13	1201856	330724	0	4	0	0	37	27	13665060	2.93E-01	1
ZBTB8OS	1441027	144	28	493416	111072	0	1	0	0	50	54	20815676	2.94E-01	1
LYSMD1	1562281	184	33	594876	184764	0	4	0	0	5	6	1818092	2.94E-01	1
TAS2R14	363803	644	2	789608	228196	0	3	1	0	50	61	20572172	2.94E-01	1
RAB34	1695792	208	40	883948	244928	0	2	1	0	50	81	25518436	2.94E-01	1
PRAP1	386585	472	13	399432	106800	0	1	1	0	10	5	3076908	2.94E-01	1
IGSF1	42910	NaN	15	3472068	972948	0	11	4	0	50	59	19502036	2.94E-01	1
RASSF3	450247	476	17	625848	156996	0	2	0	0	50	62	20810692	2.94E-01	1
GTPBP1	1291659	272	73	1679608	514064	0	5	1	0	50	42	16818864	2.94E-01	1
PDE7A	167106	425	37	1356360	342828	0	5	1	0	50	67	22724192	2.94E-01	1
SPESP1	373553	452	33	886440	242436	0	4	0	0	47	44	17980848	2.95E-01	1
PNRC1	307937	375	29	794236	260948	0	3	0	0	50	34	20041020	2.95E-01	1
C16orf46	259514	284	8	1013888	291208	0	5	0	0	50	41	18618444	2.95E-01	1
MED11	1272092	222	28	300108	88644	0	2	0	0	50	74	23892584	2.95E-01	1

LRRN3	65006	840	2	1766828	504808	0	7	0	0	37	42	16801420	2.95E-01	1
DIS3L	530074	307	44	2725180	725528	0	8	1	0	34	30	17301956	2.95E-01	1
LRBA	207641	848	17	7401952	2013536	0	21	2	0	10	8	4560716	2.95E-01	1
GBP5	379427	834	-4	1529732	393736	0	6	2	0	50	62	19610616	2.95E-01	1
PKLR	1574928	194	45	1436460	460308	0	6	2	0	50	57	20795740	2.95E-01	1
RARS2	236344	534	28	1543260	397296	0	6	1	0	33	29	11460352	2.95E-01	1
CRYBB2	174680	535	-35	547884	135636	0	2	1	0	50	76	19885092	2.96E-01	1
FBXO4	113456	795	23	1025992	270560	0	5	0	0	50	47	19282028	2.96E-01	1
TEPP	856192	158	9	769316	220720	0	3	0	0	50	56	19314424	2.96E-01	1
AP3B1	262852	592	40	2860816	758636	0	9	1	0	27	18	8882912	2.96E-01	1
DSG2	284905	450	1	2831624	813460	0	9	3	0	50	61	24671868	2.96E-01	1
ZNF521	24828	599	-1	3378796	853688	0	17	6	0	15	24	7552896	2.96E-01	1
COL16A1	1126016	197	37	4142772	1298688	0	13	2	0	8	9	3875772	2.96E-01	1
BPGM	388879	392	23	663584	178000	0	1	0	0	50	38	20327600	2.96E-01	1
IGF2	837088	579	38	592384	184052	0	3	0	0	50	46	19328664	2.96E-01	1
CORO1C	451218	217	40	1246712	317908	0	5	0	0	33	18	10547924	2.96E-01	1
ZNF180	743733	511	-55	1800648	436812	0	10	0	0	3	7	2228916	2.96E-01	1
BIN1	324544	324	17	1592388	440016	0	5	2	0	11	11	5228572	2.96E-01	1
CXorf56	617823	NaN	30	597012	147384	0	2	0	0	50	75	22656552	2.96E-01	1
PHTF2	246673	730	25	2001432	540408	0	7	2	0	17	19	6589916	2.96E-01	1
TAAR8	178165	618	22	850484	246352	0	4	0	0	42	38	17775792	2.96E-01	1
C6orf222	891439	192	38	1670352	468852	0	10	0	0	2	4	1620868	2.96E-01	1
GTF2H4	2223866	223	25	1190108	348880	0	3	2	0	50	73	23168480	2.96E-01	1
LZTS1	157405	592	9	1474908	446424	0	4	0	0	23	18	10899652	2.97E-01	1
TRIP4	764707	198	40	1523680	394448	0	6	0	0	2	2	2322900	2.97E-01	1
CDH4	437973	867	-61	2321476	682808	0	16	2	0	13	22	5201872	2.97E-01	1
RALYL	28254	1435	-22	802424	208972	0	5	0	0	21	16	5960152	2.97E-01	1
PSMA5	1073027	302	26	644716	166964	0	4	0	0	50	52	20758716	2.97E-01	1
MAT1A	365795	598	44	1013176	289784	0	4	1	0	50	60	20195880	2.97E-01	1
ANGPT4	824412	579	36	1291212	358848	0	5	2	0	50	49	19535144	2.97E-01	1
CDH2	43239	931	-16	2320408	649700	0	14	4	0	50	62	20808556	2.97E-01	1
TYW1	423966	491	32	1940556	472056	0	5	0	0	9	1	2258108	2.97E-01	1
C7orf50	769674	346	35	489144	151656	0	2	0	0	50	35	18959492	2.97E-01	1

LRP2	420134	683	14	12212224	3038816	0	39	6	0	4	8	4434692	2.98E-01	1
UBE2D1	153069	326	-12	402280	97544	0	2	0	0	50	59	23816400	2.98E-01	1
AP2S1	1217560	186	44	378784	96476	0	1	1	0	22	32	9588860	2.98E-01	1
GPHB5	147817	637	-12	224636	74404	0	2	1	0	4	3	1349240	2.98E-01	1
(IAA0319I	956500	451	27	2705244	744396	0	12	2	0	4	12	5851572	2.98E-01	1
PSMA4	505832	375	46	703812	169812	0	3	1	0	41	51	15870836	2.98E-01	1
HOXB6	946208	524	29	563904	165540	0	4	1	0	50	60	17564684	2.98E-01	1
LDOC1L	216551	425	14	567464	201496	0	2	1	0	50	58	21676128	2.98E-01	1
LGR6	674939	297	37	2515852	813104	0	8	2	0	50	56	22223656	2.98E-01	1
OOEP	732413	446	18	377360	111784	0	2	1	0	12	13	3339992	2.98E-01	1
TCEB2	1717244	210	42	426844	117836	0	1	0	0	50	80	24105472	2.98E-01	1
KRCC1	169985	543	5	679248	158064	0	5	1	0	28	31	10475300	2.98E-01	1
MINA	158573	723	-2	1204704	326808	0	7	1	0	5	8	2006060	2.99E-01	1
CLDN19	735401	426	26	689572	170168	0	2	0	0	50	74	27615988	2.99E-01	1
PRKRIP1	579276	202	61	494484	121752	0	2	1	0	50	62	18465720	2.99E-01	1
RASAL2	168177	435	-9	3304392	911004	0	16	2	0	16	21	7639404	2.99E-01	1
MKRN3	94496	904	-55	1261664	365968	0	8	2	0	34	37	11827744	2.99E-01	1
MT1H	661565	143	26	173372	35956	0	1	0	0	50	43	20327956	2.99E-01	1
CLDN5	770036	298	22	716628	261660	0	3	1	0	50	72	23922488	2.99E-01	1
RSL1D1	558203	294	37	1264512	344964	0	6	1	0	50	41	18471772	2.99E-01	1
CNOT2	156657	609	-17	1433256	364188	0	4	1	0	50	44	23827080	2.99E-01	1
FASTK	622098	373	41	1342120	460664	0	5	2	0	32	43	16522672	2.99E-01	1
LYPD6	105128	631	-8	446424	121752	0	2	0	0	50	43	19883668	2.99E-01	1
EBI3	2132502	244	39	576364	179780	0	1	0	0	50	48	20510584	2.99E-01	1
LCN15	1898901	190	39	489856	130652	0	1	1	0	50	67	24888672	2.99E-01	1
JDP2	758517	239	44	440372	133144	0	2	0	0	50	52	22293432	2.99E-01	1
EIF2A	436939	347	24	1536140	399076	0	3	0	0	30	20	9670384	2.99E-01	1
CDCA7L	687019	581	-1	1187260	309008	0	7	0	0	5	6	2504104	2.99E-01	1
TRH	329991	389	43	603064	184052	0	3	0	0	50	53	21555444	3.00E-01	1
THRSP	556958	209	38	373800	103596	0	3	1	0	25	31	8327908	3.00E-01	1
CLIP3	980636	320	19	1389824	421504	0	4	1	0	24	26	11494528	3.00E-01	1
LOX	69939	707	0	1056964	307940	0	5	0	0	40	45	16381340	3.00E-01	1
FBLN5	333910	440	29	1176580	304736	0	2	0	0	50	35	23272432	3.00E-01	1

KRTAP12-2	874480	461	25	361696	111428	0	1	0	0	50	76	22638396	3.00E-01	1
EMID1	854820	251	51	1125672	358848	0	5	1	0	50	60	20958788	3.00E-01	1
ELMOD2	125708	752	7	801356	174796	0	3	0	0	50	77	23962716	3.01E-01	1
FAM162A	416095	478	34	413672	102172	0	2	1	0	19	11	5724124	3.01E-01	1
FTHL17	77068	NaN	8	460308	131364	0	3	0	0	50	91	26528408	3.01E-01	1
HOXC6	1185281	279	54	593452	166964	0	4	2	0	14	21	4635120	3.01E-01	1
SLC4A4	216580	975	-4	3041664	803136	0	10	2	0	50	63	21170964	3.01E-01	1
HBEGF	683517	143	39	528304	162692	0	1	1	0	34	31	12849108	3.01E-01	1
EXOC5	176601	944	13	1894632	449628	0	6	0	0	16	12	5780372	3.01E-01	1
UCHL5	288979	433	-18	898188	201852	0	3	1	0	50	53	19227560	3.01E-01	1
OSBP	432625	353	6	2070140	574228	0	5	3	0	50	42	19696056	3.01E-01	1
MYH11	435762	283	29	5215044	1343544	0	19	5	0	27	33	11942376	3.01E-01	1
NOL4	10823	966	-14	1654332	435744	0	7	1	0	50	82	24136444	3.01E-01	1
FAM110C	123809	705	NaN	761840	274120	0	4	1	0	15	17	7089740	3.01E-01	1
PGD	712477	383	24	1260240	343896	0	5	1	0	50	75	29160672	3.02E-01	1
CDH22	553454	583	44	2046644	652192	0	10	4	0	26	32	10484556	3.02E-01	1
TIGD3	4112809	242	50	1143116	373444	0	2	1	0	27	30	12483852	3.02E-01	1
RPL18A	1216364	212	37	458172	130296	0	1	0	0	50	49	17585332	3.02E-01	1
MPZL1	546300	319	16	690640	195800	0	3	1	0	50	66	20961636	3.02E-01	1
ZNF28	557083	1008	-39	1724108	414028	0	8	3	0	50	82	22084104	3.02E-01	1
NELL1	45732	989	-19	2152376	527236	0	9	4	0	11	17	5000376	3.02E-01	1
ZFAND1	158801	681	12	767180	178000	0	4	1	0	50	67	18347172	3.02E-01	1
TMEM50B	884788	182	63	429336	105732	0	2	0	0	50	40	16445776	3.02E-01	1
KCNRG	337743	357	44	769316	218584	0	3	2	0	27	28	10846252	3.02E-01	1
MVP	NaN	NaN	NaN	2228560	702032	0	8	1	0	50	117	40347616	3.03E-01	1
SNW1	442490	359	42	1474196	377716	0	6	1	0	50	45	20762988	3.03E-01	1
HSF4	1101399	150	46	1287296	358492	0	4	0	0	7	2	3466372	3.03E-01	1
ZNF644	223798	843	-7	3400868	875404	0	14	2	0	13	16	7704196	3.03E-01	1
SSBP3	695802	369	25	1039164	279816	0	3	1	0	50	56	23000448	3.03E-01	1
FARSB	267637	308	18	1547532	413316	0	5	0	0	9	9	5009988	3.03E-01	1
EIF4B	1726226	177	38	1583132	437524	0	4	0	0	4	1	2378080	3.03E-01	1
CST11	188567	750	-16	373444	84728	0	2	1	0	11	14	3491648	3.03E-01	1
CYP4A22	364960	414	3	1343544	373800	0	5	3	0	50	53	20385984	3.04E-01	1

PVALB	1248441	537	35	302600	70132	0	2	0	0	50	96	28965228	3.04E-01	1
PAH	23724	961	-3	1190820	311856	0	9	1	0	50	94	20550456	3.04E-01	1
FAM47B	6541	NaN	-14	1586692	485228	0	10	2	0	50	93	25240756	3.04E-01	1
OR5AC2	200002	811	-15	765044	226060	0	4	1	0	50	87	21088372	3.04E-01	1
MRPL47	397411	520	10	661092	170880	0	2	0	0	50	51	19040304	3.04E-01	1
FCN3	866116	188	28	775368	217872	0	4	0	0	50	83	26057420	3.04E-01	1
ZNF829	308757	976	-38	1342832	331792	0	8	2	0	50	98	20471068	3.04E-01	1
ANO4	245388	937	-16	2450704	606980	0	12	4	0	50	70	16647984	3.04E-01	1
ACTA1	243405	361	29	967252	269492	0	4	1	0	50	41	20173452	3.04E-01	1
ING3	105792	514	7	1121044	283376	0	5	1	0	50	62	21516284	3.04E-01	1
FERMT3	1602391	278	57	1708800	491280	0	11	2	0	50	67	20864092	3.04E-01	1
PHB2	2704950	211	47	770740	224636	0	3	0	0	1	0	1215740	3.04E-01	1
OR51I2	99042	1030	1	766468	240656	0	2	0	0	50	49	19819232	3.05E-01	1
AGTPBP1	227838	613	9	3110728	799220	0	10	2	0	19	21	10301928	3.05E-01	1
IL10RB	861800	317	87	856180	216092	0	5	1	0	38	46	17976932	3.05E-01	1
OR2A2	221024	812	-11	780708	239232	0	5	1	0	50	70	20067720	3.05E-01	1
ZNF84	NaN	NaN	NaN	1927740	452832	0	9	4	0	26	44	12029952	3.05E-01	1
MEPE	423426	538	1	1360276	337844	0	8	0	0	50	86	25028936	3.05E-01	1
IFNA4	137739	498	1	479176	131720	0	4	1	0	8	11	3212900	3.05E-01	1
ACTR1A	871726	178	41	975440	275188	0	4	1	0	50	63	21586416	3.05E-01	1
TP53INP1	418153	395	48	650768	159844	0	4	1	0	22	28	9035280	3.06E-01	1
ACAT1	417904	695	19	1106448	313992	0	4	1	0	20	21	7720572	3.06E-01	1
KLRC4	378365	334	0	416876	105376	0	2	0	0	50	50	21701404	3.06E-01	1
LRTM1	29081	629	24	862588	256676	0	2	0	0	50	45	22431916	3.06E-01	1
GJB3	548013	580	19	668568	203988	0	3	2	0	49	58	16973724	3.06E-01	1
C2orf48	881918	220	41	409044	116412	0	1	0	0	50	71	24529112	3.06E-01	1
MSTN	508463	478	28	957640	255608	0	5	2	0	14	13	6396252	3.06E-01	1
GFRAL	28347	729	-14	1058032	243860	0	5	0	0	11	19	6549332	3.06E-01	1
ST13	894425	269	44	993596	238876	0	6	0	0	50	69	23669372	3.06E-01	1
AGRP	1082466	153	49	335352	105732	0	2	0	0	50	61	21399872	3.06E-01	1
EIF2S1	484235	291	32	830904	210396	0	4	0	0	50	43	17771164	3.06E-01	1
TTC14	182757	1031	2	2035608	511572	0	6	0	0	21	15	9560380	3.06E-01	1
PHTF1	298251	398	10	1991820	525456	0	7	1	0	50	52	23351464	3.06E-01	1

TEKT5	235538	242	-1	1245644	341404	0	3	0	0	50	43	21046364	3.07E-01	1
MYO9A	1084708	386	22	6619820	1722328	0	23	3	0	2	4	2998588	3.07E-01	1
DAB2	167695	374	-7	1965476	563548	0	6	1	0	25	23	12261708	3.07E-01	1
NARS2	302979	569	17	1247780	339268	0	5	0	0	25	29	17259236	3.07E-01	1
PDE4A	1956832	207	38	2587764	795660	0	11	4	0	50	73	23497424	3.07E-01	1
CCAR1	859993	256	45	3028492	761840	0	8	1	0	20	19	8320432	3.07E-01	1
TEC	253162	404	42	1694560	402992	0	8	1	0	50	58	19873344	3.07E-01	1
COG5	492317	753	18	2230696	619796	0	11	1	0	50	41	17036380	3.07E-01	1
MRPS18B	2080582	271	32	662160	193308	0	4	0	0	24	27	11479576	3.08E-01	1
ASF1A	116680	856	16	432896	118192	0	3	1	0	37	41	12020696	3.08E-01	1
TEX13A	9759	NaN	2	1013532	310788	0	3	0	0	22	18	8716304	3.08E-01	1
SLAMF9	812025	365	13	759704	182272	0	4	1	0	50	64	17355000	3.08E-01	1
COL1A1	1277832	210	26	3666444	1241016	0	12	1	0	12	10	5442172	3.08E-01	1
PTPN21	154907	686	-6	2982924	854400	0	9	3	0	50	67	25200172	3.08E-01	1
EDC3	635020	206	51	1290500	365968	0	4	1	0	50	57	20067008	3.08E-01	1
CST9	189307	772	-47	412248	104664	0	1	0	0	50	80	23329748	3.08E-01	1
STARD6	99256	451	41	582416	145960	0	2	0	0	50	80	26137876	3.08E-01	1
ACSL5	184750	409	-4	1938064	518336	0	6	0	0	40	30	16363896	3.08E-01	1
AKT1S1	1673246	205	37	627984	212532	0	2	1	0	50	63	21064520	3.08E-01	1
CHEK2	515703	219	-8	1533292	411536	0	7	1	0	50	72	22733092	3.08E-01	1
FUBP1	373296	256	1	1678184	471700	0	5	0	0	9	9	5883612	3.08E-01	1
EPHA6	89830	1046	-18	3004996	816308	0	13	2	0	50	55	21079116	3.09E-01	1
DGKB	24487	1141	-13	2157716	537916	0	10	3	0	29	49	17363544	3.09E-01	1
SLAMF7	1188881	618	29	860452	243860	0	5	0	0	50	82	21947756	3.09E-01	1
OR4B1	400891	509	-27	765756	226416	0	6	1	0	50	74	19622364	3.09E-01	1
B3GNT2	316454	412	38	1009260	270204	0	5	1	0	50	68	20351808	3.09E-01	1
MREG	925953	501	19	561056	144892	0	4	0	0	50	55	15772224	3.10E-01	1
FBXO40	289905	613	25	1798868	488788	0	4	1	0	50	65	25835988	3.10E-01	1
GLYATL1	368006	741	-11	877184	220720	0	6	0	0	50	59	18180920	3.10E-01	1
SOSTDC1	291856	699	-19	524388	143112	0	2	1	0	50	75	20552236	3.10E-01	1
IBSP	423426	538	1	839448	205056	0	4	0	0	50	86	25028936	3.10E-01	1
SAP130	541058	248	36	2697056	861520	0	10	1	0	3	1	1569960	3.10E-01	1
CLPTM1	1003719	196	6	1722328	482024	0	8	2	0	50	42	17116124	3.10E-01	1

MRPL51	2764720	179	42	334284	87576	0	1	0	0	50	84	25936380	3.10E-01	1
ARMC7	1511891	168	25	490212	155928	0	3	0	0	50	53	18659384	3.11E-01	1
PRPF4B	182705	353	18	2618024	673552	0	7	1	0	35	32	11880788	3.11E-01	1
MCRS1	2227486	224	53	1237812	355644	0	7	1	0	50	79	22778304	3.11E-01	1
TAS2R50	365490	676	-2	752584	212888	0	3	1	0	50	58	17850552	3.11E-01	1
SPRED1	96035	704	-21	1164832	286580	0	7	1	0	50	79	22496708	3.11E-01	1
ZCCHC13	404685	NaN	26	417588	119616	0	2	0	0	50	58	24565068	3.11E-01	1
NEIL2	470737	360	34	850484	238876	0	5	0	0	50	50	17460376	3.11E-01	1
GSTA1	539666	498	11	591316	148808	0	4	0	0	50	73	21581432	3.11E-01	1
AQP2	1033911	220	48	653972	232468	0	4	1	0	50	62	21015036	3.11E-01	1
CTNNBL1	720832	507	48	1493776	379496	0	5	1	0	50	50	22371040	3.12E-01	1
PRSS1	285408	636	11	638664	177288	0	2	0	0	50	67	24216544	3.12E-01	1
ITM2B	340844	457	36	697760	179068	0	5	0	0	50	64	19850916	3.12E-01	1
HLA-F	484408	755	34	1131724	320756	0	5	0	0	50	51	18249272	3.12E-01	1
AGFG2	1651125	242	60	1209688	381632	0	4	2	0	46	58	16249976	3.12E-01	1
DNAI2	341939	427	-2	1590964	409400	0	7	2	0	50	59	21065232	3.12E-01	1
NKAIN4	1005137	285	50	541120	156284	0	2	0	0	50	62	19950240	3.12E-01	1
GOLPH3	440207	197	16	752584	218228	0	2	0	0	50	50	21090864	3.12E-01	1
MYL5	639827	489	12	555004	139196	0	2	0	0	50	63	18978004	3.12E-01	1
HIST1H2BI	227588	622	29	317196	96120	0	4	1	0	14	21	3578512	3.12E-01	1
CEBPB	598211	143	41	828412	280172	0	2	1	0	50	43	18237168	3.13E-01	1
GYPC	176465	753	-3	334996	91136	0	1	1	0	14	10	3351384	3.13E-01	1
AKR7A2	810462	448	40	909580	271628	0	3	0	0	40	38	14129640	3.13E-01	1
CCDC54	101063	575	-12	836244	220008	0	5	0	0	50	67	26164576	3.13E-01	1
C4orf19	124686	814	32	798864	218940	0	4	0	0	50	59	22922840	3.13E-01	1
TBX5	88840	794	-25	1385908	380564	0	8	3	0	26	35	9107904	3.13E-01	1
SEMA6B	2111443	278	43	2170176	744396	0	9	2	0	50	77	21475344	3.13E-01	1
LGR4	345497	337	-18	2446076	676756	0	6	3	0	50	53	20888300	3.13E-01	1
HIST3H2BI	626588	351	7	314348	96832	0	2	1	0	17	22	7678564	3.13E-01	1
WDR93	880974	190	35	1801716	467784	0	8	1	0	50	61	22286312	3.13E-01	1
PGM5	127057	681	-4	1440732	421860	0	8	3	0	42	58	16781128	3.13E-01	1
HRCT1	853975	298	55	276968	96832	0	2	0	0	50	61	17185188	3.14E-01	1
ZNF658	17308	1040	-46	2747964	665364	0	6	1	0	3	1	2386980	3.14E-01	1

COL12A1	359824	486	-8	7849800	2244936	0	21	5	0	20	20	10641196	3.14E-01	1
POMZP3	846738	301	66	494128	134924	0	2	1	0	19	29	7882908	3.14E-01	1
KDM3A	625501	424	22	3431128	911360	0	13	2	0	31	47	17987968	3.14E-01	1
TEAD2	2183690	309	30	1145964	336420	0	5	2	0	50	56	21603148	3.14E-01	1
LSG1	370452	255	34	1725176	441796	0	8	0	0	11	16	4908528	3.14E-01	1
AMN	758150	286	50	1119976	383768	0	5	0	0	22	21	8130684	3.14E-01	1
SERPINB1[illegible]	174293	370	-5	1021364	267712	0	4	0	0	50	45	22271360	3.14E-01	1
C4orf26	461415	491	19	334284	91848	0	2	0	0	50	63	20447928	3.14E-01	1
TP53I11	131819	609	16	490568	143824	0	2	1	0	18	25	7064464	3.15E-01	1
UBE2R2	783515	187	33	626204	156640	0	3	1	0	50	47	19906452	3.15E-01	1
GALNT3	104274	888	-1	1648636	421148	0	6	0	0	50	52	21592112	3.15E-01	1
FBXO30	77282	736	0	1882884	515844	0	6	0	0	45	42	22653704	3.15E-01	1
SVOPL	256342	480	9	1261664	377716	0	4	2	0	50	52	20429416	3.15E-01	1
OR4C46	68	1239	-43	770740	220364	0	4	2	0	4	11	3381644	3.15E-01	1
C11orf73	366108	283	24	512996	138484	0	3	0	0	50	77	26264612	3.15E-01	1
WBSCR27	1099366	208	37	611252	198292	0	2	0	0	50	44	16326160	3.15E-01	1
DIP2B	736380	262	49	4018884	1191888	0	13	2	0	18	14	6399812	3.15E-01	1
PIGO	782447	350	42	2649352	884660	0	9	2	0	36	48	20387764	3.16E-01	1
OSGEP	464497	395	17	863656	255608	0	5	0	0	50	58	21385632	3.16E-01	1
SLC39A1	2028800	222	34	770384	283732	0	5	1	0	50	66	18282736	3.16E-01	1
KIAA1429	534075	405	36	4678196	1286584	0	16	3	0	13	14	5288024	3.16E-01	1
UPB1	546252	171	51	999648	272340	0	5	1	0	50	61	18447208	3.16E-01	1
UBE2J1	408992	574	51	825920	226060	0	4	0	0	27	20	8907120	3.16E-01	1
GRN	1062294	172	38	1536140	418300	0	5	0	0	40	55	20243940	3.16E-01	1
CCDC14	242712	450	20	2331444	642936	0	5	0	0	6	1	2109300	3.16E-01	1
RPTN	486922	468	25	2054832	468852	0	7	2	0	8	13	5121772	3.16E-01	1
LNP1	376655	410	13	470276	116056	0	1	1	0	22	27	9990784	3.16E-01	1
TADA2A	520126	171	25	1283024	305092	0	6	0	0	16	14	5436832	3.16E-01	1
DCP2	274112	439	25	1126740	264864	0	6	1	0	50	56	20147464	3.17E-01	1
PNLDC1	652619	453	26	1395876	350304	0	7	3	0	17	19	6396608	3.17E-01	1
DDC	180088	561	11	1254900	346032	0	4	0	0	50	46	20520552	3.17E-01	1
KRT72	1280474	464	36	1306876	374156	0	4	0	0	38	51	16451116	3.17E-01	1
CDH10	30232	737	-29	2008552	562124	0	16	4	0	18	34	6362432	3.17E-01	1

SIRT7	3798594	211	19	1011040	314348	0	3	0	0	34	28	11428668	3.17E-01	1
SLC29A2	1963418	182	55	1150236	360984	0	6	1	0	50	53	20984776	3.17E-01	1
HERC6	434791	389	32	2510868	646140	0	8	2	0	24	20	9249948	3.17E-01	1
ING5	1323203	406	32	646140	158064	0	4	1	0	50	66	20935292	3.17E-01	1
ESRRG	15599	1132	-28	1181920	314348	0	6	2	0	38	94	20207272	3.17E-01	1
CLDN25	148947	534	4	557496	179424	0	2	1	0	50	49	17384548	3.17E-01	1
EOMES	165387	429	1	1700612	521896	0	5	1	0	50	59	21496704	3.17E-01	1
HNF4A	769430	376	16	1364904	387684	0	6	2	0	50	63	21119700	3.17E-01	1
C1orf123	527317	391	27	443576	104308	0	1	0	0	50	64	21238960	3.17E-01	1
BRIX1	241905	675	21	947672	229264	0	3	0	0	50	44	20410904	3.17E-01	1
SLMAP	788284	196	35	2156648	530440	0	5	2	0	50	45	16343248	3.17E-01	1
CIR1	385177	419	24	1215384	270204	0	5	0	0	50	52	25814628	3.18E-01	1
ASTN1	6547	940	-9	3326820	920616	0	14	5	0	49	78	25022528	3.18E-01	1
SMPD4	349797	593	22	2207556	651480	0	6	3	0	40	41	18115772	3.18E-01	1
PTPRC	100900	412	0	3468864	858672	0	15	5	0	15	17	6258124	3.18E-01	1
CDK2AP2	1392213	215	53	318264	101460	0	2	0	0	50	41	15455384	3.18E-01	1
SYCE2	1886809	335	40	600928	149876	0	2	0	0	50	62	18190176	3.18E-01	1
MPND	2122615	220	27	1288008	375936	0	7	3	0	21	45	12772568	3.18E-01	1
ENPP5	62118	819	16	1224640	319688	0	6	0	0	16	11	5933808	3.18E-01	1
TLR5	361954	297	-8	2153800	602708	0	10	1	0	50	55	23363924	3.18E-01	1
HSD3B2	409405	720	3	938060	269848	0	5	2	0	39	38	14123944	3.19E-01	1
CNTD2	1005838	400	30	740836	265220	0	2	2	0	39	36	14339680	3.19E-01	1
PTRF	2209303	160	31	956572	300464	0	6	2	0	50	69	23521988	3.19E-01	1
MCEE	355097	470	40	445712	134212	0	1	0	0	50	68	19843796	3.19E-01	1
TSPAN12	106040	717	3	802780	207548	0	2	0	0	50	50	21163844	3.19E-01	1
TMTC4	123356	622	28	1953372	557496	0	7	1	0	50	33	19953088	3.19E-01	1
C9orf78	600401	187	44	776080	189392	0	4	0	0	50	71	21419808	3.19E-01	1
PYCRL	2183482	235	37	705236	237808	0	3	0	0	50	40	19636960	3.20E-01	1
PAFAH1B3	792230	218	42	586688	182272	0	3	0	0	50	65	24510600	3.20E-01	1
BCL2L11	NaN	NaN	NaN	620152	168032	0	2	0	0	50	116	39813616	3.20E-01	1
COG8	1202748	125	20	1508372	481312	0	7	2	0	20	22	7617688	3.20E-01	1
ZNF596	89938	794	10	1313996	325384	0	4	0	0	31	30	13788592	3.20E-01	1
ATP2A1	1167826	218	55	2553588	758280	0	11	2	0	7	11	4186916	3.20E-01	1

EIF2AK2	603425	281	27	1467432	365256	0	7	1	0	50	57	19166684	3.20E-01	1
GTF3C2	1245306	182	52	2301896	697048	0	7	1	0	50	41	22639464	3.20E-01	1
CYP4B1	658240	475	8	1331796	360984	0	5	1	0	17	14	6420104	3.20E-01	1
SPR	450159	421	23	631900	218228	0	1	0	0	50	54	23586068	3.20E-01	1
ERLEC1	NaN	NaN	NaN	1277684	328588	0	5	2	0	50	118	39974172	3.20E-01	1
FOXD4L3	118303	887	1	1003564	337844	0	5	2	0	12	7	3792824	3.21E-01	1
PSPC1	309425	481	34	1343900	369172	0	5	0	0	25	23	10093668	3.21E-01	1
HMP19	101001	533	23	442508	125668	0	2	1	0	25	22	10314388	3.21E-01	1
FNIP2	171083	428	10	2849424	791388	0	8	0	0	3	0	1565688	3.21E-01	1
AGPS	378489	434	26	1736212	458528	0	4	2	0	13	9	4967624	3.21E-01	1
ZNF280A	201933	683	23	1373092	370952	0	8	2	0	24	23	9152404	3.21E-01	1
TSNAX	291493	439	14	766824	186900	0	2	0	0	50	52	20960212	3.21E-01	1
SPTY2D1	637558	457	28	1733008	486296	0	4	0	0	22	27	10043472	3.21E-01	1
34GALNT:	496097	246	32	2566048	715916	0	11	3	0	50	60	19222220	3.22E-01	1
CASP7	200770	587	0	1009972	182984	0	4	0	0	50	45	20932800	3.22E-01	1
SPTLC3	64005	571	-59	1431832	386972	0	8	2	0	29	52	11801400	3.22E-01	1
KCNK1	135142	419	12	836956	251336	0	3	1	0	50	57	20671852	3.22E-01	1
CERKL	216512	380	20	1461380	387328	0	7	1	0	50	49	22354664	3.22E-01	1
OAF	323731	198	31	681028	209684	0	4	1	0	50	65	22659400	3.22E-01	1
RAPGEF5	747975	629	4	1967256	473124	0	4	1	0	11	4	3545404	3.22E-01	1
RAB11FIP:	660779	379	37	3158076	979356	0	13	3	0	37	49	19758712	3.22E-01	1
HIPK3	545645	393	18	3102184	862232	0	12	2	0	23	33	12329348	3.22E-01	1
RBM39	1401606	125	51	1405488	364188	0	6	2	0	36	51	16263148	3.22E-01	1
POLR1D	350423	583	50	645428	163048	0	3	0	0	36	26	11061632	3.22E-01	1
TRPM1	277407	497	28	4142772	1107516	0	13	5	0	17	19	6628008	3.22E-01	1
PRKAG1	1754222	212	60	878252	238876	0	3	0	0	50	67	25068096	3.23E-01	1
OR2S2	853975	298	55	783200	246352	0	3	1	0	50	61	17185188	3.23E-01	1
ING2	204597	227	15	726952	177644	0	4	0	0	50	50	16885792	3.23E-01	1
MAN2C1	814680	296	43	2660744	783556	0	8	1	0	35	47	15238224	3.23E-01	1
CLOCK	235221	384	22	2231052	568176	0	5	1	0	19	19	10446464	3.23E-01	1
SFRP4	100826	683	10	896052	237096	0	3	0	0	50	75	26634496	3.23E-01	1
C9orf64	460176	343	2	866148	242436	0	4	0	0	20	18	8075504	3.23E-01	1
RPL6	715909	201	31	741904	209684	0	3	1	0	50	39	17563260	3.23E-01	1

UPK3A	439942	277	32	718052	228196	0	2	1	0	50	57	21098696	3.24E-01	1
HINFP	1324478	195	58	1355648	342472	0	6	2	0	21	32	10115740	3.24E-01	1
LRRC49	252612	404	11	1803496	461732	0	7	2	0	50	64	24196608	3.24E-01	1
ZCCHC6	292716	429	7	3922408	981848	0	18	3	0	39	37	14366380	3.24E-01	1
RNF125	417722	442	28	605200	162692	0	2	1	0	8	6	2811332	3.25E-01	1
ZC3HAV1	553735	243	29	2314712	631900	0	8	4	0	12	15	5440748	3.25E-01	1
LONRF2	94009	703	5	1896768	569244	0	6	0	0	12	10	5613408	3.25E-01	1
PXK	574675	218	36	1526172	403704	0	5	2	0	50	55	23421596	3.25E-01	1
COL13A1	591993	561	26	1875052	592028	0	5	2	0	50	58	22151032	3.25E-01	1
BPNT1	638729	613	-6	798508	225704	0	3	0	0	50	71	21864452	3.25E-01	1
XRCC6BP1	844463	298	22	649344	165540	0	4	0	0	50	54	20630200	3.25E-01	1
MRPS28	194118	410	45	469564	143468	0	1	0	0	50	66	23293792	3.25E-01	1
C14orf177	157823	938	-42	324672	87576	0	1	0	0	50	71	16861584	3.25E-01	1
ZNF224	544061	558	-31	1857964	429692	0	7	0	0	41	40	13624120	3.25E-01	1
SMARCA4	1984564	190	40	4327536	1214316	0	19	4	0	39	57	18397368	3.25E-01	1
SLC16A3	2959536	193	27	1131724	378428	0	2	0	0	30	33	13425472	3.25E-01	1
KPNA1	413070	404	34	1410116	372376	0	6	2	0	50	39	17499536	3.26E-01	1
ABCB10	255826	277	20	1817736	603420	0	6	0	0	13	14	7152396	3.26E-01	1
PPBP	230379	408	2	329300	94696	0	2	1	0	8	11	4168048	3.26E-01	1
SH3D19	211734	335	23	2011756	582416	0	10	1	0	11	13	4738716	3.26E-01	1
LMX1A	292562	797	-13	998224	263084	0	3	0	0	33	35	10093312	3.26E-01	1
TMPRSS1	274061	981	-64	2689580	683164	0	9	0	0	19	13	7380592	3.26E-01	1
KCNA10	435777	589	29	1270564	372020	0	5	2	0	23	27	12775772	3.26E-01	1
CLDN10	228898	397	50	761128	222500	0	2	0	0	18	11	6442532	3.26E-01	1
GRIA4	86500	920	-22	2558572	688148	0	15	3	0	21	27	8126412	3.26E-01	1
TGM3	555761	399	27	1783916	493060	0	8	1	0	50	54	22027500	3.26E-01	1
CUEDC1	873797	222	6	995732	282664	0	3	2	0	50	66	19357500	3.26E-01	1
C6	252021	888	-14	2453196	615168	0	10	3	0	50	55	19252124	3.26E-01	1
MAPK1IP1	837637	144	39	585976	215024	0	1	0	0	50	47	20764412	3.26E-01	1
MPP1	1741051	NaN	3	1238524	319688	0	2	0	0	31	36	14621276	3.26E-01	1
CHAC2	NaN	NaN	NaN	473480	129940	0	3	0	0	50	116	39775524	3.27E-01	1
UPK1B	222933	588	0	692420	173728	0	2	1	0	47	47	17454680	3.27E-01	1
RLN3	1013197	231	32	352796	111784	0	2	0	0	50	62	21554376	3.27E-01	1

C1orf95	608933	509	12	359916	107868	0	2	0	0	50	64	22211196	3.27E-01	1
KRTAP5-8	586193	626	50	475616	128872	0	1	0	0	41	39	13806036	3.27E-01	1
ACAP3	1744013	273	28	2161276	614456	0	9	2	0	9	14	4108596	3.27E-01	1
SRBD1	73148	419	28	2590612	683876	0	6	2	0	39	24	15730572	3.27E-01	1
GZMK	221322	558	1	675688	192596	0	2	0	0	50	55	20812828	3.27E-01	1
GIMAP8	557261	584	38	1689932	461020	0	7	2	0	50	72	24022168	3.27E-01	1
DCUN1D4	70055	766	17	806340	175152	0	2	0	0	50	42	19699260	3.28E-01	1
TRIM68	212267	935	-3	1244220	338556	0	4	2	0	48	67	19685376	3.28E-01	1
ZNF410	502038	155	50	1237456	339980	0	3	0	0	50	57	19811400	3.28E-01	1
KRTAP19-	14979	1035	-68	221788	67640	0	2	1	0	27	40	7047376	3.28E-01	1
PRND	477570	485	26	448560	122820	0	2	0	0	50	50	22571824	3.28E-01	1
RP1L1	99570	456	15	5856556	1849064	0	24	5	0	44	50	17827056	3.28E-01	1
PPDPF	1262904	298	54	295836	85440	0	1	1	0	21	30	7391984	3.28E-01	1
DNAJC14	1909014	256	38	1758640	519404	0	9	0	0	16	13	5358156	3.28E-01	1
C18orf8	329604	164	60	1737636	453900	0	6	0	0	23	31	10604528	3.28E-01	1
ERC2	121781	694	17	2489152	637952	0	13	2	0	50	62	19951664	3.28E-01	1
PPP2R3A	181711	590	2	3086520	795660	0	13	1	0	23	22	10027808	3.28E-01	1
C2orf43	491470	393	16	851552	218584	0	5	0	0	50	66	21261032	3.28E-01	1
BANF1	3854361	208	57	232824	64080	0	1	0	0	50	60	21621304	3.28E-01	1
TREML4	249047	607	38	517624	147740	0	1	0	0	50	46	16839512	3.29E-01	1
CRYGD	327858	440	13	452120	119260	0	1	0	0	50	63	21332232	3.29E-01	1
TMED9	1316033	475	42	603420	171948	0	1	0	0	25	32	14321524	3.29E-01	1
NAA10	2341816	NaN	26	627272	160912	0	3	0	0	50	75	29231160	3.29E-01	1
CYP27A1	1077853	296	40	1339272	399432	0	5	3	0	35	64	16686788	3.29E-01	1
SHKBP1	820124	226	40	1788188	555004	0	7	2	0	50	54	21183780	3.29E-01	1
CHRND	279084	156	34	1322540	386260	0	8	2	0	50	73	20747324	3.29E-01	1
GCNT1	107632	603	-2	1087936	286580	0	5	0	0	11	15	5511236	3.29E-01	1
EXT1	190467	586	18	1926316	509792	0	8	4	0	18	15	5204720	3.29E-01	1
TMEM151A	2133752	236	53	1118908	388040	0	6	1	0	50	77	24481764	3.30E-01	1
RBM14	1343990	194	62	1598796	556428	0	8	0	0	13	11	5250288	3.30E-01	1
HMGCS1	504216	449	20	1342120	365612	0	7	2	0	50	48	17318332	3.30E-01	1
VEZF1	854628	182	27	1317912	375936	0	5	0	0	40	38	14872256	3.30E-01	1
SBDS	485411	355	34	655040	166252	0	5	2	0	25	28	9221468	3.30E-01	1

KCTD17	699535	379	44	770028	215736	0	4	1	0	50	63	26596760	3.30E-01	1
CEP70	198996	515	16	1615528	368816	0	5	2	0	50	61	20383492	3.30E-01	1
ZNF558	560313	537	-60	1050556	262016	0	5	0	0	2	43	12280576	3.30E-01	1
CLTC	1083057	398	23	4358152	1144184	0	17	1	0	2	5	3049140	3.30E-01	1
SERPINA1[illegible]	278548	858	13	1061236	287648	0	7	3	0	42	49	13225400	3.30E-01	1
ATP6V1B1	504483	623	25	1327880	376648	0	6	3	0	14	10	3081892	3.30E-01	1
APBB3	706371	319	29	1258460	371308	0	3	2	0	42	35	18761200	3.30E-01	1
H2BFWT	439642	NaN	30	433964	140620	0	1	0	0	50	63	20255688	3.30E-01	1
QRFP	727815	241	22	327520	110360	0	2	0	0	50	66	20777940	3.31E-01	1
ESX1	354255	NaN	47	993596	325384	0	6	0	0	50	58	20023576	3.31E-01	1
PAQR3	170032	699	18	803848	217160	0	3	0	0	50	57	17489568	3.31E-01	1
CSRP2BP	782027	479	-6	1998584	548596	0	8	4	0	45	53	14371008	3.31E-01	1
PEX2	66156	757	2	777148	207548	0	1	0	0	50	41	21510944	3.31E-01	1
TPRG1L	250027	288	16	686012	207904	0	2	1	0	50	45	18928876	3.31E-01	1
USH1G	1045089	223	25	1130656	358136	0	5	2	0	50	66	19891500	3.31E-01	1
NKAPL	273319	502	35	1022788	270560	0	7	2	0	38	40	12254588	3.31E-01	1
EXOSC9	324357	506	29	1200432	315060	0	7	1	0	42	48	16152432	3.31E-01	1
COX17	288657	595	42	172304	43432	0	1	0	0	50	57	18639092	3.32E-01	1
PDGFC	39685	595	-24	889288	240656	0	4	1	0	50	66	19440448	3.32E-01	1
ARHGEF3[illegible]	609620	185	37	1708800	508368	0	6	2	0	50	49	20150668	3.32E-01	1
RABL2B	266017	295	32	654684	152724	0	2	0	0	50	60	20376016	3.32E-01	1
KRT20	1036346	358	5	1093988	299752	0	7	2	0	50	61	13481008	3.32E-01	1
MIA3	225124	392	-1	4943772	1286940	0	12	1	0	29	26	13495604	3.33E-01	1
GPAT2	792288	366	33	2010688	629408	0	5	1	0	50	53	22171324	3.33E-01	1
BEST1	2389859	185	53	1709868	481668	0	3	0	0	7	1	2498052	3.33E-01	1
YPEL5	141747	692	18	310788	88644	0	2	0	0	50	61	18526240	3.33E-01	1
MTNR1A	351103	675	-26	868996	264152	0	5	1	0	50	78	22212976	3.33E-01	1
ALAD	390186	297	36	859028	248488	0	4	1	0	50	42	19391676	3.33E-01	1
ARAP3	771583	353	25	3885740	1201144	0	8	4	0	50	53	22992260	3.33E-01	1
DPM3	1801444	194	32	294768	103596	0	1	0	0	50	85	19936000	3.33E-01	1
PPP1R16B	264783	548	32	1453192	408332	0	6	1	0	50	59	20456472	3.33E-01	1
HLA-DPA1	1479000	431	28	665364	190104	0	5	0	0	50	69	17902884	3.33E-01	1
ANGPT1	50022	671	-19	1318980	313992	0	4	1	0	50	81	23751252	3.33E-01	1

CRX	881316	336	39	736564	237452	0	4	0	0	2	0	778928	3.34E-01	1
TROAP	2137968	175	37	2002856	659668	0	10	1	0	9	15	4736936	3.34E-01	1
ZNF493	166321	1140	-56	2062308	498756	0	10	1	0	49	66	19365688	3.34E-01	1
MST1R	1289886	170	38	3449284	1120688	0	16	8	0	11	20	5670724	3.34E-01	1
FAM160B1	208967	183	16	2009264	542188	0	9	1	0	5	3	1525460	3.34E-01	1
C11orf57	389805	179	31	783556	179780	0	3	1	0	37	39	12636576	3.34E-01	1
POU6F2	180662	897	4	1723396	536492	0	6	3	0	50	60	18744112	3.34E-01	1
PWWP2B	152612	271	-1	1435748	485584	0	6	0	0	7	7	3771464	3.34E-01	1
FAM134A	773232	177	36	1338204	438948	0	7	0	0	12	9	4375240	3.34E-01	1
LRGUK	239801	634	8	2163412	566396	0	7	3	0	26	34	10889684	3.35E-01	1
KPNA5	244987	581	14	1426848	363120	0	5	2	0	48	65	18929944	3.35E-01	1
C15orf41	74456	598	-16	761840	188680	0	2	0	0	50	61	22792188	3.35E-01	1
ALG1L2	NaN	NaN	NaN	563904	158064	0	3	1	0	50	117	39803648	3.35E-01	1
E2F6	342110	196	26	733004	196156	0	2	0	0	50	62	26104412	3.35E-01	1
TXLNB	192994	504	14	1767184	466004	0	5	0	0	17	17	7303696	3.35E-01	1
KCTD10	434920	420	38	808120	225704	0	5	0	0	50	68	22062744	3.35E-01	1
SCYL3	181109	624	5	1908872	527236	0	9	2	0	13	9	4214684	3.35E-01	1
DRGX	NaN	NaN	NaN	680316	201852	0	2	0	0	50	116	39847436	3.35E-01	1
ZNF595	NaN	NaN	NaN	1644008	445000	0	9	0	0	50	116	40090584	3.36E-01	1
RDH10	259225	340	19	867572	249556	0	4	1	0	50	53	22172392	3.36E-01	1
MRPL30	489016	633	27	430048	110360	0	2	0	0	50	52	21570752	3.36E-01	1
WARS	410981	215	34	1239592	315416	0	4	1	0	47	43	17641580	3.36E-01	1
PSG1	394104	283	-23	1106092	315416	0	5	0	0	50	79	23984076	3.36E-01	1
RFC2	997452	202	51	930228	252048	0	4	0	0	17	16	5434696	3.36E-01	1
P2RX2	819418	332	35	1291924	342116	0	6	0	0	44	63	20559712	3.36E-01	1
RPP40	119393	391	24	957640	242792	0	3	0	0	15	7	6717008	3.36E-01	1
CAPZA1	560292	325	23	774300	183696	0	3	0	0	50	45	22664384	3.36E-01	1
PLCXD3	219266	928	-15	823428	218940	0	4	2	0	45	53	14228252	3.36E-01	1
TPP2	195408	521	20	3252772	872912	0	16	4	0	24	23	8781452	3.36E-01	1
CDK7	423022	261	47	918836	239944	0	4	0	0	50	52	19542976	3.36E-01	1
MTRF1L	159618	677	-1	975796	270560	0	2	0	0	50	65	20954516	3.36E-01	1
LZTFL1	252915	426	42	803848	195800	0	3	1	0	50	70	22768692	3.37E-01	1
C11orf24	833157	233	52	1083308	367036	0	7	0	0	50	61	19015740	3.37E-01	1

TYR	28803	973	-28	1360632	356712	0	8	1	0	50	89	23440108	3.37E-01	1
FAM46A	121389	396	-18	1112856	315060	0	4	0	0	50	35	19326172	3.37E-01	1
TMOD1	523548	420	35	940908	250980	0	3	0	0	50	62	22763352	3.37E-01	1
ZCCHC3	1010597	348	24	983628	316128	0	7	3	0	9	7	2560352	3.37E-01	1
PFN4	269570	385	38	340336	93272	0	1	1	0	14	14	3955872	3.37E-01	1
TRPV5	294033	676	2	1857964	542900	0	7	1	0	49	57	17327232	3.37E-01	1
HTRA2	1019591	315	50	1103956	396584	0	6	3	0	39	63	16896116	3.37E-01	1
KLC3	1280459	215	23	1269852	401568	0	4	1	0	50	68	21192680	3.37E-01	1
TAX1BP1	393690	180	16	2101824	497688	0	7	2	0	50	45	22689660	3.37E-01	1
MXD1	844060	221	37	585264	147384	0	3	0	0	50	49	17507368	3.37E-01	1
ZRANB3	542328	676	16	2821300	724460	0	9	2	0	41	42	16635880	3.37E-01	1
EGF	215298	503	9	3148108	820580	0	13	5	0	31	39	14122164	3.38E-01	1
SERPINA1(	203903	744	-1	1129588	313280	0	3	1	0	50	44	20062024	3.38E-01	1
EFCAB3	344431	362	8	1309368	312924	0	4	0	0	50	49	21352168	3.38E-01	1
PCDH9	19605	831	-11	3089368	890000	0	18	5	0	29	44	15021064	3.38E-01	1
HLCS	425948	486	61	1837672	525812	0	11	2	0	7	10	3456048	3.38E-01	1
ARHGAP5	153921	392	1	3840172	996800	0	11	2	0	49	51	24381372	3.38E-01	1
PGPEP1	1294357	210	39	542188	149876	0	3	0	0	50	67	22067728	3.38E-01	1
LPP	148376	685	6	1537208	465292	0	7	1	0	50	64	25941008	3.38E-01	1
OCIAD2	256817	372	61	427556	96832	0	1	0	0	50	65	20959856	3.38E-01	1
DMRTB1	552240	385	25	834108	279816	0	4	2	0	17	18	7503768	3.38E-01	1
DEFB114	70183	1258	-7	186900	40584	0	1	0	0	50	45	19480676	3.38E-01	1
ZNF207	679038	286	30	1271632	361340	0	5	0	0	23	20	9499860	3.38E-01	1
NHLRC1	487819	397	-1	951588	319332	0	5	1	0	50	54	19544044	3.38E-01	1
IL17A	313277	556	11	391244	119260	0	2	1	0	45	48	18141048	3.38E-01	1
USPL1	373046	369	63	2776800	759348	0	11	2	0	50	59	20908236	3.39E-01	1
GNB4	405715	600	20	895340	235672	0	4	2	0	44	47	14078732	3.39E-01	1
TPMT	487819	397	-1	664296	158064	0	2	0	0	50	54	19544044	3.39E-01	1
KCNMB1	55692	614	41	485228	142756	0	3	0	0	50	44	16236448	3.39E-01	1
TPR	271754	338	-19	6197248	1592744	0	23	4	0	29	35	14089412	3.39E-01	1
PQLC1	361911	444	7	693844	199004	0	2	1	0	50	48	18275616	3.39E-01	1
OR52I2	212267	935	-3	863656	265220	0	3	0	0	50	68	21342556	3.39E-01	1
ZIK1	495857	828	-34	1252408	326096	0	8	0	0	31	36	11096164	3.39E-01	1

MSH4	146770	535	-10	2436464	648988	0	9	1	0	11	19	6012840	3.39E-01	1
CRISP1	70111	1258	-4	672840	158064	0	3	0	0	50	41	17359984	3.40E-01	1
DDX26B	236195	NaN	25	2220016	610184	0	9	1	0	15	32	11175552	3.40E-01	1
ZNF99	83716	1439	-72	2726248	624068	0	14	2	0	13	18	5386280	3.40E-01	1
UNC13C	17740	1075	-30	5760436	1464584	0	19	2	0	1	2	1963696	3.40E-01	1
CASP9	636573	188	34	1061592	310788	0	4	1	0	50	60	20520196	3.40E-01	1
OR5AP2	NaN	NaN	NaN	790320	225348	0	4	1	0	50	117	39870932	3.40E-01	1
IL6	948251	304	14	547172	152368	0	4	0	0	50	72	20119696	3.40E-01	1
ICAM3	1802015	191	21	1354224	431472	0	4	0	0	11	17	6150612	3.40E-01	1
MIDN	2214581	234	29	1140980	387328	0	3	1	0	50	68	20485664	3.40E-01	1
CEP192	326004	417	52	6527972	1791748	0	20	7	0	49	61	22900056	3.40E-01	1
STAG3L4	349765	556	55	397652	103240	0	1	0	0	50	50	14609172	3.41E-01	1
SNTN	155962	905	-12	387328	101816	0	2	0	0	50	65	25062756	3.41E-01	1
HOMEZ	1171456	330	20	1361344	408332	0	6	1	0	50	49	21656192	3.41E-01	1
ZNF148	377619	174	43	2043084	529728	0	9	0	0	19	18	7066244	3.41E-01	1
LRRC14	1755818	291	7	1181920	409400	0	4	0	0	13	17	7333956	3.41E-01	1
LARS	396074	366	11	3126748	776792	0	10	1	0	16	21	10013568	3.41E-01	1
KLHDC8B	1812695	203	45	864368	290140	0	6	1	0	50	80	19955580	3.41E-01	1
RLN2	385172	605	16	567464	144892	0	1	0	0	50	50	21817816	3.42E-01	1
ZNF737	NaN	NaN	NaN	1390892	342472	0	8	3	0	26	43	11919592	3.42E-01	1
PHKG1	738325	642	4	1006768	276968	0	3	0	0	18	12	6589560	3.42E-01	1
SLCO1A2	481259	873	-33	1751520	458172	0	9	2	0	50	57	17101172	3.42E-01	1
TIMP4	348401	238	23	582772	155216	0	2	1	0	6	4	3576020	3.42E-01	1
C2CD3	561035	225	45	4994680	1426136	0	16	1	0	4	2	2649708	3.42E-01	1
UTP11L	445132	232	42	678180	167676	0	1	0	0	50	34	17924600	3.43E-01	1
C16orf72	401555	253	7	696692	200428	0	2	0	0	50	74	26834212	3.43E-01	1
ASNSD1	418427	511	16	1620512	455680	0	6	1	0	50	64	23586780	3.43E-01	1
ST8SIA3	298355	599	9	971524	262016	0	3	0	0	50	58	22855200	3.43E-01	1
DCAF13	430771	673	28	1577436	433608	0	7	1	0	11	7	4066588	3.43E-01	1
CMKLR1	437015	597	27	936992	269848	0	4	1	0	22	26	11070888	3.43E-01	1
HLF	147479	526	-54	749736	211464	0	4	1	0	50	71	16849124	3.43E-01	1
SGIP1	116994	601	-8	2146680	611964	0	8	3	0	50	55	19777936	3.43E-01	1
PLEKHF2	385365	347	60	634748	170524	0	3	1	0	50	53	18794664	3.44E-01	1

GSTK1	379894	399	14	724816	209684	0	2	0	0	50	67	22890088	3.44E-01	1
GPR101	252008	NaN	13	1269140	360628	0	6	1	0	50	69	25151756	3.44E-01	1
ANKLE2	779125	601	32	2370604	691352	0	9	3	0	50	68	23731672	3.44E-01	1
FAM193A	637151	209	46	3151312	846212	0	16	6	0	8	16	4645800	3.44E-01	1
PABPC4	978867	358	29	1710224	471700	0	8	2	0	50	47	19090144	3.44E-01	1
CD7	2856070	193	24	579212	205768	0	3	0	0	50	47	19854120	3.44E-01	1
FUZ	1673246	205	37	1034180	353152	0	6	2	0	13	23	7061972	3.44E-01	1
EIF4H	1022182	190	51	648632	176932	0	3	2	0	10	13	2780716	3.44E-01	1
ALDH18A1	369001	454	26	2036676	586332	0	4	1	0	7	4	3529384	3.44E-01	1
EHMT2	2485614	188	36	3090436	907088	0	11	3	0	43	37	17295904	3.44E-01	1
GADD45G	163029	568	52	408688	116768	0	1	1	0	10	12	4245656	3.44E-01	1
CEMP1	2085452	321	49	686012	188680	0	3	0	0	50	76	21050280	3.44E-01	1
TBC1D4	196931	480	17	3335720	911716	0	12	6	0	25	23	8441828	3.44E-01	1
VAT1	1507064	201	26	974016	309720	0	3	0	0	22	18	8144924	3.44E-01	1
YWHAZ	1074893	213	50	648988	160556	0	4	1	0	50	63	18970884	3.44E-01	1
TULP3	494496	336	35	1318624	358136	0	6	0	0	50	57	21192324	3.44E-01	1
PDCD1	559742	487	29	717340	227840	0	3	1	0	30	65	17928160	3.45E-01	1
SPRY2	44501	729	60	783912	232824	0	4	1	0	50	47	20026780	3.45E-01	1
CSRP2	182028	705	-11	511928	129940	0	2	0	0	50	57	27619548	3.45E-01	1
KHDRBS1	1216050	133	33	1111432	345320	0	9	0	0	9	9	2859036	3.45E-01	1
CNOT10	464002	493	28	1967968	495908	0	5	0	0	21	13	8184796	3.45E-01	1
FST	202521	488	-5	903172	232112	0	3	1	0	11	8	3569256	3.45E-01	1
HDAC1	1427362	210	43	1294060	311144	0	4	1	0	50	69	25771552	3.45E-01	1
ERLIN2	619400	326	9	938416	237452	0	3	0	0	50	74	28852376	3.45E-01	1
PEX13	635177	350	33	1008904	300464	0	2	0	0	19	19	12566444	3.45E-01	1
C5orf56	543074	264	63	338912	80812	0	2	0	0	50	49	15794652	3.45E-01	1
HEATR5A	375544	154	20	4430776	1315064	0	11	1	0	9	6	6246020	3.45E-01	1
TNFRSF10I	694327	213	48	1127808	319332	0	5	2	0	50	54	16389884	3.45E-01	1
KDM5A	305933	480	27	4364916	1168392	0	15	4	0	26	23	9167000	3.46E-01	1
VCX3A	41698	NaN	2	473836	129584	0	2	0	0	50	64	26003308	3.46E-01	1
MME	72464	650	-12	2016384	483804	0	13	2	0	8	16	5186564	3.46E-01	1
ZNHIT2	3267059	207	46	949808	348880	0	5	0	0	34	42	13459292	3.46E-01	1
KRT83	936407	213	42	1256680	362408	0	5	1	0	50	55	23702836	3.46E-01	1

CTH	339263	379	-3	1063016	284800	0	5	1	0	50	50	20384204	3.46E-01	1
CCDC30	644678	461	21	2091144	484872	0	8	0	0	2	0	841940	3.46E-01	1
MSGN1	135007	1245	10	470988	149520	0	2	0	0	50	52	22598524	3.47E-01	1
RAPGEF4	397338	345	16	2687800	697760	0	7	2	0	31	31	12883284	3.47E-01	1
KIF13A	495244	363	1	4704184	1269140	0	15	5	0	48	46	19656540	3.47E-01	1
WBSCR22	1139944	214	53	747956	204700	0	5	0	0	50	75	25067740	3.47E-01	1
CAB39L	373856	343	50	896052	221076	0	3	1	0	50	58	19232544	3.47E-01	1
OR51E2	207755	972	-1	781420	251336	0	6	0	0	50	83	21165624	3.47E-01	1
DHRS3	340106	294	27	755788	236384	0	4	2	0	7	8	2032048	3.47E-01	1
TBX15	87793	927	-4	1262020	356000	0	4	0	0	18	22	6948408	3.47E-01	1
C11orf31	921177	497	8	311144	95764	0	2	0	0	50	51	17208328	3.48E-01	1
GMPPB	1551582	271	48	980068	292988	0	6	0	0	50	72	21923548	3.48E-01	1
OR4M1	275024	858	-57	771096	233892	0	6	2	0	27	46	10634076	3.48E-01	1
DUPD1	231745	298	44	558920	157708	0	4	1	0	21	31	9214704	3.48E-01	1
CST1	160568	881	-48	364188	101460	0	3	0	0	50	62	15884008	3.48E-01	1
MAPK3	NaN	NaN	NaN	1013532	299040	0	6	2	0	50	118	39944624	3.48E-01	1
COQ9	749108	159	39	825564	234960	0	3	0	0	50	52	18373516	3.48E-01	1
TAF6L	2405109	248	44	1532224	508724	0	4	2	0	50	58	19762984	3.48E-01	1
ATG10	156562	717	25	592384	145604	0	1	0	0	50	131	45941444	3.48E-01	1
CA8	206353	885	-14	763620	202920	0	2	0	0	50	65	23190908	3.48E-01	1
PHF20	1341124	136	44	2647216	671060	0	14	3	0	50	67	20965908	3.49E-01	1
DGCR8	587146	218	50	1985768	545392	0	8	2	0	50	52	19020368	3.49E-01	1
MED4	324467	567	34	714848	181204	0	1	0	0	50	44	21492432	3.49E-01	1
EIF4E2	286805	211	36	656464	159488	0	4	2	0	18	21	4682468	3.49E-01	1
C9orf24	478815	302	38	796728	186900	0	4	1	0	49	40	16308004	3.49E-01	1
CCDC117	571394	219	-10	712356	201852	0	3	0	0	50	80	22835976	3.49E-01	1
ANAPC2	1722012	202	36	2071920	620508	0	12	2	0	3	3	2037744	3.49E-01	1
SLC46A2	514712	498	10	1148100	389820	0	6	3	0	9	15	3922764	3.49E-01	1
SIGLEC9	355132	632	1	1149168	365256	0	4	3	0	11	16	4784640	3.49E-01	1
SIRT1	321700	296	-2	1903532	531508	0	8	0	0	12	20	8243536	3.50E-01	1
CCDC73	408431	441	4	2877904	655040	0	8	1	0	50	40	19464656	3.50E-01	1
BBC3	1350139	232	33	794592	57672	0	3	0	0	50	65	19950596	3.50E-01	1
CIB1	876945	154	52	504096	136704	0	2	1	0	19	41	9745856	3.50E-01	1

TCEA2	1092168	208	49	780708	218940	0	5	1	0	50	68	21614896	3.50E-01	1
EDDM3B	956361	577	8	391244	87220	0	3	0	0	50	92	23350396	3.50E-01	1
MAB21L1	NaN	NaN	NaN	892136	261304	0	3	1	0	50	117	39906888	3.50E-01	1
ZCCHC8	866834	187	42	1837316	486652	0	7	0	0	41	31	15207608	3.50E-01	1
DNAJC24	83554	879	-19	404060	93628	0	1	0	0	50	59	21145332	3.51E-01	1
DENND4A	522427	240	42	4943772	1298688	0	14	1	0	2	1	2056968	3.51E-01	1
HMGB4	131426	852	9	477752	123532	0	1	0	0	50	67	21034972	3.51E-01	1
ZNF484	599194	463	39	2224288	525812	0	6	0	0	21	25	11451808	3.51E-01	1
GPR142	362227	433	-8	1126028	370240	0	5	2	0	50	67	25396328	3.51E-01	1
ZDHHC19	1042183	210	51	785336	237808	0	2	1	0	50	66	16491344	3.52E-01	1
NSUN2	253355	585	6	2017096	522608	0	8	1	0	50	62	20875840	3.52E-01	1
MATR3	838943	177	35	2203996	568532	0	9	1	0	50	55	20798588	3.52E-01	1
RHOT2	1207790	194	33	1587404	474904	0	3	1	0	17	19	6751184	3.52E-01	1
TNPO3	821362	361	52	2388404	666076	0	10	3	0	50	81	24169196	3.52E-01	1
MAP3K8	81826	185	39	1201500	327876	0	5	2	0	25	30	9383448	3.52E-01	1
MCOLN1	609287	308	36	1481316	435744	0	8	4	0	9	11	2630484	3.53E-01	1
TTC12	107070	600	13	1865796	488076	0	3	2	0	50	57	22574316	3.53E-01	1
BST1	174344	327	66	828412	227840	0	3	1	0	50	66	21234688	3.53E-01	1
MS4A1	333232	899	13	774300	206124	0	5	0	0	50	69	19779360	3.53E-01	1
FKBP5	668286	238	27	1276616	293344	0	7	0	0	8	15	3761852	3.53E-01	1
TPSD1	1150864	215	36	614456	183340	0	3	1	0	50	52	19119336	3.53E-01	1
STX2	218767	440	-19	846568	207548	0	5	0	0	50	62	20177012	3.53E-01	1
SMOC2	121125	883	-32	1202924	315772	0	7	0	0	50	70	18195872	3.53E-01	1
ARHGEF3	380604	450	43	1641872	428980	0	6	0	0	40	49	18460736	3.53E-01	1
DCAF17	514096	271	38	1365260	361696	0	5	2	0	16	12	5469228	3.53E-01	1
OTUD7B	1115711	451	51	2133864	617304	0	6	4	0	38	43	16687144	3.53E-01	1
ACSF2	1332860	200	23	1590608	447136	0	7	1	0	50	72	24178452	3.53E-01	1
ACTR3C	NaN	NaN	NaN	557852	148096	0	1	1	0	50	117	39793680	3.53E-01	1
TAF4B	173138	460	31	2140272	684588	0	6	3	0	50	62	24315512	3.53E-01	1
FGF1	293119	481	28	408688	103952	0	2	0	0	50	51	19516632	3.54E-01	1
TCFL5	1044503	208	48	1236744	391956	0	7	1	0	50	65	20186624	3.54E-01	1
TAB2	431448	177	42	1752588	496620	0	8	1	0	50	49	17618084	3.54E-01	1
MDK	817153	212	45	378072	99324	0	2	0	0	50	67	22436900	3.54E-01	1

RHOBTB2	740323	241	48	1908516	553224	0	4	1	0	41	36	17891492	3.54E-01	1
RGS6	101461	823	26	1280176	303668	0	5	2	0	17	18	6940576	3.54E-01	1
MLLT4	138570	643	-2	4804220	1311148	0	13	2	0	2	2	2404780	3.54E-01	1
[illegible]	1993535	162	27	2222508	574584	0	10	1	0	20	37	10243188	3.54E-01	1
ZNF80	410007	614	10	699540	178356	0	5	1	0	50	78	21467868	3.54E-01	1
SLC1A6	961949	546	4	1395164	453544	0	5	0	0	5	1	1464940	3.54E-01	1
FHIT	36866	1126	5	394448	105376	0	4	1	0	8	11	3150244	3.54E-01	1
CTNNA1	1432876	380	39	2342124	636528	0	5	0	0	7	7	3775380	3.54E-01	1
GJD4	153189	598	-2	883236	309720	0	4	1	0	50	45	21159216	3.54E-01	1
SGOL1	100093	761	9	1487368	366680	0	7	1	0	50	76	28594988	3.55E-01	1
ZNF57	1119793	197	35	1437172	357068	0	5	0	0	50	56	20657968	3.55E-01	1
OR8K3	10317	969	-43	772164	228552	0	7	2	0	8	13	3063024	3.55E-01	1
AKAP5	670466	221	54	1078680	292632	0	4	0	0	50	68	23232204	3.55E-01	1
HDLBP	1287793	317	29	3285168	891780	0	13	2	0	35	32	14161680	3.55E-01	1
ITIH1	1193949	272	49	2360992	652904	0	8	2	0	50	63	22512372	3.55E-01	1
OAS1	621550	527	22	1201144	313280	0	5	1	0	50	61	19678968	3.55E-01	1
[illegible]	NaN	NaN	NaN	2295844	747956	0	11	2	0	50	118	40393540	3.55E-01	1
ERC1	516677	470	25	2906028	745464	0	6	1	0	22	19	9249948	3.55E-01	1
EIF5	941484	175	37	1144896	277680	0	4	0	0	50	68	23528752	3.55E-01	1
SLC26A7	79500	987	-11	1768608	472056	0	5	3	0	27	39	13485992	3.56E-01	1
OTX2	191584	1012	-7	744040	219296	0	3	1	0	50	43	20366760	3.56E-01	1
DNAH11	118223	671	8	11752272	3091860	0	46	13	0	50	71	25857348	3.56E-01	1
[illegible]	65396	NaN	34	2536856	766468	0	7	0	0	5	3	3156296	3.57E-01	1
ZNF286A	730837	431	-16	1364192	325384	0	3	0	0	14	14	6050932	3.57E-01	1
SAT2	2069567	213	34	452476	116768	0	3	0	0	50	68	18449700	3.57E-01	1
[illegible]	30244	1055	-70	209684	57316	0	2	0	0	50	78	14431172	3.57E-01	1
NPHS2	585744	613	-14	968320	294056	0	4	1	0	50	44	17868352	3.57E-01	1
PGBD1	273319	502	35	2086516	538628	0	9	0	0	7	3	2515496	3.57E-01	1
MBNL2	110548	419	23	1036672	292988	0	3	1	0	50	50	20472136	3.57E-01	1
RNF128	149556	NaN	17	1423288	412604	0	6	2	0	50	83	20519128	3.57E-01	1
CDH11	23628	1085	-56	2030268	566040	0	14	6	0	16	27	6639400	3.57E-01	1
CCL13	40425	871	-32	258456	69420	0	2	0	0	50	85	19967328	3.57E-01	1
LIM2	368509	610	-9	549308	159844	0	1	1	0	50	39	14321524	3.57E-01	1

TMCC1	614377	357	40	1640804	467428	0	7	1	0	41	53	20666156	3.57E-01	1
MEGF9	551753	185	15	1600576	479888	0	6	0	0	17	11	7114304	3.58E-01	1
HMGCLL1	28261	691	-14	955504	271628	0	4	1	0	39	66	19980856	3.58E-01	1
USF2	767246	472	7	892492	257744	0	2	0	0	50	41	16380272	3.58E-01	1
TXNIP	739922	172	39	1008548	277324	0	13	4	0	1	6	833752	3.58E-01	1
ABHD15	802576	353	23	1121400	387684	0	5	1	0	50	56	21184136	3.58E-01	1
ZNF683	1021101	217	22	1252764	391956	0	4	0	0	6	1	1611612	3.58E-01	1
PDP1	319459	423	5	1499116	420080	0	5	1	0	50	50	18608476	3.58E-01	1
RHOH	486675	348	48	473124	142044	0	3	1	0	50	51	20582496	3.58E-01	1
DKKL1	2183690	309	30	608404	189392	0	3	1	0	50	56	21603148	3.58E-01	1
ISM1	117713	704	-35	1187616	325740	0	4	0	0	15	30	8991492	3.58E-01	1
WNT16	97501	535	-14	989680	269492	0	4	0	0	18	29	9770064	3.58E-01	1
ZCCHC14	587162	363	21	2377368	719832	0	5	2	0	50	51	23515580	3.58E-01	1
PABPC3	436184	650	30	1564620	462444	0	5	2	0	21	18	6334308	3.58E-01	1
TAGLN3	262689	336	14	526524	131364	0	2	0	0	50	44	19588188	3.59E-01	1
JAK3	1216364	212	37	2840880	861876	0	10	4	0	41	31	13971220	3.59E-01	1
USP29	311003	924	-64	2348888	612676	0	13	0	0	10	8	4126040	3.59E-01	1
LIPG	243067	352	56	1301180	342472	0	5	0	0	50	43	14948084	3.59E-01	1
IL22RA2	124672	436	50	706660	164828	0	3	0	0	50	62	25049940	3.59E-01	1
PRR14	2255010	158	60	1436460	490212	0	5	2	0	50	74	23170972	3.59E-01	1
SMAD4	156084	422	41	1428984	385548	0	7	1	0	50	69	24802164	3.59E-01	1
NOP2	2764720	179	42	2055900	600216	0	7	3	0	32	46	13936332	3.59E-01	1
POLE	724450	495	35	5923484	1611256	0	22	5	0	17	23	8788572	3.59E-01	1
TICAM1	765978	187	52	1713072	571380	0	8	1	0	44	54	16628048	3.59E-01	1
SSPO	455147	615	28	13065556	3884672	0	33	7	0	16	20	9253508	3.59E-01	1
ZP2	402663	575	41	1947320	521896	0	6	3	0	50	69	22447580	3.59E-01	1
SERTAD1	1010633	202	34	566396	195088	0	2	0	0	50	62	18103668	3.60E-01	1
PRKAR2B	231025	644	17	1092564	292632	0	5	0	0	50	48	21470716	3.60E-01	1
ATP12A	467468	619	40	2697412	747956	0	15	5	0	50	82	24130392	3.60E-01	1
NAA30	150623	939	16	903172	275900	0	2	0	0	50	49	19772596	3.60E-01	1
DAZAP2	494861	194	40	792456	167676	0	3	0	0	50	55	17528372	3.60E-01	1
COL14A1	262056	839	-5	4645800	1312572	0	21	3	0	2	5	1924180	3.60E-01	1
CDR1	18661	NaN	-31	674620	168032	0	4	1	0	50	103	19610260	3.60E-01	1

SESN1	385493	465	26	1446784	361340	0	9	2	0	11	13	5118924	3.60E-01	1
TROVE2	288840	433	-13	1449276	374868	0	5	0	0	14	11	6525124	3.60E-01	1
WDFY1	307044	251	22	1095768	270204	0	2	0	0	50	41	23605648	3.60E-01	1
SULT2A1	881316	336	39	762908	174796	0	4	0	0	50	70	22504896	3.60E-01	1
COL11A1	37620	742	-20	4793896	1445360	0	24	4	0	19	25	8389496	3.60E-01	1
ODF3L1	569836	231	44	694556	199360	0	3	1	0	50	44	20007912	3.60E-01	1
SMC5	196227	395	-11	2922404	710932	0	12	2	0	32	31	14226828	3.61E-01	1
BCL10	348743	384	3	586688	171592	0	3	0	0	50	52	20151380	3.61E-01	1
OR9Q2	637499	828	-25	770384	243148	0	4	2	0	50	51	16635524	3.61E-01	1
HIST1H4H	390169	544	57	248844	86508	0	2	0	0	50	61	13862640	3.61E-01	1
UBE2B	929843	170	46	411180	100392	0	2	0	0	50	67	23256412	3.61E-01	1
TAF9B	549810	NaN	14	652548	182628	0	2	0	0	50	83	23441888	3.61E-01	1
ATF7	1542915	154	52	1247780	385192	0	4	1	0	41	37	15406256	3.61E-01	1
TCHP	927742	323	37	1325744	322180	0	9	0	0	3	16	3707740	3.61E-01	1
RGS16	597206	812	-3	523320	144180	0	3	0	0	50	60	22449360	3.62E-01	1
ETFB	368509	610	-9	897832	298328	0	3	2	0	50	39	14321524	3.62E-01	1
UPF3A	612511	283	46	1239592	329300	0	3	0	0	50	47	21694284	3.62E-01	1
SPINK7	72574	418	-9	232824	57672	0	1	0	0	50	57	21768688	3.62E-01	1
ABCC4	200377	596	30	3466728	960132	0	10	1	0	6	6	3771820	3.62E-01	1
IL17RC	950574	170	54	1992176	624424	0	6	2	0	39	56	16713132	3.62E-01	1
IDH3G	2103463	NaN	30	1066932	311856	0	2	0	0	16	21	8064112	3.62E-01	1
RAB1B	2133752	236	53	525100	145604	0	1	0	0	50	77	24481764	3.62E-01	1
OR1L3	409923	553	12	800288	239944	0	3	0	0	50	56	16898964	3.62E-01	1
MTHFD2L	180212	330	15	891424	255608	0	4	0	0	50	52	20371744	3.62E-01	1
LRRC39	294945	518	11	881100	229620	0	2	0	0	50	53	21667940	3.63E-01	1
MRPL32	163131	744	-6	479532	136704	0	2	1	0	45	40	17682520	3.63E-01	1
NFATC1	213864	598	6	2440380	781776	0	11	2	0	16	13	6597036	3.63E-01	1
PSG7	282702	238	-37	1064084	305092	0	7	2	0	22	51	10502000	3.63E-01	1
GLRX2	288840	433	-13	526880	149164	0	2	0	0	50	38	18047420	3.63E-01	1
OR10T2	135429	902	5	764332	242792	0	4	1	0	50	50	16503092	3.63E-01	1
OR7D4	398878	582	-50	761484	241368	0	5	0	0	50	116	31105500	3.63E-01	1
HK3	1129866	222	54	2303676	733716	0	9	3	0	21	43	11441128	3.63E-01	1
WBP1	1069803	343	45	669992	207904	0	2	1	0	50	68	19848780	3.63E-01	1

RPS19	1360684	454	23	380920	108224	0	1	0	0	50	69	18698900	3.64E-01	1
BTNL3	893236	602	29	1196872	329300	0	3	2	0	50	80	23793260	3.64E-01	1
KCNIP1	52578	618	31	702388	158420	0	3	1	0	4	2	1707732	3.64E-01	1
LONRF3	571717	NaN	27	1928452	551444	0	3	0	0	20	21	11136392	3.64E-01	1
TRMT5	216411	578	-1	1302960	350304	0	6	1	0	50	47	18400216	3.64E-01	1
ARHGAP2'	754492	194	21	1420796	402280	0	5	0	0	50	57	21830632	3.64E-01	1
OTUD4	192885	1089	33	2716992	734784	0	10	1	0	28	33	12383460	3.64E-01	1
STK31	367849	441	14	2712008	658600	0	12	2	0	48	49	18565400	3.64E-01	1
SPPL2A	329469	225	20	1365260	363832	0	3	1	0	50	38	22235048	3.64E-01	1
PIGF	603816	252	35	635460	177288	0	2	0	0	50	45	22018244	3.64E-01	1
GKAP1	468451	384	5	988612	234248	0	4	1	0	50	47	21887948	3.64E-01	1
HIST1H3D	398813	636	53	333216	112140	0	2	1	0	50	45	13638004	3.64E-01	1
FIP1L1	120581	470	32	1593812	418300	0	6	1	0	50	55	24278132	3.64E-01	1
TRMT6	301031	346	13	1297620	340692	0	5	2	0	35	39	14251392	3.64E-01	1
YWHAG	850568	195	53	629408	169456	0	4	0	0	50	69	21293072	3.64E-01	1
SNX29	477277	758	18	1127808	302244	0	5	1	0	50	40	17715272	3.64E-01	1
TMTC2	61571	1375	-11	2138848	589892	0	7	2	0	21	26	10906060	3.64E-01	1
AIPL1	249769	359	6	976152	280884	0	3	1	0	50	56	19275264	3.64E-01	1
ZNF827	110708	1103	30	2745116	766468	0	8	2	0	50	47	22796104	3.65E-01	1
KNDC1	318075	452	-11	4384852	1347104	0	12	2	0	2	2	2177296	3.65E-01	1
KTN1	687929	308	23	3705604	840872	0	10	1	0	9	21	8278780	3.65E-01	1
ZNF423	105426	876	-16	3293712	857604	0	17	5	0	21	28	8882912	3.65E-01	1
GLA	374364	NaN	37	1117128	288360	0	3	0	0	25	27	11161312	3.65E-01	1
TIMD4	135146	1235	16	968320	282308	0	5	2	0	11	12	3809200	3.65E-01	1
MSL1	1573630	202	28	919192	242792	0	3	0	0	50	61	21397024	3.65E-01	1
RAB11A	701874	296	36	561412	150944	0	1	0	0	50	58	21529100	3.65E-01	1
PHLDA1	604083	438	17	988968	305448	0	3	0	0	50	67	21773672	3.65E-01	1
CETN3	83403	824	19	455680	99680	0	2	0	0	50	53	21560072	3.65E-01	1
C19orf53	767214	289	6	257032	74048	0	2	1	0	16	26	6349972	3.65E-01	1
TAAR1	125852	605	19	865436	223924	0	4	2	0	6	9	3325396	3.65E-01	1
DOPEY1	151511	697	7	6353532	1705596	0	19	5	0	47	71	26472160	3.65E-01	1
DAND5	1886809	335	40	454968	160200	0	1	0	0	50	62	18190176	3.65E-01	1
CCDC120	833689	NaN	41	1608052	572804	0	5	1	0	50	74	21714932	3.65E-01	1

IARS2	651419	613	-1	2635468	704168	0	6	0	0	4	0	1641516	3.66E-01	1
CCDC85A	63834	739	-15	1393028	403348	0	5	1	0	30	46	16987252	3.66E-01	1
APOBEC3I	1386387	372	71	1011752	253828	0	3	1	0	50	61	16059872	3.66E-01	1
ZBTB2	337954	409	23	1293704	364900	0	4	0	0	50	37	21182356	3.66E-01	1
LPL	203407	316	8	1237812	326808	0	4	1	0	50	51	20141412	3.66E-01	1
SLMO2	948552	126	25	509436	136704	0	2	1	0	42	78	18923892	3.66E-01	1
CXADR	100910	366	-17	939128	259168	0	3	1	0	28	24	10681780	3.66E-01	1
DNAJC15	56984	249	51	395516	109648	0	2	0	0	50	57	19444720	3.66E-01	1
DALRD3	1799657	241	41	1354580	439660	0	5	3	0	27	51	14314048	3.67E-01	1
HRNR	486922	468	25	6851932	2291216	0	29	4	0	8	13	5121772	3.67E-01	1
SMR3B	264684	915	25	195800	69064	0	1	0	0	50	42	17431540	3.67E-01	1
NT5C1B	39949	1075	-7	1553228	442864	0	5	1	0	26	23	10458924	3.67E-01	1
LRIG1	82885	629	14	2762560	821648	0	11	4	0	50	50	17681096	3.68E-01	1
RGR	216056	869	-4	750092	226060	0	4	1	0	50	58	19593172	3.68E-01	1
PRRT2	NaN	NaN	NaN	830548	274832	0	4	0	0	50	116	39920416	3.68E-01	1
FAM83E	1597109	242	31	1147744	406196	0	6	1	0	50	57	20781500	3.68E-01	1
OR4C13	11675	1171	-40	773944	223568	0	4	1	0	3	3	1385908	3.68E-01	1
FAM24A	290558	619	14	275900	72268	0	2	0	0	18	28	7230360	3.68E-01	1
NUDCD2	226025	696	12	410468	110716	0	1	1	0	50	71	18478892	3.69E-01	1
MBTPS1	522600	435	8	2712008	751516	0	10	3	0	8	18	6329680	3.69E-01	1
ADAMTS9	90552	783	-10	5058760	1312928	0	23	5	0	29	43	14023552	3.69E-01	1
ADH4	166160	760	1	979000	278036	0	2	1	0	31	27	12969080	3.69E-01	1
XPO5	1249985	194	51	3148820	844432	0	10	2	0	38	36	15344312	3.69E-01	1
C3orf67	146530	837	19	1478468	379852	0	6	0	0	41	41	18636244	3.69E-01	1
IL1RAP	139316	398	0	2099332	561056	0	5	1	0	50	49	23900416	3.69E-01	1
MTRF1	309426	300	61	1177648	289784	0	5	0	0	50	68	21950960	3.69E-01	1
EPHA7	18346	1054	-25	2589188	679960	0	10	2	0	25	61	15716688	3.69E-01	1
KIAA1328	99312	893	-1	1497692	394804	0	3	0	0	50	54	22392400	3.69E-01	1
POLR2D	588745	248	39	379140	93984	0	2	0	0	50	66	22074848	3.69E-01	1
ZMIZ2	1289776	164	55	2299760	728020	0	10	4	0	47	52	18688220	3.69E-01	1
NUP43	425807	234	39	987900	264864	0	4	2	0	28	20	10059848	3.69E-01	1
RIMKLA	573046	574	22	983628	289428	0	2	0	0	14	10	5975460	3.69E-01	1
UBXN2B	197666	638	26	856892	236740	0	4	1	0	45	47	17427624	3.70E-01	1

EDAR	358833	424	14	1164120	321468	0	5	0	0	32	35	11353552	3.70E-01	1
PARP9	410045	404	35	2250988	585620	0	8	3	0	50	47	20160992	3.70E-01	1
PRDM11	145644	592	14	1312216	358136	0	6	0	0	14	19	8330756	3.70E-01	1
IIST1H2AI	227588	622	29	306516	108936	0	2	1	0	14	21	3578512	3.70E-01	1
RSPRY1	717872	149	48	1510864	395516	0	6	0	0	16	11	4261676	3.70E-01	1
INADL	274287	469	-3	4660396	1292636	0	14	5	0	47	45	18117552	3.70E-01	1
BDH2	350185	454	27	655752	170880	0	2	0	0	29	26	15759408	3.70E-01	1
PPP2R3B	NaN	NaN	NaN	1480960	415808	0	4	2	0	50	118	40061392	3.70E-01	1
TINAGL1	1061386	197	37	1208264	338200	0	4	1	0	50	49	17656888	3.70E-01	1
SP8	109058	730	11	1214316	422928	0	4	0	0	50	79	26321216	3.70E-01	1
RRP1B	892193	157	72	1961916	534000	0	7	2	0	50	49	20805352	3.70E-01	1
GRM2	759916	456	37	2126388	687792	0	8	3	0	50	51	19214744	3.70E-01	1
LMNB1	237464	579	37	1510508	412960	0	4	0	0	15	13	7340008	3.71E-01	1
VSNL1	135361	1255	-2	508368	119616	0	2	0	0	50	39	17853756	3.71E-01	1
TMEM132I	222789	740	-3	2388404	805984	0	10	4	0	31	27	10851236	3.71E-01	1
NARF	2583801	179	20	1310792	347812	0	4	0	0	50	62	26646600	3.71E-01	1
SRA1	706371	319	29	599148	177288	0	2	0	0	50	44	23563640	3.71E-01	1
OR4K17	383274	768	-37	850484	251692	0	3	1	0	49	56	18627344	3.71E-01	1
CYP27C1	354277	226	19	950520	274476	0	3	0	0	31	26	13802476	3.71E-01	1
NRIP2	495532	443	28	713780	213244	0	2	0	0	11	20	7171976	3.71E-01	1
PPFIA1	514596	530	44	3131020	843008	0	11	2	0	28	27	9320080	3.71E-01	1
CORO1B	1392213	215	53	1244220	368460	0	2	2	0	50	41	15455384	3.71E-01	1
MCM9	116680	856	16	1011396	270204	0	6	0	0	50	53	18718480	3.71E-01	1
RBM15	449729	379	28	2363840	780352	0	8	3	0	42	49	16820644	3.71E-01	1
ING4	2816983	210	61	683520	149520	0	3	0	0	28	58	17156708	3.71E-01	1
MMAB	436841	428	38	655396	185120	0	2	1	0	50	62	20971960	3.72E-01	1
PRDX6	426891	390	12	573516	166608	0	4	1	0	33	50	15760120	3.72E-01	1
PDK1	417145	365	23	1135640	309364	0	3	1	0	50	41	20266724	3.72E-01	1
ADAMTS3	70065	633	-15	3146328	809544	0	13	1	0	14	14	7105048	3.72E-01	1
MRPL55	702621	391	5	419724	131364	0	2	0	0	50	46	17014308	3.72E-01	1
PCDHGA8	NaN	NaN	NaN	2308660	733004	0	9	2	0	50	118	40378588	3.72E-01	1
C1QTNF3	273505	817	1	827344	221432	0	2	0	0	50	46	19280248	3.72E-01	1
VAPA	405142	172	42	767180	203632	0	3	0	0	50	45	16608112	3.73E-01	1

ELL3	784327	286	37	1034892	283020	0	3	0	0	17	14	6234272	3.73E-01	1
KLK8	536101	606	25	793524	210396	0	2	1	0	50	50	19945968	3.73E-01	1
RPS6KC1	226813	600	0	2750100	728376	0	14	2	0	29	25	10009652	3.73E-01	1
CAPRIN2	286973	420	-8	2902112	784624	0	9	1	0	13	9	5584928	3.73E-01	1
OR4K2	431654	854	-49	782488	226772	0	7	1	0	50	78	18107940	3.73E-01	1
KIAA0907	1988461	174	32	1562484	465648	0	6	4	0	24	42	9441476	3.73E-01	1
PDCD6	935283	530	28	510148	128516	0	3	0	0	50	64	18094768	3.73E-01	1
GPRASP1	123687	NaN	23	3538996	933788	0	9	1	0	22	14	7859768	3.73E-01	1
FAM131A	1447013	268	44	908156	289072	0	3	1	0	50	77	28073092	3.73E-01	1
RNF10	1583841	209	40	2101112	568888	0	7	2	0	50	66	21210124	3.73E-01	1
MSRA	114315	831	6	629764	163760	0	2	0	0	50	69	19688224	3.74E-01	1
MYLK	284041	449	32	4897492	1370600	0	14	4	0	50	60	22813904	3.74E-01	1
PCDH20	3527	1075	-43	2336784	717696	0	14	5	0	13	22	4734800	3.74E-01	1
NEU2	410526	294	33	931296	292632	0	3	1	0	50	54	19620940	3.74E-01	1
TMEM74	275834	891	-14	758280	226416	0	4	0	0	50	55	18296264	3.74E-01	1
CLSPN	1224684	303	25	3532944	865080	0	12	2	0	49	48	19403068	3.74E-01	1
TAS2R41	371260	379	1	758992	227840	0	3	1	0	50	51	20287016	3.74E-01	1
RNF17	486857	629	29	4284460	1070492	0	15	4	0	24	30	11572136	3.74E-01	1
HIST2H3D	681585	560	28	326096	110716	0	2	1	0	15	26	7902488	3.74E-01	1
MYOCD	113594	848	-54	2512292	718408	0	9	2	0	11	15	4366696	3.74E-01	1
HNRNPC	730651	357	37	803848	209684	0	2	0	0	50	54	23555096	3.74E-01	1
C12orf49	228972	411	-2	540052	137060	0	2	1	0	42	37	17963404	3.74E-01	1
PTDSS1	219234	671	13	1258104	313992	0	7	1	0	50	74	23505256	3.75E-01	1
OAZ2	847146	269	42	489500	137416	0	1	0	0	50	76	26244676	3.75E-01	1
CEPT1	388536	627	27	1072984	297260	0	2	0	0	50	58	29222260	3.75E-01	1
ASB11	201763	NaN	43	929516	264508	0	3	0	0	50	65	24059548	3.75E-01	1
C15orf53	98316	561	20	447492	135636	0	2	0	0	50	51	20027492	3.75E-01	1
APOM	2517236	193	20	497688	131364	0	2	0	0	50	63	27465044	3.75E-01	1
MMP28	647610	144	6	1307588	403348	0	3	1	0	50	44	20788264	3.75E-01	1
GNPTG	1211641	215	40	808832	218584	0	3	1	0	50	64	21318348	3.75E-01	1
RBM47	247828	341	64	1472772	447492	0	10	3	0	11	16	4404076	3.75E-01	1
ZFP57	484408	755	34	1368464	364900	0	5	2	0	50	51	18249272	3.75E-01	1
VARS2	2223866	223	25	2787480	847992	0	11	2	0	50	73	23168480	3.75E-01	1

SAP30	249666	413	23	556784	164116	0	3	0	0	50	49	18449700	3.75E-01	1
NOTCH3	630378	381	11	5794612	1783916	0	12	2	0	5	2	3166264	3.75E-01	1
GIP	793101	174	29	401924	112852	0	2	0	0	50	65	22956304	3.75E-01	1
HSPB11	467936	174	27	394804	93272	0	2	0	0	50	71	22598168	3.75E-01	1
IFNW1	137739	498	1	488788	139196	0	2	1	0	8	11	3212900	3.75E-01	1
RGS1	269711	604	-12	554648	137416	0	2	0	0	50	38	18560772	3.76E-01	1
F7	789162	285	14	1188328	344252	0	7	0	0	37	59	17973016	3.76E-01	1
UBXN11	1021101	217	22	1341764	389464	0	4	1	0	12	5	2705244	3.76E-01	1
OSMR	193286	381	-10	2589188	661804	0	8	2	0	36	28	15880804	3.76E-01	1
PFDN6	1456953	262	30	341760	96120	0	2	0	0	50	71	20945260	3.76E-01	1
EBPL	425192	296	62	521896	156284	0	1	0	0	50	48	18413032	3.76E-01	1
CLEC9A	351158	445	9	654684	146316	0	3	0	0	50	55	20981572	3.76E-01	1
DNAJB12	822407	145	45	1045928	301888	0	3	2	0	50	63	23074140	3.76E-01	1
PIGZ	754196	306	29	1373804	490924	0	2	0	0	46	36	22808920	3.76E-01	1
CYP4Z1	366455	479	4	1338204	332148	0	4	1	0	50	52	19874412	3.76E-01	1
C8orf22	15071	631	-19	224636	55180	0	1	0	0	50	46	19123252	3.76E-01	1
LILRA5	742939	507	-9	838736	242080	0	3	1	0	50	62	17969100	3.77E-01	1
SDPR	158359	484	4	1070136	299040	0	6	0	0	50	57	17303736	3.77E-01	1
CPLX3	764533	195	46	409400	110716	0	3	1	0	11	14	2513716	3.77E-01	1
ABCB6	773232	177	36	2117132	660736	0	6	1	0	12	9	4375240	3.77E-01	1
CTAGE1	162557	417	-9	1881816	508368	0	6	3	0	50	51	20812472	3.77E-01	1
LEMD1	662921	249	41	286224	75828	0	2	0	0	9	16	4452848	3.77E-01	1
FAM81A	613635	188	39	977932	238520	0	2	0	0	50	45	20606704	3.77E-01	1
SLC25A31	220342	437	-6	801356	232468	0	3	1	0	50	46	20279540	3.77E-01	1
WIF1	344729	671	-9	997868	258100	0	3	1	0	50	56	20297696	3.77E-01	1
STX12	726175	202	30	742616	179068	0	3	0	0	50	40	18909296	3.77E-01	1
C11orf58	466700	635	-21	493060	113564	0	2	0	0	50	43	17511996	3.77E-01	1
ZNF33B	204176	890	-35	2038812	474192	0	8	2	0	20	18	5830212	3.77E-01	1
GLIS1	598664	391	27	1530444	493416	0	7	2	0	14	14	5594184	3.77E-01	1
TBC1D5	103389	701	9	2161988	548596	0	7	3	0	50	73	29919308	3.77E-01	1
ADH1B	332785	854	8	961912	279104	0	4	1	0	50	70	20070924	3.77E-01	1
NEDD9	228484	418	30	2171600	616948	0	6	3	0	45	55	22672216	3.77E-01	1
RELL2	771583	353	25	771096	231756	0	3	1	0	50	53	22992260	3.77E-01	1

DEFB135	467502	451	22	199360	54824	0	1	0	0	50	58	22225080	3.78E-01	1
RAD51	1017781	161	47	991460	269848	0	5	0	0	50	49	19609192	3.78E-01	1
DYX1C1	298473	405	1	1218588	272340	0	3	1	0	50	51	20321192	3.78E-01	1
PCNA	497537	385	25	682096	178712	0	4	0	0	6	4	2221084	3.78E-01	1
RAPGEF2	136300	480	3	3860108	1044148	0	14	1	0	6	4	2477760	3.78E-01	1
ACVR1	81110	935	2	1323608	348880	0	5	0	0	30	17	9593844	3.78E-01	1
GAB2	404872	408	19	1719124	490568	0	6	0	0	28	23	12087624	3.78E-01	1
MAGEE2	117293	NaN	-10	1312928	365968	0	4	1	0	50	75	26941724	3.78E-01	1
GLB1L3	270350	623	21	1716632	459952	0	10	1	0	3	2	1141336	3.78E-01	1
LY6G5B	2517236	193	20	505876	149876	0	2	0	0	50	63	27465044	3.78E-01	1
SLC13A3	293732	642	28	1521188	464224	0	6	1	0	34	40	15928864	3.78E-01	1
MYBL1	329076	140	37	1979004	497688	0	6	2	0	50	56	19224712	3.79E-01	1
UPK1A	1162794	390	27	664652	190816	0	2	1	0	50	66	20625572	3.79E-01	1
SSBP1	200522	844	14	402992	101104	0	2	1	0	5	4	1324676	3.79E-01	1
SGTB	414857	242	34	824852	195088	0	3	0	0	50	59	20100116	3.79E-01	1
ARSH	218545	NaN	-4	1433256	403704	0	5	2	0	29	28	10056288	3.79E-01	1
TP53INP2	1047752	147	47	542900	178000	0	2	0	0	50	49	23319068	3.79E-01	1
GNPDA1	696239	321	48	763620	191172	0	3	1	0	50	50	19214032	3.79E-01	1
CATSPERI	394439	483	26	2938068	749736	0	10	2	0	8	10	5757232	3.79E-01	1
EAPP	517394	165	13	759704	178000	0	4	0	0	50	54	17852332	3.79E-01	1
SIX3	133317	566	23	805984	265220	0	4	0	0	50	54	20448284	3.80E-01	1
MSMP	922448	190	48	355644	101460	0	2	0	0	50	73	21892576	3.80E-01	1
ABI1	342602	447	30	1342476	394092	0	5	0	0	50	42	23330104	3.80E-01	1
LRG1	2111443	278	43	835176	286224	0	5	1	0	50	77	21475344	3.80E-01	1
ADCY2	60911	1014	-22	2826640	776792	0	18	7	0	16	53	10642264	3.80E-01	1
GTF2B	284162	537	1	813816	227484	0	2	1	0	50	57	19403424	3.80E-01	1
IFNG	268405	717	-2	445712	102172	0	2	0	0	50	64	22567908	3.80E-01	1
FPR2	436280	690	-24	857248	274832	0	5	2	0	50	53	16569308	3.80E-01	1
INO80E	NaN	NaN	NaN	618728	196156	0	3	0	0	50	116	39841740	3.80E-01	1
PTTG1	197504	619	30	525812	148096	0	2	0	0	10	11	3393392	3.80E-01	1
OR6B1	222080	812	-11	767180	232468	0	5	2	0	11	16	3490580	3.80E-01	1
GLIPR1L1	112974	692	-33	615880	153080	0	2	0	0	50	102	25765500	3.81E-01	1
SLC13A2	1517567	225	24	1609476	496620	0	4	2	0	50	49	17603488	3.81E-01	1

OSM	548674	211	43	625848	195444	0	3	0	0	50	54	19921048	3.81E-01	1
GAN	316209	160	14	1546108	412604	0	5	1	0	50	42	19446500	3.81E-01	1
FAM198B	123627	849	-14	1378788	412248	0	7	0	0	33	46	16700672	3.81E-01	1
PCGF1	1019591	315	50	695980	175508	0	2	0	0	50	69	21835972	3.81E-01	1
UBD	403531	798	18	417588	118548	0	2	1	0	8	6	2025640	3.81E-01	1
GNG2	204232	471	27	190460	48772	0	1	0	0	50	49	18536564	3.81E-01	1
PRR3	2080582	271	32	477040	141332	0	1	1	0	25	33	11740168	3.81E-01	1
TAL2	215119	773	-2	270916	80456	0	1	0	0	50	37	21261388	3.81E-01	1
CD22	767113	472	9	2189044	583484	0	6	1	0	32	25	10097228	3.81E-01	1
IL23A	2869106	184	52	477396	144180	0	1	0	0	50	84	25337232	3.81E-01	1
VTA1	169555	426	-15	808120	210752	0	4	0	0	50	50	19754084	3.82E-01	1
SPG20	137590	850	-16	1695628	475616	0	6	2	0	25	38	11906420	3.82E-01	1
TMEM8B	847663	227	54	1335000	426132	0	5	1	0	50	66	20654408	3.82E-01	1
PTMS	2816186	232	48	286580	60520	0	1	1	0	5	24	5354240	3.82E-01	1
MXRA7	913566	191	26	559276	171236	0	3	0	0	50	73	22697492	3.82E-01	1
RBL1	822888	309	42	2782140	742260	0	10	3	0	36	45	15768664	3.82E-01	1
CYP7B1	8894	1018	22	1299044	346744	0	4	1	0	50	55	21157080	3.82E-01	1
CAND1	237289	810	-13	3131376	872556	0	12	3	0	45	62	16895760	3.82E-01	1
COL8A1	226950	419	-2	1766116	629408	0	3	1	0	50	45	20219020	3.82E-01	1
ADAMTSL	112410	888	-7	4369544	1175512	0	16	5	0	28	43	14694968	3.82E-01	1
TRMU	493681	260	39	1103600	291208	0	2	0	0	50	40	19939560	3.82E-01	1
CHAF1A	2078211	278	37	2458536	669636	0	8	3	0	50	53	23615972	3.82E-01	1
MESDC2	265620	516	-2	596656	166964	0	3	1	0	50	59	19197300	3.82E-01	1
C1orf131	383866	603	14	767536	204344	0	2	0	0	50	71	23732384	3.82E-01	1
ACIN1	1261207	185	41	3504464	1031332	0	15	2	0	2	2	1796020	3.82E-01	1
IGF1	186749	752	-3	581348	143824	0	2	0	0	50	44	21730240	3.82E-01	1
ZFP41	1657385	389	19	505876	131720	0	3	0	0	50	72	20208696	3.83E-01	1
BCL9	NaN	NaN	NaN	3502684	1095056	0	14	4	0	50	120	40740640	3.83E-01	1
EPHB2	568787	464	29	2524040	705592	0	8	2	0	36	44	17671128	3.83E-01	1
CCDC43	1297937	254	27	588468	151656	0	1	0	0	50	53	19790752	3.83E-01	1
NRSN1	349698	825	-21	491636	144892	0	3	0	0	50	61	17342184	3.83E-01	1
MAGEC1	17504	NaN	-11	2828420	839092	0	20	3	0	3	12	2826996	3.83E-01	1
ACOT6	606300	169	39	529372	143468	0	2	0	0	50	45	20608840	3.83E-01	1

CCT5	623675	325	5	1400860	378428	0	6	1	0	50	72	28009724	3.83E-01	1
GCM1	414446	329	20	1123892	293344	0	4	0	0	13	10	6164496	3.83E-01	1
ASB6	794813	198	40	1099328	274120	0	4	0	0	43	45	15816012	3.83E-01	1
CYP3A43	1535089	438	36	1336424	333928	0	6	1	0	19	25	6683188	3.83E-01	1
WNT3A	688142	391	0	891068	254896	0	2	0	0	50	41	18571096	3.83E-01	1
PLRG1	59883	1034	20	1356004	356000	0	5	0	0	50	39	19125032	3.83E-01	1
NCALD	362848	629	41	512996	121396	0	1	1	0	50	62	19739844	3.83E-01	1
NRG1	28018	952	-32	3055192	875048	0	11	0	0	2	2	1568536	3.83E-01	1
CYB5R1	421206	389	30	780352	236384	0	3	2	0	32	42	11558252	3.84E-01	1
CSMD3	9197	1295	-29	9668604	2579220	0	60	9	0	1	9	2892500	3.84E-01	1
PRKAA1	211936	496	-11	1493064	389820	0	6	0	0	14	10	5635836	3.84E-01	1
NXPH2	69057	672	-4	665364	187968	0	3	0	0	50	69	21528388	3.84E-01	1
C8orf4	10573	360	3	270916	74048	0	1	0	0	50	35	20692500	3.84E-01	1
PKIB	139315	1035	-16	205768	55892	0	1	0	0	50	50	20414820	3.84E-01	1
L3MBTL2	1301913	179	53	1870780	494840	0	5	1	0	37	34	14111128	3.84E-01	1
MARK1	544426	520	-9	2064088	558920	0	10	2	0	50	56	17907868	3.84E-01	1
MAGEA6	203033	NaN	-1	782844	230688	0	3	0	0	50	57	23840964	3.84E-01	1
KLC4	1239021	301	42	1685304	494484	0	4	0	0	3	9	3514432	3.84E-01	1
EIF3D	1184089	359	38	1462092	356712	0	5	0	0	16	20	6049864	3.84E-01	1
CLDN6	1443513	234	49	527592	184764	0	1	0	0	50	48	18953440	3.85E-01	1
AMZ2	764688	237	11	940552	239588	0	5	0	0	50	65	21730952	3.85E-01	1
NEUROD4	115336	828	15	830904	237096	0	3	0	0	50	48	19378504	3.85E-01	1
FAM50A	2277674	NaN	40	935212	207548	0	2	0	0	50	46	20570392	3.85E-01	1
MAP3K2	435982	175	26	1632260	420436	0	5	2	0	43	69	20985132	3.85E-01	1
PPP3R2	112839	1008	-11	444644	114988	0	2	0	0	50	58	22010056	3.85E-01	1
KLHL5	497247	209	46	1938776	526168	0	5	0	0	10	10	4782504	3.85E-01	1
SLC40A1	446631	511	-2	1433612	428980	0	5	2	0	46	63	19923896	3.85E-01	1
ZNF700	719617	678	-1	1934860	458528	0	9	3	0	19	18	5988632	3.86E-01	1
CGGBP1	123235	894	-3	418656	123888	0	2	0	0	50	48	17285580	3.86E-01	1
PSMC6	314095	313	11	1076544	273408	0	5	1	0	50	46	19692852	3.86E-01	1
ATF1	665195	250	42	695268	201852	0	2	1	0	17	22	7365996	3.86E-01	1
TCEAL3	490345	NaN	31	517268	131008	0	1	0	0	50	66	22114364	3.86E-01	1
AEN	393645	605	14	812392	244928	0	4	1	0	50	62	21951672	3.86E-01	1

SLC10A1	610490	333	47	874336	264152	0	2	1	0	50	50	20003640	3.86E-01	1
MEI1	1452842	180	49	3261672	953724	0	8	2	0	25	27	10904992	3.86E-01	1
WIPF2	1491292	165	30	1071916	370952	0	6	1	0	16	39	10107908	3.86E-01	1
PDCD4	340524	254	9	1243152	326808	0	5	1	0	50	47	22267800	3.86E-01	1
CYB5B	1161561	NaN	46	399432	103596	0	2	0	0	50	61	20730592	3.86E-01	1
MIER1	331801	635	-9	1652552	379852	0	10	0	0	9	7	2520124	3.86E-01	1
CYP3A5	1395063	430	34	1312928	352084	0	7	3	0	29	34	10155968	3.86E-01	1
TMED7	NaN	NaN	NaN	564260	165184	0	3	0	0	50	116	39810768	3.86E-01	1
RWDD2A	153997	734	6	749380	193664	0	2	0	0	50	75	24558660	3.86E-01	1
AKR1C2	343868	608	-13	892492	238520	0	2	1	0	50	33	15973720	3.86E-01	1
DPM1	386595	367	36	687792	182628	0	3	0	0	50	59	21407704	3.86E-01	1
RBM28	906031	255	22	2008908	503028	0	6	2	0	50	54	19048492	3.87E-01	1
ZBED2	188186	406	-14	549664	152012	0	2	0	0	50	47	21927820	3.87E-01	1
DEFB116	562665	594	-29	269136	64080	0	1	0	0	50	50	17157064	3.87E-01	1
LRRC7	298237	695	-26	3967264	1065152	0	21	7	0	14	30	7398036	3.87E-01	1
PCDHGC3	665835	568	9	2401932	779640	0	7	0	0	7	4	3452488	3.87E-01	1
USMG5	545643	147	45	160556	41296	0	1	0	0	50	60	18601000	3.87E-01	1
SAE1	1351246	165	36	998936	226060	0	5	2	0	11	18	6484896	3.87E-01	1
GJD2	521214	306	-1	794236	241724	0	4	1	0	50	69	23359652	3.87E-01	1
TRAM2	421188	378	33	971168	260236	0	5	0	0	50	59	19057036	3.87E-01	1
C22orf29	NaN	NaN	NaN	882168	287292	0	3	2	0	50	118	39932876	3.88E-01	1
FBXO42	788646	186	42	1807056	531864	0	12	4	0	20	31	7497716	3.88E-01	1
KIF15	434707	426	26	3693500	902104	0	14	1	0	21	15	8254928	3.88E-01	1
NKAP	772178	NaN	38	1076188	290852	0	5	1	0	50	59	22592472	3.88E-01	1
CSDE1	600480	472	3	2199012	589536	0	7	1	0	28	25	9556108	3.88E-01	1
DNAJB6	318088	174	22	888220	233180	0	3	2	0	12	15	6298352	3.88E-01	1
C10orf113	NaN	NaN	NaN	436812	108936	0	1	0	0	50	116	39754520	3.88E-01	1
IRF7	1774224	236	57	1295840	420436	0	4	0	0	4	3	2271636	3.88E-01	1
EMP2	228675	175	8	439660	115700	0	2	1	0	50	56	20127528	3.88E-01	1
VAMP3	364696	422	22	271628	69064	0	1	0	0	50	53	25431216	3.88E-01	1
PSMD4	1256374	184	31	979356	272340	0	4	1	0	50	80	21461104	3.89E-01	1
LIPE	704668	235	34	2624788	864368	0	10	4	0	29	30	11887908	3.89E-01	1
C12orf43	1225663	380	40	678180	187968	0	6	0	0	9	17	4080828	3.89E-01	1

KIF1B	712477	306	25	5904616	1554296	0	16	4	0	18	29	11993996	3.89E-01	1
VPS45	1181361	309	32	1488792	400500	0	3	0	0	8	6	5439680	3.90E-01	1
PDE6H	380047	476	8	224280	57672	0	1	0	0	50	53	20585700	3.90E-01	1
TMEM63B	1267618	203	51	2165904	601284	0	6	0	0	35	27	14162036	3.90E-01	1
DNAJB4	373296	256	1	859740	231756	0	2	0	0	50	64	25505620	3.90E-01	1
HNRNPH1	1600541	229	28	1197584	297616	0	5	0	0	50	69	21106528	3.90E-01	1
AKR7L	799326	448	35	904240	185120	0	2	0	0	50	58	24547268	3.91E-01	1
PARD6B	480540	161	43	939484	264152	0	4	0	0	50	41	16432248	3.91E-01	1
MYL6B	2878044	171	40	552156	147384	0	1	1	0	42	54	17538340	3.91E-01	1
TRIM27	161027	558	30	1318268	358492	0	5	2	0	50	63	22594608	3.91E-01	1
HTR4	89834	578	10	1235320	350660	0	5	1	0	50	61	24091944	3.91E-01	1
TMEM167B	989913	215	18	196512	54468	0	1	0	0	50	45	17234672	3.91E-01	1
IL11RA	731413	298	51	1069424	339268	0	3	1	0	50	42	16261368	3.91E-01	1
ZNF300	944830	396	32	1609476	401568	0	4	0	0	41	29	14463924	3.91E-01	1
PPIG	345417	404	38	2016028	454256	0	8	1	0	50	58	21563632	3.92E-01	1
UBE3B	430972	428	37	2766832	769316	0	11	5	0	50	70	22063100	3.92E-01	1
CD34	320873	450	22	980068	304736	0	3	2	0	50	92	29080216	3.92E-01	1
MAN1A2	358073	526	6	1661096	447136	0	6	2	0	50	50	19700328	3.92E-01	1
FAM9C	209806	NaN	58	468140	96832	0	2	1	0	50	63	24610280	3.92E-01	1
TAAR9	178165	618	22	868284	249912	0	2	0	0	42	38	17775792	3.92E-01	1
PROX1	347543	799	-22	1867932	513708	0	11	4	0	50	51	15458588	3.92E-01	1
ADH1A	250334	786	3	969032	271984	0	4	0	0	50	52	20458608	3.92E-01	1
HAPLN2	2049505	180	37	843008	270916	0	3	1	0	50	64	19075904	3.93E-01	1
PAF1	1748498	252	32	1424712	335352	0	5	1	0	27	41	14226472	3.93E-01	1
CYP2C18	250078	725	1	1274124	333216	0	6	0	0	50	63	23371400	3.93E-01	1
ZFP37	492947	575	-4	1641516	395160	0	14	3	0	4	6	1415456	3.93E-01	1
MRPS7	1723762	171	31	622288	173372	0	3	1	0	50	81	19557572	3.93E-01	1
CSTB	919787	170	68	259168	66572	0	1	0	0	50	49	19469996	3.93E-01	1
FSCB	131880	932	-58	2006416	640088	0	5	0	0	27	27	10754404	3.93E-01	1
UTRN	183454	750	11	9044536	2271992	0	38	10	0	50	55	20787908	3.94E-01	1
FOXJ2	139049	167	22	1470280	414740	0	6	1	0	50	47	21582856	3.94E-01	1
CEP63	222647	321	12	1952304	470988	0	6	0	0	3	1	2055900	3.94E-01	1
STXBP6	259495	800	-3	560700	136704	0	2	0	0	50	42	20138920	3.94E-01	1

PCMTD2	442044	440	43	926668	254540	0	3	1	0	50	70	24395256	3.94E-01	1
BRSK1	1032110	209	12	1968680	604132	0	7	2	0	17	18	6309744	3.94E-01	1
PRKCSH	1255985	265	32	1428272	361696	0	6	0	0	33	38	12400192	3.94E-01	1
ATP6V1D	484235	291	32	655752	173016	0	1	0	0	50	43	17771164	3.94E-01	1
DHDDS	1046693	181	33	868284	239232	0	5	0	0	50	62	18137132	3.94E-01	1
ADAM17	691902	278	38	2186196	534000	0	8	1	0	50	73	22633768	3.95E-01	1
DCYAP1R	364216	631	7	1245288	323604	0	6	2	0	50	75	19738776	3.95E-01	1
UBXN7	1165640	231	37	1284092	330724	0	5	1	0	50	41	16631252	3.95E-01	1
MUC7	264556	861	26	891068	328588	0	4	1	0	50	64	23077700	3.95E-01	1
BCO2	389593	362	2	1504456	400856	0	6	1	0	24	19	7621604	3.95E-01	1
CDKL1	655106	279	33	945180	243504	0	2	0	0	50	63	24808928	3.95E-01	1
C16orf62	340032	578	15	2551096	667856	0	10	1	0	9	20	6815976	3.95E-01	1
FGD6	370367	544	32	3728388	941976	0	11	0	0	4	3	2808484	3.95E-01	1
IBTK	124946	466	-1	3530096	923464	0	10	3	0	50	51	19329020	3.95E-01	1
LMO3	149303	802	-15	384480	96120	0	1	0	0	50	74	22237540	3.95E-01	1
GRM8	60921	1082	-8	2350312	656108	0	9	3	0	50	71	23716008	3.96E-01	1
LIFR	159760	271	-2	2860816	738344	0	9	1	0	50	51	20741272	3.96E-01	1
RORC	1014595	212	33	1340340	374868	0	6	1	0	50	65	17876896	3.96E-01	1
ATP2C2	646239	396	5	2449636	697760	0	7	3	0	50	46	17529084	3.96E-01	1
CEP170	180288	1024	0	4069080	1122468	0	7	1	0	19	10	8053432	3.96E-01	1
ATP6V1C1	470948	438	47	1029552	248844	0	5	0	0	50	60	24426940	3.96E-01	1
PHKA1	385429	NaN	18	3185132	868996	0	12	1	0	13	9	3784280	3.96E-01	1
ACSBG1	421357	314	52	1861168	519404	0	5	0	0	4	1	1828416	3.96E-01	1
CRIP2	689089	298	41	554648	147028	0	2	0	0	50	46	21105816	3.96E-01	1
IMPA2	336901	181	48	732648	223212	0	3	1	0	50	46	17764044	3.96E-01	1
CDK9	1194942	179	58	960488	260236	0	5	0	0	28	49	17284512	3.96E-01	1
WIPF1	463489	400	39	1215740	428980	0	6	2	0	21	38	10225388	3.97E-01	1
MRPL28	1549307	207	37	656108	188680	0	1	0	0	36	55	20238956	3.97E-01	1
JOSD2	762018	517	35	462800	159844	0	2	0	0	50	59	22902548	3.97E-01	1
PIK3R3	1116382	318	20	1235676	287292	0	6	1	0	50	65	24285608	3.97E-01	1
GNLY	1099615	181	41	385904	108580	0	2	0	0	50	61	19294844	3.98E-01	1
PLEKHG4	1201743	142	56	2972956	931652	0	13	4	0	11	22	6211844	3.98E-01	1
PRSS48	203766	298	16	827700	241368	0	3	0	0	50	33	15273468	3.98E-01	1

PADI4	457546	210	34	1719124	474548	0	6	1	0	44	53	19075192	3.98E-01	1
ZNF335	677949	209	55	3405496	1012820	0	10	4	0	50	68	23270296	3.98E-01	1
OR2G2	204354	957	-25	773944	243860	0	7	2	0	21	47	9325064	3.98E-01	1
PDXP	1136064	180	65	703456	254540	0	1	0	0	40	37	15616296	3.98E-01	1
OR51D1	212267	935	-3	784624	260948	0	3	1	0	50	68	21342556	3.98E-01	1
SLC25A4	317559	530	42	756856	213956	0	2	0	0	50	49	15741252	3.98E-01	1
PIH1D1	2173457	290	44	757924	210752	0	4	1	0	50	67	19850560	3.98E-01	1
POLDIP3	914105	189	69	1067644	320756	0	5	1	0	50	48	20403072	3.98E-01	1
FAU	3267059	207	46	342828	105732	0	2	0	0	50	74	20482816	3.98E-01	1
JAZF1	292385	474	14	627984	170880	0	3	0	0	50	50	18228980	3.99E-01	1
AKT3	208930	954	-4	1330016	314704	0	5	2	0	50	64	20173808	3.99E-01	1
FGF23	295315	860	33	627628	188324	0	3	2	0	15	12	4899628	3.99E-01	1
PSTK	274189	673	11	903884	238876	0	3	1	0	50	64	18840944	3.99E-01	1
KCNE2	410767	518	61	320756	80812	0	2	0	0	50	51	13682860	3.99E-01	1
PHF21A	513122	344	32	1782492	499824	0	4	0	0	9	3	3284456	3.99E-01	1
PLXNA4	314659	746	9	5088308	1448920	0	20	8	0	50	62	21495280	3.99E-01	1
GHR	248127	909	-12	1655400	430404	0	6	0	0	9	5	2969040	3.99E-01	1
PCNP	549032	445	14	467784	124956	0	2	0	0	50	55	21197664	3.99E-01	1
DDX58	426359	362	-12	2453196	586332	0	6	0	0	27	19	11313680	3.99E-01	1
CAT	653478	256	23	1383060	362052	0	8	0	0	29	37	11153836	3.99E-01	1
PFKM	484445	255	31	2220728	615880	0	7	0	0	9	8	3839460	4.00E-01	1
BIRC7	1005137	285	50	758636	224992	0	4	2	0	28	31	9962660	4.00E-01	1
PRAMEF2	118495	792	12	1193312	341404	0	5	2	0	50	47	18244288	4.00E-01	1
OPHN1	186481	NaN	-13	2133152	542188	0	7	2	0	50	43	17338624	4.00E-01	1
C6orf120	233648	447	3	467428	147740	0	2	0	0	50	61	23526260	4.00E-01	1
EXOSC4	2720766	211	27	593096	205768	0	3	1	0	25	30	10299080	4.00E-01	1
CYGB	1016866	197	24	485228	139552	0	4	2	0	14	31	6757236	4.00E-01	1
AKAP12	340429	383	11	4468512	1285872	0	12	6	0	50	39	20902896	4.00E-01	1
HPGD	62916	536	-11	708440	172660	0	2	1	0	30	44	14163460	4.01E-01	1
P2RX6	577297	192	58	1137776	327520	0	3	1	0	50	77	21004712	4.01E-01	1
TFAM	152882	326	-7	655752	161268	0	1	1	0	50	60	23413764	4.01E-01	1
C1orf127	702484	307	33	2020656	670704	0	4	1	0	46	51	22404860	4.01E-01	1
IGSF10	36173	606	6	6545060	1934860	0	24	8	0	29	44	14912128	4.01E-01	1

ST3GAL5	1164530	216	51	1097548	288716	0	4	0	0	50	69	22410200	4.01E-01	1
NR1H4	191698	787	-6	1245288	308652	0	3	0	0	48	29	18900752	4.01E-01	1
ATN1	2704950	211	47	2859036	991104	0	13	0	0	0	0	991104	4.01E-01	1
BTNL8	783856	602	14	1435748	368104	0	6	0	0	21	31	11171992	4.01E-01	1
PSMC3IP	1884163	177	38	596300	136348	0	1	0	0	50	68	23826724	4.01E-01	1
ATP5E	948552	126	25	142044	35244	0	1	0	0	50	80	21891152	4.01E-01	1
RHBDL3	671605	271	22	1024924	309008	0	3	0	0	50	55	19760848	4.01E-01	1
TSPAN31	1160849	344	39	546460	150944	0	3	2	0	2	11	1409404	4.02E-01	1
FRZB	330424	450	6	830548	235316	0	4	0	0	50	52	17649412	4.02E-01	1
JKAMP	168237	606	12	805984	219296	0	2	0	0	50	50	18757640	4.02E-01	1
PGAP3	1284992	226	38	817376	243148	0	2	0	0	50	64	21003644	4.02E-01	1
BIRC5	683401	191	24	540052	118904	0	2	0	0	50	56	23959868	4.02E-01	1
ZNF394	1327454	288	60	1425424	383768	0	9	0	0	21	29	7233564	4.02E-01	1
ACTN2	427180	682	-10	2354940	598080	0	8	2	0	50	46	14893616	4.02E-01	1
OR52E8	203856	922	-5	777148	239588	0	3	1	0	50	39	17006476	4.02E-01	1
PCDHA11	NaN	NaN	NaN	2347108	751160	0	7	3	0	50	119	40396744	4.02E-01	1
CHFR	944795	674	10	1806700	500180	0	8	3	0	13	21	7354248	4.02E-01	1
TXLNA	1460758	218	29	1414744	380564	0	5	3	0	20	18	7618044	4.02E-01	1
ZNF813	361546	876	-58	1600576	392312	0	7	1	0	50	64	19075904	4.02E-01	1
CBLN4	236365	941	-74	495908	159844	0	3	1	0	50	80	19146748	4.02E-01	1
GPX6	255783	592	26	579568	150944	0	2	0	0	50	62	25311600	4.02E-01	1
RRH	212757	462	28	876472	234248	0	4	1	0	50	47	18097260	4.02E-01	1
NCSTN	987855	526	28	1824500	520828	0	9	2	0	50	69	19516276	4.02E-01	1
TC2N	368236	478	21	1283380	336776	0	4	0	0	9	14	4745836	4.02E-01	1
FKBP3	234268	565	11	600216	148452	0	2	0	0	50	42	21031768	4.02E-01	1
FNDC3A	321392	424	47	3118916	841228	0	12	0	0	16	9	5781440	4.02E-01	1
NRCAM	441225	682	1	3434332	904952	0	13	1	0	12	8	4053772	4.03E-01	1
STAT3	2140948	160	32	2055900	515844	0	10	1	0	50	67	20340772	4.03E-01	1
MLXIPL	1197825	214	52	2122472	681028	0	6	2	0	7	5	2787836	4.03E-01	1
RNF185	699351	218	41	506588	137416	0	2	0	0	50	57	23250004	4.03E-01	1
EHBP1	160013	618	8	3230344	815240	0	17	1	0	9	8	3916000	4.03E-01	1
MFSD6	651647	371	30	1983988	579212	0	8	2	0	9	12	4311516	4.03E-01	1
DNMT3L	1128093	219	76	1015312	276968	0	2	0	0	50	45	20503464	4.03E-01	1

PAX3	191783	682	11	1406200	384836	0	7	3	0	32	51	13683216	4.03E-01	1
MDP1	974559	220	41	471344	123532	0	2	0	0	50	62	24565780	4.03E-01	1
OTP	276866	584	42	787472	265576	0	2	1	0	50	60	19334360	4.03E-01	1
SMARCB1	637355	196	52	1003920	267000	0	3	0	0	24	21	8565716	4.03E-01	1
L3MBTL3	68020	844	23	2083668	508368	0	6	0	0	50	40	19288436	4.03E-01	1
MYPN	407663	251	19	3346756	965828	0	13	4	0	23	30	9823464	4.03E-01	1
ADAT2	299410	495	7	506588	132076	0	2	0	0	50	58	20836324	4.03E-01	1
DLL1	374629	631	-23	1843368	519048	0	8	2	0	36	50	14372788	4.03E-01	1
PHF20L1	195342	773	-4	2763272	686368	0	11	0	0	5	4	1938420	4.03E-01	1
S100G	294904	NaN	28	213956	50908	0	1	0	0	50	73	25991204	4.03E-01	1
ASNA1	1959466	226	29	902816	243148	0	3	1	0	50	74	23429072	4.03E-01	1
PPIL4	434611	150	40	1330728	302244	0	6	0	0	50	48	18535140	4.04E-01	1
RNASEL	597206	812	-3	1882528	516200	0	9	1	0	50	60	22449360	4.04E-01	1
CLCF1	1269888	177	51	555716	176932	0	2	0	0	50	45	20698908	4.04E-01	1
DCTN2	1347795	303	44	1048420	296192	0	5	0	0	40	78	26763012	4.04E-01	1
ZBTB11	548328	445	16	2695276	724460	0	9	2	0	37	47	14390944	4.04E-01	1
RUFY2	740263	294	23	1818804	435744	0	4	2	0	50	69	24135020	4.04E-01	1
HIST1H1B	228884	634	40	552156	179424	0	4	4	0	12	15	2172668	4.04E-01	1
GIGYF1	1884430	247	60	2643300	774300	0	10	3	0	22	30	9880780	4.04E-01	1
F5	294416	415	11	5721276	1510152	0	20	5	0	50	45	22176308	4.04E-01	1
PCDH11Y	0	0	0	3375948	999648	0	9	1	0	39	23	17595656	4.04E-01	1
SERPINI2	135686	933	-18	1074052	260948	0	5	1	0	50	78	21467156	4.04E-01	1
OR13H1	81234	NaN	4	756144	232824	0	4	1	0	48	76	26426236	4.05E-01	1
FBXL5	190822	327	37	1803496	457460	0	5	1	0	50	55	18011464	4.05E-01	1
STAT4	529053	318	19	2002144	494840	0	9	0	0	19	23	9872236	4.05E-01	1
ARHGAP2(	156598	456	38	2159140	548240	0	8	2	0	50	77	23703192	4.05E-01	1
PVRL3	79394	878	-15	1383416	400144	0	4	2	0	19	27	8785012	4.05E-01	1
ZNF14	635741	545	-30	1674268	398720	0	9	2	0	50	54	18667928	4.05E-01	1
PLSCR1	123426	671	-12	843008	213244	0	3	0	0	50	58	26015412	4.05E-01	1
PDHX	663753	258	6	1372736	419368	0	4	0	0	15	14	6734452	4.05E-01	1
IAPP	NaN	NaN	NaN	228552	68352	0	1	0	0	50	116	39713936	4.05E-01	1
WDR66	814234	162	38	3030628	804560	0	11	3	0	50	37	15422632	4.05E-01	1
CSNK1A1	607110	152	32	978644	236740	0	4	0	0	50	53	20083028	4.05E-01	1

RGS19	1032302	312	51	564616	155216	0	3	0	0	50	69	20843800	4.06E-01	1
ZFP28	135553	817	-69	2230696	585620	0	5	0	0	9	9	6477776	4.06E-01	1
ST7L	561127	355	19	1535428	409400	0	4	1	0	37	37	14993652	4.06E-01	1
PAK6	829569	167	31	1683168	536136	0	4	1	0	13	18	5586708	4.06E-01	1
MAB21L2	214274	848	18	885016	268424	0	4	1	0	50	46	18957356	4.06E-01	1
EFHD2	554165	212	29	617660	169456	0	2	1	0	29	58	14632312	4.06E-01	1
PDHA2	16119	968	-25	966184	282308	0	6	1	0	50	82	23328680	4.06E-01	1
PIP5K1A	1409327	184	32	1462448	405484	0	5	0	0	34	43	14948796	4.06E-01	1
UBR1	634287	366	32	4662888	1142760	0	10	3	0	29	29	15916048	4.06E-01	1
CLEC7A	396940	445	-1	696692	157708	0	3	1	0	50	48	19709940	4.06E-01	1
FGF5	61128	903	-3	679248	191172	0	4	1	0	50	55	19632332	4.06E-01	1
TACC1	623231	286	35	2064444	569244	0	4	2	0	9	3	3650424	4.06E-01	1
SEC23B	414545	392	23	1988972	552868	0	5	1	0	50	46	21908240	4.07E-01	1
SET	1865107	197	38	852264	190104	0	3	0	0	50	70	23922844	4.07E-01	1
MYL12B	551966	328	55	460664	106444	0	2	0	0	50	53	19696056	4.07E-01	1
CANX	1624288	256	44	1577080	382700	0	4	2	0	50	82	25179524	4.07E-01	1
SPTLC1	485523	340	32	1252408	335708	0	4	0	0	50	43	17352864	4.07E-01	1
PKDCC	210167	164	-5	1202924	405484	0	4	1	0	50	43	20630200	4.07E-01	1
ISG20	393645	605	14	453544	142400	0	2	1	0	34	45	15673256	4.07E-01	1
DPPA4	22565	1096	-19	786048	218940	0	3	1	0	50	68	19038524	4.07E-01	1
OR9G1	NaN	NaN	NaN	750092	228196	0	4	1	0	50	117	39873780	4.07E-01	1
TMC5	522510	369	13	2873988	771096	0	11	1	0	15	24	8746920	4.07E-01	1
SFPQ	822666	380	36	1774660	534356	0	3	1	0	27	21	9984020	4.07E-01	1
HTR3C	1423268	375	38	1148456	323248	0	5	0	0	5	2	2092924	4.07E-01	1
TOR3A	345937	301	4	1011040	285512	0	4	1	0	50	52	22070220	4.08E-01	1
ZG16B	1717244	210	42	525456	159132	0	1	0	0	50	80	24105472	4.08E-01	1
GNE	857846	257	46	1923824	541120	0	8	1	0	50	67	23320136	4.08E-01	1
AZU1	2168802	198	28	621220	205412	0	2	2	0	29	50	13937044	4.08E-01	1
AUTS2	52369	997	12	3276624	990036	0	15	3	0	50	68	22448292	4.08E-01	1
OR12D2	329776	872	7	761128	229976	0	3	0	0	50	77	21945976	4.08E-01	1
KCNJ4	1466610	215	23	1100040	333216	0	4	3	0	28	19	7961584	4.08E-01	1
BARHL2	82474	897	-4	961200	292632	0	3	2	0	50	62	21504536	4.08E-01	1
TMEM215	610744	401	-18	587400	173016	0	5	2	0	4	5	1442156	4.09E-01	1

LRRC61	315538	514	32	614100	218940	0	1	0	0	50	70	24657628	4.09E-01	1
SHFM1	170353	1045	10	198648	37380	0	1	0	0	40	46	17514488	4.09E-01	1
SPTLC2	500852	320	40	1445360	405484	0	5	1	0	50	46	16767956	4.09E-01	1
KHDRBS3	16039	1124	-24	897120	248844	0	5	0	0	14	26	6203656	4.09E-01	1
ZNF382	635596	889	-38	1430408	347812	0	7	1	0	35	44	12365304	4.09E-01	1
ZNF662	301212	447	21	1254900	325740	0	6	0	0	50	67	24596752	4.09E-01	1
RUNX2	54989	416	8	1328948	392668	0	3	0	0	50	48	20863380	4.09E-01	1
RAB11FIP[illegible]	100461	912	-1	1323252	337488	0	5	1	0	50	54	19390252	4.09E-01	1
FGF9	298481	648	37	528660	149520	0	3	0	0	50	54	17576788	4.09E-01	1
IZUMO1	1635420	190	44	920972	244216	0	3	1	0	50	73	21072352	4.09E-01	1
CCL26	1012112	255	51	251336	65860	0	1	0	0	50	45	16982624	4.10E-01	1
DIAPH3	80757	890	22	3178368	783912	0	7	2	0	50	51	24160652	4.10E-01	1
SNPH	441449	468	32	1224284	378784	0	2	0	0	42	27	15577492	4.10E-01	1
TMEM65	330177	445	14	607336	190460	0	3	1	0	50	47	17346456	4.10E-01	1
MYT1L	54881	901	-24	3115356	764688	0	19	6	0	24	34	8747632	4.10E-01	1
AATF	668463	384	16	1470992	373444	0	3	1	0	50	42	20432264	4.10E-01	1
DNAJC9	829738	249	44	678180	177288	0	2	1	0	25	35	10890040	4.10E-01	1
SLC26A9	690729	431	33	2281604	655396	0	8	3	0	50	63	24215832	4.10E-01	1
SH3BP5	516326	303	32	1178716	320756	0	3	2	0	50	50	19664372	4.10E-01	1
HOXA11	473864	394	13	775368	237096	0	4	0	0	44	70	23677204	4.10E-01	1
WFDC8	902945	438	27	655396	147740	0	2	1	0	50	69	22419456	4.10E-01	1
MARK4	1318362	215	31	1748672	533644	0	5	4	0	43	64	16024628	4.10E-01	1
MS4A4A	469977	882	8	621932	174796	0	2	1	0	50	69	21814256	4.11E-01	1
CYP2C19	254705	831	-17	1267716	338556	0	5	0	0	50	62	20004352	4.11E-01	1
KBTBD7	314264	337	66	1702036	492704	0	7	2	0	50	73	22716004	4.11E-01	1
SPDYA	481869	238	32	843364	200072	0	4	0	0	50	50	18398080	4.11E-01	1
FLI1	187976	647	20	1180496	305092	0	4	2	0	12	10	4463172	4.11E-01	1
BARD1	310874	948	11	2001788	533644	0	8	0	0	18	17	8493448	4.11E-01	1
CCBL2	316278	598	5	1204348	307940	0	4	0	0	50	59	20056684	4.11E-01	1
PUS3	611330	414	14	1231048	326096	0	3	1	0	50	49	20321548	4.11E-01	1
TSSC1	255965	619	-1	1013888	263440	0	3	0	0	50	58	19622364	4.11E-01	1
GSS	968413	236	40	1211468	361696	0	4	3	0	32	43	15816012	4.12E-01	1
ENOSF1	418492	425	32	1318624	323960	0	3	0	0	6	2	2910300	4.12E-01	1

SLC15A1	334185	409	41	1878968	488788	0	4	1	0	50	72	26506692	4.12E-01	1
ZNF415	392290	946	-49	1460312	356356	0	5	1	0	50	50	16685008	4.12E-01	1
CCDC63	888826	474	46	1487368	368816	0	6	0	0	50	54	22978020	4.12E-01	1
RLN1	398278	629	13	471344	128872	0	2	1	0	30	31	11991860	4.12E-01	1
OCEL1	865138	212	38	669280	203276	0	2	0	0	50	59	18864796	4.12E-01	1
PPL	702265	206	19	4470292	1250984	0	19	3	0	50	67	23167056	4.12E-01	1
TOM1L2	1093302	173	22	1311860	375580	0	9	1	0	4	6	1993244	4.12E-01	1
HOXA13	473864	394	13	943400	309364	0	4	1	0	50	80	25624168	4.13E-01	1
TNNT1	1036976	266	-1	767180	182272	0	4	1	0	50	46	14741248	4.13E-01	1
ARHGEF1(	248448	483	21	1807768	526880	0	8	3	0	15	16	6357092	4.13E-01	1
OAS2	605960	433	54	1924180	512996	0	10	3	0	22	57	10513748	4.13E-01	1
MRPL53	1069803	343	45	281596	89000	0	2	1	0	11	18	4496636	4.13E-01	1
C19orf44	815495	258	44	1653976	487364	0	5	0	0	19	22	9318300	4.13E-01	1
BTBD7	321756	407	31	2906384	840160	0	8	0	0	24	19	11059496	4.13E-01	1
ACCS	345807	680	3	1305808	360272	0	3	3	0	19	21	7155600	4.13E-01	1
ZNF608	39167	575	14	3797096	1084732	0	13	4	0	50	60	23181652	4.13E-01	1
KIN	199265	447	4	1060524	255252	0	4	0	0	16	9	5151320	4.13E-01	1
SLCO1B3	151869	521	-24	1831264	480956	0	12	1	0	9	15	3941988	4.13E-01	1
HIST1H2B/	272695	635	29	317908	94340	0	2	1	0	24	35	8484904	4.13E-01	1
RRAGB	59904	NaN	23	996444	247776	0	3	1	0	50	55	19193384	4.13E-01	1
ZCCHC5	263052	NaN	20	1187260	337844	0	5	0	0	9	7	3634760	4.14E-01	1
STEAP2	132955	1140	6	1274124	352440	0	5	2	0	50	57	19537280	4.14E-01	1
DCLK3	374894	617	-14	1644008	448204	0	4	1	0	50	31	15467488	4.14E-01	1
RPL10	2277674	NaN	40	563548	150944	0	2	0	0	50	46	20570392	4.14E-01	1
DIDO1	961230	336	40	5634056	1740484	0	36	12	0	48	80	21420520	4.14E-01	1
ZNF770	432995	382	2	1778220	443220	0	5	0	0	50	42	17923532	4.14E-01	1
SLC17A1	293308	635	30	1207908	342828	0	3	1	0	17	18	6532244	4.14E-01	1
SCAMP4	1944774	239	36	599860	162692	0	3	0	0	50	67	22320132	4.14E-01	1
PCDHGB3	662513	568	2	2304744	715560	0	10	3	0	50	85	24954176	4.14E-01	1
ZNF543	432083	869	-17	1541836	396584	0	8	1	0	50	60	18121112	4.15E-01	1
IDI2	338115	407	30	591316	156284	0	2	0	0	50	47	22055980	4.15E-01	1
PRICKLE2	163412	502	3	2155580	581704	0	8	3	0	45	41	14191584	4.15E-01	1
TCP10L2	NaN	NaN	NaN	902460	260592	0	2	1	0	50	117	39906176	4.15E-01	1

NOB1	1410481	NaN	39	1058744	300820	0	3	2	0	32	65	21411264	4.15E-01	1
LUZP1	859705	169	25	2680324	774656	0	8	1	0	4	1	1791392	4.15E-01	1
PIGA	201763	NaN	43	1220724	354576	0	3	1	0	50	65	24059548	4.15E-01	1
CALCB	277848	692	-18	328588	98612	0	2	0	0	50	78	20277404	4.16E-01	1
LDLR	1808766	233	33	2254548	576720	0	7	1	0	25	31	10760100	4.16E-01	1
CYP3A7	1430454	438	42	1316488	350660	0	6	1	0	3	9	3276268	4.16E-01	1
TCP11L2	204762	396	18	1345324	359204	0	7	1	0	50	46	15722384	4.16E-01	1
IER2	1948449	221	41	535068	182628	0	3	1	0	50	60	21446864	4.16E-01	1
C7orf34	294033	676	2	375224	105376	0	2	1	0	50	59	17403416	4.16E-01	1
B3GALT2	NaN	NaN	NaN	1065864	289428	0	3	1	0	50	117	39935012	4.16E-01	1
DDHD1	306643	448	20	2320408	644360	0	9	0	0	6	7	3966552	4.16E-01	1
IKBKAP	336944	471	9	3515144	909580	0	11	1	0	13	12	6541500	4.16E-01	1
SH3BP1	1136064	180	65	1772880	548952	0	3	2	0	40	37	15616296	4.16E-01	1
CRCT1	325831	700	20	246352	78320	0	1	0	0	50	63	27856288	4.16E-01	1
ECHDC1	120722	889	-4	813816	196512	0	2	0	0	50	65	21953808	4.16E-01	1
ARID4B	550877	297	22	3461744	843364	0	10	1	0	3	2	2066936	4.17E-01	1
CACNB4	279730	592	21	1407624	383412	0	2	1	0	35	25	16795012	4.17E-01	1
UTP6	705344	300	21	1614816	380208	0	6	2	0	50	67	23717076	4.17E-01	1
AKAP3	277828	874	18	2141340	605556	0	7	1	0	43	40	17734496	4.17E-01	1
STYXL1	1072825	218	49	828768	210396	0	2	1	0	50	64	21496704	4.17E-01	1
ZCCHC16	65327	NaN	3	776080	220364	0	3	0	0	50	66	30273528	4.17E-01	1
PSMD9	831519	195	45	582772	156284	0	3	0	0	50	66	19935288	4.17E-01	1
VPS29	922262	206	43	489144	129228	0	2	0	0	36	42	16644780	4.17E-01	1
GPR174	14321	NaN	62	836244	236028	0	4	0	0	50	74	24189132	4.18E-01	1
IL17REL	895187	271	36	872556	258456	0	4	0	0	50	75	27202316	4.18E-01	1
LMOD1	743998	168	27	1511220	422928	0	4	0	0	26	27	12270252	4.18E-01	1
CYP2A7	1187304	275	31	1281956	348880	0	9	4	0	12	14	3907100	4.18E-01	1
ZNF107	200262	1062	-11	2034184	486296	0	9	2	0	14	22	8076572	4.18E-01	1
COPS5	393936	518	12	882524	220720	0	2	0	0	50	60	21748396	4.18E-01	1
PLEKHA4	1766061	211	34	1957644	622644	0	10	3	0	13	17	6498068	4.18E-01	1
KLF11	922437	234	42	1265580	393024	0	4	1	0	50	68	25318008	4.18E-01	1
SPSB4	245950	574	22	656820	225348	0	2	0	0	50	40	20918916	4.18E-01	1
PEX3	318754	401	13	991104	254184	0	3	0	0	37	49	16650476	4.18E-01	1

NID2	234008	432	20	3485952	1012464	0	12	1	0	10	8	4026716	4.18E-01	1
GNB3	2822601	232	32	887152	243860	0	5	1	0	50	62	20662240	4.18E-01	1
KCNK6	1467279	253	0	741904	272696	0	2	0	0	18	15	5595252	4.18E-01	1
HHIP	143634	1116	-6	1812752	484516	0	7	3	0	50	47	19781852	4.18E-01	1
SUGT1	307262	469	26	993596	234604	0	3	0	0	50	55	21223296	4.18E-01	1
GRTP1	812721	237	53	864368	247420	0	5	0	0	50	61	19550452	4.19E-01	1
BLVRB	1010633	202	34	514776	165540	0	1	1	0	50	62	18103668	4.19E-01	1
GRPEL2	422727	380	28	568888	168032	0	2	0	0	50	43	19341480	4.19E-01	1
KIAA0586	290827	454	33	3821304	1022076	0	8	2	0	50	50	22364632	4.19E-01	1
NEU3	762277	454	36	1145252	343540	0	5	1	0	50	57	20954516	4.19E-01	1
CABP4	1392213	215	53	693844	213956	0	3	0	0	11	7	3262740	4.19E-01	1
MECR	390697	395	22	953724	285156	0	3	1	0	50	43	22573604	4.19E-01	1
LYN	329738	405	50	1361344	333572	0	5	0	0	50	66	21903256	4.19E-01	1
TTC31	1019591	315	50	1323608	398008	0	5	1	0	50	69	21835972	4.19E-01	1
CCDC126	389945	361	23	356712	99324	0	1	0	0	50	38	20744120	4.19E-01	1
HKR1	228816	939	-63	1677116	454612	0	5	1	0	30	29	10459280	4.19E-01	1
MS4A5	383569	899	18	523676	141688	0	2	0	0	50	45	18398080	4.20E-01	1
FCGR2A	741254	448	41	826276	220364	0	3	1	0	50	66	22531596	4.20E-01	1
LIMCH1	319991	391	31	2864376	749736	0	8	2	0	50	41	21299480	4.20E-01	1
SNX6	516968	165	10	1121044	280172	0	5	0	0	50	63	22195888	4.20E-01	1
CNR1	172187	799	37	1173020	346744	0	4	2	0	50	66	20591752	4.20E-01	1
TBL1X	119145	NaN	-3	1507304	408688	0	5	1	0	3	1	1154152	4.20E-01	1
CDKN2AII	235218	163	16	1433612	436456	0	4	1	0	50	44	18474620	4.20E-01	1
NBR1	1341175	172	25	2503748	679960	0	11	2	0	50	70	21371748	4.20E-01	1
CD300LD	698491	504	10	662516	192952	0	2	0	0	50	63	19807128	4.20E-01	1
CCT6B	268361	579	-14	1377008	381988	0	6	2	0	50	46	18612392	4.20E-01	1
GLIPR1	513848	606	-10	692064	184764	0	3	0	0	50	49	18252832	4.20E-01	1
SLC41A2	759566	294	30	1469212	408332	0	5	2	0	50	56	22524832	4.20E-01	1
FAM96B	942986	142	34	420436	126380	0	2	0	0	50	65	21541916	4.20E-01	1
LCE3A	285984	752	17	226416	60876	0	1	0	0	50	58	22596388	4.20E-01	1
SLC25A16	782017	294	35	856892	244216	0	3	0	0	50	82	24510956	4.20E-01	1
ERO1L	314095	313	11	1270920	295836	0	3	0	0	50	46	19692852	4.20E-01	1
MSRB3	324747	580	3	571024	158420	0	1	0	0	50	57	19941340	4.20E-01	1

RPL23A	1695792	208	40	410112	112140	0	2	0	0	50	81	25518436	4.21E-01	1
TCTE3	233648	447	3	524744	129940	0	2	0	0	50	61	23526260	4.21E-01	1
DCT	123751	906	0	1425424	384836	0	9	3	0	6	4	1470636	4.21E-01	1
CLEC3B	479578	326	41	516556	144536	0	2	0	0	50	48	19950952	4.21E-01	1
PLCL2	105775	400	15	2854764	776436	0	13	2	0	39	41	14488844	4.21E-01	1
DMRTA1	160489	448	-26	1214672	407620	0	5	0	0	33	61	18425848	4.21E-01	1
MATN4	1005559	233	36	1463160	440016	0	8	2	0	50	49	17548664	4.21E-01	1
COL19A1	93520	947	15	2999300	876472	0	13	2	0	42	53	17309788	4.21E-01	1
HSD17B12	432669	791	0	819868	225704	0	2	0	0	50	55	19760136	4.21E-01	1
HIST1H2BI	390169	544	57	312212	96832	0	2	2	0	15	17	2533296	4.21E-01	1
ZNF773	459366	828	-31	1146676	285512	0	6	0	0	33	27	10916384	4.21E-01	1
OR14J1	176114	872	4	783556	247064	0	6	2	0	10	15	3512296	4.21E-01	1
EYA2	306082	522	36	1401572	389464	0	5	2	0	38	28	13583180	4.21E-01	1
RERGL	21334	1070	-35	541476	137772	0	3	0	0	50	121	22337220	4.21E-01	1
ID2	215150	559	11	345676	95408	0	1	0	0	50	48	22997956	4.21E-01	1
OR13G1	191710	995	-32	762196	224636	0	4	1	0	50	116	25277780	4.21E-01	1
CLEC4D	321790	564	13	579568	133856	0	3	0	0	50	64	21703896	4.21E-01	1
RNASEH1	254257	NaN	15	754720	196868	0	3	0	0	50	52	20982640	4.22E-01	1
DARS2	442819	385	11	1683524	454612	0	6	0	0	4	6	2101824	4.22E-01	1
ZMYND12	571804	519	24	969388	233180	0	4	0	0	50	62	19023928	4.22E-01	1
TRAM1	240060	535	28	985052	259168	0	3	1	0	50	48	18331864	4.22E-01	1
ATP6AP2	456094	NaN	63	906732	254184	0	4	1	0	50	49	18942760	4.22E-01	1
OSR2	113665	228	33	800288	211108	0	4	1	0	50	60	19864444	4.22E-01	1
ITGAE	500884	176	28	3055548	855468	0	11	4	0	13	21	8207224	4.22E-01	1
G6PC3	916863	198	23	864368	268780	0	3	1	0	50	69	17825988	4.22E-01	1
LMOD2	86849	536	-10	1380568	383768	0	6	2	0	34	43	15813876	4.23E-01	1
LIN7B	2011861	267	47	528660	165540	0	1	0	0	50	71	20637320	4.23E-01	1
ERGIC1	502164	179	47	772520	198292	0	2	0	0	50	48	19182348	4.23E-01	1
FSHR	59778	1096	-9	1778932	489500	0	5	4	0	46	69	21913936	4.23E-01	1
GALNTL5	309129	476	12	1161628	295124	0	6	1	0	50	55	20641236	4.23E-01	1
GPAM	166177	679	-13	2157004	584552	0	5	0	0	6	3	4186560	4.24E-01	1
SLC43A2	1362107	198	29	1451412	430404	0	5	2	0	50	71	22263884	4.24E-01	1
CCS	1343990	194	62	706660	206480	0	1	1	0	50	53	20002216	4.24E-01	1

TTC36	1348757	167	48	466004	151300	0	2	0	0	50	63	22637684	4.24E-01	1
XG	202643	NaN	23	544324	129584	0	2	1	0	25	31	10822756	4.24E-01	1
CRYBA4	161795	547	-4	516912	135636	0	3	0	0	50	73	22494216	4.24E-01	1
EPB41L4A	164575	671	28	1832332	462800	0	4	1	0	50	34	18381704	4.25E-01	1
NR1I3	731911	229	39	1024212	281952	0	3	1	0	11	18	6488100	4.25E-01	1
PDZD9	643750	326	44	533644	133856	0	1	1	0	23	27	8781808	4.25E-01	1
AGPAT9	186399	436	21	1129944	310788	0	3	1	0	50	51	18717768	4.25E-01	1
ZSCAN1	1187584	694	-42	1011396	311856	0	4	2	0	50	67	17473904	4.25E-01	1
AKAP11	183147	411	51	4849788	1291212	0	11	2	0	10	4	3684244	4.25E-01	1
CCDC65	1696692	214	56	1270208	313636	0	5	1	0	50	56	19121116	4.25E-01	1
SENP8	1132082	352	26	547528	139196	0	2	0	0	50	50	20825288	4.25E-01	1
RAB20	495660	278	39	588112	169100	0	2	0	0	50	44	18578216	4.25E-01	1
ATP6V0E1	489007	309	40	220720	56960	0	1	0	0	50	45	17333640	4.25E-01	1
PSMD11	657957	438	15	1114636	296192	0	3	1	0	50	56	19207980	4.25E-01	1
GTF2A1	206731	456	-16	988968	253116	0	3	0	0	50	46	16194796	4.25E-01	1
RALY	873171	398	61	780352	237452	0	4	0	0	50	61	20893284	4.25E-01	1
CSN1S1	9744	991	0	550376	110716	0	2	0	0	50	64	17401280	4.26E-01	1
ANKRD23	822597	392	46	784980	231756	0	4	0	0	50	86	27656572	4.26E-01	1
MDN1	372470	603	34	14470332	3893928	0	37	10	0	49	35	20900404	4.26E-01	1
FANCM	234454	565	8	5311520	1349596	0	17	6	0	50	62	22971256	4.26E-01	1
CSRNP2	564607	155	40	1352444	407620	0	3	2	0	50	43	19587832	4.26E-01	1
ATXN7	156786	683	8	2424360	728376	0	5	0	0	13	23	10900008	4.26E-01	1
FAM195A	1115707	213	39	407976	124956	0	2	0	0	50	53	16969808	4.26E-01	1
PPP1R12B	770535	377	32	2738352	754008	0	10	2	0	40	58	19454332	4.26E-01	1
IFNA8	242654	411	-6	481312	129584	0	1	0	0	50	34	19573236	4.26E-01	1
CHD4	2790851	179	51	4999308	1296552	0	23	5	0	23	47	14312980	4.26E-01	1
GABRP	216977	618	19	1148100	303312	0	3	0	0	31	21	9847316	4.26E-01	1
TAF1L	567793	334	5	4594180	1261664	0	16	3	0	34	36	16518400	4.26E-01	1
HSPA13	155439	938	7	1193312	338200	0	5	0	0	28	24	10999332	4.26E-01	1
OR2L3	20939	1015	-27	770028	232824	0	4	1	0	50	119	22654060	4.27E-01	1
GZMM	1647685	255	-8	631188	214668	0	3	1	0	50	56	18766896	4.27E-01	1
KRT19	1445853	325	-22	995732	310432	0	7	4	0	3	8	1320048	4.27E-01	1
KCNJ1	194422	602	1	987900	274476	0	4	1	0	50	53	20284524	4.27E-01	1

HKDC1	726042	168	42	2348888	665008	0	8	3	0	50	69	20753732	4.27E-01	1
ZNF555	1287128	291	27	1625140	402992	0	6	2	0	50	60	21755516	4.27E-01	1
IRF2BP1	883734	203	39	1383416	490924	0	5	4	0	50	63	19718840	4.27E-01	1
MAMDC2	196190	405	-10	1794952	461732	0	5	0	0	23	23	12462848	4.27E-01	1
TUBA1B	2141275	214	56	1133148	327876	0	4	2	0	50	78	23413408	4.27E-01	1
SLC24A4	368522	642	27	1575300	436812	0	7	1	0	4	5	2882176	4.27E-01	1
C1orf116	451418	155	25	1466720	474904	0	6	0	0	11	14	4812764	4.28E-01	1
CYP39A1	113763	596	0	1240660	312212	0	3	0	0	31	19	13249964	4.28E-01	1
CYB5RL	465615	313	26	814172	223924	0	3	1	0	50	63	18381348	4.29E-01	1
MPP7	245887	473	-2	1531512	381276	0	6	1	0	34	33	13547224	4.29E-01	1
ZHX3	383198	262	7	2377368	693132	0	8	1	0	50	71	29215496	4.29E-01	1
CTSD	NaN	NaN	NaN	1038808	318620	0	5	1	0	50	117	39964204	4.29E-01	1
EIF3M	408431	613	7	1003208	245284	0	6	1	0	50	68	19100468	4.29E-01	1
TRAT1	107009	961	-15	503740	116768	0	3	1	0	13	24	6630144	4.29E-01	1
UBASH3A	366755	400	71	1709512	473480	0	8	1	0	17	21	6055560	4.29E-01	1
RBM46	73501	1021	-18	1363124	364900	0	5	0	0	31	41	13756908	4.29E-01	1
HAUS4	1240941	185	38	957284	247420	0	3	1	0	50	66	21938144	4.29E-01	1
PXDN	55677	536	-18	3742984	1095056	0	13	7	0	50	69	20643016	4.29E-01	1
CLDN9	1443513	234	49	507656	190816	0	2	1	0	50	48	18953440	4.29E-01	1
GUCY2C	339860	720	4	2830912	721256	0	13	2	0	36	48	13646548	4.29E-01	1
CYB561D2	843769	206	51	533644	193664	0	2	0	0	50	68	20472848	4.30E-01	1
ZFYVE27	535311	138	44	1083664	302600	0	3	1	0	50	47	17131076	4.30E-01	1
RBPMS2	803907	264	47	539340	163404	0	2	0	0	50	50	20007556	4.30E-01	1
ZDHHC22	531874	235	59	645072	209328	0	3	1	0	50	65	20921052	4.30E-01	1
ANK3	213806	634	-8	11343940	3220376	0	35	10	0	42	46	23187348	4.30E-01	1
AKIRIN2	235638	346	33	520116	150588	0	1	0	0	50	51	19368536	4.30E-01	1
DAPK2	678778	261	45	985052	250624	0	2	0	0	35	36	16052396	4.30E-01	1
RIPK1	437184	268	41	1750096	441440	0	5	1	0	50	44	17864792	4.30E-01	1
SF1	2261775	230	61	2086516	563192	0	8	2	0	50	77	23145696	4.30E-01	1
KCTD16	38690	643	-2	1080460	298328	0	4	0	0	45	58	18926028	4.30E-01	1
NHLRC2	266343	515	18	1857964	514064	0	6	0	0	50	48	20148176	4.31E-01	1
EZH2	564321	434	13	2006416	484160	0	5	3	0	42	45	16803912	4.31E-01	1
SYP	724395	NaN	44	800288	233536	0	3	1	0	50	48	21089440	4.31E-01	1

RFX7	204396	432	-2	3665020	1050200	0	8	2	0	50	47	21644800	4.31E-01	1
MAGEB18	33808	NaN	-25	870420	240300	0	4	1	0	50	122	28429092	4.31E-01	1
FOXD4	230380	398	-4	1045572	364188	0	3	0	0	50	40	20962704	4.31E-01	1
SLCO1B1	518292	799	-25	1801360	475616	0	8	2	0	50	56	16996864	4.31E-01	1
LRRC3	893345	411	43	616236	210396	0	3	0	0	50	74	24237192	4.31E-01	1
CDC20	1088321	252	27	1276260	370596	0	4	1	0	28	33	12199408	4.31E-01	1
PTP4A2	1200691	128	37	452120	103240	0	3	1	0	6	14	3647576	4.31E-01	1
OR4X2	400891	509	-27	748312	225704	0	5	2	0	23	30	6925980	4.31E-01	1
SLC17A6	51732	865	-14	1504100	410824	0	7	2	0	28	53	15922100	4.31E-01	1
GCA	70731	856	-5	587044	141332	0	1	1	0	50	70	21216532	4.31E-01	1
PLAUR	441738	209	6	944824	257744	0	5	2	0	14	16	4113936	4.31E-01	1
CD52	1021101	217	22	162336	42720	0	1	0	0	50	66	18554720	4.32E-01	1
GNA12	764046	392	30	959420	277324	0	3	0	0	37	45	15777564	4.32E-01	1
RIC8B	230956	396	11	1334644	368816	0	4	0	0	48	65	24424804	4.32E-01	1
FAM86B1	475976	625	-1	751516	227840	0	2	0	0	50	75	22680048	4.32E-01	1
C18orf54	109698	451	33	977932	247064	0	2	0	0	47	61	23463604	4.32E-01	1
FCER1G	728669	234	37	233180	64792	0	1	0	0	39	44	15898604	4.32E-01	1
CYP27B1	1160849	344	39	1247780	417232	0	7	0	0	6	9	2599512	4.32E-01	1
ARL15	83263	721	5	536492	139552	0	3	0	0	50	68	24803588	4.32E-01	1
TNFRSF17	571257	441	64	474192	127092	0	2	0	0	50	53	19474268	4.32E-01	1
RHBDL2	603263	318	27	789964	209684	0	3	1	0	50	41	18959136	4.33E-01	1
CCR8	159083	507	38	896052	248844	0	3	1	0	50	75	21803932	4.33E-01	1
AKR1C4	630098	678	-21	850840	223568	0	4	0	0	50	58	17840940	4.33E-01	1
HSPA1L	2509866	183	32	1586336	474904	0	7	0	0	4	3	2300472	4.33E-01	1
VAV1	739481	272	31	2244936	578856	0	8	2	0	41	60	20240736	4.33E-01	1
SCG3	415519	247	27	1257748	291920	0	4	0	0	29	46	15403764	4.33E-01	1
DSG4	260152	412	-4	2760068	768604	0	8	2	0	50	35	20426212	4.33E-01	1
HSPA6	741254	448	41	1572808	490568	0	6	2	0	50	66	22531596	4.33E-01	1
PROCA1	1695792	208	40	866148	228552	0	4	1	0	50	81	25518436	4.33E-01	1
DKK1	94567	519	-8	673908	194376	0	2	2	0	12	13	5276632	4.34E-01	1
AP2B1	715620	196	9	2458180	681740	0	11	1	0	27	29	12335756	4.34E-01	1
KLHL2	171218	540	5	1597728	407976	0	6	0	0	14	12	5229996	4.34E-01	1
LYVE1	755456	391	25	822716	233536	0	1	1	0	50	63	27469316	4.34E-01	1

SPAG6	177548	462	14	1312216	364544	0	3	0	0	21	20	9504488	4.34E-01	1
TCHHL1	527465	349	35	2278400	629764	0	9	3	0	50	62	21594604	4.34E-01	1
SPATA5	100687	528	11	2280180	650412	0	10	1	0	45	62	24193048	4.34E-01	1
A2M	348389	364	17	3808132	1067288	0	11	4	0	30	41	16277744	4.34E-01	1
GLTPD2	1272092	222	28	695624	257032	0	2	0	0	50	74	23892584	4.34E-01	1
CCDC137	3745109	205	30	742616	210040	0	1	0	0	33	25	10695308	4.34E-01	1
PROSC	651180	379	15	719832	196512	0	3	0	0	50	48	21015748	4.34E-01	1
HTR1D	956630	169	10	911004	300108	0	4	0	0	50	56	20074484	4.34E-01	1
LRRTM1	10982	1577	-18	1282668	397296	0	8	5	0	11	12	3017812	4.34E-01	1
ARHGAP2[illegible]	466144	327	-9	3286948	850484	0	11	3	0	38	40	14690340	4.35E-01	1
ZNF774	1110178	139	33	1253476	310076	0	6	1	0	50	72	20041376	4.35E-01	1
SMPD3	794719	279	46	1625852	501604	0	5	2	0	50	56	20638032	4.35E-01	1
IL1A	578617	298	38	716984	180136	0	1	0	0	50	43	18472484	4.35E-01	1
CNTNAP2	4892	1115	-31	3464592	901392	0	20	6	0	20	49	8153112	4.35E-01	1
UFM1	126181	926	-9	232468	64436	0	1	0	0	50	61	23706752	4.35E-01	1
EID2	1148741	322	25	566752	192596	0	2	0	0	50	44	18982632	4.35E-01	1
NOL12	1136064	180	65	548240	160912	0	2	1	0	24	18	7824168	4.35E-01	1
OR10C1	404210	854	21	741548	260236	0	3	2	0	12	14	5210416	4.35E-01	1
SULT6B1	545763	253	35	714492	165540	0	3	0	0	50	60	22530884	4.35E-01	1
IL12RB2	454381	466	-4	2224288	604844	0	7	1	0	22	23	8102204	4.35E-01	1
ABRA	113560	708	18	964048	266288	0	4	0	0	50	51	19230052	4.35E-01	1
UGT3A1	155890	614	-4	1396944	393024	0	5	2	0	50	47	22782220	4.35E-01	1
ASB14	399197	454	29	1537920	420792	0	2	0	0	50	37	23351820	4.35E-01	1
C9	168073	424	-8	1466364	372732	0	6	2	0	50	45	20095488	4.35E-01	1
FTH1	2389859	185	53	483092	119260	0	4	0	0	50	70	19465368	4.36E-01	1
PCDHB11	915569	724	-11	1938064	618728	0	11	0	0	0	0	618728	4.36E-01	1
IL20RA	133090	449	24	1407980	392668	0	4	1	0	50	54	18719904	4.36E-01	1
ZEB1	323935	635	16	2897484	756144	0	12	0	0	8	9	4998240	4.36E-01	1
LCE3D	285984	752	17	233536	68708	0	1	1	0	10	6	2606988	4.36E-01	1
ASPA	403115	442	6	822004	209684	0	3	1	0	50	47	17754432	4.36E-01	1
PRKRA	159623	482	29	850484	217516	0	3	0	0	50	60	23941356	4.36E-01	1
SSB	338901	489	35	1140980	260236	0	3	0	0	50	36	15621992	4.36E-01	1
PSMD12	486488	285	-1	1205060	304024	0	4	1	0	50	64	24095148	4.36E-01	1

EED	443833	159	26	1181920	283376	0	4	1	0	39	61	18906804	4.36E-01	1
OR6C75	667726	802	-14	772164	229620	0	3	0	0	50	83	19448636	4.36E-01	1
SNAI1	575200	181	51	657532	202208	0	3	0	0	50	63	20381712	4.36E-01	1
BAX	1927185	211	43	642224	192952	0	1	1	0	50	57	20562560	4.36E-01	1
C1QL1	1037808	191	34	627984	206124	0	3	0	0	50	53	17876540	4.36E-01	1
PRSS12	161497	917	1	2229984	630120	0	8	2	0	50	51	17698184	4.36E-01	1
F13A1	44522	696	22	1898192	510148	0	6	1	0	50	52	21251420	4.37E-01	1
C6orf58	169285	807	-9	865792	218228	0	3	0	0	50	67	21086592	4.37E-01	1
PRDM16	248448	632	16	3234260	927736	0	12	2	0	7	8	3723048	4.37E-01	1
AR	106188	NaN	-7	2318272	686012	0	6	1	0	50	51	20492784	4.37E-01	1
TEX9	208204	604	13	1065152	244216	0	4	1	0	50	61	19072700	4.37E-01	1
MYLPF	2312884	165	47	466716	107868	0	2	0	0	50	59	19035320	4.37E-01	1
SS18L2	350455	445	42	208616	51976	0	1	0	0	50	64	22168832	4.37E-01	1
KIAA1324	994979	215	25	2617668	720900	0	9	2	0	50	81	21774028	4.37E-01	1
EPS15	568927	251	11	2456044	625136	0	10	2	0	31	55	17860876	4.37E-01	1
MYO10	275988	622	-15	5371328	1396588	0	13	4	0	8	6	3349960	4.37E-01	1
C17orf80	205112	251	-15	1564976	433252	0	4	0	0	8	8	3508024	4.37E-01	1
SLC7A6	864889	281	38	1297976	393736	0	3	0	0	37	55	23126828	4.37E-01	1
ERICH1	106263	889	-34	1148100	297972	0	3	0	0	50	66	16358912	4.37E-01	1
PF4V1	233432	444	6	267000	80100	0	1	0	0	50	46	21504892	4.37E-01	1
IQCE	777967	226	44	1803496	516200	0	4	0	0	38	40	20296628	4.38E-01	1
DUSP11	1051432	404	37	993240	254184	0	5	0	0	5	6	1679608	4.38E-01	1
P4HTM	1799657	241	41	1417948	423284	0	4	2	0	50	76	22684320	4.38E-01	1
FLOT1	2157341	147	43	1111432	311144	0	4	1	0	50	59	18860524	4.38E-01	1
PRTN3	2168802	198	28	636884	205768	0	3	0	0	50	73	22578944	4.38E-01	1
CYP4X1	388889	479	4	1335712	345320	0	4	0	0	5	3	2130660	4.38E-01	1
TRIAP1	1573849	216	44	210396	44856	0	1	0	0	50	64	21461816	4.38E-01	1
ZC3H6	593451	449	24	3046292	813460	0	12	2	0	25	30	12173776	4.38E-01	1
SPATA22	365088	515	14	960132	240300	0	3	0	0	50	54	23562928	4.38E-01	1
MAGEA12	NaN	NaN	NaN	778928	234604	0	3	1	0	50	117	39880188	4.38E-01	1
CDCA4	854493	205	30	597724	186188	0	3	1	0	50	86	25553680	4.38E-01	1
ECE2	1453600	241	47	2722688	786760	0	10	1	0	11	9	3774312	4.38E-01	1
TGFB1I1	839076	558	11	1180496	351016	0	4	1	0	50	75	22592472	4.38E-01	1

TMEM5	222270	460	-17	1151660	294412	0	4	0	0	50	69	21300192	4.38E-01	1
CD82	211161	551	20	705592	187256	0	2	0	0	50	34	18377432	4.39E-01	1
LSM14B	838629	288	-13	983984	286936	0	5	1	0	33	38	11186588	4.39E-01	1
TUBB2A	444075	435	41	1131012	312924	0	6	1	0	50	79	25740224	4.39E-01	1
CEP55	458453	249	17	1218588	305448	0	6	2	0	47	55	20630556	4.39E-01	1
ASB4	176832	333	14	1148456	309364	0	5	1	0	50	46	19395948	4.39E-01	1
STARD4	181104	636	26	546460	134924	0	2	0	0	49	49	19377080	4.39E-01	1
ZPBP	135352	917	-13	918836	243148	0	5	0	0	50	64	21012544	4.39E-01	1
HCCS	80480	NaN	30	710932	176576	0	3	0	0	50	53	22365700	4.39E-01	1
LRRK2	31336	994	12	6625872	1687440	0	36	9	0	3	13	4058044	4.39E-01	1
ZNF577	435461	817	-45	1250628	323604	0	3	0	0	31	33	10043472	4.39E-01	1
KTI12	NaN	NaN	NaN	848704	290852	0	4	2	0	50	118	39936436	4.39E-01	1
FAM81B	316501	511	4	1205416	286580	0	6	1	0	50	57	21156368	4.39E-01	1
BAIAP2L1	609918	249	36	1339984	356000	0	3	1	0	50	59	22786492	4.40E-01	1
XRCC4	230543	717	8	892848	216804	0	3	0	0	31	20	10179464	4.40E-01	1
PARL	1434591	408	37	990392	267712	0	6	0	0	21	25	7903912	4.40E-01	1
SYCP2L	269928	483	20	2207912	520828	0	6	1	0	50	51	24288100	4.40E-01	1
TRMT2A	545126	218	57	1571740	485228	0	7	1	0	33	49	14596356	4.40E-01	1
MAP6	528316	227	45	1991108	634036	0	8	4	0	6	6	2939492	4.40E-01	1
FSTL1	382340	375	23	831972	198648	0	1	0	0	50	40	20692500	4.40E-01	1
TBC1D28	729405	435	-2	561056	143824	0	2	0	0	50	43	18400572	4.40E-01	1
LAMA2	67993	771	-6	8108256	2171244	0	28	5	0	32	37	13785032	4.40E-01	1
DDIT4	808655	145	40	566040	189036	0	2	1	0	50	45	18089784	4.40E-01	1
SLC6A6	484954	242	47	1605204	446424	0	4	1	0	50	40	18304096	4.40E-01	1
HSD17B1(	920771	NaN	70	651836	211108	0	1	0	0	50	62	20770820	4.40E-01	1
TRAF3IP3	220312	429	18	1454260	378428	0	4	0	0	49	54	21711728	4.40E-01	1
RP9	376764	459	15	590604	144180	0	2	1	0	50	41	17280952	4.40E-01	1
KITLG	144795	419	-29	737988	178356	0	4	1	0	50	92	23051356	4.40E-01	1
TAF1B	909213	197	43	1578860	370240	0	5	1	0	46	49	19690360	4.40E-01	1
KLHL6	712779	593	32	1583132	437524	0	9	3	0	8	18	6035624	4.41E-01	1
RNMTL1	662733	483	10	1050912	312924	0	4	0	0	5	8	3196168	4.41E-01	1
RCN2	335583	614	27	846568	197936	0	3	0	0	50	65	26816412	4.41E-01	1
CCL5	661778	235	20	237452	67996	0	1	0	0	50	59	19655472	4.41E-01	1

TMEM31	485629	NaN	43	513352	117836	0	2	0	0	50	50	18367108	4.41E-01	1
NRIP3	595760	357	33	622644	178356	0	2	0	0	50	60	26308044	4.41E-01	1
C3AR1	142724	300	23	1191888	359916	0	4	2	0	50	43	20540844	4.41E-01	1
CYP26A1	558273	470	18	1254188	373444	0	6	0	0	43	54	16544744	4.41E-01	1
GRPEL1	459652	426	70	559632	153792	0	3	1	0	50	67	19324392	4.41E-01	1
ADORA3	374092	330	27	1486300	419012	0	4	0	0	39	31	18074832	4.41E-01	1
PHACTR2	346938	363	2	1657892	479176	0	5	1	0	31	32	12102932	4.41E-01	1
C2orf61	738157	206	39	658244	181204	0	2	1	0	6	8	4933092	4.41E-01	1
HSD17B13	474615	417	8	773944	218228	0	3	1	0	50	77	27451160	4.41E-01	1
CENPB	860065	241	43	1466008	456392	0	7	1	0	50	63	24054564	4.41E-01	1
CRMP1	101380	643	14	1780712	521896	0	6	3	0	50	47	17869420	4.42E-01	1
FAM196A	92819	848	-36	1197940	352796	0	5	0	0	5	4	2529024	4.42E-01	1
FTO	215906	604	-13	1312928	342472	0	3	0	0	44	37	16611316	4.42E-01	1
FBXW4	764146	385	29	1041300	316128	0	3	2	0	50	54	20470712	4.42E-01	1
CASQ1	841605	386	29	1053404	261304	0	5	0	0	50	48	19038524	4.42E-01	1
MIPEP	262509	678	46	1865796	500892	0	9	1	0	43	41	13563956	4.42E-01	1
RIOK3	311512	215	57	1384484	332860	0	4	1	0	50	59	20443300	4.42E-01	1
QRSL1	227264	174	40	1360632	377004	0	4	1	0	50	60	16841648	4.42E-01	1
SAMD4A	653012	376	38	1917060	551088	0	7	0	0	13	16	6539364	4.42E-01	1
BUD31	1327454	288	60	390176	91492	0	2	0	0	50	58	17222924	4.43E-01	1
ATP5A1	388169	300	67	1402640	425776	0	5	0	0	50	62	22125044	4.43E-01	1
OGFOD1	523951	285	13	1448920	344252	0	3	0	0	14	9	5932384	4.43E-01	1
ICOS	122253	693	5	531152	128872	0	2	0	0	50	61	23739504	4.43E-01	1
CDH15	1307930	254	38	2015672	653260	0	7	3	0	50	57	20720624	4.43E-01	1
YRDC	445096	375	33	681740	234604	0	1	1	0	50	50	19791820	4.43E-01	1
POU2F3	323392	198	36	1134216	319332	0	2	3	0	27	23	8094372	4.43E-01	1
CRYBG3	123806	723	0	2673204	683520	0	8	2	0	28	19	11284132	4.43E-01	1
XRN2	288961	638	-12	2547892	625136	0	6	1	0	41	32	16020356	4.43E-01	1
CRIPT	603816	252	35	284444	61588	0	1	0	0	50	45	22018244	4.44E-01	1
FEZF1	61242	1007	-15	1192244	347812	0	7	1	0	50	79	22213332	4.44E-01	1
SLC16A1	601487	276	16	1250984	371308	0	3	3	0	8	13	4602012	4.44E-01	1
SYT4	4302	1101	-41	1079748	297972	0	8	1	0	4	8	1814888	4.44E-01	1
TRAM1L1	24934	NaN	-31	925956	259524	0	7	1	0	24	56	11857648	4.44E-01	1

FOXR1	1457410	180	55	752584	209684	0	1	0	0	19	13	6716296	4.44E-01	1
E2F5	99174	1450	3	899968	243860	0	3	1	0	50	42	21193748	4.44E-01	1
CA10	36963	832	-68	868996	219296	0	5	0	0	50	80	22721344	4.44E-01	1
COX7A2L	251919	375	16	292988	86152	0	1	0	0	50	44	19285944	4.44E-01	1
CEP164	440623	360	35	3774312	1039164	0	11	3	0	25	23	8756176	4.44E-01	1
FUT2	1616265	190	38	847636	258812	0	4	0	0	43	53	17176644	4.44E-01	1
T6GALNA(	319154	834	-17	858316	238520	0	4	0	0	50	60	18905736	4.44E-01	1
ATP1B4	484939	NaN	23	946960	232112	0	3	0	0	50	50	16394156	4.45E-01	1
ADAT1	449343	354	18	1298688	351372	0	6	2	0	50	60	21330452	4.45E-01	1
BRS3	374989	NaN	23	993596	298684	0	3	0	0	50	47	23054916	4.45E-01	1
KIAA1033	237030	387	34	3139920	760416	0	11	1	0	8	4	2420800	4.45E-01	1
MAPRE3	1222544	290	33	745108	184052	0	3	0	0	50	54	20146040	4.45E-01	1
KCTD5	1803069	200	47	604844	171592	0	1	0	0	50	64	19321900	4.45E-01	1
MRPL34	842088	211	43	227128	75116	0	1	0	0	50	61	21639816	4.45E-01	1
ZCWPW1	1454967	172	57	1712716	435032	0	7	0	0	9	5	2948392	4.45E-01	1
SSSCA1	3945819	202	45	499112	158776	0	2	0	0	19	25	9711324	4.45E-01	1
PSG6	282702	238	-37	1136708	327520	0	7	2	0	22	51	10502000	4.45E-01	1
SPRED2	545446	346	36	1090072	294056	0	5	0	0	50	64	21907884	4.45E-01	1
KIAA0319	417813	373	19	2769324	786048	0	6	1	0	27	34	12201188	4.45E-01	1
PAQR4	1443513	234	49	652548	233892	0	1	2	0	46	45	16352504	4.45E-01	1
SURF6	1846659	416	36	901036	278036	0	3	2	0	50	51	15758696	4.45E-01	1
YIF1A	2133752	236	53	744040	229976	0	2	0	0	50	77	24481764	4.46E-01	1
TM6SF1	308479	635	29	952656	274476	0	4	1	0	37	46	17166320	4.46E-01	1
ZNF501	433000	541	28	701320	170168	0	1	0	0	50	49	17644428	4.46E-01	1
TAS2R16	54952	972	-23	733360	206480	0	3	2	0	6	11	3438248	4.46E-01	1
TMEM63C	549191	394	47	2114996	564616	0	8	3	0	33	42	15619856	4.46E-01	1
CARKD	504689	278	33	979712	313636	0	3	2	0	49	55	19089076	4.46E-01	1
SLC6A17	552043	400	33	1843012	536492	0	5	1	0	31	30	13431524	4.46E-01	1
SPIN1	126591	495	-6	683164	176576	0	2	2	0	5	7	2663592	4.46E-01	1
HEPN1	175852	743	14	222144	63012	0	1	0	0	50	63	23580016	4.46E-01	1
SEL1L2	116952	820	-29	1844792	448204	0	9	1	0	50	77	20583208	4.46E-01	1
CEACAM7	1249321	499	27	677824	193664	0	3	1	0	50	82	23862680	4.47E-01	1
RGMA	243187	484	-19	1145608	357068	0	5	3	0	31	56	14244628	4.47E-01	1

FZD10	59974	465	-39	1439308	425420	0	7	1	0	36	50	16348944	4.47E-01	1
SLC8A2	797690	276	38	2268432	724104	0	9	3	0	50	64	22709952	4.47E-01	1
GTF2H1	601817	252	37	1453904	364900	0	4	0	0	50	58	23220812	4.47E-01	1
ACAA1	302137	411	32	1076900	331792	0	3	0	0	50	54	20798588	4.47E-01	1
WDR31	408268	248	31	965828	251692	0	4	1	0	50	55	20490292	4.47E-01	1
TAF13	989913	215	18	336420	79032	0	1	1	0	13	20	4444660	4.47E-01	1
CD200R1	245141	639	11	952300	243860	0	2	0	0	48	57	23192332	4.47E-01	1
ESD	272205	820	63	755788	185120	0	3	0	0	50	52	20497412	4.47E-01	1
LYZL6	703538	424	31	391244	98968	0	3	1	0	2	5	1582064	4.47E-01	1
CD53	424743	764	24	577432	157352	0	1	1	0	18	24	8633712	4.47E-01	1
CD14	774470	356	31	887508	321468	0	2	0	0	19	25	9696372	4.47E-01	1
SLC46A1	1410113	190	28	1119620	374512	0	5	2	0	50	55	21380292	4.47E-01	1
DCTPP1	2338593	142	59	422928	135636	0	2	0	0	50	74	22010768	4.47E-01	1
BAHD1	888588	192	47	1895344	632612	0	7	2	0	38	56	17300888	4.48E-01	1
LRRIQ3	58597	1188	-18	1636176	394092	0	6	1	0	50	81	23402372	4.48E-01	1
CLEC3A	44717	1261	-26	510860	134212	0	3	0	0	50	82	20582140	4.48E-01	1
AOX1	572062	160	23	3496988	940552	0	9	2	0	50	54	19999012	4.48E-01	1
PCCB	229049	484	25	1448564	411892	0	4	2	0	50	38	16047412	4.48E-01	1
ZNF213	1626425	234	44	1145608	345320	0	5	1	0	50	77	22657976	4.48E-01	1
FRS2	540769	350	7	1289076	363120	0	6	1	0	50	60	22275988	4.48E-01	1
DNAL1	600464	191	47	518336	123532	0	2	0	0	50	62	20887588	4.48E-01	1
FGB	59883	1034	20	1293348	315060	0	5	0	0	50	39	19125032	4.48E-01	1
CARD14	674340	219	22	2692784	786760	0	6	3	0	36	55	18118976	4.48E-01	1
RBMXL1	316278	598	5	951944	300820	0	5	1	0	50	59	20056684	4.48E-01	1
FOXF2	114944	481	14	1082240	347812	0	2	2	0	50	46	20334720	4.48E-01	1
JTB	2028800	222	34	385192	102884	0	2	0	0	50	66	18282736	4.48E-01	1
UBE2N	380369	364	15	395160	107868	0	4	1	0	8	12	2497340	4.48E-01	1
FSIP1	516737	433	0	1541480	370240	0	4	0	0	50	46	18845572	4.48E-01	1
ZNF354A	395801	765	5	1558924	399788	0	10	2	0	14	16	5120704	4.48E-01	1
ANKRD7	72287	922	-13	674620	168032	0	3	0	0	50	73	25381732	4.48E-01	1
FYTTD1	517022	210	47	849416	235672	0	3	0	0	50	42	18318336	4.49E-01	1
ZFP30	484296	888	-51	1359208	323960	0	7	0	0	28	34	9636920	4.49E-01	1
KIFAP3	181109	743	2	2115708	506232	0	10	2	0	12	16	6171972	4.49E-01	1

PSMG2	434233	206	35	688148	188680	0	1	1	0	50	50	20395596	4.49E-01	1
CCDC97	1193025	249	33	868640	250624	0	4	0	0	50	52	16514128	4.49E-01	1
PLA1A	288657	595	42	1182632	326452	0	4	0	0	50	57	18639092	4.49E-01	1
TTK	150632	841	-2	2295844	547172	0	7	0	0	1	0	883592	4.49E-01	1
RFC3	84617	580	33	969744	252048	0	1	0	0	50	40	22011836	4.49E-01	1
CLEC4A	142724	300	23	633324	150588	0	2	0	0	50	43	20540844	4.49E-01	1
GDPD1	896167	379	4	946604	239944	0	5	0	0	28	26	8427232	4.49E-01	1
SEC61A1	817705	419	42	1214672	360628	0	4	2	0	50	55	19414460	4.49E-01	1
ARSB	203299	452	50	1361700	412248	0	5	0	0	7	7	3158432	4.50E-01	1
CDCA8	445023	375	27	739768	203276	0	2	2	0	37	40	13106496	4.50E-01	1
RAB22A	841935	330	18	515488	134924	0	3	1	0	11	11	4136364	4.50E-01	1
NR2F2	94682	983	-22	1080816	311856	0	4	0	0	41	94	25592128	4.50E-01	1
LHFPL3	324628	895	5	593452	174440	0	1	0	0	50	73	22089800	4.50E-01	1
VDAC1	615558	269	56	738344	205768	0	3	1	0	50	58	21133584	4.50E-01	1
EPC1	511962	189	15	2151664	589892	0	9	0	0	2	2	1928808	4.50E-01	1
KCNJ14	1418188	209	41	1059100	349592	0	5	0	0	50	91	32574000	4.50E-01	1
RAP2C	152540	NaN	18	469208	128872	0	2	0	0	50	46	15568592	4.50E-01	1
C4BPB	434877	155	24	681384	156996	0	3	1	0	43	68	22256052	4.50E-01	1
MORC4	177448	NaN	31	2451060	622644	0	7	1	0	35	41	15323664	4.50E-01	1
RAB10	591333	135	44	534356	131008	0	2	0	0	50	54	19203708	4.50E-01	1
EPYC	20238	574	-33	831616	228908	0	3	0	0	50	96	21164912	4.51E-01	1
UCHL1	224086	190	39	596300	157708	0	3	1	0	50	60	17223280	4.51E-01	1
EBLN2	NaN	NaN	NaN	687080	187612	0	1	0	0	50	116	39833196	4.51E-01	1
SHE	1711793	337	45	1242084	370596	0	5	3	0	32	37	11254228	4.51E-01	1
CSNK1E	1550040	181	63	1072984	305804	0	3	2	0	50	58	19090856	4.51E-01	1
C6orf62	422850	362	40	603420	150588	0	2	0	0	50	54	24131104	4.51E-01	1
NNAT	790368	549	52	213600	66216	0	1	0	0	50	68	24056344	4.51E-01	1
IMPG1	187724	674	-13	2078328	546816	0	7	1	0	50	77	28104064	4.51E-01	1
RBMS2	2807863	166	57	1070136	292632	0	4	0	0	33	61	19161344	4.51E-01	1
RRM2	805187	220	47	1169816	312568	0	4	0	0	40	48	16930292	4.51E-01	1
ATP4B	723091	370	36	767536	195800	0	1	0	0	50	65	24677208	4.51E-01	1
CAPN6	132204	NaN	23	1670352	437880	0	4	2	0	50	35	16861584	4.52E-01	1
MNAT1	261238	578	5	822360	200784	0	3	0	0	50	65	21090864	4.52E-01	1

DCAKD	831501	191	28	575652	180492	0	3	0	0	50	88	23819960	4.52E-01	1
RASD2	378214	227	-30	676756	187256	0	3	1	0	50	90	20097268	4.52E-01	1
GNPAT	380550	569	8	1779644	466360	0	6	1	0	50	71	20460388	4.52E-01	1
TREH	1449531	161	45	1514424	417588	0	3	0	0	35	53	17505944	4.52E-01	1
TTC7B	367726	570	20	2152020	633324	0	11	1	0	4	6	2117488	4.52E-01	1
PRNP	556280	447	11	645072	177288	0	1	0	0	8	5	2916352	4.52E-01	1
CPNE6	992350	355	19	1444292	411892	0	5	2	0	19	27	8529760	4.52E-01	1
ARL5B	74299	539	-5	477396	120684	0	2	0	0	50	63	22442240	4.52E-01	1
RNF19A	1009773	212	41	2148460	578144	0	11	0	0	9	6	3113220	4.52E-01	1
FXYD4	388267	387	-2	256676	61588	0	1	0	0	50	47	18985836	4.52E-01	1
SCN4B	707709	293	56	581348	169456	0	1	0	0	50	54	16045632	4.52E-01	1
APPL2	233879	387	30	1761488	454612	0	6	1	0	27	27	12632660	4.52E-01	1
PIP5KL1	1278941	201	51	995020	311144	0	1	2	0	30	20	11368860	4.52E-01	1
DCAF8L1	10501	NaN	25	1505524	420080	0	8	1	0	32	42	13430812	4.52E-01	1
MCTP1	148501	629	22	2625144	735852	0	7	4	0	50	50	22065948	4.52E-01	1
PAK3	109883	NaN	18	1530444	399432	0	7	0	0	50	59	19823148	4.52E-01	1
ZNF805	NaN	NaN	NaN	1609832	415096	0	5	0	0	50	116	40060680	4.52E-01	1
CYLC1	19326	NaN	-45	1701680	408688	0	7	0	0	11	17	6558944	4.52E-01	1
TEX264	759916	456	37	778928	244216	0	3	1	0	50	51	19214744	4.53E-01	1
ARNT2	365740	542	8	1874696	502672	0	8	1	0	50	52	20350028	4.53E-01	1
MAN1C1	693953	443	29	1609832	458884	0	5	1	0	50	54	19708160	4.53E-01	1
FCRL6	801453	497	6	1112856	321468	0	5	2	0	50	57	19094772	4.53E-01	1
DARS	440321	410	30	1339984	332504	0	4	2	0	50	62	21310160	4.53E-01	1
SPAG11B	NaN	NaN	NaN	908512	234248	0	2	0	0	50	116	39879832	4.53E-01	1
ZDHHC11	843639	408	28	1079392	295124	0	2	1	0	50	66	25580380	4.53E-01	1
GAS2L1	849693	251	36	1628700	580992	0	5	2	0	50	58	21612760	4.53E-01	1
EXPH5	369515	500	13	5029212	1368108	0	20	2	0	15	14	7006792	4.53E-01	1
TATDN1	456351	544	44	822004	186188	0	2	0	0	50	66	20641948	4.53E-01	1
HDGF	1649536	208	53	805272	209328	0	2	1	0	50	69	21716356	4.53E-01	1
ELMO2	543201	612	48	1908872	490924	0	6	2	0	50	56	20294848	4.53E-01	1
YY1AP1	2262876	167	29	2064088	598436	0	7	2	0	50	73	25473936	4.53E-01	1
GOLGA2	1813435	201	46	2619092	703456	0	12	1	0	13	13	4284104	4.54E-01	1
ATP1A2	835734	386	23	2602716	762552	0	9	2	0	50	73	27174548	4.54E-01	1

MX2	241283	506	46	1865084	484516	0	6	3	0	50	49	18139624	4.54E-01	1
TLK1	439214	379	38	2070496	521540	0	5	2	0	9	7	3055904	4.54E-01	1
ANKRA2	403014	289	42	814884	221076	0	2	0	0	50	67	21722052	4.54E-01	1
JRKL	272651	489	14	1325744	356356	0	7	1	0	50	50	17174152	4.54E-01	1
SLC35E4	723012	330	56	813816	315060	0	3	0	0	35	28	11658644	4.54E-01	1
ZDHHC8	503106	277	64	2024928	699540	0	4	1	0	23	18	8463544	4.54E-01	1
ZBTB38	405071	178	40	3024576	809544	0	9	2	0	50	48	18839164	4.54E-01	1
HAUS8	945446	386	30	1060880	298684	0	2	1	0	50	43	19029624	4.54E-01	1
COMMD6	197100	362	35	267712	68708	0	1	0	0	50	62	21338284	4.54E-01	1
DBNDD1	1056657	221	35	480244	143468	0	2	0	0	50	47	16394156	4.55E-01	1
SHBG	2069567	213	34	1011040	314348	0	4	1	0	21	25	6102908	4.55E-01	1
MIOS	213895	446	16	2255260	594164	0	16	5	0	7	17	4155944	4.55E-01	1
MYBPC1	348152	448	6	3154516	797084	0	12	1	0	29	25	9759028	4.55E-01	1
PRR16	8721	1054	-19	691352	220720	0	4	1	0	50	82	21782216	4.55E-01	1
TGDS	149137	803	14	945180	226416	0	3	0	0	50	48	17425844	4.55E-01	1
SCML1	70191	NaN	15	855824	230332	0	2	0	0	50	70	22627360	4.55E-01	1
CMTM7	306655	592	19	445356	135636	0	1	0	0	50	38	27163868	4.55E-01	1
BTN3A1	384310	375	47	1464940	395516	0	5	1	0	50	51	17347168	4.55E-01	1
PRL	13319	831	-2	584552	167320	0	2	0	0	50	54	22244660	4.55E-01	1
GPC5	12353	1007	-9	1458532	407264	0	17	4	0	1	4	720544	4.55E-01	1
TAZ	2277674	NaN	40	787116	194376	0	5	2	0	11	18	4530812	4.55E-01	1
OR5T1	10317	969	-43	809900	237808	0	6	2	0	8	13	3063024	4.55E-01	1
SPANXN2	4811	NaN	-27	466004	120328	0	3	0	0	50	131	28720656	4.55E-01	1
ANAPC4	233480	611	44	2177652	538272	0	8	1	0	43	47	15187672	4.55E-01	1
HPGDS	129148	311	14	535068	127092	0	2	0	0	50	46	17889356	4.56E-01	1
TREM2	227466	611	41	585976	173372	0	3	0	0	50	50	15425836	4.56E-01	1
PDIA6	691715	507	50	1153440	312924	0	5	1	0	50	51	19636604	4.56E-01	1
OXNAD1	188483	517	34	808476	224280	0	4	1	0	50	62	20323684	4.56E-01	1
TMEM184C	71637	343	9	1141336	303668	0	4	0	0	32	34	15588172	4.56E-01	1
RABEPK	951081	168	46	958708	266288	0	5	0	0	25	28	10521936	4.56E-01	1
CHMP5	856503	167	38	592740	142044	0	1	0	0	50	62	21222584	4.56E-01	1
RAB12	241286	451	11	635460	170880	0	2	0	0	50	48	21642664	4.56E-01	1
PRSS16	161388	645	24	1281244	417944	0	4	2	0	50	40	20718132	4.56E-01	1

WBP5	448080	NaN	32	285512	58384	0	1	0	0	50	52	16837020	4.56E-01	1
PIAS3	NaN	NaN	NaN	1586336	486652	0	5	0	0	50	116	40132236	4.56E-01	1
SERPINE1	1066804	272	39	1035248	289072	0	4	1	0	50	83	23088024	4.56E-01	1
ZBP1	181790	562	-4	1158424	329300	0	3	1	0	50	59	19947392	4.57E-01	1
NMNAT2	769970	465	13	877896	228552	0	2	1	0	50	45	16269556	4.57E-01	1
ARHGAP1[illegible]	359237	346	25	2233900	556784	0	5	1	0	50	42	23283112	4.57E-01	1
CYP26B1	72898	411	-5	1267360	397652	0	5	2	0	50	55	20502752	4.57E-01	1
ART3	507519	322	41	1045928	250624	0	5	0	0	50	49	18868712	4.57E-01	1
NPM2	576057	222	41	581348	141688	0	2	0	0	50	38	18787544	4.57E-01	1
TNFSF8	241285	318	-9	599504	166252	0	2	1	0	50	48	20055616	4.57E-01	1
KLHL20	440130	417	5	1562128	439304	0	3	0	0	27	16	9168780	4.57E-01	1
SEC14L4	655714	288	38	1075120	282308	0	4	1	0	50	60	17814952	4.57E-01	1
DDX47	742991	129	27	1175868	334284	0	3	1	0	50	55	22305180	4.57E-01	1
RICTOR	193512	336	-2	4451068	1184768	0	14	2	0	20	19	9884696	4.57E-01	1
LCE2A	169158	818	24	271272	75828	0	1	0	0	50	53	20184132	4.57E-01	1
SERPINB6	446135	201	44	982560	250980	0	4	1	0	50	48	18310148	4.58E-01	1
LIF	548674	211	43	506232	152724	0	2	0	0	50	54	19921048	4.58E-01	1
PTPN2	435935	152	46	1113568	279104	0	2	1	0	50	37	16366032	4.58E-01	1
RHGAP11	220858	693	10	695268	189036	0	3	0	0	50	60	20067720	4.58E-01	1
NECAB2	494952	460	3	1011040	282308	0	2	1	0	50	40	17879032	4.58E-01	1
DSTN	568191	347	-5	437524	107156	0	1	1	0	50	56	19901468	4.58E-01	1
KRT24	953708	269	13	1326812	390532	0	5	2	0	50	60	20121832	4.58E-01	1
SCPEP1	333715	254	-10	1188684	316128	0	4	2	0	50	46	20300900	4.58E-01	1
CIC	790787	299	37	3865804	1372736	0	13	5	0	18	15	6929540	4.58E-01	1
UGT2B17	107080	748	-5	1378076	348880	0	6	2	0	20	20	8316516	4.58E-01	1
COL7A1	1386275	212	37	7435772	2500900	0	29	4	0	0	4	2500900	4.58E-01	1
ZNF251	1841573	401	17	1712004	458172	0	8	0	0	3	2	1449988	4.58E-01	1
SCG2	276568	364	-7	1584200	400144	0	10	3	0	7	10	3298340	4.58E-01	1
SHMT2	2162032	238	58	1275904	389108	0	3	1	0	50	84	24087316	4.58E-01	1
MICAL1	328699	559	47	2714144	810256	0	8	1	0	34	32	14602764	4.58E-01	1
C6orf47	2517236	193	20	699184	245996	0	2	1	0	17	7	4698132	4.58E-01	1
PPT2	2119974	392	28	799220	226060	0	3	1	0	50	62	23774036	4.58E-01	1
TCF4	92114	936	12	1750808	483448	0	7	2	0	50	62	21986560	4.58E-01	1

DNAJB11	592490	436	40	939484	249200	0	4	1	0	50	85	29356116	4.58E-01	1
RUSC2	1081620	300	28	3705960	1201500	0	12	0	0	0	0	1201500	4.59E-01	1
LRMP	250089	700	17	1336424	338200	0	4	0	0	42	47	17380276	4.59E-01	1
CDCA5	3267059	207	46	634748	197224	0	4	1	0	50	74	20482816	4.59E-01	1
TOX4	707390	470	30	1554652	473480	0	5	2	0	31	43	15582120	4.59E-01	1
AGPAT1	2119974	392	28	712712	222856	0	2	2	0	10	13	4219668	4.59E-01	1
SEPP1	343552	626	-12	1058032	283376	0	2	0	0	50	33	15396644	4.59E-01	1
TSNAXIP1	1039476	143	49	1735856	437524	0	6	0	0	37	34	16030680	4.59E-01	1
TMEM115	843769	206	51	836956	295124	0	3	1	0	50	68	20472848	4.59E-01	1
C6orf165	222811	556	26	1622648	424708	0	4	0	0	19	14	7121424	4.59E-01	1
NKAIN3	138003	904	-16	520116	135636	0	2	0	0	50	70	23225796	4.59E-01	1
PSMA1	366243	381	-3	764688	197580	0	3	0	0	22	19	8463900	4.60E-01	1
DBF4	202733	758	14	1779288	430404	0	7	2	0	50	67	22089444	4.60E-01	1
ATP6AP1L	146069	476	1	571736	166252	0	2	1	0	7	3	1387332	4.60E-01	1
GDA	285414	207	11	1200076	313280	0	6	3	0	7	11	3291932	4.60E-01	1
MMP19	1909014	256	38	1291924	373088	0	4	1	0	50	69	23288452	4.60E-01	1
MSRB2	126042	469	5	464580	138840	0	3	2	0	8	10	1363836	4.60E-01	1
TGIF2LX	8315	NaN	-35	607692	174084	0	5	0	0	50	118	24111880	4.60E-01	1
TBC1D10A	622382	211	50	1291924	375224	0	5	0	0	27	25	10479216	4.60E-01	1
CAPZA2	503859	185	8	774300	184764	0	2	0	0	50	59	21138212	4.60E-01	1
ZNF675	451758	1464	-69	1487724	348168	0	5	0	0	18	36	8523352	4.60E-01	1
CCM2	1090292	235	55	1224996	342828	0	5	2	0	22	47	12176980	4.60E-01	1
UTP18	758183	431	-15	1453548	388752	0	5	2	0	50	68	21197308	4.60E-01	1
NTN1	128482	632	-7	1532580	431472	0	5	0	0	50	54	26562584	4.60E-01	1
NOP58	386034	270	50	1409404	348524	0	4	0	0	50	50	19608836	4.60E-01	1
FAT4	15815	726	-10	12366372	3664308	0	63	18	0	3	22	5345340	4.60E-01	1
HIC2	NaN	NaN	NaN	1517628	464580	0	5	2	0	50	118	40110164	4.60E-01	1
SOX7	110362	481	9	944468	308296	0	4	1	0	50	59	22403080	4.60E-01	1
MARCO	152902	553	-2	1354936	382700	0	5	2	0	16	18	6387352	4.61E-01	1
TGIF1	444864	153	17	1034892	344964	0	4	1	0	50	55	24721708	4.61E-01	1
GMPS	345838	388	4	1800292	489500	0	4	2	0	50	64	22200160	4.61E-01	1
NCF4	1261877	537	50	1082952	302244	0	3	1	0	23	31	11720588	4.61E-01	1
HPCAL1	732179	270	44	507300	127092	0	2	1	0	50	56	21004712	4.61E-01	1

PLA2G7	141205	551	-1	1171596	291564	0	5	1	0	50	52	20657968	4.61E-01	1
GPR132	857767	198	43	928804	296192	0	4	0	0	50	58	20111152	4.61E-01	1
SEC13	742508	333	57	851552	219652	0	2	2	0	17	14	6689240	4.61E-01	1
PTPN7	649164	297	34	1016024	299752	0	1	0	0	33	33	17024988	4.61E-01	1
DNAJC12	286530	441	-5	548952	132432	0	2	0	0	50	56	21624864	4.61E-01	1
GRHL3	893155	198	22	1608408	426132	0	5	2	0	50	51	16219716	4.61E-01	1
GPR52	NaN	NaN	NaN	901392	260592	0	2	1	0	50	117	39906176	4.61E-01	1
VPS33B	753718	413	31	1650416	423640	0	4	1	0	42	60	20298408	4.61E-01	1
LRRC59	1335838	196	24	781420	231044	0	2	1	0	50	70	22959864	4.61E-01	1
RPL22L1	211469	610	16	331792	77252	0	2	0	0	14	16	4137788	4.61E-01	1
ACOX2	551037	265	33	1751876	495196	0	9	2	0	19	31	7602736	4.61E-01	1
RHAG	66931	1248	-6	1058744	295480	0	2	0	0	50	43	17490992	4.61E-01	1
PAPPA2	39980	863	-10	4592044	1293704	0	20	7	0	9	15	4863672	4.61E-01	1
OR52I1	212267	935	-3	794592	245640	0	3	2	0	30	45	12142448	4.61E-01	1
ATCAY	2187414	201	34	983984	259168	0	2	0	0	40	53	20047072	4.61E-01	1
RBM24	249340	560	-6	614812	194732	0	1	0	0	50	63	20553304	4.61E-01	1
SYNJ2BP	499052	591	37	374156	106444	0	2	0	0	50	72	22615968	4.61E-01	1
OR51B2	100115	1056	1	776792	230332	0	3	1	0	30	18	7873652	4.62E-01	1
RPL35A	612221	410	33	293344	81524	0	1	0	0	50	48	19887940	4.62E-01	1
NUDT13	539834	354	40	916344	248844	0	2	1	0	50	53	18779000	4.62E-01	1
CTBS	245445	585	-1	1001072	263440	0	3	0	0	50	48	16615588	4.62E-01	1
KCTD20	994811	148	35	1092564	283020	0	4	1	0	50	78	24184860	4.62E-01	1
POLG2	1306314	184	23	1247068	344252	0	6	0	0	50	75	23771544	4.62E-01	1
GPR89A	NaN	NaN	NaN	1208620	312212	0	5	1	0	50	117	39957796	4.62E-01	1
BTG4	383632	485	15	596300	142756	0	2	0	0	50	61	25250368	4.62E-01	1
ABCD2	70113	596	-16	1888936	523676	0	7	1	0	50	60	22563636	4.63E-01	1
LRRC1	256818	437	30	1354580	383056	0	4	1	0	50	58	22682184	4.63E-01	1
PI3	842540	324	25	300464	87220	0	1	0	0	50	49	22812836	4.63E-01	1
CSH2	1446139	380	27	595588	169100	0	1	1	0	50	63	18745892	4.63E-01	1
EIF2B2	875817	222	53	902816	257032	0	4	2	0	50	56	18192668	4.63E-01	1
MTCH2	989876	214	28	806696	218584	0	3	2	0	14	9	4489160	4.63E-01	1
PGF	875817	222	53	446068	127448	0	2	0	0	50	56	18192668	4.63E-01	1
RUFY3	262894	589	25	2002500	526524	0	6	2	0	50	56	24274572	4.63E-01	1

PSMD5	645542	212	30	1284092	374512	0	3	0	0	6	3	2392676	4.63E-01	1
EID1	NaN	NaN	NaN	479176	125312	0	2	0	0	50	116	39770896	4.63E-01	1
MMP10	510838	567	-16	1249204	319688	0	7	2	0	50	58	18555432	4.63E-01	1
SCCPDH	439136	282	3	1112500	312212	0	3	1	0	50	62	24415548	4.63E-01	1
ZBTB26	407115	430	26	1134216	286224	0	2	0	0	26	23	10651520	4.63E-01	1
ART1	537999	403	23	816664	251336	0	3	2	0	6	7	3702756	4.63E-01	1
TAAR6	178165	618	22	856892	249556	0	3	2	0	40	38	16712776	4.64E-01	1
GSX2	77093	472	20	742260	241368	0	2	2	0	8	10	3561424	4.64E-01	1
VPS37C	914160	531	42	868640	289072	0	3	0	0	50	61	25159232	4.64E-01	1
SGSM3	469297	355	56	1931656	556784	0	5	2	0	50	56	19318696	4.64E-01	1
PTPRU	365922	395	24	3713436	1074408	0	8	2	0	50	34	19843796	4.64E-01	1
TOLLIP	1373249	311	46	701320	201140	0	3	0	0	50	84	29587872	4.64E-01	1
TIGD5	2183482	235	37	1521544	538628	0	5	1	0	50	40	19636960	4.65E-01	1
SLCO4C1	36388	870	-5	1851200	522964	0	6	1	0	50	62	21494924	4.65E-01	1
TTC1	199079	458	31	789964	178712	0	2	0	0	50	74	25793624	4.65E-01	1
PROKR2	504598	489	14	958352	278392	0	5	2	0	25	33	10440412	4.65E-01	1
GRM3	123690	1072	-18	2211828	624780	0	7	4	0	18	22	10262768	4.65E-01	1
HSD17B8	1474628	377	24	666788	208972	0	4	1	0	50	90	23548688	4.65E-01	1
NEK5	407938	432	42	1904956	452120	0	8	3	0	15	18	7055920	4.65E-01	1
GDF2	NaN	NaN	NaN	1066932	315060	0	6	3	0	26	43	11892180	4.65E-01	1
CD5	805103	546	37	1273768	359204	0	3	2	0	50	44	18355004	4.65E-01	1
LIN37	1151198	321	30	655396	174440	0	1	0	0	50	47	19100468	4.65E-01	1
PSD3	129089	682	-9	2723400	735852	0	9	2	0	50	72	29578972	4.65E-01	1
NT5DC1	260580	640	19	1208264	301888	0	3	0	0	28	21	9338948	4.65E-01	1
GAPDH	2764720	179	42	865080	245640	0	4	2	0	32	46	13936332	4.65E-01	1
HIST1H3F	390169	544	57	332860	110360	0	2	1	0	50	61	13862640	4.66E-01	1
TBPL1	102500	359	6	493416	131364	0	2	0	0	50	53	18464296	4.66E-01	1
TBC1D14	474439	259	58	1795664	488788	0	4	1	0	38	37	13832736	4.66E-01	1
ADAMTS1	686411	396	0	2336072	740836	0	9	2	0	50	42	18408760	4.66E-01	1
TMEM126A	325720	554	-19	504452	142756	0	1	0	0	50	100	24725980	4.66E-01	1
ZBTB32	1151198	321	30	1199364	381276	0	7	2	0	4	6	2126744	4.66E-01	1
CPA1	419615	298	27	1088292	300108	0	4	1	0	19	12	6105756	4.66E-01	1
ALDH1L2	244603	334	33	2402288	652192	0	6	2	0	50	50	18695696	4.66E-01	1

C21orf59	265057	182	52	769672	190460	0	4	2	0	15	15	6084752	4.66E-01	1
C20orf27	860065	241	43	513708	148452	0	3	1	0	16	30	7457844	4.66E-01	1
BPI	562486	350	45	1278040	347456	0	4	2	0	11	11	3477052	4.66E-01	1
CUTA	1441897	236	31	535780	175508	0	3	1	0	50	72	19862664	4.67E-01	1
ZNF337	482230	636	-21	1928096	498400	0	5	4	0	26	20	8832716	4.67E-01	1
FAM71F1	875359	304	30	885372	247776	0	3	1	0	38	44	13792864	4.67E-01	1
GLTP	876814	323	39	542188	147740	0	6	3	0	2	4	575296	4.67E-01	1
SHD	2132502	244	39	867572	248488	0	3	1	0	50	48	20510584	4.67E-01	1
KPNA4	444964	408	21	1386620	354220	0	4	1	0	50	54	23186992	4.67E-01	1
HGD	312906	391	5	1184768	299752	0	4	1	0	50	65	21094780	4.67E-01	1
LRP12	36110	1085	15	2183704	597368	0	11	2	0	25	27	10353904	4.67E-01	1
SGK2	345777	303	4	1106092	312212	0	4	2	0	50	52	21278120	4.67E-01	1
THBS1	467443	433	-5	3053768	787828	0	9	3	0	50	57	19876192	4.67E-01	1
TSPAN16	1385739	197	31	625492	188324	0	2	0	0	50	60	22230420	4.67E-01	1
USP24	447636	230	9	6869020	1814888	0	15	1	0	1	1	2116776	4.67E-01	1
EGLN2	1187304	275	31	1002852	321468	0	4	1	0	50	50	17463580	4.67E-01	1
CDS1	94589	895	5	1213604	317908	0	5	0	0	50	51	18783628	4.68E-01	1
BBS9	233203	759	-6	2319696	619440	0	11	3	0	2	4	1726244	4.68E-01	1
DNAJC30	1197825	214	52	543968	185476	0	2	1	0	7	5	2787836	4.68E-01	1
OR6K3	175690	966	2	784624	225704	0	6	2	0	7	11	2797804	4.68E-01	1
DEDD	NaN	NaN	NaN	804560	234604	0	5	2	0	26	42	11811724	4.68E-01	1
ABI2	415048	298	23	1216808	346744	0	6	0	0	20	26	9373836	4.68E-01	1
ZNF630	582390	NaN	18	1703460	417588	0	4	0	0	24	23	10930624	4.68E-01	1
B9D2	1193025	249	33	446068	130652	0	2	0	0	50	52	16514128	4.68E-01	1
TFE3	834140	NaN	41	1419372	464580	0	5	2	0	14	22	6888244	4.68E-01	1
SFTA2	2223866	223	25	202208	61588	0	1	0	0	50	73	23168480	4.68E-01	1
BCHE	28918	1215	-18	1541836	402992	0	7	3	0	18	27	6534024	4.68E-01	1
3GALNAC	450604	563	-9	1404420	360984	0	6	1	0	31	27	9204024	4.69E-01	1
WDR83	1975767	409	1	814528	240656	0	3	1	0	50	74	27829232	4.69E-01	1
NLGN1	34593	1079	-18	2071564	585620	0	9	1	0	23	24	9141724	4.69E-01	1
AGGF1	261159	333	30	1875052	471344	0	6	3	0	50	47	17142468	4.69E-01	1
ATP6V0B	875223	214	39	531864	158064	0	2	1	0	50	67	19898264	4.69E-01	1
RHOT1	675374	312	21	1846572	456036	0	5	1	0	50	57	25651936	4.69E-01	1

SHOC2	313830	252	16	1488080	409756	0	4	1	0	50	51	20971960	4.69E-01	1
SPRY1	98850	613	21	805628	223924	0	1	0	0	50	55	21478904	4.69E-01	1
HLA-DRA	1070559	595	14	651124	185120	0	3	0	0	50	75	22719564	4.69E-01	1
UGT8	32108	966	2	1372024	385904	0	6	1	0	50	63	23229712	4.69E-01	1
CA3	100997	1460	-18	682096	181916	0	2	0	0	50	50	20070212	4.70E-01	1
C15orf26	266324	650	22	786048	209328	0	2	0	0	28	16	9927416	4.70E-01	1
ANKS1A	664697	304	26	2892144	842652	0	9	2	0	13	17	8161656	4.70E-01	1
WNT5A	28683	425	20	974016	263796	0	2	0	0	50	53	20067720	4.70E-01	1
MARS2	642112	684	32	1440376	464936	0	3	0	0	50	30	19571812	4.70E-01	1
HOXA5	356045	385	28	682808	191884	0	2	0	0	50	46	22711376	4.70E-01	1
SV2B	457413	624	1	1778220	460308	0	6	1	0	45	54	18672556	4.70E-01	1
NKIRAS2	2081077	206	23	521540	110716	0	4	1	0	32	62	12704216	4.70E-01	1
DLX2	534370	295	34	811324	251336	0	3	0	0	50	51	21776876	4.70E-01	1
MCOLN3	425053	623	4	1469924	356356	0	6	2	0	50	63	19835252	4.70E-01	1
CNGB1	822498	125	10	3278404	899612	0	9	1	0	16	14	6688528	4.70E-01	1
SDCCAG8	182671	1011	0	1930944	448560	0	6	1	0	46	45	17800000	4.70E-01	1
ZFAND6	188862	557	38	551800	139196	0	2	0	0	50	48	17953436	4.70E-01	1
CETN1	483873	521	28	442864	113564	0	2	1	0	45	52	16547592	4.71E-01	1
JSRP1	1603571	240	27	825564	265932	0	4	1	0	50	66	21932448	4.71E-01	1
C20orf194	1015129	338	40	3127816	802424	0	11	1	0	19	33	8562868	4.71E-01	1
B4GALT3	728669	234	37	969032	318976	0	2	1	0	39	44	15898604	4.71E-01	1
ARHGAP3[illegible]	195815	546	-11	5262392	1517272	0	17	8	0	24	35	11870820	4.71E-01	1
TMEM219	1994393	238	52	598080	198648	0	1	0	0	50	61	20191252	4.71E-01	1
MYOZ2	241167	683	1	690284	180136	0	3	0	0	50	62	21470716	4.71E-01	1
BHMT2	236664	526	38	945892	252404	0	5	0	0	50	56	18448988	4.71E-01	1
DNALI1	445473	274	30	720900	202920	0	1	0	0	50	61	20995456	4.71E-01	1
CCDC151	1255985	265	32	1534716	426132	0	8	3	0	50	60	18512000	4.71E-01	1
ITGA6	381919	371	23	2935576	787472	0	7	2	0	50	37	21159572	4.71E-01	1
SHMT1	957114	254	9	1242084	355644	0	4	1	0	50	59	18638380	4.72E-01	1
CHCHD3	595524	677	11	606268	154148	0	1	0	0	50	68	21687520	4.72E-01	1
TNS1	500812	399	23	4349964	1331796	0	16	1	0	1	1	1563908	4.72E-01	1
CXorf38	467563	NaN	63	829480	222500	0	3	1	0	50	48	18696052	4.72E-01	1
ELK4	721075	230	34	1188684	357780	0	5	0	0	11	17	5616968	4.72E-01	1

C1orf198	446793	592	12	821648	244216	0	4	0	0	50	74	21733444	4.72E-01	1
F2RL1	248362	418	25	976508	302956	0	4	0	0	16	12	5006784	4.72E-01	1
FASTKD3	65781	1013	-18	1680676	464936	0	10	1	0	23	39	11558608	4.72E-01	1
CYP2C9	278564	721	-15	1273768	335708	0	4	1	0	50	56	17374580	4.72E-01	1
GPBP1	177326	375	19	1271988	316128	0	4	0	0	48	53	19258176	4.72E-01	1
THEM5	966708	212	36	640444	175508	0	3	0	0	50	62	18268140	4.72E-01	1
WDR73	341520	263	32	964048	282308	0	3	2	0	30	36	12417280	4.73E-01	1
OVGP1	397926	461	18	1715208	505164	0	5	2	0	50	56	21248572	4.73E-01	1
TTLL4	1055730	269	42	3058396	863300	0	8	3	0	50	76	23587492	4.73E-01	1
ANXA5	311234	440	9	851908	227840	0	3	1	0	50	48	19987620	4.73E-01	1
MS4A10	744779	621	23	690996	197580	0	3	0	0	50	74	23297708	4.73E-01	1
AFAP1L2	303560	299	6	2118912	582060	0	7	0	0	4	2	2405492	4.73E-01	1
LDB3	352918	192	27	2102536	642224	0	4	0	0	16	16	10528344	4.73E-01	1
SPDYC	3317462	168	54	756144	210396	0	3	1	0	50	56	19574304	4.73E-01	1
NANOS3	754888	241	31	466004	156640	0	2	1	0	50	63	19916776	4.73E-01	1
GRIN2A	109137	922	-9	3745476	999648	0	11	5	0	50	67	24296288	4.73E-01	1
C11orf53	209532	617	16	587400	184764	0	4	2	0	12	11	2900332	4.73E-01	1
SEMA3D	17511	646	-28	2048780	514420	0	17	5	0	7	16	2096128	4.74E-01	1
PLSCR2	122649	611	-16	598436	148096	0	3	1	0	10	9	3129952	4.74E-01	1
PRSS21	1717244	210	42	799220	231400	0	2	1	0	50	80	24105472	4.74E-01	1
FAR1	171781	639	-7	1348884	351372	0	4	1	0	50	49	25572192	4.74E-01	1
INTS8	430887	389	38	2627280	674976	0	9	1	0	9	14	4459968	4.74E-01	1
DAP	630950	435	29	272696	70132	0	1	0	0	50	51	20828136	4.74E-01	1
COG6	73269	761	11	1741908	470988	0	3	0	0	50	60	23132168	4.74E-01	1
C5orf46	137041	470	2	235316	61588	0	1	1	0	4	2	691708	4.74E-01	1
GNAI3	1022796	187	33	939484	232112	0	5	1	0	50	62	17876540	4.74E-01	1
ZNF563	1744621	671	-8	1249560	291564	0	5	1	0	50	86	23980872	4.74E-01	1
PAAF1	544325	159	44	1023500	284800	0	5	2	0	25	27	8474224	4.75E-01	1
SERPINA5	316239	858	10	1031332	289784	0	5	1	0	50	75	18471772	4.75E-01	1
JARID2	86503	782	34	3164484	903528	0	9	2	0	21	21	10896448	4.75E-01	1
ROBO1	72749	790	-19	4290512	1205416	0	14	2	0	13	15	5937012	4.75E-01	1
PLXNA2	258618	627	10	4802796	1401216	0	16	5	0	44	48	18989040	4.75E-01	1
ABLIM1	267957	257	13	2179076	573160	0	7	3	0	50	50	19995096	4.75E-01	1

ARMCX5	95798	NaN	27	1397300	393736	0	4	1	0	50	63	24065600	4.76E-01	1
ARFGAP3	722786	187	60	1371668	348880	0	6	0	0	29	43	9912464	4.76E-01	1
PLAC9	399614	626	2	247776	79032	0	1	0	0	50	60	22966628	4.76E-01	1
SLC6A14	82720	NaN	4	1680320	435388	0	6	2	0	50	76	26718512	4.76E-01	1
RFPL4B	96263	432	0	660024	185832	0	3	0	0	50	53	22214044	4.76E-01	1
LTV1	355969	455	5	1265580	302244	0	4	0	0	31	30	11408020	4.76E-01	1
TUBD1	957047	216	26	1196516	292276	0	5	1	0	50	74	20292000	4.76E-01	1
COG4	925955	131	47	2056968	553224	0	9	4	0	42	60	19810688	4.76E-01	1
BCAS1	163555	415	2	1495912	425420	0	5	1	0	50	45	20863736	4.76E-01	1
ZNF587	884988	781	-38	1479536	374512	0	7	3	0	50	70	16966604	4.76E-01	1
FARP1	494775	574	35	2913860	784624	0	6	3	0	22	30	9129264	4.76E-01	1
SLC9A2	75782	858	-5	2054832	597012	0	5	4	0	50	70	20934224	4.77E-01	1
SRRT	1339207	433	48	2290504	600572	0	8	1	0	17	24	8713456	4.77E-01	1
NFRSF10I	640871	237	45	983628	292632	0	4	1	0	50	63	22177020	4.77E-01	1
PFDN2	689033	250	23	404416	112496	0	2	0	0	50	68	19070564	4.77E-01	1
GNL2	445473	274	30	1915280	496264	0	5	1	0	50	61	20995456	4.77E-01	1
TNNI3K	87070	1135	-16	2517988	648632	0	5	0	0	9	5	4210768	4.77E-01	1
COPS8	336171	411	17	555004	147740	0	2	0	0	50	60	23292724	4.77E-01	1
TGFBI	337362	298	32	1741552	518336	0	3	1	0	50	55	20111508	4.77E-01	1
IAP1LC3B	292439	337	1	320400	86508	0	1	0	0	50	49	24328328	4.77E-01	1
SCRG1	249791	553	8	260236	65504	0	1	0	0	50	60	23005076	4.77E-01	1
RAB37	929990	484	15	837312	236028	0	3	1	0	26	30	7658272	4.77E-01	1
TEX2	1372704	292	15	2869360	814172	0	8	2	0	35	69	20590328	4.77E-01	1
TBX4	312575	408	-11	1377008	404416	0	4	1	0	45	36	19469284	4.77E-01	1
ASPN	NaN	NaN	NaN	998580	252048	0	2	0	0	50	116	39897632	4.78E-01	1
MRGPRX2	483720	695	-1	807052	257744	0	3	0	0	13	12	4534728	4.78E-01	1
MANEA	29596	1315	-6	1186904	313636	0	4	1	0	50	48	20482104	4.78E-01	1
SLC26A10	1329939	344	52	1399080	465648	0	4	1	0	50	73	24653712	4.78E-01	1
INPP5F	656842	199	43	2947680	763620	0	10	4	0	35	40	15069124	4.78E-01	1
TRIM63	748547	221	31	940196	228196	0	2	0	0	50	47	17463224	4.78E-01	1
PHF11	422169	338	53	890712	211464	0	4	1	0	50	57	20409124	4.78E-01	1
PRAMEF4	118495	792	12	1206484	341048	0	6	0	0	19	26	7005724	4.78E-01	1
TTC33	211936	496	-11	680672	178000	0	3	0	0	50	63	20505244	4.78E-01	1

FAM103A1	452169	683	19	338200	53756	0	1	0	0	50	66	24613128	4.78E-01	1
TNFRSF9	381467	207	24	676044	174084	0	2	0	0	50	49	21387056	4.78E-01	1
OR6C65	667726	802	-14	779640	221076	0	3	0	0	50	83	19448636	4.78E-01	1
SPIN3	145082	NaN	13	653260	180848	0	3	1	0	50	63	19011112	4.78E-01	1
SPN	NaN	NaN	NaN	933788	355288	0	2	0	0	50	116	40000872	4.78E-01	1
GLUD1	397715	144	35	1442156	400144	0	5	2	0	50	55	18139268	4.78E-01	1
RALGPS1	419512	353	41	1470992	393736	0	5	1	0	50	47	21724188	4.78E-01	1
NDUFAB1	536544	561	43	408688	113564	0	2	1	0	33	45	12892540	4.79E-01	1
CCL28	500147	320	6	335708	85084	0	1	0	0	50	56	25094796	4.79E-01	1
BTF3	439732	289	38	529728	154860	0	2	0	0	50	51	17986900	4.79E-01	1
LEPROT	239577	511	-3	338912	96832	0	2	1	0	6	6	1879680	4.79E-01	1
C22orf31	759735	335	-19	734072	208972	0	2	1	0	50	74	21518776	4.79E-01	1
PGAM4	549429	NaN	24	633324	183696	0	2	0	0	50	46	18656536	4.79E-01	1
TMEM14B	263716	468	14	303312	86508	0	1	0	0	50	41	17401280	4.79E-01	1
GPR87	NaN	NaN	NaN	912784	248132	0	3	0	0	50	116	39893716	4.79E-01	1
SDCBP2	417756	427	36	748668	224280	0	2	0	0	50	68	21321908	4.79E-01	1
KRTAP4-9	NaN	NaN	NaN	537204	138840	0	2	1	0	50	117	39784424	4.79E-01	1
CCNG2	421623	562	33	901748	231400	0	3	1	0	50	47	18868356	4.79E-01	1
RAB5B	2080397	229	31	566396	147028	0	3	1	0	50	68	20292000	4.79E-01	1
YARS2	302215	238	-4	1185480	365256	0	5	1	0	50	49	20721692	4.79E-01	1
CHMP1B	NaN	NaN	NaN	504808	135992	0	2	0	0	50	116	39781576	4.79E-01	1
OR8B4	149616	857	0	761128	229976	0	8	2	0	2	3	683164	4.79E-01	1
ZNF93	525535	761	-55	1610188	392312	0	11	1	0	48	68	15977636	4.80E-01	1
PPM1D	288701	328	15	1515848	447136	0	5	1	0	50	50	22587844	4.80E-01	1
NEUROD6	48298	883	-4	857248	229976	0	4	0	0	50	66	22538360	4.80E-01	1
KCTD4	613630	276	60	653972	179068	0	3	1	0	50	54	19315136	4.80E-01	1
FBLN7	NaN	NaN	NaN	1111432	328232	0	5	2	0	50	118	39973816	4.80E-01	1
DCAF12L1	11311	NaN	-16	1124248	366680	0	5	1	0	50	105	24974468	4.80E-01	1
PTH	264102	625	-6	300820	79388	0	1	0	0	50	57	25441540	4.80E-01	1
MGRN1	729674	157	41	1528664	437524	0	5	1	0	50	62	22211552	4.80E-01	1
RPL4	507061	219	40	1100396	313636	0	3	0	0	25	13	7594904	4.80E-01	1
SH3BP4	133390	408	-10	2386624	714848	0	9	1	0	50	53	22859116	4.80E-01	1
PPP1R13B	712218	320	39	2762560	801356	0	6	1	0	13	10	6673220	4.80E-01	1

CTSO	44515	745	-2	842652	221076	0	2	1	0	12	13	5449648	4.80E-01	1
ESYT2	286663	344	1	2286232	669992	0	6	3	0	28	30	15075888	4.80E-01	1
TBX2	292199	414	-9	1728024	582060	0	5	1	0	47	39	21536932	4.81E-01	1
PLEKHA3	170323	555	37	795660	198648	0	3	1	0	50	50	18288788	4.81E-01	1
MRPL17	424776	548	8	437524	134924	0	2	1	0	50	65	19789684	4.81E-01	1
PTCHD3	345745	581	2	1918128	555360	0	10	5	0	9	16	4991476	4.81E-01	1
ANKRD34A	NaN	NaN	NaN	1162340	430048	0	5	2	0	50	118	40075632	4.81E-01	1
ANKRD46	1144761	214	67	577788	168744	0	2	0	0	50	52	20627352	4.81E-01	1
SRP68	1611239	218	22	1639024	439304	0	9	3	0	34	72	18207620	4.81E-01	1
ZFY	0	0	0	2092568	506944	0	5	1	0	50	39	21173456	4.81E-01	1
RPS26	2693176	215	38	299752	86864	0	1	0	0	50	53	18137132	4.81E-01	1
PPP1R14C	346933	439	30	421148	123532	0	1	0	0	50	44	25615268	4.82E-01	1
HSD17B7	336864	396	12	887152	244928	0	2	0	0	50	70	25034632	4.82E-01	1
AMHR2	1712232	191	56	1472416	413672	0	3	1	0	50	69	22534088	4.82E-01	1
SMARCE1	1042327	196	19	1098260	264508	0	2	0	0	3	0	893560	4.82E-01	1
KCNJ13	325071	328	33	923108	242080	0	3	1	0	50	45	19117556	4.82E-01	1
KCNMB4	148192	547	-7	536492	148096	0	3	1	0	50	63	19751948	4.82E-01	1
SLC35F3	277114	649	1	1237812	365256	0	6	3	0	5	4	1597372	4.82E-01	1
NKD1	307084	646	-6	1201856	345676	0	4	0	0	50	53	23195180	4.82E-01	1
RGL4	610486	266	30	1201856	359560	0	4	2	0	50	71	20499548	4.82E-01	1
ADD1	669373	264	45	2001788	562480	0	12	5	0	2	7	1564264	4.82E-01	1
CYB5R3	858524	189	74	894628	239588	0	3	0	0	50	49	21719560	4.83E-01	1
FAM168B	461529	331	16	497332	156284	0	2	2	0	17	17	3531876	4.83E-01	1
HPSE2	147172	933	2	1541124	450696	0	5	2	0	22	25	8837700	4.83E-01	1
SAGE1	246199	NaN	26	2352092	626560	0	9	2	0	50	66	25385292	4.83E-01	1
NCAM2	14306	1515	-61	2193672	563904	0	15	3	0	18	37	8943788	4.83E-01	1
SNX20	294632	792	1	892136	284800	0	4	2	0	27	33	11357112	4.83E-01	1
PSMB11	1261207	185	41	723036	243504	0	2	0	0	50	68	21572888	4.83E-01	1
MLX	1884163	177	38	784268	205768	0	2	1	0	50	68	23826724	4.83E-01	1
CDYL	116037	445	26	1392316	379496	0	4	0	0	8	6	4164844	4.83E-01	1
SSX1	603602	NaN	5	525100	110360	0	2	0	0	50	57	19263872	4.83E-01	1
FMOD	552574	325	40	936992	279460	0	3	0	0	31	28	11098300	4.83E-01	1
RMND1	336608	468	30	1182276	306516	0	5	1	0	45	38	16219360	4.83E-01	1

TPRX1	881316	336	39	959776	366680	0	3	0	0	2	0	778928	4.84E-01	1
PARP4	340065	606	39	4473140	1193668	0	17	4	0	50	46	18253900	4.84E-01	1
GDPD3	NaN	NaN	NaN	829480	235316	0	4	1	0	50	117	39880900	4.84E-01	1
TIMM44	960059	320	23	1184768	317908	0	4	1	0	50	63	19420512	4.84E-01	1
AVL9	371440	595	10	1681744	461732	0	6	2	0	50	70	21482464	4.84E-01	1
ALG2	203560	410	37	1024568	317908	0	5	2	0	4	6	1822720	4.84E-01	1
PIGU	1057035	190	51	1128520	315416	0	4	2	0	50	72	19313356	4.85E-01	1
SYNE1	79159	579	3	23033912	5872576	0	84	15	0	6	20	7316512	4.85E-01	1
FFAR3	767113	472	9	841940	274120	0	3	0	0	32	25	10097228	4.85E-01	1
GEMIN8	121538	NaN	35	634748	156640	0	2	1	0	50	47	20728812	4.85E-01	1
DMRT3	83374	703	9	1151304	370596	0	5	0	0	50	77	31222980	4.85E-01	1
ZNF518B	218121	1074	15	2694208	750092	0	7	0	0	5	2	2500900	4.85E-01	1
SHOX	NaN	NaN	NaN	782844	223212	0	4	0	0	50	116	39868796	4.85E-01	1
TMEM192	170385	595	0	699540	195444	0	2	0	0	50	45	20023576	4.85E-01	1
ZNF695	385851	535	-5	1352088	313992	0	7	1	0	9	8	3458896	4.85E-01	1
PLD2	1465720	208	27	2394812	699184	0	4	1	0	36	34	12888624	4.85E-01	1
ZNF836	460879	901	-41	2410120	602708	0	7	3	0	45	56	16926020	4.85E-01	1
ALMS1	777501	590	22	10442904	3005352	0	41	9	0	8	25	8117512	4.85E-01	1
DHX29	280901	417	31	3562492	938060	0	9	1	0	11	9	6191196	4.86E-01	1
CCL20	161901	296	7	261660	64080	0	1	0	0	50	47	18616308	4.86E-01	1
GPNMB	505224	169	25	1474908	403704	0	8	4	0	18	17	5313300	4.86E-01	1
OR5W2	4214	1074	-46	768960	225348	0	6	3	0	3	3	1071916	4.86E-01	1
CXCR5	1488276	175	54	904596	294768	0	2	0	0	20	24	8840192	4.86E-01	1
E4F1	2179404	224	39	1967256	605556	0	3	3	0	50	46	19527312	4.86E-01	1
SNCAIP	142971	684	-6	2332156	658244	0	8	3	0	47	59	21746972	4.86E-01	1
IL16	251183	742	17	3326820	1022076	0	13	1	0	6	3	2200080	4.86E-01	1
ARFGEF1	307618	642	26	4853704	1236032	0	13	2	0	25	25	12302648	4.86E-01	1
NEK9	919043	222	47	2519412	710220	0	6	1	0	25	41	16456812	4.86E-01	1
GPR22	500763	753	12	1083664	311144	0	3	1	0	50	65	21876200	4.86E-01	1
FAM84A	58286	1274	-10	721612	221432	0	3	2	0	22	27	10008228	4.86E-01	1
GPR162	2822601	232	32	1429340	488788	0	6	2	0	50	62	20662240	4.86E-01	1
SLC6A16	2237411	309	35	1866152	542188	0	5	1	0	50	44	20641948	4.86E-01	1
PGLYRP4	1140044	401	32	955148	277324	0	3	1	0	50	58	17066640	4.86E-01	1

NDUFA12	369755	560	25	384124	98612	0	1	0	0	50	54	25254284	4.86E-01	1
IL1R2	215759	556	12	1026348	288360	0	3	1	0	20	14	7314020	4.86E-01	1
C10orf99	216191	860	-17	210752	62656	0	1	0	0	50	78	25295580	4.86E-01	1
SPAG7	1416161	231	30	609472	148808	0	1	0	0	50	65	18681100	4.87E-01	1
ZSCAN21	1503181	254	41	1212536	318976	0	4	1	0	50	81	27223676	4.87E-01	1
PRAMEF1[illegible]	NaN	NaN	NaN	2397304	699896	0	9	3	0	50	119	40345480	4.87E-01	1
AS3MT	711862	192	45	990392	258100	0	3	2	0	33	40	13181968	4.87E-01	1
MOCOS	393505	591	40	2263448	648988	0	9	2	0	50	66	21522692	4.87E-01	1
GNB2L1	1034999	415	35	815596	235316	0	3	0	0	15	19	5268088	4.87E-01	1
ANAPC16	764632	140	40	283020	85440	0	1	0	0	50	51	21069148	4.87E-01	1
PIGK	404438	673	-17	1053048	260592	0	5	0	0	50	51	17159556	4.87E-01	1
HSPB8	139110	779	3	492348	147384	0	2	1	0	24	23	8111816	4.87E-01	1
UHRF1BP1	1047481	242	24	3665376	1037028	0	12	6	0	36	50	17203344	4.87E-01	1
WARS2	163335	948	-5	939840	271272	0	2	0	0	50	42	16793944	4.88E-01	1
VAC14	857297	299	26	2006416	579212	0	8	2	0	14	19	5529036	4.88E-01	1
TIE1	1091212	264	30	2869360	876116	0	12	4	0	11	11	3861532	4.88E-01	1
CELA2A	554165	212	29	695268	203988	0	2	1	0	29	58	14632312	4.88E-01	1
PPFIBP1	488829	369	7	2698480	689216	0	5	2	0	42	46	20787196	4.88E-01	1
CFHR5	217857	853	-24	1489504	375224	0	6	2	0	50	67	19868716	4.88E-01	1
MAGEB4	74310	NaN	16	858316	253472	0	2	0	0	50	67	20445436	4.89E-01	1
ZNF101	768855	418	-10	1127096	288004	0	4	0	0	50	54	17193020	4.89E-01	1
BFSP2	342402	497	14	1047352	311144	0	4	1	0	50	58	22195888	4.89E-01	1
RFXAP	191525	731	-15	681740	201496	0	2	1	0	26	45	11321868	4.89E-01	1
AP4E1	343136	355	34	2950528	783200	0	7	0	0	2	1	1429340	4.89E-01	1
OLAH	255576	571	28	836600	215380	0	3	0	0	50	59	22169188	4.89E-01	1
ZNF560	557413	642	-52	2056968	511572	0	10	4	0	36	84	24661544	4.89E-01	1
CIAPIN1	749108	159	39	807052	229976	0	3	1	0	50	52	18373516	4.89E-01	1
SPATA2	589377	181	48	1280888	394804	0	4	1	0	50	58	18928164	4.89E-01	1
BTLA	304354	636	35	747600	198648	0	3	0	0	26	25	6830216	4.89E-01	1
CD226	64726	630	40	875404	229976	0	3	0	0	38	35	14466416	4.89E-01	1
IL18R1	235438	588	7	1418660	358492	0	3	2	0	32	31	14469264	4.89E-01	1
FSTL3	1832767	218	10	663584	201496	0	2	0	0	29	50	17004696	4.89E-01	1
CHST8	487426	623	-26	1046640	327876	0	4	1	0	50	45	17394872	4.89E-01	1

TRAPPC2L	860087	252	37	375936	95052	0	3	1	0	8	11	2710228	4.89E-01	1
MOCS1	45558	966	22	1084376	324316	0	4	3	0	20	25	7501276	4.89E-01	1
FAM163A	458564	479	-8	416520	130296	0	2	0	0	50	70	22484604	4.89E-01	1
UNC119B	1741363	349	42	643648	180848	0	3	1	0	50	64	18828840	4.89E-01	1
ABCG1	289449	464	75	1872204	544680	0	6	3	0	41	52	15391660	4.90E-01	1
FANCL	197920	617	9	1053760	257744	0	2	0	0	19	12	9703492	4.90E-01	1
VMA21	247003	NaN	-16	260236	75116	0	1	0	0	50	56	18211180	4.90E-01	1
APOBEC3H	1225154	223	75	551088	126024	0	3	1	0	5	4	1745824	4.90E-01	1
TMEM132E	492460	860	-17	2721264	772164	0	12	4	0	50	61	17050620	4.90E-01	1
DEFB115	562665	594	-29	229976	59452	0	1	0	0	50	50	17157064	4.90E-01	1
GINS3	695011	385	-1	646140	186900	0	2	0	0	50	40	18092276	4.90E-01	1
DYNLL1	1552470	216	46	245640	55536	0	1	0	0	50	64	22266732	4.90E-01	1
F8	1741051	NaN	4	6075496	1594880	0	20	4	0	6	7	4213972	4.90E-01	1
SNAI3	802673	189	41	712712	236740	0	3	0	0	50	65	20750172	4.90E-01	1
ETS2	382323	151	5	1230692	311500	0	4	2	0	50	51	18508796	4.90E-01	1
HIGD1C	569122	220	40	254896	66572	0	1	0	0	50	55	19943476	4.90E-01	1
MS4A14	433906	899	24	1725532	470276	0	8	2	0	15	12	4671076	4.90E-01	1
MRPL40	650407	300	16	531508	146672	0	3	1	0	9	15	4249216	4.90E-01	1
OR6V1	314565	676	-1	761840	243148	0	2	1	0	50	59	16752292	4.90E-01	1
CARD17	NaN	NaN	NaN	288004	80456	0	1	0	0	50	116	39726040	4.91E-01	1
PPM1A	281503	630	36	1206484	300464	0	5	2	0	50	43	15820640	4.91E-01	1
TMEM139	379894	399	14	531864	171948	0	2	0	0	50	67	22890088	4.91E-01	1
AMOT	25417	NaN	10	2696344	822716	0	6	0	0	2	1	1456040	4.91E-01	1
PPIF	326131	300	53	532220	157708	0	2	0	0	50	51	18466432	4.91E-01	1
FGF13	14292	NaN	-5	857248	220364	0	4	2	0	50	60	19015028	4.91E-01	1
TMEM194A	2026922	199	41	1149168	312924	0	3	1	0	50	67	21271000	4.91E-01	1
RFX4	247645	482	-1	2085804	559632	0	7	1	0	32	37	13294464	4.91E-01	1
TMX2	922344	354	11	770028	213600	0	4	0	0	50	75	13734124	4.91E-01	1
LAMB3	211663	300	4	2991112	861164	0	8	1	0	22	10	7584936	4.91E-01	1
TMEM19	259684	393	-11	850840	250268	0	2	0	0	50	34	20198728	4.91E-01	1
ESM1	205256	603	13	476684	126736	0	2	0	0	50	61	19133220	4.91E-01	1
APOLD1	767161	129	35	669636	241368	0	2	0	0	50	48	22604220	4.91E-01	1
CXorf40B	280868	NaN	34	400144	117836	0	1	0	0	50	40	17736988	4.91E-01	1

PMP22	228633	345	-72	413672	121396	0	2	0	0	50	107	29414500	4.91E-01	1
LCE5A	325831	700	20	300108	85440	0	1	0	0	50	63	27856288	4.91E-01	1
MYNN	400732	689	2	1585980	401568	0	6	1	0	20	23	8190848	4.92E-01	1
QTRT1	2174644	224	32	1025280	311856	0	3	2	0	50	77	23947408	4.92E-01	1
C16orf92	NaN	NaN	NaN	366680	89356	0	1	0	0	50	116	39734940	4.92E-01	1
H2AFZ	202808	682	36	328944	101460	0	1	0	0	50	66	21102256	4.92E-01	1
UGT2B11	18988	929	1	1367040	354576	0	2	0	0	4	3	3027780	4.92E-01	1
NAP1L5	177679	486	8	466004	120328	0	2	0	0	50	67	20305884	4.92E-01	1
ADARB2	284080	633	8	1810260	599148	0	8	4	0	50	70	20533368	4.92E-01	1
PLCL1	627125	907	10	2784276	748668	0	13	3	0	50	63	21810340	4.92E-01	1
HR	584373	219	34	2951952	937704	0	5	0	0	5	0	2362772	4.92E-01	1
OR5B21	190469	812	-10	761484	229620	0	4	1	0	50	71	21027140	4.92E-01	1
ADRB2	298390	408	22	1027772	298684	0	3	1	0	50	44	21127176	4.92E-01	1
SRGN	766231	183	55	406196	113920	0	2	0	0	50	84	21063808	4.92E-01	1
CNNM4	936233	332	49	1924536	589536	0	8	3	0	50	82	22478908	4.92E-01	1
FCRL2	113186	829	26	1315420	364544	0	4	2	0	50	40	19626636	4.92E-01	1
RAB18	313003	549	0	551444	139552	0	3	1	0	50	70	21942772	4.92E-01	1
IFNA16	193208	498	-3	480244	130652	0	2	0	0	50	58	18347172	4.92E-01	1
SLC35B1	880183	361	30	831260	239944	0	3	0	0	50	53	20258180	4.93E-01	1
RANGAP1	1301913	179	53	1507304	440728	0	4	2	0	37	34	14111128	4.93E-01	1
RNPEP	751933	168	31	1652196	478464	0	3	0	0	13	9	5941284	4.93E-01	1
MAPK11	939384	257	70	953012	265576	0	3	1	0	50	46	19591036	4.93E-01	1
OR13A1	126925	808	26	813104	245284	0	2	0	0	50	38	20112220	4.93E-01	1
SCARB2	515813	403	39	1252764	328944	0	4	1	0	50	85	27607800	4.93E-01	1
ETAA1	36002	401	-3	2381640	609828	0	6	1	0	50	53	21722052	4.93E-01	1
CDC42SE1	1614665	165	40	216092	53044	0	1	0	0	50	62	20369964	4.93E-01	1
SCLY	435787	356	32	1142048	336064	0	2	1	0	50	37	15650116	4.93E-01	1
UBC	1090586	206	33	1698476	505876	0	5	2	0	50	63	17866216	4.93E-01	1
EIF2B1	871640	315	40	792100	222500	0	1	0	0	50	59	21342912	4.93E-01	1
AMDHD1	366272	436	8	1091496	310788	0	3	1	0	50	55	19720976	4.93E-01	1
MALT1	331493	198	55	2148460	563192	0	7	2	0	50	51	18885088	4.93E-01	1
STOML2	945481	350	41	902816	281596	0	4	0	0	24	35	10864052	4.93E-01	1
EYA3	827177	316	28	1513712	398008	0	4	0	0	22	22	10038132	4.93E-01	1

PCDHGA5	NaN	NaN	NaN	2285164	731936	0	11	3	0	50	119	40377520	4.93E-01	1
CCNDBP1	843516	333	42	949808	251692	0	2	0	0	25	24	10413000	4.93E-01	1
CPEB4	164298	381	43	1862236	515132	0	4	0	0	6	2	2412256	4.94E-01	1
ARHGAP1[illegible]	272834	185	22	2259532	649700	0	5	1	0	29	35	14403404	4.94E-01	1
STAMBP	1124858	404	43	1100752	299396	0	4	0	0	50	76	19904316	4.94E-01	1
SEMA3F	905995	196	40	2002856	592384	0	6	2	0	50	72	22424796	4.94E-01	1
C7	211302	644	5	2206844	569956	0	4	0	0	23	11	9665400	4.94E-01	1
RASGRP2	2042840	230	60	1574588	448204	0	3	1	0	50	73	23729892	4.94E-01	1
PODXL2	679358	336	45	1512288	461376	0	6	1	0	12	11	5764352	4.94E-01	1
MRPL11	1941577	194	51	551444	170524	0	2	0	0	50	57	20121476	4.94E-01	1
ADAD1	227942	612	-14	1505880	391956	0	4	1	0	50	46	18207264	4.94E-01	1
RXFP1	151976	534	-10	2003212	502316	0	7	1	0	9	17	5391620	4.94E-01	1
VPS52	1456953	262	30	1861880	538984	0	8	3	0	50	71	20945260	4.94E-01	1
MAPK4	243391	442	21	1467432	433608	0	5	2	0	50	65	21114360	4.94E-01	1
RBM34	592746	168	26	1152016	294056	0	2	1	0	50	42	17770096	4.94E-01	1
LRRC2	283343	528	42	984696	241368	0	5	0	0	50	62	18868356	4.94E-01	1
IMP4	369510	593	29	748312	223568	0	4	1	0	4	3	1551092	4.95E-01	1
ZNF786	598458	279	31	1982920	538628	0	5	4	0	30	41	13865132	4.95E-01	1
LAYN	418517	326	21	968676	258456	0	3	1	0	16	11	6918504	4.95E-01	1
THAP7	577297	192	58	763620	244572	0	4	2	0	47	75	19222576	4.95E-01	1
ITPK1	326946	326	35	1111432	310076	0	3	1	0	50	38	19108300	4.95E-01	1
ZNF350	452542	838	-61	1371312	349236	0	6	1	0	50	69	16927444	4.95E-01	1
DMTF1	169233	1011	4	1981852	524744	0	6	2	0	50	57	21922836	4.95E-01	1
KLRF1	226318	648	33	626204	143824	0	3	0	0	50	62	18130012	4.95E-01	1
PINX1	116190	574	16	866860	217160	0	3	0	0	50	52	20040664	4.95E-01	1
ZNF761	361546	876	-58	1944116	463156	0	13	1	0	6	9	2344260	4.95E-01	1
GDPD2	270766	NaN	38	1512288	449628	0	5	1	0	43	50	16418720	4.95E-01	1
ALK	202198	882	19	4104324	1208976	0	11	3	0	35	33	15082652	4.96E-01	1
PPP1CC	796153	347	50	850484	213244	0	2	0	0	50	62	19622364	4.96E-01	1
PRRT1	2119974	392	28	728376	268068	0	1	0	0	50	62	23774036	4.96E-01	1
LEAP2	686574	200	66	203276	57316	0	1	0	0	50	52	16038868	4.96E-01	1
PLA2G2A	490325	466	25	383056	94340	0	1	0	0	50	36	17418368	4.96E-01	1
C10orf11	29386	1001	3	520828	140264	0	2	0	0	50	65	21416248	4.96E-01	1

CCDC69	916138	407	56	782844	202920	0	2	0	0	50	59	17889712	4.96E-01	1
HIST1H2AI	NaN	NaN	NaN	314704	107156	0	4	2	0	2	6	963692	4.96E-01	1
GMDS	160107	647	20	977932	259880	0	3	1	0	50	50	19795380	4.96E-01	1
KRT38	1321356	424	-6	1154508	335352	0	5	2	0	50	49	16872976	4.96E-01	1
SRRM4	116468	835	2	1548600	463512	0	6	3	0	30	36	10425104	4.96E-01	1
SMPD2	328699	559	47	1086868	312212	0	2	0	0	50	46	17980492	4.97E-01	1
UBQLN1	481151	422	-3	1519408	413672	0	2	1	0	50	58	20655120	4.97E-01	1
LCMT1	273088	418	23	889288	226772	0	1	1	0	50	39	17977288	4.97E-01	1
APOBEC2	145501	611	37	571736	157708	0	2	1	0	50	56	20220800	4.97E-01	1
HIAT1	292450	530	4	1253476	366680	0	4	1	0	50	75	23594612	4.97E-01	1
SERPINB3	174293	370	-5	1026704	255964	0	3	2	0	9	11	4391972	4.97E-01	1
GP9	1155221	379	52	422216	152368	0	2	0	0	50	73	19769036	4.97E-01	1
TTC23	273585	361	-7	1161272	316840	0	3	1	0	31	26	14227896	4.97E-01	1
RAB33A	339869	NaN	58	599148	167676	0	1	1	0	50	61	17320468	4.97E-01	1
PAX7	520847	598	28	1406912	420436	0	5	1	0	17	19	9301212	4.97E-01	1
OR2T11	116628	850	-20	774300	241368	0	3	1	0	50	57	19537280	4.97E-01	1
HIST4H4	340728	578	-4	249556	87932	0	2	1	0	4	5	737988	4.97E-01	1
MAD2L1	106139	1069	-5	532220	144892	0	2	0	0	50	55	18853404	4.97E-01	1
MTHFS	221773	711	9	522252	142044	0	2	0	0	50	64	21697488	4.97E-01	1
CLEC5A	198115	856	-6	513708	117480	0	2	0	0	50	61	21007204	4.98E-01	1
MKLN1	258333	283	35	2002856	467428	0	4	1	0	50	48	18913568	4.98E-01	1
RDH8	1555841	413	0	771096	249912	0	3	0	0	50	64	22283108	4.98E-01	1
BDNF	380275	534	-24	922040	253828	0	3	0	0	33	46	13575704	4.98E-01	1
HOXB3	946208	524	29	1050912	337488	0	4	1	0	50	60	17564684	4.98E-01	1
OTUD7A	155907	445	13	2291572	723392	0	6	1	0	45	49	20161704	4.98E-01	1
FAM60A	317612	468	6	587756	144892	0	2	0	0	50	54	20581072	4.98E-01	1
NTNG1	78332	877	-17	1496980	401924	0	6	1	0	38	48	17472124	4.98E-01	1
SPRY4	394296	573	28	800644	242792	0	3	0	0	50	63	22212620	4.98E-01	1
CCL3	744143	565	28	244216	64436	0	1	0	0	50	68	23933524	4.98E-01	1
GON4L	2156898	179	33	5829856	1607696	0	24	5	0	2	6	2277332	4.98E-01	1
PCDHGA2	662513	568	2	2307592	741548	0	7	4	0	50	85	24954176	4.98E-01	1
KLK5	581408	606	29	754008	213600	0	3	1	0	50	56	18321184	4.98E-01	1
CHI3L2	400425	627	41	1028840	268780	0	3	1	0	50	66	20484952	4.98E-01	1

UGT1A3	NaN	NaN	NaN	1345324	385904	0	5	0	0	50	116	40031488	4.98E-01	1
KLHL31	306333	404	20	1586692	456392	0	3	1	0	5	6	3893216	4.98E-01	1
NELL2	79685	930	-19	2383064	583840	0	8	1	0	23	28	9930620	4.98E-01	1
SMC1A	920771	NaN	70	3256688	801712	0	11	2	0	6	10	3616248	4.98E-01	1
DEFB112	65301	1242	-3	296192	72268	0	2	1	0	4	6	1594168	4.99E-01	1
GDF15	1294357	210	39	734428	262016	0	1	1	0	50	67	22067728	4.99E-01	1
STARD5	245396	787	28	556072	153080	0	2	0	0	50	56	20277404	4.99E-01	1
C15orf59	313132	435	29	720900	225348	0	2	1	0	35	31	15192656	4.99E-01	1
LSAMP	121424	967	-6	855468	256320	0	4	1	0	50	64	19201572	4.99E-01	1
SPAG4	1423559	169	53	1122112	330368	0	5	2	0	50	53	18644076	4.99E-01	1
NOS1	201056	259	13	3676768	1036316	0	14	5	0	50	57	17797508	4.99E-01	1
CAMK1G	204559	405	-3	1247780	331792	0	3	0	0	50	37	21331164	4.99E-01	1
TIMP3	259538	398	-69	553224	143112	0	1	1	0	50	107	27994060	4.99E-01	1
LANCL1	159584	453	3	1043080	276968	0	2	1	0	50	43	17836312	4.99E-01	1
OLIG3	210173	486	29	662160	212532	0	3	2	0	20	29	8755820	4.99E-01	1
UBE2H	456399	226	34	497332	117836	0	2	0	0	50	56	20586056	4.99E-01	1
P2RX3	923815	191	-3	1049488	276968	0	4	1	0	50	67	17960912	4.99E-01	1
CD109	706356	215	15	3760428	1009260	0	14	5	0	50	57	20366760	5.00E-01	1
PHYHD1	1580914	198	21	911004	114276	0	5	0	0	50	84	22627360	5.00E-01	1
CLRN2	271399	912	37	583484	175864	0	2	1	0	50	49	17602776	5.00E-01	1
FOXS1	1015946	241	37	794948	267712	0	4	1	0	50	52	17181628	5.00E-01	1
ZNF845	NaN	NaN	NaN	2514428	609472	0	10	2	0	50	118	40255056	5.00E-01	1
SCTR	211577	497	30	1145252	318976	0	5	2	0	43	54	18229692	5.00E-01	1
ZNF202	386097	609	0	1654688	450340	0	5	1	0	50	70	24114016	5.00E-01	1
CHODL	272100	981	-58	713780	185476	0	2	0	0	50	51	18771168	5.00E-01	1
MAPT	369065	469	19	1946252	603064	0	6	4	0	50	66	24993692	5.00E-01	1
ATXN2	523776	302	42	3326108	986476	0	5	1	0	11	6	5005716	5.00E-01	1
SLC25A3	487754	218	21	1030620	296904	0	1	0	0	6	1	2386980	5.00E-01	1
PALLD	173564	359	-4	3410124	1007124	0	9	2	0	50	50	24631284	5.00E-01	1
AMIGO3	NaN	NaN	NaN	1201144	416876	0	8	5	0	8	21	4342844	5.00E-01	1
SFTPB	1158965	170	43	1016024	293344	0	3	2	0	36	48	15827404	5.00E-01	1
KLK14	490795	632	22	673196	206836	0	3	1	0	50	58	16739120	5.00E-01	1
UTS2	375594	415	23	440372	112852	0	2	1	0	6	6	2414748	5.00E-01	1

H2AFJ	340728	578	-4	308296	108224	0	2	1	0	15	22	4762924	5.01E-01	1
IL7	52129	920	6	480244	111428	0	1	0	0	50	50	23625228	5.01E-01	1
TMEM182	63440	913	-3	594520	159488	0	3	0	0	50	72	19428344	5.01E-01	1
PPP1R3D	73597	856	-36	711644	249556	0	3	0	0	50	68	20110084	5.01E-01	1
EGFL7	1918290	190	56	705236	206836	0	2	1	0	50	46	19629484	5.01E-01	1
MASTL	386910	555	43	2270568	595944	0	9	1	0	50	43	17116480	5.01E-01	1
SHARPIN	2720766	211	27	956572	325028	0	2	1	0	31	38	13524084	5.01E-01	1
CLDN23	174979	307	34	699896	238876	0	1	1	0	50	59	18757996	5.01E-01	1
OR2B6	225015	562	24	761840	242080	0	2	0	0	50	45	21563988	5.01E-01	1
CCDC86	794900	522	47	882880	286580	0	3	1	0	50	51	21913580	5.01E-01	1
REEP2	1196831	209	49	651124	189392	0	2	0	0	50	63	23144984	5.01E-01	1
ELAC1	189022	332	51	914208	264864	0	2	0	0	50	48	17856248	5.01E-01	1
FBXL20	1467668	179	20	1147388	312568	0	3	1	0	50	60	18894344	5.01E-01	1
REL	635308	439	34	1606984	422216	0	4	1	0	35	32	14206180	5.01E-01	1
OR52B2	465433	806	-1	784624	252404	0	2	1	0	8	7	2668576	5.01E-01	1
E2F4	1109491	150	46	1058388	308652	0	2	0	0	6	2	3107880	5.01E-01	1
UBTD1	560569	169	41	569600	169456	0	2	0	0	50	57	20350384	5.01E-01	1
CLDN22	NaN	NaN	NaN	537916	170168	0	2	1	0	50	117	39815752	5.02E-01	1
IFI30	1249693	226	42	645428	186544	0	1	0	0	50	80	23882616	5.02E-01	1
TBCA	251029	581	40	297616	64436	0	1	0	0	50	41	17733784	5.02E-01	1
ANKRD42	308108	414	18	1034536	253472	0	3	0	0	27	29	15630180	5.02E-01	1
EVL	453415	234	27	1097904	302244	0	4	0	0	8	4	1823788	5.02E-01	1
RAP1GAP	529490	297	34	1988972	535780	0	3	1	0	50	51	21530168	5.02E-01	1
CDK16	819569	NaN	38	1479180	433608	0	7	2	0	50	54	16576428	5.02E-01	1
GPRIN2	NaN	NaN	NaN	1098972	375936	0	3	2	0	50	118	40021520	5.02E-01	1
PBX3	491989	545	36	1123892	304024	0	3	0	0	38	47	16552576	5.02E-01	1
SERP1	503198	347	15	181916	41296	0	1	0	0	50	86	22944912	5.02E-01	1
LYZL1	172530	439	3	511572	132432	0	2	0	0	50	51	17170948	5.02E-01	1
SLC22A10	1307417	572	17	1365616	409400	0	5	2	0	50	74	21257472	5.02E-01	1
GOLPH3L	1611525	178	42	722324	211108	0	3	0	0	50	66	20744120	5.03E-01	1
WDR6	1799657	241	41	2800652	911716	0	10	2	0	41	61	18921044	5.03E-01	1
MOGAT2	528291	418	45	848704	244928	0	3	1	0	50	74	25033920	5.03E-01	1
JPH2	526607	344	21	1707732	560700	0	5	1	0	50	50	22619528	5.03E-01	1

C5orf15	568918	269	60	666076	194732	0	3	1	0	50	48	17593164	5.03E-01	1
SCN3A	103604	683	-8	5254204	1370600	0	17	6	0	50	63	24570764	5.03E-01	1
KIF3C	597475	166	40	1993956	579924	0	7	1	0	50	52	21949536	5.03E-01	1
TRIM14	484638	272	48	1112856	336420	0	2	1	0	50	49	20385272	5.03E-01	1
MBIP	81955	627	-10	911716	229976	0	3	0	0	50	60	23250004	5.03E-01	1
COL22A1	5667	846	-22	4153808	1332508	0	17	10	0	50	71	18999008	5.03E-01	1
TRIM47	1727802	178	35	1558568	512284	0	6	0	0	3	5	1935216	5.03E-01	1
SIM1	103376	626	0	1953372	546816	0	11	5	0	6	5	1509084	5.04E-01	1
CLEC14A	13985	544	-50	1172664	400500	0	4	0	0	4	2	1921688	5.04E-01	1
MSL3	53978	NaN	41	1443936	363120	0	6	1	0	50	65	22023228	5.04E-01	1
THUMPD2	117341	739	-8	1317200	338200	0	3	1	0	50	57	23301980	5.04E-01	1
ITGA5	653663	190	42	2690292	799932	0	7	1	0	47	47	19516632	5.04E-01	1
TNR	177437	738	4	3460676	983272	0	12	5	0	50	59	21318348	5.04E-01	1
C1QA	389456	541	29	602708	194020	0	2	0	0	50	56	21614896	5.04E-01	1
PACSIN3	1240468	184	31	1102176	297972	0	4	1	0	50	80	21424080	5.04E-01	1
STX5	2405109	248	44	923820	259524	0	2	1	0	50	58	19762984	5.04E-01	1
GSTZ1	554839	418	37	572092	157352	0	2	0	0	50	65	23475708	5.05E-01	1
CARD16	NaN	NaN	NaN	538984	135992	0	2	1	0	50	117	39781576	5.05E-01	1
CATSPER2	774279	286	51	1414744	377360	0	6	2	0	50	57	19297692	5.05E-01	1
ACAD11	210777	598	19	2042728	540764	0	6	0	0	14	12	5685320	5.05E-01	1
SPINT2	1410213	301	-1	650768	185476	0	2	0	0	50	46	17588892	5.05E-01	1
RPE	181785	778	-2	651124	170168	0	1	0	0	50	33	21367476	5.05E-01	1
MYH10	646488	488	10	5171968	1333220	0	19	5	0	4	7	2835184	5.05E-01	1
HRH3	861466	288	-13	1073340	364188	0	4	2	0	50	72	19241088	5.05E-01	1
PFKFB1	244981	NaN	0	1244576	325384	0	5	0	0	50	79	28474304	5.05E-01	1
ABCE1	189309	1139	32	1594168	404060	0	6	0	0	46	44	19455756	5.05E-01	1
KIAA1467	697100	196	7	1566400	483092	0	5	1	0	50	63	21337928	5.06E-01	1
TIPRL	475438	391	16	742616	182272	0	3	0	0	50	58	20535148	5.06E-01	1
SNAP23	552242	269	39	567464	141688	0	2	0	0	50	56	20950244	5.06E-01	1
CTXN1	952640	320	27	201140	69064	0	1	0	0	50	74	20932088	5.06E-01	1
ZNF334	416360	642	19	1781424	417588	0	6	1	0	50	53	17563616	5.06E-01	1
CCDC80	255516	510	24	2397660	679248	0	6	2	0	28	31	12789300	5.06E-01	1
ECSIT	1152381	272	31	1111076	302956	0	4	1	0	45	39	14328288	5.06E-01	1

DUXA	311003	924	-64	547528	132788	0	3	1	0	50	74	18807836	5.06E-01	1
TSTD2	506644	420	36	1354224	340692	0	4	1	0	50	64	21883676	5.06E-01	1
ZNF205	1626425	234	44	1396588	411536	0	5	0	0	13	10	4565700	5.06E-01	1
SNAP25	168916	372	-41	672484	150944	0	3	0	0	50	72	21416960	5.07E-01	1
KIAA0430	538786	282	29	4459256	1239592	0	11	4	0	50	55	19349668	5.07E-01	1
HSPA5	953574	153	47	1651484	477040	0	9	2	0	50	61	22462532	5.07E-01	1
XRCC1	416043	209	-10	1633684	468140	0	6	3	0	19	23	8087964	5.07E-01	1
C1orf106	545933	437	12	1630124	537916	0	6	1	0	10	8	4377376	5.07E-01	1
GAGE12J	547555	NaN	25	321112	76184	0	1	0	0	50	45	20590684	5.07E-01	1
FH	171676	533	-20	1304028	373800	0	6	1	0	42	98	25875860	5.07E-01	1
OGT	749568	NaN	26	2714144	730156	0	6	1	0	40	72	27399540	5.08E-01	1
SYT2	555792	497	34	1092920	286936	0	5	2	0	47	84	24751256	5.08E-01	1
ARHGAP3[illegible]	1706542	291	12	2789616	829836	0	7	1	0	4	2	2274840	5.08E-01	1
ZNF534	548808	989	-53	1743688	431828	0	6	0	0	32	30	11866192	5.08E-01	1
POSTN	135260	641	-38	2207200	568532	0	8	0	0	2	3	1649704	5.08E-01	1
SEC14L3	657636	288	48	1060524	273408	0	4	1	0	50	58	19652624	5.08E-01	1
CCDC160	217139	NaN	19	846924	199716	0	2	0	0	50	56	20766192	5.08E-01	1
RNF103	621664	442	36	1757216	453544	0	3	0	0	11	10	6118572	5.08E-01	1
SERPINB5	200746	166	24	976864	253472	0	3	1	0	50	43	19107232	5.08E-01	1
TCTA	1585328	201	32	261660	80100	0	2	1	0	2	3	559988	5.08E-01	1
NOMO1	518442	343	19	3153448	895340	0	9	1	0	17	18	8162724	5.08E-01	1
UGT1A9	320853	284	24	1345680	374868	0	2	0	0	20	18	9508760	5.08E-01	1
HMSD	114875	671	26	367392	93984	0	1	0	0	50	39	19658676	5.08E-01	1
TESK2	902221	176	27	1448564	426844	0	5	1	0	50	72	23250360	5.08E-01	1
NAMPT	246330	370	27	1286228	332860	0	4	0	0	50	43	21194816	5.08E-01	1
ADORA1	469029	453	37	806340	249912	0	4	0	0	50	65	19729876	5.09E-01	1
EREG	174005	402	7	445356	116412	0	1	1	0	4	2	1256324	5.09E-01	1
PPP1R1C	197289	370	6	299040	70488	0	1	0	0	50	40	17058808	5.09E-01	1
COX6C	282641	537	29	202208	54112	0	1	0	0	50	77	21669720	5.09E-01	1
PDLIM3	168463	422	4	1088648	315772	0	3	0	0	50	34	17193020	5.09E-01	1
IL17B	422727	380	28	458884	133856	0	1	0	0	50	43	19341480	5.09E-01	1
CNOT3	735613	439	-53	1946608	541832	0	9	3	0	18	36	7425804	5.09E-01	1
HMGA1	1074764	233	36	285156	77964	0	1	0	0	50	48	16846276	5.09E-01	1

TERF2IP	449343	354	18	996444	293700	0	4	1	0	50	60	21330452	5.09E-01	1
NUP62	1679101	205	45	1249204	430760	0	5	1	0	50	76	20785416	5.09E-01	1
C20orf195	1262904	298	54	777148	251336	0	3	2	0	21	30	7391984	5.10E-01	1
RPS9	717623	458	-6	492348	151656	0	2	0	0	50	58	18028196	5.10E-01	1
EGFLAM	159917	497	-12	2609124	729444	0	14	1	0	3	14	4240672	5.10E-01	1
FAM133B	744909	334	24	691352	148096	0	2	0	0	50	67	27513104	5.10E-01	1
PLEKHA7	511749	446	13	2865088	825920	0	11	4	0	47	56	19808196	5.10E-01	1
CREB3L3	2256881	244	42	1157712	360984	0	5	3	0	38	42	15813876	5.10E-01	1
UBLCP1	126518	284	33	858672	206124	0	3	2	0	15	20	5484180	5.10E-01	1
OR52N5	139577	922	2	802068	243504	0	3	1	0	50	48	17113632	5.10E-01	1
MORC3	281776	347	63	2475624	606624	0	9	2	0	50	53	18921756	5.10E-01	1
FIGLA	596622	629	24	559632	166608	0	2	0	0	50	44	16827408	5.10E-01	1
IGF2BP1	820571	174	28	1500184	411536	0	6	3	0	50	53	19076972	5.10E-01	1
GPR19	791330	136	44	1041656	295480	0	2	1	0	50	52	20311224	5.10E-01	1
APOC4	1003719	196	6	330012	90780	0	1	0	0	50	42	17116124	5.10E-01	1
ARL6IP5	264082	443	20	482024	132076	0	2	1	0	8	7	2888228	5.10E-01	1
CDKL4	391680	375	16	833040	209328	0	1	0	0	50	61	22212976	5.11E-01	1
FOLH1	11046	1009	-19	1965120	517980	0	9	1	0	7	17	4193324	5.11E-01	1
C15orf43	706600	569	31	597724	138128	0	2	0	0	50	70	25355032	5.11E-01	1
OR6B3	315906	569	21	806340	256320	0	2	0	0	50	47	24388136	5.11E-01	1
KCNN3	1958370	559	34	1852980	543612	0	5	3	0	50	49	18763336	5.11E-01	1
TMEM116	816439	317	43	642224	175864	0	1	1	0	50	60	21025004	5.11E-01	1
SEC24D	248929	623	24	2667508	736208	0	6	2	0	35	30	15255312	5.11E-01	1
CCDC66	178940	477	24	2523684	595944	0	5	2	0	50	33	17933144	5.11E-01	1
VPS33A	885125	222	47	1539344	424708	0	7	3	0	4	13	4742632	5.11E-01	1
ACBD6	543108	445	12	739056	197580	0	3	0	0	26	23	9718800	5.11E-01	1
KIFC1	1441897	236	31	1668928	533288	0	7	2	0	50	72	19862664	5.11E-01	1
TM4SF4	558016	159	6	521184	146316	0	2	0	0	50	60	20498480	5.11E-01	1
KIR3DL3	1155455	625	-10	1041300	307584	0	3	0	0	3	2	1551448	5.11E-01	1
ATP13A2	678965	221	52	3175876	981848	0	11	2	0	25	22	10637992	5.12E-01	1
CDIPT	NaN	NaN	NaN	545392	161624	0	2	0	0	50	116	39807208	5.12E-01	1
GRAMD1C	442573	363	36	1757572	441440	0	7	1	0	20	17	8206156	5.12E-01	1
DPP4	153730	528	9	2068360	495908	0	9	0	0	8	20	5836620	5.12E-01	1

ZNF326	176748	215	12	1542192	374868	0	3	1	0	50	59	20023576	5.12E-01	1
DPH3	205091	437	26	223568	50908	0	1	0	0	50	61	18386332	5.12E-01	1
KRT15	1445853	325	-22	1153440	340692	0	5	2	0	50	58	19098688	5.12E-01	1
ELF4	384451	NaN	48	1632260	525100	0	5	2	0	50	70	23957732	5.12E-01	1
OR2A12	222080	812	-11	767180	228196	0	4	3	0	9	13	2789616	5.12E-01	1
TMEM132A	798818	512	52	2457112	869708	0	8	0	0	13	8	4255624	5.12E-01	1
LTB	2517285	220	35	608048	191884	0	2	0	0	50	55	21824936	5.12E-01	1
ANKRD34B	321394	545	38	1287652	366680	0	5	2	0	39	38	14607036	5.12E-01	1
EMB	72996	1229	-3	866860	218228	0	2	0	0	50	37	19144256	5.12E-01	1
GXYLT2	144572	420	14	1123536	324672	0	5	1	0	50	58	19788260	5.13E-01	1
FAM71C	100701	906	0	611964	167676	0	2	0	0	50	50	19840236	5.13E-01	1
MAP3K4	80018	860	-2	4162352	1103956	0	17	2	0	50	68	23205860	5.13E-01	1
AQP4	65361	639	-33	819868	239588	0	5	0	0	50	81	21907884	5.13E-01	1
TBC1D10C	1269888	177	51	1105380	365256	0	3	1	0	31	22	11381320	5.13E-01	1
STX4	1398193	247	40	790676	206836	0	3	1	0	50	79	26328336	5.13E-01	1
CYLC2	13384	1215	-31	926312	211108	0	5	0	0	50	105	25680416	5.13E-01	1
RNF4	816930	179	39	533644	103952	0	2	0	0	50	48	16896472	5.13E-01	1
SF3A3	445132	232	42	1358140	318620	0	4	1	0	50	34	17924600	5.13E-01	1
FEZ1	363322	629	21	1041656	258100	0	2	0	0	50	49	18911788	5.13E-01	1
GPM6A	123418	931	-23	734784	195444	0	3	2	0	9	22	3607704	5.13E-01	1
FANCC	165520	457	38	1449988	400856	0	7	1	0	50	76	24964500	5.13E-01	1
NFIC	2180542	222	37	1296908	386260	0	2	1	0	50	52	23783648	5.13E-01	1
SLC25A36	209562	727	18	788896	236384	0	1	1	0	50	57	20338636	5.13E-01	1
OR51L1	97256	1024	-11	767180	243148	0	3	0	0	50	75	25324772	5.13E-01	1
SHC1	1612870	224	35	1470636	450696	0	5	2	0	50	56	18488504	5.13E-01	1
PDE1A	359828	401	-5	1489148	373444	0	7	1	0	7	9	3027424	5.14E-01	1
NFATC3	882378	152	43	2869004	862588	0	8	1	0	29	34	14862288	5.14E-01	1
C9orf16	1762678	201	44	214668	58740	0	1	0	0	50	65	19259600	5.14E-01	1
DENR	1042095	227	42	543968	123532	0	2	1	0	20	20	7505904	5.14E-01	1
OR6M1	92984	637	-1	764332	240656	0	5	3	0	4	3	960488	5.14E-01	1
NUSAP1	897414	237	24	1153440	305448	0	6	1	0	50	50	15496680	5.14E-01	1
CBR1	219580	318	66	690996	208260	0	2	1	0	50	70	20787908	5.14E-01	1
ZNF804B	3101	1030	-20	3443232	892848	0	13	2	0	15	11	5157016	5.14E-01	1

PPP4R2	128046	483	17	1104312	271272	0	2	1	0	50	49	20149956	5.14E-01	1
TMPRSS12	594068	250	43	884304	253116	0	3	1	0	35	34	15024268	5.14E-01	1
SEMA3A	61350	601	-27	2032404	509436	0	10	3	0	50	91	20761920	5.14E-01	1
GNRH2	835590	288	54	304380	98256	0	1	1	0	12	13	2338920	5.14E-01	1
WWP1	193108	1112	-6	2438956	616592	0	4	0	0	27	17	11045968	5.14E-01	1
WDR20	1492356	237	43	1493420	421504	0	6	2	0	25	42	12994712	5.14E-01	1
FGFBP2	175145	435	33	557852	164116	0	2	0	0	37	55	19726316	5.14E-01	1
SLFN5	753178	505	16	2253480	621576	0	9	0	0	2	1	1459244	5.15E-01	1
MAP1LC3C	172086	419	-12	388040	101104	0	1	0	0	50	57	23588916	5.15E-01	1
MS4A6E	433906	899	24	375936	113208	0	1	0	0	50	36	18309436	5.15E-01	1
U2AF1L4	1151198	321	30	623712	114276	0	2	0	0	50	47	19100468	5.15E-01	1
SDR16C5	435368	613	-7	802424	216448	0	3	1	0	50	41	14552212	5.15E-01	1
IK	774470	356	31	1515492	353508	0	4	0	0	19	25	9696372	5.15E-01	1
CA2	100997	1460	-18	682096	179780	0	2	1	0	19	17	8272016	5.15E-01	1
MAP4	1023686	249	35	4025648	1203280	0	9	1	0	8	10	5237828	5.15E-01	1
SPINK2	595179	572	12	225704	61588	0	1	0	0	50	65	20162772	5.15E-01	1
TIAF1	1586181	236	33	280528	91136	0	1	0	0	50	54	20950244	5.15E-01	1
STX6	217489	398	22	678892	171236	0	2	0	0	50	51	19719908	5.15E-01	1
CDKL2	485383	451	31	1303316	326452	0	4	0	0	15	11	6447160	5.15E-01	1
VN1R2	427680	921	-46	982560	290496	0	3	1	0	50	59	17329368	5.15E-01	1
TFCP2L1	240173	568	9	1265936	333928	0	3	1	0	50	58	22422304	5.15E-01	1
PGLYRP1	997099	261	31	492704	149164	0	2	1	0	14	17	5940216	5.15E-01	1
HHIPL1	375738	250	21	2022080	628696	0	5	2	0	37	38	16980844	5.15E-01	1
MAPK9	1623084	362	25	1229980	306872	0	4	0	0	32	34	10649028	5.16E-01	1
OR8D1	154391	921	-14	757568	231400	0	4	1	0	8	10	3157720	5.16E-01	1
ICT1	1111289	171	31	543612	143112	0	4	2	0	5	6	1244576	5.16E-01	1
ADAP2	594135	209	20	1004988	261660	0	2	1	0	50	57	19186976	5.16E-01	1
SBNO2	2398425	234	17	3550744	1076900	0	5	3	0	50	57	27012212	5.16E-01	1
GABRB3	21132	1430	-54	1280888	361696	0	7	1	0	13	29	6781800	5.16E-01	1
PRDM14	268840	727	10	1464584	398008	0	5	0	0	50	66	20633760	5.16E-01	1
ALKBH5	918230	192	23	979000	299396	0	5	1	0	50	73	17814952	5.16E-01	1
PRKG2	10566	894	-1	1999652	519760	0	7	2	0	25	29	11126424	5.16E-01	1
DHDH	1927185	211	43	848704	254540	0	3	1	0	50	57	20562560	5.16E-01	1

SMAD9	189427	731	7	1187260	333572	0	3	1	0	50	72	23943848	5.17E-01	1
FLRT3	NaN	NaN	NaN	1612680	474192	0	6	2	0	50	118	40119776	5.17E-01	1
OSTM1	303865	459	38	857248	239588	0	2	0	0	50	55	16635168	5.17E-01	1
NUDCD1	134762	731	17	1555720	391244	0	4	0	0	14	11	5293720	5.17E-01	1
CPEB1	567143	566	19	1456396	410468	0	7	1	0	12	13	5114652	5.17E-01	1
RNF138	409393	481	23	661092	159132	0	2	0	0	50	67	28936392	5.17E-01	1
TLE6	1119793	197	35	1500896	403348	0	2	2	0	32	37	13356764	5.17E-01	1
C3orf36	548078	520	28	389464	142400	0	2	1	0	12	23	6107892	5.17E-01	1
IL27RA	1013197	231	32	1601288	495196	0	4	3	0	8	10	3617672	5.17E-01	1
C12orf50	152672	507	-36	1119264	257388	0	6	1	0	25	36	10138524	5.17E-01	1
KEAP1	1996822	207	30	1569960	453900	0	6	3	0	50	70	19094772	5.17E-01	1
C4orf27	173367	574	17	919904	223924	0	3	0	0	50	38	15814588	5.17E-01	1
AGTR1	271257	825	-24	898544	259168	0	3	2	0	50	74	18613460	5.17E-01	1
ZNF808	546511	1005	-53	2328596	580636	0	9	2	0	29	27	10802108	5.17E-01	1
CPNE1	1423559	169	53	1574944	444644	0	7	2	0	50	53	18644076	5.18E-01	1
SGOL2	555530	248	24	3336076	760772	0	10	0	0	5	2	2099332	5.18E-01	1
NQO2	430294	268	42	608048	160912	0	2	0	0	45	45	18149948	5.18E-01	1
OR52E4	262387	914	-13	769316	231400	0	3	0	0	50	51	17452900	5.18E-01	1
BARX1	448425	479	25	626204	203632	0	1	0	0	14	13	5185496	5.18E-01	1
IL3RA	NaN	NaN	NaN	1027416	270204	0	4	3	0	8	19	4196172	5.18E-01	1
BMP3	10953	1083	4	1181208	342828	0	3	0	0	33	26	13786812	5.18E-01	1
HAP1	1617880	323	9	1594880	462088	0	4	2	0	23	22	9524424	5.18E-01	1
HMGCS2	420500	581	22	1294416	377004	0	6	0	0	50	70	22730956	5.18E-01	1
OTOL1	236814	792	-13	1192956	349236	0	4	1	0	50	65	18764404	5.18E-01	1
TLCD1	1695792	208	40	661804	204344	0	3	1	0	38	64	19088364	5.18E-01	1
SERPINB4	174293	370	-5	1032044	250624	0	3	2	0	9	11	4391972	5.18E-01	1
NKAIN1	586763	234	28	540764	151300	0	1	0	0	50	93	24549048	5.18E-01	1
ZNF599	932476	607	-52	1513000	386972	0	10	1	0	50	132	30538036	5.18E-01	1
SH3GL3	179993	757	-3	919904	229264	0	3	0	0	50	44	21730240	5.18E-01	1
MFAP1	788307	250	36	1159492	284444	0	5	1	0	50	65	23012908	5.18E-01	1
POLN	765279	332	34	2362060	623000	0	8	2	0	50	49	21406992	5.18E-01	1
TMIE	298503	495	41	400500	115344	0	2	0	0	50	70	18972664	5.19E-01	1
CXXC5	659730	244	42	790320	248844	0	1	0	0	47	60	22577520	5.19E-01	1

INTS9	396951	403	42	1709868	476328	0	5	1	0	50	45	23805720	5.19E-01	1
OR5AU1	730598	357	36	892848	269136	0	3	0	0	20	18	8578888	5.19E-01	1
PSMB2	1113527	325	34	525812	142756	0	2	0	0	50	52	17637308	5.19E-01	1
ITK	266925	1180	30	1639736	420436	0	4	0	0	50	34	21714576	5.19E-01	1
OTUD6A	166632	NaN	16	725884	202208	0	3	3	0	10	6	2726604	5.19E-01	1
ESRRA	1613996	257	65	1035960	348168	0	3	0	0	36	53	16019644	5.19E-01	1
GLI3	41720	589	-12	3958008	1167324	0	17	6	0	30	48	15405900	5.19E-01	1
GRINA	2580629	247	28	935212	282308	0	3	0	0	50	62	28229376	5.19E-01	1
SOX14	85683	880	-2	590604	185832	0	2	0	0	50	62	22060964	5.19E-01	1
TSEN54	1907659	195	31	1338560	394804	0	4	1	0	50	90	21830276	5.19E-01	1
MCCD1	2514366	220	34	300464	90424	0	1	0	0	50	60	23287028	5.19E-01	1
ETV5	780547	397	36	1329660	358848	0	3	2	0	50	63	24114728	5.20E-01	1
PGRMC1	613514	NaN	19	491280	145248	0	1	1	0	50	40	17974796	5.20E-01	1
PIWIL2	635115	144	37	2523684	690996	0	7	0	0	2	2	1465296	5.20E-01	1
ATG2B	327434	501	14	5377380	1458888	0	16	4	0	35	44	16091200	5.20E-01	1
WWTR1	558006	263	18	1021720	288716	0	4	1	0	50	56	21033548	5.20E-01	1
CAPN13	131345	692	5	1785696	454968	0	6	2	0	50	61	24046020	5.20E-01	1
NDST4	26862	1055	-20	2249920	600572	0	10	3	0	50	72	20288440	5.20E-01	1
DPP8	620582	187	39	2362416	603420	0	7	2	0	50	43	20303392	5.20E-01	1
EIF4E	225403	415	11	731224	184052	0	2	0	0	50	48	22352528	5.20E-01	1
BEST3	499701	394	14	1710580	471344	0	8	3	0	19	19	7571408	5.20E-01	1
FRG2B	50016	525	-16	709864	197936	0	3	1	0	48	72	21755872	5.20E-01	1
AMPD1	600650	472	11	2051272	519404	0	9	1	0	6	5	2131728	5.20E-01	1
ZNF716	NaN	NaN	NaN	1286228	315772	0	5	2	0	50	118	39961356	5.20E-01	1
IFNA2	232155	466	-14	478464	129228	0	1	0	0	50	45	18385976	5.20E-01	1
ARMCX2	300391	NaN	31	1525104	503028	0	5	1	0	50	58	25085896	5.20E-01	1
EFCAB5	652131	303	15	4069436	1019584	0	18	1	0	2	2	1282668	5.20E-01	1
GABRQ	203033	NaN	-1	1620512	446068	0	5	2	0	50	57	23840964	5.20E-01	1
TOR1B	600401	187	44	855468	243504	0	2	0	0	50	71	21419808	5.20E-01	1
FAM101A	1197100	504	34	340692	103596	0	2	1	0	2	2	602352	5.20E-01	1
C9orf3	250013	432	35	1862592	503028	0	8	1	0	50	54	19833116	5.21E-01	1
TRIM9	373925	516	17	1846572	521184	0	5	2	0	50	66	24314088	5.21E-01	1
TGM4	466410	380	31	1780712	471700	0	8	2	0	50	53	18679320	5.21E-01	1

PGS1	980896	209	26	1394808	430404	0	3	0	0	18	29	8859772	5.21E-01	1
ZNF557	748801	470	21	1122112	284444	0	4	2	0	50	64	18326524	5.21E-01	1
WDR59	724091	165	28	2545044	687792	0	4	3	0	41	38	18668996	5.21E-01	1
CD33	352763	629	1	929516	279460	0	4	2	0	50	65	22440104	5.21E-01	1
TRPC7	283872	588	23	2212184	599860	0	7	2	0	50	53	25081624	5.21E-01	1
TTC30A	380902	434	27	1664300	469564	0	4	0	0	39	31	17848416	5.21E-01	1
SHB	486918	551	16	1269496	385904	0	3	1	0	44	52	17428692	5.21E-01	1
OR13C2	147494	920	-31	793880	227128	0	5	3	0	10	11	2804924	5.22E-01	1
GPD2	116871	496	10	1885376	515488	0	6	1	0	25	36	12341452	5.22E-01	1
TMEM59L	1397040	222	33	868996	262016	0	1	2	0	49	52	19848424	5.22E-01	1
RUSC1	1668953	177	44	2317204	760772	0	11	7	0	6	15	2951596	5.22E-01	1
CASD1	446033	469	3	2101824	527592	0	4	1	0	50	46	18769032	5.22E-01	1
HLA-DRB[illegible]	1070559	595	14	687080	191884	0	1	0	0	50	75	22719564	5.22E-01	1
ZNF565	950699	326	-10	1295128	323960	0	7	0	0	50	52	17608116	5.22E-01	1
CACNA1F	724395	NaN	44	5079764	1462804	0	11	2	0	16	16	8111460	5.22E-01	1
PPTC7	890955	144	38	772520	226060	0	3	0	0	50	61	20817100	5.22E-01	1
SOAT1	437024	336	3	1457464	372020	0	5	1	0	50	52	21333656	5.22E-01	1
OCLN	502559	312	32	1352800	357068	0	4	1	0	50	39	16974436	5.22E-01	1
MATK	2172842	204	36	1361344	404060	0	4	2	0	50	49	23587492	5.22E-01	1
PIK3CB	281611	424	26	2801364	717696	0	6	2	0	43	46	18444004	5.22E-01	1
IGLON5	368509	610	-9	838380	271272	0	3	0	0	50	39	14321524	5.22E-01	1
OR9K2	341315	828	-2	826988	248488	0	2	2	0	42	52	16635880	5.22E-01	1
CPA6	232126	890	15	1150592	295480	0	8	2	0	22	35	9241404	5.23E-01	1
RAPGEF6	314836	627	30	4582432	1180496	0	11	2	0	25	27	10521936	5.23E-01	1
ACADS	1741363	349	42	1050200	311500	0	6	2	0	43	52	12767228	5.23E-01	1
COQ10B	681973	428	29	609472	175508	0	2	1	0	50	49	19314780	5.23E-01	1
SRR	780080	271	29	866148	256320	0	3	2	0	17	24	9019972	5.23E-01	1
FAM50B	271690	208	30	818444	230332	0	4	2	0	23	30	9160236	5.23E-01	1
RHPN1	1882843	237	35	1680320	529372	0	6	1	0	50	67	21836684	5.23E-01	1
CHRNA10	537999	403	23	1089360	374868	0	4	1	0	50	51	22444732	5.23E-01	1
TBCE	531741	178	21	1388756	371308	0	6	2	0	50	56	19303744	5.23E-01	1
OR52B6	128694	895	9	819512	254896	0	3	0	0	50	58	21404856	5.23E-01	1
NUB1	604407	282	42	1584556	404060	0	4	0	0	20	17	9787864	5.23E-01	1

ANKRD28	345831	435	29	2741912	750448	0	8	2	0	18	14	7942004	5.23E-01	1
TMEM190	1028808	209	19	461732	129940	0	2	0	0	3	0	758992	5.23E-01	1
DRG1	737051	237	38	948384	264864	0	4	1	0	29	35	12516604	5.24E-01	1
NLRP3	293118	825	-17	2651132	709864	0	11	6	0	29	29	8739800	5.24E-01	1
PPM1K	432936	304	25	955148	265576	0	4	1	0	50	49	16500956	5.24E-01	1
RNF121	737476	311	53	860808	224280	0	3	2	0	33	20	8285900	5.24E-01	1
CCDC27	249153	341	24	1705596	448560	0	8	4	0	14	20	6017824	5.24E-01	1
DSC1	214084	492	-7	2353516	623000	0	8	3	0	35	31	14424764	5.24E-01	1
RTN1	298900	639	-2	1982564	610540	0	6	1	0	49	46	17808544	5.24E-01	1
TNNC1	1078407	229	62	446780	95764	0	2	1	0	11	14	4151672	5.24E-01	1
FANK1	207005	738	-8	907800	243504	0	3	1	0	42	47	18364616	5.24E-01	1
AMH	1603571	240	27	1298688	515844	0	2	0	0	11	6	4169472	5.24E-01	1
BTG3	101069	479	-5	771096	201852	0	1	0	0	50	70	23406644	5.25E-01	1
SH2B1	1146511	221	56	1987548	639732	0	6	3	0	16	35	10080852	5.25E-01	1
NSDHL	211215	NaN	5	957640	270560	0	3	0	0	50	55	23944204	5.25E-01	1
MRGPRE	583782	421	36	740836	265220	0	3	2	0	9	11	4180152	5.25E-01	1
MYLIP	159273	596	27	1145252	309364	0	3	1	0	50	47	22634124	5.26E-01	1
PRR4	365490	676	5	437880	126024	0	2	2	0	4	5	1258104	5.26E-01	1
TSPAN7	99411	NaN	23	649344	183696	0	2	0	0	50	42	17506656	5.26E-01	1
ZNF792	903994	549	-57	1621580	419368	0	6	1	0	36	106	25699640	5.26E-01	1
OXER1	343875	378	28	1004632	356000	0	3	2	0	16	15	7444672	5.26E-01	1
KIF3B	920221	146	58	1907804	522964	0	7	1	0	27	35	11622688	5.26E-01	1
TMPRSS2	266466	454	61	1387688	372376	0	5	2	0	50	79	23245376	5.26E-01	1
KCTD9	178615	515	22	1021720	274832	0	1	0	0	50	48	21259964	5.26E-01	1
MAP7	302036	376	16	1918840	556784	0	7	1	0	3	3	1969036	5.26E-01	1
CER1	160131	882	-10	674976	190104	0	2	0	0	50	74	30175272	5.26E-01	1
ARAP1	637045	234	54	3678548	1111432	0	10	1	0	4	6	3798164	5.26E-01	1
CXorf66	66755	NaN	14	918480	252048	0	4	1	0	50	74	21999376	5.26E-01	1
NFIB	135826	563	-17	1085444	297616	0	4	0	0	50	60	25014696	5.27E-01	1
CAMK1D	326642	556	35	1049844	264864	0	3	1	0	50	44	19718128	5.27E-01	1
CNPY2	2869106	184	52	479888	127804	0	2	0	0	50	84	25337232	5.27E-01	1
KCNK15	890596	168	46	784980	281952	0	3	1	0	50	63	21028564	5.27E-01	1
WDR13	817501	NaN	29	1221792	369528	0	4	1	0	50	77	20781500	5.27E-01	1

ZNF578	547660	989	-53	1542548	363832	0	4	1	0	34	33	12611656	5.27E-01	1
NOX4	30048	1071	-25	1534004	393736	0	8	3	0	43	110	23842744	5.27E-01	1
SUN1	642960	310	29	2173024	615524	0	5	1	0	50	53	23511664	5.27E-01	1
TAC4	1152179	238	30	299752	82592	0	1	0	0	50	53	20182708	5.27E-01	1
GIN1	205281	565	14	1359920	345676	0	3	2	0	50	46	18575368	5.27E-01	1
USP53	227905	753	2	2805636	695268	0	9	1	0	50	58	23763712	5.27E-01	1
CDS2	500170	482	24	1169104	311144	0	3	1	0	33	33	12298020	5.27E-01	1
RTKN	1069803	343	45	1481672	451408	0	5	1	0	50	68	19848780	5.27E-01	1
PRKAA2	39631	514	-5	1427916	380208	0	6	0	0	27	39	13414080	5.27E-01	1
SSBP2	159943	864	23	974016	254184	0	2	0	0	50	70	27318728	5.28E-01	1
RAD54L	690472	205	31	1935928	537560	0	9	2	0	49	42	17582840	5.28E-01	1
HECTD2	288529	384	4	2072276	528304	0	5	1	0	41	42	17824208	5.28E-01	1
POLR2F	1370748	203	50	339268	87932	0	3	1	0	3	6	1253476	5.28E-01	1
SLC26A11	684326	219	17	1506592	506588	0	4	2	0	50	52	19290216	5.28E-01	1
PAPD7	253408	557	28	1392316	398720	0	7	3	0	8	7	2887516	5.28E-01	1
SCNN1A	2670962	198	46	1870424	532576	0	10	1	0	4	7	2824504	5.28E-01	1
ZNF474	69953	707	2	907800	261660	0	3	0	0	50	44	18647636	5.28E-01	1
HOXA10	473864	394	13	1000360	335708	0	4	0	0	14	15	6307608	5.28E-01	1
OR52N4	139577	922	2	797084	238876	0	3	1	0	50	48	17113632	5.28E-01	1
RAD1	241905	675	21	737632	190460	0	2	0	0	50	44	20410904	5.29E-01	1
PPM1B	448266	420	32	1284804	341760	0	8	0	0	2	0	470276	5.29E-01	1
ACTL7B	336944	471	9	1019940	317196	0	3	0	0	34	34	14709564	5.29E-01	1
DBI	203559	497	35	496976	128872	0	2	1	0	42	52	16309428	5.29E-01	1
TSPAN9	523539	476	24	631544	167320	0	2	0	0	50	44	16570020	5.29E-01	1
KDM2B	650760	244	46	3498056	970456	0	10	2	0	14	18	7881840	5.29E-01	1
UXS1	125109	844	2	1125316	285512	0	2	0	0	50	49	19189824	5.29E-01	1
FNDC7	743461	225	9	1868644	534356	0	6	1	0	50	72	24119356	5.29E-01	1
PLD3	1006719	289	35	1248492	371664	0	4	2	0	50	73	21939212	5.29E-01	1
PRG2	923815	191	-3	578144	157708	0	3	1	0	50	67	17960912	5.29E-01	1
KLHL30	428658	356	29	1430408	454612	0	3	0	0	9	3	3567120	5.29E-01	1
MIP	2793663	184	61	641868	218940	0	3	1	0	42	73	21769400	5.29E-01	1
TSPAN3	319089	441	40	656464	182984	0	3	2	0	6	19	4110020	5.29E-01	1
PAWR	230993	395	-8	854756	259168	0	2	1	0	50	45	23122556	5.29E-01	1

ZNF547	432083	869	-17	1040944	263084	0	3	1	0	50	60	18121112	5.29E-01	1
INCA1	1407967	231	28	614456	170524	0	3	1	0	50	70	21740208	5.30E-01	1
ARL6IP6	275277	716	6	562836	177288	0	2	1	0	50	59	20198016	5.30E-01	1
KRIT1	707455	454	27	1943760	485940	0	10	4	0	16	21	6298708	5.30E-01	1
POF1B	105434	NaN	-3	1577080	381632	0	3	2	0	33	45	15328648	5.30E-01	1
ZXDC	295496	600	35	2119268	677824	0	5	1	0	31	21	10346072	5.30E-01	1
MPP4	466105	534	28	1713428	420436	0	2	0	0	10	6	3716284	5.30E-01	1
OR5H15	200002	811	-15	778928	224992	0	4	1	0	50	87	21088372	5.30E-01	1
OR5L2	4183	1091	-36	761128	238520	0	8	2	0	14	26	3895352	5.30E-01	1
IYD	286182	334	29	920616	212532	0	3	1	0	50	55	19430480	5.30E-01	1
SP1	1707021	145	57	1945184	596656	0	11	5	0	12	21	4913512	5.31E-01	1
HERC5	442906	295	29	2695988	682096	0	6	1	0	34	23	13756196	5.31E-01	1
PEX16	427820	291	48	927736	291920	0	1	0	0	50	52	20813896	5.31E-01	1
IL10	464449	357	29	473836	118904	0	2	1	0	3	4	1132436	5.31E-01	1
NAA50	441530	419	24	454968	106800	0	1	0	0	50	57	20353232	5.31E-01	1
CKAP4	168486	445	23	1484876	453544	0	4	1	0	37	42	14520884	5.31E-01	1
TIMM8A	374364	NaN	37	268780	69776	0	1	0	0	50	61	21564700	5.31E-01	1
SLC10A5	158801	681	12	1082240	322180	0	4	0	0	50	67	18347172	5.31E-01	1
CD93	199022	554	-50	1586336	510148	0	8	3	0	19	25	7971196	5.31E-01	1
NDEL1	761259	427	18	933788	250624	0	4	0	0	50	72	23239324	5.32E-01	1
COMMD2	449009	263	21	520472	139552	0	2	0	0	50	60	19623788	5.32E-01	1
ZBTB10	239826	369	30	2198656	616592	0	3	0	0	25	18	9184800	5.32E-01	1
MFRP	1054477	263	40	1480960	428624	0	5	3	0	50	80	23473572	5.32E-01	1
ZNF155	498673	419	-36	1404064	339980	0	7	0	0	50	66	18850912	5.32E-01	1
GLS	537377	292	36	1738348	480956	0	3	0	0	16	9	8381664	5.32E-01	1
DGCR14	487560	277	18	1210400	358492	0	4	0	0	36	33	13333268	5.32E-01	1
LBH	136617	581	18	280172	67996	0	1	0	0	50	51	18086936	5.32E-01	1
EPHB1	74866	856	9	2513360	706660	0	12	2	0	50	69	23991196	5.32E-01	1
OR7C2	961949	546	4	777860	246352	0	5	0	0	50	101	24125052	5.32E-01	1
USP18	521791	289	11	988612	244928	0	2	0	0	38	57	20964840	5.32E-01	1
HPCAL4	974845	388	36	506588	121396	0	2	1	0	50	62	16757632	5.32E-01	1
NMU	247026	456	28	474548	124600	0	3	1	0	8	8	2091856	5.33E-01	1
IL5	713465	320	66	349236	98256	0	1	0	0	50	41	19744828	5.33E-01	1

PTH2	2173457	290	44	236740	95408	0	1	1	0	50	67	19850560	5.33E-01	1
NECAP2	813996	191	40	781776	209328	0	3	1	0	50	47	17210108	5.33E-01	1
SLC28A2	494393	471	23	1698120	485940	0	4	0	0	23	24	10629092	5.33E-01	1
YIPF5	39627	428	1	671060	176932	0	2	0	0	50	47	20521620	5.33E-01	1
GNAL	281108	361	18	1325032	351728	0	4	0	0	8	11	5969408	5.33E-01	1
KRTAP23-	4608	1035	-69	162692	50908	0	1	0	0	50	77	14168444	5.33E-01	1
BZW2	326035	468	-15	1112144	280528	0	3	1	0	50	62	19985484	5.33E-01	1
DSCR4	78525	448	1	302244	87576	0	1	0	0	50	44	18242152	5.33E-01	1
NLRP9	1024341	362	-13	2543620	671060	0	12	2	0	30	37	10224676	5.34E-01	1
ATP5J	534575	641	29	321468	76896	0	1	0	0	50	49	20134292	5.34E-01	1
C10orf32	711862	192	45	289428	76896	0	1	0	0	50	62	22441884	5.34E-01	1
PPAPDC1E	763369	277	42	756500	211108	0	1	1	0	50	65	22314080	5.34E-01	1
TMEM98	443753	833	-6	576008	176932	0	2	0	0	50	62	19613108	5.34E-01	1
LNX1	116784	492	26	1926672	552156	0	3	1	0	50	39	21850212	5.34E-01	1
CCDC64B	1443513	234	49	1251696	415452	0	3	1	0	14	11	5039180	5.34E-01	1
SMOC1	607548	307	41	1135284	308652	0	2	0	0	16	8	7111100	5.34E-01	1
AGA	44980	837	-8	901748	244216	0	5	1	0	46	76	21257116	5.34E-01	1
HIST1H2B(	390169	544	57	313636	96476	0	3	2	0	15	17	2533296	5.34E-01	1
UGT1A5	NaN	NaN	NaN	1338916	392312	0	3	2	0	50	118	40037896	5.35E-01	1
MOGAT3	1022626	258	39	866504	254896	0	4	0	0	50	67	19705312	5.35E-01	1
IFRD2	843769	206	51	1264868	408688	0	4	1	0	50	68	20472848	5.35E-01	1
CCDC116	521773	194	37	1521188	463156	0	4	1	0	12	11	5081544	5.35E-01	1
TUBA3E	349797	593	22	1133860	332504	0	3	1	0	50	45	22118992	5.35E-01	1
MYCT1	188249	600	-9	583484	179068	0	2	1	0	50	78	26690388	5.35E-01	1
SDHAF1	NaN	NaN	NaN	271628	102172	0	1	0	0	50	116	39747756	5.35E-01	1
AKR1C1	343868	608	-13	843364	228908	0	2	0	0	50	33	15973720	5.35E-01	1
PDILT	212375	823	0	1524036	397296	0	4	2	0	14	10	5780728	5.35E-01	1
S100A16	1454773	186	26	276612	65148	0	1	0	0	50	56	19376724	5.35E-01	1
CPA3	306125	544	-14	1108228	275900	0	6	2	0	50	77	21753736	5.35E-01	1
KRT80	473704	198	46	1189040	336064	0	5	2	0	45	37	15400916	5.35E-01	1
NLRP10	105341	679	6	1650772	459596	0	6	2	0	50	63	24898284	5.35E-01	1
HOXD10	242935	874	15	866860	232112	0	2	1	0	50	65	19479964	5.35E-01	1
CXCL14	493676	608	26	291920	79744	0	1	0	0	50	58	22046368	5.35E-01	1

SSU72	1690623	248	29	513708	128160	0	2	1	0	8	12	4331808	5.36E-01	1
C2orf88	450974	478	21	247776	59808	0	1	0	0	16	16	5626936	5.36E-01	1
TOB2	1480984	187	73	849060	260592	0	2	2	0	41	44	12897168	5.36E-01	1
SHANK2	544815	678	36	4538644	1373804	0	10	3	0	26	18	10788224	5.36E-01	1
KLRC3	378365	334	0	723748	178712	0	2	1	0	50	50	21701404	5.36E-01	1
N4BP2	540527	260	45	4572108	1166256	0	16	6	0	11	14	5465668	5.36E-01	1
TGS1	324398	409	17	2244224	543256	0	9	1	0	15	19	7370624	5.36E-01	1
RPAP3	429377	382	5	1770032	432184	0	3	0	0	48	32	18159560	5.36E-01	1
RPTOR	782748	381	22	3451064	970456	0	11	4	0	50	79	29828884	5.37E-01	1
TCP11L1	415951	215	25	1310792	361696	0	6	0	0	10	9	3642948	5.37E-01	1
METTL2A	330776	370	5	997868	252760	0	5	2	0	9	16	4728748	5.37E-01	1
STX1B	1398193	247	40	772164	192240	0	1	1	0	50	79	26328336	5.37E-01	1
THEMIS	191535	553	-9	1733364	474192	0	7	2	0	50	70	23075920	5.37E-01	1
EID2B	1148741	322	25	387328	133856	0	1	0	0	50	44	18982632	5.37E-01	1
GBP4	344056	834	-2	1663944	432540	0	8	2	0	43	54	17079100	5.37E-01	1
PPIE	975799	388	39	903528	218940	0	3	2	0	50	81	18001852	5.37E-01	1
SOX12	879317	185	41	759348	253116	0	3	0	0	50	64	20950244	5.37E-01	1
DSP	825560	218	12	7362792	1937352	0	31	9	0	50	67	20744476	5.37E-01	1
ACSM1	175989	641	11	1491996	413316	0	2	0	0	9	4	3793892	5.37E-01	1
FAM126A	1063802	413	26	1349596	365612	0	3	2	0	50	77	26007224	5.37E-01	1
OR6C74	411183	816	-1	771096	230688	0	3	0	0	50	58	19503104	5.37E-01	1
KCTD11	1911735	206	36	549664	196868	0	2	1	0	50	69	22072000	5.38E-01	1
TRIM60	169984	637	5	1201500	315060	0	4	1	0	13	17	5631208	5.38E-01	1
PANK3	413558	432	32	950520	263796	0	4	1	0	50	55	19572168	5.38E-01	1
FMO4	421557	891	-1	1443580	381632	0	8	2	0	50	76	21940992	5.38E-01	1
HOXD13	242935	874	15	836600	271984	0	3	2	0	33	42	10580676	5.38E-01	1
GPR1	332753	487	25	891780	253116	0	3	0	0	50	57	21975880	5.38E-01	1
ZNF311	143617	569	25	1731228	427200	0	3	1	0	50	40	18953796	5.38E-01	1
FBP1	260812	350	38	867216	248844	0	3	0	0	35	38	13111124	5.38E-01	1
DNAJC17	1011507	172	46	812748	211464	0	3	0	0	30	31	11416920	5.38E-01	1
COL5A3	1386309	413	1	4411908	1466364	0	14	7	0	50	79	25890812	5.38E-01	1
PPIL6	328699	559	47	890712	226416	0	1	0	0	50	46	17980492	5.38E-01	1
DAK	1394229	286	57	1439308	474548	0	5	3	0	50	56	17921040	5.38E-01	1

REP15	346550	376	-4	598436	163048	0	2	1	0	13	13	5178732	5.38E-01	1
S100A14	1454773	186	26	275544	73692	0	1	0	0	50	56	19376724	5.38E-01	1
UGT2A1	19584	1065	-8	1354580	360628	0	4	1	0	50	65	23148900	5.38E-01	1
PAQR6	2266205	211	33	1216096	281240	0	3	0	0	50	61	22146048	5.38E-01	1
INHBE	1347802	194	32	839092	289784	0	4	0	0	50	70	20818880	5.38E-01	1
XKRX	363715	NaN	37	1125672	324672	0	4	0	0	36	40	16063788	5.38E-01	1
AQP9	242649	602	4	745820	223924	0	2	0	0	50	41	18036384	5.38E-01	1
PEX7	253053	408	33	850484	228196	0	3	0	0	23	23	8726272	5.39E-01	1
CRY1	169569	473	18	1525104	404772	0	4	1	0	50	43	20357504	5.39E-01	1
OR13C8	147494	920	-31	788184	239232	0	4	0	0	50	80	20017168	5.39E-01	1
OR2M4	18846	936	-35	766468	232112	0	3	0	0	50	93	20010048	5.39E-01	1
PDZK1IP1	364960	414	3	297260	84016	0	1	0	0	50	53	20385984	5.39E-01	1
INTS2	350722	380	2	3115356	850128	0	10	3	0	27	30	10835216	5.39E-01	1
SAMSN1	157937	898	56	989680	240656	0	4	0	0	50	45	16631964	5.39E-01	1
CNFN	792230	218	42	293700	81168	0	1	0	0	50	65	24510600	5.39E-01	1
PLTP	725555	209	59	1258816	388040	0	6	0	0	27	40	9561092	5.39E-01	1
HDAC8	366313	NaN	39	1060524	278748	0	3	0	0	50	67	20763700	5.39E-01	1
SSFA2	180253	346	18	3299408	914920	0	6	1	0	4	4	2835896	5.39E-01	1
OPTC	529605	314	28	838736	258100	0	2	2	0	48	64	19367112	5.40E-01	1
MIXL1	739974	278	21	554292	195444	0	2	0	0	43	57	19098688	5.40E-01	1
SLC16A14	377632	648	26	1284448	369884	0	2	1	0	9	10	6066596	5.40E-01	1
RPSA	160739	595	32	756144	217872	0	2	0	0	50	47	22330100	5.40E-01	1
ZMAT3	371562	807	-9	746532	203988	0	3	0	0	50	62	21058112	5.40E-01	1
ARV1	376655	616	12	714136	180848	0	3	1	0	50	64	20436536	5.40E-01	1
MTMR14	899035	348	39	1692068	470632	0	5	0	0	7	4	3016032	5.40E-01	1
ECH1	1989663	219	30	852264	242436	0	4	0	0	50	70	21843448	5.40E-01	1
CSF1	785919	216	30	1399080	413316	0	6	3	0	50	74	21989764	5.40E-01	1
LCLAT1	140581	648	21	1084020	271272	0	3	1	0	50	48	22483892	5.40E-01	1
NHLRC3	150239	998	11	889644	250980	0	3	2	0	22	32	8661480	5.40E-01	1
ZNF641	809696	412	16	1210400	327520	0	3	1	0	50	60	19819232	5.40E-01	1
CLK1	635570	201	31	1412252	327520	0	7	1	0	50	61	17116836	5.40E-01	1
NANOG	175700	467	16	800644	192596	0	3	0	0	50	63	21166336	5.40E-01	1
TNIP1	892080	382	27	1660028	453544	0	5	0	0	24	27	9916380	5.41E-01	1

DPH2	875223	214	39	1178716	412604	0	6	2	0	50	67	19898264	5.41E-01	1
MYOZ1	1051799	153	43	761484	221076	0	2	0	0	50	57	24499920	5.41E-01	1
TMEM225	89099	736	-8	588468	148452	0	2	0	0	50	74	25706404	5.41E-01	1
ATG4B	1268313	406	27	1066932	269136	0	3	1	0	50	59	18539056	5.41E-01	1
NUP35	349831	524	11	845500	237452	0	3	0	0	50	47	18939200	5.41E-01	1
ARHGEF2	1880483	213	32	2498764	753296	0	7	2	0	17	27	7552540	5.41E-01	1
ZNF117	216422	994	-9	1263088	296192	0	2	0	0	50	54	21329740	5.41E-01	1
SIRPB1	421731	424	39	1929876	588468	0	7	1	0	40	48	16033172	5.41E-01	1
KRTAP13-[illegible]	4608	1035	-69	425776	130652	0	3	0	0	11	5	1333576	5.41E-01	1
MOAP1	325787	222	35	874336	253472	0	6	0	0	1	0	373088	5.41E-01	1
PITX3	1020192	169	44	734428	249200	0	2	1	0	18	20	9534392	5.41E-01	1
C1D	210519	537	2	381632	90424	0	1	0	0	50	48	21668652	5.41E-01	1
DNAJA1	953054	286	0	1049844	259524	0	5	1	0	50	53	17598860	5.41E-01	1
CBWD2	553274	555	22	1068000	260592	0	3	0	0	32	23	11661492	5.41E-01	1
CBX7	1063921	223	78	638664	192240	0	2	1	0	41	45	17422640	5.42E-01	1
OR5M8	NaN	NaN	NaN	776080	223568	0	4	2	0	26	42	11800688	5.42E-01	1
GPR137C	356488	313	14	1075120	328232	0	4	0	0	3	2	1220012	5.42E-01	1
PXDNL	62919	1068	-16	3719844	1066932	0	17	3	0	50	68	20781500	5.42E-01	1
FANCB	111662	NaN	21	2216100	573516	0	4	2	0	50	58	21189476	5.42E-01	1
ANXA6	926280	300	41	1803496	462800	0	6	2	0	50	73	23722772	5.42E-01	1
EPO	1483965	336	46	480600	158064	0	2	2	0	9	15	4840176	5.42E-01	1
KCNK10	131280	834	-23	1453548	414384	0	4	1	0	50	79	18934216	5.42E-01	1
IFNA5	232155	466	-14	485940	127092	0	1	0	0	50	45	18385976	5.42E-01	1
ZNF554	1287128	291	27	1383416	360628	0	3	1	0	50	60	21755516	5.42E-01	1
SAMD14	1237828	210	23	1120688	351016	0	5	0	0	28	38	13129636	5.42E-01	1
LBX2	1019591	315	50	464224	164828	0	2	1	0	40	63	17658668	5.42E-01	1
NXF1	2405109	248	44	1676404	452120	0	8	1	0	50	58	19762984	5.42E-01	1
SCAI	951391	290	31	1663232	432184	0	7	1	0	50	70	19790752	5.42E-01	1
IQCG	612221	410	33	1188328	276968	0	5	0	0	5	0	874692	5.42E-01	1
EIF3L	1385064	203	66	1501608	362052	0	4	1	0	50	52	18523392	5.43E-01	1
ZSCAN22	1621097	406	-25	1231760	353152	0	5	1	0	50	63	19879752	5.43E-01	1
OR4C16	1141	1100	-37	775724	218584	0	6	0	0	50	92	19702464	5.43E-01	1
HMCN1	260400	509	-19	14461432	4049144	0	67	19	0	14	38	9299788	5.43E-01	1

ING1	560003	274	51	1161984	351372	0	2	0	0	29	25	10055220	5.43E-01	1
DOCK3	395621	756	25	5336084	1395520	0	10	1	0	8	8	5630496	5.43E-01	1
ZIC4	20725	1070	-30	1055896	305804	0	7	1	0	50	122	25186644	5.43E-01	1
ANXA3	243621	351	21	871844	217516	0	3	0	0	50	54	20771888	5.43E-01	1
PPRC1	1029829	187	42	4040244	1352088	0	18	4	0	6	16	5941284	5.43E-01	1
COL9A2	627703	373	18	1761488	583840	0	3	0	0	22	17	9075152	5.43E-01	1
SENP1	457498	255	30	1705240	430760	0	4	0	0	27	17	8787148	5.43E-01	1
ZNF664	903375	539	36	687436	152012	0	2	0	0	50	46	18738416	5.43E-01	1
TMEM53	563553	252	45	682452	218940	0	2	0	0	28	30	12632304	5.43E-01	1
MED1	1434634	214	17	4003220	1133860	0	20	4	0	17	33	9306908	5.44E-01	1
AAGAB	312039	320	45	835532	215380	0	5	1	0	30	35	9988648	5.44E-01	1
CREM	179129	346	15	1117484	298684	0	2	0	0	50	55	20218664	5.44E-01	1
RAB9B	472103	NaN	26	515132	136348	0	2	1	0	50	79	25392412	5.44E-01	1
TMUB1	622098	373	41	587756	212176	0	4	2	0	2	4	1207196	5.44E-01	1
DDTL	NaN	NaN	NaN	336064	107156	0	1	0	0	50	116	39752740	5.44E-01	1
GTF3C3	404194	370	14	2319340	597368	0	6	3	0	50	59	23575388	5.44E-01	1
RBBP7	289514	NaN	39	1127452	284444	0	4	1	0	50	82	21778656	5.44E-01	1
CCRN4L	332509	361	32	1063372	329300	0	1	2	0	50	40	18887936	5.44E-01	1
SOX1	67826	793	-33	934856	323248	0	3	1	0	25	42	10226812	5.44E-01	1
PPAP2A	345749	277	33	864724	239588	0	4	1	0	50	60	20518060	5.44E-01	1
RAP1B	439623	356	37	496620	126024	0	2	1	0	20	35	10689256	5.44E-01	1
LPAR4	16059	NaN	22	921684	271272	0	2	1	0	50	57	20357504	5.44E-01	1
DCN	24351	591	-19	928448	254896	0	4	0	0	33	29	10682848	5.44E-01	1
ERAL1	1693126	177	31	1116772	327164	0	4	2	0	50	79	19478896	5.44E-01	1
E2F3	138711	568	7	1161984	356712	0	2	1	0	50	57	20908592	5.45E-01	1
PDIA5	468830	360	44	1387688	346744	0	6	1	0	50	51	17356424	5.45E-01	1
CREBL2	791567	157	32	322180	78320	0	1	0	0	50	66	23408068	5.45E-01	1
HIST1H2BI	398813	636	53	318976	96476	0	7	3	0	10	17	1340340	5.45E-01	1
CASKIN1	2179404	224	39	3487376	1184056	0	9	2	0	50	46	19527312	5.45E-01	1
CYB561D1	1056159	272	37	747600	197580	0	3	0	0	50	61	17976932	5.45E-01	1
MEF2C	98604	1192	32	1339272	369528	0	4	0	0	50	50	20204424	5.45E-01	1
GTDC1	60793	692	12	1201856	307228	0	3	0	0	50	57	21672924	5.45E-01	1
SUPT5H	1410725	230	39	2845508	768604	0	9	4	0	50	81	27705344	5.45E-01	1

CLEC1A	396940	445	-1	738700	185120	0	2	0	0	50	48	19709940	5.45E-01	1
ZFAND2A	774288	346	36	397296	87576	0	1	0	0	50	36	18514848	5.45E-01	1
CD164	347811	446	41	546816	161268	0	2	0	0	50	66	21277764	5.45E-01	1
SERTAD3	1010633	202	34	481668	153792	0	2	0	0	50	62	18103668	5.45E-01	1
MUM1	2285841	209	31	1810616	526168	0	5	1	0	50	60	22146760	5.45E-01	1
DPF3	317807	599	41	937348	245996	0	1	0	0	50	45	17709220	5.45E-01	1
SMR3A	264684	915	25	323604	117480	0	1	0	0	50	42	17431540	5.46E-01	1
TRIM67	381758	603	8	1950524	601996	0	5	1	0	39	57	16597432	5.46E-01	1
OPA3	1107927	194	44	766112	247420	0	3	1	0	50	55	18375296	5.46E-01	1
RAB28	134712	704	22	715916	174796	0	3	0	0	50	133	48571928	5.46E-01	1
IRF1	713465	320	66	866860	216092	0	2	0	0	50	41	19744828	5.46E-01	1
OR51G1	65798	1006	-15	782488	247064	0	3	0	0	50	67	21104748	5.46E-01	1
EXTL2	193606	859	-3	853688	223924	0	3	0	0	50	59	20822796	5.46E-01	1
BLID	143859	453	4	265220	84016	0	1	0	0	50	49	16541896	5.46E-01	1
BIRC3	462955	280	5	1565688	406908	0	4	1	0	50	68	23275992	5.46E-01	1
ST18	155128	793	-15	2724468	718764	0	13	3	0	18	42	9318300	5.46E-01	1
OSGIN2	251472	419	35	1396944	386616	0	4	1	0	48	47	18562552	5.46E-01	1
ZNF790	352375	976	-36	1671776	386260	0	5	0	0	17	38	9834856	5.46E-01	1
ITFG1	489472	809	-5	1613748	425064	0	2	1	0	50	57	20424432	5.46E-01	1
C1QTNF5	NaN	NaN	NaN	589892	200428	0	2	0	0	50	116	39846012	5.46E-01	1
ST3GAL4	349521	514	31	864724	235316	0	1	1	0	48	58	21642308	5.46E-01	1
LRIT1	216124	810	-10	1514780	499468	0	7	2	0	50	72	21473208	5.46E-01	1
SDCBP	200469	608	11	783912	211464	0	2	0	0	50	52	22903972	5.46E-01	1
TFDP2	488711	410	27	1263088	336776	0	4	2	0	50	72	22568976	5.47E-01	1
MAFB	308177	550	-29	794948	243148	0	4	2	0	19	34	8704200	5.47E-01	1
IL12B	129528	415	27	861164	218584	0	2	1	0	50	38	20134292	5.47E-01	1
CTRL	879834	158	50	671060	205768	0	3	0	0	50	85	23764780	5.47E-01	1
TYMP	1075525	207	42	1186548	407976	0	4	0	0	6	6	2468504	5.47E-01	1
DNAJB8	829484	317	50	582416	164116	0	3	0	0	50	61	19655472	5.47E-01	1
TCL1B	256627	582	30	333216	95052	0	1	0	0	50	57	21579296	5.47E-01	1
HACE1	108074	924	-9	2396236	617660	0	6	0	0	16	28	9983664	5.47E-01	1
FAM135B	6187	989	-37	3567476	1021720	0	21	4	0	1	4	1335000	5.47E-01	1
ZNF160	395233	946	-48	2112860	528304	0	11	2	0	50	54	17306228	5.47E-01	1

RELA	4131934	202	53	1383416	427912	0	4	0	0	24	26	11885772	5.47E-01	1
EGFR	423489	336	-13	3378796	892136	0	9	3	0	44	36	17124312	5.47E-01	1
NFE2	753625	205	59	907088	299752	0	3	0	0	31	42	11449672	5.47E-01	1
SP100	478720	559	38	2890364	653260	0	13	1	0	19	21	7686752	5.47E-01	1
PLIN2	500437	324	-7	1119976	313280	0	3	0	0	50	72	22846300	5.47E-01	1
KIAA1143	433853	426	27	405840	101460	0	1	1	0	6	8	3605924	5.48E-01	1
CACNA2D[illegible]	240778	593	28	2998944	805272	0	7	1	0	8	8	5073000	5.48E-01	1
NLGN4Y	0	0	0	2202216	601284	0	4	0	0	39	23	17595656	5.48E-01	1
SMARCD1	1003045	211	56	1327524	377004	0	4	2	0	25	40	9711324	5.48E-01	1
COPS3	910162	218	11	1126028	281596	0	6	0	0	19	33	9655432	5.48E-01	1
DHRS4L2	984811	442	23	591672	184764	0	2	1	0	50	76	25828868	5.48E-01	1
LHFP	153723	939	4	503384	149164	0	2	0	0	50	58	17558276	5.48E-01	1
BBS10	563668	507	-10	1815244	510860	0	6	2	0	50	62	19644080	5.48E-01	1
PSMB1	425191	598	-16	623356	176576	0	2	0	0	50	46	17122888	5.48E-01	1
B3GNT9	1093307	150	47	948740	342472	0	2	0	0	11	6	4687808	5.48E-01	1
MTR	407246	773	-13	3317208	875760	0	7	3	0	47	52	17999360	5.48E-01	1
MYEOV	558031	443	37	751516	263084	0	1	0	0	50	71	25824596	5.48E-01	1
KLHDC3	1200368	259	40	996444	273408	0	4	0	0	39	44	15846272	5.49E-01	1
GABRG2	8332	869	-14	1341764	354220	0	4	1	0	35	69	20870856	5.49E-01	1
LYRM1	260596	545	33	313992	90780	0	1	0	0	50	57	21545120	5.49E-01	1
BBOX1	178328	527	-18	1018872	254184	0	5	2	0	27	56	15715264	5.49E-01	1
SLC6A20	286183	385	43	1506948	435744	0	4	3	0	50	53	19140340	5.49E-01	1
PKN2	280301	449	0	2577440	668212	0	7	1	0	50	54	24221528	5.49E-01	1
GALNS	836710	188	44	1351376	381988	0	3	0	0	13	5	4023512	5.49E-01	1
YTHDF1	1005137	285	50	1419016	392312	0	4	0	0	50	62	19950240	5.49E-01	1
SYTL2	354036	435	1	4522268	1199008	0	13	3	0	26	30	11520516	5.49E-01	1
ATP5SL	1104034	376	38	732648	160200	0	2	1	0	50	74	17589960	5.49E-01	1
BIRC6	388754	517	24	12356760	3520128	0	52	8	0	6	12	5149896	5.49E-01	1
PSMC2	355264	620	17	1142404	297260	0	3	2	0	11	8	4841956	5.50E-01	1
C1orf56	1614665	165	40	823428	278748	0	2	2	0	14	12	4709880	5.50E-01	1
SPARC	725286	496	43	808832	203632	0	2	0	0	50	56	25411992	5.50E-01	1
ZNF613	452542	838	-61	1596304	398720	0	6	3	0	25	40	8703844	5.50E-01	1
NDFIP2	154294	370	50	862944	248844	0	1	0	0	38	33	13055588	5.50E-01	1

APIP	650532	399	1	658244	164116	0	2	0	0	50	45	18155644	5.50E-01	1
POU3F2	171189	927	-7	1084376	338200	0	4	0	0	50	51	19879396	5.50E-01	1
AGAP3	616572	337	39	2339988	726240	0	8	4	0	11	15	4977236	5.50E-01	1
TRIM24	272427	552	10	2719128	725172	0	9	1	0	34	34	14976564	5.50E-01	1
ENO1	800628	171	28	1129588	311144	0	2	2	0	50	54	21005424	5.50E-01	1
POLR3C	NaN	NaN	NaN	1398368	377716	0	5	1	0	50	117	40023300	5.50E-01	1
FUNDC1	91432	NaN	20	409044	107868	0	1	0	0	50	57	24001876	5.50E-01	1
LHX8	162631	959	-23	922752	255252	0	5	0	0	50	109	25474292	5.50E-01	1
TSTA3	2183482	235	37	848704	225704	0	2	0	0	50	40	19636960	5.50E-01	1
FBXO38	82911	459	1	3088300	810968	0	6	1	0	17	11	6237476	5.50E-01	1
TSPAN11	315469	555	-5	655040	188680	0	1	1	0	50	54	16395936	5.50E-01	1
SLC17A5	713348	132	31	1268784	363120	0	4	1	0	50	45	21015036	5.51E-01	1
OR4K13	383274	768	-37	748668	226416	0	1	2	0	32	40	12755836	5.51E-01	1
CHRM5	409900	384	2	1322540	389464	0	3	0	0	44	43	16471764	5.51E-01	1
SSTR3	845055	230	67	1000360	346388	0	3	0	0	4	3	2346040	5.51E-01	1
TRPC1	414031	437	20	1971172	510860	0	7	0	0	20	30	14421560	5.51E-01	1
ZFYVE26	484591	344	49	6452856	1860456	0	22	7	0	50	51	19049204	5.51E-01	1
ARMC8	199119	358	18	1799224	477752	0	3	3	0	31	31	10929556	5.51E-01	1
SERAC1	572602	209	40	1715208	450696	0	5	1	0	3	3	1684948	5.51E-01	1
BMP15	15214	NaN	27	981136	286580	0	4	1	0	50	66	21104392	5.51E-01	1
PRMT6	75609	921	-16	912428	294412	0	3	3	0	6	12	3762920	5.51E-01	1
DLG1	751489	417	36	2477404	640088	0	8	3	0	50	74	25235772	5.51E-01	1
ZC4H2	98165	NaN	-21	631544	126736	0	2	0	0	50	74	17661872	5.51E-01	1
CLDN2	148608	NaN	22	557140	182984	0	2	1	0	50	128	42308820	5.51E-01	1
MOB2	859301	426	39	718052	187612	0	2	0	0	50	47	19040304	5.51E-01	1
TLX3	269453	434	25	714848	231400	0	2	0	0	21	18	8332892	5.51E-01	1
TRIP13	812469	491	34	1134928	305804	0	4	1	0	50	60	20635184	5.52E-01	1
FLCN	910162	218	11	1615884	456036	0	5	0	0	18	27	9154896	5.52E-01	1
RRP15	75487	488	-20	747956	178000	0	5	2	0	2	6	1047352	5.52E-01	1
MYO3B	324392	844	28	3650424	929160	0	11	2	0	9	6	3461744	5.52E-01	1
PLEKHN1	1971475	239	15	1563196	502316	0	5	2	0	12	27	8570344	5.52E-01	1
SYPL2	1056159	272	37	700252	195800	0	3	0	0	50	61	17976932	5.52E-01	1
CD81	911893	584	36	620508	168744	0	2	0	0	50	50	18764760	5.52E-01	1

SDAD1	486576	399	44	1855472	438592	0	5	4	0	15	15	5064812	5.52E-01	1
CRISPLD1	137515	703	-22	1336424	328588	0	5	0	0	16	20	6223236	5.52E-01	1
AOAH	247838	600	15	1861880	434320	0	7	3	0	10	11	4189764	5.52E-01	1
TDP2	419582	373	8	945180	245640	0	3	0	0	50	71	26744144	5.52E-01	1
TMED6	1202748	125	20	620864	166252	0	5	2	0	6	6	1573164	5.52E-01	1
ZNF704	143727	501	1	1061592	295836	0	4	1	0	50	46	18102956	5.52E-01	1
ZNF460	419642	859	-40	1434680	377716	0	7	2	0	50	68	17222212	5.52E-01	1
SLC6A1	154960	444	36	1546108	431828	0	6	3	0	27	40	11357112	5.52E-01	1
PLEKHA6	554014	370	33	2668576	782132	0	11	3	0	19	17	7723420	5.52E-01	1
RASSF1	843769	206	51	987900	297972	0	2	2	0	50	68	20472848	5.53E-01	1
FAM177B	221712	236	-4	427200	99324	0	2	1	0	4	5	1144896	5.53E-01	1
MBOAT1	127205	592	1	1287652	352796	0	2	1	0	50	58	24809640	5.53E-01	1
SRC	685195	571	60	1370244	397296	0	4	0	0	6	1	1663232	5.53E-01	1
IFIT3	253413	213	22	1262376	322536	0	3	2	0	20	24	9226452	5.53E-01	1
FBXO22	550701	339	47	1057676	299752	0	5	3	0	21	19	6114300	5.53E-01	1
ZNF257	75568	1305	-69	1466720	353152	0	6	3	0	50	85	16338620	5.53E-01	1
CCDC24	875223	214	39	779996	241012	0	3	1	0	50	67	19898264	5.54E-01	1
SLC25A39	1036092	172	39	917412	283020	0	3	2	0	4	6	2271992	5.54E-01	1
NUDT9	530607	537	23	916344	238164	0	3	0	0	50	47	17651192	5.54E-01	1
POLI	98827	436	44	1914568	500180	0	5	1	0	24	31	11733404	5.54E-01	1
FAM171B	264318	538	-9	2088296	591316	0	7	1	0	37	37	16586040	5.54E-01	1
CD3E	805791	211	41	561412	139196	0	2	0	0	50	62	21266728	5.54E-01	1
HOXC5	1185281	279	54	566040	154860	0	2	0	0	50	59	19280960	5.54E-01	1
CSTA	534078	602	44	259880	65860	0	1	0	0	50	70	22369260	5.54E-01	1
SH2B3	466447	479	47	1407980	467428	0	4	0	0	7	6	3855480	5.54E-01	1
SLC38A10	2679984	197	27	3000012	947316	0	11	3	0	21	28	10249596	5.54E-01	1
TRHR	151352	616	18	1001072	283732	0	8	3	0	7	9	1871492	5.54E-01	1
STOML1	337765	439	34	986120	320044	0	3	0	0	50	54	20944548	5.54E-01	1
ETV6	233493	629	31	1163052	318264	0	7	1	0	6	7	1301180	5.54E-01	1
FBXO6	682357	264	32	754364	208972	0	1	0	0	50	62	21513436	5.54E-01	1
KBTBD2	373657	264	15	1580996	426844	0	6	2	0	45	38	15985468	5.54E-01	1
NAA35	320125	381	21	1962272	457816	0	5	0	0	11	10	5992904	5.54E-01	1
TARS	349408	674	4	1924892	471700	0	8	1	0	50	58	19970532	5.54E-01	1

PCDHGC4	665835	568	9	2416528	786404	0	7	1	0	11	13	5171968	5.54E-01	1
MRPS14	176818	828	4	327520	96476	0	1	0	0	50	65	19765832	5.54E-01	1
MRPS26	835590	288	54	514776	160200	0	2	0	0	50	59	17488856	5.54E-01	1
PDPN	64295	864	9	595944	193308	0	2	0	0	50	67	24632708	5.55E-01	1
TMBIM4	243040	696	5	615168	178356	0	2	0	0	50	59	21205496	5.55E-01	1
KCNK2	363438	818	-28	1114992	323604	0	5	0	0	50	63	18080528	5.55E-01	1
MOGAT1	251851	308	19	870420	231756	0	2	0	0	17	11	6296572	5.55E-01	1
HP1BP3	641625	206	30	1432900	393380	0	3	0	0	6	3	2392676	5.55E-01	1
TAGLN2	803074	403	7	518692	139196	0	2	0	0	50	49	15043848	5.55E-01	1
THSD4	312827	505	3	2614464	720900	0	6	2	0	29	33	12375984	5.55E-01	1
OR7A10	963401	546	14	762196	231044	0	2	2	0	37	53	12940600	5.55E-01	1
SLC25A44	2294665	211	39	785336	236740	0	2	1	0	50	46	21184848	5.55E-01	1
MAGEA3	201810	NaN	-3	781776	231756	0	3	1	0	14	10	4013188	5.55E-01	1
MPRSS11	170820	716	-19	1097548	276968	0	3	0	0	15	16	7705620	5.55E-01	1
CD180	36320	392	14	1671420	458172	0	6	1	0	50	58	19989044	5.56E-01	1
AKR7A3	810462	448	40	846212	245284	0	1	0	0	50	48	16954856	5.56E-01	1
RNF44	607190	318	49	1087224	342828	0	2	2	0	50	44	17331860	5.56E-01	1
OR52L1	432250	877	-13	800288	261304	0	2	1	0	50	63	18144964	5.56E-01	1
CCDC74A	455705	540	29	960844	285512	0	3	0	0	46	48	16849480	5.56E-01	1
SLC12A7	639628	424	31	2751880	821648	0	10	4	0	50	49	17595300	5.56E-01	1
MRC1	NaN	NaN	NaN	7734812	1851556	0	24	8	0	50	124	41497140	5.56E-01	1
RPS11	2126800	210	42	420080	110716	0	1	0	0	50	63	21387412	5.56E-01	1
IPCEF1	100494	765	9	1187260	286580	0	4	0	0	50	66	27099432	5.56E-01	1
GPT	1755818	291	7	1249204	385904	0	3	1	0	24	29	10834860	5.56E-01	1
POLR2C	760307	159	42	730868	189748	0	3	0	0	50	66	21727748	5.56E-01	1
TUFT1	1379189	176	30	1042012	264152	0	4	1	0	50	76	23715296	5.56E-01	1
KRTAP13-	4608	1035	-69	431472	134568	0	3	0	0	11	5	1333576	5.56E-01	1
NR1H2	1072052	528	26	1165900	345320	0	3	1	0	50	63	18767608	5.56E-01	1
KCNJ8	501891	984	-9	1060524	309720	0	5	0	0	50	74	20750528	5.56E-01	1
UBA6	154681	604	33	2804924	707728	0	8	2	0	50	47	21728460	5.56E-01	1
IFI44	85210	732	-5	1151660	308296	0	3	0	0	16	8	5174816	5.56E-01	1
IL18RAP	230715	671	4	1556788	408332	0	6	2	0	50	52	19350024	5.56E-01	1
TTC7A	785914	242	38	2212184	621220	0	8	1	0	5	4	2249208	5.57E-01	1

AADACL3	145424	454	23	895340	248488	0	3	0	0	50	54	19660812	5.57E-01	1
UHMK1	334603	248	3	1120332	308652	0	2	1	0	50	54	22683608	5.57E-01	1
AFTPH	532219	332	21	2401932	641868	0	7	1	0	48	45	22040316	5.57E-01	1
OR1G1	445889	579	-8	772520	233536	0	4	2	0	42	39	11853020	5.57E-01	1
NVL	626336	139	16	2229628	612320	0	5	3	0	50	41	19100468	5.57E-01	1
FBXO46	929036	203	43	1447496	491992	0	3	2	0	33	37	14234660	5.57E-01	1
PDLIM4	451605	191	40	846568	241724	0	2	0	0	50	63	18692136	5.57E-01	1
TNFRSF21	206419	389	1	1635464	487720	0	5	1	0	50	56	26659060	5.57E-01	1
SERPINC1	443726	385	13	1188684	328944	0	4	0	0	16	13	6358516	5.57E-01	1
TARS2	1532790	163	43	1825568	552868	0	9	1	0	50	76	23299844	5.57E-01	1
OR52N2	203856	922	-5	796016	234604	0	2	1	0	50	39	17006476	5.58E-01	1
CDC6	1480458	191	23	1445716	398720	0	8	0	0	2	5	1437528	5.58E-01	1
NHS	70191	NaN	12	4121412	1244220	0	13	1	0	1	2	1914924	5.58E-01	1
ARHGAP2[illegible]	241496	281	-28	1621224	422928	0	7	0	0	18	23	6781800	5.58E-01	1
AEBP2	152349	339	-21	1337136	360984	0	2	0	0	48	46	20494920	5.58E-01	1
OR13C9	147494	920	-31	794948	226060	0	4	0	0	50	80	20017168	5.58E-01	1
WDR74	2342595	181	71	994664	291208	0	2	0	0	50	77	24280268	5.58E-01	1
HAUS7	971101	NaN	7	965828	254896	0	2	0	0	50	42	13736972	5.58E-01	1
PDZD11	215874	NaN	52	379496	97900	0	1	0	0	50	56	22682540	5.58E-01	1
DMC1	1160654	238	38	916344	231756	0	3	0	0	50	52	18166324	5.58E-01	1
NOD2	294632	792	1	2588476	793880	0	7	4	0	27	33	11357112	5.58E-01	1
SLC7A10	380625	270	-9	1306520	417232	0	4	0	0	29	29	12084420	5.59E-01	1
PDYN	461602	543	28	639020	186544	0	3	0	0	20	21	6282332	5.59E-01	1
SBK1	1139917	221	55	1021364	353152	0	2	0	0	22	41	14686780	5.59E-01	1
RABIF	422793	318	28	313636	90068	0	1	0	0	50	56	20577156	5.59E-01	1
HSPB9	2171374	206	37	388752	126024	0	1	0	0	50	38	17832396	5.59E-01	1
TSC22D2	514603	176	19	1877188	637952	0	5	2	0	50	47	19320120	5.59E-01	1
CIB3	761374	298	42	501604	124244	0	2	0	0	50	57	18801428	5.59E-01	1
CC2D2B	515809	798	33	1072628	258100	0	3	1	0	50	29	15313696	5.59E-01	1
PLEKHO2	556478	205	50	1214316	380208	0	5	0	0	50	48	18457888	5.59E-01	1
PRLR	247066	854	17	1600220	430048	0	5	1	0	50	47	19048492	5.59E-01	1
TOP3A	941112	223	13	2574948	712356	0	9	2	0	38	60	19722400	5.59E-01	1
MAP2K2	2276561	180	36	1029552	297972	0	3	0	0	50	55	23481760	5.59E-01	1

RRP9	1013177	370	39	1235320	353864	0	4	2	0	50	85	19762272	5.59E-01	1
APOH	135537	178	-23	907088	234604	0	2	1	0	50	60	18369600	5.59E-01	1
RIC3	244487	555	19	949096	256676	0	2	0	0	50	34	19662236	5.59E-01	1
PCGF2	1158356	207	39	884660	261304	0	3	1	0	50	41	15942036	5.60E-01	1
MITF	48041	436	-15	1560348	400500	0	6	1	0	50	60	22282396	5.60E-01	1
SLC9A7	686372	NaN	45	1861880	536848	0	4	2	0	50	47	21094068	5.60E-01	1
ESCO2	406636	229	23	1570316	401212	0	2	0	0	12	3	4890372	5.60E-01	1
CXCL6	233432	444	6	285512	95764	0	1	1	0	9	6	2595952	5.60E-01	1
MYH7B	968413	236	40	5086172	1454260	0	16	6	0	50	62	22730956	5.60E-01	1
NCOA2	265128	742	35	3776092	1007480	0	10	1	0	3	2	1917416	5.60E-01	1
EPN2	658567	227	6	1589184	501960	0	3	0	0	14	9	7175536	5.60E-01	1
SMC2	114849	953	-33	3153448	787472	0	14	1	0	10	10	4610912	5.60E-01	1
MGST3	321831	408	0	398008	113564	0	1	0	0	50	57	21890440	5.60E-01	1
PADI2	610093	194	45	1720192	479888	0	6	1	0	50	72	21551172	5.60E-01	1
CYP4F2	825892	575	15	1346392	373088	0	5	2	0	25	33	9024600	5.61E-01	1
LAS1L	321325	NaN	10	1909584	503028	0	7	1	0	50	67	23589984	5.61E-01	1
SKP2	174910	599	-4	1238524	349592	0	4	0	0	50	57	22147116	5.61E-01	1
FTMT	70878	920	-16	597368	183340	0	2	0	0	50	63	20452556	5.61E-01	1
ZNF366	447936	344	28	1896056	508012	0	5	1	0	28	27	11147428	5.61E-01	1
SCGB2A2	3058870	450	42	253116	58740	0	1	0	0	50	51	16305156	5.61E-01	1
NFKBIE	1037256	312	54	1199720	428980	0	4	1	0	50	68	19769036	5.61E-01	1
NDUFA9	277828	874	18	979712	276256	0	4	2	0	4	6	2002144	5.61E-01	1
AMTN	264427	861	27	538272	168744	0	2	1	0	34	56	17305872	5.61E-01	1
C1orf64	886121	197	43	412604	138484	0	1	0	0	50	55	20099048	5.61E-01	1
OR8B8	171093	824	-10	772520	226060	0	3	2	0	7	13	3002860	5.61E-01	1
BICD2	573438	393	45	2140628	638308	0	6	3	0	21	26	10140660	5.61E-01	1
PITPNA	1362942	220	31	743684	171592	0	1	0	0	50	63	19643724	5.61E-01	1
CCDC25	380493	229	27	583484	120328	0	1	0	0	50	66	26294872	5.61E-01	1
ZNF667	135115	713	-50	1585980	388752	0	7	1	0	10	32	8144212	5.61E-01	1
BRPF1	955408	348	34	3103252	864368	0	8	2	0	8	11	5725192	5.62E-01	1
KRTCAP3	1244404	179	39	592740	207192	0	2	1	0	50	61	21622372	5.62E-01	1
RAB8A	761374	298	42	558208	138128	0	2	0	0	50	57	18801428	5.62E-01	1
CCRL2	243983	625	36	891424	256676	0	3	0	0	50	49	17267068	5.62E-01	1

TNFAIP1	1405196	214	10	810968	226060	0	3	1	0	50	74	22499912	5.62E-01	1
CCDC110	173409	422	20	2192960	506944	0	6	2	0	50	51	19200504	5.62E-01	1
PLXNB2	939384	257	70	4667872	1373804	0	15	5	0	5	9	3295492	5.62E-01	1
KCNA6	277089	903	16	1283024	415096	0	6	3	0	23	39	11011792	5.62E-01	1
TFB2M	389456	409	4	1024924	276968	0	3	1	0	50	50	20658324	5.62E-01	1
SPAG17	192120	741	-7	5859404	1471348	0	27	4	0	7	9	3873280	5.62E-01	1
LRP5L	155932	535	-31	642224	181204	0	2	2	0	4	9	1789612	5.62E-01	1
PMS2	NaN	NaN	NaN	2233544	593452	0	7	1	0	50	117	40239036	5.63E-01	1
DMRT2	71695	845	4	1448208	428268	0	4	0	0	10	7	3347824	5.63E-01	1
TOPBP1	406166	396	37	3954092	1040944	0	14	1	0	1	1	1408692	5.63E-01	1
TMEM107	1381225	202	37	376648	111428	0	1	0	0	50	76	28183808	5.63E-01	1
NINL	613303	454	-26	3527604	1001784	0	13	2	0	32	36	12571072	5.63E-01	1
NOC3L	242278	482	12	2125676	528304	0	9	0	0	22	20	7593836	5.63E-01	1
ATF2	347113	641	24	1299400	373088	0	2	1	0	50	49	22377092	5.63E-01	1
GPX1	1661046	201	34	527236	178712	0	2	1	0	42	56	17031752	5.63E-01	1
C2orf40	99910	798	0	386616	103596	0	1	0	0	50	55	21162064	5.63E-01	1
PIWIL1	224466	621	-26	2251700	595588	0	10	3	0	23	52	12737680	5.63E-01	1
PPP3CA	84583	574	5	1378432	349592	0	6	0	0	8	9	3705960	5.63E-01	1
TP53I3	269570	385	38	833040	255252	0	3	1	0	50	60	20133936	5.63E-01	1
OR1K1	433740	487	10	754720	259880	0	2	1	0	50	48	19317628	5.63E-01	1
OR10A2	427462	681	8	741904	232112	0	4	2	0	9	7	2352448	5.63E-01	1
ECE1	744763	196	27	2125676	580636	0	5	1	0	21	24	11283776	5.63E-01	1
MTMR1	288573	NaN	9	1730872	467072	0	7	0	0	15	14	5514084	5.63E-01	1
ENTHD1	818387	503	24	1545396	423996	0	7	2	0	35	44	14686424	5.64E-01	1
MASP1	401220	445	31	2609836	702032	0	7	3	0	21	18	10490608	5.64E-01	1
GSTO1	293891	376	8	639376	161624	0	2	0	0	50	51	20513788	5.64E-01	1
CLDN11	372782	377	30	505520	169456	0	1	0	0	50	40	20393104	5.64E-01	1
SLC25A14	301928	NaN	28	851552	231400	0	1	0	0	50	75	26309824	5.64E-01	1
MS4A3	336373	691	4	553936	160556	0	3	2	0	12	16	3983640	5.64E-01	1
FIBIN	176115	648	-17	527592	151656	0	1	0	0	50	64	25560444	5.64E-01	1
TACC3	867402	350	39	2134220	616948	0	6	3	0	50	61	21170964	5.65E-01	1
WDR47	942575	216	19	2399796	633324	0	8	1	0	26	24	11645828	5.65E-01	1
MUC21	2483101	261	36	1362768	462444	0	4	0	0	24	19	9039552	5.65E-01	1

SIRT3	1463610	437	24	991460	317908	0	2	0	0	6	2	2533652	5.65E-01	1
QSER1	408746	172	23	4368120	1241016	0	13	1	0	0	1	1241016	5.65E-01	1
SNRNP35	1090769	424	50	633324	180492	0	2	0	0	50	69	22305892	5.65E-01	1
NUDT7	79181	1194	-24	607336	173372	0	4	1	0	4	9	1560704	5.65E-01	1
SV2C	171171	858	16	1889292	495552	0	6	2	0	50	50	17421928	5.65E-01	1
POTEA	236622	755	-37	1331440	320756	0	2	0	0	32	52	15813520	5.65E-01	1
EPHX4	260329	242	1	938060	250624	0	2	1	0	50	39	21744124	5.65E-01	1
PCDH8	248822	638	27	2539348	900680	0	13	5	0	23	23	7127832	5.65E-01	1
RALGPS2	364466	434	-2	1567112	385192	0	4	0	0	35	29	14994720	5.65E-01	1
BNIP1	483076	397	34	707728	189392	0	2	0	0	50	49	20240736	5.65E-01	1
SNRPD1	301496	165	46	309008	88288	0	1	0	0	50	46	17182340	5.65E-01	1
WNT2B	561338	355	21	1077612	335352	0	3	1	0	50	57	24552964	5.66E-01	1
CCDC125	434060	261	49	1371668	313636	0	3	1	0	50	55	20040308	5.66E-01	1
ANKRD27	369667	455	-38	2741912	745108	0	12	2	0	50	66	17430472	5.66E-01	1
TAS2R40	379894	399	14	802068	234960	0	3	1	0	50	67	22890088	5.66E-01	1
CHMP6	1660463	211	27	541120	138128	0	2	1	0	33	46	14482792	5.66E-01	1
OAZ1	1603571	240	27	583128	170880	0	2	0	0	50	66	21932448	5.66E-01	1
HSP90B1	1190703	281	40	2120692	527948	0	10	4	0	15	23	6446448	5.66E-01	1
APOL2	1237332	331	42	844788	250980	0	5	2	0	8	28	6483472	5.66E-01	1
TIPIN	505508	307	46	798508	199004	0	2	0	0	50	49	21873708	5.66E-01	1
TREM1	270628	607	34	592028	175864	0	2	0	0	34	31	10799972	5.66E-01	1
PEX5L	307645	761	5	1631904	436812	0	5	1	0	50	70	24161720	5.66E-01	1
OTUD3	437659	348	22	1041656	266644	0	1	0	0	50	42	18701748	5.66E-01	1
TTC13	407409	595	14	2254904	599860	0	9	1	0	50	67	22042096	5.66E-01	1
P2RY13	67416	548	15	892848	250980	0	3	1	0	50	59	22461820	5.67E-01	1
MLNR	321392	424	51	974016	351372	0	3	0	0	38	47	18334712	5.67E-01	1
WNT5B	270921	254	34	913140	257388	0	3	0	0	34	29	10410864	5.67E-01	1
DAZAP1	2352825	221	38	1132436	321112	0	2	1	0	50	55	18329728	5.67E-01	1
XAF1	936146	438	5	800288	192952	0	3	1	0	50	45	14808532	5.67E-01	1
EPHB3	1325702	411	47	2510512	754364	0	10	4	0	9	14	4999664	5.67E-01	1
HIST1H2AI	408919	685	43	313992	107868	0	1	0	0	50	57	16881520	5.67E-01	1
NCR2	338294	510	40	694556	214312	0	2	1	0	50	50	17742328	5.67E-01	1
NPC1	347696	164	64	3289440	911004	0	9	2	0	21	28	10525852	5.67E-01	1

OR8K1	10293	963	-36	792456	230688	0	6	0	0	50	115	24676496	5.67E-01	1
NDUFS6	387770	635	11	322536	92916	0	1	0	0	50	63	21058112	5.67E-01	1
MAP2K1	506284	172	43	1029196	278036	0	2	0	0	50	52	17050264	5.67E-01	1
SLAIN2	310987	328	55	1454260	440372	0	5	0	0	14	10	3880400	5.67E-01	1
HOXB1	946208	524	29	743684	232468	0	5	3	0	16	19	4118564	5.68E-01	1
DHX9	673873	517	23	3290152	897476	0	17	1	0	0	1	897476	5.68E-01	1
SLC9A4	184297	671	6	2038456	568532	0	11	4	0	2	4	1069424	5.68E-01	1
LRRN4	301252	351	-6	1771456	619796	0	4	2	0	23	18	10367788	5.68E-01	1
SUFU	872436	165	36	1253476	356000	0	4	2	0	50	52	20342196	5.68E-01	1
SLC22A14	301168	437	22	1503744	443220	0	3	3	0	46	67	19788972	5.68E-01	1
LIMA1	1008076	144	57	1970816	510148	0	6	5	0	22	26	7897860	5.68E-01	1
RAB3B	701660	353	22	567108	152724	0	5	1	0	1	3	843720	5.68E-01	1
NTHL1	1747301	183	46	787116	239232	0	2	1	0	50	75	19137492	5.68E-01	1
COASY	1884163	177	38	1479180	466716	0	4	1	0	50	68	23826724	5.68E-01	1
DDX19A	1126886	134	44	1258104	325740	0	4	1	0	50	59	22331524	5.68E-01	1
PLCB1	85844	872	-34	3320768	825208	0	15	2	0	33	34	12189796	5.68E-01	1
CXCR6	NaN	NaN	NaN	861164	242080	0	3	0	0	50	116	39887664	5.68E-01	1
ITGA2B	1062294	172	38	2656828	801356	0	10	3	0	50	71	23775460	5.68E-01	1
ACTR3B	190917	535	5	1098260	291208	0	2	0	0	50	53	20518060	5.68E-01	1
OR4K14	420010	827	-51	767180	228196	0	2	0	0	42	60	13737684	5.68E-01	1
S1PR1	190734	597	4	934500	292632	0	8	4	0	3	6	1120332	5.68E-01	1
CA6	840956	264	31	808476	213600	0	1	0	0	50	62	20541556	5.68E-01	1
TPTE2	288628	778	-16	1414388	346744	0	5	0	0	38	41	12380612	5.68E-01	1
IFIT5	253827	213	17	1227844	323960	0	4	0	0	44	34	14070544	5.68E-01	1
GDAP1	230695	529	-14	929872	245996	0	4	2	0	50	67	21547968	5.68E-01	1
MAP1LC3I	583365	363	27	333216	83304	0	1	0	0	50	48	19133220	5.69E-01	1
SLC39A7	1474628	377	24	1170172	365612	0	6	0	0	22	42	9267748	5.69E-01	1
FBXO5	164387	538	-5	1144540	307940	0	3	1	0	50	71	23025368	5.69E-01	1
UBE2Q2	560951	339	55	1123892	275188	0	4	0	0	50	55	20947040	5.69E-01	1
RASGEF1(	1740168	432	23	1214672	337132	0	4	2	0	50	39	15917472	5.69E-01	1
TFAP2D	17771	1090	-15	1143472	337844	0	3	1	0	50	83	24114728	5.69E-01	1
DLX4	1160590	191	12	639376	210752	0	2	1	0	36	34	13080508	5.69E-01	1
SDR42E1	207928	635	13	976508	294412	0	3	1	0	50	63	25699996	5.69E-01	1

C9orf50	886775	205	39	1055184	354576	0	5	0	0	50	69	20923544	5.69E-01	1
KIAA1586	431412	348	29	2019944	517624	0	4	0	0	12	4	4275560	5.69E-01	1
ADK	622159	598	40	981848	243148	0	2	1	0	50	73	25680060	5.69E-01	1
DCLRE1B	335231	545	13	1325388	395160	0	6	1	0	50	60	19779004	5.70E-01	1
C5orf22	235664	649	4	1152016	301532	0	3	0	0	50	46	21343980	5.70E-01	1
TDRD1	279026	477	22	3139208	780352	0	8	3	0	50	61	22629140	5.70E-01	1
MAP3K12	1712232	191	56	2140628	676756	0	11	1	0	6	6	2296912	5.70E-01	1
SLC4A1AP	1116452	205	37	2061596	547528	0	6	3	0	16	14	5946980	5.70E-01	1
PRRG2	2080142	210	41	515132	160912	0	2	0	0	50	75	21834192	5.70E-01	1
ADAM22	178742	722	17	2511580	626204	0	7	3	0	50	49	19395948	5.70E-01	1
BDP1	365874	516	16	6788208	1784628	0	30	3	0	4	11	3837680	5.70E-01	1
ZNF141	427337	756	5	1243152	293700	0	5	0	0	50	68	24799316	5.70E-01	1
ZNF420	262327	1018	-64	1790680	429692	0	6	0	0	19	13	6434700	5.70E-01	1
SLC25A23	865106	219	33	1180852	360272	0	5	1	0	50	76	20954160	5.70E-01	1
IGF2BP2	928734	367	38	1558212	430404	0	5	1	0	29	29	10129624	5.70E-01	1
DLGAP5	764452	146	54	2302252	588824	0	9	2	0	43	74	18012532	5.70E-01	1
ZNF718	270777	858	0	1248136	299396	0	5	0	0	50	55	20432264	5.70E-01	1
SHROOM2	157955	NaN	0	3972960	1248492	0	9	0	0	0	0	1248492	5.70E-01	1
ATPAF1	612038	266	23	847992	240300	0	3	1	0	50	52	18788968	5.70E-01	1
CYP3A4	1430454	438	42	1321472	344608	0	7	2	0	3	9	3276268	5.70E-01	1
YES1	398625	360	44	1416524	373444	0	5	1	0	50	49	21581076	5.71E-01	1
ZNF431	173248	1093	-42	1511932	353864	0	5	0	0	11	14	5145980	5.71E-01	1
EPS8L2	1788356	193	48	1864372	515132	0	8	4	0	10	12	3053056	5.71E-01	1
POTEC	NaN	NaN	NaN	1441444	341048	0	6	3	0	26	43	11918168	5.71E-01	1
C15orf40	452169	683	19	869352	237096	0	3	1	0	34	45	17843788	5.71E-01	1
VCX2	30870	NaN	-19	351728	103240	0	1	0	0	50	66	19882956	5.71E-01	1
POU3F3	165707	869	-17	1199364	404772	0	3	0	0	31	55	18221860	5.71E-01	1
SRM	577167	408	34	788540	214312	0	2	1	0	50	47	21558292	5.71E-01	1
LAIR1	742939	507	-9	745820	215380	0	3	1	0	50	62	17969100	5.71E-01	1
C14orf105	150716	939	15	783912	189036	0	3	1	0	50	53	18773304	5.71E-01	1
CA14	1213150	197	38	883236	242436	0	1	0	0	50	58	21071640	5.72E-01	1
SDCCAG3	1303729	233	36	1112856	322536	0	3	0	0	49	69	21284528	5.72E-01	1
ZDHHC14	272499	402	35	1239948	360984	0	4	3	0	34	28	11230732	5.72E-01	1

TRPC3	324047	634	-3	2363840	641512	0	6	2	0	50	55	19154580	5.72E-01	1
ZNF764	2214934	142	54	1001784	317196	0	4	0	0	50	72	21947756	5.72E-01	1
FBXO8	64734	647	-5	837312	209328	0	2	0	0	50	45	20526604	5.72E-01	1
ANKRD2	564805	169	40	915632	275188	0	3	0	0	50	62	20233616	5.72E-01	1
NR1I2	422482	474	24	1221792	333216	0	3	2	0	8	9	3838036	5.72E-01	1
HSPA12A	198263	480	-15	1720548	492348	0	6	2	0	34	36	10838064	5.72E-01	1
FRAS1	244829	726	0	10436852	2812756	0	29	9	0	50	61	25037480	5.72E-01	1
COX18	255276	747	14	826632	264864	0	1	0	0	50	63	21904680	5.72E-01	1
DEFB129	1010597	348	24	468496	127448	0	2	0	0	50	85	21827784	5.72E-01	1
TPPP	683895	411	28	552512	165184	0	2	1	0	31	37	12084064	5.72E-01	1
ZMAT4	42867	678	-14	605556	156996	0	2	1	0	18	27	9385940	5.72E-01	1
HIGD2A	576954	347	45	262728	86508	0	2	2	0	3	5	710932	5.73E-01	1
C1orf115	447403	520	-11	344252	118192	0	1	0	0	50	71	22983716	5.73E-01	1
PSAT1	248468	637	-20	965472	259524	0	4	2	0	16	22	5553600	5.73E-01	1
SIT1	1202125	248	37	493772	156640	0	1	0	0	50	57	20786840	5.73E-01	1
CYP2A6	1187304	275	31	1271632	350660	0	7	3	0	17	19	5409776	5.73E-01	1
PALMD	272208	540	-15	1433256	365256	0	7	0	0	47	86	24147124	5.73E-01	1
CTGF	169902	699	-4	893204	245284	0	4	0	0	19	23	8560376	5.73E-01	1
HVCN1	827901	194	41	711644	191884	0	2	1	0	50	44	18392740	5.73E-01	1
DPEP1	1092575	211	38	1047708	310788	0	2	1	0	50	48	15460724	5.74E-01	1
RNF215	696089	214	57	931652	313636	0	4	1	0	47	74	22296992	5.74E-01	1
IMPDH2	1799657	241	41	1337848	372020	0	7	3	0	25	50	13524440	5.74E-01	1
PRMT8	227736	768	15	1043436	260592	0	3	0	0	50	47	19037812	5.74E-01	1
IFNA1	242654	411	-6	479888	131008	0	1	0	0	50	34	19573236	5.74E-01	1
RUNDC3B	287293	839	-3	1239592	326096	0	3	0	0	50	54	19694632	5.74E-01	1
FABP12	186546	595	0	369528	92916	0	1	0	0	50	46	20081248	5.74E-01	1
KCNQ1	812471	589	39	1708800	531864	0	3	0	0	18	11	6741572	5.74E-01	1
C11orf84	1320765	201	47	956216	289072	0	3	0	0	50	62	23459332	5.74E-01	1
RNF135	585832	178	25	1160560	317552	0	4	2	0	38	52	18401640	5.74E-01	1
FKBP2	1613996	257	65	371308	108224	0	2	1	0	19	29	6446448	5.74E-01	1
DACT1	303507	489	32	2055900	638664	0	5	2	0	50	53	20004352	5.74E-01	1
SELENBP1	1322568	174	42	1220012	344608	0	5	2	0	50	77	23375672	5.75E-01	1
CCT6A	738325	642	4	1377364	382700	0	4	0	0	18	12	6589560	5.75E-01	1

GPRC5C	491117	504	7	1203280	378428	0	2	2	0	50	50	18209400	5.75E-01	1
TSFM	1160849	344	39	941264	282664	0	3	0	0	50	87	20175588	5.75E-01	1
TAF12	816470	183	25	427200	113208	0	1	0	0	50	51	20622368	5.75E-01	1
POLR1C	1278255	230	56	987900	278748	0	4	1	0	50	54	19955936	5.75E-01	1
CD300C	698491	504	10	562836	170880	0	2	0	0	50	63	19807128	5.75E-01	1
DSCR3	349267	263	58	770384	214312	0	3	1	0	50	62	20638388	5.75E-01	1
KBTBD3	86519	753	-16	1572808	399788	0	5	2	0	22	34	10023536	5.75E-01	1
BOC	437186	634	21	2803856	847636	0	12	4	0	49	59	18144964	5.75E-01	1
BARHL1	1522970	581	37	794592	264864	0	3	2	0	4	6	2003212	5.75E-01	1
HCFC1R1	1443513	234	49	353508	111072	0	1	1	0	46	45	16352504	5.75E-01	1
PGR	23389	962	-25	2289080	733360	0	8	2	0	23	42	14013584	5.75E-01	1
ARID5A	872383	360	47	1476332	457816	0	5	2	0	50	94	27272092	5.76E-01	1
ACSM2A	181483	823	6	1491640	415808	0	5	1	0	50	56	18359276	5.76E-01	1
PRR23A	NaN	NaN	NaN	629764	225704	0	2	1	0	50	117	39871288	5.76E-01	1
ARHGAP2[illegible]	29589	739	13	2043084	516912	0	4	0	0	34	30	15562184	5.76E-01	1
LMF1	1167662	304	17	1433968	430760	0	3	2	0	50	67	23509172	5.76E-01	1
DBN1	1079488	261	50	1736212	502316	0	4	1	0	50	44	16518044	5.76E-01	1
HADH	230614	771	21	861520	236384	0	3	1	0	50	62	22670080	5.76E-01	1
GUCY2F	131317	NaN	29	2846932	786404	0	6	0	0	31	27	18312640	5.76E-01	1
RAI2	61797	NaN	26	1309012	396584	0	3	0	0	35	42	14749436	5.76E-01	1
TCTEX1D[illegible]	1164213	231	36	381632	93628	0	1	0	0	50	38	16464644	5.76E-01	1
HIST1H2A[illegible]	228884	634	40	314704	109292	0	1	2	0	13	15	2405492	5.76E-01	1
GPR88	348181	846	-6	883948	353864	0	2	1	0	50	66	21387056	5.77E-01	1
ADRA2C	326572	592	36	1089716	395872	0	3	2	0	25	25	11882924	5.77E-01	1
TECTA	243065	769	17	5500912	1498760	0	17	5	0	37	33	15863004	5.77E-01	1
PARG	340607	454	28	2558572	644360	0	4	0	0	3	1	2304032	5.77E-01	1
GIMAP4	563029	671	24	841228	224636	0	2	0	0	25	19	9400180	5.77E-01	1
GAS7	91570	604	-30	1304384	337132	0	6	0	0	50	111	24746628	5.77E-01	1
OR4D5	107524	830	-5	774656	247420	0	3	1	0	50	68	19655828	5.77E-01	1
ARMC10	594757	579	34	870776	257032	0	3	0	0	50	70	23421240	5.77E-01	1
PRPF38B	743461	225	9	1410472	363476	0	3	1	0	50	72	24119356	5.77E-01	1
ACER3	279729	351	36	722324	179068	0	2	1	0	40	34	14093328	5.77E-01	1
PPP1R14A	1410213	301	-1	374156	114988	0	1	0	0	50	46	17588892	5.77E-01	1

PAK1IP1	262380	410	14	1041300	258456	0	3	1	0	50	53	22408420	5.77E-01	1
PSMC5	1446139	380	27	1058744	296548	0	3	1	0	50	63	18745892	5.77E-01	1
PIM2	834040	NaN	38	773588	247420	0	1	0	0	50	43	14994364	5.77E-01	1
MIER3	201203	393	41	1458176	357424	0	5	0	0	2	0	803136	5.77E-01	1
AGBL1	5624	980	-56	2769680	747244	0	8	3	0	29	36	11390932	5.78E-01	1
CASQ2	287179	639	8	1076544	247776	0	5	1	0	50	70	19878328	5.78E-01	1
SLAIN1	131557	900	38	896764	250268	0	4	0	0	50	64	20840240	5.78E-01	1
SLC3A1	433205	522	29	1787120	451408	0	2	0	0	26	13	9889680	5.78E-01	1
RRAGA	500397	450	-27	789608	216448	0	3	1	0	50	61	18541548	5.78E-01	1
DSN1	794301	231	64	949808	236740	0	3	0	0	15	15	6166988	5.78E-01	1
ERAS	823539	NaN	29	569244	180492	0	2	0	0	50	93	24512736	5.78E-01	1
CSHL1	1446139	380	27	583484	154504	0	2	1	0	50	63	18745892	5.78E-01	1
RAD23B	255220	467	1	1051268	300820	0	2	0	0	50	69	25106544	5.79E-01	1
ELAVL2	7648	719	-40	926312	252760	0	4	0	0	49	97	27978040	5.79E-01	1
PRB2	175992	673	-18	996444	354576	0	3	0	0	15	35	9917804	5.79E-01	1
MYH2	75573	738	-49	5090088	1294416	0	20	4	0	13	33	9377040	5.79E-01	1
UGCG	598588	172	14	1033112	266644	0	3	0	0	50	48	19178076	5.79E-01	1
PPIP5K2	204934	549	16	3206848	833396	0	9	1	0	7	5	3173740	5.79E-01	1
BCAP31	1716845	NaN	44	807408	228552	0	2	0	0	50	73	19103672	5.79E-01	1
PPP1CB	483830	229	30	865080	220008	0	4	1	0	50	56	17934568	5.79E-01	1
AMFR	498079	442	-9	1646500	472412	0	3	1	0	35	43	14703512	5.79E-01	1
FOXR2	89607	NaN	10	797796	201852	0	3	1	0	50	70	21092288	5.79E-01	1
OTX1	240308	505	12	871132	274832	0	5	1	0	34	43	12973708	5.79E-01	1
PNMA1	600464	191	47	875404	258812	0	3	2	0	41	49	16938124	5.79E-01	1
EVC	101111	641	18	2537924	729088	0	8	0	0	1	0	1128520	5.80E-01	1
CCDC141	163273	839	12	2287656	570312	0	13	1	0	18	27	7588496	5.80E-01	1
TREX2	971101	NaN	7	563904	198648	0	2	0	0	50	42	13736972	5.80E-01	1
ST8SIA2	288853	578	-30	953724	272340	0	5	2	0	50	109	25194476	5.80E-01	1
ZFYVE9	791018	448	19	3680328	970812	0	18	4	0	50	64	20020728	5.81E-01	1
KYNU	48515	369	20	1254544	323960	0	2	0	0	24	32	12243552	5.81E-01	1
DIABLO	846994	187	29	620152	170168	0	3	1	0	50	91	26931756	5.81E-01	1
WDR41	269224	518	36	1207552	317552	0	4	0	0	42	36	14163460	5.81E-01	1
FBXO27	1653595	282	32	705236	226060	0	3	2	0	30	33	10703140	5.81E-01	1

DDX18	194969	760	-7	1746892	460664	0	7	1	0	13	16	6240680	5.81E-01	1
MED6	243988	706	29	661804	161624	0	2	0	0	50	52	20427636	5.81E-01	1
UGT2A3	NaN	NaN	NaN	1354580	360628	0	5	2	0	50	118	40006212	5.81E-01	1
SLC25A46	127798	846	-7	1068000	306516	0	3	1	0	50	72	20427636	5.81E-01	1
NEIL1	814680	296	43	991104	300108	0	2	0	0	50	63	19824928	5.81E-01	1
EPB41L4B	337644	371	15	2457112	696692	0	7	2	0	20	22	10181600	5.81E-01	1
MAGIX	724395	NaN	44	845856	293700	0	2	0	0	32	28	13371716	5.81E-01	1
ITM2C	1020678	519	45	679960	200072	0	2	0	0	50	73	27243968	5.81E-01	1
ZNF713	757446	584	-6	1131012	264864	0	5	1	0	50	101	23105112	5.81E-01	1
NRG3	5107	1174	-53	1941268	586688	0	8	2	0	43	75	19849492	5.81E-01	1
MAP3K1	200595	412	38	3819168	1109652	0	10	3	0	11	16	6069088	5.81E-01	1
ACAT2	648197	420	22	1012820	296548	0	2	0	0	50	70	24941360	5.81E-01	1
CWC22	35642	927	7	2408696	580636	0	4	1	0	36	37	20048140	5.81E-01	1
WIBG	1994705	229	35	546816	159132	0	3	1	0	17	35	8442184	5.81E-01	1
STK38L	555677	345	37	1251340	294056	0	5	0	0	50	62	21306956	5.82E-01	1
HRH4	144714	390	16	986120	277324	0	3	1	0	50	46	17547596	5.82E-01	1
WISP2	890596	168	46	615524	201496	0	2	0	0	50	63	21028564	5.82E-01	1
POU2AF1	238940	567	19	632612	207904	0	1	0	0	50	28	20439740	5.82E-01	1
NLK	1261475	410	-3	1355292	379140	0	6	0	0	20	26	8342504	5.82E-01	1
NOL9	636407	203	41	1775728	525812	0	6	1	0	45	46	19251768	5.82E-01	1
MAP2K4	45834	583	-52	1054828	271628	0	4	0	0	11	11	4650072	5.82E-01	1
COL4A3BI	253484	500	37	1958356	535424	0	6	2	0	25	26	9666824	5.82E-01	1
PLXNC1	218710	580	31	4043448	1111788	0	10	4	0	50	49	21067012	5.82E-01	1
SERPINB7	159863	441	-11	993596	257032	0	2	1	0	18	22	7562508	5.82E-01	1
FITM1	974559	220	41	700964	246352	0	2	1	0	50	62	24565780	5.82E-01	1
ASB17	140995	603	-11	750092	208972	0	2	0	0	50	62	22943844	5.82E-01	1
QTRTD1	426794	504	26	1068712	298328	0	4	0	0	16	18	7312596	5.82E-01	1
PRKAG2	600611	437	20	1476332	414028	0	2	1	0	50	69	22394892	5.82E-01	1
CD36	120451	351	-28	1244576	324316	0	3	0	0	6	4	2489508	5.83E-01	1
ZNF784	1077402	254	-10	780708	263796	0	4	0	0	8	8	2559640	5.83E-01	1
GAB3	1737597	NaN	10	1502320	422216	0	2	0	0	7	6	4198664	5.83E-01	1
RRAGC	524940	458	24	1040232	269136	0	1	1	0	50	45	18683236	5.83E-01	1
GLI2	183735	374	12	3898200	1239948	0	13	7	0	14	25	7691024	5.83E-01	1

UQCRB	217849	671	12	305448	66216	0	1	0	0	50	75	24887960	5.83E-01	1
CASP3	281208	471	28	740124	176220	0	2	1	0	50	43	17634816	5.83E-01	1
HTR7	98852	510	-4	1205772	350304	0	5	1	0	48	69	22203720	5.83E-01	1
PTOV1	1673246	205	37	1061236	321824	0	3	2	0	13	23	7061972	5.83E-01	1
PLA2G5	528859	466	24	365968	96476	0	1	0	0	50	43	18821008	5.83E-01	1
SNX31	1141276	196	50	1181564	286936	0	5	1	0	41	56	16967316	5.83E-01	1
PIR	193887	NaN	37	772164	198648	0	1	1	0	50	59	18336848	5.83E-01	1
SCAMP5	840042	199	44	620864	160912	0	2	0	0	50	60	20575376	5.83E-01	1
PLOD3	1022626	258	39	1892852	551800	0	4	4	0	50	67	19705312	5.84E-01	1
RSAD2	40919	1132	10	938060	243148	0	3	0	0	50	63	24613128	5.84E-01	1
R3HDM1	561193	558	20	2855120	771808	0	10	1	0	14	14	5721632	5.84E-01	1
COQ4	1813435	201	46	660380	217516	0	1	1	0	50	69	20850564	5.84E-01	1
PRAMEF1	141239	571	18	1194024	342828	0	4	1	0	50	46	17600996	5.84E-01	1
PNPLA1	839966	358	30	1345680	420792	0	5	0	0	50	56	19422292	5.84E-01	1
EXOSC7	479578	326	41	757568	212176	0	2	0	0	50	48	19950952	5.84E-01	1
PAIP2	835535	177	41	351728	71200	0	1	0	0	50	51	18654044	5.84E-01	1
EIF2AK3	118199	366	9	2865444	781776	0	8	3	0	50	55	22512728	5.84E-01	1
GNG5	238252	685	-2	176932	52688	0	1	1	0	4	3	1008192	5.84E-01	1
TRPM6	95662	726	-16	5328608	1362412	0	18	5	0	25	46	12519452	5.84E-01	1
VAMP4	415434	718	3	397296	87576	0	1	0	0	50	55	19543332	5.84E-01	1
NUBP1	257980	356	37	851196	222144	0	1	0	0	50	52	17944536	5.84E-01	1
SLC25A20	1790488	140	38	778572	223212	0	3	0	0	50	72	22035688	5.84E-01	1
IMMT	872251	420	42	1941624	550020	0	7	0	0	20	23	7842324	5.85E-01	1
CDK8	96507	578	22	1236744	306516	0	5	2	0	23	28	9885052	5.85E-01	1
OR52M1	190769	879	-1	762552	255252	0	1	1	0	50	51	19817096	5.85E-01	1
DYRK4	276521	874	24	1365972	350304	0	6	2	0	24	36	11118592	5.85E-01	1
TLX1	469702	221	52	804916	264152	0	2	0	0	50	47	19691784	5.85E-01	1
NME5	1044706	377	41	561768	142044	0	3	0	0	50	71	18121112	5.85E-01	1
PSG5	307971	178	-25	850484	247420	0	2	1	0	50	84	21330452	5.85E-01	1
SEMA3B	843769	206	51	1884308	590248	0	5	0	0	19	14	5772184	5.85E-01	1
OR10K1	177013	929	7	757212	246708	0	4	2	0	5	4	1493420	5.85E-01	1
POU4F1	41876	1004	-1	1006768	343184	0	4	1	0	50	65	17985120	5.85E-01	1
ATP8B1	340613	247	36	3274488	852264	0	12	3	0	21	16	7776464	5.85E-01	1

WDR61	447425	371	54	811324	211820	0	1	0	0	50	56	19480320	5.85E-01	1
C10orf128	NaN	NaN	NaN	287648	77608	0	1	1	0	8	17	4003576	5.85E-01	1
ZNF430	156264	1079	-42	1487724	358848	0	7	1	0	24	39	9980460	5.85E-01	1
INSL3	1216364	212	37	319332	110004	0	1	0	0	50	49	17585332	5.85E-01	1
LRRC37A3	802924	241	8	4071928	1213604	0	14	9	0	47	70	20437604	5.86E-01	1
RAB31	441061	148	43	522964	130652	0	1	0	0	50	42	17584264	5.86E-01	1
ISOC2	1048909	399	24	550020	182628	0	2	1	0	50	71	20869076	5.86E-01	1
ZNF486	375734	904	-57	1213960	289784	0	7	0	0	11	16	4456408	5.86E-01	1
CDK3	1639614	194	22	782844	227484	0	4	1	0	50	92	24769768	5.86E-01	1
C4orf32	167195	334	20	330368	100036	0	1	0	0	50	57	20459320	5.86E-01	1
VPS8	1340797	626	37	3818100	956928	0	11	0	0	0	0	956928	5.86E-01	1
OR10J3	285980	737	-6	807052	248132	0	4	2	0	4	7	1763268	5.86E-01	1
XRN1	502945	403	29	4514792	1131724	0	13	2	0	2	7	2719840	5.86E-01	1
RHOXF1	NaN	NaN	NaN	470632	132788	0	1	0	0	50	116	39778372	5.86E-01	1
AIM2	97976	866	8	888932	234604	0	3	1	0	50	66	22415540	5.86E-01	1
RASEF	119712	813	-45	1928096	514420	0	5	0	0	8	4	2686020	5.86E-01	1
ZP3	870785	220	59	1076188	319688	0	2	0	0	11	8	5429712	5.86E-01	1
SLTM	532170	212	23	2705244	698472	0	9	3	0	30	35	12037072	5.86E-01	1
TREML1	227466	611	41	770028	250980	0	2	0	0	14	16	4588484	5.87E-01	1
CLTA	857985	257	46	656820	166608	0	2	0	0	50	67	23320136	5.87E-01	1
CXCL5	230379	408	2	292276	89000	0	1	1	0	8	11	4168048	5.87E-01	1
FAM171A1	211657	716	8	2224644	662160	0	11	0	0	0	0	662160	5.87E-01	1
H2AFY	583238	414	41	1033112	297616	0	4	0	0	50	86	28633792	5.87E-01	1
NUDT5	328303	227	34	593808	149520	0	1	2	0	14	15	4453916	5.87E-01	1
ZNF526	790787	299	37	1639736	518692	0	7	2	0	50	73	25484972	5.87E-01	1
MICALL2	706729	345	23	2260956	707016	0	5	0	0	4	3	2581356	5.87E-01	1
TRABD	944000	242	49	960132	286224	0	4	0	0	50	67	23711024	5.87E-01	1
EXTL3	403198	327	42	2275196	689572	0	6	1	0	31	26	13553988	5.87E-01	1
OLFM2	1143339	469	-13	1141336	339980	0	5	2	0	50	57	17645140	5.87E-01	1
HOXB8	946208	524	29	612676	173372	0	4	2	0	16	19	4118564	5.87E-01	1
ADAMTSL	1869593	315	35	2448568	674264	0	5	4	0	32	36	12446472	5.87E-01	1
XKR9	241667	562	22	959776	247064	0	2	0	0	50	36	19597088	5.87E-01	1
MIB1	246867	325	47	2609124	702744	0	7	3	0	27	35	11754764	5.87E-01	1

PDZD4	2103463	NaN	30	1915280	581704	0	5	1	0	16	21	8064112	5.87E-01	1
OR2A25	222080	812	-11	760416	234960	0	3	1	0	50	70	20067720	5.87E-01	1
TP53TG5	1037999	382	35	752940	200784	0	4	2	0	50	68	17427980	5.87E-01	1
ZNF230	533136	558	-35	1244220	294768	0	6	1	0	50	58	16442928	5.87E-01	1
ANKAR	408508	452	20	3724828	971168	0	14	1	0	6	5	2836964	5.87E-01	1
C4orf29	231217	284	16	1089716	282664	0	3	1	0	50	46	19400576	5.87E-01	1
CRY2	429658	271	34	1588116	467784	0	5	1	0	50	58	18989396	5.87E-01	1
CCDC60	151508	727	1	1448564	372376	0	4	1	0	50	50	19734148	5.88E-01	1
TNFRSF1E	516231	263	32	1172664	348168	0	5	0	0	50	61	19795024	5.88E-01	1
JD7-PLA2(	874211	259	40	2592036	758280	0	7	1	0	25	29	15107928	5.88E-01	1
PAFAH1B2	426428	228	46	785692	199004	0	2	0	0	50	39	17316908	5.88E-01	1
PCDHB4	915569	724	-11	1922756	627628	0	10	0	0	0	0	627628	5.88E-01	1
DCAF16	271201	1073	21	534000	161268	0	1	0	0	50	33	21067012	5.88E-01	1
TAS2R3	200522	844	14	784980	234960	0	3	0	0	50	60	17687504	5.88E-01	1
DBR1	190056	464	9	1412964	363120	0	3	2	0	50	61	22322980	5.88E-01	1
CD1A	120861	833	3	843008	229264	0	4	2	0	37	48	13642632	5.88E-01	1
FAM159A	663153	261	19	488788	135992	0	1	1	0	33	35	12992220	5.89E-01	1
TSSK6	868278	284	7	658600	219296	0	3	0	0	50	74	19156360	5.89E-01	1
C19orf73	2011861	267	47	298328	118192	0	1	0	0	50	71	20637320	5.89E-01	1
PGP	2179404	224	39	770028	268068	0	2	1	0	50	46	19527312	5.89E-01	1
ZNF844	NaN	NaN	NaN	1721260	428624	0	6	2	0	50	118	40074208	5.89E-01	1
GTSF1L	395176	374	38	382700	96832	0	1	0	0	50	55	22722056	5.89E-01	1
MMP11	637355	196	52	1222148	376648	0	2	0	0	24	21	8565716	5.89E-01	1
CHCHD7	343112	552	19	356356	63368	0	2	1	0	6	8	1962628	5.89E-01	1
SOS1	805852	351	18	3455692	914564	0	20	2	0	3	3	1284804	5.89E-01	1
EPCAM	806315	246	37	828768	214668	0	1	0	0	50	58	20490648	5.89E-01	1
MUT	64105	1139	-14	1919552	537916	0	7	0	0	40	62	23707464	5.89E-01	1
TINF2	915114	220	38	1137064	347456	0	3	1	0	50	69	21077692	5.89E-01	1
ADAMTS1:	1843278	263	33	3595600	1099328	0	7	1	0	18	12	6692088	5.89E-01	1
ZBTB9	1397665	236	31	1139912	383056	0	3	1	0	50	78	20353232	5.89E-01	1
MRPL33	1044890	146	30	254540	50908	0	1	0	0	50	70	19048848	5.89E-01	1
LCOR	989428	512	43	1103244	309720	0	3	0	0	50	71	27165648	5.89E-01	1
SNX7	42534	903	-21	1186904	297616	0	5	0	0	10	11	4585992	5.90E-01	1

SORL1	199734	650	21	5798884	1500896	0	13	3	0	15	16	6162716	5.90E-01	1
NME3	1604603	288	36	428980	132788	0	1	0	0	50	49	20717064	5.90E-01	1
BMX	188273	NaN	36	1810616	432184	0	6	3	0	50	64	16201560	5.90E-01	1
MCM5	366628	224	3	1891784	531508	0	5	0	0	34	37	15707076	5.90E-01	1
CCKAR	165749	636	36	1075832	315772	0	2	0	0	29	33	11418344	5.90E-01	1
S100A9	1140044	401	32	303312	73692	0	1	1	0	15	16	5467448	5.90E-01	1
IL27	1133167	269	63	601996	199004	0	2	1	0	17	21	6723060	5.90E-01	1
C11orf74	96246	531	-26	598436	136348	0	1	0	0	50	102	22901836	5.90E-01	1
ALG10B	42332	589	-38	1199364	327876	0	4	0	0	32	39	13919244	5.90E-01	1
TWF1	132402	425	-5	1003208	266644	0	3	1	0	50	55	21812832	5.90E-01	1
SLC36A4	219463	580	-18	1308300	354576	0	3	1	0	22	33	12280932	5.90E-01	1
OCA2	154909	1141	-61	2140984	645428	0	12	7	0	18	29	6864036	5.90E-01	1
CCDC68	89155	373	-5	908868	214668	0	2	0	0	50	59	24312308	5.90E-01	1
FAM53B	300236	352	32	1053404	314704	0	1	1	0	50	43	19201928	5.90E-01	1
USP14	527535	689	-11	1332152	317908	0	4	1	0	50	62	17535136	5.90E-01	1
PTPN1	621979	153	51	1148812	286580	0	3	1	0	50	61	17650836	5.90E-01	1
ITGA7	1746452	384	9	3109660	928448	0	7	2	0	22	26	10692816	5.90E-01	1
ADAM2	12694	576	-2	1977580	468140	0	8	0	0	6	13	3579580	5.91E-01	1
DBNDD2	1037999	382	35	467428	132788	0	2	0	0	50	68	17427980	5.91E-01	1
CCL21	712216	220	49	349236	98256	0	1	0	0	50	47	18490640	5.91E-01	1
ARMCX1	329999	NaN	8	1121400	333216	0	4	0	0	50	59	21254980	5.91E-01	1
ABHD3	269726	244	57	1055896	291920	0	4	1	0	50	64	20293068	5.91E-01	1
FAM21A	NaN	NaN	NaN	3504820	925244	0	10	2	0	50	118	40570828	5.91E-01	1
HMGA2	274514	562	-6	350660	97900	0	1	0	0	50	55	18846996	5.91E-01	1
PUS7	403383	192	37	1745112	440016	0	4	0	0	34	39	15057376	5.91E-01	1
SLC22A4	531594	238	44	1385196	421860	0	3	1	0	48	36	18730228	5.91E-01	1
XAGE3	476232	NaN	26	300820	77252	0	1	0	0	50	80	25507400	5.91E-01	1
EVI5L	836245	270	24	2073700	592028	0	6	1	0	26	36	12639424	5.91E-01	1
IL1R1	232947	522	20	1493776	375224	0	3	1	0	50	49	21598164	5.91E-01	1
T6GALNA(	1278941	201	51	767180	224992	0	2	0	0	50	53	22309808	5.91E-01	1
FAM102A	1604109	201	61	985408	290852	0	4	1	0	50	69	19892212	5.91E-01	1
RNF186	559698	259	28	542544	187968	0	2	1	0	49	68	20089792	5.91E-01	1
LYSMD2	440341	247	34	532220	168388	0	2	0	0	50	66	19788616	5.92E-01	1

KIF21B	552427	490	32	4149536	1197940	0	14	7	0	40	78	20043512	5.92E-01	1
GGT7	1040878	205	48	1651484	534712	0	5	2	0	50	64	21062384	5.92E-01	1
DAPP1	204991	693	19	742972	193664	0	2	1	0	16	13	4120700	5.92E-01	1
CHPF2	610106	290	32	1848352	641156	0	5	1	0	50	52	23170260	5.92E-01	1
DDHD2	734603	277	44	1923824	494128	0	7	1	0	22	25	7515516	5.92E-01	1
PPP1R10	2080582	271	32	2369180	722680	0	9	2	0	24	27	11479576	5.92E-01	1
OR51A7	65798	1006	-15	774300	226416	0	3	0	0	50	67	21104748	5.92E-01	1
POLR3G	84917	780	16	621220	126380	0	1	0	0	50	44	20122188	5.92E-01	1
TANK	207004	370	26	1126384	301532	0	3	1	0	50	55	22457192	5.92E-01	1
G3BP1	584468	406	46	1212180	335352	0	2	1	0	50	66	24436196	5.92E-01	1
PFKFB3	560233	399	38	1379500	368816	0	5	4	0	10	15	3481680	5.92E-01	1
OR7A5	963401	546	14	783556	241724	0	4	1	0	47	68	17530508	5.93E-01	1
UFSP2	173409	422	20	1228556	324316	0	4	1	0	50	51	19200504	5.93E-01	1
SLC2A12	102277	359	16	1528664	468496	0	3	2	0	50	52	19521616	5.93E-01	1
GH1	1446139	380	27	578856	138840	0	2	1	0	50	63	18745892	5.93E-01	1
H1F0	1385064	203	66	479888	144892	0	1	0	0	50	52	18523392	5.93E-01	1
ZNF441	904926	521	-1	1810616	425776	0	9	1	0	22	30	7232496	5.93E-01	1
PIGB	243817	487	11	1456040	371308	0	5	1	0	50	53	19444008	5.93E-01	1
DCAF7	934619	247	-1	880032	245640	0	3	1	0	50	54	17706016	5.93E-01	1
GRHL2	424003	399	36	1664300	405484	0	5	2	0	50	64	21336504	5.93E-01	1
IL17RD	426285	332	37	1899616	524744	0	4	1	0	50	42	19333648	5.93E-01	1
HTR5A	186338	603	-1	874692	278748	0	3	2	0	10	8	3230344	5.94E-01	1
SLC25A41	865106	219	33	926668	289784	0	4	1	0	50	76	20954160	5.94E-01	1
NMT1	831501	191	28	1301536	339980	0	4	1	0	50	88	23819960	5.94E-01	1
PSG4	310387	125	-33	1065864	303312	0	6	2	0	39	78	15097248	5.94E-01	1
TMEM38B	214951	773	-8	751160	205768	0	2	1	0	25	26	11426532	5.94E-01	1
SDR9C7	2103486	286	26	783912	234960	0	3	0	0	28	47	16026408	5.94E-01	1
SGK3	391086	405	25	1327880	328588	0	4	2	0	9	12	4307244	5.94E-01	1
BHLHE22	9265	1050	-1	900324	323604	0	3	1	0	50	58	20627352	5.94E-01	1
SEMA3E	74057	955	-30	2034540	520116	0	12	2	0	50	100	23101196	5.94E-01	1
ZNF676	58109	1305	-63	1537208	358492	0	8	2	0	44	98	21167760	5.94E-01	1
NAA38	72287	922	-13	254540	69064	0	1	1	0	12	16	4728036	5.94E-01	1
BCL2L13	413666	221	-23	1214672	368104	0	5	3	0	38	66	16349300	5.94E-01	1

KRR1	513610	524	-10	1017448	247064	0	2	1	0	50	68	22647652	5.94E-01	1
ACTR2	606183	343	31	1039520	280528	0	2	0	0	32	31	16950940	5.94E-01	1
PEA15	841605	386	29	343540	89000	0	1	0	0	50	48	19038524	5.94E-01	1
CPO	169586	723	2	982560	257388	0	3	0	0	50	63	23766204	5.94E-01	1
TMEM184I	1631137	184	67	1047352	294056	0	1	0	0	15	28	11661492	5.95E-01	1
AKAP1	413131	258	-6	2259532	679604	0	6	2	0	50	65	24306968	5.95E-01	1
C1S	2495896	211	52	1781780	472768	0	6	1	0	27	33	9879000	5.95E-01	1
RNF39	1820240	496	30	1016024	349948	0	4	1	0	50	54	18891140	5.95E-01	1
GBP7	340715	745	0	1661452	428624	0	5	0	0	50	62	26016124	5.95E-01	1
OR1D2	457580	465	8	765400	236384	0	2	0	0	50	57	20535504	5.95E-01	1
ATG5	181529	390	32	740836	173372	0	3	1	0	27	40	11859072	5.95E-01	1
SPA17	172228	604	18	407976	96120	0	2	1	0	14	17	4070148	5.95E-01	1
ZNF189	112947	898	-22	1634040	383412	0	5	1	0	12	13	4649716	5.95E-01	1
TSGA10IP	3854361	208	57	1395876	422928	0	5	1	0	29	35	13479584	5.95E-01	1
QSOX1	440831	254	9	1875764	581704	0	5	2	0	26	44	13168796	5.95E-01	1
CSPG4	578030	231	39	5560364	1923112	0	17	2	0	2	3	2376656	5.96E-01	1
FAM118A	461688	277	26	932364	248844	0	2	2	0	50	71	20236464	5.96E-01	1
MAGOHB	407094	432	4	401924	94696	0	1	0	0	50	44	18906092	5.96E-01	1
SUPT4H1	782135	399	22	315416	81880	0	1	0	0	50	85	29222972	5.96E-01	1
TMEM165	235221	348	8	808476	254184	0	1	0	0	50	59	18369244	5.96E-01	1
ADAMTS1	546305	566	37	3115000	906020	0	8	1	0	3	2	2082244	5.96E-01	1
ICAM2	1455216	404	25	693488	207904	0	1	0	0	50	68	18709936	5.96E-01	1
USP20	552898	184	42	2380928	653260	0	5	3	0	50	52	18059880	5.96E-01	1
PDX1	321222	612	38	687080	227128	0	2	0	0	50	43	16590312	5.96E-01	1
VPS39	625465	394	35	2307948	601284	0	5	1	0	8	4	2355652	5.97E-01	1
PLLP	701043	125	43	454968	144180	0	1	1	0	32	29	12193356	5.97E-01	1
DKK2	48475	1014	-18	663584	182272	0	3	2	0	8	10	3191896	5.97E-01	1
NNMT	162422	499	-4	668568	191172	0	3	0	0	13	14	5164848	5.97E-01	1
CACNA1B	286606	675	29	5937368	1752232	0	16	5	0	41	37	17443288	5.97E-01	1
PITPNM2	1122735	196	44	3417600	1010328	0	8	3	0	17	25	8803168	5.97E-01	1
MFAP3	174087	592	16	909936	257388	0	4	0	0	50	47	15545096	5.97E-01	1
FRMD6	238905	442	22	1613036	408688	0	5	0	0	24	35	11575696	5.97E-01	1
LCMT2	823006	415	50	1663588	537560	0	7	3	0	50	74	24588920	5.97E-01	1

CCL14	779228	525	19	295480	71912	0	1	0	0	50	69	21358220	5.97E-01	1
ZNF510	327128	491	14	1792104	416520	0	6	2	0	50	51	18992956	5.97E-01	1
ARL9	662749	594	27	320044	87932	0	1	0	0	50	64	23234340	5.97E-01	1
PREX2	14825	990	1	4447864	1114280	0	22	5	0	3	7	1963340	5.97E-01	1
GORAB	72379	883	-2	1016024	270916	0	3	0	0	50	58	21687164	5.97E-01	1
KCNJ2	31424	614	-44	1073696	297616	0	7	1	0	6	13	2516920	5.97E-01	1
SIRPB2	397239	386	42	861520	254540	0	2	0	0	50	49	22689304	5.97E-01	1
MDM4	420144	185	39	1289432	326452	0	2	1	0	50	51	19267432	5.97E-01	1
HNF1B	553994	403	28	1426136	395872	0	3	1	0	50	51	18666860	5.98E-01	1
GPX7	647361	355	12	473836	139196	0	1	0	0	50	62	25476784	5.98E-01	1
TNK2	934412	285	48	2750812	892136	0	6	3	0	50	71	24124340	5.98E-01	1
C1orf194	989913	215	18	415808	109648	0	1	0	0	50	45	17234672	5.98E-01	1
ZNF581	1077402	254	-10	485228	153436	0	2	0	0	50	67	17041008	5.98E-01	1
TRAF2	1950713	176	39	1292280	358848	0	5	2	0	50	66	22923196	5.98E-01	1
C1orf111	363646	391	1	668924	181204	0	2	1	0	50	54	19589612	5.98E-01	1
OR8B3	149616	857	0	779284	224636	0	2	0	0	50	56	23031420	5.98E-01	1
CELA1	504747	239	33	669280	192596	0	2	0	0	50	57	19104384	5.98E-01	1
MUC16	551668	537	-61	34960980	11879364	0	166	43	0	2	43	12280576	5.98E-01	1
TRMT1	1948449	221	41	1679252	505876	0	7	2	0	50	60	21446864	5.98E-01	1
SUSD3	504920	448	61	641156	198292	0	2	0	0	16	18	6280196	5.99E-01	1
TMEM117	140202	874	-1	1345324	334640	0	4	1	0	24	20	8845176	5.99E-01	1
PKIA	46989	984	-2	201496	53756	0	1	0	0	50	86	17958420	5.99E-01	1
KLHL18	872055	242	49	1463872	416876	0	5	1	0	50	62	26356816	5.99E-01	1
TUBA3D	455705	540	29	1130656	333572	0	4	1	0	46	48	16849480	5.99E-01	1
QDPR	271816	901	31	627628	182984	0	2	0	0	50	57	19505952	5.99E-01	1
ACE2	179295	NaN	32	2132084	524032	0	3	3	0	42	50	19673628	5.99E-01	1
FAM21C	105248	558	33	3447504	908868	0	10	1	0	27	17	9527272	5.99E-01	1
COG3	763957	379	50	2183704	568532	0	7	4	0	29	40	12884352	5.99E-01	1
MPV17L2	1178047	210	43	518692	163760	0	2	0	0	50	86	22294856	5.99E-01	1
USP43	78043	204	-50	2792820	868284	0	7	1	0	9	10	4351744	5.99E-01	1
C15orf39	840096	203	46	2482032	884304	0	14	1	0	0	1	884304	5.99E-01	1
SLC15A2	516880	677	28	1910296	518336	0	5	1	0	4	1	1959424	5.99E-01	1
SDR39U1	898202	411	17	724816	240656	0	1	0	0	40	55	15489560	5.99E-01	1

WNT2	331093	392	5	926668	247064	0	3	1	0	50	58	21373172	5.99E-01	1
DPP3	1941577	194	51	1882884	557496	0	6	0	0	7	6	3210052	5.99E-01	1
ATP8A1	189404	856	28	3149888	819868	0	9	2	0	50	67	22739144	5.99E-01	1
SCUBE2	623689	357	31	2686732	706304	0	8	2	0	21	24	10563944	6.00E-01	1
IMMP1L	124843	916	-4	442508	116056	0	1	0	0	50	52	18245712	6.00E-01	1
TMEM211	363566	415	-21	327164	100036	0	1	0	0	50	61	20841664	6.00E-01	1
WDR18	2292363	206	44	1092208	335708	0	2	0	0	24	28	9676436	6.00E-01	1
PRR5	320559	447	36	969032	307228	0	6	3	0	9	12	2882176	6.00E-01	1
HMOX1	364892	241	2	727664	217516	0	2	0	0	50	59	22984428	6.00E-01	1
GNB1L	690032	204	10	817376	259168	0	3	2	0	50	74	22993328	6.00E-01	1
HSPA14	255871	306	35	1319336	370240	0	5	0	0	50	52	19352872	6.00E-01	1
ETS1	215125	644	28	1344612	340692	0	5	0	0	12	14	5407996	6.00E-01	1
KLK6	581408	606	29	632968	173372	0	2	0	0	50	56	18321184	6.00E-01	1
NCKAP1	350474	419	10	3011048	757924	0	9	2	0	50	73	24138936	6.00E-01	1
SRD5A3	235221	348	8	808120	233180	0	3	1	0	50	59	18369244	6.00E-01	1
KRTAP21-[illegible]	30244	1055	-70	196512	57672	0	1	0	0	50	78	14431172	6.00E-01	1
MARCKS	217954	608	16	803136	268068	0	2	0	0	50	53	22511304	6.00E-01	1
GIMAP6	682986	689	20	728732	216448	0	3	2	0	6	8	2107876	6.00E-01	1
GIPC3	2160126	269	39	787828	238520	0	3	1	0	50	60	21798948	6.00E-01	1
PCBP4	1029408	224	38	1012820	332860	0	3	1	0	50	44	16191592	6.00E-01	1
KLHL7	792869	218	25	1582064	406552	0	4	1	0	39	53	17584264	6.01E-01	1
BACE2	197460	530	-6	1306520	390532	0	4	1	0	40	42	17438660	6.01E-01	1
CD164L2	847257	188	26	449984	126736	0	1	0	0	50	53	18526596	6.01E-01	1
GTPBP8	279709	639	18	728376	208260	0	4	2	0	5	11	2891432	6.01E-01	1
FAM71B	166999	1198	18	1499828	448204	0	4	1	0	50	43	19254260	6.01E-01	1
KRTAP27-[illegible]	4608	1035	-69	528304	142400	0	3	1	0	50	77	14168444	6.01E-01	1
ALPL	547986	297	42	1356360	368460	0	4	1	0	14	10	5523696	6.01E-01	1
UBE2D3	380953	471	41	478464	115344	0	2	1	0	10	18	5255984	6.01E-01	1
GBX2	134179	453	6	852976	271628	0	3	2	0	4	4	1457820	6.01E-01	1
NKX2-5	483508	458	33	885016	300464	0	3	1	0	50	51	20532300	6.02E-01	1
A1BG	1621097	406	-25	1223928	397296	0	3	2	0	50	63	19879752	6.02E-01	1
KDM1B	477756	397	1	1550024	411892	0	4	3	0	13	12	4986136	6.02E-01	1
MED17	307453	532	10	1691000	445000	0	6	0	0	3	5	3072280	6.02E-01	1

PAICS	662749	594	27	1135284	294768	0	2	0	0	50	64	23234340	6.02E-01	1
RAB26	1938163	224	40	658600	199004	0	2	1	0	50	61	21257472	6.02E-01	1
NCOA4	406207	579	41	1712360	428980	0	5	0	0	50	76	22964848	6.02E-01	1
SERPINB1(	133459	522	19	1042724	255964	0	1	1	0	50	50	20467152	6.02E-01	1
CABLES2	1190359	252	33	1191176	384124	0	3	0	0	15	16	5673928	6.02E-01	1
ZCCHC7	517331	433	32	1435036	342116	0	5	0	0	18	16	7076924	6.02E-01	1
PLEKHH2	503591	421	29	3888944	1021720	0	14	2	0	1	4	2153444	6.02E-01	1
CD84	1164343	634	35	910292	230332	0	2	0	0	50	83	21889016	6.02E-01	1
VSTM2A	255140	678	-34	608760	167676	0	3	1	0	50	100	25193764	6.02E-01	1
TOP1	383646	129	14	2077972	461732	0	5	0	0	23	14	8993984	6.02E-01	1
WFDC12	865186	324	46	292276	77252	0	1	0	0	50	92	27552620	6.02E-01	1
MRPS9	168425	599	-3	1049488	267356	0	1	0	0	50	51	20449352	6.02E-01	1
DLAT	403342	179	27	1628344	503384	0	4	0	0	10	16	8530828	6.02E-01	1
PRAMEF1(	118495	792	12	1196872	339980	0	3	1	0	50	47	18244288	6.02E-01	1
ATP13A4	144678	776	-2	3116068	843008	0	8	0	0	1	0	1179428	6.03E-01	1
TACR2	704483	285	50	996800	298684	0	2	0	0	50	54	17315484	6.03E-01	1
C1orf43	2028928	185	35	659312	184408	0	3	0	0	50	68	16958060	6.03E-01	1
CCDC50	100733	768	12	1284092	310432	0	4	2	0	50	55	21296632	6.03E-01	1
KCNIP2	865397	335	45	888932	234604	0	4	0	0	50	84	26858420	6.03E-01	1
ELOVL6	204465	522	23	696336	173016	0	2	0	0	50	51	19768324	6.03E-01	1
ACOT2	606300	169	39	1172664	386616	0	3	0	0	50	45	20608840	6.03E-01	1
GLRA1	496476	528	28	1185480	316128	0	5	1	0	14	17	5190836	6.03E-01	1
JUNB	1937827	226	32	823072	291920	0	3	0	0	47	69	20609908	6.03E-01	1
H2AFV	1200579	164	55	350660	103240	0	2	1	0	5	14	2994672	6.03E-01	1
C2orf49	198223	381	8	598080	163404	0	2	0	0	50	54	18850912	6.03E-01	1
FABP2	218603	824	-1	354220	86864	0	1	0	0	50	51	19615600	6.03E-01	1
PRAM1	726124	214	1	1670352	516912	0	7	1	0	50	67	22455056	6.03E-01	1
CAPS2	110847	692	-32	1501964	358492	0	4	0	0	5	4	2236392	6.03E-01	1
RPLP2	1799017	232	51	304736	85084	0	1	0	0	50	57	18763692	6.03E-01	1
SLC20A1	613047	214	57	1716632	500536	0	8	2	0	50	71	21130736	6.04E-01	1
FCAR	914047	573	-19	819512	194020	0	3	2	0	19	46	8972980	6.04E-01	1
SPINT4	777303	359	16	270560	62656	0	1	0	0	50	71	19735928	6.04E-01	1
TMEM60	253754	795	28	337132	96476	0	1	0	0	50	57	20418736	6.04E-01	1

LILRA6	717623	458	-6	1210044	364188	0	3	1	0	50	58	18028196	6.04E-01	1
ITM2A	13949	NaN	29	679248	187968	0	2	0	0	50	72	25915020	6.04E-01	1
MLN	1565139	428	28	308296	80456	0	1	0	0	50	71	18646212	6.04E-01	1
GRAP2	842645	411	34	859740	230688	0	3	0	0	50	54	22048504	6.04E-01	1
UBA52	1394856	259	48	340336	90068	0	5	2	0	1	2	217160	6.04E-01	1
SYBU	133930	623	11	1682456	488788	0	11	0	0	1	0	563904	6.04E-01	1
NDUFAF2	205876	502	11	453544	106088	0	1	0	0	50	53	24898640	6.04E-01	1
GHRL	742508	333	57	374156	102172	0	1	0	0	50	38	14951288	6.05E-01	1
C10orf12	1008851	512	25	3126748	871844	0	10	0	0	4	4	2579932	6.05E-01	1
ATOH1	58522	802	24	859740	277680	0	2	0	0	50	43	18780424	6.05E-01	1
BLOC1S1	1814311	384	26	333216	87576	0	1	0	0	50	44	15945952	6.05E-01	1
NFX1	909394	141	38	3053768	762196	0	8	1	0	12	11	5265240	6.05E-01	1
ANAPC5	959300	258	39	1965832	524744	0	11	0	0	0	0	524744	6.05E-01	1
CLPP	943949	407	31	700964	211108	0	2	1	0	50	47	18083732	6.05E-01	1
DBP	1597109	242	31	774656	282664	0	2	0	0	42	46	18285940	6.05E-01	1
TMEM51	379959	432	16	627628	190460	0	2	1	0	50	59	20325820	6.05E-01	1
IRF6	220312	429	18	1209688	319688	0	3	3	0	17	17	5893224	6.05E-01	1
RAB40B	855855	243	21	700252	215024	0	2	0	0	50	47	17866928	6.05E-01	1
OR2C1	1361831	303	29	761128	245996	0	3	1	0	50	74	21539424	6.05E-01	1
ZWINT	65824	1191	-19	731936	195088	0	3	2	0	5	8	2099332	6.05E-01	1
OR1B1	407749	646	9	765756	255252	0	1	0	0	50	67	20364980	6.06E-01	1
DCC	2849	995	-34	3693144	1065864	0	26	7	0	8	33	6589560	6.06E-01	1
CAPRIN1	770116	279	28	1876832	491992	0	4	0	0	25	33	13371360	6.06E-01	1
CHMP7	490797	288	41	1159492	333572	0	3	3	0	9	5	3213612	6.06E-01	1
FBXO39	936146	438	5	1137064	295124	0	3	0	0	31	17	8079064	6.06E-01	1
PTPN22	326668	406	15	2154156	542544	0	11	4	0	21	27	8646172	6.06E-01	1
PDCD10	145528	766	-10	570312	137772	0	2	1	0	15	23	7478848	6.06E-01	1
HAS2	25994	485	-22	1404776	377716	0	5	0	0	3	4	1838384	6.06E-01	1
SLC35B3	294217	804	-11	1044504	286224	0	3	0	0	13	16	4709880	6.06E-01	1
HAGH	1604603	288	36	788184	236028	0	2	0	0	50	49	20717064	6.06E-01	1
SMTNL2	1239739	318	24	1148456	363832	0	3	0	0	43	38	15910708	6.06E-01	1
LRRC47	249433	341	28	1415812	483092	0	3	1	0	50	57	18706732	6.06E-01	1
MAOB	66825	NaN	22	1354580	374512	0	2	0	0	40	44	16364608	6.06E-01	1

RRBP1	580543	347	0	2539704	696336	0	10	1	0	50	58	21907172	6.06E-01	1
CDCP1	513877	326	46	2106452	615880	0	6	1	0	24	21	9380956	6.06E-01	1
DLK1	383705	323	2	967964	279460	0	3	1	0	50	68	24664036	6.06E-01	1
SPINK8	1054170	263	37	270204	60876	0	1	0	0	50	63	18179140	6.06E-01	1
TOMM20L	290701	552	46	407620	103952	0	1	1	0	18	22	7982232	6.06E-01	1
RIN3	447158	623	42	2437532	760060	0	9	4	0	50	75	23586424	6.06E-01	1
FAM83C	1290513	170	45	1798868	610540	0	6	2	0	36	55	15300168	6.06E-01	1
PCGF5	190999	410	3	695980	161624	0	1	0	0	50	43	21117564	6.06E-01	1
SMCP	59999	804	12	311144	67996	0	1	0	0	50	65	21559004	6.07E-01	1
C16orf90	805495	246	39	453188	141688	0	1	0	0	50	45	22037468	6.07E-01	1
BTBD9	295241	821	32	1666436	448204	0	3	1	0	50	46	19749456	6.07E-01	1
STOML3	181679	1010	-2	767180	227128	0	2	1	0	13	11	5049860	6.07E-01	1
OR56A1	432250	877	-13	781776	244572	0	3	1	0	50	63	18144964	6.07E-01	1
TM4SF19	1164213	231	36	519404	169456	0	1	1	0	6	8	2119624	6.07E-01	1
KANK4	280646	378	0	2482744	746888	0	7	2	0	17	21	10868324	6.07E-01	1
SYPL1	327117	336	29	667856	190816	0	2	1	0	22	20	7801740	6.07E-01	1
RBM45	234602	603	21	1238524	323960	0	3	1	0	11	8	4753312	6.07E-01	1
BAZ2A	2782714	164	50	4810272	1416168	0	12	3	0	5	29	7463896	6.07E-01	1
PARK7	387339	207	26	491636	142756	0	2	1	0	13	18	4984712	6.07E-01	1
TMEFF1	238688	591	-11	1002140	257032	0	3	1	0	50	49	19310152	6.07E-01	1
ZNF85	157327	1055	-68	1555364	367036	0	8	2	0	10	7	1313996	6.08E-01	1
APPL1	427002	389	33	1893564	473124	0	5	0	0	13	5	3556440	6.08E-01	1
KRT6C	1194800	389	29	1422220	424352	0	6	1	0	50	64	18116128	6.08E-01	1
SURF2	1827885	416	28	668568	180492	0	2	0	0	50	47	17145316	6.08E-01	1
BNIPL	1614665	165	40	915276	270204	0	3	0	0	50	62	20369964	6.08E-01	1
RCCD1	793838	291	37	933788	299752	0	3	0	0	18	20	7402664	6.08E-01	1
TRIP6	1339207	433	48	1183700	378784	0	2	2	0	17	24	8713456	6.08E-01	1
VCAM1	277299	899	-6	1886444	518692	0	12	4	0	3	7	1893920	6.08E-01	1
SNX14	393321	384	19	2530092	623712	0	7	2	0	50	61	23125404	6.08E-01	1
SYCP1	601139	601	2	2673916	588824	0	8	1	0	6	7	3569612	6.08E-01	1
OR2M5	19998	935	-32	771452	230332	0	8	2	0	7	15	2404780	6.08E-01	1
EEFSEC	858998	410	48	1484520	456036	0	6	2	0	50	75	23112588	6.08E-01	1
GABRA3	237855	NaN	-32	1264156	353864	0	4	2	0	50	91	20918560	6.08E-01	1

RBPJL	1005559	233	36	1315776	395160	0	5	4	0	20	21	7219324	6.09E-01	1
ADD45GII	1886809	335	40	548952	174084	0	2	1	0	50	62	18190176	6.09E-01	1
AFAP1L1	386336	380	25	1991464	549308	0	4	0	0	36	22	15847696	6.09E-01	1
ATP5I	639827	489	12	190816	50552	0	1	1	0	3	5	1246712	6.09E-01	1
DAP3	2240116	152	32	1056252	273408	0	4	1	0	50	72	23667592	6.09E-01	1
CCNI2	717412	356	59	935568	275544	0	1	0	0	50	46	17209040	6.09E-01	1
TDRD7	489355	386	36	2834828	754720	0	8	5	0	50	60	19681460	6.09E-01	1
CCDC89	325720	554	-19	950164	251336	0	4	1	0	50	100	24725980	6.09E-01	1
NPPA	519155	207	43	381632	116056	0	1	1	0	23	18	8123564	6.09E-01	1
RIMS3	678456	421	20	777504	238164	0	2	2	0	50	65	22572536	6.09E-01	1
SRPK3	2103463	NaN	30	1464940	416876	0	5	2	0	16	21	8064112	6.09E-01	1
P2RX5	504736	354	20	1104668	299752	0	2	1	0	50	62	23395252	6.09E-01	1
CHORDC1	132433	1152	-21	898188	211464	0	3	1	0	16	21	7212204	6.09E-01	1
TMC2	778654	450	41	2345328	644004	0	8	0	0	9	7	3340704	6.09E-01	1
ZNF140	558706	834	17	1192600	291920	0	3	0	0	37	24	11504496	6.10E-01	1
CDHR2	593410	318	45	3338924	996088	0	11	2	0	13	12	4640104	6.10E-01	1
SECISBP2	163101	627	15	2214320	595588	0	5	1	0	9	3	3168756	6.10E-01	1
GLOD4	662733	483	10	792456	203988	0	2	0	0	28	22	9770420	6.10E-01	1
CLEC2B	295505	634	25	405484	92204	0	1	0	0	50	49	21223652	6.10E-01	1
CTSK	1818753	188	35	862232	223924	0	3	1	0	50	99	23376028	6.10E-01	1
PCYOX1	669606	164	36	1291212	353508	0	2	1	0	50	57	21567192	6.10E-01	1
B3GNT3	1216364	212	37	908156	295480	0	1	1	0	50	49	17585332	6.10E-01	1
PELI2	315208	818	10	1068712	301532	0	4	2	0	50	61	18646568	6.10E-01	1
STAM	488136	364	7	1420084	368816	0	3	1	0	50	60	25802880	6.10E-01	1
CHURC1	515337	275	41	310432	69776	0	1	1	0	2	1	551088	6.10E-01	1
CDC45	710221	298	19	1598796	404772	0	5	3	0	10	9	3384492	6.10E-01	1
LYSMD3	86012	780	7	784980	207192	0	2	1	0	50	52	20605992	6.10E-01	1
MAGEF1	1301642	485	36	760060	226772	0	1	0	0	50	92	26331896	6.10E-01	1
FAM83H	2182434	229	28	2835184	962624	0	13	2	0	50	80	25006864	6.10E-01	1
UCMA	246179	354	35	376648	87932	0	1	0	0	50	50	18895768	6.10E-01	1
RASL10B	675201	196	3	508368	153792	0	1	0	0	50	52	20535860	6.10E-01	1
EEF1D	2183482	235	37	1619444	490924	0	2	0	0	50	40	19636960	6.10E-01	1
DGCR6L	605373	292	43	557496	169812	0	1	0	0	30	25	15207964	6.10E-01	1

NSUN7	238024	202	47	1836960	513708	0	5	0	0	26	25	9545784	6.11E-01	1
ACTRT1	938	NaN	-35	932720	275188	0	3	0	0	16	26	4889304	6.11E-01	1
EXOC7	1611239	218	22	1927384	514064	0	7	3	0	50	86	24078416	6.11E-01	1
TCP10L	265057	182	52	549664	158420	0	2	0	0	50	53	17819580	6.11E-01	1
ZMYM6	719158	490	30	3428992	883592	0	9	2	0	33	43	16452184	6.11E-01	1
MGAT2	563227	409	-4	1113568	321824	0	2	2	0	50	55	20194100	6.11E-01	1
BCAP29	512690	753	26	940908	211464	0	2	1	0	49	38	18677184	6.11E-01	1
GPSM3	2119974	392	28	402992	129940	0	1	0	0	50	62	23774036	6.11E-01	1
CTDSPL2	697417	534	37	1222504	325028	0	4	0	0	1	3	2052340	6.11E-01	1
SLC12A5	630342	381	51	2959072	860096	0	15	6	0	27	45	11933120	6.11E-01	1
DUSP16	748543	157	30	1673912	481312	0	6	0	0	17	17	7943072	6.11E-01	1
GJA3	405200	472	50	1058744	342472	0	3	1	0	50	66	25477852	6.11E-01	1
MAPK14	892075	407	36	1110720	239232	0	1	1	0	50	39	18159560	6.11E-01	1
PRKD2	1153249	224	32	2231052	657888	0	7	5	0	34	39	12948076	6.11E-01	1
CGB5	2070141	200	35	404416	138128	0	2	1	0	12	19	4170896	6.12E-01	1
OR6A2	427462	681	8	799932	250980	0	4	1	0	50	71	18227912	6.12E-01	1
AMIGO2	150007	380	-12	1292636	387328	0	4	0	0	29	36	13688556	6.12E-01	1
BARX2	287214	440	-19	703456	208616	0	2	1	0	50	53	19869784	6.12E-01	1
FGGY	60575	728	4	1501252	412604	0	5	3	0	12	13	4522268	6.12E-01	1
IQCH	275948	360	31	2782852	733004	0	7	2	0	50	38	19125388	6.12E-01	1
RARA	1350242	191	23	1307232	382344	0	5	0	0	50	69	21148536	6.12E-01	1
PPP2R5E	237658	445	20	1247068	307940	0	4	0	0	50	64	22834196	6.12E-01	1
AFMID	605209	191	26	819156	217872	0	2	1	0	15	16	7419040	6.12E-01	1
KIAA0232	500642	179	50	3555016	947672	0	10	1	0	2	2	1984700	6.12E-01	1
R5-ARHG/	310964	487	28	1456396	413672	0	3	1	0	45	49	17567532	6.12E-01	1
KAAG1	390563	612	-3	311500	69776	0	1	0	0	50	64	20353944	6.12E-01	1
FUT8	412062	875	38	1577792	409756	0	5	0	0	50	38	15463216	6.12E-01	1
OSBPL5	513529	333	53	2255616	653616	0	6	2	0	50	54	18972308	6.12E-01	1
ZNF540	382032	888	-56	1722684	412248	0	6	2	0	50	63	18541548	6.12E-01	1
CRHR1	408198	563	16	1154864	326452	0	2	1	0	27	29	11423328	6.12E-01	1
SPG11	666585	441	35	6362076	1637244	0	13	3	0	5	4	4145264	6.12E-01	1
CCDC78	1207790	194	33	1125316	336776	0	4	0	0	17	19	6751184	6.12E-01	1
ZNF454	95669	845	10	1364904	323604	0	3	1	0	50	71	23161360	6.12E-01	1

C8G	1979517	188	34	519048	159132	0	2	0	0	50	78	21046364	6.12E-01	1
CKAP2	345539	356	45	1756148	469564	0	4	2	0	50	53	18811040	6.12E-01	1
OR2T33	18846	936	-35	787116	240300	0	5	3	0	6	7	1703816	6.12E-01	1
KRT16	1494501	323	11	1192956	355644	0	5	1	0	20	34	10955900	6.12E-01	1
CENPK	424356	444	25	735140	168388	0	2	1	0	50	63	24355384	6.13E-01	1
PEX11B	NaN	NaN	NaN	647920	217160	0	2	1	0	50	117	39862744	6.13E-01	1
CTAGE9	NaN	NaN	NaN	1953728	538984	0	7	3	0	50	119	40184568	6.13E-01	1
CD46	321738	372	8	1136352	262728	0	4	1	0	50	50	19979076	6.13E-01	1
AFF1	270752	518	48	3130308	881100	0	8	2	0	50	50	19488152	6.13E-01	1
DNAJA4	447425	371	54	1164832	291920	0	2	1	0	50	56	19480320	6.13E-01	1
AQP5	1033911	220	48	643292	221788	0	2	1	0	50	62	21015036	6.13E-01	1
SPIRE1	483118	292	49	1955508	540408	0	3	2	0	50	41	19655472	6.13E-01	1
LARS2	439463	363	38	2347108	634748	0	4	0	0	16	18	8041684	6.13E-01	1
DHX35	262965	581	31	1839808	507656	0	6	1	0	50	55	21096204	6.13E-01	1
GABRE	127993	NaN	-10	1304740	356000	0	5	2	0	50	75	26343644	6.13E-01	1
OR8B12	171574	746	-1	762552	231756	0	7	1	0	6	10	1750808	6.14E-01	1
DNASE2B	235919	769	-13	943044	242436	0	3	1	0	50	76	18267428	6.14E-01	1
ARPC1A	903923	263	51	978288	248844	0	2	2	0	10	14	5285176	6.14E-01	1
ST3GAL2	935790	134	42	895340	250624	0	3	0	0	50	60	24635556	6.14E-01	1
SLC39A3	1354357	291	25	835532	291208	0	2	0	0	50	54	21517708	6.14E-01	1
PKHD1L1	134643	732	6	10942728	2983992	0	33	13	0	50	76	24883332	6.14E-01	1
PIGS	1517567	225	24	1403352	425064	0	10	0	0	2	5	1431476	6.14E-01	1
RPH3AL	231893	591	2	807408	239232	0	2	0	0	50	44	18925672	6.14E-01	1
WDR26	519341	256	18	1698832	477752	0	5	2	0	50	44	18352512	6.14E-01	1
CLCN3	186100	464	12	2243156	590248	0	5	1	0	50	53	21919632	6.14E-01	1
OR2F2	368507	732	-11	769316	248488	0	3	2	0	50	64	17819224	6.14E-01	1
RPRD1A	370331	430	22	813816	216804	0	2	1	0	50	60	25742360	6.14E-01	1
C17orf74	1906324	206	33	1239236	381988	0	4	2	0	50	87	23542992	6.14E-01	1
MON2	299830	359	-1	4416180	1233540	0	10	1	0	14	13	7829152	6.14E-01	1
PPIAL4A	NaN	NaN	NaN	1260240	338556	0	2	0	0	50	116	39984140	6.14E-01	1
TUBB3	1131847	221	40	1143472	316484	0	5	1	0	50	53	16265996	6.15E-01	1
XIRP1	163147	452	44	4474564	1437884	0	14	6	0	28	41	14241424	6.15E-01	1
ALX4	301098	575	9	1024212	308652	0	3	1	0	50	54	20785416	6.15E-01	1

TMEM9B	548433	243	21	506944	147740	0	1	0	0	50	57	20741984	6.15E-01	1
RIPPLY1	148608	NaN	22	388396	113564	0	1	0	0	50	128	42308820	6.15E-01	1
OR10Q1	637499	828	-25	777860	251692	0	3	0	0	50	51	16635524	6.15E-01	1
RELN	291011	954	7	9008936	2353516	0	30	6	0	25	35	13142808	6.15E-01	1
HPX	424837	471	17	1194380	329656	0	2	1	0	50	55	20339348	6.15E-01	1
NAGLU	1993535	162	27	1816312	588824	0	4	0	0	14	15	6959800	6.15E-01	1
FZD7	363643	441	36	1394452	449984	0	7	4	0	41	58	15814944	6.15E-01	1
TAF6	1299015	205	52	1697408	536848	0	4	1	0	35	32	12810304	6.15E-01	1
TMEM176[illegible]	734961	689	14	686368	210752	0	2	0	0	50	53	19509868	6.15E-01	1
PLS1	435882	406	28	1658960	423640	0	6	1	0	34	50	15129288	6.15E-01	1
BCL2L12	1733181	195	36	819868	279104	0	2	1	0	9	8	4296208	6.15E-01	1
KHDC1L	NaN	NaN	NaN	333572	90424	0	1	0	0	50	116	39736008	6.15E-01	1
CCDC37	295496	600	35	1616952	412248	0	5	2	0	14	12	4828784	6.16E-01	1
MMAA	224882	1168	19	1063372	304736	0	3	1	0	50	36	17247488	6.16E-01	1
PTCD1	1327454	288	60	1744044	531864	0	6	3	0	50	58	17222924	6.16E-01	1
EXOC8	377235	569	2	1797088	531152	0	7	2	0	50	74	24456844	6.16E-01	1
RNPC3	NaN	NaN	NaN	370952	108580	0	1	0	0	50	116	39754164	6.16E-01	1
RHOV	1036414	172	51	582060	185832	0	2	1	0	50	67	20324396	6.16E-01	1
RTN4IP1	223177	271	40	1019584	286580	0	4	0	0	50	67	19271348	6.16E-01	1
CTCFL	181464	562	10	1735856	434320	0	3	1	0	50	49	22374600	6.16E-01	1
TWSG1	499362	387	43	591672	143112	0	2	1	0	41	49	16034240	6.16E-01	1
ENPP6	256949	424	-5	1132436	310432	0	2	1	0	50	34	20546184	6.16E-01	1
CDK17	248700	405	26	1423288	362408	0	5	0	0	38	43	16252112	6.16E-01	1
CCR4	511282	469	17	900324	260592	0	3	1	0	50	50	16803556	6.16E-01	1
MEOX1	1180329	165	16	657176	170524	0	2	0	0	38	38	13184816	6.16E-01	1
ZNF347	392290	946	-49	2166260	545392	0	7	2	0	50	50	16685008	6.16E-01	1
SH2D1A	216982	NaN	-14	334284	91848	0	1	1	0	50	55	16552576	6.16E-01	1
DNAJB13	533576	196	41	828056	217516	0	3	1	0	50	52	16080164	6.17E-01	1
GAS2L2	675201	196	3	2147036	699184	0	5	5	0	50	52	20535860	6.17E-01	1
ZNF222	533136	558	-35	1312928	302956	0	4	0	0	50	58	16442928	6.17E-01	1
FAM13A	160978	576	8	2712008	700252	0	7	1	0	50	49	20735932	6.17E-01	1
SPRY3	NaN	NaN	NaN	710932	215024	0	2	0	0	50	116	39860608	6.17E-01	1
COTL1	575674	182	24	372376	98612	0	1	0	0	50	65	23856628	6.17E-01	1

FKBP1A	394062	427	40	288716	77608	0	1	1	0	19	31	9934892	6.17E-01	1
SARS	1000045	287	32	1339628	355288	0	5	0	0	5	13	3974740	6.17E-01	1
SERPINB9	457673	201	41	969744	263796	0	3	0	0	50	47	16458236	6.17E-01	1
TMEM40	487514	176	35	635460	161268	0	2	0	0	50	61	20316208	6.17E-01	1
ENAH	744021	207	2	1525104	431472	0	5	0	0	10	10	3617672	6.17E-01	1
OR7G3	311030	471	-63	757568	244216	0	3	1	0	50	102	27434784	6.17E-01	1
ARMCX3	329999	NaN	8	946604	270916	0	1	1	0	50	59	21254980	6.17E-01	1
MRPL1	159162	508	15	865080	213600	0	2	1	0	50	55	20732016	6.17E-01	1
EPHA3	27204	NaN	-18	2562488	684232	0	15	4	0	6	13	2867580	6.17E-01	1
CHAF1B	289668	352	52	1442868	406908	0	5	2	0	50	44	16524096	6.17E-01	1
DNER	253305	456	-3	1891784	524032	0	4	2	0	50	50	20503108	6.18E-01	1
CALM2	811122	132	35	411892	90068	0	1	0	0	50	47	19691072	6.18E-01	1
CMTM2	777342	214	9	631544	179068	0	2	0	0	50	68	21478904	6.18E-01	1
LYPLA2	903441	254	32	603776	178000	0	2	0	0	50	57	18158136	6.18E-01	1
ARNTL2	544579	377	40	1691000	421504	0	6	2	0	50	66	19666508	6.18E-01	1
CCDC34	336836	378	-21	1052336	257032	0	3	0	0	50	56	21832768	6.18E-01	1
BMP7	207872	617	-21	1105736	304024	0	3	0	0	33	50	19143900	6.18E-01	1
FASTKD1	358154	404	30	2205420	569244	0	6	3	0	22	23	9071948	6.18E-01	1
CAMP	1113209	193	40	435744	127092	0	1	0	0	50	47	15907504	6.18E-01	1
PNPO	1183803	203	21	677112	187968	0	2	1	0	50	60	21372816	6.18E-01	1
CCL15	779228	525	19	300108	77964	0	1	0	0	50	69	21358220	6.18E-01	1
ATOH7	459384	251	17	370952	119260	0	1	1	0	45	51	19708160	6.18E-01	1
SCNN1B	520509	611	32	1660384	444644	0	4	2	0	50	67	23209420	6.18E-01	1
HOXC10	1257445	274	23	861520	243860	0	3	0	0	50	62	22406640	6.18E-01	1
AHSA2	704383	371	36	366324	92916	0	1	0	0	50	69	26054572	6.18E-01	1
INO80B	1069803	343	45	876828	288360	0	2	0	0	50	68	19848780	6.18E-01	1
EIF4EBP1	672015	258	29	303312	88644	0	1	0	0	50	81	22003292	6.19E-01	1
PPIL2	447011	252	56	1448920	332504	0	3	0	0	50	50	17980848	6.19E-01	1
LYRM4	128806	502	24	469208	131008	0	1	0	0	50	44	21281324	6.19E-01	1
ORM1	206775	425	4	532932	135636	0	1	0	0	50	33	18679320	6.19E-01	1
OR7A17	963401	546	14	755788	235316	0	2	2	0	37	53	12940600	6.19E-01	1
NR2F1	263987	505	-1	1049844	317196	0	3	1	0	42	47	17046704	6.19E-01	1
POU2F2	855248	369	32	1194736	349592	0	2	3	0	35	38	12991152	6.19E-01	1

CRNKL1	230275	685	10	2218236	563904	0	9	4	0	42	58	16975504	6.19E-01	1
MRPL24	1649536	208	53	559632	159132	0	2	0	0	50	69	21716356	6.19E-01	1
CREB3L4	2028800	222	34	1008904	298328	0	5	1	0	30	41	11118948	6.19E-01	1
CES2	942986	142	34	1582064	464224	0	5	4	0	27	45	13673248	6.19E-01	1
HES4	1971475	239	15	586332	221076	0	2	0	0	12	27	8570344	6.19E-01	1
STX8	84658	634	-10	626204	163048	0	2	1	0	29	29	9743720	6.19E-01	1
GABRG3	20652	1307	-54	1216096	321824	0	5	2	0	28	69	16104372	6.20E-01	1
POP7	1483965	336	46	342472	113564	0	1	0	0	50	89	28030016	6.20E-01	1
CDC37	1956832	207	38	1004988	239232	0	4	0	0	50	73	23497424	6.20E-01	1
OPRD1	765350	208	27	904952	298684	0	3	1	0	50	59	21238248	6.20E-01	1
COX15	422578	304	12	1092564	331080	0	3	0	0	18	19	8553256	6.20E-01	1
ZFPL1	3267059	207	46	778928	247420	0	3	1	0	50	74	20482816	6.20E-01	1
POU2F1	399046	569	0	1863660	584196	0	5	2	0	21	29	10300148	6.20E-01	1
DUSP1	537128	180	44	904596	287292	0	4	0	0	50	59	18403064	6.20E-01	1
HSD17B4	302141	472	27	1933792	525812	0	5	0	0	33	25	12692824	6.20E-01	1
MADCAM1	1509324	255	-16	919192	327164	0	1	0	0	50	52	17766892	6.20E-01	1
OR51B6	100115	1056	1	771096	230688	0	2	1	0	30	18	7873652	6.20E-01	1
MTIF3	306964	570	60	739056	195444	0	3	0	0	37	36	9927772	6.20E-01	1
ZNF143	566722	135	27	1662164	449272	0	6	1	0	32	25	10470672	6.20E-01	1
LTF	266701	565	34	1841944	506588	0	9	0	0	2	1	858672	6.20E-01	1
TYROBP	1060028	321	47	292632	91848	0	1	0	0	50	67	19297692	6.20E-01	1
OR14I1	141426	710	-10	761128	238520	0	3	2	0	4	7	2673204	6.20E-01	1
ZNF70	610486	266	30	1143828	292632	0	4	3	0	12	20	5342848	6.20E-01	1
C9orf9	1952019	575	33	447848	112852	0	1	1	0	50	48	18580708	6.20E-01	1
IQSEC3	241073	592	27	2984704	945536	0	8	4	0	50	56	22665808	6.21E-01	1
C3orf38	123481	894	0	836956	228908	0	2	0	0	50	44	19702820	6.21E-01	1
MCM2	679358	336	45	2317204	648632	0	5	2	0	12	11	5764352	6.21E-01	1
S100A7	1140114	279	38	275900	61588	0	1	0	0	50	63	17848772	6.21E-01	1
ACHE	1339207	433	48	1592388	537204	0	5	3	0	17	24	8713456	6.21E-01	1
DNAJB2	694442	193	44	845500	243860	0	3	0	0	50	75	22905396	6.21E-01	1
NUS1	190019	639	3	746888	212176	0	2	0	0	50	54	20822796	6.21E-01	1
ZSCAN4	532349	849	-37	1108940	290140	0	3	1	0	50	57	17028192	6.21E-01	1
GAP43	43284	622	-2	720188	203632	0	2	1	0	50	54	20390256	6.21E-01	1

PUS10	635242	350	33	1417592	353152	0	5	1	0	50	78	28107624	6.21E-01	1
GGTLC1	159782	930	-48	576008	169456	0	3	1	0	9	6	1765404	6.21E-01	1
SVEP1	211688	369	1	9174120	2471352	0	19	5	0	41	46	23222592	6.21E-01	1
NIPBL	341972	479	11	7333244	1896412	0	14	4	0	10	11	5774676	6.21E-01	1
CCT2	484023	382	5	1397656	385904	0	4	0	0	12	8	4239248	6.21E-01	1
INSM1	226483	808	-9	1201856	435388	0	2	0	0	7	4	3870788	6.21E-01	1
HDX	20850	NaN	-20	1780356	469920	0	5	0	0	10	12	5059828	6.22E-01	1
PSMA3	290719	552	26	695624	169456	0	2	0	0	50	62	21857688	6.22E-01	1
NR1D2	395355	211	2	1513356	374868	0	5	0	0	50	59	21121836	6.22E-01	1
TIMM17B	834040	NaN	38	573872	170524	0	1	0	0	50	43	14994364	6.22E-01	1
CASC5	958415	139	47	6111096	1506948	0	23	3	0	43	52	21170608	6.22E-01	1
ACOT7	643590	176	26	1183344	344964	0	3	0	0	6	3	2497340	6.22E-01	1
DSTYK	715889	277	39	2363484	669636	0	7	2	0	50	68	23980516	6.22E-01	1
RIMS4	942798	168	40	682096	204344	0	2	0	0	50	73	23693224	6.22E-01	1
DUOXA2	667418	544	27	975440	290140	0	3	0	0	50	85	27585372	6.22E-01	1
KRTAP19-[illegible]	14979	1035	-68	179068	53756	0	3	2	0	6	5	494840	6.22E-01	1
PYGM	2261775	230	61	2179432	602708	0	8	3	0	50	77	23145696	6.23E-01	1
KIAA0513	517155	273	32	1088648	278392	0	4	2	0	50	59	18983344	6.23E-01	1
TM9SF3	790373	642	24	1552160	398008	0	6	2	0	50	74	24523060	6.23E-01	1
ZNF528	548808	989	-53	1633328	399076	0	4	1	0	32	30	11866192	6.23E-01	1
SPATA9	305999	216	35	657176	176932	0	2	1	0	50	49	17197648	6.23E-01	1
TDO2	44515	745	-2	1089716	263440	0	3	1	0	12	13	5449648	6.23E-01	1
EDN3	831202	402	-9	645428	183340	0	2	2	0	13	14	4879336	6.23E-01	1
C4orf50	109875	686	16	700252	215024	0	3	2	0	41	51	13854096	6.23E-01	1
AKIRIN1	681587	318	30	496620	138840	0	1	0	0	50	44	22595320	6.23E-01	1
SLU7	197504	619	30	1583844	360984	0	9	2	0	10	11	3393392	6.23E-01	1
GDF5	1521661	167	53	1216808	395872	0	4	1	0	26	29	11106844	6.24E-01	1
RNASEH2[illegible]	4131934	202	53	410824	130652	0	1	0	0	27	30	12483852	6.24E-01	1
COBL	133439	509	-22	3132088	962624	0	10	6	0	50	108	26967000	6.24E-01	1
HIST1H3A	408919	685	43	331436	109648	0	1	0	0	50	57	16881520	6.24E-01	1
DPYD	64696	807	-6	2681392	743684	0	11	5	0	41	55	17108292	6.24E-01	1
AKAP10	545359	348	-11	1733364	450696	0	4	1	0	50	57	21314788	6.24E-01	1
CBY1	1056349	262	45	332860	91136	0	1	1	0	31	51	16710284	6.24E-01	1

COQ2	344601	218	40	1042368	335352	0	1	0	0	50	50	18924960	6.24E-01	1
MRPS5	172221	647	11	1130656	297260	0	2	0	0	50	64	20536928	6.24E-01	1
LYZ	675242	361	13	389108	103240	0	1	0	0	50	46	17692132	6.24E-01	1
RND3	53679	438	5	641512	164828	0	1	0	0	50	38	18241796	6.24E-01	1
PROX2	884998	232	41	1473484	439304	0	2	1	0	50	64	26283480	6.24E-01	1
TSC22D1	205312	350	38	2713788	871488	0	8	2	0	17	20	7247804	6.24E-01	1
CALHM2	542721	170	51	788896	253472	0	3	2	0	4	7	1946964	6.24E-01	1
CLMN	207561	612	30	2573168	691708	0	11	3	0	13	12	4060180	6.25E-01	1
ATR	564756	403	24	6874360	1796732	0	25	3	0	14	13	6044524	6.25E-01	1
L3MBTL4	236747	877	7	1664656	411536	0	4	2	0	50	61	20322260	6.25E-01	1
FBXO45	1149194	156	30	708084	220008	0	1	0	0	50	74	19186976	6.25E-01	1
SIGLEC8	393294	583	17	1238168	391600	0	5	2	0	13	19	6561080	6.25E-01	1
DCBLD1	189294	465	9	1536496	413672	0	3	1	0	50	65	22672572	6.25E-01	1
ATP1B2	2069567	213	34	769316	188680	0	4	1	0	29	47	10244968	6.25E-01	1
NDUFB6	474275	362	-7	341404	84728	0	1	0	0	50	60	19977652	6.25E-01	1
WDR55	774470	356	31	972592	283376	0	3	2	0	50	59	20786128	6.25E-01	1
GPX3	923608	382	22	579212	165184	0	2	1	0	50	82	23494220	6.25E-01	1
CLDN15	1022626	258	39	572804	178000	0	2	0	0	50	67	19705312	6.25E-01	1
MYH4	77401	731	-50	5098276	1281956	0	17	5	0	13	33	9377040	6.25E-01	1
ARL13B	85478	1310	-13	1134216	283020	0	3	0	0	50	61	24243244	6.25E-01	1
CNOT4	579978	407	19	1721972	481312	0	5	1	0	50	55	22011124	6.25E-01	1
CEP135	585468	376	18	3021728	740836	0	6	1	0	15	16	6257768	6.25E-01	1
ALB	315782	613	8	1613748	402636	0	7	2	0	50	74	22650500	6.25E-01	1
BIN2	499804	194	37	1462092	404772	0	3	3	0	9	8	3043088	6.25E-01	1
HYAL2	843769	206	51	1168748	362764	0	4	0	0	35	37	12054872	6.25E-01	1
RBM43	215149	617	17	912428	247420	0	4	1	0	18	13	4237112	6.26E-01	1
LPPR2	1385739	197	31	1118552	367036	0	2	1	0	50	60	22230420	6.26E-01	1
COMMD5	1454261	384	20	543968	181204	0	2	0	0	50	96	23337580	6.26E-01	1
SULF2	266328	588	41	2287656	588468	0	8	3	0	14	12	3821660	6.26E-01	1
TIGD7	1397628	303	32	1401928	360272	0	8	2	0	50	92	23311236	6.26E-01	1
AANAT	1202221	197	29	625136	198292	0	2	0	0	50	70	20748392	6.26E-01	1
MYBL2	382727	276	24	1789256	512284	0	6	1	0	50	81	26257136	6.26E-01	1
COPA	939853	358	27	3216104	874336	0	11	5	0	43	56	16809964	6.26E-01	1

ARHGEF1!	824719	186	40	2008196	628696	0	5	4	0	50	48	17520896	6.26E-01	1
OR2Z1	536422	487	-47	758992	249200	0	3	1	0	50	119	30937112	6.26E-01	1
APOOL	83775	NaN	-12	705592	192596	0	1	0	0	50	73	23486744	6.26E-01	1
KIF1A	1094176	446	29	4373104	1241372	0	12	4	0	21	31	10772916	6.26E-01	1
ARHGAP2:	385975	470	20	1710580	445712	0	5	3	0	50	70	28571136	6.27E-01	1
SGCE	393749	462	4	1276616	331792	0	2	1	0	50	52	19426208	6.27E-01	1
PPP2R3C	640994	321	45	1222860	283020	0	5	0	0	18	20	7609144	6.27E-01	1
CNIH4	628752	256	18	377716	90068	0	1	0	0	50	58	19280604	6.27E-01	1
PSMG1	383292	235	30	750804	200784	0	2	0	0	50	68	25776536	6.27E-01	1
CDCA3	2822601	232	32	674264	208972	0	2	1	0	50	62	20662240	6.27E-01	1
NOS3	698665	373	33	3258468	1009260	0	12	5	0	7	19	5376312	6.27E-01	1
TRIP12	321955	722	21	5150964	1405488	0	13	4	0	29	49	18048132	6.27E-01	1
C11orf65	405487	579	12	846568	195800	0	3	1	0	50	67	19690004	6.27E-01	1
TFEC	236823	538	-14	905308	239588	0	3	1	0	50	57	18201568	6.27E-01	1
NUP188	1517799	203	29	4503044	1289788	0	21	7	0	12	17	5050928	6.27E-01	1
XBP1	571394	219	-10	1021364	233536	0	2	0	0	50	80	22835976	6.27E-01	1
KCNJ16	32643	589	-53	1055896	290852	0	7	3	0	2	3	875760	6.27E-01	1
ABCG2	432576	209	-7	1702036	463868	0	8	0	0	4	11	3271996	6.27E-01	1
SLC13A4	471922	312	2	1603068	469920	0	4	0	0	26	34	12846260	6.27E-01	1
ALG5	176745	650	16	851196	230688	0	2	1	0	50	55	19294132	6.27E-01	1
BRDT	311662	242	9	2507664	606624	0	9	1	0	50	50	19831692	6.28E-01	1
TMED2	987941	424	47	520828	139196	0	1	0	0	50	77	26168848	6.28E-01	1
BICC1	156609	497	-22	2506952	702388	0	8	1	0	13	17	6362076	6.28E-01	1
ATG3	302697	636	41	851552	204700	0	3	0	0	13	13	3413684	6.28E-01	1
UBASH3B	660955	261	6	1679252	458884	0	6	2	0	50	83	28996556	6.28E-01	1
PPM1F	471638	379	52	1139912	347812	0	4	0	0	30	39	14874748	6.28E-01	1
KIR3DL1	987531	620	-23	1141336	320756	0	6	0	0	22	41	9340728	6.28E-01	1
OR5AS1	4262	1044	-40	804560	234604	0	6	3	0	5	12	1863660	6.28E-01	1
GTF2F1	943949	407	31	1367396	343540	0	4	2	0	50	47	18083732	6.28E-01	1
ERCC6	NaN	NaN	NaN	3834120	1038096	0	11	2	0	50	118	40683680	6.28E-01	1
COBLL1	76781	413	-4	2975092	819512	0	9	1	0	50	53	20309444	6.28E-01	1
SDS	444479	276	36	829124	254896	0	2	2	0	18	16	7704552	6.28E-01	1
UTP14A	379883	NaN	35	2001076	534356	0	8	3	0	11	12	4377376	6.28E-01	1

IQCK	289773	639	13	762196	194732	0	2	0	0	50	74	27480708	6.28E-01	1
PRODH2	1105613	321	38	1334288	433252	0	4	1	0	50	70	19299472	6.28E-01	1
CHPF	548994	487	35	1839808	659312	0	6	2	0	50	86	24911100	6.28E-01	1
MAN2B2	501896	278	33	2545400	769672	0	9	4	0	50	56	19768680	6.28E-01	1
CSTL1	188567	750	-16	381632	96832	0	1	0	0	50	74	22064524	6.28E-01	1
HCST	1060028	321	47	235672	78320	0	1	1	0	6	12	3110728	6.28E-01	1
MRAP	436753	379	8	526168	153080	0	1	0	0	50	69	24300916	6.28E-01	1
RALB	221264	444	37	542900	137416	0	1	0	0	50	64	22775100	6.28E-01	1
CC2D1B	815523	448	26	2200080	650412	0	8	1	0	39	49	18550448	6.28E-01	1
ADAMTS1[illegible]	373086	875	7	4108240	1102532	0	22	9	0	23	34	8683908	6.28E-01	1
SRP19	242849	340	34	386972	96832	0	1	0	0	50	48	18096548	6.29E-01	1
CACNB3	1655612	214	57	1252052	353152	0	3	1	0	50	64	19943476	6.29E-01	1
ACSM4	1157790	439	27	1504456	408332	0	4	2	0	50	66	20642304	6.29E-01	1
ZNF114	1052191	391	32	1081172	270916	0	4	0	0	41	37	11983316	6.29E-01	1
C19orf52	2009333	193	41	629408	213244	0	2	1	0	50	71	21822800	6.29E-01	1
EBP	726793	NaN	2	585264	171948	0	2	0	0	50	78	22575384	6.29E-01	1
ATPAF2	886959	193	17	747244	211820	0	2	0	0	50	52	20036748	6.29E-01	1
RSF1	577731	354	33	3741560	942688	0	14	1	0	32	47	18090496	6.29E-01	1
ZNF680	199707	1110	-18	1495912	347456	0	4	0	0	32	31	15179840	6.29E-01	1
XAGE2	NaN	NaN	NaN	583840	143468	0	3	2	0	8	18	4069436	6.29E-01	1
TMEM130	1040178	526	34	1102888	323960	0	5	1	0	50	72	20241448	6.29E-01	1
NDUFS7	2285841	209	31	557496	162336	0	1	0	0	50	60	22146760	6.29E-01	1
PHLDA3	842013	209	26	309720	104664	0	6	2	0	1	2	239588	6.29E-01	1
ITFG3	1304158	199	40	1395876	422928	0	6	2	0	50	80	23363924	6.29E-01	1
FAM167B	1427362	210	43	396584	133144	0	1	0	0	50	69	25771552	6.29E-01	1
APOE	1003719	196	6	782844	248844	0	2	0	0	50	42	17116124	6.29E-01	1
OR4D10	430021	507	-1	757924	241724	0	2	1	0	50	65	21241096	6.30E-01	1
SLC2A3	122883	305	13	1253476	377360	0	6	4	0	19	28	7056988	6.30E-01	1
B2M	673122	428	36	315416	84016	0	2	1	0	5	10	1894632	6.30E-01	1
CCDC101	NaN	NaN	NaN	765756	216804	0	2	0	0	50	116	39862388	6.30E-01	1
DHX36	94558	921	-11	2665372	672128	0	8	0	0	6	7	3588836	6.30E-01	1
HIST1H2B.	227588	622	29	315416	95764	0	2	1	0	14	21	3578512	6.30E-01	1
GSDMD	2183482	235	37	1219300	377360	0	3	0	0	50	40	19636960	6.30E-01	1

ATP1A4	841605	386	29	2641164	748668	0	8	4	0	46	48	17341116	6.30E-01	1
NR1D1	1573630	202	28	1718768	457816	0	3	0	0	19	18	8498788	6.30E-01	1
GLYAT	285823	620	9	781064	194020	0	3	1	0	50	55	20943480	6.30E-01	1
NDE1	495419	283	26	994308	257388	0	9	0	0	2	0	378072	6.30E-01	1
KRTAP10-:	867542	461	28	673908	201852	0	2	0	0	50	64	21746972	6.30E-01	1
TMEM18	144358	557	-14	377716	93272	0	1	0	0	50	63	23811060	6.30E-01	1
IPHOSPH1	355097	470	40	1813820	416164	0	6	0	0	29	47	12822052	6.30E-01	1
MED8	1088321	252	27	782844	218940	0	2	0	0	50	56	22185208	6.31E-01	1
TRIM16L	752804	391	-6	901392	233892	0	3	0	0	50	50	17232180	6.31E-01	1
HOXC11	1257445	274	23	750092	231400	0	2	0	0	50	62	22406640	6.31E-01	1
OR52J3	97256	1024	-11	753652	236384	0	2	0	0	50	75	25324772	6.31E-01	1
GNPTAB	345225	190	14	3272352	840516	0	6	3	0	50	44	19957716	6.31E-01	1
HIRA	611613	300	1	2608056	756144	0	6	3	0	50	73	23945272	6.31E-01	1
TMEM45A	346650	411	7	714848	190816	0	2	1	0	50	51	17913920	6.31E-01	1
DENND2C	626568	394	8	2274128	588112	0	6	2	0	50	53	19309440	6.31E-01	1
CDK14	127937	1027	7	1187972	315772	0	4	0	0	50	65	23228288	6.31E-01	1
SPCS3	128128	550	11	464936	132076	0	1	1	0	33	43	15998284	6.31E-01	1
PTF1A	124603	469	1	787828	270560	0	2	0	0	50	53	19494916	6.31E-01	1
ZBTB37	443726	385	13	1316132	381988	0	4	0	0	8	5	2938424	6.31E-01	1
ISLR2	492755	317	46	1771456	623000	0	5	1	0	50	52	22095852	6.31E-01	1
TXNL4B	414449	479	9	394448	98968	0	1	0	0	50	53	20136784	6.31E-01	1
LHPP	305373	430	22	690284	205768	0	2	1	0	50	60	21135720	6.31E-01	1
TMED8	551151	418	50	830192	234604	0	2	1	0	50	92	24426584	6.31E-01	1
SPAG5	1651143	225	31	3074416	851552	0	11	0	0	1	1	1128876	6.31E-01	1
VKORC1L	404213	365	30	439304	138484	0	2	2	0	5	4	1395164	6.32E-01	1
OR6C68	802038	706	3	791744	224992	0	1	0	0	50	71	20539776	6.32E-01	1
PRKCZ	1013925	293	28	1574232	398364	0	3	0	0	31	38	15125728	6.32E-01	1
C17orf67	306329	420	-58	303668	79744	0	1	0	0	50	115	29933548	6.32E-01	1
C16orf87	469144	217	12	410468	101104	0	3	1	0	10	22	4155588	6.32E-01	1
ABL2	328468	301	6	3089368	917768	0	7	1	0	1	1	1499828	6.32E-01	1
ABI3	816202	346	24	907444	298328	0	2	1	0	50	56	24378524	6.32E-01	1
RBBP9	414549	436	23	493772	124600	0	1	0	0	50	57	25166708	6.32E-01	1
CA5B	170565	NaN	42	825920	222856	0	3	0	0	50	71	21993680	6.32E-01	1

AQP11	603969	401	30	671416	208616	0	3	2	0	13	10	3550388	6.32E-01	1
MAGEB1	74310	NaN	16	867928	251336	0	3	1	0	50	67	20445436	6.32E-01	1
KCNA7	2070141	200	35	1087580	380920	0	3	0	0	24	30	8450372	6.33E-01	1
GPR119	301748	NaN	37	815596	265220	0	3	0	0	50	61	18917128	6.33E-01	1
PDP2	942986	142	34	1315064	387328	0	3	1	0	50	65	21541916	6.33E-01	1
ZNF777	588536	467	17	2082600	604488	0	9	2	0	50	68	19904316	6.33E-01	1
IQCD	417427	252	35	883948	239588	0	2	0	0	50	52	19240376	6.33E-01	1
MAT2A	1241734	170	39	1018872	284088	0	4	0	0	33	39	13452884	6.33E-01	1
NETO2	542002	532	7	1367752	351728	0	5	1	0	50	71	21375308	6.33E-01	1
ZNF594	1391000	384	20	2102180	490924	0	11	3	0	50	83	22837756	6.33E-01	1
PARP16	762474	223	53	824852	234604	0	1	2	0	21	29	9522644	6.33E-01	1
SEC14L1	777991	192	20	1871136	506232	0	7	1	0	50	58	22457548	6.33E-01	1
MED20	429349	341	34	544680	150588	0	1	0	0	50	42	17067708	6.33E-01	1
SLC25A2	800016	708	-15	742616	224992	0	6	1	0	50	156	25349336	6.33E-01	1
FLT3	346092	614	42	2627992	657176	0	8	3	0	50	57	19179500	6.33E-01	1
ESR1	221347	568	0	1515136	428624	0	4	2	0	36	28	11382032	6.33E-01	1
ACTR8	128709	552	39	1620512	435388	0	6	2	0	50	59	17062012	6.33E-01	1
PLA2G15	910198	281	30	1034892	311856	0	2	1	0	50	63	19207980	6.34E-01	1
MAPK8IP3	1598087	288	36	3459608	973660	0	11	3	0	27	27	12385240	6.34E-01	1
PDE8A	393930	366	27	2177296	571736	0	4	1	0	50	39	20168468	6.34E-01	1
C5	628273	379	37	4409060	1137064	0	9	0	0	6	5	3853344	6.34E-01	1
ZNF488	117080	445	21	816664	275900	0	2	1	0	50	54	20315496	6.34E-01	1
ESRP1	467699	426	31	1777508	496264	0	6	2	0	50	60	22214756	6.34E-01	1
KIAA1715	237188	967	11	1120332	305448	0	1	0	0	50	48	19923540	6.34E-01	1
CCDC84	1457410	180	55	887864	223924	0	2	0	0	19	13	6716296	6.34E-01	1
NCF2	618153	407	31	1396232	356356	0	4	2	0	29	29	9311180	6.34E-01	1
ANKFY1	816229	357	26	3082604	870064	0	7	0	0	0	0	870064	6.34E-01	1
IVL	59999	804	12	1499828	381988	0	3	1	0	50	65	21559004	6.34E-01	1
TMEM89	1386275	212	37	383412	132432	0	1	0	0	50	75	27156392	6.34E-01	1
PTX3	NaN	NaN	NaN	944112	288360	0	4	2	0	50	118	39933944	6.34E-01	1
BTBD11	206629	764	16	2858324	862588	0	7	4	0	50	45	18384908	6.34E-01	1
ULBP3	402308	382	51	630120	166608	0	2	2	0	11	11	2658608	6.35E-01	1
KLF17	865527	167	38	977576	284800	0	3	0	0	50	61	20286304	6.35E-01	1

GDNF	176444	628	4	534712	152012	0	4	1	0	2	4	960132	6.35E-01	1
CCDC6	137718	555	0	1224996	331080	0	3	0	0	50	47	20113288	6.35E-01	1
DNTTIP1	742720	180	48	873624	239232	0	4	2	0	43	45	12440420	6.35E-01	1
HIST1H2AI	390169	544	57	312568	109292	0	2	2	0	18	19	3237820	6.35E-01	1
KDM3B	1254629	209	42	4432200	1311504	0	18	2	0	0	2	1311504	6.35E-01	1
FAM114A1	370538	352	63	1463516	399076	0	4	1	0	50	59	20806064	6.35E-01	1
FCGRT	2080142	210	41	912072	286224	0	3	2	0	17	20	5347832	6.35E-01	1
MALL	NaN	NaN	NaN	390888	115344	0	1	0	0	50	116	39760928	6.35E-01	1
INTS4	577731	361	32	2495560	689216	0	4	0	0	9	13	5857980	6.35E-01	1
KRTAP1-1	977588	404	10	453544	118904	0	2	1	0	50	68	14276668	6.35E-01	1
B3GALT5	286705	689	-70	783912	214668	0	3	1	0	50	117	33315904	6.35E-01	1
FGF6	291341	908	23	516200	166252	0	1	0	0	50	46	20758360	6.35E-01	1
SPRN	485067	472	8	346744	144536	0	1	0	0	50	60	20562916	6.35E-01	1
ALOX5	127792	737	36	1758996	461376	0	4	0	0	3	1	1106804	6.36E-01	1
LRRC66	91954	815	7	2218592	619084	0	17	4	0	2	9	1654332	6.36E-01	1
ADRB1	272214	502	25	1122112	411536	0	3	2	0	38	39	16048124	6.36E-01	1
DHRS1	915114	220	38	797796	242436	0	1	0	0	50	69	21077692	6.36E-01	1
HOXA9	473864	394	13	674976	206124	0	2	0	0	50	80	25624168	6.36E-01	1
PURG	339429	549	-11	962980	268424	0	3	1	0	50	55	19089432	6.36E-01	1
SMPD1	424837	471	17	1580996	508012	0	5	2	0	50	55	20339348	6.36E-01	1
TSPAN1	908945	190	29	633680	171592	0	2	1	0	50	82	25648732	6.36E-01	1
NRARP	1724821	399	43	281240	87220	0	1	1	0	50	60	16636592	6.36E-01	1
DLG2	57955	1000	-20	2742624	712356	0	10	4	0	50	87	22619884	6.36E-01	1
SORCS3	14547	986	-29	3138140	891424	0	18	5	0	31	58	11005384	6.37E-01	1
MRPL18	652619	453	26	458528	134212	0	1	0	0	50	49	21402008	6.37E-01	1
TAB1	872660	250	73	1354580	398008	0	5	1	0	50	50	20133936	6.37E-01	1
TM4SF18	529009	254	8	525812	144892	0	2	1	0	17	43	11386660	6.37E-01	1
EFNB3	2066943	232	27	824496	285156	0	4	1	0	50	93	23452924	6.37E-01	1
MAFK	595899	243	38	383768	127804	0	1	0	0	50	61	22104040	6.37E-01	1
VSIG4	317695	NaN	42	1023856	289784	0	3	2	0	34	33	12069824	6.37E-01	1
EHD1	3218288	209	61	1342832	388396	0	4	1	0	50	89	25050652	6.37E-01	1
YARS	1339617	195	26	1369176	377004	0	4	2	0	50	75	22599948	6.37E-01	1
SYT3	612522	593	35	1453548	478464	0	5	2	0	50	69	23489948	6.37E-01	1

GATS	1382416	198	55	413672	128872	0	1	0	0	50	42	17341828	6.37E-01	1
SLC6A4	558157	298	25	1613748	459240	0	5	1	0	50	46	19048136	6.37E-01	1
BCL9L	1488276	175	54	3665376	1170528	0	10	1	0	10	11	4967624	6.37E-01	1
UBE2D2	641116	244	39	399076	100748	0	1	0	0	50	81	26068456	6.37E-01	1
GSTM5	927739	201	22	592740	140976	0	2	1	0	50	50	14380264	6.37E-01	1
LCTL	553330	219	58	1488080	383056	0	8	2	0	50	70	21308024	6.37E-01	1
ARF4	703082	422	29	474548	126736	0	1	1	0	19	17	7060904	6.37E-01	1
MORN1	644518	302	30	1281600	371664	0	2	0	0	50	52	21858044	6.38E-01	1
NAGA	1321127	363	65	1066932	287292	0	2	1	0	50	55	15728436	6.38E-01	1
RBM42	1162794	390	27	1179784	401924	0	5	2	0	50	66	20625572	6.38E-01	1
CYTH1	986469	250	32	1077256	254540	0	3	0	0	50	60	22466804	6.38E-01	1
NAALADL	3267059	207	46	1870424	578500	0	6	1	0	34	42	13459292	6.38E-01	1
ATHL1	1463610	437	24	1845148	574940	0	4	1	0	6	2	2533652	6.38E-01	1
C1orf159	2134483	234	21	518692	153080	0	2	0	0	50	82	21419808	6.38E-01	1
MFSD2A	974339	387	27	1387688	410824	0	4	0	0	4	8	2945900	6.38E-01	1
BIRC8	427680	921	-46	603064	158420	0	2	0	0	50	59	17329368	6.39E-01	1
DPY19L4	449158	380	32	1914924	481668	0	5	1	0	36	29	13039568	6.39E-01	1
HYAL4	87602	443	-10	1223216	333928	0	4	0	0	50	64	23412340	6.39E-01	1
PITPNC1	648233	365	3	984696	247776	0	2	1	0	50	49	19163124	6.39E-01	1
DLD	470337	486	12	1320048	369528	0	5	2	0	50	55	19417664	6.39E-01	1
HOXD1	245316	874	13	789964	268424	0	2	0	0	24	30	6968700	6.39E-01	1
PTPMT1	1001413	219	34	619084	153080	0	2	1	0	50	61	18105804	6.39E-01	1
DNHD1	425139	491	23	11789652	3622656	0	10	0	0	0	0	3622656	6.39E-01	1
PLIN1	880974	190	35	1292992	416876	0	1	0	0	13	10	5919212	6.39E-01	1
RALGAPA	258743	457	12	5426508	1432188	0	11	2	0	15	12	6870444	6.40E-01	1
MPHOSPH	1064138	201	37	2684240	703456	0	12	0	0	4	3	1841232	6.40E-01	1
ADH7	332944	854	12	1065508	296192	0	4	0	0	50	57	17161692	6.40E-01	1
CKM	1280459	215	23	988256	265576	0	6	3	0	5	13	2335360	6.40E-01	1
CDK19	322538	480	29	1326456	338556	0	2	1	0	40	43	16563256	6.40E-01	1
HOXB2	946208	524	29	859384	288716	0	3	1	0	50	60	17564684	6.40E-01	1
GRWD1	1418188	209	41	1120332	337488	0	2	2	0	31	58	18583556	6.40E-01	1
DOK5	139298	847	-37	805628	207904	0	3	0	0	50	63	20079112	6.40E-01	1
POMT1	651666	272	34	1934504	543256	0	1	0	0	4	0	3784280	6.40E-01	1

RTN4RL2	923775	197	9	997868	358492	0	3	2	0	50	53	18462160	6.40E-01	1
ESYT1	2878044	171	40	2844796	855824	0	8	4	0	42	54	17538340	6.40E-01	1
KIAA1210	556208	NaN	8	4316500	1217876	0	12	3	0	4	8	3768260	6.40E-01	1
RANBP6	350338	339	24	2780360	765400	0	7	2	0	18	19	8731968	6.40E-01	1
PSME1	974559	220	41	746176	187256	0	2	1	0	50	62	24565780	6.40E-01	1
IPO8	239796	580	-19	2740488	687792	0	9	4	0	9	18	4682824	6.40E-01	1
LEPR	178338	528	-8	3237820	818444	0	9	2	0	5	6	3092572	6.40E-01	1
MBD5	119685	955	7	3736932	1091496	0	8	1	0	12	10	6535804	6.40E-01	1
FAM169B	149919	473	-53	505164	134568	0	2	1	0	50	76	17954860	6.41E-01	1
SGPP1	321254	311	18	1077612	349236	0	2	1	0	50	46	24415904	6.41E-01	1
OGFRL1	132568	459	-14	1172664	301176	0	7	1	0	1	4	1165188	6.41E-01	1
SETMAR	96339	400	-20	1734432	473124	0	2	0	0	12	10	6226796	6.41E-01	1
ZACN	1611239	218	22	1040588	318976	0	2	1	0	50	86	24078416	6.41E-01	1
GLRX3	84315	854	-23	890000	231400	0	3	0	0	34	59	14018212	6.41E-01	1
ADIPOQ	449980	384	25	611252	182272	0	2	0	0	18	26	8166284	6.41E-01	1
MPP5	483147	260	32	1773236	443932	0	6	0	0	17	19	6370976	6.41E-01	1
T6GALNAC	1278941	201	51	849060	246708	0	2	0	0	50	53	22309808	6.41E-01	1
PNMA5	249931	NaN	35	1107160	331436	0	2	0	0	6	6	2788192	6.41E-01	1
ZSWIM7	943249	350	-33	495908	121396	0	1	1	0	50	81	22749824	6.41E-01	1
ZNF329	1308496	619	-64	1383772	357068	0	8	1	0	50	132	31228320	6.41E-01	1
IMPAD1	137667	562	-5	901748	268780	0	3	0	0	50	65	20366048	6.41E-01	1
DNAJC5	1157316	206	36	520116	134568	0	1	0	0	50	49	18613460	6.41E-01	1
G6PD	2163759	NaN	42	1411184	389464	0	3	2	0	50	72	22102972	6.41E-01	1
N4BP1	156698	392	1	2265940	633680	0	6	2	0	50	49	24281336	6.41E-01	1
UGT1A7	NaN	NaN	NaN	1340696	377716	0	2	0	0	50	116	40023300	6.41E-01	1
C21orf58	940047	624	53	798508	266288	0	3	0	0	25	46	14228964	6.41E-01	1
SLC27A1	930383	202	34	1616596	505520	0	6	3	0	50	68	22072712	6.42E-01	1
HTR1B	11897	1123	-35	943044	309720	0	4	0	0	5	6	1763624	6.42E-01	1
CS	2878071	171	46	1193312	345676	0	4	1	0	33	67	18798224	6.42E-01	1
GPR180	149137	803	14	1146320	302956	0	2	0	0	7	6	2859392	6.42E-01	1
IL34	884849	184	33	618728	181204	0	2	0	0	50	82	24395612	6.42E-01	1
AADACL4	145424	454	23	1018516	301532	0	3	1	0	50	54	19660812	6.42E-01	1
NOS1AP	372116	410	2	1335000	383412	0	4	0	0	16	22	6917436	6.42E-01	1

ARHGAP30	689033	250	23	2749744	828056	0	12	2	0	12	19	4867588	6.42E-01	1
BBX	224591	699	2	2457112	625136	0	7	2	0	25	32	12112544	6.42E-01	1
ME1	173617	711	-13	1485944	405484	0	5	1	0	50	71	24695364	6.42E-01	1
USP8	325514	203	30	2911368	755076	0	11	3	0	38	49	18779356	6.42E-01	1
RIT1	2096440	179	32	572448	153792	0	2	0	0	50	65	18558992	6.42E-01	1
HTR3D	1423268	375	38	1293704	377716	0	3	0	0	5	2	2092924	6.42E-01	1
WSB1	147758	292	-21	1095056	291208	0	3	1	0	50	49	20077332	6.42E-01	1
CEACAM4	1249321	499	27	615524	195088	0	2	1	0	50	82	23862680	6.43E-01	1
IRX4	387770	635	11	1258460	424708	0	4	0	0	2	2	1692780	6.43E-01	1
LINGO3	1553589	240	29	1379500	524744	0	5	1	0	50	57	18912144	6.43E-01	1
OR10H4	843515	575	12	782132	232468	0	3	0	0	50	77	21682536	6.43E-01	1
TMOD4	1562281	184	33	915632	233536	0	3	0	0	5	6	1818092	6.43E-01	1
GPR61	1056159	272	37	1082596	365612	0	4	2	0	50	61	17976932	6.43E-01	1
PDE4C	1178047	210	43	1927384	577076	0	5	2	0	3	5	2142052	6.43E-01	1
FAR2	104924	674	-4	1348528	351728	0	3	0	0	21	28	8973336	6.43E-01	1
LDOC1	18788	NaN	-20	369172	101816	0	1	0	0	50	76	19909300	6.43E-01	1
FGF7	273513	370	-7	508012	125312	0	1	0	0	50	42	20108304	6.43E-01	1
NFKBIZ	550073	421	16	1835180	515488	0	4	2	0	50	52	20308020	6.43E-01	1
ZNF281	264057	431	9	2250632	624424	0	6	0	0	7	7	3624080	6.43E-01	1
MIF	649838	202	61	295836	86508	0	1	1	0	28	38	9653652	6.43E-01	1
TTC9	244305	523	41	553580	169456	0	2	2	0	39	52	14247832	6.43E-01	1
HAPLN3	562979	560	7	898544	275188	0	1	0	0	12	17	6029572	6.43E-01	1
N6AMT1	312901	480	6	548240	164116	0	3	3	0	6	14	2210760	6.44E-01	1
CALY	386585	472	13	555004	164828	0	1	0	0	50	46	19712788	6.44E-01	1
GMCL1	857542	180	44	1370600	338200	0	3	0	0	27	25	9871880	6.44E-01	1
KCTD15	680604	598	-26	722680	223568	0	2	1	0	50	56	18372804	6.44E-01	1
FBXO25	173778	838	-19	1011396	210396	0	1	0	0	50	59	20999728	6.44E-01	1
DVL2	1782958	316	21	1855116	566040	0	7	2	0	39	39	14081580	6.44E-01	1
CHRNA6	594393	454	34	1264156	345320	0	1	1	0	50	55	25462900	6.44E-01	1
FEZ2	451487	318	-3	1001428	255608	0	2	0	0	12	23	6725908	6.44E-01	1
ZDHHC20	383737	430	34	952656	231756	0	3	0	0	50	60	19911436	6.44E-01	1
OR5H2	414958	885	-14	786404	222856	0	3	2	0	50	65	18540480	6.44E-01	1
SEC22C	352808	445	32	823784	222856	0	2	0	0	50	51	21713152	6.44E-01	1

SNX17	1245306	182	52	1222504	346388	0	5	2	0	32	27	12262064	6.44E-01	1
STK33	314196	551	13	1366684	330368	0	4	1	0	50	66	22391688	6.44E-01	1
TMF1	263910	443	9	2841592	731936	0	7	0	0	6	4	2993248	6.44E-01	1
POLM	1151124	282	56	1242440	388396	0	7	3	0	4	8	1887868	6.44E-01	1
RXRB	1474628	377	24	1327880	421504	0	4	2	0	50	90	23548688	6.44E-01	1
GRK1	785061	NaN	36	830548	232112	0	2	0	0	50	59	23348616	6.45E-01	1
WRB	383982	346	44	459596	120328	0	1	0	0	50	50	21398804	6.45E-01	1
SNTG2	78833	824	-24	1415812	382700	0	6	4	0	25	53	11809944	6.45E-01	1
STRAP	323662	534	22	925244	237808	0	4	1	0	2	4	1266292	6.45E-01	1
LDLRAD2	502881	181	28	669992	223924	0	2	2	0	9	21	7483120	6.45E-01	1
COL4A5	118458	NaN	19	4251352	1375940	0	5	3	0	33	28	16316192	6.45E-01	1
PDCD2	425191	598	-16	912784	244928	0	1	0	0	50	46	17122888	6.45E-01	1
SLC6A15	20920	1015	-34	1966900	537560	0	11	1	0	9	31	6894296	6.45E-01	1
ZNF735	NaN	NaN	NaN	1079392	254540	0	3	1	0	50	117	39900124	6.45E-01	1
MC3R	254407	729	-58	801712	236384	0	4	1	0	50	93	23205148	6.45E-01	1
RBP3	117080	445	21	3006420	1004988	0	10	4	0	50	54	20315496	6.45E-01	1
PRKCE	172588	465	32	1915992	508368	0	4	2	0	50	63	23943136	6.45E-01	1
AHCYL2	776656	420	44	1696696	460664	0	4	1	0	50	64	22600660	6.45E-01	1
SMARCD3	610106	290	32	1290500	361696	0	3	1	0	50	52	23170260	6.46E-01	1
SEMA6A	184843	1024	4	2636536	743684	0	4	1	0	11	8	5592760	6.46E-01	1
THAP5	171249	838	6	1016736	262728	0	1	2	0	22	19	7302984	6.46E-01	1
PARVA	447343	318	-4	979712	268780	0	1	1	0	50	61	21123616	6.46E-01	1
OR2M3	19998	935	-32	768960	232824	0	5	3	0	7	15	2404780	6.46E-01	1
TEKT2	853740	282	27	1104668	316840	0	3	1	0	50	63	23628076	6.46E-01	1
SIL1	1351545	442	35	1177292	341404	0	2	1	0	30	40	12632304	6.46E-01	1
MICA	NaN	NaN	NaN	986476	271628	0	1	0	0	50	116	39917212	6.46E-01	1
ANKMY1	452566	485	21	2629416	746532	0	5	3	0	13	13	4432912	6.46E-01	1
SENP5	766452	258	33	1967968	492704	0	7	1	0	41	59	17564328	6.46E-01	1
RTN2	1183590	164	41	1349596	442508	0	3	1	0	50	61	18576080	6.46E-01	1
ITPRIPL2	799976	275	20	1250272	467072	0	5	2	0	50	58	19469284	6.46E-01	1
RAD9A	1269888	177	51	1001072	299752	0	2	1	0	31	22	11381320	6.46E-01	1
PMEPA1	197938	488	15	730156	216092	0	2	0	0	50	47	17164896	6.47E-01	1
NFYC	552400	323	24	933432	246708	0	1	1	0	50	50	20705316	6.47E-01	1

SLC22A9	1061448	478	28	1407624	405840	0	3	0	0	25	32	11950564	6.47E-01	1
TPD52L2	1140529	206	40	600216	170880	0	2	1	0	50	55	17291632	6.47E-01	1
KNTC1	917926	215	37	5859404	1490572	0	15	4	0	15	16	6966920	6.47E-01	1
PSMB5	1251074	185	39	727308	222144	0	2	0	0	50	62	21672924	6.47E-01	1
C3orf18	603887	293	52	410824	127448	0	1	1	0	14	10	4594180	6.47E-01	1
FAM92A1	221692	552	-4	761128	199004	0	2	0	0	50	52	19445076	6.47E-01	1
METAP1	218494	549	21	1016024	266644	0	2	0	0	50	37	18026772	6.47E-01	1
FBXL14	333350	216	28	1015312	333572	0	3	3	0	3	7	2513716	6.47E-01	1
PNCK	1716845	NaN	44	1142760	328944	0	5	1	0	50	73	19103672	6.47E-01	1
APOBEC3(	1386387	372	71	504096	121752	0	2	0	0	50	61	16059872	6.47E-01	1
ARSD	191640	NaN	18	1630836	474192	0	5	0	0	34	36	12348928	6.47E-01	1
C16orf78	18490	907	-25	701676	169812	0	3	0	0	50	88	20997592	6.47E-01	1
RAMP1	571081	382	23	373800	112140	0	1	1	0	15	19	7180164	6.47E-01	1
LSP1	871765	481	43	898900	233180	0	2	0	0	50	50	21769756	6.47E-01	1
PPID	188459	429	14	983272	243860	0	3	0	0	50	66	21358932	6.48E-01	1
NKRD20A	NaN	NaN	NaN	2173380	526524	0	5	2	0	50	118	40172108	6.48E-01	1
FAM3A	2163759	NaN	42	617304	161268	0	2	0	0	50	72	22102972	6.48E-01	1
FAIM3	467839	385	39	979356	307584	0	3	1	0	50	72	22657976	6.48E-01	1
LHCGR	75021	934	-16	1776796	510860	0	6	1	0	31	43	12769364	6.48E-01	1
TMEM87A	595635	394	35	1522612	386972	0	4	1	0	50	51	20941344	6.48E-01	1
CDC73	285984	442	-12	1401572	371308	0	4	0	0	50	46	18043504	6.48E-01	1
ZNF614	436768	901	-53	1513712	376648	0	5	0	0	29	36	9985444	6.48E-01	1
TMEM123	491236	312	16	520472	166252	0	3	2	0	16	23	4655412	6.48E-01	1
PGM1	135416	330	3	1700968	470276	0	5	3	0	20	18	6057340	6.48E-01	1
SLC10A7	60942	1043	16	917768	267712	0	3	0	0	50	48	19111504	6.48E-01	1
NGFR	971109	303	31	1065152	327520	0	2	1	0	50	72	22431560	6.48E-01	1
ADAP1	737267	310	41	984696	259524	0	2	1	0	15	6	3945904	6.48E-01	1
PRKD3	482178	231	38	2319340	608048	0	6	1	0	31	18	11177688	6.48E-01	1
REN	557652	370	34	1048776	295836	0	3	2	0	9	11	3453200	6.48E-01	1
NPEPL1	1026997	504	15	1187616	375936	0	4	0	0	5	7	2286944	6.48E-01	1
LMTK2	457962	375	19	3788908	1085444	0	15	4	0	50	53	18947032	6.48E-01	1
PI15	149375	824	-13	667500	183696	0	2	0	0	50	80	23346836	6.48E-01	1
NR1H3	1274054	198	38	1148456	325384	0	3	2	0	50	62	18403064	6.48E-01	1

UNC13B	1081701	432	37	4167692	1095412	0	9	2	0	6	2	2528668	6.49E-01	1
ZNF443	1754294	593	0	1746180	419724	0	5	0	0	6	7	3661460	6.49E-01	1
C1GALT1C	388105	NaN	7	827700	202920	0	2	0	0	50	56	20001504	6.49E-01	1
CCT8	316438	178	79	1430408	390532	0	3	1	0	24	34	10688544	6.49E-01	1
RPS15	2352825	221	38	374868	110004	0	1	1	0	21	20	7617688	6.49E-01	1
TMEM38A	980514	362	41	764688	220008	0	4	1	0	50	85	21686096	6.49E-01	1
C6orf136	2115149	171	41	1198296	415452	0	4	2	0	50	71	23126828	6.49E-01	1
ASCC3	103404	825	2	5806360	1530800	0	17	7	0	45	50	16309784	6.49E-01	1
CRYGS	676664	501	35	469208	114988	0	2	1	0	2	1	779996	6.49E-01	1
KLHL35	587975	205	39	1409048	483448	0	3	0	0	8	10	4402652	6.49E-01	1
KRT40	977588	404	10	1108584	304380	0	5	1	0	50	68	14276668	6.49E-01	1
PRCC	1649536	208	53	1221792	380208	0	4	0	0	15	17	6672508	6.49E-01	1
OR2D3	403341	827	6	814172	245284	0	4	1	0	23	38	8913172	6.50E-01	1
RNASE4	709113	577	11	373444	105020	0	1	0	0	50	80	24629148	6.50E-01	1
SYNM	271694	361	-3	3844088	1185124	0	7	0	0	6	4	4005712	6.50E-01	1
EVX2	242935	874	15	1135284	399432	0	3	0	0	8	6	2472776	6.50E-01	1
KCNQ4	428440	188	32	1739416	548240	0	3	2	0	10	9	3535080	6.50E-01	1
FAM78A	745091	213	50	709864	204344	0	2	1	0	50	53	18357496	6.50E-01	1
DMXL2	380843	422	11	7805300	2107876	0	33	8	0	50	80	26239336	6.50E-01	1
ZNF154	680410	849	-50	1123892	288004	0	3	2	0	50	69	18021076	6.50E-01	1
HBE1	109545	1056	-1	376292	106444	0	1	0	0	50	50	17996156	6.50E-01	1
MCAM	1087523	214	48	1662164	477040	0	7	5	0	8	11	3051276	6.50E-01	1
GPATCH3	1025094	188	34	1317556	395516	0	4	1	0	50	62	18463584	6.50E-01	1
ZNF841	NaN	NaN	NaN	2378080	602708	0	6	1	0	50	117	40248292	6.50E-01	1
PTAR1	288169	640	-11	1040944	282308	0	2	1	0	50	44	23027148	6.50E-01	1
NHLH2	503800	639	9	331792	108224	0	1	0	0	50	64	20102608	6.51E-01	1
EPHB6	284406	645	18	2539704	802068	0	9	2	0	46	48	19769392	6.51E-01	1
ELF1	291800	476	39	1563552	452832	0	2	3	0	26	26	8817408	6.51E-01	1
ZNF527	228816	939	-63	1602356	369172	0	4	0	0	27	22	9756892	6.51E-01	1
UROD	939872	310	20	936636	280884	0	3	1	0	50	62	19779716	6.51E-01	1
NPDC1	1951939	202	40	805272	273408	0	3	1	0	50	61	21392396	6.51E-01	1
TPD52L3	355544	469	16	415452	123888	0	1	0	0	50	45	18541548	6.51E-01	1
RPL10L	10737	1057	-48	541120	152012	0	2	2	0	4	5	1223928	6.51E-01	1

APLF	227462	617	22	1341764	337132	0	4	1	0	7	8	4014612	6.51E-01	1
CHUK	1070291	198	25	1987548	490212	0	6	1	0	50	78	19641944	6.51E-01	1
MTF1	445066	303	33	1901396	557140	0	6	0	0	10	6	3345688	6.51E-01	1
)N1-GTF2/	75084	934	-3	3017456	817732	0	7	1	0	9	16	4807424	6.51E-01	1
WDR76	792287	375	34	1620512	439660	0	5	1	0	50	62	24020032	6.52E-01	1
DYRK1B	1289328	526	23	1559992	500180	0	4	0	0	17	31	9835924	6.52E-01	1
ANKDD1A	705779	185	57	1348528	389108	0	4	2	0	50	75	20194100	6.52E-01	1
PAX4	411207	619	-7	856180	280172	0	4	2	0	7	7	2091856	6.52E-01	1
OPN5	173532	829	-4	905308	259880	0	3	1	0	50	61	18813888	6.52E-01	1
UPK2	1457410	180	55	452476	159488	0	1	0	0	50	46	18727024	6.52E-01	1
ZNF451	372718	348	26	2794956	667500	0	9	3	0	4	5	2547536	6.52E-01	1
CMTM6	436595	592	31	463868	138484	0	1	0	0	50	59	24824236	6.52E-01	1
FUT3	1217326	294	35	890000	274120	0	3	1	0	50	56	18189820	6.52E-01	1
MFAP5	297035	320	19	478464	117480	0	1	0	0	50	52	22957016	6.52E-01	1
ZNF589	1083690	193	38	929516	254896	0	3	1	0	50	49	17416588	6.52E-01	1
OR5M3	NaN	NaN	NaN	768248	216448	0	2	3	0	8	19	4142416	6.52E-01	1
SRPK2	452775	468	34	1877188	474548	0	6	2	0	50	41	18391672	6.52E-01	1
TSPYL4	260690	640	16	1023144	308652	0	3	1	0	20	27	9815632	6.52E-01	1
GHITM	216194	860	-26	881100	261660	0	4	2	0	50	77	19237528	6.52E-01	1
SOX6	126866	989	-18	2228560	587756	0	8	2	0	35	52	15092620	6.52E-01	1
TRIM39	2043435	430	32	1311148	381632	0	4	1	0	50	49	19085516	6.52E-01	1
CDK6	697669	464	26	834820	238520	0	4	3	0	9	12	3424720	6.52E-01	1
SMYD1	175471	543	6	1291924	321824	0	4	1	0	48	61	22357512	6.52E-01	1
LY75	325669	405	36	4572820	1095056	0	13	2	0	21	20	8167708	6.52E-01	1
ZNF572	550341	596	20	1391604	315060	0	7	1	0	7	10	2823080	6.52E-01	1
CD27	2702142	198	50	673552	184052	0	4	1	0	4	24	5194396	6.52E-01	1
SETD4	219580	318	66	1175512	313280	0	5	0	0	50	70	20787908	6.52E-01	1
DIRAS1	1354357	291	25	498756	143112	0	1	1	0	50	54	21517708	6.52E-01	1
AGPAT5	130932	475	32	949808	249556	0	1	0	0	50	67	24458624	6.53E-01	1
IL6R	1724679	319	39	1193312	347812	0	4	0	0	10	18	6483116	6.53E-01	1
ACOXL	NaN	NaN	NaN	1516204	417944	0	3	1	0	50	117	40063528	6.53E-01	1
CREB3	922448	190	48	951944	274120	0	6	4	0	4	8	1514068	6.53E-01	1
FIBCD1	764789	241	29	1149168	360984	0	3	2	0	50	80	24081976	6.53E-01	1

KCNS3	143212	1197	6	1238524	342116	0	4	1	0	50	58	19093704	6.53E-01	1
MRPS10	524386	273	43	537204	137772	0	1	0	0	50	56	22267444	6.53E-01	1
PMF1	2280435	211	36	534356	144892	0	1	1	0	4	1	1493420	6.53E-01	1
OSTN	124914	878	7	333216	102528	0	1	0	0	50	64	20044224	6.53E-01	1
MEFV	1445318	253	37	1961560	584552	0	8	4	0	5	6	2317916	6.54E-01	1
ABT1	411992	498	24	677112	208260	0	2	1	0	1	2	1415456	6.54E-01	1
CHRM2	72122	1001	-25	1166256	334284	0	4	2	0	50	106	25519148	6.54E-01	1
WNK3	352631	NaN	25	4595604	1268784	0	10	0	0	0	0	1268784	6.54E-01	1
SNCA	65544	475	-7	372732	100392	0	1	0	0	50	60	20713860	6.54E-01	1
SFTPA2	478276	NaN	11	626916	187968	0	2	1	0	50	83	24692872	6.54E-01	1
MTMR9	499514	671	36	1423644	377004	0	2	0	0	15	14	6600240	6.54E-01	1
MEF2D	2141203	201	41	1309012	406196	0	2	2	0	50	61	21808916	6.54E-01	1
HERPUD2	189678	636	4	1053048	280884	0	3	1	0	6	2	1680320	6.54E-01	1
GP2	243266	848	-5	1393384	377360	0	4	1	0	50	54	19846644	6.54E-01	1
PMCH	229712	552	12	432896	111784	0	1	0	0	50	54	22837756	6.54E-01	1
OR1A1	506514	628	0	760060	231044	0	3	2	0	13	18	5228216	6.54E-01	1
NRN1	49114	693	17	364900	101816	0	1	1	0	50	57	19565760	6.54E-01	1
PADI3	490967	168	38	1719124	477752	0	4	5	0	38	35	11720944	6.54E-01	1
SPATA18	137116	878	9	1401572	376648	0	5	1	0	50	63	21726324	6.54E-01	1
TSPAN13	360860	385	-12	524744	153436	0	1	0	0	50	41	21229704	6.55E-01	1
SLC24A2	493733	635	-12	1689220	472412	0	5	2	0	50	34	15038508	6.55E-01	1
EVX1	473864	394	13	977932	337844	0	3	1	0	50	80	25624168	6.55E-01	1
SLC10A4	310162	396	52	1055540	356356	0	2	0	0	50	60	20167400	6.55E-01	1
RDX	137601	158	10	1558924	367748	0	4	0	0	50	52	21970540	6.55E-01	1
LSM14A	655485	284	-13	1216808	340336	0	3	2	0	50	72	19162768	6.55E-01	1
SNX25	295066	528	30	2230340	541120	0	5	1	0	28	28	12727356	6.55E-01	1
CYP1A1	746043	199	41	1293348	375936	0	3	1	0	18	22	7453928	6.55E-01	1
PDE4B	103928	596	-8	2229272	578500	0	5	2	0	50	53	19697124	6.55E-01	1
CHRDL2	521541	204	40	1173376	323960	0	4	1	0	50	47	14958052	6.55E-01	1
SVOP	574926	NaN	32	782132	229264	0	2	1	0	48	63	24015404	6.55E-01	1
OR11H12	141957	747	-11	808832	236740	0	3	1	0	5	7	1889648	6.55E-01	1
RAP1A	348519	394	29	498756	123888	0	1	0	0	50	57	25593552	6.55E-01	1
ZFR	386475	357	27	2744048	783556	0	4	0	0	12	6	6765780	6.55E-01	1

KRBA1	471461	615	29	2533296	833040	0	4	3	0	32	36	14158832	6.55E-01	1
ATP13A3	324325	269	27	3221088	842652	0	11	3	0	19	32	9495944	6.55E-01	1
CCDC138	366787	474	8	1760776	435032	0	4	1	0	23	24	9242116	6.56E-01	1
PATE2	607531	493	3	310432	69776	0	1	1	0	6	7	2338564	6.56E-01	1
FGF14	82526	937	-2	823072	215024	0	3	2	0	50	75	20368896	6.56E-01	1
NMI	215149	617	17	820224	196512	0	1	1	0	18	13	4237112	6.56E-01	1
PCDHGA1(	NaN	NaN	NaN	2387692	749024	0	9	3	0	50	119	40394608	6.56E-01	1
FCHSD2	475608	318	46	1956576	498756	0	3	0	0	27	25	12460356	6.56E-01	1
NAV3	23591	1077	-25	6021028	1715564	0	25	7	0	23	62	15398424	6.56E-01	1
SLC5A3	823351	574	88	1776084	527592	0	6	2	0	21	19	7765784	6.56E-01	1
OR5M11	NaN	NaN	NaN	751516	227840	0	2	0	0	50	116	39873424	6.56E-01	1
PPP2R2C	480192	540	50	1233184	315416	0	4	3	0	26	26	9280208	6.56E-01	1
CYTH3	NaN	NaN	NaN	1075120	257744	0	2	2	0	26	42	11834864	6.56E-01	1
UGT2B10	99797	764	14	2700972	708084	0	4	1	0	50	47	20739492	6.56E-01	1
C11orf30	268174	391	43	3320412	1003920	0	12	3	0	16	21	6360652	6.56E-01	1
SLC5A4	498785	580	-34	1693136	485584	0	5	3	0	50	51	16560764	6.56E-01	1
CHCHD10	637355	196	52	354932	118192	0	1	0	0	50	63	20521620	6.57E-01	1
GH2	1446139	380	27	881100	202920	0	3	0	0	50	63	18745892	6.57E-01	1
PAMR1	646177	410	0	1896412	517268	0	3	2	0	50	39	17770096	6.57E-01	1
MARVELD	490595	314	41	1422576	394092	0	3	1	0	50	43	18722040	6.57E-01	1
GRM4	1013590	629	35	2276620	687080	0	7	5	0	48	57	16902168	6.57E-01	1
DHODH	438866	372	19	984696	320400	0	1	0	0	50	58	21008628	6.57E-01	1
HARBI1	914919	176	39	873268	252404	0	3	0	0	50	74	21923192	6.57E-01	1
HBB	109545	1056	-1	370596	114276	0	1	0	0	50	50	17996156	6.57E-01	1
USP38	182738	678	3	2626568	753652	0	7	1	0	20	20	8266320	6.57E-01	1
SMYD3	219137	939	-6	1132436	289072	0	1	0	0	50	46	18302316	6.57E-01	1
IFITM5	1463610	437	24	320756	111784	0	1	0	0	50	72	18233964	6.57E-01	1
OR5R1	NaN	NaN	NaN	801356	237808	0	3	2	0	26	42	11814928	6.57E-01	1
STK10	329023	272	49	2521904	659668	0	5	2	0	50	41	18569672	6.58E-01	1
AMT	1585328	201	32	1040588	317908	0	3	1	0	14	11	4719136	6.58E-01	1
ZSCAN5A	486579	522	-32	1257748	347456	0	3	1	0	50	64	16936344	6.58E-01	1
FER1L6	453048	561	15	4853348	1270564	0	14	6	0	50	59	19966616	6.58E-01	1
ICAM1	1729517	200	31	1301180	432184	0	6	1	0	50	94	24020032	6.58E-01	1

LILRA1	978578	625	-1	1233896	374512	0	5	1	0	50	108	22158508	6.58E-01	1
PHAX	235652	347	15	1025636	259168	0	3	1	0	50	55	18930300	6.58E-01	1
MMP16	27285	939	-34	1705240	445712	0	10	0	0	3	4	1146676	6.58E-01	1
SLC35A3	287540	530	9	831616	242792	0	2	0	0	50	55	22041028	6.58E-01	1
P2RY10	14218	NaN	50	858672	234960	0	2	1	0	50	43	18270276	6.58E-01	1
CC2D2A	163271	420	27	4421876	1140268	0	9	2	0	9	7	4304752	6.58E-01	1
RNASE13	937278	320	20	399432	107868	0	1	0	0	50	63	19474980	6.58E-01	1
UBE2Q1	1896659	403	41	1108584	300108	0	2	0	0	50	61	17758348	6.58E-01	1
IKBKE	504324	300	4	1855116	527592	0	4	2	0	50	73	25409144	6.58E-01	1
C1orf189	2028928	185	35	280884	63012	0	1	0	0	50	68	16958060	6.58E-01	1
ACADVL	1782958	316	21	1688508	496620	0	3	1	0	39	39	14081580	6.58E-01	1
CA12	449769	275	47	927024	253116	0	2	0	0	50	57	22268868	6.58E-01	1
PEX12	756039	227	14	892848	269136	0	5	3	0	9	13	3221088	6.58E-01	1
KRTAP13-4	14979	1035	-68	396940	116768	0	2	0	0	50	77	14168444	6.58E-01	1
EIF5AL1	342919	284	39	389464	109292	0	1	0	0	50	47	17143536	6.59E-01	1
FAIM	198181	515	26	607336	148808	0	1	0	0	50	39	20230768	6.59E-01	1
POLR2H	1447013	268	44	401924	101104	0	1	0	0	50	77	28073092	6.59E-01	1
APOL3	1228837	331	13	993596	308296	0	1	0	0	10	14	5496284	6.59E-01	1
RPL28	1038859	194	22	702388	203276	0	1	0	0	17	8	4536152	6.59E-01	1
OR3A1	506514	628	0	761484	250980	0	1	2	0	13	18	5228216	6.59E-01	1
CST2	159399	889	-45	364188	101460	0	1	0	0	50	66	16827052	6.59E-01	1
RASGRP3	124045	680	9	1811328	466716	0	5	1	0	50	74	26184868	6.59E-01	1
NFATC4	897133	265	40	2388760	816308	0	6	0	0	1	0	995020	6.59E-01	1
CPSF6	758784	353	3	1373804	433252	0	3	1	0	50	62	19068072	6.59E-01	1
MYD88	302137	411	32	817732	218228	0	2	0	0	50	54	20798588	6.59E-01	1
CIZ1	1762678	201	44	2297624	651124	0	6	0	0	3	2	1485588	6.59E-01	1
LPIN2	583114	425	44	2335360	619796	0	7	1	0	19	23	8192984	6.59E-01	1
SEC11A	341258	214	52	470276	127804	0	1	0	0	50	46	19417664	6.59E-01	1
RGS20	266629	345	18	1037384	308296	0	2	0	0	44	43	19227560	6.60E-01	1
HDDC2	155442	373	-19	537560	140620	0	1	0	0	50	54	21922836	6.60E-01	1
ABCC9	529911	984	-17	4139568	1136352	0	17	5	0	50	63	17526236	6.60E-01	1
ARHGDIA	3798594	211	19	538628	139552	0	1	0	0	34	28	11428668	6.60E-01	1
ZUFSP	247712	479	10	1524392	369172	0	3	2	0	50	50	20262808	6.60E-01	1

PIGG	562684	624	19	2446076	757924	0	7	3	0	38	51	14207960	6.60E-01	1
SLC38A11	80659	427	-3	989324	282664	0	1	1	0	50	59	20497056	6.60E-01	1
CASK	531097	NaN	45	2456756	626560	0	7	1	0	19	31	11548996	6.60E-01	1
HDAC9	65395	684	-6	2883956	792100	0	7	4	0	48	63	22503472	6.60E-01	1
DNLZ	1194160	258	28	433964	150232	0	1	0	0	50	42	16832036	6.60E-01	1
PRPH	2154742	171	49	1188684	356712	0	4	2	0	50	57	20003284	6.60E-01	1
GOT1L1	660779	379	37	974372	261304	0	2	0	0	40	53	23201232	6.60E-01	1
TRPM3	135903	707	-9	4499484	1204704	0	17	1	0	1	2	1478824	6.60E-01	1
CLNK	214087	1111	17	1177648	273764	0	2	0	0	50	31	18582844	6.60E-01	1
ARMCX6	329999	NaN	8	756856	207548	0	2	0	0	50	59	21254980	6.60E-01	1
C19orf43	1959466	226	29	440372	137416	0	1	0	0	50	74	23429072	6.60E-01	1
ASCC2	849790	182	33	1981852	527948	0	4	2	0	50	83	25009356	6.60E-01	1
NUP205	521421	326	14	5206856	1424356	0	21	7	0	39	53	15819216	6.60E-01	1
CPNE2	745404	154	48	1436816	386260	0	5	0	0	8	5	2176228	6.60E-01	1
GLYCTK	1113485	229	49	1301536	394448	0	4	2	0	50	60	20776872	6.61E-01	1
EFNB2	167697	1267	2	856536	232824	0	4	2	0	4	5	1691356	6.61E-01	1
DNAJA3	753259	148	40	1263444	353508	0	3	0	0	27	23	10410508	6.61E-01	1
TTC39A	526044	281	10	1587404	401212	0	5	1	0	46	69	25278136	6.61E-01	1
GATM	465759	445	4	1098616	294056	0	3	0	0	50	40	18428696	6.61E-01	1
WT1	356326	749	9	1324676	373444	0	3	2	0	50	75	22939928	6.61E-01	1
NCR1	840563	573	-15	786404	218584	0	2	0	0	50	100	21516284	6.61E-01	1
MGAT3	812764	467	76	1300824	413316	0	3	1	0	19	24	9501640	6.61E-01	1
RPS4X	374992	NaN	53	683876	191884	0	4	2	0	27	28	5668232	6.61E-01	1
RASSF2	467742	485	32	855112	235316	0	3	1	0	50	61	20205136	6.61E-01	1
RAMP3	1003768	291	50	377360	110716	0	1	0	0	50	66	19072700	6.61E-01	1
ANKRD53	380551	561	27	1435392	427200	0	3	0	0	28	28	13715256	6.61E-01	1
HIST1H2BI	228884	634	40	312568	96476	0	2	2	0	13	15	2405492	6.62E-01	1
POLR2B	570731	443	33	3074416	797084	0	9	1	0	50	47	20860532	6.62E-01	1
KRTAP5-7	586193	626	50	417944	116056	0	1	0	0	41	39	13806036	6.62E-01	1
OR5AR1	NaN	NaN	NaN	770740	223568	0	2	0	0	50	116	39869152	6.62E-01	1
PEX26	516479	239	27	761128	240656	0	2	2	0	28	40	9045248	6.62E-01	1
PALB2	544239	561	38	3026712	827700	0	14	0	0	0	0	827700	6.62E-01	1
ZNF90	NaN	NaN	NaN	1561772	379852	0	5	0	0	50	116	40025436	6.62E-01	1

ZCCHC24	334525	284	46	615880	172304	0	1	0	0	50	53	19904672	6.62E-01	1
BAG2	314024	348	23	543968	143824	0	1	0	0	50	46	22560076	6.62E-01	1
RPL10A	683460	280	36	573872	150232	0	3	1	0	15	26	5361716	6.62E-01	1
FGF3	783792	561	37	579212	198292	0	1	0	0	50	41	18513780	6.62E-01	1
CD37	2183690	309	30	725884	207548	0	2	2	0	4	6	1626920	6.62E-01	1
PRAMEF14	NaN	NaN	NaN	2227848	636528	0	6	4	0	50	120	40282112	6.62E-01	1
ZNF679	NaN	NaN	NaN	1080104	259168	0	3	0	0	50	116	39904752	6.62E-01	1
NRBP2	2376841	247	22	1323608	357424	0	3	0	0	50	67	29170640	6.63E-01	1
TMEM200C	269994	959	-10	1471348	525812	0	4	0	0	3	3	2312220	6.63E-01	1
MBOAT2	438336	469	25	1363480	357068	0	4	0	0	18	20	8243892	6.63E-01	1
DPRX	737115	684	-48	493772	134212	0	2	0	0	50	77	17808544	6.63E-01	1
OR2H2	403531	798	18	757212	249912	0	1	1	0	50	36	17308008	6.63E-01	1
DOK1	1019591	315	50	1188328	375224	0	3	2	0	40	63	17658668	6.63E-01	1
CREG2	475096	278	19	725884	221432	0	1	2	0	5	7	2417240	6.63E-01	1
NSUN4	597667	255	28	963692	291208	0	1	0	0	5	5	2618024	6.63E-01	1
FCRL3	180228	829	21	1873984	540764	0	5	3	0	44	38	17330080	6.63E-01	1
FOXD4L1	528113	595	15	971524	338912	0	3	1	0	22	35	8797472	6.63E-01	1
ZNF254	384073	1381	-62	1713784	413672	0	7	1	0	22	49	10308692	6.63E-01	1
KCNMB2	144490	882	-8	610540	162692	0	1	0	0	50	85	30349356	6.63E-01	1
OR8S1	1494035	421	25	875404	281240	0	2	1	0	50	75	18513780	6.63E-01	1
TTR	294098	541	20	371308	115700	0	1	1	0	12	9	3640812	6.63E-01	1
AKAP8	494792	481	28	1797088	480956	0	3	0	0	8	5	3331092	6.63E-01	1
DPPA5	732413	446	18	302956	82592	0	1	1	0	12	13	3339992	6.64E-01	1
SEPN1	746382	372	24	1504456	442508	0	5	0	0	9	10	4139924	6.64E-01	1
USP49	429516	341	40	1584556	486296	0	4	1	0	50	45	22966628	6.64E-01	1
EPRS	630371	608	-8	3958364	1021720	0	10	2	0	26	31	11972280	6.64E-01	1
SPEF2	140884	598	-5	4813476	1203636	0	15	4	0	41	45	18862304	6.64E-01	1
PPM1E	469298	448	11	1913856	534000	0	5	3	0	50	70	24450436	6.64E-01	1
POMGNT1	908945	190	29	1728024	479532	0	7	1	0	50	82	25648732	6.64E-01	1
C5orf24	877491	696	39	471344	138484	0	1	0	0	50	61	24328328	6.64E-01	1
EBF3	85621	947	-28	1442868	391956	0	5	0	0	7	11	3369540	6.64E-01	1
POLR2J3	NaN	NaN	NaN	306516	84372	0	1	1	0	26	41	11661492	6.64E-01	1
OXGR1	111002	623	-10	843008	244216	0	2	1	0	37	34	14099024	6.64E-01	1

GPLD1	399209	465	7	2225000	615880	0	7	1	0	25	24	9720580	6.64E-01	1
YWHAB	952219	282	32	652192	160556	0	2	0	0	50	67	20264944	6.65E-01	1
PPP1R2	762316	331	42	555004	126380	0	1	0	0	50	48	21214752	6.65E-01	1
MDM1	354120	575	16	1993244	533644	0	8	2	0	8	22	6123200	6.65E-01	1
FGFR4	1070874	201	46	2060172	660024	0	6	2	0	50	65	22893292	6.65E-01	1
OR1L6	433740	487	10	766112	231400	0	2	2	0	48	44	16675396	6.65E-01	1
STRADB	468064	491	26	1096836	292632	0	1	1	0	48	56	21587840	6.65E-01	1
CCR7	1130946	203	25	946960	278036	0	2	2	0	30	44	12265624	6.65E-01	1
NAA25	771475	200	42	2566760	651124	0	11	1	0	1	2	1007124	6.65E-01	1
OR5K3	416592	885	-4	808120	222500	0	3	2	0	50	71	20173096	6.65E-01	1
GPATCH1	420460	212	-7	2415816	651480	0	7	1	0	35	43	14923520	6.65E-01	1
TAGLN	426428	228	46	528660	135636	0	1	1	0	50	39	17316908	6.65E-01	1
NUDT3	983693	233	31	452832	118548	0	1	0	0	50	47	19037456	6.65E-01	1
TRAK1	313554	321	29	2727672	803136	0	8	2	0	28	37	11120728	6.65E-01	1
BCL2A1	234803	702	28	504096	128160	0	1	0	0	50	49	21774384	6.65E-01	1
PPP2R1B	436031	242	25	1735144	495908	0	5	4	0	38	45	13909988	6.65E-01	1
TERF1	155308	637	20	1167680	282664	0	3	0	0	50	51	20402004	6.65E-01	1
BAZ1A	615028	184	24	4060180	1039520	0	9	2	0	20	22	9450020	6.65E-01	1
EIF1B	195546	675	10	303312	74760	0	1	1	0	22	40	9855148	6.65E-01	1
IIST1H2BM	267617	634	32	314348	94696	0	3	2	0	12	20	2202928	6.66E-01	1
SSX7	250163	NaN	25	520828	114632	0	1	0	0	50	72	24232920	6.66E-01	1
CTBP2	283236	508	20	2453196	807408	0	5	2	0	50	58	22733092	6.66E-01	1
HIST1H4F	390169	544	57	249912	85440	0	1	0	0	50	61	13862640	6.66E-01	1
RARB	216662	812	-14	1160560	307940	0	5	1	0	50	80	20997236	6.66E-01	1
SPACA1	176962	684	32	761128	209684	0	2	1	0	29	42	13500944	6.66E-01	1
SH3YL1	304765	624	-18	891780	248844	0	2	1	0	50	45	17740904	6.66E-01	1
CXorf65	759088	NaN	27	492348	120684	0	1	0	0	50	74	25911460	6.66E-01	1
NMT2	236087	620	23	1311504	336420	0	4	0	0	15	13	5818108	6.66E-01	1
NIT1	689033	250	23	975440	299752	0	2	1	0	50	68	19070564	6.66E-01	1
PYGO2	1612870	224	35	987544	325028	0	3	1	0	50	56	18488504	6.66E-01	1
INSL5	335045	692	-16	348880	93272	0	1	1	0	50	58	17879032	6.66E-01	1
GRIK4	298412	678	31	2444652	698472	0	7	3	0	24	21	9953760	6.66E-01	1
CALCA	344274	627	-9	501248	143824	0	1	0	0	50	38	17139264	6.66E-01	1

WDR36	205349	738	6	2471708	672484	0	13	0	0	0	0	672484	6.67E-01	1
QRFPR	268761	809	-2	1102532	302956	0	3	1	0	50	50	19637316	6.67E-01	1
CHRNA9	442913	347	53	1226064	331080	0	2	2	0	50	52	18993668	6.67E-01	1
OR2L8	27938	1015	-28	771808	231044	0	4	1	0	50	127	24860904	6.67E-01	1
TRPS1	55597	711	-10	3274844	895696	0	14	4	0	17	46	14581404	6.67E-01	1
C7orf43	1299015	205	52	1429340	474904	0	4	2	0	35	32	12810304	6.67E-01	1
IGDCC4	642731	280	45	3094352	995020	0	6	1	0	3	2	2328952	6.67E-01	1
OR51G2	65798	1006	-15	765044	244216	0	5	3	0	7	10	2021012	6.67E-01	1
IFNAR2	861800	317	87	1479180	383412	0	2	0	0	17	15	7867956	6.67E-01	1
GLUL	349818	783	5	968320	255608	0	3	1	0	20	31	10297300	6.67E-01	1
ARHGEF18	613770	308	30	2964412	878252	0	7	1	0	50	52	21930312	6.67E-01	1
SLC38A6	220020	527	-2	1447852	383768	0	2	1	0	35	35	13922448	6.68E-01	1
EIF3J	667670	534	30	697760	161980	0	2	0	0	50	70	23140000	6.68E-01	1
GPR55	1020678	519	45	796016	229264	0	2	1	0	50	73	27243968	6.68E-01	1
TAT	446645	530	10	1181208	323604	0	2	1	0	50	64	19453620	6.68E-01	1
CCNYL1	330620	210	26	951588	240300	0	2	0	0	50	51	23774392	6.68E-01	1
ATP1A1	616485	464	18	2652200	737632	0	9	1	0	19	20	8006440	6.68E-01	1
KRTAP4-4	996799	468	-7	430760	106444	0	1	1	0	50	60	17141400	6.68E-01	1
TMEM150C	712807	493	17	654684	176220	0	2	1	0	50	53	16909288	6.68E-01	1
OR2K2	348469	501	19	775724	238876	0	2	0	0	31	45	15250684	6.68E-01	1
WDR34	1891397	197	41	1335356	423640	0	2	2	0	50	54	22151388	6.68E-01	1
IL15	74660	679	5	446068	101816	0	1	0	0	50	62	23746980	6.68E-01	1
ZNF461	627070	922	-29	1489860	338556	0	5	2	0	50	70	18261376	6.68E-01	1
CARD11	635400	558	24	2982924	815952	0	8	3	0	50	65	21272780	6.68E-01	1
OR8G5	154391	921	-14	860452	250268	0	3	0	0	50	57	19362484	6.68E-01	1
RCSD1	520615	405	14	1051268	310432	0	3	0	0	7	11	3336076	6.69E-01	1
ZC3H14	169650	686	-1	2259888	583128	0	9	1	0	27	32	12385952	6.69E-01	1
NUDT21	504003	442	5	597012	159132	0	1	0	0	50	53	21046364	6.69E-01	1
PSMD3	1652518	216	28	1369176	391956	0	5	2	0	50	67	22696068	6.69E-01	1
NIPAL1	225089	474	38	1037028	303312	0	2	0	0	43	74	19036032	6.69E-01	1
PGBD5	286328	478	10	1171596	317196	0	3	1	0	50	48	22392044	6.69E-01	1
P4HA1	539936	354	44	1475976	378072	0	4	0	0	9	9	3435756	6.69E-01	1
HS2ST1	332598	515	0	938060	233536	0	3	1	0	50	74	21718136	6.69E-01	1

ATG4D	2036812	226	23	1195448	367036	0	7	3	0	24	50	10294452	6.69E-01	1
ARSI	954044	220	46	1393028	437524	0	2	2	0	11	10	4811340	6.69E-01	1
OR9G4	384896	828	-34	803848	245996	0	3	0	0	50	70	19076260	6.69E-01	1
ATF7IP	339265	882	12	3191540	940552	0	12	2	0	20	25	7326836	6.69E-01	1
FOXF1	70370	416	3	931296	292632	0	2	1	0	50	39	17333284	6.69E-01	1
EFNA5	78424	628	21	596656	154148	0	1	0	0	50	47	21193036	6.69E-01	1
CDC25A	1116598	151	36	1375940	365968	0	4	1	0	50	69	23782580	6.70E-01	1
GYG1	356219	442	8	997156	219296	0	3	2	0	18	24	5782864	6.70E-01	1
PLAGL2	1071799	262	51	1219656	377004	0	5	0	0	5	3	1539344	6.70E-01	1
KLHDC7B	1075525	207	42	1397656	508724	0	4	2	0	42	47	16742324	6.70E-01	1
RECQL5	1912060	198	37	2596664	751516	0	9	1	0	30	41	14104364	6.70E-01	1
FN3K	783773	243	18	789964	226772	0	2	1	0	50	50	19020012	6.70E-01	1
TLR10	304012	437	42	2060528	545392	0	9	1	0	50	77	23183788	6.70E-01	1
EDEM1	260923	447	14	1674624	480600	0	4	2	0	50	53	20200864	6.70E-01	1
LMLN	607598	410	30	1808480	482380	0	6	1	0	50	49	18579640	6.70E-01	1
DCDC2B	1460758	218	29	880744	274832	0	2	1	0	50	52	21684672	6.70E-01	1
C7orf31	254281	319	21	1537208	394804	0	3	0	0	9	5	4251708	6.70E-01	1
ANGPT2	NaN	NaN	NaN	1315064	311500	0	2	1	0	50	117	39957084	6.70E-01	1
PRM2	632841	308	37	263440	70844	0	1	1	0	9	8	2023504	6.70E-01	1
GTF3C1	282478	597	25	5404792	1511576	0	15	6	0	31	37	16508076	6.70E-01	1
KL	209854	595	23	2533296	731580	0	7	2	0	50	49	21487092	6.70E-01	1
EFNB1	141847	NaN	19	870420	258456	0	2	1	0	50	48	16561476	6.71E-01	1
KRTAP3-2	977588	404	10	244572	74760	0	1	0	0	50	68	14276668	6.71E-01	1
HLA-DQA1	1292851	535	17	651480	187968	0	2	0	0	50	68	20377084	6.71E-01	1
GPR63	130400	1336	-5	1036672	309008	0	2	0	0	50	40	20137852	6.71E-01	1
RANBP1	503106	277	64	539340	129228	0	1	0	0	50	40	17277036	6.71E-01	1
C1orf52	348743	384	3	475260	121752	0	1	0	0	50	52	20151380	6.71E-01	1
C9orf131	782447	350	42	2656828	826988	0	6	3	0	36	48	20387764	6.71E-01	1
OR5D13	4183	1091	-36	781776	226416	0	4	2	0	14	26	3895352	6.71E-01	1
INSL6	351963	629	20	547172	147028	0	1	0	0	50	54	20206204	6.71E-01	1
MPG	1005761	238	36	761128	241724	0	2	0	0	50	46	16624132	6.71E-01	1
CCDC155	2178574	290	37	1457108	427912	0	4	2	0	50	53	21503468	6.71E-01	1
MIA	1127178	201	27	337488	100392	0	1	0	0	50	68	19652268	6.71E-01	1

IGFBPL1	234360	688	16	676400	236740	0	2	0	0	50	56	18543328	6.71E-01	1
TOR1A	600401	187	44	857604	228552	0	2	2	0	13	24	6188704	6.71E-01	1
RING1	1474628	377	24	1003208	326452	0	2	1	0	50	90	23548688	6.72E-01	1
OR56A4	432250	877	-13	893916	276612	0	3	2	0	50	63	18144964	6.72E-01	1
IQCF1	1013177	370	39	526880	150232	0	2	0	0	50	85	19762272	6.72E-01	1
ABHD4	975314	380	49	876116	250624	0	1	0	0	50	81	24089452	6.72E-01	1
KCNB2	110047	904	-11	2278756	651836	0	15	6	0	7	15	3783924	6.72E-01	1
CBWD1	230380	398	-4	1110364	273764	0	2	1	0	50	40	20962704	6.72E-01	1
GNA15	908206	241	21	964048	263084	0	3	1	0	50	56	18551872	6.72E-01	1
CASC1	255116	633	18	2057324	516556	0	7	0	0	2	1	954436	6.72E-01	1
CNGB3	191008	1171	2	2114996	555004	0	6	2	0	50	41	16639796	6.72E-01	1
HSPB7	886121	197	43	424352	132076	0	1	0	0	50	55	20099048	6.72E-01	1
CD300LF	NaN	NaN	NaN	746888	212176	0	2	1	0	50	117	39857760	6.72E-01	1
NDRG4	678092	320	0	1032756	287292	0	3	1	0	50	61	20996880	6.72E-01	1
NOL11	692622	197	7	1887512	494128	0	5	1	0	50	62	21309448	6.72E-01	1
LMBR1	338947	615	6	1290500	351016	0	5	0	0	50	62	19739132	6.72E-01	1
PITPNB	112113	310	-54	753296	167320	0	1	0	0	50	84	21168828	6.72E-01	1
DGKE	320339	324	-38	1474196	392668	0	7	1	0	50	92	24556168	6.72E-01	1
FAM3B	224593	506	24	626916	159132	0	1	0	0	50	51	19918200	6.73E-01	1
CRYZ	103167	1070	-17	852976	238520	0	2	0	0	50	58	23167768	6.73E-01	1
ZMYM1	767910	390	22	2955512	745108	0	9	3	0	50	94	32507784	6.73E-01	1
PRR22	990266	225	26	1018160	347812	0	3	0	0	28	33	11164872	6.73E-01	1
SCG5	244857	383	1	555004	148808	0	1	0	0	50	53	26379956	6.73E-01	1
UBTF	930750	175	41	2054120	482380	0	7	1	0	43	57	18154220	6.73E-01	1
TAS2R30	371136	676	4	795660	229620	0	2	0	0	50	58	20325464	6.73E-01	1
MEP1B	342655	617	11	1841232	469920	0	5	1	0	8	10	3854412	6.73E-01	1
PDCL3	235908	579	9	632612	157708	0	1	0	0	50	47	22759792	6.73E-01	1
ZFP91	284768	644	2	1466008	406196	0	2	0	0	50	42	18244644	6.73E-01	1
CPSF2	337384	415	29	2042016	526524	0	2	1	0	50	56	24697500	6.73E-01	1
IRAK2	744536	224	49	1602712	456392	0	4	1	0	50	61	20524112	6.73E-01	1
PDZRN3	82249	888	-7	2693852	765400	0	8	1	0	39	49	20806776	6.73E-01	1
MBTPS2	93093	NaN	16	1322184	386616	0	2	0	0	45	47	18140336	6.73E-01	1
ZNF585B	248427	984	-56	1983632	496264	0	7	2	0	50	47	18194092	6.73E-01	1

FAM129C	961640	206	42	1830196	593096	0	3	1	0	21	19	8583160	6.73E-01	1
CHST4	443039	600	18	965116	279104	0	4	1	0	50	67	18330440	6.73E-01	1
DENND3	360825	204	51	3087588	847992	0	7	5	0	31	27	11628740	6.73E-01	1
GPRC5A	719556	169	17	889288	270560	0	2	1	0	50	52	20692856	6.73E-01	1
BCL7B	955146	171	48	522608	149164	0	1	0	0	50	59	22598168	6.73E-01	1
VAV3	132139	554	-11	2297980	558920	0	6	1	0	29	37	14181972	6.74E-01	1
MAOA	62832	NaN	13	1383060	368460	0	4	1	0	25	33	12611300	6.74E-01	1
BRD4	557083	381	20	3473848	988256	0	10	4	0	50	56	21965556	6.74E-01	1
CCDC38	396472	400	6	1517628	355644	0	5	1	0	50	64	23039608	6.74E-01	1
SPIN4	90301	NaN	-19	620864	180136	0	2	1	0	50	84	21849144	6.74E-01	1
LMBRD2	174910	599	-4	1839808	462800	0	5	2	0	50	57	22147116	6.74E-01	1
FANCF	52076	970	-4	902816	300820	0	3	1	0	50	84	18756928	6.74E-01	1
C21orf91	113501	686	26	784624	187256	0	2	1	0	50	126	46192068	6.74E-01	1
CD40LG	314961	NaN	37	685300	173372	0	2	0	0	50	62	19178788	6.74E-01	1
OR2A1	NaN	NaN	NaN	750448	243860	0	2	1	0	50	117	39889444	6.74E-01	1
RAB30	307557	285	13	531864	138840	0	1	0	0	50	41	19709228	6.74E-01	1
NUMB	567659	180	41	1647924	483804	0	3	1	0	50	55	20651916	6.74E-01	1
RNASE7	937278	320	20	397296	110004	0	1	0	0	50	63	19474980	6.74E-01	1
OR2W1	155147	738	1	799220	229264	0	4	1	0	4	3	1290500	6.74E-01	1
HMG20B	2160126	269	39	828768	228552	0	3	0	0	50	60	21798948	6.74E-01	1
CRYGN	612907	196	36	483448	119972	0	1	0	0	50	52	21833836	6.74E-01	1
KRTAP3-1	977588	404	10	247064	72268	0	1	0	0	50	68	14276668	6.74E-01	1
FAM63B	524797	311	28	1585624	441440	0	4	0	0	13	16	5646872	6.74E-01	1
MMADHC	102377	521	1	775724	205768	0	2	0	0	50	55	21361424	6.74E-01	1
LIX1	382123	614	38	732292	195800	0	2	0	0	50	67	23046728	6.74E-01	1
GRB10	185646	602	-4	1561060	411536	0	4	1	0	50	50	20416244	6.75E-01	1
DSG3	275713	450	-18	2522972	745108	0	6	1	0	27	25	8036700	6.75E-01	1
MVD	802673	189	41	1009616	315772	0	3	1	0	50	65	20750172	6.75E-01	1
LRRC4C	4203	1185	-34	1584200	473836	0	7	2	0	16	25	5675352	6.75E-01	1
DUSP18	723012	330	56	475972	133856	0	1	0	0	50	37	15331496	6.75E-01	1
SMARCC2	2878044	171	40	3207560	904240	0	15	2	0	4	7	2028132	6.75E-01	1
DHRS11	468055	548	23	665008	196868	0	2	1	0	50	60	18803564	6.75E-01	1
UGT3A2	173490	614	-9	1340340	364188	0	6	2	0	6	8	2537924	6.75E-01	1

SPOCK1	216344	646	26	1161272	289072	0	3	0	0	46	45	17127160	6.75E-01	1
OSBP2	752472	303	37	2315780	677824	0	5	2	0	5	9	4714864	6.75E-01	1
AKNAD1	801073	236	8	2185840	556784	0	11	4	0	50	68	20779720	6.75E-01	1
STIM2	116073	934	37	1987904	528304	0	3	2	0	50	66	21682180	6.75E-01	1
VGLL4	186722	488	35	829480	237452	0	3	0	0	3	7	1809548	6.75E-01	1
TXN2	1199592	195	21	419724	126024	0	1	0	0	50	59	21111868	6.75E-01	1
MMP13	374035	482	-10	1239236	313636	0	3	1	0	50	62	25006508	6.75E-01	1
ARMC4	125789	454	-4	2694208	735140	0	7	3	0	50	62	22808208	6.75E-01	1
CTPS2	295447	NaN	30	1557500	400144	0	2	1	0	50	57	22686812	6.75E-01	1
MRPL54	2144458	206	34	354576	101460	0	1	0	0	50	63	19224000	6.75E-01	1
NPFFR1	552278	225	44	1051980	342828	0	2	0	0	50	43	19472844	6.75E-01	1
TEF	1407073	187	64	816308	246352	0	2	1	0	50	53	18994380	6.75E-01	1
ISG20L2	1851672	180	37	886084	256676	0	3	0	0	50	84	23475352	6.75E-01	1
MDH1B	206970	816	1	1356716	355288	0	5	1	0	50	64	21632340	6.75E-01	1
RET	454068	563	-19	2839100	846568	0	9	1	0	21	24	8752616	6.75E-01	1
MS4A12	333232	899	13	686368	197936	0	2	0	0	50	69	19779360	6.75E-01	1
PPP4C	NaN	NaN	NaN	804560	216448	0	2	1	0	50	117	39862032	6.75E-01	1
POTEH	66885	909	-64	1444292	348880	0	5	2	0	50	75	22229352	6.75E-01	1
AADACL2	91028	611	-5	1025636	279460	0	2	0	0	27	26	13265272	6.76E-01	1
PLA2G12E	587592	291	43	508012	134924	0	1	0	0	50	52	22573248	6.76E-01	1
POLK	253484	500	54	2295132	555360	0	10	2	0	25	32	10469960	6.76E-01	1
LRRC43	808864	187	12	1645788	510504	0	6	1	0	50	65	20660104	6.76E-01	1
SLC16A6	764688	237	11	1315776	384480	0	4	2	0	50	65	21730952	6.76E-01	1
NUDT4	416655	419	18	471700	128516	0	1	0	0	50	48	19395236	6.76E-01	1
ASTE1	313779	586	19	1736924	458884	0	3	0	0	48	41	27620616	6.76E-01	1
CBLN2	12878	1030	-35	551088	178356	0	2	1	0	7	9	2527244	6.76E-01	1
XPNPEP3	969480	269	43	1306876	361340	0	2	0	0	11	8	5825940	6.76E-01	1
OVCH2	97859	823	0	1479892	389108	0	2	0	0	50	60	22175240	6.76E-01	1
FBXO36	352105	601	28	488788	131720	0	1	0	0	50	56	24268520	6.76E-01	1
ORM2	206775	425	4	532932	135636	0	1	1	0	22	16	8233568	6.76E-01	1
SLC7A1	222765	467	9	1584200	481312	0	8	4	0	17	23	6093652	6.76E-01	1
VPREB1	433352	612	35	370596	103596	0	1	0	0	50	68	22190548	6.76E-01	1
STUB1	1207790	194	33	794948	206836	0	3	2	0	36	64	16344316	6.76E-01	1

C10orf54	NaN	NaN	NaN	784268	241012	0	2	0	0	50	116	39886596	6.77E-01	1
YTHDF3	137401	619	7	1489860	403704	0	2	1	0	50	53	20427280	6.77E-01	1
EPS8L1	1125216	432	-26	1880392	576008	0	4	1	0	32	39	14270616	6.77E-01	1
SRP72	662749	594	27	1778576	453544	0	6	1	0	50	64	23234340	6.77E-01	1
F2RL3	901964	305	31	892848	348168	0	3	2	0	50	87	22448292	6.77E-01	1
SULT1C2	361432	609	-10	880388	206836	0	2	0	0	50	35	14338612	6.77E-01	1
SIGLEC12	360870	583	-4	1546108	473480	0	7	2	0	10	11	3776804	6.77E-01	1
SNRK	153482	399	30	1924180	547172	0	3	2	0	17	19	9931688	6.77E-01	1
SMYD2	465993	505	-11	1159136	280528	0	2	1	0	50	70	23026792	6.77E-01	1
PLCG2	217457	451	36	3359572	833396	0	7	2	0	49	63	22812480	6.77E-01	1
CLCA4	279475	692	-14	2363840	641512	0	10	1	0	16	16	4428284	6.77E-01	1
WNT3	522194	254	23	899968	257744	0	1	1	0	50	54	16440080	6.77E-01	1
MKNK1	608423	266	25	1220012	324316	0	4	1	0	50	58	19047424	6.77E-01	1
TOP2A	1220026	191	23	4061604	992172	0	11	0	0	1	0	1247780	6.78E-01	1
SMAP2	718633	438	37	1123180	295124	0	2	0	0	14	24	7644744	6.78E-01	1
FOXN2	254318	752	23	1108940	296548	0	8	4	0	3	8	1333220	6.78E-01	1
SYT10	93167	905	-41	1340696	363832	0	7	4	0	14	25	3398732	6.78E-01	1
OR1F1	1493009	253	42	765044	236740	0	2	1	0	50	77	26841332	6.78E-01	1
PAQR7	746382	372	24	849060	267000	0	3	2	0	21	28	9671096	6.78E-01	1
WRAP53	2068255	213	31	1393028	414028	0	4	0	0	2	0	803848	6.78E-01	1
ZNF600	536540	1010	-40	1866508	456392	0	13	1	0	2	6	1314352	6.78E-01	1
KRTAP4-7	NaN	NaN	NaN	395872	103952	0	1	0	0	50	116	39749536	6.78E-01	1
SLC7A6OS	819579	281	46	795304	219296	0	2	0	0	50	67	24041036	6.78E-01	1
VPS37D	1197825	214	52	604844	215380	0	1	0	0	50	52	22245372	6.78E-01	1
PURA	830974	290	48	790676	246352	0	3	2	0	14	29	8881844	6.78E-01	1
ZCCHC18	NaN	NaN	NaN	1003564	290852	0	1	0	0	50	116	39936436	6.78E-01	1
CLGN	134135	752	4	1637600	379852	0	5	1	0	50	60	19749100	6.79E-01	1
TXNDC9	386109	630	24	597368	147028	0	1	0	0	50	51	23299132	6.79E-01	1
ME3	346701	316	1	1545040	453188	0	6	0	0	3	1	1096836	6.79E-01	1
PRSS45	298503	495	41	585264	161268	0	2	0	0	50	70	18972664	6.79E-01	1
C2CD2	251773	366	76	1755792	532932	0	7	3	0	27	37	10835216	6.79E-01	1
NIF3L1	635570	201	31	985052	251692	0	4	1	0	50	61	17116836	6.79E-01	1
ANKRD39	822597	392	46	461732	142756	0	1	0	0	50	86	27656572	6.79E-01	1

RNF208	1724821	399	43	624780	214668	0	2	2	0	50	60	16636592	6.79E-01	1
MTHFR	530804	196	39	1682100	469920	0	5	3	0	40	43	12524436	6.79E-01	1
ARAF	775214	NaN	38	1555720	453188	0	2	2	0	50	61	23561148	6.79E-01	1
CCDC42	473874	604	17	832328	208972	0	3	0	0	45	70	18199432	6.79E-01	1
CMIP	334492	428	21	2012824	576008	0	5	2	0	50	50	22328320	6.79E-01	1
HGFAC	547964	412	46	1656468	505164	0	3	2	0	50	60	22528392	6.79E-01	1
NXNL1	899127	211	26	522608	166252	0	1	1	0	50	49	18107584	6.79E-01	1
OR5D16	4214	1074	-46	810256	241724	0	3	0	0	25	38	10956256	6.79E-01	1
ID3	1050775	159	32	296548	96476	0	1	0	0	50	68	18237524	6.80E-01	1
FAM133A	33770	NaN	-11	647920	150944	0	2	0	0	50	124	35207332	6.80E-01	1
NTRK1	1505782	208	45	2192604	653616	0	5	3	0	46	60	20885808	6.80E-01	1
RERE	916698	436	27	3923120	1187260	0	14	2	0	3	12	4918140	6.80E-01	1
C15orf27	374349	405	22	1355648	391600	0	3	0	0	50	45	21903612	6.80E-01	1
RLIM	289586	NaN	13	1562128	453188	0	5	3	0	43	54	14944880	6.80E-01	1
PIGC	113155	450	11	728732	226060	0	2	0	0	50	71	23190908	6.80E-01	1
FOXN3	111760	684	20	1258816	339980	0	3	1	0	50	52	20976232	6.80E-01	1
JRK	384374	412	23	1467432	453900	0	3	2	0	32	19	11392712	6.80E-01	1
ROCK1	185443	407	-6	3644016	834108	0	8	1	0	50	41	20257112	6.80E-01	1
CLDN12	140224	1085	-8	597724	191528	0	1	0	0	50	46	23330816	6.80E-01	1
FBXO32	535498	203	19	937348	237452	0	2	1	0	50	45	19740556	6.80E-01	1
PNMA2	194700	452	14	901036	268424	0	2	1	0	50	58	22105464	6.80E-01	1
DOCK1	97878	856	-32	4953740	1244932	0	8	0	0	8	4	4126040	6.80E-01	1
ENTPD5	443041	158	51	1128164	301888	0	3	0	0	50	55	19172380	6.80E-01	1
PRB4	311837	673	-1	601996	207548	0	2	0	0	50	60	16426552	6.80E-01	1
TRIM43	394116	611	16	1173020	284800	0	3	0	0	50	45	20194812	6.80E-01	1
CGN	1385164	176	31	3042376	900680	0	6	3	0	50	72	22882968	6.81E-01	1
RGS11	1340678	205	43	1231404	342828	0	1	0	0	32	34	15239292	6.81E-01	1
ATP5G2	1616455	189	50	490924	163760	0	1	1	0	50	62	21036040	6.81E-01	1
FAM163B	NaN	NaN	NaN	411180	127092	0	1	0	0	50	116	39772676	6.81E-01	1
BANK1	80568	482	-7	2078328	508368	0	6	4	0	50	65	21971608	6.81E-01	1
KIDINS22(	280134	559	14	4547544	1253832	0	16	2	0	1	3	1488080	6.81E-01	1
OR13C5	147494	920	-31	789252	230688	0	3	1	0	50	80	20017168	6.81E-01	1
PON2	137217	333	-1	917056	254540	0	2	1	0	17	17	7429720	6.81E-01	1

DDX50	897697	246	45	1902820	521540	0	4	0	0	22	34	13431524	6.81E-01	1
ZNF616	460879	901	-41	2012112	506232	0	7	3	0	50	67	18693560	6.81E-01	1
C22orf23	1370748	203	50	574228	149876	0	1	0	0	50	37	18381704	6.81E-01	1
NOL7	277395	425	37	687436	173372	0	2	1	0	50	65	20454336	6.81E-01	1
GLS2	2793663	184	61	1576012	428624	0	4	1	0	29	60	17503096	6.81E-01	1
GSTM2	927739	201	22	608048	141688	0	2	2	0	12	20	4092220	6.81E-01	1
LEF1	231204	771	22	1132080	295836	0	3	1	0	50	56	21017528	6.81E-01	1
UBE2F	507348	433	27	508368	126024	0	1	0	0	50	65	22767268	6.82E-01	1
PCNXL2	146659	581	-1	5449292	1541836	0	18	4	0	15	17	7268096	6.82E-01	1
MFSD2B	243145	226	33	1233896	412960	0	3	3	0	50	59	18078748	6.82E-01	1
CEACAM3	1360684	454	23	642936	195444	0	2	0	0	50	69	18698900	6.82E-01	1
SPATA2L	1090549	219	38	1008192	360984	0	3	1	0	50	51	16551864	6.82E-01	1
ADAM33	942842	353	32	2081532	618372	0	4	0	0	7	3	3281608	6.82E-01	1
C14orf93	1240941	185	38	1339628	408688	0	3	0	0	16	13	6672864	6.82E-01	1
ZNF624	1055714	499	-26	2247784	548240	0	6	0	0	7	4	3120340	6.82E-01	1
SOX8	1131712	304	22	1091140	349592	0	3	2	0	13	7	3546116	6.82E-01	1
SLC16A4	403650	479	31	1252408	345320	0	2	0	0	37	21	12040632	6.82E-01	1
ZNF793	257166	914	-56	1058388	262728	0	3	1	0	50	53	18226488	6.82E-01	1
PTPRK	107062	714	4	3760428	1003920	0	14	1	0	0	1	1003920	6.83E-01	1
HINT2	847663	227	54	412248	134568	0	1	0	0	50	66	20654408	6.83E-01	1
VTCN1	322193	566	12	725884	204344	0	3	2	0	21	24	6588136	6.83E-01	1
DENND5A	672390	330	31	3318632	902104	0	6	2	0	8	5	3294068	6.83E-01	1
VWC2	53908	941	-13	815952	238164	0	2	0	0	50	75	26171340	6.83E-01	1
GGT6	1252775	318	22	1164476	433252	0	3	0	0	50	49	18259952	6.83E-01	1
SYT17	766632	275	7	1207552	344252	0	2	1	0	50	86	27421256	6.83E-01	1
MANSC1	698798	242	20	1065864	331080	0	2	0	0	39	46	14308352	6.83E-01	1
PLEKHB2	461881	331	16	589892	154504	0	2	1	0	33	39	10825604	6.83E-01	1
APLP2	624855	203	15	2010688	509792	0	5	2	0	50	42	19945968	6.83E-01	1
SPATA4	134663	504	-4	788896	212888	0	6	3	0	2	6	1294416	6.83E-01	1
G3BP2	485383	451	31	1251340	343184	0	3	1	0	50	54	22354308	6.83E-01	1
RBM25	456437	274	42	2223932	557140	0	7	0	0	5	7	3021372	6.83E-01	1
FAM126B	646602	167	33	1351732	392312	0	2	1	0	50	61	20826000	6.83E-01	1
KRTAP10-[illegible]	874480	461	25	909224	265576	0	3	0	0	50	76	22638396	6.83E-01	1

SCARA3	350607	232	30	1529020	473480	0	4	0	0	43	53	22780796	6.83E-01	1
OR8D4	89099	736	-8	776436	230688	0	2	0	0	34	50	17740192	6.83E-01	1
ST8SIA4	24350	1122	36	920972	251692	0	2	1	0	50	57	23360008	6.83E-01	1
RFK	106798	603	-10	410468	102172	0	1	0	0	50	66	23428716	6.83E-01	1
HAO1	67871	715	-23	946960	273764	0	5	0	0	14	22	5641888	6.83E-01	1
GRIN2C	1027517	320	20	3020304	984696	0	10	5	0	10	17	5209348	6.83E-01	1
GTPBP10	138505	1085	-5	1019584	264152	0	1	0	0	50	49	18619868	6.83E-01	1
SLCO2B1	752269	449	48	1783560	546816	0	3	1	0	50	59	22513796	6.83E-01	1
SCN2B	707709	293	56	552156	152724	0	1	0	0	50	54	16045632	6.83E-01	1
FSCN2	3623687	204	19	1140624	363120	0	3	1	0	40	39	14920316	6.84E-01	1
MYO7B	528013	229	36	5447512	1531868	0	13	4	0	36	31	13816716	6.84E-01	1
CBFB	1068656	125	48	526524	130296	0	1	0	0	50	46	20351096	6.84E-01	1
FMO2	440278	1004	-6	1228556	317908	0	4	1	0	50	72	23262464	6.84E-01	1
COPE	1390116	198	20	803492	227128	0	3	0	0	50	73	19047780	6.84E-01	1
MAN2B1	1975767	409	1	2578508	764332	0	8	3	0	50	74	27829232	6.84E-01	1
SHPRH	93507	878	7	4415824	1148456	0	11	3	0	31	31	14902872	6.84E-01	1
GPCPD1	556687	225	3	1785340	452120	0	6	0	0	20	22	8672160	6.84E-01	1
HADHB	642780	250	34	1237812	348168	0	2	0	0	13	11	7197608	6.84E-01	1
USP37	841802	168	42	2585984	652192	0	7	1	0	10	14	5863676	6.84E-01	1
PSME3	1633092	190	33	722680	178712	0	2	0	0	24	22	8134244	6.84E-01	1
COMMD1	563800	420	26	485584	137060	0	1	0	0	50	63	23156376	6.84E-01	1
ISCA2	749329	384	31	392668	118904	0	1	0	0	50	65	22029992	6.84E-01	1
KRTAP19-[illegible]	14979	1035	-68	208616	62656	0	1	1	0	27	40	7047376	6.84E-01	1
TPBG	131014	486	-3	1005700	343184	0	4	2	0	20	29	8569988	6.84E-01	1
ERGIC3	1515751	169	58	1040232	263796	0	1	0	0	36	47	16385256	6.84E-01	1
PLA2G1B	1621662	187	56	398720	95764	0	1	0	0	50	72	23682188	6.84E-01	1
COL9A1	126322	633	5	2435396	755788	0	8	3	0	29	34	10808160	6.85E-01	1
SIGLEC5	443199	644	-23	1385196	417588	0	4	0	0	27	27	10304064	6.85E-01	1
UBA3	264082	443	20	1240304	321112	0	5	2	0	8	7	2888228	6.85E-01	1
CRYBB1	162003	462	-9	646140	187968	0	5	2	0	3	2	712712	6.85E-01	1
EHD4	830035	224	39	1374516	385548	0	2	3	0	41	44	15672544	6.85E-01	1
FAM199X	354255	NaN	47	1002140	265576	0	3	0	0	50	58	20023576	6.85E-01	1
TFDP1	729832	370	53	1076900	286936	0	3	0	0	50	58	19838100	6.85E-01	1

PPP6C	951081	168	46	886440	242436	0	3	0	0	25	28	10521936	6.85E-01	1
C1orf87	66197	942	-13	1427204	372376	0	4	3	0	17	30	8887896	6.85E-01	1
ZNF776	680410	849	-50	1341764	329656	0	4	1	0	50	69	18021076	6.85E-01	1
THADA	428893	408	38	5068372	1350308	0	11	2	0	6	6	3640456	6.85E-01	1
PARS2	578299	343	23	1168748	360628	0	3	1	0	50	44	21555444	6.85E-01	1
C11orf45	NaN	NaN	NaN	370596	110004	0	1	1	0	26	41	11687124	6.85E-01	1
GLDN	317532	443	10	1402640	404416	0	4	0	0	50	42	18573588	6.85E-01	1
OR10S1	107524	830	-5	805272	258456	0	3	3	0	13	13	3654340	6.85E-01	1
TMCO1	321853	406	-2	496976	134212	0	1	0	0	50	54	20372100	6.85E-01	1
PBX4	818567	284	-1	953368	279104	0	3	0	0	50	58	19983348	6.85E-01	1
CDH13	94658	929	-26	1813108	530084	0	9	4	0	20	41	7917440	6.85E-01	1
HOXD12	242935	874	15	654328	218228	0	2	1	0	50	65	19479964	6.85E-01	1
SQRDL	201356	490	1	1155220	328232	0	1	1	0	50	57	22840604	6.86E-01	1
NUP37	232146	467	9	862588	221432	0	2	0	0	48	53	21168472	6.86E-01	1
TRPV3	441141	442	-2	2047000	559988	0	5	3	0	50	42	19085160	6.86E-01	1
BAZ1B	929895	162	48	3846936	988968	0	12	3	0	20	28	10522648	6.86E-01	1
DUT	333686	381	-34	656108	203632	0	2	1	0	50	82	24242532	6.86E-01	1
CRABP2	1851672	180	37	363120	95052	0	1	0	0	50	84	23475352	6.86E-01	1
CDC20B	222731	430	2	1344968	368104	0	2	2	0	40	47	17209040	6.86E-01	1
HPS1	127652	581	10	1823432	535780	0	3	2	0	50	52	22253916	6.86E-01	1
LCAT	879834	158	50	1106804	331792	0	2	2	0	43	70	20481748	6.86E-01	1
FAM151A	723136	415	20	1451056	460664	0	5	1	0	50	74	23149256	6.87E-01	1
PLA2G4D	680403	360	46	2089008	618372	0	2	0	0	18	10	6924200	6.87E-01	1
LUZP2	1913	1282	-32	928092	230688	0	5	1	0	31	64	15414088	6.87E-01	1
MAK16	91648	507	12	819156	187968	0	2	1	0	50	66	24660120	6.87E-01	1
CCDC106	1077402	254	-10	715560	206124	0	3	0	0	8	8	2559640	6.87E-01	1
ATL2	808394	366	25	1618020	423996	0	4	0	0	42	43	19220440	6.87E-01	1
LACTB2	240331	562	31	758636	195088	0	2	1	0	50	69	23240392	6.87E-01	1
RASL10A	849693	251	36	494484	169812	0	1	1	0	50	58	21612760	6.87E-01	1
ATP6V1C2	744427	428	42	1123180	303668	0	3	1	0	50	69	24253924	6.87E-01	1
CPB2	354901	679	53	1128164	275188	0	5	1	0	10	11	2131372	6.87E-01	1
DENND1C	865106	219	33	2046644	619084	0	7	2	0	50	76	20954160	6.87E-01	1
OBP2A	327501	539	37	456036	118548	0	1	1	0	41	41	15650828	6.87E-01	1

TSEN34	735613	439	-53	763976	249556	0	2	0	0	18	36	7425804	6.87E-01	1
SNRPD3	480889	194	48	328588	91136	0	1	1	0	7	4	1654688	6.87E-01	1
CHST9	48644	872	-24	1162340	281596	0	3	0	0	19	36	9570348	6.87E-01	1
SLC22A25	1852481	572	32	1383416	406552	0	4	0	0	28	25	11288760	6.87E-01	1
NECAB1	108662	842	-13	956572	222500	0	3	0	0	50	100	31419492	6.87E-01	1
NPY5R	74427	1197	-18	1122112	311144	0	5	2	0	37	62	15371012	6.87E-01	1
RPL32	508965	269	39	353508	95052	0	1	1	0	13	13	4086880	6.87E-01	1
SLC29A3	619612	487	40	1176936	379140	0	3	1	0	50	71	27440836	6.87E-01	1
SPRYD4	2793663	184	61	514064	158776	0	1	0	0	29	60	17503096	6.88E-01	1
SPRYD3	1726226	177	38	1149168	315060	0	3	0	0	9	18	5240676	6.88E-01	1
IFT20	1405196	214	10	395160	105732	0	1	0	0	50	74	22499912	6.88E-01	1
PLAC8L1	396247	406	-2	461376	123888	0	1	0	0	50	55	18144608	6.88E-01	1
ASGR2	1682013	348	15	828412	205412	0	2	1	0	50	56	20137140	6.88E-01	1
SNAP47	595623	459	1	1237100	378784	0	3	2	0	50	38	16917832	6.88E-01	1
NME1	NaN	NaN	NaN	461020	126380	0	1	1	0	50	117	39771964	6.88E-01	1
RCAN1	337408	491	39	724816	195800	0	2	1	0	50	52	17638732	6.88E-01	1
ARHGAP2(	146681	501	-5	3036680	842296	0	13	3	0	17	20	6267024	6.88E-01	1
ITGB6	227667	212	2	2050560	541476	0	5	0	0	30	23	12382748	6.88E-01	1
SIVA1	711772	247	36	443932	134924	0	2	1	0	10	13	2962988	6.88E-01	1
MYLK3	443420	253	-10	2062664	615880	0	7	0	0	3	2	1637956	6.88E-01	1
PLEKHA1	255946	174	10	1069068	275544	0	1	1	0	50	53	18916416	6.88E-01	1
TMEM196	187539	874	-4	438592	130652	0	1	0	0	50	56	18458956	6.88E-01	1
KBTBD6	314264	337	66	1674980	487720	0	4	1	0	50	73	22716004	6.89E-01	1
BIN3	761528	224	49	683520	166608	0	1	1	0	50	53	19833472	6.89E-01	1
TYRO3	744461	166	44	2251700	679960	0	6	3	0	50	61	20671140	6.89E-01	1
TEAD3	683460	280	36	1134216	313992	0	5	2	0	15	26	5361716	6.89E-01	1
CAV3	99517	555	-9	393024	106800	0	1	1	0	50	60	19567184	6.89E-01	1
PQLC2	810462	448	40	733360	232112	0	2	2	0	7	5	2296912	6.89E-01	1
SPATA8	93099	986	-46	271272	76896	0	1	1	0	13	17	4106104	6.89E-01	1
EML5	161619	704	1	5162000	1354936	0	9	2	0	27	23	12060924	6.89E-01	1
GOLGA6B	1113552	419	37	1756504	449984	0	2	0	0	5	2	2293352	6.89E-01	1
TNIP2	628294	228	46	1084376	321112	0	4	0	0	12	17	5904260	6.89E-01	1
LPO	838530	401	15	1811684	524032	0	8	1	0	27	38	11195132	6.89E-01	1

TBC1D16	660356	237	21	1935216	572448	0	7	3	0	50	66	18046708	6.90E-01	1
APBB1	424837	471	17	1801004	532576	0	5	0	0	7	3	2390896	6.90E-01	1
OR5M9	NaN	NaN	NaN	767892	226416	0	2	1	0	50	117	39872000	6.90E-01	1
COG2	379098	568	3	1929164	511216	0	4	0	0	5	9	4171252	6.90E-01	1
ZNF407	190381	1127	23	5794256	1578148	0	14	5	0	50	43	21305888	6.90E-01	1
LIN7C	371553	477	-10	509436	144180	0	1	0	0	50	60	24983012	6.90E-01	1
DYNC1LI1	434251	568	35	1355648	376648	0	4	0	0	11	17	5121416	6.90E-01	1
TET1	824682	168	41	5365632	1524036	0	17	5	0	50	54	19092636	6.90E-01	1
CSNK1A1I	156140	631	31	858316	224636	0	2	1	0	24	23	9893240	6.90E-01	1
WBP2NL	1328069	323	66	770740	245996	0	2	2	0	50	50	16070196	6.91E-01	1
MMP21	216532	351	-6	1435392	416520	0	3	2	0	29	27	12476732	6.91E-01	1
LSS	775666	624	68	1918484	526168	0	9	0	0	0	0	526168	6.91E-01	1
SPIN2A	145137	NaN	18	653972	180136	0	2	0	0	50	49	14659724	6.91E-01	1
KCNIP3	246871	498	14	774300	195444	0	2	1	0	50	55	18159204	6.91E-01	1
VNN1	127166	569	2	1311504	363120	0	3	1	0	50	64	25911104	6.91E-01	1
MRPS18A	1626879	278	43	504452	150232	0	1	0	0	50	72	24531960	6.91E-01	1
GUCY1A3	43073	764	1	1780712	481312	0	9	3	0	4	9	2265228	6.91E-01	1
DCP1A	782208	167	43	1469568	436812	0	3	2	0	41	57	19274908	6.91E-01	1
RSAD1	1332860	200	23	1094344	361340	0	3	0	0	50	72	24178452	6.91E-01	1
FKBP6	902769	422	52	881812	250268	0	3	0	0	50	86	23744844	6.91E-01	1
SLC7A5	604095	133	34	1260240	407976	0	4	0	0	50	49	19160632	6.91E-01	1
GPHA2	3151458	209	52	328588	98612	0	1	0	0	50	83	23753744	6.91E-01	1
CD83	210380	594	40	527236	149876	0	1	0	0	50	49	18477468	6.91E-01	1
C1orf141	444524	673	-5	1054472	255964	0	8	0	0	5	7	1360632	6.92E-01	1
HLA-DMB	1276842	431	40	672128	195088	0	2	1	0	19	22	6752608	6.92E-01	1
GDF9	702301	200	62	1138488	323604	0	5	2	0	50	64	17001136	6.92E-01	1
ANKRD52	2878071	171	46	2743336	824852	0	9	1	0	13	15	6175532	6.92E-01	1
CLK3	635020	206	51	1625496	473124	0	2	1	0	38	35	15894688	6.92E-01	1
GGA2	525761	628	36	1590608	447136	0	4	1	0	50	62	19047424	6.92E-01	1
DNAJC21	241905	675	21	1553940	346032	0	4	0	0	50	44	20410904	6.92E-01	1
PAX6	172494	631	-11	1131724	315416	0	1	0	0	50	54	24314800	6.92E-01	1
MRPL38	1697896	165	28	970812	284088	0	3	2	0	26	52	10930624	6.92E-01	1
FRG2	59973	1059	-12	712000	196868	0	2	1	0	16	30	9186580	6.92E-01	1

PCSK1	525524	288	24	1987904	529372	0	6	2	0	27	22	9083696	6.92E-01	1
FIS1	1022626	258	39	397652	111784	0	1	1	0	50	67	19705312	6.92E-01	1
OR52W1	466636	728	4	752584	273764	0	1	2	0	24	21	8464256	6.92E-01	1
ZNF267	263122	850	-18	1939488	457104	0	5	0	0	19	25	7327904	6.92E-01	1
GFPT1	481090	144	17	1799580	462444	0	5	2	0	50	43	20692856	6.92E-01	1
PLEKHJ1	1603571	240	27	399788	104308	0	1	0	0	50	66	21932448	6.92E-01	1
DSCC1	294228	677	12	1038808	257744	0	2	0	0	50	63	19367112	6.92E-01	1
RGS7BP	78559	688	-3	668212	179780	0	2	0	0	50	62	18106516	6.92E-01	1
REM2	1177534	225	34	841228	270560	0	2	0	0	50	44	16175572	6.92E-01	1
GBP6	441714	820	-2	1648636	425420	0	4	2	0	16	19	5357444	6.92E-01	1
GDF10	NaN	NaN	NaN	1161272	381988	0	4	0	0	50	116	40027572	6.93E-01	1
PDGFRL	462541	220	4	955148	273052	0	1	2	0	20	18	6619464	6.93E-01	1
YY1	412002	237	37	1051624	295124	0	2	0	0	50	44	19346464	6.93E-01	1
REEP6	2352825	221	38	469920	139908	0	1	0	0	50	55	18329728	6.93E-01	1
COL2A1	374433	246	47	3742272	1251696	0	11	1	0	0	1	1251696	6.93E-01	1
ZNF576	441738	209	6	431472	124956	0	1	0	0	50	64	22145336	6.93E-01	1
TG	237421	677	9	7080128	1995736	0	25	8	0	50	62	22618816	6.93E-01	1
PIP5K1C	2156221	206	32	1721260	496976	0	7	1	0	18	26	7576036	6.93E-01	1
GCLM	558028	275	9	714136	192596	0	2	1	0	26	48	15139968	6.93E-01	1
CSNK1G2	1944774	239	36	1080104	295480	0	4	2	0	8	8	3166620	6.93E-01	1
FAM107B	256093	327	36	793168	207548	0	2	1	0	46	50	17301600	6.93E-01	1
PHACTR1	251440	714	17	1508372	408688	0	4	2	0	17	20	6217540	6.93E-01	1
FHL5	116593	1376	3	767180	167320	0	3	1	0	15	12	3880400	6.93E-01	1
DDB1	1259416	286	50	2958004	808832	0	8	1	0	15	15	6420816	6.93E-01	1
ADCK5	2284602	200	32	1472416	451052	0	5	2	0	50	61	21451136	6.93E-01	1
PVRL2	920101	196	-3	1646856	541476	0	5	1	0	4	3	1628344	6.94E-01	1
TMEM181	646528	306	46	1583132	451408	0	5	3	0	28	30	11722368	6.94E-01	1
MUC17	1124475	272	43	10683204	3768972	0	52	16	0	33	68	17122176	6.94E-01	1
CD47	288227	650	5	857248	227840	0	2	2	0	19	17	7092944	6.94E-01	1
SAMD8	215938	188	39	1101108	291564	0	2	0	0	50	65	18393096	6.94E-01	1
FSTL4	599945	623	42	2146324	618728	0	8	2	0	50	63	22132876	6.94E-01	1
RB1CC1	97322	559	-1	4171608	1032756	0	15	5	0	26	33	11860852	6.94E-01	1
CCDC7	602870	482	2	1327524	305448	0	4	2	0	50	46	16397360	6.94E-01	1

CCL22	716661	153	38	243148	68708	0	2	1	0	1	2	469208	6.94E-01	1
IREB2	506215	435	33	2491288	687080	0	8	2	0	10	7	2993960	6.94E-01	1
HIST1H3G	390169	544	57	331080	110004	0	1	1	0	50	61	13862640	6.94E-01	1
SKIV2L2	344578	395	38	2762204	692776	0	9	2	0	32	38	14493828	6.94E-01	1
VSX2	652027	358	47	907088	271984	0	1	0	0	26	16	11798908	6.94E-01	1
KRT33A	1321356	424	-6	1027772	297616	0	3	0	0	18	19	6810636	6.94E-01	1
ATP8A2	331788	648	20	3122120	841228	0	12	4	0	13	21	6233204	6.94E-01	1
RAB40AL	208589	NaN	36	686012	210040	0	2	1	0	50	54	16148872	6.94E-01	1
ZNF331	605686	808	-54	1211824	287648	0	4	2	0	50	72	17053468	6.94E-01	1
MYPOP	874987	198	41	938772	351372	0	3	1	0	50	67	20862312	6.94E-01	1
SPANXN4	2776	NaN	-19	267000	61944	0	1	0	0	50	96	22070576	6.94E-01	1
RARS	413838	432	30	1734788	445000	0	6	1	0	24	27	10474944	6.94E-01	1
TAS2R7	408176	517	12	799932	226416	0	2	0	0	50	54	19041016	6.94E-01	1
IL31	NaN	NaN	NaN	418300	121040	0	2	2	0	2	6	977576	6.95E-01	1
PSMB3	1198833	207	37	541476	144180	0	2	1	0	7	7	2216100	6.95E-01	1
RNF133	53042	1027	6	946604	263440	0	2	0	0	50	55	23106180	6.95E-01	1
FUBP3	529622	192	35	1507304	405484	0	3	1	0	50	57	18712428	6.95E-01	1
RNF214	436617	360	36	1806700	508724	0	3	0	0	10	11	5321132	6.95E-01	1
PANX2	944000	242	49	1644364	536492	0	3	1	0	50	67	23711024	6.95E-01	1
AGT	422372	568	6	1193668	380564	0	4	3	0	50	70	22303400	6.95E-01	1
SMTNL1	923607	249	32	1314708	358848	0	5	2	0	50	61	18117196	6.95E-01	1
ALG13	124127	NaN	20	3133512	777504	0	5	0	0	11	10	6234272	6.95E-01	1
CCNC	239824	619	10	782844	174084	0	2	0	0	50	61	21893644	6.95E-01	1
CHMP4C	158801	681	12	609472	157352	0	2	0	0	50	67	18347172	6.95E-01	1
RNFT1	962470	223	14	1136708	296548	0	3	0	0	44	53	19823504	6.95E-01	1
DDO	248863	469	20	925244	279460	0	1	1	0	50	63	22854132	6.95E-01	1
PLEC	2478735	247	25	12038852	3850852	0	34	10	0	20	45	17672196	6.95E-01	1
ZFP1	647304	162	19	1068000	252048	0	1	0	0	50	54	22779728	6.95E-01	1
NET1	740293	446	8	1603780	457460	0	3	2	0	50	45	16733424	6.95E-01	1
SLMO1	533826	240	48	462444	122820	0	1	0	0	50	50	20246432	6.95E-01	1
LCN1	327501	539	37	467072	126736	0	1	0	0	50	46	18874052	6.95E-01	1
OR2T4	104321	936	-39	868996	249200	0	7	2	0	9	22	2442516	6.95E-01	1
LOXL3	1019591	315	50	1925960	547528	0	6	3	0	40	63	17658668	6.95E-01	1

HEATR1	461836	603	0	5573536	1487012	0	14	4	0	50	69	23199452	6.95E-01	1
FEM1C	352735	498	23	1548956	435388	0	4	2	0	9	6	3756512	6.95E-01	1
GCNT4	479847	634	32	1154152	300464	0	3	2	0	50	58	21360000	6.95E-01	1
CHCHD1	1009357	191	42	306872	85084	0	2	1	0	1	1	459240	6.95E-01	1
MGLL	587659	313	51	796372	239588	0	2	0	0	50	52	18414456	6.95E-01	1
CCNL1	365148	610	22	1347104	384124	0	2	0	0	22	21	7975112	6.95E-01	1
SHQ1	140728	510	21	1498404	396228	0	3	1	0	50	38	19715992	6.95E-01	1
ENTPD7	422578	304	12	1546464	443220	0	4	1	0	50	65	25074504	6.96E-01	1
L1CAM	2328608	NaN	27	3249924	896052	0	7	1	0	13	17	8460340	6.96E-01	1
LPIN3	383021	309	5	2165904	645072	0	3	0	0	12	13	5412268	6.96E-01	1
THYN1	270350	623	21	607336	146672	0	2	0	0	3	2	1141336	6.96E-01	1
YEATS4	675242	361	13	611252	144892	0	1	0	0	50	46	17692132	6.96E-01	1
IP6K3	1558308	328	34	1042368	291564	0	3	1	0	50	61	18849132	6.96E-01	1
PLXND1	754944	210	53	4852992	1469568	0	11	7	0	50	51	17829192	6.96E-01	1
ARSG	745529	225	-8	1339272	388752	0	4	3	0	16	25	6612700	6.96E-01	1
NUDT16L1	717235	194	35	517980	171948	0	1	1	0	50	55	22982648	6.96E-01	1
OR5M1	NaN	NaN	NaN	779640	232824	0	2	1	0	50	117	39878408	6.96E-01	1
FOXC2	47545	445	10	1222504	390176	0	3	1	0	50	53	22234336	6.96E-01	1
ELL2	316216	247	51	1653264	447492	0	2	0	0	16	12	6345344	6.96E-01	1
GNAI2	846254	199	44	946604	237808	0	3	1	0	39	49	14244272	6.96E-01	1
RORB	95756	913	-21	1211468	300820	0	4	1	0	7	10	4011764	6.96E-01	1
HIRIP3	NaN	NaN	NaN	1422932	387328	0	7	3	0	26	43	11964448	6.96E-01	1
TBC1D8	333394	406	17	2952308	784624	0	6	3	0	23	32	12061636	6.96E-01	1
THAP1	594393	454	34	574584	119616	0	1	0	0	50	55	25462900	6.96E-01	1
IMPACT	144714	390	16	870420	200784	0	2	1	0	50	46	17547596	6.96E-01	1
LHX9	98527	448	-18	1030264	296192	0	2	2	0	50	44	18122892	6.96E-01	1
C11orf40	190769	879	-1	557140	153080	0	1	0	0	50	51	19817096	6.96E-01	1
IRAK1BP1	198307	832	-13	659312	189748	0	2	1	0	43	74	19217948	6.96E-01	1
MRGPRX3	631291	436	20	784624	254540	0	2	1	0	50	76	24807148	6.96E-01	1
DTX3L	410045	404	35	1896768	500892	0	5	3	0	50	47	20160992	6.96E-01	1
DDX46	893816	193	36	2706312	694200	0	9	1	0	34	40	16186964	6.96E-01	1
STAT2	2869106	184	52	2223220	604844	0	4	2	0	23	47	14312980	6.96E-01	1
CCNJL	197942	448	33	1093988	328588	0	1	2	0	50	73	26144284	6.96E-01	1

OR5B17	475497	835	-29	775724	231400	0	3	1	0	50	60	16320820	6.96E-01	1
TRIM74	826724	337	62	638308	182984	0	1	1	0	50	46	17329012	6.97E-01	1
C8orf76	545561	171	22	989680	254540	0	1	2	0	50	60	20198016	6.97E-01	1
PACS1	2403161	236	49	2477048	709864	0	7	0	0	0	0	709864	6.97E-01	1
ECT2	301474	239	11	2328240	600216	0	5	1	0	34	35	14146016	6.97E-01	1
NCKAP5L	1560163	231	56	3208628	1115704	0	8	4	0	32	42	13923516	6.97E-01	1
PDZD3	1258103	195	57	1259528	406552	0	1	0	0	46	54	22055268	6.97E-01	1
PSD4	630071	479	37	2664660	786048	0	6	0	0	10	9	4743344	6.97E-01	1
OLA1	389793	418	26	1055540	257032	0	2	1	0	22	18	9008580	6.97E-01	1
GSR	488347	242	40	1339272	389820	0	2	1	0	50	30	16338620	6.98E-01	1
ZNF721	553434	644	19	2380928	588112	0	10	1	0	50	60	18021788	6.98E-01	1
ATF7IP2	199617	364	9	1771456	459596	0	6	2	0	50	71	20969468	6.98E-01	1
UBA2	707545	355	5	1689576	434676	0	3	2	0	50	60	23415544	6.98E-01	1
NAGPA	635794	302	9	1297264	396584	0	3	1	0	50	65	26718156	6.98E-01	1
C17orf59	1381225	202	37	836600	310432	0	2	0	0	50	76	28183808	6.98E-01	1
PTPN12	306142	489	24	2051984	525100	0	5	1	0	50	54	24154244	6.98E-01	1
OR5H1	200002	811	-15	778216	225704	0	3	2	0	50	87	21088372	6.98E-01	1
RPS5	1900140	270	-14	525456	153792	0	1	0	0	50	64	20061668	6.98E-01	1
ABHD6	611196	221	43	879320	237808	0	2	0	0	50	58	20830272	6.98E-01	1
ZNF692	411701	567	-4	1338204	390888	0	3	0	0	2	1	1058388	6.98E-01	1
RPAIN	1145030	544	24	642580	156284	0	2	0	0	50	77	22011836	6.98E-01	1
TIAL1	823029	217	22	1048420	257744	0	2	0	0	34	25	11162380	6.98E-01	1
GMNN	422850	362	40	557496	140976	0	1	0	0	50	54	24131104	6.98E-01	1
IL1RL2	238921	541	8	1499828	392668	0	4	0	0	9	10	4062672	6.98E-01	1
KRT85	936407	213	42	1286584	375224	0	3	2	0	50	55	23702836	6.99E-01	1
WDR25	410676	215	23	1364548	407264	0	4	1	0	6	4	2315068	6.99E-01	1
TRIM31	1820240	496	30	1113212	285868	0	3	1	0	50	54	18891140	6.99E-01	1
TM2D1	174892	296	-9	540408	151656	0	1	1	0	34	33	14375636	6.99E-01	1
MRPS30	87489	1075	-7	1106092	322892	0	3	0	0	39	41	15783616	6.99E-01	1
MAP4K1	1503183	363	27	2225000	645784	0	6	3	0	50	60	18913568	6.99E-01	1
VRK1	313555	715	13	1072272	250980	0	3	1	0	50	59	19630908	6.99E-01	1
MED7	166999	1198	18	605556	148452	0	1	0	0	50	43	19254260	6.99E-01	1
GPA33	287664	611	-1	826988	223924	0	2	0	0	50	52	19348956	6.99E-01	1

GRIK1	71816	904	-14	2508376	675332	0	11	0	0	4	8	2661812	6.99E-01	1
TCEAL8	455954	NaN	17	307584	74760	0	1	1	0	32	42	10453940	6.99E-01	1
STAR	705837	277	46	737632	204344	0	1	1	0	50	54	23183788	6.99E-01	1
STAC2	1426821	212	31	1058032	304736	0	3	1	0	50	55	19657964	6.99E-01	1
CCL27	731413	298	51	294412	82592	0	1	1	0	12	11	3186200	6.99E-01	1
ZNF586	680410	849	-50	1035248	264508	0	2	1	0	50	69	18021076	6.99E-01	1
BHLHE40	274112	183	33	1035604	304736	0	4	2	0	50	61	17470344	6.99E-01	1
WDR62	980636	320	19	3880756	1138844	0	10	1	0	24	26	11494528	6.99E-01	1
TMEM133	23524	920	-24	320400	96120	0	1	0	0	50	80	20784348	6.99E-01	1
C3orf30	197435	589	-3	1360988	370240	0	4	2	0	50	54	19335784	6.99E-01	1
MTL5	712475	354	47	1311860	359560	0	3	0	0	50	46	20924612	6.99E-01	1
AM120AO	422600	547	26	728020	210752	0	1	0	0	18	25	6338936	6.99E-01	1
GJA8	NaN	NaN	NaN	1071916	325028	0	3	0	0	50	116	39970612	7.00E-01	1
KAT5	4131934	202	53	1399436	404416	0	3	0	0	24	26	11885772	7.00E-01	1
KRT31	1321356	424	-6	1056608	307228	0	3	1	0	50	49	16872976	7.00E-01	1
PIK3AP1	794261	622	28	2079040	571736	0	5	1	0	16	15	7205084	7.00E-01	1
AVPR2	2328608	NaN	27	907088	314704	0	2	1	0	50	67	26342932	7.00E-01	1
B3GNTL1	303219	483	14	953724	259524	0	2	0	0	50	47	19817452	7.00E-01	1
RTAP10-1	874480	461	25	740124	220008	0	2	0	0	50	76	22638396	7.00E-01	1
DYRK1A	314682	366	47	2049848	537916	0	6	2	0	50	58	19398084	7.00E-01	1
CSAD	1729009	191	55	1342120	391244	0	2	1	0	50	55	18282736	7.00E-01	1
RASAL1	518680	387	34	2052340	612320	0	7	2	0	50	55	20506668	7.00E-01	1
FHDC1	227983	342	25	2834828	875404	0	7	3	0	42	39	17178780	7.00E-01	1
C9orf172	1921909	176	44	2285164	844076	0	7	0	0	4	4	2535076	7.00E-01	1
GPR18	NaN	NaN	NaN	831972	236028	0	2	1	0	50	117	39881612	7.00E-01	1
RTEL1	1110751	172	45	3346044	963336	0	10	3	0	11	11	4997884	7.00E-01	1
RNF126	1832767	218	10	796016	237808	0	2	0	0	29	50	17004696	7.00E-01	1
LARP7	252277	692	39	1548956	370240	0	6	1	0	50	66	20369252	7.00E-01	1
TPM1	431470	321	19	1201500	295836	0	2	0	0	7	6	3053412	7.00E-01	1
RAB3IL1	1998419	212	69	974728	290852	0	2	0	0	50	69	27604952	7.00E-01	1
IQCA1	112916	599	11	2169108	544680	0	6	1	0	50	49	20578936	7.01E-01	1
MAP2K3	415581	517	-43	918836	243148	0	6	3	0	7	15	2788548	7.01E-01	1
NIT2	378824	361	26	734428	193664	0	1	0	0	50	35	21164200	7.01E-01	1

HUNK	341416	624	12	1808480	525100	0	7	2	0	50	63	18144252	7.01E-01	1
UNC5B	608276	559	34	2383420	715916	0	8	1	0	21	30	11994352	7.01E-01	1
ZNF208	87537	1261	-66	3340704	776436	0	14	2	0	13	23	5123908	7.01E-01	1
GOLGA8A	542657	234	22	1583132	416164	0	2	1	0	50	46	18844148	7.01E-01	1
OASL	1225663	380	40	1331084	342472	0	4	2	0	50	68	17858740	7.01E-01	1
UBN2	519766	345	29	3364556	1027060	0	11	3	0	50	55	19811044	7.01E-01	1
FAM162B	244987	581	14	405840	129228	0	1	0	0	50	65	21736648	7.01E-01	1
POLD1	1055164	491	28	2800652	861520	0	6	1	0	14	21	7268096	7.01E-01	1
DPY30	375703	295	27	268780	68708	0	1	1	0	4	3	1058388	7.01E-01	1
IGF2R	653159	460	18	6440040	1745112	0	13	6	0	14	14	6496288	7.01E-01	1
ALDH4A1	642600	453	27	1447496	423640	0	4	0	0	12	11	5041316	7.01E-01	1
ACTR6	184664	650	9	1064084	252760	0	2	0	0	50	80	23899348	7.01E-01	1
ACADSB	256010	673	17	1128164	301888	0	3	1	0	50	53	18719548	7.01E-01	1
TNNI2	916434	481	33	508368	123888	0	2	1	0	6	7	2022792	7.01E-01	1
AP3S1	380598	759	6	514420	128516	0	1	0	0	50	68	24737372	7.01E-01	1
AES	1092464	195	30	706660	201140	0	3	0	0	50	80	18913212	7.01E-01	1
FZD3	306800	256	45	1693136	465292	0	5	1	0	50	62	21059536	7.02E-01	1
TMLHE	272469	NaN	-2	1215384	315060	0	1	1	0	50	58	21976592	7.02E-01	1
TOPORS	474275	362	-7	2620872	739056	0	8	2	0	18	24	8352472	7.02E-01	1
GPC6	42298	1135	-25	1428628	386972	0	6	4	0	21	40	8701352	7.02E-01	1
CTRB2	612638	171	16	672484	203276	0	1	0	0	50	41	19443652	7.02E-01	1
PCOLCE	1767777	242	60	1120688	357424	0	2	0	0	17	23	11241768	7.02E-01	1
ACOT4	606300	169	39	1033112	327520	0	2	0	0	50	45	20608840	7.02E-01	1
CEL	1828532	402	50	1872204	600216	0	6	3	0	50	62	18209756	7.02E-01	1
DDX23	1655612	214	57	2113928	582772	0	8	2	0	50	64	19943476	7.02E-01	1
PSMB7	492165	406	37	714136	208616	0	2	1	0	50	75	22681828	7.03E-01	1
WNT4	434362	307	28	884660	260236	0	2	0	0	41	37	14867272	7.03E-01	1
LTBP2	782985	242	42	4661464	1325744	0	9	1	0	5	4	3000012	7.03E-01	1
LDLRAP1	562107	424	19	802068	222144	0	4	3	0	3	9	1595592	7.03E-01	1
TMEM110	1107657	264	52	763620	213600	0	2	0	0	50	69	21773316	7.03E-01	1
RPS27A	964970	349	25	420436	103952	0	1	0	0	50	65	19655116	7.03E-01	1
CALML6	1434423	233	27	493416	113208	0	1	0	0	50	61	20095132	7.03E-01	1
SERBP1	454341	466	-6	1049132	291208	0	4	1	0	50	63	20567188	7.03E-01	1

SYTL1	884974	188	30	1403708	423640	0	3	0	0	28	60	17682876	7.03E-01	1
DDX1	186504	1386	10	1995024	485940	0	6	1	0	50	56	22216892	7.03E-01	1
CDADC1	329994	399	53	1358852	329656	0	2	0	0	50	64	21335436	7.03E-01	1
TBC1D29	488195	329	28	386616	116412	0	1	0	0	50	53	16914984	7.03E-01	1
RABGAP1I	235125	938	7	2253836	567820	0	6	2	0	50	55	22336864	7.03E-01	1
MCF2L	789438	285	25	3133512	882168	0	7	2	0	39	52	20692500	7.03E-01	1
C2orf76	193588	467	26	346744	83660	0	2	1	0	3	1	509080	7.04E-01	1
MPO	838530	401	15	1871492	565684	0	4	3	0	50	70	19096196	7.04E-01	1
PCDHA12	NaN	NaN	NaN	2300472	739056	0	10	7	0	22	43	10314032	7.04E-01	1
RIBC1	920771	NaN	70	1017448	260948	0	1	1	0	50	62	20770820	7.04E-01	1
C22orf42	426151	580	-16	674976	170880	0	2	0	0	50	56	16800352	7.04E-01	1
GSTCD	292053	357	8	1640448	437880	0	3	0	0	50	58	20260316	7.04E-01	1
TSHR	214327	492	-10	2065868	567820	0	5	1	0	31	30	14046336	7.04E-01	1
UBE2M	2673870	276	11	486652	124244	0	3	2	0	3	7	1419728	7.04E-01	1
TMEM108	418705	773	10	1382348	484516	0	3	2	0	50	71	23415188	7.04E-01	1
ZNF605	1014082	674	11	1756148	412960	0	6	0	0	19	19	9013208	7.04E-01	1
FRMD3	494570	610	-26	1571740	399788	0	5	0	0	50	47	17790388	7.04E-01	1
MAG	767180	472	8	1593100	495908	0	4	2	0	50	43	16821356	7.04E-01	1
LYPD4	1360684	454	23	625848	180492	0	2	0	0	50	69	18698900	7.04E-01	1
MSL2	206721	398	16	1440020	416164	0	4	1	0	50	49	17863724	7.04E-01	1
TAPBP	1456953	262	30	1241016	417588	0	4	1	0	50	71	20945260	7.04E-01	1
LRAT	73491	1036	-3	570668	176932	0	2	2	0	8	18	4906748	7.04E-01	1
CEP68	647041	340	38	1856540	597724	0	8	1	0	6	10	2940916	7.05E-01	1
CRBN	61304	1017	-8	1177292	286936	0	3	0	0	50	64	21277052	7.05E-01	1
PRKAR2A	1694388	196	44	1058744	281596	0	4	0	0	37	60	17081236	7.05E-01	1
MTNR1B	55111	742	-18	887864	280528	0	1	2	0	50	80	23488880	7.05E-01	1
DNASE1L3	685716	150	45	814172	202564	0	4	2	0	2	4	814528	7.05E-01	1
C2orf54	1187158	297	30	1108940	358492	0	3	0	0	50	60	21100120	7.05E-01	1
CUX2	493539	608	39	3707740	1146320	0	11	6	0	48	63	19897552	7.05E-01	1
ZNF831	876466	202	15	4034904	1358496	0	18	8	0	6	9	2732656	7.05E-01	1
OR2L2	20939	1015	-27	776792	230332	0	4	1	0	50	119	22654060	7.05E-01	1
RFX6	206546	730	-19	2406916	648632	0	9	0	0	0	0	648632	7.05E-01	1
ZNF124	374414	704	-12	765756	176220	0	2	1	0	50	64	19760848	7.05E-01	1

ZNF19	443039	600	18	1187260	300464	0	3	1	0	50	67	18330440	7.06E-01	1
ISY1	1142757	352	44	769316	189748	0	1	1	0	50	78	19071632	7.06E-01	1
CLDN18	160932	572	6	845144	252760	0	2	0	0	50	56	20671140	7.06E-01	1
RPS4Y2	0	0	0	681028	190460	0	1	0	0	50	39	21173456	7.06E-01	1
CTSB	470951	301	42	898544	229264	0	2	0	0	38	43	17149232	7.06E-01	1
GBA	1574928	194	45	1377720	394092	0	4	1	0	50	57	20795740	7.06E-01	1
ARL3	878626	180	27	481668	126024	0	1	0	0	50	59	20336144	7.06E-01	1
TCEAL1	490345	NaN	31	412248	104664	0	1	0	0	50	66	22114364	7.06E-01	1
RPL11	960799	225	28	476684	122464	0	1	0	0	50	55	20574664	7.06E-01	1
OR56A3	262387	914	-13	770740	239588	0	2	2	0	44	44	14880444	7.06E-01	1
TBC1D1	156977	578	48	3014608	812036	0	14	7	0	8	15	3445012	7.06E-01	1
NFKBIB	1989663	219	30	963336	255252	0	3	1	0	50	70	21843448	7.06E-01	1
CIB4	1039210	428	29	504096	119616	0	1	0	0	50	58	21568616	7.06E-01	1
C16orf71	717235	194	35	1310436	397296	0	3	3	0	31	31	12704572	7.06E-01	1
CACNA1H	1275017	294	21	5845164	1842300	0	8	2	0	4	2	2970820	7.06E-01	1
SLC16A2	357915	NaN	14	1523324	465292	0	3	1	0	50	64	23309456	7.06E-01	1
TCTEX1D1	335045	692	-16	475260	118548	0	1	0	0	50	58	17879032	7.06E-01	1
PMM2	401569	247	-2	661804	161624	0	1	2	0	3	4	1412608	7.06E-01	1
KRT10	836683	269	-10	1464584	446068	0	5	2	0	22	26	7229292	7.06E-01	1
UBE3A	241743	1263	-34	2298692	580636	0	10	0	0	4	12	3595600	7.06E-01	1
CLEC4G	688482	384	31	764688	213600	0	2	1	0	50	67	22649432	7.06E-01	1
ITGAM	1163323	478	7	2975092	848348	0	11	6	0	22	30	9171272	7.06E-01	1
SYDE1	703673	419	1	1765404	624780	0	2	0	0	50	38	17686080	7.07E-01	1
SERPINF2	1447501	189	32	1256680	368816	0	2	0	0	44	62	19487440	7.07E-01	1
DOCK9	435646	494	27	5621952	1448208	0	14	2	0	21	16	8794980	7.07E-01	1
NLRP6	1463610	437	24	2163412	726596	0	4	0	0	6	2	2533652	7.07E-01	1
FAHD2B	515149	535	38	804916	234248	0	2	1	0	50	66	22337220	7.07E-01	1
KRT18	1635177	181	43	1088292	323604	0	3	0	0	37	46	15752644	7.07E-01	1
LTA	2517285	220	35	507300	165540	0	1	0	0	50	55	21824936	7.07E-01	1
DHRS2	1299548	595	26	798508	200072	0	3	1	0	50	74	20710300	7.07E-01	1
SPINK5	68058	390	-10	2986484	677824	0	7	1	0	24	25	10751912	7.07E-01	1
TIMM23	404636	579	39	541832	156640	0	1	1	0	50	55	20910372	7.07E-01	1
NT5C2	562038	150	47	1500540	372732	0	2	2	0	50	48	17844500	7.07E-01	1

AFM	331900	594	3	1605204	379140	0	5	2	0	50	56	21463596	7.07E-01	1
RPUSD3	953772	264	43	870420	293700	0	2	1	0	50	72	27173124	7.07E-01	1
RNF38	564580	248	40	1315064	385192	0	1	0	0	16	15	8610572	7.07E-01	1
PSMB4	1322568	174	42	677468	199360	0	2	1	0	50	77	23375672	7.07E-01	1
BTBD17	362227	433	-8	1145608	397652	0	1	2	0	50	67	25396328	7.07E-01	1
RBM6	1079795	152	39	2924184	762552	0	9	1	0	14	30	9290532	7.07E-01	1
SLC25A26	85846	616	0	551800	158420	0	1	0	0	50	54	20785416	7.07E-01	1
CD8A	330931	543	19	586332	191172	0	2	1	0	11	11	3300476	7.07E-01	1
ZNF699	401242	705	-65	1676048	401212	0	7	2	0	50	125	30656940	7.08E-01	1
IL5RA	59117	1039	-15	1128876	288360	0	4	0	0	26	36	10639060	7.08E-01	1
SFT2D2	442995	299	18	430404	117480	0	1	0	0	50	57	19929948	7.08E-01	1
ABI3BP	459170	548	-1	2829132	765756	0	9	1	0	16	25	8890744	7.08E-01	1
LPCAT2	119965	430	-29	1401928	399788	0	3	0	0	13	31	7830576	7.08E-01	1
TRIM25	320339	324	-38	1610900	447136	0	5	3	0	43	83	19922828	7.08E-01	1
RAB39B	371405	NaN	-13	535068	154860	0	1	0	0	50	60	21240384	7.08E-01	1
ENTPD8	1591716	213	30	1239592	388040	0	4	1	0	50	64	20302324	7.08E-01	1
ZNF233	618477	671	-31	1755080	411892	0	4	1	0	50	57	18064864	7.08E-01	1
TOM1L1	131125	869	-61	1268784	322536	0	4	1	0	50	51	18003276	7.08E-01	1
CA9	1202125	248	37	1153796	364900	0	3	1	0	50	57	20786840	7.08E-01	1
MTMR2	285979	390	-1	1673200	449984	0	4	1	0	50	52	23754812	7.08E-01	1
SGK1	97901	129	15	1692068	440728	0	4	4	0	34	41	12078012	7.08E-01	1
FLRT1	NaN	NaN	NaN	1626920	540052	0	3	1	0	50	117	40185636	7.09E-01	1
NKIRAS1	399346	319	6	500892	130296	0	1	0	0	50	53	22341848	7.09E-01	1
C16orf45	544403	200	30	594164	147028	0	2	0	0	50	82	20960568	7.09E-01	1
RHBDF2	1202221	197	29	2187264	631188	0	4	1	0	7	6	3655052	7.09E-01	1
DVL1	1744013	273	28	1703104	508724	0	4	0	0	9	14	4108596	7.09E-01	1
NRTN	1217326	294	35	459596	179068	0	1	0	0	50	56	18189820	7.09E-01	1
TMPRSS4	579148	447	50	1152016	302600	0	5	4	0	4	12	2537924	7.09E-01	1
SIGLEC11	1679101	205	45	1746892	537560	0	3	1	0	4	3	2137424	7.09E-01	1
RTKN2	111669	382	9	1600220	403348	0	4	2	0	50	55	22389196	7.09E-01	1
LGALS12	887496	459	31	860096	257032	0	2	0	0	50	61	22549396	7.09E-01	1
GMIP	768855	418	-10	2436820	759704	0	8	2	0	29	30	10641908	7.09E-01	1
CENPP	558433	574	45	758636	199360	0	2	0	0	50	60	20281676	7.09E-01	1

STAP1	128996	689	35	789964	194732	0	2	0	0	50	68	23467520	7.09E-01	1
HS6ST2	126179	NaN	16	1280888	346744	0	2	0	0	50	76	24196608	7.09E-01	1
PPP5C	1079866	357	39	1320048	335352	0	6	2	0	50	75	18027128	7.09E-01	1
FILIP1L	267848	573	10	2922760	760772	0	6	2	0	50	46	20143192	7.09E-01	1
ATP5G1	757963	239	29	350660	107512	0	1	1	0	13	21	5325404	7.09E-01	1
BRD2	1276842	431	40	2028844	587756	0	17	4	0	3	7	1166256	7.09E-01	1
MBD2	98718	437	6	1200432	368460	0	1	2	0	50	42	16743392	7.10E-01	1
CCDC82	272687	372	14	1451412	324672	0	4	0	0	43	56	20415532	7.10E-01	1
C11orf70	420989	358	-16	605200	153080	0	1	0	0	50	45	21324756	7.10E-01	1
SNAI2	16597	598	-22	684588	185832	0	2	0	0	50	63	19278468	7.10E-01	1
SLC4A7	110819	413	-3	3137072	860452	0	8	2	0	50	55	22816040	7.10E-01	1
PIGW	416822	556	23	1246712	375580	0	4	1	0	50	74	27633432	7.10E-01	1
CSRNP1	213514	500	30	1461380	446068	0	3	0	0	21	31	10103992	7.10E-01	1
EPOR	1385739	197	31	1255968	404772	0	3	1	0	50	60	22230420	7.10E-01	1
RCOR1	1468114	190	42	1262732	331792	0	2	0	0	50	94	31610308	7.10E-01	1
MED15	474817	286	44	2040948	561768	0	7	0	0	1	0	856892	7.10E-01	1
WBP11	340728	578	-4	1625496	478464	0	8	0	0	1	1	700252	7.10E-01	1
KRT32	1445853	325	-22	1138488	327876	0	3	1	0	50	58	19098688	7.10E-01	1
PPM1M	1114737	181	52	769316	218584	0	2	0	0	50	71	22668656	7.10E-01	1
GATA2	937778	274	52	1187260	375224	0	3	0	0	38	43	15428328	7.10E-01	1
OR5K2	390532	796	2	785692	228908	0	4	1	0	2	2	680316	7.10E-01	1
BCCIP	206845	738	-4	1022788	259880	0	2	0	0	23	17	8027088	7.10E-01	1
GPR31	149346	696	40	759348	264864	0	3	3	0	13	19	4900696	7.11E-01	1
SPERT	813295	613	47	1118908	330368	0	3	0	0	50	51	22342560	7.11E-01	1
SH3PXD2A	539433	300	35	2771460	827700	0	8	2	0	27	33	11343584	7.11E-01	1
RPS4Y1	0	0	0	682452	193308	0	1	0	0	50	39	21173456	7.11E-01	1
ZNF780A	1181917	521	22	1829484	438948	0	6	0	0	49	74	22707460	7.11E-01	1
LHFPL4	858637	425	36	614812	188324	0	1	0	0	50	51	22245372	7.11E-01	1
CDH19	6200	1015	-45	1996804	522608	0	10	2	0	29	51	11619484	7.11E-01	1
OR4D9	430021	507	-1	767180	239944	0	2	2	0	50	65	21241096	7.11E-01	1
PLXDC1	1452583	212	26	1306164	354576	0	2	0	0	20	9	6232492	7.11E-01	1
GEN1	135007	1245	10	2373452	594520	0	6	1	0	50	52	22598524	7.11E-01	1
RALGDS	1852158	322	51	2332512	695268	0	4	4	0	50	60	19926388	7.11E-01	1

SEC22B	738180	388	24	561768	147384	0	1	0	0	50	68	29309480	7.11E-01	1
TNFRSF14	339388	228	38	734428	205412	0	1	1	0	50	44	17226128	7.11E-01	1
OR51F2	66543	987	-3	840516	256320	0	3	2	0	50	67	17575720	7.12E-01	1
SH2D2A	1649536	208	53	1010684	307228	0	3	1	0	50	69	21716356	7.12E-01	1
SPANXB1	NaN	NaN	NaN	540408	140976	0	1	0	0	50	116	39786560	7.12E-01	1
LCN9	362222	524	29	461732	119260	0	2	1	0	3	3	1017804	7.12E-01	1
MRI1	767214	289	6	978288	330012	0	1	2	0	50	92	27852728	7.12E-01	1
GRM1	92600	941	-13	3032764	897476	0	15	3	0	5	12	2901756	7.12E-01	1
HERC4	337983	246	12	2767544	724816	0	6	0	0	5	3	2464232	7.12E-01	1
CHMP2B	57851	415	-19	569956	137060	0	1	0	0	50	45	17728800	7.12E-01	1
RETSAT	975949	211	18	1537564	462800	0	4	1	0	9	4	2652200	7.12E-01	1
KRT28	836683	269	-10	1184412	337488	0	4	1	0	22	26	7229292	7.12E-01	1
SECTM1	2856070	193	24	622644	192240	0	1	0	0	50	47	19854120	7.12E-01	1
TOMM34	952219	282	32	799932	218940	0	4	3	0	6	9	2311152	7.12E-01	1
OR11G2	371813	563	-29	846212	260236	0	3	1	0	50	77	20304460	7.12E-01	1
SVIL	138938	504	1	5656484	1589896	0	25	6	0	8	11	3212900	7.12E-01	1
SUZ12	717248	310	27	1943404	491636	0	4	2	0	37	35	18723108	7.12E-01	1
OR10AD1	511392	352	32	786760	232112	0	2	1	0	50	55	18729516	7.12E-01	1
ANKRD30A	53742	1067	-43	3609840	837312	0	16	2	0	4	7	2518700	7.12E-01	1
NHEJ1	798168	177	37	775368	213600	0	2	1	0	50	46	15760120	7.12E-01	1
SERTAD4	178673	440	-8	909580	247064	0	2	0	0	50	49	20032476	7.12E-01	1
JAKMIP2	102729	642	-4	2150596	529016	0	3	1	0	50	43	21155656	7.12E-01	1
TBC1D15	219208	563	-9	1956220	470276	0	7	2	0	50	69	22507032	7.12E-01	1
SLFNL1	341851	171	29	1008904	311144	0	3	1	0	6	8	3210052	7.12E-01	1
TTC9B	1005838	400	30	583484	197224	0	1	1	0	50	48	18018940	7.12E-01	1
NPHP1	NaN	NaN	NaN	1935216	499824	0	6	0	0	50	116	40145408	7.12E-01	1
ARL4C	121224	608	15	474548	143824	0	2	2	0	7	9	2255260	7.12E-01	1
INTS6	219283	371	38	2309372	615880	0	6	0	0	5	9	3657188	7.13E-01	1
AK1	1278941	201	51	505876	144536	0	1	0	0	50	53	22309808	7.13E-01	1
FAM47C	71087	NaN	-10	2497340	824140	0	8	2	0	40	78	26924280	7.13E-01	1
ZNF17	451095	869	-24	1717344	415452	0	6	1	0	50	68	17938840	7.13E-01	1
FBXO17	1794625	282	21	718764	225348	0	2	0	0	28	34	11289116	7.13E-01	1
MPPED2	29166	1075	-21	816664	224636	0	3	0	0	50	82	20482104	7.13E-01	1

INPP5A	155331	452	12	1118908	272696	0	5	2	0	6	16	4149180	7.13E-01	1
C9orf85	284245	358	5	430404	88644	0	1	1	0	24	28	9605948	7.13E-01	1
HIST1H4I	227588	622	29	248844	86508	0	6	3	0	4	8	497688	7.13E-01	1
PRR7	1038665	261	59	663940	221432	0	2	1	0	12	17	5103260	7.13E-01	1
UGT1A1	NaN	NaN	NaN	1340340	389820	0	2	1	0	50	117	40035404	7.13E-01	1
LILRB5	717623	458	-6	1490572	457460	0	5	1	0	50	58	18028196	7.13E-01	1
RPN2	787991	404	44	1816312	548240	0	3	2	0	50	64	22911804	7.13E-01	1
LDHC	599909	176	42	861164	235672	0	3	2	0	5	12	3796384	7.13E-01	1
YIPF4	396035	325	23	622288	184052	0	1	0	0	50	48	23365704	7.13E-01	1
HIST1H3H	267617	634	32	333216	110004	0	3	2	0	12	20	2202928	7.14E-01	1
MRS2	399209	465	7	1144896	320400	0	3	0	0	50	48	18933504	7.14E-01	1
SLC45A2	268032	863	8	1346748	398364	0	3	3	0	50	67	19262804	7.14E-01	1
KRT17	1494501	323	11	1094700	322536	0	4	0	0	20	34	10955900	7.14E-01	1
TMC4	735613	439	-53	1761844	582416	0	7	3	0	18	36	7425804	7.14E-01	1
UBFD1	528849	628	48	797796	221076	0	2	0	0	44	46	16041716	7.14E-01	1
NUAK2	664542	178	30	1562128	478820	0	5	1	0	3	2	1248492	7.14E-01	1
ARHGAP1[illegible]	945635	397	26	1291212	343896	0	5	2	0	50	76	23133592	7.14E-01	1
TAS2R1	349235	840	-18	743328	217872	0	2	1	0	50	69	19638384	7.14E-01	1
CXCR3	947642	NaN	52	1002496	351728	0	3	0	0	38	47	15669696	7.14E-01	1
TIGD6	1070181	149	44	1319692	357068	0	1	1	0	50	53	22084104	7.14E-01	1
RIN2	213905	508	0	2267720	647920	0	6	3	0	22	29	10278432	7.14E-01	1
MYL10	653305	246	45	601284	155928	0	1	0	0	50	55	22143556	7.14E-01	1
ASCL3	548433	243	21	456036	133500	0	1	0	0	50	57	20741984	7.14E-01	1
FXR2	2013497	194	29	1635820	487364	0	5	1	0	42	59	19296980	7.14E-01	1
TCTEX1D[illegible]	574317	191	43	520116	199716	0	1	0	0	50	62	20422296	7.14E-01	1
UBAP2	777874	170	29	2866868	841228	0	9	2	0	3	2	1578148	7.14E-01	1
ABCB1	287293	822	0	3300476	919192	0	8	3	0	50	49	19764764	7.15E-01	1
KLK10	490795	632	22	691708	220364	0	2	1	0	50	58	16739120	7.15E-01	1
EIF2S3	528219	NaN	41	1219300	345320	0	4	0	0	50	78	28181316	7.15E-01	1
IKZF4	2693176	215	38	1474552	432896	0	2	0	0	42	42	15617720	7.15E-01	1
TAB3	81645	NaN	13	1798512	515844	0	5	1	0	4	4	1980428	7.15E-01	1
FBXL4	237914	891	-6	1582776	440016	0	3	1	0	50	55	20394528	7.15E-01	1
GCN1L1	1586296	225	52	6757948	2048780	0	17	4	0	9	16	6486676	7.15E-01	1

C14orf169	NaN	NaN	NaN	1550736	505164	0	5	1	0	50	117	40150748	7.15E-01	1
PLEKHM3	331278	370	20	1945540	521540	0	4	2	0	50	50	24634132	7.15E-01	1
ZFC3H1	259684	393	-11	5176596	1344612	0	11	1	0	13	7	5790696	7.15E-01	1
IL26	325920	623	5	459952	110360	0	1	0	0	50	71	21956656	7.15E-01	1
PUF60	2279638	229	25	1421152	422216	0	4	2	0	50	72	27190568	7.15E-01	1
SCN3B	681725	451	6	566396	151300	0	1	0	0	50	49	18920688	7.15E-01	1
UBXN2A	240935	226	33	691352	167320	0	2	0	0	50	61	18110788	7.15E-01	1
PPP1R12A	222118	398	-8	2690648	719476	0	5	0	0	5	3	3220376	7.15E-01	1
UBE2U	175464	384	0	620864	140620	0	1	0	0	50	52	24719928	7.16E-01	1
RFC1	668832	299	43	3026356	754364	0	6	1	0	37	25	15124304	7.16E-01	1
ASB7	290809	374	-9	812748	228552	0	1	0	0	24	13	9389856	7.16E-01	1
GALNT14	94332	876	6	1446072	385548	0	3	0	0	22	24	10767576	7.16E-01	1
SFRP2	142627	793	-3	753296	203632	0	4	2	0	6	9	2473132	7.16E-01	1
DUSP26	91655	610	11	526168	165896	0	1	0	0	50	61	21651564	7.16E-01	1
CAMKV	1298303	170	38	1279820	371308	0	4	0	0	23	34	10991856	7.16E-01	1
SP140	504037	626	37	2446076	568888	0	10	1	0	50	81	22477128	7.16E-01	1
GTPBP2	1221714	194	46	1508728	472412	0	6	2	0	50	87	24616332	7.16E-01	1
RNF152	96352	799	-12	495552	158064	0	1	0	0	50	80	25910748	7.16E-01	1
MS4A13	337606	859	15	402280	109292	0	1	0	0	50	56	17876896	7.16E-01	1
MGAT4A	319617	696	36	1508372	373444	0	5	0	0	24	27	11399832	7.17E-01	1
OR2A4	129668	852	9	754364	241012	0	2	0	0	50	67	21034972	7.17E-01	1
EFCAB7	133143	401	7	1664656	408332	0	3	1	0	50	38	19140696	7.17E-01	1
DNM1	1788057	201	45	2250632	637240	0	6	1	0	13	10	4217888	7.17E-01	1
TMEM57	613155	230	24	1733720	439660	0	5	1	0	6	12	5474924	7.17E-01	1
DYDC2	340132	598	-7	472056	111072	0	1	0	0	50	52	15550080	7.17E-01	1
ZNF668	1398193	247	40	1575300	497688	0	5	1	0	50	79	26328336	7.17E-01	1
UAP1	320314	193	10	1318980	340692	0	3	2	0	50	51	19713856	7.17E-01	1
RUNDC1	1507064	201	26	1510152	478464	0	4	0	0	22	18	8144924	7.17E-01	1
AIP	1392213	215	53	846568	235316	0	2	0	0	11	7	3262740	7.17E-01	1
OR2H1	404210	854	21	765400	250268	0	2	2	0	12	14	5210416	7.17E-01	1
ABCD3	459824	348	-8	1777152	464580	0	5	1	0	22	21	8508400	7.17E-01	1
ZNF445	366436	418	42	2641164	686724	0	6	3	0	49	45	21387412	7.17E-01	1
RBBP5	655446	431	37	1408692	373800	0	3	0	0	34	34	14715260	7.17E-01	1

RNF113B	470049	470	20	814172	227128	0	2	0	0	50	50	17280596	7.17E-01	1
C4orf3	218603	824	-1	504452	148096	0	1	0	0	50	51	19615600	7.17E-01	1
PCDHGA1[illegible]	NaN	NaN	NaN	2304388	735140	0	8	5	0	26	45	12312260	7.18E-01	1
TRRAP	1040178	470	42	9834144	2739420	0	32	6	0	26	36	14837724	7.18E-01	1
BTAF1	366076	306	22	4802440	1285160	0	12	2	0	7	7	4248148	7.18E-01	1
PDE12	703082	422	29	1506948	456036	0	5	1	0	50	58	21505604	7.18E-01	1
PTGES3	2757565	164	43	456392	89356	0	1	1	0	25	34	10546856	7.18E-01	1
CRP	737948	602	-2	565328	159844	0	2	2	0	6	7	2167684	7.18E-01	1
KLF3	294258	364	39	883948	245996	0	3	0	0	4	3	1517272	7.18E-01	1
TIMP2	681697	157	24	575296	149876	0	1	1	0	50	50	21912512	7.18E-01	1
PHEX	92340	NaN	14	2000364	492348	0	6	1	0	17	37	10589220	7.18E-01	1
CREG1	481959	506	15	545036	178000	0	1	0	0	50	60	19997588	7.18E-01	1
SEH1L	436900	355	69	1102176	300108	0	1	1	0	50	58	19829556	7.18E-01	1
SLC26A3	511053	515	19	1975444	561056	0	8	4	0	16	22	5595252	7.18E-01	1
MFSD6L	423397	636	11	1410828	469920	0	5	3	0	50	71	20685024	7.18E-01	1
EVI5	488549	506	4	2144188	529016	0	7	1	0	50	55	21434404	7.18E-01	1
RINL	1989663	219	30	1123892	361696	0	3	0	0	34	48	16044920	7.18E-01	1
HSPBAP1	424676	412	39	1268784	327876	0	4	2	0	41	61	18865508	7.18E-01	1
WDR49	145736	911	-20	1839808	456392	0	8	2	0	8	16	4877912	7.18E-01	1
ADAMTS1[illegible]	61971	1102	-26	3155940	853332	0	17	5	0	50	127	27636280	7.19E-01	1
BTN3A3	384310	375	47	1489504	423284	0	7	4	0	17	22	5974748	7.19E-01	1
STX18	140686	633	44	897832	223568	0	2	0	0	34	32	11430448	7.19E-01	1
LRRC31	404285	689	13	1430408	377716	0	10	0	0	1	2	705592	7.19E-01	1
VGLL2	181822	589	-25	790676	241012	0	1	0	0	14	45	11037424	7.19E-01	1
CLASP2	463864	393	9	3899624	1086868	0	10	0	0	0	0	1086868	7.19E-01	1
PFKFB2	434877	155	24	1387688	365968	0	3	1	0	22	32	10203316	7.19E-01	1
GLB1L2	270422	686	5	1659672	458172	0	5	3	0	12	19	5967628	7.19E-01	1
PA2G4	2785610	165	39	1054116	262728	0	3	0	0	36	47	15674324	7.19E-01	1
RAX2	2144458	206	34	443220	158064	0	1	0	0	50	63	19224000	7.19E-01	1
VAT1L	44717	1259	-28	1084732	297260	0	6	2	0	50	111	22577876	7.19E-01	1
OR4L1	383274	768	-37	776436	226416	0	2	2	0	32	40	12755836	7.19E-01	1
F9	69360	NaN	0	1207908	304380	0	3	0	0	38	47	18112924	7.19E-01	1
ATL1	483990	295	43	1470280	376292	0	3	0	0	19	26	11475660	7.19E-01	1

ZNF121	706527	642	-58	1011040	250268	0	2	1	0	50	120	30635224	7.19E-01	1
GATSL3	548674	211	43	836244	255252	0	2	1	0	50	54	19921048	7.19E-01	1
SENP3	1957426	194	24	1458176	427912	0	2	0	0	17	20	7764360	7.19E-01	1
SATB1	67460	883	-6	1956932	533644	0	5	1	0	50	56	23209064	7.19E-01	1
G2E3	354135	695	-10	1873628	451408	0	5	1	0	50	57	21194816	7.19E-01	1
T	141864	323	-23	1107872	318976	0	6	3	0	6	7	1578860	7.20E-01	1
SMOX	784685	231	26	1483808	423640	0	5	2	0	50	77	22406996	7.20E-01	1
UTY	0	0	0	3725896	987188	0	6	0	0	0	0	987188	7.20E-01	1
CCDC127	1040169	530	24	666788	178000	0	2	0	0	50	65	18657604	7.20E-01	1
TARDBP	702484	307	33	1065864	285156	0	2	1	0	5	5	3216460	7.20E-01	1
ZNF432	436768	901	-53	1691000	414028	0	7	3	0	50	58	17193732	7.20E-01	1
C10orf53	NaN	NaN	NaN	463512	124956	0	1	0	0	50	116	39770540	7.20E-01	1
MST1	1551582	271	48	1875408	527592	0	6	4	0	43	65	18594592	7.20E-01	1
NASP	873800	161	27	2117488	554648	0	8	0	0	3	4	1792816	7.20E-01	1
ZNF684	693560	457	26	999648	231756	0	3	0	0	50	57	20934580	7.20E-01	1
FCRLA	598281	504	20	959064	289428	0	3	1	0	50	64	18701036	7.20E-01	1
FAM102B	615760	313	-3	949452	252048	0	3	0	0	50	78	24723488	7.20E-01	1
TYW1B	NaN	NaN	NaN	1870424	458884	0	7	2	0	50	118	40104468	7.20E-01	1
TMEM119	437636	399	40	681028	233180	0	1	1	0	7	13	3206492	7.20E-01	1
SGMS1	295850	573	28	1059456	284088	0	3	1	0	50	70	23338292	7.21E-01	1
IFNB1	144026	601	5	482736	121752	0	1	0	0	50	56	18608832	7.21E-01	1
DOCK6	1691034	233	32	5184784	1579928	0	13	1	0	4	7	3765768	7.21E-01	1
MKL2	185822	323	12	2652912	775368	0	6	0	0	11	6	4235332	7.21E-01	1
GARNL3	673881	270	47	2642944	721256	0	8	3	0	50	58	23032488	7.21E-01	1
MYBPC2	1038277	491	30	2970108	806340	0	6	4	0	50	64	20883672	7.21E-01	1
ASPHD2	163136	553	-46	922040	271984	0	3	1	0	50	80	20251060	7.21E-01	1
TRPV2	829707	254	8	1934148	572448	0	5	3	0	50	75	21935652	7.21E-01	1
MYH9	1223952	226	49	5111804	1342120	0	27	9	0	3	11	2314000	7.21E-01	1
TBP	425191	598	-16	875404	239588	0	2	0	0	50	46	17122888	7.21E-01	1
USP25	74958	477	30	2795668	686012	0	7	4	0	13	13	4745480	7.21E-01	1
GRIPAP1	834590	NaN	41	2271280	590960	0	5	0	0	11	9	4312584	7.21E-01	1
SMPDL3A	138284	1073	-1	1179784	304736	0	3	1	0	50	41	15762612	7.21E-01	1
EGLN1	362960	518	3	1068356	316840	0	3	0	0	37	51	17896476	7.21E-01	1

ZNF232	1391000	384	20	1137064	303668	0	4	1	0	50	83	22837756	7.21E-01	1
NAP1L2	196410	NaN	-3	1174800	302244	0	4	1	0	10	9	3260960	7.21E-01	1
ADRBK1	1369264	177	44	1822720	473480	0	5	4	0	41	55	16974792	7.21E-01	1
LYG1	386109	630	24	506944	139196	0	2	2	0	3	2	896052	7.21E-01	1
MORN3	743586	194	50	642936	150588	0	2	0	0	50	60	16334704	7.21E-01	1
PPP2R2D	123624	470	-21	1104668	270916	0	4	0	0	50	80	22720632	7.22E-01	1
FOXRED2	1191841	195	30	1708800	519048	0	4	2	0	7	7	3809200	7.22E-01	1
KIR2DL1	NaN	NaN	NaN	897120	253116	0	1	2	0	26	42	11830236	7.22E-01	1
SLC5A7	122653	911	-16	1459244	436456	0	6	4	0	7	10	2725892	7.22E-01	1
DRG2	899154	183	23	957284	265576	0	3	0	0	50	50	14179124	7.22E-01	1
ANKRD45	432542	423	4	699184	177644	0	1	0	0	50	39	18573588	7.22E-01	1
CYP20A1	417679	298	23	1217520	317196	0	3	1	0	28	30	11332192	7.22E-01	1
CALCRL	61297	453	1	1231404	300108	0	5	2	0	4	7	2374876	7.22E-01	1
SALL1	332955	935	-42	3275912	980068	0	16	5	0	50	65	15613448	7.22E-01	1
TTC29	34493	970	-9	1263088	309008	0	4	3	0	4	4	1536852	7.22E-01	1
TM9SF4	1055549	262	36	1689576	443220	0	8	3	0	7	11	2339276	7.22E-01	1
CPT1A	619314	303	45	2045576	553936	0	5	3	0	46	43	19313356	7.22E-01	1
AP1G2	1250373	553	35	1987548	620508	0	7	2	0	50	82	25432996	7.22E-01	1
SBF2	530606	482	12	4807780	1286228	0	12	3	0	5	3	2520836	7.22E-01	1
ILDR2	261602	681	-9	1626564	464580	0	4	1	0	50	60	25885472	7.22E-01	1
HSPA2	662511	188	36	1574944	475616	0	5	3	0	24	27	10666472	7.23E-01	1
C2orf80	321897	440	5	522252	133500	0	1	0	0	50	50	16889352	7.23E-01	1
RERG	354506	469	3	512996	140620	0	1	1	0	33	33	13518388	7.23E-01	1
GNMT	1200368	259	40	756856	215024	0	2	2	0	47	62	20023576	7.23E-01	1
HPS4	163136	553	-46	1807412	541120	0	6	3	0	7	12	3713792	7.23E-01	1
SLC35F5	518912	544	19	1377008	365968	0	3	0	0	24	27	9341084	7.23E-01	1
AP1S2	170722	NaN	47	416876	104308	0	1	1	0	50	65	20146396	7.23E-01	1
TANC2	778921	453	-27	5005716	1478112	0	16	4	0	50	64	20635184	7.23E-01	1
KIAA1161	478815	302	38	1730160	564972	0	4	2	0	50	40	17367460	7.23E-01	1
GRID1	89265	965	-18	2571388	730868	0	11	4	0	50	74	21986204	7.23E-01	1
CRK	1380844	220	18	766468	219296	0	3	1	0	50	73	21304464	7.23E-01	1
SSX4	NaN	NaN	NaN	1130656	206480	0	1	0	0	50	116	39852064	7.23E-01	1
BTC	110870	606	3	466360	127448	0	1	0	0	50	61	22065592	7.23E-01	1

KCNH3	2227486	224	53	2654336	878608	0	9	5	0	50	79	22778304	7.23E-01	1
BTK	374364	NaN	37	1757928	433608	0	5	0	0	25	27	11161312	7.24E-01	1
SEL1L	192523	330	-11	2090076	542544	0	9	0	0	2	1	1065152	7.24E-01	1
C10orf2	1020226	247	40	1701324	528660	0	5	1	0	50	62	20159924	7.24E-01	1
MTMR11	1115711	451	51	1849064	533644	0	5	3	0	50	61	22950252	7.24E-01	1
PCDH18	87218	686	-17	2844796	807764	0	8	1	0	6	9	4607708	7.24E-01	1
CHI3L1	507603	453	31	995020	273764	0	2	0	0	28	30	12709912	7.24E-01	1
COLEC11	253814	371	14	801000	184764	0	2	1	0	50	69	23313728	7.24E-01	1
DNAJB1	1008795	204	26	858672	244572	0	3	1	0	50	74	20546896	7.24E-01	1
ZSCAN18	1248040	619	-53	1405132	439304	0	4	0	0	5	4	2453552	7.24E-01	1
GADD45A	446714	228	-7	425420	119260	0	1	1	0	25	34	11032084	7.24E-01	1
CYP2E1	563924	525	-10	1272700	346388	0	4	2	0	50	61	17772588	7.25E-01	1
GRID2	4518	1143	-17	2571744	720900	0	9	5	0	17	22	6256700	7.25E-01	1
DDIT3	1347795	303	44	429692	123532	0	1	0	0	50	94	29615284	7.25E-01	1
ATP5O	948739	419	44	553224	162336	0	1	0	0	50	67	21864452	7.25E-01	1
OSBPL10	373732	604	7	1939488	558564	0	4	3	0	50	78	21206564	7.25E-01	1
GDAP1L1	664405	367	19	936636	265932	0	2	0	0	50	60	23006500	7.25E-01	1
RAET1G	423805	245	34	849772	244928	0	2	0	0	50	59	20035324	7.25E-01	1
HNRNPF	368953	270	7	1046640	290496	0	2	1	0	39	45	19219016	7.25E-01	1
OXR1	124540	888	-2	2358144	630120	0	6	3	0	26	24	9158812	7.25E-01	1
LHB	2070141	200	35	352084	115700	0	1	0	0	50	68	18142828	7.25E-01	1
RBMS1	191666	366	5	1080816	278748	0	2	0	0	47	39	18272768	7.25E-01	1
C19orf12	223109	552	-46	381988	116768	0	2	2	0	4	7	1407624	7.25E-01	1
EML4	251363	292	17	2559640	680672	0	5	2	0	29	22	10440056	7.25E-01	1
CALML3	769552	446	17	375224	105376	0	1	1	0	15	16	4289444	7.25E-01	1
ZNF226	554986	634	-28	2138492	514420	0	6	2	0	50	52	17611320	7.25E-01	1
MADD	1231960	198	41	4251708	1178004	0	10	2	0	4	6	4285528	7.25E-01	1
CRIM1	267484	414	-1	2677832	715204	0	7	0	0	10	9	4743700	7.25E-01	1
TPM2	1202125	248	37	906020	219652	0	2	1	0	50	57	20786840	7.25E-01	1
PAOX	435826	472	10	1391960	364900	0	3	2	0	50	50	20101896	7.25E-01	1
ZNF566	645107	833	-56	1113212	249556	0	4	1	0	50	67	17388464	7.25E-01	1
CD70	836909	212	21	470988	159132	0	1	0	0	50	46	16886860	7.26E-01	1
ZNF341	718430	161	58	2149884	629052	0	8	5	0	21	33	7584936	7.26E-01	1

ERMN	78933	837	8	786404	181204	0	2	1	0	50	73	23743776	7.26E-01	1
NPR3	477035	487	-15	1363836	399432	0	5	1	0	5	9	2415460	7.26E-01	1
WFIKKN2	1152451	161	15	1430052	422928	0	5	2	0	34	34	10998264	7.26E-01	1
C1orf177	521961	249	25	1143116	311500	0	1	1	0	12	7	3277336	7.26E-01	1
TXLNG	289514	NaN	39	1386620	348880	0	4	1	0	50	82	21778656	7.26E-01	1
HDC	308338	282	4	1691000	480244	0	3	2	0	50	49	25082692	7.26E-01	1
SCARF2	606391	351	4	2142408	690996	0	4	1	0	28	35	13311552	7.26E-01	1
FAM170A	296413	644	-2	850128	224280	0	2	1	0	50	52	17495976	7.26E-01	1
NAA16	302803	283	51	2356720	531152	0	4	1	0	50	44	19326172	7.26E-01	1
NINJ1	504920	448	61	389820	115344	0	1	0	0	50	67	19457180	7.27E-01	1
SNX2	144132	532	31	1382348	345676	0	3	0	0	50	62	25955960	7.27E-01	1
EXD1	933164	175	39	1328948	359560	0	5	0	0	16	20	6877208	7.27E-01	1
MANBA	379516	303	32	2299404	590604	0	3	1	0	31	24	11928848	7.27E-01	1
OR5T3	10317	969	-43	845856	245640	0	4	2	0	8	13	3063024	7.27E-01	1
ASPHD1	NaN	NaN	NaN	935568	325740	0	3	3	0	26	43	11902860	7.27E-01	1
CDRT4	405431	452	-68	389820	105732	0	1	0	0	50	113	28837424	7.27E-01	1
SRD5A1	253440	585	-1	662872	187256	0	1	1	0	50	46	17146028	7.27E-01	1
AIFM3	577297	192	58	1590608	451408	0	3	1	0	26	48	11427244	7.27E-01	1
PPP1R16A	1755818	291	7	1319692	413672	0	3	0	0	13	17	7333956	7.27E-01	1
IM6-TRIM	128694	895	9	2156292	571380	0	4	0	0	2	1	1225352	7.27E-01	1
ZNF518A	686240	857	37	3755800	995732	0	6	2	0	7	6	5350324	7.27E-01	1
ZRSR2	170722	NaN	47	1294772	299752	0	2	2	0	50	65	20146396	7.27E-01	1
TSHZ2	102643	626	-18	2611260	713424	0	8	4	0	12	13	5118568	7.28E-01	1
IL17RE	950574	170	54	1679252	529372	0	2	2	0	39	56	16713132	7.28E-01	1
SEC14L5	635794	302	9	1781780	515488	0	5	1	0	50	65	26718156	7.28E-01	1
ZNF839	1615110	214	35	2273772	731580	0	8	0	0	0	0	731580	7.28E-01	1
NAT1	370301	569	-10	903528	240300	0	2	0	0	50	39	15102944	7.28E-01	1
RAB23	314024	348	23	633324	154860	0	1	0	0	50	46	22560076	7.28E-01	1
RPRD2	1460825	163	36	3609128	1117840	0	11	3	0	34	75	22729532	7.28E-01	1
STX19	85529	1378	-18	766468	185120	0	2	1	0	42	52	15985468	7.28E-01	1
SCN2A	119701	879	-9	5265596	1375228	0	18	6	0	9	16	5446800	7.28E-01	1
SORD	724004	565	19	910648	270560	0	2	0	0	4	3	1975088	7.28E-01	1
CIB2	414873	271	56	501604	122108	0	1	0	0	50	44	17114700	7.28E-01	1

KLHL24	1124894	463	32	1533292	417944	0	5	3	0	40	53	16060228	7.28E-01	1
PEBP4	718204	366	44	604488	151656	0	1	0	0	50	59	26221892	7.28E-01	1
SH3KBP1	340334	NaN	58	1788188	489856	0	6	2	0	5	5	1975444	7.28E-01	1
YWHAE	1417492	215	22	671060	170524	0	2	0	0	50	67	19106876	7.28E-01	1
DNTTIP2	558028	275	9	1952660	502672	0	4	2	0	50	71	25235060	7.28E-01	1
PLEKHG2	1410725	230	39	3392324	1128520	0	9	1	0	1	1	1641516	7.29E-01	1
THOP1	1320743	291	26	1771100	490924	0	5	1	0	50	53	22696780	7.29E-01	1
ACOT12	114956	831	16	1453904	389464	0	5	3	0	8	7	2938068	7.29E-01	1
CST5	159399	889	-45	372376	96476	0	1	0	0	50	66	16827052	7.29E-01	1
SNRPC	995432	242	24	427556	106444	0	1	0	0	50	58	18985480	7.29E-01	1
SKA2	843250	507	9	449984	92560	0	1	0	0	50	55	18982276	7.29E-01	1
BCDIN3D	1291800	231	45	731224	213956	0	2	1	0	50	88	26715664	7.29E-01	1
PSMC3	1022438	245	47	1145964	312924	0	3	0	0	50	66	23883684	7.29E-01	1
SAFB	879991	295	21	2388760	633680	0	10	0	0	1	0	719832	7.29E-01	1
CCDC18	657064	412	9	3776804	881812	0	7	1	0	13	9	5729464	7.29E-01	1
RNF183	426351	312	26	472412	150232	0	1	0	0	50	53	18446852	7.29E-01	1
TAS2R4	200522	844	14	735496	225704	0	2	0	0	50	60	17687504	7.29E-01	1
HEATR4	602175	149	42	2503392	700608	0	7	3	0	50	49	19416240	7.29E-01	1
RSBN1L	262077	795	25	2154156	591672	0	4	3	0	32	32	12246756	7.29E-01	1
ZNF568	265140	1008	-41	1690288	393380	0	6	2	0	50	86	20457540	7.29E-01	1
ZNF705A	172474	438	26	793168	190460	0	2	0	0	13	15	4762924	7.29E-01	1
CR1L	386490	361	13	1477756	397652	0	4	1	0	5	7	3327888	7.29E-01	1
INSM2	313860	434	11	1336068	480600	0	3	0	0	44	49	19533720	7.29E-01	1
VMO1	1272092	222	28	537204	164472	0	1	1	0	14	13	5818820	7.29E-01	1
MACROD2	37709	920	-12	1239236	271984	0	4	1	0	50	78	25344708	7.30E-01	1
SPTBN4	915378	177	37	6445024	2021012	0	18	7	0	13	19	7876500	7.30E-01	1
LEMD3	299752	577	7	2300116	675332	0	6	1	0	31	37	12774348	7.30E-01	1
GJB7	222729	596	22	571380	150588	0	1	1	0	36	33	14766168	7.30E-01	1
GJA10	383829	567	24	1365260	375580	0	3	0	0	50	66	27209080	7.30E-01	1
SAMD11	1851188	467	3	1676760	566040	0	4	0	0	4	6	3383780	7.30E-01	1
WSCD1	283312	593	-11	1437528	437880	0	5	0	0	3	1	1044860	7.30E-01	1
THAP4	1213423	426	21	1449632	432184	0	3	1	0	50	69	19805704	7.30E-01	1
ARFGAP1	1278630	332	62	1113568	278036	0	4	1	0	50	61	17105444	7.30E-01	1

IRS4	110759	NaN	29	3054480	980424	0	9	4	0	8	17	5419032	7.30E-01	1
EIF2AK1	NaN	NaN	NaN	1639736	441796	0	5	1	0	50	117	40087380	7.30E-01	1
RPL13	1094600	211	38	536492	164116	0	1	0	0	50	48	16202984	7.30E-01	1
RABAC1	1150724	438	33	469920	145248	0	1	0	0	50	64	21926752	7.30E-01	1
CUL3	255624	463	20	2021368	506588	0	5	0	0	5	2	2096840	7.30E-01	1
TBC1D19	116288	735	17	1417236	356712	0	4	0	0	29	25	11829524	7.30E-01	1
C10orf76	932053	319	42	1853692	463868	0	2	2	0	50	67	21448644	7.30E-01	1
REST	590352	569	37	2788904	744040	0	5	1	0	15	22	8393768	7.31E-01	1
NME7	270055	491	12	1001072	255964	0	2	0	0	50	54	20457184	7.31E-01	1
C2orf68	1158965	170	43	430048	119972	0	1	1	0	36	48	15827404	7.31E-01	1
OPTN	244367	372	16	1532936	378784	0	7	4	0	7	8	2219304	7.31E-01	1
MFAP2	678965	221	52	503028	120684	0	1	0	0	50	57	20959500	7.31E-01	1
ABCA9	263023	602	-42	4250284	1118552	0	14	1	0	1	2	1352800	7.31E-01	1
MCM10	246179	354	35	2276976	610896	0	8	3	0	10	9	3885740	7.31E-01	1
KRTAP9-9	1149433	468	-14	433608	113208	0	1	0	0	50	53	16834528	7.31E-01	1
KRT34	1321356	424	-6	1115348	312568	0	3	1	0	50	49	16872976	7.31E-01	1
MEX3B	229948	844	-8	1364548	466004	0	2	2	0	50	70	22189480	7.31E-01	1
FOLR3	715293	267	62	646852	158420	0	1	0	0	50	46	18093700	7.31E-01	1
TUBB	2115149	171	41	1125316	313280	0	3	1	0	50	71	23126828	7.31E-01	1
CCDC15	409881	760	21	2493780	620508	0	5	0	0	18	14	8707048	7.31E-01	1
SEC23IP	450354	404	39	2584916	701320	0	11	1	0	12	17	6055204	7.32E-01	1
UCP2	533576	196	41	775012	243860	0	2	1	0	50	52	16080164	7.32E-01	1
SUSD5	496534	256	21	1559992	475616	0	4	1	0	50	47	18103668	7.32E-01	1
ZFP36	1579611	230	36	768604	285512	0	2	1	0	50	51	17214024	7.32E-01	1
HMBOX1	382811	465	40	1103244	284088	0	4	1	0	50	76	23547264	7.32E-01	1
SNIP1	445473	274	30	996444	290496	0	2	0	0	50	61	20995456	7.32E-01	1
ATP5B	2757565	164	43	1322540	414028	0	3	1	0	32	38	15422276	7.32E-01	1
SOCS7	1035666	181	22	1439308	463868	0	3	1	0	26	33	10159172	7.32E-01	1
ANKRD40	1585602	196	18	926668	272696	0	4	1	0	50	100	24286320	7.32E-01	1
NFATC2IP	1189140	257	55	1034892	342828	0	3	1	0	50	73	21972320	7.32E-01	1
RNF165	452638	289	33	889288	254540	0	2	0	0	50	50	18341476	7.32E-01	1
TUBA1C	2154742	171	49	1131012	326808	0	2	0	0	8	8	2941984	7.32E-01	1
FCGR2C	693228	503	23	835532	230332	0	3	1	0	50	68	21398092	7.32E-01	1

TNFAIP8L2	1562281	184	33	452120	144892	0	1	1	0	50	64	20964128	7.33E-01	1
PNLIPRP1	128161	704	-16	1231760	318976	0	8	4	0	7	15	2983992	7.33E-01	1
LPAR3	340292	614	-7	887508	255252	0	3	3	0	24	23	6614124	7.33E-01	1
MAGEA1	607570	NaN	18	762908	234604	0	1	1	0	50	39	20146752	7.33E-01	1
SMPDL3B	891090	272	20	1256680	357068	0	5	2	0	32	41	12160248	7.33E-01	1
FSCN3	411207	619	-7	1261664	360628	0	3	2	0	50	41	15048476	7.33E-01	1
MRPL13	223063	877	-16	479176	119972	0	1	0	0	50	70	24196252	7.33E-01	1
CPNE8	87865	674	-4	1509796	383768	0	5	2	0	50	67	21411976	7.33E-01	1
SH3BP2	643291	228	43	1625852	498400	0	2	2	0	50	58	21966268	7.33E-01	1
C1orf74	220312	429	18	655040	214312	0	1	0	0	50	55	21775452	7.33E-01	1
ZMAT2	774470	356	31	547528	116768	0	1	0	0	50	59	20786128	7.34E-01	1
SBK2	1094560	399	4	835888	289784	0	2	0	0	27	25	7431500	7.34E-01	1
DPP7	1722012	202	36	1254544	378428	0	3	0	0	3	3	2037744	7.34E-01	1
ACBD3	756400	214	24	1357428	369528	0	4	0	0	23	34	13760112	7.34E-01	1
OR51E1	212267	935	-3	774300	252048	0	2	1	0	50	68	21342556	7.34E-01	1
USP13	361278	589	12	2270924	584908	0	6	3	0	50	66	18819584	7.34E-01	1
POGK	258555	773	-7	1552516	423284	0	4	2	0	12	17	7100776	7.34E-01	1
SH3BP5L	411701	567	-4	977576	306160	0	3	0	0	2	1	1058388	7.34E-01	1
STARD7	850399	251	48	959064	259524	0	2	2	0	14	26	9280208	7.34E-01	1
ATAD1	335357	169	45	952300	245996	0	2	0	0	50	49	18258528	7.35E-01	1
GGCT	624959	400	36	493060	125312	0	1	1	0	13	15	4297276	7.35E-01	1
ATP6V1E2	587710	213	36	572092	158420	0	1	0	0	50	49	20644440	7.35E-01	1
OR2D2	403341	827	6	744396	244572	0	2	0	0	50	71	21769756	7.35E-01	1
CST4	189028	819	-21	364544	101104	0	1	1	0	7	7	2401932	7.35E-01	1
TNIK	146464	805	25	3555372	941976	0	9	3	0	50	39	17190884	7.35E-01	1
C12orf10	1689542	166	53	949808	288004	0	3	0	0	19	22	7674648	7.35E-01	1
HNRNPUL2	2425755	248	51	1926672	525456	0	8	1	0	3	1	1126740	7.35E-01	1
FOXA3	883734	203	39	845500	283376	0	2	3	0	12	25	5236404	7.35E-01	1
SUCNR1	200470	526	2	843008	238876	0	2	2	0	9	6	2714856	7.35E-01	1
KIF14	361992	370	7	4316500	1086512	0	12	3	0	50	51	19244648	7.35E-01	1
TMEM99	836683	269	-10	632256	197580	0	2	1	0	50	63	16537268	7.35E-01	1
NLRC4	394223	276	21	2619804	697404	0	11	5	0	30	40	15258516	7.35E-01	1
PPM1J	559669	325	27	1253120	408688	0	3	1	0	50	65	23160292	7.35E-01	1

FUT1	1635420	190	44	890712	286224	0	2	2	0	22	26	9150980	7.35E-01	1
TOP1MT	1770114	220	27	1566044	422572	0	5	1	0	37	70	17757636	7.36E-01	1
FGF21	1635420	190	44	508368	173016	0	1	1	0	50	73	21072352	7.36E-01	1
SLC25A43	655775	NaN	34	845144	267712	0	1	0	0	15	14	7762580	7.36E-01	1
CHGB	310620	346	13	1761132	428268	0	11	3	0	9	14	3442876	7.36E-01	1
ZNF217	142853	451	-30	2646504	731580	0	10	5	0	18	40	11130696	7.36E-01	1
ESRP2	910198	281	30	1775728	584552	0	6	1	0	6	12	2828064	7.36E-01	1
KIF17	632832	208	35	2594172	765756	0	9	2	0	50	62	22215824	7.36E-01	1
BRF1	899979	260	32	1751876	493060	0	7	1	0	8	8	2676408	7.36E-01	1
FUNDC2	NaN	NaN	NaN	485228	140620	0	1	0	0	50	116	39786204	7.36E-01	1
LYPLAL1	82669	735	-6	622644	159132	0	1	0	0	50	60	26851300	7.36E-01	1
ARHGEF1[illegible]	601834	218	54	5050572	1650060	0	12	3	0	8	11	5649364	7.36E-01	1
CDNF	256031	306	32	487364	129940	0	1	0	0	50	56	19659388	7.36E-01	1
TLE1	36728	629	-33	1992532	558920	0	8	3	0	25	42	8468884	7.36E-01	1
EMCN	231787	807	-5	695980	190460	0	1	1	0	50	40	19193028	7.36E-01	1
ITIH4	1193949	272	49	2399796	683520	0	7	1	0	11	9	4817392	7.37E-01	1
SLC38A3	846254	199	44	1310436	371664	0	3	2	0	39	49	14244272	7.37E-01	1
RC3H2	407115	430	26	3110016	875760	0	6	0	0	1	0	1161984	7.37E-01	1
CACNA1A	1604988	497	12	6494152	1856540	0	23	9	0	43	55	19818520	7.37E-01	1
ZFPM2	46151	998	-25	2935932	787116	0	11	1	0	22	44	10612004	7.37E-01	1
VCX3B	28829	NaN	-28	559276	144536	0	2	0	0	50	122	25026088	7.37E-01	1
TEX101	357202	182	-39	678892	201140	0	1	2	0	19	46	9630512	7.37E-01	1
KRTAP26-[illegible]	6302	1011	-69	516912	159132	0	2	1	0	50	66	13353560	7.37E-01	1
UBP1	483698	352	9	1436104	365612	0	4	1	0	50	73	27359668	7.37E-01	1
RAN	214809	440	-6	574228	146672	0	1	1	0	50	48	20748748	7.37E-01	1
C4orf33	98087	645	-2	535780	126380	0	1	0	0	50	54	20805708	7.37E-01	1
TSPAN8	233516	489	-31	627984	164472	0	2	0	0	50	93	22219028	7.37E-01	1
PDE1C	124074	828	6	1681744	425420	0	12	3	0	2	3	1061236	7.37E-01	1
USP31	576886	701	22	3375592	1023500	0	7	3	0	40	35	15583544	7.37E-01	1
COL15A1	199434	148	18	3529384	1096124	0	9	2	0	22	15	6998960	7.38E-01	1
PPP1R9B	1277832	210	26	2016384	640800	0	2	1	0	50	71	23861612	7.38E-01	1
POTEG	148332	835	-50	1348172	326452	0	3	1	0	9	6	3224292	7.38E-01	1
NDUFA6	1321127	363	65	398364	108936	0	1	0	0	50	55	15728436	7.38E-01	1

PRKAR1A	662487	313	-28	995732	270916	0	4	2	0	50	85	21558648	7.38E-01	1
POLR3GL	NaN	NaN	NaN	582416	149164	0	1	1	0	50	117	39794748	7.38E-01	1
LTA4H	364666	436	16	1611612	428268	0	4	1	0	41	37	15411240	7.38E-01	1
MAP2K5	449735	377	31	1217164	311144	0	3	1	0	50	44	17096544	7.38E-01	1
TBC1D9B	1626799	359	34	3201864	898188	0	8	5	0	50	46	18304452	7.38E-01	1
GSTT2B	699286	202	63	621220	185120	0	2	1	0	47	61	15440432	7.38E-01	1
PACS2	892593	289	40	2310440	652192	0	4	0	0	18	23	12290900	7.38E-01	1
ZKSCAN5	1460222	332	56	2168752	548240	0	9	3	0	50	70	18313708	7.38E-01	1
ZNF550	459366	828	-31	965828	262372	0	2	0	0	33	27	10916384	7.38E-01	1
FEN1	1239228	212	59	950876	274120	0	2	0	0	43	58	22262460	7.38E-01	1
OLFM4	207557	842	2	1289788	364544	0	3	1	0	50	57	20775448	7.39E-01	1
CCNY	158899	591	2	896052	238164	0	2	0	0	50	54	19372808	7.39E-01	1
MTA2	2695768	217	42	1723040	493060	0	6	0	0	17	27	9232504	7.39E-01	1
TTC27	358024	636	17	2210404	574940	0	3	1	0	50	54	22541564	7.39E-01	1
PHACTR4	778726	182	29	1840520	518692	0	5	1	0	3	1	1089004	7.39E-01	1
OR5D14	4183	1091	-36	776792	231400	0	4	1	0	14	26	3895352	7.39E-01	1
KRTAP10-[illegible]	874480	461	25	725528	215380	0	2	0	0	50	76	22638396	7.39E-01	1
DNAJC5G	1242113	182	45	506588	123532	0	1	0	0	50	76	21110800	7.39E-01	1
DNAH5	148607	674	-17	12001472	3152380	0	56	16	0	16	36	10797124	7.39E-01	1
CDK5R2	922162	386	35	865080	313992	0	2	1	0	50	52	18596728	7.39E-01	1
TRIM48	981	1138	-38	599148	144180	0	2	0	0	50	95	21188052	7.39E-01	1
GRHPR	534559	337	36	837668	252760	0	2	1	0	50	61	21069860	7.39E-01	1
DNAJC8	779314	213	33	692776	157352	0	1	0	0	50	49	18363192	7.39E-01	1
RTP4	377182	466	33	625492	172304	0	1	1	0	50	37	17546172	7.39E-01	1
B9D1	630769	332	11	536136	146316	0	1	0	0	50	72	28508124	7.39E-01	1
C3orf58	34520	1009	-6	1092564	331080	0	4	1	0	50	71	18987972	7.39E-01	1
AKR1B1	328321	415	9	840160	216092	0	2	0	0	50	67	23011484	7.39E-01	1
ZFP3	1399774	343	26	1299400	316484	0	3	0	0	50	79	21015392	7.39E-01	1
TYMS	416109	486	19	905664	237096	0	2	1	0	50	60	21957368	7.39E-01	1
TOR1AIP2	470940	364	5	1525460	419368	0	4	1	0	50	51	22478552	7.39E-01	1
EFCAB6	306296	627	2	3934156	1008548	0	11	3	0	50	52	19697836	7.39E-01	1
ZC3H15	269890	299	10	1143116	263440	0	2	0	0	50	38	17890780	7.39E-01	1
TSKS	1699718	192	47	1489860	457104	0	8	1	0	4	11	3068008	7.39E-01	1

TTC4	615595	343	19	1027060	256676	0	2	0	0	50	53	22535156	7.39E-01	1
SLC8A1	39449	706	-11	2566048	712712	0	7	2	0	18	43	13970152	7.40E-01	1
CCT7	536944	452	20	1389468	400500	0	4	2	0	50	54	17210108	7.40E-01	1
PCDH15	2832	1222	-41	6411204	1764336	0	29	7	0	2	9	2297980	7.40E-01	1
ZNF317	311030	471	-63	1538276	396940	0	5	1	0	9	11	2373096	7.40E-01	1
G6PC2	173915	724	7	917412	242436	0	2	0	0	50	64	23708176	7.40E-01	1
CNBP	1130979	189	50	479176	114632	0	1	0	0	50	66	20268860	7.40E-01	1
CCDC136	830283	310	35	3037036	736208	0	6	3	0	50	76	22960220	7.40E-01	1
JAM2	534549	723	26	782132	214312	0	1	0	0	8	2	2225000	7.40E-01	1
PTPRD	15780	1275	-26	4976524	1371668	0	29	4	0	24	47	11320444	7.40E-01	1
C11orf16	548433	243	21	1174444	350660	0	5	3	0	5	4	1456040	7.40E-01	1
SIGLEC1	942842	353	32	4166268	1398012	0	9	2	0	7	3	3281608	7.40E-01	1
VPS13C	115338	586	14	9856216	2560352	0	26	3	0	0	3	2560352	7.40E-01	1
CCDC115	360632	593	5	454968	142044	0	1	1	0	50	63	20334720	7.40E-01	1
CRLF1	1397040	222	33	1056608	332860	0	3	1	0	50	56	22349324	7.40E-01	1
ZBTB34	367308	322	48	1259884	349592	0	3	1	0	50	60	21727036	7.40E-01	1
OR2F1	368507	732	-11	778572	244572	0	2	1	0	50	64	17819224	7.40E-01	1
AKAP14	772178	NaN	38	538628	126736	0	1	0	0	50	59	22592472	7.40E-01	1
DPPA3	237142	672	14	415096	112496	0	1	1	0	38	49	13251388	7.40E-01	1
FBXL21	489402	443	19	1106804	305092	0	3	0	0	50	52	16320820	7.40E-01	1
GPR82	531498	NaN	45	849416	234604	0	1	0	0	50	59	24984080	7.40E-01	1
SMURF1	918974	379	46	1973308	532220	0	5	4	0	29	52	16181268	7.40E-01	1
SYF2	613835	460	36	652192	157352	0	1	0	0	50	56	23795752	7.41E-01	1
ZNF607	553692	829	-48	1824144	426132	0	9	1	0	7	8	2624076	7.41E-01	1
PEX11G	609287	308	36	591316	203276	0	1	0	0	50	64	23561504	7.41E-01	1
ZADH2	73586	993	4	912784	302600	0	2	1	0	50	66	23179160	7.41E-01	1
DMD	31871	NaN	-12	9956964	2477760	0	32	7	0	17	40	10617344	7.41E-01	1
VIM	546890	465	11	1190108	340336	0	6	4	0	3	4	1120332	7.41E-01	1
C1QTNF9	270913	589	44	830548	252404	0	3	1	0	50	54	16310140	7.41E-01	1
PPEF1	141559	NaN	17	1737280	422216	0	5	2	0	50	77	21383852	7.41E-01	1
CHD6	378513	502	-1	6981872	1873984	0	23	9	0	43	57	19317984	7.41E-01	1
SSR1	755548	283	24	747600	201852	0	2	0	0	47	68	25663684	7.41E-01	1
UBTD2	391482	182	42	600928	160556	0	1	0	0	50	45	17551868	7.41E-01	1

ADSSL1	681504	208	29	1439664	428268	0	3	0	0	11	9	4689944	7.41E-01	1
TRIM22	139577	922	2	1290500	338200	0	3	0	0	17	11	5272004	7.41E-01	1
ABHD11	1082063	214	54	1008904	316484	0	3	1	0	46	78	21680756	7.41E-01	1
ADSS	710088	534	2	1197584	317908	0	4	1	0	50	89	24819964	7.41E-01	1
SPP2	288133	558	9	576364	134924	0	1	0	0	50	60	22433340	7.41E-01	1
APRT	813333	189	51	449984	149164	0	1	1	0	50	71	20606704	7.41E-01	1
PERP	329471	484	24	488788	141332	0	2	1	0	4	6	1605916	7.41E-01	1
INSRR	NaN	NaN	NaN	3285168	995376	0	9	3	0	50	119	40640960	7.41E-01	1
IQSEC1	516922	406	48	2873632	879320	0	7	3	0	22	26	11074804	7.41E-01	1
GIMAP1	734961	689	14	748668	243504	0	6	3	0	4	5	941264	7.41E-01	1
BRD9	875343	491	30	1643296	343184	0	4	2	0	50	62	19703532	7.42E-01	1
KLHL32	123818	1288	-2	1592744	439660	0	3	1	0	50	39	19497764	7.42E-01	1
NAV2	164305	496	9	6243172	1824500	0	18	4	0	1	4	2165548	7.42E-01	1
ALDH9A1	321831	408	0	1328592	377004	0	5	0	0	1	0	490568	7.42E-01	1
PRMT2	530282	377	28	1133504	299752	0	1	0	0	47	55	19319052	7.42E-01	1
OSBPL7	1118277	217	27	2179432	615524	0	6	2	0	50	61	18316556	7.42E-01	1
KCNK9	62115	562	5	936992	273052	0	3	2	0	7	9	2890720	7.42E-01	1
WNT9A	673663	406	-4	906020	283732	0	2	0	0	50	48	18273836	7.42E-01	1
IRF9	974559	220	41	1011040	284444	0	2	1	0	50	62	24565780	7.42E-01	1
ZBTB25	670466	221	54	1108940	298684	0	1	0	0	50	68	23232204	7.42E-01	1
MAPK12	941692	217	60	960488	265576	0	1	0	0	15	7	6050220	7.42E-01	1
CACNB2	74282	408	4	1967612	530440	0	5	0	0	13	10	4788556	7.42E-01	1
FRS3	429684	341	47	1220724	380208	0	3	0	0	46	51	18574300	7.42E-01	1
HLA-DQA	1373102	417	37	654328	185120	0	3	0	0	11	17	4212192	7.42E-01	1
MAGEB16	3857	NaN	-24	815596	229976	0	1	1	0	50	126	29477156	7.42E-01	1
INHBB	218512	446	15	991816	321824	0	3	2	0	13	20	6623024	7.42E-01	1
MGST1	266051	641	-23	398008	114632	0	1	0	0	50	91	25580024	7.42E-01	1
ASMTL	NaN	NaN	NaN	1656112	480956	0	5	3	0	50	119	40126540	7.43E-01	1
MYL6	2878044	171	40	441440	106444	0	1	0	0	50	64	20725608	7.43E-01	1
MOSPD2	111964	NaN	15	1396232	326452	0	4	0	0	50	74	23618108	7.43E-01	1
KSR1	171783	444	-9	1960136	561412	0	5	0	0	1	2	1472416	7.43E-01	1
FKBP14	661477	560	23	556428	135636	0	1	0	0	50	81	25997256	7.43E-01	1
BLMH	516668	184	26	1217876	290140	0	5	1	0	50	83	21999732	7.43E-01	1

HEPH	282730	NaN	4	3134224	837668	0	10	1	0	14	14	5370616	7.43E-01	1
SOCS5	630448	252	34	1359564	365256	0	2	0	0	15	12	7630148	7.43E-01	1
PDS5A	606929	175	42	3543268	913496	0	8	2	0	50	56	21538000	7.43E-01	1
RASGEF1A	424273	492	-11	1267360	328232	0	3	1	0	50	78	25563648	7.43E-01	1
TEKT1	1077116	438	14	1085088	289428	0	3	3	0	27	31	6642604	7.44E-01	1
IFIT1	253827	213	17	1224996	313992	0	3	0	0	44	34	14070544	7.44E-01	1
NIPAL4	266195	1087	41	1152016	365612	0	2	0	0	9	6	2436108	7.44E-01	1
SAMD10	1152034	208	48	510504	156996	0	1	0	0	50	78	24047088	7.44E-01	1
EIF3F	135129	587	4	896052	283020	0	2	1	0	50	63	23312304	7.44E-01	1
UNC5C	25813	910	-12	2370604	679604	0	10	5	0	8	18	5692440	7.44E-01	1
PFAS	870950	218	18	3354944	1050556	0	7	2	0	21	15	9161304	7.44E-01	1
ALS2CR12	554245	411	31	1218232	270560	0	3	1	0	50	58	20665088	7.44E-01	1
SERINC1	140855	973	-19	1180140	315060	0	2	1	0	50	87	22963780	7.44E-01	1
OR4F15	215780	643	-16	774300	226416	0	2	1	0	42	59	17722748	7.44E-01	1
MRAS	201973	408	19	552512	138484	0	2	1	0	24	24	7548624	7.44E-01	1
YWHAH	531528	344	-1	626560	169100	0	1	0	0	50	54	19869072	7.44E-01	1
PLXNB3	2103463	NaN	30	4847652	1549668	0	9	2	0	14	18	7222884	7.44E-01	1
AGTR2	224760	NaN	7	911360	259168	0	3	0	0	22	26	8958740	7.44E-01	1
STAT5B	2101162	190	31	2054120	547528	0	5	2	0	14	20	7266316	7.45E-01	1
SYTL5	106836	NaN	19	1965120	515844	0	4	0	0	34	30	16596720	7.45E-01	1
GABRA5	22005	1346	-56	1199008	322892	0	5	0	0	27	62	15565032	7.45E-01	1
MRPS15	581032	347	31	676044	182628	0	1	0	0	33	33	16739476	7.45E-01	1
LAMA3	317706	216	60	8776824	2410476	0	32	6	0	15	24	7679632	7.45E-01	1
TOE1	994992	249	29	1326812	378784	0	3	0	0	40	40	17539408	7.45E-01	1
CCDC3	202131	444	13	674264	202564	0	1	1	0	50	49	21382784	7.45E-01	1
CD58	692758	418	19	659668	168032	0	1	1	0	30	39	13798204	7.45E-01	1
MDGA2	14033	1195	-46	2459604	674976	0	9	2	0	12	26	6462468	7.45E-01	1
FGL2	312362	639	30	1127452	288716	0	3	1	0	24	27	9725920	7.45E-01	1
PTPRO	367328	560	-14	3184420	825920	0	8	2	0	16	15	5969764	7.45E-01	1
HDAC4	66632	584	22	2770748	812392	0	5	4	0	50	49	21234688	7.45E-01	1
MON1B	60048	1058	-19	1325744	451408	0	2	2	0	50	67	20710300	7.45E-01	1
PIK3R2	1249693	226	42	1829840	565684	0	4	2	0	28	50	15908216	7.45E-01	1
ITGA9	477042	601	18	2692428	744396	0	9	4	0	31	45	10756184	7.45E-01	1

ZNF830	268361	579	-14	938772	260592	0	3	3	0	14	10	3349248	7.46E-01	1
SLC17A7	2173457	290	44	1425424	419012	0	3	3	0	50	67	19850560	7.46E-01	1
.MMECR1	588745	248	39	791744	230332	0	2	2	0	5	7	2853340	7.46E-01	1
CARS	547495	237	43	2263092	557496	0	3	2	0	50	41	18867288	7.46E-01	1
C20orf196	365507	199	12	528304	140264	0	1	0	0	50	53	21209768	7.46E-01	1
ACO1	290567	424	-18	2302964	634036	0	6	1	0	50	44	18681456	7.46E-01	1
ACAP2	431673	561	43	2084024	505876	0	5	2	0	50	61	20525536	7.46E-01	1
MAP1B	410285	427	30	6167700	1768608	0	19	3	0	5	5	3234972	7.46E-01	1
AGAP4	136259	NaN	34	1680676	472412	0	3	2	0	13	9	5164492	7.47E-01	1
MMP12	468929	506	-21	1237456	311144	0	3	0	0	5	4	2495560	7.47E-01	1
DTWD2	305376	554	8	764332	217160	0	1	1	0	50	69	24092300	7.47E-01	1
POU3F4	12933	NaN	-23	892848	269136	0	3	1	0	50	114	26838484	7.47E-01	1
GPC4	66980	NaN	40	1445360	373444	0	3	1	0	50	72	22173460	7.47E-01	1
KCNN1	1328847	217	35	1354224	427200	0	5	1	0	46	63	18587828	7.47E-01	1
HS6ST1	406221	635	24	1015312	311144	0	2	0	0	46	44	19164548	7.47E-01	1
RPN1	1216952	303	49	1536496	452120	0	3	1	0	50	62	19157072	7.47E-01	1
RNH1	2088403	221	50	1162340	360628	0	1	1	0	50	63	21242164	7.47E-01	1
PTH2R	221399	671	0	1435748	380920	0	6	3	0	22	24	7140292	7.47E-01	1
PSMD1	1175473	345	36	2508020	651124	0	6	0	0	2	2	1417592	7.47E-01	1
NPAT	411907	695	26	3639744	1010328	0	10	3	0	8	9	4479904	7.47E-01	1
ALDH8A1	152922	364	19	1238524	352796	0	3	1	0	50	56	19542620	7.47E-01	1
CWC15	305262	504	-10	611252	150232	0	1	0	0	50	57	24306256	7.47E-01	1
C8orf33	454161	440	8	583484	172660	0	1	0	0	50	71	24081620	7.47E-01	1
VN1R4	427680	921	-46	746176	220364	0	2	1	0	50	59	17329368	7.47E-01	1
ZSCAN10	1626425	234	44	1786764	558564	0	6	3	0	50	77	22657976	7.47E-01	1
KRTAP4-1	NaN	NaN	NaN	500892	129228	0	1	0	0	50	116	39774812	7.47E-01	1
CNTN1	22448	1105	-22	2662524	700608	0	11	6	0	48	81	19416240	7.47E-01	1
NTF3	28523	735	17	676756	197936	0	1	0	0	50	47	20476408	7.47E-01	1
ASAP2	661229	340	34	2641164	700608	0	8	4	0	14	22	7821676	7.47E-01	1
FBXO21	206608	213	20	1637600	426844	0	4	3	0	15	16	4887880	7.47E-01	1
TCF19	3364017	401	19	838380	278748	0	2	0	0	45	41	13756552	7.47E-01	1
CRIP3	1293543	363	51	547172	139552	0	1	0	0	50	71	24456132	7.48E-01	1
TRMT12	489180	476	13	1102888	335708	0	5	0	0	3	7	2269856	7.48E-01	1

ANGPTL6	1675365	272	15	1155220	377360	0	3	0	0	1	0	604132	7.48E-01	1
NEUROG3	824126	519	31	509792	182272	0	1	0	0	50	58	21443660	7.48E-01	1
ZBTB17	856018	170	31	2071208	566752	0	6	3	0	50	100	28094452	7.48E-01	1
NEK2	391935	167	9	1154152	304736	0	4	0	0	16	19	6368840	7.48E-01	1
OR2T10	116628	850	-20	777860	223924	0	5	3	0	4	8	1241728	7.48E-01	1
FBXO16	295355	204	47	778216	194732	0	1	0	0	50	50	19317272	7.48E-01	1
CLDN7	1782958	316	21	546816	145248	0	1	2	0	16	13	4887168	7.48E-01	1
ERO1LB	498153	632	9	1255968	307584	0	3	0	0	3	1	1010684	7.48E-01	1
NEXN	367341	256	1	1805988	411180	0	3	0	0	9	9	5883612	7.48E-01	1
CCDC17	873800	161	27	1533292	514064	0	4	0	0	3	4	1792816	7.48E-01	1
SEPHS1	246468	433	33	1011752	281596	0	2	0	0	50	59	23759084	7.48E-01	1
CDKN3	332892	474	6	567108	145248	0	1	0	0	50	46	19065224	7.48E-01	1
MARK3	937868	161	36	1962984	525456	0	4	1	0	50	65	22886884	7.48E-01	1
TPCN1	429315	206	36	2328952	639020	0	4	3	0	50	54	20956652	7.48E-01	1
BNIP3L	194595	376	26	579212	147028	0	1	0	0	50	53	22298416	7.48E-01	1
YY2	93077	NaN	16	940552	254540	0	2	0	0	50	55	20174520	7.48E-01	1
ABCC12	229669	628	-27	3494496	982560	0	13	7	0	35	65	16450048	7.48E-01	1
ZFP42	19937	551	-33	784624	216092	0	3	2	0	23	41	9150268	7.48E-01	1
SRPR	385871	426	29	1638668	464224	0	3	0	0	34	32	16734848	7.49E-01	1
UCN3	711034	519	-1	399432	123888	0	1	0	0	50	96	24023592	7.49E-01	1
ZBTB33	588956	NaN	28	1690644	469920	0	5	1	0	28	28	10349276	7.49E-01	1
ITIH5	199273	528	-3	2499476	721612	0	5	2	0	29	27	10231440	7.49E-01	1
LHFPL2	214373	434	44	569600	168388	0	1	1	0	50	70	24437264	7.49E-01	1
XDH	99156	725	18	3453200	972592	0	8	2	0	50	47	20118628	7.49E-01	1
TRAPPC6A	1230654	243	41	447848	133144	0	1	1	0	50	79	24609568	7.49E-01	1
MKKS	166085	252	-14	1426136	418300	0	3	0	0	50	65	26436560	7.49E-01	1
TDG	1190703	281	40	1086156	269136	0	4	0	0	50	90	26642684	7.49E-01	1
PRDM1	162338	278	44	2093992	589892	0	6	3	0	50	71	19761204	7.49E-01	1
OLFML3	365516	596	7	1001784	310788	0	3	1	0	50	72	21295920	7.49E-01	1
JAK1	269434	396	-5	3044512	758636	0	4	1	0	43	34	18422288	7.49E-01	1
ZBTB8A	1429703	230	21	1130656	297260	0	4	0	0	4	4	1288720	7.49E-01	1
RABGGTA	915114	220	38	1451056	439304	0	5	3	0	50	69	21077692	7.49E-01	1
OR52K2	264090	862	-7	767180	246352	0	3	2	0	3	4	1513356	7.49E-01	1

LMO7	197158	342	46	4467444	1135284	0	11	4	0	24	28	9198684	7.49E-01	1
FGF19	465152	561	38	537560	166252	0	1	0	0	50	60	20189828	7.49E-01	1
CACNB1	1426821	212	31	1739060	515488	0	3	3	0	50	55	19657964	7.49E-01	1
KRT8	1781225	288	27	1233540	348168	0	3	1	0	13	20	5681404	7.50E-01	1
TRAPPC4	1457410	180	55	559632	162336	0	1	0	0	50	46	18727024	7.50E-01	1
AP3B2	509813	608	16	2707024	788540	0	10	5	0	32	52	12801404	7.50E-01	1
VPS72	1562281	184	33	908868	284088	0	4	1	0	5	6	1818092	7.50E-01	1
PER1	1381225	202	37	3192608	1033468	0	6	1	0	3	1	1651128	7.50E-01	1
VANGL1	71378	604	7	1318624	389108	0	4	1	0	50	74	22692152	7.50E-01	1
ARG1	147816	864	7	822360	244572	0	2	0	0	50	56	19662592	7.50E-01	1
TMEM56	228305	452	-8	692776	178712	0	1	1	0	50	48	20399156	7.50E-01	1
SUSD1	536415	164	32	1930588	537560	0	6	1	0	49	65	19455400	7.50E-01	1
HAS3	1179391	201	24	1456752	446424	0	6	3	0	11	18	4871148	7.50E-01	1
SLC25A15	270527	470	44	773944	219296	0	2	0	0	50	78	23838472	7.50E-01	1
IDO1	11280	520	7	1051980	280884	0	2	0	0	14	16	7354960	7.50E-01	1
PALM	1914670	200	23	978644	298684	0	3	1	0	50	80	21759432	7.50E-01	1
ADAM9	389317	428	22	2161276	557852	0	5	1	0	50	56	26781168	7.50E-01	1
VHL	825905	170	39	532576	161624	0	1	1	0	50	47	17447560	7.50E-01	1
GOT2	572798	188	2	1108584	310788	0	1	0	0	50	64	20692144	7.50E-01	1
HES3	643428	205	30	462800	151300	0	1	0	0	50	47	15784328	7.50E-01	1
CD99	NaN	NaN	NaN	505164	129228	0	1	0	0	50	116	39774812	7.50E-01	1
ZNF492	51041	1399	-76	1389824	327520	0	7	2	0	50	84	16653324	7.50E-01	1
ANGPTL3	NaN	NaN	NaN	1212536	292276	0	3	0	0	50	116	39937860	7.50E-01	1
SIRT5	277510	425	37	833040	245640	0	2	1	0	50	65	20454336	7.50E-01	1
ESF1	123437	617	-10	2286232	499112	0	7	0	0	23	26	9689608	7.50E-01	1
SLC25A38	160739	595	32	768960	233892	0	1	1	0	49	47	20476764	7.50E-01	1
C10orf82	195359	618	-17	410112	103596	0	1	1	0	5	12	3265588	7.51E-01	1
MGMT	83542	1073	-25	592740	192240	0	2	0	0	50	133	29128632	7.51E-01	1
BACH2	294466	601	13	2110012	604844	0	10	5	0	24	42	8683196	7.51E-01	1
LINGO1	400940	503	38	1505880	490212	0	4	2	0	50	51	18811396	7.51E-01	1
NLRP4	959325	484	-56	2545400	681028	0	13	5	0	50	132	30544088	7.51E-01	1
PSD2	714699	223	64	1958356	574940	0	11	2	0	3	4	1256324	7.51E-01	1
DCAF6	505920	417	13	2291216	614812	0	6	2	0	50	51	19688936	7.51E-01	1

MED25	1673246	205	37	1865796	607692	0	5	1	0	13	23	7061972	7.51E-01	1
TNFSF9	836909	212	21	599504	228196	0	1	2	0	24	20	7359944	7.51E-01	1
RNF149	442031	259	20	1007480	302956	0	3	1	0	50	53	19261024	7.51E-01	1
SYCP2	73470	842	-30	4138856	950164	0	18	1	0	0	1	950164	7.51E-01	1
SLC22A16	248863	469	20	1464940	419012	0	4	1	0	50	63	22854132	7.51E-01	1
ABCA13	93300	878	-11	12878300	3410836	0	40	14	0	4	17	4860824	7.51E-01	1
ATP6AP1	2277674	NaN	40	1186192	363476	0	2	0	0	50	46	20570392	7.51E-01	1
LCK	1427362	210	43	1326456	359916	0	3	1	0	50	69	25771552	7.51E-01	1
SERINC5	362413	673	36	1241372	328588	0	1	1	0	50	65	23237900	7.51E-01	1
GABBR2	398895	584	32	2406560	688504	0	4	2	0	16	8	5855488	7.51E-01	1
ASAH2	377258	409	14	2025284	560344	0	6	0	0	7	3	1954440	7.51E-01	1
ZSCAN5B	486579	522	-32	1249916	352084	0	4	0	0	33	32	9602744	7.51E-01	1
CMBL	625000	192	9	649344	160200	0	1	0	0	50	55	21476056	7.51E-01	1
SFXN5	453374	421	27	883592	264508	0	2	1	0	50	68	23944560	7.51E-01	1
ROR1	138354	492	-3	2372740	673196	0	6	2	0	18	25	7273792	7.52E-01	1
ANO1	763592	286	35	2591324	677824	0	8	5	0	26	37	11273808	7.52E-01	1
UNC93A	254009	704	1	1147388	349948	0	2	3	0	14	31	8777180	7.52E-01	1
RAD51AP2	135410	1311	2	3014252	715204	0	10	1	0	9	5	2831980	7.52E-01	1
ZBTB44	658636	276	18	1157000	318976	0	2	0	0	20	18	8570344	7.52E-01	1
CAMK2D	166680	632	21	1432544	347812	0	3	0	0	50	56	23081616	7.52E-01	1
TMEM202	1116695	366	41	706304	188680	0	3	1	0	50	77	17950232	7.52E-01	1
PAFAH2	774988	221	31	1023144	278748	0	3	1	0	50	72	22183072	7.52E-01	1
PTGDR	405022	533	18	865080	294768	0	2	1	0	42	61	17033532	7.52E-01	1
KRTAP4-1[illegible]	832368	459	-9	513352	135992	0	1	0	0	50	50	15460012	7.52E-01	1
FGF12	34070	937	0	642224	172660	0	3	3	0	2	8	1378076	7.52E-01	1
TMEM41B	669286	170	20	758636	217516	0	1	0	0	50	57	25092660	7.52E-01	1
NF1	462449	396	26	7422244	1983632	0	30	9	0	5	11	2812756	7.52E-01	1
TRADD	1093307	150	47	758636	261304	0	1	0	0	41	31	16107220	7.52E-01	1
ATP6V0A2	953707	315	40	2238172	588824	0	6	1	0	11	21	5673216	7.53E-01	1
METTL8	515299	342	49	1079392	266288	0	2	0	0	40	40	15863716	7.53E-01	1
SEMA4G	1020226	247	40	2222508	646140	0	5	5	0	50	62	20159924	7.53E-01	1
TESC	208178	290	16	576008	142756	0	1	0	0	50	39	15876176	7.53E-01	1
ASCL1	21203	964	3	583128	180492	0	1	0	0	50	64	23226508	7.53E-01	1

NAALADL	58421	1139	-15	2055900	552156	0	6	1	0	50	68	25840260	7.53E-01	1
PLEKHM1	748554	327	12	2640096	793524	0	5	4	0	50	71	23705684	7.53E-01	1
ADCY1	287931	686	11	2793888	877896	0	6	3	0	50	69	20748036	7.53E-01	1
GSTA2	539666	498	11	592740	147384	0	1	0	0	50	73	21581432	7.53E-01	1
BBS12	134067	172	14	1776084	506232	0	5	2	0	50	56	18910364	7.53E-01	1
LGALS9	186737	462	-5	923464	259880	0	2	1	0	48	47	17360696	7.53E-01	1
PCDHA7	896676	606	-12	2282672	747244	0	13	6	0	43	105	19890076	7.53E-01	1
DYSF	324429	533	10	5626936	1545752	0	15	4	0	2	5	2627280	7.53E-01	1
PDE7B	283639	392	6	1208264	288004	0	2	1	0	50	57	18822432	7.53E-01	1
SLC4A8	527734	331	43	2876480	786760	0	7	3	0	27	22	10515884	7.53E-01	1
CRCP	501759	345	35	477040	117836	0	1	0	0	50	59	20074840	7.53E-01	1
GPX8	222731	430	2	534356	149164	0	1	0	0	50	56	22132164	7.53E-01	1
GJB2	405200	472	50	571380	160200	0	1	0	0	50	66	25477852	7.54E-01	1
CDC123	328303	227	34	920972	211108	0	2	1	0	50	51	17923176	7.54E-01	1
SLC20A2	582397	459	34	1628700	501960	0	3	2	0	22	17	8757244	7.54E-01	1
GDI1	2277674	NaN	40	1171240	306872	0	2	1	0	50	46	20570392	7.54E-01	1
TSC22D4	1479297	186	60	943400	338200	0	2	1	0	50	47	17722036	7.54E-01	1
WDR78	334231	692	-14	2261668	592028	0	8	3	0	50	56	17878676	7.54E-01	1
PLAA	116221	568	-17	2055188	553936	0	3	1	0	50	59	25347556	7.54E-01	1
GYS1	1927185	211	43	1893920	534712	0	9	0	0	9	15	4237468	7.54E-01	1
GRAP	652784	198	21	562836	154860	0	1	0	0	50	67	23515580	7.54E-01	1
DKC1	1739324	NaN	7	1353156	356712	0	1	0	0	32	49	18514492	7.54E-01	1
OR1L8	407749	646	9	760772	232468	0	2	1	0	50	67	20364980	7.54E-01	1
ZDHHC9	370406	NaN	69	943044	260592	0	3	2	0	3	2	907444	7.54E-01	1
CHRD	1433862	268	46	2380928	776080	0	7	2	0	10	15	5067660	7.54E-01	1
DDX53	86947	NaN	6	1589896	437168	0	2	0	0	4	1	2064444	7.54E-01	1
ANKRD29	331927	138	67	770740	235316	0	2	0	0	20	36	11223612	7.54E-01	1
DAPK3	2173603	207	30	1155220	334640	0	2	1	0	38	56	17113632	7.54E-01	1
GPR153	643428	205	30	1458888	512640	0	3	0	0	10	5	3474916	7.54E-01	1
ZDHHC3	472994	290	36	1072984	275900	0	2	1	0	46	41	16711352	7.54E-01	1
AMN1	313930	228	11	670348	185120	0	2	2	0	4	4	1621224	7.54E-01	1
RNF169	620831	160	37	1747960	547172	0	4	2	0	50	46	20412328	7.54E-01	1
CLNS1A	603969	401	30	631544	158776	0	1	0	0	50	50	19753728	7.54E-01	1

H1FOO	779764	210	59	860096	273052	0	2	0	0	37	48	15447552	7.54E-01	1
ADAM18	175645	764	4	1979004	475260	0	4	1	0	50	59	20839172	7.54E-01	1
ENPP3	127852	852	9	2342480	568888	0	3	3	0	50	66	21455052	7.54E-01	1
IMP3	586224	231	34	443576	149164	0	1	1	0	4	3	1514780	7.54E-01	1
SPDYE5	785864	337	70	884660	228196	0	2	1	0	30	37	13235368	7.54E-01	1
CASP8AP2	326497	580	22	5059828	1280888	0	12	3	0	50	49	25310532	7.55E-01	1
NEFM	182761	573	-2	2314000	636884	0	5	2	0	50	51	19246428	7.55E-01	1
ST7	507440	389	19	1622648	423640	0	6	2	0	50	67	20723472	7.55E-01	1
ARL2BP	717872	149	48	440372	106444	0	1	0	0	50	58	17873692	7.55E-01	1
SLC2A4RG	1126597	202	36	949808	325384	0	2	0	0	50	50	18382060	7.55E-01	1
CYP1B1	716797	384	25	1332152	418300	0	2	2	0	20	24	12974420	7.55E-01	1
UGT1A10	NaN	NaN	NaN	1347104	373444	0	4	2	0	50	118	40019028	7.55E-01	1
PDIA4	598458	279	31	1669284	438948	0	2	0	0	50	54	22068084	7.55E-01	1
MAML2	272830	409	6	2902824	821292	0	6	1	0	50	51	20287728	7.55E-01	1
RNF26	1054477	263	40	1024924	365612	0	1	0	0	22	27	8116800	7.55E-01	1
C8A	39104	713	-15	1527952	389108	0	5	2	0	43	77	21766196	7.55E-01	1
ERF	790787	299	37	1322184	449628	0	3	1	0	50	73	25484972	7.55E-01	1
PSMD6	161890	582	19	1016736	264864	0	2	1	0	50	43	17827412	7.55E-01	1
PITX2	64585	758	-15	977220	279816	0	3	1	0	19	28	8464612	7.55E-01	1
SNRNP40	643810	234	30	933432	254184	0	2	0	0	38	47	15273824	7.55E-01	1
B3GAT2	178636	733	7	794592	258456	0	4	2	0	7	9	2255616	7.56E-01	1
DUSP19	350474	524	20	562480	148808	0	1	1	0	8	18	3228208	7.56E-01	1
PSMD7	459751	433	-7	851908	215024	0	2	0	0	37	50	16053464	7.56E-01	1
GNAQ	328481	671	-24	940552	240656	0	3	1	0	50	76	20106880	7.56E-01	1
WDTC1	854040	162	27	1751520	481668	0	3	2	0	50	55	19142832	7.56E-01	1
NKX2-8	49455	583	-20	571024	202208	0	1	0	0	50	49	19936356	7.56E-01	1
OR8B2	149616	857	0	776436	228552	0	5	1	0	2	3	683164	7.56E-01	1
RGL1	493691	597	14	2097552	555360	0	3	3	0	50	66	20501684	7.56E-01	1
PARM1	175795	775	18	757212	252048	0	1	0	0	50	40	20901828	7.56E-01	1
KLC2	2133752	236	53	1597728	466716	0	3	1	0	50	77	24481764	7.56E-01	1
AQP7	982082	163	21	865080	265932	0	3	0	0	35	56	13851248	7.56E-01	1
COL21A1	263671	483	-11	2477404	715916	0	7	0	0	4	1	1204348	7.56E-01	1
DUSP22	224772	684	46	498400	124244	0	1	0	0	50	42	14392368	7.57E-01	1

DGKK	43036	NaN	7	3305816	888220	0	11	1	0	13	28	8623388	7.57E-01	1
ZNF229	725569	528	-49	2122472	536848	0	13	0	0	0	0	536848	7.57E-01	1
CCNI	394078	520	52	969388	267356	0	4	0	0	20	28	6391624	7.57E-01	1
LAPTM5	348498	411	35	686724	187968	0	2	0	0	50	58	19503104	7.57E-01	1
PPP1R12C	1036976	266	-1	1973308	627272	0	2	2	0	48	41	14115044	7.57E-01	1
POU5F1B	NaN	NaN	NaN	875760	277680	0	2	1	0	50	117	39923264	7.57E-01	1
UBR4	787493	414	33	13317248	3742984	0	39	13	0	38	46	18796800	7.57E-01	1
PCSK4	2352825	221	38	1891784	592384	0	3	1	0	45	46	17083016	7.57E-01	1
RWDD3	172385	582	-13	707728	184052	0	1	0	0	50	56	23011484	7.57E-01	1
PGAM2	1151124	282	56	643292	183340	0	3	1	0	4	8	1887868	7.57E-01	1
OR6B2	315906	569	21	759348	241368	0	1	0	0	50	47	24388136	7.57E-01	1
DIRAS2	94332	706	13	509792	135280	0	1	0	0	50	53	19803212	7.57E-01	1
IRGC	452493	192	-2	1114636	378428	0	2	1	0	50	48	20632336	7.57E-01	1
KLHL14	213836	913	-12	1596304	453188	0	4	1	0	50	51	18688932	7.57E-01	1
XPO4	413321	385	42	2993604	793524	0	10	0	0	0	0	793524	7.57E-01	1
TCAP	1284992	226	38	415808	128872	0	1	1	0	36	47	13340032	7.57E-01	1
VWCE	1124603	480	43	2393032	751160	0	7	3	0	17	31	11995064	7.58E-01	1
SYN2	330718	424	21	1527596	463156	0	3	0	0	39	37	18347884	7.58E-01	1
UBA5	210898	768	21	1069068	275544	0	2	1	0	50	58	22555804	7.58E-01	1
KDELC2	383210	579	11	1304384	355288	0	3	0	0	50	50	18048488	7.58E-01	1
DPEP3	863919	158	54	1274836	414740	0	5	3	0	2	7	1254544	7.58E-01	1
PMPCB	375620	620	8	1275904	347456	0	5	2	0	50	70	20312292	7.58E-01	1
STC2	471196	519	41	767180	216448	0	2	1	0	50	79	23438684	7.58E-01	1
MLEC	1741363	349	42	736564	219296	0	2	1	0	50	64	18828840	7.58E-01	1
SUSD4	464408	581	-4	1378076	392668	0	2	2	0	50	59	20933868	7.58E-01	1
CHRNB2	1896659	403	41	1253476	379496	0	5	4	0	3	6	1545396	7.58E-01	1
PTPRM	66398	696	-17	3808488	1025280	0	14	3	0	5	12	4284816	7.58E-01	1
CDC14B	351543	349	27	1362768	346032	0	4	1	0	7	8	3276980	7.58E-01	1
GPHN	262406	846	19	1992532	568532	0	5	2	0	50	50	21709236	7.58E-01	1
GUCY2D	1528802	272	26	2704888	913496	0	6	1	0	12	8	5211484	7.58E-01	1
KLRC2	378365	334	0	614100	152724	0	3	3	0	7	5	1263088	7.58E-01	1
NPRL3	1005761	238	36	1452836	427912	0	3	0	0	19	18	7980096	7.58E-01	1
IRX5	100202	762	-46	1168036	391244	0	8	2	0	4	13	2505172	7.58E-01	1

GPT2	482346	362	12	1341052	384836	0	2	1	0	46	75	22343984	7.58E-01	1
SLC4A10	229857	820	0	2986840	802424	0	6	2	0	50	48	19273484	7.59E-01	1
AMZ1	767440	392	30	1230692	389464	0	3	0	0	41	52	17224704	7.59E-01	1
KLHL15	528219	NaN	41	1534004	412960	0	5	0	0	10	25	8236060	7.59E-01	1
TNFSF13	1957426	194	24	642580	192596	0	2	1	0	50	81	21975524	7.59E-01	1
DAGLB	NaN	NaN	NaN	1743332	472768	0	6	5	0	8	21	4398736	7.59E-01	1
TMEM131	362239	708	36	4852636	1354580	0	17	3	0	13	17	6528328	7.59E-01	1
PRRX2	702852	198	42	623000	203632	0	1	1	0	50	65	22678624	7.59E-01	1
PPFIA4	430455	453	42	1795308	522252	0	5	0	0	23	30	10347496	7.59E-01	1
FXYD5	745516	357	-9	469920	135636	0	1	1	0	50	54	17856960	7.59E-01	1
GALNT2	305688	439	16	1506592	392312	0	4	2	0	50	53	19925320	7.59E-01	1
TTL	536287	433	32	976864	259880	0	2	0	0	32	35	13667908	7.59E-01	1
CLN5	213768	637	24	1037028	285156	0	2	0	0	50	50	20313716	7.59E-01	1
STAU2	261922	447	16	1608764	427912	0	4	1	0	26	34	12393072	7.59E-01	1
TTC3	351773	343	47	5354240	1329304	0	21	3	0	24	25	8941652	7.59E-01	1
OR4S1	278638	642	-40	763976	228196	0	5	2	0	7	18	3234972	7.60E-01	1
DLL3	1410725	230	39	1510864	512996	0	4	0	0	16	38	12814220	7.60E-01	1
ZNF181	932476	607	-52	1491996	353508	0	4	0	0	12	11	4459256	7.60E-01	1
PNLIPRP3	127796	855	-19	1237100	311500	0	1	1	0	50	67	23232204	7.60E-01	1
KRTAP5-4	NaN	NaN	NaN	567820	168032	0	1	0	0	50	116	39813616	7.60E-01	1
DNPEP	604261	335	38	1250628	366324	0	2	0	0	11	15	4791048	7.60E-01	1
REG3G	21996	1408	-29	465292	119972	0	2	1	0	42	91	16713132	7.60E-01	1
LRP10	1177534	225	34	1723396	589892	0	5	3	0	15	22	6086176	7.60E-01	1
ST6GAL2	52131	1042	-22	1400148	412248	0	4	0	0	6	12	3313292	7.60E-01	1
ZNF283	464802	286	11	1776440	417232	0	5	1	0	50	60	22964136	7.61E-01	1
TSPY2	0	0	0	795660	217872	0	1	0	0	50	39	21173456	7.61E-01	1
ABHD13	71759	952	-32	847636	239588	0	4	1	0	50	89	18161696	7.61E-01	1
ABHD14B	1045639	224	36	517980	170880	0	1	0	0	50	51	17801068	7.61E-01	1
EPS8L3	933010	190	27	1546108	437168	0	3	3	0	50	77	22647296	7.61E-01	1
CYTIP	79169	895	18	938772	246708	0	2	0	0	50	63	22717428	7.61E-01	1
POLR2E	2387949	206	26	557496	150588	0	2	1	0	3	10	2596664	7.61E-01	1
ZNF287	993246	383	-15	1971172	487364	0	4	2	0	50	56	18211892	7.61E-01	1
LRFN3	984653	303	21	1461380	562480	0	4	3	0	11	20	5855844	7.61E-01	1

ALPK2	204534	290	67	5481688	1525460	0	16	1	0	2	2	2047000	7.61E-01	1
AP3S2	952242	212	33	508368	134568	0	2	2	0	18	34	7073720	7.61E-01	1
ATF6B	2170336	224	32	1776440	553936	0	7	3	0	19	28	8032784	7.61E-01	1
XKR8	891090	272	20	947672	331792	0	2	1	0	50	57	20821372	7.61E-01	1
STS	61317	NaN	23	1493776	415808	0	4	1	0	15	16	6403728	7.61E-01	1
ALDOB	112947	898	-22	938060	265576	0	3	2	0	33	48	12765092	7.61E-01	1
WWOX	51599	1260	-34	1116772	311144	0	4	1	0	14	26	6687460	7.62E-01	1
TTC8	162049	703	-6	1365260	349948	0	2	0	0	21	20	10724144	7.62E-01	1
LRRC55	925743	431	-21	838736	261304	0	2	0	0	35	50	16577140	7.62E-01	1
WDR3	204177	596	-5	2480608	655040	0	7	3	0	50	49	19709940	7.62E-01	1
TBC1D2	490665	254	52	2309728	682808	0	5	2	0	50	37	17178424	7.62E-01	1
RAB3C	142186	420	9	603420	146316	0	1	0	0	50	49	19883312	7.62E-01	1
'MPRSS11l	210733	767	22	1083308	299752	0	1	2	0	28	33	10325780	7.62E-01	1
RPL8	1454261	384	20	645428	204700	0	2	0	0	50	96	23337580	7.62E-01	1
THNSL1	157790	417	-10	1873272	514776	0	3	1	0	50	52	22882612	7.62E-01	1
SRCIN1	1028227	232	33	2915284	955148	0	6	1	0	18	20	9455004	7.62E-01	1
CXorf21	81961	NaN	21	767536	203276	0	1	1	0	43	51	19043152	7.62E-01	1
CLLU1	NaN	NaN	NaN	312924	77964	0	1	1	0	8	17	4003932	7.62E-01	1
TAF8	490949	186	35	803136	229620	0	2	1	0	50	63	19697836	7.63E-01	1
TMEM95	1911735	206	36	482380	138128	0	1	0	0	50	69	22072000	7.63E-01	1
ZNF324B	2179183	270	-4	1347104	411892	0	4	1	0	50	76	25634848	7.63E-01	1
C14orf79	854493	205	30	824140	237452	0	2	1	0	50	86	25553680	7.63E-01	1
ANP32B	466644	269	42	679960	153080	0	2	0	0	20	30	8142432	7.63E-01	1
BVES	108905	702	-1	945180	241368	0	1	2	0	16	18	5289448	7.63E-01	1
CPN1	1067171	188	51	1179428	325384	0	4	1	0	50	72	19313356	7.63E-01	1
ADD3	349258	424	3	1833044	491992	0	4	2	0	50	54	21183780	7.63E-01	1
OR5A2	417582	672	-4	801356	237808	0	2	2	0	35	51	14743384	7.63E-01	1
ZZEF1	717943	314	23	7611636	2111436	0	22	8	0	7	21	7787144	7.63E-01	1
KIAA0895I	1109491	150	46	1168036	369884	0	1	0	0	6	2	3107880	7.63E-01	1
CBX1	1205950	255	20	493060	119972	0	1	0	0	50	81	26318368	7.63E-01	1
ST8SIA5	292667	608	15	970456	263084	0	2	2	0	11	13	5594184	7.63E-01	1
TOMM20	583355	168	26	385192	99680	0	3	1	0	1	2	393736	7.63E-01	1
SIRPA	467799	486	41	1271276	380920	0	3	3	0	26	34	12097948	7.63E-01	1

GFAP	1101543	172	28	1202568	356712	0	5	2	0	50	74	17295192	7.63E-01	1
[illegible]TAMBPL	175317	289	20	1150948	291920	0	2	1	0	50	42	18559704	7.63E-01	1
KIAA1024	261814	789	13	2317204	633680	0	6	2	0	29	39	12143872	7.64E-01	1
APP	529745	793	-13	2019944	522964	0	6	3	0	50	56	17547952	7.64E-01	1
ULK4	310078	648	15	3333940	908156	0	11	2	0	15	18	7245312	7.64E-01	1
PPP2CA	838413	147	48	801712	217160	0	2	1	0	50	76	23078412	7.64E-01	1
RNF13	536427	340	14	1005344	257032	0	4	0	0	19	34	9718088	7.64E-01	1
ZNF674	420626	NaN	33	1521188	360628	0	3	0	0	50	42	17867284	7.64E-01	1
FGF4	465152	561	38	501248	170524	0	1	0	0	50	60	20189828	7.64E-01	1
CDKN2D	2036812	226	23	402636	140976	0	1	0	0	50	81	22329032	7.64E-01	1
RUNX3	566264	443	27	1077612	336420	0	2	1	0	50	54	20654408	7.64E-01	1
ARPP21	3796	1075	-21	2126744	566752	0	6	1	0	2	1	1104668	7.64E-01	1
CADPS	76552	1010	3	3594888	945180	0	15	3	0	50	61	19617024	7.64E-01	1
PSEN2	428197	373	1	1150592	330724	0	2	2	0	50	42	17764044	7.65E-01	1
SFTPC	587455	196	50	504808	155216	0	1	0	0	50	67	20087300	7.65E-01	1
GADL1	78546	487	-12	1393028	341404	0	3	0	0	29	47	18704952	7.65E-01	1
CHKA	1168866	251	63	1197584	316840	0	2	0	0	50	71	20685736	7.65E-01	1
TAS1R3	1744013	273	28	2071564	681740	0	9	5	0	17	31	7122136	7.65E-01	1
TNFAIP8	305890	296	55	520116	126024	0	1	0	0	50	51	17665788	7.65E-01	1
SMYD4	1458897	153	26	2044864	577076	0	6	2	0	50	63	21449712	7.65E-01	1
GRIK3	361850	626	30	2347108	664652	0	11	5	0	3	10	2124608	7.65E-01	1
ACSF3	1277584	239	40	1440020	447136	0	4	1	0	50	67	22562212	7.65E-01	1
VPS37A	251352	387	6	1050200	269848	0	2	0	0	50	43	17427268	7.65E-01	1
VGF	1022626	258	39	1476332	501604	0	5	2	0	50	67	19705312	7.65E-01	1
TMEM81	655446	431	37	630120	190104	0	1	0	0	50	47	19772952	7.65E-01	1
APLNR	929334	250	-20	936992	283732	0	3	2	0	50	75	20906100	7.65E-01	1
CHRNB3	603002	507	32	1167324	324672	0	4	2	0	45	82	22015040	7.65E-01	1
SNAPC1	246220	410	26	990036	230688	0	2	2	0	33	35	12608452	7.65E-01	1
OMA1	301227	564	-3	1362056	354220	0	3	0	0	2	0	869352	7.65E-01	1
KAL1	16107	NaN	-30	1738348	499112	0	7	0	0	0	0	499112	7.65E-01	1
IL1RN	672307	479	25	535068	134568	0	1	0	0	50	59	20875128	7.65E-01	1
RBM4B	1432488	187	46	901748	264508	0	2	1	0	31	42	14892904	7.65E-01	1
PPP2CB	365415	346	28	801712	217160	0	1	0	0	50	42	21823156	7.65E-01	1

LIN7A	27634	987	-4	595588	173372	0	2	0	0	50	80	17902172	7.65E-01	1
LRRC40	297942	492	-14	1567468	424352	0	3	2	0	50	65	18887224	7.65E-01	1
PLK3	574317	191	43	1627632	507300	0	9	4	0	6	14	2995384	7.65E-01	1
ZNF362	726025	413	29	1061948	321112	0	2	1	0	50	50	19681104	7.65E-01	1
PPIH	650376	461	21	490212	120684	0	1	0	0	50	60	20231480	7.66E-01	1
HECW1	496063	617	13	4121056	1147388	0	14	5	0	50	58	18687508	7.66E-01	1
COLQ	507200	256	40	1245644	367036	0	3	0	0	2	0	794236	7.66E-01	1
EFNA2	2214581	234	29	533288	165184	0	1	0	0	50	68	20485664	7.66E-01	1
IFNA17	193208	498	-3	478820	132076	0	1	1	0	50	58	18347172	7.66E-01	1
PSMD8	1467279	253	0	887508	264864	0	2	1	0	50	59	19987620	7.66E-01	1
GALNT11	286605	586	15	1574232	423996	0	4	2	0	24	48	12212580	7.66E-01	1
EDNRA	71235	430	-6	1114636	286580	0	3	2	0	50	52	20317988	7.66E-01	1
RPS6KB1	959758	216	20	1383060	363120	0	3	1	0	22	18	7908896	7.66E-01	1
FRMD7	151115	NaN	25	1857608	480244	0	3	1	0	45	29	15288776	7.66E-01	1
MAS1	653091	460	26	805984	240656	0	2	2	0	17	18	5741568	7.66E-01	1
CELA3A	448600	190	29	704168	198292	0	2	2	0	19	30	8333248	7.66E-01	1
MYLK2	1015946	241	37	1536496	427556	0	5	3	0	16	18	6273076	7.66E-01	1
NDUFB9	456351	544	44	476328	119616	0	1	1	0	50	66	20641948	7.66E-01	1
MATN1	286775	493	27	1250984	373444	0	3	0	0	48	43	17380276	7.67E-01	1
TELO2	1254229	256	34	2084380	686012	0	6	1	0	16	22	6593476	7.67E-01	1
GDE1	349996	491	21	851908	233180	0	2	1	0	36	51	16404124	7.67E-01	1
RAD52	473725	289	44	1094344	295124	0	2	0	0	14	19	8476004	7.67E-01	1
RGS17	158803	677	1	558208	134924	0	1	0	0	50	55	21110444	7.67E-01	1
PTGS1	343200	635	27	1541480	423640	0	2	0	0	29	28	16671836	7.67E-01	1
CPZ	92046	471	35	1653976	485228	0	3	1	0	39	58	18034960	7.67E-01	1
A2ML1	300254	407	12	3773600	1038808	0	11	5	0	45	62	22152812	7.67E-01	1
MAGEC3	17504	NaN	-11	1957644	528660	0	7	1	0	3	12	2826996	7.67E-01	1
PHKB	320760	885	-3	3011404	790676	0	7	2	0	50	62	18921756	7.67E-01	1
ALDH1A2	187239	674	-1	1348172	370240	0	5	3	0	11	17	3843732	7.67E-01	1
SECISBP2I	372654	382	9	2731588	723392	0	7	1	0	50	60	26255000	7.67E-01	1
RNGTT	202476	672	37	1589540	390532	0	3	0	0	8	11	3992896	7.67E-01	1
RBBP8	254087	158	40	2432904	528660	0	8	3	0	17	26	6631924	7.67E-01	1
TBPL2	729264	159	37	955148	275188	0	1	0	0	15	19	5697068	7.67E-01	1

KRT79	1781225	288	27	1350308	403348	0	5	2	0	13	20	5681404	7.67E-01	1
TPST1	551438	412	26	944112	264864	0	2	0	0	50	59	21400584	7.67E-01	1
ALDH1A1	344512	542	1	1306876	354932	0	4	2	0	50	83	24697500	7.67E-01	1
C12orf65	1014179	201	29	424352	119260	0	1	0	0	50	76	21287376	7.67E-01	1
ACSS1	572146	563	-18	1749384	516912	0	4	1	0	25	35	10964088	7.67E-01	1
ABHD1	1214097	238	50	1025636	313636	0	2	1	0	50	60	22743060	7.67E-01	1
CDK4	1160849	344	39	765400	238520	0	2	2	0	10	12	4058400	7.68E-01	1
NOC2L	1851188	467	3	1935572	546460	0	6	2	0	50	77	24957380	7.68E-01	1
CCNJ	518423	857	35	980068	271628	0	1	0	0	50	26	17177712	7.68E-01	1
ATP1A3	1150724	438	33	2597376	745464	0	8	5	0	37	48	14815652	7.68E-01	1
SLC39A6	365879	399	35	1959424	514064	0	4	1	0	38	39	16349656	7.68E-01	1
DLX6	99426	991	-15	746888	203632	0	1	0	0	50	65	20345400	7.68E-01	1
HPCA	1019374	232	15	508012	126380	0	1	0	0	24	29	8401244	7.68E-01	1
SLC22A1	653494	449	3	1431120	389820	0	2	2	0	50	45	16706368	7.68E-01	1
RIPK3	897133	265	40	1319336	381988	0	4	2	0	5	12	4197596	7.68E-01	1
HSH2D	761374	298	42	881812	270560	0	2	0	0	50	57	18801428	7.68E-01	1
GATA3	182932	675	-2	1104312	338556	0	6	4	0	3	6	894628	7.68E-01	1
ZNF91	410897	1524	-57	3074772	757212	0	18	3	0	17	38	8115732	7.69E-01	1
SLC38A8	494952	460	3	1086868	348524	0	2	3	0	12	9	4469224	7.69E-01	1
SLC30A2	748547	221	31	949096	275900	0	2	0	0	37	34	12359964	7.69E-01	1
TBX10	1391063	393	56	967964	300820	0	1	2	0	18	26	7431856	7.69E-01	1
MMP8	530224	567	-10	1230336	309720	0	3	1	0	50	50	17183764	7.69E-01	1
OR2A14	221024	812	-11	760060	235316	0	2	1	0	50	70	20067720	7.69E-01	1
ANKRD13I	1369264	177	44	1545752	455680	0	5	1	0	44	60	18796088	7.69E-01	1
MYO5C	534690	195	24	4625508	1129944	0	11	4	0	43	61	18228624	7.69E-01	1
MKRN2	351796	280	34	1092920	275188	0	2	1	0	50	50	19374944	7.69E-01	1
NLRP13	802494	561	-68	2680680	707016	0	7	5	0	50	113	28863056	7.69E-01	1
VEGFB	1613996	257	65	525456	168744	0	1	0	0	50	66	21324400	7.69E-01	1
LRRC48	971820	193	20	1431120	352440	0	2	0	0	6	3	2823792	7.69E-01	1
PNPLA8	366326	803	-6	2017096	530084	0	3	1	0	33	39	14597780	7.69E-01	1
SAMD4B	1748498	252	32	1739416	534356	0	7	3	0	7	16	3674988	7.69E-01	1
CATSPER4	896535	217	30	1222148	330724	0	3	1	0	50	84	26408792	7.69E-01	1
ARL13A	414180	NaN	45	788540	208972	0	2	1	0	50	53	16106508	7.69E-01	1

GAS2	52076	981	1	820580	217516	0	3	0	0	50	67	19149596	7.69E-01	1
KLRG2	568414	428	26	998580	334284	0	2	1	0	50	59	22632344	7.69E-01	1
AURKB	870950	218	18	886796	252760	0	2	1	0	50	57	18595660	7.70E-01	1
CYR61	296507	345	13	973304	267712	0	5	2	0	9	13	3181216	7.70E-01	1
SLC6A12	321580	557	28	1586692	443576	0	6	0	0	18	32	8257776	7.70E-01	1
ANLN	332583	329	21	2992892	808120	0	10	0	0	1	2	1241016	7.70E-01	1
NDUFA13	868278	284	7	383412	102528	0	1	0	0	50	74	19156360	7.70E-01	1
XIAP	337935	NaN	14	1294416	326808	0	3	1	0	50	59	21683248	7.70E-01	1
GGA1	1136064	180	65	1694204	493060	0	5	0	0	14	11	4469224	7.70E-01	1
FZR1	2160126	269	39	1269496	378428	0	3	1	0	50	60	21798948	7.70E-01	1
AGAP6	NaN	NaN	NaN	1747604	483448	0	5	0	0	50	116	40129032	7.70E-01	1
DTL	452008	399	15	1885376	516556	0	3	0	0	10	6	3378440	7.70E-01	1
ACTR3	402980	629	15	1101108	288360	0	2	0	0	50	51	21246080	7.70E-01	1
TBCC	960153	187	36	854400	259524	0	2	1	0	50	76	23812128	7.70E-01	1
KRT6A	1194800	389	29	1419372	425064	0	4	2	0	50	64	18116128	7.70E-01	1
FGD3	478130	463	43	1859388	535068	0	3	1	0	26	31	11908200	7.70E-01	1
KRTAP10-[illegible]	860604	461	30	547884	167676	0	1	1	0	50	62	22093360	7.70E-01	1
GTF2IRD1	896611	194	52	2474912	712000	0	7	2	0	49	53	18848064	7.70E-01	1
FMO5	NaN	NaN	NaN	1504812	390888	0	4	1	0	50	117	40036472	7.70E-01	1
MMP17	364888	206	17	1502320	473480	0	3	1	0	50	45	20819236	7.70E-01	1
MIS12	1145030	544	24	534000	130296	0	1	0	0	50	77	22011836	7.71E-01	1
WDFY3	92480	950	-1	9118228	2503748	0	31	6	0	10	17	5964780	7.71E-01	1
C7orf25	163131	744	-6	1189396	354932	0	2	1	0	50	42	19437244	7.71E-01	1
KIF27	464240	285	5	3629064	935568	0	10	3	0	50	67	24803944	7.71E-01	1
TCEA3	1050778	163	27	922396	238520	0	4	1	0	50	94	20813184	7.71E-01	1
PAPD5	294222	418	24	1788188	504808	0	4	1	0	13	10	4828428	7.71E-01	1
FKBP4	495532	443	28	1197940	314348	0	5	0	0	11	20	7171976	7.71E-01	1
KRTAP4-5	996799	468	-7	465648	119616	0	1	0	0	50	60	17141400	7.71E-01	1
THBS4	354204	746	36	2504816	667144	0	4	2	0	50	46	18418372	7.71E-01	1
PRRT3	950574	170	54	2302608	856536	0	7	3	0	39	56	16713132	7.71E-01	1
IFNA14	193208	498	-3	488788	122108	0	1	1	0	36	47	13510556	7.71E-01	1
MFN1	408187	600	11	1947320	502672	0	6	3	0	50	74	21239672	7.71E-01	1
HAPLN4	940952	232	17	974372	336064	0	2	1	0	41	56	18509864	7.71E-01	1

ZNF574	886688	438	31	2158072	720188	0	8	1	0	0	1	720188	7.71E-01	1
VPS26A	749325	145	48	880744	204344	0	3	0	0	50	53	17331148	7.71E-01	1
OR8J3	10101	1007	-38	772164	238164	0	6	2	0	3	5	1002140	7.71E-01	1
ED7-TICA	359255	611	45	600572	172660	0	1	0	0	50	51	18890428	7.71E-01	1
SLC30A7	173324	727	-4	993240	261660	0	3	2	0	35	36	12140312	7.71E-01	1
OTUB2	200224	560	38	613032	161268	0	1	0	0	50	46	19261024	7.71E-01	1
PDLIM5	137154	349	18	1660740	472056	0	4	3	0	7	7	2069428	7.71E-01	1
IRF2BP2	503819	149	31	1412252	478108	0	4	1	0	50	65	20660104	7.71E-01	1
C1orf94	28616	907	16	1497336	447492	0	4	0	0	50	71	23319068	7.71E-01	1
TGM1	915114	220	38	2066936	613744	0	7	2	0	50	69	21077692	7.72E-01	1
MXRA8	1744013	273	28	1111432	348524	0	2	1	0	9	14	4108596	7.72E-01	1
PHYHIPL	115861	812	-3	1006056	250980	0	3	0	0	9	12	3962636	7.72E-01	1
SNAPC3	201198	650	5	1075476	280884	0	2	0	0	50	50	20204068	7.72E-01	1
LITAF	649939	209	30	480956	144892	0	1	0	0	50	53	16597076	7.72E-01	1
EFCAB1	16699	533	-28	573160	131720	0	4	2	0	8	14	1998228	7.72E-01	1
NEK4	1205825	272	48	2165548	596300	0	4	1	0	25	21	10678220	7.72E-01	1
LZIC	757819	183	29	512996	124600	0	1	0	0	50	66	22774032	7.72E-01	1
SCP2	609222	386	23	1481316	362052	0	3	1	0	50	66	25149976	7.72E-01	1
NCK2	209853	689	15	968320	265220	0	3	3	0	5	7	2430768	7.72E-01	1
TXNL1	82448	867	15	770028	189036	0	2	1	0	50	61	18446496	7.72E-01	1
LDHB	502803	826	-13	871132	232112	0	2	0	0	50	58	17456104	7.72E-01	1
C7orf57	127890	364	24	774300	207192	0	2	2	0	2	3	1002140	7.72E-01	1
PCID2	759371	229	38	1080460	263084	0	3	1	0	47	50	18676472	7.72E-01	1
NUMA1	747702	311	59	5328608	1557856	0	11	7	0	39	29	12526572	7.72E-01	1
FBXO33	314889	405	13	1365260	428980	0	2	0	0	7	9	3582428	7.72E-01	1
C17orf62	2583801	179	20	492348	135636	0	2	1	0	2	1	614812	7.72E-01	1
OBP2B	1875784	397	51	458172	116412	0	1	0	0	50	62	18361056	7.72E-01	1
PTRH2	1084897	398	21	446780	132076	0	1	0	0	50	68	19817452	7.72E-01	1
SYN3	259538	520	-72	1485944	426844	0	6	1	0	15	17	4919920	7.73E-01	1
HMBS	1390854	195	58	943756	271628	0	1	0	0	1	0	845144	7.73E-01	1
POU6F1	496692	176	40	754364	234604	0	2	0	0	50	57	17262440	7.73E-01	1
CXCL11	489826	399	43	256320	63012	0	1	1	0	5	4	867572	7.73E-01	1
LDHAL6B	NaN	NaN	NaN	952300	273764	0	2	2	0	50	118	39919348	7.73E-01	1

WWC2	244213	295	-3	3074772	841584	0	6	3	0	50	45	22860540	7.73E-01	1
ZNF385A	664708	190	45	950876	321112	0	2	1	0	50	62	22308028	7.73E-01	1
ITPKA	820644	237	37	1140268	367748	0	3	2	0	50	57	18076256	7.73E-01	1
SLC25A17	854552	191	47	790320	230688	0	1	1	0	50	68	22364988	7.73E-01	1
TYW3	104029	1055	-14	692776	165896	0	4	1	0	3	7	1605204	7.73E-01	1
KLHL28	228561	481	-22	1446784	400856	0	6	1	0	50	100	25336876	7.74E-01	1
ZNF682	468677	845	-59	1304740	309008	0	3	2	0	50	65	17028904	7.74E-01	1
SLC27A6	39183	962	-10	1593100	431828	0	5	0	0	50	79	26451868	7.74E-01	1
ZNF286B	NaN	NaN	NaN	1351376	337132	0	2	1	0	50	117	39982716	7.74E-01	1
CDC42BPA	290976	481	-2	4514436	1145964	0	11	5	0	46	54	19460740	7.74E-01	1
CCNB2	748599	329	34	1035604	276968	0	1	0	0	50	49	23289876	7.74E-01	1
NTF4	2070141	200	35	497688	182628	0	1	0	0	44	57	16789316	7.74E-01	1
CTBP1	858194	391	33	1112500	344252	0	2	0	0	50	51	21376020	7.74E-01	1
SLC7A3	824487	NaN	-9	1548600	488076	0	4	2	0	50	80	18280956	7.74E-01	1
TSN	227057	517	22	589536	165540	0	1	1	0	50	56	21861604	7.74E-01	1
YPEL4	923511	354	15	336776	86152	0	1	1	0	15	21	4964064	7.74E-01	1
APOBEC1	237142	672	14	624068	154504	0	1	0	0	50	67	22681828	7.74E-01	1
SULT1A1	1141256	269	53	907088	229264	0	2	0	0	50	68	22561856	7.74E-01	1
F10	774267	229	26	1270564	330368	0	4	2	0	50	74	21866944	7.74E-01	1
PTGS2	275352	552	-19	1564264	412604	0	5	2	0	9	18	4567480	7.74E-01	1
PLS3	231348	NaN	3	1672844	412960	0	5	0	0	8	10	4092576	7.74E-01	1
MTMR7	317985	345	7	1731940	441440	0	5	1	0	50	64	21435116	7.75E-01	1
SEZ6L2	NaN	NaN	NaN	2295844	737276	0	8	0	0	7	14	4425436	7.75E-01	1
LPAR2	768855	418	-10	841584	292632	0	2	0	0	50	54	17193020	7.75E-01	1
YTHDC2	171693	686	10	3715928	997156	0	13	2	0	19	26	8579244	7.75E-01	1
C6orf10	1754032	479	27	1268072	316840	0	3	1	0	50	43	15982264	7.75E-01	1
EEF1G	2695768	217	42	1143828	300108	0	3	0	0	17	27	9232504	7.75E-01	1
ZNF575	416043	209	-10	601996	194732	0	1	0	0	50	64	21352880	7.75E-01	1
DCLRE1C	255711	396	38	1811684	466360	0	5	1	0	29	35	12433300	7.75E-01	1
HNMT	82611	496	-3	954792	233892	0	2	1	0	50	66	23018248	7.75E-01	1
FUT4	341083	589	-7	1263088	438236	0	4	2	0	46	50	14524444	7.75E-01	1
ARHGEF9	108759	NaN	-13	1405844	327520	0	3	0	0	50	61	24015404	7.75E-01	1
NUDT1	814176	430	38	478108	122108	0	1	1	0	41	37	12492040	7.75E-01	1

ZNF782	313097	491	17	1824144	435744	0	4	1	0	35	29	14087988	7.75E-01	1
UBE2E2	385056	530	2	530440	138128	0	1	0	0	50	80	23662608	7.75E-01	1
AMBN	262055	689	14	1163408	325384	0	3	1	0	50	59	19443296	7.75E-01	1
SLC27A3	1687837	175	33	1782136	600572	0	6	0	0	2	6	1657536	7.75E-01	1
RXFP3	268032	863	8	1130656	375224	0	3	3	0	37	46	14390944	7.75E-01	1
FDPS	1574928	194	45	1086868	302600	0	3	0	0	50	57	20795740	7.75E-01	1
BCAS2	652485	394	6	604488	147384	0	1	0	0	50	52	20091572	7.75E-01	1
UBAP1	726384	125	47	1483808	404416	0	3	0	0	9	5	2918132	7.75E-01	1
MTO1	720190	139	29	1929876	554292	0	3	1	0	43	33	18388112	7.75E-01	1
TMEM52	1434423	233	27	514064	178000	0	1	0	0	50	61	20095132	7.76E-01	1
COPZ2	1205950	255	20	561412	146672	0	1	0	0	50	81	26318368	7.76E-01	1
PIAS4	2296242	207	29	1302604	379496	0	3	1	0	50	68	25796828	7.76E-01	1
PGBD4	409752	384	1	1472060	407620	0	4	0	0	44	44	17065216	7.76E-01	1
KRTAP9-2	996799	468	-7	445712	117124	0	1	0	0	50	60	17141400	7.76E-01	1
SERPINA1[illegible]	278548	858	13	1056252	316128	0	3	2	0	50	55	16626980	7.76E-01	1
SPCS2	767563	454	24	595232	153436	0	1	0	0	50	66	22116144	7.76E-01	1
SPAG16	38908	1199	-3	1680676	425420	0	6	3	0	12	18	5605576	7.76E-01	1
IGFBP7	544216	314	32	699540	226416	0	1	0	0	50	54	21635900	7.76E-01	1
NIPSNAP3[illegible]	194451	718	-21	647920	170168	0	2	0	0	50	93	21652988	7.76E-01	1
PRDM13	225399	799	2	1702392	578856	0	4	1	0	50	49	22063456	7.76E-01	1
IL17C	802673	189	41	480244	164828	0	1	1	0	50	65	20750172	7.76E-01	1
RASGRP1	97743	604	-5	2066224	558920	0	5	1	0	23	26	10841980	7.76E-01	1
SGPP2	283423	596	13	984696	312924	0	2	2	0	33	49	10883632	7.76E-01	1
ZNF367	355218	400	23	877540	264152	0	1	0	0	50	37	22078764	7.76E-01	1
ATP6V0E[illegible]	438832	686	26	589180	211820	0	1	0	0	11	10	4417960	7.76E-01	1
MITD1	504421	770	21	666432	162336	0	1	0	0	50	44	18823144	7.76E-01	1
PCDHA9	896676	606	-12	2414392	787472	0	16	4	0	50	135	24247872	7.77E-01	1
CYP11B1	447193	438	36	1277328	373800	0	4	0	0	1	0	749380	7.77E-01	1
KLHDC4	594175	251	28	1348528	368816	0	5	2	0	42	63	16307292	7.77E-01	1
GPX2	521616	387	35	481668	136704	0	1	0	0	50	69	21146044	7.77E-01	1
BBS2	543900	285	20	1858320	523320	0	4	1	0	44	47	19608124	7.77E-01	1
SALL4	166846	214	-9	2600224	791744	0	6	3	0	22	22	11229664	7.77E-01	1
RAPGEF3	395089	320	14	2378436	699540	0	5	2	0	50	52	22689304	7.77E-01	1

DKK4	535273	459	37	574228	161624	0	1	1	0	50	58	21295208	7.77E-01	1
ZNF69	729638	633	-1	413316	86508	0	1	1	0	8	7	2329664	7.77E-01	1
STMN4	360513	561	6	582060	138840	0	1	0	0	50	71	22890088	7.77E-01	1
ST14	622463	276	19	2212184	607336	0	5	2	0	28	33	11617348	7.77E-01	1
C2orf42	677043	143	42	1462448	414028	0	1	0	0	13	12	5948760	7.77E-01	1
TBC1D21	358897	439	26	907800	216804	0	1	0	0	18	16	9194768	7.77E-01	1
GUCY1B3	43576	643	-1	1615528	426488	0	5	0	0	8	8	3410480	7.77E-01	1
FOXD3	146780	565	-4	1131368	403348	0	1	1	0	50	67	21689300	7.77E-01	1
RPF1	238252	685	-2	918836	238876	0	2	1	0	50	57	18845928	7.77E-01	1
VKORC1	1296884	351	35	435744	98256	0	1	1	0	12	24	6411204	7.77E-01	1
YIPF6	201571	NaN	12	612676	174440	0	1	0	0	50	61	18944896	7.77E-01	1
LUM	23173	523	-18	855468	239232	0	2	0	0	39	59	16633032	7.77E-01	1
HSF5	818916	384	23	1486300	447848	0	3	0	0	22	31	14264208	7.78E-01	1
EXD3	1632320	208	30	2212540	689216	0	5	0	0	6	12	3901048	7.78E-01	1
OXSR1	301294	386	31	1390536	373800	0	2	0	0	50	39	22178088	7.78E-01	1
ATP13A1	768855	418	-10	3029560	940196	0	6	2	0	19	23	8318652	7.78E-01	1
SOD2	643775	604	19	583840	152012	0	1	1	0	50	53	20025356	7.78E-01	1
GPSM1	1194160	258	28	1843368	554292	0	5	1	0	50	42	16832036	7.78E-01	1
TTLL12	624112	241	48	1662164	462088	0	5	2	0	50	66	22537648	7.78E-01	1
IPO4	974559	220	41	2745828	844788	0	7	1	0	7	5	2187620	7.78E-01	1
KLHDC1	627751	342	18	1098972	258456	0	2	0	0	50	47	19771172	7.78E-01	1
ANKMY2	291955	612	-13	1165188	289428	0	2	0	0	9	6	3485240	7.78E-01	1
BCL6B	1696302	360	24	1195804	376292	0	3	1	0	50	49	16256384	7.78E-01	1
IFT74	116568	447	-12	1701680	376648	0	2	1	0	50	64	23869088	7.78E-01	1
PTGIR	1139694	347	28	923464	325028	0	2	1	0	10	11	4186916	7.78E-01	1
ADPRH	289679	595	40	905664	254184	0	2	2	0	15	13	4953740	7.78E-01	1
TJP2	224404	525	24	3264164	918124	0	9	1	0	20	17	8052008	7.79E-01	1
GSTP1	1374022	393	53	545392	159488	0	1	0	0	50	76	21858400	7.79E-01	1
PCSK6	372683	406	-1	2888940	811680	0	8	1	0	16	15	5500912	7.79E-01	1
TMC1	395593	486	10	2031336	492348	0	5	2	0	50	44	19918200	7.79E-01	1
BMP8B	975799	388	39	1021720	319688	0	2	1	0	50	81	18001852	7.79E-01	1
MTSS1L	880781	184	34	1862236	594164	0	4	2	0	50	66	22674352	7.79E-01	1
TMEM61	454766	217	13	512640	171948	0	3	2	0	3	5	1042368	7.79E-01	1

ATP6V1E1	431401	243	-32	614812	146672	0	2	1	0	50	96	21207632	7.79E-01	1
CHN2	249263	599	5	1370600	360628	0	2	0	0	13	9	5616968	7.79E-01	1
KSR2	639801	685	-6	2369180	668212	0	8	2	0	10	20	5464956	7.79E-01	1
TP53AIP1	195815	580	-2	269492	84016	0	1	1	0	2	2	684232	7.79E-01	1
GZMA	222027	430	1	674976	189036	0	1	0	0	50	55	23634840	7.79E-01	1
CFHR1	205744	852	-29	854400	227484	0	3	1	0	17	30	7614128	7.79E-01	1
DGKD	397457	227	33	3169468	875048	0	8	2	0	24	21	8698148	7.79E-01	1
OLFM1	199862	446	28	1212892	316484	0	2	2	0	7	11	3551812	7.79E-01	1
OR6Q1	NaN	NaN	NaN	774656	243148	0	3	3	0	8	19	4169116	7.79E-01	1
SLC23A2	476336	464	37	1658604	487008	0	3	3	0	50	71	21447932	7.79E-01	1
C17orf75	679038	286	30	1051980	260592	0	2	0	0	50	56	21353948	7.79E-01	1
LIPA	254295	263	31	1053404	265576	0	2	1	0	50	55	18781492	7.79E-01	1
FNBP1L	621313	224	2	1648992	380208	0	3	0	0	5	5	3328600	7.79E-01	1
AZIN1	689560	171	54	1186548	294768	0	5	1	0	50	79	19000432	7.79E-01	1
IKZF1	160478	NaN	37	1346036	349948	0	5	4	0	29	47	9257424	7.79E-01	1
ZNF253	550586	609	-52	1296196	320756	0	3	1	0	37	99	25776892	7.79E-01	1
KIAA0391	586870	321	38	1499828	396940	0	4	0	0	7	9	2967972	7.79E-01	1
CPSF3	658161	278	37	1819516	452120	0	5	3	0	34	48	13006104	7.80E-01	1
RRAS	1733181	195	36	554648	170524	0	1	2	0	4	5	1157712	7.80E-01	1
NOX3	108543	628	13	1479536	400144	0	5	0	0	16	20	6825232	7.80E-01	1
RAB6C	342211	671	4	631900	185120	0	2	1	0	12	15	3773956	7.80E-01	1
MAP3K7	74576	643	17	1610188	402992	0	3	1	0	50	58	18976580	7.80E-01	1
ARID5B	81587	388	20	3014252	833752	0	7	4	0	36	39	15542248	7.80E-01	1
SATB2	104340	978	2	1872204	522252	0	6	2	0	50	57	18316200	7.80E-01	1
ADAM29	49942	889	-36	2085448	545036	0	12	2	0	4	9	1643296	7.80E-01	1
PAX1	283661	885	-41	1283024	450340	0	6	1	0	24	32	7441824	7.80E-01	1
ALX1	35916	925	-34	839804	222856	0	5	2	0	21	43	6896788	7.80E-01	1
CD1B	128145	833	4	849060	242436	0	3	2	0	28	39	9447172	7.80E-01	1
LGALS14	1243326	439	23	453544	118904	0	1	1	0	38	41	11948784	7.80E-01	1
PCYT2	3798594	211	19	1070492	292276	0	2	0	0	34	28	11428668	7.80E-01	1
ELP2	390096	591	41	2180144	563548	0	5	2	0	50	64	21297344	7.80E-01	1
BTN2A1	384310	375	47	1361700	386616	0	4	2	0	50	51	17347168	7.80E-01	1
FAM65A	1082466	153	49	3015320	983272	0	5	4	0	34	29	12620200	7.81E-01	1

UBE2K	621887	145	45	529016	140620	0	1	0	0	50	61	20374236	7.81E-01	1
ZNF333	1032767	448	29	1722684	458172	0	2	2	0	50	65	24090164	7.81E-01	1
U2AF1	687433	279	59	697404	182628	0	1	0	0	47	50	18430476	7.81E-01	1
FPR1	436280	690	-24	854756	274120	0	2	2	0	50	53	16569308	7.81E-01	1
PFKFB4	1480689	223	45	1232116	331436	0	1	1	0	50	66	22607068	7.81E-01	1
DSG1	255599	412	15	2670356	753652	0	7	1	0	4	3	2311152	7.81E-01	1
N4BP2L2	237148	455	47	3142056	737988	0	12	1	0	8	17	5284108	7.81E-01	1
DUSP21	66992	NaN	15	472768	139196	0	1	1	0	44	68	20182352	7.81E-01	1
PRX	1008676	202	34	3522264	1244220	0	15	5	0	8	10	3459252	7.81E-01	1
PIK3IP1	667559	221	43	673908	193308	0	1	0	0	50	51	21792540	7.81E-01	1
SCGN	260299	630	23	754364	175864	0	1	0	0	19	12	6945916	7.81E-01	1
MC4R	44259	929	3	830548	238520	0	1	1	0	18	14	7680344	7.81E-01	1
ZNF32	310272	630	-6	704168	182272	0	1	0	0	50	50	20373168	7.81E-01	1
KCNH7	70298	965	-6	3122120	847636	0	8	3	0	47	50	17358560	7.81E-01	1
LRRC8B	360067	647	5	2015672	568888	0	5	2	0	50	73	22210128	7.81E-01	1
SELO	944000	242	49	1648992	534000	0	2	1	0	50	67	23711024	7.81E-01	1
XPA	484322	358	36	722680	176576	0	1	0	0	50	49	17917836	7.81E-01	1
C1orf105	135244	400	11	492348	122820	0	1	0	0	50	62	19992960	7.82E-01	1
ALOX15	1226703	318	25	1684236	497688	0	4	0	0	13	18	5723056	7.82E-01	1
COL23A1	533179	634	18	1394452	458528	0	4	0	0	23	31	9739448	7.82E-01	1
MSX2	30602	599	14	649700	215380	0	1	0	0	50	63	23587848	7.82E-01	1
GSX1	338630	610	36	641156	216448	0	1	0	0	50	50	21149604	7.82E-01	1
HOXA4	356045	385	28	776436	258456	0	1	0	0	50	46	22711376	7.82E-01	1
ZNF324	2179183	270	-4	1357784	430048	0	4	1	0	50	76	25634848	7.82E-01	1
ALPP	307666	204	37	1341052	419012	0	3	3	0	50	41	16352860	7.82E-01	1
YOD1	434877	155	24	862944	261660	0	2	0	0	22	32	10203316	7.82E-01	1
DUSP27	287664	611	-1	2919200	811324	0	7	5	0	13	16	5255984	7.82E-01	1
ZNF25	140131	959	-27	1207908	277680	0	4	1	0	50	101	25523420	7.82E-01	1
ACTL7A	336944	471	9	1076188	320756	0	2	2	0	50	49	19942052	7.82E-01	1
MYRIP	195545	917	9	2208268	615524	0	2	0	0	6	1	2691716	7.82E-01	1
HS3ST3A1	116063	727	-76	982204	326096	0	3	1	0	50	88	21898628	7.82E-01	1
TPPP2	971417	320	12	445356	117480	0	1	1	0	37	52	10249952	7.82E-01	1
NRD1	707583	382	16	3228208	819512	0	13	2	0	9	14	5452852	7.82E-01	1

TMPRSS11[illegible]	108503	748	7	1105380	293700	0	5	2	0	4	5	1709868	7.82E-01	1
TFEB	458237	251	49	1211112	351372	0	2	1	0	50	38	17579280	7.82E-01	1
OR52H1	99343	946	-2	791032	236384	0	3	2	0	50	83	21652988	7.82E-01	1
PRKAB1	782827	498	17	703456	190460	0	3	2	0	2	3	1028128	7.82E-01	1
FBXL22	754243	197	47	587044	194732	0	1	0	0	50	51	18910364	7.83E-01	1
ARHGAP1[illegible]	73209	550	26	1745112	444288	0	5	0	0	7	7	3591684	7.83E-01	1
HIPK4	1006719	289	35	1531868	457816	0	7	3	0	20	41	9222180	7.83E-01	1
SDC3	392864	269	48	1052336	386260	0	2	1	0	50	54	20471068	7.83E-01	1
GALNT7	249234	381	15	1716276	441084	0	4	1	0	50	56	21942416	7.83E-01	1
DHFRL1	85529	1378	-18	480600	126024	0	1	0	0	50	62	20142480	7.83E-01	1
XPC	308100	258	33	1526528	430048	0	4	0	0	18	15	6102552	7.83E-01	1
NXPH3	1007676	348	26	628340	186544	0	1	0	0	50	66	18748384	7.83E-01	1
KLHL23	342296	393	35	1435036	368816	0	4	2	0	50	59	20786484	7.83E-01	1
SERPINB2	152044	522	12	1082240	280528	0	2	0	0	50	65	26208720	7.83E-01	1
ZNF799	1754294	593	0	1676048	400144	0	5	1	0	50	80	24610280	7.83E-01	1
RAB3D	1385739	197	31	567464	154504	0	1	0	0	50	60	22230420	7.83E-01	1
NLRP5	631041	561	-72	3035968	871844	0	11	4	0	15	60	16264572	7.83E-01	1
SPAM1	87688	541	-19	1346392	359204	0	2	1	0	50	66	21591400	7.83E-01	1
CHCHD2	738325	642	4	381276	120684	0	1	0	0	50	103	24262112	7.83E-01	1
ELMO3	1109491	150	46	1977580	585620	0	2	1	0	6	2	3107880	7.83E-01	1
ZC3H7A	585294	214	41	2578152	625848	0	5	2	0	7	7	2417596	7.83E-01	1
BEST4	574317	191	43	1189752	365256	0	3	1	0	50	62	20422296	7.83E-01	1
EPB41L5	218487	406	27	2110368	539340	0	5	0	0	32	30	12969080	7.83E-01	1
ZNF354B	204252	845	6	1592744	388396	0	2	1	0	50	57	19290928	7.83E-01	1
APOO	509881	NaN	41	535780	135992	0	1	0	0	50	81	26944928	7.83E-01	1
LOXL2	484892	288	41	2008196	530440	0	6	1	0	30	34	11783956	7.83E-01	1
STAT6	2122802	172	52	2178720	627984	0	7	0	0	6	7	2714856	7.83E-01	1
RNASE1	956361	577	8	393736	113564	0	1	0	0	50	92	23350396	7.83E-01	1
CMTM5	1220624	456	31	400144	119972	0	1	1	0	20	38	9710256	7.84E-01	1
TUBGCP2	362932	393	1	2313644	652192	0	5	5	0	34	35	12937752	7.84E-01	1
AASDH	639545	594	5	2809908	771096	0	9	1	0	4	6	2392320	7.84E-01	1
TADA3	953772	264	43	1106092	317552	0	2	0	0	50	72	27173124	7.84E-01	1
KIRREL	120128	589	13	1908160	580280	0	10	4	0	2	5	1226420	7.84E-01	1

VPS53	486092	514	7	2178720	593808	0	2	2	0	50	59	19809976	7.84E-01	1
ACSL1	311713	515	30	1829128	495908	0	3	1	0	50	75	23657268	7.84E-01	1
VEZT	371223	395	30	2002500	543612	0	4	3	0	33	33	11394136	7.84E-01	1
KCNV2	158557	682	-5	1348528	407264	0	3	0	0	12	16	5906396	7.84E-01	1
NEBL	71095	484	-19	3074416	689216	0	9	3	0	50	59	18867288	7.84E-01	1
PPAT	651147	594	16	1333220	369172	0	3	0	0	6	14	4229992	7.84E-01	1
VCPIP1	372262	213	38	3036324	890712	0	8	1	0	33	30	14246764	7.84E-01	1
FAM105A	514082	380	28	937704	236028	0	3	3	0	6	6	1702036	7.84E-01	1
NOL3	1109491	150	46	631544	96832	0	2	1	0	2	2	991104	7.84E-01	1
FAM160B2	584373	219	34	1723752	515844	0	3	0	0	8	6	3997880	7.84E-01	1
FAM118B	493677	341	28	915276	242436	0	3	0	0	15	28	7604160	7.84E-01	1
RAD18	302992	473	18	1320760	321824	0	2	0	0	17	17	9148844	7.84E-01	1
KDELR2	NaN	NaN	NaN	696336	190104	0	1	0	0	50	116	39835688	7.84E-01	1
PMVK	1695232	224	37	496620	138840	0	1	0	0	12	18	6034912	7.84E-01	1
KCNJ5	194422	602	1	1055184	299040	0	2	1	0	50	53	20284524	7.84E-01	1
NAP1L3	33770	NaN	-11	1296908	327520	0	3	0	0	4	7	2344260	7.84E-01	1
ACTC1	521214	306	-1	965116	271628	0	2	1	0	50	69	23359652	7.84E-01	1
SORCS2	279342	555	36	2943052	884660	0	6	1	0	3	3	2259888	7.84E-01	1
VRK3	1661159	187	40	1242084	334284	0	4	1	0	50	74	21280612	7.84E-01	1
RPA2	891090	272	20	714492	187968	0	1	0	0	50	57	20821372	7.84E-01	1
EXOSC2	505506	211	24	763620	214668	0	2	0	0	25	34	10186940	7.85E-01	1
RRAS2	428878	363	-3	545392	138128	0	1	0	0	50	53	18346104	7.85E-01	1
KCNK4	1613996	257	65	956216	331792	0	2	4	0	19	29	6446448	7.85E-01	1
OTOF	787643	408	41	5434340	1510864	0	19	6	0	33	41	14192652	7.85E-01	1
PPP2R5C	1400431	346	28	1723040	432184	0	4	0	0	50	70	19832760	7.85E-01	1
NR0B1	73561	NaN	28	1156644	358848	0	2	0	0	50	64	26759452	7.85E-01	1
GTF2F2	613630	276	50	661804	169100	0	1	0	0	50	49	18713496	7.85E-01	1
RCE1	1628594	187	29	823072	266288	0	3	0	0	50	79	20457184	7.85E-01	1
SYNPO2	267272	589	2	3194032	981848	0	14	2	0	2	3	1477756	7.85E-01	1
KATNAL1	288583	374	48	1278040	337844	0	1	2	0	44	50	17206904	7.85E-01	1
DNAH8	229301	845	25	11765088	3004284	0	41	11	0	16	37	10762236	7.85E-01	1
PMM1	1467336	183	66	690284	184408	0	4	3	0	6	9	1870780	7.85E-01	1
AMELX	NaN	NaN	NaN	537204	148452	0	1	0	0	50	116	39794036	7.85E-01	1

METTL4	247615	485	-5	1229268	316128	0	3	0	0	19	22	8226804	7.85E-01	1
AMPD2	989433	201	29	2273416	658244	0	7	3	0	50	79	23548688	7.85E-01	1
LCA5	300263	564	0	1800648	461376	0	6	0	0	7	4	2150952	7.85E-01	1
NRG4	467195	406	28	316484	76540	0	1	1	0	1	4	1037028	7.85E-01	1
FNBP1	559377	228	38	1661452	386972	0	4	1	0	50	55	21540492	7.85E-01	1
HEBP1	701221	196	10	487720	133856	0	1	0	0	50	75	22618816	7.85E-01	1
HAT1	537965	177	33	1123536	267000	0	4	1	0	50	76	21530880	7.85E-01	1
SH3RF1	241771	244	24	2197232	698116	0	8	1	0	8	8	3431840	7.85E-01	1
FCRL1	113186	829	26	1171952	318976	0	3	2	0	18	20	6578880	7.86E-01	1
ZRANB2	107389	955	-15	909224	234604	0	2	0	0	50	73	23083752	7.86E-01	1
POLR1E	523221	234	35	1105024	289784	0	3	1	0	50	50	18959848	7.86E-01	1
GALE	903441	254	32	903172	257744	0	2	0	0	50	57	18158136	7.86E-01	1
EPHX2	336774	361	36	1475976	384480	0	4	2	0	50	57	18999008	7.86E-01	1
ABCC10	1270265	230	49	3556440	1218588	0	7	3	0	36	41	15982976	7.86E-01	1
CRLS1	301141	290	4	766112	238876	0	1	1	0	50	52	25209428	7.86E-01	1
NRBP1	1244404	179	39	1409404	380564	0	4	1	0	50	61	21622372	7.86E-01	1
LIMK1	1047202	219	52	1666080	474192	0	7	0	0	4	6	1929520	7.86E-01	1
CYCS	254010	319	11	280172	67996	0	1	1	0	2	3	773232	7.86E-01	1
NEB	248944	641	13	22791476	5411200	0	45	8	0	3	10	6549688	7.86E-01	1
SLC10A3	2163759	NaN	42	1131368	400144	0	3	2	0	50	72	22102972	7.86E-01	1
RIN1	2048585	182	54	1891428	658956	0	3	3	0	50	70	23587492	7.86E-01	1
KLHL22	542099	326	28	1593100	467072	0	3	3	0	22	36	10953764	7.86E-01	1
R3HDM2	1862761	172	55	2531160	690996	0	5	0	0	7	5	2754728	7.86E-01	1
VAMP2	1381225	202	37	308652	85440	0	1	1	0	2	1	591672	7.86E-01	1
ITGA3	1197823	210	20	2810620	835532	0	8	1	0	9	9	3119272	7.86E-01	1
GFM2	418815	581	30	2057680	561056	0	3	1	0	22	16	7517296	7.86E-01	1
PROM2	238302	558	8	2120336	653260	0	3	2	0	50	60	22920704	7.87E-01	1
CPNE7	1094600	211	38	1631904	467784	0	3	0	0	4	1	1545396	7.87E-01	1
KLF8	111210	NaN	1	914208	260592	0	2	0	0	50	58	16395936	7.87E-01	1
FOXM1	495532	443	28	2004280	603776	0	10	4	0	7	16	3959432	7.87E-01	1
ZDHHC15	101818	NaN	-11	975796	237452	0	2	0	0	50	78	27065968	7.87E-01	1
COPB1	365537	228	11	2486304	660024	0	5	1	0	50	55	21429420	7.87E-01	1
TRIM17	654512	278	7	1332508	388040	0	3	2	0	50	76	28604244	7.87E-01	1

MFSD5	1677275	191	49	1339272	454968	0	5	1	0	2	9	2217524	7.87E-01	1
C17orf85	505430	215	30	1625140	417944	0	4	0	0	24	27	9391636	7.87E-01	1
DYNC2LI1	544941	480	35	1041300	254184	0	2	0	0	50	81	24603160	7.87E-01	1
OR5AN1	417582	672	-4	775012	223568	0	2	2	0	35	51	14743384	7.87E-01	1
GAD2	101835	680	-1	1553940	391956	0	7	5	0	4	12	2138492	7.87E-01	1
CFLAR	617625	205	39	1300824	340692	0	2	0	0	25	25	11232512	7.87E-01	1
NDOR1	1724821	399	43	1527240	473124	0	3	3	0	50	60	16636592	7.87E-01	1
TOMM40L	731911	229	39	793168	231044	0	1	0	0	50	46	23778308	7.87E-01	1
ARTN	877978	214	35	598792	238520	0	1	0	0	50	53	19748388	7.87E-01	1
CROCC	681579	247	45	4993256	1628344	0	9	2	0	10	10	6451432	7.87E-01	1
PRAMEF20	NaN	NaN	NaN	2387336	697048	0	6	3	0	50	119	40342632	7.87E-01	1
CREBZF	325720	554	-19	849772	287648	0	3	0	0	10	19	5029568	7.87E-01	1
TM4SF1	529009	254	8	517980	149520	0	1	0	0	50	86	27295232	7.87E-01	1
CH25H	255590	367	34	670704	206124	0	1	0	0	50	45	17350016	7.87E-01	1
CNN2	2387949	206	26	799932	218940	0	3	2	0	2	9	2446076	7.87E-01	1
TUBGCP6	944000	242	49	4546120	1389824	0	9	4	0	33	52	17836668	7.87E-01	1
FAM129A	349492	479	3	2394100	637952	0	7	1	0	5	8	3103964	7.87E-01	1
KCNK5	181911	634	29	1246712	372376	0	5	0	0	8	9	2528668	7.87E-01	1
XIRP2	7405	1014	-18	10391284	2674628	0	47	15	0	13	24	6259548	7.88E-01	1
TMEM102	1906324	206	33	1205772	433608	0	2	0	0	8	11	3438248	7.88E-01	1
ZNF398	611542	254	24	1610544	470988	0	4	1	0	13	15	5571400	7.88E-01	1
TNN	169074	612	14	3316140	925956	0	16	5	0	5	7	2198300	7.88E-01	1
FMO1	427825	968	-4	1369888	372020	0	2	2	0	50	76	22201584	7.88E-01	1
SLITRK6	18898	822	-27	2110724	591316	0	8	1	0	40	83	18266004	7.88E-01	1
PKIG	791812	171	44	199360	55892	0	1	1	0	3	4	684588	7.88E-01	1
PDCD7	678641	189	61	1176224	400144	0	3	0	0	8	6	2592748	7.88E-01	1
EFS	1220624	456	31	1341052	480956	0	5	1	0	6	13	4269864	7.88E-01	1
ACTN3	1343990	194	62	2334648	642936	0	6	1	0	13	11	5250288	7.88E-01	1
OR56B1	139577	922	2	794236	247064	0	1	1	0	50	48	17113632	7.88E-01	1
LDHD	681970	162	22	1270208	402280	0	2	2	0	50	55	23748760	7.88E-01	1
CNTN6	6599	831	-23	2673560	717340	0	8	5	0	50	73	18765828	7.88E-01	1
TNFAIP3	315946	256	51	2029556	538984	0	5	2	0	50	51	19565404	7.88E-01	1
MPZL2	805791	211	41	556784	156640	0	1	0	0	50	62	21266728	7.89E-01	1

PCYOX1L	422727	380	28	1238524	370952	0	3	1	0	15	16	5771116	7.89E-01	1
SPON2	789524	319	29	826276	258812	0	1	0	0	50	39	20243228	7.89E-01	1
CDK2	2080397	229	31	757212	226416	0	1	0	0	11	12	5452140	7.89E-01	1
SCML4	294918	581	36	1045216	314348	0	2	2	0	10	9	3850140	7.89E-01	1
ZKSCAN4	273319	502	35	1392672	373800	0	2	0	0	11	6	3929884	7.89E-01	1
APPBP2	372707	242	7	1535072	393736	0	1	1	0	50	54	22426576	7.89E-01	1
MRPL3	276806	843	15	909936	246708	0	2	1	0	50	51	17071624	7.89E-01	1
TMEM120A	1072825	218	49	913852	235316	0	2	0	0	50	64	21496704	7.89E-01	1
NIPSNAP3A	194451	718	-21	644004	171948	0	2	0	0	50	93	21652988	7.89E-01	1
FXYD3	745516	357	-9	535068	144180	0	1	0	0	50	54	17856960	7.89E-01	1
SUN2	1291659	272	73	1828416	544680	0	2	2	0	50	42	16818864	7.89E-01	1
NAAA	483325	429	44	929872	273764	0	2	0	0	50	72	24127900	7.89E-01	1
DCXR	3814891	211	28	620864	198292	0	1	0	0	32	25	10015348	7.89E-01	1
FBLIM1	752403	174	29	1133148	343896	0	2	1	0	50	55	22856980	7.89E-01	1
NES	1851672	180	37	3974384	1235320	0	15	7	0	50	84	23475352	7.89E-01	1
ZNF532	352047	299	51	3271640	934144	0	10	3	0	50	52	19357856	7.89E-01	1
OR11H4	371637	528	-29	794948	245284	0	2	0	0	50	73	20208696	7.89E-01	1
ZNFX1	886628	261	50	4885388	1318624	0	17	4	0	50	75	24073432	7.89E-01	1
PBK	406636	229	23	853688	211108	0	1	0	0	50	46	20251772	7.90E-01	1
PAXIP1	162706	628	-6	2795312	720544	0	6	0	0	50	53	26297008	7.90E-01	1
PPA1	458949	217	39	790320	181560	0	1	0	0	50	34	16665428	7.90E-01	1
AGK	227799	887	16	1119264	300108	0	1	1	0	50	80	24757308	7.90E-01	1
IPO5	496805	532	2	2945544	745464	0	6	2	0	46	69	21303396	7.90E-01	1
PRPH2	975176	194	36	874692	245640	0	2	1	0	50	75	23617396	7.90E-01	1
SYAP1	295989	NaN	32	936636	228552	0	2	0	0	37	50	16971232	7.90E-01	1
C5orf38	18983	986	-29	342828	115344	0	1	0	0	50	109	22870864	7.90E-01	1
CGB8	2070141	200	35	404416	138128	0	1	0	0	50	68	18142828	7.90E-01	1
GAS2L3	197479	849	-12	1749740	513352	0	3	0	0	6	4	2122472	7.90E-01	1
DCTN3	731413	298	51	576720	153792	0	1	1	0	50	42	16261368	7.90E-01	1
ICAM4	1729517	200	31	830904	152724	0	1	0	0	50	94	24020032	7.90E-01	1
SLC25A11	1416161	231	30	793880	245284	0	2	0	0	50	65	18681100	7.90E-01	1
DNAJC10	332624	421	7	2120692	512996	0	6	1	0	50	48	17749448	7.90E-01	1
INTS7	469325	370	16	2487016	679604	0	8	1	0	11	14	4679620	7.90E-01	1

CDH3	777628	299	24	2110724	612676	0	6	4	0	17	33	11530484	7.90E-01	1
ITGA1	173759	379	9	3077620	824852	0	9	0	0	2	2	1306876	7.90E-01	1
ANKRD12	434324	391	56	5348188	1312928	0	18	2	0	10	13	4568548	7.90E-01	1
TEDDM1	349818	783	5	678892	199004	0	2	2	0	10	16	4089728	7.91E-01	1
NCAN	940952	232	17	3294424	1001072	0	10	2	0	3	3	1638312	7.91E-01	1
RSPO1	445248	274	29	677468	185476	0	1	0	0	50	53	22093004	7.91E-01	1
ARSK	310860	359	29	1385908	364544	0	2	1	0	50	36	19058104	7.91E-01	1
ACTL6B	1884430	247	60	1117484	306160	0	6	3	0	3	8	1530800	7.91E-01	1
NUDT19	420201	410	-36	916344	296904	0	1	1	0	50	81	19347176	7.91E-01	1
ZNF653	1204183	265	32	1547888	459952	0	4	1	0	50	53	17472836	7.91E-01	1
ITPKC	1127178	201	27	1712004	507300	0	5	3	0	30	43	10578184	7.91E-01	1
STAB1	1114087	192	48	6623380	1908872	0	17	4	0	13	15	5893224	7.91E-01	1
FKBP11	1737773	214	55	559632	169812	0	1	0	0	50	50	18572876	7.91E-01	1
AGPAT3	842264	294	67	966184	275900	0	3	2	0	18	28	7699924	7.91E-01	1
TMEM80	1781290	193	53	421148	139552	0	1	1	0	8	9	2748676	7.91E-01	1
VLDLR	192846	541	-1	2310796	566396	0	5	0	0	17	14	6986500	7.91E-01	1
LTB4R2	915114	220	38	838380	328944	0	2	1	0	50	69	21077692	7.91E-01	1
C19orf45	609287	308	36	1278752	378784	0	3	2	0	23	22	8935600	7.91E-01	1
NINJ2	456582	197	40	470988	149520	0	1	1	0	34	39	11902148	7.91E-01	1
NR3C2	73880	914	-15	2483456	706660	0	5	2	0	31	37	12881148	7.91E-01	1
ADAMTS2[illegible]	126632	906	-21	5032416	1251696	0	16	2	0	3	6	2747252	7.92E-01	1
HAUS5	1162794	390	27	1604848	505520	0	5	3	0	50	66	20625572	7.92E-01	1
YIF1B	1438746	253	-1	885728	264508	0	2	0	0	17	15	5386992	7.92E-01	1
SFMBT1	1008757	282	50	2264872	594164	0	3	1	0	50	62	19950240	7.92E-01	1
GNB5	540700	195	21	1048064	274120	0	2	0	0	50	45	18900752	7.92E-01	1
EIF4A1	1957426	194	24	1063728	284088	0	4	1	0	50	81	21975524	7.92E-01	1
KIAA1919	364757	424	30	1289788	385904	0	2	0	0	50	52	24685396	7.92E-01	1
EEF1B2	332753	487	25	605556	150588	0	1	0	0	50	57	21975880	7.92E-01	1
HIF1A	252998	315	30	2157716	551800	0	4	3	0	50	50	17520540	7.92E-01	1
ZNF564	2026532	472	-13	1440020	347812	0	4	1	0	50	70	21595672	7.92E-01	1
FAM19A5	11714	952	-88	414740	123532	0	1	0	0	50	77	15919252	7.92E-01	1
ZNF296	1135715	208	25	1172664	365256	0	4	1	0	20	33	8378460	7.92E-01	1
PNMAL1	1099113	393	25	1121044	320756	0	6	3	0	2	8	1231404	7.92E-01	1

MRAP2	54672	813	-16	532220	140620	0	1	0	0	50	75	25182728	7.92E-01	1
TMPRSS5	124189	613	12	1175512	343184	0	1	0	0	50	69	22012192	7.92E-01	1
TRIM7	1034999	415	35	1363836	432540	0	5	1	0	15	19	5268088	7.92E-01	1
IGFBP6	1726226	177	38	590248	196868	0	1	0	0	26	39	13050248	7.92E-01	1
HNRNPL	1989663	219	30	1514424	429336	0	4	1	0	50	70	21843448	7.92E-01	1
TSPYL5	387608	610	-13	1019228	320044	0	1	0	0	23	8	6292300	7.93E-01	1
SCARB1	1090766	206	35	1420796	392668	0	2	1	0	50	46	16572512	7.93E-01	1
INSIG1	263493	NaN	14	863656	222500	0	3	3	0	7	10	1883952	7.93E-01	1
LDLRAD1	430138	201	31	537916	145604	0	1	0	0	50	52	17828480	7.93E-01	1
ZNF7	1454261	384	20	1773236	447136	0	4	1	0	19	57	13071252	7.93E-01	1
CYB5D2	753798	357	30	656108	205768	0	1	0	0	50	58	21286664	7.93E-01	1
TLE3	300053	417	28	1999652	558208	0	4	1	0	50	54	24344348	7.93E-01	1
CTTN	511118	530	43	1807056	464580	0	9	5	0	7	11	2601292	7.93E-01	1
RFPL2	462468	580	-25	1021364	295480	0	2	1	0	50	53	18796088	7.93E-01	1
DGAT2L6	163833	NaN	14	865792	244928	0	2	0	0	50	62	22960220	7.93E-01	1
NEDD1	51429	882	-6	1746536	457816	0	2	0	0	50	68	24674004	7.93E-01	1
YLPM1	878419	232	43	5410132	1554296	0	10	3	0	50	66	24386356	7.93E-01	1
TADA2B	459652	426	70	1053048	300108	0	3	1	0	50	67	19324392	7.93E-01	1
C15orf57	892776	192	48	508724	133144	0	1	0	0	50	76	24292372	7.93E-01	1
KIF16B	108352	867	-26	3447860	881812	0	11	4	0	50	71	17734140	7.93E-01	1
PMS1	424679	385	20	2436464	604132	0	5	1	0	34	29	14533344	7.94E-01	1
GSPT2	126151	NaN	16	1567824	447492	0	4	2	0	50	76	24196608	7.94E-01	1
OR10H5	808269	590	18	766468	244928	0	2	1	0	41	68	19792888	7.94E-01	1
LPIN1	219334	431	19	2307236	628696	0	7	3	0	14	11	4836972	7.94E-01	1
AKR1B10	403585	319	21	838736	215380	0	1	1	0	50	49	23084464	7.94E-01	1
SLC30A8	496501	456	8	960844	263084	0	3	3	0	7	8	2802432	7.94E-01	1
PLB1	478034	172	32	3913508	1042012	0	12	2	0	33	36	11939884	7.94E-01	1
ACADL	190249	696	2	1121400	302244	0	2	0	0	40	45	17502384	7.94E-01	1
ZBTB7A	2296242	207	29	1428272	454612	0	5	3	0	3	5	1522612	7.94E-01	1
NPTX1	755391	222	23	1075476	328944	0	3	2	0	50	66	22712088	7.94E-01	1
ANXA13	492587	192	12	948384	247776	0	3	3	0	7	12	2382708	7.94E-01	1
RRM1	392720	601	31	2084736	532932	0	4	0	0	37	37	20111508	7.94E-01	1
SHROOM3	570950	418	17	4902476	1538632	0	18	5	0	2	7	2471708	7.94E-01	1

ANAPC7	964525	206	50	1551804	416520	0	5	1	0	50	64	19341480	7.94E-01	1
SNTA1	539120	237	49	1236744	414384	0	3	3	0	34	37	12806388	7.94E-01	1
IP6K2	1646019	212	45	1572096	425064	0	2	0	0	5	3	2853696	7.94E-01	1
SLIT1	1008851	470	26	3966552	1105380	0	10	4	0	6	8	4075132	7.94E-01	1
FAM122C	293030	NaN	27	986476	264152	0	2	1	0	50	78	26755892	7.94E-01	1
KIAA0195	1928925	195	19	3457116	1018872	0	6	4	0	22	30	10939524	7.94E-01	1
PGRMC2	277451	189	8	610184	192952	0	1	0	0	50	49	18995804	7.94E-01	1
DHX57	850010	297	25	3563204	979000	0	9	2	0	25	28	12292680	7.95E-01	1
VHLL	2266205	211	33	342116	106444	0	1	1	0	6	7	1890360	7.95E-01	1
NLRP8	802494	561	-68	2671068	730512	0	17	6	0	6	24	4383072	7.95E-01	1
CATSPERG	1467279	253	0	3036324	799932	0	8	0	0	0	0	799932	7.95E-01	1
DOCK7	222018	474	4	5507676	1459956	0	15	2	0	10	11	6439684	7.95E-01	1
VWC2L	52906	1018	-1	585264	142044	0	1	0	0	50	75	22628784	7.95E-01	1
HLA-G	514408	685	38	862588	249200	0	2	1	0	26	31	10504136	7.95E-01	1
VCAN	253331	529	0	8485616	2458180	0	31	7	0	2	7	2961208	7.95E-01	1
CLDN16	163494	373	9	772520	224992	0	2	1	0	50	75	20810692	7.95E-01	1
CSRP1	842013	209	26	521896	134924	0	1	0	0	50	52	17165964	7.95E-01	1
MET	558228	262	2	3613400	986476	0	16	6	0	33	52	14315472	7.95E-01	1
NUDT6	126682	414	19	799932	234960	0	2	2	0	3	6	2385200	7.95E-01	1
AASS	57655	988	-13	2417952	650412	0	8	2	0	16	33	9643684	7.95E-01	1
GLP1R	188770	730	34	1203280	336776	0	3	1	0	50	57	20964484	7.95E-01	1
ZNF385B	65074	927	-3	1227488	347812	0	2	3	0	50	85	21890440	7.95E-01	1
FNDC8	516190	504	23	819868	236384	0	2	1	0	43	54	16171300	7.95E-01	1
CTLA4	148748	632	18	582060	148452	0	1	1	0	50	50	16978352	7.95E-01	1
DPPA2	22565	1096	-19	791388	200784	0	2	1	0	50	68	19038524	7.95E-01	1
CPOX	364371	640	6	1146320	337132	0	4	1	0	50	71	19922472	7.95E-01	1
LAP3	271232	912	35	1344612	372732	0	3	1	0	50	51	18233608	7.96E-01	1
RIPK2	251042	447	5	1408336	367748	0	6	0	0	1	0	559276	7.96E-01	1
EAF1	507426	256	49	696692	186544	0	1	0	0	50	44	18728804	7.96E-01	1
HS3ST4	36636	1012	-35	1111076	357424	0	3	1	0	18	50	9866896	7.96E-01	1
HERC1	706823	369	40	12359964	3546828	0	24	7	0	17	25	11701720	7.96E-01	1
TRPV1	472939	354	9	1877544	528660	0	4	4	0	9	16	4275560	7.96E-01	1
EDEM2	1346435	168	46	1482028	420080	0	5	4	0	17	32	7653644	7.96E-01	1

SPATA7	140042	800	-13	1571028	400500	0	7	2	0	2	20	4075488	7.96E-01	1
HSPA12B	860065	241	43	1711648	540764	0	4	2	0	50	63	24054564	7.96E-01	1
KIAA1279	860888	246	52	1590608	427912	0	5	2	0	50	61	18490996	7.96E-01	1
KLK13	490795	632	22	693488	216448	0	3	2	0	5	7	1231048	7.96E-01	1
CHKB	1073094	203	43	1027772	283732	0	4	2	0	2	5	1253120	7.96E-01	1
GOLGA1	1116965	142	30	2037032	509080	0	9	2	0	34	48	11748356	7.96E-01	1
ZNF570	234564	939	-52	1404776	332860	0	4	1	0	50	52	17537628	7.96E-01	1
CACNG8	813774	360	-33	1022788	358136	0	3	2	0	50	87	22426932	7.97E-01	1
SYNPO2L	1042853	166	43	2403712	853688	0	4	1	0	13	21	9192988	7.97E-01	1
PIP4K2B	1198833	207	37	1094700	279816	0	2	1	0	50	46	18180920	7.97E-01	1
DOK4	771506	159	45	844788	232824	0	2	1	0	50	64	18678964	7.97E-01	1
RNF167	1416161	231	30	889644	269136	0	2	0	0	50	65	18681100	7.97E-01	1
LATS2	446900	192	41	2707736	807052	0	6	3	0	50	49	17570024	7.97E-01	1
PSMA7	861466	288	-13	645072	178356	0	1	2	0	15	16	4860112	7.97E-01	1
GORASP1	213514	500	30	1108228	341048	0	2	1	0	50	57	21099764	7.97E-01	1
MYOG	430455	453	42	559632	169812	0	1	1	0	30	34	12036716	7.97E-01	1
FAM178A	1007780	247	35	3098980	823784	0	8	2	0	21	22	9378464	7.97E-01	1
PDXDC1	535071	214	23	2060172	561768	0	5	4	0	10	13	4468868	7.97E-01	1
ZNF174	1361831	303	29	1104312	301176	0	3	1	0	50	74	21539424	7.97E-01	1
TXNRD1	1237281	168	29	1695272	470632	0	5	0	0	1	1	930584	7.97E-01	1
PIK3C2B	482894	188	34	4172676	1202568	0	10	7	0	20	34	9024600	7.97E-01	1
SEC23A	314158	376	-7	1996448	538984	0	5	1	0	6	7	2698480	7.97E-01	1
FGFR2	204200	386	-9	2336428	634748	0	11	4	0	3	6	1945540	7.97E-01	1
RBKS	933934	279	32	828768	238164	0	2	1	0	50	70	20114356	7.97E-01	1
HTR2A	129425	885	34	1328948	364900	0	3	2	0	50	73	23908248	7.97E-01	1
OPRK1	147912	861	-14	954436	279104	0	4	2	0	2	5	1555364	7.97E-01	1
FAM63A	1621279	172	30	1319692	382700	0	5	2	0	33	60	14315472	7.97E-01	1
RNF207	627248	240	31	1635108	469920	0	3	1	0	50	61	18409472	7.97E-01	1
SH3GLB1	315130	393	-12	958352	248488	0	1	1	0	50	32	18801072	7.97E-01	1
NMNAT3	224111	848	9	552512	152368	0	1	0	0	50	55	19046356	7.97E-01	1
TEAD1	339042	432	-1	1129232	285868	0	2	1	0	50	57	21442592	7.97E-01	1
RBM41	226335	NaN	39	1084020	277680	0	1	1	0	50	62	18781848	7.98E-01	1
PRPSAP2	792064	224	1	967964	260236	0	2	1	0	50	72	20640524	7.98E-01	1

CCNB1	420987	273	30	1109652	315060	0	3	1	0	30	42	14095108	7.98E-01	1
SLFN13	763419	456	18	2273416	620864	0	5	3	0	50	58	19205844	7.98E-01	1
BCAT2	1700740	181	39	1006412	297616	0	2	1	0	44	58	19937068	7.98E-01	1
HSDL2	458381	341	17	1110364	276968	0	3	1	0	50	62	19477828	7.98E-01	1
DHX32	206845	738	-4	1927384	499112	0	6	0	0	11	10	4561784	7.98E-01	1
NUDT22	1602391	278	57	745108	252404	0	3	3	0	9	21	4572108	7.98E-01	1
MEN1	2261775	230	61	1530800	481312	0	3	4	0	15	29	8712388	7.98E-01	1
RNF40	1632278	158	61	2545400	746176	0	5	3	0	47	62	18440088	7.98E-01	1
SNTG1	2777	1189	-32	1374872	359560	0	5	4	0	10	25	4036684	7.98E-01	1
MMP15	856192	158	9	1675336	509792	0	4	1	0	50	56	19314424	7.98E-01	1
ZNF134	532349	849	-37	1108940	270916	0	3	1	0	50	57	17028192	7.98E-01	1
EPHX1	925960	168	8	1177292	317908	0	2	0	0	50	49	18850556	7.98E-01	1
PKN3	1865107	197	38	2240308	700964	0	2	4	0	50	70	23922844	7.98E-01	1
SLC7A2	462453	253	2	1883240	556072	0	3	1	0	50	67	23716720	7.98E-01	1
GABBR1	443970	755	26	2568896	757924	0	6	3	0	8	16	4473496	7.98E-01	1
OAZ3	1062482	255	29	715204	206480	0	1	0	0	50	50	22423016	7.98E-01	1
A4GNT	190056	464	9	863300	237808	0	2	2	0	14	15	5086528	7.99E-01	1
VIP	188249	600	-9	457104	116412	0	1	1	0	5	9	1360988	7.99E-01	1
DMWD	929036	203	43	1625140	556784	0	4	2	0	33	37	14234660	7.99E-01	1
HABP4	354931	349	36	1050556	305804	0	1	0	0	12	9	5079764	7.99E-01	1
TARBP1	336295	173	15	4138500	1182276	0	7	1	0	13	11	6292656	7.99E-01	1
LDB1	1018634	187	38	1088648	274120	0	2	1	0	50	54	21305176	7.99E-01	1
TCEAL2	132641	NaN	4	593096	141688	0	1	0	0	50	53	17321536	7.99E-01	1
SLC37A3	354350	587	28	1441444	363476	0	6	1	0	6	7	2035252	7.99E-01	1
GPR84	664708	190	45	963692	312568	0	2	1	0	50	62	22308028	7.99E-01	1
PARVB	158082	393	34	1137776	318976	0	4	0	0	6	11	3568900	7.99E-01	1
CNDP2	162468	969	7	1233896	338200	0	4	1	0	50	59	19429056	7.99E-01	1
ARHGAP3(	65931	NaN	13	1385196	413316	0	3	1	0	21	28	10204384	7.99E-01	1
ALPK3	351352	213	28	4654344	1514424	0	13	4	0	5	16	5242456	8.00E-01	1
HTR2C	23811	NaN	-5	1153440	334284	0	2	1	0	50	56	17919260	8.00E-01	1
HPSE	335397	224	27	1397656	400856	0	3	0	0	15	19	7763648	8.00E-01	1
CCDC134	1384410	199	52	602352	160200	0	1	1	0	33	28	11235360	8.00E-01	1
STARD8	172747	NaN	15	2771104	825920	0	5	0	0	2	0	1312572	8.00E-01	1

PANK4	339388	228	38	1984344	574584	0	5	3	0	30	30	12300512	8.00E-01	1
MAPK10	77738	549	-4	1249560	301176	0	8	3	0	3	9	1584200	8.00E-01	1
ENKUR	159854	276	8	681384	163404	0	1	1	0	50	45	19226492	8.00E-01	1
H1FX	948414	355	44	517980	167676	0	1	1	0	50	85	24887248	8.00E-01	1
OR51M1	99042	1030	1	802780	249200	0	1	1	0	50	49	19819232	8.00E-01	1
C9orf152	203263	245	32	596656	176576	0	1	1	0	50	50	17872268	8.00E-01	1
SAFB2	873075	295	17	2471708	670348	0	6	1	0	10	9	2899976	8.00E-01	1
TIGIT	369332	614	19	616592	183340	0	1	0	0	50	53	20325464	8.00E-01	1
PRICKLE4	429684	341	47	972948	286224	0	2	1	0	50	57	19608480	8.00E-01	1
CLYBL	308985	612	45	874336	254540	0	2	3	0	42	48	14897888	8.01E-01	1
KIRREL2	1060028	321	47	1784272	594164	0	4	1	0	50	67	19297692	8.01E-01	1
PIGM	820944	365	15	1038808	319688	0	1	1	0	50	71	19111504	8.01E-01	1
UQCRH	690472	205	31	252048	57672	0	1	1	0	3	8	1466720	8.01E-01	1
CXCL2	199590	368	13	272696	86152	0	1	1	0	3	8	1586692	8.01E-01	1
PATL1	431271	409	27	1982920	564260	0	3	1	0	31	35	13655092	8.01E-01	1
PLCD1	318742	457	24	2028844	562124	0	3	1	0	50	68	24477492	8.01E-01	1
ZNF3	1503181	254	41	1260596	324316	0	3	1	0	50	81	27223676	8.01E-01	1
MRPL27	1335838	196	24	377716	116768	0	1	1	0	8	12	3275556	8.01E-01	1
MAZ	NaN	NaN	NaN	1323964	447848	0	3	4	0	26	44	12024968	8.01E-01	1
FASTKD5	NaN	NaN	NaN	1904244	546816	0	5	1	0	50	117	40192400	8.01E-01	1
SGSM2	780080	271	29	2715212	753652	0	6	3	0	50	67	22879052	8.01E-01	1
YJEFN3	868278	284	7	745464	245640	0	2	1	0	50	74	19156360	8.01E-01	1
STEAP3	176742	558	14	1220724	395160	0	3	0	0	15	20	7438264	8.01E-01	1
CNIH2	2133752	236	53	427556	109648	0	1	1	0	8	10	2971532	8.01E-01	1
DLGAP3	603922	534	28	2409764	768604	0	9	2	0	50	79	23921064	8.01E-01	1
ATP8B2	1695430	236	31	3274844	898900	0	9	3	0	50	72	22163848	8.02E-01	1
GREB1	219132	360	21	5035976	1455328	0	13	8	0	30	34	12735188	8.02E-01	1
UCKL1	1157316	206	36	1410472	410468	0	3	0	0	26	29	11317952	8.02E-01	1
INPP1	682366	401	33	1015668	287292	0	2	0	0	12	15	5881832	8.02E-01	1
DFNB31	260520	290	19	2197944	758280	0	5	1	0	12	13	6435768	8.02E-01	1
CHRNB1	1906324	206	33	1273056	380208	0	5	1	0	50	87	23542992	8.02E-01	1
TOR2A	1098647	172	32	983984	245284	0	1	2	0	46	62	15244988	8.02E-01	1
TXNRD3	NaN	NaN	NaN	1658248	469208	0	2	0	0	50	116	40114792	8.02E-01	1

CRLF3	617388	264	30	1135284	313992	0	3	1	0	50	70	20323328	8.02E-01	1
NKX2-4	283501	638	-3	846924	296904	0	1	1	0	50	44	18575724	8.02E-01	1
NARS	304947	352	38	1438952	375580	0	3	3	0	49	45	16084436	8.02E-01	1
TRIM42	108667	896	10	1859388	477396	0	11	5	0	6	15	2583848	8.02E-01	1
CENPE	344088	569	3	7211848	1652552	0	19	5	0	11	22	7009640	8.02E-01	1
TINAG	58216	617	4	1266648	304380	0	4	2	0	10	11	3327532	8.02E-01	1
RGAG4	582710	NaN	53	1414388	411892	0	2	1	0	19	28	8635492	8.02E-01	1
ZNF436	1032187	163	25	1222504	299396	0	5	2	0	50	98	21204072	8.02E-01	1
ZXDB	80578	NaN	-18	1949456	626560	0	5	0	0	10	11	4506604	8.02E-01	1
MTHFSD	70370	416	3	1068356	322180	0	1	0	0	21	14	8766856	8.02E-01	1
CHRNA5	505832	375	46	1212892	313280	0	3	0	0	50	59	20749460	8.03E-01	1
OR3A2	506514	628	0	783556	252404	0	2	2	0	13	18	5228216	8.03E-01	1
OR6C6	411183	816	-1	780708	226416	0	2	1	0	14	15	4464240	8.03E-01	1
WDR11	242834	597	-7	3181216	863300	0	11	5	0	14	22	7647948	8.03E-01	1
WBP4	305794	417	76	1012108	234248	0	2	0	0	3	3	1345680	8.03E-01	1
DTX1	562320	387	44	1530444	493416	0	3	2	0	50	51	21834548	8.03E-01	1
SPIC	305133	649	-1	647920	160556	0	1	0	0	50	61	20043868	8.03E-01	1
NCKAP5	36731	826	18	4813120	1383416	0	21	10	0	20	30	7425804	8.03E-01	1
ZBTB5	534559	337	36	1696340	480244	0	5	0	0	12	11	4772892	8.03E-01	1
C10orf62	NaN	NaN	NaN	562836	154860	0	1	1	0	50	117	39800444	8.03E-01	1
SFRP1	248918	632	-4	804204	213600	0	1	1	0	50	55	23632704	8.03E-01	1
UHRF2	251045	431	23	2075836	561056	0	6	1	0	10	10	4335368	8.03E-01	1
HMGCR	357296	490	17	2294420	635104	0	6	0	0	2	3	1895700	8.03E-01	1
MBNL1	194580	402	27	1116772	279104	0	1	0	0	42	36	17893984	8.03E-01	1
ITGA11	370132	531	41	3086164	849416	0	9	3	0	50	57	18621648	8.03E-01	1
DCBLD2	337232	411	6	1989684	560700	0	4	3	0	50	51	17204056	8.03E-01	1
ZNF687	1256374	184	31	3016388	984340	0	9	7	0	28	47	11804604	8.03E-01	1
ZMAT1	262044	NaN	16	1676048	394804	0	5	1	0	2	3	1604848	8.03E-01	1
DDX55	929791	224	44	1568892	414384	0	3	0	0	44	46	23376028	8.03E-01	1
SH3TC1	172983	242	51	3302968	1053404	0	7	5	0	50	48	19092992	8.03E-01	1
PJA2	203821	583	15	1850132	459952	0	4	0	0	15	9	4384140	8.03E-01	1
NOSTRIN	169372	724	12	1523324	356356	0	2	2	0	50	70	22312656	8.03E-01	1
B3GALT1	67289	1010	-14	826988	220720	0	2	1	0	28	33	10819552	8.03E-01	1

CCDC51	1401411	263	32	1013176	319688	0	3	1	0	50	79	21907884	8.03E-01	1
SH3BGR	294735	503	5	634748	161980	0	1	0	0	50	56	21853060	8.03E-01	1
GLT6D1	362222	524	29	716272	188324	0	3	1	0	3	3	1017804	8.03E-01	1
FAM161B	443041	158	51	1642940	469564	0	4	1	0	50	55	19172380	8.03E-01	1
HELLS	251937	675	12	2236036	541832	0	4	2	0	50	77	23990128	8.04E-01	1
TAS2R13	363803	644	2	762196	211820	0	1	1	0	50	61	20572172	8.04E-01	1
TSPAN4	1799017	232	51	614812	180848	0	1	1	0	50	57	18763692	8.04E-01	1
GBAS	760564	642	11	770028	190104	0	1	0	0	50	58	23044592	8.04E-01	1
TLL1	81353	538	-19	2673204	661092	0	6	2	0	50	63	21229348	8.04E-01	1
KDSR	201946	278	77	859740	245640	0	2	1	0	50	72	21821732	8.04E-01	1
SSBP4	1384225	259	42	1018160	291208	0	2	0	0	50	68	24078060	8.04E-01	1
ABLIM3	339889	423	17	1826280	463512	0	2	2	0	50	58	21711372	8.04E-01	1
ANO5	51558	935	-7	2422580	595588	0	7	1	0	7	9	3208984	8.04E-01	1
KPNA2	779255	283	1	1354936	385904	0	4	1	0	50	64	20733796	8.04E-01	1
PAK1	502767	479	27	1493420	372376	0	2	0	0	10	4	3244584	8.04E-01	1
ACSM3	211686	580	23	1665724	446780	0	3	1	0	50	45	20285236	8.04E-01	1
ZNF426	706527	642	-58	1443224	356356	0	3	3	0	44	113	28426244	8.04E-01	1
ZKSCAN2	189953	551	12	2467792	659312	0	8	4	0	11	9	4062316	8.04E-01	1
MBNL3	143759	NaN	34	995020	281240	0	2	1	0	11	15	6849084	8.04E-01	1
GSTA4	477320	400	17	589892	150232	0	1	0	0	50	57	19796448	8.04E-01	1
OR52E2	97256	1024	-11	803492	239944	0	2	2	0	15	18	5687456	8.04E-01	1
TEX15	352456	346	11	7135664	1816312	0	25	3	0	24	26	11757612	8.04E-01	1
ZNF75A	1397628	303	32	782844	181560	0	2	0	0	50	92	23311236	8.05E-01	1
ALKBH6	980636	320	19	679604	203632	0	1	1	0	4	5	2374164	8.05E-01	1
SPAG1	806617	270	36	2426852	620152	0	5	3	0	50	66	21746972	8.05E-01	1
C14orf37	285319	683	3	1948744	564260	0	6	1	0	23	35	9612000	8.05E-01	1
SLC39A2	1005555	429	4	754008	252048	0	2	1	0	50	67	17732716	8.05E-01	1
BRPF3	821543	358	31	3030272	885016	0	9	3	0	50	51	18277752	8.05E-01	1
ZNF645	91162	NaN	2	1067644	299396	0	2	1	0	50	62	22640176	8.05E-01	1
LRRC15	344707	269	34	1440732	451764	0	4	3	0	25	28	9645108	8.05E-01	1
GPR149	95537	925	-8	1822364	540052	0	6	3	0	32	45	16255316	8.05E-01	1
HS3ST1	74823	1201	3	759704	227128	0	1	1	0	41	39	16267064	8.05E-01	1
HAAO	344647	338	34	747956	212176	0	1	1	0	28	26	10970496	8.05E-01	1

AIDA	222206	392	1	823428	198648	0	1	0	0	50	57	27572556	8.05E-01	1
PTK2	466681	356	35	2847644	729088	0	7	2	0	50	55	18446852	8.05E-01	1
RFXANK	940952	232	17	669636	200784	0	1	0	0	41	56	18509864	8.05E-01	1
CLEC2D	251092	648	8	598436	127804	0	1	0	0	50	64	22393468	8.05E-01	1
ASNS	455051	636	0	1468856	378784	0	2	0	0	26	42	12160604	8.05E-01	1
TBK1	459373	411	-22	1945896	478464	0	6	0	0	12	13	4692792	8.05E-01	1
RPS6KA6	19108	NaN	-22	1980428	501604	0	10	3	0	7	13	3394460	8.05E-01	1
FAM9B	47340	NaN	-11	527592	103596	0	1	1	0	24	48	12822052	8.05E-01	1
GABPB1	314083	161	5	1047708	302244	0	2	1	0	50	55	20718844	8.05E-01	1
RAB19	359405	521	24	559988	153436	0	1	0	0	50	82	27745928	8.05E-01	1
TMTC1	99753	510	-15	2004636	551088	0	4	3	0	8	15	4203292	8.05E-01	1
MEPCE	1479297	186	60	1661452	562124	0	3	2	0	45	39	16383476	8.06E-01	1
DRD2	108041	610	5	1131012	321468	0	4	1	0	50	64	18536564	8.06E-01	1
RBM4	1432488	187	46	908156	274120	0	1	1	0	31	42	14892904	8.06E-01	1
SLC30A3	1238919	230	39	962268	313992	0	2	1	0	50	56	18256392	8.06E-01	1
RAG1	97499	412	-17	2627280	721968	0	4	2	0	37	33	14693900	8.06E-01	1
NAF1	72763	1218	-7	1371312	393024	0	3	0	0	24	16	7770768	8.06E-01	1
TACO1	934619	247	-1	750092	221788	0	2	2	0	33	40	10957324	8.06E-01	1
LRRC8C	300032	514	7	2020300	564260	0	4	1	0	37	33	14740892	8.06E-01	1
EP400	408569	269	27	7842324	2385912	0	20	7	0	4	11	4232128	8.06E-01	1
UIMC1	1162907	218	47	1880748	485940	0	4	0	0	8	7	3769684	8.06E-01	1
DHX33	1145030	544	24	1785340	532220	0	4	5	0	18	23	7154176	8.06E-01	1
DCAF11	992350	355	19	1426492	385904	0	4	1	0	50	70	21277764	8.06E-01	1
NOSIP	2080142	210	41	772164	230688	0	2	2	0	17	20	5347832	8.06E-01	1
GAB1	183119	699	18	1853692	511928	0	5	2	0	50	60	18561840	8.06E-01	1
COL3A1	205492	515	-5	3692788	1221080	0	9	4	0	18	17	7065176	8.06E-01	1
PLEKHG3	608112	186	46	2917776	871488	0	5	4	0	50	67	20041376	8.06E-01	1
WDR35	482678	390	24	3113932	788540	0	6	1	0	19	24	9109684	8.06E-01	1
BCL7A	796948	211	31	606624	158064	0	1	0	0	50	69	22212976	8.06E-01	1
SSX2	NaN	NaN	NaN	1317912	273408	0	1	0	0	50	116	39918992	8.06E-01	1
PIK3R6	423397	636	11	1922756	576364	0	4	2	0	50	71	20685024	8.06E-01	1
RAB27A	212605	573	-20	585620	147028	0	1	1	0	43	51	17548308	8.06E-01	1
SIKE1	600310	596	-5	559988	138484	0	1	0	0	50	67	20940632	8.06E-01	1

RECK	866055	155	44	2547892	653972	0	7	2	0	50	57	23712092	8.06E-01	1
PIAS1	325771	194	31	1678540	468140	0	2	0	0	13	16	6765068	8.06E-01	1
LYPLA1	268612	450	31	609116	165184	0	1	1	0	50	60	23006144	8.06E-01	1
NEK8	1695792	208	40	1744400	535780	0	4	0	0	5	3	1668216	8.06E-01	1
RNASEH2I	137500	621	34	876116	206836	0	1	0	0	50	48	19838100	8.06E-01	1
SLC35D1	387355	673	-4	920616	267000	0	2	2	0	45	47	15541180	8.06E-01	1
MSLNL	1187310	273	39	2567116	873980	0	6	1	0	30	36	11469608	8.06E-01	1
EPX	851306	401	14	1788900	556428	0	5	0	0	1	1	969388	8.07E-01	1
OR5B3	475497	835	-29	781420	225704	0	2	2	0	50	60	16320820	8.07E-01	1
ULBP1	423805	245	34	637240	166964	0	1	0	0	50	59	20035324	8.07E-01	1
HNRNPH3	615189	250	20	913852	236384	0	2	0	0	50	57	19504528	8.07E-01	1
PSMD2	1447013	268	44	2332156	667856	0	6	5	0	19	29	8493092	8.07E-01	1
KCTD8	73476	1109	-10	1170884	352084	0	3	0	0	15	27	8485260	8.07E-01	1
OXSM	217009	717	-3	1135284	349236	0	2	0	0	32	44	15912844	8.07E-01	1
HPRT1	270801	NaN	45	588824	147028	0	1	0	0	50	74	23163852	8.07E-01	1
RBX1	1044535	269	43	302244	66216	0	1	1	0	2	2	734784	8.07E-01	1
IRF4	208360	631	48	1171596	310788	0	3	1	0	50	55	17448272	8.07E-01	1
CDK20	207783	628	5	964760	218584	0	1	0	0	50	51	21413756	8.07E-01	1
SPATA16	222219	577	-15	1497692	371308	0	3	0	0	44	36	16986896	8.07E-01	1
WAS	824559	NaN	27	1254544	406196	0	3	1	0	50	75	24392764	8.07E-01	1
FOXL2	345888	340	21	901748	306160	0	5	2	0	3	5	968320	8.07E-01	1
ZNF655	1460222	332	56	1726244	423640	0	5	0	0	25	25	8683196	8.07E-01	1
ZNF768	2214934	142	54	1347104	390532	0	7	3	0	5	13	2288012	8.07E-01	1
IWS1	471209	229	30	2169820	512996	0	4	2	0	50	53	18478892	8.08E-01	1
DUSP2	850399	251	48	761840	262372	0	1	1	0	50	61	24385644	8.08E-01	1
ZFR2	2201226	204	37	2475268	788540	0	7	0	0	0	0	788540	8.08E-01	1
PCDHGC5	665835	568	9	2419732	805628	0	4	1	0	7	4	3452488	8.08E-01	1
PAX5	429504	542	34	1000360	294056	0	1	1	0	50	36	17098324	8.08E-01	1
ME2	212718	332	61	1531512	406908	0	1	1	0	50	50	19362484	8.08E-01	1
KIAA2013	519155	207	43	1514068	529016	0	3	0	0	7	3	2103604	8.08E-01	1
PRR25	1187310	273	39	939484	359204	0	2	0	0	50	70	23301268	8.08E-01	1
OR5AK2	474417	617	-38	773232	217872	0	3	2	0	15	32	6556096	8.08E-01	1
CDKAL1	132584	883	10	1517984	400144	0	6	3	0	15	24	5567840	8.08E-01	1

UBAP2L	1841112	185	32	2819164	833396	0	9	3	0	50	97	23540856	8.08E-01	1
DLGAP2	99986	671	-37	2467436	706660	0	5	3	0	4	10	3400512	8.08E-01	1
C17orf70	3665223	205	12	2128880	733360	0	3	3	0	41	41	15183400	8.09E-01	1
KANK1	136434	548	16	3405140	976864	0	10	3	0	6	10	3182640	8.09E-01	1
TAF1C	582429	364	12	2131016	712000	0	5	2	0	37	46	17599928	8.09E-01	1
VAPB	962331	385	24	636884	166252	0	2	2	0	5	16	2579932	8.09E-01	1
C5orf51	113456	795	23	771808	194732	0	1	0	0	50	47	19282028	8.09E-01	1
CLPTM1L	612621	541	30	1414032	383412	0	4	2	0	50	76	24615976	8.09E-01	1
CDC7	269978	454	-6	1505880	387684	0	4	1	0	50	51	19657608	8.09E-01	1
LIN28A	1046693	181	33	538984	146672	0	1	1	0	50	62	18137132	8.09E-01	1
SLC12A6	498240	193	16	3059464	859028	0	7	2	0	50	49	19929948	8.09E-01	1
KLF4	255934	434	-14	1172308	382700	0	2	1	0	50	45	17800000	8.09E-01	1
CPT2	527317	391	27	1647924	480600	0	5	0	0	3	2	1393384	8.09E-01	1
APEH	1551582	271	48	1904600	533644	0	4	3	0	50	72	21923548	8.09E-01	1
KIF22	NaN	NaN	NaN	1674624	519048	0	5	0	0	9	16	4876488	8.09E-01	1
PI4K2A	514755	143	54	1220368	353864	0	2	3	0	19	24	6368484	8.09E-01	1
UPF1	1302747	189	27	2867224	816308	0	10	6	0	5	16	3122120	8.09E-01	1
RAPSN	1022438	245	47	1057676	295480	0	2	0	0	50	66	23883684	8.09E-01	1
NSD1	948717	227	42	6834132	1901040	0	18	4	0	12	15	5674284	8.09E-01	1
STOX1	916932	216	43	2510868	680316	0	5	0	0	28	29	14802480	8.10E-01	1
CHSY3	12401	1069	-8	2210760	626916	0	6	1	0	35	44	16485292	8.10E-01	1
POLR3E	483001	235	50	1856896	500180	0	1	1	0	50	38	18234320	8.10E-01	1
C9orf84	540873	264	17	3911728	969032	0	9	1	0	13	12	4893932	8.10E-01	1
SMNDC1	449754	359	18	631900	153080	0	1	1	0	50	63	20954516	8.10E-01	1
SEZ6	1793486	220	36	2510868	759348	0	5	0	0	2	2	1385552	8.10E-01	1
ZNF823	904926	521	-1	1583488	386972	0	8	1	0	22	30	7232496	8.10E-01	1
F2R	257923	372	46	1051624	317552	0	2	1	0	50	58	20299120	8.10E-01	1
FAM117B	549913	515	26	1457820	464580	0	2	3	0	34	38	11870820	8.10E-01	1
HTATIP2	168576	547	-6	792100	233180	0	1	0	0	38	48	16625200	8.10E-01	1
FIGF	200632	NaN	40	930228	232824	0	2	0	0	17	28	9467108	8.10E-01	1
SORT1	1030626	302	28	2148104	600928	0	6	2	0	17	23	9014632	8.10E-01	1
PLEKHG6	2670962	198	46	2006416	639020	0	7	3	0	3	7	1833400	8.10E-01	1
NFIA	113571	399	-8	1473484	422216	0	3	0	0	39	36	15507716	8.10E-01	1

PRPS1L1	89480	675	-1	795304	231044	0	4	2	0	4	12	2417240	8.10E-01	1
STK19	2314693	203	34	961912	304736	0	2	2	0	3	2	1410472	8.11E-01	1
PPP2R1A	478211	896	-38	1497336	452832	0	4	3	0	50	69	18103668	8.11E-01	1
TAF7	729604	645	-10	891068	230332	0	1	0	0	50	112	24591768	8.11E-01	1
VSTM1	798576	338	-46	632612	160912	0	6	4	0	2	6	670348	8.11E-01	1
PPOX	728669	234	37	1195804	389108	0	5	4	0	9	17	3362420	8.11E-01	1
CISH	583785	302	50	697760	222856	0	1	0	0	50	43	17510216	8.11E-01	1
ENPEP	81828	684	-3	2496272	654328	0	7	2	0	32	50	12817780	8.11E-01	1
OIT3	635147	291	38	1391248	392312	0	4	0	0	7	8	3131732	8.11E-01	1
CSTF2T	NaN	NaN	NaN	1496980	482024	0	4	3	0	50	119	40127608	8.11E-01	1
ULK2	553377	220	-11	2687444	750448	0	7	0	0	1	0	952300	8.11E-01	1
BMS1	420649	787	-27	3359928	844788	0	10	2	0	6	6	2807416	8.11E-01	1
PDE4DIP	NaN	NaN	NaN	7374896	2024572	0	16	7	0	50	123	41670156	8.11E-01	1
SEMA3C	114018	419	-27	1967968	514064	0	5	2	0	50	94	22861964	8.11E-01	1
TTC37	314679	431	26	4123192	1060880	0	9	1	0	1	1	1231048	8.12E-01	1
CTSC	148974	526	-10	1303672	346388	0	4	1	0	6	6	2496984	8.12E-01	1
EVC2	69455	688	19	3340348	945536	0	10	3	0	50	54	20208696	8.12E-01	1
CD200R1L	240755	616	6	697048	195800	0	1	2	0	21	19	8165572	8.12E-01	1
SRL	825667	154	32	1208976	333216	0	3	2	0	50	71	23327968	8.12E-01	1
PIPOX	1752742	236	34	998224	284444	0	3	3	0	9	18	3667156	8.12E-01	1
HAPLN1	220863	552	-9	903528	250980	0	2	0	0	50	67	22173104	8.12E-01	1
MRPL16	395993	494	8	635816	184408	0	1	0	0	50	53	19654760	8.12E-01	1
ZDHHC4	946573	387	40	886440	246708	0	2	1	0	50	70	19201216	8.12E-01	1
RAG2	96246	531	-26	1336068	359916	0	4	2	0	50	102	22901836	8.12E-01	1
LIG1	973756	431	26	2378436	684588	0	5	0	0	1	1	1081172	8.12E-01	1
BSN	1657622	271	40	9451088	3173740	0	22	4	0	20	27	13246404	8.12E-01	1
PNMAL2	1099113	393	25	1540056	497688	0	5	2	0	50	66	20824932	8.12E-01	1
PLEKHH1	483892	311	46	3476340	1016736	0	4	1	0	26	22	12725932	8.12E-01	1
C1QTNF4	989876	214	28	784268	277324	0	1	0	0	50	74	23251428	8.12E-01	1
KCTD7	615008	246	43	730156	216092	0	1	1	0	50	57	22742348	8.12E-01	1
ZFP82	714733	758	-61	1391604	333216	0	6	0	0	11	18	3804928	8.12E-01	1
CTSE	468286	400	32	1113924	238164	0	2	0	0	47	49	18606340	8.12E-01	1
RAB6B	530925	520	25	559632	139908	0	1	1	0	50	50	16137836	8.12E-01	1

DYRK2	232901	596	-9	1496268	441084	0	2	0	0	2	1	1689932	8.12E-01	1
CNPY4	1299015	205	52	652548	167676	0	1	1	0	35	32	12810304	8.12E-01	1
HTR3E	1453689	334	42	1198296	343896	0	4	1	0	10	20	5986140	8.13E-01	1
DLX1	534370	295	34	656820	171948	0	1	1	0	24	27	8869384	8.13E-01	1
KCNC1	499802	582	19	1479180	445356	0	5	1	0	4	4	1623360	8.13E-01	1
PRMT7	819579	281	46	1808836	486296	0	6	0	0	5	6	2560352	8.13E-01	1
SIPA1	4131934	202	53	2531872	873980	0	4	4	0	27	30	12483852	8.13E-01	1
MRPS23	873797	222	6	494128	137060	0	1	0	0	50	66	19357500	8.13E-01	1
GPR160	396829	426	27	863300	227128	0	1	0	0	50	44	22887596	8.13E-01	1
WASL	86898	536	-13	1286228	377716	0	1	0	0	16	33	11253160	8.13E-01	1
DUSP10	37303	599	2	1206484	353864	0	3	1	0	34	55	19620584	8.13E-01	1
SKA3	447305	240	43	1116416	291208	0	2	0	0	50	47	18943116	8.13E-01	1
GJB5	548013	580	19	681028	201140	0	4	0	0	2	2	669280	8.13E-01	1
HNRNPH2	374364	NaN	37	1135996	310076	0	2	0	0	25	27	11161312	8.13E-01	1
HAVCR2	166999	1198	18	772876	220364	0	1	0	0	50	43	19254260	8.13E-01	1
HSPA9	1188562	205	52	1754368	492704	0	4	1	0	50	59	22765844	8.13E-01	1
ACRBP	2816983	210	61	1398368	385192	0	2	0	0	25	55	15777920	8.13E-01	1
SNTB1	154339	819	2	1339272	413316	0	3	0	0	23	30	9884696	8.13E-01	1
JAGN1	950574	170	54	463156	130652	0	1	0	0	50	71	20154584	8.13E-01	1
MPPED1	354735	618	-6	736920	208260	0	1	1	0	50	47	16691772	8.13E-01	1
TMEM175	675007	256	45	1251696	409044	0	2	2	0	36	37	16261012	8.13E-01	1
NME6	1054170	263	37	506232	142044	0	1	0	0	50	63	18179140	8.13E-01	1
ZBED1	NaN	NaN	NaN	1727668	506588	0	5	2	0	50	118	40152172	8.13E-01	1
HEY1	147129	428	30	760772	246352	0	1	0	0	43	52	20861600	8.13E-01	1
SLC25A1	487560	277	18	791032	242792	0	1	0	0	36	33	13333268	8.14E-01	1
ZNF234	554986	634	-28	1826280	436812	0	5	0	0	50	52	17611320	8.14E-01	1
CD1C	120861	833	3	855824	235672	0	2	2	0	37	48	13642632	8.14E-01	1
LEFTY2	923085	239	20	897120	291564	0	2	2	0	26	35	11269180	8.14E-01	1
SNRPA1	372683	406	2	670348	186188	0	1	0	0	50	53	20605636	8.14E-01	1
WDR44	131060	NaN	25	2381996	627628	0	7	0	0	7	8	3551812	8.14E-01	1
CCND2	305146	860	35	736208	210040	0	1	0	0	50	51	18886868	8.14E-01	1
FAM90A1	172474	438	26	1141692	365256	0	5	5	0	8	10	2385200	8.14E-01	1
KCNG3	254123	368	24	1073340	331080	0	1	1	0	50	52	22214400	8.14E-01	1

SEMA4A	2294665	211	39	1923468	577788	0	6	4	0	17	18	6366348	8.14E-01	1
SEMA6D	140187	583	-58	2798872	762908	0	10	3	0	7	10	2655404	8.14E-01	1
PSAP	765338	182	40	1391960	362764	0	4	1	0	37	42	13222196	8.14E-01	1
ALDH1A3	378556	374	0	1318980	375936	0	2	1	0	50	41	18549024	8.14E-01	1
C5orf42	355603	213	29	8298004	2166260	0	21	3	0	1	3	2555724	8.14E-01	1
SUPT6H	1673468	208	36	4537932	1149168	0	14	5	0	13	23	7061972	8.14E-01	1
C17orf64	436889	204	1	619440	163404	0	1	0	0	50	53	19269924	8.14E-01	1
ZNF197	435137	541	40	2700260	665008	0	8	1	0	32	41	12650816	8.14E-01	1
NDUFV1	1374022	393	53	1176936	353508	0	3	1	0	31	38	12874384	8.14E-01	1
EXTL1	748547	221	31	1674624	537204	0	4	1	0	37	34	12359964	8.14E-01	1
ZNF319	856192	158	9	1443936	423996	0	3	1	0	50	56	19314424	8.14E-01	1
INO80D	290764	492	23	2576728	755432	0	8	3	0	50	66	23182008	8.14E-01	1
CMTM1	777342	214	9	716984	217516	0	2	2	0	16	36	9379888	8.14E-01	1
TRIM45	331822	566	12	1463516	419368	0	3	0	0	11	9	3044156	8.14E-01	1
BMP10	404215	470	27	1072628	293344	0	3	1	0	7	9	3626216	8.14E-01	1
TRMT2B	418391	NaN	37	1301536	367748	0	2	0	0	50	52	22193752	8.15E-01	1
NUBPL	335822	583	-7	834108	236028	0	2	0	0	20	20	6247444	8.15E-01	1
DMKN	954618	367	35	1500184	430760	0	1	1	0	19	21	8718796	8.15E-01	1
UNCX	748119	384	40	1260952	451052	0	2	0	0	5	13	6885040	8.15E-01	1
BPTF	772730	293	1	7757240	2131372	0	27	6	0	6	8	3912084	8.15E-01	1
CD276	312591	374	29	1341052	411536	0	1	1	0	50	34	20103320	8.15E-01	1
FAM26E	261450	479	19	782132	217516	0	1	0	0	50	57	23330104	8.15E-01	1
HSD17B3	293976	504	20	815240	226060	0	1	1	0	50	57	22265664	8.15E-01	1
ALG1	552298	419	3	1198296	344964	0	2	1	0	50	50	19837032	8.15E-01	1
PML	375211	344	41	2997876	943044	0	7	0	0	0	0	943044	8.15E-01	1
PAPOLG	370439	436	18	1932368	518692	0	4	0	0	17	20	8360660	8.15E-01	1
ZNF530	532349	849	-37	1532580	398364	0	3	0	0	22	27	8358168	8.15E-01	1
ADAMTS4	728669	234	37	2051984	667144	0	5	2	0	39	44	15898604	8.15E-01	1
GPR20	219865	239	41	846924	307584	0	2	2	0	16	21	6325052	8.15E-01	1
CLCNKA	886121	197	43	1755792	529728	0	4	3	0	50	55	20099048	8.15E-01	1
POLE2	612579	342	25	1413676	359204	0	1	1	0	37	28	16443996	8.15E-01	1
ZNF701	546511	1005	-53	1372736	348880	0	3	0	0	29	27	10802108	8.15E-01	1
ABO	1865433	416	45	897476	266644	0	2	0	0	50	64	17966608	8.15E-01	1

GAS6	716976	465	45	1730872	504452	0	3	0	0	33	37	15963752	8.15E-01	1
BEND5	25250	886	-8	1081528	296192	0	2	0	0	50	83	29777620	8.15E-01	1
TMEM140	464513	594	20	446068	154148	0	1	1	0	50	57	15046340	8.15E-01	1
SLC4A2	622098	373	41	3088300	985052	0	5	3	0	3	5	2744048	8.15E-01	1
GABRB1	106512	689	24	1222504	333572	0	6	3	0	3	3	1166612	8.16E-01	1
TES	445405	363	-9	1116416	261304	0	2	0	0	15	21	8614488	8.16E-01	1
PLCG1	383568	362	13	3381644	890356	0	9	2	0	5	7	3327888	8.16E-01	1
FUK	930873	125	44	2699192	875404	0	5	2	0	50	63	24667952	8.16E-01	1
PNO1	197504	594	14	654684	183696	0	1	0	0	50	55	18711004	8.16E-01	1
LRFN4	NaN	NaN	NaN	1480604	561412	0	3	0	0	9	16	4918852	8.16E-01	1
P2RY1	224331	861	8	921328	276968	0	2	1	0	50	58	18917840	8.16E-01	1
SORBS1	295509	445	17	3492004	993596	0	9	3	0	23	28	12872960	8.16E-01	1
TCP1	648197	420	22	1430052	401568	0	3	2	0	50	70	24941360	8.16E-01	1
LIAS	648710	177	43	980068	259880	0	2	1	0	50	66	22117568	8.16E-01	1
LGI2	293829	582	26	1404776	376648	0	2	1	0	50	67	27511324	8.16E-01	1
CPSF7	1394229	286	57	1292992	401924	0	1	2	0	50	56	17921040	8.16E-01	1
GRAMD3	234274	201	10	1369888	301532	0	3	1	0	50	51	18116840	8.16E-01	1
FCN2	229567	450	26	808832	229264	0	2	3	0	5	7	1620868	8.16E-01	1
IDE	363888	236	23	2704176	668568	0	3	0	0	13	9	7559660	8.16E-01	1
HEXIM2	806811	207	32	713780	214312	0	1	0	0	50	63	22248576	8.16E-01	1
CHRM4	817153	212	45	1159492	380564	0	2	0	0	30	36	13043484	8.16E-01	1
PEX10	561270	302	24	852976	282308	0	1	0	0	50	37	18961984	8.16E-01	1
TPP1	424523	491	17	1426492	433964	0	1	2	0	50	62	22561500	8.17E-01	1
FAM35A	403850	144	27	2143476	564972	0	4	1	0	29	28	15292692	8.17E-01	1
CPT1C	1716449	192	41	2042728	610184	0	8	0	0	12	19	5666808	8.17E-01	1
KIF25	142012	702	-17	966184	303668	0	2	1	0	40	76	23394540	8.17E-01	1
ZNF284	533136	558	-35	1559636	360628	0	6	1	0	50	58	16442928	8.17E-01	1
CHP2	517081	598	33	518692	138128	0	1	1	0	50	61	19252836	8.17E-01	1
OR2G3	204354	957	-25	757212	234960	0	3	2	0	21	47	9325064	8.17E-01	1
E2F2	1050775	159	32	1092208	336776	0	3	0	0	1	0	433252	8.17E-01	1
GFOD1	258422	407	37	963336	293700	0	2	2	0	31	36	11478864	8.17E-01	1
FKBPL	2170336	224	32	854756	273052	0	2	0	0	19	28	8032784	8.17E-01	1
ZNF438	89942	603	20	2092568	590248	0	6	1	0	50	66	20225072	8.17E-01	1

NFIX	1914702	221	41	1125316	331436	0	2	1	0	50	57	21745904	8.17E-01	1
CDC16	612511	283	46	1665368	401212	0	3	1	0	50	47	21694284	8.17E-01	1
MZF1	2673870	276	11	1799936	576364	0	5	3	0	11	16	4808492	8.17E-01	1
KIAA0556	349212	620	12	4139924	1164832	0	10	4	0	14	12	5410844	8.17E-01	1
ABCA7	2387949	206	26	5322200	1749028	0	9	7	0	12	24	8124276	8.17E-01	1
DDX21	860888	246	52	2034184	539696	0	7	2	0	50	61	18490996	8.17E-01	1
PYROXD2	142644	581	4	1489504	441440	0	1	1	0	50	67	24359656	8.17E-01	1
KRT78	1781225	288	27	1321472	384124	0	4	1	0	13	20	5681404	8.17E-01	1
UCK1	609539	272	36	740836	177644	0	1	0	0	50	50	21880116	8.17E-01	1
FYN	245639	317	21	1531868	409756	0	3	2	0	13	13	5621596	8.17E-01	1
ZBTB1	666489	188	45	1886088	447492	0	6	0	0	18	19	7262756	8.17E-01	1
GPR83	299664	704	-13	1058388	312924	0	2	1	0	50	57	18892208	8.17E-01	1
RAB11FIP5	489748	460	18	1597016	515488	0	3	4	0	27	27	8658276	8.17E-01	1
KCNK16	190767	749	31	1094344	333572	0	2	0	0	50	54	20647288	8.18E-01	1
OR1J1	373314	646	3	790320	243504	0	1	0	0	50	63	22349324	8.18E-01	1
BID	395559	280	-18	617660	179068	0	1	1	0	50	67	23231848	8.18E-01	1
BEST2	1959466	226	29	1279108	389108	0	3	0	0	50	74	23429072	8.18E-01	1
PYCR2	923085	239	20	800288	253828	0	1	1	0	50	52	17968032	8.18E-01	1
DCUN1D1	232043	574	13	707016	153792	0	1	0	0	50	43	17563616	8.18E-01	1
PLA2G3	731144	218	40	1279108	380564	0	4	2	0	2	2	975796	8.18E-01	1
SASH1	77939	454	-8	3164840	914920	0	8	2	0	50	55	19870852	8.18E-01	1
SGCA	1277832	210	26	973660	310076	0	2	0	0	50	71	23861612	8.18E-01	1
HTR3A	175442	586	1	1324676	389464	0	2	0	0	47	45	19577508	8.18E-01	1
FEM1A	798897	203	23	1618376	528304	0	3	2	0	50	51	20192320	8.18E-01	1
SPRED3	1467279	253	0	1022432	358492	0	2	1	0	18	15	5595252	8.18E-01	1
SNX32	3779084	287	42	1050556	295124	0	5	1	0	2	2	703456	8.18E-01	1
SLC25A45	4112809	242	50	727308	224280	0	1	1	0	27	30	12483852	8.18E-01	1
NXF5	272029	NaN	23	988968	240300	0	2	1	0	50	72	24362148	8.18E-01	1
CTDSPL	408788	457	22	724816	194732	0	1	1	0	50	57	23271720	8.18E-01	1
CSF2RA	NaN	NaN	NaN	1351376	330724	0	3	2	0	50	118	39976308	8.18E-01	1
ABHD8	842088	211	43	1076544	350304	0	2	2	0	50	61	21639816	8.18E-01	1
ZGPAT	1126597	202	36	1319692	410468	0	4	3	0	15	17	5909600	8.18E-01	1
U2AF2	1077402	254	-10	1225708	346388	0	4	1	0	8	8	2559640	8.19E-01	1

TRIM56	1066804	272	39	1801360	624068	0	5	2	0	50	83	23088024	8.19E-01	1
MLC1	909819	215	25	965472	292632	0	3	3	0	7	11	2349244	8.19E-01	1
SACM1L	319452	385	45	1580996	384124	0	3	1	0	50	56	20846292	8.19E-01	1
TMIGD1	475180	232	26	690640	177644	0	3	2	0	28	50	10398048	8.19E-01	1
ZEB2	56402	621	16	3127460	803848	0	13	5	0	28	37	10396980	8.19E-01	1
STRA6	530359	220	45	1725532	542900	0	2	0	0	21	12	7934528	8.19E-01	1
PPP2R5B	3184873	209	57	1277684	369172	0	4	4	0	11	22	4645800	8.19E-01	1
ZNF711	90533	NaN	1	1981852	489500	0	4	0	0	7	7	2914216	8.19E-01	1
TIAM1	210411	924	-24	4077268	1130300	0	15	6	0	45	92	20411972	8.19E-01	1
ADAL	823006	415	50	965828	245284	0	2	0	0	50	74	24588920	8.19E-01	1
ACPT	652250	593	27	1048064	364900	0	2	1	0	50	64	22467160	8.19E-01	1
SPG7	1107401	203	39	2145256	633680	0	5	4	0	21	23	6867240	8.19E-01	1
SOX17	240943	554	-8	1009260	324672	0	2	1	0	50	63	21777588	8.19E-01	1
USP34	841742	471	34	9380244	2323968	0	29	3	0	1	4	2886092	8.19E-01	1
KIAA1755	499325	245	60	2964412	939128	0	6	1	0	12	10	5388060	8.19E-01	1
REPS1	310511	613	16	2053764	580992	0	4	0	0	37	39	21180932	8.20E-01	1
PALM2	276166	485	4	1073696	284800	0	2	0	0	12	18	7117152	8.20E-01	1
FADS3	1998419	212	69	1158780	317196	0	2	2	0	9	15	4289444	8.20E-01	1
RNF139	472766	476	28	1657180	477752	0	3	0	0	17	13	5575672	8.20E-01	1
GSDMA	1652518	216	28	1141692	334284	0	2	0	0	50	67	22696068	8.20E-01	1
ABHD2	639252	388	22	1106804	296548	0	2	0	0	50	72	26774404	8.20E-01	1
HAX1	1653296	236	29	726952	195800	0	1	0	0	50	69	22962712	8.20E-01	1
AWAT1	161347	NaN	27	853332	228552	0	1	0	0	50	47	22645516	8.20E-01	1
BGN	971101	NaN	7	942332	269848	0	2	1	0	50	42	13736972	8.20E-01	1
BUB1B	876373	161	42	2756508	707016	0	7	3	0	50	50	20747680	8.20E-01	1
AADAC	200470	526	2	1017448	283376	0	2	2	0	9	6	2714856	8.20E-01	1
HDAC2	217826	610	14	1316488	305804	0	3	1	0	32	43	13809596	8.20E-01	1
LMNA	2150035	180	39	1686728	513352	0	5	3	0	4	8	2049848	8.20E-01	1
ZNF551	606379	849	-44	1695272	411892	0	6	2	0	50	65	17258880	8.20E-01	1
EGR3	765839	325	52	962624	284800	0	2	2	0	22	17	6163784	8.20E-01	1
ZNF304	432083	869	-17	1698832	424352	0	5	0	0	47	53	17112920	8.20E-01	1
XKR4	93897	925	-13	1613392	483092	0	4	1	0	50	69	23504188	8.20E-01	1
AUH	124246	342	24	869352	260592	0	1	0	0	27	24	10683916	8.21E-01	1

SIDT2	426428	228	46	2176228	601640	0	2	2	0	50	39	17316908	8.21E-01	1
SCRT1	2525749	200	28	836244	286224	0	1	0	0	50	56	26181308	8.21E-01	1
DNASE2	1937827	226	32	914208	263796	0	2	1	0	50	71	21091932	8.21E-01	1
SLITRK3	26494	1315	-17	2412256	725528	0	9	4	0	23	27	8431148	8.21E-01	1
DDX24	200295	605	47	2181212	608404	0	8	4	0	33	44	12874740	8.21E-01	1
ADAM30	390094	560	9	1994312	540052	0	7	2	0	50	69	20372456	8.21E-01	1
P2RX7	1061231	396	46	1559992	402992	0	3	2	0	50	66	18904312	8.21E-01	1
NT5DC3	1181243	358	15	1449632	367036	0	4	2	0	50	79	22436188	8.21E-01	1
RAB32	56448	901	-33	576008	156640	0	2	2	0	28	48	9227164	8.21E-01	1
TAS2R38	198115	856	-6	824852	249556	0	1	1	0	50	61	21007204	8.21E-01	1
SLC5A8	261778	828	-15	1566400	451052	0	5	0	0	26	33	10061984	8.21E-01	1
NEK3	397134	432	30	1359564	328944	0	2	1	0	30	29	13552564	8.21E-01	1
NAE1	868790	142	24	1487012	354220	0	4	1	0	50	59	19451484	8.21E-01	1
KPRP	64902	818	18	1422576	440016	0	11	1	0	2	2	609828	8.21E-01	1
NR2E3	1039394	440	25	1038452	311500	0	2	0	0	2	0	815952	8.21E-01	1
CACNG2	1202913	359	42	816664	234248	0	3	2	0	18	29	6160936	8.21E-01	1
FBXW9	1967616	218	15	1143472	367748	0	3	3	0	5	16	3885740	8.21E-01	1
BEND4	263825	496	24	1339628	391600	0	2	0	0	50	47	19155292	8.21E-01	1
SLC17A9	961230	336	40	1117484	333928	0	1	1	0	50	82	23029640	8.21E-01	1
MYH8	80361	731	-49	5091512	1280176	0	16	1	0	2	10	3856548	8.21E-01	1
PPP2R5A	485902	298	16	1294060	317552	0	3	0	0	13	12	4540424	8.21E-01	1
CRIPAK	1018381	507	41	1075120	357068	0	2	0	0	32	50	18344324	8.22E-01	1
PI4KB	1256374	184	31	2098264	606980	0	9	1	0	4	14	2831624	8.22E-01	1
UPP2	115042	838	-4	973660	264152	0	3	1	0	50	65	19143544	8.22E-01	1
EXOSC1	724294	188	40	520828	139196	0	1	1	0	16	27	8144924	8.22E-01	1
CABIN1	749515	202	40	5615544	1654332	0	7	2	0	1	2	2048780	8.22E-01	1
SLC36A2	821078	530	48	1220012	369172	0	2	2	0	50	54	22128960	8.22E-01	1
ODF4	772200	257	20	655752	179424	0	1	0	0	50	52	17300532	8.22E-01	1
OR4N5	371813	563	-29	759704	229264	0	1	2	0	15	16	5073712	8.22E-01	1
EID3	NaN	NaN	NaN	845144	224992	0	2	2	0	26	42	11802112	8.22E-01	1
ZNF397	260556	599	7	1634040	400500	0	2	0	0	50	41	21849856	8.22E-01	1
PCDH7	28650	682	6	3156296	962980	0	7	3	0	50	55	25571480	8.23E-01	1
CLEC16A	378690	430	37	2715568	755432	0	7	3	0	50	72	21806068	8.23E-01	1

EBF1	123440	728	39	1537564	423284	0	4	1	0	50	62	18899328	8.23E-01	1
APOC3	421374	323	39	261304	71912	0	2	1	0	1	2	262372	8.23E-01	1
DOLK	1517799	203	29	1284092	442864	0	2	2	0	50	43	19078752	8.23E-01	1
SEC16B	103475	499	-10	2736572	769672	0	7	1	0	16	25	8369560	8.23E-01	1
CHST12	792353	251	35	1024568	309364	0	3	1	0	50	73	22042808	8.23E-01	1
SHC2	1370962	423	-24	1451412	467784	0	3	0	0	7	6	2945900	8.23E-01	1
LGI3	587455	196	50	1372736	416164	0	4	2	0	50	67	20087300	8.23E-01	1
SLC47A1	558023	414	2	1458176	441796	0	3	2	0	50	52	18951660	8.23E-01	1
SIDT1	466309	468	21	2171600	583840	0	7	2	0	43	39	13685708	8.23E-01	1
ALLC	252769	496	5	1026704	276256	0	1	1	0	50	54	24675428	8.23E-01	1
HABP2	189589	596	2	1487368	362408	0	2	1	0	50	48	20896488	8.24E-01	1
LOH12CR1	707022	279	27	508724	135280	0	3	2	0	2	5	1013888	8.24E-01	1
ARHGEF1[illegible]	264872	312	25	4064452	1058744	0	10	2	0	10	10	4549680	8.24E-01	1
MAGEA4	125659	NaN	-21	782132	241012	0	2	1	0	37	49	12693536	8.24E-01	1
PRKCH	312210	434	11	1776084	470988	0	4	1	0	50	56	21714932	8.24E-01	1
ZNF276	1162469	219	39	1560704	454612	0	6	3	0	12	13	3733372	8.24E-01	1
TUBA3C	193250	922	-15	1132436	333928	0	3	2	0	34	35	10148492	8.24E-01	1
TSPAN2	595238	555	4	581704	161624	0	1	1	0	50	89	28246820	8.24E-01	1
ZMYM4	916800	406	19	4058044	1030976	0	12	1	0	0	1	1030976	8.24E-01	1
KCNA3	318208	730	22	1398012	447492	0	4	4	0	3	11	2881464	8.24E-01	1
CNNM3	879415	332	48	1701680	598792	0	4	2	0	50	74	25443320	8.24E-01	1
DUSP15	1015946	241	37	595944	185832	0	1	0	0	50	52	17181628	8.24E-01	1
GBX1	611046	337	38	867928	301532	0	2	0	0	11	15	4791048	8.24E-01	1
CCPG1	267069	396	11	1987192	471344	0	2	0	0	42	54	21897204	8.24E-01	1
TP73	249590	288	20	1717344	422928	0	3	3	0	5	4	2676408	8.24E-01	1
RHOQ	595763	213	36	532932	144180	0	1	2	0	10	12	3712368	8.24E-01	1
SYT12	1643265	152	47	1068712	313280	0	3	1	0	50	72	20561848	8.24E-01	1
GP6	1125216	432	-26	1601288	420436	0	2	1	0	46	55	18459668	8.24E-01	1
TRIM16	471003	431	-19	1442156	389464	0	1	2	0	50	54	19765476	8.24E-01	1
IRF5	845100	361	49	1300112	384124	0	3	2	0	50	80	25321924	8.24E-01	1
DSEL	18159	527	-33	3096132	822360	0	12	2	0	19	31	7153820	8.24E-01	1
LAD1	857879	209	26	1300112	398008	0	5	0	0	3	2	847636	8.25E-01	1
GPR152	1392213	215	53	1117840	395516	0	2	1	0	11	7	3262740	8.25E-01	1

KRT75	1194800	389	29	1392316	412604	0	4	1	0	50	64	18116128	8.25E-01	1
KRTAP11-	31443	1030	-60	395872	131720	0	1	0	0	13	13	3304392	8.25E-01	1
PTGFRN	760797	540	24	2192248	661448	0	4	1	0	31	58	19038168	8.25E-01	1
AGMAT	656850	162	31	870776	285868	0	1	0	0	33	29	14413372	8.25E-01	1
CHTF8	1179391	201	24	308652	95052	0	1	1	0	5	13	2574948	8.25E-01	1
SLC37A4	1424132	180	57	1142760	344964	0	1	0	0	6	7	2842304	8.25E-01	1
TGFB1	1193025	249	33	979000	299396	0	3	0	0	50	52	16514128	8.25E-01	1
OR1N2	407749	646	9	806696	252760	0	2	1	0	50	67	20364980	8.25E-01	1
OLFML1	108110	869	11	1015668	284088	0	3	3	0	10	22	4294428	8.25E-01	1
SPTA1	177547	940	1	6368484	1603068	0	36	8	0	5	15	3331804	8.25E-01	1
GOLGA4	409750	283	20	5961220	1387688	0	17	7	0	11	22	6459620	8.25E-01	1
HYI	1044061	239	35	708796	213956	0	1	0	0	50	52	18519832	8.25E-01	1
WNT11	265144	359	38	889288	265220	0	2	0	0	21	22	6791412	8.25E-01	1
EEF2K	522343	235	44	1904600	494128	0	7	0	0	2	2	1237456	8.25E-01	1
HSF1	2525749	200	28	1352088	397296	0	5	3	0	4	8	1894276	8.25E-01	1
DOC2A	NaN	NaN	NaN	1022432	305092	0	2	2	0	50	118	39950676	8.25E-01	1
FAM151B	320158	516	35	730156	178712	0	1	1	0	17	12	5536868	8.25E-01	1
SMARCC1	843941	200	39	2892500	766468	0	9	2	0	50	59	19702108	8.25E-01	1
EVI2B	NaN	NaN	NaN	1108940	333928	0	2	0	0	50	116	39979512	8.25E-01	1
DERL1	470881	412	33	661448	175864	0	1	0	0	50	52	20461456	8.25E-01	1
PKD1L2	290329	327	3	6273432	1859388	0	8	4	0	35	28	15576780	8.25E-01	1
CDHR1	216191	860	-17	2340700	696692	0	8	2	0	50	78	25295580	8.26E-01	1
PABPN1L	860087	252	37	734784	218940	0	1	0	0	50	60	24489240	8.26E-01	1
KCNJ10	829863	386	17	926668	292988	0	2	1	0	50	71	21371748	8.26E-01	1
LGMN	423482	499	48	1150948	295124	0	2	0	0	50	51	19702820	8.26E-01	1
TKT	782559	192	54	1607340	451764	0	2	0	0	2	0	1246000	8.26E-01	1
AQP1	402058	568	10	873268	281240	0	2	1	0	50	61	18066288	8.26E-01	1
RASSF4	75634	905	25	847992	226416	0	1	0	0	50	52	22092648	8.26E-01	1
UBOX5	872596	192	54	1342476	411180	0	3	0	0	28	26	9830228	8.26E-01	1
RAD9B	922262	206	43	1145252	279460	0	2	1	0	36	42	16644780	8.26E-01	1
COL6A5	202934	533	11	6516224	1746892	0	11	0	0	3	2	2930592	8.26E-01	1
HIP1R	977561	184	37	2766476	793168	0	6	4	0	50	67	22668656	8.26E-01	1
VPS16	766497	396	44	2182992	610896	0	7	0	0	4	3	1699544	8.26E-01	1

SEC14L2	676863	214	52	1144540	299396	0	2	1	0	50	63	21810340	8.26E-01	1
DNAJB9	171249	838	6	587756	138484	0	1	1	0	36	35	12002896	8.26E-01	1
OR10A4	427462	681	8	773944	242792	0	2	0	0	50	71	18227912	8.26E-01	1
OR10A5	427462	681	8	782132	236740	0	2	1	0	50	71	18227912	8.26E-01	1
ROR2	457045	572	30	2354940	703812	0	7	4	0	24	28	7763292	8.26E-01	1
FRMD4A	212541	732	24	2679968	754720	0	7	4	0	13	16	5393400	8.26E-01	1
URB2	299238	340	15	3782500	1142048	0	7	1	0	7	7	4475276	8.26E-01	1
USP17L2	475312	625	3	1328236	373088	0	3	2	0	50	62	20793248	8.27E-01	1
VCP	782447	350	42	2063732	590248	0	4	3	0	50	63	24855920	8.27E-01	1
HOXD11	242935	874	15	826276	264152	0	2	0	0	50	65	19479964	8.27E-01	1
PDHA1	216523	NaN	39	1132436	311500	0	3	2	0	17	28	8194764	8.27E-01	1
CWH43	246165	439	49	1813464	493416	0	5	3	0	14	24	8547916	8.27E-01	1
TMEM11	416965	517	-27	474192	148452	0	1	0	0	50	71	19737708	8.27E-01	1
PNLIPRP2	161760	618	-16	1242084	321468	0	3	2	0	19	24	7721640	8.27E-01	1
MAGEA1(	191719	NaN	-20	924176	267712	0	2	2	0	14	11	3714148	8.27E-01	1
CDR2L	1078189	171	28	1157712	352440	0	2	3	0	50	72	16166672	8.27E-01	1
SIX2	74076	508	23	715916	223924	0	1	1	0	37	34	14192652	8.27E-01	1
OR51B4	100115	1056	1	765044	230332	0	1	1	0	30	18	7873652	8.27E-01	1
NSFL1C	397239	386	42	962268	260592	0	5	2	0	1	2	515132	8.27E-01	1
OR2T35	141426	710	-10	788896	249200	0	1	0	0	50	70	30005816	8.27E-01	1
TRIT1	996021	270	31	1216452	327876	0	4	2	0	15	20	6653284	8.27E-01	1
ADCY3	284877	296	37	2919912	836244	0	4	3	0	50	44	18472128	8.27E-01	1
CYP8B1	301212	447	21	1245288	363120	0	2	2	0	43	60	20254976	8.27E-01	1
CEBPG	302640	510	-21	385192	102884	0	1	1	0	10	44	8616624	8.27E-01	1
MECP2	2370576	NaN	29	1252764	390888	0	2	1	0	50	69	29055296	8.28E-01	1
PSMB9	1253457	401	26	555716	172660	0	1	0	0	50	63	19067004	8.28E-01	1
PEX11A	880974	190	35	621932	183340	0	1	0	0	50	61	22286312	8.28E-01	1
TTF1	1431869	429	34	2347108	598436	0	5	3	0	50	60	18365328	8.28E-01	1
STT3B	270559	511	-4	2145256	566396	0	4	1	0	23	19	8534388	8.28E-01	1
SCRIB	2182434	229	28	4090796	1370956	0	14	4	0	50	80	25006864	8.28E-01	1
ZNF860	NaN	NaN	NaN	1635820	392312	0	5	0	0	50	116	40037896	8.28E-01	1
ABCD1	1910154	NaN	37	1829484	599148	0	4	2	0	50	67	22713868	8.28E-01	1
DENND2D	400425	627	41	1229980	329300	0	3	1	0	50	66	20484952	8.28E-01	1

BAAT	112947	898	-22	1042012	313280	0	3	1	0	12	13	4649716	8.28E-01	1
PPAPDC3	745091	213	50	654684	221076	0	1	0	0	50	53	18357496	8.28E-01	1
DIO3	1315103	577	-26	741548	235672	0	2	0	0	50	77	19026776	8.28E-01	1
DPP9	860250	203	29	2317204	629408	0	6	1	0	35	67	19910368	8.28E-01	1
PIP	321366	568	-9	384124	103952	0	1	1	0	9	11	2748676	8.28E-01	1
UNC45B	634684	498	20	2373452	693844	0	8	4	0	50	64	19807840	8.28E-01	1
DNAJB14	202808	682	36	1003564	243860	0	2	0	0	50	66	21102256	8.28E-01	1
N4BP2L1	226626	435	53	716984	161980	0	1	0	0	50	62	24158872	8.28E-01	1
OR7E24	398878	582	-50	841584	247776	0	2	1	0	50	116	31105500	8.28E-01	1
ARMC2	414039	431	32	2246360	607336	0	3	1	0	5	2	2568540	8.28E-01	1
HERPUD1	820472	125	47	1005344	280528	0	2	1	0	50	77	25691452	8.28E-01	1
ISPD	285724	921	-20	1164120	322536	0	3	0	0	11	30	6047372	8.28E-01	1
ZNF200	1493009	253	42	1022788	263084	0	2	1	0	50	77	26841332	8.29E-01	1
JMJD6	827288	206	22	1075832	281596	0	2	2	0	50	41	14517680	8.29E-01	1
ATOH8	1105419	181	46	763620	276612	0	1	2	0	21	27	10587440	8.29E-01	1
SHROOM1	718028	356	58	2041660	721256	0	4	1	0	37	29	12992220	8.29E-01	1
RNF114	589377	181	48	599860	157352	0	1	1	0	50	58	18928164	8.29E-01	1
MPZL3	805791	211	41	607692	171948	0	1	1	0	50	62	21266728	8.29E-01	1
ERI1	147910	440	44	918480	228552	0	1	0	0	24	35	12300868	8.29E-01	1
NOXO1	1747301	183	46	924888	317196	0	2	1	0	50	75	19137492	8.29E-01	1
SLAMF6	1145424	590	26	863300	235672	0	2	0	0	50	77	21751956	8.29E-01	1
TMEM59	438316	201	29	840516	227484	0	2	1	0	26	33	9053080	8.29E-01	1
A1CF	113129	613	19	1631904	459240	0	4	2	0	50	64	20119340	8.29E-01	1
SURF4	1827885	416	28	693844	192596	0	1	1	0	50	47	17145316	8.29E-01	1
NDST3	150671	985	-20	2262736	588824	0	7	3	0	50	78	20142124	8.29E-01	1
HCRTR1	1061386	197	37	1058032	332504	0	2	1	0	50	49	17656888	8.29E-01	1
POMC	319647	280	37	671060	196156	0	1	1	0	40	37	14099736	8.29E-01	1
NAB2	2026922	199	41	1290856	420080	0	1	1	0	50	67	21271000	8.29E-01	1
LIN52	541052	236	43	319688	78676	0	1	1	0	2	4	1058032	8.29E-01	1
KRT73	1441383	464	29	1371312	398364	0	4	4	0	10	11	3503396	8.29E-01	1
POLR1A	983860	391	46	4430064	1224996	0	15	6	0	50	77	22499556	8.29E-01	1
FCGR1A	NaN	NaN	NaN	986832	272340	0	2	0	0	50	116	39917924	8.30E-01	1
RTN4R	530222	292	44	1117484	405484	0	2	1	0	5	5	2629416	8.30E-01	1

NFXL1	186668	602	41	2411188	604844	0	7	1	0	7	5	2114284	8.30E-01	1
SPI1	1106152	245	46	706304	182272	0	1	0	0	50	78	25933888	8.30E-01	1
ARSF	217524	NaN	-11	1511220	425064	0	3	1	0	50	64	23203012	8.30E-01	1
AKT2	1005838	400	30	1265936	333928	0	2	0	0	38	34	14222556	8.30E-01	1
B3GNT8	1104034	376	38	940908	338556	0	3	0	0	42	61	14196212	8.30E-01	1
FBLN1	448796	195	3	2233544	598792	0	8	0	0	12	12	4270220	8.30E-01	1
MED29	1748498	252	32	559632	166608	0	1	0	0	50	78	23410916	8.30E-01	1
CYP17A1	883280	188	44	1292992	367748	0	3	0	0	28	27	10116096	8.30E-01	1
SDHB	678965	221	52	738344	191884	0	1	1	0	50	57	20959500	8.30E-01	1
ACER2	496889	421	24	712356	193308	0	1	1	0	50	58	23254276	8.31E-01	1
PRDM15	279380	366	35	3912796	1051268	0	7	2	0	32	19	10777544	8.31E-01	1
PLIN5	2111443	278	43	1148812	367748	0	1	2	0	50	77	21475344	8.31E-01	1
HIST1H2BI	267617	634	32	315060	96120	0	1	3	0	8	13	1153440	8.31E-01	1
UBE2C	742720	180	48	597368	164116	0	1	0	0	50	49	15404476	8.31E-01	1
FBXO10	511883	338	34	2409052	699896	0	4	2	0	50	53	19482100	8.31E-01	1
RAB5C	2150553	206	33	562480	154148	0	1	1	0	50	72	21985492	8.31E-01	1
ALDH1B1	272380	622	3	1271276	392668	0	2	1	0	50	46	19139984	8.31E-01	1
TRAF4	1695792	208	40	1192244	342472	0	3	0	0	6	7	2121760	8.31E-01	1
GALK1	1885370	198	27	971524	319688	0	2	2	0	50	68	21651920	8.31E-01	1
CIDEB	915114	220	38	555004	171236	0	1	0	0	50	69	21077692	8.31E-01	1
SIGLEC10	393294	583	17	1745468	533644	0	9	5	0	3	8	1627988	8.31E-01	1
S1PR4	908206	241	21	899968	335708	0	2	3	0	21	23	7152040	8.31E-01	1
CEP250	1521661	167	53	6205792	1755080	0	15	9	0	26	29	11106844	8.31E-01	1
PCSK9	447775	230	13	1721616	545748	0	5	2	0	50	77	25253928	8.31E-01	1
TMEM30A	394127	423	-3	932364	253116	0	1	2	0	50	59	20882960	8.31E-01	1
CABP1	1678287	311	38	1099328	361696	0	2	1	0	50	55	20293424	8.31E-01	1
MRPL15	268859	464	31	748668	222144	0	1	1	0	50	48	20317988	8.31E-01	1
SSTR4	199022	554	-50	926312	324316	0	2	1	0	19	25	7971196	8.31E-01	1
KIF7	804572	319	27	3338924	1044148	0	4	0	0	2	2	2957292	8.32E-01	1
IQSEC2	886836	NaN	77	3780008	1189396	0	6	2	0	11	11	5843384	8.32E-01	1
LZTR1	577297	192	58	2173736	607336	0	8	3	0	50	77	21004712	8.32E-01	1
SCARA5	413257	154	32	1237100	386260	0	2	3	0	50	57	18399148	8.32E-01	1
EIF4E1B	579630	359	40	635816	172660	0	1	1	0	15	19	6442532	8.32E-01	1

CAPN9	438898	550	7	1830196	467072	0	5	0	0	10	14	5482044	8.32E-01	1
CDHR3	337002	408	29	2273416	642224	0	3	2	0	31	38	15093332	8.32E-01	1
ARRDC5	777835	173	42	862944	244572	0	2	2	0	49	74	22044944	8.32E-01	1
NARFL	1207790	194	33	1223572	349592	0	1	1	0	50	77	22508812	8.32E-01	1
PPEF2	482292	429	45	1967968	516200	0	4	3	0	45	53	19423360	8.32E-01	1
MSR1	53956	947	-48	1221080	314704	0	4	1	0	50	62	17031752	8.32E-01	1
CDH6	133952	902	-26	2014248	562836	0	11	2	0	50	104	21117920	8.32E-01	1
MEIS3	797690	276	38	1093276	310076	0	2	2	0	50	64	22709952	8.32E-01	1
UGGT1	449913	419	24	4108596	1047708	0	11	3	0	25	25	9939876	8.32E-01	1
PRPF31	735613	439	-53	1293704	363832	0	4	3	0	50	119	29395632	8.33E-01	1
ULK1	374096	180	23	2655760	826988	0	7	4	0	8	18	5872576	8.33E-01	1
GATA6	230941	437	-1	1443224	491992	0	2	0	0	50	45	21833836	8.33E-01	1
MINPP1	470910	190	25	1282668	336420	0	5	1	0	5	3	1165544	8.33E-01	1
DQX1	1019591	315	50	1786408	561056	0	3	2	0	50	69	21835972	8.33E-01	1
AQP3	1001877	166	4	729088	233180	0	1	0	0	50	43	17029972	8.33E-01	1
LRRC16A	247903	576	28	3590260	959420	0	10	1	0	24	19	8732324	8.33E-01	1
TRIM73	NaN	NaN	NaN	638308	182984	0	1	1	0	50	117	39828568	8.33E-01	1
PEF1	1093701	197	37	718052	212176	0	1	0	0	50	47	18470348	8.33E-01	1
STXBP3	772267	191	9	1593812	385192	0	4	1	0	50	68	20925680	8.33E-01	1
NOX1	352830	NaN	29	1472060	389464	0	3	0	0	47	59	22369260	8.33E-01	1
GZF1	188823	688	-22	1815244	483092	0	4	2	0	37	66	17618440	8.33E-01	1
HSD3B1	422140	672	7	942332	268780	0	3	3	0	9	7	2352448	8.33E-01	1
TRDN	23697	1090	-16	2077260	434676	0	5	0	0	18	29	7929188	8.33E-01	1
CDON	611194	249	10	3208272	925956	0	9	4	0	40	59	20371744	8.33E-01	1
CAPNS2	NaN	NaN	NaN	621932	178000	0	1	1	0	50	117	39823584	8.34E-01	1
UBR5	504282	414	39	7232140	1988972	0	23	7	0	31	55	17655108	8.34E-01	1
SYMPK	883734	203	39	3221800	974372	0	9	3	0	50	63	19718840	8.34E-01	1
DISP2	872646	184	42	3374880	1149168	0	6	2	0	39	36	15292336	8.34E-01	1
HAL	366272	436	8	1707732	485940	0	5	4	0	20	26	6505544	8.34E-01	1
HELT	335039	582	29	805272	257388	0	1	0	0	50	58	22976596	8.34E-01	1
CAGE1	761240	283	15	2319696	540408	0	5	0	0	13	15	6262752	8.34E-01	1
HCFC1	2355024	NaN	25	4939144	1691000	0	6	1	0	1	1	2253836	8.34E-01	1
RAET1L	402308	382	51	629408	175864	0	1	1	0	50	64	22330812	8.34E-01	1

TASP1	121103	796	-26	1101464	302956	0	3	2	0	50	81	21275272	8.34E-01	1
MRPS17	760564	642	11	328588	99680	0	2	2	0	2	2	452476	8.34E-01	1
MGEA5	817474	335	37	2386624	615524	0	9	0	0	0	0	615524	8.34E-01	1
ZNF862	438832	686	26	2924184	854400	0	6	2	0	11	10	4417960	8.34E-01	1
CENPQ	64168	1198	-6	724816	171236	0	1	0	0	50	47	18347172	8.34E-01	1
NFYB	1222031	156	17	563548	132788	0	1	0	0	50	57	17603844	8.34E-01	1
MRPL19	138530	559	2	762196	200072	0	1	0	0	50	67	24679344	8.34E-01	1
LONP1	935129	225	23	2419020	731580	0	9	4	0	50	48	15409460	8.34E-01	1
ECHS1	386585	472	13	764332	200072	0	1	0	0	50	46	19712788	8.34E-01	1
RAB24	942017	227	53	551088	136704	0	1	1	0	50	65	19830268	8.34E-01	1
LFNG	783535	226	47	1197228	363120	0	2	1	0	50	52	20797164	8.35E-01	1
ZNF697	421423	599	13	1362412	395516	0	3	0	0	39	52	17563260	8.35E-01	1
ZNF131	506232	537	21	1538988	381276	0	4	2	0	50	66	21136788	8.35E-01	1
IFNA21	137739	498	1	479176	131720	0	1	2	0	8	11	3212900	8.35E-01	1
CFTR	116609	480	-7	3825932	1030264	0	14	2	0	4	8	2961208	8.35E-01	1
CDK11A	NaN	NaN	NaN	2097908	519760	0	8	4	0	26	44	12096880	8.35E-01	1
ILF2	1656449	183	40	1023856	286580	0	2	0	0	27	29	10394844	8.35E-01	1
SH2D4B	302345	686	-11	965472	254184	0	1	1	0	50	56	21159572	8.35E-01	1
COG1	221646	251	-11	2479540	725528	0	6	2	0	27	27	11526568	8.35E-01	1
BTRC	649315	421	27	1578504	422928	0	3	1	0	50	47	18228624	8.35E-01	1
AGPAT4	74993	874	-5	982560	265932	0	2	1	0	50	70	22423372	8.35E-01	1
TTNBP2N	559406	335	23	1596304	471344	0	2	2	0	50	40	21444728	8.35E-01	1
IGHMBP2	575839	253	48	2483456	763264	0	5	1	0	3	2	1894276	8.35E-01	1
FAM122B	293030	NaN	27	778928	195088	0	1	0	0	50	78	26755892	8.35E-01	1
UST	169694	476	8	1053048	280884	0	2	1	0	50	67	20598872	8.35E-01	1
PHC1	296906	416	12	2510156	769672	0	3	1	0	31	34	15035660	8.35E-01	1
MLLT6	1117878	207	41	2713076	875404	0	5	0	0	1	0	989324	8.35E-01	1
CLCNKB	886121	197	43	1873984	567464	0	5	4	0	35	41	14362820	8.35E-01	1
CDH26	73597	856	-36	2152376	610540	0	7	2	0	8	10	3553236	8.35E-01	1
LYL1	1948449	221	41	672840	240300	0	1	0	0	50	60	21446864	8.36E-01	1
WSB2	639801	584	-3	1042724	291208	0	2	0	0	12	17	6241036	8.36E-01	1
PDLIM1	302337	444	6	850484	232468	0	1	0	0	28	21	10474232	8.36E-01	1
PTPN5	605685	562	26	1463160	410112	0	1	1	0	50	62	22631632	8.36E-01	1

DCAF10	477149	524	35	1383772	438236	0	4	0	0	24	23	7950904	8.36E-01	1
UPF3B	762240	NaN	61	1300824	294768	0	3	1	0	34	38	12790368	8.36E-01	1
INTS12	293906	417	3	1168036	342116	0	2	1	0	50	56	21183068	8.36E-01	1
KAZALD1	469702	221	52	752584	241724	0	1	0	0	50	47	19691784	8.36E-01	1
GPR65	115758	976	-36	855824	227128	0	3	0	0	50	108	24423380	8.36E-01	1
ACER1	943949	407	31	691352	181204	0	1	0	0	50	47	18083732	8.36E-01	1
THAP6	461415	491	19	589536	142044	0	1	0	0	50	63	20447928	8.36E-01	1
HOXC12	1257445	274	23	682808	226060	0	1	0	0	50	62	22406640	8.36E-01	1
ITPR3	1401905	205	38	6916368	1888224	0	10	9	0	50	71	27027164	8.36E-01	1
CDC34	1647685	255	-8	606624	169812	0	1	1	0	50	56	18766896	8.36E-01	1
GPR37	34802	462	-24	1492352	479176	0	6	2	0	50	71	17173440	8.36E-01	1
CNGA1	220829	474	40	1969036	502316	0	4	1	0	50	77	23317288	8.36E-01	1
ACOT8	742720	180	48	829124	233536	0	3	0	0	2	3	781064	8.37E-01	1
TBX21	1118277	217	27	1335000	403704	0	1	0	0	33	34	11473880	8.37E-01	1
CLCN2	1447013	268	44	2283740	694912	0	6	1	0	4	10	2959072	8.37E-01	1
CBX2	633863	209	31	1590964	505520	0	2	3	0	50	64	19219728	8.37E-01	1
SLC19A1	422182	618	40	1423644	494484	0	3	3	0	50	61	20576800	8.37E-01	1
GLG1	499497	209	36	3161280	811680	0	6	0	0	0	0	811680	8.37E-01	1
MLKL	586358	173	30	1271276	316840	0	3	3	0	11	18	4635120	8.37E-01	1
SHISA2	96652	584	12	726952	227840	0	1	0	0	50	61	23431564	8.37E-01	1
ZFAND3	303392	630	32	595588	156284	0	1	1	0	50	56	16262436	8.37E-01	1
MRPS6	785767	574	70	321468	90780	0	2	1	0	1	1	338556	8.37E-01	1
VWA5A	177269	744	-11	2040592	555716	0	5	2	0	15	20	7197964	8.37E-01	1
NOV	261913	519	-14	911716	252404	0	2	1	0	50	86	25453644	8.37E-01	1
STRC	784339	198	44	4347828	1464228	0	6	0	0	1	0	1579572	8.37E-01	1
TMEM222	884974	188	30	544680	148452	0	1	1	0	50	101	28643048	8.37E-01	1
STARD10	529647	207	50	752228	204700	0	2	2	0	3	4	1508372	8.37E-01	1
PPA2	217228	603	29	901392	221076	0	2	0	0	13	15	5206856	8.37E-01	1
LRPPRC	455568	429	26	3684244	945536	0	7	2	0	6	5	2676764	8.37E-01	1
SULT1E1	9735	1009	4	797084	178000	0	1	0	0	50	57	22716716	8.38E-01	1
CD74	949599	212	40	795304	194732	0	1	0	0	50	73	24033560	8.38E-01	1
SLC3A2	2342595	181	71	1668572	506944	0	2	1	0	50	77	24280268	8.38E-01	1
SETD3	340080	429	-11	1583488	421148	0	3	1	0	50	59	22500980	8.38E-01	1

TAOK2	2038342	238	60	3788552	1226776	0	10	2	0	1	3	1674980	8.38E-01	1
MSI2	801767	507	-7	961200	247776	0	3	1	0	50	59	15911064	8.38E-01	1
SPON1	414198	644	-10	2096484	555360	0	4	0	0	15	13	5082256	8.38E-01	1
ZKSCAN1	1503181	254	41	1437528	390888	0	3	0	0	18	21	7410140	8.38E-01	1
VARS	2509866	183	32	3196880	982204	0	5	1	0	5	4	3282676	8.38E-01	1
PNPLA4	43100	NaN	-9	649700	189748	0	1	1	0	40	84	20456828	8.38E-01	1
BTNL2	1582117	580	27	1154864	327520	0	2	2	0	21	47	10638704	8.38E-01	1
NAT10	769733	358	33	2662524	744396	0	7	3	0	50	54	20584276	8.38E-01	1
LGI1	488719	532	4	1444648	373088	0	3	1	0	50	65	23021096	8.38E-01	1
KCNS2	440962	550	28	1187616	348168	0	6	4	0	9	13	3009268	8.38E-01	1
MYOF	458453	380	16	5412268	1420796	0	13	5	0	14	14	4963708	8.38E-01	1
SLC25A19	1723762	171	31	815240	238876	0	2	1	0	50	81	19557572	8.38E-01	1
ZNF835	174439	849	-68	1395876	400500	0	4	0	0	10	9	3531876	8.38E-01	1
CXXC4	18498	982	18	495196	148808	0	1	2	0	20	32	7273080	8.38E-01	1
OR52B4	363779	797	-8	783912	229620	0	1	1	0	31	35	13623052	8.38E-01	1
SLC35A1	229577	534	27	865792	249200	0	1	0	0	17	10	5337152	8.38E-01	1
MAP3K13	953031	397	36	2480608	673196	0	10	2	0	6	6	1986124	8.38E-01	1
RRP1	835438	194	66	1204348	329300	0	2	2	0	7	4	2342124	8.38E-01	1
CEP152	418237	340	6	4366696	1044860	0	9	3	0	50	53	22315148	8.39E-01	1
CCT8L2	107592	852	-83	1339984	447848	0	5	3	0	50	76	18817448	8.39E-01	1
DPP10	7721	1178	-22	2279112	563904	0	12	5	0	3	7	1505168	8.39E-01	1
ONECUT2	300344	480	15	1231760	392668	0	2	1	0	50	46	18182344	8.39E-01	1
ITFG2	495532	443	28	1155220	327164	0	2	2	0	7	16	3959432	8.39E-01	1
ZNF34	1647917	356	18	1447496	371308	0	4	1	0	37	49	15171296	8.39E-01	1
ACAD8	270350	623	21	1077256	300464	0	3	0	0	3	2	1141336	8.39E-01	1
ALDH7A1	235232	191	19	1413320	389464	0	2	1	0	50	41	19182704	8.39E-01	1
NIPA1	352778	529	-8	810612	265932	0	1	0	0	50	53	22668300	8.39E-01	1
RPH3A	651570	527	28	1829128	483092	0	8	4	0	4	12	2618380	8.39E-01	1
FGF11	1906324	206	33	566396	174796	0	1	0	0	50	87	23542992	8.39E-01	1
CPSF4	1327454	288	60	720188	174796	0	2	2	0	12	13	3472424	8.39E-01	1
HPN	738570	419	-34	1068712	321824	0	3	1	0	31	49	12641916	8.39E-01	1
LCP2	79392	629	34	1430408	365968	0	2	0	0	14	13	6571048	8.39E-01	1
ZNF496	352232	742	-15	1491996	421860	0	4	2	0	50	50	15613092	8.39E-01	1

TMEM72	75634	905	25	689928	213600	0	1	2	0	16	17	6943780	8.39E-01	1
DDX54	468053	252	35	2232832	679604	0	6	1	0	41	44	15877600	8.39E-01	1
TSSK4	974559	220	41	860808	240300	0	1	1	0	50	62	24565780	8.39E-01	1
CHERP	815495	258	44	2360636	652192	0	7	2	0	50	65	20976232	8.39E-01	1
DSE	261273	541	30	2428988	665008	0	4	0	0	10	12	5622664	8.39E-01	1
RAB4B	1157241	186	29	556428	159132	0	1	2	0	4	5	1782136	8.39E-01	1
CEP350	446036	211	16	8035988	2112148	0	24	4	0	5	6	2825572	8.39E-01	1
RBMX	275533	NaN	28	1116772	234248	0	2	0	0	50	59	21499908	8.39E-01	1
PSG3	386041	417	-19	1092920	307228	0	8	2	0	1	2	616592	8.40E-01	1
COMP	1351321	189	26	1980072	527592	0	5	3	0	50	68	21923548	8.40E-01	1
KCNMB3	412428	641	14	883236	234960	0	1	1	0	50	59	23508816	8.40E-01	1
ARHGAP4	2328608	NaN	27	2496628	761840	0	3	0	0	7	3	3174096	8.40E-01	1
HUS1	147364	445	21	748668	181560	0	1	2	0	7	13	2683172	8.40E-01	1
LIMK2	667559	221	43	1995024	553224	0	4	2	0	50	51	21792540	8.40E-01	1
CCDC70	353099	349	69	612676	141332	0	1	0	0	50	63	20681108	8.40E-01	1
SLC45A4	277190	239	57	1977224	612676	0	3	3	0	50	63	20197304	8.40E-01	1
PPIL1	836025	244	43	432540	115344	0	1	1	0	20	33	7678920	8.40E-01	1
AKTIP	247554	403	8	771096	206124	0	1	1	0	50	47	20614180	8.40E-01	1
CHAT	NaN	NaN	NaN	1943404	567464	0	3	3	0	50	119	40213048	8.40E-01	1
TNS4	1185295	203	19	1796376	548952	0	4	1	0	9	9	3119272	8.40E-01	1
BMP8A	1026100	352	33	1000004	316840	0	2	1	0	50	65	20172028	8.40E-01	1
PTPN11	704590	167	43	1573876	393380	0	3	0	0	50	61	22148540	8.40E-01	1
ACRC	848605	NaN	39	1830908	437524	0	3	0	0	20	19	6479556	8.40E-01	1
LY9	1188881	618	29	1769320	511928	0	6	4	0	21	35	7939156	8.40E-01	1
PPHLN1	174675	545	14	1377364	365612	0	4	0	0	12	19	6802092	8.40E-01	1
IL1RL1	240161	588	9	1462092	377004	0	2	0	0	44	43	20577868	8.41E-01	1
NLRP1	1016229	605	10	3771820	1097192	0	9	4	0	32	43	13687488	8.41E-01	1
SPDEF	890476	207	34	859740	238164	0	1	0	0	50	68	21500264	8.41E-01	1
FBXO31	576709	311	18	1363480	402992	0	2	0	0	25	26	11217204	8.41E-01	1
OR10G7	158396	830	-10	751872	247776	0	3	2	0	7	15	3266300	8.41E-01	1
OR4C12	11630	1171	-38	770028	227484	0	2	0	0	50	94	21047432	8.41E-01	1
MPI	764533	195	46	1080816	309720	0	4	4	0	11	14	2513716	8.41E-01	1
NFU1	488690	285	23	676400	174796	0	1	0	0	43	37	13861216	8.41E-01	1

KIR2DS4	987531	620	-23	770028	215736	0	1	0	0	50	135	24266740	8.41E-01	1
CXCL16	1272092	222	28	685300	216092	0	1	1	0	50	74	23892584	8.41E-01	1
GPS1	3850694	197	34	1402284	393024	0	6	0	0	0	0	393024	8.41E-01	1
MRPS27	414939	342	29	1093988	280528	0	1	0	0	50	44	21968760	8.41E-01	1
STX17	244386	519	14	790320	210396	0	1	3	0	15	30	6220744	8.41E-01	1
HNRNPUL	1186597	214	24	2197232	608404	0	8	3	0	13	22	5265952	8.41E-01	1
FAM109A	473383	479	42	626204	224992	0	1	0	0	50	65	22007208	8.41E-01	1
MAP4K4	325378	593	24	3463880	928804	0	6	1	0	17	27	11950920	8.41E-01	1
CRYZL1	909314	375	58	935924	236740	0	2	1	0	50	61	18140692	8.41E-01	1
KRT77	1441383	464	29	1468500	422928	0	5	2	0	50	83	20991896	8.41E-01	1
SPOPL	85990	638	15	1045928	255964	0	2	2	0	32	36	12165588	8.42E-01	1
TTC26	562181	345	38	1503744	347100	0	3	2	0	50	59	20736644	8.42E-01	1
CPXM1	796251	438	41	1862592	550020	0	4	2	0	50	52	19055612	8.42E-01	1
ATP10B	170108	904	11	3742628	1035604	0	5	1	0	11	5	5046656	8.42E-01	1
EXOC3L2	1356264	243	38	1007836	344252	0	2	0	0	31	51	16516976	8.42E-01	1
EEA1	296393	427	17	3756512	887152	0	10	1	0	15	16	7552896	8.42E-01	1
GPR148	626077	372	11	830904	286224	0	1	0	0	50	57	22417676	8.42E-01	1
ADAMTSL	313133	527	-18	4465664	1304740	0	15	2	0	1	3	1694204	8.42E-01	1
TTC19	954548	370	-32	1289788	359204	0	3	1	0	12	18	4747260	8.42E-01	1
GGT1	451217	194	36	1436816	443932	0	4	4	0	12	26	6095432	8.43E-01	1
APOB	222162	698	-11	11539740	3202932	0	31	9	0	21	27	11621976	8.43E-01	1
EXO1	173100	325	-16	2181212	588112	0	5	3	0	11	14	4788556	8.43E-01	1
MEX3A	2005405	213	39	1242796	430760	0	4	2	0	30	49	13721664	8.43E-01	1
KIAA1549	482191	289	18	4787132	1547176	0	8	2	0	5	5	2819876	8.43E-01	1
SLC5A6	1238919	230	39	1590964	506588	0	4	0	0	13	18	6024232	8.43E-01	1
SNX30	573618	453	19	1125316	312212	0	3	3	0	36	49	13306924	8.43E-01	1
EIF3E	187290	965	6	1196872	285512	0	4	3	0	7	8	2473488	8.43E-01	1
ZNF391	143920	645	19	925244	230332	0	1	0	0	50	54	19682172	8.43E-01	1
ASB13	761737	299	21	707728	209684	0	1	0	0	50	72	24124696	8.43E-01	1
GALNT1	486812	562	61	1466720	372376	0	4	0	0	7	5	2431124	8.43E-01	1
CTAG2	2090264	NaN	31	613744	217160	0	1	0	0	15	20	6233916	8.43E-01	1
CYP2A13	1004808	354	10	1268428	353864	0	4	2	0	50	71	13386668	8.43E-01	1
PEMT	958193	236	12	603064	184052	0	1	0	0	50	71	22026432	8.43E-01	1

NLRX1	1258103	195	57	2500188	840516	0	6	4	0	21	28	9010004	8.43E-01	1
ALOX12B	1528802	272	26	1794240	514776	0	2	0	0	12	8	5211484	8.43E-01	1
GRIP1	228672	944	-6	2762560	786404	0	10	5	0	6	10	3022440	8.43E-01	1
CRYBA1	1533931	159	32	584196	131364	0	1	1	0	50	69	21586416	8.44E-01	1
CYP1A2	746043	199	41	1294416	387684	0	3	2	0	45	57	17713136	8.44E-01	1
ANXA2	431518	299	14	962624	242080	0	1	1	0	14	13	5067304	8.44E-01	1
BBS5	370890	418	22	917056	227840	0	1	0	0	50	53	25210496	8.44E-01	1
COMTD1	208687	275	38	651124	221432	0	1	1	0	50	61	19380284	8.44E-01	1
LYPD3	368705	182	-29	852620	276256	0	2	1	0	50	84	21649072	8.44E-01	1
MC1R	1207036	221	45	756500	262372	0	2	0	0	8	21	5465312	8.44E-01	1
AMPD3	777397	378	27	2028488	544324	0	4	2	0	50	63	26181308	8.44E-01	1
CLEC11A	652250	593	27	798508	252404	0	1	1	0	50	64	22467160	8.44E-01	1
WFDC13	777303	359	16	262372	51620	0	2	1	0	2	1	197224	8.44E-01	1
ZNF71	174439	849	-68	1231760	342472	0	1	2	0	50	78	23279908	8.44E-01	1
CD300A	491117	504	7	762196	224636	0	1	0	0	50	50	18209400	8.44E-01	1
APEX1	464497	395	17	805272	233892	0	1	0	0	50	58	21385632	8.44E-01	1
P2RY6	578209	176	56	784624	273764	0	2	0	0	27	43	11447892	8.45E-01	1
CTU1	NaN	NaN	NaN	810968	315772	0	1	0	0	50	116	39961356	8.45E-01	1
CASP6	211934	473	25	773588	196156	0	1	0	0	50	40	16217224	8.45E-01	1
PRPF38A	815523	448	26	833040	208260	0	1	1	0	50	62	23467164	8.45E-01	1
TMCO3	768219	318	50	1716276	507300	0	7	0	0	2	2	956216	8.45E-01	1
ZNF746	588536	467	17	1595948	499468	0	4	2	0	50	68	19904316	8.45E-01	1
PRPF6	1152034	208	48	2431836	671772	0	8	2	0	50	78	24047088	8.45E-01	1
ABCC8	454503	598	14	4039176	1191888	0	10	5	0	50	72	20591396	8.45E-01	1
LBR	660503	319	0	1597372	431828	0	5	0	0	5	6	2511936	8.45E-01	1
ESPNL	428658	356	29	2426140	831260	0	2	0	0	7	2	3167688	8.45E-01	1
ANKRD1	103854	510	4	839092	220364	0	1	0	0	25	21	8382376	8.45E-01	1
ILK	424523	491	17	1194024	312924	0	2	0	0	35	41	15296964	8.45E-01	1
COL4A1	429137	434	12	4221448	1347104	0	12	3	0	7	19	6535092	8.45E-01	1
NRL	992350	355	19	567464	203632	0	1	1	0	19	27	8529760	8.45E-01	1
EBF2	277181	616	-1	1500184	411536	0	2	4	0	11	13	4340352	8.45E-01	1
ZNF749	451095	869	-24	2013536	490924	0	4	1	0	32	34	10625888	8.45E-01	1
PYGB	607993	348	-8	2186552	598792	0	8	6	0	9	16	3367404	8.45E-01	1

USP21	728669	234	37	1407624	457104	0	5	2	0	17	26	6686748	8.45E-01	1
ALKBH3	437017	676	6	761128	196868	0	1	0	0	50	52	16264928	8.46E-01	1
LRRTM2	1371519	442	31	1294772	365968	0	4	0	0	26	40	11585308	8.46E-01	1
NANOS2	866240	261	42	340692	106800	0	1	1	0	4	6	1282312	8.46E-01	1
ODF3L2	1370962	423	-24	697760	244216	0	1	0	0	47	54	17671484	8.46E-01	1
KRTAP10-[illegible]	874480	461	25	642936	194376	0	1	0	0	50	76	22638396	8.46E-01	1
MAGI1	74202	843	-10	3936648	1092564	0	13	3	0	32	46	15605616	8.46E-01	1
KLF10	528975	178	42	1207552	351728	0	3	0	0	32	35	10556468	8.46E-01	1
TPD52L1	124606	243	-26	550020	132432	0	1	0	0	50	67	19055256	8.46E-01	1
SRCAP	1632278	158	61	7786076	2698480	0	33	7	0	0	7	2698480	8.46E-01	1
PCDHGB6	662513	568	2	2320408	721256	0	8	0	0	0	0	721256	8.46E-01	1
IDI1	338115	407	30	739056	191172	0	1	1	0	27	30	12219700	8.46E-01	1
CSF1R	1127889	228	50	2504460	698472	0	9	1	0	11	14	4436116	8.46E-01	1
JUND	1178047	210	43	819868	295124	0	2	1	0	50	86	22294856	8.46E-01	1
ERN2	517081	598	33	2443940	769672	0	8	6	0	50	61	19252836	8.46E-01	1
ZNF688	2214934	142	54	799932	262728	0	1	2	0	50	72	21947756	8.46E-01	1
TOP3B	471565	474	58	2228916	610896	0	6	2	0	9	11	3506600	8.46E-01	1
CD300LB	698491	504	10	615168	165540	0	1	0	0	50	63	19807128	8.46E-01	1
KIF5B	360559	166	29	2575660	619796	0	6	2	0	3	3	1731584	8.46E-01	1
VPRBP	549963	415	40	3729812	1030264	0	8	1	0	10	25	10556112	8.46E-01	1
MANEAL	445096	375	33	1152372	330012	0	2	1	0	50	50	19791820	8.46E-01	1
GJC3	1584669	391	36	686724	214668	0	1	0	0	50	53	18917840	8.47E-01	1
THBS2	172423	720	-32	3056972	791032	0	10	3	0	50	100	24861616	8.47E-01	1
METTL13	419860	718	-5	1766116	506588	0	7	1	0	13	17	4328960	8.47E-01	1
CD38	174323	327	55	792812	201496	0	1	1	0	50	44	18052048	8.47E-01	1
PCNT	991065	606	52	8511960	2376300	0	27	10	0	16	31	10775052	8.47E-01	1
PBX1	61567	624	-24	1113212	301888	0	3	0	0	16	23	6970124	8.47E-01	1
VASH1	308800	471	39	923820	274476	0	2	1	0	50	58	16606688	8.47E-01	1
VAMP5	1158965	170	43	296904	88644	0	1	1	0	7	8	1535784	8.47E-01	1
DAAM1	156241	405	32	2846932	716984	0	9	3	0	2	3	1524748	8.47E-01	1
ECHDC2	626387	311	30	660736	215024	0	1	2	0	11	12	4605572	8.47E-01	1
BYSL	437075	225	41	1093632	335352	0	1	0	0	50	36	18443292	8.47E-01	1
C14orf166	176784	471	22	654684	160200	0	1	0	0	50	55	19163124	8.48E-01	1

ALG11	385945	461	41	1248136	346388	0	3	0	0	50	79	22488876	8.48E-01	1
TEKT3	266888	359	-53	1256680	346388	0	3	1	0	36	61	14178412	8.48E-01	1
OAS3	613755	433	38	2740488	811680	0	7	0	0	0	0	811680	8.48E-01	1
MRC2	200276	280	-18	3835544	1030264	0	8	3	0	32	36	14985108	8.48E-01	1
CORO2A	494263	272	50	1353512	376648	0	2	2	0	50	41	18058100	8.48E-01	1
C18orf21	399000	585	15	573160	152012	0	1	0	0	50	73	22047436	8.48E-01	1
WDR64	172691	227	-16	2857968	719832	0	7	2	0	11	11	4439320	8.48E-01	1
CNOT8	523671	378	41	774300	190104	0	1	1	0	37	41	12356760	8.48E-01	1
LIME1	1126597	202	36	716984	252760	0	1	0	0	50	50	18382060	8.48E-01	1
ATP5H	1111289	171	31	439304	103240	0	1	0	0	50	75	17336132	8.48E-01	1
PTCD3	971062	420	46	1862236	451052	0	5	1	0	50	79	25822816	8.48E-01	1
PWP1	122461	805	19	1336068	334284	0	2	0	0	24	25	11216848	8.48E-01	1
TNC	194891	374	0	5569264	1597016	0	16	6	0	7	18	7056632	8.48E-01	1
PLCB2	851107	167	36	3108592	823784	0	5	3	0	50	48	19638028	8.48E-01	1
WASF3	149391	643	10	1273412	372376	0	3	2	0	50	69	21943128	8.48E-01	1
CCR3	212198	634	46	936280	280172	0	2	1	0	50	50	14767948	8.48E-01	1
IPMK	153476	357	-28	1075120	282308	0	3	0	0	50	74	20179148	8.48E-01	1
SEC62	398638	553	20	1066220	247420	0	2	2	0	9	34	8128904	8.48E-01	1
RGN	830380	NaN	52	778216	208616	0	1	0	0	50	59	18783628	8.48E-01	1
CDC25B	860065	241	43	1501252	424352	0	3	2	0	16	30	7457844	8.49E-01	1
AXIN1	1444992	205	40	2168040	639732	0	4	0	0	8	21	7077636	8.49E-01	1
ZNF736	NaN	NaN	NaN	1129232	254896	0	2	0	0	50	116	39900480	8.49E-01	1
SMARCA5	183412	821	9	2782496	689572	0	3	0	0	6	2	2846220	8.49E-01	1
LRFN1	1793696	252	31	1808124	669636	0	5	2	0	22	30	9916736	8.49E-01	1
RNF8	485024	426	37	1343188	344252	0	3	1	0	50	66	22029280	8.49E-01	1
KEL	294033	676	2	1894276	531152	0	6	1	0	49	57	17327232	8.49E-01	1
MTTP	318764	787	11	2309728	633680	0	11	5	0	3	7	1624428	8.49E-01	1
ZNF77	1119793	197	35	1405132	357068	0	1	2	0	20	24	8194408	8.49E-01	1
LRFN5	8637	1309	-33	1800648	523320	0	12	5	0	10	19	3585988	8.49E-01	1
STK17B	140953	373	14	972236	252760	0	2	0	0	50	59	18579284	8.49E-01	1
GPM6B	135235	NaN	35	938060	241012	0	1	0	0	44	52	20068432	8.49E-01	1
AIMP1	158895	711	5	877184	232468	0	1	1	0	50	61	23547620	8.49E-01	1
SIGLEC14	443199	644	-23	995020	302600	0	1	0	0	27	27	10304064	8.49E-01	1

ADRA1D	805921	428	14	1361700	480600	0	3	0	0	9	10	4022088	8.49E-01	1
ZNF646	1398193	247	40	4515860	1365616	0	10	4	0	50	79	26328336	8.49E-01	1
LRRTM3	2412	967	-44	1454260	419012	0	7	3	0	7	9	2336428	8.49E-01	1
CARD9	1194160	258	28	1428272	398008	0	2	3	0	50	42	16832036	8.50E-01	1
RRP8	424523	491	17	1157712	334284	0	2	2	0	21	26	9803528	8.50E-01	1
FOSL1	3779084	287	42	668924	215380	0	1	0	0	2	1	623712	8.50E-01	1
GPR25	545933	437	12	829480	330368	0	1	0	0	10	8	4377376	8.50E-01	1
MNX1	387871	369	24	1007836	349592	0	1	0	0	50	42	22575740	8.50E-01	1
PCDH19	128045	NaN	-15	2838744	864012	0	9	3	0	16	21	6110384	8.50E-01	1
KIF26A	655915	468	32	4488804	1604136	0	7	2	0	26	18	11173772	8.50E-01	1
SARS2	1794625	282	21	1393028	420436	0	1	1	0	28	34	11289116	8.50E-01	1
HELB	237928	773	5	2805992	735496	0	6	1	0	50	72	23996892	8.50E-01	1
PNN	314417	166	13	1871492	465292	0	4	0	0	12	11	4914936	8.50E-01	1
LAIR2	819283	601	-4	390888	116412	0	1	0	0	50	111	22449716	8.50E-01	1
ZSWIM1	725555	209	59	1207196	354220	0	4	0	0	27	40	9561092	8.50E-01	1
TRIM29	325456	214	31	1509440	411892	0	3	1	0	37	52	19834540	8.50E-01	1
ACCSL	389604	680	1	1484164	398720	0	4	0	0	8	9	3168756	8.50E-01	1
NDUFS8	1263224	251	47	548596	153080	0	1	1	0	15	29	8327196	8.50E-01	1
ANKS1B	149256	941	-9	3465660	946248	0	9	6	0	21	23	8092592	8.50E-01	1
FAM8A1	470794	339	13	1012464	331080	0	3	0	0	6	18	5216468	8.51E-01	1
IL1RAPL2	17392	NaN	-4	1787476	456392	0	4	1	0	13	11	4467444	8.51E-01	1
TTF2	331822	566	12	2994316	828056	0	6	5	0	47	57	18652264	8.51E-01	1
DHRS7C	77786	204	-62	793880	229264	0	2	1	0	40	65	16189100	8.51E-01	1
TACR3	101583	931	-24	1166612	343540	0	5	3	0	12	29	4677128	8.51E-01	1
SLC4A5	1065336	272	45	2934864	826632	0	7	2	0	50	78	24840968	8.51E-01	1
MAD1L1	814176	544	26	1862592	513708	0	2	1	0	50	75	25739156	8.51E-01	1
GALR1	90366	614	-14	860096	269848	0	2	2	0	5	11	3222512	8.51E-01	1
TMEM101	920082	241	29	639020	202564	0	1	0	0	50	50	17463580	8.51E-01	1
NLGN4X	13333	NaN	-22	2049492	585264	0	3	1	0	1	1	1610188	8.51E-01	1
CKB	934253	225	36	975440	278392	0	2	0	0	50	57	18756928	8.51E-01	1
TRPV6	284406	645	18	1855472	530440	0	5	3	0	10	13	4455340	8.51E-01	1
CD80	290702	599	38	749024	198292	0	1	0	0	50	49	19375656	8.51E-01	1
ARL4D	1147224	172	1	487008	164472	0	1	2	0	5	9	2637248	8.51E-01	1

RGS2	307992	477	-15	567820	130652	0	1	0	0	50	79	22972680	8.51E-01	1
RASSF9	22408	552	-27	1116416	284800	0	5	1	0	50	100	20673632	8.51E-01	1
C21orf2	1153513	308	65	640088	208972	0	1	0	0	50	59	19026776	8.51E-01	1
CHST7	646939	NaN	40	1149880	414740	0	2	1	0	50	88	27404168	8.51E-01	1
ZNF611	536540	1010	-40	1830908	443932	0	10	2	0	2	6	1314352	8.51E-01	1
RASA1	154501	726	24	2727316	743684	0	5	0	0	9	94	31273888	8.51E-01	1
AVPR1A	250995	521	-32	1039164	307584	0	3	2	0	50	84	20593888	8.51E-01	1
PLEKHA5	149622	577	-29	3206848	820580	0	13	4	0	50	112	26330116	8.51E-01	1
ISYNA1	1384225	259	42	1392672	441084	0	2	1	0	50	68	24078060	8.51E-01	1
NDUFS2	728669	234	37	1219656	341760	0	3	1	0	39	44	15898604	8.51E-01	1
ATXN1	109777	623	30	1981140	637596	0	5	3	0	6	7	2477048	8.51E-01	1
SLC35F2	392825	538	12	956216	275188	0	1	1	0	50	55	20139632	8.51E-01	1
SERPINI1	145987	567	-12	1085088	265932	0	2	1	0	4	5	1744756	8.51E-01	1
NAA40	1322584	277	42	640444	152012	0	1	0	0	50	83	26795052	8.51E-01	1
TRPT1	1602391	278	57	657532	199004	0	1	0	0	50	67	20864092	8.52E-01	1
PDDC1	1788356	193	48	573872	166252	0	1	0	0	50	64	19157072	8.52E-01	1
VGLL3	55913	332	-18	827700	232824	0	1	0	0	37	35	15501664	8.52E-01	1
IGFBP3	146330	623	8	744396	227484	0	1	0	0	50	55	20455404	8.52E-01	1
LRIT2	216191	860	-17	1353868	422216	0	3	2	0	50	78	25295580	8.52E-01	1
MYSM1	303727	517	-2	2214320	527236	0	5	1	0	13	9	4131380	8.52E-01	1
SPINT1	1036414	172	51	1358140	382700	0	3	2	0	50	67	20324396	8.52E-01	1
TBC1D8B	149206	NaN	19	2996096	749380	0	9	0	0	0	0	749380	8.52E-01	1
ADORA2A	611616	186	54	1002496	325028	0	1	2	0	50	77	20208340	8.52E-01	1
SQSTM1	1610057	359	38	1123536	321468	0	2	3	0	11	17	4550036	8.52E-01	1
TBCEL	308922	724	25	1097904	293700	0	2	1	0	23	30	10752980	8.52E-01	1
HBS1L	152992	353	26	3010692	827700	0	4	2	0	36	35	15395220	8.52E-01	1
TAGAP	509192	447	41	1862948	537916	0	4	4	0	29	44	15984400	8.52E-01	1
DCUN1D3	252367	503	32	770384	211108	0	1	1	0	28	30	11000044	8.52E-01	1
ZIC2	323784	500	35	1310792	405484	0	2	1	0	50	43	17731648	8.52E-01	1
OR8G1	154391	921	-14	858672	250980	0	2	1	0	8	10	3157720	8.52E-01	1
CSH1	1446139	380	27	776080	205412	0	1	1	0	50	63	18745892	8.52E-01	1
POLR3F	414549	436	23	845144	204700	0	1	1	0	50	57	25166708	8.52E-01	1
XRCC3	765678	266	43	857960	279460	0	1	3	0	12	18	3996812	8.52E-01	1

BSDC1	1529661	216	41	1156644	323604	0	2	0	0	50	65	23479268	8.52E-01	1
FOXB2	180535	713	-10	1039876	347456	0	1	2	0	9	10	4747260	8.53E-01	1
AP1B1	849693	251	36	2435752	702032	0	10	6	0	4	10	1766828	8.53E-01	1
TRAF3IP1	295666	345	32	1822720	462800	0	1	0	0	50	47	19908588	8.53E-01	1
APBB1IP	236125	536	19	1708800	481668	0	7	3	0	3	4	1203636	8.53E-01	1
HIST1H2A(	398813	636	53	314348	109648	0	2	0	0	4	8	638308	8.53E-01	1
FAM43B	573203	429	30	785692	271628	0	1	1	0	50	55	21704964	8.53E-01	1
CYP2S1	1098360	209	13	1236744	417588	0	3	2	0	20	20	6505188	8.53E-01	1
IL4R	227872	560	25	2094704	607336	0	4	2	0	37	30	13554344	8.53E-01	1
SLC2A7	840956	264	31	1280176	410468	0	3	1	0	50	62	20541556	8.53E-01	1
RFC5	639801	584	-3	901748	242080	0	2	0	0	50	91	25169556	8.53E-01	1
PSKH1	879834	158	50	1036316	333928	0	2	3	0	13	27	6013552	8.53E-01	1
PTPRH	1036194	199	2	2833404	825564	0	7	3	0	50	57	16886504	8.53E-01	1
FAM120C	610328	NaN	28	2756864	845500	0	8	0	0	0	0	845500	8.53E-01	1
NTAN1	564013	463	22	815952	221076	0	1	1	0	50	49	18364972	8.53E-01	1
ZFYVE21	765678	266	43	612676	168032	0	2	0	0	7	12	2444296	8.53E-01	1
C7orf60	157620	814	-14	1040944	276968	0	4	2	0	14	33	7901420	8.53E-01	1
TTC17	355422	919	-7	2976872	782488	0	10	3	0	50	58	19428700	8.53E-01	1
CLCN4	168498	NaN	33	1919552	565684	0	4	1	0	11	17	6770764	8.53E-01	1
TGFB3	226588	582	34	1056964	291920	0	1	2	0	16	10	6190128	8.53E-01	1
ADRA2A	285613	547	-1	1110720	382344	0	2	1	0	30	33	11494172	8.53E-01	1
DMGDH	215159	434	45	2222508	621576	0	4	2	0	17	27	9981884	8.53E-01	1
PPP1R15A	1766061	211	34	1658960	514420	0	5	0	0	7	10	3608772	8.53E-01	1
ZNF681	445524	1441	-58	1684948	397652	0	7	2	0	29	56	14236796	8.54E-01	1
EXOSC8	176745	650	16	740124	192240	0	1	0	0	50	55	19294132	8.54E-01	1
RAET1E	423805	245	34	682096	180848	0	1	2	0	18	21	7899284	8.54E-01	1
CLRN3	434702	806	-25	573872	161980	0	1	1	0	50	58	17294124	8.54E-01	1
MAP4K5	603138	207	43	2279468	562480	0	5	0	0	1	0	800288	8.54E-01	1
UBE2O	1412378	183	26	3272352	945180	0	6	4	0	50	53	20215460	8.54E-01	1
PNMT	1284992	226	38	697048	220364	0	1	1	0	50	64	21003644	8.54E-01	1
ABHD12B	411016	431	41	779284	203276	0	1	0	0	50	64	24599600	8.54E-01	1
PRSS33	1717244	210	42	680316	241368	0	1	1	0	50	80	24105472	8.54E-01	1
ADD2	659649	592	24	2087940	543612	0	8	0	0	1	0	639020	8.54E-01	1

CLIC2	NaN	NaN	NaN	649344	166608	0	1	0	0	50	116	39812192	8.54E-01	1
CHRNB4	485971	455	45	1248136	372020	0	2	0	0	4	3	1981140	8.54E-01	1
RHOXF2	NaN	NaN	NaN	1478824	398720	0	1	0	0	50	116	40044304	8.54E-01	1
SFN	1022828	188	30	621576	178356	0	1	1	0	50	67	19034252	8.54E-01	1
IGDCC3	646843	280	40	2007128	659668	0	4	3	0	5	8	3117136	8.54E-01	1
KCNC2	175202	822	-31	1630836	508368	0	5	1	0	50	74	21376376	8.54E-01	1
TBKBP1	1198427	217	24	1526884	480956	0	5	3	0	14	21	6144560	8.54E-01	1
DET1	212296	630	4	1415456	406552	0	2	0	0	40	36	16548304	8.54E-01	1
TPST2	162211	501	-14	935212	297260	0	1	0	0	5	8	4713796	8.54E-01	1
INHBC	1347802	194	32	859028	276256	0	1	0	0	50	70	20818880	8.54E-01	1
PRTG	322073	254	-3	2928100	843008	0	8	1	0	3	4	1757572	8.54E-01	1
PAPOLB	NaN	NaN	NaN	1592032	452120	0	4	2	0	50	118	40097704	8.54E-01	1
ZNF354C	718142	781	-4	1455328	339980	0	3	2	0	9	11	4295852	8.54E-01	1
REEP1	696855	425	37	676756	122108	0	1	1	0	9	19	6194400	8.54E-01	1
ZNF629	1632278	158	61	2171956	624068	0	5	3	0	47	62	18440088	8.54E-01	1
KLK11	490795	632	22	779284	228908	0	1	0	0	49	56	16666496	8.55E-01	1
LYNX1	427585	373	34	546460	161624	0	1	0	0	50	60	17573228	8.55E-01	1
NFKBID	1060028	321	47	775012	265220	0	1	0	0	50	67	19297692	8.55E-01	1
PRG3	923815	191	-3	594164	153436	0	1	0	0	50	67	17960912	8.55E-01	1
GCH1	673378	380	40	734072	202564	0	1	1	0	30	44	16904660	8.55E-01	1
C11orf42	466636	728	4	808120	274832	0	1	1	0	50	57	22924620	8.55E-01	1
GPR150	307041	359	32	998580	395160	0	1	0	0	50	41	18933504	8.55E-01	1
TRAK2	511735	479	26	2335004	656464	0	4	2	0	9	5	3235328	8.55E-01	1
ANK1	574508	533	36	5189768	1532224	0	17	4	0	4	10	3325752	8.55E-01	1
SPTBN1	714370	430	22	6315796	1620512	0	15	9	0	50	58	22011124	8.55E-01	1
DNMT3B	675780	173	42	2253124	615524	0	8	0	0	2	1	978644	8.55E-01	1
ETHE1	416043	209	-10	647920	196868	0	1	0	0	50	64	21352880	8.55E-01	1
EIF3G	1675365	272	15	850840	226772	0	1	0	0	1	0	604132	8.56E-01	1
CLDND1	390532	796	2	726240	186900	0	1	0	0	50	54	19516632	8.56E-01	1
TBC1D7	258474	423	35	775368	196512	0	1	0	0	50	48	18607764	8.56E-01	1
ALG10	23464	1025	-29	1199364	327876	0	6	1	0	24	45	7647592	8.56E-01	1
IGF1R	203046	470	-27	3516568	954080	0	10	3	0	26	53	14891836	8.56E-01	1
GRM6	434745	839	2	2148816	702744	0	7	4	0	15	15	4822732	8.56E-01	1

OR5H14	200002	811	-15	775368	221076	0	2	2	0	50	87	21088372	8.56E-01	1
OR51I1	99042	1030	1	779284	234248	0	1	0	0	50	49	19819232	8.56E-01	1
BRD8	1085933	314	45	3435756	941976	0	9	0	0	1	2	1443224	8.56E-01	1
FPGS	1194942	179	58	1482028	463868	0	3	0	0	28	49	17284512	8.56E-01	1
SYT7	1339753	274	55	1044860	285868	0	2	0	0	48	47	15205116	8.56E-01	1
PRPF40B	2010227	265	53	2281604	616948	0	5	3	0	50	68	21090864	8.56E-01	1
IDH3B	792941	450	48	1138488	318264	0	2	1	0	50	75	25450084	8.56E-01	1
KIF1C	1399774	343	26	2788548	838380	0	5	3	0	50	79	21015392	8.56E-01	1
CTRC	554165	212	29	698828	197224	0	1	0	0	50	81	24526264	8.56E-01	1
GFER	1747301	183	46	519404	151300	0	1	0	0	50	75	19137492	8.56E-01	1
LRRC4B	762018	517	35	1710580	585620	0	4	4	0	45	54	18323320	8.56E-01	1
NRK	18768	NaN	2	4082964	1108584	0	4	0	0	8	4	3862956	8.56E-01	1
MAPK15	2190348	229	30	1360632	443220	0	3	2	0	45	64	20057040	8.56E-01	1
CAPZB	811050	429	41	722324	186544	0	1	1	0	50	54	19588188	8.56E-01	1
CHRNE	1416161	231	30	1256324	379852	0	3	1	0	50	65	18681100	8.56E-01	1
IL2RB	668971	289	53	1386976	420080	0	2	0	0	28	17	7339296	8.56E-01	1
CSE1L	862939	155	50	2558572	658244	0	10	4	0	24	38	10888616	8.56E-01	1
ZNRF3	693562	326	-13	2080108	633680	0	5	0	0	0	0	633680	8.56E-01	1
GLE1	1917147	160	39	1831620	487008	0	2	1	0	50	81	26625596	8.56E-01	1
MRPL9	1062482	255	29	699184	211820	0	1	1	0	12	10	4320060	8.57E-01	1
SEC31A	775541	268	38	3153804	871488	0	7	4	0	50	53	22497420	8.57E-01	1
KRT25	836683	269	-10	1149880	327164	0	3	1	0	22	26	7229292	8.57E-01	1
ITGAV	266463	373	7	2784276	752940	0	10	6	0	15	20	5707748	8.57E-01	1
C17orf47	892839	376	10	1395164	438592	0	6	3	0	50	67	14367804	8.57E-01	1
LAG3	2816186	232	48	1289788	427556	0	2	2	0	6	24	6345344	8.57E-01	1
OR4N4	156689	855	-31	779284	235316	0	1	0	0	50	68	20763700	8.57E-01	1
THNSL2	180956	612	6	1232472	355644	0	1	1	0	50	41	17753008	8.57E-01	1
PSMB6	1368906	195	28	607692	186900	0	1	1	0	50	66	19691784	8.57E-01	1
GYLTL1B	427820	291	48	1803852	564972	0	3	0	0	24	32	10815992	8.57E-01	1
MN1	107535	256	-110	3200084	1036672	0	6	1	0	19	13	6542924	8.57E-01	1
KLF13	204790	324	35	700964	229264	0	1	1	0	50	54	18706732	8.58E-01	1
PQBP1	834040	NaN	38	698828	185476	0	1	0	0	50	43	14994364	8.58E-01	1
CDH18	4731	1195	-17	2039168	537916	0	13	5	0	19	34	6534380	8.58E-01	1

CLIC3	1979517	188	34	600928	184052	0	2	1	0	16	30	5786424	8.58E-01	1
OR1M1	311030	471	-63	763620	241368	0	2	3	0	20	24	5614120	8.58E-01	1
MS4A7	433906	899	24	618728	179068	0	1	1	0	16	14	4743700	8.58E-01	1
PELI1	379419	441	35	1072628	295480	0	2	0	0	27	32	10577116	8.58E-01	1
MAPK8IP1	427820	291	48	1788188	540052	0	3	2	0	50	52	20813896	8.58E-01	1
CASR	557700	602	41	2725180	785336	0	5	1	0	50	70	22732736	8.58E-01	1
PXMP4	714390	161	56	537560	157708	0	1	0	0	50	83	20010760	8.58E-01	1
ZNF280B	201933	683	23	1381992	365256	0	2	0	0	50	138	46792640	8.58E-01	1
ELOVL2	270200	422	22	785336	196156	0	1	1	0	35	33	13099020	8.58E-01	1
FZD4	195696	281	-12	1338560	389464	0	2	0	0	50	60	24260332	8.58E-01	1
PPFIA2	20298	1132	-25	3294068	868996	0	12	2	0	8	18	3972960	8.58E-01	1
INF2	651235	241	21	3133512	999648	0	8	6	0	46	56	16932784	8.58E-01	1
PODN	491749	391	14	1631548	534356	0	4	4	0	12	10	3951956	8.58E-01	1
RNPS1	2220466	233	46	777504	228552	0	1	1	0	50	67	22076272	8.58E-01	1
CCNL2	1736268	273	28	1337136	402636	0	5	1	0	1	1	562480	8.58E-01	1
MAMSTR	1635420	190	44	1021720	349592	0	2	1	0	50	73	21072352	8.58E-01	1
PNLIP	128161	704	-16	1223216	321112	0	4	4	0	7	15	2983992	8.58E-01	1
SYNJ1	355341	345	65	4180508	1160560	0	11	1	0	8	11	4629068	8.58E-01	1
GATSL2	NaN	NaN	NaN	847636	243860	0	1	0	0	50	116	39889444	8.58E-01	1
C2CD4B	NaN	NaN	NaN	835176	338556	0	1	0	0	50	116	39984140	8.58E-01	1
HIF1AN	1127095	264	39	923820	229620	0	2	2	0	50	74	20907168	8.58E-01	1
MGAT4B	1610057	359	38	1535428	439304	0	3	1	0	50	48	18056676	8.59E-01	1
CD55	500581	191	20	1146320	309364	0	2	1	0	50	52	18195160	8.59E-01	1
NLRP7	840563	573	-15	2636892	731580	0	10	4	0	50	100	21516284	8.59E-01	1
ARFGAP2	1206881	273	24	1369176	369528	0	2	0	0	50	49	20651560	8.59E-01	1
PCSK7	431523	199	41	2009620	572804	0	5	1	0	17	16	6704548	8.59E-01	1
ORAI2	579276	202	61	624424	201140	0	1	2	0	30	42	9572128	8.59E-01	1
B4GALT1	901836	181	28	993952	305804	0	2	0	0	50	89	27149984	8.59E-01	1
LIN54	667566	169	45	1888224	566040	0	4	0	0	3	2	1625496	8.59E-01	1
GHDC	2129732	206	30	1372736	457816	0	3	0	0	15	27	8388428	8.59E-01	1
AVPR1B	755856	400	10	1017448	348524	0	3	4	0	11	13	3503752	8.59E-01	1
GALNT13	21842	1035	-13	1458176	369172	0	4	0	0	40	65	19663304	8.59E-01	1
INHBA	42008	671	-27	1074408	297972	0	4	1	0	50	80	18444716	8.59E-01	1

KIAA1524	214268	867	2	2372740	615524	0	5	0	0	2	2	1460668	8.59E-01	1
TIMELESS	2793663	184	61	3136716	856536	0	9	7	0	15	27	7752256	8.59E-01	1
ZNF302	910826	607	-54	1055896	242792	0	3	2	0	50	132	31086632	8.59E-01	1
KCND3	320241	623	23	1642940	488788	0	3	4	0	19	16	6985788	8.59E-01	1
KRT82	1065603	213	35	1306876	374156	0	3	2	0	50	54	16033884	8.59E-01	1
RBM22	1107515	211	54	1092564	299040	0	2	0	0	21	38	12090472	8.59E-01	1
TBX22	26689	NaN	-26	1346748	358848	0	4	2	0	50	120	30826752	8.59E-01	1
OR52A1	109516	1024	-15	774656	226060	0	1	0	0	50	47	18695696	8.59E-01	1
ECD	653488	233	44	1767896	466360	0	3	3	0	9	12	3920984	8.59E-01	1
MTX2	239999	888	11	709508	174796	0	1	1	0	50	76	20894708	8.59E-01	1
FAM58A	940418	NaN	24	629052	184764	0	1	0	0	50	66	17527304	8.59E-01	1
NR3C1	138106	270	41	2024216	559276	0	6	1	0	31	50	13893968	8.59E-01	1
SLC39A13	1022438	245	47	934144	300464	0	1	2	0	50	66	23883684	8.59E-01	1
TBC1D26	730837	431	-16	672840	173016	0	1	0	0	50	68	22280260	8.59E-01	1
LSM12	916863	198	23	510148	134924	0	1	1	0	50	69	17825988	8.59E-01	1
DDX28	863919	158	54	1276260	459240	0	1	1	0	50	81	22845944	8.59E-01	1
MYLK4	470344	285	28	1043792	249556	0	2	2	0	30	30	9906768	8.59E-01	1
HS3ST5	217498	675	-8	871844	246352	0	3	2	0	2	2	895340	8.60E-01	1
ZBTB7C	110354	212	61	1540056	452832	0	1	1	0	50	61	21476768	8.60E-01	1
ZNF506	575637	609	-50	1160560	278036	0	3	1	0	50	113	30023972	8.60E-01	1
NUP160	931839	158	14	3744764	1011040	0	8	4	0	50	49	22919280	8.60E-01	1
MYT1	812501	312	48	2897128	785336	0	8	3	0	50	65	20645152	8.60E-01	1
ARIH1	1093921	432	37	1495200	348168	0	4	0	0	13	16	5215400	8.60E-01	1
GALR2	1611239	218	22	923464	328232	0	4	4	0	6	25	4671788	8.60E-01	1
S1PR5	2036812	226	23	917768	364900	0	2	2	0	50	81	22329032	8.60E-01	1
SLC35E3	534146	294	25	787828	235316	0	1	2	0	50	50	16078384	8.60E-01	1
KRT27	836683	269	-10	1169104	336776	0	1	2	0	50	63	16537268	8.60E-01	1
GABRD	1252791	279	38	1145608	342116	0	2	1	0	50	65	22176664	8.60E-01	1
MUC5B	1400716	311	46	13841280	4840176	0	33	9	0	5	13	7063040	8.60E-01	1
CD3EAP	1259254	191	39	1254544	393380	0	2	1	0	50	63	21808204	8.60E-01	1
PUM1	498145	189	36	3029916	869352	0	10	2	0	0	2	869352	8.60E-01	1
CNDP1	162886	941	13	1304384	370240	0	3	2	0	50	73	24982656	8.60E-01	1
PXN	1615004	187	57	1516916	426844	0	3	0	0	14	12	4982220	8.60E-01	1

PTRH1	1098647	172	32	524032	186188	0	1	0	0	50	71	18037452	8.60E-01	1
WTIP	707545	355	5	1051980	358848	0	1	1	0	50	60	23415544	8.60E-01	1
SLC25A34	683349	174	22	740124	254184	0	1	0	0	50	63	23254276	8.60E-01	1
ACTBL2	160055	456	3	933432	276612	0	1	1	0	50	43	18277396	8.60E-01	1
STK36	1033606	269	43	3313648	1013888	0	9	2	0	3	7	2924184	8.60E-01	1
MAGEA11	NaN	NaN	NaN	1100396	306160	0	2	0	0	50	116	39951744	8.61E-01	1
AXL	1180169	214	15	2279112	669636	0	6	1	0	20	25	8335384	8.61E-01	1
CECR1	305072	574	-55	1354936	356000	0	2	1	0	50	114	30165304	8.61E-01	1
LUC7L	1267639	220	36	967252	265220	0	2	1	0	50	56	16678244	8.61E-01	1
PAQR9	397648	422	17	913140	297972	0	1	2	0	50	45	19439380	8.61E-01	1
HSPA1A	2509866	183	32	1573876	483092	0	3	2	0	7	9	3981504	8.61E-01	1
MAP3K3	934952	247	3	1714496	462088	0	3	0	0	3	2	1410116	8.61E-01	1
CPNE3	192772	1166	22	1426492	361340	0	5	3	0	10	12	3772176	8.61E-01	1
HOXB9	909944	509	28	626560	181916	0	1	0	0	50	58	17672908	8.61E-01	1
IGSF9B	270106	534	-8	3343196	1063372	0	8	4	0	41	45	18251052	8.61E-01	1
SLCO5A1	145499	674	16	2132796	621576	0	9	6	0	8	18	3258468	8.61E-01	1
SSH1	474364	293	45	2935220	842296	0	3	2	0	31	33	15053104	8.61E-01	1
FTSJ2	814176	430	38	621576	189036	0	3	3	0	4	9	1531512	8.62E-01	1
LSM11	243382	987	31	868284	303312	0	1	0	0	50	48	18799648	8.62E-01	1
POLDIP2	1405196	214	10	950520	273408	0	3	0	0	3	2	766468	8.62E-01	1
FRMD5	929260	504	33	1482740	402280	0	4	2	0	50	60	17397364	8.62E-01	1
C1orf27	277882	399	-14	1208620	304736	0	1	1	0	50	30	18183768	8.62E-01	1
POC1A	1096747	193	52	1055184	299040	0	4	2	0	20	29	6954816	8.62E-01	1
OR1C1	164924	995	-27	771096	236028	0	2	1	0	50	130	29092676	8.62E-01	1
STX16	1026997	504	15	846924	231756	0	2	1	0	42	57	14881868	8.62E-01	1
GPR179	1101194	504	10	5832704	1799224	0	20	4	0	15	20	6907824	8.62E-01	1
USP10	494151	258	31	2027064	588468	0	5	2	0	50	60	19276332	8.62E-01	1
DAB1	22463	1004	-12	1447496	389464	0	2	2	0	50	80	23854136	8.62E-01	1
CBFA2T3	967879	252	41	1632972	509436	0	4	0	0	5	9	3798876	8.62E-01	1
RACGAP1	1018478	211	52	1643652	452832	0	5	2	0	50	59	16479240	8.62E-01	1
GANAB	2560761	217	47	2495560	707372	0	9	3	0	8	13	4768620	8.62E-01	1
CHD9	228561	675	-9	7410496	1981496	0	23	3	0	0	3	1981496	8.63E-01	1
TBRG1	171901	604	9	1054828	299396	0	1	0	0	14	15	8435776	8.63E-01	1

SENP7	555098	622	1	2768612	697048	0	5	4	0	50	103	28199828	8.63E-01	1
SLC7A7	1153069	351	36	1289788	389108	0	3	2	0	50	63	17715984	8.63E-01	1
SESN2	766835	170	29	1218944	360628	0	2	0	0	4	3	1922044	8.63E-01	1
DLG3	306369	NaN	30	2354228	648988	0	3	2	0	50	60	23823520	8.63E-01	1
MED12L	98951	632	9	5552532	1504812	0	15	6	0	50	73	23333664	8.63E-01	1
CLDN3	1082063	214	54	524032	184052	0	1	1	0	13	28	7303340	8.63E-01	1
P2RY14	130485	632	8	854400	236028	0	2	3	0	4	3	873980	8.63E-01	1
NUCB2	522189	387	19	1157356	241724	0	2	0	0	37	43	15205116	8.63E-01	1
MGAM	196155	846	-7	4857976	1295840	0	12	4	0	50	65	21414468	8.63E-01	1
MUC13	364165	212	42	1326456	360984	0	2	2	0	50	49	19028200	8.63E-01	1
SIGLEC7	355132	632	1	1172308	354932	0	2	0	0	50	65	22303756	8.63E-01	1
HADHA	641003	274	38	1970816	558208	0	4	1	0	46	69	19778648	8.63E-01	1
ABR	1346718	270	22	2359568	638308	0	5	3	0	50	58	20111864	8.63E-01	1
CA11	1597109	242	31	831616	256676	0	1	1	0	50	57	20781500	8.63E-01	1
FAM73B	1473097	203	29	1501964	461020	0	2	0	0	50	40	18400572	8.63E-01	1
PPAP2C	233160	578	-13	817732	257744	0	1	0	0	50	46	18336136	8.63E-01	1
DHCR24	465622	249	27	1322896	367748	0	2	0	0	5	2	1196872	8.63E-01	1
BSCL2	2425755	248	51	1178716	349592	0	3	0	0	2	0	601284	8.63E-01	1
LYSMD4	173729	507	16	753296	218584	0	1	0	0	50	47	18551516	8.63E-01	1
BATF2	3151458	209	52	664652	224992	0	1	0	0	50	83	23753744	8.63E-01	1
CAPN2	528883	407	2	1858320	478464	0	3	1	0	50	48	20497056	8.63E-01	1
CACYBP	176818	828	4	608048	147028	0	1	0	0	50	65	19765832	8.63E-01	1
TNK1	1911735	206	36	1625496	546816	0	2	0	0	8	13	4624440	8.63E-01	1
ZIC1	20725	1070	-30	1114992	328944	0	12	3	0	1	4	634748	8.63E-01	1
PIP5K1B	184422	543	27	1415100	371664	0	2	0	0	27	24	10409084	8.63E-01	1
PDE9A	411822	331	69	1700612	371308	0	4	2	0	50	66	21754448	8.63E-01	1
SEZ6L	196333	672	-62	2587764	764688	0	11	3	0	3	5	1483808	8.63E-01	1
CALCR	197869	823	-10	1352800	337844	0	8	3	0	5	9	1946964	8.64E-01	1
ARHGEF1[illegible]	772200	257	20	2060172	701676	0	5	1	0	21	24	8505196	8.64E-01	1
PRAMEF1[illegible]	NaN	NaN	NaN	1197228	337488	0	3	3	0	26	43	11914608	8.64E-01	1
UBAC1	753354	352	37	1051624	287648	0	1	1	0	50	47	22131096	8.64E-01	1
GLI1	1347802	194	32	2723400	870420	0	10	4	0	50	70	20818880	8.64E-01	1
RXFP4	1880483	213	32	883236	319332	0	2	3	0	27	52	11843052	8.64E-01	1

VN1R1	451095	869	-24	887508	246708	0	2	0	0	50	68	17938840	8.64E-01	1
OR10X1	179404	940	1	794948	243148	0	2	2	0	6	15	3888232	8.64E-01	1
PBRM1	1217701	231	47	4281256	1081172	0	21	3	0	2	12	2685664	8.64E-01	1
F3	411634	258	-7	787472	182272	0	1	1	0	50	63	23803228	8.64E-01	1
COPS7A	2816186	232	48	700252	213956	0	1	0	0	6	24	6345344	8.64E-01	1
MYH15	257473	874	3	5130316	1285160	0	17	3	0	50	65	21007916	8.64E-01	1
SLC25A35	870950	218	18	870064	229976	0	1	1	0	50	57	18595660	8.64E-01	1
UBE2J2	2050211	196	28	716984	195088	0	1	1	0	50	71	21421944	8.64E-01	1
RPS6KA3	242944	NaN	36	1957644	506232	0	7	0	0	0	0	506232	8.64E-01	1
DNAJC18	832127	177	48	942688	239588	0	2	0	0	40	65	18544396	8.64E-01	1
LRRN2	401240	221	35	1699544	588112	0	2	2	0	33	22	12231804	8.64E-01	1
CBX5	753625	205	59	513708	118548	0	1	1	0	33	48	11939884	8.64E-01	1
HMHA1	2387949	206	26	2871852	865080	0	6	2	0	50	78	32398136	8.64E-01	1
TWISTNB	168714	941	-11	868284	232824	0	2	1	0	16	19	5176240	8.65E-01	1
FAM153B	592877	609	19	1077612	257388	0	2	0	0	21	22	7004656	8.65E-01	1
OR7G2	311030	471	-63	844076	262372	0	2	2	0	50	102	27434784	8.65E-01	1
TMEM209	414437	266	34	1474196	386260	0	4	0	0	50	59	18847352	8.65E-01	1
TMEM156	435367	152	49	779996	197224	0	1	0	0	50	49	18176648	8.65E-01	1
P4HA3	641945	201	37	1388044	411536	0	2	1	0	48	49	20114356	8.65E-01	1
TUB	189808	555	12	1481672	412960	0	2	0	0	50	49	21584280	8.65E-01	1
TMC7	850990	171	18	1877900	505876	0	4	3	0	10	19	5365988	8.65E-01	1
TTC25	1969457	191	28	1589540	406552	0	4	1	0	50	66	20772956	8.65E-01	1
MTMR6	330431	538	31	1643652	404772	0	4	2	0	43	63	20749104	8.65E-01	1
BCL2L10	540700	195	21	494484	168744	0	1	2	0	3	3	1109296	8.65E-01	1
FOXO4	759088	NaN	27	1229268	400500	0	2	1	0	50	74	25911460	8.65E-01	1
CLDN4	1099366	208	37	503028	169812	0	3	2	0	3	7	1131012	8.65E-01	1
ADCY6	1715886	328	38	2943052	892136	0	10	2	0	5	7	2313288	8.65E-01	1
CLEC12A	305376	565	19	707372	166252	0	1	1	0	5	5	2335716	8.65E-01	1
FKBP9	375055	235	13	1452124	415808	0	5	3	0	4	5	1737280	8.65E-01	1
DEPDC1B	166902	679	13	1386976	356000	0	4	1	0	50	69	18171664	8.65E-01	1
ZNF672	411701	567	-4	1090072	361340	0	2	1	0	2	1	1058388	8.65E-01	1
TECTB	170055	567	-5	872556	223212	0	2	1	0	50	65	18974444	8.65E-01	1
OR10G2	658946	624	20	746888	248488	0	1	0	0	49	65	23465740	8.65E-01	1

TSNARE1	163885	685	12	1310792	391600	0	4	3	0	44	64	15549012	8.65E-01	1
PDZD2	440207	557	6	7025660	2171956	0	19	5	0	15	21	7322564	8.65E-01	1
SH3GLB2	1517799	203	29	1017092	294412	0	1	2	0	50	43	19078752	8.65E-01	1
GARS	504898	433	42	1915992	523320	0	4	1	0	17	25	10088328	8.65E-01	1
MCTP2	46228	457	-17	2371316	615880	0	6	4	0	29	30	9156676	8.65E-01	1
C11orf54	290677	527	0	702032	180136	0	1	0	0	50	59	19845576	8.65E-01	1
TNNT3	827096	481	53	817376	168388	0	1	1	0	50	76	25042464	8.65E-01	1
CLIP2	938020	179	51	2619804	803136	0	7	2	0	26	34	11429736	8.65E-01	1
FASLG	116331	506	7	708440	207904	0	1	1	0	50	69	21465732	8.65E-01	1
NR4A1	468067	234	41	1465652	479176	0	1	0	0	40	21	13369580	8.65E-01	1
DOK3	1120311	347	41	1481316	508368	0	1	0	0	13	16	5959084	8.65E-01	1
ZNF446	2179183	270	-4	1119264	351372	0	2	1	0	50	76	25634848	8.65E-01	1
TBCD	651944	456	14	3098624	886084	0	10	6	0	8	13	3523688	8.65E-01	1
MAN1A1	105366	702	6	1676760	465648	0	3	0	0	21	23	10942016	8.65E-01	1
IFT122	848687	163	58	3388408	887864	0	7	2	0	50	62	20910016	8.65E-01	1
CARD10	985853	176	51	2581000	812036	0	5	3	0	50	63	19932440	8.66E-01	1
MAEA	938287	391	37	1028484	279816	0	2	1	0	50	57	18627700	8.66E-01	1
RASL12	688371	189	44	669636	202920	0	1	1	0	50	72	23489592	8.66E-01	1
DYM	241451	414	57	1749028	466004	0	4	3	0	50	61	21908596	8.66E-01	1
XKR3	106864	720	-97	1178004	308652	0	3	1	0	50	87	22203720	8.66E-01	1
LSR	767246	472	7	1626564	495552	0	5	5	0	13	13	4190476	8.66E-01	1
DHX8	1124329	147	20	3139208	868996	0	11	1	0	0	1	868996	8.66E-01	1
PITPNM1	1392213	215	53	3122120	965116	0	7	1	0	11	7	3262740	8.66E-01	1
CEP120	173569	467	12	2574592	673196	0	4	0	0	3	3	2175516	8.66E-01	1
GZMB	853527	610	-12	635816	178000	0	2	1	0	50	132	24594616	8.66E-01	1
BRD7	303953	520	14	1745112	417588	0	6	3	0	4	10	2039168	8.66E-01	1
NAT6	843769	206	51	730868	265576	0	1	1	0	50	68	20472848	8.66E-01	1
RILPL1	1039355	424	49	1035960	284088	0	2	0	0	50	81	25992984	8.66E-01	1
KRTAP15-[illegible]	14979	1035	-68	346744	97544	0	1	1	0	40	56	9651872	8.66E-01	1
ZNF211	532349	849	-37	1492708	372020	0	4	0	0	22	27	8358168	8.66E-01	1
KCNN4	452493	192	-2	1060524	344964	0	3	3	0	8	13	3599872	8.66E-01	1
CYSLTR2	404943	430	46	859740	254184	0	1	1	0	50	55	23510596	8.66E-01	1
SLC39A5	2878071	171	46	1301180	477040	0	2	1	0	13	15	6175532	8.67E-01	1

HMGXB4	359646	425	-49	1545040	426488	0	5	1	0	30	51	13034584	8.67E-01	1
TPD52	211901	353	43	668212	178712	0	1	0	0	50	58	18686440	8.67E-01	1
GDAP2	204311	596	-6	1336780	360272	0	2	2	0	50	56	22798952	8.67E-01	1
PTK7	1273888	301	39	2727316	785336	0	7	1	0	17	40	12635508	8.67E-01	1
RNF113A	772178	NaN	38	873268	231044	0	1	0	0	50	59	22592472	8.67E-01	1
PI16	832054	220	41	1144896	367392	0	2	1	0	50	65	22074136	8.67E-01	1
CARNS1	1269888	177	51	2276620	808832	0	4	1	0	50	45	20698908	8.67E-01	1
ADAMTSL	2122824	221	35	1189040	365968	0	2	0	0	27	22	8391632	8.67E-01	1
CUL4A	787509	214	46	2020300	498044	0	3	2	0	31	37	13246404	8.67E-01	1
PRICKLE3	724395	NaN	44	1552516	457460	0	2	0	0	32	28	13371716	8.67E-01	1
OR1A2	506514	628	0	769316	223924	0	1	1	0	50	74	22952032	8.67E-01	1
C19orf26	2214581	234	29	1099328	370240	0	2	1	0	50	68	20485664	8.67E-01	1
GLYATL2	285727	673	-11	786760	179780	0	3	2	0	7	8	1745112	8.68E-01	1
ENTPD6	605543	305	-18	1262020	357068	0	2	0	0	12	23	6007144	8.68E-01	1
ASPG	622747	468	20	1449276	477396	0	3	1	0	50	61	20058108	8.68E-01	1
RLBP1	649712	400	25	827344	221432	0	5	3	0	2	8	1368108	8.68E-01	1
ANKRD16	649866	305	28	930228	261660	0	1	0	0	50	54	23924980	8.68E-01	1
ZNF440	739660	633	-2	1540056	382344	0	6	3	0	4	6	1213960	8.68E-01	1
PLEKHA2	487952	201	38	1105380	306516	0	2	0	0	25	22	7699568	8.68E-01	1
RGPD4	NaN	NaN	NaN	4535084	1196872	0	16	1	0	7	15	4885032	8.68E-01	1
DLGAP1	368461	215	24	2515140	717696	0	7	3	0	6	8	2537568	8.68E-01	1
RNF145	123474	225	42	1776796	485228	0	4	4	0	29	49	12154552	8.68E-01	1
CLPX	678641	189	61	1630480	458528	0	4	1	0	8	6	2592748	8.68E-01	1
FAM46C	344088	557	16	981492	278748	0	1	0	0	50	55	25972692	8.68E-01	1
RP1	148553	735	-8	5478484	1445360	0	16	3	0	8	11	4692792	8.68E-01	1
DISP1	236778	210	5	3879332	1038808	0	10	5	0	50	59	21006848	8.68E-01	1
TBRG4	1070050	291	53	1576368	491280	0	6	0	0	10	18	4766484	8.68E-01	1
TTC30B	380902	434	27	1668928	467072	0	5	2	0	5	7	2744404	8.68E-01	1
ENOX2	138723	NaN	23	1617664	395516	0	2	0	0	50	36	17510572	8.68E-01	1
CNTN4	52413	1215	-22	2657184	727308	0	10	5	0	19	30	7174468	8.68E-01	1
TCIRG1	1168866	251	63	2094704	651124	0	4	1	0	16	16	6547552	8.68E-01	1
CAMK2A	978175	220	47	1302604	344252	0	2	2	0	36	38	15465352	8.68E-01	1
CTR9	794670	261	20	3091148	772876	0	6	0	0	4	2	2081888	8.68E-01	1

OR4D11	430021	507	-1	757212	242436	0	3	3	0	8	24	4424012	8.68E-01	1
SQLE	518053	572	19	1379144	398008	0	4	1	0	3	4	1311504	8.68E-01	1
EPHB4	1339207	433	48	2465656	768248	0	7	0	0	4	7	3076552	8.68E-01	1
NMBR	152447	513	-28	975440	288004	0	3	1	0	50	123	25680772	8.68E-01	1
GAPVD1	954577	218	46	3834120	1044504	0	7	1	0	10	6	4446084	8.68E-01	1
PRDX2	1937827	226	32	652192	188324	0	1	1	0	50	71	21091932	8.68E-01	1
PISD	538801	268	47	950520	279816	0	1	1	0	33	30	12881860	8.69E-01	1
OR5D18	4183	1091	-36	767536	237452	0	6	4	0	4	12	1169816	8.69E-01	1
HIST1H1T	398813	636	53	510148	160556	0	1	2	0	33	36	8882912	8.69E-01	1
LRRC10	499701	394	14	675688	215024	0	1	1	0	50	62	19775088	8.69E-01	1
WISP3	159324	296	16	966184	248132	0	1	0	0	50	38	15373504	8.69E-01	1
TMEM47	7188	NaN	-16	453544	138128	0	1	1	0	39	88	19402000	8.69E-01	1
SLC41A1	717622	377	37	1279108	406196	0	2	1	0	50	68	25178456	8.69E-01	1
XPO6	879658	233	39	2903892	802068	0	8	3	0	42	47	17338624	8.69E-01	1
DDX42	1259976	346	12	2427920	653260	0	8	0	0	10	13	4595960	8.69E-01	1
DAOA	13092	1235	-28	527236	103952	0	1	0	0	50	101	22823516	8.69E-01	1
SCFD1	386053	583	2	1735856	428980	0	5	0	0	30	50	16047768	8.69E-01	1
ADH1C	332785	854	8	963336	277680	0	2	0	0	50	70	20070924	8.69E-01	1
AKT1	711772	247	36	1269140	327520	0	3	2	0	50	66	18830620	8.69E-01	1
CDC5L	958127	443	34	2092212	544680	0	4	1	0	50	47	18885800	8.69E-01	1
DERL3	637355	196	52	692064	209328	0	1	1	0	50	63	20521620	8.69E-01	1
FAM83B	25624	495	-15	2562844	698828	0	6	0	0	1	0	765044	8.69E-01	1
RCL1	238716	573	26	949452	283020	0	1	0	0	50	50	23431564	8.69E-01	1
NOS2	474206	541	-22	2985060	823428	0	9	3	0	17	23	6725908	8.69E-01	1
TBC1D2B	435470	232	54	2469572	670348	0	4	1	0	50	51	18436528	8.70E-01	1
DYNC1I2	354433	344	33	1686016	433964	0	3	0	0	35	29	13090832	8.70E-01	1
PADI1	524388	182	42	1719124	474548	0	6	4	0	5	7	2373808	8.70E-01	1
CSRP3	132848	504	16	513708	132432	0	1	1	0	9	12	3421516	8.70E-01	1
DGUOK	1036341	284	45	723392	195088	0	1	0	0	50	73	24714944	8.70E-01	1
MTX3	319253	795	31	815596	223568	0	1	1	0	16	12	5360648	8.70E-01	1
ENC1	293932	567	26	1473128	425776	0	3	1	0	50	63	24367132	8.70E-01	1
PNMA3	265470	NaN	16	1134216	352440	0	1	0	0	50	50	25261048	8.70E-01	1
TAF5L	299238	340	15	1487724	423996	0	2	1	0	50	56	23073428	8.70E-01	1

NETO1	32720	916	-26	1432544	359560	0	3	4	0	15	25	4648292	8.70E-01	1
KRTAP1-5	977588	404	10	448560	114276	0	1	1	0	50	68	14276668	8.70E-01	1
TMEM14C	263716	468	14	303312	84372	0	1	1	0	4	6	1045928	8.70E-01	1
DGKI	196508	861	-7	2792108	765400	0	7	1	0	7	8	3880756	8.70E-01	1
PUS7L	132735	558	-16	1830196	453188	0	4	2	0	7	6	3077976	8.70E-01	1
LRRC32	269123	379	26	1577792	555004	0	3	0	0	50	54	22933164	8.70E-01	1
STRN4	1166804	224	36	1945896	572448	0	4	1	0	50	50	18884020	8.70E-01	1
RPP30	103854	510	4	864012	240300	0	1	0	0	25	21	8382376	8.70E-01	1
PTCHD1	397667	NaN	-2	2216812	642224	0	7	2	0	23	39	11684276	8.71E-01	1
NNT	439246	597	0	2736216	836244	0	7	2	0	50	66	22769760	8.71E-01	1
ELK1	772893	NaN	41	1039876	356000	0	1	1	0	50	65	24254280	8.71E-01	1
STARD13	157153	583	41	2956936	826988	0	5	1	0	9	5	3754376	8.71E-01	1
RSPH3	514213	348	42	1414744	412604	0	2	1	0	38	37	15269908	8.71E-01	1
TEX10	246976	476	12	2387336	665008	0	3	0	0	1	0	1193312	8.71E-01	1
RSBN1	318105	406	18	2019944	580636	0	4	1	0	22	28	11935968	8.71E-01	1
RABGEF1	539697	302	55	1294060	316484	0	3	3	0	14	14	4379868	8.71E-01	1
TNS3	192490	488	16	3658968	1085088	0	13	3	0	6	10	3369896	8.71E-01	1
FBXW12	1401411	263	32	1252764	321468	0	1	1	0	50	79	21907884	8.71E-01	1
HAUS2	534369	269	40	625848	155928	0	1	0	0	22	24	7399816	8.71E-01	1
IGSF5	285788	762	-70	1051980	290496	0	3	1	0	50	96	22399520	8.71E-01	1
USP5	2822601	232	32	2229984	607692	0	3	2	0	50	62	20662240	8.71E-01	1
LUC7L3	1462233	148	16	1151304	278748	0	3	0	0	24	42	11846256	8.71E-01	1
WSCD2	451405	644	29	1440376	402992	0	2	2	0	30	33	13631240	8.71E-01	1
MT1A	653985	187	28	174796	34532	0	1	1	0	6	1	255608	8.71E-01	1
ETV3L	934232	423	32	902104	274832	0	1	0	0	50	48	19649776	8.71E-01	1
SP110	473233	643	31	1964764	489500	0	3	4	0	14	14	3886096	8.71E-01	1
TMX3	70753	920	-24	1228912	292988	0	2	0	0	39	65	16515908	8.71E-01	1
STEAP4	155853	454	-6	1148812	342116	0	2	1	0	50	55	19200504	8.71E-01	1
AGAP5	NaN	NaN	NaN	1747248	485940	0	4	1	0	50	117	40131524	8.71E-01	1
KLHL1	4513	1341	-27	1916704	525812	0	13	5	0	11	25	3528672	8.71E-01	1
TMEM68	319097	538	-3	672128	171592	0	1	1	0	50	54	17729868	8.71E-01	1
DCX	131531	NaN	13	1147388	310432	0	2	1	0	42	49	17013596	8.72E-01	1
TEX13B	184382	NaN	40	779996	231400	0	1	1	0	50	69	19292352	8.72E-01	1

ENSA	1543587	149	37	522964	132788	0	1	0	0	17	33	8290528	8.72E-01	1
SIRT6	2256881	244	42	886084	286580	0	1	0	0	50	51	21490652	8.72E-01	1
OR2AG1	427462	681	8	772520	247420	0	1	0	0	50	71	18227912	8.72E-01	1
CEP72	683895	411	28	1661808	463512	0	3	0	0	8	7	3458540	8.72E-01	1
OR6C4	1393447	546	-13	763264	227840	0	1	0	0	27	23	8982592	8.72E-01	1
TMTC3	149643	429	-9	2362416	624780	0	6	0	0	0	0	624780	8.72E-01	1
AREG	172071	NaN	10	1322896	341048	0	1	0	0	29	24	15581408	8.72E-01	1
PEG10	393749	462	4	2498408	24208	0	2	0	0	50	52	19426208	8.72E-01	1
OXTR	158952	534	3	944112	309720	0	1	1	0	50	40	17926024	8.72E-01	1
SLC2A4	1782958	316	21	1256324	420436	0	3	2	0	39	39	14081580	8.72E-01	1
FOXG1	52980	1173	-46	1191888	378072	0	4	3	0	10	25	6182296	8.72E-01	1
RAB40A	458015	NaN	22	687080	207904	0	1	0	0	50	57	19034964	8.72E-01	1
GOLGA7B	543507	369	12	512640	104664	0	1	1	0	10	20	5305468	8.72E-01	1
HEYL	976856	358	33	801712	271628	0	1	0	0	37	40	15215084	8.72E-01	1
ASCC1	761166	140	40	952300	233180	0	1	0	0	50	52	21413756	8.72E-01	1
SEC24A	899027	175	40	2794600	804560	0	6	6	0	50	70	22324404	8.72E-01	1
ITGB7	1703142	191	52	2021012	598792	0	3	0	0	7	10	4059824	8.72E-01	1
TECPR2	1666122	191	37	3592040	1027060	0	9	5	0	15	29	8383444	8.72E-01	1
ATXN7L2	1056159	272	37	1801360	559988	0	5	3	0	50	61	17976932	8.72E-01	1
MESP2	934542	212	37	950164	329300	0	1	1	0	50	65	20979792	8.73E-01	1
RCBTB1	430735	338	67	1372380	379140	0	2	1	0	50	49	19642300	8.73E-01	1
CBLL1	513169	515	28	1244220	353508	0	3	2	0	50	63	20041732	8.73E-01	1
CPAMD8	947295	386	29	4890728	1479892	0	10	6	0	50	43	18675404	8.73E-01	1
DHRS12	252271	384	33	859384	225704	0	1	1	0	50	47	18986904	8.73E-01	1
PFDN5	1677275	191	49	418656	100392	0	1	1	0	5	17	3210408	8.73E-01	1
CDC27	955548	395	18	2170176	569244	0	4	1	0	3	3	2097552	8.73E-01	1
YIPF2	2009333	193	41	803136	250980	0	2	2	0	8	15	3918848	8.73E-01	1
RHBG	2199280	201	27	1148456	363832	0	3	1	0	50	86	24636980	8.73E-01	1
RPIA	103964	309	18	806696	229264	0	1	1	0	50	49	19268856	8.73E-01	1
LHX2	306688	342	19	1018516	302600	0	2	2	0	12	14	4791760	8.73E-01	1
FAM169A	424900	581	31	1743332	457816	0	4	0	0	14	13	4964776	8.73E-01	1
FBXW8	208959	359	16	1526172	437880	0	3	0	0	7	7	2310440	8.73E-01	1
SMAD2	108087	185	42	1220012	322180	0	3	0	0	50	76	20349672	8.73E-01	1

UHRF1	789691	248	32	2070140	578500	0	2	1	0	50	55	20315852	8.73E-01	1
DTNA	116061	552	-20	2029556	541120	0	5	0	0	8	14	5508388	8.73E-01	1
OCIAD1	257328	372	63	699184	163760	0	1	0	0	50	65	20895420	8.73E-01	1
MRGPRF	557282	416	51	840160	274832	0	2	2	0	9	18	4515148	8.73E-01	1
ZNF214	418263	881	-4	1585980	367392	0	4	0	0	49	69	19482456	8.73E-01	1
ZNF264	407201	924	-63	1601644	423284	0	4	0	0	26	28	9636564	8.73E-01	1
USP39	1158965	170	43	1470992	395872	0	3	0	0	8	11	3234616	8.73E-01	1
ALDOC	1651143	225	31	926312	277324	0	1	1	0	50	69	21609556	8.73E-01	1
PRPF40A	285742	716	16	2499120	592740	0	6	0	0	9	8	4154164	8.73E-01	1
ERCC8	199321	599	6	1053048	268068	0	1	1	0	50	40	20381000	8.73E-01	1
UBN1	704578	195	26	2859392	849772	0	5	3	0	16	24	11753340	8.73E-01	1
IL6ST	317374	438	51	2393744	614812	0	7	0	0	11	23	7600244	8.73E-01	1
TCF3	2396187	262	31	1854404	572092	0	3	1	0	50	60	26523068	8.73E-01	1
STXBP4	132396	845	-50	1456752	386616	0	3	1	0	17	15	5742636	8.73E-01	1
SAMD9L	362550	625	8	4048788	1033824	0	15	2	0	12	25	5903904	8.74E-01	1
CHRNA4	1278630	332	62	1541124	492348	0	4	3	0	50	61	17105444	8.74E-01	1
CPA5	422275	212	28	1182988	268424	0	3	1	0	9	9	2932372	8.74E-01	1
UEVLD	630607	341	24	1236744	324672	0	1	1	0	50	40	23340072	8.74E-01	1
SKA1	277376	392	52	681028	164828	0	1	2	0	11	19	6446448	8.74E-01	1
NPNT	262174	361	7	1585980	433608	0	3	1	0	50	53	18278108	8.74E-01	1
AFP	331900	594	3	1613392	402992	0	5	0	0	10	14	3999660	8.74E-01	1
FBXO9	450160	386	26	1196160	293700	0	2	1	0	50	53	19531584	8.74E-01	1
PLIN3	765978	187	52	1096480	327164	0	6	4	0	2	7	1252408	8.74E-01	1
APCDD1	219220	604	2	1295484	371664	0	2	1	0	50	51	19400220	8.74E-01	1
MTFR1	146207	407	16	952656	267000	0	3	2	0	5	11	2007840	8.74E-01	1
LAMB4	487451	326	4	4590976	1195448	0	12	3	0	50	62	22591048	8.74E-01	1
THSD7A	138176	906	-11	4306888	1116416	0	12	3	0	9	15	4608420	8.74E-01	1
DLGAP4	874457	360	46	2582068	771452	0	6	2	0	50	99	27581456	8.74E-01	1
AOC2	1631295	190	28	1845860	594520	0	3	1	0	50	77	21382784	8.74E-01	1
GRSF1	263233	553	29	1230692	351016	0	2	0	0	50	67	22623800	8.74E-01	1
LACE1	346225	491	37	1267004	328588	0	2	1	0	50	50	18596016	8.74E-01	1
TTC39C	292559	189	54	1528308	398364	0	3	0	0	50	60	20945616	8.74E-01	1
TATDN3	331599	445	22	744752	199360	0	1	0	0	50	84	29810016	8.74E-01	1

STT3A	391073	595	23	1829128	505520	0	3	1	0	50	54	21894000	8.74E-01	1
GSK3A	790787	299	37	1206128	387328	0	2	2	0	50	73	25484972	8.74E-01	1
FJX1	629751	396	-15	1022076	381276	0	1	1	0	50	49	19793600	8.74E-01	1
R3HDML	813168	376	10	652192	178712	0	1	1	0	50	60	16040648	8.74E-01	1
AMMECR1	157872	NaN	34	833396	260236	0	1	0	0	20	26	10875444	8.74E-01	1
KIAA0753	462930	285	10	2505528	672840	0	8	2	0	4	7	2323968	8.74E-01	1
SLC35D3	177175	449	21	996800	343540	0	2	3	0	26	29	8897864	8.75E-01	1
IFI35	1507064	201	26	728020	223568	0	1	0	0	50	50	17618084	8.75E-01	1
KCNAB2	593369	271	22	977932	265220	0	1	2	0	20	18	8138160	8.75E-01	1
ZNF524	1077402	254	-10	633680	219652	0	1	1	0	50	67	17041008	8.75E-01	1
SIX5	929036	203	43	1716276	663228	0	3	2	0	33	37	14234660	8.75E-01	1
DRD4	1774224	236	57	991816	368816	0	1	0	0	4	3	2271636	8.75E-01	1
CERK	404043	265	-1	1398012	377004	0	2	0	0	50	62	24407004	8.75E-01	1
DERL2	1145030	544	24	629408	167320	0	1	1	0	50	77	22011836	8.75E-01	1
UQCRC1	1386275	212	37	1234964	359560	0	2	2	0	50	75	27156392	8.75E-01	1
GOPC	189169	465	9	1187260	330368	0	2	2	0	50	65	22672572	8.75E-01	1
PIGL	905220	163	0	656464	181916	0	1	1	0	50	54	16613452	8.75E-01	1
PCSK2	536476	528	-40	1640092	454256	0	5	3	0	50	63	17722036	8.75E-01	1
USF1	689033	250	23	812036	227128	0	2	0	0	12	19	4867588	8.75E-01	1
MMP25	1534969	234	46	1398724	443576	0	3	2	0	50	70	19650844	8.75E-01	1
ZNF98	38548	1322	-77	1491996	356712	0	5	1	0	2	1	702032	8.75E-01	1
HPD	829168	195	44	1052692	270560	0	2	1	0	13	13	4295140	8.75E-01	1
MYH7	1220544	456	29	5035620	1329660	0	16	5	0	5	12	3841240	8.75E-01	1
SMS	92980	NaN	23	975796	244928	0	1	0	0	13	8	4100052	8.75E-01	1
MSX1	132550	558	37	724460	253828	0	1	0	0	50	62	20678972	8.75E-01	1
C8orf58	757216	224	45	902460	295836	0	1	0	0	48	49	21352880	8.75E-01	1
CSN2	9753	991	-4	582416	172660	0	1	0	0	50	76	18672912	8.75E-01	1
OR8J1	10293	963	-36	770384	243148	0	1	2	0	5	2	1310080	8.75E-01	1
PRSS36	1296884	351	35	2117488	687080	0	5	2	0	16	27	8122140	8.75E-01	1
KRT71	1280474	464	36	1334644	380564	0	5	2	0	3	5	1590964	8.75E-01	1
RPL3L	1756887	183	42	1048064	299752	0	2	1	0	50	63	19992604	8.76E-01	1
AZI2	68916	925	9	1115704	266288	0	6	2	0	1	2	397652	8.76E-01	1
AGFG1	194117	353	23	1510152	426132	0	1	0	0	24	31	10153832	8.76E-01	1

CIT	905544	436	24	5285888	1408336	0	19	6	0	50	79	25908612	8.76E-01	1
TJP1	194015	515	-46	4460324	1258816	0	8	2	0	0	2	1258816	8.76E-01	1
TRAF7	2179404	224	39	1754724	480600	0	2	2	0	50	46	19527312	8.76E-01	1
CYBRD1	486534	352	34	789964	142400	0	1	0	0	50	46	17563260	8.76E-01	1
GUCA1C	90728	971	-15	556072	131720	0	2	2	0	2	10	1882172	8.76E-01	1
NRXN2	1897094	411	56	4432556	1430764	0	11	4	0	31	35	12682856	8.76E-01	1
MYBPH	507603	453	31	1212536	363832	0	2	2	0	50	72	22933520	8.76E-01	1
LECT1	288742	469	24	868996	229976	0	1	0	0	50	67	23526972	8.76E-01	1
SLC30A10	623319	641	-12	1195092	374868	0	2	0	0	29	39	14135336	8.76E-01	1
ADPGK	1106545	526	43	1240304	379852	0	2	0	0	50	70	26051368	8.76E-01	1
OTOP3	1045089	223	25	1459956	477396	0	3	1	0	50	66	19891500	8.76E-01	1
MCM8	301031	346	13	2185128	586332	0	8	0	0	14	16	5157372	8.76E-01	1
METTL2B	893183	199	45	997512	253116	0	2	1	0	29	50	14161324	8.76E-01	1
CEBPA	355217	510	-9	857248	292988	0	1	1	0	50	58	23777240	8.76E-01	1
MICAL3	415161	288	11	5690660	1642228	0	6	0	0	2	1	2416528	8.76E-01	1
GLI4	1657385	389	19	938772	281952	0	4	4	0	4	6	1374872	8.76E-01	1
CCDC159	1385739	197	31	807408	194376	0	1	0	0	50	60	22230420	8.76E-01	1
TMEM8C	1858670	315	39	566396	166252	0	1	2	0	14	26	5231420	8.76E-01	1
CLU	346863	361	29	1306164	344964	0	1	0	0	24	18	11650812	8.77E-01	1
OR14A16	164924	995	-27	764332	228908	0	2	0	0	50	130	29092676	8.77E-01	1
SYCE1	563924	525	-10	981848	241012	0	2	2	0	42	51	14206180	8.77E-01	1
MCHR1	726133	165	54	1026704	332860	0	3	2	0	50	90	19997588	8.77E-01	1
KCNK7	3945819	202	45	808476	294768	0	1	2	0	19	25	9711324	8.77E-01	1
OTUD6B	104917	935	1	850484	215380	0	1	0	0	50	66	21354660	8.77E-01	1
CARD8	1052191	391	32	1404776	368104	0	3	0	0	41	37	11983316	8.77E-01	1
ABCB7	183021	NaN	-5	1935572	548596	0	3	0	0	50	48	19458960	8.77E-01	1
ILVBL	703673	419	1	1580284	511928	0	2	2	0	50	38	17686080	8.77E-01	1
PRPS2	208595	NaN	46	823784	233536	0	1	1	0	50	83	24786856	8.77E-01	1
GJA1	60722	773	-23	959776	271628	0	2	0	0	13	18	5367768	8.77E-01	1
G6PC	1629499	190	23	909224	257032	0	2	1	0	50	91	23722772	8.77E-01	1
NEO1	328878	705	14	3733016	1070848	0	10	2	0	15	19	7763648	8.77E-01	1
HOOK2	1959466	226	29	1868288	532576	0	4	0	0	3	1	1302248	8.77E-01	1
PHKG2	1632278	158	61	1077256	312212	0	2	1	0	47	62	18440088	8.77E-01	1

ARCN1	1359480	167	47	1321472	357424	0	4	1	0	11	21	5243524	8.77E-01	1
LPGAT1	465080	281	13	973660	244928	0	2	1	0	13	29	8325060	8.77E-01	1
SLC19A2	294526	487	16	1229980	386972	0	2	2	0	50	48	17944536	8.77E-01	1
SCN8A	575393	464	27	5101124	1357072	0	10	4	0	50	56	23518428	8.77E-01	1
ACRV1	564542	595	13	693132	171948	0	1	0	0	50	72	22698560	8.77E-01	1
SRRM3	851224	195	50	1610544	541476	0	1	0	0	33	36	13007528	8.77E-01	1
PLA2G6	1578246	197	69	2051272	602708	0	3	2	0	33	38	9574264	8.77E-01	1
CITED2	187396	504	14	687792	184764	0	1	0	0	50	62	20256756	8.77E-01	1
RXFP2	223402	693	5	1991464	502316	0	4	0	0	1	0	772876	8.77E-01	1
NANP	547844	515	-31	630120	174084	0	1	0	0	50	55	16283440	8.78E-01	1
PTGER1	954144	204	43	925600	376292	0	1	0	0	22	20	9379532	8.78E-01	1
SERPINF1	1447501	189	32	1058388	313992	0	2	1	0	44	62	19487440	8.78E-01	1
CD79B	1455216	404	25	604844	158776	0	1	1	0	50	68	18709936	8.78E-01	1
RAP1GAP2	518031	418	12	1923468	523320	0	3	1	0	3	3	1798512	8.78E-01	1
BHMT	164477	578	36	1051980	281952	0	1	1	0	50	53	19761560	8.78E-01	1
KLHL17	1911331	239	9	1600220	506944	0	7	3	0	5	9	2282672	8.78E-01	1
LIX1L	NaN	NaN	NaN	840516	265932	0	1	0	0	50	116	39911516	8.78E-01	1
CYP4F3	852124	605	29	1346392	373088	0	2	0	0	6	7	2396236	8.78E-01	1
NFYA	145501	611	37	894272	259168	0	1	1	0	50	56	20220800	8.78E-01	1
ALKBH8	234842	643	-2	1734076	443576	0	3	0	0	25	19	7791416	8.78E-01	1
GPR137B	483624	632	8	1011396	297972	0	2	1	0	50	71	22022516	8.78E-01	1
IRAK1	2355024	NaN	25	1779288	562836	0	2	0	0	2	3	2559640	8.78E-01	1
FAM179A	471053	404	28	2547180	797796	0	7	0	0	1	3	1758284	8.78E-01	1
ZCCHC11	663395	394	20	4297988	1099684	0	14	2	0	7	13	4336080	8.78E-01	1
TAS2R19	365490	676	-2	739056	221076	0	1	1	0	50	58	17850552	8.78E-01	1
HSF2	140855	973	-19	1415812	356000	0	4	2	0	50	87	22963780	8.78E-01	1
AOC3	1629499	190	23	1873984	586688	0	5	0	0	2	2	1071204	8.78E-01	1
GCM2	263829	483	14	1286584	357068	0	4	3	0	20	20	5316148	8.78E-01	1
FOXH1	1899637	200	22	878964	302244	0	1	0	0	17	18	8072656	8.79E-01	1
DEPDC7	415951	215	25	1379144	348880	0	2	0	0	10	9	3642948	8.79E-01	1
CD2	697845	431	21	903884	243148	0	1	0	0	50	69	23474640	8.79E-01	1
CD163L1	931883	567	13	3735508	1006412	0	12	7	0	13	23	5442884	8.79E-01	1
PCDH12	696239	321	48	2892144	917412	0	7	4	0	50	50	19214032	8.79E-01	1

CHML	NaN	NaN	NaN	1657536	447492	0	3	2	0	50	118	40093076	8.79E-01	1
CCR1	211405	639	48	879676	265220	0	1	1	0	50	45	15842000	8.79E-01	1
FCN1	237562	489	35	851196	232824	0	3	3	0	3	4	1205060	8.79E-01	1
MAF	10920	1003	-30	986476	314348	0	3	0	0	8	19	3368116	8.79E-01	1
CEACAM1	393294	583	17	1008192	287292	0	1	1	0	13	19	6561080	8.79E-01	1
DEF8	1056657	221	35	1394808	383412	0	4	3	0	20	22	5927400	8.79E-01	1
ZNF282	639444	254	30	1689932	493060	0	3	1	0	34	48	13028532	8.79E-01	1
PTER	104989	791	-7	888576	249912	0	1	0	0	10	22	8211852	8.79E-01	1
CHST13	289633	508	52	813460	290852	0	1	2	0	20	25	7817404	8.79E-01	1
PSMC4	1335888	522	35	1092564	294768	0	2	0	0	50	92	28057784	8.79E-01	1
PIK3R4	198723	430	17	3488800	946604	0	8	4	0	50	56	19741624	8.79E-01	1
PDIA2	1340678	205	43	1325388	406908	0	2	1	0	50	57	21858756	8.79E-01	1
GPR155	405127	409	30	2243512	611252	0	7	3	0	31	36	12221480	8.79E-01	1
HTR6	776287	266	32	1035604	385904	0	5	3	0	4	5	1164120	8.79E-01	1
GIT2	939215	254	46	2019944	534712	0	3	3	0	50	62	26978036	8.80E-01	1
USP6NL	300268	388	22	2182636	596300	0	2	2	0	34	27	16563612	8.80E-01	1
PDZRN4	15244	1060	-21	2708092	730868	0	10	4	0	50	85	21381716	8.80E-01	1
SLC35C2	543487	612	42	924176	282664	0	2	2	0	19	36	9570348	8.80E-01	1
APTX	833170	286	-5	949452	236028	0	2	0	0	50	65	18547600	8.80E-01	1
ARHGEF3	NaN	NaN	NaN	1218588	339624	0	2	2	0	50	118	39985208	8.80E-01	1
SYNPR	152983	976	-5	755788	209684	0	1	0	0	50	52	18613104	8.80E-01	1
MED13L	186212	605	-12	5630140	1584200	0	10	4	0	29	25	11262772	8.80E-01	1
TMEM87B	564343	466	36	1488792	369528	0	2	2	0	50	55	23724196	8.80E-01	1
CELF6	1128261	260	47	1226776	370952	0	2	0	0	23	29	10172700	8.80E-01	1
FGD1	299313	NaN	18	2419732	735140	0	6	2	0	35	49	16509500	8.80E-01	1
ZNF597	1361831	303	29	1100396	274120	0	2	2	0	50	74	21539424	8.80E-01	1
IGBP1	166319	NaN	1	885016	225704	0	1	0	0	50	67	24685396	8.80E-01	1
CRTC2	2028800	222	34	1721616	559632	0	7	4	0	25	39	8426876	8.80E-01	1
C9orf171	1522970	492	36	812036	244216	0	3	3	0	5	9	1904956	8.80E-01	1
CP	388273	312	10	2799228	693132	0	5	4	0	7	8	3570680	8.80E-01	1
LRRC46	1071489	214	29	823072	238520	0	2	2	0	12	13	4017104	8.80E-01	1
RPGRIP1	722324	263	39	3342840	881100	0	7	1	0	18	36	14750148	8.80E-01	1
TAC1	343278	854	-15	359560	82592	0	1	1	0	6	8	1624072	8.80E-01	1

SPATA20	1532661	200	20	2041660	601640	0	4	3	0	50	83	22594252	8.80E-01	1
C1orf86	849779	293	25	866860	186188	0	2	2	0	5	9	2624788	8.80E-01	1
DBX1	175444	453	-5	928092	311856	0	1	2	0	50	45	19304812	8.80E-01	1
RFX5	1322568	174	42	1527596	487720	0	4	0	0	6	11	3531520	8.80E-01	1
ARHGEF1	1255704	364	28	2392320	702744	0	6	2	0	41	43	16154568	8.81E-01	1
WWP2	1410481	NaN	42	2272348	618728	0	4	2	0	24	30	12332196	8.81E-01	1
CBR3	261889	232	56	700964	200428	0	1	2	0	22	28	9427236	8.81E-01	1
UAP1L1	1951939	202	40	1262732	401212	0	2	1	0	50	61	21392396	8.81E-01	1
ZNF732	NaN	NaN	NaN	1517984	364900	0	3	1	0	50	117	40010484	8.81E-01	1
TJP3	2144458	206	34	2400152	735496	0	3	4	0	13	11	5053064	8.81E-01	1
ZNF18	42879	557	-52	1416168	371664	0	2	0	0	8	6	3291576	8.81E-01	1
BSPRY	390186	297	36	1008904	305804	0	1	1	0	50	42	19391676	8.81E-01	1
NBPF1	675669	259	40	3019592	738700	0	10	4	0	14	21	5843384	8.81E-01	1
CUEDC2	975992	178	39	751160	205768	0	1	0	0	50	84	25980880	8.81E-01	1
TMEM198	548994	487	35	856536	317196	0	1	1	0	50	86	24911100	8.81E-01	1
NTSR1	1286411	208	27	1024212	331080	0	2	2	0	36	61	17235384	8.81E-01	1
TMEM55B	464497	395	17	721256	217516	0	1	3	0	14	7	2572812	8.81E-01	1
TSPAN5	217859	797	12	712000	179780	0	1	0	0	50	58	20050988	8.81E-01	1
SLC2A9	222993	916	37	1426136	430048	0	3	1	0	50	62	19126456	8.81E-01	1
TRNAU1AI	831495	183	26	770028	189036	0	1	0	0	50	53	18469280	8.81E-01	1
DCAF5	680338	289	33	2364908	690640	0	1	1	0	11	4	6311880	8.81E-01	1
TBX20	154022	926	5	1133148	331080	0	2	0	0	3	0	739768	8.81E-01	1
TNFRSF11I	179358	381	9	1048064	257032	0	2	1	0	50	67	20002216	8.81E-01	1
NPL	651460	626	22	850484	224992	0	1	0	0	50	47	17472480	8.81E-01	1
BCKDHA	1104034	376	38	1146320	318976	0	4	4	0	12	25	4126040	8.81E-01	1
ZNF442	1744621	671	-8	1637600	391600	0	6	1	0	50	86	23980872	8.81E-01	1
PDCD6IP	293448	409	9	2254192	618728	0	4	2	0	50	55	23177380	8.81E-01	1
CYP51A1	691564	461	26	1312572	359916	0	2	0	0	50	58	21780436	8.81E-01	1
ZNF449	255033	NaN	36	1335712	344252	0	2	0	0	6	5	2594884	8.81E-01	1
SF3B5	321795	374	17	223568	55180	0	2	1	0	1	1	206124	8.82E-01	1
PANK2	712060	170	37	1410116	449272	0	3	3	0	35	46	14263140	8.82E-01	1
FIG4	335902	637	29	2389828	613388	0	5	0	0	5	5	2979008	8.82E-01	1
GFI1B	1887171	461	34	855112	231044	0	1	1	0	50	44	17804984	8.82E-01	1

ISL1	27991	1083	-28	902816	239944	0	3	2	0	9	32	6472436	8.82E-01	1
CYP4V2	371112	606	-2	1371668	356356	0	3	1	0	25	27	9814208	8.82E-01	1
CHD8	710667	380	34	6600596	1840876	0	14	7	0	15	20	7348196	8.82E-01	1
BBS4	1110048	380	40	1381636	350660	0	2	1	0	50	73	18018584	8.82E-01	1
FPR3	435870	690	-35	877896	260592	0	2	1	0	50	83	20552236	8.82E-01	1
ABCC5	1425631	375	43	3712368	1063728	0	14	3	0	0	3	1063728	8.82E-01	1
RMND5A	431437	538	16	1027772	262372	0	2	0	0	50	60	20755156	8.82E-01	1
PRR21	315906	569	21	913852	334640	0	1	1	0	35	41	15467488	8.82E-01	1
TPRN	1722012	202	36	1624784	559276	0	4	3	0	4	6	2597020	8.82E-01	1
SPEF1	860065	241	43	611608	173372	0	1	1	0	16	30	7457844	8.82E-01	1
CEACAM:	1341589	499	16	1768608	522252	0	5	3	0	50	67	19496340	8.82E-01	1
HIP1	785864	326	62	2728028	728020	0	7	1	0	7	14	4382004	8.82E-01	1
NGRN	1036233	139	38	729088	217160	0	1	0	0	50	83	27989788	8.82E-01	1
TOM1	362269	241	-24	1272344	370240	0	4	1	0	28	46	12143872	8.82E-01	1
FA2H	612822	173	27	949096	273764	0	1	1	0	50	39	16913204	8.82E-01	1
GBP3	316278	598	5	1550736	401568	0	3	1	0	50	59	20056684	8.82E-01	1
HCLS1	289905	613	25	1275904	337844	0	5	3	0	3	7	1618020	8.82E-01	1
RCC2	399978	210	28	1342120	386972	0	2	0	0	28	38	15120032	8.82E-01	1
RCBTB2	387005	404	41	1426848	393024	0	2	2	0	13	16	6737656	8.83E-01	1
SEL1L3	247887	575	54	2939136	789252	0	6	1	0	34	26	11140664	8.83E-01	1
GLCCI1	179344	405	24	1359564	426132	0	2	0	0	19	21	8617336	8.83E-01	1
WDR24	1207790	194	33	1990040	580636	0	3	2	0	17	19	6751184	8.83E-01	1
TEKT4	167705	749	-6	1100396	322180	0	1	1	0	50	42	19395948	8.83E-01	1
TNFAIP8L:	285978	427	26	741192	208260	0	3	2	0	2	5	1160204	8.83E-01	1
OSTF1	94966	640	7	589892	137416	0	1	0	0	50	95	25771552	8.83E-01	1
ATP1B1	231221	416	19	793524	201852	0	1	1	0	50	49	18870136	8.83E-01	1
TRMT61A	900930	225	38	707728	234248	0	1	0	0	50	68	21985848	8.83E-01	1
BIRC2	463959	312	15	1604136	409044	0	3	1	0	50	55	19641232	8.83E-01	1
NEGR1	23816	1136	-23	909224	255964	0	2	1	0	50	84	21257116	8.83E-01	1
GABPB2	1614665	165	40	1135284	335352	0	2	2	0	36	47	15006468	8.83E-01	1
VSIG8	803074	403	7	1038452	316840	0	2	2	0	50	49	15043848	8.83E-01	1
FSTL5	9944	1215	-23	2220728	560344	0	11	4	0	17	35	6852288	8.83E-01	1
RGS12	570419	412	48	3690296	1096480	0	9	4	0	12	21	6353532	8.83E-01	1

OR5I1	4262	1044	-40	778572	229620	0	2	2	0	5	12	1863660	8.83E-01	1
SPNS1	1189140	257	55	1300112	446068	0	5	2	0	7	10	2316848	8.83E-01	1
ZNF23	383757	648	-2	1677472	394448	0	4	2	0	50	51	16919968	8.83E-01	1
OR9Q1	690075	744	-13	767180	233536	0	4	4	0	10	25	3703824	8.83E-01	1
OR6S1	709113	577	11	799576	264152	0	1	1	0	50	80	24629148	8.83E-01	1
SPATA13	255006	569	36	3218240	923464	0	3	0	0	1	0	1267716	8.83E-01	1
MAN1B1	1836975	202	38	1781780	514420	0	4	1	0	50	63	21774384	8.83E-01	1
SLC22A17	1220624	456	31	1310792	452476	0	1	1	0	6	13	4269864	8.83E-01	1
ANKS3	717235	194	35	1693492	479888	0	2	2	0	50	55	22982648	8.83E-01	1
YKT6	1492561	412	68	521896	141332	0	1	0	0	50	62	15008604	8.83E-01	1
SPSB3	1604603	288	36	904952	261304	0	1	1	0	50	49	20717064	8.84E-01	1
CD68	1957426	194	24	879676	279104	0	1	0	0	17	20	7764360	8.84E-01	1
PDCD11	544182	147	48	4777876	1369532	0	8	3	0	6	7	3223936	8.84E-01	1
KCNQ2	1314528	298	49	2248496	685300	0	4	0	0	0	0	685300	8.84E-01	1
CPNE4	238347	974	8	1473840	377004	0	3	3	0	30	30	12322228	8.84E-01	1
ASMT	NaN	NaN	NaN	968320	270560	0	1	0	0	50	116	39916144	8.84E-01	1
TMPRSS13	527704	576	45	1442868	430404	0	3	3	0	50	65	20508092	8.84E-01	1
TBX3	32369	548	-30	1839808	573872	0	8	1	0	11	21	4230348	8.84E-01	1
RPRD1B	582740	137	45	837312	236028	0	2	3	0	8	8	2316492	8.84E-01	1
SUCLG2	66926	891	7	1243508	340336	0	2	0	0	50	59	21334012	8.84E-01	1
CCDC109B	211934	479	26	880032	229620	0	1	0	0	50	43	17069132	8.84E-01	1
ZDHHC23	441946	487	29	1036316	298684	0	1	1	0	31	22	11529772	8.84E-01	1
IL12A	422983	616	22	658244	181204	0	1	0	0	50	54	15877956	8.84E-01	1
OGDHL	251871	545	15	2607344	724816	0	8	5	0	13	24	7619468	8.84E-01	1
PHF14	139683	990	-2	2342836	574940	0	4	2	0	50	62	19591392	8.84E-01	1
USP16	316514	178	57	2192960	519760	0	4	1	0	50	51	19604564	8.84E-01	1
TRPM8	297512	527	14	2887160	760060	0	6	3	0	50	59	19292352	8.84E-01	1
TPX2	890615	237	23	1960492	504452	0	6	0	0	10	11	3556796	8.84E-01	1
C2CD2L	1390854	195	58	1750452	573516	0	5	0	0	0	0	573516	8.84E-01	1
GPR135	168237	606	12	1153084	432896	0	2	0	0	4	3	1691356	8.85E-01	1
VPS4A	1202748	125	20	1137776	308296	0	2	1	0	20	22	7617688	8.85E-01	1
JPH1	236544	478	-15	1666080	478464	0	4	3	0	50	73	21645868	8.85E-01	1
SETBP1	15027	678	31	4141348	1196516	0	9	5	0	50	52	21407704	8.85E-01	1

FOXI2	447201	806	-35	763620	262728	0	1	0	0	50	62	17479956	8.85E-01	1
MAT2B	225938	681	11	898188	234960	0	1	0	0	50	64	19585340	8.85E-01	1
GRIK2	10349	1062	-25	2490932	674620	0	11	4	0	50	142	30413080	8.85E-01	1
MERTK	NaN	NaN	NaN	2568540	712356	0	6	2	0	50	118	40357940	8.85E-01	1
WDR5	307164	368	38	900324	230688	0	1	1	0	50	42	17032108	8.85E-01	1
ZNF707	2198262	229	33	947672	261304	0	1	1	0	50	57	21610980	8.85E-01	1
GOLGB1	300562	591	28	8458560	2076192	0	29	13	0	35	39	13273460	8.85E-01	1
LRCH3	520915	322	36	1834112	529372	0	6	0	0	0	0	529372	8.85E-01	1
VTN	1405196	214	10	1256324	310432	0	2	0	0	4	2	1076900	8.85E-01	1
C2orf57	994385	202	43	962268	308652	0	2	0	0	4	8	2679612	8.85E-01	1
SMEK1	340189	467	40	2140984	553580	0	4	1	0	50	72	21076268	8.85E-01	1
NIN	445811	257	34	5692084	1425068	0	14	4	0	37	51	14725584	8.85E-01	1
KIF9	816399	242	46	2075836	543968	0	4	1	0	50	52	21285952	8.85E-01	1
FN1	585272	533	13	6371688	1793172	0	19	11	0	45	58	17413028	8.85E-01	1
AGAP2	1160849	344	39	3054480	1019940	0	4	7	0	12	13	5013548	8.85E-01	1
CLTB	576954	347	45	603064	155216	0	1	1	0	9	9	2972600	8.85E-01	1
RNF20	112827	872	-13	2561064	647208	0	1	0	0	8	7	5043808	8.85E-01	1
ARSA	1073094	203	43	1237456	424352	0	2	2	0	41	49	17129652	8.85E-01	1
GALNT5	78933	837	8	2406204	649344	0	5	0	0	6	6	3381288	8.86E-01	1
OLFML2B	407279	388	15	1874696	561412	0	3	2	0	11	17	5260612	8.86E-01	1
PDGFB	1046938	205	60	637596	186900	0	1	2	0	20	21	6546128	8.86E-01	1
CACHD1	213591	581	-6	3185132	847636	0	8	4	0	29	35	12212580	8.86E-01	1
KIF5A	1347795	303	44	2721620	707728	0	6	0	0	3	4	2021724	8.86E-01	1
SIK3	421847	300	34	3258112	892136	0	5	2	0	50	47	18002564	8.86E-01	1
CNTLN	31539	761	-22	3732660	914208	0	13	2	0	4	9	2996096	8.86E-01	1
C4orf17	331308	847	9	934500	253116	0	2	1	0	50	70	18591744	8.86E-01	1
CPXCR1	1148	NaN	-19	769316	202564	0	2	1	0	50	99	22169900	8.86E-01	1
SYT9	97813	941	-3	1255612	346388	0	3	0	0	14	21	6157376	8.86E-01	1
ARID3A	2292363	206	44	1469212	463868	0	1	1	0	27	33	10566080	8.86E-01	1
BLK	476916	493	55	1308656	362764	0	2	2	0	50	61	14342172	8.86E-01	1
FGFRL1	689217	247	58	1239592	406196	0	2	1	0	41	49	16148160	8.86E-01	1
EPB41L2	122847	707	17	2607700	696692	0	5	3	0	50	58	20138208	8.86E-01	1
PRSS50	298503	495	41	968676	291564	0	2	0	0	50	70	18972664	8.86E-01	1

SLC37A1	440393	284	67	1389824	398008	0	2	1	0	41	47	18261020	8.86E-01	1
SMAP1	169371	764	11	1210044	332148	0	2	0	0	45	56	19159920	8.86E-01	1
MDM2	541243	228	32	1317556	320756	0	2	1	0	50	54	19475692	8.86E-01	1
HOXD4	245316	874	13	627984	196512	0	1	2	0	41	61	15014300	8.86E-01	1
GPR62	1013177	370	39	821648	360628	0	2	3	0	50	85	19762272	8.86E-01	1
PVRL4	689033	250	23	1265936	405484	0	3	4	0	17	24	6314728	8.86E-01	1
ATRX	548517	NaN	8	6552892	1579928	0	19	2	0	6	12	4451068	8.86E-01	1
CNTN3	5646	949	-21	2660388	728376	0	4	3	0	50	63	20612044	8.86E-01	1
EPDR1	100826	683	10	855112	260948	0	1	1	0	50	75	26634496	8.86E-01	1
CHST11	1030269	419	34	896408	243148	0	2	3	0	15	18	4919208	8.86E-01	1
PLD6	910162	218	11	612320	204700	0	1	1	0	50	78	21744124	8.86E-01	1
CRB1	212549	428	-23	3611264	943756	0	17	6	0	2	9	2333936	8.86E-01	1
TEX11	615286	NaN	4	2581712	603064	0	4	1	0	32	41	14463924	8.87E-01	1
LXN	196969	940	-12	593452	142400	0	1	1	0	30	30	8434352	8.87E-01	1
EFHD1	301517	211	37	605200	176576	0	1	0	0	17	19	5445732	8.87E-01	1
EFR3A	99296	999	6	2160564	567108	0	5	3	0	50	59	23627008	8.87E-01	1
EFNA3	1696720	214	44	607336	177644	0	1	0	0	50	84	23988348	8.87E-01	1
FOXP4	461476	388	43	1735144	515132	0	3	1	0	45	45	17266712	8.87E-01	1
NIPAL2	612198	462	32	960132	269136	0	1	1	0	50	53	24244668	8.87E-01	1
ECT2L	336688	610	20	2375944	605912	0	7	5	0	10	11	3534368	8.87E-01	1
LMX1B	106522	480	39	1072984	285512	0	2	0	0	42	72	20606704	8.87E-01	1
OR8K5	10101	1007	-38	765400	220364	0	4	2	0	3	5	1002140	8.87E-01	1
TBX1	728313	211	8	1481316	468852	0	3	0	0	29	37	13825260	8.87E-01	1
IL13RA1	221322	NaN	44	1139912	276256	0	2	1	0	50	81	24965924	8.87E-01	1
DPH1	1415256	294	27	1130300	345676	0	2	2	0	50	68	21536932	8.87E-01	1
MEGF8	792230	218	42	6878276	2196520	0	15	5	0	39	50	19147816	8.87E-01	1
TSSK2	487560	277	18	892492	257744	0	1	1	0	36	33	13333268	8.87E-01	1
TSPAN6	182582	NaN	-2	648276	170880	0	1	1	0	50	64	18681100	8.87E-01	1
GGN	1467279	253	0	1515136	587756	0	1	0	0	1	0	1387688	8.87E-01	1
WDR46	1456953	262	30	1556432	461020	0	4	3	0	50	71	20945260	8.87E-01	1
SLC38A1	752720	334	9	1288720	338912	0	2	1	0	50	69	22957372	8.87E-01	1
SLFN12L	NaN	NaN	NaN	1589896	405128	0	2	3	0	26	43	11982248	8.87E-01	1
RNPEPL1	837485	485	35	1253476	370952	0	2	1	0	50	53	18987616	8.87E-01	1

ROBO3	175852	743	14	3462456	1096836	0	6	1	0	8	8	4227856	8.87E-01	1
USP9Y	0	0	0	6674288	1703104	0	10	2	0	39	23	17595656	8.87E-01	1
MUC20	907437	365	47	1294416	449628	0	4	3	0	25	54	15178772	8.87E-01	1
WIPF3	400552	579	17	1160916	415452	0	2	0	0	14	19	6598104	8.87E-01	1
SLC34A1	1038665	261	59	1657536	539340	0	5	0	0	0	0	539340	8.87E-01	1
IRGQ	428891	209	-2	1447140	560700	0	2	2	0	50	54	21487092	8.87E-01	1
GK5	459081	395	25	1408336	353864	0	1	1	0	15	22	7005724	8.87E-01	1
SYT8	916434	481	33	994664	329656	0	2	2	0	45	53	15935628	8.87E-01	1
QPCTL	939821	180	44	929872	322892	0	4	0	0	3	7	1483096	8.87E-01	1
UTP23	483166	504	23	639376	172304	0	2	2	0	7	11	2315068	8.87E-01	1
UBR2	1007026	194	42	4795676	1180852	0	14	4	0	2	5	1640092	8.88E-01	1
SCUBE1	473937	440	30	2587408	673196	0	6	2	0	17	18	7161652	8.88E-01	1
USP26	88462	NaN	12	2329308	600216	0	4	3	0	19	19	8565360	8.88E-01	1
MEMO1	342215	385	18	793880	195088	0	1	1	0	50	67	25053144	8.88E-01	1
ABCG5	544941	480	35	1649704	490568	0	4	3	0	41	61	20014320	8.88E-01	1
RRAGD	408382	574	54	1042012	268424	0	1	0	0	8	6	1849776	8.88E-01	1
CYB5R4	87867	870	-12	1395520	341048	0	1	1	0	8	21	6467096	8.88E-01	1
CHM	57906	NaN	-6	1737992	441796	0	1	0	0	24	34	12642628	8.88E-01	1
PSG9	312803	148	-41	1086512	305092	0	2	1	0	15	37	6807788	8.88E-01	1
THOC6	1443513	234	49	893560	257744	0	1	1	0	50	48	18953440	8.88E-01	1
KCNK13	300869	604	12	1008904	305804	0	2	0	0	50	73	20592108	8.88E-01	1
AKR1A1	873800	161	27	838380	242436	0	1	2	0	50	63	21320128	8.88E-01	1
ASS1	502828	260	35	1098616	284444	0	1	1	0	50	62	22656196	8.88E-01	1
MFSD7	639827	489	12	1430052	476328	0	3	1	0	40	48	16311920	8.88E-01	1
SH3PXD2I	495571	267	42	2311864	661448	0	5	6	0	10	13	3358860	8.89E-01	1
DDX43	723493	232	21	1704172	443576	0	2	2	0	50	68	20465372	8.89E-01	1
YBX1	650376	461	21	834820	236384	0	1	0	0	50	60	20231480	8.89E-01	1
MTMR10	279767	591	22	2032048	529016	0	3	1	0	41	41	20057396	8.89E-01	1
ANKRD17	265492	747	19	6518716	1965476	0	13	6	0	38	38	17495264	8.89E-01	1
CLEC4F	596622	629	24	1522612	395516	0	3	1	0	50	44	16827408	8.89E-01	1
LRIT3	212757	462	28	1570672	472412	0	5	3	0	13	17	4637968	8.89E-01	1
HSD17B1	1884163	177	38	804916	270560	0	1	1	0	50	68	23826724	8.89E-01	1
NEDD4	259242	271	-1	3697772	966184	0	6	4	0	39	33	14654384	8.89E-01	1

ZNF610	526380	960	-32	1200788	299752	0	5	2	0	50	87	21714576	8.89E-01	1
TARSL2	255024	469	2	2123540	528304	0	4	2	0	50	57	23664744	8.89E-01	1
C8B	38776	713	-12	1548956	396940	0	3	1	0	15	40	12821696	8.89E-01	1
MAD2L2	682357	264	32	564616	148808	0	1	1	0	8	13	3283032	8.89E-01	1
CD99L2	287647	NaN	12	699540	185832	0	1	1	0	50	74	19695700	8.89E-01	1
TNXB	2242514	203	33	12062704	3833408	0	26	7	0	4	7	4563564	8.89E-01	1
FDXR	1027517	320	20	1246712	398008	0	3	2	0	10	17	5209348	8.89E-01	1
FOXE3	193133	347	8	738700	286580	0	1	2	0	28	42	11461064	8.89E-01	1
AK7	328475	560	14	1914212	480244	0	5	6	0	14	22	5637972	8.89E-01	1
OR2AE1	1535089	438	36	797440	240656	0	1	0	0	50	61	21736292	8.89E-01	1
PREX1	752875	360	47	4313296	1174088	0	11	3	0	50	60	23469300	8.89E-01	1
CTDSP2	1051368	243	40	702032	199360	0	1	0	0	50	63	19246784	8.89E-01	1
IGFBP4	1202660	149	21	645784	196868	0	1	1	0	50	67	18633040	8.89E-01	1
KRTAP13-	4608	1035	-69	434676	120684	0	1	1	0	39	55	9409080	8.90E-01	1
FBXO7	371339	401	3	1350664	400856	0	2	1	0	50	51	19098332	8.90E-01	1
HCN3	1574928	194	45	1882528	632612	0	4	3	0	50	57	20795740	8.90E-01	1
SLC22A11	1970283	411	54	1364904	438948	0	3	1	0	50	67	19338632	8.90E-01	1
ADAR	1927515	403	36	3117136	873980	0	11	1	0	2	5	1751164	8.90E-01	1
FBXL16	1207790	194	33	1168036	391244	0	2	1	0	17	19	6751184	8.90E-01	1
LANCL3	108134	NaN	13	1105024	335708	0	1	0	0	6	3	2249564	8.90E-01	1
NDRG1	234084	322	0	1045928	283732	0	1	0	0	50	63	28611720	8.90E-01	1
SLCO2A1	547213	487	21	1636176	482736	0	5	1	0	50	66	17217228	8.90E-01	1
PCDHGB4	662513	568	2	2284808	700252	0	8	4	0	36	67	17833108	8.90E-01	1
SNX11	1195543	255	11	697404	196512	0	1	0	0	31	39	12335044	8.90E-01	1
HIST1H1D	390169	544	57	540052	175508	0	1	2	0	36	34	7770412	8.90E-01	1
LILRA2	819283	601	-4	1217520	365256	0	3	3	0	23	60	10718448	8.90E-01	1
DLG4	1732485	316	18	2022436	562124	0	4	1	0	17	14	5459260	8.90E-01	1
ARHGAP1	75227	678	22	2087584	527948	0	2	0	0	14	14	5768624	8.90E-01	1
KCNN2	7106	987	0	1463160	425064	0	5	2	0	14	26	6280196	8.90E-01	1
UBE2L6	923607	249	32	398364	110004	0	2	2	0	3	7	991816	8.90E-01	1
PPAP2B	39932	563	7	799220	221788	0	1	1	0	50	80	27848456	8.90E-01	1
TPI1	2822601	232	32	739056	208260	0	1	1	0	50	62	20662240	8.90E-01	1
TRIM2	222401	350	14	1971528	551088	0	4	3	0	50	59	21507740	8.90E-01	1

LETM1	851373	239	44	1881104	545392	0	2	2	0	50	72	23546552	8.90E-01	1
COL6A3	423594	399	10	7980452	2435752	0	28	13	0	2	19	5477772	8.90E-01	1
KCTD3	82180	597	-30	2102892	584196	0	8	2	0	50	91	19692496	8.90E-01	1
TAS2R46	371136	676	4	775724	217516	0	1	1	0	50	58	20325464	8.91E-01	1
C1QL4	2137968	175	37	573872	196156	0	1	2	0	8	15	4118564	8.91E-01	1
DLK2	1278255	230	56	959420	292276	0	1	0	0	50	54	19955936	8.91E-01	1
ZC3H12A	446254	357	25	1473840	469920	0	5	3	0	2	4	1202212	8.91E-01	1
OR2C3	272980	927	-14	786404	246352	0	3	3	0	9	13	2441804	8.91E-01	1
LARP6	250598	339	10	1297264	379496	0	2	1	0	50	54	19786124	8.91E-01	1
ZC3H12B	321325	NaN	10	2122472	578500	0	4	0	0	9	13	5384144	8.91E-01	1
SLC5A1	451895	458	-26	1704528	485940	0	4	3	0	21	26	7887892	8.91E-01	1
PDK4	195488	393	-3	1075832	286936	0	1	0	0	50	50	22095852	8.91E-01	1
SLITRK1	9008	874	-31	1717700	515488	0	7	1	0	50	87	19973380	8.91E-01	1
NAT2	322678	627	-26	736208	200428	0	1	0	0	31	56	15832744	8.91E-01	1
OR1J4	373314	646	3	762552	242436	0	1	0	0	50	63	22349324	8.91E-01	1
OR5P2	103028	823	-1	790320	244572	0	1	2	0	21	26	9293380	8.91E-01	1
DGAT1	2525749	200	28	1275192	359916	0	3	2	0	4	8	1894276	8.91E-01	1
KLHDC10	428896	266	37	1139556	318264	0	1	0	0	50	39	17425488	8.91E-01	1
HHLA2	281982	859	7	1088648	275188	0	2	1	0	50	73	21583212	8.91E-01	1
DHCR7	507631	626	51	1202924	352084	0	2	1	0	18	11	4290868	8.91E-01	1
SDK1	48060	954	-23	5585284	1696340	0	14	5	0	9	19	5457480	8.91E-01	1
ZNF396	306655	499	43	865436	221788	0	2	1	0	50	78	19811756	8.91E-01	1
FHOD3	336896	728	61	3672140	1046284	0	6	2	0	26	39	13026396	8.91E-01	1
PHYH	246468	433	33	894628	227840	0	1	1	0	50	59	23759084	8.91E-01	1
TRIM44	605264	411	-6	909580	212888	0	1	0	0	50	48	19517344	8.91E-01	1
SHROOM4	36907	NaN	11	3755800	1067288	0	7	1	0	5	13	6103620	8.92E-01	1
NCOA5	595034	381	53	1461380	426844	0	4	4	0	17	37	7854428	8.92E-01	1
TJAP1	1278255	230	56	1398368	431116	0	2	0	0	50	54	19955936	8.92E-01	1
USP2	1054477	263	40	1651484	500536	0	4	3	0	50	80	23473572	8.92E-01	1
ZNF778	1307930	254	38	1855116	500892	0	4	1	0	50	57	20720624	8.92E-01	1
GK	82798	NaN	34	1496624	383056	0	2	0	0	40	44	17244640	8.92E-01	1
GPAA1	2720766	211	27	1518340	523676	0	3	2	0	25	30	10299080	8.92E-01	1
KRT5	1280474	464	36	1482740	445000	0	6	2	0	3	5	1590964	8.92E-01	1

SLC16A9	135396	527	-16	1286228	369172	0	2	2	0	17	16	6540788	8.92E-01	1
NFE2L1	1205950	255	20	1908160	585620	0	3	3	0	5	6	2531160	8.92E-01	1
C2orf44	243145	226	33	1798868	527236	0	5	1	0	33	43	11192996	8.92E-01	1
AP3M1	776926	227	49	1085088	291564	0	1	1	0	50	44	19288436	8.92E-01	1
ABCB4	225315	837	8	3316852	924176	0	9	1	0	40	46	16648696	8.92E-01	1
NWD1	947233	293	35	3553236	1092564	0	11	4	0	18	31	9222536	8.92E-01	1
TAS2R5	200522	844	14	743684	217516	0	1	1	0	50	60	17687504	8.92E-01	1
TRUB1	188808	343	9	887508	265932	0	3	2	0	6	19	3921340	8.92E-01	1
CALCOCO	1501666	272	56	1780356	496620	0	4	1	0	25	33	11784668	8.92E-01	1
TSR1	780080	271	29	2091856	549308	0	4	0	0	6	7	3452132	8.92E-01	1
TRPM4	2151496	267	43	3043088	954436	0	7	3	0	50	72	21458256	8.92E-01	1
EIF3H	483437	504	11	930228	232824	0	1	0	0	50	62	20131444	8.92E-01	1
VSTM2L	604196	440	54	510860	158776	0	1	1	0	24	57	11546504	8.92E-01	1
SELE	269222	597	12	1606984	406196	0	5	2	0	50	64	18409116	8.92E-01	1
LGR5	250555	516	-34	2316492	667500	0	10	6	0	7	13	2280536	8.92E-01	1
PNPT1	501257	354	17	2071564	557852	0	7	0	0	0	0	557852	8.92E-01	1
ATP6V1B2	158991	511	-4	1320760	375224	0	1	0	0	3	0	1182632	8.92E-01	1
API5	349378	924	-23	1379856	357780	0	4	2	0	50	95	21664380	8.93E-01	1
CHGA	325312	387	30	1175868	321468	0	1	1	0	50	42	21567904	8.93E-01	1
B3GNT7	1124713	359	42	984696	307584	0	2	2	0	50	75	18162764	8.93E-01	1
FLVCR1	326908	445	13	1387688	432184	0	2	3	0	50	47	18388112	8.93E-01	1
B3GNT6	368988	395	37	922752	316128	0	1	3	0	8	5	2893924	8.93E-01	1
TAF2	272257	591	13	3169468	782132	0	8	3	0	50	67	18638736	8.93E-01	1
CADPS2	61224	1046	-3	3443232	883236	0	9	3	0	50	48	16856956	8.93E-01	1
RPUSD1	1187310	273	39	777504	246708	0	1	0	0	50	70	23301268	8.93E-01	1
PRKACB	205640	919	-13	1107516	274476	0	2	0	0	50	59	18889360	8.93E-01	1
PRMT1	1733181	195	36	987188	249556	0	1	1	0	9	8	4296208	8.93E-01	1
NEK6	483948	406	41	991460	250624	0	2	2	0	12	13	4070860	8.93E-01	1
MLLT1	1294183	417	20	1438952	402280	0	3	1	0	50	60	18011108	8.93E-01	1
PCDHA10	NaN	NaN	NaN	2431836	774300	0	8	5	0	26	45	12351420	8.93E-01	1
PRDM7	822636	221	26	1334288	288004	0	2	1	0	50	59	21945976	8.93E-01	1
RAD51AP1	275215	908	30	933432	236028	0	1	0	0	50	52	20960924	8.93E-01	1
COL27A1	224550	425	3	4672856	1545040	0	6	5	0	37	26	14881512	8.93E-01	1

SLC22A13	301168	437	22	1379144	430048	0	4	0	0	4	6	2137068	8.93E-01	1
STC1	321715	555	14	632256	175152	0	1	2	0	13	21	5172680	8.93E-01	1
NTRK3	124519	730	-57	2399796	646140	0	5	3	0	18	41	11566796	8.93E-01	1
CYP2B6	1041792	375	14	1255256	357424	0	7	5	0	20	36	5344628	8.93E-01	1
TAS2R43	371136	676	4	784268	213244	0	1	1	0	50	58	20325464	8.93E-01	1
PDHB	567081	265	35	938060	255964	0	1	0	0	50	52	21882608	8.93E-01	1
PDLIM2	757216	224	45	1007480	292276	0	1	1	0	48	49	21352880	8.93E-01	1
MCL1	1475649	152	31	885728	259168	0	2	3	0	7	11	2967260	8.93E-01	1
ZNF260	644122	889	-48	1070848	252404	0	3	0	0	3	4	1145252	8.93E-01	1
HAVCR1	138350	1207	11	919192	284444	0	1	0	0	50	51	21035328	8.93E-01	1
OR5B12	190469	812	-10	784624	228908	0	1	0	0	50	71	21027140	8.93E-01	1
MED16	2168802	198	28	2197588	679604	0	3	3	0	50	73	22578944	8.93E-01	1
GNAT3	118115	385	-32	938060	227128	0	2	0	0	50	87	21817816	8.93E-01	1
HRC	2011861	267	47	1841588	424708	0	7	4	0	6	12	2720552	8.93E-01	1
MTIF2	964970	349	25	1888936	501248	0	8	1	0	11	18	4282680	8.93E-01	1
C12orf56	475083	294	-12	1611256	436100	0	3	2	0	50	51	19738776	8.93E-01	1
ANO3	20260	970	-20	2629416	627984	0	6	1	0	18	23	7387000	8.93E-01	1
SAC3D1	3267059	207	46	847636	310076	0	1	0	0	34	42	13459292	8.93E-01	1
C3	740979	259	28	4315432	1191176	0	17	8	0	7	14	3839816	8.94E-01	1
CACNG1	300354	491	-30	569600	159844	0	1	1	0	50	89	22343272	8.94E-01	1
MTHFD1	640692	221	45	2426852	687436	0	6	2	0	50	64	22356088	8.94E-01	1
ZNF404	464802	286	11	1436104	330368	0	6	0	0	3	5	1651128	8.94E-01	1
CFHR3	200038	941	-27	866504	217516	0	2	1	0	50	99	23208708	8.94E-01	1
MBD3	2122824	221	35	754720	204344	0	1	1	0	50	54	18221504	8.94E-01	1
CHD7	248521	572	45	7673936	2085448	0	26	10	0	43	54	14720600	8.94E-01	1
CEACAM1	749323	335	-51	766824	227484	0	2	1	0	50	84	18078392	8.94E-01	1
ADAM20	333836	706	26	1968680	525100	0	2	1	0	50	48	24682192	8.94E-01	1
KRT14	1494501	323	11	1198652	348880	0	2	0	0	20	34	10955900	8.94E-01	1
ASAH1	366238	474	20	1181920	305804	0	2	0	0	7	11	3334296	8.94E-01	1
JMJD7	NaN	NaN	NaN	816664	231044	0	1	1	0	50	117	39876628	8.94E-01	1
PASD1	153115	NaN	3	2025640	518336	0	3	2	0	26	25	12104712	8.94E-01	1
LHX4	463883	254	13	1001784	272340	0	3	2	0	9	28	6325764	8.94E-01	1
FAM124A	175682	518	37	1440376	444644	0	6	3	0	8	14	2479184	8.94E-01	1

SMAD7	160419	191	79	1049844	331080	0	3	2	0	9	19	4240672	8.94E-01	1
GLB1	520588	372	29	1762912	473480	0	3	2	0	50	65	22749824	8.94E-01	1
PGAM5	724450	495	35	765044	242080	0	1	2	0	19	29	9337168	8.94E-01	1
SUCLA2	322899	636	35	1206840	324672	0	2	1	0	34	29	10524072	8.94E-01	1
SCNN1G	547445	631	23	1683168	450696	0	3	2	0	45	44	16098320	8.94E-01	1
REPIN1	315538	514	32	1520120	493060	0	2	0	0	12	10	4964420	8.94E-01	1
HRFAM7	313450	686	-8	1064796	292632	0	2	0	0	50	59	21348252	8.94E-01	1
GSG2	NaN	NaN	NaN	1961560	600572	0	4	0	0	7	14	4288732	8.94E-01	1
PIM1	705207	236	50	801000	226416	0	1	0	0	50	60	20782924	8.94E-01	1
RELB	1135715	208	25	1433968	462800	0	3	3	0	30	44	12265624	8.94E-01	1
OR13F1	146816	930	-33	784268	238876	0	2	0	0	50	86	17955928	8.94E-01	1
EPM2AIP1	392784	473	21	1515848	432184	0	5	0	0	5	11	3117848	8.94E-01	1
TANC1	311739	405	27	4720204	1357784	0	12	8	0	17	24	7772192	8.94E-01	1
PRKCG	831741	360	-41	1801360	507656	0	5	4	0	32	51	10483488	8.94E-01	1
CHMP4B	761783	146	53	589536	148452	0	1	1	0	18	29	6608072	8.95E-01	1
STK16	694442	193	44	784624	221432	0	1	1	0	50	75	22905396	8.95E-01	1
C9orf91	285717	202	14	872200	260948	0	1	0	0	31	25	10464976	8.95E-01	1
GNL1	2080582	271	32	1547888	447136	0	2	1	0	24	27	11479576	8.95E-01	1
TAS1R2	636814	532	25	2097196	616592	0	4	4	0	24	51	14505576	8.95E-01	1
SLC6A9	875223	214	39	1819516	539696	0	5	2	0	50	67	19898264	8.95E-01	1
UNC93B1	1357582	356	31	1500540	459240	0	3	2	0	50	65	20667936	8.95E-01	1
PDE8B	276128	451	26	2320764	609828	0	4	1	0	7	6	2434328	8.95E-01	1
REXO4	1827885	416	28	1088292	296904	0	1	1	0	50	47	17145316	8.95E-01	1
RAD23A	1886809	335	40	935568	264864	0	3	3	0	22	36	7729472	8.95E-01	1
SLC22A7	1293543	363	51	1336068	463512	0	3	3	0	31	44	12099728	8.95E-01	1
IQUB	89539	779	-21	2071564	517268	0	5	2	0	50	67	19760848	8.95E-01	1
PMPCA	1413299	233	44	1344968	395872	0	2	1	0	50	69	23563996	8.95E-01	1
LAT2	997452	202	51	664296	168744	0	1	2	0	14	15	4682112	8.95E-01	1
MYC	406323	204	23	1136352	330012	0	2	2	0	29	35	10794632	8.95E-01	1
ZNF691	803442	334	27	722324	195088	0	2	1	0	4	8	1741196	8.95E-01	1
CSNK1D	2856070	193	24	1096480	312212	0	3	3	0	4	3	1196516	8.95E-01	1
WDR33	605495	187	43	3748324	1059812	0	12	3	0	50	70	22042452	8.95E-01	1
SIPA1L2	94875	598	-14	4365272	1240660	0	13	3	0	5	11	3222512	8.95E-01	1

PDE6B	639827	489	12	2250632	580636	0	5	3	0	40	48	16311920	8.95E-01	1
TBC1D24	2085452	321	49	1392672	403704	0	1	1	0	50	76	21050280	8.95E-01	1
JPH4	1250373	553	35	1512288	520116	0	3	2	0	50	82	25432996	8.95E-01	1
MCF2	68546	NaN	-2	2770036	646496	0	7	2	0	3	2	1375228	8.95E-01	1
MBTD1	787991	267	-10	1664656	414740	0	6	0	0	20	25	6652572	8.95E-01	1
WDR70	302307	592	15	1738348	437168	0	4	3	0	33	51	15660440	8.95E-01	1
SLA	NaN	NaN	NaN	822360	234960	0	1	0	0	50	116	39880544	8.96E-01	1
SLC39A9	857161	395	40	768960	243504	0	1	1	0	50	64	20875484	8.96E-01	1
C17orf104	1297937	254	27	2440380	642936	0	6	1	0	50	53	19790752	8.96E-01	1
PLCH2	339388	228	38	3547540	1082240	0	8	2	0	30	30	12300512	8.96E-01	1
CYP4F11	843515	575	12	1361344	370952	0	4	2	0	6	10	3199728	8.96E-01	1
ANO8	842088	211	43	3057684	965472	0	8	3	0	50	61	21639816	8.96E-01	1
DYNC1I1	184459	747	-7	1692424	445712	0	2	1	0	5	8	2959072	8.96E-01	1
MYBPHL	988224	302	29	902460	271272	0	1	0	0	27	37	12111476	8.96E-01	1
PLCXD2	188186	322	-3	1010684	286936	0	1	2	0	27	29	11820268	8.96E-01	1
ZBTB41	222912	614	-19	2379148	577076	0	8	0	0	0	0	577076	8.96E-01	1
KIAA0020	137713	763	16	1728380	423640	0	5	0	0	6	7	2629416	8.96E-01	1
LOXL1	337765	439	34	1419728	448204	0	2	2	0	50	54	20944548	8.96E-01	1
ZNF639	410660	641	2	1282668	291564	0	2	1	0	50	64	22262104	8.96E-01	1
SUSD2	744077	202	44	2077972	620864	0	3	0	0	13	12	6175888	8.96E-01	1
CCBE1	257439	491	24	1048064	300820	0	1	0	0	50	52	20269216	8.96E-01	1
ZNF880	526380	960	-32	1498760	368104	0	3	1	0	8	12	3141700	8.96E-01	1
FOXD4L6	NaN	NaN	NaN	1001784	337488	0	1	0	0	50	116	39983072	8.96E-01	1
FSCN1	1965381	192	56	1233540	366324	0	5	3	0	8	14	3336076	8.96E-01	1
COCH	428678	454	11	1419016	393380	0	3	1	0	50	67	22131452	8.96E-01	1
NDUFB10	1747301	183	46	463868	105376	0	1	2	0	6	6	1391960	8.96E-01	1
RNF41	2878057	171	43	817020	223212	0	1	0	0	40	74	21948824	8.96E-01	1
SEC24C	1009357	191	42	2763272	839092	0	8	3	0	2	4	1298332	8.96E-01	1
LIPT1	504421	770	21	952300	251336	0	1	0	0	38	36	14850184	8.96E-01	1
TXNDC2	431025	168	34	1398724	386972	0	3	0	0	20	32	9707764	8.96E-01	1
GTPBP3	842088	211	43	1269140	445000	0	2	1	0	50	61	21639816	8.96E-01	1
MDC1	2115149	171	41	5112516	1643652	0	11	6	0	31	37	12513044	8.96E-01	1
EARS2	528849	628	48	1316488	398720	0	2	0	0	41	43	15675036	8.96E-01	1

PVR	749323	335	-51	1072272	325740	0	4	0	0	2	2	835176	8.97E-01	1
RRAD	942986	142	34	766112	245284	0	1	0	0	50	65	21541916	8.97E-01	1
GPI	671792	292	-2	1562128	430760	0	3	2	0	50	67	21104036	8.97E-01	1
HSDL1	550248	435	13	842296	235316	0	1	0	0	43	46	16971944	8.97E-01	1
PITPNM3	255838	313	8	2479184	725884	0	3	2	0	50	54	20769752	8.97E-01	1
TOP2B	216871	636	-6	4279120	1067288	0	6	2	0	7	15	7396968	8.97E-01	1
PAQR8	372545	431	33	872556	269136	0	3	2	0	4	10	2361348	8.97E-01	1
ANO9	2206786	344	58	2038812	566040	0	5	4	0	27	50	14299808	8.97E-01	1
SLC1A1	228047	813	19	1340340	388752	0	3	2	0	5	3	1122824	8.97E-01	1
ANKH	503265	410	8	1259172	367392	0	2	1	0	50	76	26025024	8.97E-01	1
PCDHGA9	NaN	NaN	NaN	2331088	735140	0	5	4	0	50	120	40380724	8.97E-01	1
UGT1A6	NaN	NaN	NaN	1349952	374868	0	2	0	0	50	116	40020452	8.97E-01	1
SNX15	3209258	207	49	866148	264864	0	2	1	0	50	90	24481408	8.97E-01	1
SLC25A25	1898149	195	32	1607340	464580	0	2	1	0	50	88	21996172	8.97E-01	1
KLC1	816643	204	41	1687796	400144	0	1	3	0	50	56	19472844	8.97E-01	1
PLEKHH3	1740597	177	36	1918128	677112	0	8	1	0	0	1	677112	8.97E-01	1
ATG16L1	427221	155	31	1591676	428980	0	4	2	0	39	43	13588164	8.97E-01	1
GCAT	1385064	203	66	1117840	351728	0	1	0	0	6	2	1790680	8.97E-01	1
ZNF195	560134	470	28	1649348	390532	0	2	0	0	43	44	20071636	8.97E-01	1
KLK3	655673	589	25	734784	221076	0	1	1	0	50	77	24644100	8.97E-01	1
THAP8	980636	320	19	662160	231756	0	1	0	0	50	64	18556500	8.97E-01	1
PGM2L1	629890	143	29	1625852	425776	0	2	2	0	50	39	16620216	8.97E-01	1
UBIAD1	582356	524	31	817020	273408	0	2	2	0	19	36	8595264	8.97E-01	1
ABCC1	409366	264	33	3912440	1124248	0	11	7	0	50	59	18384908	8.97E-01	1
DUSP7	1045639	224	36	1029196	327164	0	3	4	0	17	18	4270932	8.97E-01	1
SPATC1	2650698	211	27	1422932	493060	0	2	0	0	11	12	5322556	8.98E-01	1
ELN	1047492	240	54	1823076	654684	0	6	1	0	10	25	6330036	8.98E-01	1
B4GALT6	293258	554	32	1009260	252048	0	1	0	0	50	66	25208004	8.98E-01	1
ERCC5	196444	475	33	3048784	816308	0	6	1	0	1	1	1547176	8.98E-01	1
SLC6A19	657890	541	24	1613392	470276	0	6	4	0	7	23	4939856	8.98E-01	1
NAP1L4	550048	221	38	1031332	237452	0	1	1	0	50	55	20617740	8.98E-01	1
ACVR1C	79510	911	7	1265224	351728	0	2	1	0	50	56	19494204	8.98E-01	1
IRX6	130838	706	-26	1102176	351372	0	4	3	0	5	8	1938776	8.98E-01	1

BCL2L1	864206	213	21	585264	168744	0	1	1	0	12	11	3125324	8.98E-01	1
RIMS1	35104	964	-12	4474208	1263088	0	12	3	0	32	51	17702456	8.98E-01	1
PRKCB	513265	610	21	1919196	474192	0	7	2	0	32	41	10903568	8.98E-01	1
SFTA3	58829	624	-18	246352	70844	0	1	1	0	1	2	381632	8.98E-01	1
RUFY1	1583299	229	17	1857252	487008	0	4	2	0	17	51	11199760	8.98E-01	1
STAG1	245682	512	22	3349248	825564	0	18	5	0	2	7	1961204	8.98E-01	1
EIF2B3	839426	185	24	1211824	323960	0	1	0	0	21	12	6622668	8.98E-01	1
EXT2	291324	616	7	2019232	551444	0	2	2	0	50	65	22851284	8.98E-01	1
ZBED4	700457	276	-4	2906740	848348	0	8	4	0	50	67	21014680	8.98E-01	1
EDA2R	28261	NaN	-6	770740	207548	0	1	0	0	50	70	21390972	8.98E-01	1
DPCR1	2483101	261	36	3396596	1078324	0	1	0	0	9	0	2968328	8.98E-01	1
FEZF2	213716	935	-3	1140268	350660	0	2	1	0	50	69	21505960	8.98E-01	1
TSPAN33	787448	408	51	733360	206480	0	1	1	0	50	85	25701064	8.98E-01	1
NLRC3	775547	224	39	2644012	845144	0	5	1	0	5	4	2473132	8.98E-01	1
C8orf74	110362	481	9	741548	218584	0	3	3	0	11	22	4377376	8.98E-01	1
ANKRD6	403943	609	43	1845148	531152	0	4	1	0	50	65	20118272	8.98E-01	1
NEK7	114791	402	-13	808476	200784	0	4	1	0	1	1	528304	8.99E-01	1
CCDC150	165315	393	3	2946256	699896	0	3	0	0	15	10	7212916	8.99E-01	1
FYB	171734	367	-6	2165904	570312	0	4	1	0	35	31	14601696	8.99E-01	1
COL17A1	455010	271	19	3872924	1161628	0	7	3	0	31	44	14032452	8.99E-01	1
USP11	807577	NaN	47	2483456	690640	0	5	4	0	18	18	7274148	8.99E-01	1
SEC24B	202883	438	21	3227496	938772	0	8	2	0	24	34	10648316	8.99E-01	1
PLD1	180676	440	23	2841236	714136	0	7	4	0	23	27	8577820	8.99E-01	1
BEND3	208838	417	30	2068004	600928	0	8	0	0	2	5	1715564	8.99E-01	1
WDR81	1447501	189	32	4768264	1558568	0	7	3	0	4	4	3275200	8.99E-01	1
PTGES2	1898149	195	32	945180	291564	0	2	2	0	50	88	21996172	8.99E-01	1
ZNF677	427680	921	-46	1534716	352440	0	4	0	0	36	51	13351780	8.99E-01	1
DLC1	73786	559	-28	3961568	1092208	0	15	6	0	50	94	20608484	8.99E-01	1
NOM1	386240	474	22	2194028	609472	0	9	1	0	3	8	2335360	8.99E-01	1
C16orf59	2085452	321	49	1073340	357780	0	2	2	0	50	76	21050280	8.99E-01	1
HNRNPR	1032187	163	25	1644008	439660	0	4	1	0	50	98	21204072	8.99E-01	1
COLEC12	587478	572	14	1898192	520828	0	3	2	0	36	45	12892540	8.99E-01	1
WBSCR17	38269	1094	-34	1525816	436100	0	6	1	0	7	10	2347820	8.99E-01	1

TMCC3	242132	576	26	1206840	337488	0	4	2	0	4	4	1510152	8.99E-01	1
CYFIP2	266925	1129	33	3285880	860096	0	7	0	0	3	1	1440732	8.99E-01	1
AGXT2	243997	675	23	1342120	363476	0	2	2	0	37	36	14953780	8.99E-01	1
DHX16	2115149	171	41	2663236	791744	0	7	0	0	3	8	3342128	8.99E-01	1
GSG1L	592259	575	10	849416	242080	0	2	0	0	50	69	20967688	9.00E-01	1
PAN2	2869106	184	52	3096844	860096	0	14	7	0	5	14	3286236	9.00E-01	1
DDRGK1	909603	255	55	806696	238876	0	1	2	0	38	44	13303008	9.00E-01	1
LZTS2	1020226	247	40	1602712	561056	0	3	2	0	50	62	20159924	9.00E-01	1
MRPL22	516166	461	38	548952	142044	0	1	2	0	16	22	5399452	9.00E-01	1
GZMH	897577	582	3	629408	181204	0	1	0	0	50	79	22165272	9.00E-01	1
WDR77	397926	461	18	885372	254184	0	1	1	0	50	56	21248572	9.00E-01	1
LCORL	271221	1064	26	1694560	458528	0	1	0	0	50	30	21937076	9.00E-01	1
TMEM45B	632501	231	-2	713780	191884	0	1	1	0	50	67	20666512	9.00E-01	1
CDK5R1	636808	587	14	748668	238164	0	1	2	0	19	22	7552184	9.00E-01	1
SNX27	1294954	173	29	1376652	367392	0	4	0	0	11	25	5995752	9.00E-01	1
MYO1H	438867	470	39	2709516	696336	0	5	2	0	50	70	21741988	9.00E-01	1
ZNF660	435137	541	40	855112	208616	0	1	0	0	50	60	18467144	9.00E-01	1
DCST2	1654795	214	40	1949456	596656	0	5	4	0	50	87	24762648	9.00E-01	1
FUT11	1009357	191	42	1216096	374156	0	2	0	0	5	12	4589196	9.00E-01	1
GPRC5B	258899	641	9	988968	317196	0	1	0	0	50	53	21947756	9.00E-01	1
HAUS1	396070	187	62	741904	188324	0	1	0	0	50	78	22105820	9.00E-01	1
PLCD4	986502	179	45	1987904	520828	0	4	1	0	10	9	3842308	9.00E-01	1
MARVELD	446645	530	10	1503744	478464	0	3	2	0	50	64	19453620	9.00E-01	1
KDM4B	993857	258	26	2792464	812036	0	3	2	0	50	51	20205136	9.00E-01	1
EEF2	2173603	207	30	2187620	626560	0	4	5	0	33	49	14189804	9.00E-01	1
CHRDL1	108458	NaN	14	1218944	298684	0	2	0	0	30	47	15672544	9.00E-01	1
TIFA	182788	351	37	478464	118548	0	1	1	0	7	11	2551096	9.00E-01	1
MAGT1	549266	NaN	20	962624	257032	0	2	0	0	19	32	7840900	9.01E-01	1
TMEM185A	206117	NaN	9	898900	251336	0	1	1	0	50	63	23523768	9.01E-01	1
TM7SF3	408250	320	26	1462448	414028	0	2	0	0	18	19	8077640	9.01E-01	1
PGC	429684	341	47	1247068	363476	0	2	2	0	50	57	19608480	9.01E-01	1
HNRNPM	726124	214	1	1893564	514776	0	2	3	0	50	67	22455056	9.01E-01	1
PAFAH1B1	612041	184	14	1078324	276968	0	1	0	0	50	48	19587120	9.01E-01	1

LONP2	200017	467	-14	2163412	631544	0	6	5	0	30	33	9936672	9.01E-01	1
TAPT1	165112	581	29	1481316	396228	0	2	1	0	50	55	21676484	9.01E-01	1
RGPD3	NaN	NaN	NaN	4532592	1199364	0	13	2	0	9	18	5556804	9.01E-01	1
TBX19	410552	299	20	1134928	335708	0	4	1	0	4	11	2765052	9.01E-01	1
OS9	1245394	344	46	1728736	471344	0	3	2	0	32	63	15576424	9.01E-01	1
MTUS2	267724	784	-5	3521552	1017448	0	8	3	0	5	6	3202932	9.01E-01	1
SLC18A1	158991	511	-4	1368108	377004	0	1	0	0	3	0	1182632	9.01E-01	1
GLRA4	485629	NaN	43	1250628	328944	0	1	1	0	50	50	18367108	9.01E-01	1
VWA3A	577505	269	45	3136004	803848	0	6	1	0	15	16	7625876	9.01E-01	1
TLE2	1106128	195	32	1968680	573160	0	4	3	0	50	69	18666504	9.01E-01	1
FBLN2	375523	199	36	2059460	559276	0	5	1	0	27	34	11735540	9.01E-01	1
RFX2	1209062	392	33	1859388	532932	0	2	1	0	17	18	6366704	9.01E-01	1
TSSK1B	267968	620	13	915988	263084	0	3	2	0	16	26	5661824	9.01E-01	1
ITGA10	NaN	NaN	NaN	2959072	909224	0	5	2	0	50	118	40554808	9.01E-01	1
NGEF	404109	215	33	1933436	520828	0	3	1	0	42	39	16643000	9.01E-01	1
MLLT10	128481	488	16	2650420	739412	0	6	0	0	8	8	3453556	9.01E-01	1
HOXD8	242935	874	15	724460	212176	0	1	1	0	50	65	19479964	9.01E-01	1
OMG	473619	396	27	1100396	316840	0	3	2	0	9	18	4624440	9.01E-01	1
WDR60	338838	241	3	2801720	719476	0	5	2	0	50	54	22811412	9.01E-01	1
PRF1	549339	573	40	1355292	438948	0	5	3	0	4	6	1783916	9.01E-01	1
MDFIC	38349	520	15	922040	278392	0	1	0	0	50	54	21535152	9.01E-01	1
TMEFF2	69320	677	0	996088	243860	0	6	3	0	2	5	1026348	9.01E-01	1
OR8A1	171574	746	-1	804560	242080	0	4	2	0	6	10	1750808	9.01E-01	1
ZNF619	195638	546	22	1599152	422572	0	2	2	0	50	48	20952736	9.01E-01	1
OR5F1	4262	1044	-40	772520	235672	0	4	2	0	5	12	1863660	9.02E-01	1
HIST2H2BI	462959	519	19	673196	203632	0	1	2	0	14	13	3281608	9.02E-01	1
APOL1	1237332	331	42	1058388	296904	0	3	2	0	8	28	6483472	9.02E-01	1
RALGAPB	531076	440	45	3851920	1061948	0	11	4	0	4	7	2477760	9.02E-01	1
KNG1	463823	395	37	1739060	454612	0	4	1	0	50	74	23993688	9.02E-01	1
TMEM135	159214	587	-7	1212892	317552	0	2	1	0	50	66	21158148	9.02E-01	1
NUP210	359039	285	48	4756516	1461380	0	13	10	0	22	26	9351764	9.02E-01	1
MUTYH	1002765	249	29	1449632	434320	0	2	0	0	49	55	22400232	9.02E-01	1
TMEM171	447937	584	21	808832	247420	0	1	1	0	50	58	20189472	9.02E-01	1

SEMA7A	640438	204	54	1707732	489144	0	3	1	0	34	37	15457164	9.02E-01	1
XRCC5	597243	384	33	1952304	481668	0	1	1	0	20	20	8441828	9.02E-01	1
PLCH1	326198	765	-9	4363848	1175868	0	11	6	0	20	28	8470664	9.02E-01	1
CNTN5	3647	1135	-26	2834116	791744	0	12	5	0	24	57	10123572	9.02E-01	1
SLC22A3	545081	363	-12	1394452	435032	0	2	2	0	50	55	20845580	9.02E-01	1
RPS6KA4	1610483	257	51	1939132	608048	0	3	1	0	40	75	19957360	9.02E-01	1
UBE2Z	775532	169	29	901748	263440	0	1	0	0	50	61	22704256	9.02E-01	1
MTA3	343875	378	27	1364548	346388	0	1	0	0	50	42	24595684	9.02E-01	1
ICK	463740	386	22	1630836	448560	0	2	2	0	50	53	22580724	9.02E-01	1
LRP1B	8988	1120	-11	12201188	2921692	0	48	12	0	33	57	18501676	9.02E-01	1
CCDC74B	348702	548	13	964760	288004	0	1	0	0	50	61	19664372	9.02E-01	1
MAPK13	855485	410	35	965472	256320	0	1	0	0	50	54	22437968	9.02E-01	1
PRPF4	426351	312	26	1368108	365256	0	3	0	0	12	15	4898560	9.03E-01	1
EIF3B	795806	251	43	2139204	551088	0	5	1	0	16	26	6910672	9.03E-01	1
OR12D3	329776	872	7	786760	231044	0	1	1	0	50	77	21945976	9.03E-01	1
PCCA	139575	733	33	1920976	512996	0	4	0	0	14	15	6308320	9.03E-01	1
SPNS3	1297591	273	30	1275904	416876	0	2	0	0	27	36	12283780	9.03E-01	1
SCFD2	170883	852	18	1707732	521184	0	3	3	0	11	10	4043804	9.03E-01	1
GATA1	823539	NaN	29	1000716	347100	0	2	2	0	50	93	24512736	9.03E-01	1
RNF130	1665304	424	17	1072984	306872	0	2	0	0	29	58	16958060	9.03E-01	1
COQ10A	2878071	171	46	657888	198648	0	1	0	0	33	67	18798224	9.03E-01	1
TRIM32	68232	682	-15	1605916	496976	0	5	2	0	21	37	9152760	9.03E-01	1
LRIG3	21039	678	-29	2925252	804204	0	8	0	0	0	0	804204	9.03E-01	1
OR51A2	65798	1006	-15	771096	232824	0	1	1	0	47	66	18360344	9.03E-01	1
TLR4	58763	944	-8	2117488	582416	0	6	5	0	3	7	1508728	9.03E-01	1
GATAD2A	899226	214	6	1577792	500536	0	5	6	0	12	14	3559288	9.03E-01	1
CCDC59	53465	1263	-24	639376	150944	0	1	1	0	25	36	8963724	9.03E-01	1
AARS	1335968	139	42	2487372	702744	0	5	4	0	50	76	22539428	9.03E-01	1
ADH5	192327	549	11	966896	270916	0	1	0	0	50	55	22804648	9.03E-01	1
ETV7	942911	192	46	881456	244216	0	2	1	0	2	5	1095412	9.03E-01	1
PANX3	171574	746	-1	972592	299396	0	3	2	0	6	10	1750808	9.03E-01	1
FAM117A	993241	361	26	1121044	365612	0	2	0	0	5	6	2347464	9.03E-01	1
UGGT2	175188	834	34	4029564	993240	0	11	4	0	40	52	17438660	9.03E-01	1

ACTB	1906749	195	34	951232	274832	0	17	4	0	0	4	274832	9.03E-01	1
CNTNAP3	51498	1010	-36	3277692	950520	0	8	0	0	3	3	2091500	9.03E-01	1
GPRASP2	123687	NaN	23	2101112	587044	0	3	0	0	22	14	7859768	9.03E-01	1
PRSS27	1803069	200	47	736208	217516	0	1	1	0	50	64	19321900	9.03E-01	1
MAMDC4	1921909	176	44	2878972	880388	0	6	4	0	50	56	20791468	9.03E-01	1
SIRT4	1621662	187	56	785692	235316	0	1	0	0	50	72	23682188	9.03E-01	1
RNMT	142674	697	42	1276972	294056	0	2	0	0	50	65	20366048	9.03E-01	1
DNAJC22	2137968	175	37	834820	265220	0	1	0	0	50	68	23225440	9.03E-01	1
PARP11	273563	859	31	915632	200428	0	1	0	0	50	70	26185936	9.04E-01	1
EML2	950605	194	45	1675692	485940	0	7	0	0	0	0	485940	9.04E-01	1
SLC25A28	383678	436	18	900680	281596	0	1	2	0	50	57	21945264	9.04E-01	1
PPM1L	585349	509	-5	922396	249200	0	2	2	0	50	74	18195872	9.04E-01	1
BRCC3	NaN	NaN	NaN	851196	213600	0	1	0	0	50	116	39859184	9.04E-01	1
ADAMTS6	275284	480	5	2941628	738700	0	6	1	0	6	10	3200440	9.04E-01	1
SGCB	114535	815	8	830904	214668	0	1	0	0	50	63	22288804	9.04E-01	1
CALB1	272948	534	25	715204	166964	0	4	2	0	4	12	1651484	9.04E-01	1
NFASC	388939	440	33	3545404	989324	0	8	6	0	9	12	4514436	9.04E-01	1
PARP1	805195	409	9	2654692	691352	0	9	2	0	13	13	3997524	9.04E-01	1
ISX	234416	597	-76	614456	186544	0	1	0	0	49	107	27622752	9.04E-01	1
JAKMIP3	146849	592	-12	2216456	580636	0	3	1	0	49	50	22022516	9.04E-01	1
ADIPOR2	259057	403	29	1001072	268780	0	1	1	0	50	63	26825668	9.04E-01	1
ZNF766	495542	909	-36	1220724	296904	0	2	1	0	50	69	18343256	9.04E-01	1
STRN	580141	370	0	2043084	534000	0	4	0	0	50	49	20006132	9.04E-01	1
TFF1	313101	464	90	222500	60520	0	1	1	0	3	3	451764	9.04E-01	1
TNFRSF19	181889	614	32	1097904	306516	0	1	1	0	42	40	15676104	9.04E-01	1
ARHGEF4	547548	369	13	1860100	501248	0	3	4	0	13	21	6063748	9.04E-01	1
HSD17B14	1766061	211	34	679604	222856	0	1	2	0	5	6	2154868	9.04E-01	1
CACNG6	806175	338	-40	629764	219296	0	1	1	0	50	83	19101180	9.04E-01	1
STK35	505026	539	32	1301892	425064	0	2	2	0	50	62	21344336	9.04E-01	1
SPOCD1	1213493	183	35	3010692	952656	0	5	2	0	29	24	12224684	9.04E-01	1
FAM187B	767246	472	7	925600	268424	0	1	0	0	50	41	16380272	9.04E-01	1
ALDH1L1	203027	610	30	2324324	662872	0	3	0	0	11	9	5231776	9.04E-01	1
FIGNL1	177267	585	25	1686372	480600	0	2	1	0	44	38	18149592	9.05E-01	1

WDR4	439191	276	57	1069068	303312	0	1	1	0	50	47	17637664	9.05E-01	1
MFN2	518052	207	39	1968680	532576	0	5	4	0	42	44	13163456	9.05E-01	1
KHSRP	865106	219	33	1821652	540764	0	2	0	0	14	12	6196180	9.05E-01	1
BHLHB9	162942	NaN	27	1371312	384480	0	2	2	0	19	27	10235356	9.05E-01	1
FANCG	782447	350	42	1562484	489144	0	2	1	0	36	48	20387764	9.05E-01	1
PEX14	819616	392	27	970812	276612	0	1	1	0	50	67	27507052	9.05E-01	1
PDE3B	364855	539	-14	2824860	805272	0	9	4	0	9	25	7174112	9.05E-01	1
FBP2	261413	427	41	859740	257388	0	1	1	0	50	80	21776876	9.05E-01	1
CNKSR1	896535	217	30	1828060	547172	0	4	3	0	50	84	26408792	9.05E-01	1
TMEM199	1405196	214	10	527948	167320	0	1	2	0	27	46	10403388	9.05E-01	1
ST3GAL1	198193	357	24	874336	243860	0	2	2	0	2	3	800644	9.05E-01	1
DRD3	418141	589	23	1007836	304736	0	1	1	0	50	51	21092644	9.05E-01	1
IFT81	1034390	181	52	1915636	447848	0	6	2	0	33	46	12832732	9.05E-01	1
GSDMB	1602321	292	18	1090784	292276	0	1	1	0	4	3	1229624	9.05E-01	1
FCGR2B	598281	504	20	810612	217872	0	1	0	0	50	64	18701036	9.05E-01	1
FITM2	813168	376	10	656108	195088	0	1	0	0	50	60	16040648	9.05E-01	1
FBXO24	1651125	242	60	1597016	470632	0	2	3	0	46	58	16249976	9.05E-01	1
ADCY9	740126	140	19	3390900	990036	0	7	1	0	34	37	14687848	9.05E-01	1
VAX2	504483	623	25	694912	248132	0	3	0	0	4	3	825208	9.05E-01	1
ITGB3BP	130856	477	5	497332	108224	0	2	1	0	7	7	1190108	9.05E-01	1
C1GALT1	357040	748	14	945180	233892	0	1	1	0	50	59	20500616	9.05E-01	1
VPS36	365031	318	37	1032756	264864	0	1	1	0	50	49	21269576	9.05E-01	1
OPRL1	1032302	312	51	898900	302600	0	2	3	0	22	39	8929904	9.06E-01	1
ANKRD44	744603	444	27	2411544	642936	0	4	3	0	50	55	21454696	9.06E-01	1
TCEANC	138133	NaN	57	975796	256676	0	1	0	0	50	58	23047796	9.06E-01	1
RASSF6	351984	573	28	995732	234604	0	3	2	0	5	7	1863660	9.06E-01	1
ENDOD1	273779	513	16	1229980	379496	0	2	1	0	50	67	22599236	9.06E-01	1
HLA-DPB1	1479000	431	28	661448	191884	0	1	0	0	50	69	17902884	9.06E-01	1
RPUSD2	892776	192	48	1336068	421860	0	2	0	0	4	12	4703116	9.06E-01	1
PARP3	1013177	370	39	1403708	376648	0	2	2	0	50	85	19762272	9.06E-01	1
NOXA1	1591716	213	30	1221080	389464	0	2	1	0	50	64	20302324	9.06E-01	1
PLK1	530660	509	35	1525104	450696	0	4	2	0	50	89	25757312	9.06E-01	1
SP3	296444	376	27	1946608	584552	0	2	3	0	50	42	22176664	9.06E-01	1

ADCK2	354697	567	38	1530800	508012	0	2	1	0	50	48	20059176	9.06E-01	1
TMEM179I	2405109	248	44	545392	180848	0	2	3	0	5	10	1698832	9.06E-01	1
TULP2	1846623	211	38	1346748	374868	0	2	1	0	50	62	21964132	9.06E-01	1
BMP1	587455	196	50	2635468	702032	0	4	3	0	42	48	15501664	9.06E-01	1
ATG16L2	528497	281	50	1588116	473124	0	1	1	0	50	37	16556492	9.06E-01	1
CHAD	1332860	200	23	890356	278036	0	2	2	0	8	12	3275556	9.06E-01	1
MFHAS1	164853	395	34	2536856	845500	0	7	4	0	7	14	4451780	9.06E-01	1
NFAT5	1302986	NaN	41	3953736	1072272	0	10	1	0	0	1	1072272	9.06E-01	1
SHF	667418	544	27	1074052	314348	0	3	2	0	14	30	7432212	9.06E-01	1
VDR	387225	207	44	1105024	300464	0	1	1	0	50	46	19163480	9.06E-01	1
MLXIP	776345	NaN	34	2321476	694556	0	4	2	0	50	63	22458616	9.07E-01	1
MRPL10	1071489	214	29	655040	203632	0	1	1	0	12	13	4017104	9.07E-01	1
LRRC34	404285	689	13	1228200	327876	0	2	2	0	50	66	21327960	9.07E-01	1
ZBTB49	146158	506	40	1966188	517980	0	9	3	0	13	29	6297640	9.07E-01	1
HDAC6	823539	NaN	29	3076908	938772	0	10	1	0	15	22	7055208	9.07E-01	1
PSKH2	191789	1214	-25	961200	281952	0	2	1	0	50	94	23368552	9.07E-01	1
TRIOBP	1114524	180	74	6037760	1957288	0	10	2	0	4	3	2759712	9.07E-01	1
ZNF500	706891	195	33	1196516	365968	0	1	0	0	16	14	6945916	9.07E-01	1
DBX2	126551	805	-18	834108	268068	0	1	2	0	32	42	12628744	9.07E-01	1
FBXL8	1093307	150	47	887508	326808	0	1	3	0	6	5	2474200	9.07E-01	1
SEC22A	338654	421	48	797440	215024	0	1	1	0	50	72	24009352	9.07E-01	1
PSRC1	988224	302	29	938416	231044	0	1	0	0	50	71	22043520	9.07E-01	1
GAS8	939646	221	31	1252052	325384	0	3	2	0	31	47	11896096	9.07E-01	1
STYK1	408572	432	0	1086868	306872	0	1	2	0	50	56	20777940	9.07E-01	1
NOP56	792941	450	48	1532936	424708	0	4	3	0	8	13	3871856	9.07E-01	1
ACTN1	609757	344	44	2395880	625492	0	7	0	0	2	2	1119264	9.07E-01	1
KIAA0101	766091	263	33	321112	77252	0	1	1	0	2	5	812036	9.07E-01	1
FAM135A	114193	606	11	3991116	1034892	0	4	3	0	50	52	19406272	9.07E-01	1
NR2C2	483299	400	43	1593100	440372	0	4	0	0	4	3	1158424	9.07E-01	1
HSPA1B	2509866	183	32	1573876	483092	0	2	1	0	50	56	22464668	9.07E-01	1
DDB2	1274054	198	38	1106092	303668	0	3	5	0	4	13	1874340	9.07E-01	1
CDCA2	178565	618	22	2627636	710932	0	4	2	0	42	38	17775792	9.07E-01	1
SLC22A8	2447828	398	48	1368820	417944	0	5	2	0	24	40	8624812	9.07E-01	1

MYOZ3	1107515	211	54	641156	190816	0	1	2	0	12	23	6739080	9.07E-01	1
SLC37A2	428178	762	30	1342120	375224	0	1	2	0	15	3	2294776	9.07E-01	1
RRN3	564013	463	22	1720904	442864	0	3	3	0	50	49	18364972	9.07E-01	1
ZBTB24	337228	559	33	1839808	475616	0	3	1	0	45	64	23750184	9.07E-01	1
KRT23	1036346	358	5	1096124	289072	0	1	2	0	50	61	13481008	9.07E-01	1
FRMPD2	112127	451	35	3365980	950876	0	6	3	0	50	66	22591760	9.07E-01	1
CAPN7	511283	367	30	2149884	543612	0	2	3	0	50	66	22193040	9.08E-01	1
PCYT1A	1103198	207	43	965828	247420	0	1	0	0	34	35	13970152	9.08E-01	1
FAM57B	NaN	NaN	NaN	686012	212176	0	1	2	0	26	42	11789296	9.08E-01	1
SLC44A4	2485614	188	36	1839452	526168	0	3	5	0	13	13	3768616	9.08E-01	1
NPHP3	211862	768	24	3455336	922396	0	11	3	0	16	19	6776104	9.08E-01	1
TRIM6	NaN	NaN	NaN	1315420	370952	0	1	3	0	26	43	11948072	9.08E-01	1
HEPACAM	283718	743	13	1031688	330012	0	1	1	0	50	60	22507744	9.08E-01	1
KCMF1	926772	202	23	975084	274476	0	2	1	0	50	68	17629476	9.08E-01	1
ZNF227	618477	671	-31	2082956	497332	0	4	1	0	50	57	18064864	9.08E-01	1
EIF6	1290513	170	45	741904	194732	0	2	3	0	2	8	1208264	9.08E-01	1
CC2D1A	930314	231	32	2434328	735496	0	6	2	0	9	21	4861892	9.08E-01	1
REXO2	98814	612	-12	631544	156640	0	1	1	0	5	7	2025996	9.08E-01	1
CNOT6L	152352	493	18	1452836	377716	0	2	0	0	23	26	10220048	9.08E-01	1
LRRC3B	5998	918	-24	651124	181916	0	1	0	0	50	80	20784348	9.08E-01	1
FKBP7	170323	555	37	586688	140620	0	1	2	0	11	13	2651488	9.08E-01	1
RBMX2	301748	NaN	37	842296	216092	0	1	1	0	50	61	18917128	9.08E-01	1
RGL3	1255985	265	32	1813108	563192	0	4	0	0	4	6	2083668	9.08E-01	1
PODXL	236261	220	27	1384484	440728	0	1	2	0	50	48	19559352	9.08E-01	1
GCNT3	521844	172	33	1112500	298328	0	2	2	0	26	35	11001468	9.08E-01	1
MIA2	314590	300	21	1693492	426488	0	3	0	0	5	7	3414752	9.08E-01	1
MPRSS11	212678	809	4	1105736	284800	0	2	2	0	50	60	18569316	9.08E-01	1
GCC2	362408	478	6	4446796	1045928	0	12	1	0	0	1	1045928	9.08E-01	1
SLC22A23	465372	453	27	1702748	536848	0	3	1	0	5	3	1365972	9.08E-01	1
MEST	325887	298	31	881100	242436	0	1	1	0	48	54	17663652	9.08E-01	1
CABYR	233052	360	52	1731228	517980	0	3	0	0	9	4	2145256	9.08E-01	1
INHA	548994	487	35	866504	313636	0	1	3	0	19	44	12076944	9.08E-01	1
BCOR	377184	NaN	42	4390192	1295840	0	9	3	0	50	57	25006152	9.09E-01	1

CPM	645106	228	28	1159492	297260	0	2	1	0	44	62	17868708	9.09E-01	1
RILP	1361271	198	27	997156	320756	0	1	0	0	50	60	20575376	9.09E-01	1
ZNF467	471461	615	29	1471348	455324	0	2	0	0	32	36	14158832	9.09E-01	1
ANPEP	934542	212	37	2492000	690640	0	6	2	0	50	65	20979792	9.09E-01	1
BMPR2	369483	417	30	2641520	738700	0	7	4	0	18	25	8689960	9.09E-01	1
TBX6	2171952	164	62	1080816	353508	0	2	0	0	19	38	10731976	9.09E-01	1
FCHSD1	771583	353	25	1766472	530796	0	2	0	0	28	26	13202616	9.09E-01	1
IDH2	851518	192	38	1175868	320400	0	2	2	0	50	59	18318692	9.09E-01	1
JHRF1BP1	184820	559	7	3817744	985052	0	9	5	0	50	57	22009344	9.09E-01	1
INSC	161776	781	-17	1475620	436100	0	4	2	0	47	78	19540484	9.09E-01	1
LRRFIP1	542668	347	33	3085452	786048	0	3	0	0	7	4	3744764	9.09E-01	1
RRP12	892254	188	39	3330024	969744	0	9	3	0	50	71	21552240	9.09E-01	1
TXNDC11	593829	214	40	2419020	704880	0	6	0	0	1	0	773588	9.09E-01	1
ERBB4	20702	1173	-5	3415820	895696	0	9	2	0	5	6	2427564	9.09E-01	1
UROC1	289633	508	52	1904600	544324	0	5	4	0	20	25	7817404	9.09E-01	1
FUT6	1217326	294	35	892136	265576	0	1	1	0	50	56	18189820	9.09E-01	1
RABL2A	522522	603	16	626916	145248	0	1	1	0	50	76	19682528	9.09E-01	1
ASZ1	262404	394	-1	1259172	319332	0	1	2	0	46	46	23425512	9.09E-01	1
APCDD1L	1027182	485	24	1204348	416876	0	2	0	0	19	28	9283412	9.09E-01	1
KRT36	1445853	325	-22	1170884	354220	0	4	0	0	2	3	1035960	9.09E-01	1
AP2A1	1686482	172	42	2487728	744040	0	4	0	0	1	0	1031688	9.09E-01	1
ZNF146	923127	419	-26	758636	180136	0	4	2	0	1	2	441440	9.09E-01	1
NBPF15	NaN	NaN	NaN	1781780	432184	0	3	1	0	50	117	40077768	9.09E-01	1
TBCB	978272	326	6	629764	176576	0	1	2	0	32	46	10310116	9.10E-01	1
SNED1	1332160	278	33	3627996	1033824	0	7	2	0	1	2	1605916	9.10E-01	1
SLC6A11	225460	545	30	1636888	451052	0	2	1	0	50	52	20886520	9.10E-01	1
BCL2L14	331421	489	26	915632	245284	0	1	1	0	41	51	16729152	9.10E-01	1
KCNAB1	502198	782	4	1491284	410824	0	4	3	0	4	6	1677828	9.10E-01	1
SHANK1	632386	593	31	5200092	1823076	0	15	7	0	50	58	19947748	9.10E-01	1
LCP1	400791	682	47	1646856	429336	0	4	3	0	12	10	3796384	9.10E-01	1
WNK2	479663	408	49	5419744	1806344	0	8	2	0	8	9	6221812	9.10E-01	1
OR51F1	207755	972	-1	762552	239232	0	1	2	0	12	12	3866160	9.10E-01	1
KCTD19	1201743	142	56	2369180	671416	0	8	3	0	11	22	6211844	9.10E-01	1

AURKA	254679	672	-43	1045216	283376	0	2	0	0	33	67	17771520	9.10E-01	1
SEPSECS	338354	506	32	1297976	353152	0	1	1	0	50	49	20148532	9.10E-01	1
SEC31B	1153157	264	50	2998944	888576	0	8	1	0	2	2	1274480	9.10E-01	1
CINP	1659098	214	32	616236	145248	0	2	1	0	4	14	2498764	9.10E-01	1
TUBG2	1740597	177	36	1165900	325028	0	4	3	0	4	17	2862596	9.10E-01	1
PANX1	367552	774	-16	1082240	302956	0	2	1	0	50	61	16199780	9.10E-01	1
SLC9A6	248961	NaN	24	1810616	511216	0	2	1	0	18	23	11089756	9.10E-01	1
PIK3C2A	513121	436	10	4380936	1158780	0	13	3	0	3	6	2138848	9.10E-01	1
B3GAT3	2695768	217	42	814884	280884	0	1	1	0	17	27	9232504	9.10E-01	1
GAA	663665	237	27	2399796	734784	0	5	0	0	0	0	734784	9.10E-01	1
TAF5	545643	147	45	2022436	588824	0	2	1	0	50	60	18601000	9.10E-01	1
POU5F1	3364017	401	19	896052	279816	0	1	1	0	45	41	13756552	9.10E-01	1
PROZ	759371	229	38	1032044	284800	0	1	1	0	47	50	18676472	9.10E-01	1
C6orf203	214917	417	22	637952	167320	0	1	2	0	13	18	5048080	9.11E-01	1
ARID3C	731413	298	51	1015668	332148	0	3	3	0	12	11	3186200	9.11E-01	1
WDR12	596746	392	31	1122824	286936	0	1	1	0	50	51	21099764	9.11E-01	1
RAB3IP	561550	488	-11	1258104	312924	0	2	1	0	50	60	19572168	9.11E-01	1
TMEM161A	1260413	215	35	1192956	394092	0	1	0	0	30	40	9461768	9.11E-01	1
JMJD4	595623	459	1	1235320	370952	0	1	2	0	50	38	16917832	9.11E-01	1
KATNB1	712945	168	34	1686372	492348	0	3	2	0	50	57	21352524	9.11E-01	1
LRRC23	2704950	211	47	1042368	285156	0	1	1	0	1	1	1276260	9.11E-01	1
LINGO4	1062482	255	29	1403708	503740	0	2	2	0	12	10	4320060	9.11E-01	1
TBC1D13	1768145	201	34	1059456	272340	0	1	1	0	26	29	9773980	9.11E-01	1
DENND2A	356082	503	31	2564268	744396	0	10	5	0	7	13	3679260	9.11E-01	1
ANXA11	366637	626	7	1297264	383768	0	3	1	0	50	75	19965548	9.11E-01	1
CLK4	492630	675	17	1297620	295836	0	2	1	0	50	64	22444376	9.11E-01	1
DDN	1737773	214	55	1691000	596656	0	4	0	0	0	0	596656	9.11E-01	1
BCAR1	568174	171	16	2435752	797084	0	4	2	0	42	36	16524808	9.11E-01	1
HLA-DRB1	1074109	595	29	682808	196156	0	1	1	0	50	76	18146032	9.11E-01	1
STK11IP	548994	487	35	2758288	870776	0	6	2	0	40	56	20065584	9.11E-01	1
NCAPD2	2764720	179	42	3597024	1027416	0	10	3	0	19	23	8223600	9.11E-01	1
ZC3H3	2168942	235	34	2317916	771808	0	3	1	0	30	24	12078012	9.11E-01	1
CLINT1	227687	916	44	1631192	421504	0	3	0	0	6	7	2923116	9.11E-01	1

CCDC144A	1101297	499	-18	3760784	889288	0	7	1	0	5	3	2066224	9.11E-01	1
TTYH2	321651	535	3	1368820	402992	0	3	4	0	5	12	2806348	9.11E-01	1
RAB3GAP2	651142	476	4	3652204	959420	0	7	1	0	2	3	2152020	9.11E-01	1
MMP1	510838	567	-16	1238524	310076	0	2	1	0	50	58	18555432	9.11E-01	1
WISP1	235882	481	12	935212	265220	0	3	2	0	1	2	793524	9.11E-01	1
C19orf54	1127178	201	27	861520	287648	0	1	1	0	50	68	19652268	9.11E-01	1
SLC5A5	1291250	204	37	1596660	526524	0	3	3	0	50	53	16788604	9.11E-01	1
NBPF11	NaN	NaN	NaN	4183000	1039520	0	9	3	0	50	119	40685104	9.11E-01	1
RGS4	315405	691	-14	804204	187968	0	1	1	0	50	56	17889000	9.11E-01	1
CGNL1	189519	561	10	3356724	894984	0	5	2	0	50	47	23187704	9.12E-01	1
AWAT2	166632	NaN	16	861520	240656	0	1	0	0	50	73	21348964	9.12E-01	1
OR1L1	409923	553	12	766468	227840	0	1	1	0	50	56	16898964	9.12E-01	1
SULT1C3	354622	708	-17	818088	183696	0	1	0	0	50	49	14515188	9.12E-01	1
FBXL3	218827	551	34	1093988	296548	0	3	2	0	19	26	7166636	9.12E-01	1
USP40	351003	307	35	3227140	822716	0	6	2	0	50	42	18327948	9.12E-01	1
TBXAS1	485631	679	36	1502320	419012	0	2	2	0	50	51	19055256	9.12E-01	1
TAS2R42	311688	617	7	786404	222856	0	1	1	0	50	66	20956296	9.12E-01	1
ZIC3	12849	NaN	-2	1166256	341760	0	3	1	0	33	55	12652952	9.12E-01	1
CDRT1	464501	419	-27	1943048	516556	0	2	1	0	43	54	16909288	9.12E-01	1
LNX2	327769	583	60	1755436	496976	0	3	3	0	48	48	12817424	9.12E-01	1
ZNF517	1454261	384	20	1221080	375580	0	3	4	0	7	18	3447148	9.12E-01	1
PGBD2	113516	547	-2	1502676	405840	0	2	0	0	50	68	24078772	9.12E-01	1
NSF	525541	203	12	1964764	507656	0	6	1	0	4	10	2496272	9.12E-01	1
LRRC8E	952640	320	27	1951948	610184	0	3	4	0	18	34	7729828	9.12E-01	1
BTNL9	893236	602	29	1354580	405484	0	4	0	0	4	6	1552160	9.12E-01	1
PLA2G4F	655295	394	36	2166260	640444	0	5	5	0	9	15	4733020	9.12E-01	1
TUBGCP5	347364	564	-32	2744404	681740	0	4	3	0	50	71	19747320	9.12E-01	1
VPS4B	206996	278	92	1156288	312212	0	2	1	0	50	67	19860172	9.12E-01	1
SIGIRR	2206786	344	58	1026704	324316	0	2	1	0	50	92	24128968	9.12E-01	1
SRFBP1	70781	809	-4	1123180	286580	0	4	0	0	2	6	1247780	9.13E-01	1
GJC1	1162976	172	29	1001784	274476	0	1	0	0	16	18	5718072	9.13E-01	1
NRBF2	219747	715	8	741904	193664	0	3	2	0	1	2	631544	9.13E-01	1
HOXD3	245316	874	13	1064796	331080	0	2	1	0	50	66	17973016	9.13E-01	1

UNC45A	833957	291	42	2390184	718764	0	5	3	0	50	63	21711728	9.13E-01	1
SLC25A18	431029	390	-37	804916	241724	0	1	1	0	50	76	20635896	9.13E-01	1
IL9R	NaN	NaN	NaN	1324320	382344	0	2	1	0	50	117	40027928	9.13E-01	1
GYG2	197004	NaN	17	1289076	362052	0	2	1	0	50	73	23563284	9.13E-01	1
OR6N1	103627	985	1	763264	238520	0	6	3	0	3	9	983984	9.13E-01	1
ISLR	492755	317	46	1033468	345320	0	1	1	0	50	52	22095852	9.13E-01	1
HHATL	369022	329	34	1288008	379140	0	1	0	0	50	41	20048140	9.13E-01	1
GPR182	2103486	286	26	982916	318976	0	1	2	0	50	85	24401308	9.13E-01	1
AHCYL1	660515	373	29	1401928	367748	0	2	2	0	3	5	1719836	9.13E-01	1
SAAL1	631291	436	20	1264512	308652	0	2	0	0	50	76	24807148	9.13E-01	1
CD177	345698	182	-48	1111076	328588	0	4	1	0	23	48	10296232	9.13E-01	1
GPBP1L1	913416	157	28	1231760	332860	0	2	2	0	50	81	24195896	9.13E-01	1
PPM1H	298536	426	0	1295484	393024	0	1	1	0	50	54	21635188	9.13E-01	1
RNF43	782135	399	22	1934504	611608	0	3	0	0	17	38	14673608	9.13E-01	1
ASB1	246085	513	27	833752	262016	0	1	1	0	50	51	17467852	9.13E-01	1
KIF26B	609429	825	-5	5199736	1617308	0	13	5	0	20	19	6768272	9.13E-01	1
RER1	561270	302	24	519760	137060	0	1	2	0	3	3	1062304	9.13E-01	1
SPAG8	847663	227	54	1447496	426844	0	2	0	0	2	0	838380	9.13E-01	1
ZNF625	1025728	728	-15	788540	199360	0	3	0	0	50	149	23540500	9.13E-01	1
GALT	731413	298	51	985408	276968	0	1	0	0	45	36	14802836	9.13E-01	1
TACSTD2	301269	564	-5	775012	263084	0	1	1	0	50	54	16901812	9.13E-01	1
SV2A	798975	560	17	1883952	543612	0	5	2	0	21	23	6253852	9.13E-01	1
APBA1	209495	673	-5	2139204	597012	0	3	2	0	50	55	23316932	9.13E-01	1
PLA2G2F	567393	501	23	554292	144180	0	1	2	0	8	12	2829844	9.13E-01	1
CADM4	441738	209	6	984340	298328	0	2	2	0	14	16	4113936	9.13E-01	1
BAG1	856503	167	38	880388	255964	0	1	1	0	50	62	21222584	9.13E-01	1
ZNF689	2255010	158	60	1232828	380920	0	2	1	0	50	74	23170972	9.14E-01	1
PER3	370145	415	23	3076552	860096	0	9	1	0	5	5	2330020	9.14E-01	1
CDH24	1261207	185	41	2012824	661448	0	3	0	0	2	2	1796020	9.14E-01	1
TRUB2	1813435	201	46	840516	255252	0	1	1	0	50	69	20850564	9.14E-01	1
EPN3	1532661	200	20	1556432	505876	0	4	3	0	50	83	22594252	9.14E-01	1
MRE11A	320374	589	-10	1883952	464580	0	3	2	0	50	38	15707076	9.14E-01	1
CARM1	2031768	193	35	1570672	444644	0	5	2	0	6	17	3124968	9.14E-01	1

KBTBD4	995645	214	31	1344256	389108	0	2	0	0	14	14	5095784	9.14E-01	1
PGGT1B	237444	619	9	984696	262728	0	1	0	0	32	41	16069840	9.14E-01	1
RPLP0	1608347	225	59	808476	242436	0	1	0	0	50	60	16943820	9.14E-01	1
NFRSF13I	1122696	237	2	748668	212532	0	1	0	0	50	59	17313348	9.14E-01	1
USP45	256853	638	15	2146680	537204	0	3	1	0	20	31	14347156	9.14E-01	1
KCTD1	173654	429	31	2145256	646496	0	2	0	0	5	9	3927392	9.14E-01	1
FAM46B	929681	336	34	1014600	354576	0	4	0	0	0	0	354576	9.14E-01	1
ZFP90	765949	421	34	1650060	405840	0	4	1	0	50	68	22402012	9.14E-01	1
BAG5	867608	225	39	1240304	334996	0	2	1	0	50	63	19588900	9.14E-01	1
PRDX5	1613996	257	65	535068	175152	0	1	1	0	19	29	6446448	9.14E-01	1
ALG12	771339	208	6	1208976	396228	0	2	2	0	50	76	22468940	9.14E-01	1
AP2A2	1509286	246	48	2400864	700608	0	4	1	0	2	2	1460312	9.14E-01	1
NRAP	195180	587	1	4574600	1148812	0	12	5	0	21	21	8579956	9.14E-01	1
ERCC1	1259254	191	39	870420	258456	0	2	0	0	8	11	2860460	9.14E-01	1
MTRR	66042	1013	-9	1865796	526524	0	4	2	0	50	63	21157792	9.14E-01	1
DDX20	333198	440	25	2115708	570312	0	11	5	0	1	6	1192956	9.14E-01	1
TFAP2C	280847	442	-44	1129944	340692	0	3	3	0	7	14	3512296	9.14E-01	1
NSMCE1	227767	448	27	712356	173016	0	4	2	0	1	2	501960	9.14E-01	1
ST8SIA1	401092	910	-22	911004	249912	0	2	1	0	50	80	17976576	9.14E-01	1
EHHADH	775543	587	35	1810260	537204	0	3	1	0	13	12	4893932	9.14E-01	1
BCKDK	1296884	351	35	1051268	316840	0	1	1	0	50	76	22416964	9.14E-01	1
IPP	972928	146	29	1597016	420436	0	5	0	0	6	8	1941624	9.14E-01	1
RPAP1	744461	166	44	3437180	1131724	0	10	3	0	12	23	7923848	9.14E-01	1
RFFL	439420	504	19	934856	257032	0	1	0	0	50	60	19451840	9.15E-01	1
LEKR1	364723	522	10	1832332	439304	0	3	0	0	6	5	1975444	9.15E-01	1
CIRH1A	1175833	201	34	1789612	482024	0	3	2	0	46	51	17751584	9.15E-01	1
ABCA4	512482	370	-2	5859760	1635464	0	11	6	0	50	42	16929580	9.15E-01	1
FXR1	182655	1058	-2	1635820	425420	0	4	3	0	13	10	3725540	9.15E-01	1
MELK	394473	231	38	1726244	435388	0	2	1	0	50	44	20119340	9.15E-01	1
ARX	252498	NaN	11	1349952	470988	0	1	1	0	36	36	17122888	9.15E-01	1
SLC39A10	104678	431	3	2161988	542188	0	5	0	0	5	5	1875764	9.15E-01	1
SNX1	742159	223	45	1380568	357068	0	1	0	0	28	26	13033160	9.15E-01	1
SLC2A5	865627	264	31	1398724	429692	0	2	2	0	50	56	19512004	9.15E-01	1

RTTN	64673	504	37	5752960	1589540	0	15	4	0	19	26	8741580	9.15E-01	1
PRAMEF18	NaN	NaN	NaN	2397304	699896	0	5	4	0	50	120	40345480	9.15E-01	1
GRK4	675364	336	51	1544684	374512	0	2	0	0	50	48	16882588	9.15E-01	1
PHF23	1782958	316	21	1005700	305804	0	1	0	0	39	39	14081580	9.15E-01	1
CDR2	483001	235	50	1162340	312568	0	1	0	0	50	38	18234320	9.15E-01	1
PROKR1	260004	596	12	977220	287292	0	1	2	0	50	62	18294484	9.15E-01	1
ROM1	2695768	217	42	833040	303312	0	1	0	0	17	27	9232504	9.15E-01	1
SLC16A10	362574	424	28	1288008	386616	0	1	0	0	50	49	24777244	9.15E-01	1
RECQL4	1755818	291	7	3000012	962268	0	5	5	0	24	29	10834860	9.15E-01	1
WBP2	1727802	178	35	687436	181916	0	1	0	0	25	44	12389512	9.15E-01	1
GFRA1	33734	913	-25	1206128	325384	0	3	1	0	50	89	21235400	9.15E-01	1
PRDM10	631721	248	1	3012116	829480	0	7	1	0	1	1	1209688	9.15E-01	1
PABPC5	8224	NaN	-28	953012	274120	0	3	3	0	18	47	8523708	9.15E-01	1
RPS8	574317	191	43	550376	144892	0	1	1	0	6	14	2995384	9.15E-01	1
CLIC4	518507	198	30	664296	170880	0	1	1	0	50	93	23750540	9.15E-01	1
MBL2	68687	786	-26	628696	184052	0	1	1	0	50	87	21573244	9.15E-01	1
KCNK18	202100	640	4	969388	271628	0	1	0	0	50	54	19534788	9.15E-01	1
PKP3	1830414	344	53	1961560	648632	0	2	0	0	8	14	5014972	9.15E-01	1
HSPH1	194024	518	35	2258108	566752	0	7	0	0	12	18	5832348	9.15E-01	1
PUM2	532255	184	29	2720908	774656	0	7	2	0	34	53	17017156	9.15E-01	1
RDH12	482264	186	44	793880	247420	0	1	1	0	50	59	18220436	9.15E-01	1
PHF10	233648	447	3	1320760	329300	0	1	2	0	10	6	4111800	9.15E-01	1
GTF3C4	1570209	581	46	2071920	582060	0	6	3	0	9	12	3932376	9.16E-01	1
CTNNAL1	337834	433	14	1926316	507656	0	4	2	0	15	15	5207924	9.16E-01	1
FAAH	597667	255	28	1465652	454612	0	2	1	0	5	5	2618024	9.16E-01	1
ARHGAP2	NaN	NaN	NaN	1741552	536492	0	3	2	0	50	118	40182076	9.16E-01	1
ANXA10	55532	402	-16	881456	206836	0	1	1	0	36	34	12824544	9.16E-01	1
KCNMA1	116099	807	3	3357436	902816	0	7	6	0	25	35	8714880	9.16E-01	1
MFSD10	680459	336	49	1122824	389464	0	1	1	0	50	44	17555428	9.16E-01	1
SLC27A4	1928973	188	64	1626564	488076	0	3	0	0	2	1	1070492	9.16E-01	1
NLRP14	418263	881	-4	2828420	723748	0	6	4	0	50	72	19642656	9.16E-01	1
UBE2S	1048909	399	24	546816	180492	0	2	2	0	2	7	1253120	9.16E-01	1
MICALCL	445785	318	-1	1747960	516200	0	3	2	0	50	64	22681472	9.16E-01	1

DMPK	929036	203	43	1811328	475260	0	2	0	0	29	28	12459288	9.16E-01	1
TAP1	1253457	401	26	1976156	658600	0	3	2	0	17	20	6165208	9.16E-01	1
TMUB2	930750	175	41	787472	261304	0	1	1	0	50	69	21793252	9.16E-01	1
KIAA0226	560122	252	50	2551452	699540	0	4	1	0	13	16	5680336	9.16E-01	1
BAIAP3	1150864	215	36	3038104	909224	0	4	0	0	5	2	2170176	9.16E-01	1
ZNF212	667346	385	37	1245288	363120	0	1	1	0	50	65	25807508	9.16E-01	1
TTLL10	2356409	196	27	1734432	515844	0	2	0	0	19	29	12817424	9.16E-01	1
KRT9	1494501	323	11	1569604	461732	0	3	1	0	20	34	10955900	9.16E-01	1
ACE	874574	267	-20	3535080	975084	0	9	1	0	5	11	3489868	9.16E-01	1
OR4D2	851306	401	14	746888	238876	0	1	1	0	50	68	18120400	9.16E-01	1
USP1	239359	394	5	2041660	510860	0	3	0	0	26	24	10291960	9.16E-01	1
TYRP1	77286	530	-31	1373092	380564	0	3	0	0	8	12	3835188	9.16E-01	1
CHD5	620866	240	30	5110736	1330372	0	15	12	0	10	18	4330384	9.17E-01	1
CPNE9	955408	348	34	1461736	394448	0	3	1	0	37	52	16256384	9.17E-01	1
EWSR1	854820	251	51	1796020	500180	0	4	0	0	13	21	6778952	9.17E-01	1
SRGAP1	320926	460	-13	2837676	733716	0	6	2	0	50	52	20150668	9.17E-01	1
SIRT2	1989663	219	30	1039164	274476	0	1	0	0	50	70	21843448	9.17E-01	1
ZNF670	379967	612	-15	1016024	246352	0	1	1	0	50	38	15803196	9.17E-01	1
FCHO2	449417	440	18	2149172	556072	0	1	1	0	50	40	16621996	9.17E-01	1
RASSF5	504163	288	21	1150592	345676	0	1	0	0	11	9	4776096	9.17E-01	1
APOBEC3[illegible]	1413709	372	44	1004988	254184	0	4	0	0	0	0	254184	9.17E-01	1
WNK1	467405	289	45	7100420	2254192	0	20	6	0	16	18	7482052	9.17E-01	1
SDSL	444479	276	36	825920	261304	0	1	1	0	50	61	20196236	9.17E-01	1
CDK5RAP[illegible]	1183803	203	21	1318624	363476	0	3	2	0	11	11	3448216	9.17E-01	1
KLHL8	271051	327	43	1597016	426844	0	2	1	0	50	54	18892564	9.17E-01	1
SEMA3G	1078407	229	62	1955864	616948	0	3	1	0	7	6	2946256	9.17E-01	1
IDO2	11054	414	0	1090428	301176	0	1	1	0	50	46	20992964	9.17E-01	1
TNFSF11	188787	595	67	811680	224280	0	1	0	0	45	49	15618788	9.17E-01	1
CSF3R	581032	347	31	2385912	562836	0	4	3	0	13	12	6160936	9.17E-01	1
KRT4	1781225	288	27	1503388	437168	0	3	2	0	13	20	5681404	9.17E-01	1
TRIM23	421173	406	28	1547176	409400	0	5	0	0	0	0	409400	9.17E-01	1
PHKA2	182760	NaN	35	3213968	882880	0	8	3	0	9	12	3889300	9.17E-01	1
WHSC1L1	802555	253	46	3819880	976508	0	9	0	0	1	1	1437528	9.17E-01	1

MAGEB2	74310	NaN	16	795304	234248	0	1	0	0	50	67	20445436	9.17E-01	1
JMY	166218	588	35	2487728	723748	0	4	2	0	50	53	17127160	9.18E-01	1
BAG3	731222	199	48	1404776	453544	0	2	1	0	5	4	1536140	9.18E-01	1
OR6K2	175690	966	2	799932	241368	0	1	4	0	5	11	1978292	9.18E-01	1
PSMF1	524116	527	14	695624	205768	0	1	0	0	50	74	20142480	9.18E-01	1
DDX17	1313632	177	31	1864016	533644	0	5	1	0	24	36	10446464	9.18E-01	1
PLK4	230666	331	9	2518700	656464	0	10	0	0	6	17	4651496	9.18E-01	1
ALS2CR11	436378	491	24	4848364	1207196	0	2	1	0	3	2	5115720	9.18E-01	1
KIR2DL4	987531	620	-23	972592	269492	0	3	0	0	50	135	24266740	9.18E-01	1
LUC7L2	545432	339	30	1034892	262728	0	1	0	0	50	53	20830984	9.18E-01	1
GOT1	277366	575	-5	1058032	302600	0	1	0	0	4	2	1659316	9.18E-01	1
HS3ST3B1	92906	944	-54	950164	306872	0	1	1	0	50	51	16644780	9.18E-01	1
ST3GAL3	939679	230	33	1166256	310788	0	2	2	0	50	64	18800004	9.18E-01	1
OR10G4	107524	830	-5	755788	241724	0	1	1	0	50	68	19655828	9.18E-01	1
MAGEA8	275638	NaN	NaN	792100	234248	0	1	1	0	50	75	22641244	9.18E-01	1
GPR183	NaN	NaN	NaN	911360	252760	0	1	0	0	50	116	39898344	9.18E-01	1
KRT74	1280474	464	36	1343188	391244	0	2	1	0	39	54	16629472	9.18E-01	1
AP1M1	814131	255	26	1144184	304024	0	1	1	0	50	74	24987640	9.18E-01	1
ARHGEF1[illegible]	958228	350	36	4011052	1167680	0	9	5	0	30	49	14496676	9.18E-01	1
STRADA	1097630	319	10	1179072	316128	0	3	2	0	50	76	16837376	9.18E-01	1
GIPC1	981470	204	34	829124	266644	0	1	1	0	50	72	19535500	9.18E-01	1
ETFDH	171388	429	1	1598440	432896	0	2	0	0	50	56	21491720	9.19E-01	1
KCNE1	381338	502	16	324316	96476	0	1	1	0	1	3	615880	9.19E-01	1
PRSS3	912856	220	33	802780	240656	0	1	0	0	50	68	19088364	9.19E-01	1
ZNF648	126238	679	-1	1405132	422216	0	2	1	0	50	66	22135368	9.19E-01	1
TCTE1	1037256	312	54	1240660	386972	0	2	2	0	44	60	16584260	9.19E-01	1
NPAS3	24372	929	-23	2397304	703100	0	4	0	0	7	6	3072280	9.19E-01	1
ZGLP1	1802015	191	21	669992	214312	0	1	1	0	50	98	25785436	9.19E-01	1
FBXW2	645088	212	21	1173376	310076	0	3	3	0	10	12	3383424	9.19E-01	1
AGBL5	1257014	290	34	2279824	691352	0	7	1	0	13	18	5211128	9.19E-01	1
ZNF418	1252385	750	-19	1744044	437880	0	7	0	0	8	15	3337500	9.19E-01	1
P2RY8	NaN	NaN	NaN	866504	291208	0	1	2	0	50	118	39936792	9.19E-01	1
ASB5	134663	504	-4	845856	239232	0	1	0	0	22	37	10362804	9.19E-01	1

OPN4	352918	192	27	1233896	378784	0	1	0	0	41	43	21038888	9.19E-01	1
TRMT61B	481869	238	32	1217520	341760	0	4	4	0	7	10	2260956	9.19E-01	1
ATXN2L	1146511	221	56	2890720	898544	0	6	2	0	18	35	13346440	9.19E-01	1
RHBDD2	1054488	218	59	881456	302956	0	1	0	0	9	10	3309020	9.19E-01	1
OR2T12	18846	936	-35	786048	241368	0	3	2	0	20	38	5983648	9.19E-01	1
PTPN14	464456	404	-8	3039884	839092	0	7	1	0	4	12	3782856	9.19E-01	1
FOSB	1259254	191	39	825208	273764	0	2	3	0	12	19	4145620	9.19E-01	1
TRIM3	424837	471	17	1862236	571736	0	2	0	0	7	3	2390896	9.19E-01	1
C12orf40	72641	714	-20	1741908	401568	0	5	0	0	7	10	3170892	9.20E-01	1
GRIN1	1722012	202	36	2517276	719832	0	5	1	0	3	3	2037744	9.20E-01	1
SMG1	790295	125	37	9421896	2576016	0	17	4	0	8	9	5309740	9.20E-01	1
CD72	1202125	248	37	932720	254896	0	1	0	0	50	57	20786840	9.20E-01	1
ADORA2B	931951	350	-33	810968	260236	0	1	1	0	50	81	22960220	9.20E-01	1
NEIL3	53396	746	-20	1571740	408332	0	3	3	0	14	20	6477776	9.20E-01	1
EPB42	834640	330	43	1833756	532932	0	3	1	0	42	56	19474980	9.20E-01	1
ROBO2	22183	1224	-17	3604144	920972	0	17	9	0	14	23	4833768	9.20E-01	1
SLC9A3	787824	367	29	2092924	650768	0	5	6	0	8	11	2447144	9.20E-01	1
SLC12A9	1339207	433	48	2195452	791744	0	4	1	0	17	24	8713456	9.20E-01	1
FASTKD2	206970	816	1	1845148	479888	0	3	1	0	50	64	21632340	9.20E-01	1
ZNF562	776094	617	-51	1114636	274832	0	3	0	0	50	124	30214788	9.20E-01	1
RECQL	518837	836	-23	1724108	418300	0	4	1	0	50	62	16740900	9.20E-01	1
MAPK7	630769	332	11	1983276	660024	0	3	2	0	50	72	28508124	9.20E-01	1
TLN1	1062287	163	43	6427936	1955864	0	12	3	0	9	13	6219320	9.20E-01	1
TCTN2	871640	315	40	1817380	491636	0	8	1	0	2	2	919192	9.20E-01	1
CES1	317734	581	-28	1465296	413316	0	3	2	0	31	61	17363900	9.20E-01	1
ZNF654	123235	894	-3	1498404	370596	0	2	0	0	50	48	17285580	9.20E-01	1
BRAP	591444	191	32	1553584	393380	0	3	2	0	50	64	19456468	9.20E-01	1
PAPPA	69253	553	-19	4148112	1157712	0	11	6	0	50	55	19799652	9.21E-01	1
SAG	419346	227	28	1077612	287292	0	1	0	0	50	57	24239328	9.21E-01	1
LENG1	735613	439	-53	662872	205412	0	1	1	0	50	119	29395632	9.21E-01	1
PHF13	628180	259	38	765044	212176	0	1	1	0	46	63	18237524	9.21E-01	1
SCRN2	1071489	214	29	1079392	330368	0	2	3	0	12	13	4017104	9.21E-01	1
TSPAN15	660613	400	40	761128	213956	0	2	2	0	5	16	3262028	9.21E-01	1

GRB7	1448852	226	36	1367752	399788	0	2	0	0	50	82	28058140	9.21E-01	1
REV3L	343769	434	41	8015340	2148816	0	18	6	0	16	23	9150624	9.21E-01	1
CNTFR	843127	370	55	940552	287648	0	1	0	0	41	39	13720596	9.21E-01	1
KLF15	181307	600	34	1022076	322536	0	1	0	0	50	52	18741620	9.21E-01	1
COL4A3	253951	211	10	4295496	1355292	0	10	3	0	28	29	10585660	9.21E-01	1
HDGFRP2	2094827	278	40	1737992	485584	0	3	1	0	50	65	21272780	9.21E-01	1
FBL	1289328	526	23	818088	249912	0	1	1	0	50	79	21241096	9.21E-01	1
RNF170	513134	475	32	847280	231400	0	1	0	0	50	79	25125412	9.21E-01	1
DDX3Y	0	0	0	1740840	449628	0	1	0	0	50	39	21173456	9.21E-01	1
SLC4A1	1009890	175	40	2292284	710932	0	3	0	0	2	1	1480248	9.21E-01	1
KPNA6	1351816	218	27	1394808	383412	0	2	1	0	50	66	22560432	9.21E-01	1
HSD11B2	1115833	153	42	979000	338912	0	1	1	0	50	65	24004012	9.21E-01	1
SFXN3	744964	188	46	841584	245640	0	1	1	0	28	30	10248884	9.21E-01	1
DCHS2	63258	1024	3	9134248	2693852	0	34	9	0	4	11	4155232	9.21E-01	1
CPT1B	1073094	203	43	2012824	545036	0	2	1	0	9	11	4009272	9.21E-01	1
DRP2	417357	NaN	26	2494848	668568	0	4	2	0	50	57	24871228	9.21E-01	1
ANKLE1	865138	212	38	1502676	505164	0	2	0	0	14	13	5317572	9.21E-01	1
FAM65B	430274	256	26	2759356	770384	0	4	3	0	13	20	6987212	9.21E-01	1
KCNH5	68312	704	-32	2556792	690996	0	10	2	0	8	12	2975804	9.21E-01	1
SCNN1D	1744013	273	28	1776440	544324	0	4	2	0	9	14	4108596	9.21E-01	1
IFT80	427333	617	9	2058392	515488	0	6	0	0	1	1	693844	9.22E-01	1
ANGPTL1	NaN	NaN	NaN	1271988	321468	0	2	1	0	50	117	39967052	9.22E-01	1
NBPF9	NaN	NaN	NaN	2493780	613032	0	3	2	0	50	118	40258616	9.22E-01	1
STK39	135182	980	-1	1441444	380564	0	2	0	0	7	11	4453204	9.22E-01	1
TFAP2E	1049488	388	34	1072628	372376	0	2	1	0	14	17	4776096	9.22E-01	1
CCHCR1	3364017	401	19	2208980	657532	0	11	6	0	4	9	1440732	9.22E-01	1
MMP20	530118	436	17	1256680	334640	0	2	2	0	50	55	17536916	9.22E-01	1
FOXI3	NaN	NaN	NaN	996800	355288	0	1	1	0	50	117	40000872	9.22E-01	1
C5orf49	65781	1013	-18	375224	107512	0	1	1	0	2	4	754720	9.22E-01	1
CMAS	605789	955	-13	1116416	309364	0	1	3	0	5	9	2369892	9.22E-01	1
RPS6KA5	410309	521	27	2097196	548240	0	7	2	0	3	5	1833044	9.22E-01	1
NSMCE4A	249185	396	25	1010328	271272	0	1	0	0	23	21	8816340	9.22E-01	1
MDGA1	359430	461	50	2400508	730868	0	4	1	0	40	62	21993680	9.22E-01	1

CCDC142	1044697	315	48	1819160	598792	0	4	1	0	50	77	21769756	9.22E-01	1
APOF	2869106	184	52	801356	252760	0	1	0	0	23	47	14312980	9.22E-01	1
IFIT1B	253827	213	17	1211824	316484	0	1	0	0	44	34	14070544	9.22E-01	1
SPZ1	316792	617	34	1114636	266288	0	1	0	0	25	34	12940244	9.22E-01	1
WDR48	226944	500	25	1789968	461376	0	2	1	0	50	41	18734500	9.22E-01	1
CSTF2	341945	NaN	21	1476688	432896	0	5	2	0	1	3	1165900	9.22E-01	1
PARP15	419536	311	44	1799580	498756	0	2	1	0	50	52	23137864	9.22E-01	1
SNX21	742720	180	48	964760	308296	0	1	1	0	50	49	15404476	9.22E-01	1
BMP2K	234707	489	30	3022440	803136	0	8	1	0	5	11	3023864	9.22E-01	1
SUV420H2	1028808	209	19	1129588	383768	0	2	0	0	2	0	629052	9.23E-01	1
ASB16	930750	175	41	1314352	254540	0	2	1	0	50	69	21793252	9.23E-01	1
MRPS2	298554	539	14	743328	223212	0	1	1	0	50	72	20768328	9.23E-01	1
MEF2B	1260413	215	35	1053048	272340	0	2	1	0	50	63	16455388	9.23E-01	1
NUFIP1	685617	360	32	1302604	327164	0	2	0	0	9	16	5761148	9.23E-01	1
LPCAT1	557296	390	37	1359208	410468	0	3	2	0	21	32	8425808	9.23E-01	1
KCNG2	323907	509	-22	1095056	404416	0	2	2	0	33	73	17692844	9.23E-01	1
NEFL	180048	521	-2	1372024	386972	0	2	2	0	21	14	6475284	9.23E-01	1
RRM2B	504282	507	33	1148812	297260	0	2	0	0	50	86	24565068	9.23E-01	1
DOCK4	133354	718	-18	5177308	1342832	0	15	5	0	12	24	7108964	9.23E-01	1
CSTF1	254679	672	-43	1101464	304024	0	2	0	0	33	67	17771520	9.23E-01	1
LRRC25	1384225	259	42	743328	241368	0	1	2	0	21	32	9187648	9.23E-01	1
INVS	246802	735	19	2726604	757212	0	6	2	0	48	60	22374956	9.23E-01	1
CYP19A1	263003	535	17	1317556	335708	0	1	1	0	42	40	19095128	9.23E-01	1
CYP11A1	641004	222	46	1332508	374156	0	1	1	0	50	62	22671504	9.23E-01	1
KREMEN2	1443513	234	49	1182632	334996	0	1	0	0	14	11	5039180	9.23E-01	1
PKD2	435445	367	21	2476336	690284	0	4	1	0	50	51	21623440	9.23E-01	1
BAIAP2	1727896	243	16	1507304	417232	0	3	2	0	50	93	26971628	9.23E-01	1
GPR68	251273	462	42	888576	284088	0	4	3	0	5	7	1318624	9.23E-01	1
GSPT1	564730	294	51	1659316	444644	0	2	0	0	2	0	920616	9.23E-01	1
FAM124B	310772	460	12	1205416	359204	0	1	0	0	45	37	17693912	9.23E-01	1
CCNO	217401	417	22	845856	287292	0	1	0	0	8	10	3136004	9.23E-01	1
PLCD3	819156	170	30	1985768	604132	0	5	3	0	50	86	25371052	9.23E-01	1
REPS2	291898	NaN	23	1699188	491280	0	3	0	0	3	8	2605208	9.23E-01	1

OR4D6	430021	507	-1	770740	237452	0	2	3	0	8	24	4424012	9.23E-01	1
ATP6V0D2	192441	1158	-20	920972	235672	0	1	0	0	50	69	21910020	9.23E-01	1
ZNF709	1754294	593	0	1679608	390176	0	3	0	0	19	33	11978688	9.24E-01	1
CLCA2	248812	762	-13	2417240	662872	0	4	1	0	34	37	11546860	9.24E-01	1
CMYA5	263588	684	38	10210792	2882888	0	23	9	0	49	58	21072352	9.24E-01	1
ANO6	313473	719	1	2642588	669280	0	6	1	0	15	30	10215776	9.24E-01	1
SIK2	426569	296	19	2354228	675688	0	5	2	0	3	3	1435748	9.24E-01	1
ESRRB	243524	616	39	1291568	373444	0	3	1	0	13	17	4618744	9.24E-01	1
NYX	590228	NaN	54	1125672	422928	0	2	1	0	12	24	6562860	9.24E-01	1
DYTN	269047	832	14	1495200	406908	0	3	2	0	50	51	16973724	9.24E-01	1
GNS	441663	484	28	1436104	391244	0	1	1	0	30	20	11449672	9.24E-01	1
EMILIN1	1214097	238	50	2439312	849060	0	4	0	0	6	4	2593460	9.24E-01	1
ATP6V0A4	310803	385	7	2206844	573160	0	8	0	0	0	0	573160	9.24E-01	1
TXK	219787	358	39	1411896	341760	0	6	3	0	3	11	1857964	9.24E-01	1
PKNOX1	434727	131	65	1137776	305092	0	2	1	0	50	78	21346116	9.24E-01	1
SLC16A13	1696302	360	24	1007124	373800	0	1	1	0	50	49	16256384	9.24E-01	1
HEPHL1	363497	702	-19	3010692	787116	0	13	3	0	12	12	3883960	9.24E-01	1
BSND	454766	217	13	817732	223568	0	1	0	0	50	78	23577168	9.24E-01	1
TMEM214	1257014	290	34	1758640	520472	0	3	5	0	10	13	3564984	9.24E-01	1
ZDHHC18	1022828	188	30	974016	304380	0	1	0	0	40	52	15503088	9.24E-01	1
GRIN2D	1448469	209	38	3227496	1107516	0	7	7	0	12	33	9799256	9.24E-01	1
ANKRD49	341083	589	-7	615168	162336	0	1	2	0	27	33	7208644	9.24E-01	1
SLC9A5	1155617	142	51	2247784	692420	0	6	2	0	1	2	946248	9.24E-01	1
DCTN1	1086740	343	42	3284100	956928	0	6	2	0	13	15	5534020	9.24E-01	1
CAMSAP1	721697	446	46	4057332	1147032	0	8	3	0	50	58	23359652	9.24E-01	1
ARHGEF5	189369	838	0	3979012	1200788	0	7	1	0	17	14	7283404	9.24E-01	1
TIA1	667934	164	33	1037384	253828	0	1	0	0	50	55	21301616	9.24E-01	1
CBWD5	176193	NaN	-8	939128	228196	0	1	0	0	50	72	23175244	9.24E-01	1
UTS2R	2598845	179	36	895696	352796	0	1	1	0	50	59	20440452	9.24E-01	1
SCMH1	287585	346	25	1807056	498756	0	4	3	0	21	24	8498788	9.24E-01	1
CCDC132	260737	785	8	2608412	643648	0	8	0	0	1	2	1084376	9.24E-01	1
PABPC1	1141833	276	41	1650772	448916	0	5	2	0	21	45	11348924	9.25E-01	1
HFE	408919	685	43	900324	239232	0	4	1	0	1	1	411536	9.25E-01	1

MSH6	488225	587	32	3448216	955148	0	9	3	0	15	21	5630496	9.25E-01	1
KIAA1217	136799	839	-15	4920276	1393740	0	14	4	0	12	23	7186572	9.25E-01	1
CHRNA1	414231	577	21	1246356	341760	0	2	1	0	50	72	22389196	9.25E-01	1
ACTR5	358558	551	35	1538988	445356	0	2	2	0	16	12	5068728	9.25E-01	1
OR11H6	371813	563	-29	807052	252404	0	1	0	0	47	62	18578928	9.25E-01	1
ENTPD3	195830	611	18	1369532	371308	0	3	2	0	34	31	9178748	9.25E-01	1
FAM181B	303563	599	-7	998224	369884	0	1	1	0	25	29	11152412	9.25E-01	1
KCTD13	NaN	NaN	NaN	817020	261660	0	2	3	0	8	19	4187628	9.25E-01	1
EFTUD2	1101543	172	28	2546112	686724	0	6	5	0	50	74	17295192	9.25E-01	1
SLC5A10	661956	198	16	1538632	487364	0	4	0	0	2	5	1405488	9.25E-01	1
ATAD3C	1725369	187	27	1058032	309008	0	2	0	0	50	87	21566480	9.25E-01	1
ALDH3B1	1357582	356	31	1174088	360628	0	2	0	0	50	65	20667936	9.25E-01	1
KLF1	1937827	226	32	872912	300820	0	1	0	0	38	53	16981556	9.25E-01	1
MMP2	129512	538	-28	1723040	451408	0	7	4	0	13	40	7496648	9.25E-01	1
NOBOX	185754	838	-7	1429340	442864	0	3	2	0	50	64	21600656	9.25E-01	1
ALG6	133567	477	2	1336424	346744	0	3	0	0	2	1	848704	9.25E-01	1
TBX18	28205	623	-12	1511576	466360	0	3	3	0	32	51	15456096	9.25E-01	1
ZNF30	903994	549	-57	1620512	399076	0	4	1	0	36	106	25699640	9.25E-01	1
PIAS2	152679	618	42	1673200	455324	0	1	0	0	8	4	3779652	9.25E-01	1
SLC2A8	700547	270	42	1183700	388396	0	1	0	0	16	21	7354960	9.25E-01	1
PTGR2	502038	155	50	925244	241012	0	1	0	0	50	57	19811400	9.25E-01	1
PAK7	93375	786	-46	1830196	510860	0	3	1	0	7	5	2489508	9.25E-01	1
IL2RA	613453	373	40	730868	173728	0	1	1	0	8	13	3958364	9.25E-01	1
PRSS54	742919	385	13	1011040	279104	0	2	2	0	10	13	3310088	9.25E-01	1
OR51V1	109545	1056	-1	794592	236028	0	3	3	0	5	3	786048	9.25E-01	1
RPS6KA2	235623	646	9	2019588	555360	0	5	4	0	9	11	2928100	9.25E-01	1
IQGAP3	2095354	166	39	4189052	1200076	0	10	4	0	11	21	6972972	9.25E-01	1
PAX8	633218	385	36	1127096	231400	0	1	1	0	23	20	9556108	9.25E-01	1
DIS3L2	497951	464	30	2287300	636884	0	4	0	0	9	7	3130308	9.25E-01	1
PDGFRB	1065098	228	49	2820588	820224	0	4	1	0	8	5	2610192	9.25E-01	1
EFHC2	75979	NaN	21	1976156	488788	0	3	1	0	50	57	20924968	9.26E-01	1
SLC16A5	1311590	159	28	1248136	394448	0	3	3	0	42	79	20822440	9.26E-01	1
PEX1	750974	369	28	3305104	909224	0	9	2	0	24	29	11433652	9.26E-01	1

SCD	1162653	171	42	919904	254896	0	1	1	0	50	67	22016464	9.26E-01	1
OTOP1	178901	584	34	1517272	470276	0	2	1	0	50	56	20578936	9.26E-01	1
SLC44A3	329970	274	-13	1699900	457460	0	3	1	0	50	55	20286660	9.26E-01	1
GRK6	1038665	261	59	1556432	407620	0	3	1	0	50	73	23403440	9.26E-01	1
OTOA	520295	466	45	2981500	836600	0	2	2	0	16	15	7900708	9.26E-01	1
KIF11	347309	312	20	2754728	721612	0	3	0	0	6	7	4777164	9.26E-01	1
EPHA8	407931	541	25	2661100	813104	0	9	6	0	6	9	2709516	9.26E-01	1
NLGN3	759088	NaN	27	2105028	640800	0	3	1	0	47	69	24709248	9.26E-01	1
NDUFS1	311488	433	24	1886088	523320	0	4	0	0	3	3	1603780	9.26E-01	1
ZNF318	1262275	273	43	5652568	1693136	0	16	5	0	27	43	12797844	9.26E-01	1
OR5L1	4183	1091	-36	763620	236028	0	1	3	0	14	26	3895352	9.26E-01	1
FOXC1	145123	487	20	1338560	436456	0	1	2	0	49	44	19665440	9.26E-01	1
OR9A2	314565	676	-1	769672	226772	0	1	0	0	50	59	16752292	9.26E-01	1
CDC40	132627	636	19	1542192	378072	0	3	1	0	50	62	20629844	9.26E-01	1
GPKOW	833689	NaN	41	1209332	363832	0	2	0	0	50	74	21714932	9.26E-01	1
BMP2	44205	385	-10	992884	287648	0	1	1	0	41	48	18142472	9.26E-01	1
SH2D3A	736129	272	27	1407268	479888	0	2	2	0	50	67	24524840	9.26E-01	1
ETV2	1162794	390	27	864724	262016	0	1	0	0	50	66	20625572	9.26E-01	1
DNAH7	105357	518	-3	10462128	2709516	0	34	10	0	10	20	7344992	9.26E-01	1
SMO	811174	420	54	1963340	608404	0	5	2	0	50	74	21498128	9.26E-01	1
ATP8B3	2180359	206	29	3385916	949096	0	6	6	0	38	58	16575360	9.27E-01	1
CTAGE4	215477	711	-8	3907456	1082240	0	2	0	0	6	2	3916000	9.27E-01	1
ZNF638	328513	459	19	5112516	1339272	0	13	2	0	7	12	5061608	9.27E-01	1
C17orf50	675201	196	3	438948	132432	0	1	2	0	5	8	1611612	9.27E-01	1
ITSN1	921622	370	44	4498060	1190108	0	13	3	0	50	80	26037840	9.27E-01	1
BECN1	1633092	190	33	1197228	296904	0	1	0	0	24	22	8134244	9.27E-01	1
ABCB8	622098	373	41	1777864	592028	0	3	2	0	50	65	25140720	9.27E-01	1
RHBDD3	854820	251	51	914920	346388	0	1	0	0	50	60	20958788	9.27E-01	1
FKBP15	448462	456	9	3147396	876828	0	7	2	0	15	19	7552896	9.27E-01	1
OR4K15	420010	827	-51	847636	268424	0	1	2	0	50	72	16673972	9.27E-01	1
HGSNAT	277447	559	6	1640448	472056	0	3	3	0	50	72	22875492	9.27E-01	1
FARS2	127934	845	28	1161272	312568	0	1	1	0	35	45	16983336	9.27E-01	1
LILRA4	742939	507	-9	1254900	379140	0	3	1	0	50	62	17969100	9.27E-01	1

LRCH1	284604	798	49	2077972	571736	0	3	1	0	30	23	10011076	9.27E-01	1
AP4B1	335231	545	13	1885020	530796	0	4	1	0	50	60	19779004	9.27E-01	1
MAGEH1	140844	NaN	-23	543612	161268	0	1	1	0	50	113	23871224	9.27E-01	1
LTK	782552	166	40	2191892	660736	0	2	2	0	50	49	16541184	9.27E-01	1
GBE1	54244	720	0	1849420	467072	0	2	1	0	38	33	15329004	9.27E-01	1
KCTD18	542097	378	26	1074408	319332	0	1	1	0	39	34	14275956	9.27E-01	1
DNMT3A	330147	286	43	2475268	677468	0	6	3	0	50	59	20174164	9.27E-01	1
MTPAP	91344	331	12	1495200	409044	0	2	0	0	50	59	21246792	9.27E-01	1
ICA1L	600089	387	25	1293348	318264	0	5	3	0	9	17	3601652	9.27E-01	1
FAM71D	479620	324	19	1085444	299752	0	1	1	0	6	6	3123188	9.27E-01	1
CLPB	755787	258	56	1814888	524032	0	1	3	0	50	60	20431552	9.27E-01	1
CDH5	479049	262	-20	1992532	569600	0	8	5	0	10	23	4474208	9.27E-01	1
NR4A2	117105	440	4	1516560	428268	0	4	3	0	24	22	6529396	9.28E-01	1
RASSF10	267963	398	10	1475264	502672	0	1	0	0	50	58	23954172	9.28E-01	1
TULP1	683460	280	36	1400148	401568	0	1	1	0	50	60	24068092	9.28E-01	1
TMEM8A	1549307	207	37	1917772	606980	0	3	2	0	36	55	20238956	9.28E-01	1
CAST	394245	345	33	2216812	585620	0	3	1	0	29	22	9653652	9.28E-01	1
ITLN1	1048810	583	27	824140	211820	0	2	3	0	21	34	6982584	9.28E-01	1
SRMS	1262904	298	54	1222860	378072	0	5	0	0	3	6	1057320	9.28E-01	1
TK1	527017	217	27	604844	173728	0	2	2	0	9	36	5477416	9.28E-01	1
SLC30A5	420762	273	29	2045220	567108	0	1	1	0	50	64	24108320	9.28E-01	1
WHAMM	476426	631	23	2036320	597368	0	3	0	0	3	1	1278752	9.28E-01	1
PPCDC	887844	262	43	530440	147740	0	1	2	0	5	6	1521188	9.28E-01	1
STK17A	655380	310	23	1065864	289428	0	1	0	0	50	63	25441896	9.28E-01	1
STAP2	2122615	220	27	1164476	332860	0	2	3	0	21	45	12772568	9.28E-01	1
KCNB1	1002860	392	54	2139560	616948	0	6	3	0	50	78	21344692	9.28E-01	1
ZNF727	NaN	NaN	NaN	1306164	306516	0	2	0	0	50	116	39952100	9.28E-01	1
DEF6	682809	264	39	1624072	445712	0	3	0	0	50	86	25287392	9.28E-01	1
GJD3	1220026	191	23	689572	255608	0	1	0	0	14	22	5859760	9.28E-01	1
SVIP	52085	1031	2	207904	56960	0	1	1	0	1	2	292632	9.28E-01	1
CCIN	858124	257	46	1462448	426844	0	2	2	0	50	67	23320136	9.28E-01	1
CHRM3	52023	1031	-25	1472772	425064	0	4	2	0	50	127	28184520	9.28E-01	1
CSTF3	588753	326	16	1987548	493416	0	3	0	0	28	31	12066976	9.28E-01	1

GPR12	173793	619	18	810612	267000	0	1	1	0	50	48	14299096	9.28E-01	1
PELI3	1941577	194	51	1159848	374868	0	1	0	0	26	24	10661844	9.29E-01	1
OR6C1	667726	802	-14	776792	224992	0	2	3	0	8	13	2615888	9.29E-01	1
ZAN	1483965	336	46	7206508	2073344	0	19	8	0	10	24	9680352	9.29E-01	1
KRT26	836683	269	-10	1205060	329656	0	3	1	0	22	26	7229292	9.29E-01	1
LSM2	2509866	183	32	257032	67640	0	2	1	0	1	1	198648	9.29E-01	1
COL9A3	1044503	208	48	1730872	598436	0	1	2	0	50	65	20186624	9.29E-01	1
MAP2K7	952640	320	27	1087580	302956	0	3	2	0	18	34	7729828	9.29E-01	1
SLC22A15	589216	538	9	1394808	407976	0	2	1	0	50	62	20343264	9.29E-01	1
WDR27	197890	593	-17	2215032	636528	0	2	0	0	4	2	2213964	9.29E-01	1
ARNTL	296688	414	13	1654688	437524	0	3	1	0	16	24	7786076	9.29E-01	1
PRKDC	902699	459	27	10754760	2838744	0	27	7	0	5	9	3861176	9.29E-01	1
ZNF571	279768	914	-61	1591320	375936	0	3	1	0	50	63	18808548	9.29E-01	1
TTC16	1098647	172	32	2233900	624068	0	7	1	0	4	5	1521544	9.29E-01	1
PNKD	732698	197	35	1265224	397652	0	1	1	0	50	47	21819596	9.29E-01	1
AHRR	808969	399	34	1830196	571736	0	3	0	0	28	36	15015012	9.29E-01	1
SDF4	2356409	196	27	1035960	272340	0	1	1	0	39	53	20819592	9.29E-01	1
SLC6A2	130705	565	-32	1710224	487720	0	4	1	0	50	106	22384568	9.29E-01	1
KCTD2	1111289	171	31	665364	203988	0	1	0	0	50	75	17336132	9.29E-01	1
SPDYE6	NaN	NaN	NaN	1050556	270560	0	1	0	0	50	116	39916144	9.29E-01	1
WIZ	524410	481	33	1921688	655396	0	4	1	0	9	22	7155956	9.29E-01	1
TMPPE	513008	372	33	1096836	357780	0	1	1	0	50	53	21203360	9.29E-01	1
UBA7	1306720	191	39	2566048	779996	0	5	1	0	5	7	2686376	9.29E-01	1
PAX2	1089188	481	19	1221080	322180	0	2	3	0	26	24	7561084	9.29E-01	1
ADRA2B	792336	366	41	1077256	358136	0	1	2	0	50	64	25935668	9.29E-01	1
ACAA2	290591	530	35	1020652	292988	0	1	1	0	50	50	19363908	9.29E-01	1
LIPJ	160462	318	32	983984	229264	0	1	0	0	50	54	20094064	9.29E-01	1
TRAF6	98751	412	-8	1358496	342828	0	1	1	0	50	46	20659036	9.29E-01	1
ZNF821	468594	163	28	939484	270560	0	1	0	0	14	22	6992908	9.29E-01	1
ENOX1	99614	523	36	1694916	428268	0	4	2	0	50	66	20138564	9.29E-01	1
LDB2	55867	983	2	1070136	276612	0	6	2	0	1	3	512284	9.29E-01	1
SOX3	24994	NaN	-22	1058388	373800	0	1	0	0	12	17	6367060	9.29E-01	1
FAM188B	410182	538	21	1949812	553580	0	5	3	0	9	30	7540792	9.29E-01	1

NPEPPS	1229549	176	18	2394456	647208	0	5	0	0	5	3	1870068	9.30E-01	1
PTHLH	315469	581	-4	449984	135280	0	3	3	0	5	8	873268	9.30E-01	1
GRK7	426724	406	30	1396944	390888	0	3	3	0	15	15	4383072	9.30E-01	1
FAM13C	121813	707	-15	1534716	400500	0	3	1	0	27	63	18602424	9.30E-01	1
JAK2	255685	634	28	2997164	731224	0	6	1	0	6	10	2672136	9.30E-01	1
UBXN1	2425755	248	51	799220	233536	0	2	2	0	8	12	2683884	9.30E-01	1
KY	221323	459	13	1716988	448916	0	2	1	0	50	55	20313004	9.30E-01	1
NXPH4	2162032	238	58	744396	249912	0	1	0	0	50	84	24087316	9.30E-01	1
ZNF74	606391	351	4	1633684	449984	0	2	2	0	50	57	22041384	9.30E-01	1
PCK2	992350	355	19	1682100	506232	0	2	0	0	5	5	2775732	9.30E-01	1
FGG	59962	1036	-4	1215384	291564	0	6	3	0	1	4	915632	9.30E-01	1
RNF175	143076	685	18	868284	220008	0	1	1	0	50	60	18833468	9.30E-01	1
SGK223	132314	199	43	3426144	1086156	0	7	5	0	50	69	20107236	9.30E-01	1
MCM3AP	857856	624	60	5005004	1459600	0	16	3	0	0	3	1459600	9.30E-01	1
RPAP2	363408	559	0	1611968	406552	0	3	2	0	32	48	14701732	9.30E-01	1
TSPAN32	990037	630	25	819512	247420	0	2	0	0	50	84	21586060	9.30E-01	1
OR11H1	69794	916	-70	808476	237096	0	3	3	0	7	31	4401940	9.30E-01	1
KRT6B	1194800	389	29	1422576	423996	0	3	1	0	50	64	18116128	9.30E-01	1
C6orf89	836025	244	43	926312	241012	0	2	2	0	20	33	7678920	9.30E-01	1
DES	604261	335	38	1194736	350660	0	1	1	0	50	56	19025352	9.30E-01	1
ADAMTS8	693280	382	-6	2199368	686368	0	5	1	0	3	5	1922756	9.30E-01	1
PGLYRP2	524410	481	33	1390892	477040	0	2	2	0	50	79	23624160	9.30E-01	1
RIMBP2	225009	632	-35	2660032	786404	0	10	8	0	5	13	2487016	9.30E-01	1
SNAP91	181148	734	-28	2320052	708796	0	5	0	0	2	10	3389476	9.31E-01	1
KIRREL3	311236	849	9	2067648	608760	0	3	4	0	50	70	18721684	9.31E-01	1
DST	403209	446	23	17452544	4428640	0	37	12	0	7	19	7453572	9.31E-01	1
LHX5	444269	468	25	1013176	295124	0	1	0	0	16	19	7720572	9.31E-01	1
KIAA1257	1149132	379	52	1073696	269848	0	2	0	0	50	74	19642300	9.31E-01	1
TRIM46	1801444	194	32	1876832	598792	0	6	3	0	50	85	19936000	9.31E-01	1
PAPSS2	379125	169	46	1596660	441084	0	2	3	0	50	40	16055244	9.31E-01	1
AHCTF1	422581	405	4	5833060	1610900	0	10	3	0	39	24	14074460	9.31E-01	1
MRPL2	1239021	301	42	763264	242792	0	1	0	0	50	105	29539812	9.31E-01	1
SLC12A3	785707	125	37	2656828	755432	0	3	2	0	8	9	5309740	9.31E-01	1

SNX3	336217	451	42	421148	113920	0	2	1	0	1	3	513352	9.31E-01	1
NOL8	527573	574	45	3058752	751872	0	8	1	0	27	32	10821332	9.31E-01	1
EYA1	50979	967	-3	1579572	421860	0	4	3	0	50	90	21137500	9.31E-01	1
CHRM1	2342595	181	71	1113924	367392	0	1	0	0	32	47	15722384	9.31E-01	1
STK24	513130	468	59	1192956	319332	0	2	0	0	19	23	6959800	9.31E-01	1
IGSF3	691376	359	23	3053056	882524	0	5	0	0	1	2	1573520	9.31E-01	1
PTGER3	130184	907	-19	1211824	370952	0	5	1	0	4	8	1823432	9.31E-01	1
MBD4	917611	355	56	1506592	384836	0	4	3	0	50	67	16013236	9.31E-01	1
FANCE	683460	280	36	1334644	424352	0	2	2	0	15	26	5361716	9.31E-01	1
CAD	1238919	230	39	5579588	1736212	0	11	5	0	13	18	6024232	9.31E-01	1
CAPN12	1697047	322	45	1850132	542188	0	2	2	0	50	82	22226860	9.31E-01	1
MAGEE1	55359	NaN	-19	2312932	756500	0	5	1	0	10	24	6209352	9.31E-01	1
SLC12A8	372775	150	47	1803852	542544	0	2	2	0	50	38	16603128	9.31E-01	1
GRAMD1E	684637	439	15	1954796	494128	0	3	2	0	50	56	19966260	9.31E-01	1
KCNS1	865186	324	46	1275548	425776	0	2	3	0	20	39	11157396	9.31E-01	1
RBM12B	221692	552	-4	2519412	695268	0	6	0	0	2	2	1297976	9.32E-01	1
MTCH1	832054	220	41	950164	291920	0	1	1	0	50	65	22074136	9.32E-01	1
FMR1	96476	NaN	-6	1742264	354220	0	1	1	0	50	73	22840960	9.32E-01	1
CYP2J2	60418	824	-6	1297976	349948	0	2	0	0	5	6	2479540	9.32E-01	1
UGT2B7	21319	782	12	1368820	352796	0	1	1	0	50	51	21244300	9.32E-01	1
EGR2	176901	852	32	1148812	388040	0	1	2	0	43	60	20312292	9.32E-01	1
CTNS	504736	354	20	1055540	294412	0	1	1	0	50	62	23395252	9.32E-01	1
MBOAT7	735613	439	-53	1200432	347100	0	3	2	0	50	119	29395632	9.32E-01	1
MYCBPAF	1432761	200	22	2529380	705592	0	9	2	0	2	5	1209332	9.32E-01	1
NCAPH2	1075525	207	42	1625496	440016	0	2	0	0	5	6	2060528	9.32E-01	1
EIF4ENIF1	750597	313	19	2509800	729444	0	9	2	0	2	5	1343900	9.32E-01	1
C1RL	2422791	224	38	1236032	351016	0	2	2	0	14	22	6719500	9.32E-01	1
HCFC2	1209623	156	26	2034184	567464	0	3	0	0	11	12	5088664	9.32E-01	1
TBCK	176206	606	7	2350312	620864	0	6	0	0	0	0	620864	9.32E-01	1
FRMD4B	222480	432	12	2698836	711288	0	4	0	0	1	0	885728	9.32E-01	1
USP3	617922	178	42	1389824	339268	0	4	2	0	3	6	1752232	9.32E-01	1
SH2D4A	145909	265	-11	1228556	314704	0	3	1	0	4	4	1390536	9.32E-01	1
MR1	201966	253	23	881812	237452	0	2	3	0	6	11	2681392	9.32E-01	1

DDR1	2223866	223	25	2307236	713068	0	3	0	0	18	18	8274152	9.32E-01	1
OR10G9	107524	830	-5	755788	241724	0	1	1	0	50	68	19655828	9.32E-01	1
KRT2	1441383	464	29	1603780	483092	0	4	3	0	50	83	20991896	9.32E-01	1
TCEB3	960799	225	28	2049136	555716	0	3	1	0	50	55	20574664	9.32E-01	1
NPTXR	1317460	358	54	1204704	417588	0	2	2	0	50	65	18953440	9.32E-01	1
CHRNG	286805	211	36	1309012	399788	0	2	1	0	15	19	4104324	9.32E-01	1
SF3A1	696089	214	57	2044864	565328	0	4	4	0	23	37	9595268	9.33E-01	1
FNTA	304258	559	12	1008548	245284	0	1	0	0	50	59	21217244	9.33E-01	1
DGKG	664709	533	32	2107164	528660	0	12	4	0	2	4	958352	9.33E-01	1
BOP1	NaN	NaN	NaN	1889648	572092	0	5	5	0	26	45	12149212	9.33E-01	1
ZNF275	840610	NaN	19	849772	224636	0	2	1	0	2	2	797084	9.33E-01	1
IFT172	1218835	179	39	4581008	1231048	0	15	1	0	4	6	2618380	9.33E-01	1
DTX4	542222	686	9	1542548	478108	0	3	3	0	11	18	5612340	9.33E-01	1
GAB4	156217	639	-95	1462804	417944	0	5	3	0	21	35	7979740	9.33E-01	1
CD48	1156068	634	26	628340	168388	0	1	2	0	29	48	11074448	9.33E-01	1
KRTAP10-[illegible]	874480	461	25	927024	268068	0	2	3	0	8	6	1620512	9.33E-01	1
MAST2	1003923	416	24	4500196	1385552	0	9	1	0	1	3	2286232	9.33E-01	1
PRMT5	1177534	225	34	1712360	467428	0	4	0	0	4	6	2075480	9.33E-01	1
FZD9	904644	399	48	1422576	474192	0	2	3	0	48	71	23446160	9.33E-01	1
KCNG1	449718	474	23	1251696	403704	0	2	1	0	9	11	3779652	9.33E-01	1
SNX5	756104	499	29	1087580	267712	0	1	0	0	50	68	23326188	9.33E-01	1
FHL2	198241	215	13	748312	170168	0	1	3	0	7	6	1887868	9.33E-01	1
ZDHHC2	223323	568	3	988968	241368	0	1	0	0	50	51	18682880	9.34E-01	1
ROS1	168682	310	-18	6082972	1619444	0	21	2	0	2	7	3435756	9.34E-01	1
SYNJ2	478315	208	44	3801724	1105736	0	9	2	0	11	11	4009984	9.34E-01	1
NOL10	804107	344	32	1846928	446068	0	3	1	0	50	50	19580356	9.34E-01	1
OR11L1	87551	993	-35	788896	245996	0	2	1	0	31	88	16315480	9.34E-01	1
WDR43	478715	398	26	1785340	461732	0	4	0	0	2	0	582416	9.34E-01	1
OR2M7	18846	936	-35	774300	227484	0	3	2	0	20	38	5983648	9.34E-01	1
GLRA2	78081	NaN	18	1239236	323248	0	3	3	0	9	13	3195812	9.34E-01	1
ZMYM3	728738	NaN	25	3522620	991816	0	7	2	0	23	43	13991156	9.34E-01	1
RAPGEFL1	1502125	165	37	1184768	334996	0	2	3	0	15	40	11108268	9.34E-01	1
PREB	1214097	238	50	1045216	328232	0	1	0	0	50	60	22743060	9.34E-01	1

TMEM39B	1242874	218	24	1245288	368460	0	2	2	0	14	25	7848020	9.34E-01	1
CRISPLD2	444452	243	21	1315776	339624	0	1	2	0	28	19	9715240	9.34E-01	1
GKN2	340835	327	14	486652	127448	0	1	2	0	3	4	1019228	9.34E-01	1
PLA2R1	260653	401	24	3890724	930228	0	13	2	0	6	6	2532940	9.34E-01	1
NCEH1	314731	235	20	1122824	332860	0	3	4	0	6	10	2866156	9.34E-01	1
GAPDHS	1081638	390	41	1041300	311856	0	2	1	0	50	73	18185192	9.34E-01	1
TRIP11	336377	375	30	5165916	1263444	0	9	1	0	13	14	6578524	9.34E-01	1
ADRA1B	206270	458	31	1255968	417588	0	1	0	0	38	48	19035320	9.34E-01	1
COL4A2	521715	478	32	4296208	1393028	0	8	2	0	4	7	3588480	9.34E-01	1
OR8D2	154391	921	-14	762908	235672	0	1	1	0	8	10	3157720	9.34E-01	1
CSMD1	10896	1290	-36	9055216	2462096	0	25	6	0	4	7	3455336	9.34E-01	1
RTL1	356726	585	-24	3352452	1006056	0	7	1	0	5	4	2397304	9.34E-01	1
KRT39	977588	404	10	1255612	348524	0	4	2	0	50	68	14276668	9.34E-01	1
UGT2B15	106841	NaN	-2	2749744	702032	0	5	1	0	8	9	3725184	9.34E-01	1
PCDHGB1	662513	568	2	2292640	714848	0	6	4	0	50	85	24954176	9.34E-01	1
PIP4K2C	1347795	303	44	1097192	297616	0	6	3	0	2	3	717340	9.35E-01	1
SLFN12	763419	456	18	1491284	376648	0	2	1	0	50	58	19205844	9.35E-01	1
SRRM2	1717244	210	42	6529396	2351024	0	30	14	0	37	66	18539056	9.35E-01	1
NEU1	2485614	188	36	1034180	320044	0	1	2	0	36	35	13374208	9.35E-01	1
NUP214	759063	213	46	5194040	1655044	0	10	2	0	7	10	4578516	9.35E-01	1
DDX6	1493911	154	36	1269140	332860	0	3	0	0	9	25	6129608	9.35E-01	1
SNTB2	1187512	NaN	32	1332508	428624	0	2	1	0	39	44	14280584	9.35E-01	1
EPM2A	76860	748	-3	858672	252048	0	1	1	0	8	12	4123548	9.35E-01	1
IGJ	262444	609	19	416876	108580	0	1	1	0	1	1	443932	9.35E-01	1
SLC23A1	832127	177	48	1522256	471700	0	1	2	0	50	77	20769396	9.35E-01	1
TESK1	1202125	248	37	1518340	529016	0	2	1	0	50	57	20786840	9.35E-01	1
HOXD9	242935	874	15	854400	283020	0	1	0	0	50	65	19479964	9.35E-01	1
FZD2	1261037	254	16	1375940	437524	0	2	3	0	12	21	7147768	9.35E-01	1
ACO2	1474160	162	69	1999652	577432	0	4	4	0	19	17	5722700	9.35E-01	1
P2RY2	554585	189	59	902104	313280	0	1	2	0	50	78	20291288	9.35E-01	1
GDPD5	567353	205	40	1542904	462800	0	2	2	0	50	55	19678612	9.35E-01	1
BRWD3	150153	NaN	-20	4719492	1228200	0	7	2	0	5	9	4658616	9.35E-01	1
DNAJC25	NaN	NaN	NaN	925956	247776	0	1	0	0	50	116	39893360	9.35E-01	1

ZNF789	1327454	288	60	1156288	268424	0	2	0	0	50	58	17222924	9.35E-01	1
ZC3H7B	1354493	173	58	2530804	696692	0	6	1	0	2	3	1612680	9.35E-01	1
CCDC91	96900	922	-5	1180140	285156	0	1	0	0	50	49	18542260	9.35E-01	1
MAGI2	17425	1055	-24	3687804	1066932	0	14	6	0	50	120	29031088	9.35E-01	1
TNKS	106755	854	20	3378796	989324	0	4	0	0	13	12	8005728	9.35E-01	1
FBXO41	536944	452	20	2135644	718052	0	5	3	0	50	54	17210108	9.35E-01	1
TBC1D17	1673246	205	37	1655400	492348	0	3	1	0	13	23	7061972	9.36E-01	1
NDRG3	902649	176	61	1005344	263440	0	1	1	0	50	46	16998644	9.36E-01	1
KIAA0895	342379	285	17	1363480	362408	0	1	0	0	10	11	6055916	9.36E-01	1
SLITRK5	20779	1253	-27	2360992	715916	0	12	6	0	5	11	1927028	9.36E-01	1
RBBP4	1452351	195	34	1133504	280528	0	3	3	0	2	3	881456	9.36E-01	1
GANC	604999	342	37	2412612	617304	0	5	3	0	34	46	13753704	9.36E-01	1
ADRBK2	149272	580	-13	1854404	438592	0	2	1	0	50	49	22609204	9.36E-01	1
N4BP3	821697	248	39	1327168	436100	0	3	0	0	1	0	534712	9.36E-01	1
KBTBD11	NaN	NaN	NaN	1463160	536136	0	2	0	0	9	16	4893576	9.36E-01	1
POPDC2	288657	595	42	898900	283376	0	1	0	0	50	57	18639092	9.36E-01	1
FKRP	1166804	224	36	1168036	425420	0	1	1	0	50	50	18884020	9.36E-01	1
TBC1D22E	516467	376	38	1323964	348524	0	2	1	0	50	66	20539064	9.36E-01	1
OR6C70	802038	706	3	774300	226416	0	1	1	0	50	71	20539776	9.36E-01	1
ARRDC4	32065	851	-57	1058388	313992	0	1	0	0	17	18	6539364	9.36E-01	1
CCDC57	3405115	181	31	2323256	679960	0	6	0	0	0	0	679960	9.36E-01	1
TRIM55	277437	480	30	1494488	401212	0	3	3	0	19	24	8134600	9.36E-01	1
POLD2	1151124	282	56	1191888	358848	0	2	0	0	16	20	5937724	9.36E-01	1
POU4F3	388550	463	20	841584	253116	0	1	0	0	14	24	6888956	9.36E-01	1
ITGA2	193331	354	4	3087944	823072	0	5	3	0	50	51	21159572	9.36E-01	1
GALK2	315919	528	8	1206484	328232	0	1	1	0	50	56	21924260	9.36E-01	1
TMOD3	511166	132	27	934500	234960	0	1	1	0	50	63	20727744	9.36E-01	1
CECR6	244431	574	-79	1338560	516556	0	1	0	0	11	14	3797452	9.37E-01	1
FBXO15	149669	952	-6	1315776	362052	0	1	0	0	47	46	17533356	9.37E-01	1
ALDH2	691258	242	44	1325388	385548	0	1	0	0	23	20	10569640	9.37E-01	1
PPP1R15B	517418	230	28	1775372	516556	0	3	3	0	25	44	10490252	9.37E-01	1
CCNT2	288841	434	22	1895700	482736	0	4	1	0	37	46	15637656	9.37E-01	1
OR10H3	878287	605	13	781776	231756	0	1	0	0	50	75	21080184	9.37E-01	1

CALR	1886809	335	40	1118908	256676	0	1	3	0	22	36	7729472	9.37E-01	1
MAP3K6	884974	188	30	3228208	1021364	0	7	5	0	50	101	28643048	9.37E-01	1
HAUS6	500417	324	-17	2505884	625492	0	8	0	0	0	0	625492	9.37E-01	1
NAV1	778347	223	27	4759008	1440732	0	10	3	0	8	14	5668944	9.37E-01	1
DDX19B	1317982	139	47	1258816	326096	0	3	2	0	21	41	9045604	9.37E-01	1
LDHAL6A	611782	176	31	866504	230332	0	1	0	0	36	48	13303008	9.37E-01	1
FAM20A	662487	313	-28	1374516	404772	0	5	1	0	2	4	1016736	9.37E-01	1
LRRC4	NaN	NaN	NaN	1598440	501248	0	2	3	0	50	119	40146832	9.37E-01	1
THOC3	537044	631	37	907800	241368	0	1	0	0	50	69	22538004	9.37E-01	1
ZNF765	374515	876	-62	1370244	321468	0	2	1	0	50	58	17533000	9.37E-01	1
HUWE1	888642	NaN	47	11155616	3207916	0	23	9	0	35	59	17549732	9.37E-01	1
OAT	280862	543	16	1128520	319688	0	1	0	0	37	48	19006128	9.37E-01	1
SPATA17	87153	966	-21	970100	234604	0	1	1	0	50	90	26055640	9.37E-01	1
ACAD9	1117839	382	54	1609120	456392	0	4	2	0	50	75	20430128	9.37E-01	1
WDR82	1114111	181	50	830548	209684	0	1	0	0	50	72	20057396	9.37E-01	1
PTPDC1	449197	349	25	2117132	598792	0	7	1	0	11	12	3610552	9.37E-01	1
FBXO18	649866	305	28	2842304	762196	0	6	4	0	3	5	1793528	9.37E-01	1
FMNL3	1919377	265	60	2657540	742972	0	4	2	0	27	33	11775056	9.37E-01	1
ATG9B	622098	373	41	2256328	763976	0	3	0	0	2	2	1758996	9.37E-01	1
EPT1	698050	346	36	1112856	309720	0	2	0	0	1	0	578856	9.37E-01	1
SLC39A14	683723	144	32	1487724	435744	0	1	0	0	50	51	21613828	9.37E-01	1
RAVER2	269434	456	-10	1739416	485228	0	1	1	0	50	42	19719908	9.37E-01	1
PHC3	396500	426	32	2508376	744752	0	3	0	0	3	2	1939844	9.37E-01	1
LMAN2	942017	227	53	916700	257032	0	3	3	0	13	20	4127820	9.37E-01	1
TYK2	1802015	191	21	3013896	890712	0	9	6	0	2	6	1467432	9.37E-01	1
PEPD	302640	598	-23	1280532	362052	0	1	0	0	3	2	1774304	9.37E-01	1
ACSS2	1004645	205	44	1861880	505876	0	2	3	0	12	15	6442176	9.37E-01	1
DAG1	1657622	236	28	2154868	720188	0	4	1	0	2	1	1166968	9.37E-01	1
PES1	653792	330	28	1537208	411892	0	1	0	0	37	30	15384540	9.37E-01	1
NKD2	692388	484	29	1154508	334284	0	1	1	0	50	54	21721340	9.37E-01	1
DFNB59	170323	555	37	908512	248132	0	1	2	0	11	13	2651488	9.37E-01	1
SMC1B	461206	199	17	3275200	789608	0	6	2	0	43	48	18109008	9.37E-01	1
SLC45A3	747509	281	33	1310436	477396	0	4	3	0	14	23	5793544	9.37E-01	1

C9orf142	1979517	188	34	518336	166252	0	1	1	0	16	30	5786424	9.37E-01	1
MEGF11	708482	458	49	2728740	713424	0	4	0	0	0	0	713424	9.37E-01	1
TTLL7	157542	957	-14	2337496	588824	0	8	2	0	3	8	2086516	9.38E-01	1
C19orf18	1252385	750	-19	574584	140976	0	1	1	0	50	145	23910384	9.38E-01	1
SERPINA6	203903	744	-1	1027416	290496	0	4	2	0	3	5	1280532	9.38E-01	1
SART3	437636	399	40	2514072	651480	0	6	0	0	0	0	651480	9.38E-01	1
RNF157	1737490	208	23	1765404	492348	0	4	3	0	7	17	4960504	9.38E-01	1
SLC46A3	167695	629	37	1178004	336420	0	2	0	0	26	35	9892172	9.38E-01	1
PAN3	265548	616	57	2268076	653972	0	5	1	0	21	21	6697784	9.38E-01	1
ADAM32	347510	612	-5	2111792	515488	0	5	1	0	25	29	9007512	9.38E-01	1
BEGAIN	402460	304	5	1472416	456392	0	1	2	0	50	61	23805008	9.38E-01	1
KIF3A	717412	356	59	1849776	461376	0	2	0	0	40	33	14637296	9.38E-01	1
ODF2	1916608	160	35	2270924	597724	0	6	3	0	50	88	22837400	9.38E-01	1
FLNC	830283	310	35	6865460	2069428	0	18	9	0	47	71	22027144	9.38E-01	1
IKBKG	2163759	NaN	42	1270564	333572	0	2	0	0	50	72	22102972	9.38E-01	1
CLEC4M	719851	329	21	1063372	266288	0	3	2	0	3	15	3114644	9.38E-01	1
NRXN3	21523	905	-32	3016388	826276	0	8	4	0	50	84	17166676	9.38E-01	1
OR4C3	278638	642	-40	806340	248844	0	2	2	0	7	18	3234972	9.38E-01	1
MAPK6	583597	153	23	1861168	469208	0	3	1	0	50	52	19123964	9.38E-01	1
ATP8B4	278209	408	-21	3137784	799932	0	13	8	0	3	10	1681032	9.38E-01	1
DNAJA2	498873	362	13	1082596	274832	0	2	0	0	26	50	13805680	9.38E-01	1
MAVS	740484	241	42	1318268	440728	0	3	2	0	4	3	1253832	9.38E-01	1
PMFBP1	443927	479	0	2733012	646140	0	9	1	0	3	4	1242440	9.38E-01	1
PORCN	726793	NaN	2	1189752	350304	0	1	0	0	24	24	10365296	9.38E-01	1
KIF20B	202048	401	23	4723408	1118552	0	9	4	0	12	14	5216112	9.38E-01	1
NADK	1511678	243	30	1155932	323248	0	2	1	0	50	69	18623784	9.38E-01	1
PPARA	536661	179	24	1203636	324672	0	1	2	0	50	76	22770472	9.38E-01	1
GMEB2	1094905	193	54	1322896	416876	0	1	0	0	8	6	3583852	9.38E-01	1
AJAP1	5695	921	4	1007836	333572	0	2	2	0	3	3	1196160	9.38E-01	1
ZNF669	379967	612	-15	1182276	320400	0	1	1	0	50	38	15803196	9.38E-01	1
EDC4	879834	158	50	3544336	1067288	0	8	5	0	50	85	23764780	9.38E-01	1
SERPINE3	202753	434	28	1081884	307584	0	1	0	0	34	43	14320456	9.38E-01	1
UBL7	640438	204	54	972236	291208	0	1	1	0	50	66	22913228	9.38E-01	1

ARL6IP4	1093113	196	47	925600	303668	0	1	0	0	50	70	23094788	9.38E-01	1
ACTR10	291132	545	21	1090072	301532	0	2	2	0	8	9	2706668	9.38E-01	1
GPR32	652250	593	27	861876	280884	0	2	3	0	11	12	2724112	9.39E-01	1
HNF1A	1225663	380	40	1569604	495908	0	9	4	0	5	12	2076904	9.39E-01	1
ZC3H12C	137968	266	8	2243156	612676	0	4	3	0	50	59	20309800	9.39E-01	1
ST5	519265	369	26	2877548	849772	0	4	2	0	15	8	4429708	9.39E-01	1
PDK2	1197823	210	20	1062304	287648	0	1	1	0	50	73	23672220	9.39E-01	1
OR5K4	414958	885	-14	799932	229620	0	1	1	0	50	65	18540480	9.39E-01	1
CYP4F8	852124	605	29	1339984	378428	0	4	4	0	4	6	1391604	9.39E-01	1
CBLC	866184	178	-12	1202568	364188	0	2	2	0	50	72	19151732	9.39E-01	1
ZDHHC16	556333	188	42	973660	280172	0	1	1	0	50	54	18863728	9.39E-01	1
SERPINA3	316239	858	10	1070492	305092	0	2	1	0	50	75	18471772	9.39E-01	1
ERBB2IP	400865	189	32	3573172	920972	0	8	1	0	3	5	1873628	9.39E-01	1
HS1BP3	580167	278	28	994664	292276	0	1	0	0	34	42	13866200	9.39E-01	1
SCN9A	93566	908	-9	5122128	1326456	0	17	3	0	7	17	5214688	9.39E-01	1
FKBP10	1760409	175	35	1467432	438948	0	2	2	0	18	37	7890028	9.39E-01	1
ASRGL1	3053973	413	49	787116	232824	0	2	3	0	16	13	2613396	9.39E-01	1
MFAP3L	136790	831	-4	1019940	297972	0	1	2	0	50	55	17384192	9.39E-01	1
DBH	765804	481	31	1572096	457104	0	3	3	0	12	19	6466740	9.39E-01	1
ZMYM2	329235	391	43	3608772	904596	0	6	1	0	5	6	2966192	9.39E-01	1
GPRIN3	108672	672	-1	1901752	587756	0	3	2	0	50	61	19723112	9.39E-01	1
RNF14	696239	321	48	1241016	310788	0	1	1	0	50	50	19214032	9.39E-01	1
PACRG	81261	1273	-28	767536	209684	0	1	0	0	50	110	24171332	9.39E-01	1
APOA1BP	2049505	180	37	727664	221788	0	2	3	0	3	5	1179072	9.39E-01	1
OR7D2	311030	471	-63	765756	237096	0	1	2	0	50	102	27434784	9.39E-01	1
STX10	1948449	221	41	653260	176576	0	1	2	0	6	8	2024216	9.39E-01	1
SERPINE2	314351	346	11	1059456	291564	0	1	1	0	50	57	21233620	9.39E-01	1
PRSS35	185011	557	-30	1042012	288716	0	2	2	0	50	102	24837764	9.40E-01	1
NAIF1	1898149	195	32	794948	260236	0	1	1	0	50	88	21996172	9.40E-01	1
CWF19L1	1070791	198	40	1419372	365256	0	2	0	0	16	15	4935228	9.40E-01	1
DPYSL3	97519	827	-10	1479180	405840	0	2	1	0	50	81	26726700	9.40E-01	1
SYT6	760371	596	13	1091852	302956	0	1	2	0	2	2	854756	9.40E-01	1
F2	965022	176	48	1613036	442864	0	2	3	0	9	13	3752596	9.40E-01	1

CDSN	NaN	NaN	NaN	1281956	422572	0	2	3	0	26	43	11999692	9.40E-01	1
SYNGR2	527017	217	27	569600	166252	0	3	3	0	5	26	2872564	9.40E-01	1
TRIM8	878626	180	27	1389468	400500	0	4	0	0	2	1	755432	9.40E-01	1
SERPINA4	316239	858	10	1078680	309720	0	2	1	0	50	75	18471772	9.40E-01	1
SLC30A4	207518	299	10	1096480	306872	0	1	2	0	18	16	5485604	9.40E-01	1
SCO1	59713	749	-45	770028	221076	0	1	1	0	23	51	13509844	9.40E-01	1
CCR9	222879	519	54	940908	253116	0	1	1	0	21	22	8029580	9.40E-01	1
KLHL13	57664	NaN	-2	1895700	499824	0	5	3	0	14	31	4959436	9.40E-01	1
ERCC4	125854	251	11	2333936	648988	0	4	1	0	41	53	18388468	9.40E-01	1
ATG9A	773232	177	36	2118556	632612	0	4	3	0	50	56	19964124	9.40E-01	1
ARPC1B	903923	263	51	972236	261304	0	1	2	0	10	14	5285176	9.40E-01	1
34GALNT4	1454043	344	48	2599868	815596	0	4	2	0	8	21	8495940	9.40E-01	1
MYO5A	517214	386	19	4871860	1245644	0	11	3	0	21	29	10209724	9.40E-01	1
PPP1R7	1363901	233	35	953724	243504	0	1	1	0	50	73	23887244	9.40E-01	1
OR1S1	637499	828	-25	804916	238520	0	2	4	0	12	16	3304748	9.40E-01	1
TTLL2	254009	704	1	1495556	415096	0	1	3	0	14	31	8777180	9.40E-01	1
PPFIBP2	104307	853	12	2286944	621220	0	3	3	0	50	70	20469644	9.40E-01	1
ITGB4	1885370	198	27	4784996	1398724	0	13	8	0	27	40	12343944	9.40E-01	1
ANXA4	864962	193	50	857604	225348	0	1	1	0	50	77	22354308	9.40E-01	1
OR2W3	87551	993	-35	756856	250268	0	2	2	0	4	10	1329304	9.40E-01	1
DDX27	886628	261	50	2084736	556428	0	2	1	0	50	75	24073432	9.40E-01	1
MOXD1	188578	673	1	1592032	424352	0	2	1	0	50	61	22625580	9.40E-01	1
IRX3	99177	509	-42	1204348	416876	0	1	2	0	6	6	3221800	9.40E-01	1
FARSA	1886809	335	40	1312928	371308	0	2	1	0	50	62	18190176	9.40E-01	1
SASH3	370406	NaN	69	969388	281240	0	1	0	0	50	66	22247152	9.41E-01	1
COPS7B	955894	365	41	684588	190104	0	1	0	0	50	89	21316212	9.41E-01	1
SRP54	629868	171	32	1369532	312568	0	2	2	0	50	66	20974452	9.41E-01	1
CCDC114	1265470	217	34	1720548	484872	0	4	3	0	50	73	18591388	9.41E-01	1
PDE6A	1032925	135	43	2291572	556784	0	2	0	0	3	1	1675692	9.41E-01	1
SUV39H2	255711	396	38	909580	232112	0	1	1	0	50	62	20607060	9.41E-01	1
VASP	1107927	194	44	978644	293344	0	1	1	0	50	55	18375296	9.41E-01	1
SUN5	461222	461	40	1019584	253472	0	1	1	0	50	77	24604940	9.41E-01	1
TTC21B	100856	599	-2	3469576	869708	0	9	1	0	5	7	3191184	9.41E-01	1

DZIP1L	175494	464	8	1980428	551800	0	4	2	0	30	38	11679648	9.41E-01	1
ACOT9	473183	NaN	48	1209688	292988	0	1	0	0	50	48	19909300	9.41E-01	1
SLC11A1	734742	197	37	1392672	432540	0	1	2	0	42	49	17001136	9.41E-01	1
PTPRF	996816	182	33	4814544	1435392	0	12	5	0	5	7	3076196	9.41E-01	1
RYK	538368	306	16	1583844	433608	0	1	3	0	19	26	8325060	9.41E-01	1
MAP2	109927	678	-9	4830208	1368464	0	11	4	0	50	60	24955956	9.41E-01	1
POLG	636068	420	24	3161992	900680	0	6	2	0	7	13	4963708	9.41E-01	1
WDR72	15464	1008	-29	2863308	751872	0	9	5	0	26	48	8409076	9.41E-01	1
TMEM39A	270591	599	29	1245288	355644	0	1	1	0	30	37	11842340	9.41E-01	1
IMPA1	160074	681	-1	916344	201852	0	2	2	0	2	3	1055540	9.41E-01	1
HSCB	571394	219	-10	625492	154148	0	2	2	0	2	2	589536	9.41E-01	1
TTLL6	727820	415	24	2385912	622644	0	4	1	0	25	45	16791452	9.41E-01	1
SI	13371	1355	-23	4837328	1218232	0	19	4	0	5	10	3117492	9.41E-01	1
MSTO1	2230401	185	32	1446428	440728	0	2	1	0	50	72	23683256	9.41E-01	1
PIH1D2	389805	179	31	873268	224636	0	1	1	0	37	39	12636576	9.41E-01	1
SMG5	2266205	211	33	2618736	729444	0	9	5	0	6	7	1890360	9.41E-01	1
ATRIP	1441050	223	39	1991464	601640	0	3	0	0	4	6	3126748	9.41E-01	1
CCNE1	167528	609	-33	1093988	271984	0	2	0	0	50	100	24916796	9.41E-01	1
RUNDC3A	1009890	175	40	1169816	328588	0	1	0	0	17	28	10725568	9.41E-01	1
COL10A1	NaN	NaN	NaN	1604848	585620	0	2	1	0	50	117	40231204	9.42E-01	1
AMICA1	707709	293	56	1037740	282308	0	1	0	0	35	34	11396628	9.42E-01	1
DOT1L	1658089	199	28	3857260	1187972	0	21	12	0	7	21	3803860	9.42E-01	1
SMU1	953054	286	0	1343544	350304	0	4	0	0	7	14	3185844	9.42E-01	1
CXorf23	339910	NaN	56	1797444	433608	0	3	1	0	16	15	5251000	9.42E-01	1
GNAT1	846254	199	44	922396	235316	0	1	1	0	39	49	14244272	9.42E-01	1
ACTL9	536422	487	-47	994308	343896	0	4	3	0	5	15	1866508	9.42E-01	1
ADSL	445026	427	47	1267004	342472	0	1	0	0	15	20	9024244	9.42E-01	1
CILP2	868278	284	7	2791752	947316	0	7	2	0	31	41	11001112	9.42E-01	1
USP54	1072614	150	40	4308668	1181920	0	9	3	0	6	19	5606644	9.42E-01	1
KCNJ11	454503	598	14	952656	300108	0	2	3	0	4	11	2547892	9.42E-01	1
THRB	370625	518	-12	1206484	307940	0	1	2	0	50	69	23081260	9.42E-01	1
ZNF92	300914	894	18	1536496	357068	0	1	1	0	50	54	21619168	9.42E-01	1
LMAN1	292594	549	35	1343900	346744	0	1	1	0	50	41	19123964	9.42E-01	1

KIAA0355	662469	216	-1	2697056	785692	0	5	3	0	50	60	20026424	9.42E-01	1
LYPD5	464802	286	11	633680	192952	0	1	1	0	7	12	2949104	9.42E-01	1
PPP2R2A	194762	353	13	1229980	287648	0	1	1	0	50	61	20706384	9.42E-01	1
FBF1	1667990	194	22	2894280	858672	0	13	2	0	0	2	858672	9.42E-01	1
TRAP1	611617	242	43	1822008	515844	0	1	0	0	14	15	5350680	9.42E-01	1
PRCP	306726	371	-6	1336068	362052	0	2	1	0	10	12	4032412	9.42E-01	1
MED24	1613074	202	28	2555012	728020	0	5	3	0	50	64	20738780	9.42E-01	1
ZMYND15	1272092	222	28	1857608	578500	0	1	2	0	50	74	23892584	9.42E-01	1
TBCCD1	676664	501	35	1420440	397296	0	1	0	0	17	17	7760088	9.42E-01	1
KIAA1109	325000	770	8	12909272	3486664	0	34	9	0	3	10	4298344	9.42E-01	1
SMARCAL	590685	384	31	2435396	694912	0	4	3	0	40	45	17232180	9.42E-01	1
CERCAM	1928973	188	64	1505168	453544	0	5	1	0	6	10	2403356	9.42E-01	1
SMCR8	957114	254	9	2345328	664296	0	8	1	0	1	2	1019940	9.43E-01	1
NPC1L1	1577451	363	49	3383424	1057320	0	10	4	0	12	22	5892868	9.43E-01	1
MAPKAPK	755663	242	42	1260240	313992	0	1	0	0	5	4	2579576	9.43E-01	1
CELA3B	448600	190	29	698472	201852	0	2	4	0	5	7	931296	9.43E-01	1
TFCP2	530649	155	40	1339628	331792	0	2	1	0	50	50	17804628	9.43E-01	1
TRIM33	727314	264	13	2902468	794948	0	4	1	0	4	4	3009268	9.43E-01	1
LRP8	563155	310	23	2488796	676756	0	5	1	0	1	1	959064	9.43E-01	1
ADCK4	1022809	201	31	1388756	417232	0	2	1	0	35	42	13429032	9.43E-01	1
SLC2A13	70254	909	-18	1630480	489500	0	2	1	0	17	21	7364928	9.43E-01	1
TNRC6B	502506	484	34	4765416	1336068	0	10	3	0	23	43	14054524	9.43E-01	1
ZNF678	606105	575	-6	1515848	358492	0	3	0	0	49	70	21063808	9.43E-01	1
PPM1N	1107927	194	44	1038808	359204	0	1	0	0	18	23	8456780	9.43E-01	1
FZD5	335586	341	26	1411896	465648	0	1	1	0	50	45	22065948	9.43E-01	1
ADAM12	214443	485	-29	2437176	685656	0	6	3	0	50	98	22668656	9.43E-01	1
PLSCR4	119025	361	-10	866860	224636	0	1	1	0	12	18	5852996	9.43E-01	1
METTL14	238966	636	31	1198652	308296	0	4	0	0	4	5	1092920	9.43E-01	1
DDX60	83741	350	-10	4512300	1129944	0	8	2	0	1	4	1954084	9.43E-01	1
PSTPIP2	386983	300	54	930584	204700	0	1	0	0	50	60	19433684	9.43E-01	1
REXO1	2226995	214	26	3011048	970456	0	7	2	0	50	75	24188776	9.43E-01	1
VPS41	139398	862	3	2320764	540408	0	5	1	0	50	58	19640520	9.43E-01	1
FAM83F	712106	370	28	1239948	384480	0	3	3	0	3	6	2127456	9.43E-01	1

SLC4A11	1086365	338	49	2446076	724816	0	6	2	0	50	68	19319764	9.43E-01	1
PCNX	262398	420	40	5952676	1700612	0	11	5	0	33	46	14204044	9.43E-01	1
TBR1	219774	676	10	1699900	509792	0	3	2	0	50	63	19999724	9.43E-01	1
CNTROB	1794630	337	33	2351736	694200	0	6	2	0	45	62	18104736	9.44E-01	1
ZFP14	784358	758	-67	1399792	326096	0	4	1	0	50	76	18074120	9.44E-01	1
DUSP4	307440	267	42	1103600	347812	0	1	2	0	50	47	17510572	9.44E-01	1
ZCCHC9	148763	777	23	718764	174084	0	1	2	0	5	6	1922756	9.44E-01	1
RHD	642678	270	36	1061948	317908	0	1	1	0	50	58	20611688	9.44E-01	1
ZNF280C	320988	NaN	38	1961560	479888	0	3	0	0	3	3	1343900	9.44E-01	1
ADAMDEC	57540	741	5	1243864	320756	0	1	1	0	50	63	24260688	9.44E-01	1
HIVEP2	248800	596	25	6110028	1751520	0	19	2	0	18	26	10069460	9.44E-01	1
NAPB	188695	688	-19	812392	188324	0	1	1	0	50	79	22836332	9.44E-01	1
LRTM2	245027	593	30	914208	287292	0	7	3	0	2	3	465292	9.44E-01	1
CCNG1	226025	696	12	766468	205412	0	1	0	0	50	71	18478892	9.44E-01	1
DYNC1LI2	804191	145	10	1278396	354576	0	1	1	0	50	63	22381720	9.44E-01	1
CXCL1	233432	444	6	273408	87576	0	1	2	0	3	3	454968	9.44E-01	1
AP4M1	1401098	205	46	1163408	353152	0	1	0	0	50	62	24927476	9.44E-01	1
HEXA	1126741	366	48	1378788	374868	0	2	0	0	5	4	1817024	9.44E-01	1
ITIH3	1193949	272	49	2330020	616592	0	4	2	0	50	63	22512372	9.44E-01	1
DCAF15	930314	231	32	1531868	447136	0	2	3	0	24	40	9354256	9.44E-01	1
METAP2	441602	328	18	1259172	318264	0	3	0	0	8	6	2232120	9.44E-01	1
ACTR1B	362146	520	25	969388	281240	0	1	1	0	27	40	13121448	9.44E-01	1
ZNF358	609287	308	36	1366328	463156	0	2	1	0	14	18	5583504	9.44E-01	1
AP3M2	580110	382	45	1079748	296904	0	1	2	0	8	9	3483460	9.45E-01	1
SLC16A7	22532	899	-36	1207908	343896	0	3	3	0	4	5	1651484	9.45E-01	1
CCDC148	133882	759	-5	1637600	368104	0	2	1	0	18	15	6115012	9.45E-01	1
LEMD2	1565139	428	28	1250628	400500	0	1	0	0	3	0	862944	9.45E-01	1
ZBTB3	2405109	248	44	1383772	462800	0	5	1	0	4	7	1804564	9.45E-01	1
DIP2A	895243	477	47	4043804	1265224	0	4	5	0	50	53	22697492	9.45E-01	1
PKP4	212889	532	5	3020660	891424	0	6	3	0	50	58	21474276	9.45E-01	1
ZNF853	NaN	NaN	NaN	1640448	482736	0	2	0	0	50	116	40128320	9.45E-01	1
FMO3	395042	1004	-11	1381992	359916	0	10	3	0	2	3	640444	9.45E-01	1
DUSP8	NaN	NaN	NaN	1507660	519404	0	2	1	0	50	117	40164988	9.45E-01	1

OR10J1	347588	699	-5	789964	242792	0	2	3	0	12	18	3300832	9.45E-01	1
PSMC1	318736	622	14	1150592	307228	0	1	0	0	50	63	24033204	9.45E-01	1
LILRB1	978578	625	-1	1656824	495196	0	3	3	0	50	108	22158508	9.45E-01	1
LYAR	146135	584	35	1021008	230688	0	1	0	0	50	50	17705304	9.45E-01	1
MOV10	559669	325	27	2540416	766112	0	5	2	0	50	65	23160292	9.45E-01	1
FOXP3	547555	NaN	25	1098972	332148	0	1	1	0	42	42	16120392	9.45E-01	1
COMT	645345	217	24	678536	207904	0	2	3	0	1	4	647564	9.45E-01	1
TRIM50	902769	422	52	1219656	369528	0	2	0	0	50	86	23744844	9.45E-01	1
SLC43A1	923775	197	9	1427560	426488	0	2	1	0	50	53	18462160	9.45E-01	1
SLC39A11	276039	538	-34	865436	271984	0	1	0	0	50	69	19703532	9.45E-01	1
PLG	181774	624	-9	2120336	557140	0	3	2	0	50	68	26918584	9.45E-01	1
IFT57	289544	711	11	1134572	285868	0	1	0	0	50	61	19059172	9.45E-01	1
CCDC113	785059	347	15	993596	253828	0	2	2	0	9	13	3363132	9.45E-01	1
PM20D1	699823	377	35	1295840	369172	0	2	3	0	16	28	8483836	9.45E-01	1
TDRD5	599875	613	-9	2526176	688504	0	5	3	0	50	65	21156368	9.45E-01	1
AAMP	730654	197	33	1123536	315060	0	1	0	0	24	27	9730548	9.45E-01	1
CUL5	421044	572	14	2085804	493416	0	5	1	0	50	63	19373876	9.45E-01	1
PGA5	1124603	480	43	993596	289072	0	1	1	0	50	76	25756956	9.45E-01	1
TNNT2	857879	209	26	868996	176576	0	1	2	0	39	38	11559676	9.45E-01	1
VSX1	598947	563	-8	969032	320044	0	1	0	0	50	58	18814956	9.45E-01	1
AIFM2	484729	425	25	945892	286580	0	2	0	0	4	2	874336	9.45E-01	1
DEFB125	86	430	NaN	391244	116056	0	1	1	0	6	9	1389824	9.45E-01	1
S1PR2	1729517	200	31	843008	295480	0	1	1	0	50	94	24020032	9.45E-01	1
ZAR1	310162	396	52	1032756	341760	0	1	0	0	50	60	20167400	9.45E-01	1
WDR54	1069803	343	45	854044	258812	0	1	1	0	50	68	19848780	9.46E-01	1
NKRF	663615	NaN	48	1786052	488788	0	3	0	0	16	23	6519784	9.46E-01	1
KRT7	742854	178	53	1186192	353864	0	2	2	0	50	77	19170956	9.46E-01	1
NUP210L	2028800	184	37	4845872	1375228	0	15	3	0	0	3	1375228	9.46E-01	1
FAM155A	73040	1144	-37	1153440	325740	0	6	1	0	1	5	630832	9.46E-01	1
TBL2	955146	171	48	1133504	327520	0	1	2	0	14	15	4918852	9.46E-01	1
ZFAT	35738	963	15	3191184	858672	0	5	4	0	50	72	22511304	9.46E-01	1
OTC	125559	NaN	36	928092	247776	0	1	0	0	50	56	17194444	9.46E-01	1
TAOK1	822855	224	26	2633688	657888	0	10	1	0	3	5	1645076	9.46E-01	1

C11orf80	1534660	164	38	1748316	494484	0	3	0	0	22	37	12351776	9.46E-01	1
F2RL2	NaN	NaN	NaN	933432	272340	0	2	3	0	8	19	4198308	9.46E-01	1
NUFIP2	1348501	128	32	1762912	482024	0	3	0	0	12	14	5358868	9.46E-01	1
C12orf42	568754	897	2	913852	264152	0	1	2	0	5	8	2673560	9.46E-01	1
CHST14	888588	192	47	900324	307584	0	1	1	0	38	56	17300888	9.46E-01	1
TRAF5	298152	424	2	1470636	358848	0	1	1	0	50	54	21836684	9.46E-01	1
ABCA3	2220466	233	46	4315432	1275548	0	7	3	0	18	22	8844820	9.46E-01	1
ACTRT2	308009	634	19	928448	284800	0	1	2	0	16	20	6999672	9.46E-01	1
RALBP1	472280	369	46	1712716	427556	0	3	3	0	13	14	3951244	9.46E-01	1
LRRC45	3814891	211	28	1713784	504452	0	2	1	0	32	25	10015348	9.46E-01	1
CD19	1189140	257	55	1438596	411180	0	4	3	0	7	10	2316848	9.46E-01	1
DPY19L1	112453	935	0	1789612	466004	0	3	0	0	41	52	18697476	9.46E-01	1
DENND4B	2028800	222	34	3687092	1224640	0	17	5	0	25	39	8426876	9.46E-01	1
GALM	860461	176	27	870420	254184	0	2	3	0	4	4	1187616	9.46E-01	1
PTCHD2	759100	482	25	3467084	1081528	0	11	8	0	8	14	4359932	9.46E-01	1
THEG	901402	578	-17	971524	278036	0	2	2	0	50	99	20859820	9.46E-01	1
WNT7B	498719	270	28	884660	249556	0	1	2	0	24	27	8489176	9.46E-01	1
SEMA4B	876945	154	52	2098264	642224	0	5	2	0	50	83	23818892	9.46E-01	1
CLEC17A	1022856	198	29	779640	198648	0	1	1	0	50	85	21567548	9.46E-01	1
GTF2IRD2	875900	173	54	2461740	646140	0	5	0	0	1	1	1105380	9.46E-01	1
SOX18	1152034	208	48	910648	327164	0	1	0	0	50	78	24047088	9.46E-01	1
LRWD1	642828	230	65	1635464	502672	0	2	1	0	6	4	2203640	9.46E-01	1
PTPRZ1	64760	971	-15	5910668	1633684	0	18	7	0	30	52	13576772	9.47E-01	1
ADAM8	339279	452	-12	2185840	468140	0	3	0	0	30	32	12924580	9.47E-01	1
WFS1	418118	599	48	2215388	672484	0	3	2	0	38	34	13572144	9.47E-01	1
BEND7	241076	532	25	1233896	351016	0	2	1	0	7	10	3391612	9.47E-01	1
PATZ1	723505	237	57	1952304	569244	0	4	2	0	50	75	22402368	9.47E-01	1
LAMB2	1898051	203	36	4542204	1354224	0	12	3	0	0	3	1354224	9.47E-01	1
SUCLG1	293337	672	-17	886440	259524	0	1	0	0	50	74	19405560	9.47E-01	1
HIPK2	545030	522	37	3015676	885728	0	4	3	0	50	68	27916452	9.47E-01	1
TTBK1	1293543	363	51	3228564	1066932	0	6	4	0	44	65	22785424	9.47E-01	1
PPP1R8	717145	218	26	894984	260592	0	2	2	0	2	2	822360	9.47E-01	1
OR52D1	99343	946	-2	773588	252760	0	1	2	0	50	83	21652988	9.47E-01	1

SSX2IP	335727	572	-6	1643652	382344	0	2	0	0	12	12	3298696	9.47E-01	1
CENPI	426524	NaN	26	2016384	496620	0	2	1	0	50	50	22981580	9.47E-01	1
NUDT12	201826	521	0	1192244	316840	0	1	0	0	38	43	17213312	9.47E-01	1
PIGN	125694	442	28	2455688	650056	0	1	0	0	19	9	7707756	9.47E-01	1
TRIM49	151171	1107	-28	1189396	287648	0	3	2	0	50	137	28003316	9.47E-01	1
DDOST	631461	208	35	1169104	337844	0	1	0	0	50	63	21866232	9.47E-01	1
DHPS	1975767	409	1	956928	267000	0	3	4	0	5	13	2513360	9.47E-01	1
MYOM2	104051	570	-26	3836256	1014600	0	11	5	0	50	97	21899340	9.47E-01	1
TET2	188424	632	24	5156660	1394452	0	8	3	0	50	42	19519124	9.47E-01	1
FAM43A	406498	421	39	1018160	340336	0	1	0	0	50	65	22418032	9.47E-01	1
SLC25A12	443171	185	35	1754724	495552	0	5	1	0	9	16	3941988	9.47E-01	1
PTGIS	1117465	380	52	1270920	374868	0	3	1	0	50	75	19539416	9.47E-01	1
SFMBT2	169112	363	12	2325392	627628	0	5	2	0	50	66	20980148	9.47E-01	1
TAAR2	125852	605	19	894272	237808	0	1	0	0	50	67	19971956	9.47E-01	1
RPE65	201619	477	-16	1406556	362052	0	2	1	0	50	71	22141420	9.47E-01	1
MYO1D	502828	818	5	2631908	686368	0	6	3	0	10	16	5810632	9.47E-01	1
PLEKHM2	683349	174	22	2600580	748668	0	5	2	0	36	49	19723468	9.47E-01	1
OPLAH	2720766	211	27	3196524	1048776	0	5	4	0	25	30	10299080	9.47E-01	1
FBXO3	755929	424	15	1246356	318264	0	2	2	0	3	3	1430052	9.47E-01	1
SLC5A12	52729	921	-8	1584556	458528	0	2	1	0	50	68	24203016	9.47E-01	1
APCS	492825	646	-4	562480	161624	0	3	3	0	1	3	391956	9.47E-01	1
OTUD5	834315	NaN	40	1438240	435032	0	2	0	0	35	44	13840212	9.47E-01	1
POM121C	806294	337	66	2396236	816308	0	4	4	0	8	10	3306172	9.47E-01	1
PROC	443349	175	30	1199364	315060	0	2	1	0	16	16	4827360	9.48E-01	1
TTC22	578299	343	23	1484520	475260	0	1	1	0	50	44	21555444	9.48E-01	1
AFG3L2	492344	214	26	2051628	573516	0	6	1	0	4	5	1786052	9.48E-01	1
ARRB2	1272092	222	28	1073340	300108	0	1	0	0	50	74	23892584	9.48E-01	1
EME1	1335838	196	24	1484876	420436	0	2	1	0	50	70	22959864	9.48E-01	1
WDR75	413405	503	12	2199012	551088	0	3	1	0	2	1	987544	9.48E-01	1
PNKP	1673246	205	37	1335712	407264	0	2	3	0	13	23	7061972	9.48E-01	1
ADAMTS2	1496298	677	-4	3148464	904596	0	7	4	0	34	54	14663284	9.48E-01	1
L1TD1	274287	422	-1	2225356	553580	0	2	0	0	18	15	8095796	9.48E-01	1
CBX6	1317460	358	54	1000716	341760	0	1	1	0	50	65	18953440	9.48E-01	1

TRAF3	719158	178	49	1490216	375580	0	3	2	0	50	57	15090840	9.48E-01	1
IQGAP2	229166	675	34	4144196	1054828	0	10	2	0	32	49	14971224	9.48E-01	1
STRN3	431319	370	14	2071564	557852	0	5	0	0	0	0	557852	9.48E-01	1
XRCC6	1475100	183	50	1604492	401212	0	2	0	0	26	34	12579616	9.48E-01	1
REC8	974559	220	41	1411896	421860	0	8	5	0	3	6	1006412	9.48E-01	1
ACTG2	1036341	284	45	976508	267712	0	1	0	0	50	73	24714944	9.48E-01	1
TTC21A	213514	500	30	3453200	902104	0	6	5	0	34	47	15558980	9.48E-01	1
SEMA4C	822597	392	46	2080108	651836	0	5	5	0	13	20	5944132	9.48E-01	1
KRTAP8-1	30244	1055	-70	161268	45924	0	1	1	0	2	1	160912	9.48E-01	1
ARHGAP2[illegible]	164987	309	-3	4985424	1393740	0	13	2	0	9	8	3680684	9.48E-01	1
RPA1	1473497	224	18	1620156	425064	0	1	3	0	50	90	23672932	9.48E-01	1
COL24A1	263693	863	-12	4442168	1306876	0	14	3	0	8	9	3638676	9.48E-01	1
LPPR4	100692	868	-15	1913144	560344	0	4	5	0	6	15	3497700	9.49E-01	1
GFI1	370328	580	1	1059456	321468	0	1	2	0	50	67	22579300	9.49E-01	1
GLT8D2	1193959	281	37	925244	234604	0	1	2	0	44	53	16574648	9.49E-01	1
ADAMTS5	33106	441	-79	2307948	704880	0	7	3	0	32	40	9945928	9.49E-01	1
TAS2R8	408176	517	12	779640	212532	0	1	3	0	5	3	1250628	9.49E-01	1
MYCN	169653	1491	-12	1134928	359204	0	6	4	0	2	7	1326456	9.49E-01	1
ALOXE3	1455014	202	31	2139916	628340	0	3	0	0	15	15	6988280	9.49E-01	1
CCDC88B	1610483	257	51	3657188	1188328	0	8	2	0	5	14	5199380	9.49E-01	1
UBQLNL	99343	946	-2	1189396	335708	0	2	2	0	50	83	21652988	9.49E-01	1
FOXK2	2114072	155	26	1615172	536848	0	5	5	0	2	6	1430408	9.49E-01	1
ZNF785	2214934	142	54	1013888	297616	0	4	4	0	5	13	2288012	9.49E-01	1
WIPI2	1750164	417	34	1207196	336064	0	1	0	0	50	47	17226484	9.49E-01	1
TDRD10	1804226	337	43	987544	272696	0	1	1	0	50	73	19734148	9.49E-01	1
UNC13D	1727802	178	35	2795668	832328	0	6	3	0	25	44	12389512	9.49E-01	1
SRRM1	466068	348	27	2250632	717340	0	4	0	0	2	1	958708	9.49E-01	1
ZBTB48	628180	259	38	1760776	491636	0	4	3	0	46	63	18237524	9.49E-01	1
LRPAP1	475773	609	54	925600	253472	0	2	1	0	6	7	1274124	9.49E-01	1
RASA3	434290	406	58	2209692	563904	0	4	4	0	20	21	7325056	9.49E-01	1
SLC25A5	617823	NaN	30	747956	222856	0	1	1	0	28	51	12210800	9.49E-01	1
FMNL2	289066	611	8	2855832	752940	0	5	3	0	50	66	24219036	9.49E-01	1
DNM3	435280	643	6	2236036	617660	0	4	2	0	50	51	16943820	9.49E-01	1

IRX2	18983	986	-29	1151304	378072	0	3	3	0	42	88	16235024	9.49E-01	1
DHX37	1085697	229	46	2947324	876116	0	5	4	0	50	54	20728100	9.49E-01	1
MCM6	469154	395	29	2129948	574228	0	5	3	0	50	78	22581436	9.49E-01	1
TAF1A	228041	467	-2	1213604	274120	0	1	1	0	32	31	12819560	9.49E-01	1
SH2D3C	1194942	179	58	2251344	692064	0	5	2	0	17	32	8660768	9.49E-01	1
LIG3	362649	525	15	2653268	725884	0	4	2	0	50	58	23206928	9.49E-01	1
SLC26A4	515284	579	37	2008552	579212	0	8	5	0	2	5	908512	9.49E-01	1
MFSD3	1755818	291	7	960488	381988	0	1	4	0	7	11	3540420	9.49E-01	1
MAPKAP1	850799	475	38	1387332	352440	0	1	1	0	50	40	18879748	9.49E-01	1
KIAA1211	460708	489	17	3077976	909936	0	6	2	0	50	62	21782928	9.49E-01	1
C6orf118	31222	466	-31	1212536	329656	0	3	1	0	50	98	20698552	9.49E-01	1
LIPN	175532	327	31	1052336	258100	0	1	0	0	50	58	19686800	9.50E-01	1
CHEK1	477807	595	18	1271988	311856	0	3	0	0	25	32	6874360	9.50E-01	1
TRIM35	353563	561	8	1243864	362408	0	1	2	0	50	61	22463244	9.50E-01	1
GNRHR	209065	767	35	885728	176932	0	2	2	0	5	9	2150952	9.50E-01	1
OR4A5	68	1280	-38	786048	230688	0	2	1	0	1	1	543968	9.50E-01	1
LLGL2	1907659	195	31	2653268	789964	0	6	4	0	50	90	21830276	9.50E-01	1
STK32C	148625	280	4	1256680	350660	0	2	0	0	2	0	610184	9.50E-01	1
SIX4	352813	482	5	1913500	602708	0	5	0	0	0	0	602708	9.50E-01	1
TAS2R39	321366	568	-9	845856	239232	0	2	3	0	8	11	2366332	9.50E-01	1
NCAPG	271201	1073	21	2641520	701320	0	2	0	0	19	14	9068032	9.50E-01	1
CCDC130	767214	289	6	1026348	279816	0	1	2	0	50	92	27852728	9.50E-01	1
ZNF804A	18097	1171	4	3079756	809900	0	17	7	0	3	12	1924536	9.50E-01	1
UBE2D4	872910	239	44	401568	100392	0	1	1	0	2	6	913496	9.50E-01	1
XPNPEP2	358809	NaN	48	1764692	483448	0	2	2	0	50	64	23182008	9.50E-01	1
ANTXR2	62176	905	6	1309724	370240	0	1	0	0	25	26	11416920	9.50E-01	1
ABCF1	2080582	271	32	2239596	575652	0	3	0	0	24	27	11479576	9.50E-01	1
PDK3	512129	NaN	34	1093276	288716	0	1	2	0	50	78	21432980	9.50E-01	1
NCKIPSD	1646019	212	45	1797088	572804	0	1	0	0	5	3	2853696	9.50E-01	1
BTBD3	59672	322	-61	1333220	355288	0	3	2	0	50	68	18866220	9.50E-01	1
CDC37L1	228353	608	21	901392	207192	0	1	0	0	4	6	2162344	9.50E-01	1
RANBP3	1166723	392	36	1478824	409400	0	3	0	0	0	0	409400	9.50E-01	1
BMPER	20466	507	-17	1797088	460664	0	4	1	0	50	73	20882604	9.50E-01	1

SP5	318434	709	28	952656	330012	0	1	1	0	11	13	4395888	9.50E-01	1
ETF1	1202314	205	51	1151304	294768	0	1	1	0	23	28	9944504	9.50E-01	1
GNAT2	989433	201	29	938416	231044	0	1	0	0	50	79	23548688	9.50E-01	1
GORASP2	414976	406	36	1129588	360272	0	1	0	0	48	56	21071640	9.50E-01	1
IVD	880617	184	45	1104668	310432	0	1	1	0	44	61	19363196	9.50E-01	1
CASKIN2	1918292	195	25	2939136	992172	0	6	1	0	15	21	7661832	9.50E-01	1
GMPR2	915114	220	38	948384	261660	0	1	1	0	50	69	21077692	9.51E-01	1
CNOT6	625675	485	17	1453904	380920	0	2	0	0	22	29	9407656	9.51E-01	1
FRY	221552	488	40	7786076	2127100	0	26	11	0	9	24	5424372	9.51E-01	1
TST	699535	379	44	731580	231756	0	4	2	0	2	4	695268	9.51E-01	1
GRAMD2	1132082	352	26	940196	246352	0	1	2	0	24	29	8168420	9.51E-01	1
IL20RB	137627	421	9	804560	220720	0	2	2	0	4	8	1727312	9.51E-01	1
SPATA5L1	340172	254	10	1822008	623712	0	1	1	0	50	45	20758360	9.51E-01	1
CSF3	1652518	216	28	520828	162692	0	1	2	0	4	17	2215032	9.51E-01	1
SELPLG	448698	280	40	957996	341760	0	1	0	0	50	58	19104740	9.51E-01	1
NUAK1	143018	473	7	1668216	478464	0	6	4	0	13	20	4272356	9.51E-01	1
ZSCAN29	823006	415	50	2169108	580992	0	4	1	0	50	74	24588920	9.51E-01	1
AGXT	1187158	297	30	996444	305448	0	1	1	0	10	14	4259540	9.51E-01	1
ASB2	200153	600	30	1472772	441084	0	2	1	0	25	33	10893600	9.51E-01	1
KCNT1	404991	494	38	3180504	909936	0	7	4	0	50	54	17973016	9.51E-01	1
NUP88	1179795	474	24	1928096	519760	0	3	1	0	42	58	18912500	9.51E-01	1
FREM1	254826	882	-17	5645092	1544684	0	15	7	0	29	38	12294104	9.51E-01	1
RCN1	251970	683	-1	865792	219296	0	1	0	0	50	65	18605272	9.51E-01	1
FAM149A	373525	536	-13	1229980	364544	0	1	2	0	50	71	21317280	9.51E-01	1
COX7B2	86349	938	11	211108	55892	0	1	1	0	1	3	306516	9.51E-01	1
PRAMEF11	NaN	NaN	NaN	1101108	307584	0	3	4	0	8	20	4233552	9.51E-01	1
TUT1	2695768	217	42	2229984	733716	0	4	2	0	17	27	9232504	9.51E-01	1
TECPR1	464632	375	26	2995028	843364	0	8	2	0	6	4	1829484	9.51E-01	1
BMP4	252916	645	-3	1015312	303668	0	1	3	0	9	11	3310444	9.51E-01	1
MARS	1347799	194	38	2320052	658600	0	2	1	0	20	34	11604176	9.51E-01	1
RABGAP1	340132	482	34	2818452	716628	0	4	1	0	50	40	17123600	9.51E-01	1
RUNX1T1	5155	1075	-30	1556076	436812	0	6	3	0	50	124	25218684	9.51E-01	1
DNAJC28	884788	182	63	1005700	244928	0	4	0	0	1	0	350660	9.51E-01	1

SIAE	172228	604	18	1344612	372732	0	2	1	0	50	48	13956624	9.51E-01	1
ITPRIP	293891	376	8	1349240	410824	0	1	2	0	50	51	20513788	9.51E-01	1
FAM131B	379715	379	29	924888	257388	0	2	2	0	3	7	1614104	9.51E-01	1
NLRP2	982889	432	-21	2700260	756856	0	10	6	0	7	19	4696352	9.51E-01	1
ALDH16A1	2173457	290	44	1965476	675688	0	8	6	0	14	25	5097208	9.51E-01	1
WBSCR16	733800	220	50	1168392	364188	0	1	0	0	26	28	9856572	9.51E-01	1
ISOC1	38196	985	-2	746176	231044	0	1	1	0	50	84	17946672	9.51E-01	1
ENPP1	168112	699	-5	2468148	603420	0	5	2	0	24	35	11212932	9.51E-01	1
UNC5CL	134733	565	32	1305808	391244	0	1	0	0	50	55	22917856	9.51E-01	1
GRM5	158962	944	-23	3033120	887508	0	19	7	0	10	25	3681752	9.51E-01	1
NR2F6	865138	212	38	981848	328588	0	1	1	0	50	59	18864796	9.51E-01	1
OR52R1	66543	987	-3	771096	240300	0	1	1	0	50	67	17575720	9.52E-01	1
KRT35	1445853	325	-22	1153440	339624	0	3	0	0	2	3	1021364	9.52E-01	1
FARP2	1221130	383	31	2736216	755076	0	6	2	0	14	17	5950184	9.52E-01	1
MRPL37	465615	313	26	1070848	315416	0	4	4	0	11	19	3532232	9.52E-01	1
PHACTR3	137383	818	-29	1441088	404416	0	5	4	0	3	6	1672132	9.52E-01	1
CASP14	990597	524	16	630832	171236	0	1	1	0	27	45	9464260	9.52E-01	1
D2HGDH	960711	380	30	1291568	419368	0	1	0	0	50	47	19676120	9.52E-01	1
CSNK1G1	743794	263	43	1109652	288360	0	3	0	0	4	6	1495556	9.52E-01	1
RORA	231490	883	13	1782848	451408	0	3	2	0	50	67	19482100	9.52E-01	1
MGAT5B	1010920	228	17	2103960	605556	0	7	6	0	14	17	4263100	9.52E-01	1
CUL9	1308754	363	35	6358160	1880392	0	21	7	0	12	24	6411204	9.52E-01	1
CCDC102A	771506	159	45	1368820	426488	0	2	0	0	6	10	3627640	9.52E-01	1
FYCO1	224232	519	54	3761496	1045572	0	4	3	0	39	37	16129648	9.52E-01	1
SAP30BP	1898715	198	32	826276	208616	0	1	2	0	17	30	7129968	9.52E-01	1
PRPSAP1	1622536	209	24	989324	285868	0	1	1	0	50	86	23974464	9.52E-01	1
KIAA1147	213663	781	17	1166968	328232	0	1	0	0	17	17	6826300	9.52E-01	1
MAP7D1	800052	190	31	2103604	662516	0	6	1	0	2	4	1583488	9.52E-01	1
C19orf35	1603571	240	27	1106804	424708	0	1	1	0	50	66	21932448	9.52E-01	1
SSH2	724760	390	21	3621588	1002852	0	11	2	0	10	19	6398388	9.52E-01	1
SCYL2	184701	578	3	2417952	634392	0	4	2	0	50	44	17871200	9.52E-01	1
PHGDH	425891	581	19	1337492	420436	0	2	2	0	32	42	12159536	9.52E-01	1
CEP290	150984	429	-18	6651860	1520476	0	7	2	0	27	18	11005384	9.52E-01	1

MIB2	1666776	248	31	2670000	846924	0	3	2	0	50	64	21384564	9.53E-01	1
DMRT1	85434	622	8	939484	275900	0	4	2	0	2	4	907088	9.53E-01	1
HCN2	1740226	218	1	2183704	699896	0	2	1	0	6	7	4213972	9.53E-01	1
GABRA2	69760	992	3	1176580	310076	0	3	2	0	6	9	2222508	9.53E-01	1
GALNT6	504747	239	33	1589896	445712	0	3	3	0	50	57	19104384	9.53E-01	1
GTF3C5	1828532	402	50	1363480	372020	0	2	0	0	50	62	18209756	9.53E-01	1
.NKRD20A	NaN	NaN	NaN	2169464	530440	0	4	2	0	50	118	40176024	9.53E-01	1
FAM91A1	483934	269	10	2206132	582416	0	4	4	0	14	35	8258132	9.53E-01	1
SLC10A6	266749	603	40	953368	279104	0	1	1	0	50	48	15603480	9.53E-01	1
MKS1	851306	401	14	1513712	412960	0	2	1	0	15	17	5719852	9.53E-01	1
FAM161A	758678	249	34	1706664	434676	0	10	3	0	1	5	1129232	9.53E-01	1
CCDC112	273705	493	20	1410828	325740	0	1	0	0	50	49	22158864	9.53E-01	1
RASGRF1	420514	488	42	3303680	893560	0	6	2	0	4	9	2910656	9.53E-01	1
PLCB3	1613996	257	65	3164840	920260	0	10	7	0	19	29	6446448	9.53E-01	1
MAP3K9	247989	426	43	2793888	842652	0	9	2	0	5	8	2008552	9.53E-01	1
FGFR1OP	169081	639	35	1054828	280172	0	1	0	0	26	27	9675012	9.53E-01	1
DUSP9	1716845	NaN	44	931652	314704	0	1	1	0	50	73	19103672	9.53E-01	1
FNDC1	805575	596	23	4662532	1503032	0	10	5	0	5	25	7169128	9.53E-01	1
SNX4	319722	132	35	1208264	294412	0	1	1	0	50	46	17844856	9.53E-01	1
HECTD1	431156	164	26	6692800	1852268	0	20	5	0	3	9	3300832	9.53E-01	1
GATAD2B	1697153	161	36	1506592	438236	0	2	1	0	34	56	17060588	9.53E-01	1
HLA-DOB	1373102	417	37	700964	200428	0	1	0	0	11	17	4212192	9.53E-01	1
COL11A2	1474628	377	24	4431844	1489148	0	14	6	0	22	42	9267748	9.53E-01	1
CUZD1	301441	588	12	1563196	419012	0	2	0	0	12	19	4510164	9.53E-01	1
SBNO1	1090982	125	37	3631200	963336	0	13	3	0	19	43	11852308	9.53E-01	1
AP2M1	1456939	257	45	1173376	267356	0	1	0	0	27	40	13815648	9.53E-01	1
C2orf71	471053	404	28	3173740	960488	0	7	3	0	24	46	13239640	9.53E-01	1
PICK1	1478013	200	76	1089716	294412	0	1	1	0	50	52	18287364	9.53E-01	1
PDCD2L	689668	292	2	925244	254896	0	1	3	0	8	8	2930236	9.53E-01	1
DNAH3	335315	549	29	10637280	2813112	0	39	17	0	19	32	8714880	9.53E-01	1
DISC1	236101	622	-3	2921692	752228	0	3	0	0	13	7	4344624	9.53E-01	1
ZNF33A	139504	916	-19	2128524	490212	0	4	2	0	9	15	4259540	9.54E-01	1
ACVR1B	493265	371	27	1398724	394448	0	1	1	0	7	4	2520480	9.54E-01	1

SLC6A18	657890	541	24	1586336	475972	0	3	3	0	33	54	17107224	9.54E-01	1
BIK	624112	241	48	410468	122464	0	1	2	0	4	7	1169460	9.54E-01	1
CD4	2819394	232	40	1178716	330368	0	2	2	0	36	48	14117536	9.54E-01	1
HBP1	249919	627	12	1355292	337488	0	1	0	0	20	30	13166304	9.54E-01	1
FRYL	287083	428	52	7840188	2077260	0	17	9	0	29	35	14612732	9.54E-01	1
RPS7	253814	371	14	514776	137772	0	2	2	0	3	6	869708	9.54E-01	1
SNAPC4	1194160	258	28	3645440	1160560	0	4	5	0	50	42	16832036	9.54E-01	1
CTSA	725555	209	59	1303672	361340	0	2	1	0	27	40	9561092	9.54E-01	1
PZP	352696	301	19	3830560	1072628	0	13	4	0	3	8	2497696	9.54E-01	1
KIF4B	516166	461	38	3116068	840872	0	6	3	0	34	40	13986172	9.54E-01	1
VEGFC	145765	585	-14	1093632	280884	0	2	1	0	4	7	2060172	9.54E-01	1
RMI1	458954	343	14	1584912	425064	0	4	1	0	29	48	13606676	9.54E-01	1
HDAC10	944000	242	49	1676048	551800	0	2	3	0	50	67	23711024	9.54E-01	1
GAL3ST2	598219	487	29	970456	320756	0	1	0	0	50	85	26481416	9.54E-01	1
GLCE	367968	218	35	1570672	422216	0	4	1	0	4	7	2213252	9.54E-01	1
LRRC42	430138	201	31	1100752	303668	0	1	2	0	50	52	17828480	9.54E-01	1
SIM2	451019	447	47	1780712	548596	0	3	3	0	23	30	10320796	9.54E-01	1
NONO	728362	NaN	26	1231760	323248	0	1	0	0	50	84	28751272	9.54E-01	1
PTPRG	223467	890	7	3762920	993952	0	11	7	0	18	32	8246740	9.54E-01	1
ANKRD33	570716	438	22	1195448	342472	0	1	0	0	22	25	10128200	9.54E-01	1
OSBPL9	586185	288	18	2066580	504096	0	3	0	0	13	18	6838404	9.54E-01	1
USP30	592814	487	20	1339984	370952	0	3	0	0	23	33	8751904	9.54E-01	1
OR14C36	104321	936	-39	765756	234960	0	5	4	0	9	22	2442516	9.54E-01	1
SOX4	117516	676	1	1132792	389108	0	2	2	0	14	18	4975812	9.54E-01	1
NMUR2	261658	955	4	1046284	299396	0	1	1	0	50	87	24234700	9.54E-01	1
ITGB5	330119	254	40	2061240	561768	0	3	2	0	50	50	18077324	9.54E-01	1
RASSF7	1970020	236	42	952656	332148	0	1	1	0	50	66	20259248	9.54E-01	1
EHD3	102236	884	15	1357428	381276	0	2	3	0	40	51	14956272	9.54E-01	1
GCLC	363341	337	24	1681032	427200	0	6	3	0	2	5	1410116	9.55E-01	1
OMD	537639	574	47	1093988	266644	0	1	0	0	50	52	17802848	9.55E-01	1
ENPP4	64703	791	12	1164832	302600	0	1	0	0	50	52	20330092	9.55E-01	1
HAS1	436280	690	-24	1399080	475260	0	2	1	0	50	53	16569308	9.55E-01	1
IRS2	217826	438	4	3176588	1117840	0	2	2	0	14	12	5974036	9.55E-01	1

RELT	561848	218	56	1079036	344608	0	1	2	0	50	64	21419452	9.55E-01	1
ZBTB16	162787	529	-6	1712004	473124	0	2	2	0	50	65	22986564	9.55E-01	1
RBPJ	178487	597	36	1356716	358492	0	2	0	0	18	21	6595968	9.55E-01	1
SHPK	504736	354	20	1187972	373444	0	1	2	0	50	62	23395252	9.55E-01	1
ILDR1	524127	715	38	1275192	362052	0	1	0	0	27	30	12290188	9.55E-01	1
KCNAB3	1794630	337	33	1046996	306160	0	1	0	0	45	62	18104736	9.55E-01	1
PRG4	265627	419	-24	3444300	1110720	0	10	2	0	2	9	2288724	9.55E-01	1
OR52E6	203856	922	-5	776436	227484	0	1	2	0	5	6	1890360	9.55E-01	1
ARRDC2	1291557	226	34	1199720	416164	0	2	1	0	50	74	18619868	9.55E-01	1
GAK	679769	256	41	3339636	979356	0	10	6	0	12	19	4883964	9.55E-01	1
FAM53C	1312427	273	36	962624	309364	0	1	0	0	50	73	22485672	9.55E-01	1
ZNF519	134820	933	-24	1413676	328232	0	3	2	0	50	105	25777960	9.55E-01	1
SERPING1	923607	249	32	1262376	374868	0	2	3	0	12	17	4985780	9.55E-01	1
DIO2	47674	533	-44	777860	230332	0	1	1	0	14	22	6013196	9.55E-01	1
BCKDHB	131029	869	-9	1018872	285156	0	1	0	0	50	94	31502440	9.55E-01	1
FAM69C	162468	969	7	1043080	317552	0	1	0	0	43	51	17208684	9.55E-01	1
DPP6	32056	1019	-25	2380572	620508	0	5	2	0	29	67	15637656	9.55E-01	1
CSK	746043	199	41	1173020	323248	0	6	4	0	2	7	1086868	9.55E-01	1
HOXC4	1185281	279	54	669992	189748	0	1	1	0	14	21	4635120	9.55E-01	1
OXCT2	975799	388	39	1259528	402280	0	2	0	0	4	5	1799580	9.55E-01	1
WNK4	1633092	190	33	3068364	996444	0	7	4	0	24	22	8134244	9.55E-01	1
FATE1	125659	NaN	4	476328	132432	0	1	2	0	9	14	2346396	9.55E-01	1
GPATCH2	42648	1015	-18	1379500	356000	0	2	1	0	50	70	20423720	9.55E-01	1
IFT52	370278	303	11	1164476	294412	0	1	1	0	6	7	2787836	9.55E-01	1
FLNA	2356905	NaN	27	6688528	1992176	0	12	9	0	13	23	8346776	9.55E-01	1
NKPD1	1230654	243	41	2017096	664652	0	3	3	0	50	79	24609568	9.55E-01	1
FBN1	399245	304	-1	7624452	1850844	0	24	7	0	4	11	3718064	9.55E-01	1
APOBEC3C	1386387	372	71	1018160	245284	0	1	2	0	50	61	16059872	9.55E-01	1
FETUB	508316	436	45	969388	285512	0	6	4	0	2	6	933788	9.55E-01	1
HECA	202042	674	13	1363836	391956	0	2	0	0	38	51	13998988	9.55E-01	1
NPTX2	545145	560	20	1061948	339268	0	1	2	0	50	59	19225780	9.56E-01	1
KCTD14	556958	209	38	641156	185476	0	1	2	0	4	11	2266652	9.56E-01	1
DACH2	49531	NaN	-32	1547176	433964	0	1	1	0	32	63	14783968	9.56E-01	1

GPATCH8	1121044	166	30	3763988	1083664	0	13	2	0	1	2	1186904	9.56E-01	1
POTEF	347607	560	4	2801364	710220	0	7	2	0	50	71	22357512	9.56E-01	1
RNF180	56176	941	-5	1556432	396940	0	4	2	0	50	80	19418020	9.56E-01	1
CYP4F22	772367	543	20	1363124	391600	0	2	2	0	6	2	1187616	9.56E-01	1
CCR2	228487	625	39	1044148	315416	0	1	1	0	50	50	15010028	9.56E-01	1
AFAP1	166375	260	38	2088296	595588	0	6	5	0	5	12	2193316	9.56E-01	1
SLC12A4	871877	158	52	2965124	856180	0	8	4	0	50	83	23981940	9.56E-01	1
GFRA3	1219794	273	43	1018516	296192	0	1	1	0	50	87	28035356	9.56E-01	1
SOHLH2	81451	880	-44	1110008	299752	0	2	1	0	25	29	7285540	9.56E-01	1
KREMEN1	794944	335	-4	1271988	358848	0	2	0	0	1	0	594876	9.56E-01	1
DZIP3	172270	941	0	3231056	779284	0	8	2	0	27	41	14292332	9.56E-01	1
EFHC1	396934	431	34	1667148	437880	0	2	2	0	50	59	19996876	9.56E-01	1
USP46	115480	870	12	991460	236740	0	1	0	0	50	64	20216884	9.56E-01	1
PCLO	42656	1075	-27	12827036	3769684	0	45	11	0	5	18	5027076	9.56E-01	1
IL1B	578617	298	38	716628	174084	0	2	3	0	3	6	1248492	9.56E-01	1
PCOLCE2	398812	422	15	1074764	292276	0	1	1	0	50	54	17910716	9.56E-01	1
ZNF132	2179183	270	-4	1816668	457104	0	3	1	0	50	76	25634848	9.56E-01	1
RTF1	820644	237	37	1877900	474904	0	2	0	0	12	10	4741208	9.56E-01	1
DYNC2H1	134215	883	-19	11304780	2900688	0	19	4	0	2	7	4393396	9.56E-01	1
KLHL36	567037	187	33	1538632	455324	0	3	3	0	16	27	7619468	9.56E-01	1
LRSAM1	784133	209	45	1917772	504452	0	6	3	0	7	9	2007484	9.56E-01	1
TM7SF2	3267059	207	46	1043792	337132	0	1	2	0	50	74	20482816	9.56E-01	1
MAP1S	1181745	246	21	2516564	909580	0	4	1	0	30	42	15709212	9.56E-01	1
AK5	484699	536	-8	1479180	380208	0	4	3	0	38	41	11751560	9.56E-01	1
ZNF175	328446	627	-26	1850488	443576	0	3	3	0	31	56	15991520	9.57E-01	1
SS18L1	861466	288	-13	1049488	267356	0	1	1	0	25	32	9886476	9.57E-01	1
DEAF1	1774224	236	57	1426492	433964	0	1	1	0	50	45	17867640	9.57E-01	1
EIF5B	387331	612	25	3239600	770740	0	10	3	0	3	6	1983276	9.57E-01	1
CKMT1B	794398	269	37	1048776	324672	0	1	0	0	18	30	10348564	9.57E-01	1
ZBTB43	247985	252	46	1194024	309720	0	1	0	0	50	55	20675056	9.57E-01	1
ERRFI1	387339	207	26	1152728	343540	0	1	1	0	21	24	9239624	9.57E-01	1
MAST1	1937827	226	32	3902472	1239948	0	12	8	0	6	10	2714856	9.57E-01	1
TFPT	735613	439	-53	634392	205056	0	1	0	0	18	36	7425804	9.57E-01	1

LRCH4	1651125	242	60	1695984	570312	0	3	2	0	46	58	16249976	9.57E-01	1
GLP2R	76286	212	-53	1420796	405484	0	2	1	0	26	43	12639780	9.57E-01	1
FABP1	180956	612	6	344608	82592	0	1	1	0	1	2	438236	9.57E-01	1
MMRN1	39671	703	-11	3128172	841584	0	7	3	0	21	46	14661148	9.57E-01	1
LILRB2	717623	458	-6	1510508	464224	0	2	3	0	50	58	18028196	9.57E-01	1
FOXO1	237876	449	55	1628344	482024	0	2	1	0	43	62	21325468	9.57E-01	1
SNX13	139013	585	-4	2547180	629052	0	4	0	0	8	10	4328248	9.57E-01	1
RHBDF1	1005761	238	36	2176228	639020	0	3	2	0	50	46	16624132	9.57E-01	1
GPN3	943393	206	47	937348	222500	0	1	0	0	17	22	6683188	9.57E-01	1
CAMLG	910140	193	33	745108	219296	0	3	3	0	1	3	400500	9.57E-01	1
SREBF1	1129921	260	21	2881820	973660	0	4	0	0	0	0	973660	9.57E-01	1
ANGPTL5	416999	514	-21	1033112	247420	0	2	0	0	8	15	3825576	9.57E-01	1
GLTSCR2	812020	262	36	1207552	382700	0	1	1	0	50	64	23225796	9.57E-01	1
PAK4	1652029	286	26	1446072	484872	0	1	0	0	5	2	1706308	9.57E-01	1
ARR3	188610	NaN	39	1029552	287292	0	1	0	0	38	58	18272768	9.57E-01	1
CNKSR3	109534	588	14	1424000	408688	0	6	3	0	1	3	707372	9.57E-01	1
RGP1	922448	190	48	1079748	341760	0	1	2	0	31	42	12963384	9.57E-01	1
SLC6A8	1716845	NaN	44	1614104	474904	0	3	1	0	41	64	16780772	9.57E-01	1
RAD50	715524	320	48	3491292	818088	0	8	0	0	1	1	1009260	9.57E-01	1
ADCY7	304156	520	21	2746540	819512	0	4	5	0	7	14	3940208	9.57E-01	1
PAM	206242	550	9	2553944	672484	0	3	2	0	24	31	12015356	9.57E-01	1
FAT2	731456	563	27	10783952	3249568	0	28	12	0	16	26	10130692	9.57E-01	1
TUBE1	152199	296	5	1253120	321112	0	1	0	0	2	0	580636	9.57E-01	1
RHBDL1	1207790	194	33	1085800	348524	0	1	0	0	17	19	6751184	9.57E-01	1
CENPJ	481837	639	18	3495208	863300	0	11	3	0	50	62	17657956	9.57E-01	1
ABCF3	1460188	257	49	1837316	522964	0	3	2	0	50	68	20724896	9.58E-01	1
DNAH10	1032254	482	39	11562524	3096844	0	27	16	0	47	58	21039244	9.58E-01	1
KRT3	1444461	422	38	1576724	474904	0	3	3	0	20	23	7791060	9.58E-01	1
EBNA1BP2	1068006	294	30	957640	244928	0	2	2	0	2	2	829124	9.58E-01	1
CASZ1	809310	433	27	4405500	1312572	0	8	5	0	50	58	23799668	9.58E-01	1
HYDIN	592186	723	1	13169152	3616604	0	21	4	0	9	10	6657556	9.58E-01	1
AZGP1	1584669	391	36	768960	203988	0	1	2	0	14	22	5504472	9.58E-01	1
RPS6KL1	884998	232	41	1353512	425776	0	1	0	0	50	64	26283480	9.58E-01	1

KIF2A	176808	575	33	1969036	503384	0	4	0	0	5	8	3110728	9.58E-01	1
C14orf28	228479	577	-32	810612	201852	0	1	0	0	50	99	24622028	9.58E-01	1
GGT5	729749	200	28	1457820	473124	0	1	1	0	26	25	11596344	9.58E-01	1
LIG4	71759	952	-32	2318984	607336	0	8	1	0	10	23	5472788	9.58E-01	1
MED19	923511	354	15	493060	146672	0	1	2	0	5	10	1552160	9.58E-01	1
ATF4	721040	467	72	891068	247420	0	2	3	0	5	7	1638312	9.58E-01	1
ZNF219	937278	320	20	1721260	612320	0	1	0	0	15	13	6796752	9.58E-01	1
DMAP1	962892	211	29	1197584	351016	0	1	0	0	50	61	21094068	9.58E-01	1
VAV2	674833	504	29	2355296	587044	0	5	3	0	50	66	21147468	9.58E-01	1
PGK1	549810	NaN	14	1081172	300820	0	1	1	0	50	83	23441888	9.58E-01	1
WDR53	1180405	156	30	899968	260948	0	1	1	0	50	69	20130020	9.58E-01	1
CDCP2	465615	313	26	1110720	343896	0	2	4	0	19	27	6430784	9.58E-01	1
ZNF584	2179183	270	-4	1080104	284800	0	1	0	0	50	76	25634848	9.58E-01	1
MICALL1	1370748	203	50	2174092	660380	0	6	5	0	3	6	1253476	9.58E-01	1
SEC63	290010	481	37	2038812	484872	0	6	3	0	5	5	1460312	9.58E-01	1
ZNF81	583623	NaN	19	1718412	415452	0	2	0	0	9	8	3648644	9.58E-01	1
ACP2	1274054	198	38	1108940	325384	0	1	2	0	50	62	18403064	9.58E-01	1
GABRR3	189744	702	0	1226420	306160	0	1	1	0	50	62	22807852	9.58E-01	1
KIF19	362227	433	-8	2515140	766824	0	6	1	0	2	5	1534716	9.58E-01	1
SLC6A3	606209	490	30	1580996	468496	0	6	4	0	8	11	2509088	9.59E-01	1
CXCL13	405364	627	10	293700	75828	0	2	1	0	1	2	254184	9.59E-01	1
RAPGEF1	629872	355	32	2824148	798508	0	3	3	0	8	9	5362784	9.59E-01	1
KDM5C	877207	NaN	80	4017104	1193668	0	4	2	0	13	12	6357804	9.59E-01	1
ZBTB20	86308	644	10	1849776	540408	0	3	3	0	28	41	12932056	9.59E-01	1
BMP6	830850	327	8	1294416	378072	0	3	3	0	19	32	7513024	9.59E-01	1
PIGQ	1115707	213	39	1951948	595232	0	4	2	0	39	42	13074456	9.59E-01	1
VRK2	198322	479	8	1394808	336420	0	1	0	0	38	48	16210816	9.59E-01	1
BCORL1	389645	NaN	38	4357796	1420084	0	9	8	0	10	20	5184428	9.59E-01	1
INSR	558979	505	21	3529028	991816	0	10	7	0	33	40	10272380	9.59E-01	1
CA7	868790	142	24	687080	187612	0	1	2	0	3	6	1558924	9.59E-01	1
NUPL1	331109	381	37	1522612	463868	0	2	2	0	50	68	21157436	9.59E-01	1
GPC1	409918	407	20	1395164	430048	0	1	2	0	50	50	21954164	9.59E-01	1
SRF	1308754	363	35	1239592	416876	0	2	0	0	12	24	6411204	9.59E-01	1

FAM134C	1884163	177	38	1177292	356356	0	1	0	0	50	68	23826724	9.59E-01	1
OBSCN	698626	316	9	20750884	6518360	0	52	13	0	0	13	6518360	9.59E-01	1
ELF2	358887	261	30	1542904	454256	0	1	1	0	50	76	27327628	9.59E-01	1
TMIGD2	2127558	220	33	718764	207192	0	1	0	0	1	2	881812	9.59E-01	1
CHID1	1694280	232	52	1091852	306160	0	1	2	0	50	58	19804992	9.59E-01	1
PPWD1	424356	444	25	1686728	431116	0	3	1	0	4	5	2019944	9.59E-01	1
FBXL17	73947	913	4	1747604	535780	0	3	0	0	8	8	2851916	9.59E-01	1
ZNF556	1287128	291	27	1175156	304024	0	4	3	0	4	7	1704884	9.59E-01	1
FAH	201157	561	32	1106804	296548	0	2	3	0	5	12	2074056	9.59E-01	1
EDEM3	370601	493	4	2443584	626916	0	5	1	0	2	2	1304384	9.59E-01	1
RNF31	974559	220	41	2711296	814172	0	4	1	0	8	6	3001792	9.59E-01	1
KPNA3	422339	296	51	1380212	360628	0	2	0	0	45	51	16335060	9.59E-01	1
DMP1	500313	623	-7	1358852	309364	0	2	0	0	15	24	7044884	9.59E-01	1
AMPH	167080	639	7	1811684	505876	0	3	4	0	17	26	7410852	9.59E-01	1
FGFR1	841062	381	47	2446076	700252	0	5	2	0	50	87	27457924	9.59E-01	1
KCNT2	37679	1035	-24	2990044	767180	0	8	4	0	50	116	28340804	9.59E-01	1
ZNF462	259213	625	-7	6370620	1713072	0	13	3	0	4	6	3854768	9.59E-01	1
SSRP1	926574	191	-11	1871848	467072	0	8	2	0	1	3	719832	9.60E-01	1
SUPV3L1	732419	168	40	2029200	556428	0	3	2	0	25	28	10275584	9.60E-01	1
TCEB3CL	120831	639	34	3960856	1292636	0	1	1	0	19	11	8108968	9.60E-01	1
IARS	521598	409	42	3314004	873624	0	4	1	0	5	3	2087228	9.60E-01	1
CDAN1	610808	453	26	3075128	974728	0	3	2	0	28	24	12713828	9.60E-01	1
TH	837088	579	38	1338204	414384	0	1	1	0	50	46	19328664	9.60E-01	1
OR6T1	107524	830	-5	780352	256676	0	2	0	0	4	6	1246000	9.60E-01	1
INPP5B	445083	232	38	2410832	615880	0	3	1	0	34	26	12915324	9.60E-01	1
TUFM	1146511	221	56	1143472	356000	0	1	1	0	16	35	10080852	9.60E-01	1
SLC7A11	141546	570	-1	1273412	381988	0	1	0	0	50	55	21820308	9.60E-01	1
C19orf57	842601	185	32	1573520	500536	0	2	2	0	50	79	23810704	9.60E-01	1
SULT2B1	1590428	242	33	966540	261660	0	2	3	0	4	5	1298332	9.60E-01	1
UGT2B28	21304	997	-3	1367752	353864	0	3	1	0	50	73	18832756	9.60E-01	1
EVPL	1611239	218	22	5071932	1534716	0	11	8	0	37	76	19948816	9.60E-01	1
FRRS1	276548	540	-5	1643296	427556	0	4	0	0	5	7	2201504	9.60E-01	1
XPO1	842390	442	42	2847644	689572	0	5	4	0	50	46	17728800	9.60E-01	1

PLAGL1	358761	422	8	1144184	346744	0	1	0	0	50	56	20606704	9.60E-01	1
SYDE2	367677	468	1	3011404	845144	0	3	1	0	29	40	14586388	9.60E-01	1
NFIL3	150560	369	18	1158424	325028	0	1	2	0	50	53	18420508	9.60E-01	1
DLEC1	310440	411	28	4682824	1305452	0	13	6	0	19	25	8834496	9.60E-01	1
CACNA2D1	29637	816	12	2923116	735852	0	5	2	0	50	60	21954876	9.60E-01	1
SERPINA1	201688	843	9	1061236	298328	0	1	1	0	50	70	22072712	9.60E-01	1
RANBP10	1098330	136	50	1600220	445000	0	3	1	0	3	5	1787832	9.60E-01	1
NPLOC4	3705166	205	21	1594880	424708	0	2	0	0	27	21	7365640	9.60E-01	1
ARG2	481915	260	42	899612	267712	0	1	2	0	18	19	5599880	9.60E-01	1
NACA	2637730	154	47	4833412	1866152	0	3	0	0	0	0	1866152	9.60E-01	1
RAB14	611254	365	27	580992	140976	0	1	0	0	1	1	323960	9.60E-01	1
XK	115395	NaN	11	1110720	325740	0	1	1	0	50	65	20429060	9.61E-01	1
UNG	595266	487	20	890000	253828	0	1	2	0	50	72	19690360	9.61E-01	1
ANXA8	NaN	NaN	NaN	1766472	429336	0	2	0	0	50	116	40074920	9.61E-01	1
KIAA1683	1178047	210	43	3291932	1106092	0	8	2	0	3	5	2142052	9.61E-01	1
VCL	843223	125	52	2892856	835532	0	4	0	0	0	0	835532	9.61E-01	1
TSPYL6	619188	448	9	1015668	301176	0	2	3	0	13	11	2655760	9.61E-01	1
KLK12	490795	632	22	713424	171948	0	2	2	0	5	7	1231048	9.61E-01	1
NRP2	278279	567	5	2646860	736564	0	4	3	0	50	70	23039252	9.61E-01	1
PCDHGA11	NaN	NaN	NaN	2349956	746176	0	5	0	0	7	14	4434336	9.61E-01	1
SERPINA9	278548	858	13	1097904	316128	0	1	2	0	50	55	16626980	9.61E-01	1
ZSCAN2	341520	263	32	1645788	420792	0	2	2	0	50	63	20933512	9.61E-01	1
CBX8	633863	209	31	958352	310432	0	1	0	0	1	3	815952	9.61E-01	1
CECR5	305072	574	-55	1095412	337844	0	2	3	0	17	25	5876848	9.61E-01	1
FAM65C	568493	153	55	2403712	720188	0	3	5	0	42	57	14421204	9.61E-01	1
MYO9B	945446	330	36	5506608	1587048	0	17	5	0	1	5	1909228	9.61E-01	1
MYO1C	1380844	220	18	2781072	783912	0	4	4	0	48	69	20951668	9.61E-01	1
SDC2	230943	740	-2	518336	145960	0	1	1	0	2	5	1089716	9.61E-01	1
ZNF345	352375	976	-36	1266648	304380	0	2	0	0	17	38	9834856	9.61E-01	1
EXOC6	557904	421	36	2230340	536848	0	3	2	0	50	59	22205500	9.61E-01	1
KLHL21	628180	259	38	1454616	476328	0	2	3	0	46	63	18237524	9.61E-01	1
EFHB	103479	720	-15	2149172	574228	0	8	3	0	13	28	6838404	9.61E-01	1
PIGR	467960	385	26	1926316	567464	0	5	3	0	7	6	1940912	9.61E-01	1

MAST3	1249693	226	42	3269860	1038452	0	5	1	0	2	3	1790680	9.61E-01	1
SLC38A9	340223	390	24	1477756	382700	0	2	2	0	2	4	1807412	9.61E-01	1
CDKL3	910029	148	49	1569960	383412	0	2	0	0	6	12	2883600	9.61E-01	1
FLNB	760236	182	43	6701344	1934504	0	21	9	0	9	25	5997532	9.61E-01	1
HSPA4	637803	223	39	2219304	552156	0	2	1	0	24	26	12535116	9.61E-01	1
SHISA3	197282	646	24	583128	186900	0	1	2	0	2	4	849060	9.61E-01	1
TRIM36	219228	619	-1	2106452	532576	0	6	1	0	3	3	1274124	9.61E-01	1
MAML1	1617173	256	41	2504816	770740	0	3	1	0	50	55	22803224	9.61E-01	1
PROM1	174831	435	37	2280536	605200	0	3	1	0	50	67	21898984	9.61E-01	1
ABCA6	240031	493	-39	4263100	1083308	0	14	1	0	3	5	1859388	9.61E-01	1
OR4C15	1141	1100	-37	923820	262728	0	6	3	0	8	19	2180856	9.61E-01	1
C1orf101	716278	590	1	2497340	644716	0	3	1	0	5	2	2069428	9.61E-01	1
FAM98B	97405	643	-15	1186548	338556	0	1	0	0	50	68	24653356	9.61E-01	1
TPM3	2028928	185	35	1093632	268068	0	1	2	0	6	17	3124968	9.61E-01	1
SIPA1L1	161803	523	34	4573532	1295128	0	9	3	0	26	33	10537244	9.61E-01	1
CASS4	254881	551	-25	1959068	583840	0	2	3	0	4	10	3284456	9.62E-01	1
INTS3	1687837	175	33	2735860	729800	0	6	4	0	44	72	16821000	9.62E-01	1
RAD54B	544647	412	23	2369536	609116	0	2	1	0	46	45	21814968	9.62E-01	1
PTPRT	17956	1018	-72	3756512	1056964	0	16	7	0	20	25	3997168	9.62E-01	1
33GALNT:	536528	350	16	1300112	352084	0	2	1	0	50	78	20983352	9.62E-01	1
GSN	579825	326	42	2022436	593096	0	1	1	0	23	20	10843760	9.62E-01	1
RARRES3	854193	303	56	425776	117836	0	1	2	0	8	9	1189396	9.62E-01	1
OR6C3	667726	802	-14	772520	226060	0	1	1	0	50	83	19448636	9.62E-01	1
BCAM	920101	196	-3	1580640	498756	0	2	0	0	4	3	1628344	9.62E-01	1
IRF8	407770	461	27	1098972	302244	0	1	1	0	6	7	2876480	9.62E-01	1
PKD2L2	946917	396	47	1655044	396584	0	2	0	0	50	72	24090520	9.62E-01	1
SLC1A2	643540	263	15	1453192	431828	0	3	0	0	0	0	431828	9.62E-01	1
LNPEP	393651	469	46	2657184	702744	0	4	2	0	50	63	23913232	9.62E-01	1
PGM3	152280	734	8	1411540	379496	0	2	1	0	50	89	26599608	9.62E-01	1
PRAMEF1:	NaN	NaN	NaN	2407984	687080	0	4	5	0	26	45	12264200	9.62E-01	1
RAB36	261227	555	48	870776	244216	0	2	1	0	2	2	731224	9.62E-01	1
GCDH	1886809	335	40	1183344	344964	0	3	3	0	22	36	7729472	9.62E-01	1
RIOK1	789838	250	17	1538988	352440	0	2	1	0	50	56	22022516	9.62E-01	1

MEX3D	2122824	221	35	1574588	583840	0	1	0	0	12	9	4358508	9.62E-01	1
APEX2	245504	NaN	8	1300468	385904	0	2	3	0	13	18	4812408	9.62E-01	1
IGLL5	223193	631	29	529372	168032	0	1	2	0	9	16	2050204	9.62E-01	1
RANBP17	214548	691	12	2861528	745108	0	6	7	0	28	39	9869744	9.62E-01	1
CMPK2	41287	1132	-4	1095412	363476	0	1	1	0	50	72	22675776	9.62E-01	1
CLCN7	1232935	209	37	2057324	627628	0	1	3	0	50	50	16330788	9.62E-01	1
PCDHAC1	931701	643	-10	2369892	755076	0	8	1	0	0	1	755076	9.62E-01	1
BTN2A2	378286	330	51	1328948	379852	0	4	0	0	0	0	379852	9.62E-01	1
ZNF559	401242	705	-65	1399436	344608	0	3	0	0	8	13	3433976	9.62E-01	1
ETNK1	206703	644	2	1237812	348168	0	3	2	0	5	7	1848352	9.62E-01	1
CXorf57	148900	NaN	12	2209336	588824	0	5	2	0	7	7	2713076	9.62E-01	1
GPR176	731008	387	17	1269496	392312	0	1	2	0	32	44	15443636	9.63E-01	1
SLIT3	401911	706	20	3942700	1089716	0	9	4	0	6	7	3194744	9.63E-01	1
ZNF76	682809	264	39	1469924	415096	0	3	3	0	27	46	11443620	9.63E-01	1
GCC1	411207	619	-7	1926316	564260	0	4	1	0	12	10	3643304	9.63E-01	1
ISM2	504548	359	47	1487368	373088	0	2	2	0	35	45	14248544	9.63E-01	1
APOL5	1161568	376	-43	1084732	322892	0	3	1	0	7	14	3048072	9.63E-01	1
HJURP	301500	439	13	1902108	534000	0	3	0	0	17	16	5323268	9.63E-01	1
MPP2	1025836	241	19	1403708	419368	0	4	3	0	9	14	3060176	9.63E-01	1
CPVL	112083	634	7	1259884	319688	0	2	2	0	7	8	2541128	9.63E-01	1
CDH9	39282	1140	-26	2044152	534000	0	8	3	0	23	48	8829512	9.63E-01	1
MUM1L1	48678	NaN	-10	1767896	465292	0	2	0	0	41	76	24383152	9.63E-01	1
TRPM5	833749	589	47	2942696	893560	0	3	4	0	50	48	23387064	9.63E-01	1
LAMC2	764899	413	19	3095776	830192	0	7	3	0	50	81	24834916	9.63E-01	1
STXBP5	94997	696	-5	2992892	813460	0	4	4	0	24	29	9729836	9.63E-01	1
EPC2	91724	642	7	2097552	548952	0	4	0	0	2	3	1221792	9.63E-01	1
FOXN4	438714	519	35	1299400	398720	0	1	0	0	50	52	19818520	9.63E-01	1
KRT37	1321356	424	-6	1144540	325028	0	1	0	0	18	19	6810636	9.63E-01	1
PLXNA1	422395	494	48	4750464	1455684	0	7	6	0	50	51	20682532	9.63E-01	1
ZNF417	1252385	750	-19	1479536	374512	0	3	0	0	23	60	10985804	9.63E-01	1
MAPKAPK	583785	302	50	1001072	268780	0	2	3	0	2	3	744752	9.63E-01	1
PRKACA	1034381	321	36	954436	239588	0	1	3	0	5	11	1929164	9.63E-01	1
PRKACG	168122	498	36	882168	247776	0	2	0	0	6	14	2839456	9.63E-01	1

KLHL3	406828	409	26	1534360	409400	0	1	0	0	15	14	6957308	9.63E-01	1
FBXL12	1069900	579	-26	788184	268068	0	1	0	0	50	87	20502040	9.63E-01	1
DGKQ	675007	256	45	2366332	751160	0	2	2	0	36	37	16261012	9.63E-01	1
FRMPD4	191024	NaN	22	3351028	956216	0	6	3	0	50	148	45674800	9.63E-01	1
KRT12	1036346	358	5	1245644	372376	0	2	1	0	38	46	10303352	9.63E-01	1
NOTUM	3814891	211	28	1267004	368104	0	2	3	0	14	15	3835188	9.63E-01	1
ZNF708	170282	1121	-57	1468856	351016	0	2	1	0	40	51	14856948	9.63E-01	1
ARSE	191640	NaN	18	1502320	430760	0	2	2	0	50	72	20725252	9.63E-01	1
ZNF239	310244	585	-36	1186192	288716	0	2	0	0	50	86	20571104	9.63E-01	1
VIL1	739473	144	37	2148460	585620	0	2	3	0	50	47	20845580	9.64E-01	1
SORBS2	157889	374	-1	3760072	1038452	0	4	3	0	47	50	24365708	9.64E-01	1
TBC1D12	231059	361	22	1966900	570668	0	3	0	0	10	16	5819532	9.64E-01	1
STIP1	1602391	278	57	1446428	352084	0	4	4	0	9	21	4572108	9.64E-01	1
LSM10	636943	212	28	307940	93628	0	1	1	0	1	3	412604	9.64E-01	1
CD44	685695	222	28	1990040	463156	0	2	1	0	43	46	17262440	9.64E-01	1
PRRG3	125659	NaN	4	581704	174440	0	1	2	0	9	14	2346396	9.64E-01	1
DUOX2	695711	544	23	3954804	1149168	0	8	7	0	10	24	6997892	9.64E-01	1
THOC2	333292	NaN	4	4250640	1021008	0	8	4	0	50	58	18547600	9.64E-01	1
AHNAK2	854493	205	30	14201196	4394820	0	45	20	0	39	74	21382784	9.64E-01	1
SFXN1	184629	674	15	848704	228908	0	1	0	0	50	65	17130720	9.64E-01	1
SMAD6	580112	197	46	1222504	421148	0	2	3	0	25	37	9194056	9.64E-01	1
SCML2	82842	NaN	17	1816312	489500	0	2	3	0	23	21	8206868	9.64E-01	1
SACS	180800	625	14	11584240	3124256	0	33	12	0	22	31	10585304	9.64E-01	1
HIF3A	1079866	357	39	1705596	528660	0	6	4	0	7	13	2821656	9.64E-01	1
ANKRD50	47412	616	-24	3563916	1030620	0	10	1	0	14	17	5839468	9.64E-01	1
STX1A	1082063	214	54	794592	171948	0	1	2	0	8	23	5638684	9.64E-01	1
FHL1	248739	NaN	23	1031332	231044	0	1	1	0	14	16	5405860	9.64E-01	1
KCNQ5	648013	975	15	2401932	708084	0	11	4	0	3	7	1721972	9.64E-01	1
CCDC13	369284	309	33	1839808	518336	0	3	3	0	7	10	3747968	9.64E-01	1
TMEM201	491496	268	31	1653264	546816	0	2	1	0	50	61	19818876	9.64E-01	1
ALDH3B2	1408104	424	60	989324	281596	0	1	1	0	50	64	18269920	9.64E-01	1
LRP5	830388	241	53	4079048	1194736	0	8	6	0	50	71	20784704	9.64E-01	1
ASB18	121194	481	8	1142760	373800	0	1	1	0	22	32	9982952	9.64E-01	1

ZPLD1	68082	621	-10	1122468	306516	0	1	1	0	48	64	22241812	9.64E-01	1
KLHL4	34243	NaN	-31	1902464	516556	0	4	4	0	11	12	3516568	9.64E-01	1
ZNF480	526380	960	-32	1392672	341760	0	3	0	0	8	12	3141700	9.64E-01	1
WRN	283981	708	2	3802792	933788	0	6	4	0	27	44	14510204	9.64E-01	1
IL12RB1	1291557	226	34	1809904	522608	0	3	4	0	50	74	18619868	9.64E-01	1
CDC42EP4	193467	212	-15	864368	279460	0	1	3	0	2	6	1091140	9.64E-01	1
DMBX1	592811	255	23	942332	299752	0	6	3	0	1	6	872200	9.64E-01	1
ZCCHC12	234959	NaN	44	1000004	291208	0	1	2	0	32	52	15643708	9.64E-01	1
LARP1B	232013	314	13	2512292	635104	0	3	1	0	4	2	1724464	9.64E-01	1
INPPL1	744547	182	58	3178724	970456	0	10	8	0	20	31	7048800	9.65E-01	1
TRNT1	59117	1039	-15	1137064	286580	0	1	1	0	50	60	20398800	9.65E-01	1
RGMB	141802	729	11	1190464	361340	0	1	1	0	50	76	24974824	9.65E-01	1
SYNGAP1	1419781	236	31	3356368	1026704	0	4	4	0	39	58	15951292	9.65E-01	1
CASP10	590374	386	39	1470280	407264	0	1	1	0	50	64	21368544	9.65E-01	1
ZC3H12D	434842	139	40	1289076	423996	0	1	2	0	50	51	19481744	9.65E-01	1
SORBS3	757216	224	45	1733008	505520	0	1	1	0	48	49	21352880	9.65E-01	1
ZNF649	444002	817	-53	1307944	326096	0	3	2	0	50	71	16382052	9.65E-01	1
IEPACAM	311972	785	12	1233896	336064	0	1	1	0	50	63	22339000	9.65E-01	1
NLE1	516190	504	23	1244932	365612	0	1	2	0	43	54	16171300	9.65E-01	1
CLN8	100540	544	-13	713780	210040	0	1	2	0	4	8	2027064	9.65E-01	1
CACNA1D	514645	578	42	5781084	1530444	0	12	6	0	50	71	20599584	9.65E-01	1
CLP1	923511	354	15	1044860	328588	0	1	1	0	50	73	18938488	9.65E-01	1
PBXIP1	1612870	224	35	1847640	540408	0	3	0	0	0	0	540408	9.65E-01	1
TBC1D3B	739634	565	19	1420440	396228	0	3	0	0	3	3	1177292	9.65E-01	1
KDM5D	0	0	0	4017104	1131724	0	4	0	0	0	0	1131724	9.65E-01	1
PILRA	1430637	204	54	779996	219652	0	4	0	0	0	0	219652	9.65E-01	1
EIF2S2	898361	357	55	898188	206124	0	1	1	0	50	63	16230752	9.65E-01	1
MRPL43	1020226	247	40	1092208	274832	0	1	0	0	50	62	20159924	9.65E-01	1
ZNF346	1195948	218	40	778928	194020	0	2	2	0	5	16	2615176	9.65E-01	1
AMDHD2	2085452	321	49	1617308	481312	0	3	2	0	50	76	21050280	9.65E-01	1
SH3TC2	350600	254	15	3245296	953012	0	2	2	0	32	30	13753704	9.65E-01	1
ZNF433	975609	728	-8	1743332	428980	0	7	0	0	6	25	3938072	9.65E-01	1
ZNF549	459366	828	-31	1619444	401212	0	3	1	0	50	62	16455032	9.65E-01	1

EEF1A2	1262904	298	54	1174088	342472	0	2	3	0	21	30	7391984	9.65E-01	1
DMRTC2	1360684	454	23	914564	298684	0	1	1	0	50	69	18698900	9.65E-01	1
CPXM2	147419	771	10	1942336	538628	0	2	1	0	39	46	15577492	9.65E-01	1
BCL11A	151287	732	7	2201504	600928	0	5	4	0	14	25	7135664	9.65E-01	1
MAGEC2	16386	NaN	-29	932720	269848	0	4	3	0	6	24	2913148	9.65E-01	1
MYCBP2	236609	713	59	12051312	3290508	0	31	9	0	25	34	11592072	9.65E-01	1
PCDHGA6	NaN	NaN	NaN	2285876	747244	0	6	7	0	22	43	10322220	9.65E-01	1
GPSM2	817825	236	19	1783916	466360	0	5	0	0	3	7	1527240	9.65E-01	1
MAGEL2	94496	904	-55	2977584	1027416	0	5	3	0	24	28	8834852	9.65E-01	1
GLDC	213479	411	26	2648996	724816	0	7	5	0	7	10	2561420	9.65E-01	1
ADAM19	261911	1079	45	2378080	662516	0	4	5	0	50	48	16798572	9.65E-01	1
ZDHHC17	181943	605	-12	1683168	413316	0	1	0	0	30	26	13086204	9.65E-01	1
PCDHA8	896676	606	-12	2353516	758636	0	9	6	0	43	105	19890076	9.65E-01	1
SERGEF	645462	595	17	1186192	331436	0	1	0	0	14	18	6596324	9.65E-01	1
DDX51	608356	528	26	1662876	536136	0	2	1	0	50	70	22588912	9.65E-01	1
FAM71F2	875359	304	30	783200	229264	0	2	3	0	4	9	1731584	9.66E-01	1
DOK6	50550	968	-4	877540	216092	0	1	2	0	50	84	18756928	9.66E-01	1
COX11	131464	869	-43	766824	210396	0	1	2	0	9	11	2264872	9.66E-01	1
IKBIP	488021	461	18	1610188	419012	0	5	0	0	5	5	1487012	9.66E-01	1
PARP6	1129780	260	47	1671776	439660	0	2	3	0	43	62	21156368	9.66E-01	1
RPS6KB2	1331051	161	52	1243152	366324	0	1	3	0	24	23	8804948	9.66E-01	1
RNF6	96737	562	25	1718056	492704	0	3	1	0	1	1	897120	9.66E-01	1
PRKG1	75025	995	-15	2037388	522608	0	6	0	0	0	0	522608	9.66E-01	1
ADCY5	372027	458	42	3179080	951944	0	10	7	0	6	15	4100052	9.66E-01	1
SNAPC2	952640	320	27	804916	287648	0	1	2	0	18	34	7729828	9.66E-01	1
GNPDA2	62624	1009	11	735496	177644	0	2	2	0	1	3	733004	9.66E-01	1
MSN	325257	NaN	47	1524392	378784	0	1	1	0	50	61	21526252	9.66E-01	1
MOV10L1	934225	242	31	3212188	855824	0	8	2	0	3	10	2241376	9.66E-01	1
PRR5L	196876	169	-1	941976	274476	0	1	2	0	6	6	1972952	9.66E-01	1
HIST2H2AB	798975	560	17	312924	108936	0	2	2	0	3	5	431116	9.66E-01	1
OSBPL11	269912	188	31	1936284	511572	0	4	0	0	1	1	745820	9.66E-01	1
CATSPER3	725156	226	27	1046640	265932	0	2	3	0	7	8	2101112	9.66E-01	1
PCDHB5	915569	724	-11	1918840	631544	0	15	6	0	1	12	1262376	9.66E-01	1

XPR1	378102	589	11	1808480	484516	0	6	5	0	7	15	3090436	9.66E-01	1
GPATCH4	2049505	180	37	1013176	242792	0	1	0	0	50	64	19075904	9.66E-01	1
OSBPL6	150530	526	16	2556792	661092	0	5	1	0	1	1	901748	9.66E-01	1
GOLGA6C	NaN	NaN	NaN	1828772	469564	0	2	0	0	50	116	40115148	9.66E-01	1
CNP	2081077	206	23	1047708	317196	0	1	0	0	3	5	1159492	9.66E-01	1
PYROXD1	500770	836	-29	1319336	332860	0	2	1	0	50	63	17056316	9.66E-01	1
MYOC	411007	718	10	1264156	364544	0	5	4	0	2	10	1381280	9.66E-01	1
CFI	212346	417	26	1550380	372020	0	3	1	0	12	12	3963348	9.66E-01	1
PUSL1	1744013	273	28	757212	248844	0	1	2	0	17	31	7122136	9.66E-01	1
TP53BP1	794398	368	43	5048792	1404064	0	20	4	0	1	6	1762200	9.66E-01	1
POMT2	554839	418	37	1927028	564616	0	2	0	0	7	7	3455692	9.66E-01	1
ZNF184	162358	606	37	1954796	471700	0	6	3	0	16	20	4747972	9.67E-01	1
DGCR2	474521	277	16	1405844	398008	0	1	0	0	35	30	12357116	9.67E-01	1
TWF2	1114737	181	52	903528	252048	0	1	2	0	13	22	5456056	9.67E-01	1
SLC9A9	209532	954	7	1685304	448560	0	1	1	0	39	34	17260304	9.67E-01	1
STON2	203328	364	-1	2273060	644716	0	2	1	0	50	59	28536604	9.67E-01	1
PTPRE	424354	683	-15	1889292	479532	0	3	1	0	48	45	15284504	9.67E-01	1
SERPINA7	23303	NaN	21	1058744	291208	0	1	2	0	50	64	19839524	9.67E-01	1
TOX	192821	695	19	1351020	371664	0	6	4	0	2	7	1077256	9.67E-01	1
TBC1D22A	310166	538	-25	1351732	359204	0	2	2	0	11	19	5021024	9.67E-01	1
CTDP1	275544	498	-3	2448568	684944	0	2	0	0	3	3	1800648	9.67E-01	1
CCL3L3	691971	684	13	496976	131008	0	1	1	0	6	7	1275904	9.67E-01	1
SKAP2	1041630	540	14	987900	216804	0	1	2	0	34	52	12925292	9.67E-01	1
MFSD1	181588	972	2	1349952	366324	0	3	2	0	7	11	2661100	9.67E-01	1
PARD6G	161542	NaN	38	907088	309364	0	1	0	0	50	71	18774372	9.67E-01	1
PTPN6	2704950	211	47	1691356	452120	0	1	1	0	1	1	1443224	9.67E-01	1
NOX5	370981	394	26	2021012	569956	0	6	4	0	2	4	1183700	9.67E-01	1
TOX2	639806	396	14	1377008	422572	0	2	4	0	5	7	1608408	9.67E-01	1
UNC119	1517567	225	24	658600	187256	0	2	3	0	1	6	697048	9.67E-01	1
SYT11	2096440	179	32	1079748	317196	0	2	4	0	5	8	2110012	9.67E-01	1
OSCAR	767094	317	-50	805272	281952	0	1	1	0	50	88	18435460	9.67E-01	1
KIAA2022	251853	NaN	7	3848360	1024924	0	12	3	0	1	3	1131724	9.67E-01	1
PC	1649909	187	38	2956936	903884	0	6	5	0	24	32	10055220	9.67E-01	1

MAPKAPK	477806	261	32	1086868	296192	0	1	2	0	50	60	19078752	9.67E-01	1
PWWP2A	199338	417	21	1979716	598436	0	3	1	0	25	32	9996836	9.67E-01	1
KRBA2	772200	257	20	1258460	327520	0	1	1	0	50	52	17300532	9.67E-01	1
FKBP8	1394856	259	48	1038452	322180	0	2	0	0	1	0	449272	9.67E-01	1
TYSND1	471839	217	32	1353156	476328	0	2	1	0	50	69	19518412	9.67E-01	1
DRD1	178213	729	14	1095056	337132	0	1	1	0	50	66	21791472	9.67E-01	1
PHIP	209556	739	0	4762212	1242084	0	9	1	0	1	2	1555364	9.67E-01	1
TPM4	803963	298	26	872200	218228	0	1	1	0	8	18	4645088	9.67E-01	1
FBXW10	752804	391	-6	2709516	718764	0	5	4	0	50	50	17232180	9.67E-01	1
KIF18B	1037808	191	34	2130304	675332	0	3	1	0	3	2	1585268	9.67E-01	1
CLCN6	524980	196	41	2341412	550732	0	6	5	0	10	15	3457828	9.67E-01	1
SLC17A4	272695	635	29	1272344	370240	0	2	2	0	21	31	7635132	9.67E-01	1
SELP	294306	478	6	2168040	563904	0	4	4	0	22	31	9368140	9.67E-01	1
ASTN2	63839	937	-17	3453912	1009260	0	9	2	0	10	17	5943776	9.67E-01	1
DPY19L3	308903	531	-54	1871848	502316	0	5	1	0	14	19	4772536	9.67E-01	1
MTFMT	697075	170	51	1000004	285868	0	1	1	0	50	53	14851608	9.67E-01	1
EYS	28896	1167	-23	8103272	2097196	0	8	1	0	2	2	2740132	9.67E-01	1
MON1A	1289886	170	38	1597728	515844	0	2	1	0	21	30	9711324	9.68E-01	1
WRNIP1	473112	285	31	1661096	498400	0	1	2	0	50	47	18385620	9.68E-01	1
PRKX	104714	NaN	11	933788	250624	0	1	2	0	28	48	13159896	9.68E-01	1
DHRS4	984811	442	23	708084	217872	0	2	2	0	3	9	1530800	9.68E-01	1
ELMOD3	1021843	180	29	1178716	334640	0	2	3	0	19	36	8553256	9.68E-01	1
FBXW11	380827	400	36	1474196	388396	0	1	0	0	41	50	19219016	9.68E-01	1
ANKRD24	2194691	244	41	2856188	904240	0	3	1	0	39	34	15527652	9.68E-01	1
CYP2U1	144587	735	6	1368464	396940	0	1	0	0	18	29	9541512	9.68E-01	1
GOLGA6D	NaN	NaN	NaN	1830196	468140	0	2	1	0	50	117	40113724	9.68E-01	1
PHLDB3	392374	166	-19	1625852	491992	0	2	0	0	9	8	3174096	9.68E-01	1
USP19	1898051	203	36	3331448	997156	0	5	3	0	13	27	9263832	9.68E-01	1
PELP1	1249398	222	26	2773240	920972	0	4	1	0	16	17	7261688	9.68E-01	1
CHD1L	NaN	NaN	NaN	2350312	623000	0	3	2	0	50	118	40268584	9.68E-01	1
LIPI	95960	991	-18	1274480	310432	0	2	0	0	28	43	11687836	9.68E-01	1
NT5DC2	1114087	192	48	1545396	433608	0	2	3	0	50	74	21371748	9.68E-01	1
CCT3	2232742	187	30	1417236	387684	0	4	3	0	10	23	4939500	9.68E-01	1

HOMER3	1390116	198	20	919904	278392	0	1	1	0	50	73	19047780	9.68E-01	1
ELP3	417570	338	41	1428984	386616	0	2	2	0	3	5	1956220	9.68E-01	1
ANKRD26	390573	459	24	4558224	1064796	0	13	3	0	2	6	2315424	9.68E-01	1
TM9SF2	310978	472	69	1739772	456036	0	3	1	0	20	29	8270948	9.68E-01	1
KIAA1407	438696	487	29	2467792	602708	0	7	4	0	5	6	1596304	9.68E-01	1
SLC34A3	1724821	399	43	1471704	505164	0	2	2	0	50	60	16636592	9.68E-01	1
NFKB1	376672	311	41	2507664	698472	0	2	1	0	7	6	3930596	9.69E-01	1
IL17RA	183789	639	-103	2173736	655396	0	3	1	0	40	51	17554716	9.69E-01	1
SCUBE3	654416	207	36	2595596	681028	0	2	0	0	2	3	1858320	9.69E-01	1
CELA2B	636573	188	34	699540	199716	0	3	2	0	2	7	1049132	9.69E-01	1
CEP78	249135	554	-32	1870068	510504	0	4	0	0	12	16	4412620	9.69E-01	1
SEPHS2	2338593	142	59	1084732	353864	0	1	1	0	50	74	22010768	9.69E-01	1
PPP1R3F	547555	NaN	25	1910296	667856	0	1	1	0	12	10	3450352	9.69E-01	1
SLC24A5	219744	588	-38	1282312	361340	0	2	0	0	29	52	12899660	9.69E-01	1
SLC25A42	1293709	215	30	797084	254896	0	1	3	0	23	43	9649380	9.69E-01	1
INCENP	2712305	253	52	2346396	674976	0	6	1	0	8	25	6609496	9.69E-01	1
SLC11A2	566864	155	40	1603068	467784	0	1	1	0	50	43	19587832	9.69E-01	1
MYO19	416822	556	23	2549672	721612	0	5	0	0	1	1	1097192	9.69E-01	1
PRLHR	613868	617	8	873980	318976	0	1	3	0	1	6	1536852	9.69E-01	1
ZNF365	157974	731	-3	1797800	495196	0	5	1	0	8	12	2978652	9.69E-01	1
ITGB2	746455	191	74	1980072	551088	0	6	6	0	6	9	2297624	9.69E-01	1
XYLT2	1335838	196	24	2144900	672484	0	3	3	0	50	70	22959864	9.69E-01	1
CHD3	1971623	337	29	5400164	1477756	0	11	4	0	24	28	10940592	9.69E-01	1
DHX34	1132395	232	41	2842660	886796	0	10	2	0	0	2	886796	9.69E-01	1
OR4K1	420010	827	-51	783200	220720	0	1	1	0	50	72	16673972	9.69E-01	1
CSF2RB	750264	469	44	2236392	692064	0	2	2	0	50	54	22237184	9.69E-01	1
LILRB4	978578	625	-1	1153084	332504	0	2	2	0	50	108	22158508	9.69E-01	1
CTCF	1074126	147	45	1902464	472768	0	16	4	0	0	4	472768	9.69E-01	1
PTPRN	694442	193	44	2460672	775368	0	4	2	0	44	64	21097984	9.69E-01	1
RNF5	2119974	392	28	470632	130652	0	1	2	0	4	7	976152	9.69E-01	1
KRT13	1445853	325	-22	1161628	341048	0	1	1	0	50	58	19098688	9.69E-01	1
PTK2B	326685	491	30	2659676	704524	0	5	2	0	27	30	11271316	9.69E-01	1
HMGB2	249666	413	23	563548	126380	0	3	3	0	2	4	521540	9.69E-01	1

XKR6	125681	704	26	1569604	498044	0	1	2	0	50	127	45340516	9.69E-01	1
ORAI3	1508459	247	46	705948	246708	0	1	3	0	4	13	2794600	9.69E-01	1
ITPKB	508274	407	19	2337852	721968	0	4	1	0	14	19	6888244	9.69E-01	1
GBF1	987676	141	41	4750108	1375940	0	14	2	0	0	2	1375940	9.69E-01	1
MYB	150100	410	32	1979716	525812	0	4	0	0	2	3	1524748	9.69E-01	1
SNRNP70	2041001	200	41	1106448	335352	0	1	0	0	50	75	23316220	9.69E-01	1
GREM2	47375	688	-30	423640	121040	0	2	2	0	1	6	637596	9.69E-01	1
OR2G6	112460	903	-20	774300	241368	0	1	2	0	6	14	2846932	9.69E-01	1
SCAF1	1733181	195	36	3148820	1100752	0	5	0	0	4	3	2087940	9.69E-01	1
HLTF	369132	386	8	2672136	670704	0	3	0	0	6	6	3584564	9.69E-01	1
ENG	1236941	179	55	1685304	511572	0	2	3	0	7	14	3338924	9.69E-01	1
ANGPTL2	NaN	NaN	NaN	1248492	351372	0	1	2	0	50	118	39996956	9.69E-01	1
LIMS2	603350	218	42	1030264	269492	0	1	3	0	2	5	1133148	9.70E-01	1
EPSTI1	109052	417	33	1095768	274476	0	1	0	0	3	2	1231048	9.70E-01	1
RHCG	747784	363	22	1234252	348524	0	4	4	0	4	7	1191532	9.70E-01	1
C9orf72	93979	434	-12	1249560	339624	0	2	2	0	7	10	2833404	9.70E-01	1
CX3CR1	159083	507	38	973660	275900	0	2	2	0	10	16	3452488	9.70E-01	1
SLC9A8	649593	165	52	1510864	417944	0	4	7	0	6	17	2456044	9.70E-01	1
MTUS1	457439	237	-5	3515144	949096	0	8	1	0	0	1	949096	9.70E-01	1
SUN3	147364	445	21	957284	236740	0	2	2	0	7	13	2683172	9.70E-01	1
KIR2DL3	1155455	625	-10	882880	244928	0	2	1	0	50	146	23999028	9.70E-01	1
ALAS2	245504	NaN	8	1567112	440728	0	2	2	0	50	78	23195536	9.70E-01	1
WNT6	1087669	386	42	886796	298684	0	1	1	0	50	74	18604204	9.70E-01	1
PDE10A	46364	571	-26	2123896	545036	0	4	3	0	50	93	20218664	9.70E-01	1
AAAS	1701810	166	58	1387332	431472	0	4	0	0	0	0	431472	9.70E-01	1
NTN5	1597109	242	31	1199008	396584	0	1	0	0	3	2	1342120	9.70E-01	1
C2orf69	214653	674	14	976864	264152	0	1	0	0	50	62	16806048	9.70E-01	1
SLC1A7	509533	391	21	1399436	440728	0	1	0	0	10	15	6355668	9.70E-01	1
ADA	801801	171	42	956572	256676	0	2	3	0	7	18	3520128	9.70E-01	1
TMEM44	370452	255	34	1269496	334640	0	1	2	0	50	55	18948812	9.70E-01	1
ADAM7	56052	741	6	2024216	485584	0	2	0	0	34	52	18932080	9.70E-01	1
ZNF627	1065261	394	15	1191532	301532	0	3	0	0	8	15	3051988	9.70E-01	1
MTMR8	56100	NaN	0	1838384	478108	0	3	2	0	7	9	2998588	9.70E-01	1

THOC1	402181	689	-26	1775016	420792	0	3	0	0	50	69	19550808	9.70E-01	1
ANAPC1	NaN	NaN	NaN	5059116	1373448	0	13	2	0	9	18	5730888	9.70E-01	1
INA	522257	125	41	1217164	395516	0	1	0	0	26	23	8384512	9.70E-01	1
BEND2	66906	NaN	11	2091144	555360	0	4	1	0	5	5	2259532	9.70E-01	1
TSPYL2	884583	NaN	77	1795664	453544	0	1	0	0	11	11	5843384	9.70E-01	1
AGBL4	16483	1041	-18	1333932	336420	0	1	0	0	50	78	21636968	9.70E-01	1
PKN1	954144	204	43	2407272	751872	0	1	0	0	8	7	4677128	9.70E-01	1
UGDH	658220	165	48	1285516	347456	0	2	1	0	50	60	15242496	9.70E-01	1
FMN1	238197	520	5	3054480	849060	0	2	0	0	21	15	9349984	9.70E-01	1
IKZF5	274189	673	11	1078680	281952	0	1	0	0	50	64	18840944	9.70E-01	1
PHF19	645995	270	39	1634752	454256	0	2	1	0	50	78	24143564	9.70E-01	1
PIK3CD	533533	204	35	2686020	756144	0	5	0	0	0	0	756144	9.71E-01	1
MAGED1	127069	NaN	23	2112148	618728	0	2	1	0	50	36	16563612	9.71E-01	1
CALU	852821	304	33	1027772	221788	0	1	0	0	50	94	24312664	9.71E-01	1
PKD1L1	239031	377	12	7277352	2093280	0	14	9	0	50	67	22345052	9.71E-01	1
ZNF671	680410	849	-50	1365972	363120	0	8	6	0	3	10	1245288	9.71E-01	1
SCN1B	738570	419	-34	864012	244572	0	1	1	0	50	80	19399508	9.71E-01	1
TNKS2	333272	331	15	3014964	835176	0	10	1	0	0	1	835176	9.71E-01	1
DIP2C	406596	632	12	3969400	1175156	0	11	2	0	9	12	4006780	9.71E-01	1
QSOX2	936497	244	40	1758996	529728	0	2	0	0	8	16	5966560	9.71E-01	1
TPH1	645462	571	16	1171240	295124	0	2	2	0	8	9	2418308	9.71E-01	1
TTLL1	627533	202	46	1108940	288004	0	2	3	0	14	28	6756524	9.71E-01	1
PKHD1	237026	946	0	10390928	2986840	0	29	15	0	27	45	12292680	9.71E-01	1
SEMA5A	45422	891	-2	2780360	753652	0	5	5	0	25	29	10425104	9.71E-01	1
TSPAN14	308181	588	-12	713424	189036	0	2	3	0	2	3	690640	9.71E-01	1
ANP32D	904892	390	11	333572	88288	0	1	2	0	2	5	609472	9.71E-01	1
ZNF138	213856	1035	-19	969744	218940	0	1	1	0	50	80	21697488	9.71E-01	1
SLC2A6	1858670	315	39	1241372	426844	0	3	4	0	14	26	5231420	9.71E-01	1
ZC3H4	1263858	179	37	3251348	986476	0	8	2	0	12	19	6006788	9.71E-01	1
MYH6	1220624	456	31	5048792	1327168	0	13	4	0	6	13	4269864	9.71E-01	1
HERC3	219535	452	13	2742980	726952	0	3	0	0	11	11	5542208	9.71E-01	1
SENP2	943739	367	46	1560704	398008	0	2	0	0	50	75	23064528	9.71E-01	1
LENG9	807048	535	-7	1179072	431472	0	2	2	0	50	83	18359276	9.71E-01	1

DNAH6	NaN	NaN	NaN	10811008	2839100	0	12	2	0	0	2	2839100	9.71E-01	1
ZNF100	144970	1235	-65	1415456	341404	0	3	0	0	16	27	6560368	9.71E-01	1
PHLDB1	1404506	161	46	3417600	1091496	0	8	3	0	22	37	11314748	9.71E-01	1
ZNF274	1390072	581	-58	1676760	447492	0	4	2	0	50	127	30675096	9.71E-01	1
NUP85	1723762	171	31	1723040	461020	0	7	4	0	7	23	3592396	9.71E-01	1
GPC3	96913	NaN	25	1564976	404416	0	1	0	0	9	8	3315784	9.72E-01	1
MLYCD	387177	550	-6	1212536	389464	0	2	1	0	4	6	1756148	9.72E-01	1
APBA3	2144458	206	34	1426136	462088	0	1	0	0	13	11	5053064	9.72E-01	1
OR2M2	19998	935	-32	862588	250268	0	2	3	0	7	15	2404780	9.72E-01	1
NOG	268027	457	-44	562480	184052	0	2	2	0	3	7	965828	9.72E-01	1
[illegible]GALNAC[illegible]	171847	356	17	1370244	363120	0	1	2	0	50	50	18852692	9.72E-01	1
SCN5A	366751	578	25	5197244	1483096	0	17	5	0	1	5	1748316	9.72E-01	1
NAPSA	1072052	528	26	1044860	342472	0	2	3	0	4	8	1979004	9.72E-01	1
MTX1	1801444	194	32	1150236	375936	0	2	0	0	1	0	479532	9.72E-01	1
CELSR3	1516147	212	41	8089744	2670356	0	20	7	0	2	7	3185132	9.72E-01	1
ATP9B	91487	731	13	2986484	817732	0	4	1	0	50	52	21721340	9.72E-01	1
LRRC16B	992350	355	19	3514432	1051268	0	6	1	0	5	5	2775732	9.72E-01	1
SPATA21	813996	191	40	1208976	343896	0	1	3	0	20	19	6157376	9.72E-01	1
ZFX	615356	NaN	50	2145612	529728	0	4	2	0	50	63	18195160	9.72E-01	1
FOXRED1	385871	426	29	1238168	367036	0	2	3	0	5	7	2062664	9.72E-01	1
THAP9	778608	268	42	2302964	610540	0	3	4	0	11	17	5686032	9.72E-01	1
ZNF384	2816983	210	61	1474196	416164	0	2	0	0	25	55	15777920	9.72E-01	1
PJA1	136194	NaN	39	1610900	452476	0	3	2	0	50	77	18855184	9.72E-01	1
NACC1	1948449	221	41	1324676	388396	0	1	1	0	50	60	21446864	9.72E-01	1
LRRC14B	836250	543	14	1223572	432896	0	2	3	0	25	47	10315812	9.72E-01	1
CCNT1	1705947	328	30	1846572	519048	0	4	2	0	12	18	4998596	9.72E-01	1
FGD5	489640	490	39	3695280	1073340	0	8	2	0	13	18	6930608	9.72E-01	1
ZER1	1816626	165	36	1978292	543256	0	3	4	0	39	73	17627340	9.72E-01	1
TRPC6	70247	847	-24	2410832	626560	0	7	2	0	27	49	11532264	9.72E-01	1
TAF4	801904	411	-25	2619448	919904	0	5	1	0	1	5	2398016	9.72E-01	1
GPR17	NaN	NaN	NaN	894628	291920	0	2	4	0	2	8	1148456	9.72E-01	1
GMEB1	817267	128	27	1475976	406908	0	1	1	0	50	63	23807144	9.72E-01	1
RHPN2	458398	356	-12	1769676	493416	0	1	2	0	34	30	15785040	9.72E-01	1

SPDYE3	1430637	204	54	1419728	385192	0	4	1	0	1	1	604844	9.72E-01	1
ALS2	438202	523	29	4330028	1204348	0	4	3	0	22	10	6876852	9.72E-01	1
FBXO47	1256859	143	31	1198296	295836	0	1	1	0	50	76	22095496	9.72E-01	1
NR4A3	280636	538	8	1660740	496620	0	1	1	0	50	63	23469656	9.72E-01	1
OR2L13	24438	1015	-27	779284	227840	0	1	0	0	47	108	21910732	9.72E-01	1
NOC4L	608356	528	26	1323252	397296	0	1	1	0	50	70	22588912	9.73E-01	1
ZNF592	344313	214	42	3170536	926312	0	3	1	0	5	9	3184064	9.73E-01	1
TLR2	143076	685	18	1977936	541476	0	3	3	0	50	60	18833468	9.73E-01	1
KIF2C	574317	191	43	1915992	495552	0	4	5	0	6	14	2995384	9.73E-01	1
CNN1	1152381	272	31	771808	208616	0	1	2	0	8	13	2728740	9.73E-01	1
PCNXL3	4038877	202	49	5048080	1617308	0	7	7	0	24	26	11885772	9.73E-01	1
ZSWIM4	754888	241	31	2444296	778928	0	4	4	0	50	63	19916776	9.73E-01	1
CD247	478690	558	10	445712	112852	0	1	2	0	3	9	1203636	9.73E-01	1
ATXN7L1	406187	454	33	2229272	678892	0	2	0	0	19	16	7534384	9.73E-01	1
SLCO1C1	91874	501	-24	1969392	484872	0	11	7	0	4	16	2107520	9.73E-01	1
KCND1	834590	NaN	41	1590964	506588	0	2	0	0	50	75	21713152	9.73E-01	1
TMPRSS6	699535	379	44	2075124	599148	0	5	0	0	2	2	1062660	9.73E-01	1
POFUT1	1013932	190	56	1029196	292988	0	1	1	0	50	69	20279184	9.73E-01	1
CUX1	554381	461	45	4565344	1305452	0	9	2	0	8	11	5331812	9.73E-01	1
TFAP2B	17044	1081	-14	1151660	351016	0	3	3	0	5	10	2154512	9.73E-01	1
SLC19A3	164044	353	-10	1257392	356356	0	1	1	0	22	30	9433644	9.73E-01	1
CRTAC1	530090	369	12	1728736	452120	0	2	2	0	50	74	22251780	9.73E-01	1
PARP12	462854	639	35	1804920	491280	0	2	1	0	50	71	22248576	9.73E-01	1
ZNF24	306655	499	43	939840	255252	0	1	0	0	50	78	19811756	9.73E-01	1
HCN4	314300	642	16	2908876	978644	0	4	4	0	38	40	17576076	9.73E-01	1
MAP3K11	3945819	202	45	2066580	688860	0	2	1	0	19	25	9711324	9.73E-01	1
ESR2	528074	387	46	1432900	392312	0	2	3	0	8	9	2790684	9.73E-01	1
BICD1	387398	460	23	2497696	669992	0	3	2	0	9	25	9750484	9.73E-01	1
SPATS2L	476225	433	24	1449632	388396	0	2	3	0	6	9	2080108	9.73E-01	1
INTS1	595899	243	38	5518356	1702392	0	12	5	0	31	45	14566808	9.74E-01	1
SLC22A18	552601	256	34	1041300	360984	0	1	2	0	12	15	4721272	9.74E-01	1
CHRNA3	495901	373	46	1432544	387328	0	1	2	0	46	55	17838804	9.74E-01	1
ACVR2B	337567	514	28	1314708	373800	0	2	1	0	12	12	3515144	9.74E-01	1

COL1A2	461036	499	-14	3435400	1162340	0	8	3	0	5	6	2373808	9.74E-01	1
SIN3B	854784	305	35	2979364	830192	0	4	4	0	49	77	22853064	9.74E-01	1
ESYT3	201756	408	19	2266296	669636	0	3	3	0	26	24	8426876	9.74E-01	1
MIER2	901402	578	-17	1407624	399432	0	3	1	0	50	99	20859820	9.74E-01	1
HARS2	774470	356	31	1308300	367392	0	1	0	0	19	25	9696372	9.74E-01	1
LYST	438216	401	9	9812072	2587408	0	29	6	0	2	19	6110028	9.74E-01	1
KANK3	752886	204	7	1986124	690284	0	2	1	0	23	34	12142092	9.74E-01	1
KLHL26	1397040	222	33	1506236	478108	0	1	3	0	50	56	22349324	9.74E-01	1
OR4Q3	275024	858	-57	774300	229620	0	3	4	0	3	9	929160	9.74E-01	1
GCK	1321842	282	62	1285872	350304	0	1	3	0	6	8	1864728	9.74E-01	1
INMT	463251	489	22	659312	197224	0	1	2	0	3	6	1442156	9.74E-01	1
MAP3K14	789809	207	23	2396592	703812	0	3	3	0	43	50	19051340	9.74E-01	1
CENPF	457503	186	-7	8073724	1987904	0	16	9	0	50	63	20559712	9.74E-01	1
ATP2A2	969445	178	26	2677476	762552	0	7	5	0	50	83	22052064	9.74E-01	1
MINK1	1440940	208	29	3232836	921684	0	4	4	0	50	56	21043872	9.74E-01	1
MCC	271270	620	16	2678900	729088	0	7	2	0	3	4	1710580	9.74E-01	1
WDR1	223391	873	42	1576724	427912	0	3	4	0	24	30	7611992	9.74E-01	1
TATDN2	743522	224	53	1922756	548596	0	2	0	0	14	17	6359584	9.74E-01	1
RBCK1	802744	165	41	1333576	370952	0	1	2	0	50	55	17464292	9.74E-01	1
GUF1	62787	1009	2	1738348	476684	0	2	1	0	50	59	20161348	9.74E-01	1
TFF2	313101	464	90	341760	87576	0	1	1	0	3	3	451764	9.74E-01	1
OR5J2	10101	1007	-38	770384	230332	0	2	1	0	3	5	1002140	9.74E-01	1
OLIG2	824830	554	61	769316	273052	0	1	0	0	2	1	601996	9.74E-01	1
MAP4K2	2261775	230	61	2144544	620508	0	3	1	0	36	57	17760128	9.74E-01	1
GPR15	390532	796	2	890000	264508	0	2	1	0	2	2	680316	9.74E-01	1
IGSF9	807549	310	10	2887872	974016	0	5	5	0	50	80	24723488	9.74E-01	1
WDR63	443738	623	11	2383776	568176	0	4	0	0	4	10	3002504	9.74E-01	1
MAGEB6	34143	NaN	-38	1006412	305092	0	5	4	0	2	8	1099684	9.74E-01	1
NBEAL2	537135	371	45	6839116	2214320	0	8	3	0	9	10	5252424	9.74E-01	1
SARDH	720319	481	29	2331088	698828	0	2	4	0	50	63	24101912	9.75E-01	1
ZBTB39	2065204	199	34	1783916	504808	0	4	3	0	18	28	6101484	9.75E-01	1
ATP10A	104149	1350	-49	3756156	1135284	0	17	8	0	10	23	4422944	9.75E-01	1
USP42	1663156	229	43	2163056	587044	0	4	1	0	5	7	2676764	9.75E-01	1

SNX9	255375	169	40	1592744	389464	0	2	0	0	17	21	6573184	9.75E-01	1
WEE2	200522	844	14	1472772	394092	0	2	2	0	50	60	17687504	9.75E-01	1
LAMC1	767936	374	17	4175168	1098616	0	10	1	0	4	4	2174448	9.75E-01	1
TCERG1	377370	463	14	2841236	771808	0	3	2	0	48	40	17922108	9.75E-01	1
ANGEL2	315884	412	12	1412252	368104	0	1	0	0	49	65	24874788	9.75E-01	1
IPO11	176850	735	18	2694208	684944	0	3	0	0	24	18	7786076	9.75E-01	1
HSPD1	681973	428	29	1467076	419012	0	5	0	0	1	1	594520	9.75E-01	1
STARD3	1224964	213	33	1201856	300820	0	1	3	0	18	23	6688172	9.75E-01	1
FADS2	1618824	212	64	1177648	295124	0	1	0	0	34	55	17629832	9.75E-01	1
UGT1A4	NaN	NaN	NaN	1340696	392668	0	1	0	0	50	116	40038252	9.75E-01	1
CFP	775214	NaN	38	1202212	344252	0	1	0	0	5	7	2499476	9.75E-01	1
TRPC4AP	1128095	236	45	2069428	566396	0	4	4	0	42	84	23723128	9.75E-01	1
PTPRR	157760	458	-26	1705240	458528	0	6	4	0	7	25	5204008	9.75E-01	1
SMAD3	444194	194	45	1170528	311856	0	7	4	0	3	8	1107872	9.75E-01	1
KLHL25	159493	592	-11	1458888	440016	0	1	2	0	24	28	10013212	9.75E-01	1
MYOM1	593610	424	50	4385564	1174444	0	11	6	0	13	27	6017112	9.75E-01	1
C16orf89	594046	302	6	1226420	360628	0	1	3	0	5	9	2842660	9.75E-01	1
ZBTB4	1906324	206	33	2398372	859028	0	5	3	0	8	11	3438248	9.75E-01	1
JPH3	594064	251	23	1844436	572448	0	2	3	0	16	17	6268448	9.75E-01	1
PTPRS	1002562	262	26	4932736	1469924	0	10	5	0	50	50	20143904	9.75E-01	1
KALRN	315141	549	32	7875076	2109656	0	25	12	0	8	17	4937720	9.75E-01	1
PNPLA6	610314	205	34	3462812	1089004	0	5	3	0	21	22	10213640	9.75E-01	1
ELAC2	93345	606	-52	2152020	595944	0	2	0	0	1	0	867572	9.75E-01	1
DHX58	2171374	206	37	1732296	493416	0	3	3	0	4	5	1887512	9.75E-01	1
DUOXA1	667418	544	27	1305096	380208	0	1	2	0	50	85	27585372	9.75E-01	1
AHDC1	738004	217	25	3801368	1346392	0	5	1	0	3	6	2931660	9.75E-01	1
SLC25A6	NaN	NaN	NaN	776792	233536	0	1	3	0	8	19	4159504	9.75E-01	1
GUSB	434728	365	31	1664656	471344	0	1	2	0	50	41	17099748	9.76E-01	1
SYTL4	199096	NaN	47	1765048	456392	0	3	1	0	4	5	1364548	9.76E-01	1
RPL36	879991	295	21	276968	77608	0	1	1	0	2	5	463512	9.76E-01	1
CCNK	345332	429	2	1469568	434676	0	1	0	0	15	24	8144568	9.76E-01	1
MKNK2	1853815	239	37	1295840	352084	0	1	0	0	50	68	23613124	9.76E-01	1
MUC15	21485	951	-18	913852	263084	0	4	4	0	1	8	947316	9.76E-01	1

ZNRF4	932785	365	19	1029196	352796	0	3	0	0	0	0	352796	9.76E-01	1
SGCZ	3398	1175	-34	804204	228552	0	1	1	0	15	25	5464244	9.76E-01	1
HIPK1	350373	545	10	3119272	935924	0	8	1	0	0	1	935924	9.76E-01	1
NCL	1052303	216	43	1857964	475616	0	2	0	0	12	14	5327540	9.76E-01	1
LMAN1L	764533	195	46	1328592	417588	0	4	3	0	11	14	2513716	9.76E-01	1
LRCH2	210224	NaN	-8	2014604	527236	0	2	0	0	5	13	5668588	9.76E-01	1
SLC38A7	572798	188	2	1166256	355644	0	1	2	0	50	64	20692144	9.76E-01	1
DSC2	213526	521	-12	2347820	651124	0	5	3	0	8	9	3134224	9.76E-01	1
SDC1	532338	320	37	765400	248132	0	3	3	0	1	3	470988	9.76E-01	1
MYEF2	219744	588	-38	1594524	399432	0	3	1	0	50	80	20921764	9.76E-01	1
SLC7A9	475532	451	-24	1247068	367748	0	1	3	0	13	17	5519424	9.76E-01	1
LMOD3	264082	443	20	1447852	358136	0	2	3	0	7	7	2245292	9.76E-01	1
THRA	1573630	202	28	1403708	332860	0	1	1	0	50	61	21397024	9.76E-01	1
EXOC6B	148260	625	-1	2151308	542188	0	2	1	0	15	18	7854072	9.76E-01	1
TRIM41	1034999	415	35	1663232	482380	0	3	5	0	9	11	2951240	9.76E-01	1
PLP1	472103	NaN	26	709864	206480	0	1	0	0	6	5	1299400	9.76E-01	1
ZAP70	358719	582	36	1596660	441084	0	1	3	0	22	30	11620552	9.76E-01	1
STK32B	66595	613	12	1103600	275188	0	11	2	0	0	2	275188	9.76E-01	1
PRPF39	234083	565	14	1780712	421504	0	3	0	0	2	2	920972	9.76E-01	1
RNF19B	858954	233	33	1868644	539696	0	2	0	0	26	25	9195480	9.76E-01	1
ACACB	528828	519	29	6314016	1784628	0	16	6	0	19	26	8300496	9.76E-01	1
PCDHGB2	662513	568	2	2290860	719832	0	3	3	0	50	85	24954176	9.76E-01	1
CDC14A	387265	622	6	1665012	444288	0	2	1	0	50	65	19494204	9.76E-01	1
DNAJC13	210456	538	14	5904616	1520120	0	20	9	0	5	16	3536504	9.76E-01	1
FMNL1	798310	207	28	2812044	826632	0	5	4	0	50	80	24627368	9.76E-01	1
GTPBP4	338115	407	30	1680320	424708	0	1	1	0	50	47	22055980	9.76E-01	1
SLC34A2	218417	631	35	1755436	509792	0	3	2	0	32	32	9783236	9.76E-01	1
SBF1	1028405	207	46	4771824	1467432	0	8	2	0	9	14	5484892	9.76E-01	1
SMURF2	937631	151	-8	1972952	503740	0	5	2	0	11	20	4990052	9.76E-01	1
FURIN	833957	291	42	2001432	605556	0	6	5	0	9	26	5816328	9.76E-01	1
C10orf90	122926	478	-50	1770744	506232	0	10	5	0	3	12	1438240	9.76E-01	1
ZNF479	13773	995	-29	1366684	332504	0	9	5	0	2	12	971524	9.76E-01	1
ZCCHC4	233874	611	49	1368108	332148	0	1	1	0	50	54	18959492	9.76E-01	1

ULK3	764533	195	46	1225708	353864	0	1	1	0	50	66	21528032	9.76E-01	1
TPCN2	729282	416	50	1942692	572448	0	3	3	0	50	81	23665100	9.76E-01	1
IFT140	1366033	256	35	3762920	1048420	0	8	2	0	4	10	2737284	9.76E-01	1
GATA5	1190564	205	22	975796	325028	0	2	1	0	4	4	1076544	9.76E-01	1
DOCK10	123941	521	11	5776100	1466008	0	13	4	0	27	40	14668268	9.76E-01	1
TSPAN10	3745109	205	30	846212	305092	0	1	2	0	14	15	3835188	9.76E-01	1
ROCK2	495833	170	25	3715216	871844	0	14	3	0	5	7	1864016	9.76E-01	1
MOGS	1069803	343	45	2012824	689216	0	3	3	0	50	68	19848780	9.77E-01	1
ANO2	349024	712	13	2619804	689928	0	4	2	0	16	18	6469944	9.77E-01	1
SOBP	288292	592	36	2150596	675332	0	1	0	0	6	7	2795312	9.77E-01	1
RLTPR	1065785	147	41	3615536	1161628	0	7	3	0	6	12	4408348	9.77E-01	1
OR2T6	104321	936	-39	769316	220720	0	3	2	0	9	22	2442516	9.77E-01	1
CYP26C1	558273	470	18	1251340	444644	0	1	3	0	50	65	19245716	9.77E-01	1
HIST1H1A	408919	685	43	524032	172304	0	1	0	0	1	1	411536	9.77E-01	1
MURC	234309	702	-21	928092	247776	0	1	2	0	10	9	2379148	9.77E-01	1
LAMP1	815646	237	53	1059100	314348	0	1	3	0	15	22	5794968	9.77E-01	1
FSD1	2122615	220	27	1289788	353864	0	1	2	0	50	78	23857340	9.77E-01	1
PCDHGA3	662513	568	2	2322188	746176	0	10	5	0	7	35	5782152	9.77E-01	1
KDM2A	1502318	162	42	2994316	817376	0	5	4	0	50	77	27193060	9.77E-01	1
SMARCD2	1446139	380	27	1352444	403348	0	1	3	0	31	46	12420840	9.77E-01	1
TEK	116738	387	-28	2919556	778928	0	9	2	0	2	4	1575300	9.77E-01	1
KLHDC8A	662921	249	41	874336	267356	0	1	2	0	8	16	3499480	9.77E-01	1
ZXDA	42394	NaN	-21	1941980	621220	0	1	3	0	47	76	22268512	9.77E-01	1
NUP54	525212	403	38	1314708	359916	0	1	2	0	50	70	20834544	9.77E-01	1
MAL	171487	647	-1	386972	121396	0	1	1	0	2	3	495196	9.77E-01	1
ADAMTS1(	89385	683	-23	3148108	870776	0	11	9	0	6	13	1941980	9.77E-01	1
MORC2	763716	303	37	2514072	695268	0	4	1	0	7	13	4508384	9.77E-01	1
CACNA1E	111356	832	10	5844808	1627988	0	14	9	0	24	43	11138172	9.77E-01	1
IDUA	675007	256	45	1625852	527236	0	1	0	0	5	6	3102896	9.77E-01	1
ATG2A	3218288	209	61	4840888	1542548	0	8	7	0	50	89	25050652	9.77E-01	1
TTPA	137738	789	-12	709508	203632	0	1	1	0	2	5	731580	9.77E-01	1
CTNNA3	14523	1168	-41	2321832	621576	0	3	2	0	3	3	1385908	9.77E-01	1
EZH1	1740597	177	36	1988972	488788	0	4	4	0	17	36	7482408	9.77E-01	1

GART	896855	182	65	2591324	744040	0	4	1	0	2	1	1094700	9.77E-01	1
NDST1	1109777	188	60	2238528	646140	0	2	4	0	18	15	6385572	9.77E-01	1
CUL7	1239021	301	42	4479192	1348884	0	9	4	0	50	105	29539812	9.78E-01	1
STAB2	907417	599	10	6689240	1777864	0	14	3	0	11	13	6274144	9.78E-01	1
BBS1	1642783	194	56	1509796	463868	0	1	0	0	6	4	2325748	9.78E-01	1
GPRIN1	579630	359	40	2397660	839448	0	2	0	0	11	9	4573176	9.78E-01	1
MTSS1	481244	561	39	1915992	561768	0	3	3	0	50	73	21012900	9.78E-01	1
TACR1	451907	644	9	1029552	296904	0	1	0	0	11	13	3810980	9.78E-01	1
CKAP5	1075464	145	38	5278056	1419372	0	13	7	0	32	56	17907868	9.78E-01	1
TMEM168	160383	814	-16	1755792	493416	0	3	0	0	1	0	589536	9.78E-01	1
UNC13A	1029781	246	37	4480972	1153796	0	8	3	0	33	30	11261704	9.78E-01	1
LRP4	1227153	268	38	4934160	1330728	0	8	4	0	40	45	16842716	9.78E-01	1
PEX6	1200368	259	40	2375232	838380	0	3	3	0	39	44	15846272	9.78E-01	1
ZNF490	2001150	409	-6	1380212	334996	0	1	2	0	50	65	22643380	9.78E-01	1
LRP3	406033	270	-8	1888936	609116	0	1	1	0	50	56	22286668	9.78E-01	1
TGM6	611435	477	30	1800648	517980	0	2	2	0	5	10	2094348	9.78E-01	1
SLC17A8	188107	693	-3	1529020	408332	0	5	3	0	3	5	1050200	9.78E-01	1
CYHR1	2043455	200	36	1168392	365256	0	2	2	0	2	13	2164836	9.78E-01	1
TERF2	1197858	125	33	1281956	361696	0	3	2	0	13	27	5427932	9.78E-01	1
GALNT10	557034	451	31	1553940	428268	0	1	0	0	50	57	22260324	9.78E-01	1
SNX19	130522	523	7	2466012	758280	0	6	4	0	2	5	1513712	9.78E-01	1
CTSZ	1009250	152	30	776436	218940	0	1	2	0	15	26	5327896	9.78E-01	1
GALNT12	221241	164	17	1472772	430404	0	1	2	0	23	20	7447164	9.78E-01	1
KIF2B	930	1125	-86	1677828	481668	0	5	4	0	50	87	13483500	9.78E-01	1
LARGE	31722	968	-69	1949812	535424	0	5	1	0	8	24	4287664	9.78E-01	1
PCDHA6	896676	606	-12	2332868	744040	0	11	7	0	6	28	4240316	9.78E-01	1
RNF34	650760	244	38	972236	260236	0	1	1	0	29	52	13287344	9.78E-01	1
DUS3L	990266	225	26	1639380	501960	0	2	2	0	50	73	21365340	9.78E-01	1
ACBD4	806811	207	32	1009616	156640	0	1	2	0	6	3	1157000	9.78E-01	1
ZNF780B	1181917	521	22	2170888	518336	0	3	2	0	50	77	22919280	9.78E-01	1
C12orf29	152325	507	-27	864012	206124	0	1	0	0	50	109	24231496	9.78E-01	1
OSBPL1A	150936	360	35	2545044	617304	0	4	0	0	11	13	4596316	9.78E-01	1
TAF1	729010	NaN	32	4954096	1274480	0	7	2	0	5	6	3201864	9.78E-01	1

TEP1	415850	401	-7	6625516	2025284	0	14	10	0	5	16	4430420	9.78E-01	1
ZNF598	1747301	183	46	2244580	706304	0	3	1	0	1	1	857604	9.78E-01	1
CIITA	299383	410	38	2822012	882880	0	5	4	0	43	56	17455748	9.78E-01	1
TCF20	1257937	305	43	4893220	1428272	0	12	5	0	13	33	8750124	9.78E-01	1
GPR35	837485	485	35	736208	257032	0	1	3	0	3	6	1495912	9.78E-01	1
SIAH2	348288	544	27	798152	247420	0	2	2	0	3	6	1243152	9.78E-01	1
PLCZ1	82407	911	-29	1615528	393380	0	5	2	0	50	88	17017156	9.78E-01	1
MAP1A	794398	269	37	6875784	2116776	0	15	4	0	10	21	7868312	9.78E-01	1
LYPD6B	132203	698	-3	553224	138840	0	1	2	0	2	4	821648	9.78E-01	1
PLOD1	518604	207	41	1885732	525812	0	2	2	0	50	50	16403768	9.78E-01	1
SERINC3	781823	276	46	1223572	337844	0	9	4	0	3	10	1219300	9.78E-01	1
GRIA3	226353	NaN	-6	2417596	641156	0	5	0	0	0	0	641156	9.78E-01	1
DPY19L2	195841	681	-28	1996092	525456	0	6	1	0	18	43	10534040	9.78E-01	1
GLIS2	944378	161	33	1277684	427912	0	1	1	0	37	46	14908212	9.78E-01	1
PNPLA7	1453700	213	35	3421516	1026704	0	7	2	0	6	6	2500188	9.79E-01	1
ZMYM5	314256	363	44	1828060	462800	0	3	0	0	8	9	2877192	9.79E-01	1
AGPAT6	365713	496	30	1191888	319332	0	2	1	0	18	28	7972976	9.79E-01	1
DICER1	215900	674	16	4955520	1316844	0	14	4	0	20	17	5995752	9.79E-01	1
MAPK8IP2	1073094	203	43	2087584	695624	0	2	0	0	1	2	1119976	9.79E-01	1
RHOXF2B	800534	NaN	23	1478824	398720	0	1	0	0	50	70	25377104	9.79E-01	1
TPPP3	1115833	153	42	457104	122820	0	1	2	0	2	6	841940	9.79E-01	1
FCRL5	234921	764	25	2453196	748668	0	5	4	0	50	65	18961628	9.79E-01	1
USP44	442533	328	13	1821296	484516	0	1	0	0	4	3	2248140	9.79E-01	1
VNN2	127166	569	2	1325032	372020	0	1	3	0	16	14	4555732	9.79E-01	1
E2F1	710350	161	55	1075832	353152	0	1	2	0	50	88	20870500	9.79E-01	1
CNTNAP1	1740597	177	36	3495208	1040588	0	8	5	0	17	36	7482408	9.79E-01	1
ZNF468	557083	1008	-39	1357072	331436	0	6	1	0	1	4	745464	9.79E-01	1
ARFGEF2	808420	174	49	4671432	1213248	0	14	5	0	11	18	5035976	9.79E-01	1
PDZD8	203138	607	5	2862596	855112	0	5	2	0	7	7	2981500	9.79E-01	1
FBXO11	486669	587	27	2525108	636172	0	6	1	0	2	1	756856	9.79E-01	1
LGI4	745516	357	-9	1320760	437168	0	3	0	0	0	0	437168	9.79E-01	1
CLSTN3	1872466	224	33	2441448	697404	0	4	1	0	26	35	10588864	9.79E-01	1
LPA	287006	545	-24	5240676	1465296	0	16	2	0	3	8	2642588	9.79E-01	1

EPHA4	54488	1025	5	2541484	691352	0	2	1	0	2	4	1526884	9.79E-01	1
PHC2	668735	413	31	2145256	664652	0	4	0	0	2	4	1569960	9.79E-01	1
ATRNL1	45264	1002	-20	3624080	917056	0	8	2	0	9	14	4617676	9.79E-01	1
HEATR6	972352	235	18	2993960	876472	0	6	4	0	27	31	10094024	9.79E-01	1
RPS6KA1	901637	184	32	2015316	580992	0	5	1	0	1	1	762196	9.79E-01	1
SCARF1	1361271	198	27	2137780	569600	0	3	0	0	9	20	5743704	9.79E-01	1
ABCC6	385823	188	23	3815252	1218232	0	6	4	0	29	38	14579624	9.79E-01	1
GOLGA8B	NaN	NaN	NaN	1582776	416520	0	1	0	0	50	116	40062104	9.79E-01	1
FZD8	144247	708	-26	1663944	564972	0	3	2	0	37	58	16508076	9.79E-01	1
ZBBX	69051	928	-23	2136356	506944	0	5	2	0	46	76	19312644	9.79E-01	1
FSD2	490130	631	17	1953372	503028	0	5	2	0	50	74	19009688	9.79E-01	1
CCDC61	931670	261	36	1347104	417232	0	1	0	0	4	4	1800292	9.79E-01	1
MYO15A	911350	192	29	8869740	2712720	0	19	8	0	25	50	15465352	9.79E-01	1
FOXD2	190214	478	16	1153796	435388	0	1	0	0	5	9	3000368	9.79E-01	1
RGPD2	177375	633	3	9065896	2385200	0	5	0	0	0	0	2385200	9.79E-01	1
MLPH	468096	440	11	1549312	440372	0	3	0	0	1	3	1150948	9.79E-01	1
USHBP1	865138	212	38	1752588	554292	0	2	2	0	50	59	18864796	9.79E-01	1
PIWIL3	399624	395	-4	2303676	610896	0	4	2	0	16	25	7548980	9.79E-01	1
MRPL39	533414	822	75	975440	252760	0	2	2	0	3	4	1064796	9.80E-01	1
IFNGR1	117339	309	35	1259172	337488	0	1	2	0	50	65	19546536	9.80E-01	1
HTR1E	169399	752	-17	902460	270204	0	1	3	0	8	12	2471708	9.80E-01	1
NSUN5	902769	422	52	1204704	352440	0	1	1	0	50	86	23744844	9.80E-01	1
SLC2A11	643596	182	57	1312928	414028	0	1	0	0	50	84	21648360	9.80E-01	1
PDPK1	2083323	321	42	1451768	388396	0	2	0	0	50	76	19852696	9.80E-01	1
SLC36A3	871795	530	57	1306876	376292	0	4	3	0	4	12	1804564	9.80E-01	1
ADAMTS1[illegible]	38096	1075	-16	3124612	839804	0	5	5	0	20	31	8975472	9.80E-01	1
POLL	843302	421	19	1443580	436100	0	1	1	0	50	65	21446508	9.80E-01	1
CRB2	346407	389	44	3128172	1045572	0	3	3	0	29	30	13297668	9.80E-01	1
LRP2BP	224125	422	25	915632	229264	0	1	0	0	2	2	919548	9.80E-01	1
ZYG11B	652910	232	28	1918840	523676	0	6	2	0	17	41	8399820	9.80E-01	1
SMTN	726734	202	32	2417596	766112	0	2	1	0	10	12	5233912	9.80E-01	1
XKR5	131291	564	40	1709868	517980	0	1	0	0	14	10	4934872	9.80E-01	1
MKL1	578345	228	55	2297980	739412	0	3	1	0	25	38	12633372	9.80E-01	1

NOLC1	1041024	187	45	1804564	489500	0	3	0	0	1	1	671416	9.80E-01	1
ZW10	148947	534	4	2037744	527592	0	1	2	0	42	34	13987240	9.80E-01	1
AKNA	218424	360	14	3572816	1126384	0	8	1	0	1	4	1677472	9.80E-01	1
PIGT	1037999	382	35	1453548	450696	0	2	4	0	50	68	17427980	9.80E-01	1
IL20	467839	385	39	462444	123888	0	1	2	0	5	11	1456752	9.80E-01	1
PDZD7	1020226	247	40	1283736	410112	0	1	0	0	35	46	14845556	9.80E-01	1
GPR78	96216	471	31	855824	318976	0	1	4	0	7	9	2233900	9.80E-01	1
EPPK1	2376841	247	22	5780372	1978648	0	9	7	0	50	67	29170640	9.80E-01	1
JAKMIP1	366026	678	41	2226780	600216	0	4	3	0	6	9	3068008	9.80E-01	1
UNC5A	1056724	222	44	2119624	641156	0	2	2	0	50	61	21713864	9.80E-01	1
KIF5C	87934	682	0	2477760	615168	0	4	0	0	2	0	706304	9.80E-01	1
MED23	147816	864	7	3590972	943756	0	3	2	0	13	15	6244952	9.80E-01	1
RGPD1	NaN	NaN	NaN	9034568	2365264	0	3	0	0	0	0	2365264	9.80E-01	1
C1QTNF9B	314992	678	51	853332	226416	0	1	1	0	21	23	5433984	9.80E-01	1
SMYD5	536944	452	20	1112144	283732	0	1	3	0	18	24	6456416	9.80E-01	1
RADIL	1009587	523	13	2644012	863300	0	4	4	0	15	28	6816332	9.80E-01	1
NCAPH	907236	316	46	1945184	506944	0	2	4	0	12	26	7976180	9.80E-01	1
POLR3A	362530	184	31	3599160	985764	0	11	4	0	3	6	1604492	9.80E-01	1
OCRL	330789	NaN	22	2392676	595588	0	2	1	0	50	55	24691448	9.80E-01	1
TTC5	371637	528	-29	1135284	318264	0	1	1	0	50	73	20208696	9.80E-01	1
FCER1A	285980	737	-6	679604	168388	0	3	2	0	2	5	650768	9.80E-01	1
SRPK1	761613	404	28	1735500	430404	0	1	1	0	50	64	25956672	9.80E-01	1
RFWD2	66809	929	-3	1930944	495552	0	4	2	0	14	23	6387352	9.80E-01	1
CDK11B	1569768	214	33	2109656	521896	0	2	0	0	3	2	1529020	9.80E-01	1
C16orf86	1157184	136	50	777860	253828	0	3	0	0	0	0	253828	9.80E-01	1
ATP2C1	313762	475	19	2596308	676044	0	3	2	0	11	13	5820244	9.81E-01	1
ZNF703	587619	452	3	1383060	514776	0	1	1	0	30	32	11551488	9.81E-01	1
TMEM37	203559	497	35	468140	150232	0	1	3	0	2	7	839448	9.81E-01	1
ZNF408	965022	176	48	1790680	570668	0	2	1	0	6	7	1921332	9.81E-01	1
ZNF652	748320	256	25	1563552	398364	0	2	1	0	7	14	4020308	9.81E-01	1
OR2T1	104321	936	-39	913140	271272	0	4	2	0	9	22	2442516	9.81E-01	1
OPN1MW	NaN	NaN	NaN	1859032	526880	0	1	1	0	50	117	40172464	9.81E-01	1
FAM13B	992763	396	39	2514072	611964	0	6	1	0	3	4	1552872	9.81E-01	1

OR2T34	116628	850	-20	784980	237096	0	2	3	0	4	8	1241728	9.81E-01	1
HSD17B2	192405	635	2	984696	277680	0	4	3	0	2	5	838024	9.81E-01	1
TRAIP	1306720	191	39	1234964	330724	0	1	2	0	50	70	21778300	9.81E-01	1
THBD	199022	554	-50	1398368	447136	0	3	0	0	2	4	1281600	9.81E-01	1
DDX31	1546589	581	42	2186552	631900	0	4	0	0	9	12	3932376	9.81E-01	1
PDLIM7	1120311	347	41	1285516	361340	0	1	1	0	50	73	19933508	9.81E-01	1
LMTK3	1500967	182	37	3587412	1250628	0	6	4	0	21	39	13236080	9.81E-01	1
CCDC93	195266	578	-2	1705240	417944	0	1	1	0	50	49	18798580	9.81E-01	1
TSC2	1722112	181	44	4583856	1386264	0	9	9	0	21	31	8039548	9.81E-01	1
PSMB10	879834	158	50	684944	227128	0	1	3	0	8	20	3682108	9.81E-01	1
SLC33A1	284462	473	4	1376296	407264	0	2	3	0	14	27	7457844	9.81E-01	1
TRIM4	1559879	391	36	1278040	352796	0	1	0	0	19	26	8437912	9.81E-01	1
PLAG1	343641	552	19	1259172	350304	0	2	4	0	5	7	1375940	9.81E-01	1
DHX40	1081217	417	25	2038812	532932	0	4	0	0	2	3	1081172	9.81E-01	1
MAMLD1	311047	NaN	16	2847644	855112	0	2	1	0	28	36	16012168	9.81E-01	1
MMP14	1177534	225	34	1485944	420436	0	2	3	0	15	22	6086176	9.81E-01	1
BCAN	1851672	180	37	2322188	732292	0	4	2	0	43	61	19959496	9.81E-01	1
TEAD4	506329	267	35	1135284	305448	0	3	2	0	5	14	2426852	9.81E-01	1
CLRN1	353086	635	10	635460	173016	0	3	3	0	1	7	854400	9.81E-01	1
EPB41L1	1300256	235	50	2278400	637240	0	5	5	0	5	19	4169828	9.81E-01	1
CHST1	160913	609	18	996800	327520	0	2	3	0	10	12	2584916	9.81E-01	1
NR5A1	492584	353	24	1156288	349592	0	1	0	0	9	9	2987196	9.81E-01	1
DCUN1D5	166625	585	-9	646852	145604	0	2	1	0	3	6	917768	9.81E-01	1
ZSWIM5	1015582	422	26	2954800	900680	0	9	2	0	0	2	900680	9.81E-01	1
AKAP4	56301	NaN	5	2188688	572092	0	2	3	0	20	23	9124992	9.81E-01	1
FAM186B	2227486	224	53	2269144	623000	0	4	0	0	4	10	2584560	9.81E-01	1
DNAI1	811768	370	49	1859388	464580	0	4	3	0	18	33	8770060	9.82E-01	1
TKTL2	74744	1164	-26	1540768	468140	0	5	2	0	6	13	2391252	9.82E-01	1
UGT2B4	18715	1044	-22	1358140	360272	0	2	3	0	50	110	25751972	9.82E-01	1
ZNF48	2338593	142	59	1509084	480600	0	1	2	0	50	74	22010768	9.82E-01	1
TRMT11	142337	297	11	1230692	307228	0	2	3	0	17	29	6295504	9.82E-01	1
RNF148	53042	1027	6	766112	214312	0	2	3	0	1	3	477752	9.82E-01	1
NCDN	1049488	388	34	1769320	599504	0	3	1	0	14	17	4776096	9.82E-01	1

CAMK2B	1467518	412	65	1746180	492348	0	4	6	0	19	25	5655060	9.82E-01	1
MAP7D3	314502	NaN	20	2319340	586688	0	2	1	0	31	33	12341096	9.82E-01	1
USH2A	82180	967	-25	13285920	3696348	0	34	7	0	2	8	4033836	9.82E-01	1
SUOX	2080397	229	31	1335000	427200	0	1	3	0	7	8	2941984	9.82E-01	1
C12orf60	340728	578	-4	633680	158776	0	2	2	0	4	5	737988	9.82E-01	1
PHF2	421433	612	29	2817028	787472	0	2	3	0	10	15	7918508	9.82E-01	1
PARD3B	51582	973	-1	3075484	882524	0	5	4	0	27	50	11997556	9.82E-01	1
SLC38A4	518041	415	-10	1434680	380920	0	1	0	0	28	37	13064844	9.82E-01	1
ERI2	211686	580	23	2109656	535780	0	2	0	0	16	14	5815972	9.82E-01	1
SHC3	207531	633	8	1506948	446424	0	1	1	0	50	57	22178088	9.82E-01	1
AQR	468734	288	-5	3896064	1010328	0	8	2	0	24	37	12202968	9.82E-01	1
APOBEC3I	1386387	372	71	1095412	274832	0	1	1	0	50	61	16059872	9.82E-01	1
POTEM	NaN	NaN	NaN	1347104	327520	0	1	1	0	50	117	39973104	9.82E-01	1
CABLES1	270708	198	72	1621580	505876	0	1	2	0	26	42	14142812	9.82E-01	1
SPTBN2	1432488	187	46	6025300	1789256	0	11	6	0	31	42	14892904	9.82E-01	1
CSMD2	137660	907	16	8957316	2515140	0	30	13	0	9	23	5400876	9.82E-01	1
LRRC56	1970020	236	42	1335000	451764	0	1	2	0	50	66	20259248	9.82E-01	1
ZMYND11	223637	625	3	1634752	377360	0	1	1	0	10	7	3556796	9.82E-01	1
DCDC2	362257	711	-12	1338916	336776	0	1	2	0	50	65	19879396	9.82E-01	1
LARP1	549658	179	27	2626212	720900	0	4	1	0	27	42	13622696	9.82E-01	1
ATXN3L	295243	NaN	40	915988	224636	0	1	2	0	21	35	7221816	9.82E-01	1
ANKRD36I	390850	455	29	3648288	873624	0	2	1	0	43	33	21068436	9.82E-01	1
RBM19	198554	673	11	2511936	669636	0	5	2	0	50	64	20156008	9.82E-01	1
TNFRSF10	633411	240	42	1191176	349948	0	4	2	0	1	2	598792	9.82E-01	1
SCAPER	393531	675	22	3700620	956928	0	10	5	0	2	5	1723040	9.82E-01	1
FAM129B	857814	182	46	1926316	543968	0	4	0	0	1	1	652192	9.82E-01	1
SPAG9	1009546	138	-3	3447148	912428	0	8	3	0	5	8	2186196	9.82E-01	1
NBEAL1	478657	384	31	7047020	1814176	0	13	2	0	4	4	2650776	9.82E-01	1
TRIM37	678139	530	13	2547536	648988	0	8	1	0	0	1	648988	9.82E-01	1
TRIM26	1932849	486	48	1365972	389820	0	1	1	0	50	60	20279184	9.82E-01	1
SLC9A3R2	1747301	183	46	843364	265220	0	1	0	0	1	0	416520	9.82E-01	1
RGS14	942017	227	53	1429696	446780	0	5	1	0	6	8	1593456	9.82E-01	1
TMPRSS3	339928	400	80	1247780	347812	0	1	2	0	47	54	17291276	9.82E-01	1

PPARD	670775	267	35	1157356	310076	0	5	3	0	4	12	1621936	9.82E-01	1
ZNF772	451095	869	-24	1276616	310432	0	1	0	0	32	34	10625888	9.82E-01	1
KRT76	1444461	422	38	1619800	463868	0	4	4	0	9	15	3373456	9.82E-01	1
DNAJC2	375620	620	8	1676404	386972	0	2	2	0	50	70	20312292	9.83E-01	1
DOK7	547964	412	46	1299400	346388	0	1	0	0	23	36	11802112	9.83E-01	1
KCNH4	2129732	206	30	2514784	810968	0	4	4	0	15	27	8388428	9.83E-01	1
FAM73A	368215	207	1	1665012	429336	0	1	3	0	11	14	6172684	9.83E-01	1
TCOF1	949599	212	40	3759004	1147388	0	5	4	0	50	73	24033560	9.83E-01	1
ALPK1	185655	467	42	3194744	858316	0	6	2	0	3	3	1700256	9.83E-01	1
OR4C6	1295	1091	-20	765400	225704	0	5	4	0	2	8	660736	9.83E-01	1
ZNF185	235275	NaN	23	1997872	565328	0	2	2	0	4	4	2053408	9.83E-01	1
FES	833957	291	42	2105740	608048	0	3	2	0	50	63	21711728	9.83E-01	1
TNKS1BP1	929334	250	-20	4249216	1340696	0	8	5	0	6	11	3788908	9.83E-01	1
XYLT1	30248	996	-2	2405492	719476	0	6	5	0	50	74	17194800	9.83E-01	1
NCLN	905626	241	30	1430052	436812	0	1	2	0	50	57	19345752	9.83E-01	1
TRIM72	1203752	478	17	1175156	381988	0	1	2	0	50	54	16147804	9.83E-01	1
TET3	1029946	250	44	4067300	1288720	0	8	3	0	8	12	4633696	9.83E-01	1
FBXL19	1508459	247	46	1692068	577432	0	2	1	0	50	71	20582852	9.83E-01	1
SP2	1127646	178	25	1515492	477396	0	2	3	0	34	49	12154196	9.83E-01	1
NDNL2	242355	885	-39	750804	226416	0	1	3	0	18	28	5264172	9.83E-01	1
TMEM26	84084	468	-19	926668	276968	0	4	3	0	1	4	457104	9.83E-01	1
MPDZ	77490	688	-36	5238184	1494488	0	15	3	0	3	7	2801008	9.83E-01	1
COL20A1	1278630	332	62	3177656	1027060	0	4	4	0	50	61	17105444	9.83E-01	1
CCDC64	1390014	218	43	1452124	423284	0	2	2	0	7	10	2923472	9.83E-01	1
SLC41A3	210410	610	32	1512288	477396	0	2	0	0	1	1	607692	9.83E-01	1
UNC5D	34554	1152	-45	2455688	669280	0	6	6	0	10	17	4394820	9.83E-01	1
VWF	1716132	481	15	7239972	1991820	0	15	4	0	7	15	6077276	9.83E-01	1
DCAF8L2	NaN	NaN	NaN	1597016	432184	0	1	1	0	50	117	40077768	9.84E-01	1
DAB2IP	448984	475	22	2920624	881456	0	5	1	0	6	8	3173740	9.84E-01	1
CCDC149	280003	621	34	1377008	372376	0	2	1	0	16	18	4360644	9.84E-01	1
CHN1	389626	701	12	1218944	306160	0	3	3	0	9	20	3810268	9.84E-01	1
PRSS53	1347539	227	37	1382704	435032	0	1	0	0	50	69	22509168	9.84E-01	1
CLCN5	75681	NaN	26	2080108	588824	0	3	3	0	48	63	19445432	9.84E-01	1

GOLGA6A	412657	344	49	1831620	466716	0	1	0	0	7	6	2794956	9.84E-01	1
DLL4	1049616	176	50	1742620	498044	0	3	3	0	27	42	10668252	9.84E-01	1
PLIN4	2111443	278	43	3225716	1146676	0	3	2	0	10	14	5030280	9.84E-01	1
LRRK1	402246	363	12	5115364	1480604	0	10	4	0	5	8	4008204	9.84E-01	1
GRIA2	44831	925	-34	2389116	634392	0	8	5	0	21	43	6896788	9.84E-01	1
NRF1	459189	253	32	1275548	385192	0	2	3	0	5	5	1521188	9.84E-01	1
TMPRSS9	1553941	267	33	2615888	847636	0	5	3	0	36	43	12243908	9.84E-01	1
B4GALT7	1316033	475	42	828768	245640	0	2	2	0	1	2	417588	9.84E-01	1
SH3GL2	28189	392	-31	931652	233536	0	1	1	0	35	62	12630880	9.84E-01	1
PCDH10	9477	1355	-31	2592748	821648	0	12	9	0	4	16	2154512	9.84E-01	1
VASN	754886	161	41	1564976	598792	0	1	1	0	21	21	8108256	9.84E-01	1
BCL3	812266	188	-22	1104668	389464	0	1	1	0	8	17	4475632	9.84E-01	1
WDR19	604829	230	49	3566764	890000	0	7	4	0	50	64	20686448	9.84E-01	1
ATL3	1058363	170	50	1416880	370952	0	1	0	0	50	66	21187340	9.84E-01	1
WHSC1	815091	239	43	3594176	949096	0	16	6	0	8	14	3011404	9.84E-01	1
BAHCC1	3216151	268	24	6243172	2050916	0	7	2	0	0	2	2050916	9.84E-01	1
NGF	537568	558	6	597724	181916	0	2	2	0	2	6	734784	9.84E-01	1
CRYL1	346767	584	28	834108	223212	0	2	3	0	3	6	1078680	9.84E-01	1
SCLT1	98655	645	8	1857252	435744	0	3	0	0	10	19	5505540	9.84E-01	1
NEURL4	1911735	206	36	3905676	1223928	0	8	6	0	50	69	22072000	9.84E-01	1
UBE4A	963668	192	48	2800652	721612	0	6	1	0	2	1	927380	9.84E-01	1
DNMT1	1702441	200	23	4271644	1131368	0	9	5	0	50	95	25984796	9.84E-01	1
RTN3	1291649	168	46	2674628	748312	0	9	2	0	0	2	748312	9.84E-01	1
DDX41	1120311	347	41	1623716	442864	0	2	1	0	50	73	19933508	9.84E-01	1
CDH23	641879	524	41	8835208	2648996	0	14	5	0	4	7	4630136	9.84E-01	1
NRM	2115149	171	41	635104	222500	0	2	2	0	1	4	637952	9.84E-01	1
SF3B4	798975	560	17	1045572	339624	0	3	0	0	1	1	458528	9.84E-01	1
ANP32A	437749	562	32	669992	156640	0	4	3	0	3	4	516556	9.84E-01	1
TTC39B	237773	710	-17	1818448	475616	0	3	1	0	1	1	550732	9.84E-01	1
TSHZ3	8215	1022	-74	2704532	768604	0	13	8	0	38	56	8770060	9.84E-01	1
ZIC5	323784	500	35	1589540	542188	0	1	3	0	22	20	7687108	9.84E-01	1
BNC1	206688	679	11	2522972	684232	0	4	4	0	40	58	16229684	9.84E-01	1
SS18	167310	632	37	1129944	257388	0	1	1	0	50	62	16815660	9.84E-01	1

CLASP1	238179	612	21	4005356	1143472	0	10	3	0	2	4	1674624	9.84E-01	1
TSGA10	504327	770	19	1856896	451052	0	2	0	0	12	9	4118564	9.84E-01	1
PARP2	415850	401	-7	1550024	387328	0	2	0	0	5	16	4430420	9.84E-01	1
PRMT3	162124	694	-1	1432900	335708	0	1	0	0	28	35	12313684	9.84E-01	1
PDE2A	741790	193	55	2572812	680316	0	2	0	0	7	9	3320056	9.84E-01	1
ZNF75D	255033	NaN	36	1331084	323248	0	7	3	0	1	3	667500	9.85E-01	1
USP32	913326	361	6	4192968	1090428	0	9	6	0	50	60	14447548	9.85E-01	1
FBXL13	720636	510	30	1940556	492348	0	2	1	0	50	69	21619168	9.85E-01	1
EML3	2695768	217	42	2254548	709152	0	3	2	0	17	27	9232504	9.85E-01	1
CD300LG	1025836	241	19	933076	286580	0	1	0	0	9	14	3060176	9.85E-01	1
CLIC6	284989	406	43	1703460	519048	0	1	2	0	50	65	23149612	9.85E-01	1
SLC22A2	653647	326	-9	1414032	410112	0	5	3	0	2	11	1543616	9.85E-01	1
PDE4D	91124	682	10	2367756	640800	0	1	0	0	3	2	1340696	9.85E-01	1
RSPH10B	NaN	NaN	NaN	4631916	1111788	0	4	0	0	0	0	1111788	9.85E-01	1
SLC15A3	802735	546	56	1424000	472768	0	1	2	0	50	83	25238620	9.85E-01	1
SRGAP2	472089	300	6	2550384	689928	0	4	0	0	0	0	689928	9.85E-01	1
MGAT4C	15683	881	-41	1234964	314704	0	3	2	0	2	4	1039876	9.85E-01	1
HECW2	166718	447	13	4042024	1117484	0	11	7	0	13	20	6304760	9.85E-01	1
ESPN	644193	198	33	2104316	686368	0	2	3	0	50	58	20455048	9.85E-01	1
PCDHB16	915569	724	-11	1876832	612676	0	12	5	0	1	11	1243508	9.85E-01	1
HS6ST3	105227	954	-3	1186192	332504	0	1	2	0	50	77	21022868	9.85E-01	1
ADCY4	906124	173	39	2717348	843364	0	4	3	0	50	64	20790400	9.85E-01	1
CNTNAP5	1260	1308	-14	3380932	904952	0	12	6	0	2	8	1703460	9.85E-01	1
FAS	187494	375	39	899612	215380	0	1	2	0	4	5	1278396	9.85E-01	1
RASAL3	524410	481	33	2483456	831616	0	4	2	0	9	22	7155956	9.85E-01	1
RHOJ	143550	637	-13	561412	144536	0	1	1	0	2	2	522608	9.85E-01	1
CDK5RAP1	464431	395	39	1519052	420436	0	5	4	0	7	14	2542196	9.85E-01	1
TMEM150A	1158965	170	43	686724	210396	0	1	2	0	7	8	1535784	9.85E-01	1
NECAB3	710350	161	55	1023856	295124	0	1	0	0	23	40	8652580	9.85E-01	1
TRPM2	1023429	308	54	3867940	1085444	0	10	5	0	46	61	17048128	9.85E-01	1
ILF3	2128709	224	33	2359568	674620	0	5	2	0	1	2	881812	9.85E-01	1
FERMT2	309280	188	12	1829484	451764	0	1	2	0	50	40	18195516	9.85E-01	1
NCAPG2	267997	565	-31	3000368	780352	0	2	1	0	19	33	8965860	9.85E-01	1

TMEM2	235852	312	-7	3561424	971168	0	4	3	0	50	53	22425152	9.85E-01	1
LLGL1	925111	223	18	2672136	835176	0	3	2	0	37	44	15624484	9.85E-01	1
CHRNA2	326685	448	42	1320048	399432	0	5	2	0	2	5	803136	9.85E-01	1
ACAD10	599161	203	33	2778580	806696	0	6	4	0	9	17	4747972	9.85E-01	1
LRRTM4	7531	1175	-21	1493064	413316	0	6	4	0	1	4	726596	9.86E-01	1
ZNF512B	1154675	189	42	2254548	674976	0	1	2	0	17	20	6007856	9.86E-01	1
TAP2	1313279	295	31	1764692	572092	0	1	0	0	11	14	5772540	9.86E-01	1
ZNF221	464210	419	-38	1616240	380920	0	4	0	0	1	2	817732	9.86E-01	1
CDYL2	161731	507	-16	1286584	363476	0	1	2	0	17	25	6797820	9.86E-01	1
APAF1	488214	461	11	3298696	814172	0	6	1	0	0	1	814172	9.86E-01	1
PKD2L1	1071291	198	56	2072276	574228	0	10	2	0	0	2	574228	9.86E-01	1
MAP2K6	58848	397	-42	890000	230332	0	1	2	0	6	11	2529024	9.86E-01	1
PLCB4	77680	715	-57	3267012	758280	0	8	2	0	20	51	13352136	9.86E-01	1
WDR90	1161748	194	36	4396956	1379856	0	6	5	0	14	16	6811704	9.86E-01	1
SEMA4D	163051	596	34	2667864	759348	0	2	0	0	23	21	9825956	9.86E-01	1
SLC44A1	265154	490	0	1699544	472768	0	1	1	0	50	57	23594968	9.86E-01	1
ZSWIM3	734138	136	53	1746180	491280	0	3	2	0	50	87	20836324	9.86E-01	1
PIK3R5	359348	636	10	2218236	681384	0	3	4	0	50	67	20081604	9.86E-01	1
QRICH2	1650351	209	21	4157724	1250628	0	10	5	0	28	67	17270984	9.86E-01	1
TTC38	519654	205	31	1224996	338556	0	1	0	0	43	59	16573224	9.86E-01	1
ADAM15	1696720	214	44	2216812	647564	0	7	1	0	0	1	647564	9.86E-01	1
DYRK3	491162	261	34	1498760	421504	0	1	2	0	34	54	15229324	9.86E-01	1
GFPT2	1453124	362	19	1785340	482024	0	5	4	0	5	25	5384500	9.86E-01	1
CACNG4	293409	491	-23	822004	243860	0	3	4	0	5	12	1555008	9.86E-01	1
ZNF573	623089	829	-44	1843724	440728	0	2	1	0	4	7	1831264	9.86E-01	1
SLC6A13	316827	557	28	1555008	436812	0	2	2	0	33	45	12576056	9.86E-01	1
C10orf88	282374	619	13	1138844	311500	0	4	2	0	9	18	2581356	9.86E-01	1
LONRF1	119955	694	3	1959068	569956	0	2	1	0	2	1	1004276	9.86E-01	1
IGSF22	616892	562	25	3399444	946248	0	6	1	0	8	12	4953384	9.86E-01	1
CNBD1	148474	1191	-31	1170172	276968	0	5	4	0	2	7	1064796	9.86E-01	1
DOCK11	207684	NaN	38	5497708	1371668	0	7	1	0	1	2	1593100	9.86E-01	1
KLHL34	93001	NaN	7	1531156	535424	0	2	3	0	17	26	6913876	9.86E-01	1
KIR3DL2	987531	620	-23	1159136	338200	0	2	2	0	50	135	24266740	9.86E-01	1

ACTN4	1596646	322	36	2393388	616236	0	9	1	0	0	1	616236	9.86E-01	1
CDC23	1127160	314	50	1571384	410824	0	1	1	0	50	62	19912860	9.86E-01	1
CBWD3	114607	887	-9	2134932	523320	0	1	0	0	17	37	15510564	9.86E-01	1
RTN4RL1	1445978	224	22	1076544	346032	0	1	0	0	4	4	1288720	9.86E-01	1
TUBGCP4	823006	415	50	1731584	478108	0	1	1	0	50	74	24588920	9.86E-01	1
EPHA10	445060	375	30	2530804	810968	0	4	4	0	50	55	18360344	9.86E-01	1
CABP5	993450	433	26	461732	117124	0	1	2	0	1	3	513708	9.86E-01	1
IRAK3	239871	696	4	1563196	398720	0	6	1	0	1	1	509792	9.86E-01	1
FASN	3850694	197	34	6251360	1976512	0	10	2	0	0	2	1976512	9.86E-01	1
OR5T2	10209	969	-40	892848	255252	0	1	0	0	50	99	20331516	9.86E-01	1
SLITRK4	4706	NaN	-30	2097196	592028	0	7	5	0	7	14	2985416	9.86E-01	1
ZNF280D	229613	626	9	2676052	653972	0	2	1	0	34	39	16221140	9.86E-01	1
SPACA3	286646	837	-10	553224	160200	0	1	3	0	2	6	860452	9.86E-01	1
KARS	449343	354	18	1694560	443576	0	9	4	0	4	8	1316844	9.86E-01	1
DSCAML1	413931	624	44	5318284	1593812	0	13	5	0	50	60	18511288	9.86E-01	1
PPARGC1B	908037	285	49	2572812	762552	0	3	0	0	4	17	6737656	9.86E-01	1
NCOA7	184468	179	18	2450348	635104	0	4	4	0	15	13	4244232	9.86E-01	1
TCP11	625705	198	29	1368108	382344	0	1	2	0	50	50	15609888	9.86E-01	1
ELFN2	969938	179	53	1992176	638308	0	3	3	0	50	63	19671848	9.86E-01	1
MAP7D2	263236	NaN	38	1998228	550020	0	2	1	0	45	55	17959132	9.86E-01	1
MSH3	319054	752	36	2954088	792456	0	3	2	0	50	51	20448996	9.86E-01	1
TDRD9	694566	406	28	3619096	963692	0	4	3	0	37	43	14873324	9.87E-01	1
CAPN11	1176811	216	49	1975800	491280	0	2	2	0	50	72	23213336	9.87E-01	1
UBQLN4	2005405	213	39	1516560	457104	0	1	1	0	50	71	21768332	9.87E-01	1
SLC36A1	736209	598	22	1209688	361340	0	1	3	0	5	21	6043456	9.87E-01	1
CCDC157	696089	214	57	1880036	577432	0	2	0	0	7	10	3486308	9.87E-01	1
DIS3	257535	685	2	2565692	669280	0	5	2	0	24	36	10307980	9.87E-01	1
AVIL	1106109	238	40	2134932	571380	0	6	2	0	0	2	571380	9.87E-01	1
IL17RB	475623	578	42	1305452	351016	0	1	0	0	50	71	20802860	9.87E-01	1
CD248	2133752	236	53	1816668	614100	0	1	4	0	50	77	24481764	9.87E-01	1
TARBP2	1590803	191	55	923464	286580	0	1	3	0	3	3	1044504	9.87E-01	1
NAGS	920082	241	29	1306164	433608	0	1	0	0	16	24	6853000	9.87E-01	1
SPEN	823686	169	28	9026736	2775732	0	25	11	0	11	23	6371688	9.87E-01	1

ZC3H11A	521302	280	32	2085804	576720	0	2	0	0	50	57	19465368	9.87E-01	1
PCDHGA1	662513	568	2	2329308	720900	0	9	10	0	6	33	5059116	9.87E-01	1
THOC5	713942	190	39	1812396	460308	0	7	1	0	0	1	460308	9.87E-01	1
NEK1	245919	399	12	3346400	828412	0	5	0	0	16	25	9024956	9.87E-01	1
RASIP1	1635420	190	44	2321476	814172	0	4	3	0	50	73	21072352	9.87E-01	1
GRIN3A	111816	1063	-27	2798516	811324	0	5	3	0	50	146	30649820	9.87E-01	1
PADI6	428762	210	31	1802784	489144	0	1	0	0	25	22	9346424	9.87E-01	1
SLC25A22	1788356	193	48	811680	260592	0	1	0	0	3	4	1065864	9.87E-01	1
NHSL2	623448	NaN	41	3002504	953368	0	1	0	0	3	2	2712364	9.87E-01	1
H3F3C	271605	394	20	328588	109292	0	1	2	0	1	3	343540	9.87E-01	1
SLC22A6	2447828	398	48	1399792	445712	0	2	3	0	24	40	8624812	9.87E-01	1
ARHGAP3[illegible]	1151198	321	30	3129596	1082596	0	7	4	0	3	4	1745468	9.87E-01	1
LRRC19	116568	447	-12	957996	247776	0	2	1	0	1	2	624424	9.87E-01	1
CFH	198896	941	-26	3250992	803136	0	11	5	0	22	59	10777188	9.87E-01	1
ITGB1	390742	205	-15	2322188	553936	0	3	2	0	50	64	19593528	9.87E-01	1
FOXA2	49625	627	-66	1148456	342472	0	2	3	0	17	39	7383796	9.87E-01	1
KRT1	1441383	464	29	1619088	483804	0	1	1	0	16	20	6903908	9.87E-01	1
FLYWCH1	1490697	189	48	1773236	559276	0	1	0	0	43	47	17733784	9.87E-01	1
UMODL1	220293	430	68	3636184	1089716	0	8	7	0	13	24	6173396	9.87E-01	1
FOXK1	436950	446	18	1765404	622644	0	1	2	0	41	34	14098668	9.87E-01	1
CILP	720558	183	57	2963700	862944	0	4	5	0	50	70	19785056	9.87E-01	1
CHTF18	1187310	273	39	2439668	783556	0	4	4	0	32	39	12013220	9.87E-01	1
HYOU1	1390854	195	58	2589188	721612	0	2	1	0	1	1	1295128	9.87E-01	1
VPS11	1390854	195	58	2431124	652192	0	2	1	0	1	1	1225708	9.87E-01	1
SENP6	428787	364	24	2949104	715204	0	3	0	0	0	0	715204	9.87E-01	1
TSHZ1	69176	993	4	2564624	749380	0	3	3	0	50	64	23012908	9.87E-01	1
GRIP2	501828	342	52	2864732	895696	0	6	1	0	0	1	895696	9.87E-01	1
GPN1	1192074	306	43	1231404	322536	0	1	1	0	50	104	28814640	9.87E-01	1
SPHK1	1622536	209	24	1181208	396228	0	1	0	0	5	13	3574596	9.88E-01	1
ZDHHC13	194027	625	11	1644008	420436	0	1	0	0	3	1	1108584	9.88E-01	1
PLBD1	339369	778	7	1439664	378072	0	1	0	0	50	74	23296640	9.88E-01	1
CTSG	897577	582	3	640800	198648	0	3	3	0	3	20	1909940	9.88E-01	1
RREB1	664661	310	35	4315076	1312216	0	11	5	0	0	5	1312216	9.88E-01	1

RBM15B	538437	536	39	2143476	713424	0	2	2	0	50	76	25835276	9.88E-01	1
FUCA1	903441	254	32	1198296	330012	0	1	0	0	7	7	2389828	9.88E-01	1
XAGE5	476232	NaN	26	295836	72624	0	2	2	0	2	2	193308	9.88E-01	1
TMBIM6	1828526	265	67	775724	211108	0	3	2	0	1	5	549308	9.88E-01	1
KANK2	1749900	233	33	2107876	694556	0	5	4	0	10	18	4181576	9.88E-01	1
PRDM8	60915	903	-4	1676404	547172	0	1	1	0	50	56	20692500	9.88E-01	1
INPP5D	430468	259	37	2664660	733716	0	2	5	0	47	36	15994012	9.88E-01	1
LPCAT3	2600423	211	49	1268428	346388	0	1	2	0	8	13	3527960	9.88E-01	1
PNPLA2	1799017	232	51	1245288	406908	0	1	0	0	2	1	824496	9.88E-01	1
UQCRFS1	158775	892	-73	669280	216092	0	3	2	0	4	20	1896412	9.88E-01	1
PLEKHG5	644634	203	44	2745472	825920	0	5	2	0	50	75	21467868	9.88E-01	1
TRO	244459	NaN	-8	3666088	1127096	0	5	1	0	2	2	1604492	9.88E-01	1
C5orf34	490107	369	10	1664656	433964	0	4	0	0	0	0	433964	9.88E-01	1
ERAP1	394409	345	36	2475624	652548	0	1	2	0	17	11	6306184	9.88E-01	1
ARMC5	839076	558	11	2420444	933076	0	5	2	0	35	62	15935984	9.88E-01	1
STAT5A	2072592	190	33	2061596	562480	0	2	2	0	22	23	5687812	9.88E-01	1
PHRF1	1872122	236	49	4056264	1299756	0	10	4	0	3	5	2124252	9.88E-01	1
TRIL	67820	570	21	1896412	705236	0	3	5	0	18	26	7124272	9.88E-01	1
TTLL11	285948	541	24	2053764	627984	0	1	0	0	2	1	1353512	9.88E-01	1
GGA3	1723762	171	31	1916348	567820	0	3	3	0	50	81	19557572	9.88E-01	1
ATAD2B	233701	431	27	3794604	995376	0	4	2	0	15	11	5588844	9.88E-01	1
GRM7	4936	1156	-6	2381284	674264	0	11	8	0	4	11	1749740	9.88E-01	1
TUBAL3	711034	519	-1	1120332	326808	0	1	2	0	50	96	24023592	9.88E-01	1
ZNF277	169396	550	-8	1216808	275188	0	4	4	0	4	7	1358852	9.88E-01	1
BTG1	114728	510	7	433252	122108	0	1	3	0	2	5	613388	9.88E-01	1
AAK1	639466	309	35	2462096	705592	0	3	3	0	11	15	6007856	9.88E-01	1
RTN4	913521	362	11	3027068	867928	0	8	2	0	2	5	1520120	9.88E-01	1
ZDHHC5	923511	354	15	1785340	551444	0	3	4	0	15	21	4964064	9.88E-01	1
OR6N2	103627	985	1	783556	234248	0	4	3	0	3	9	983984	9.88E-01	1
LCT	493727	403	28	4877556	1370244	0	12	5	0	19	41	11702076	9.88E-01	1
LMF2	1075525	207	42	1746180	579924	0	2	1	0	5	6	2060528	9.88E-01	1
XRRA1	743843	365	29	2031336	582060	0	2	1	0	5	12	2983992	9.88E-01	1
PCDHB6	915569	724	-11	1923112	624068	0	13	7	0	1	13	1254900	9.88E-01	1

POLRMT	1832767	218	10	3079044	954792	0	5	6	0	7	9	3061600	9.88E-01	1
GRHL1	913916	234	40	1624072	423284	0	8	3	0	0	3	423284	9.88E-01	1
RGL2	1456953	262	30	1917416	647920	0	5	1	0	1	4	961912	9.88E-01	1
KIF24	596029	186	43	3453200	984340	0	6	3	0	50	69	22074848	9.88E-01	1
XPNPEP1	248752	644	-31	1756504	466004	0	2	2	0	50	90	24758376	9.88E-01	1
ARNT	1787393	188	33	2053764	567108	0	6	1	0	2	3	809544	9.88E-01	1
REG3A	10958	1389	-22	465648	123888	0	3	2	0	2	7	338912	9.88E-01	1
THEM4	966708	212	36	629764	163760	0	1	2	0	1	2	339268	9.88E-01	1
FRK	230837	693	-4	1311504	339624	0	3	0	0	6	11	2236748	9.88E-01	1
EXOC2	157724	679	36	2451416	627628	0	5	5	0	18	24	6542924	9.88E-01	1
RGS3	408310	330	38	3604500	1059456	0	3	0	0	0	0	1059456	9.88E-01	1
UVRAG	520479	503	32	1800648	501960	0	2	1	0	50	89	24618824	9.88E-01	1
SEMA6C	1562281	184	33	2360280	802068	0	6	4	0	5	6	1818092	9.88E-01	1
FBXO2	682357	264	32	763620	211464	0	1	2	0	7	11	2008552	9.88E-01	1
FAM46D	102816	NaN	-24	995376	258456	0	1	1	0	50	121	24775108	9.88E-01	1
SOX9	159643	606	-41	1270208	372376	0	3	4	0	2	5	1133148	9.89E-01	1
FERMT1	302334	351	-16	1778932	453188	0	4	3	0	3	7	1439308	9.89E-01	1
CDT1	813333	189	51	1351020	442152	0	1	3	0	50	71	20606704	9.89E-01	1
POGZ	1356854	163	37	3555016	1054472	0	5	2	0	25	42	14160612	9.89E-01	1
SHANK3	669934	295	41	4245300	1449276	0	1	0	0	0	0	1449276	9.89E-01	1
SYN1	775214	NaN	38	1756860	556428	0	2	1	0	5	7	2499476	9.89E-01	1
ZNF157	792781	NaN	29	1306520	332860	0	2	0	0	1	0	445712	9.89E-01	1
TLR9	1114737	181	52	2499120	814884	0	3	5	0	16	23	7041324	9.89E-01	1
WDR37	334592	460	42	1282668	358848	0	2	2	0	8	15	3013540	9.89E-01	1
CD163	665695	608	13	2981500	793880	0	10	2	0	0	2	793880	9.89E-01	1
SLC2A10	292810	636	33	1291568	464224	0	5	6	0	15	19	2776800	9.89E-01	1
LAMC3	815585	293	36	3998592	1168392	0	8	6	0	50	78	24498852	9.89E-01	1
CCNB3	45831	NaN	1	3579224	940552	0	8	3	0	14	16	4934160	9.89E-01	1
DIXDC1	416878	221	22	1830196	468140	0	3	1	0	1	1	610184	9.89E-01	1
TCF7	694434	227	60	1126384	321824	0	1	1	0	2	1	741548	9.89E-01	1
PRPF18	234408	635	32	911716	225704	0	4	3	0	13	18	2251700	9.89E-01	1
MMP9	630342	381	51	1789256	532576	0	4	5	0	6	20	3294424	9.89E-01	1
INTS5	2425755	248	51	2399796	874692	0	9	8	0	8	12	2683884	9.89E-01	1

JAG1	165910	431	-28	3223936	788540	0	10	5	0	2	6	1366684	9.89E-01	1
ZFHX3	122465	495	-11	9234284	2671780	0	20	11	0	7	16	5664316	9.89E-01	1
RASGEF1E	9755	972	9	1269852	304380	0	4	2	0	1	2	617660	9.89E-01	1
GIT1	719319	383	30	1979716	576008	0	6	6	0	8	13	2253480	9.89E-01	1
ZNF536	106911	926	-73	3242092	943400	0	21	11	0	5	29	3364200	9.89E-01	1
NOTCH4	2119974	392	28	5013192	1535784	0	12	3	0	7	10	3195456	9.89E-01	1
AMIGO1	1056159	272	37	1212892	374156	0	1	3	0	25	37	9031364	9.89E-01	1
KLKB1	371112	606	-2	1683880	422216	0	1	0	0	17	21	8299072	9.89E-01	1
SP140L	535116	642	36	1580996	359560	0	1	0	0	50	56	19137492	9.89E-01	1
ABTB2	769733	368	32	2144544	615168	0	2	1	0	37	53	17650836	9.89E-01	1
ZNF8	1546372	406	-39	1459600	398720	0	1	1	0	47	62	18114704	9.89E-01	1
CPEB2	105433	624	23	2576016	796728	0	1	0	0	28	23	12719524	9.89E-01	1
RPP38	254900	620	21	710220	206124	0	3	0	0	0	0	206124	9.89E-01	1
TRIM28	2673870	276	11	2111436	635460	0	4	1	0	3	6	1930944	9.89E-01	1
CDH20	45961	896	-6	2045932	568532	0	1	2	0	50	57	21439744	9.89E-01	1
ABCG8	501561	480	30	1722328	490568	0	1	3	0	37	56	17515200	9.89E-01	1
SMG6	1171119	368	30	3601652	1027060	0	3	2	0	8	11	4548256	9.89E-01	1
VWA2	290609	437	21	1797800	575296	0	1	1	0	50	60	22942064	9.89E-01	1
OR10H2	878287	605	13	767180	249556	0	2	2	0	1	2	481312	9.89E-01	1
NFRKB	635581	248	-3	3324328	1023500	0	7	1	0	0	1	1023500	9.89E-01	1
CDC25C	1312427	273	36	1263088	311144	0	2	4	0	3	8	1774660	9.89E-01	1
ABCC11	216539	578	-22	3546472	1008548	0	12	6	0	1	11	2648640	9.89E-01	1
MGAT5	177903	761	17	1945184	498400	0	2	3	0	20	19	6079768	9.89E-01	1
SREBF2	1336623	243	62	2849068	886796	0	6	1	0	0	1	886796	9.89E-01	1
CALCOCO	757963	239	29	1217876	265576	0	1	2	0	13	21	5325404	9.89E-01	1
PTDSS2	2206786	344	58	1260240	350304	0	1	0	0	46	82	22476060	9.89E-01	1
ERCC6L	NaN	NaN	NaN	3156652	857960	0	6	0	0	7	14	4546120	9.90E-01	1
GLTSCR1	788433	258	33	3708452	1348528	0	4	1	0	1	2	1841232	9.90E-01	1
RALGAPA	239875	869	6	4855840	1315064	0	12	3	0	50	67	21249640	9.90E-01	1
ABCA2	1951939	202	40	6265600	1919552	0	9	3	0	12	16	6825232	9.90E-01	1
ZFYVE1	370279	341	43	1980428	555004	0	2	3	0	5	7	2662168	9.90E-01	1
GPR137	1613996	257	65	1369888	420080	0	2	2	0	19	29	6446448	9.90E-01	1
CORO7	754886	161	41	2347108	735140	0	3	3	0	50	65	19876192	9.90E-01	1

PEAR1	1362027	244	38	2679968	739768	0	1	0	0	10	12	6577100	9.90E-01	1
USO1	482408	363	51	2405492	608404	0	2	0	0	7	11	4789624	9.90E-01	1
TGM7	825765	415	44	1818448	512996	0	1	0	0	33	42	14598492	9.90E-01	1
TMEM104	1005466	320	19	1252408	378428	0	1	2	0	9	16	4922056	9.90E-01	1
DOCK8	193637	462	2	5637260	1507660	0	14	11	0	6	14	3425788	9.90E-01	1
MRGPRD	557282	416	51	779640	250980	0	3	4	0	1	6	525812	9.90E-01	1
PCDHB14	800016	708	-15	1931656	628340	0	11	5	0	6	22	2779292	9.90E-01	1
COL8A2	853740	282	27	1622292	639732	0	2	0	0	5	8	2512292	9.90E-01	1
E2F7	106320	887	-11	2292284	678892	0	6	2	0	6	25	5893580	9.90E-01	1
HLA-DOA	1276842	431	40	629408	194020	0	1	2	0	3	7	1166256	9.90E-01	1
ANKRD13A	950689	254	54	1555364	398008	0	2	1	0	17	21	5484536	9.90E-01	1
MID2	167346	NaN	49	1892496	504096	0	4	1	0	6	11	2833404	9.90E-01	1
TMEM63A	924499	239	8	2107164	579924	0	2	2	0	50	59	18688932	9.90E-01	1
FUT9	76115	1328	-21	920616	237096	0	2	2	0	2	5	985052	9.90E-01	1
MYO1G	1110535	235	57	2592392	764332	0	6	5	0	11	30	8518368	9.90E-01	1
SLC35A5	279107	502	33	1092564	294768	0	1	3	0	8	9	2540416	9.90E-01	1
GALC	83365	976	-37	1843724	489856	0	4	2	0	9	22	3619808	9.90E-01	1
MYO5B	276079	530	40	4838040	1252764	0	9	6	0	50	49	16508432	9.90E-01	1
ALKBH1	455772	384	47	997512	277680	0	1	2	0	16	22	4877200	9.90E-01	1
FLII	925111	223	18	3288016	907088	0	2	1	0	7	7	4320416	9.90E-01	1
PSG11	263820	178	-32	853688	244216	0	3	0	0	0	0	244216	9.90E-01	1
MMS19	556333	188	42	2643300	788184	0	4	0	0	3	5	2086872	9.90E-01	1
TTYH3	769920	259	35	1329304	405128	0	1	0	0	10	14	4479192	9.90E-01	1
CLCA1	279518	762	-8	2330020	657176	0	5	4	0	10	20	6328612	9.90E-01	1
SNRNP200	904852	213	43	5517288	1517628	0	7	3	0	26	28	11982604	9.90E-01	1
COL6A2	742075	538	10	2870784	860808	0	5	7	0	10	12	3254196	9.90E-01	1
TIAM2	120675	362	27	4341776	1213960	0	20	8	0	3	9	1756504	9.90E-01	1
NPHS1	1060028	321	47	3117848	981136	0	9	7	0	6	12	3110728	9.90E-01	1
TUBB8	9202	NaN	NaN	1125316	313280	0	1	0	0	10	47	9932044	9.90E-01	1
WDR91	464513	594	20	1916704	541832	0	3	5	0	20	26	6396964	9.90E-01	1
IPO13	875223	214	39	2453908	715916	0	3	3	0	50	67	19898264	9.90E-01	1
TRIB2	49360	996	-2	861876	248844	0	4	4	0	2	12	1468144	9.90E-01	1
KIF20A	1127160	314	50	2298692	632968	0	3	1	0	39	55	16930648	9.90E-01	1

FBXL2	496632	352	15	1115348	307228	0	5	4	0	3	5	818088	9.90E-01	1
TRIM5	134136	838	5	1464584	399076	0	2	0	0	3	2	901036	9.90E-01	1
TCTN3	370056	547	31	1556432	447136	0	1	0	0	21	39	11759036	9.90E-01	1
IGF2BP3	494999	197	26	1500540	417588	0	4	1	0	2	1	538272	9.90E-01	1
OR10K2	135429	902	5	760416	240300	0	1	3	0	10	17	2925608	9.90E-01	1
TMC3	245396	787	28	2844084	773232	0	4	5	0	27	36	9419760	9.90E-01	1
MPRIP	783403	214	11	2720196	746532	0	3	3	0	50	80	24314800	9.90E-01	1
ACACA	526151	346	13	6227864	1704172	0	16	8	0	26	50	13441492	9.90E-01	1
LRIG2	641888	455	13	2743692	744396	0	5	2	0	11	14	4319348	9.90E-01	1
ADAD2	582429	364	12	1636176	540408	0	1	0	0	0	0	540408	9.90E-01	1
DDX11	335562	591	-4	2589900	637596	0	9	3	0	1	4	1235676	9.90E-01	1
CST9L	189307	772	-47	383768	98968	0	3	3	0	1	3	203632	9.90E-01	1
ST8SIA6	514060	523	-4	1022076	286224	0	2	3	0	7	13	2309728	9.90E-01	1
PANK1	212241	345	18	1516560	460308	0	2	4	0	8	8	2313644	9.90E-01	1
RPL7	259225	340	19	658244	166252	0	2	1	0	1	2	415808	9.90E-01	1
ELSPBP1	881809	433	29	611608	131720	0	2	2	0	1	9	899256	9.91E-01	1
NLN	407983	149	33	1840876	469208	0	2	2	0	16	19	5852640	9.91E-01	1
ACP1	304765	624	-18	578144	120328	0	1	2	0	3	4	725528	9.91E-01	1
ANKK1	107636	610	12	1907804	578500	0	1	0	0	4	6	2722688	9.91E-01	1
TAS2R31	365490	676	-2	772876	224636	0	3	4	0	3	7	880388	9.91E-01	1
VPS35	432970	253	-24	2088296	535780	0	4	4	0	18	30	6953748	9.91E-01	1
SLC4A9	683517	143	39	2408340	759348	0	4	1	0	3	3	1425068	9.91E-01	1
NPAS2	339144	406	14	2134932	593808	0	4	6	0	6	12	2632620	9.91E-01	1
MMP3	468929	506	-21	1240304	331792	0	1	1	0	50	104	26781880	9.91E-01	1
ADNP2	477656	572	4	2783208	856536	0	2	3	0	18	21	9174476	9.91E-01	1
FBXL6	2525749	200	28	1316844	451764	0	2	3	0	4	8	1894276	9.91E-01	1
FBXL18	1906749	195	34	1750452	572448	0	2	0	0	0	0	572448	9.91E-01	1
CDK5	622098	373	41	774300	211464	0	2	3	0	1	5	803492	9.91E-01	1
TRIM69	673122	428	36	1300112	332860	0	4	3	0	5	10	1894632	9.91E-01	1
ATRN	971216	421	27	3751172	975796	0	4	3	0	2	4	2056968	9.91E-01	1
ZNF561	776094	617	-51	1254544	322892	0	1	0	0	16	21	5938792	9.91E-01	1
MAP9	44097	944	-5	1734076	397652	0	5	0	0	0	0	397652	9.91E-01	1
SOX30	260667	1079	41	1871848	561056	0	1	0	0	2	1	1160916	9.91E-01	1

BRSK2	1234040	426	43	1733720	490924	0	2	1	0	7	11	3317208	9.91E-01	1
ESCO1	333266	221	35	2179788	548952	0	4	1	0	1	1	668568	9.91E-01	1
LRRC8D	180057	257	13	2169820	586688	0	3	3	0	50	62	18767608	9.91E-01	1
FLT1	198131	693	41	3710232	978288	0	6	4	0	50	67	19959852	9.91E-01	1
DUSP13	223189	298	40	1202924	374512	0	1	0	0	6	9	2642232	9.91E-01	1
MAPKBP1	816015	259	37	3828780	1153440	0	6	1	0	0	1	1153440	9.91E-01	1
DPF1	1410213	301	-1	1122824	288004	0	1	2	0	3	2	852976	9.91E-01	1
DSPP	500313	623	-7	3566408	622288	0	8	2	0	15	24	7044884	9.91E-01	1
DVL3	1453689	334	42	1831264	525812	0	2	1	0	10	20	5986140	9.91E-01	1
DBF4B	1261173	175	29	1615172	459952	0	1	1	0	6	11	3459608	9.91E-01	1
WDR7	101104	885	38	3801368	1091140	0	7	4	0	50	70	21403788	9.91E-01	1
PREP	105381	510	12	1868288	469564	0	1	0	0	49	64	24390272	9.91E-01	1
MYO6	362445	482	17	3440740	824852	0	5	2	0	1	2	1459956	9.91E-01	1
MAN2A2	833957	291	42	2913504	866148	0	4	4	0	50	63	21711728	9.91E-01	1
SUPT7L	1192074	306	43	1058388	292632	0	4	2	0	3	9	1304028	9.91E-01	1
CCDC88C	271632	469	47	5147760	1479180	0	9	5	0	24	35	13100088	9.91E-01	1
FAM47A	7968	NaN	-15	1938776	600928	0	5	4	0	4	6	1434324	9.91E-01	1
DLST	884998	232	41	1171596	344964	0	1	2	0	2	3	1210044	9.91E-01	1
CELF5	903046	241	39	1276972	334640	0	1	2	0	5	8	2437176	9.91E-01	1
TCTN1	859648	194	32	1660740	483804	0	1	2	0	50	88	26880492	9.91E-01	1
MTMR3	752285	253	29	3097912	853688	0	8	1	0	3	11	2602004	9.91E-01	1
MUS81	3779084	287	42	1388756	443932	0	1	0	0	2	1	852264	9.91E-01	1
EHD2	812020	262	36	1356716	407620	0	5	4	0	3	9	1472416	9.91E-01	1
ARL14	493112	396	8	484516	133856	0	1	2	0	2	4	598080	9.91E-01	1
ASXL3	7579	1014	-10	5611984	1642940	0	17	4	0	2	6	2323256	9.91E-01	1
COL6A6	198659	527	16	5786424	1619088	0	21	8	0	7	15	3282320	9.91E-01	1
USP51	92250	NaN	8	1785696	499824	0	1	2	0	40	78	22198736	9.91E-01	1
MAML3	351118	591	8	2846932	810968	0	4	2	0	50	65	20955584	9.91E-01	1
LRRC27	150194	280	13	1537564	398720	0	1	0	0	25	35	10389860	9.91E-01	1
NOTCH2	429750	510	14	6350328	1710936	0	11	5	0	9	14	5493080	9.92E-01	1
TMC6	527017	217	27	2032404	631188	0	5	7	0	9	36	5477416	9.92E-01	1
OFD1	135235	NaN	48	2680680	658956	0	8	1	0	0	1	658956	9.92E-01	1
PLEKHG1	284652	263	27	3535436	969388	0	4	3	0	9	8	3733728	9.92E-01	1

C9orf117	1098647	172	32	1344968	362764	0	1	2	0	50	71	18037452	9.92E-01	1
GLMN	377839	559	-1	1607340	375936	0	2	1	0	11	16	4333232	9.92E-01	1
SOHLH1	362222	524	29	996088	320756	0	1	1	0	3	3	1017804	9.92E-01	1
ATIC	530312	531	12	1538276	425776	0	2	4	0	23	33	8340368	9.92E-01	1
ATP2B4	513211	256	35	3219664	919904	0	13	7	0	6	14	2592392	9.92E-01	1
ACVR2A	90992	951	-2	1346036	343540	0	1	2	0	50	81	20491360	9.92E-01	1
PLK2	125347	446	5	1787120	466360	0	4	0	0	2	3	1003564	9.92E-01	1
GPR161	476316	391	10	1323252	396228	0	7	2	0	1	3	493060	9.92E-01	1
SLC12A1	263035	455	-25	2949460	788540	0	4	0	0	1	1	1022788	9.92E-01	1
NBPF14	NaN	NaN	NaN	2443228	602708	0	2	2	0	50	118	40248292	9.92E-01	1
IL23R	453952	581	-4	1650060	411180	0	1	0	0	7	7	2473844	9.92E-01	1
PCDH17	8275	1095	-28	2848356	881100	0	12	8	0	10	28	3966196	9.92E-01	1
CAMKK1	505430	215	30	1412964	400500	0	1	1	0	44	57	16214732	9.92E-01	1
SGK494	1651143	225	31	1065864	300108	0	2	3	0	3	13	2353516	9.92E-01	1
IL24	467839	385	39	573160	116768	0	1	1	0	5	11	1456752	9.92E-01	1
SULT1A2	1141256	269	53	779284	201140	0	1	3	0	8	10	1683524	9.92E-01	1
HSPA8	709511	307	8	1646856	460308	0	10	3	0	0	3	460308	9.92E-01	1
ACADM	152734	578	-13	1119264	292632	0	1	2	0	4	5	1462092	9.92E-01	1
SOAT2	1727618	177	46	1344256	393380	0	1	1	0	50	73	19535500	9.92E-01	1
FGL1	399891	253	-16	841940	190816	0	1	2	0	8	15	2877192	9.92E-01	1
FSIP2	44989	953	4	17835956	4678552	0	16	3	0	0	3	4678552	9.92E-01	1
TACC2	233439	340	9	7332532	2284808	0	15	12	0	37	43	14565740	9.92E-01	1
TRPC5	113544	NaN	10	2489152	674264	0	7	5	0	1	14	2302252	9.92E-01	1
FAM193B	1120311	347	41	1970460	593808	0	2	0	0	7	14	4379512	9.92E-01	1
ABCC2	1035115	171	30	3965484	1120332	0	10	4	0	45	65	18031756	9.92E-01	1
CCDC146	312362	639	35	2543620	596300	0	3	1	0	6	5	2189400	9.92E-01	1
SPEG	581956	335	35	7943428	2708804	0	13	9	0	26	35	13221128	9.92E-01	1
CNGA2	126208	NaN	-2	1682456	473836	0	5	4	0	4	10	2035608	9.92E-01	1
HIATL1	229346	486	34	1290856	380564	0	2	1	0	5	8	1894276	9.92E-01	1
OR6K6	103627	985	1	851552	250624	0	4	3	0	3	9	983984	9.92E-01	1
PHF8	830265	NaN	30	2872920	793524	0	4	3	0	50	70	21609912	9.92E-01	1
SIPA1L3	688670	605	-17	4425792	1364904	0	11	7	0	50	87	21088728	9.92E-01	1
WDR88	406033	270	-8	1229268	331080	0	1	3	0	5	9	2449280	9.92E-01	1

PARP10	2580629	247	28	2479184	850840	0	5	3	0	1	3	1133148	9.92E-01	1
PTPRA	807310	396	46	2105028	557496	0	2	3	0	50	84	27392064	9.92E-01	1
SLC35F1	200805	959	-18	1029196	311144	0	2	4	0	5	13	1932012	9.92E-01	1
UBQLN3	99343	946	-2	1596304	509792	0	2	1	0	12	27	7334312	9.92E-01	1
BNC2	37330	534	-14	2776800	773232	0	10	8	0	4	14	2238884	9.92E-01	1
SPG21	705779	185	57	814884	209328	0	1	2	0	7	19	3412616	9.92E-01	1
SIN3A	775031	296	47	3277692	889644	0	9	1	0	3	8	2300116	9.92E-01	1
SNX8	806717	291	44	1197584	342472	0	1	3	0	5	12	2974736	9.92E-01	1
RHOBTB1	99491	403	-13	1791748	479888	0	4	2	0	4	9	2119980	9.92E-01	1
KLHL38	517269	192	22	1466008	411536	0	2	0	0	2	2	875404	9.92E-01	1
SETDB2	413604	343	39	1893208	469208	0	2	0	0	4	10	3625504	9.92E-01	1
ZNF182	550680	NaN	20	1680676	409400	0	2	1	0	19	32	7840900	9.92E-01	1
MATN3	501661	150	25	1236032	356356	0	3	1	0	2	1	477040	9.92E-01	1
KIF18A	316347	482	-11	2356008	592740	0	7	4	0	1	4	1039520	9.92E-01	1
RINT1	454408	172	36	2060172	540408	0	6	1	0	0	1	540408	9.92E-01	1
KLK1	655673	589	25	677824	186188	0	1	2	0	4	6	897120	9.92E-01	1
APBB2	227095	394	39	1962628	536492	0	1	0	0	10	12	5338932	9.92E-01	1
VPS13D	490718	340	28	11231800	3125324	0	28	16	0	13	25	6936660	9.92E-01	1
ZNF628	1048909	399	24	2497696	890000	0	3	4	0	50	71	20869076	9.92E-01	1
ADAMTS1[illegible]	622624	214	-14	2810976	828768	0	7	2	0	2	5	1343544	9.92E-01	1
INTS10	207502	242	10	1877900	468496	0	1	1	0	20	27	9567500	9.92E-01	1
SPATA6	27215	691	-19	1286940	331080	0	1	2	0	50	79	21309448	9.92E-01	1
TMEM164	216187	NaN	42	752228	223924	0	2	3	0	4	9	1275548	9.92E-01	1
PKP1	758588	193	23	1926316	530084	0	1	1	0	50	56	22830280	9.93E-01	1
KIFC2	2043455	200	36	2044508	714136	0	6	6	0	2	13	2164836	9.93E-01	1
PSMA2	163131	744	-6	617304	167676	0	1	3	0	3	6	981492	9.93E-01	1
GJA4	548013	580	19	810256	264152	0	2	0	0	1	2	468140	9.93E-01	1
EIF4G1	1447013	268	44	4052704	1206128	0	13	3	0	4	10	2959072	9.93E-01	1
VANGL2	987855	526	28	1294772	403348	0	2	4	0	11	14	2917064	9.93E-01	1
TMEM207	163494	373	9	377360	112852	0	1	2	0	1	3	337844	9.93E-01	1
GGCX	1241734	170	39	1934860	556784	0	5	4	0	5	9	2228204	9.93E-01	1
MUC6	1429028	466	43	5994328	1962272	0	8	4	0	10	16	7065176	9.93E-01	1
TTPAL	781823	276	46	857604	258456	0	3	3	0	3	10	1219300	9.93E-01	1

RBM44	570382	407	24	2752592	685300	0	1	0	0	3	4	3274844	9.93E-01	1
CHST6	488582	225	6	935568	337488	0	1	3	0	9	15	2647216	9.93E-01	1
NEU4	598219	487	29	1191532	416876	0	3	3	0	3	8	1676048	9.93E-01	1
SEMA4F	964055	364	41	1906024	619796	0	2	1	0	26	39	11425464	9.93E-01	1
FAM111A	542222	686	9	1582064	391600	0	4	3	0	3	9	1530444	9.93E-01	1
RAF1	408529	172	32	1692780	454968	0	3	2	0	3	4	1220012	9.93E-01	1
SH3RF3	266032	464	13	1882172	626560	0	1	0	0	4	3	1726244	9.93E-01	1
ABCC3	1585602	196	18	3909948	1203636	0	11	5	0	29	77	18114348	9.93E-01	1
ERMAP	811934	327	28	1209332	358492	0	1	2	0	4	4	1433968	9.93E-01	1
DCHS1	424523	491	17	7819896	2831268	0	19	10	0	35	41	15296964	9.93E-01	1
FAM114A2	171379	592	5	1316132	360628	0	1	0	0	2	1	787472	9.93E-01	1
DNAH9	42730	859	-75	11500936	3168044	0	27	6	0	1	6	3481324	9.93E-01	1
MPP3	1053443	192	27	1534716	419724	0	2	1	0	50	89	20520196	9.93E-01	1
BACH1	315733	289	60	1875764	502672	0	2	0	0	26	31	8614132	9.93E-01	1
TLR1	310020	437	58	2003924	521896	0	4	1	0	13	22	5227860	9.93E-01	1
ADCY10	534611	238	13	4260252	1038096	0	16	5	0	19	42	9864404	9.93E-01	1
RAD54L2	647863	387	39	3710232	1082952	0	5	6	0	12	15	4881828	9.93E-01	1
USH1C	442495	619	12	2400508	677468	0	2	2	0	6	13	4877912	9.93E-01	1
LIPF	145689	327	40	1054116	262728	0	5	4	0	2	7	946604	9.93E-01	1
ANKFN1	184382	511	-53	2000720	519760	0	7	5	0	5	16	2206488	9.93E-01	1
SCYL1	3988328	197	46	2069428	597368	0	1	1	0	29	33	14530852	9.93E-01	1
SPPL2B	1503606	267	31	1516916	470632	0	1	0	0	0	0	470632	9.93E-01	1
GTF2IRD2B	774871	220	56	2460316	647564	0	3	3	0	50	72	21683960	9.93E-01	1
TNRC18	1893797	256	14	7230360	2401932	0	9	2	0	2	7	4265592	9.93E-01	1
CCDC28A	402265	482	22	703812	198648	0	5	3	0	1	3	357780	9.93E-01	1
ENPP7	633863	209	31	1150592	343540	0	5	5	0	1	8	849060	9.93E-01	1
ALPI	271362	191	32	1334288	405484	0	2	4	0	2	7	1221436	9.93E-01	1
ZNF266	408298	705	-45	1421864	357424	0	3	0	0	15	28	5497352	9.93E-01	1
SOS2	778308	166	46	3463168	903884	0	6	2	0	5	8	2506596	9.93E-01	1
TDP1	296799	607	12	1605560	409756	0	5	5	0	11	23	3901404	9.93E-01	1
PLCE1	299843	633	2	6181228	1635464	0	8	3	0	7	8	4461392	9.93E-01	1
SYNCRIP	393389	384	14	1648992	440016	0	1	0	0	6	9	3223936	9.93E-01	1
KIAA0408	151114	811	-10	1795664	448204	0	1	0	0	10	10	4169472	9.93E-01	1

SLC16A12	249560	164	13	1297264	384836	0	1	0	0	1	1	745464	9.93E-01	1
CHDH	475623	578	42	1481316	454968	0	1	2	0	50	71	20802860	9.93E-01	1
MYO18B	198261	712	-37	6521208	1886088	0	15	8	0	28	51	14604188	9.93E-01	1
CDC42BPE	824377	286	43	4432912	1206128	0	6	7	0	50	70	20120408	9.93E-01	1
PTPN23	866535	215	46	4064096	1285516	0	3	1	0	0	1	1285516	9.93E-01	1
NOVA1	15854	883	-41	1263800	411892	0	2	2	0	2	4	1039876	9.93E-01	1
ZNF44	1474345	671	-10	1725888	418656	0	4	1	0	50	125	23446160	9.94E-01	1
FAF2	592072	239	47	1165188	308652	0	3	0	0	0	0	308652	9.94E-01	1
KDELR3	1466610	215	23	599860	166964	0	2	3	0	1	6	500180	9.94E-01	1
EN1	154049	576	-8	943756	319688	0	3	4	0	5	10	1472772	9.94E-01	1
LAMA1	241772	228	-37	7948768	2173736	0	25	11	0	6	20	3839816	9.94E-01	1
XKR7	1006109	386	37	1394096	472768	0	7	7	0	4	11	1044504	9.94E-01	1
PRKD1	23615	1041	-39	2362772	635104	0	7	3	0	1	3	948384	9.94E-01	1
AGAP1	134111	614	3	2189756	631900	0	4	4	0	16	16	4088304	9.94E-01	1
DENND1B	206539	480	-21	2087584	536492	0	2	1	0	14	34	9878288	9.94E-01	1
SLC12A2	257189	531	24	3111440	886084	0	6	3	0	3	7	1915280	9.94E-01	1
C7orf26	NaN	NaN	NaN	1119620	343540	0	2	5	0	2	9	1200076	9.94E-01	1
TFIP11	162673	501	-30	2189400	551088	0	2	0	0	4	4	1895344	9.94E-01	1
ZNF606	1219985	694	-30	2076904	489500	0	7	3	0	50	120	21391328	9.94E-01	1
C17orf53	930750	175	41	1611256	503384	0	1	1	0	43	57	18154220	9.94E-01	1
SEMG1	842540	324	25	1191176	302956	0	6	3	0	3	6	850484	9.94E-01	1
KIAA0922	188087	454	39	4208632	1100396	0	9	4	0	50	81	23911452	9.94E-01	1
SLC47A2	547418	219	-8	1532224	468140	0	1	2	0	50	80	22806784	9.94E-01	1
PDE3A	92407	514	-28	2876480	848704	0	9	9	0	7	16	2515140	9.94E-01	1
UBE4B	749198	240	25	3358860	931296	0	8	8	0	10	20	4854772	9.94E-01	1
CRAMP1L	1534704	320	36	3152736	997512	0	5	1	0	1	2	1289076	9.94E-01	1
C11orf1	437513	221	22	402636	93984	0	1	2	0	2	2	310076	9.94E-01	1
KIFC3	691782	148	29	2118200	644716	0	2	0	0	0	0	644716	9.94E-01	1
GLRB	44131	733	-34	1295840	338200	0	1	0	0	11	15	4186916	9.94E-01	1
MRVI1	755456	391	21	2306524	673196	0	2	2	0	50	90	30937824	9.94E-01	1
DDX56	1462615	191	56	1389468	421860	0	1	0	0	0	0	421860	9.94E-01	1
PCDHB3	987049	724	-11	1920620	632968	0	9	5	0	3	22	2510868	9.94E-01	1
KLHL10	1809123	171	34	1562840	407620	0	2	1	0	50	96	21744124	9.94E-01	1

ABCB9	1093113	196	47	1908872	595588	0	1	1	0	42	53	19208336	9.94E-01	1
STAG3	1382416	198	55	3201864	871488	0	7	3	0	3	4	1330372	9.94E-01	1
PTPRN2	248205	792	-33	2570676	778572	0	8	8	0	7	14	3141344	9.94E-01	1
LINGO2	8941	927	-34	1500540	448560	0	3	4	0	19	38	6396252	9.94E-01	1
PRUNE2	178730	851	-15	7855496	2118556	0	13	6	0	18	26	9600252	9.94E-01	1
SETDB1	1688656	172	31	3315784	913496	0	12	8	0	7	23	3592396	9.94E-01	1
SAMD9	362550	625	8	4063384	1035248	0	12	8	0	34	49	12981540	9.94E-01	1
CELF2	96767	864	5	1420796	380920	0	5	4	0	1	5	758636	9.94E-01	1
ANKRD30[illegible]	NaN	NaN	NaN	3713080	899612	0	4	1	0	9	17	5257052	9.94E-01	1
NBPF6	NaN	NaN	NaN	1743688	459596	0	1	0	0	50	116	40105180	9.94E-01	1
HDAC5	923807	171	32	2858680	849416	0	3	2	0	4	5	2289792	9.94E-01	1
ZNF473	1643218	222	35	2251700	559276	0	6	1	0	0	1	559276	9.94E-01	1
CCNA2	324357	506	29	1107872	309364	0	2	2	0	3	6	1258816	9.94E-01	1
CHST5	488582	225	6	978644	341404	0	1	2	0	9	15	2647216	9.94E-01	1
NTRK2	17424	692	1	2244224	592384	0	7	4	0	1	4	905664	9.94E-01	1
PLEKHG4[illegible]	836250	543	14	3184776	965472	0	8	3	0	10	23	5652568	9.94E-01	1
C2orf78	1124858	404	43	2274128	691708	0	3	4	0	50	76	19904316	9.94E-01	1
REG1A	10958	1389	-22	449272	107156	0	6	2	0	2	7	338912	9.94E-01	1
ADPRHL1	808201	223	49	917412	247776	0	1	3	0	5	7	1598084	9.94E-01	1
MOS	344171	552	20	829836	280884	0	2	1	0	3	8	1127452	9.94E-01	1
PAPLN	525540	269	45	3183352	934856	0	5	2	0	7	11	3741204	9.94E-01	1
TM6SF2	940952	232	17	966540	285156	0	2	3	0	1	4	621220	9.94E-01	1
HPS5	601817	252	37	2930236	784268	0	2	2	0	50	58	23220812	9.94E-01	1
SKI	788773	262	30	1782848	580636	0	1	2	0	50	66	22647652	9.94E-01	1
SCN10A	304612	680	21	4988628	1394808	0	15	5	0	0	5	1394808	9.94E-01	1
GEMIN5	519919	378	40	3894996	1057320	0	7	5	0	23	33	8239264	9.94E-01	1
HIVEP1	210548	453	33	6756880	1985768	0	16	4	0	2	7	2444652	9.94E-01	1
PCDHA5	896676	606	-12	2364196	754364	0	6	4	0	50	135	24247872	9.94E-01	1
GRIN3B	2387949	206	26	2485948	895340	0	2	0	0	2	7	3122476	9.94E-01	1
DOPEY2	267887	232	65	5830568	1689220	0	24	12	0	3	17	2855476	9.94E-01	1
POLA1	255855	NaN	22	3873280	970100	0	4	1	0	12	11	5438612	9.94E-01	1
SLC15A4	34588	1027	-23	1425068	458884	0	1	2	0	50	107	24998320	9.94E-01	1
C16orf58	826452	688	-17	1200788	357424	0	3	0	0	13	53	7539724	9.94E-01	1

PABPC1L	952219	282	32	1578148	454256	0	1	2	0	50	67	20264944	9.94E-01	1
LMBRD1	42273	983	-2	1426136	371308	0	1	2	0	50	90	19887228	9.94E-01	1
AP3D1	1712606	216	30	3153448	836600	0	8	4	0	5	11	2872920	9.94E-01	1
ANKHD1	NaN	NaN	NaN	6468520	1931300	0	1	0	0	0	0	1931300	9.95E-01	1
SYVN1	3292261	168	50	1564264	479888	0	1	2	0	50	69	20640168	9.95E-01	1
ZNF16	1539444	440	23	1749740	447136	0	3	5	0	4	9	1628344	9.95E-01	1
TRANK1	391968	523	0	7455708	2013180	0	21	6	0	6	18	5452140	9.95E-01	1
HERC2	219134	1011	-32	12305852	3578512	0	28	13	0	8	29	7457844	9.95E-01	1
RARG	1677275	191	49	1279108	372020	0	14	6	0	0	6	372020	9.95E-01	1
FLT4	855370	515	13	3508024	994664	0	9	8	0	15	25	6479912	9.95E-01	1
CCNF	2161773	309	44	2015316	576720	0	6	6	0	13	22	4142772	9.95E-01	1
C16orf93	1632278	158	61	771096	208260	0	1	3	0	2	7	1266648	9.95E-01	1
FGD4	357808	378	-9	2005704	515844	0	3	0	0	0	0	515844	9.95E-01	1
ETV3	895723	423	31	1306164	386616	0	1	0	0	4	8	2274128	9.95E-01	1
FBXW5	1979517	188	34	1420796	430048	0	1	1	0	50	78	21046364	9.95E-01	1
NPR1	1656449	183	40	2670356	822004	0	3	1	0	12	12	4249216	9.95E-01	1
MYO7A	442776	450	42	5747264	1592032	0	12	5	0	50	66	24494224	9.95E-01	1
LTBP3	3945819	202	45	3347112	946248	0	4	4	0	19	25	9711324	9.95E-01	1
NLGN2	1906324	206	33	2032760	673552	0	2	3	0	50	87	23542992	9.95E-01	1
OR10AG1	4262	1044	-40	751160	215380	0	1	2	0	5	12	1863660	9.95E-01	1
PCDHA13	NaN	NaN	NaN	2335716	755076	0	9	9	0	4	13	2059816	9.95E-01	1
HOOK3	478917	475	25	1933792	459596	0	1	2	0	5	8	3434688	9.95E-01	1
ACSBG2	1332100	417	40	1745824	451052	0	5	1	0	0	1	451052	9.95E-01	1
CDKL5	66108	NaN	25	2667864	718764	0	2	2	0	17	19	7678564	9.95E-01	1
PTCH2	656648	191	36	2993604	975084	0	2	2	0	29	35	14305504	9.95E-01	1
CLSTN1	614159	177	31	2542908	680316	0	4	3	0	35	47	12765448	9.95E-01	1
NOVA2	866240	261	42	1176936	417588	0	2	2	0	4	6	1282312	9.95E-01	1
IGSF8	829863	386	17	1466720	526168	0	1	0	0	1	1	819156	9.95E-01	1
BDKRB2	330952	500	10	975796	288716	0	2	3	0	1	4	791744	9.95E-01	1
COL4A6	171617	NaN	31	4233908	1390180	0	5	3	0	29	33	12825968	9.95E-01	1
ZFPM1	626557	184	37	2439312	827700	0	1	1	0	36	33	15230036	9.95E-01	1
USP6	1391000	384	20	3668936	961912	0	9	5	0	50	83	22837756	9.95E-01	1
PITRM1	266524	438	16	2723756	715204	0	1	1	0	14	11	6479556	9.95E-01	1

FN3KRP	855855	243	21	797440	219296	0	1	3	0	5	7	1242796	9.95E-01	1
[illegible]NFRSF11[illegible]	145370	264	42	1570316	447136	0	1	1	0	50	80	20614536	9.95E-01	1
ERMP1	314189	313	25	2301896	659668	0	1	1	0	3	10	4558580	9.95E-01	1
PIK3C2G	24982	986	-31	3814896	950520	0	7	2	0	11	22	5432204	9.95E-01	1
SLC44A2	2082774	226	33	1880392	511928	0	9	2	0	0	2	511928	9.95E-01	1
AIRE	1153513	308	65	1599508	510860	0	1	1	0	18	20	6199384	9.95E-01	1
ARHGEF6	306872	NaN	39	2049848	529372	0	6	1	0	4	7	1479892	9.95E-01	1
PACSIN2	627533	222	56	1289432	313636	0	3	3	0	1	5	814172	9.95E-01	1
FAM76B	298879	467	2	917768	210040	0	1	2	0	1	3	532576	9.95E-01	1
AIM1	220264	271	41	4379512	1225352	0	10	3	0	1	3	1511932	9.95E-01	1
TALDO1	1788356	193	48	876472	238520	0	3	4	0	3	8	1043792	9.95E-01	1
ZBTB40	440712	555	28	3184776	860808	0	5	8	0	26	24	7881128	9.95E-01	1
CCDC62	1009828	184	40	1844792	454612	0	4	3	0	1	3	783200	9.95E-01	1
ABCB11	223582	560	1	3412260	938772	0	4	1	0	2	3	1559992	9.95E-01	1
FOXL1	47545	445	10	824852	283732	0	1	2	0	1	3	673908	9.95E-01	1
GLB1L	694442	193	44	1688152	478820	0	1	1	0	50	75	22905396	9.95E-01	1
USP36	854363	214	27	2852984	825208	0	4	4	0	50	72	20992252	9.95E-01	1
LHFPL5	621095	404	34	556784	160912	0	3	3	0	2	3	315772	9.95E-01	1
AEBP1	1151124	282	56	2958360	840516	0	5	3	0	50	90	23246444	9.95E-01	1
OR13J1	847663	227	54	749024	252760	0	1	3	0	2	3	664296	9.95E-01	1
CAPZA3	82407	911	-29	763976	199360	0	1	2	0	9	19	2976872	9.95E-01	1
ZNF516	113406	813	18	2854052	893560	0	7	4	0	1	4	1292992	9.95E-01	1
VILL	318742	457	24	2213252	611608	0	5	6	0	5	16	3672140	9.95E-01	1
PRAMEF8	62200	792	6	3545760	1058388	0	3	1	0	17	22	10083344	9.96E-01	1
CYLD	279624	792	2	2486660	647920	0	2	2	0	40	43	15605972	9.96E-01	1
COL18A1	513400	481	41	4413688	1498760	0	5	5	0	50	60	23189484	9.96E-01	1
TGM2	565545	141	41	1791748	498044	0	1	3	0	12	13	4660752	9.96E-01	1
C14orf159	240206	471	35	1579572	464580	0	1	4	0	18	25	7372048	9.96E-01	1
ABCA5	52620	314	-50	4335012	1091496	0	8	4	0	14	23	6143492	9.96E-01	1
POTEE	462125	NaN	11	2798872	710576	0	4	3	0	50	94	26072372	9.96E-01	1
OR51A4	65798	1006	-15	768960	234960	0	1	4	0	4	8	1185480	9.96E-01	1
HIVEP3	190045	457	27	5849436	1883952	0	19	7	0	15	20	5422592	9.96E-01	1
ZNF43	123927	1235	-66	2111080	496976	0	5	3	0	34	64	11960532	9.96E-01	1

RBM12	1423559	169	53	2278044	715560	0	1	1	0	15	12	5442528	9.96E-01	1
FIBP	3779084	287	42	948384	259524	0	1	1	0	2	2	667856	9.96E-01	1
RAP2B	59808	598	-2	456036	133500	0	1	3	0	1	6	633324	9.96E-01	1
TMEM132I	32086	969	-33	2763984	794592	0	16	8	0	4	15	2233188	9.96E-01	1
ANKS6	298496	375	12	2165548	690284	0	1	3	0	50	56	22224368	9.96E-01	1
POLR2A	1931875	194	29	4960148	1474552	0	11	4	0	16	31	8361372	9.96E-01	1
KCNH8	25033	1018	-14	2834116	779996	0	14	7	0	1	9	1265224	9.96E-01	1
ZNF250	1539444	440	23	1441800	377004	0	4	3	0	4	9	1628344	9.96E-01	1
RGAG1	100181	NaN	21	3346044	1112856	0	4	4	0	27	38	12767228	9.96E-01	1
ADARB1	596496	250	42	1874696	557140	0	2	3	0	4	5	1750808	9.96E-01	1
PTPRB	159250	454	-26	5737652	1567468	0	13	7	0	28	55	14822060	9.96E-01	1
PRR12	1906661	195	38	4859044	1723040	0	4	3	0	18	19	8233568	9.96E-01	1
ESPL1	1677275	191	49	5261680	1662164	0	16	7	0	2	9	2217524	9.96E-01	1
PCDHB12	915569	724	-11	1932724	617660	0	10	6	0	1	12	1248492	9.96E-01	1
ODF2L	218105	874	-18	1883240	420436	0	1	1	0	46	72	20795384	9.96E-01	1
ALOX15B	1528802	272	26	1715920	508724	0	1	2	0	50	58	18618800	9.96E-01	1
NRIP1	103852	526	8	2914928	798508	0	6	4	0	7	15	3877908	9.96E-01	1
TRHDE	66026	995	-40	2636536	726596	0	11	4	0	5	13	1971172	9.96E-01	1
VPS18	1036414	172	51	2391252	746532	0	3	1	0	10	13	3963348	9.96E-01	1
GCNT2	254228	404	13	2568540	704880	0	1	2	0	36	44	18231472	9.96E-01	1
NBAS	186706	1405	10	6161648	1658248	0	9	4	0	35	44	15288064	9.96E-01	1
PSORS1C1	3178076	283	21	392312	114988	0	3	1	0	0	1	114988	9.96E-01	1
ZDHHC1	1115833	153	42	1219656	380208	0	3	3	0	2	6	841940	9.96E-01	1
CTSW	3779084	287	42	980068	268424	0	1	1	0	2	2	676756	9.96E-01	1
CCR10	1740597	177	36	838380	331080	0	1	4	0	2	13	1860456	9.96E-01	1
ZHX1	545561	171	22	2203284	601284	0	1	2	0	50	60	20198016	9.96E-01	1
SF3B2	3581017	208	47	2320052	642580	0	5	4	0	3	6	1404064	9.96E-01	1
MLH3	897430	222	50	3738712	971168	0	5	3	0	48	62	21588908	9.96E-01	1
SARM1	1407654	189	19	1777508	579568	0	1	1	0	20	27	8197256	9.96E-01	1
TRAPPC1(	860750	202	62	3244940	886084	0	7	2	0	0	2	886084	9.96E-01	1
GAL3ST3	3581017	208	47	1035248	353152	0	1	1	0	3	6	1404064	9.96E-01	1
OSGIN1	387177	550	-6	1383772	439304	0	1	2	0	4	6	1756148	9.96E-01	1
AATK	1737949	243	22	3352808	1110364	0	5	1	0	3	9	2959428	9.96E-01	1

MPPE1	323758	148	39	1026348	284088	0	2	3	0	1	4	813460	9.96E-01	1
B4GALT4	248430	588	3	892492	234248	0	3	3	0	1	3	495908	9.96E-01	1
TIPARP	407941	502	22	1698120	431472	0	5	1	0	0	1	431472	9.96E-01	1
SLC39A4	2043455	200	36	1676760	573516	0	2	0	0	2	7	2024216	9.96E-01	1
MMP24	1290513	170	45	1436104	402992	0	1	3	0	27	43	10380248	9.96E-01	1
HSPG2	481189	179	27	11063412	3420804	0	24	10	0	3	10	3634760	9.96E-01	1
CAPN5	403200	395	38	1644720	460308	0	3	4	0	1	12	1880392	9.96E-01	1
ATP13A5	141358	851	1	3184064	843364	0	7	6	0	7	12	2939136	9.96E-01	1
SNX22	743794	251	43	508724	140620	0	3	4	0	1	5	297260	9.96E-01	1
ARFIP1	218185	385	33	974372	258100	0	1	2	0	8	17	2775376	9.96E-01	1
MCPH1	128440	702	9	2169820	572804	0	4	0	0	4	7	2039880	9.96E-01	1
C1R	2459343	195	45	1546464	417588	0	1	0	0	2	4	1577080	9.96E-01	1
KCNU1	7048	474	-36	2988620	809188	0	8	6	0	5	12	2263092	9.96E-01	1
DNAH1	1095946	229	55	10992212	3007132	0	24	11	0	18	42	10916028	9.96E-01	1
TRIM65	1727802	178	35	1274480	408688	0	1	2	0	17	36	7482408	9.96E-01	1
TAS1R1	628180	259	38	2157716	563548	0	2	5	0	26	45	12018560	9.97E-01	1
ADAM28	55817	565	5	2133864	506232	0	1	1	0	50	69	25885472	9.97E-01	1
SERINC2	748895	262	28	1164120	335352	0	1	3	0	6	13	2986128	9.97E-01	1
PIGV	1022828	188	30	1210044	385548	0	3	4	0	3	8	1202924	9.97E-01	1
CCDC108	872634	324	36	5059116	1410828	0	13	6	0	12	33	7794264	9.97E-01	1
RAB3A	1178047	210	43	573160	149876	0	2	3	0	1	4	445000	9.97E-01	1
ENGASE	592461	285	15	1872916	568532	0	3	2	0	0	2	568532	9.97E-01	1
OLFML2A	1076828	207	26	1633684	488432	0	1	2	0	50	76	19654760	9.97E-01	1
EHF	1039672	320	12	815596	182984	0	3	3	0	6	17	1717700	9.97E-01	1
HEATR5B	614688	281	16	5233912	1554296	0	13	3	0	1	6	1925604	9.97E-01	1
STIM1	409663	476	31	1763980	480956	0	1	0	0	1	0	614456	9.97E-01	1
CTNND2	15425	1060	-13	3090080	927736	0	13	8	0	1	8	1241016	9.97E-01	1
HLX	177316	564	-2	1197228	382344	0	2	4	0	4	8	1575300	9.97E-01	1
BTBD16	218081	303	-3	1346748	343896	0	1	3	0	10	14	3539708	9.97E-01	1
ZNF256	1252385	750	-19	1619800	402992	0	3	4	0	17	37	7252076	9.97E-01	1
FANCI	642890	400	25	3509804	908512	0	11	4	0	6	14	3241736	9.97E-01	1
VIPR1	355160	527	21	1187616	331080	0	1	3	0	4	8	1746180	9.97E-01	1
TRPV4	825887	423	41	2206488	651480	0	5	5	0	5	10	2463164	9.97E-01	1

SLCO3A1	70107	867	-8	1849776	557496	0	2	4	0	6	12	3107880	9.97E-01	1
NLRP11	991833	362	-35	2637960	713424	0	7	2	0	10	16	3583852	9.97E-01	1
MFSD4	694640	268	35	1287296	403348	0	2	2	0	9	18	3063380	9.97E-01	1
ZMYND19	1315685	404	41	590604	161268	0	2	0	0	0	0	161268	9.97E-01	1
CR1	442029	361	18	6453212	1721260	0	11	2	0	3	5	2300828	9.97E-01	1
CELSR1	467110	260	11	7464608	2340700	0	19	12	0	13	36	7963720	9.97E-01	1
ZNF83	563874	1010	-41	1335356	321112	0	1	0	0	3	6	1635464	9.97E-01	1
CLCN1	379715	379	29	2527244	739768	0	8	4	0	3	7	1614104	9.97E-01	1
RBMY1E	NaN	NaN	NaN	3865092	1053048	0	2	0	0	0	0	1053048	9.97E-01	1
CPSF3L	1744013	273	28	1566400	429692	0	1	1	0	9	14	4108596	9.97E-01	1
C6orf15	2992134	283	23	785692	263084	0	2	2	0	1	3	378072	9.97E-01	1
KIAA0141	696239	321	48	1309012	394448	0	2	3	0	3	9	1813820	9.97E-01	1
AKAP13	230754	454	17	7237124	2007484	0	14	8	0	28	30	11215424	9.97E-01	1
DNAJC11	623016	259	34	1455684	404772	0	4	3	0	1	3	770028	9.97E-01	1
ACSL4	209488	NaN	44	1851912	484872	0	1	1	0	50	79	25041040	9.97E-01	1
MEGF6	250204	251	15	4008204	1086156	0	4	5	0	50	49	18153152	9.97E-01	1
AGBL2	984490	155	23	2372028	595944	0	5	1	0	15	38	8802456	9.97E-01	1
DDX60L	134820	238	-11	4507672	1115348	0	9	3	0	4	6	2191180	9.97E-01	1
ZFP64	145657	427	-8	2741556	734784	0	1	2	0	50	47	20178080	9.97E-01	1
INPP4A	212473	623	41	2683172	747244	0	3	1	0	0	1	747244	9.97E-01	1
CWF19L2	147499	853	-10	2388760	553580	0	3	0	0	1	2	909580	9.97E-01	1
CACNA2D[illegible]	639042	259	57	3038104	820580	0	2	2	0	50	63	21319772	9.97E-01	1
VPS13A	186200	696	-2	8458560	2149884	0	11	3	0	4	5	3171604	9.97E-01	1
ANK2	237324	911	14	10067680	2879684	0	44	12	0	0	12	2879684	9.97E-01	1
TBC1D9	146110	621	8	3263808	881100	0	6	6	0	10	14	4065876	9.97E-01	1
ADAMTSL	1475649	152	31	2722688	874336	0	5	3	0	12	30	7579240	9.97E-01	1
MCHR2	258745	719	-16	873268	240656	0	2	4	0	3	6	885728	9.97E-01	1
EPN1	1050871	254	-11	1698476	552868	0	1	3	0	8	8	2559640	9.97E-01	1
COL6A1	764197	590	23	2655048	789252	0	2	4	0	50	92	27167784	9.97E-01	1
NCBP1	495483	358	36	2111080	517268	0	2	1	0	50	59	18737348	9.97E-01	1
WLS	331085	524	-20	1526528	389464	0	1	1	0	14	40	9423676	9.97E-01	1
MCM3	313097	427	23	2086516	573872	0	1	2	0	39	44	16330076	9.97E-01	1
CCNA1	197057	880	1	1184768	342472	0	4	3	0	1	3	568176	9.97E-01	1

TBC1D25	772147	NaN	16	1686372	542544	0	2	1	0	3	3	1075832	9.97E-01	1
VWA1	1736268	273	28	1077256	360272	0	1	4	0	5	11	2076904	9.97E-01	1
BZRAP1	810333	399	19	4624084	1461380	0	9	9	0	15	25	7565712	9.97E-01	1
FAM110B	190462	601	15	913852	274832	0	5	2	0	0	2	274832	9.97E-01	1
TRPC4	72221	573	-19	2516564	675688	0	18	6	0	0	6	675688	9.97E-01	1
FRMD1	142012	702	-17	1416524	402280	0	1	1	0	9	20	4699200	9.97E-01	1
VDAC2	208687	275	38	846568	222500	0	3	2	0	1	3	443932	9.97E-01	1
DIAPH1	696745	502	14	3292288	901748	0	5	1	0	0	1	901748	9.97E-01	1
TBC1D10E	2312884	165	47	1956220	672128	0	1	2	0	44	52	17331148	9.97E-01	1
STXBP2	688482	384	31	1544684	439660	0	1	0	0	3	2	995020	9.97E-01	1
METTL7A	569122	220	40	615168	174084	0	2	3	0	2	3	382344	9.97E-01	1
RABEP1	1246822	382	24	2275908	561768	0	2	2	0	50	69	21505960	9.97E-01	1
PCDHA2	861651	606	2	2374520	771808	0	8	6	0	5	30	3429704	9.97E-01	1
ENTPD1	370896	631	30	1417948	359204	0	1	3	0	2	8	1413320	9.97E-01	1
ADNP	386595	367	36	2806704	740124	0	6	2	0	1	2	922752	9.97E-01	1
OR2Y1	831824	515	18	754720	244928	0	1	1	0	1	2	469564	9.97E-01	1
BCL6	88374	374	24	1796732	502672	0	3	1	0	0	1	502672	9.97E-01	1
SLC1A5	1166804	224	36	1311148	460664	0	1	3	0	5	7	1788900	9.97E-01	1
ELAVL1	967479	369	19	838380	230688	0	6	4	0	1	4	268068	9.97E-01	1
TXNRD2	648548	197	18	1352800	408332	0	1	3	0	2	3	1040588	9.97E-01	1
ACSL3	261505	361	14	1872560	497332	0	2	0	0	0	0	497332	9.97E-01	1
FLVCR2	656924	248	50	1330372	396584	0	4	5	0	3	8	1345324	9.97E-01	1
KDM4A	939679	209	34	2772884	729088	0	6	6	0	9	15	3433264	9.97E-01	1
PDE6C	461114	305	19	2276976	564972	0	2	1	0	1	1	871844	9.97E-01	1
KIF4A	243320	NaN	45	3248500	830192	0	3	2	0	50	71	23272788	9.97E-01	1
BBS7	324357	506	29	1890004	484160	0	6	2	0	3	6	1258816	9.97E-01	1
PHLPP2	451025	366	17	3343196	973660	0	5	5	0	35	46	13940248	9.97E-01	1
IRS1	146164	493	-3	3023508	965472	0	7	4	0	12	18	4361712	9.97E-01	1
USP9X	734477	NaN	38	6693868	1727312	0	11	3	0	2	5	2392676	9.97E-01	1
GGH	137738	789	-12	846568	209684	0	2	2	0	2	5	731580	9.97E-01	1
RNASET2	225834	633	18	686368	173372	0	1	2	0	3	2	421860	9.98E-01	1
TUBB1	978901	125	28	1144896	316128	0	1	3	0	21	41	7643676	9.98E-01	1
SYNE2	525935	430	41	18196940	4477768	0	47	13	0	26	41	15430820	9.98E-01	1

VPS13B	209453	862	27	10492032	2861172	0	28	14	0	19	38	11482780	9.98E-01	1
TBL3	1747301	183	46	2056256	629764	0	4	2	0	1	2	781064	9.98E-01	1
DNAJC16	636573	188	34	2029912	538628	0	9	4	0	2	7	1049132	9.98E-01	1
CUBN	529248	641	-4	9405520	2489864	0	47	14	0	0	14	2489864	9.98E-01	1
RUVBL2	1998663	190	39	1202924	347812	0	1	4	0	7	15	3171960	9.98E-01	1
CD3G	963668	192	48	493416	120684	0	1	2	0	2	2	326452	9.98E-01	1
TF	489558	523	26	1830552	477396	0	4	5	0	14	18	3692432	9.98E-01	1
KIAA1462	184440	520	12	3376304	993952	0	7	5	0	5	8	2404424	9.98E-01	1
LTBR	2686552	198	48	1108228	327164	0	3	2	0	0	2	327164	9.98E-01	1
CACNA1G	1559131	196	19	6182652	1877544	0	14	3	0	9	29	7424024	9.98E-01	1
EIF2AK4	806826	256	26	4311516	1139556	0	11	5	0	4	11	2631196	9.98E-01	1
TLR6	316028	437	75	2030980	526880	0	4	2	0	4	6	1656112	9.98E-01	1
SLC7A4	577297	192	58	1524392	530440	0	2	5	0	18	35	6734096	9.98E-01	1
TLN2	315754	568	15	6490236	1892496	0	10	9	0	18	29	9811360	9.98E-01	1
XPO7	489191	222	26	2883956	775012	0	6	2	0	2	2	895696	9.98E-01	1
F12	1038665	261	59	1573164	458172	0	3	6	0	3	8	1205416	9.98E-01	1
CALHM1	542721	170	51	844788	271272	0	1	3	0	3	6	999292	9.98E-01	1
ZNF552	884988	781	-38	1053404	262372	0	3	3	0	2	7	1077612	9.98E-01	1
ABLIM2	172573	230	36	1758996	451764	0	1	2	0	50	66	18353936	9.98E-01	1
LAMP2	475981	NaN	26	1291924	365612	0	2	1	0	2	1	486296	9.98E-01	1
GNAS	1025539	416	11	3822372	503028	0	6	3	0	50	58	13537968	9.98E-01	1
RNF219	58504	954	4	1875764	477040	0	5	2	0	0	2	477040	9.98E-01	1
MYBPC3	1189866	245	45	3285880	939128	0	4	2	0	2	2	1305096	9.98E-01	1
KCNC3	1072052	528	26	1830908	614812	0	1	3	0	50	63	18767608	9.98E-01	1
PTX4	1254229	256	34	1129232	396940	0	1	4	0	4	10	2143476	9.98E-01	1
JAG2	872033	260	25	3186200	890356	0	1	0	0	11	8	4424724	9.98E-01	1
DRD5	207324	923	30	1173376	358136	0	2	4	0	1	5	980780	9.98E-01	1
GNAZ	NaN	NaN	NaN	891068	253828	0	2	4	0	2	8	1110364	9.98E-01	1
SLCO4A1	1254192	192	24	1797444	566040	0	4	4	0	1	4	821648	9.98E-01	1
TCF7L2	156660	435	-9	1793884	489500	0	1	4	0	1	6	1400504	9.98E-01	1
DPEP2	863919	158	54	1222504	380564	0	2	3	0	2	7	1254544	9.98E-01	1
SON	908921	375	68	6191552	1908160	0	7	2	0	0	2	1908160	9.98E-01	1
LRRIQ1	25069	946	-28	4558224	1075476	0	19	3	0	5	13	2446432	9.98E-01	1

IDS	179393	NaN	3	1430764	400856	0	5	1	0	0	1	400856	9.98E-01	1
MB	936853	205	-13	406196	105376	0	1	3	0	1	4	358136	9.98E-01	1
OBSL1	548994	487	35	4748328	1573164	0	6	4	0	40	56	20065584	9.98E-01	1
HECTD3	939872	310	20	2196520	652904	0	5	3	0	1	4	933788	9.98E-01	1
CLN6	376180	365	33	792456	232824	0	3	2	0	0	2	232824	9.98E-01	1
PPP1R13L	1269856	191	31	2037388	669992	0	2	4	0	32	56	13921736	9.98E-01	1
ANKRD13C	295772	396	-1	1411540	376292	0	4	4	0	3	8	1379856	9.98E-01	1
CCR5	243983	625	36	881456	253828	0	4	4	0	1	4	510504	9.98E-01	1
MKX	283178	535	0	905308	251336	0	1	4	0	4	7	1072628	9.98E-01	1
C10orf71	NaN	NaN	NaN	3610552	1084376	0	3	2	0	50	118	40729960	9.98E-01	1
ZNF136	1114898	721	-14	1406912	341404	0	3	2	0	50	149	24457912	9.98E-01	1
CBX4	647412	242	-2	1395876	418656	0	8	4	0	0	4	418656	9.98E-01	1
TULP4	669285	403	41	3828780	1173732	0	14	6	0	3	9	2036320	9.98E-01	1
NOD1	623997	400	37	2393032	706304	0	5	3	0	5	11	2478828	9.98E-01	1
MFGE8	562979	560	7	995376	277680	0	5	4	0	1	4	552868	9.98E-01	1
FNDC3B	327135	466	25	3110016	857604	0	11	4	0	1	4	1076188	9.98E-01	1
ENDOU	395089	320	14	1097192	258100	0	1	3	0	4	7	1579572	9.98E-01	1
FAT3	32222	1011	-33	11407308	3301188	0	46	15	0	1	16	3838748	9.98E-01	1
AMY1A	NaN	NaN	NaN	4035972	1021008	0	2	0	0	0	0	1021008	9.98E-01	1
EHMT1	977227	583	38	3388408	936992	0	2	5	0	50	43	18730940	9.98E-01	1
ACLY	1857838	191	33	2853340	797084	0	6	9	0	7	24	3634048	9.98E-01	1
PCDHA3	861651	606	2	2376300	765756	0	5	2	0	0	2	765756	9.98E-01	1
PPIP5K1	794398	269	37	3788196	1062660	0	3	2	0	10	21	7868312	9.98E-01	1
RSPH10B2	974099	506	41	4631916	1111788	0	4	1	0	19	27	11907844	9.98E-01	1
SCN7A	68529	918	-11	4383072	1111788	0	7	3	0	43	66	22503472	9.98E-01	1
TRAPPC9	327071	689	16	3170536	920972	0	2	2	0	27	35	13514116	9.98E-01	1
KRTCAP2	1801444	194	32	413672	129940	0	1	2	0	2	4	372376	9.98E-01	1
CKAP2L	595832	214	47	1907804	518692	0	1	1	0	18	30	9369920	9.98E-01	1
ITPR1	290645	395	19	7207576	1888580	0	11	2	0	1	3	2122828	9.98E-01	1
GTSE1	506667	205	35	1836248	581704	0	6	5	0	1	5	788540	9.98E-01	1
UQCRC2	646330	424	43	1176580	333572	0	5	2	0	0	2	333572	9.98E-01	1
HMMR	225981	603	11	1954796	443932	0	3	1	0	0	1	443932	9.98E-01	1
PER2	349323	252	28	3220020	898188	0	6	5	0	3	13	2886804	9.98E-01	1

KCNA1	260795	903	12	1234608	354576	0	9	6	0	1	7	588824	9.98E-01	1
ACAN	561599	560	-1	6134948	2047000	0	15	11	0	13	23	6879700	9.98E-01	1
ZNF814	NaN	NaN	NaN	2216100	535068	0	1	1	0	50	117	40180652	9.98E-01	1
PRAMEF6	NaN	NaN	NaN	2397304	684944	0	1	1	0	50	117	40330528	9.98E-01	1
EGFL6	196762	NaN	49	1460668	364544	0	2	0	0	0	0	364544	9.98E-01	1
ZNF142	1033606	269	43	4196528	1241728	0	5	4	0	50	68	23381368	9.98E-01	1
NDUFA10	271696	569	17	941620	239588	0	3	2	0	1	3	473836	9.98E-01	1
NOMO3	368918	346	15	3148820	897832	0	1	0	0	4	3	2248852	9.98E-01	1
DENND1A	314875	517	32	2636892	801000	0	1	4	0	19	24	8270236	9.98E-01	1
RASGRF2	214755	766	29	3217884	859740	0	5	7	0	10	16	4143128	9.98E-01	1
ZCCHC2	164923	286	44	2925252	907800	0	3	2	0	10	12	4498416	9.98E-01	1
GOLGA3	861960	601	21	3930596	1111432	0	14	4	0	0	4	1111432	9.98E-01	1
ITGAD	839076	558	11	2988620	860452	0	4	3	0	35	62	15935984	9.98E-01	1
MEX3C	146631	570	45	1583844	535068	0	2	0	0	2	3	939128	9.98E-01	1
MXRA5	223878	NaN	-28	6974040	2115708	0	19	8	0	5	19	4933448	9.98E-01	1
HELQ	330595	378	40	2847288	756144	0	3	2	0	2	2	959064	9.98E-01	1
IQGAP1	1142649	150	37	4348184	1124248	0	8	5	0	1	6	1490216	9.98E-01	1
DOCK5	178665	512	14	4952672	1259884	0	4	3	0	14	13	5964068	9.98E-01	1
URGCP	872910	239	44	2324680	684944	0	5	5	0	2	6	913496	9.98E-01	1
PGAP1	549386	370	17	2429700	638664	0	4	2	0	0	2	638664	9.98E-01	1
TFR2	1884430	247	60	2021368	623000	0	1	0	0	0	0	623000	9.98E-01	1
EXOG	373229	578	28	944824	260948	0	2	1	0	0	1	260948	9.98E-01	1
CACNA1I	725838	466	60	5580300	1699188	0	8	4	0	11	13	4505892	9.98E-01	1
KIAA1958	459178	374	11	1789968	520116	0	2	1	0	1	4	1230692	9.98E-01	1
YEATS2	1447754	410	26	3614824	1072628	0	10	5	0	4	15	2888940	9.98E-01	1
SLC7A8	1257542	168	41	1348884	411180	0	2	4	0	2	4	681384	9.98E-01	1
SH3RF2	396248	406	-7	1803496	569600	0	2	5	0	7	19	4186916	9.99E-01	1
TTN	172693	713	23	92721624	26303772	0	301	89	0	12	99	30308772	9.99E-01	1
PCDHGB7	662513	568	2	2287656	715560	0	3	1	0	0	1	715560	9.99E-01	1
NFKB2	975992	178	39	2271280	709508	0	1	4	0	14	24	7562508	9.99E-01	1
POM121L1[illegible]	10922	1116	-24	714492	239232	0	7	4	0	0	4	239232	9.99E-01	1
KIAA1598	202512	533	-14	1689220	404060	0	1	0	0	12	18	4860468	9.99E-01	1
CAPN1	3317462	168	54	1871492	508012	0	2	5	0	11	22	4645800	9.99E-01	1

DBNL	1096873	226	52	1158068	305092	0	2	3	0	2	5	896408	9.99E-01	1
REG1B	10958	1389	-22	448560	107868	0	4	3	0	2	7	338912	9.99E-01	1
ARMC9	1157442	394	41	1759708	459596	0	2	4	0	28	45	9794272	9.99E-01	1
C6orf25	2517236	193	20	683876	160912	0	1	1	0	1	1	292276	9.99E-01	1
DACT3	1139694	347	28	1474908	558564	0	1	1	0	1	1	651480	9.99E-01	1
POLR1B	563286	166	34	2907452	792100	0	3	2	0	32	43	11993640	9.99E-01	1
NEFH	723458	203	35	2543264	742972	0	4	1	0	0	1	742972	9.99E-01	1
NLRP12	783610	486	-58	2742624	803136	0	4	6	0	43	115	27683628	9.99E-01	1
OTUB1	1322584	277	42	715204	181916	0	1	2	0	2	2	396940	9.99E-01	1
ADAMTS7	507164	413	46	4194036	1309368	0	5	3	0	12	16	5722700	9.99E-01	1
SYT14	223343	547	-5	1616240	410824	0	6	4	0	1	4	609828	9.99E-01	1
ABCA8	295848	637	-41	4138500	1088292	0	16	8	0	4	18	2486304	9.99E-01	1
ZYX	379715	379	29	1430052	447492	0	2	1	0	3	7	1614104	9.99E-01	1
INTU	211890	437	-11	2425428	661092	0	3	1	0	2	3	1202212	9.99E-01	1
OR4F17	250228	708	5	755432	224992	0	1	0	0	0	0	224992	9.99E-01	1
ARID3B	655453	206	59	1407624	423996	0	5	4	0	2	5	804204	9.99E-01	1
OR2T8	87551	993	-35	763264	237452	0	1	3	0	4	10	1329304	9.99E-01	1
ZBTB22	1456953	262	30	1516204	522608	0	10	7	0	3	16	1502676	9.99E-01	1
C4A	NaN	NaN	NaN	8346776	2480608	0	1	0	0	0	0	2480608	9.99E-01	1
OPN3	172337	341	-13	991460	312568	0	1	3	0	2	5	952300	9.99E-01	1
ETV1	46434	989	-16	1329660	320400	0	10	4	0	1	7	629052	9.99E-01	1
MAP3K15	222229	NaN	38	3402648	927024	0	3	5	0	5	8	2542552	9.99E-01	1
LENG8	807048	535	-7	2039168	591316	0	2	3	0	50	83	18359276	9.99E-01	1
CECR2	424899	390	-46	3646864	1051268	0	7	2	0	4	7	2375588	9.99E-01	1
KRT81	742854	178	53	1283024	374512	0	1	0	0	2	4	1093988	9.99E-01	1
FER1L5	906473	234	41	5495572	1435748	0	5	1	0	0	1	1435748	9.99E-01	1
ELOVL5	379445	287	27	790320	200784	0	2	1	0	1	2	269492	9.99E-01	1
OR2A7	209930	767	-5	755432	239944	0	1	2	0	1	4	643648	9.99E-01	1
ZNF235	661800	671	-34	1927740	457104	0	4	4	0	2	6	1366328	9.99E-01	1
ATXN3	356163	415	27	989324	215380	0	5	2	0	1	2	290852	9.99E-01	1
AARS2	1037256	312	54	2456400	794592	0	2	3	0	44	60	16584260	9.99E-01	1
RYR3	140241	806	-13	12692112	3356724	0	48	18	0	2	20	4075488	9.99E-01	1
HM13	741997	309	-33	1154152	341048	0	1	1	0	0	1	341048	9.99E-01	1

CLDN14	391816	428	78	576720	196512	0	1	4	0	2	6	660380	9.99E-01	1
OR10G8	158396	830	-10	753652	245996	0	3	3	0	1	5	493772	9.99E-01	1
NTS	22180	677	-32	454256	106444	0	5	2	0	0	2	106444	9.99E-01	1
SGSM1	365627	308	-21	2984704	803492	0	2	2	0	50	67	22639820	9.99E-01	1
MYH14	1215275	528	26	5139216	1566756	0	6	6	0	38	60	19974804	9.99E-01	1
MRPL20	1736268	273	28	385548	110004	0	1	3	0	1	3	269848	9.99E-01	1
ZNF248	142662	979	-22	1520476	354932	0	2	3	0	4	6	995376	9.99E-01	1
ZNF20	1025728	728	-15	1389468	331080	0	2	0	0	14	59	8376680	9.99E-01	1
CTNNA2	10595	1614	-24	2153800	599504	0	22	8	0	0	8	599504	9.99E-01	1
C2	2400153	188	35	2184416	596656	0	1	0	0	4	5	2119624	9.99E-01	1
ZNF609	841050	278	43	3533300	1024924	0	4	2	0	24	40	12377052	9.99E-01	1
PCDHB15	800016	708	-15	1892496	632256	0	8	5	0	6	22	2779292	9.99E-01	1
GBA2	922448	190	48	2378080	663584	0	1	2	0	31	42	12963384	9.99E-01	1
T6GALNA(	259709	989	-11	802424	207904	0	3	3	0	1	4	442152	9.99E-01	1
TMED3	259115	736	19	548596	160556	0	1	1	0	0	1	160556	9.99E-01	1
PRAMEF5	NaN	NaN	NaN	2399440	682808	0	2	0	0	7	14	4370968	9.99E-01	1
NAIP	329526	581	16	3771464	991816	0	2	2	0	9	21	6698496	9.99E-01	1
ASPM	221779	614	-25	8929904	2328952	0	24	6	0	2	9	2971888	9.99E-01	1
GCKR	1193266	306	39	1623004	461732	0	5	3	0	1	4	646140	9.99E-01	1
ANO7	1333138	317	35	2451060	719832	0	4	6	0	11	25	5699916	9.99E-01	1
FAM166B	1108352	300	27	556428	162336	0	1	1	0	0	1	162336	9.99E-01	1
SLC5A2	832764	558	-3	1680676	533288	0	2	4	0	34	63	13258864	9.99E-01	1
NRG2	749094	290	57	2184416	677824	0	1	1	0	18	19	5902480	9.99E-01	1
GPRC6A	232554	730	-15	2363840	631900	0	5	1	0	3	3	1000360	9.99E-01	1
DCAF12L2	11596	NaN	-29	1117484	369172	0	8	4	0	1	9	701676	9.99E-01	1
PCM1	381765	253	2	5334304	1311860	0	3	1	0	14	15	8425452	9.99E-01	1
OR4C11	1141	1100	-37	777860	216448	0	2	4	0	2	7	697760	9.99E-01	1
RNF123	1551582	271	48	3389120	986476	0	12	9	0	3	16	2340700	9.99E-01	1
LPAR1	247269	624	21	914564	264508	0	3	1	0	0	1	264508	9.99E-01	1
BRD1	644644	276	-18	2673916	766112	0	5	5	0	14	29	6698852	9.99E-01	1
ASH2L	671169	258	34	1652196	431472	0	1	1	0	1	1	699184	9.99E-01	1
POR	1063656	218	54	1753300	492704	0	1	3	0	19	38	9233572	9.99E-01	1
SYNPO	1119691	198	51	2766476	950164	0	2	3	0	49	69	19376012	9.99E-01	1

GIPR	950605	194	45	1186548	365256	0	4	4	0	0	4	365256	9.99E-01	1
KDM6B	2148615	337	24	4104680	1373092	0	5	2	0	6	7	2623008	9.99E-01	1
ITPR2	359658	548	22	7086180	1810260	0	27	9	0	0	9	1810260	9.99E-01	1
OSGEPL1	398590	452	25	1062660	301176	0	1	0	0	0	0	301176	9.99E-01	1
PRAMEF7	NaN	NaN	NaN	2364196	705236	0	1	2	0	50	118	40350820	9.99E-01	1
GNAO1	465845	448	-26	1234608	308652	0	1	3	0	5	12	2050204	9.99E-01	1
HIST1H2BI	398813	636	53	312568	96476	0	1	4	0	2	8	432184	9.99E-01	1
POLR3B	247626	539	7	2972244	776436	0	3	2	0	6	10	3349960	9.99E-01	1
CASP2	379804	372	21	1182276	311856	0	2	3	0	1	4	725172	9.99E-01	1
DECR1	272948	534	25	867572	249556	0	2	6	0	3	9	765400	9.99E-01	1
ANKRD32	54375	399	20	2788548	687792	0	2	0	0	9	15	5105040	9.99E-01	1
KIAA0100	1651143	225	31	5709528	1619088	0	21	8	0	3	13	2353516	9.99E-01	1
RYR1	1481209	353	17	12899660	3693856	0	36	16	0	25	56	15039220	9.99E-01	1
ELFN1	NaN	NaN	NaN	1990396	665720	0	1	0	0	7	14	4353880	9.99E-01	1
NLRC5	804832	154	53	4768620	1414032	0	12	7	0	18	35	7943428	9.99E-01	1
SLC5A11	280701	277	18	1720904	511216	0	2	3	0	3	6	1631548	9.99E-01	1
CCDC121	1192074	306	43	1111432	305804	0	2	2	0	3	9	1304028	9.99E-01	1
NUP98	449630	310	25	4648292	1333576	0	9	5	0	14	22	4970828	9.99E-01	1
DMBT1	335358	404	2	6194044	1762556	0	13	7	0	1	7	2112860	9.99E-01	1
SLC43A3	923815	191	-3	1249916	373444	0	1	5	0	3	7	961556	9.99E-01	1
KCNH6	934619	247	-1	2497340	752584	0	10	7	0	3	11	1475620	9.99E-01	1
UBR3	340876	631	32	4945196	1269496	0	4	1	0	2	4	2137068	9.99E-01	1
CA4	973133	347	10	809188	225704	0	2	3	0	3	11	1162340	9.99E-01	1
DNAH12	455990	454	29	8133532	2070140	0	5	2	0	7	6	4083676	9.99E-01	1
ADAMTS1	33367	441	-72	2441804	693844	0	2	1	0	7	9	2880040	9.99E-01	1
CCDC67	234882	649	-20	1628700	365256	0	1	3	0	8	16	3798520	9.99E-01	1
AACS	894670	306	13	1765760	463156	0	1	4	0	12	17	4043448	9.99E-01	1
PTAFR	794194	213	28	854756	248488	0	1	4	0	1	8	1075120	9.99E-01	1
SLC2A14	149291	305	14	1316844	395160	0	2	3	0	6	13	2105028	9.99E-01	1
EEPD1	357816	522	18	1425068	426844	0	3	5	0	2	7	1048776	9.99E-01	1
ALS2CL	298503	495	41	2451060	712356	0	2	3	0	50	70	18972664	9.99E-01	1
TERT	657890	541	24	2765408	930940	0	2	2	0	12	28	8581736	9.99E-01	1
HLA-C	3244502	401	38	933076	274832	0	2	3	0	5	6	755788	9.99E-01	1

GEMIN4	662733	483	10	2612684	784624	0	1	2	0	5	8	3196168	9.99E-01	1
NUP153	474858	267	12	3710232	1108584	0	10	4	0	0	4	1108584	9.99E-01	1
TBC1D3F	711283	NaN	16	2835540	797796	0	1	0	0	18	29	11185520	9.99E-01	1
ASPRV1	844060	221	37	836600	265576	0	2	4	0	2	4	524032	9.99E-01	1
ANKRD10	506421	402	22	1065864	304380	0	1	2	0	1	2	568888	9.99E-01	1
PLEKHO1	1202779	309	41	1030620	306516	0	3	2	0	0	2	306516	9.99E-01	1
MYH1	76487	723	-50	5099700	1278396	0	12	7	0	1	12	2560352	9.99E-01	1
OR10H1	808269	590	18	779996	246352	0	1	3	0	2	5	715916	9.99E-01	1
NR2C1	365826	544	27	1645076	424708	0	4	2	0	1	4	672128	9.99E-01	1
SETD8	1167786	177	45	913140	249912	0	2	3	0	5	9	1079036	9.99E-01	1
NEDD4L	321507	522	58	2612684	674620	0	2	1	0	6	8	2703464	9.99E-01	1
CNNM2	711862	233	38	2208624	670704	0	9	6	0	1	7	935568	9.99E-01	1
FLAD1	1612870	224	35	1569604	491636	0	8	4	0	2	5	590248	9.99E-01	1
TBC1D23	378718	265	25	1812396	454968	0	3	3	0	1	3	792456	9.99E-01	1
CD6	802735	546	56	1699544	495196	0	2	6	0	7	12	2144900	9.99E-01	1
PGM2	136995	603	38	1606272	415452	0	2	4	0	9	18	2898552	9.99E-01	1
GLIS3	170537	983	-1	2315780	709864	0	5	4	0	2	6	1083664	9.99E-01	1
CNTN2	655446	431	37	2637604	791744	0	8	5	0	5	10	1894632	9.99E-01	1
SEMG2	842540	324	25	1505880	372732	0	5	3	0	3	6	850484	9.99E-01	1
MMRN2	396293	164	36	2345328	724104	0	1	2	0	28	35	11815284	9.99E-01	1
CELSR2	994135	287	31	7213272	2298336	0	13	9	0	7	15	4734444	9.99E-01	1
INPP5E	1413299	233	44	1592032	512996	0	1	3	0	2	4	1077256	9.99E-01	1
ZNHIT3	416822	556	23	419368	99680	0	3	1	0	0	1	99680	9.99E-01	1
AICDA	291883	422	15	515488	141332	0	1	3	0	1	4	375580	9.99E-01	1
TMC8	527017	217	27	1799580	593808	0	4	3	0	9	36	5477416	9.99E-01	1
PLXNA3	2220716	NaN	41	4706676	1427916	0	6	6	0	50	64	18875120	9.99E-01	1
CYP7A1	200469	608	11	1300112	341404	0	7	6	0	1	6	552868	9.99E-01	1
FRMPD1	498002	434	27	3958364	1164832	0	12	7	0	7	13	2870072	9.99E-01	1
AKAP8L	509601	481	30	1693848	438948	0	5	4	0	6	23	3086520	9.99E-01	1
CDK5RAP[illegible]	386697	450	10	4952316	1274124	0	8	2	0	7	11	3463168	9.99E-01	1
CDC42BP[illegible]	2740031	204	61	3894284	1232116	0	5	4	0	30	61	17975864	9.99E-01	1
CAND2	508965	269	39	3033476	991816	0	1	4	0	31	29	10881496	9.99E-01	1
CNGA3	228601	630	29	1755080	501604	0	6	5	0	8	16	1709512	9.99E-01	1

RGPD8	NaN	NaN	NaN	4541492	1212892	0	4	1	0	7	15	4901052	9.99E-01	1
FOXD4L4	0	1096	-26	2000720	671416	0	3	2	0	11	31	6042744	9.99E-01	1
ABL1	505506	211	29	2931660	884304	0	6	6	0	17	36	7115016	9.99E-01	1
SLC14A2	374175	438	-8	2344972	687080	0	4	6	0	2	10	1454972	9.99E-01	1
SETD1A	1508459	247	46	4204716	1344612	0	6	7	0	49	70	18790748	9.99E-01	1
PLA2G4A	265620	592	-18	1987904	487720	0	1	3	0	20	30	8034564	9.99E-01	1
STOX2	169045	458	-14	2316492	666432	0	1	1	0	31	38	11875804	9.99E-01	1
MED14	479033	NaN	63	3718420	1071560	0	3	1	0	3	3	1719124	9.99E-01	1
C4B	NaN	NaN	NaN	8346776	2480608	0	3	2	0	0	2	2480608	9.99E-01	1
MC5R	131146	815	8	805984	238520	0	3	4	0	1	4	453188	9.99E-01	1
CYP21A2	NaN	NaN	NaN	2481320	770740	0	2	0	0	7	14	4458900	9.99E-01	1
PCDHB7	915569	724	-11	1907804	636172	0	10	5	0	1	11	1267004	9.99E-01	1
ATP7A	549429	NaN	19	3801368	1101820	0	7	6	0	3	8	1625140	9.99E-01	1
PPP1R9A	419387	534	-15	3580292	932008	0	6	3	0	0	3	932008	9.99E-01	1
LBP	563754	350	47	1248492	357780	0	2	2	0	6	10	1454260	9.99E-01	1
ABCA1	195112	637	-18	5854064	1602712	0	12	4	0	2	5	1857964	9.99E-01	1
RGS22	290819	449	22	3359216	809188	0	13	8	0	3	11	1732296	1.00E+00	1
NOTCH1	1489379	212	43	6494152	1836248	0	13	5	0	0	5	1836248	1.00E+00	1
VSIG1	193027	NaN	32	1082952	305448	0	2	3	0	2	6	1006056	1.00E+00	1
SH2D7	434458	271	63	1131368	338200	0	2	3	0	2	5	814884	1.00E+00	1
EPHA1	375488	379	15	2459248	745820	0	1	5	0	21	25	7747628	1.00E+00	1
CYP24A1	158398	386	-31	1337136	359916	0	4	5	0	1	5	587044	1.00E+00	1
SMC3	438920	177	46	3257756	764332	0	5	1	0	1	2	973304	1.00E+00	1
RENBP	2355024	NaN	25	1110364	305804	0	4	2	0	0	2	305804	1.00E+00	1
CSPP1	353616	640	25	3327532	830192	0	10	3	0	0	3	830192	1.00E+00	1
RBM33	213580	646	10	2966192	858316	0	1	0	0	3	2	1454260	1.00E+00	1
CAMK1	954590	264	39	1085088	257388	0	2	3	0	1	4	651480	1.00E+00	1
PPP2R2B	309460	509	1	1292992	313280	0	2	3	0	1	3	651480	1.00E+00	1
SERPINB8	114875	671	26	992528	246352	0	4	2	0	1	2	340336	1.00E+00	1
TLR8	208025	NaN	64	2626568	718408	0	6	4	0	7	21	3953380	1.00E+00	1
SLC4A3	547550	487	29	3129952	1005344	0	4	2	0	7	12	3506956	1.00E+00	1
AGRN	1971475	239	15	5064456	1642584	0	9	5	0	1	5	1863660	1.00E+00	1
GNG3	2425755	248	51	198648	52332	0	3	1	0	0	1	52332	1.00E+00	1

JUP	1760409	175	35	1882172	567820	0	4	7	0	6	22	3387340	1.00E+00	1
NCAPD3	270385	601	14	3901404	1046640	0	6	2	0	0	2	1046640	1.00E+00	1
ZC3H10	2878044	171	40	1055540	342472	0	1	5	0	4	10	1466364	1.00E+00	1
RFX1	971756	231	32	2459960	765400	0	1	4	0	50	60	18946676	1.00E+00	1
PLAU	988012	191	43	1414032	359916	0	1	0	0	0	0	359916	1.00E+00	1
[illegible]OLGA6L1	NaN	NaN	NaN	2467436	670348	0	1	0	0	7	14	4358508	1.00E+00	1
EFTUD1	373597	701	13	2908520	764332	0	2	2	0	8	10	3317920	1.00E+00	1
BCR	384444	575	43	3250992	918480	0	1	2	0	50	43	17722036	1.00E+00	1
ATP4A	1081638	390	41	2638672	772520	0	3	7	0	26	46	9651160	1.00E+00	1
ATP11C	66616	NaN	18	3032052	793524	0	3	3	0	3	5	1403352	1.00E+00	1
ZBTB46	1125169	202	40	1469212	438236	0	2	4	0	5	7	1182988	1.00E+00	1
ASAP3	1050777	159	29	2360636	662872	0	4	6	0	14	28	5192260	1.00E+00	1
TTBK2	611976	453	31	3146328	902460	0	10	4	0	0	4	902460	1.00E+00	1
MTMR12	384742	160	27	1969036	491636	0	7	3	0	0	3	491636	1.00E+00	1
HRH2	238233	642	19	970812	310788	0	1	3	0	1	3	612676	1.00E+00	1
ARHGEF1[illegible]	100967	551	-24	3481680	947316	0	5	4	0	26	53	13220060	1.00E+00	1
ASPH	423823	377	14	2677120	634748	0	2	2	0	3	5	2028488	1.00E+00	1
MKRN1	359405	521	24	1249204	330368	0	1	2	0	3	4	863656	1.00E+00	1
MUC1	1801444	194	32	798508	140264	0	1	2	0	3	6	512640	1.00E+00	1
SLITRK2	1640	NaN	-34	2101468	617660	0	3	6	0	12	28	4996816	1.00E+00	1
CYP2F1	1016551	354	11	1245644	369172	0	5	6	0	7	20	1926672	1.00E+00	1
NIPAL3	604127	325	29	1054472	292276	0	3	2	0	0	2	292276	1.00E+00	1
PHF12	1834230	220	37	2563912	741548	0	3	6	0	4	10	1975444	1.00E+00	1
SLC8A3	527365	409	28	2430412	691352	0	5	7	0	2	13	2457468	1.00E+00	1
NTN4	461515	237	7	1629412	424352	0	3	2	0	4	9	1529376	1.00E+00	1
THBS3	1801444	194	32	2483456	678892	0	2	2	0	50	85	19936000	1.00E+00	1
RFT1	907827	192	39	1369176	420792	0	1	5	0	3	11	2165192	1.00E+00	1
DUSP28	645026	485	28	425776	149876	0	1	1	0	0	1	149876	1.00E+00	1
WNT10B	1737773	214	55	962980	303668	0	4	3	0	1	5	431472	1.00E+00	1
PPARG	372685	222	25	1307944	341048	0	12	5	0	0	5	341048	1.00E+00	1
KCNJ12	405028	480	-60	1075476	319332	0	2	1	0	0	1	319332	1.00E+00	1
MAP1LC3[illegible]	990458	214	54	376648	89000	0	3	2	0	1	3	154860	1.00E+00	1
TTLL8	895187	271	36	2116776	613032	0	1	1	0	14	24	5955524	1.00E+00	1

PCDHB2	987049	724	-11	1932724	627272	0	4	6	0	3	22	2510868	1.00E+00	1
TCEB3C	NaN	NaN	NaN	2640808	862232	0	1	1	0	9	17	5219672	1.00E+00	1
ABCA10	137274	415	-43	4076200	1024568	0	7	4	0	10	17	3527604	1.00E+00	1
PDE1B	643658	392	33	1462092	385548	0	3	3	0	1	3	510504	1.00E+00	1
OR8I2	9992	1007	-32	765400	229976	0	7	4	0	2	14	692064	1.00E+00	1
CCDC8	1099113	393	25	1313996	412960	0	2	3	0	2	8	1231404	1.00E+00	1
TMEM187	2355024	NaN	25	629052	210396	0	3	2	0	0	2	210396	1.00E+00	1
OXCT1	109769	687	11	1365616	372020	0	1	3	0	5	12	2120692	1.00E+00	1
TRIM11	682437	316	7	1154152	369884	0	3	4	0	1	7	830192	1.00E+00	1
NBPF10	NaN	NaN	NaN	9399468	2342124	0	4	1	0	0	1	2342124	1.00E+00	1
APOA1	421374	323	39	683164	190460	0	2	1	0	1	2	262372	1.00E+00	1
GPX5	247624	576	27	594876	135636	0	2	1	0	0	1	135636	1.00E+00	1
KAT2B	102581	583	4	2153444	588112	0	4	7	0	7	15	2051272	1.00E+00	1
STH	365925	469	18	321468	96120	0	2	2	0	0	2	96120	1.00E+00	1
ANKRD36	NaN	NaN	NaN	5282328	1260240	0	8	1	0	7	15	4948400	1.00E+00	1
DNAH17	999172	188	25	11480288	3160924	0	33	17	0	50	82	21484956	1.00E+00	1
TRIM59	435184	513	16	1035604	263084	0	4	2	0	0	2	263084	1.00E+00	1
ID1	741997	309	-33	395516	134212	0	3	3	0	0	3	134212	1.00E+00	1
CA5A	612550	133	27	791744	216448	0	3	2	0	0	2	216448	1.00E+00	1
OLFM3	66875	991	-15	1183344	308652	0	9	3	0	0	3	308652	1.00E+00	1
C2orf70	931476	428	35	515132	147028	0	2	1	0	0	1	147028	1.00E+00	1
TRIM38	392745	685	34	1199008	315416	0	3	4	0	2	10	1178004	1.00E+00	1
AMBRA1	880494	212	45	3020304	925956	0	9	5	0	0	5	925956	1.00E+00	1
OR2T2	112460	903	-20	791388	249912	0	1	6	0	3	10	959776	1.00E+00	1
NTSR2	219483	336	21	990036	341760	0	1	5	0	2	6	866148	1.00E+00	1
ADAMTS1[illegible]	143599	755	-36	2796024	807408	0	2	4	0	14	36	8696012	1.00E+00	1
GRIN2B	153586	825	-10	3770752	1038452	0	9	2	0	1	4	1286228	1.00E+00	1
DYNC1H1	1456679	196	38	11940952	3276980	0	23	10	0	29	60	19922828	1.00E+00	1
CBL	1172813	183	53	2305456	664652	0	5	3	0	0	3	664652	1.00E+00	1
CYFIP1	352283	539	-19	3589548	961200	0	7	7	0	5	9	2170532	1.00E+00	1
GALNT8	283327	862	24	1639380	449628	0	6	6	0	2	9	1034180	1.00E+00	1
CES3	993495	135	41	1468144	417944	0	6	4	0	0	4	417944	1.00E+00	1
MID1	117200	NaN	14	1709512	469208	0	6	4	0	0	4	469208	1.00E+00	1

NR5A2	120174	616	-8	1395164	371308	0	5	2	0	0	2	371308	1.00E+00	1
DUOX1	667418	544	27	3962992	1150592	0	2	4	0	50	85	27585372	1.00E+00	1
LRP1	2190357	238	60	11820980	3117136	0	31	11	0	41	73	20645864	1.00E+00	1
UNC80	NaN	NaN	NaN	8369560	2343548	0	2	1	0	0	1	2343548	1.00E+00	1
EPB41	619053	227	30	2282316	597012	0	3	1	0	6	16	3362064	1.00E+00	1
PRPF8	1361271	198	27	6038828	1625140	0	7	6	0	50	60	20575376	1.00E+00	1
NYNRIN	897133	265	40	4642240	1476332	0	13	9	0	1	9	1655044	1.00E+00	1
FAF1	237738	691	-5	1719480	443220	0	3	4	0	1	4	782844	1.00E+00	1
PCDHA4	861651	606	2	2306880	754008	0	7	8	0	5	30	3429704	1.00E+00	1
SLC6A5	148079	842	1	2033116	587756	0	4	3	0	2	9	1656824	1.00E+00	1
PARVG	201266	327	30	877184	237808	0	1	3	0	1	3	495908	1.00E+00	1
DSCAM	25449	971	-72	5118568	1467788	0	20	9	0	21	48	7019964	1.00E+00	1
TMED4	1462615	191	56	584908	164828	0	2	2	0	0	2	164828	1.00E+00	1
TMEM97	1405196	214	10	448916	126736	0	1	3	0	1	3	232468	1.00E+00	1
ADRA1A	259233	692	3	1384840	420080	0	5	5	0	1	6	654328	1.00E+00	1
SYNRG	559565	201	27	3443588	939484	0	8	5	0	13	32	5999668	1.00E+00	1
HDAC7	391157	182	29	2493780	791388	0	2	3	0	1	3	1079748	1.00E+00	1
RPRM	22780	737	-11	266288	86152	0	3	2	0	0	2	86152	1.00E+00	1
SPAST	378148	197	28	1589540	455680	0	5	3	0	0	3	455680	1.00E+00	1
SCN4A	1455216	404	25	4704896	1281244	0	9	9	0	21	37	8446812	1.00E+00	1
PLD4	855494	247	41	1271276	393736	0	1	2	0	0	2	393736	1.00E+00	1
DGKZ	811789	212	44	3222512	967252	0	3	6	0	12	18	4998240	1.00E+00	1
FAM98C	1467279	253	0	859384	294056	0	2	3	0	0	3	294056	1.00E+00	1
MATN2	616574	272	28	2508376	634748	0	8	4	0	0	4	634748	1.00E+00	1
KCNV1	102383	890	-18	1238524	375224	0	6	4	0	0	4	375224	1.00E+00	1
ZFYVE19	1023960	172	48	1204348	350660	0	4	3	0	0	3	350660	1.00E+00	1
OR8H2	9992	1007	-32	769672	231044	0	6	4	0	2	14	692064	1.00E+00	1
COL5A1	229207	421	31	4691012	1478824	0	15	10	0	2	14	2226068	1.00E+00	1
AIFM1	379257	NaN	58	1698476	487720	0	1	2	0	21	24	4310448	1.00E+00	1
SALL3	47663	635	-65	3144548	1032400	0	3	9	0	16	39	7197252	1.00E+00	1
GPR4	1029266	194	44	882168	280884	0	3	4	0	1	5	462800	1.00E+00	1
ANGEL1	308800	471	39	1694560	493772	0	1	5	0	5	12	2383776	1.00E+00	1
CLUL1	449991	486	23	1244220	290496	0	3	1	0	0	1	290496	1.00E+00	1

PCDHB10	915569	724	-11	1935572	630832	0	10	6	0	0	6	630832	1.00E+00	1
ATP2B3	940418	NaN	24	3218596	943400	0	3	6	0	44	62	14837724	1.00E+00	1
DCP1B	232517	618	29	1561416	454968	0	4	1	0	4	6	866148	1.00E+00	1
PCDHB1	987049	724	-11	2006416	619796	0	6	5	0	3	22	2510868	1.00E+00	1
MAST4	36320	621	7	6652216	2013536	0	8	10	0	22	39	12527284	1.00E+00	1
ANKRD13I	719319	383	30	1594524	474192	0	1	1	0	7	11	2172668	1.00E+00	1
TMEM106C	374433	246	47	651124	182984	0	4	2	0	0	2	182984	1.00E+00	1
SPTB	584151	204	38	6028148	1652908	0	8	7	0	24	30	9230012	1.00E+00	1
NPR2	885056	190	51	2718772	802424	0	6	6	0	2	9	1739772	1.00E+00	1
ALG9	437477	182	28	1607696	440728	0	1	0	0	1	2	639020	1.00E+00	1
NCK1	181954	195	20	974728	249200	0	2	3	0	1	4	493772	1.00E+00	1
KCNK3	1198614	300	41	957996	311856	0	2	3	0	0	3	311856	1.00E+00	1
SLC39A12	94785	360	-9	1779288	489144	0	12	7	0	1	8	713780	1.00E+00	1
WDSUB1	386986	265	29	1242084	328944	0	1	2	0	2	6	935568	1.00E+00	1
GRAMD1A	821282	419	-46	1860100	543968	0	2	3	0	10	23	3877196	1.00E+00	1
RPL26L1	488219	181	36	379852	100748	0	2	2	0	0	2	100748	1.00E+00	1
KIF13B	333305	417	42	4706320	1316132	0	6	2	0	3	8	2781428	1.00E+00	1
FANCA	1234390	219	41	3773956	1079036	0	14	9	0	1	9	1265580	1.00E+00	1
DNAJB5	676166	394	23	1051980	309720	0	2	3	0	1	5	605556	1.00E+00	1
LAMB1	481432	406	11	4674992	1191532	0	10	4	0	0	4	1191532	1.00E+00	1
RWDD2B	316591	303	34	831616	212888	0	2	1	0	0	1	212888	1.00E+00	1
PFKP	221025	438	11	2029912	577076	0	4	4	0	0	4	577076	1.00E+00	1
UBA1	819569	NaN	38	2716992	780708	0	1	1	0	4	9	2805636	1.00E+00	1
NOMO2	707984	675	17	3268436	933076	0	1	2	0	3	5	2171956	1.00E+00	1
ZNF878	975609	728	-8	1492708	379496	0	2	3	0	5	25	3319344	1.00E+00	1
USP15	239150	456	-26	2532940	608048	0	10	4	0	0	4	608048	1.00E+00	1
MYH3	60385	749	-41	5083680	1301892	0	15	6	0	4	11	2394100	1.00E+00	1
PELO	158780	388	0	962980	278036	0	1	2	0	0	2	278036	1.00E+00	1
PTK6	1262904	298	54	1149524	330724	0	2	4	0	3	10	1009972	1.00E+00	1
CCDC129	34323	943	-1	2676408	727308	0	6	3	0	1	6	899968	1.00E+00	1
SLC44A5	178229	935	-21	2010688	510860	0	2	1	0	0	1	510860	1.00E+00	1
DCAF4	354097	341	41	1327524	320400	0	3	2	0	0	2	320400	1.00E+00	1
KIF23	307077	298	19	2528668	644360	0	8	3	0	0	3	644360	1.00E+00	1

LMBR1L	1955973	214	60	1251696	386616	0	1	4	0	1	5	834820	1.00E+00	1
KIAA2026	349735	317	26	5199736	1571384	0	15	7	0	0	7	1571384	1.00E+00	1
FGF8	817474	335	37	622644	183696	0	3	2	0	0	2	183696	1.00E+00	1
FADS1	1239228	212	59	1297264	358136	0	2	3	0	1	3	416876	1.00E+00	1
PI4KA	436102	288	42	5341780	1441088	0	11	4	0	0	4	1441088	1.00E+00	1
LAMA5	1090360	252	21	9305484	2876124	0	14	12	0	0	12	2876124	1.00E+00	1
KLB	648710	177	43	2623008	744396	0	6	6	0	2	7	1136352	1.00E+00	1
KIAA0196	489847	520	25	3036324	795660	0	6	3	0	3	8	1393740	1.00E+00	1
ERN1	1464674	379	16	2516208	707016	0	1	0	0	1	16	4400872	1.00E+00	1
DLG5	364785	259	27	4875420	1410828	0	11	7	0	2	13	2497340	1.00E+00	1
KDM4C	153801	838	14	2975804	752584	0	8	5	0	2	6	1178360	1.00E+00	1
PCDHB9	915569	724	-11	1927740	625848	0	5	6	0	1	12	1256680	1.00E+00	1
HHIPL2	227631	467	-6	1833044	526168	0	9	4	0	0	4	526168	1.00E+00	1
CYP4F12	865205	605	21	1362056	372376	0	7	5	0	0	5	372376	1.00E+00	1
NBPF3	563178	283	37	1668572	418300	0	5	2	0	0	2	418300	1.00E+00	1
SLC18A2	202102	624	0	1340696	373444	0	1	5	0	3	7	1114992	1.00E+00	1
GPR45	184828	497	0	899256	295836	0	4	3	0	0	3	295836	1.00E+00	1
PTPRJ	601164	223	-10	3425432	971524	0	7	6	0	4	8	1750808	1.00E+00	1
RNLS	193044	396	33	947316	256320	0	4	5	0	1	8	561768	1.00E+00	1
FUT10	96386	500	5	1221436	329300	0	4	2	0	0	2	329300	1.00E+00	1
CALN1	17685	956	-29	690996	174084	0	1	3	0	2	10	813104	1.00E+00	1
TNPO2	1959466	226	29	2350668	631188	0	4	6	0	3	7	1400860	1.00E+00	1
DTX3	1338867	303	48	852264	280884	0	4	3	0	0	3	280884	1.00E+00	1
TRIM58	87551	993	-35	1215384	368460	0	1	4	0	4	10	1329304	1.00E+00	1
USP22	431615	382	-19	1386976	349592	0	1	3	0	2	4	791032	1.00E+00	1
SIX1	352813	482	5	703812	213600	0	3	3	0	0	3	213600	1.00E+00	1
UNK	1806586	178	31	2253480	656820	0	1	2	0	50	93	20102964	1.00E+00	1
MYBBP1A	1252775	318	22	3396596	1013176	0	3	8	0	3	9	2258820	1.00E+00	1
ATP2A3	617494	268	33	2694564	820224	0	4	4	0	1	7	1224996	1.00E+00	1
ADCK1	427928	517	24	1344256	377360	0	3	3	0	1	4	446068	1.00E+00	1
DUS1L	3850694	197	34	1235320	338912	0	4	4	0	0	4	338912	1.00E+00	1
ALDH5A1	407627	461	18	1376652	427200	0	1	4	0	2	7	1186548	1.00E+00	1
FDXACB1	437513	221	22	1582064	439660	0	1	1	0	0	1	439660	1.00E+00	1

CASC3	1537878	156	32	1773592	537560	0	1	2	0	5	22	3656476	1.00E+00	1
CD209	719851	329	21	1044504	280884	0	3	4	0	1	6	547172	1.00E+00	1
PASK	1394664	278	35	3309376	1005344	0	3	6	0	17	39	9146352	1.00E+00	1
ARF3	1737773	214	55	472412	127804	0	1	2	0	0	2	127804	1.00E+00	1
ZP4	14616	1051	-30	1384484	398008	0	5	5	0	0	5	398008	1.00E+00	1
PAQR5	366429	210	29	854756	231400	0	1	2	0	0	2	231400	1.00E+00	1
TTC9C	2415432	248	47	435388	124244	0	1	1	0	0	1	124244	1.00E+00	1
SNF8	793101	174	29	678536	183340	0	2	3	0	1	3	296192	1.00E+00	1
CDK10	1090549	219	38	987900	274476	0	2	3	0	1	3	373088	1.00E+00	1
PSG2	263820	178	-32	852620	247420	0	7	5	0	0	5	247420	1.00E+00	1
RNF11	503560	305	7	394804	110360	0	1	1	0	0	1	110360	1.00E+00	1
PCDHGB5	662513	568	2	2311152	723036	0	2	2	0	0	2	723036	1.00E+00	1
TCF25	1207036	221	45	1754012	489856	0	5	6	0	2	11	1302960	1.00E+00	1
DNMBP	1100254	221	32	3986132	1138132	0	8	4	0	2	11	2301896	1.00E+00	1
BTBD1	384974	679	27	1236032	341404	0	1	1	0	0	1	341404	1.00E+00	1
RAB9A	135235	NaN	48	514776	136704	0	1	2	0	0	2	136704	1.00E+00	1
NDST2	1009357	191	42	2210760	672840	0	2	4	0	2	5	1132080	1.00E+00	1
MOCS3	386595	367	36	1093632	385548	0	7	6	0	0	6	385548	1.00E+00	1
PCDHB13	915569	724	-11	1934504	625492	0	7	6	0	1	12	1256324	1.00E+00	1
ERH	911402	431	39	285512	65860	0	2	1	0	0	1	65860	1.00E+00	1
DONSON	908921	375	68	1427916	429336	0	4	3	0	0	3	429336	1.00E+00	1
SLC24A3	161681	913	-20	1672132	462800	0	6	4	0	0	4	462800	1.00E+00	1
ARMC6	1327004	215	24	1192244	365968	0	4	3	0	0	3	365968	1.00E+00	1
ARHGEF10	375252	279	30	3263808	956928	0	3	2	0	0	2	956928	1.00E+00	1
FECH	304947	352	38	1119264	301176	0	2	3	0	1	6	676756	1.00E+00	1
NPHP4	508577	370	13	3592396	1103600	0	3	6	0	2	7	2149528	1.00E+00	1
GLRA3	62161	797	-15	1215028	313280	0	4	2	0	0	2	313280	1.00E+00	1
SDK2	182941	571	-22	5510168	1640092	0	2	5	0	10	20	6075140	1.00E+00	1
MTOR	577167	416	32	6583864	1829840	0	5	6	0	27	28	10197620	1.00E+00	1
SPIRE2	1220713	219	43	1802072	550732	0	4	5	0	1	5	737276	1.00E+00	1
SRGAP3	373293	469	22	2882532	768960	0	4	5	0	1	6	1378432	1.00E+00	1
OR8H3	9992	1007	-32	771808	231044	0	4	6	0	2	14	692064	1.00E+00	1
PKDREJ	519654	205	31	5574248	1647568	0	6	7	0	43	59	16573224	1.00E+00	1

ATP9A	171625	419	0	2709160	763976	0	6	4	0	0	4	763976	1.00E+00	1
PLOD2	118043	471	-12	2009620	503384	0	4	2	0	1	3	646140	1.00E+00	1
LRRC8A	1644028	201	14	2000720	606268	0	1	7	0	5	17	3102540	1.00E+00	1
C12orf66	472096	296	-17	1127096	312568	0	5	3	0	0	3	312568	1.00E+00	1
C2orf47	237591	673	22	739056	215736	0	2	2	0	0	2	215736	1.00E+00	1
TSLP	205349	738	6	416164	109292	0	1	1	0	0	1	109292	1.00E+00	1
SERPIND1	435437	288	41	1275904	343184	0	6	4	0	0	4	343184	1.00E+00	1
CENPL	441912	417	9	995732	278392	0	2	3	0	0	3	278392	1.00E+00	1
SCN11A	276625	707	18	4639392	1208976	0	10	9	0	2	12	2178364	1.00E+00	1
VEPH1	202651	739	5	2262736	586688	0	5	5	0	2	6	966540	1.00E+00	1
NUF2	130027	852	-9	1258104	287292	0	2	3	0	1	5	643292	1.00E+00	1
LSM7	1503606	267	31	280528	69776	0	1	1	0	0	1	69776	1.00E+00	1
TXNDC16	396835	518	14	2166616	561056	0	9	5	0	1	5	657532	1.00E+00	1
ZMIZ1	319778	442	39	2727316	784268	0	8	8	0	2	11	1496624	1.00E+00	1
EPAS1	375634	213	28	2247428	607336	0	5	4	0	0	4	607336	1.00E+00	1
TPTE	30271	1268	-81	1482740	375580	0	12	8	0	0	8	375580	1.00E+00	1
OR10P1	1678592	523	-8	744752	260236	0	1	3	0	1	5	440372	1.00E+00	1
EIF4G2	793205	266	21	2388760	610184	0	8	4	0	0	4	610184	1.00E+00	1
DCTN5	544239	561	38	484160	125668	0	1	1	0	0	1	125668	1.00E+00	1
NTM	9703	1115	-5	991816	284444	0	4	5	0	0	5	284444	1.00E+00	1
RCAN2	55610	819	8	502672	144536	0	2	2	0	0	2	144536	1.00E+00	1
THUMPD3	703818	404	32	1317912	348168	0	3	3	0	0	3	348168	1.00E+00	1
WIPI1	699214	233	-15	1163052	322536	0	2	4	0	0	4	322536	1.00E+00	1
PLXNB1	1401411	263	32	5300128	1697408	0	8	6	0	13	30	6945916	1.00E+00	1
TPH2	223598	619	-18	1277684	338200	0	3	4	0	0	4	338200	1.00E+00	1
DOCK2	94542	680	33	4887524	1199008	0	9	7	0	1	7	1582064	1.00E+00	1
GRIK5	886688	438	31	2454620	767536	0	11	7	0	0	7	767536	1.00E+00	1
LTBP4	869282	226	37	4341420	1311504	0	9	5	0	0	5	1311504	1.00E+00	1
KRI1	2036812	226	23	1894988	458884	0	8	8	0	4	19	1627988	1.00E+00	1
ACSL6	331471	484	25	1954796	535780	0	1	4	0	3	5	1080816	1.00E+00	1
ZIM2	221899	902	-70	1366684	339980	0	1	3	0	8	24	3006420	1.00E+00	1
EPS15L1	793775	258	19	2258108	609472	0	7	5	0	0	5	609472	1.00E+00	1
KIAA1468	133045	374	40	3144904	873980	0	6	6	0	1	8	1326456	1.00E+00	1

GALNTL6	5263	1023	-13	1572452	403348	0	10	4	0	0	4	403348	1.00E+00	1
RNF150	134725	790	-13	1113924	318264	0	2	2	0	0	2	318264	1.00E+00	1
KDM1A	715760	153	29	2250632	644716	0	7	5	0	0	5	644716	1.00E+00	1
NAALAD2	131926	1141	-22	1944828	506232	0	4	5	0	3	7	985408	1.00E+00	1
POC5	265325	605	19	1499828	406552	0	2	2	0	0	2	406552	1.00E+00	1
ENO2	2704950	211	47	1121400	319332	0	2	2	0	0	2	319332	1.00E+00	1
BTN3A2	378286	330	51	864012	243504	0	2	3	0	0	3	243504	1.00E+00	1
CLTCL1	488349	222	11	4258472	1138132	0	7	6	0	9	19	3907812	1.00E+00	1
DAXX	1456953	262	30	1898192	569956	0	6	6	0	2	9	980068	1.00E+00	1
CDK18	679311	268	34	1312572	369528	0	2	6	0	2	9	787116	1.00E+00	1
OR2J2	162844	808	12	766112	237808	0	3	4	0	0	4	237808	1.00E+00	1
ERAP2	393863	469	58	2499832	651836	0	2	3	0	3	8	1797088	1.00E+00	1
IGFN1	572258	333	23	9173764	2808128	0	3	3	0	23	20	11862632	1.00E+00	1
ZNF41	791516	NaN	27	2023504	488432	0	2	2	0	2	4	853332	1.00E+00	1
C12orf57	2704950	211	47	324316	95408	0	1	3	0	0	3	95408	1.00E+00	1
ETV4	1093430	148	21	1257392	347812	0	4	4	0	0	4	347812	1.00E+00	1
LDLRAD3	240340	434	-10	886440	245640	0	2	3	0	0	3	245640	1.00E+00	1
AP1G1	460697	NaN	24	2156292	588468	0	6	4	0	1	6	825564	1.00E+00	1
IHH	823105	324	37	996444	334284	0	1	3	0	1	5	517980	1.00E+00	1
ADRM1	990360	252	9	1034892	311856	0	5	5	0	0	5	311856	1.00E+00	1
RPL3	932556	250	69	1052692	284444	0	1	4	0	1	4	503028	1.00E+00	1
RIMBP3	616215	NaN	23	3962992	1291568	0	2	2	0	4	7	2545044	1.00E+00	1
SYT5	1036976	266	-1	969388	304736	0	5	4	0	0	4	304736	1.00E+00	1
FICD	437636	399	40	1135284	343896	0	1	3	0	1	3	443220	1.00E+00	1
FAM84B	203378	592	-8	763264	237452	0	3	3	0	0	3	237452	1.00E+00	1
UMOD	243266	848	-5	1634752	463868	0	2	5	0	1	6	841228	1.00E+00	1
C1orf21	258985	642	-7	337132	70844	0	2	1	0	0	1	70844	1.00E+00	1
BLOC1S2	1071291	198	56	383768	93628	0	3	2	0	0	2	93628	1.00E+00	1
UBL3	322680	335	21	316840	78320	0	1	1	0	0	1	78320	1.00E+00	1
IL4I1	1676173	205	41	1484876	453544	0	5	4	0	0	4	453544	1.00E+00	1
RAP2A	343135	533	28	466360	127448	0	2	2	0	0	2	127448	1.00E+00	1
HIST1H1C	408919	685	43	519048	170880	0	6	5	0	0	5	170880	1.00E+00	1
CPSF1	2043455	200	36	3703468	1085444	0	6	5	0	2	13	2164836	1.00E+00	1

OTOP2	1045089	223	25	1395164	434320	0	5	4	0	0	4	434320	1.00E+00	1
LRRC30	38085	336	-37	737276	229264	0	1	3	0	1	7	534356	1.00E+00	1
TTC32	479338	390	9	398720	96832	0	1	1	0	0	1	96832	1.00E+00	1
ALDH3A1	550749	220	-13	1172308	325028	0	4	3	0	0	3	325028	1.00E+00	1
PCDHB8	915569	724	-11	1939844	627628	0	10	8	0	1	14	1258460	1.00E+00	1
DPAGT1	1390854	195	58	1036316	308296	0	6	5	0	0	5	308296	1.00E+00	1
FABP6	198270	448	38	484160	116056	0	1	1	0	0	1	116056	1.00E+00	1
AP1M2	2036812	226	23	1111432	296192	0	2	6	0	4	19	1627988	1.00E+00	1
ZNF512	1192074	306	43	1494488	383056	0	1	4	0	3	9	1304028	1.00E+00	1
HNRNPA0	427110	409	28	739056	241368	0	2	3	0	0	3	241368	1.00E+00	1
CENPA	1334693	378	40	356000	112852	0	1	2	0	0	2	112852	1.00E+00	1
KCNF1	668276	514	23	1217876	368104	0	5	4	0	0	4	368104	1.00E+00	1
VWA3B	361135	716	28	3372388	892136	0	13	6	0	0	6	892136	1.00E+00	1
STK40	636943	212	28	1120688	318976	0	1	2	0	1	3	412604	1.00E+00	1
DEFA5	124400	715	-3	236384	74404	0	1	2	0	0	2	74404	1.00E+00	1
RPL24	548150	419	13	421148	110716	0	2	2	0	0	2	110716	1.00E+00	1
HTR1A	3636	1003	-20	1021720	332504	0	6	5	0	0	5	332504	1.00E+00	1
FAM53A	902379	350	33	971524	319688	0	2	3	0	0	3	319688	1.00E+00	1
HCK	1039298	386	21	1384840	363476	0	5	4	0	0	4	363476	1.00E+00	1
APC2	2352825	221	38	5475280	1966544	0	3	4	0	17	18	6317576	1.00E+00	1
GPRC5D	710389	169	14	859740	255252	0	1	2	0	0	2	255252	1.00E+00	1
ENPP2	255492	552	7	2512292	615880	0	8	7	0	1	7	686012	1.00E+00	1
AADAT	132912	831	-13	1133860	286580	0	2	2	0	0	2	286580	1.00E+00	1
ZBTB6	407115	430	26	1086512	279460	0	2	2	0	0	2	279460	1.00E+00	1
ATXN10	498533	172	15	1235320	336776	0	1	3	0	0	3	336776	1.00E+00	1
RDM1	703538	424	31	808120	208616	0	1	2	0	1	3	307584	1.00E+00	1
C1orf216	1177566	325	35	571024	170168	0	1	2	0	0	2	170168	1.00E+00	1
DFNA5	254601	464	21	1263088	367748	0	5	4	0	0	4	367748	1.00E+00	1
ST6GAL1	447917	433	32	1050912	274476	0	3	5	0	2	5	402992	1.00E+00	1
PIWIL4	367057	478	6	2229272	584908	0	4	4	0	0	4	584908	1.00E+00	1
LCA5L	339359	346	25	1743332	438592	0	2	2	0	0	2	438592	1.00E+00	1
MEIG1	256253	571	35	241012	52688	0	1	1	0	0	1	52688	1.00E+00	1
CAPS	1166723	392	36	487008	143112	0	1	2	0	0	2	143112	1.00E+00	1

HTT	661693	464	41	8035276	2316848	0	13	10	0	1	11	2731588	1.00E+00	1
TMEM208	1109491	150	46	451764	127092	0	1	2	0	0	2	127092	1.00E+00	1
WNT9B	526881	366	16	881100	280884	0	2	5	0	1	7	468140	1.00E+00	1
KCNA4	30326	940	-38	1628700	466716	0	6	4	0	1	8	771808	1.00E+00	1
PIP4K2A	196977	577	25	1072628	269848	0	4	3	0	0	3	269848	1.00E+00	1
GPN2	1025094	188	34	781064	232468	0	4	4	0	0	4	232468	1.00E+00	1
HSD17B6	2637730	154	47	804204	231756	0	1	2	0	0	2	231756	1.00E+00	1
CACNA1C	457513	765	26	6085820	1721260	0	10	8	0	5	10	2550384	1.00E+00	1
DAGLA	1235604	208	58	2607700	815240	0	4	6	0	1	6	873980	1.00E+00	1
AHNAK	2746294	204	49	14780764	4226432	0	47	21	0	3	22	4867232	1.00E+00	1
BCL11B	316817	629	-40	2193672	686724	0	3	5	0	4	18	2486304	1.00E+00	1
ANKRD11	1263742	322	40	6702056	1880392	0	21	11	0	0	11	1880392	1.00E+00	1
HSD11B1L	879991	295	21	920972	299752	0	1	4	0	2	5	463512	1.00E+00	1
HN1L	1591571	320	37	490568	140620	0	1	2	0	0	2	140620	1.00E+00	1
IGFL1	1150094	196	22	297260	73336	0	2	2	0	0	2	73336	1.00E+00	1
AGR2	361606	385	-7	474192	119616	0	2	3	0	0	3	119616	1.00E+00	1
CENPO	299133	374	39	775012	219296	0	1	4	0	1	4	327876	1.00E+00	1
MMP7	524316	359	17	694912	187256	0	1	2	0	0	2	187256	1.00E+00	1
FCGBP	1312608	522	29	13328640	4143840	0	24	17	0	45	71	21546188	1.00E+00	1
MYO1E	773032	386	43	2935932	732648	0	7	7	0	1	7	1076544	1.00E+00	1
TTC23L	245995	558	-4	955860	246708	0	1	5	0	1	5	445712	1.00E+00	1
TRAFD1	730140	239	40	1510864	404060	0	3	3	0	0	3	404060	1.00E+00	1
C1QTNF1	592461	285	15	720188	196156	0	5	4	0	0	4	196156	1.00E+00	1
ZNF285	715346	528	-44	1537208	369172	0	5	4	0	0	4	369172	1.00E+00	1
LRRC52	262572	574	-22	777860	234604	0	1	6	0	1	7	468852	1.00E+00	1
PRKCD	845193	192	46	1794596	447136	0	6	6	0	2	8	786048	1.00E+00	1
SLC26A6	1386275	212	37	1958712	602352	0	3	5	0	0	5	602352	1.00E+00	1
RSPH6A	906385	203	41	1812040	509792	0	7	5	0	0	5	509792	1.00E+00	1
PYHIN1	97254	914	6	1297264	347456	0	6	4	0	0	4	347456	1.00E+00	1
MSH5	2509866	183	32	2204352	631188	0	2	4	0	1	4	762196	1.00E+00	1
STAC	151436	827	-17	1065508	270560	0	4	3	0	0	3	270560	1.00E+00	1
BCAR3	595695	173	9	2093992	599504	0	4	7	0	1	7	759704	1.00E+00	1
NKRD20A	NaN	NaN	NaN	4340352	1059456	0	1	1	0	7	15	4747616	1.00E+00	1

STX11	298896	322	23	724104	202920	0	2	3	0	0	3	202920	1.00E+00	1
CHST15	149497	632	19	1448920	377360	0	6	4	0	0	4	377360	1.00E+00	1
SP7	1701810	166	58	1045216	347456	0	2	4	0	0	4	347456	1.00E+00	1
KCNJ9	829863	386	17	962980	303668	0	1	3	0	0	3	303668	1.00E+00	1
PLSCR5	124275	768	-20	710220	191172	0	1	2	0	0	2	191172	1.00E+00	1
PCDHA1	861651	606	2	2331444	758280	0	9	11	0	1	17	1530088	1.00E+00	1
MRPL41	1315685	404	41	346032	103596	0	1	3	0	0	3	103596	1.00E+00	1
GML	447193	438	36	413672	108580	0	1	2	0	0	2	108580	1.00E+00	1
GALP	500375	480	-61	322180	74048	0	2	2	0	0	2	74048	1.00E+00	1
ACOX1	1667990	194	22	1855116	498756	0	4	6	0	0	6	498756	1.00E+00	1
PYGL	388112	431	28	2215032	583128	0	1	2	0	0	2	583128	1.00E+00	1
MRTO4	788190	448	30	641512	157352	0	1	2	0	0	2	157352	1.00E+00	1
[illegible]AN-P2RY	1675365	272	15	1058388	296904	0	1	4	0	0	4	296904	1.00E+00	1
ZNF320	557083	1008	-39	1322540	324316	0	4	7	0	1	10	738344	1.00E+00	1
LHX1	625114	462	21	1026704	296548	0	4	4	0	0	4	296548	1.00E+00	1
NIPSNAP1	713942	190	39	758280	195444	0	2	4	0	0	4	195444	1.00E+00	1
HPR	426658	372	14	895340	242080	0	2	3	0	0	3	242080	1.00E+00	1
NCKAP1L	643138	151	35	2950884	793524	0	8	7	0	0	7	793524	1.00E+00	1
NFATC2	363545	482	28	2400508	735140	0	7	9	0	0	9	735140	1.00E+00	1
SLC28A1	355337	291	25	1723040	505876	0	1	3	0	0	3	505876	1.00E+00	1
ACR	669934	295	41	1044504	328944	0	1	3	0	0	3	328944	1.00E+00	1
CLEC18B	475713	278	44	1192956	319332	0	2	3	0	0	3	319332	1.00E+00	1
PCBP3	724898	611	38	973304	274120	0	1	3	0	0	3	274120	1.00E+00	1
RTP2	88374	374	24	574228	154148	0	2	3	0	0	3	154148	1.00E+00	1
MYADM	849708	486	-50	766468	272696	0	1	4	0	0	4	272696	1.00E+00	1
AKAP7	165730	682	15	979000	228908	0	1	2	0	0	2	228908	1.00E+00	1
MAP3K10	980250	400	23	2321832	776436	0	3	5	0	1	7	942688	1.00E+00	1
ZNF135	1187584	694	-42	1851912	476328	0	1	5	0	2	10	1277684	1.00E+00	1
TNNC2	742720	180	48	444288	95052	0	2	2	0	0	2	95052	1.00E+00	1
ZNF12	721477	506	39	1823076	430404	0	6	6	0	0	6	430404	1.00E+00	1
APBA2	161108	927	-21	1933792	520472	0	8	7	0	0	7	520472	1.00E+00	1
C8orf59	99038	1463	13	269492	62656	0	1	2	0	0	2	62656	1.00E+00	1
SHCBP1	422519	419	-38	1761844	447848	0	5	5	0	0	5	447848	1.00E+00	1

USP35	549791	208	26	2516564	791032	0	4	8	0	3	9	1278040	1.00E+00	1
ACBD7	254900	620	21	242792	57316	0	3	3	0	0	3	57316	1.00E+00	1
NPY2R	49938	944	-5	945536	282664	0	2	4	0	0	4	282664	1.00E+00	1
TMOD2	443665	165	27	927736	238520	0	2	3	0	0	3	238520	1.00E+00	1
NSMCE2	328239	420	38	663940	156284	0	1	2	0	0	2	156284	1.00E+00	1
KCNH1	153525	862	-7	2508376	706304	0	2	7	0	2	7	1133148	1.00E+00	1
KCNG4	582429	364	12	1274124	398364	0	2	4	0	0	4	398364	1.00E+00	1
CCDC40	663665	237	27	2946612	798864	0	7	7	0	0	7	798864	1.00E+00	1
HEXDC	2591323	179	28	1467788	461020	0	4	5	0	0	5	461020	1.00E+00	1
PUS1	383304	269	30	1074408	318264	0	2	4	0	0	4	318264	1.00E+00	1
C19orf40	481910	448	-16	549308	159844	0	1	3	0	0	3	159844	1.00E+00	1
NAT9	983414	484	17	551444	140620	0	2	3	0	0	3	140620	1.00E+00	1
DERA	328516	534	10	826632	229620	0	1	3	0	0	3	229620	1.00E+00	1
PRR11	834161	507	4	927736	267356	0	3	4	0	0	4	267356	1.00E+00	1
ERCC3	428616	175	23	2033116	537560	0	7	5	0	0	5	537560	1.00E+00	1
SLC45A1	971531	321	25	1839452	590248	0	7	9	0	0	9	590248	1.00E+00	1
CETP	842366	125	52	1294416	354576	0	2	5	0	0	5	354576	1.00E+00	1
RPS20	344871	489	59	397652	108580	0	1	3	0	0	3	108580	1.00E+00	1
QARS	1898051	203	36	2020300	568532	0	5	5	0	0	5	568532	1.00E+00	1
QKI	81134	1131	1	965472	264864	0	1	3	0	0	3	264864	1.00E+00	1
RBP4	450249	305	20	531508	137060	0	3	4	0	0	4	137060	1.00E+00	1
MYH13	83322	731	-47	5103972	1277328	0	14	9	0	0	9	1277328	1.00E+00	1
GPR173	878367	NaN	63	914564	288004	0	2	5	0	0	5	288004	1.00E+00	1
GMPR	117199	561	26	891068	251692	0	2	4	0	0	4	251692	1.00E+00	1
34GALNT	1329939	344	52	1319336	433252	0	1	4	0	0	4	433252	1.00E+00	1
STMN2	147465	518	-12	476684	119260	0	1	3	0	0	3	119260	1.00E+00	1
SNRPD2	950605	194	45	310432	81524	0	1	3	0	0	3	81524	1.00E+00	1
ITGAX	1122895	481	-2	2995740	859740	0	2	4	0	0	4	859740	1.00E+00	1
SNX18	68402	643	7	1767540	521184	0	3	5	0	0	5	521184	1.00E+00	1
LPAR6	NaN	NaN	NaN	869708	235672	0	6	9	0	0	9	235672	1.00E+00	1
SLC2A1	918944	334	35	1230336	387684	0	2	5	0	0	5	387684	1.00E+00	1
SH3GL1	2100413	216	32	960132	260592	0	5	6	0	0	6	260592	1.00E+00	1
LRRC37A	502280	340	12	4256692	1253120	0	1	4	0	3	14	3471000	1.00E+00	1

KHNYN	897668	265	29	1684948	520472	0	2	5	0	0	5	520472	1.00E+00	1
RASGRP4	1459788	221	4	1715920	511928	0	1	4	0	0	4	511928	1.00E+00	1
ATP2B2	673500	458	50	3177656	897832	0	9	8	0	0	8	897832	1.00E+00	1
GRB2	1869080	157	24	574584	140976	0	1	3	0	0	3	140976	1.00E+00	1
PKD1	1696923	224	41	10435072	3547184	0	7	11	0	15	40	10600968	1.00E+00	1
C5AR1	1132395	232	41	844788	285156	0	3	5	0	0	5	285156	1.00E+00	1
ZNF425	605000	242	27	1929164	496264	0	10	8	0	0	8	496264	1.00E+00	1
CD40	559725	526	55	767536	159488	0	2	3	0	0	3	159488	1.00E+00	1
DCST1	1696720	214	44	1814532	521184	0	5	7	0	0	7	521184	1.00E+00	1
DNAH2	2107779	232	25	11396628	3151668	0	18	11	0	7	23	5245304	1.00E+00	1
SSTR1	49574	544	-46	950876	305092	0	3	7	0	0	7	305092	1.00E+00	1
THPO	1447013	268	44	1007480	143824	0	4	2	0	0	2	143824	1.00E+00	1
PRKCA	206987	603	-27	1787476	439304	0	3	5	0	0	5	439304	1.00E+00	1
GSTA5	572117	455	9	593096	147028	0	1	4	0	0	4	147028	1.00E+00	1
RGS9	391807	516	-16	1820584	475616	0	5	8	0	0	8	475616	1.00E+00	1
MYO18A	1560056	159	33	5276276	1483096	0	7	12	0	2	15	2152020	1.00E+00	1
PILRB	1430637	204	54	665720	81880	0	2	2	0	0	2	81880	1.00E+00	1
CAMK2G	998685	191	42	1568892	412248	0	2	6	0	1	7	497332	1.00E+00	1
CFL1	3779084	287	42	433964	113920	0	1	5	0	0	5	113920	1.00E+00	1
ADAM11	1224408	175	31	2008552	569600	0	2	10	0	1	11	867572	1.00E+00	1
SLC1A4	653388	340	36	1301180	436456	0	1	7	0	0	7	436456	1.00E+00	1
KCNH2	775233	547	25	3161992	1019228	0	4	9	0	0	9	1019228	1.00E+00	1
LRRIQ4	404285	689	13	1410828	403704	0	1	6	0	0	6	403704	1.00E+00	1
FZD1	64493	946	-2	1573520	502672	0	2	9	0	0	9	502672	1.00E+00	1
PCDHAC2	931701	643	-10	2520124	814172	0	4	11	0	0	11	814172	1.00E+00	1
CACNA1S	522609	490	31	4846940	1341052	0	12	16	0	0	16	1341052	1.00E+00	1
A2BP1	2474	1138	-47	1215384	321468	0	0	0	0	28	48	14145304	1	1
A2LD1	NaN	NaN	NaN	370952	126736	0	0	0	0	50	119	40864172	1	1
A4GALT	808038	168	72	861164	277324	0	0	1	0	47	52	18983344	1	1
AARSD1	1507064	201	26	1573876	423284	0	0	0	0	22	18	8144924	1	1
AASDHPPT	86519	753	-16	807408	207192	0	0	0	0	50	81	23487456	1	1
ABAT	402778	358	-10	1324320	344964	0	0	0	0	16	12	6492016	1	1
ABCD4	652027	358	47	1580284	441440	0	0	2	0	50	51	20215460	1	1

ABCF2	610106	290	32	1660028	440728	0	0	1	0	50	52	23170260	1	1
ABHD14A	1045639	224	36	660380	228196	0	0	0	0	50	51	17801068	1	1
ABHD5	103561	437	33	898900	248132	0	0	1	0	50	68	24633420	1	1
ABP1	763140	604	19	1864728	563904	0	0	0	0	0	0	563904	1	1
ABTB1	679358	336	45	1227844	356000	0	0	1	0	50	52	21051348	1	1
ACAP1	1911735	206	36	1900684	565328	0	0	2	0	50	69	22072000	1	1
ACCN1	34504	1007	-15	1885020	555360	0	0	0	0	4	2	1396232	1	1
ACCN2	1003045	211	56	1487368	401924	0	0	0	0	1	2	778928	1	1
ACCN3	622098	373	41	1469212	402992	0	0	0	0	2	2	1758996	1	1
ACCN4	554323	403	33	1653620	521896	0	0	0	0	26	23	10254224	1	1
ACCN5	43787	643	-2	1329304	332504	0	0	0	0	45	57	18464296	1	1
ACN9	146007	987	-9	321824	86152	0	0	0	0	50	40	20438316	1	1
ACOT1	NaN	NaN	NaN	1022788	337844	0	0	0	0	50	116	39983428	1	1
ACOT13	421216	362	24	367392	107868	0	0	1	0	0	1	107868	1	1
ACP6	NaN	NaN	NaN	1112144	302956	0	0	1	0	50	117	39948540	1	1
ACPL2	333903	352	22	1225708	336776	0	0	0	0	33	29	13439356	1	1
ACY1	1045639	224	36	1071560	298684	0	0	0	0	50	51	17801068	1	1
ACYP1	919043	222	47	336064	81524	0	0	0	0	50	76	25981236	1	1
ACYP2	619188	448	11	265932	67284	0	0	0	0	50	61	19308728	1	1
ADAT3	NaN	NaN	NaN	828056	299752	0	0	0	0	50	116	39945336	1	1
ADC	756118	264	21	1174088	341404	0	0	0	0	31	33	11578544	1	1
ADCYAP1	272034	531	-13	447492	137772	0	0	0	0	50	63	21974100	1	1
ADI1	254699	390	16	473480	118192	0	0	0	0	50	49	21518776	1	1
ADIG	525955	539	39	215024	50908	0	0	0	0	50	78	25602808	1	1
ADM2	1075525	207	42	353864	127804	0	0	1	0	11	15	4417248	1	1
ADO	176901	852	32	650768	217516	0	0	1	0	43	60	20312292	1	1
ADPRHL2	853740	282	27	912428	277324	0	0	2	0	14	17	5956948	1	1
ADRB3	672861	356	25	950520	364188	0	0	0	0	34	29	15405544	1	1
AG2	NaN	NaN	NaN	300464	93628	0	0	0	0	50	116	39739212	1	1
AGAP11	401785	164	45	1384840	397652	0	0	0	0	18	9	5793188	1	1
AGAP7	NaN	NaN	NaN	1680676	472412	0	0	0	0	50	116	40117996	1	1
AGAP8	NaN	NaN	NaN	1683524	469564	0	0	0	0	50	116	40115148	1	1
AGBL3	NaN	NaN	NaN	2420088	599148	0	0	0	0	7	14	4287308	1	1

AGER	2119974	392	28	1099684	273764	0	0	2	0	27	33	11704212	1	1
AGPHD1	506023	375	39	966896	253828	0	0	0	0	50	65	19264228	1	1
AGXT2L1	104349	499	21	1323252	334284	0	0	0	0	50	39	19491000	1	1
AGXT2L2	672705	448	46	1168748	327520	0	0	0	0	50	73	25444744	1	1
AHCY	962066	151	50	1116772	309008	0	0	1	0	50	68	22738432	1	1
AHSP	826452	688	-17	266644	71912	0	0	1	0	5	18	2545756	1	1
AIF1	2517285	220	35	565328	148096	0	0	0	0	50	55	21824936	1	1
AIF1L	829407	293	36	480956	111784	0	0	1	0	48	83	24890096	1	1
AIG1	298429	699	13	623356	165896	0	0	1	0	50	59	19534432	1	1
AK2	807536	233	27	627272	174796	0	0	3	0	4	10	1786052	1	1
AK3	231882	608	30	575296	172304	0	0	0	0	11	13	3441452	1	1
AK3L1	280010	351	-9	578144	156640	0	0	0	0	50	40	21132516	1	1
AKAP2	NaN	NaN	NaN	2354584	700964	0	0	0	0	7	14	4389124	1	1
AKD1	345757	686	21	5087952	1219656	0	0	0	0	0	0	1219656	1	1
ALDH3A2	544087	332	-2	1325744	365968	0	0	1	0	50	67	23348260	1	1
ALDH6A1	495634	236	40	1370956	395516	0	0	1	0	50	35	16762616	1	1
ALG14	275272	425	-9	543256	166964	0	0	0	0	50	42	21086948	1	1
ALG3	1460188	257	49	1148812	351728	0	0	0	0	14	11	5039180	1	1
ALG8	551110	208	23	1414744	394448	0	0	1	0	28	36	12193000	1	1
ALKBH2	595266	487	20	658600	194732	0	0	1	0	50	72	19690360	1	1
ALKBH4	611052	174	63	740480	241012	0	0	1	0	50	68	17004696	1	1
ALKBH7	943949	407	31	539340	186900	0	0	1	0	50	47	18083732	1	1
ALOX12	1645511	360	14	1700612	484516	0	0	1	0	25	37	12739104	1	1
ALOX5AP	381672	451	76	422216	116056	0	0	0	0	50	60	18444360	1	1
ALPPL2	307666	204	37	1337848	414740	0	0	1	0	20	21	6327188	1	1
ALS2CR4	435399	534	25	1080816	302244	0	0	0	0	13	19	4474564	1	1
ALS2CR8	543912	392	33	1877900	508012	0	0	0	0	44	43	19528380	1	1
ALX3	571576	400	29	841228	275900	0	0	0	0	49	55	19542620	1	1
AMAC1	753178	505	16	793168	295124	0	0	0	0	33	33	12656868	1	1
AMAC1L2	499514	671	36	791744	296548	0	0	0	0	50	54	19916064	1	1
AMAC1L3	NaN	NaN	NaN	799220	293344	0	0	0	0	50	116	39938928	1	1
AMBP	244063	558	0	923464	245996	0	0	3	0	2	5	977220	1	1
AMD1	321448	282	31	890712	216804	0	0	0	0	50	66	20005064	1	1

AMELY	0	0	0	497688	142044	0	0	1	0	4	1	883592	1	1
AMY1B	NaN	NaN	NaN	4035972	1021008	0	0	1	0	7	15	4709168	1	1
AMY1C	24508	1029	-14	4035972	1021008	0	0	1	0	2	3	1819516	1	1
ANAPC10	182708	1139	20	489144	123888	0	0	0	0	50	43	21505960	1	1
ANAPC11	3798594	211	19	530796	113208	0	0	0	0	34	28	11428668	1	1
ANAPC13	254024	188	16	201496	47348	0	0	0	0	50	41	17953436	1	1
ANG	NaN	NaN	NaN	368104	110360	0	0	0	0	50	116	39755944	1	1
ANGPTL7	NaN	NaN	NaN	899968	228908	0	0	2	0	26	42	11806028	1	1
ANKRD18A	NaN	NaN	NaN	2616956	630832	0	0	0	0	7	14	4318992	1	1
ANKRD20A	NaN	NaN	NaN	6509816	1589896	0	0	0	0	0	0	1589896	1	1
ANKRD22	175425	289	26	503028	133500	0	0	0	0	50	42	20400580	1	1
ANKRD31	NaN	NaN	NaN	4887168	1219656	0	0	0	0	0	0	1219656	1	1
ANKRD33B	NaN	NaN	NaN	1200432	398364	0	0	0	0	50	116	40043948	1	1
ANKRD34C	NaN	NaN	NaN	1315064	406552	0	0	0	0	50	116	40052136	1	1
ANKRD37	173409	422	20	416876	112852	0	0	1	0	4	6	1787120	1	1
ANKRD43	718028	356	58	1268072	494128	0	0	0	0	32	24	12039208	1	1
ANKRD5	194301	442	-34	1984344	543612	0	0	0	0	11	15	4131736	1	1
ANKRD56	411278	462	26	1909228	634748	0	0	0	0	6	7	2876480	1	1
ANKRD57	191347	329	22	1260596	424708	0	0	0	0	5	6	2713432	1	1
ANKRD58	649508	NaN	73	744396	270204	0	0	0	0	1	3	517624	1	1
ANKRD9	1673146	191	42	737632	287648	0	0	0	0	45	80	22534444	1	1
ANP32C	NaN	NaN	NaN	600216	151656	0	0	0	0	50	116	39797240	1	1
ANTXR1	338803	288	14	1531512	429336	0	0	1	0	50	41	20521264	1	1
ANUBL1	111914	603	36	1852268	514420	0	0	0	0	6	3	1678184	1	1
ANXA8L1	NaN	NaN	NaN	1766472	429336	0	0	2	0	50	118	40074920	1	1
ANXA8L2	NaN	NaN	NaN	883236	214668	0	0	0	0	50	116	39860252	1	1
AP1AR	181002	351	22	814172	195088	0	0	0	0	50	52	21104748	1	1
AP1S1	1044715	199	39	420792	105732	0	0	0	0	50	49	18578928	1	1
AP1S3	263604	197	16	416520	97188	0	0	0	0	50	41	18108652	1	1
AP4S1	432061	238	23	542544	131364	0	0	1	0	21	16	5943064	1	1
APH1B	450620	272	49	657532	192596	0	0	1	0	50	54	21410908	1	1
APITD1	771199	383	28	559988	138484	0	0	0	0	50	65	24479272	1	1
APLN	340263	NaN	46	195088	63368	0	0	0	0	50	58	20655476	1	1

APOB48R	NaN	NaN	NaN	2678188	830192	0	0	0	0	7	14	4518352	1	1
APOBEC3A	1413709	372	44	521896	135992	0	0	3	0	0	3	135992	1	1
APOD	794351	315	38	490924	134924	0	0	0	0	50	61	22903972	1	1
APOL4	1233084	331	28	948740	232468	0	0	2	0	8	10	4440032	1	1
APOL6	936853	205	-13	857960	252760	0	0	1	0	50	64	17894340	1	1
AQP12A	1077835	446	25	717696	245640	0	0	0	0	50	79	25908612	1	1
AQP12B	1077835	446	25	738700	258812	0	0	0	0	50	79	25908612	1	1
AQPEP	372067	763	17	2569964	686368	0	0	0	0	8	4	3405852	1	1
ARC	300961	567	26	976508	295480	0	0	2	0	1	3	721256	1	1
ARF1	702621	391	5	473480	126736	0	0	0	0	50	46	17014308	1	1
ARF6	680412	174	41	434676	129228	0	0	1	0	17	21	6831284	1	1
ARFIP2	424988	469	20	868996	256676	0	0	0	0	31	40	13844484	1	1
ARGLU1	169398	1237	11	694200	196512	0	0	0	0	50	61	19830980	1	1
ARHGAP1	939971	176	44	1152016	309008	0	0	3	0	5	3	1077256	1	1
ARHGAP2[illegible]	NaN	NaN	NaN	3439316	1129588	0	0	0	0	0	0	1129588	1	1
ARHGAP4[illegible]	NaN	NaN	NaN	1714496	510148	0	0	0	0	9	16	4867588	1	1
ARHGAP4[illegible]	NaN	NaN	NaN	2313644	590248	0	0	0	0	7	14	4278408	1	1
ARHGAP8	NaN	NaN	NaN	1255612	359204	0	0	0	0	50	116	40004788	1	1
ARHGDIB	360900	426	8	533644	134924	0	0	1	0	50	56	20049920	1	1
ARHGDIG	1340678	205	43	571736	175864	0	0	0	0	50	57	21858756	1	1
ARHGEF3[illegible]	NaN	NaN	NaN	2246004	610896	0	0	0	0	7	14	4299056	1	1
ARHGEF3[illegible]	233591	486	15	578144	139552	0	0	1	0	39	36	12641560	1	1
ARL1	283715	649	2	482736	121752	0	0	0	0	50	47	18604916	1	1
ARL10	563059	347	37	605200	194732	0	0	0	0	50	66	22259256	1	1
ARL11	425192	296	62	482380	153080	0	0	1	0	50	48	18413032	1	1
ARL17A	NaN	NaN	NaN	1138132	303668	0	0	0	0	50	116	39949252	1	1
ARL17B	372453	231	22	569244	151656	0	0	0	0	50	51	21352880	1	1
ARL2	3151458	209	52	474548	137416	0	0	0	0	50	83	23753744	1	1
ARL5A	269131	610	15	479176	118904	0	0	0	0	50	61	21553664	1	1
ARL5C	NaN	NaN	NaN	475616	121396	0	0	0	0	50	116	39766980	1	1
ARMC1	145894	455	4	731224	201140	0	0	1	0	50	50	17452900	1	1
ARMS2	252955	279	5	265932	86508	0	0	0	0	50	51	21633052	1	1
ARPC3	964525	206	50	490212	111072	0	0	0	0	50	64	19341480	1	1

ARPC5L	1125524	207	41	394448	113920	0	0	0	0	50	59	18818160	1	1
ARPM1	397180	820	-9	933788	265576	0	0	0	0	9	6	3514076	1	1
ART4	340728	578	-4	798152	221788	0	0	1	0	50	47	15743388	1	1
ART5	537999	403	23	730156	222500	0	0	0	0	50	51	22444732	1	1
ASAH2B	131929	508	36	435744	119616	0	0	0	0	50	65	20578224	1	1
ASAM	713809	216	13	940552	283376	0	0	0	0	50	65	24429788	1	1
ASB10	611046	337	38	1350664	461732	0	0	4	0	21	35	9435068	1	1
ASB12	57924	NaN	2	761840	235672	0	0	1	0	50	63	26324776	1	1
ASB3	NaN	NaN	NaN	1352444	360628	0	0	0	0	50	116	40006212	1	1
ASB9	204248	NaN	17	789608	214312	0	0	1	0	50	70	21277052	1	1
ASCL2	NaN	NaN	NaN	448916	176932	0	0	0	0	50	116	39822516	1	1
ASCL4	119393	805	22	415096	142400	0	0	1	0	16	14	6831284	1	1
ASF1B	1034381	321	36	514776	148452	0	0	0	0	50	92	22111516	1	1
ASIP	962066	151	50	335352	101460	0	0	0	0	50	68	22738432	1	1
ASPDH	762018	517	35	735140	238876	0	0	0	0	50	59	22902548	1	1
ATAD3A	1714469	248	27	1624784	475972	0	0	3	0	1	4	970456	1	1
ATF3	418310	224	22	520116	142044	0	0	0	0	50	47	18982276	1	1
ATF5	1679101	205	45	671060	239944	0	0	0	0	50	76	20785416	1	1
ATG12	384075	759	1	359204	105376	0	0	0	0	50	61	23231848	1	1
ATG4A	201673	NaN	25	1072984	256676	0	0	1	0	50	59	19172380	1	1
ATP1B3	467997	345	28	734428	188324	0	0	2	0	3	4	937704	1	1
ATP5D	2214581	234	29	417944	142756	0	0	0	0	50	68	20485664	1	1
ATP5J2	1327454	288	60	284088	64080	0	0	0	0	50	58	17222924	1	1
ATP5L	963668	192	48	264152	77608	0	0	0	0	50	58	20862668	1	1
ATP5L2	NaN	NaN	NaN	244216	79388	0	0	0	0	50	116	39724972	1	1
ATP6V0C	2085452	321	49	380564	127804	0	0	1	0	50	76	21050280	1	1
ATP6V0D1	1099149	153	45	915988	243860	0	0	1	0	50	49	19815672	1	1
ATP6V1F	845100	361	49	301888	89000	0	0	0	0	50	80	25321924	1	1
ATP6V1G1	288977	221	10	312212	77608	0	0	1	0	1	2	311856	1	1
ATP6V1G[illegible]	105821	404	-16	378072	69420	0	0	0	0	50	47	20170248	1	1
ATPBD4	189209	811	-14	877540	218228	0	0	0	0	50	84	23079836	1	1
ATPIF1	779314	213	33	316840	80456	0	0	0	0	50	49	18363192	1	1
ATXN1L	NaN	NaN	NaN	1657180	553580	0	0	0	0	9	16	4911020	1	1

ATXN7L3I	NaN	NaN	NaN	244216	69776	0	0	0	0	50	116	39715360	1	1
AVEN	409752	499	0	911360	275188	0	0	2	0	50	63	22123976	1	1
AVP	835590	288	54	414384	127092	0	0	0	0	50	59	17488856	1	1
AVPI1	514755	143	54	371664	111072	0	0	0	0	50	73	19766188	1	1
AZI1	1748003	243	28	2757932	812392	0	0	0	0	0	0	812392	1	1
B3GALNT	340975	583	-20	848348	219652	0	0	1	0	50	76	22958440	1	1
B3GALT6	2356409	196	27	776436	283020	0	0	0	0	50	61	26748416	1	1
B3GALTL	183118	606	27	1325744	332860	0	0	0	0	24	22	11398052	1	1
B3GAT1	270422	686	5	822360	272340	0	0	2	0	12	19	5967628	1	1
B3GNT1	1963418	182	55	1015668	321468	0	0	0	0	44	48	19173092	1	1
B4GALT2	875223	214	39	935568	285156	0	0	1	0	50	67	19898264	1	1
B4GALT5	1040657	132	55	1011752	268780	0	0	1	0	50	63	21322264	1	1
BAALC	461588	584	15	371308	105020	0	0	0	0	50	64	19808908	1	1
BACE1	436617	360	36	1267716	374868	0	0	2	0	50	59	19286656	1	1
BAD	1613996	257	65	491636	139552	0	0	0	0	50	66	21324400	1	1
BAGE	NaN	NaN	NaN	120684	32040	0	0	0	0	50	116	39677624	1	1
BAGE2	NaN	NaN	NaN	302244	86508	0	0	0	0	50	116	39732092	1	1
BAGE3	NaN	NaN	NaN	302244	86508	0	0	0	0	50	116	39732092	1	1
BAGE4	NaN	NaN	NaN	120684	32040	0	0	0	0	50	116	39677624	1	1
BAGE5	30235	1262	-79	120684	32040	0	0	0	0	50	73	13278800	1	1
BAI1	296361	567	25	3971536	1230692	0	0	0	0	1	1	1656468	1	1
BAI2	1169755	173	36	3979368	1234608	0	0	0	0	0	0	1234608	1	1
BAI3	11277	1175	-22	3982928	1024924	0	0	0	0	2	1	1923468	1	1
BAIAP2L2	1528130	197	73	1351376	402280	0	0	0	0	9	10	3205424	1	1
BANF2	594840	432	1	262728	64080	0	0	0	0	50	44	17649768	1	1
BAT1	2515825	220	35	1123180	294056	0	0	0	0	50	55	21824936	1	1
BAT2	2517260	193	28	5260968	1781424	0	0	0	0	0	0	1781424	1	1
BAT2L1	651666	272	34	5625512	1649704	0	0	0	0	4	0	3784280	1	1
BAT2L2	406372	793	2	7013200	2041304	0	0	0	0	1	0	2228204	1	1
BAT3	2517236	193	20	2821656	913140	0	0	0	0	3	0	1778576	1	1
BAT4	2517236	193	20	872912	279460	0	0	0	0	50	63	27465044	1	1
BAT5	2517236	193	20	1591676	454612	0	0	0	0	6	2	2321832	1	1
BATF	691659	175	49	323604	88644	0	0	1	0	1	3	464224	1	1

BCAS4	421783	191	36	607692	173016	0	0	1	0	9	12	3967620	1	1
BCL2	207116	522	60	605912	202564	0	0	1	0	50	60	14793224	1	1
BCL2L2	1171456	330	20	472056	158064	0	0	0	0	50	49	21656192	1	1
BCL7C	1546514	207	48	545392	176576	0	0	0	0	50	59	22090512	1	1
BCMO1	302391	288	1	1424712	373800	0	0	0	0	50	57	27106196	1	1
BCORL2	NaN	NaN	NaN	460308	117480	0	0	0	0	50	116	39763064	1	1
BCS1L	1033606	269	43	1056608	321112	0	0	0	0	50	68	23381368	1	1
BDH1	685310	263	39	876472	251336	0	0	2	0	25	42	10709192	1	1
BEAN	NaN	NaN	NaN	644716	205412	0	0	0	0	50	116	39850996	1	1
BET1	467883	483	0	314704	79388	0	0	0	0	50	66	21215108	1	1
BET1L	1463610	437	24	402636	117480	0	0	0	0	50	72	18233964	1	1
BET3L	NaN	NaN	NaN	474904	127448	0	0	0	0	50	116	39773032	1	1
BEX2	455954	NaN	17	424708	99680	0	0	0	0	50	69	22928180	1	1
BEX4	466184	NaN	-5	313636	78320	0	0	1	0	2	6	1876476	1	1
BEX5	119562	NaN	22	289428	73692	0	0	0	0	50	49	18250696	1	1
BHLHA15	464632	375	26	456036	156996	0	0	0	0	50	53	18981920	1	1
BHLHA9	NaN	NaN	NaN	538984	216092	0	0	0	0	50	116	39861676	1	1
BHLHE23	821863	447	61	532220	194020	0	0	1	0	50	60	19521260	1	1
BIVM	195534	475	24	1328236	343184	0	0	0	0	50	40	16969452	1	1
BLCAP	790368	549	52	216448	65504	0	0	0	0	50	68	24056344	1	1
BLOC1S3	1230654	243	41	469208	185476	0	0	0	0	50	79	24609568	1	1
BNIP2	521844	172	33	1130656	306872	0	0	0	0	22	27	9079068	1	1
BOD1	255069	371	31	487720	152012	0	0	1	0	38	30	14418356	1	1
BOD1L	133545	511	27	7791416	2093992	0	0	0	0	2	0	3169468	1	1
BOK	1214632	426	30	521184	178356	0	0	3	0	1	4	606980	1	1
BOLA2	NaN	NaN	NaN	766112	235672	0	0	0	0	50	116	39881256	1	1
BOLA2B	1906000	253	52	766112	235672	0	0	0	0	50	58	18919264	1	1
BPHL	444075	435	41	749736	213600	0	0	0	0	50	79	25740224	1	1
BPIL1	488576	461	43	1154508	380208	0	0	0	0	26	30	11873312	1	1
BPIL2	371339	401	3	1321472	365968	0	0	0	0	26	36	12156688	1	1
BPIL3	515931	491	46	1175156	338200	0	0	0	0	37	51	18754436	1	1
BPY2	NaN	NaN	NaN	842652	237096	0	0	0	0	50	116	39882680	1	1
BPY2B	NaN	NaN	NaN	842652	237096	0	0	0	0	50	116	39882680	1	1

BPY2C	NaN	NaN	NaN	842652	237096	0	0	0	0	50	116	39882680	1	1
BRD3	495575	398	40	1850488	521540	0	0	4	0	18	38	8431860	1	1
BRI3	566928	294	41	359204	110716	0	0	0	0	50	37	18400216	1	1
BRMS1L	188271	400	10	880032	196512	0	0	0	0	50	66	22056692	1	1
BRP44	535270	290	14	340336	91136	0	0	0	0	50	57	21284172	1	1
BRP44L	199596	556	18	285512	78676	0	0	0	0	50	35	16755496	1	1
BSG	1647685	255	-8	1016024	293344	0	0	0	0	49	53	18640160	1	1
BSPH1	NaN	NaN	NaN	370952	78676	0	0	0	0	50	116	39724260	1	1
BST2	899127	211	26	462088	134924	0	0	0	0	50	49	18107584	1	1
BTBD12	745599	224	39	4550392	1384484	0	0	0	0	6	4	3766836	1	1
BTBD18	NaN	NaN	NaN	1749384	543612	0	0	0	0	9	16	4901052	1	1
BTBD19	NaN	NaN	NaN	723748	241724	0	0	0	0	50	116	39887308	1	1
BTBD6	927925	289	40	1204704	369528	0	0	1	0	50	58	26260696	1	1
BTD	433102	235	32	1375228	381632	0	0	1	0	50	48	19509512	1	1
C10orf10	NaN	NaN	NaN	515132	171592	0	0	1	0	50	117	39817176	1	1
C10orf105	NaN	NaN	NaN	325028	108580	0	0	0	0	50	116	39754164	1	1
C10orf107	79162	310	-21	563548	131720	0	0	0	0	50	50	18827772	1	1
C10orf111	254900	620	21	387684	116412	0	0	0	0	0	0	116412	1	1
C10orf114	133573	250	-10	326808	116412	0	0	0	0	50	60	25863756	1	1
C10orf116	401785	164	45	202208	53044	0	0	0	0	50	45	17084796	1	1
C10orf118	275620	477	24	2378792	565684	0	0	0	0	25	32	12235720	1	1
C10orf119	516746	281	42	1686016	438236	0	0	0	0	0	0	438236	1	1
C10orf120	321415	530	1	867216	222144	0	0	1	0	50	73	23964852	1	1
C10orf122	NaN	NaN	NaN	482024	129940	0	0	0	0	50	116	39775524	1	1
C10orf125	386585	472	13	409044	122820	0	0	0	0	50	46	19712788	1	1
C10orf129	321487	512	1	1245644	338200	0	0	0	0	10	10	3674988	1	1
C10orf131	NaN	NaN	NaN	483092	109648	0	0	0	0	50	116	39755232	1	1
C10orf137	216532	351	-6	3151312	807764	0	0	0	0	4	3	2596308	1	1
C10orf140	128481	409	22	2226780	685656	0	0	0	0	21	17	9633716	1	1
C10orf18	679964	303	35	6139220	1726600	0	0	0	0	0	0	1726600	1	1
C10orf25	75634	905	25	310076	88288	0	0	0	0	50	52	22092648	1	1
C10orf26	883280	188	44	938060	274120	0	0	0	0	50	54	19097264	1	1
C10orf27	521475	579	30	903528	258456	0	0	0	0	33	39	13441492	1	1

C10orf28	247144	581	10	1991820	529728	0	0	0	0	44	45	19335784	1	1
C10orf46	597231	564	19	967608	252048	0	0	0	0	12	14	4828072	1	1
C10orf47	370730	134	19	1046640	363120	0	0	0	0	50	46	22757656	1	1
C10orf55	NaN	NaN	NaN	488788	122108	0	0	1	0	50	117	39767692	1	1
C10orf57	432591	650	-2	324316	87932	0	0	0	0	50	73	20895776	1	1
C10orf58	340132	598	-7	593096	165184	0	0	0	0	50	52	15550080	1	1
C10orf68	493605	708	-15	1701324	399432	0	0	0	0	38	37	13109344	1	1
C10orf72	NaN	NaN	NaN	917768	271984	0	0	0	0	50	116	39917568	1	1
C10orf78	401935	324	2	652548	154860	0	0	0	0	50	73	25648020	1	1
C10orf79	379380	324	16	4417248	1078680	0	0	0	0	1	0	1281244	1	1
C10orf81	261759	545	-4	975084	239232	0	0	0	0	2	1	901036	1	1
C10orf84	179655	787	4	636884	142756	0	0	0	0	50	52	20318700	1	1
C10orf91	152612	271	-1	363476	114988	0	0	0	0	50	44	20690364	1	1
C10orf93	330124	590	-13	1063016	274120	0	0	0	0	50	38	15628756	1	1
C10orf95	871726	178	41	604488	228552	0	0	0	0	50	63	21586416	1	1
C10orf96	118426	896	-15	725528	138484	0	0	0	0	50	65	24282048	1	1
C11orf10	1239228	212	59	212532	58740	0	0	0	0	50	69	25687536	1	1
C11orf17	548433	243	21	539696	157708	0	0	0	0	50	57	20741984	1	1
C11orf2	3267059	207	46	1919908	629408	0	0	0	0	14	9	4229992	1	1
C11orf20	1613996	257	65	522608	140620	0	0	0	0	50	66	21324400	1	1
C11orf21	NaN	NaN	NaN	446780	145960	0	0	0	0	50	116	39791544	1	1
C11orf34	NaN	NaN	NaN	536848	142400	0	0	0	0	50	116	39787984	1	1
C11orf35	1970020	236	42	1580284	514064	0	0	0	0	6	5	2263092	1	1
C11orf41	826778	524	19	4590620	1437172	0	0	0	0	0	0	1437172	1	1
C11orf46	28896	1021	-17	676756	172304	0	0	0	0	50	72	21290224	1	1
C11orf48	2425755	248	51	694912	178712	0	0	0	0	50	68	20007556	1	1
C11orf51	715293	267	62	341048	66928	0	0	0	0	50	46	18093700	1	1
C11orf52	437513	221	22	327876	84372	0	0	0	0	50	49	18660452	1	1
C11orf59	NaN	NaN	NaN	417944	118192	0	0	0	0	50	116	39763776	1	1
C11orf61	168652	622	14	1262020	380564	0	0	0	0	20	27	9447172	1	1
C11orf66	1415260	274	58	1114992	305448	0	0	0	0	47	49	15868700	1	1
C11orf67	577731	361	35	318264	90780	0	0	0	0	50	54	24420176	1	1
C11orf68	3779084	287	42	709508	238876	0	0	1	0	1	1	408332	1	1

C11orf75	240034	598	-25	149164	47348	0	0	0	0	50	100	26107260	1	1
C11orf82	307255	371	0	2547892	669992	0	0	0	0	7	7	4586348	1	1
C11orf83	2425755	248	51	234604	72980	0	0	0	0	50	68	20007556	1	1
C11orf86	NaN	NaN	NaN	287292	90780	0	0	0	0	50	116	39736364	1	1
C11orf87	32039	927	-12	468140	166252	0	0	1	0	50	73	26064540	1	1
C11orf9	1239228	212	59	2953732	878252	0	0	0	0	1	0	936992	1	1
C11orf90	NaN	NaN	NaN	511572	145248	0	0	0	0	50	116	39790832	1	1
C11orf91	NaN	NaN	NaN	462088	163760	0	0	0	0	50	116	39809344	1	1
C11orf92	209532	617	16	309364	91136	0	0	0	0	50	58	22491012	1	1
C11orf93	NaN	NaN	NaN	393024	107868	0	0	0	0	50	116	39753452	1	1
C11orf94	427820	291	48	243860	86152	0	0	0	0	50	52	20813896	1	1
C11orf95	NaN	NaN	NaN	1643652	551088	0	0	0	0	9	16	4908528	1	1
C12orf11	409433	327	21	1864016	469564	0	0	0	0	20	16	8845888	1	1
C12orf12	20238	574	-33	1024568	279460	0	0	0	0	50	96	21164912	1	1
C12orf23	156112	473	21	292632	90780	0	0	0	0	50	46	19326884	1	1
C12orf24	922262	206	43	720900	182628	0	0	0	0	36	42	16644780	1	1
C12orf26	54295	1263	-20	1570316	414028	0	0	0	0	19	14	7483476	1	1
C12orf34	786670	423	45	1081528	378428	0	0	0	0	50	71	23130032	1	1
C12orf35	230869	442	27	4439320	1174088	0	0	0	0	1	0	1503032	1	1
C12orf36	548501	509	5	353864	100036	0	0	0	0	50	64	21640172	1	1
C12orf39	518837	836	-23	313280	85084	0	0	0	0	50	62	16740900	1	1
C12orf41	1696008	421	21	1270208	347812	0	0	0	0	1	2	684944	1	1
C12orf44	468067	234	41	548952	161268	0	0	0	0	50	28	16136768	1	1
C12orf48	229712	552	12	1300468	336776	0	0	0	0	50	54	22837756	1	1
C12orf51	704098	239	40	10758320	3160924	0	0	0	0	1	0	3383780	1	1
C12orf52	417427	252	35	646852	226772	0	0	0	0	50	52	19240376	1	1
C12orf53	2816186	232	48	682808	245284	0	0	0	0	6	24	6345344	1	1
C12orf59	397577	398	18	412248	126024	0	0	0	0	50	59	21415536	1	1
C12orf61	299026	359	3	306516	118548	0	0	0	0	50	56	22855556	1	1
C12orf62	1040456	192	58	149520	40584	0	0	0	0	50	72	21686808	1	1
C12orf63	152762	629	-4	3128884	822716	0	0	0	0	15	10	7298712	1	1
C12orf64	127493	587	-16	6267380	1491640	0	0	0	0	1	0	1590964	1	1
C12orf68	511392	352	32	478108	146672	0	0	0	0	50	55	18729516	1	1

C12orf69	340728	578	-4	567108	161268	0	0	0	0	50	47	15743388	1	1
C12orf70	NaN	NaN	NaN	912072	226416	0	0	0	0	50	116	39872000	1	1
C12orf72	313930	228	11	669992	181204	0	0	0	0	50	58	20419804	1	1
C12orf73	NaN	NaN	NaN	186544	52688	0	0	0	0	50	116	39698272	1	1
C12orf74	208773	509	-1	477040	153080	0	0	1	0	29	28	8042396	1	1
C12orf75	NaN	NaN	NaN	180492	45924	0	0	0	0	50	116	39691508	1	1
C12orf76	1009535	193	57	356356	92204	0	0	0	0	50	72	20505600	1	1
C12orf77	251399	819	7	373800	106800	0	0	0	0	50	54	18499184	1	1
C13orf1	342767	321	50	518336	129940	0	0	0	0	50	56	19537280	1	1
C13orf15	264598	508	43	364188	95052	0	0	0	0	50	63	18833112	1	1
C13orf16	335348	633	41	406552	101816	0	0	0	0	50	63	17209040	1	1
C13orf18	383104	791	50	1715208	460308	0	0	0	0	40	33	13426896	1	1
C13orf23	165959	998	5	2276264	802780	0	0	0	0	5	2	2034540	1	1
C13orf26	362646	555	39	770384	186544	0	0	0	0	50	51	20112576	1	1
C13orf27	195534	475	24	600216	151656	0	0	0	0	50	40	16969452	1	1
C13orf28	191446	610	-22	527948	127804	0	0	0	0	50	87	26475364	1	1
C13orf30	175913	538	37	359204	102172	0	0	0	0	50	57	17926380	1	1
C13orf31	139965	346	45	1110720	294768	0	0	0	0	22	27	8854788	1	1
C13orf33	369916	547	59	775368	215736	0	0	0	0	26	23	5589200	1	1
C13orf34	257535	685	2	1470992	370240	0	0	0	0	2	2	1219656	1	1
C13orf35	572556	389	44	302600	92560	0	0	0	0	50	51	21299124	1	1
C13orf36	194859	868	-12	259880	86152	0	0	0	0	50	72	27083056	1	1
C13orf37	257353	685	-9	211464	63012	0	0	0	0	50	51	23339360	1	1
C13orf38	NaN	NaN	NaN	735140	179068	0	0	0	0	50	116	39824652	1	1
C13orf39	195502	521	34	677824	186188	0	0	0	0	50	58	20187692	1	1
C14orf1	639204	372	46	363120	105732	0	0	0	0	50	44	21978016	1	1
C14orf101	137131	961	2	1826636	505876	0	0	0	0	2	2	1337848	1	1
C14orf102	318736	622	14	2997876	792456	0	0	0	0	4	1	1567112	1	1
C14orf104	587903	318	10	2050560	645072	0	0	0	0	15	10	7515872	1	1
C14orf106	234595	448	8	2974024	724460	0	0	0	0	3	2	1812396	1	1
C14orf109	325787	222	35	429336	119616	0	0	0	0	50	47	17896832	1	1
C14orf115	604423	384	45	1695984	559632	0	0	0	0	34	33	15742320	1	1
C14orf118	251674	586	36	1282312	350660	0	0	0	0	11	4	4756516	1	1

C14orf119	1261207	185	41	351728	103240	0	0	0	0	50	68	21572888	1	1
C14orf126	408399	362	20	429336	122820	0	0	0	0	50	57	23476776	1	1
C14orf129	328104	560	12	360272	95764	0	0	0	0	50	59	21341488	1	1
C14orf135	296496	516	2	2390540	656464	0	0	0	0	0	0	656464	1	1
C14orf138	747266	166	51	595232	165184	0	0	0	0	50	69	18038164	1	1
C14orf142	325787	222	35	260236	71912	0	0	2	0	1	2	191528	1	1
C14orf143	297843	598	3	442864	106088	0	0	0	0	50	65	23251428	1	1
C14orf145	127660	629	-21	2893568	713068	0	0	0	0	2	2	1967256	1	1
C14orf147	517820	249	16	187968	49128	0	0	0	0	50	55	21660108	1	1
C14orf148	551151	418	50	970812	275544	0	0	0	0	2	1	962980	1	1
C14orf149	168237	606	12	878964	277680	0	0	0	0	4	3	1691356	1	1
C14orf153	867608	225	39	549664	131720	0	0	0	0	50	63	19588900	1	1
C14orf156	455772	384	47	297260	76540	0	0	0	0	50	62	21471784	1	1
C14orf166B	357783	370	41	1288364	333928	0	0	0	0	23	14	10174124	1	1
C14orf174	551151	418	50	1718412	452832	0	0	0	0	2	1	962980	1	1
C14orf176	NaN	NaN	NaN	551800	172304	0	0	0	0	50	116	39817888	1	1
C14orf178	429207	388	37	327164	89356	0	0	0	0	50	72	23377808	1	1
C14orf179	227740	582	35	645072	166608	0	0	0	0	50	44	20816744	1	1
C14orf180	625253	450	20	393380	139552	0	0	0	0	50	62	21152808	1	1
C14orf181	543449	259	46	384836	150232	0	0	0	0	22	18	8559664	1	1
C14orf182	689076	154	46	283732	75116	0	0	0	0	50	47	16670056	1	1
C14orf183	747266	166	51	822360	234960	0	0	1	0	50	69	18038164	1	1
C14orf184	396915	483	32	346744	130652	0	0	0	0	50	36	15818860	1	1
C14orf21	915114	220	38	1575300	510504	0	0	0	0	35	38	14278804	1	1
C14orf38	NaN	NaN	NaN	2135644	493772	0	0	0	0	50	116	40139356	1	1
C14orf4	429724	206	52	1902820	650768	0	0	0	0	6	8	2885024	1	1
C14orf43	535648	191	50	2585628	808476	0	0	0	0	1	1	1037028	1	1
C14orf45	469337	158	45	1418660	326452	0	0	0	0	50	45	16890776	1	1
C14orf49	202508	475	32	2466724	730868	0	0	0	0	0	0	730868	1	1
C14orf50	662511	188	36	1129232	277324	0	0	0	0	50	58	22626648	1	1
C14orf68	412002	237	37	758280	255252	0	0	0	0	50	44	19346464	1	1
C14orf73	956863	211	36	1778576	582772	0	0	0	0	0	0	582772	1	1
C14orf80	689089	298	41	1059100	366680	0	0	0	0	36	23	13861928	1	1

C15orf17	764533	195	46	496264	158420	0	0	0	0	50	66	21528032	1	1
C15orf2	179638	1121	-44	2763628	943400	0	0	0	0	3	2	2005348	1	1
C15orf23	872646	184	42	874336	219296	0	0	0	0	50	55	20353232	1	1
C15orf24	409752	384	1	615880	181916	0	0	0	0	50	53	19269924	1	1
C15orf29	431919	242	7	820224	195444	0	0	0	0	50	78	24326904	1	1
C15orf32	288049	578	-30	461020	121040	0	0	0	0	50	109	25194476	1	1
C15orf33	239848	385	-11	1381992	312924	0	0	0	0	50	40	19775088	1	1
C15orf38	969942	239	29	582416	166252	0	0	0	0	50	61	20193032	1	1
C15orf42	804572	319	27	4787844	1424712	0	0	0	0	1	0	2468860	1	1
C15orf44	552209	185	35	1343188	366680	0	0	0	0	21	21	7694228	1	1
C15orf48	214586	299	16	229620	56604	0	0	0	0	50	37	17513064	1	1
C15orf54	251181	384	-14	462444	127092	0	0	0	0	50	31	18047420	1	1
C15orf55	542657	234	22	2774308	881456	0	0	0	0	5	3	2162700	1	1
C15orf56	NaN	NaN	NaN	380208	145248	0	0	0	0	50	116	39790832	1	1
C15orf58	876945	154	52	930228	312924	0	0	0	0	20	41	10581388	1	1
C15orf60	294157	472	16	684232	199004	0	0	0	0	50	57	20981216	1	1
C15orf61	NaN	NaN	NaN	392312	120328	0	0	0	0	50	116	39765912	1	1
C15orf62	NaN	NaN	NaN	421148	142756	0	0	0	0	50	116	39788340	1	1
C15orf63	784327	286	37	337488	91848	0	0	0	0	50	69	23862324	1	1
C16orf11	1115707	213	39	1341408	499824	0	0	0	0	4	2	1457108	1	1
C16orf13	1115707	213	39	623712	134568	0	0	0	0	50	53	16969808	1	1
C16orf3	NaN	NaN	NaN	286224	93984	0	0	0	0	50	116	39739568	1	1
C16orf42	1181253	215	38	784980	243504	0	0	0	0	50	49	18614528	1	1
C16orf48	1111484	129	46	861520	275900	0	0	0	0	31	22	11565016	1	1
C16orf5	751633	160	39	526524	160200	0	0	0	0	50	50	17444712	1	1
C16orf52	NaN	NaN	NaN	416520	130296	0	0	0	0	50	116	39775880	1	1
C16orf53	NaN	NaN	NaN	631544	194020	0	0	0	0	50	116	39839604	1	1
C16orf54	NaN	NaN	NaN	532932	190104	0	0	0	0	50	116	39835688	1	1
C16orf55	1090549	219	38	360628	98612	0	0	0	0	50	51	16551864	1	1
C16orf57	856192	158	9	689572	190460	0	0	0	0	50	56	19314424	1	1
C16orf61	231500	284	4	222144	46992	0	0	0	0	50	47	21594604	1	1
C16orf63	411695	191	34	459952	119972	0	0	0	0	50	57	21453628	1	1
C16orf68	403747	542	-11	1039520	298684	0	0	0	0	38	49	15187316	1	1

C16orf7	1090549	219	38	1580640	504096	0	0	0	0	1	0	602708	1	1
C16orf73	1685537	214	37	1253476	314348	0	0	0	0	12	18	6034912	1	1
C16orf74	390985	250	38	210396	49128	0	0	0	0	50	44	18864440	1	1
C16orf75	663123	342	28	359560	121040	0	0	0	0	9	7	4896068	1	1
C16orf79	2179404	224	39	573516	181560	0	0	0	0	50	46	19527312	1	1
C16orf80	898142	292	14	507656	135280	0	0	0	0	50	60	19167396	1	1
C16orf82	202160	672	-9	384480	114276	0	0	0	0	50	78	30843128	1	1
C16orf88	320648	639	18	1178004	309720	0	0	0	0	50	55	26101564	1	1
C16orf91	1211641	215	40	881456	277324	0	0	1	0	50	64	21318348	1	1
C17orf100	663953	424	9	269848	108224	0	0	0	0	50	52	18304452	1	1
C17orf101	2598845	179	36	971524	281240	0	0	0	0	50	59	20440452	1	1
C17orf102	222789	740	-3	406908	137772	0	0	0	0	50	58	22213332	1	1
C17orf103	416965	517	-27	185120	62656	0	0	0	0	50	71	19737708	1	1
C17orf105	NaN	NaN	NaN	445000	105020	0	0	0	0	50	116	39750604	1	1
C17orf106	NaN	NaN	NaN	317196	89712	0	0	0	0	50	116	39735296	1	1
C17orf107	NaN	NaN	NaN	457816	162692	0	0	0	0	50	116	39808276	1	1
C17orf108	632198	535	-24	213600	52332	0	0	0	0	50	55	19046712	1	1
C17orf28	1045089	223	25	2009620	595232	0	0	0	0	0	0	595232	1	1
C17orf37	1284992	226	38	301176	85440	0	0	0	0	50	64	21003644	1	1
C17orf39	886959	193	17	756500	229264	0	0	0	0	50	52	20036748	1	1
C17orf42	594135	209	20	920972	250624	0	0	0	0	50	57	19186976	1	1
C17orf46	789809	207	23	976152	278748	0	0	0	0	32	34	14564672	1	1
C17orf48	59041	801	-49	892492	219296	0	0	0	0	11	10	4812052	1	1
C17orf49	1696302	360	24	492348	147384	0	0	0	0	50	49	16256384	1	1
C17orf51	NaN	NaN	NaN	540408	175152	0	0	0	0	50	116	39820736	1	1
C17orf55	2679984	197	27	636528	216804	0	0	0	0	50	50	22269224	1	1
C17orf56	2679984	197	27	1315064	419368	0	0	0	0	11	12	5322556	1	1
C17orf57	1135425	196	23	2611972	602708	0	0	0	0	0	0	602708	1	1
C17orf58	854444	293	-12	357068	101104	0	0	0	0	50	70	19156004	1	1
C17orf60	1306314	184	23	246352	58028	0	0	0	0	50	75	23771544	1	1
C17orf61	1906324	206	33	292988	89356	0	0	0	0	50	87	23542992	1	1
C17orf63	1694459	152	36	72980	21004	0	0	0	0	13	23	6206504	1	1
C17orf65	930750	175	41	602708	18868	0	0	0	0	50	69	21793252	1	1

C17orf66	620019	235	8	1496268	393024	0	0	0	0	43	53	21666160	1	1
C17orf68	870950	218	18	3039884	958708	0	0	0	0	11	8	5694932	1	1
C17orf71	834161	507	4	2468860	724460	0	0	0	0	0	0	724460	1	1
C17orf72	NaN	NaN	NaN	485228	147028	0	0	0	0	50	116	39792612	1	1
C17orf76	829707	254	8	906376	211820	0	0	0	0	50	75	21935652	1	1
C17orf77	698491	504	10	605200	180848	0	0	0	0	50	63	19807128	1	1
C17orf78	498943	171	21	716272	195800	0	0	0	0	50	63	21901120	1	1
C17orf79	668593	374	12	480600	124956	0	0	0	0	50	47	18123960	1	1
C17orf81	1782958	316	21	901036	258812	0	0	0	0	39	39	14081580	1	1
C17orf82	292199	414	-9	583128	224280	0	0	0	0	47	39	21536932	1	1
C17orf87	1279084	384	24	390888	96120	0	0	0	0	50	70	19173092	1	1
C17orf89	2679984	197	27	189748	61232	0	0	0	0	50	50	22269224	1	1
C17orf90	3745109	205	30	363476	117124	0	0	0	0	33	25	10695308	1	1
C17orf95	827288	206	22	494840	134212	0	0	0	0	50	41	14517680	1	1
C17orf96	NaN	NaN	NaN	877896	339624	0	0	0	0	50	116	39985208	1	1
C17orf97	248876	591	1	242436	86508	0	0	0	0	50	51	18116484	1	1
C17orf98	1198833	207	37	395516	111784	0	0	0	0	50	46	18180920	1	1
C17orf99	NaN	NaN	NaN	678180	193308	0	0	0	0	50	116	39838892	1	1
C18orf1	247407	688	28	850128	232824	0	0	0	0	50	50	21301616	1	1
C18orf10	174235	834	53	779640	210396	0	0	0	0	50	45	16618792	1	1
C18orf19	161398	676	44	688860	198648	0	0	0	0	36	36	12650104	1	1
C18orf22	438786	506	20	931296	196512	0	0	0	0	50	57	23157088	1	1
C18orf26	152164	427	5	531508	155216	0	0	0	0	50	41	18377788	1	1
C18orf34	13428	1050	-27	2343904	526880	0	0	0	0	2	0	960488	1	1
C18orf45	299157	244	65	797796	213600	0	0	0	0	40	60	20352520	1	1
C18orf55	150380	981	4	642224	176932	0	0	0	0	50	60	19204420	1	1
C18orf56	NaN	NaN	NaN	389108	119260	0	0	0	0	50	116	39764844	1	1
C18orf62	63196	1063	-18	266288	71200	0	0	0	0	50	59	18488148	1	1
C19orf10	921603	203	35	452832	128160	0	0	0	0	50	61	21420164	1	1
C19orf2	163583	776	-30	1404776	357424	0	0	0	0	50	89	22589268	1	1
C19orf20	NaN	NaN	NaN	688504	250268	0	0	0	0	50	116	39895852	1	1
C19orf21	1914670	200	23	1662164	533644	0	0	0	0	14	20	6799600	1	1
C19orf22	2230583	191	36	684232	209684	0	0	0	0	50	56	24250720	1	1

C19orf24	NaN	NaN	NaN	320044	117836	0	0	0	0	50	116	39763420	1	1
C19orf25	2352825	221	38	294412	95408	0	0	1	0	18	20	6511952	1	1
C19orf28	2160126	269	39	1358496	442152	0	0	0	0	50	60	21798948	1	1
C19orf29	2167983	269	31	1926672	550020	0	0	0	0	14	11	6637264	1	1
C19orf33	NaN	NaN	NaN	286580	73336	0	0	0	0	50	116	39718920	1	1
C19orf36	1853815	239	37	632256	154860	0	0	0	0	50	68	23613124	1	1
C19orf38	NaN	NaN	NaN	594164	173728	0	0	0	0	50	116	39819312	1	1
C19orf39	1385739	197	31	547528	195800	0	0	0	0	50	60	22230420	1	1
C19orf41	1458117	445	27	578144	163048	0	0	0	0	50	69	17838092	1	1
C19orf42	980514	362	41	215736	46992	0	0	0	0	50	85	21686096	1	1
C19orf46	984653	303	21	999648	330012	0	0	0	0	19	27	9494164	1	1
C19orf47	1006719	289	35	892136	278392	0	0	2	0	20	41	9222180	1	1
C19orf48	655673	589	25	282664	95408	0	0	0	0	50	77	24644100	1	1
C19orf50	1394856	259	48	457104	127092	0	0	0	0	50	73	26137876	1	1
C19orf51	1036976	266	-1	1463160	473124	0	0	0	0	1	2	1100396	1	1
C19orf55	1151198	321	30	1060168	349592	0	0	0	0	14	10	6958732	1	1
C19orf56	NaN	NaN	NaN	295124	69064	0	0	0	0	50	116	39714648	1	1
C19orf59	688482	384	31	510504	119616	0	0	0	0	50	67	22649432	1	1
C19orf6	2387949	206	26	1554296	478108	0	0	0	0	50	78	32398136	1	1
C19orf60	1395948	185	41	502672	161624	0	0	0	0	50	75	28293812	1	1
C19orf61	452493	192	-2	1338916	385904	0	0	0	0	50	48	20632336	1	1
C19orf62	865138	212	38	864012	231756	0	0	0	0	50	59	18864796	1	1
C19orf63	1038277	491	30	722324	247420	0	0	0	0	50	64	20883672	1	1
C19orf66	1615603	272	8	759348	208260	0	0	0	0	15	15	6218964	1	1
C19orf69	NaN	NaN	NaN	327520	98612	0	0	0	0	50	116	39744196	1	1
C19orf70	879991	295	21	307940	86152	0	0	0	0	50	57	20179860	1	1
C19orf71	2160126	269	39	530440	159488	0	0	0	0	50	60	21798948	1	1
C19orf75	352763	629	1	508012	143468	0	0	0	0	50	65	22440104	1	1
C19orf76	1733181	195	36	366324	131364	0	0	0	0	9	8	4296208	1	1
C19orf77	NaN	NaN	NaN	345676	89000	0	0	0	0	50	116	39734584	1	1
C1QB	389456	541	29	626560	195800	0	0	2	0	3	2	875760	1	1
C1QBP	1145030	544	24	720544	209684	0	0	0	0	50	77	22011836	1	1
C1QC	389456	541	29	599148	197580	0	0	0	0	50	56	21614896	1	1

C1QL2	169866	574	10	697760	229264	0	0	1	0	50	46	19603496	1	1
C1QL3	96710	791	-1	626916	197580	0	0	0	0	50	51	25206936	1	1
C1QTNF6	668971	289	53	709508	199360	0	0	0	0	50	45	17293056	1	1
C1QTNF8	1154740	294	20	614456	208972	0	0	0	0	50	63	24684684	1	1
C1orf100	706923	537	-1	388396	105020	0	0	0	0	50	92	23798244	1	1
C1orf103	406255	715	28	1930232	549664	0	0	0	0	11	12	7102912	1	1
C1orf104	NaN	NaN	NaN	556072	203276	0	0	0	0	50	116	39848860	1	1
C1orf107	221666	464	7	1964764	509792	0	0	0	0	15	19	7305120	1	1
C1orf109	445023	375	27	521184	149520	0	0	2	0	13	13	4332164	1	1
C1orf113	685495	212	32	1923824	569956	0	0	0	0	3	6	1906380	1	1
C1orf114	286300	491	7	1334288	317908	0	0	0	0	7	8	2818808	1	1
C1orf122	445096	375	33	275188	93272	0	0	0	0	50	50	19791820	1	1
C1orf124	377235	569	2	1285160	337132	0	0	0	0	29	41	12912120	1	1
C1orf125	495704	593	-2	2746184	655396	0	0	0	0	3	6	2673204	1	1
C1orf128	903441	254	32	552512	148096	0	0	0	0	50	57	18158136	1	1
C1orf129	385644	965	-9	2375588	608404	0	0	0	0	1	1	1019940	1	1
C1orf130	468063	348	28	260592	82236	0	0	0	0	50	59	20065584	1	1
C1orf135	746382	372	24	912784	242792	0	0	0	0	50	64	28906488	1	1
C1orf14	757638	424	20	1674980	458884	0	0	0	0	12	19	5715936	1	1
C1orf144	783285	176	42	402636	100392	0	0	0	0	50	73	23177024	1	1
C1orf150	204354	957	-25	368816	86152	0	0	0	0	50	111	25819256	1	1
C1orf151	775439	271	32	217160	50908	0	0	0	0	50	62	21875844	1	1
C1orf156	244851	614	10	948028	249200	0	0	0	0	9	14	6536160	1	1
C1orf161	591845	538	12	929516	254896	0	0	0	0	50	63	21307668	1	1
C1orf162	397300	348	28	402992	118192	0	0	0	0	50	30	19906096	1	1
C1orf163	678946	261	27	605912	148096	0	0	0	0	50	72	23244664	1	1
C1orf172	1025094	188	34	982916	308296	0	0	0	0	1	1	703812	1	1
C1orf173	101813	1070	-14	3901048	1064084	0	0	0	0	0	0	1064084	1	1
C1orf174	248975	412	20	616948	177644	0	0	1	0	4	2	1133148	1	1
C1orf175	652892	415	15	3344976	993240	0	0	0	0	0	0	993240	1	1
C1orf182	2199280	201	27	324672	89712	0	0	0	0	50	86	24636980	1	1
C1orf183	346154	440	31	751160	224992	0	0	0	0	50	56	23952748	1	1
C1orf185	NaN	NaN	NaN	523676	134212	0	0	0	0	50	116	39779796	1	1

C1orf187	682357	264	32	878252	268780	0	0	0	0	4	4	964048	1	1
C1orf190	908945	190	29	588112	187256	0	0	0	0	50	82	25648732	1	1
C1orf192	751212	293	30	455324	134212	0	0	0	0	50	50	22076272	1	1
C1orf201	748641	197	26	746532	203988	0	0	0	0	50	64	24787212	1	1
C1orf204	NaN	NaN	NaN	597368	181204	0	0	0	0	50	116	39826788	1	1
C1orf210	1091212	264	30	280528	93272	0	0	0	0	50	55	21358220	1	1
C1orf212	NaN	NaN	NaN	234604	67640	0	0	0	0	50	116	39713224	1	1
C1orf213	1032187	163	25	380208	119616	0	0	0	0	50	98	21204072	1	1
C1orf223	NaN	NaN	NaN	514776	152724	0	0	0	0	50	116	39798308	1	1
C1orf226	363646	391	1	776792	248488	0	0	0	0	50	54	19589612	1	1
C1orf227	326908	445	13	264152	63724	0	0	0	0	50	47	18388112	1	1
C1orf228	NaN	NaN	NaN	1144184	315772	0	0	0	0	50	116	39961356	1	1
C1orf229	NaN	NaN	NaN	542900	219652	0	0	0	0	50	116	39865236	1	1
C1orf230	NaN	NaN	NaN	252048	63012	0	0	0	0	50	116	39708596	1	1
C1orf25	344321	458	-3	1908872	502672	0	0	0	0	48	64	22686100	1	1
C1orf26	333911	535	-7	2385200	578500	0	0	0	0	21	17	8629440	1	1
C1orf31	247580	299	10	330724	83660	0	0	0	0	50	52	20001860	1	1
C1orf38	804118	218	23	1623716	463156	0	0	0	0	29	27	10167360	1	1
C1orf49	341211	348	-10	738344	174796	0	0	0	0	50	41	19634824	1	1
C1orf50	820426	426	30	515488	144536	0	0	0	0	50	65	22644448	1	1
C1orf51	1213150	197	38	957996	295836	0	0	0	0	29	24	10186584	1	1
C1orf53	98527	448	-18	366680	111784	0	0	0	0	50	44	18122892	1	1
C1orf55	923085	239	20	1159136	316840	0	0	0	0	50	52	17968032	1	1
C1orf57	141149	615	-2	486296	144892	0	0	0	0	50	50	20340416	1	1
C1orf58	221959	236	-1	1095768	277680	0	0	0	0	50	48	21277052	1	1
C1orf59	679610	225	3	1014244	278036	0	0	0	0	50	60	22708884	1	1
C1orf63	613835	460	36	718052	231400	0	0	0	0	50	56	23795752	1	1
C1orf65	368078	600	-10	1539700	460664	0	0	0	0	6	3	1747248	1	1
C1orf66	1750604	179	45	1223572	331436	0	0	0	0	1	0	482736	1	1
C1orf68	NaN	NaN	NaN	621220	182984	0	0	0	0	50	116	39828568	1	1
C1orf69	702358	341	15	846924	307584	0	0	0	0	11	7	3192252	1	1
C1orf70	NaN	NaN	NaN	448204	124244	0	0	0	0	50	116	39769828	1	1
C1orf77	1656449	183	40	614456	204700	0	0	0	0	50	74	21280612	1	1

C1orf83	446494	225	28	539696	147028	0	0	0	0	50	61	20216172	1	1
C1orf84	1088321	252	27	456748	135992	0	0	0	0	50	56	22185208	1	1
C1orf85	2266205	211	33	996444	331080	0	0	0	0	50	61	22146048	1	1
C1orf88	398726	559	15	510504	128160	0	0	0	0	50	61	21076268	1	1
C1orf89	824719	186	40	642580	204344	0	0	0	0	50	48	17520896	1	1
C1orf9	116362	506	10	3255264	864012	0	0	0	0	0	0	864012	1	1
C1orf91	1460758	218	29	359560	113564	0	0	0	0	50	52	21684672	1	1
C1orf92	1172126	244	39	1433256	420792	0	0	0	0	17	17	5732668	1	1
C1orf93	323457	335	30	510504	154860	0	0	0	0	50	41	20137140	1	1
C1orf96	285692	361	9	683520	197580	0	0	0	0	50	71	23765848	1	1
C20orf103	74540	807	-42	726952	194732	0	0	0	0	18	18	8531540	1	1
C20orf106	264053	455	-17	444288	119616	0	0	0	0	50	65	20944192	1	1
C20orf107	273023	455	-27	439304	120328	0	0	0	0	50	100	24792196	1	1
C20orf108	254679	672	-43	467784	161268	0	0	0	0	33	67	17771520	1	1
C20orf11	961230	336	40	599148	151656	0	0	0	0	50	82	23029640	1	1
C20orf111	515643	367	28	734072	217516	0	0	0	0	33	42	14272396	1	1
C20orf112	677178	310	61	1107160	322892	0	0	0	0	50	38	17449696	1	1
C20orf114	478384	482	27	1245288	372732	0	0	0	0	8	0	2332512	1	1
C20orf117	802783	231	67	4159148	1289788	0	0	0	0	0	0	1289788	1	1
C20orf118	817783	225	47	561768	158064	0	0	0	0	50	68	25624524	1	1
C20orf12	414892	436	2	1961916	536136	0	0	0	0	32	27	13442204	1	1
C20orf123	NaN	NaN	NaN	1344256	483092	0	0	0	0	50	116	40128676	1	1
C20orf132	808071	360	45	2697412	756500	0	0	0	0	1	0	1299044	1	1
C20orf134	NaN	NaN	NaN	583840	204344	0	0	0	0	50	116	39849928	1	1
C20orf135	1123741	206	44	1118196	389820	0	0	0	0	13	15	6638332	1	1
C20orf141	796251	438	41	391600	146672	0	0	1	0	50	52	19055612	1	1
C20orf144	NaN	NaN	NaN	373800	128160	0	0	0	0	50	116	39773744	1	1
C20orf151	1190462	205	28	1653976	532220	0	0	0	0	4	3	2923472	1	1
C20orf152	1308823	144	53	1504456	380564	0	0	0	0	50	49	21453628	1	1
C20orf160	1022704	386	29	1081172	345676	0	0	0	0	50	54	19949528	1	1
C20orf165	725555	209	59	555716	183340	0	0	0	0	27	40	9561092	1	1
C20orf166	1191147	NaN	20	304024	84728	0	0	0	0	50	50	20525536	1	1
C20orf173	NaN	NaN	NaN	532932	138840	0	0	0	0	50	116	39784424	1	1

C20orf177	73597	856	-36	951588	283020	0	0	0	0	50	68	20110084	1	1
C20orf185	515931	491	46	1188684	401568	0	0	0	0	15	9	6738012	1	1
C20orf186	515931	491	46	1538632	498044	0	0	0	0	12	7	6045592	1	1
C20orf197	73705	930	-58	322892	88288	0	0	0	0	50	51	18383484	1	1
C20orf20	1044503	208	48	539340	136704	0	0	0	0	50	65	20186624	1	1
C20orf201	NaN	NaN	NaN	568176	210396	0	0	0	0	50	116	39855980	1	1
C20orf202	NaN	NaN	NaN	309008	91492	0	0	0	0	50	116	39737076	1	1
C20orf203	NaN	NaN	NaN	477040	156284	0	0	0	0	50	116	39801868	1	1
C20orf24	1019481	176	62	486296	126736	0	0	0	0	50	44	18338984	1	1
C20orf26	228680	750	-3	3252416	859384	0	0	0	0	5	7	2546468	1	1
C20orf29	740484	241	42	485228	167320	0	0	0	0	4	3	1253832	1	1
C20orf3	545344	676	-29	1055184	317196	0	0	0	0	50	56	19251056	1	1
C20orf30	494904	385	26	462088	146672	0	0	0	0	50	60	20022864	1	1
C20orf4	1278077	449	44	967964	278392	0	0	0	0	18	25	8306904	1	1
C20orf43	255083	551	-7	809188	208616	0	0	0	0	50	52	20618452	1	1
C20orf46	442604	527	22	613032	214668	0	0	0	0	50	83	28195912	1	1
C20orf54	764796	557	31	1139912	383056	0	0	0	0	43	62	21007560	1	1
C20orf7	125337	703	-6	936636	238164	0	0	0	0	24	33	11910336	1	1
C20orf70	418196	491	31	648988	186188	0	0	0	0	50	44	17537984	1	1
C20orf71	478384	482	27	671060	171592	0	0	0	0	50	51	25529828	1	1
C20orf72	756104	499	29	891068	231400	0	0	0	0	50	68	23326188	1	1
C20orf79	256301	699	4	395160	111072	0	0	0	0	50	62	22399876	1	1
C20orf94	165910	412	-17	1056608	281596	0	0	0	0	50	37	20003996	1	1
C20orf96	1010597	348	24	973660	235316	0	0	3	0	7	7	1817380	1	1
C21orf29	860604	461	30	1685660	510148	0	0	0	0	0	0	510148	1	1
C21orf33	879237	219	61	681740	207904	0	0	0	0	50	43	18931724	1	1
C21orf45	436753	379	8	610184	158776	0	0	0	0	50	69	24300916	1	1
C21orf56	758870	488	39	836244	269136	0	0	0	0	1	0	395872	1	1
C21orf57	940047	624	53	457104	124956	0	0	0	0	50	58	20984420	1	1
C21orf63	268842	162	39	1133504	312568	0	0	0	0	50	54	16442572	1	1
C21orf7	316263	146	77	640800	167676	0	0	0	0	50	67	21678264	1	1
C21orf70	746455	191	74	583840	179780	0	0	0	0	41	42	16971588	1	1
C22orf13	480889	194	48	627984	165540	0	0	0	0	50	44	18325456	1	1

C22orf15	637355	196	52	384836	113920	0	0	0	0	50	63	20521620	1	1
C22orf24	531528	344	-1	411892	112496	0	0	0	0	50	54	19869072	1	1
C22orf25	587146	218	50	726240	195444	0	0	0	0	50	52	19020368	1	1
C22orf26	NaN	NaN	NaN	323248	128516	0	0	0	0	50	116	39774100	1	1
C22orf28	392443	401	-6	1315420	352796	0	0	0	0	6	18	4575668	1	1
C22orf30	547970	268	36	5433272	1495912	0	0	0	0	0	0	1495912	1	1
C22orf32	1321127	363	65	270204	86508	0	0	0	0	50	55	15728436	1	1
C22orf33	724899	379	44	731936	191884	0	0	0	0	50	67	27206944	1	1
C22orf36	NaN	NaN	NaN	756144	271272	0	0	0	0	50	116	39916856	1	1
C22orf39	650407	300	16	406908	116412	0	0	0	0	50	53	20360352	1	1
C22orf40	519654	205	31	322180	85796	0	0	0	0	50	72	19238596	1	1
C22orf41	NaN	NaN	NaN	239232	52332	0	0	0	0	50	116	39697916	1	1
C22orf43	604420	399	31	638664	145248	0	0	0	0	50	47	18688220	1	1
C22orf46	NaN	NaN	NaN	605912	182272	0	0	0	0	50	116	39827856	1	1
C22orf9	417645	225	9	1122824	301888	0	0	0	0	18	22	8061976	1	1
C2CD4A	125995	446	20	845856	343896	0	0	0	0	50	54	19716704	1	1
C2CD4C	NaN	NaN	NaN	1002140	354220	0	0	0	0	50	116	39999804	1	1
C2CD4D	NaN	NaN	NaN	809900	326452	0	0	0	0	50	116	39972036	1	1
C2orf18	1266654	231	40	925244	290140	0	0	0	0	33	55	17466784	1	1
C2orf24	773232	177	36	1025992	320756	0	0	0	0	12	9	4375240	1	1
C2orf27A	NaN	NaN	NaN	501960	160200	0	0	0	0	50	116	39805784	1	1
C2orf27B	NaN	NaN	NaN	511928	165184	0	0	0	0	50	116	39810768	1	1
C2orf28	1238919	230	39	721256	217516	0	0	0	0	50	56	18256392	1	1
C2orf29	413646	262	19	1282668	382344	0	0	0	0	30	38	12130344	1	1
C2orf3	136392	523	4	2062308	511572	0	0	0	0	2	0	771096	1	1
C2orf34	414128	685	26	853332	227484	0	0	0	0	17	14	9265256	1	1
C2orf39	751542	408	42	1965476	479176	0	0	0	0	9	11	4922412	1	1
C2orf50	466103	384	26	409756	125312	0	0	1	0	39	47	15635520	1	1
C2orf51	120457	497	2	468140	124600	0	0	0	0	50	38	16927088	1	1
C2orf53	1214097	238	50	972592	350660	0	0	0	0	15	24	8286612	1	1
C2orf55	493189	692	29	2354940	768960	0	0	0	0	11	2	2750812	1	1
C2orf56	488101	253	42	1139912	316840	0	0	0	0	24	18	8787504	1	1
C2orf60	226122	602	18	844788	208260	0	0	0	0	50	38	20562204	1	1

C2orf62	734742	197	37	1007124	276612	0	0	0	0	12	17	6139220	1	1
C2orf63	967446	333	25	1550380	377360	0	0	0	0	0	0	377360	1	1
C2orf64	260302	696	39	201852	49128	0	0	0	0	50	65	21358576	1	1
C2orf65	996981	278	50	1357784	386260	0	0	0	0	50	62	20826000	1	1
C2orf66	404194	370	14	299396	87220	0	0	2	0	2	5	781064	1	1
C2orf67	190249	712	1	2572812	652548	0	0	0	0	16	15	8521216	1	1
C2orf7	536944	452	20	487364	137416	0	0	0	0	50	54	17210108	1	1
C2orf72	NaN	NaN	NaN	692776	264152	0	0	0	0	50	116	39909736	1	1
C2orf73	659505	429	8	746532	195444	0	0	1	0	50	54	19842728	1	1
C2orf74	NaN	NaN	NaN	506232	131364	0	0	0	0	50	116	39776948	1	1
C2orf77	345692	393	35	1480960	327164	0	0	0	0	33	34	14103652	1	1
C2orf79	299133	374	39	351728	108580	0	0	0	0	50	53	17494552	1	1
C2orf81	NaN	NaN	NaN	1421152	478820	0	0	0	0	50	116	40124404	1	1
C2orf82	NaN	NaN	NaN	293700	110004	0	0	0	0	50	116	39755588	1	1
C2orf83	186199	353	25	467428	130652	0	0	1	0	0	1	130652	1	1
C2orf84	320978	385	35	547884	136704	0	0	0	0	50	47	18904312	1	1
C2orf85	521265	487	30	1393384	451052	0	0	0	0	1	4	890000	1	1
C2orf86	334800	642	3	1975800	517980	0	0	0	0	45	48	16760480	1	1
C2orf89	725789	275	5	1141336	346388	0	0	0	0	50	83	28220476	1	1
C3orf1	290702	599	38	733716	208260	0	0	0	0	50	49	19375656	1	1
C3orf10	825905	170	39	202920	51264	0	0	0	0	50	47	17447560	1	1
C3orf15	368404	568	31	2016740	517624	0	0	0	0	22	40	13435440	1	1
C3orf16	NaN	NaN	NaN	1381280	386260	0	0	0	0	50	116	40031844	1	1
C3orf19	561517	424	39	1239592	304736	0	0	0	0	50	75	26834568	1	1
C3orf21	431673	536	46	975084	300108	0	0	0	0	50	58	20550100	1	1
C3orf22	289633	508	52	357424	110360	0	0	0	0	50	55	23322984	1	1
C3orf23	319459	418	27	1362412	326096	0	0	0	0	50	54	21600656	1	1
C3orf24	825905	170	39	448916	129940	0	0	0	0	50	47	17447560	1	1
C3orf26	267848	573	10	765756	184764	0	0	0	0	50	46	20143192	1	1
C3orf27	1046071	282	53	359560	121040	0	0	0	0	50	66	18818160	1	1
C3orf31	229249	423	35	826988	216448	0	0	0	0	50	60	21365340	1	1
C3orf32	93870	581	8	914564	258100	0	0	0	0	50	59	21381716	1	1
C3orf33	304724	473	0	655396	169100	0	0	0	0	50	67	25006864	1	1

C3orf34	818753	446	45	443220	103596	0	0	0	0	50	63	24450080	1	1
C3orf35	471302	442	16	533644	163760	0	0	0	0	50	53	17635884	1	1
C3orf37	1033808	136	49	917768	245284	0	0	0	0	50	52	20231480	1	1
C3orf39	331914	493	28	1406556	454968	0	0	0	0	10	8	3334652	1	1
C3orf43	1180405	156	30	547884	149520	0	0	0	0	50	69	20130020	1	1
C3orf45	843769	206	51	410112	133500	0	0	0	0	50	68	20472848	1	1
C3orf52	268780	440	28	859028	238876	0	0	1	0	50	52	20365692	1	1
C3orf54	1306720	191	39	703100	226060	0	0	0	0	50	70	21778300	1	1
C3orf55	147326	810	-11	505876	96476	0	0	0	0	50	73	21755872	1	1
C3orf57	240601	602	-4	201140	49840	0	0	0	0	50	54	18332932	1	1
C3orf59	34577	631	8	1235676	344964	0	0	0	0	50	77	24846308	1	1
C3orf63	209166	363	34	4395176	1121044	0	0	0	0	4	6	3103608	1	1
C3orf64	263737	581	-2	1192600	281240	0	0	0	0	15	7	4170184	1	1
C3orf71	1838219	241	33	670704	261660	0	0	0	0	50	64	19644436	1	1
C3orf72	345888	340	21	421504	150944	0	0	0	0	50	44	21655124	1	1
C3orf75	884829	197	44	688148	192952	0	0	0	0	50	57	20048140	1	1
C3orf77	NaN	NaN	NaN	4386276	1121400	0	0	0	0	0	0	1121400	1	1
C3orf78	NaN	NaN	NaN	183696	50196	0	0	0	0	50	116	39695780	1	1
C3orf79	55177	914	-4	265932	68352	0	0	0	0	50	60	19400576	1	1
C4orf14	570731	443	33	1731584	537916	0	0	0	0	50	47	20860532	1	1
C4orf21	219601	647	30	5452496	1407268	0	0	0	0	1	0	1715564	1	1
C4orf23	108213	416	43	1920264	555360	0	0	0	0	22	25	9635852	1	1
C4orf31	106601	931	-7	1438240	396584	0	0	0	0	22	29	11102928	1	1
C4orf34	645570	165	40	265932	71556	0	0	0	0	50	40	20506312	1	1
C4orf35	264684	915	25	979356	291564	0	0	0	0	50	42	17431540	1	1
C4orf36	268001	603	36	310788	84372	0	0	1	0	4	5	1072628	1	1
C4orf37	69244	1096	-24	1196872	321824	0	0	0	0	8	21	4860824	1	1
C4orf39	NaN	NaN	NaN	377004	131364	0	0	0	0	50	116	39776948	1	1
C4orf40	181352	975	12	536848	185120	0	0	0	0	50	57	17976932	1	1
C4orf41	154470	254	4	2996096	761128	0	0	0	0	3	2	1538988	1	1
C4orf43	75022	1136	-3	555360	123888	0	0	0	0	50	57	20887588	1	1
C4orf44	612481	464	32	697404	205056	0	0	0	0	50	53	22900056	1	1
C4orf46	154316	534	-11	279816	89712	0	0	0	0	50	71	24690736	1	1

C4orf47	NaN	NaN	NaN	818088	207192	0	0	0	0	50	116	39852776	1	1
C4orf48	NaN	NaN	NaN	315060	115344	0	0	0	0	50	116	39760928	1	1
C4orf49	384151	208	24	601640	183340	0	0	0	0	50	49	21368188	1	1
C4orf52	NaN	NaN	NaN	178356	48060	0	0	0	0	50	116	39693644	1	1
C4orf6	57657	589	10	245996	63724	0	0	0	0	50	74	25586788	1	1
C4orf7	191457	928	7	227128	63368	0	0	0	0	50	63	21710660	1	1
C5orf13	118271	617	34	308296	76184	0	0	0	0	50	44	18965188	1	1
C5orf20	625030	509	47	592740	192240	0	0	0	0	50	53	24138580	1	1
C5orf23	477035	487	-15	305804	85084	0	0	0	0	50	81	22714936	1	1
C5orf25	532154	427	25	1174444	327164	0	0	0	0	24	24	9815276	1	1
C5orf30	203042	531	16	511216	156284	0	0	0	0	50	59	20715640	1	1
C5orf32	788704	185	45	252048	70488	0	0	0	0	50	74	21727748	1	1
C5orf33	184013	476	2	1141336	325028	0	0	0	0	25	34	13105784	1	1
C5orf35	201203	393	41	776080	212888	0	0	0	0	50	66	22083036	1	1
C5orf36	162976	515	3	3348536	823072	0	0	0	0	8	7	3543268	1	1
C5orf39	488323	520	21	479532	142044	0	0	0	0	50	69	22356800	1	1
C5orf4	525975	293	35	871132	231044	0	0	0	0	50	62	20636608	1	1
C5orf40	NaN	NaN	NaN	562836	162336	0	0	0	0	50	116	39807920	1	1
C5orf41	486042	309	37	1684236	428268	0	0	0	0	6	4	2157716	1	1
C5orf43	202017	385	13	194020	50552	0	0	0	0	50	58	21154232	1	1
C5orf44	417990	406	31	1110720	283020	0	0	0	0	22	19	8191204	1	1
C5orf47	NaN	NaN	NaN	452476	131720	0	0	0	0	50	116	39777304	1	1
C5orf48	235652	347	15	354220	91136	0	0	0	0	50	55	18930300	1	1
C5orf52	NaN	NaN	NaN	407976	115344	0	0	0	0	50	116	39760928	1	1
C5orf53	812666	260	50	139552	33464	0	0	0	0	50	63	20948820	1	1
C5orf54	197504	619	30	1513712	396940	0	0	0	0	6	6	2982212	1	1
C5orf55	787542	399	37	288360	96120	0	0	0	0	50	58	23058832	1	1
C5orf60	NaN	NaN	NaN	698472	215736	0	0	0	0	50	116	39861320	1	1
C5orf62	NaN	NaN	NaN	151300	48416	0	0	0	0	50	116	39694000	1	1
C6orf1	1074764	233	36	398364	127092	0	0	1	0	45	43	14096176	1	1
C6orf103	NaN	NaN	NaN	4377732	1118196	0	0	0	0	0	0	1118196	1	1
C6orf105	117356	372	24	647208	176220	0	0	0	0	50	49	20253552	1	1
C6orf106	920583	132	31	769316	205768	0	0	1	0	50	55	18393808	1	1

C6orf108	1308754	363	35	494128	157352	0	0	0	0	38	56	16581768	1	1
C6orf114	NaN	NaN	NaN	347456	91492	0	0	0	0	50	116	39737076	1	1
C6orf115	289502	640	16	212888	58384	0	0	0	0	24	30	12471748	1	1
C6orf125	1551477	328	40	338556	85440	0	0	0	0	50	60	24086604	1	1
C6orf126	621095	404	34	264152	72268	0	0	0	0	50	56	22245372	1	1
C6orf127	621095	404	34	321112	82592	0	0	0	0	50	56	22245372	1	1
C6orf129	498007	434	43	259880	69064	0	0	0	0	50	67	24906116	1	1
C6orf130	145501	611	37	410112	101460	0	0	0	0	50	56	20220800	1	1
C6orf132	NaN	NaN	NaN	2813824	1012820	0	0	0	0	0	0	1012820	1	1
C6orf134	2097865	171	37	854400	260592	0	0	0	0	50	76	22825296	1	1
C6orf138	100851	907	-18	2116064	608404	0	0	0	0	6	4	3006420	1	1
C6orf141	NaN	NaN	NaN	584196	200784	0	0	0	0	50	116	39846368	1	1
C6orf142	104005	635	-1	1190820	331080	0	0	0	0	50	46	21341488	1	1
C6orf145	336205	311	17	596656	163760	0	0	0	0	50	54	24945276	1	1
C6orf146	182705	353	18	1313996	342472	0	0	0	0	35	32	11880788	1	1
C6orf150	723493	232	21	1321472	373444	0	0	0	0	18	27	7193692	1	1
C6orf153	1200368	259	40	677112	183696	0	0	0	0	50	74	22706748	1	1
C6orf154	1278255	230	56	782132	254896	0	0	0	0	50	54	19955936	1	1
C6orf162	222902	596	14	255252	67284	0	0	0	0	44	46	15301948	1	1
C6orf163	NaN	NaN	NaN	872200	204344	0	0	0	0	50	116	39849928	1	1
C6orf167	91667	1258	2	3245652	842652	0	0	0	0	3	2	1803140	1	1
C6orf168	259352	595	-11	1065508	269492	0	0	0	0	50	49	17950588	1	1
C6orf170	60566	895	-10	3339992	825208	0	0	0	0	2	2	2114640	1	1
C6orf174	157237	811	-9	2349244	713780	0	0	0	0	6	3	3105388	1	1
C6orf182	390388	492	33	1236032	283732	0	0	0	0	50	35	17230044	1	1
C6orf186	183030	586	22	926668	266288	0	0	0	0	50	40	20933512	1	1
C6orf191	72955	603	26	345676	89000	0	0	0	0	50	48	24186640	1	1
C6orf192	129252	443	10	1173020	346744	0	0	0	0	38	54	17263152	1	1
C6orf201	181759	353	22	369884	94696	0	0	1	0	9	17	4129244	1	1
C6orf204	129707	1029	8	2230340	552868	0	0	0	0	17	15	8042396	1	1
C6orf211	336608	468	30	1144896	288360	0	0	0	0	50	39	19548316	1	1
C6orf221	732413	446	18	543612	165540	0	0	0	0	50	56	20226496	1	1
C6orf223	1407986	199	47	665008	174440	0	0	0	0	50	56	23257836	1	1

C6orf225	145073	296	-5	208616	59452	0	0	0	0	50	56	21849856	1	1
C6orf26	2509866	183	32	461732	131008	0	0	0	0	50	56	22464668	1	1
C6orf27	2509866	183	32	2196876	728376	0	0	0	0	4	3	2300472	1	1
C6orf35	213310	374	21	360628	107156	0	0	0	0	50	49	21364628	1	1
C6orf48	2485614	188	36	201140	53044	0	0	0	0	50	45	21269576	1	1
C6orf52	NaN	NaN	NaN	412248	97188	0	0	0	0	50	116	39742772	1	1
C6orf57	119480	618	15	280884	79032	0	0	0	0	50	68	21955944	1	1
C6orf64	188770	730	34	446424	147384	0	0	0	0	50	57	20964484	1	1
C6orf70	233648	447	3	1775728	476684	0	0	0	0	25	36	14685356	1	1
C6orf72	435829	150	41	858672	236028	0	0	0	0	50	47	17518048	1	1
C6orf81	621095	404	34	924532	280172	0	0	0	0	3	0	516200	1	1
C6orf94	NaN	NaN	NaN	588112	156284	0	0	0	0	50	116	39801868	1	1
C6orf97	301605	535	7	1861524	477396	0	0	0	0	21	19	8722000	1	1
C7orf10	120126	802	-13	1137420	318264	0	0	0	0	50	74	23915368	1	1
C7orf11	221361	636	20	440728	142400	0	0	0	0	15	12	5997532	1	1
C7orf16	26433	889	1	416520	100392	0	0	0	0	50	40	20787908	1	1
C7orf23	182644	1011	28	310432	83660	0	0	0	0	50	35	17531220	1	1
C7orf27	783535	226	47	2016740	672484	0	0	0	0	11	13	5447512	1	1
C7orf28A	2208864	358	32	1306164	303312	0	0	0	0	50	54	20921052	1	1
C7orf28B	700380	514	57	1306164	303312	0	0	0	0	7	15	4038464	1	1
C7orf29	NaN	NaN	NaN	583484	175864	0	0	0	0	50	116	39821448	1	1
C7orf30	500943	169	27	595232	174796	0	0	0	0	50	73	23018960	1	1
C7orf36	221842	487	5	585976	152012	0	0	0	0	50	63	23879412	1	1
C7orf41	656945	411	31	347812	83660	0	0	0	0	50	47	17476040	1	1
C7orf42	490088	228	41	811324	223568	0	0	0	0	50	29	16514840	1	1
C7orf44	654530	310	38	393736	98612	0	0	0	0	50	62	22151032	1	1
C7orf45	414437	266	34	644360	151300	0	0	0	0	50	59	18847352	1	1
C7orf46	378677	441	19	781064	204700	0	0	0	0	50	60	23233984	1	1
C7orf47	1479297	186	60	615524	215380	0	0	0	0	45	39	16383476	1	1
C7orf49	464513	594	20	398720	116056	0	0	1	0	50	57	15046340	1	1
C7orf51	1479297	186	60	2016740	706660	0	0	0	0	10	5	3847648	1	1
C7orf52	1022626	258	39	887152	311144	0	0	0	0	50	67	19705312	1	1
C7orf53	171853	470	-5	339980	95764	0	0	0	0	50	56	18542972	1	1

C7orf55	522451	339	35	281952	89712	0	0	0	0	50	59	20493140	1	1
C7orf58	99302	534	-3	2717348	709864	0	0	0	0	6	15	6018536	1	1
C7orf59	1299015	205	52	253828	81524	0	0	0	0	50	42	20842020	1	1
C7orf61	1479297	186	60	515488	156284	0	0	1	0	50	47	17722036	1	1
C7orf63	134871	1140	2	2496628	615524	0	0	0	0	8	3	2613396	1	1
C7orf64	750974	369	28	946604	251692	0	0	0	0	50	55	20378508	1	1
C7orf65	237253	211	21	391600	106088	0	0	0	0	50	52	19936000	1	1
C7orf66	75279	982	-24	301176	79032	0	0	0	0	50	99	27060984	1	1
C7orf68	902782	246	34	158420	50908	0	0	0	0	50	68	20282388	1	1
C7orf69	NaN	NaN	NaN	326096	78676	0	0	0	0	50	116	39724260	1	1
C7orf70	NaN	NaN	NaN	635460	201852	0	0	0	0	50	116	39847436	1	1
C7orf71	NaN	NaN	NaN	437880	115344	0	0	0	0	50	116	39760928	1	1
C7orf72	NaN	NaN	NaN	1133504	307228	0	0	0	0	50	116	39952812	1	1
C8orf30A	2713803	210	26	1898192	654328	0	0	0	0	12	12	5430424	1	1
C8orf31	821065	470	4	349236	98256	0	0	0	0	50	37	16863364	1	1
C8orf38	409865	395	57	865792	240656	0	0	0	0	50	66	21800372	1	1
C8orf40	627586	464	26	291920	66928	0	0	0	0	50	48	19882244	1	1
C8orf41	91648	507	12	1268072	392668	0	0	0	0	39	45	17606692	1	1
C8orf42	152113	904	-20	469208	135280	0	0	0	0	50	70	21670076	1	1
C8orf44	372262	213	38	410468	110716	0	0	0	0	50	49	19494204	1	1
C8orf45	392325	393	22	1791392	477040	0	0	0	0	11	5	3808132	1	1
C8orf46	285889	151	36	528304	159488	0	0	0	0	50	57	18510932	1	1
C8orf47	630526	229	39	940196	269848	0	0	0	0	22	26	11248176	1	1
C8orf48	NaN	NaN	NaN	805272	220008	0	0	0	0	50	116	39865592	1	1
C8orf55	427585	373	34	491280	182628	0	0	0	0	50	60	17573228	1	1
C8orf73	2183482	235	37	1730160	634392	0	0	0	0	19	15	8775400	1	1
C8orf79	111627	643	-11	1165900	303668	0	0	0	0	50	54	24871940	1	1
C8orf80	409228	222	34	2084380	546104	0	0	0	0	11	12	5138860	1	1
C8orf83	NaN	NaN	NaN	233180	61588	0	0	0	0	50	116	39707172	1	1
C8orf84	161465	570	18	678536	187612	0	0	0	0	50	42	17036736	1	1
C8orf85	495171	346	16	388752	117480	0	0	0	0	50	87	23711736	1	1
C9orf100	1202125	248	37	855112	255608	0	0	0	0	50	57	20786840	1	1
C9orf102	260804	471	25	1849776	490212	0	0	0	0	22	17	8211140	1	1

C9orf103	493702	567	-8	367036	100748	0	0	0	0	50	50	16413736	1	1
C9orf106	1220935	283	48	569956	182984	0	0	0	0	50	79	25104408	1	1
C9orf11	116649	454	-34	782488	196868	0	0	0	0	50	89	22655840	1	1
C9orf114	1768145	201	34	983628	275544	0	0	0	0	26	29	9773980	1	1
C9orf116	298554	539	14	368104	81524	0	0	0	0	50	72	20768328	1	1
C9orf119	1813435	201	46	589892	183340	0	0	0	0	50	69	20850564	1	1
C9orf123	73228	1033	-33	278392	90068	0	0	0	0	50	132	25244672	1	1
C9orf125	112814	872	-15	991104	307584	0	0	0	0	50	76	29064552	1	1
C9orf128	847663	227	54	1037740	276968	0	0	0	0	50	66	20654408	1	1
C9orf129	439538	426	41	497332	150944	0	0	0	0	50	75	25974116	1	1
C9orf135	243260	478	-8	609828	152724	0	0	0	0	50	42	19534076	1	1
C9orf139	1951939	202	40	458528	157708	0	0	0	0	50	61	21392396	1	1
C9orf140	1951939	202	40	951588	335352	0	0	0	0	50	61	21392396	1	1
C9orf144B	NaN	NaN	NaN	3300832	992528	0	0	0	0	0	0	992528	1	1
C9orf150	77482	482	-32	570312	167676	0	0	0	0	50	92	19644080	1	1
C9orf163	1413299	233	44	485228	168388	0	0	0	0	50	69	23563996	1	1
C9orf167	1724821	399	43	999648	363120	0	0	0	0	50	60	16636592	1	1
C9orf169	NaN	NaN	NaN	364188	104664	0	0	0	0	50	116	39750248	1	1
C9orf170	123702	421	-8	305804	91492	0	0	0	0	50	45	21090508	1	1
C9orf173	1724821	399	43	780708	232824	0	0	0	0	50	60	16636592	1	1
C9orf21	322237	430	26	580636	170168	0	0	0	0	50	50	22560076	1	1
C9orf23	731413	298	51	388040	141688	0	0	0	0	50	42	16261368	1	1
C9orf25	629612	302	40	490568	126736	0	0	0	0	50	41	19141052	1	1
C9orf30	245284	516	0	715204	177644	0	0	0	0	50	52	19148884	1	1
C9orf37	1197708	478	45	425776	143468	0	0	0	0	50	83	28003316	1	1
C9orf4	338668	272	18	850840	271628	0	0	0	0	11	12	6064460	1	1
C9orf46	373599	629	18	402280	89000	0	0	0	0	50	53	21135364	1	1
C9orf5	337902	387	13	2212540	681740	0	0	0	0	10	8	4666448	1	1
C9orf57	NaN	NaN	NaN	429692	108580	0	0	0	0	50	116	39754164	1	1
C9orf6	337389	433	12	483092	123532	0	0	0	0	50	62	22004004	1	1
C9orf66	219110	462	-10	693844	254540	0	0	1	0	50	46	18998296	1	1
C9orf68	226049	673	17	885372	229620	0	0	0	0	50	49	16026052	1	1
C9orf69	799089	244	42	321824	118192	0	0	0	0	50	64	23158512	1	1

C9orf7	1858670	315	39	443220	130296	0	0	0	0	50	66	20649424	1	1
C9orf71	127057	578	-20	424352	129940	0	0	0	0	50	63	23745200	1	1
C9orf79	206257	613	8	3547184	1102888	0	0	0	0	10	8	6150968	1	1
C9orf80	525036	382	18	280528	72980	0	0	0	0	50	59	19929592	1	1
C9orf82	104669	632	-28	935568	245640	0	0	0	0	50	107	24606008	1	1
C9orf86	1921909	176	44	2057324	603064	0	0	0	0	30	35	13642988	1	1
C9orf89	504920	448	61	470276	140620	0	0	0	0	50	67	19457180	1	1
C9orf93	150665	600	-9	3499124	857248	0	0	0	0	1	1	1036316	1	1
C9orf95	95091	623	0	553936	121040	0	0	0	0	50	45	21801440	1	1
C9orf96	1827885	416	28	1761132	495552	0	0	0	0	0	0	495552	1	1
C9orf98	1939494	575	33	1252408	336776	0	0	0	0	50	47	18980496	1	1
CA13	99038	1463	13	683876	184408	0	0	0	0	0	0	184408	1	1
CABC1	411156	481	5	1665724	470276	0	0	0	0	7	8	3761496	1	1
CABP2	1392213	215	53	569244	166608	0	0	0	0	50	41	15455384	1	1
CABP7	848649	195	37	562124	149164	0	0	1	0	50	54	19150308	1	1
CALB2	402231	648	0	755076	161268	0	0	0	0	50	58	17908224	1	1
CALHM3	NaN	NaN	NaN	839448	274476	0	0	0	0	50	116	39920060	1	1
CALM3	1139694	347	28	409044	92916	0	0	0	0	50	46	21360712	1	1
CALML4	375804	365	28	514776	133500	0	0	0	0	50	29	19439380	1	1
CALML5	769552	446	17	362764	108224	0	0	1	0	15	16	4289444	1	1
CAMK2N1	573203	429	30	200784	56604	0	0	0	0	50	55	21704964	1	1
CAMK2N2	NaN	NaN	NaN	202564	58028	0	0	0	0	50	116	39703612	1	1
CAMSAP1L	492001	374	12	3809200	997868	0	0	0	0	0	0	997868	1	1
CANT1	612708	177	22	1001072	299752	0	0	1	0	50	46	18646212	1	1
CAPN10	837485	485	35	1804564	562124	0	0	1	0	36	44	16158840	1	1
CAPN14	NaN	NaN	NaN	1818804	465648	0	0	0	0	50	116	40111232	1	1
CAPN8	NaN	NaN	NaN	1566400	411536	0	0	0	0	50	116	40057120	1	1
CAPNS1	978272	326	6	716272	191528	0	0	0	0	50	68	15103300	1	1
CARTPT	456944	601	19	292988	90424	0	0	1	0	30	41	9147064	1	1
CAV1	554882	159	0	463868	120328	0	0	0	0	50	58	17599572	1	1
CAV2	548426	187	-5	482024	89356	0	0	0	0	50	62	21417316	1	1
CBARA1	473220	212	33	1258816	321824	0	0	0	0	50	61	19642300	1	1
CBLN1	6672	907	-33	480956	149164	0	0	1	0	33	58	10564300	1	1

CBLN3	897133	265	40	489856	178712	0	0	0	0	50	54	25824596	1	1
CBS	428442	156	63	1429340	407620	0	0	0	0	9	8	4281612	1	1
CBWD6	NaN	NaN	NaN	1066576	262016	0	0	0	0	50	116	39907600	1	1
CBX3	915230	445	43	496620	114276	0	0	0	0	50	54	23143916	1	1
CBY3	NaN	NaN	NaN	595232	191884	0	0	0	0	50	116	39837468	1	1
CCBP2	335379	396	26	953368	284444	0	0	0	0	50	45	26016480	1	1
CCDC103	1101543	172	28	607336	184052	0	0	1	0	50	74	17295192	1	1
CCDC104	618209	382	27	927380	212176	0	0	0	0	6	4	3198304	1	1
CCDC105	990597	524	16	1254188	375580	0	0	1	0	10	15	3457828	1	1
CCDC107	1202125	248	37	705948	222144	0	0	0	0	50	57	20786840	1	1
CCDC109A	635147	218	34	897832	262016	0	0	0	0	50	55	21174524	1	1
CCDC11	211344	300	35	1372024	310076	0	0	0	0	50	59	18884732	1	1
CCDC111	290265	471	30	1490572	358136	0	0	0	0	50	48	21442948	1	1
CCDC12	489602	371	43	477396	127092	0	0	0	0	50	51	19584628	1	1
CCDC122	139965	346	45	735852	159132	0	0	0	0	50	58	18845216	1	1
CCDC123	478721	448	-20	2063020	527948	0	0	0	0	2	2	1246000	1	1
CCDC124	1366136	217	36	573872	160912	0	0	0	0	50	77	24277420	1	1
CCDC135	712945	168	34	2291572	584552	0	0	0	0	37	30	15703872	1	1
CCDC144B	728759	435	-5	1915992	451764	0	0	0	0	25	27	10843760	1	1
CCDC144N	463169	398	-24	569244	154860	0	0	1	0	50	70	23608496	1	1
CCDC147	281526	415	2	2319340	550376	0	0	0	0	6	7	3121052	1	1
CCDC152	NaN	NaN	NaN	708796	144536	0	0	0	0	50	116	39790120	1	1
CCDC153	1258103	195	57	557140	148808	0	0	1	0	6	15	3269504	1	1
CCDC154	NaN	NaN	NaN	1698476	512284	0	0	0	0	9	16	4869724	1	1
CCDC19	803074	403	7	1454260	363476	0	0	0	0	22	21	6788920	1	1
CCDC21	958818	217	26	1969036	531152	0	0	0	0	10	8	4384140	1	1
CCDC22	635975	NaN	35	1585980	494484	0	0	0	0	5	7	2878260	1	1
CCDC41	181169	690	25	1872560	439660	0	0	0	0	50	131	45924712	1	1
CCDC42B	NaN	NaN	NaN	774300	245640	0	0	0	0	50	116	39891224	1	1
CCDC45	1036796	184	8	2175872	541120	0	0	0	0	23	17	7927764	1	1
CCDC46	138097	778	-38	2714856	634392	0	0	0	0	3	10	3064448	1	1
CCDC48	1155221	379	52	1461736	487364	0	0	0	0	24	31	9912108	1	1
CCDC52	458321	566	22	2209336	603776	0	0	0	0	6	4	2222864	1	1

CCDC53	234580	467	7	452832	123888	0	0	1	0	12	17	5692440	1	1
CCDC55	605144	298	24	1488080	330724	0	0	0	0	50	44	21663312	1	1
CCDC56	1633092	190	33	258100	91136	0	0	0	0	50	66	20371388	1	1
CCDC58	475086	478	39	393380	90424	0	0	0	0	50	74	22270648	1	1
CCDC72	1401411	263	32	185120	38092	0	0	0	0	50	79	21907884	1	1
CCDC75	603425	281	27	714492	152724	0	0	0	0	50	57	19166684	1	1
CCDC76	293698	518	8	1270920	320400	0	0	0	0	50	49	21769400	1	1
CCDC79	NaN	NaN	NaN	1943760	460308	0	0	0	0	50	116	40105892	1	1
CCDC85B	3779084	287	42	480956	169456	0	0	0	0	1	1	408332	1	1
CCDC85C	NaN	NaN	NaN	1029196	337844	0	0	0	0	50	116	39983428	1	1
CCDC9	1350139	232	33	1354580	396940	0	0	3	0	50	65	19950596	1	1
CCDC90A	276494	496	27	914564	275188	0	0	0	0	50	45	18741264	1	1
CCDC94	2132502	244	39	840160	229976	0	0	2	0	14	14	5282684	1	1
CCDC99	96768	495	19	1599864	388752	0	0	0	0	50	42	20180216	1	1
CCK	338797	473	29	288360	91848	0	0	0	0	50	46	21775452	1	1
CCL1	40425	871	-32	256676	64792	0	0	0	0	50	85	19967328	1	1
CCL11	40425	871	-32	251692	70844	0	0	0	0	50	85	19967328	1	1
CCL17	749108	159	39	246708	70488	0	0	0	0	50	52	18373516	1	1
CCL18	779228	525	19	236384	62656	0	0	0	0	50	69	21358220	1	1
CCL19	731413	298	51	254184	75828	0	0	0	0	50	42	16261368	1	1
CCL24	1013258	255	46	306872	84016	0	0	0	0	50	61	24609568	1	1
CCL25	954819	369	13	394448	106444	0	0	0	0	50	71	15374572	1	1
CCL3L1	NaN	NaN	NaN	496976	131008	0	0	0	0	50	116	39776592	1	1
CCL4	744143	565	28	242080	66572	0	0	0	0	50	68	23933524	1	1
CCL4L1	NaN	NaN	NaN	479888	137416	0	0	0	0	50	116	39783000	1	1
CCL4L2	691971	684	13	479888	137416	0	0	0	0	50	57	20760140	1	1
CCL8	40425	871	-32	260236	68708	0	0	1	0	9	9	2480608	1	1
CCNB1IP1	393743	401	-18	715560	187968	0	0	1	0	50	45	19157784	1	1
CCR6	149346	696	40	941620	268424	0	0	1	0	50	68	21350744	1	1
CCRL1	210898	768	21	883948	244928	0	0	0	0	50	58	22555804	1	1
CCT4	688730	226	34	1377720	407976	0	0	1	0	50	51	20523044	1	1
CD151	1799017	232	51	658244	185476	0	0	1	0	50	57	18763692	1	1
CD24	NaN	NaN	NaN	185832	73692	0	0	0	0	50	116	39719276	1	1

CD302	361333	405	34	618728	151300	0	0	0	0	50	40	19566116	1	1
CD320	778783	209	14	696692	229264	0	0	0	0	50	66	22945268	1	1
CD3D	963668	192	48	442152	128160	0	0	0	0	21	25	8075148	1	1
CD59	755929	424	15	337488	88644	0	0	0	0	50	60	19185196	1	1
CD69	226318	648	33	533644	126380	0	0	1	0	18	26	5237472	1	1
CD8B	330931	543	19	803848	190460	0	0	0	0	50	55	23650504	1	1
CD97	996589	183	32	2154512	605200	0	0	0	0	4	6	2810620	1	1
CDA	631461	208	35	381276	102528	0	0	1	0	6	8	3603076	1	1
CDC26	426351	312	26	225348	58740	0	0	0	0	37	41	12733408	1	1
CDC42EP1	991778	176	51	933788	326452	0	0	0	0	40	48	15032456	1	1
CDC42EP2	3946871	242	49	505876	170168	0	0	0	0	27	30	12483852	1	1
CDC42EP3	477159	404	31	632612	184408	0	0	3	0	7	5	1248492	1	1
CDC42EP5	NaN	NaN	NaN	347456	134212	0	0	0	0	50	116	39779796	1	1
CDC42SE2	313468	414	34	230688	54468	0	0	0	0	50	34	18270276	1	1
CDHR4	1306720	191	39	2007840	601284	0	0	0	0	5	7	2686376	1	1
CDK1	109701	396	-21	790676	204700	0	0	1	0	50	42	17725596	1	1
CDK2AP1	1014179	201	29	299396	85084	0	0	0	0	50	76	21287376	1	1
CDKN1C	552601	256	34	752228	280528	0	0	0	0	50	59	21410552	1	1
CDRT15	96214	975	-61	462444	149520	0	0	0	0	33	27	10757964	1	1
CDV3	391516	327	36	682096	184052	0	0	0	0	50	52	21749108	1	1
CDX1	1002307	228	49	644360	218584	0	0	0	0	50	53	19888652	1	1
CDX2	309835	614	47	766468	248132	0	0	1	0	50	55	18324388	1	1
CDX4	221528	NaN	-13	714492	206124	0	0	0	0	50	44	16162400	1	1
CDY1	NaN	NaN	NaN	2839456	787472	0	0	0	0	7	14	4475632	1	1
CDY1B	NaN	NaN	NaN	2839456	787472	0	0	0	0	7	14	4475632	1	1
CDY2A	NaN	NaN	NaN	2726248	755432	0	0	0	0	7	14	4443592	1	1
CDY2B	NaN	NaN	NaN	2726248	755432	0	0	0	0	7	14	4443592	1	1
CEACAM1	812266	188	-22	1045572	344964	0	0	0	0	8	17	4475632	1	1
CEACAM2	761897	511	-61	1513000	452120	0	0	1	0	19	80	19760136	1	1
CEBPD	918236	460	15	646496	220720	0	0	0	0	3	1	690996	1	1
CEBPE	1261207	185	41	684944	222856	0	0	3	0	0	3	222856	1	1
CENPBD1	1056657	221	35	472412	129940	0	0	1	0	20	22	5927400	1	1
CENPC1	128864	689	7	2482032	619440	0	0	0	0	22	33	14099380	1	1

CENPM	1335011	323	67	497688	148452	0	0	1	0	50	49	15999352	1	1
CENPV	881521	169	4	684588	209328	0	0	0	0	50	44	16627336	1	1
CENPW	142596	704	-1	227128	66572	0	0	0	0	50	58	21173100	1	1
CEP110	610711	365	34	6129964	1499828	0	0	0	0	13	7	8569632	1	1
CES7	348857	581	-30	1612324	461732	0	0	0	0	26	34	11173772	1	1
CES8	1044004	135	48	1536496	406196	0	0	0	0	24	17	9327912	1	1
CFB	2314693	203	34	2002144	525812	0	0	0	0	22	25	13143520	1	1
CFC1	NaN	NaN	NaN	1137064	345320	0	0	0	0	50	116	39990904	1	1
CFC1B	569565	NaN	12	1137064	345320	0	0	0	0	22	24	9259204	1	1
CFD	2168802	198	28	623356	209684	0	0	0	0	50	73	22578944	1	1
CFDP1	507054	133	17	799576	187256	0	0	0	0	50	43	20298408	1	1
CGB	2070141	200	35	404416	138128	0	0	1	0	12	19	4170896	1	1
CGB1	2070141	200	35	380208	130296	0	0	0	0	50	68	18142828	1	1
CGB2	2070141	200	35	396940	139196	0	0	0	0	50	68	18142828	1	1
CGB7	2070141	200	35	404416	138128	0	0	1	0	12	19	4170896	1	1
CGREF1	1214097	238	50	1014600	307584	0	0	1	0	50	60	22743060	1	1
CHAC1	1049616	176	50	651124	208616	0	0	2	0	27	42	10668252	1	1
CHADL	NaN	NaN	NaN	1839096	689928	0	0	0	0	7	14	4378088	1	1
CHCHD4	312564	463	27	432896	101104	0	0	0	0	50	51	19996876	1	1
CHCHD5	590285	166	36	288360	80100	0	0	0	0	50	42	19807840	1	1
CHCHD6	250457	604	39	619440	168744	0	0	1	0	28	30	10153476	1	1
CHCHD8	555075	214	47	229620	56604	0	0	0	0	50	56	20095488	1	1
CHIC1	319167	NaN	11	590604	151656	0	0	0	0	50	64	23168124	1	1
CHIC2	92904	472	19	446780	106444	0	0	0	0	50	43	19076260	1	1
CHMP1A	1090549	219	38	762908	56248	0	0	0	0	50	51	16551864	1	1
CHMP4A	974559	220	41	692064	181560	0	0	0	0	50	62	24565780	1	1
CHP	956634	214	44	522608	131008	0	0	0	0	25	25	11307628	1	1
CHRNA7	199973	778	-9	1291568	360628	0	0	0	0	32	25	12483496	1	1
CHST10	226558	642	9	924176	241012	0	0	1	0	50	59	23206572	1	1
CHST2	319752	591	25	1260240	441084	0	0	0	0	32	53	21609556	1	1
CHST3	762207	169	54	1176580	365612	0	0	2	0	50	92	21141416	1	1
CIDEC	950574	170	54	606268	180848	0	0	0	0	50	71	20154584	1	1
CISD1	153256	357	-17	286580	71200	0	0	0	0	50	46	21905748	1	1

CISD2	373094	471	33	368816	75472	0	0	0	0	50	42	18599220	1	1
CISD3	NaN	NaN	NaN	325384	99680	0	0	0	0	50	116	39745264	1	1
CITED1	370863	NaN	50	543256	172304	0	0	0	0	50	63	22275632	1	1
CITED4	NaN	NaN	NaN	426132	166608	0	0	0	0	50	116	39812192	1	1
CKLF	688682	214	-1	401568	115344	0	0	0	0	50	64	21771180	1	1
CKMT1A	774279	286	51	1048776	324672	0	0	0	0	21	17	5977596	1	1
CKS1B	1612870	224	35	216804	48060	0	0	1	0	2	5	590248	1	1
CLCC1	880200	216	19	1442868	375936	0	0	0	0	22	20	9174832	1	1
CLDN20	NaN	NaN	NaN	536848	168032	0	0	0	0	50	116	39813616	1	1
CLDN24	NaN	NaN	NaN	538984	166964	0	0	0	0	50	116	39812548	1	1
CLDN8	10231	994	-69	560700	163404	0	0	0	0	18	26	5070864	1	1
CLDND2	368509	610	-9	427200	126024	0	0	0	0	50	39	14321524	1	1
CLEC12B	305376	565	19	747600	179424	0	0	0	0	50	34	26162796	1	1
CLEC18A	1511555	162	36	1165188	313992	0	0	0	0	8	23	5592048	1	1
CLEC18C	1353954	139	37	1165544	313636	0	0	0	0	50	63	21892220	1	1
CLEC2A	NaN	NaN	NaN	473124	106800	0	0	0	0	50	116	39752384	1	1
CLEC2L	557910	522	32	541832	164116	0	0	0	0	50	98	28041408	1	1
CLECL1	251092	648	8	433252	109292	0	0	1	0	19	24	7035628	1	1
CLPS	621095	404	34	291564	81168	0	0	0	0	50	56	22245372	1	1
CMA1	898202	411	17	627272	188680	0	0	2	0	1	2	429336	1	1
CMC1	84882	793	10	291564	64080	0	0	0	0	50	61	22171680	1	1
CMTM3	777342	214	9	466004	141688	0	0	0	0	50	68	21478904	1	1
CMTM4	790767	145	9	597368	174796	0	0	1	0	50	59	21196952	1	1
CMTM8	306655	590	11	441440	128872	0	0	0	0	50	66	21077336	1	1
CNGA4	466636	728	4	1439664	429336	0	0	1	0	50	57	22924620	1	1
CNIH	399536	371	17	386616	96120	0	0	0	0	50	69	21693572	1	1
CNN3	282666	333	-6	863300	219652	0	0	1	0	23	23	10227880	1	1
CNO	501020	201	62	529728	170880	0	0	0	0	43	51	15731284	1	1
CNOT7	237338	387	4	760416	185832	0	0	1	0	50	39	20285948	1	1
CNPY1	267612	599	-5	255608	55180	0	0	0	0	50	61	19878684	1	1
CNPY3	1143209	203	41	717340	197936	0	0	0	0	50	59	17728800	1	1
CNRIP1	204433	586	15	468496	131720	0	0	0	0	50	47	17734140	1	1
CNTD1	1633092	190	33	840516	245640	0	0	0	0	24	22	8134244	1	1

COBRA1	1724821	399	43	1468500	444288	0	0	0	0	18	19	5825228	1	1
COLEC10	180181	380	2	729800	184408	0	0	0	0	50	44	24256416	1	1
COMMD1(	312789	981	8	537560	140620	0	0	0	0	50	53	20400224	1	1
COMMD3	195915	462	23	524744	135280	0	0	0	0	50	54	17596724	1	1
COMMD4	814680	296	43	521896	150944	0	0	1	0	50	63	19824928	1	1
COMMD7	661510	132	45	546816	131364	0	0	2	0	6	7	1361700	1	1
COMMD8	87789	528	23	486296	122464	0	0	0	0	50	46	20065584	1	1
COPG	1119201	189	55	2286588	617304	0	0	0	0	3	0	2294420	1	1
COPG2	298918	433	16	1071916	270560	0	0	0	0	7	6	4045584	1	1
COPS6	1503181	254	41	862944	228552	0	0	2	0	21	30	10491676	1	1
COPZ1	664708	190	45	483448	123176	0	0	1	0	50	62	22308028	1	1
COQ7	861035	199	8	564616	157352	0	0	0	0	50	60	19206200	1	1
CORT	829921	392	32	386616	117480	0	0	0	0	50	57	23206928	1	1
COX16	500896	549	32	284800	70844	0	0	1	0	9	9	1606272	1	1
COX19	765060	346	34	251336	50908	0	0	0	0	50	53	20860888	1	1
COX4I1	381135	298	28	454612	106088	0	0	0	0	50	64	24463608	1	1
COX4I2	837796	213	18	455680	112496	0	0	0	0	50	55	18907516	1	1
COX4NB	381135	298	28	545392	147740	0	0	0	0	50	64	24463608	1	1
COX5B	362146	520	25	334996	98612	0	0	1	0	1	2	379852	1	1
COX6A2	839076	558	11	248132	78676	0	0	0	0	50	75	22592472	1	1
COX6B1	1162794	390	27	239588	51976	0	0	0	0	50	66	20625572	1	1
COX6B2	1028808	209	19	243148	54824	0	0	0	0	50	60	18195872	1	1
COX7A1	978272	326	6	212888	58384	0	0	0	0	50	68	15103300	1	1
COX7C	84088	829	-14	169456	46280	0	0	0	0	7	8	2503392	1	1
COX8A	1322584	277	42	167676	63012	0	0	0	0	50	83	26795052	1	1
COX8C	NaN	NaN	NaN	177288	63012	0	0	0	0	50	116	39708596	1	1
CP110	349996	491	21	2577796	656108	0	0	0	0	12	10	5660756	1	1
CPLX2	473039	624	32	358492	86864	0	0	0	0	50	71	25529472	1	1
CPLX4	293564	549	20	431472	92916	0	0	0	0	42	44	18840944	1	1
CPNE5	844979	244	43	1570316	420436	0	0	1	0	50	60	23128964	1	1
CPPED1	75434	797	-3	804560	217516	0	0	1	0	48	51	18537276	1	1
CPSF4L	NaN	NaN	NaN	491280	111072	0	0	0	0	50	116	39756656	1	1
CRB3	865106	219	33	356000	123532	0	0	0	0	50	76	20954160	1	1

CREB1	184506	198	25	873624	256320	0	0	0	0	50	47	19904316	1	1
CREB3L1	801520	158	36	1326812	386260	0	0	1	0	50	45	19214032	1	1
CRELD2	842221	271	15	1055540	278392	0	0	1	0	50	69	23287384	1	1
CRH	277437	480	30	470276	165184	0	0	2	0	6	10	2710940	1	1
CRHBP	245514	418	17	832684	227840	0	0	1	0	50	48	20406988	1	1
CRIP1	689089	298	41	221076	50196	0	0	0	0	50	46	21105816	1	1
CRISP2	67596	1248	-6	653260	158420	0	0	1	0	50	43	17490992	1	1
CRKL	523674	192	49	754008	228552	0	0	1	0	50	50	19418376	1	1
CRTAP	513008	372	33	1035604	278036	0	0	0	0	50	53	21203360	1	1
CRYAA	687433	279	59	435388	132788	0	0	0	0	50	52	19003280	1	1
CRYAB	437513	221	22	442864	131720	0	0	0	0	50	49	18660452	1	1
CRYBB3	247839	525	-31	555360	145248	0	0	1	0	50	85	21194816	1	1
CRYGA	321897	440	5	453544	119972	0	0	0	0	50	50	16889352	1	1
CRYGC	327858	440	13	453900	119616	0	0	0	0	50	63	21332232	1	1
CSAG2	NaN	NaN	NaN	650768	182272	0	0	0	0	50	116	39827856	1	1
CSAG3	202421	NaN	-2	650768	182272	0	0	0	0	44	50	16755496	1	1
CSDA	410051	455	-4	939484	294056	0	0	0	0	50	83	25154248	1	1
CSDC2	1467336	183	66	380564	121396	0	0	0	0	50	56	20734864	1	1
CSF2	362855	407	21	375936	103596	0	0	0	0	50	41	21683248	1	1
CSNK2A2	862670	292	15	938772	232824	0	0	1	0	36	57	15421564	1	1
CSNK2B	2517236	193	20	581348	136348	0	0	0	0	50	63	27465044	1	1
CST3	189028	819	-21	366324	115344	0	0	0	0	50	70	17124312	1	1
CST6	3854361	208	57	377004	114276	0	0	0	0	29	35	13479584	1	1
CT45A1	NaN	NaN	NaN	499468	126380	0	0	0	0	50	116	39771964	1	1
CT45A2	NaN	NaN	NaN	501248	124600	0	0	0	0	50	116	39770184	1	1
CT45A3	NaN	NaN	NaN	500180	125668	0	0	0	0	50	116	39771252	1	1
CT45A4	NaN	NaN	NaN	1001784	249912	0	0	0	0	50	116	39895496	1	1
CT45A5	246199	NaN	26	500892	124956	0	0	0	0	50	66	25385292	1	1
CT45A6	NaN	NaN	NaN	500536	125312	0	0	0	0	50	116	39770896	1	1
CT47A1	NaN	NaN	NaN	7116440	2228560	0	0	0	0	0	0	2228560	1	1
CT47A10	NaN	NaN	NaN	6404796	2005704	0	0	0	0	0	0	2005704	1	1
CT47A11	NaN	NaN	NaN	6404796	2005704	0	0	0	0	0	0	2005704	1	1
CT47A2	NaN	NaN	NaN	6404796	2005704	0	0	0	0	0	0	2005704	1	1

CT47A3	NaN	NaN	NaN	6404796	2005704	0	0	0	0	0	0	2005704	1	1
CT47A4	NaN	NaN	NaN	6404796	2005704	0	0	0	0	0	0	2005704	1	1
CT47A5	NaN	NaN	NaN	6404796	2005704	0	0	0	0	0	0	2005704	1	1
CT47A6	NaN	NaN	NaN	7116440	2228560	0	0	0	0	0	0	2228560	1	1
CT47A7	NaN	NaN	NaN	711644	222856	0	0	0	0	50	116	39868440	1	1
CT47A8	NaN	NaN	NaN	6404796	2005704	0	0	0	0	0	0	2005704	1	1
CT47A9	NaN	NaN	NaN	6404796	2005704	0	0	0	0	0	0	2005704	1	1
CT62	276800	462	10	348168	99324	0	0	0	0	50	45	22716716	1	1
CTAG1A	NaN	NaN	NaN	866504	314704	0	0	0	0	50	116	39960288	1	1
CTAG1B	NaN	NaN	NaN	866504	314704	0	0	0	0	50	116	39960288	1	1
CTAGE5	314800	300	16	2163056	545392	0	0	0	0	21	20	9386652	1	1
CTAGE6P	453170	544	-7	1955508	539340	0	0	0	0	2	3	1217520	1	1
CTNNBIP1	770684	193	34	218228	57316	0	0	0	0	50	47	19426208	1	1
CTPS	341851	171	29	1562484	411180	0	0	0	0	17	21	9135316	1	1
CTRB1	612638	171	16	673196	202564	0	0	0	0	50	41	19443652	1	1
CTSH	421272	488	45	902104	223568	0	0	3	0	1	4	432540	1	1
CTSL1	122983	577	26	885016	215024	0	0	0	0	50	39	19738776	1	1
CTSL2	339842	464	24	884660	218584	0	0	0	0	28	45	15439720	1	1
CTSS	1818753	188	35	868996	223568	0	0	2	0	30	59	12923512	1	1
CTU2	802673	189	41	1330016	387328	0	0	1	0	50	65	20750172	1	1
CTXN2	NaN	NaN	NaN	210752	56248	0	0	0	0	50	116	39701832	1	1
CWC25	1198833	207	37	1120688	282664	0	0	0	0	50	46	18180920	1	1
CXCL12	33976	922	-34	466004	126736	0	0	0	0	50	89	19323680	1	1
CXCL3	199590	368	13	272696	86152	0	0	0	0	50	60	21369968	1	1
CXCR1	718028	301	27	857248	271628	0	0	1	0	50	49	26873016	1	1
CXCR2	694517	301	27	877896	283020	0	0	0	0	50	45	24502056	1	1
CXCR7	121886	599	10	904240	263084	0	0	0	0	47	48	17306940	1	1
CXorf1	1641	NaN	-34	285156	73692	0	0	0	0	50	124	22337220	1	1
CXorf22	3828	NaN	-13	2528668	667856	0	0	0	0	0	0	667856	1	1
CXorf26	102519	NaN	16	619796	151300	0	0	0	0	50	75	23903264	1	1
CXorf27	106141	NaN	28	304380	75828	0	0	0	0	50	59	27275296	1	1
CXorf30	NaN	NaN	NaN	1668928	422216	0	0	0	0	50	116	40067800	1	1
CXorf40A	187616	NaN	16	410824	118904	0	0	0	0	50	63	20686092	1	1

CXorf41	229024	NaN	26	576364	138128	0	0	0	0	50	61	21116140	1	1
CXorf48	228366	NaN	23	682096	190460	0	0	0	0	50	50	19392388	1	1
CXorf49	NaN	NaN	NaN	2551096	789608	0	0	0	0	7	14	4477768	1	1
CXorf49B	NaN	NaN	NaN	2551096	789608	0	0	0	0	7	14	4477768	1	1
CXorf51	NaN	NaN	NaN	554648	160912	0	0	0	0	50	116	39806496	1	1
CXorf58	528624	NaN	37	878252	222856	0	0	0	0	50	50	22018244	1	1
CXorf59	3834	NaN	-17	1325388	328944	0	0	0	0	20	56	11817064	1	1
CXorf61	82720	NaN	4	287292	84372	0	0	0	0	50	76	26718512	1	1
CXorf64	NaN	NaN	NaN	730156	234248	0	0	0	0	50	116	39879832	1	1
CYB561	874574	267	-20	626560	202208	0	0	1	0	50	76	20536572	1	1
CYB5A	152675	989	26	376292	89356	0	0	0	0	50	37	19320120	1	1
CYB5R2	100503	853	13	731936	189748	0	0	0	0	50	69	20074128	1	1
CYBASC3	1394229	286	57	656108	198292	0	0	0	0	50	56	17921040	1	1
CYBB	105298	NaN	9	1483808	399076	0	0	1	0	50	83	24688956	1	1
CYP2D6	1296288	363	43	1237456	396584	0	0	1	0	9	18	3861532	1	1
CYP2W1	765060	346	34	1188684	420792	0	0	1	0	50	53	20860888	1	1
CYS1	863812	214	44	381276	136704	0	0	0	0	50	61	22507388	1	1
CYSLTR1	486217	NaN	-9	856180	233180	0	0	1	0	50	50	20394884	1	1
CYTL1	76400	557	36	343896	110004	0	0	0	0	50	58	21214040	1	1
CYTSA	702019	200	38	2866868	779284	0	0	0	0	0	0	779284	1	1
CYTSB	302887	339	-13	2771104	784268	0	0	0	0	2	1	1062660	1	1
CYYR1	345442	626	-75	395872	113564	0	0	0	0	50	116	27785444	1	1
CYorf15A	0	0	0	351016	84728	0	0	0	0	50	39	21173456	1	1
CYorf15B	0	0	0	474904	121040	0	0	0	0	50	39	21173456	1	1
DACT2	NaN	NaN	NaN	1870424	627628	0	0	0	0	7	14	4315788	1	1
DAD1	975314	380	49	287648	88288	0	0	0	0	50	81	24089452	1	1
DAO	494991	393	49	902460	255252	0	0	0	0	50	69	24144988	1	1
DAPL1	270644	361	14	283732	77252	0	0	0	0	50	59	21427284	1	1
DARC	99569	785	14	825564	289428	0	0	0	0	50	43	18472484	1	1
DAZ1	NaN	NaN	NaN	1964408	537916	0	0	0	0	9	16	4895356	1	1
DAZ2	NaN	NaN	NaN	2922404	775012	0	0	0	0	7	14	4463172	1	1
DAZ3	NaN	NaN	NaN	3452132	918124	0	0	0	0	5	8	3290152	1	1
DAZ4	NaN	NaN	NaN	3989336	1074052	0	0	0	0	0	0	1074052	1	1

DBC1	3696	1010	-25	1951236	520116	0	0	0	0	18	21	6810636	1	1
DCD	615189	494	31	291564	83304	0	0	0	0	50	83	25232212	1	1
DCI	2199935	194	42	757924	240656	0	0	0	0	50	61	21477480	1	1
DCTN6	452029	297	47	507656	132076	0	0	1	0	50	54	21508808	1	1
DDAH2	2517236	193	20	695268	246708	0	0	3	0	1	3	378072	1	1
DDT	NaN	NaN	NaN	300464	95764	0	0	0	0	50	116	39741348	1	1
DDX25	611330	414	14	1269140	328588	0	0	0	0	4	5	2325748	1	1
DDX39	954144	204	43	1118196	295836	0	0	0	0	22	20	9379532	1	1
DDX49	1390116	198	20	1236388	367748	0	0	3	0	29	40	11150988	1	1
1-Dec	117692	604	-17	196156	48416	0	0	0	0	35	38	17416232	1	1
DECR2	1549307	207	37	737988	232824	0	0	0	0	36	55	20238956	1	1
DEDD2	790787	299	37	784624	280172	0	0	0	0	18	15	6929540	1	1
DEFA1	NaN	NaN	NaN	730156	208616	0	0	0	0	50	116	39854200	1	1
DEFA1B	132154	699	-1	730156	208616	0	0	0	0	21	26	10670388	1	1
DEFA3	132154	699	-1	243860	69064	0	0	0	0	50	63	21418740	1	1
DEFA4	131884	628	11	247420	75116	0	0	0	0	50	58	19951664	1	1
DEFA6	131884	628	11	257744	72268	0	0	1	0	2	1	194376	1	1
DEFB1	131884	628	11	178356	46992	0	0	0	0	50	58	19951664	1	1
DEFB103A	NaN	NaN	NaN	360272	96832	0	0	0	0	50	116	39742416	1	1
DEFB103B	NaN	NaN	NaN	360272	96832	0	0	0	0	50	116	39742416	1	1
DEFB104A	NaN	NaN	NaN	387328	97544	0	0	0	0	50	116	39743128	1	1
DEFB104B	NaN	NaN	NaN	387328	97544	0	0	0	0	50	116	39743128	1	1
DEFB105A	NaN	NaN	NaN	423640	97544	0	0	0	0	50	116	39743128	1	1
DEFB105B	NaN	NaN	NaN	423640	97544	0	0	0	0	50	116	39743128	1	1
DEFB106A	NaN	NaN	NaN	357424	82592	0	0	0	0	50	116	39728176	1	1
DEFB106B	NaN	NaN	NaN	357424	82592	0	0	0	0	50	116	39728176	1	1
DEFB107A	NaN	NaN	NaN	379496	92560	0	0	0	0	50	116	39738144	1	1
DEFB107B	NaN	NaN	NaN	379496	92560	0	0	0	0	50	116	39738144	1	1
DEFB108B	727046	465	37	192240	48060	0	0	0	0	50	66	22765488	1	1
DEFB110	70183	1258	-7	308296	59096	0	0	0	0	50	45	19480676	1	1
DEFB113	70183	1258	-7	212888	48772	0	0	0	0	50	45	19480676	1	1
DEFB118	591819	594	-83	316128	87576	0	0	0	0	50	104	27802888	1	1
DEFB119	591819	594	-83	445356	104664	0	0	1	0	50	104	27802888	1	1

DEFB121	598822	450	-79	220720	58028	0	0	0	0	50	111	28209084	1	1
DEFB126	215351	430	25	287292	80100	0	0	1	0	5	5	857604	1	1
DEFB127	215351	430	25	256676	67996	0	0	2	0	4	5	628340	1	1
DEFB130	475312	625	0	408688	112496	0	0	0	0	50	72	22627360	1	1
DEFB131	176573	885	12	189748	40940	0	0	0	0	50	73	20658680	1	1
DEFB132	1010597	348	24	247420	66572	0	0	0	0	50	85	21827784	1	1
DEFB133	NaN	NaN	NaN	165540	37380	0	0	0	0	50	116	39682964	1	1
DEFB134	467502	451	22	177288	40584	0	0	0	0	50	58	22225080	1	1
DEFB136	467502	451	22	203632	51620	0	0	0	0	50	58	22225080	1	1
DEFB4A	69060	693	-5	169100	45568	0	0	0	0	50	71	21235400	1	1
DEGS1	638932	174	13	833752	212888	0	0	0	0	50	52	21216888	1	1
DEGS2	405420	237	39	797440	251336	0	0	1	0	50	35	18495980	1	1
DEM1	701723	457	31	929516	273052	0	0	0	0	50	59	24104048	1	1
DEPDC6	244861	712	8	1073340	274476	0	0	0	0	23	14	7214340	1	1
DEXI	339622	430	41	228908	78676	0	0	0	0	50	75	24230784	1	1
DFFB	249345	412	26	872556	239232	0	0	1	0	50	57	20727032	1	1
DGAT2	534135	418	46	991460	284800	0	0	1	0	50	59	22557940	1	1
DGCR6	621867	545	-1	557140	168032	0	0	0	0	50	79	22714224	1	1
DHDPSL	564805	169	40	826632	249912	0	0	0	0	50	62	20233616	1	1
DHFR	319054	626	35	501604	122108	0	0	1	0	22	22	6814908	1	1
DHH	1770671	214	65	942332	338200	0	0	3	0	0	3	338200	1	1
DHRS13	1834230	220	37	915632	312568	0	0	1	0	50	67	23772612	1	1
DHRS4L1	992350	355	19	725172	216804	0	0	0	0	50	70	21277764	1	1
DIMT1L	176808	575	33	830548	222500	0	0	0	0	50	56	21434048	1	1
DIO1	505734	319	24	647564	168388	0	0	2	0	1	2	389108	1	1
DIRAS3	331080	524	-25	572448	168744	0	0	3	0	2	5	781776	1	1
FZP781G0	NaN	NaN	NaN	1797444	456036	0	0	0	0	50	116	40101620	1	1
KFZp761E1	3834148	287	55	1897836	737988	0	0	0	0	0	0	737988	1	1
DLEU7	136985	649	31	381276	139908	0	0	0	0	50	49	21266728	1	1
DLX5	99426	991	-15	724816	212888	0	0	0	0	50	65	20345400	1	1
DMRTA2	147973	639	-7	828412	316484	0	0	2	0	11	6	4120344	1	1
DMRTC1	NaN	NaN	NaN	969032	314704	0	0	0	0	50	116	39960288	1	1
DMRTC1E	NaN	NaN	NaN	969032	314704	0	0	0	0	50	116	39960288	1	1

DNAH14	NaN	NaN	NaN	11868328	3000368	0	0	2	0	0	2	3000368	1	1
DNAJB3	NaN	NaN	NaN	369528	100392	0	0	0	0	50	116	39745976	1	1
DNAJB7	NaN	NaN	NaN	795304	197936	0	0	1	0	50	117	39843520	1	1
DNAJC19	182678	1094	-4	316840	81524	0	0	0	0	50	35	18626632	1	1
AJC25-GN	513721	212	17	372732	133500	0	0	0	0	50	51	20911084	1	1
DNAJC27	270621	296	35	721612	184052	0	0	2	0	8	7	2623364	1	1
DNAJC4	1608193	179	61	616592	182272	0	0	1	0	50	67	20204068	1	1
DNAJC7	2081077	206	23	1323608	317908	0	0	2	0	50	79	22998668	1	1
DNAL4	1291659	272	73	279104	69064	0	0	0	0	50	42	16818864	1	1
DNASE1	611617	242	43	733360	208616	0	0	0	0	50	54	23753744	1	1
DNASE1L1	2277674	NaN	40	768604	232112	0	0	1	0	50	46	20570392	1	1
DNASE1L2	2179404	224	39	756144	230688	0	0	1	0	50	46	19527312	1	1
DND1	774470	356	31	865436	283732	0	0	0	0	19	25	9696372	1	1
DOC2B	NaN	NaN	NaN	771808	235316	0	0	0	0	50	116	39880900	1	1
DOHH	2172733	210	36	742260	245640	0	0	0	0	50	54	24098352	1	1
DOLPP1	1428394	206	30	618016	179780	0	0	1	0	50	55	20767616	1	1
DOM3Z	2314693	203	34	997868	301888	0	0	0	0	50	60	23217608	1	1
DPCD	843302	421	19	542900	136348	0	0	0	0	50	65	21446508	1	1
DPH3B	NaN	NaN	NaN	202920	50196	0	0	0	0	50	116	39695780	1	1
DPH5	153041	727	-6	749736	196512	0	0	0	0	31	28	12783960	1	1
DPM2	1441525	201	56	217872	67284	0	0	0	0	50	40	17334352	1	1
DPT	358217	440	9	538272	123888	0	0	1	0	50	57	21132160	1	1
DPYSL5	1261383	378	36	1439308	422216	0	0	1	0	25	34	10393776	1	1
DR1	699815	223	13	457460	118192	0	0	1	0	8	15	2897840	1	1
DRAM1	340236	238	17	619084	172304	0	0	0	0	50	49	23432276	1	1
DRAP1	3779084	287	42	541120	144536	0	0	0	0	2	1	552868	1	1
DSCR6	414773	378	76	475972	148808	0	0	0	0	50	59	19569676	1	1
DTD1	262495	613	6	547884	146316	0	0	0	0	50	44	19160632	1	1
DTHD1	NaN	NaN	NaN	2025996	536136	0	0	0	0	9	16	4893576	1	1
DTYMK	1323203	406	32	543256	156284	0	0	0	0	50	66	20935292	1	1
DULLARD	1782958	316	21	636172	178712	0	0	0	0	39	39	14081580	1	1
DUS2L	859239	138	55	1281244	365612	0	0	0	0	4	7	2455688	1	1
DUS4L	512690	753	26	835176	209328	0	0	1	0	49	38	18677184	1	1

DUSP23	801453	497	6	364900	127448	0	0	0	0	50	57	19094772	1	1
DUSP3	1081050	192	35	474192	132432	0	0	1	0	50	58	18400572	1	1
DUSP5	493408	157	51	944112	302244	0	0	1	0	50	52	20126460	1	1
DUX4	NaN	NaN	NaN	2278400	840160	0	0	0	0	7	14	4528320	1	1
DYDC1	352963	598	18	488076	110004	0	0	0	0	50	52	25894016	1	1
DYNLL2	857807	352	11	242792	54112	0	0	1	0	0	1	54112	1	1
DYNLRB1	990458	214	54	259880	65860	0	0	1	0	1	3	154860	1	1
DYNLRB2	149480	465	-30	259880	65860	0	0	0	0	50	106	22824228	1	1
DYSFIP1	3733473	NaN	19	386616	120684	0	0	0	0	39	38	14805328	1	1
EBAG9	134329	623	8	570312	140976	0	0	0	0	50	56	20322260	1	1
EBF4	NaN	NaN	NaN	1527596	464224	0	0	0	0	50	116	40109808	1	1
ECEL1	271362	191	32	1976156	581704	0	0	2	0	43	56	15064496	1	1
ECSCR	806824	NaN	41	195088	54824	0	0	0	0	50	65	20236108	1	1
EDARADD	482656	589	11	605556	145248	0	0	0	0	50	69	21799304	1	1
EDDM3A	956361	577	8	389464	89000	0	0	0	0	50	92	23350396	1	1
EDF1	1921909	176	44	417588	116412	0	0	0	0	50	56	20791468	1	1
EFCAB2	675692	505	10	473124	112140	0	0	0	0	50	62	20840596	1	1
EFCAB4A	1799017	232	51	742972	232112	0	0	0	0	50	57	18763692	1	1
EFCAB4B	241693	793	18	2070140	550732	0	0	0	0	36	29	14343240	1	1
EFCAB9	NaN	NaN	NaN	526168	122108	0	0	0	0	50	116	39767692	1	1
EFEMP2	3779084	287	42	1154864	310432	0	0	1	0	2	2	718764	1	1
EFHA1	345194	422	31	1159848	283020	0	0	0	0	50	50	23860188	1	1
EFHA2	191406	621	-37	1386620	374512	0	0	0	0	22	44	10538312	1	1
EFNA1	1801444	194	32	538272	138840	0	0	2	0	2	4	372376	1	1
EFNA4	1696720	214	44	666788	184408	0	0	0	0	50	84	23988348	1	1
EFR3B	NaN	NaN	NaN	2129948	584908	0	0	0	0	7	14	4273068	1	1
EGFL8	2119974	392	28	757212	218940	0	0	1	0	50	62	23774036	1	1
EGLN3	163649	564	0	611252	174796	0	0	0	0	50	52	19769392	1	1
EI24	391073	595	23	921328	212888	0	0	1	0	24	23	10416916	1	1
EIF1AD	3854361	208	57	444644	108580	0	0	0	0	29	35	13479584	1	1
EIF1AX	262207	NaN	37	403704	86508	0	0	0	0	50	54	19347532	1	1
EIF1AY	0	0	0	404060	86152	0	0	0	0	50	39	21173456	1	1
EIF2C1	973030	273	12	2209692	616236	0	0	0	0	4	4	2222508	1	1

EIF2C2	451419	238	46	2219660	614812	0	0	0	0	16	9	5474212	1	1
EIF2C3	966499	282	25	2217524	618016	0	0	0	0	7	13	4826292	1	1
EIF2C4	1098857	273	19	2218948	615524	0	0	0	0	23	44	13858012	1	1
EIF3C	NaN	NaN	NaN	4786776	1244220	0	0	0	0	0	0	1244220	1	1
EIF3CL	1139917	269	56	4786776	1244220	0	0	0	0	0	0	1244220	1	1
EIF4EBP3	706371	319	29	299752	34532	0	0	0	0	50	44	23563640	1	1
EIF5A2	199827	640	26	404060	106444	0	0	0	0	50	52	19158852	1	1
ELF5	685229	295	5	715560	162336	0	0	0	0	50	78	27692172	1	1
ELK3	267884	525	23	1004276	320044	0	0	0	0	17	21	8937024	1	1
ELL	1389540	259	45	1569960	472056	0	0	1	0	17	23	8910680	1	1
ELMOD1	300861	676	-3	904952	215380	0	0	0	0	50	63	15971584	1	1
ELOF1	1152381	272	31	226060	55892	0	0	0	0	50	48	19593884	1	1
ELOVL3	1041024	187	45	699184	181916	0	0	1	0	32	41	18107940	1	1
ELOVL4	151698	913	-14	824496	206124	0	0	0	0	50	54	19664016	1	1
ELTD1	74049	903	-17	1807412	466360	0	0	0	0	6	3	3284812	1	1
EME2	1604603	288	36	1084732	372020	0	0	0	0	12	7	3876840	1	1
EMG1	2704950	211	47	624780	180492	0	0	0	0	1	0	1171596	1	1
EMID2	653305	280	43	1092208	365612	0	0	0	0	50	50	22575028	1	1
EML6	NaN	NaN	NaN	5077272	1372380	0	0	0	0	0	0	1372380	1	1
EMP1	636324	203	-3	408688	114632	0	0	0	0	50	72	21753736	1	1
EMR1	748613	383	27	2324680	604844	0	0	0	0	17	16	8272372	1	1
EMR2	1032767	448	29	2128524	592740	0	0	0	0	7	6	3335720	1	1
EMR3	1036916	384	31	1698120	458172	0	0	0	0	28	26	9799256	1	1
EMX1	450159	421	23	703812	237096	0	0	2	0	2	5	1503032	1	1
EMX2	130702	632	4	646852	172304	0	0	2	0	10	17	3232480	1	1
EN2	267699	599	-4	806340	268068	0	0	0	0	6	4	1628700	1	1
ENDOG	1768145	201	34	718408	247064	0	0	0	0	26	29	9773980	1	1
ENHO	843127	370	55	185476	61232	0	0	0	0	50	48	16293408	1	1
ENTPD4	476980	332	37	1588116	440016	0	0	2	0	50	49	19188044	1	1
ENY2	135626	732	20	281952	64080	0	0	0	0	50	137	47881288	1	1
EPB49	584373	219	34	1069780	298328	0	0	0	0	47	53	20310512	1	1
ERLIN1	1070291	198	25	928448	236740	0	0	0	0	50	78	19641944	1	1
ERP27	341752	426	8	718764	186900	0	0	2	0	24	34	8550052	1	1

ERV3	216422	994	-9	1509440	428980	0	0	0	0	8	3	4441100	1	1
ERVFRDE1	NaN	NaN	NaN	1319692	407264	0	0	0	0	50	116	40052848	1	1
ESAM	168652	622	14	957996	320400	0	0	1	0	50	50	19749100	1	1
ETFA	307633	186	35	871132	248132	0	0	0	0	29	34	9748348	1	1
ETNK2	557652	370	34	999292	270560	0	0	2	0	9	11	3453200	1	1
EVPLL	NaN	NaN	NaN	791032	219296	0	0	0	0	50	116	39864880	1	1
EXD2	821714	373	39	1267004	355288	0	0	1	0	50	53	21084456	1	1
EXOC3L	1109491	150	46	1825924	625136	0	0	0	0	5	1	3011048	1	1
EXOSC3	489340	453	23	694200	207192	0	0	0	0	50	50	17617728	1	1
EXOSC6	1353954	139	37	626560	250268	0	0	0	0	50	63	21892220	1	1
F8A1	NaN	NaN	NaN	867216	326808	0	0	0	0	50	116	39972392	1	1
F8A2	NaN	NaN	NaN	2603784	980424	0	0	0	0	0	0	980424	1	1
F8A3	NaN	NaN	NaN	867216	326808	0	0	0	0	50	116	39972392	1	1
FAAH2	91305	NaN	-6	1368108	382344	0	0	0	0	26	33	13034228	1	1
FABP3	708176	262	29	350660	93628	0	0	0	0	50	70	24308392	1	1
FABP4	191177	595	1	353152	87932	0	0	0	0	50	53	20427636	1	1
FABP5	231632	463	42	362408	88288	0	0	0	0	50	80	21960928	1	1
FABP7	138284	1073	-1	352440	86508	0	0	0	0	50	41	15762612	1	1
FADS6	1027517	320	20	875404	272696	0	0	1	0	50	57	19874768	1	1
FAHD1	1604603	288	36	791388	225348	0	0	1	0	50	49	20717064	1	1
FAIM2	1291800	231	45	821292	241368	0	0	1	0	50	88	26715664	1	1
FAM100A	742112	160	46	447492	131364	0	0	0	0	50	60	18215452	1	1
FAM100B	1678166	209	19	421504	115700	0	0	0	0	50	115	26690388	1	1
FAM104B	214019	NaN	18	437524	96476	0	0	0	0	50	59	20814964	1	1
FAM105B	511502	422	16	913140	243504	0	0	0	0	50	55	17947028	1	1
FAM107A	534993	383	31	373444	103952	0	0	0	0	50	51	19608836	1	1
FAM108A1	2226995	214	26	893204	283732	0	0	0	0	50	75	24188776	1	1
FAM108B1	284075	358	4	761128	208616	0	0	0	0	50	55	22803580	1	1
FAM108C1	334777	313	7	812748	253116	0	0	0	0	50	58	24713520	1	1
FAM109B	1321127	363	65	636884	200428	0	0	0	0	50	55	15728436	1	1
FAM110A	824412	579	36	688860	263796	0	0	1	0	50	49	19535144	1	1
FAM113A	766497	396	44	1143828	343896	0	0	0	0	50	66	23498136	1	1
FAM113B	261073	398	0	1055540	336064	0	0	0	0	24	21	9975832	1	1

FAM115A	453936	618	-5	2292284	695980	0	0	0	0	0	0	695980	1	1
FAM115C	433430	463	-7	2571744	779640	0	0	0	0	2	1	1201144	1	1
FAM116A	718677	332	30	1611612	422928	0	0	0	0	0	0	422928	1	1
FAM116B	939384	257	70	1385196	382344	0	0	0	0	5	9	3295492	1	1
FAM119A	183578	198	24	554292	160200	0	0	0	0	50	47	20425856	1	1
FAM119B	1160849	344	39	687436	201140	0	0	0	0	50	87	20175588	1	1
FAM122A	NaN	NaN	NaN	704168	218584	0	0	1	0	50	117	39864168	1	1
FAM123A	331169	650	34	1625852	529372	0	0	0	0	3	2	1648992	1	1
FAM123B	57924	NaN	2	2810264	833752	0	0	0	0	1	1	1069424	1	1
FAM123C	607528	203	17	2082244	687080	0	0	0	0	2	1	1339272	1	1
FAM125A	899127	211	26	698472	215736	0	0	0	0	50	49	18107584	1	1
FAM125B	112775	646	33	835532	232468	0	0	0	0	50	59	22884748	1	1
FAM127C	304745	NaN	42	282664	84728	0	0	0	0	50	59	20553304	1	1
FAM128A	455705	540	29	389464	130652	0	0	0	0	50	54	19959852	1	1
FAM128B	349797	593	22	388396	131720	0	0	0	0	50	45	22118992	1	1
FAM131C	876105	197	40	722324	203632	0	0	0	0	50	65	21358576	1	1
FAM136A	671278	370	38	367392	88644	0	0	0	0	50	76	25574328	1	1
FAM136B	NaN	NaN	NaN	356000	87220	0	0	0	0	50	116	39732804	1	1
FAM149B1	NaN	NaN	NaN	1504100	419368	0	0	0	0	50	116	40064952	1	1
FAM150A	105913	682	-11	333928	99680	0	0	1	0	14	20	7447520	1	1
FAM153A	1267336	553	18	868284	209328	0	0	0	0	50	70	20646220	1	1
FAM153C	961008	363	32	325028	77608	0	0	0	0	50	53	18751944	1	1
FAM154A	500270	450	-18	1206128	328588	0	0	0	0	0	0	328588	1	1
FAM154B	382946	701	18	1023856	265220	0	0	0	0	50	79	28686124	1	1
FAM155B	74378	NaN	23	1162340	363832	0	0	2	0	50	51	18505236	1	1
FAM156A	NaN	NaN	NaN	1053760	317552	0	0	0	0	50	116	39963136	1	1
FAM156B	NaN	NaN	NaN	1053760	317552	0	0	0	0	50	116	39963136	1	1
FAM157A	NaN	NaN	NaN	966540	289428	0	0	0	0	50	116	39935012	1	1
FAM157B	NaN	NaN	NaN	965472	293700	0	0	0	0	50	116	39939284	1	1
FAM158A	974559	220	41	537560	156640	0	0	0	0	50	62	24565780	1	1
FAM159B	NaN	NaN	NaN	415452	108936	0	0	0	0	50	116	39754520	1	1
FAM160A1	NaN	NaN	NaN	2635468	742616	0	0	0	0	7	14	4430776	1	1
FAM164A	49383	969	2	846212	234604	0	0	0	0	50	59	22499912	1	1

FAM164C	919043	222	47	1154508	318264	0	0	0	0	25	41	16456812	1	1
FAM165B	410767	518	61	163404	38448	0	0	0	0	50	51	13682860	1	1
FAM167A	483148	520	43	530084	163048	0	0	1	0	50	62	21466088	1	1
FAM168A	521861	311	48	606624	179424	0	0	0	0	50	50	21501688	1	1
FAM170B	NaN	NaN	NaN	719832	196512	0	0	0	0	50	116	39842096	1	1
FAM171A2	NaN	NaN	NaN	1973308	706304	0	0	0	0	7	14	4394464	1	1
FAM173A	1207790	194	33	568176	209328	0	0	0	0	50	77	22508812	1	1
FAM174B	281213	478	-17	384124	137060	0	0	0	0	50	87	23793616	1	1
FAM175A	275241	378	29	1085800	264152	0	0	0	0	50	47	22805716	1	1
FAM176A	224734	608	8	386616	112140	0	0	0	0	50	54	23225084	1	1
FAM176B	685495	212	32	403348	137060	0	0	0	0	50	43	18907872	1	1
FAM178B	677234	392	46	1762912	517268	0	0	0	0	42	30	17765112	1	1
FAM180B	NaN	NaN	NaN	456748	143468	0	0	0	0	50	116	39789052	1	1
FAM183A	1068006	294	30	362408	87220	0	0	0	0	50	45	20299476	1	1
FAM184B	NaN	NaN	NaN	2740844	731224	0	0	0	0	7	14	4419384	1	1
FAM185A	NaN	NaN	NaN	996088	292988	0	0	0	0	50	116	39938572	1	1
FAM186A	NaN	NaN	NaN	5877204	1690644	0	0	0	0	0	0	1690644	1	1
FAM189A1	NaN	NaN	NaN	1338560	434320	0	0	0	0	50	116	40079904	1	1
FAM18A	258562	356	36	564972	148452	0	0	0	0	50	51	18027128	1	1
FAM18B	832902	284	-7	551088	136704	0	0	0	0	50	64	19453264	1	1
FAM18B2	457998	452	-34	839092	232112	0	0	0	0	50	67	19233256	1	1
FAM190A	104011	1180	-4	2312220	645072	0	0	0	0	7	6	3797096	1	1
FAM190B	215927	942	1	2161632	556428	0	0	0	0	13	33	8832360	1	1
FAM192A	731638	149	48	671416	171236	0	0	2	0	18	13	4999308	1	1
FAM194A	359484	397	30	1743688	443576	0	0	0	0	50	51	23959156	1	1
FAM194B	NaN	NaN	NaN	1840164	448560	0	0	0	0	50	116	40094144	1	1
FAM196B	NaN	NaN	NaN	1336068	389820	0	0	0	0	50	116	40035404	1	1
FAM198A	NaN	NaN	NaN	1412252	450340	0	0	0	0	50	116	40095924	1	1
FAM19A1	121492	954	-14	354932	91492	0	0	1	0	1	1	326096	1	1
FAM19A3	559669	325	27	461732	98968	0	0	0	0	50	65	23160292	1	1
FAM200A	1460222	332	56	1467076	376292	0	0	0	0	42	54	14660080	1	1
FAM200B	NaN	NaN	NaN	1679252	428980	0	0	0	0	50	116	40074564	1	1
FAM20C	195067	593	-4	694556	237808	0	0	0	0	50	57	20503464	1	1

FAM21B	109302	804	30	6545060	1729804	0	0	0	0	0	0	1729804	1	1
FAM22A	409986	144	20	2134576	707372	0	0	0	0	14	10	8104696	1	1
FAM22D	490965	332	22	1367040	446424	0	0	0	0	6	3	2543264	1	1
FAM22F	219475	445	31	1843012	610184	0	0	0	0	6	3	2037032	1	1
FAM22G	309543	582	27	1838384	610540	0	0	0	0	8	18	4384496	1	1
FAM23A	278936	286	5	1747960	537560	0	0	0	0	50	54	23607072	1	1
FAM24B	290558	619	14	244572	68352	0	0	0	0	18	28	7230360	1	1
FAM25A	NaN	NaN	NaN	236028	65148	0	0	0	0	50	116	39710732	1	1
FAM25B	NaN	NaN	NaN	708084	195444	0	0	0	0	50	116	39841028	1	1
FAM25C	NaN	NaN	NaN	708084	195444	0	0	0	0	50	116	39841028	1	1
FAM25G	102837	642	23	708084	195444	0	0	0	0	50	41	21078048	1	1
FAM26F	261319	541	34	779284	243860	0	0	0	0	50	48	19636960	1	1
FAM32A	787753	219	34	305092	71912	0	0	0	0	50	45	17714204	1	1
FAM36A	694721	405	3	313992	80100	0	0	0	0	50	40	17173440	1	1
FAM38A	NaN	NaN	NaN	6353532	1940556	0	0	0	0	0	0	1940556	1	1
FAM38B	90278	610	30	7185148	1853336	0	0	0	0	2	0	2160920	1	1
FAM40A	660515	373	29	2182636	589892	0	0	0	0	3	5	1719836	1	1
FAM40B	625985	286	38	2190112	595232	0	0	0	0	10	12	4697420	1	1
FAM47E	NaN	NaN	NaN	1020296	274120	0	0	0	0	50	116	39919704	1	1
FAM48A	166443	631	23	2088652	522608	0	0	0	0	24	26	11347500	1	1
FAM48B1	644763	NaN	34	2156648	687436	0	0	0	0	7	1	4598808	1	1
FAM48B2	644763	NaN	34	1983632	636172	0	0	0	0	7	1	4598808	1	1
FAM54A	283639	240	15	997512	269136	0	0	0	0	43	49	16499176	1	1
FAM54B	746382	372	24	783912	180492	0	0	0	0	50	64	28906488	1	1
FAM55A	103922	612	-15	1040944	272696	0	0	0	0	50	51	23797176	1	1
FAM55B	NaN	NaN	NaN	1449276	368460	0	0	0	0	50	116	40014044	1	1
FAM55C	549022	421	12	1408336	402992	0	0	0	0	1	0	660380	1	1
FAM55D	109030	716	-17	1395164	372376	0	0	0	0	4	11	3451064	1	1
FAM58B	137061	607	1	625848	186900	0	0	0	0	50	49	20648000	1	1
FAM59A	268182	781	-5	2183348	646852	0	0	0	0	9	9	6348904	1	1
FAM59B	NaN	NaN	NaN	2092924	731936	0	0	0	0	7	14	4420096	1	1
FAM5B	5755	900	-14	1968324	573516	0	0	0	0	16	24	8691740	1	1
FAM5C	6711	1261	-26	1961560	525812	0	0	0	0	3	2	1318268	1	1

FAM64A	249769	359	6	601996	185120	0	0	0	0	50	56	19275264	1	1
FAM69A	609398	372	13	1110008	283732	0	0	0	0	36	41	14510204	1	1
FAM69B	1898901	190	39	1054472	348880	0	0	0	0	50	67	24888672	1	1
FAM70A	526128	NaN	22	895340	264508	0	0	0	0	50	50	19240732	1	1
FAM70B	782003	465	45	818444	267712	0	0	0	0	50	51	21146400	1	1
FAM71E1	1038277	491	30	576720	185832	0	0	1	0	50	64	20883672	1	1
FAM71E2	NaN	NaN	NaN	2266652	733360	0	0	0	0	7	14	4421520	1	1
FAM72A	783744	442	11	396584	96832	0	0	0	0	50	56	17077320	1	1
FAM72B	398623	NaN	14	396940	96476	0	0	0	0	50	58	19600292	1	1
FAM72D	NaN	NaN	NaN	395516	97900	0	0	0	0	50	116	39743484	1	1
FAM75A1	NaN	NaN	NaN	6668948	1998940	0	0	0	0	0	0	1998940	1	1
FAM75A2	18945	NaN	-38	6668948	1998940	0	0	0	0	2	0	2282672	1	1
FAM75A3	17308	1040	-46	3334296	999648	0	0	0	0	3	1	2386980	1	1
FAM75A5	NaN	NaN	NaN	10002532	2999300	0	0	0	0	0	0	2999300	1	1
FAM75A6	NaN	NaN	NaN	3325396	995732	0	0	0	0	0	0	995732	1	1
FAM75A7	NaN	NaN	NaN	10002532	2999300	0	0	0	0	0	0	2999300	1	1
FAM75C1	NaN	NaN	NaN	2922048	902460	0	0	0	0	5	8	3274488	1	1
FAM82A1	664248	420	27	1942336	488432	0	0	0	0	7	6	3242448	1	1
FAM82A2	1011507	172	46	1208976	351372	0	0	0	0	15	14	6826656	1	1
FAM82B	192988	1166	15	830548	217160	0	0	0	0	50	37	15740540	1	1
FAM83A	519858	171	22	1132436	352084	0	0	0	0	50	65	19013604	1	1
FAM86A	552298	419	3	838024	254540	0	0	0	0	50	50	19837032	1	1
FAM86B2	NaN	NaN	NaN	838736	251692	0	0	0	0	50	116	39897276	1	1
FAM86C	726153	465	35	500536	141332	0	0	0	0	50	52	23166700	1	1
FAM89A	376655	616	12	445712	153436	0	0	1	0	50	64	20436536	1	1
FAM89B	3945819	202	45	507300	116412	0	0	0	0	19	25	9711324	1	1
FAM90A1(	NaN	NaN	NaN	1138488	363120	0	0	0	0	50	116	40008704	1	1
FAM90A1:	NaN	NaN	NaN	3412972	1091852	0	0	0	0	0	0	1091852	1	1
FAM90A1‹	NaN	NaN	NaN	3412972	1091852	0	0	0	0	0	0	1091852	1	1
FAM90A1ǫ	NaN	NaN	NaN	2277688	725528	0	0	0	0	7	14	4413688	1	1
FAM90A1ǫ	NaN	NaN	NaN	2277688	725528	0	0	0	0	7	14	4413688	1	1
FAM90A2(	NaN	NaN	NaN	1137420	364188	0	0	0	0	50	116	40009772	1	1
FAM90A5	NaN	NaN	NaN	1137776	363832	0	0	0	0	50	116	40009416	1	1

FAM90A7	NaN	NaN	NaN	2275552	729800	0	0	0	0	7	14	4417960	1	1
FAM90A8	NaN	NaN	NaN	1137420	364188	0	0	0	0	50	116	40009772	1	1
FAM90A9	NaN	NaN	NaN	1138132	363476	0	0	0	0	50	116	40009060	1	1
FAM92B	553233	273	36	792812	214312	0	0	1	0	0	1	214312	1	1
FAM9A	13530	NaN	-19	895340	210040	0	0	0	0	50	85	21689656	1	1
FBRS	2255010	158	60	1127808	391956	0	0	0	0	12	15	4690300	1	1
FBRSL1	NaN	NaN	NaN	2544332	875404	0	0	0	0	7	14	4563564	1	1
FBXL15	975992	178	39	717340	259880	0	0	0	0	50	84	25980880	1	1
FBXO44	682357	264	32	749736	126024	0	0	1	0	8	13	3283032	1	1
FCAMR	467960	385	26	1467076	419012	0	0	0	0	7	6	1940912	1	1
FCER2	688482	384	31	852264	222144	0	0	1	0	50	67	22649432	1	1
FCF1	886320	184	43	544680	127092	0	0	1	0	50	49	19127880	1	1
FCGR1B	285005	NaN	8	727308	192240	0	0	0	0	50	56	22934944	1	1
FCGR3A	693228	503	23	748668	200784	0	0	0	0	50	68	21398092	1	1
FCRLB	598281	504	20	1045572	346032	0	0	3	0	28	32	9012496	1	1
FDFT1	470737	360	34	1084732	284444	0	0	0	0	50	50	17460376	1	1
FDX1	137098	403	6	457460	148096	0	0	0	0	50	34	17565396	1	1
FDX1L	1802015	191	21	463512	145248	0	0	0	0	50	98	25785436	1	1
FEM1B	376557	437	37	1571384	447136	0	0	2	0	50	62	19016808	1	1
FEV	922162	386	35	588468	185832	0	0	0	0	50	52	18596728	1	1
FFAR1	767113	472	9	698116	270560	0	0	0	0	32	25	10097228	1	1
FFAR2	827597	367	29	793524	267000	0	0	2	0	32	37	12083352	1	1
FGF17	584373	219	34	559988	154504	0	0	0	0	50	59	21330096	1	1
FGF18	300840	347	30	533288	150232	0	0	1	0	50	43	20088724	1	1
FGF2	139927	184	18	689216	247420	0	0	1	0	50	50	19092636	1	1
FGF20	176882	719	-38	527592	160200	0	0	1	0	23	39	12397700	1	1
FGF22	1832767	218	10	409044	146316	0	0	0	0	29	50	17004696	1	1
FGFBP1	175145	435	33	591672	165540	0	0	1	0	50	69	24440468	1	1
FGFBP3	355265	331	24	616592	217516	0	0	0	0	50	45	21612048	1	1
FGFR1OP2	408842	320	24	682096	164828	0	0	0	0	50	58	22459684	1	1
FHAD1	NaN	NaN	NaN	3735508	919904	0	0	0	0	5	8	3291932	1	1
FHL3	445132	232	42	732292	189392	0	0	0	0	50	34	17924600	1	1
FIGNL2	570716	438	22	1517628	579924	0	0	0	0	15	19	8128192	1	1

FIZ1	1077402	254	-10	1186192	414740	0	0	2	0	8	8	2559640	1	1
FKBP1B	243145	226	33	323604	86508	0	0	0	0	50	59	18078748	1	1
FKSG83	161388	645	24	485228	123532	0	0	0	0	50	40	20718132	1	1
FKTN	215248	637	-5	1214672	304024	0	0	0	0	50	48	24787568	1	1
FLJ10357	937278	320	20	3718064	1252408	0	0	0	0	4	0	2192248	1	1
FLJ25363	NaN	NaN	NaN	257388	68352	0	0	0	0	50	116	39713936	1	1
FLJ35220	748790	222	29	796016	247420	0	0	0	0	50	66	20942056	1	1
FLJ36031	276499	628	22	553936	202208	0	0	0	0	8	4	3221088	1	1
FLJ37543	166738	437	-1	341048	91492	0	0	0	0	50	61	23208708	1	1
FLJ43859	NaN	NaN	NaN	2274484	681740	0	0	0	0	7	14	4369900	1	1
FLJ43860	153254	382	38	3399800	952300	0	0	0	0	1	0	1261664	1	1
FLJ44082	NaN	NaN	NaN	2274484	681740	0	0	0	0	7	14	4369900	1	1
FLJ44606	NaN	NaN	NaN	519048	130296	0	0	0	0	50	116	39775880	1	1
FLJ44635	582710	NaN	53	368104	87932	0	0	0	0	50	77	18724888	1	1
FLJ46321	37702	928	-45	3912084	1157712	0	0	0	0	4	1	2283740	1	1
FLT3LG	2173457	290	44	595588	194732	0	0	0	0	50	67	19850560	1	1
FLYWCH2	1537882	210	47	349236	111072	0	0	0	0	50	56	20433332	1	1
FNDC4	1193266	306	39	594164	184408	0	0	1	0	1	4	646140	1	1
FNDC5	1019374	232	15	423284	109648	0	0	0	0	50	66	21117564	1	1
FNTB	501431	387	39	1136708	313636	0	0	3	0	17	33	6506612	1	1
FOLH1B	116674	1062	-16	1172664	297972	0	0	0	0	50	67	24992268	1	1
FOLR1	744547	182	58	677468	168388	0	0	0	0	49	67	18790748	1	1
FOLR2	744547	182	58	677468	164116	0	0	1	0	50	69	18856964	1	1
FOLR4	424538	765	-12	629764	163760	0	0	0	0	50	54	17810324	1	1
FOXB1	573015	427	14	795660	255252	0	0	0	0	50	52	20013252	1	1
FOXD1	476449	473	34	1079392	410468	0	0	0	0	45	45	18751944	1	1
FOXD4L2	NaN	NaN	NaN	2000720	671416	0	0	0	0	7	14	4359576	1	1
FOXI1	94287	650	24	944824	275900	0	0	0	0	50	46	20691432	1	1
FOXJ1	1796815	218	28	1017448	338912	0	0	1	0	50	79	21856264	1	1
FOXO3	346225	428	34	1652552	515488	0	0	1	0	50	55	21562564	1	1
FRA10AC1	471978	399	17	891780	174084	0	0	1	0	50	57	20017168	1	1
FRAT1	903121	397	31	643292	253828	0	0	0	0	50	44	16656884	1	1
FRAT2	903121	397	31	538984	210752	0	0	0	0	50	44	16656884	1	1

FREM3	NaN	NaN	NaN	5298704	1587760	0	0	0	0	0	0	1587760	1	1
FRG1	49764	1059	-15	706304	159844	0	0	2	0	5	11	2707736	1	1
FSD1L	NaN	NaN	NaN	1494488	361696	0	0	0	0	50	116	40007280	1	1
FSHB	28139	1007	-29	341048	84016	0	0	0	0	50	117	22416964	1	1
FTSJ1	726793	NaN	2	868996	241724	0	0	1	0	50	78	22575384	1	1
FTSJD1	420704	736	3	1961916	512640	0	0	0	0	25	23	9439340	1	1
FTSJD2	498007	434	43	2205420	571380	0	0	0	0	2	1	969388	1	1
FUT7	1951939	202	40	828412	279104	0	0	0	0	50	61	21392396	1	1
FXC1	425139	491	23	266288	77608	0	0	0	0	0	0	77608	1	1
FXN	196497	491	27	594164	145960	0	0	0	0	50	50	21521980	1	1
FXYD1	745516	357	-9	263796	64080	0	0	1	0	3	2	401568	1	1
FXYD2	516252	624	54	448916	126736	0	0	0	0	16	13	3406564	1	1
FXYD7	745516	357	-9	225348	57672	0	0	0	0	50	54	17856960	1	1
G0S2	218767	300	12	254184	83304	0	0	0	0	50	47	18085156	1	1
ABARAPI	397577	398	18	316840	74048	0	0	0	0	50	59	21415536	1	1
ABARAPI	449343	354	18	315772	75116	0	0	0	0	50	60	21330452	1	1
GABRR2	415409	541	41	1274124	335352	0	0	2	0	50	64	19924252	1	1
GADD45B	1604277	267	35	414740	113920	0	0	0	0	50	58	22805716	1	1
GAGE1	493560	NaN	3	400144	96476	0	0	0	0	50	56	21881896	1	1
GAGE10	547555	NaN	25	315416	78676	0	0	0	0	50	45	20590684	1	1
GAGE12B	NaN	NaN	NaN	978644	226060	0	0	0	0	50	116	39871644	1	1
GAGE12C	NaN	NaN	NaN	1895344	446780	0	0	0	0	50	116	40092364	1	1
GAGE12D	NaN	NaN	NaN	1950880	453188	0	0	0	0	50	116	40098772	1	1
GAGE12E	NaN	NaN	NaN	1950880	453188	0	0	0	0	50	116	40098772	1	1
GAGE12F	NaN	NaN	NaN	915276	221076	0	0	0	0	50	116	39866660	1	1
GAGE12G	NaN	NaN	NaN	1627632	378072	0	0	0	0	50	116	40023656	1	1
GAGE12H	NaN	NaN	NaN	1301892	301176	0	0	0	0	50	116	39946760	1	1
GAGE12I	NaN	NaN	NaN	324672	75828	0	0	0	0	50	116	39721412	1	1
GAGE13	NaN	NaN	NaN	322536	76896	0	0	0	0	50	116	39722480	1	1
GAGE2A	NaN	NaN	NaN	1278396	308652	0	0	0	0	50	116	39954236	1	1
GAGE2B	NaN	NaN	NaN	315772	77252	0	0	0	0	50	116	39722836	1	1
GAGE2C	NaN	NaN	NaN	1277684	308296	0	0	0	0	50	116	39953880	1	1
GAGE2D	NaN	NaN	NaN	959776	231044	0	0	0	0	50	116	39876628	1	1

GAGE2E	NaN	NaN	NaN	1050912	257388	0	0	0	0	50	116	39902972	1	1
GAGE4	NaN	NaN	NaN	324672	75828	0	0	0	0	50	116	39721412	1	1
GAGE5	NaN	NaN	NaN	324672	75828	0	0	0	0	50	116	39721412	1	1
GAGE6	NaN	NaN	NaN	648988	152012	0	0	0	0	50	116	39797596	1	1
GAGE7	NaN	NaN	NaN	324672	75828	0	0	0	0	50	116	39721412	1	1
GAGE8	541043	NaN	12	1053048	257388	0	0	0	0	50	69	23629500	1	1
GAL	780720	354	49	321824	94696	0	0	0	0	50	63	21444016	1	1
GALNT4	NaN	NaN	NaN	1450344	404772	0	0	0	0	50	116	40050356	1	1
GALNT9	838016	534	14	1526528	451408	0	0	0	0	10	22	5822024	1	1
GALNTL1	909703	431	38	1451412	403704	0	0	0	0	0	0	403704	1	1
GALNTL2	221698	444	17	1632260	456748	0	0	0	0	31	34	13237148	1	1
GALNTL4	715278	720	16	1563196	427556	0	0	0	0	2	1	1767896	1	1
GALR3	1385064	203	66	863300	327520	0	0	0	0	6	2	1790680	1	1
GAMT	2319333	196	35	796372	242792	0	0	0	0	50	55	21725612	1	1
GAS1	129795	431	10	821648	286936	0	0	0	0	50	62	21266372	1	1
GAST	1617880	323	9	261304	76184	0	0	0	0	50	72	24149616	1	1
GATA4	474662	360	27	1080104	360628	0	0	0	0	4	1	1047352	1	1
GATAD1	708388	401	27	681740	200428	0	0	0	0	50	69	27239696	1	1
GATSL1	NaN	NaN	NaN	847636	243860	0	0	0	0	50	116	39889444	1	1
GBGT1	1875784	397	51	877540	258812	0	0	0	0	50	62	18361056	1	1
GBP2	337373	745	2	1546820	392668	0	0	1	0	50	62	23640892	1	1
GCET2	268780	440	28	483092	118192	0	0	0	0	50	52	20365692	1	1
GCFC1	389063	482	69	2494848	642936	0	0	0	0	1	0	924176	1	1
GCG	95439	554	7	485228	116056	0	0	1	0	1	4	651480	1	1
GCHFR	1011507	172	46	221432	61588	0	0	0	0	50	64	23154596	1	1
GCNT7	264053	455	-17	1097904	297972	0	0	0	0	50	65	20944192	1	1
GCOM1	171839	487	11	1542548	384124	0	0	0	0	29	35	12755124	1	1
GDF1	NaN	NaN	NaN	861520	339980	0	0	0	0	50	116	39985564	1	1
GDF7	580167	278	28	1058744	392668	0	0	1	0	50	63	19729164	1	1
GEFT	1329939	344	52	1664656	493772	0	0	0	0	0	0	493772	1	1
GEM	518471	543	24	761128	207548	0	0	0	0	22	20	6782512	1	1
GEMIN6	843382	297	31	436100	112852	0	0	0	0	50	65	20544760	1	1
GFOD2	1157184	136	50	946604	298684	0	0	6	0	0	6	298684	1	1

GFRA2	333006	607	7	1190464	333572	0	0	0	0	50	61	20737356	1	1
GFRA4	942842	353	32	752940	225348	0	0	0	0	50	61	21305176	1	1
GGPS1	513285	188	22	777860	199360	0	0	2	0	29	30	10398048	1	1
GHRH	787991	404	44	289072	75116	0	0	0	0	50	64	22911804	1	1
GINS2	390985	250	38	489856	125312	0	0	0	0	50	44	18864440	1	1
GIPC2	412519	265	-6	803848	229976	0	0	2	0	11	15	5315792	1	1
GIYD1	NaN	NaN	NaN	1379144	440728	0	0	0	0	50	116	40086312	1	1
GIYD2	1906000	253	52	1379144	440728	0	0	0	0	8	8	4018528	1	1
GJB1	728738	NaN	25	695624	218584	0	0	1	0	50	87	31895108	1	1
GJB4	548013	580	19	653260	202208	0	0	5	0	0	5	202208	1	1
GJB6	402703	472	36	663584	180136	0	0	0	0	50	45	18027128	1	1
GJC2	702358	341	15	1043436	370596	0	0	0	0	4	3	2210048	1	1
GLIPR2	858124	257	46	416164	99680	0	0	0	0	50	67	23320136	1	1
GLRX	315156	242	52	274120	77252	0	0	0	0	50	58	20747324	1	1
GLRX5	209037	509	35	387684	122820	0	0	0	0	50	60	18652620	1	1
GLT25D1	961640	206	42	1572452	472768	0	0	0	0	21	19	8583160	1	1
GLT25D2	348183	732	11	1609120	446780	0	0	0	0	7	14	4443236	1	1
GLTPD1	1744013	273	28	527592	169812	0	0	0	0	9	14	4108596	1	1
GLYATL3	NaN	NaN	NaN	735140	212176	0	0	0	0	50	116	39857760	1	1
GM2A	871795	530	57	608404	187256	0	0	1	0	50	74	24521636	1	1
GMFG	1748498	252	32	392668	91136	0	0	0	0	50	78	23410916	1	1
GNA11	1000335	173	25	929516	249556	0	0	3	0	5	28	5031704	1	1
GNA14	180930	608	-10	930584	235672	0	0	2	0	3	6	895340	1	1
GNB2	1884430	247	60	880744	250268	0	0	1	0	22	30	9880780	1	1
GNG10	527624	264	17	179780	51976	0	0	0	0	50	52	19780428	1	1
GNG13	1187310	273	39	184408	42008	0	0	0	0	50	70	23301268	1	1
GNG7	1472723	262	33	178356	51264	0	0	0	0	50	85	21265304	1	1
GNG8	1139694	347	28	178356	52332	0	0	1	0	1	1	145248	1	1
GNGT1	228127	480	-12	201852	49128	0	0	0	0	50	63	21073420	1	1
GNGT2	868792	346	21	189748	43076	0	0	0	0	50	55	19730588	1	1
GOLGA2B	184712	650	16	376292	105376	0	0	0	0	50	57	19871208	1	1
GOLGA6L[illegible]	NaN	NaN	NaN	1814532	365256	0	0	0	0	50	116	40010840	1	1
GOLGA6L[illegible]	NaN	NaN	NaN	2045932	394448	0	0	0	0	50	116	40040032	1	1

GOLGA6L[illegible]	NaN	NaN	NaN	2232120	615168	0	0	0	0	7	14	4303328	1	1
GOLGA7	284397	522	28	379496	89356	0	0	1	0	24	26	8408364	1	1
GOLM1	320201	489	20	1053404	273052	0	0	0	0	34	41	17022852	1	1
GOLT1A	557652	370	34	341048	102172	0	0	1	0	9	11	3453200	1	1
GOLT1B	518837	836	-23	363832	98612	0	0	0	0	50	62	16740900	1	1
GOSR1	488195	329	28	674620	170168	0	0	1	0	50	53	16914984	1	1
GOSR2	618151	366	28	704524	182984	0	0	1	0	15	18	6359940	1	1
GP1BB	NaN	NaN	NaN	467784	201852	0	0	0	0	50	116	39847436	1	1
GP5	324325	269	27	1330728	470988	0	0	6	0	4	10	2004280	1	1
GPER	774288	346	36	909224	295480	0	0	0	0	19	9	5654348	1	1
GPIHBP1	1104860	446	20	469920	137772	0	0	0	0	50	69	22671148	1	1
GPR109A	966471	227	38	905664	262728	0	0	0	0	50	53	19484948	1	1
GPR109B	1004283	227	40	961912	281240	0	0	0	0	50	55	20718844	1	1
GPR110	203250	291	-5	2364552	665364	0	0	0	0	4	3	2447144	1	1
GPR111	176427	691	7	1627632	466716	0	0	0	0	34	40	18003276	1	1
GPR112	380446	NaN	28	7585292	2384488	0	0	0	0	3	0	3002504	1	1
GPR113	644558	346	30	2877904	913496	0	0	0	0	8	9	4503756	1	1
GPR114	749405	135	43	1333576	408332	0	0	0	0	37	36	14548296	1	1
GPR115	176427	691	7	1780356	488076	0	0	0	0	23	18	12284136	1	1
GPR116	168285	392	0	3466728	934500	0	0	0	0	1	0	2182992	1	1
GPR120	450249	305	20	917056	306872	0	0	0	0	0	0	306872	1	1
GPR123	296872	499	-10	1372024	446780	0	0	0	0	50	55	24864820	1	1
GPR124	655980	379	26	3259536	1085088	0	0	0	0	3	2	1815244	1	1
GPR125	101844	818	16	3349604	962980	0	0	0	0	10	3	3851564	1	1
GPR126	177409	421	-26	3319344	844788	0	0	0	0	0	0	844788	1	1
GPR128	342000	509	12	2056256	564616	0	0	0	0	6	8	4229992	1	1
GPR133	218974	468	0	2265228	640800	0	0	0	0	6	4	2777512	1	1
GPR139	254650	827	-17	888576	252048	0	0	2	0	50	62	19142120	1	1
GPR143	125267	NaN	2	1018160	313636	0	0	0	0	50	64	19327952	1	1
GPR144	NaN	NaN	NaN	2372740	796016	0	0	0	0	7	14	4484176	1	1
GPR146	765060	346	34	794236	278036	0	0	0	0	48	49	19862664	1	1
GPR157	890298	264	31	804916	286580	0	0	1	0	50	58	18535852	1	1
GPR172A	2525749	200	28	1042724	399076	0	0	0	0	7	8	5183360	1	1

GPR172B	1399774	343	26	1054116	397296	0	0	0	0	50	79	21015392	1	1
GPR21	NaN	NaN	NaN	873624	246708	0	0	0	0	50	116	39892292	1	1
GPR26	208204	808	17	814884	278748	0	0	0	0	50	37	21011832	1	1
GPR27	86859	605	6	872200	331436	0	0	1	0	50	74	20739492	1	1
GPR3	847257	188	26	782132	282664	0	0	0	0	19	9	4778588	1	1
GPR34	531498	NaN	45	963692	268780	0	0	1	0	50	59	24984080	1	1
GPR44	794900	522	47	931296	341760	0	0	0	0	50	51	21913580	1	1
GPR56	727305	168	41	1766828	528304	0	0	0	0	11	12	5101836	1	1
GPR64	197855	NaN	25	2636536	739412	0	0	0	0	0	0	739412	1	1
GPR77	1132395	232	41	798152	289072	0	0	0	0	0	0	289072	1	1
GPR81	1042095	227	42	860808	250980	0	0	0	0	50	59	20574308	1	1
GPR89C	NaN	NaN	NaN	1673912	451408	0	0	0	0	50	116	40096992	1	1
GPR97	712945	168	34	1390536	419724	0	0	0	0	42	42	18149948	1	1
GPR98	115632	755	-2	16022492	4567480	0	0	0	0	0	0	4567480	1	1
GPX4	2398425	234	17	768604	200072	0	0	0	0	50	57	27012212	1	1
GRAMD4	415189	265	7	1520476	409400	0	0	2	0	16	26	7371336	1	1
GRAPL	NaN	NaN	NaN	310788	83304	0	0	0	0	50	116	39728888	1	1
GRASP	468067	234	41	969032	331792	0	0	0	0	50	28	16136768	1	1
GREB1L	NaN	NaN	NaN	4924904	1374160	0	0	0	0	0	0	1374160	1	1
GRID2IP	NaN	NaN	NaN	3013184	959776	0	0	0	0	5	8	3331804	1	1
GRINL1A	168063	487	12	945892	249200	0	0	0	0	50	57	22156372	1	1
GRLF1	1201856	207	29	3784636	1042724	0	0	0	0	4	3	2923472	1	1
GRP	280782	512	53	389108	98968	0	0	0	0	50	54	16867280	1	1
GRPR	217879	NaN	19	956216	285868	0	0	1	0	50	55	20713504	1	1
GRRP1	NaN	NaN	NaN	641156	234604	0	0	0	0	50	116	39880188	1	1
GRXCR2	394778	568	-4	640444	165896	0	0	4	0	0	4	165896	1	1
GSC2	487560	277	18	492348	175152	0	0	1	0	50	61	20002216	1	1
GSTM4	958586	201	25	610540	145604	0	0	1	0	50	82	20730948	1	1
GSTT1	748735	202	66	609116	182272	0	0	0	0	11	9	4537220	1	1
GSTT2	699286	202	63	1240304	370240	0	0	1	0	3	4	1004988	1	1
GTF2A1L	NaN	NaN	NaN	1268784	339624	0	0	0	0	50	116	39985208	1	1
GTF2H2	370823	581	25	1067644	265220	0	0	0	0	50	49	25438692	1	1
GTF2H2C	NaN	NaN	NaN	1068356	264508	0	0	0	0	50	116	39910092	1	1

GTF2H2D	514523	312	23	1068356	264508	0	0	0	0	39	41	17165964	1	1
GTF2H5	617881	209	40	191172	48060	0	0	0	0	50	61	23000448	1	1
GTF3A	304169	414	59	989680	245996	0	0	2	0	44	69	19976228	1	1
GTF3C6	320358	372	33	576364	132788	0	0	0	0	50	43	19942052	1	1
GTPBP5	861466	288	-13	1005700	323960	0	0	0	0	25	32	9886476	1	1
GUCA1A	524386	273	43	527592	132432	0	0	4	0	2	4	399788	1	1
GUCA2A	418399	579	24	297616	84728	0	0	0	0	50	41	18603492	1	1
GUCA2B	418399	579	24	284800	87932	0	0	1	0	16	16	6770052	1	1
GXYLT1	172471	684	-9	1142760	300108	0	0	1	0	50	62	29653732	1	1
GYPE	166879	959	-9	211464	56604	0	0	0	0	50	51	21567904	1	1
H2AFB1	NaN	NaN	NaN	823428	297972	0	0	0	0	50	116	39943556	1	1
H2AFB2	NaN	NaN	NaN	548952	198648	0	0	0	0	50	116	39844232	1	1
H2AFB3	494257	NaN	-7	823428	297972	0	0	0	0	43	47	19241800	1	1
H2AFY2	484729	425	25	955504	273764	0	0	1	0	50	56	21866944	1	1
H2BFM	NaN	NaN	NaN	390888	116412	0	0	0	0	50	116	39761996	1	1
HAGHL	1207790	194	33	883592	257032	0	0	1	0	50	77	22508812	1	1
HAND2	251136	719	-10	541832	160912	0	0	0	0	50	65	26127552	1	1
HBA1	1267639	220	36	354220	114632	0	0	1	0	31	44	9790000	1	1
HBA2	1267639	220	36	354220	114632	0	0	0	0	50	56	16678244	1	1
HBG2	109545	1056	-1	378072	108936	0	0	0	0	50	50	17996156	1	1
HBM	1267639	220	36	357068	110716	0	0	0	0	50	56	16678244	1	1
HBQ1	1267639	220	36	348524	120328	0	0	0	0	50	56	16678244	1	1
HBXIP	403650	479	31	438948	133500	0	0	0	0	50	40	19131440	1	1
HBZ	1267639	220	36	355288	113564	0	0	1	0	31	44	9790000	1	1
HCP5	NaN	NaN	NaN	114632	29548	0	0	0	0	50	116	39675132	1	1
HCRT	2129732	206	30	308652	122820	0	0	0	0	50	83	22131452	1	1
HDAC3	771583	353	25	1149880	286580	0	0	2	0	7	9	3981148	1	1
HDHD1A	55330	NaN	19	742260	211464	0	0	0	0	9	7	3781788	1	1
HDHD2	119441	561	-11	669992	188680	0	0	0	0	50	64	21463952	1	1
HEATR2	545367	390	14	2091500	706660	0	0	0	0	5	5	2186908	1	1
HEATR7A	2550138	210	26	4248504	1330728	0	0	0	0	0	0	1330728	1	1
HEATR7B2	211261	644	-5	4171608	1087224	0	0	0	0	1	0	1391248	1	1
HEBP2	421773	590	19	535424	137416	0	0	0	0	50	54	21692148	1	1

HEMGN	467637	269	40	1256324	314704	0	0	1	0	50	47	18389892	1	1
HEMK1	583785	302	50	873624	253116	0	0	0	0	50	43	17510216	1	1
HES2	643751	198	22	420080	152368	0	0	0	0	50	68	23732028	1	1
HES5	339388	228	38	419724	126024	0	0	0	0	50	44	17226128	1	1
HES6	349323	252	28	545392	188324	0	0	1	0	3	13	2886804	1	1
HES7	1381225	202	37	559276	195800	0	0	0	0	50	76	28183808	1	1
HESX1	454806	389	37	486296	122464	0	0	0	0	50	59	21368544	1	1
HFE2	NaN	NaN	NaN	1030264	350660	0	0	3	0	26	43	11927780	1	1
HGC6.3	NaN	NaN	NaN	405840	145248	0	0	0	0	50	116	39790832	1	1
HHAT	87459	754	1	1345680	386616	0	0	2	0	18	13	6480268	1	1
HHEX	359162	317	17	680672	202564	0	0	0	0	50	56	24663324	1	1
HHLA1	NaN	NaN	NaN	1390180	378428	0	0	0	0	50	116	40024012	1	1
HHLA3	296134	396	5	421148	81880	0	0	0	0	50	56	20386340	1	1
HIBADH	427440	372	10	859740	249912	0	0	1	0	50	74	28610652	1	1
HIBCH	499049	471	24	1039164	256320	0	0	0	0	25	22	9983308	1	1
HIC1	1415256	294	27	1754368	601640	0	0	0	0	10	8	3417244	1	1
HIGD1A	369545	396	31	288360	74760	0	0	0	0	50	43	19115064	1	1
HIGD1B	1101543	172	28	259880	73336	0	0	0	0	50	74	17295192	1	1
HINT1	310375	228	15	323960	91492	0	0	0	0	50	52	18023212	1	1
HINT3	150315	177	15	475616	127804	0	0	0	0	50	45	16944888	1	1
IIST1H2A/	272695	635	29	320044	107156	0	0	0	0	50	55	17264220	1	1
HIST1H3J	228884	634	40	330724	110360	0	0	0	0	50	48	14804260	1	1
HIST1H4B	408919	685	43	248488	86864	0	0	0	0	50	57	16881520	1	1
HIST1H4G	390169	544	57	240656	78676	0	0	1	0	36	34	7770412	1	1
IST2H2AA	NaN	NaN	NaN	632256	215736	0	0	0	0	50	116	39861320	1	1
IST2H2AA	NaN	NaN	NaN	632256	215736	0	0	0	0	50	116	39861320	1	1
HIST2H3A	NaN	NaN	NaN	660024	226416	0	0	0	0	50	116	39872000	1	1
HIST2H3C	NaN	NaN	NaN	660024	226416	0	0	0	0	50	116	39872000	1	1
HIST2H4A	NaN	NaN	NaN	502672	172304	0	0	0	0	50	116	39817888	1	1
HIST2H4B	NaN	NaN	NaN	502672	172304	0	0	0	0	50	116	39817888	1	1
HIST3H3	626588	351	7	330012	111072	0	0	0	0	50	72	28650524	1	1
HLA-DMA	1276842	431	40	669280	189392	0	0	0	0	6	11	2998944	1	1
HMGB3	226983	NaN	-8	539340	121752	0	0	0	0	50	68	26849520	1	1

HMGCL	903441	254	32	831616	249200	0	0	1	0	50	57	18158136	1	1
HMGN2	1003175	167	31	251692	61232	0	0	1	0	2	7	1400504	1	1
HMGN3	233858	624	0	276968	64792	0	0	0	0	50	60	19892568	1	1
HMGN4	426020	498	34	228908	66928	0	0	0	0	50	36	18178784	1	1
HMGN5	141842	NaN	-22	767180	160912	0	0	0	0	50	73	22104752	1	1
HMGXB3	NaN	NaN	NaN	3253484	970456	0	0	0	0	0	0	970456	1	1
HMHB1	142005	404	33	111072	27768	0	0	1	0	0	1	27768	1	1
HMX3	237830	708	23	856180	294056	0	0	2	0	3	5	1280888	1	1
HN1	1511891	168	25	521896	98612	0	0	0	0	50	53	18659384	1	1
INRNPA1L	324687	462	32	808120	224636	0	0	0	0	50	50	19140696	1	1
HNRPDL	684394	387	46	1080104	298684	0	0	0	0	50	53	22010768	1	1
HNRPLL	859042	294	24	1420084	384836	0	0	0	0	7	8	3191184	1	1
HOMER2	457445	683	24	934500	237096	0	0	2	0	1	6	825564	1	1
HOPX	597691	572	16	356356	94340	0	0	0	0	50	70	20269572	1	1
HORMAD2	655208	178	30	834108	195444	0	0	0	0	7	6	2529380	1	1
HOXB13	727820	415	24	695980	221432	0	0	2	0	4	6	2224644	1	1
HOXB5	946208	524	29	672484	199004	0	0	0	0	50	60	17564684	1	1
HOXB7	946208	524	29	546104	157708	0	0	0	0	50	60	17564684	1	1
HP	438866	372	19	1052692	279104	0	0	2	0	4	8	2763272	1	1
HPDL	1010538	355	28	876116	315772	0	0	0	0	28	31	10911044	1	1
HPS6	1018634	187	38	1802428	686012	0	0	3	0	48	50	19195164	1	1
HRASLS2	854193	303	56	406196	131008	0	0	2	0	8	9	1189396	1	1
HRK	NaN	NaN	NaN	216448	82592	0	0	0	0	50	116	39728176	1	1
HRNBP3	NaN	NaN	NaN	811680	238164	0	0	0	0	50	116	39883748	1	1
HS3ST6	1766472	214	39	742616	261304	0	0	0	0	31	55	16867280	1	1
HSBP1L1	NaN	NaN	NaN	202920	50196	0	0	0	0	50	116	39695780	1	1
HSD11B1	219540	222	15	762196	204344	0	0	0	0	50	49	17060944	1	1
HSD3B7	1453326	247	43	927380	283732	0	0	1	0	50	71	24760156	1	1
HSF2BP	878395	518	73	869708	233536	0	0	1	0	50	58	21958080	1	1
HSFX1	NaN	NaN	NaN	2100400	633680	0	0	0	0	7	14	4321840	1	1
HSFX2	NaN	NaN	NaN	2100400	633680	0	0	0	0	7	14	4321840	1	1
HSFY1	NaN	NaN	NaN	2199368	609472	0	0	0	0	7	14	4297632	1	1
HSFY2	NaN	NaN	NaN	2199368	609472	0	0	0	0	7	14	4297632	1	1

HSPB6	1151198	321	30	387684	138840	0	0	0	0	50	47	19100468	1	1
HSPC159	336405	452	17	448560	122820	0	0	0	0	50	53	21582856	1	1
HSPE1	681973	428	29	265220	77608	0	0	1	0	1	2	253116	1	1
HTN3	34716	965	-1	157708	30260	0	0	0	0	50	89	21005780	1	1
HTRA1	252955	279	5	1198652	376648	0	0	0	0	50	51	21633052	1	1
HTRA4	421614	428	42	1187972	374512	0	0	0	0	50	56	22426932	1	1
HYAL1	843769	206	51	1079392	330368	0	0	0	0	50	68	20472848	1	1
HYAL3	843769	206	51	1026704	325384	0	0	2	0	50	68	20472848	1	1
HYLS1	NaN	NaN	NaN	748312	217160	0	0	1	0	50	117	39862744	1	1
IAH1	691902	278	38	641868	179424	0	0	1	0	39	61	17489568	1	1
ICOSLG	1128093	219	76	781420	215024	0	0	0	0	50	45	20503464	1	1
ID4	111172	864	-23	390176	137416	0	0	0	0	50	84	22456836	1	1
IER3	2199534	167	44	374512	134924	0	0	0	0	50	60	20858752	1	1
IER3IP1	148377	504	8	212888	63724	0	0	1	0	0	1	63724	1	1
IER5L	1452033	257	39	964760	332860	0	0	0	0	50	72	22966272	1	1
IFFO1	2764720	179	42	1422576	425064	0	0	1	0	33	46	14927436	1	1
IFFO2	NaN	NaN	NaN	1318624	375224	0	0	0	0	50	116	40020808	1	1
IFI27	200295	605	47	299752	101816	0	0	0	0	50	57	19244648	1	1
IFI27L1	200295	605	47	269492	88288	0	0	0	0	50	57	19244648	1	1
IFI6	732210	217	24	360984	101460	0	0	1	0	2	3	627984	1	1
IFITM1	1454043	344	48	313636	94340	0	0	0	0	50	96	29119376	1	1
IFITM2	1454043	344	48	334640	97900	0	0	0	0	50	96	29119376	1	1
IFITM3	1454043	344	48	333928	101816	0	0	0	0	50	96	29119376	1	1
IFLTD1	200328	517	1	1150592	297616	0	0	0	0	45	51	19665796	1	1
IFNA10	193208	498	-3	478108	132788	0	0	0	0	50	58	18347172	1	1
IFNA13	232155	466	-14	485228	131008	0	0	1	0	24	24	6810636	1	1
IFNA6	232155	466	-14	478108	128516	0	0	1	0	24	24	6810636	1	1
IFNA7	193208	498	-3	481668	129228	0	0	4	0	4	6	646852	1	1
IFNAR1	857199	179	81	1467076	363476	0	0	1	0	50	47	20588548	1	1
IFT27	1235005	537	19	493416	131364	0	0	0	0	50	67	19039236	1	1
IFT46	1359480	167	47	954792	232824	0	0	0	0	50	64	22940284	1	1
IGFALS	1604603	288	36	1522968	546816	0	0	1	0	27	29	13226112	1	1
IGFBP2	582017	536	19	793880	263440	0	0	0	0	32	42	12298376	1	1

IGFBP5	573046	620	16	690640	196868	0	0	1	0	50	71	20876552	1	1
IGFL2	1177162	249	28	364188	92916	0	0	0	0	50	46	17997580	1	1
IGLL1	604420	399	31	538628	155572	0	0	0	0	50	47	18688220	1	1
IGSF6	NaN	NaN	NaN	624424	174440	0	0	0	0	50	116	39820024	1	1
IL11	1028808	209	19	467428	190460	0	0	0	0	2	0	629052	1	1
IL13	717582	349	30	375224	110716	0	0	2	0	1	2	316484	1	1
IL13RA2	166070	NaN	19	1014600	244572	0	0	1	0	50	44	15622704	1	1
IL15RA	650283	305	32	669992	214312	0	0	1	0	29	31	15717756	1	1
IL17D	385825	434	29	485940	173016	0	0	1	0	6	8	2391608	1	1
IL18	389593	362	2	527592	115344	0	0	0	0	50	48	17867640	1	1
IL1F5	672307	479	25	401212	115700	0	0	0	0	50	59	20875128	1	1
IL1F6	611416	469	33	411536	108580	0	0	0	0	50	62	26386364	1	1
IL1F7	590466	420	46	640088	156640	0	0	0	0	50	70	24279556	1	1
IL1F8	641862	469	29	626916	152724	0	0	0	0	50	60	22667588	1	1
IL1F9	611416	469	33	442864	118904	0	0	0	0	50	62	26386364	1	1
IL2	227942	612	-14	402636	105732	0	0	0	0	50	46	18207264	1	1
IL22	383435	623	12	468852	127092	0	0	0	0	50	61	20003996	1	1
IL25	1220624	456	31	455324	131008	0	0	0	0	50	78	24903980	1	1
IL28A	1838894	286	29	508724	160912	0	0	0	0	50	70	23062392	1	1
IL28B	1838894	286	29	487720	161624	0	0	0	0	50	70	23062392	1	1
IL28RA	840519	235	31	1321828	375224	0	0	0	0	50	73	22939928	1	1
IL29	1838894	286	29	498400	164828	0	0	0	0	50	70	23062392	1	1
IL3	365169	484	35	398364	111072	0	0	0	0	50	43	18137132	1	1
IL4	717412	356	59	399432	108936	0	0	0	0	50	46	17209040	1	1
IL8	273344	444	13	265932	69420	0	0	0	0	50	46	18441512	1	1
IL9	489402	443	19	383768	100036	0	0	0	0	50	52	16320820	1	1
ILKAP	388991	248	29	1029908	276256	0	0	1	0	1	1	564616	1	1
IMMP2L	67842	871	-7	459952	125312	0	0	0	0	50	84	28978400	1	1
IMP5	369065	563	15	1650772	546104	0	0	0	0	9	6	3472780	1	1
IMPDH1	902782	246	34	1547176	460664	0	0	3	0	48	66	19144256	1	1
INO80C	368170	474	38	590248	169100	0	0	1	0	26	24	9180528	1	1
INPP5J	731144	218	40	1624428	476328	0	0	0	0	11	11	6390912	1	1
INPP5K	1371893	220	24	1169460	316128	0	0	1	0	50	74	22937080	1	1

INS	NaN	NaN	NaN	276968	89356	0	0	0	0	50	116	39734940	1	1
INS-IGF2	837088	579	38	494840	166252	0	0	0	0	50	46	19328664	1	1
IQCF3	849777	453	39	404060	107512	0	0	0	0	50	44	18199076	1	1
IQCF5	NaN	NaN	NaN	384836	101104	0	0	0	0	50	116	39746688	1	1
IQCF6	NaN	NaN	NaN	271272	79032	0	0	0	0	50	116	39724616	1	1
IQCJ	170382	930	-11	456036	122820	0	0	1	0	20	23	6898212	1	1
IRGM	NaN	NaN	NaN	451408	129584	0	0	0	0	50	116	39775168	1	1
ISCA1	311398	417	8	334284	95052	0	0	0	0	50	58	21260676	1	1
ISCA1P1	177047	815	-3	332148	100392	0	0	0	0	50	45	16884012	1	1
ISCU	437636	399	40	380208	99324	0	0	0	0	50	64	24423380	1	1
ISG15	1971475	239	15	405128	133144	0	0	2	0	0	2	133144	1	1
ISL2	289294	229	39	912072	264864	0	0	1	0	50	56	17124668	1	1
ITGB1BP3	2173603	207	30	603420	162336	0	0	0	0	50	75	21194460	1	1
ITIH5L	257678	NaN	19	3219664	1043792	0	0	0	0	0	0	1043792	1	1
JHDM1D	462584	624	29	2471708	629764	0	0	0	0	10	19	8477784	1	1
JMJD5	227767	448	27	1158780	328944	0	0	0	0	27	27	10397336	1	1
JMJD8	1207790	194	33	726240	228552	0	0	0	0	17	19	6751184	1	1
JOSD1	1056349	262	45	525812	137416	0	0	1	0	50	70	24305544	1	1
JUB	1240941	185	38	1335356	421504	0	0	0	0	9	7	3338924	1	1
KAZ	163077	802	9	2169108	646140	0	0	0	0	3	0	1858676	1	1
KBTBD10	370890	418	22	1543616	424708	0	0	0	0	50	53	25210496	1	1
KBTBD12	727461	390	49	1602356	416164	0	0	0	0	50	64	20779008	1	1
KBTBD13	688371	189	44	1085088	385548	0	0	1	0	50	72	23489592	1	1
KBTBD5	369022	329	34	1548956	467428	0	0	0	0	32	25	14158120	1	1
KBTBD8	99545	940	13	1544684	399076	0	0	1	0	50	75	23167768	1	1
KCNE1L	203975	NaN	36	340692	117480	0	0	0	0	50	52	15315832	1	1
KCNE4	279069	585	3	424352	127804	0	0	2	0	2	3	623712	1	1
KCNK12	791803	516	25	1021364	363832	0	0	0	0	50	76	24983724	1	1
KCNK17	190767	749	31	941264	285868	0	0	0	0	50	54	20647288	1	1
KCP	NaN	NaN	NaN	4184780	1203280	0	0	0	0	0	0	1203280	1	1
KCTD12	202176	734	11	778572	265932	0	0	1	0	50	59	19546892	1	1
KDELR1	1478750	217	36	545748	153792	0	0	1	0	50	89	31278516	1	1
KDM4DL	NaN	NaN	NaN	1261664	362764	0	0	0	0	50	116	40008348	1	1

KHK	1214097	238	50	933076	268424	0	0	1	0	50	60	22743060	1	1
KIAA0040	NaN	NaN	NaN	255252	65148	0	0	0	0	50	116	39710732	1	1
KIAA0090	788190	448	30	2541128	741904	0	0	0	0	0	0	741904	1	1
KIAA0146	918018	606	6	2346040	672128	0	0	0	0	8	1	1829128	1	1
KIAA0174	471197	218	31	925244	269848	0	0	0	0	44	48	15751932	1	1
KIAA0182	422186	213	37	3027068	941620	0	0	0	0	2	3	1948388	1	1
KIAA0240	1023102	187	38	2722332	784980	0	0	0	0	3	4	1850844	1	1
KIAA0247	600813	299	44	758636	236740	0	0	0	0	35	28	16633032	1	1
KIAA0284	855494	247	41	3822728	1236388	0	0	0	0	0	0	1236388	1	1
KIAA0317	900799	207	42	2134932	582060	0	0	0	0	0	0	582060	1	1
KIAA0406	599934	303	49	2720908	801356	0	0	0	0	3	0	1348172	1	1
KIAA0415	479282	523	22	2002144	657176	0	0	0	0	6	2	2037744	1	1
KIAA0427	99954	406	51	1543616	428980	0	0	0	0	5	4	3007132	1	1
KIAA0467	1066191	239	31	6379876	1974020	0	0	0	0	1	0	2525820	1	1
KIAA0494	615653	417	20	1290500	341404	0	0	0	0	12	10	4601656	1	1
KIAA0495	249153	341	24	487720	163760	0	0	0	0	50	59	21371392	1	1
KIAA0528	213736	811	-9	2608412	701320	0	0	0	0	6	3	2743692	1	1
KIAA0562	249714	412	32	2414036	642580	0	0	0	0	2	1	1001072	1	1
KIAA0564	259442	557	50	4938788	1360276	0	0	0	0	0	0	1360276	1	1
KIAA0649	298554	539	14	2949104	932008	0	0	0	0	3	4	1996804	1	1
KIAA0652	914919	176	39	1346392	381632	0	0	0	0	7	13	3919560	1	1
KIAA0664	561252	218	18	3335720	968320	0	0	0	0	2	0	1602356	1	1
KIAA0748	246749	748	11	1358140	356000	0	0	0	0	21	38	10326848	1	1
KIAA0754	NaN	NaN	NaN	3447504	1127808	0	0	0	0	0	0	1127808	1	1
KIAA0776	105478	1376	-1	2086872	539340	0	0	0	0	4	2	2033472	1	1
KIAA0802	276661	394	22	3981148	1167680	0	0	0	0	7	7	4229636	1	1
KIAA0831	739675	256	39	1277684	342472	0	0	0	0	5	9	3977944	1	1
KIAA0892	920442	232	2	1599508	446780	0	0	0	0	3	2	1410116	1	1
KIAA0913	1009357	191	42	4576736	1437172	0	0	0	0	0	0	1437172	1	1
KIAA0947	89016	648	-14	5733024	1607340	0	0	0	0	2	0	2221796	1	1
KIAA1009	51818	807	-15	3738712	870776	0	0	0	0	0	0	870776	1	1
KIAA1012	416975	489	28	3735508	985052	0	0	0	0	2	1	1512288	1	1
KIAA1107	NaN	NaN	NaN	3462456	913140	0	0	0	0	5	8	3285168	1	1

KIAA1191	549164	427	28	784268	226060	0	0	1	0	50	60	21788980	1	1
KIAA1199	265840	460	2	3546472	936992	0	0	0	0	4	4	2322900	1	1
KIAA1239	NaN	NaN	NaN	4382716	1227488	0	0	0	0	0	0	1227488	1	1
KIAA1244	341233	545	21	5537580	1581708	0	0	0	0	1	0	1674268	1	1
KIAA1267	388537	357	21	2764696	836600	0	0	0	0	2	1	1372736	1	1
KIAA1274	549709	487	39	2189044	637952	0	0	0	0	11	19	7465320	1	1
KIAA1310	889428	234	44	2211828	694200	0	0	0	0	1	0	822360	1	1
KIAA1370	421095	345	21	2772884	728020	0	0	0	0	24	17	9626596	1	1
KIAA1377	418167	411	-19	2874700	750092	0	0	0	0	0	0	750092	1	1
KIAA1383	134159	644	-2	2600580	757212	0	0	0	0	2	2	2404780	1	1
KIAA1409	321756	678	21	6369908	1709512	0	0	0	0	0	0	1709512	1	1
KIAA1430	316425	530	37	1360632	364188	0	0	0	0	50	41	17128584	1	1
KIAA1432	311104	473	36	3539708	953368	0	0	0	0	3	1	1608052	1	1
KIAA1486	17102	517	-4	1614460	502316	0	0	0	0	19	24	10236424	1	1
KIAA1529	378658	528	34	4351388	1087936	0	0	0	0	0	0	1087936	1	1
KIAA1530	1018381	507	41	1813820	516556	0	0	0	0	32	50	18344324	1	1
KIAA1539	1108514	411	41	1305096	451764	0	0	0	0	17	25	6408356	1	1
KIAA1543	611341	384	33	3124256	1046284	0	0	0	0	3	2	2243512	1	1
KIAA1609	584311	285	14	1170528	323604	0	0	0	0	0	0	323604	1	1
KIAA1632	385282	380	41	6645096	1807056	0	0	0	0	1	0	2075836	1	1
KIAA1644	218812	280	16	515132	143824	0	0	0	0	50	50	18401996	1	1
KIAA1671	NaN	NaN	NaN	4460324	1374160	0	0	0	0	0	0	1374160	1	1
KIAA1704	685405	245	40	900680	222856	0	0	0	0	50	72	25667600	1	1
KIAA1712	63527	647	11	1137420	281952	0	0	0	0	50	59	21722408	1	1
KIAA1731	NaN	NaN	NaN	6716296	1746536	0	0	0	0	0	0	1746536	1	1
KIAA1737	520205	235	60	993952	300464	0	0	0	0	7	7	3171604	1	1
KIAA1751	1343607	233	33	1975444	541832	0	0	0	0	23	39	10806736	1	1
KIAA1797	125322	525	20	4659328	1297976	0	0	0	0	6	7	5055200	1	1
KIAA1804	146614	392	5	2566760	794236	0	0	0	0	5	0	1607696	1	1
KIAA1826	84555	890	-19	887152	225704	0	0	0	0	8	11	3265588	1	1
KIAA1949	2115149	171	41	1488792	491280	0	0	0	0	16	25	8100068	1	1
KIAA1967	757216	224	45	2339988	705948	0	0	0	0	4	2	2156648	1	1
KIAA1984	1910405	176	41	1412252	361696	0	0	0	0	50	62	23187704	1	1

KILLIN	NaN	NaN	NaN	427912	145604	0	0	0	0	50	116	39791188	1	1
KIR3DP1	1071493	620	-17	832684	243860	0	0	0	0	50	141	24027152	1	1
KISS1	557652	370	34	338912	114988	0	0	1	0	9	11	3453200	1	1
KISS1R	2292363	206	44	947672	347812	0	0	0	0	24	28	9676436	1	1
KLF12	32882	755	41	1017804	303312	0	0	1	0	50	56	18501676	1	1
KLF14	241201	399	35	765756	274476	0	0	0	0	23	19	6565708	1	1
KLF16	2226995	214	26	594164	222856	0	0	0	0	50	75	24188776	1	1
KLF2	785109	258	12	843720	307584	0	0	0	0	50	74	23231492	1	1
KLF7	207002	598	20	762908	220720	0	0	1	0	43	37	16174148	1	1
KLF9	167186	278	1	611252	178000	0	0	2	0	1	2	403704	1	1
KLHDC5	349689	387	11	1254900	377004	0	0	0	0	50	69	26720292	1	1
KLHDC9	689033	250	23	915632	238876	0	0	0	0	12	19	4867588	1	1
KLHL29	NaN	NaN	NaN	2020656	625848	0	0	0	0	7	14	4314008	1	1
KLHL33	NaN	NaN	NaN	1283736	442152	0	0	0	0	50	116	40087736	1	1
KLK2	655673	589	25	699896	198292	0	0	2	0	4	6	897120	1	1
KLK4	581408	606	29	649344	184764	0	0	0	0	50	56	18321184	1	1
KLK7	581408	606	29	646140	189036	0	0	1	0	50	56	18321184	1	1
KLK9	490795	632	22	636884	186544	0	0	1	0	50	58	16739120	1	1
KLRAQ1	168167	780	24	2066580	529728	0	0	0	0	0	0	529728	1	1
KLRC1	382960	334	1	619796	155572	0	0	0	0	50	54	22790052	1	1
KLRD1	406053	331	1	492704	109648	0	0	0	0	50	61	22130028	1	1
KLRK1	378365	334	0	598436	126736	0	0	0	0	50	50	21701404	1	1
KMO	171933	382	-17	1297264	325028	0	0	0	0	50	41	21048144	1	1
KNCN	608423	266	25	255252	80100	0	0	0	0	50	58	19047424	1	1
KPNA7	NaN	NaN	NaN	1309012	385904	0	0	0	0	50	116	40031488	1	1
KRT86	839631	175	48	1238880	357780	0	0	0	0	8	13	4005000	1	1
KRTAP1-3	977588	404	10	428268	112140	0	0	1	0	50	68	14276668	1	1
KRTAP10-[illegible]	860604	461	30	699896	208972	0	0	2	0	0	2	208972	1	1
KRTAP10-1[illegible]	819099	391	35	604488	185832	0	0	0	0	50	61	23447584	1	1
KRTAP10-[illegible]	860604	461	30	636528	185832	0	0	1	0	50	62	22093360	1	1
KRTAP10-[illegible]	860604	461	30	1000716	289428	0	0	1	0	50	62	22093360	1	1
KRTAP12-[illegible]	874480	461	25	243860	71200	0	0	1	0	8	6	1620512	1	1
KRTAP19-[illegible]	14979	1035	-68	199360	63368	0	0	0	0	50	77	14168444	1	1

⟨RTAP19-(	22916	1048	-79	147384	43788	0	0	0	0	50	84	16257808	1	1
⟨RTAP19-'	22916	1048	-79	159132	44856	0	0	0	0	50	84	16257808	1	1
⟨RTAP19-ξ	200531	972	-45	165184	44144	0	0	0	0	50	66	17353576	1	1
KRTAP2-1	NaN	NaN	NaN	315772	99680	0	0	0	0	50	116	39745264	1	1
KRTAP2-2	NaN	NaN	NaN	301888	95408	0	0	0	0	50	116	39740992	1	1
KRTAP2-4	NaN	NaN	NaN	315416	100036	0	0	0	0	50	116	39745620	1	1
⟨RTAP20-2	28000	1055	-80	162336	48060	0	0	0	0	50	90	15947376	1	1
⟨RTAP20-3	NaN	NaN	NaN	116768	29548	0	0	0	0	50	116	39675132	1	1
⟨RTAP21-3	NaN	NaN	NaN	159132	34176	0	0	0	0	50	116	39679760	1	1
⟨RTAP22-2	NaN	NaN	NaN	121040	28480	0	0	0	0	50	116	39674064	1	1
⟨RTAP25-1	NaN	NaN	NaN	268780	65504	0	0	0	0	50	116	39711088	1	1
KRTAP3-3	977588	404	10	242792	76540	0	0	0	0	50	68	14276668	1	1
KRTAP4-1	996799	468	-7	328944	87576	0	0	2	0	8	3	991104	1	1
KRTAP4-2	996799	468	-7	350660	90424	0	0	0	0	50	60	17141400	1	1
KRTAP4-3	996799	468	-7	505520	122464	0	0	0	0	50	60	17141400	1	1
KRTAP4-8	NaN	NaN	NaN	476328	121752	0	0	0	0	50	116	39767336	1	1
⟨RTAP5-1(	586193	626	50	504096	148452	0	0	0	0	40	36	13709560	1	1
KRTAP5-2	NaN	NaN	NaN	444644	127804	0	0	0	0	50	116	39773388	1	1
KRTAP5-3	NaN	NaN	NaN	599860	168032	0	0	0	0	50	116	39813616	1	1
KRTAP5-5	NaN	NaN	NaN	590960	173728	0	0	0	0	50	116	39819312	1	1
KRTAP5-6	NaN	NaN	NaN	326808	91848	0	0	0	0	50	116	39737432	1	1
KRTAP5-9	586193	626	50	429692	114988	0	0	0	0	41	39	13806036	1	1
KRTAP6-1	22916	1048	-79	177644	55180	0	0	0	0	50	84	16257808	1	1
KRTAP6-3	22916	1048	-79	276256	81524	0	0	1	0	43	66	10445752	1	1
KRTAP7-1	NaN	NaN	NaN	223212	60876	0	0	0	0	50	116	39706460	1	1
KRTAP9-3	996799	468	-7	409756	105020	0	0	0	0	50	60	17141400	1	1
KRTDAP	827597	367	29	271984	74048	0	0	0	0	50	50	19263872	1	1
KTELC1	280646	599	33	1044860	259168	0	0	0	0	40	25	13091188	1	1
L3MBTL	321277	399	-2	2089008	561768	0	0	0	0	2	0	1046284	1	1
LAGE3	2163759	NaN	42	347100	122820	0	0	0	0	50	72	22102972	1	1
LALBA	1494035	421	25	383056	90068	0	0	2	0	1	3	371308	1	1
LAPTM4B	609859	175	39	798152	246352	0	0	0	0	50	44	20926036	1	1
LASS1	1346431	176	24	885016	269492	0	0	0	0	50	70	20858752	1	1

LASS2	1621279	172	30	996444	270204	0	0	0	0	33	60	14315472	1	1
LASS3	239951	474	-31	1027772	244216	0	0	0	0	50	93	22407708	1	1
LASS4	826957	209	15	1014600	292632	0	0	0	0	50	62	22292720	1	1
LASS5	1040456	192	58	1029552	270204	0	0	0	0	5	3	1138488	1	1
LASS6	192358	937	21	1007124	264864	0	0	0	0	50	38	20880824	1	1
LAT	1148899	257	51	781776	225348	0	0	3	0	1	4	349592	1	1
LBX1	572649	418	43	685300	222500	0	0	1	0	50	61	24181300	1	1
LBXCOR1	305591	319	21	2263804	728732	0	0	0	0	3	2	2719128	1	1
LCE1A	62450	804	15	276612	77964	0	0	0	0	50	49	19136780	1	1
LCE1B	64902	818	18	296192	85084	0	0	0	0	50	55	20674344	1	1
LCE1D	64902	818	18	292988	81880	0	0	2	0	0	2	81880	1	1
LCE1E	64902	818	18	302244	83304	0	0	1	0	13	28	5373820	1	1
LCE2C	169158	818	24	280884	79032	0	0	0	0	50	53	20184132	1	1
LCE3B	285984	752	17	238520	67996	0	0	0	0	50	58	22596388	1	1
LCE3C	285984	752	17	241012	67640	0	0	0	0	50	58	22596388	1	1
LCE3E	285984	752	17	234248	67996	0	0	0	0	50	58	22596388	1	1
LCE4A	169158	818	24	255964	68708	0	0	0	0	50	53	20184132	1	1
LCE6A	NaN	NaN	NaN	207192	56604	0	0	0	0	50	116	39702188	1	1
LCN10	1898901	190	39	515488	152012	0	0	0	0	50	67	24888672	1	1
LCN12	1979517	188	34	501960	142044	0	0	1	0	16	30	5786424	1	1
LCN6	1898901	190	39	425776	123176	0	0	0	0	50	67	24888672	1	1
LCNL1	1979517	188	34	408688	128516	0	0	0	0	50	78	21046364	1	1
LDHA	599909	176	42	1027416	263796	0	0	0	0	50	55	21406992	1	1
LEFTY1	924499	239	8	898544	292276	0	0	0	0	7	5	3057684	1	1
LELP1	822908	602	28	255608	65860	0	0	0	0	50	85	27475012	1	1
LENEP	1612870	224	35	154504	50552	0	0	0	0	50	56	18488504	1	1
LEP	925335	373	30	422216	124600	0	0	0	0	50	50	19541196	1	1
LEPRE1	820426	426	30	1903888	537560	0	0	0	0	11	7	4652208	1	1
LEPREL1	165372	399	3	1819516	511928	0	0	0	0	17	12	7975824	1	1
LEPREL2	2822601	232	32	1830196	592028	0	0	0	0	14	18	6079412	1	1
LEPROTL1	387693	231	51	526880	157708	0	0	0	0	50	42	19128948	1	1
LETM2	841740	381	49	1019228	286936	0	0	0	0	50	76	25273152	1	1
LEUTX	NaN	NaN	NaN	425776	122108	0	0	0	0	50	116	39767692	1	1

LGALS1	1136064	180	65	356000	96832	0	0	1	0	24	18	7824168	1	1
LGALS2	991778	176	51	351728	89356	0	0	0	0	50	60	20105812	1	1
LGALS3	801045	143	47	709508	208972	0	0	0	0	50	68	21306600	1	1
LGALS3BI	632955	177	28	1458176	440728	0	0	0	0	1	0	1286228	1	1
LGALS7	1697047	322	45	348168	107868	0	0	0	0	50	82	22226860	1	1
LGALS7B	1697047	322	45	348168	107868	0	0	0	0	50	82	22226860	1	1
LGALS8	464894	556	5	946604	246352	0	0	0	0	50	64	22818176	1	1
LGALS9B	384584	413	-6	929160	256320	0	0	0	0	11	24	6624448	1	1
LGALS9C	928601	254	0	933076	255608	0	0	1	0	50	52	17516624	1	1
LGTN	504002	288	38	1514068	420080	0	0	0	0	32	22	11458216	1	1
LHFPL1	25206	NaN	7	560344	160556	0	0	1	0	50	77	25634136	1	1
LHX6	220092	528	30	1053048	307584	0	0	1	0	50	54	20193388	1	1
LILRA3	730281	458	-8	1099328	340336	0	0	0	0	9	12	3447148	1	1
LIMD2	935285	319	8	341404	85796	0	0	0	0	50	76	15668628	1	1
LIMS3	NaN	NaN	NaN	613744	159488	0	0	0	0	50	116	39805072	1	1
LIN28B	108630	702	-4	639732	177288	0	0	0	0	50	63	20781856	1	1
LIN9	739974	278	21	1472060	378784	0	0	1	0	43	57	19098688	1	1
LINS1	290809	374	-9	1940912	512284	0	0	0	0	24	13	9389856	1	1
LIPK	160610	327	36	1051268	262372	0	0	1	0	50	63	20127172	1	1
LIPM	NaN	NaN	NaN	1111788	283020	0	0	0	0	50	116	39928604	1	1
LIPT2	NaN	NaN	NaN	548952	200784	0	0	0	0	50	116	39846368	1	1
LLPH	243040	696	5	348880	76184	0	0	2	0	1	2	254540	1	1
LMO1	245012	535	10	414384	101460	0	0	0	0	50	63	23938152	1	1
)C1001280	NaN	NaN	NaN	307584	111072	0	0	0	0	50	116	39756656	1	1
)C1001285	NaN	NaN	NaN	399432	121752	0	0	0	0	50	116	39767336	1	1
)C1001302	NaN	NaN	NaN	1029908	383056	0	0	0	0	50	116	40028640	1	1
)C1001309	NaN	NaN	NaN	193664	46636	0	0	0	0	50	116	39692220	1	1
)C1001309	NaN	NaN	NaN	162336	43788	0	0	0	0	50	116	39689372	1	1
)C1001318	NaN	NaN	NaN	209684	45568	0	0	0	0	50	116	39691152	1	1
)C1001322	NaN	NaN	NaN	2760780	898188	0	0	0	0	5	8	3270216	1	1
)C1001329	NaN	NaN	NaN	257032	76184	0	0	0	0	50	116	39721768	1	1
)C1001338	NaN	NaN	NaN	863656	239588	0	0	0	0	50	116	39885172	1	1
)C1002874	NaN	NaN	NaN	962624	318976	0	0	0	0	50	116	39964560	1	1

)C1002877	NaN	NaN	NaN	574940	156640	0	0	0	0	50	116	39802224	1	1
)C1002887	NaN	NaN	NaN	369528	124956	0	0	0	0	50	116	39770540	1	1
)C1003026	223391	572	-10	1433968	391244	0	0	0	0	14	11	6258836	1	1
LOC15078	456915	540	2	632256	184764	0	0	0	0	50	92	26952404	1	1
LOC15332	471108	443	23	405840	117480	0	0	0	0	50	57	22870864	1	1
LOC20003	NaN	NaN	NaN	4183000	1039520	0	0	0	0	0	0	1039520	1	1
LOC20072	269047	832	14	466360	125312	0	0	0	0	50	51	16973724	1	1
LOC22171	251884	409	26	227484	71556	0	0	0	0	50	57	20107948	1	1
LOC28399	NaN	NaN	NaN	924888	353508	0	0	0	0	50	116	39999092	1	1
LOC28503	904852	213	43	306160	94340	0	0	0	0	50	63	24111168	1	1
LOC28623	126536	629	8	290496	75828	0	0	0	0	50	60	20316920	1	1
LOC33904	NaN	NaN	NaN	1911008	532576	0	0	0	0	9	16	4890016	1	1
LOC34234	NaN	NaN	NaN	2861528	860452	0	0	0	0	7	14	4548612	1	1
LOC36003	NaN	NaN	NaN	509436	111072	0	0	0	0	50	116	39756656	1	1
LOC37519	NaN	NaN	NaN	881100	201852	0	0	0	0	50	116	39847436	1	1
LOC38858	NaN	NaN	NaN	207548	56248	0	0	0	0	50	116	39701832	1	1
LOC38894	NaN	NaN	NaN	563192	150232	0	0	0	0	50	116	39795816	1	1
LOC38933	NaN	NaN	NaN	2369180	888220	0	0	0	0	5	8	3260248	1	1
LOC38949	NaN	NaN	NaN	241368	79032	0	0	0	0	50	116	39724616	1	1
LOC39059	NaN	NaN	NaN	928092	315060	0	0	0	0	50	116	39960644	1	1
LOC39132	NaN	NaN	NaN	301888	101816	0	0	0	0	50	116	39747400	1	1
LOC40069	NaN	NaN	NaN	398008	103952	0	0	0	0	50	116	39749536	1	1
LOC40105	944872	170	42	297616	87932	0	0	0	0	50	62	23148188	1	1
LOC40109	NaN	NaN	NaN	570312	224280	0	0	0	0	50	116	39869864	1	1
LOC40138	NaN	NaN	NaN	2203996	576008	0	0	0	0	7	14	4264168	1	1
LOC40264	NaN	NaN	NaN	408332	101104	0	0	0	0	50	116	39746688	1	1
LOC40277	NaN	NaN	NaN	324672	99324	0	0	0	0	50	116	39744908	1	1
LOC44056	NaN	NaN	NaN	742972	201140	0	0	0	0	50	116	39846724	1	1
LOC44105	NaN	NaN	NaN	4553240	1677472	0	0	0	0	0	0	1677472	1	1
LOC44129	368082	372	-17	1953728	540052	0	0	0	0	5	1	1889648	1	1
LOC44186	NaN	NaN	NaN	925600	366680	0	0	0	0	50	116	40012264	1	1
LOC55908	1691034	233	32	489856	162692	0	0	0	0	50	70	21150672	1	1
LOC64258	199789	427	-2	253116	85440	0	0	0	0	50	45	22610984	1	1

LOC642597	NaN	NaN	NaN	184764	43788	0	0	0	0	50	116	39689372	1	1
LOC643008	NaN	NaN	NaN	197936	60520	0	0	0	0	50	116	39706104	1	1
LOC643596	NaN	NaN	NaN	377716	129584	0	0	0	0	50	116	39775168	1	1
LOC643677	NaN	NaN	NaN	18000784	4704896	0	0	0	0	0	0	4704896	1	1
LOC644538	NaN	NaN	NaN	198292	70844	0	0	0	0	50	116	39716428	1	1
LOC645961	NaN	NaN	NaN	2790684	860808	0	0	0	0	7	14	4548968	1	1
LOC646498	1812695	203	45	524032	146672	0	0	0	0	50	80	19955580	1	1
LOC646627	NaN	NaN	NaN	190816	65504	0	0	0	0	50	116	39711088	1	1
LOC646851	NaN	NaN	NaN	1534360	363476	0	0	0	0	50	116	40009060	1	1
LOC647309	NaN	NaN	NaN	861876	230688	0	0	0	0	50	116	39876272	1	1
LOC649330	118495	792	12	746176	200072	0	0	0	0	50	47	18244288	1	1
LOC653486	NaN	NaN	NaN	250980	69420	0	0	0	0	50	116	39715004	1	1
LOC653543	NaN	NaN	NaN	2364908	863656	0	0	0	0	7	14	4551816	1	1
LOC653544	NaN	NaN	NaN	6835200	2520480	0	0	0	0	0	0	2520480	1	1
LOC653545	NaN	NaN	NaN	6835200	2520480	0	0	0	0	0	0	2520480	1	1
LOC653548	NaN	NaN	NaN	6835200	2520480	0	0	0	0	0	0	2520480	1	1
LOC728392	NaN	NaN	NaN	287292	97188	0	0	0	0	50	116	39742772	1	1
LOC728410	NaN	NaN	NaN	5696000	2100400	0	0	0	0	0	0	2100400	1	1
LOC728819	NaN	NaN	NaN	805272	207192	0	0	0	0	50	116	39852776	1	1
LOC729020	NaN	NaN	NaN	574940	158776	0	0	0	0	50	116	39804360	1	1
LOC729991	NaN	NaN	NaN	328232	81880	0	0	0	0	50	116	39727464	1	1
729991-MI	NaN	NaN	NaN	962980	239588	0	0	0	0	50	116	39885172	1	1
LOC730755	NaN	NaN	NaN	315416	100036	0	0	0	0	50	116	39745620	1	1
LOC81691	244139	580	32	2011756	552512	0	0	0	0	11	12	4334300	1	1
LOR	836876	556	24	732292	274832	0	0	0	0	44	89	28132900	1	1
LOXHD1	NaN	NaN	NaN	5935232	1566400	0	0	0	0	0	0	1566400	1	1
LPAR5	2816983	210	61	867572	327520	0	0	0	0	25	55	15777920	1	1
LPCAT4	542657	234	22	1318268	421504	0	0	2	0	50	46	18844148	1	1
LPHN1	1036713	321	25	3682464	1141692	0	0	0	0	1	0	1308656	1	1
LPHN2	55308	1274	-19	3604500	975084	0	0	0	0	4	1	2875412	1	1
LPHN3	11170	1494	-18	3762208	1041656	0	0	0	0	1	0	1354936	1	1
LPPR1	135891	952	-29	833040	241368	0	0	0	0	50	98	23524480	1	1
LPPR3	2168802	198	28	1809192	611964	0	0	1	0	50	73	22578944	1	1

LPXN	237619	644	-4	1041300	264864	0	0	0	0	50	56	23443312	1	1
LRDD	1793686	176	50	2209692	777504	0	0	0	0	1	0	901392	1	1
LRP11	423793	234	42	1230692	400144	0	0	1	0	50	37	17400924	1	1
LRRC10B	NaN	NaN	NaN	679960	258812	0	0	0	0	50	116	39904396	1	1
LRRC20	551999	225	45	471344	138484	0	0	0	0	50	39	20343976	1	1
LRRC24	1755818	291	7	1218588	447492	0	0	1	0	24	29	10834860	1	1
LRRC26	1722012	202	36	763976	318976	0	0	0	0	3	3	2037744	1	1
LRRC29	1109491	150	46	558208	180848	0	0	0	0	50	47	19075192	1	1
LRRC33	1117984	250	31	1677116	551800	0	0	0	0	6	4	4027428	1	1
LRRC37A	372453	231	22	4253132	1256680	0	0	0	0	5	0	3225360	1	1
LRRC50	566339	315	12	1857252	520116	0	0	0	0	18	22	9182664	1	1
LRRC58	384022	358	6	909936	296904	0	0	1	0	50	46	20144260	1	1
LRRC67	393936	518	12	597368	157708	0	0	0	0	50	60	21748396	1	1
LRRC69	NaN	NaN	NaN	909580	237452	0	0	0	0	50	116	39883036	1	1
LRRN4CL	2425755	248	51	569600	200428	0	0	0	0	50	68	20007556	1	1
LRTOMT	731498	267	60	1208620	385904	0	0	2	0	31	33	12046684	1	1
LSM1	705837	277	46	347456	94696	0	0	1	0	10	15	3794960	1	1
LSM3	303635	258	39	269136	73692	0	0	0	0	50	49	17059520	1	1
LSM4	1294357	210	39	364900	100748	0	0	0	0	50	67	22067728	1	1
LSM5	371992	639	6	249200	64792	0	0	0	0	50	69	19642656	1	1
LSM6	82460	1023	21	218228	54112	0	0	0	0	50	48	21101900	1	1
LSMD1	2148615	337	24	420080	143824	0	0	0	0	50	73	27995840	1	1
LST-3TM1[illegible]	487942	773	-31	1661096	441796	0	0	0	0	9	5	3775736	1	1
LTB4R	915114	220	38	823072	312212	0	0	1	0	50	69	21077692	1	1
LUZP4	226836	NaN	8	811680	209328	0	0	0	0	50	69	23846304	1	1
LUZP6	NaN	NaN	NaN	144892	44144	0	0	0	0	50	116	39689728	1	1
LY6D	427585	373	34	322892	101104	0	0	0	0	50	60	17573228	1	1
LY6E	795935	470	13	335352	104664	0	0	1	0	1	2	333216	1	1
LY6G5C	2517236	193	20	388752	103596	0	0	0	0	50	63	27465044	1	1
LY6G6C	2517236	193	20	318976	95408	0	0	0	0	50	63	27465044	1	1
LY6G6D	2517236	193	20	335708	102172	0	0	1	0	1	1	233536	1	1
LY6G6F	2517236	193	20	751160	227128	0	0	0	0	50	63	27465044	1	1
LY6K	384374	412	23	561412	137060	0	0	1	0	11	7	4009272	1	1

LY86	133091	314	29	430404	111072	0	0	0	0	50	58	21463952	1	1
LYPD1	46944	848	8	365612	97900	0	0	1	0	0	1	97900	1	1
LYPD2	427585	373	34	313636	102884	0	0	0	0	50	60	17573228	1	1
LYRM2	394472	603	35	234248	61588	0	0	0	0	50	48	22834552	1	1
LYRM5	260142	633	19	243504	56604	0	0	0	0	48	40	16567528	1	1
LYRM7	312101	294	34	286936	68708	0	0	0	0	50	45	18246424	1	1
LYZL4	342219	527	19	387328	100748	0	0	0	0	50	67	22278836	1	1
M6PR	299430	416	11	719476	195800	0	0	0	0	50	48	22394892	1	1
MACROD1	1324858	278	53	834820	252404	0	0	0	0	26	28	8291596	1	1
MAFA	2154403	237	32	854044	280172	0	0	1	0	50	62	21812476	1	1
MAFF	1604692	184	68	398364	138840	0	0	1	0	30	52	11756544	1	1
MAFG	3798594	211	19	397652	133144	0	0	0	0	34	28	11428668	1	1
MAGEA2	NaN	NaN	NaN	1561416	465648	0	0	0	0	50	116	40111232	1	1
MAGEA2E	202421	NaN	-2	1561416	465648	0	0	0	0	4	2	2027420	1	1
MAGEA5	117300	NaN	-16	305448	99324	0	0	0	0	50	63	19912860	1	1
MAGEA9	NaN	NaN	NaN	1595592	437880	0	0	0	0	50	116	40083464	1	1
MAGEA9E	224617	NaN	8	1595592	437880	0	0	0	0	50	75	25320500	1	1
MAGEB3	74310	NaN	16	879676	236384	0	0	0	0	50	67	20445436	1	1
MAGED4	NaN	NaN	NaN	3754376	1096480	0	0	0	0	0	0	1096480	1	1
MAGED4E	NaN	NaN	NaN	3776448	1100040	0	0	0	0	0	0	1100040	1	1
MAL2	247332	442	-1	441084	142044	0	0	0	0	50	50	21146400	1	1
MANF	538437	536	39	480600	130296	0	0	2	0	5	8	2237460	1	1
MAP1D	537616	126	34	882168	234960	0	0	0	0	50	50	18405556	1	1
MAP6D1	1441187	410	25	483092	168388	0	0	0	0	50	75	18513780	1	1
MAPK1	458136	299	47	945536	245284	0	0	2	0	50	43	19892924	1	1
MAPKSP1	203900	633	28	338912	87220	0	0	0	0	50	48	18832756	1	1
1-Mar	56946	1136	-17	844076	227128	0	0	0	0	50	61	21100120	1	1
10-Mar	200669	205	-17	2082244	552512	0	0	0	0	0	0	552512	1	1
11-Mar	160001	888	-24	999292	304736	0	0	0	0	28	45	11180536	1	1
2-Mar	726556	150	0	613388	195088	0	0	0	0	50	51	19031048	1	1
3-Mar	298349	603	27	637240	193664	0	0	0	0	50	59	25326908	1	1
4-Mar	590758	342	27	1024924	304736	0	0	0	0	18	17	7206508	1	1
5-Mar	429135	317	31	718052	197224	0	0	0	0	50	34	17182340	1	1

6-Mar	627256	164	17	2360636	665008	0	0	0	0	13	7	4966200	1	1
7-Mar	363925	374	28	1796732	500536	0	0	0	0	36	19	16653324	1	1
8-Mar	128745	725	34	768960	192240	0	0	0	0	50	64	21685740	1	1
9-Mar	1160849	344	39	845856	278748	0	0	0	0	50	87	20175588	1	1
ɪARCKSL	1478512	210	42	485584	146672	0	0	2	0	0	2	146672	1	1
ɪARVELD	NaN	NaN	NaN	411180	146316	0	0	0	0	50	116	39791900	1	1
MAX	501431	387	39	719476	174440	0	0	0	0	50	76	23893652	1	1
MBD3L1	560313	537	-60	489856	139196	0	0	0	0	50	122	29063128	1	1
MBD3L2	NaN	NaN	NaN	510148	165896	0	0	0	0	50	116	39811480	1	1
MBD3L3	NaN	NaN	NaN	512640	163404	0	0	0	0	50	116	39808988	1	1
MBD3L4	NaN	NaN	NaN	512284	163760	0	0	0	0	50	116	39809344	1	1
MBD3L5	NaN	NaN	NaN	511216	164828	0	0	0	0	50	116	39810412	1	1
MBLAC2	83822	824	25	688148	213244	0	0	2	0	8	13	3461388	1	1
MBOAT4	NaN	NaN	NaN	1086512	321112	0	0	0	0	50	116	39966696	1	1
MC2R	133238	815	-2	736920	217872	0	0	1	0	16	18	5864744	1	1
MCART1	485791	524	28	743328	217872	0	0	0	0	15	19	6871868	1	1
MCART2	415934	554	29	736564	220364	0	0	0	0	50	60	22710308	1	1
MCART6	357831	NaN	45	751516	239588	0	0	0	0	50	50	20582852	1	1
MCAT	624112	241	48	973304	294412	0	0	1	0	50	66	22537648	1	1
MCFD2	809702	242	33	548596	133856	0	0	0	0	50	57	18549380	1	1
MCOLN2	373596	649	-5	1503744	368460	0	0	0	0	22	24	6973328	1	1
MDFI	486789	331	52	625848	186900	0	0	2	0	0	2	186900	1	1
MDH2	1072825	218	49	858316	264152	0	0	0	0	50	64	21496704	1	1
MEAF6	446254	357	25	545748	133500	0	0	0	0	50	40	16603128	1	1
MED10	253175	763	-12	364188	86508	0	0	1	0	23	24	8536168	1	1
MED18	754355	170	25	535780	142400	0	0	0	0	50	68	26747348	1	1
MED22	1827885	416	28	517268	152368	0	0	2	0	0	2	152368	1	1
MED30	139000	574	0	460664	125668	0	0	1	0	0	1	125668	1	1
MED31	663953	424	9	355644	80100	0	0	0	0	50	52	18304452	1	1
MESDC1	265620	516	-2	856892	306160	0	0	0	0	16	17	7192980	1	1
MESP1	880974	190	35	634748	233536	0	0	0	0	50	61	22286312	1	1
METRN	1207790	194	33	704524	252404	0	0	1	0	50	77	22508812	1	1
METRNL	222738	483	14	762196	250268	0	0	1	0	50	34	17488856	1	1

METT10D	678213	167	19	1448208	391956	0	0	0	0	50	49	22829212	1	1
METT11D1	1005555	429	4	1261308	332148	0	0	0	0	30	29	8819900	1	1
METT5D1	271729	616	-14	1085800	296192	0	0	0	0	22	15	7104336	1	1
METTL1	1160849	344	39	749736	161268	0	0	0	0	50	87	20175588	1	1
METTL10	294304	352	42	750804	213600	0	0	0	0	50	57	18328304	1	1
METTL11A	886775	205	39	574228	156284	0	0	0	0	50	69	20923544	1	1
METTL11B	NaN	NaN	NaN	737988	189036	0	0	0	0	50	116	39834620	1	1
METTL7B	1678592	523	-8	609116	180136	0	0	2	0	7	8	2744404	1	1
METTL9	479469	466	40	819156	220008	0	0	2	0	29	41	12985100	1	1
MFAP4	630769	332	11	663584	180136	0	0	0	0	50	72	28508124	1	1
MFNG	979927	179	51	817732	251336	0	0	0	0	50	67	19634824	1	1
MGC26647	NaN	NaN	NaN	639732	176220	0	0	0	0	50	116	39821804	1	1
MGC29506	832127	177	48	474548	149164	0	0	0	0	50	77	20769396	1	1
MGC42105	505867	537	26	1100752	307940	0	0	0	0	2	0	428624	1	1
MGC70857	1755818	291	7	520828	182984	0	0	0	0	24	29	10834860	1	1
MGC87042	NaN	NaN	NaN	913140	242436	0	0	0	0	50	116	39888020	1	1
MGP	341752	426	8	279104	67996	0	0	0	0	50	50	19251768	1	1
MGST2	382197	256	12	383768	109648	0	0	0	0	50	70	26196616	1	1
MICB	2514366	220	34	973304	278392	0	0	2	0	16	15	4268440	1	1
MID1IP1	91133	NaN	31	458528	131008	0	0	0	0	50	54	22551532	1	1
MIIP	518052	207	39	973660	311144	0	0	2	0	42	44	13163456	1	1
MIOX	1075525	207	42	758992	195800	0	0	2	0	11	15	4417248	1	1
MKI67IP	225849	358	31	766112	203632	0	0	0	0	50	45	19401288	1	1
MLANA	337967	183	27	312212	86152	0	0	0	0	50	62	26807156	1	1
MLF1IP	299322	515	32	1118552	277324	0	0	0	0	50	69	23222592	1	1
MLF2	2816186	232	48	651480	180492	0	0	0	0	6	24	6345344	1	1
MLL	1338034	192	49	9940944	2930592	0	0	0	0	1	0	3080468	1	1
MLL2	1770671	214	65	13597420	4372748	0	0	0	0	0	0	4372748	1	1
MLL3	261375	616	16	12498804	3487020	0	0	0	0	0	0	3487020	1	1
MLL4	1151198	321	30	6654708	2201148	0	0	0	0	0	0	2201148	1	1
MLL5	443176	381	40	4728748	1334288	0	0	0	0	3	2	2219660	1	1
MLLT11	1614665	165	40	232824	63012	0	0	0	0	50	62	20369964	1	1
MMD	147492	526	-59	629408	161980	0	0	0	0	50	86	18568604	1	1

MMGT1	253719	NaN	20	333572	102172	0	0	0	0	24	22	9536172	1	1
MMP23B	NaN	NaN	NaN	971524	315416	0	0	0	0	50	116	39961000	1	1
MMP26	97256	1024	-11	689928	173016	0	0	1	0	15	18	5687456	1	1
MND1	202295	238	27	568176	123888	0	0	0	0	50	38	18733788	1	1
MNS1	211810	681	12	1354936	272696	0	0	0	0	50	71	18983344	1	1
MNT	752972	271	28	1401928	487364	0	0	0	0	18	26	10374552	1	1
MOBKL1A	265400	638	25	579568	137060	0	0	0	0	50	48	20795028	1	1
MOBKL1E	1025255	172	44	578856	137772	0	0	0	0	43	61	23133236	1	1
MOBKL2A	1853815	239	37	556784	150232	0	0	0	0	50	68	23613124	1	1
MOBKL2E	117319	434	-22	561412	142400	0	0	0	0	50	64	21389904	1	1
MOBKL2C	608423	266	25	688860	187968	0	0	0	0	50	58	19047424	1	1
MOBKL3	667169	428	25	611964	144180	0	0	0	0	50	72	22561500	1	1
MOCS2	204924	419	-5	682808	150232	0	0	0	0	50	36	19447212	1	1
MOG	484408	755	34	784624	220364	0	0	0	0	50	51	18249272	1	1
MORF4	260919	785	-29	593096	161980	0	0	0	0	50	79	21017884	1	1
MORF4L2	485629	NaN	43	727664	202564	0	0	2	0	13	6	3222868	1	1
MORN2	NaN	NaN	NaN	211820	50908	0	0	0	0	50	116	39696492	1	1
MORN4	564805	169	40	383768	104308	0	0	0	0	50	62	20233616	1	1
MORN5	220092	528	30	438948	99324	0	0	1	0	6	10	3510872	1	1
MOSC1	319790	564	-2	840872	267712	0	0	0	0	14	10	3606636	1	1
MOSC2	319790	564	-2	859028	247420	0	0	0	0	14	10	3606636	1	1
MOSPD1	296514	NaN	17	555004	150944	0	0	0	0	50	45	22514152	1	1
MOSPD3	1884430	247	60	577432	200072	0	0	1	0	22	30	9880780	1	1
MPST	699535	379	44	783912	247776	0	0	1	0	50	63	26596760	1	1
MPV17	1245306	182	52	461376	135636	0	0	0	0	50	41	22639464	1	1
MPV17L	545296	212	36	523320	120684	0	0	0	0	50	58	20390968	1	1
MRC1L1	75804	350	-2	7734812	1851556	0	0	0	0	0	0	1851556	1	1
MRFAP1L1	501020	201	62	328232	86152	0	0	2	0	1	2	257032	1	1
MRGPRG	NaN	NaN	NaN	673552	253472	0	0	0	0	50	116	39899056	1	1
MRGPRX4	631291	436	20	780708	256320	0	0	1	0	50	76	24807148	1	1
MRO	240974	355	12	778572	201852	0	0	0	0	50	73	21837752	1	1
MRP63	447305	240	43	259168	75116	0	0	0	0	50	47	18943116	1	1
MRPL12	3745109	205	30	499468	155216	0	0	0	0	33	25	10695308	1	1

MRPL14	1358424	203	53	365968	110360	0	0	1	0	34	28	11204032	1	1
MRPL23	827096	481	53	401568	111072	0	0	0	0	50	76	25042464	1	1
MRPL36	394241	608	6	253116	84372	0	0	0	0	50	75	20915000	1	1
MRPL4	1729517	200	31	831260	257032	0	0	2	0	29	50	11342516	1	1
MRPL42	380369	364	15	420080	88288	0	0	0	0	50	68	27055288	1	1
MRPL45	1101194	504	10	745108	195800	0	0	0	0	50	61	15954140	1	1
MRPL46	212296	630	4	708084	206124	0	0	0	0	50	56	21422656	1	1
MRPL48	553005	214	42	568532	145960	0	0	0	0	50	46	18683948	1	1
MRPL49	3267059	207	46	422572	127448	0	0	0	0	50	74	20482816	1	1
MRPL52	1165302	225	35	353508	67284	0	0	1	0	5	12	2536144	1	1
MRPS11	212296	630	4	495196	150944	0	0	0	0	50	56	21422656	1	1
MRPS12	1794625	282	21	339624	114276	0	0	1	0	11	10	3517992	1	1
MRPS16	829738	249	44	346744	103952	0	0	1	0	1	2	281240	1	1
MRPS25	532551	395	45	456036	114276	0	0	0	0	50	63	21310516	1	1
MRPS33	354283	679	10	279104	72268	0	0	0	0	50	66	23073784	1	1
MRRF	319049	597	19	695624	172660	0	0	1	0	26	20	10394132	1	1
MS4A15	744779	621	23	610896	186900	0	0	0	0	50	74	23297708	1	1
MS4A8B	559288	756	16	635816	192952	0	0	0	0	50	40	17570024	1	1
MSI1	1597755	182	50	961200	263796	0	0	0	0	4	10	2645080	1	1
MSL3L2	NaN	NaN	NaN	889644	254184	0	0	0	0	50	116	39899768	1	1
MSMB	406207	579	41	335352	48060	0	0	0	0	50	76	22964848	1	1
MST4	151066	NaN	32	1098972	281952	0	0	0	0	14	15	5940216	1	1
MT1B	653985	187	28	171948	37380	0	0	0	0	50	41	16409108	1	1
MT1E	653985	187	28	170524	36668	0	0	0	0	50	41	16409108	1	1
MT1F	653985	187	28	171948	35244	0	0	0	0	50	41	16409108	1	1
MT1G	661565	143	26	173728	35600	0	0	0	0	50	43	20327956	1	1
MT1M	653985	187	28	170168	37024	0	0	0	0	50	41	16409108	1	1
MT1X	661565	143	26	173016	36312	0	0	0	0	50	43	20327956	1	1
MT2A	653985	187	28	173728	35600	0	0	0	0	50	41	16409108	1	1
MT3	653985	187	28	190460	39160	0	0	0	0	50	41	16409108	1	1
MT4	598942	187	24	180136	34532	0	0	0	0	43	58	19598156	1	1
MTAP	223932	357	-28	738700	201140	0	0	0	0	50	86	24121136	1	1
MTCP1	NaN	NaN	NaN	292988	74404	0	0	1	0	8	17	4000372	1	1

MTCP1NB	985719	NaN	6	187968	41652	0	0	0	0	50	40	15864784	1	1
MTERF	512495	617	-7	1017448	272696	0	0	0	0	17	27	7871516	1	1
MTERFD1	217849	671	12	1086156	285156	0	0	0	0	50	75	24887960	1	1
MTERFD2	1394664	278	35	983272	255608	0	0	0	0	50	94	24308036	1	1
MTERFD3	156112	473	21	979000	262016	0	0	0	0	50	46	19326884	1	1
MTG1	485067	472	8	866504	249556	0	0	0	0	27	41	12590296	1	1
MTMR15	280093	591	19	2627992	713780	0	0	0	0	14	7	5907464	1	1
MTP18	657636	288	48	543612	153792	0	0	0	0	50	58	19652624	1	1
MTPN	387359	588	-9	307228	86864	0	0	1	0	50	43	13940604	1	1
MUC12	NaN	NaN	NaN	12680720	4462816	0	0	0	0	0	0	4462816	1	1
MUC2	1467711	466	43	7097216	2130304	0	0	0	0	1	4	4092576	1	1
MUDENG	167619	944	19	1259172	343896	0	0	0	0	27	15	9596692	1	1
MUL1	573203	429	30	880744	263084	0	0	2	0	17	21	6692444	1	1
MUSTN1	1193949	272	49	213244	61232	0	0	0	0	50	63	22512372	1	1
MUTED	578469	368	13	502316	119260	0	0	0	0	50	69	20657612	1	1
MVK	442710	465	38	1006412	312568	0	0	1	0	50	62	19254616	1	1
MXD3	942017	227	53	593096	178000	0	0	0	0	50	65	19830268	1	1
MXI1	403252	359	12	822004	227840	0	0	0	0	6	11	4844092	1	1
MYADML2	NaN	NaN	NaN	728376	260592	0	0	0	0	50	116	39906176	1	1
MYCBP	524940	458	24	283732	68708	0	0	0	0	50	45	18683236	1	1
MYCL1	996021	270	31	1076544	328944	0	0	0	0	26	31	10740876	1	1
MYEOV2	340416	482	28	645072	184764	0	0	0	0	50	52	22426576	1	1
MYL1	186926	511	3	550376	138484	0	0	0	0	50	49	20830628	1	1
MYL3	427952	376	40	524032	131720	0	0	0	0	50	63	25265676	1	1
MYL4	960078	160	22	526880	139552	0	0	1	0	50	82	21390260	1	1
MYL7	1151124	282	56	477040	116768	0	0	1	0	4	8	1887868	1	1
MYL9	858383	190	46	458884	108224	0	0	1	0	25	34	12171996	1	1
MYOD1	391398	626	17	782844	254184	0	0	1	0	50	41	18571096	1	1
MYST1	1296884	351	35	1266648	344964	0	0	0	0	16	27	8122140	1	1
MYST2	1129239	238	27	1604492	416164	0	0	0	0	50	64	21704608	1	1
MYST3	608794	464	31	5134944	1353156	0	0	0	0	2	1	1827348	1	1
MYST4	246599	335	42	5309028	1400148	0	0	0	0	0	0	1400148	1	1
NAA11	103025	821	-5	586688	154504	0	0	3	0	4	9	1143828	1	1

NAA20	225789	502	12	494840	120328	0	0	0	0	50	57	21416248	1	1
NACAD	NaN	NaN	NaN	3745476	1294416	0	0	0	0	0	0	1294416	1	1
NACC2	809002	262	46	1444292	461020	0	0	1	0	50	59	19879040	1	1
NANOS1	680734	411	-1	686012	254896	0	0	0	0	50	41	17641580	1	1
NANS	484638	272	48	922752	254184	0	0	2	0	50	49	20385272	1	1
NAPRT1	2183482	235	37	1330728	451764	0	0	0	0	50	40	19636960	1	1
NARG2	230111	266	23	2546468	667144	0	0	0	0	6	6	2565336	1	1
NAT14	NaN	NaN	NaN	481312	190460	0	0	0	0	50	116	39836044	1	1
NAT15	1083663	246	34	629764	170168	0	0	0	0	50	59	19712432	1	1
NAT8B	978005	466	31	552868	179780	0	0	0	0	50	67	22206212	1	1
NBL1	775439	271	32	552868	157352	0	0	0	0	50	62	21875844	1	1
NBPF16	NaN	NaN	NaN	3563204	864724	0	0	0	0	7	14	4552884	1	1
NBPF4	NaN	NaN	NaN	1669640	437524	0	0	0	0	50	116	40083108	1	1
NBPF7	415110	581	26	1106092	274832	0	0	0	0	25	17	10230016	1	1
NCBP2	754196	306	29	414028	103952	0	0	0	0	50	41	23872292	1	1
NCCRP1	1652029	286	26	680316	227484	0	0	0	0	5	2	1706308	1	1
NCF1	895858	125	49	1021008	276612	0	0	0	0	50	71	23498848	1	1
NCR3	2517285	220	35	594520	194732	0	0	0	0	50	55	21824936	1	1
NCS1	650366	231	30	531864	127092	0	0	2	0	1	2	381276	1	1
NDFIP1	606399	371	40	584908	156284	0	0	0	0	50	63	21927464	1	1
NDP	73234	NaN	13	335352	98256	0	0	1	0	8	11	3906388	1	1
NDUFA1	772178	NaN	38	189392	48772	0	0	1	0	4	4	1150592	1	1
NDUFA11	1192025	294	35	362052	107868	0	0	0	0	50	52	17132856	1	1
NDUFA2	774470	356	31	276612	79032	0	0	0	0	50	59	20786128	1	1
NDUFA3	735613	439	-53	231756	57672	0	0	0	0	50	119	29395632	1	1
NDUFA4	142078	1098	-11	220008	55536	0	0	1	0	3	4	1014956	1	1
NDUFA4L[illegible]	2162032	238	58	233180	61588	0	0	0	0	50	84	24087316	1	1
NDUFAF4	123845	1308	1	455680	118904	0	0	1	0	7	3	2288368	1	1
NDUFB1	356163	415	27	276968	75472	0	0	0	0	50	54	24970908	1	1
NDUFB11	819569	NaN	38	417588	120684	0	0	0	0	50	54	16576428	1	1
NDUFB3	644877	205	39	263440	62300	0	0	0	0	50	62	23017892	1	1
NDUFB7	1008795	204	26	358492	96476	0	0	0	0	50	74	20546896	1	1
NDUFB8	1153157	264	50	496264	124244	0	0	1	0	18	20	7416192	1	1

NDUFC1	387523	299	20	200428	59096	0	0	1	0	4	15	3530808	1	1
NDUFC2	556958	209	38	305804	87220	0	0	0	0	50	53	19579288	1	1
NDUFS3	989876	214	28	683876	197224	0	0	0	0	50	74	23251428	1	1
NDUFS5	682309	208	26	281952	69420	0	0	0	0	50	58	23038184	1	1
NDUFV3	441011	206	66	1173376	360272	0	0	1	0	4	4	2079396	1	1
NELF	1591716	213	30	1395164	380920	0	0	0	0	20	24	9013920	1	1
NENF	416515	283	18	427556	141688	0	0	1	0	50	60	19934576	1	1
NEURL	532306	170	44	1401572	462088	0	0	0	0	19	18	7146344	1	1
NEURL1B	NaN	NaN	NaN	1328592	469920	0	0	0	0	50	116	40115504	1	1
NEURL2	725555	209	59	685300	235316	0	0	0	0	27	40	9561092	1	1
NEUROG1	496830	608	34	564972	197580	0	0	0	0	50	66	23468944	1	1
NEUROG2	186926	647	22	649344	228552	0	0	0	0	50	50	20264944	1	1
NFAM1	967833	185	53	679248	212532	0	0	0	0	50	62	18302672	1	1
NFKBIL1	2517285	220	35	944112	292632	0	0	0	0	50	55	21824936	1	1
NFKBIL2	2043455	200	36	3445012	1082240	0	0	0	0	0	0	1082240	1	1
NGB	554839	418	37	382344	117480	0	0	0	0	50	65	23475708	1	1
NHEDC1	357993	363	26	1527240	403704	0	0	0	0	50	46	23956664	1	1
NHEDC2	350752	454	26	1361700	409044	0	0	0	0	14	15	7726268	1	1
NHLH1	987855	526	28	316128	113208	0	0	0	0	50	69	19516276	1	1
NHP2	821697	248	39	407620	98612	0	0	0	0	50	45	23405932	1	1
NHP2L1	1475100	183	50	329656	97544	0	0	0	0	50	47	18945964	1	1
NHSL1	NaN	NaN	NaN	4115004	1281600	0	0	0	0	0	0	1281600	1	1
NICN1	1585328	201	32	547172	161980	0	0	1	0	50	56	18997584	1	1
NKX1-2	NaN	NaN	NaN	733716	268068	0	0	0	0	50	116	39913652	1	1
NKX2-3	278426	497	13	894628	279104	0	0	1	0	50	51	16901100	1	1
NKX2-6	NaN	NaN	NaN	524388	185832	0	0	0	0	50	116	39831416	1	1
NKX3-1	378220	544	16	579212	180136	0	0	0	0	50	67	24898996	1	1
NKX3-2	134006	592	24	799576	276968	0	0	1	0	50	51	20316920	1	1
NKX6-1	95574	895	-5	887508	304380	0	0	0	0	50	57	20885096	1	1
NKX6-2	278505	581	-12	676044	227484	0	0	0	0	50	37	17495620	1	1
NMB	341389	197	42	391244	119260	0	0	1	0	50	48	19895416	1	1
ME1-NME	817798	267	-4	696692	194020	0	0	0	0	50	65	20140344	1	1
NME2	817798	267	-4	400500	108936	0	0	0	0	50	65	20140344	1	1

NMNAT1	744954	240	24	725884	188324	0	0	1	0	50	68	23004008	1	1
NMS	226558	642	9	432540	103596	0	0	0	0	50	59	23206572	1	1
NMUR1	1052303	216	43	1029908	346744	0	0	1	0	50	70	25384580	1	1
NOTCH2NI	NaN	NaN	NaN	617660	158776	0	0	0	0	50	116	39804360	1	1
NOTO	NaN	NaN	NaN	611252	204700	0	0	0	0	50	116	39850284	1	1
NPAS1	1175364	207	35	1447140	489144	0	0	1	0	50	53	19463232	1	1
NPBWR1	99395	697	-19	774656	278392	0	0	5	0	1	6	458528	1	1
NPIP	506128	214	23	904952	251692	0	0	0	0	24	25	10120012	1	1
NPIPL3	499242	417	49	2735860	933788	0	0	0	0	9	9	6682476	1	1
NPM1	300840	347	30	812748	186900	0	0	0	0	50	43	20088724	1	1
NPPB	519155	207	43	333216	110004	0	0	0	0	50	49	18705664	1	1
NPTN	310307	416	20	1026348	286224	0	0	2	0	15	18	8067672	1	1
NPVF	254553	444	32	512284	129584	0	0	0	0	50	61	25227228	1	1
NPW	1747301	183	46	380208	155928	0	0	0	0	50	75	19137492	1	1
NQO1	1410481	NaN	39	715560	186900	0	0	0	0	50	80	29234008	1	1
NR2E1	334933	436	35	987544	283376	0	0	2	0	50	52	19543688	1	1
NR6A1	892645	345	29	1251696	327876	0	0	1	0	50	64	20722760	1	1
NRGN	168652	622	14	199716	61944	0	0	0	0	50	50	19749100	1	1
NRN1L	879834	158	50	404416	138128	0	0	0	0	50	85	23764780	1	1
NSA2	418815	581	30	683520	176220	0	0	1	0	25	19	8118580	1	1
NSL1	365714	364	26	824852	199360	0	0	1	0	2	1	796728	1	1
NT5C	1511891	168	25	514064	152368	0	0	0	0	50	53	18659384	1	1
NT5C3	376609	359	13	925956	234960	0	0	0	0	7	8	3892860	1	1
NT5C3L	1760409	175	35	795660	205056	0	0	0	0	35	50	15900028	1	1
NT5M	900505	220	15	578500	172304	0	0	0	0	50	63	22306960	1	1
NTN3	2085452	321	49	1401572	483448	0	0	0	0	5	6	2042728	1	1
NUCKS1	732566	281	35	656108	151300	0	0	0	0	50	68	23701056	1	1
NUDCD3	1535714	363	52	928804	252404	0	0	1	0	50	96	25773332	1	1
NUDT10	112254	NaN	3	417588	120684	0	0	0	0	50	81	25225804	1	1
NUDT15	324467	567	34	422572	114632	0	0	0	0	50	44	21492432	1	1
NUDT18	584373	219	34	787116	261660	0	0	0	0	50	59	21330096	1	1
NUDT2	478815	302	38	381632	101104	0	0	0	0	50	40	17367460	1	1
NUDT8	1374022	393	53	336776	125668	0	0	0	0	50	76	21858400	1	1

NUMBL	918439	226	34	1504812	488076	0	0	2	0	50	67	19969464	1	1
NUP62CL	227679	NaN	32	492348	130296	0	0	1	0	16	22	5951964	1	1
NUPR1	1133167	269	63	253472	78676	0	0	0	0	50	70	20905744	1	1
NUTF2	959655	143	49	350660	76540	0	0	0	0	50	65	23996180	1	1
NXF2	NaN	NaN	NaN	3382000	815240	0	0	0	0	7	14	4503400	1	1
NXF2B	95507	NaN	6	3382000	815240	0	0	0	0	1	0	1200788	1	1
NXN	941400	376	15	1096124	332860	0	0	2	0	50	69	16529792	1	1
NXT1	188823	688	-22	358848	97188	0	0	0	0	50	91	27388860	1	1
NXT2	133688	NaN	30	520828	132788	0	0	0	0	50	52	25221176	1	1
OBFC1	529915	300	29	976152	244572	0	0	1	0	50	50	18714564	1	1
OBFC2A	160466	461	20	538272	139908	0	0	0	0	50	42	18256036	1	1
OBFC2B	2878071	171	46	546460	158420	0	0	0	0	33	67	18798224	1	1
OC90	100916	999	6	1245644	341404	0	0	0	0	14	18	6873292	1	1
OCLM	277882	399	-14	115700	28480	0	0	0	0	50	30	18183768	1	1
OCM	2208864	358	32	298328	69064	0	0	0	0	50	54	20921052	1	1
OCM2	451365	522	25	296548	68708	0	0	1	0	17	31	6456060	1	1
ODF3	1233553	437	17	647564	195088	0	0	0	0	50	63	18496336	1	1
ODF3B	1075525	207	42	633324	208260	0	0	0	0	50	60	21418028	1	1
ODZ1	121229	NaN	-13	6973684	1917416	0	0	0	0	1	0	2235680	1	1
ODZ2	71443	880	-10	6993620	1988260	0	0	0	0	2	2	3277692	1	1
ODZ3	144640	636	-20	6835200	1928808	0	0	0	0	2	2	3870076	1	1
ODZ4	78290	871	0	6989704	2013536	0	0	0	0	2	0	2381996	1	1
OGFOD2	1093113	196	47	721256	232468	0	0	1	0	50	70	23094788	1	1
OGFR	1044503	208	48	1681744	518336	0	0	1	0	38	49	16027832	1	1
OLIG1	801943	554	66	624780	247776	0	0	0	0	2	1	611608	1	1
OLR1	397577	398	18	739768	159488	0	0	1	0	50	59	21415536	1	1
ONECUT3	NaN	NaN	NaN	1164120	425064	0	0	0	0	50	116	40070648	1	1
OPALIN	799460	684	38	386616	105732	0	0	2	0	3	7	1326100	1	1
OPN1MW2	2438903	NaN	17	1859032	526880	0	0	0	0	11	7	4822376	1	1
OPRM1	44509	878	0	1838740	508724	0	0	0	0	50	42	21841668	1	1
OR10A3	105341	679	6	773944	234248	0	0	1	0	50	63	24898284	1	1
OR10A7	411183	816	-1	781776	232824	0	0	0	0	50	58	19503104	1	1
OR10G3	707623	533	22	754364	250624	0	0	0	0	50	70	23763000	1	1

OR10W1	604763	835	-19	739768	240656	0	0	0	0	50	68	16404124	1	1
OR13C4	146816	930	-33	782844	237096	0	0	2	0	28	39	9194412	1	1
OR13D1	161420	821	-14	857248	253472	0	0	0	0	50	86	23751608	1	1
OR1E1	365088	515	14	774300	234960	0	0	0	0	50	54	23562928	1	1
OR1E2	365088	515	14	796016	241012	0	0	1	0	50	54	23562928	1	1
OR1I1	990597	524	16	869352	269136	0	0	0	0	10	15	3457828	1	1
OR1L4	409923	553	12	768604	231044	0	0	2	0	3	4	974016	1	1
OR1N1	373314	646	3	765756	233892	0	0	0	0	50	63	22349324	1	1
OR1Q1	407749	646	9	779996	227128	0	0	1	0	50	67	20364980	1	1
OR2A42	199649	767	-2	750448	243860	0	0	0	0	50	37	24768344	1	1
OR2A5	222080	812	-11	762196	236384	0	0	1	0	50	70	20067720	1	1
OR2T29	116628	850	-20	765400	227840	0	0	0	0	50	57	19537280	1	1
OR2T5	112460	903	-20	779996	232468	0	0	1	0	50	74	22093716	1	1
OR2W5	272980	927	-14	782488	248132	0	0	0	0	50	59	17172372	1	1
OR3A3	365088	515	14	777148	253472	0	0	1	0	50	54	23562928	1	1
OR4C45	NaN	NaN	NaN	768604	215024	0	0	0	0	50	116	39860608	1	1
OR4D1	851306	401	14	758636	235672	0	0	3	0	0	3	235672	1	1
OR4E2	658946	624	20	770384	233536	0	0	2	0	30	32	13383108	1	1
OR4F16	NaN	NaN	NaN	1682456	515488	0	0	0	0	9	16	4872928	1	1
OR4F21	89938	794	10	766824	233892	0	0	0	0	50	66	22789696	1	1
OR4F29	NaN	NaN	NaN	1682456	515488	0	0	0	0	9	16	4872928	1	1
OR4F3	NaN	NaN	NaN	1682456	515488	0	0	0	0	9	16	4872928	1	1
OR4F4	185501	643	-9	755788	224636	0	0	1	0	50	55	27208368	1	1
OR4F5	369933	594	NaN	756144	224280	0	0	0	0	50	55	24344704	1	1
OR4M2	156689	855	-31	778928	231400	0	0	0	0	50	68	20763700	1	1
OR51T1	65798	1006	-15	869352	267000	0	0	0	0	1	1	499824	1	1
OR52A4	109516	1024	-15	752940	228552	0	0	0	0	50	47	18695696	1	1
OR52N1	203856	922	-5	791032	236384	0	0	0	0	50	39	17006476	1	1
OR56A5	NaN	NaN	NaN	762196	241724	0	0	3	0	8	19	4167692	1	1
OR5B2	475497	835	-29	772876	224636	0	0	3	0	13	14	4428996	1	1
OR5C1	433740	487	10	765400	262016	0	0	0	0	50	48	19317628	1	1
OR5P3	103028	823	-1	762908	235672	0	0	0	0	40	52	18180920	1	1
OR6C2	802038	706	3	776436	226416	0	0	4	0	5	8	1326100	1	1

OR6C76	802038	706	3	767180	234604	0	0	2	0	16	13	4515504	1	1
OR6P1	NaN	NaN	NaN	773944	243860	0	0	0	0	50	116	39889444	1	1
OR6X1	92984	637	-1	758636	242080	0	0	0	0	50	47	21991544	1	1
OR7G1	311030	471	-63	822360	252048	0	0	0	0	9	11	2373096	1	1
OR8G2	196141	921	-12	749736	225348	0	0	0	0	50	60	20258892	1	1
OR8U8	12744	946	-38	241724	56248	0	0	0	0	50	111	19272060	1	1
OR9A4	198115	856	-6	777860	231400	0	0	1	0	50	61	21007204	1	1
OR9G9	252874	882	-38	750092	228196	0	0	0	0	50	67	16937056	1	1
ORAI1	720229	228	56	741192	237096	0	0	0	0	50	83	23210132	1	1
ORC1L	815523	448	26	2190824	639376	0	0	0	0	10	13	4824512	1	1
ORC2L	641949	201	29	1547176	373088	0	0	0	0	11	6	3543268	1	1
ORC3L	236047	435	28	1896412	471344	0	0	0	0	6	3	3634404	1	1
ORC4L	90907	937	4	1175512	280172	0	0	0	0	50	40	22486384	1	1
ORC5L	152701	927	0	1238524	316484	0	0	0	0	50	55	20707096	1	1
ORC6L	443420	253	-10	658600	179780	0	0	0	0	50	67	21403788	1	1
ORMDL1	398590	452	25	394092	112140	0	0	3	0	0	3	112140	1	1
ORMDL2	1909014	256	38	390176	114988	0	0	0	0	50	69	23288452	1	1
ORMDL3	1602321	292	18	392668	113564	0	0	0	0	50	82	23538364	1	1
OSBPL2	990360	252	9	1278040	314348	0	0	0	0	0	0	314348	1	1
OST4	NaN	NaN	NaN	102172	27056	0	0	0	0	50	116	39672640	1	1
OSTBETA	688371	189	44	331792	90068	0	0	0	0	50	72	23489592	1	1
OSTC	160719	574	14	388752	106800	0	0	0	0	50	46	20184844	1	1
OSTalpha	1042183	210	51	854756	271984	0	0	0	0	40	55	13677164	1	1
OTOR	76240	856	-21	342472	85796	0	0	0	0	50	67	21170608	1	1
OTOS	340416	482	28	233892	63012	0	0	0	0	50	52	22426576	1	1
OTUD1	NaN	NaN	NaN	1156288	388040	0	0	0	0	50	116	40033624	1	1
OVCA2	1415256	294	27	551800	185120	0	0	0	0	50	68	21536932	1	1
OVOL1	3834148	287	55	681740	189748	0	0	3	0	0	3	189748	1	1
OVOL2	764857	499	-9	702388	194732	0	0	3	0	7	13	2373808	1	1
OXA1L	1153069	351	36	1250272	377360	0	0	0	0	11	13	4543984	1	1
OXT	835590	288	54	320044	96476	0	0	2	0	10	11	1775372	1	1
P2RX1	561462	215	32	1044148	284444	0	0	0	0	50	57	20132868	1	1
P2RX4	1003980	396	45	1017804	275544	0	0	0	0	50	73	21384920	1	1

P2RY11	1675365	272	15	18868	3560	0	0	0	0	0	0	3560	1	1
P2RY12	51795	505	10	855824	247420	0	0	1	0	50	64	25325484	1	1
P2RY4	161347	NaN	27	876472	296192	0	0	2	0	18	20	8351404	1	1
P4HA2	371617	246	35	1453192	392312	0	0	1	0	35	25	11825964	1	1
'ABPC1L2.	NaN	NaN	NaN	500892	143112	0	0	0	0	50	116	39788696	1	1
'ABPC1L2I	NaN	NaN	NaN	500892	143112	0	0	0	0	50	116	39788696	1	1
PABPC4L	NaN	NaN	NaN	1086156	292632	0	0	0	0	50	116	39938216	1	1
PACRGL	42454	1252	22	578144	163048	0	0	0	0	50	47	20860888	1	1
PAEP	327501	539	37	484516	125312	0	0	0	0	50	46	18874052	1	1
PAG1	185952	441	18	1109296	303668	0	0	0	0	50	55	18831332	1	1
PAGE2	214019	NaN	18	301888	76184	0	0	0	0	50	59	20814964	1	1
PAGE2B	214019	NaN	18	301176	75828	0	0	0	0	50	59	20814964	1	1
.LM2-AKA	250166	485	18	2784632	798508	0	0	2	0	50	48	20858396	1	1
PALM3	NaN	NaN	NaN	1666080	514776	0	0	0	0	9	16	4872216	1	1
PAPL	1652812	254	29	1133148	320400	0	0	1	0	34	41	15057376	1	1
PARN	519767	367	31	1760420	392668	0	0	0	0	32	27	13342880	1	1
PATE1	607531	493	3	349236	76896	0	0	1	0	6	7	2338564	1	1
PATE3	NaN	NaN	NaN	266288	61588	0	0	0	0	50	116	39707172	1	1
PATE4	NaN	NaN	NaN	266288	61588	0	0	0	0	50	116	39707172	1	1
PATL2	NaN	NaN	NaN	1410828	396228	0	0	0	0	50	116	40041812	1	1
PCBD1	327428	592	29	277680	71556	0	0	0	0	50	51	23052424	1	1
PCBD2	727517	696	49	338912	95764	0	0	1	0	7	10	2830556	1	1
PCBP2	1712232	191	56	969032	266644	0	0	1	0	50	69	22534088	1	1
PCDP1	309897	441	17	1478468	368104	0	0	0	0	20	21	10460348	1	1
PCGF3	672497	312	27	658600	154148	0	0	0	0	50	53	25717440	1	1
PCGF6	533950	125	43	914208	250980	0	0	1	0	50	45	17906088	1	1
PCMT1	424800	234	40	740124	208260	0	0	0	0	50	35	18596016	1	1
PCOTH	314992	678	51	605556	160200	0	0	0	0	20	20	5337508	1	1
PCP4	177752	829	-71	173016	39516	0	0	0	0	50	77	21869436	1	1
PCP4L1	735152	293	41	184052	45568	0	0	0	0	50	46	22289160	1	1
PCSK1N	NaN	NaN	NaN	602352	244572	0	0	0	0	50	116	39890156	1	1
PCTP	56508	631	-60	567108	143112	0	0	1	0	50	104	24466100	1	1
PCYT1B	424049	NaN	31	1054116	268068	0	0	0	0	43	44	15155276	1	1

PDAP1	1115689	263	56	485584	118904	0	0	1	0	0	1	118904	1	1
PDC	278089	492	-7	648632	155572	0	0	0	0	50	59	25737020	1	1
PDCL2	237456	456	32	656820	143112	0	0	1	0	50	73	26751620	1	1
PDE6D	979792	193	33	401212	99680	0	0	0	0	50	65	18656536	1	1
PDE6G	3745109	205	30	231756	63012	0	0	0	0	50	50	17890424	1	1
PDF	1202748	125	20	584908	203276	0	0	0	0	20	22	7617688	1	1
PDGFA	598406	318	9	563548	155216	0	0	0	0	50	80	29320872	1	1
PDPR	1339196	137	19	2276976	615168	0	0	5	0	9	11	2905672	1	1
PDRG1	1006109	386	37	357780	88644	0	0	2	0	4	11	1044504	1	1
PDZK1	NaN	NaN	NaN	1331796	368460	0	0	1	0	50	117	40014044	1	1
PEBP1	637549	608	17	480956	134212	0	0	1	0	18	23	7133172	1	1
PECAM1	1304459	178	16	24208	7832	0	0	0	0	0	0	7832	1	1
PECI	180813	452	27	1039520	275188	0	0	0	0	36	36	13624476	1	1
PECR	603452	419	35	784624	221432	0	0	0	0	50	58	22205500	1	1
PET112L	146825	477	14	1439308	401924	0	0	0	0	20	15	7870448	1	1
PET117	782027	479	-6	211108	58028	0	0	0	0	50	53	15933848	1	1
PEX5	1322142	267	27	1684948	482024	0	0	0	0	6	5	2677476	1	1
PF4	230379	408	2	257744	79744	0	0	0	0	50	43	23670796	1	1
PFDN1	764742	185	39	328944	80100	0	0	0	0	50	52	16695688	1	1
PFDN4	78451	492	-28	365256	80100	0	0	0	0	50	102	21816036	1	1
PFKL	1153513	308	65	2002856	591316	0	0	2	0	50	59	19026776	1	1
PFN2	622464	490	17	449272	119972	0	0	0	0	50	68	19536924	1	1
PFN3	1038665	261	59	328232	118192	0	0	0	0	50	73	23403440	1	1
PGA3	1023217	531	47	993596	289072	0	0	0	0	50	86	29296664	1	1
PGA4	NaN	NaN	NaN	993596	289072	0	0	0	0	50	116	39934656	1	1
PGAM1	892254	188	39	649700	182272	0	0	1	0	50	71	21552240	1	1
PGBD3	122000	224	15	1495556	411892	0	0	0	0	13	8	4717000	1	1
PGCP	235137	741	-10	1202924	342472	0	0	0	0	46	63	22952744	1	1
PGK2	67972	1246	2	1034892	304380	0	0	2	0	1	3	540052	1	1
PGLS	961640	206	42	631900	217160	0	0	2	0	3	3	1578860	1	1
PGLYRP3	836876	556	24	877896	247776	0	0	2	0	5	13	2804212	1	1
PGPEP1L	217299	248	-20	495196	153080	0	0	1	0	50	59	22018956	1	1
PHB	733027	256	23	686368	213956	0	0	2	0	35	41	13634444	1	1

PHF15	909404	177	51	1982564	592384	0	0	0	0	1	2	1395520	1	1
PHF16	830380	NaN	45	2140272	542544	0	0	0	0	29	17	10428664	1	1
PHF17	163287	544	26	2183704	586688	0	0	0	0	11	7	4399448	1	1
PHF21B	350834	525	-5	1342476	417588	0	0	1	0	26	32	11695312	1	1
PHF5A	1480984	187	73	300108	70488	0	0	0	0	50	55	16540828	1	1
PHGR1	NaN	NaN	NaN	206836	67640	0	0	0	0	50	116	39713224	1	1
PHLDA2	552601	256	34	378428	118192	0	0	0	0	50	59	21410552	1	1
PHOSPHO	763613	346	27	757212	240300	0	0	1	0	27	34	13858724	1	1
PHOX2A	744547	182	58	687436	234248	0	0	1	0	50	69	18856964	1	1
PHPT1	1921909	176	44	384124	91136	0	0	0	0	50	56	20791468	1	1
PHYHIP	587455	196	50	848704	228908	0	0	0	0	50	67	20087300	1	1
PID1	105238	675	-12	668568	176220	0	0	0	0	50	66	28734540	1	1
PIF1	556478	205	50	1575300	532932	0	0	1	0	50	48	18457888	1	1
PIGH	483892	311	46	480956	139552	0	0	0	0	50	46	20345400	1	1
PIGP	353934	358	51	414384	107868	0	0	1	0	42	44	14645840	1	1
PIGX	818753	446	45	714848	198292	0	0	0	0	50	63	24450080	1	1
PIGY	449479	295	29	458884	144536	0	0	1	0	50	41	15076956	1	1
PIM3	842221	271	15	808476	260592	0	0	1	0	50	69	23287384	1	1
PIN1	1069900	579	-26	428268	112140	0	0	0	0	50	87	20502040	1	1
PIN4	374992	NaN	53	486296	129940	0	0	1	0	50	50	16164536	1	1
PINK1	631461	208	35	1424712	472056	0	0	1	0	50	63	21866232	1	1
PION	309983	573	35	2312220	557496	0	0	0	0	6	3	2056968	1	1
PIRT	58616	911	-49	339268	102884	0	0	1	0	2	2	730868	1	1
PITX1	725156	226	27	781064	236740	0	0	1	0	50	83	24477492	1	1
PKD1L3	NaN	NaN	NaN	4441812	1229268	0	0	0	0	0	0	1229268	1	1
PKM2	1130931	192	36	1494132	435744	0	0	0	0	16	19	7218256	1	1
PKMYT1	1443513	234	49	1280532	391956	0	0	1	0	14	11	5039180	1	1
PL-5283	NaN	NaN	NaN	130652	31684	0	0	0	0	50	116	39677268	1	1
PLA2G10	519767	367	45	422572	124244	0	0	0	0	50	58	18288076	1	1
PLA2G12A	211934	473	25	486652	134924	0	0	1	0	3	3	749024	1	1
PLA2G16	854193	303	56	423996	115344	0	0	1	0	50	62	16526588	1	1
PLA2G2C	608368	501	27	394092	98256	0	0	0	0	50	65	19952732	1	1
PLA2G2D	567393	501	23	387328	95408	0	0	0	0	50	58	17485652	1	1

PLA2G4B	874211	259	40	2002856	585976	0	0	0	0	10	18	9759740	1	1
PLA2G4E	733131	267	42	2165192	603064	0	0	2	0	8	11	2939492	1	1
PLAC1L	336373	691	4	411536	110716	0	0	0	0	50	70	21435828	1	1
PLAC4	187016	530	-16	352084	131720	0	0	0	0	50	77	26022532	1	1
PLAC8	361292	169	58	310076	78676	0	0	0	0	50	62	21214752	1	1
PLBD2	442841	251	37	1495912	443576	0	0	1	0	50	36	17266712	1	1
PLCXD1	NaN	NaN	NaN	819868	243860	0	0	4	0	2	8	1100396	1	1
PLDN	200904	369	3	458172	113208	0	0	0	0	50	39	22335796	1	1
PLEK2	484235	291	32	915988	254540	0	0	2	0	2	2	637952	1	1
PLEKHA9	168990	630	-14	987900	268068	0	0	0	0	50	50	23471080	1	1
PLEKHF1	222973	612	-63	690640	210752	0	0	0	0	50	115	29520588	1	1
PLGLB1	NaN	NaN	NaN	532576	121040	0	0	0	0	50	116	39766624	1	1
PLGLB2	NaN	NaN	NaN	532576	121040	0	0	0	0	50	116	39766624	1	1
PLK1S1	302588	632	-13	1768252	443576	0	0	0	0	34	16	11197624	1	1
PLSCR3	1911735	206	36	734428	239588	0	0	0	0	50	69	22072000	1	1
PLUNC	478384	482	27	644004	213600	0	0	0	0	8	0	2332512	1	1
PM20D2	376032	446	27	1102532	325384	0	0	1	0	3	3	1452836	1	1
PMAIP1	200064	434	48	139552	40940	0	0	1	0	0	1	40940	1	1
PMP2	191177	595	1	353508	87576	0	0	0	0	50	53	20427636	1	1
PNMA6A	NaN	NaN	NaN	1925248	637952	0	0	0	0	7	14	4326112	1	1
PNRC2	NaN	NaN	NaN	362764	90068	0	0	0	0	50	116	39735652	1	1
POC1B	236022	460	-6	1249204	332504	0	0	0	0	50	47	18158848	1	1
PODNL1	930314	231	32	1247424	447492	0	0	1	0	50	60	18484588	1	1
POFUT2	596236	334	45	1221792	338556	0	0	1	0	50	56	22040316	1	1
POLB	524868	327	41	915276	216804	0	0	0	0	50	46	18041012	1	1
POLD4	1269888	177	51	281952	76896	0	0	0	0	50	45	20698908	1	1
POLR2G	2405109	248	44	462800	121396	0	0	2	0	7	12	2285876	1	1
POLR2J	642828	230	65	312212	80812	0	0	0	0	50	41	15606684	1	1
POLR2J2	NaN	NaN	NaN	304380	84372	0	0	1	0	26	41	11661492	1	1
POLR2K	810757	396	25	169100	32752	0	0	0	0	50	67	28712824	1	1
POLR2L	1799017	232	51	172304	51976	0	0	1	0	2	2	469564	1	1
POLR3D	586506	193	43	1014244	298328	0	0	0	0	50	73	21834904	1	1
POLR3K	712205	238	36	286224	73692	0	0	0	0	50	62	19943476	1	1

POM121L2	NaN	NaN	NaN	2495916	825564	0	0	0	0	7	14	4513724	1	1
POMP	167695	629	37	381988	96476	0	0	0	0	50	62	16797860	1	1
POP4	223167	612	-63	585264	150588	0	0	3	0	3	8	1236744	1	1
POP5	1615212	311	34	433608	113208	0	0	0	0	50	52	20694992	1	1
POTEB	NaN	NaN	NaN	2173736	528304	0	0	0	0	9	16	4885744	1	1
POTED	42480	1066	-58	1552160	369172	0	0	0	0	10	19	4540780	1	1
POU1F1	58819	470	-15	824140	216092	0	0	0	0	50	67	23598528	1	1
POU5F2	263452	587	3	794592	261660	0	0	0	0	50	58	21330096	1	1
PPAN	NaN	NaN	NaN	1058388	296904	0	0	0	0	50	116	39942488	1	1
PPAPDC1A	261792	849	5	699184	197936	0	0	1	0	50	66	20411616	1	1
PPAPDC2	224825	673	12	705236	245284	0	0	1	0	50	77	24742712	1	1
PPIA	1200579	164	55	438948	112140	0	0	0	0	50	70	24613484	1	1
PPIAL4B	NaN	NaN	NaN	1260240	338556	0	0	0	0	50	116	39984140	1	1
PPIAL4C	397277	779	29	840160	225704	0	0	0	0	38	25	14914976	1	1
PPIAL4D	NaN	NaN	NaN	833752	227840	0	0	1	0	50	117	39873424	1	1
PPIAL4E	NaN	NaN	NaN	416876	113920	0	0	0	0	50	116	39759504	1	1
PPIAL4F	216744	582	41	833752	227840	0	0	0	0	50	54	20450420	1	1
PPIAL4G	119504	375	34	419724	111072	0	0	0	0	50	59	20323328	1	1
PPIC	183387	519	12	540764	158776	0	0	1	0	5	8	2404424	1	1
PPIL3	635570	201	31	532220	129940	0	0	0	0	50	61	17116836	1	1
PPIL5	563227	409	-4	1081528	260948	0	0	0	0	50	55	20194100	1	1
PPME1	628196	280	38	1029908	267712	0	0	1	0	50	64	18575012	1	1
PPP1R14B	1613996	257	65	373800	113208	0	0	1	0	19	29	6446448	1	1
PPP1R14D	1036414	172	51	553224	110004	0	0	1	0	39	55	14892192	1	1
PPP1R1A	643658	392	33	451764	124956	0	0	0	0	50	58	20886164	1	1
PPP1R3G	NaN	NaN	NaN	852620	297616	0	0	0	0	50	116	39943200	1	1
PPP2R4	1440213	206	34	946960	245996	0	0	0	0	50	83	27113316	1	1
PPPDE1	718652	540	-1	505164	136704	0	0	0	0	50	92	22613120	1	1
PPPDE2	1471218	183	58	436456	128516	0	0	0	0	50	56	19858392	1	1
PPT1	873408	387	23	807052	210752	0	0	0	0	50	79	27832436	1	1
PPY	920082	241	29	239944	78320	0	0	0	0	50	50	17463580	1	1
PPYR1	NaN	NaN	NaN	922396	286580	0	0	0	0	50	116	39932164	1	1
PRAC	873680	509	26	147740	44500	0	0	0	0	50	70	19254972	1	1

PRAF2	833689	NaN	41	431116	153080	0	0	0	0	50	74	21714932	1	1
PRAMEF1[illegible]	NaN	NaN	NaN	1195092	339624	0	0	0	0	50	116	39985208	1	1
PRAMEF1[illegible]	NaN	NaN	NaN	1194736	337844	0	0	0	0	50	116	39983428	1	1
PRAMEF2[illegible]	61945	NaN	7	2387336	697048	0	0	0	0	2	2	1422576	1	1
PRAMEF2[illegible]	NaN	NaN	NaN	2403356	704524	0	0	0	0	7	14	4392684	1	1
PRAMEF3	NaN	NaN	NaN	1201856	353152	0	0	0	0	50	116	39998736	1	1
PRAMEF9	60626	NaN	8	2407984	687080	0	0	0	0	0	0	687080	1	1
PRB1	175992	673	-18	805272	273408	0	0	0	0	15	35	9917804	1	1
PRB3	311837	673	-1	750448	262016	0	0	0	0	8	4	2073344	1	1
PRCD	1016866	197	24	149520	40584	0	0	0	0	50	75	20563984	1	1
PRDM12	505506	211	24	920972	277324	0	0	0	0	25	34	10186940	1	1
PRDM6	NaN	NaN	NaN	1484164	455324	0	0	0	0	50	116	40100908	1	1
PRELID1	942017	227	53	564616	157352	0	0	2	0	13	20	4127820	1	1
PRELID2	342210	568	-3	517980	124956	0	0	2	0	5	7	862944	1	1
PRELP	529605	314	28	943400	292276	0	0	1	0	50	66	20381000	1	1
PRH2	NaN	NaN	NaN	419724	131364	0	0	0	0	50	116	39776948	1	1
PRHOXNE[illegible]	309835	614	47	421860	139908	0	0	0	0	50	55	18324388	1	1
PRIC285	1178905	193	54	6458908	2131016	0	0	0	0	0	0	2131016	1	1
PRIM1	2637730	154	47	1145964	256320	0	0	0	0	11	12	6165920	1	1
PRIM2	177580	847	-16	1370956	327164	0	0	1	0	47	54	17740192	1	1
PRIMA1	311378	676	19	390532	119972	0	0	1	0	2	4	1441800	1	1
PRKAB2	457805	737	33	714492	190104	0	0	0	0	50	47	19391320	1	1
PRKAG3	1077853	296	40	1247068	376292	0	0	1	0	50	83	24994048	1	1
PRKCDBP	444453	625	-7	621220	224636	0	0	0	0	50	51	16375288	1	1
PRLH	492799	440	11	210396	74760	0	0	0	0	50	68	23707108	1	1
PRM3	632841	308	37	259524	70488	0	0	0	0	50	60	21937788	1	1
PRMT10	71970	263	20	2181568	576008	0	0	0	0	9	11	4957656	1	1
PROCR	1346435	168	46	592740	185832	0	0	1	0	17	32	7653644	1	1
PRODH	513620	343	5	1533648	449628	0	0	0	0	50	57	23414832	1	1
PROK1	403650	479	31	271272	79032	0	0	1	0	2	1	557852	1	1
PROK2	86859	605	6	334284	95052	0	0	0	0	50	74	20739492	1	1
PROP1	961008	363	32	556072	179780	0	0	0	0	50	53	18751944	1	1
PRR13	1712232	191	56	381988	108224	0	0	0	0	50	69	22534088	1	1

PRR15	313344	579	-1	318976	97544	0	0	0	0	50	57	16687500	1	1
PRR18	199596	556	18	678892	269492	0	0	0	0	50	35	16755496	1	1
PRR19	792230	218	42	861520	292988	0	0	0	0	50	65	24510600	1	1
PRR20A	NaN	NaN	NaN	2680680	918480	0	0	0	0	5	8	3290508	1	1
PRR20B	NaN	NaN	NaN	2680680	918480	0	0	0	0	5	8	3290508	1	1
PRR20C	NaN	NaN	NaN	2680680	918480	0	0	0	0	5	8	3290508	1	1
PRR20D	NaN	NaN	NaN	2680680	918480	0	0	0	0	5	8	3290508	1	1
PRR20E	NaN	NaN	NaN	2680680	918480	0	0	0	0	5	8	3290508	1	1
PRR23C	NaN	NaN	NaN	624780	217872	0	0	0	0	50	116	39863456	1	1
PRR24	NaN	NaN	NaN	328232	132076	0	0	0	0	50	116	39777660	1	1
PRRG1	81601	NaN	3	628340	171592	0	0	1	0	50	74	27381740	1	1
PRRT4	NaN	NaN	NaN	2136712	763976	0	0	0	0	7	14	4452136	1	1
PRSS2	NaN	NaN	NaN	338912	101104	0	0	0	0	50	116	39746688	1	1
PRSS22	1537882	210	47	793524	248844	0	0	0	0	50	56	20433332	1	1
PRSS23	230637	198	-7	960132	274476	0	0	1	0	50	48	20491360	1	1
PRSS37	199781	856	-13	613388	159844	0	0	0	0	50	63	22945624	1	1
PRSS38	672760	406	5	813104	251692	0	0	1	0	50	43	16613096	1	1
PRSS41	NaN	NaN	NaN	788184	249912	0	0	0	0	50	116	39895496	1	1
PRSS55	78691	633	3	895696	256676	0	0	0	0	50	43	20021796	1	1
PRSS8	1296884	351	35	858672	264864	0	0	2	0	12	24	6411204	1	1
PRSSL1	1832767	218	10	693844	235316	0	0	0	0	29	50	17004696	1	1
PRTFDC1	164255	142	18	613744	146672	0	0	1	0	31	26	11572492	1	1
PRY	NaN	NaN	NaN	765400	208616	0	0	0	0	50	116	39854200	1	1
PRY2	NaN	NaN	NaN	765400	208616	0	0	0	0	50	116	39854200	1	1
PSCA	384374	412	23	294412	84728	0	0	1	0	4	5	1087224	1	1
PSENEN	1151198	321	30	267356	72268	0	0	1	0	4	5	1817736	1	1
PSMD10	201673	NaN	25	585264	161268	0	0	1	0	50	59	19172380	1	1
PSMD13	1463610	437	24	1074408	271272	0	0	0	0	6	2	2533652	1	1
PSMD14	210764	520	13	831260	207904	0	0	0	0	50	67	22276700	1	1
PSME2	974559	220	41	652904	163048	0	0	1	0	50	62	24565780	1	1
PSMG3	547999	271	44	307584	95052	0	0	1	0	26	33	9317588	1	1
PSMG4	NaN	NaN	NaN	470988	135636	0	0	0	0	50	116	39781220	1	1
PSORS1C2	3364017	401	19	333572	109648	0	0	1	0	4	9	1440732	1	1

PSTPIP1	327336	436	33	1107516	288360	0	0	2	0	28	31	11642624	1	1
PTBP1	2041736	198	25	1433968	413672	0	0	2	0	50	82	22041028	1	1
PTCRA	1086050	203	41	674976	243504	0	0	3	0	7	10	2401220	1	1
PTGDS	1979517	188	34	499112	138484	0	0	1	0	16	30	5786424	1	1
PTGES	600401	187	44	380208	120684	0	0	0	0	50	71	21419808	1	1
PTGR1	499818	309	16	886796	242080	0	0	0	0	50	51	18603848	1	1
PTP4A1	256545	154	15	454968	119616	0	0	0	0	50	40	18105804	1	1
PTPLA	488883	383	4	744040	209684	0	0	0	0	50	41	18269564	1	1
PTPLAD1	602545	187	27	964760	241012	0	0	0	0	13	14	6055560	1	1
PTPLAD2	144026	601	5	619084	155216	0	0	0	0	50	56	18608832	1	1
PTPLB	306542	274	38	657176	187612	0	0	0	0	50	54	17275612	1	1
PTPN18	369510	593	29	1188684	350304	0	0	1	0	4	3	1551092	1	1
PTPN20A	NaN	NaN	NaN	2286232	511928	0	0	0	0	9	16	4869368	1	1
PTPN20B	157964	642	23	2286232	511928	0	0	0	0	16	18	8077284	1	1
PTPRCAP	1392213	215	53	490568	179068	0	0	1	0	50	41	15455384	1	1
PTPRQ	NaN	NaN	NaN	5526188	1467076	0	0	0	0	0	0	1467076	1	1
PTS	383407	362	9	396228	95052	0	0	0	0	50	54	24713876	1	1
PTTG2	NaN	NaN	NaN	471344	142756	0	0	0	0	50	116	39788340	1	1
PVRIG	1465817	204	59	789964	274832	0	0	1	0	50	49	18736636	1	1
PWP2	879237	219	61	2363840	671416	0	0	0	0	5	2	2802432	1	1
PYY	920082	241	29	245284	79388	0	0	0	0	50	50	17463580	1	1
ProSAPiP1	909603	255	55	1625496	542544	0	0	0	0	4	7	2147036	1	1
R3HCC1	NaN	NaN	NaN	640800	190104	0	0	0	0	50	116	39835688	1	1
RAB11FIP3	1422744	207	37	1929164	569956	0	0	1	0	50	84	28578612	1	1
RAB15	521616	387	35	551088	146316	0	0	0	0	50	69	21146044	1	1
RAB17	492799	440	11	550020	153792	0	0	0	0	50	68	23707108	1	1
RAB1A	640694	343	41	541476	139908	0	0	0	0	50	54	22755520	1	1
RAB27B	105818	372	-13	576720	146316	0	0	1	0	3	7	1585624	1	1
RAB2A	234041	512	24	572092	140264	0	0	0	0	50	49	20263520	1	1
RAB2B	707390	470	30	580280	149164	0	0	1	0	31	43	15582120	1	1
RAB33B	392237	299	32	582416	158776	0	0	2	0	8	11	3821660	1	1
RAB35	1564246	225	45	558564	110004	0	0	0	0	50	65	21843804	1	1
RAB39	421657	536	15	546816	158064	0	0	0	0	50	52	19313356	1	1

RAB40C	1115707	213	39	724104	205056	0	0	2	0	0	2	205056	1	1
RAB41	215874	NaN	52	597012	146316	0	0	0	0	50	56	22682540	1	1
RAB43	1142757	352	44	537560	153436	0	0	2	0	4	6	1197228	1	1
RAB4A	285692	361	9	582772	148808	0	0	0	0	50	71	23765848	1	1
RAB7A	1090310	277	54	547172	140620	0	0	1	0	6	5	1515848	1	1
RAB7L1	717622	377	37	534000	136704	0	0	0	0	50	68	25178456	1	1
RABL5	838595	278	46	498400	119972	0	0	0	0	50	76	26049944	1	1
RAC2	845055	230	67	500180	143824	0	0	1	0	1	1	490212	1	1
RAD21L1	NaN	NaN	NaN	1492352	349948	0	0	0	0	50	116	39995532	1	1
RAD51L1	446885	404	37	1026704	290140	0	0	0	0	50	82	23472148	1	1
RAD51L3	516190	504	23	972948	315060	0	0	0	0	50	59	21526608	1	1
RAG1AP1	1801444	194	32	592740	142044	0	0	0	0	50	85	19936000	1	1
RAGE	1531739	237	40	1101464	291208	0	0	0	0	50	73	25009356	1	1
RALA	221320	445	20	545748	134568	0	0	0	0	50	65	22028924	1	1
RAMP2	1633092	190	33	448204	130652	0	0	0	0	24	22	8134244	1	1
RANGRF	NaN	NaN	NaN	642936	150588	0	0	0	0	50	116	39796172	1	1
RARRES2	315538	514	32	423996	122820	0	0	1	0	14	18	6003584	1	1
RASA4	NaN	NaN	NaN	2055900	607692	0	0	0	0	7	14	4295852	1	1
RASD1	915530	220	-3	707016	200784	0	0	0	0	50	67	16916408	1	1
RASL11A	284055	265	46	608404	182984	0	0	0	0	50	59	21531948	1	1
RAVER1	1802015	191	21	1843012	635816	0	0	1	0	11	17	6150612	1	1
RAX	293564	549	20	826988	293344	0	0	0	0	50	49	26609220	1	1
RBM11	99517	964	-30	734428	188324	0	0	1	0	50	92	21719560	1	1
RBM16	146505	412	17	3228920	927736	0	0	0	0	3	5	2098620	1	1
RBM20	NaN	NaN	NaN	3097200	894984	0	0	0	0	5	8	3267012	1	1
RBM3	817501	NaN	29	419012	112852	0	0	0	0	50	77	20781500	1	1
RBM38	181292	604	9	398720	127804	0	0	1	0	7	2	2238172	1	1
RBM9	1264586	388	-26	1247424	297972	0	0	0	0	50	61	20904320	1	1
RBMXL3	NaN	NaN	NaN	2636536	787472	0	0	0	0	7	14	4475632	1	1
RBMY1A1	NaN	NaN	NaN	3865092	1053048	0	0	0	0	0	0	1053048	1	1
RBMY1B	NaN	NaN	NaN	5153456	1404064	0	0	0	0	0	0	1404064	1	1
RBMY1D	NaN	NaN	NaN	5153456	1404064	0	0	0	0	0	0	1404064	1	1
RBMY1F	NaN	NaN	NaN	2579576	699184	0	0	0	0	7	14	4387344	1	1

RBMY1J	NaN	NaN	NaN	2579576	699184	0	0	0	0	7	14	4387344	1	1
RBP5	2422791	224	38	358136	92560	0	0	0	0	50	48	17746956	1	1
RBP7	744954	240	24	367036	80456	0	0	2	0	3	12	1418660	1	1
RBPMS	473452	363	27	702032	200428	0	0	0	0	8	4	2161632	1	1
RCAN3	470059	348	28	633324	159132	0	0	1	0	50	61	20596024	1	1
RCC1	NaN	NaN	NaN	1156288	337844	0	0	1	0	50	117	39983428	1	1
RCN3	2080142	210	41	844432	235316	0	0	1	0	50	75	21834192	1	1
RCVRN	63518	366	-41	521540	131008	0	0	0	0	50	79	20220088	1	1
RD3	384781	399	5	490212	149520	0	0	1	0	50	53	21479972	1	1
RDBP	2314693	203	34	985408	278036	0	0	0	0	50	60	23217608	1	1
RDH11	479937	260	39	812036	237808	0	0	3	0	6	9	2430768	1	1
RDH13	1125216	432	-26	831616	257744	0	0	1	0	50	58	18866576	1	1
RDH14	39949	1075	-7	816664	267356	0	0	0	0	26	23	10458924	1	1
RDH5	1814311	384	26	773944	265220	0	0	0	0	50	44	15945952	1	1
REEP5	248831	340	44	498044	127804	0	0	1	0	7	9	2818452	1	1
REG4	415110	581	26	625848	159132	0	0	0	0	50	55	20927460	1	1
RELL1	137374	764	41	698472	200784	0	0	1	0	50	68	19642656	1	1
RESP18	NaN	NaN	NaN	597368	161980	0	0	0	0	50	116	39807564	1	1
RETN	688482	384	31	281240	80812	0	0	2	0	3	4	636172	1	1
RETNLB	129526	941	-3	290852	78676	0	0	0	0	50	77	20664376	1	1
REXO1L1	NaN	NaN	NaN	3295136	1036672	0	0	0	0	0	0	1036672	1	1
RFNG	3850694	197	34	829480	266288	0	0	0	0	25	26	10294096	1	1
RFPL1	723458	203	35	794592	228552	0	0	3	0	0	3	228552	1	1
RFPL3	413546	556	-16	792456	230688	0	0	1	0	50	71	22153880	1	1
RFPL4A	NaN	NaN	NaN	728020	201140	0	0	0	0	50	116	39846724	1	1
RFX8	NaN	NaN	NaN	1244220	321468	0	0	0	0	50	116	39967052	1	1
RG9MTD1	549735	614	11	1039520	259168	0	0	0	0	50	69	21364272	1	1
RG9MTD2	331308	847	9	910648	212888	0	0	0	0	50	70	18591744	1	1
RG9MTD3	489340	453	23	842296	207548	0	0	0	0	50	50	17617728	1	1
RGNEF	279980	577	39	4499840	1203280	0	0	0	0	6	5	3195812	1	1
RGPD5	341807	568	18	13830244	3682820	0	0	0	0	1	0	4205784	1	1
RGPD6	281803	568	17	9288752	2469928	0	0	0	0	0	0	2469928	1	1
RGS13	306250	526	-13	436812	92916	0	0	1	0	32	50	13827752	1	1

RGS8	650082	702	23	548952	143112	0	0	1	0	24	18	10033860	1	1
RGS9BP	380440	455	-45	549308	206836	0	0	1	0	50	79	19821724	1	1
RGSL1	NaN	NaN	NaN	2850492	687792	0	0	0	0	7	14	4375952	1	1
RHEBL1	1770671	214	65	489144	130296	0	0	1	0	1	4	468496	1	1
RHOC	559669	325	27	499824	138840	0	0	0	0	50	65	23160292	1	1
RHOD	1615307	162	47	538628	156640	0	0	2	0	5	12	2001788	1	1
RHOF	826816	195	43	538984	159488	0	0	0	0	50	49	18963764	1	1
RHOG	425863	258	23	472768	146672	0	0	0	0	50	55	19846644	1	1
RIBC2	460724	199	8	993952	243860	0	0	1	0	50	68	20463236	1	1
RICH2	91679	790	-62	2109300	600216	0	0	0	0	15	12	6602376	1	1
RILPL2	1129277	177	47	535780	156284	0	0	0	0	50	58	19591036	1	1
RIMBP3B	NaN	NaN	NaN	7932036	2577084	0	0	0	0	0	0	2577084	1	1
RIMBP3C	572259	221	26	7932036	2577084	0	0	0	0	1	0	3128528	1	1
RMND5B	821697	248	39	1015668	285156	0	0	3	0	0	3	285156	1	1
RNASE11	637784	493	22	514776	130296	0	0	0	0	50	49	16434740	1	1
RNASE12	637784	493	22	382700	95764	0	0	0	0	50	49	16434740	1	1
RNASE2	1005555	429	4	410112	113208	0	0	0	0	50	67	17732716	1	1
RNASE6	956361	577	8	385904	102172	0	0	0	0	50	92	23350396	1	1
RNASE8	937278	320	20	384836	109648	0	0	0	0	50	63	19474980	1	1
RNASEH2A	1937827	226	32	771452	221788	0	0	0	0	50	71	21091932	1	1
RNASEK	1696302	360	24	257388	68352	0	0	0	0	50	49	16256384	1	1
RNASEN	191847	649	-9	3616960	929516	0	0	0	0	12	16	7648660	1	1
RND2	1507064	201	26	574940	172660	0	0	0	0	50	50	17618084	1	1
RNF115	638533	230	36	799932	211464	0	0	1	0	50	57	21960928	1	1
RNF122	91655	610	11	420080	101104	0	0	1	0	3	6	1552516	1	1
RNF144B	322018	573	-11	790676	213244	0	0	0	0	50	41	14789664	1	1
RNF146	113326	889	-6	884660	268780	0	0	0	0	50	63	21878692	1	1
RNF151	1747301	183	46	618728	186544	0	0	0	0	50	75	19137492	1	1
RNF160	316591	303	34	4697064	1237812	0	0	0	0	0	0	1237812	1	1
RNF166	802673	189	41	617304	170880	0	0	0	0	50	65	20750172	1	1
RNF187	NaN	NaN	NaN	587044	181916	0	0	0	0	50	116	39827500	1	1
RNF212	739370	247	43	818088	175152	0	0	0	0	1	1	331792	1	1
RNF222	NaN	NaN	NaN	526168	186188	0	0	0	0	50	116	39831772	1	1

RNF24	683635	170	33	459596	110716	0	0	0	0	50	60	21495636	1	1
RNF32	299200	635	-10	944824	252404	0	0	0	0	50	42	20786840	1	1
ROBLD3	2005405	213	39	320400	96120	0	0	0	0	50	71	21768332	1	1
ROD1	510989	242	31	1431832	409400	0	0	0	0	5	3	1690288	1	1
ROGDI	706891	195	33	754364	213244	0	0	1	0	50	50	19489932	1	1
ROMO1	1423559	169	53	205768	59096	0	0	0	0	50	53	18644076	1	1
ROPN1B	215425	583	27	549308	154504	0	0	1	0	1	1	268424	1	1
RP2	705104	NaN	42	907800	233892	0	0	0	0	42	43	15994368	1	1
RPA4	53531	NaN	36	653260	186188	0	0	1	0	50	53	21213684	1	1
RPL12	784133	209	45	442508	119260	0	0	1	0	7	9	2007484	1	1
RPL13A	2173457	290	44	532220	153436	0	0	0	0	50	67	19850560	1	1
RPL14	195734	546	20	567820	155216	0	0	1	0	39	26	13865132	1	1
RPL15	399346	319	6	514064	155572	0	0	0	0	50	53	22341848	1	1
RPL17	236267	352	55	496976	123532	0	0	0	0	50	49	16950940	1	1
RPL18	1597109	242	31	485228	150232	0	0	2	0	3	4	1095768	1	1
RPL19	1426821	212	31	522608	134212	0	0	0	0	50	55	19657964	1	1
RPL27	1507064	201	26	361696	96476	0	0	0	0	50	50	17618084	1	1
RPL27A	338882	406	24	388752	105732	0	0	0	0	50	43	21578228	1	1
RPL29	1045639	224	36	410112	115344	0	0	0	0	50	51	17801068	1	1
RPL30	625892	272	35	314348	76540	0	0	0	0	50	58	22975884	1	1
RPL35	1125524	207	41	318976	95408	0	0	1	0	2	2	446780	1	1
RPL36AL	563227	409	-4	277324	71912	0	0	0	0	50	55	20194100	1	1
RPL37A	590718	460	37	249200	65860	0	0	0	0	50	49	23263888	1	1
RPL38	297367	535	-8	199360	47348	0	0	0	0	50	41	19792532	1	1
RPL39	762240	NaN	61	143112	34176	0	0	0	0	50	57	19802144	1	1
RPL39L	475647	452	36	136704	34176	0	0	0	0	50	55	18333288	1	1
RPL41	2878044	171	40	81880	14240	0	0	0	0	32	45	13371716	1	1
RPL7A	1827885	416	28	692776	196868	0	0	1	0	50	47	17145316	1	1
RPL9	648710	177	43	515132	132076	0	0	0	0	50	66	22117568	1	1
RPLP1	307077	298	19	297260	86152	0	0	2	0	0	2	86152	1	1
RPP14	596733	221	40	332148	89712	0	0	1	0	0	1	89712	1	1
RPP21	2023315	430	29	406196	111784	0	0	0	0	50	50	19288792	1	1
RPP25	792240	199	45	471700	169100	0	0	2	0	1	2	284444	1	1

RPRML	618151	366	28	284088	103596	0	0	4	0	1	5	286580	1	1
RPS10	892621	233	25	433964	119260	0	0	0	0	50	50	16023560	1	1
RPS14	1087686	188	71	388040	118192	0	0	0	0	50	45	17687504	1	1
RPS15A	728854	279	21	348524	90424	0	0	0	0	50	67	24093012	1	1
RPS16	1410725	230	39	377360	114988	0	0	1	0	1	1	627984	1	1
RPS19BP1	721040	467	72	345676	106088	0	0	2	0	1	5	353508	1	1
RPS21	1190359	252	33	234604	58028	0	0	0	0	50	49	16039936	1	1
RPS23	146308	572	30	371308	105020	0	0	0	0	50	51	20418024	1	1
RPS24	361024	184	30	777860	214312	0	0	0	0	5	9	2785344	1	1
RPS25	1457410	180	55	340336	89000	0	0	0	0	50	46	18727024	1	1
RPS27	2028800	222	34	234604	54824	0	0	1	0	19	26	5582792	1	1
RPS27L	451944	230	36	231400	53756	0	0	0	0	50	52	20650492	1	1
RPS29	563227	409	-4	196868	45568	0	0	0	0	50	55	20194100	1	1
RPS3	587975	205	39	619084	190460	0	0	0	0	50	62	20480680	1	1
RQCD1	939398	179	48	764688	225348	0	0	1	0	33	45	15865852	1	1
RRP7A	969686	196	65	722324	205768	0	0	2	0	18	15	5362428	1	1
RS1	65885	NaN	26	592028	152368	0	0	0	0	50	65	19781140	1	1
RSL24D1	195231	721	-36	454968	91848	0	0	0	0	50	97	26051724	1	1
RSPH9	1626879	278	43	707016	201852	0	0	0	0	50	72	24531960	1	1
RSPO3	106114	826	-9	720900	170880	0	0	0	0	50	56	17887932	1	1
RSPO4	779522	586	27	602352	169812	0	0	1	0	50	82	26677928	1	1
RTBDN	1937827	226	32	656820	208260	0	0	0	0	50	71	21091932	1	1
RTCD1	333324	526	7	987188	277324	0	0	0	0	50	49	19790752	1	1
RTDR1	223123	573	53	886796	259168	0	0	0	0	50	53	18614172	1	1
RUFY4	671005	344	28	1463872	411536	0	0	1	0	17	14	7258128	1	1
RUNDC2A	570118	441	51	981136	255608	0	0	0	0	50	88	25391344	1	1
RWDD4A	150717	254	-3	511572	119616	0	0	0	0	50	49	21644088	1	1
S100A1	NaN	NaN	NaN	249556	63368	0	0	0	0	50	116	39708952	1	1
S100A10	769085	225	29	264152	58384	0	0	0	0	50	63	21033548	1	1
S100A11	527465	349	35	273764	74404	0	0	0	0	50	62	21594604	1	1
S100A12	1140044	401	32	243860	62656	0	0	0	0	50	58	17066640	1	1
S100A13	1555611	183	33	262372	65504	0	0	3	0	0	3	65504	1	1
S100A3	1454773	186	26	268424	66928	0	0	0	0	50	56	19376724	1	1

S100A4	1454773	186	26	272340	63012	0	0	0	0	50	56	19376724	1	1
S100A7L2	1140114	279	38	308296	66572	0	0	0	0	50	63	17848772	1	1
S100A8	1140044	401	32	253472	58384	0	0	0	0	50	58	17066640	1	1
S100B	530282	377	28	249912	55536	0	0	0	0	50	60	21149960	1	1
S100P	501655	278	47	247420	66572	0	0	1	0	7	5	1681032	1	1
S100Z	246938	418	21	269492	63724	0	0	1	0	2	2	343540	1	1
S1PR3	248480	646	4	929160	285156	0	0	2	0	30	23	10629448	1	1
SAA4	601955	371	17	345676	86864	0	0	0	0	50	58	21345760	1	1
SAMD1	1023789	215	34	1048064	355288	0	0	1	0	50	55	16427620	1	1
SAMD12	190644	575	17	540764	145960	0	0	0	0	50	35	16404480	1	1
SAMD5	97528	850	-17	424352	137416	0	0	0	0	50	73	23876564	1	1
SAP25	NaN	NaN	NaN	494128	165896	0	0	0	0	50	116	39811480	1	1
SAP30L	581156	416	29	480244	122108	0	0	0	0	50	52	20192320	1	1
SAPS1	1035412	209	5	2269856	654328	0	0	0	0	17	10	5854776	1	1
SAPS2	960335	257	60	2395168	683876	0	0	0	0	0	0	683876	1	1
SAPS3	832270	272	48	2376300	592740	0	0	0	0	16	16	5489164	1	1
SAR1A	458949	217	39	527592	135636	0	0	0	0	50	34	16665428	1	1
SAR1B	887915	177	46	527236	135992	0	0	1	0	50	63	20304460	1	1
SARNP	1861663	256	32	577432	143468	0	0	0	0	50	59	21018240	1	1
SART1	3854361	208	57	2057324	592384	0	0	1	0	6	2	2582780	1	1
SASS6	292450	530	4	1737992	438592	0	0	0	0	34	41	15575000	1	1
SAT1	491139	NaN	46	466004	106444	0	0	0	0	50	59	22607424	1	1
SATL1	82752	NaN	-16	1636532	408688	0	0	0	0	13	22	7121780	1	1
SAV1	458995	259	31	980068	267356	0	0	0	0	50	64	19838100	1	1
SBSN	1081638	390	41	1482028	426488	0	0	1	0	36	55	13682504	1	1
SC4MOL	171541	540	6	781420	181916	0	0	0	0	50	65	21971252	1	1
SC5DL	173979	744	28	781776	196512	0	0	0	0	50	58	21524828	1	1
SC65	1760409	175	35	1118196	315060	0	0	0	0	35	50	15900028	1	1
SCAMP1	222123	434	39	887152	232112	0	0	0	0	50	76	21837040	1	1
SCAMP2	764533	195	46	846924	246708	0	0	1	0	15	22	5290516	1	1
SCAND3	239465	576	27	3383424	877896	0	0	0	0	0	0	877896	1	1
SCD5	740142	473	21	1007124	288360	0	0	0	0	50	67	18469992	1	1
SCGB1A1	3053973	413	49	241724	63724	0	0	0	0	50	76	19971956	1	1

SCGB1C1	1003496	437	11	250980	69420	0	0	0	0	50	55	15012164	1	1
SCGB1D1	2778889	450	53	235316	66928	0	0	1	0	8	11	1470992	1	1
SCGB1D4	3058870	450	42	218940	60876	0	0	0	0	50	51	16305156	1	1
SCGB2A1	2778889	450	53	258812	59452	0	0	2	0	3	6	422928	1	1
SCGB3A1	855370	515	13	254184	92916	0	0	0	0	50	68	19116844	1	1
SCGB3A2	137041	470	2	238520	73336	0	0	0	0	50	55	18068068	1	1
SCGBL	790899	512	-33	251336	70132	0	0	0	0	50	60	18225064	1	1
SCHIP1	94850	870	-8	1261664	333928	0	0	0	0	50	83	29420552	1	1
SCIN	119569	510	-1	1869000	489144	0	0	1	0	50	63	21709236	1	1
SCO2	1075525	207	42	646140	215736	0	0	3	0	2	6	852264	1	1
SCOC	186461	708	3	484872	123888	0	0	0	0	50	57	19551520	1	1
SCRN1	549429	560	23	1132436	295480	0	0	0	0	34	24	13509132	1	1
SCRT2	622277	480	42	749024	242080	0	0	0	0	50	60	28148208	1	1
SCT	NaN	NaN	NaN	298684	107156	0	0	0	0	50	116	39752740	1	1
SCXA	NaN	NaN	NaN	965472	337488	0	0	0	0	50	116	39983072	1	1
SCXB	NaN	NaN	NaN	965472	337488	0	0	0	0	50	116	39983072	1	1
SDC4	1005559	233	36	509792	147028	0	0	0	0	50	49	17548664	1	1
SDCCAG1	661668	174	27	2904604	684944	0	0	0	0	2	0	1470636	1	1
SDF2	1651143	225	31	532932	156996	0	0	1	0	3	13	2353516	1	1
SDF2L1	521773	194	37	548596	173372	0	0	0	0	50	70	19466792	1	1
SDHD	389805	179	31	402992	124600	0	0	3	0	3	5	597724	1	1
SEBOX	1405196	214	10	799932	220008	0	0	0	0	3	2	766468	1	1
SEC61B	203560	410	37	246708	79032	0	0	0	0	50	60	20776872	1	1
SEC61G	277856	325	-11	189748	44144	0	0	0	0	50	41	18400216	1	1
SELK	128709	552	39	256320	65148	0	0	0	0	50	59	17062012	1	1
SELL	293592	597	13	1032756	238164	0	0	0	0	50	66	18679320	1	1
SELM	731144	218	40	368104	118904	0	0	0	0	50	54	25122564	1	1
SELS	372683	406	2	496976	139552	0	0	0	0	50	53	20605636	1	1
SELT	370681	397	34	516556	134924	0	0	0	0	50	46	19851272	1	1
SELV	1148741	322	25	821292	313992	0	0	1	0	23	18	9021752	1	1
15-Sep	333484	410	-6	446424	102528	0	0	0	0	50	56	20168824	1	1
1-Sep	2312884	165	47	964404	261660	0	0	0	0	50	59	19035320	1	1
10-Sep	191347	329	22	1211824	288716	0	0	0	0	50	51	19327596	1	1

11-Sep	402678	462	39	1142760	273408	0	0	0	0	50	74	20914288	1	1
12-Sep	706891	195	33	929160	259524	0	0	0	0	50	50	19489932	1	1
14-Sep	841217	627	-10	1155220	270560	0	0	0	0	50	155	25723492	1	1
2-Sep	1242448	383	24	969388	241724	0	0	0	0	50	71	21719916	1	1
3-Sep	1335011	323	67	988256	247420	0	0	0	0	50	49	15999352	1	1
4-Sep	874267	376	16	1319336	358492	0	0	0	0	8	12	3552168	1	1
5-Sep	728313	211	8	989324	243148	0	0	0	0	50	70	22450784	1	1
6-Sep	656561	NaN	61	1161984	293700	0	0	0	0	6	2	2085092	1	1
7-Sep	355149	577	33	1185124	267356	0	0	0	0	30	41	17890424	1	1
8-Sep	717720	356	59	1378788	336420	0	0	0	0	40	33	14637296	1	1
9-Sep	673808	391	24	1882172	545392	0	0	0	0	0	0	545392	1	1
SEPW1	812020	262	36	241368	61944	0	0	1	0	3	9	1472416	1	1
SEPX1	1766472	214	39	310432	77252	0	0	0	0	50	74	24152108	1	1
SERF1A	NaN	NaN	NaN	717696	170880	0	0	0	0	50	116	39816464	1	1
SERF1B	NaN	NaN	NaN	717696	170880	0	0	0	0	50	116	39816464	1	1
SERF2	784327	286	37	168744	34176	0	0	0	0	50	69	23862324	1	1
SERINC4	784327	286	37	719120	200428	0	0	1	0	50	69	23862324	1	1
SERP2	190099	161	43	178000	42008	0	0	0	0	50	61	17683944	1	1
SERPINB1	469210	229	37	984340	258812	0	0	0	0	50	42	19101536	1	1
SERPINB1[illegible]	190401	339	20	1059812	264508	0	0	0	0	50	49	19965548	1	1
SETD1B	NaN	NaN	NaN	4721984	1513000	0	0	0	0	0	0	1513000	1	1
SF3A2	1603571	240	27	1145964	378072	0	0	1	0	50	66	21932448	1	1
SF3B14	243145	226	33	334640	81880	0	0	0	0	50	59	18078748	1	1
SF4	930697	232	10	1685304	442152	0	0	0	0	9	7	4173032	1	1
SFRP5	555867	209	33	794236	233180	0	0	3	0	3	4	1045216	1	1
SFRS1	854628	182	27	663584	202564	0	0	0	0	50	49	18253544	1	1
SFRS11	296676	445	-11	1243152	362052	0	0	0	0	50	48	19751948	1	1
SFRS12	388631	437	33	1616240	433252	0	0	0	0	20	23	9134960	1	1
SFRS12IP1	81822	632	-1	434320	82592	0	0	0	0	50	53	21422300	1	1
SFRS13A	NaN	NaN	NaN	737276	196156	0	0	0	0	50	116	39841740	1	1
SFRS13B	376032	446	27	665720	190816	0	0	0	0	21	15	11169500	1	1
SFRS14	1327004	215	24	2730520	779996	0	0	0	0	0	0	779996	1	1
SFRS15	291511	621	26	2899264	860096	0	0	0	0	5	5	2599512	1	1

SFRS16	1135715	208	25	1746536	501604	0	0	0	0	7	11	2611260	1	1
SFRS17A	NaN	NaN	NaN	1741196	505876	0	0	0	0	9	16	4863316	1	1
SFRS18	259457	638	13	2111792	513352	0	0	0	0	4	11	6783936	1	1
SFRS2	827288	206	22	525456	190104	0	0	0	0	50	41	14517680	1	1
SFRS2B	273779	513	16	657888	245640	0	0	0	0	50	67	22599236	1	1
SFRS2IP	817865	602	11	3760784	989680	0	0	0	0	0	0	989680	1	1
SFRS3	889664	178	40	423996	126024	0	0	0	0	50	67	21004000	1	1
SFRS4	494473	235	21	1260240	347100	0	0	0	0	16	10	5736940	1	1
SFRS5	610490	333	47	704880	199716	0	0	0	0	50	50	20003640	1	1
SFRS6	316530	559	-45	865080	261660	0	0	0	0	35	63	15111488	1	1
SFRS7	850854	184	26	605200	190460	0	0	0	0	50	52	18448988	1	1
SFRS8	364490	367	7	2426852	695980	0	0	0	0	3	3	2104672	1	1
SFRS9	1563159	216	45	562124	161980	0	0	0	0	50	63	22226860	1	1
SFT2D1	199596	556	18	431116	113564	0	0	0	0	50	35	16755496	1	1
SFT2D3	NaN	NaN	NaN	482736	209328	0	0	0	0	50	116	39854912	1	1
SFTPA1	478276	NaN	11	678180	192240	0	0	3	0	1	4	380208	1	1
SFTPD	570627	613	29	940908	293700	0	0	0	0	50	62	22394892	1	1
SGCD	48800	1181	3	812036	229264	0	0	2	0	3	3	814884	1	1
SGCG	189677	771	-4	756856	206480	0	0	0	0	50	43	24303408	1	1
SGEF	94659	975	-13	2041660	583484	0	0	0	0	6	8	4783216	1	1
SGK196	304258	559	12	884304	248844	0	0	0	0	50	59	21217244	1	1
SGK269	311464	502	30	4367052	1243152	0	0	0	0	2	3	2292284	1	1
SGSH	674340	219	22	1268428	377360	0	0	1	0	36	55	18118976	1	1
SGTA	1354357	291	25	837668	215380	0	0	0	0	50	54	21517708	1	1
SH2B2	574073	361	45	1442156	475972	0	0	1	0	3	5	2755084	1	1
SH3BGRL	137219	NaN	-22	304024	77252	0	0	1	0	17	28	5925620	1	1
SH3BGRL2	205695	824	2	289072	69776	0	0	0	0	50	62	21965912	1	1
SH3BGRL3	1021101	217	22	241368	68352	0	0	0	0	50	66	18554720	1	1
SH3D20	748554	327	12	620152	229976	0	0	0	0	50	71	23705684	1	1
SHISA4	736064	208	24	499824	151656	0	0	0	0	50	72	25735952	1	1
SHISA6	NaN	NaN	NaN	1363124	426844	0	0	0	0	50	116	40072428	1	1
SHISA7	NaN	NaN	NaN	1265580	474192	0	0	0	0	50	116	40119776	1	1
SHISA9	NaN	NaN	NaN	1152728	338200	0	0	0	0	50	116	39983784	1	1

SIAH1	170149	392	3	783200	227128	0	0	0	0	50	39	21914292	1	1
SIAH3	820469	632	51	673552	197936	0	0	0	0	50	66	24211916	1	1
SIGLEC15	384767	380	42	806696	268780	0	0	0	0	50	39	21640884	1	1
SIGMAR1	731413	298	51	557496	175152	0	0	1	0	50	42	16261368	1	1
SIK1	807666	312	65	1941980	625492	0	0	0	0	7	9	3077976	1	1
SILV	2080397	229	31	1643296	522608	0	0	0	0	11	12	5452140	1	1
SIP1	314287	166	3	747956	192952	0	0	0	0	50	56	19917132	1	1
SIX6	325446	618	0	604488	191172	0	0	0	0	50	64	21583212	1	1
SLAMF1	1160206	634	30	861520	240656	0	0	2	0	13	20	4124260	1	1
SLBP	884891	350	36	719120	179068	0	0	0	0	50	73	24227224	1	1
SLC13A1	99560	955	-15	1533292	438236	0	0	2	0	14	28	7454996	1	1
SLC15A5	NaN	NaN	NaN	1478112	414384	0	0	0	0	50	116	40059968	1	1
SLC16A11	1696302	360	24	1095768	431472	0	0	0	0	10	3	2758288	1	1
SLC16A8	1478013	200	76	1191888	443220	0	0	0	0	8	10	2911368	1	1
SLC1A3	376343	436	11	1386264	407976	0	0	1	0	50	58	21458968	1	1
LC22A18A	552601	256	34	622288	204344	0	0	0	0	50	59	21410552	1	1
SLC22A20	3317462	168	54	865792	279104	0	0	0	0	50	56	19574304	1	1
SLC22A24	NaN	NaN	NaN	1388756	416164	0	0	1	0	50	117	40061748	1	1
SLC22A5	543074	264	63	1400504	425776	0	0	0	0	20	15	7288744	1	1
SLC24A6	441204	251	37	1468144	470276	0	0	0	0	19	12	6287672	1	1
SLC25A10	3745109	205	30	739056	228552	0	0	1	0	33	25	10695308	1	1
SLC25A21	68926	481	-44	793168	210752	0	0	0	0	9	12	4318992	1	1
SLC25A29	412002	237	37	726240	260592	0	0	2	0	23	21	8168064	1	1
SLC25A32	430771	673	28	851908	240656	0	0	0	0	21	16	9275580	1	1
SLC25A33	749351	268	33	815240	242080	0	0	3	0	6	11	1883596	1	1
SLC25A37	474894	409	36	849772	249200	0	0	1	0	50	70	23889024	1	1
SLC25A40	237024	758	6	901748	227128	0	0	1	0	50	75	26036772	1	1
SLC26A1	675007	256	45	1737636	627984	0	0	0	0	5	6	3102896	1	1
SLC26A2	1070181	149	44	1838740	540764	0	0	0	0	1	1	897832	1	1
SLC31A1	437406	312	17	499824	129228	0	0	2	0	7	5	1034892	1	1
SLC31A2	448462	456	9	366324	107868	0	0	0	0	50	64	19509512	1	1
SLC35A2	834040	NaN	38	1117840	339980	0	0	1	0	50	43	14994364	1	1
SLC35B4	286704	493	14	870420	231756	0	0	0	0	50	52	17857672	1	1

SLC35C1	431496	381	20	881456	292276	0	0	1	0	50	54	21067368	1	1
SLC35D2	340302	400	21	880388	251692	0	0	0	0	50	49	24353960	1	1
SLC35E1	815495	258	44	1014600	325740	0	0	2	0	15	13	6426868	1	1
SLC35E2	1569768	214	33	1676048	527236	0	0	0	0	3	2	1529020	1	1
SLC38A5	726793	NaN	2	1226776	351728	0	0	0	0	24	24	10365296	1	1
SLC7A13	192785	1147	3	1165188	358848	0	0	3	0	5	8	2516564	1	1
SLC9A10	268687	508	22	3120696	773232	0	0	0	0	2	2	1977580	1	1
SLC9A11	426813	335	9	2984348	735496	0	0	0	0	3	2	2078684	1	1
SLC9A3R1	983414	484	17	899612	271984	0	0	0	0	0	0	271984	1	1
SLFN14	NaN	NaN	NaN	2317204	623000	0	0	0	0	7	14	4311160	1	1
SLN	322905	676	-5	83660	23140	0	0	0	0	50	57	16410888	1	1
SLPI	842540	324	25	353508	87576	0	0	0	0	50	49	22812836	1	1
SLURP1	427585	373	34	270204	73692	0	0	1	0	12	11	4451424	1	1
SMAD5	284497	449	34	1206128	305092	0	0	0	0	50	53	20837392	1	1
SMAGP	494861	194	40	294056	59452	0	0	1	0	16	23	4828072	1	1
SMCR7	925111	223	18	1201500	306516	0	0	0	0	46	58	19081600	1	1
SMCR7L	766902	467	74	1135996	367748	0	0	0	0	1	3	615168	1	1
SMN1	NaN	NaN	NaN	1541480	417232	0	0	0	0	50	116	40062816	1	1
SMN2	NaN	NaN	NaN	1571384	423640	0	0	0	0	50	116	40069224	1	1
SMPX	93024	NaN	0	238164	64080	0	0	0	0	50	60	20571816	1	1
SMUG1	798393	279	48	658600	218228	0	0	1	0	50	49	17808188	1	1
SNAPC5	507061	219	40	263084	69064	0	0	0	0	50	40	15710992	1	1
SNAPIN	1656449	183	40	341760	112140	0	0	0	0	50	74	21280612	1	1
NHG3-RC(	846521	183	26	1077256	313280	0	0	0	0	26	27	10005736	1	1
SNN	602364	214	40	220720	68708	0	0	0	0	50	57	21732376	1	1
SNRNP25	1005761	238	36	348880	94340	0	0	0	0	50	46	16624132	1	1
SNRPF	426671	400	5	234960	56604	0	0	0	0	50	36	19949172	1	1
SNRPG	671278	370	38	215380	46280	0	0	0	0	50	76	25574328	1	1
SNUPN	594867	165	39	948384	242436	0	0	0	0	50	44	21001508	1	1
SNURF	233460	1194	-44	192952	52688	0	0	1	0	1	2	286936	1	1
SNX24	154483	532	18	454968	115344	0	0	0	0	50	51	21559360	1	1
SOCS1	632841	308	37	504096	179424	0	0	0	0	50	60	21937788	1	1
SOCS2	359700	369	30	493060	148808	0	0	0	0	50	40	21698556	1	1

SOCS3	962609	209	27	550732	177644	0	0	1	0	50	76	22461464	1	1
SOCS4	795059	144	37	1125672	291564	0	0	1	0	50	42	18424424	1	1
SOD3	269537	617	31	582416	194020	0	0	0	0	8	6	2499476	1	1
SOLH	1205944	213	38	2661812	867928	0	0	0	0	8	6	3183352	1	1
SOST	1081050	192	35	524388	167676	0	0	0	0	50	58	18400572	1	1
SOX11	10248	1198	-17	1094344	323960	0	0	0	0	37	63	15382048	1	1
SOX13	560073	314	31	1609832	440728	0	0	0	0	50	53	21764416	1	1
SOX15	1957426	194	24	564260	189748	0	0	1	0	50	81	21975524	1	1
SOX21	154313	759	41	661448	228196	0	0	0	0	50	63	18377076	1	1
SP6	1071489	214	29	916344	295836	0	0	0	0	50	58	18849844	1	1
SP9	NaN	NaN	NaN	1157356	400856	0	0	0	0	50	116	40046440	1	1
SPACA4	1597109	242	31	299396	101104	0	0	1	0	13	24	5400164	1	1
SPACA5	NaN	NaN	NaN	851552	216448	0	0	0	0	50	116	39862032	1	1
SPACA5B	566535	NaN	19	851552	216448	0	0	0	0	15	27	7730896	1	1
SPAG11A	69060	693	-5	279816	82236	0	0	0	0	50	71	21235400	1	1
SPANXA1	NaN	NaN	NaN	522608	131008	0	0	0	0	50	116	39776592	1	1
SPANXA2	NaN	NaN	NaN	522608	131008	0	0	0	0	50	116	39776592	1	1
SPANXB2	NaN	NaN	NaN	540408	140976	0	0	0	0	50	116	39786560	1	1
SPANXC	23523	NaN	-15	256320	66216	0	0	0	0	50	88	22177732	1	1
SPANXE	28355	NaN	-20	258812	65860	0	0	0	0	50	79	20617028	1	1
SPANXF1	NaN	NaN	NaN	540408	140976	0	0	0	0	50	116	39786560	1	1
SPANXN1	2363	NaN	-22	195444	42720	0	0	1	0	7	27	2635824	1	1
SPATA1	241849	585	-1	1224640	302600	0	0	0	0	50	48	16741612	1	1
SPATA24	NaN	NaN	NaN	557140	128516	0	0	0	0	50	116	39774100	1	1
SPATA3	NaN	NaN	NaN	482736	148452	0	0	0	0	50	116	39794036	1	1
SPC24	1808766	233	33	517268	138484	0	0	1	0	13	21	5810276	1	1
SPC25	173915	724	7	608760	137772	0	0	0	0	50	64	23708176	1	1
SPCS1	1217701	272	47	429336	130296	0	0	0	0	50	99	29167792	1	1
SPDYE2	NaN	NaN	NaN	2101112	541120	0	0	0	0	9	16	4898560	1	1
SPDYE2L	NaN	NaN	NaN	2101112	541120	0	0	0	0	9	16	4898560	1	1
SPDYE4	NaN	NaN	NaN	624424	161624	0	0	1	0	50	117	39807208	1	1
SPEM1	1906324	206	33	768960	237096	0	0	0	0	50	87	23542992	1	1
SPHAR	285692	361	9	165896	39160	0	0	0	0	50	71	23765848	1	1

SPIN2B	145137	NaN	18	657176	180136	0	0	0	0	50	49	14659724	1	1
SPINK1	137041	470	2	216448	54824	0	0	0	0	50	55	18068068	1	1
SPINK13	72574	418	-9	263440	58028	0	0	0	0	50	57	21768688	1	1
SPINK14	63265	390	-8	263796	63012	0	0	0	0	50	59	22095140	1	1
SPINK4	856503	167	38	234248	61588	0	0	1	0	8	3	1779644	1	1
SPINK6	63265	390	-8	219296	53044	0	0	0	0	50	59	22095140	1	1
SPINK9	79210	459	-15	239588	54112	0	0	1	0	0	1	54112	1	1
SPINLW1	1069725	438	36	398720	89356	0	0	0	0	50	84	21025004	1	1
SPINT3	NaN	NaN	NaN	235316	59452	0	0	0	0	50	116	39705036	1	1
SPNS2	1252775	318	22	1364904	448560	0	0	1	0	50	49	18259952	1	1
SPOCK2	757700	169	40	1157356	297260	0	0	0	0	26	27	9094376	1	1
SPPL3	1410077	392	45	987544	288716	0	0	1	0	7	16	3810980	1	1
SPRR1A	426723	748	28	230332	62300	0	0	1	0	5	4	1424000	1	1
SPRR2B	687290	704	30	195444	50196	0	0	0	0	50	34	21429064	1	1
SPRR2E	687290	704	30	187968	50196	0	0	0	0	50	34	21429064	1	1
SPRR2G	822908	602	28	190460	50908	0	0	1	0	4	3	756856	1	1
SPRR3	426723	748	28	421504	127448	0	0	0	0	50	44	20259960	1	1
SPRR4	426723	748	28	208616	51976	0	0	0	0	5	4	1424000	1	1
SPRYD5	4214	1074	-46	1185480	291564	0	0	0	0	25	38	10956256	1	1
SPSB1	980939	281	32	672128	210040	0	0	0	0	50	70	22411268	1	1
SR140	361096	467	23	2750456	667144	0	0	0	0	3	1	2013892	1	1
SRCRB4D	870785	220	59	1431476	456748	0	0	0	0	11	8	5429712	1	1
SRP14	846041	256	23	357068	101104	0	0	0	0	50	54	19897196	1	1
SRP9	927421	168	9	326096	82948	0	0	0	0	50	55	20151380	1	1
SRPX	141319	NaN	17	1192244	336064	0	0	1	0	50	77	21383852	1	1
SRPX2	190839	NaN	23	1199720	331792	0	0	1	0	50	145	46592212	1	1
SRRD	163136	553	-46	875760	243504	0	0	0	0	7	12	3713792	1	1
SRRM5	NaN	NaN	NaN	1831976	501604	0	0	0	0	50	116	40147188	1	1
SRXN1	622277	480	42	332860	115700	0	0	0	0	50	60	28148208	1	1
SRY	0	0	0	524032	132788	0	0	0	0	50	39	21173456	1	1
SSC5D	NaN	NaN	NaN	3790332	1312572	0	0	0	0	0	0	1312572	1	1
SSNA1	1722012	202	36	308652	84372	0	0	3	0	0	3	84372	1	1
SSR2	1880483	213	32	465648	143112	0	0	1	0	50	94	23803584	1	1

SSR3	545591	679	10	485584	129584	0	0	1	0	9	14	5232132	1	1
SSR4	2103463	NaN	30	453544	129584	0	0	0	0	50	72	20936716	1	1
SST	124494	431	13	287648	91492	0	0	0	0	50	61	22197312	1	1
SSTR2	238181	336	-7	917412	272340	0	0	2	0	5	4	3155228	1	1
SSTR5	1154740	294	20	864012	311856	0	0	1	0	50	63	24684684	1	1
SSX2B	NaN	NaN	NaN	1063728	207192	0	0	0	0	50	116	39852776	1	1
SSX3	660654	NaN	14	562836	128160	0	0	2	0	5	15	2177652	1	1
SSX4B	660654	NaN	14	1130656	206480	0	0	0	0	50	64	21317280	1	1
SSX5	590474	NaN	11	624068	147028	0	0	0	0	50	57	20146040	1	1
[illegible]6GALNA[illegible]	1016866	197	24	951232	284444	0	0	2	0	50	75	20563984	1	1
STAG3L1	NaN	NaN	NaN	542188	143468	0	0	0	0	50	116	39789052	1	1
STAG3L2	745065	173	55	364544	93628	0	0	0	0	50	95	21608488	1	1
STAG3L3	661753	332	50	366324	91848	0	0	0	0	50	54	17812104	1	1
STARD9	NaN	NaN	NaN	11776836	3424008	0	0	0	0	0	0	3424008	1	1
STBD1	416594	436	26	899256	255252	0	0	0	0	16	16	7691380	1	1
STK11	2214581	234	29	1119976	309008	0	0	1	0	50	68	20485664	1	1
STK25	1215841	426	39	1105736	309364	0	0	2	0	19	27	7164856	1	1
STMN1	801428	221	31	522608	134212	0	0	0	0	50	70	21975880	1	1
STRA13	3814891	211	28	171236	48772	0	0	0	0	50	47	17845212	1	1
STX7	180943	618	12	705236	172660	0	0	1	0	50	45	19218304	1	1
STYX	310977	213	19	624424	140264	0	0	0	0	50	36	19973380	1	1
SUB1	487935	442	22	347100	79032	0	0	0	0	50	53	21518420	1	1
SUDS3	635715	823	32	888220	212888	0	0	0	0	50	33	21562208	1	1
SULT1A3	NaN	NaN	NaN	1554296	402280	0	0	0	0	50	116	40047864	1	1
SULT1A4	1906000	253	52	1554296	402280	0	0	0	0	8	8	4018528	1	1
SULT4A1	157623	473	13	749024	189748	0	0	2	0	6	6	2239952	1	1
SUMF1	155099	172	10	958352	279460	0	0	0	0	39	43	15324376	1	1
SUMF2	738325	642	4	1059100	282308	0	0	0	0	18	12	6589560	1	1
SUMO2	1511891	168	25	260592	59808	0	0	1	0	11	3	1507304	1	1
SUMO3	690574	213	62	279816	66216	0	0	2	0	2	4	515844	1	1
SUMO4	NaN	NaN	NaN	245640	64080	0	0	0	0	50	116	39709664	1	1
SUV39H1	824559	NaN	27	1053760	292988	0	0	2	0	5	13	2870072	1	1
SWAP70	545476	179	25	1559280	367392	0	0	0	0	44	68	20999728	1	1

SYCE1L	NaN	NaN	NaN	653616	167676	0	0	0	0	50	116	39813260	1	1
SYCN	1652029	286	26	331080	110004	0	0	1	0	36	43	13904292	1	1
SYNC	1452351	195	34	1278040	354932	0	0	0	0	43	68	24275284	1	1
SYNGR1	932556	250	69	760772	218584	0	0	0	0	50	47	20307308	1	1
SYNGR3	1747301	183	46	562480	187256	0	0	1	0	50	75	19137492	1	1
SYNGR4	1478750	217	36	591672	176220	0	0	1	0	50	89	31278516	1	1
SYS1	1005559	233	36	391956	123888	0	0	0	0	50	49	17548664	1	1
SYT13	173203	637	14	1061236	328232	0	0	0	0	20	24	9624816	1	1
TADA1	258555	773	-7	866504	239944	0	0	0	0	50	53	24325480	1	1
TAF10	424523	491	17	546460	178712	0	0	0	0	50	62	22561500	1	1
TAL1	364960	414	3	799220	276256	0	0	1	0	50	53	20385984	1	1
TAPBPL	2702142	198	50	1163052	367392	0	0	0	0	5	24	6185500	1	1
TARM1	NaN	NaN	NaN	683164	209684	0	0	0	0	50	116	39855268	1	1
TARP	164254	382	6	445000	111428	0	0	0	0	50	38	17892560	1	1
TAS2R10	408176	517	12	776792	210040	0	0	0	0	50	54	19041016	1	1
TAX1BP3	504736	354	20	319688	93628	0	0	0	0	50	62	23395252	1	1
TBC1D3	NaN	NaN	NaN	2835540	797796	0	0	0	0	7	14	4485956	1	1
TBC1D3C	609181	684	12	2840880	792456	0	0	0	0	3	0	1438952	1	1
TBC1D3G	493184	556	12	1419728	396940	0	0	0	0	0	0	396940	1	1
TBC1D3H	NaN	NaN	NaN	1420796	395872	0	0	0	0	50	116	40041456	1	1
TBXA2R	2164055	269	35	1003208	367036	0	0	0	0	50	44	20043868	1	1
TCEA1	267995	392	29	810968	195088	0	0	0	0	50	55	25888676	1	1
TCEAL4	490345	NaN	31	571380	129228	0	0	0	0	50	66	22114364	1	1
TCEAL5	455954	NaN	17	536136	131364	0	0	0	0	50	69	22928180	1	1
TCEAL6	132641	NaN	4	475972	117836	0	0	0	0	50	53	17321536	1	1
TCEAL7	455954	NaN	17	267000	60876	0	0	0	0	50	69	22928180	1	1
TCEB1	258047	417	25	299752	75116	0	0	0	0	50	45	17237876	1	1
TCERG1L	2679	871	-27	1476332	453544	0	0	0	0	2	0	887152	1	1
TCF15	617464	404	39	473124	174084	0	0	0	0	50	70	23044592	1	1
TCF7L1	973960	394	18	1476332	459952	0	0	1	0	2	2	1066576	1	1
TCL1A	256627	582	30	301532	82948	0	0	0	0	50	57	21579296	1	1
TDRD12	NaN	NaN	NaN	1066932	253116	0	0	0	0	50	116	39898700	1	1
TENC1	1726226	177	38	3620520	1131012	0	0	0	0	4	1	2378080	1	1

TEX12	389593	362	2	331792	82592	0	0	0	0	50	48	17867640	1	1
TEX19	2598845	179	36	415808	112852	0	0	0	0	50	59	20440452	1	1
TEX261	380551	561	27	508724	143824	0	0	0	0	50	69	23313372	1	1
TEX28	NaN	NaN	NaN	1035960	300108	0	0	0	0	50	116	39945692	1	1
TFAP4	944378	161	33	861520	250268	0	0	1	0	50	70	21042448	1	1
TFDP3	76255	NaN	28	1020296	282664	0	0	0	0	48	63	26284904	1	1
TFPI	50680	404	-4	931652	216448	0	0	0	0	50	49	18533360	1	1
TGFA	715918	463	13	419368	117836	0	0	0	0	50	53	19361772	1	1
TGFB2	75157	386	-18	1145964	303312	0	0	0	0	50	41	18575012	1	1
TGIF2LY	0	0	0	469208	131008	0	0	1	0	5	2	1014600	1	1
TH1L	1009250	152	30	1537208	418300	0	0	0	0	2	2	688148	1	1
THAP2	259684	393	-11	589180	153080	0	0	0	0	50	34	20198728	1	1
THAP3	628180	259	38	464936	120328	0	0	1	0	46	63	18237524	1	1
THOC4	3798594	211	19	658244	214312	0	0	0	0	34	28	11428668	1	1
THOC7	153669	826	-6	559632	127092	0	0	0	0	50	61	19340056	1	1
TIGD1	NaN	NaN	NaN	1495912	402992	0	0	0	0	50	116	40048576	1	1
TIMM10	923775	197	9	240656	59452	0	0	0	0	50	53	18462160	1	1
TIMM13	1604277	267	35	249556	66572	0	0	0	0	50	58	22805716	1	1
TIMM16	849632	161	37	333216	91848	0	0	0	0	50	44	18897548	1	1
TIMM22	1306390	270	27	493416	146316	0	0	0	0	50	50	19552232	1	1
TIMM50	1410725	230	39	1150236	356712	0	0	3	0	1	3	869708	1	1
TIMM8B	389805	179	31	257032	68708	0	0	0	0	50	59	22578232	1	1
TIMM9	290701	552	46	244216	56960	0	0	0	0	50	48	18596016	1	1
TIMP1	775214	NaN	38	542544	147384	0	0	0	0	50	61	23561148	1	1
TIRAP	385871	426	29	603420	186900	0	0	1	0	5	7	2062664	1	1
TK2	600023	214	-11	738344	189748	0	0	0	0	50	80	23111164	1	1
TLCD2	NaN	NaN	NaN	639020	222856	0	0	0	0	50	116	39868440	1	1
TLX1NB	469702	221	52	295836	98256	0	0	0	0	50	47	19691784	1	1
TLX2	1019591	315	50	676756	244928	0	0	2	0	39	63	16896116	1	1
TM2D2	421614	428	42	625848	180492	0	0	0	0	50	56	22426932	1	1
TM2D3	322884	469	-9	636172	181916	0	0	0	0	50	52	22455056	1	1
TM4SF20	248187	164	18	588468	163404	0	0	0	0	50	45	19359992	1	1
TM4SF5	1272092	222	28	498044	157708	0	0	1	0	14	13	5818820	1	1

TM7SF4	40736	1032	14	1174800	347100	0	0	0	0	50	65	24287032	1	1
TM9SF1	974559	220	41	1540768	469208	0	0	0	0	8	5	2656828	1	1
TMBIM1	730654	197	33	809900	236740	0	0	0	0	50	45	18509152	1	1
TMCO2	627703	373	18	465292	129584	0	0	0	0	50	58	21496704	1	1
TMCO5A	92801	921	-59	788184	180492	0	0	0	0	50	55	17871912	1	1
TMCO7	925521	253	30	2770392	812748	0	0	0	0	7	7	3551100	1	1
TMED10	884650	154	47	564260	159844	0	0	0	0	50	71	22625936	1	1
TMED5	608924	412	10	653260	153080	0	0	1	0	48	49	20217240	1	1
TMEM100	59099	585	-58	333928	102884	0	0	1	0	6	10	2271636	1	1
TMEM106A	1341175	172	25	677824	194732	0	0	0	0	50	70	21371748	1	1
TMEM106B	114769	532	-9	723392	187612	0	0	0	0	21	26	9451800	1	1
TMEM109	794900	522	47	581348	213244	0	0	0	0	50	51	21913580	1	1
TMEM111	944872	170	42	692420	176932	0	0	0	0	50	62	23148188	1	1
TMEM114	NaN	NaN	NaN	304380	96120	0	0	0	0	50	116	39741704	1	1
TMEM121	689089	298	41	755432	269848	0	0	0	0	36	23	13861928	1	1
TMEM125	1091212	264	30	506944	197936	0	0	0	0	50	55	21358220	1	1
TMEM126B	325720	554	-19	522964	138128	0	0	0	0	50	100	24725980	1	1
TMEM127	904852	213	43	581348	192952	0	0	1	0	50	63	24111168	1	1
TMEM128	146135	584	35	376292	95764	0	0	0	0	50	50	17705304	1	1
TMEM129	867402	350	39	894272	281596	0	0	0	0	15	10	5965136	1	1
TMEM132C	NaN	NaN	NaN	2742624	844788	0	0	1	0	9	17	5202228	1	1
TMEM134	1392213	215	53	508724	147028	0	0	0	0	50	41	15455384	1	1
TMEM136	290558	173	28	386972	98968	0	0	0	0	50	56	24592480	1	1
TMEM138	1394229	286	57	421504	113564	0	0	0	0	50	56	17921040	1	1
TMEM141	1898901	190	39	295836	72624	0	0	0	0	50	67	24888672	1	1
TMEM146	990266	225	26	2095772	558208	0	0	0	0	11	8	4396244	1	1
TMEM147	1081638	390	41	588824	161980	0	0	1	0	50	73	18185192	1	1
TMEM149	1151198	321	30	860096	299752	0	0	0	0	14	10	6958732	1	1
TMEM14A	531039	498	18	260948	76540	0	0	0	0	50	62	18527308	1	1
TMEM14E	194580	402	35	312568	93272	0	0	1	0	15	24	6088668	1	1
TMEM150B	1028808	209	19	594876	180492	0	0	6	0	1	6	235316	1	1
TMEM151B	NaN	NaN	NaN	1377720	447492	0	0	0	0	50	116	40093076	1	1
TMEM154	165925	507	44	495552	119616	0	0	1	0	50	68	21089440	1	1

TMEM155	326547	440	18	335352	97188	0	0	0	0	50	63	25522708	1	1
TMEM158	NaN	NaN	NaN	689572	274832	0	0	0	0	50	116	39920416	1	1
TMEM160	1175364	207	35	454256	164116	0	0	0	0	50	53	19463232	1	1
TMEM161I	96521	1090	3	1264868	345676	0	0	1	0	50	36	16918544	1	1
TMEM163	261980	649	5	739056	222144	0	0	0	0	50	50	20062380	1	1
TMEM167/	230539	717	8	197580	49128	0	0	0	0	50	52	20884384	1	1
TMEM17	303217	553	14	511216	139196	0	0	0	0	50	63	23133948	1	1
TMEM170/	490399	133	16	358136	117124	0	0	1	0	21	19	7032068	1	1
TMEM170I	126262	429	32	333572	101104	0	0	0	0	50	60	24666172	1	1
TMEM173	781521	204	35	940552	300464	0	0	0	0	42	37	13738752	1	1
TMEM174	447937	584	21	604844	183340	0	0	2	0	7	11	2169464	1	1
TMEM176/	749051	604	16	598080	183696	0	0	0	0	50	78	22402724	1	1
TMEM177	337284	441	29	747956	255964	0	0	2	0	7	5	2923828	1	1
TMEM178	155474	624	-7	738700	231044	0	0	0	0	50	60	26628800	1	1
TMEM179	625253	450	20	496976	148096	0	0	0	0	50	62	21152808	1	1
TMEM180	871726	178	41	1275192	418656	0	0	0	0	48	58	21114360	1	1
TMEM183/	NaN	NaN	NaN	971524	270560	0	0	0	0	50	116	39916144	1	1
TMEM183I	421206	389	30	971524	270560	0	0	0	0	50	58	20614536	1	1
TMEM186	401809	358	-9	531864	160200	0	0	1	0	50	53	20820660	1	1
TMEM188	270030	594	-12	382700	103240	0	0	0	0	50	41	18369244	1	1
TMEM189	NaN	NaN	NaN	694200	195444	0	0	0	0	50	116	39841028	1	1
M189-UBI	571130	136	58	953368	265220	0	0	1	0	50	87	17843076	1	1
TMEM194I	NaN	NaN	NaN	1086512	289072	0	0	0	0	50	116	39934656	1	1
TMEM195	17876	1102	-22	1170884	309364	0	0	0	0	5	10	2783920	1	1
TMEM20	497832	589	1	909580	273764	0	0	0	0	50	72	24067024	1	1
TMEM200I	598249	235	19	709152	281952	0	0	0	0	50	55	18402708	1	1
TMEM203	1723416	200	39	326096	112852	0	0	2	0	0	2	112852	1	1
TMEM205	1385739	197	31	479532	144180	0	0	1	0	50	60	22230420	1	1
TMEM212	NaN	NaN	NaN	493060	150944	0	0	0	0	50	116	39796528	1	1
TMEM216	1394229	286	57	384836	113920	0	0	0	0	50	56	17921040	1	1
TMEM217	626559	236	44	598436	151300	0	0	0	0	50	57	22705680	1	1
TMEM218	428178	762	30	285868	98612	0	0	0	0	50	45	18518764	1	1
TMEM22	194047	195	27	1035248	292276	0	0	0	0	50	42	19034252	1	1

TMEM220	59041	801	-49	417944	119260	0	0	1	0	14	14	5326828	1	1
TMEM223	2405109	248	44	480600	176220	0	0	1	0	43	54	16227904	1	1
TMEM229A	NaN	NaN	NaN	910292	310432	0	0	0	0	50	116	39956016	1	1
TMEM229B	488731	311	39	418300	124244	0	0	1	0	39	29	12549712	1	1
TMEM231	488582	225	6	914208	230688	0	0	2	0	9	15	2647216	1	1
TMEM232	NaN	NaN	NaN	1737992	425776	0	0	0	0	50	116	40071360	1	1
TMEM233	NaN	NaN	NaN	283376	77608	0	0	0	0	50	116	39723192	1	1
TMEM25	1359480	167	47	943756	302600	0	0	1	0	11	21	5243524	1	1
TMEM27	170317	NaN	28	583128	152724	0	0	0	0	50	56	24571832	1	1
TMEM30B	351491	448	-11	852976	274832	0	0	0	0	50	55	22840248	1	1
TMEM33	272281	261	25	641868	178356	0	0	0	0	50	58	22426220	1	1
TMEM35	422603	NaN	29	406552	135992	0	0	0	0	50	44	19018944	1	1
TMEM41A	953031	366	31	664296	203988	0	0	0	0	50	52	20219732	1	1
TMEM42	466410	380	31	399076	124244	0	0	1	0	1	3	595944	1	1
TMEM43	312564	463	27	1030264	301532	0	0	0	0	50	51	19996876	1	1
TMEM48	535585	319	24	1761488	473836	0	0	0	0	2	1	904240	1	1
TMEM49	1018847	361	22	1070848	280172	0	0	0	0	12	18	5887172	1	1
TMEM54	1019374	232	15	552512	183340	0	0	2	0	26	35	8711676	1	1
TMEM64	184765	746	8	904596	324672	0	0	1	0	50	71	22626292	1	1
TMEM66	387693	231	51	860808	249912	0	0	0	0	50	42	19128948	1	1
TMEM69	953032	157	30	619084	184052	0	0	1	0	50	86	24486748	1	1
TMEM70	258047	417	25	655396	200072	0	0	1	0	50	45	17237876	1	1
TMEM82	683349	174	22	819512	304024	0	0	1	0	50	63	23254276	1	1
TMEM85	453823	242	11	479888	128872	0	0	0	0	50	82	26130400	1	1
TMEM86A	616892	562	25	589536	193308	0	0	0	0	50	61	21289512	1	1
TMEM86B	1035412	209	5	552156	183696	0	0	0	0	50	50	17199784	1	1
TMEM88	2148615	337	24	370596	148452	0	0	1	0	32	35	14422984	1	1
TMEM88B	NaN	NaN	NaN	372376	156284	0	0	0	0	50	116	39801868	1	1
TMEM9	572258	333	23	475616	135280	0	0	0	0	50	45	23164208	1	1
TMEM90A	604423	384	45	594520	184052	0	0	0	0	50	47	21806424	1	1
TMEM90B	178967	927	-37	655752	186900	0	0	0	0	50	84	15628756	1	1
TMEM91	1193025	249	33	512996	153436	0	0	0	0	50	52	16514128	1	1
TMEM93	504736	354	20	264864	95052	0	0	0	0	50	62	23395252	1	1

MPRSS11B	NaN	NaN	NaN	283732	68708	0	0	0	0	50	116	39714292	1	1
TMSB10	848135	275	12	125668	27056	0	0	0	0	50	83	24831356	1	1
TMSB15A	96151	NaN	0	128872	27056	0	0	0	0	15	12	5373464	1	1
TMSB15B	439642	NaN	30	128872	27056	0	0	0	0	50	63	20255688	1	1
TMSB4X	NaN	NaN	NaN	126380	26344	0	0	0	0	50	116	39671928	1	1
TMSL3	NaN	NaN	NaN	118548	27768	0	0	0	0	50	116	39673352	1	1
TNFAIP2	952836	211	40	1626208	515132	0	0	1	0	50	66	22779372	1	1
TNFAIP8L	921603	203	35	450696	152724	0	0	0	0	50	61	21420164	1	1
NFRSF12	1443513	234	49	324316	107156	0	0	0	0	50	48	18953440	1	1
TNFRSF18	2356409	196	27	836600	228196	0	0	0	0	50	61	26748416	1	1
TNFRSF4	2356409	196	27	699896	218584	0	0	2	0	6	12	3767192	1	1
TNFRSF6E	1126597	202	36	731936	249556	0	0	0	0	50	50	18382060	1	1
TNFSF12	NaN	NaN	NaN	621576	209328	0	0	1	0	50	117	39854912	1	1
SF12-TNF	1957426	194	24	850128	257388	0	0	0	0	17	20	7764360	1	1
TNFSF14	745830	259	29	593096	196156	0	0	0	0	50	62	22972324	1	1
TNFSF15	249550	286	-8	641156	179068	0	0	2	0	0	2	179068	1	1
TNFSF18	251425	492	0	517268	133144	0	0	0	0	50	56	21520912	1	1
TNNI3	1036976	266	-1	545392	157352	0	0	0	0	50	46	14741248	1	1
TOMM22	1056349	262	45	364900	108224	0	0	0	0	50	70	24305544	1	1
TOMM40	961910	196	2	923108	281596	0	0	0	0	50	53	16742680	1	1
TOMM5	511883	338	34	264864	75828	0	0	1	0	11	19	4786420	1	1
TOMM6	NaN	NaN	NaN	191884	56960	0	0	0	0	50	116	39702544	1	1
TOMM7	956904	381	19	152724	37380	0	0	0	0	50	63	20553660	1	1
TP53RK	292810	636	33	617660	200428	0	0	0	0	7	10	1621224	1	1
TP53TG3	NaN	NaN	NaN	896052	318264	0	0	0	0	50	116	39963848	1	1
TP53TG3B	NaN	NaN	NaN	896052	318264	0	0	0	0	50	116	39963848	1	1
TPRA1	682035	336	42	1009616	231400	0	0	4	0	4	6	1095056	1	1
TPSAB1	1275017	294	21	693132	210396	0	0	0	0	50	54	21927108	1	1
TPSB2	1275017	294	21	690284	214312	0	0	0	0	50	54	21927108	1	1
TPSG1	1275017	294	21	786760	270560	0	0	0	0	50	54	21927108	1	1
TRAF1	645995	270	39	1055540	306160	0	0	0	0	50	78	24143564	1	1
TRAPPC1	1794630	337	33	376292	106444	0	0	0	0	50	73	19666152	1	1
TRAPPC2	135235	NaN	48	478108	108224	0	0	0	0	50	57	19943832	1	1

TRAPPC3	800052	190	31	471700	127448	0	0	0	0	50	71	22071644	1	1
TRAPPC5	688482	384	31	457460	154504	0	0	0	0	50	67	22649432	1	1
TREML2	227466	611	41	800644	248132	0	0	3	0	3	6	896408	1	1
TRIB1	322911	193	40	912072	291564	0	0	2	0	50	58	19804992	1	1
TRIM34	NaN	NaN	NaN	1309724	338200	0	0	0	0	50	116	39983784	1	1
TRIM40	1932849	486	48	592740	161268	0	0	0	0	50	60	20279184	1	1
TRIM43B	NaN	NaN	NaN	1172664	285156	0	0	0	0	50	116	39930740	1	1
TRIM49L	NaN	NaN	NaN	2357432	596656	0	0	0	0	7	14	4284816	1	1
TRIM52	1034999	415	35	772876	186188	0	0	0	0	50	49	14891124	1	1
TRIM54	1245306	182	52	1037028	285156	0	0	1	0	32	27	12262064	1	1
TRIM61	170469	732	22	544680	136704	0	0	1	0	7	96	28550132	1	1
TRIM64	NaN	NaN	NaN	1165900	297260	0	0	0	0	50	116	39942844	1	1
TRIM64B	NaN	NaN	NaN	1163764	299396	0	0	0	0	50	116	39944980	1	1
TRIM66	NaN	NaN	NaN	3111440	868996	0	0	0	0	7	14	4557156	1	1
TRIM77	NaN	NaN	NaN	1171240	292988	0	0	0	0	50	116	39938572	1	1
TRNP1	NaN	NaN	NaN	520828	211820	0	0	0	0	50	116	39857404	1	1
TRYX3	207881	645	-7	630832	168032	0	0	0	0	50	51	27456500	1	1
TSC22D3	189575	NaN	51	609472	175508	0	0	0	0	50	53	21768688	1	1
TSEN15	372562	761	8	464580	122820	0	0	0	0	50	75	24832424	1	1
TSGA14	419615	298	27	979000	264152	0	0	0	0	38	45	16850548	1	1
TSHB	605838	601	1	359916	95052	0	0	0	0	50	87	24852004	1	1
TSPAN17	579630	359	40	862588	238520	0	0	1	0	50	54	21554376	1	1
TSPAN19	22140	974	-27	672840	159132	0	0	0	0	50	91	23528396	1	1
TSPO	624112	241	48	545036	97900	0	0	1	0	22	29	9517660	1	1
TSPO2	145501	611	37	420080	140620	0	0	2	0	8	15	2453196	1	1
TSPY1	NaN	NaN	NaN	796016	217516	0	0	0	0	50	116	39863100	1	1
TSPY3	NaN	NaN	NaN	2401220	650056	0	0	0	0	7	14	4338216	1	1
TSPY4	NaN	NaN	NaN	2489864	679960	0	0	0	0	7	14	4368120	1	1
TSR2	296262	NaN	5	500536	133856	0	0	0	0	50	50	17196224	1	1
TSSC4	833749	589	47	793880	267712	0	0	0	0	50	48	23387064	1	1
TSTD1	NaN	NaN	NaN	324672	96120	0	0	0	0	50	116	39741704	1	1
TTC15	255812	502	9	1857608	547528	0	0	0	0	33	31	13432592	1	1
TTC18	838780	249	45	2935576	774656	0	0	0	0	5	2	2535788	1	1

TTC24	2049505	180	37	1467076	443576	0	0	1	0	50	64	19075904	1	1
TTC28	NaN	NaN	NaN	6199384	1846928	0	0	0	0	0	0	1846928	1	1
TTC35	188664	962	21	816308	183340	0	0	0	0	50	36	20404140	1	1
TTLL13	956589	139	45	1194736	317552	0	0	0	0	50	59	25416976	1	1
TUBA4A	694442	193	44	1129232	328588	0	0	0	0	50	75	22905396	1	1
TUBA8	519135	179	19	1134572	326452	0	0	1	0	50	47	18843792	1	1
TUBB2C	1724821	399	43	1129944	316128	0	0	0	0	50	60	16636592	1	1
TUBB4	851008	212	27	1120332	318264	0	0	0	0	5	6	1991108	1	1
TUBB4Q	59973	1059	-12	1093632	309720	0	0	0	0	50	72	25435132	1	1
TUSC1	NaN	NaN	NaN	501960	180492	0	0	0	0	50	116	39826076	1	1
TUSC2	843769	206	51	275900	88288	0	0	0	0	50	68	20472848	1	1
TUSC3	50254	1013	-41	929516	250624	0	0	0	0	50	97	23911096	1	1
TWIST1	103347	812	-15	498044	156640	0	0	1	0	6	5	1796020	1	1
TXN	207910	302	8	292276	66572	0	0	0	0	50	50	20275980	1	1
TXNDC17	663953	424	9	328588	81524	0	0	0	0	50	52	18304452	1	1
TXNDC3	103411	646	13	1585624	369884	0	0	0	0	50	48	18056676	1	1
TXNDC5	748889	288	10	1106092	321824	0	0	0	0	42	79	24788992	1	1
TXNDC6	199119	358	18	706660	177644	0	0	0	0	50	53	18163120	1	1
TXNDC8	210246	302	7	326096	71200	0	0	0	0	50	49	19313712	1	1
TXNL4A	399915	506	36	377716	89000	0	0	1	0	19	23	7453216	1	1
TXNRD3IT	283619	508	43	338200	99680	0	0	0	0	50	65	18431188	1	1
UBAC2	333101	390	55	1060880	247420	0	0	1	0	50	62	20369252	1	1
UBB	881521	169	4	568888	172304	0	0	2	0	30	26	8257420	1	1
UBE2CBP	164732	607	-6	1017092	270916	0	0	0	0	50	70	26293092	1	1
UBE2E1	400750	319	10	510504	132432	0	0	0	0	50	50	21924260	1	1
UBE2G1	1040590	292	26	453544	115700	0	0	0	0	50	70	22622732	1	1
UBE2G2	754837	213	48	448560	106800	0	0	0	0	50	50	16454676	1	1
UBE2NL	5046	NaN	-19	385904	109648	0	0	0	0	50	90	21252488	1	1
UBE2QL1	NaN	NaN	NaN	407620	115700	0	0	0	0	50	116	39761284	1	1
UBE2V1	571130	136	58	477752	114988	0	0	0	0	50	87	17843076	1	1
UBE2W	257905	407	34	445000	105020	0	0	0	0	50	50	19337564	1	1
UBL4B	571576	400	29	433964	128872	0	0	0	0	49	55	19542620	1	1
UBL5	1069900	579	-26	205768	48416	0	0	0	0	50	87	20502040	1	1

UBR7	325787	222	35	1154864	252760	0	0	0	0	1	0	372376	1	1
UBTFL1	NaN	NaN	NaN	1023144	238164	0	0	1	0	50	117	39883748	1	1
UBXN10	649343	508	30	690996	213600	0	0	0	0	50	72	24127900	1	1
UBXN8	365415	346	28	705236	194020	0	0	2	0	1	2	411180	1	1
UCK2	321876	403	1	683520	181560	0	0	3	0	0	3	181560	1	1
UCN	1245306	182	52	294056	112852	0	0	0	0	50	41	22639464	1	1
UCN2	1433482	183	41	268780	97544	0	0	0	0	50	91	30129348	1	1
UCP3	523442	225	51	792812	237808	0	0	1	0	50	45	21449000	1	1
UFD1L	650407	300	16	827700	206124	0	0	2	0	2	3	469208	1	1
UFSP1	1339207	433	48	347456	110716	0	0	1	0	1	4	647920	1	1
UGT2A2	19584	1065	-8	1353512	362764	0	0	0	0	49	60	22864456	1	1
ULBP2	423805	245	34	632612	178000	0	0	0	0	50	59	20035324	1	1
UNC50	260302	696	39	669992	184408	0	0	1	0	50	65	21358576	1	1
UNKL	1211641	215	40	1281956	391600	0	0	0	0	18	25	8945212	1	1
UPF0639	NaN	NaN	NaN	1338560	339268	0	0	0	0	50	116	39984852	1	1
UPK3B	882992	220	56	929516	201496	0	0	0	0	50	51	18156712	1	1
UPK3BL	NaN	NaN	NaN	658244	211108	0	0	0	0	50	116	39856692	1	1
UPP1	108417	409	26	801000	225348	0	0	0	0	50	38	21206920	1	1
UQCC	1308738	170	50	794948	204700	0	0	0	0	50	46	20035324	1	1
UQCR11	2259505	213	33	148452	44856	0	0	0	0	50	56	21477124	1	1
UQCRQ	686574	200	66	216804	57672	0	0	0	0	50	52	16038868	1	1
URB1	NaN	NaN	NaN	5751180	1692780	0	0	0	0	0	0	1692780	1	1
URM1	1928973	188	64	432896	128872	0	0	0	0	50	61	23763356	1	1
USP17	NaN	NaN	NaN	7962652	2238884	0	0	0	0	0	0	2238884	1	1
USP27X	NaN	NaN	NaN	1111788	294768	0	0	1	0	50	117	39940352	1	1
USP50	325027	203	27	877184	221788	0	0	0	0	50	59	24680412	1	1
UTF1	NaN	NaN	NaN	793168	309008	0	0	0	0	50	116	39954592	1	1
UTP15	403014	289	42	1349596	364544	0	0	0	0	50	67	21722052	1	1
UTS2D	112669	773	9	321468	80100	0	0	0	0	50	66	26969136	1	1
UXT	770573	NaN	45	446780	121396	0	0	1	0	4	2	1344256	1	1
VAMP1	2702142	198	50	335708	89356	0	0	0	0	50	83	26876220	1	1
VAMP7	NaN	NaN	NaN	781420	157352	0	0	1	0	50	117	39802936	1	1
VASH2	315884	412	12	914920	251336	0	0	0	0	49	65	24874788	1	1

VAX1	202295	640	-14	918124	311144	0	0	1	0	31	34	13339676	1	1
VBP1	371405	NaN	-13	532220	125668	0	0	0	0	50	60	21240384	1	1
VCX	43100	NaN	-9	526524	140976	0	0	0	0	50	96	29130412	1	1
VCY	NaN	NaN	NaN	632968	195800	0	0	0	0	50	116	39841384	1	1
VCY1B	NaN	NaN	NaN	632968	195800	0	0	0	0	50	116	39841384	1	1
VDAC3	535273	459	37	751516	195800	0	0	0	0	50	58	21295208	1	1
VENTX	339279	452	-12	629052	211464	0	0	2	0	2	2	954436	1	1
VGLL1	358658	NaN	43	655040	191884	0	0	0	0	50	60	22953100	1	1
VIPAR	527850	359	49	1323964	337844	0	0	0	0	32	44	15803552	1	1
VMAC	1166723	392	36	406552	144536	0	0	0	0	50	67	17965184	1	1
VN1R5	352232	742	-15	891068	254896	0	0	0	0	50	50	15613092	1	1
VOPP1	947740	541	23	445356	126024	0	0	0	0	50	74	21057044	1	1
VPREB3	610486	266	30	310432	93272	0	0	0	0	50	71	20499548	1	1
VPS24	617233	424	18	595232	140620	0	0	0	0	50	62	23532668	1	1
VPS25	1633092	190	33	474904	115700	0	0	0	0	24	22	8134244	1	1
VSIG10L	NaN	NaN	NaN	2079040	740480	0	0	0	0	7	14	4428640	1	1
VSTM2B	NaN	NaN	NaN	695268	238164	0	0	0	0	50	116	39883748	1	1
VWA5B1	NaN	NaN	NaN	3069788	915988	0	0	0	0	5	8	3288016	1	1
VWA5B2	NaN	NaN	NaN	3023864	1035604	0	0	0	0	0	0	1035604	1	1
VWDE	NaN	NaN	NaN	4165200	1051980	0	0	0	0	0	0	1051980	1	1
WASH1	332463	398	NaN	1185480	350304	0	0	0	0	29	40	14411236	1	1
WBSCR28	1099366	208	37	649700	215380	0	0	2	0	12	12	3580292	1	1
WDFY2	246186	384	46	1062304	269492	0	0	1	0	50	55	20167756	1	1
WDFY4	NaN	NaN	NaN	8122140	2343192	0	0	0	0	0	0	2343192	1	1
WDR16	79661	336	-24	1611256	436100	0	0	0	0	8	9	2543620	1	1
WDR45L	1250099	217	26	908156	235672	0	0	0	0	50	67	21838464	1	1
WDR52	440896	672	24	4892508	1210044	0	0	0	0	0	0	1210044	1	1
WDR5B	416095	478	34	826632	233892	0	0	0	0	50	33	16216156	1	1
WDR65	1068006	294	30	1788544	496976	0	0	0	0	32	32	15081584	1	1
WDR67	482518	203	27	2811688	698828	0	0	0	0	2	0	819512	1	1
WDR69	161693	425	4	1103956	282308	0	0	0	0	50	36	17719900	1	1
WDR8	250027	288	16	1191888	332148	0	0	0	0	36	30	13749432	1	1
WDR85	1315685	404	41	1160204	325384	0	0	0	0	0	0	325384	1	1

WDR86	608657	190	39	929160	300108	0	0	0	0	50	44	20451132	1	1
WDR87	NaN	NaN	NaN	7284828	1946964	0	0	0	0	0	0	1946964	1	1
WDR89	320174	221	27	982560	264864	0	0	0	0	50	71	27035352	1	1
WDR92	190719	594	5	933076	245996	0	0	0	0	50	43	17972304	1	1
WDYHV1	544059	225	35	552156	129228	0	0	0	0	50	54	19128592	1	1
WFDC1	585154	396	8	563548	170168	0	0	0	0	50	58	22662604	1	1
WFDC10A	NaN	NaN	NaN	206124	56604	0	0	0	0	50	116	39702188	1	1
WFDC10B	777303	359	16	411536	82948	0	0	0	0	50	71	19735928	1	1
WFDC11	736165	438	18	244928	49840	0	0	0	0	50	60	20623436	1	1
WFDC5	865186	324	46	325384	86864	0	0	0	0	50	92	27552620	1	1
WFDC6	1069725	438	36	233180	56248	0	0	0	0	50	84	21025004	1	1
WFDC9	736165	438	18	245640	55536	0	0	0	0	50	60	20623436	1	1
WFIKKN1	1115707	213	39	1335000	430404	0	0	1	0	39	42	13074456	1	1
WHSC2	747944	321	38	1351732	421148	0	0	0	0	2	3	2275196	1	1
WNT1	1737773	214	55	907088	294412	0	0	1	0	50	50	18572876	1	1
WNT10A	1087669	386	42	1030264	321824	0	0	2	0	50	74	18604204	1	1
WNT8A	1044706	377	41	917056	234248	0	0	1	0	50	71	18121112	1	1
WNT8B	1153157	264	50	887508	261660	0	0	0	0	50	61	20699264	1	1
XAGE1A	NaN	NaN	NaN	1304740	169100	0	0	0	0	50	116	39814684	1	1
XAGE1B	NaN	NaN	NaN	1304740	169100	0	0	0	0	50	116	39814684	1	1
XAGE1C	NaN	NaN	NaN	1304740	169100	0	0	0	0	50	116	39814684	1	1
XAGE1D	NaN	NaN	NaN	1304740	169100	0	0	0	0	50	116	39814684	1	1
XAGE1E	NaN	NaN	NaN	1304740	169100	0	0	0	0	50	116	39814684	1	1
XAGE2B	NaN	NaN	NaN	583840	143468	0	0	0	0	50	116	39789052	1	1
XCL1	389559	440	15	290496	88644	0	0	0	0	50	53	17696404	1	1
XCL2	389559	440	15	290140	89000	0	0	0	0	50	53	17696404	1	1
XCR1	225584	551	54	816308	258100	0	0	1	0	50	52	16537980	1	1
XKRY	NaN	NaN	NaN	588824	167320	0	0	0	0	50	116	39812904	1	1
XKRY2	NaN	NaN	NaN	588824	167320	0	0	0	0	50	116	39812904	1	1
YBX2	1782958	316	21	897476	308296	0	0	0	0	39	39	14081580	1	1
YDJC	521773	194	37	780352	276968	0	0	0	0	12	11	5081544	1	1
YPEL1	447011	252	56	320756	76540	0	0	1	0	8	9	3332160	1	1
YPEL2	1000345	452	16	318620	78676	0	0	0	0	50	57	16009320	1	1

YPEL3	2261614	201	56	405840	113208	0	0	0	0	50	76	22107600	1	1
YSK4	297611	434	28	3421160	877540	0	0	0	0	11	5	3532944	1	1
ZAK	219235	429	16	2447856	608760	0	0	0	0	5	6	2331088	1	1
ZAR1L	NaN	NaN	NaN	815952	230688	0	0	0	0	50	116	39876272	1	1
ZBED3	NaN	NaN	NaN	553224	203988	0	0	0	0	50	116	39849572	1	1
ZBED5	NaN	NaN	NaN	1740484	483092	0	0	0	0	50	116	40128676	1	1
ZBED6	NaN	NaN	NaN	2470996	666788	0	0	0	0	7	14	4354948	1	1
ZBTB42	NaN	NaN	NaN	1018160	341404	0	0	0	0	50	116	39986988	1	1
ZBTB8B	NaN	NaN	NaN	1251340	350660	0	0	0	0	50	116	39996244	1	1
ZC3H8	591586	348	29	798152	171592	0	0	1	0	50	51	21061672	1	1
ZC3HAV1I	553735	243	29	760416	223212	0	0	0	0	50	84	23437972	1	1
ZCCHC10	654533	223	38	449628	113208	0	0	1	0	11	18	5207924	1	1
ZCCHC17	675993	215	30	651124	154148	0	0	1	0	50	52	16586396	1	1
ZCRB1	174511	545	18	594876	133500	0	0	0	0	50	51	19585696	1	1
ZDHHC12	1865107	197	38	662160	215736	0	0	1	0	50	70	23922844	1	1
ZDHHC24	1343990	194	62	669992	251692	0	0	1	0	50	53	20002216	1	1
ZFP106	570116	284	37	4789980	1323252	0	0	0	0	0	0	1323252	1	1
ZFP112	705123	671	-38	2387692	557852	0	0	0	0	0	0	557852	1	1
ZFP161	52395	1017	-6	1158424	291920	0	0	0	0	33	38	12798912	1	1
ZFP62	NaN	NaN	NaN	2321120	573160	0	0	0	0	9	16	4930600	1	1
ZFP92	NaN	NaN	NaN	1036672	311144	0	0	1	0	50	117	39956728	1	1
ZFYVE20	564079	395	44	2008552	549308	0	0	0	0	25	31	12087980	1	1
ZG16	NaN	NaN	NaN	425420	127804	0	0	0	0	50	116	39773388	1	1
ZMAT5	848649	195	37	450340	118904	0	0	0	0	50	54	19150308	1	1
ZMYND10	843769	206	51	1142404	317552	0	0	2	0	50	68	20472848	1	1
ZMYND17	847821	249	45	1153084	349592	0	0	0	0	50	67	24394188	1	1
ZNF167	419284	467	45	1949456	510148	0	0	0	0	3	4	1774304	1	1
ZNF169	219475	445	31	1524748	427556	0	0	1	0	50	67	27019332	1	1
ZNF177	401242	705	-65	1259172	306516	0	0	0	0	8	13	3433976	1	1
ZNF187	273319	502	35	1244932	304736	0	0	0	0	27	25	8631220	1	1
ZNF192	202009	514	34	1480248	396228	0	0	0	0	8	11	4628000	1	1
ZNF193	237664	486	34	1018160	260236	0	0	0	0	5	9	1798512	1	1
ZNF2	223452	616	24	1094700	290496	0	0	1	0	48	53	21341844	1	1

ZNF22	79469	905	15	583840	141332	0	0	1	0	50	68	20748036	1	1
ZNF238	220918	795	-11	1345680	363120	0	0	0	0	3	3	1262732	1	1
ZNF259	419339	326	36	1208620	322892	0	0	0	0	50	47	21154588	1	1
ZNF268	334913	834	6	517268	111784	0	0	1	0	50	71	19917132	1	1
ZNF295	241619	430	84	2671780	751160	0	0	0	0	3	3	1683524	1	1
ZNF321	425659	984	-56	430048	102884	0	0	0	0	50	49	18465008	1	1
ZNF322A	397964	619	14	1047352	243860	0	0	0	0	50	54	21261744	1	1
ZNF322B	308580	548	17	1046284	244928	0	0	0	0	50	55	26358596	1	1
ZNF323	273498	502	33	1053760	267356	0	0	0	0	35	30	12904644	1	1
ZNF389	NaN	NaN	NaN	703100	166252	0	0	0	0	50	116	39811836	1	1
ZNF397OS	260556	599	7	1297976	320044	0	0	0	0	50	41	21849856	1	1
ZNF414	726124	214	1	974372	321112	0	0	1	0	50	67	22455056	1	1
ZNF416	459366	828	-31	1527596	391600	0	0	1	0	50	62	16455032	1	1
ZNF428	441738	209	6	552868	163760	0	0	0	0	50	64	22145336	1	1
ZNF434	1361831	303	29	1247068	322892	0	0	0	0	40	53	17390600	1	1
ZNF444	500375	480	-61	793880	269848	0	0	0	0	0	0	269848	1	1
ZNF469	NaN	NaN	NaN	9384516	3198660	0	0	0	0	0	0	3198660	1	1
ZNF497	1621097	406	-25	1226064	377004	0	0	3	0	50	63	19879752	1	1
ZNF498	1395063	430	34	1378076	389464	0	0	0	0	41	52	15693904	1	1
ZNF503	173705	547	13	1515492	561768	0	0	2	0	18	22	8155604	1	1
ZNF541	NaN	NaN	NaN	3407632	1028840	0	0	0	0	0	0	1028840	1	1
ZNF579	1094560	399	4	1331440	476684	0	0	0	0	27	25	7431500	1	1
ZNF618	354541	591	-3	2221440	598080	0	0	1	0	12	12	4555376	1	1
ZNF621	195638	546	22	1118196	308652	0	0	0	0	50	48	20952736	1	1
ZNF642	701723	457	31	1377720	332148	0	0	0	0	50	59	24104048	1	1
ZNF643	701723	457	31	1385196	346032	0	0	0	0	50	59	24104048	1	1
ZNF673	194312	NaN	27	505520	113920	0	0	0	0	50	54	18733788	1	1
ZNF696	1657385	389	19	927024	283020	0	0	1	0	50	72	20208696	1	1
ZNF705D	475312	625	3	796372	189392	0	0	0	0	50	62	20793248	1	1
ZNF705G	NaN	NaN	NaN	793168	189392	0	0	0	0	50	116	39834976	1	1
ZNF706	1084949	371	46	210396	49128	0	0	0	0	50	66	19910012	1	1
ZNF717	NaN	NaN	NaN	2373808	573872	0	0	0	0	9	16	4931312	1	1
ZNF720	NaN	NaN	NaN	343540	80456	0	0	0	0	50	116	39726040	1	1

ZNF740	1729009	191	55	516200	126736	0	0	1	0	35	36	12318668	1	1
ZNF747	2214934	142	54	454968	164472	0	0	0	0	50	72	21947756	1	1
ZNF763	719617	678	-1	1044148	243860	0	0	0	0	50	85	22511660	1	1
ZNF771	NaN	NaN	NaN	785692	245996	0	0	0	0	50	116	39891580	1	1
ZNF775	315538	514	32	1314352	417944	0	0	1	0	50	70	24657628	1	1
ZNF787	565708	480	-66	941264	297616	0	0	0	0	0	0	297616	1	1
ZNF79	742340	209	43	1287296	328588	0	0	1	0	50	64	25586076	1	1
ZNF816A	425659	984	-56	1695628	406196	0	0	0	0	47	43	17882236	1	1
ZNF826	NaN	NaN	NaN	450340	119972	0	0	0	0	50	116	39765556	1	1
ZNF828	612511	283	46	2008908	603420	0	0	0	0	12	13	5849792	1	1
ZNF837	NaN	NaN	NaN	1280888	427912	0	0	0	0	50	116	40073496	1	1
ZNF843	NaN	NaN	NaN	828056	290140	0	0	0	0	50	116	39935724	1	1
ZNF879	NaN	NaN	NaN	1462804	361340	0	0	0	0	50	116	40006924	1	1
ZNF883	519671	575	-11	973660	241724	0	0	0	0	50	46	17403772	1	1
ZNRD1	1820240	496	30	336776	87220	0	0	0	0	50	54	18891140	1	1
ZNRF1	731807	160	27	571736	175864	0	0	0	0	50	53	23106536	1	1
ZSCAN12	NaN	NaN	NaN	1580996	392668	0	0	0	0	50	116	40038252	1	1
ZSCAN16	213170	562	23	901748	229264	0	0	1	0	50	42	19286656	1	1
ZSCAN23	NaN	NaN	NaN	1000360	262016	0	0	1	0	50	117	39907600	1	1
ZSWIM6	NaN	NaN	NaN	3052344	899256	0	0	0	0	5	8	3271284	1	1
ZYG11A	NaN	NaN	NaN	1954440	536136	0	0	0	0	9	16	4893576	1	1
