# Supplementary material for: An Epithelial-Mesenchymal Transition (EMT) Preoperative Nomogram for Prediction of Lymph Node Metastasis in Bladder Cancer (BLCA)
Source: Dis Markers. 2020 Nov 3;2020:8833972. doi: 10.1155/2020/8833972 (PMC7656235; doi:10.1155/2020/8833972)
Supplement: Supplementary 5 — Supplementary Table S5: differentially mutated genes based on LN metastasis status. [file 8833972.f5.docx]

| Symbol | Fisher test p value |
| --- | --- |
| C3orf70 | 0.013517206 |
| TP53 | 0.027352667 |
| FGFR3 | 0.037310566 |
| RHOB | 0.049965145 |
| ZFP36L1 | 0.076662265 |
| RB1 | 0.208741189 |
| FBXW7 | 0.246109015 |
| FOXQ1 | 0.272862142 |
| NCOR1 | 0.360262769 |
| KDM6A | 0.38696192 |
| PIK3CA | 0.41974614 |
| STAG2 | 0.432186131 |
| PSIP1 | 0.465467856 |
| ARID1A | 0.528331626 |
| CDKN1A | 0.573715987 |
| CDKN2A | 0.625728123 |
| KLF5 | 0.639071443 |
| RBM10 | 0.649964584 |
| EP300 | 0.77186367 |
| TSC1 | 0.841494133 |
| ELF3 | 1 |
| CREBBP | 1 |
| PARD3 | 1 |
| RHOA | 1 |
| HRAS | 1 |
