# Supplementary material for: An Epithelial-Mesenchymal Transition (EMT) Preoperative Nomogram for Prediction of Lymph Node Metastasis in Bladder Cancer (BLCA)
Source: Dis Markers. 2020 Nov 3;2020:8833972. doi: 10.1155/2020/8833972 (PMC7656235; doi:10.1155/2020/8833972)
Supplement: Supplementary 6 — Supplementary Table S6: selected features with nonzero coefficients in the LASSO logistic regression model. [file 8833972.f6.docx]

| Supplementary Table S6: Selected features with nonzero coefficients in the LASSO logistic regression model. | | | |
| --- | --- | --- | --- |
|  |  |  |  |
| Features | Coeffcient |  | EMT-LN signature = ACTA2*(-9.507734e-04)+ANPEP*(4.855212e-04)+COMP*(-3.336651e-04)+DAB2*(8.354169e-03)+ELN*(7.837673e-03)+FOXC2*(-2.707915e-02)+IGFBP2*(-3.429749e-03)+MGP*(1.656966e-06)+MMP1*(-5.033370e-05)+MYL9*(-1.244071e-04)+OXTR*(3.535212e-03)+PRSS2*(-1.303686e-04)+PTX3*(3.235470e-03)+RGS4*(3.024129e-02)+SPP1*(4.988541e-05)+TAGLN*(1.122562e-03)+WNT5A*(-1.098872e-03) |
| (Intercept) | -8.08E-01 |  |  |
| ACTA2 | -9.51E-04 |  |  |
| ANPEP | 4.86E-04 |  |  |
| COMP | -3.34E-04 |  |  |
| DAB2 | 8.35E-03 |  |  |
| ELN | 7.84E-03 |  |  |
| FOXC2 | -2.71E-02 |  |  |
| IGFBP2 | -3.43E-03 |  |  |
| MGP | 1.66E-06 |  |  |
| MMP1 | -5.03E-05 |  |  |
| MYL9 | -1.24E-04 |  |  |
| OXTR | 3.54E-03 |  |  |
| PRSS2 | -1.30E-04 |  |  |
| PTX3 | 3.24E-03 |  |  |
| RGS4 | 3.02E-02 |  |  |
| SPP1 | 4.99E-05 |  |  |
| TAGLN | 1.12E-03 |  |  |
| WNT5A | -1.10E-03 |  |  |
