# Supplementary material for: An Epithelial-Mesenchymal Transition (EMT) Preoperative Nomogram for Prediction of Lymph Node Metastasis in Bladder Cancer (BLCA)
Source: Dis Markers. 2020 Nov 3;2020:8833972. doi: 10.1155/2020/8833972 (PMC7656235; doi:10.1155/2020/8833972)
Supplement: Supplementary 7 — Supplementary Table S7: multivariable logistic regression analysis for preoperative features. [file 8833972.f7.docx]

| Univariable logistic regression |  |  |  |  |  |
| --- | --- | --- | --- | --- | --- |
|  | β | Odds ratio | Lower 95%CI | Upper 95%CI | P value |
| (Intercept) | -0.894 |  |  |  | 0.000 |
| EMT-LN signature | 1.073 | 2.924 | 1.914 | 4.706 | 0.000 |
| FGFR3 | -0.320 | 0.726 | 0.327 | 1.518 | 0.410 |
| C3orf70 | -2.068 | 0.126 | 0.007 | 0.658 | 0.049 |
| TP53 | 0.356 | 1.428 | 0.890 | 2.297 | 0.140 |
|  |  |  |  |  |  |
|  |  |  |  |  |  |
| Multivariable logistic regression |  |  |  |  |  |
|  | β | Odds ratio | Lower 95%CI | Upper 95%CI | P value |
| (Intercept) | -0.775 |  |  |  | 0.000 |
| EMT-LN signature | 1.128 | 3.089 | 2.027 | 4.950 | 0.000 |
| C3orf70 | -2.071 | 0.126 | 0.007 | 0.657 | 0.049 |
