# Supplementary figures and images for: An Epithelial-Mesenchymal Transition (EMT) Preoperative Nomogram for Prediction of Lymph Node Metastasis in Bladder Cancer (BLCA)
Source: Dis Markers. 2020 Nov 3;2020:8833972. doi: 10.1155/2020/8833972 (PMC7656235; doi:10.1155/2020/8833972)

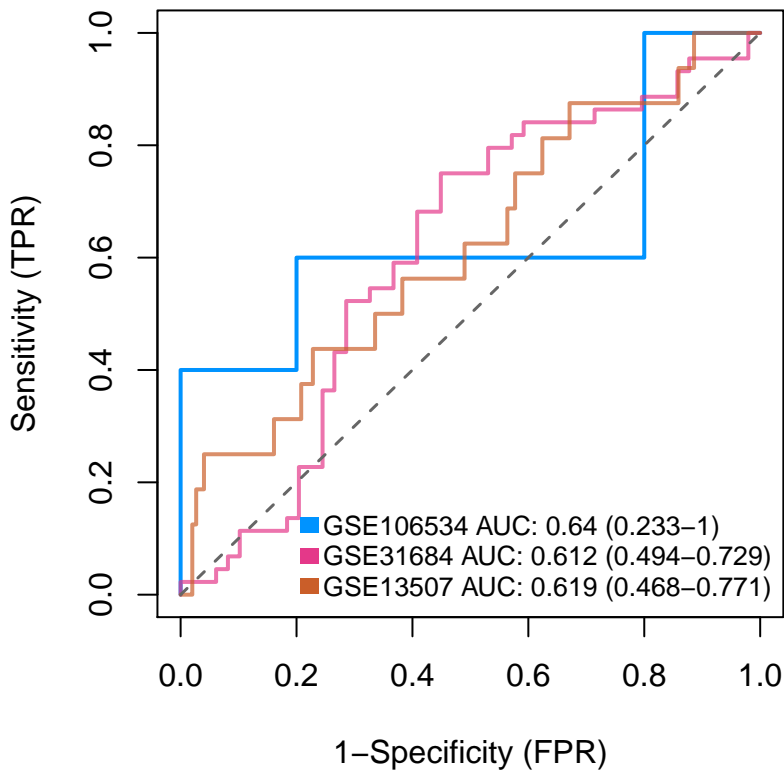

Supplement: Supplementary 8 — Figure S1: the design and workflow of the study. [file 8833972.f8.pdf]
