# Supplementary material for: An Epithelial-Mesenchymal Transition (EMT) Preoperative Nomogram for Prediction of Lymph Node Metastasis in Bladder Cancer (BLCA)
Source: Dis Markers. 2020 Nov 3;2020:8833972. doi: 10.1155/2020/8833972 (PMC7656235; doi:10.1155/2020/8833972)
Supplement: Supplementary 9 — Figure S2: ROC curves showed the prediction accuracy of EMT-LN signature in prediction of the LN metastasis in GSE13507, GSE31684, and GSE106534 cohorts. [file 8833972.f9.pdf]

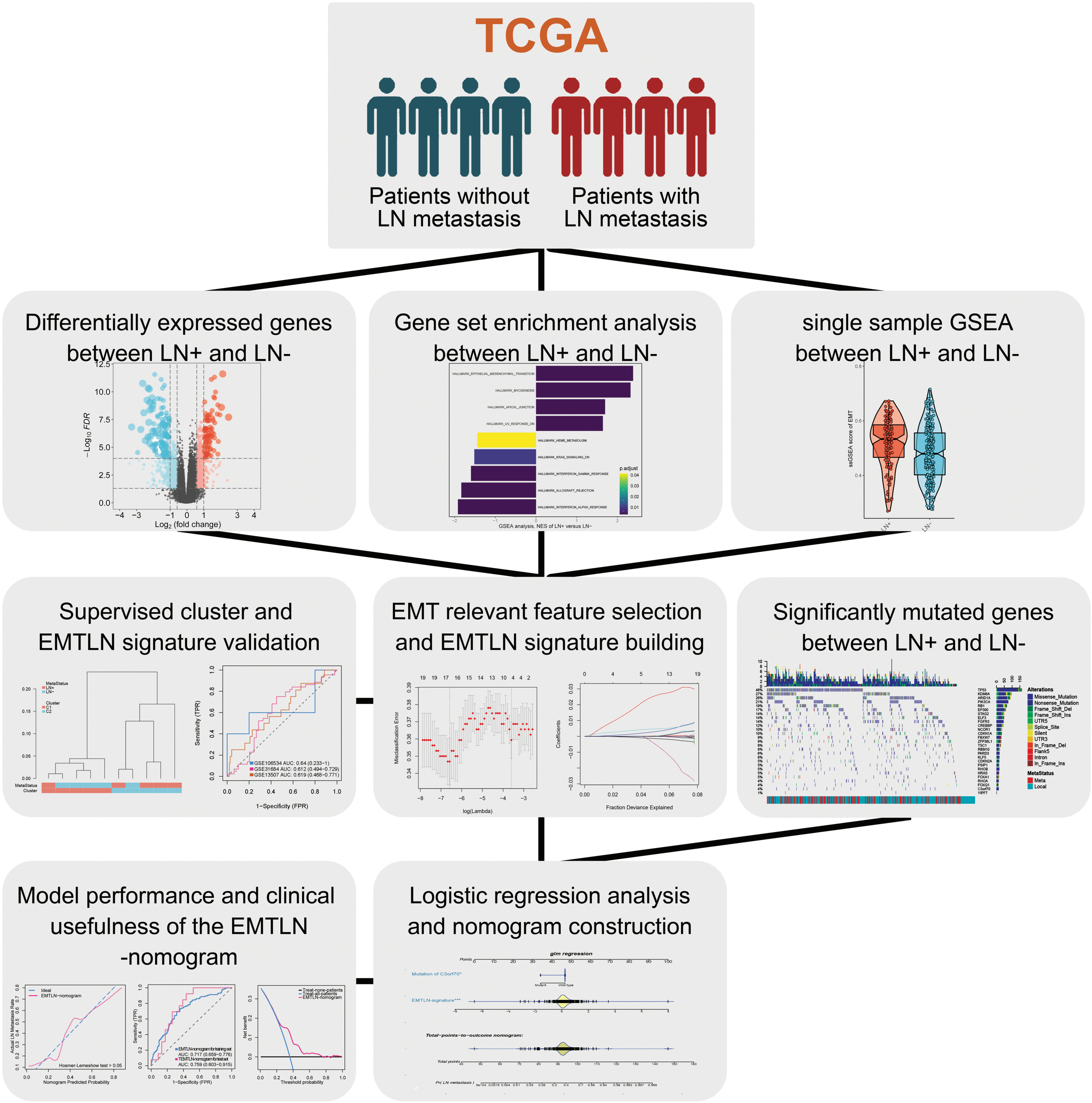

TCGA
Patients without LN metastasis
Patients with LN metastasis
Differentially expressed genes between LN+ and LN-
Gene set enrichment analysis between LN+ and LN-
single sample GSEA between LN+ and LN-
Supervised cluster and EMTLN signature validation
EMT relevant feature selection and EMTLN signature building
Significantly mutated genes between LN+ and LN-
Model performance and clinical usefulness of the EMTLN -nomogram
Logistic regression analysis and nomogram construction
